# Extensive intron gain in the ancestor of placental mammals

**DOI:** 10.1186/1745-6150-6-59

**Published:** 2011-11-23

**Authors:** Dušan Kordiš

**Affiliations:** 1 Department of Molecular and Biomedical Sciences; Josef Stefan Institute; Ljubljana, Slovenia

## Abstract

**Background:**

Genome-wide studies of intron dynamics in mammalian orthologous genes have found convincing evidence for loss of introns but very little for intron turnover. Similarly, large-scale analysis of intron dynamics in a few vertebrate genomes has identified only intron losses and no gains, indicating that intron gain is an extremely rare event in vertebrate evolution. These studies suggest that the intron-rich genomes of vertebrates do not allow intron gain. The aim of this study was to search for evidence of *de novo *intron gain in domesticated genes from an analysis of their exon/intron structures.

**Results:**

A phylogenomic approach has been used to analyse all domesticated genes in mammals and chordates that originated from the coding parts of transposable elements. Gain of introns in domesticated genes has been reconstructed on well established mammalian, vertebrate and chordate phylogenies, and examined as to where and when the gain events occurred. The locations, sizes and amounts of *de novo *introns gained in the domesticated genes during the evolution of mammals and chordates has been analyzed. A significant amount of intron gain was found only in domesticated genes of placental mammals, where more than 70 cases were identified. *De novo *gained introns show clear positional bias, since they are distributed mainly in 5' UTR and coding regions, while 3' UTR introns are very rare. In the coding regions of some domesticated genes up to 8 *de novo *gained introns have been found. Intron densities in Eutheria-specific domesticated genes and in older domesticated genes that originated early in vertebrates are lower than those for normal mammalian and vertebrate genes. Surprisingly, the majority of intron gains have occurred in the ancestor of placentals.

**Conclusions:**

This study provides the first evidence for numerous intron gains in the ancestor of placental mammals and demonstrates that adequate taxon sampling is crucial for reconstructing intron evolution. The findings of this comprehensive study slightly challenge the current view on the evolutionary stasis in intron dynamics during the last 100 - 200 My. Domesticated genes could constitute an excellent system on which to analyse the mechanisms of intron gain in placental mammals.

**Reviewers: **this article was reviewed by Dan Graur, Eugene V. Koonin and Jürgen Brosius.

## Background

Spliceosomal introns are one of the major eukaryote-specific genome components, and their evolution has been studied extensively during the last decade [for recent reviews see [1-11]]. The availability of numerous eukaryotic genomes has enabled genome-wide studies of the intron loss and gain dynamics [reviewed in [2,4,5] and [12]]. These studies have been limited mostly to comparisons of intron positions across highly conserved, orthologous or paralogous genes from often very distant species. Two main approaches - maximum parsimony (MP) and maximum likelihood (ML) - have been used for inferring intron evolution from the patterns of intron position conservation [reviewed in [4]].

Maximum likelihood reconstruction of intron gain and loss in eukaryotes has revealed a significant excess of losses and a nonuniform distribution of gains and losses [2,4,5,12-14]. A substantial excess of intron gains has been detected only for those intervals of eukaryotic evolution that are associated with major evolutionary innovations, such as the origin of eukaryotes and animals [5,12-14]. It appears that intron losses, although rare, occur at a measurable rate in most eukaryotes [2,4,12-15] whereas intron gains, at least in the last 100 - 200 Myrs, appear to be restricted to specific taxonomic groups [5,12-14,16-21]. Overall, many more loss than gain events have been inferred and documented [2,4,12-14]. The large-scale comparisons of the evolutionary dynamics of introns have demonstrated surprising evolutionary stasis in the intron dynamics over the last 100 - 200 My [13,14].

In some large taxonomic groups, such as in vertebrates and especially in mammals, no intron gain was observed in the genome-wide comparisons [15,22,23]. Large-scale intron studies in orthologous mammalian genes have indicated that very little intron turnover has occurred, with convincing evidence only for loss of introns [15,22]. Similarly, the analysis of a few fish genomes and the fish-mammals comparisons identified only intron losses and no gains, indicating that intron gain is an extremely rare event in vertebrate evolution [23]. These genome-wide studies suggest that most of the introns in the extant vertebrates are likely to be of ancient origin [15,22-24], and that the intron-rich genomes of vertebrates might not allow intron gain [23].

Such absence of intron gain in »recent« evolutionary history might be real, but could also be artefactual, the consequence of inadequate taxon sampling or inadequate comparisons, since only the »old« orthologous genes have been compared. To test the claims on the absence of intron gain in some taxonomic groups such as mammals [15,22] and in recent evolutionary history (in the last 100 - 200 Mya) [13,14], we need a quite simple and robust »gene model« that is independent of the inference procedures about intron gain. If the ancestral intron state is definitely known, as in the cases of horizontally transferred bacterial genes into Entamoeba [25] or of plastid genes into the nuclei of plants [26], where the ancestral intron state was intronless, the intron gain can be easily recognized. Such an approach, coupled with the known ancestral state (intronless), has been used in this study for evaluation of the hypotheses on the absence of intron gain in the recent evolutionary past [13,14], and especially in mammals [15,22].

Domesticated genes [27-31], originating from retroelements or from DNA transposons, constitute an ideal system for testing the hypothesis on the absence of intron gain in mammals. Since single copy domesticated genes [29] originated from the intronless multicopy transposable elements (TEs) [32], the ancestral intron state for domesticated genes is zero. Therefore, any intron present in these genes will constitute a *de novo *gained intron. The prerequisite for recognizing the origin, extent and timing of *de novo *gained introns is reliable and wide taxon sampling [33]. In the past few years a quite large and dense collection of vertebrate, and especially mammalian, genomes has been accumulated. For some of these taxa a number of well annotated genomes and genes exist, human and mouse genomes and transcriptomes being especially useful, with the full-length mRNAs that enabled reconstructions of the complete gene structures in these species [34,35]. By using annotated human or mouse introns we can trace their origin in mammals through genome wide comparisons of orthologous genes in placentals, marsupials and monotremes.

In this study a phylogenomic approach [36] has been used to analyse all domesticated genes in mammals and chordates that originated from the coding parts of TEs. The aim was to look for evidence of *de novo *intron gain in domesticated genes of mammals from an analysis of their exon/intron structures. The location, size and amount of *de novo *gained introns in domesticated genes of mammals and chordates have been analyzed. Surprisingly, a burst of intron gain was found only in domesticated genes of placental mammals. *De novo *gained introns shows clear positional bias since they are distributed mainly in 5' UTR and coding regions, and rarely in 3' UTRs. Up to 8 *de novo *gained introns have been found in the coding regions of some domesticated genes, with intron density of 4 introns per kb. Surprisingly, the majority of intron gains have taken place in the ancestor of placentals. None of these cases of intron gain have been observed in any of the previous genome-wide analyses [15,22] as a consequence of biased comparisons of a limited number of evolutionarily younger placental species that belong only to the superorder Boreoeutheria [37-39].

## Results

### The ancestral intron state in domesticated genes is known and is intronless

In the majority of previous genome-wide studies of the intron gain there was a remarkable degree of uncertainty concerning the ancestral intron states. Single copy domesticated genes originated from multicopy TEs, their parts or from their remains [27-31]. Since the TEs are without introns the ancestral intron state for domesticated genes is known and is intronless. Therefore any intron found in domesticated genes will have been gained *de novo*, either in the coding or untranslated regions of the gene. An extensive phylogenomic analysis of all domesticated genes in chordate and mammalian genomes has therefore been made. The exon/intron structures of domesticated genes were studied in detail throughout mammals, vertebrates and chordates. The most important part of this study was to find the transition point where and when TEs were transformed into domesticated genes, allowing *de novo *gain of introns to be precisely pinpointed in these genes.

### Phylogenomic analysis of domesticated genes in chordates and mammals

The rich collection of numerous mammalian genomes, belonging to all three major extant mammalian lineages, Eutheria (placentals), Metatheria (marsupials) and Prototheria (monotremes), is a major advantage in studying the origin and evolution of domesticated genes. In the placental mammals, genomes of four superorders (Afrotheria, Xenarthra, Laurasiatheria and Euarchontoglires) are well represented. In addition to the mammalian genomes, all other available vertebrate and chordate genomes were analyzed in order to find the transition point from TEs to domesticated genes. Until now only a few studies of TE-derived domesticated genes have been published [27-31], but due to the limited availability of genome data at the time of study, the origin of domesticated genes in the vast majority of cases has remained unknown.

By the phylogenomic analysis of all available domesticated genes in mammalian, vertebrate and chordate genomes, unequivocal data about their origins (when and in which taxonomic group they originated) and numerous gene-related data (exon/intron structure, genome location, chromosomal position etc.) have been obtained. Crucial information about the transition points from TEs to domesticated genes has been obtained in this study. Even more importantly, the gene structures of domesticated genes has provided direct evidence for extensive intron gain in placental mammals. An extensive phylogenomic analysis of these genes will be presented elsewhere (Kokosar and Kordis, manuscript in preparation).

### The majority of mammal-specific domesticated genes contain *de novo *gained introns

The analysis of all known domesticated genes in chordates and mammals (Additional file 1, Tables 1 and 2) shows that both retroelement- and DNA transposon-derived genes contain introns. In the case of retroelement-derived genes the exon/intron structures are simple, since in these cases the process of gene fusion or exon shuffling to the pre-existing »normal« genes is almost always absent (one such exception is the SCAND3 gene).

**Table 1:** DNA transposon-derived genes with *de novo *gained introns

TE group (progenitor of the novel gene)	Gene name (ID)	Presence of introns	Location of introns	Number of introns
Tc1/mariner/pogo	JRK	yes	5' UTR/coding	2
	JRKL	yes	coding	2
	TIGD 1-7	yes	5' UTR	1
	POGZ	yes	coding	5-13
	POGK	yes	coding	5
	SETMAR	yes	coding	2
Transib	RAG1	yes	coding	1-3
hAT	ZBED1	yes	5' UTR	1
	ZBED4	yes	5' UTR	1-2
	ZBED5	yes	5' UTR/coding	2
	ZMYM6	yes	coding	14
	ZNF862	yes	coding	7
	C5ORF54 (Buster3)	yes	5' UTR	1
	GTF2IRD2	yes	5' UTR/coding	15
	PRKRIR (THAP0)	yes	coding	4
*P*-element	THAP 1-11	yes	5' UTR/coding	diverse
PIF/Harbinger	HARBI1	yes	5' UTR/coding	2
	NAIF1	yes	coding	1
piggyBac	PGBD1	yes	5' UTR/coding	1-6
	PGBD2	yes	5' UTR/coding	2
	PGBD3	yes	5' UTR/coding	1-4
	PGBD4	yes	coding	1
	PGBD5	yes	coding	8

**Table 2:** Retroelement (Metaviridae and ERV)-derived genes with *de novo *gained introns

TE group (progenitor of the novel gene)	Gene name (ID)	Presence of introns	Location of introns	Number of introns
**gag**				
Chromovirus (sushi): MART	RGAG1	yes	5' UTR/coding	3
	ZCCHC16	yes	5' UTR	2-6
	ZCCHC5	yes	5' UTR	1
	PEG10	yes	5' UTR or coding	1-2
	LDOC1L	yes	5' UTR	1
	RGAG4	yes	3' UTR	1
	C22ORF29	yes	5' UTR	2
	FAM127b	yes	coding	1
Barthez: PNMA	PNMA2	yes	5' UTR	2
	PNMA3	yes	5' UTR	1
	MOAP1	yes	5' UTR	1-2
	PNMA5	yes	5' UTR or 3' UTR	3
	PNMA6A	yes	5' UTR or coding	1-2
	PNMA6B	yes	5' UTR	1
	ZCCHC12	yes	5' UTR	3
	ZCCHC18	yes	5' UTR	2
	CCDC8	yes	coding	1
	PNMAL1	yes	5' UTR	2
	PNMAL2	yes	coding	1
Osvaldo: ARC	ARC	yes	3' UTR	2
**rve**	Gin1	yes	5' UTR/coding	7
	SCAND3	yes	coding	3
	KRBA2	yes	5' UTR or coding	1-3
	NYNRIN	yes	5' UTR/coding	8
ERV: **env**	ERVFRD-1	yes	5' UTR	1
	ERVW-1	yes	5' UTR	1
	syncytin b	yes	5' UTR	2

However, the situation in the case of DNA transposon-derived genes is more complicated, since these genes can originate by three different routes: a) from the entire DNA transposon, b) by a complete DNA transposon being fused to the »normal« gene in the form of a single long exon that is 3' end located, and c) the most prevalent case, by gene fusion or exon shuffling of DNA binding domains (DBD) of DNA transposons with »normal« genes (where the exonization is necessary before the gene fusion). Therefore, in the case of DNA transposon-derived genes, intron gain can be recognized easily only in the first case, while the second and a third cases are much more difficult for inferring intron gain in these genes. In the majority of the cases of fused entire transposases or just the DBDs, the newly recruited exons remain intact as very long or relatively short exons, but they are mostly without any intron. Therefore, in the case of DNA transposon-derived genes, these fused transposases and DBDs have been excluded from the analysis of intron gain. Progenitors of novel domesticated genes were from diverse DNA transposon superfamilies, such as from Tc1/*mariner*, Transib, hAT, P-element, PIF/Harbinger and from piggyBac [29,31].

Of the 36 analyzed orthologous DNA transposon-derived genes, only 11 have been used for the inference and timing of intron gain (POGK, ZBED1, ZNF862, Buster3, PRKRIR, THAP9, Harbi1, Naif1, PGBD1, PGBD2 and PGBD5) (Table 1). In these cases the number of newly gained introns varies from 1 to 7, and they are located in the coding and 5' UTR regions.

The situation regarding intron gain in retroelement-derived genes is definitely much simpler and less problematic, since no fusion genes have originated from retroelements (except SCAND3). 34 orthologous retroelement-derived genes were analyzed and intron gain was found in 27 genes (Table 2). Among cases without intron gain are RTL1 and LDOC1 genes that originated from the *gag *gene of chromoviruses [27,28,40-42]. The number of gained introns in these cases varies greatly, from 1 to 8, their prevailing locations being 5' UTR and coding regions, with only three cases with 3' UTR locations of gained introns (PNMA5, RGAG4 and ARC).

### The burst of intron gain in domesticated genes was in the ancestor of placental mammals (Eutheria)

The analysis of all domesticated genes in chordates and mammals has shown that by far the greatest amount of intron gain occurred in the ancestor of placentals (Table 3). 20 intron-containing domesticated genes originated in the ancestor of placentals, 18 of them being retroelement-derived and only 2 DNA transposon-derived genes. Interestingly, a recent study reported that 11 retrogenes with newly gained 5' UTR introns also originated in the ancestor of placentals [19]. In the case of retrogenes they found 18 intron gains, 17 into the 5' UTR and a single gain in the 3' UTR. In the case of domesticated genes, I found 49 to 57 cases of *de novo *gained introns in the ancestor of placentals. In retroelement-derived genes 42 to 50 cases of intron gain have been found, while in DNA transposon-derived genes only 7 cases were found. Collectively, the 20 domesticated genes and 11 retrogenes provide evidence for at least 50 to 70 cases of intron gain in the ancestor of placentals. This finding contrasts strongly with previous studies [15,22], in which no intron gain could be found in mammals.

**Table 3:** Phylogenomic analysis of domesticated genes: insight into their origin and timing of intron gains

Gene name	Origin in the LCA of Chordata	Origin in the LCA of Gnathostomata	Origin in the LCA of Tetrapoda	Origin in the LCA of Amniota	Origin in the LCA of Theria	Origin in the LCA of placentals
RGAG1						▲
ZCCHC16						▲
ZCCHC5						▲
PEG10					▲	
LDOC1L						▲
RGAG4						▲
C22ORF29						▲
PNMA2						▲
PNMA3						▲
MOAP1						▲
PNMA5						▲
PNMA6A						▲
PNMA6B						▲
ZCCHC12						▲
ZCCHC18						▲
CCDC8						▲
PNMAL1						▲
PNMAL2						▲
ARC			▲			
SCAND3					▲	
KRBA2						▲
NYNRIN					▲	
POGK				▲		
ZBED1			▲			
ZNF862					▲	
Buster3						▲
PRKRIR		▲				
THAP 9		▲				
HARBI1		▲				
NAIF1		▲				
PGBD1						▲
PGBD2					▲	
PGBD5	▲					

Although these genes represent a very small proportion of placental gene innovations, the observed extent of intron gain most probably represents just the tip of the iceberg. Regardless of the situation with the normal mammalian and vertebrate genes (»old genes«), there was a large scale gene origination in the ancestor of placentals (evolutionarily young genes), at least in some classes of transcription factors (e.g. in C2H2 ZNFs). To test the extent of intron gain in some other placental-specific gene families I analyzed the presence of intron gain in KRAB and SCAN ZNF genes [43,44], especially in those orthologous genes that originated in the ancestor of placentals (>150 orthologous genes were analyzed). The analysis shows that the amount of intron gain in these genes is not as high as in the case of TE-derived domesticated genes and retrogenes, but a number of cases with intron gain can, even so, be recognized. The ancestral state for KRAB-ZNF, SCAN-ZNF and SCAN-KRAB-ZNF genes can easily be reconstructed, and is 1 intron in the coding region of SCAN-ZNF, 1 - 2 introns in the coding region of KRAB-ZNF and 2 - 3 in the coding region of SCAN-KRAB-ZNF genes. In those genes SCAN, KRAB and ZNF domains are encoded as single exons (or sometimes in two exons, in KRAB domain only). Therefore any serious deviations from these numbers of introns in coding regions most probably represent newly gained introns (Additional file 2).

### Characteristics of *de novo *gained introns in domesticated genes: numbers of introns per gene, intron densities, sizes of introns and preferred locations of *de novo *gained introns

#### a) Numbers of gained introns per gene

The numbers of *de novo *gained introns in domesticated genes vary greatly. In the case of DNA transposon-derived genes (Table 1), three orthologous genes exist with a single intron gain, either in the 5' UTR (ZBED1, Buster3) or in the coding region (NAIF 1). Two cases show from 1 to 6 introns per orthologous gene (Rag1 and PGBD1), while the other genes (PRKRIR, Thap9, POGK, Znf862, PGBD5) show stable numbers of introns in orthologous genes that range from 4 to 7 gained introns. In the case of retroelement-derived genes the variability in the numbers of gained introns is smaller, with the majority containing 1 to 2 *de novo *gained introns in their 5' UTR or coding regions (Table 2). In the case of gag-derived genes, the number of gained introns reaches up to 6 gains per gene at most (ZCCHC16). In the case of integrase-derived genes, up to 7 (Gin1) and 8 (NYNRIN) introns have been gained *de novo*.

#### b) Intron densities

**Figure 1:**
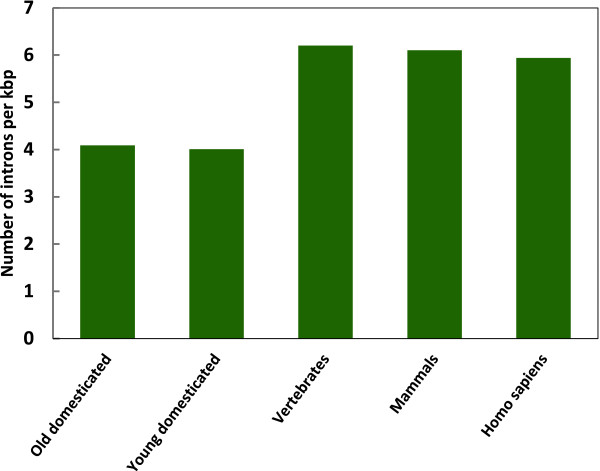
**Intron densities in domesticated genes compared with vertebrate, mammalian and human normal genes**. The intron density is given as the number of introns per kb of coding sequence for vertebrate, mammalian and human normal genes, values were taken from literature [13]. The intron density of old domesticated genes is given as the number of introns per kb of coding sequence (introns in CDS) while the intron density of young Eutheria-specific domesticated genes is given as number of introns per kb of 5' UTR sequence (5' UTR introns).

They were calculated separately for Eutheria-specific domesticated genes and for much older domesticated genes that originated early in vertebrates (Figure 1). A major difference between these older and younger domesticated genes is in the position of introns. In older genes they are located mainly in coding regions, but in younger genes in 5' UTRs. In both cases the intron positions are conserved. The average intron densities for Eutheria-specific domesticated genes are 4.01 intron per kb of 5' UTR. Intron densities for older domesticated genes that originated early in vertebrates (e.g. Gin-1 and PGBD5) are 4.09 intron per kb of CDS. This comparison shows that intron densities are similar in domesticated genes, the major difference however, being in the position of introns. Intron densities in Eutheria-specific domesticated genes and in older domesticated genes that originated early in vertebrates are therefore lower than the intron densities for normal mammalian and vertebrate genes [13,14].

#### c) Size of introns per gene

The sizes of the gained introns in domesticated genes are highly variable (Additional file 3). They mostly range from a few hundred to a few thousand base pairs. DNA transposon-derived genes contain longer introns than retroelement-derived genes - it is also evident that older domesticated genes (e.g. originating in the ancestor of chordates) contain much longer introns than evolutionarily younger domesticated genes that originated in the ancestor of placentals. Surprisingly, the longest introns exist in the gag-derived ZCCHC16 gene. The human ZCCHC16 gene contains two introns, the first of 47.8 kb and the second of 323.3 kb. Both introns are located in the 5' UTR region. In the mouse genome, this gene contains 6 introns, where they are again located in the 5' UTR. The second intron in the mouse ZCCHC16 gene is also the longest intron observed in the chordate domesticated genes, being longer than 410 kb. This gene resembles the mammalian retrogenes with very long introns that are restricted to the 5' UTRs [19].

#### d) Preferred locations of de novo gained introns

In all the orthologous domesticated genes analyzed, the preferred locations of *de novo *gained introns are the 5' UTRs and the coding region, while newly gained introns in the 3' UTR locations are very rare (Tables 1 and 2). These preferred intron locations are similar to those in the »normal« chordate genes [45]. However, the preferred locations of *de novo *gained introns in domesticated genes differ from the recently reported case of mammalian retrogenes, in which newly gained introns are located preferentially in the 5' UTRs [19].

### Intron positions in domesticated genes are highly conserved

The extensive data on intron position conservation (Additional file 4) has been collected from the genomic alignments for all placental-specific intron gains. Such an approach has the advantage over the presentation in a table or figure, since one can readily see the genes and their exons and introns compared at the nucleotide level over quite large evolutionary distances (at least 80 - 100 Myr) and, what is surprising, to see the remarkable level of conservation at the nucleotide level. The human orthologous gene was always aligned with at least one representative of the placental superorders Afrotheria, Xenarthra, Laurasiatheria and Euarchontoglires. In such a way a direct insight into the extent of conserved intron positions can be seen as well as in the several cases of highly conserved introns. Genomic alignments have shown that the vast majority of intron positions in placental-specific domesticated genes are highly conserved. All or the vast majority of gained introns in domesticated genes have been fixed in the eutherian ancestor, as demonstrated by their presence and sequence conservation in all eutherian superorders (Additional file 4, Table 4).

**Table 4:** Intron position conservation in Eutheria-specific domesticated genes

Gene name	Euarchontoglires	Laurasiatheria	Xenarthra	Afrotheria
RGAG1	●	●	●	●
RGAG4	●	●	incomplete	●
ZCCHC5	●	●	●	●
LDOC1L	●	●	●	●
C22OF29	●	●	●	●
PNMA2	●	●	●	●
PNMA3	●	●	incomplete	●
MOAP1	●	●	●	●
PNMA5	●	●	●	●
PNMA6A	●	?	?	●
ZCCHC12	●	●	●	●
PNMAL1	●	●	●	●
KRBA2	●	●	●	●
PGBD1	●	●	●	●
Buster3	●	●	●	●

●: conserved intron position, ?: poor sequence quality, incomplete: only parts of the intron or intron positions can be seen in the current assembly.

### Alternative splicing in domesticated genes

In the ASTD database (Alternative Splicing and Transcript Diversity 1.1) [46] I have checked all domesticated genes for the presence of alternative splicing and several examples have been found (Table 5). It is interesting that DNA transposon-derived genes possess a larger amount of alternative splicing than the retroelement-derived genes. In some genes (e.g. in the human PNMA2 gene) up to 14 alternative splicing events have been documented in the ASTD database. It is interesting that the majority of these alternative splicing events are found in humans. Alternative splicing events can be traced only in a limited number of mammals (human, mouse and rat), since for any other placental lineage no data are available. There are many possible causes of alternative splicing events, from weaker splicing sites to diverse intronic and exonic splicing silencers or enhancers [47]. It has been demonstrated that *Alu *SINEs can generate alternative splicing in humans [48]. Since most of the alternative splicing events in domesticated genes are limited to humans the involvement of *Alu *SINEs is among the most interesting possibilities. The presence of alternative splicing events in humans indicates that these events might be quite recent. Genomic alignments of orthologous genes can provide the precise timing of these events (Additional file 5). In the case of PNMA2 gene such data has shown that the intron containing several *Alu *repeats is highly conserved in Simiiformes, but not in the tarsiers or prosimians. Alternative splicing has also been included in the intronization model of intron gain [6,7], however, this model does not appear to explain the observed situation. Although the gained introns in domesticated genes have been fixed in the eutherian ancestor, the alternative splicing events can be found, in the majority of cases, only in humans and, possibly, in primates. Such a distribution pattern may indicate very recent, and probably regulatory, adaptations in the human or primate lineages.

**Table 5:** Alternative splicing in Eutheria-specific domesticated genes

Gene name	human	mouse	number of splicing events	splicing event details
PNMA2	●		14	EI, CE
PNMA3	●		3	II, IR
MOAP1	●		1	IR
ZCCHC12		●	7	EI, CE, II, IR
ZCCHC18		●	9	II, IR, EI
POGK	●		5	EI, CE, II
POGK		●	3	CE
PRKRIR		●	1	CE
THAP9	●		7	EI, CE, II
Buster3	●		1	CE
PGBD1	●		2	EI, II
PGBD2	●		2	CE, II
PGBD5		●	1	IR

●: alternative splicing is present. Splicing events: intron isoform (II), intron retention (IR), exon isoform (EI) and casette exon (CE).

### Ongoing gain and loss of introns in domesticated genes: evidence from comparative genomics of mammals

The availability of RefSeq genes [34,35] and numerous mammalian genomes (at NCBI WGS site and at the Ensemble site) has enabled the conservation of intron positions and numbers to be analyzed. Even comparison of the well studied genomes of human and mouse has shown differences in the locations (5' UTR or coding regions), sizes and numbers of introns in orthologous genes (Additional file 6). Some older orthologous genes (e.g. Gin1, PGBD5), show stable numbers of introns per gene throughout chordates, and only the intron sizes differ greatly between orthologues. In contrast, evolutionarily much younger placental-specific domesticated genes show diversity in the numbers and positions of introns in orthologous genes (Additional file 6). This indicates that some introns have not been fixed yet, or that the intron gain was more active in some species. Comparative genome data therefore provides evidence for ongoing gain and loss of introns in domesticated genes of placental mammals. For example, while human ZCCHC16 gene contains two introns, the same gene contains 6 introns in the mouse genome.

### Sequences of *de novo *gained introns are highly conserved in placental mammals

Although the positions of many introns are highly conserved in evolution, their sizes and nucleotide sequences are generally not conserved. I examined whether *de novo *gained introns were evolutionarily conserved in mammals. The intron sequences of all human domesticated genes were compared to sequences obtained from WGS genomic sequences from the representatives of all placental superorders. Surprisingly, intron sequences were highly conserved between human and Afrotheria in only 9 out of 19 retroelement-derived genes, showing an overall conservation of 70 - 75% between the representatives of all four superorders of placental mammals analyzed (Additional file 7). These genes are RGAG1, ZCCHC16, ZCCHC5, RGAG4, ZCCHC12, PEG10, LDOC1L, PNMA2, and PNMAL1. The first five are located on the X chromosome, while the others are autosomal genes. To find out whether such conservation is restricted to *de novo *gained introns alone, the sequence conservation of the entire domesticated genes between humans and representatives of all placental superorders has been examined. In addition to the conserved *de novo *gained introns, a high level of sequence consevation in the whole domesticated genes has been found. The analysis of intron conservation in other randomly selected genes indicates that intron sequences between humans and the representatives of all placental superorders may be more highly conserved than generally acknowledged (Additional file 7).

### Lineage-specific enrichment of intron sequences with TEs in diverse placental superorders

Searching for TEs in the long introns with RepeatMasker has shown the presence of species-specific repeats in the analyzed species (Additional file 8). Up to 50% of the intron sizes can be occupied by diverse TEs. Different species of placental mammals (e.g. human, mouse, cow, elephant and sloth) show unique lineage- or species-specific TE content in these introns. This finding indicates that these TEs have been inserted in the particular lineages (e.g. Afrotheria, Laurasiatheria, Rodentia, Primates, etc.). It is evident that the majority of intron origination events in domesticated genes have occurred in the eutherian ancestor, but that they were later independently bombarded with lineage-specific TEs in all three eutherian sister groups Afrotheria, Xenarthra and Boreoeutheria. Independent TE bombardment of introns occurred also inside Boreoeutheria, as evidenced by the large differences in TE repertoires in these introns between Laurasiatheria and Euarchontoglires, as well as between rodents and primates.

### The number of *de novo *gained introns in domesticated genes is among the highest in eukaryotes

The analysis of placental-specific domesticated genes, retrogenes and placental-specific transcription factors (~200 were analyzed) has shown that numerous intron gains occurred in the ancestor of placentals and that intron gain is still ongoing in mammals. At least 50 - 70 cases of intron gain can be documented from the analysis of >30 domesticated genes and retrogenes, and a few more cases can be documented also for placental-specific transcription factors (KRAB-ZNFs, SCAN-ZNFs and SCAN-KRAB-ZNFs). Up to 100 cases of intron gain are recognized from the analysis of ~200 orthologous genes. These intron gains have occurred at different time points of placental evolution, the vast majority of them in the ancestor of placentals and the others in diverse lineages or species of placental mammals.

## Discussion

This study provides clear evidence for extensive intron gain in the ancestor of placental mammals. Vertebrate, and especially mammalian, genomes contain a number of genes that have originated from transposable elements (TEs) or their remains [27-31]. Since vertebrate retroelements and DNA transposons do not contain introns [32], the ancestral state for TE-derived genes is intronless. During the transition process from a multicopy TE to the single copy domesticated gene, intron gain can occur, meaning that any intron present in the domesticated gene will constitute a *de novo *intron gain. The availability of numerous mammalian and vertebrate genomes was crucial for tracing the origin of these genes, the origin of specific exon/intron structures and for determining the extent of intron gain.

It has been shown that intron losses outnumber intron gains in eukaryotic orthologous genes [13,14]. Genome-wide comparisons of closely related species in numerous intron-rich lineages have shown that recent intron gains are indeed very rare [13,14,16,25]. Comparison of orthologous genes from mammalian genomes failed to reveal any gains at all, suggesting that all introns currently contained in mammalian genes were already present at the time of radiation of mammalian orders [15,22]. However, in contrast to previous observations, this study has demonstrated (based on the analysis of >200 orthologous genes) quite extensive intron gain, mainly in the ancestor of placental mammals. The placental mammals can therefore now be added to the list of taxonomic groups with significant amounts of intron gain arising in the relatively recent evolutionary past (100 - 200 Mya).

Rates of intron gain in the past tens to hundreds of million years in diverse eukaryotes have been very low [4,12-14]. Studies of closely related species have shown that diverse eukaryotic lineages experienced surprisingly few intron gains in this period [reviewed in [4] and 12]. The highest rate of recent intron gain yet observed in genome-wide ortholog comparisons was in Oikopleura, where 4260 newly acquired introns have been detected [49]. As this study shows, the extent of intron gain in chordate, lower vertebrate, amniote, mammalian and therian ancestors has been much smaller. The domesticated genes have finally provided evidence for the numerous intron gains in the ancestor of placental mammals, more than 160 My ago [50,51]. At least 50 - 100 cases of intron gain have been observed in this ancestor. This extent of *de novo *gained introns is similar to that reported in diverse eukaryotic lineages [12,16,17,20]. The comparative genomics of eutherian domesticated genes show differences in the numbers of introns, indicating that intron gain is still ongoing.

An extensive phylogenomic analysis of all the domesticated genes in chordate and mammalian genomes has provided crucial information as to where and when TEs were transformed into domesticated genes, allowing *de novo *gain of introns in these genes to be pinpointed precisely. The number of gained introns in these genes varies greatly, from 1 to 8. Domesticated genes in placentals and chordates accumulated a large number of introns, such that their density in these genes has become close to that in »normal« genes [10]. The average intron density for Eutheria-specific domesticated genes is 4.01 intron per kb of 5' UTR. Intron density for older domesticated genes that originated early in vertebrates (e.g. Gin-1 and PGBD5) is 4.09 intron per kb of CDS. This comparison indicates that intron densities are similar in domesticated genes, the major difference however being in their position. Intron densities in Eutheria-specific domesticated genes and in older domesticated genes that originated early in vertebrates are therefore lower than those for normal mammalian and vertebrate genes [13,14]. The emerging pattern of evolution of eukaryote gene architecture is simple: whenever an intronless gene is added to an intron-rich genome via any route, it is rather quickly saturated by introns until an intron density close to that in "old" genes is reached. In this respect the results of this work extend and generalize the previous observations on chloroplast-derived genes in plants [26]. The selective and/or neutral factors affecting this saturating insertion of introns remain unknown and appears to be of great interest, as are the molecular mechanisms of intron insertion.

The sizes of the gained introns in domesticated genes are highly variable, ranging from a few hundred to a few thousand base pairs. DNA transposon-derived genes contain longer introns than retroelement-derived genes, just as evolutionarily older domesticated genes contain much longer introns than evolutionarily younger domesticated genes. Surprisingly, the longest introns exist in the gag-derived ZCCHC16 gene, and the second intron in the mouse (~410 kb long) is also the longest intron in the chordate domesticated genes. This gene resembles mammalian retrogenes with very long introns [19]. The preferred locations of *de novo *gained introns in domesticated genes are the 5' UTRs and coding regions, while 3' UTR locations are very rare. These preferred intron locations are similar to those of the »normal« chordate genes [45]. However, the preferred locations of *de novo *gained introns in domesticated genes differ from the recently reported case of mammalian retrogenes, where newly gained introns are preferentially located in the 5' UTRs [19].

The extensive information on intron position conservation collected from the genomic alignments for all placental-specific intron gains has shown that the great majority of intron positions in placental-specific domesticated genes are highly conserved. The great majority of gained introns in domesticated genes have been fixed in the eutherian ancestor, as demonstrated by their presence and sequence conservation in all eutherian superorders. From the genomic alignments we can readily trace the genes and their exons and introns compared at the nucleotide level over quite large evolutionary distances (at least 80 - 100 Myr) and, what is surprising, see remarkable level of conservation at the nucleotide level.

The rate of sequence divergence in introns is very high, therefore many introns are less conserved in sequence between organisms than their associated exons [4,52]. The sequence conservation of *de novo *gained introns has been analyzed, and a striking conservation of the intron sequences in their entire length was found in 9 out of 19 retroelement-derived domesticated genes, showing 70 - 75% nucleotide identity between humans and Afrotheria, Xenarthra and Laurasiatheria. Comparison of the entire domesticated genes between human and the representatives of all placental superorders also shows ~75% nucleotide identity between humans and Afrotheria, Xenarthra and Laurasiatheria. Conservation of intronic sequences has been observed in some other Boreoeutheria genes [52], however only several short regions were shown to be highly conserved. Most of the unusually conserved introns are located in the 5' UTR regions. It is possible that some of these introns are so highly conserved because they may have some conserved regulatory role in enhancing expression, in mRNA localization, stability or efficiency of translation [47,53]. It has been demonstrated that some of the domesticated genes are evolving under negative selection [27], therefore the level of unusual conservation is not limited to the exons but may also include the introns. Some of these genes are located on the X chromosomes, which may cause unusual patterns of evolution, such as lower mutation rates than on the autosomes [54]. The mutation rate on human X chromosome is indeed low and X-linked genes evolving mainly under negative selection are therefore evolving slowly [54,55]. The analysis of intron conservation in other randomly selected genes indicates that intron sequences in all placental superorders may be more highly conserved than is generally acknowledged. Such a high level of conservation of intron sequences may reflect their functional significance for the expression and regulation of domesticated and some other genes [47,53].

The analysis of domesticated genes in ASTD database has shown the presence of alternative splicing. Up to 14 alternative splicing events can be seen per domesticated gene. It is interesting that more alternative splicing events can be seen in human than in mouse orthologous genes. Alternative splicing in domesticated genes may have originated by mutations in splicing sites (evolution of weaker splice sites), by sequence changes in the intronic and exonic splicing silencers or enhancers (generating lower or higher densities) or by accumulation of *Alu *SINEs that can change the mode of splicing of the flanking exons [47,48]. Comparison of the orthologous introns in placental superorders has shown the presence of species- or lineage-specific enrichment of TEs and highly dynamic evolution of TE content in placental mammals. These findings indicate that introns in each species are under constant bombardment with TEs [56,57]. By such accumulation of lineage-specific SINEs they may influence the alternative splicing of the flanking exons in some species [47,48].

The presence of ~100 intron gains in placental mammals is remarkable, and clearly represents just the tip of the iceberg, the number of *de novo *gained introns in the ancestor of placental mammals probably being much higher. This study pointed to the serious problems arising from comparison of orthologous introns in coding regions only and from sparse taxon sampling in the genome-wide analyses of intron gain [15,22,23]. None of the cases reported here were observed in the previous studies of closely related (human, mouse, rat and dog as an outgroup) [15] or distantly related (fish vs mammals) species [23]. In the closely related mammalian species analyzed [15,22] intron gains occurred before those species originated. In comparisons of distantly related vertebrate species [23] only »old« orthologous genes have been compared, and evolutionary novelties were excluded from such analyses, however the neglected intron gains occurred after the analyzed species originated. Therefore, as this study shows, the overall extent of intron gain in eukaryotes could be much higher than reported in previous studies. The solution to the above problems is to analyse the highly neglected evolutionary gene novelties at particular time points (like in the ancestor of placentals). This study provides a further cautionary example in using only closely or distantly related species and sophisticated statistical methods in directionalizing intron loss/gain events, and underscores the importance of using appropriately selected taxa and evolutionary gene novelties for accurate inferences of genome evolution. All future studies of intron dynamics should be made in a correct taxonomic context in order to infer the real extent of intron gain during eukaryotic evolution.

To obtain a genome-wide insight into the extent of intron gain in placental mammals, highly accurate exon/intron structures (including 5' UTR, with 5' UTR introns) of genes from marsupials and platypus are needed, as well as from the key Afrotheria (African mammals) and Xenarthra (South American mammals) species. The major problem in comparative genomics of mammals is the quality of sequence data for just 2× genome covered species [58]. Therefore, for the large scale genome-wide based analysis of intron gain in mammals, the key basal placental taxa should be compared with the marsupials and platypus. Such data could provide a definitive answer to the real extent of intron gain in placental mammals.

## Conclusions

The first evidence for intron gain in the ancestor of placental mammals has been provided. A phylogenomic analysis of domesticated genes in mammals and chordates has provided evidence for *de novo *intron gain from the analysis of their exon/intron structures. *De novo *gained introns show clear positional bias, since they are distributed mainly in 5' UTR and coding regions, while 3' UTR located introns are very rare. Large numbers of *de novo *gained introns have been found in the coding regions of some domesticated genes, reaching as many as 8 introns per gene. Surprisingly, the majority of intron gains have occurred in the ancestor of placentals, more than 160 Mya. None of these cases of intron gain were observed in earlier genome-wide analyses of limited numbers of evolutionarily younger placental species. All previous claims for the absence of intron gain in mammals, or in the last 100 - 200 My of eukaryotic evolution, were the consequence of inadequate taxon sampling and the comparison of only the »old« orthologous genes. Thus, as this study has demonstrated, adequate taxon sampling is crucial for the reconstruction of intron dynamics and evolution.

## Methods

### Data mining

The databases analyzed were the Ensembl http://www.ensembl.org and the nonredundant (NR), EST, GSS, HTGS, WGS, as well as the diverse taxon-specific (mammalian, chordate and metazoan) genome databases at the National Center for Biotechnology Information (NCBI) http://www.ncbi.nlm.nih.gov. Comparisons were performed using the diverse BLAST tools [59] with the E-value cutoff set to 10^-5 ^and other parameters to default settings. Domesticated genes, as well as diverse gag, integrase and transposase domains, have been used as queries. DNA sequences were translated using the Translate program http://www.expasy.org/tools/dna.html. The reference set of human representatives of the domesticated genes is available in Additional file 1. Orthologs of domesticated genes were identified in Ensembl and WGS.

### Phylogenomic analysis of domesticated genes and establishment of their exon/intron structures

The locations, sizes and numbers of *de novo *gained introns in domesticated genes of mammals and chordates have been analyzed. The availability of RefSeq genes [34,35] and numerous mammalian genomes (at the Ensembl and the NCBI WGS sites) has enabled the conservation of intron sequences, positions and numbers to be analyzed. The use of annotated human or mouse introns has enabled their origin in mammals to be traced by genome-wide comparisons of orthologous genes in placentals, marsupials and monotremes. The comparison of orthologous human and mouse genes has shown differences in the locations (5' UTR or coding regions), sizes and numbers of introns.

### Phylogenetic analysis

All the nonredundant representatives of the gag-, integrase- and DNA-transposon-derived domesticated genes have been included in the analyses. Protein or nucleotide sequences were aligned using Clustal W2 [60]. All the available correction models were tested, but the complex ones were outperformed by the simple correction models. Phylogenetic trees were reconstructed using the neighbor-joining (NJ) [61] and maximum likelihood (ML) methods [62]. The reliability of the resulting topologies was evaluated by 1000 bootstrap replications. Diverse representatives of the Metaviridae and DNA transposases were used as outgroups. Phylogenetic analyses were performed with the programs MEGA5 [63] and PhyML 3.0 [62].

### Conservation of intron positions

The extensive information on intron position conservation has been collected from the Ensembl genomic alignments for all placental-specific intron gains. By this approach the genes and their exons and introns can be compared at the nucleotide level over quite large evolutionary distances (at least 80 - 100 Myr) and a remarkable level of conservation at the nucleotide level can be demonstrated. The human orthologous gene was always aligned with at least one representative of the placental superorders Afrotheria, Xenarthra, Laurasiatheria and Euarchontoglires. The intron density of a gene is given as the number of introns per kb of coding sequence (introns in CDS) or as number of introns per kb of 5' UTR sequence (5' UTR introns).

### Alternative splicing in domesticated genes

All domesticated genes have been checked in the ASTD database (Alternative Splicing and Transcript Diversity 1.1) [46] for the presence of alternative splicing. Alternative splicing events can be traced only in a limited number of mammals (human, mouse and rat); no data are available for any other placental lineage.

### Lineage-specific enrichment of intron sequences with TEs in diverse placental superorders

TE content was analyzed using the RepeatMasker web site http://www.repeatmasker.org[64].

## Abbreviations

DBD: DNA binding domain; LCA: last common ancestor; Mya: million years ago; My(r): million year; TE: transposable element; UTR: untranslated region; ZNF: zinc finger.

## Competing interests

The author declares that they have no competing interests.

## Authors' contributions

DK designed and executed the analyses, and wrote the manuscript.

## Reviewer's Comments

### Reviewer report 1

Dan Graur, University of Houston, United States of America

No comments. An interesting idea.

### Reviewer report 2

Eugene V. Koonin, NCBI, NLM, NIH, United States of America

Kordiš reports apparent gain of a large number of introns in "young" vertebrate genes, primarily in genes that evolved from domesticated transposable elements. A burst of intron insertion is mapped to the origin of placental mammals. Clearly, this is a very interesting and important finding that runs against the numerous published observations of the (near) lack of intron gain throughout the evolution of mammals or even the evolution of vertebrates.

*Author's response: I agree with the above statement*.

However, the "refutation" of the intron stasis scenario should be taken in perspective. This entire study involves approximately 200 "young" genes, and not all of them have gained many introns. However interesting, this finding pertains to a small fraction of genes in any given animal genome, so quantitatively this is a small correction to the stasis picture. There is no need to over-dramatize the revision of the existing views. This is not to deny the importance of the result. To me, the emerging pattern of evolution of eukaryote gene architecture is simple: whenever an intron-less gene is added to an intron-rich genome via any route, it is rather quickly saturated by introns until intron density close to that in "old" genes is reached. In this respect the results of this work extend and generalize the previous observations on chloroplast-derived genes in plants (Ref. 26) The selective and/or neutral factors affecting this saturating insertion of introns remain unknown and seem to be of great interest, and so are the molecular mechanisms of intron insertion. One has to agree with the author of the article that domesticated genes are an excellent model to study these fascinating and fundamental problems.

*Author's response: I really don't like to refute your intron stasis scenario and to overdramatize the revision of the existing views about the extent of intron gain. As this study pointed out, in the previous genome-wide analyses of intron gain (e.g. in mammals or in vertebrates) the serious problem was the comparison of orthologous introns in coding regions only and the sparse taxon sampling. None of the cases reported here were observed in the previous studies of closely related (human, mouse, rat and dog as an outgroup) or distantly related species (fish vs mammals). In the closely related mammalian species analysed, intron gains occurred before those species originated. In comparisons of distantly related vertebrate species only »old« orthologous genes have been compared, and evolutionary novelties were excluded from such analyses, however the neglected intron gains occurred after the analysed species originated. Therefore, as this study shows, the overall extent of intron gain could be much higher than reported in previous studies. As this study demonstrated, the solution to the above problems is to analyse the highly neglected evolutionary gene novelties at particular time points (like here, in the ancestor of placentals). This study therefore provides a further cautionary example in using only closely or distantly related species and sophisticated statistical methods in directionalizing intron loss/gain events, and underscores the importance of using appropriately selected taxa and evolutionary gene novelties for accurate inferences of genome evolution*.

The article as it stands now presents many omissions and lost opportunities some of which seem to be a must for revision whereas others are extensions of the work done that perhaps could be postponed until subsequent publications. Here are several specific areas where the submitted manuscript appears to be amiss. The information on intron position conservation probably should be much more detailed and specific and should be presented in the main paper not in an additional file, probably in the form of a table or figure.

*Author's response: As requested I have added the extensive information on intron position conservation in a large supplementary file that contains genomic alignments for all placental-specific intron gains. This has the advantage over presentation in a table or figure, since one can readily see the genes and their exons and introns compared at the nucleotide level over quite large evolutionary distances (at least 80 -100 million years) and, what is surprising, see the remarkable level of conservation at the nucleotide level. The human gene is always aligned with at least one representative of the placental superorders Afrotheria, Xenarthra, Laurasiatheria and Euarchontoglires. In this way a direct insight into the extent of conserved intron positions can be seen, as well as the several cases of highly conserved introns. It should be noted that most of the mammalian genomes are now available as low-coverage (~2×) assemblies. Sequencing errors in some of these genomes can still have surprising effects for apparent lineage-specific insertions of introns. A careful reinspection of such a data is always needed to exclude sequencing errors*.

The inferred number of gained introns should be given specifically, claiming a "remarkable number" is not enough.

*Author's response: This suggestion is accepted and the inferred numbers of gained introns are now given specifically*.

In the same vein, I believe that specific numbers should be presented on intron densities in the domesticated genes (per kilobase not per gene) and compared to the intron densities in other genes.

*Author's response: I calculated intron densities for all Eutheria-specific intron gains as well as for some cases of older intron gains that occurred during the evolution of vertebrates. I also compared these data with the intron densities in other genes. The requested figure showing the histograms of intron densities in gained introns compared to other genes has been added to the MS*.

Further, it is unclear to me why not performing a formal, maximum-likelihood reconstruction of the history of intron gains and losses in the analyzed genes; this can be easily done using the COUNT software (Csurös M. Count: evolutionary analysis of phylogenetic profiles with parsimony and likelihood. Bioinformatics. 2010 Aug 1;26(15):1910-2). Such a reconstruction would yield a more robust and more informative evolutionary scenario.

*Author's response: I have added genomic alignments for all placental-specific intron gains. These data clearly demonstrate that the massive intron gain has occurred only at the particular time point, namely in the ancestor of placental mammals. The numbers of additional intron gains or losses are indeed very small and are restricted to the particular species or to the small taxonomic groups. Therefore maximum likelihood analysis was not practical because of the small number of potential intron gains and losses*.

This suggestion also brings up the issue of illustration. The current manuscript contains no figures which is strange considering the subject. For example, it is easy to imagine a figure showing the phylogenetic tree of vertebrates with the ML estimates of intron gains and losses in the analyzed genes mapped to specific branches.

*Author's response: I know that it is strange that the current version of manuscript contained no figures. As explained above the requested phylogenetic tree of vertebrates with the ML estimates of intron gains and losses in the analyzed genes mapped to specific branches was not practical because of the small number of potential intron gains and losses. This MS clearly demonstrates that the only case of massive intron gain in the domesticated genes occurred only at one particular time point, namely in the ancestor of placental mammals. However, outside this evolutionary time point the additional intron gains or losses are very small and are restricted to particular species or to small taxonomic groups*.

Other figures are easy to come up with as well, for example, histograms of intron densities. Some interesting possibilities are not pursued at all, e.g. comparing the structures of the splice sites for the new and older introns.

*Author's response: The requested figure showing the histograms of intron densities in gained introns compared to those in other genes has been added to the MS. Intron densities are indeed not as high as in the typical mammalian genes, but still they are remarkable with 4 introns/kb of exonic sequence. I checked also the structure of the splice sites for the new and older introns but no differences in the conservation of splicing signals can be found, as already reported for some other cases of intron gains. All gained introns use canonical splice sites without any deviations*.

Also, do any of these genes undergo alternative splicing? Knowing this could provide a clue to the evolutionary factors behind intron saturation.

*Author's response: In the ASTD database (Alternative Splicing and Transcript Diversity 1.1) I checked all domesticated genes for the presence of alternative splicing and found several examples of alternative splicing. It is interesting that larger amounts of alternative splicing are observed in DNA transposon-derived genes than in the retroelement-derived genes. In some genes (e.g. human PNMA2 gene) up to 14 alternative splicing events have been documented in the ASTD database. It is interesting that the majority of these alternative splicing events can be found only in humans but not in mouse. Alternative splicing events can be traced only in a limited number of mammals (human, mouse and rat) - no data are available for any other placental lineage. There are numerous possible causes of alternative splicing events, from the intronic silencers or enhancers, exonic silencers or enhancers, to the diverse repetitive elements. In humans, it has been demonstrated that Alu SINEs can generate alternative splicing. Since most of the alternative splicing events are limited to humans the involvement of Alu SINEs is among the most interesting possibilities. The presence of alternative splicing events exclusively in humans indicates that these events are indeed quite recent, but the absence of comparative data cannot provide their precise timing. However, since human and mouse belong to the same eutherian superorder Euarchontoglires, the timing of these events can be more restricted to the primates or to the younger lineages within primates (e.g. to Hominoidea). Alternative splicing has also been implied in the intronization model of intron gain, however, I believe that this model cannot explain the observed situation. As is evident, all (or the vast majority) of gained introns in domesticated genes have been fixed in the eutherian ancestor, as demonstrated by their presence and sequence conservation in all eutherian superorders. But the alternative splicing events can be found, in the majority of cases, only in humans. This may indicate very recent, probably regulatory, adaptation in the human lineage*.

Finally, I find the conservation of the actual sequences of introns illustrated in Additional File 8 extremely unexpected, almost to the extent of being shocking. A more careful discussion of this finding in light of the current knowledge of the rates of neutral sequence evolution seems necessary.

*Author's response: The requested discussion of the conservation of the intron sequences has been added to the MS*.

### Reviewer report 3

Jürgen Brosius, University of Muenster, Germany

This is an interesting approach towards detection of relatively novel events of intron generation. The author is using genes that were derived from, by nature intronless, transposable DNA elements or transposed retroelements. This has the advantage that events that occurred over the past 100 to 200 million years could be detected. My main problem with the current version is the terminology of intron gain. Looking at the events, it turns out that in many cases no novel sequences actually were gained but existing sequences converted to introns. Perhaps the term intronization would fit better and I would recommend the author to search PubMed using that term. There are 10 matches and the first use of the term intronization is from Gromoll et al. (2007). The paper merely describes the disuse of one of the exons in the LH receptor type I and type II genes. A second early and relevant paper describes an Alu element induced intronization and could be cited as well (Sela et al. 2007).

*Author's response: From the literature data we can only see a few cases of intronized sequence in the normal gene that already contained introns (Irimia M, Rukov JL, Penny D, Vinther J, Garcia-Fernandez J, Roy SW. Origin of introns by 'intronization' of exonic sequences. Trends Genet. 2008 24:378-81.; Roy SW. Intronization, de-intronization and intron sliding are rare in Cryptococcus. BMC Evol Biol. 2009 9:192). All such introns were quite short (~50 bp) in both Caenorhabditis (Irimia et al., 2008) and Cryptococcus (Roy 2009). For me it is highly unlikely that several quite long introns in domesticated genes have been generated by the intronization of coding sequences. The main reasons for this are the sizes of the ancestral gag and integrase domains, which are in the range of 250-400 amino acids. The sizes of the domesticated genes are in the range from ~100 (contraction) to ~2000 (expansion) amino acids. Therefore it is highly unlikely that existing sequences have been converted to introns in domesticated genes. It was estimated recently (Roy 2009) that intronization may not be a major overall contributor to modern day gene structures and that intronization is unlikely to explain a large fraction of modern introns. I think that nonsense-mediated decay (NMD) is the more likely mechanism to enable intron gain*.

In several parts of the manuscript the author mentions a "significant amount of intron gain" or the "remarkable number of de novo gained introns etc. This is relative and it would be better to provide at least approximate numbers instead.

*Author's response: This suggestion is accepted and most cases were changed into the approximate numbers*.

At the end of the background section the author mentions studies restricted to the superorder Boreoeutheria. The original publication concerning the classification is Springer et al. (2001).

*Author's response: This suggestion is accepted and the original citation has been added to the MS*.

How can the author rule out that young genes with introns in fact encode non-protein coding RNAs that acquired introns or simply feature insertions that are not being removed by splicing? It would be useful to provide information on how many of the transcripts are supported by bona fide messenger RNAs, or at least ESTs, or even better by protein sequence data. In additional file 4 entitled "ongoing gain and loss of introns in domesticated genes" I detect very little information concerning messenger RNAs; one of the few examples where such information is given, refers to the ZCCHC16 gene.

*Author's response: All these domesticated genes are protein coding genes and a few may also encode non-protein coding RNAs. Domesticated genes are also highly expressed in particular tissues (e.g. placenta, brain, testis, etc.). Rich collections of EST/expression profiles for all these genes are present in the literature as well as in the NCBI Genes section, at the Gene Expression Atlas and in many additional transcriptomic databases. In any case, for the annotation of the well covered taxa (such as human and mouse) numerous ESTs and cDNAs exist and helped before in establishing their exon/intron structures at the Ensembl and NCBI Genes as well as for the recognition of some cases of alternative splicing events*.

The frequently mentioned fact that novel introns can be quite large is not surprising. Once an intron had been generated, the floodgates are open for constant bombardment with transposed elements (Brosius, 1999) potentially increasing the sizes of such introns to a great extent over the past 100 to 200 million years.

*Author's response: I agree with this statement. Some of the gained introns are indeed large or even huge. I checked the TE repertoires of some of these introns in different species by RepeatMasker. Up to 50% of the intron size can be occupied by diverse TEs. Different species of placental mammals (e.g. human, mouse, cow, elephant and sloth) shows unique lineage- or species-specific TE content in these introns. This finding indicates that introns in each species are indeed under constant bombardment with TEs*.

In general, it would be interesting to obtain information as to the origin of the sequences that now are being used as introns. My guess is that most of the sequences will be derived from pre-existing sequences at the corresponding loci previously serving as exons or intergenic regions. As mentioned above, one would expect additional TEs that were inserted after the initial intronization. Should the initial intronic sequence be derived from sequences outside the genes' loci, one might obtain clues about events including mobile element insertions that contributed to such novel introns.

*Author's response: Despite a rigorous search I could not identify homologous parental origin for any novel intron elsewhere within the placental and marsupial genomes, consistent with the other studies about the intron gain. Homology searching has shown just orthologous introns in placental mammals, but the sources of these sequences are still unknown. Searching for TEs in the long introns with RepeatMasker has shown the presence of species-specific repeats in the analysed species. This means that these TEs has been inserted in the particular lineages (e.g. Afrotheria, Laurasiatheria, Rodentia, Primates etc.). Regarding the current division of Eutheria into three sister groups Afrotheria, Xenarthra and Boreoeutheria it is evident that the majority of intron origination events in domesticated genes has occurred in the eutherian ancestor, but they were later independently bombarded with lineage-specific TEs in all three eutherian sister groups and also inside Boreoeutheria in Laurasiatheria and in Euarchontoglires (as evident from the big differences in TE repertoires in these introns between rodents and primates). I agree that it is possible that some intergenic regions might also serve as a source of introns. This will be more extensively explained in a separate MS (Kokosar and Kordis, MS in preparation) by the analysis of conserved synteny in Eutheria-specific domesticated genes. It should be noted that by homology searching with introns of domesticated genes in three marsupial genomes no homologous sequences can be found. Since these genes originated in the LCA of placental mammals no orthologs (except a few cases: e.g. PEG10) can be found in marsupials. Knowing the limits of nucleotide homology-searching for unconstrained sequences it is impossible to infer the origin of intron sequences in domesticated genes*.

In any event, as mentioned before, the terminology "intron gain" leads to the expectation that there is perhaps a general mechanism in placental mammals leading to the de novo generation of intron sequences. A more thorough investigation will probably show that most events will be due to "bricolage" or tinkering (Jacob, 1982) via random mutations including small or large indels leading to jury-rigged outcomes including intronization.

*Author's response: It has been demonstrated recently by the Lynch group (Li W, Tucker AE, Sung W, Thomas WK, Lynch M: Extensive, recent intron gains in Daphnia populations. Science 2009, 326:1260-1262.; Catania F, Lynch M: Where do introns come from? PLoS Biol 2008, 6:e283.; Catania F, Gao X, Scofield DG: Endogenous mechanisms for the origins of spliceosomal introns. J Hered 2009, 100:591-596.; Gao X, Lynch, M: Ubiquitous internal gene duplication and intron creation in eukaryotes. Proc Natl Acad Sci USA 2009, 106:20818-20823.;) and others (e.g.: Farlow A, Meduri E, Dolezal M, Hua L, Schlötterer C: Nonsense-mediated decay enables intron gain in Drosophila. PLoS Genet 2010, 6:e1000819.) that the cellular surveillance systems have played a crucial role in the endogenous origin of introns throughout the eukaryotes. I think that these explanations are more appropriate than those suggested by the reviewer*.

In the paragraph titled "ongoing gain and loss of introns in domesticated genes: evidence from comparative genomics in mammals" the author refers to the great diversity in the numbers and positions of introns in younger orthologous placental specific domesticated genes and states that this indicates that some introns have not been fixed yet. This would agree with phylogenetic studies using transposed element insertions showing that during periods of rapid speciation of placental mammals such markers can be polymorphic (unfixed) and phylogenetically unreliable due to incomplete lineage sorting (Churakov et al., 2009).

*Author's response: I agree with this comment*.

Often it is mentioned that the majority of intron gains have occurred in the ancestor of placental mammals (see also Table 3). This bias could well be due to better gene annotations and much more transcriptome information in that group.

*Author's response: I think that this explanation is not appropriate. Even if we have 1 monotreme and 3 marsupial genomes they are mostly without the domesticated genes, that are indeed specific for placental mammals. Even in the other potential cases of intron gain the problem still persists since monotreme and marsupial genomes are not as well sequenced and annotated as the human and mouse genomes. For the majority of gene structures in these more basal mammals the 5' UTRs and 3' UTRs are not known due to the incomplete nature of their cDNAs*.

There are more precise numbers concerning the dating of the common ancestor of placental mammals namely after the therian divergence 148 million years ago as shown in Figure 1 of Warren et al. (2009).

*Author's response: Very recently (Luo ZX, Yuan CX, Meng QJ, Ji Q: A Jurassic eutherian mammal and divergence of marsupials and placentals. Nature 2011, 476:442-445.) a fossil of a Jurassic eutherian mammal (Juramaia sinensis) was found. This fossil is 160 My old and provides new estimates for the divergence of marsupials and placentals. It extends the first appearance of the eutherian-placental clade by about 35 Myr from the previous record, it also has reduced and resolved a discrepancy between the previous fossil record and the molecular estimate for the placental-marsupial divergence. Because Juramaia is unambiguously placed on the placental side of the marsupial-placental divergence, the marsupial-placental divergence must have occurred before Juramaia. Therefore this new fossil serves to reset the minimal age at 160 Myr for the basal-most diversification of marsupials and placentals. This new eutherian fossil age is now similar to the age of placentals at 160 Myr with 95% posterior distribution from 143 to 178 Myr by the latest molecular estimate*.

The tables in the main section need further explanation: for example, what is the significance of a range for intron numbers such as 5 to 13 for the POGZ gene etc., Table 1? In Table 2 under location of introns, the entry often says "5' UTR or coding". I presume the precise location is unclear which would call into question the precise annotation of the corresponding genes and/or transcripts.

Author's response: Intron numbers are variable in some genes or in some species, this is connected to the loss or additional gain of introns. »5' UTR or coding«: precise location is always clear, either in 5' UTR or in coding region. These genes in human or mouse have been really correctly annotated (at the Ensembl or at the NCBI Genes), but in some other low coverage genomes erroneous annotations can still be found. Explanations:

*-5' UTR/coding: introns are present both in 5' UTR and in coding region (for example: Homo RGAG1 gene contains 2 introns in 5' UTR and one intron in coding region)*,

*-5' UTR or coding: introns are present either in 5' UTR or in coding region (for example ZCCHC12 gene: in humans three introns are present in 5' UTR while in chimp 5 introns in coding region were reported (erroneous annotation that was corrected very recently)*,

*-5' UTR or 3' UTR: introns are present in one species in 5' UTR and in the other species in 3' UTR (example: PNMA5 gene: in human 5' UTR intron, while in mouse intron is located in 3' UTR)*.

The fact that introns are readily being generated in the 5' flanking regions of retrogenes has been discussed as early as 1992 by Brosius and Gould. This could be cited.

*Author's response: This reference has been added to the MS*.

Where the author mentions that "the highest rate of recent intron gain yet observed in genome-wide ortholog comparisons was in ascomycetous fungi" he could add the publication of Denoeud et al. (2010) on massive intron gain in the tunicate Oikopleura dioica.

*Author's response: The citation about the massive intron gain in Oikopleura genome has been added to the MS*.

Also, recent work on the SCAN domain, which is derived from the C-terminal portion of the gag capsid (CA) protein from the Gmr1-like family of Gypsy/Ty3-like retrotransposons could be cited (Emerson and Thomas, 2011). This paper and others (Baertsch et al, 2008) show that parts of retrogenes contribute to novel protein domains to existing genes, giving an alternative explanation to some of the "intronization" events.

*Author's response: The requested reference has been added to the MS*.

The last part of the last sentence of the first paragraph in the results section is unclear and should be modified.

*Author's response: The unclear sentence has been clarified*.

References that the author could cite:

Baertsch R, Diekhans M, Kent WJ, Haussler D, Brosius J. Retrocopy contributions to the evolution of the human genome. BMC Genomics. 2008 Oct 8;9:466.

Brosius J, Gould SJ. On "genomenclature": a comprehensive (and respectful) taxonomy for

pseudogenes and other "junk DNA". Proc Natl Acad Sci U S A. 1992 Nov 15;89(22):10706-10.

Brosius J. Genomes were forged by massive bombardments with retroelements and retrosequences. Genetica. 1999;107(1-3):209-38.

Churakov G, Kriegs JO, Baertsch R, Zemann A, Brosius J, Schmitz J. Mosaic retroposon insertion patterns in placental mammals. Genome Res. 2009 May;19(5):868-75.

Emerson RO, Thomas JH. Gypsy and the birth of the SCAN domain. J Virol. 2011 Aug 24.

Denoeud F, et al. Plasticity of animal genome architecture unmasked by rapid evolution of a pelagic tunicate. Science. 2010 Dec 3;330(6009):1381-5.

Gromoll J, Lahrmann L, Godmann M, Müller T, Michel C, Stamm S, Simoni M. Genomic checkpoints for exon 10 usage in the luteinizing hormone receptor type 1 and type 2. Mol Endocrinol. 2007 Aug;21(8):1984-96.

Jacob F: The Possible and the Actual (Pantheon Books, New York 1982).

Sela N, Mersch B, Gal-Mark N, Lev-Maor G, Hotz-Wagenblatt A, Ast G. Comparative analysis of transposed element insertion within human and mouse genomes reveals Alu's unique role in shaping the human transcriptome. Genome Biol. 2007;8(6):R127.

Springer MS, de Jong WW. Phylogenetics. Which mammalian supertree to bark up? Science. 2001 Mar 2;291(5509):1709-11.

Warren WC et al. Genome analysis of the platypus reveals unique signatures of evolution. Nature. 2008 May 8;453(7192):175-83.

*Author's response: Some of these references have been added to the MS*.

## Supplementary Material

Additional file 1 **Reference set of human domesticated genes**. Click here for file

Additional file 2 **Intron gain in Eutheria-specific KRAB-ZNF, SCAN-ZNF and KRAB-SCAN-ZNF genes**. Intron numbers are shown for the well annotated human genes. The ancestral state for KRAB-ZNF genes is 1 to 2 introns in the coding region and 1 intron in the 5' UTR. The ancestral state for SCAN-ZNF genes is 1 intron in the coding region and 1 intron in the 5' UTR. The ancestral state for SCAN-KRAB-ZNF genes is 2 to 3 introns in the coding region and 1 intron in the 5' UTR. Click here for file

Additional file 3 **Intron sizes in human domesticated genes**. Click here for file

Additional file 4 **Conservation of intron positions in domesticated genes**. Click here for file

Additional file 5 **Alternative splicing in PNMA2**. Click here for file

Additional file 6 **Ongoing gain and loss of introns in domesticated genes**. Click here for file

Additional file 7 **Highly conserved introns in domesticated genes of placental mammals**. Click here for file

Additional file 8 **Lineage-specific accumulation of TEs in orthologous introns in four placental superorders**. Click here for file

## References

[B1] Rogozin IB Sverdlov AV Babenko VN Koonin EV Analysis of evolution of exon-intron structure of eukaryotic genes Brief Bioinform 2005 6 118 134 10.1093/bib/6.2.118 15975222

[B2] Jeffares DC Mourier T Penny D The biology of intron gain and loss Trends Genet 2006 22 16 22 10.1016/j.tig.2005.10.006 16290250

[B3] Rodríguez-Trelles F Tarrío R Ayala FJ Origin and evolution of spliceosomal introns Annu Rev Genet 2006 40 47 76 10.1146/annurev.genet.40.110405.090625 17094737

[B4] Roy SW Gilbert W The evolution of spliceosomal introns: patterns, puzzles and progress Nat Rev Genet 2006 7 211 221 16485020 10.1038/nrg1807

[B5] Koonin EV Intron-dominated genomes of early ancestors of eukaryotes J Hered 2009 100 618 623 10.1093/jhered/esp056 19617525 PMC2877545

[B6] Catania F Lynch M Where do introns come from? PLoS Biol 2008 6 e283 10.1371/journal.pbio.0060283 19067485 PMC2586383

[B7] Catania F Gao X Scofield DG Endogenous mechanisms for the origins of spliceosomal introns J Hered 2009 100 591 596 10.1093/jhered/esp062 19635762 PMC2877546

[B8] Lynch M Kewalramani A Messenger RNA surveillance and the evolutionary proliferation of introns Mol Biol Evol 2003 20 563 571 10.1093/molbev/msg068 12654936

[B9] Lynch M Intron evolution as a population-genetic process Proc Natl Acad Sci USA 2002 99 6118 6123 10.1073/pnas.092595699 11983904 PMC122912

[B10] Lynch M The origins of eukaryotic gene structure Mol Biol Evol 2006 23 450 468 16280547 10.1093/molbev/msj050

[B11] Lynch M Richardson AO The evolution of spliceosomal introns Curr Opin Genet Dev 2002 12 701 710 10.1016/S0959-437X(02)00360-X 12433585

[B12] Roy SW Irimia M Mystery of intron gain: new data and new models Trends Genet 2009 25 67 73 10.1016/j.tig.2008.11.004 19070397

[B13] Carmel L Wolf YI Rogozin IB Koonin EV Three distinct modes of intron dynamics in the evolution of eukaryotes Genome Res 2007 17 1034 1044 10.1101/gr.6438607 17495008 PMC1899114

[B14] Csuros M Rogozin IB Koonin EV A detailed history of intron-rich eukaryotic ancestors inferred from a global survey of 100 complete genomes PLoS Comput Biol 2011 7 e1002150 10.1371/journal.pcbi.1002150 21935348 PMC3174169

[B15] Coulombe-Huntington J Majewski J Characterization of intron loss events in mammals Genome Res 2007 17 23 32 17108319 10.1101/gr.5703406 PMC1716263

[B16] Nielsen CB Friedman B Birren B Burge CB Galagan JE Patterns of intron gain and loss in fungi PLoS Biol 2004 2 e422 10.1371/journal.pbio.0020422 15562318 PMC532390

[B17] Li W Tucker AE Sung W Thomas WK Lynch M Extensive, recent intron gains in Daphnia populations Science 2009 326 1260 1262 10.1126/science.1179302 19965475 PMC3878872

[B18] Ragg H Kumar A Köster K Bentele C Wang Y Frese MA Prib N Krüger O Multiple gains of spliceosomal introns in a superfamily of vertebrate protease inhibitor genes BMC Evol Biol 2009 9 208 10.1186/1471-2148-9-208 19698129 PMC2746811

[B19] Fablet M Bueno M Potrzebowski L Kaessmann H Evolutionary origin and functions of retrogene introns Mol Biol Evol 2009 26 2147 2156 10.1093/molbev/msp125 19553367

[B20] Farlow A Meduri E Dolezal M Hua L Schlötterer C Nonsense-mediated decay enables intron gain in Drosophila PLoS Genet 2010 6 e1000819 10.1371/journal.pgen.1000819 20107520 PMC2809761

[B21] Gao X Lynch M Ubiquitous internal gene duplication and intron creation in eukaryotes Proc Natl Acad Sci USA 2009 106 20818 20823 10.1073/pnas.0911093106 19926850 PMC2791625

[B22] Roy SW Fedorov A Gilbert W Large-scale comparison of intron positions in mammalian genes shows intron loss but no gain Proc Natl Acad Sci USA 2003 100 7158 7162 10.1073/pnas.1232297100 12777620 PMC165846

[B23] Loh YH Brenner S Venkatesh B Investigation of loss and gain of introns in the compact genomes of pufferfishes (Fugu and Tetraodon) Mol Biol Evol 2008 25 526 535 10.1093/molbev/msm278 18089580

[B24] Putnam NH Srivastava M Hellsten U Dirks B Chapman J Salamov A Terry A Shapiro H Lindquist E Kapitonov VV Jurka J Genikhovich G Grigoriev IV Lucas SM Steele RE Finnerty JR Technau U Martindale MQ Rokhsar DS Sea anemone genome reveals ancestral eumetazoan gene repertoire and genomic organization Science 2007 317 86 94 10.1126/science.1139158 17615350

[B25] Roy SW Irimia M Penny D Very little intron gain in *Entamoeba histolytica *genes laterally transferred from prokaryotes Mol Biol Evol 2006 23 1824 1827 10.1093/molbev/msl061 16847043

[B26] Basu MK Rogozin IB Deusch O Dagan T Martin W Koonin EV Evolutionary dynamics of introns in plastid-derived genes in plants: saturation nearly reached but slow intron gain continues Mol Biol Evol 2008 25 111 119 17974547 10.1093/molbev/msm234

[B27] Brandt J Veith AM Volff JN A family of neofunctionalized Ty3/gypsy retrotransposon genes in mammalian genomes Cytogenet Genome Res 2005 110 307 317 10.1159/000084963 16093683

[B28] Campillos M Doerks T Shah PK Bork P Computational characterization of multiple Gag-like human proteins Trends Genet 2006 22 585 589 10.1016/j.tig.2006.09.006 16979784

[B29] Feschotte C Pritham EJ DNA transposons and the evolution of eukaryotic genomes Annu Rev Genet 2007 41 331 368 10.1146/annurev.genet.40.110405.090448 18076328 PMC2167627

[B30] Volff JN Turning junk into gold: domestication of transposable elements and the creation of new genes in eukaryotes Bioessays 2006 28 913 922 10.1002/bies.20452 16937363

[B31] Sinzelle L Izsvák Z Ivics Z Molecular domestication of transposable elements: from detrimental parasites to useful host genes Cell Mol Life Sci 2009 66 1073 1093 10.1007/s00018-009-8376-3 19132291 PMC11131479

[B32] Kazazian HH Jr Mobile elements: drivers of genome evolution Science 2004 303 1626 1632 10.1126/science.1089670 15016989

[B33] Heath TA Hedtke SM Hillis DM Taxon sampling and the accuracy of phylogenetic analyses J Syst Evol 2008 46 239 257

[B34] Pruitt KD Tatusova T Klimke W Maglott DR NCBI Reference Sequences: current status, policy and new initiatives Nucleic Acids Res 2009 37 D32 D36 10.1093/nar/gkn721 18927115 PMC2686572

[B35] Maglott D Ostell J Pruitt KD Tatusova T Entrez Gene: gene-centered information at NCBI Nucleic Acids Res 2007 35 D26 D31 10.1093/nar/gkl993 17148475 PMC1761442

[B36] Eisen JA Fraser CM Phylogenomics: intersection of evolution and genomics Science 2003 300 1706 1707 10.1126/science.1086292 12805538

[B37] Springer MS de Jong W Phylogenetics. Which mammalian supertree to bark up? Science 2001 291 1709 1711 10.1126/science.1059434 11253193

[B38] Nishihara H Maruyama S Okada N Retroposon analysis and recent geological data suggest near-simultaneous divergence of the three superorders of mammals Proc Natl Acad Sci USA 2009 106 5235 5240 10.1073/pnas.0809297106 19286970 PMC2655268

[B39] Churakov G Kriegs JO Baertsch R Zemann A Brosius J Schmitz J Mosaic retroposon insertion patterns in placental mammals Genome Res 2009 19 868 875 10.1101/gr.090647.108 19261842 PMC2675975

[B40] Gorinsek B Gubensek F Kordis D Evolutionary genomics of chromoviruses in eukaryotes Mol Biol Evol 2004 21 781 798 10.1093/molbev/msh057 14739248

[B41] Gorinsek B Gubensek F Kordis D Phylogenomic analysis of chromoviruses Cytogenet Genome Res 2005 110 543 552 10.1159/000084987 16093707

[B42] Kordis D A genomic perspective on the chromodomain-containing retrotransposons: Chromoviruses Gene 2005 347 161 173 10.1016/j.gene.2004.12.017 15777633

[B43] Tadepally HD Burger G Aubry M Evolution of C2H2-zinc finger genes and subfamilies in mammals: species-specific duplication and loss of clusters, genes and effector domains BMC Evol Biol 2008 8 176 10.1186/1471-2148-8-176 18559114 PMC2443715

[B44] Emerson RO Thomas JH Gypsy and the birth of the SCAN domain J Virol 2011 85 12043 12052 10.1128/JVI.00867-11 21865395 PMC3209298

[B45] Hong X Scofield DG Lynch M Intron size, abundance, and distribution within untranslated regions of genes Mol Biol Evol 2006 23 2392 2404 10.1093/molbev/msl111 16980575

[B46] Koscielny G Le Texier V Gopalakrishnan C Kumanduri V Riethoven JJ Nardone F Stanley E Fallsehr C Hofmann O Kull M Harrington E Boué S Eyras E Plass M Lopez F Ritchie W Moucadel V Ara T Pospisil H Herrmann A G Reich J Guigó R Bork P Doeberitz MK Vilo J Hide W Apweiler R Thanaraj TA Gautheret D ASTD: The Alternative Splicing and Transcript Diversity database Genomics 2009 93 213 220 10.1016/j.ygeno.2008.11.003 19059335

[B47] Kim E Goren A Ast G Alternative splicing: current perspectives Bioessays 2008 30 38 47 10.1002/bies.20692 18081010

[B48] Lev-Maor G Ram O Kim E Sela N Goren A Levanon EY Ast G Intronic *Alu*s influence alternative splicing PLoS Genet 2008 4 e1000204 10.1371/journal.pgen.1000204 18818740 PMC2533698

[B49] Denoeud F Henriet S Mungpakdee S Aury JM Da Silva C Brinkmann H Mikhaleva J Olsen LC Jubin C Cañestro C Bouquet JM Danks G Poulain J Campsteijn C Adamski M Cross I Yadetie F Muffato M Louis A Butcher S Tsagkogeorga G Konrad A Singh S Jensen MF Cong EH Eikeseth-Otteraa H Noel B Anthouard V Porcel BM Kachouri-Lafond R Nishino A Ugolini M Chourrout P Nishida H Aasland R Huzurbazar S Westhof E Delsuc F Lehrach H Reinhardt R Weissenbach J Roy SW Artiguenave F Postlethwait JH Manak JR Thompson EM Jaillon O Du Pasquier L Boudinot P Liberles DA Volff JN Philippe H Lenhard B Roest Crollius H Wincker P Chourrout D Plasticity of animal genome architecture unmasked by rapid evolution of a pelagic tunicate Science 2010 330 1381 1385 10.1126/science.1194167 21097902 PMC3760481

[B50] Luo ZX Yuan CX Meng QJ Ji Q A Jurassic eutherian mammal and divergence of marsupials and placentals Nature 2011 476 442 445 10.1038/nature10291 21866158

[B51] Meredith RW Janečka JE Gatesy J Ryder OA Fisher CA Teeling EC Goodbla A Eizirik E Simão TL Stadler T Rabosky DL Honeycutt RL Flynn JJ Ingram CM Steiner C Williams TL Robinson TJ Burk-Herrick A Westerman M Ayoub NA Springer MS Murphy WJ Impacts of the Cretaceous Terrestrial Revolution and KPg extinction on mammal diversification Science 2011 334 521 524 10.1126/science.1211028 21940861

[B52] Hare MP Palumbi SR High intron sequence conservation across three mammalian orders suggests functional constraints Mol Biol Evol 2003 20 969 978 10.1093/molbev/msg111 12716984

[B53] Cenik C Derti A Mellor JC Berriz GF Roth FP Genome-wide functional analysis of human 5' untranslated region introns Genome Biol 2010 11 R29 20222956 10.1186/gb-2010-11-3-r29 PMC2864569

[B54] Vicoso B Charlesworth B Evolution on the X chromosome: unusual patterns and processes Nat Rev Genet 2006 7 645 653 10.1038/nrg1914 16847464

[B55] Schaffner SF The X chromosome in population genetics Nat Rev Genet 2004 5 43 51 10.1038/nrg1247 14708015

[B56] Brosius J Gould SJ On "genomenclature": a comprehensive (and respectful) taxonomy for pseudogenes and other "junk DNA" Proc Natl Acad Sci USA 1992 89 10706 10710 10.1073/pnas.89.22.10706 1279691 PMC50410

[B57] Brosius J Genomes were forged by massive bombardments with retroelements and retrosequences Genetica 1999 107 209 238 10.1023/A:1004018519722 10952214

[B58] Milinkovitch MC Helaers R Depiereux E Tzika AC Gabaldon T 2X genomes - depth does matter Genome Biol 2010 11 R16 10.1186/gb-2010-11-2-r16 20144222 PMC2872876

[B59] Gertz EM Yu YK Agarwala R Schäffer AA Altschul SF Composition-based statistics and translated nucleotide searches: improving the TBLASTN module of BLAST BMC Biol 2006 4 41 10.1186/1741-7007-4-41 17156431 PMC1779365

[B60] Larkin MA Blackshields G Brown NP Chenna R McGettigan PA McWilliam H Valentin F Wallace IM Wilm A Lopez R Thompson JD Gibson TJ Higgins DG Clustal W and Clustal X version 2.0 Bioinformatics 2007 23 2947 2948 10.1093/bioinformatics/btm404 17846036

[B61] Saitou N Nei M The neighbor-joining method: a new method for reconstructing phylogenetic trees Mol Biol Evol 1987 4 406 425 3447015 10.1093/oxfordjournals.molbev.a040454

[B62] Guindon S Dufayard JF Lefort V Anisimova M Hordijk W Gascuel O New algorithms and methods to estimate maximum-likelihood phylogenies: assessing the performance of PhyML 3.0 Syst Biol 2010 59 307 321 10.1093/sysbio/syq010 20525638

[B63] Tamura K Peterson D Peterson N Stecher G Nei M Kumar S MEGA5: Molecular Evolutionary Genetics Analysis Using Maximum Likelihood, Evolutionary Distance, and Maximum Parsimony Methods Mol Biol Evol 2011 28 2731 2739 10.1093/molbev/msr121 21546353 PMC3203626

[B64] Huda A Jordan IK Analysis of transposable element sequences using CENSOR and RepeatMasker Methods Mol Biol 2009 537 323 336 10.1007/978-1-59745-251-9_16 19378152

