# Supplementary material for: Extensive intron gain in the ancestor of placental mammals
Source: Biol Direct. 2011 Nov 23;6:59. doi: 10.1186/1745-6150-6-59 (PMC3257199; doi:10.1186/1745-6150-6-59)
Supplement: Additional file 1 — Reference set of human domesticated genes. [file 1745-6150-6-59-S1.PDF]

Additional file 1 - Reference set of human domesticated genes

Gene name	GeneID	Chromosomal position	Presence of introns	Introns absent in humans but present in other species	Exon/intron structure in humans
**DNA transposon derived domesticated genes**					
JRK (jerky)	8629	8q24.3	yes		3/2 or 2/1
JRKL	8690	11q21	no	yes	1/0
TIGD1	200765	2q37.1	no	no	1/0
TIGD2	166815	4q22.1	no	yes	1/0
TIGD3	220359	11q13.1	yes		2/1
TIGD4	201798	4q31.3	yes		2/1
TIGD5	84948	8q24.3	no	yes	1/0
TIGD6	81789	5q32	yes		2/1
TIGD7	91151	16p13.3	no	yes	1/0
POGZ	23126	1q21.3	yes		17/16, 18/17 or 19/18
POGK	57645	1q24.1	yes		6/5
SETMAR	6419	3p26.1	yes		3/2
RAG1	5896	11p13	yes		2/1
ZBED1	9189	Xp22.33;Yp11	yes		2/1
ZBED4	9889	22q13.33	yes		2/1
ZBED5	58486	11p15.3	yes		3/2
ZMYM6	9204	1p34.2	yes		16/15
ZNF862	643641	7q36.1	yes		8/7
C5orf54 (Buster3)	63920	5q33.3	yes		2/1
GTF2IRD2	84163	7q11.23	yes		16/15
PRKRIR	5612	11q13.5	yes		5/4
THAP1	55145	8p11.21	yes		3/2 or 2/1
THAP2	83591	12q21.1	yes		3/2
THAP3	90326	1p36.31	yes		6/5 or 5/4
THAP4	51078	2q37.3	yes		6/5 or 5/4
THAP5	168451	7q31.1	yes		3/2
THAP6	152815	4q21.1	yes		5/4
THAP7	80764	22q11.2	yes		5/4 or 4/3
THAP8	199745	19q13.12	yes		4/3
THAP9	79725	4q21.22	yes		5/4
THAP10	56906	15q23	yes		3/2
THAP11	57215	16q22.1	no	no	1/0
HARBI1	283254	11p11.2	yes		3/2
NAIF1	203245	9q34.11	yes		2/1

PGBD1	84547	6p22.1	yes		7/6
PGBD2	267002	1q44	yes		3/2
PGBD3	267004	10q11	yes		2/1
PGBD4	161779	15q14	no	yes	1/0
PGBD5	79605	1q42.13	yes		7/6
**Retroelement-derived domesticated genes**					
RGAG1	57529	Xq23	yes		4/3
RGAG4	340526	Xq13.1	yes		2/1
ZCCHC5	203430	Xq21.1	yes		2/1
ZCCHC16	340595	Xq23	yes		3/2
PEG10	23089	7q21	yes		2/1
RTL1	388015	14q32.2	no	yes	1/0
LDOC1	23641	Xq27	no	no	1/0
LDOC1L	84247	22q13.31	yes		2/1
C22orf29	79680	22q11.21	yes		3/2
FAM127A	8933	Xq26	no	no	1/0
FAM127B	26071	Xq26.3	yes		1/0 or 2/1
FAM127C	441518	Xq26	no	yes	1/0
PNMA1	9240	14q24.3	no	yes	1/0
PNMA2	10687	8p21.2	yes		3/2
PNMA3	29944	Xq28	yes		2/1
MOAP1	64112	14q32	yes		3/2
PNMA5	114824	Xq28	yes		4/3, 3/2 or 2/1
PNMA6A	84968	Xq28	yes		2/1
PNMA6B	728513	Xq28	no	no	1/0
ZCCHC12	170261	Xq24	yes		4/3
ZCCHC18	644353	Xq22.2	yes		3/2
CCDC8	83987	19q13.32	no	yes	1/0
PNMAL1	55228	19q13.32	yes		3/2
PNMAL2	57469	19q13.32	no	yes	1/0
ARC	23237	8q24.3	yes		3/2
ASPRV1	151516	2p13.3	no	yes	1/0
GIN1	54826	5q21.1	yes		8/7
SCAND3	114821	6p22.1	yes		4/3
KRBA2	124751	17p13.1	yes		2/1
NYNRIN (KIAA1305)	57523	14q12	yes		9/8
ERVFRD-1 (syncytin-2)	405754	6p24.1	yes		2/1
ERVW-1 (syncytin-1)	30816	7q21-q22	yes		2/1 or 1/0
