# Supplementary material for: Extensive intron gain in the ancestor of placental mammals
Source: Biol Direct. 2011 Nov 23;6:59. doi: 10.1186/1745-6150-6-59 (PMC3257199; doi:10.1186/1745-6150-6-59)
Supplement: Additional file 2 — Intron gain in Eutheria-specific KRAB-ZNF, SCAN-ZNF and KRAB-SCAN-ZNF genes. Intron numbers are shown for the well annotated human genes. The ancestral state for KRAB-ZNF genes is 1 to 2 introns in the coding region and 1 intron in the 5' UTR. The ancestral state for SCAN-ZNF genes is 1 intron in the coding region and 1 intron in the 5' UTR. The ancestral state for SCAN-KRAB-ZNF genes is 2 to 3 introns in the coding region and 1 intron in the 5' UTR. [file 1745-6150-6-59-S2.PDF]

Additional file 2 - Intron gain in Eutheria-specific KRAB-ZNF, SCAN-ZNF and KRAB-SCAN-ZNF genes. Intron numbers are shown for the well annotated human genes. The ancestral state for KRAB-ZNF genes is 1 to 2 introns in the coding region and 1 intron in the 5' UTR. The ancestral state for SCAN-ZNF genes is 1 intron in the coding region and 1 intron in the 5' UTR. The ancestral state for SCAN-KRAB-ZNF genes is 2 to 3 introns in the coding region and 1 intron in the 5' UTR.

Gene name	Total number of introns	Number of introns in coding region
**KRAB-ZNF genes**		
ZNF436	3	2
ZNF777	5	4
ZNF746	5	5
ZNF641	6	5
ZNF398	5 or 6	3 or 5
ZNF3	5	3
ZNF282	7	7
ZNF212	4	4
**Eutheria-specific KRAB-ZNF genes**		
ZNF643	4	2
ZNF642	5	4
ZNF684	4	3
ZNF514	4	2
ZNF2	3 or 4	2 or 3
ZNF621	4	3
ZNF662	4	3
ZNF300	5	3
ZNF454	4	3
ZNF184	5	4
ZNF311	6	5
ZFP57	5	4
RBAK	4	3
ZNF12	4	3
ZNF713	3	3
ZNF789	3 or 4	2 or 3
ZNF498	7	4
ZNF596	5	4
ZNF707	6	3
ZNF7	4	3
ZNF250	5	4
ZNF658	4	3
ZNF484	3 or 4	2 or 3
ZNF510	5	4
ZNF782	5	4
ZNF189	2 or 3	1 or 2
ZFP37	3	3
ZNF79	4	4
ZNF248	4	3

ZNF25	5	4
ZNF33A	3	3
ZNF33B	4	3
ZNF37A	5	3
ZNF214	2	1
ZNF705A	4	4
ZNF26	3	3
ZNF140	4	4
ZNF10	4	3
ZNF205	6	5
ZNF75A	5	2
ZNF597	3	2
ZNF688	2	2
ZNF764	2	2
ZNF689	2	2
ZNF23	5	2
ZNF19	5	3
ZFP1	3	2
ZNF286A	5	4
ZNF624	5	4
ZNF554	4	4
ZNF555	3	3
ZNF57	3	3
ZNF558	5	5
ZNF699	4	4
ZNF559	5	3
ZNF177	8	4
ZNF333	11	10
ZNF599	3	3
ZNF565	4	3
ZFP14	4	3
ZFP82	4	3
ZNF566	4	3
ZNF382	4	2
ZNF461	5	4
ZNF567	3	2
ZNF790	4	3
ZNF568	6	4
ZNF585A	5	2
ZNF585B	4	3
ZNF383	4	3
HKR1	5	3
ZNF527	4	3
ZNF569	5	3
ZNF570	4	3
ZNF793	7	3
ZNF571	3	2
ZFP30	5	3
ZNF546	6	4
ZNF283	6	3

ZNF404	2	2
ZNF234	5	3
ZNF226	5	3
ZNF227	5	3
ZNF235	4	3
ZFP112	3 or 4	2 or 3
ZNF806	2	2
ZNF180	4	4
ZNF114	4	2
ZNF473	4	3
ZNF577	6	4
ZNF649	4	3
ZNF613	5	2 or 3
ZNF350	4	3
ZNF615	5	3
ZNF677	4	2
ZNF331	4 to 6	2
ZNF582	4	3
ZNF583	4	3
ZNF667	4	3
ZFP28	7	7
ZNF470	5	3
ZIM2	10	8
ZNF543	3	3
ZNF772	3 or 4	3 or 4
ZNF419	2 to 4	2 to 4
ZNF773	3	3
ZNF671	3	3
ZNF606	6	5
ZNF274	4 or 6 or 7	3 or 5 or 6
ZNF8	3	3
ZNF584	3	3
ZNF132	2	2
ZNF324	3	2
ZNF343	5	3
ZNF133	3 or 6	2
ZNF334	4 or 5	2 or 3
ZNF74	4	4
ZNF157	3	3
ZNF630	4	3
ZNF275	3	2
**SCAN-ZNF genes**		
ZSCAN23	3	2
ZSCAN29	4	4
ZSCAN2	2	1
ZSCAN10	4	4
ZNF24	3	2
**Eutheria-specific SCAN-ZNF genes**		

ZNF193	3	2
ZNF323	3 or 6 or 7	2
ZSCAN12	5	3
ZSCAN21	3	2
ZNF434	5	2
ZNF174	2	2
ZNF232	4	3
ZNF397	3 or 5	2 or 4
ZNF397OS	3	2
ZNF444	4	3
ZSCAN5A	4	3
PEG3	8	5
ZSCAN4	4	2
ZSCAN18	5 to 6	4 to 6
ZSCAN22	2	1
MZF1	5	4
ZNF449	4	3
**SCAN-KRAB-ZNF genes**		
ZKSCAN2	6	6
ZSCAN20	7	6
ZNF496	8	6
ZNF184	5	4
ZNF192	5	4
ZKSCAN5	6	5
ZKSCN1	5	4
ZNF202	8	5
ZNF213	5	4
ZNF263	5	5
ZNF18	8	5
**Eutheria-specific SCAN-KRAB-ZNF genes**		
ZNF445	7	5
ZNF167	5	4
ZNF197	5	4
ZKSCAN3	5	4
ZKSCAN4	4	4
ZNF394	2	2
ZNF483	5	4
ZNF215	6	4
ZNF287	5	4
ZNF446	6	5
ZNF75D	6	4
ZNF498	7	4
ZNF274	7	7
