# Supplementary material for: Extensive intron gain in the ancestor of placental mammals
Source: Biol Direct. 2011 Nov 23;6:59. doi: 10.1186/1745-6150-6-59 (PMC3257199; doi:10.1186/1745-6150-6-59)
Supplement: Additional file 3 — Intron sizes in human domesticated genes. [file 1745-6150-6-59-S3.PDF]

Additional file 3 - Intron sizes in human domesticated genes.

Gene name	Intron sizes (from 5' to the 3' end of the gene) in bp	Differences between humans and placentals or vertebrates (in the case of the RAG1 gene)
**Retroelement-derived genes**		
RGAG1	26018 – 5299 – 536	
ZCCHC16	47864 – 323398	
ZCCHC5	611	
PEG10	6752	Dog: 4867 – 537
LDOC1L	231	
RGAG4	452	Dog: 1015 – 636 – 567 – 496 – 38
C22ORF29	279	
PNMA1	Intronless in human	Dog: 145 – 133
PNMA2	4137 – 305	
PNMA3	310	
MOAP1	95 – 195	
PNMA5	99 (in 5' UTR)	Mus: 835 (in 3' UTR)
PNMA6A	326	
ZCCHC12	332 – 197 – 416	
ZCCHC18	192 – 186	
CCDC8	Intronless in human	Mus: 419; Dog: 3025 – 84
PNMAL1	221 – 2535	
PNMAL2	Intronless in human	Dog: 346 – 145 – 110 – 3220
ARC	230 – 249	
ASPRV1	Intronless in human	Dog: 1788 – 173
Gin1	11348 – 1658 – 1868 – 6758 98 – 486 – 8367	
SCAND3	6877 – 3134 – 915	
KRBA2	921	Chimp: 5029 – 925 – 513
KIAA1305	142 – 8423 – 123 – 868 – 146 – 1545 – 216 – 1177	
Syncytin-2 (primate-specific)	6045	
Syncytin B (rodent-specific)	-	Mus: 749 – 150
**DNA-transposon derived genes**		
POGK	1349 – 5522 – 754 – 1344 – 3160	
RAG1	5167	Danio: 87 – 847
ZBED1	10046	
ZNF862	5988 – 1413 – 1478 – 1729 – 10082 – 855 – 1613	
C5ORF54 (Buster3)	4098	
PRKRIR	14774- 4787 – 5300 – 2822	
THAP9	3852 – 1391 – 1156 – 9007	
Harbi1	741 – 1657	

Naif1	689	
PGBD1	1856 – 1340 – 1371 – 9646 – 948 – 2731	
PGBD2	7472 – 2721	
PGBD4	Intronless in human	Macaca: 222
PGBD5	20175 – 5800 – 13668 – 4065 – 7427 – 1688	
