# Supplementary material for: Extensive intron gain in the ancestor of placental mammals
Source: Biol Direct. 2011 Nov 23;6:59. doi: 10.1186/1745-6150-6-59 (PMC3257199; doi:10.1186/1745-6150-6-59)
Supplement: Additional file 5 — Alternative splicing in PNMA2. [file 1745-6150-6-59-S5.PDF]

Additional file 5 - Alternative splicing in PNMA2

>**Homo PNMA2 gene** (chromosome:GRCh37:8:26361617:26372208:-1)
CCCCCACACCGACCTGGTCGTTGATGTTCTGCAGGGCTTCTTTGCCCGAGGCCCTCCGGA
TCTCCACCTTCCGGCCCGATTCAACAGATGGCTCTCCGCCGGCGCGCCGGCCCTGCCGCG
AGTACGGCGGCTGCGGGTCCGGGCCCAAGTGGGCGACGTCCCGCTGCTGAGCCACCACCT
GCCCCGAGCCCCGGCCCACCGACAGCGAGTCGAAGTCGATGGTCTTGTTCTCCGAGGACC
CTTCCACCGGGCAGAACATGCCACAGAATTTCTGCCGGGGCTTGGGGCTGCCGGAGCCCA
GGTTTTTAAAAAAAGCAGGGACCTCTTCGTCCTCTGCCGCCTTCCCGGATTGCTTTCTCT
CGGCCATGGCTCGGGGGCTCGGGTAGGGTCCTCGCCGTCCGTCCGTCCGTCTGCCAGTCC
CTAAGTCTCCCTCGCTGTGTGCACAAGCCCCCTAGCTGCTGCCGCGGCTGCCGTTCGCCG
CCCACCGCAAGGTGCACAGAAAGGAAGGAGGGCTGGCGAGCCGGCGACCCACCCTCCCGG
TCCCTCCCCCTGACCCCGCTTCTCCCCTCCCTGCCCACGCCCCCCGCGGCTACAGCGCGA
GCTCGGGGAAGCGGCAAAGTTGGATCTGGGAGGGCGTCCGCTGCGGCTGCGGGAGCCGAA
AGGCTGCCAGTTCCCGGCAGCGCTGGGAGGAGGGAGGCGCGGGCGGGGCGCGGCGCCGGG
GAGGGGAGCGGTGCTCAGGGGAGGGCTGGAGGGGAGGGAAGGAGAGAGAGAGGGGAGGGC
GGCACCGCCCCTAGCCCCGCGCTCCGGAAGTGAAGCGGCCAGACCACCAGCTAATGGATG
CGGAGCGGAGGGCCCGCTGACCGCTCTCCGCGCCTGGGTAAGTATCGGGCAGGGCACGCG
GAGTGAGGACCGGAGGACGCAGCCCCCAGCCCCAGTGCCCCTCTCCTCCACCTGCCTGGC
CCAGGCGCCTCTGCCCCGCCGCATGGCGGAGGTGGGCCGGGGGCGAGGGCGCACTGTGGA
TAGGGGTCCCGCCCCGTTACCCTGGGTGTGAGCTGATGGATGGGAAAAGGGGACAAGCCG
GGCGCCAGGCTACTCAGGGTGTGGGAAGCGGGATGGCACTGAAGATGAGCAAGGATAGCA
CAGGCAGGGTGGACAGGAGGGGGGCAGCCAAGACGCTGGACACCCCAGCTTCAGTTTGTC
CACTCGCCTTGGCAGGCTGCAGGGAGCAGAGACTGAAACGAGAAGGTTCCCGCTTCGTGC
ATCCACTTTGGTGTGGTTGGGGTGACGGGGGAGTGGGGGGGGGCACACAACATGGCGGG
AAGAAATTCAGAGGGGCGTAGCAGATGGGAGAGGAATATAGAGAGAGACGCAGGTCTGCG
GTCTATGGGGGTGTAGCAAAGAGTGCAGTCGGGAATTCCAGCCCAGGGCCACGACTTAAA
GACGGGGTGCAGACCGACTCTATTGCGCAAGGCGCCATGGACCGTGTCTGCGCCACTGCG
CCAAGAAGGGCGCAGGAACGTGTCGCGATTTGGTTTATTCTGCTTCCTCCACCAAAAAAA
AAAAAAAAAAAAAAAAAAGAGAGAAAAAACAAACTGAAACCCCCTTCCCAAATCTCCAGC
TTTCCCCTGCGCCCCAGAGCCCCGCCTGGTTCCCCCGCCCCTGTTGCGCACTGCGCC**ACA**
GTCGCAGCGTTTCCCGGGTTACCAGATGCCACGTGTTTGGGGAGTAAGTGCGGGTGCGCA
TCTGGTGCGGAGTAGGAGGTAGGATGTGGGCTGTGAGTGCCATGAATTGTGTGGGGCCAA
GCAGGGGAAAGGTGTGTAGGAACTTGGTGTGTGTGTGCAGTAGCTGAGCCACGGCCACCG
GGTTGGTAAGTAACCAGGGGGCAATTAGATTTGTGTGCCTAACACGGTCAATGCTGGTCC
TTCATTGCTTATTCCCATCTCCACGTCCTTTCCGCGTATCAGTCCCTGCCTCTGCGCGAT
GACAGCTCCGCCTCTCTTTCCACAGTGGCAAAATCCTGCAGAAATGGTGCCCCTCCTTTG
GTGGCCCGGGAGGGAGTTCTGGGCATGCTCAGTAACCAGGGTGGGTTTGGGCTGAGAGGC
CGTTTACCGCAGGGCCACCGCGTAGGGCCGTGTAACCAGGGATCCAGTCCCATTGTCAGC
CCAGGATTAAATGCGGTTACCAACCACCCCCACAGTCCAATTGTGAGAGATAGTTTGGTG
AATATAGTTACATCTGCTGGACAATCAGTCGCAATTTGAAGATCAGCCCATCTTGATAGG
TGGAAAGGCGTCTCCGGATGAGAAACAGACTGGGGAAGAAGAATATAGGAGTGGAGGGGC
TGAGAGAACCGGAGGGGGCATTTTGGACTTAGCGGCAGATGTGCTTTGCATTTGAATAAA
GCATTTGAAAAAAGCGCTTCCAACTGAATTACATCATTTCCTACTCACAGCCCCACTGTG
AGATGCGTGCCTTATCAGCACTGCTTTCCAGGTTAGGAAACAGATTTAGAGAGCTTGGGG
CGCATAGCCAAGGTTGGGTATTTAGTTAAATGATCCAGCAATTCCAGTGGGAGTCTTCCA
CCTCAAAGTCTGGCTTCTTTTCCAGCTTGTCCTGTGACCCCCTTTATGTGAGAAAATACA
AGGTTCTTCTATTTCTACCGTATCCAGATATTTCCATCCATGGCCCTATTGTATATCAAG
GGTGTTTCTTGCCATGTTTTTATTTGAAGGTAACACTGCAGGTCACACTGGGAGGAAGAG
TCATCTGAAGATAGCCTAACTTCAGCATAGCAGTCATTCTGTAGACCATAGAGATGCTTT
TTCCTGGGCAGTGTCTTTTTACATTCCCTGTTTCCTCTAAATATCCCAGTGGGGAGGAAG
GTTGCCCCCAGCTGGTTTTTTTATTTTTGTTTTTGTTTTCAG**ACAGAGTCTCACACTGTC**
ACCCAGGCTGGAATGCAATGGTGCAAACGCAACTCACTGTAGCCTTGACCCACTCGGCTC
AAGCAGTCCTCCCTCCTCAGCCTCCCAAGTAGTTGGGACTACAGGCGAGTGCCACACGCC
CAGCTAATTTTTTTATTTTTCCTTTTTGTAGAGACAGGGTCGCACTATGTTGCTGGTCTC
AAACTCCTGGGCTCAAGTGATCCTCCCGCTTTGGCCTCCCAAAGTGCTGGGTTTACAGGT
GTAAGCCACTGCACCTGGCCCTCCCCCACAGTCCCCACATTTTAGAAAGGAAAAGTCAGG
CATGGAGAAGTCAGCAGTCTCAGGTCTCTGGGGCTCCTGGGCACAGGGGTGAAGCAGCAT
TTTGCCCTCCTGGCTCTCTGGCACCCCTGGTGCCTTCAGCACTTCCCAGAAGACTCAGGA
GATCTTCCCACAGCAGGAGCACTGCTGATAGAGTTACAGGACAGCACTGTGGCGTATGGC
CCCCATTCGAATTGATGAAGATGTTTAGGCCAAGGGGGAAATGGTTGTAGCCCCTTAACA
CACACTCCGCAGCAGTAACTGGGAGGTATTAGGAACTCTAACTTTTTTTTTTTTTTTTTT
TGAGACAGAGTCTTGCTCTGTTACTCAGGTTAGAGTGCAGTGGTGCAATCGTGGCTCACT
GCAACTTCCGTCTCCCAGGTTTAAGCGATTCTCCTGCCTCAGCCTCCCAAGTAGCTGGGA
TTACAGGCACGTGCCACCACACCCAGATAATTTTTGTATTTTTGGTAGAGACGGGGTTTG
GCCATGTTGGCCAGGCTGGTTTCGAATTCCTGACCTCAGTGATCTACCCGCCTTGGCTTC
CCAAAGTGCTGGGATTACAGGCATGAGTCACCACGCCTGGCCAACTCTAACTTTTTTACG
TTCTCTAATCCTCACAGGTTCTCCAGAACAAAATCCTTGATAGGTGTGCTTCCTCCTTTT
TTCTTTTTTCTCAAATGATGGGTTAAAGTTCAAATGTTAGGAAAGGCTGGGTGTGGTGAT
TCAAGTGTGTAATTCAGCACTGTGAGAGGCTGTGGCGGGAGGATTGCTTGAGCCCAGGAG
TTTGAGACCAGGCTGAACAACGTAAGGAGACTGCGTCTCTACACAAAATTTAAAAAACTA
GCCAGGCATGGTGGCATGTGCCTGTGGTCCCAGGTACTCAGGAGGTCGAGGTGGGAGGAT
TGCTTGAGCCCAGGAGAGATCAAGGCTACTACAGTGAGCCGTGTTTACACCACTGCATTC
CAGCCTCGGCAACAGAGTGAGACCCTGTCTCAAAAAACAGAAACAGAAACAAATGCTTGG
AGAGTGTTGTGTTAGTAACTTATTGTCTTAGTCTATTTTGTGTTGCTATAAAGGAATACC
TGAGGCTGGATAATTTATAAAGAAAAGAGGTTTATTTGGGTCACAGTTCTGCAGGCTGTA
CAAGAAGTATGGTGCTGACATCTGCATCTGGTGACCCCTCAGGAAGCTTCCAGTCATAGC
AGAAAGCAAAGAGGGAGCAGATGCGGCAAGAAAGGGAGCAAGAGTGAGGGGAGGGAGGTG
GCAGTCTGTTTTTAACAATCAGATCTCGAGGAAGCTAATAGAACAAGAACCCACTCATTA
CCCCAAGAATGGCACCAAGCCATTCATAAGGGATCCACTCTCATGACCCAAACACATCCC
ACCAGGCCCCACCTCCAACACTGGGGATCACATTTCAGCATGAGATTTGGAGGGGACAAA
CCTTCAAACTATTTCACTTACCATTCATAGCAAAAGCAAAGCCCAAATGTGTGTTTGCAA
AGCCTTCCAGACACAACAAAAGAGAATTAATTCATAATTCCCGATTTAACTTAGTCGGCC
CTGAGATTTTTTTCCCTGAATTGTTATACCTAGATTAAACTATTTTTAAACGTTTGAGTG
ATTCCTCATTAGTTTTGTTAATCAAAGAGGTAAAGCAACTTAATATTGAGTAGTATTGTG
AAGGATTATCATATTTTTGAATAAGTGCATTAACGGTCTTCCTACTGAATATTAATGAAG
CATAAATACCTGTTCTTATTGTTTATGTGTAACAG**AGCAGCTTGGCTTGGCTGGAGCTAA**
GAGCCAGACACACCACTGTGTGGAGGTGGGTGATGTCTTCCTGTGCTAAAAGGTGAATAA
ATAAGCTCCTCACCTCTCGCGGAACACTCGGGAACACATCAACAGGTATTTAGCCACATC
TCCTGATTTCATCTTTCAATGGCAAAGACCTTGAATCCTGTCCTTAACTGGGGCTCAGAC
ATTAATCAACAGGTCTGATGAGCATATGGTGGGGACACCAGATTTCATTGGAGTCCCCAA
GAGAAAAAAAAGTCACAGTGAGCAAAGAAGGGGTCTGACAGAGTGGGACAAATGCAAGCG
CAGTGTAAAGCAGAACCGCCATCTGCCCCCAGTTCCATTCCAGATTAGAGTATGGATGCT

```
TCCAAGGAAAGAGAGTGCTGTGTAGTAAAACTGAACATTGTTTCACCAGGGGTCCAAGCC
GCCCTGCTGGGAGGCTTCTCTTCAAGAGTTCTGGGTCCCAGAGTGGAAGGCATTTTCCCA
TCAACTGGAGAGAGACGAAACATCAGAGACCAGGAGGCTGTGGAGAAAGCAGCTGTCCCA
GGTGCCTCAACTATCAGAGAAGGGTCAGCGTCACGTGGCTGCCAGCATCTTTGAGAAAAT
CACTGGCAATCGGACTTCAGAGCTGCGGGCACAGGTGTGGTTAGAACTGAGATACGACCT
GCCCACCTGGGTCAGGCCTAAAGACAAGAAGTCCTGAGTTCTTGCCACTGAGTAGGCCAG
GGTCATTTGTCCAGAAAACTTTGTGACTGTCTTTGAGTGACCTAGTCTGGGACCCATTCA
TTGGTGGGTTCTAAGGTTAGAAGCTCATCCAGGATATTTTCAATATTAAGTCAGTGCATA
GCTGCACCACTAACAAATTGGTGCCTGTAGAGTCAGAGTGGGTCAATTCTTAGGACAATG
GCGCTGGCACTGTTAGAGGACTGGTGCAGGATAATGAGTGTGGATGAGCAGAAGTCACTG
ATGGTTACGGGGATACCGGCGGACTTTGAGGAGGCTGAGATTCAGGAGGTCCTTCAGGAG
ACTTTAAAGTCTCTGGGCAGGTATAGACTGCTTGGCAAGATATTCCGGAAGCAGGAGAAT
GCCAATGCTGTCTTACTAGAGCTTCTGGAAGATACTGATGTCTCGGCCATTCCCAGTGAG
GTCCAGGGAAAGGGGGGTGTCTGGAAGGTGATCTTTAAGACCCCTAATCAGGACACTGAG
TTTCTTGAAAGATTGAACCTGTTTCTAGAAAAAGAGGGGCAGACGGTCTCGGGTATGTTT
CGAGCCCTGGGGCAGGAGGGCGTGTCTCCAGCCACAGTGCCCTGCATCTCACCAGAATTA
CTGGCCCATTTGTTGGGACAGGCAATGGCACATGCGCCTCAGCCCCTGCTACCCATGAGA
TACCGGAAACTGCGAGTATTCTCAGGGAGTGCTGTCCCAGCCCCAGAGGAAGAGTCCTTT
GAGGTCTGGTTGGAACAGGCCACGGAGATAGTCAAAGAGTGGCCAGTAACAGAGGCAGAA
AAGAAAAGGTGGCTGGCGGAAAGCCTGCGGGGCCCTGCCCTGGACCTCATGCACATAGTG
CAGGCAGACAACCCGTCCATCAGTGTAGAAGAGTGTTTGGAGGCCTTTAAGCAAGTGTTT
GGGAGCCTAGAGAGCCGCAGGACAGCCCAGGTGAGGTATCTGAAGACCTATCAGGAGGAA
GGAGAGAAGGTCTCAGCCTATGTGTTACGGCTAGAAACCCTGCTCCGGAGAGCGGTGGAG
AAACGCGCCATCCCTCGGCGTATTGCGGACCAGGTCCGCCTGGAGCAGGTCATGGCTGGG
GCCACTCTTAACCAGATGCTGTGGTGCCGGCTTAGGGAGCTGAAGGATCAGGGCCCGCCC
CCCAGCTTCCTTGAGCTAATGAAGGTAATACGGGAAGAAGAGGAGGAAGAGGCCTCCTTT
GAGAATGAGAGTATCGAAGAGCCAGAGGAACGAGATGGCTATGGCCGCTGGAATCATGAG
GGAGACGACTGAAAACCACCTGGGGGCAGGACCCACAGCCAGTGGGCTAAGACCTTTAAA
AAATTTTTTTCTTTAATGTATGGGACTGAAATCAAACCATGAAAGCCAATTATTGACCTT
CCTTCCTTCCTTCCTTCCCTCCCTTCCTCCTTCTCTCCTTCTCTCCTCCTCTCTCCTCTC
CTCTCCTCTCTTTCCTTCCTTCCTTCCTTTTTTCTTTTTCTCTTTCTTCTTTATTTCTTG
GGTCTCACTCTCATCACCCAGGCTAGAGTGCAGTGGCACAAAAATCTCGGCTCACTGCAG
CCTTGACTTCCCAGGCTCAGGCTCAGGTGATCCTCACACCTTAGCCTCCCAAGTACCTGG
GACTACAGGCACGCACCACCATGCCTAGCTATTCTTTTGTATTTTTGGTAGAGACAGGGT
TTTGCTGTGTTGCTCAGGCTGGTCTGGAACCCCTAGGCTCAAATGATGTGCCCAACTCGG
CCTCCCAAAGTGCTGGGATTACAGGCATGAACCGCCATGCCTGGCCCTTGATTTTTCTTT
TTAAGAAAAAAATATCTAGGAGTTTCTTAGACCCTATGTAGATTATTAATGAACAAAAGA
TTAAACTCCAAATATTAAATAGTAAGCCTGAAGGAATCTGAAACACTTGTACTTCCAATT
TTCTTTAAATAATCCCAAATAGACCAGAATTGGCCCATACCATAGAAGAAAGAATTGGCA
GTCAAAAAAAAAAATACCTTTTGTAATGTTTGAAAAATAAAGCTGTTTGACTTGTCAGGT
GTTTTCCTTTCTCAAATCAGCAAATTCTCTCTGAGTGCCTGGCTTTGTGAGACACTGTAC
AAGGAGTTACAAGACTACAGCTATAACCTGCAGTTGAGCAGTTATAAACCTACAAAATGG
GCCCTGCCCTCAGAGAGGTTCCAGTCTAGATGAGGAGCTGATCTAGACAGGTAAAAGGCT
AACTAACCCTTTGTGTAAATAAGTTCATCACCCCAGTAAAAGTGTCATCACCCAGTGAAT
AGGACCACCTCTGCCTGCAGATTTTTGTTGTTGTTGTTGTCATTGTTGTTGTTGTTTTAA
CCTGGGAAGTGTTCTTCCTGCCTTTCTGCTAGGTGTCAGATAGATGGTCCCAGAGCTAGG
TGCTGTGTCAGGCCCTGAAGACACAGATGACTCAACCTAAGCTTTACTTTCCAGAGGTCC
ACAGCCTGAGAGGTGTCCCCAAAGAAAGGGGGACATGAGGGGACTGCATGCTTGAGAGCA
GGGTTGTTTAGGGCAGGTTTGGATTTAGTGAGCAGGCTGGTTTGCTTAGAGAAGGCTTTT
AGTGGCAACAAAGGATGAAGAGGAGAGAAAAGGAACTCACATTTATTGAGGGCCTACTGT
GTGCAAAGTGTTTCATGTATATCTCATTGAATGTATACAGCCACCCTGTTGTGGTATAAT
TTTGCTCTTTATAAAGAGAAAGACCGAAGCTCAGATGAGTTAAGTGGTCTCCTCAACACC
AAAATGCCAAGAAGTGATGGAGCCTAGACAGAAGCCCAGAACTTTCTGACTCACACTAGT
CCATCCTCTACCATCACGATGACTTTCAAATTGTGCTCTGCAGTTCTGCAGATTTTCTAG
CAGTGCCATCTCCAAAATGTGTTTTAAACTCTTTATTTTTTTAATTATTATTAGTATTAT
TTTGAGACTGAGTCTTGCTCTATCACCCAGGCTGGAGTGCAGTGGTGCAATCTCAGCTCA
CTGCAACCTCCGCCTCCCAGGTTCAAGCGATTTCGTGCCTCAGCCTCCCGAGTAGCTGGG
ATTACAGGCACCCACCACCACGCCCAGCTAATTTTTGTATTTTTAGTAGAAATGGGGTTT
CACCATGTTGGCCAGGCTGGTCTCGAACTCCTGACCTCAAGTGATCCACTCACCTCGGCC
TCCCAAAGTGCTGGGATTACAGGTGTGAGCCACCATGCCTGGGCTAAACTCTTTAAGTCT
CTAGTAAATGCAGCTAGATTCAAATGGGCTGATAACCAAATTTTAACACATCAGCATTCA
CCACCAGGTTTACTTTTATTTTCAGATTGGCTCATTTTGTGCAGACCTTAGAGCAAAGTT
TCCTTTATGGTATCTGTGTACGTATCCAAACTTCTTTTAATTGTTCACAGATTTTAAAAG
CGGTAGCACCACATGGTTGTGTAGATCAGACCTGTGTATTTAGATCAGACCTGTGTATCA
CGTAAGTGTGTGAGTGCAGTGCAGATGAGCACCATTTAGTTATATGTGCTAGGCAAATCT
CCAACACAGTTGATGTGTAGTCTTGTGGTAGATTTGTGCATACTGTAAGCAAATTGCTTA
GCTTCTCTAGACATCAGTTTCCACATCTGAAAAATAAGAAGATGAGAGTACACGGTTGTT
ATGAACAAATGACTTAATGCTTTTTAAGCACGTTGCATGACATCTGGAACACAGAAAGCC
CTCAATACATTGAAGCTCTTAGGATTTTCACGATGTTCCTGTCTGCTCAATGCATGCTTT
CTTTATTGTTCTGACAGTTGTGTGGTAACAAGCTAATATGCTTCCAGTTGACTTCCAGTC
TACCCTGGTGTTAGAAACCGTTTCATCTCTTATTGTAAATTTGAGTGCTTGTTGTTTTTT
ATATTTGTGATGACTCTTCCAGCAGTTGTTGACAATTGTTAGAGGTTTGACTTTTAAATA
ATTACTTATTTTTTCTGATTGTGGTTCAGTTTAACTGAAGAATATCCTGAGATTGTAAGA
AAAGCATTTTTTAAAAGGTATCACTTGTGATCATTTATCTTTCTAAATTCTATTTTTAAT
ACTGTTCCACCAAAGTGATGCAGTGGTTACCATGACACCCTAATTTCATGTGTTTTTGTA
TTTATGAAAATAGTTTCATTGTCATTTATTGGCGGTATACAAAGTAAAATGTTATAAATG
TGAAGTTATAAAATAAATATATGCTAATAAAATCCTGAGTTTTTCTGTTTCCTATATCGT
GGTCTCTATAAAGTACTGCTTGAAGAAGGAGCTTTGTTACTGAAAAATTTTGAGAGATAA
GTTGAGAAAATCAAGCTAAAGCCAAAAGGTCTTAGAATCCTGTAGGCAAGAAAACCTGAA
AGCAAAAAAAATGCTTGAACAAGAATACTTGAACTATGTCTCTGGGGACAAGGGAGAGCTC
GGTCCACCCATTATCGATTTCTCTTTTAAAATATACAGAAACAACAGATGGAGGTCAGTT
GCCTCAGAGACCCCCCAGCTCATTTGCTTAGGAAAGTAACTGTACAGATAAATTCCCAGA
CTCTATTCTGCCAAGGGTGAATGACCCTGAAGGTCGAATATTGCTTTTCTGGGCTGAAGT
CACCCTTTTTCTTCTTCTTCTTCTTCTTTTTTTTTGAGACAAGGTGTCACCCAGGCTGGAG
TATAGTGGCACAATCTCGGCTCACTGCAAACTCTGCCTACCAGCTTCAAGTGATTCTCAT
GCCTCAGCTTCCCGAGTAGCTGGGATTACAGGGGTGCACCACCACGTTCCAGCTAAGTTT
TTGTATTTTTAGTAGAGACAGGGTTTCACTAT
```

Repetitive sequences (including *Alu* SINEs) in human PNMA2 gene

```
  SW    perc perc perc  query            position in query     matching    repeat             position in repeat
score    div. del. ins.  sequence         begin end    (left)    repeat     class/family     begin   end     (left)   ID
```

22	50.0	0.0	0.0	UnnamedSequence	696	717	(9875)	+	GC_rich	Low_complexity	1	22	(0)	1
184	13.9	0.0	6.9	UnnamedSequence	718	779	(9813)	+	G-rich	Low_complexity	16	73	(0)	2
264	10.0	0.0	0.0	UnnamedSequence	1554	1593	(8999)	+	(A)n	Simple_repeat	1	40	(0)	3
357	34.1	9.1	1.7	UnnamedSequence	2384	2604	(7988)	C	MIRc	SINE/MIR	(13)	255	19	4
1790	14.8	2.3	2.0	UnnamedSequence	2896	3200	(7392)	C	AluJb	SINE/Alu	(6)	306	1	5
2187	11.3	0.3	0.0	UnnamedSequence	3523	3822	(6770)	C	AluSx	SINE/Alu	(11)	301	1	6
1623	18.4	0.3	2.0	UnnamedSequence	3945	4253	(6339)	+	AluJr	SINE/Alu	1	304	(8)	7
2461	15.5	3.1	0.0	UnnamedSequence	4284	4696	(5896)	C	MSTB	LTR/ERVL-MaLR	(0)	426	1	8
3488	31.7	1.0	0.7	UnnamedSequence	5837	6952	(3640)	+	MamGyp-int	LTR/Gypsy	4	1122	(3981)	9
405	15.4	1.1	2.2	UnnamedSequence	7137	7229	(3363)	+	CT-rich	Low_complexity	87	178	(0)	10
1694	16.4	1.9	3.5	UnnamedSequence	7230	7546	(3046)	C	AluJb	SINE/Alu	(0)	312	1	11
226	25.7	1.2	16.7	UnnamedSequence	7817	7985	(2607)	C	L2a	LINE/L2	(10)	3416	3250	12
246	6.2	0.0	0.0	UnnamedSequence	8065	8096	(2496)	+	(TTG)n	Simple_repeat	2	33	(0)	13
495	29.3	11.5	1.2	UnnamedSequence	8373	8607	(1985)	C	MIRb	SINE/MIR	(7)	261	3	14
549	33.2	4.5	3.6	UnnamedSequence	8647	8671	(1921)	+	Charlie15b	DNA/hAT-Charlie	85	109	(952)	15
2233	9.3	0.3	0.7	UnnamedSequence	8672	8982	(1610)	C	AluSx1	SINE/Alu	(0)	312	3	16
549	33.2	4.5	3.6	UnnamedSequence	8983	9312	(1280)	+	Charlie15b	DNA/hAT-Charlie	110	443	(618)	15
473	27.1	7.0	2.8	UnnamedSequence	9329	9499	(1093)	+	MIRb	SINE/MIR	85	262	(6)	17
545	29.3	8.1	1.7	UnnamedSequence	9522	9854	(738)	+	Charlie15b	DNA/hAT-Charlie	440	793	(268)	18
602	26.8	2.3	1.8	UnnamedSequence	9859	10075	(517)	+	Charlie15b	DNA/hAT-Charlie	837	1054	(7)	19
1254	15.0	3.9	0.5	UnnamedSequence	10386	10592	(0)	C	AluSx3	SINE/Alu	(2)	310	97	20

Genomic alignment of PNMA2 genes (for tracing the timing of *Alu* SINE insertions)

```
Homo sapiens             CCCCCACACCGACCTGGTCGTTGATGTTCTGCAGGGCTTCTTTGCCCGAGGCCCTCCGGATCTCCACCTTCCGGCCCGATTCAACAGATGGCTCTCCGCCGGCGCGCCGGCCCTGCCGCG
Pan troglodytes          CCCCCACACCGACCTGGTCGTTGATGTTCTGCAGGGCTTCTTTGCCCGAGGCCCTCCGGATCTCCACCTTCCGGCCCGATTCAACAGATGGCTCTCCGCCGGCGCGCCGGCCCTGCCGCG
Gorilla gorilla          CCCCCACACCGACCTGGTCGTTGATGTTCTGCAGGGCTTCTTTGCCCGAGGCCCTCCGGATCTCCACCTTCCGGCCCGATTCAACAGATGGCTCTCCGCCGGCGCGCCGGCCCTGCCGCG
Pongo abelii             CCCCCACACCGACCTGGTCGTTGATGTTCTGCAGGGCTTCTTTGCCCGAGGCTCTCCGGATCTCCACCTTCCGGCCCGATTCAACAGATGGCTCCCCGCCGGCTCGCCGGCCCTGCCGCG
Macaca mulatta           CCCCCACACCGACCTGGTCGTAGATGTTCTGCAGGGCTTCCTTGCCCGAGGCCCTCCGGATCTCCACCTTCCGGCTCGATTCAACAGATGGCTCCCCGCCGGCGCGCCGGCCCTGCCGCG
Callithrix jacchus       CCCCCACACCGACCTGGTCGTAGATGTTCTGCAGGGCTTCCTTGCCCGAGGCCCTCCGGATCTCCACCTTCCGGCCCGATTCAACAGATGGCTCCCCGCCGGCGCGCCGGCCCTGCCGCG
Mus musculus             CCCCCATACCAACCTGATCATAGATGTTCTGCAGGGCTTCCTTGCCCGAGGCCCGCCGGATCTCCACCTTCCGGCTCGATTCAACAGATGGCTCCCCACTGGCACGCCGGCTCTGCCG--
Rattus norvegicus        CCCCCATACCAACCTGATCGTAGATGTTCTGCAGAGCTTCCTTGCCCGAGGCCCGCCGGATCTCCACCTTCCGGCCCGATTCAACAGATGGCTCCCCGCTGGCACGCCGGCTCTGCCG--
Bos taurus               CCCCCATACCAACCTGGTCGTTGATGTTCTGCAGAGCTTCCTTGCCCGAGGCCCGCCGGATCTCCACCTTCCTGCTCGATTCAACAGATGGCTCCCCGCCGGCGCGCCGGCCCTGCCGGG
Sus scrofa               CCTCCATACCAACCTGGTCGTTGATGTTCTGCAGGGCTTCCTTACCGGAGGCCCGCCGGATCTCCACCTTCCGGCCCGATTCAACAGATGGCTCCCCGCCGGCGCGCCGACCCTGCCGGG
Canis lupus familiaris   CCCCCACACCAACCTGGTCGTAGATGTTCTGCAGCGCTTCCTTGCCCGAGGCCCGCCGGATCTCCACCTTCCGGCTCGATTCAACAGATGGCTCCCCGCCGGCGCGCCGGCCCTGCCGGG
Equus caballus           CCCCCATACCAACCTGGTCGTTGAGGTTCTGCAGGGCTTCCTTGCCCGAGGCCCGCCGGATCTCCACCTTCCGGCTCGATTCAACAGATGGCTCCCCGCCGGCGCGCCGGCCCTGCCGGG

Homo sapiens             AGTACGGCGGCTGCGGGTCCGGGCCCAAGTGGGCGACGTCCCGCTGCTGAGCCACCACCTGCCCCGAGCCCCGGCCCACCGACAGCGAGTCGAAGTCGATGGTCTTGTTCTCCGAGGACC
Pan troglodytes          AGTACGGCGGCTGCGGGTCCGGGCCCAAGTGGGCGACGTCCCGCTGCTGAGCCACCACCTGCCCCGAGCCCCGGCCCACCGACAGCGAGTCGAAGTCGATGGTCTTGTTCTCCGAGGACC
Gorilla gorilla          AGTACGGCGGCTGCGGGTCCGGGCCCAAGTGGGCGACGTCCCGCTGCTGAGCCACCACCTGCCCCGAGCCCCGGCCCACCGACAGCGAGTCGAAGTCGATGGTCTTGTTCTCCGAGGACC
Pongo abelii             AGTACGGCGGCTGCGGGTCCGGGCCCAAGTGGGCGACGTCCCGCTGCTGAGCCACCACCTGCCCCGAGCCCCGGCCCACCGACAGCGAGTCGAAGTCGATGGTCTTGTTCTCCGAGGACC
Macaca mulatta           AGTACGGCGGCTGCGGGTCCGGACCCAAGTGGGCGACGTCCCGCTGCTGAGCCACCACTTGCCCCGAGCCCCGGCCCACCGACAGCGAGTCGAAGTCGATGGTCTTGTTCTCCGAGGACC
Callithrix jacchus       AGTACGGCGGCTGTGGGTCCGGGCCCAAGTGGGCGACGTCCCGCTGCTGAGCTACCACCTGCCCCGAGCCCCGGCCCACCGACAGCGAGTCGAAGTCGATGGTCTTGTTCTCCGAGGACC
Mus musculus             ----------GTGCGGGTCGGGGCCCAAGTGGGCGACATCGCGCTGCTGAGCCACCACTTGCCCCGAGCCCCGGCCCACTGAAAGCGAGTCGAAGTCGATGGTCTTGTTCTCCGAGGACC
Rattus norvegicus        ----------GTGCGGGTCCGGGCCCAAGTGGGCGACATCCCGCTGCTGAGCCACCACCTGCCCCGAGCCCCGGCCCACTGAAAGCGAGTCGAAATCGATGGTCTTGTTCTCCGAGGATC
Bos taurus               AGTAGGGCGTCTGCGGGTCGGGGCCCAAGTGCGCGACGTCCCGCTGCTGGGCCACCACCTGCCCCGAGCCCCGGCCCACCGTCAGCGAGTCGAAGTCGATGGTCTTGTTCTCCGAGGACC
Sus scrofa               AGTACGGCGGCTGCGGGTCCGGGCCCAAGTGGGCGACGTCCCGCTGCTGAGCCACCACCTGCCCCGAGCCCCGGCCCACCGACAGCGAGTCGAAGTCGATGGTCTTGTTCTCCGAGGACC
Canis lupus familiaris   GGTGCGGCTGCTGCGGGTCCGGGCCCAGGTGGGCGACGTCCCGCTGCTGAGCCACCACCTGCCCCGAGCCCCGGCCCACAGACAGCGAGTCAAAGTCGATGGTCTTGTTCTCGGAGGACC
Equus caballus           AGTACGGCGGCTGCGGGTCCGGGCCCAAGTGGGCGACGTCCCGCTGCTGAGCCACCACCTGCCCCGAGCCCCGGCCCACCGACAGCGAGTCGAAGTCGATGGTCTTGTTCTCCGAGGACC

Homo sapiens             CTTCCACCGGGCAGAACATGCCACAGAATTTCTGCCGGGGCTTGGGGCTGCCGGAGCCCAGGTTTTTAAAAAAAGCAGGGACCTCTTCGTCCTCTGCCGCCTTCCCGGATTGCTTTCTCT
Pan troglodytes          CTTCCACCGGGCAGAACATGCCACAGAATTTCTGCCGGGGCTTGGGGCTGCCGGACCCCAGGTTTTTAAAAAAAGCAGGGACCTCTTCGTCCTCTGCCGCCTTCCCGGATTGCTTTCTCT
Gorilla gorilla          CTTCCACCGGGCAGAACATGCCACAGAATTTCTGCCGGGGCTTGGGGCTGCCGGAGCCCAGGTTTTTAAAAAAAGCAGGGACCTCTTCGTCCTCTGCCGCCTTCCCGGATTGCTTTCTCT
Pongo abelii             CTTCCACCGGGCAGAACATGCCACAGAATTTCTGCCGGGGCTTGGGGCTGCCGGAGCCCAGGTTTTTAAAAAAGGCAGGGACCTCTTCGTCCTCTGCCGCCTTCCCGGATTGCTTTCTCT
Macaca mulatta           CTTCCACCGGGCAGAACATGCCACAGAATTTCTGCCGGGGCTTGGGGCTGCCGGAGCCCAGGTTTTTAAAAAAGGCAGGGACCTCTTCGTCCTCTGCCGCCTTCCCGGATTGCTTTCTCT
Callithrix jacchus       CTTCCACCGGGCAGAACATGCCACAGAATTTCTGCCGGGGCTTGGGGCTGCCTGAGCCCAGGTTTTTAAAAAAGGCAGGGACCTCTTCGTCCTCTGCCGCCTTCCCAGACTGCTTTTTCT
Mus musculus             CTTCCACAGGGCAGAACATGCCGCAGAATTTCTGCCGCGGCTTGGGACTGCCGGAGCCCAGGTTTTTAAAAAAGGCAGGGACCTCTTCGTCCTCTGCTGCCTTTCCAGATTGCTTTCTCT
Rattus norvegicus        CTTCCACGGGGCAGAACATGCCGCAGAATTTCTGCCGCGGCTTCGGACTGCCCGAACCCAGGTTTTTAAAAAAGGCAGGGACCTCTTCGTCCTCTGCTGCCTTCCCAGATTGCTTTCTCT
Bos taurus               CCTCCACCGGGCAGAACATGCCACAGAATTTCTGCCGAGGCTTGGGGCTGCCTGAGCCCAGGTTTTTAAAAAAGGCAGGGACCTCTTCGTCCTCTGCCGCCTTCCCGGATTGCTTCCTCT
Sus scrofa               CTTCCACCGGGCAGAACATGCCACAGAATTTCTGTCGAGGCTTGGGGCTACCGGAGCCCAGGTTTTTAAAAAAGGCAGGGACCTCTTCGTCCTCTGCCGCCTTCCCGGATTGCTTCCTCT
Canis lupus familiaris   CTTCCACTGGGCAGAACATGCCACAGAATTTCTGCCGGGGCTTGGGGCTGCCCGAGCCCAGGTTTTTAAAAAAGGCAGGGACTTCTTCATCCTCTGCCGGCTTCCCGGACTGCTTCCTCT
Equus caballus           CTTCCACGGGGCAGAACATGCCACAGAATTTCTGCCGGGGCTTGGGGCTGCCTGAGCCCAGGTTTTGAAAAAGGCAGGGACCTCTTCGTCCTCTGCCGCCTTCCCGGATTGCTTCCTCT

Homo sapiens             CGGCCATGGCTCGGGG---GCTCGGGTAGGGTCCTCGCCGTCCGTCCGTCCGTCTGCCAGTCCCTAAGTCTCCCTCGCT--GTGTGCACAAGCCCC-----CTAGCTGCTGCCGCGGCTG
Pan troglodytes          CGGCCATGGCTCGGGG---GCTCGGGTAGGGTCCTCGCCGTCCGTCCGTCCGTCTGCCAGTCCCTAAGTCTCTCTCGCT--GTGTGCACAAGCCCC-----CTAGCTGCTGCCGCGGCTG
Gorilla gorilla          CGGCCATGGCTCGGGG---GCTCGGGTAGGGTCCTCGCCGTCCGTCCGTCCGTCTGCCAGTCCCTAAGTCTCCCTCGCT--GTGTGCACAAGCCCC-----CTAGCTGCTGCCGCGGCTG
Pongo abelii             CGGCCATGGCTCGGGG---GCTCGGTTAGGGTCCTCGCCGTCCGTCCGTCCGTCTGCCAGTCCCTAAGTCTCCCTCGCT--GTGTGCACAAGCCCC-----CTAGCTGCTGCCGCGGCTG
Macaca mulatta           CGGCCATGGCTCGGGG---GCTCAGGGAGGGTCCTCGCCGTCCGTCCGTCCGTCTGCCAGTCCCTAACTCTCCCTTGCT--GTGTGCACAAGCCCC-----CTAGCTGCTGCCGCGGCTG
Callithrix jacchus       CGGCCATGGCTCGGGG---GCTCGGAGAGGGTCCTCGCCGTCCGTCCGTCCGTCTGCCAGTCCCTAAGTCTCCCTCGCG--GTGTGCACAAGCCCC-----CTAGCTGCTGCAGCGGCTG
Mus musculus             CGGCCATGGCTCAGGGGGCGCT-GGGGAAGGTCCTGTCTGTTCGTCCGTCCGTCCGTGGTTTCCTAAGTCTCCCTAGCTGGGTGTGCACTAGCCT------CTTGCTGCCGCCGCGGCTG
Rattus norvegicus        CGGCCATGGCTGGGGGTGCGCT-AGGGAAGGTTCTGTCTGTCTGTCCGTCCGTCCGTGGTTCCCTAAATCTCCCTGGTTGGGTGTGCACTAGCCT------CTTGCTGCTGCCGCGGCTG
Bos taurus               CAGCCATGGCGTGGGG---GCTCGGGGAGGGTCCTTGCCGTTCGTCCGTCCGTCTGCTGGTCCCTACGTCTGCCTGTCT--CTAGGCACAAACCCCCTGCCCCGGCTGCAGCCGCGGCTG
Sus scrofa               CTGCCATGGCTTGGGG---GCTTGGGGAGTGTCCTCTCTGTCCGTTCTTTCCTCAGCGGGTCCCTAAGTCTGCCTGTCT--CTGTGCAAAAATCCCC---CCCGGCTGCTGCCTCGGCTG
Canis lupus familiaris   CGGCCATGGCGCGGGA---GCTCAGGGAGGGTCCTCGCGG----TGCGTCCGTCTGCAGGTCCCTAAGTCGGCCTCT----CTGTGCACAAACCCC-----CGGGCTGCCGCCGAGGCTG
Equus caballus           CGGCCATGGCGCGGGG---GCTCGAGGAGGGTCCTCTCCGTCAGTCCGTCCGTCTGCCGGTCCCTAAGTCTGCCTCTCT--CTGTGCACAAACCCC-----CTGGCTGCAGCCGCCGCTA

Homo sapiens             CCGTTCGCCGCCCACCGCAAGGTGCACAGAAAGGAAGGAGGGCTGGCGAGCCGGCGACCCACC-CTCCCGGTCCCTCCCCCTGACCCCGCTT-CTCCCCTCCCTGCCCACGCCCCCCGCG
Pan troglodytes          CCGTTCGCCGCCCACCGCAAGGTGCACAGAAAGGAAGGAGGGCTGGCGAGCCGGCGACCCACC-CTCCCGGTCCCTCCCCCTGACCCCGCTT-CTCCCCTCCCTGCCCACGCCCCCCGCG
Gorilla gorilla          CCGTTCGCCGCCCACCGCAAGGTGCACAGAAAGGAAGGAGGGCTGGCGAGCCGGCGACCCACC-CTCCCGGTCCCTCCCCCTGACCCCGCTT-CTTCCCTCCCTGCCCACGCCCCCCGCG
Pongo abelii             CCGTTCGCCGCCCACCGCAAGGTGCACAGAAAGGAAGGAGGGCTGGCGAGCCGGCGACCCACC-CTCCCGGTCCCTCCCCCTGACCCCGCTT-CTCCCCTCCCTGCCCACGCCCCCCGCG
Macaca mulatta           CCGCTCGCCGCCCACCGCAAGGTGCACAGAAAGGAAGGAGGGCTGGCGAGCCGGCGACCCACC-CTCCAGGTCCCTCCCCCTGACCCCGCTT-CTCCCCTCCCTGCCCACGCCCCCCGCG
Callithrix jacchus       CCGCTCGCTGCCCACCGCAAGGTGCACAGAAAGGAAGGAGGGCTGGCGAGCCGGCGACCCACC-CTCCCGGTCCCTCCCCCTGACCCCGCTT-CTCCCCTCCCTGCCCACGCCCCCCGCG
Mus musculus             CCTCTCCCTGCCTACCGCAAGGTGCACAGAAAGGATGGAGAGCTGGCGAACCGGCGACCCACCCCTCGCGGTCCCTCCTCCTGACCCCGCTT-TCTCCCTCCCTGCCCACGCCCCCCGGC
Rattus norvegicus        CTACTCCCTGCCTACCGCAAGGTGCACAGAAAGGATGGAGGGCTGGCGAACCGGCGACCCACCCCTCGCAGTCCCTCCTCCTGACCCGGCTT-TCTCCCTCCCTGCCCACGCCCCCCGGC
Bos taurus               CCGCTCTCTGCCTACCGCAAGGTGTACAGAAAGGAAGGAGGGCTGGCGAACGAGCGACCCACCCCTCTCGGTCCCTCCCCCTGACCCCGCTTTCTCCCCTCCCTGCCCACGCCCCCCG--
Sus scrofa               CTGCTCTCTGCCTACCGCAAGGTGTACAGAAAGGAACGAGGGCTGGCGAGCGAGCGACCCACCCCTCCCGGTCCCTCCCCCTGACCCCGCTTTCTCCCCTCCCTGCCCACGCCCCCCGCA
Canis lupus familiaris   CCGCTCTCTGCCTACCGCAAGGTGTACAGAAAGGAAGGAGGGCTGGCGAGCAG-CGGCCCACCCCTCCCGGCCCCTCCCTCTGACCCCGCTTTCTCCCCTCCCTGCCCACGCCCCCCGCC
Equus caballus           CCGCTCTGTGCCTACCGCAAGGTGTACAGAAAGGAAGGAGGGCTTGCGAGCGGACGACCCGCCCCTCCCGGTCCCTCCCCCTGACCCCGCTTTCTCCCCTCCCTGCCCACGCCCCCCGCC

Homo sapiens             GCTACAGCGCGAGCTCGGGGAAGCGGCAAAGTTGGATCTGGGA-GGGCGTCCGCTGCGGCTGCGGGAGCCGAAAGGCTGCCAGTT-C-CCGGCAGCGC-TGGGAGG---AGG--------
Pan troglodytes          GCTACAGCGCGAGCTCGGGGAAGCGGCAAAGTTGGATCTGGGA-GGGCGTCCGCTGCGGCTGCGGGAGCCGAGAGGCTGCCAGTT-C-CCAGCAGCGC-TGGGAGG---AGG--------
Gorilla gorilla          GCTACAGCGCGAGCTCGGGGAAGCGGCAAAGTTGGATCTGGGA-GGGCGTCCGCTGCGGCTGCGGGAGCCGAGAGGCTGCCAGTT-C-CCGGCAGCGC-TGGGAGG---AGG--------
Pongo abelii             GCTACAGCGCGAGCTCGGGGAAGCGGCAAAGTTGGATCTGGGA-GGGCGTCCGCTGCGACTGCGGGAGCCGAGAGGCTGCCAGTT-C-CCGGCAGCGC-GGGGAGG---AGG--------
Macaca mulatta           GCTACAGCGCGAGCTCGGGGAAGCGGCAAAGTTGGATCTGGGA-GGGCGTCCGCGGCGGCTGCGGGAGCCGAGAGGCTGCCAGTT-C-CCGGCAGCGC-GGGGAGG---AGG--------
Callithrix jacchus       GCCACCGCGCGAGCTCGGGCGAGCGGCAAAGTTGGATCTGGGA-GGGCGGCCGCTGCGGCTGCGGGAACGGAGAGGCTGCCAGTT-C-CCGGCAGCGC-GGGGAGG---AGG--------
Mus musculus             GCCGCCACGCCACCTCCGA------GCAAAGTTGGATCTGAGC-GGGCGGTCGCTGCGGC-----------AGAGGATGCCAGTT-C-CCGGCAACGC-GGGGCGG---G--GCGGAGTG
Rattus norvegicus        GCCGCCACGCCACCTCCGA------GCAAAGTTGGATCTGAGCTGGGCGGTCGCTGCGGC-----------AGAGGATGCCAGTT-C-CCGGCAGCGC-GGGGAGG---G---------
Bos taurus               ---------CCAGCTCCCGCGAGCAGCAAAGTTGGGTCTGGGC-GGGTGGCCGCTGCGGCAGCGAGAGCGGAGAGGCTGCCAGTT-C-CCGGCAGCTG-CGGGAGG---AGC--------
Sus scrofa               GC---CTCGCGAGCTCCCGCGAGCGGCAAAGTTGGGGTTGGGA-GGGTGGCCGCTGCGGCAGCAAGAACCGAGAGGCTGCCAGTT-A-CCAGCAGCGG-CGGGAGGTGGGGG--------
Canis lupus familiaris   GC---CGCGCCAGCGCCCGCGAGCAGCAAAGTTGGGTCTGGGA-GGGCGGCCGCTGCGGCAGCAGGAG--GAGAGGCTGCCAGCTTC-CCGGCAGCGG-CGGCGGC---AGG--------
Equus caballus           GC---CTCGCGAGCTTGGGTGAGCGG-AAAGTTGGGTCTGGGA-GGGCGGCCGCTGCGGCAGCGGGAGCGGAGAGGCTGCCAGTTTCCCCGGCGGCGGGCGGGAGG---AGG--------

Homo sapiens             --GAGGCGCGGGCGG-----------GGCGCGGCGCCG---GGGAGGGGAGCGGT--GCT-------------C-AGGGGAG--GGCTGGAGGGGAGGGAAGGAG------AGA--GA--
Pan troglodytes          --GAGGCGCGGGCGG-----------GGCGCGGCGCCG---GGGAGGGGAGCGGT--GCG-------------C-AGGGGAG--GGCTGGAGGGGAGGGAAGGAG------CGA--GA--
Gorilla gorilla          --GAGGCGCGGGCGG-----------GGCGCGGCGCCG---GGGAGGGGAACGGC--GCG-------------C-AGGGGAG--GGCTGGAGGGGAGGGAAGGAG------AGA--GA--
Pongo abelii             --GAGGCGCGGGCGG-----------GGCGCGGCGCCG---GGGAGGGGAGGGGA--GGG-------------C-AGGGGAG--GGCTGGAGGGGAGGGAAGGAG------AGA--GA--
Macaca mulatta           --GAGGCGCGGGTGG-----------GGCGCGGCGCCG---GGGAGGGGAGCAGC--GCG-------------C-AGGGGAG--GGCTGGAGGGGAGGGAAGGAG------AGA--GA--
Callithrix jacchus       --GAGGCGCGGGCGGGGCGCAGGCGGGGCGCGGCGCCA---GGGAGGGGAGCGGC--GCG-------------C-AGGGGAG--GGCAGGAGGGGAGGGAAGGAG------AGAGAGA--
Mus musculus             GGGCGGGGCAGGAGG-----------AACGCAGGATCAGGAGGGAGGGGACAAGG--AGG-------------G-AGAGGACCCAGAAGGAGGAGACGGA-GAAG------GGA--GG--
Rattus norvegicus        --GCGGGGCGGGCGG-----------AACGCGCGATCAGAAGGGAGGGGACAAGA--AGG-------------G-AGGGGACCCAGAAGGAGGAGACGGA-GAAG------GGA--GG--
Bos taurus               --GAGGCGCGGGCGG-----------GGCG--G--------AGGCGGGGCGCGGC--ACG-------------C-CGGGGAG--G--------GGAGCGA-GGAG------CCG--GGGA
Sus scrofa               --GAGGCGCGGGCGG-----------GGCG--G--------GGGCGGGGCGCGGC----G-------------C-TGGGGAG--G--------GGAGCTC-AGCG------CTG--GGGA
```

```
Canis lupus familiaris  --TAGGCGCGTGCGG-----------G-CG--G--------GGGCGGGGCGGGGCGCCCGCCCGGAGGGGAGGTGCGGGCCC--C--------GGAGCGG-AGCGCGGAC-CCG--GGAG
Equus caballus          --GGGGAGGGAGGAG-----------GGAG--G--------AGG-GAGGATGGAGGCGCG-------------TGCGGGGCG--G--------GGGGCGG-GGCGCGTCCTCCG--GGAG

Homo sapiens            -------GAGGGGAGGGCGG-------------------CACCGCCCCTAGCCCC-GCGCTCCGGAAGTGAAGCGGCCAGACCACCAGCTAATGGATGCGGAGCGGAGGGCCCGCTGACC
Pan troglodytes         -------GAGGGGAGGGCGG-------------------CACCGCCCCTAGCCCC-GCGCTCCGGAAGTGAAGCGGCCAGACCACCAGCTAATGGATGCGGAGCGGAGGGCCCGCTGACC
Gorilla gorilla         -------GAGGGGAGGGCGG-------------------CACCGCCCCTAGCCCC-GCGCTCCGGAAGTGAAGCGGCCAGACCACCAGCTAATGGATGCGGAGCGGAGGGCCCGCTGACC
Pongo abelii            -------GAGGGGAGGGCGG-------------------CACCGCCCCTAGCCCC-GCGCTCCGGAAGTGAAGCGGCCAGACCACCAGCTAATGGATGCGGAGCGGAGGGCCCGCTGACC
Macaca mulatta          -------GAGGGGAGGGCGG-------------------CACCGCCCCTAGCCCC-GCGCTCCGGAAGTGAAGCGGCCAGACCACCAGCTAATGGATGCGGAGCGGAGGGCCTGCTGACC
Callithrix jacchus      -------GAGGGGAGGGCGG-------------------CACCGCCCCTAGCCCA-GAGCTCCGGAAGTGAAGCGGCCAGACCACCGGCTAATGGATGCGGAGCGGAGGGCTAGCTGACT
Mus musculus            --GTACGAGGGGGTGGGGGG----------------------CGCCCGGAG-CCT-GCGCCCCGGAAGTAAAGCGGACACTCCACCCGCTAATGGACGCGCGGCAGCCGCCCGACAGACC
Rattus norvegicus       --GTACGAAGGGGTGGGGGG----------------------CGCCCTAAG-CCC-GCGCTCCGGAAGTGAAGCGGCCACTCCACCCGCTAATGGACGCACAGCAGCCGCCGGAGAAACC
Bos taurus              GG-----CCAGGGAGGGC------------------------------------C-TCGCTCCGGAAGTGAAGCTGCCAGACCACCGGCTAATGGA-GCGCAGCGGAGGGCCCGCTGACC
Sus scrofa              AG-----CTGGGGAGGGCCG-------------------CGCCGCCCAGA-CCCC-ACGCTCCGGAAGTGAAGCGGCCAGACCCCCGGCTAATGGATGCGGAGCAGAAAGCCCGCTGACC
Canis lupus familiaris  GG-----GAGAGCAGGGCCG-------------------GGCCGCCGAGAGCCCC-GCGCTCCGGAAGTGGAGCGGCCAGACCACCCGCTAATGGATGCGGAGCGCAGGGCCCGCCGACC
Equus caballus          AG-----GCGCGCAGCGCAGGGGAGGGAGCGACCGCCGCCGCCGCCGAGAGCCCCGGCGCTCCGGAAGTGAAGTGGCCAGACCACCGGCTAATGGATGCGGAGCGGAGGGCCCGCTGACT

Homo sapiens            GCTCTCCGCGCCTGGGTAAGTATCGGGCAGGGCACGCG-GAGTGAGGACCGGAGGACGCAGCCCCCAGCCCCAGTGCCCCTCTCCTCCACCTGCCTGGCCCAGGCGCCTCTGCCCCGCCG
Pan troglodytes         GCTCTCCGCGCCTGGGTAAGTATCGGGCAGGGCACGCG-GAGTGAGGACCCCAGGACGCAGCCCCAAGCCCCAGTGCCCCTCTCCTCCACCTGCCTGGCCCAGGCGCCTCTGCCCCGCCG
Gorilla gorilla         GCTCTCCGCGCCTGGGTAAGTATCGGGCAGGGCACGCG-GAGTGAGGACCGGAGGACGCAGCCCCCAGCCCCAGTGCCCCTCTCCTCCACCCGCCTGTCCCAAGCGCCTCTGCCCCACCG
Pongo abelii            GCTCTCCGCGCCTGGGTAAGTATCGGGCAGGGCACGCG-GAGTGAGGACCCGAGGACGCAGCCCCCGGCCCCAGTGCCCTTCTCCTCCACCTGCCTGGCCCAGGCGCCTCTGCCCCGCCG
Macaca mulatta          GCTCTCCGCGCCTGGGTAAGTATTGGGCAGGGCACGCG-GAGTGAGGACCCGAGGGCGCAGCCTTCTGCCCCAGTGCCCCTCTCCTCCACCTGCCTGGTCCAGGCGCCTCTGCCCCGTCG
Callithrix jacchus      GCTCTCCGCGCCTGGGTAAGTATCGGGCAGGGCACGCG-GGGTGAGGACCGGAGGGCGCAGCCCCCGGCCCCTGTGCCCCTCTCCCCCACCTGCCCGGCCCAGGCACCTCTACC-TGTAG
Mus musculus            TCTCTCGGCGCCAGGGTGAGTGTCGGATAGCTATAGCC-GGGCAAGGACCCGAAGGCTCAGACCCTAGT-CCAGAGCCCCTCTCCCCGACCTGCCT-------------------CACTG
Rattus norvegicus       GCTCTCGGCGCCAGGGTGAGTGTCCGAGAGATACAGCT-GGGCAAAGACCGGAAAGAGCAGATCCTGGC-CCAGAGTCCCACTCCCCAACCTGCCT-------------------CACTG
Bos taurus              GCTCTAGGCGCCACGGTGAGTACCCGGCAGGGCACGCGGGGGCGAAGATCGTAGGGCGCAGTCCCTGGTCCCCGCGCCCCTCTCCCGGACCTGCCCGTCCCAGGCGCCTCTTCCTCACCG
Sus scrofa              GCTCTCGGCCCCAGGGTAAGTATGGGGCAGGGCACGCTGGGGCGAAGATCCGAGGGCGCCATCCCAGGCCCCCGCTCCCCTCTCCCCGAACTGCCCGGCCCAGGCACCTCTCTCCCACCG
Canis lupus familiaris  GCTCTCCGCTCCGGGGTAAGTGTGGGGCAGCGCACGCAGGGGCGGAGACCCGAGGGCGCAGTCCCTGGCCTCCGCGCCCCTGTCCCCCAC----------------------CCCCACCG
Equus caballus          GCTCTCGGCTTCACCGTAAGTATGGGGCAGGGCACGCGGGGGCGAAGACCTGAGGGCGCAGCCCCTGGCCCCGGCGCCCTTCTCCCC----------------------------CACCA

Homo sapiens            CATGGCGGAGGTGGGCCGGGGGCGAGGGCGCACTGTGGATAGGGGTCCCGCCCCGTTACCCTGGGTGTGAGCTGATGGATGGGAAA---AGGG-GACAAGCCG---GGCGCCAGGCTACT
Pan troglodytes         CATGGCGGAGGTGGGCCGGGGGCGAGGGCGCACTGTGGATAGGGGTCCCGCCCCGTTATCCTGGGTGTGAGCTGATGGATGGGAAA---AGGG-GACAAGCCG---GGCGCCAGGCTACT
Gorilla gorilla         CATGGCGGAGGTGGGCCGAGGGCGAGGGCGCACTGTGGATAGGGGTCCCGCCCCGTTACCCTGGGTGTGAGCTGATGGATGGGAAA---AGGC-GACAAGCCG---GGCGCCAGGCTACT
Pongo abelii            CATGGCGGAGGTGGGCGGGGGGCGAGGGCGCACTGTGGATAGGGGTTCCTCTCCGTTACCCTGGGTGTGAGCTGATGGATGGGAAA---AGGG-GACAAGCCG---GGCGCCACGCTACC
Macaca mulatta          CATGGCGGAGGTGGGCCGGGGGCGAGGGCGCACTGCGGATAGGGCTCCCGCCGCGTTACCCTGGGTGTGAGCTGATGGATGGGAAA---AGGG-GACAAGCCA---TGCGCCAGGCTACT
Callithrix jacchus      TGAGGCTGTGGTCGGCCGGGGTCGAGGGCGCACTGTGGCTACGGCTCCCTCCCCGTTACCCTGGGTGTGAGCTGATGGATGGGAAA---AGGG-GACCAGCCG---GGCGCCAGCCTACT
Mus musculus            CATTGCTAAGGTGGCCCTGGGGCT---------------------------CCCCTGGCCTCGGGTGAGAGCTGAGGGTTGAGGGAGCCAAGG-TTCAAGCCT---G-------------
Rattus norvegicus       CATTGCTAAGGTGGCCCTGGAGCT---------------------------CACCTAGCCTC-GGTGAGGGCTGAGGGTTGGGGGAGCCAAGG-GCCAAGCCG---G-------------
Bos taurus              CATTGCGGAGGTGGGCCCACGACGAGTGCCCACCGTGGGGAGGGCTCCCTCCCCCTGCCCC-GGGTGTGAGCTGATGGGTGGGGAA---AGTGGCACACTCTGGCGGGCTCCAGGATACC
Sus scrofa              CAATGCAGAGCTGGCCCAGGGGCGAGGGCCCACTGTGTGGAGGGTTCCCTCCCCCTTACCC-GAGTGCGAGCTAATGGATAGGAAA---AGGGGCAGAAACTGGTAGACT-CAGGACACT
Canis lupus familiaris  CATTGCAGAGGTGGCCCCGGGGCGAGG------------GAGGGCGCCCTCCCCCTTACGCGGGGTGTGAGCTGATGGATGGGCAG---AGAGGGGCGAGCCCAGTGAGGCCCGGACACC
Equus caballus          CATTGCGGAGGTGGGCCAAGGGCTAGGGCCCACCGTGGGGAGGGCTTCCTCCCCCTTAACCGGGATGTGAACTGATGGATGGGCAA---AGGGGGACAAACTGACTGGCGCCAGGACACC

Homo sapiens            CAGGGTGTGGG--------------AAGC------GGGATGGCACTGAAGATGAGCAAG-GATAGCACAGGCAGGGTGGACAGGAGGG-----------GGGCAGCCAAGACGCTG-GAC
Pan troglodytes         CAGGGTGTGGG--------------AAGC------GGGATGGCACTGAAGATGAGCAAG-GATAGCACAGGCAGGGTGGACAGGAGGG-----------GGGCAGCCAAGACGCTG-GAC
Gorilla gorilla         CAGGGTGTGGG--------------AAGC------GGGATGGCACTGAAGATGAATAAG-GATAGCACAGGCAGGGTGGACAGAAGGG-----------GGCCAGCCAAGACGCTG-GAT
Pongo abelii            CAGGGTGTGGG--------------AAGG------GGGATGGCACTGAAGATGGACAAG-GATAGCACAGGCAGGGTGGACAGGAGGG-----------GAGCAGCCAAGACGCTG-GAC
Macaca mulatta          CAGGGTATGAG--------------AAGG------GGGTTGACACTGAAGATGAACAAGGGGTAGCACACGCAGGGTGGACAGGAGGG-----------GGGCAGCCAAGGCGCTG-GAC
Callithrix jacchus      CCAGGTGTGGG--------------AAGG------GGGGTGGCACTGAAGATGAACAAGGGGTGAC-------------ACAGGACGG-----------GGGCAGCCGAGACGCTG-GAC
Mus musculus            AAGGCTCTTGG--------------AAACTGGGCAGGGGTGACCCTGGAAAC-AGCAA------------------------------AGTCTACTCCGGGATAACCAAGTAT-CC-GGC
Rattus norvegicus       CAGGCTCTGGG--------------AAGCTGGGGAGGGGTGACCCTGGAAAC-AGCAA-----------------------------------------GGGCATCCAAGTATTCT-GGC
Bos taurus              CATGGTGGAGGGGCGGGGGAGAGAAGG-A------GGGGCGGCACTGGGGATGGGGAAGAGATGGCGCAGGCCGCGCGGAAAAGGGGG-----------GACCATAAGAGGCGCGGGGAC
Sus scrofa              CAGCGGGAAGG--------------TA-A------GGGGCGGCACTCAGGACGGAGAAAGGATGGCGCAGGCTGCGCAGAGTGGTGGG-----------GGCCATATAGGGCGC------
Canis lupus familiaris  CGCGGGCAGGG--------------GA-A------GGGGCGGCGCTGACGATGGGGAAGGGGTGGCGCAGTCGGTTTAGGAAGGTGGG-----------GGCCTTACGAGGCACGG-GAC
Equus caballus          CAGGGGGAGGG--------------GA-A------GGGGCGGCGCTGGGGACGGGGAAGGGATGGCGCAGGCCGCGCGGAGAGGTGGG-----------GGCCAAACAAGGCGCGG-AAC

Homo sapiens            ACCCCAGCTTCAGTTTGTCCACT-------CGCCTTGGC-AGG--CTGCAGGGAGCAGA--GACTGAAACGAGAAGGTTCC---CGCTTCGTGCATCCACTTTGGTGTGGTTGGGGTGAC
Pan troglodytes         ACCCCAGCTTCAGTTTGTCCACT-------CGCCTTGGC-AGG--CTGCAGGGAGCAGA--GACTGAAACGAGAAGGTTCC---CGCTTCGTGCATCCACTTTGGTGTGGTTGGGGTGAC
Gorilla gorilla         ACCCCAGCTTCAGTTTGTCCACT-------CGCCTTGGC-AGG--CTGCAGGGAGCAGA--GACTGAAACGAGAAGGTTCC---CGCTTCGTGCATCCACTTTGGTGTGGTTGGGGTGAC
Pongo abelii            ACCCAAGCTTCAGTTTGTCCACT-------CGTCTTGGC-AGG--CTGCAGGGAGCAGA--GACTGAAACGAGAAGGTTCC---CGCTTCGTGCATCCACTTTGGTGTGGTTGGGGTGAC
Macaca mulatta          ACCCAAGCTTCAGTTTGCCCACT-------CGCCTTGGC-AGG--CTGCACGGAGCAGA--GACTGAAACGAGAAGGTTCC---CGCTTCGTGCATCCACTTTGGTGTGGTTGGGGTGAC
Callithrix jacchus      ACCCAAGCTTCAGTTTGCCCACT-------CGCCTTGGC-AGG--CTGCAGGGAGCAGAGAGACTAAAACCGTAAGATTCC---CGCTTCGTGCATCCACTTTGGTGTGGTTGAGGTGGC
Mus musculus            ACCAATGCTTCAGTTTACCCACGACCTGGGCGCCAGGGC-GCGCACTGCAAGGAGCAGA--A--GGAAAGGAGAATGTTCT---CCCTGCTTGCAGCCCCT-----GTGGTTGGGA----
Rattus norvegicus       ACCAATGCTTAAGTTTACCTACGATCTAGGGGCCGAGGC-GTGCACTGCAAGAAGCAAA--A--GGAAAGGAGAATGTTCT---TCCCGCTTGCAGACGCT-----GTGGTTGGGA----
Bos taurus              ACTAGAGCTTCGGTCTAACCACC-------GGCCGTGGT-GGA--ATACTGGGAGCAGA--GATTGAAACGA---GATTCT---CACTTACTGCTCCCACAGTAGTG-------------
Sus scrofa              -----------GGTCTATCCATT-------GGCCTTGGT-GGG--CTACTGGGAGCAGA--GATTGAAACAA---GTTCCT---CATTTACTATACCCACTGTGGTG-------------
Canis lupus familiaris  ACCGAGCCTT---GTTAGCCACG-------GGCCTGGGGGGGG--CCAC-----GCAGA--GATCGAAACGCAAAGGTTCTCCTCACTCGCAGCACCCACAGTGGTG-------------
Equus caballus          ACCGAAGCTTTATTTTCCCCATG-------GGCCATGGT-GGG--CTACTGGGAGCAGA--GATTGAAACGAGAAGGTTCT---CACTTTGTGCATCCACAGTGTTG-------------

Homo sapiens            GGGGG--AGTGG--G--GGGGGGGCACACAACATGGCGGGA--AGAAATTCAGAGGGGCGTAGCAGATGGG--------AGAGGA-------------------ATATA----------G
Pan troglodytes         GAGGG--AGTGG--G--G-GGGGGCACACAACATGGCGGGA--AGAAATTCAGAGGGGCGTAGCAGATGGG--------AGAGGA-------------------ATATA----------G
Gorilla gorilla         GGGGG--AGTGG--G--G-GGGGGCACACAACATGGCGGGA--AGAAATGCAGAGGGGCGTAGCAGATGGG--------AGAGGA-------------------ATATA----------T
Pongo abelii            GGGGG--AGTGG--G-----GGGGCACAAAACATGGCGGGA--AGAAATGCAGAGGGGCGTAGCAGATGGG--------AGAGAA-------------------ATATA----------T
Macaca mulatta          GGGGG--AGTTG--G-----GGGACACACAACATGGGGGGA--AGAAATGCAGAGGGGCGTAGCAGATGGG--------AGAGGA-------------------ACATA----------G
Callithrix jacchus      GGGTGGTGGTTG--G-----GGGGGGCACAACATGGCGGGA--AGAAATGCAGAGTGGCGTAGCAGATGGG--------AGACGA-------------------GTATA----------G
Mus musculus            --------------GGT---GGGGCTCACAACATGGCGGGGGCGAAAAAGCAGAGGAGCATAGCAAATGCA--------AGCGGTGACCGGGG--------------------------G
Rattus norvegicus       --------------GGT---GGGGCTCACAACATGGCGGGGGCAAAAAAGCAGAGGGACGTAGCAAATGGA--------AGCGATGACCGGGGGTGGGGGGTGGGGGTG----------G
Bos taurus              ------------ATG-----GTGGGGCGCATTATGGCCGGG--AGAGACACAGAGGGGCGTAGCAAATACGA----AGTGGGTGG-------------------GTGTA----------G
Sus scrofa              --------------G-----GGGGCGCGCAGCGTGGCGGAG--AGAAATGCAGAGAGGCATAGCAAATGCGGAGGAAGTCGGTAT-------------------ATATA----------G
Canis lupus familiaris  --------------G-----GGGGCGCCCAGCCAGGCGGGG--GGAAATACAGAGGGGCGTAGCCCAGT--------GTGTGTGC-------------------GTGGAGGTGGGGGGGG
Equus caballus          --------------G-----GAGAGGCACGGCATGGCGGGG--AGAAATGCAGAGGGGCGTAGCAAATG--------GTGGGAGC-------------------GTATA----------G

Homo sapiens            AGAGAGA-CGCAGGTCTGCGGTCTATGG-----------GGGTGTAGCAAAGAGTGCAGTCGG-GAA-TTCCAGCCCAGGGCCACGACTTA----------AAGACGGGGTGCA-GACCG
Pan troglodytes         AGAGAGA-CGCAGGTCTTTGGTCTATGG-----------GGGTGTAGCAAAGAGTGCAGTCGG-GAA-TTCCAGCCCAGGGCCACGACTTA----------AAGAGGGGGTGCA-GACCG
Gorilla gorilla         AGAGAGA-CGCAGGTCTGCGGTCTATGG-----------GGGTGTAGCAAAGAGTGCAGTCGG-GAA-TTCCAGCCCAGGGCCACGACTTA----------AAGAGGGGGTGCA-GACCG
Pongo abelii            AGAGAGA-CGCAGGTCTGCGGTCTATGG-----------GGGTGTAGCAAAGAGTGCAGTCAG-GAA-TTCCAGCCCAGTGCCACGACTTA----------AAGAGGGGGTGCA-GACCG
Macaca mulatta          AGAGAGA-CGCAGGTCTGTGGTCTATGG-----------GGGTGTAGCAAAGGGTGCAGTCGG-GAA-TTCCAGCCCAGTGCCACGACTTA----------GAGAGGGGGTGCA-GACCT
Callithrix jacchus      AGAGAGA-CGCAGGTCTGAGGTCTATAA-----------GGGTGTAGCAAAGAGTGCGGTGCG-GAA-TTCCAGCCCAGTGCCACGTCTTA----------GAAAGGCGGTGCA-GACCC
Mus musculus            AGAAAGC-AGCAAGCCTGCGGTGCGGGGGGGGGGGGGGGGGGCATAGTAAAGAATGCAGACTGAGGA-TT--------GCGCTTCAGCATA----------GAAACTGGG-----GACCC
Rattus norvegicus       GGGGAGC-AGCAACCCTGTAGTGCTAGA-----------GGGAATAGTAAAGAATGCAGGCTGTGGA-TT--------GAGCCTCAGCATA----------GAGACTGGG-----AACCC
Bos taurus              AGACAGAAAATAGATCTACAGTCTGTGC-----------AAGTGTAGCAAGGCGAGGAGTTGG-CAG-GTACAGCCCAGCGCGGCAACTTAAGGATATAGTGAGAGGGGACACCGGTG-C
Sus scrofa              AGAGAGA-CATAGGTCCACGGTCTGTGG-----------GAGTGTAGCAAAGAGTGTAGTCTG-CAAATTCCAGCCCAGCGCGGCAACTTGGATGTATAGTGAGAGAGGGAGCA-GAC-C
Canis lupus familiaris  ATAAAGA-CATAGGCCTGCGGGCTGTGG-----------GAGTGTAGCAAAAAGTGCAGTCTG-CAA-ATTCTGGCACGGACGGCAACTCG------TAGTAAGAGTGGGTAGG-GACCT
Equus caballus          AGAGAGA-CATAGGTCCACGGTTCGTGG-----------GAGTGTAGCAAAGAGTGCAGTCTG-CAG-T--CT-----GCACGGCAACTTA--TATATAGTGGGAGAGCGCGCA-AACCC

Homo sapiens            ACTCTATTGCGC-AAG-GCGCCATGGA-CCGTGTCTGCGCCACTGCGCCAAGAAGGGCGCAGGAACGTGTCGCGATTTGGTTTATTCTGCTTCCTCC-----ACCAAAAAAAAAAAAAAA
Pan troglodytes         ACTCTATTGCGC-AAG-GCGCCATGGA-CCGTGTCTGCGCCACTGCGCCAAGAAGGGCGCAGGAACGTGTCGCGATTTGGTTTATTCTGCTTCCTCC-----ACCAAAAAAAAAAAA---
Gorilla gorilla         ACTCTATTGCGC-AAG-GCGCCATGGA-CCGTGTCTGCGCCACTGCGCCAAGAAGGGCGCAGGAACGTGTCGCGATTTGGTTTATTCTGCTTCCCCC-----ACCAAAAAAAAAAAAAAA
Pongo abelii            GCTCTATTGCGC-AAG-GCGCCATGGA-CCGTGTCTGCGCCACTGCTCCAAGAAGGGCGCAGGAACGTGTCGCGATTTGGTTTATTCTGCTTCCCTC-----ACCAAAAAAACAAAAACA
Macaca mulatta          GCTCTATTGCCC-AAG-GCGCCATGGA-CCATGTCTGCGCCACTGCACCAAGAAGGGCGCAGGAACGTGTCGCGGTTTGGTTTATTCTGCTTCCCCC-----ACCAAAAAAACAAAAA--
Callithrix jacchus      GCTCTATTGCCC-AAGGGCGCCATGGA-CCCTTTCTGCACCACTGCGCCAAAAAGGACGCAGGAACGTGTCGCGATTTGGTTTCTTCTGCTTCCCCC-----CC-AAA-AAATAATA---
Mus musculus            GTAACATCATCTGAAA-AGGCCATGCC-CTGCACATCAGCCATTGCCTCAGAAAAGGCTCAGAAGAGTC-CTGGATTTGATATCTCCAATCCCTC----------AAAA-CACACACAC-
Rattus norvegicus       TCAACATTATCTGAAA-GGGCCATGTCTGTGCACGCACCCCAATGACTCTTGAAGGGTTCA-ACGAGTC-CGGGATTTGATATCTCCCACCCCCC----------CACC-C---------
Bos taurus              GCGGTATTGGCCGAGG-GCGCCGTGTA-CCGTCGCTGTGCCACTGCGCCAAGAAGGGTCCGGCAGAAGGTCTTCATCTGATATCTTCCGCCCCTCCC-TCCTGCCAA-------------
Sus scrofa              GCTACACTGGC------GCGCCGTATA-CCGTAATTGCGCCACAGCGCCAAGAAAAGCCCAGCAGAATGC-----------ATCTTCCCCCGCCCCCAACCCGGCAAAGA----------
Canis lupus familiaris  GCTCTCGTGCCCGAAG-GCGCCGCGTG-CCGTAGCTGCGCCACTGCACCAAGAAGGGCCCGGGAGAATGCCTTCATTTGCTATCTTCCACCTTCCCT-----GCCAAAAA----------
Equus caballus          GCTATATTGCCCGAGG-GCGCCATGCA-CCA-------GTGACTGCGCCAAGAAGAGCCCAGGAGACTGTCTTCATTTGGTATCTTCCGCCC---GC-----ACCCAAAG----------

Homo sapiens            A-AAAAAAAAAGAGA--GAAAAAA---CAAACTGAAACCCCCTT-CCCAAAT--------------------------------------------CTCCAGCTT-T-CCCCTGCGCCCC
Pan troglodytes         --AAAAAAAGAGAGA--GAAAAAA---CAAACTGAAACCCCCTC-CCCAAAT--------------------------------------------CTCCAGCTT-T-CCCCTGCGCCCC
Gorilla gorilla         AAAAAAAAAATGAGA--GAAAAAA---CAAACTGAAACCCCCTC-CCCAAAT--------------------------------------------CTCCAGCTT-T-CCCCTGCGCCCC
Pongo abelii            A-----AAACAAAAA-CAAACAAA---CAAATTGAAACCCCCTC-CCCAAAT--------------------------------------------CTCCAGCTT-T-TCCCCGCGCCCC
Macaca mulatta          --ACAAAAAGTGGGC--GAAAAAA---CAAATAGAAACCCCCTC-CCCAAAT--------------------------------------------CTCCAGCTT-C-CC---TCGCCCC
Callithrix jacchus      --ATAAAAAGGGGGG--GGAGAAA---CAAATTGAAACCCCCTC-CCCAAAT--------------------------------------------CTCCAGCTG-T-TCCCGGCGCCCC
Mus musculus            --ACACACACACACAC-ACACACA---CACACACACACACACAC-CCAAAACGGAAAATGGTTGGGGACTAAGCAGACTGAAGCACAC------TCATCTTGCTT-T-C----------A
Rattus norvegicus       -----------CCCAC-ACACACCCTGGTTACCCTCACTCCCAC-CCAA-------AATGGTTGGGGTCTAAGCAGACCGAAGCACACTCGGACTCATCTTGCTTTT-C----------A
Bos taurus              ----------AGGGGG-AAAGATA---CACATGGAAGCCCACCCGGCCAAAT--------------------------------------------CCCCAGCCC-T-ACCCGGGGCCAC
Sus scrofa              ----------AGGGAG-AAAGACA---TTCTTTGAAGCCCACTC-CCCAAAT--------------------------------------------CTCCAGCCC-T-ACCCAGCGCCCA
Canis lupus familiaris  ----------AGAGGG-TAAGACG---CAGACTGAAGCCCATTC-CCCAAGT--------------------------------------------CCCCG-------------------
Equus caballus          ----------GGGGAG-AAAGCCG---CAGATTAAAGCCCACTC-GCCAAAT--------------------------------------------CTCGAGTCC-TGACCCGGCGCCCC

Homo sapiens            A-G-A----------------------------------------------------GCCCCGCCTGGTTCC----CC-CGCCCCTGTTGCGCACTGCGCCACAGTCGCAGCGTTTCCCG
Pan troglodytes         A-G-A----------------------------------------------------GCCCCTCCTGGTTCC----CC-CGCCCCTGTTGCGCACTGCGCCACAGTCGCAGCGTTTCCCG
Gorilla gorilla         A-G-A----------------------------------------------------GCCCCGCCTGGTTCC----CC-CGCCCCTGTTGCGCACTGCGCCACAGTCGCAGCGTTTCCCG
```

```
Pongo abelii            A-G-A----------------------------------------------------GCCCCGCCTGGTTCC----CC-CGCCCCTGTTGCGCACTGCGCCACAGCCGCGGTGTTTCCCG
Macaca mulatta          A-G-T----------------------------------------------------GCCCCGCCTGATTCC----CC-CGCCCCTGTTGCGCACTGCGCCACAGCCGCGGCGTTTCCCA
Callithrix jacchus      A-G-C----------------------------------------------------GCCCCGCCTGGCTCC----CC-CGCCCCTGTTGCGCACTGCGCCACAGACGCGGCGTTTCCCA
Mus musculus            A-GTC----------------------------------------------------GCCCCGCCTAGCTTC----CC-CGCCCCTGTTGCACGCTGCGCAGGAGGCCC-GGG------A
Rattus norvegicus       A-GTC----------------------------------------------------GC-CTGTCTGGCTCC----CCTAGCCCCTGTTGCACGCTGCGCCAGAGGCCCCGCG------A
Bos taurus              TGG-C----------------------------------------------------GCCCCGCCTCATTCT----AC-AGCCCATGTAGCGCACTGCGCCAGATCCGCGGGGTTTCCTA
Sus scrofa              GGG-C----------------------------------------------------GCCCCGCCTCATTTC----CC-CGCCC-TGCTGCGCACTGCGCCAGAACCACGGGGTTTCCTA
Canis lupus familiaris  ------------------------------------GCCCTTCCTCGCGCCCGGAGCGCCCCGCCTCGATCC----CC-CGCCCGCGTTGCGCACTGCGCCAGAGCCGCGGCGTCCCCCA
Equus caballus          GGG-GGCCCCGCCTCGCCCCCTCCACCCCCACCCCGCCCCCTCCAC----CCCCACCCCGCCCCCTCCACCCCCACCC-CGCCACTGTTGCGCAGTGCGCCAGAGCCGCGGGGTTTCCCA

Homo sapiens            GGTTACCAGATGCCACGTGTTTGGG-GAGTA-AGTGC--GGGTGCGCATCTGGTGCGG-AGTAGGAGGTAGGATGTGG-----------GCTGTGA----GTGCC--ATGAATTGTGT-G
Pan troglodytes         GGTTACCAGATGCCACGTGTTTGTG-GAGTA-AGTGC--GGGTGCGCATCTGGTGCGG-AGTAGGAGGTAGGATGTGG-----------GCTGCGA----GTGCC--ATGAATTGTGT-G
Gorilla gorilla         GGTTACCAGATGCCACGTGTTTGGG-GAGTA-AGTGC--GGGTGCGCATCTGGTGCGG-AGTAGGAGGTAGGATGTGG-----------GCTGTGA----GTGCC--ATGAATTGTGT-G
Pongo abelii            GGTTACCAGATGCCACGTGTTTGGG-GAGTA-AGTGC--GGGTGCGCATCTGGTGCGG-AGTAGGAGGTAGGATGTGG-----------GCTGTGA----GTGTC--ATGAATTGTGT-G
Macaca mulatta          GGTTACCAGATGCCACGTGTTTGGG-GAGTG-AGTGC--GGGTGCGTATCTGGTGCGG-AGAAGGAGGTAGGATGTGG-----------GCTGTGA----GTGCC--ATGAATTGTGT-G
Callithrix jacchus      GGTTACCAGATGCCACGTGTTTGG--GAGTG-AGCGC--GGATGCGCATCTGGTGAAG-AGTAGGAGGTAGGT------------------TGTGA----GTACC--GTGAATTGTGT-G
Mus musculus            GTTTGCCAGATGTTCCTTCTCTAGG-GCCGC-AGC-----GGTTTGCA-----TGGAG-GGTA-GAGGTAGATTGCTGTTCAGGGGAGGGTTAC------ATGCTGTGTGAGTTGTGG-G
Rattus norvegicus       GTTTGCCCGATGATCCTTCTCTAGGGGCCGC-AGT-----GGTCTGTA-----TGGAG-GGTA-GAGGTAGACCGCTGTTTTGGGGAAGGTTAC------ATGTT--GTGAATTGTGGGG
Bos taurus              GGCTGCCAGAAGCCGCGTGCCTGG--GCGCT-AGTGC--G--CGCGAGTCCAGTACAGGATCAGCATGTGGGGCGC-G-----------GCTGTGATG-GTTGCC--TTGAATTGTGT-G
Sus scrofa              GGTTGCCAGAAGCCTCGTGCCTGAG-GCCTT-CTTGC--G--CGCGCGGCCAGTGCAGGGTAAGGAGATGGGGTGCCA-----------GCTGTGATG-AGTG----AAGAACTGTGT-G
Canis lupus familiaris  GGTGGCCCGAAGCCCAGTGCCTGGG-ATGTAAAGTGCTGGGGCGCGAGTCCGCTGATGGGGTGGGAGATCGGGTGCCG-----------CCTGTGACT--GTGCC--TTGAATGGTGT-G
Equus caballus          GGTTGCCGGACGACCCGTGCCTGCG-GCGTA-AGTGC--GCGCGCGCATCCGGTGGCG-GGTAGAAGGTGGGGTGCTG-----------TCTGTGATTGAGTACC--TTGGACTGTGT-G

Homo sapiens            GGGCCAAGCAGGGGAAAGGTGTGTAGGAACTTGGTGTGTGTGTGC--A-----GTAGCTGAGCCACGGCCA---CCGGGTTGGTAAGTA---ACCAGGGGGCAATTAGATTTG-------
Pan troglodytes         GGGCCAAGCAGGGGAAAGGTGTGTAGGAACTTGGTGTGTGTGTGC--A-----GTAGCTGAGCCACGGCCA---CCGGGTTGGTAAGTA---ACCAGGGGGCAATTAGATTTG-------
Gorilla gorilla         GGGCCAAGCAGGGGAAAGGTGTGTAGGAACTTGGTGTGTGTGTGC--A-----GTAGCTGAGCCACGGCCA---CCGGGTTGGTAAGTA---ACCAGGGGGCAATTAGATTTG-------
Pongo abelii            GGGCCAAGCAGGGGAAAGGTGTGTAGGAACTTGGTGTGTGTGTGC--G-----GTAGCTGAGCCACGGCCA---CCAGGTTGATAAGTA---ACCAGGGGGCAATTAGATTTG-------
Macaca mulatta          GGGCCAAGCGGGGGAAAGGTGTGTAGGAACTTGGTGTGTGTGTGC--AG----GTAGCTGAGCCACGGCCA---CAGGG-TGGTAAGTTTGCACCAGAGGGCAATTAGATTTG-------
Callithrix jacchus      GAGCCAAGCGGAGGAAAGGTACGTAGGAACTTGGTGTGCGTGTGC--AG----GTAGTGGAGCTACTGCCA---CCGAGGTGGTAAGGG---G-GGGGGGCAATTGGATTTG-------
Mus musculus            CAGCCAAA------------GTGTGGGGATTTGGAGTGTGTGCGCGCGTGCACACGGTTCAGCCACGACCG---CCAGGTTCGA------------------------------------
Rattus norvegicus       CAGCCAAA------------GTGTGGGGATTCGGAGTGTGTGTGC--ATGCACACGGTTCAGCCACAGCCG---CCAGGTTCGA------------------------------------
Bos taurus              AGGCCAAGCAGCGGAAAGGTGTATGGGGACGTGGTGTGTATGCAC--GA----GTAGATCAGCCTCAGCCA---TGGAATTAGTGGACA---TCCAGGGGGCATTTGATGTAACGCCAG
Sus scrofa              AAATCCAGCAGGAGAATGAGGTATGGGGACATGGTGTGCATGCGC--CA----GTAGACTAGCCACAGCCACCACCGGTTTAGTGGGCA---TCCAGAGGGCATTTTAATGTA-------
Canis lupus familiaris  AGGCCACGCGA-----GGGTGCGTGGGGCTG-GGTGTGTGCGGGC--GG----CTAAACCAGCCACAGCTA---CTGGGTTGGCGGGCA---GATAAGGGGCATTTAGATTTG-------
Equus caballus          AGGCCAAGCTGGGGAAAGGTGTGTGGGGACT-TGTGTGTATGTGC--GG----GTAGACCAGCCACAGCCA---CAGGGTAGGAGGGCA---TCCAGGGGACACTTTGATTTG-------

Homo sapiens            -------TGTGCCTAACACGGTCAATGCTGGTCCTTCATTGCTTATTCCCAT-CTCCACGTCCTTTCCGCGTATCAGTCCCTGCCTCTGCGCGATGACAGCTCCGCCTCTCTTTCCACAG
Pan troglodytes         -------TGTGCCTAACACGGTCAATGCTGGTCCTTCATTGCTTATTCCCAT-CTCCACGTCCTTTCCGCGTATCAGTCCCTGCCTCTGCGCGATGACAGCTCCGCCTCTCTTTCCACAG
Gorilla gorilla         -------TGTGCCTAACACGGTCAATGCTGGTCCTTCATTGCTTATTCCCAT-CTCCACGTCCTTTCCGCGTATCAGTCCCTGCCTCTGCGCGATGACAGCTCCGCCTCTCTTTCCACAG
Pongo abelii            -------TGTGCCTAACATGGTCAATGCTGGTCCTTCATTGCTTTTTCCCAT-CCCCACGTCCTTTCCGCTTATCAGTCCCTGCCTCTGCGCGATGACAGCTCCGCCTCTCTTTCCACAG
Macaca mulatta          -------TGTG-CTAACACGGTCAATGCGGGTCCTTCATTACTTATTCCCAT-CTCCACGTCCTTTCCGCGTATCAGTCCCTGCCTCTGCGCGATGACAGCTCCGCCTCTCTTTCCACAG
Callithrix jacchus      -------C-TGCGTAACGCGGTCAGTGCGGGTTCTTCATTGCTTAGTCCCAT-CTCCACGTCCTTTCTGCGTATCAGACCTTGCCTCTGCCGGATGACAGCCCCTCCTCTCTTTCCACAG
Mus musculus            -----------------------------GGCTTTTCCTTGCTTAGTCCCAC-CTCCTGGTCCTGGCT------------------TGGTTGCATTTAAGCCCCTCCTGTTTTTCCAGAG
Rattus norvegicus       -----------------------------G----------GCTTAGTCCCAT-CTCCTGG----------------------------AGTGCTTTAAAGCCCCTCCCGTTTTTCCCGAG
Bos taurus              TACATCAGGCCAGTAACACGGCCTCCAAGGACCCTTCATTGCTTACTTCTATTCCCCGTGTCTTTTCCACGTCTCAGTCCCTGCCCAGGGTTGATTACAGCCCCGCCCCTCTCTCCACAG
Sus scrofa              -------TGCCAGTAACACAGTCTCTGAGGGCTCTTTATTACTTACACCTAT-CCCTCTGTCCTTTCCACGTCTCAGTCCCTGCCCATGATTGATTACAGCCCTGCCTTTCTCTCTTCAG
Canis lupus familiaris  -------TGCCGGGAACGCGGTCTCTGAGAGTCCTTCATTGCTTGTTCCTGT-CCCCACGTCCTTTCCATGCATCAGTCCCTGCCTCTGGCCGATTACAACCCCGCCCCTCTTTCCATAG
Equus caballus          -------TGCCAGTGGCACGGTCTCTGAGGGTTCTTCATGGCTTACTCCCAA-CCCCGCGTCCTTTCCATGTATCAGTCCCTGCCTCTGGTCGATTACC-CCCCACCCCTCTTTCCACGC

Homo sapiens            TGGCAAAATCCTGCAGAAATGGTGCCCCTCCTTTGGTGGCCCGGGAGGGAGTTCTGGGCATGCTCAGTA-ACCAGGGTGGGTTTGGGCT--GAGAG--GCCGTTTACCGCAGGGCCACCG
Pan troglodytes         TGGCAAAATCCTGCAGAAATGGTGCCCCTCCTTTGGTGGCCCGGGAGGGAGTTCTGGGCATGCTCAGTA-ACCAGGGTGGGTTTGGGCT--GAGAG--GCCGTTTACCGCAGGGCCACCG
Gorilla gorilla         TGGCAAAATCCTGCAGAAATGGTGCCCCTCCTTTGGTGGCCCGGGAGGGAGTTCTGGGCATGCTCAGTA-ACCAGGGTGGGTTTGGGCT--GAGAG--GCCGTTTACCGCAGGGCCACCG
Pongo abelii            TGGCAAAATCCTGCAGAAATGGTGCCCCTCCTTTGGTTGCCCGGGAGGGAGTTCTGGGCATGCTCAGTA-ACCAGGGTGGGTTTGGGCT--GAGAG--GCCGTTTACCGCAGGGCCACCG
Macaca mulatta          TGGCAAAATCCTGCAGAAATGGTGCCCCTCCTTTGGTGGCCCGGGAGGGAGGTCTGGGCATGCTCAGTAAACCAGGGTGGGTTTGGGCT--GAGAG--GCCATTTACCGCAGGGCGACCG
Callithrix jacchus      TGGCAAGATCCTGCGGAAATGGTGCCCCTCCTTTGGCGGCCCGGGAGGGAGGTCTGGGCATGCTCAGTA-ACCTGGGTGGGTTTGGGCT--GAGAG--GCCGTTTCCCGCAAGGCCACTG
Mus musculus            TGGCTGAAAGCTGCAAAAGTGTTGCCCATCCTTTAGGGGTTCTGGAGCGAGATCTGAGCATGCTCACTA-AGATGGATGGGTTTGGTCTAGGAGAG--GCAGTTTACTGCAGCGTGTTCT
Rattus norvegicus       TGGCTGAAACCTGAAGAAGTGTTGCCCATCCTTTAGGGGTTCTGGATGGAGATCTGAGCATGCTCACGA-AGATGGATGGGTTTGGTCTAGGAGAG--GCAGTTTACTGCAGCATGTTCT
Bos taurus              TGGCAAAACCCTGCAGAAACGATGCCCATCCTTTGGCAGCTCGAGAAGGAGCTCTGGGCATGCTCAGTG-ATCAGGGTGGGTTTAGACT--GGGAA--ACTGTTAACCACAGGAGCGTCG
Sus scrofa              TGGCAAAATCCTGCAGAAATGATGCCCATCCTTTGGCTGCTGGAGAAGGAGCTCTGGGCATGCTCACTA-AGCTGGGTGGGT-TGGACT--GGAAA--ACACTCTACCATAGGAGTGTCG
Canis lupus familiaris  TGGCAAAATCCTGCAGAAATGATGCCCATCCTTTGGCGACTGGAGACGGAGATCTGGGCATGCTCAGTA-ACCAGGGTGGGTTTGGGCT--GGGAGAAGCTGTTTACCGCAAGACCCGCG
Equus caballus          TGGCAAAATCCTGCAGAAATGGTGCCCATCCTCTGGCGGCTGGAGAGGGAGGTCTGGGCATGCTCAGTA-ACCAGGGTGGGTTTGGGCT--AAGAG--GT------------------AG

Homo sapiens            -CGTAGGGCCGTGTAACCAGGGATCCAGTCCCATTGTCAGCCC-----------AGGATTAAATGCGGTTACCAACCACCC-----CCACAGTCCAATTGTGAGAGATAGTTTGGTGAAT
Pan troglodytes         -CGTAGGGCCGTGTAACCAGGGATCCAGTCCCATTGTCAGCCC-----------AGGATTAAATGCGGTTACCAACCACCC-----CCACAGTCCAATTGTGAGAGATAGTTTGGTGAAT
Gorilla gorilla         -CGTAGGGCCGTGTAACCAGGGATCCAGTCCCATTGTCAGCCC-----------AGGATTAAATGCGGTTACCAACCACCC-----CCACAGTCCAATTGTGAGAGAGAGTTTGGTGAAT
Pongo abelii            -CGTAGGGCCATGTAACCAGGGATCCAGTCCCATTGTCAGCCC-----------AGGATTAAATGCGGTTACCAACCACCC-----CCACAGTCCAATTGTGAGAGATAGTTTGGTGAAT
Macaca mulatta          -CGCAGGGCCGTGTAACCAGGAATCCAATCCCATTGTCAGCCC-----------AGGATTAAATGCGGTTACCAACCACCC-----CCACCATCCAATTGTGAGAGATAGTTTGGTGAAT
Callithrix jacchus      -CGTAGGGCCTTGTGACCAGGAATCCTGTCCCATTGTCAGCCC-----------AGGAGTAAATGCGGTTACCAACCACTC-----CCACCGTTCAATTGT-AGGGATAGTTTGGTGAAT
Mus musculus            -CACAGGGCGTTGTGACCAAGAAATCAGTCCCATTGTCAGCCTTTGCTTAGGTAACGATTGCTTGCAGATATCAACGCCCC-------------CAGTTGCCCGAGATAGCTTGATTGAT
Rattus norvegicus       -CACAGGGCGTTGTGACCAAGAAATCAGACCCATTGTCAGCCTTAGCTTAGGGAACGATTGCATGCGGATACCAACGCCCC-------------CTGTTGCTGGAGATAGCTTGATGGAT
Bos taurus              -TGCCAGGCTGAGCAACCAAAGATTCGGTCCCATTGTCAGCCCTTGCCCTTGGAAGGACTAAATGCGGATACCCAGCCCTCCTCCA--------CAAATGCCAGGGAAAG----GTGAAT
Sus scrofa              TTGCTGGGCAGAGTAACCAAAGATCCGATCCCATTGTCATCCCTCACCCCTGGAAGGGTTAAATGTGGATACCCTGCCTC--T-CC--------CAATTGTGATGGATAGTTTGGTAAAT
Canis lupus familiaris  -TGCAGGGCAGCGTGACCAAGGATTCACTCCCATTGTCAGCTCTTGCCCCTGCAAGGATTAAGTTCGGATACGCTCCCCCACCCC--------CAATCGTGAGGGACAGTTTGGGGAAT
Equus caballus          -CGCTGGGCAGTGTGACTGAGGATTCCGTCCCATTGTCAACCGTTGCACCTGGAAGGATTAAATGCGGGTACCCACC------CCC--------TAATTGTGAGGGGTAGTTTGGTGAAT

Homo sapiens            ATAGTTACATCTGCTGG----------------------------------------------------------------------------------ACAATCA-----GTCGCAATT
Pan troglodytes         ATAGTTACATCTGCTGG----------------------------------------------------------------------------------ACAACCA-----GTCGCAATT
Gorilla gorilla         ATAGTTACATCTGCTGG----------------------------------------------------------------------------------ACAACCA-----GTCGCAATT
Pongo abelii            ATAGTTACATCTGGTGG----------------------------------------------------------------------------------ACAACCA-----GTCGCAATT
Macaca mulatta          AGAGTTACATCTGGTTG----------------------------------------------------------------------------------ACAACCA-----GTCGCAGTT
Callithrix jacchus      AGAGTTACATCTAGTGG----------------------------------------------------------------------------------ACCACCA-----GTTGCAGTT
Mus musculus            GGAGTCATAATTTGTGC----------------------------------------------------------------------------------GCACACA-----CACACAATC
Rattus norvegicus       GGAGTCTCTCTCTCTTCTCTCTCTCTTCTCTCTCTCTCTCTCTCACACACACACACACACACACACACACACACACACACACACACACACACACCACACACACGCCCCACACCATC
Bos taurus              GGAGTTAAACTTAGAGG----------------------------------------------------------------------------------ACAACCA-----ATCCCAGTT
Sus scrofa              GGAGTTAAATTCTGAGG----------------------------------------------------------------------------------ACAACCA-----GTCCCAGTT
Canis lupus familiaris  GGAGTTGCATTTGGAGG----------------------------------------------------------------------------------ACAACCG-----GTCACAGTT
Equus caballus          GGAGGTAAATTTAGAGA----------------------------------------------------------------------------------ACCG-CA-----GTCGTAGTT

Homo sapiens            TGAAGATCAGCCCATCTTGATAGGTGGAAAGGCGTCTCCGGATGAGAAAC--AGACTGGGGA-----AGAAGAATATAGGAGTGGAGGGGCTGAGAGAACC-----GGAGG-GGGCATTT
Pan troglodytes         TGAAGATCAGCCCATCTTGATAGGTGGAAAGGCGTCTCCGGATGAGAAAC--AGACTGGGGA-----AGAAGAATATAGGAGTGGAGGGGCTGAGAGAACC-----GGAGG-GGGCATTT
Gorilla gorilla         TGAAGATCAGCCCATCTTGATAGGTGGAAAGGCGTCTCCGGATGAGAAAC--AGACTGGGGA-----AGAAGAATATAGGAGTGG--GGGCTGAGAGAACC-----GGAGG-GGGCATTT
Pongo abelii            TGAAGATCAGCCCACCTTGATCGGTGGAAAGGCGTCTCCGGATGAGAAAC--AGACTGGGGA-----AGAAGAATATAGCAGTGGAGGGGCTGAGAGAACA-----GGAGG-GGGCATTT
Macaca mulatta          TGAAGATCAGCCCACCGTGATAGGTGGAAAGGCGTCTCCGGATGAGAAAGAGAGACTGAGGA--------AGAATATAGGAGTGGAGGGGCTGAGAGAACC-----CGAGG-GCGCATTT
Callithrix jacchus      TGAAGATCAGCCCACCATGAT-GGTAGAAAGTCGTCTCCAGATGAGAAAGGGAGACTGAGGA-----AGAAGAATATAGGAGTGGAGGGGCTGAGAGAACC-----CGACG-G-------
Mus musculus            ---------------------------------------------------------------------------------CTGTAGGAGCCGAAAG-------------GAGGGCCTAT
Rattus norvegicus       ---------------------------------------------------------------------------------CTGTAGGAGCTGAAAGGACTCCATGGGAGGAGAGCCGAT
Bos taurus              GGAAGATAAGCCCACCATGATAGG----------------------------ATGAGGAGGAAGGACAGAGGAACGTACCTGTGCA---GATGAGAGAG-C-----AGAGA-CGGACGTT
Sus scrofa              TGAAGATAAGCCCACCATGATAGGTGGGAAGGCATCTGGGGATGAGAAGGAGAGGAGGAGGGAGGAGAGAGGAATTTACATGTGCAGGGGATGAGAGAACC-----AGAGG-CAGGCATT
Canis lupus familiaris  TGAAGACGAGCCCACCATGGCAGGTGGGAAGGAGGACCCTGCTGAGAACGGGAGACTGAGGAAGG--AGAGGAATATTTGTGTGGAGGGGCCA---------------------------
Equus caballus          TGAAGATGAGCCCACCGTGCTAGGTGGGAAGGAG--------------------------GAAGG--AGAGGAATATGCGGGTGGAGGGACTGAGAGATCC-----AGATG-CAGGCTTT

Homo sapiens            TGGACTTAGCGGCAGATGTGCTTTGCATTTGAATAAAGCATTTGAAA------AAAGCGCTTCC---------------AACTGAATTACATCATTTCCTACTCACAGCCCCACTGTGAG
Pan troglodytes         TGGACTTAGCGGCAGATGTGCTTTGCATTTGAATAAAGCATTTGAAA------AAAGCGCTTCC---------------AACTGAATTACATCATTTCCTACTCACAGCCCCACTGTGAG
Gorilla gorilla         TGGACTTAGCGGCAGATGTGCTTTGCATTTGAATAAAGCATTTGAAA------AAAGCGCTTCC---------------AACTGAATTACATCATTTCCTACTCACAGCCCCACTGTGAG
Pongo abelii            TGGACTTAGCGCCAGATGAGCTTTGCATTTGAATAAAGCATTTGAAA------AAAGCGCTTCC---------------AACTGAATTACATCATTTCCTACTCACAGCCCCACTGTGAG
Macaca mulatta          TGGACTTAGCGCCAGATGTGCTTTGCATTTGAATAAAGCATTTGAAA------AAAGCGCTTCCTTCTTCATTCCAAGGAAATGAATTATATCATATCCTCCTCACAGCCCCT--CTGAG
Callithrix jacchus      ----------GCCAGATGTGCTTTGCATTTGAATAAAGCATTTGAAAAAAAAAAAAAGCGATTCC---------------AACTGAATTACATCATTTCCCACTCACAGCCCC-CTGTAAG
Mus musculus            GGACCTTAGCACCAGAAATGCTTGGAATTTTCTTATAA-----------------A--GTGTCC---------------ATTGGAATTG--TCAATGCCACACTAAAGCCC-----TGAG
Rattus norvegicus       GGACCTTAGCACCAGATGTGCTTGACATTTTAATAGAA-----------------A--TTTTCC---------------ACTGGAATTG--TCAATGCTGCACTAAAGTCC-----TGAG
Bos taurus              TGGACCCAGCACCAGATGTGCTTTGCATTTGAATAA-------------------AGTGCCTCC---------------ACACGAATT---CCATTTCCTACTCCTAGCAGACCCCTGGG
Sus scrofa              TGGACTGAGCACCAGATGTGCTTCACATTTGAATAA-------------------AATGCTTCC---------------ACACGAATCACGCCATTTCCTACTGCCAGCAGCCCCCTGAG
Canis lupus familiaris  -GGACTTTGCACCAGATGTGCTTCGCATTTGAATAA-------------------AGTGCTTCC---------------ACACGAATTACATCATTTCCTACCCAGAGCAGGCACGCGAG
Equus caballus          CGGACTTAGCACCAGCTGTGCTTGGCGTTTGAAGAA-------------------AGTGCTTCC---------------ACACGGATGATGTCACTTCCTGCTCACAGCAGCCCGGGGAG

Homo sapiens            A--TGC-GTGCC-TTATCAGCACTGCT--------TTCCAGGTTAGGAAAC--AGATTTAGAGAGCTTGGGGCGCATAGCCAAGGTTGGGTATTTAGTTAAATGATCCAGCAATTCCAGT
Pan troglodytes         A--TGC-GTGCC-TTATCAGCACTGCT--------TTCCAGGTTAGGAAAC--AGATTTAGAGAGCTTGGGGCGCATAGCCAAGGTTGGGTATTTAGTTAAATGATCCAGCAATTCCAGT
Gorilla gorilla         A--TGC-GTGCC-TTATCAGCACTGCT--------TTCCAGGTTAGGAAAC--AGATTTAGAGAGCTTGGGGCGCATAGCCAAGGTTGGGTATTTAGTTAAATAATCCAACAATTCCAGT
Pongo abelii            A--TGC-GTGCC-TTATCGGCACTGCT--------TTCCAGGTTAGGAAAC--AGATTTAGAGAGCTTGAGGTGCATAGCCAAGGTTGGGTATTTAGTTAAATGATCCAACAATTCCAGG
Macaca mulatta          A--TGC-GTGCC-TTATCAGCATTGCT--------TTCCAGGTTAAGAAAC--AGATTAAGAGAGCTTGGGGTGCATCGCCAAGGTTGGGTATTTAGTTAAATGATCCAACAATTCCAGT
Callithrix jacchus      A--TTC-ATGC--TTATCAGCACTGCT--------TTCCAGGTTGGGAAAC--AGATTTAGAGAGCTTGG--------TCCAAGGTTGGGTATCTACTTAAATGATTCAACAATTCCAGT
Mus musculus            -------GTCTT-TCATCCTCCCTGCC--------TTCCAAGTTAGGACAC--CAGTTTATGAGTCATTA------CGCCTAAAGTCATGTGTCTAGTTGTCCAGGCC------TTT---
Rattus norvegicus       G--TGAGGTCTT-TCAACCTCTCTGCC--------TTCCAAGTTAGAACAC--CAGTGTAGGAGGCCTTA------TGCCTATGGTCATGTGTCTAATTGAACTACCCGGTAATTCT---
Bos taurus              A--TGA-GTGCT-TCCTTATCACCACT--------TTCCAGGGTAGGAAAC--AGAGTTAGAT----------GCTTTGCCCAAGGTGACTATATGGTTAAATGATCCAACAAATCCAGT
```

```
Sus scrofa              A--TGA-GTGTT-TCATTGTCACCACTTCCCAGGATTCCAGGGTAGAAAAC--AGATGTAGAG----------GCTTTGCCCAAGGTAACTGTCTAGTTAAATGATCCAGCAATTCCAGT
Canis lupus familiaris  ATAGGA-GTGCTTTTATCATCACCCAT--------TTCCAGAGCAGGAAAC--AGATCTAGAGAGATTGAAGCACTTTGGCTAAGGTCACTATGTAGCTAAATGATCCAGCCAA--CAAC
Equus caballus          A--AGA-GTGCT-TTATCATCCCTGCT--------TTCTGGGGTAGGAAACTGAGATTTAGAGCCATTGAGGAGCTTTGCCCAAGGTCACTATCTGGCTAAATGATCCAGCAGTTGCAGT

Homo sapiens            GG----------------------------------------------------------------------------------------------------------------------
Pan troglodytes         GG----------------------------------------------------------------------------------------------------------------------
Gorilla gorilla         GG----------------------------------------------------------------------------------------------------------------------
Pongo abelii            GA----------------------------------------------------------------------------------------------------------------------
Macaca mulatta          GA----------------------------------------------------------------------------------------------------------------------
Callithrix jacchus      GG----------------------------------------------------------------------------------------------------------------------
Mus musculus            ------------------------------------------------------------------------------------------------------------------------
Rattus norvegicus       ------------------------------------------------------------------------------------------------------------------------
Bos taurus              GG----------------------------------------------------------------------------------------------------------------------
Sus scrofa              GA----------------------------------------------------------------------------------------------------------------------
Canis lupus familiaris  GACACCTGAAAATCTGAGTCCTTTTTTTCTTTTTAAGATTTATTTATTTCTTCATGAGAGACACACACAGAGAGAGGCAGAGACACAGGCTGAGGGAGAAGCAGGCTCCATGCAGGGAG
Equus caballus          GG----------------------------------------------------------------------------------------------------------------------

Homo sapiens            ----------------------------------------------------------------------------GAGTCTTCCACCTCAAAGTCTGGCTTCTTTTCCAGCTTGTCCTG
Pan troglodytes         ----------------------------------------------------------------------------GAGTCTTCCACCTCAAAGTCTGGCTTCTTTTCCAGCTTGTCCTG
Gorilla gorilla         ----------------------------------------------------------------------------GAGTCTTCCACCTCAAAGTCTGGCTTCTTTTCCAGCTTGTCCTG
Pongo abelii            ----------------------------------------------------------------------------GGGTCTTCCACCTCAAAGTCTGGCTTCTTTTCCAGCTTGTCCTG
Macaca mulatta          ----------------------------------------------------------------------------GG-GCTTACACCTCAAAGTCTGGCTTCTTTTCCAGCTTGTCCTG
Callithrix jacchus      ----------------------------------------------------------------------------G-GTCTTCCACTTCAAAGTCTGGCTTCTTTTCCAGCTTGTCCTG
Mus musculus            --------------------------------------------------------------------------------G----CCCCAAGAGCCTGGTTCCTTCAACAGGTTATCCTA
Rattus norvegicus       --------------------------------------------------------------------------------GCCCCCCCCCCCAGCCTGGTTCCTTCAACAGGTTATCCTG
Bos taurus              ----------------------------------------------------------------------------G-GTCTTCCACCTGAAAGTCTAGTTCTTTTTCCACTTTATCCTA
Sus scrofa              ----------------------------------------------------------------------------G-GTCTTCCACCTGGAAGTCTGGTTCCTTTTTGACCTTATCCTG
Canis lupus familiaris  CCCCATGTGGGACTCGATCCCTGGTTTCCAGGATCACACCCTGGACTGAAGGCAGCGCTAAACACCTGAGCCACCCG-GGCTGC--CCGGAAAATCTGATTCCTTTTCCAACTTATCCCA
Equus caballus          ----------------------------------------------------------------------------G-GTCTTCCACCTGAAAGTCTGGCTTCTTTTCCAACTTATCATA

Homo sapiens            TGACCCCCTTTATGTGAGAA-AATACAAGGTTCTT-------------------------------------------------------------------------------------
Pan troglodytes         TGACCCCCTTTATGTGAGAA-AATACAAGGTTCTT-------------------------------------------------------------------------------------
Gorilla gorilla         TGACCCCCTTTATGTGAGAA-AATACAAGGTTCTT-------------------------------------------------------------------------------------
Pongo abelii            TGACCCCCTTTATGTGAGAA-AATACAAGGTTCTT-------------------------------------------------------------------------------------
Macaca mulatta          TGACCCCCTTTATGTGAGAA-AATACAAGGTTCTT-------------------------------------------------------------------------------------
Callithrix jacchus      TGACCCCCTTTATGTGAGAA-AATACAAGGTTCTTCTTTTTTTTTTTTTTTTTTTTGAGACGGAGTTTCGCTCTTGTTACCTAGGCTGGAGTGCAATGGCGCGATCTCGGCTCACCGCAA
Mus musculus            TAGGCCTCTTTA-GTGAGAAAAGTACAAAATCCTT-------------------------------------------------------------------------------------
Rattus norvegicus       TAGGCCCCTTTA-GTGAGAAAAATACAAAATCCTT-------------------------------------------------------------------------------------
Bos taurus              T------CTTTATGTGAG-----------ATTTTC-------------------------------------------------------------------------------------
Sus scrofa              T------CTTTATAGGAGAA-AGGACAAGATTTTC-------------------------------------------------------------------------------------
Canis lupus familiaris  T------CTTTACCTAAGAA-AATACAAAATTTT--------------------------------------------------------------------------------------
Equus caballus          T------CTTTATGTGAGAA-AATACGAGATTTTC-------------------------------------------------------------------------------------

Homo sapiens            ------------------------------------------------------------------------------------------------------------------------
Pan troglodytes         ------------------------------------------------------------------------------------------------------------------------
Gorilla gorilla         ------------------------------------------------------------------------------------------------------------------------
Pongo abelii            ------------------------------------------------------------------------------------------------------------------------
Macaca mulatta          ------------------------------------------------------------------------------------------------------------------------
Callithrix jacchus      CCTCCGCCTCCTGGGTTCAGGCAATTTTCCTGCCTCAGCCTCCTGAGTAGCTGGGATTACAGGCACGCGCTACCATGCCCAGCTGATTTTTTGTATTTTTAGTAGAGACGGGGTTTCACC
Mus musculus            ------------------------------------------------------------------------------------------------------------------------
Rattus norvegicus       ------------------------------------------------------------------------------------------------------------------------
Bos taurus              ------------------------------------------------------------------------------------------------------------------------
Sus scrofa              ------------------------------------------------------------------------------------------------------------------------
Canis lupus familiaris  ------------------------------------------------------------------------------------------------------------------------
Equus caballus          ------------------------------------------------------------------------------------------------------------------------

Homo sapiens            ---------------------------------------------------------------------------------------------------------CTATT---TCTACCG
Pan troglodytes         ---------------------------------------------------------------------------------------------------------CTATT---TCTACCG
Gorilla gorilla         ---------------------------------------------------------------------------------------------------------CTATT---TCTACCG
Pongo abelii            ---------------------------------------------------------------------------------------------------------CTATT---TCTACCG
Macaca mulatta          ---------------------------------------------------------------------------------------------------------CTATT---TCTACCG
Callithrix jacchus      ATGTTGACCAGGATGGTCTCAATCTCTGGACCTCGTGATCCACCCGCCTCTGCCTCCCAAAGTGGGATTACAGGCTTGAGCCACCGCGCCCGGCCCAAGGTTCTTCTATT---TCTACC-
Mus musculus            ---------------------------------------------------------------------------------------------------------GCATT-CT---TCCA
Rattus norvegicus       ---------------------------------------------------------------------------------------------------------GCATT-CTTCCTCCA
Bos taurus              ---------------------------------------------------------------------------------------------------------ACATTT--TTTCCCC
Sus scrofa              ---------------------------------------------------------------------------------------------------------ACATTT--TCCCCTA
Canis lupus familiaris  ---------------------------------------------------------------------------------------------------------ACTTTT--TT-----
Equus caballus          ---------------------------------------------------------------------------------------------------------ACGTTT--TC-----

Homo sapiens            TATCCAGATATTTCCA--------------------------------------------------------------------------------------------------------
Pan troglodytes         TATCCAGATATTTCCA--------------------------------------------------------------------------------------------------------
Gorilla gorilla         TATCCAGATATTTCCA--------------------------------------------------------------------------------------------------------
Pongo abelii            TGTCCAGATATTTCCA--------------------------------------------------------------------------------------------------------
Macaca mulatta          TATCCAGATATTTCCA--------------------------------------------------------------------------------------------------------
Callithrix jacchus      -------ATATTTCCA--------------------------------------------------------------------------------------------------------
Mus musculus            TACCCAAGCATTTCCC--------------------------------------------------------------------------------------------------------
Rattus norvegicus       TACCCGAATATTTCCT--------------------------------------------------------------------------------------------------------
Bos taurus              TATCCAAATATTTCCAGTCTTGGTCTTATCCTGAAGCACCCAGGTGGCTCAGACCATAAAGAATCAGCCTGCAATGCAAGAGACCCAGGTTCCATCCCTGGGTGGGGAAGATACCCTGGA
Sus scrofa              TATTCAAATATTTCCA--------------------------------------------------------------------------------------------------------
Canis lupus familiaris  ------------------------------------------------------------------------------------------------------------------------
Equus caballus          --------------CA--------------------------------------------------------------------------------------------------------

Homo sapiens            ----------------------------------------------------------------------------------------------------------------------TC
Pan troglodytes         ----------------------------------------------------------------------------------------------------------------------TC
Gorilla gorilla         ----------------------------------------------------------------------------------------------------------------------TC
Pongo abelii            ----------------------------------------------------------------------------------------------------------------------TC
Macaca mulatta          ----------------------------------------------------------------------------------------------------------------------TC
Callithrix jacchus      ----------------------------------------------------------------------------------------------------------------------TC
Mus musculus            ----------------------------------------------------------------------------------------------------------------------T-
Rattus norvegicus       ----------------------------------------------------------------------------------------------------------------------T-
Bos taurus              GAAGGAAATTGCAACCCATTCCAGTATTCTTGCCTCAGAAATCCCATGGACAGAGGAGCCTGGAGGGCTGTAGTCCATGAGGTCACAAAGAGTGGGACATGACCAGTGACTAACACTTTC
Sus scrofa              ----------------------------------------------------------------------------------------------------------------------TC
Canis lupus familiaris  ------------------------------------------------------------------------------------------------------------------------
Equus caballus          ----------------------------------------------------------------------------------------------------------------------TC

Homo sapiens            CATGGCCCTATTGTATATCAAGGGTGTTTC--TT--GCCA--TGTT--------T-----------------------------------------------------------------
Pan troglodytes         CATGGCCCTATTGTATATCAAGGGTGTTTC--TT--GCCA--TGTT--------T-----------------------------------------------------------------
Gorilla gorilla         CATGGCCCTATTGTATATCAAGGGTGTTTC--TT--GCCA--TGTT--------T-----------------------------------------------------------------
Pongo abelii            CGTGGCCCTATTGTATATCAAGGATGTTTC--TT--GCCA--TGTT--------T-----------------------------------------------------------------
Macaca mulatta          CATGGCCCTATTGTATATCAAGGGTGTTTT--TT--GCCA--TGTT--------T-----------------------------------------------------------------
Callithrix jacchus      TGTGGCCCTATTGTATATCAAGGGTATTTC--TT--GCCA--TGTT--------T-----------------------------------------------------------------
Mus musculus            GGTGGTTCTTTTGTACATCAAGGGTGTTTC--ATTTACAG--TCTTAAAATCTTTTTTGGTTAACCTTCATT-TATGTATATGGATGTTTTGCTTGAATGTTTGTACATGCACTGTGTAC
Rattus norvegicus       AGTGGCTCT-TTGTACATTAAGGGTGTTTC--ACTTATGG--TTTTAAAATTATTTTTGATTAACTTTCATTTTATGTATGTATGTATCTTGCTGGAATATATGCACATGCACCGTGTAC
Bos taurus              ACTGGCCTTACGGTGTACCAGGGATGTTTCATTT--GCCA--TATT--------T-----------------------------------------------------------------
Sus scrofa              TGTGGCTTTATTGTGTGTCAGGAATGTTTCACTC--GCCTTTTATT--------T-----------------------------------------------------------------
Canis lupus familiaris  ------------------------------------------TTTT--------T-----------------------------------------------------------------
Equus caballus          TGTGACCCTGTCGTGTATCAAGGACACGTTACTT--GCCA--CCTT--------T-----------------------------------------------------------------

Homo sapiens            ------------------------------------------------------------------------------------------------------------------------
Pan troglodytes         ------------------------------------------------------------------------------------------------------------------------
Gorilla gorilla         ------------------------------------------------------------------------------------------------------------------------
Pongo abelii            ------------------------------------------------------------------------------------------------------------------------
Macaca mulatta          ------------------------------------------------------------------------------------------------------------------------
Callithrix jacchus      ------------------------------------------------------------------------------------------------------------------------
Mus musculus            AGGCCTGGGGCCATTGGCGGCCAGACAAAAGTGCCAGATTCCCCTGGAATTGAAGTTACAGAGTAAGCCAACTTGTGGGTGCTAGTAACCAAACCTGTGCATTTCACCACTAAGCAATCC
Rattus norvegicus       ATGCCCGGGGCTGTTGAAGGCCAGACGAAAGTGCCAGATGC-CCTGGGATTGAAGTCACAGAGTAAGCCAACATGTGGGTGCTAGGAACCAAACCTGTGCTTTTAACCACTGAGCAGTCT
Bos taurus              ------------------------------------------------------------------------------------------------------------------------
Sus scrofa              ------------------------------------------------------------------------------------------------------------------------
Canis lupus familiaris  ------------------------------------------------------------------------------------------------------------------------
Equus caballus          ------------------------------------------------------------------------------------------------------------------------

Homo sapiens            --------------------TTATT---------------------TG----AAGGTAACACTGCAGGT------CACACTGGGAGGAAG-AGTCATC----------------------
Pan troglodytes         --------------------TTATT---------------------TG----AAGGTCACACTGCAGGT------CACACTGGGAGGAAG-AGTCATC----------------------
```

```
Gorilla gorilla        --------------------TTATC---------------------TG----AAGGTCACACTGCAGGT------CACACTGGGAGGAAG-AGTCATC----------------------
Pongo abelii           --------------------TTATT---------------------TG----AAGGTCACACTGCAGGT------GACATTGGGAGGAAG-AGTCATC----------------------
Macaca mulatta         --------------------TTATT---------------------TG----AATGTCACATTGCAGGT------CACATTGGCAGGAAG-AGTCATC----------------------
Callithrix jacchus     --------------------TTATT---------------------AG----AAAGTCACACTACAGAT------CACATTAGGAGGAAG-AGGCATG----------------------
Mus musculus           CTCCAGATGTGTGAGGCCTT------------------------------------------------------------------------------AGTTAATTGAAGAGCAACCCTT
Rattus norvegicus      CTCCAGACACGTGGGGCCTT----------------------------------------------------------------------------------------------------
Bos taurus             --------------------CTATTT--------------------GG----AAGGTTGCATTACAGATTGAGATAGCCTTTGGAGGAAGAAGCCACT----------------------
Sus scrofa             --------------------TTATTTTTATTTTTTTTAACTTGAAGG----TAGGTTGCATTGCAGGTAGAGACTGGTTTTGGAAGAAGAAGACACC----------------------
Canis lupus familiaris --------------------TTAAT---------------------GTGCCAATATTTTCATTGCAGGTAGAGATTGCCTTTGGAAGAAGCAGCCACC----------------------
Equus caballus         --------------------TTATT---------------------GG----AAGGTCACACTGCATGTAGAGATTGCCTTTGCAGGAAGAGGCCGCC----------------------

Homo sapiens           --------------------------------TGAAGATAGCCTAACTTCAGCATAGCAGTCATTCTGTA-GACCAT-AGAG-----ATGCTTTTTCCTGGGC----AGTGTCTTTTTAC
Pan troglodytes        --------------------------------TGAAGATAGCCTAACTTCAGCATAGCAGTCATTCTGTA-GACCAT-AGAG-----ATGCTTTTTCCTGGGC----AGTGTCTTTTTAC
Gorilla gorilla        --------------------------------TGAAGATAGCCTAACTTCAGCATAGCAGTCATTCTGTA-GACCGT-AGAG-----ATGCTTTTTCCTGGGC----AGTGTCTTTTTAC
Pongo abelii           --------------------------------TGAAGACAGCCTAACTTCAGCATAGCAGTCATTCTGTA-GACCGT-AGAG-----ATGCTTTTTCCCAGGC----AGTATCTTTTCAC
Macaca mulatta         --------------------------------CGAAGACAGCCTAACTTCAGCATGGCAGTCATTCTGTA-GACCGT-AGAG-----ATGCTTTTTCCTGGGC----AGTATCTTTTTAC
Callithrix jacchus     --------------------------------GGAAGATAGCCTAACTTCAGCGTAGAGGTCATTCTGTA-GACCAT-AGAG-----ATGCTTCTTTCTGGGC----AGTGTCTTTTTAC
Mus musculus           GTAGGTATGGTTACCATTGGGTTAAAGGTCTCAAAGGCTAG-CAGATTTCAGCATGACAATTTTTCTGTG-AATC-T-GGACATTTGGTGTTTTTTGTTCATT----TGTTTGTATTTGT
Rattus norvegicus      --------------------------------TAAGACTAG-TGGATTTTCACGTGACAATTTTTTTCTG-GATC-T-GGGCATCCAGTGCCATTGGT---------TGGCTGTGTTTTT
Bos taurus             --------------------------------CAAAGGTAGCCTAACCTGAGCAGGACAGTTA---TGTAAGACTTTAAGAG-----GTGTTTCTTCCTGGGTATGCAATGTCTTC-AAC
Sus scrofa             --------------------------------CAAAGATAGCCTAACTTCAGCATGACCATTATTATGTA-GACCTT-AGAG-----CTGTTTCTTCCTGGGTACCCAGTGTCTTTTTCC
Canis lupus familiaris --------------------------------CACAGGTAGCCTAACCTCAGCATGGCCGTTATTATGTA-GACCTT-GGAG-----ATGTTTCTTCCTGGGCATTCAGTGCCTTTTTAC
Equus caballus         --------------------------------CAAAGGTAGCCTGACCTCGACATGACGGTTACTGTGGA-GACCTT-GGAG-----AAGTTTGTTCCTGGGCATTCGATGCCTTTTTAC

Homo sapiens           ATTCCCTGTTTCCTCTAAATATCCCAGTGGGGAGGAAGGTT---------------------------------------------------GCCCCCAG------CTGGTT-T-TTTTA
Pan troglodytes        ATTCTCTGTTTCCTCTAAATATCCCAGTGGGGAGGGAGGTT---------------------------------------------------GCCCCCAG------CTGTTT-T-TTTTG
Gorilla gorilla        ATTCCCTGTTTCCTCTAAATATCCCAGTGGGGAGGAAGGTT---------------------------------------------------GCCCCCAG------CTGTTT-TTTTTTA
Pongo abelii           ATTCCCTGTTTTCTCTAAATATCCCAGTGGGGAGGAAGGTT---------------------------------------------------GCCCCCAG------CTGTTT---TTTTA
Macaca mulatta         ATTCCCTGTTTCCTCTAAATATCCCAGTGGGGAGGAAGGTT---------------------------------------------------GCCCCCAG------TTGTTT---TTTTA
Callithrix jacchus     ATT--CTGTTTCCTCTAAATATCCCGGTGGGGG---ATATTTAGTGTTGTGTTCACAGATGTGAGCCACTGCACCTGGCCCTCCCCCACCGGCCCCCATATTTTGTTGTGT---TTTTG
Mus musculus           CTTTTTTGTTTGTTC---------------------------------------------------------------------------------------------GTTCG--TTTTC
Rattus norvegicus      CTTTCTTGTTTGT-------------------------------------------------------------------------------------------------TCG--TTTTC
Bos taurus             GTCTCTTGTTTCCTCTAAATATTATAACCGG---GAAGGTC---------------------------------------------------ACC-------------------------
Sus scrofa             ACCTTATGTTTCCTCT----ATTGCAACAGGGAGGAAGGTC---------------------------------------------------ACT-------------------------
Canis lupus familiaris ATCTCGTATTTCCTTTAAATATCACAA-GGAAAGGAATGTT---------------------------------------------------ACC-------------------------
Equus caballus         ATCTCCTGTTTCCTCTAAATATTACAGTGGGGAGGAAGGTC---------------------------------------------------ACC-------------------------

Homo sapiens           TTT-TTGTTTTTGTTTTC--AGACAGAGTCTCACACTGTCA---CCCAGGCTGGAATGCAAT----GGTGCAAACGCAACTCACTGTAGCCTTGACCCACTCGGCTCAAGCAGTCCTCCC
Pan troglodytes        TTT-TTGTTTTTGTTTTC--AGACAGAGTCTCACACTGTCA---CCCAGGCTGGAATGCAAT----GGTGCAAACGCAACTCACTGTAGCCTTGACCCACTCAGCTCAAGCAGTCCTCCC
Gorilla gorilla        TTT-TTGTTTTTGTTTTC--AGACAGAGTCTCACTCTGTCA---CCCAGGCTGGAATGCAAT----GGTGCAAACGCAACTCACTGTAGCCTTGACCCACTCAGCTCAAGCAGTCCTCCC
Pongo abelii           TTT-TTATTTTTGTTTTC--AGACAGAGTCTCACACTATCA---CCCAGGCTGGAATGCAAT----GGTGCAAACGCAGCTCACTGCAGCCTTGACCCACTCTGCTCAAGCAGTCCTCCC
Macaca mulatta         TTT-TTGTTTTTGTTTTC--AGACAGAGTCTCACACTGTCA---CCTAGGCTGGAATGCAAT----GGTGCAAACGCAGCTTACTGTAGCCTTTATCCGTTCGGC----------TCCC
Callithrix jacchus     TTT-TTGTTTTAGTTTTC--AGGCAGAGTCTTCCACTGTGA---CCCAGGCTGGAATGCAGT----GGTGCAAACGCAGCTTACTGTAGCCTTGACCTGCTGGACTCAAGCAATCATCCC
Mus musculus           TGT-TTGTTTCGGTTTTTCAAGAGAGGGTTTCTCCGTGTGACAGCTCTGGCTGTCCTAGAACATACTCTGTAGAC------CAGGCTGGCCTGGA---ACTCA-------GAGTTCTGCC
Rattus norvegicus      TGTTTTGTTTCAGTTTTTCAAGATAGGATTTCTCTGTGTAAAAACCCTGGCTGTCCTGGAACTTACCCTGTAGAC------CAGGCTAGCCTGGA---ACTCA-CTCACGGAGTTCTACC
Bos taurus             ------------------------------------------------------------------------------------------------------------------------
Sus scrofa             ------------------------------------------------------------------------------------------------------------------------
Canis lupus familiaris ------------------------------------------------------------------------------------------------------------------------
Equus caballus         ------------------------------------------------------------------------------------------------------------------------

Homo sapiens           T-CCTCAGCCTCCCAAGTAGTTGGGACTACAGGCGAGTGCCACA-C-GCCCAGCTAAT-TTT----TTTAT---TTTTCCTTTTTGTAGAGACAGGGTCGCACTATGTT-G------CTG
Pan troglodytes        T-CCTCAGCCTCCCAAGTAGTTGGGACTACAGGCGAGTGCCACA-C-GCCCAGCTAAT-TTT----TTTAT---TTTTCCTTTTTGTAGAGACAGGGTCGCACTATGTT-G------CTG
Gorilla gorilla        T-CCTCAGCCTCCCAAGTAGTTAGGACTACAGGCGAGTGCCACA-C-GCCCAGCTAAT-TTT----TTTAT---TTTTCCTTTTTGTAGAGACAGGGTTGCACTATGTT-G------CTG
Pongo abelii           T-CCTCAGCCTCCCAAGTAGTTGGGACTACAGGCGAGTGCCACA-C-ACCCAGCTAAT-TTT----TTAAT---TTTTCCTTTTTGTAGAGACAGAGTCGCACTGTGTT-G------CTG
Macaca mulatta         T-CCTCAGCCTCCCAAGTAGTTGGGACTACAGGCGACTGCCACA-C-GCCCAGCTAAT-TTT----TTTAT---T-TTCCTTTTTGTAGAGACAGGGTCTCACTGTGTT-GCCCAGGCTG
Callithrix jacchus     T-CCTCAGCCTCCCAAGTACTTGGGACTACACACGAGTACCACA-A-GCTCAGCTAAT-TTT----TTTATATTTTTTTCTTTTTGTAGAGGCAGGGTCCTACTATGTT-GTCCAGGCTG
Mus musculus           TGCCTCTGCTTCCCAAGT-GCTAGGATTACAGGCATGCGCCACCACTACCCAGCATGTCGCTTGTGTTTGT---TTTTGCTCTTTCT-GAGACAAGGTCC-TGTTTGTAAGCCCAGGCTG
Rattus norvegicus      TGCCTCTGCCTTCCAAAT-GCTAGGATTAAAGGCACATGACACCATTGCCTGGTATGTCTCT----TTTGT---TTTTGCTCTTTCT-GAGAAAAGGTCC-TTTATGTAACCCCAGGCTG
Bos taurus             ------------------------------------------------------------------------------------------------------------------------
Sus scrofa             ------------------------------------------------------------------------------------------------------------------------
Canis lupus familiaris ------------------------------------------------------------------------------------------------------------------------
Equus caballus         ------------------------------------------------------------------------------------------------------------------------

Homo sapiens           GTCTCAAACTCCTG-------------GGCTCAAG---TGATCCTCCCGCTTTGGCCTCCCAAAGTGCTGGGTTTACAGGTGTAAGCCACTGCACCTGGCCCTCCC-C-CA-CAG-----
Pan troglodytes        GTCTCAAACTCCTG-------------GGCTCAAG---TGATCCTCCCGCTTTGGCCTCCCAAAGTGCTGGGTTTACAGGTGTAAGCCACTTCACCTGGCCCTCCC-C-CA-CAG-----
Gorilla gorilla        GTCTCAAACTCCTG-------------GGCTCAAG---TGATCCTCCCGCTTTGGCCTCCCAAAGTGCTGGGTTTACAGGTGTAAGCCACTGCACCTGGCCCTCCCCC-CA-CAG-----
Pongo abelii           GTCTCAAACTCCTG-------------GGCTCAAG---TGATCCTCCCGCCTTGGCCTCCCAAAGTGCTGGGTTTACAGGTGTAAGCCACTGCACCTGGCCCTCCC-C-CA-CAG-----
Macaca mulatta         GTCTCAAACTCCCG-------------GGCTCAAG---TGATCCTCCCGCCTTGGCCTCCCAAAGTGCTAGGTTTACAGGTGTGAGCCACTGTACCTGCCCCTCCC-C-CA-CAG-----
Callithrix jacchus     GTCTCAAACTCCTA-------------GGCTCAAG---TGATCCTCCCACCTTGGTCTTCCAAAGTGCTGTGTTCACAGATGTGAACCACTGCACCTGGCCTTCCC-CTCACCGG-----
Mus musculus           ACCTCAAACTCCCGATGTAGCCAAGGGAGTTAAACTCCTGGCTCACTTACCTTCAC-CACTTAAGTGCTGGGGTAATGGGTATGTGCCACGGCGCCTGGCTTA------TG-CAGTGCTT
Rattus norvegicus      GCCTCAAACTCCCTATGTAGCCTAGTGAGTTAAACTTCTGATCCACCTACCTTCAC-TACCTAAGTGCTGGGGTAATGGGCATACGCCACGATGTCTGGTTTG------CA-CAGTGCTG
Bos taurus             ------------------------------------------------------------------------------------------------------------------------
Sus scrofa             ------------------------------------------------------------------------------------------------------------------------
Canis lupus familiaris ------------------------------------------------------------------------------------------------------------------------
Equus caballus         ------------------------------------------------------------------------------------------------------------------------

Homo sapiens           ------------------------------------------------------------------------------------------------------------------------
Pan troglodytes        ------------------------------------------------------------------------------------------------------------------------
Gorilla gorilla        ------------------------------------------------------------------------------------------------------------------------
Pongo abelii           ------------------------------------------------------------------------------------------------------------------------
Macaca mulatta         ------------------------------------------------------------------------------------------------------------------------
Callithrix jacchus     ------------------------------------------------------------------------------------------------------------------------
Mus musculus           GGGATCTAACTCAAGGCTTTGTACATAGTGACA-GCACTCTGCCAACTAGGCTACAGTCCTAGCCCCTCCAGAGCTC--TTCACATCTCATTCCTCGAAACAACTCCAAGCA--------
Rattus norvegicus      GGGATCCGACTCAAGGCTTTGTGCATAGTGACAAGCACTCGACCAACTGAGCTACAGTCTTA-CCCCTGCAGTGCTCATTTCATCTCTCATTCCTCCAAGTGACTCCAAGCGAATGAATA
Bos taurus             ------------------------------------------------------------------------------------------------------------------------
Sus scrofa             ------------------------------------------------------------------------------------------------------------------------
Canis lupus familiaris ------------------------------------------------------------------------------------------------------------------------
Equus caballus         ------------------------------------------------------------------------------------------------------------------------

Homo sapiens           ---------------TCCCCACATTTTAGAAAGGAAAAGTCAGGCATGG---AGAAG-TCAGCAGTCTCAGGTCTCTGGGGCTCCTGGGCACAGGGGTGAAGCAGCATTTTGCCCTCCTG
Pan troglodytes        ---------------TCCCCACATTTTAGAAAGGAAAAGTCAGGCATGG---AGAAG-TCAGCAGTCTCAGGTCTCTGGGGCTCCTGGGCACAGGGGTGAAGCAGCATTTTGCCTTCCTG
Gorilla gorilla        ---------------TCCCCACATTTTAGAAAGGAAAAGTCAGGCATGG---AGAAG-TCAGCAGTCTCGGGTCTCTGGGAATCCTGGGCACAGGGGTGAAGCAGCATTTTGCCCTCCTG
Pongo abelii           ---------------TCCCCACATTTTAGAAAGGAAAAGTCAGGCATGG---AGAAG-TCAGCAGTCTCAGGTCTCTGGGGTTCCTGGGCACGGGGGTGAAGCAGCATTTTGCCCTCCTG
Macaca mulatta         ---------------CCCCCACATTTTAGAAAGGAAATGTCAGGCATGG---AGAAG-TCAGCAGTCTCAGGTCTGTGAGACTCCTGGGCACAG-GGTGAAGCAGTATTTTGCCCTCCTG
Callithrix jacchus     ---------------CCCCCGTATTTTAGAAAGGAAAAGTCAGGCATAG---AGAAG-TCAGAAGTCTCAGGTCCCTGGGGCTCCTGGGCACAGAGGTGAAGTAGCATTTTGCCCTCCTG
Mus musculus           ----GAGGAAGTTCACTCCCATATTTTACAA-GGGAATCTCAGGCCTAG---ATTGG-TCTGAAGTTCCGGG-CTCCCATGCTGCCAAGCACAG-GGTGAGACAGCATCTTGCTCTCCTG
Rattus norvegicus      TGGGGAGGAAGTTCATTCCCATATTTTACAA-GGAGATCTCAGGCCTGG---GTTGG-TCTGAAGTTCTGGG-CCCCCATTCTGTCAGACACAG-GGTGAGGTAGCACCTTGCTCTCCCG
Bos taurus             -----------------CCTATATCTTAGAAGAGGGTCATGAGGCATGAGGGAGGAG-TCAATGTACTCAGATCCCTGGGGCTGCTGGGCAAGGACTCAATCCAGCATTTTGCCCTCCCT
Sus scrofa             -----------------CCCACGTCTTAGAAGAGGAACAGGAGGCATGA---TGAAG-TCCCTGGGTTCAGATCCCTGGGGCTGCAGGGCAAGGACGTGATCCAGCATTTTGCCCTTCTG
Canis lupus familiaris -----------------CCCGTATCTCTGAAG-GGAGAGTGCAGCATGG---AGAAGCTCACTGGGCTCAGATCCCTGGGGCTGCTGGTTGAAGGTGGACCCAGTATTTTGCTCTCTTG
Equus caballus         -----------------CCTGGATCTTAGAAGAGGAAGGTGAGGTATGG---TGGAG-TCTCTGGGCTCAGATCCCTGGGCCTGCTGGG-GAGGGCCTGATCCAGCATTTTGCCCTTCTG

Homo sapiens           GCTCTCTGGCACCCCTG--GT-GCCTTCAGCACTTCCCAG-AA-GACTCAGGAGATCTTCCCACAGCAGGAGCA--CTG--CTGATAGAGTTACAGGACAGCA-CTGTGGCGTATGGCCC
Pan troglodytes        GCTCTCTGGCACCCCTG--GT-GCCTTCAGCACCTCCCAG-AA-GACTCAGGAGATGTTCCCACAGCAGGAGCA--CTG--CTGATAGAGTTACAGGACAGCA-CTGTGGCGTATGGCCC
Gorilla gorilla        GCTCTCTGGCACCCCTG--GT-GCCTTCAGCACCTCCCAG-AA-GACTCAGGAGATGTTCCCACAGCAGGAGCA--CTG--CTGATAGAGTTACAGGACAGCA-CTGTGGCATATGGCCC
Pongo abelii           GCTCTCTGGCACCCCTG--GT-GCCTTCAGCACCTCCCAG-AA-GACTCAGGAGATGTGCCCACAGCAGGAGCA--CTG--CTGATAGATTTACAGGACAGCA-CTGTGGCGTATGGCCC
Macaca mulatta         GCTCTCTGGCACCGCTG--GT-GCCTTCAGCACCTCCTAG-AA-GACTCAGGAGATGTGCCCACAGCAGGAGCA--CTG--CTGATAGAGTTACAGGACAGCA-CTGTGGCGTATGGCCC
Callithrix jacchus     GCTCTCTGGCACC-CTA--GT-GTCTTCAGCACCTGCCTA-AA-GATTCTAGAGATGTACGCACAGCGGGAGCA-GCTG--CTGATAGAGGTACAGGACAGCA-TTGAGGCATATGGCCC
Mus musculus           GTT--CTGACACAGTGGAACA-GCCTTCAACACCTTCCAGGAA-GACC--GGATCTGCTCCT-----------G--CTG--CTGCCATAGTTA-AGACCAGCATCTGTGGCATACAACCT
Rattus norvegicus      GTT--TTGGCACAGAGGCCCA-GTCTTCAGCACCTTCCAGGAA-GAGT--GGATCTGCTCCT-----------A--CTG--CTGCCATAGTTA-AGGCCAGCATCTATGGCATATGGCCT
Bos taurus             GCCCTCTGGCACACGCA--CCTCCTTTCAGCACCTTCTAG-A---ACTCAGGATATGTGCCTACAGCAGGAGCAG-CTCCCCCGCTTTAGCTGCAAGTTGGTGTCTGTGGCATATGGACC
Sus scrofa             ACCCTCTGGCACATGCC--CC-ACTTTCAGCACCTTCCAG-AAGGACTCAGGAAATGTGCCTGCAGCAAGAACAG-CTG--CCACTGTAGCTGCAGGTTGGCATCTGTGGCATATGGACC
Canis lupus familiaris GCCCTCCGGCACACCCC--AG-GCCTTCAGCACCTTCTAG-AAGGACTTGGAAAATGTGTCTGCAGCAGGAGCAG-CTG--CCTCTATAGCTGCAAGTTGATGTCTGGGGCATATGAACC
Equus caballus         GCCCTCTGGTTCTC-GC--GT-GCCTTCAGCACCTTCCAG-AAAGACTCAGGAAATGTACCTGCAGCAGGAGCAG-CTG--CCACCATAACCGCAGGTGGACATCTGTGGCATAGGGACC

Homo sapiens           CCATTCGAATTGAT-GAAGAT-GTTTAGGCCAAGGGGGAAATGGTTGTAGCCCCTT---AACACACACTC-------CGCAGCAGTAACTGGGAGGTATTAGGAACTCTAACTTTTT---
Pan troglodytes        CCATTCGAATTGAT-GAAGAT-GTTTAGGCCAAGGGGGAAATGGTTGTAGCCCCTT---AACACACACTC-------TGCAGCAGTAACTGGGAGGTATTAGGAACTCT------TT---
Gorilla gorilla        CCATTCAAATTGAT-GAAGAT-GTTTAGGCCAAGGGGGAAATGGTTGTAGCCCCTT---AACACACACTC-------CGCAGCAGTAACTAGGAGGTATTAGGAACTCTAACTTTTTTTT
Pongo abelii           CCATTCGAATTGAT-GAAGAT-GTTTAGGCCAAGGGGGAAATGGTTGTAGCCCCTT---AACACACACTC-------CACAGCAATAACTGGGAGGTATCAGGAACTCTAACTTTTTTTT
Macaca mulatta         CCATTCGAGNNNNN-NNNNNN-NNNNNNNNNNNNNNNNNNNNNNNNNNNNNNNNNN---NNNNNNNNNNN-------NNNNNNNNNNNNNNNNNNNNNNNNNNNNNNNNNNNNNNNNNNNN
Callithrix jacchus     CCATTCGAATTGAT-GAAGAT-GTTTAGGCAAAAAGGGAAATGACTGCAGCCCCCT---AACACACACTA-------TGCAGTGGTAACTGGGAGGTATTAGGAACTCTAACTTTTT---
Mus musculus           CCT-TCAGAGTGATGGGAGAT-GTTCAAACCAAGGAGGAAGTGGATATAGCACCCT---AACACACCCTC-------TACA--AGTCTCTGAGAGGCAC---------------------
Rattus norvegicus      CCT-TCAGAGTCATGGGAAAT-GTCCAGGCCAAAGGGGG-ATGGTTGTAGCACCCT---AACATACCCTC-------TACA--AGTGTCTGAGAGGCAC---------------------
```

```
Bos taurus               CCATTTGGACTGAT-GGAAAA-TTTTAGG-----GCAAGAATGATCGTATCCACTT---AATAC----TC-------CATGACAATAACTC-AAGA---TAGAAGCTCTTC---------
Sus scrofa               CCATTTGGAGTGAT-GGAGAT-TTTTAGG-----GAAAAAATAGTTGTGGCACCTT---AACACACACTCCACGATGCATGACAGCAACGGGATGA---TAGACAGTCTTC---------
Canis lupus familiaris   CCGTTTGGATGGAT-GGGGATCTTTGAGACCTACAGAAGAATATTCGTAGCCCCTT---AACACATACCC-------CATAGCTGTAACTGGGAGACAGTAGGAGCTCTTC---------
Equus caballus           CCATTCGGACTGAA-GGAGAC-TTTCAGGCCTGGGGAAGAATGGTCATGGCTCCTGCCCTAGACACACTC-------CACACCAGTAGCTGGGAGACA-TAGGAGCTGTCC---------

Homo sapiens             -----TTTTTTTTTTTTTTGAGACAGA-GTCTTGCTCTGTTACTCAGGTTAGAGTGCAGTGGTGCAATCGTGGCTCACTGCAACTTCCGTCTCCCAGGTTTAAGCGA-TTCTCCT-GCCT
Pan troglodytes          -----TTTTTTTTTTTTTTGAGACAGA-GTCTTGCTCTGTTACTCAGGTTAGAGTGCAGTGGTGCAATCGTGGCTCACTGCAACTTCCGTCTCCCAGGTTTAAGCGA-TTCTCCT-GCCT
Gorilla gorilla          TTTTTTTTTTTTTTTTTTTGAGACAGA-GTCTTGCTCTGTTACTCAGGTTAGAGTGCAGTGGTGCAATCGTGGCTCACTGCGACTTCCGTCTCCCAGGTTTAAGCGA-TTCTCCT-GCCT
Pongo abelii             TTT--TTTTTTTTTTTTTTGAGACAGA-ATCTTGCTCTGTTACTCAGGTTAGAGTGCAGTTGTGCAATCTCGGCTCATTGCAACTTCTGCCTCCCAGGTTCAAGCAA-TTCTCCT-GCCT
Macaca mulatta           NNN--NNNNNNNNNNNNNNNNNNNNNN-NNNNNNNNNNNNNNNNNNNNNNNNNNNNNNNNNNNNNNNNNNNNNNNNNNNNNNNNNNNNNNNNNNNNNNNNNNNNNNNNNNNNNNNNNNNNNNN
Callithrix jacchus       -----------TCTTTTTTGAGGCAGAAATCTCACTCTGTCACTCAGACTAGAGTGCGGTGGTACAATCTCAGCTCATTGCAACTTCCACCTTCCAGGTTCAAGCAA-TTCTCCT-GC--
Mus musculus             ------------------------------------------------------------------------------------------------------------------------
Rattus norvegicus        ------------------------------------------------------------------------------------------------------------------------
Bos taurus               ------------------------------------------------------------------------------------------------------------------------
Sus scrofa               ------------------------------------------------------------------------------------------------------------------------
Canis lupus familiaris   ------------------------------------------------------------------------------------------------------------------------
Equus caballus           ------------------------------------------------------------------------------------------------------------------------

Homo sapiens             CAGCCTCCCAAGTAGCTGGGATTACAGGCACGTGCCACCACACCCAGATAATTTTTGTATTTT-TGGTAGAGACGGGGTTTGG-CCATGTTGGCCAGGCTGGTTTCGAATTCCTGACCTC
Pan troglodytes          CAGCCTCCCAAGTAGCTGGGATTACAGGCACGTGCCACCACACCCAGATAATTTTTGTATTTT-TGGTAGAGACGGGGTTTGG-CCATGTTGGCCAGGCTGGTTTCGAACTCCTGACCTC
Gorilla gorilla          CAGCCTCCCAAGTAGCTGGGATTACAGGCACGTGCCACCACACCCAGATAATTTTTGTATTTT-TGGTAGAGACGGGGTTTGG-CCATGTTGGCCAGGCTGGTTTCGAACTCCTGACCTC
Pongo abelii             CAGCCTCCCAAGTAGCTGGGATTACAGGCACTTGCCACCACACCCAGCTAATTTTTGTATTTT-TGGTAGAGACGGGGTTTGG-CCATGTTGGCCAGGCTGGTTTCAAACTCCTGACCTC
Macaca mulatta           NNNNNNNNNNNNNNNNNNNNNNNNNNNNNNNNNNNNNNNNNNNNNNNNNNNNNNNNNNNNNNNNNNNNNNNNNNNNNNNNNNNNNNNNNNNNNNNNNNNNNNNNNNNNNNNNNNNNNNNN
Callithrix jacchus       ----CTCCCAAGTAGCTGGGATTACAGGCATGTGCCACCACACCGGGCTAATTTTTGTATTTT-TACTAGAAACGAGGTTTTG-CCATGTTGGTCAGGCTGGTCTTGAATTCCTGACCTC
Mus musculus             ------------------------------------------------------------------------------------------------------------------------
Rattus norvegicus        ------------------------------------------------------------------------------------------------------------------------
Bos taurus               ------------------------------------------------------------------------------------------------------------------------
Sus scrofa               ------------------------------------------------------------------------------------------------------------------------
Canis lupus familiaris   ------------------------------------------------------------------------------------------------------------------------
Equus caballus           ------------------------------------------------------------------------------------------------------------------------

Homo sapiens             A-GTGATC---TACCCGCCTTGGCTTCCCAAAGTGCTGGGATTACAGGCATGAGTCACCACGCCTGGCCAA-CTCTAA------------------------------------------
Pan troglodytes          AGGTGATC---TACCCGCCTTGGCTTCCCAAAGTGCTGGGATTACAGGCATGAGTCACCACGCCTGGCCAA-CTCTAA------------------------------------------
Gorilla gorilla          AGGTGATC---TACCCGCCTTGGCTTCCCAAAGTGCTGGGATTACAGGCATGAGTCACCATGCCTGGCCAA-CTCTAA------------------------------------------
Pongo abelii             AGGTGATC---TACCCGCCTCGGCTTCCCAAAGTGCTGAGATTACAGGCATGAGTCACCACGCCTGGCCAA-CTCTAA------------------------------------------
Macaca mulatta           NNNNNNNNNNNNNNNNNNNNNNNNNNNNNNNNNNNNNNNNNNNNNNNNNNNNNNNNNNNNNNNNNNNNNNN-NNNNNN------------------------------------------
Callithrix jacchus       AGGTGATC---CACCCGCCTTGGCCTCCCAAAGTGCTGGGATTACAAGCGTGAGTCCCCATGCCCAGCCAA-CTCTAA------------------------------------------
Mus musculus             -----------------------------------------------------------------AGTCAGGCACCAAATTACAACATTTCAGTTGACATAACACATGTGCCCTATAAGA
Rattus norvegicus        -----------------------------------------------------------------GGCCAAGCTCCAAATTACAACATATCAGGTAGCATACCACATGGGCCCCATAAGA
Bos taurus               ------------------------------------------------------------------------------------------------------------------------
Sus scrofa               ------------------------------------------------------------------------------------------------------------------------
Canis lupus familiaris   ------------------------------------------------------------------------------------------------------------------------
Equus caballus           ------------------------------------------------------------------------------------------------------------------------

Homo sapiens             --------------------------------------------------------------------------------------------------------CTTTTTTACGTTCTCT
Pan troglodytes          --------------------------------------------------------------------------------------------------------CTTTTTTACGTTCTCT
Gorilla gorilla          --------------------------------------------------------------------------------------------------------CTTTTTTATGTTCTCT
Pongo abelii             --------------------------------------------------------------------------------------------------------CTTTTTTATGTTCTCT
Macaca mulatta           --------------------------------------------------------------------------------------------------------NNNNNNNNNNNNNNNN
Callithrix jacchus       --------------------------------------------------------------------------------------------------------CTTTTTTACATTCTCT
Mus musculus             ATGCATGGTCTAGTGATGGCACTCT--GACATTCAGCCAATTAGGAAGCGGCTTCTCTGGAGGCATCCTCATGACTAAGTGACCACACTGTCACTGGGAGCACTCCCTTTTACATTCTCC
Rattus norvegicus        ACGCACAGTCTAGTGTTGGCACTCTGTGACTTTTAGCCAATTAGGAAGCTGCTTCTCTGAAGGCATCCTCGTGGCTAAGGGGTCACACTGTCACTGGGAGCATCCCCTTTTACACTTCTA
Bos taurus               --------------------------------------------------------------------------------------------------------CCTTTTTAAATCAGCC
Sus scrofa               --------------------------------------------------------------------------------------------------------CCTTTTTACATTATTC
Canis lupus familiaris   --------------------------------------------------------------------------------------------------------CCTTTCTAGGTTACCC
Equus caballus           --------------------------------------------------------------------------------------------------------CCTTG-TCACTTACCC

Homo sapiens             AATCCTCACAGGTTCTCCAGAACAAAA----------------TCCT----TGATAGGTGTGCTTCCTCCTT---TTTTCTTTT-TTCTCAAATGATGGGTTAAAGTTCAAATGTTAGGA
Pan troglodytes          AATCCTCACAGGTTCTCCAGAACAAAA----------------TCCT----TGATAGGTGTGCTTCCTCCTT---TTTTCTTTT-TTCTCAAATGATGGGTTAAAGTTCAAATGTTAGGA
Gorilla gorilla          AATCCTCACAGGTTCTCCAGAACAAAA----------------TCCT----TGATAGGTGTGCTTCCTCCTT---TTTTCTTTT-TTCTCAAATGATGGGTTAAAGTTCAAATGTTAGGA
Pongo abelii             AATCCTCACAGGTTCTCCAGAACAAAA----------------TCCT----TGATAGGTGTGCTTCCTCCTT---TTTTCTTTT-TTCTCAAATGATGGGTTAAAGTTCAAATGTTAGGA
Macaca mulatta           NNNNNNNNNNNNNNNNNNNNNNNNNNN----------------NNNN----NNNNNNNNNNNNNNNNNNNNN---NNNNNNNNN-NNNNNNNNNNNNNNNNNNNNNNNNNNNNNNNNNNN
Callithrix jacchus       AATCTCCACAGGCTATCCAGAACAAAC----------------TCCT----TGATAAGTGAGCTTCCTCCTTTTTTTTTCTTTT-TTCTCAAATGATGGGTTAAAGTTCCAATGCTAGGA
Mus musculus             AGTCTC-CCCAGCCCTGCAGAGCAAGCACGCTTGGTGCACACATCACTTTGCAGTGGATATGTGTTCTGTCT----TTGCCCTT-TTCTCAACGGCCCAGTTAAATTT-AAATGACA---
Rattus norvegicus        GTTCTC-CCCAGCCCTTCAAAGCAAGCA----TGGGGCACACATCACTTAGTGGTGAATATGTTTTCTGCCT---TTTGTCTTT-TTCTCAATGGCCCAGTCAAATTT-AAATGACA---
Bos taurus               GGTCTCCACCGCTTC-------------------------------T----TCACAGATGTGCCTCCTGTCT---TTTTCTTTGCTTTTCAAATGACCCATTCAAGTTCAGATGCTAGGA
Sus scrofa               AGTCTCCACCGGCTC-------------------------------A----CCACAGATGTGCCTCCTCGAT---TTTTCTTTG-TCCTCAAATCACCCATTCAAGTTCAGATGCTATGA
Canis lupus familiaris   AGACTCCACCA-CTC-------------------------------T----CCATGGATGGGCTTCCTG--T---TTTTCTTTT-TCTTCAAATGACCCATTCAAGCCTAAATGCTATGA
Equus caballus           AGTCTCTGCCC-CCC-------------------------------T----CCACAGGTGTGCTTCCTCTCT---TCTCCTTTT-TCCTCAAATGACCCATTCAAGTCCAAATGCTAAGA

Homo sapiens             AAGGCTGGGTGTGGTGATTCAAGTGTGTAATTCAGCACTGTGAGAGGCTGTGGCGGGAGGATTGCTTGAGCCCAGGAGTTTGAGACCAGGCTGAACAACGTAAGGAGACTGCGTCTCTAC
Pan troglodytes          AAGGCTGGGTGTGGTGGTTCAAGTGTGTAATTCAGCACTGTGAGAGGCTGTGGCGGGAGGATTGCTTGAGCCCAGGAGTTTGAGACCAGGCTGAACAACGTAAGGAGACTGCGTCTCTAC
Gorilla gorilla          AAGGCTGGGTGTGGTGGTTCAAGTGTGTAACTCAGCACTGTGAGAGGCTGTGGCAGGAGGATTGCTTGAGCCCAGGAGTTTGAGACCAGGCTGAACAACGTAAGGAGACTGCGTCTCTAC
Pongo abelii             AAGGCTGGGTGTGGTGGTTCAAGTGTGTAATTCAGCACTGCGAGAGGCTGTGGTGGGAGGATTGCTTGAGCCCAGGAGTTTGAGACCAGGCTGAACAACGTAAGGAGACTCCATCTCTAC
Macaca mulatta           NNNNNNNNNNGTGGTGGTTGATGTGTATAATTC---ACTGTGAGAGGCTGTGGTGGGATGATTGCTTGAACCCAGGAGTTTGAGACCAGGCTGGACAACGTAGGGAGACTCCATCTCTAT
Callithrix jacchus       GAGGCTGGGCATTGTGTTCCATGCGTGTAGTTCAGTACTGTGGGTGGCTGTAGCAGGAGGACTGCTTGAGCCCAGGAGTTTGAGACCAGGCTGAACAACACAGGGAGACTCTGTCTCCAC
Mus musculus             ------------------------------------------------------------------------------------------------------------------------
Rattus norvegicus        ------------------------------------------------------------------------------------------------------------------------
Bos taurus               AG----------------------------------------------------------------------------------------------------------------------
Sus scrofa               AG----------------------------------------------------------------------------------------------------------------------
Canis lupus familiaris   AG----------------------------------------------------------------------------------------------------------------------
Equus caballus           AG----------------------------------------------------------------------------------------------------------------------

Homo sapiens             ACAAAATTTAAAAAACTAGCCAGGCATGGTGGCATGTGCCTGTGGTCCCAGGTACTCAGGAGGTCGAGGTGGGAGGATTGCTTGAGCCCAGGAGAGATCAAGGCTACTACAGTGAGCCGT
Pan troglodytes          ACAAAATTAAAAAAACTAGCCAGGCATGGTGGCATGTGCCTGTGGTCCCAGGTACTCAGGAGGTCGAGGTGGGAGGATTGCTTGAGCCCAGGAGAGATCAAGGCT---ACAGTGAGCCGT
Gorilla gorilla          ACAAAATTAAAAAAACTAGACAGGCATGGTGGCATGTGCCTGTGGTCCCAGGTACTCAGGAGGTCGAGGTGGGAGGATTGCTTGAGCCCAGGAGAGATCAAGGCT---ACAGTGAGCCGT
Pongo abelii             ACAAAATTAA-AAAACTAGCCAGGCATGGTGGCATGTGCCTGTGGTCCCAGGTACTCAGGAGGTCAAGGTGGGAGGATTGCTTGAGCCCAGGAGAGATCAAGGCT---ACAGTGAGCCGT
Macaca mulatta           GCAAAATTTA-AAAACTAGCCAGGCATGGTGGCATGTGCCTGTGGTCCCAGGTAGTCAGGAGGTTGAGGTGGAAGGATTGCTTGAGCCCAGGAGAGGTCAAGGCT---ACAGTGAGCCGT
Callithrix jacchus       CAAAAATTAA-AATACTAGCCAAGAATGGTGGCATGTGCCTTTGGTCCCAGATACTTAGCAGGTTGAGGTTGGAAGATTGCTTTAGCCCAGGAGAGGTCAAGGCT---ACAGTGAGCCAT
Mus musculus             ------------------------------------------------------------------------------------------------------------------------
Rattus norvegicus        ------------------------------------------------------------------------------------------------------------------------
Bos taurus               ------------------------------------------------------------------------------------------------------------------------
Sus scrofa               ------------------------------------------------------------------------------------------------------------------------
Canis lupus familiaris   ------------------------------------------------------------------------------------------------------------------------
Equus caballus           ------------------------------------------------------------------------------------------------------------------------

Homo sapiens             GTTTACACCACTGCATTCCAGCCTCGGCAACAGAGTGAGACCCTGTCTCAAAAAACAGAAACAGAAACAAATGCTTGGAG--AGTGTTGTGTTAGTAACTTATTGTCTT-AGTCTATTTT
Pan troglodytes          GTTTACACCACTGCATTCCAGCCTCTGCAACAGAGTGAGACCCTGTCTCAAAAAACAGAAACAGAAACAAATGCTTGGAG--AGTGTTGTGTTAGTAACTTATTGTCTT-AGTCTATTTT
Gorilla gorilla          GTTTACACCACTGCATTCCAGCCTCGGCAACAGAGTGAGACCCTGTCTCAAAAAACAGAAACAGAAACAAATGCTTGGAG--AGTGTTGTGTTAGTAACTTATTGTCTT-AGTCTATTTT
Pongo abelii             GTTTACACCACTGCATTCCAGCCTCGGCAACAGAGTGAGACCCTGTCTCAAAAAACAGAAACAGAAACAAATGCCTGGAG--AGTGTTGTGTTAGTAACTTATTGTCTT-AGTCTATTTT
Macaca mulatta           GTTTACACAACTGCATTCCAGCCTAGGCAACAGAGTGAGACCCTGTCTCAAAAAACAGAAACAGAAACAAATGCTTGGGG--AGTGTTGTGTTAGTAACTTACTGTCTTAAGTCTATTTT
Callithrix jacchus       GTTTACACCACTGCATTCCAGCCTAGGCAGCAGAGTGAGACCCTGTCTCAAAAAACAAAAACAGAAACAAATGCTAGGAGAAAGAGTTGTGCTAGTAACTTACTGTCTT-AGTCTATTTT
Mus musculus             -----------------------------------------------------------------------------------ATGTCAAGT------TTAAATGTCTT-AGTTACTTTT
Rattus norvegicus        -----------------------------------------------------------------------------------ATGTTAAAT------TTAAACATCTT-AGTTACCTTT
Bos taurus               ------------------------------------------------------------------------------------------------------------------------
Sus scrofa               ------------------------------------------------------------------------------------------------------------------------
Canis lupus familiaris   ------------------------------------------------------------------------------------------------------------------------
Equus caballus           ------------------------------------------------------------------------------------------------------------------------

Homo sapiens             GTGTTGCTATAAAGGAATACCTGAGGCTGGATAATTTATAAAGAAAAGAGGTTTATTTGGG-TCACAGTTCTGCAGGCTGTA----CAA------------GAAGTATGGTGCTGACATC
Pan troglodytes          GTGTTGCTATAAAGGAATACCTGAGGCTGGATAATTTATAAAGAAAAGAGGTTTATTTGGG-TCACAGTTCTGCAGGCTGTA----CAA------------GAAGTATGGTGCTGACAGC
Gorilla gorilla          GTGTTGCTATAAAGGAATACCTGAGGCTGGATAATTTATAAAGAAAAGAGGTTTATTTGGG-TCACAGTTCTGCAGGCTGTA----CAA------------GAAGTATGGTGCTGACATC
Pongo abelii             GTGTTGCTATAAAGGAATACCTGAGGCTGGATAATTTATAAAGAAAAGAGGTTTATTTGGG-TCACAGTTCTGCAGGCTGTA----CAA------------GAAGTATGGTGCTGACATC
Macaca mulatta           GTGTTTCTATAAAGGAATACCTGAGGCTGGATAATTTATAAA-----GAGGCTTATTTGGG-TCACAGTTCGGCAGGCTGTA----CAA------------GAAGTATGGTGCTGGCATC
Callithrix jacchus       GTGTTACTGTAAAGGAATACCTGAGGCTGGGTAATTTATAAAGAAAAGAGGTTTATTTGGC-TCACAGTTCTGCAGGCTGTG----CAA---------------GCATGGTGCTGGCATC
Mus musculus             CTATTTCTCTGGCAGAGCACCATGACCAAGGCAGCTTATAGAAGAAAGCATTTGATGGGGGCTTACCGTCTCAGAGGATGAGTTCATAACCAGTGTGGGCAGGAATATGGC---------
Rattus norvegicus        CTATTTCTTTGCTAAAGCACCATGACCAAGGCAACTTATAGAAGAAAGCGTTTAATGGGGGCTTCCAGTCTCGGAGGGGAAATTCATATCCATGCTGGCCAGGAGTATGGC---------
Bos taurus               ------------------------------------------------------------------------------------------------------------------------
Sus scrofa               ------------------------------------------------------------------------------------------------------------------------
Canis lupus familiaris   ------------------------------------------------------------------------------------------------------------------------
Equus caballus           ------------------------------------------------------------------------------------------------------------------------

Homo sapiens             TGCATCTGGTGACCCCTCAGGAAGCTTCCAGTCATAGCAGAAAGCAAAGAGGGAGCAGATGCGGCA---AGAAAGGGAGCAAGAGTGAGGGGAGGGAGGTGGCAGTCTGTTTTTAACAAT
```

```
Pan troglodytes         TGCATCTGGTGACCCCTCAGGAAGCTTCCAGTCATAGCAGAAAGCAAAGAGGGAGCAGATGTGGCA---AGAAAGGGAGCAAGAGTGAGGGGAGGGAGGTGGCAGTCTGTTTTTAACAAT
Gorilla gorilla         TGCATCTGGTGACCCCTCAGGAAGCTTCCAGTCATAGCAGAAAGCAAAGAGGGAGCAGATGTGGCA---AGAAAGGGAGCAAGAGTGAGGGGAGGGAGG------TCTGTTTTTAACAAT
Pongo abelii            TGTACCTGGTGACCCCTCAGGAAGCTTCCAGTCATAGCAGAAAGCAAAGAGGGAGCAGATGTGGCA---AGAAAGGGAGCAAGAGTGAGGGGAGGGAGGTGGTAGTCTGTTTTTAACAAT
Macaca mulatta          TGCGTCTGGTGACCCCTCAGGAAGCTTCCAGTCATAGCAGAAGGCAAAGGGGGAGCAGATGTGGCA---AGAAAGGGAGCAAGAGTGAGGGGAGGGAGGTGCCAGTCTGTTTTTAACAAT
Callithrix jacchus      TGCATCTGGTGACCCCTCAGGAAGCTTCCAATCATAGCAGAAGGCAAAAGTGGAGCAGATGTGGCA---AGAGAGGGAGCAAGAATGAAGGGAAGGAGGTGCCAGTCTCTTTTTAACAGT
Mus musculus            ------------------------------------------------------------------AGCAGGCAGGGAGGCATGGAG-----------GTGG------------------
Rattus norvegicus       ---------------------------------------------------------------------AGACAGGGAGGTACAGTG-----------CTGG------------------
Bos taurus              ------------------------------------------------------------------------------------------------------------------------
Sus scrofa              ------------------------------------------------------------------------------------------------------------------------
Canis lupus familiaris  ------------------------------------------------------------------------------------------------------------------------
Equus caballus          ------------------------------------------------------------------------------------------------------------------------

Homo sapiens            CAGAT----------------------------CTCGAGGA--AGCTAATAGAACAAGAAC-------------CCACTCATTACCCCAAGAATGGCACCAAGCCATTCATAAGGGATCC
Pan troglodytes         CAGAT----------------------------CTCGAGGA--AGCTAATAGAACAAGAAC-------------CCACTCATTACCCCAAGAATGGCACCAAGCCATTCATAAGGGATCC
Gorilla gorilla         CAGAT----------------------------CTCGAGGA--AGCTAATAGAACAAGAAC-------------CCACTCATTGCCCCAAGAATGGCACCAAGCCGTTCATAAGGGATCC
Pongo abelii            CAGAT----------------------------CTTGAGGA--AGCTAATAGAACAAGAAC-------------CCACTCATTACCCCAAGAATGGCACCAAGCCATTCATAAGGGATCC
Macaca mulatta          CAGAT----------------------------CTCGAGGA--AACTAATAGAACAAGAAC-------------CCACTCATTACTCCAAGAATGGCACCAGGCCATTCATAAGGGATCC
Callithrix jacchus      CAGAT----------------------------CTGGAGGA--AACTAATATAGCAAGAAC-------------CCACTCATTACCCCAAGGATGGCACCAACCCATTCATAAGGGGTCC
Mus musculus            -----AGCA------GAGAGCTGATATCCGATCCACAA---GCACAGGACAGAGACT----GACAGTGAGAAGGGCA-------------------------------------------
Rattus norvegicus       -----AGCAGTAGCTGAGAGCTTATATCTGATCCACAA---GCATGGGACAGAGATT----GACCATGGGAATGGTA-------------------------------------------
Bos taurus              ------------------------------------------------------------------------------------------------------------------------
Sus scrofa              ------------------------------------------------------------------------------------------------------------------------
Canis lupus familiaris  ------------------------------------------------------------------------------------------------------------------------
Equus caballus          ------------------------------------------------------------------------------------------------------------------------

Homo sapiens            ACTCTCATGACCCAAACACATCCCACCAGGCCCCACCTCCAACACTGGGGATCACATTTCAGCATGAGATTTGGAG----------------GGG---------------ACA---A-AC
Pan troglodytes         ACTCTCATGACCCAAACACATCCCACCAGGCCCCACCTCCAACACTGGGGATCACATTTCCGCATGAGATTTGGAG----------------GGG---------------ACA---A-AC
Gorilla gorilla         ACTCTCATGACCCAAACACATCCCACCAGGCCCCACCTCCAACACTGGGGATCACATTTCAGCATGAGATTTGGAG----------------GGG---------------ACA---A-AC
Pongo abelii            ACTCTCATGACCCAAACACATCCCACCAGGCCCCACCTCCAACACTGGGGATCACATTTCAGCATGAGATTTGGAG----------------GGG---------------ACA---A-AC
Macaca mulatta          ACTCTCATGACACAAACACATCCCACCAGGCCCCACCTCCAACACTGGGGATCACATTTCACCATGAGATTTGGAG----------------GGG---------------ACA---A-AC
Callithrix jacchus      ACGCCCATGACCCAAACACATCCCACCAGGCCCCACCTCCAACGCTGGGGATCACATTTCAGCACGAGATTTGGAG----------------GGG---------------ACA---AAAT
Mus musculus            ----------------------------------------------------------------------------TGGCCTTCTGAAATCTTAAAGCCCACCCCCAGTGGCACACC-TC
Rattus norvegicus       ----------------------------------------------------------------------------TGGACTTTTGAAATCTTAAAGCCCACTGTCAGTGATGCACC-GC
Bos taurus              ----------------------------------------------------------------------------------------------------------------A---A-AG
Sus scrofa              ----------------------------------------------------------------------------------------------------------------A---A-AG
Canis lupus familiaris  ----------------------------------------------------------------------------------------------------------------C---A-TT
Equus caballus          ----------------------------------------------------------------------------------------------------------------A---A-AG

Homo sapiens            CTTCAAACTATTTCACTTACCATTCATAGCAAAAGC-AA---------------AGCCCAA-----------------------------------------------------------
Pan troglodytes         CTTCAAACTCTTTCACTTACCATTCATAGCAAAAGC-AA---------------AGCCCAA-----------------------------------------------------------
Gorilla gorilla         CTTCAAACTATTTCACTTACCATTCACAGCAAAAGC-AA---------------AGCCCAA-----------------------------------------------------------
Pongo abelii            CTTCAAACTATTTCACTTACCATTCATAGCAAAAGC-AA---------------AGCCCAA-----------------------------------------------------------
Macaca mulatta          ATTCAAACTATTTCAC-AACTGTTCATAGCAAAAGC-AA---------------AGCCCAA-----------------------------------------------------------
Callithrix jacchus      ATTCAAACTATTTTACTTACCATTCATAGCAAAAGC-AA---------------AACCCAA-----------------------------------------------------------
Mus musculus            CTCCAAAATCCTTCTCAAACAGTTC----CACCAGCTAA---------------GGACCAAGCATTTGAACACATGAGCTTATGGGGCCCGCTCTCCTTCAAAGCACCACAGGAAGAGCC
Rattus norvegicus       CTCCGAAATCCTTCTCAAACAGTTC----CACAAGCTAA---------------GGACCAAGCATTTGAATACATGAGCTCATGGGGCCCATTCTCATTCAAAGCACCACAGGGGGAGTT
Bos taurus              CATTGTGTTAC-TATCTTAACATTTATAGCAAAAGA-AAA--------------CCCCTAG-----------------------------------------------------------
Sus scrofa              CATCATGTTAC-TAATGAAACATTTATAGCAAGA-A-AGG--------------CCCCTAC-----------------------------------------------------------
Canis lupus familiaris  ---------------TTTAACATTAATCGCAAAAAA-AA-ACAAAAAAAAACAAAAAAAAA-----------------------------------------------------------
Equus caballus          TATCCTGTTAG-TAACTTACCATTCATAGCAAGAGA-AG---------------CATCTAG-----------------------------------------------------------

Homo sapiens            ----------------ATGTGTG---TTTGC-AAAGCCTT-CCAGACACAA---CAAAAGAGAATTAATTCATAATTCCCGATTTAACTTAGTCGGCCCTG--AGATTT---TTTTCC--
Pan troglodytes         ----------------ATGTGTG---TTTGC-AAAGCCTT-CCAGACACAA---CAAAAGAGAATTAATTCATAATTCCCGATTTAACTTAGTCGGCCCTG--AGATTT---TTTTCC--
Gorilla gorilla         ----------------ATGTGTG---TTTGC-AAAGCCTT-CCAGACACAA---CAAAAGAGAATTAATTCATAATTCCCGATTTAACTTAGTCGGCCCTG--AGATTT---TTTTCC--
Pongo abelii            ----------------ATGTGTG---TTTGC-AAAGCCTT-CCAGACACAA---CAAAAGAGAATTAATTCATAATACCCAATTTAACTTAGTCGGCCCTG--AGATTT---TTTTCC--
Macaca mulatta          ----------------ATGTGTG---TTTGC-AAAACCTT-CCAGAGACAA---CAAGAGAGAATTAATTCATAATTCCCAATTTAACTTAGTCGGCCCTG--AGGTTT---TTTTCC--
Callithrix jacchus      ----------------ATGTGTG---TTTGC-AAAGCCTT-CCAGACACAA---CAAAAGAGAATTAATTCATAATTCCAAATTTAACTTAGTCTTACCTG--AGATTT---TTTTCC--
Mus musculus            GGGTTGGTAACTTACCATGCATGGCCCTTGA-AAAGCCTGGCTAGACACAG---CCACAGAAGACTGACTCCTAACTCCAAAGTGTACTTCTTT--TTCTG--AGATTTGAATTTTCT--
Rattus norvegicus       GGATGGGTGACTTTCCATGTGTGGCCCTTGAAAAAGTCTGGCTAGACACAAAACCTGAAGAGGACTGACTTGTAACTCCAAAGTGTACTTCTTT--TTCTG--AGATTTGAATTTTCT--
Bos taurus              ----------------TTGTACA---TTTGC-AAAGCATAACCAGGCATAA---CCAAAGAGAATTAATTCATAACTTCAAGTTTAATTTATTTGGCTCTG--AGTTTT---TA--GTAG
Sus scrofa              ----------------TTGTGCA---TTTTC-AAAACCTGACCAGGCATAA---CCATAGGGAA----TTCATAATTCCAAGTTTAACTTATTTGGTTCTACAAGTTTT---TT--CTA-
Canis lupus familiaris  ----------------CATTGCA---TTTGT-AAAGCCTGCCCAGACATAA---CAAAAGGGAATTGACTTGTAATTCCAGGTTTGATTTATTTGGTCCA-----ATTT---TTTAAAAA
Equus caballus          ----------------TTTTGCT---TTTGC-AAGGCCTGACCAGCCATAA---CTAAAGAGACTTAATTCATAATTTTAAGTTTAATTGATTTGGTCCTG--AGGTTT---TTTA---A

Homo sapiens            -------------------------------------------------------------------------------CTGAATTGTTATACCTAGATTAAACTATTTTTAAACGTTTG
Pan troglodytes         -------------------------------------------------------------------------------CCGAATTGTTATACCTAGATTAAACTATTTTTAAACGTTTG
Gorilla gorilla         -------------------------------------------------------------------------------CCGAATTGTTATACCTAGATTAAACTATTTTTAAACATTTG
Pongo abelii            -------------------------------------------------------------------------------CTGAATTGTTATACCTAGATTAAACTATTTTTAAACGTTTG
Macaca mulatta          ------------------------------------------------------------------------------CCCGAACTGTTATACCTAGATTAAACTATTTTTAAACGTTTG
Callithrix jacchus      ------------------------------------------------------------------------------CCTGAATTGTTATACCTAGATTAAACTATTTTTAAATGTTTG
Mus musculus            -------------------------------------------------------------------------------C-----CGTTCTACCCATTTTAA-GTACTCGTAAGTGTTTG
Rattus norvegicus       -------------------------------------------------------------------------------C-----TGCTATACCCATGTTTAGTTAGTCTTAAGTGTTTG
Bos taurus              ATAGTAAAACCTATATTAAGTTGT---ATTTATG-----------------TTAGTATATTAAAGCATATTATGCAAG-TTATATTGCTAAACCCATATTAAGTTGATTTTAAATGTTTG
Sus scrofa              ATTGTTAAACCTATATTAAATTTT---ACCTATA-----------------ATGTTATATTAAAGTGTATTAT--ATG-TTATATT-TTATACCTGTGTTCAGTTGGTTTTAAATGTTTG
Canis lupus familiaris  ATTGTTAAACCTATACTAAATTATTATGCCTATATTACTTTATATTATACTATATTATATTAAAGTGTAGTATGTAAA-TTAAATTGTTATACCTATAT-----TGGTTTTAAATGTTTG
Equus caballus          ATTTTTATACTTCTATTAAGTTGTAATACCTATATTA------------TTATGTCATATTAAAGTATATTATATAAA-TTATATTGTTATGCCTATATTAAATTGTTTTTAAATGTTTG

Homo sapiens            AGTGATTCCTCATTAGTTTT-GTT------------------------------------------------------------------------------------------------
Pan troglodytes         AGTGATTCCTCATTAGTTTT-GTT------------------------------------------------------------------------------------------------
Gorilla gorilla         AGTGATTCCTCATTAGTTTT-GTT------------------------------------------------------------------------------------------------
Pongo abelii            AGTGATTCCTCATTAGTTTT-GTT------------------------------------------------------------------------------------------------
Macaca mulatta          AGTGATTCCTAATTAGTTTT-GTT------------------------------------------------------------------------------------------------
Callithrix jacchus      AGTGATTCCTAATTAGTTTT-GTT------------------------------------------------------------------------------------------------
Mus musculus            AATG-ATTCTGTTTAGTTTT-GTTTGTGTCTTCTTTTGGAGACATGATTTCTCTATATAACCCAGATTGACTTCAAACTTGCAGTCCTCCTGCCTCAACTTCACAAATGCTGGGAGTGCT
Rattus norvegicus       AATGGATTCTGTTTAGTTTT-GTTTGTGTCTTCTTTT-GAGACATGATGTCTCTATGTAGCCCAGATTCACTTCAAACTTGCAATCCTCCTGCCTCAACTTCACAAATGCTGGGAGTGCC
Bos taurus              AATGATT-CTAATTGGATTT--TT------------------------------------------------------------------------------------------------
Sus scrofa              AGTGATTTCTGATTGGATTT-TTT------------------------------------------------------------------------------------------------
Canis lupus familiaris  AGTGATTTCTAACTGGATTTTTTT------------------------------------------------------------------------------------------------
Equus caballus          AGTGATTTCTA-------------------------------------------------------------------------------------------------------------

Homo sapiens            ------------------------------------------------------------------------------------------------------------------------
Pan troglodytes         ------------------------------------------------------------------------------------------------------------------------
Gorilla gorilla         ------------------------------------------------------------------------------------------------------------------------
Pongo abelii            ------------------------------------------------------------------------------------------------------------------------
Macaca mulatta          ------------------------------------------------------------------------------------------------------------------------
Callithrix jacchus      ------------------------------------------------------------------------------------------------------------------------
Mus musculus            ACCAAATACAATTCAGTTGGATTTGTTTTTTAAAGATGGTTCTCATGTAGCCCAGGCTGGCCTCCCACTCCCTATGAGGTTAAAAAGGCTCTTAAACCCCTGATTCTCCTGTCTCCAGCT
Rattus norvegicus       ACCAAACACAATTCGGTTGGATTTGTTT-TTAAAGATGGTTCTCATGTAGCCCACACTGGC-TCACACTCCCTGT--GGCTAAGAATGATCTGAAACTCCTGATCCTCCTGTCTCCAGCT
Bos taurus              ------------------------------------------------------------------------------------------------------------------------
Sus scrofa              ------------------------------------------------------------------------------------------------------------------------
Canis lupus familiaris  ------------------------------------------------------------------------------------------------------------------------
Equus caballus          ------------------------------------------------------------------------------------------------------------------------

Homo sapiens            ------------------------------------------------------------------------------------------------------------------------
Pan troglodytes         ------------------------------------------------------------------------------------------------------------------------
Gorilla gorilla         ------------------------------------------------------------------------------------------------------------------------
Pongo abelii            ------------------------------------------------------------------------------------------------------------------------
Macaca mulatta          ------------------------------------------------------------------------------------------------------------------------
Callithrix jacchus      ------------------------------------------------------------------------------------------------------------------------
Mus musculus            TTCAGGGGCTGGTGTTACAGGGAGGGAGCACCA-----TACCCAGTTTATACGGTACTGGAAATTGAATGCCAGGCTTTCTAAAGGCTTCTTCTAGGCACGCATTCTGCAAAGTGAGGTG
Rattus norvegicus       TTCCAGTGTGGGGATTACAGGGATGTATTACCAATGATTGCTCAATTTGTATGGTGCTG-AAATTGGACTCTAGGCTGCCTAAAGGCTTCTTCCAGGCAAGCACTCTGCTAACTGAGGAG
Bos taurus              ------------------------------------------------------------------------------------------------------------------------
Sus scrofa              ------------------------------------------------------------------------------------------------------------------------
Canis lupus familiaris  ------------------------------------------------------------------------------------------------------------------------
Equus caballus          ------------------------------------------------------------------------------------------------------------------------

Homo sapiens            --------------------------AATCAAAGAGGTAAAGCAACTTAATATTGAGTAGTA------------TTGTGAAGGAT-----------------------------------
Pan troglodytes         --------------------------AATCAAAGAGGTAAAGCAACTTAATATTGAGTAGTA------------TTGTGAAGGAT-----------------------------------
Gorilla gorilla         --------------------------AATCAAAGAGGTAAAGCAACTTAATATTGAGTAGTA------------TTGTGAAGGAT-----------------------------------
Pongo abelii            --------------------------AATCAAAGAGGTAAAGCAACTTAATATTGAGTAGTA------------TTGTGAAGGAT-----------------------------------
Macaca mulatta          --------------------------AATCAAAGAGGTAAAGCAACTTAATACTGAGTAGTA------------TTGTGAAAGAT-----------------------------------
Callithrix jacchus      --------------------------CAACAAAGAAGTAAAGCAACTTCATATTGAGTAGCA------------TTGTGAAGGAC-----------------------------------
Mus musculus            CGTTCCCCGAACTACCTGGGCTTTTTCTTCTAAGAAGTATGGTGACTCTGTCCTGTTTAGGATGCTTTATTAAATTCTG-AGGAT-----------------------------------
```

```
Rattus norvegicus          CATCCCCTGAACAA------CTCTTTCTTCAAAGAAGTGTAGTGACACTGTCCTGTTTAGGTTGCTCCTTTAAATTCAG-AGGAC-----------------------------------
Bos taurus                 --------------------------AATCAAAGGAATGAACTAACTCAATATTGTCTAGTAGCATTAATTAGATTATGAAGGATTTGGGGGATTTTTTTTTTTTTGGCTTCATGGCAAT
Sus scrofa                 --------------------------AATCAAAGAAATGAAGCAACTCACTATTGTTTAGTAGATTTAATTAAATTGTGAAGGAT-----------------------------------
Canis lupus familiaris     --------------------------AATTAAAGAAATGCAGCAACTTGATATCGATTCGTAGATTTAACTAAGTTGTAAAAGAC-----------------------------------
Equus caballus             --------------------------AATTAAAGAAATGAAGCAACTCAATACTGTTTAGTAGATTTAATTAAATTTTGAAGGAT-----------------------------------

Homo sapiens               ------------------------------------------------------------------TATCATAT--------------------------TTTTGAATAAGTGCATTAAC
Pan troglodytes            ------------------------------------------------------------------TATCATAT--------------------------TTTTGAATGAGTGCATTAAC
Gorilla gorilla            ------------------------------------------------------------------TATCATAT--------------------------TTTTGAATAAGTGCATTAAC
Pongo abelii               ------------------------------------------------------------------TATCATAT--------------------------TTTTGAATAAGTGCATTAAC
Macaca mulatta             ------------------------------------------------------------------TGTCCTAT--------------------------TTTTGAATAAGCGCATTAAC
Callithrix jacchus         ------------------------------------------------------------------TGTCATAT--------------------------TTTTGAATAAGTGCATTAAT
Mus musculus               ------------------------------------------------------------------TGTCATTCCTTTCTTTCTTTACTAAACTGTTACGTTTTAATAAGTGCATTAAT
Rattus norvegicus          ------------------------------------------------------------------GGTCATTTCTTTCATCCTTTACTGAACTGTTACGTCTTTAATAAGGGCATTAAT
Bos taurus                 GCTTGTGGGCCTGGCAGTGAAAGCACCAAGTCTCAACCACTGGACCGCAAGAAAATTTCCAAGAATTCCTGTAT--------------------------TTTTCAATAAGTGTATTAAC
Sus scrofa                 ------------------------------------------------------------------TGCCATGT--------------------------TTTGGAAAAAGTGTATTAAC
Canis lupus familiaris     ------------------------------------------------------------------TGTCATTT--------------------------TTTTTAATAAGTGCATTAAC
Equus caballus             ------------------------------------------------------------------TGTCATAT--------------------------TTTTGAATAAGTGCCTTAAC

Homo sapiens               GGTCTTCCT-AC------------------------------------------------------------------------------------------------------------
Pan troglodytes            GGTCTTCCT-AC------------------------------------------------------------------------------------------------------------
Gorilla gorilla            GGTCTTCCT-AC------------------------------------------------------------------------------------------------------------
Pongo abelii               GGTCTTCCT-AC------------------------------------------------------------------------------------------------------------
Macaca mulatta             AGTCTTCCT-GC------------------------------------------------------------------------------------------------------------
Callithrix jacchus         GGTCTTCTT-AG------------------------------------------------------------------------------------------------------------
Mus musculus               A--TTTGCT-AC------------------------------------------------------------------------------------------------------------
Rattus norvegicus          C--TTTGTT-AC------------------------------------------------------------------------------------------------------------
Bos taurus                 AGTGTTCAC-CCTGGGTATTGTCATTGTTGAGTCTATAAGTCATGTCTGCTTCCCCATGGACTGTAACCTGCCAGGGTCCTTTGTCCACAGGATTTCCCAGGCAAGAATACTGGAGTGAG
Sus scrofa                 AGTCTTCAC-TT------------------------------------------------------------------------------------------------------------
Canis lupus familiaris     AGTCCTCATCAC------------------------------------------------------------------------------------------------------------
Equus caballus             AGTCTTCATCAT------------------------------------------------------------------------------------------------------------

Homo sapiens               -------------------------TGAATATTAATGAAGCATAAATACCTGTTCT-TATTGTTTATGTGTAACAGAGCAGCTTGGCTTGGCTGGAGCTAAG----AGCCAGACACACCA
Pan troglodytes            -------------------------TGAATATTAATGAAGCATAAATACCTGTTCT-TATTGTTTATGTGTAACAGAGCAGCTTGGCTTGGCTGGAGCTAAG----AGCCAGACACACCA
Gorilla gorilla            -------------------------TGAATATTAATGAAGCATAAATACCTGTTCT-TATTGTTTATGTGTAACAGAGCAGCTTGGCTTGGCTGGAGCTAAG----AGCCAGACACACCA
Pongo abelii               -------------------------TGAATATTAATGAAGCATAAATACCTGTTCT-TATTGTTTATGTGTAACAGGGCAGCTTGGCTTGGCTGGAGCTAAG----AGCCAGACACACCA
Macaca mulatta             -------------------------TGAATATTAATGAAGCATAAATACCTGTT-T-TCTTATTTATGTGTAACAGGGCAGCTTGGCTTGGCTGGAGCTAAG----AGCCAGACACACCA
Callithrix jacchus         -------------------------TGAATATTAATGATGCATAAGTACCTATTCT-TATTATGTATGTGTAACAGGGCAGCTTGGCTTGGCTGAAGCTAAG----ACTCAGATACATCA
Mus musculus               -------------------------TAGGTTTTA----GACCTAAAAAGATGCTAT-TCATGTTTGTGTGTAACAGGGCAGCTTGACTCGACTGAAATTGAG----ACACATACATCCTA
Rattus norvegicus          -------------------------TAGATTTTA----GGCCTAAAAACATGTTAT-TCATGTTT--GTATAACAGGACAGCTTGACTTGGCTGAAGGTAAG----ACACATGTATACTA
Bos taurus                 TTGCCATTTCCTCCTCCAGGGACATTGGATATTATC--GTCATAAAAATACACTATTTACTATATTTAT---ACAGGGCAGCTTGGCTTGGCTGGAGCAGAG--ACACCCAGACACACCA
Sus scrofa                 -------------------------TGGATATCATTAGGTCATAAAATTACACTAC-AATTATATTTAT---ACAGGACAGCGTGGCTTGGCTGGCGCTGAGAAACACCCAGACATGCCG
Canis lupus familiaris     -------------------------TGGATATTATCAAGCCATAAATATATACTCT-TATTATCTATGTGTAACAGGGCAGTTTGGCTTGGCTGGAGCTAAGAAAGACCCAGACACACCA
Equus caballus             -------------------------TGGGTATTGTCAAGCCATAAAAATACACTCT-TATTATTTATGTGTAACAGGGAAGCTTGGCTTGGCTGAAGCTAA----GACCCAGACACACCA

Homo sapiens               CTGTGTGGAGGTGGGTGATGTCTTCCTGTGCTAAAAGGTGA----ATAAATAAGCTCCTCACCTCTCGCGGAACACTCGGGAACACATCAACAGGTATTTAGCCA-C-ATCTCCTGATTT
Pan troglodytes            CTGTGTGGAGGTGGGTGATGTCTTCCTGTGCTAAAAGGTGA----ATAAATAAGCTCCTCACCTCTCGCGGAACACTCAGGAACACATCAACAGGTATTTAGCCA-C-ATCTCCTGATTT
Gorilla gorilla            CTGTGTGGAGGTGGGTGATGTCTTCCTGTGCTACAAGGTGA----ATAAATAAGCTCCTCACCTCTCGCGGAACACTCGGGAACACATCAACAGGTATTTAGCCA-C-ATCTCCTGATTT
Pongo abelii               CTGTGTGGAGGTGGGTGACGTCTTCCCGTGCTAAAAGGTGA----ATAAATAAGCTCCTCACCTCTCGCGGAACACTCGAGAACACATCAACAGGTATTTAGCCA-C-ATCTCCTGATTT
Macaca mulatta             TTGTGTGGAGGTGGGTGACGTCTTCCTGTGCTAAAAGGTGAATGAATAAATAAGCTCCTCACCTCTTGCGGAACACTTGGGAACACATAAACAGGTATTTAGCCC-C-ATCTCCTGATTT
Callithrix jacchus         TTGTGTGGAGGTGGGTGACGTCTTCCTGTGCTAGAAGGTGA----AAAAATAAACTCCTCAACTCTTGCAGAACACCCGGGAACACATAAACAGGTATTTAGCCA-C-GTCTCCTGATTT
Mus musculus               TTGTATAGAGATGTGTGACATCTTCCTGATCTGGAAGCTGA----GTACTT-GGCTCCTTGGTTCTTGAAAAACATTCTGGAACACATGAACAGGTATTTAACTA-C-ATCTCCTAATTC
Rattus norvegicus          CTGTATAGAGGTGTGTGACATCTTCCTGATCTGGAAGCTGA----GTACGT-GGCTCCTTGACTCTTGAAGAACATTCTGGAACACATAAACAGGTATTTTACTA-C-ATCTGCTAATTC
Bos taurus                 TTGTGCTGAGT-GGGTGACATCTTCTTGTACTAGAAGCTGA----ATACATAAGCGCCTTGGCTGTCACAGAACATTCTGGAACA----AACAGGTACCTAACAAACAGCCTCCCGATTT
Sus scrofa                 TTGTGTGGCATGGAGTGACATCT---TGTGCTAGAAGCTGA----ATACATAAGCTCCTTGGCTGTCATGGGATGATTTGAAACACATAAACAGGTATCTGGCCA-C-ATCTCCTGATGT
Canis lupus familiaris     TTGTGTGGAGTTGGGTGACATCTTCCTTTTCTAGAAGCTGA----ATAAATAAGCTCA-TGACTCTCACGGAACAAT-TGGATCGCGT-AACAGGTATCTAGCCA-C-A---CCTGATTT
Equus caballus             TTGGGCAGAGTTGGGTGACATCATCCTGTGCTAGAAGCTGA----ATAAATAAGCTCCTTGACTCTTATGGAACATTCTGGAATGCGTAAACAGGTATCTAGCCA-C-ATTTCCTGATTT

Homo sapiens               CATCTTTC----A-ATGGCAAAGACCTTGAATCCTGTCCTTAACTGGGGCTCAGACATTAATCAACAGGTCTGATGAGCATATGGTGGGGACACCAGATTTCATTGGAGTCCCCAAGAGA
Pan troglodytes            CATCTTTC----A-ATGGCAAAGACCTTGAATCCTGTCCTTAACTGGAGCTCAGACATTAATCAACAGGTCTGATGAGCATATGGTGGGGACACCAGATTTCACTGGAGTCCTCGAGAGA
Gorilla gorilla            CATCTTTC----A-ATGGCAAAGACCTTGAATCCTGTCCTTAACTGGGGCTCAGACATTAATCAACAGGTTTGATGAGCATATGGTGGGGACACCAGATTTCATTGGAGTCCTCTAGAGA
Pongo abelii               CATCTTTC----A-ATGGCAAAGACCTTGAATCCTGTCCTTAAATGGGGCTCAGACATTAATCAACAGGTCTGATGAGCATATGGTGGGAACACCAGATTTCATTGGAGTCCTCAAGAGA
Macaca mulatta             CATCTTTC----ACATGGCAAAGACCTTGAATCCTGTCCTTAACTGGGGCTCAGACATTAATCAACAGGTCTGATGAGCATATGGTGGGAACGCCAGATTTCACTGGAGTCCTCAGGAGA
Callithrix jacchus         CATCTTTC----ACATGGCAAAGACCTTGAATCCTGTCCTTAACTGGGGCTCAGACATTAATCAACAGGTCTGACAAGGTTTTAGTGGGGGCGCCAAGTTTCACTGGAGTCCTAGAGAGG
Mus musculus               CATCTTTT---GA-ATGGTGAAGGTCTTGAATCCTGCTCACTGGTAGGGCTT---------------------------TCAGGGTGGAAAGACTGGATTTTAGCTG-GTTCTCACCGG-
Rattus norvegicus          ATCCTTTTGATGA-AGGGTGAAGGTCTTGAATCCTGCTCACTGACGGGGTTT---------------------------TCAGGGTGGAAAGACTGGATTTTATCTG-GTCCACACAGG-
Bos taurus                 CACTTTTC----ATGTGAGAAAGATCTTGAGTCCTGTCCATCACTGGAGCTC---CATTAACTGGCATGCCTGATGAGTATATGGTGGGGAAGCCCAATTGCAGGAGGGTCCCCAAGAGA
Sus scrofa                 CATTCTGC----ATGTGGGAAAGATCT----TCCTGTCCACAACTAGGGCTC---CATTAATCTGCATGCCTGGTGAGCATAGGGTGGGAAAGCCAGCTTGCAGGAAAGTCCCCAAGAGA
Canis lupus familiaris     TGCTCTTA----ACATGGGGAACATCTTGAGTCCTGTCCACAATTGGGGCTCAGACATTAATTGGCATGTCTAATGAGCAAATGGTGGGGAAGCCAGATTGCAGGAGGGTCCCCAAGAGA
Equus caballus             CATTTTTC----ATGTGGGAAAGATCTTGAGTCTTGTCCACAACTGGGGCTCAGACATTAATCAGCATGTCTGATGAGCGTGTGGTGGGGAAGCCAGCTTGCAGGAGGGTCCCCAAGAGA

Homo sapiens               A--------AAAA---AAGTCACAGTGAGCAAAGA-AGGGGTCT-GAC-AGAGTGGGACAAATGCAAGCGCAGTGTAAAGCAGAACCGCC-----ATCTGCCCC--CAGTTCCATTCCAG
Pan troglodytes            A--------A-AA---AAGTCACAGTGAGCAAAGA-AGGGGTCT-GAC-AGAGTGGGACAAATGCAAGCGCAGTGTAAAGCAGAACCGCC-----ATCTGCCCC--CAGTTCCATTCCAG
Gorilla gorilla            A--------A-AA---AAGTCACAGTGAGCAAAGA-AGGGGTCT-GAC-AGAGTGGGACAAATGCAAGCATAGTGTAAAGCAGAACCGCC-----ATCTGCCCC--CAGTTCCATTCCAG
Pongo abelii               A--------A-AA---AAGTCACAGTGAGCAAAGA-AGGGGTCT-GAC-AGAGTGGGACAAATGCAAGCACAGTGTAAAGTAGAACCACC-----ATCTGCCCC--CAGTTCCATTCCAG
Macaca mulatta             A--------A-AA---AAGTCACAGTAAGCAAACA-AGGGGTGT-GACAAGAGTGGGATGAATGCAAGCACAGTGTGAAGCAGAACCCCT-----ATCTGCCCC--CAGTTCCATTCCAG
Callithrix jacchus         A--------A-AA---GAGTCACAGTAAGCAAAGA-AGGGGTGT-GAC-AGAGTGGGGCAGATGCAAGCACACTGTGAAACAGAACCTTC-----ATCTTCCCC--CAGCTCCATTGCAA
Mus musculus               G--------G-AA---AAG-GACAGTTGGCAAAGAAAAGGGATTCACC-AGAGTGGGGTAGACACAGGCACAGTGGGAGTCAGAGCCCTT----GGTTCACCCC--TAGTCCCAC-----
Rattus norvegicus          G--------A-AA---AAG-TACAGTCGGCAAAAA-AGGGGATTCACC-AGAGTGGGGTAGGCACAAGCACAGTGGGAGTTAGAGCCCTT----GGTCCACTCC--TAGTTTCACCCCAC
Bos taurus                 AT-------A-AA---AAGTCACTGTAAGCAAAGA-AGAGCGAT-G---------------ATTCAACTATAATATAAGGCAGTCTCCCT----GTTCCCCCCTTCCGCTTCCATCCCTC
Sus scrofa                 ACAACAACAA-AA---AAATCACTATAAGCAAAGA-AGAGGTCT-G---------------ACTCAAATACAGTGTGGGGCAGAATCCCT----GGTTTACCCT--CACTTCTGTCCCTA
Canis lupus familiaris     A--------A-AA---AAGTCACCCTATGCAAAGA-AGAGGTCT-AAC---AGTGGGGTAGGTGCAAATCCAATGTGAGATGGAA---------------CCCC--TACTTCCATCCTGA
Equus caballus             A--------A-AAACAATGTCACTATAAGCAAAG----AGATCT-GAT-GGAGTGGGATAGACACAAATATAATGTGAGGCAAATACAATGTGGGGTCTACACC--CGTGTCCATCCACA

Homo sapiens               ATTAGAGTAT------------------------------------GGATGCTTCCAAGGAAAGAGAGTGCTGTGTAGTAAAACTGAACAT-TGT-TTCACCAGGGGTCCAAGCCGCCCT
Pan troglodytes            ATTAGAGTAT------------------------------------GGATGCTTCCAAGGAAAGAGAGTGCTGTGTAGTAAAACTGAACAT-TGT-TTCACCAGGGGTCCAAGCCGCCCT
Gorilla gorilla            ATTAGAGTAT------------------------------------GGATGCTTCCAAGGAAAGAGAGTGCTGTGTAGTAAAACTGAACAT-TGTTTTCACCAGGGGTCCAAGCCGCCCT
Pongo abelii               ACTAGAGTGT------------------------------------GGATGCTTCCAGGGAAAGAGAGTGCTGTATAGTAAAACTGAACAT-TGT-TTCACCAGGGGTCCAAGCCGCCCT
Macaca mulatta             ACTAGAGTAT------------------------------------GGATGCTTCCAGGGAAAGAGAGCACTATGTAGTAAAACTGAACAT-TGT-TTCACCAGGGTTCCAAGCCACCCT
Callithrix jacchus         ACTAGAGCAT------------------------------------GGATGCTTCCTGGGAAAGAGAGTGCTGCGTGGTAAAACTGAACCT-TGT-TTCACTAGGGGTCCAAGGCGCCCT
Mus musculus               -CTAGTGTGTGTGTGTGTGTGTGTGTGTGTGTGTGTGTGTGTGTGTGTGTCCCCTGGGAAGTAGAGGGCTGTGTAGTCAAAATGAGCCTCTGT-TTCCCTAGGAGTCCTTGCCATCTC
Rattus norvegicus          ACTAGAGTGT------------------------------------GGGTGTACCCTGGGACATAGAGGGCTATGTA--TAAAGTGAGTCTCTGT-TTCCCTAGGAGTCCTTGCT-TCCT
Bos taurus                 ACCAGGT-AC------------------------------------CCATGCTTCCCAGAAATGAGAGAGCTGTGTGGTAAAACTGAGCCT-TGT-TTCCCTAGGAGCCCAAGCCACTCT
Sus scrofa                 ACCAGGT-AC------------------------------------AGGTGCTTCCTGGGAAGGAGAAAGCTGTGTGGTAAAACTGGCCAT-TGT-TTCCCCAAGAGCCCAAGCCACGCT
Canis lupus familiaris     ACCAAAG-CA------------------------------------CGATGCTCCCCCAGAAGGAAAGGGCTATGTGGTAAAACTGGCCAT-TGT-TTCACTAGGAGTCCAAGCACCCCT
Equus caballus             AGTGGGG-TA------------------------------------CGATGCTCCCTGGGAAGGAGAGG--TGTGTGGTAAAACTGACCGT-TCT-TTCACTAGGAGTCCAGGGCACTCT

Homo sapiens               GCTGGGAGGCTTCTCTTCAAGAGTTCTGGGTCCCAGAGTGGAAGGCATTTT-CCCATCAACTGGAGA---------G-----AGACGAAACATCAGAGACC-AGGAGGCTGTGGAGAAAG
Pan troglodytes            GCTGGGAGGCTTCTCTTCAAGAGTTCTGGGTGCCAGAGTGGAAGGCATTTT-CCCATCAACTGGAGA---------G-----AGACGAAACATCAGAGACC-AGGAGGCTGTGGAGAAAG
Gorilla gorilla            GCTGGGAGGCTTCTCTTCAAGAGTTCTGGGTGCCAGAGTGGAAGGCATTTT-CCCATCAACTGGAGA---------G-----AGACGAAACATCAGAGACC-AGGAGGCTGAGGAGAAAG
Pongo abelii               GCTGGGAGGCTTTTCTTCAAGAGTTCTGGGTGCCAGAGTGGAAGGCATTTT-CCCATCAACTGGAGA---------G-----AGACGAAACATCTGAGACC-AGGAGGCTGTGGAGAAAG
Macaca mulatta             GCTGGGAGGCTTCTCTCCAAGAATTCTGGGTACCAGAGTGGAAGGCATTTT-CCCACCAACTGGAGA---------G-----AGACGAAACATCGGAGACC-AGGAGGCTGTGGAGAAAG
Callithrix jacchus         GCTGGGAGACTTCTCTCCAAGAGTTCTGGGTGCCATAGTGGAAGGCATTTT-CTCACCAACTGGAGA---------G-----AGGCGAAACATCAGAGACC-AGGAGGCTGTGGAGAAAG
Mus musculus               ACTGTGTGACTCCCC----AGAGT--TGGGTGCTAGAGTGGAAGCCACCTCCCCCATTGACTAGAGA---------G-----AGGCGAAGTGACAGAAACC-AGGAGATGGTGGAAAAG
Rattus norvegicus          GCTGTGTGAGTCCCC----AGCGT--CGGGTGCTAGAGTGGACACCACCTA-CCCACTGATTAGAGAGA-------G-----AGGTGAGGTGACAGAAACC-AGGAGACTG---------
Bos taurus                 GCTGGGAAACTTCCCTCCAAGAGTTCTGGCTGCCAGAGTGGA-GGCATCTC-CCCACTGGCTAGAGA---------G-----AGGTGAGGAGGAAGAGACC-CAGAGA--GGGGTGAAAG
Sus scrofa                 GCTGGGAGACTTCCCTCCAGGAGTTCTGGGTGCCTGAGTGGA-GGCGTCTC-CCCACCTGCTAGA-----------G-----TGGTGAGGTGCCAGAGCCC-CAGAGC--ACTCTGAAAG
Canis lupus familiaris     GCGGTGACACTTCTCTCCAGGAGTTCTGGGTGCCAGAGTGGA-GGCCTCT--CCCAGTGACTAGTGC--GAGAGGAGAGGTGAAGCGAGGCAGCCAAGACTTGGGAGA--GTGGCAAAAG
Equus caballus             GCAGTGAGACGTCTCTCCAGGACTTCTGAGAGCTGGAGTGGA-GGTGTTTC-CCCATTGACTAGAGA---------G-----AAGTGAAGCAGCACAGACG-GGGAAA--GTGGTGAAAA

Homo sapiens               CAGCTGTCCCAGGTGCCTCA-ACTATCAGAGAAGGGTCAGCGT---CACGTGGCTGCCAGCA---TCTTTGAGAAAATCACTGGCAATCGGA---CTTC------AGAGCTGCGGGCACA
Pan troglodytes            CAGCTGTCCCAGGTGCCTCA-ACTATCAGAGGAGGGTCAGCGT---CACGTGGCTGCCAGCA---TCTTTGAGAAAATCACTGGCAATCGGA---CTTC------AGAGCTGCGGGCACA
Gorilla gorilla            CAGCTGTCCCAGGTGCCTCA-ACTATCAGAGAAGGGTCAGCGC---CACGTGGCTGCCAGCA---TCTTTGAGAAAATCATTGGCAACCGGA---CTTC------AGAGCTGTGGGCACA
Pongo abelii               CAGCTGTCCCAGGTGCCTCA-ACTATCAGAGAAGGGTCAGCGC---CACGTGGCTGCCAGCA---TCTTTGAGAAAATCATTGGCAACCGGA---CTTC------AGAGCTGCAGGCAGA
Macaca mulatta             CAGTTGACCCAGGTGCCTCA-ACTATCCAGAAGGGTCAGCGC---CACGTGGCTGCCAGCA---TCTTTGAGAAAATCATCCGCAACCAAA---CTTC------AGAGCTGCGGACAGA
Callithrix jacchus         CAGCTGTCCCAAGTGCCTCA-ACTGTCAGAGAAGGGTC-GTGC---CGCCTGGCTGCCAGCA---TTTTTGAGAAGATCATCTGCAACCAGA---CTTC------AGAGCTGCCGGCAGA
Mus musculus               CGATTGTTCCAGGTGCCTCC--CGAT--GACAAGAGTCAGCAC---CACGCAGTTACAACAACCAGCCCTCGGAA--GCAGCAGCGACCCCA---CTGC------AGAGCAACAGCCC--
Rattus norvegicus          ---TTGTTCCAGGTGCCTCA--CAGT--GACCAGAGTCAACAC---CACACAGCTACAACAA---ACCCTCGGAA--ACAGCAGCAAACGTA---CTGC------AGAGCAACAGCCC--
Bos taurus                 GAACTAC--CACGTGTTTCCGG----------TGGGGCAGGTCC-CCACCCGGCCTC----------CT---------------CAACGAGA---CTGC------AGAGCCTCTGGCCAT
Sus scrofa                 CAGCTGCTTCTCACTTTTCCAG----------TGGGGCAGTCCCACCACGTGGCTTC----------AG---------------GAGGGAACCGGCTCTGGAGACACAGGCGGGGGCTGG
Canis lupus familiaris     CAGCCCTTGCAGGTGCCTCGGGCAGTCGGAGGAGGGTCAACACCACCACGTGGCTGCTGGCA---TCAG-GAGAGCCACAGCAGCCACTGGA---CCGC------AGAGCAACAGGCAGT
Equus caballus             CAGCTCTCGAAGGTGCCTTGGGCGGTCAGAGAAGGGTCAGCACCACCAAGTGGCTGCTAGCA---TTGGCGAGTGAGACAGCAGCCACCAGA---CTGC------AGAGCCACTGACACT
```

```
Homo sapiens              GGTGTGGTTAGAACTGAGATA-----------CGACCTGCCCACC-TGGGTCAGGCCTAAAGA------CAAGAAGTCCTGAGTTCTTGCCACTGAGTA---GG-CCAGGGTCATTTGTC
Pan troglodytes           GGTGTGGTTAGAACTGAGATA-----------CGACCTGCCCACC-TGGGTCAGGCCTAAAGA------CAAGAAGTCCTGAGTTCTTGCCACTGAGTA---GG-CCAGGGTCATTTGTC
Gorilla gorilla           GGTGTGGTTAGAACTGAGATA-----------CGACCTGCCCACC-TGGGTCAGGCCTAAAGA------CAAGAAGTCCTGAGTTCTTGCCACTGAGTA---GG-CCAGGGTCATTTGTC
Pongo abelii              GGTGTGGTTAGAACTGAGATA-----------CGACCTGCCCACC-TGGGTCAGGCCTAAAGA------CAAGAAGTCCTGAGTTCTTGCCACTGAGTA---GG-CCAGGGTCATTTGTC
Macaca mulatta            GGTGTGGTTAGAACTGAGATA-----------CAACCTGCCCACC-TGGGTCAGGTCTAAAGA------CAAGAAGTCCTGAGTTCTTGCCACTGAGGA---GG-CCAGGGTCATTTGTC
Callithrix jacchus        GGTGTGGTTAGAACTGAC--------------------------------TTAGGTCTAAAGACC---ACAGGAAGTCCTGAGTTCTTGCCACTGGGTA---GG-GCAGGGTTATTTGTC
Mus musculus              -----------ACCCTACGTACGCTGCAGGACCCGTCAGCCTTGC-T---GTGGGTCTGAAGACA--GACAGGGAGTGTCTGGTTCTTGTGACTGGGTAAAGGG-ACAGAGCATTTGGCC
Rattus norvegicus         -----------ACCTTACCTA-----------CCATCAGCCTTGC-T---GTGGGTCTGAAGACA--GACAGGGAGCGTCTGGTTCTTGCTACTGGGTGAAGAG-ACAAGGCATTTGGTC
Bos taurus                GGTCCTGTCATAACTGAGATA-----------CTAAC--------CTGGGGCAGGTCTGGAGACCTC-ACGGAAAGGGCCAAGTTCTTGCCACTGAGCA---G--CAAGGGTGATTGGTC
Sus scrofa                GGTGCTGTC----CTGAGATA-----------CTAACTGCTCACCTTGGGTCAGGTGTGGAGACCTA-ACAGAAAGAGCCGAGTTCTGGCCACTGGGCA---G--TAAGGGTTATTGTTC
Canis lupus familiaris    GGCACGGTCATAACCTTGATA-----------GGAACCACCTGCCTTGGGTCAGGTCTAGAGACATA-ACTGGAAGTGCTGAGTTCTTGCCACTGGGCA---A-GTAAGGGTTATTGGTC
Equus caballus            AC-------------------------------CAGCCAC----C-----CCAGGTCTAGAGACGTA-ACAGGAGGTTCTGAGTTCTTGCCACTGGGCA---A--TAAGGGTTATTGGTC

Homo sapiens              CAGAAAACTTTGTGACTG-TCTTTGAGTGACCTAGTCTGGGAC-----CCATTCATTGGTGGGTT---CTAAGGTTAGAAGCTCATCCAGGATATTTTCAATATTAAGTCAGTGCATAGC
Pan troglodytes           CAGAAAACTTTGTGACTG-TCTTTGAGTGACCTAGTCTGGGAC-----CCATTCATTGGTGGGTT---CTAAGGTTAGAAGCTCATCCAGGATATTTTCAATATTAAGTCAGTGCATAGC
Gorilla gorilla           CAGAAAACTTTGTGACTG-TCTTTGAGTGACCTAGTCTGGGAC-----TCATTCATTGGTGGGTT---CTAAGGTTAGAAGCTCATCCAGGATATTTTCAATATTAAGTCAGTGCATAGC
Pongo abelii              CAGAAAACTTTGTGACTG-TCTTTGAGTGTCCTAGTCTGGGAC-----CCATTCATTGGTGGGTT---CTAAGGTTAGAAGCTCATCCAGGATATTTTCAATATTAAGTCAGTGCATAGC
Macaca mulatta            CAGAAAACTTTGAGACTG-TCTTTGAGTGACCTAGTCTGGGAC-----CCATTCACTGGTGGGTT---CTAAGGTTAGACGCTCATTCAGGATATTTTCAATACTAAGTCAGTGCATAGC
Callithrix jacchus        CAGAAAACTTCGTGACTA-TCTTGGGGTGACCTAGCCTGGGAC-----CCATTCACTGGTGGGTT---CTAAGGTTAGAAGCTCATCCAGGATACTTTCAATACTAAGTCAGTGCATAGC
Mus musculus              CAAGAGGCTTTGAGAC-C-TCTTGGA--GACCAAGTCCAGAAG-----------------------------------------------------------------------------
Rattus norvegicus         CACAAGACTTTGAG-C-G-GCTTGGA--GACCAAGTGCAAAAG-----------------------------------------------------------------------------
Bos taurus                CAG-------------------------------------GAC-----CTGTTAACTGGTGAGTT---AGAAGTCTAGAAGCTCATCTGGGATATTCTCAATTTGAACTCAGCGCCAGTC
Sus scrofa                CAGAAGCCTCTGATGCTGCTGTTGGAATGACCCAG-----CAC-----CCATTCACTGGTGGGTT---ATCAGTTGAGAAGCACATCCAGGATCTTCTCAATCCTAAGGCAGTCTCTATT
Canis lupus familiaris    CAGAAGCCTTTGGGACTGCTGTTGGAGTGGCCAG------GCC-----CCAGTCGCTGGTAGAGTATAATAAGATCAGGAGCTCACCCAGGATACTCTGAGTCCTAAGGCAGTGCCTCGC
Equus caballus            CAGAAGACT--GAGACTGCTCTTGGAGTGACCTG------ACCCAGGTCCATTCACTGGTGGGTT---GTAAGTTTAGAAGCTC-CTCAGGATAGTCTCCATCCTAAGGCAGTGCCCATC

Homo sapiens              TGCACCACTAACAAATT-GGTGCCTGTAGAGTCAGAGTGGGTCAATTCTTAGGACAATGGCGCTGGCACTGTTAGAGGACTGGTGCAGGATAATGAGTGTGGATGAGCAGAAGTCACTGA
Pan troglodytes           TGCACCACTAACAAATT-GGTGCCTGTAGAGTCAGAGTGGGTCAATTCTTAGGACAATGGCGCTGGCACTGTTAGAGGACTGGTGCAGGATAATGAGTGTGGATGAGCAGAAGTCACTGA
Gorilla gorilla           TGCACCACTAACAAATT-GGTGCCTGTAGAGTCAGAGTGGGTCAATTCTTAGGACAATGGCGCTGGCACTGTTAGAGGACTGGTGCAGGATAATGAGTGTGGATGAGCAGAAGTCACTGA
Pongo abelii              TGCACCACTAACAAATT-GGTGCCTGTAGAGTCAGAGTGGGTCAATTCTTAGGACAATGGCGCTGGCACTGTTAGAGGACTGGTGCAGGATAATGAGTGTGGATGAGCAGAAGTCACTGA
Macaca mulatta            TGCCCCACTAACAAATT-GGTGACTGTAGAGTCAGAGCGGGTCAGTTCTTGGGACAATGGCGCTGGCGCTGTTAGAGGACTGGTGCAGGATAATGAGTGTGGATGAGCAGAAGTCACTGA
Callithrix jacchus        A--ACCACTAACAGATT-GGTACCCGTAGAGTCAGAGTGGGTCAGTTCTTGGGACAATGGCACTGGCACTCTTAGAGGACTGGTGCAGGATAATGAGTGTGGATGAGCAGAAGTCACTAA
Mus musculus              ------------------------------------------CATATCCTGGGACAATGGCGGTGGCCCTGCTGGAGGAGTGGTGTAAGATCATGGGTGTAGACGTCCAGAAGTCACTGC
Rattus norvegicus         ------------------------------------------CACACCCTGGGACAATGGCAGTGGCCCTGCTGGAGGAGTGGTGCAAGATCATGGGTGTGGATGTCCAGAAGTCCCTGC
Bos taurus                TGTCCCATAAGCTAATC-AGTAGCCATAGAGTTCCAGCCAGTCAGTATCCAG--CAATGGCGCTGGCCCTGTTGGAGGACTGGTGTAGGATTCTGAGTGTGGATGATCAGAAATCACTGA
Sus scrofa                GGCCCCACAAACTGATCCGGTACCCAC--ACTTAGAGCGAGTCAATATCTGGGACCATGGCGCGGGCACTGTTGGAGGACTGGTGTAGAATCATGAGTGCAGATGATCAGAAATCACTGA
Canis lupus familiaris    CACACCACAAACTATTG-GGTGGCCAGAGAGTTAGGGTGAGTGATAATT--GGACGATGGCGCTGGCGCTGTTGGAGGACTGGTGCAGGATCATGAGCGTGGATGAACAGAAATCACTGG
Equus caballus            TGCA-------CTAATT-AGTACCCATAGAGTCAGAGCGAGTAAATATTCAGGACGATGGCGCTGGCACTGCTGGAGGACTGGTGCAGGATAATGAGCGTGGATGATCAGAAATCGCTGA

Homo sapiens              TGGTTACGGGGATACCGGCGGACTTTGAGGAGGCTGAGATTCAGGAGGTCCTTCAGGAGACTTTAAAGTCTCTGGGCAGGTATAGACTGCTTGGCAAGATATTCCGGAAGCAGGAGAATG
Pan troglodytes           TGGTTACGGGGATACCGGCGGACTATGAGGAGGCTGAGATTCAGGAGGTCCTTCAGGAGACTTTAAAGTCTCTGGGCAGGTATAGACTGCTTGGCAAGATATTCCGGAAGCAGGAGAATG
Gorilla gorilla           TGGTTACGGGGATACCGGCAGACTATGAGGAGGCTGAGATTCAGGAGGTCCTTCAGGAGACTTTAAAGTCTCTGGGCAGGTATAGACTGCTTGGCAAGATATTCCGGAAGCAGGAGAATG
Pongo abelii              TGGTTACGGGGATACCGGCGGACTATGAGGAAGCTGAGATTCAGGAGGTCCTTCAGGAGACTTTAAAGTCTCTGGGCAGGTATAGACTGCTTGGCAAGATATTCCGGAAGCAGGAGAATG
Macaca mulatta            TGGTTACGGGGATACCGGTGGACTATGAGGAGGCTGAGATTCAGGAGGTCCTTCAGGAGACTTTAAAGTCTCTGGGCAGTTATAGACTGCTTGGCAAGATATTCCGGAAGCAGGAGAATG
Callithrix jacchus        TGGTTACAGGGATACCCGTGGAGTATGAGGAGGCTGAGATTCAGGAGGTCCTTCAGGAGACTTTAAAGTCTCTGGGCAGGTATAGATTGCTTGGCAAGATATTCCGGAAGCAGGAGAATA
Mus musculus              TGGTGGTTGACATTCCGGTGGACTGTGGTGAACCTGAGATCCAGACAGTCCTCCAGGAGGCTCTGAAGTGTGTCGGCAGCTACCGGCTACTAGGCAAGATATTCCAGAAGCAGGACAACA
Rattus norvegicus         TGGTGATTGGCATTCCTGTGAACTGTGGTGAGCTTGAGGTCCAGACAGTCCTACAGGAGGCGCTGAAGTGTGTGGGCAGCTACCGGCTACTGGGCAAAATACTCCAGAAGCAAGACAATA
Bos taurus                TGGTTATGGGGATCCCAGTGGACTGCAGTGAGGATGAGATTCAGGAGGTCCTGCAGGAGACTTTAAAGCCTTTGGGCAGGTATAAACTCCTTGGCAAAATATTCCGGAAGCAGGAGAATG
Sus scrofa                TGGTTATGGGGATACCGGTGGACTGTGATGAGGCTGAGATTCAGGAGGTCCTCCAGGAGACTTTAAAGTCGCTGGGCAGGTACAGACTGCTCGGCAAAATATTCCGGAAGCAGGAGAACG
Canis lupus familiaris    TGGTTATGGGGATACCGGTGGACTGTGATGAGGCTGAGATTCAGGAGGTTCTCCAGGAGACCCTAAAGTCTCTGGGCAGGTATAGACTTCTTGGCAAAATATTCAGGAAGCAGGACAATG
Equus caballus            TGGTTATGGGGATACCAGTGGACTGTGATGAGGCTGAGATTCAGGAGGTTCTCCAGGAGACTTTGAAGTCTCTGGGCAGGTATAGACTGCTTGGCAAAATATTCAGGAAGCAGGAGAATG

Homo sapiens              CCAATGCTGTCTTACTAGAGCTTCTGGAAGATACTGATGTCTCGGCCATTCCCAGTGAGGTCCAGGGAAAGGGGGGTGTCTGGAAGGTGATCTTTAAGACCCCTAATCAGGACACTGAGT
Pan troglodytes           CCAATGCTGTCTTACTAGAGCTTCTGGAAGATACTGATGTCTCGGCCATTCCCAGTGAGGTCCAGGGAAAGGGGGGTGTCTGGAAGGTGATCTTTAAGACCCCTAATCAGGACACTGAGT
Gorilla gorilla           CCAATGCTGTCTTACTAGAGCTTCTGGAAGATACTGATGTCTCAGCCATTCCCAGTGAGGTCCAGGGAAAGGGGGGTATCTGGAAGGTGATCTTTAAGACCCCTAATCAGGACACTGAGT
Pongo abelii              CCAATGCTGTTTTACTAGAGCTTCTGGAAGATACTGATGTCTCGGCCATTCCCAGTGAAGTCCAGGGAAAGGGGGGTGTCTGGAAGGTGATCTTTAAGACCCCTAATCAGGACACTGAGT
Macaca mulatta            CCAATGCTGTCTTACTAGAGCTTCTGGAAGATACTGATGTCTCGGCCATTCCCAGTGAGGTCCAGGGAAAGGGGGGTGTCTGGAAGGTGGTCTTTAAGACCCCTAATCAGGACACTGAGT
Callithrix jacchus        CCAATGCTGTCTTACTAGAGCTCCTGGAAGATACTGATGTCTCAGCCATCCCCAGTGAGGTCCAGGGAAAGGGGGGTGTCTGGAAGGTGATCTTCAAGACCCCTAATCAGGACACTGAGT
Mus musculus              CCAGTGTTGTCTTAGTGGAACTTATGGAGGACACAGATATGTCTGTGGTCCCCAGTGAGGTACAGGGCAAGGGAGGTGTCTGGAAAGTGATCTTCAAGACCCCTAATCAGGACACTGAGT
Rattus norvegicus         CCAGTGCTGTCTTACTAGAACTTATGGAGGACACGGATATGTCTGTGGTTCCCAGTGAGGTGCAGGGAAAGGGAGGTGTCTGGAAAGTGATCTTCAAGACCCCTAATCAGGACACTGAGT
Bos taurus                CCAACGCGGTGCTGTTAGAGTTGCTGGAGGACCCTGAGGTCTCTGTGATCCCCAGCAAGGTTCAGGGCAAGGGGGGCATCTGGAAAGTCATCTTCAAGACCCCTAACCAGGACACAGAAT
Sus scrofa                CCAACGCTGTGCTGTTAGAGTTTCTGGAGGATACCGATGTCTCCATGATCCCCAATGAGATTCAAGGAAAAGGGGGTATTTGGAAAGTGATCTTTAAGACCCCTAACCAGGACACTGAAT
Canis lupus familiaris    CCAATGCTGTCTTGTTGGAGCTTATGGAGGATGTTGACGTCTCGGTGATCCCCAGTGAGGTACGGGGAAAGGGGGGTATCTGGAAAGTCATCTTTAAGACCCCTAACCAGGACACTGAGT
Equus caballus            CCAATGCTGTGTTATTAGAGCTCATGGAGGACATTGATGTCTCAGTGATCCCCAGTGAGGTTCAGGGAAGGGGGGGCATCTGGAAAGTGATCTTTAAGACCCCTAACCAAGACACTGAAT

Homo sapiens              TTCTTGAAAGATTGAACCTGTTTCTAGAAAAAGAGGGGCAGACGGTCTCGGGTATGTTTCGAGCCCTGGGGCAGGAGGGCGTGTCTCCAGCCACAGTGCCCTGCATCTCACCAGAATTAC
Pan troglodytes           TTCTTGAAAGATTGAACCTGTTTCTAGAAAAAGAGGGGCAGACGGTCTCGGGTATGTTTCGAGCTCTGGGGCAGGAGGGCGTGTCTCCAGCCACAGTGCCCTGCATCTCACCAGAATTAC
Gorilla gorilla           TTCTTGAAAGATTGAACCTGTTTCTAGAAAAAGAGGGACAGACGGTCTCGGGTATGTTTCGAGCCCTGGGGCAGGAGGGCGTGTCTCCAGCCACAGTGCCCTGCATCTCACCAGAATTAC
Pongo abelii              TTCTTGAAAGATTGAACCTGTTTCTAGAAAAAGAGGGGCAGACGGTCTCGGGTATGTTTCGAGCCCTGGGGCACGAGGGTGTGTCTTCAGCCACAGTGCGCTGCATCTCACCAGAATTAC
Macaca mulatta            TTCTGGAAAGACTGAACCTCTTTCTAGAAAAAGAGGGGCAGACGGTCTCAGGTATGTTTCGAGCCCTGGGGCACGAGGGCATGTCTCCAGCCACAGTGCCCTGCATCTCACCAGAATTAC
Callithrix jacchus        TTCTGGAAAGGCTGAACCTCTTTCTAGAAAAAGAGGGACAGACCATCTCCGGTATGTTTCGAGCCCTGGGGCATGAGGGCGTGTCTCCAGCCACCGTGCCCTGCATCTCACCAGAATTAC
Mus musculus              TTCTCCAAAGACTGAACCTCTTTCTAGAAAAAGAAGGGCAGACGGTCGCAGGGATGTTCCGAGCCCTTAAGCATGAGGGAGTGTCTCCAGCCACCCCACCCTGCACGTCCCCTGAGTTAT
Rattus norvegicus         TTCTCCAAAGACTGAACCTCTTTCTAGAAAAAGAAGGGCAGACGGTCGCAGGGATGTTCCGAGCCCTGAAGCATGAGGGAGTGTCTCCAGCCACCACACCCTGCACATCCCCCGAGTTAT
Bos taurus                TTCTGGAAAGACTGAACCTCTTTCTAGAAAAAGAGGGACAGACGGTCTCGGGAATGTTCCGGGCACTTGGGCACCAGGGGTTGTCTCCAGCAGCCATGCCCTGCATCTCACCAGAGTTAC
Sus scrofa                TTCTTGAGAGACTGAACCTCTTTCTAGAAAAAGAGGGCCAGACGGTCTCAGGAATGTTCCGAGCGTTGGGGCATGAGGGATTGTCTCCAGCGGCAGTGCCCTGTGTCTCACCGGAATTAT
Canis lupus familiaris    TTCTGGAACGACTGAACCTCTTTCTGGAAAAAGAGGGGCAGACGGTCTCAGGTATGTTCCGAGCCCTCGGGCATGAGGGAATGTCTCCAGCCACAGTGCCCTGCGTATCACCAGAGCTGT
Equus caballus            TTCTTGAAAGACTGAACCTCTTTCTAGAAAAAGAGGGGCAGACAGTCTCCGGGATGTTCCGGGCCCTTGGGCATGAGGGAGTGTCTCCAGCCGCAGTGCCCTGCGTATCACCGGAGTTAT

Homo sapiens              TGGCCCATTTGTTGGGACAGGCAATGGCACATGCGCCTCAGCCCCTGCTACCCATGAGATACCGGAAACTGCGAGTATTCTCAGGGAGTGCTGTCCCAGCCCCAGAGGAAGAGTCCTTTG
Pan troglodytes           TGGCCCATTTGTTGGGACAGGCAATGGCACACGCGCCTCAGCCCCTGCTACCCATGAGATACCGGAAACTGCGAGTATTCTCAGGGAGTGCTGTCCCAGCCCCAGAGGAAGAGTCCTTTG
Gorilla gorilla           TGGCCCATTTGTTGGGACAGGCAATGGCACACGCGCCTCAGCCCCTGCTACCCATGAGATACCGGAAACTGCGAGTATTCTCAGGGAGTGCTGTCCCAGCCCCAGAGGAAGAGCCCTTTG
Pongo abelii              TGGCCCATTTGTTGGGACAGGCAATGGCACACGCGCCTCAGCCCCTGCTACCCATGAGATACCGGAAACTGCGAGTATTCTCGGGGAGTGCTGTCCCAGCCCCAGAGGAAGAGCCCTTTG
Macaca mulatta            TGGCCCATTTGTTGGGACAGGCAATGGCACATGCGCCTCAGCCCCTGCTACCTATGAGATACCGGAAACTGCGAGTGTTCTCTGGGAGTGCTGTCCCAGCCCCAGAGGAAGAGCCCTTTG
Callithrix jacchus        TGGCCCATCTGTTGGGACAGGCAATGGCACACGCGCCTCAGCCCCTGCTACCCATGAGATACCGGAAACTGCGAGTGTTCTCGGGGAGTGCCGAGCCAGCCCCGGAGGAAGAGCCTTTTG
Mus musculus              TGGCCCATTTGACGGGGCAGGCAATGGTTCACGGCCAGAGGCCTCTGTTGCCTGTGAAGTACTGTAAGATGAGAATATTCTCTGGGAGCACAGCAGCTGCCCCAGAGGAGGAGCCCTTTG
Rattus norvegicus         TGGCCCACTTGGTAGGGCAGGCAATGGTTCACGCCCAGAGGCCTCTGTTGCCTGTGAGGTACTGTAAGCTGAGAGTATTCTCTGGGAGCACAGTAGCTGCCCCAGAGGAGGAGCCCTTTG
Bos taurus                TGGCCCACGTGTTGGGACAGGTGATAGCACATCCCCCTCAGCCTCTGCTCCCCATGAGGTACCGGAAGCTGAGAGTGTTCTCAGGGAGCGCAGTGCCTGCCCCGGAGGAAGAGCCCTTTG
Sus scrofa                TGGCCCATTTGTTGGGACAGGCAATAGCGCACGCCCCCCAGCCTCTGCTCCCGATGAGGTACCGGAAACTGAGAGTGTTTTCCGGGAGCGCGGTGCCAGCCCCAGAGGAAGAGCCCTTTG
Canis lupus familiaris    TGGCTCATTTGTTGGGACAGGCGATGGCACATGCCCCTCAGCCTCTGCTCCCCATGAGATACCGGAAACTGAGAGTGTTCTCAGGGAGCGCTGTGCCTGCCCCGGAAGAAGAGCCCTTTG
Equus caballus            TGGCCCATTTGTTGGGCCAGGCAATAGCACATGCCCCTCAGCCTCTGCTCCCCATGAGATACCGGAAACTGAGAGTGTTCTCCGGGAGTGCTGTGCCGGCGCCAGAGGAAGAGCCCTTTG

Homo sapiens              AGGTCTGGTTGGAACAGGCCACGGAGATAGTCAAAGAGTGGCCAGTAACAGAGGCAGAAAAGAAAAGGTGGCTGGCGGAAAGCCTGCGGGGCCCTGCCCTGGACCTCATGCACATAGTGC
Pan troglodytes           AGGTCTGGTTGGAACAGGCCACGGAGATAGTCAAAGAGTGGCCAGTAACAGAGGCAGAAAAGAAAAGGTGGCTGGCGGAAAGCCTGCGGGGCCCTGCCCTGGACCTCATGCACATAGTGC
Gorilla gorilla           AGGTCTGGTTGGAACAGGCCACGGAGATAGTCAAAGAGTGGCCAGTAACGGAGGCAGAAAAGAAAAGGTGGCTGGCGGAAAGCCTGCGGGGCCCCGCCCTGGACCTCATGCACATAGTGC
Pongo abelii              AGGTCTGGTTGGAACAGGCCACCGAGATAGTCAAAGAGTGGCCAGTAACAGAGGCAGAAAAGAAAAGGTGGCTGGCGGAAAGCCTGCGGGGCCCTGCCCTGGACCTCATGCACATAGTGC
Macaca mulatta            AGGTCTGGTTGGAACAGGCCACGGAGATAGTCAAAGAGTGGCCAGTAACAGAGGCAGAAAAGAAAAGGTGGCTGGCAGAAAGCCTGCGGGGCCCTGCCCTGGACCTCATGCACATAGTGC
Callithrix jacchus        AGGTCTGGTTGGAACAGGCCGTGGAGATAGTCAAAGAGTGGCCGGTAACGGAGGCAGAAAAGAAGAGGTGGCTGGCGGAGAGCCTGCGGGGCCCTGCCCTGGACCTCATGCACATAGTTC
Mus musculus              AGGTCTGGTTGGAACAGGCCACTGAGATAGCCAAGGAATGGCCCATCCCTGAAGCAGAAAAGAAAAGGTGGGTGGCAGAGAGCCTCCGTGGCCCTGCCCTGGACCTCATGCACATAGTAC
Rattus norvegicus         AGGTTTGGTTGGAACAGGCCACTGAGATAGCCAAAGAGTGGCCCATCCCTGAGACAGAAAAGAAAAGGTGGGTGACAGAGAGCCTCCGTGGCCCTGCCCTGGACCTCATGCACATAGTAC
Bos taurus                AAGTCTGGTTGGAACAGGCCACTGAGATAGTCAAAGAGTGGCCGGTAGCAGAGGCAGAAAAGAAGAGATGGTTGATGGAAAGCCTTCGCGGGCCAGCCCTGGACCTCATGCACATAGTCC
Sus scrofa                AAATCTGGTTGGAACAGGCCACCGAGATAGTCAAAGAGTGGCCGGTTGCAGAGGCAGAAAAGAAAAGGTGGTTGATGGAAAGCCTCCGTGGCCCTGCCCTGGACCTCATGCACATCGTAC
Canis lupus familiaris    AAATCTGGTTGGAACAGGCCACTGAGATCGTCAAAGAGTGGCCAGTTGCAGAGGCAGAAAAGAAAAGGTGGTTGATGGAAAGCCTGCGTGGCCCTGCCCTGGACCTCATGCATATAGTAC
Equus caballus            AAATCTGGTTGGAACAGGCCACTGAGATAGTCAAAGAGTGGCCGGTAGCCGAGGCAGAAAAGAAAAGGTGGTTGATGGAAAGCCTGCGTGGCCCTGCCCTGGACCTCATGCATATAGTGC

Homo sapiens              AGGCAGACAACCCGTCCATCAGTGTAGAAGAGTGTTTGGAGGCCTTTAAGCAAGTGTTTGGGAGCCTAGAGAGCCGCAGGACAGCCCAGGTGAGGTATCTGAAGACCTATCAGGAGGAAG
Pan troglodytes           AGGCAGACAACCCGTCCATCCGTGTAGAAGAGTGTTTGGAGGCCTTTAAGCAAGTGTTTGGGAGCCTAGAGAGCCGCAGGACAGCCCAGGTGAGGTATCTGAAGACCTATCAGGAGGAAG
Gorilla gorilla           AGGCAGACAACCCGTCCATTAGTGTAGAAGAGTGTTTGGAGGCCTTTAAGCAAGTGTTTGGGAGCCTAGAGAGCCGCAGGACAGCCCAGGTGAGGTATCTGAAGACCTATCAAGAGGAAG
Pongo abelii              AGGCAGACAACCCGTCCATCAGTGTAGAAGAGTGTTTGGAGGCCTTTAAGCAAGTGTTTGGGAGCCTAGAGAGCCGCAGGGCAGCCCAGGTGAGGTATCTGAAGACCTATCAGGAGGAAG
Macaca mulatta            AGGCAGACAACCCGTCCATCAGTGTAGAAGAGTGTTTGGAGGCCTTTAAGCAGGTGTTTGGGAGCCTAGAGAGCCGCAGGACAGCCCAGGTGAGGTATCTGAAGACCTATCAGGAGGAAG
Callithrix jacchus        AGGCAGACAACCCGTCCATCAGTGTGGAAGAGTGTTTGGAGGCCTTTAAGCAGGTGTTTGGGAGCCTGGAGAGCCGCAGGACCTCCCAGGTGAGGTACCTGAAGACCTATCAGGAGGAAG
Mus musculus              AGGCAGACAACCCTTCCATCAGTGTGGGGGAATGTCTGGAGGCCTTTAAGCAGGTGTTTGGGAGCACGGAGAGCCGTAGAACCTCACAGGTAAAATACCTAAGAACCTATCAGCAAGAAG
Rattus norvegicus         AAGCAGACAACCCTTCTATCAGTGCAGAAGAATGTCTGGAGGCCTTTAAGCAGGTGTTTGGGAGCATGGAGAGCCGTAGAACCTCACAGGTCAAATACCTAAAAACCTATCAGCAAGAAG
Bos taurus                AGGCAGACAATCCATCCATCAGCGTGGAGGAGTGTTTGGAAGCATTTAAGCAAGTGTTTGGGAACCTGGAGAGCCGCCGGACCTCCCAGGTGAAGTACCTGAAAACCTATCAGGAGGAAG
Sus scrofa                AGGCAGACAACCCGTCCATAAGTGTGGAGGAGTGTCTGGAGGCTTTTAAGCAAGTGTTTGGAAGCCTGGAGAGCCGCAGGACCTCCCAGGTGAAATACCTGAAGACCTATCAGGAGGAAG
Canis lupus familiaris    AAGCAGACAATGCGGCCATAAGCGTGGAAGAGTGTTTGGAGGCGTTTAAGCAAGTGTTTGGAAGCCTCGAGAGCCGCAGGACCTCCCAGGTGAAGTACCTGAAGACCTATCAGGAGGAGG
Equus caballus            AGGCGGACAATCCGTCCATCAGTGTGGAGGAGTGTTTGGAGGCGTTTAAGCAAGTGTTTGGAAGCCTGGAGAGCCGCAGGACCTCCCAGGTGAAGTATCTGAAGACCTATCAGGAGGAAG

Homo sapiens              GAGAGAAGGTCTCAGCCTATGTGTTACGGCTAGAAACCCTGCTCCGGAGAGCGGTGGAGAAACGCGCCATCCCTCGGCGTATTGCGGACCAGGTCCGCCTGGAGCAGGTCATGGCTGGGG
Pan troglodytes           GAGAGAAGGTCTCAGCCTATGTGTTACGGTTAGAAACCCTGCTCCGGAGAGCGGTGGAGAAACGCGCCATCCCTCGGCGTATTGCGGACCAGGTCCGCCTGGAGCAGGTCATGGCTGGGG
Gorilla gorilla           GAGAGAAGGTCTCAGCCTATGTGTTACGGTTAGAAACCCTGCTCCGGAGAGCGGTGGAGAAACGCGCCATCCCTCGGCGTATTGCGGACCAGGTCCGCCTGGAGCAGGTCATGGCTGGGG
Pongo abelii              GAGAGAAGGTCTCAGCCTATGTGTTACGGTTAGAAACCCTGCTCCGGAGAGCGGTGGAGAAACGCGCCATCCCTCGGCGTATCGCGGACCAGGTCCGCCTGGAGCAGGTCATGGCTGGGG
Macaca mulatta            GAGAGAAAGTCTCAGCCTATGTGTTACGGTTAGAAACCCTGCTCCGAAGAGCCGTGGAGAAACGCGCCATCCCTCGGCGTATTGCGGACCAGGTCCGCCTGGAGCAGGTCATGGCTGGGG
Callithrix jacchus        GAGAGAAGGTGTCGGCCTATGTGTTAAGGTTAGAAACCCTGCTCCGGAGAGCGGTGGAGAAACGCGCCATCCCGCGGCGCATCGCGGACCAGGTCCGCCTGGAGCAGGTCCTGGCTGGGG
```

```
Mus musculus               GGGAGAAAATCTCAGCCTACGTGCTCCGGCTGGAAACTCTGCTCCGGAGAGCTGTGGAAAAAAGGGCCATCCCCCGGAACATTGCTGACCAGGTACGCCTGGAGCAGGTCATGGCTGGTG
Rattus norvegicus          GAGAGAAAATCTCAGCCTATGTGCTCCGGCTGGAGACTCTGCTCCGGAGAGCTGTGGAAAAAAGGGCCATCCCCCGGAACATTGCTGACCAGGTACGTCTGGAGCAGGTCATGGCTGGAG
Bos taurus                 GAGAGAAAGTCTCAGCCTACGTGTTACGGTTAGAAACCCTGCTGCGGAGAGCGGTGGAGAAGCGGGCGATCCCCAGGAACATCGCTGATCAAATCCGCCTGGAGCAGGTCATGGCCGGGG
Sus scrofa                 GAGAGAAAGTCTCGGCCTACGTGTTACGGTTAGAAACTCTGCTGCGGAGAGCGGTGGAGAAGCGGGCCATCCCGAGGAATATCGCCGATCAGGTCCGCTTGGAGCAAGTCATGGCCGGGG
Canis lupus familiaris     GGGAGAAGGTCTCAGCCTACCTGTTGCGGTTAGAAACCCTGCTCCGGCGGGCCGTGGAGAAACGAGCCATCCCCAGGAATATCGCAGATCAGGTCCGGCTGGAGCAGGTCATGGCAGGGG
Equus caballus             GAGAGAAGGTCTCTGCCTATGTGTTGCGGTTAGAAACCCTGCTCCGGAGAGCTGTGGAGAAGCGGGCCATCCCGAGGAACATCGCGGATCAGGTCCGCTTGGAGCAGGTCATGGCTGGGG

Homo sapiens               CCACTCTTAACCAGATGCTGTGGTGCCGGCTTAGGGAGCTGAAGGATCAGGGCCCGCCCCCCAGCTTCCTTGAGCTAATGAAGGTAATACGGGAAGAAG---AGGAGGAAGAGGCCTCCT
Pan troglodytes            CCACTCTTAACCAGATGCTGTGGTGCCGGCTTAGGGAGCTGAAGGATCAGGGCCCGCCCCCCAGCTTCCTTGAGCTAATGAAGGTAATACGGGAAGAAG---AGGAGGAAGAGGTCTCCT
Gorilla gorilla            CCACTCTTAACCAGATGCTGTGGTGCCGGCTTAGGGAGCTGAAGGATCAGGGCCCGCCCCCCAGCTTCCTTGAGCTAATGAAGGTAATACGGGAAGAAG---AGGAGGAAGAGGCCTCCT
Pongo abelii               CCACTCTTAACCAGATGCTGTGGTGCCGGCTTAGGGAGCTGAAGGATCAGGGCCCGCCCCCCAGCTTCCTTGAGCTAATGAAGGTAATACGGGAAGAAG---AGGAGGAAGAGGCCTCCT
Macaca mulatta             CCACTCTTAACCAGATGCTGTGGTGCCGGCTTAGGGAGCTGAAGGATCAGGGCCCGCCCCCCAACTTCCTTGAGCTAATGAAGGTAATACGGGAAGAAG---AGGAGGAAGAGGCCTCCT
Callithrix jacchus         CCACTCTTAACCAGACGCTGTGGTGTCGGCTCAGGGAGATGAAGGATCAGGGCCCACCCCCCAGCTTCCTTGAGCTAATGAAGGTAATACGGGAAGAGG---AGGAGGAAGAGGCCTCCT
Mus musculus               CCAACCTCGGCAACGTTCTGTGGTGCAGGCTACAGGAGCTGAAAGATCAGGGCCCACTCCCCACCTTTTTGCAGCTGATGAAGGTGATACGTGAGGAAGAAGAAGAAGAAGACGCCTACT
Rattus norvegicus          CCAACCTTAGCAACGTTCTTTGGTGCAGGCTACGGGAGCTGAAGGATCAGGGCCCACTGCCCACGTTTTTGCAGCTGATGAAGGTGGTCCGTGAGGAGG---AAGAAGAAGAGGCCTACT
Bos taurus                 CAAGCCTCAGTGAGGTACTCTGGTGTCGACTTAGGGAGCTGAAAGACCAGGGCCGACCTCCCAGCTTCCTGCAGTTAATGAAGGTCATCCGGGAAGAAG---AGGAGGAAGAGGCCACTT
Sus scrofa                 CGAGCCTCAGCGAAATCCTCTGGTGTAGGCTGAGGGAGCTGAAAGATCAGGGGCCGCCTCCCAGCTTCCTGGAGTTGATGAAGGTGATCCGCGAGGAAG---AAGAGGAAGAGGCCGCCT
Canis lupus familiaris     CCAGCCTTAGCGAAGTCCTTTGGTGCAGGCTGAGGGAGCTGAAGGATCAGGGCCCGCCTCCCAACTTCCTTGAGTTAATGAAGATAATCCGGGAGGAAG---AGGAGGAAGAGGCCTCTT
Equus caballus             CGAGCCTTAGCGAGATCCTCTGGTGTAGGCTCAGGGAGCTGAAAGACCAGGGGCCGCCTCCCAGCTTCCTCGAGTTGATGAAGGTGATCCGGGAAGAGG---AGGAGGAAGAGGCCTCTT

Homo sapiens               TTGAGAATGAGAGTATCGAAGAGCCAGAGGAACGAGATGGCTATGGCCGCTGGAATCATGAGGGAGACGACTGAAAACCACCTGGGGGCAGGACCCACAGCCAGTGG-------------
Pan troglodytes            TTGAGAATGAGAGTATCGAAGAGCCAGAGGAACGAGATGGCTATGGCCGCTGGAATCATGAGGGAGACGACTGAAAACCACCTGGGGGCAGGACCCACAGCCAGTGG-------------
Gorilla gorilla            TTGAGAATGAGAGTATCGAAGAGCCAGAGGAACGAGATGGCTATGGCCGCTGGAATCATGAGGGAGACGACTGAAAACCACCTGGGGGCAGGACCCACAGCCAGTGG-------------
Pongo abelii               TTGAGAATGAGAGTATCGAAGAGCCAGAGGAAGGAGATGGCTATGGCCGCTGGAATCATGAGGGAGACGACTGAAAACCACCTGGGGGCAGGACCCACAGCCAGTGG-------------
Macaca mulatta             TTGAGAATGAGAGTATCGAAGAGCCAGAGGAAGGAGATGGCTATGGCGGCTGGAATCATGAAGGGGACGACTGAAAACCACCTGGGGGCAGGACCCATAGCCAGTGG-------------
Callithrix jacchus         TTCAGAATGAGAATATCGAAGAGCCAGAGGTAGGAGATGGCTATGGCCGCTGGGATCATGAGGGGGAGGACTGAAAACCACCTGAGTGCAGGACCCACAGCCAGTGGGCCCAGGGACAT-
Mus musculus               TTGAACAAGAGAGCAGGGAGGAGCCGGGGGAAAGGGAGGGCTCTGGCTGCTGGAACAATTCAAGAAACAACTGAGAGATATCCCCACT-CCGTCCCTGATCTGGTG-------------G
Rattus norvegicus          TTGAACACGAGAGCAGAGAGGAGCCAGGGGAAAGGGAGGGCTCTGGCCGCTGGGACAATTCAAGGAACAACTGACAGACATCCCTACT-CCATTTCTGATCTGGTA--------------
Bos taurus                 TCGAAAATGAGAATACTGAAGAGCCGGAGGGAGGGGATGGCTATGGCCACTGGGGTAACGAGGCCAATGACTAAAACCCAACTCCGAGGGCGACCCACAGATGGTGGTTGGAGGGACAG-
Sus scrofa                 TCGAAAACGAGAATATTGAAGAGCCGGATGAAGGGGATGGCTATGGCCACTGGGATCTTGAGGCGGATGAGTAAAGCCCATCCCAGAGGGGACCCACAGCCGGCAG--GGTAGGGCCT-
Canis lupus familiaris     TTGAAAATGAGAATATTGAAGAGCCAGATGAAGGGGATGGCTATGGCCACTGGGATGACGAGGCAGATGACTAAAAACCAGCTTGGAGCCA-GTGCACAGCCAGTGCACCTCGGGACAT-
Equus caballus             TTGAAAACGAGAATATTGAAGAGCCAGATGAAGGGGACGGCTATGGCCACTGGGACAATGAGGCAGATGACTGAAAACCAACTCAGAGGGGGGCCCACCGCCACTGGGCTTGGGGCCGT-

Homo sapiens               ---------------------------------GCTAAG------------------------ACCTTTAAAAAATTT------------------------------------------
Pan troglodytes            ---------------------------------GCTAAG------------------------ACCTTTAAAAAATTT------------------------------------------
Gorilla gorilla            ---------------------------------GCTAAG------------------------ACCTTTAAAAAATTT------------------------------------------
Pongo abelii               ---------------------------------GCTAAG------------------------ACCTTTAAAAAATT-------------------------------------------
Macaca mulatta             ---------------------------------GCCTAGGGACATTCACTTTACTAGGCTAAGNNNNNNNNNNNNNNNNNNNNNNNNNNNNNNNNNNNNNNNNNNNNNNNNNNNNNNNNN
Callithrix jacchus         ---------------------GCGCCTTACTAGGCTAAG------------------------ACTTTTAAAGAATCT------------------------------------------
Mus musculus               GCGATGCTTGACAAGAGACG-ATGCTTGACAAGGCTAAC------------------------CCCATTACAGTGGAT------------------------------------------
Rattus norvegicus          ---------------------ATGCTTGACAAGGCTAAT------------------------CCCATTCTAGTGCAC------------------------------------------
Bos taurus                 ---------------------CCACGTTATTAGGCTAAG------------------------ACCTTTTCAGTCTTT------------------------------------------
Sus scrofa                 ---------------------CTGCCTTACCAGGCTAAG------------------------ACCTTTTCAGATATT------------------------------------------
Canis lupus familiaris     --------------------TTCCTTTTATCCGGCTAAG------------------------ACCGGGTGGCTATTT------------------------------------------
Equus caballus             ---------------------TCGCTTTACCAGGCTAAG------------------------ACCTTGTCAATATGT------------------------------------------

Homo sapiens               --------------------------------------------------------------TTTT---CTTTA-ATGTATGGGACTGAAATCAAACC------ATGAAAGCCAATTATT
Pan troglodytes            --------------------------------------------------------------TTTT---CTTTA-ATGTGTGGGACTGAAATCAAACC------ATGAAAGCCAATTATT
Gorilla gorilla            --------------------------------------------------------------TTTT---CTTTA-ACGTATGGGACTGAAATCAAACC------ATGAAAGCCAATTATT
Pongo abelii               --------------------------------------------------------------TTTT---CTTTA-ATGTATGGGACTGAAATCAAAGC------ATGAAAGCCAATTATT
Macaca mulatta             NNNNNNNNNNNNNNNNNNNNNNNNNNNNNNNNNNNNNNNNNNNNNNNNNNNNNNNNNNNNNNNNNN---NNNNN-NNNNNNNNNNNNNNNNNNNNNNN------NNNNNNNNNNNNNNNN
Callithrix jacchus         --------------------------------------------------------------TTTT---CTTTA-ATGTATGGGACTGATATCAAGGC------ATGAAACCCAATTATT
Mus musculus               --------------------------------------------------------------TCTT---CTT---AAGTGCAGGACTGAAGCTAAACATTAGGACTGAAGG----TTTTT
Rattus norvegicus          --------------------------------------------------------------TTTG---CTTTA-AAGTGCAGGACTGAAGTCAAGCATTAGGAATGAAGG----TTTTG
Bos taurus                 --------------------------------------------------------------TTTTCT-CTATA-ATTTGCACAGTTGAAATCAAAGC------ATTCAAGCCAGGTTTT
Sus scrofa                 --------------------------------------------------------------TTTTCTCCTTTA-ATGTATGCATTTGAAATGACAGC------ATTTGAGCCAGATCTT
Canis lupus familiaris     --------------------------------------------------------------TTGT---CTTTAAATGGATGCAACTGAAATCGAAGC------TTTCAAGCCAGGTCTT
Equus caballus             --------------------------------------------------------------TTTT---CTTTA-CTGTACGCAATTGAAATCAAGGC------GTTCAAGCCAGGTCTT

Homo sapiens               GAC-CTTCCTTCCTTCCTTCCT---T----------------------------------------------------------------------------------------------
Pan troglodytes            GAC-CTTCCTTCCTT----CCT---T----------------------------------------------------------------------------------------------
Gorilla gorilla            GAC-CTTCCTTCCTTCCTTCCT---T----------------------------------------------------------------------------------------------
Pongo abelii               GAC-CTTCCTTCCTT----CCT---T----------------------------------------------------------------------------------------------
Macaca mulatta             NNN-NNNNNNNNNNNNCTTCCT---T----------------------------------------------------------------------------------------------
Callithrix jacchus         GAC-CTTCCTTCCTT----CCT---T----------------------------------------------------------------------------------------------
Mus musculus               GTC-TTT-----------------------------------------------------------------------------------------------------------------
Rattus norvegicus          GGG-TTT-----------------------------------------------------------------------------------------------------------------
Bos taurus                 ATTTTGTTTTTCTTT----CATCTTTTTGGCTGCATCGTGGGGTATGTGGGATCTTAGTCCCCCAACCAGGGATTGAATTCATGTGCCCTGCATTGAAAGTGGAGCCTTAACCTCTGAGT
Sus scrofa                 GATTCTTCCTTCCTT----CCT---T----------------------------------------------------------------------------------------------
Canis lupus familiaris     G-----------------------------------------------------------------------------------------------------------------------
Equus caballus             G-----------------------------------------------------------------------------------------------------------------------

Homo sapiens               -----------------------------CC--------CTCCCTTCCTCCTTCTCTCCTTCTCTCCTCCTCTCTCCTCTCCTCTCCTCTCTTTCCTTCCTTCCTTCCT-TTTTTCTTT-
Pan troglodytes            -----------------------------CC--------CTCCCTTCCTCCTTCTCTCCTTCTCTCCTCCTCTCTCCTCTCCTCTCCTCTCTTTCCTTCCTTCCTTCCT-TTTTTCTTT-
Gorilla gorilla            -----------------------------CC--------TTCCTTCCCTCCCTCCCTCCTTCTCTCCTTCTCTC----CTCCTCTCCTCTCTTTCCTTCCTTCCTTCCT-TTTTTCTTT-
Pongo abelii               -----------------------------CC--------CTCCCTCCCTCCTTCTCT--------CCT----------CTCCTCTCCT----GTCCTTCCTTCCTTCCT-TTTTTCTTT-
Macaca mulatta             -----------------------------CCTTCCTTCCTTCCTTCCTTCCTTC-------------------------------------CTTCCTGCCTTCCTTCCTATTTTTCTTT-
Callithrix jacchus         -----------------------------TT--------TTTCTTCCTTCTTTCTTTCCTTCCTTCCTTTTTC--------TCTTCCTTCCTTCCTTCCTTCCTTCCT-TCCCTCCCTT
Mus musculus               ------------------------------------------------------------------------------------------------------------------------
Rattus norvegicus          ------------------------------------------------------------------------------------------------------------------------
Bos taurus                 GGCCAGGGAAGTCCCTCAAGCAAGGAAGATC--------TT-------------------------------------------------------------------------------
Sus scrofa                 -----------------------------CC--------TT-------------------------------------------------------------------------------
Canis lupus familiaris     ------------------------------------------------------------------------------------------------------------------------
Equus caballus             ------------------------------------------------------------------------------------------------------------------------

Homo sapiens               --TTCTCTTTCTTCTTTATTTC--TTGGG---------------------------------------------------TCTCACTCTCATCACCCAGGCTAGAGTGCAGTGGCACAAA
Pan troglodytes            --TTCTCTTTCTTCTTTATTTC--TTGGG---------------------------------------------------TCTCACTCTCATCACCCAGGCTAGAGTGCAGTGGCACAAA
Gorilla gorilla            --TTCTCTTTCTTCTTTATTTC--TTGGG---------------------------------------------------TCTCACTCTCATCACCCAGGCTAGAGTGCAGTGGCACAAA
Pongo abelii               --TTCTCTTTCTTCTTTATTTC--TTGGG---------------------------------------------------TCTCACTCTCATCACCCAGGCTGGAGTGCAGTGGTGCAAA
Macaca mulatta             --TTCTCTTTCTTCTTTATTTC--GTGGG---------------------------------------------------TCTCACTCTCATCACCCAGGCTGGAGTGCAGTG-------
Callithrix jacchus         CCTTCCCTCCCTTCCTTCCCTCCCTTCCTTCCCTCCTTCCCTCCTTCATATTTTTCTTTTTTCTGTCTTTCTTTCTTGGGTCTCACTCTCATTACCCAGGCTGGAGTGCAGACACACAAC
Mus musculus               -----TCT----------------------------------------------------------------------------------------------------------------
Rattus norvegicus          -----TTT----------------------------------------------------------------------------------------------------------------
Bos taurus                 ------------------------------------------------------------------------------------------------------------------------
Sus scrofa                 ------------------------------------------------------------------------------------------------------------------------
Canis lupus familiaris     ------------------------------------------------------------------------------------------------------------------------
Equus caballus             ------------------------------------------------------------------------------------------------------------------------

Homo sapiens               AATCTCGGCTCACTGCAGCCTTGACTTCCCAGGCTCAGGCTCAGGTGATCCTCACACCTTAGCCTCCCAAGTACCTGGGACTACAGGCACGCACCACCATGCCTAGCTATTCTTT-----
Pan troglodytes            AATCTCGGCTCACTGCAGCCTTGACTTCCCAGGCTCAGGCTCAGGTGATCCTCACACCTTAGCCTTCCAAGTACCTGGGACTACAGGCACGCACCACCATGCCTAGCTATTCTTT-----
Gorilla gorilla            AATCTCGGCTCACTGCAGCCTTGACTTCCCAGGCTCAGGCTCAGGTGATCCTCACACCTTAGCCTCCCAAGTACCTGGGACTACAGGCACGCACCACTATGCCTAGCTATTCTTT-----
Pongo abelii               AATCTCGGCTCACTGCAGCCTTGACTTCCCAGGCTCAGGCTCAGGTGATCCTCACACCTTAGCCTCCCAAGTTCCTGGGACTACAGACCCGCACCACCATGCCTAGCTATTCTTT-----
Macaca mulatta             -------GCTCACTGCAGCCTTGACTTCCCAGGCTCAGGCTCAGGTGATCCTCACACCTTAGCCTCCCAAGTACCTGGGACTACAGGCATGGACCACCACGCCTAGCTATTCTTT-----
Callithrix jacchus         AATCTTGGCTCCCTGCAGCCTTGACTGCCCAGGCTCAGGCTCAGGTGCTCCTCACACCTTAGCCTCCCAAGTAGCTGGGACTACAGGCATGCACCACCATGCCTAGCTTTTTTTTTTTT
Mus musculus               -----------------------------------------------------------------------------------------------------------TT-----------
Rattus norvegicus          ---------------------------------------------------------------------------------------------------------GTTTTTCC-------
Bos taurus                 ------------------------------------------------------------------------------------------------------------------------
Sus scrofa                 ------------------------------------------------------------------------------------------------------------------------
Canis lupus familiaris     ------------------------------------------------------------------------------------------------------------------------
Equus caballus             ------------------------------------------------------------------------------------------------------------------------

Homo sapiens               --------TGTATTTTTGGTAGAGACAGGGTTTTGCTGTGTTGCTCAGGCTGGTCTGGAACCCCTAGGCTCAAATGATGTGCCCAACTCGGCCTCCCAA------AGTGCTGGGATTACA
Pan troglodytes            --------TGTATTTTTGGTAGAGACAGGGTTTTGCTGTGTTGCTCAGGCTGGTCTGGAACTCCTAGGCTCAAATGATGTGCCCAACTCGGCCTCCCAA------AGTGCTGGGATTACA
Gorilla gorilla            --------TGTATTTTTGGTAGAGACAGGGTTTTGCTGTGTTGCTCAGGCTGGTCTGGAACTCCTAGGCTCAAATGATGTGCCCAACTCGGCCTCCCAA------AGTGCTGGGATTACA
Pongo abelii               --------TGTATTTTTGGTAGAGACAGGGTTTTGCTGTGTTGCTCAGGCTGGTCTGGAACTCCTAGGCTCAAATGATGTGCCCAACTTGGCCTCCCAA------AGTGCTGGGATTACA
Macaca mulatta             --------TGT-TTCTTGGTAGAGACAGGGTTTT---GTGTTGCTCAGGCTGGTCTGGAACTCCTAGGCTCAAATGATGTGCCCAACTCGGCCTCCCGA------AGTGCTGGGATTATA
Callithrix jacchus         TTTTTTTCTGTATTTTTAGTAGAGACAGGGTTTTACTATGTTGCTCAGGCTGGTCTGGAACACCTAGGCTCAAGCGATATGCCCAGCTCAGCCTCCCAA------AGTGCTAGGATTACA
Mus musculus               --------CTTTTCTTTTGTTTTGATAGGGTCTCACCTTATAGCCCAAGTTGGCCTTGAACTGACAT----------TCTTCCTGCCTCAACCTCCCAAATTGAGAGTTCTGGGATTAGA
Rattus norvegicus          --------TTTTTCTTTGGTTTTGATAGGTTCTCACCTTATAGCCCAGGCTGGCCTTGAACTGGTAT----------TCTTCCTGCCTCAGCCTTCCAAGTTTGGAGTTCTGGGATTAGA
Bos taurus                 ------------------------------------------------------------------------------------------------------------------------
Sus scrofa                 ------------------------------------------------------------------------------------------------------------------------
Canis lupus familiaris     ------------------------------------------------------------------------------------------------------------------------
Equus caballus             ------------------------------------------------------------------------------------------------------------------------
```

```
Homo sapiens             GGCATGAACCGCCATGCCTGGCC---CTTGATTTTTCTTTTTAAG------------------------------------------------------AAAAAA----A---TATCTAG
Pan troglodytes          GGCATGAACCGCCATGCCTGGCC---CTTGATTTTTCTTTTTAAG------------------------------------------------------AAAAAA----A---TATCTAG
Gorilla gorilla          GGCATGAACCGCCATGCCTGGCC---CTTGATTTTTCTTTTTAAG------------------------------------------------------AAAAAA----A---TATCTAG
Pongo abelii             GGCATGAACAACCATGCCTGGCC---CTTGATTTTTCTTTTTAAG------------------------------------------------------AAAAAA----A---TATCTAG
Macaca mulatta           GGCATGAACCACCATGCCTGGCC---CTTGATTTTTCTTTTTAAG------------------------------------------------------AAA-AA----A---TATCTAG
Callithrix jacchus       GGCCTGAGCCACCATGCCCAGCC---CCTGATTTTTCTTTTTTAA------------------------------------------------------AAAAAT----A---TAGCTAC
Mus musculus             GGAACATCCCACCACTAGTTCCCCATCTCGGTTTTTCTTTCTGAG------------------------------------------------------AAAAAA----A----------
Rattus norvegicus        GGAATATACTACCACTAGAGCTCCATCTT-GTTTTTCTTTCTGAGGTAAAAAAAAAAAAAAAAACCTGGAGTTTGAGGGACTCTAACTAGATTGTTAAAAAAAAA----A----------
Bos taurus               -----------------------------GATTCTTCTTCATAAG------------------------------------------------------GAAAAAAAAAAA---ATATGA
Sus scrofa               -----------------------------GATTCTTCTTTCTAAG------------------------------------------------------AA-------------------
Canis lupus familiaris   ------------------------------ATTCTTCTTTCTAAG------------------------------------------------------AAAAAAAAAAAAAGTATCCCA
Equus caballus           ------------------------------ATTCTTCTTTCTAAA------------------------------------------------------AAAAA----------GCCTGA

Homo sapiens             GAGTTTCTTAGACCCTATGTAGAT------------------------------------------------------------------------------------------------
Pan troglodytes          GAGTTTCTTAGACCCTATGTAGAT------------------------------------------------------------------------------------------------
Gorilla gorilla          GAGTTTCTTAGACCCTATGTAGAT------------------------------------------------------------------------------------------------
Pongo abelii             GAGTTTCTTAGACCCTATGTAGAT------------------------------------------------------------------------------------------------
Macaca mulatta           GAGTTTCTTAGACCCTTTGTAGAT------------------------------------------------------------------------------------------------
Callithrix jacchus       GAGTTTCTTAGACTCTATGTAGAT------------------------------------------------------------------------------------------------
Mus musculus             ------------------------------------------------------------------------------------------------------------------------
Rattus norvegicus        ------------------------------------------------------------------------------------------------------------------------
Bos taurus               GAGCTTCTTGGACTCTA-------CATAGATAATTGCTAAGTCTCTTCAGTCGTGTTTGATTCTGTGCAACCCTGACCTAATGGACTATAGCCTACCGGGCTCCTCTGTCCATGGGATTC
Sus scrofa               ------------------------------------------------------------------------------------------------------------------------
Canis lupus familiaris   GAGTTTCTTGGACCCTATATAGAT------------------------------------------------------------------------------------------------
Equus caballus           GGGTTTCTTGGGCCCTTCATAGAT------------------------------------------------------------------------------------------------

Homo sapiens             ----------------------------------------------------------------------------------------TATTAATGAACAAAAGATTAAACTCCAAATAT
Pan troglodytes          ----------------------------------------------------------------------------------------TATTAATGAACAAAAGATTAAACTCCAAATAT
Gorilla gorilla          ----------------------------------------------------------------------------------------TATTAATGAACAAAAGATTAAACTCCAAATAT
Pongo abelii             ----------------------------------------------------------------------------------------TATTAATGAACAAAAGATTAAACTCCAAATAT
Macaca mulatta           ----------------------------------------------------------------------------------------TATTAATGAACAAAAGATTAAACTCCAAATAT
Callithrix jacchus       ----------------------------------------------------------------------------------------TATTAATGAACAAGAGATTAGACTCCAAATAT
Mus musculus             ---------------------------------------------------------------------------------------------AAAAATACAAGACTAAGTTCCAAT---
Rattus norvegicus        ---------------------------------------------------------------------------------------------AAAAATACAAGACTAAGTTCCAAT---
Bos taurus               TCCAGGTAAGAATACTGGAGTGGGTTGCCATGCCCTCCTTCAGGGGATCTTCCCGATCCAGGGACCAACCCTGCGATTCACAGATGATTATTAATAAACAAAAACTTAAATTCCAAATAG
Sus scrofa               ------------------------------------------------------------------------------------------------------------------------
Canis lupus familiaris   ----------------------------------------------------------------------------------------GATGACTAAACACTGGATTCAATTCCAAATAT
Equus caballus           ----------------------------------------------------------------------------------------TATGAACAAACAAAAGATTAAATTCCAAATGT

Homo sapiens             TAAATAGTAAGCCTGAAGGAATCTGAAACACTTGTACTTCCAATTTTCTTT---------------------------------------------------------------------
Pan troglodytes          TAAATAGTAAGCCTGAAGGAATCTGAAACACTTGTACTTCCAATTTTCTTT---------------------------------------------------------------------
Gorilla gorilla          TAGATAGTAAGCCTGAAGGAATCTGAAACACTTGTACTTCCAATTTTCTTT---------------------------------------------------------------------
Pongo abelii             TAGATAGTAAGCCTGAAGGAATCTGAAACACTTGTACTTCCAATTTTCTTT---------------------------------------------------------------------
Macaca mulatta           TAGATAGTAAGCCTGAAGGAATCTGAAACACTTGTACTTCCAATTTTCTTT---------------------------------------------------------------------
Callithrix jacchus       TAGATAGTAAATCTGAAGGAATCTGAAACACTTGTACTTCCAATTTTCTTT---------------------------------------------------------------------
Mus musculus             --------------------ACCTAAAACTTCTGAACTT-TAGTTTCCTTTAAGTACACCAGGGTTGGCCAGACAGCTCGGTTGATCAAGTTACTTACCATTCTCTTCAGAGGGAGCAAT
Rattus norvegicus        --------------------ACCTAAAACTCCTGAATCT-TAGTTTCCT------ACACAAGGGTTGGCCAGACAGCTTGGTTGGTCAAGTTGCTTGTCAGTCTCTTCAGACGGAGTGAT
Bos taurus               GAGGTCAGAAGCCTAAAGGAATCTGAACCAG-----------GTGTCCTTT---------------------------------------------------------------------
Sus scrofa               ------------------------------------------------------------------------------------------------------------------------
Canis lupus familiaris   GCGATAGGAAGTCTGAAGGAATCTGAAGCACTTGA------AGTTT--------------------------------------------------------------------------
Equus caballus           GAGACAGGAAGCCTAAAGGAATCTGAAGCACTTCA------GGTTTCCTTT---------------------------------------------------------------------

Homo sapiens             ------------------------------------------------------------------------------------------------------------------------
Pan troglodytes          ------------------------------------------------------------------------------------------------------------------------
Gorilla gorilla          ------------------------------------------------------------------------------------------------------------------------
Pongo abelii             ------------------------------------------------------------------------------------------------------------------------
Macaca mulatta           ------------------------------------------------------------------------------------------------------------------------
Callithrix jacchus       ------------------------------------------------------------------------------------------------------------------------
Mus musculus             AATGTGAACAGGAGATAGGAATTGGCCAGGCAGGGGCTGGAGGGATGCT---CAGCCATTAAGCACATTTGCTATTCTTTTGGAGGAGTTCAGATCGCAGCACCCATGTAGTGGTTCACA
Rattus norvegicus        AATGTGAACAGGAGATAGAAACTGGCCAGGCAGGGGCTGAAGAGCTACTCGTCAGCAACTAAACACACTTGCTACTCTTTTGAAGG------GATCCCAGTACCCATGCGGTGGTTCCCA
Bos taurus               ------------------------------------------------------------------------------------------------------------------------
Sus scrofa               ------------------------------------------------------------------------------------------------------------------------
Canis lupus familiaris   ------------------------------------------------------------------------------------------------------------------------
Equus caballus           ------------------------------------------------------------------------------------------------------------------------

Homo sapiens             ------------------------------------------------------------------------------------------------------------------------
Pan troglodytes          ------------------------------------------------------------------------------------------------------------------------
Gorilla gorilla          ------------------------------------------------------------------------------------------------------------------------
Pongo abelii             ------------------------------------------------------------------------------------------------------------------------
Macaca mulatta           ------------------------------------------------------------------------------------------------------------------------
Callithrix jacchus       ------------------------------------------------------------------------------------------------------------------------
Mus musculus             ATTCTCTATAAC----------GGGGATCCAACACCTTCTTCTGGTCTCTTTGGGTACTTTGCATATGTGGTACACATGCATACATGCAGACCATACACATAAAATAAAAACAAATAAAT
Rattus norvegicus        ACTATCAATAACTCTTGTTTCAGGGGATTCAACCTCTTCTTCTGGTCTCTTTAAGCACTGCACACATGTGGCACATACACATACATGCAGACCAGACACATAAAATAAAAACAA----AA
Bos taurus               ------------------------------------------------------------------------------------------------------------------------
Sus scrofa               ------------------------------------------------------------------------------------------------------------------------
Canis lupus familiaris   ------------------------------------------------------------------------------------------------------------------------
Equus caballus           ------------------------------------------------------------------------------------------------------------------------

Homo sapiens             ------------------------------------------------------------------------------------------------------------------------
Pan troglodytes          ------------------------------------------------------------------------------------------------------------------------
Gorilla gorilla          ------------------------------------------------------------------------------------------------------------------------
Pongo abelii             ------------------------------------------------------------------------------------------------------------------------
Macaca mulatta           ------------------------------------------------------------------------------------------------------------------------
Callithrix jacchus       ------------------------------------------------------------------------------------------------------------------------
Mus musculus             CTTTAAGAAGAAAAGAACCTGGCTAGGCAGTTTCCAGGCATTCCAAAAGTGTCCCTGGTCTTCTGGTGCATGAACCTCACAAGGAGCTCTGATTTCCCATTGTCCCCTTCCTGAAGGTTA
Rattus norvegicus        CCTTAGGAAGGAAAGAAACAGGCCGGGCAGTTTCTAGGCATTCCCAATGTATCCCTGGTTCTCTGGTCC-------------------------------TGTCCCCTTCCTAAGGGTTA
Bos taurus               ------------------------------------------------------------------------------------------------------------------------
Sus scrofa               ------------------------------------------------------------------------------------------------------------------------
Canis lupus familiaris   ------------------------------------------------------------------------------------------------------------------------
Equus caballus           ------------------------------------------------------------------------------------------------------------------------

Homo sapiens             ------------------------------------------------------------------------------------------------------------------------
Pan troglodytes          ------------------------------------------------------------------------------------------------------------------------
Gorilla gorilla          ------------------------------------------------------------------------------------------------------------------------
Pongo abelii             ------------------------------------------------------------------------------------------------------------------------
Macaca mulatta           ------------------------------------------------------------------------------------------------------------------------
Callithrix jacchus       ------------------------------------------------------------------------------------------------------------------------
Mus musculus             CACTTGAGGGTGGGACCTTCAGTCAGACACTAATCCATTTGTGGATAGTGTTAGAATTAGGTCCATAGATTT-TTCATGATAAAATGGCTTATATCTGTTCCCCAGTTTTAGCCTAATCT
Rattus norvegicus        CACTTGAGGGTGGGACCTTTAGCCAGACACTAATCCATTTGTGGATAGCTGTAGAATTAGGTCCATAGATTTTTTCATGATAAAATGGCTTATATCTGTTCCCCAATTTTAGCCC-ATCT
Bos taurus               ------------------------------------------------------------------------------------------------------------------------
Sus scrofa               ------------------------------------------------------------------------------------------------------------------------
Canis lupus familiaris   ------------------------------------------------------------------------------------------------------------------------
Equus caballus           ------------------------------------------------------------------------------------------------------------------------

Homo sapiens             ------------------------------------------------------------------------------------------------------------------------
Pan troglodytes          ------------------------------------------------------------------------------------------------------------------------
Gorilla gorilla          ------------------------------------------------------------------------------------------------------------------------
Pongo abelii             ------------------------------------------------------------------------------------------------------------------------
Macaca mulatta           ------------------------------------------------------------------------------------------------------------------------
Callithrix jacchus       ------------------------------------------------------------------------------------------------------------------------
Mus musculus             CCTATATGATTTTAGGGGTAGCTCATCAGAGGGGGAAATTTACAGTAGCCTTGATCAGGACTTTTCTGAGTCTCATTAAGAGAAAACCATTGCCTTCTGGTCAGAAAAATGGAATCTACC
Rattus norvegicus        CCTAAATGATTTTAGGGTTACCTCATCAGAGGGGAGAATTTACAGTAGCCTTAGTCAGGGCTCTTCTGAATCTCATTCAGAGAAAACCGTTGCCTTCTGGTCAGAAGAATGG--------
Bos taurus               ------------------------------------------------------------------------------------------------------------------------
Sus scrofa               ------------------------------------------------------------------------------------------------------------------------
Canis lupus familiaris   ------------------------------------------------------------------------------------------------------------------------
Equus caballus           ------------------------------------------------------------------------------------------------------------------------

Homo sapiens             ------------------------------------------------------------------------------------------------------------------------
Pan troglodytes          ------------------------------------------------------------------------------------------------------------------------
Gorilla gorilla          ------------------------------------------------------------------------------------------------------------------------
Pongo abelii             ------------------------------------------------------------------------------------------------------------------------
Macaca mulatta           ------------------------------------------------------------------------------------------------------------------------
```

```
Callithrix jacchus         ------------------------------------------------------------------------------------------------------------------------
Mus musculus               TCTTGCCTAGGCCGAGACCTACCATTGCAAGCTAACCAAAACATAAGCGTTAATCAAGTCGGGCAGGTCCATCACCAGGCTAATGGACTGCCCATTAGGAAGGCAGATTCACCTAACTCT
Rattus norvegicus          -----TCTAAGCAGAGACATACCATTGCAGGCTAACCACAGCATGA--GTTAATCCAGTTGGACAGGTTCATCGCCTGGCTAGTGGACTGCCCATCAGGAAGGCAGATTCACCT-TCTCT
Bos taurus                 ------------------------------------------------------------------------------------------------------------------------
Sus scrofa                 ------------------------------------------------------------------------------------------------------------------------
Canis lupus familiaris     ------------------------------------------------------------------------------------------------------------------------
Equus caballus             ------------------------------------------------------------------------------------------------------------------------

Homo sapiens               ------------------------------------------------------------------------------------------------------------------------
Pan troglodytes            ------------------------------------------------------------------------------------------------------------------------
Gorilla gorilla            ------------------------------------------------------------------------------------------------------------------------
Pongo abelii               ------------------------------------------------------------------------------------------------------------------------
Macaca mulatta             ------------------------------------------------------------------------------------------------------------------------
Callithrix jacchus         ------------------------------------------------------------------------------------------------------------------------
Mus musculus               ATGCTAATGAGATGGAAGGAATAATTATCCCTTTAATGAAGTAATGTCCTTTTCCTTCCCAGAGTCCCTGCTATCTGTACAGTCACTACAGAACTACCATGAAAAGTAATTTTCACTTTT
Rattus norvegicus          ATGCTAATGAGATGGGAAGAATAACTATCCCTTTAATGAGGTCATATACTTTTCCTTCCCAGAATCCCTGCTATCTATACAGCCACTACAGAACCACCATGAAATGTCATTTTCACTTTT
Bos taurus                 ------------------------------------------------------------------------------------------------------------------------
Sus scrofa                 ------------------------------------------------------------------------------------------------------------------------
Canis lupus familiaris     ------------------------------------------------------------------------------------------------------------------------
Equus caballus             ------------------------------------------------------------------------------------------------------------------------

Homo sapiens               ------------------------------------------------------------------------------------------------------------------------
Pan troglodytes            ------------------------------------------------------------------------------------------------------------------------
Gorilla gorilla            ------------------------------------------------------------------------------------------------------------------------
Pongo abelii               ------------------------------------------------------------------------------------------------------------------------
Macaca mulatta             ------------------------------------------------------------------------------------------------------------------------
Callithrix jacchus         ------------------------------------------------------------------------------------------------------------------------
Mus musculus               TCTTTTTCTTTTT----------GCTGGCCCAGGCCTAAGCGCACATTCTTTAGTACTTTGAGCTGTTTCACTCTCTCCATGTGGCTGCATGCTAACCCTCTACCCACTCAAAAGAAAGC
Rattus norvegicus          TCCTTTTCGTTTTCTGCTTTGGCACTGGCCCAGGTTTAAGTACACACTCTCTAGTATTCTGGGCTGTTTCTCTTTCTCCATGTGGCTACATGCTTACCTTCTACCTACTCAAAA---ACA
Bos taurus                 ------------------------------------------------------------------------------------------------------------------------
Sus scrofa                 ------------------------------------------------------------------------------------------------------------------------
Canis lupus familiaris     ------------------------------------------------------------------------------------------------------------------------
Equus caballus             ------------------------------------------------------------------------------------------------------------------------

Homo sapiens               ------------------------------------------------------------------------------------------------------------------------
Pan troglodytes            ------------------------------------------------------------------------------------------------------------------------
Gorilla gorilla            ------------------------------------------------------------------------------------------------------------------------
Pongo abelii               ------------------------------------------------------------------------------------------------------------------------
Macaca mulatta             ------------------------------------------------------------------------------------------------------------------------
Callithrix jacchus         ------------------------------------------------------------------------------------------------------------------------
Mus musculus               GTGGCTTTCTCTCTTCTTTTTCTCTATATCCCTCTTCCAGGTATGTTTGATCCCCATAAGCCATAGGGTAGAAGGAGAGAACCAATTCCTGCAAGCTGTCATTTTACAC---ACACACAC
Rattus norvegicus          GTGGCT-TCTCTCTTCCTTTTCTCTAT-TCCTTCTTCCGGGCAGTTTTGATCCCCATAACCCATGGGGTAGAAGAAGAAAACCACTTCCTGCAAGCTGTCATTACACACCACACACACAC
Bos taurus                 ------------------------------------------------------------------------------------------------------------------------
Sus scrofa                 ------------------------------------------------------------------------------------------------------------------------
Canis lupus familiaris     ------------------------------------------------------------------------------------------------------------------------
Equus caballus             ------------------------------------------------------------------------------------------------------------------------

Homo sapiens               ------------------------------------------------------------------------------------------------------------------------
Pan troglodytes            ------------------------------------------------------------------------------------------------------------------------
Gorilla gorilla            ------------------------------------------------------------------------------------------------------------------------
Pongo abelii               ------------------------------------------------------------------------------------------------------------------------
Macaca mulatta             ------------------------------------------------------------------------------------------------------------------------
Callithrix jacchus         ------------------------------------------------------------------------------------------------------------------------
Mus musculus               ATGTACAAATA-------------------------------------------------------------------------------------------------------------
Rattus norvegicus          ACACACACACACACACACACACACACATACACACACACACATTCATGTAATTTTTTTTTTTTTTTTGGTTCTTTTTTTCGGAGCTGGGGACCGAACCCAGGGCCTTGCGCTTCCTAGGTA
Bos taurus                 ------------------------------------------------------------------------------------------------------------------------
Sus scrofa                 ------------------------------------------------------------------------------------------------------------------------
Canis lupus familiaris     ------------------------------------------------------------------------------------------------------------------------
Equus caballus             ------------------------------------------------------------------------------------------------------------------------

Homo sapiens               ----------------------------------------------AAATAATCCCAAATAGACCAGAATTGGCCCATACCA----TAGAAGAAAGAATTGGC---AGTCAAAAA-----
Pan troglodytes            ----------------------------------------------AAATAATCCCAAAAAGACCAGAATTGGCCCATACCA----TAGAAGAAAGAATTGGC---AGTCAAAAAA----
Gorilla gorilla            ----------------------------------------------AAATAATCCCAAATAGACCAGAATTGGCCCATACCA----TAGAAGAAAGAATTGGC---AGTCAAAAAA----
Pongo abelii               ----------------------------------------------AAATAATCCCAAATAGACCAGAATTGGCCCATACCA----TAGAAGAAAGAATTAGC---AGTCAAAAA-----
Macaca mulatta             ----------------------------------------------ACATAATCCCAAGTAGACCAGAATTGGCCCATACCA----TAGAAGAAAGAATTGGC---AGTCAAAAAA----
Callithrix jacchus         ----------------------------------------------AAGTAATCCCAAGTAGACTAGAGTTGGCCCATACCATACATAGAAGAAAGAATTGGC---AGTCAAAAAC-AAA
Mus musculus               ---------------------------------ATGTAATT-TTTAAAACCACAACAAGTAGACCAATGTTTACTCATACCT----TTGAAAAATGGACTGACCTTATTTAAAAAAA---
Rattus norvegicus          AGCGCTCTACCATTGAGCTAAATCCCCAGCCCCATGTAATTTTTTAAAACCACGCCAAGTAGACCAATGTTTACTCATACCA----CAGAAGGATAAACTGACCTTATTTAAAACAA---
Bos taurus                 ----------------------------------------------AAATCCCTCCCAATAGACCAA----------------------AAGAAAGAAATGAT---G-TGAGAAAA----
Sus scrofa                 ------------------------------------------------------------------------------------------------------------------------
Canis lupus familiaris     -----------------------------------------------------CCCCAATAGACCAAAGTTTACCCATACCA----TGAAAGAAAGAAATGAC---A-TGAGGA------
Equus caballus             ----------------------------------------------AAATACCCCCAAACAGACCAAAGTTTACCCACACCA----TAGAAGAAAGAAATGAT---G-TGAGAA------

Homo sapiens               ---------AA--AAAATACCTTTTGTAA---TGTTTGAA-AAATAAAGCTGTTTGACT-TGTCAGGTG-TTTTCCTTTCTCAAATCAGCAAATT--CTCTCTGAGTGCCTGGCTTTGTG
Pan troglodytes            ---------AA--AAAATACCTTTTGTAA---TGTTTGAA-AAATAAAGCTGTTTGACT-TGTCAGGTG-TTTTCCTTTCTCAAATAAGCAAATT--CTCTCTGAGTGCCTGGCTTTGTG
Gorilla gorilla            ---------AA--AAAATACCTTTTGTAA---TGTTTGAA-AAATAAAGCTGTTTGACT-TGTCAGGTG-TTTTCCTTTCTCAAATCAGCAAATT--CTCTCTGAGTGCCTGGCTTTGTG
Pongo abelii               ----------A--AAAATACCTTTTGTAA---TGTTTCAA-AAATAAAGCTGTTTGACT-TGTCAGGTG-TTTTCCTTTCTCAAATCAGCAAATT--CTCTCTGAGTGCCTGGCTTTGTG
Macaca mulatta             -----AAAAAA--AAAATGCCTTTTGTAAT-ATGTTTGAA-AAATAAAGCTGTTTGACT-TGTCAGGTG-TTTCGCTTCCTTAAATCAGCAAATT--CTCCCTGAGTACCTGGCTTTGTG
Callithrix jacchus         CAAAC----AA--AAAAAACCTTTTGTAATTATGTTTGAA-AAATAAAGCTATTTGACTTTGTCAGCTG-TTTTCCTTTCTCAAATCAGCAAATT--C----TGAGTCCAGAA-ATTCT-
Mus musculus               ------------------AAATTATACAA-------------------------------------------------------------------------------------------
Rattus norvegicus          ---------CA--ACAACAACTTACGTGA-------------------------------------------------------------------------------------------
Bos taurus                 ---------AA--AAAATCTCTTTTGTCAT-CTATTTAAAAAATCCCAGCTGTTTGAAT-TAA--TTTG-TTTTCCTTTCTCAAATCAGCAAGCTGTGTCTCTGAGTACC-----ATATA
Sus scrofa                 ------------------------------------------------------------------------------------------------------------------------
Canis lupus familiaris     -----------ACAAAATATCTTTTGTAAT-CTATTTAAA-AATCCGAGCTTTTTGACT-TGTCAGGTGGATTTCCTTTCTCAAATCAGCACATT--CTTTCTGAGTGCCTAGCAGTGTG
Equus caballus             -------------GAAATATCTTTTGTAAT-CTATTTAAA-AATCTGAGCTCTTTGACT-TGTTGGGTG-TTTTCCTTTCTCAAATCAGCAGATT--CCCTCTGAGTCCCCAGCAGCATG

Homo sapiens               AGACACTGTACAAGGA-------GTTACAAGACTACAGCTATAACCTGCAGTTGAGCAGTTATAAACCTACAAA-ATGGGCCCTGCCCTCAGAGAGGT-TCCAGTCTAGATGAGGAGCTG
Pan troglodytes            AGACACTGTACAAGGA-------GTTACAAGACTACAGCTATAACCTGCAGTTGAGCAGTTATAAACCTACAAA-ATGGGCCCTGCCCTCAGAGAGGT-TCCAATCTAGATGAGGAGCTG
Gorilla gorilla            GGACACTGTACAAAGA-------GTTACAAGACTACAGCTATAACCTGCAGTTCAGCAGTTACAAACCTACAAA-ATGGGCCCTGCCCTCAGAGAGGT-TGCAGTCTAGATGAGGAGCTG
Pongo abelii               AGACACTGCACAGGGA-------GTTACAAGACTACAGCTATAACCTGCAGTTGAGCAGTTATAAACCTACAAA-ATGGGCCCTGCCCTCAGAGAGTT-TCCAGTCTAGATGAGGAGATG
Macaca mulatta             AGACACAGTTCAAGGA-------GTTACAAGACTACAGCTATAACCTACAGTTGAGCAGTTATAAACCAACAAA-ATGGGCCCTGCCCTCAGACAGGT-TCCAGTCTAGATGAGGAGATG
Callithrix jacchus         -CTCTCTGTACGAGGA-------GTTACAAGACTACAGCTCTAACCTGCAGTCGAGCAGTTATAAACCTACAAA-ATGGGCCCTGCCCTCAGAGTGGT-TCCAGTCTACATGAGAAGATG
Mus musculus               ------------------------------------------------------------------------------------------------------------------------
Rattus norvegicus          ------------------------------------------------------------------------------------------------------------------------
Bos taurus                 AGACAGTGTTCAGAGAGCTAACC-----------------------------------ATCATAAACCTACCAGCCT-GGCCCTGCTC-----------------------------ATG
Sus scrofa                 ------------------------------------------------------------------------------------------------------------------------
Canis lupus familiaris     AAGCACTGGTCAAGCAGGCAAGA-----------------------------------GTCCTACACCCAGGAGCATGGGCCATGCTCTCAAGGAGCTTTACTGCCTCATTGAGGATGTG
Equus caballus             AGGCACTGGTCAAGGAGAACACA-----------------------------------GTCATAAACCCACGAGCAGGGGCCGTGCTCTCAGGGCACC-TGCAGTCTCGTTGAGGACGTG

Homo sapiens               ATCTAGACAGGTAAAAGGCTAACT----AACCCTTTGTGTAAA-TAAGTTCATCACC---------------------------CCAGTAAAAGTGTCATCACCCAGTGAATAGGACCAC
Pan troglodytes            ATCTAGACAGGTAAAAGGCTAACT----AACCCTTTGTGTAAA-TAAGTTCATCACC---------------------------CCAGTAAAAGTGTCATCACCCAGTGAATAGGACCAC
Gorilla gorilla            ATCTAGACAGGTAAAAGGCTAACT----AAGCCTTTGTGTAAA-TAAGTTCATCACC---------------------------CCAGTAAAAGTGTCATCACCCAGTGAATAGGACCAC
Pongo abelii               ATCTAGACAGGTAAAAGGCTAACT----AACCCTTCGTGTAAA-TAAGTTCATCACC---------------------------CCAGTAAAAGTGTCATCACCCAGTGAATAGGACCAT
Macaca mulatta             ATCTAGACAGGTAAAAGGCTAACT----AATCCTTTGTGTGAATTAAGTTCATCACC---------------------------CCAATAAAAGTGTCATCACCCAGTGAATAGGACCAT
Callithrix jacchus         GTCTAGACAGGT------------------------------------------------------------------------------------------------------------
Mus musculus               ------------------------------------------------------------------------------------------------------------------------
Rattus norvegicus          ------------------------------------------------------------------------------------------------------------------------
Bos taurus                 GCCTCTGAATATGAAAAGCTAAATAAATAACCTTGTGTGTAAA-GAAG---------GAAGCACACACTGGAAGCCAGTAGAAGCCAAGAGAAGTGTCGTTGCTGAATGAGTATGACTCC
Sus scrofa                 ------------------------------------------------------------------------------------------------------------------------
Canis lupus familiaris     GCCTCTGCATGGGAGAAGCTAAAT----GACCTAATGTGTAAA-TAAGT-CACAACC---------------------------CCAATAGAAGCGCCATTGCCAAATGAATACAACCAC
Equus caballus             ACCTTGACAAGTGAAAAGCTAAATATGTAACCTCATGTGTAAA-TAAGTTCATGATG---------------------------CCAATAGAAGCGTCATTGCCAAGTGAGTACAGCTGT

Homo sapiens               CTCTGCC---TGCAGATTTTTGTTGTTGTTGTTGTCATTGTTGTTGTTGTTTTAACCTGGGAAGTGTTCTTCCTGCCTTTCTGCTAGGTGTCAGATAGATGGTCCCAGAGCTAGGTGCTG
Pan troglodytes            CTCTGCC---TGCAGATTTTTGTTGTTGTTGTTGTCATTGTTGTTGTTGTTTTAACCTGGGAAGTGTTCTTCCTGCCTTTCTGCTAGGTGTCAGATAGATGGTCCCAGAGCTAGGTGCTG
Gorilla gorilla            CTCTGCC---TGCAGATTTTTGTTGTTGTTGTTGTCGT---TGTTGTTGTTTTAACCTGGGAAGTGTTCTTCCTGCCTTTCTGCTAGGTGTCAGATAGATGGTCCCAGAGCTAGGTGCTG
Pongo abelii               CTCTGCC---TGCAGATTTTTGTTGTTGTTGTTGTTGTTGTTGTTGTTGTTTTAACCTGGGAAGTGTTCTTCCTGCTTTTCTGCTAGGTGTCAGATAGATGGTCCCAGAACTAGGTGCTG
Macaca mulatta             CTCCGCT---TGCAGGTTTTTGTTGATATTGTCGTTATTG----TGTTGTTTTAACCTGGGAAGTGTTCTTCCTGCCTTTCTGCTAGGTGTCAGATAGATGGTCCCAGAACTAGGTGCTG
Callithrix jacchus         ------------------------------------------------------------------------------------CAGGTGTCAGATAGATGGTCCCAGAACTAGGTGCTG
Mus musculus               ------------------------------------------------------------------------------------------------------------------------
Rattus norvegicus          ------------------------------------------------------------------------------------------------------------------------
Bos taurus                 TCCATCC---TGG--ATTCCT-----------------------CTCTTTCCTTTCTTGGGAAGTACTCT--CCACGTCTCTGCTGGGTGTCAGATAGATGGTTCCAG------GCAGCA
Sus scrofa                 ------------------------------------------------------------------------------------------------------------------------
Canis lupus familiaris     TTCCGCT---ATG--ATGTTT-----------------------CTTTTTCTTTACTTCGGATGCACTCCT-CTACTTGTCCGCTAGGTGTCAGATGGTTGGTTCCAG------GCACTA
```

```
Equus caballus            CTCTGCCGTTCTG--TCTTTT-----------------------TCTTTTCCTGACTTGGAGAGCGTGCCTCCTGCTTCCCCAGGAGGTGTCGGCTGGACAGTTCCAG------GCACTG

Homo sapiens              TGTCAGGCCCTGAAGACACAGATGACTCAACCTAAGCTTTACTTTCCAGAGGTCCACAGCCTGAGAGGTGTCCCCAAAGAAAGGGGGACATGAGG-GGACTGCATGCTTGAGAGCAGGGT
Pan troglodytes           TGTCGGCCCTGAAGACACAGAGGACTCAACCTAAGCTTTACTTTCCAGAGGTCCACAGCCTGAGAGGTGTCCCCAAAGAAAGGGGGACATGAGG-GGACTGCATGCTTGAGAGCAGGGT
Gorilla gorilla           TGTCGGCCCTGAAGACACAGATGACTCAACCTAAGCTTTACTTTCCAGAGGTCCACAGCCTGAGAGGTGTCCCCAAAGAAAGGGGGACATGAGGGGGACTGCATGCTTGAGAGCAGGGG
Pongo abelii              TGTCAGGCCCTGAAGACACAGATGACTCAACCTAAGCTTTGCTTTCCAGAGGTCCACAGCCTGAGAGGTGTCCCCAAAGAAAGGGGGACATGAGG-GGACAGCATGCTCGAGAGCAGGGG
Macaca mulatta            TGTCGGCCCTGAAGACACAGATGACTCAATCTAAGCTCTGCTTTCAAGA---------GCCTGAGAGGTGTCCCCAGAGAAAGGGGGACATAAGG-GGACAGCATGCTCAAGAGCAGAGG
Callithrix jacchus        TGTCAGGCCCTGAAGACACAGATGACTCAACCTAAGCTCTGCTTTCAAGAAGTCCCCAGCCTTGGAAAAATAC-----------------------------------------------
Mus musculus              ------------------------------------------------------------------------------------------------------------------------
Rattus norvegicus         ------------------------------------------------------------------------------------------------------------------------
Bos taurus                TGTTAC-CCCCAAGGCCAC----------------------------AGAAGTCCATGGTGTGAGGGACATTCTCAAGAAAGGGTAGACATCACA-GGAGGGCCTGGAG--GAGCAGGGA
Sus scrofa                ------------------------------------------------------------------------------------------------------------------------
Canis lupus familiaris    TGATAGGCCCTCAAGATACCCATGACTCAGCCTAATCCCTGCTTTCAAGAGATCTACAGTGTGCAAGACACCTTCAAAAAAGTGTACACATTATG-GGAAGGCATGCTAGAGAGGGAGGA
Equus caballus            TGCTAGGCCCTGAAGACACAGATGACTCAGCCTAACCCTTGCTTTCAAGAGGTCTGCAGTGTGACAGGCGTGCTCCGAGAAG-GGAGACATTAGA-AGAGACGGTGCTGGAGAGTGGGGA

Homo sapiens              TGTTTAGGGCAGGTTTGGATTTAGTGAGCAGGCTGGTTTGCTTAGAG---AAGGCTTTTAGTGGCAACAAAGGATGAAGAGGAGAGAAAAGGAACTCA-CATTTATTGAGGGCCTACTGT
Pan troglodytes           TGTTTAGGGCAGGTTTGTATTTAGTGAGCAGGCTGGTTTGCTTAGAG---AAGGCTTTTAGTGGCAACAAAGGACGAAGCGGAGAGAAAAGGAACTCA-CATTTATTGAGGGCCTACTGT
Gorilla gorilla           TGTTTAGGGCAGGTTTGGATTTAGTGAGCAGGCTGGTTTGCTTAGAG---AAGGCTTTTAGTGGCAACAAAGGACGAAGAGGAGAGAAAAGGAACTCA-CATTTATTGAGGGCCTACTGT
Pongo abelii              TGTTTAGGGCAGGTTTGGATTTAGTGAGCAGGCTGGTTTGCTTAGAG---AAGGTTTTTAGTGGCAACAAAGGACAAAGAGGAGAAAAAAGGAACTCA-CATTTATTGAGGGCCTACTAT
Macaca mulatta            TGTTTAGGGCAGGTTTGGATTTAGTGAGCAGGCTGGTTTGCTTACAG---AAGATTTTTAGTGGCAACAGAGGACAAAGAGGAGAGAAAAGGAACTCA-CATTTATTGAGGGCCTACTGT
Callithrix jacchus        ------------------------------------------------------------------------------------------------------------------------
Mus musculus              ------------------------------------------------------------------------------------------------------------------------
Rattus norvegicus         ------------------------------------------------------------------------------------------------------------------------
Bos taurus                CGCACTGACCATGTTTTGATT-------CAGGCCAG--TGGTTAGAGAAGAAGATTTTTAGTGACGACAAAAGACAAAGAAGAGAGGAAGTGAGCTGA-TAGTTACTGAGAAAATGCTCT
Sus scrofa                ------------------------------------------------------------------------------------------------------------------------
Canis lupus familiaris    --------------TTGGATTTTATGAGCAGACCATTCTGATCAGAG---AATATTTTTAGTGGTGACAAAGGGGACAGAAGAGAGGAATGGAGCTCGTTATTTATTGAGGGTCTATTAT
Equus caballus            CACACAGGCCGTGTTTGGATTTGGTGAGCAGGCTGGTCTGGTTAGAGAGGAAGATCTTTCATGGTGACAGAGGGCAAAGAGCAGAGGAAGGGAACTCA-CATTTGCTGAGGGCCTACTAT

Homo sapiens              GTGCAAAGTGTTTCATGTATATCTCATTGAATGTATACAGCCACCCT------------------------------------------------------------------GTTGTGG
Pan troglodytes           GTGCAAAGTGTTTCATGTATATCTCATTGAATGTATACAGCCACCCT------------------------------------------------------------------GTTGTGG
Gorilla gorilla           GTGCAAAGTGTTTCATGTATATCTCATTGAATGTATACAGCCACCCT------------------------------------------------------------------GTTGTGG
Pongo abelii              GTGCAAAGTGTTTCATGTATATCTCATTCAATGTATACAGCCACCCT------------------------------------------------------------------GTTGTGG
Macaca mulatta            GTGCAAGGTGTTTCATGTATATCTCACTGAATGTATACAGCCACCCT------------------------------------------------------------------GTTGTGG
Callithrix jacchus        ------------------------------------------------------------------------------------------------------------------------
Mus musculus              ------A-----ACACATATATCCTACTTAATGTGTGCAACAACCTACTCTTTATAGGGTGTGTGTGTGTGTGCGCTTGCATGCATGTATGTGTGTGTGGAGGAGGGGTGGGGCTTGTGG
Rattus norvegicus         ------A-----ACACATATATCC-------TGTGTGCAGCAATCTACTCGTTATAGGGTGTGTGTGTGT----GCTTGCATGCATGTGTGTGTGTGTGGAGGAGGGGCAGTGCTTGTGA
Bos taurus                GCGCCAGGCATCTTACATGTATTTCCTTTCATGTGTACAGCATCCCC------------------------------------------------------------------AGTGTGG
Sus scrofa                ------------------------------------------------------------------------------------------------------------------------
Canis lupus familiaris    GTGCCAGGTGTTGTACACATATCTCATTTAATGTAGACAGCAACATC------------------------------------------------------------------AGTA-GG
Equus caballus            GTGCCAGATGCTTTACGCAGATCTCTTTTAACATGTCCAGCAGCCCC------------------------------------------------------------------AGTATGG

Homo sapiens              TATAATTTTGCTCTTTATAA-AG-------------------------------------------------------------------------------------------------
Pan troglodytes           TATAATTTTGCTCTTTATAA-AG-------------------------------------------------------------------------------------------------
Gorilla gorilla           TATAATTTTGCTCTTTATAA-AG-------------------------------------------------------------------------------------------------
Pongo abelii              TATAATTTTGCTCTTTATAA-AG-------------------------------------------------------------------------------------------------
Macaca mulatta            TATAATTTTGCTCTTTATAA-AG-------------------------------------------------------------------------------------------------
Callithrix jacchus        ------------------------------------------------------------------------------------------------------------------------
Mus musculus              TA--------CACTTCATAA-AG-------------------------------------------------------------------------------------------------
Rattus norvegicus         CA--------CACCTCACAA-CA-------------------------------------------------------------------------------------------------
Bos taurus                GATAATTATGCTCATTTTAAAAGAGAGGGGGGACTTCCCTGATGTTCCAGTGGTTAGGACTCCACAGTTCCAAAAATAAAAAAGCAGGGGTTGAAGATTTGATCCCTGGTTGGAGAGCTA
Sus scrofa                ------------------------------------------------------------------------------------------------------------------------
Canis lupus familiaris    TATCATTTTGCCCTTTTTGAAGG-------------------------------------------------------------------------------------------------
Equus caballus            TATGATTTTGCTCTTTTTGAAAG-------------------------------------------------------------------------------------------------

Homo sapiens              ----------------------------------------------------------------------------------------AGAAAG--ACCGAAGCTCAGATGAGTTAAGTG
Pan troglodytes           ----------------------------------------------------------------------------------------AGAAAGAAACCGAAGCTCAGATGGGTTAAATG
Gorilla gorilla           ----------------------------------------------------------------------------------------AGAAAGAAACTGAAGCTCAGATGGGTTAAGTG
Pongo abelii              ----------------------------------------------------------------------------------------AGAAAGAAACTGAAGCTCAGATGGGTTAAGTG
Macaca mulatta            ----------------------------------------------------------------------------------------AGAAAGAAACTGAAGCTCAGATGGGTTAAGTG
Callithrix jacchus        ------------------------------------------------------------------------------------------------------------------------
Mus musculus              ----------------------------------------------------------------------------------------GGAAAGATGC-AAAGGTCAACTAAGTTAAATG
Rattus norvegicus         ----------------------------------------------------------------------------------------AGAAAGAAGC-AAAGCTCTACTGAGTTAAATG
Bos taurus                AGATCCCACATTGCCAAATGGCACAGCCAAAACTAAACAAATTATAATTTTTAAAAATATCTCCCAAATAAAAATAACTAGATAAGTGAGAAAGAAAAAAAAGTCCTCATGGGTTACATG
Sus scrofa                ------------------------------------------------------------------------------------------------------------------------
Canis lupus familiaris    ----------------------------------------------------------------------------------------AGAACGAAGACAAAACTCAGAAGAGTCAAGTG
Equus caballus            ----------------------------------------------------------------------------------------ACAAAGAAACTGAAGCTCAGATGGGTTAAGTG

Homo sapiens              GTCTCCTCAACACCAAAA-TGCCAAGAAGTGATGGAGCCTAGACAGAAGCCCA-------------------------------------------------------------------
Pan troglodytes           GTCTCCTCAGCACCAAAA-TGCTGAGAAGTGATGGAGCCTAGACAGAAGCCCA-------------------------------------------------------------------
Gorilla gorilla           GTCTCCTCAACACCAAAA-TGCTGAGAAGTGATGGAGCCTAGACAGAAGCCCA-------------------------------------------------------------------
Pongo abelii              GGCTCCTCAACACCAAAA-TGCTAAGAAATGATGGAGCCTAGACAGAAGCCCA-------------------------------------------------------------------
Macaca mulatta            GTCTCCTCAACACCAAAA-TGCTAAGAAGTAATGGAGCCTAGACAGAAACCCA-------------------------------------------------------------------
Callithrix jacchus        ------------------------------------------------------------------------------------------------------------------------
Mus musculus              GC---CTCAGCACCAAAA-TGCTCAGAAGTGAA---ACCAGAGCAGAGGCCCA---------------------------------------------------------------GAAC
Rattus norvegicus         GT---CTCAGCACCAAAAGTGCTCAGAAGTGAT---ACCC----------------------------------------------------------------------------GAAC
Bos taurus                GTCTGCTCAACATCAAAA-CGCTAACAGGTGGCAGAGCACA-ACGGAAGTCGGGACCTCTGTGACTCTCCCTAATCCGCTCTCCACCGTGATCGCAGTTTTCAAATCGTGCTTTGT----
Sus scrofa                ------------------------------------------------------------------------------------------------------------------------
Canis lupus familiaris    GTCTCGTCAATATCGAAA-TCCTGACAAGCAGCAGAGCGGACACAGAA----------CTCTGACTCTGGCTAATCCTCTTGCCACTACAATCATGATTTTCAAGTCGTGCTGTAT----
Equus caballus            GCTTTCTCAGTATCAAAC-TGCTGACGAGGGGCAGAGCTGAGACCGGAGTCCAGAGCTCTCTGACTTTCACTAGTCCTCTCTCCACTGTCACTGTGGTTTTCGAACTCTGCTGTGC----

Homo sapiens              ---------------------------------------------------GAACTTTCTGACTCACACTAGTCCATCCTCTACCATCACGATGACTTTCAAATTGTGCTCTGCAGTTCT
Pan troglodytes           ---------------------------------------------------TAACTTTCTGACTCACACTAGTCCATCCTCTACCATCACGATGACTTTCAAATTGTGCTCTGCAGTTCT
Gorilla gorilla           ---------------------------------------------------GAACTTTCTGACTCACACTAGTCCATCCTCTACCATCACGATGACTTTCAAATTGTGCTCTGCAGTTCT
Pongo abelii              ---------------------------------------------------GAACTTTCTCACTCACACTAGTCCATCCTCTACCATCATGATGACTTTCAAATTGTGCTCTGCAGTTCT
Macaca mulatta            ---------------------------------------------------GAACTT--TGACTCACACTAGTCCACCCTCCACCATCTTGATGACTTTCACATTGTGCTCTGCAGTTCT
Callithrix jacchus        ------------------------------------------------------------------------------------------------------------------------
Mus musculus              AAGACTTTAGGGATCCACCCTC---TGTGCGTCGCTCAAATGTGTTTTCTT---------------------------------------------------------------AGTTCT
Rattus norvegicus         AAGACTTTAGGAATCCACCCTTCGTTGTGTGTCACTCCAGTGTGTTTTCTC---------------------------------------------------------------AGTTCT
Bos taurus                ------------------------------------------------------------------------------------------------------------------AGTTCT
Sus scrofa                ------------------------------------------------------------------------------------------------------------------------
Canis lupus familiaris    ------------------------------------------------------------------------------------------------------------------AGTTCT
Equus caballus            ------------------------------------------------------------------------------------------------------------------AGGTCT

Homo sapiens              GCAG-------A-TTTTCTAGCAGTGCCATCTCCAAAATGTGTTTTAAACTCTTTATTTTTT---TA-ATTATTA---TTAGTATTATTTTGAGACTGAGTCTTGCTCTATCACCCAGGC
Pan troglodytes           GCAG-------A-TTTTCTAGCAGTTCCATCTCCAAAATGTATTTTAAACTCTTTATTTTTT---TA-ATTATTATTATTATTATTATTTTGAGACTGAGTCTTGCTCTATCACCCAGGC
Gorilla gorilla           GCAG-------A-TTTTCTAGCAGTTCCATCTCCAAAATGTATTTTAAACTCTTTATTTTTT---AA-ATTATTA---TTATTATTATTTTGAGACTGAGTCTTGCTCTATCACCCAGGC
Pongo abelii              GCAGAG-----A-TTTTCTAGCAGTTCCATCTCCAAAATGTATTTTAAACTCTTTATTTTTT---AAAATTATTA---TTATTATTATTTTGAGATTGAGTCTTGCTCTATCGCCCAGGC
Macaca mulatta            GCAGAG-----A-TTTTCCAGTAGTTCCATCTCCAAAATGTATTTTAAACTCTTTATTTTTTTTTTA-ATTGTTA---TTATTATTATTTTGAGACTGAGTCTTGCTCTATCGCCCAGGC
Callithrix jacchus        ------------------------------------------------------------------------------------------------------------------------
Mus musculus              GTAGAGGACTGA-TTT-TAAGCA-------------CTAGTGTTTTAAACTC--------------------------------------------------------------------
Rattus norvegicus         GTAGAGGATTGA-TTTTTAGGCA-------------AGTGTATTTTAAACTC--------------------------------------------------------------------
Bos taurus                GCAGGG-----A-CATTTCAGCAATTCCATGTTGAAATTGTATTTTACACTC--------------------------------------------------------------------
Sus scrofa                ------------------------------------------------------------------------------------------------------------------------
Canis lupus familiaris    GCAGAT-----TTTTTTTCAGCAGTTCCATCTTTAAAATGTATTTTAAACTC--------------------------------------------------------------------
Equus caballus            GCAGCG-----A-TTTTTCAGCAGTTCCATCTTTAAAGTGTACTTTAAACTC--------------------------------------------------------------------

Homo sapiens              TGGAGTGCAGTGGTGCAATCTCAGCT-CACTGCAACCTCCGCCTCCCAGGTTCAAGCGATT-TCGTGCCTCAGCCTCCCGAGTAGCTGGGATTACAGGCACCCACCACCACGCCCAGCTA
Pan troglodytes           TGGAGTGCAGTGGTGCAATCTCAGCT-CACTGCAACCTCCGCCTCCCAGGTTCAAGCGATTTCGTGCCTCAGCCTCCCGAGTAGCTGGGATTACAGGCACCCACCACCACGCCCAGCTA
Gorilla gorilla           TGGAGTGCAGTGGTNNNNNNNNNNNNNNNNNNNNNNNNNNGCCTCCCAGGTTCAAGCGATTCTCGTGCCTCAGCCTCCCGAGTAGCTGGGATTACAGGCACCCACCACCATGCCCAGCTA
Pongo abelii              TGGAGTGCAGTGGCGCAATCTTAGCT-CACTGCAACCTCCACCTCCCAGGTTCAAGTGATTCTCGTGCCTCAGCCTCCCGAGTAGCTGGGATTACAGGCACCCACCACCACGCCCAGCTA
Macaca mulatta            TGGAGTACAGTGGCACAATCTCAGCT-CACTGAAACCTCTGCCTCCCAGGTTCAAGTGATTCTCATGCCTCAGCCTCCCAAGTAGCTGGGATTACAGGCGCCCGCCACCATGCCCGACTA
Callithrix jacchus        ------------------------------------------------------------------------------------------------------------------AAAATA
Mus musculus              ------------------------------------------------------------------------------------------------------------------------
Rattus norvegicus         ------------------------------------------------------------------------------------------------------------------------
Bos taurus                ------------------------------------------------------------------------------------------------------------------------
Sus scrofa                ------------------------------------------------------------------------------------------------------------------------
Canis lupus familiaris    ------------------------------------------------------------------------------------------------------------------------
Equus caballus            ------------------------------------------------------------------------------------------------------------------------

Homo sapiens              ATTTTTGTATTTTTAGTAGAAATGGGGTTTCACCATGTTGGCCAGGCTGGTCTCGAACTCCTGACCTCAAGTGATCCACTCACCTCGGCCTCCCAAAGTGCTGGGATTACAGGTGTGAGC
Pan troglodytes           ATTTTTGTATTTTTAATAGAAATGGGGTTTCACCATGTTGGCCAGGCTGGTCTCGAACTCCTGACCTCAAGTGATCCACTCACCTCGGCCTCCCAAAGTGCTGGGATTACAGGTGTGAGC
Gorilla gorilla           ATTTTTGTATTTTTAGTAGAAATGGGGTTTCACCATGTTGGCCAGGCTGGTCTCGAACTCCTGACCTCAAGTGATCCACTCACCTCAGCCTCCCAAAGTGCTGGGATTACAGGTGTGAGC
Pongo abelii              ATTTTTGTATTTTTAGTAGAAACGGGGTTTTACCATGTTGGCCAGGCTGGTCTCGAACTCCTGACCTCAAGTGATCCACTCACCTCAGCCTCCCAAAGTGCTAGGATTACAGGTGTGAGC
```

```
Macaca mulatta              ATTTTTGTATTTTTAGTAGAAACGGGGTTTCGCCATGTTGGCCAGGCTGGTCTCAAACTCCTGACCTCAAGTGATCCGCTCACCTCAGCCTCCCAAAGTGCTGGGATTACAGGTGTGAGC
Callithrix jacchus          ATTTTTGTATTTTTAGTAGAAACTGGGTTTCACCGTGTTGGCCAGGCTGGTCTTGAACTCCTGACCTCAAGTGATCCACTCACCTCAGCCTCCCAAAGCGCTGGGATTACAGGCATGAGC
Mus musculus                ------------------------------------------------------------------------------------------------------------------------
Rattus norvegicus           ------------------------------------------------------------------------------------------------------------------------
Bos taurus                  ------------------------------------------------------------------------------------------------------------------------
Sus scrofa                  ------------------------------------------------------------------------------------------------------------------------
Canis lupus familiaris      ------------------------------------------------------------------------------------------------------------------------
Equus caballus              ------------------------------------------------------------------------------------------------------------------------

Homo sapiens                CACCATGCCTGGGCTAAACTCTTTAAGTCTCTAGTAAAT-GCAGC----TAGATTCAAATGG------GCTGATAACCAAATTTTAACACATCAGCATTCACCACCAGGTTTACTTTTAT
Pan troglodytes             CACCATGCCTGGGCTAAACTCTTTAAATCTCTAGTAAAT-GCAGC----TAGATTCAAATGG------GCTGATAACCAAATTTTAACACATCAGCATTCACCACCAGGTTTACTTTTAT
Gorilla gorilla             CACCATGCCTGGGCTAAACTCTTTAAATCTCTAGTAAAT-GCAGC----TAGATTCAAATGG------GCTGATAACCAAATTTTAACTCATCAGCATTCACCACCAGGTTTACTTTTAT
Pongo abelii                CACCGTGCCTGGGCTAAACTCTTTAAATCTCTAGTAAATAGCAGC----TAGATTCAAATGG------GCTGATAACCAAATTTTAACACATCAGCATTCACCACCAGGTTTACTTTTAT
Macaca mulatta              CACCACGCCCGGCCTAAACTCTTTAAATCTCTAGTAAATAACAGC----TAGATTCAAATGG------GCTGATAACCAAATTTTAACACAGAAGCATTCACCACCAGGTTTGCGTTTAT
Callithrix jacchus          CCCTGCACCCAGCCTAAAGTCTTTGACTTTCTAGTGAATAACAGC----TAGATTAAAATGG------GCTGATAACCAAATTTGGGCACATTAGCATTCACCACCAGGTTTGCTTTTGT
Mus musculus                ---------------------CTTACATCTCAGACACCCAGCAGG----TAGTATCAAGTGT------GCTAATGACCAAGTCTCAGTATCTCCGTGTTCA---CCAAGCTTGCTTTTGT
Rattus norvegicus           ---------------------CTTAAGTCTCGAACACCCAGCAGG----CAGTATCAAGTGC------GCTAGT-ATCAGGTCTCAGGATGTCAGTGTTCA---CCAGGCTTGCTTTTGT
Bos taurus                  ---------------------TTTAAATCTCTATTAGATAACAGGTAGACAGACACAAGTGT------GCTGATATCCAGATTTTAACATACTGGCATTTGCCAGCAGGCTCACTGTTGT
Sus scrofa                  -----------------------------------------------AACATATCTGAGTGTTTCTTGGCCCCTACATAGATTTTAACAGACTGGCATTTACCAACAGGCTCTTCTTTGT
Canis lupus familiaris      ---------------------TTCACATCTCTACTGAAGGACAGG----TAGATTCAAGTGC------GTCGATATCCAGATGTTCACACATTGGCATTCATCCGCAGGCTCACTTATGT
Equus caballus              ---------------------TTTAAATCTCTAATAAATGACAGA----TAGACTCAAGTCT------G---TTATCCAAATTTTAACACATTGGCATTCCCCAGCAGACTTGCTTTTGA

Homo sapiens                TTT---------------------------------------------------------------------------------------------------------------------
Pan troglodytes             TTT---------------------------------------------------------------------------------------------------------------------
Gorilla gorilla             TTT---------------------------------------------------------------------------------------------------------------------
Pongo abelii                TTC---------------------------------------------------------------------------------------------------------------------
Macaca mulatta              TTC---------------------------------------------------------------------------------------------------------------------
Callithrix jacchus          TTC---------------------------------------------------------------------------------------------------------------------
Mus musculus                TTC---------------------------------------------------------------------------------------------------------------------
Rattus norvegicus           TTC---------------------------------------------------------------------------------------------------------------------
Bos taurus                  TTC---------------------------------------------------------------------------------------------------------------------
Sus scrofa                  TTT---------------------------------------------------------------------------------------------------------------------
Canis lupus familiaris      TTCCAGATTTTTTTTTAAGATTTTATTTATTTATTCATGAGAGACAGAGAGAGATAGAGAGAGAGAGGCAGAGACACAGGCAGAGGGAGAAGCAGGCTCCATGCAGGGAGAGCAATGTG
Equus caballus              TTC---------------------------------------------------------------------------------------------------------------------

Homo sapiens                ---------------------------------------------------------------------------------------CAGATTGGCTCATTTTGTGCAGACCTTAGAGCA
Pan troglodytes             ---------------------------------------------------------------------------------------CAGATTGGCTCGTTTTGTGCAGACCTTAGAGCA
Gorilla gorilla             ---------------------------------------------------------------------------------------CAGATTGGCTCATTTTGTGCAGACCTTAGAGCA
Pongo abelii                ---------------------------------------------------------------------------------------CAGATTGGCTCATTTTGGGCAGACCTTAGAGCA
Macaca mulatta              ---------------------------------------------------------------------------------------CAGATGGGCTCATTTTGTGTAGACCTTAGAGCA
Callithrix jacchus          ---------------------------------------------------------------------------------------CAGATTGGCTCATTTTGTGTAGACCTTGGAGCA
Mus musculus                ---------------------------------------------------------------------------------------CAAACTGACCCAG--------GACTGCAGAACA
Rattus norvegicus           ---------------------------------------------------------------------------------------CAGACTCACCCAT--------GGCTGCAGAACA
Bos taurus                  ---------------------------------------------------------------------------------------CAGATTGACTCATTTTGTGCAGACCTTGGAAAA
Sus scrofa                  ---------------------------------------------------------------------------------------CAGGTTGACTTATTTTGTGCAGATGTTGGAAGA
Canis lupus familiaris      GGACTCGATCCTGGGACTCCAGGATCACTCCCTGGGCCCAAGGCAGGCACTCAACCACTGAGCCACCCAGGGATCCCCTTATGTTTCCAGATTGACTTGTTTTGTGCAGGCCTTGGAAGT
Equus caballus              ---------------------------------------------------------------------------------------GAGATTGACTCATTTTGTGCAGACCTTGGAGAA

Homo sapiens                AAGTTTCCTTTATGGTATCTGTGTACGTAT-CCAAACTTCTTTTAATTGTTCACAGATTTTAAAAGCGGTAGCACCACATGGTTG--TGTAGATCAGACCTGTGTATTTAGATCAGACCT
Pan troglodytes             AAGTTTCCTTTATGGTATCTGTGTACGTAT-CCAAACTTCTTTTAATTGTTCACAGATTTTAAAAGCGGTAGCACCACATGGCTG--TGTAGATCAGACCTGTGTATTTAGATCAGACCT
Gorilla gorilla             AAGTTTCCTTTATGGTATCTGTGTACGTAT-CCAAACTTCTTTTAATTGTTCACAGATTTTAAAAGCGGTAGCACCACATGGCTG--TGTAGATCAGACCTGTGTATTTACATCAGACCT
Pongo abelii                AAGTTTCGTTTATGGTATCTGCGTACGTAT-TCGAACTTCTTTTAATTGTTCATAGATTTTAAAAGCGGTAGCACCACATGGTTC--TTTAGATCAGACCTGTGTATTTAGATCAGACCT
Macaca mulatta              AAGTTTCCTTTATGGTGTCTGTGTACGTAT-CTGAACT---TTTAATTGTTCATAGATTTTAAAAGCGGTAGTACCACATGGCTG--TTTAGATCAGACCTGTGTATTTAGGTCAGACCT
Callithrix jacchus          AAGCTTCCTTCATGGTATCTGTGTACGTAT-CCAAACTTCTTTTAACTGTTCATAGATT-TAAAAGTAGTGGCACCACGTGGGTG--TTTAGATGAGACCTGTGTATGT----C------
Mus musculus                AAGCTCCCT-GCTGGCAATGGTGAGCCTATTCCAAAAGCCCTGTAATTGTGCACAGACTTGAAAAGCCATAGCGCCTTGTGAGTA--TG-------------------------------
Rattus norvegicus           AAGCTGCCT-GCTGGCAATGGTGTGCACATTCCAACCAGCCTGTGGCTGTGCACAGCCTTGAACAGC--TAGCATC--GTGAGCACGTG-------------------------------
Bos taurus                  AAGATTCCCTGGTGAGATCCAAGCACACAG-CTAAACTTCTACTCATTCTTCGTAGATTAAGGAAGTCATAGCACTTCAGAGGTG--TTTCATTCAGGCTTATGCATGT--------TCT
Sus scrofa                  AAAGTTCTCTTGCAATATCTGAGCACATAT-CCAAACTTACTTTAACTTTCCATAGATTAAGGAAGCCATAGCACCTCATGGGTA--TTTAAATCAGACCTGTGCTTGT--------TCT
Canis lupus familiaris      AAGGTTCCCTTGTGATATCTGAGCACATAT-CTGGACTTCTCTTAATTGTTCATAGATTTAAGGAGTTATGGCACCTGACAGGTG--TTTAAATCATGTCCATGCATGC--------CCT
Equus caballus              AAGGTTCCCTTGAGATATCTGAGTGCATAT-CAGAAGTTCTTTTAATCATGCACAGACTAAGGAAGTGATAGCACCTTGTGGGTG--TTTAAATCAGGCCCATGCCTGT--------GCT

Homo sapiens                GTGTATCACGT-AAGTGTGTGAGTGCAGTGCAGATGAGCACCATTTAGTTATA-TGTGCTAGGCAAATCTCCAACACAGTTGATGTGTAGTCTTGTGGTAGATTTGTGCATACTGTAAGC
Pan troglodytes             GTGTATCACGT-AAGTGTGTGAGTGCAGTGCAGATGAGCACCATTTAGTTATA-TGTGCTAGGCAAAGCTCCAACACTGTTGATGTGTAGTCTTGTGGTAGATTTGTGCATACTGTAAGC
Gorilla gorilla             GTGTATCACGT-AAGTGTGTGAGTGCAGTGCAGATGAGCACCATTTAGTTATA-CGTGCTAGGCAAAGCTCCAACACAGTTGATGTGTAGTCTTGTGGTAGATTTGTGCATACTGTAAGC
Pongo abelii                GTGTATCACCT-AAGTGTGTGAGTGCAGTGCAGATGAGCACCATTTAGTTATA-TGTGCTAGGCAAAGCTCCAACACAGTTGATGTGTAGTCTTGTGGTAGATTTGTGCCTACTGTAAGC
Macaca mulatta              GTGTATCACAT-AAGTGTGTGAGTGCAGTGCAGATGAGCACCATTTAGTTATA-TGTGCTAGGCAAAGCTCCAACACAGTTGATGTGTAGTCTCGTGGTAGATTTGTGCATACTGTAAGC
Callithrix jacchus          ATGTATCACGT-AAGTGTGTGAGTGCAGTGCAGAGGCGCACCATTCAGTTATA-CGTGCTAGGCGAAGCTCCAACACAATGGACGTATAGTCTCGTGGTAGATTTGCGCATACTGCGAGT
Mus musculus                -TGTGCCAGGTCAAGAGTACTAGGGCACCATAAACAAACACTGCTTAGTTG---CCTGCCAGGCAGAGCTCGGGCACAGTTAGTGTGCAGCCT-GTGGTAGATCCCTGTGTG--GTTAGC
Rattus norvegicus           -TGTGCCAAGTCAAGTGCACTAGGGCACCATACACAAATGCTGCTTAGTTA--ACATGCCAGACAGAGCTCAGGCACAGCTAATGTACAGCCT-GTGGTAGATCCCTGTGCACCATAAGC
Bos taurus                  GT----GATGCCAAGTGTGCT--TCAAGCATAAATGAGCATCATTCAGTTA---AGCACTACCCAAGGCTCCAACACAATCGATGTCTCACATCCTGGCAGCTTTGTATATACAACAAAC
Sus scrofa                  AT----GATGTGAAGTGTGTT--TCAGGTATAAACGAGCATCATTCAGTTA---AATGCTAGGCAAGGTTCCAACACAACTGATATATAGCATCATAGCAGCTTTGTATATACAATAAAC
Canis lupus familiaris      GT----GATGTAATGTGTGCTAATAGAGCATAAGTGAGCACCATTCAGTA----AATGCTAGT------------------TGATATACAGCATCACGGTAGCTTTTGTATACAACAA--
Equus caballus              GT----GATGTGAAGTGTGCCAGCAGAGCATAAATGGGCACCATTCAGTTC---ACTGCTAGACAAGGCTCCCACACAG-TGACAGATAGCATCCTGGAAGCTTCGTGTATCCAATAAAC

Homo sapiens                AAATTGCTTAGCTTCTCTAG----ACATCAGTT-TCCACATCTGAA--AAATAAGAAGATGAGAGTACACGGTTG---TTAT---GAACAAATGACTTAATGCTTTTTAAGCACGTTGCA
Pan troglodytes             AAATTGCTTAGCTTCTCTAG----ACATCAGTT-TCCACATCTGAA--AAATAAGAAGATGAGAGTACACGGTTG---TTAT---GAACAAATGACTTAATGCTTTTTAAGCACGTTGCA
Gorilla gorilla             AAATTACTTAGCTTCTCTGG----ACATCAGTT-TCCACATCTGAA--AAATAAGAAGATGAGAGTACACGGTTG---TTAT---GAACAAATGACTTAATGCTTTTTAAGCACGTTGCA
Pongo abelii                AAATTGCTTAGCTTCTCTAG----ACATCAGTT-TCCACATCTGAA--AAATAAGAAGATGAGAGTACACGGTTG---TTAT---GAACAAATGACTTAATGCTTTTTAAGCATGTTGCA
Macaca mulatta              AAATTGCTTAGCTTCTCTAG----ACATCAGTT-TCCACATCTGAA--AAATAAGAAGATGAGAGTACACGGTTG---TTAT---GAACAAATGACTTAATGCTTTTAAAGCACGTTGCA
Callithrix jacchus          AAATTGCTTAGCTTATCTAGATAGACGTCAGTT-TCCGCCTCTGAA--AAATTAGAAGATGAGAGTACACAGTTG---TTAGGAGGATCAGATGACTCAATGCTTTTTAATCACTTCGCA
Mus musculus                AAGCTGCTTGGTTTCTCTGA----ACATCCGTT-TCCTTATCTAAA---AATGGG--------------CTGCCA---TTAG---GATCAAGTGGCACAATGCTTTTTAAGGGCTTGGAG
Rattus norvegicus           AAGTTGTTTAGCTTCTTTGA----ACGTCAGTT-TCCTTATCTAAA--AAATGGG--------------CTGCCA---TTAG---GATCAAATG--------------------------
Bos taurus                  AATTGGGCTAGCTTCTCTAA----GTATCAGTTTTCCTCATCAGAA--AAATGGGAGGCCTATAGAATAGGGTTGTTGTTAG---GATCAAATGACATAATGCCTTTTAAGCACTTAACG
Sus scrofa                  AAGTGGCTTAACTTTTCTAA----ACATCTGTT-TCTGCATCAGAAAGAAATGGGAAGATAATAGAATAGGGTTATTGGTAG---GATCAAATGGCATCATGCCTTTTAAGCACTTAGCA
Canis lupus familiaris      ----------ACTTCTCTAA----ACATCAGTT-TCCTCATCAGGA--AAATGGGAACATAACAAAATAGGGTTGTTGTTAG---GCTCCAATGACATAATGCCTTTGAATCACTTAGTA
Equus caballus              AAGTTGCTCAACTTCTCTAA----ACATCAGCT-TCTTCATCAGAA--AAATGGGAAAAGAATAGAATCAGGTTGCTGTTAG---GATCAGATGCCCTCGAGCCTTTTCAGCACTTAGCG

Homo sapiens                TGACATCTGGAACACAGAAAGCCCTCAATACATTGAAGCTCTTA----GGATTTTCACGATG-TTCCTGTCTGCTC-----AATGCATGCTTTCTTTATTGTTCTGACAGT---TGTGTG
Pan troglodytes             TGACATCTGGAACACAGAAAGCCCTCAATACATTGAAGCTCTTA----GGATTTTCACGATG-TTCCTGTCTGCTC-----AATGCATGCTTTCTTTATTGTTCTGACAGT---TGTGTG
Gorilla gorilla             TGACATCTGGAACACAGAAAGCCCTCAATACATTGAAGCTCTTA----GGATTTTCACGATG-TTCCTGTCTGCTC-----AATGCATGCTTTCTTTATTGTTCTGACAGT---TGTGTG
Pongo abelii                TGACATCTGGAACACAGAAAGCCCTCAATACATTGAAGCTCTTA----GGATTTTCACGATG-TTCCTGTCTGCTC-----AATGCATGCTTTCTTTATTGTTCTGACAGT---TGTGTG
Macaca mulatta              TGACATCTGGAACATGGAAAGCCCTCAATACATTGAAGCGCTTA----GGATTTTCATGATG-TTGCTGTCTGCTCAATTCAATGCATGCTTTCTTTATTGTCCTGACAGT---TGTGCG
Callithrix jacchus          TGACATCTGGAACACAGAAAGCCCTCAGTACATTGACAC---TA----AGATTTCCGTGATG-TTCCTATCTGCTC-----AATGCATGCCTTCTTTATTGTTCCAACAGT---TGTGTG
Mus musculus                TAACGTTTGGAACTTAGAAGCCCCTCCATATACTGAGGATCTTT----GTATTTTTGTGATA-GT--TGTCAGCTC-----AATGCATGTTCTCTTGATTGTTTC------CATTGTATG
Rattus norvegicus           ------------------------------------------------GTATTTTTGTGATA-GT--TGTCAGCTC-----AATGCATGGTCTCCTGACTGTTCT---------CGGGTG
Bos taurus                  TGGTGTCCGGAACACAGAAAGCCCTCAATACGTGGAGGCTCTTTCTCTT-ATCTTTCCCATGCTTATTGTCTGATC-----AGTGCATGTTCTCTT-CTTTTTCTA--AGT---CCTGCA
Sus scrofa                  TGAAGTCTGGAACATAGAAAGCCCTCAATGCATTGAGACTCTTTTTATTTATTTTCACAGTGCTTGTTGTCTGGTA-----AGTGCATGCTCTCTTTCTTGTTCTAACAGT---CCTGCA
Canis lupus familiaris      TGATGTCTGGAACATAGAAAGTCTTCAATATATTAAGACTC---A---TGGTTTTCATGATACTTACTGTCTGTTC-----AACGCATGTTCTCTTTATTGTTCTAACAGT---TATGCA
Equus caballus              TGATGTCTGGGACGTAGAAAGCCCTCGATGCGTTGAGACTCGTTA---TGATTTTCATGATGCTTATTGTCTGTTC-----GATGCATGCTCTCT-TATTGTTCTAACACT---TGTGCC

Homo sapiens                GTAACAAGCTA-------ATATG----CTTCCAGTTGACTTCCAGTCT-----ACCCTGGTGTTAGAAACCGTTTCATCTCTTATTGTAAATTTGAGTGC--TTGTT-GTTTTTTATATT
Pan troglodytes             GTAACAAGCTA-------ATATG----CTTCCAGTTGACTTCCAGTCT-----ACCCTGGTGTTAGAAACCGTTTCAGCTCTTATTGTAAATTTGAGTGC--ATGTT-GTTTTTTATATT
Gorilla gorilla             GTAACAAGCTA-------ATATG----CTTCCAGTTGACTTCCAGTCT-----ACCCTGGTGTCAGAAACCGTTTCAGCTCTTATTGTAAATTTGAGTGC--TTGTT-GTTTTTTATATT
Pongo abelii                GTAACAAGTTA-------ATATG----TTTCCAGTTGACTTCCAGTCT-----ACCCTGGTGTTAGAAACCGTTTCAGCTCTTATTGTAAATTTGAGTGC--TTG----TTTTTTATATT
Macaca mulatta              GTCACAAGTTA-------ATATG----CTTCCAGTTGACTTCCAGTCT-----ACCGTGGTGTTAGAAACCGTTTCAGCTCTTATTGTAAATTTGAGTGC--TTGTT-GTTTTTTATATT
Callithrix jacchus          CTAACAAGTTA-------ATATG----CTCCCAGTTGACTCCCAGGCT-----ACCTTGGTGTTAGAAACCATTTCTGCTCTTACTGTAAATGTGA--GC--TTGTT-GTT-TTTATATT
Mus musculus                GAAACAAGTCACAAAGTAACAGA----CTTACA------TTCCAGTCGGGATGCTCCTGCAGTGAGGAAGAACTTTGGCT-----TGGAAGTAAGTGTGCACTAGCTGGCCGTCCACATT
Rattus norvegicus           GAAACAGGTCACAATGTAACAGA----CTTCTG------TTGCAGTCATGATGCTCCTGCAGTCAGAAAGCATGTTGGTTCTTTTTGGAAATCAGTGTGCAACAGCTGGTTGTCCATACT
Bos taurus                  ATAATGAGTAA-------ATAGGTAATCTGTCCG----GTTGC-GACA-----A-CATTGCATTAGAAACCATT-AAGTACTTGTGGTAAGTTGGCGCGC--TTATT-GC--ATTATCTT
Sus scrofa                  CTAACAAGTAA-------ATATATAATCTGTCAGTCAATTTGT-GACA-----A-CTTTGCATTAGAAACCATTTTAGCACTTGTGGTAAGTTCGAACGT--TTGTT-GTCTATTACGTT
Canis lupus familiaris      GTAATAAGTAA-------ATATGTCAGCTGTCAGTCAAGTTGT-GACG-----A-CGTTGCTTTAGAAACCATTTTAGCTTCTCTGGTATATTCCAGTGC--TTGCT-GCCTGTTACCTT
Equus caballus              ATAACGAGTAA-------ATATGTATGCTGCCAGTCAAGTTGT-GAC---------ATCGCATTAGAAACCGTTTTGGCTCTTGT-GTAAGTCTGAGTGC--TTG-T-GTCTGTTATCTT

Homo sapiens                TGTGATGACTCTTCCAGCAGTTGTTGACAATTGTTAG-AGGTTTGACTTTTAAATAATTACTTATTTTTTCTGATTGTGGTTCA--GTTTAACTGAAG-AATATCCTGAGATTGTAAGAA
Pan troglodytes             TGTGATGACTCTTCCAGCAGTTGTTGACAATTGTTAG-AGGTTTGACTTTTAAATAATTACTTATTTTTTCTGATTGTGGTTCA--GTTTAACTGAAG-AATATCCTGAGATTGTAAGAA
Gorilla gorilla             TGTGATGACTCTTCCAGCAGTTGTTGACAATTGTTAG-AGGTTTGACTTTTAAATAATTACTTATTTTTTCTGATTGTGGTTCA--GTTTAACTGAAG-AATATCCTGAGATTGTAAGAA
Pongo abelii                TGTGATGACTCTTCCAGCAGTTGTTGACAATTGTTAG-AGGTTTGACTTTTAAATAATTACTTATTTTTTCTGATTGTGGTTCGACGTTTAACTGAAG-AATATCCTGAGATTGTAAGAA
Macaca mulatta              TGTGATGACTCTTCCAGCAGTTGTTGACAATTGTGAG-AGGTTTGACTTTTAAATAATTACTTATTTTTTCTGATTGTGGTTCA--GTTTAACTGAAG-AATAGCCTGAGATTGTAAGAA
Callithrix jacchus          TGTGATGAGTCTTCCAGCAGCTGTTGACAATTGTTAG-AGGTTTGACTTTTAAATAGTCACTTATTTTTTCTGATTGTGGTTTG--GTTTATCTAAAG-AATATCCTGACATTGTAAGAA
Mus musculus                TGTGGTAAGTATTCCAGAAGTAGTTGGTGACTGTTAATCGACTTGACTTTGGAATGATGACCTGTTCTTCCGGGTTGTGGGTTA--GTTTAACTGAAG-AATAA---GAAGTTCTAAGAA
Rattus norvegicus           TTTGGTAAGTGTTCCAGAAGTGGTTGGTAA---TTAGTTGACTTAACTTTGAAACAATGACCTGTTCTTCTGGGTTGTGGGTGA--GTTTAATTGAAG-AATAA---GAAGTTGTAAGAG
Bos taurus                  TGTGATCAGTTTTCCAGCTATTGTTGACTATTGTTAG-TGATT------ATAAGAAATAAATTATTCTTTCTGATGGTAAATAA--TTTTAACTGAAGGAATATCTTGAGATTGTAAGAA
Sus scrofa                  TCTGATAAAATTGCCAGCAATTGTTGACTATTACTAG-TGATG------ATAACAAATAA---ATTCTTTCTGATCATGGATAA--TTTTAACTGAAG-AACATCCTGGGGATGTTAGAA
```

```
Canis lupus familiaris   TGCGATAAGCATTCCAGCAGCTGTTGACTATTGTTAG-TGATTTGACTTGGAAACAATACATTATTCTTTCTGATTGTGGATAA--TTTTAAAGGAAG-AATATCCTGAGATTGTAAGAA
Equus caballus           TGTGATAAGAATTCCAGCAG---TTGACTATTACTAG-TGATTTGACTCTGAAACAATAAATTATTCTTTCTGATCATGGATAA--TTTTAACTG----AATATCCTGAGAGTATAAGAA

Homo sapiens             AAGCATTT-------T-TTAA------A--AGGTATCACTTGTGATCATTTATCTTTCTAAATTCTA-----------------------------------------------------
Pan troglodytes          AAGCATTT-------T-TTAA------A--AGGTATCACTTGTGATCATTTATCTTTCTAAATTCTA-----------------------------------------------------
Gorilla gorilla          AAGCATTT-------T-TTAA------A--AGGTATCACTTGTGATCATTTATCTTTCTAAATTATA-----------------------------------------------------
Pongo abelii             AAGCATTT-------T-TTAA------A--AGGTATCACTTGTGATCATTTATCTTTCTAAATTATA-----------------------------------------------------
Macaca mulatta           AAGCATTT-------T-TTTA------A--AGGTATCACTTGTGATCATTTATCTTTCTAAATTATA-----------------------------------------------------
Callithrix jacchus       AAGCATTT-------T-TTAA------A--AGGTATCATTTGTGATCAATTATCTTTCTAAATTGTA-----------------------------------------------------
Mus musculus             AGGTGGTTTGTTTGTTTATTT------A--AGTCATCATCTGTGATGGTTTATCTTTCGGAAATATA-----------------------------------------------------
Rattus norvegicus        TGGTATTT-------TTTTTA------A--AGTCATCATCTGTGATGGTTTATCTCTCGAAACTATA-----------------------------------------------------
Bos taurus               AAGGCTTT-------TATTTAAAAAAAAAAATGTACCACTTGTGAGTACTTACCTTTACAAATAATAGTTCAA-----------------------------------------------
Sus scrofa               AAGCATTT-------T-TTTAAGAAAGA--AGATACCCTATGTGATCATTTATCTTTCTAAGTTAT------A-----------------------------------------------
Canis lupus familiaris   AAGTATTT-------TTGTTAAA--------ACTACCATTTGTAATCATT------TCTAAGTTATAA----------------------------------------------------
Equus caballus           AAGTATTT-------TTGTTAAA----A--AGGTACCATTTGTGATCATTTATCTTTCTAAGTTATAT-----TTTTTTTTTTAAAGATTTATTTTTTTCCTCTTTCTCCCCAAAGCC

Homo sapiens             ------------------------------------------------------------------------------------------------------------------------
Pan troglodytes          ------------------------------------------------------------------------------------------------------------------------
Gorilla gorilla          ------------------------------------------------------------------------------------------------------------------------
Pongo abelii             ------------------------------------------------------------------------------------------------------------------------
Macaca mulatta           ------------------------------------------------------------------------------------------------------------------------
Callithrix jacchus       ------------------------------------------------------------------------------------------------------------------------
Mus musculus             ------------------------------------------------------------------------------------------------------------------------
Rattus norvegicus        ------------------------------------------------------------------------------------------------------------------------
Bos taurus               ------------------------------------------------------------------------------------------------------------------------
Sus scrofa               ------------------------------------------------------------------------------------------------------------------------
Canis lupus familiaris   ------------------------------------------------------------------------------------------------------------------------
Equus caballus           CCCTGGTACATAGTTGTATATTCTTCGTTGTGGGTCCTTCTAGTTGTGGCATGTGGGACGCTGCCTCAGCGTGGTTTGATGAGCAGTGCCATGTCCGCGCCCAGGATTCGAACCAACGAA

Homo sapiens             -----------------------------------------------------------------------TTT-TTAATACTGTTCCACCAAAGTGATGC---------AGTGGT----
Pan troglodytes          -----------------------------------------------------------------------TTT-TTAATACTGTTCCACCAAAGTGATGC---------AGTGGT----
Gorilla gorilla          -----------------------------------------------------------------------TTT-TTAATACTGTTCCACCAAAGTGATGC---------AGTGGT----
Pongo abelii             -----------------------------------------------------------------------TTT-TTAATACTGTTCCACCAAAGTGATGC---------AGTGGT----
Macaca mulatta           -----------------------------------------------------------------------TTT-TTAATACTGTTCGACCAAAGTGATGC---------GGTGGT----
Callithrix jacchus       -----------------------------------------------------------------------GTT-TTAATACTGTCTGACCAAAGTGATAC---------AGTTGT----
Mus musculus             -----------------------------------------------------------------------TTTCTCAACACTGTCCAGCCACAGTGATGTCAGTGATGGGATTGTCCTT
Rattus norvegicus        -----------------------------------------------------------------------TTTCTCAACACTGTCCAGCCACAGCACTGT---------GATTGTCCCT
Bos taurus               -----------------------------------------------------------------------TTTTTCAATACTGT-CAAACAAAGTG-----------------------
Sus scrofa               -----------------------------------------------------------------------TTTTTCAATACTGTTCAATCAAGATGCTAA---------AACT------
Canis lupus familiaris   -----------------------------------------------------------------------TTTTTCAATGCTGTTCAACCAAAACGATAC---------AGTTGT----
Equus caballus           ACACTGGGCCGCCTGCAGCGGAGCGCGCGAACCCAACCACTCGGCCACGGGGCCAGCCCCTCTAAGTTATGTTTTTCAATACTGTTC----AAAGTGATAC---------AGTTGT----

Homo sapiens             TA-CCATGACACCCTAATTTCATGTG---TTTTTGTATTTATGAAAATAGTTTCA---TTG---TCATTTATTGGCGGTATACAAAGTAAAATGTTATAAATGTGAAGTTATAAAATAAA
Pan troglodytes          TA-CCATGACACCCTAATTTCATGTG---TTTTTGTATTTATAAAAATAGTTTCA---TTG---TCATTTATTGACGGTATACAAAGTAAAATGTTATAAATGTGAAGTTATAAAATAAA
Gorilla gorilla          TA-CCATGACACCCTAATTTCATGTG---TTTTTGTATTTATGAAAATAGTTTCA---TTG---TCATTTATTGACAGTATACAAAGTAAAATGTTATAAATGTGAAGTTATAAAATAAA
Pongo abelii             TA-CCATGACACCCTAATTTCATGTG---TTTTTGTATTTATAAAAATGGTTTCA---TTG---TCATTTATTGACAGTATACAAAGTAAAATGTTATAAATGTGAAGTTATAAAATAAA
Macaca mulatta           TA-CCATGACTCCCTAATTTCATGTG---CTTTTGTATTTATGAAAATGGTTTCA---TTG---TCATTTATTGACCGTATACAAAGTAAAATGTTATAAATGTGAAGTTATAAAATAAA
Callithrix jacchus       TA-CCGTGACACTCTGATTTCATGTG---TTTTTGTATTTATGAAAGTGGTCTC----TTG---TCATTTCTTGACAGTATACAAAGTAAAATGTTATAAATGTGAAGTTATAAAATAAA
Mus musculus             GA-CCACTCTAGCCTGACTCCACGTG---GCATTATGTTCCTGGGAATAGCTTTGCCGTTG---T-ACTTACCAACAGAATACAGAGTTAAATGTTACCACTGCGAAGTTACCAAATAAA
Rattus norvegicus        GA-CCATTCTATGCTGATGCCGTGTG---GTCTTGTGTTCCTGGGAATGGCTTTGCCGTTG---T-ATTTACCAACAGACTTCAGAGTTAAATGTTACCACTGTGAAGTTACCAAATAAA
Bos taurus               ------ATACACCCGGATTTCATGTGT--TTTTTGTATTTGTAAAAATGGCCTCA---TTGTTCTCATGTATTCTCAGT------AGTAAAGTGTTATAAATGTGAAGTTATAAAATAAA
Sus scrofa               CATCCAATACACCCTGATTTTATGCATTTTTTTGTATTCATGAACATGGCCTCA---TTGTTCTCATGAATTGACACTATACAAAGTAAAATGTTATAAATGTGAAGTTGTAAAATAAA
Canis lupus familiaris   ---CCAGTATACCATAATTTCATGT----TTTTTGTATTCATGAAAATGGCA--------------------------TGTACAAAGTAAAATGTTATAAACATGAAGTTATAAAATAAA
Equus caballus           CATCCAATGGACCCTGATTTCATGTG---TTTTTGTATTCATGAAAATGGCCTCA---TTGTTCTCATTTATTGCCAGTAAACAAAGTGCAATGTTATAAGTGTGAAGTTATAAAATAAA

Homo sapiens             TATATGCTAATAAAATCCTGAGTT---TT-----TCTGTTTCCTATATCGTGGT--CTCTATAAAGTACTGCTTGAA-----GAAGGAGCTTTGTTACTGAAAAA---------------
Pan troglodytes          TATATGCTAATAAAATCCTGAGTT---TT-----TCTGATTCCTATATCGTGGC--CTCTATAAACTACTGCTTGAA-----GAAGGAGCTTTGTTACTGAAAAA---------------
Gorilla gorilla          TATATGCTAATAAAATCCTGAGTT---TT-----TCTGATTCCTATATCGTGGC--CTCTATAAACTACTGCTTGAA-----GAAGGAGCTTTGTTACTGAAAAA---------------
Pongo abelii             TATATGCTAATAAAATCCTGAGTT---TT-----TCTGATTCCTATATCATGGC--CTCTATAAACTACTGCTTGAA-----GAAGGAGCTTTGTTACTGAAAAA---------------
Macaca mulatta           TATATGCTAATAAAATCCTAAGTT---TT-----TCTGATTCCTGTATTGTGGC-CCTCTATAAACTACTGCCTGAA-----GAAGGAGCTTTGTTACTGAAAAA---------------
Callithrix jacchus       TATATCCTAATAGAATCCTGAGTT---TT-----TCTGATTCGTAAATCGTGGC--CTGTACAAACTACTACTTGAA-----GAAGGAGCTCCATCACTGAAAAT---------------
Mus musculus             TCTGAGCTAGTGAAATCGTGAGTT-GTTT-----TCTGCTTCCTATAT----------------------GCCTGAGT--TTTAAGGAGCTTCATTACTAAAA-G---------------
Rattus norvegicus        TCTGAGTGAGTGAGATCCTGAGTT-ATTT-----TCTGATTCCTCTAT----------------------GTCTGAGTGATCGAAGGAGCTTCGTTACTAAAAAA---------------
Bos taurus               TCCATTCTGGTAAGATTCTGAATTT--TT-----TGTGCTTTGTGTATTATGGTT-CTGTATAAACTTTTGCTTAAC-----AAAGGGATTTAATTACTAAAAAAAAATTT--------T
Sus scrofa               TACCTGCTGATAATATTCTGAGTTT--TT-----TCTGCTTCATGTATTGTGGTT-CTGTACAAACCATCGCCTAAA-----TAGGGCATTTCATTACTAAAAAAA----T--------T
Canis lupus familiaris   TACAAGCTAATAAAATTCTGAGGTT--TTTTTTGTGTATTTCATGTATTGTGATT-CTGTATTAGCTATTGCTTGAA-----GAA---GTTTCATTACTAAAAA-------AAAAAAAAA
Equus caballus           TGCATGCTAATAAAATTCTGAGTTT--TC-----TCTGTTTCATGTATTGTGGTT-CTGTGTAAACTATTGCTTGAG-----GAAGGAATTTCGTTACTAAAAA---------------A

Homo sapiens             TTTTGAGAGATA-AGTTGAGAAAATCAAGCTAAAGC------------------------------------------------------------------------------------
Pan troglodytes          TTTTGAGAGATA-AGTTGAGAAAATCAAGCTAAAGC------------------------------------------------------------------------------------
Gorilla gorilla          TTTTGAGAGATA-AGTTGAGAAGATCAAGCTAAAGC------------------------------------------------------------------------------------
Pongo abelii             TTTTGAGAGATA-AGTTGAGAAAATCAAGCTAAAGC------------------------------------------------------------------------------------
Macaca mulatta           TTTTGAGAGATA-ACTTGAGAAAATCAAGCTAAAGT------------------------------------------------------------------------------------
Callithrix jacchus       TTTTGAGAGATA-AGTTGA-AAAATCAAGCTAAAGC------------------------------------------------------------------------------------
Mus musculus             C----------A-A---AACAAAATCAAGCTAAAGGGGCTGGAGAAATGGCTCCGTGGTTAAGAGCACTTGCTGATCTTCCAGAGGTCCTGAGCCCAGTTTCTAGCACCTACATCTGGTG
Rattus norvegicus        C----------A-A---AACCAAAGCAGACTGAAGGGGCTGGAGAGGTGACT--GTGGTTTAGGGCACTTGCTGATCTTCCAGAGAACCTGAGCTCAGTTTCTAGCACCTACATAGGGTG
Bos taurus               TTTTGAGAGATAGAGATTAGAAAATCAAGCTAAAAC------------------------------------------------------------------------------------
Sus scrofa               TTTTGAGAGGTA-AGATTAGAAAATCAAGCTAAAGC------------------------------------------------------------------------------------
Canis lupus familiaris   AATTAATGGACA-AGGTTAGAAAATCAAACCAAAGC------------------------------------------------------------------------------------
Equus caballus           TTTTGAGAGGTA-AGGTTAGAA-ATCAG------TT------------------------------------------------------------------------------------

Homo sapiens             ------------------------------------------------------------------------------------------------------------------------
Pan troglodytes          ------------------------------------------------------------------------------------------------------------------------
Gorilla gorilla          ------------------------------------------------------------------------------------------------------------------------
Pongo abelii             ------------------------------------------------------------------------------------------------------------------------
Macaca mulatta           ------------------------------------------------------------------------------------------------------------------------
Callithrix jacchus       ------------------------------------------------------------------------------------------------------------------------
Mus musculus             GCTCCTAAGTATCTGTAACTCCAATTCCAAG-----------------------------------------------------------------------------------------
Rattus norvegicus        GCTCATAAGTACCTGTAACTCCAATTCCAAAGGATCTAACACCTTCTTCTGGCCTCTCAGGGTACCAGCATGTACACGGTATTCACACGCACTTAAAAATGAATCTTTAAGCCAGGAGGT
Bos taurus               ------------------------------------------------------------------------------------------------------------------------
Sus scrofa               ------------------------------------------------------------------------------------------------------------------------
Canis lupus familiaris   ------------------------------------------------------------------------------------------------------------------------
Equus caballus           ------------------------------------------------------------------------------------------------------------------------

Homo sapiens             ------------------------------------------------------------------------------------------------------------------------
Pan troglodytes          ------------------------------------------------------------------------------------------------------------------------
Gorilla gorilla          ------------------------------------------------------------------------------------------------------------------------
Pongo abelii             ------------------------------------------------------------------------------------------------------------------------
Macaca mulatta           ------------------------------------------------------------------------------------------------------------------------
Callithrix jacchus       ------------------------------------------------------------------------------------------------------------------------
Mus musculus             ------------------------------------------------------------------------------------------------------------------------
Rattus norvegicus        GGTGGCGCATGCCTTTGATCCCAAAACTCGGGAGGCAGAGACAGTGCTCTGAGTTTGAGGCCAGCCTGGTCTGCAGAGTGAGTTCTGGGACAGCTACACAGAGAAGCTCTCGTCTTGAAA
Bos taurus               ------------------------------------------------------------------------------------------------------------------------
Sus scrofa               ------------------------------------------------------------------------------------------------------------------------
Canis lupus familiaris   ------------------------------------------------------------------------------------------------------------------------
Equus caballus           ------------------------------------------------------------------------------------------------------------------------

Homo sapiens             ----------------------------------------------------------C--AAAAGGTCTTAGA--ATCCTGTAGGCAA---------GAAAACCTGAAAGCAAAAAAAT
Pan troglodytes          ----------------------------------------------------------C--AAAAGGTCTTAGA--ATCCTGTAGGCAA---------GAAAACCTGAAAGCAAAAAAAT
Gorilla gorilla          ----------------------------------------------------------C--AAAAGGTCTTAGA--ATCCTGTAGGCAA---------GAAAACCTGAAAGCAAAAAAAT
Pongo abelii             ----------------------------------------------------------C--AAAAGGTCTTAGA--ATCCTCTAGGCAA---------GAAAACCTGAAAGCAAAAAAAT
Macaca mulatta           ----------------------------------------------------------C--AAAAGGTCTTAGA--ATCCTCTAGGCAA---------GAAAACCTGAAAGCAAA-AAAT
Callithrix jacchus       ----------------------------------------------------------C--AA-ATGTCTTGAA--CTCCCCTAGGCAA---------GAAAACCTGAAAGCAAAAAAAT
Mus musculus             ------------------------------------------------------------------------------------------------------------------------
Rattus norvegicus        AACCAAATAAAAACATAATAAATAAAACTTAAATAAATCAATAAATCGTTAAGTAAAAG--AAAAGGTCTTTAA--CTCTTCTAGGCAA---------GAAAACCTGAGCACAGGGA---
Bos taurus               ----------------------------------------------------------CACAAAAGATCTTGAA--TTCTTCTAGG---AAAAAA---AAAAAACCGAGAGCAAA---AT
Sus scrofa               ----------------------------------------------------------C--CAAAGGTCTTAAA--CTCTTTGAG----AGAGAA---AAAAACCTGAGAGCAAA---AT
Canis lupus familiaris   ----------------------------------------------------------C--AAAAGGTCTTGAA--CTCATCTAGA---------TAGGAAAACCTAAGAACAAA---AT
Equus caballus           ----------------------------------------------------------C--AAAAGGTCTTGAACACTCTTCTAGA------------GAAACCATAAAAGCAAA---AT

Homo sapiens             GCTTGAAC-AAGAATACTTGAACTATGTCTCTGGGGACAA--GGGAGAGCTCGGTCCACCCATTATCGATTTCTCTTTTAAAATATAC-AGAAACAACAGATGGAGGTCAGTTGCCTCAG
Pan troglodytes          GCTTGAAC-AAGAATACTTGAACTATGTCTCTGGGGACAA--GGGAGAGCTCGGTCCACCCATCATCGATTTCTCTTTTAAAATATAC-AGAAACAACAGATGGAGGTCAGTTGCCTCAG
Gorilla gorilla          GCTTGAAC-AAGAATACTTGAACTATGTCTCTGGGGACAA--GGGAGAGCTCGGTCCACCCATCATTGATTTCTCTTTTAAAAGATAC-AGAAACAACAGATGGAGGTCATTTGCCTCAG
```

```
Pongo abelii            GCTTCAAC-AAGAATACTTGAACTATGTCTCCGGGAACAA--GGGAGAGCTCGGTCCGCCCATCATCGATTTCTCTTTTAAAATATAC-AGAAACAACAGATGGAGGTCAGTTGCCTCAG
Macaca mulatta          GCTTGAAT-AAGAATACTTGAACTATGTCTCTGGGGACAA--GAGAGAGCTTGATCCACCCATCATCGATTTCTCTTTTAAAATATAC-AGAAACAAAAGATGGAGGTCAGTTGCCTCAG
Callithrix jacchus      GATTGAGT-AAG------------------------ACAA--GAGAGAGCTCAGTCC-CCCATTATTGATTTCTCTTTTAAAATAGAT-AGAAACAACAGAGGGAGGCCAGGTCTCTCAG
Mus musculus            ------------------------------------------------------------------------------------------------------------------------
Rattus norvegicus       CCTAAAGT-AA--GTACTCGTGTGCTGTCTCCAGGAACAA--A--ACAGCTCTGCCCACCCACCATTGATTTCCTTATCA--ATGCAC-AGGACC-AGAAATGGA---------------
Bos taurus              GCTGGGAT-GGAAATACTTGGACTTGGTCTCCAAAGATGA--GAGGGAGCTTAGTTCACCCATCACTGATTTCCCTTTTAACCTACA--AGAAATGACAGATGGAGGCCGGGTCCCTTGG
Sus scrofa              GCTGGAATCAGGAGTACTTGGGCTTTGTCTCCAAGGATGA--GTGAGAGCTCAGCCCATCCTTCATTGATTTCTCCTTTAAAATATA--AGAAATGACAGATGGAGGCCAGTTTCCTCAG
Canis lupus familiaris  GCTGA-------AATACTTGAGCTTTGTCTCCAGGGCTGAGAGAGAGAGCACAGTCCAC-CATGATCGATTTTCCTTTTTAAATACA-TGGAGACAACATATGGAGGCCTATTCCCTCAG
Equus caballus          GCTGGGA--AAGAATACTGGGGCTTTGTCTCCAGGGAGGA--GAAAGAACTCAGTCCACCCACCATTGATTTCCCTTTTAAAATATA--AGAAACGACAGATGGAGGCCAGAACCCTCAG

Homo sapiens            AGACCCCCCAGCTCATTTGCTTAGGAAAGTAACTGTACAGATAAATTC-CCAGACTCTATTCTGCCAAGGGTGAATGACCCTGAAGGTCGAATATTGCTTTTCTGGGCTGAAGTCAC--C
Pan troglodytes         AGACCCCCCAGCTCATTTGCTTAGGAAAGTAACTGTATAGATAAATTC-CCAGACTCTATTCTGCCAAGGGTGAATGACCCTGAAGGTAGAATATTGCTTTTCTGGGCTGAAGTCAC--C
Gorilla gorilla         AGACCCCCCAGCTCATTTGCTTAGGAAAGTAACTGTACAGATAAATTC-CCAGGCTCTATTCTGCCAAGGGTGAATGACCCTGAAGGTAGAATATTGCTTTTCTGGGCTGAAGTCAC--C
Pongo abelii            AGACCCCCCAGCTCATTTTCTTAGGAAAGTAACTGTACAGATAAATTC-CCAGGCTCTATTCTGCCAAGGGTGAATGACCCTGAAGGTAGAATATTGCTTTTCTGGGCTGAAGTCAC--C
Macaca mulatta          AGACCCCCCAGCTCATTTGCTTAGGAAAGTAACCGTACAGATAAATTT-CCAGGCTCTATTCTACCAAGGGTGAATGACCCTGAAGGTAGAATATTGCTTTTCTGGACTGAAGTAAC--C
Callithrix jacchus      AGACTG-CCAACTCATTTGCTTAGGAAAATAACTGTACAGATAAATTC-CCAGGCTCTATTCCGCTTAGGGTGAATGACCCTTAAGGCAGAATATTGCTTTTCTAGGCTGAAGTCCCTCC
Mus musculus            ------------------------------------------------------------------------------------------------------------------------
Rattus norvegicus       -------TCAACTCACTTGCTCAGGAAAGTAAAGTCTCAGACTAGCTCTTCAGCCTGAGCTCGGCCATGGGTGA----------------------------------------------
Bos taurus              AGACCT-CCAGCCCATTTGCTTAGACAAGTAACTGTACCTTGACATTCACCAGCCTCTATTCTGCCAAAGAGGAAGTGACCCAAAGGTGAAATACTGCATTTCTTGGATGAAGTC-----
Sus scrofa              AGACCT-CCCACTCCTTTGCCTAGACAAGTAACTGTACAGAGGCATTCACCAGCCTCTATTCTGCCAAGAGTCAAATGACCTAAAGGTGAAATACTACATTTCTTGGTTGGAGTC-----
Canis lupus familiaris  AGACCT-CCAACTCAT---CTTGGGCAAGTTACTGTACAGATAAATTCGCCA-TGTCTATTTTGCCAAGAGTGAAATAACCTAGAGGTGAAATATTGTGTCTTTTGGATGAAGTC-----
Equus caballus          AGACCT-CCAACTCATTTGCTCAAGCAAGGAACTGTACAGACAATTTCACCACCCTCTATTCTGCCAAGGGTGAAATGAGTGAAAGGTGAACTATTACACTTTTTGGATGAANNN-----

Homo sapiens            CTTTTTCTTC---TTCTTC------TTCTTCTTTTTTTTGAGACAAGGTGTCACCCAGGCTGGAGTATAGTGGCACAATCTCGGCTCACTGCAAACTCTGCCTACCAGCTTCAAGTGATT
Pan troglodytes         CTTTTTCTTC---TT------------CTTCTTTTTTTTGAGACAAGGTGTCACCCAGGCTGGAGTATAGTGGCACAATCTCGGCTCACTGCAAACTCTGCCTACCAGCTTCAAGTTATT
Gorilla gorilla         CTTTTTCTTC---TTT---------TTTTTTTTTTTTTTGAGACAAGGTGTCACCCAGGCTGGAGTATAGTGGCACAATCTCAGCTCACTGCAAACTCTGCCTACCAGCTTCAAGTGATT
Pongo abelii            CTTTTTCTTC---TTCTTCTTCTTTTTTTTTTTTTTTTTGAGCCAAGGTGTCACCCAGGCTGGAGTATAGTGGCGCAACCTCGGCTCACTGCAAACTCTGCCTACTGGCTTCAAGTGATT
Macaca mulatta          CTTTTTTTCT---TTTT---TCTTTTTTCTTTTTTTTTTGAGACAAGGTGTCACCCATGCTGGAGTACAGTGGTGCAATCTCGGCTCACTGCAAACTCTGCCTACTGGCTTCAAGTGATT
Callithrix jacchus      CTTTTTTTCCCTGTTTTTTTTTTTTTTTTTTTTTTTTTTGAGATAAGGTGTCACCCAGGCTAGAGTGTAGTCACACGATCATGGCTCACTGCAAAT--TGCCTACCAAATTTGAGTGATT
Mus musculus            ------------------------------------------------------------------------------------------------------------------------
Rattus norvegicus       ------------------------------------------------------------------------------------------------------------------------
Bos taurus              ------------------------------------------------------------------------------------------------------------------------
Sus scrofa              ------------------------------------------------------------------------------------------------------------------------
Canis lupus familiaris  ------------------------------------------------------------------------------------------------------------------------
Equus caballus          ------------------------------------------------------------------------------------------------------------------------

Homo sapiens            CTCATGCCTCAGCTTCCCGAGTAGCTGGGATTACAGGGGTGCACCACCACGTTCCAGCTAAGTTTTTGTATTTTTAGTAGAGACAGGGTTTCACTAT
Pan troglodytes         CTCATGCCTCAGCTTCCCGAGTAGCTGGGATTACAGGGGTGCACCACCACGTTCCAGCTAAGTTTTTGTATTTTTAGTAGAGACAGGGTTTCACTAT
Gorilla gorilla         CTCATGCCTCAGCTTCCCGAGTAGCTGGGATTACAGGGGTGCACCACCACGTTCCAGCTAAGTTTTTGTATTTTTAGTAGACACAGGGTTTCACTAT
Pongo abelii            CTCATGCCTCAGCTTCCCAAGTAGCTGGGATTACAGGGGTGCACCACCATGTTCCAGCTAAGTTTTTGTATTTTTAGTAGAGACAGGGCTTCACTAT
Macaca mulatta          CTCATGCCTCAGCCTCCCGAGTAGCTGGGATTACAGAGGTGTACCACCACGGTCCAGCTAAGTTTTTGTATTTTTAGTAGAGATGGGGTTTCACTCT
Callithrix jacchus      CTTAGGCCTCAGCCTCTCGGGTAGCTGGGATTACAGGGCTGCGCCACCACAGCCCAACTAA-TTTTTGTATTTTTAGTAGACGTGGGGTTTCACTGT
Mus musculus            -------------------------------------------------------------------------------------------------
Rattus norvegicus       -------------------------------------------------------------------------------------------------
Bos taurus              -------------------------------------------------------------------------------------------------
Sus scrofa              -------------------------------------------------------------------------------------------------
Canis lupus familiaris  -------------------------------------------------------------------------------------------------
Equus caballus          -------------------------------------------------------------------------------------------------
```
