# Supplementary material for: Extensive intron gain in the ancestor of placental mammals
Source: Biol Direct. 2011 Nov 23;6:59. doi: 10.1186/1745-6150-6-59 (PMC3257199; doi:10.1186/1745-6150-6-59)

Additional file 6 - Ongoing gain and loss of introns in domesticated genes

PNMA6A-like gene

>gi|239746976|ref|XP_001127211.3| PREDICTED: similar to paraneoplastic antigen MA1 [Homo sapiens] gene name: LOC649201 similar to paraneoplastic antigen MA1 (this is PNMA6A-like gene)

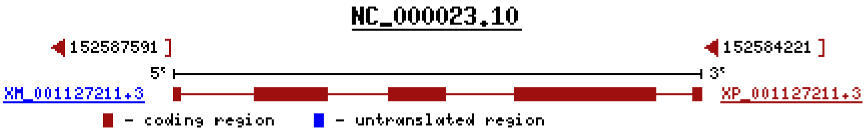

>LOC473826 similar to paraneoplastic antigen like 6A [Pan troglodytes]

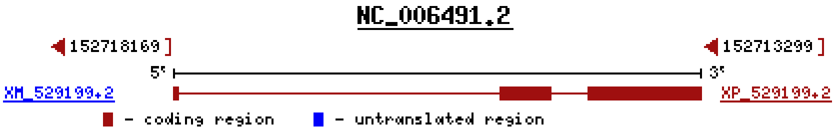

>LOC526946 similar to rCG43868 [Bos taurus]

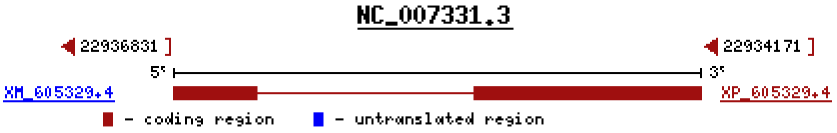

>RGD1559982 similar to paraneoplastic antigen like 6A [Rattus norvegicus]

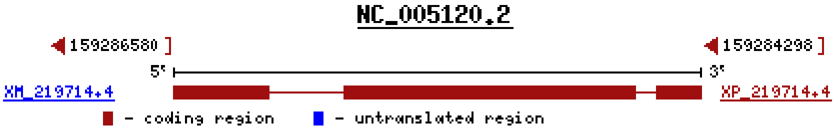

>LOC649238 similar to hCG1645335 [Homo sapiens]

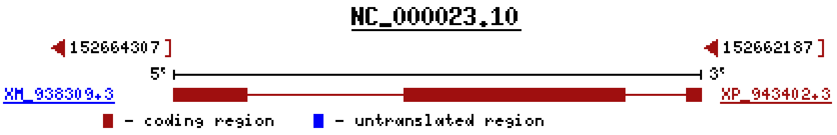

>LOC696694 similar to paraneoplastic antigen MA3 [Macaca mulatta]

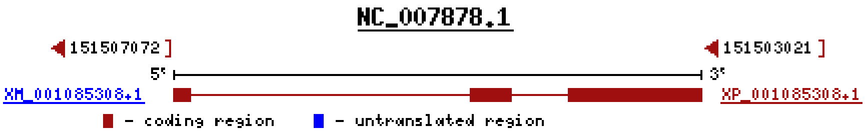

>LOC100147440 similar to mCG1032934 [Equus caballus]

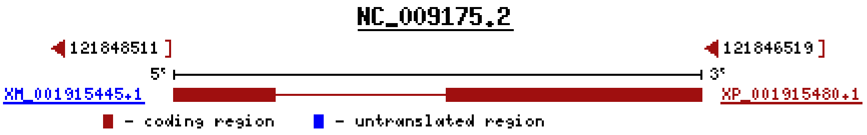

>PNMA6A paraneoplastic antigen like 6A [Canis lupus familiaris]

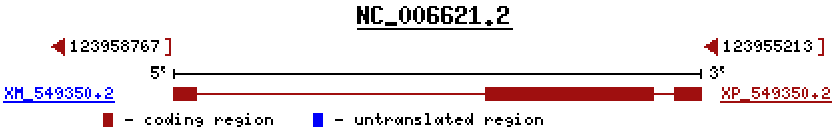

>LOC100058475 similar to rCG43868 [Equus caballus]

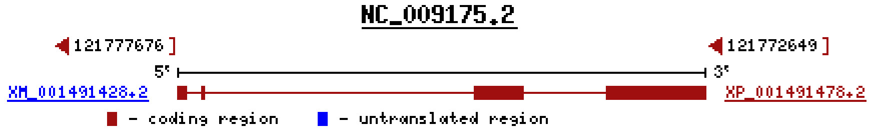

>OC100057070 similar to hCG1645335 [Equus caballus]

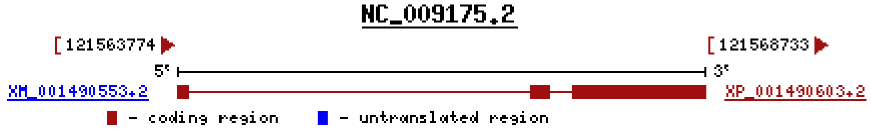

>PNMA6A paraneoplastic antigen like 6A [Homo sapiens] only 45% amino acid identity with the human PNMA6-like sequence

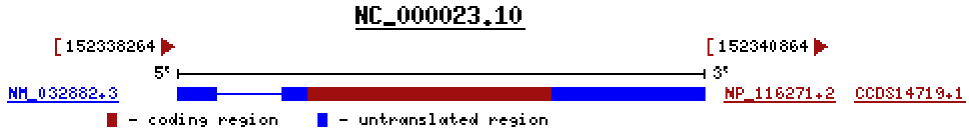

>RGD1559982 similar to paraneoplastic antigen like 6A [Rattus norvegicus]

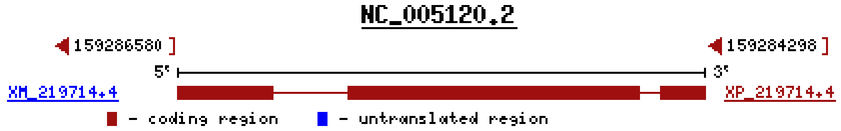

>LOC100128960 similar to Putative paraneoplastic antigen-like protein 6B-like protein [Homo sapiens]

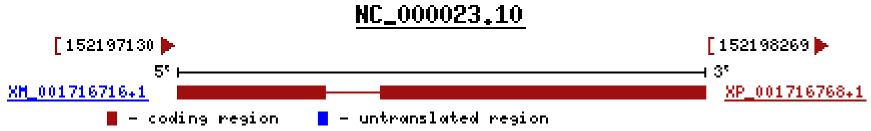

ZCCHC18 gene

>ZCCHC18 zinc finger, CCHC domain containing 18 [Homo sapiens]

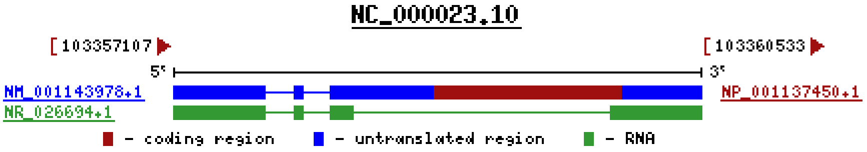

>Zcchc18 zinc finger, CCHC domain containing 18 [Mus musculus]

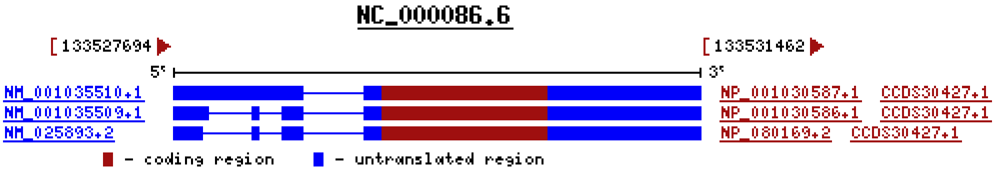

>LOC679126 similar to SMAD-interacting zinc finger protein 2 [Rattus norvegicus]
(96% amino acid identity with Mus ZCCHC18 gene, but new intron in the CDS)

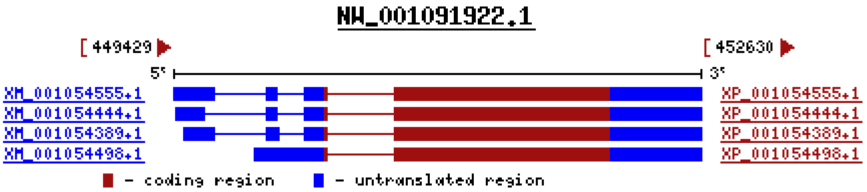

ZCCHC12 gene

>ZCCHC12 zinc finger, CCHC domain containing 12 [Homo sapiens]

NC_000023.10

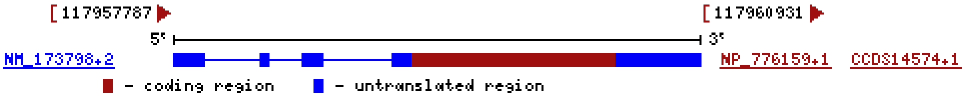

>ZCCHC12 zinc finger, CCHC domain containing 12 [Bos taurus]

NW_001508678.1

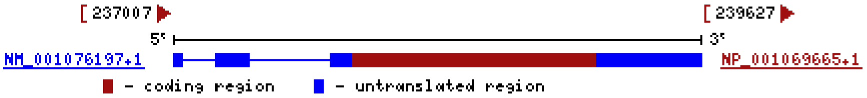

>Zcchc12 zinc finger, CCHC domain containing 12 [Mus musculus]

NC_000086.6

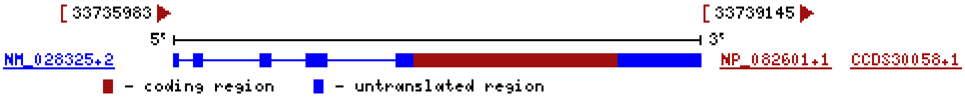

>Zcchc12 zinc finger, CCHC domain containing 12 [Rattus norvegicus]

NC_005120.2

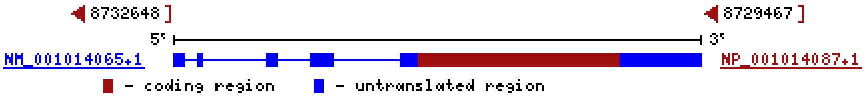

PNMA5 gene

>PNMA5 paraneoplastic antigen like 5 [Homo sapiens]

NC_000023.10

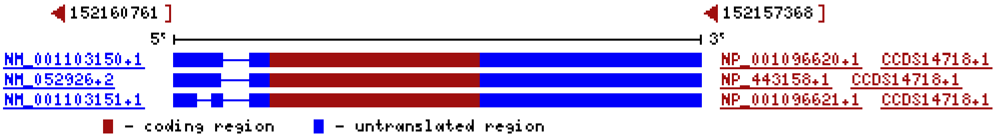

>Pnma5 paraneoplastic antigen family 5 [Mus musculus]

NC_000086.6

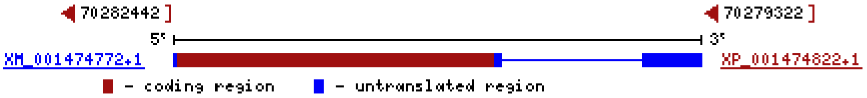

PNMA3 gene

>PNMA3 paraneoplastic antigen MA3 [Homo sapiens]

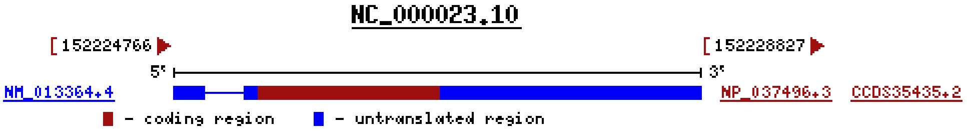

>Pnma3 paraneoplastic antigen MA3 [Mus musculus]

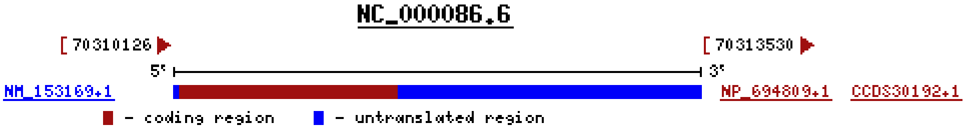

MOAP1 gene

>MOAP1 modulator of apoptosis 1 [Homo sapiens]

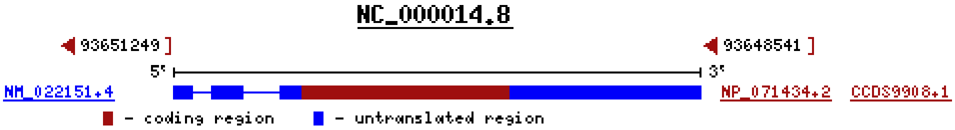

>Moap1 modulator of apoptosis 1 [Mus musculus]

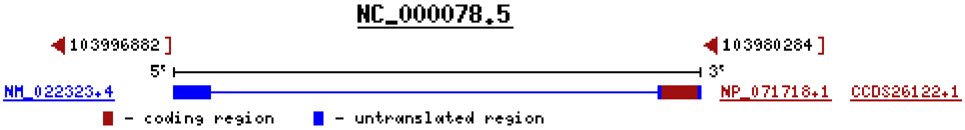

CCDC8 gene

>CCDC8 coiled-coil domain containing 8 [Homo sapiens]

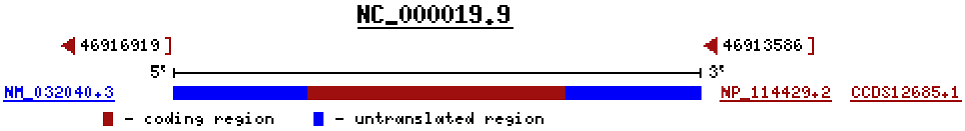

>Ccdc8 coiled-coil domain containing 8 [Rattus norvegicus]

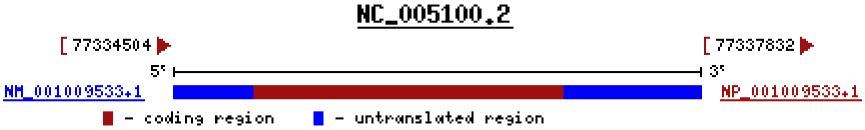

>CCDC8 coiled-coil domain containing 8 [Bos taurus]

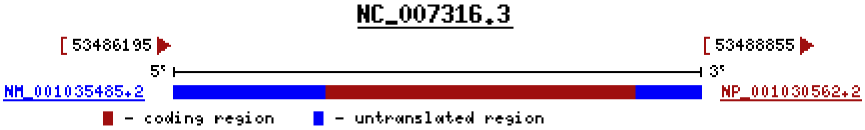

Exceptions:
Equus: 2 exons/1 intron
Rabbit: 2 exons/1 intron

PNMAL1 gene

>PNMAL1 PNMA-like 1 [Homo sapiens]

NC_000019.9

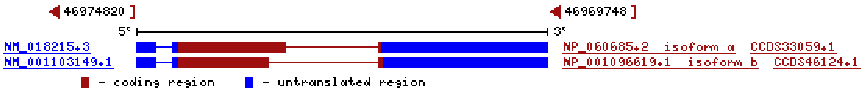

>Pnmal1 PNMA-like 1 [Mus musculus]

NC_000073.5

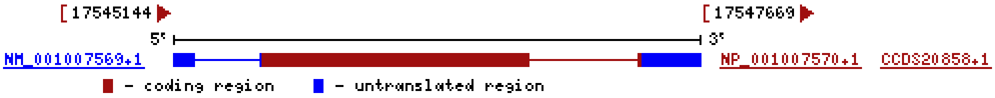

>PNMAL1 PNMA-like 1 [Bos taurus]

NC_007316.3

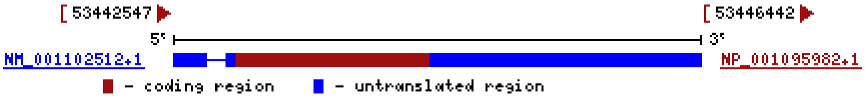

PNMAL2 gene

>PNMAL2 PNMA-like 2 [Homo sapiens]

NC_000019.9

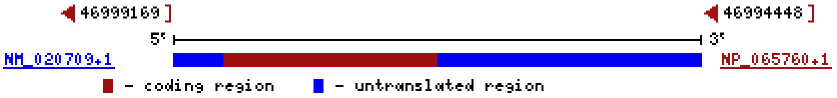

>PNMAL2 PNMA-like 2 [Canis lupus familiaris]: This record was discontinued !

NC_006583.2

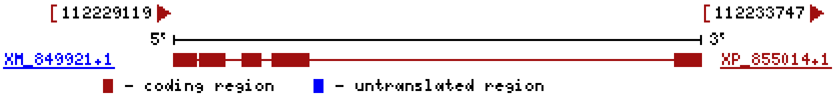

HomoPNMAL2 (RefSeq): intronless vs HomoPNMAL2 (Ensembl): 1 intron in CDS
Genomic alignments at Ensembl: this intron is highly conserved in placentals.

KRBA2 gene

>KRBA2 KRAB-A domain containing 2 [Homo sapiens]

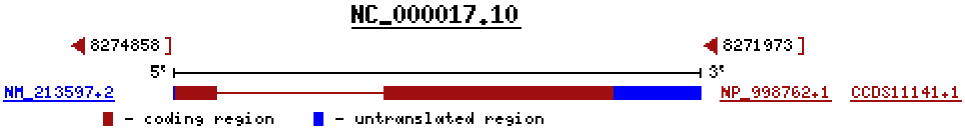

>KRBA2 KRAB-A domain containing 2 [Pan troglodytes]

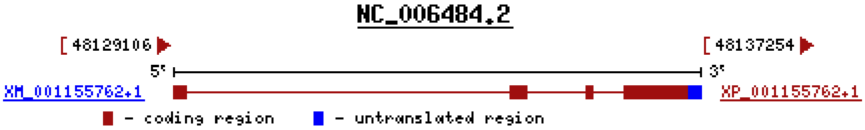

>KRBA2 KRAB-A domain containing 2 [Macaca mulatta]

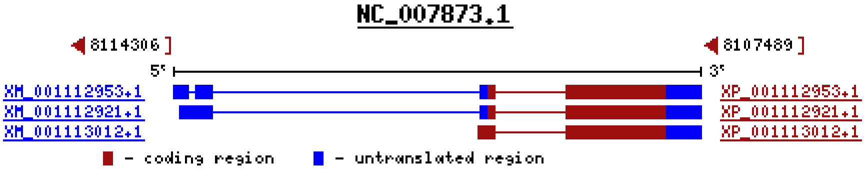

>KRBA2 KRAB-A domain containing 2 [Equus caballus]

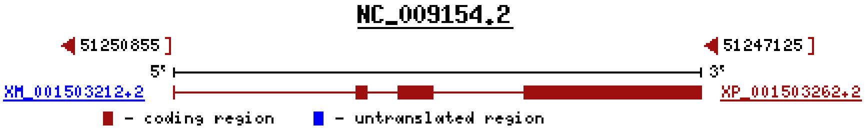

NYNRIN (KIAA1305) gene

>NYNRIN NYN domain and retroviral integrase containing [Homo sapiens]

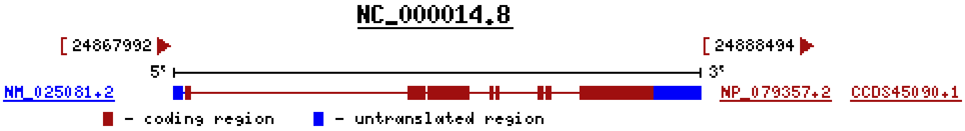

>Nynrin NYN domain and retroviral integrase containing [Mus musculus]

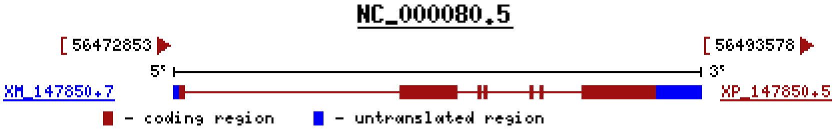

ZCCHC16 gene

>ZCCHC16 zinc finger, CCHC domain containing 16 [Homo sapiens] (374.22kb long), 2 introns

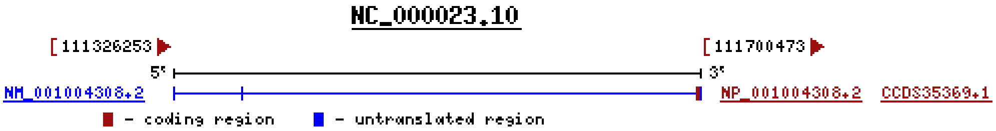

mRNA: join 1..93,47959..48077,371476..374221

>Zcchc16 zinc finger, CCHC domain containing 16 [Mus musculus] (431.96kb long), 6 introns

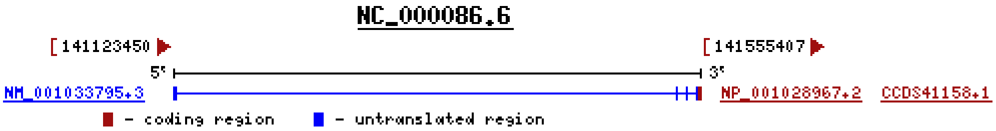

mRNA: join 1..145,1514..1613,412255..412335,420525..420579, 427949..428060,429022..429084,430427..431958

PEG10 gene

>PEG10 paternally expressed 10 [Homo sapiens]

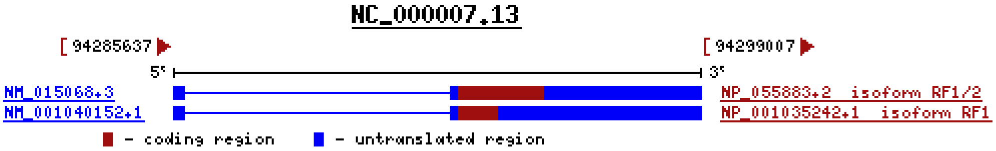

>PEG10 paternally expressed 10 [Bos taurus]

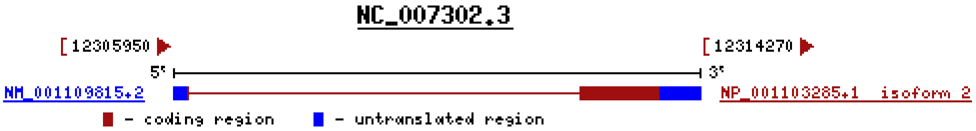

>LOC678843 similar to paternally expressed 10 [Rattus norvegicus]

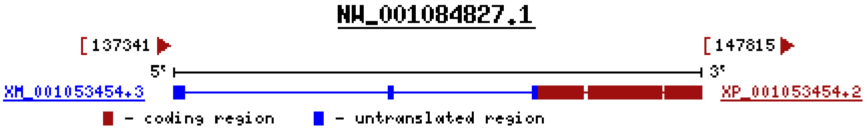

>Peg10 paternally expressed 10 [Mus musculus]

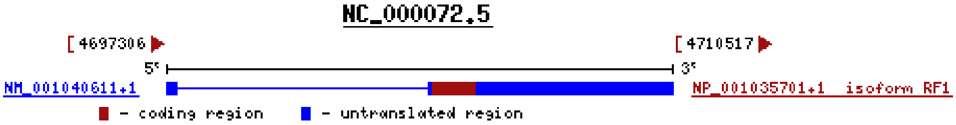

LDOC1L gene

>LDOC1L leucine zipper, down-regulated in cancer 1-like [Homo sapiens]

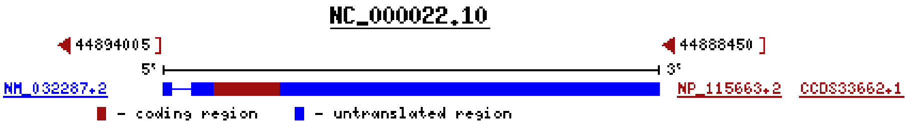

>Ldoc1l leucine zipper, down-regulated in cancer 1-like [Mus musculus]

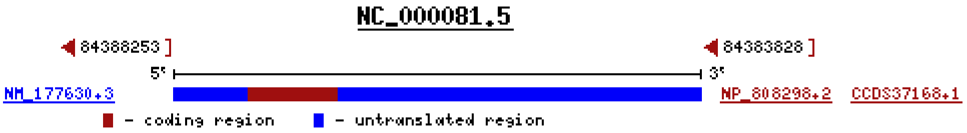

ZNF862

>ZNF862 zinc finger protein 862 [Homo sapiens]

NC_000007.13

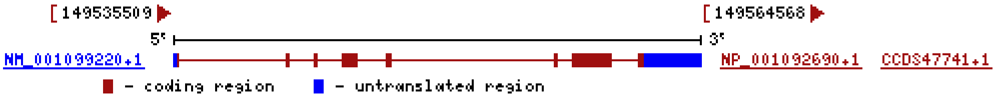

>ZNF862 zinc finger protein 862 [Pan troglodytes]

NC_006474.2

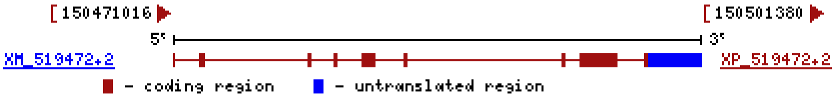

>ZNF862 zinc finger protein 862 [Macaca mulatta]

NC_007860.1

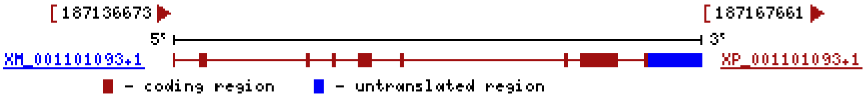

>ZNF862 zinc finger protein 862 [Bos taurus]

NC_007302.3

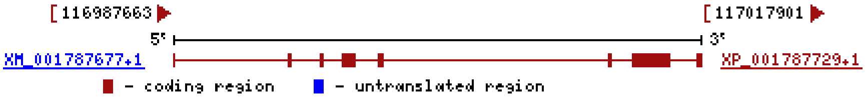

C5orf54 gene

>C5orf54 chromosome 5 open reading frame 54 [Homo sapiens]

NC_000005.9

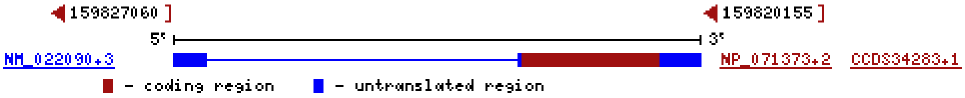

>LOC539846 transposon-derived Buster3 transposase-like [Bos taurus]

NC_007305.3

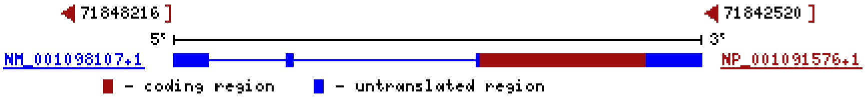

PGBD1 gene

>PGBD1 piggyBac transposable element derived 1 [Homo sapiens]

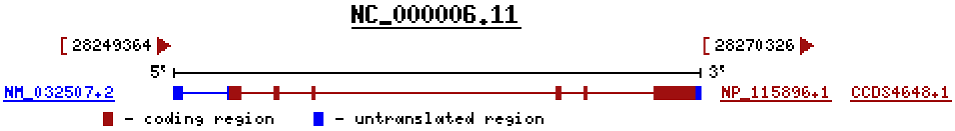

>PGBD1 piggyBac transposable element derived 1 [Pan troglodytes]

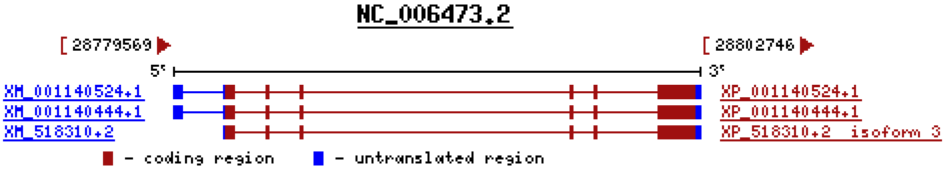

>LOC100157732 similar to piggyBac transposable element derived 1 [Sus scrofa]

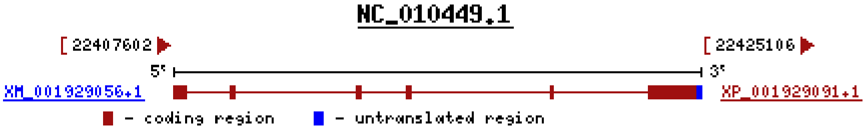

>LOC100061599 similar to cerebral protein-4 [Equus caballus]/this is PGBD1 gene

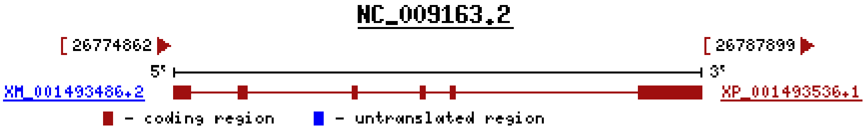

>LOC100347064 piggyBac transposable element derived 1 [Oryctolagus cuniculus]

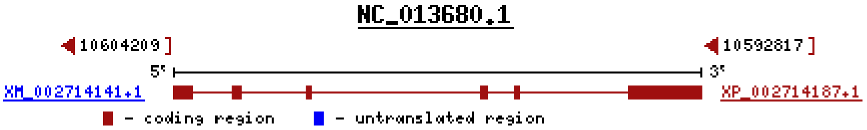

PGBD2 gene

>PGBD2 piggyBac transposable element derived 2 [Homo sapiens]

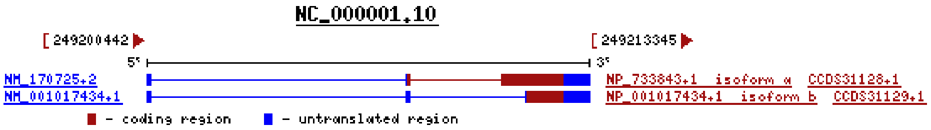

>LOC469779 similar to PiggyBac transposable element derived 2 [Pan troglodytes]

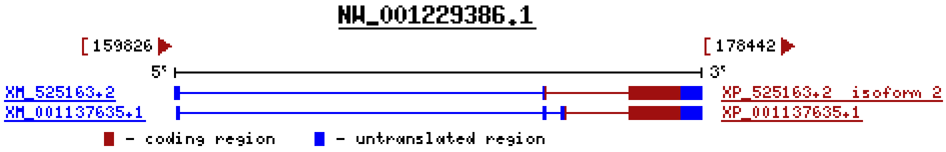

>LOC540000 hypothetical LOC540000 [Bos taurus]/this is PGBD2 gene

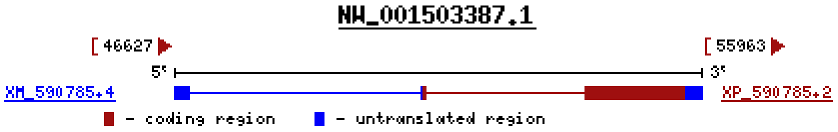

>LOC100055456 hypothetical protein LOC100055456 [Equus caballus]/this is PGBD2 gene

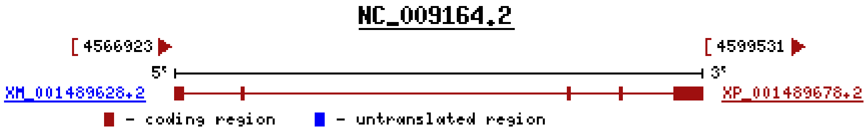

>LOC475503 similar to piggyBac transposable element derived 2 isoform a [Canis lupus familiaris]

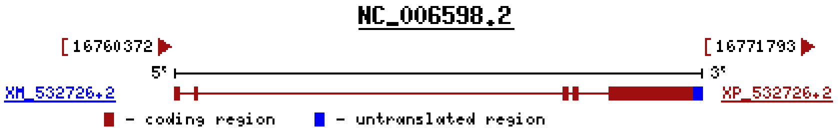

Supplement: Additional file 6 — Ongoing gain and loss of introns in domesticated genes. [file 1745-6150-6-59-S6.PDF]
