# Supplementary material for: Extensive intron gain in the ancestor of placental mammals
Source: Biol Direct. 2011 Nov 23;6:59. doi: 10.1186/1745-6150-6-59 (PMC3257199; doi:10.1186/1745-6150-6-59)
Supplement: Additional file 7 — Highly conserved introns in domesticated genes of placental mammals. [file 1745-6150-6-59-S7.PDF]

Additional file 7 - Highly conserved introns in domesticated genes of placental mammals

A. Typical examples of highly conserved introns in domesticated genes of placental mammals

a) Query was intron 1 (located in the 5'UTR) of the Homo ZCCHC5 gene

>gb|AAGU03047692.1| Loxodonta africana cont3.47691, whole genome shotgun sequence

```
Length=17341

 Score =  527 bits (584),  Expect = 6e-147
 Identities = 546/717 (76%), Gaps = 55/717 (7%)
 Strand=Plus/Plus

Query  1     ATTTACACATTTCCCTCCTCAGCAATGGGTACACAGACAGCCAAGCTCCTGGACTAAGTC  60
              ||| ||||| ||||||||||||  |||| |  ||||||||| |||||||||||||| | |
Sbjct  5359  ATTCACACACTTCCCTCCTCAGTGATGGTTGTACAGACAGCTAAGCTCCTGGACTACGGC  5418

Query  61    TCTGAATGGTAACGTACATTTATAAAGATTATTTTCTTAACTATTTTGAGGTATTTTATA  120
              ||||||||||||| || | ||||||||||||||||||||| |||||||||| ||||||||
Sbjct  5419  TCTGAATGGTAACATATACTTATAAAGATTATTTTCTTAAATATTTTGAGGAATTTTATA  5478

Query  121   AATAATATGCATGTGTCTTCTGAGGGCatatatatatatatatatatatTTA------  174
              ||||||||||||||||||||||||  |||||||||  || ||| | | ||| ||
Sbjct  5479  AATAATATGCATGTGTCTTCTGAG--CATATATATGCATGTATGTGTGTATGTATAACTT  5536

Query  175   -------------GT-----------------CGACTTTTGCTTGTTTTCTCCTGAGGCT  204
                           ||                 | || ||||  |||||||||| ||||||
Sbjct  5537  TTGCTTGTTTTCTGTACACACACACACGCCTGCAACATTTGTGTGTTTTCTCCCGAGGCT  5596

Query  205   GGTCTTGTGTGGTTACTTATGTGGTATGATTTCTGAGGTGTCAGAAAGAGCTCCCTCCTA  264
              | |||| || | |||||||  |||||| |||||||||||||||||||| | ||||  | |
Sbjct  5597  GATCTTATGCGTTTACTTACATGGTATAATTTCTGAGGTGTCAGAAAGGGTTCCCCTCCA  5656

Query  265   CTTCCTACCACCCTAGTCACCCAAAAATATCTCTTGGGCTGGCCCACCGCAGAGCCTAAA  324
              | ||||   |||| ||| ||| |||||||| ||||||| |||||||   ||| |||||||
Sbjct  5657  CCTCCT---ACCCCAGTTACCAAAAAATAT-TCTTGGGTTGGCCCATTACAGGGCCTAAA  5712

Query  325   ATTCCCACATTGCTCAAACATTTGATTTAGTCACTGAAAGCAGCTGTTAAAATCCAAATG  384
              || ||||| |||| ||||||| ||||||||||| ||||| | |||||| |||||||
Sbjct  5713  ATCCCCACGTTGCCCAAACATATGATTTAGTCATTGAAAACTGCTGTT-AAATCCA---T  5768

Query  385   TTACATTGGGATATTAGGCAGATATTCAAGATTAAAGACTATATAAAACAGAAAATAATA  444
              | || || |||| ||| ||||  |||  | |||||||||||| |||||||||  | ||||
Sbjct  5769  TAACTTTTGGATGTTAAGCAGCCATTAGAAATTAAAGACTATGTAAAACAGAGGAGAATA  5828

Query  445   TTAAAAAGTTGCTTTTGATATAATGTTAAGTTTCAAAGGATAAATGCAAAATGACATGTG  504
              |  | || |||||| |||||||||||||| |||  || |   ||||||||| ||||
Sbjct  5829  TGGAGAAATTGCTTCTGATATAATGTTAACTTTTGAAAG---AATGCAAAACGACACACA  5885

Query  505   CAGTTTGGGACAGATGCTGTTCTTAAAATGTCGTGGAGTCAGATCCAGCATATATACCCC  564
               ||| |||| ||| |||||||||||||||  |  ||   |||| |||||| ||||  |||
Sbjct  5886  TAGTGTGGGTCAGGTGCTGTTCTTAAAATTCCACGGGACCAGAGCCAGCAAATATGTCCC  5945

Query  565   AGCTCATGAACATAAGCCTATGGTAAAAGAAACCCAGCATTGTTGGGCAGATTT-----T  619
              ||||||||||| | |||   || | |||||||| ||||| |||  |||| ||||     |
Sbjct  5946  AGCTCATGAACTTGAGCTCTTGATGAAAGAAACTCAGCACTGTCAGGCAAATTTAATTCT  6005

Query  620   GTTTTATGTTGAATGTGTGCTTTCCACAGTTTCTGATCCTCAGCT-CCCACTCTCTT  675
              | ||||| || |||| || |||||||||||||||||||||||||| |||||||||||
Sbjct  6006  GATTTATTTTAAATGAGTACTTTCCACAGTTTCTGATCCTCAGCTCCCCACTCTCTT  6062
```

>gb|ABRP01216211.1| Pteropus vampyrus cont1.216210, whole genome shotgun sequence

```
Length=5874

 Score =  765 bits (848),  Expect = 0.0
 Identities = 581/685 (84%), Gaps = 19/685 (2%)
 Strand=Plus/Minus

Query  1     ATTTACACATTTCCCTCCTCAGCAATGGGTACACAGACAGCCAAGCTCCTGGACTAAGTC  60
             ||| ||||| ||||||||||||| ||| |||| ||||||||| | |||||||||||||||
Sbjct  3056  ATTCACACACTTCCCTCCTCAGCGATGAGTACGCAGACAGCCGATCTCCTGGACTAAGTC  2997

Query  61    TCTGAATGGTAACGTACATTTATAAAGATTATTTTCTTAACTATTTTGAGGTATTTTATA  120
             |||||||||||| |||||||||||||||||||| ||||||||||||||||  ||||||||
Sbjct  2996  TCTGAATGGTAATGTACATTTATAAAGATTATTCTCTTAACTATTTTGAGCCATTTTATA  2937

Query  121   AATAATATGCATGTGTCTTCTGAGGGCatatatatatatatatatatatatTTAGTCGAC  180
             ||||| || ||||||||| |||        |  | | ||||| | |||||||||||||| ||
Sbjct  2936  AATAAAATACATGTGTCTGCTG--------TGCACATATACACATATATATTTAGTCAAC  2885

Query  181   TTTTGCTTGTTTTCTCCTGAGGCTGGTCTTGTGTGGTTACTTATGTGGTATGATTTCTGA  240
             ||||||||||||||| ||||||||||||||||||||||| ||||||||| ||||||||||
Sbjct  2884  TTTTGCTTGTTTTCTTCTGAGGCTGGTCTTGTGTGGTTATTTATGTGGTCTGATTTCTGA  2825

Query  241   GGTGTCAGAAAGAGCTCCCTCCTACTTCCTACCACCCTAGTCACCCAAAAATATCTCTTG  300
             ||||||||||||||||||| ||||| ||||   |||| || ||||||||||||||| || 
Sbjct  2824  GGTGTCAGAAAGAGCTCCCCCCTACCTCCT---ACCCCAGCCACCCAAAAATATCTTTTA  2768

Query  301   GGCTGGCCCACCGCAGAGCCTAAAATTCCCACATTGCTCAAACATTTGATTTAGTCACTG  360
             | |||||||||| |||||||||||   |||||||||||||||||||||| ||||||| ||
Sbjct  2767  GACTGGCCCACCACAGAGCCTAAATGCCCCACATTGCTCAAACATTTGAGTTAGTCATTG  2708

Query  361   AAAGCAGCTGTTAAAATCCAAATGTTACATTGGGATATTAGGCAGATATTCAAGATTAAA  420
             ||||||||||||||||||||| |||||||||| ||||||||||||| |    | ||||||
Sbjct  2707  AAAGCAGCTGTTAAAATCCAAGTGTTACATTGAGATATTAGGCAGACA---GAAATTAAA  2651

Query  421   GACTATATAAAACAGAAAATAATATTAAAAAGTTGCTTTTGATATAATGTTAAGTTTCAA  480
             |||||| |||||||||||| |||||  | || |||||| | |||||||||||||||||||
Sbjct  2650  GACTATGTAAAACAGAAAAAAATATGGAGAAATTGCTTCTAATATAATGTTAAGTTTCAA  2591

Query  481   AGGATAAATGCAAAATGACATGTGCAGTTTGGGACAGATGCTGTTCTTAAAATGTCGTGG  540
             | ||  |||||||||| ||||   |||| |||| |||||| |||||  |||||||| |||
Sbjct  2590  AAGAAGAATGCAAAATTACATACACAGTCTGGGTCAGATGATGTTCACAAAATGTCATGG  2531

Query  541   AGTCAGATCCAGCATATATACCCCAGCTCATGAACATAAGCCTATGGTAAAAGAAACCCA  600
             ||| ||| |||||||||||  |||| |||||||| || | ||||| |||| ||||| |||
Sbjct  2530  AGTGAGAGCCAGCATATATGACCCAACTCATGAATATGACCCTATAGTAAGAGAAATCCA  2471

Query  601   GCATTGTTGGGCAGATTTTGT-----TTTATGTTGAATGTGTGCTTTCCACAGTTTCTGA  655
             ||| | ||||| || || |||     ||||| ||||||| |||||||| || ||||||||
Sbjct  2470  GCACTATTGGGTAGGTTCTGTTCTTATTTATTTTGAATGAGTGCTTTCTACTGTTTCTGA  2411

Query  656   TCCTCAGCTCCCACTCTCTTCGCAG  680
             ||||||| | ||||||||||| |||
Sbjct  2410  TCCTCAGATTCCACTCTCTTCACAG  2386
```

b) Query was intron 3 (located in the CDS) of the Homo RGAG1 gene

>gb|AAGU03056054.1| Loxodonta africana cont3.56053, whole genome shotgun sequence
Length=37961

```
 Score =  529 bits (586),  Expect = 2e-147
 Identities = 496/621 (79%), Gaps = 20/621 (3%)
 Strand=Plus/Minus

Query  111    TAGAATTGGGAAGAGGCAAGGGGGAGGCATGGAGCTCCTAGACCATTAAACCCCA----G  166
              ||||||  ||||| ||||| |||||||| | ||| ||| |||| || ||||||||    |
Sbjct  21276  TAGAATAAGGAAGGGGCAATGGGGAGGCGTTGAGGTCCCAGACTAT-AAACCCCACACTG  21218

Query  167    CAGTTTCATCAAGTGAGCATGGCTCTGCAGTTATTTTGGGTATATTAACTACTTTGAGGA  226
               |||||||||| |||| || ||||   |||||||||||||| ||||||| | |||||||
Sbjct  21217  GAGTTTCATCA-GTGATCACGGCTACACAGTTATTTTGGGTGTATTAACCATTTTGAGGG  21159

Query  227    GCAATGTCCTGCCCAAGTTACTGAGAACGTGGGATAGTTGAAGTACTAATTCTTCTCATT  286
              |||||| ||||||||| ||||| ||    |||||| ||| ||| ||||| |||    |||
Sbjct  21158  GCAATGCCCTGCCCAAATTACTTAGGGACTGGGATGGTTAAAGAACTAAATCTG---ATT  21102

Query  287    ATGAGGGAACTGGATGGGCTCAGTTCCAATTCTCCCCTTGTTCCAAGTCCAATTCTAATA  346
              ||||||||| ||| |||||||||  ||| |||||| ||| |||||||| || ||||||
Sbjct  21101  ATGAGGGAATTGGCTGGGCTCAGCCCCAGTTCTCCACTTATTCCAAGTTCAGTTCTAAGG  21042

Query  347    AATGGGTCTT----TTGGGAGCTGGGGCCCATCTACAGCA-ACATG-TGTTGTGGAGTCA  400
              ||   || ||    ||||||| |||| ||||  || |||  | ||| |||| |||| |||
Sbjct  21041  AAGCAGTATTCATTTTGGGAGGTGGGTCCCACTTATAGCCCATATGGTGTTATGGAATCA  20982

Query  401    GGTCACTAGGGAATTAGAATAGCCTACTCCTTGGTCACACTGTATGCAAGTGA-GATAAA  459
              ||||| ||||||||| |  ||||| |  | |||||| | || | | ||||| | ||||||
Sbjct  20981  GGTCATTAGGGAATTTGGTTAGCCCAGCCTTTGGTCTCCCTTTGTACAAGTTAAGATAAA  20922

Query  460    TTAGCAAAGTGTCTTGATCTGCTAAGTCACAGAACTTATCCAGGCAGAACACCCGTTGCC  519
              ||||| ||||  ||||||||||||| |  | ||| |||| |||||||||||||  ||| |
Sbjct  20921  TTAGCCAAGTTCCTTGATCTGCTAATTG-CTGAATTTATTCAGGCAGAACACCTCTTGTC  20863

Query  520    ATAGTAAGTGTGGCTCTTACCTCTGTTGTCTTTTTCTCACTCCCAGCACCAGCATGTTTC  579
              | ||||||||||| | ||||  ||| ||| |||||||  || | |||||||||||||| |
Sbjct  20862  ACAGTAAGTGTGGTTTTTACTGCTGCTGTGTTTTTCTTTCTACTAGCACCAGCATGTTCC  20803

Query  580    CAAACGCTGTTACTACCTGAAAGAGCATGGAGACCCCCAAGAAGGTCTTCATGATCACCT  639
              ||||||||||||||||||||||||||||||||||||||||||||||||||| ||||||||
Sbjct  20802  CAAACGCTGTTACTACCTGAAAGAGCATGGAGACCCCCAAGAAGGTCTTCACGATCACCT  20743

Query  640    TGGACAGAGCACAGGCCATCATCAGAAGGCCCATACCAACAAGTAAAACTCCATGGAATC  699
              | ||||||||||||||   |||| ||||||||  ||  ||||||||  ||| ||||| ||
Sbjct  20742  TCGACAGAGCACAGGC---CATCCGAAGGCCCCCACTGACAAGTAATGCTCAATGGATTC  20686

Query  700    TTCTCCTGTGATATCTGACTC  720
              |||||||||| |||| |||||
Sbjct  20685  TTCTCCTGTGGTATCCGACTC  20665
```

c) Q was intron 1 (26 kb long) of the Homo RGAG1 gene

```
>gb|AAGU03056056.1|  Loxodonta africana cont3.56055, whole genome shotgun sequence
Length=18534

                                                          Sort alignments for this subject
sequence by:
                                                            E value  Score  Percent identity
                                                            Query start position  Subject start
position

 Score = 2078 bits (2304),  Expect = 0.0
 Identities = 1755/2136 (82%), Gaps = 67/2136 (3%)
 Strand=Plus/Minus

Query  685    aaattaaacaaaagaaataaaaagcataaaaaGCTAAGTTTTTATTGGGTATTTACTATG  744
              |||| ||||||||  ||||||| || ||| | | ||| |||||| || ||  ||||||||
Sbjct  13794  AAATCAAACAAAAAGAATAAAACGCCTAAGAGGTTAACTTTTTACTGAGTGCTTACTATG  13735

Query  745    TGCCAGGAACTGTTCTAAGTGTTCTACACAGATTATCTCATTTAATCCTCATCATAACCC  804
              ||||| |||||||||||||||||||||| ||||||||||||||||  |||| ||  |||
Sbjct  13734  TGCCAAGAACTGTTCTAAGTGTTCTACACTGATTATCTCATTTAATTTTCATAATGGCCC  13675

Query  805    AATGACGTCAACTCTATTATTAGCTCCATTTAACAGATGAAGGAATTGAGGCTCTGGGAT  864
              ||||| ||  | |||||| |||||||||||| | |||||| |||||||||||||| |||
Sbjct  13674  AATGATGTAGATTCTATTTTTAGCTCCATTTTATAGATGAGGGAATTGAGGCTCTGAGAT  13615

Query  865    GTTAAGAAAAGTGCCCAAGGTCACACAGCTAGCAAGAGACAGAACCAAAAGTTGAACTCA  924
              || ||| || |  |||||| |||| ||||| |    ||| ||||||||||||||||| ||
Sbjct  13614  GTGAAGCAAGGGTCCCAAGATCACCCAGCTTGTGGGAGGCAGAACCAAAAGTTGAACCCA  13555

Query  925    AGGCAGTCTGAGTCCA-AGTCCACAAACTAAATCATCTATGCCAC-CTGCAGCCCCCACC  982
              |||||||||||||||  ||||||    | || ||||    ||||| | ||||||     ||
Sbjct  13554  AGGCAGTCTGAGTCCCTAGTCCATGCAATACATCACGGATGCCTCTCTGCAG-----ACA  13500

Query  983    TCATGTTACCCTACACCTCCCACGCCACCCCCAGGCACGATTCTACTACAGCACTGGCCA  1042
                ||| | || | || | ||    | ||| ||  | |  || |           |||   ||
Sbjct  13499  GGATGCTTCCTTGCAGCCCCT--GGCACACCTTGCCCAGAAT----------ACTCATCA  13452

Query  1043   CATTGCAGAACAGGGGTTTGTTTACTTG--TCTGTCCTTCCTACTAGACGAGGAGCTTCT  1100
              ||||| || ||||||||||||||||     ||||||||||| |||||||    |||| |||
Sbjct  13451  CATTGTAGTACAGGGGTTTGTTTACTCTCATCTGTCCTTCCCACTAGACTGAGAGCCTCT  13392

Query  1101   CAAGTGCAGAGACTGGATCTTATTCATTTTTGCTTCCCCTGTGCCTGGAAGAGAGCTTGT  1160
               ||||| || |||    |||||    |||||||||||||||| |   | ||  ||||||||
Sbjct  13391  AAAGTGTAGGGACC--ATCTT---CATTTTTGCTTCCCCTGCG--TAGAGTAGAGCTTGT  13339

Query  1161   TCTGGAATAGCTAACCAAGGAATATTCGTTGAATGAGCAAATGAGTGAGGGAGTAAGTGA  1220
              ||  || || |  ||| |  |||||| ||||| |||    ||||| ||||        |||
Sbjct  13338  TCCAGAGTA-CGGACCGATCAATATTTGTTGACTGAATGAATGAATGAG--------TGA  13288

Query  1221   ATCCATATTCCTTAATATTTTCAGGCTTCAGGCTTATGGGCTCATGTGTATTTCCCTGGT  1280
              |||||| ||||||   |   ||| |||||||| |||||||||||| |||  |||||||||||
Sbjct  13287  ATCCATGTTCCTTTTTTTCTTTTAGGCTTCAAGCTTATGGGCTCGTGTTCATTTCCCTGGT  13228

Query  1281   CAA-------GCATAGCACTGGGCACAAGGGTATTCTGGTGCAAGAGGTGCCTCTCACCA  1333
                |       |  |||||||||| |||||||| | |||||||||||| | |||  |||||
Sbjct  13227  TTATATTAACGTGTAGCACTGGGGACAAGGGTGTGCTGGTGCAAGAGATCCCTTGCACCA  13168

Query  1334   CAGGGTACAAGAAGGACTAGCTCTGGCTGGGTAAACCAAAGCAGTGTTCCACAGAGCAAG  1393
                ||||| ||  ||||| ||||||| ||||  ||||||||||| ||||| | ||||||||
Sbjct  13167  TGGGGTAAAAAGAGGACCAGCTCTGACTGGTAAAACCAAAGCAATGTTCTATAGAGCAAG  13108

Query  1394   GGGCTTGATCCAGAAGGTTTCATTTTGTCATCGGTCTAAAAAGTAGCTGGACCTGTTTAG  1453
              | ||||||| || | |||||||||||||| ||||| || ||||||||||||||||||||
Sbjct  13107  GCGCTTGATTCAAAGGGTTTCATTTTGTCGTCGGTTTAGAAAGTAGCTGGACCTGTTTAG  13048

Query  1454   GACTTAAACAAACTTTCAGTGTTCCAATCCCATCTCAAATCCTTTGCAGAATGAGGCAGG  1513
              ||||||||||||||||| ||||| ||||||||||||||||||  ||||||||||| ||
Sbjct  13047  GACTTAAACAAACTTTCAATGTTCAAATCCCATCTCAAATCCTTCTCAGAATGAGGCGGG  12988

Query  1514   GTGTGGGGAG-GTGG--TGGTGGGGCAGTTATAAATAAACAAATACCCACAAACCGGCTG  1570
               ||||||| | | ||    |   | || |||||||||||||||||||||||||||||| |||
Sbjct  12987  TTGTGGGGGGAGAGGCAAGAGGAGGGAGTTATAAATAAACAAATACCCACAAACCGCCTG  12928

Query  1571   GTTTTTTAAACAGAATTTGCTTCAGTCAGTGCTGCACCACATTTTCCCAACTGGCCTCCG  1630
              ||||||||||||||||| | ||| |||| ||||| | |||| |||||||||||||| |
```

```
Sbjct  12927  GTTTTTTAAACAGAATTTACCTCAATCAGCGCTGCGCTACATCTTCCCAACTGGCCTCTG  12868

Query  1631   TCGTGAGAACAAGTGGGAACTTTCATACTGAATGGCTTTA---TTTTTATTCTGACCAGA  1687
              || ||||||||||||||||| |||||||||||||||||||   ||||| ||| |||||||
Sbjct  12867  TCATGAGAACAAGTGGGAACGTTCATACTGAATGGCTTTATTTTTTTTTTTCCGACCAGA  12808

Query  1688   TGAATGGCAGGAGTTTTGGCTCGGGAGGCAGGTTTCCGATCCTCAGCACACAGTTTGCTG  1747
              ||||||||||||||||||||||| ||||||||||||  |||||   ||||||||||||||
Sbjct  12807  TGAATGGCAGGAGTTTTGGCTCGAGAGGCAGGTTTCAAATCCTTGACACACAGTTTGCTG  12748

Query  1748   CCCTACTGTGCCCCGTCCATCACACACAGCGCCCTGCACAACATACCCAGTCATTGCATC  1807
              |||||||||||||| ||| |||||||||| || |||||||||||||||||||||  ||||
Sbjct  12747  CCCTACTGTGCCCCATCCGTCACACACAGTGCACTGCACAACATACCCAGTCATCACATC  12688

Query  1808   TACTGCCTCAGCCCTTGAAAATAATTGGGTCATTGTTTTATTACACCAGCGAGAGAGCTG  1867
              | |||| |||| || | |||| || |||||||||||||||||||||||||||||||||||
Sbjct  12687  TGCTGCTTCAGTCC-TCAAAAAAACTGGGTCATTGTTTTATTACACCAGCGAGAGAGCTG  12629

Query  1868   CAAGCCTGTTAGGATTATGTCATTGATATTTGCGTGAAAAGGCAATCATGATATGCTGGT  1927
              |||||||| || |||||||||||||| |||||||||||||||||||||||||||||||||
Sbjct  12628  CAAGCCTGCTACGATTATGTCATTGACATTTGCGTGAAAAGGCAATCATGATATGCTGGT  12569

Query  1928   CATTTAACACAGCTGGGAATGCAACTGTGTTGTCATTGTTTAAGGTTCTCATCC-AGCCC  1986
              |||||||||||||||||||||||||||||||||||||||| ||||||||||||| | | |
Sbjct  12568  CATTTAACACAGCTGGGAATGCAACTGTGTTGTCATTGTTCAAGGTTCTCATCCAAACTC  12509

Query  1987   CTCTGACTTTTTTGCACAGCCCAGCCCGTGAAAATGGATCACTCTGATGTATGTCACCTT  2046
               ||||||||  ||   |||||| || ||| || |||||| ||||||||||||| |||||
Sbjct  12508  TTCTGACTTCGTTTTCCAGCCCAACCAGTGTAAGTGGATCGCTCTGATGTATGTAACCTT  12449

Query  2047   T-AAAATGCTTTCTAG-CCCTTCAAGGGACCAAGCAGGATTTAACTCTTTTCATGCACTT  2104
              | ||| || ||| ||| ||||||||  ||||||||||||| || |||||| |||||||||
Sbjct  12448  TGAAAGTGTTTTTTAGCCCCTTCAAAAGACCAAGCAGGATCTACCTCTTTCCATGCACTT  12389

Query  2105   GGACTTTGCTGCTTGTAGGCACAGCAGTCTGAAGGTGAGAGATGCTTGCTTTTCATACTT  2164
              ||||||   |||||||||||||||| ||||||||||||||||||||||| |||||| | |
Sbjct  12388  GGACTT---TGCTTGTAGGCACAGCCGTCTGAAGGTGAGAGATGCTTGC-TTTCATGCGT  12333

Query  2165   GTGGGAGTTGTACACCCTAGTGGCAGGATGCTGCCATACAGTGTCATTTGTTCACTAACT  2224
              |||||||||||||||||||||||||||||||||| ||||||||||||||||||||||| |
Sbjct  12332  GTGGGAGTTGTACACCCTAGTGGCAGGATGCTGCAATACAGTGTCATTTGTTCACTAAGT  12273

Query  2225   GCTGATCAGAGCCAAGGACAATTTTGGCTCAAGAACAAATGAGACCTAA---TGTCTGTG  2281
              ||| |||||||||||||||| ||||||||||| ||||||||||  ||||   ||||||||
Sbjct  12272  GCTAATCAGAGCCAAGGACAGTTTTGGCTCAAAAACAAATGAGGGCTAATGTTGTCTGTG  12213

Query  2282   AGGGAAGACTATGTAGATAAGTCACGGGTCAGAGTTGTGTTGCGACTTGTATTGTGTGCT  2341
               ||||||| ||| |||| || ||| |  ||||| || ||||||||||| ||||||||| |
Sbjct  12212  GGGGAAGATTATTTAGACAATTCATGAATCAGAATTCTGTTGCGACTTCTATTGTGTGTT  12153

Query  2342   AGTTTCAGTAGGACTTGTGCTTTCATTGCAATTGTCAGTATGCTTAAGCAGGGGTCTCTG  2401
              ||||| |||||||||||  |||  |||||| |||||||| |||||| |||||||||||||
Sbjct  12152  AGTTTAAGTAGGACTTGCACTTGGATTGCAGTTGTCAGTGTGCTTATGCAGGGGTCTCTG  12093

Query  2402   CTAATGAAATTCGAGCATATTTGCATTTATCGACAGGTATTACTGATGGTAGAACATCTT  2461
              |||||||||||| |||||||||||||||||| |||||| ||| || ||| |||| | |||
Sbjct  12092  CTAATGAAATTCAAGCATATTTGCATTTATCAACAGGTGTTAATGTTGGGAGAAAAGCTT  12033

Query  2462   ATTTCCACACAAGGCAATGAGTAACATTGATTATAGAAGTGTAAACAAAGCTCAAATTCA  2521
              |||| || |||||| ||||| || | |||  ||||||| | |||||  |||||||| |||
Sbjct  12032  ATTTTCAAACAAGGAAATGATTAGCGTTGGCTATAGAA-TATAAACGGAGCTCAAAGTCA  11974

Query  2522   TGTGCCAATTTGTCTATTTCAACTCATCGATCCTCTTCTTCC-ACTGGCATGCTAGCCTG  2580
              ||||| ||||| ||| || |||||| ||  || ||| || ||  | |||| | ||||  |
Sbjct  11973  TGTGCTAATTTTTCTGTTGCAACTCTTC--TCTTCTCCTACCGGCAGGCACGTTAGCTGG  11916

Query  2581   AGTTTTATGGCATAAGGCCATTAAAGTATTCTTCAAAGGAATCTGTTGCAGACACCTGTT  2640
              |||||||||||||  | |||||||  |||||||| ||||||||| |||||| ||||||
Sbjct  11915  AGTTTTATGGCATGTGACCATTAATTTATTCTTCGAAGGAATCTATTGCAGGCACCTGGC  11856

Query  2641   CATCCCTCTTCTGGTCCCGGTCCTTGCCAAACCAGCTC--TGCCTTTGCCAACCACCTTG  2698
              ||||| | |||| ||||| |||| || |||||||||||  |||| |||||| ||||||||
Sbjct  11855  CATCCATTTTCTAGTCCCAGTCCCTGGCAAACCAGCTCTGTGCCCTTGCCAGCCACCTTG  11796

Query  2699   AGGTGTGACAGGAAAAGCTGACCCAATAAGGGAGTCAACTGAAGGGATGGAGTGGCTTAG  2758
              |||||||||||| | || || |||||||| ||||||| |||||| |||||  |||  |||
Sbjct  11795  AGGTGTGACAGGGAGAGTTGGCCCAATAAAGGAGTCAGCTGAAGAGATGGTCTGGGGTAG  11736
```

```
Query  2759   ATGCAAATGGCCAAATCCCAATGGCCATGGCTATAA  2794
              |||| ||   |||| ||||||| ||| |||||||||
Sbjct  11735  ATGCCAACCTCCAAGTCCCAATCGCCCTGGCTATAA  11700
```

Score = 2008 bits (2226), Expect = 0.0
Identities = 2451/3322 (73%), Gaps = 272/3322 (8%)
Strand=Plus/Minus

```
Query  11174  TACTAACATTTATTGAGCAATGATTATGTGTCAGGTATCATTCTAAGTGCTTTGTGTGTA  11233
              |||||||||||||||| || | | |||||| |||||| |||||||||| ||||||||||||
Sbjct  4733   TACTAACATTTATTGAACATTTACTATGTGCCAGGTACCATTCTAAGTACTTTGTGTGTA  4674

Query  11234  TTAACTCATTTAATTCTCACTATGATGCTGTGAGGTAGGTTACTATTATTATCTTCACAT  11293
              |||| |||||||||||||| |||  | |  |||||||||| || |||||| ||   |  |
Sbjct  4673   TTAATTCATTTAATTCTCAGTATACTTCA-TGAGGTAGGT-ACCATTATTGTCCCAATTT  4616

Query  11294  TACAGAGGAGGCACCAGACTCAGACAGGCTGAATAACTTGGCCAAGGTCACATAGCTAGT  11353
              || ||||| |||||||||| |||||||||||| | |||||||||||||||||||| ||||| |
Sbjct  4615   TAGAGAGGTGGCACCAGGCTCAGACAGGCTAAGTAACTTGGCCAAGGTCACACAGCTAAT  4556

Query  11354  GAATGGCGGAACACAGATATGAACCCAGGCACCCTAGAGACACATCTACTTACCACCTTG  11413
               |||||  ||||   |||||||||| ||||| ||| ||| ||||| ||  | ||| | ||
Sbjct  4555   AAATGGTAGAACTTGGATATGAACCTAGGCAACCTGGAGCCACATTTAACTGCCATCCTG  4496

Query  11414  TATTGGAGAAGAGGTCAACAGTAGTGACAGAAGCAATAGAGTGGAAATTTTCAGTACTGC  11473
              ||||| |||||||  ||||||||||||||| |||||| ||||||| | ||||||||| | |
Sbjct  4495   TATTGAAGAAGAGAGCAACAGTAGTGACAGCAGCAATTGAGTGGACAATTTCAGTATTAC  4436

Query  11474  CAAAA---CTATGAGAGCCATATAGTCCACACCAGGCATGAAAAGGAAGCTGGCATGGTC  11530
              | ||    |   |||||||| | || ||||||||||||||  |||| |  ||||||| ||
Sbjct  4435   CGAAGGGGCAGCGAGAGCCACACAGGCCACACCAGGCATGGGAAGGGACTTGGCATGATC  4376

Query  11531  TTGTGTTGTCAGTTAACCCTTGTATATAGGGTTTTCATTTCCT--AACTAGAGAAGGAGG  11588
              || | |||  ||||||||||||||| |||  ||||  ||||||  || ||||  |||||
Sbjct  4375   TTATATTGGTAGTTAACCCTTGTATGTAGTTTTTT--TTTCCTCCAAATAGAATAGGAGC  4318

Query  11589  AGTTTGAAAGCACGAACCATGAATTACAGCTCTTTGAAATGCTCAACATAGATTTGCTGA  11648
              |||||||||| | |||||||  ||||||||||||||||| |||||| |||||||||||||
Sbjct  4317   AGTTTGAAAGTAGGAACCATAGATTACAGCTCTTTGAAACGCTCAATATAGATTTGCTGA  4258

Query  11649  GTGAATAAATGACTGTTTTGAAATTAAACACAGGAGTGCTTTGAATAAATTACTTTGCAT  11708
              |||||||||||| | ||||||||||||||||| |||| ||||||| ||| ||||||  |||
Sbjct  4257   GTGAATAAATGAGTCTTTTGAAATTAAACACATGAGTACTTTGAGTAAGTTACTTCACAT  4198

Query  11709  CTTGTATGTCAAACTGAAATTGGTGAGACATCTTT-CACCTTGTGTTAAAAACTAAAAAT  11767
              ||||| |||||||| |||||||||||| | | |||| ||| |||| |||||| || || |
Sbjct  4197   CTTGTGTGTCAAATTGAAATTGGTGACAAAGCTTTTCACTTTGTTTTAAAACCTGAAGAC  4138

Query  11768  CCCTAGATGTGGTCATAACATGGCTGGTTATAATGACACCTTGCATTTGTACTTGTGTAT  11827
               ||||||| |||| |||||||||| |||| ||| |||||||||||| |||||||  ||||||
Sbjct  4137   ACCTAGATATGGCCATAACATGTCTGGGTATGATGACACCTTGCGTTTGTACCAGTGTAT  4078

Query  11828  GATACTCAGGACTCCCTGAGAAAAGGAATGAAGAAACTTGGATTTTGCCTTCATAGGCCC  11887
              |                  ||| ||||  ||||||| || |||| |||||||||||||||
Sbjct  4077   GG-----------------GAATAGGACAGAAGAAAGTTAGATTCTGCCTTCATAGGCCC  4035

Query  11888  TGAGGCTGTCAGGAACCAAGGGCCACTGTGTTTGTAATCCATTCCTTTGGCTGCTCTCCA  11947
              || |||||||||||||| | || ||||  ||||||||||||||| || | ||  | ||||
Sbjct  4034   TGGGGCTGTCAGGAACCTAAGGTCACTAGGTTTGTAATCCATTCTTTGGCCTTATGTCCA  3975

Query  11948  AATCTAGGAAGACCTGGAGGAAAGGGGGGCTTTATTTTCT---ATCTTTGCCAAGATCAG  12004
              ||||||||| ||||| ||| ||||| || ||| |||| ||   ||| |||||||  | ||
Sbjct  3974   AATCTAGGAGGACCTAGAGAAAAGGAGGACTTCATTTGCTCTTATCCTTGCCAAACT-AG  3916

Query  12005  GAAATAATTTG----TGAACTCTCAGT---AA--ACATTCTGGGGGCCTTCCCTCTAACA  12055
              ||   ||||||    || |||||| ||   ||  | || || |     |||  |||| |
Sbjct  3915   GATCGAATTTGAGTGTGGACTCTCTGTTGGAATGAAATCCTAGT----TTCTATCTATCT  3860

Query  12056  CTG-----CTTTGG------TGGAATAATATCTCCAGTTTTCACCA----ATCTTG--GT  12098
               ||     | |||       |  || || ||  || |   | |||     ||| ||  ||
Sbjct  3859   GTGGATATCATTGAGACACCTCCAAAAACATACCCTGAAATTACCTTTAGATCCTGAAGT  3800

Query  12099  TCAATATTTACCTGGGGGTTTGCAGTGATCA-------TGGGGACAGGGCTCCAACATGA  12151
              ||||  ||||| |||||||| ||||||| ||       ||||| ||||||||||  | ||
Sbjct  3799   TCAACCTTTACTTGGGGGTTCGCAGTGAGCAACTAGCTTGGGGGCAGGGCTCCAGAAGGA  3740
```

```
Query  12152  AGTGAAGGTTCATGA--GAAGGAAAATGAGGGGAAGGCtgtgtgtgtgtgtgtgtgtgtg  12209
              || | |     |||   ||| | || ||  |||| ||| ||   ||| | | ||||||||
Sbjct  3739   AGAGGA-----ATGGAGGAATGGAATTG--GGGAGGGCCGT---TGTCTATATGTGTGTG  3690

Query  12210  tgtgtgtgtgCTGGCTGAGGGATGAAGGTGGGAGGAATACCAGGATTCTGAAGCAATGAG  12269
              ||| ||||| |||| |||||  ||   | ||| ||    |||| ||  ||||||||||||
Sbjct  3689   TGTATGTGTACTGGGTGAGGAGTG---GGGGGTGGGTGCCCAG-ATAATGAAGCAATGAG  3634

Query  12270  AGTGGGATTGGGAGAGAGAGCTCATGTTGAA-GAAGAAATAACTCACCAGTTCACATGGG  12328
              |||||||||||||  ||| |     ||   | ||||||||||||  || |||||  |||
Sbjct  3633   AGTGGGATTGGGATGGAGTGGGAGGGTACCATGAAGAAATAACTTCCCGGTTCATTTGGT  3574

Query  12329  CTCCTTTTCAAGGGTTTTTCTTTATATAAACAATTTGCCTGGTTCCAAT--TAGAAGCCC  12386
               || |||||||||| || |||||    |||||| ||||||||||| | |  |||||||||
Sbjct  3573   -TCATTTTCAAGGGCTTCTCTTTGCTGAAACAACTTGCCTGGTTCTAGTCCTAGAAGCCC  3515

Query  12387  ACATTAGGCAGTTAGCTCCAATTTGGCAGTTCATTCTTAATTTGGCTTTCCTTATGGGTG  12446
              | ||| |||||||||  || |       ||||||    |||||| ||| | ||||  |||
Sbjct  3514   AGATTGGGCAGTTAGTCCCTAAG-----GTTCAT----AATTTG-CTTGCTTTATATGTG  3465

Query  12447  GGAGGGAGGGCTGACAGTTAAAAGGTTGATGGGAAAAACCTTGGTATCCTGGTGTGAAGA  12506
              || | ||||      | |||| ||| ||| |||||  |  |||| |||||||| ||||||
Sbjct  3464   GGGGTGAGGTT----AATTAAGAGGGTGAAGGGAAGGATTTTGGGATCCTGGTATGAAGA  3409

Query  12507  CAGACCCTGGGATCAGAAGGTTGCCCTGATGACTTTG-CCAATGTGTTCATTGCCCCTG-  12564
               ||               ||||    ||||  || |  |||||||   |||  ||| ||
Sbjct  3408   AAG---------------GGTT----TGATTTCTCAGACCAATGTTGGCATGTCCCTTGG  3368

Query  12565  -TCCTGCTGGGGCGCATACAGCAGGCC-----CTTCTTTCTCATACGCCC----TTGACA  12614
               ||  |      || |  | | | |  |     |||| ||    | | | |    |  | |
Sbjct  3367   ATCAAGAATTTGCCCTGATAACTGTGCTATTACTTCATTGAGCTCCCCACCGAGTCCATA  3308

Query  12615  GACCAGGACTCACTGCTAGAAAACCCCTGGGG-AAGTCATTGCTGAAGACTCTAGTGTGG  12673
              |||||||||| |||   || ||| | |||||| |||  |||| |||||||| || |||||
Sbjct  3307   GACCAGGACTAACTCTAAGGAAATCTCTGGGGGAAGAGATTGTTGAAGACTTTAATGTGG  3248

Query  12674  GGCACATTAGGTTTGAGGAGCAGGGGAAACATTCAGGCAGCTGGCAAGGTCTGGGCTACC  12733
              | |||||| ||||||||||||| ||| | || | ||||||||||  ||||||||| |
Sbjct  3247   GACACATTGGGTTTGAGGAGCATGGGGACCACTTAGGCAGCTGGGGAGGTCTGGG-TGGA  3189

Query  12734  AGGAGAGATTTGGGAGTTAGAAGCATATTAGGTGAGAGTTGACACTGTCCAAAGGGAGTA  12793
              | ||||||  ||||||  || ||||||| ||| ||||||||| ||||   ||||| ||||
Sbjct  3188   AAGAGAGACATGGGAGCCAGGAGCATAT-AGGGGAGAGTTGAAACTGCTTAAAGGAAGTA  3130

Query  12794  TATAGAAAGAAAAGCAGAGGAAAGGGCTCATGGAGTCATTACAGCCTGTGAGGTGGGAAC  12853
              || ||||||||||||||||||||||||| |||| || |||  || ||| || |||| |||
Sbjct  3129   TAGAGAAAGAAAAGCAGAGGAAAGGGCTTATGGGGTTATTGTAGTCTGAGAAGTGGAAAC  3070

Query  12854  AGGGTTTGGGAGTACTAAGGGTTTGGGGAGGGTTTATGTGTTTATTCACCCATTCATTTA  12913
              |||| ||||||||| |||||||||| |||||||   | |||| || || |||   ||| |
Sbjct  3069   AGGGCTTGGGAGTAGTAAGGGTTTGTGGAGGGTCCGT-TGTTCATCCATCCAACTATTCA  3011

Query  12914  TTCAACATTTCTTGAGCTTTATGGCAGATGCTGAGTTGAAGAGGAAGCTGGGGATTTAAG  12973
              |||| | ||| |||||||||| | ||||| | ||||||||||||||| |  |||||||||
Sbjct  3010   TTCATCCTTTATTGAGCTTTAGGCCAGATACAGAGTTGAAGAGGAAGTTAAGGATTTAAG  2951

Query  12974  AGGGAGAAGAGTTAAGAGGGGATCCCTGGGTGGATCCAGATGAAGGAGAAGGGAGGGGCT  13033
              | ||||||| |||||||||||||||||||||||||||||||||||||||||||||||||
Sbjct  2950   AAGGAGAAGGGTTAAGAGGGGATCCCTGGGTGGATCCAGATGAAGGAGAAGGGAGGGGCC  2891

Query  13034  AAGTGCCTAGGCTCGCTCTTGCCAGGAGAGCACATCTGTTAACACAAAGCTGCATCTGTA  13093
               ||||||| ||||| |||||||||||||| ||||||||||| ||||||||||||||||||
Sbjct  2890   GAGTGCCTGGGCTCCCTCTTGCCAGGAGAACACATCTGTTAGCACAAAGCTGCATCTGTA  2831

Query  13094  TCTCAGCCAGCTCTCTTCCATTTCATCTCCTCCTCTCTACCCACACTTGCAAGACGGGGA  13153
              || |||||||||||| | |  |||||| |||||||||||||||| |   |||||| ||||
Sbjct  2830   TCCCAGCCAGCTCTCCTTCCCTTCATCCCCTCCTCTCTACCCACCCCCACAAGACAGGGA  2771

Query  13154  GCCACATGGCGGCTTCCTACTCCCTTGTACCACTACACCCCCTGCCGGGTTCTTTCTAGG  13213
              |||||||||||||||||||||||| ||| ||      |||||  |   ||||||||||||
Sbjct  2770   GCCACATGGCGGCTTCCTACTCCCCTGTTCC------CCCCCCCCAATGTTCTTTCTAGG  2717

Query  13214  CATTTAGCTGTGGCATCAGCAGCACCCCTTCTGAGAGATCAGGCAATCCCCAAGGGTGAG  13273
              || |||||||||||  | ||| ||||||||||||||| |||||||||||||| ||| | |
Sbjct  2716   CACTTAGCTGTGGCGCCGGCACCACCCCTTCTGAGAGCTCAGGCAATCCCCAGGGGCGGG  2657
```

```
Query  13274  TGGTAGTGGTAGTGGTGGGGAGGGTTGATAGCTTCGAGAGAAGAGCTGGAGAAGGCG---  13330
              || |       | || |||  |||||||  | |  ||||||| ||| |||| ||| |
Sbjct  2656   TGAT-------GAGGGGGGCGGGGTTGA-CGGTCTGAGAGAAAAGCCGGAGTAGGAGCTG  2605

Query  13331  -CTGTCTGGCTGTAATTAGCCCCAGCTGCTGTTGTGAGGCAGCGTTGGGGGCCGTCAGGA  13389
               |||||| |||||||||||  ||||||||||||  | ||||||||||||||| ||||||
Sbjct  2604   TCTGTCTGTCTGTAATTAGCTTCAGCTGCTGTTGCCA-GCAGCGTTGGGGGCCATCAGGA  2546

Query  13390  GTGTACATGCATGTGTATGAACCTGTGAGCGTGGCACCTCATTGCTATCACTCTATTTTT  13449
              ||||  |||||||| |||||||||| | |||||||  |||||  |||||||||||||||
Sbjct  2545   GTGTGAATGCATGTATATGAACCTGAGTGCGTGGCGTCTCATCACTATCACTCTATTTTT  2486

Query  13450  CACTGTATTGTGTGCTGGCTGCAGCCTCGCTGCTCTGAAACCCCTCCTGTCCCTGAGGAT  13509
              | | |||||||| ||||||||  ||||||||||| || |||| ||||||||||||||||
Sbjct  2485   CTCCGTATTGTGCGCTGGCTGATGCCTCGCTGCTGTGCAACCTCTCCTGTCCCTGAGGAT  2426

Query  13510  CCCGAAAGACAGAGTGTTCCACAGGCAGCACCAGCTTGAATCACTAATGCTATTAAGCAG  13569
              |||  | ||||||||||||||| ||||||||||||||||||||||||||  |||||||
Sbjct  2425   CCCAGAGGACAGAGTGTTCCACAAGCAGCACCAGCTTGAATCACTAATGCTGCTAAGCAG  2366

Query  13570  GAGGACAACAAATATAGGCCCCCATATGTCCTGGATATGCAAGTCCCTGCTCTCTCTTTC  13629
               | |||||||||||| |||||||||| | | || |||  |||| ||||| || | |  ||
Sbjct  2365   AAAGACAACAAATATCGGCCCCCATACGCCTTGAATA-CCAAGCCCCTGTTC-CCCCCTC  2308

Query  13630  CCCACCCCTGCTTCCTGAATGTTTCCAAGTGAGTGAAGTCCCAGCACTTGAAATTAGCAA  13689
              | | ||||  |||||||||| ||||| ||||||||||| |||||  ||||||||||||||
Sbjct  2307   CACTCCCCCACTTCCTGAATATTTCCTAGTGAGTGAAGCCCCAGTCCTTGAAATTAGCAA  2248

Query  13690  CACCTC--ACTTCTGCACAGCCTTTTGGCAAAGAGCTTTCACAGCTGCCATGTCATGCAT  13747
              ||||||  |  ||||||||| ||||||||||||||||||| || || |||| || ||||
Sbjct  2247   CACCTCATATCTCTGCACAGACTTTTGGCAAAGAGCTTTCAGAGATGTCATGCCAGGCAT  2188

Query  13748  CCTCAGAC-GCTTCTAGGAGAAGCATTTTATAGGTGAGGACCCTGTGGTTCAGGGAGGTG  13806
              |||||||| |||     || | |||||| || |||||||||||||| || |||| || |
Sbjct  2187   CCTCAGACAGCTCTGGGGGGCAGCATTCTACAGGTGAGGACCCTGAGGGTCAGAGAAGCA  2128

Query  13807  GAGAGATTTGCACAGGCTAACCCAGACAGTCAGTGGCAGACTTGTGTCCAGGAAGCAAGT  13866
              ||||||||||| |||||                            ||||||| ||| |
Sbjct  2127   GAGAGATTTGCTCAGGC----------------------------ATCCAGGATGCAGGC  2096

Query  13867  CCTCGACCTCCTGGCCTGGTGGCCCATCCCCAGCAGAGCACTGCCAAGAGGGCTGCTCCC  13926
              ||| |  ||||||    | ||| ||  |||| ||||||||||||||    | |
Sbjct  2095   CCTTGGTCTCCTGAGGTTGTGCCCTGTCCCTAGCAGAGCACTGCCTCAATG---------  2045

Query  13927  GGGTGGCTCCAGCTGCTGCTGTCTGTGAGCTCGTGAGAACCTGTCAGCTGTGGCTCCAAG  13986
                        |||    ||| ||| || ||| ||||  |||| | |||  || |||||||
Sbjct  2044   ----------AGC----GCTCTCTCTGTGCTTGTGAATACCTATGAGCCCTGACTCCAAG  1999

Query  13987  ACCTGCCTCAGTGATTGCTGAAAACGCTGAGAGTGCTGAAAACCAGGA-CCCTCAAGCCG  14045
              || ||||||||  ||  |||| || ||||| | |||| | ||| |||| |||| ||| |
Sbjct  1998   ACTTGCCTCAGCAATCACTGAGAAAGCTGATACTGCTAATAACTAGGACCCCTGAAGTCA  1939

Query  14046  AGCCTGACGTCACATAGTTGATTGAGTACCTGCTATGTGCCCACCACTGTGCTGGTGCTT  14105
              ||||   | | | | || |  || ||              |||           ||||||
Sbjct  1938   AGCCCAGCCTAAAAGAGCTTCTTTAG--------------CCA-----------GTGCTT  1904

Query  14106  ATGGGAGACA-AAACAGGGGTAGTGCATAGCCCGTATCCTCAAAGAAGTGATAACTTGAA  14164
              ||||| |||| ||| ||| || |    | ||||| | ||||||||||| |||||||||||
Sbjct  1903   ATGGGGGACACAAAAAGGAGTGGAATA-AGCCCCTGTCCTCAAAGAAATGATAACTTGAG  1845

Query  14165  TGGGGAAACAACTCTTAGATACCAAGTCCAAAGGCAATTCCAAGCACTTCTCTCTCTTCC  14224
              |||| | ||||  | |||| |||||||||||||||||||||  | ||||| |||||
Sbjct  1844   TGGGAAGACAAACCCTAGACAACAAGTCCAAAGGCAATTCCAAGAGCATCTCTTTCTTC-  1786

Query  14225  ACTAAGCAGTCTGTTGTCAGTGACAATGACACAGGAACCCCTTCTGAATTCA--TCTGTC  14282
                               ||| ||||||||| |||  ||| |||||| |||||  ||||||
Sbjct  1785   -----------------CAGAGACAATGACTCAGAGACCACTTCTGCATTCATTTCTGTC  1743

Query  14283  TTATCGCAGGCCTGGGGTaggagaggatgggaggatgactaggaagggtaatgaggaagg  14342
               |||| ||| |||| |   ||  |||  || ||| | |  ||| |  |||||   ||||
Sbjct  1742   CTATCACAGCCCTGTG--TGGGCAGG--GGTAGG-TAA--AGGGA-AGTAATAGTGAAGA  1691

Query  14343  gcagggagatgggggaaggaagggaaAAACTGGAGCTCCTAATAAACACCAGGCACATTG  14402
              |  || |||||   | | ||| |||| |||| ||| ||||| || |  ||||||||| ||
Sbjct  1690   G--GGAAGATGAAAGGAAGAAAGGAAGAACTAGAGTTCCTACTACATGCCAGGCACAGTG  1633

Query  14403  TGAAGTTTATATGTTATACATA  14424
```

```
              || ||  ||||||| ||| |||
Sbjct  1632   TGGAG--TATATGTCATATATA  1613


 Score = 1819 bits (2016),  Expect = 0.0
 Identities = 1989/2621 (75%), Gaps = 170/2621 (6%)
 Strand=Plus/Minus

Query  5259   TAGGTGCTTTGAACCCAGCTACCAGTTGTAGAGTTCAAACCCAGGCCTGCATGAGTCCCT  5318
              ||||||||||  |  ||||| | | || ||  | | ||||| || |||  ||||||||
Sbjct  9260   TAGGTGCTTTATATTCAGCTGCTAATTATAATGCTGAAACCAAGACCTATATGAGTCCAG  9201

Query  5319   TG--CACCCATTCTACTTTTCTGCACTGCTTGTAATGT-AACACAGGAGGTATGGGACAT  5375
              ||  | ||||| | | ||||||  |  ||||||| ||| ||||||||||  |||||| ||
Sbjct  9200   TGTGCTCCCATGCCATTTTTCTATATCGCTTGTATTGTTAACACAGGAGTCATGGGATAT  9141

Query  5376   CCATAACTCAGGTAGGTAATTGACTTCAAGCTTAACATTCAC---TCACCAATCATTTAT  5432
                | || |  || || |  |||||| | |||| |||   ||    ||| |||   ||||
Sbjct  9140   ATACAATTTGGGGAGATTGTTGACTGCTAGCTCAACTACCAACATTCATCAAATGTTTAC  9081

Query  5433   TGAGCACATATTAGATAAGCAACCTATGGCTCCTGCCCTGGAGTTGTTCATAAGACCTTC  5492
              |||||| ||| ||||| | ||| |||| ||||||||| |  || || |||||||||||||
Sbjct  9080   TGAGCATATACTAGATGAACAAGCTATAGCTCCTGCCTTCCAGCTGCTCATAAGACCTTC  9021

Query  5493   CAGGCAGACAGCTATGGGACGAATACACTTGAA-----TTTATTAAATCTACCTCAGGCA  5547
               |   |||||| ||||| | ||||| | |  ||     ||| || ||| | ||| | | |
Sbjct  9020   AAAAGAGACAGATATGGAAAGAATAAATTAAAAAAAACTTTTTTTAATTTGCCTGATGTA  8961

Query  5548   AAAAACCAAGCATGTAATTATAAGTAGTATATCAGAGTATGTGTAAATGGGGTTCTGTAA  5607
              |||| | | | | | |  |||||  |||||||||||| || || |||  |        ||
Sbjct  8960   AAAA-CTAGGTACG-ACCTATAATCAGTATATCAGAGAATCTGAAAACAGAC------AA  8909

Query  5608   AATGTTTGAAAGAGGCAGGCCTGGGTTAGTGGTGAGAACAAGGATCAAGAAGGCTCTGCC  5667
              |  | ||||||||||||||||   ||||| ||||||| ||| |  |||| ||| |||||
Sbjct  8908   AGGGCTTGAAAGAGGCAGGCCTACTTTAGTAGTGAGAATAAGAAGAAAGA-GGCCCTGCC  8850

Query  5668   ACCAAATAACAACATGAGTGTTG-----TCCCGTTAATCTCTCTGGGCTTCTGTTTCCAG  5722
                 ||||| | || | ||  | |     |||| |||| ||||||||||| |||||||||
Sbjct  8849   GTTAAATAGCTACGTAAGCATAGGTAAGTCCC-TTAAACTCTCTGGGCTCCTGTTTCCAC  8791

Query  5723   ATCAAGGACCTCAAAATAATAGTCATAGTCACCATTTATTGAGTGCTTTACTGTTAACCA  5782
              | |  | ||||||||| |||||| | || |||  |||||||||  ||| |||||  || |
Sbjct  8790   ACCTGGAACCTCAAAAGAATAGTAACAGCCACTGTTTATTGAGCACTT-ACTGTGCACTA  8732

Query  5783   GGCAGTATGCTAGGTGGTTTTCATTTAGTCTTGACAACAGTATTCTCGCTTTCCCTTTTC  5842
              ||||  |  | |  ||| || | || |||| ||||||| |  || |||||||||||||||
Sbjct  8731   GGCACCAAACAAT-TGGCTTCCGTTCAGTCCTGACAACCGCTTTGTCGCTTTCCCTTTTC  8673

Query  5843   TACAGCTGAGACTCAGAGACATTAGGTAATGT---CTAGGTCACACAGCTCTTCAGTGAC  5899
              ||||||  || ||||||||   ||||||| ||   ||||||||||||||| |||| | |
Sbjct  8672   TACAGCGCAGGCTCAGAGAGGATAGGTAAAGTGCTCTAGGTCACACAGCTCATCAGCGGC  8613

Query  5900   CAGTTGTTTCTATGTC-AGAATCCTCTTATAATCACTGAGTTGTATCGCTTCCCTTGCCT  5958
              || |||||||||| || |||||||||| |||| || | |||||||| || || |||  ||
Sbjct  8612   CAATTGTTTCTATCTCTAGAATCCTCTCATAACCATTAAGTTGTATTGCCTCTCTTATCT  8553

Query  5959   CCTG-CTC----CCAAGTCAAGTATTCTCTACTATGTCGCCTGAAAATTAAAATTAAAAT  6013
              |||| ||     ||||||| |||||||||| |||||| |||| ||| |||||||||||||
Sbjct  8552   CCTGACTGAACTCCAAGTCCAGTATTCTCTGCTATGTTGCCTTAAAGTTAAAATTAAAAT  8493

Query  6014   CAAGGTTAATCTTGTTCAACTGAGCCAAGAGGATAAACATTAGAAATAGCAATTAGTAGG  6073
              ||||| ||||||||||||||||||||||||||||||||||||||||||| ||||||||||
Sbjct  8492   CAAGGGTAATCTTGTTCAACTGAGCCAAGAGGATAAACATTAGAAATAGTAATTAGTAGG  8433

Query  6074   TAGAAATGTTGCCAGGAAGAGCTATACAAAAGCATAGAAAGGCAACCAAGGGAGTTAA--  6131
              ||||||| ||||||||| ||||||||||| ||    ||||| || |||||  ||||||
Sbjct  8432   TAGAAATTTTGCCAGGAGGAGCTATACAAGAGTGCGGAAAGACACCCAAGTCAGTTAACA  8373

Query  6132   -TGATGGGGGTGTGGCAGGGGTTGCATGTTGATGGGTAATTGGATCCTGGCCAGCTCTTA  6190
               |||||||||||  || ||||||||||||||| || ||||||| |||  |||||| | ||
Sbjct  8372   ATGATGGGGGTGCAGC-GGGGTTGCATGTTGAAGGTTAATTGGTTCCCTGCCAGCCCGTA  8314

Query  6191   CCTTCTTCCTTCACCTTCCCACTCCGCAATTTCTCAGGCTTCTCTAGAGAGCACAACCCA  6250
              ||||| |||||||||||||  || || |        | || ||||| ||| ||   ||||
Sbjct  8313   CCTTCCTCCTTCACCTTCCTGCTACGAA--------GCCTGCTCTAAAGAACAAGGCCCA  8262

Query  6251   CCTAGAGACAGCCTGTGTTGCCACAGGAGGGCACCAGGCCAGGAATGGCAGGCCTAGGGC  6310
```

```
                 || |||||||||| | | | ||||||  |||| |                       |||
Sbjct  8261   CCAAGAGACAGCCGGAGAT-CCACAGAGGGGCTCA----------------------GGC  8225

Query  6311   AGTGGGGCTCTGGGCTGGGAACCATAAATAACCACTTTAATGAAAACCAGCTTTGGCCAA  6370
              |||||||||||||||||||||||||||||||| | ||||||||| ||||||| ||  |
Sbjct  8224   AGTGGGGCTCTGGGCTGGGAACCATAAATAACCGCCTTAATGAAAGCCAGCTTTTGCTTA  8165

Query  6371   A-AGCCTTATCATTTCTTAAGAGGTTCTGTGCTTCTTCTAAT---GACCTAGTTACTTGA  6426
              | |||||| |||||||||||||  || | |||  ||||||||   ||| || ||||||||
Sbjct  8164   AGAGCCTTGTCATTTCTTAAGAAATTTTTTGC--CTTCTAATATGGACTTAATTACTTGA  8107

Query  6427   GAAAGGCCTAGCCTAACATATACAACAACAATAATA------------GTTGCCCTATAG  6474
              ||||| |||||||||   |||||||||||||||||             ||||  || | |
Sbjct  8106   GAAAG-CCTAGCCTACTCTATACAACAACAATAATAATAATAATGGCAGTTGATCTTTGG  8048

Query  6475   TGAGTACTTACTATGGGCCAGGGCCTGTGCTAAACACTCACTAATTCATTTAATCCTCAC  6534
              |||| || |||||||||| |    | ||       |||  |||||| ||||||||| |||
Sbjct  8047   TGAGCACCTACTATGGGCTAATACTTG------ACATACACTAACTCATTTAATTCTCCT  7994

Query  6535   ACAGCCCTACAGGAGCAAGTACTATTATTTCCCCCACGTTACAGAGCAGGCATCAGGCTG  6594
               |||||||    | |||  || ||||||||| |||    ||||| || ||||||||||||
Sbjct  7993   GCAGCCCTTTGAGGGCACATATTATTATTTCTCCCGTTTTACACAGGAGGCATCAGGCTC  7934

Query  6595   AGAGAGGCTAAGTCATTTGCTCAAGATCATATAGCTGGTTGGAGCTGGAATTTGAAGCCT  6654
              ||||||||||||| | ||||||||| ||||| ||||||||||||||||||||||||| |
Sbjct  7933   AGAGAGGCTAAGTAACTTGCTCAAGGTCATACAGCTGGTTGGAGCTGGAATTTGAAGCTT  7874

Query  6655   GAAGCATCTAGTTCCAGGGCCTGAGCTCTTTAATTCCTATATTTAATGAATGTCTGTCGA  6714
              ||||||||| ||||||||||| ||| |||| ||||||| |||||||| ||||||||| |
Sbjct  7873   GAAGCATCTGGTTCCAGGGCCAGAGTTCTT-AATTCCTGTATTTAATAAATGTCTGTTGG  7815

Query  6715   AGGAATAAACAGATGAATGAATAGACAAATGTATT----CATTTCATTTCACTTTAACAA  6770
              || |||||||||||||||||| ||||||||||||     |||| || || |||| | |||
Sbjct  7814   AGTAATAAACAGATGAATGAATGGACAAATGTATTAATTCATTACAGTTTACTTCAGCAA  7755

Query  6771   ACATTTATTGAGTGCTCTGT----GTCAGCTCCTGTGCTAGGCATTGAGGATTTATAAGT  6826
              ||||||||||||||||||||    ||||||| ||||||||||||||||||||||    ||
Sbjct  7754   ACATTTATTGAGTGCTCTGTATATGTCAGCTTCTGTGCTAGGCATTGAGGATTTAAGTGT  7695

Query  6827   TTAGTGCGTGCGTGCGCGcacacacacacacacacaaacgcacacacacacacacaca  6886
              |||                        ||||||||| || ||||||||||||||| |
Sbjct  7694   TTA------------------------ACACACACACACTCACACACACACACACCC---  7662

Query  6887   TCCTGCATGGCCCTTGCTCTCCAGGGGTTCACAAAAG--CATTCTCTAT--TGTGGCTTG  6942
               ||  |||||||| ||    |  ||  | | ||||||  |||||||| |  ||| || ||
Sbjct  7661   -CCCACATGGCCCCTGTCTTTGAGAAGCTTACAAAAAAACATTCTCTCTAATGTAGCATG  7603

Query  6943   ATTTCTTCTGGAGAGAAGTAATTTGAATGTCTTGAACAGCAGTTTTTAATGATGCTATTA  7002
              |||||||  || || ||||||||||   | ||||||| || ||| || |||||||||||||
Sbjct  7602   ATTTCTTTGGGGGAAAAGTAATTTGCGTTTCTTGAATAGTAGTCTT-AATGATGCTATTA  7544

Query  7003   AATTTAACCTAAAGCGGGGGTTTTGCACCGGTGAGCAAAGTGTTTCAAATGAGAATGTGT  7062
              ||||||||  |||| ||||||| |||||||||||||||||| ||||||||  ||||  |||
Sbjct  7543   AATTTAACTCAAAGTGGGGGTTGTGCACCGGTGAGCAAAGAGTTTCAAAACAGAACATGT  7484

Query  7063   CTTTGATTAATCATCGATTCAGCCAGAGAATAGTTACTACCAGAATG-GAGCTGCAACTC  7121
              |||||||||||||| ||||||| |||| ||| ||||||||||||| | | ||||||||||
Sbjct  7483   CTTTGATTAATCATTGATTCAGGCAGAAAATGGTTACTACCAGAAAGAGGGCTGCAACTC  7424

Query  7122   CTATTATATTAGCTAACTCTTCTTCCATCTATCATGTCTCTATCTTGTCGAACCCTCCCA  7181
              |||| | ||||||||||||||||||||||||| |||||| |||    || ||||||||||
Sbjct  7423   CTATAACATTAGCTAACTCTTCTTCCATCTATTATGTCTGTAT----TCTAACCCTCCCA  7368

Query  7182   CGATACAAAAGTTATCTGTACCCTAATGGACAATTATCCCATCCATCATTCCTGCTGGTC  7241
              ||  |||  |||||||||||| ||||||||||||||||| ||||||||||  |||||| |
Sbjct  7367   CGGCACAGTAGTTATCTGTACTCTAATGGACAATTATCCTATCCATCATTTGTGCTGGCC  7308

Query  7242   TTGTTGATTGCTGGTAGTCTGCTTGCCCACGGAGGAGACTTACAAATAGTCATGACCAGT  7301
               || |||||||||| | ||| || |||| | |||||||||||||| |||||| ||||  |
Sbjct  7307   CTGCTGATTGCTGGCAATCTACTCGCCCGCAGAGGAGACTTACAA-TAGTCACGACCGAT  7249

Query  7302   GACGCACTGGAAAAACTGCTCATTTTTAGACATTTTAAATCTATGTAGAATGAATTATTTT  7361
              || ||| ||||||| |||| ||||||| ||||||||||||| ||  ||||||||| |||
Sbjct  7248   GATGCAGTGGAAAAATTGCTGATTTTTACACATTTTAAATCTGTGCCGAATGAATTCTTTC  7189

Query  7362   TCAGGCTGTACTT-AAGCTGCATTTGCTTTGTTGGTTATGTAAGAAGAGAT-ATGAATTC  7419
               |||||||  ||| |||||||||||| |||  ||||||| |||||  || | |||||||
```

```
Sbjct  7188  GCAGGCTGCCCTTTAAGCTGCATTTGTTTTTCTGGTTATATAAGAGCAGGTTATGAATTT  7129

Query  7420  CTTACCTATGCGTGAGACATGGCGTCTG--AATATTTTTAAGTT--------CCTGTGGG  7469
              ||||||    |||| |  |||  ||||  |||||||  |||          ||| | ||
Sbjct  7128  TTTACCTGCATGTGAAATGTGGACTCTGTGAATATTTCAAAGGAAAAAAAACCCTCTAGG  7069

Query  7470  CAGCAGCCCTATACATATTTGTTAAAATGATGAAAGAATAAAGTAAACATACCTAGAGTA  7529
             ||||||||  ||| |||||||||||||||  |   ||   || |||||| |  | | |||
Sbjct  7068  CAGCAGCCTCATAAATATTTGTTAAAATGCGGGCTGATCGAAATAAACACA--TGGGGTA  7011

Query  7530  ATTCCAGAGGAGCAGGCTTCATACCTGGAGCTCGGGTGGGTGGCCATCATGTCAGGGTAG  7589
             ||   |||||||||||   ||    | | | || || || ||| |||||  |||| ||
Sbjct  7010  ATAAAAGAGGAGCAGGTCCCAAGATTAGGGTTCAGGGGGCTGGTCATCACATCAGAGTGA  6951

Query  7590  TCACC-GTCTTCCTTTTCCTTTCTAAGTACTTTGAGGAGAATCCCAAGTTGATTAGGGTC  7648
              |||| ||||||||||||||||| | ||| ||||| ||||| ||||||||||| ||| ||
Sbjct  6950  CCACCAGTCTTCCTTTTCCTTTCCAGGTATTTTGAAGAGAAACCCAAGTTGATGAGGATC  6891

Query  7649  AAGGTGAGGACTCATGGTGCTGAAAATAGGGATTGAGTGGACAATGAGAGATGCCTGTTG  7708
              ||||||||||||  ||| |||||| ||| | ||||||||  | ||||||||||||||||
Sbjct  6890  CAGGTGAGGACTCTGGGTACTGAAAGTAGTGGTTGAGTGGGGAGTGAGAGATGCCTGTTG  6831

Query  7709  AATTCCCTGGGACTATTGCTATGAGATTTGAGGGCAGAAATGC-CCCCCTC----TC---  7760
             |||||||||||| |||||||||||||||||||| ||||||||| | |||||    ||
Sbjct  6830  AATTCCCTGGGAGTATTGCTATGAGATTTGAGGACAGAAATGCTCTCCCTCCATGTCCCA  6771

Query  7761  --CCATGTCCTACCCTAAGCCTGGTAACACATTTAACTTTT  7799
               |||||||| | || | || |||||||||| |||||||||
Sbjct  6770  CTCCATGTCCCATCCCAGGCATGGTAACACACTTAACTTTT  6730


 Score = 1121 bits (1242),  Expect = 0.0
 Identities = 924/1116 (82%), Gaps = 15/1116 (1%)
 Strand=Plus/Minus

Query  2802   AGGTTCCTGAAGTTTTCTTGGCAACTAGCTCTAACTGCTTTGCCTTCTGAGTG-CTTTGG  2860
              |||||||| || ||||||||||||||   || |||||  ||| ||| || |   ||||||
Sbjct  11541  AGGTTCCTAAATTTTTCTTGGCAACTGATTCCAACTGTCTTGTCTTTTGGGGAACTTTGG  11482

Query  2861   TATTTGTTTTAT-TCTAAGTTCCCAGTTTTGATCTATTTTTGCCTTATGGCTGCAGATTC  2919
              |||||||||  | |||  |||||  |||||||||| | ||| |||| ||||||  | ||
Sbjct  11481  TATTTGTTTATTCTCTCTGTTCCTGGTTTTGATCTGTCTTTTCCTTCTGGCTGTGGTTTT  11422

Query  2920   CCTTTCTGGAAAATGGGGCTCTGGCTGGGATCAAATCAGAGAACACACCCAGA---CTTG  2976
              |||||||| ||||||||||| | ||||| |||||||   |||||  |||||||   |  |
Sbjct  11421  CCTTTCTGTAAAATGGGGCTTTTGCTGGAATCAAAT---AGAACGTACCCAGAAGACCCG  11365

Query  2977   GCATAGTGCCTGGCACAGAGTAAGTGCTCAACAAATGGTAGCTGTTGTCTCTTTTTCCCT  3036
              ||| |||||||| |||||||||||||||| ||||||||||| ||||||||  ||||||||
Sbjct  11364  GCACAGTGCCTGACACAGAGTAAGTGCTCCACAAATGGTAGTTGTTGTCTTCTTTTCCCT  11305

Query  3037   GTCCAGGTTTCTTCTGGTTCTGTTCTCTGTACCTCCAGTATTGGCCCAGAACCAGGAACT  3096
              || |||||||||||||| || |||||||| ||||||| ||||||||| | ||  ||  |
Sbjct  11304  GTACAGGTTTCTTCTGGGTCAGTTCTCTGCACCTCCAATATTGGCCCTGGACTGGGGCCA  11245

Query  3097   GTTCCCTCCCCTGACTGGCAAGTCAGATTACCTGGCCCTCAGACAAGCTGGGCTCCCTTT  3156
              ||||| |||| || |||| ||| ||| | || ||||||||| |||||||| |||||| ||
Sbjct  11244  GTTCCTTCCC-TGGCTGGGAAGCCAGGTCACTTGGCCCTCAAACAAGCTGAGCTCCCCTT  11186

Query  3157   TCTGGCAGCCTTTTGTTGTCCTTACCTGTTGCCATGTGCCACTCAGTGTAACCACAGGCA  3216
              |||||||||||| ||||||||||||||||  || |||| ||||||  |||||||||||||
Sbjct  11185  TCTGGCAGCCTTCTGTTGTCCTTACCTGTCACCGTGTGGCACTCACAGTAACCACAGGCA  11126

Query  3217   GACCTCTGCCAATTGCTGACATTAACCTATTGAATCCTGGCAGAGAAGAATAAGTTTGCA  3276
              |||||||||||  |||| |||||||||| |||||| || ||||||||||| || ||||||
Sbjct  11125  GACCTCTGCCAGCTGCTAACATTAACCTGTTGAATTCTAGCAGAGAAGAACAACTTTGCA  11066

Query  3277   GACTGGAATTTGCATGTCTGGGCCAGATACGAGACCAGGAGGCAAAGCCATATTT-ACCT  3335
              ||| |||||||||||||||||||||||||| |||||| ||||||| ||| ||||| | ||
Sbjct  11065  GACAGGAATTTGCATGTCTGGGCCAGATACAAGACCAAGAGGCAAGGCCGTATTTTATCT  11006

Query  3336   GTTCTCCCCAGTTGTG-GCAAATCACCCACATTCTGGTTAAAACCAGATGTTTCACACCC  3394
              |  ||||||| ||||| || |||||| ||| |||||||| ||||||||||||||||||||
Sbjct  11005  GCCCTCCCCAATTGTGAGCGAATCACACACCTTCTGGTTCAAACCAGATGTTTCACACCC  10946

Query  3395   ATGACTGCAATGCCACACATCAAACAGTTGCTTACAAAGAACACAAAGTGGTTTTCACAT  3454
              ||||||||||||| ||||||||||||||||||||||| |||||   | ||||||||||||
```

```
Sbjct  10945  ATGACTGCAATGCTACACATCAAACAGTTGCTTACAAGGAACATGGAATGGTTTTCACAT  10886

Query  3455   CGCAAATGTAACTTGCAGGTCACTTCTCAAATTTCCTCGCAGCAGCTACCATGCCTGGGT  3514
              |  |||||||||||| |||||||||||||||||| |   || |||||||||| |||| |
Sbjct  10885  CAGAAATGTAACTTGTAGGTCACTTCTCAAATTTCTTAAAAGTAGCTACCATGTCTGGAT  10826

Query  3515   ATTTTTCCCTTAAAAATGACAACCTTGTTGGGTCTCTTGTAGGGACTTTCATTTGGAAGC  3574
              |||||||| ||  | ||||||||  |||||||||||||| ||||  ||| ||||||||||
Sbjct  10825  ATTTTTCCTTTGGAGATGACAACTCTGTTGGGTCTCTTGAAGGGGTTTTAATTTGGAAGC  10766

Query  3575   CCTGGGAGAACTTATATGCTCTTTCCAAGAGGGTCTCTGATGGCTTTAATTCACATGGAG  3634
              |||||||  | |||| |||| |||||||| ||||||||||  |||||||||   | ||||
Sbjct  10765  CCTGGGAACAATTATCTGCTTTTTCCAAGGGGGTCTCTGAATGCTTTAATTTGTAGGGAG  10706

Query  3635   TTAATATATTGGAAAAGAAAAGAGATCTTCCCCAAACTCACCTGCTCCTTGAAAATGCTA  3694
              ||||  | ||||| |||| |||||||||||||||||||||||||||| |||||| |||||
Sbjct  10705  TTAAAGTGTTGGAGAAGATAAGAGATCTTCCCCAAACTCACCTGCTCTTTGAAAGTGCTA  10646

Query  3695   TTCTGTTGCCTTGGCGAACAGCCAATCCAAACATTAAAAAAGAGATAGAGAATGCAGGAA  3754
              ||| ||||||||||| | ||||||||||||||| ||||||||||| ||||||||||||||
Sbjct  10645  TTCAGTTGCCTTGGCTAGCAGCCAATCCAAACACTAAAAAAGAGACAGAGAATGCAGGAA  10586

Query  3755   TCCTAAATGCTATTCTCAGTGCATGTCCAAACAAGAAC--TGGGGGCTGAGGGGCAAAGA  3812
              |||| |||| ||||||||  |||||||||||||||||    |||||   | ||||   | |
Sbjct  10585  TCCTGAATGTTATTCTCATCGCATGTCCAAACAAGAACAACGGGGGGGGGGGGGGGGACA  10526

Query  3813   ACAGAAAGAGGAGAAAAG--AGTTTTTGTAGCCTAGGCCGTGAGTTGATCCCACCCCGTC  3870
              | | ||||||||||||||    ||||||| |||| |||||    |||||| | | ||
Sbjct  10525  AGAAAAAGAGGAGAAAAGGCGGTTTTTGCAGCCCAGGCTGTGAGCCCATCCCATCTCATC  10466

Query  3871   ATGTCCTGTACCCTTCACGCTCATAAAGAAGACAGA  3906
               |||||  | |    ||| ||| |||||| || |||
Sbjct  10465  GTGTCCCATTCTACGCACACTCCTAAAGAGGAGAGA  10430


 Score =  502 bits (556),  Expect = 1e-137
 Identities = 539/707 (76%), Gaps = 53/707 (7%)
 Strand=Plus/Minus

Query  1      TAAGTCCATTCATCTCTCTGGGCTTTCCATTTCCTCAGCTTCAAAATGCATGTACTAATC  60
              ||||||  || |||||| ||  ||||||||||||||| ||||||||| ||||    ||||
Sbjct  14733  TAAGTCTCTTAATCTCTGTGA-CTTTCCATTTCCTCATCTTCAAAATACATGCCTTAATT  14675

Query  61     CTGCCGGCTTTACCTCCCTCCTAGGGCTCTGTGTCTCAGCCACACTGGTCTTTCAATTTC  120
              ||||| |||||||    ||| | |||||| | |||||| ||||  ||  ||||||||||||
Sbjct  14674  CTGCCTGCTTTACTCACCTACCAGGGCTGTATGTCTCTGCCATGCTATTCTTTCAATTTC  14615

Query  121    TGCAACATTCTCG--GTCTTTTCTCGCCCCAGGGCCTTTGTTC-----GTGCTCCTGCCT  173
              ||  ||||||      ||||||||  |||    || ||||||  |      || || ||||||
Sbjct  14614  TGGGACATTCCAAAAGTCTTTTCCTGCCTTGGGTCCTTTGCACATACTGTTCTTCTGCCT  14555

Query  174    GGACTGCCCTTCCTCTTGCTTTTGGCATG---GAAGGCTCCTTCCTAACCTTTCAGTCTC  230
               |||||       ||||||||||| |||     | ||| | ||||    ||||||||||||
Sbjct  14554  AGACTGT------TCTTGCTTTTGTCATCAGAGGAGGTTTCTTCTCTCCCTTTCAGTCTC  14501

Query  231    AGCTTAA-CGTTACCTCCTGAGAAGAGCCTTCTCTGACCACCCACAGTTAGATGCCTCCT  289
              ||||||| |||||||||||| |||| ||||||||||||| || | |||||   | ||||||
Sbjct  14500  AGCTTAAACGTTACCTCCTGCGAAGTGCCTTCTCTGACCGCCTATAGTTAATTCCCTCCT  14441

Query  290    GTTCCTCTCTTACATTTCTTTTATTAGCAATGCTTCCTTAACCCCATTTACTGCTCTGGG  349
              |  |||| || | | | |||||||| | || |||||||| | | || ||||  | |||||
Sbjct  14440  GCCCCTCCCTAATACTCCTTTTATTGGAAAGGCTTCCTTCATCACAATTACCAC-CTGGG  14382

Query  350    TAATTATGTCTATTTATATTTGTCTATTTATGGTATGTCTCTCCCACTGGAATGTAAGCT  409
              ||||| | ||| |||||||||     |  |||         |||      || | | ||||||
Sbjct  14381  TAATTGTATCTGTTTATATTTACAGACATAT--------TCT-----TGAAGTATAAGCT  14335

Query  410    CCAAGAGGACAGGGACCTTGTCTGTGTCACTCAGCAATGTGTCCCTCTCCTCTAGAGTGG  469
              ||||||||||||||||||||||||| |  |||||||              | ||| ||||||
Sbjct  14334  CCAAGAGGACAGGGACCTTGTCTGTATTGCTCAGCAC-----------CATCTGGAGTGG  14286

Query  470    TGCTTGGCACATAGTAGGCTGACATAAGCAAAACCATCTGGAAAATATTTTCTTCCTGAG  529
              ||||||||||||||||||||||| | |||||||||||||| |  ||||| || ||||||
Sbjct  14285  TGCTTGGCACATAGTAGGCTGACACA-GCAAAACCATCTGGGATGTATTTCCTCCCTGAG  14227

Query  530    GTTCTTGCCAGACATATGGTTGCTGAGGGAAAAATTATGGCAGAACTGTGTCATTCCATA  589
              ||| ||||||||||| || ||||||||||| |||| ||||||||||||||||||||||||
```

```
Sbjct  14226  GTTTTTGCCAGACATTTGTTTGCTGAGGGAGAAATCATGGCAGAACTGTGTCATTCCATA  14167

Query  590    -GGaaaaaaaa------TCAGGCTAAAAGGGAAGAGTTTGTTGAAAACAGTTCTGTTTTA  642
                |||||||||      ||||||||||||||| |||||  ||||||||||||||||| ||
Sbjct  14166  CTGAAAAAAAAAAAAAATTCAGGCTAAAAGGGAGGAGTTGCTTGAAAACAGTTCTGTTGTA  14107

Query  643    GAAACCAGTCCTTGGAGTCTCATCGCCAGACACA--GTCTTATGaaa  687
              |||||||| | |||||||||  || |||||||||   ||||| ||||
Sbjct  14106  GAAACCAGCCTTTGGAGTCTACTCTCCAGACACAACTTCTTAGGAAA  14060


 Score =  479 bits (530),  Expect = 1e-130
 Identities = 667/914 (72%), Gaps = 33/914 (3%)
 Strand=Plus/Minus

Query  4311   TGCTAGGGTTAATTGCTCAGCCTGAGAGGCTCTCCAGGCTGGCAGGTCACACTAGAGCCA  4370
              ||| ||||| || |||  || |||||||| |||| |   ||||||| ||||| |  ||||
Sbjct  10410  TGCCAGGGTCAAGTGCGTAGGCTGAGAGGTTCTCTAATTTGGCAGGCCACACCAAGGCCA  10351

Query  4371   AGAGTGAAGGGTCCATTCAGTAAGGTCACTGCCATGATCAGTTCTCAACCACTTTAACTC  4430
              |||| |||||  |  | |||| ||| || |   ||||||||||||||||  |||| |||
Sbjct  10350  AGAGCGAAGGTGCAGTCCAGTGAGGCCAGTATAATGATCAGTTCTCAACTGCTTTGACTT  10291

Query  4431   AGTTTTTCTGTTGCTGCTTTGTTGGTTAGTGGTTTGTTTGTTTCTACCCATTTGGGAGAA  4490
               |||||||||||     ||  ||||||  |||| | |||||||| ||||||||||||  |
Sbjct  10290  GGTTTTTCTGTTTTGAATTCTTTGGTTCTTGGTGTATTTGTTTCAACCCATTTGGGAAGA  10231

Query  4491   GGGTAGGAAAGGAACTAAGGTGATCAACTATTCCATATGTGTCAGGCCCTGTACTTGT-C  4549
              ||||||| ||| | ||||  ||||  |||||| | |||||| ||||| | || ||||| |
Sbjct  10230  GGGTAGGGAAGAAGCTAACATGATTGACTATTTCGTATGTGCCAGGCTCAGTGCTTGTAC  10171

Query  4550   TTTTCAAACACTTATTCACACTCTGAGGGACTCTCTCAGGTCCAGCAACTGTGTAGCTAT  4609
              ||||| |||||| |  || |  |||| ||| | | | |  | |||||||||||| | |||
Sbjct  10170  TTTTC-AACACTCAAGCATA--CTGAAGGA-TTTTTGACCTTCAGCAACTGTGTGGTTAT  10115

Query  4610   GATGTGAAATAGCAGCTTCTCTCTATGCATTCACTGGGAATGGTTTGTTCTGATCACACA  4669
               ||||||||  |    |  | |||||  |||| ||||||| ||||||||||||| || |
Sbjct  10114  AATGTGAAACTG----TGGTTTCTATATATTCCCTGGGAAAGGTTTGTTCTGATGACTCT  10059

Query  4670   ATTTTTTCCAGTTCTTCCGACGTTAGGTGTTGCATGGTAAGAAACAGGTGGTTAATTATT  4729
              |  |||||| ||  ||   || ||  ||  |  ||||| ||| |||||| ||||| | ||
Sbjct  10058  A-ATTTTCCCGTGTTTTGAACATTGAGTAGTAGATGGTGAGAGACAGGTTGTTAAGTGTT  10000

Query  4730   GAGACAGTTCTTATTTGGAATTGTCTCTTCCTCCTTCTCCTCTCAGGCAGATGCTAAAGG  4789
               |  |||| |||||| ||| ||  |||||| || |||   | ||| |||  || | ||
Sbjct  9999   TA-CCAGTACTTATTGGGAGTTACCTCTTCTTCTTTCAGATTTCATGCAAGTGGTCAACA  9941

Query  4790   ATGTCTGTAGTTT-TTTCATGGCATTTTGCTAAAGATGCACATTCATTAATTCATCAAAT  4848
               |||||| | ||| ||| |||  |||||  |  || ||||| ||||| |||||||| ||||
Sbjct  9940   GTGTCTGGAATTTCTTTTATGCTATTTTATTGAAAATGCATATTCACTAATTCATAAAAT  9881

Query  4849   AGCtttttttGAGACATTATTATGA---GTGAGGTGTTGTGCTATGATATGCTTTATATA  4905
               ||  | |||||| |||||| ||||   |  | || |||||  || | |||||||| |||
Sbjct  9880   CGC-CTCTTTGAGTCATTATGATGATATGCTACGTTTTGTG--ATTA-ATGCTTTACATA  9825

Query  4906   CCATATCTTATTTCATCCTCACCACAAAGCTATGAGGTAGGTACTATCATTATCTTAATT  4965
               |||||||||||| ||| ||||||| ||||| | ||||||||||| |||||| ||| |||
Sbjct  9824   TCATATCTTATTTTATCTTCACCACCAAGCTGTCAGGTAGGTACTGTCATTAGCTTCATT  9765

Query  4966   TTAC-AATGAAGAGACTGAGGCTCAGGAAACATAATGCAAGGTCACACTCAGTTAATAAG  5024
              || | ||||| ||||  || ||||||  |  |||| |||||  ||||  |||| ||||||
Sbjct  9764   TTCCAAATGAGGAGATCGAAGCTCAGAGATTATAACGCAAGACCACA--CAGTCAATAAG  9707

Query  5025   TTGAGAGCTAGGATTCAAACCCAGGT-TTATCTGACTCAGTTACACTACACTTCCTCCAC  5083
              | | || || |||||||||  ||| | |||   ||  ||  | || || |||||||||||
Sbjct  9706   TGGGGAACTGGGATTCAAA--CAGCTGTTA---GATGCAACTGCATTATACTTCCTCCAC  9652

Query  5084   ATAGCACATTGTGGGCCCGTCACAGCTCTTGAGGACAAAATTCATCCTTAGAAAAGTTTT  5143
               ||    ||| ||||||| ||||||  |||||| ||||||||  |||||||  ||  |||
Sbjct  9651   GTACTGTATTTTGGGCCCCTCACAGGCCTTGAGAACAAAATTTCTCCTTAGGGAA-ATTT  9593

Query  5144   CTTACTCCACTTTTtgctgatact----actgctgctgttgctgctgctactactattac  5199
              | ||||| || ||||||| ||| |    ||  | ||||   || || |||||||||||
Sbjct  9592   CCTACTCTACATTTTGCTAATAGTACGAACAACCGCTGCCTCTACTACTACTACTATTGT  9533

Query  5200   tactactgctactg  5213
              |||| || ||||||
```

```
Sbjct  9532   TACTGCTACTACTG  9519


 Score =  405 bits (448),  Expect = 2e-108
 Identities = 406/522 (77%), Gaps = 25/522 (4%)
 Strand=Plus/Minus

Query  10658  tttttgttagtatcttgttctttggcaatgttttcaaaatcttttgttttttAATACAT  10717
              |||| | ||||| | |||||| |||  |||||||||||||  ||||     |||||||||
Sbjct  5898   TTTTCTATTAGTGTGTTGTTCCTTGCTAATGTTTTCAAAAATTTT-----TTAATACAT  5844

Query  10718  GTAAAACTTATTTTATATTTTATATCACCTAATTGCAATACCTGAGGTCTTTGAAGACCT  10777
              ||| |||||||||||||| ||||       |||| |||  |||| |||||||| || ||
Sbjct  5843   GTAGAACTTATTTTATATTATATA-------ATTGTAATGTCTGAAGTCTTTGAGGATCT  5791

Query  10778  GATTCTG-TTTTCATTGTTTCTGGCTTCATCGTGTTCTTTGTAATTTTGGATTATGAGTT  10836
               |||||| ||||  ||||||||||||||||  | || ||||||||||| ||||||||||
Sbjct  5790   AATTCTGCTTTTTGTTGTTTCTGGCTTCAT--TATT-TTTGTAATTTTAGATTATGAGTT  5734

Query  10837  CATGTTCAACCTGACTTTATCCTTGGTAATCCTATGAAAACTGGGTGGTGGGCATGTTCT  10896
              |||||||| || ||||| |||| ||| |||  ||||| |||||| | | | |  |||||
Sbjct  5733   CATGTTCAGCCAGACTTCATCCATGGCAATTGTATGAGAACTGGATTGAGAGTGTGTTCC  5674

Query  10897  TCCAGGGAGGATTTGTGTTTGGTTCTTCCAAGTGCATCAGGGCACTATTAATCCAGGAGT  10956
              ||||| ||| |||| |||||| |||||||| |||  ||  ||||||||||| | |||| |
Sbjct  5673   TCCAGAGAGAATTTTGTTTGCTTCTTCCAGGTG-TTCCAGGCACTATTAACCAAGGACT  5615

Query  10957  TCTTCCAGTGAATTGTTTAGTCCCAAAGGCAAGGGCCATGTCTTCTTTGGCTATATGATC  11016
               |||  |||||||||||||| |||||||||| ||| |||| |||  ||| ||||| || |
Sbjct  5614   ACTTAGAGTGAATTGTTTAGCCCCAAAGGCAGGGGTCATGGCTTACTTGTCTATAGGACC  5555

Query  11017  CCAGGGCTTAACTCAGGGA-------CTTGTAGATGCTCAACATATATTTGCTGATTGAG  11069
              |||| ||||| ||||||||       |||  || | | ||||| ||||||||||||||
Sbjct  5554   CCAGCGCTTAGCTCAGGGACTGGCCCCTTTCAGGTCCGCAACAAATATTTGCTGATTGAA  5495

Query  11070  TGGATGAATGAATGTGGTACTTTACC-GCTTCTTTTGGAAGCCCAATAAGAATTGTGACC  11128
              ||| |||||| ||  || | | |||| ||||| |||||||||| |||||||||||| |||
Sbjct  5494   TGGTTGAATGGATAAGGCATTCTACCAGCTTCGTTTGGAAGCCAAATAAGAATTGTAACC  5435

Query  11129  TCTGGTATTAACAAAGAACAGTAGGCACATCAGGAAAATAAT  11170
              ||||||  || || ||   |||||    || |||||||||||
Sbjct  5434   TCTGGTGATAGCATAGGGTAGTAGATGAATAAGGAAAATAAT  5393


 Score =  374 bits (414),  Expect = 3e-99
 Identities = 785/1174 (66%), Gaps = 156/1174 (13%)
 Strand=Plus/Minus

Query  14584  AATGAGTGTTAACTAGCCTTTCTTCTTATCATGATCATCTTCATTGTCATTATCGGCAT-  14642
              ||| | ||||||||    ||||||| ||||||  ||||| | |||   |||||| ||||
Sbjct  1144   AATAAATGTTAACTTTTTTTTCTTCGTATCATCGTCATCATTATT---ATTATCTGCATG  1088

Query  14643  ---ATTATATAAATTATCTCATTTATTTCTTCTAACTACAGGA-----------CT----  14684
                 |||||||| ||||||||| ||| | ||| ||||| ||| |           ||
Sbjct  1087   AATATTATATACATTATCTCAATTAATCCTTGTAACTCCAGAAAAAAAAAAAAACTCCAG  1028

Query  14685  ----TAGCAACTATTATTTTTATTTCCAGTTTACAAATGAGGAAACCAGTATCTTATTTT  14740
                  | |||  ||||||||| ||  |||||||| |||||||||||||| |||||| || |
Sbjct  1027   AAGGTGGCATTTATTATTTTCATCCCCAGTTTATAAATGAGGAAACCAATATCTTGTTGT  968

Query  14741  ATCCAAGCTTGTGGGTACTGAGAATATAAAGAGGAATTCAATATGGTTCTAGGCAAACTT  14800
              ||| |||| || |||||||||||||| ||||  |||  ||||||||| | |||| |  ||
Sbjct  967    ATCTAAGCCTGAGGGTACTGAGAATACAAAGGAGAACCCAATATGGTCCCAGGCCACTTT  908

Query  14801  TGGGG-TCTTCCCTATCTAGTGGGAGGGACCGAGGCACACATAAGCCCTCATGGTCCAAT  14859
              ||||| ||| | || ||||||||||||||| |||||| |||||   |||| ||||||||
Sbjct  907    TGGGGATCTCCTCT-TCTAGTGGGAGGGACCAAGGCACCCATAAATACTCA-GGTCCAAT  850

Query  14860  AGGTGCTGTGGTAGAGGA-------------------------------ACGTATCTGGG  14888
               |||||||   ||||||                                |||||||||||
Sbjct  849    GGGTGCTGTAACAGAGGACTGTACCAGTGCAACTGAGGACTGGATGACCACGTATCTGGG  790

Query  14889  GAAGTTGAGGAAGTCTCCACGAATGAAGGAGGC------TTGAGCAGCATCTTGAGGGAT  14942
              ||||| | ||||| ||    |    |||| ||          |||||| | ||||   |||
Sbjct  789    GAAGTAG-GGAAGGCTTTGCAGGTGAAAGACATACATATTTGAGCTGAATCTAATGGGT-  732

Query  14943  GGCAGGTTATGTCAGGAGGAACAGTGGAGG-----CAGGGGAAGACATTCCAGGCAAAGT  14997
```

```
               |||||   ||  ||||||| |||||||||     ||||| | | |||||||| ||
Sbjct  731    -GCAGGGCTTGCAAGGAGGAGCAGTGGAGGAGGGGCAGGGTAGGGCATTCCAGTCAGGCG  673

Query  14998  GAGCAGTATTTGTGTGACAGGGCCTAGTGCATGTTTCAAAGGGCAGGACA-ATTGTAGCT  15056
              ||   | ||| | ||||||| ||   ||||  | |    |||| ||||   ||  | | |
Sbjct  672    GAAGTGCATT-GCGTGACAGTGCACGGTGCTGGCTCTTCAGGGAAGGATGCATCTTTGGT  614

Query  15057  GGG-TGCACCACTGGCAGCAGCAGGAGGGGAGTGGACGAGTATTTGGGGTCAGCT-ATTC  15114
              ||| ||    | |||| ||| ||| |||| ||||||  ||    |  |||||||| ||||
Sbjct  613    GGGCTGTGTGATTGGCGGCACCAGAAGGGAAGTGGAAAAGGTGGTCAGGTCAGCTTATTC  554

Query  15115  AGAGCCCTATTGGCCAAGCTGAAGGTTT-----TGTTCTGTGAGCAAGAGGGAGCCACTG  15169
              || ||  |  ||||||||||||  ||||     |||||||||||||| |||||| |||
Sbjct  553    AGGGCTTTGCTGGCCAAGCTGAGAGTTTACACTTGTTCTGTGAGCAAAAGGGAGTCACCC  494

Query  15170  GAGCTTTTTA---TTAAGGCAGATTCGGGACAGACTAGG------------AAGACAGTC  15214
               || ||||||   ||||  ||||   | ||||||| |||            |||||| |
Sbjct  493    AAGGTTTTTAAAATTAAAACAGAGGAGTGACAGACCAGGTTTAGGTTGAGGAAGACAATT  434

Query  15215  CTTAC-----CAGGGTGTTGAAAGATTGGAGGGAAGAGAGGCCAGCTGCATGCGGACCAA  15269
              | |||     |||| |   ||| |  ||||||| | |||||| |   ||| |  ||  |
Sbjct  433    CCTACAGCACCAGGATAAGGAAGGGCTGGAGGGGAAAGAGGCTAAAAGCAGGGAGATGAG  374

Query  15270  TTGGGAGACCATCTCCAGGGTCTGGGCAAGAAGTGGAACAGGCTCTGAACCACGACCGTG  15329
              || | || ||||||| || |||  |||||| ||||         || |         |||
Sbjct  373    TTAGAAGGCCATCTCAAGTGTCCAGGCAAGTAGTG---------CTAA---------GTG  332

Query  15330  GCAGTGAGAGCCGAGAGGAGGGGATGACTCAAGGCACATGTCAACAGGTAGAAACCTCAG  15389
               ||| |||           |||| |||||| ||| ||||||||| |||||||| ||  ||
Sbjct  331    -CAGGGAG-----------GGGGGTGACTCCAGGTACATGTCAATAGGTAGAAGCCATAG  284

Query  15390  GGCTGGCTGTCATGGGG----TCAAGGAGAGGGAAAAGGTGACAAGGACCCTGAGGTTCC  15445
              |||||  ||||||||||    |||| |||| ||||||||||||| | ||| |||||| |
Sbjct  283    GGCTGTGTGTCATGGGGGGGGTCAAAGAGAAGGAAAAGGTGACGATGACTCTGAGGCTTT  224

Query  15446  AGCTTGGGTCCGTGGTGATACTGTCAACTGAGGCA----------GGGAACACAAGACTG  15495
               ||||||||  ||| ||||||||  |||  || ||          |||  |  ||||||
Sbjct  223    GGCTTGGGTAAGTGATGATACTGGTAACCAAGACATTGAGGAGAGGGGGCCGTAAGACTA  164

Query  15496  TTAGTAGAGAGGGAGAGTGAGGGGAGACAGATGACAAGGTCTGTTTGGACTTGCTGGGTT  15555
              ||||||| ||||| || |||| | |||||| ||||||| || |   |||| || ||||||
Sbjct  163    TTAGTAGGGAGGGGGAATGAGTGCAGACAGGTGACAAGTTCAGCCAGGACATGTTGGGTT  104

Query  15556  TGGAGTGC---TGG---AT---AGCGGACATGTCCAGCAAGAAACTGGGAATCTTGGCTT  15606
              ||| ||||   |||   ||   || |||||||||||||| ||| |||| ||||| |||||
Sbjct  103    TGGGGTGCCAGTGGGACATCCAAGTGGACATGTCCAGCAGGAAGCTGGAAATCTGGGCTT  44

Query  15607  TGAGCCCAGCTGAGACATGGGGGCTGGAAAGCCA  15640
               ||| |||| ||||| |||||| |||| ||||||
Sbjct  43     AGAGGCCAGATGAGAGATGGGGTCTGGGAAGCCA  10


 Score = 89.7 bits (98),  Expect = 2e-13
 Identities = 103/139 (74%), Gaps = 5/139 (3%)
 Strand=Plus/Minus

Query  7844  GCCCAGGGCAAAAACCCAAGAGTTGTAGTTCACTCATCTTTATTTCT-----CACCTCTA  7898
             || || || ||||||||||   ||||  ||||||||||  |||| ||     ||||||||
Sbjct  6454  GCTCAAGGTAAAAACCCAAAGATTGTCTTTCACTCATCCCTATTCCTTACCTCACCTCTA  6395

Query  7899  CATTCAGCCAGTCACTAAGCCCTGCCAAGTATATTTCCTAAGCATTCCTTGCATGTCACC  7958
             ||| | || |||| |||||||||||||||  |  |||||||  ||| ||   || | |||
Sbjct  6394  CATCCCGCTAGTCCCTAAGCCCTGCCAAGCCTCCTTCCTAAATATTACTCAAATTTGACC  6335

Query  7959  TCTTTTCTCCATTCCTACT  7977
               |||| ||||||||| ||
Sbjct  6334  CATTTTTTCCATTCCTTCT  6316


 Score = 66.2 bits (72),  Expect = 2e-06
 Identities = 89/120 (74%), Gaps = 3/120 (2%)
 Strand=Plus/Minus

Query  11235  TAACTCATTTAATTCTCACTATGATGCTGTGAGGTAGGTTACTATTATTATCT-TCACAT  11293
              ||||||||||||||||| ||      || |||||     || ||||||| |||  |   |
Sbjct  8012   TAACTCATTTAATTCTC-CTGCAGCCCTTTGAGGGCACATATTATTATT-TCTCCCGTTT  7955
```

```
Query  11294  TACAGAGGAGGCACCAGACTCAGACAGGCTGAATAACTTGGCCAAGGTCACATAGCTAGT  11353
              |||| |||||||| ||| |||||| ||||| | |||||||  |||||||| | |||| ||
Sbjct  7954   TACACAGGAGGCATCAGGCTCAGAGAGGCTAAGTAACTTGCTCAAGGTCATACAGCTGGT  7895


 Score = 62.6 bits (68),  Expect = 3e-05
 Identities = 86/117 (73%), Gaps = 5/117 (4%)
 Strand=Plus/Minus

Query  6516  TAATTCATTTAATCCTCA-CACAGCCCTACAGGA-GCAAGTACTATTATTTCCCCCACGT  6573
             ||||||||||||| ||||  | |   || || || | | |||| ||||||  ||| |  |
Sbjct  4672  TAATTCATTTAATTCTCAGTATA---CTTCATGAGGTAGGTACCATTATTGTCCCAATTT  4616

Query  6574  TACAGAGCAGGCATCAGGCTGAGAGAGGCTAAGTCATTTGCTCAAGATCATATAGCT  6630
             || ||||  |||| |||||| ||| ||||||||| | |||  |||| ||| | ||||
Sbjct  4615  TAGAGAGGTGGCACCAGGCTCAGACAGGCTAAGTAACTTGGCCAAGGTCACACAGCT  4559


 Score = 59.0 bits (64),  Expect = 3e-04
 Identities = 117/171 (68%), Gaps = 3/171 (1%)
 Strand=Plus/Minus

Query  725   TTTATTGGGTATTTACTATGTGCCAGGAACTGTTCTAAGTGTTCTACACAGATTATCTCA  784
             |||||||   ||||||||||||||||| ||  ||||||||  | |      ||||  |||
Sbjct  4725  TTTATTGAACATTTACTATGTGCCAGGTACCATTCTAAGTACTTTGTGTGTATTAATTCA  4666

Query  785   TTTAATCCTCATCATAACCCAATGACGTCAACTCTATTATTAGCTCCATTTAACAGATGA  844
             |||||| ||||  || ||   |||| ||     | ||||||  | | |||| | ||| |
Sbjct  4665  TTTAATTCTCAGTAT-ACTTCATGAGGTAGGTACCATTATTGTCCCAATTTTAGAGAGGT  4607

Query  845   AGGAATTGAGGCTCTGGGATGTTAAGAAAAGTGCCCAAGGTCACACAGCTA  895
              | |    |||||| |  | | |||| ||  || |||||||||||||||||
Sbjct  4606  GGCA--CCAGGCTCAGACAGGCTAAGTAACTTGGCCAAGGTCACACAGCTA  4558
```

B. Typical example of highly conserved nucleotide sequences of the entire domesticated genes in placental mammals

Alignment of Procavia ZCCHC12 gene (including promoter, CDS and 3'flanking regions) with human orthologous gene

```
>gb|AADB02037198.1|  Homo sapiens chromosome X CRA_219000002708586, whole genome shotgun
sequence
Length=1063140

 Score = 1918 bits (2126),  Expect = 0.0
 Identities = 2418/3301 (73%), Gaps = 274/3301 (8%)
 Strand=Plus/Minus

Query  2       CCGCCCTCGCGCCCCTCTGATTGGCTGTACCACCCCGCTCTGTCACGCTCTTTTGTCTCA  61
               |||||| ||| |  | ||||||||| | || | ||||||| |||||||||||||||||||
Sbjct  118596  CCGCCCCCGCACTTCGCTGATTGGCCGCACTAGCCCGCTC-GTCACGCTCTTTTGTCTCA  118538

Query  62      GTGGGTAGAGGCTAAAAGCAGCGGCGGCTGCCGTGGGAACCGCGTTGCCTCCACTCGGCT  121
               |  || ||||| ||||||| || ||||||||| | ||||| ||| ||||||  ||| |||
Sbjct  118537  GCTGGCAGAGGATAAAAGCCGCCGCGGCTGCCTTAGGAACGGCGCTGCCTCGTCTCTGCT  118478

Query  122     ACTT-TGGCTGTGCGGCGGTGCGAAGCAGCTCAGGCGTGCGGCCCCGGGTAACTTCCTAG  180
               ||   ||| || |||||  |||||||||||||   || || |||||||||  |||   ||
Sbjct  118477  ACCCCTGGTTGGGCGGCCCTGCGAAGCAGCTCCTTCGGGCAGCCCCGGGTCGCTT---AG  118421

Query  181     TTACCGAGGAGGCGCCCGT-CTTTCTCTCTTTGCAGGACGGCGGCGGCCAGGACGCGGAC  239
                  || |||||||  | || ||||  | | |    | | ||||| | |||| | |||||
Sbjct  118420  CGGCCAAGGAGGCTTCAGTTCTTTGCCGCCT----GCAAGGCGGAGACCAGAAGGCGGAA  118365

Query  240     TCCACAGCTATAGATGCCGT---------TCTCCATCACCAGGTC-CAGGTAGAGAAAGG  289
               |||||||||   || || |          | |||| || | |  | |||||| |||| ||
Sbjct  118364  TCCACAGCTGGCGACGCGGGAGCATCTGCTGTCCACCAGCGGAGCACAGGTAAAGAATGG  118305

Query  290     GG-TGCGATTGCCTAGCACCGAA---GGTG------------------CTGGGTGGCTCC  327
               || || |||||||  ||   ||    ||||                  ||||| ||||||
Sbjct  118304  GGGTGTGATTGCCCGGCGGGGAGAGGGGTGGGGGAAGACCCACCAGTACTGGGCGGCTCC  118245

Query  328     CCGCTTGG-----------------GGCA-----AAGAGCACTCCAGTAGCTGGGCTGCA  365
               | ||||||                 ||||     | ||| ||||  || ||  ||||||
Sbjct  118244  CAGCTTGGAGAATCGGTGACTGTGAGGCAGGGGTAGGAGTACTCTCGTGGCCCGGCTGCG  118185

Query  366     --GAGAGAGGAGGTAGGGTTCAACTGGATGCTGCCGAATGGGTAGGGTTTGCCTGAATTG  423
                 ||| |||||||| ||||||| |||||| |||| |  | |||||| |||||||| |||
Sbjct  118184  CTGAGGGAGGAGGTGGGGTTCACCTGGATACTGCGGGCTAGGTAGGATTTGCCTGGATT-  118126

Query  424     AGAATGGGGGAGAATTTGGGGGCAGGGGCAGGCTTGGAAAGATGGAGGGTTGAAATCGCG  483
                   ||||||      |||| | ||||||  |   ||  ||  || ||   | |||| ||
Sbjct  118125  ----TGGGGG------TGGGAGGAGGGGCGAG---GGGTAGCGGGGGGA--GGAATCTCG  118081

Query  484     AACTGCTGCCTTA-AACCCCTTTTGCCTCTGCTCATCTCGTCCTGGAGAAAATATGTGAA  542
               | ||| |  |||  |||  | ||||| ||| |||||||| | ||||    ||||||||
Sbjct  118080  AGCTGTTTGCTTCGAACATCCTTTGCATCTCCTCATCTCTTTCTGGGAGGAATATGTGGG  118021

Query  543     TGG------CCGGCTCATTTTCCCTTTCTCCGTGACAGGTCATCGAAGCCAAAGCCGAAG  596
               |||      ||  ||| | ||||||||||||||| |||| |||| |||||  | | |||
Sbjct  118020  TGGGTGTGTCCAACTCCTATTCCCTTTCTCCGTGGCAGGCCATCAAAGCCGCATCTGAAC  117961

Query  597     TTAAGCTCCGTGTGGCTGATTTCAGAGCTGGTAGGCCCACGGTTCCTCGGGGAGGGGGGC  656
               || |  || |||  ||||||| ||||||||||||||| |||| | | |||||
Sbjct  117960  TTGAATTCTGTGCAGCTGATTGCAGAGCTGGTAGGCCGACGGCTTCCCGGGG--------  117909

Query  657     GGTGGGATTTAATGGAGGAGGGA------CTCG-CTTTGGCTTGCATTCAGCAGGTGGGA  709
                  |  || |||||  ||||||       || | || ||||||  | |||| ||| || |
Sbjct  117908  ---GAAATGTAATGCTGGAGGGTGGGGGGCTGGTCTCTGGCTTATAGTCAGGAGGGGGCA  117852

Query  710     GTGGAAATTTTTCACCTGGCGACAGCAGAGGTGACTGGGATGGGGGTG--GGG---AGGT  764
               | |  |||||||| |||||| ||||| ||||||   || |   || ||  |||   || |
Sbjct  117851  GAGAGAATTTTTCGCCTGGCAACAGCGGAGGTGGTGGGTAGCAGGATGACGGGTGAAGCT  117792

Query  765     GGGAATGAGGCTGCCCTTTTCT---CTAC--CACAGTG---CAGTCTTGGACGCTGTGAG  816
               |    | |  || ||||| |||   || |  | | |||   || ||||| |  ||   ||
Sbjct  117791  GCTTCTAACCCTTCCCTTCTCTGGCCTTCTCCGCTGTGGCGCAATCTTGAAACCTCA-AG  117733
```

```
Query  817     GACCCTAATCCGCGAACCTCAGTGGACAGAGGCCTACAGGACCTTGAGGCTG-GATTTCT  875
               |||||  ||| |||| || | |||||||||||   || | |||  |  |  | | |||||
Sbjct  117732  GACCCGGATCTGCGACCCCCTGTGGACAGAGGTTGACCGTACCCCGGAGAGGAGCTTTCT  117673

Query  876     CA-GCACTG-ACTGAAGGCAGAGGCTGGAACTGAAACAAAGGCGTACACTTGTTCCTGAG  933
               || | |  | ||||   ||||||||||||| ||||| |||| ||  | |||||| | |||
Sbjct  117672  CACGGAGGGCACTGGTTGCAGAGGCTGGAAGTGAAATAAAGACGCGCTCTTGTTTCAGAG  117613

Query  934     ATCACGTAAGAATC--AGAAATGAGAAT---------------------TTTGAGAAGGG  970
                ||  |||||||||  |||||| | ||                      |||||||||||
Sbjct  117612  TTCGTGTAAGAATCTGAGAAATAACAAGGGGGACGGGCGTGGGGGGCACTTTGAGAAGGG  117553

Query  971     GGTGTGGTGGAGACGTTAGGCCTTGAGATTAATGAGATTTTATTT-ATTTTCACAAATCT  1029
               |||||||| |||||   || || |||| |||||||||| |||||| |||| |||||||||
Sbjct  117552  GGTGTGGTTGAGACCAGAGACCATGAGGTTAATGAGATCTTATTTTATTTCCACAAATCT  117493

Query  1030    ---ATCAGATTTAGACCGTGACACTCTACTTAGAGAGCCCAGTCTCAGCTCCACACACCA  1086
                  ||||   ||     ||    ||||| || ||||||||| | ||||||||| ||||
Sbjct  117492  GCGATCA---TT-----GT---ACTCTATTTGGAGAGCCCAATATCAGCTCCAGACACTG  117444

Query  1087    TTACACCTAGAATGTGAAGCAACAGTTGCCCTCTGTATTGGAGGATTAGCTCGCGGAGAT  1146
                |||||||||||| ||||||||||||| || ||  | |||||||| | |   | || |||
Sbjct  117443  CTACACCTAGAATTTGAAGCAACAGTTACCGTCCCTCTTGGAGGACTGGTGGGAGG-GAT  117385

Query  1147    AAATGCGGCTCTGAAAAGCACCGTACTGGCCAAGGGACACTGGCTATTGCTCTG--AGGC  1204
                 ||| ||||   ||||||||| | || ||     | ||  || ||  ||||||  |||
Sbjct  117384  GGATGTGGCTGCAAAAAGCACCTTGCTAGCACGCAGGCATCGGGTA--GCTCTGGGAGGT  117327

Query  1205    CGTAGTTGTCTTTTTCTTGCAGGGCG-TTTTTAGTGCAGTGGGCAGATACAAAATGCAGT  1263
                |||  ||||  | || | ||||    ||||||| | |||||||| ||| |||||| | |
Sbjct  117326  TGTAAGTGTCAATCTCCTACAGGATTATTTTTAGGGTAGTGGGCAAATATAAAATGTACT  117267

Query  1264    GTAGGGTATTCAGAAAGGGAGAGCACTGGGCTGCAGATTGCCACTGTGTAGCCTGCTACT  1323
               | ||| |||||||  ||| |||| |      ||||| |         | |||| ||| |
Sbjct  117266  GCAGGATATTCAGGTAGGAAGAGAA------TGCAGCT---------GCAGCCCGCTCCC  117222

Query  1324    TTACTGACCCCCTTCCCCTTTTTTCCCTTTTCTTTACAGTTCCTACTGAGATACAAGGGA  1383
               ||| | |||  |  |||||||     |||||  ||||||  ||| ||||||||   || |
Sbjct  117221  TTATTAACCAGCC-CCCCTTT-----CTTTT--TTACAGCCCCTGCTGAGATA---GGAA  117173

Query  1384    GGCAGAGCCCTGGAACATTGCTTTGGAGATTCCTGGTGCAAACTAGGCTTTTCTGCAAAA  1443
               |||||||||     || |  || |     | ||   |||| | || ||||||||  |||
Sbjct  117172  GGCAGAGCC-----ACCTC-CTCTCCTC-TCCCACCTGCAGATTAAGCTTTTCTAAAAAG  117120

Query  1444    CCTGAGCATTTTGTTATATTCAGATAACCAGTCATTGTCAGTCATGGCTAGCATCATTGC  1503
               |||  |||| || ||||||||||||| ||  || | ||||||||||||||||||||||||
Sbjct  117119  CCTAGGCATCTTCTTATATTCAGATACCCTATCGTCGTCAGTCATGGCTAGCATCATTGC  117060

Query  1504    ACATATGTTGAACAGTCGGGGCCAGAATGCACCCTTGCCACCTTGGGCCCATTCCATGCT  1563
               || | |    ||||| ||| | | ||||||||||||||| ||||||||||||||||||||
Sbjct  117059  ACGTGTCGGTAACAGCCGGCGGCTGAATGCACCCTTGCCGCCTTGGGCCCATTCCATGCT  117000

Query  1564    GAGGTCCCTGGGGAGGAGCCTTGGTCCCTTAATGGCCCACTTGGCAGAGAGAAACATAAA  1623
               ||||||||||||||| || || |||||  ||||||||  | ||||||| |||||||| ||
Sbjct  116999  GAGGTCCCTGGGGAGAAGTCTCGGTCCTATAATGGCCAGCATGGCAGACAGAAACATGAA  116940

Query  1624    GTTGTTCTCGGGAAGGGTGGTGCCAGTCCAGGGGGAAGAAACCTTTGAAAACTGGCTGAA  1683
               |||||||||||| ||||||||||||| ||| ||||||||||||||||||||||||||||
Sbjct  116939  GTTGTTCTCGGGGAGGGTGGTGCCAGCCCAAGGGGAAGAAACCTTTGAAAACTGGCTGAC  116880

Query  1684    TCAAGTCAATGGGATCTTGCCAGATTGGAATACCTCTGAGGAGGAAAAACTCAAGCGCTT  1743
                |||||||||||  || |||||||||||||||  |||||||||||||| |||||||||||
Sbjct  116879  CCAAGTCAATGGCGTCCTGCCAGATTGGAATATGTCTGAGGAGGAAAAGCTCAAGCGCTT  116820

Query  1744    GATGCAGACCCTTAGGGGCCCTGCCAGGGAGGTCATGCGCTTGCTTCAGGCAGCCAACCC  1803
               |||| | |||||||||||||||||| | |||||||||||  ||||||||||  |||||||
Sbjct  116819  GATGAAAACCCTTAGGGGCCCTGCCCGCGAGGTCATGCGTGTGCTTCAGGCGACCAACCC  116760

Query  1804    CAGCCTAAGTGTGGCAGATTTCTTGCGGGCCATGAAACTGGTGTTTGGGGAGTCTGAAAG  1863
                | |||||||||||||||||||||||| ||||||||| ||||||||||||||||||||||
Sbjct  116759  TAACCTAAGTGTGGCAGATTTCTTGCGAGCCATGAAATTGGTGTTTGGGGAGTCTGAAAG  116700

Query  1864    TAGCGTGACTGCCCATGGTAAATTTTTTAACACCCTGCAGGCGCAAGGAGAGAAAACATC  1923
                || |||||||||||||||||||||||||||||||| || || ||||| |||||| | ||
Sbjct  116699  CAGTGTGACTGCCCATGGTAAATTTTTTAACACCCTACAAGCTCAAGGGGAGAAAGCCTC  116640

Query  1924    CCTGTATGTGATCCGTTTAGAGGTGCAGCTCCAGAATGCTATTCAGGCAGGAGTCGTAGC  1983
```

```
                 ||| |||||||||||||||||||||||||||||||| ||||||||||||||   |  ||||
Sbjct  116639  CCTTTATGTGATCCGTTTAGAGGTGCAGCTCCAGAACGCTATTCAGGCAGGCATTATAGC  116580

Query  1984    TGAGAAAGATGCAAACCAGACTCGCTTGCACCAACTCCTTCTAGGGGCTGAGCTGAACAG  2043
               ||||||||||||||||| |||||||||||| || |||||| |||| | ||||||||  ||
Sbjct  116579  TGAGAAAGATGCAAACCGGACTCGCTTGCAGCAGCTCCTTTTAGGCGGTGAGCTGAGTAG  116520

Query  2044    GGACCTGCGCTTCAGGCTTAAGGAGCTTCTCAGGATGTATGCAAATGAGCAGGAGTGTCT  2103
               |||||| ||  |||| ||||||||  |||||||||||||||||||||||||||||| | ||
Sbjct  116519  GGACCTCCGACTCAGACTTAAGGATTTTCTCAGGATGTATGCAAATGAGCAGGAGCGGCT  116460

Query  2104    TCCCGGTTTCCTGGAGCTAATCAGAATGATAAGGGCTGAAGAGGATTGGGATGGCACTTT  2163
               ||||   || |||||| ||||||||||| ||||||  ||||||||||||||||   ||||
Sbjct  116459  TCCCAACTTTCTGGAGTTAATCAGAATGGTAAGGGAGGAAGAGGATTGGGATGATGCTTT  116400

Query  2164    TATTAAACGAAAGCGACCCAAAAGATCTGAGTCAGTTGTGGAGAGGGCAGCTAACCCTGT  2223
               ||||||||| ||||| || ||||| ||||||||| | |||||||||||||   | ||||||
Sbjct  116399  TATTAAACGGAAGCGTCCAAAAAGGTCTGAGTCAATGGTGGAGAGGGCAGTCAGCCCTGT  116340

Query  2224    GGCATCGCAGGGCCTGCAGCCGATAGTGACTGGCAATGCTAATTGCAACTTGATAGAGAT  2283
               |||||  ||||||    |  |||||||||||  |||| |||| | |||||  ||||||||||
Sbjct  116339  GGCATTTCAGGGCTCCCCACCGATAGTGATCGGCAGTGCTGACTGCAATGTGATAGAGAT  116280

Query  2284    CGATGATTCCCTTGATGATTCAGATGAGGATGTGATCTTGGTGGAGCCTCAGGACCCCTCC  2343
                |||||| |||| || || || ||||||||||||||| |||||||| |||||||||||||
Sbjct  116279  AGATGATACCCTCGACGACTCCGATGAGGATGTGATCCTGGTGGAGTCTCAGGACCCCTCC  116220

Query  2344    AGTCATATCGGTGGCTTCTCCTCCCCCTGTGTG-CAGGGCCAGACCTCAGGATCAGGTGC  2402
               | |   |||    || | | ||||||| |   | | |||||||||||||||||||| | ||||
Sbjct  116219  ACTTCCATCCTGGGGTGCCCCTCCCC-TCAGAGACAGGGCCAGACCTCAGGATGAAGTGC  116161

Query  2403    TGGTCAATGATTCCCCCAACAATTCCTGGGCCCGGTTTCCTTCCACCAGTGGTGGTTCTG  2462
               |||||| ||||||||||| |||||||| |||| | |||||||||||||||||||||||||
Sbjct  116160  TGGTCATTGATTCCCCCCACAATTCCAGGGCTCAGTTTCCTTCCACCAGTGGTGGTTCTG  116101

Query  2463    GGCATCAGAGTGATGGTCCTGGGGATAAGCGTAAGGCCAGGAAGCGAAAATACACAATCC  2522
               |  || ||| | | ||||||||||| | |||||  ||||||||||||||| |||||||||
Sbjct  116100  GCTATAAGAATAACGGTCCTGGGGAGATGCGTAGAGCCAGGAAGCGAAAACACACAATCC  116041

Query  2523    GCTGTTTATTTTGTGGTGAAGAGGGCCACACAAAAGAAATTTGTG---GTGAAAACAACA  2579
               ||||||   | ||||||||| || |||||| |||||||||  ||||      || |   |||
Sbjct  116040  GCTGTTCGTATTGTGGTGAGGAAGGCCACTCAAAAGAAACCTGTGACAACGAGAGTGACA  115981

Query  2580    AAGCCCAGGTTTTTGAGAATCTCATTATCACCCTTCAGGAACTGACACATACAGAGGAGG  2639
               | ||||||||||||||||| | || ||||| || ||||| ||||| ||||| |||  ||
Sbjct  115980  AGGCCCAGGTTTTTGAGAATTTGATCATCACTCTCCAGGAGCTGACCCATACTGAGATGG  115921

Query  2640    AGGAGACAAAAGAGGTCTCTGGTGGACGCAATGACCTCTCTGAGCTGCGGAAAGGAGCTA  2699
               ||  | ||| || || | |||| | |  ||||||| |||||||||   | | ||||   | |
Sbjct  115920  AGAGGTCAAGAGTGGCCCCTGGCGAATACAATGACTTCTCTGAGCCACTGTAAGGGACCA  115861

Query  2700    GCCCCCAGCCCTAAATGAACCGTTGTCTATATTTAGCGTACAGTGGGGGGAAGACTGGGG  2759
                |||||||    | | |||||| ||   ||||||| ||| | |||| | ||||| |||||||
Sbjct  115860  -CCCCCAGGTTTCAGTGAACCCTTACCTATATTCAGCATCCAGTAGTGGGAAAACTGGGG  115802

Query  2760    AGGGG-------------TTTTCATTGCATGCATTAATCCTCAGAGCAACTTTCTTTTGG  2806
                ||||              || | | |||||| |||||||| || |||   || ||||||||
Sbjct  115801  TGGGGGTGGGGGTGGGACTTCTAACTGCATGAATTAATCCACAAAGCGGCTATCTTTTGG  115742

Query  2807    GGTGGAGGACAAAAAGCTTTTGATGCCAGCACAGGGGAGGGGAATGGCCTGACCTTTCTG  2866
               ||||||| | |||   |   || ||| ||||||||  ||||||  || ||||||||  ||||
Sbjct  115741  GGTGGAGTAGAAAGGGTCTTGGATACCAGCACATTGGAGGGAGATAGCCTGACC--TCTG  115684

Query  2867    TCTCTGCTC--TCTCCCT---ACCTAAGGGTCCATTTTCTGTGTGTGTCTAATTCTTTGA  2921
               ||  |||||  |||||||     |||| |||||  ||||||||||||| | | ||| ||||
Sbjct  115683  TCCTTGCTCCTTCTCCCTGCAGCCTACGGGTCTGTTTTCTGTGTGTGCCCATTTCCTTGA  115624

Query  2922    TGGGTTTACTCTCTTTGTGAAACTGGTGCAA------------------------------  2952
                 | ||||   ||||||||||| ||||  ||
Sbjct  115623  CAGCTTTA--TTCTTTGTGAAAGTGGTATAATTTATTGTTAAATATTTGAACAATAAAAA  115566

Query  2953    --GTACAAAAACTAATGTAC-AGCTACCTGAAACCCTCCACCCATATATAATCATTGTCA  3009
                 ||||||||| | | |||| |  ||||  || | |||||||| ||||||||||||||||
Sbjct  115565  AGGTACAAAAAGTGAAGTACAAATTACCCAAATCTCTCCACCCTTATATAATCATTGTCA  115506

Query  3010    AGCCTTTGATGAATGCCTTTCTAGATACTTCCCTATACCTGTGTACCCAGATAGATTTAT  3069
               | |||||||||| |          |||| |||||||||||| |||||||||||||| |||
```

```
Sbjct  115505  ACCCTTTGATGAGT---------GATATTTCCCTATACCTATGTACCCAGATAGATATAT  115455

Query  3070    GTATAGATAAAAAGTGATCAAATAAAAAGTGCTGTTGTATATTGTGTATTTTTCACCAAA  3129
               | |||||| ||||||||| |||| | |||||||||| |||  ||| | ||||||||||||
Sbjct  115454  GCATAGAT-AAAAGTGATGAAAT-ATAAGTGCTGTTCTAT-CTGTAT-TTTTTCACCAAA  115399

Query  3130    C  3130
               |
Sbjct  115398  C  115398
```

C. Highly conserved introns in placental mammals are not limited to domesticated genes but can be found in diverse genes located either on autosomes or sex chromosomes

Query was Homo ZFX intron 1 (located in the 5'UTR)

>gb|AAGU03060905.1| Loxodonta africana (AFROTHERIA) cont3.60904, whole genome shotgun sequence

```
Length=100138

 Score = 1348 bits (1494),  Expect = 0.0
 Identities = 1463/1929 (75%), Gaps = 191/1929 (9%)
 Strand=Plus/Minus

Query  1      TGAGTCCCGGGTGCCGCCGCGGCCGCGGGGCCTAGTGCGCGCACAGCGGCCTGGTCTCGG  60
              ||||||||||| || |        |||||||||||  ||||| ||||||||||||| |||
Sbjct  84575  TGAGTCCCGGGCGCTG--------GCGGGGCCTAGCACGCGCGCAGCGGCCTGGTCCCGG  84524

Query  61     CCTGGTcggcggcccgcaaggcgccctcccgcgctaggccgggcggcgtggcgcgcggcg  120
              ||||||||||||||||||||||||| ||||||||||||||||||||||||||||||||||
Sbjct  84523  CCTGGTCGGCGGCCCGCAAGGCGCCTTCCCGCGCTAGGCCGGGCGGCGTGGCGCGCGGCG  84464

Query  121    ccgAGCAGGCCCCGAGGAGGCCGCAGTTAGGCCCGGGGAGGAGCCCGGCTGCCCCGAGCG  180
              ||||||||||||||||||||||||||||||||||||||||||||||||||||||||| ||
Sbjct  84463  CCGAGCAGGCCCCGAGGAGGCCGCAGTTAGGCCCGGGGAGGAGCCCGGCTGCCCCGATCG  84404

Query  181    GCGGCGGAGGCGCGCTCCGTAAGCGGGCGGGGGTTGGGGGAGGGTCGCCCGGTTGTGCCG  240
              | |||||||||||| |||||||||||||||||||||||||||||||||||||| |  |
Sbjct  84403  GTGGCGGAGGCGCGTTCCGTAAGCGGGCGGGGGTTGGGGGAGGGTCGCCCGGTAGCCCGT  84344

Query  241    GAGGCGGTCGAGGGGCCCCGGGTCGGCTCCGCGGCCGACCTGGGGCACCGCGCCGGGGTG  300
              |||||||  ||||||| |||||||| | || |||||| ||  |||||| |||| |||  |
Sbjct  84343  GAGGCGGCTGAGGGGCTCCGGGTCGACGCCACGGCCGGCCCTGGGCACTGCGCGGGGTCG  84284

Query  301    AGGCCTGGCGAGGAGGCGAAGGCTGCAGGCGTGAGGTGAAGGCCGCAGGCCGGCCGGGCC  360
              |||||||| |||||||||||||||||||||||||||||||||||||||||||||||||||
Sbjct  84283  AGGCCTGGTGAGGAGGCGAAGGCTGCAGGCGTGAGGTGAAGGCCGCAGGCCGGCCGGGCC  84224

Query  361    GATTTTCGCTATGTAAATATCGGTGAggggggggggagggacgggggACAAGATGGCGGCG  420
              ||||||||||||||||||||||||||  ||||||||||||||||||||||||||||||||
Sbjct  84223  GATTTTCGCTATGTAAATATCGGTGA--GGGGGGGAGGACGGGGGACAAGATGGCGGCG  84166

Query  421    GCTCGGCGCCTGCTGCAGGGGACGATAGAGGGGGTTGCCGGGAGGGGGAGCCGCCATCTT  480
              |||||||||||||||||||||||| | |||||||||||||||||||||||||||||||||
Sbjct  84165  GCTCGGCGCCTGCTGCAGGGGACGGTTGAGGGGGTTGCCGGGAGGGGGAGCCGCCATCTT  84106

Query  481    GGAGGCGGTGTCTGGAGAGAAAATTCCGCTACAGCCCGTGAgggggggtgggagagcggg  540
              | |||||||||| |||||||||||||||||||||||||||||||||||| ||||||||
Sbjct  84105  GAAGGCGGTGTCGGGAGAGAAAATTCCGCTACAGCCCGTGAGGGGGGGTCGGAGAGCG--  84048

Query  541    cggcggcggcagcggcgccggtgacgggccccggaggcccggcgcggcggtgtgcgcg  600
                  ||||| |||||||||||||| |||||| ||||||||||||||  ||||||||||||
Sbjct  84047  ----GGCGGTAGCGGCGCCGGTGAAGGGCCCGGGAGGCCCGGCGCGATGGCGTGTGCGCG  83992

Query  601    cggaggggcgTGCTCGCTCCCCGTGGCGGCCATTGCCCTTGCCGCCATGATGAGCGCTCG  660
              |||||||||||||||| |||||   |||||||||||||| |||||||||||||| | | |
Sbjct  83991  CGGAGGGGCGTGCTCGTTCCCCACAGCGGCCATTGCCCTCGCCGCCATGATGAGAGTTTG  83932

Query  661    GGCTCCAGGCGCTCGGCGGCAGCGCCACCTTCCTGCCTTGCCTCCCGCAGCCCCGTGACT  720
              |||||||||  |||||||||||||||||||||||| | ||||| ||||| ||||||||||
Sbjct  83931  GGCTCCAGGTCCTCGGCGGCAGCGCCACCTTCCTGTCCTGCCTGCCGCAACCCCGTGACT  83872

Query  721    GGCTGCAGTTTCCGCTGCGTTTCAGCTGAGCTGCCGCAGGCGGC---CACCGCCGCCGCC  777
              | |||| ||||||||||| ||| | |||||||||||||||||||   | | ||||| |||
Sbjct  83871  GACTGCTGTTTCCGCTGCATTTTAACTGAGCTGCCGCAGGCGGCCGTCGCTGCCGCTGCC  83812

Query  778    CGGACGCCGGGACCGTTTCACCCTCAGCGCCCCTGGCCCTGCGCCTTCCCCCGCGCCTGT  837
              | | ||||| | |||           | |    |||           || | |||  |||
Sbjct  83811  CAGGCGCCGAGCCCG-----------GGGGTGTTGG----------GCCACTGCGTTTGT  83773

Query  838    AGCCACCCGAGGGCAGTCGGGGCAGGTGGCATTCCGGACACCTGGGCTTACCAGGGCATA  897
              |||          || ||           | |  ||| || || | || |||| |||| |
Sbjct  83772  AGC---------TCACTC-----------CGTATCGG-CATCTCGCCTCACCAAGGCAGA  83734

Query  898    CGGGACCCCAGGAAATGTTATTTTTGCTTAAGTCAAATCATATGTGGCTGCTAAGGTGCT  957
```

```
               |||||  || ||||| |||              || | ||  ||| ||  || ||||||
Sbjct  83733  TGGGACATCAAGAAATTTTA--------------AAGTTATGAGTGCCTCTTAGGGTGCT  83688

Query  958    TTCGATGCAGTTGTTTTTCCAACAATCTTTGCCGCCCCGAGTATCTGAATAAAGGAAGAA  1017
               ||  ||   ||| ||||||||||||| ||| |  | | ||||||||||||||| || ||
Sbjct  83687  CTCTGTGTGCTTG-TTTTCCAACAATCATTGTCTTCTCCAGTATCTGAATAAAGAAATAA  83629

Query  1018   AAATAAAGATTGATGGGA---AAGTTTTTGCCATTCTTTTGTCCTATAG-GGaaaa----  1069
              |||| ||  ||||||| |   ||||| |   || |||||||||| ||||  |||||
Sbjct  83628  AAATGAAATTTGATGGCAAAGAAGTTCTAATCACTCTTTTGTCCAATAGAAGAAAAGTGC  83569

Query  1070   aaaTTGCACAACACGAGAGCGGAGGAATAAAATT-TTGAATC-ATTCGTACTCATTTATG  1127
              | |||||||||||| | ||||||||||||||||| | || ||  ||  |  || |||||
Sbjct  83568  AGATTGCACAACACCAAAGCGGAGGAATAAAATTGTGGAGTCTTTTTATTGTCGTTTATT  83509

Query  1128   TGGAAACAACTTTCTTGA-----------AAAACGATTTAAGTA-----GTGAT-CTGTT  1170
              |||||||| || | ||||            |||| | |||| ||      | || |||||
Sbjct  83508  TGGAAACAGCTGTGTTGACGAGCTTCTTTTAAACTA-TTAATTAATCCCCTTATACTGTT  83450

Query  1171   GG-----------------TGGTAttt----ttttAAATCG--AGGAATAAG-GGCAGAC  1206
               |                 |  |||||    ||||  |  |  | ||| |||    ||
Sbjct  83449  TGCTCTTTAATGAGTTTCATACTATTTACTGTTTTTCAGGGTTAAGAAAAAGACAAAGGT  83390

Query  1207   TTTTTTATTTTCTTATTGGAGGACAACTTAGTGTGGTGCA-TATTTATTGTGCATTTGCC  1265
              ||||||||||| | |||| |||||| | || | |  || | |||||||||| | | | ||
Sbjct  83389  TTTTTTATTTT-TAATTGAAGGACAGCATAATCTAATGAAGTATTTATTGTTCCTGTACC  83331

Query  1266   TGTCATGGTATTTCGATTTTTCATTTGTGAAGTTTTAAGGCGGAGTTTCATCCCCTAACG  1325
              |||||||||||||  |||    |||||  ||||||||||   ||||  |||||| |||||
Sbjct  83330  TGTCATGGTATTTAAATT----ATTTGAAAAGTTTTAAGATTGAGTACCATCCCATAACG  83275

Query  1326   TACTTTTTTGATTGCACACTGTGCAACTTTCTTGATAAACTCGGAGCCTACTGTTAGCGA  1385
              |||  | |||||||| || | | | |||||| || || | |||||||| |||||| ||||
Sbjct  83274  TAC--TGTTGATTGCGCAGTATACCACTTTCCTGGTACAGTCGGAGCCCACTGTTTGCGA  83217

Query  1386   TGCACCGTGCTAGATGCTAAGAAACATTAAAAAGGTTCTTGTAATGCAGACGGTGGCTGC  1445
              |||||  |||||| || | ||||| ||||||| |||| | ||||||||||||||||||||
Sbjct  83216  TGCACTCTGCTAGGTG-TTAGAAAGATTAAAACGGTTGTGGTAATGCAGACGGTGGCTGC  83158

Query  1446   AAGAAGTCTC-ATGTTTAGAGGTAGGTGGGGCAAGGTGAAATGCAGTAAAGTG-------  1497
              |||||| |||  |||  ||| |||| || | | |||||||||||| | |||||
Sbjct  83157  AAGAAG-CTCGTTGTCCAGAAGTAGATGAGACCAGGTGAAATGCACT-AAGTGCTGCGGA  83100

Query  1498   AGAGGTACAACTAATTACTGGGATTTCCGAGGGAAGCAAGGTTGCCA-TTGGAGGATCAG  1556
              | ||||||| | | ||||||||  | | || | | ||  ||||   | |||||| ||||
Sbjct  83099  AAAGGTACACCCAGTTACTGGGGGTCCAGACGAAGGCGGGGTTTTTATTTGGAGCATCA-  83041

Query  1557   GGAGGGAC-GGGTTCCTGAAGTATTAATAGGT-GGCATTTTGAGAAGGTTACCGAGGGAT  1614
                  |||| |||||||||  |||      ||| |||     ||||||||| | || ||||
Sbjct  83040  ----GGACAGGGTTCCTGGTGTA-----GGGTAGGC-----GAGAAGGTTTCTGAAGGAT  82995

Query  1615   GGGTAGGACTTTGTTAGGTAGAGGTGGAGGTGGAGAAAGGACGTTTCAAGGGTGAAGCTT  1674
              |||||||||||||| |||||||||||| ||||| | |||| | |||||||||||| ||||
Sbjct  82994  GGGTAGGACTTTGTGAGGTAGAGGTGG-GGTGGGGGAAGGGCATTTCAAGGGTGAGGCTT  82936

Query  1675   CTTCAGTAAACAGTAAGTATTTACAGTTTGGCAAAAGTGGATGGTATAGGTAGGGGAGTG  1734
              |||||| |||||| |||| ||||| |||||||   | | ||||||||||
Sbjct  82935  CTTCAGAAAACAGCAAGTGTTTACCGTTTGGCTGGAATCGATGGTATAG-----------  82887

Query  1735   GTGGGAATTCAGGCTAGAAAAGTTAGATTGCAGAAGGGCTTGCTTGCCAGGT-GGAGGGG  1793
                       || |||||||| | ||| ||||  ||| ||| || ||| | || |||||||
Sbjct  82886  --------ACATGCTAGAAATGGTAG-TTGCCAAAGAGCTGGCCTGCTATGTGGGAGGGG  82836

Query  1794   CTTATACTCAATGGGGTAG---GCAGCGGGGGTGCCCTCAACTAGAAGAGTGATGGCACA  1850
              ||| |||||   | || ||   ||||||  || ||| || || ||  |||||| |  |||
Sbjct  82835  CTTCTACTCTGCGTGGGAGAGAGCAGCGAAGG-GCCTTCTACCAGCGGAGTGAAGATACA  82777

Query  1851   CCTGAGCTG  1859
              |||||||||
Sbjct  82776  CCTGAGCTG  82768
```

>gb|AAGV020292018.1| Dasypus novemcinctus (XENARTHRA) cont2.292017, whole genome shotgun sequence

Length=2385

Sort alignments for this subject sequence by:

E value Score Percent identity
Query start position Subject start position

```
 Score =  495 bits (548),  Expect = 6e-137
 Identities = 629/844 (74%), Gaps = 51/844 (6%)
 Strand=Plus/Plus

Query  523    ggggggtgggagagcgggcggcggcggcagcggcgccggtgacgggcccc-ggaggcccg  581
              ||||||||| |||||||||| | |||||||||   || |||||||| ||| |||| ||||
Sbjct  3      GGGGGGTGG-AGAGCGGGCG-CAGCGGCAGCG---CCCGTGACGGGTCCCAGGAGACCCG  57

Query  582    gcgcggcggcgtgtgcgcgcggaggggcgTGCTC-GCTCCCCGTGGCGGCCATTGCCCTT  640
               ||||   ||||| |||||| ||| ||||||||| |||||||      | ||||||||||
Sbjct  58     -CGCGC--GCGTGAGCGCGCAGAGAGGCGTGCTCCGCTCCCCCACCGCGACATTGCCCTT  114

Query  641    GCCGCCATGATGAGCGCTCGGGCTCCAGGCGCTCGGCGGCAGCGCCACCTTCCTGCCTTG  700
              |||||||||||||||||||| | |||||||||||||||||||||||||||| ||||| ||
Sbjct  115    GCCGCCATGATGAGCGCTCGAGTTCCAGGCGCTCGGCGGCAGCGCCACCTTTCTGCCCTG  174

Query  701    CCTCCCGCAGCCCCGTGACTGGCTGCAGTTTCCGCTGCGTTTCAGCTGAGCTGCCGCAGG  760
              ||| || ||||| ||||||||||||| | ||||||||| ||| | |||||||||||||||
Sbjct  175    CCTTCCCCAGCCGCGTGACTGGCTGCTGCTTCCGCTGCATTTTAACTGAGCTGCCGCAGG  234

Query  761    CGGCCACCGCCGCCGCCCGGACG--CCGGGACCGTTTCACCCTCAGCGCCCCTGGCCCTG  818
              | |   |||||| ||| ||| ||  ||| | |   |   ||||| | | ||| ||||| |
Sbjct  235    CAG---CCGCCGTCGCTCGGGCGTCCCGCGCCAACTCGTCCCTCCGTGTCCCCGGCCCCG  291

Query  819    CGCCTTCCCCCGCGCCTGTAG-CCACCCGAGGGCAGTCGGGGCAGGTGGCATTCCGGACA  877
              |||| |  ||||||  ||||| |||| ||||||| ||| |||| | ||||| ||||| ||
Sbjct  292    CGCC-TGTCCCGCGTTTGTAGCCCACGCGAGGGCCGTCTGGGCGGTTGGCAATCCGGGCA  350

Query  878    CCTGGGCTTACCAGGGCATACGGGACCC-CAGGAAATGTTATTTTTGCTTAAGTCAAATC  936
               ||||||| | |   ||  | || ||||  ||||||| || |||||  || | | ||   |
Sbjct  351    TCTGGGCTCATCGATGCCAAAGGAACCCTGAGGAAATTTTTTTTTTTTTTTAATAAAGGC  410

Query  937    ATATGTGGCTGCTAAGGTGCTTTCGATGCAGTTGTTTTTCCAACAATC----TTTGCCGC  992
              ||| |||||||||| ||||   | ||||| |||| ||||| | |||||     ||||| |
Sbjct  411    ATACGTGGCTGCTAGGGTGTCCTTGATGCGGTTG-TTTTCTAGCAATCACTGATTGCCCC  469

Query  993    CCCGAGTA----TCTGAATAAAGGAAGAAAAATAAAGATTGATGGGAAAGTTTTTGCCAT  1048
              | |  |||    ||||||||| ||| |||||| ||  |||| |||||||  |||  |||
Sbjct  470    CTCATGTACCAGCCTGAATAAAAGAACAAAAATGAAATTTGACGGGAAAGAATTTCTCAT  529

Query  1049   -TCTTTTGTCCTATAGGGaaaaaa------aTTGCACAACACGAGAGCGGAGGAATAAAA  1101
                || |||||| ||||| | | ||      |||||||  || || |||||||||||||||
Sbjct  530    CACTCTTGTCCAATAGGAACAGAAGTGCAGATTGCACTGCAAGAAAGCGGAGGAATAAAA  589

Query  1102   TTTTGAATCA---TTCGTACTCATTTATGTGGAAACAACTTTCTTGAAAAA--CGATTTA  1156
              |||||||  |   || || ||| ||||| ||||||||||||||||| ||||  | |||||
Sbjct  590    TTTTGAAGGAGTCTTTGTTCTCGTTTATTTGGAAACAACTTTCTTGGAAAACTCCATTTA  649

Query  1157   ----AGTAGTGATCTGTTGGTGGTAtttttttAAATCGAGG-----AATAAGGGCAGACT  1207
                  | || ||||||     |  |||||  ||   |||| |      | |||| |  | |
Sbjct  650    AAGTATTACTGATCTCCCTATCCTATTTACTTTTTTCGACGGTTAAGAAAAGGACGAAGT  709

Query  1208   TTTTTATTTTCTTATTGGAGGACAACTTAGTGTGGTGC-ATATTTATTGTGCATTTGCCT  1266
              |||| ||||||| |||| || ||| | || |||| ||| ||||||||||| ||| | |||
Sbjct  710    TTTTCATTTTCTAATTGAAGCACATCATAATGTGATGCAATATTTATTGTTCATATACCT  769

Query  1267   GTCATGGTATTTCGATTTTTCATTTGTGAAGTTTTAAGGCGGAGTTTCATCCCCTAACGT  1326
              |||| |||| ||  ||| |  |||  | |||||||||||  |||||||| ||| | | ||
Sbjct  770    GTCACGGTACTTAAATTCTCTATT--TTAAGTTTTAAGGTTGAGTTTCAACCCTTGAAGT  827

Query  1327   ACTT  1330
              ||||
Sbjct  828    ACTT  831


 Score =  235 bits (260),  Expect = 9e-59
 Identities = 286/386 (74%), Gaps = 43/386 (11%)
 Strand=Plus/Plus

Query  1418   AGGTTCTTGTAATGCAGACGGTGGCTGCAAGAAGTCTCATGTTTA-GAGGT-AGGTGGGG  1475
              |||||||| ||||||||||||||    |||||| | || | ||   ||||| || || |
```

```
Sbjct  1363  AGGTTCTTATAATGCAGACGGTG----CAAGAATT-TCGTTTTCCTGAGGTTAGATGAGA  1417

Query  1476  CAAGGTGAAATGCAGTAAA------GTGAGAGGTACAACTAATTACTGGGATTTCCGAGG  1529
             |||||||||||||| ||||      |  ||||||||| | ||||||||||||||| ||||
Sbjct  1418  CAAGGTGAAATGCACTAAATACCGCGGAAGAGGTACAGCCAATTACTGGGATTTCAGAGG  1477

Query  1530  GAAGCAAGGTTGCCATT-GGAGGATCAGGGAGGGACGGGTTCCTGAAGTATTAATAGGTG  1588
              | ||| | || ||||| |||| |||||| | |     |||||||| |||      ||||
Sbjct  1478  AAGGCAGGTTTTCCATTTGGAGCATCAGGAAAGA----GTTCCTGATGTA------GGTG  1527

Query  1589  GCATTTTGAGAAGGTTACCGAGGGATGGGTAGGACTTTGTTAGGTAGAGGTGGAGGTGGA  1648
             |||||||||||||||||  || ||| ||| |||||||||||||| | |||||| |||||
Sbjct  1528  GCATTTTGAGAAGGTTAGTGAAGGACGGGAAGGACTTTGTTAGGCAAAGGTGGGGGTGGG  1587

Query  1649  GAAAGGACGTTTCAAGGGTGAAGCTTCTTCAGTAAACAGTAAGTATTTACAGTTTGGCAA  1708
             |||||| | ||||||| |       | || || |||||| ||||||||||| ||||||
Sbjct  1588  GAAAGGGCATTTCAAGAG-------TTTTTAGAAAACAGCAAGTATTTACAATTTGGCTG  1640

Query  1709  AAGTGGATGGTATAGGTAGGGGAGTGGTGGGAATTCAGGCTAGAAAAGTTAGATTGCAGA  1768
              ||||||| |||||||||| | |   |||| |||||          || ||| |||   |
Sbjct  1641  TAGTGGATAGTATAGGTAGTGAA---GTGGCAATTC---------CAGGTAGGTTGTGAA  1688

Query  1769  AGGGCTTGCTTGCCAGGTGGAGGGGC  1794
             || ||||||||||||||||||| |||
Sbjct  1689  AGAGCTTGCTTGCCAGGTGGAGAGGC  1714
```

 Score = 100 bits (110), Expect = 5e-18
 Identities = 81/97 (83%), Gaps = 2/97 (2%)
 Strand=Plus/Plus

```
Query  1331  TTTTGATTGCACACTGTGCAACTTTCTTGATAAACTC--GGAGCCTACTGTTAGCGATGC  1388
             ||| ||||||||| | |||||||||| || |||||||  |||| | | |||||||||
Sbjct  1138  TTTCGATTGCACATTATGCAACTTTCATGGTAAACTCCCGGAGGCCAGTGTTAGCGACCT  1197

Query  1389  ACCGTGCTAGATGCTAAGAAACATTAAAAAGGTTCTT  1425
              | |||||||||||||||||| |||||||||||||||
Sbjct  1198  GCTGTGCTAGATGCTAAGAAATATTAAAAAGGTTCTT  1234
```

>gb|AAEX02025623.1| Canis familiaris (LAURASIATHERIA) cont2.025622, whole genome shotgun sequence
Length=145932

 Score = 1454 bits (1612), Expect = 0.0
 Identities = 1505/1958 (76%), Gaps = 166/1958 (8%)
 Strand=Plus/Minus

```
Query  1       TGAGTCCCGGGTGCCGCCGCGGCCGCGGGGCCTAGTGCGCGCACAGCGGCCTGGTCTCGG  60
               ||||| ||||| ||||||||||||| ||||||||| |||||| ||||||||||||| |||
Sbjct  141612  TGAGTTCCGGGCGCCGCCGCGGCCGTGGGGCCTAGCGCGCGCGCAGCGGCCTGGTCCCGG  141553

Query  61      CCTGGTcggcggcccgcaaggcgccctcccgcgctaggccgggcggcgtggcgcgcggcg  120
               ||||||||||||||||| ||||||||| |||||||||||||||||| | |||||| ||||
Sbjct  141552  CCTGGTCGGCGGCCCGCGAGGCGCCCTTCCGCGCTAGGCCGGGCGGTGCGGCGCGTGGCG  141493

Query  121     ccgAGCAGGCCCCGAGGAGGCCGCAGTTAGGCCCGGGGAGGAGCCCGGCTGCCCCGAGCG  180
               ||||||||||||||||||||||||||||||||||||||||||||||||||||||||||||
Sbjct  141492  CCGAGCAGGCCCCGAGGAGGCCGCAGTTAGGCCCGGGGAGGAGCCCGGCTGCCCCGAGCG  141433

Query  181     GCGGCGGAGGCGCGCTCCGTAAGCGGGCGGGGGTTGGGGGAGGGTCGCCCGGTTGTGCCG  240
                ||||||||||||||||||| | |||||||||||||||||||||||||||||| |  | |
Sbjct  141432  CCGGCGGAGGCGCGCTCCGTGAACGGGCGGGGGTTGGGGGAGGGTCGCCCGGTGGCCCGG  141373

Query  241     GAGGCGGTCGAGGGGCCCCGGGTCGGCTCCGCGGCCGACCTGGGGCACCGCGCCGGGGTG  300
               |||||||  |||||||  ||||||||| ||||||||  || |||||||||||| |||| |
Sbjct  141372  GAGGCGGCGGAGGGGCGTCGGGTCGGCGCCGCGGCCTGCCCGGGGCACCGCGCGGGGGCG  141313

Query  301     AGGCCTGGCGAGGAGGCGAAGGCTGCAGGCGTGAGGTGAAGGCCGCAGGCCGGCCGGGCC  360
               ||||||||| || |||||||||||||||||||||||||||||||||||||||||||||||
Sbjct  141312  AGGCCTGGCAAGAAGGCGAAGGCTGCAGGCGTGAGGTGAAGGCCGCAGGCCGGCCGGGCC  141253

Query  361     GATTTTCGCTATGTAAATATCGGTGAgggggggggaggacggggggACAAGATGGCGGCG  420
               ||||||||||||||||||||||| ||  ||||||||||||||||||||||||||||||||
Sbjct  141252  GATTTTCGCTATGTAAATATCGGCGA--GGGGGGGAGGACGGGGGGACAAGATGGCGGCG  141195

Query  421     GCTCGGCGCCTGCTGCAGGGGACGATAGAGGGGGTTGCCGGGAGGGGGAGCCGCCATCTT  480
               |||||||||||||||||||| ||||| |||||||||| ||||||||||||||||||||||
```

```
Sbjct  141194  GCTCGGCGCCTGCTGCAGGGAACGATTGAGGGGGTTGTCGGGAGGGGGAGCCGCCATCTT  141135

Query  481     GGAGGCGGTGTCTGGAGAGAAAATTCCGCTACAGCCCGTGAgggggggtgggagagcggg  540
               | |||||| |||| ||||||||||||||||||||||||||||||||||| ||||||||||
Sbjct  141134  GAAGGCGGCGTCTGAAGAGAAAATTCCGCTACAGCCCGTGAGGGGGGGTCGGAGAGCGGG  141075

Query  541     cggcggcggcagcggcgccggtgacgggccccggaggcccggcgcggcggcgtgtgcgcg  600
               ||||||||   ||||| ||||||||||||||| |||||||||| |||| || ||||||||
Sbjct  141074  CGGCGGCG---GCGGCTCCGGTGACGGGCCCCCGAGGCCCGGCACGGCAGCCTGTGCGCG  141018

Query  601     cgga-ggggcgTGCTCGCTCCCCGTGGCGGCCATTGCCCTTGCCGCCATGATGAGCGCTC  659
               |||| |||||||||| |||||||   |||||||||||||||||||||||||||| ||||||
Sbjct  141017  CGGAGGGGGCGTGCTTGCTCCCCACAGCGGCCATTGCCCTTGCCGCCATGATGGGCGCTC  140958

Query  660     GGGCTCCAGGCGCTCGGCGGCAGCGCCACCTTCCTGCCTTGCCTCCCGCAGCCCCGTGAC  719
                ||||| ||||||||||||||||||||||||| ||||| ||||||||||||||||||||
Sbjct  140957  AGGCTCTAGGCGCTCGGCGGCAGCGCCACCTTGCTGCCCTGCCTCCCGCAGCCCCGTGAC  140898

Query  720     TGGCTGCAGTTTCCGCTGCGTTTCAGCTGAGCTGCCGCAGGCGGCCACCGCCGCCGCCCG  779
               ||||||| | ||||||||| ||| | |||||||||||||||||||| |
Sbjct  140897  TGGCTGCTGCTTCCGCTGCATTTTAACTGAGCTGCCGCAGGCGGCCATC-----------  140849

Query  780     GACGCCGGGACCGTTTCACCCTCAGCGCCCCTGGCCCTGCGCC-TTCCCCCGCGCCTGTA  838
                           |||    ||||  ||| |||||||||||||||| ||| ||||| |||| |
Sbjct  140848  ----------TCGT----CCCTGGGCGTCCCTGGCCCTGCGCCTTTCTCCCGCTCCTGGA  140803

Query  839     GCCACCCGAGGGCAGTCGGGGCAGGTGGCATTCCGGACACCTGGGCTTACCAGGGCATAC  898
               || |||||||||  |||||| | |||||||  || | || |||||||  | | |||| |
Sbjct  140802  GCAACCCGAGGGATGTCGGGACGGGTGGCAGCCCAGGCATCTGGGCTCGCTACGGCAAAT  140743

Query  899     GGGACCCCAGGAAATGTTATTTTTGCTTAAGTCAAATCATATGTGGCTGCTAAGGTGCTT  958
               || |||||| || || |||||||| ||     ||  || || ||||||  || |||||
Sbjct  140742  GGAACCCCACGACATTTTATTTTTACT----CCACGTCGTACGTGGCTATTATGGTGCCC  140687

Query  959     TCGATGCAGTTGTTTTTCCAACAATCTTTGCCGCCCCGAGTATCTGAATAAAGGAAGAAA  1018
               |||||||    | |||| || || ||| |||   | | |||| | |||||||| || |||
Sbjct  140686  TCGATGC----GGTTTTGCAGCACTCTCTGCTTTCTCTAGTACCCGAATAAAGAAATAAA  140631

Query  1019    AATAAAGATTGATGGGAA-AGTTTTTGCCATTCTTTTGTCCTA--TAGGGaaaa----aa  1071
               ||||||  | ||| |||| |||| |   ||  ||||| ||| |   |||| |||    ||
Sbjct  140630  AATAAAATTCGATAGGAACAGTTCTCATCACGCTTTTATCCAACGGAGGGGAAAGTGTAA  140571

Query  1072    aTTGCACAACACGAGAGCGGAGGAATAAAATTTT----GAATCATTCGTACTCATTTATG  1127
                ||||||||||||| | | ||| | |||| ||||    || || || | |||||||||
Sbjct  140570  TTTGCACAACACGAAAACAGAGCAGTAAATTTTTTAAGGAGTCTTTTGAACTCATTTATT  140511

Query  1128    TGGAAACAACTTTCTTGAAAA--ACGATTTAAG----TAGTGATCTGTTGGTGGTA----  1177
               || || ||||||||| |||||   | ||||||||    ||||||||||||||  ||
Sbjct  140510  TGAAAGCAACTTTCTAGAAAAGGTCCATTTAAGCTATTAGTGATCTGTTGGTATTAACTT  140451

Query  1178    ---tttttttAAATCGAGGAATA---AGGGCAGACTTTTTTATTTTCTTATTGGAGGACA  1231
                  |||||||   | ||||| ||   ||| ||||    ||||||| ||||
Sbjct  140450  TTTTTTTTTTTTTTTGAGGAGTAAGGAGGTCAGA-AAGTTTATTTCCTTA----------  140402

Query  1232    ACTTAGTGTGGTGCA-TATTTATTGTGCATTTGCCTGTCATGG--TATTTCGATTTTTCA  1288
                      || ||||| ||||||||   |||| ||| || ||||||  | ||| | || || |
Sbjct  140401  -------GTCGTGCAGTATTTATT---CATCTGCTTGCCATGGTTTTTTTAGTTTCTTTA  140352

Query  1289    TTTG---TG--AAGTTTTAAGGCGGAGTTTCATCCCCTAACGTACTTTTTTGATTGCACA  1343
               ||||   ||  || || | ||| ||  || | ||||  |  | ||  ||||||||||||||
Sbjct  140351  TTTGACTTGAAAAATTCTTAGGTGGCATTCCGTCCCTGAGAGGAC--TTTTGATTGCACA  140294

Query  1344    CTGTGCAACTTTCTTGATAAACTCGGAGCCTACTGTTAGCGATGCACCGTGCTAGATGCT  1403
               |||   ||||  | || |||||| ||| || | ||||||||||||||| ||||| | || |
Sbjct  140293  CTGCAGAACTCCCCTG-TAAACTTGGAACCCAGTGTTAGCGATGCACTGTGCTCGCTGAT  140235

Query  1404    -AAGAAACATTAAAAAGGTTCTTGTAATGCAGACGGTGGCTGCAAGAAGTCTCATGTTTA  1462
                |||||| |||| |||||||||||  ||||||||||||||||||||||||    |||| |
Sbjct  140234  AAAGAAAGATTAGAAAGGTTCTTGGGATGCAGACGGTGGCTGCAAGAAGTTCGTTGTTCA  140175

Query  1463    GAGGTAGGTGGGGCAAGGTGAAATGCAGTAAAGTG-------------AGAGGTACAACT  1509
               ||||  |  | | |  |||||| |||| | |||||             | ||||||| |
Sbjct  140174  GAGGCGGAGGAGTCCTGGTGAATTGCACTCAAGTGCTGCTGGGGGGAAAAAGGTACAGCC  140115

Query  1510    AATTACTGGGATTTCCGAGGGAAGCAAGGTTGCCATTGGAGGATCAGGGAGGGACGGGTT  1569
               |||||| ||| || | ||||  | |||  ||| |||||   ||| | | |||| | || ||
Sbjct  140114  AATTACGGGGGTTCCAGAGGAAGGCAGAGTTTCCATT--TGGAGC-GTGAGGAAAGGTTT  140058
```

```
Query  1570    CCTGAAGTATTAATAGGTGGCATTTTGAGAAGGTTACCGAGGGATGGGTA---GGACTTT  1626
               |||||||      |||||| ||||||    |||||||  | ||||||| |   || ||||
Sbjct  140057  CCTGAAG------TAGGTGACATTTT--TGAGGTTACTTAAGGATGGGGAGGGGGGCTTT  140006

Query  1627    GTT-AGGTAGAGGT-GGAGGTGGAGAAAGGACGTTTCAAGGGTGAAGCTTCTTC----AG  1680
               ||| | | | | || |  ||||| |||||| | |||||||||||||| ||||||    ||
Sbjct  140005  GTTAAAGCATAAGTAGCCGGTGGGGAAAGGGCATTTCAAGGGTGAAGTTTCTTCACAAAG  139946

Query  1681    T---------------------AAACAGTAAGTATTTACAGTTTGGCAAAAGTGGATGGT  1719
               |                     || |||  |||||||  |||||||||  ||| ||||||
Sbjct  139945  TAGCAAGTATTTGAGAAAAACAAACCAGCCAGTATTTGTAGTTTGGCAGGAGTCGATGGT  139886

Query  1720    ATAGGTAGGGGAGTGGTGGGAATTCAGGCTAGAAAAGTTAGATTGCAGAAGGGCTTGCTT  1779
                 ||||||||  ||| || || |||  |||||| |||      |||  ||||| ||||||
Sbjct  139885  GAAGGTAGGGAGGTGATGAGATTTCTTGCTAGAGAAG-----GTGC--AAGGGTTTGCTT  139833

Query  1780    GCCAGGTGGAGGGGCTTATACTCAATGGGGTAGGCAGCGGGGGTGCCCTCAACTAGAAGA  1839
               ||||||||||||||||||||  |  ||| |  |||  | |||  |||||    ||||||
Sbjct  139832  GCCAGGTGGAGGGGCTTATA--CGTTGGAGGTGGC--CAGGGCAGCCCTTGCGTAGAAGG  139777

Query  1840    GTGATGGCACACCTGAGCTGCACCTTGTTATTTACTTA  1877
                ||||   ||||||||||||   |||  |||| |||||
Sbjct  139776  ATGATAATACACCTGAGCTG-GGCTTTATATTCACTTA  139740
```

>gb|AAGW02040882.1| Oryctolagus cuniculus (EUARCHONTOGLIRES) cont2.40881, whole genome shotgun sequence
Length=2750

```
 Score = 1041 bits (1154),  Expect = 0.0
 Identities = 1284/1717 (74%), Gaps = 121/1717 (7%)
 Strand=Plus/Minus

Query  175   CGAGCGGCGGCGGAGGCGCGCTCCGTAAGCGGGCGGGGGTTGGGGGAGGGTCGCCCGGTT  234
             ||| | |||||| ||||| | ||  ||| ||||||| ||||| ||||||||||| | |
Sbjct  2675  CGATCTGCGGCGTAGGCGTGTTCA-TAAACGGGCGGTGGTTGCGGGAGGGTCGCACAGCG  2617

Query  235   GTGCCGGAGGCGG-TCGAGGGGCCCCGGGTC-GGCTCCGCGGCC-GACCTGGGGCACCGC  291
             |  |  ||||||| | |||||||||||| || |||  ||| ||| | || | ||||| |
Sbjct  2616  GGTCTTGAGGCGGGTTGAGGGGCCCCGGATCAGGCGTCGCAGCCTGTCCCGAGGCACTGA  2557

Query  292   GCCGGGGTGAGGCC-TGGCGAGGAGGCGAAG-GCTGCAGGCGTGAGGTGAAGGCCGCAGG  349
             |  |||||||| || |||||||||||||||| ||||||||||||||||||||||||||||
Sbjct  2556  GAGGGGGTGAGACCCTGGCGAGGAGGCGAAGAGCTGCAGGCGTGAGGTGAAGGCCGCAGG  2497

Query  350   CCGGCCGGGCCGATTTTCGCTATGTAAATATCGGTGAgggggggggagggacgggggACA  409
             ||||||||||||||||||||||||||||||||||  ||||||||||||||||||||||||
Sbjct  2496  CCGGCCGGGCCGATTTTCGCTATGTAAATATCGG--AGGGGGGGGGAGGGACGGGGGACA  2439

Query  410   AGATGGCGGCGGCTCGGCGCCTGCTGCAGGGGACGATAGAGGGGGTTGCCGGGAGGGGGA  469
             |||||||||||||||||||| |||||||||||||||  ||||||||||||||||||||||
Sbjct  2438  AGATGGCGGCGGCTCGGCGCTTGCTGCAGGGGACGACTGAGGGGGTTGCCGGGAGGGGGA  2379

Query  470   GCCGCCATCTTGGAGGCGGTGTCTGGAGAGAAAATTCCGCTACAGCCCGTGAgggggggt  529
             |||||||||||| |||||| ||||||||||||||||||||||||||||||||||||||||
Sbjct  2378  GCCGCCATCTTGAAGGCGGCGTCTGGAGAGAAAATTCCGCTACAGCCCGTGAGGGGGGGT  2319

Query  530   gggagagcgggcggcggcggcagcggcgccggtgacgggccccggaggcccggcgcggcg  589
             |||||||||||| || ||||| |||||||||||||||||| |||||||||| |||| |||
Sbjct  2318  GGGAGAGCGGGCTGCAGCGGCGGCGGCGCCGGTGACGGGCACCGGAGGCCCTGCGCAGCG  2259

Query  590   gcgtgtgcgcgcggaggggcgTGCTCGCTCCCCGTGGCGGCCATTGCCCTTGCCGCCATG  649
             |||||||||||||||||||||||||||||||||  |||||||||||||   |||||||||
Sbjct  2258  GCGTGTGCGCGCGGAGGGGCGTGCTCGCTCCCCACGGCGGCCATTGCCTCGGCCGCCATG  2199

Query  650   ATGAGCGCTCGGGCTCCAGGCGCTCGGCGGCAGCGCCACCTTCCTGCCTTGCCTCCCGCA  709
             |||||| ||||||||| | |||||||||||||||||||||||||||||||||||||||||
Sbjct  2198  ATGAGCTCTCGGGCTCTAAGCGCTCGGCGGCAGCGCCACCTTCCTGCCTTGCCTCCCGCA  2139

Query  710   GCCCCGTGACTGGCTGCAGTTTCCGCTGCGTTTCAGCTGAGCTGCCGCAGGCGGCCACCG  769
             ||||||||||||||||| | ||||| ||||||| ||||||||||||||||||||||||||
Sbjct  2138  GCCCCGTGACTGGCTGCTGCTTCCGTTGCGTTTTAGCTGAGCTGCCGCAGGCGGCCACCG  2079

Query  770   CCGCCGCCCGGACGCCGGGACCGTTTC-ACCCTCAGCGCCCCTGGCCCTGCGC-CTTCCC  827
             ||| ||||||| |||| |  || | ||  ||||||||||||| || || ||||  ||  |
Sbjct  2078  CCGGCGCCCGGGCGCCAGAGCCCTCTCGCCCCTCAGCGCCCCGGGGCCCGCGCTTTTTTC  2019
```

```
Query  828   CCGCGCCTGTAGCCACCCGAGGGCAGTC-GGGGCAGGTGGCATTCCGGACACCTGGGCTT  886
               |||| ||  ||  |||||||||  || ||||| |||||||   || ||||| | ||||
Sbjct  2018  TTGCGCTTGCCGCGCCCCGAGGGCCATCGGGGGCCGGTGGCAGCCCAGACACTTAGGCTC  1959

Query  887   ACCAGGGCATACGGGACCCCAGGAAATGTTATTTTTGCTTAAGTCAAATCA--TATGTGG  944
              ||  |||  | |||||||  |||| | ||| ||||| |     | ||||||  || ||||
Sbjct  1958  CCCGAGGCGGAAGGGACCCTGGGAATTTTTAATTTTGGT----TTAAATCAGTTAAGTGG  1903

Query  945   CTGCTAAGGTGCTTTCGATGCAGTTGTTTTTCCAACAATCTTTGCCGCCCCGAGTATCTG  1004
             ||   | | |||  ||  ||| |||| ||| ||   | ||| |||||   | ||||| || |
Sbjct  1902  CTTTCAGGATGCCCTCTGTGCGGTTG-TTTGCCTGCCATCCTTGCCTTCTCGAGTGTCGG  1844

Query  1005  AATAAAGGAAGAAAAATAAAGATTGATGGGAAAGTTTTTGCCATTCTTTTGTCCTATAGG  1064
             |||          ||||||  | | ||||||||||     ||| |||  ||||| |||||
Sbjct  1843  AAT----------AAATAAAAGTGGGTGGGAAAGTTCCCACCACTCTCCTGTCCAATAGG  1794

Query  1065  G---aaaaaaaTTGCACAACACGAGAGCGGAGGAATAAAATT----TTGAATCATTCGTA  1117
             |   ||  |  |||||||  |||| |||||| || |||  ||      || ||||| |||
Sbjct  1793  GGGAAAGTATGTTGCACAGAACGAAAGCGGAAGAGTAAGGTTCGGAGGGAGTCATTTGTA  1734

Query  1118  CTCATTTATGTGGAAACAACTTTCTTGAAAAACGATTTAAGTAGTGA-TCTGTTGGTGGT  1176
              ||| ||  |||||||||||||||||||| |     |  ||  |||  ||||| |  ||
Sbjct  1733  GTCACTTGGGTGGAAACAACTTTCTTGAAAAGC----TGCGT--TGACGCTGTTAG-CGT  1681

Query  1177  AtttttttAAATCGAGGAATAAG-GGCAGAC-TTTTTTATTTTCTTATTGGAGGACAACT  1234
             |  |   |      ||||||||||  |  |||  |||| ||||||||| | ||||| ||
Sbjct  1680  AGCTGGCT---CTGAGGAATAAGAAGAGGACAGTTTTAATTTTCTTACT-GAGGAAAA--  1627

Query  1235  TAGTGTGGTG-CATATTTATTGTGCATTTGCCTGTCATGGTATTTCGATTTTTCATTTGT  1293
               |  ||||||  |||||||||| ||||| | ||||||||  |||| ||||||  ||| |
Sbjct  1626  -AACGTGGTGAGGTATTTATTGTTCATTTACTTGTCATGGCGTTTC-ATTTTTTTTTTTT  1569

Query  1294  GAAGTTTTAAGGCGGAGTTTCATCCCCTAACGTACTTTTTTGATTGCACACTGTGCAACT  1353
                 ||||     | ||||||  ||  |  ||      |||||| |  || |||  ||||
Sbjct  1568  ----TTTTTTTTTGAAGTTTC--CCGGTGGCG------TTTGATGG--CAGTGTAGAACT  1523

Query  1354  TTCTTGATAAACTCGGAGCCTACTGTTAGCGATGCACCGTGCTAGATGC---TAAGAAAC  1410
             ||  || |  ||||||||||   ||| |||   |||  || || | |||   | ||||
Sbjct  1522  TTGCTGGT-GACTCGGAGCCCGGTGTGAGCCGGGCATTGTACTGGGTGCTGGTCAGAA--  1466

Query  1411  ATTAAAAAGGTTCTTGTAATGCAGACGGTGGCTGCAAGAAG-TCTCATGTTTAGAGGTAG  1469
             || | |  |||||| |  | || |||  |||| || ||  | ||    ||| || | |||
Sbjct  1465  ATGACAGGGGTTCTGGAGACGCGGACCCTGGCCGCCAGGGGCTCCGTGGTTCAGGGTTAG  1406

Query  1470  GTGGGGCAAGGTGAAATGCAGTAAAGTG-AGAGGTACAACTAATTACTGGGATTTCCGAG  1528
              ||  ||||||||  ||| |  |||||  | ||   |   | | ||   |  | ||| |
Sbjct  1405  ATGACTCAAGGTGAGGTGC-GGCAAGTGTCGCGGGGAAGGGACTGAC---GTGTCCCGCG  1350

Query  1529  GGAAGCAAGGTTGCCATTGGAGGATCAGGGAGGGACGGGTTCCTGAAGTATTAATAGGTG  1588
             |     |||| |||  | ||||| ||||||             ||||      | | ||||
Sbjct  1349  G-----AAGGCTGCGTTCGGAGGCTCAGGG-----------TCTGA------ACTTGGTG  1312

Query  1589  GCATTTTGAGAAGGTTACCGAGGGATGGGTAGGACTTTGTTAGGTAGAGGTGGAGGTGGA  1648
             |||||||  ||||||| ||       |||||||||||||||||| ||| || | ||||
Sbjct  1311  GCATTTTCGGAAGGTTGCC-------GGGTAGGACTTTGTTAGGTGGAGTTGAAAGTGGG  1259

Query  1649  GAAAGGACGTTTCAAGGGTGAAGCTTCTTCAGTAAACAGTAAGTATTTACAGTTTGGCAA  1708
             |||||| ||||||||||||||||||||||       | | |||| ||  |||  ||||
Sbjct  1258  GAAAGGGCGTTTCAAGGGTGAAGCTTCTT-------CCGAAAGTGTT--CAGCGTGGCTC  1208

Query  1709  AAGTGGATGGTATAGGTAGGGGAGTGGTGGGAATTCAGGCTAGAAAAGTTAGATTGCAGA  1768
              |||||  | |||||||| |||||||| || |||     ||||||||| | |||||||||
Sbjct  1207  CAGTGGGAGCTATAGGTA-GGGAGTGGTAGGGATTGCACCTAGAAAAGGTGGATTGCAGA  1149

Query  1769  AGGGCTTGCTTGCCAGGTGGAGGGGCTTATACTCAATGGGGTAGGCAGCGGGGGTGCCCT  1828
              | ||| || || |||||||| ||||||||| ||| ||||| |   |||||||| |||||
Sbjct  1148  GGAGCTGGCGTG-CAGGTGGAAGGGCTTATA-TCAGTGGGGGACATAGCGGGGGAGCCCT  1091

Query  1829  CAACTAGAAGAGTGATGGCACACCTGAGCTGCACCTT  1865
             | ||||| ||||||  | |||| |||  |||  ||||
Sbjct  1090  CGACTAGCAGAGTGGGGACACATCTGTCCTGTGCCTT  1054
```

Q was intron 1 from the Homo ZNF436 (KRAB-ZNF) gene, located on the Chr.1 (autosomal)

>gb|AAGU03057289.1| Loxodonta africana cont3.57288, whole genome shotgun sequence

```
Length=158909

 Score =  944 bits (1046),  Expect = 0.0
 Identities = 1018/1330 (76%), Gaps = 56/1330 (4%)
 Strand=Plus/Minus

Query  4      CAGGAGGTAAGTCTGTTCGTCCCCCACTCGGGTTTTCGGCTTCCTACCTTCAACCGGACA  63
              |||||||||||||||||| |  || ||||||||||||||||| | ||||||| |||||||
Sbjct  95677  CAGGAGGTAAGTCTGTTCATTTCCAACTCGGGTTTTCGGCTTTCCACCTTCAGCCGGACA  95618

Query  64     GGGACTGCTAACATCCTGAAGGCCCGCCCGGATCAGGGCCTCAGACGCCCAGGGTCCCTT  123
              || |||| ||| |         ||| || |  |||||  |||||| ||||        ||
Sbjct  95617  GG-ACTGTTAATA---------CCCACCAGTCTCAGGCTCTCAGAGGCCC--------TT  95576

Query  124    CGGTGCGGGAGGCCGCTGGGAGCCCCTGTAGCAGACAAGCCCTCTGGGACGCTAAGGAGG  183
              | |||| || ||||||  | | |   | |||||||||| |||   |||| | |||||| |
Sbjct  95575  CTGTGCTGGGGGCCGCCAGAAACAATTTTAGCAGACAAACCCCAAGGGAGGATAAGGATG  95516

Query  184    GCCCGccccccccGCAGGGGCCTGGCTATCTCCTCCTTACACGCC----CTACAC---CG  236
              ||||  |||  || | |||  |||||| |||||||  | ||  ||    |||| |   ||
Sbjct  95515  GCCCTTCCCTGCC-CTGGGCTCTGGCTCTCTCCTCTCTGCATACCTCTCCTACCCTCCCG  95457

Query  237    CCACCC---GCTTCTGAGTCCCC-CAGGGTTTTCGCCTCCCCGCCGCTGGACGCCACTCA  292
              | || |   ||||||| |||||| |||||||||| |   ||| || |||| | | ||||
Sbjct  95456  CTACTCCCGGCTTCTGGGTCCCCTCAGGGTTTTCACTCTCCCTCCTCTGGGCACGACTCT  95397

Query  293    GCCCGCCGCTCGA-GGGGCGCCCCCTTGAGAAGGACGCAGTCGCACGCCCCTACAGGGTT  351
              ||||| | ||| | ||||||||| | | |||||||||| ||    || ||  ||||||||
Sbjct  95396  GCCCGTCCCTCAAAGGGGCGCCCTCCTCAGAAGGACGCGGTTAGCCGTCCTGACAGGGTT  95337

Query  352    AA-AGCGCCGAAGCCAAGACACCGCCAGGCTCTACTGCCGACCGCACTCCCGAGTCGCCC  410
              || ||||  |  |||  | ||| | |||||   |  |||| | |     |||||||  ||
Sbjct  95336  AACAGCGATGGGGCCGGGGCACTGTCAGGCCTAAACGCCGGCTGT----CCGAGTCAGCC  95281

Query  411    CGGCGCGGGGCGGCGCGGGGCCTCTCGGGAGTTGTATTGCGTACAGAGCCGCGCCG---C  467
               | | |  ||  |     ||||  |||||||||||| |  | ||||    | ||||   |
Sbjct  95280  GGACCCTTGGTAG-----GGCCAGTCGGGAGTTGTAGTATGCACAGCAGGGTGCCGATGC  95226

Query  468    CGCAGGCGCTGTTGGGAGATGTAGTGCGTGGGCGGGGCTCAAAGTTCCAGCGCGGCCTCC  527
              |||||||||||  |||||||||||| ||| ||||||||||||||||||||||| ||  ||
Sbjct  95225  CGCAGGCGCTGCCGGGAGATGTAGT-CGTAGGCGGGGCTCAAAGTTCCAGCGCTGCTGCC  95167

Query  528    GAGTCTCCCCTGGGGAGTGGGCGGCTGCCGCTTCCCTCTCCAGCCTGTGCTGTCAAG-CA  586
              ||||| |||| ||||||||||||||||| |||||||| ||  ||||| | ||||||  ||
Sbjct  95166  GAGTCGCCCCCGGGGAGTGGGCGGCTGCTGCTTCCCTGTCACGCCTGGGTTGTCAAATCA  95107

Query  587    GGGGCTCATTCTAGCTCAGCTCAACACAGGCCAGCGCCTAAATTGGC-CTGACTTCGCAG  645
               || |  || | |||  |||||||| | |||| |||||||||| ||  | ||  |||| |
Sbjct  95106  AGGTCA-ATCCCAGCAGAGCTCAACTCCGGCCGGCGCCTAAATGGGGTCGGATGTCGCGG  95048

Query  646    ATAATAAGGCCCGACTCAAGAGAAGCTTCTAGGGACGTGAGGCGAAGCAGGGCAGCCTCT  705
               |||   || ||||||||| |||||||||||||||||||||| || | |||||||| ||
Sbjct  95047  TTAA---GGTCCGACTCAAAAGAAGCTTCTAGGGACGTGAGGAGAGGGAGGGCAGCTTCC  94991

Query  706    A--GCTTAATCGTGCTGCAG-TCTCCATAAGAAAAAAGCGAGA-ATACTCCGCCCTGACT  761
                 ||| |||||  |||  | ||||||  |||| ||||||| | ||||||||||||| ||
Sbjct  94990  TCGGCTGAATCGAACTGGGGGTCTCCAAGAGAAGAAAGCGAAAGATACTCCGCCCTGGCT  94931

Query  762    GGGAGGACGCGCTTACTTTTTCACCCTTTTGTTCTTTTCCCTCCTTGGCTTTTGGCCTCT  821
               ||||||| | ||   || |||||||||||||||||||||||||  ||||||||||||||
Sbjct  94930  AGGAGGACACACTGCTTTCTTCACCCTTTTGTTCTTTTCCCTCCCCGGCTTTTGGCCTCT  94871

Query  822    GGCTAGCTTTTGTACGCGGGAGTGGAACTCCAACTCCCGGCAGGCCATGGGGGCAGGCAC  881
              | ||  | |||||||||  || ||||||||||| ||||||||||||| ||| |||||| |
Sbjct  94870  GACTGTCCTTTGTACGCCCGAATGGAACTCCAAGTCCCGGCAGGCCACGGGAGCAGGCGC  94811

Query  882    AGGCGCACTAGAGGCTCTTGGCCAGGCTGTGGGAGGCGGGCATAGTGTTCCGCCTCTGAT  941
              |||||   ||   |  || ||||| ||    |||||||||| |||||  | |   || ||
Sbjct  94810  AGGCGTGATACTCGTGCTCGGCCACGCCCCAGGAGGCGGGCCTAGTGGCCGGTAGCTAAT  94751

Query  942    TGGCAGAGTGGTTCACGAACTTCGCTCGCTGATTGGCTGAGGCAGCTGCCGGAAAGGACC  1001
              |||||||  | |    || | ||||||  ||||||||||   ||||||||||||||| ||
Sbjct  94750  TGGCAGAACGTTATGTGAGCATCGCTCTTTGATTGGCTGGTCCAGCTGCCGGAAAGGGCC  94691

Query  1002   CGGCCTCGAGATGGCAGAGCGGAGGCGTTCCCTGGGCTGAGCTGAGCACCTCCTCCCTGA  1061
              |||||||| ||||| ||||| |||||||||  |||| || ||| ||  ||||||||||||
Sbjct  94690  CGGCCTCGGGATGGAAGAGCTGAGGCGTTCTGTGGGTTGGGCTAAGAGCCTCCTCCCTGA  94631
```

```
Query  1062   GACAGCAGCGGTCTAACCCCCGGCGCGGGAGGGACCCAGCCAGGGGGCTGTCCTAGTAGA  1121
              ||||| |||||  | ||||| ||| ||||||||||| ||| |||||||||||||  || |
Sbjct  94630  GACAGAAGCGGCGTGACCCCTGGCCCGGGAGGGACCGAGCTAGGGGGCTGTCCTCCTAAA  94571

Query  1122   TTCCGGCATTGCCTCCACCCCGCTGTGGCGGAGACGCAGAGGGCGGGTCGCGCTCGAGGG  1181
              ||||| || ||||||| |||||| ||||||||||||||||||||||||  ||||| ||||
Sbjct  94570  TTCCGACAGTGCCTCCGCCCCGCGGTGGCGGAGACGCAGAGGGCGGGTTTCGCTCAAGGG  94511

Query  1182   TCAGGGAGGAGAG-TGACCCGGCCCTGGTCCAGGGCCCTCCCTGCCCGCCTTGGGGCCAG  1240
              ||||| ||||||| |||||| |||| ||| |||||||||||| || || |||| ||||||
Sbjct  94510  TCAGGAAGGAGAGTTGACCCAGCCCCGGTGCAGGGCCCTCCCCGCTCGTCTTGCGGCCAG  94451

Query  1241   AACTCCAGCCCTGGGGAGTGGGCAGGGCCTGTTTTCCGTGCCTCACGCCCTGCTTTTCGC  1300
              |||||||||||||||||||||||||||||||||||||||||||||||||||||||||| |
Sbjct  94450  AACTCCAGCCCTGGGGAGTGGGCAGGGCCTGTTTTCCGTGCCTCACGCCCTGCTTTTCCC  94391

Query  1301   CTCCTTCAGC  1310
              ||||||||||
Sbjct  94390  CTCCTTCAGC  94381
```
