# Supplementary material for: Extensive intron gain in the ancestor of placental mammals
Source: Biol Direct. 2011 Nov 23;6:59. doi: 10.1186/1745-6150-6-59 (PMC3257199; doi:10.1186/1745-6150-6-59)
Supplement: Additional file 8 — Lineage-specific accumulation of TEs in orthologous introns in four placental superorders. [file 1745-6150-6-59-S8.PDF]

Additional file 8 - Lineage-specific accumulation of TEs in orthologous introns in four placental superorders.

Lineage-specific accumulation of TEs in orthologous introns of Gin-1 (a typical example of domesticated genes) is shown in four placental superorders Afrotheria, Xenarthra, Laurasiatheria and Euarchontoglires. Marsupial Gin-1 gene was also analysed. Exons are in red. TE contents and amounts were produced by RepeatMasker.

Afrotheria

>Loxodonta Gin-1 (supercontig:loxAfr3:scaffold_1:15860097:15880892:-1)
GCTCATCTGTAGTTAAGAAGAGTATATGAAATCTGGCAGAGCTAGACGTCTTTTCTGTTA
TTTGCCATTTGAACATTTCCAAAGTATATATTTCTCATATATGATTTGAATAGTTGTTAT
TATTTTATTTTTTTTGTTTTGTTTTTGCATTTTGCAGTAACCATGAATATGGTTGGTAGT
GATTTAAAATTATATTTTCTTGCCTTATCATTTATTGTCATTTATGTAAGTGTCACATAA
CAGGACCATCCTATGTAAGTGACATTGCAGTTGATGGCAATAGGAGAGACTTTAACAAGA
TTTTTCAAAGTGTACTACTTGTTCATTTCCTTTTTTTTCTCCCTGATATGTGGAAAATTTT
CAGCATTTCTTTTCACATGGAAAGAGACAAGATAATTTGTCATGCTGTATCTCAAAAATA
GCATTTGAATATTTTAGCTCATATGCATCACTCTATTCCTTTTCTGTACTGCAATTAACC
AACCCCCTCAGATAATATTCTGGGAAAGCTAATGTCATATGTAAGTGGAAGCCTGAACTG
AAATAGCAATGAATTAGCTAATTCAAATTCTATTTTTAAATACCGTTACCTAGGTTAACA
ATGGTCCGTAGTGGAAAAAAATGGTGACCTTCATCTTAAGCAGATTGCATATTATAAACGA
ACTGGTGAATATCATCCAACTACACTGCCAAGTGAGAGAAGTGGCATAAGAAGAGCAGCA
AAAAAATTTGTCTTTAAAGGTAAAATTAATGTGCACAACCTTGGGTACAGAATTAATATA
GATGTGTCTCCAAATGTGTTCTTTGTGAAATCCTGAGAATCTGTATTGTTGGATTGGTTA
TTGCTTAACATTTAATGGTAAAATTGATGATGCCACTCAGTCAAAAGCAAAATAAAAATT
ACACTGAAGCTAAGTGAAGGCCAGAAGTATGGAAAGGGCAGTTTAAATTACAAAAACTGG
TACCATGAAAGGAAACAAAACAGCTAAGTTAACTTCTCATGCTTTTACAACCTGACGTGG
TTCCTACTCTAATGGTGCAGTTTCATGCACTGAGACTGCTATTAATGAGAAACGGATTTG
GTGGTTAATATGAAAATGTTGAAAAGTTAACTAATTTGTTTACCGAAGTTAAAATAGAAG
GAATGATTATATTTATGTGTATGATCTGTCTTCCCACTGCCATTAAATATTCTTAGAATT
GTATTTATTTTTGGACCCTAAGTCCCCCTGAATGGAATGTTTGAAAGGTGGTAGAATGCG
TATCTTGAAAGGTTATGATAATAGTTTATCCTTCTTATCCCTCCTTCAAAGGAGAAGAAA
AGTTTGGGGTGAGATGAGCAGCAAGTACAATCATAGATAACTAAAGGAATGTTGGTGCTA
TTATTGGGAGTTTTGCCTGTAGGTTCAGAATGAAGATTTCTCCAAAGGAGGTCAAGAAAA
TTGTAAGATGAGAAATGCCTATTCATTTCTCCCTGTGTACACTTGTCCCTATAACTTCCT
TTTGCCAAAAAATTAGCATTTATTACCTCTTAGACTAAAGTGAGGAGTCATTCATTCATT
TATTTACTCATTTATGCAACATATGTTTACCGAGTCCCTACTATGTGCCAATCACATTCT
AGGCCTCAGGGATGCGTTGATGAGTAAAATAGGTAAGATTGAAGTAAACACTATTCCACT
ACACATGTGTTTAGGCACTCTAGTAGCTTGGTAGTATATTCTGAAGTTTGAATAGCTGCC
CCATCTACATTGGAGGGGTGGTGGTTCTTTCCATTTCCTAGTTGCCTCCCAGCTATAGAT
TCTTCTCGGTGTCCTCCCAAAAAGGAAAGCAAAAAATTATCGTGAAGATTAAGGCTCTCC
CACTTTCCCACTCTTCCTTCCACACCTCCATCTCAGATCCAGAAGAACCTGAGAAAGATG
GTTGCTTTAAAAAAATGTAACTATGATGAAACTATTTGGGAAAATTCTGTTCCAAGGTGT
TGCTTATCTCTACGTAATTCATTTACTAATTCATACCATTTTTAGGAGGTACCTTCAAAT
ATTACTTCTAATTCAAGTGCTCAACAAAGGTGATTTCATACTCCCATAGTTATTTCAGGG
GCCCTGGTGGTGCAGTGGTTAAGAGCTACGGCATGCTATGGCTGCTAACCAAAAGATCGG
TAGTTCAAATCCACCAGCCATCCGTTGGAAACCCTATGGAGCAGTTCTGCTCTGTCCTGT
GGGGTCACTATAAGTCAGCATCAACTCGACGGCAAAAGGTTTTAATGAATGTAGTTATTG
CATGCTTTGGTGAACAACCCAGCTCTACTTGTATTCATAACTAGTAAAATTATAGCAGCT
AGATATCTAGTACTTATAACAGCTGAGAACCTTTCAGATTGTGAAAGGTCATTACAAATA
GATATTCTGTCTTAGGGGAAGAAAAGCTTTATTAAAGAACAAATGACATGGTTAGAAGAT
AAGGGGATGATTATCTTAAAACTTTATCGATAGCTAAAAATTCCAATTCTATCTTTTTGG
TATCATTGTTCCAG**AAAAAAAGCTATTTTATGTTGGAAAAGACAGAAAACAAAATCGTTT**
GGTAATTGTTTCAGAAGAGGAAAAAAAGAAAGTCCTGAGAGAATGCCATGAAAATGACAC
TGGAAGCCATCATGGCATATCCAGAACTCTCACTCTAGTGGAATCCAGCTACTATTGGAC
TTCTGTGACCAATGATGTCAAACAGTGGGTATGGCTTATGTATTTAGAATATTTTGAATA
ATGTCTTTGTAATAGATATCCAAAACTCATTTTTGCTACAGATTACTCAGGCTATAACTT
CTCATTAAGTACTTTTTGTATGGGTATTTTTTATACAAAAACCTTGAGAGGTAGTTTTAT
TTCACTTCACAATTTTTCATTAATATCTCTAATGCATAATTTGAACTAGAAAGAAGTATT
ATTTAAATATTTTAAAGTACCAGATTTAGATAGGAAATTATTTAGAACTAGCTTTTCATT
GTTGAACTCTTTTGGTGTAGGTAGAATTTGAATGAAGTTCAAATTCATTTGACTAAATCC
TTATGTCTTTGATTGCAGTCCAAGTCAGGCCTATTGTGTGCTCTCATCTTTGCGTCAACT
CTATCTTGTGGTAGAAAATGTCAGATATGTAGAAATGCAAAACTAACTAAATAAAAATCA
AGTTGTAATGACAAAGGAATTAGCCTGATTTTTGTAACTTTATAATCTATTTGAGCCTTC
AATCTCTCCTAATAGAAAATTCACCTAGGGTTTTGCAGGTCAGCTGATCCCCACTCTTTC
TGGTGTACTTTCTTTGCCAAATCGTAGACATCCCTTGGTATGTACATGTAATAGGGCATC
ACAAAGACTGATATTTTAGCCGTAAAGGATTAAACCAACATTTAACCAAGTATCATTTGT
GAGTTATCCATTTGTGAGTTATTCCAAATACCTGTATATTTGAATTTGCCTTCAGAACCC
GCCACACATTCAGTGAAAATTTTACCAGATGTTTATCATTTGAAAATGGATATGATTTTT
GGAAATAATCAGAAGTTATTTGTGTTGAAAACTAGTGAATGATTTTGAAAGTAAAAATTT
TGATAAAAAGCAACTGTGGCTGTAATGTAATAAGATGGTTTTAAAAATTTTATACAGAAG
TTGGTTTGAGAGTAGTTTCCAAAGATTCCAGAAATATATTGAGCAGTGGCACACCATCAA
TAAGTTGGTCAATGGGTGACTTCTCCCCTTACTCAAATGAAAGCTCCATGACTGTTTTTG
TTCACTCTTTTTTCCCAGGACCTAGAGCAATTTCTGGCATCTATAAATATTGTTGGAATA
AAATTATATTTTAATAAATATTATTGGAATAGATGGATGAGTAAACTTCTTCGGGTGATA
ACTTGGAAAGAAAAAATCTCTTACATATAGTAATTTTTGTATTTTTACATACCAATTCAA
AAATTATAGCAAACTTATTTCAAGATCTATTTATTTAATATGTATTGAACGTGAACCAGA
ATTATATTCCATGTGAATAAGACCCTTAAAGTGGTTATTTAATAGTAGTAGCAACTAGTA
TTTGCAGCCTAACTATAATGATCTATTTTCAGTCCAGAAGCTCTGGTTCTAAACAGAGTA
CCTGTGAGCCATTTTAAAATTCAGATTTATAATATTGCTTTATTATGTTGACTATATTAA
CATTTATGTATTTTATTTTTATTATGTGCCTATTTTGCAG**GTGTATGCTTGTCAGCATTG**
CCAAGTGGCAAAAAATACAGTTACTCTAGCACCTAAACAGCACCTTCTCAAGGTGGAAAA
TCCATGGAGTATAGTTACTGTTGATCTGATGGGACCTTTTCACACAAGCAACAGAAGTCA
TGTATATGCCATAATCATGACAGATTTGTTCACAAAATGGGTTGTGATTTTGCCTCTATG
TGATGTTTCAGCATCTGAAATTTCTAAAGCTATTATCAATATATTTTTCTTATATGGACC
TCCTCAGAAAATAATAATGGACCAAAGAGATGAGTTCATTAATCAGGTAAGAAAGCTGTT

CAGATCTTACTTATCTTTCTTTGCCCATCGCCAAGTGCCCTACACGTTATAGGATGCTTA
ATGAGTACAGATGTATTGCATTGGGTAAATCTGCTGCAAAAGACCTCTTTCAAGTGTTAA
AAAGCAAAGATGTCACCTTGAGGACTAAGGTGCACCTGACTCAACTCATGGTATTTCAGT
CACCTCATAGGCATGTGAAAACTGGAGAATGAATAAGGAGGACCAAAGAAGAGTTGATGC
CTTTGAATTATGGTGTTGGTGAAAAATATTGAATATGCCATAGACTGCCAGAAGAATGAA
CAAATCTGTCTTGGAAAAAGTACAGCCAGAATGCTCCTTAGAAGCGAGGATGGCGAGACT
TTGTCTCGCTTACTTTGGACATATTATAGAGGAACCAGTCCCTGGAGAAGGACATCATGC
TTGGTAAAGCAGAGGGTCAGCAAAAAAGAGGAAGGCCCGTAAGAGACGGATTGACACAGT
GGCTGCAACAGTGGGCTCAAACATAGAAACAGTTGTGAGGATGGCGCAGGACTGAGCAGT
GATTCTGTTGTGCATGGGGTTGCTGTGAGTCAGAACCAACTCAATGGCACCTGACAACAA
CAACTATAGTTAAATTGCTACAGATATAGAGGTATAACATATATTACAAAGGTGGTTGGA
CTAAAAATTAGGAAACCAGAATATTAGTACTTATTGTGCCACTAAAAAATTGTAAAATTA
TCTTCAATTATCCTCAAATTAAAATGAGAGATTGGGCCAAATTTCTGTTTTTTATGTACT
TCTAGTTTAAAAATTCTATGATTGACATATAAAGAGGGACTTGACTAAATGGAGAAATAC
ATTATGTTGATGAATGAGAATACTTTATTGAAAGATGCTGATTCATCCCAAATCAAAATT
AATTTATATTATTAATGCATTTTAATTCAGCATTAAAAAGGAGGAAGCTTTTTCTTTGCT
CAAGTCTTTTTCCAGATTACCCTGAAAGAACAAGCAGGGATGGATGTATTTACAAATTCT
GAAAAAGAAATGTAAAGGTTACTAGACTTAAGGTGTATTTAAAATTTTGTATAATTAATA
AAGCACTGGTAATAATCAGAGATAGCCAGACTATTGGTTCACAGCACACTTAGTATCTCA
TTTCAAAAGAAATATCTGAAGTTTATTAAGTAGTTAGGTCCTGTGTCTCTGGTCCCCAGT
ACCACCCCAGGTTCAATGATTCACTAGGAGTACTTGGCATATTGTCATACTCATGGCTAA
AATGTATTATACCAAAAGTATACAAAGCAAAATCAGCAAAGGGAAAAGGTGCATGGGAAA
AAATCTGGGAAAATCAGATGCAAACTTGCAAGAGTCTTTTACCAGTAGAGACACACAGAG
TGTACTTAATTCTTTTAGCAATGAATTTTGACAGCACACATGAAATGTTGTCTACCACGA
AGTTCATTAGAGACTCAATGCTCAAGGTTTTTCTTGGGAGCTGGTCACATAAGCAGTCTC
TGCCTAACAGGTACCGAAATTCTGCACTCCCAGAAGGAAAGCAGGTGTTCTGCATAAACT
ACATGGTTTATACAAACAGTTTAGGTGCAGCAAGCCACCCTTATCCATTAGGGAATGTTG
GTAGTGTTGTTATTAGGTGCCATCAAGTCAGCTCTAACTCATAGCAACAGAACGAAACAC
TGCCTGGTCTTCCACCATCCTCGCAATCATCGCTGTTTGAGCTCACTGTGGGCATCCACT
GTGTCAATCCATCTCATCGAGGGTCTTCATCTTTTTTGCTGACCCTCTACCAAGAGTGAT
GTCCCTCTCTAGGGACTGGTCCTCCCTGATAACATGTCCAAAGTACCCAAAATAAAGTTT
CACCATCCTTCTAAGGAACATTCTGGCTGTACTTCTTCCAAGACGGATTTGTTTATTCTT
CTGGCAATCCATGGCATATTCAGTATTCTTCACAACATCATAATTTAGAGGCATCAGTCT
TCTTCATTATTCATTGTCCAGTGGTGGTTAAGGTTGTGTTTCAACGTAGCTGGGCCACAA
TTCTCAGTGGTTTGGCAGTTATGATATAGTTTGGCAGTTGTGTGATGTGATTACTTCCAT
GACAAGATTGGATATAATGTGATCACCTCCATGATGGGATCTGCTGTGAGCAGCCAGTCA
GTTGAAAAGGAGTTTACTTGAACCTGTGGCCTCGAATATAAGTGGACATTCTGGCAAGGC
TTGGGGGCGTTTGCTTGCTCTGGATCCTGCAGCTGGCTCTTGTTGGTCCGATCTCTGGTT
CTTGGGACTTGATCTAGCAGCTTACCTGCAGTCTTGTCTGCCAACCCATCTTCAAAACCT
GTGAGCAAGAGACCTGCTCTCTGACCTGCCAATCTTCGGTTCACCCGCCCCTGTGGCTAC
ATGAATCAGGAGAAGCTTCTATCATGACCCATAGACTTGGGATATTCAAGCCTCTGCTAC
TGCATGGGCCATTTCTTTGATATAAATCTCTTTCTACATATATTTTTATGCTTTTCTGAT
TTTGCTTCTCTAAAGAACCCAGCCTAAGACACCTTCTGAAGTCTCAGTTCCTAGATACCA
GCACATGGCCAGCCTTGTAACCAGGCCTTTCAAAGGGTGACAGTAACAGTCAAATAATTT
AATAAGTGTTTATGTCCTAATCACTTAGTAGCCATTTAAAAAATAACTACAGTTACTTAC
TAATGGGATGTGTGTACCTGTTGAACATTGTTCAGCTTGTCAAACCGTGGAATGAGATTG
AATACTGCCACCTTCATTTCTTGTTCCACATTGATTTTTACACAGTAGTTACTTTTTATC
ACAGTGTCTGCCAAAAAAAACCTGTTCTGCAAAGATATTATATCGTCAAAAGGAATGCAA
CTGGCTCTAATGTTGAAACTGAACTACCTCAAGTTAGTAGTTTTTATGGTGTCCAACAGA
CATTAAGGGGGCCCCTTGGCACAGAGTTTGGTAACTGTGGTTAAAATGATAAAACTGGCA
TCACAAAGGAGTTGAATAGAGAAGAATTATTTAATTAACTTCATTAAGCATTAACAGGTA
ACAATCAGAAATAATATTTTGGGATCGTATACCATATACTAAAATTACTTTGAGGCCAAT
TATGACTTATTTTTAGTAATCAAAATATAAAATTAGTTTTAAAAAATGTAGTTGCCTTTT
TAAAAAAATCTCTAGCAATGCAGATTACATTCTAAGCTTATTAACAGTGTTAAAAATTTC
TAAAGAAAGACATCACCAATTTTGGCTGTGTAAAAATTAGAAACTTATCTATAGTAAAAT
AACCAAAACATAAAGGCAGGTAACAGACCAAGAAAAATACATAAAACAGATCCATGAGGA
AAAGGGATTTTTTAAACTACGAAATTTAGTTCGTATCAAAAGTAATTCGCAAGGGGGGAA
TAAAAAAATAAATTGGAAAAAAATGTTCAAACTCAGTAGTAATCAGAGTAATGCAAATTA
AGACACCAGTTTTTGCTTATGTAATCAGCAGAAGTAATAACAAATGCACTACAGATAGAA
TGACATAGCCTTACATTACTGGTGGCATTGCAGTGTGGAGCTAATCATTTTGTAGACCAA
TTTGTCAGAGATTCATGAAGAGCCTTACATTTTTGTCTTTCAGTTAGCAATCCTAATTCA
AACTTATTCTAAGAAAATTATCCAATGTAAAGAAATAGTCTTTTGTGACAAAACTTACCA
CAATTCTGTTTACATTCCTGAGAGATGAGAAACAACCTAAATATTAAAGAAAAAAAAAAA
AAAAGCTTCAGAAAATATGTCCAGCCACATAATGGAATGTCTTGTAGCCGTTAAAAATGC
TGTTTAAGTAAATGGAAAGGTGTTCCAAGTTCATGGATTGGGAGACTTAATATTGTTAAG
ACGTCAGTACTACCAAAAGCAATGTAGAGATTCAGCCCATTCTGGTCAAAACTCCAACAG
CTTTCTTTACAGAAAGAGACTCTCAACTTTATATGGAATGGCAAGAGACCCTGAATAGCT
AAAACAGTGTTGAAAGAGAAGAAAGTAGGAGGACCCACACTGGCTAGTTTTAAAACATAC
TGTATAGCTACTGTAATCAAAACAGTCTGGTAATAGTATAACAATAGACACATATAGATC
AATAGAATAGAATTGAGAGTCTAGAAATAAACCCATACATCTATCTGTGTCAACTGATTT
TTGACTAGGGTGCTAAGTCCATTCAATGGGGAAAGAAGAGTCTCTTCAATAAATGGTGCT
GGGAAATTTAGATTTCTACATGCAGAAAAATGAAGCAGGATTCATGCCTCACACCATACA
TAAAAACAAATTCAAAATAGATTAAGGACCTGAATGTGAAAACTAAAACCATAAAATTCT
TAAAAGAACAAGCAGGGGCAATGCTGTCAGGCTTAGCTTTCAACAGTGGATTAATAAAAG
CACAAACAGCAAAGGACAAAATAAATAAATGGTACCTCATAAAAATTAAGAACTTCTGTT
CATCAAAAGACTTTACCAAAAAAAGTGAAAAGACAAGCTACCAGTTGGGAGAATATCTTC
AGAAACCATACGTCTGACAAGAATCTAATAACCAAAATACATATAAGGTTCCAACAGCTT
AACAACAAAAAAGACAACCCAATCATAAAATGGGCAGAGGACTTGAATAGACATTTCACC
AAAGAAGACATTCAGGTAGCAACCAAACACATGAAAAGATGCTCAGCATTATTAGCCATC
AGAGATGAAAATCAAAACCATACTGAGATACTGCTTCACTCCCACTAGGATGGCTTAAAA
AAAAAAAAAAAAGGCAGAAAATAACAAATGGTGATGAAGATGTAGGAAAATTAGAACCTT
TGTCCATTGCTGGTGGGAATGCAAAATGGTACAGCCATTGTGGAAAACTGTGACAGTGCC
TCAAAAAACTAAAACTACCATAAAACCTGAAAAAAACAAACCTGTTGCCATTGAGTCGAT
TCCTACTTGTAGTGACCCTATAGCACAGAGTAGAACTGCCCCATAGACTTTCCAAGGAGT
GGCTGATGGATTCAAATTGCTGACCTTTTTGTTAGCAGCCAGGCTCTTAATCACTGTGCC
ACCAGGGCTCCAGAACTACCATATGGCCCAGCAAATCCACTCCTAGGTATATACCCAAAA
GACTTGAAAGTAGAGACTCAAAAAGAGACTTGTACACCAATGTTCATTGCAGCACTGTTC
ACAATAGCGAAAAGGTGAAGACAACCTAAATGCCCATGAACAGATGAATGGATAAACAAA
ATGTGGTACATACATACAATGGGATACTGCTCGCCAGTAGGAGAAATGAAGTCTTGATGT
AGACTACAGTATGGATGGAGCTTGAAGACATTATGCTGAGCAAAATAAGCCAACTACAAA
AGAACAAATACTGTATGACCTCACTTACATAAAAAGACAAGAAAAGGCAAATGTATAGAG
ACCAAAGTTTATTAATGGTTACCAGGAGGAGTAGGGAGGGGGAAAAGAGGGGTTAACAGA
GATGGAAAAATTGCATTGATTAAGGCTAGGGTTGCACAGCTGATTATTGTAATTGTAGTT
AATAAGTTGTTCACTTTAAAAAGTTGAGTTGGCATCTGCAGACATGTATTGGACTGGACA
ATGGGTTGGAGAGAGATGCTGGTGAGGAGTGAGCTTCTTGGATCAGGTGGACACTTGAGA

CTCTGTTGGCGTCTCCTGCCTGGAGGGGAGATGAGAGGGTGGAGGGAGTTAGAAGCTGGC
GAAGTGGACGTGAAAAGAGCGGGTGGAGGGAGAGAGCGGGCTGTCTCATTAGGGGGAGAG
TAATTGGGAGTGTGTAGCAAAGTGTATATGGGTTTTTGTGTGAGAGACTGACTTGATTTG
TCGTCTTTCACTTAAAGCACAATAAAAATTATAAAAAAAAAAAAAAAACGAGTTGGCAAAA
GTTGTGAGATAGATGTATTTAAAAATGATGACAAAAAAAAACAGTAGGTGCTTATGTAGAA
CCAAAGACTTCATGAGATTTGGTTCCTTGATTTGGAGGTTTAGGGTCCTGGTTTCATGGA
CATCCCTGTTAACTGGCCTAATAACATGTTTACTGCTTCTGGTTTATCTTCTAGTTCATT
GTGTAGTGCCAGGGGTCTTAAAAGCTAGAAAGCAGCCATCCAAAGCACAGCAATTGGGTC
TCTCTGCCTGGAGCAACAGAGAAAGGAGGAGAGTTAGAAATAGGAGGATATGAAATGTGT
GGTTAATTGCCTCTGTGAACTACTGCCTCCCTTGCCATGAGAGAAGAAGAACTGGATGGT
GCCCAGCTAGCCTTACTGAACATTTTGGTCAAAGATTTTATAGAAGAATTCTTATCAATA
GCAGGAAATTCAGAATTTCAAATTCTCATGGACTCCAGGCTTCCTGGAGCCATGGAGGCT
GAATTAACCCCTGAAACTATTGCTCTGAGATAATCTTTAAACCTTAAACTAAAAATATCC
CACGAAGTCTTCTTAAAACTATAGTTTAGCTTAACTAGTTAAAAAGGTCTGCCTTGAGCA
TTACACTCATTTAAAAGTGATGAAATTATAGATTTTTTCTTAATTTTCCAGTTTGTATGT
GTTGTTGTGTTACATTTATTATTTTGAGTGATTAAAATATCTGTTGCATTTGTGGTACAA
ATTACTAGTGGTTAGCAGTATCTAGTTCTAGCCTTCCAAACCTGGAATACTGTACTTCTA
GCCTTTTGTGTGTAATTTTCAGCCTGAGGCAATGAAAAGCTCATGCTCAATTTTTCAGTC
TCTCAGTCTTAATCTTTCTCTGGGTAATTCAGTATTCCAGATTAAAAAAAAAAAGTAACC
TGTTGCAGTCAAGTTGATTCCCACTCCAAGGATTCCATTTCCAAGGAGCAGCTGGTAGAT
TCCAGCTGCTGACCTTTGGATTAGCAGCTGAGCTCTTAACCGCTGCACCACCAGGGGTCC
CACAACACTGTGAAGTGGTAGAACCTGTCTAACCCAGCATTGCTGAGCCAACTAGTGGAA
GACAGTTGCGTTGGAGAGACATCCAGACCTGAAGCAGATTTTGCATGAGCAAGAAATAAA
ACTGTTGTGTTAAGTTGCCAAGACTTGGGGATATTTTGTAACTGCAACACGATTTAGCCT
ACCCTGACTGACACAGCAGTGGTCTATAAATTGGCGTGTGTAAGACAATCCAGTTCATCA
GTTTGTGGCAATACAGTATTACAACTTCTGTTTATATTTCTTTTTTATCTAAGAATAAAA
AAGAAGTGAAACTTTGCTAATGTGTACGGGTTGATATTAGCATCCTTAATCAACAAGCCA
GATGATCAGTGTCACATGGTATGTGAGAGATCCTGAGAGAAAAGTGAGAGTCCACAACAC
TGTGGAAGTCTACAGTGGTTCCCTCACTGCATTCCTTTGCTTTCAGCTTTCAGTCGTTAA
TCAAATTTATGAATGATATTATCTGATTTTCACCACACTGCTCATAACAGAATGGCCAGG
TAAGTAAAGGTTCTTGCAATAAAACAAGATAATACTAAAAATGTAAACACCCATCAACCA
TAAGAAAATGATAGAACTGAGACTTCTGCTTAACTCCTAGTACTAGCTCTGTGTTAGACG
CATTAATGAGGTGACAACAAATATGAACTAATCAGATCTAATAGTAAATGGGCCACCAAA
ATTCAAAATTATCAGAAGACTGTTTGAAATATTGATTTATTTCAACTTTTACTAATGAAG
AGCCTTACCCTAAGTGCATATAGTCTACAAGATATTAGCTAATGGTAGTATGATGTCATT
ATGATTAGCCAGCAACTTTTCCTAGTCATGTTTAAACTCATATGATAGTCTATACAGGAC
TTTAGAAAATTTTGCTAAACTTAATAATATTTATAAGTCTCTTGTGAATTTTCTTAACAA
AAAACAAAAAGTCACACGATTGGGGAAACTTGTTCTTTTTGCTGTGATTAAAATGGCTCA
AATATTACATAGAAAGCATAATGGTGACAAAATACTTCCTTTTGTTAGCAAATAATAACA
TAGAAAGCTGCTAAAGTACTGACATACAATGTGGGAGGTTTATTATACAGTTGGATGAAA
ATATAGATGTTTCTAATATATCTCAGCTTAGGGTCTTTGCTAAATTCTTGAAATACACAA
CTTTTTGTAAAGCTCTGAAGAAAGGAGAATAAACATTTGCAATGGAAAATTACTTTAACT
TAAAAAAAAAGTCTTATAAAAAACTACAGTTGTAACTACTGATAGAATGTCTGCTTTGAT
GGAGAAAAAAAAAAGACTGGTGGGGTAACAGTATCAAGATAGCAACCTGCAAATGTATTCA
CTGCATCATTCCTAGGCCACCTCTTGAACACAGAAGATGAATCTCTTTCCAAAAGTCAGC
CATTTAAAATCATTGATCAGGACCTCAGATTGAAGCTGACCCAAATGATACCTATTCCCT
GGGATTGTTTTTAAATTATTTTTTAATTAAAAGGAGGCAGCCTGAATTAGTACATGTATT
AGCTAGGAGTTATATTAAGCTGCAAGGAACAGAGAACTTCCCTCACCCAAAATATATGAA
CTCTATGAAGAGTTTATTTCGTATTTATGTAAAAGAAGTCTGGGTGGTCTAAGGTTGGAA
GGATAGCTGTCTTTAGGAACCCATGTTGCTAATTTTCTGTTCCACCATCTTTGAGGTATC
ACCCTAAATCTTTTGCTCACAGTATGAGCCAGAGCCCCTTTTCTACATTTTAGGCAGAGA
GAAGAGGAAGGGCCATGTACAAAAAAAAAAAAAAGCATCAGCTGAGCCTGCCCAACAATTT
TGAAGAGCCCCACAACTTCTACTTCCTTTTTTTACCGGAACTGTGCTGTATGACCACCCC
TACCTTCAAACAAGCTTGGGAAATACAGTTTGGTTTGTTGTTTTCTCCTTATTTATTAGC
TTTGCCTTTTTAGTTACCCTGATATTGTTTGCTTATAATTAAGCAACAAGAAATATAATA
TTCTTCTTTAAAAAATAGTATATCTTCCAATGGTTTACCTAATTAAAAAAACAAGGGATA
AAGTAGAAAAAATAAGTTATTTTACACTTATCAGGCCTATTGTTAGAAGTTTTCTGAACT
ATGATTTCTTTTAGGCCCTGAACATAATATAACAATAATAATAGCAATTAAGAGTTGTAA
AGTGCTTACTGCCTGCCAAGAACTGCTTTTCATGCTTTATGAATATTAACTCATTTAATT
CTCAAAAGAGTCATGAAAAGGGTCAACGAAAAAGGCAAGATTCAAATTCTTTTGAAGCTT
TTTTTTTTTTTTAACACGGTGGAAAAACTAACATTTTTAGAAGTCAACTTTGTTTTAATA
TGTTTTTTAG**ATCAATGTAGAACTGTATGGATTGTTTGGCACAAAGCAGATTGTAATTTC**
TCATGCCTCTCAAACTGTTGATCCTACTGTAAGTACACCTAGCACAATCAGAACGTTTCT
CTCCAAACACTGTGCTGACCACCCGAACAACTGGGATGATCATCTGTCAGCTGTTTCGTT
TGCCTTCAATATAGTTCACTTGGTATGTGCCTTTTTATAATGCTGTACTTTGAAGTTGTA
ACTGATTATTTCTTGGCTAGATAAATATTTGCTGTTTATTTTCCTTAAAATAG**GAGCCTG**
CTAAAAATACACCATATTTTCAAATGTTTAATCGAAATCCTTACATGCCTGAGACTTCAG
ATAGTCTTCATGAAGTGGATGGTGATAATACAAGTATGTTTGCCAAAATTCTAGATGCAG
TTAAAGAAGCTGATAAAATAATGGCAAATAAGACAACTTCAGCGGGCCAGGTGATTCTAT
TTAATAGAAAAAAACTCTATAATCAATATAGTGATCATTTATTTTTAAGATTTTGAGGAT
ATACACACACGTTATATACTCTTAAGATAAAAATTTTCTACCAAATCTACTTGTCTTGAT
TAAACACTGTGTGTATTAGCTCTGAATTTGAGAGACAACAGTGGGAAAACAATGGATTTT
GAAATCAGGTAGAGACAGATTTAAATCCCTAGTCTTGCTGCTTTCTATTTGTTTGACTCA
GACATTATTTAACCTATCTCTACCTCAGTTACCTTACATGTAAAGGCAGTAATAACAACT
TCGAAATACTGTTTTAAGAATTAAATGGGATTCATGCCTACCTTTTACTAGCATTTCAGT
AACTAATCATTTTGTTATAATATCTGCAGTCAAGAAATTACAGCTCTTTTATACCCTGCT
CTTTTTTTTTTTTTTTCCGGTACATTTCAGTAACTAATCATTTTGTTACAATATCTGCAG
TCAAGAAATTACAGCTCTTTTATACCCTGCTCTTTTTTTTTTTTTTTTTTTGGTACTTGT
AG**CTGGAGAACAACAATTTTGATGACGTAAATAAAAGCAAGATTGTTGTTAAAAAGAAAC**
CAAAGCAGTTAAATCCATTTCATCTAAAAGTGGGTCATGAAGTTTTAAGACAAAGGAAAA
ATTGGTGGAAGGATGGTCGTTTCCAGTCCGAATGGGTTGGCCCTTGTGTCATAGACTATA
TTACAGAAAGTGGTTGTGCTGTCCTGAGAGACAACACTGGGGCTAGACTTAAAAGACCGA
TCAAAATGTCTCACCTTAAGCCCTACGTGAGAGAATCCACTGAACAAGGTAATTATCAAT
CACTTCTCTACATTTTTACTTTCTTTGAATGAGGAGAGAAGATTAAAATACAAGAGACTT
TTTCTTTTCATTTTGGTTGGGTCTTCTTTTCCTCTCTTATATTTAGAGATTTTGGTATAG
TTTTAGAAATCCCAGGTAGTCTGGTTTTTTTTTTTTTGGAATGTGTGTTCTGGAACTGTCA
GGCTTGTGAGAGGTTTTATTTTGATTTCTAATACACTTTCATAGTTCATATCGGAGGTGC
TGAACCTGTGTCACAGAAGAATTATGTCTTCTCTGTTTTGCTTTCATAATGGTCTATTTA
GTTACTTTGTGCCTCATTTTAGTAGCATGAAAAAAACAGCAACTAAGATATGTAATGCCA
AGAATATACTGTGGAATTCAGTTTAGGAAACTTAAAAAACGTGATTAATTTTTAATGTAG
TGTCACAGTCGGGCCACGGGTGGGCTCATTTGAGGGTGTGGGGTGGCTTATGTTCAGAAT
GTTATAAGGTGATACAAGGAGGTGATAGTTCCCTAAATGATGTTATTGTTAGGTGCTGCC
AGATCAATTTTTGACTGATGGTGACCCCATGTGACAAAGTAGCACTGCCCATAGTATTTT
CTAAGCTGTAATCTTTATGGGAGCAGATTGACAGGTCTTTCTCCTGCAGAGGTGCTGGGT
GGGTTTGAACTGCCAAACTTTCTGTTAGCAGCCAAGCACTTAACCATTGTATCACGAGAT

```
CTCCTTAAAACGATGTTATACTTGCCCCCAAATGTGACTGATTCTGAACAGGCAAGATAC
CCTGATCTCTCTAATCGTTTCTGTCATCGACTAAAACTAAACATTTTTGCCATGGCTTTC
AGATTAAGGCCTTCCTTACTGGGATTGTTTTATTTTGTCTTGTAAAAAAGAAAAATAAAG
CATCAGTATTATTTAAATATGAGTAACATTTGATGTGGAGGGCCAGTGAAAACAACAGAA
ACGCTCAATGAGATGGTCTGACACAGTAGCTTCAACAGTGGACTCAAACATAAAAGATCA
TGGAGACGGTGTAGGACCAGGCAACGTTATGTTCTGTTATACATATGGTCACCATTGTCA
GAGCCTAGTTGACGGCAACTAACAAAAACATTTGATGGAAAGTACCATATACTGTAATTT
AAAACAATATAAACAACTAAGTTATTCAACAAATAACCTTCACTTAGCCTATGCCATTTT
TTAGGGTATGATGACCTCAAGGGCACTAAGTGATAGTGCAACTCTGCTTTTGCTTGGGCT
GCAGTTCTTGCGTTAAAAGCTCTTTGTACCTGTTCTTCTGCTTGTGACAGACCCAATTGC
TCTAGCCAGAAAACATGTCTTGAACAGTGTTCTAATTCTTAAAACTCACAAGTACCTTCA
CTTTTTTTTTGTTAAAAGACGGGCTTTTTCATTCCTTGTACCTCTTCTCGATTCTGATCT
ACCCTTTATGGTCTCTCCCTTCTGCAACTTAAGTAGAAAAAAAAAATGTATATATATATA
TATGCATATTACACAAGTACATGTTGTTTTGTGTTTTTCAACATGATTTTCCTCGTAATC
TTTGACAGTAGGACTTGCTTCTTTCCAATATGTTACCCTTCCTTAATTAATACAAATTTT
ACATTTAAAATGTTTCTGTATCATGATGTGTTTCAACATTTTGCAATTTGCTCTTGCCAA
AAACTTGCTTAGAAAGGAGGTAATAACATTTTGCTTCTCCTGAATGTTTTAGAACCCAGT
TGGATAGAACAGTTCCTAAACAGAGGCAGTATAATTTTTGCCCCATAGTTAAATTGGCAG
CATTAGTACTGGTGTTCACATAAGAAATCTGAGATTAATAGGTGAAACAGTTCTCTTTTT
TCTTGTTAACCAAAGGAGGTATGACCAGGCCATCCTAGAATCTGGTTCTTGATTACTATT
TGGTTCTTTTGTGTGTGTGTTTTTCTCTGACTTGCAGAAATTCAAAATAAATACTATTTC
TGCCACCTTTTCCCTCAGTATCACCTGCTTTCCTGAAAATCTTCACCTCAATTTTAAAGA
GTCTGTGAGCTGAATTTACAGCGAACAAAATGTCTTTGAATAGTCATCCAACTTTTTATT
TGGTGTAAATTTGGATGAGCTCTATCCAAAAGAACTATGAATTTGTCTGTCTGTACTTGT
TGTGTCTGCTCTTACCAACCTTGAGATTTCATAGCTGTGTTAAAGTCTCACAGTCTAAAG
CAGATATGCTTCCGTTGCCAACTTCTTACCCATTCTTATCTAGGACATAAAGTAGAAGGA
GCACATTCAGTACTAGGTCTACAGTGTTTTTCAGTACTATTTTAAGAAGGCTTCTTTGTC
ATTTTGTCTAATTCCATGTGCCAAGCACCTTTTCTGAGTATTTTTTATTAAAATAATGAC
AAGAAATCTTATGACATACCACAATCTCTTGGTAATCGTGATGGCCCTCTTTGAATGGGA
AAATGATGTTATTGACCCACAATTCAAGTAGACATAGATTTTGAGTAGTTTGTTTTGTTT
CATTTTTTGGATGAGGAATATTATGATAGGTTTGTGTCTGCCTCACCAAAAGGTTTTAGT
CTGATTGTCCTTCACAGTTCAAAGTCCTCAATTGTAGAACAGTTTTTATTATTAGTTTCC
TATTGCTGCCATAACAAATTGCCACAAATTCGATGGCTTAAAACAACATAGATTTATTAT
CTTATAGTTCTGTAGGTTAGAAGCCCAATACAGGTCCCACTGACCTAAAATCAAGCATTG
GCAGAGCTGTGTTTCTTTTTAGTGGCTCTAGAGGAAAACCAGGTTCCTTGCCTTTTCCGG
CTTCTAGAGGCCACCCACATTCTTTGACGCATGGCCCTGTTTCTCCACTTTCAAAGCCAG
CAATGGTGGATTAAGTGCTTCTCACACTGCATCACTCTTGACTTCCTCTTCTGCCTGCCT
CTTCCACATTTAAGGACCATTGTGATAACATTGGGCCCACTAGGATAAACCAGAATAATC
TCCCTATTTTAAGGTCAGCTGATTAGCAACCTTAATTCCATCTGTTACCTTAACTCCCCT
TTCCCTTATAAGGTAACATATTCACAGGTTCCGGGAATTAGTGCGTGGACGTCTTTGAGG
GGCCATTGTACTGCCGACCACAAGTATAATCTACATTCATGAAATAAAGCAAAACTTAGT
CCCTATGGCATCAAGCCCATGATCTTGACTTTATTATTAATATGGTCTATGCAAAAACCT
TTCTTTTGGCCTCTTGGTTATATAATCCCTAAATTAAGACTCATCCAGTTCTCTCTGTTG
TTCATTAGAATTGGTTTCTCTGTTGCAATGATAATAATACAGTATCAAATTTTAGTTATA
AAATTGTCATTTTATCACAGGAAAGGATGTACTCGTATGGTGACACATTCTAATAGTTTT
AGGATTATCAGGCCACTTATAGGATTAGTCTAGTTATCTCTCTTCAGAATAAGAAAATTA
AGTATTTTGAAACCTTGTGCTTTTCTTTGTATGTGAAGAAAGAAATCAGATCACTAAGAC
TTTTGAGATACTTCGATTTTAATTTATTTTTCTCCTTTGGGAAATTTTAAATATTATCAG
CTGCTAACCAAAAGGTCAGCAGTTGGAATCCACCAGCTGCTCGTTGGAAGGCCAATGGGG
CAGTTCTACTCTGTCCTATAGGGTCACTATGAGTCAAAATCAAGTGGACGGCAGCGGGTT
TGGTTTTCTGGTTTTTAATCTAATTTTTTCTTCCTTAAAATTACATAAGGAAAAGTATG
TCAAAGCTGCTGATAACGTCTTCCTGAAATATCTTGAAAATATCTCACTTTTAAAGAAAC
CAAAGACAATTTATTCTGTCTGTTCAAAACTGAAACGATGCTTTCATCAACAAATGTCCT
AACTATAACAATTAGTACACATTAACCTTCCTATATAAAGATGTCCCTGCTTAGCCATCA
GCGTAACATTCATGCTTCAACATTTTCTTTCCCTAATGGAGAAGATAAATTGCTGAAAAT
AAATTGCAGTGCTGGCAAGGATGATTCCTTGTGAGTACTTTGGTATGGATTTTTTAGTTA
GACCTAAATTAAGTTCCTGAGAATTTTGGTATTAAGTCAGATTTACATGAGCTGAGCTTT
GAAGCTACTTCATTACAGCCTCGCTATTTACACATTCCTATCACATAAACCTGATCACAT
AAGCCTGATTCTAGAAGCCTTATCTTTGTATTAGTTCATAACTTCATATTGTATCAATTC
TGTTTGTTAAACTATCTTCCATGATCCCATTAATTCATCCAGAAATTCTTTATACATGCA
TATTAAGGATATTATCAACATTTTTCAAAGACAAATATAGTTTTACAATTTTTTAAAAAT
ACATACCATAATTGTCCGACTTCCTTCCTTGGCCCCATCTACCAAAGACAGGTCAAGGAA
ACAGGAAAATCTAGCTGTTTTAAAATCCTTGGTCTTTCTTGCATTCACTGTGTAATAACT
GTAAAAATTATATATTCATTCTTTCATATGTGTATTTAGTAGACAAGAGAACTTCCAAAA
GTTAATCTTGATAGTTAATTTTTATTACTTGAAATTTTGTTCCTCAATTAACATTTTTTA
GTTTTAAAATGATGTTTAATTATCATTCTAAAAAGCAAACTTATGATGTTTCTTCTTTCA
GACAGTCTGTATCTCTTACAAGGTTCAGTAGTAGCAGATCATGACTACATCGGGTTGCCT
GAAATTCCACTTGGAGCATACCAAGCAAATATTCTCGTAGAAGATGCAACTATTGCTGTA
GTTGACAATGAATTACTAACATCAACCAAGGACCGTGAACTATTAGAATATAGAAATGCT
AAAGTCTCTCCACTGATAGAAGATCGTGGTACTCTTGACAAACAGACTTTCAGTCTGTTG
GATTCTTCAAATCAAGTCCTTGAGTACTTAAGTTAGTAAATACCAAAATTTATTTAAAAT
GTTTGTTTATAATGTGTAAACCGTTGAATCTTGATATCTCATCTATCAGAAAAAGTTGTG
TAGAAGTGACTTGACACACTCTTGATAACTAAATCCTGATGTTTATTTTATAACTATTTA
TTTATCTAAAGTTGTGACCATGTTTGAATGTCAATGCATAGATATACAGGCAGAGGAAGC
ATAACATAAATAGAATTCAGTTCTTCTAAAAAGAGAATAGGCAAAGCGTACTTGCTTCTT
AGGAAGTAAATTCTTGTTCAAAACTCCTAAATCTTTGATATCTGTATTGTGTTTCAGTTT
TAATTTTTATGGAATGCCCGCCCTCTGCCCCAGTGGCATAGTGGTTAAGAGCTATGGCTG
CTAACCAAAAGGTAGGCAGTTCGAATCTACCAGGCGCTTCTTGGAAATCGTATGGGGCAG
TTCTACTCTGTCCTATAGGGTTGCTAAGAGTCGGAATTGACTTGACAGCAGTGGGCTTGT
GTAAGTAAATTCCAGCTCCCTACAATGGGATTAATTTGAATCAGCCATAACAATGAATTA
AATGAAACTCCCCTATGTGTCATTAAAGCTTGTAAA
```

```
==================================================
```

Loxodonta Gin-1

```
total length:      20796 bp  (20796 bp excl N/X-runs)
GC level:         35.81 %
bases masked:       9489 bp ( 45.63 %)
==================================================
               number of      length   percentage
               elements*    occupied  of sequence
--------------------------------------------------
```

SINEs:	13	1928 bp	9.27 %
Alu/B1	0	0 bp	0.00 %
MIRs	2	233 bp	1.12 %
LINEs:	10	2473 bp	11.89 %
LINE1	6	1946 bp	9.36 %
LINE2	2	194 bp	0.93 %
L3/CR1	0	0 bp	0.00 %
RTE	4	874 bp	4.20 %
LTR elements:	5	2067 bp	9.94 %
ERVL	2	1064 bp	5.12 %
ERVL-MaLRs	3	1003 bp	4.82 %
ERV_classI	0	0 bp	0.00 %
ERV_classII	0	0 bp	0.00 %
DNA elements:	7	1805 bp	8.68 %
hAT-Charlie	6	1552 bp	7.46 %
TcMar-Tigger	0	0 bp	0.00 %
Unclassified:	0	0 bp	0.00 %
Total interspersed repeats:		8273 bp	39.78 %
Small RNA:	0	0 bp	0.00 %
Satellites:	1	1126 bp	5.41 %
Simple repeats:	0	0 bp	0.00 %
Low complexity:	4	90 bp	0.43 %

==

SW score	perc div.	perc del.	perc ins.	query sequence	position in query begin	end	(left)		matching repeat	repeat class/family	position in repeat begin	end	(left)	ID	
374	30.1	1.1	0.0	UnnamedSequence	1566	1658	(19138)	C	L2a	LINE/L2	(0)	3426	3333	1	
909	15.3	0.0	6.6	UnnamedSequence	2102	2262	(18534)	+	AFRO_LA	SINE/tRNA-RTE	7	157	(15)	2	
21	47.6	0.0	0.0	UnnamedSequence	2937	2957	(17839)	+	AT_rich	Low_complexity	1	21	(0)	3	
488	27.5	13.0	2.1	UnnamedSequence	3459	3711	(17085)	C	MARNA	DNA/TcMar-Mariner	(170)	416	137	4	
264	17.7	19.1	1.7	UnnamedSequence	3742	3842	(16954)	+	L2a	LINE/L2	3293	3421	(5)	5	
23	69.6	0.0	0.0	UnnamedSequence	3843	3865	(16931)	+	AT_rich	Low_complexity	1	23	(0)	6	
358	8.7	0.0	0.0	UnnamedSequence	4629	4674	(16122)	+	RTE1_LA	LINE/RTE-BovB	2688	2733	(500)	7	
381	32.1	6.2	4.3	UnnamedSequence	4675	5155	(15641)	+	ART2A	SINE/RTE-BovB	38	527	(24)	8	*
464	17.9	4.7	1.1	UnnamedSequence	5076	5160	(15636)	+	SINE2-1_Pca	SINE/tRNA-RTE	253	340	(0)	9	
306	24.3	3.5	2.2	UnnamedSequence	5350	5479	(15317)	+	L1M5	LINE/L1	4465	4570	(1576)	10	
225	16.1	9.4	12.9	UnnamedSequence	5678	5741	(15055)	C	MER119	DNA/hAT-Charlie	(54)	529	468	11	
2495	18.6	3.0	0.0	UnnamedSequence	5742	6181	(14615)	C	MER68	LTR/ERVL	(0)	563	111	12	
299	19.2	19.0	1.1	UnnamedSequence	6187	6265	(14531)	C	SINE2-1_EC	SINE/tRNA-RTE	(1)	406	314	13	
1622	15.7	6.3	0.3	UnnamedSequence	6274	6560	(14236)	C	RTE1_LA	LINE/RTE-BovB	(107)	3126	2823	14	
3364	14.7	3.5	0.0	UnnamedSequence	6561	7111	(13685)	+	MLT2_LA	LTR/ERVL	2	571	(0)	15	
349	24.7	0.0	0.0	UnnamedSequence	7112	7184	(13612)	C	MER68	LTR/ERVL	(457)	106	34	12	
1474	18.4	0.9	0.9	UnnamedSequence	7191	7507	(13289)	C	MER119	DNA/hAT-Charlie	(115)	468	102	11	
302	17.8	9.5	0.8	UnnamedSequence	7960	8075	(12721)	+	L1ME3G	LINE/L1	5386	5438	(756)	16	
430	33.1	4.5	2.4	UnnamedSequence	8112	8399	(12397)	+	L1ME4c	LINE/L1	278	571	(259)	17	
1027	13.1	5.4	0.0	UnnamedSequence	8404	8571	(12225)	+	L1-3_LA	LINE/L1	5614	5790	(2319)	18	
683	32.9	4.2	5.8	UnnamedSequence	8573	9501	(11295)	+	Sat-1_TS	Satellite	1	913	(658)	19	
951	16.9	0.6	2.4	UnnamedSequence	9502	9671	(11125)	C	AFRO_LA	SINE/tRNA-RTE	(3)	169	3	20	
683	32.1	4.5	5.9	UnnamedSequence	9672	9868	(10928)	+	Sat-1_TS	Satellite	2717	2911	(325)	19	
2121	9.3	0.3	0.3	UnnamedSequence	9873	10174	(10622)	+	L1-3_LA	LINE/L1	6925	7226	(883)	21	
2371	5.1	0.0	0.0	UnnamedSequence	10175	10485	(10311)	+	L1-6_LA	LINE/L1	7274	7584	(0)	22	*
3973	12.0	4.2	0.8	UnnamedSequence	10481	11116	(9680)	+	L1-3_LA	LINE/L1	7210	7867	(242)	21	
423	18.2	23.8	0.0	UnnamedSequence	11397	11522	(9274)	C	AFRO_LA	SINE/tRNA-RTE	(16)	156	1	23	
473	21.5	2.1	0.0	UnnamedSequence	11623	11715	(9081)	+	MLT1I	LTR/ERVL-MaLR	317	411	(0)	24	
786	25.3	5.9	3.9	UnnamedSequence	11733	11987	(8809)	+	Charlie7	DNA/hAT-Charlie	35	294	(2318)	25	
1302	25.4	3.7	1.8	UnnamedSequence	11996	12372	(8424)	+	Charlie7	DNA/hAT-Charlie	328	711	(1901)	26	
818	20.5	9.1	0.8	UnnamedSequence	12374	12594	(8202)	+	Charlie7	DNA/hAT-Charlie	757	995	(1617)	27	
569	18.7	14.6	0.0	UnnamedSequence	12624	12746	(8050)	+	Charlie7	DNA/hAT-Charlie	1058	1198	(1414)	28	
454	29.3	8.6	0.8	UnnamedSequence	12722	12941	(7855)	+	Charlie7	DNA/hAT-Charlie	1174	1410	(1202)	29	*
25	72.0	0.0	0.0	UnnamedSequence	13028	13052	(7744)	+	AT_rich	Low_complexity	1	25	(0)	30	
365	34.1	9.8	6.1	UnnamedSequence	13075	13471	(7325)	C	MLT1J	LTR/ERVL-MaLR	(0)	512	102	31	
358	33.0	0.0	0.0	UnnamedSequence	13715	13820	(6976)	C	THER1_MD	SINE/MIR	(0)	274	169	32	
343	26.2	4.7	3.9	UnnamedSequence	14558	14684	(6112)	+	MIR3_MarsA	SINE/MIR	16	143	(71)	33	
21	38.1	0.0	0.0	UnnamedSequence	14913	14933	(5863)	+	AT_rich	Low_complexity	1	21	(0)	34	
1178	18.9	0.5	0.5	UnnamedSequence	15758	15964	(4832)	C	AFROSINE2	SINE/tRNA-RTE	(0)	207	1	35	
254	28.3	0.0	0.0	UnnamedSequence	16171	16230	(4566)	+	RTE1-N1b_LA	SINE/RTE-BovB	283	342	(128)	36	
446	26.6	0.9	0.9	UnnamedSequence	16235	16348	(4448)	+	SINE2-1_Pca	SINE/tRNA-RTE	227	340	(0)	37	
2237	19.6	2.3	4.4	UnnamedSequence	17870	18382	(2414)	C	MLT1D	LTR/ERVL-MaLR	(2)	503	1	38	
880	13.1	0.0	1.4	UnnamedSequence	18838	18977	(1819)	+	AFRO_LA	SINE/tRNA-RTE	34	171	(1)	39	
1067	12.5	0.0	0.0	UnnamedSequence	20548	20699	(97)	+	AFRO_LA	SINE/tRNA-RTE	7	158	(14)	40	

>Hyrax Gin-1 (genescaffold:proCap1:GeneScaffold_4075:5509:24907:-1)
ACAATCCTATTGAAGAAAGAATAGCTCACAAGTAAGGGTGTGTGAAATCGGGCACAGCTA
GACATCCTTTCTGTAATTTGACATTTGAGCATTTCTGGAGTACATATTTTTTGCATATGA
CAAGTTGGGTTTTTTTGGTTCATTTTTCAGTGACCTTGAATATCATTAGTAGCAGTATAT
AATTATATTTTCTCCCCTTATAACTTACCATTTATGTAGATGATGTATAACAGGACCGTC
CTGTATAAGTAACATTGCAGTTGATGGCAATGGGAGGGCCTTTAAGGTGGCAATGGGAGA
GACTTTTCAAAGTGTATTTGTTCATTTCCTTTGCCCTCCCCTCAGTGTGTGGAAAATTTT
CAGCATTTCTTTTCACATGGAAAGAGACAAGATAATTTGTCATGATGTGTCTCAAAAATA
GCATTTGAATATTTTAGCTGATATGCATCATTATGTCCCTTTTCTGTAGTACAATTAACC
CCTCAGATAGTATTCTGGGAAAGCTATTTGTAAGTCATTTGCAAGTGGAAGCCTGCACTG
AAATAACAGTGAATTAGCTAATTCAAATTCTATATTTAAATGCCTTTGCCTAGGTTAACG
ATGGTCCGTAGTGGAAAAAATGGTGACCTTCATCTTAAACAGATTGCATATTATAAACGA
ACTGGTGAATATCATCCAACTACATTGCC**AGTGAGAGAAGTGGTATA**AG**AGAGCAGCAAA**
AAAGTTTGTCTTTAAAGGTAAAATTAATGTACAAAATCTTGCGTATAGAATTAATATAGA
TGCTTTTCCACATGTGTCCTNNNNNNNNNNNNNNNNNNNNNNNNNNNNNNNNNNNNNNNN
NNNNNNNNNNNNNNNNNNNNNNNNNNNNNNNNNNNNNNNNNNNNNNNNNNNNNNNNNNNN
GTCAAAGGCAGAATAAAAATTACACTGAAGCTAACTGAAGTCCAGGAATATGGAAAACCA
AAACCAAAAACCCACCACTGTTGAGTCAGTTCCAGAGTATCGAAAGGGCTGTTTAAATCA
TAAAAAATGGTACTGTGAAAGGAAATGAAACATTTAGTTCCTTTCTCCTCTGTTTGCAAA
CTGAGAAGGTTCCTGCTCTCATAGTACACTTCCGTGCACACTGATGCCTCTGTCAGTGAG
AAACTGATTTGATTGTTAATATGAAAATGTCAAAAAGTTAACTAATTTGTTTACTGAAGT

TAAGTAGAAGGCATGACTGTATTTATGTCTGTGGTCTGCCTTCTCACTGCCATTAAATAA
TCATATTGGGGGGGGAACTTACGTCCCCTTGAATGTACGAGGTCTGACATTAAGTTTGCA
AACTCATCCTAGAAAAAGTGCGGCATATCTCATTGTTGAATATCACTGTGGTCACCTTTT
AAGTATTACTCTTGGGAAGCTATGTGCTGACGCCAGTGCTTAGTCCACCCTTCAAAGCAA
TTTTGAAACTCTTTTTCTGAAATGGCCATCAGAGCTACCCTTGATGTCCTGAATGTCATC
AAGATGTCTTCCTTTCAATATTTTCTATATCTTCAGGTAAAGAAAAAAAGTCACTGCGGG
CCAGATCAGGTGAGTAGGTAGGATGTTCCAATACAGTTATCTGTTTACTGGCTAAAAACT
CCCTCCCAGACAACTATAAAATTAAGTTTGCAAACTTGCCACCATTCCACCATGCACAAT
GACCATTCCAGAAAGAGTTCCAAATTTGCTTTGCAGGGTGGACCAGATGCTGATGTCAGT
GCATAGCTTCCCAAGGTGAGTACTTTGAAGGTGACTGTAGTGATACTCAGCAGTGAGGTA
TATAGCACTTAGAACAATTGAGTTTGTAACTTAGTTGTTGGACTTCAAAATATTTGAAAA
GTGGTAGATTATGTGGCTTGAAAGTTTAAGATAACTTTTAAGAAAAATATGGGGGTAAGA
TCCACAACAAATACAGTCACAGATAACTAATTGAATGTTGGTGCTGTCACTGGCAGTTTG
CCTGTAGGTTCAGAATGAAGGTTTTCTCTGGAGTACTGTAAGATGAAAAATGTCCATTCA
TTTCTCCCTGTTTACGGTTACTGTTCATCTATATGTAATTCATTTACTACTAATTCATGC
CATTTTTAGGTGGAACTTGCAAGTATTATTCCAATTAAAGTATTCAACAAGGATAATTTC
ATGCTCCCATGGTTATCTCAGGGGCTCTGATAGCACAGTGCTTGAGAGCTAAAAGGTTGG
CAGTTCAAATCTACCAGCTGCTCCTTGCAAACCCTGTGGAGTAGTTCTACTCTGTCCTAT
AGGGTCACTATGAGTCAGATTGCATGACAACAGGTTTTAACAGGTGTAGTTGTGGCATGT
ATTGGTGAACAACCTAACTCTGCTTACATATATAACCAGTAAAATTATAGCAGGTAATGA
TCCTTCTAGATATCTAGTACTTGTAATAGCTGAGCACTCTTCAGATTATGTAGTAATATC
ATTATAGGTAGATACTCTGTTTTAGGGGGAGAAATGTTTTGCTAAAAAATGAAAACCATG
GTTTGAAGATGAGGTGTTGATTGGCTTCAAACTTCATAGATAATTAAAAATTTTAATTCT
ATATTTTTGGTATTATTCCAG**AGAAAAAACTATTTTATGTTGGAAAAGACAGAAAACAAA**
ATCGTTTGGTAATTGTTTCAGAAGAGGAGAAAAAGAAAGTCCTGAGAGAATGTCACGAAA
ATGACATTGGC**AACCCATCATGGTATATCCAGAACCCTNNNNNNNNNNNNNNNNNNNNNN**
NNNNNNNNNNNNNNNNNNNNNNNNNNNNNNNNNNNNNNNNNNNNNNNNNNNNNNNNNNNN
NNNNNNNNNNNNNNNNNNNNNNNNNNNNNNNNNNNNNNNNNNNNNNNNNNNNNNNNNNNN
NNNNNNNNNNNNNNNNNNNNNNNNNNNNNNNNNNNNNNNNNNNNNNNNNNNNNNNNNNNN
NNNNNNNNNNNNNNNNNNNNNNNNNNNNNNNNNNNNNNNNNNNNNNNNNNNNNNNNNNNN
NNNNNNNNNNNNNNNNNNNNNNNNNNNNNNNNNNNNNNNNNNNNNNNNNNNNNNNNNNNN
NNNNNNNNNNNNNNNNNNNNNNNNNNNNTTCTACTTCTAACAGAAAAATCAGCTATGATT
TTTCAGCTCTGCTGATCCCTGTATCTTTCTGGTGTACTGTATTTGCCAGATGGTAGAAAT
TACTTGGTATGTCTGTGTAATAGTGCATGACAAAGACCGCCGTTGTGGCTACAAAGGATT
AATCCACCATTTAAGTATCATTTATGAGTCATCAACTTACTATTCTGTGCAAGTATTCGA
GACCTCTATACTTGGATTTGCTTTCAGAATCTACCTCATATTCTACACAAATATTCCGGG
AAAACTTTGCCAGATGTGTATCATTTGAGAATCTGTATGATTTTGGGAAATAATTAGAAG
TGACTTGGGTTGAAACTAGTAAGTTGGAAAATAAAATTTTTTATAAAAAGTTACTGACTG
TAGTGTAAAGAGTTTTAACAGTTGTTTCCAGAAGTTGATTTAAGAGTAGTTTCCTGAGAT
TTCAGAAATATATTGAGCAATAACACTGCCAATAAGTTGATCAGTGGATCATCTTTCCCC
TTACTCAAAGGAAAACTGTGACAGCTTTATTTTTGTTCACTGTCTTTTCCCAGGACTCAG
AACAGTAATATAATTTTATTCAAGCAGTATTGCTATTTAAATAAAATTTCATTTTAATAT
ATATTGTTGGAATAAATGGATGAATAGACAGAATACTTGAAAAGAAAATAATCTCTTACC
GGTAGTAATCAATTTATGCTTCTATACTATTGTATTTTCACATAGAGATTCAAATTATAG
CAAACTTCTTTTAAGATCTACTTATTTAATAAACATATAATGTAAACCCGAATTATAGTT
CATATGAATAAGACCCCTAGGATGGTTACTTAACAGTAGTAGCAACTAGTATTTGCTTTT
CATTCTGAATAGACAGCTTAACTTAGTAATCTATTTTAAGCCCAGAAGCTTCTCCTTCAA
AACAGAAGACTTATTGCTAAAACAAAATACCAAACTCATTGCCATGAAGGTGATTGCAAC
TCATAATGACCCTGTAGGAAAGAGTAGAACTGCCCTATGGAGTTTCCAGGGAGCACCTGG
TAGATTTGAACTGCTGACCTCTTGGTTAGTGTATATAGCACTTAACCACTCCACCACCAC
CATTTCCAAAGTACCCGTTAATCATTTTAAAATTCAAATTTATAAGAGTACTTTTATCAT
GTTGAATATATTAACGTTTCTGTATCTTATCTTTATTATGTGCTTGTTTTGTAG**GTGTAT**
GCTTGTCAGCATTGCCAAGTGGCAAAAAATACAGTTATTCTCACAACCAAACAACATGTT
CTCAAGGTGGAAAATCCATGGAGTATAGTGACTGTTGATCTGATGGGACCTTTTCACACA
AGCGATAGAAGTCATATGTATGCTATAATCATGACAGATTTGTTTACAAAATGGGTTGTG
ATTTTGCCTCTATGTGATGTTTCAGCATCTGAAATTTCTAAAGCTGTTATCAATATATTT
TTCTTATATGGACCTCCTCAGAAAATGATAATGGACCAAAGAGATGAGTTCATTCATCAG
GTAAGAAAACTGTTTAGATCTGACATATCTTTCATTGCCTGTTGCTAAGTGCCCTACACA
TTAGAGGATGCTTAATCATTATAGATGTAGTTAGTATAGATGTGTTAGTTACAATGGGGA
AATGTATTGCAAAAGACCTGTTTACGCTATTAAAGAACAAAGAAGTCCCCTTGTAGACTA
AGATGCACCTGACCCAAAGCATGATAATTTTTAGTCACCTCAGATGCGTATGATACTGCA
CAGTGAATAGAGAAAACCAAAGAAAGATTGATGCTTTTGAATTATGGTGTTGGTGAGAAT
ACTGCATATACCATGGACTGCATGAATAACAAACAAATTTGTCTTGAAGATGTGTGGGCA
GAATGCTCCTTAAAANNNNNNNNNNNNNNNNNNNNNNNNNNNNNNNNNNNNNNNNNNNNN
NNNNNNNNNNNNNNNNNNNNNNNNNNNNNNNNNNNNNNNNNNNNNNNNNNNNNNNAAAAA
GGCAAACCTTGAGGCGGATTGACACAGTGGCTTGCCAGCCGGGGGTCTCAAACAGCAATA
GTTGTGAGGACGGAACAGGATCGGGCAGTGTCTCTTTCTTTGTGCGTGGGGTCACTGTGA
GCCAGACCAACTCAGTGGCACCTCAGGACAGCAACAAGTATAGTTAAATTGCTACAGATA
CAGTCACCTTGCCAAAATACTAGGAAGGTGGTTGTACTAGGAATTAGGAAGACAGAATTT
TAATATTTATTGTGCCATGAAATACTTGTGAAATTGTCTTTAGTTATCCTCAAAACCAGG
AGATTAGGCAAAATCATTTTTTCTTTCTTAACTTCTGATTTAAAACTTCTATGATTGACA
CGTAAAGAGGGACTTGACTAAATGGACAAATACATTACATTGATGAATGAGAATACGTGG
TATTGAAAGATACTGAGTCTTCCTGAATCAAAATTAATTTATATTATAATGCATTTTAAT
TTGACGTTGAAAAGGAGGAAAATTATTTTTTGCTCTAATCTTTTTCCAGAGTATCCTGAA
AGAATAGGCAATCTGGATATATTCAACAAATTCTAAAAAAGAAATGTATTGTTGTCAGGT
GCTGTCAAGTTGGTTCTGACTCATAGCAACCCTATGAACAACAGAGCAGAACACTGCCCG
GTCTTGCGCCATGTCTGCAGTCATTTTTATGCTTAAACCCATTGTTGCAGCCACTGTGTC
AAACCATCTCGTTGAGGCTCTTCCTCTTTTTCACTGACCCTCCACTCTAACACGCCTTCT
CCAGGGTCTGATCCTTCCAGACAACATGTCCAAAATACGTAACACGCAGTCTTGCCACTG
TTGTTTCCAAGGAACACTCTCTTTGTGCTTCCTCCAGAACAGATCTGTTTGTTCTTTCAG
TGGTCTGTGGTACACTGAATATTTTTCTCCAATAACACAATTCAAGGGCATCAGTTCTTC
ATCTGTCTTCTTTGTTCATTGTCCAGCTTTCGCATGTGTATGATGTAACTGAAAATACCA
TGGCTTGGGTCAAGGGAACTTTGGTCTTCAAGGTGACATTTTTACTTTTCAATATTTTAA
AGAGATCCTCTGCAGCAGATTTTCCTGATACAATGCATCTTTTGATTTCTTGACTGTTGC
TTCCGTGGGCATTGATTGTGGACCCAAGTAAAATGAAATCCTTGACAACCGCAATCCTTT
CTCCATTTGTGATGATGTTGCTTACTGGTCCAGTTGTGAGGATTTGTAAGACATGTAATG
ACTACTAAAAGCACATGTAAAATTTTTTATGATTAATAGGGTACTGATAATAATCATAGA
TAGCCAGACTTCACTGGTTCTCAGTTCAAAAGAAACACCTAAAGTTTATTAAGTAGTTAG
CTCCTGTGTTAGTGATCCCCAGTACTATACCTAGGTTCAATGATTCAGTAGGAGTACTCA
GCACATTGTGATACTCATGACTAAAATACATTATAGCAAAAGTCACAAGGCAAAATTAGC
AAAGGGAAAGGGTTCTTAGGAAAAAAAATTAGGGAAACCAGAGGCAAGCTTTCAAGAGTC
TTTTCCTAGTGCAGTCACATGGGATGTGCTTAATTCCTCTAGTTATGAGTTTTGACAACA
TGTGTGAAATGTTGTCTACTAGGAAAATTGATCACAGACTCAATGCTCAGTTTTTCTTGG
GAGTGGGTCACATAGGTACTCTATCTGACATGTACTTGCCAGTCAGTTGAAAGGGAGTTT
CCTTGAAAGTGTGGCTTCAAATATAAGTGGGTATTCTGGCAAAGTTTAGGGACTTTTGCT
CAATCTAGATCCTGCAGCTGGGTCTTATTCATTTGACCTCCAGTTCTTGGGACTCCAGCT
AGCAGTTTATCTGTGGCCCTGCCCACCAGTCTATCTTTGCAGCCGGTGCACAAGAGCTCT
GCTCTCTGAACTGCTAGTGGTTGACCAGCCCCTGTGGCTACATGAATCAGGAGAAGCCCC
TATCCTGACTCACACACTTGGGTGTTCCAGCATCTACTACTGCATGGGCCACTTCCTTGA
TATAAATCTCTCTTTACATTTACTTATTTGCTTTACAGGTTGTGCTACCCTAGAGAATCC
AGCCTAAGACACCTCCTAAAATCTAAGTTCGTAAACACCAGCCCATGGCAAACCTTGTAA
GCAGGGCTTTCAAGGGGTAACAGTCACAGTTTAAATAATTTAATAAGTGTTTATATCTTA
ATCACTTGCCAGCCAAGTGCCATCGAGTCGGATCCGACTCATGGCAACCCTTAATCACCT
AGAAGCCGTTTTAAAAAAATAACCAGTTACTTACTAATGGAATGTGTATACCTGTGGAAT
GCTGCTCAGCTTGTCAAACCTTGGAATGAGACTGAATTCTGCCACCCTTATTTCCTGTTT
CATATTGATTTTTACACAATAATTGCTTTTTTATCAATGTCTGCCAAAACTCTTCTGCAG
AGATATCACATCCTGGACAGGAATGTAAGCTGTACCGGTCTTGAAACTGAACTACCTCAA
GTTAGTATTTATTTTAAATATTTTATTAGCCTTTAAGTGAAAGCTCAGACTTCAAGTCAG
TTTGCCGTATAAAACCCCACATACACACCACTAAGAATTCTGGGTGCTCTCCCCACTCTG
TGACAGTATGCACCTCATTTTTACCCTGTAGTTCTTAAATCCATTCATCCAGCTTCTGTC
CCCCAGCCCCTCAACCATCTTATTTCTCCTCTGGACAGGAGCTGTTCAGACAGCTTCATT
TATGTATTTAAGCCAAGAAGCACATTCTTTACCAGGATCACTCTCTTTCTTAGAGTCTAT
TCCAATTTTTATCTGAAGAGTTGGCATCAGTGCCGGTTTCAGCTTTGGGCTATCGATAGA
GTCTGGTGGCTATGTCCTTAGGGGACCCAAGTTAGAAGTTTTTAAGGGGGCTTCTTGGCA
CAGAGTTTGATAACTATGGTTAAAATTATAAAACTGTCATCACAAATGACTGGAATAGAG
AAGGATTATTTAATTAATTAACAGTTAATGTACCCAACCCCATTGCTGTCAATTCTGACT
CTTAGTAACTCTCTAGGACAGAGTAGAACTGCCCCATAGAGTTCCCGAGGGGTGTATGGT
GGATTTGAACTGCCAGCTTTTGATTAGTAGCTTTAGCAGTTAACCACTATACCACCAGGA
GTTCTAACAACTAACACAGGTAACAATTAGCAATAAAATGATTTTAGGACAATATACTGC
ATACCAAAATTACTTTGAGGTCAAGTAATGACTTATTTTTATTAATCAGAATGTAAAATG
AGTTAAAAAAAATTCAGTTGCCTTTTTTATAAATTCTCTAATGAAGCAGATAACATTCTG
AGTTTATTAATAGTGGCAAAAAGTACCAAGAGTAATGCAAATTAATACACCAATTTTTGT
TTATATAATCAGCAAAAATAGTTAATAGCAACAAATACACTACAGGTAGAATGACAGAAC

CTTACATTACTGGTGACATTGTAATGTGGAACTAATCTTTTGGAAAGCAATTTAGAGATT
CATGAAGAACTTTCTATTTTTTTTCTTTTGGTTAACAACCCTAATTCTAAGTTATTCTAA
GAAAATTATCCATTGTATAGAAATTATCTTTTATGGCAGTGCTACAGTGCAGCTTACATC
CATGAGTTGAGAAACCATCTAAATATTTTTAAAAAGGGAATGTTTAAGAAAAAATACATCC
AGCCACATAGCAGAATATCTTGTAGCTGTTAAAAATGATGCTTAAGTAAATGGAAAGATG
TTCCCTGCTTATGGACTAGAAGACTTAATATTGTTGAGATGTTAATACTACCCAAAGTGA
TGTGGAGATTCAGTGTAATCCTGATCAAAATTCTAGCAGCCTTTACAGAAATACAAAAAC
CAACTTTCAAGTTTCTGTGGAATGGCAAGAAAACCTGAATAGCTAAAACAGTGTTGAAAG
AGAAGTAGAAGGCTTCACACTTTCTAATTTTAAAACATACTGTTACAAGTACAGTAATCA
TGACAGCATGGTACTTGTGTAACAATAGACATATGGAACAGTGAAATAGAATTGATGGTC
CAGAAATAAAATTAGACAACTATGATCAACTGATTTTTTGATGCAAGCACTAAGACCATT
TGATGGAGAAAGAAGGATCTCTTCAATAAATGGTGCTAGGAAAAGTGGATTTCCATGTGC
AGAAAAATGAAATGTGATTCATGTCTCACATCACATACAAAGACAAATTCAAAGTGGGTT
AACGACTTAAATGTGCAAACTAAAACCATAGAACTCTTAAATAACAAGTGGGGCAATGCT
GTCAGGCCTCGCTTTCAACAATAGATTATCTAATATGGTAACAATACCAGAAAGAGCAAA
AGAAAAAGTAAATGTGACCTTATAAAAATTAAAAGCTTGTGTTCATCAAAAGACTTTACC
TGAAAATTAAAAAGACAAGCTGCTGACTAGTAGACTGTCTTAAAAAACCATATATCTGAA
AAGAACCTAATAACCAAAATATAAATAAAGTGTCAGCAACTTAACAAAAGACACATAACC
TAATCCATAAAATGAGCAAAGGACTTGAATAGACATTTCCCCAAAGAGGACATTCAGAAA
CTTGTATACCAGTGTTCACTGCAGCACTGTTCACGATAAGGAAAAGGTGAAAACAACCTG
AATGCCTGTGAATGGATGAATGGATAAACAAAGTGTAGTACTTACATACAATGGAATACT
ACTCAGCCACTTGTTAGGTGCCATTGAGTTGGTTCTGACTTACACTGATCCTGTGTATAA
CGCAAGGAAATACTGCCCAGTCCTGTGCCATGCCCACAATCGTTTTTGTGCTTAAATCCA
TTGTTGCAGCCACTGTGTCAGTCCATCTTGTTGAGGGTCTTCCTCGTGTTCGTTGACCCT
CTGTTAAGCATGATGTCATTCTCCAGGGACTGATTGCTCCTGACAACAAGTCCAAAGTAT
GTAAGACACAATCTTACCCTCATATTGTTTCAAAGGAGTGCTCTGGTTGGACTTCTTCCA
GAAGAGATCTCTTNNNNNNNNNNNNNNNNNNNNNNNNNNNNNNNNNNNNNNNNNNNNNNN
NNNNNNNNNNNNNNNNNNNNNNNNNNNNNNNNNNNNNNNNNNNNNNNNNNNNNTCAAATT
GTTTTGGTGAATTCACAACTTTTTATAAGGCTCTGAAGGAAAGAGGATAAATATTTGCCA
AGGAAAATGACTTAACTTAAAAATGTCTTATGAAAAACTATAATTGTAACCATTGATGGA
ATGTCTATTTTGATGGATGGGGAAATAAAACAACTCACAGGTCAACACTATCAAGAGACC
ATCTCACAAGTACATCCAACTACATCATTCATTCCTAGGCACTTTTTGAACAAAAAATGT
GAATCACTTTCTGAAGGTTCCAACGCCACCCCATTGCTATCGGATCAGGTCCAACTCATA
GTGACCCTATAGGTCAGAGTAGAACTGCCCCATGGAATTTCCAAGGAGTGCCTGGTGGAT
TTGAACTGCCGACCTTTTGGTTAGCACGTGTAGCACTTAACCCACTGCGCCACCATTTGA
AATCGTTGGTTTCAGCCATTTGAAATCATTAATTAGGTCCTCGATTGAAGCTAACCCAGA
TAATACCTATTCTCTGGGATTGTTTTCAAATTATTATTTAATGTAAAGGAGACAGCCTGA
ATTAGTACTTAGTATCAGCCAGCAGTTATTTGAAGCAGCAAGGAACAGAGAACTTTCCCC
TCCTCAAAATGTATAAACTCTATTGAGAGTTTATTTTGTACCCTTGTAAAAGAAGTCTGG
GTGATCCAGAGTTAGAAGGGTAGCTGTTTTTAGGAATCCATGTCCTTAACTTTCTGTCAC
CCTAAATCTTCTTCTTGGTGTGAGCCAGAGCCATTTCTTTTACATTTTAGGCGGAAAGAA
GGGGAAGAAAGATGTGCAAAAGAAGAAAAAAACTACCAGCTAAGTCTACCCAAGAATGTT
CAAGAGCCCCACATCAGCCATTTCTTTTTTTTTCCCACCACACTGTGTTGTAGGACCACT
CCCACCTGCATGCAACTTTGGGAAATACAGTTTTATTTTGTTTTCTCCTCATTTCCTAGC
TTTACCTTTTTCGTTACTCTGGTATCATTTGCTGCTTATACTTAAGCAACAGGAAATACA
GTATTCTTTAAAAAATAGTGAATCTGCCAATGGTTTATCCAATTAAAACTAAGGAGTAAA
ACAGGAAAAATGCTATTTTACATTTATCAGACATACTGTTAGAAAGTGTTCTAAACTCTG
ACTTCTTTTAGGCCCTGTATGTAACAAACGTAACAACAACAATAATACCAATTAAGAGTT
ATAAAGTGTGGACCGTCTGCTAGGAACTGGTTTTCATGCTTTAAAAACATTAACTCATAA
TCCTCAAAAGAGCGGTGAAAAGATCCAATGAAAGGGGCAAGATTCAAATTCTTTTAAAGC
TTTTTTTTTCTCATCTCAATGGGAAAACAACAGATTTTTAGAAGTTAGCTTTGTTTTAAT
ATGTTTTCTAG**ATCAATGTAGAACTATATGGCTTGTTTGGCACAAAGGAAATTGTAATTT**
CTCATGCCTCTCAAACTGTTGACCCACT**GAAAGTGCACCCAGAACAATCAAA**CA**TTTCTC**
TCCAAGCACTGTGCTGACCACCCGAACAACTGGGATGAGCATCTGTCGGCTGTTTCATTT
GCCTTCAACATAATTNNNNNNNNNNNNNNNNNNNNNNNNNNNNNNNNNNNNNNNNNNNNN
NNNNNNNNNNNNNNNNNNNNNNNNNNNNNNNNNNNNNNNNNNNNNNNNNNNNNNNNNNNN
NNNNNNNNNNNNNNNNNNNNNNNNNNNNNNNNNNNNNNNNNNNNNNNNNNNNNNNNNNNN
NNNNNNNNNNNNNNNNNNNNNNNNNNNNNNNNNNNNNNNNNNNNNNNNNNNNNNNNNNNN
NNNNNNNNNNNNNNNNNNNNNNNNNNNNNNNNNNNNNNNNNNNNNNNNNNNNNNNNNNNN
NNNNNNNNNNNNNNNNNNNNNNNNNNNNNNNNNNNNNNNNNNNNNNNNNNNNNNNNNNNN
NNNNNNNNNNNNNNNNNNNNNNNNNNNNNNNNNNNNNNNNNNNNNNNNNNNNNNNNNNNN
NNNNNNNNNNNNNNNNNNNNNNNNNNNNNNNNNNNNNNNNNNNNNNNNNNNNNNNNNNNN
NNNNNNNNNNNNNNNNNNNNNNNNNNNNNNNNNNNNNNNNNNNNNNNNNNNNNNNNNNNN
NNNNNNNNNNNNNNNNNNNNNNNNNNNNNNNNNNNNNNNNNNNNNNNNNNNNNNNNNNNN
NNNNNNNNNNNNNNNNNNNNNNNNNNNNNNNNNNNNNNNNNNNNNNNNNNNNNNNNNNNN
NNNNNNNNNNNNNNNNNNNNNNNNNNNNNNNNNNNNNNNNNNNNNNNNNNNNNNNNNNNN
NN**NNNNNNNNNNNNNNNNNNNNNNNNNNNNNNNNNNNNNNNNNNNNNNNNNNNNNNNNNN**
NNNNNNNNNNNNNNNNNNNNNNNNNNNNNNNNNNNNNNNNNNNNNNNNNNNNNNNNNNNN
NNNNNNNNNNNNNNNNNNNNNNNNNNNNNNNNNNNNNNNNNNNNNNNNNNNNNNNNNNNN
NNNNNNNNNNNNNNNNNNNNNNNNNNNNNNNNNNNNNNNNNNNNNNNNNNNNNNNNNNNN
NNNNNNNNNNNNNNNNNNNNNNNNNNNNNNNNNNNNNNN**NNNNNNNNNNNNNNNNNNNNN**
NNNNNNNNNNNNNNNNNNNNNNNNNNNNNNNNNNNNNNNNNNNNNNNNNNNNNNNNNNNN
NNNNNNNNNNNNNNNNNNNNNNNNNNNNNNNNNNNNNNNNNNNNNNNNNNNNNNNNNNNN
NNNNNNNNNNNNNNNNNNNNNNNNNNNNNNNNNNNNNNNNNNNNNNNNNNNNNNNNNNNN
NNNNNNNNNNNNNNNNNNNNNNNNNNNNNNNNNNNNNNNNNNNNNNNNNNNNNNNNNNNN
NNNNNNNNNNNNNNNNNNNNNNNNNNNNNNNNNNNNNNNNNNNNNNNNNNNNNNNNNNNN
NNNNNNNNNNNNNNNNNNNNNNNNNNNNNNNNNNNNNNNNNNNNNNNNNNNNNNNNNNNN
NNNNNNNNNNNNNNNNNNNNNNNNNNNNNNNNNNNNNNNNNNNNNNNNNNNNNNNNNNNN
NNNNNNNNNNNNNNNNNNNNNNNNNNNNNNNNNNNNNNNNNNNNNNNNNNNNNNNNNNNN
NNNNNNNNNNNNNNNNNNNNNNNNNNNNNNNNNNNNNNNNNNNNNNNNNNNNNNNNNNNN
NNNNNNNNNNNNNNNNNNNNNNNNNNNNNNNNNNNNNNNNNNNNNNNNNNNNNNNNNNNN
NNNNNNNNNNNNNNNNNNNNNNNNNNNNNNNNNNNNNNNNNNNNNNNNNNNNNNNNNNNN
NNNNNNNNNNNNNNNNNNNNNNNNNNNNNNNNNNNNNNNNNNNNNNNNNNNNNNNNNNNN
NNNNNNNNNNNNNNNNNNNNNNNNNNNNNNNNNNNNNNNNNNNNNNNNNNNNNNNNNNNN
NNNNNNNNNNNNNNNNNNNNNNNNNNNNNNNNNNNNNNNNNNNNNNNNNNNNNNNNNNNN
NNNNNNNNNNNNNNNNNNNNNNNNNNNNNNNNNNNNNNNNNNNNNNNNNNNNNNNNNNNN
NNNNNNNNNNNNNNNNNNNNNNNNNNNNNNNNNNNNNNNNNNNNNNNNNNNNNNNNNNNN
NNNNNTAGGATGGAGTCTTACGGGAGCAGATCGCCAGATCATTTGGCTGCAGAGCTGCTG
GATGGGTTCAAACTACCAAACGTTTGATTAGTGGCCAAGCTCTTAACCTTTATGCCAGTA
GGTTTCCTTAAAACCAAGTTATACTTTCCCCAAATGTGACTAATTATGAAGAGTTCAAGA
TACCTTGATATCACTAATCCTTTCTTTCACTACCTAGAGCTAAATAATATTTTTGCGACA
TCTTTCAGATTAAGGCCTACTTTATTAGGATTATTTTGTCTTGTAAAAAGAAATATAAAA
CATCAGTATTATTTGAATATGAGTAACATTTGATGGAGGGGAGCATCAGCGAAAAGGGGA
GAAACCCTCAGTAAGATGGAGTGACACAGTAGCTGTAGCAGTAGACTCAGGCACAGTGGA
AATCACGGAGGTGGCGTAGGACCAGGCGGCACTGTATTGTTATATAGAACCTACTTGACA
GCAGTTAACAAGAACGTTTCATGGAAAATGCCATATGGTATGTTTTAAACAACTAAGTTA
TGTAGCAGGCTTAAGTATTAGTAGCAACAAAAACAACAAATAACCTTCAGTTACCCTAGG
CCATTTTTAGGGTATAATGATGCCAAGGGCTGCTGAGTAATAATGCAGCTGGGCTTTAGA
TTTGGGCCACAACTCTTGTGTTAAAAGCTCTCGGTACCTGTTTTTCTGCTTGTGACAGAC
CCAGCTGTTCTAGCCAGAAACACATACCTTGAACATTGTTGTAATTCTTAAAATTCACAA
GTACCTTCATTTTCTTTGTTGGAAGATAAGCTATTTCATTCCTTGTTCTACTTTTCAGTC
CTCAGCTACCCTTTATGTTCTCTCCCTTTTATGAGTTAAGTAGGGAAAAAAAATGTAATG
TGTGTGCGTGTATACATTTGCATGTGTACATACATATATATATGTATACATGTATATATG
TGTATATAACACAAGTACGTGTTTTTCAGTATGATTCTCCTCTTTGGCAATAGGACTTCT
TTCAAGTGTGTTAGCCTTCTTTATGTATTATAAACTTTACATTGAAAATGAGGCTGCATC
ATCATGTGTTCCAAAATTTTGTGATTTGCTCTTGCCAAAAATTTTCTTAGGATGGAGATC
ATACATTTTGCTTCTCCTGAGCATTTCAGAACTGAGTTGGCTAGAACAGTCACTCCACAG
AGGCAGTATGATTTTTGCCCCCTACTTTAATTTGCTGCAGACGTTCTTTCCAGTAATATA
AGGAGATTGGCTGCCCTGGTGCTGACGTGCACATGAGAGACCTGAGATGAGTGGGGAAGA
CCGTTTTCTTTTTTCTTGTTGACTGAAGAAAGTATGACCAGACTAGCCTTGAATCTGGTT
CTTGATTACTGTTTGGTTCTTTTATGTGTTTGTGTGTGTCTCTCCTATTTGCAGAAATTT
AAAACAAATTCTGTTTCTGTCACCTTTTTCCTCAGTATCACATACTTCCCTGAAAATCTT
CACTTCAGTTTCAAGGAGTCTGAGAAACTGAATTTAAAGCAAACAAAATATCTTTGAGTA
ATCATCCATATTTTTAAATGCAAAGTTGGATGAAACTGTCCAAAAGAACTATGAATTTGT
TGTCTCTGTTCTTAGAAACCTTGCAGTTTTGTCACTGTATTGAAGTCTCACTGTCTGAAG
TAGCTATGCTTCTGTTGCTGACTTCTTACCAAGAAAATAATATAGAAGGAGTATATTCAG
TACTAGGTCTCTGATTTTTTCAGTGCTGTTTTGAGGTTTCTGTTTTACTTTGTGTATTCC
ATGCTAAGCACCTTTTCTGAGTGATTTTTACTACAATAATGACAATAAGAAATGTTACGA
TATACCACAGTGTCTCTAGGTACTCAGAATGACCCTTTTTCAATGGGAAAATGATGTGGT
TGACTCATTCTTGACACCCTGGTGGCATAGTGGTGTAGTGCAGCAGCTGCTAACCAAAAG
GTTGGCTGTTTCAAATCACCAAACACTCCTTGCAAGTCCTACGGGGCTGTTTTACTCTGT
CCTGTAGGATCACTGTGAGTTGGAATGAACTCAGCAGCAATCAGTGTTTATGTCAGTGAA
CATAGATTTAGGGTAGTTGGTTTTGTTTTATTTTTTGTATGAAGAATGTTGTGATAGATT

```
TGTTTCTGCTTCATAAAAACGTTTTAGTTTGAGTGTCNNNNNNNNNNNNNNNNNNNNNNN
NNNNNNNNNNNNNNNNNNNNNNNNNNNNNNNNNNNNNNNNNNNNNNNNNNNNNNNNNNNN
NNNNNNNNNNNNNNNNNNNNNNNNNNNNNNNNNNNNNNNNNNNNNNNNNNNNNNNNNNNN
NNNNNNNNNNNNNNNNNNNNNNNNNNNNNNNNNNNNNNNNNNNNNNNNNNNNNNNNNNNN
NNNNNNNNNNNNNNNNNNNNNNNNNNNNNNNNNNNNNNNNNNNNNNNNNNNNNNNNNNNN
NNNNNNNNNNNNNNNNNNNNNNNNNNNNNNNNNNNNNNNNNNNNNNNNNNNNNNNNNNNN
NNNNNNNNNNNNNNNNNNNNNNNNNNNNNNNNNNNNNNNNNNNNNNNNNNNNNNNNNNNN
NNNNNNNNNNNNNNNNNNNNNNNNNNNNNNNNNNNNNNNNNNNNNNNNNNNNNNNNNNNN
NNNNNNNNNNNNNNNNNNNNNNNNNNNNNNNNNNNNNNNNNNNNNNNNNNNNNNNNNNNN
NNNNNNNNNNNNNNNNNNNNNNNNNNNNNNNNNNNNNNNNNNNNNNNNNNNNNNNNNNNN
NNNNNNNNNNNNNNNNNNNNNNNNNNNNNNNNNNNNNNNNNNNNNNNNNNNNNNNNNNNN
NNNNNNNNNNNNNNNNNNNNNNNNNNNNNNNNNNNNNNNNNNNNNNNNNNNNNNNNNNNN
NNNNNNNNNNNNNNNNNNNNNNNNNNNNNNNNNNNNNNNNNNNNNNNNNNNNNNNNNNNN
NNNNNNNNNNNNNNNNNNNNNNNNNNNNNNNNNNNNNNNNNNNNNNNNNNNNNNNNNNNN
NNNNNNNNNNNNNNNNNNNNNNNNNNNNNNNNNNNNNNNNNNNNNNNNNNNNNNNNNNNN
NNNNNNNNNNNNNNNNNNNNNNNNNNNNNNNNNNNNNNNNNNNNNNNNNNNNNNNNNNNN
NNNNNNNNNNNNNNNNNNNNNNNNNNNNNNNNNNNNNNNNNNNNNNNNNNNNNNNNNNNN
NNNNNNNNNNNNNNNNNNNNNNNNNNNNNNNNNNNNNNNNTCAAGGGACTTGATTCTTGT
ATTTATTATTAATTTAGTATATGTAAAAACATTTCGTTTGGCCATCGGTTATATAATCTC
TAAGTTAAGAGTCATTCACTTCTCTCTGTTATTAGTTAAAATTTGTGGTCTATGATACTG
AGTCGACAGCATTGGGTTTCATTATAATAATGAATGGTATAAAATTTTAGTTTCAAAGTT
TTCATTTTATCACAGGAAAAGATGTACTAATATGGAAGTATGTTATAGTAGCTTTAGAAT
TATCAGATCATCTATAGGATTTGTCTAGTTATCTCTCTTCAGAATAAGAAGGTTAAGTAT
TTTGAAGCCTGTGCTTTTCCAATGTGTGTAAAGATATAAATCAGATCACTCAGACTTTTT
AGGTACTTTGACTTTATTTTTCTCCTTTGGAAATTTTTAAGTATCTGTTGATCAGCTACT
AACCAAGAGGTCAGCAGTTCAGATCCCCGAGCCACTCCTTGGAAACCCCATGGGCAGTTC
CAGTCTGTCCTGTAAGATTGGTATGAGTAGGAATTGACTGGACAGTAACCGGTTTGATTT
TTTGGTTTTTTAATCTAATTTTTTTGCTCCTTAAAATTACATAAGGAAAAGTTTGTCAAA
ACTGGTGATAACATCTTCCTGGACTGTCTTGAAAATACTGTAGTTTTAAAGAAACTAAAG
ACATAATTTATTCTGTCTTGTCAAAACTGGAACAGTGCTTTCATCAATGCATGTCATAAC
AATTAATATACATTAACCTTCACATGTAAAGATGCTTCTGCCTAGCCACCAGTTTAATAT
TCATTTTGAACATTTTCTTTCTCTAATGGAGATGATAAATTGCTCAAAATAAATCAAAAT
GCTGGCAAGAATGACTCTTTGTCCATACTTTGGAATAGGTTTTTTATTTAGATCTAAATT
AAGTTCCTGAGAACTCTGGTATTAAGTCAGACTGACATGAGCTAAGTTTTGAAGCTACTT
CACTACACCCTCCTGTGTATACATCCTTACCACATATGCCCTATTCTGGAAATTTTAGCT
TTGTATTAGCTTATAAATTTATATTGTAACAATTCTGTTTGTTAAAATAACTTCCATGAC
CCCATTAATTTACCCCGAAATTCTTTACAGAACCATATTAAGGATATTATCAATATTTTT
TCAAAGCAAATATAGTTTTGCAGTTTCAAAGAATGTGTGCCATAATTGCCCAACTTCCTT
GTTTGCCTCCACTGACCAAAGATATGTCAAGGAAACAGGAAAATCTCACTGTTTTAAAAT
CCATGATCTTTCTTGCATTTGATGTCTAAGAACTGTGAAAATTTTATATTATGTTCATTC
TTTTACATATATGTTTAGTTAGTAGCAAAGAGAACTTCCACAAGTTAATCTTGATAGTTA
ACTTTTATTGCTTGCAATTCTGTTCCTCATTTAACATTTTTGAATTTTGAAATAATTTTT
TAATTATCATCCTAAAAACAAACTTGGTGTTTTTTTTTCTTTCAGACAGTCTTTATCTCTG
CCAAGGCCCAGTAGTAGCAGATCATGACTACACTGGGTTTCCTGAAATTCCAGTTGGCAC
ATACCAAGCAAATATTCTTGTAAAAGATGCAGCTATTGCTGTAATAGATAATGAATTACT
GACATCAGCCAAGGACTGTGAACTATTAGCGTATAGACATGCCAAAGTCTCTCCATTGAC
GGAAGATCATGGTAGTCTTGACCGACAGACTTTCAGTCTCTTGGATTCTTCAAACCAAGT
TCTTGAGTACTGAAGTTAGTAAATACCTAAATGCATTTAAAATGCTTGTTTATAATGTGT
AAACCCTAGAATCTGATATCTCAACTGTCAAAAAGAGTTGTATAGGAATGTCTTGATACA
CTCTTGGTAAGTAAATCATGATGTTTATTTTAGTAACTATTTATTTATTATGACCATTTT
TGAATGTTATTGCATAAGAGGTGTGACAGTTAAGTTGGTGAACTCATCCTGGAAGAAGTG
CTACATACCTCATGACTGCACATCACTATGGCCACTGTCAACATACTCCCCTTGGGAAGC
TGTGCACCAATACCAGCACCTAGAATCTACCTTTCAAAGAAATTTTGTAGTCTTTTTCTG
GAATGGCCATCAGAGCTGTCGTTGTATTATCCTTGATGTTGTGAATGTCATTAAAATGTC
TTCCTTTCAATATTTCCTATATTTCCAGTTAAAGAAAAAAGTCATTGGGGGCCAAATCAC
GTGGTTAGTGAGTGTGTTCCAATACAGTTATTCGTTTATTGGGTAAAACCTCCCTCACAG
ACAACTGTGTGATTAAGTTTTCAAACTTGCCACCGTGCACAATGACCATTTCAGAAAGAG
TTCCAGAATTGCTTTGCAG
```

```
==================================================
**Hyrax Gin-1**
total length:      19399 bp  (15756 bp excl N/X-runs)
GC level:        36.54 %
bases masked:       6921 bp ( 35.68 %)
==================================================
               number of      length   percentage
               elements*    occupied  of sequence
--------------------------------------------------
SINEs:                12         1764 bp    9.09 %
      Alu/B1           0            0 bp    0.00 %
      MIRs             1          104 bp    0.54 %

LINEs:                 8         2354 bp   12.13 %
      LINE1            4         1288 bp    6.64 %
      LINE2            0            0 bp    0.00 %
      L3/CR1           0            0 bp    0.00 %
      RTE              5         1469 bp    7.57 %

LTR elements:          3          824 bp    4.25 %
      ERVL             2          725 bp    3.74 %
      ERVL-MaLRs       0            0 bp    0.00 %
      ERV_classI       0            0 bp    0.00 %
      ERV_classII      1           99 bp    0.51 %

DNA elements:          4         1458 bp    7.52 %
      hAT-Charlie      2          513 bp    2.64 %
      TcMar-Tigger     0            0 bp    0.00 %

Unclassified:          0            0 bp    0.00 %

Total interspersed repeats:      6400 bp   32.99 %


Small RNA:             0            0 bp    0.00 %

Satellites:            1          417 bp    2.15 %
Simple repeats:        0            0 bp    0.00 %
Low complexity:        4          104 bp    0.54 %
```

==

SW score	perc div.	perc del.	perc ins.	query sequence	position in query begin	end	(left)		matching repeat	repeat class/family	position in repeat begin	end	(left)	ID	
3738	11.0	2.3	0.2	UnnamedSequence	1309	1838	(17561)	C	Mari1_Pca	DNA/TcMar-Mariner	(4)	542	2	1	
712	13.3	10.8	0.2	UnnamedSequence	2185	2318	(17081)	+	AFRO_LA	SINE/tRNA-RTE	7	201	(17)	2	
22	48.3	0.0	0.0	UnnamedSequence	2560	2588	(16811)	+	AT_rich	Low_complexity	1	29	(0)	3	
22	72.7	0.0	0.0	UnnamedSequence	3448	3469	(15930)	+	AT_rich	Low_complexity	1	22	(0)	4	
25	65.6	0.0	0.0	UnnamedSequence	3694	3725	(15674)	+	AT_rich	Low_complexity	1	32	(0)	5	
923	18.9	0.0	0.6	UnnamedSequence	4039	4208	(15191)	C	AFRO_LA	SINE/tRNA-RTE	(3)	169	1	6	
1791	36.2	2.6	0.7	UnnamedSequence	4729	5257	(14142)	+	RTE1_LA	LINE/RTE-BovB	2695	3233	(0)	7	
21	71.4	0.0	0.0	UnnamedSequence	5550	5570	(13829)	+	AT_rich	Low_complexity	1	21	(0)	8	
478	15.7	3.6	1.2	UnnamedSequence	5689	5772	(13627)	C	SINE2-1_EC	SINE/tRNA-RTE	(1)	406	321	9	
324	33.9	7.7	6.1	UnnamedSequence	5708	6175	(13224)	C	ART2A	SINE/RTE-BovB	(40)	511	37	10	*
1306	8.8	0.0	0.0	UnnamedSequence	6176	6345	(13054)	C	RTE1_Pca	LINE/RTE-BovB	(498)	2761	2592	11	
232	18.2	12.7	0.0	UnnamedSequence	6430	6484	(12915)	C	MER119	DNA/hAT-Charlie	(54)	529	468	12	
1507	24.8	4.2	0.0	UnnamedSequence	6485	6815	(12584)	C	MER68	LTR/ERVL	(0)	563	219	13	
2160	16.4	5.3	1.2	UnnamedSequence	6818	7211	(12188)	+	MLT2b_Pca	LTR/ERVL	138	547	(0)	14	
322	4.5	2.3	0.0	UnnamedSequence	7328	7371	(12028)	C	PSINE1	SINE/tRNA-RTE	(5)	161	117	15	
989	22.0	4.3	0.8	UnnamedSequence	7397	7630	(11769)	C	MER119	DNA/hAT-Charlie	(215)	368	127	12	
1347	26.4	0.3	3.1	UnnamedSequence	7641	8008	(11391)	C	L1-4_LA	LINE/L1	(2)	7089	6732	16	
838	17.6	3.9	0.0	UnnamedSequence	8134	8286	(11113)	C	AFRO_LA	SINE/tRNA-RTE	(13)	159	1	17	
354	33.5	10.2	1.2	UnnamedSequence	8493	8864	(10535)	+	L1ME4c	LINE/L1	174	578	(252)	18	*
1079	14.4	1.6	1.1	UnnamedSequence	8849	9030	(10369)	+	L1-3_LA	LINE/L1	5603	5785	(2324)	19	
355	33.0	3.0	6.2	UnnamedSequence	9037	9336	(10063)	+	Sat-1_TS	Satellite	1811	2101	(1135)	20	
2140	13.7	1.8	0.3	UnnamedSequence	9337	9718	(9681)	+	L1-3_LA	LINE/L1	6097	6484	(1625)	21	
244	31.3	0.8	1.7	UnnamedSequence	9733	9849	(9550)	+	Sat-1_TS	Satellite	2805	2920	(316)	20	
318	28.3	2.9	2.9	UnnamedSequence	9851	9952	(9447)	C	SINE2-1_EC	SINE/tRNA-RTE	(4)	403	302	22	
1182	15.1	2.5	1.0	UnnamedSequence	9953	10153	(9246)	C	RTE1_Pca	LINE/RTE-BovB	(108)	3151	2948	23	
365	26.3	11.2	5.1	UnnamedSequence	10280	10503	(8896)	+	Charlie7	DNA/hAT-Charlie	1174	1410	(1202)	24	
893	16.1	0.6	0.6	UnnamedSequence	10520	10675	(8724)	C	AFRO_LA	SINE/tRNA-RTE	(7)	165	10	25	
236	33.6	2.2	0.0	UnnamedSequence	11439	11542	(7857)	C	MIR	SINE/MIR	(0)	274	169	26	
580	24.6	0.8	0.0	UnnamedSequence	13806	13927	(5472)	C	AFROSINE2	SINE/tRNA-RTE	(84)	123	1	27	
389	31.3	12.1	0.0	UnnamedSequence	14130	14295	(5104)	+	RTE1_Pca	LINE/RTE-BovB	3072	3257	(2)	28	
263	25.2	15.2	0.0	UnnamedSequence	14670	14768	(4631)	+	RLTR17B_Mm	LTR/ERVK	290	403	(399)	29	
791	19.7	0.7	0.7	UnnamedSequence	15733	15885	(3514)	+	AFRO_LA	SINE/tRNA-RTE	3	155	(17)	30	
683	21.2	0.7	1.4	UnnamedSequence	17453	17591	(1808)	+	AFRO_LA	SINE/tRNA-RTE	34	171	(1)	31	
2634	14.3	2.2	0.5	UnnamedSequence	18985	19399	(0)	C	Mari1_Pca	DNA/TcMar-Mariner	(0)	546	125	32	

Xenarthra

>Dasypus Gin-1 (scaffold:dasNov2:scaffold_5385:8291:27787:1)

NNNNNNNNNNNNNNNNNNNNNNNNNNNNNNNNNAAAATCATTTCTGTTCAAGAAAAAGCA
GGTCATAATATAGTTAGGAAGGGTTATGAGATCTGGCATAAATTAATATCTTCTTTATTT
GAACATTTTCAAAATATATATGTGATTTGAGTAGATTTTTTTTCCATTTTGCAGTGACCA
TGAATGTATTTAAAAGTAATATAAAACTACATTTATTTAAATGGCACATAACAGGACTAT
CCTATTAATATTTAAGTAATATTGCAGTTGATGACAATAAGAGAGACTTTAAGCAAGACT
TTTTGAAATATATTTAATCATTTCCTTTTTTTTCCGTTAGTGGAAAATTTTCAGCATTTC
TTGTCACATGGAAAGAGACAAGATAATTTGTCATGATGTATCTCAAAACAGCATTTGAAT
ATTTTAGTTCATATACATCACTCTATTCCTTTTTGTACTGCAGTTATCTTTCTCATAGAT
AATGTTCTGGGAAAGCTAAATTCACGTAAGCAGAATTATGACAGAAGCCTGAACTGAAGT
AGCAACTAAATTAGCTTATCACTTAAATTATATCTTTAAATACCTTTCCCTAGGTTAACA
ATGGTCCGTAGTGGAAAGAATGGTGACCTTCATCTTAAGCAGATTGCATATTATAAACGA
ACTGGTGAATATCATCCCACTACATTGCCAAATGAGAGAAGTGGCATAAGAAGAGCAGCA
AAAAAATTTGTCTTCAAAGGTAAAATTAATATGCTTAACCTGGAGTATGGTATGGGGTTA
ATATAGATGCTTCTGCACATGTTCTTTATGGAACCTGAAAATTTCTGTAGCAATTAGTGT
TGTTTAACAATTACTAGTTAATGAAGCCACCCAGTGAAAAACAAATAGAAATCTCACTGA
AGCTATCTGAAGGCCAGTAAGCATTTAAAGTGCTATTGAAATGATAGGCAAAAAAAAAAA
TGACACCATAAAAGAAATATAACACATCAAAATTCTGCTTTTGCTGACTCAGGTGGTTTT
TCATATAATGGTGTACTTTCGAGCATTGATGCCACTTTTAATGAGGAACTGATTTTATGC
TTCATATGAAAATGTTGAAAAAACTCATTGGTTTATTAAAGTGAAAAATGAAAGGAATTA
ACTATATTTCTATAGTTTCCCTACTCCCACTCCCATTAAATATTTTCCAGATTATATTTA
TTTTGGACCCTAAGTCTCCTGAATGAATTAAATATTTGTAAGGCAGTAGACTGTTTTTCT
TCAAAGGTTATGATCATTTCCCCCCATTTCACCCCATCCTGCAAAGAAAAAAAGAAGTAT
AAACACTAGATAAGTACATGAACAGTTGTGATATTAATGGAAGTTTTACCTATAGGAACA
GCATTAGGTTTTCCCCAAAGCAGGACAAGAAAATTGTAAGATAAGTAAATGCCTCCTCTA
TGTATACTCATCCCAATAACTCCCACAGGTTGAAGATGGTTTGCCTAAATCCATTACTGC
TTAGACTAAAGTGAGGAGTCACTCACTCATGTATTTATTCATTTATGCAACAAATATGAG
TCTCTACTATCTGCCAACCACATTTTAAGTCTTGGGGATACTTGGATAAATAAGATAGAC
AAGGTTCCTACCCTCAAGTTGTTTACCTCCAGGTCAGGAATGTTCCACTTAGCACGTCCT
TAGGATTATTAGTAGTTTAATGGCTTATTCTGGATTTTGAATAGTTGCCCCATCCACATT
GGAGAAGTGGCAATTCTTTTTCTTTCCTTGGTTTCTTCCTAGTTGCAGATTCTTCTCAGG
TTTCCTCTGAGGAAAACAAAGAACTATTTTGAAAATTAAGGTCTCCCACTTTCCCACCCT
ACCATCACCACCTCTAACTCAATCTAGAAAATCCTGAGAGTATAGTTACTTACGAAAATG
CAGCTATATGATGAAATACTATTTTAAATTCTGTTCTAGGAAGTTGCTTATTTGTCTGTA
ATTCATTTGCTAATTCATGCCATTTTTAGGAGACACCTGCAAGTATTTTTTCAGTTTGAA
TGCTCAGTAGAGATAATTTCATATTCTGGCAATATTGTGTGCTTTGGTGAACAAATCCAC
TCTACTAGTATTGATAACTATAAAATTATAGCAGCTAGTGCTCCTCCTAGACAGCTAGTA
CATATAATGGCCAAGGACCTTTCAAATTGTATGGTAGTATCATTAAAAATAAATATTTCA
TTTTAGGAGGAGAAAGATTTTGTAAAAAAATGAAAACAAAAACCTTATAGTTTTAAGATG
AAGCCATGATTTTCTTAAAACTACAGAGATCTCTAAAAAATTGTAATTCTATCTTTTTGG
TATCATTTATTTCAG**AAAAAAAGCTGTTTTATGTGGGAAAAGACAGAAAACAAAATCGTT**
TGGTAATTGTTTCAGAAGAAGATAAAAAGAAAGTCCTAAGAGAATGCCATGAAAATGACA
CTGGAGCCCATCATGGCATTTCCAGAACACTTACTCTAGTGGAATCCAGTTACTATTGGA
CTTCTGTGACCAATGATGTCAAACAGTGGGTATGGCTATGTATTTAAAGTATTTTGAATA
ATGTCTTTATAATAAATATCTGAAATTCATCAATGCTCTAGATTTATTCTGGGTATCATC
TCTCATATACTTTTTGTATAGGAAGTTTTTATACAAAACTCTTGAAGTTTTATTTCCCTT
TACAATTCTTTATTAAAATCATTGAAATATAATTTGAACAAAAAAGAATCACTAAACATA
CAGTCTTTTGAAAATACCTGATTTAGATACCTTTCTAAGTATAGAGCCAGGTTTTCATCA
TTACTTCTTTATGTGGGTAGGACTTAAAGGGAACTGAAATTCTTTTGACTAAATTATTAC
CTCTTTGACTACAGTTGAGTTTTTGTGTGTACTCATCTTTTGTGCCTATTCCATCTTGGG
TTAGAAATGTCAAATATGGCAGAAATCCAAACCAACTAAATATACAATCAAGTTTTGTTC
GTAAGGAAAAGAGACTCATTTTTGTGACTTTATAGCCTATTAAGAGCTTTAACTTTTTCT
GATAGAAAATTCCACTAGGGTTTCTGCCAGTAGCTCAACTGATCCCTATCCATTTTAATA
TTAAAGGACCAAATTCCTTGTTATGTATGTGAAAGCTCTTTAATAATTATAGTAGTGCAT
ATCAGAAAGCCTGCTATTTCATCCATAAAGGATTAAACCAACATTCAACTAAATACTGTC

TGTGAGATAGCAGTTTATGATTCTGTACAGATATTCCACAGACCTCTGTATTTGAAATTG
CCTTGAAAATGTGCCACATATTCCTTTTCAAATATTCGGTGAAATCTTTGTCAAATCTTT
TCATTTGAAAATGAATATGGTTTTTGGAAATAATCAAGTTATTTGAATTAAAACTAATGA
ATACATTAAAAAATTAAAATTTTGATCAAAAGTAGTCATGTAATAAAATGGTTTTTAAAA
TTTTATTTATAAATTGGCTTTGAAAGTAGTTTCCAAAGTGAATTCCAGAAATATATTGAG
CCATAGTTACCATCAATAAGTTGAGCAACTGATTACTTCTCACCTCATTCAAATGAAAGC
CCATGGCAGCATGAGCTTTGTTTTTGTTTGTTTTTTTGCTTACTTTTTTTTTTTTAAAGAT
TTATTTATTTATTTATTTCTCTCCCCTTCCCCTGCCCCGGTGTCTATTCTCTGTGTCTAT
TTGCTGCATGTTCTTCTTTGTGCACTTCTGTTATTGTCAGCGGCCCGGGAATATGTGTCT
CTCTTTGTTGCGTCACTTGTGTCAGCTCTCCGTATGGGCGACGCCATTCTTAGGCAGGCT
GCCCTTTCTTTTGTGCTGGGCAGCTCTCCTTATGAGGCGCACTCCTTGTGCATAGGGTTC
CCCTACGCGGGGGACACCCCTGCGTGGCAGGGCACTCCTTGCGAGCATCAGCACTGCACA
TGGGCCAGCTCCACACGAGTCAACGAGGACGGGGGTTTGAACCACAGACCTCCCATGTGG
TAGACGGACGCCCTAACCACTGGGCCAAATCCACTTCCCTGGGCTTTGTTTTTTGTTTAC
TGTTTTCCCAAGATATAGAGCAGTTTCTGGCATCTATAAATATTTTTTTAATAAAATGAT
CTTTTAATAACTTAAGAGAATAGATAAATGAATTTCTCAGTGACAGCTTTCAAAGAATGA
TCTTTCATATATAAAAATTACTGTATTTCAAATACTGATTTAAAATTATATCAAATCTGT
TTAAGGATATATTCATTTATTAAGTATTTATTAGATGTGAACCAAAAGTATGTTCTGTGT
GAATAAGACCTTCTTACAGTATGGTCATTTAATAGTAGTAATAACTAATACATGCTTATC
ATTCTGAGTAGATGTTTAACTCAGTGGTCTATTCTCAGTCCTGAATCTTCTCCATCAAAA
CACAAGTGCCTATAAATCATTTTAAAATTCAGATTCATAACATTGTTTTTATTTTGTATT
TTATCTTGATTATGTGTCTTTTTTTTTTAG**GTATATGCTTGTCAGCATTGCCAAGTGGCAA**
AAAATACAGTTATTTTAGCACCTAAACAACACCTTCTCAAGGTGGAAAATCCATGGAGTA
TAGTTACTGTTGATCTGATGGGACCTTTTCATACAAGTAACAAAAGTCATGTATATGCTA
TAATCATGACAGATTTGTTTACAAAATGGGTTGTGATTTTGCCTCTAAGTGACGTTTCAG
CATCAGAAATTACTAAAGCTATTAGCAATATATTTTTCTTATATGGACCTCCTCAGAAAA
TAATAATGGACCAAAGAGATGAGTTCATTCATCAGGTAAGAAAAACTATTTATGTCTGTG
AGTATATTTTAGTCTCCCTTCAGTGCCCAAAACAAGTGCCTTGTACATAATAAGGTACTT
AATAAGTTTAGATAAATCCTGCAATTATAAAAGATGTATTATATAGCACAAAGTAGTCAG
ACTGGAAATTAGGAAGCCAGGATTATAGTACTTATTTTTATCACTAAGTAATTGTAAAAC
TATTTTCCGGTTTCTTCAAACTAAAATGTGGTAAGATCAGATGATCTTTGTTTCCTTCCA
GCTTAAAAATTCTATGATTTACATGTTAAAGGGACTTGACTAAATGGAAAAATATATCAT
ATTCATACATGAAATTGCTTAATACTGAAAGATATTGATTCTTCCTAAATCAAAATTAAT
TTGTAAAAGTAATACCTTTCTAACTAGCATTTTAAAAGAGGGAGATTTTCCAGTTTAAAA
TTAACTTTAAAGAATAAGTAAGCATGAATATATTTGAAAATTCTAAAAAAGAAATGTAGC
AACTACTAGACTTAAGGCATATTTTTAAATTCTTACAATTGACAAAGTATGAGGATAAAA
ATAATCAGAAATAGCATTGCACTTTTTGTGTCTTAATCCAAAAAAAAACCCTAAAGTTGTG
TTTATTACAGAGTTAAGTCCTGTGTTTAAGGTCCCCAAGACCACCCCAGTTTTGATTCTG
TATGAGGACTCACAGGGCTCAGTGTGTTGTACTTATGGCTAAATTTATTATAGCCAAAGG
ATACAGAGCAAAATCAGCAAACAGAAAAAGCACATGGGGTAAAGTCCAAGGGAAACCAGG
CACCGGCTTTTAAGAAACTTCCCTCAGTGGAGTCACGTGGGACACTCTTAATTACCTGGA
CCAACAAGTTGTGACAACATGTGTGANNNNNNNNNNNNNNNNNNNNNNNNNNNNNNNNNNNNNN
NNNNNNNNNNNNNNNNNNNNNNNNNNNNNNNNNNNNNNNNNNNNNNNNNNNNNNNNNNNNNNNN
NNNNNNNNNNNNNNNNNNNNNNNNNNNNNNNNNNNNNNNNNNNNNNNNNNNNNNNNNNNNNNNN
NNNNNNNNNNNNNNNNNNNNNNNNNNNNNNNNNNNNNNNNNNNNNNNNNNNNNNNNNNNNNNNN
NNNNNNNNNNNNNNNNNNNNNNNNNNNNNNNNNNNNNNNNNNNNNNNNNNNNNNNNNNNNNNNN
NACCATTTAAAATATATAGGGAGAGAAAGAAAAAATATTTCTCTAAACAGTTAATAATGG
GATGTGTGTACCTGTTGAGTACTGCTCAACTTCTCAAACCATGGATCAGATTGGTCATTG
CCACTCTTATTTCCCTTCCACATTGATTTTTGCACAATAGTTGCTTGTTGTCACAACTTT
CACTGGAAAACCAGTTTTGCAAAGGTAGGACATCATCAAAAGGAATGTAGCTTGATCTAT
TGTTGACCCTGTGAGGTACATCAAGCTAGTAATGTTCACTGTGTCCAAGTAATGAGAACA
ACTTGACAGAGTGTGGTATCTGTGGTTATAATGATAAAGTCAGCATTACAAGTAAGTGGA
ATAGGGAAAGATTAATTAGTAAACTTAATTCACAATTAACATAGGTAACAATCAGACATA
ATATAATTTAGCATTTCAAACCATGTATCAAAATTGCATTCGGATAATTTTTTTAATATA
ACAGAAAATTAGTAGAAAATTCAGCTGACCTTTTTTTAAATCTCTAGTGTGAGAAATAAT
AGCCTTAGGAAAGTGATAAAAATTTCAAAGGAAAAAAATCACTAAATTCGGCTGCATAAA
AATTAAAAACCTAGGTGTGGTAAAATGATCAGAGCACAAAGACAGATAACAGAACAAGAA
AAATACTTATGGCAAATCCATGTTAGAAAGGACTATTGTCTTCATTGTATAAGATTCAAA
TAAATTAATAAAATTTGTCAGTAGTGTAAACAGAAAATCTATAGAAGGATAAAGCTAATA
AATATATGAAAAAACTGTTCAAACTCAGTAAAAATCAGATAAATGCAAATTAAGACACCA
TCATTTGCTTTTCTAATCAGCAAAAATATTAACATTATCAAATCGACTACTAGCACAATG
ATAACAAACATTTTTGATGGAGTTGCAACTGGGAACTAAATCTTTTATAAAGGTAGTTCA
TCAAAGATCCATAAGGAGCCAAAAATGTATTTTTGCCTTCTGACTAACAACTCTGTTTCT
AAGAATTTATTCTAAGGAGATTATACAAAGTATAAAAGCAACTATTATGAAGATACTTAA
TACAGTGCTGTTAATATTAGTGAGAGATTGGAAACAACCTGAATATTCAAAAAACAGAAG
TGTTTCAGGAAAATGTCTAGCCACATGATGAAATGTTATAGCCATTGAAAATAATATTTG
CATAGAAAAGGAAATAGCATTGAAAATATTCACAGCATAATATTAAATTAGAACTTTATT
AGAGTATCACAACTGTAATGGATATAAACTCAAAAATAATATTTTATAAGGTTATCTGTG
GAGTGTTTTTTTTTTTAAATTTAAGGATGATGGGATTATAGATTATTTCTTAATTTTCCGG
TTTGTATTATAATTTTTAAATAGTTAAAATATGCATAATCATTTCTTGCATTTGTGGTAG
AAATTGTACAAATTACTGGTGCTCAACAATCTAGTTCTTCTATCATTCTGAACCTGGAAG
GCTCTACTTCCAGCCCCTTGCAGGTGGACAAGCCTAAGTAACTAGTTCTGGCAAGAAAAA
CTGAAGGAGTGGATGTGTCGCTTCCATTCTGAGCTAGTGACAAGCCTATGCTCAATTTTT
CAGTCTTCCTATCGTGCTCTTTCTCTGGGGAATGACTGAATCTTTCAGATAATACATGCC
AGTTTGGTGGCTTTTGTCACCCAACAGGGGGACCCCAGGAAGTTTGGCTGCTTTGGATGC
CGCTGCCTTGAAGGCTCTGCAGACATGGCTGAGGTTGGGAAAGGGTACCTTTCCTCACCG
GAGGGCCTTACTGCAGGTTGTTCTCAAACACCTGGAGCCTTCCTGGTAACCAGAAGGCAC
CAAGGCTTCCCCTCTAGAACGCGGCTTGGTCTCCATTTGGGGTACTCTTGGTCGAGAGTG
CTGTGGGCCTTCTGGTCTTCTTCACCTGGTCCAGTGGGACCTGGCACCAACTTGCCCACC
CACCCACCCCATGCACAGGACGTGCCCAGACCCTCTCCTGGCAGGTAAAAAAGCCAATCA
AGGCTGCTGGGCCTGCCTTGATCCTGCAGACAGACCTAACCTACACTATCAGGAGGGCAC
GCCCTGTGGGTCTGGAGCTGGTCTTGGCGAGGGGCCACCGAGGAGCCTCTTCCTCATCAC
TCCTTCCCANNNNNNNNNNNNNNNNNNNNNNNNNNNNNNNNNNNNNNNNNNNNNNNNNNNNNNN
NNNNNNNNNNNNNNNNNNNNNNNNNNNNNNNNNNNNNNNNNNNNNNNNNNNNNNNNNNNTTGTG
ATGTTTTCTTGCTGCACATAATCCAATCTACCTTAACTGACACAGCAGTGGTCTATAAAG
TGGTATGTGTAAAGCAATGCATTTCATCATTTCATGGGAAGGCAATATTAGAACTTCTGT
AGATATTTCTTTTCCGACCTAAAAAATAAGAAAGAATTGAAGCTTTACTAATAATGTATA
CAGATTGATTCTGACTTAGATGAGAAGACAGAAGATTGTGTGTCATATGATGTGTGAGAT
ATTTTAAGAGAAAATAAGGATCCATAACATGGGAGACTACAATGGTCCCCTCACTCATTC
TTTTGCTTTCAGATTTCAGTGGTTAATTGAATTTATGAATGACATTATCTAGTTTTAACC
AAACTACTCTTTGCAAAATGGCTAGGTGGTGGAAAGTTCTTGCTACAACGCAAGATAGTA
ATAATGCATATATCTGTCAGCCACAAGAAAATGATAGAGCTGACATTTAATTCCTAGTAC
TAGATCTTTTTTAGAAACTTTAATGAGATGAAAACAAATACGAACTAGACAGATCTGATA
GAAAATTCGCCACCAAAATTCAAAATTATCAAGAAGCCTATTTGAAGTATCAATTTACTT
CCACTTTTATTAATGAAGAAGCTTACCCTAAACGAATATACTACCTCATTATATTAGCCA

ATAGTATGAAGCCATTATGATTAGCCAGAAATTTTTCCACTTGTTATTTTTCCTGTGATT
AAAATGTCTCAAATATTACTTAGCATAATAATAACAGAATAAAATGTATCTTTTTATCAG
CAAATACTAGCATAGAAAATACCACTAAAATGTTAAAATAAGATTAGAACATAATATACA
ATGTGGGAAGTTAATAAACTTCCTGTACAGTTAGATGAAATTACAAGTGTTTCTAACATA
TCTTAGCATATGCTAAATTGTTTCAATGACATGCACAAAGAATTTTTTATAAACCTCTGA
AGGAAAGAGCCTATTCAGTGGGAAAAATATGACTAATTAGACAATGTCCTCTAGAAAACT
ATAATTATAACCACTGATGGAATATCTGCTTTGATAGGGGAATAAAATAGTGATGGGGTA
AGGATGTTAAGATAGCCTTTCATAAATGTAGTCACTGTGTCATAACTTAGTCATCCTCTT
GAAACAAAGAAGCTGAATCCTTTTCCAAAGGTTAGCCATTTAAAATTATGGATCAGGACC
TCAGATTGAAGTTAATTTAAATGACACGTATTCCCTGGGATTTTTTAAAAATTATTTTTT
AATTAAAAGAAGGCATCCTGAATTAGTACATATATTGGGCTATAAGCTAGGACTTACTTG
TATTAAGCTGCAAGTAACAGAAAACTCCTCCCCTCCCCACTCCCCCAGTATATAAACTCC
AATAAGAGTTTATTTTGTCATCACCTGAAAGAATGCCAGGTAGTCCAAGACTGGAAGGGT
AGTCTGACTGTCTTTGGGACCCATGGTCTTAGCTTTCTGTTCCACCATCTTTGGTGTGAC
CCTCAGACTTGTGCTCACAATATGGCTGCCAAAACCCATGTCACCTTTTTTACGTTTCAG
GCAAAAAGAAGGAAGAGCAACGGGCAGTAAAGGCTAACAGCAGATGAGTCTACGCCCACC
AACTTTTAAGAGCCCCACAACTTCCATTTACATTTTTTTGGCCAGAACGTCATCCTATGT
CCACCACTACCTACAAGGGAGCTTGGGAAATATAATTTTGTTTTCTTTCACCCTCAATTT
TTAGCTTTATTTGATTACTCATTGTAACTCGTTATAAGCTCTTCTGTTTACAATTAAGCA
AAAAGCAACTTCTAACAGTAAGTCTAACAGGCTTACTGTTAGAAGTTTTCTAAATTCATT
TTTCCTTTTTTTTGGCCATATCTTAAAGATAATGACAGCAACAATAACCAAAATATTAGC
AGTTGAAAGTTATGAAGTGCTTACTACCTACCAAGAACTCTTTTAAATGCTATGCAGATC
TTATTTAACCTTCAAAAGATTTGTGTGAGGGTAAGAATTTCCTCATTTTAGAGATGAAGT
AACTGTGTATAGACTTCACTGACTTGGCCATGGTCATACAGCTTTTAACCAGGTTATGTG
ACATAGTATTTATTTGGGAAACTACTTAATTAAAATATTATGTGTACTCAGATTTTGGGT
AAATTGTGCTTTTACATTTCCTTGCTGTATGAATGTTTTTGGCAAGCACTTAACCTCCCA
AGGATTCAACTCTTATCTTTAAAATGAGATTAATAATACCTAAGATGGTTGTAAGATAGA
AGTGATAGCAATGTAAAGCACTTAGTTTACTAACCAGCACATAATAAGAACTCAATAAAT
GGTAAAAAAAAATATTTTATTTTGTGTAGAATAGCCGTGGCCAGTGAAAAAGGCTAGATT
CAAATTCTTTTTAAGCTTTGTAAAATCAGTGGAAAAATAATATTACAGATTTTTAAAAGT
TAGCTTTGTTTTAATATATTTTTCAG**ATCAATGTTGAACTGTATGGATTGTTTGGCACAA**
AGCATATTGTAATTTCTCATGCCTCTCAAACTGTTGATCCAACTGAGAGTACACCTAGCA
CAATCAAAACGTTCCTCTCCAAACACTGTGCTGACCACCCAAACAACTGGGATGATCACC
TATCAGCTGTTTCATTTGCCTTCAATGTAACTCACTTAGTATGTGCCGTTTTATAATTCT
ATACTTCTGAATTAAGACTCATTTCTTGCCTAGGTAAGTATTTTATTATAAACTTGCTGT
TTATTTTCCTTAAAATAG**GAGCCTGCTAAAAATACACCATATTTTCAGATGTTTAATCGA**
AATCATTATGTGCCTGAGACTTCAGATAGTCTTCATGGAGTGGATGCTGATAATACAAGT
ATGTTTGCAAAAATTTTAGATGCAATTAAAGAAGCTGATAAAATAATGGAGAATAAGACA
ACTACAGTCAGCCAGGTAATTCTATTATTAAGAAAAACTATAATCAATCAAGTGATCATT
TTTTTTATATTTTCAGTATATATCTGTGTGTATATTTATACACATACATACATATACATA
CATATACATACATATACATACCCATTCATATAGTATACCCTTAATCAAAATATTTTCCCA
TTAAATTCTACATGTCTTGATTTGATAGTATGCTAATTAGCGATGAAATTTTGCAGACAA
CAGTGGGGAGCACCTTATATGTAAAGTGAGGTCATAATACTGACTTGAAAGTGTTGTTTT
AAGAATTAAATGGGATTCATGCCTACTTCTTAATAGCTTTTGATATTATTGCGTGTATTT
GTCTTCAAGAGAGGATAGCTCATTTGTCCTTTGTTCTTTTTGAGATTGTTTTTACATTTG
TAG**GTGGAGAATGACAGTTGTGATGACCTAAATAAAGCCCAGATAATTGTTAAAAAGAAA**
CCAAAGCAGTTAAATCCATTTAATCTAAAAGTGGGCCATGAAGTTTTAAGACAAAGGAAA
AATTGGTGGAAAGATGGTCGTTTCCCGTCTGAATGGGTTGGCCCTTGTGTCATAGACTAT
ATTACAGAAAGTGGATGTGCTATTCTGAGAGACTACACTGGGACTAGACTTAAAAGACCC
ATCAAAATGTCCCACCTCAAGCCCTATATTAGGGAATCCAGTGAACAAGGTAAATATCAA
TTGTTACAATTTTACTTTTTCTGAATGAAGAGAGAAGCTAAAAATACACGAATCTCATTA
CCACCACCACCTTTGGTTAGGTTTGCTTTCTCCCTCTCAATTTTATATTTATAGAATTTG
GTATTGTTTTAGATATCCCACATAGTCTATTTAAGTAGAGTGTCTGTTATGGAACTGTCA
GGCTTGTAAGAGATTATGTTCTGATTTCAGGTGTATTTTCATAGTTCATGTGGGAGATAC
TGAACCTGTTTTACAGAAGAAAGGATTCTATCTTCTCTCATTTTACTTTTACATTATTAG
TCATTTTGTGCCTCATTTGAGCATGAAAATTCAGCAGCTAAGGTCATTAATGCCAAAAAC
AAACATACTGTCCAGAATCCAATTTTGAAGAGAAATGTATCAGGGGACTGAAAATATTAA
TTTTTGGTATAACATTATAGGCAGGTTATGAGAGGGGCAGGTGGGAGTATGGGGTGGAAT
TTACTCAGAATGCCATAAATATTAGAAAAAGTGATAGCCCCCTAAAAAGGTTATACTTTC
CCCAAAATATAAATGGTAATGAACAGTCAAGATATCCTAATCTTTCTAAGTTGTTCTTTC
ACAGATGAAAGCCAAATGACATTTTTGCCATAACTCTCAAATTAGGGCTCACTCTACATA
GACCTTTATTTTGTCCTACTATATCTATCCCCTAGAGTTTCTCTGAGATGGATAACTGAG
GGGAAGTTAATTAACACCTTAACCAGTTAATTGTGCTTTTTCTAAAAAAGAAAGATAAAG
CATCAATATTATTTGAAATTGAGTATAATTTATGGGAAATACAATATAGTATATTTGTAA
ATAATATGAACAACAGCAACTCAGTTAATAGGCGTATGTAAACAAGAATAATAGCAGGAA
CAACAGGAAATAACTTTTCCTTACCCTGTACCNNNNNNNNNNNNNNNNNNNNNNNNNNNNNNNN
NNNNNNNNNNNNNNNNNNNNNNNNNNNNNNNNNNNNNNNNNNNNNNNNNNNNNNNNNNNNNNNN
NNNNNNNNNNNNNNNNNNNNNNNNNNNNNNNNNNNNNNNNNNNNNNNNNNNNNNNNNNNNNNNN
NNNNNNNNNNNNNNNNNNNNNNNNNNNNNNNNNNNNNNNNNNNNNNNNNNNNNNNNNNNNNNNN
NNNNNNNNNNNNNNNNNNNNNNNNNNNNNNNNNNNNNNNNNNNNNNNNNNNNNNNNNNNNNNNN
NNNNNNNNNNNNNNNNNNNNNNNNNNNNNNNNNNNNNNNNNNNNNNNNNNNNNNNNNNNNNNNN
NNNNNNNNNNNNNNNNNNNNNNNNNNNNNNNNNNNNNNNNNNNNNNNNNNNNNNNNNNNNNNNN
NNNNNNNNNNNNNNNNNNNNNNNNNNNNNNNNNNNNNNNNNNNNNNNNNNNNNNNNNNNNNNNN
NNNNNNNNNNNNNNNNNNNNNNNNNNNNNNNNNNNNNNNNNNNNNNNNNNNNNNNNNNNNNNNN
NNNNNNNNNNNNNNNNNNNNNNNNNNNNNNNNNNNNNNNNNNNNNNNNNNNNNNNNNNNNNNNN
NNNNNNNNNNNNNNNNNNNNNNNNNNNNNNNNNNNNNNNNNNNNNNNNNNNNNNNNNNNNNNNN
NNNNNNNNNNNNNNNNNNNNNNNNNNNNNNNNNNNNNNNNNNNNNNNNNNNNNNNNNNNNNNNN
NNNNNNNNNNNNNNNNNNNNNNNNNNNNNNNNNNNNNNNNNNNNNNNNNNNNNNNNNNNNNNNN
NNNNNNNNNNNNNNNNNNNNNNNNNNNNNNNNNNNNNNNNNNNNNNNNNNNNNNNNNNNNNNNN
NNNNNNNNNNNNNNNNNNNNNNNNNNNNNNNNNNNNNNNNNNNNNNNNNNNNNNNNNNNNNNNN
NNNNNNNNNNNNNNNNNNNNNNNNNNNNNNNNNNNNNNNNNNNNNNNNNNNNNNNNNNNNNNNN
NNNNNNNNNNNNNNNNNNNNNNNNNNNNNNNNNNNNNNNNNNNNNNNNNNNNNNNNNNNNNNNN
NNNNNNNNNNNNNNNNNNNNNNNNNNNNNNNNNNNNNNNNNNNNNNNNNNNNNNNNNNNNNNNN
NNNNNNNNNNNNNNNNNNNNNNNNNNNNNNNNNNNNNNNNNNNNNNNNNNNNNNNNNNNNNNNN
NNNNNNNNNNNNNNNNNNNNNNNNNNNNNNNNNNNNNNNNNNNNNNNNNNNNNNNNNNNNNNNN
NNNNNNNNNNNNNNNNNNNNNNNNNNNNNNNNNNNNNNTTTATGGGATACCCATAATGTCTCTT
AGTAAATTATATTGTTCCCCTTTCGAATGAGAAAAATGATGTTATTGACGCACTATTACA
TCTGACATAGATCTATTTTTATTGTGGCAACATATACAACAAAATTTCTAATTTTAAATA
CTCTTAAGTGTACAATTCAGTGGCACTAATTATATTCACACTGCAGGCAAAGATTTTGAG
TAGTTTTATTGTTTTGTTTTAGTGGATGTATTGGGATAGCATCAACAAAAAGTTTTCATC
TCATTGGCCTCTATAGTCCCCCATTTGTGAAATATTTTTCATTATTGTATTAATTTCCTA
TTGCTGCTTTAACAAATTATCATAGATTTAGACTTGAAACACAAAAGCTTTAACCTTAAA
TTCTTTAGAAGTCCAACATGGGTCTCAGTGGGCTAGAATCAGGCTTTGGCCTGTGTTCCC
TCTGGAGGCTCTACAGGAGAATCAGTTTTCTTGCCTGTTTCAGCTTTTTGAGGCCACCTG

```
CATTCTTTGGCTTGTGGTCCTCTTCCTCTGTCTTCAAAGATAGCAATTACAGGTTGGGTC
CTTCTGACATTGCATCACTCCAGCTTCCTCTTTTGCCTCCCTCTTCTACATTTAAGAATG
GTGTGTTTACATTGGGCCCACTTGGAAATCCAGAATAATATGAGGTGGCATATTCAAAGA
TTCTAGGCAGGGATGAGTCTGTGGACATCTTGGTGGACCATTATTTTGCTTACACAATTA
TAGTCTGCATTCATGACAAAGCAAAGCTTAGTCCCTATGGCATCAAGCCCATGGTCTTGA
CTTCATTATTAATATAGTTTATGTAAAGAGCCTTCTTTTGATCTCTTACCTAATCGTTAT
TTAGTTTTCTCTATTATTAGTATTGGTTATTTATTATAAAGATAATGATGTATTATCAAT
TTTTTATTATTGCCATTTCATTATGAGGACGGATATACTAGTATGGTAAAACATTTTAAA
TAATTTTAGGATTATCAAGCTATATATATCATTTAGACTAGTCATCTCTTGAGAAATCTT
GTGCTTTTTAGTTGAAGAAATAAATAATCCAGATCACTCAACGTTTTGTGAGACTTTGTG
TTTTAGTTTCCTGACTTCTAAAGCAAGCACCAAATAATGGGTTGACGTGTGAGATTATTG
GCTCAGAGATTTAAAACAGGAGAAGTTCAAAATCAAGCATTATCAAGGTGATGCTTTCTC
CCAGAAGCCTGTGGCATTCTGGGTCTGGCTGGTGACTATCCTTGTTCCCTAGTCCTTAGC
ATTTTTGTCACATGGTAATACACACAGCGACCTCTCCCAGCTTTTCCCTTTTCTTCCCAG
TTCTGTTGATTTTCAGCTTCTGGCTGCCCCCTCTGTGGCTTTCTGTCTCTATGTGTCTAA
ATTTCATTCTGCTTTTAAAGGGCTCCAGGAATAGAATCGATTAAGATCTATCTTGATTTA
GTTGCGGTATACCTTAATTGAATAACTTCATCAAAATGTCCTATTTATAATGGGTTCATA
CCCATAGAAAGGAATTAAGTCTAAGAACACGTTTTTCTGGGGTACATTGCTCCAAGCCAC
TACACTTTGTATTTTTGTTTTTTAAAGATACTTAGATTATACAAATGTCACATAAAAATA
TATAGGGGATTCCCATATGCCCCGCTTCCCACACTTACCACATTTACATCCTTCGTTAGT
GAGGTACCTTCATTGCAATTGATGAACACATTTTGGAACATTGCCAGTAAGCATGGATTA
ATGNNNNNNNNNNNNNNNNNNNNNNNNNNNNNNNNNNNNNNNNNNNNNNNNNNNNNNNNN
NNNNNNNNNNNNNNNNNNNNNNNNNNNNNNNNNNNNNNNNNNNNNNNNNNNNNNNNNNNN
NNNNNNNNNNNNNNNNNNNNNNNNNNNNNNNNNNNNNNNNNNNNNNNNNNNNNNNNNNNN
NNNNNNNNNNNNNNNNNNNNNNNNNNNNNNNNNNNNNNNNNNNNNNNNNNNNNNNNNNNN
NNNNNNNNNNNNNNNNNNNNNNNNNNNNNNNNNNNNNNNNNNNNNNNNNNNNNNNNNNNN
NNNNNNNNNNNNNNNNNNNNNNNNNNNNNNNNNNNNNNNNNNNNNNNNNNNNNNNNNNNN
NNNNNNNNNNNNNNNNNNNNNNNNNNNNNNNNNNNNNNNNNNNNNNNNNNNNNNNNNNNN
NNNNNNNNNNNNNNNNNNNNNNNNNNNNNNNNNNNNNNNNNNNNNNNNNNNNNNNNNNNN
NNNNNNNNNNNNNNNNNNNNNNNNNNNNNNNNNNNNNNNNNNNNNNNNNNNNNNNNNNNN
NNNNNNNNNNNNNNNNNNNNNNNNNNNNNNNNNNNNNNNNNNNNNNNNNNNNNNNNNNNN
NNNNNNNNNNNNNNNNNNNNNNNNNNNNNNNNNNNNNNNNNNNNNNNNNNNNNNNNNNNN
NNNNNNNNNNNNNNNNNNNNNNNNNNNNNNNNNNNNNNNNNNNNNNNNNNNNNNNNNNNN
NNNNNNNNNNNNNNNNNNNNNNNNNNNNNNNNNNNNNNNNNNNNNNNNNNNNNNNNNNNN
NNNNNNNNNNNNNNNNNNNNNNNNNNNNNNNNNNNNNNNNNNNNNNNNNNNNNNNNNNNN
NNNNNNNNNNNNNNNNNNNNNNNNNNNNNNNNNNNNNNNNNNNNNNNNNNNNNNNNNNNN
NNNNNNNNNNNNNNNNNNNNNNNNNNNNNNNNNNNNNNNNNNNNNNNNNNNNNNNNNNNN
NNNNNNNNNNNNNNNNNNNNNNNNNNNNNNNNNNNNNNNNNNNNNNNNNNNNNNNNNNNN
NNNNNNNNNNNNNNNNNNNNNNNNNNNNNNNNNNNNNNNNNNNNNNNNNNNNNNNNNNNN
NNNNNNNNNNNNNNNNNNNNNNNNNNNNNNNNNNNNNNNNNNNNNNNNNNNNNNNNNNNN
NNNNNNNNNNNNNNNNNNNNNNNNNNNNNNNNNNNNNNNNNNNNNNNNNNNNNNNNNNNN
NNNNNNNNNNNNNNNNNNNNNNNNNNNNNNNNNNNNNNNNNNNNNNNNNNNNNNNNNNNN
NNNNNNNNNNNNNNNNNNNNNNNNNNNNNNNNNNNNNNNNNNNNNNNNNNNNNNNNNNNN
NNNNNNNNNNNNNNNNNNNNNNNNNNNNNNNNNNNNNNNNNNNNNNNNNNNNNNNNNNNN
NNNNNNNNNNNNNNNNNNNNNNNNNNNNNNNNNNNNNNNNNNNNNNNNNNNNNNNNNNNN
NNNNNNNNNNNNNNNNNNNNNNNNNNNNNNNNNNNNNNNNNNNNNNNNNNNNNNNNTTAT
CACTGCTTGTATTTCCATCATAAATATGTGCAACCTTTTACCAGCAATTTCAACCTGTTC
ACCACTTTAACATTAACAGAAATTGGTGGGGTTAAATTTGAAAAACTTCTCAAAACTAAA
ATACCAAACACACCTTGTCTGCAGAAGAACCAAATGTGCAAAACTAAAGGACCACTCAGA
TCCAAGCCCCCTTAGACCGCCTGTTGTTAGAAAGCATAGTTTTGTTTGCTCTATCAAAAT
TTGTAAGTTGGAAGTTCTTTAATACATGAACTTCTGATGTTGAAATATGCTTAATGAACT
TTTCAAATCTTAGTGCAAGGACAGATAGGGTGGGATCATGTTCTAGGATGTACTTTCAAA
TGTACCTGCTGTGAGAAACTTTCATTCATCTCTAAAGCAAATGGGCCATTAATCTTCCTT
TTATTATTTCTTGAATGTAAAATTGGTGGCCTGTTTAAACTATTAAAATCTTTAAGAGTA
TCTATTAATGTTTTTGGCCCAGGAAGTGAATTGTTGTAGGAAGAGTTAATTTTTGTAATG
AATTAAAATATCAAGTAAAAAAGAATTCTTTTATATACATTAATCCTCATATATAAAGAT
GCTGCTTGATGTTAGTTTAACATTCACTTTGACCATTTTTTTTCCTGATGGTGATGATAC
ATTTGCTCAAAGTAGAATAAAGTGCTGATAAGGAAAATACCCTGTCAGACCTTTGGTTCA
GATTTAATCTGACCTTTAAAATTTCCTGAAAATTTTGGTATTAAATCAGACTTAAATAAG
CTGAGTTTTTAAGCTTCTCCTTATAACTTCTCTACTTACACATTCCTATCACATAAACCT
GGTTCTAGGAACTGTCTTAGTTTTAGTTTATTACTTCATATTGTAGCAATTTGGTATTTT
CCTAGTTTGGGGTCTATGAAGACTGGGGAATTATCTAAAGTTGTAAATATAAAGATTTAA
CAATCCTGTTTGTTAGGTAAAAAGAACAGGAAGACCTGGATGTTTTTAAATCCTTGATGC
TTCCTTTTTATAAGTATATAAATGGTCAAAATTATATGTTTTTGTATTCATTATTTGACA
TGTTTATTTATTTAGTAGACAAGGGAGTCTCCTCTCCCCTCCCAAAGTTAATCTTAGTAG
GTAATTTTAATTATTCTTTGGCATTTGTTCCTCATTTACCATTGTTTAACTTAAAAACTT
ATGATGTTTCTTTTTTTTTCAGACAGTCTTTATCTTTTACAAGGTTCAGTAGTGGCAGAT
CATGACTACCTCGGATTGCCTGAAATTCCAGTTGGAGCATACCAAGCAAATATTCTGGTA
GAAGATGCAACTATTGGTGTAGTTAATAATGAATTACTGACATCAAGCAAAGATCATGAA
CTATTAGAATACAGAAATACCAAAATCTCTCCATTGATAGAAGATCGTGTTGCTCTTGAA
AAGCAGGCTTTTAGTCTCTTGGACTCTTCAAACCACGTTCTTGAGTACTTAAATTAGTAA
ATACCAGAATTCATTAATGTTGTTTAGAGAATATAACCCTTAAACCTTGATGTCTCAATC
TATTAAAAAAGGTCTAGGGAAGAAGTGTCTTGACACCCTTGATGACTATATCTTGAAGTT
TTTTTTAAACAACTGTTTAAAGTTGTGTCTGTTTCCATGCCAGTGCATATATATATATGA
AGTCAAAGGAAACATTATATAGAATTCACTTTTCCCAAAAAGAGAACAGGCAAAGTACTA
TTGCTTCTTAGAAAGTAAATTAATTTCCAGCTCAAAACTTTCAACCTCTTTTCTTGGTAT
TCATACTGTGTTTTCAGTTTTACTTTTTCTGAAATATTTCCCTTTTAAAAAATAAGTGAT
AACTATCTGGCAATGGGTTAATAAATATTGAATCAACCATAACAATGAGTTAAATGAAAC
TTCTCTCTTTTGTTGGACTGTATAAAAATAGTACTGTAGGAAAAATTTTTAGGGTTGTCT
AGTGAGGAACCCTGAACCCAGGAAGAAGCAAGCCCTGTGCAGGAAGGAACCTTAAACCCA
GAGAGAAACAAGACCCTGGAAGGTAGGAACCTAGGAAGCCTGAACCCTAGCAGCTGT
```

```
===================================================
Dasypus Gin-1
total length:       19497 bp  (16328 bp excl N/X-runs)
GC level:          33.84 %
bases masked:        5216 bp ( 26.75 %)
===================================================
               number of      length   percentage
               elements*    occupied  of sequence
---------------------------------------------------
```

SINEs:	6	969 bp	4.97 %
Alu/B1	0	0 bp	0.00 %
MIRs	5	566 bp	2.90 %
LINEs:	7	972 bp	4.99 %
LINE1	5	831 bp	4.26 %
LINE2	2	141 bp	0.72 %
L3/CR1	0	0 bp	0.00 %
RTE	0	0 bp	0.00 %
LTR elements:	6	1700 bp	8.72 %
ERVL	1	246 bp	1.26 %
ERVL-MaLRs	4	1365 bp	7.00 %
ERV_classI	0	0 bp	0.00 %
ERV_classII	1	89 bp	0.46 %
DNA elements:	7	1470 bp	7.54 %
hAT-Charlie	5	1262 bp	6.47 %
TcMar-Tigger	0	0 bp	0.00 %
Unclassified:	0	0 bp	0.00 %
Total interspersed repeats:		5111 bp	26.21 %
Small RNA:	1	403 bp	2.07 %
Satellites:	0	0 bp	0.00 %
Simple repeats:	1	20 bp	0.10 %
Low complexity:	4	85 bp	0.44 %

==

SW score	perc div.	perc del.	perc ins.	query sequence	position in query begin	end	(left)		matching repeat	repeat class/family	position in repeat begin	end	(left)	ID	
233	28.1	1.2	3.6	scaffold:dasNov2:scaffold_5385:8291:27787:1	1564	1648	(17849)	C	L2B_ME	LINE/L2	(31)	3416	3334	1	
311	28.0	4.5	6.1	scaffold:dasNov2:scaffold_5385:8291:27787:1	3291	3423	(16074)	C	MARNA	DNA/TcMar-Mariner	(170)	416	286	2	
264	21.8	1.8	0.0	scaffold:dasNov2:scaffold_5385:8291:27787:1	3487	3541	(15956)	C	MARNA	DNA/TcMar-Mariner	(387)	199	144	2	
238	17.2	9.1	2.9	scaffold:dasNov2:scaffold_5385:8291:27787:1	3496	3561	(15936)	+	MARNA	DNA/TcMar-Mariner	396	465	(121)	3	*
180	0.0	0.0	0.0	scaffold:dasNov2:scaffold_5385:8291:27787:1	3659	3678	(15819)	+	(TTTA)n	Simple_repeat	2	21	(0)	4	
2564	12.2	1.2	0.1	scaffold:dasNov2:scaffold_5385:8291:27787:1	3679	4081	(15416)	C	DASIIIb2	SINE/tRNA	(32)	385	1	5	
25	48.0	0.0	0.0	scaffold:dasNov2:scaffold_5385:8291:27787:1	4114	4138	(15359)	+	AT_rich	Low_complexity	1	25	(0)	6	
220	21.3	0.0	7.7	scaffold:dasNov2:scaffold_5385:8291:27787:1	4872	4927	(14570)	+	L2a	LINE/L2	3356	3407	(19)	7	
220	33.3	1.4	2.9	scaffold:dasNov2:scaffold_5385:8291:27787:1	4965	5035	(14462)	+	MIR3	SINE/MIR	16	85	(123)	8	
251	23.6	8.3	0.0	scaffold:dasNov2:scaffold_5385:8291:27787:1	5048	5119	(14378)	+	MIR3	SINE/MIR	122	199	(9)	8	
295	27.1	2.4	0.0	scaffold:dasNov2:scaffold_5385:8291:27787:1	5126	5210	(14287)	+	L1M5	LINE/L1	4484	4570	(1576)	9	
1280	19.6	3.2	0.4	scaffold:dasNov2:scaffold_5385:8291:27787:1	5481	5726	(13771)	C	MER68	LTR/ERVL	(0)	563	311	10	
1261	20.6	0.8	0.0	scaffold:dasNov2:scaffold_5385:8291:27787:1	6051	6288	(13209)	C	MER119	DNA/hAT-Charlie	(231)	352	113	11	
308	18.9	2.3	1.3	scaffold:dasNov2:scaffold_5385:8291:27787:1	6745	6840	(12657)	+	L1ME3G	LINE/L1	5368	5436	(758)	12	*
628	33.5	6.5	0.6	scaffold:dasNov2:scaffold_5385:8291:27787:1	6810	7253	(12244)	+	L1ME4c	LINE/L1	162	631	(199)	13	
21	57.1	0.0	0.0	scaffold:dasNov2:scaffold_5385:8291:27787:1	7385	7412	(12085)	+	AT_rich	Low_complexity	1	28	(0)	14	
635	27.2	7.4	1.3	scaffold:dasNov2:scaffold_5385:8291:27787:1	8377	8592	(10905)	+	Charlie7	DNA/hAT-Charlie	65	293	(2319)	15	
1243	25.2	5.1	0.5	scaffold:dasNov2:scaffold_5385:8291:27787:1	8600	8974	(10523)	+	Charlie7	DNA/hAT-Charlie	326	717	(1895)	16	
753	22.1	12.6	3.0	scaffold:dasNov2:scaffold_5385:8291:27787:1	8978	9248	(10249)	+	Charlie7	DNA/hAT-Charlie	903	1198	(1414)	17	
372	31.7	4.3	0.6	scaffold:dasNov2:scaffold_5385:8291:27787:1	9279	9440	(10057)	+	Charlie7	DNA/hAT-Charlie	1246	1413	(1199)	18	
26	42.3	0.0	0.0	scaffold:dasNov2:scaffold_5385:8291:27787:1	9517	9542	(9955)	+	AT_rich	Low_complexity	1	26	(0)	19	*
29	75.9	0.0	0.0	scaffold:dasNov2:scaffold_5385:8291:27787:1	9520	9548	(9949)	+	AT_rich	Low_complexity	1	29	(0)	20	
509	31.6	8.6	6.4	scaffold:dasNov2:scaffold_5385:8291:27787:1	9662	9999	(9498)	C	MLT1J	LTR/ERVL-MaLR	(67)	445	101	21	
482	27.0	7.6	1.1	scaffold:dasNov2:scaffold_5385:8291:27787:1	10192	10363	(9134)	C	MIRb	SINE/MIR	(0)	268	86	22	
252	25.5	6.5	3.1	scaffold:dasNov2:scaffold_5385:8291:27787:1	10436	10528	(8969)	+	MIR3_MarsB	SINE/MIR	54	149	(66)	23	
318	22.6	11.7	1.0	scaffold:dasNov2:scaffold_5385:8291:27787:1	10531	10624	(8873)	+	MIRb	SINE/MIR	154	257	(11)	24	
244	27.3	0.0	1.1	scaffold:dasNov2:scaffold_5385:8291:27787:1	11284	11372	(8125)	C	RLTR17B_Mm	LTR/ERVK	(395)	407	320	25	
252	25.0	1.6	0.0	scaffold:dasNov2:scaffold_5385:8291:27787:1	11473	11536	(7961)	+	MIRb	SINE/MIR	134	198	(70)	26	
429	15.9	4.5	0.0	scaffold:dasNov2:scaffold_5385:8291:27787:1	14236	14323	(5174)	C	L1MB4	LINE/L1	(1)	6179	6088	27	
1368	20.6	20.1	2.9	scaffold:dasNov2:scaffold_5385:8291:27787:1	14446	14873	(4624)	C	MLT1D	LTR/ERVL-MaLR	(0)	505	5	28	
2294	18.6	2.7	3.5	scaffold:dasNov2:scaffold_5385:8291:27787:1	15239	15724	(3773)	C	LTR21_DN	LTR/ERVL-MaLR	(0)	482	1	29	
320	28.4	10.1	0.6	scaffold:dasNov2:scaffold_5385:8291:27787:1	15734	15882	(3615)	C	L1MC2	LINE/L1	(20)	6324	6162	30	
245	19.6	0.0	0.0	scaffold:dasNov2:scaffold_5385:8291:27787:1	19381	19431	(66)	+	LTR73_DN	LTR/ERVL-MaLR	307	357	(190)	31	*
497	15.6	0.0	0.0	scaffold:dasNov2:scaffold_5385:8291:27787:1	19404	19493	(4)	+	LTR73_DN	LTR/ERVL-MaLR	250	339	(167)	31	

>Sloth Gin-1 (genescaffold:choHof1:GeneScaffold_4383:6884:26396:-1)

TTTCCAAGCCAGATTACTATTTTTATACACACAACCAAAAATCATTTTTGTTCAAGAAAG
AACAGGTCATATATAGTTANNNNNNNNNNNNNNNNNNNNNNNNNNNNNNNNNNNNNNNNN
NNNNNNNNNNNNNNNNNNNNNNNNNNNNNNNNNNNNNNNNNNNNNNNNNNNNNNNNNNNN
NNNNNNNNNNNNNNNNNNNNNNNNNNNNNNNNNNNNNNNNNNNNNNNNNNNNNNNNNNNN
NNNNNNNNNNNNNNNNNNNNNNNNNNNNNNNNNNNNNNNNNNNNNNNNNNNNNNNNNNNN
NNNNNNNNNNNNNNNNNNNNNNNNNNNNNNNTTTCTTTTTCCCCAGTATATGAAAATTTC
AGCATTCTTTCACATGAAAGAGACAAGATAATTGTCATGATTGTTTCAAAATAACATTGA
ATATTAGTCAAAAACATCATTCTGTTCCTTTTTGTTACTGCAATTTCTGCTCATAGATAA
TATTCTGGGAAAGCTAAATTCATTTGTAAGCAGAAATATGATAGAAGGCTAAACTGAAAT
AGCAACTGAATTAGCTTAGCACTTAAATTATATCTTTAAATACCTTTCCCTAGGTTAACA
ATGGTCCGTAGTGGAAAAAATGGTGACCTTCATCTTAAACAGATTGCATATTATAAACGA
ACTGGTGAATATCATCCAACTACACTGCCAAGTGAGAGAAGTGGTATAAGAAGAGCAGCA
AAAAAATTTGTCTTCAAAGGTAAAATTAATATGCAAACCTGGACTATATGGTTAATACAG
ATGCTTCTCCACATGTTTTGTGGAATCTGAGAATTTCTGCAGTGATTAGTGTTCCTTAAC
AATTACTAGCTAAGTTGATGCTGCCACCCAGTCAGAAGCAAAATAGAAATCTCACTGAAG
CTAACTAAAGGCCCGTAAGCATTTAAAGGGAAGTTTAAATCATAGGCAAAAAATGACACC
ATAAAAGAAAACAAAACACCAAAATTAACTTCTGCTTTTGCTGACTCAAGTGGTTCCTAC
TCTAATGGTGCACTTTCAAGCACTGATGCCACTTTCAAAGAGGAACTAATTTAATGCTTC
ACATGAAAATGTTGACAAAACTATGTGTTTATTAAAGTGAAAAATGAAAGGAATTAACTG
TATTTTTATGGTTTCCCCCTTCCACTGCCATTAAATATTCTCAGGATTATATTTATGTTT
GATCTTAAGTCTCCTGAATGTATTAAATATTTGAAAAACAGTGGGATATTTACCTTAAAA
GGTTGTGAAAATCGCCTCCCCCTTGTCCCCACTTACAAAGTAAGAGAGAGATTTAATTGC
CAGATAAGTAAATGAATGATGGTGATATTAATGGGAGTTTTGCCTGTAGGTACAGAATTA
GGTTTTCCCCAAAGCAGTTCAAGAAAATTATAAGATAAATAAATGCCTGCTCATTTCTTC
CTGTAAATACTCTCCCAATAACTTCCTTAAGCTAAAGATTATTTGCCTAAATCCATTATC
ACTTAGACTAAAATGAGAATTCACTCATTGATTTATTTACTCATTTATGCAACAATTGTT
AGTCTCTACTCTTCTGCTAATCACATCCTAGGCCTTGGGGACACATTGATGAATAAGATA
GATAAGATTTCTTCCTTCAAGTTGCTTACCTCTGATCCAAATAAGGAATGTTCCACTCAG
CACATGCTTAGGCATATTAGTAGCTTAATGCATATTCTGGAGTTTGAATAACTGCCCCAC
ATTGGAGAGATGGTGATTTTTTCCGTTCCTTGATTGCTTCCCAGCTGCACATTCTTCTCA
GGTATCCTCAGAGGAAAACAGAACTATCTTGAGGATCAAGATTCTCCCCACTTTCACACT
CTACCATCCCCACCTCTAACTCAGTCCAGAAAAACTGGAGAGAAGTGGTTGCTTACAAAA

ATGCAGCTGTGATGAAATATTGTTTTGGATTTTGTTCCAGGAAGTTGCTTATCTGTCTGT
GATTCATTTGCTGACTCATGCTATTTTTAGGAGGCACCTGCAAATATTTTTTCAGTTTTT
CTAAAAATTGCAGTTCTATCTTTTTGGTATCATTTATTTCAG**AAAAAAAGCTGTTTTATG**
TTGGAAAAGACAGAAAACAAAATCGTTTGGTAATTGTTTCAGAAGAAGAAAAAAACAAAG
TCCTAAGAGAATGCCATGAAAATGACATTGGAGCCCATCATGGCATATCCAGAACCCTTA
CTCTAGTGGAATCCAGTTATTATTGGACTTCTGTGACCAATGATGTCAAACAGTGGGTAT
GGCTTATGCATTTAGAGTATTTTGAATAATGTCTTTAATATCCAAAATTCATCAATGCTC
TAGATTTAGTCTCATAAGTACTTTTTGCCTCGGAAGTTAAACATTTAAAGGAAGCTTTAT
TCCCCTTCACAACTCTTCATTAAAATCATTAAAGTATAATTTGAACTAGAAAGAATCACT
AATTAATCATATGAACAGTCTTGAAAATACCAGATTTAGATACCTTTCTAAGTATAGAAC
CAGATTTTCATCGTTAAACTCCTTTGTGTAGGTAGAATTTAAATGGGGCTGAAATTCATT
TGACTAGATCATTACTTCTTTGATTACAGTCAGAAAAGCTTTTGCTTGAGGAAGAATATC
TTCATAATAAGTCAAGCTCAGTGTGTGCTCATCTTTTGTGACTATTCCACCTTAAAGTAG
AAATATCAAATATGTCAGAAATCCAAAACCAACTAAATGTAAAATCAAGTTGTGTTGATA
AAGAAATGAGTCTGATTTTTGTAACTTTATAGCCCCGTGGGACCTTCAATTTCTTCTAAT
AGAAAATTCACCTAGGAATTCTAGGGTTCTTGCTGGTGGAAAAACTGATCCCTATTCTTT
CTAGCATACTCTGTTTGCCAAAGGAAGGAATTCCTTGGTATGTAAATGAGAGCTTTTTAA
TAATTTTAATAGTGCATGAAAAAGACTTCCATTTCATCCATAAAGGATTAAACCAACATT
CAACTAAGTATCATTTATGAATTTGCAGTTCATGAATCTGTATAGGTATTCCAAAGACCT
CTATATTTGGATTTGCCTCCAGATCATGCCACATACTCCTTTACATTCAATGAAATCTTT
GTCAAATCTTTTTATTTTAGAATGAATACATTTTTTGGAAAATAATCAGAAGTTATTTNN
NNNNNNNNNNNNNNNNNNNNNNNNNNNNNNNNNNNNNNNNNNNNNNNNNNNNNNNNNNNNNNNN
NNNNNNNNNNNNNNNNNNNNNNNNNNNNNNNNNNNNNNNNNNNNNNNNNNNNNNNNNNNNNNNN
NNNNNNNNNNNNNNNNNNNNNNNNNNNNNNNNNNNNNNNNNNNNNNNNNNNNNNNNNNNNNNNN
NNNNNNNNNNNNNNNNNNNNNNNNNNNNNNNNNNNNNNNNNNNNNNNNNNNNNNNNNNNNNNNN
NNNNNNNNNNNNNNNNNNNNNNNNNNNNNNNNNNNNNNNNNNNNNNNNNNNNNNNNNNNNNNNN
NNNNNNNNNNNNNNNNNNNNNNNNNNNNNNNNNNNNNNNNNNNNNNNNNNNNNNNNNNNNNNNN
NNNNNNNNNNNNNNNNNNNNNNNNNNNNNNNNNNNNNNNNNNNNNNNNNNNNNNNNNNNNNNNN
NNNNNNNNNNNNNNNNNNNNNNNNNNNNNNNNNNNNNNNNNNNNNNNNNNNNNNNNNNNNNNNN
NNNNNNNNNNNNNNNNNNNNNNNNNNNNNNNNNNNNNNNNNNNNNNNNNNNNNNNNNNNNNNNN
NNNNNNNNNNNNNNNNNNNNNNNNNNNNNNNNNNNNNNNNNNNNNNNNNNNNNNNNNNNNNNNN
NNNNNNNNNNNNNNNNNNNNNNNNNNNNNNNNNNNNNNNNNNNNNNNNNNNNNNNNNNNNNNNN
NNNNNNNNNNNNNNNNNNNNNNNNNNNNNNNNNNNNNNNNNNNNNNNNNNNNNNNNNNNNNNNN
NNNNNNNNNNNNNNNNNNNNNNNNNNNNNNNNNNNN**NNNNNNNNNNNNNNNNNNNNNNNNNNNN**
NNNNNNNNNNNNNNNNNNNNNNNNNNNNNNNNNNNNTCCAATGTGGTGGAATTCA**CGCGTTC**
TCAAGGTGGAAAATCCGTGGAGTATAGTTACTGTTGATCTGATGGGACCTTTTCATACAA
GCAACAGAAGTCATGTATATGCTATAATCATGACAGATTTGTTTACAAAATGGGTTGTGA
TTTTGCCTCTATGTGATGTTTCAGCATCAGAAATTACTAAAGCCATTATCAATATATTTT
TCTTATATGGACCTCCTCAGAAAATAATAATGGACCAAAGAGATGAATTCATTCATCAGG
TAAGACAAACTATTTATGTCTGAGAGCATATCTTACTCACCTTTCAGTGCCCAGAACAAG
TGCCTTGTACATAATGGGTACTTAATAAGCTTAGATAAATGCTGCAGTTATGAGATTTAA
TATATAGTACAAAAGTACTTGGACTGGAAATTAGGAAACCAGGATTTTAATACTTGTTTT
ATCACTAAGTAATTGTAAAACTATTGTCAAGTTTCCTCAAACTAGAATAAGGAGGTAAGA
TCAGATAATCTTTGTTTCCTTAATAGCTTAAAAAATCTATGATTTACATATAAAGGGAAC
TTGTCAAAAAGGAAAAACAAATCACATTCATAAAAGAAAATAATCCTGAAAGAAACTGAT
TCTTCCTAAATCAAAATCAATTTATAAAAATAATTCTAATTAACATTTAAGAGAGCTTTT
TCTTATTTAAAATTCACTTTGAAGAATAAGTAGTCATGAATGTATTTGAAAATTCTAAAA
AATAAATGTAATGACTACTAGACAAGATATATTTTTAAATTCTTATAATTGACAAGGTAC
TGGTATAAGAATAAACAGAGATAGCATGGTACCCTTATTGTCTTCATCCAAAAGAATTAA
GTTGTGTTTATTAAGTAGTTAGGTCCTGTGTTGGAAGTCCCCAAGATCACCCCAGGTTTG
ATTCTCTGGGGGGATTTACAGGACTCAGCATATAGTCATACTTATGGCCAAGATTTTATT
ACAGTGAAAGGATACAGACCAAACTCAACAAAGAGAAAAAGCACATGGGGCAAAGTCCAG
GGAAACGAGGCAATAGCTTCCTAGAAACCTCTTTCAGTGGAATCATACAGTGTACTCTTG
ATACCCCCAGCAATGAGTTGTGGCAACATGTGTGAAATGTTGTCGATCTGGGAAGTTCAT
TTAAGATTCAGTAACCCAGAGTTTTTATTAGAGGCTGGTCACATAGGCACCCTCTGCCTG
GCATCATGTCCCAAAATTCAAGACTTCCAGAAGGAAGTAGGTGTTCAACATAAACAGTAT
TATTTATATAACAGTTTAGGCACAGTGAACCATCCTTATCAATTAGAGAATGGTGGGAAC
CTTCCCAAATTCCAAGTTCCTAGATGCCAAACAAGGGCCAAACTTATTAAGCAAACTTTT
CAAAGGATAGCAGTCCCAGGTCTGCCATCTTAACTTTTTTCTTCACAGGTCCAGACAACC
TCATAAGCATTTATGTTTTAACTTTTAGTAACCATTTTAAAAATTAATATATACTGAAAG
AAAAAATATTACTTCCTTCTTAAACAGTTAATAACAAAATATGTAAACCTATTGAGCACT
GCTCACCTTGTCAAACCTTAGATCAGATTGGACGCTGCCACTCTTATTTCCTGCTCCACA
TTGATTTTTATACAATAGTTAATTTTTATCACAACTTTCACTGAAAAACCAGTTTTGCAA
AGATACAACATCATCAAAAGGAATATAACCTTATCTAGTATTGACACTGAGCTACCTCAA
GCTAGTAATTTTCACTGTTTCCCTCCTATGTTAGGGGGACATGTTGGCACAGAATTTGGT
AACTTGNNNNNNNNNNNNNNNNNNNNNNNNNNNNNNNNNNNNNNNNNNNNNNNNNNNNNNNNNN
NNNNNNNNNNNNNNNNNNNNNNNNNNNNNNNNNNNNNNNNNNNNNNNNNNNNNNNNNNNNNNNN
NNNNNNNNNNNNNNNNNNNNNNNNNNNNNNNNNNNNNNNNNNNNNNNNNNNNNNNNNNNNNNNN
NNNNNNNNNNNNNNNNNNNNNNNNNNNNNNNNNNNNNNNNNNNNNNNNNNNNNNNNNNNNNNNN
NNNNNNNNNNNNNNNNNNNNNNNNNNNNNNNNNNNNNNNNNNNNNNNNNNNNNNNNNNNNNNNN
NNNNNNNNNNNNNNNNNNNNNNNNNNNNNNNNNNNNNNNNNNNNNNNNNNNNNNNNNNNNNNNN
NNNNNNNNNNNNNNNNNNNNNNNNNNNNNNNNNNNNNNNNNNNNNNNNNNNNNNNNNNNNNNNN
NNNNNNNNNNNNNNNNNNNNNNNNNNNNNNNNNNNNNNNNNNNNNNNNNNNNNNNNNNNNNNNN
NNNNNNNNNNNNNNNNNNNNNNNNNNNNNNNNNNNNNNNNNNNNNNNNNNNNNNNNNNNNNNNN
NNNNNNNNNNNNNNNNNNNNNNNNNNNNNNNNNNNNNNNNNNNNNNNNNNNNNNNNNNNNNNNN
NNNNNNNNNNNNNNNNNNNNNNNNNNNNNNNNNNNNNNNNNNNNNNNNNNNNNNNNNNNNNNNN
NNNNNNNNNNNNNNNNNNNNNNNNNNNNNNNNNNNNNNNNNNNNNNNNNNNNNNNNNNNNNNNN
NNNNNNNNNNNNNNNNNNNNNNNNNNNNNNNNNNNNNNNNNNNNNNNNNNNNNNNNNNNNNNNN
NNNNNNGATGATACTTATTACAGTGCTGTTTTTATCAGTGGGAGATTGGAAATAACCTGA
ATATTCAAAGAAAGGAAGAGTTCAGAAAGATGTCCAGCCATATGATGAAATATTTTATGG
CCATTAAAAATTATGTTTATGTAGAAAAGGAAATAACATCAAAAATATTTACAACATATT
GTTAAATTAGGAATCTGGATAAAGAACTTTATTATAGCATCACAACTATGCAAAAAAATG
GATATAAACTAAAAAATAATTTTTCATAAATTAACCATAATTTTTTTTTTACTTTAAGAA
TGATGGGATTATAGATTTTTCCTTAATTTTTTGGTTTATACATATTATCATGGTGGTCTG
TTATATTTTTGTGATTAAAATATGCATAATCATCTCTTGCATTTGTGGTACAAATTACTA
GTGCTCAGCAATCTCTAGTTCTCCTATTGTTCCGAACCTTGAAGACTCTATTTCCAGCTT
CTTGCAGGTGAGCAAGCCTAATTAACTAAAGAACTGCCAAGAAAATTGAATGGGTGGATG
TGTGTCACTTCTGGTCTGAGACAGTGAAAAGCCCATGTTCAATTCTTCAGTCTCCCTGTC
TTGCTCTTTCTCTGGGGAATTGACTAAATATTCTAGATAATACACGATAAGATGGTGGAA
CCTCTCTAATCCAGGTTTGCTGAATCACCAAGTAGAGGACAGTGCCTTGCAAAGACACCC
AGACTTTGTATGAGCAAGAAATAATACGTTGTGTTAAGTCACTGAGACTTCTAGGTGTTT
TGTTACTGCAGTGGTCTGCAAAGTGGCATNNNNNNNNNNNNNNNNNNNNNNNNNNNNNNNNNNN
NNNNNNNNNNNNNNNNNNNNNNNNNNNNNNNNNNNNNNNNNNNNNNNNNNNNNNNNNNNNNNNN
NNNNNNNNTCTCTCCGCTCACATTGTACTATAAGCTGCAAGGAGAAGCCAGGGAACATC
CTCCACATGTAGTCTGGAGATCTCCTCAGCTAAGTATTCCAGGTTGTTGTTTTCAAATTC
TTCCTTCCATCTGACACCAGGACCCAATTTTGCCAAATTCTCTGCCACTTTAAAACAAGG
ATCGCCTTTCTTCCAGTTCACAACAACACATTCATCATTTCTGTTCAAGACCGCATCAGA
AGTATCTTTAGAGTCCACATTTCCACAAACAGTCTCTTCAAAGCAGTTTAGGCCTTTTCT
ATCAAGCTCCTCACAGTTCTTCCAGAATCTTCCCCTTATCCATTTAAAAAGGCATTCCAA
CATGTTTGGTATTTGCAAACTCAGCAGCAAAAGCACCCCACTTCTCTGGTACCAAAATCT
GTTCTAATTTGCTAATGCTGTGGAATGCAGAACACCAGAGATGGATTGGCTTTTATAAAA
AGGGGGTTTATTTGGCTACACAGTTACAGTCTTAAGGCCATAAAGTGTCCAAGGTAACAC
ATCAGTATTCGGGTACCTTCACTGGAGGATGGCCAATGGCATCCAGAAAATCTCTGTTAG
CTGGGAAGATATGTGGCTGGCGTCTGCTCCAAAGTTCCGGCTTCAAAATGGCTTTCTCCC
AGGACGTTCCTCTCGCAAGCTTGCTCCTCTTCAAAACGTCACTCACAGCTGCACTGAATT
CCTTCTCTTTGAGTCAGCTCATTTATATGGCTCCACTGATCAAGTCCCACCCTGAATGGG
TGGGGCCATGCCTCCATGGGAATATCTCATCAGAGTCATCACCCACAGCTGGGTGAGGCA
CATCCCAAGAAAATCTAATCAGCGCCAAAATGTCTGCCCCACAAGACTACATCAAAGATA
ATGGCATTTGGGGAACACAATACATTCAAACTGGCACACTGACTCAGTCAACAAAGCAGA
AGACTGTGTGTCACATATGGTGTGTGAGAGATGTTGAGAGAAACAGAATCCACAACATGT
GGGAGTTTACAATGGTCCCCTCACTGATTCTTTTGCTTTCAGCTTTCAGTGGTTAATTGA
ATTTATGAATGAAATTATCTAGTTTTAACCCAACTATTCTTCACAGAATGTCTAGGTGGT
GGAAAGGTCCTGAAATGATGCATACACCCATTGGCCACAAGAAAATGATAGAGTTGACAC
TTCTGCTTAATTCCTAGTACTAGCCTTTGTCAAACACGTTAATGAAATAAAAACAAATAT
GAACTGGAAAGATCTGATAGGAAACTGGCCACCAAAATTAAAAGTTTTCAAGAAGACTAT
TTGAAATATTGATTCACTTCCACTTTGATTAATGAAGAAACTTAGACTAAGTGCATATAC
TACTTCAAAATACTAGCTAATCGTATGAAGCCATTATGATTAGCCAGAAATTTTTCCAGT
TATGTTTAAAGTCATGTGATACACTCAGTGCAGGTCTTTAGAAAAATTTACTAAAGTTAA
CAATAAATGTTTATAAGTCTCTTTTGTGAGATTTCTTAATAACAAAAAATAAGTCACATG
ATTTGGAAAAGTTCTTTTGCTGATTAAAATGGCTCAAATATTACTCAGAAAGCATAACAG
TGACAATATAAAATGTATTCTTTTATCAGCAGATACTGACATAGAAAACACTGCTAAAGT
GNNNNNNNNNNNNNNNNNNNNNNNNNNNNNNNNNNNNNNNNNNNNNNNNNNNNNNNNNNNNNNN
NNNNNNNNNNNNNNNNNNNNNNNNNNNNNNNNNNNNNNNNNNNNNNNNNNNNNNNNNNNNNNNN

NNNNNNNNNNNNNNNNNNNNNNNNNNNNNNNNNNNNNNNNNNNNNNNNNNNNNNNNNNNN
NNNNNNNNNNNNNNNNNNNNNNNNNNNNNNNNNNNNNNNNNNNNNNNNNNNNNNNNNNNN
NNNNNNNNNNNNNNNNNNNNNNNNNNNNNNNNNNNNNNNNNNNNNNNNNNNNNNNNNNNN
NNNNNNNNNNNNNNNNNNNNNNNNNNNNNNNNNNNNCCCCAATAAATAAACCCCAATAGGCGT
TTATTATGTACTCATGTAAAAGAATGCCAAGCAGTCCAACACTTGGAGGGTAGTCTGAAT
GTCATTGGGAATCCATGTTCCTAGCTTTGTGTTCCACCATCTTTGGTGTGACTCTCAATC
TCGTGCTCACAATATGGCCTCCAGAGCTCGTGCCACATTTCAGGCAAAAAGAAGGGGAAG
AGCAATAGGCAGTGAAGGCTAGCAACAGCTGAGTCTGCCCAACAACTTTTAAGAGCCCTG
CAACTTCCACTGACATCCCTACCTACAAGGGAACTTGGGAAATATTTTGTTTTTCCCTCA
TTTATTGGCTTTGCTTTTTTTGGTTATCCTTATGTTCTTGTCTGCTTACAATTAAGCAAC
AAGAAATAAAATATTCTTCTTTAAAAAAAATTCATCTGCCAAAGCTTTATGCAATTAAAA
TTAATAAGAAAAACAAAGTTGGAAGGGTAAGATAGGAAAATTATTTTACATTTGACAGGC
TTACTGTTGTATAAGTTTTCAAAACTCAATTTTTACTTTTTTTAGGTCGTATCCATAATG
ATGATGACAACAATAACCATAATAGTAGCAGTTTAAAATTAATAAGGTGCTTACTACCTG
CCAAGAACTGTTTTAAATGTTATGTAAACTTTATTTAACCCTCAAAAGAGTTGCATGGTG
GTAGAAATTTCCTCCTTTTACAGATGTTATAACAGTACAGAGAGAGTCACTGAGTTGGAC
AAGGTCATACAGCTGTTAACTAGATTACGTGACCTACAATTTATTTGGTAAACTATCTGC
TGATTTAAAATGTTATGTGGTTTCAGATTTGGTGTAAATTCTGCTCTTCCATTTCCTTAC
TATATGTATGTTTTGGGGAAGCACTTACCCTCTCAAGGCTTCTGCTTTCTTATCCTAAAA
ATGGGGTTACTAATATCTAAGATTGTTGTTAAATGTGATAAACATTTACATAAATGTTAA
ATGCCTAGCTTACTGACAAGCACATGATAAGGACTCAATAAATGGTAACTACTATTTTAA
ACTATTTATGTTTTTATGTAGAATTTCTGTGATCAACAAAAAAGACAAGATTCAAATTCT
TTTTAAGCTTTTTTTTTAATCAATGGAAAAATAAAGTACAGATTTTTTTAAAACTTAGC
TTTGTTTTATTATATTTTTCAG**ATCAATGTTGAACTATATAGATTGTTTGGCACAAAGCA**
TATTGTAATTTCTCATGCCTCTCA**ACTGTTGATCCAACTGAAAGTACACCTAGCACAATC**
AAAACCTTTCTTTCCAAACACTGTGCTGACTACCCAAACAACTGGGATGATCACCTATCA
GCTGTTTCATTTGCCTTCAATGTAACTCACTTAGTATGTGCCTTTTTATAATTTTATATT
TCTGAATTATGACTGGTTATTTCTTGCCTAGATAAGTATTCATTACAAACTTCCTGTTTT
CCTTAAAATAG**GAACCTACTAAAAATACACCATATTTTCAAATGTTCAATCGAAATCATT**
ACGTTCCTGAGACTTCAGATAGTCTTCATGGAGTGGATGCTGATAATACAAGTATGTTTG
CAAAAATTCTAGATGCAATTAAAGAAGCTGATAAAATAATGGAAAATAAGACAACTAGAG
CCAGCCAGGTAATTCTATTATTAAGAAAAACTATATAATCAATATAGTGACCATTTATTT
TTAAATTTTCATTTTATGTGTGCACATATACATACATACATACAGACACATATGATATAC
CCATAAGCAAACTATTTTTCTATTAAATTCTGCTTGTCTTGATTAGACAGTATTCCTGTT
AGCTATGAAATTCAGGATATAACAGTAAGATCACCCCAATCAGCTTATATATAAAGTGGA
GCACTAATAGTGACTTTGAAGTACTGTTTTAAGAATTCATGCCTACCTCTTAGCAGCATC
TCAATAAATAATTGTTCTTAGTATTATTATTTGGTATATTGTTTGTCTTCAAGAAATTAT
AACTAATTTATCCCTTTGTTCTTTCTCTTTTGCATTTGTTAG**ATGGAGAACAACAATTTT**
GATGAACTAAATAAAAGCAAGATCGTTGTTAAAAAGAAACCAAAGCAGTTAAATCCATTT
CATCTAAAAGTGGGTCATGAAGTTTTAAGACAAAGGAAAAATTGGTGGAAGGATGGTCGT
TTCCAGTCCGAATGGGTTGGCCCTTGTGTCATAGACTATATTACAGAAAATGGATGTGCT
GTCCTGAGAGACAACACTGGTTCCAGACTTAAAAGACCTATCAAAATGTCCCACCTCAAG
CCCTATATACGAGAATCCAGTGAACAAGGTAAATGTCAGTTGCTACATTTTTACTTTCTT
TAAATAAGGAGAAAGGCTAAAAATCCCACCCCCCACCACCACCACCTCTTTGGTTAGGTT
TTCTTTCTCCCTCTCATTTTTATATTTATAAAATGGGGTATTGTTTTAGATATCCCAAGT
AGTCTTTAGTAGAATGTTTGTTCTGAAACTGTCGGGCTTGTGAGAGATTGTATTCTGATT
TCCACTGTATTTTCATAGTTCATGTGGGAAATACTGAACCTGTTTTACAGAAGGATTGTA
TCTTCTCTATATTTACTTTCAGAATATCCTATTAGTCACTTTGTCTCTCATTTGAGCATG
AGAAACCAGCAACTAAAATCATTAATGCCAAAAATAAACATACTGTCCAGAATCTAGTTT
TGAGACAAATATATTAGGGGACTAAAAAATATTAATTTTTAATTATAGGCAGGTCATGGA
GTGGGGACAGATGAGAGTGTAGGGTGGGATTTACTCAGAATGCTATAAAGTGCTTATAAG
ATGGTGATAGCACCCTAAAGAGGTTATATTTGCCCCAAAATGTGAATGGCTCTGACAGTC
AAGATATCCTGATCTTTGTAAGCCTTTCTTTCATAGAGTAGAGCTAAATGACATTGGGCC
ATGACTTTCAATTAGCTCACTTTATAACGACTTTTATTTGTCTGTTTATCTATCCCCTAG
AGTTTCTCTGAGATGGATAACTTGGGGGAGTTAACTGACACTTTAACCAGTTAATTGTAC
TTTTTCTAAAAAGCAAAGATAAAGCATCATTATTATTTGAAATTGAGTAACATTTATGGG
AAATACAATATAGTAATTTTTTTTAAAGAATATGAACAGCAGCAACTAGGTTATTTTAAC
AGGCCTATGTAAATTATAACAGTAACGACAACAATAAGAAATAACTTTCACTTACCCTAT
ACCTTTTTTTTAGGGTATGAACCCCTTGGGGCTACTCAGTGATAGCTCAACTCTGCTTCT
GATCTGGGCCATAATCATGGTGTTAAAAGCTCTTAGTATCTTTCCTTCTGCTTGTGACAA
AGTCAGCTGTTCTAGCCAGACAATTTATCTTCGATTGTGTTCTAATTCTGAAAACTCACA
AGTACCTTCAGTTTCTTTGTTAGAAGATAAGCGCTTTCATTTCTTGTGCTGCTTCTCAGC
TCCAAGCTACTTTTTATAATCTCTCCCTTGTACAAGTTAAGTAGAAGAAAATGATTGTAC
AGGTACATGTTTTCCTTTCTTCTTCAGCATGATTTTCTCCTTAATCTTTGGCATCCAGAA
TTCATTTCCTTCCAATTTGTTAACTTTCCCTAATTAATATAAAAGTTATATTTAAAGTGT
TGCTGTATCAGGATCTGTTTCAACATTTTGCAATTTGCTCTTGCCGAAGACTTTCTCAGA
AAGACGACCAAATATTTTGCTTTACCTGAGTATTTCAGAACAGTTTCTAAACAAAGGCAG
TATAATTCTTGCCCTATGTTTGAATTTCTGCATATTTTTCTTTCTGTAATATAAGGAATT
TGTCAATATTAATACTGGTGTACACATAAAGGGACCTGGGATTTTACGGGTGAATTGGTT
TTCTTTTTTTCTTTTGACCAAGGGAAATGTTTATCTGTCCATCCTAGAACGTGGTTGCTG
ATTACCATTTGGTCCCTTTTTTGTGTTTCTCTTTTTGATAGAAATTCAAAATAAGTGTGA
TTTCTGCCATCTTTTTCCTCAGTATCATGTATTTTCCTGAAAATCTTCACTCACAAGCTT
AAGAGTTTTTGAGCAGAATGTAAAGCAAACACATTGTCTTTGAATAATCATGCACCTTTT
TTATTTGGCATGAATTTAGATGAGCTGAATCCAAAAGAACTAGAATTTGTGTGTGCACAT
GCCATTACAACTACCAACCTTGAGGTTTCATAACTGTATTAAAGACTTCCTGTCTAAAGC
AGATATGCTTCTGTTGCCAACTTCTTATCTATTCTTATCTAGAGTATATTGTAGAGGGAG
CACAGTACTGTCTATAATTACTATTTTAAGCAGGCCTGTTTGTCATTTCATGTAGTTCAA
TGCCAAGAACCTTTGCAGACTCATTATTTTAATGAAATCACAACAAGAACTGTTATGACA
TACCATAATGTCTCTTAATAATCATAATGTTCCCTTTTGAATGGGAAAATGATGTTATTG
GCCTACTATTCCATCAGACAGATTTTTTTTATTGTGGCAATATATACAACTTAAAATTTC
TCATTTAAATTTCTCACTTTTGTGTATACAATTCAGTGGCTCTAATTACATTCACAGTGC
AGACATAGATTTTGAATAATTTTTTTGTTTTGTTTTGGTGGAGGTATTATGATAGATTTA
TGTCTGTCTCACAAAAAAAGTTTTTATCTGATTGTCCTTCACAGTCAAAATCTCCCATTC
ATGAAATATTTTTCATTATTAATTTCCTATTGCTGCTATAATAAACTACCACAAATTTAG
ATTTGAAACACACGTATTTTGATCTTAGGTTCTTTAGAAGTCCAAATCAAATCTCAGTGA
ACTAAAACCAAGCTTTGGCCAGACTGTGTTTCCTCTGGAGGCCCTAGGAGAGAATCAGTT
TCCTTGCCTTTTTTAGCTTTTAGAGGCCACCCACATCCCTTGGTTTGTGGCCCTCTTCCT
CCATCTTCAAAGCTAGCAATTACAGGTTGAGTTCTTCTCACACTGGGTCACTCCAACTTC
CTCTTTTGCCTCCCTTTTCCACATTTAAGAGCACTGTGATTACATTGGGCCCACTGGGTA
ATCCAGGATAATGTAAGGCAGCATATTCAAAGGTTCCAGGGATTAGTCCGTGGACATCTT
AGGGGACCATAATTCTACCTACCACAATTATGGTCTGCATTCATGATACAAAGCAAAGCT
TAGTCCCTATGGCATCAAGCCCATGGTCTTGACTTTATGATTAATATAGTTTGTGTAAAG
AGCCTTCTTTTGGCCTCTTAGTTATAAGCTAATAGTCATGTCGTTTGCTCTATTGTTAAT
ATCGGTTATCAATTATAATGATAATGATTATTAGTTGTCATTTTATCATGAAGAAGGATG
TACTAGTATGGTAAAACTTTCTAAATAGTTTTAGGATTATCAAGCTATATATAGGATTAG
TCTGGTCATATCTTCAGAATAAGAAAATTAAGTATTTTGAAACCTTGTTCTTTTCTTTTT
ATTAGAATAAATAGGTAATCCAAATCATTCAGACTTTTGTGAGACTTTATGTTTTAGTTT
CTTGGCTGCTAAAGCAAACACCAAACAATGGGTTGGCTTAAATAGTGGGAATTCATTGAC
TCAGAATTTTTAGGCTAGGAGAAGTTCAGAATCAAGGCATCACCAAGGTGATACTTTCTC
CCAGAAGACTACTGTGGCATTCTGGGGCTGGCTGCTGGCTACCCTGGTTCCTTGGTCCTT
TTCTGTCACAGGGCAATGCACATGGCAGCCTCTCCTAGCTTTTCCCTTCTCTTCTAAGTT
CCGTTGACTTTCAGCTTCTGCCTACTCCCTCTGTGGCTTTCTCTCTCTGTATCTGAATTT
CATTCTACTTATAAAGAACTCCAGGAATAGGATTAAGACCCATCCTGATTCAGTTGGGCC
ACACCTTAACTGATGTAACCACATCCAAAGGTCCTATTTACAATAGGTTCACACTTACAG
GAAGAGATTAAGTTTAAAAACATGTTTTTCTGGGAGTACATAGCTCCAAGCTACCACACT
TTGGATTTAATTTTATTTTTCCTTAAGGAAATTAAAAAATCTATTTCTTAATCTCATTTT
ACTCTTTAAAACTATATAAGGACTGGGACTGTATAATTTAGTGAAACCGAGAGTGGATAG
TGATGGTGGTTAATTGTATAAATATAAGAAAGTTCTTATATGAACTAGAACAAACATATG
TCACTATTAGAAGGTGTTAATAATAGGGTGATATATGGAAAAAATACTATTAGTGTAAAC
TAAAGTCTATGGTAAACAGTAACATTGTAATACTCTTGCATTAATGGTAATAAAGACACT
ATACCAAAGCTAAATGTCAGTAATGGGGGATATAAAGGGTATGGGATTTTTTTCTTTGGA
AGCAATGAGAACGTTCTCATATTAACTCTAGTGGTGAATGCATAACTATGTGATTATACC
AAGAACCATGGATTGTACACTGAGGATGGATTGTATGGTGTGTTAAATAAAACTGTTTAA
AAAAANNNNNNNNNNNNNNNNNNNNNNNNNNNNNNNNNNNNNNNNNNNNNNNNNNNNNNN
NNNNNNNNNNNNNNNNNNNNNNNNNNNNNNNNNNNNNNNNNNNNNNNNATTGTGTAATGTAGA
AGTGTTCCTTATAATGGTATGCTTGTTTATTTCACTCTGTGTCTTGAGGGTCAAGACTGT
GTCTTGTTCATTTCTATATCCCTGATTCAGTGTAGTTCTAGAGCAGTGCTGTCCAATAGA
AATATAATGCAAAAATAGGTAATATGTAATTTTAAGTATTCTAATTATTCAAAAAGCACA
GAAATGAAAATGATTGTAATAATATATCTTATATAAACCAGTATAGCCAAAATATTATCA
TTTCAACATGTCTGTAGGCCCCAAGTTTTTTTTGTTTTTTTGTTTTTTTTTTGGTAGATA
AGCTTTATTGTAATTAAATAATATTGTGTTGAGATGTTTGTTTTGAGAGGTTGGTTTTTT
TCCTATGTAAATAGGCCTTGACATCTTCAGAGTAATTTTCCATTGCATATAAAAAGATTT
TATATAGAATTTCCTGAGTATAATCTGTTATTATTATAGTTTTAGTACAATAAGGTACTT

TAAAGTTTATCAATTACTTAAAAGAAAAAGGGTGTATGTATATATGTATGTATGTGTATG
TAATATATCACATACAGAAGCACTCCTTAAAAACAGATGAAGTTGAAGTCCACTGTAACA
AGGTCTCTGAATGCATTTCCAACAAATAAAGTTGGTAGCTTCCAACAGCTTTAAATTTAG
GCAAGTTATTCCCTGTCAATGTTATGTCTTACTGGACATTTTTTTCTAAAATAGACCTTC
ATTCTGTATCAAAAAAATATTTTGGGATTGAAAGCTCTTCATCTAAGATATCTGCAGTTA
TCTTCAGAAATTCCTTAAGAGCTATGGTGTCTGCCTTCAGTAATACTTTGCAGCATTATA
AGATCTTTAACCTATGGAACTATCCAGAAAAATTTCATCAGCAATACTTTAACCATACCT
AACCTTTTTAAGTTCCCATATAAACTACTACTTTGCCTTTTCCAGTTAACAGTAGGAGAG
TAGCCAATTTTGATGCTGAATTTTTAACAACACAACTTCTAGCTGCCCACTCTAAAAGCT
TTCAATTTCAAAGATTATTTTTTCTTCTTTCTTGATACTATTGGACATCTTTGAGAATTG
TTCTTCTTGTCCAGCAGTTCATCTCATATCAGCATATGGAAACCATTATTTTCTTACCAC
TTTCAATTATCAGTACTTTTATTTCTTCATAAATATGTGCATCTTCTTACCAGCTATTTC
ACCTGATTGCTGCTTTAGCATTAACTATAATTGGTGGATCTCAATTTGGAAAAGTTCTCA
AAACTAAATGACCAAACACACTTTGCCTTATGGAGAACTGAATGGGCAAAACTAAAGGGC
CATATAAGTCTAAGCTTACCCAGGCCATCTTTTGGCAGAAAGCATAGCTTTGTTTGATTT
ATCAAAATTTGTAAGTTGGAAGTTTTTAAATACATGAACTTCTGATACTGAAATAGGCTT
TGTGAGCTTTTTAAATCTTAGTGTGGGGATAGATAGGGTAGAAGGTTGTATTTGCAGATA
TACCTGCTAGGAGAAACTTTCATCCAGCTCTAAAGTAAATGGGCAATTAATCTTCCTTTT
GTTATTTCTTGAACATAAAATGTGCGGACTGTTTAAGCTATTAACATCTTTAAGAGCATC
TATTAATGTTTTGGGAGAAAATACAGAAAGAAAAGATATGTGTCCAGGAAGTTAATCAGC
TATGGAGGAAAAGTTCATTGTTGTAATCAATTAAAATGTCAGCTAAAAAAGAATCTTTTT
ATGTACATTAACCTTCATATATAAAAATGCTGCTGCTTTGATATTAGTTTAACATTCATT
TGAGCATTCTTTTTCTCCTGATGGTGATGAAAAATTTTCTCAAAATAGAATAAAATGCTG
ATAAGGAAACTTCCCTATCAATACTTTGGTACAGATTTAACTAGACCTTAAAAGTTCCTG
AGAATTTTGGTATTAAATCAGAATTAAATAAACTAAACTTTTAAGCTACTTCATTGTAAC
TTCTCTGCTTAACACATTGCTATCGCATAAACCTGGTTCTAGGAATTTTATCTTAGTTTT
AGTTTATTTCTTCATCATATTATAGCAATTTGGTATTATTTTCCTATTTTGGATTCTATA
AAGTTTGGGGAATTATCTGAAGTTGTAAATATAAGGATTTAACAATCCTATTTGTTAGGT
CAAAAGAACAGGAAAGCCTGCGTGTTTTAAAATCCTTGATCGTTCTTGTATTAGATGAAT
ATAAACTGTCAAAATTATATGTTCTTGTATTCAATATTTGACATGTATATTTATTTAGTA
GACAAGAACTTCTCCCCCTCCCCTGCCAAAATTAATCTTAATGGGTAATTTTTATTAATT
GGAATTTTTTCTTTATTTACCATTGTTTAACTTAAAATTTATTTTTAATTATCTTTTAAT
TATTCTAATGGACAAACTTATGATGTTTCTTTTTTCAG**ACAGTCTTTATCTCTTACAAGG**
TTCAGTAGTGGCAGATCATGACTACATTGGATTGCCTGAAATTCCAGTTGGAGCATACCA
AGCAAATATTCTGGTAGAAGATGCAACAATTGGTGTAGTCGATAATGAATTACTGACATC
AAGCAAAGATCATGAACTATTAGAATATAGACATGCCAAAATCTCTCCATTGATAGAAGA
TCATGGTACTCTTGAAAAGCAGACTTTCAGTCTTTTGGACTCTTCAAACCAAGTCCTTGA
GTACTTAAGTTAGTAAATACCAAAATTCATGTCAAGCATTTGTTTAGAGTGTATAACCCT
TGAATCTTAATGTCTCATCTATCAAAATAGGTTGTATAGAAGAAGTATGTTGACATACCC
TTGACTAAATCTTGAAGTTTTTTTTGTAACTATTTATTTATCTAAAATTGTGACCATGTT
TGAATGTCAGTGTATATATGTATAAAGTCAGAGGAAACATAATATATAGAATTCACTTTT
TCTAAAAAGAGAACAGGCAAAACACAATTGCTTCTTAGAAAGTAAATTAATTTCCAGCTC
AAAACTGTCAACTCTTTCCCTGATATCCATACAATGTTTTCAGTTTTAATTTTTCTGGGA
TACTTCTCTTTTTTAAAGAATAGGTTCTAACTGCCTAGTAGTGAATTAATAAATATTGAA
TCAACCATAACAACAAATTGAGTGAAACTTCTCTCTTTGTTGTTGCAGTGTGTAAAAATA
ATATTGTAGGAAAAATATTTAGGTTTGTCTAATGGACTCTTATAGGATATTGGTAATTTC
ACTGTGGTAGGTAGATAAGGTCTGAAACTATATTATTGTTCTGGGACCATTTGTTCATTT
CCATAATATATAT

==

Sloth Gin-1

```
total length:      19513 bp  (17133 bp excl N/X-runs)
GC level:         33.73 %
bases masked:       5914 bp ( 30.31 %)
==================================================
               number of      length   percentage
               elements*    occupied  of sequence
--------------------------------------------------
SINEs:                 3          485 bp    2.49 %
      Alu/B1           0            0 bp    0.00 %
      MIRs             3          485 bp    2.49 %

LINEs:                 6          968 bp    4.96 %
      LINE1            3          734 bp    3.76 %
      LINE2            3          234 bp    1.20 %
      L3/CR1           0            0 bp    0.00 %
      RTE              0            0 bp    0.00 %

LTR elements:          6         2993 bp   15.34 %
      ERVL             1          561 bp    2.88 %
      ERVL-MaLRs       5         2432 bp   12.46 %
      ERV_classI       0            0 bp    0.00 %
      ERV_classII      0            0 bp    0.00 %

DNA elements:          6         1257 bp    6.44 %
      hAT-Charlie      6         1257 bp    6.44 %
      TcMar-Tigger     0            0 bp    0.00 %

Unclassified:          0            0 bp    0.00 %

Total interspersed repeats:      5703 bp   29.23 %


Small RNA:             0            0 bp    0.00 %

Satellites:            0            0 bp    0.00 %
Simple repeats:        2           81 bp    0.42 %
Low complexity:        4          130 bp    0.67 %
==================================================
```

```
SW   perc perc perc  query            position in query     matching   repeat             position in repeat
score   div. del. ins.  sequence         begin end     (left)   repeat      class/family    begin  end     (left)  ID

  303   30.0  6.3  0.8  UnnamedSequence   1539  1649 (17864) C L2a        LINE/L2            (0)   3426   3310   1
  236   22.6  3.1  2.1  UnnamedSequence   4289  4348 (15165) + L2a        LINE/L2           3289   3405   (21)   2
```

281	36.1	0.0	2.3	UnnamedSequence	4392	4479	(15034)	+	MIR3	SINE/MIR	23	108	(100)	3	
1557	21.1	7.7	1.5	UnnamedSequence	4848	4886	(14627)	C	MER119	DNA/hAT-Charlie	(71)	512	468	4	
3118	17.3	1.4	1.1	UnnamedSequence	4887	5447	(14066)	C	MER68	LTR/ERVL	(0)	563	1	5	
1557	21.1	7.7	1.5	UnnamedSequence	5448	5782	(13731)	C	MER119	DNA/hAT-Charlie	(116)	467	115	4	
480	30.4	4.3	0.0	UnnamedSequence	6607	6836	(12677)	+	L1ME4c	LINE/L1	441	680	(150)	6	
22	78.0	0.0	0.0	UnnamedSequence	6842	6891	(12622)	+	AT_rich	Low_complexity	1	50	(0)	7	
385	29.9	8.4	4.5	UnnamedSequence	7004	7391	(12122)	+	MLT1J1	LTR/ERVL-MaLR	1	421	(23)	8	
1298	28.0	0.2	2.5	UnnamedSequence	7510	7919	(11594)	C	MLT1J-int	LTR/ERVL-MaLR	(1088)	401	1	9	
3839	8.8	0.4	1.4	UnnamedSequence	7920	8438	(11075)	C	LTR1C2_Cho	LTR/ERVL-MaLR	(0)	514	1	10	
383	33.6	3.2	0.0	UnnamedSequence	8439	8563	(10950)	+	Charlie7	DNA/hAT-Charlie	166	294	(2318)	11	
308	24.7	3.4	0.0	UnnamedSequence	8570	8658	(10855)	+	Charlie7	DNA/hAT-Charlie	326	417	(2195)	12	*
943	26.0	1.4	2.5	UnnamedSequence	8654	8933	(10580)	+	Charlie7	DNA/hAT-Charlie	441	717	(1895)	13	
878	19.1	10.4	1.8	UnnamedSequence	8929	9179	(10334)	+	Charlie7	DNA/hAT-Charlie	757	1028	(1584)	14	*
389	30.4	10.7	0.9	UnnamedSequence	9533	9728	(9785)	C	MLT1J	LTR/ERVL-MaLR	(67)	445	231	9	
253	31.4	7.1	6.5	UnnamedSequence	10092	10275	(9238)	C	Mon1f5	SINE/MIR	(1)	266	82	15	
594	24.1	8.0	6.5	UnnamedSequence	10344	10556	(8957)	+	MIRb	SINE/MIR	50	265	(3)	16	
22	50.0	0.0	0.0	UnnamedSequence	10900	10921	(8592)	+	AT_rich	Low_complexity	1	22	(0)	17	
202	25.5	2.0	0.0	UnnamedSequence	11235	11285	(8228)	+	(CATATA)n	Simple_repeat	3	54	(0)	18	
25	52.0	0.0	0.0	UnnamedSequence	12263	12287	(7226)	+	AT_rich	Low_complexity	1	25	(0)	19	
385	16.5	2.1	4.3	UnnamedSequence	14065	14159	(5354)	C	L1MB4	LINE/L1	(0)	6180	6088	20	
1457	19.8	18.8	1.8	UnnamedSequence	14297	14726	(4787)	C	MLT1D	LTR/ERVL-MaLR	(2)	503	1	21	
2979	13.4	0.6	2.1	UnnamedSequence	15110	15598	(3915)	C	LTR21_DN	LTR/ERVL-MaLR	(0)	482	1	22	
1297	17.5	26.1	0.2	UnnamedSequence	15676	16084	(3429)	+	L1-2_Cho	LINE/L1	2589	3103	(1988)	23	
221	25.4	11.1	0.0	UnnamedSequence	16220	16282	(3231)	+	L2c	LINE/L2	3266	3335	(52)	24	
577	14.0	8.8	3.2	UnnamedSequence	16304	16451	(3062)	C	MER33	DNA/hAT-Charlie	(0)	324	169	25	
194	3.3	6.7	0.0	UnnamedSequence	16713	16742	(2771)	+	(TATG)n	Simple_repeat	3	34	(0)	26	
26	63.6	0.0	0.0	UnnamedSequence	18572	18604	(909)	+	AT_rich	Low_complexity	1	33	(0)	27	

Laurasiatheria

>Bos Gin-1 (chromosome:UMD3.1:7:104529477:104560001:-1)

```
CGGCCACTCCTCCACACCCGGGCCGCCTTTCTCTCTGGCCACACGCCCCGCGGAGGAGCG
GCAGCCAGAGCTTTGAAACAGCTGCCCTGCGGCGGCCGCAAACCTCTGGGGACTAGGCTC
CGCATCTCGATTCTCCTCAGTTCTCGCCCCGCCCCCCCTGCTCCCCGCACCTGCCACGGC
TGCGGTCATCACGCGAACTGCAGATGTCCGAGTCCATTCAGGGGCTCCATGCCCCCCTTT
AATCCCGGGGGAGCGGGGGTTGGGGGGCGGTCAGGATTGAGGCGCCCGTCAAAGAAGACT
CCAGCTGCTAGCGGGAACGTGGGTCTCCTCTCCTCAGCAGCAGCTGAATGGCCCACAAAG
AGGTACCGAAGGTACTACCGCACTTCGCGAAGCCCGGTCACGCCTCTTCCCCTCCCTCTC
CGGGTCTCACACAGAAACCTCCCCGGGCGGAACGATTTCCGGCAAGAGCCAAACGATCCG
CGCCTAACCTTTTCTGACCGCGTCTCCGAGACACCCGCCCCCCGGCAGAAACACGCCCTT
TGTAAACCACCACGCGGATAGTTCCGCTTCCGGCAGCACCACAAAAGTCGCGAGAGGAAG
CTTAAATCTTGACGGTAGCCTTTTGGACCACTTCGGTGAGTGGTCGTCTTAGTGTGCTCT
CACAGCTGCTGATCGTTTTAAAATGAGGCGAGACCTGACTAATTTCAAAGCCGGGAGAGG
CGACCAGTTTGAAGAAGGGTGTAATTGGCTTTATGGAGATTGCGCAGGCGCCCTTGGGGC
GGGAGGGCCCTGTTTGGGGCGTTACGCTGCCGCCGCCCGCCGAGCGGCCAAGGGGCGAGT
TCCGTTGTCGGGCTGAGGGAGGCGCCTACCCTAGATAAAATTATTGGAGGAATTAAGTAA
TAAGTATTAACAGGAGTATTCACTGGTTTCATTTAATGTTTTACCAGGGACAAAGTGTAA
AAAAAAGTATATATATATATAACCAGATTGAACCCTGAGTTTTACTTAACGGGTCATTTTCT
TGCTTACGCTAAATTTAGCTGGACCTTGCCCCAGAGTTAGTTAACAAGTAACCAGAACGC
TCTGGGAATGCACAAAGTGGCATCTGAAACTGGGAGCAAACTCGGAAGTTGAGTGTGGCA
TTTGGCTGAAAGGAAAGGATTCTCTTCCCCCCAGCCCCCCTTATCCTGGTTGTCGTTTCA
GCAGATATTTCCCGGTTTTGCCGCAGATCAAAAATCTGCGGGGAGAGCAATGCGAGCTAA
CAGGAGAATCTTGTCCTTTTGTTTTAAAAGAAAATTACACCTCTGTGGGTTTTCTTTTAA
ATTACTGTGTCTGAACCAAAATGGGAAAATAGTTTTTAAAAAATTTCATTTTTCACACGT
GGCTTCATTTGCATACGGAGATGAAGGCGTTTTTAAGTGAAATTTGCACGCTGAAGCGAA
GCAGATTATAGGGAAGTGTCTTGTGTAGTTGGATTTCCCAGTCGTTTTCGTTACATAAAA
TTGTGGTCTTCCGATTTTTCAGAGATTAAAATAGAGTAATGCAGGAATAAAATTAATGAT
CCCAAGTAAAATGCTCACTGAAATATTACAGATTTTAAGTTTTAAAGTTGAAACTCGTGC
ATATTATAACCTTTTAGAGGCTTAAATTAGGTGGTCCTGATGGGTTTTGCCCTAGGTGAA
ACACAGTTAAATCCTAATTTTATTTAAAGTGGTACTTAACTGCCTGTGTTGCGCACAAGA
TAAAATTATTAAAGGAATTAAGTAATAAGTATTTGCACTTAAAAGAAGTATTCACTGGTT
TCATTTAATGTTTTACCAGGGACAAAGTGTAAAAAATAATCAGATTTGACCCTGACAGTT
CATCAATTAGGAATTGTAATCAGACAGCTTTATTGCCTTTTCTTACTCCTAGGTATTTCA
ACACACAAGCAATTATTTGTCAAAGAATTCTTGAAAATATGAGCATATCCAAAAAAAAAG
GTGTAATGTAATCTTCATCTTTATAACTCAAGCCACCTAATTCTCCCTTGTAAACGATTT
CTTTCCTTAATAATATTTGCAAACAGACATTTTGTTTATATTTGTTCTGAGTATTACAGC
TTAAAGCCATTTTCTCAAATTAACTGCTGGGATGGTTTCTAGGGACCAAGTAGTGAATGT
ACTTTCGAGAAGGGAAAAATTATTAGTTCTTTTAAGTTTCATCAATAAGTAAATAGCAAA
CACGCGCAGGAAACTTTTAGCCTTCCTCTTGGACTTCCTTGGTGACTCAGACAGTAAAGA
ATCTACCTGCAATGCAGGAGACCCGGGTTCCATTCCTGGGTTGGGAAGAACTGGAGAAGG
AACTAGCTACCCACTCCAGCATTCTGCTGGGAGAATCCTGTGGACAGAGGACACTGGTGG
GCTACAGCCCATGGCGTTGCAAAGAGGCGGACAGGACTGAGTGACGAACACTGCCACTCA
GAAGGCTCTACTTTTTGTGAACTAATTTTTCTTTCTTATGCATTTCTGTTTCTTCTTGTC
GTCTGTCTTTACTGACAGAACAGTATTAAATATTATGGATGATAAATACTTAAACATTTT
AACAGTAATTATATATTGCTGTACCGTATGCAAAGAGCATTTCAAATCCATTATCTCATT
GGTCTTTATAATTGTGAAAAAGAAAGGATAAGTAATATTTCTGTTTTGTCACACATGGAT
ACTGAGACTTAGGTAACCGTTGCATGCTAGAAATTTTGACTCCTAAAAGCCTTACCTTTT
TAGATAGGCCCTTTCTGAAGTGAAGTAATTGTCACCTTTGAAACCTGTGCTCTTTTTATA
TTAATGCTACTTCTCCTGTCAGGAAATGCAAGTAATTCTTGTTCATTTACAAATAGTTGA
ATAGCCACTGTGTCCCTATAAAAGTCGCCTTGTAGCTTTTCTAACAGAAGCCTGAACCAA
GTATCAATGTTATGAAAGCTGAGGATGAGAAATAATTAAATTAGTGGGGGAGAGGGGTTG
AGGTTGGAAAAACATGAAATTTGAAACAACAGAGACCAAGGTTTAAATGTCAATGGATGG
GCTCACTCTATTGCCTTGAGGAAAGTGGACAATCTCTCTGAAGCTACGTTTTTTTATCTC
TAAAATGTATGCAGAATTACTACCTACCCCTTAGGATGTTGTGAGGATCATCACAGTGTA
GTACCATATACATGTTTGCAGCTGTTTTTAAAATTTGCTTTTGTGGTTATTTTTAAAAAT
TAATTTTAAAAAATTGTTTCCCATCTATATAGTTTTGGCATAAAAAGAGCAGCATGTAGT
ATGTTTGTAGCCTTTTGTGTCTGGGTTTCACTTAGTCCAATAATTTTGAGGTTCGTCCCT
ATTAAATATATTTGTAGTTCCTTTTTGCTGAGGAGTATTCCGCTACGTGGATCTACCACA
ATTTGTTTATCCATTCACTTGTTGAAGAGCATTTAGATTGTTTTTAGGTTTTGGCTGTTA
CGAATAAACATTCTAGAAATATTCACATACAGATTTTTGTGTGGACATGTACTTTCATTT
CTCTTAAGTTAAAGCTGTTTTCCAACATGTTTACACCATTTTTTATTCTTACCAGCAGTG
CTTTGCATGCTTGGTAGTACTTAGTATTGGCAGATTTTTTTTTTTTGCCATTTTGATGGA
TTTGTAGTGGTATCTTATTGTGATTGAGAGCTTCCCAGGTGGCTCAGTGGTAAAGAATAC
ATCTGCAAGTGCAGGAGATGCAGGTTCGATCTCTTGTCTGGGAAGACCCCCTGCAGAAGG
AAATGGCAACCCACTCCAATATTCTTGCCTGGAAAAACCCCATAGACNNNNNNNNNNNNN
NNNNNNNNNNNNNNNNNNNNNNNNNNNNNTCCCAGCTGTGATTACTCTGTTTTCTTAACA
GTATTTTAAAAGAACATAAGTTTAATTTTTTCTTTTACGGGTGGTTCTTTATGTTTGCTA
TCTGAAGTCATTGTCTAACTTAAGCTTACAAAAATTTCTCTTTTACTGTCTTCTAGAAA
TTCTCAACCTTTAGTTTTTGCATTTAGATCTGTAAACCATCGTGAATAATTTTAATTTCT
TTGTATGGTGCAAGGTGTTTGTTGAGATTCATCTTTTTTAATGTGGATGTTTAGTTAGTT
GCTTCAGCATCATTTGTTGTAAACATTACTCTTTCTCTGTTGAGTTACCTTGGCACCTAT
GTAAAAAAATCAGTACATACCATTTCTCTGTAGCTGTATCTCTGGATACTATTCTGTTCC
ATAGATTGATGGCTATCATTTTGCCAGTACCACTGGGTCTTGATTACAATTGCAATAAGT
AAGTCTTGAAACAAGGTAATATACAAATTTTCAACTATGTTCTTGTTTTCCAAACTTGTT
TTAGCTATTGTGAGTCATCTGGTTTTCCATATTGATTTTTTTTTATTATTATTATTTTTG
TCTGTGCCACAAGGCATGCAGGATCTTAGTTCCCTGACCAGGAATTTGACATACCCCCAG
CACCACTGGACTAACATGTCAATTTTAGAATCAGCTTGTCGATTTCTACAAAAGAGCTTG
TTGGGTTTCTGTTTGCATTGCATTGATTCTGTAAATCAATTTGGGGAGAATTGACATCTT
AGTAAGTTTATACAGCTTTTTTCATTGTTAGGGTGGGGAGTGATCTTTTTTTCCACCAGG
CAGAAGCCTGTAGAAGAATTCTTTAATGCTATTTACTTCCATTGTTGAATATTATAATTT
ACCAGTCAATTTCAATCTAAATTAACCCCTTGAGGTCTTTTTTTCTTATCTGGAAAATGG
GATATTGTATGGGGTTTCTTTGAGTGCTGTTTTAAAGTATTTTTAGTCGAGATCTAATTT
TTCATTGTGTGATGTGGTATATTTAATTTTTCAAAAATCTTTTCTCTTTCCCTACTTGAA
GATTAAATATATTTTACTATCCTTTTCTATAATTGTGTGAAAGAATAGAAACAATAGAAT
AATGAAACAGAATAGAAATAATGAAATAAACTGACTCTCCTGTTAATAATAATTGGTCTT
TCAAATTAGAGAGTCTTATTTCCTGAACATTTTGGAAATTAGAAATACATGTCATCAAGA
```

ATGATGTGTTAAAGAAGTGTAAAAATTAATTTTACTAAGAGAAAATGAAATGGAATGCCT
TTTTCTTCTGCGAAATTACCACCTGCCTTATTTTCTGTATTTAGGAAGTGCTTAGGAAAG
AGATTGGAGAAGGAAATGGCAACCCACTCCAGTGTCCTTGCCTGGAGAATCCCAGGGACT
GCGGAGCTTGGTGGGCTGCTGTCTGTGGGGTCGAACAGAGTCGGACACGACTGAAGCGAC
TTAGCAGCAGCAGCATCAGCAGCAGTAGGAAAGAGATGCAAGAAAATGACCTGATAGGTG
CTTTTTGTTATACACGTAGAGAACTTAGTATTAACTTGTTTTCTGCCAGGTTTCTTTAGT
TCCAGTTAAAGAAATGCTGGAAACTTTTAGCCTGTACCTCTAGTTCAGTTCAGTTCAGTT
CAGTCCAGTTGCTTCATCGTGTCCGACTCTTCGCGACCCCATGAATCCCAGCACGCCAAG
CCTCCCTGTCCATCACCAACTCCCGGAGTTCACCCAGACTCACGTCCATCGAGTCAGTGA
TGCCATCCAGCCATCTCATCCTCTGTCGTCCCCTTCTCCTCCTGCCCCCAATCCCTCCCA
GCATCAGAGTCTTTTCCAATGAGTCAACTCTTCGCATGAGGTGGCCAAAGTACTGGAGTT
TCAGCTTTAGCATCATTCCCTCCAAAGAAATCCCAGGGCTGATCTCCTTCAGAATGGACT
GGTTGGATCTGTTGCAGTCCAAGGGATTCTCAAGAGTCTTCTCCAACACCACAGTTCAAA
AGCATCAATTCTTTGGCGCTCAGCCTTCTTCACAGTCCAACTCTCACATCCATACATGAC
CACAGGAAAAACCATAGCCTTGACTAGACGGACCTTTGTTGGCAAAGTAATGTCTCTGCT
TTTGAATATACTATCTAGGTTGGTCATCACTTTCCTTCCAAGGGGCAAGCGTCTTCTAAT
TTCATGGCTGCAGTCACCATCCGCAGTGATCTTGGAGCCCAAAAAAACAAAGTCTGACAC
TGTTTCCACTGTTTCCTCATCCATTTCCCATGAAGTGATGGGACCGGATGCCATGATCTT
CGTTTTCTGAACGTTGAGCCTTAAGCCAACTTTTTCACTCTCCACTTTCACTTTCATCAA
GAGGCTTTTGAGTTCCTCTTCACTTTCTGCCATAAGGGTGGTGTCATCTGCATATCTGAG
GTTATTGATATTTCTCCTGGCAATCTTGATTCCAGCTTGTGTTTCTTCCAGTCCAGTGTT
TCTCATGATGTACTCTGCATATAAGTTAAATAAGCAGGGTGATAATATACAGCCTTGATG
GACTCCTTTTCCTATTTGGAACCAGTCTGTTGTTCCATGTCCAGTTCTAACTGTTGCTTC
CTGACCTGCATATAGTTTTCTCAAGAGGTAGGTCAGGTGGTCTGGTATTCCCATGTCTTT
CAGAATTTTCCACAGTTTATTGTGATCCACACAGTCAAAGGCTTTGGCATAGTCAAGAAA
GCAGAAATAGATGTTTTTCTGGAACTCTCTTGCTTTTTCCATGATCCAGTGGATGTTGGC
AATTTGATCTCTGGTTCCTCTGCCTTTTCTAAAACCAGCTTGAACATCAGGAAGTTCACA
GTTCACGTATTGCTGAAGCCTGGCTTGGAGAATTTTGAGCATTACTGTACCTCTAAGTGA
CTTATAATATCAGTGTTATTAGTCTACAACCTTTTCTTAATCTCTGAAAATTTAATAAAT
CTAATTTGTGTTTACTTATTAGATGCCTAACTCACAGTTGTCAAACTCTGTGCCAAGGCT
TTCCAGGACATCATAGTAAATTCACAGGGTACCATGAAGTGTTTTAAACCTTCAAAGGAA
GCACAGCTGTATTCAACATCTATCAGATGTCTTGTGGACTACTAGTTGACAGTTTCAACA
TTAGGTCACACTACTTTCCTTTCTTCTACATCTTTGCAAAACTGGGTTTTGGATGTTGGC
TGTGGTAATAATGCAAATACTGCATGAAAACCAGTATGGAGGAAGAAAAGAAGGTGACAG
TGTTTAATCTGATTCCCAGGTTTCAGAAGTGTAACACTGACCAGAGGTGCATGCATACCG
TTAGTAAGAAACTAGAGTTACCTCAACACTATATACTGAATATTTTATTCCTCCAAAATT
CATATGTTAAAGTTCTAAGCCCTAATGAGATGGTATTAGTAGGTGGTGCCTTTGGTAGGT
GATTAGATCCTGAGGGTAGACCTCTCAGGAATGGAATAAGTGTCTTTCTAGAAGAGAACT
CTGAGAGCTCCTGAACCCCTTCTACCATGTGAGGATATAGCAAAGATGCCATTTATGAAG
CAGGAAGCAAGCTCACCAGACAGGGAATCTACCACTTGTTCTTGAACTTCCCAGCCTCTA
AAACTAGGAGAAACAAGTGTTTGTTATTATTAAGTCAGATATCTAGTCTATGATACATTG
CTACAGCAACCCAAATGGACTAAGACACCCTGGAGAAGGGAAAGGGGGAGGGGTATTGTG
GGAACTCAGAGAGGTTTTTAACTGTAAGGGCAAAATACAGACATCACTGTGCTTTTTGCA
GACATTCGTGTTTCCTCATGGCCAGTTTTTTCCTATGCTAAGTCACTTCAGTCGTTTCCG
ACTCTGTGCGACCCCATAGACGGCAGCCCACCAGGCTCCCCCGTCCCTGGGATTCTCCAG
GCAAGAACACTGGAGTGGGCTGCCATTTCCTTCTCCAATGCATGAAAGTGAAAAGTGAAA
AGTGAAGTCGCTCAATCTTGTCCGACTCTTAGCGACCCCATGGACTGCAGCCTAACAGGC
TCCTCCATCCATGGGATTTTCCAAGCAAGAGTACTGGAGTGGGGTGCCATTGCCTTCTCC
GAGTTTTTTCCTACCAGTTACAATTACTCACATCCTCCTGTTTCAGTTTGGTGAATCACA
TGGATTAATTTGCTTGTGTGGAAGAATCCTTGCTTCCCTAGTATAAACCTCTGTGTTTTA
AGGTCTGTATTTCCTTTGAGGTCTGAGAGTTTGGTCTTTTACTCCTGCAGGCTTTTTAGA
GGCCTGATGAGACTTCTTCCCCCCATTTCTTTTAATGTCCTTCAGGAAATTTGCAACGTT
TACTGGTCTCTGAAAAGAAGGCAACAGATAATTTTACATTTTAAACAAAATAAACATCTT
TCTGCAGGTACTGTGCTCTCAGTGGCTAAACTGGGATGTTTTATTAAAGTTTTCTTTTAG
ATGATTTAGAATATTGGTGGTCCCAAAAGTCAACAAGGGAAAACATGGGGTCATTGGGGG
AATGACCCCATGGGGTCACATGGGGAATGCGGGGTCATTTATAGTATATGATACATTTTA
CAGAATATAGACCATAATGATTTAAACTAAGAAAAAGCAGGTCCTTCTGGCTCTTAGACT
TGGAAGAGCTTGTGTCAGTGTATCTTAGAGAGAAATGTAAGATAGGAAAATGATTTTTGC
TTGTTTCTTGGTTGTTTTTTAAGAGTGAAGGAGAATTAATACAAAACTGGAATGACAGG
CAAATAGATTGGTAGACTGACAGTCTATACTCCCTACTTGAAACCACACACAGCTTCCTA
AATTGAAAATTGATGAAGGATGGAAGTTTGGTACAGAATCCTTTGGCAGAGGAAAGGCTG
TAGACAGGATGGATGACATGAAAGTAAAAGCATCGAGGGGTCTCCTGTCGCAGATTGAGG
TTGCGGAGCAGCGCTTCCCCACCGTCAGCTGGAAAAGCACTGAGGTTTAGAGCACGCGGA
ACCGGAGAACACCAGACGTGTCTACAGCAAGGGCTGAGTGCGCACGGGGCAGACCCTCTC
GACACGCTTCCTGTTGCCAGCTTCATCTAGGAAAACTTCTGTACACATCTTTTGATGGTC
ATACACTCATTTCACTAGGATGTAAACTTAAAAATAGAATTGCCAAGTCCTTAGGGTATG
CTTAGCTTTAGAAGAAGCTGTCAGTTTTCCAAAGTGACTGTAGCCTTTTACACTTACACA
AACCATGTGTGAGAGTATCCTCCCAACACCTGCTGTCTATGTTTTTAAATTTAGCCTTTT
TGGTAGTGTATAGTTGTAAATCATTTTGGCTTTAGTTTGCATTGCCCTGGGAGTAATGAT
GTTTGAGGACATTTTCACTTGCTTATTATCCCTTTGAGCATTCACTTTTTTTGAAATCTC
ATGTGAGGTATTTCCCTGTTTTTTTATATTTTTATCCTTTGCCTTTTGATTTTGTCATAA
TTCATTGTATTTGCTGGGTATGAGTCCTTTGTTAGATATATATATTAAAGTTATCCTCTC
CCAGTCAGTGGATTATCTTTTCCCTTTCTTAATGGTCTTTCGATGAACAGATTTTGATTT
TAATAAAATCTAACTTGTTATAATTTTTTTCTTTTATGAATAGGACTTTTTGTTCCTGTT
TCAGAAATCTTTATCTTTTCCCAAGGTTTTGAAGATCTTTTTCTATGTTTTTGTCCAGAA
GCTTTATACTTTACTTTTCATATTTAGGTTTGTGATTTATCTGGAATTTTGTAGATGTTG
GTAGATGGTTGATATTAATTTTTTTCTGATACAAATATCCAATTGCTTCAGCATCATTTA
TTGAAAAGACCATCTCTTCTCCCATTGAATCAATGCCCTTGTTGTAAAGTAATTGACTGT
ATACATGTGGATTTAATTCTGGACTCTATATTCTATTTCCATGGCCTGTTTGTCTTATGC
CAAGACTTCACTGTTGTATTTACTGTAGTTTTATAAATGGTTTTAAAGTCTGATCCATTT
ATTTTTGAGATGTTTTAGAAATTGTAATAAGGTACATTTGTTTGATTTTTGTTTGTAACC
ATTTATTACAAAATGGAAAAATTATATATTAAGAATTTAATTAGAATAAACTGTTTTATA
GAAATAATTAACCTGTCTTTTAGTTATGATAGGAGAGAATTAGTCTTACAGAACGTTTGC
TTTTAAAATAGCCTACACTTTATACTAATCTAAGGTTAAGGGATGTTAGAAACATACTGA
TATTCCAGAGTTGTGAAAAGATCAGAAAGATGGTTATTAAATGTGCAAAGTGCTGTTTCA
TTGGCAAAGAGACTTAATAGGCTATTTAAGACCTAATTCATAGGCCTAGTCTGTTCTGCT
TTGTTCTGACCTTCTCTCTGCAGAAATATCTTCTTTTATCCTTATCCTTAGATCTTCTGG
TGGTCAGTAAACGTCCATATTAATTTTGAAAATTCAAAATGTTAAGTCTTTGCATCAGTT
AACATTTCTAGTGGGAAACAGAAGATATACTCTGGTGGGATTTTAAAGAAAATTTAATAA
AGCTCCTGTTTACACACCTGATGGGAAGGGTCAAAGAAGGCAACGTGGCTCCTGGAGACT
AGCGACAGCAGAAAATAGCTACCACCTCTAGGGCTGAAGTGTCAAGAGAGGAAATGGTAC
AGACTTCCAGTGAGAGCTAGAGACACAGAAGATGGGCTTCCTGGTAAGGAGCTATGGTTG
TAGAGGGTTGCAGCTTAAACCAAGGAAGGACCTGGGAAATAAATATCCTGACGTCTTCTG
CTGCCTGATTTGACTGCTCATACCTCCTGCTGGACAAGGCCAACCAAAAAACTGTAAATG
CAGTCTGTAAGGGCCAGTCTGTTAGGGTACAGCAGCAGGGAGAGTAAGGTCAAAAATATA
TCTGGATCAGTTAGGTGGTGACAGATGGAATAACCAGCACAGAATAGTTTGTTTCAACTG
AAAATATGAAAGAAAAGATGTTTTTCTTTTAATGTAATGGTTTTTCGTAATGCTTAGGGT
CTAAAATAGAGAAGTTTTGTTTTGGCCAGTACAGCCAGACTTTTTATAACACAGAGAAAG
TATACACAGAACAAAAATCTTTTCTGTTCAAGAAAAATTAGCTTCCATGTAGTTCAGAAT
AGTGTATGAAATCTAGCATAGCTAAAATCTTCTTTGTCATTTATCATCTGAACATTTTAA
AAATGGATCTTTTTCATAAATGATTAGATTTTTGTGACCATGAATATATTTAGAAGTAAC
ATAAAACTACATTTTTCCTTTTATCACCTATCATTTATTTAAATGTCACATAAGAGAACC
GTCCTATATAAGTAACTTTTCACGTGATGTGTAATAAGAGACTTGAAGCAAAACTTTTTG
AAGTGTACTTGTTCCTTTTTTCCTAGTATGGGAAAATTTTTAAGTATTTCTTTTCACTTG
GAAAGAGACAAGATAATGTGTCATGTTGTATCTCAAAAACAGCATCTGGATATTCTAGTT
CATATACATCATACTGTTCCTTTTCTGTACTGCAGTTATTTCTTGTCACTGATAATATTC
CAGGAAAGTTCAATTCATGGATAAGCACAAATATGACACAAGCCTGAACTGAAATAGCAA
CTGAATTAGCTTATCACTTAAATTGTATCTTTAAATACCTTTTGCCTAG**GTTCCTGATGG**
TTCGTAGTGGAAAAAATGGTGACCTTCATCTTAAACAGATTGCATATTATAAACGAACTG
GTGAATATCATCCAACTACACTGCCTAGTGAGAGAAGTGGCATAAGAAGAGCAGCAAAAA
AATTTGTCTTCAAAGGTAAAATATCCCCTGTTTTTAGTATAGACTTTTATATAGGTGCTT
CCCCATGTGGAGTTCTTTGTGGAGTCCTGAGGATTTCTGTAGCAATTGGTTGTTGCTTAA
ATTGATAATGGCGTTCAGTCGAAAGCAAAATAAAAATCACGTTGAAGCTAACTGAAGGCT
AGTAAGCATGAAAGGGGCATTTTAAATCATAGACAGAAAATGATAGCATGAAAAAACCAG
CACTTGCCTGAATTAACGCCGCTCTTGTTCCTTGGCGAGGCCTACTTCGGTGGTGCACTT
GCGGGCACTCACGTCACCCTCAGTGAAGAACTGACTGGATGCCTAATGTGAAAATGTTGA
CAAAGTTAACTTATTTACTCTATTACTTCCGTGATTCCTCCCTCTCCCTGCTGTTAAATG
GTTTCAGGATCATATTTATCTTTGAACTCCTAATCTCCCTGAGTGAATTAAATGTTTGAA
AGGCAGTGGAATATTTATCTTGAAAGATTATGATCATTCTCCCCTCTTTGTCCTCCTCCT
CCAAAAGGGAAGAGAAGTTTGAGATATATAAGCAAAAAGTACAGTCACCAGATGGCTAAA
TGAATGTTGGTGTTATTAACGGGAATTTTGCCTGTAGGTACAGAATCAGGCTTTTCTCAA
AGCAGGTCAAGAAAATTCTAAAATTATAAATAAATACCTGCTTATTTCTCCCTGTGTACA
TCCCTTCCAGTAGCTCCCTTTAATTGAAGAATGCTAAGTTGCTTTAGTCACGTCCCACTC

TGCGATCCCGTAGATTGTAGCCCGCCAGGCTTCTGTGTCCATGGGACTCTCCAGGCAAGA
ATACTGGAGTGGGTTGCCATGTCCTGCAGGCAGTCTTCCCACCCAGGGACTGAACCTGCA
TCTGTCATGTCTCTTACACACCTATTACATTTTACACTAAAGAGTATTCGTTCATTCGTT
CACTTAATGATTTATGCACCAATTGTTTACTGAGTTCCTACTATGTGCCAGTCACATTTT
AGGCCTTGGGAATACACTGATGAATGAAATAGATTAGGCTCCTGGCCTTAAGCTGCTTAC
TTTCTAATTCAAGTAAGGAATGTTCCACTCAGCATATTCTTAGCTAAGCACACTAACAAT
GGCGTCTTTTTGAGTTTGAATATTCATCCACACTGGAGAAGTGGCAGTTCTCTTCATGCC
TTGATTACATTGCTCATAGACTCTTTTTAGGTGTCTCCAAAGGAAAGCAGAACTGTCCTG
AAGATTAAAGTTCTGCTACTTTCTTGGCTTTACTAACCCTATTTCCTACTCAAATCCAGA
AAAACTTAAGAGAGGTGGTGGCTTACAAAAGTACAGCTCTGATGAAATGCTCTTTTGGAA
AATTCTATTCCAGAAGTTGCTTATCTGTACATAATTTATTTGCTAATTCATGCCATTTTT
AGGAGGTGCCTGCTGCAAGTGTTTTTCTAGTTTGATTACTCCCATAGGAAGATGATTTTA
TACTACCATATTACCATGTGCTTTGGTGAACAAGTTAGCTCTACTTGATTTGATAGCTAC
AAAACTATAGCAGCTAGATCTACATATCCAGTAAGTATAACAGTGGAAGATGTTTCAGAT
TGTATGGTAGCATCATCACAAATATTCTATCTTAGAGGGAGAAAGATTTAACCAAAACAA
AAACCCTCATGGTTTGAAGATAAGGCTGTGATTATCTTAAAACTATATAGATGTCTAAAA
ATTGCAATGATATCATTTTGGTGTTTTTTATTCTAG**AAAAGAAGCTGTTTTATGTCGGAA**
AAGACAGAAAACAGGATCGCTTGGTAGTTGTTTCAGAAGAAGAAAAAAAGAAAGTCCTAA
GAGAATGCCATGAAAATGACACTGGAGCCCATCATGGCATATCCAGAACCCTTACTCTAG
TGGAATCCAGTTACTATTGGACTTCTGTGACCAATGATGTCAAACAGTGGGTATGGCTTA
TGTCTTTAGAATATTTTGAATAATGTCTTTATGGTATACATTCAAAATTCATTACTGCTT
TAGATTAACTCTAGATATCACTTCTCATTAAGTACTTTTTGTATGGTCACTTCTCAAGTA
CTTTTTGTATGGGTAGTTTTTTGTGCAGAACATTTGAAGAGAGTTTTATTTCCCTTCAAA
AGTCTTCTTTAAAATCACTGAAATATAATTTGAACTAGAACAAAACATTATTTAAATATT
TGAATTTACTACATTTAGATTATCTTGGGCACAGATTTTCATCATTGAACTATTTTGTGT
GGCAGAATTTAAATGGGGCTGAAGTTTCTTTGACTAAAAATAAGTCACTACATTTTTAAT
GACATTCATACAGTCTTTTCTTGAGAAGAGAATATGTTTGTGATAAATCAAGCCCAGTAT
GTTTCTCATCTTTTGTGCCTGTTACTGTCTTGACATAGAAAATGTCAAGTATGACAGAAA
CCCCCAACCAAAAAATGAAGTTGTGAAGATAATGAAGTTCTTCTGAGTTTTGTAACGTTA
TAACCCATTAGGACCTTCAGCTTCTTCTGATAGAAAATTCAGGGATTCTTGCTGCTAGGT
CAGCTGATCCCTACTCGTGTGCTCATGTGCTGTCTGTAGCAAATGGTAGAAATTCCTTGG
TTTATGTGTGAGGATCCTTTAATAATTGTGATAGTGCATTAAAAGGCTGTCATTTCAGGC
ATAAAAAATTAACATATTTAATTTAACCAAGTATCATTTGTGAGTCTGTGAAGGTATTCC
AAAGACCTCAGTGTTTGTATTCACCTTTAGAACATGCCACACACTCCTTTTCCAATATTC
AGTGAAGTCTTTGTCAAATCTTTATCATTGAGAAATGAATTTTGGATGTAAGGTTATTTG
TGATGAGAACTAGTTTAAGTTAGAAAATAAAAATTTAATCAAAAGTAACTATATTTCAGT
AAAATCATTTTAAAATTTTTCTTCAGGCCTTGATTTGAAAGTAGTTTCCAAAATAGAGTT
CCGGAAATATGTTGAACAATGGCATCTTCCACAAGTCGGTCAATTTATATCTTCTCATCT
CACTACAGTGAAAACACCATGACAGCATGGGCTGGATTTTTGTTCGTACTCTTTTCCTAG
GACCTAGAGGAATTTCTGGCATCTAAAAATATTGCTTGAATAAAGTTATATTTTAATAAA
TATTACTGCAGTAGATGAATGAACTTATTAGAGTGGTATCTTTAAAAGAAAAATATTTTT
TGCTTATAGAAATTATATACTCACATACTGATGTAAAATTGTAGCAAATATGTTTTGAGA
TCTATTCATTTAATAAGTATTTATTGACTGTAAACCAGAATTATGTTCCATGTGAATAAG
ACCCATTAACATGGTCATTGAATAGTTGATTTCAATTAATTAATAGCAATTAATTAATAG
CAAATATTTGATTATTCTGAATAGGTTTCATTTTATTCTGAGTACAGTCCAAAAGTTTCT
CCCTTAAAATATAAAGTACCTATATCAACCGTTACAAAGTTCAGATTTATAATACTGCTT
TTTTTATATTGGATATATTAACACTTGTCTATCTTTTCTTTATTATATGCCTGTTTTATA
G**GTATATGCTTGTCAGCATTGCCAAGTGGCAAAAAATACAGTTATTCTAGCACCTAAACA**
GCACCTTCTCAAAGTGGAGAATCCATGGAGTATAGTTACTGTTGATCTGATGGGCCCATT
TCATACAAGCAACAGAAGTCATGTATATGCTATAATCATGACAGATTTGTTCACAAAATG
GGTTGTGATTTTGCCTCTTTGTGATGTTTCAGCATCAGAAATTTCTAAAGCTATTATCAA
CATATTTTTCTTATATGGGCCTCCTCAGAAAATAATAATGGACCAAAGAGATGAATTCAT
TCATCAGGTAAGACAAATAAACTACTTAGATCTAGGAGCATATTTTACTCACTTTTCAGT
GTACAACACAGCTTATTATATGATGCTTAATAAGTTTAGATAAATTCCTACAGGTATAAA
GGTTTAATATTGTATAGTACAAAAGTAGTTGAACTGGAAATTAAGAGACCAGTATTTTAG
TATCAGTACTTACTATGCCACTAAATAATTGAAACTTATCTTTAGTTTTCTTCAAACTAA
AATGAGATGGGACCAAATAGTCTTTTTTTGCTACTAGCTTAAAAATTCTGTGATTGACAT
GTAGAGGGAGAATTGACTAAATGGAGAAGTATTATACATCATTTCATGAATGAGAATATT
CAATATTATAATATCAATTTTTCTAAAATCAAAATTAATTTATATGATTAATGGATTTCT
AATCAGCATTAAATAGGAGGAAGCATTTTCCCTTGCCCTGGCCTTTTTATCCAAATTAAA
GTTAACCTTAAAGAATAAGCAGACAGTAATATATACAAAAAATCTAACAAAGGAATATAC
TACTATATTTAGAGGTATATTATTAAATTTTTACAATTGACAAAAGACTGGTATAAATAA
CCAGACTAAGTTCACAGCACCTTTGATATCTCAATCCAGAAGAAATACCTAAAGTTATAC
TTATTAAATAGTTAGGGCTTGGGTCAGGGGGTTCCTAAGGTGATCTCCAGGTTTTATATT
AATAATTCACTAGGAGGACTCTGCAGATGGACATACTCATGCCTAAGATTTATATAATTA
AAGGATACAAAGCAAGATTAACAGAGAAAAGGCTCATGGGGATAAGTCCAGGGGAAAACC
AGGAACAAACTTGTAAGACATTCTCTCCCAGTGGAGTCTCACAGGACATGGTTAATTCTC
CCAGTGAAGAGTTGTAGCAACACATGTAAATTGTTGTCTACTTGGGAAGCTTATTACAGA
CTTGGTGCCTGGGATTTTAAATGGAAGCCGATCACATTGGCACCCAAAATTCTAGAGTCT
CTAAAGAAGTTTTCAGCATAAACCACCTTCTTTGTAAAACAGTTTAGGCACCATGAGCCA
TCCTTAATCCGTTAGGGAATGGTAGAAACCCTCACAGAATCCAGGTTCCTAGTCATCAGC
CAAGGGCCAACTTTGTAAGCAGGACTTTCACAGGATAGCAGCCTTAGTTCTGCTGTGTTA
ACTCTTCCTACACAGGTCCAAACAACTTAGTGTTTATGTCCTAATCACTTAGCCATTTAA
GAGAATAATAGTCATAAATTGAAAGAAAACTATTTTCATTTCATTCTTAAATAGCTTCAG
TTAGTTACTAATGGGATATATATGCCTGTTGACCTCTGCTCAGTTTTTCAAACCTTGGAA
TCAGATTAAACACTGTAATCATTTCCTGTTTCACAATGATTTTTATACAATTGTTGCTTT
TTATCATAGAGCGTAGAGCAAAAGTCACTTAGTCGTGTCCAACTCTTTGTGACCCCTTGG
ACGATAGCCTGCCAGGCTCTTCCGTTCATGGGATTCTCCAGGCAAGAATACTGCAGTGGG
TTGCCATTCCCTTCTCCATGGGATCTTCCTACCCCAGGGATTGAACCTAGGTCTCCCGGA
TTACAGGCAGATTCTTTACTGTCTGAGCTACTAGGAAATCATGGCACCTATCAAAGAACA
CTTCTATAGAGATACATCATTATCGAGAGAAATGTAGTCTTACCTAATCTAATGTTGAAA
CCATAAACTACCTTAAGCTAGTAGTTTCCAAGGTGTCCAACAGATGCCAAGGGGGCCATT
TGGCACAGAGTTCGGTAATTATGTTCAAAATGATAGAGCTGGTATCACAAGAGAGTGGAG
TTGGGGAAGGATTATTTAATACATTTAATTTAGTTAATAGCATAAATAAAAATTTTCTAA
TTATTTCTATTTATTTCTATAATATGATTTTAGGATTACTGCATACCAAAATTATTTTCA
GATAAATTAAAATTTTTGTGTTTTTTAATTGAAACAAACAAAAATTCAATTAACTTAAAA
AAAAAAAAAAAAAAACTCCAGAGACTGACTTGACATTCCAGGCTTAGGGAACAGAGGCAT
AAATTTGAATGGAAAAATCACCAAATTTGGGCTGTTTAAAAATTAGAAATTTGTATGTGG
TAAAATTTGTATGTGGTCAGTTTTCATTCCAATCCCAAAGAAAGGCAATGCGAAAGAATG
CTCAAACTACCACACAATTGCATGCATCTCAATGGCACCCCACTCCAGCACTCTTGCCTG
GAAAATCCCATGGATGGAGGAGCCTGGTAGGCTCCAGTCCATGGGGTCGATGAGGGTTGG
ACACGACTGAGCCACTTCACTTTCACTTTTCACTATCATGCATTGGAAAAGGAAATGGCA
ACCCACTCCAGTGTTCTTGCCTGGAGAATCTCAGGGACAGGGAAGCCTGGTGGGCTGCCG
TCTATGGGGTCGCACAGAGTCGGACACGACTGAAGTGACTGAGCAGCAGCACACACTAGT
AAAGTAATGCTCCAAATTCTCCAAGCCAGGCTTCAGCAATATGTGAACCGTGAACTTCCT
GATTTCCTGATGTTCAAGCTGGTTTTAGAAAAGGCAGAGGAACCAGAGATCAAATTGCCA
ACATCCGCTGGATCATGGAAAAAGCAAGAGAGTTCCACAAAAACATCTGTTTCTGCTTTA
TTGACTATGCCAAAGCCTTTGACTGTGTGGATCACAATAAACTGTGGACAATTAAGAAAG
AGATGGGAATACCAGACCACCTGACCTGCCTCTTGAGAAATCTGTATGCAGGTCAGGAAG
CATGGAACAACAGACTGGTTCCAGATAGGAAAAGGAGTCCATCAAGGCTGTATATTGTCA
CCCTGCTTATTTAACTTATATGCACAGTACATCATGAGAAACGCTGGACTGGAAGAAACA
CAAGCTGGAATCAAGATTGCCGGGAGAAATATCACTAACCTCAGATATGCAGATGACGCC
ACCCTTATGGCAGAAAGTGAAGAGGAACTCAAAAGCCTCTTGATGAAAGTGAAAGTGGAG
AGTGAAAAAGTTGGCTTAAAGCTCAACATTCAGAAAATGAAGATCATGGCATCCGGTCCC
ATCACTTCATGGGAAATAGATGGGGAAACAGTGGAAACAGTGTCAGACTTTATTTTTCTG
GGCTCCAAAATCACTGCAGATGGTGACTGCAGCCATGAAATTAAAAGACGCTTGCTCCTT
GGAAGGAAAGTGATGACCAACCTAGATAGTATATTGAAAAGCAGAGACATTACTTTGCCA
ACAAAGGTTTGTCTAGTCAAGGCTATGGTTTTTCCTGTGGTCATGTATGGATGTGAGAGT
TGGACTGTGAAGAAGGCTGAGCACTGAAGAATTGATGCTTTTGAACTGTGGTGTTGGAGA
AGACTTGAGAGTCCCTTGGACTGCAAGGAGATCCAACCAGTCCATTCTGAAGGAGATCAG
CCCTGGGATTTCTTTGGAAGGAATGATGCTAAAGCTGAAATTCCAGTACTTTGGCCACCT
CATGCAAAGAGTTGACTCATTGGAAAAGACTCTGATGCTGGGAGGGATTGGGGGCAGGAG
GAGAAGGGGACAACAGAGGATGAGATGGCTGGATGGCATCACTGACTCAATGGCTGTGAG
TCTGGGTGAACTCTGGGAGATGGTGATGGACAGTGAGGCCTGGCGTGCTGCGATTCATGG
GGTTGCAAAGAGTCGGACACGACTGAGCGACTGAACTGAAAATAGCCAAAACATAAAGGC
CAAGAAACAGGCCAACAAAAATAATGCAGATTCATGCAGGAAAGTGTTACTGCCTTTATT
ATAAAAGATTCAAACAAAAAACTGAGTCAGTGACAGAAATGTAATTCACAAGAGGGAATA
AAAATATGAAAAAAGATTCAAACCGAATAGTAATGAGTAAGGCACTCATTTTTTGTTTAT
CCATATCAAGAAAAATAGTAATAATAACAAAAATATCAAGTCGACTATAGATCAGAGAAT
GCGATGGCACCCCACTCCAATAATCTTGCCTGGAAAATCCCATGGACGGAGGAGCCTGGT
AGGCTGCACACACATGCATGCATATGTTTTCTGGTTAGATTAAAAATCTGGGTAAAAGAT
TTTATTACATATATTATACATACATATTTTGTGTGTATAACTATGTAAAAAATATATGGA
ATCTAGAAAGTGGTATTGATGAATCTGTCTGTAGGGCAGCAGTAGAGACAGAGACATCGA

GGACAGATTTGTAGACACAGCAGGGGAGGGAGAGAGCGGGATTGATTGATAAGAGTAGCA
CTGAAACAACACACTGCCACACGTGAAACACACACATTGCCACACGTAAGACACAGTGGG
AATTTGCTGTGTGACGCAGGGAGCTCAGCCTGGGGCACTCACACCCGTGAGGGGTGGGAT
GGGCTGGGACGTGGGAAGGAGTTTCAGGAGGGCGGGGACATATGTATACTCACGGCTGAT
TCATGCTGATGTATGGCAGAAACCAACACAACACTGTAAAGCAGTTATTCTCCAATTAAA
AATAAATACATTAAAAAAATTTTTTTTAGAAAATTAAAAAAAAATGTAAAGAAGCTAAAA
CAAAAATTTGTATGGTTAACCATACAAGTTGTATGGGGTTTTTAAGAGCAATGAGGTTAC
AGATTTGTTTTTTAAATTTTTCAGTTTGTGTTATCATTATATTACGTTATTATCAGTTTG
TGTTTCAGTATGTGTTATCATTATTAGCTTAGTGATTAAAATATGCATAACCATCGGTTG
TTTTCTGATTGGTGCTCAGCAATAACTAGTTCTCCTAGCCTTCCAAATAGGCTGTCCTAG
CCTCTTGCAGGTGAGTAAACCCAAGTAGCACTTTTGGCCAGAGAAACTAAATGAATGAAC
GTGTGACACTTTCAACTCAAAGTATTGAAAACCTCATGCTCAATTCTTCATTCTTCCAGT
CTTATTCTTTCTCTGGAGAATCAACTGAATATTTCAGGTGTGACCCTAAGCGATAGTGGA
TTTTCTCTTATCCGGGATTGTTGAGGTACCTGGGGGAGGCCAGTTGCTTTGGAGAGACAC
CCTGGCCTGTAGCTGGCCTTGTCCTAGCAAGTAGTAAAATTTCTGAGTTGAACCCCTGAG
CCTTGATTGGGTTTTGTAACTTGCCTACCTTGACTAAGGTAGCAGTCATCTGCAAAATAA
GGAATGACAATATTAGAGCTTTTGTTTATTTTTTCCATCCAATAAGAAAGAAATGAAGCT
TTTACTAATATGTATAAATTGATAATAGCCTCCTGAATCAACAAAGCAGAAGACTGTGTG
TTGCACATGGTATGGAAATGTATCTTGGGAGAAAAGTGAGAATCTGCAACATGTGGGAGT
CCATAGTGGCCCCTCACTCATTTTTTGCTTTCCTGACAGTAATCTAGTTTCTAGATATTG
TCCAGTTTTCACCAAACTACCCTTCATGGAATGGGCTTCCCAGGCTCGGCGGTAAATGCA
GGTTTGATCCATGGATCGGGAAGATCCCCTGGAGAAGGGAAATGGCAACCCACTCCAGTA
TTCTTGCCTGAGAAATCCATGGACAGAGGAGCCTAGTGGGGCTATGGTCCATGGGATGGC
AGAAGAGTTGGACACGACTTAGCCACTGACACAGCAAACAAATGGTCAAGGAAAAGGCAA
GATTTAAATTCTTTTGAAACCTTTCCATCAAGGGAAAAATAAAATACAGACTTTTAAAGT
CACTGTTGTTTTTATGTATTTTTCAG**ATCAATGTAGAGCTATGTGAATTGTTTGGCACAA**
AGCAAATTGTTATTTCTCATGCCTCCCAAACAATTAATCCAGCTGAAAGTACACCTAGCA
CTATCAAAACCTTTCTTTCCAAACATTGTGTGGACTACCCAAACGACTGGGATGACCACC
TGCCAGCTGTTTCATTTGCCTTCAACGTCACTCACTTGGTATGTATCTATTTATAATTCA
GTACTTTTGAGTTATGATTATCTCTTGGCTGCATGTTTTATTACAGATTGTTTATTTTTC
TTGATAG**GAACCTACTAAAAATACACCATATTTTCAAATGTTTAATCGAAATCCTTACAT**
GCCTGAATCTTCAGATATTCGTGAAGTGGATGGTGATAATACAAGTATGTTTGCCAAAAT
TCTAGATGCAATTAAAGAAGCTGATAAAATAATGGAGAATAAGACAACTTCAGTGGGCCA
GGTGATTCTAATAGAAAAAAACTATAAAGTCTATAAAATGATACAAAAAAGTTTTTTCA
GTATATATACACACACACATACATATGATACACACTGAAACCAAATTTTTTCTATTGAAT
TCTGCATGTCTTGATTAAACTGTGTTTATTAGCTATGAAATTCTGAGAGACATTAATAGG
ACCATGTGTTTTGGAATGAAGGTGCAGCTGAATTCAAACCCCTGGTCTTGCTTCCCAGTT
GTTTAACTTGGGCCTGTTACTTCCTCTCATATTTCTTTATAAAGTTGGGGTAATACTGAT
TTTGAAATACTATTTTAAAAAATTAAGTGGGATTCTTCCGTAGCTTTCAGTAGCATCTCA
GTAATATTTTTATTATAATATTATCTGTATTTGCCTTCAAGAAACTATAGCTGTTTCATC
CTTTTTTTTTTAATACTTGCAG**ATGGAGAACAACAATTGTCATGAATTAAATAAAAGCAA**
AATCATTGTTAAAAAGAAACCGAAGCAACAAAATCCATTTCATCTGAAAGTGGGTCATGA
AGTGTTAAGGCAGAGGAAAAACTGGTGGAAGGATGGTCGCTTCCGGTCTGAATGGGTCGG
CCCTTGTGTCATAGACTACATTACGGAGAACGGGGGCGCTGTTCTGCGAGACAGCTCTGG
GGCTAGACTTAAACGACCTATCAAGATGTCCCACCTGAAGCCCTATGTCAGAGAATCGGG
TGAACAAGGTAAACATCTGTCACCTTTCTACGTTTGTACTTTCTTTGAATGAATAGAGAA
GTTAAATTATAAGAAATCTCTCCTTCTCCTCCCTGCTTTTCCTTTGGTTAGGTTTTATTT
TCCCTTTTCATTCTTATAAAATTTGTACAGTGTTGGAGCCCAAAGAGTGTTGTTTTAAT
TAACATATGGTTTCTGAAGCTGTCAGGTTTGTAAGAGATTTTATTTTGATTTCTAGTGTA
CTTTCCACACACTTCTCCCCACCGTTCATGTGCAGGACACTGGACATCTTTCATAGAGGG
AAGAATTATGTGTTCATTATTTGCACTTTCAGAATATTTTGTCACTCTTAGCATGACATG
ATAGTAGCTAAGATAAATAATGCCAAAACAAACACTATCTGGAATTCTGTTGTGAGATAA
ATATATTAGGGGACATTTAAAAATGTGATTGATTTTTAATGTTGCATCTTAAAACGGCCA
TGGTAAATGCAGGGGAAGGTGAGTGAGAGGTTCTGAGAATGATGCGCTATTATAAGAGGT
GAACAGCACCCTAGATGATGCTACTTGCTCCACAATGTGAATCTGAATAGTTAAGATATC
CTGATCTTCTCTAATACTTTCTTTCATGGACTAGAGCTAATTGATATGGCTTTCAGTTTA
AATCCTACTTTATTGAAGCTTTTATTTTGTCCTATAGTATCTGCTTTCTAGACTTTCTTT
GAGGTAGGTAACTGAAAGGGAATAAATTGGTATCTTAGCTAGTTACTTTTGTTTTTTCTG
TAAAGAAAGATAAAATAATTCAAAATTTTTTGAATCTGAGTACCAATTAATAAAGATACA
ATTTAGTGCCTTTTAAAACAGTATAAACAACTGTGTTATTTAACAGTCTTAAGTAAATAA
TAAATAATAATTAAAAAATAATGATAATAGATGACCCTCTTTCTTAGGATATGAAGCCCT
TGAGGCTGCTCAACTGTTTCTGATCTGGGTCACAACTCTCATGTTAGAAGCTCTTAGTAC
CTTTCTTTCTGCTTGTGACAGAACCAGTTGCTCTAGTAAACTTATCTTGGACCATTCCAA
CTCAGAAAGCTCCCAGCACCTTCATTCTCTGTTAGAGGAAGCACTTTTATTCCTTGTGCC
TTCTCAGTTCTAAGCTACCCTTTATGATTTCTTCCTTCTGCCAGTTAAGTAGGAACAAAA
GATTATAAGGTGCAGGTTGTCCTTTCTTCCGATTTTCCCTTCAATCCTTGGTGTTCAGGA
CTTGATTCCTTCTAATGTGTTAACCTTCCTAAATTAATGCAAAACCTGTGTTTAAAATGT
TACTGCATCAGGATCTGTCTCACCATTTTGTAACTTGCTCTTGCCAAAGCATTTCTTAAG
GAGACCTAACATTCTGCTACAACTGAATATTTCAGAATCCAGCTGAATAGAACAATTTCT
AAACTAGCAATACAATTTTTGCTTCATGCTTAAATTCTCTACAGATTCTTCTTCCAGTAA
TACAAGGGAAATTGCAAAATTAGTTCTAGTGTACATATAAGGAACCTGAGATTGATGGGT
AAAATTGTTTTTCTTATTAATCAAAAGAAGTACTAATCCAGCTGAAGTCTGGTTCTTGTT
TACTCTGATCGGTTTTTGTGTATTTCTCTTATTTGTAGAAATTCAAAACAAGTGTGATTG
CTATCACCTTTTCCTTTGGTAGCACATATTTTCCTGAAAATCTTCACCTCAAAAGCTGAT
TTTAAAGTCTTTGAATTAAATTTAAAGTAAATATAATGCCTCTGAGTAGTCATCCCATTC
TTTTGACGTAAATTTGAACATAGTGTAGCCAAAAGGACTATGAATTTGTCTGCACTGTTG
CTCTTACCAGCCTCGAGATGTCGTAACTGCAGTAAAGCTCACCAGCCGGAACAGACATGC
TTCTGTTGCCAACTTCTCATCCATACCGAGAGGGGGCACATTCAGTACTAGGCTGGCAGT
GTTTCTCATTGCTGTTTTAAGCAGGCCTGTGTGTCTGTCTGTCTAGTTCAGTTCCAGGCA
CCTTTTCAAGACTATTTTAACAAATTTTATGCCATACCATCGTGTCTGTTCATAGTCATA
ATGTTTTCCTTTGAGTGGAAAAATGTTATTGACTCCCCATTATTGTATGTGACATAGATT
TTGAGTAATTTTGTTTTGATTGGGAATTATCGTGATCAGTATGTGTCTGCCTCCCCTAAA
GGCCCTGGTCTGATTGCCCCTCACAGTTTGAGAAATGGTTTTTATTCTTTAATAAGTTTC
CCATTGCTGCCATAACAATTTACCACAAATTTAGTGGCTTAAAACACTTACTGTCTCACA
GTTCTGTACTTAGAAATCTGGTATAGATCTCAGTGGGCTACAGTAAGGCATGGCAGGGCT
GTGTTCTTTTCTGGAGGCTCAAGAGGGTGTTTTGTGCATTTTCCACCTTCTAGAGGCTGC
TCAGATCACTTGACTTGTGGTCCTCTTCCTGTCTTAAAAGCCAGCAGTGGGGTAGAGTTC
TTCTCACATTATATCACTCCAGCCTCCTATTCTTCCTTCATCCTCTGTGTTTCAGGTCAC
CTGTGCTTGGGCCCATCCTGATAATCCAAGATATCCTCTCTACTTTAAGGTTGATTGGTC
AGTTGGTTAGCAACCTTAATTCCATCACTACCCTAATCCTCCTTTACCATGTTAGGTAAC
GTAATCACAAGGTAAATGAATTAGTACATAGCTACCATTAGGGGTCCACTATTCTGCCTA
ACAGGAGAAGGAAATGGCAACCCACTCCAGTATTCTCGCCTAGAGAATCCCGTGGACAGA
GGAGCCTGGTGTCCATAGGGTCACACAGAGTCGGACACGAATGAAGCAACGTAGCAGCAG
CAGCAGCATTCTGCCTAACACAGTTATGGTCTGCACTCATAATAGAAAACAGAGCTTAGT
CCCTATATTAAATAAAGTTTGACTTTATTGTTAATGCACAGTCATCTGTTGGTATCCACA
GGGAATCAGTTCCAGGACCCCTGTGGATACGAAAATCCACATGTGCCCAGGTCCCTTGTA
TGAAATGGCCCTGTTCTTCCCCACCTCCACATCCGTGGTGCAAACCCTGGGGTATGAACA
CCAACTGTAGTTCCTGCACAGCGCCTCCCTTTGACTTCTTAGTTATGTCATCTCAAAGTA
AGAATCATTCAGTTCTCTGTTAGTACTGGTTTATCTACTACAATAATTAAGTAGTATCAA
ACTACAGTTATTTATTTTATCATGAGAAAAGCCCTAGTATGGCAACACATCATGCTAGTT
TTAGGATATCAGACCATGTATAGGATTAGTCAAGACACTTCATTTCAGAATGAGAAAATT
GTCATGAAACCTTATATTTTTTCTATTTGAAAAAATAAGTTAATCCAAATCACTGAGACT
TTTCTGAGGCCTTGGTTTTAATACGTGGTTTTAATTTTTCTCCTTAGGGAAATTTTAATA
TTCTTACCTCACTTTGTTTTTTACTCTAAGACTAAATAAGAAAAAGTATGTCAAAACTGC
TGATAATACCTTCTTGACATGTCTTGAAAATACCTCTCCTTCTGAGAAACTAAAGACTTA
ATCAGTCAGTCTGGTCAAAACTGAAATAATGCTTTCATCAAGCATTTATATTAGTAACTA
AATCAATAGAATGAGAAAATAAAAATTAGGTAAATTCTGTCATGTAATGTGACTGAGTAG
ACTAGTCCTTTAATATGGTTTTGTTTATCTCTGTTTATAATGGGGGCAAAAACTTATCTT
GTTCTTTTCTTTATCTGTAACTCAGGATTATTCTAGATTAGTACTAATAGATATATAATG
AAAAAACTATGTAATATATAATTTAAAATTTTCTAGTAACCATGTTAAAAAAAGTAGGTG
AAAATAATTTGTTATATATTTTATAATAATCCAATATATCTAAAATAGTATTATTTCAGC
ATTCATGTAAGGCCTCAAAAATCTCTTTCTGGTAAATCTTTGGTAATCTTTTTTGAAGTT
AAAGAACATTTCTGTTGGCTGTACTAGGTTTAGAACAAAGTAGGATGGAAACATATTTGA
CCTTTCCAAATGAATGAGAATTATTTTTTCTGTACAAAGGCAACTTGAAATCTCCAGAGT
AGTTTTCCATTCCATATAGAGTAACTTTTGTAGACTTTGCTGAATATAATCCTGATTAGC
TGCAGTTTTGTCTCACTAAGTTACTTTAAAGCTTACTTAAGAAAGTGTGTGTATACATCA
GATGTAGATTGGTTACTTACATTTGGAAGATCTTGTCTTTTACAAACAAGTGAGGTTGGT
CAANNNNNNNNNNNNNNNNNNNNNNNNNNNNNNNNNNNNNNNNNNNNNNNNNNNNNNNNN
NNNNNNNNNNNNNNNNNNNNNNNNNNNNNNNNNNNNNNNNNNNGCAGAGATCCAGTTAGG
AAATACTTAACATGCTTATAGTAACCGCCCTCACTCGTACCTGGTGGCTCAGAAACCCTC
CAAGACTTATTAGACAGGTGTCATCAGCAGGTGTCATTCTTACCCCGCCTAACCTTCCAA
GTGACTACCTAGCTTTCTCTGATATTAACAGACTAAAAGCAAACTTTGATGTTGAACCTC
TAGAACACATTCTAAAGGTGTTTCAATTTCACTGATCCTTTTTTCCTCTTTCCTGCTACT

```
GTTGAGCATAGCACATATCCTGGGTTGCCTGATTTTTTTTTATCTTTCAGAATTGTTCTC
TTGTTCAGCATTTCATTGCCTACTAACATAGAAAAATCATTTTGTTATCACCTTCAATTA
TCCATACTTTTATTTCCATCATAACTATGTGTATCTTAACAGCATTTTCAATGTAATTAC
TGCTTTTGCATTAATAAGAATTGGTGAGGTTAAACTTAGAAAACCTCTTAAAACTAAAAT
ACCAGGTACACTTTCCCTAATGAAGAAGTAAATGGGCAAAACTAAATGTCCATACAAGCC
CAAGTCTACTTATGCCACCTGTTAGGAAAGAGCATAGCTTTGTTTGATCTTTTGATGTTT
GTAAATTTAAAGTTCATAAATGTGTGACTTACAGGGTTGGAATAGATTCAGTTAGCTTTT
CACATATTCAGTTCAGTTCAGTTCAGTCACTTAGTCGTGTCTGACTCTTTGCAACGCCAT
GGACTGCAGCACGCCAGGCCTCCCTGTCCATTGCCAACTCCCCAGAGTTTACTCAAACTC
ATGTCCATTGAGTCGGTGATGCCATGTAACCATCTCATCCTCTGTCATCCCCTTCTCCTC
CGCCTTCAATCTTTCTCAGCATCAGGGTCTTTTCAAATGGTCAGCTTTTCGCATCAGGTA
GCCAAAGTATTGGAGTTTCAGCTTCAGCATCAGTCCTTCCAATATTAGTATGAGGATAAG
TATTGTGGAATCATGTTCTGGGATATATTAGCACATTTATCTGCTTTGAGAAACTTTCAT
TGATCTTTAGTCTAAATGAGCTATTAATGTTCCTTTTGCTCTTTCTTGAACTTAAAATTT
GAGGGTTCTTTAAGCTATTAAAATCTTTAAGAGATTCTGTTAATGTTTTGGGAGAAAATT
CAAAAAGAGATGACAGGGGTCCAGGAAGCTAATCAGTGGTAGTAGAATAGTTCCTTGTTG
TAATGAATTAAAATACTATGTAGGGTGGAATTCTTTTATCTACATTAACCTTTGTTTATA
AAGATTCTGCTGCTTTGCCATCTGTTTAACCCTCATTTTAAACATTTTTTTTTCCCTTAC
AGTGAAGATAAATTTGCTGAAAATAGAATAAAATACTAAGAAGGAAAATTCCTTGTCAGT
ATTTTAGTATGGATTTAGCTATACCTCGAAAGTTCATGAGCATTTTAATATTAAATTAGA
CCTAAGTAAGCTAATTCACCTGATCCATTGTAGCCTGCCTACTTTCGTATACCTCTTATA
TAAACCTGATTCTAGGAACTTTATCTTCATTGTAGTTTAATCACTTCATAGTGTGACAAT
TTGATGTTATTGTCCTAATATGAATTCTATAAAGATTAAAGAATTACCTAAAGTTGTAAA
TATAAGGATTTAACAATTCCATTTGTTAGTGTATCTTTCAGGACCCCATAAATGCATCCA
GAAATGCTTTGAGATATATATTAAGGATATCATCAACATCAAAAATATAGTTTAGAACTT
TTGTTTTTAACCTATACCATGATTCTCAGCCCTTTCCACCCCCTCACTTACACCTACCAA
AGACATATCAAGAGAATACTCTGTAATGGCCTATAGGGAAAAGAACCCCAAACAAGTTGG
GTGTATGTATATGTATATAACAGATTCACTTCGCTGTGCACCTGAAACTAACACAACATT
GTAAATCAATTGTACCACAATAAAAAAATTTTTAACTTGTAAAAAAAAAACCCATCCCTT
TATATTATTCCTATTTTTTTAAAAAAGTCTGGCTTTCCTTGATCTCTCCTGAATTCTGTA
TGTATGAGCCGTCAGATTCATATATCATTTTATTCATTCTTCGACGTGTACTTATTTAGG
AGATACGAGAACCCATCAGAAATTAATCTTGATAGTTAATTTGTATTACTTGGGATTTTA
TTCCTTATTTTATATTATTTCATTTGAATGTGGTTTAATTATAGTTCTAAAAGGCAAACT
TGTGTTCCTTCTTTCAGATAGTCTCCATCTCTTACACGGTTCAGTGGTGGCGGATCATGA
CTATGTTGGAATGCCCGAGCTGCCAGTTGGAGCATACCAAGCAAGTATTCTGGTGGAAGA
TGCGGCTATTGGAGTAGACGACAGTGAGTTACTGACGTCAAGCAAGGATCGTGAACTATT
AGAATACAGAAATGCCAAAATCTCTCCATTGATGGAAGATCATAATGCTCTTGAAAAGCA
GACTTTTAGTCTCTTGGACTCTTCAAACCAAGTCCTTGAGTACTTAACTTAATAAACACA
AAAATTCATTTAAAGTGCTATTTAGAGAATACATCACCTTGAATCTTATCTTTTCCATTC
AAAAAGATTACATAGAAAAAGTATCTTGACAAACTCTTAATGACTAAATCCTGAAGGTTT
ATTTTGTAATTATTTATCTGAAGTTGTTCTTCTTTTGTATATATATATGGATATATATAT
ATTTAGGCAAAGGAAGCATGTAATATATGGATATTATTTTTTCTTCTACAAATACAACAG
GCAAAGCATGCTTGCTTCTTAGGAAGTAAACTAAATTCCAACTCAAAACTTTGGTATTGT
TACCATGTTTTCAGTTTCAATTTTTTTAAAAATATTCCCCCCTTTTAAAAACTGTTTGAA
CTACCTAGAACCGCATTAATAAATATTTATTGAATGTGTTAAATGAAACCTATTCCTGTG
TCATTGAAACATCTAAAAATATTGTAGTTACTTAATGGGCTCCTACAGTGTATTGGTAAG
TTCACCTTATAGACAGGCTAAGGATTAGGATCTGAAACTATATATTGGATTGAAAATGTG
TGCATTTCCATAGTATATATTAAAGTCAAAAGCATCCATGAATAAATTTTTTTCTGCAGT
CTTAAAAAAGTAAATTATAAAATAACTGATATTAAAGATTTTAAACTATAATTTCTATAA
TAGTTGCTGCTACTGCTGCTGGATCCAATGATCCAGCTGCTGCTGCTGCTGCTAAGTCGC
TTCAGTCGTGTCCGACTCTGTACAACCCCATAGACGGCGGCCCACCAGGCTCCTCCGTCC
CTGGGATTCTCCAGGCAAGAACACTGGAGTGGGGTGCTATTGCCTTCTCCAATGATCCAG
CAGAGGTTGGCAATTTGTTGTGTATGTGGCTTGTATGATCTATATTATAGATTATAAATC
ATATATGATCTATATATTACAGATCATACAAGCCATCTATATATTAGCAGCAGCAGCAGC
TGGATCATTGGAAAAGCAAGAGAGTTCCAGAGAAACACTTACTTA
```

```
==================================================
Bos Gin-1
total length:      30525 bp  (30383 bp excl N/X-runs)
GC level:         37.60 %
bases masked:      11504 bp ( 37.69 %)
==================================================
               number of      length   percentage
               elements*    occupied  of sequence
--------------------------------------------------
SINEs:                19         3764 bp   12.33 %
      Alu/B1           0            0 bp    0.00 %
      MIRs             4          529 bp    1.73 %

LINEs:                12         4399 bp   14.41 %
      LINE1            7         2603 bp    8.53 %
      LINE2            2          221 bp    0.72 %
      L3/CR1           0            0 bp    0.00 %
      RTE              6         2870 bp    9.40 %

LTR elements:          4         1853 bp    6.07 %
      ERVL             2         1006 bp    3.30 %
      ERVL-MaLRs       2          847 bp    2.77 %
      ERV_classI       0            0 bp    0.00 %
      ERV_classII      0            0 bp    0.00 %

DNA elements:          5         1159 bp    3.80 %
      hAT-Charlie      4         1068 bp    3.50 %
      TcMar-Tigger     1           91 bp    0.30 %

Unclassified:          0            0 bp    0.00 %

Total interspersed repeats:     11175 bp   36.61 %


Small RNA:             0            0 bp    0.00 %

Satellites:            0            0 bp    0.00 %
Simple repeats:        3           76 bp    0.25 %
Low complexity:        6          253 bp    0.83 %
==================================================
```

SW   perc perc perc query   position in query   matching   repeat   position in repeat

score	div.	del.	ins.	sequence	begin	end	(left)		repeat	class/family	begin	end	(left)	ID	
1157	15.9	2.0	0.0	UnnamedSequence	2251	2451	(28074)	+	Bov-tA2	SINE/BovA	5	209	(3)	1	
319	29.5	7.3	1.5	UnnamedSequence	2589	2712	(27813)	C	MIRc	SINE/MIR	(21)	247	117	2	
341	28.2	6.4	5.8	UnnamedSequence	2983	3170	(27355)	+	MIRb	SINE/MIR	2	190	(78)	3	
29	58.6	0.0	0.0	UnnamedSequence	3227	3255	(27270)	+	AT_rich	Low_complexity	1	29	(0)	4	
1130	21.7	23.7	1.2	UnnamedSequence	3279	3685	(26840)	C	L1ME1	LINE/L1	(252)	5927	5430	5	
839	15.1	0.7	1.4	UnnamedSequence	3687	3827	(26698)	+	Bov-tA2	SINE/BovA	4	143	(69)	6	
1199	28.2	1.9	3.1	UnnamedSequence	3922	4408	(26117)	C	L1MC	LINE/L1	(1011)	5135	4655	7	
358	22.5	2.7	1.5	UnnamedSequence	4409	4502	(26023)	C	SINE2-2_BT	SINE/tRNA-Glu	(52)	136	45	8	
623	15.1	0.9	0.0	UnnamedSequence	4515	4620	(25905)	C	L1ME1	LINE/L1	(1491)	4655	4549	5	
944	5.8	0.0	0.0	UnnamedSequence	5223	5342	(25183)	+	BOV-A2	SINE/BovA	141	260	(12)	9	
180	7.7	0.0	0.0	UnnamedSequence	5343	5368	(25157)	+	(CAG)n	Simple_repeat	2	27	(0)	10	
240	3.5	0.0	0.0	UnnamedSequence	5502	5530	(24995)	+	(CAGTT)n	Simple_repeat	2	30	(0)	11	
3800	8.0	0.4	0.4	UnnamedSequence	5531	6045	(24480)	C	ART2A	SINE/RTE-BovB	(36)	515	1	12	
6184	3.3	0.0	0.0	UnnamedSequence	6047	6770	(23755)	C	BovB	LINE/RTE-BovB	(533)	3314	2591	13	
1366	27.4	5.2	0.6	UnnamedSequence	6873	7220	(23305)	+	MER119	DNA/hAT-Charlie	2	365	(218)	14	
1673	22.1	2.1	1.8	UnnamedSequence	7230	7587	(22938)	+	MLT1A0	LTR/ERVL-MaLR	4	365	(0)	15	
2235	3.0	0.0	0.4	UnnamedSequence	7716	7980	(22545)	C	BOV-A2	SINE/BovA	(8)	264	1	16	
346	18.3	0.0	0.6	UnnamedSequence	8013	8091	(22434)	C	L1M3	LINE/L1	(2430)	6038	5978	17	
1989	24.3	6.5	1.0	UnnamedSequence	8967	9894	(20631)	C	L1M5	LINE/L1	(740)	5704	4725	18	
579	30.1	11.2	3.6	UnnamedSequence	10371	10841	(19684)	C	LTR33	LTR/ERVL	(0)	515	1	19	
958	16.7	3.6	0.0	UnnamedSequence	12451	12618	(17907)	C	Bov-tA1	SINE/BovA	(10)	217	44	20	
458	27.7	1.7	0.0	UnnamedSequence	12670	12788	(17737)	C	L2a	LINE/L2	(4)	3422	3302	21	
250	29.4	6.9	0.0	UnnamedSequence	14744	14845	(15680)	+	L2a	LINE/L2	3282	3390	(36)	22	
201	29.4	0.0	10.9	UnnamedSequence	15751	15811	(14714)	+	MIR3	SINE/MIR	30	84	(124)	23	
229	23.5	4.2	10.7	UnnamedSequence	15907	16025	(14500)	+	L1MC	LINE/L1	4489	4600	(1546)	24	
1449	19.9	7.1	1.8	UnnamedSequence	16280	16814	(13711)	C	MER68	LTR/ERVL	(0)	563	1	25	
1154	17.0	5.9	0.4	UnnamedSequence	16815	17049	(13476)	C	MER119	DNA/hAT-Charlie	(111)	472	225	26	
1352	14.3	0.0	0.0	UnnamedSequence	17063	17258	(13267)	C	Bov-tA2	SINE/BovA	(8)	204	9	27	
494	27.4	1.5	4.6	UnnamedSequence	17258	17391	(13134)	C	MER119	DNA/hAT-Charlie	(352)	231	102	26	*
26	85.3	0.0	0.0	UnnamedSequence	17471	17545	(12980)	+	AT_rich	Low_complexity	1	75	(0)	28	
25	85.1	0.0	0.0	UnnamedSequence	17582	17655	(12870)	+	AT_rich	Low_complexity	1	74	(0)	29	
601	5.3	0.0	0.0	UnnamedSequence	17775	17849	(12676)	+	BovB	LINE/RTE-BovB	2507	2581	(1266)	30	
2119	5.7	0.0	0.0	UnnamedSequence	17850	18111	(12414)	+	BOV-A2	SINE/BovA	9	270	(2)	31	
5974	2.4	2.4	1.1	UnnamedSequence	18112	18833	(11692)	+	BovB	LINE/RTE-BovB	2584	3314	(533)	30	
3998	6.9	0.6	0.4	UnnamedSequence	18835	19359	(11166)	+	ART2A	SINE/RTE-BovB	1	526	(25)	32	
593	5.4	0.0	0.0	UnnamedSequence	19615	19688	(10837)	+	BOV-A2	SINE/BovA	2	75	(197)	33	
1287	22.1	1.6	5.4	UnnamedSequence	19792	20178	(10347)	+	L1-3_BT	LINE/L1	8092	8464	(4)	34	
489	30.0	8.4	1.3	UnnamedSequence	20820	21032	(9493)	+	Charlie7	DNA/hAT-Charlie	64	291	(2321)	35	
979	13.6	0.6	1.8	UnnamedSequence	21109	21273	(9252)	+	Bov-tA2	SINE/BovA	48	210	(2)	36	
246	32.6	12.8	1.7	UnnamedSequence	22018	22173	(8352)	+	MIRb	SINE/MIR	26	198	(70)	37	
25	48.0	0.0	0.0	UnnamedSequence	22201	22225	(8300)	+	AT_rich	Low_complexity	1	25	(0)	38	
29	62.1	0.0	0.0	UnnamedSequence	23454	23482	(7043)	+	AT_rich	Low_complexity	1	29	(0)	39	
990	29.0	5.7	4.0	UnnamedSequence	24835	25323	(5202)	C	MLT1D	LTR/ERVL-MaLR	(6)	499	3	40	
789	10.7	6.6	0.0	UnnamedSequence	25324	25444	(5081)	+	BOV-A2	SINE/BovA	144	272	(0)	41	
476	23.1	1.1	0.0	UnnamedSequence	25539	25629	(4896)	C	MER2	DNA/TcMar-Tigger	(0)	345	254	42	
418	15.8	8.6	4.1	UnnamedSequence	26384	26522	(4003)	C	MER33	DNA/hAT-Charlie	(9)	315	171	43	
189	0.0	0.0	0.0	UnnamedSequence	27667	27687	(2838)	+	(CAGTT)n	Simple_repeat	4	24	(0)	44	
1725	10.7	0.8	0.8	UnnamedSequence	27688	27942	(2583)	C	ART2A	SINE/RTE-BovB	(35)	516	262	45	
380	19.3	4.4	2.2	UnnamedSequence	28858	28947	(1578)	+	L1-2_BT	LINE/L1	1918	2009	(6)	46	
21	52.4	0.0	0.0	UnnamedSequence	29891	29911	(614)	+	AT_rich	Low_complexity	1	21	(0)	47	
1064	5.3	0.0	0.0	UnnamedSequence	30222	30354	(171)	C	BOV-A2	SINE/BovA	(0)	272	140	48	
310	16.7	0.0	0.0	UnnamedSequence	30471	30524	(1)	+	BovB	LINE/RTE-BovB	2703	2756	(1091)	49	

>Canis Gin-1 (chromosome:BROADD2:3:10979668:10998144:1)
TTCTGTTTCAGAATAAGCTTATATGTAGTTGAGAAGAATATATGAGATCTAGCATAGCTA
AGTATCTTCTCTGTCATTTATCATTGAAGCATTTCCAAAATAGATCTTTCTCATAAATTA
TATGAATGGACTATTTTTTTTTTTGCATTTCACAATGACCATGAATATTTTTAGAAGGAA
AAAAACCTATATCTCCCCTTTATAACATATCATGTATTTAAATGTCACATAACAGAACCA
TCTGTATAAGTAATATTTCATTTGATGATAATGAGAGACTTGAAGCAAAACTTTTTGAAG
CGTAATTGTTTATTTTCTTTTTTCCCAGTATGTGGAAAATTTTCAACATTTCTTTTCACA
TGGAAAGAGCAAGATAATGTGTCATGATGTATCTATCTCAAAAATAGCTTTTGAGTATAT
TAGTTCATATACATCACACAGTTCCTTTTCTGTACTATATTTATCTCCATCACAGATAAT
ATTCCGGGAATGTTCAATTCATATGTAAGCAGAAATATGACAGAAGCCTGAACTAAAATA
GCAACTGAATGAGCTTATCATTTAAATTTTATCTTTAAATATCTATTGCCTAGGTACATG
ATGGTCCGTAGTGGAAAAAATGGTGACCTTCATCTTAAACAGATTGCATATTATAAACGA
ACTGGTGAATATCATCCAACTACACTACCGAGTGAAAGAAGTGGCATAAGACGAGCAGCA
AAAAAATTTATCTTCAAAGGTAACACTGGGGTGCCTGAGTGGCTCAGTTGGTTAAGCATC
TGACTCTTGATTTTGGCTCAGGTCGCAATCTCAGGGTCTTGAGATGGAGCCTCTTGTTGA
GCTCTGTGCTGGACATGGAGCCTGCTTAAGATTCTCTCTCCCTCTCTCTCTGCCCCTTTT
TTTTGCACACACACACCCCCACCTCCACTCATTTTCTTTTTTTTTTTTTTTTTTTTTGGG
TCACATTCCTTTTTGGATACCTAATCTCCCTGAATGGATTAAATATTTGAAAGGCAGTAG
ACAATTTATGTTGAAAGATTATGGTAATTTTTCTCTCTTCTCTTCCTTCAAAGGAAAGAG
AAGTTTGCAGTGAAATAAAGCAGAAAAGTCACCAGATGACTAAGTGAATGTTGGTGCTAT
TAATTAATGGGAGTTTTGCCTCCAGGTACAAAGTTAGGCTTCCCAAAAAACAAGTCAAGA
AGACTTTAAAATAATAAATAAATACGTCCTCACATCTCTCTGTGTATATATCTCCTCCTA
TAACTCTTACATTTCATAAGTATACCTGTTACCTCTTAATAAAGTTGTGATTCATTCATT
CATTTATTTAAACATTTATGCAACAAATGTCTATTAAGTTCCTACTGAATATCAGTCACA
TTTTAGGTCTTAGGAATAATACACTGATGAATAAAGTAGAGAAGGTTTCTGCCCTTAAGT
TGCTTACCAAGTAAGGAATGTTTTTCACTCAGCATATGCTTAATTAAACATATTAGTAAC
TTAATGCTGTATTTTGGAGTTTGTGTAGCAATCTCATCCACACTGGAGAGAGAGAGTGAT
TCTTTCCCCAACCTCTTGTTTTCTAGCTGTGAATTCTTCTCAGGTGCCTTCCAAGGAAAA
CAGAGAACTGTCTTAAAGGATTAAGGTTCTCTCACTTTCCTGCTTTACAAACCCCACCTC
CTGTTCAAATCTAGAAAAAGTTAACAGTGGCGGTTGCTTACAAAAATGTACTTATGGTGA
AATGCTATTTTAGAAAATTCTATTCCAGAAGTTGCTTATCTATACATAATTTATCTGCTA
ATTCATGCCATTTTTAGGAGGTGCCTACAAGTATTTTTCCAGTTTGAATACTCCCATAGA
AAGATATTATACTCCTGTAGTTATCACATGTTTTTGATGAACAAGCTAGCTCCACTGGT
ATAGATAGCTACAGAAGTATAGCAACTAGTGATCCTTCTACATATCAAGTAAGCATAACA
GCTGAGGACCTTTCAGACTGTGTGTGCATTCCAAATAAATATTGTCTTAGAGGCAAAAAA
AATTTTAAAGATTGAAATGATTTGAAGATAAGGCTCTGATTAGATTATCTTAAAACTGTA
GTGATCTCTAAAAATTGTAATCATATCTTTTGTTATTATTTATTCCAG**AAAAAAAGCTGT**
TTTATGTTGGAAAAGACAGAAAACAAAATCGTTTGGTAATTGTTTCAGAAGAAGAAAAAA
AGAAAGTCCTAAGAGAGTGCCATGAAAATGACACTGGAGCCCATCATGGCATATCCAGGA
CCCTTACTCTAGTGGAATCTAGTTATTATTGGACTTCAGTGACCAATGATGTCAAACAGT
GGGTATGGCTTATGTTTTAAGAATATTTTAAATACTATCTTTGTGGTAGATATCCAAAAT
TCATTATTGCTCCAGATTTACTCTGAATATTATTTCTTGTTAAATACTTTTTGTACTAGT
AGTGTTTGTACAGAAACGTTTAAAAGGAGTTTTATTTCCCATCAAAATTCTCCTTTGGAG
TGCCTGGGTCACTTAGTCTATTAAGCATCTGAGTCTTAATTGCACCTTAGGTCATGATCT
CAGGGTTGTGATACTGAGTCTCACTTTGGGCTATGAACTGTGGGGTGGAGCCTGCTTAAG
ATTTTCTCTCTTCCTCTCTTCCTCTATCTTTGTCCCTCCTCCCTCCCTCTCTTAAAAAAA
AAAAAAAAAGCTTTTCAAAATTCTCTTTTAAAATTACTGAAGTATAATTTGAACTAAAAC
ATTATTTAAACCTTTGAAAAATAGCATATTTAAATAGATTATTTAGACAGATTTTCATCA
TTGAACTCTTTTGTATGGGCAGAATTCTAACATTTCATCTTAATGTAGAAAATGTCAAAT
ATGGCAGAGACCCAAAATTAAAGAAGGCAAAATCAAGTTGTAAAAATAATGAAATTAGTC
TTAGTTTGAAACTTTACAACCTATTACAACCTTCGGCTTCTTATAGGATATTCATCTAGG
AATAAGTCATCTGATCCCTACTCTATGTAGTGTACTCTGTATGACAAATGGTAGGAATTC
CTTGGTATGTATGGGAGATCCTTTTAATAATTATAAAAGTACATCAAAAAGACTGTTATT
TCAGGTATAAAGAATTAAAACAACATTTAACTAAATAACATTTGAGAGTTACCAGTTTGT
AGCCTGTGCATGTATTCTAAGACCTCAGTATTTGTATTTGCCTTTAGAACTTGTCACACA
TTCCTTTATTCAGTGAAACCTTTGTCAGATCTTTATCATTTGAGAATGGGTATGACCTTT
AGAAATAATAAGATTATTTATGATGAAAGCTAGTATGTACATAAATTGGAAGATAAAACT
TTTGATTAAGAGTAACTGTAATGCAAATATAGTTTATAAAATTTTGCTCACAAGTTGATT
TGAAAGTGGTTTCAAAAGTGGAATTCTAGAAATATATTGAGCATTGGCACCATCCCGTAT
GTTACTCAGTTCTCATCCCACTCACATTAAAAGCTCTTTGACAACATGGGCTTTGTTTTT
GTTCTATTTTCCTGGGACCTTGAGGAATTTCTGGCATCTGCGTTTTTTGAATAAAATTAT
GTTTTAATAAATACTGTTCTAACAGATTGGTGAATGAATTTATTAGAATGATAACTTTAA
AAGAAAAAAATCTTTCGCTTATAGAAATGATTATAAATTCAGACACTGACTTAAAAATAT
AATGAATTTGATATAACAAATTTGTTTTGAGATTTATTCACATAATGTTTATTGAATGTA
AACTAGAATTTTTTTTTCTTTTTGAATGTAAACTAGAATTAATGTTCCATGTGAATAAGA
CCTCTGAAGGTAGTCACTGGATTGTAATAGCAAGTAATATTTGCTTATTATTCTGAACAG

AAGGTTTTATTCTATTCTGAGAATAGTGTAAAAGCTTCTCCTTCAAAACAAAGTACCTAT
TAACCATTTTAAAATTGAGATTTATAAGACTGCTTTTTTATTATGTTAAATATATTAACA
TCTCTGTATCTTTTTTTTCTTATTGTGTGCCTGTTTTATAG**GTATATGCTTGCCAGCATT**
GCCAAGTGGCAAAAAATACAGTTATTCTAGCACCTAAACAGCACCTTCTCAAGGTGGAGA
ATCCATGGAGTATAGTTACTGTTGATCTGATGGGCCCATTTCATGCAAGCAACAGAAGTC
ATGTTTATACTATAATCATGACAGATTTGTTCACAAAATGGGTTGTGATTTTGCCTCTCT
GTGATGTTTCAGCATCAGAAATTTCTAAAGCTATTATCAATATATTTTTCTTATATGGCC
CTCCTCAGAAAATAATAATGGACCAAAGAGAAGAGTTCATTCATGAGGTAAGATAAATTA
ATTAGGTCTAAGAGCATATTTTACTTACTTTCAGTACCTAGCACACTGGTGCATTATAGA
GTAATGCATTATAGAGTAAACTTAGTAAGTTTATATAAATTGCTACAGATGTAGAGGTTT
AATACTGTAGAGCACAAAGGTAGCTGGACTGGAAATTAAGAGACCAGGATTTTGTATTAA
TTGTGCAACTAAGTAATTATAAATCTATCTTCAGGTTTCCTAAAACTAAAATGAGGAAGT
TGGACCAAATAATCCTTGTTTTCTAGCTTGAAAATTCTGTAATTGGGGACCCGGGGCTGG
CTCAGTGAGAGCATCAAGAGCATGTGACTCTTGATCTCGAGGTCATAGGTTCAAACTCAT
GTTGGATGTAGAGATGACTTAAAAATAAAATCTTTAGAAACATTCTATGATTGACATACA
GAAGGAGAATTGACTAAATGGAGAAATACATCATATTTATAAATGAGAATATTCAGTATT
GAAAGGTATTGATTTTTCCCAATTCAAAATTAATTTACATAAGTAATTTCTAATCAGCAT
TAATAAAGAGGGAGCTTTTTTCCTTGCCTTTGACTTTTTCCAGAGTTGACTTGGAATATA
TGGATATTCTATCTATATATATTCTATATATATAGATATTCTTTATATATATAGAAAGAA
TTCTTTCATAGAAAGAATATATAGATATTTTTTCTATATATATTCTGTATATATATTCCA
TGAAAGAATTCTATATTCTTTCATATAGATATATATATATATATCTCATTATATAGATCA
TATATATAGATAGATCATTATATAGATCATCATATATATAGATATATATATATCTATATG
AAAGAATATTCTTTCAAGTTAACTTGAATATATATGAAAATCCTAAAAAAGTAATAGTGA
CAATAACACTTAAGGTATATTTTAAAATTTTCATGATTAAACAGAATACTAGTATAAGAA
AAAATCAGAGACAACCAGACATGTTTAATTCTGTCAACAAGGAGTTGTAGCAAACATGTA
AAATGTTATCTACCAGGGAAGCTCATTAGAGACTCTGTGTGTAGGATTTTTATTGGGAGC
TGTCACATAGGCACCCTCTGCCTGGCTTGTACCAAAATCTTAGATTCCTAAAGGAAAAAA
ATGTATTCAGCATAAACCACCTTGTTTGTTTAGTTTAGGCACTGTAAGCCACATTTATCA
GGAAACAGTGGAAAATCCCCAAATCCAAGTTCCTATACCCTGGGTGGCTCAGTCAGTTAA
GTGTTCAACTCTTGATTTCACTCAGGTCATGATCTCAGGGTCTTGGGTTTGAGCCCTGTG
TTGGCTCTCTGCTCAGTGCAGTCTGTTGTCCCTCTCCCTCCCTCTTTGTCGTCCCCCCCC
CCCCCACCATGCTCATGTGCCCTCTCTCTCAAAGAAGTAAATAAATCTTTTTAAAAAATA
ACAAACATGGAAAAGTGTTCAAATTAAATAGGAATGAATAATGCAAAGTAAGACCATTTT
AACTTGTCTAATCAACAAAAATGGTAATAATACCAAGTAGACTACAGGTAGAATGAGACA
GCCTTACCTTCCTGGCAGCATTGCAACTTGAAACTAACTCTCTCATAAAATAGTTTTCCA
AAGGTCCATTAAAAGCCATATATATATGTGTTTGTGTGTGTGTGTGTGTGTGTCTTTTGG
TTAGCAATCCTATTTCTAAGAATTTATTCTAAGGCAATTATCCAAAGTACAGAAAAAAAG
CTCTTTTATGACATGATTACCACAGTGCCATTTATATTAGCGAGAGACAGGAAACAACCT
AAAATCCCAAAAAAGAGAGTGTTTAAAAAATTGTGTCTGGCCACATGATGAGATTATTTT
GTGGCCATTAAAAATGATATTTACCCAAAAAAGTTATATTTACAAAGAAAAGGAATTAAC
AGGGGCACCTGGGTGGCTCAGTGGTTGAGCATCTGCTTTCATCTCAGGTCGTGATCCTGG
GATCAAGTCCCACATCATGCTCCCTGCGGGGAGCCTGGTTCTCTGCCTATACCTCTGCCT
CTCTCTGTGTGTCTCTCATGAATAAATAAGTAAAATATTAAAAAAAAGACATGTAAACTG
TTTTTAAGATTTTATTTATTTATTCATGAGACACAGAGAGAGAGAGGCAGAGACACAGGC
AGAGGGGAGAAGCAGGCGTCATGCAGGGAGCCCGACATGGGACTCAATCCCGGGCCTCCA
GGATCAGGCCCCGGGCCAGAGGCGGTGCCAAACCTCTGAGCCACCCAGGCTGCCTGCTCT
TAACAGTTTTTCGTGATTAAAATATACACAATCATCTGTTGCTTTTGTAATATAAATTAC
TAATGCTCAGCAATAACTAGTTCTCCTAGCCTTTCAAATGTAGAAGGCTGTCTTGTCTTC
TTGGAGGTTGGCAAGCCCAAGTAACTAGATCTAGCCAAAGAAATTGAATGGATGGATGGA
TGCCACTTTCAGACTGAGGTAGTGAAAACCATATGCTCCTTTCTTCAGTCTCCCAGTCTT
TTACTTTTTCTCTGGGGAATTGACTGAACATTCCAGATATTACATTATAGGGGGTGAAAT
TTTCTCATGTAGGATTGCTGAGGCATGTAGTCAAGAATTGTTGCCTTGAAGAGACATACT
GACCCATAGCCAAGTTTGTAGGAGCAAGCAGTAAAATTACTGCATTAAAGCTGAGACTTG
AAAGTGTTTTATAACTACCACATAACCTTGCCTGCCTTGACTGACACAGTGGTCAAAATG
AGGTGCGTGAAACAATCCTGTTTATGGGCTAACAATGTTGGAACTTTGCTTATGTTTCTT
TTTTATCTAATAAGAAAGAAAAGTTTTGCTAATGTTGTATACAGATGATTAATAGCATTC
TGAATCAACAAGGCGGGTGATTGAATGTCACACATGATGTATGAGGGTATGAAAAGAGAA
AGAATCCACAACACATAAGAGGTCTATCTACAGTTCCCTTGTTCATTCTTGGTTTTCAGC
AGTAGTTGTGTTTATAAATATCACCTAGTTTTCATCAAACTACTCCTCATGGAATAGCCA
GATGGCTTAAGTTTCCTACAGTGAAAACAAGGTACTACTAATAACGCAATCATCCATCAA
CCACAAGAAAATCATAGAGCTGGTACTTCTACTTAACTTCTGTTACTAATTCTTTTATAG
ACACATTAATGAGATGAAAACAAACATCAACTAATTAAATCTGATAGGAAATTGTTCACC
AAAATCCAAAATATCAAGAAACTGATTTGCTTCCACCACAACTAATAAAAAGCCTCATGC
TAAGTGCATGTAGTGCTTAGAGATAATAATCATGACAATATGGACTATTATGAGTCACCC
ATTTTTCCAGTTATGTTTAAAGTCACATAATAATCCAGGACTTTGGAAATTTTACCAAAG
ATAACAATGTTTATAAGCCTCTTTTGTGAGGTTTAATAGCAAAAAGCTAAAAATCACAGA
TGATTGAGAAAATTTGTTCTTTTTACTCTGATGTATGTAAATGGCTTAAATATTATACAG
TGAGTATAATGGTGACAAAATGAAATGTCTTCTTTTGTTAGCAAACACATACCAAACACT
GCTAAAGTGTTGAAATAAGATTACAATGTGGCAATGTGGATGAATTCAGTGTGTGTATTA
CACAGTTGGATGAAAATATAAATGTTTGAATACAATGCAGGAACTTTTAGTAAAGCTCTG
AAGGAAAGAAAACAAACATTTCCAAAGGAAAATCTCCTCCTTTGATTTTAAATACAGGTC
TTTAGCAAAACAGAATTGTAACCACTAATAGAAGGGTTTGATGGGAGCTAAAAGGCTCTG
GGGTTATGATATCAGGCTAGCATCCCACAAATGAATTCACTGGATCATTCCTAGGCAACC
TCTTGAAGCAAAGAAGTTGTTAGACCCTCTTCCAGAGGTCAGTCATTTAAAAGCATTGAT
TATGACCTCAGATTGAGGCTGACCCAAATAACACCAATTCTCTGAGATTTCTGTCTCTTT
TTTAAAAGATTGTATTTTTAAGTAATCTCTACACCCAGCATGGGGCTTGAACTTAAAACC
CCAAGATCAAGAGTCGCATGCTTTACCCACTGAGCCGGCTAGGCGTCCCTCTAGGATTTG
GTTGTTGTGTTTTTTGTTTTTTGTTTTTTTTTTTTTAAGTATATCTTTTATATTACTTTT
TTTTTTTTAATTTTTATTTATTTATGATAGTCACACACAGAGAGAGAGAGAGGCAGAGAC
ATAGGCAGAAGGAGAAGCAGGCTCCATGCACCGGGAGCCCGATGTGGGATTCGATCCCGG
GTCTCCAGGATCGCGCCCTGGGCCAAAGGCAGGCGCCAAACCGCTGCGCCACCCAGGGAT
CCCTATATTACTTTTGAATGAAAAGACTGAAAGGTGGGGGTAGAGCTGGTTAAGCATCCT
ACTTTTGGTTTTGGCTCAGATCATGATCTCATGGTCATGGGATTGAGCTCCCCATCGGAC
TCTGCGCTCAGTGCAGAGTATGCTTGGGATTCTCTCTCCCTTTTCTTCTACTGCCCTCTC
CCCCCAACTCATGCTCTCTCTTTCTCTCTCTTTCAAATAAATCTTTAAAAAAATTAAAAG
GAGGTCGCCAAAATTAGTACATATATCAGCTAGGAACTTTATTAGTTGCAAGGAAGAGAA
CTGCTGTCTTCTACCCAATATATATTCTGCCCAAAAGTTACAAGAAGGCTAAACTGTCCA
AGGTTGAATGTCTTTGGGAATCCAATCTTCCAGTCTTCTGTTCCACCATCTTTGAGATAC
GACTCTTAGTCTTATGTTCACAAGATGGGTACCAGAGCCTACGCCACAAAGTCTATAATT
CAGACAAAAAATGGAGAGGACAACAGACACAAAAAAACCTAGTATGAACTGAGTCTACCC
AACAACTTTTAAAAGCCGAACAACTTCCACATTTCCTTTTGACCAAAACAGTATCACATA
GCTACTCCTACCTGCAGGGTAGGTTGGGAAATGTGGTTTTGGTTTTGGTGGTTATTAGCT
TTACCTTTTTAGTTACTGTGATATGATTTGTCTTCTTACAATTAGGCAACAAGAATACAG
TCTTTTAAAAGACCTAGTTTATTAACCAAAAGTTTACCCAGTTAAAAATAATAAGAAAAG
ATAGTCTGAGGCATAAGATAGGAAACATAAGTTATTTTATGTTTTATAGGCTTATAGTTA
GAAGTTTTCTAAACTCATTTTGACTTTTCTTAGGTCCTGCAGATAATAAATATAACTATG
ATAATAATAGTAACATTACAAGTTACAAAGTGCCTACTACTTGCCAAGAACTGTTGTAGG
TGCTTCACAGATATATACTCATAATTCTCAAAAGAATCCTGGGCAGAATTATCCTCATCT
TACGCATGAGGAAACTGAGTATACAAGTTTAGTTATTTGCCAGTCTCATAACTGTTAACT
GGGTTATGTGACCTAGTATGTGTTTGAGAAATTGTCTGCTGAATTAAAATGTTACATGAA
CTCAGAGTTAGTGTTAAATTCTGTTCTTCCGTTTGCTTGCTGTATATGTATGTTTGGCAA
GGCTCCTAGCCTCCCAAAACTTTTATCTTTAAAATGGGGTTAATGGTAACTCATGATTGT
TGTAAGGTATAACAACAGCAATGTAAAGCATCTATTGTATTGAACAATGGATAATAAGCA
CTCAGTAAATGGTAATTGCTGTTTTAAATACAGTATGTGTATTTTTATATATAGAATGGC
TATGGTCAATGAAAAAGGCAAGATTCAAATTCTTTGAAACCTTTTGGTCAGGGAAAAGTA
AAATACAAATTTTAAAGTCCACTTTGTTTTATATACTTTTCAG**ATCAATGTTGAACTGTA**
TGGATTGTTTGGCACAAAGCAAATTGTAATTTCTCATAGCTCCCAAACCATTAATCCAAC
TGAAAGTACACCGAGCACAATAAAGGCATTTCTTTCTAAACACTGTGCTGACCACCCAAA
CAACTGGGATGATCACCTATCAGCTATTTCATTTGCCTTCAATGTAACTCACTTGGTATG
TGTCTTTTTATTATTCTGTACTTTTAAGTTATGACTGTTTCTTGGCTACATAAATAATTA
CAAATTGTTGTTCATTTTCCTTAAAATAG**GAGCCTACTAAAAATATACCGTATTTTCAAA**
TGTTTAATCGAAATCCATACATGACTGAGACTTCAGATATTCTCCATGAAGCAGATGGTA
ATGATACAAGTATGTTTGCCAAAATTCTAGGTGCAATTAAAGAAGCTGATAAAATAATGG
AGAATAAGACAACTTCAGCAGGACAGGTGATTCTATTTAATAGAAAACTCTATAACCTAA
AAATCCATGAAGTAATTTTAAAAATTTCACCATGCACACACATACACATGGATGCACACA
TACATATGTTATATAAGCCAAGTTTGTTTCTATTAAATTCTGCTTGTTTTGATTACATAG
TATGCTTACTAACTATAAAGTTCTGAGAGACGGTAATGGGAATACCTTGTTCTTTGGAAT
TAGGTTGAACTGAATTCACATTCCTGGTCTTGTTGCTTTCTGGTCGTTTGATTTGGGCAA
ATTACTTAACCTATCTCAGTTTAATTTCTTTGCAAAGTTGGGGTAATGGTACTGGTTTTG
AAATATTTTTCAGAATTAAATAGAGATTCATGTCTACATTGTAATAATATCTTAGTAAGT
AACCATTTTGTTATTTGCATTTACTTTGGAGAAACTATAACTTTCCTCCCTTTGTTCTCT
CTTTTTTATTTTGCTCTTATAG**ATGGAGAACAACAATCTTGATGAACTAAATAAAAGCAA**
AATCATTGTTAAAAAGAAACCAAAGCAATTAAATCCATTTCATTTAAAAGTGGGTCATGA

AGTTTTAAGACAAAGAAAAAATTGGTGGAAGGATGGTCGTTTCCAGTCAGAATGGGTTGG
TCCTTGTGTCATAGACTATATTACAGAAAGTGGATGTGCTGTTCTAAGAGACAACACTGG
GACGAGACTTAAAAGACCTATCAAAATGTCCCACCTTAAGCCTTATGTAAGAGAGTCCAG
TGAGCAAGGTAAATATCTGTTACTTCTTTACATTTTACTTTTTGTGAATGAGGAGGGAAG
TTAAAAGTAATATAAGAATCCACCCTCCACCCAACCAAAACCTCTTTTCTCAGTTAGGTT
TTCTTTTACTTTCTCATTTTTATAAAATGTGGTATAATTTTAGATCCCAGGTATTCTGTT
CTGTTAGAATGACTCTCTTGAAACTGTCAGGCTTCTAAGAGATACGGTTCTGATTTCTAG
TGTACTTTCCATACATTTACCTTATAGTTCATGTAGGAGTTATTGGACCCATTTCACAGA
AGGAAGAATTAGGTCTTCTTTATTTTTGCTTTCACAACATTTTGTCACTTTGTGCCTCAG
CTTAGCATAAGAAAATATCCACTAAGGTAAATGATGCCAAAACCAAACATACTATCCAGA
ATTCTGTTGTGCGATAAATATACTAGAGAACTTTTTTAAAAGTGTTTTTTTTATGTTATG
CCATAGTCAGGTTATGATAGATGCAAGGGAAGGTGCACGGTGGGGTGTAATCAGAATGCT
GTAAGGTGCTATGTGGAGAGGTGAAAGCCTGAACATGGTTATGCTTGCCCCCAGATGTGG
TTTTGAACAGTCAGAAGGATATCCTGATTTCTTTAACCTTTTCTTTGAGAGATTAAGAGA
GAAATGACTTTTTGCTATGGCTTTCAGATTAAATCCCACTTACTTGAGACTGGTTTGTTC
TGTCCTATTGTAACCATTCCCTAGAGTTTATATGAGGTGGATAACAGAGGTAGTGAATTG
GTACCTCAGCCAGTTTTAATTTGGCTTTTCCTATAAAGAAAAATAAAGCATCAATATTAC
TTGAAGATAATATTTATACTTATTTATATTATACAGAGTATTTATACTTAACCTTATTTC
TTTAGGGTAAAGAAGCCCTTAAGGGCTGTTTGGTAAATAGCTCAACTTTCCTTCTGATCT
GGATCAAAACTGTCAAGTTTAAAAACTCTTAGTATCAAAAAAACAAACAAAAAACCTCTT
AGTATCTTTCCTTCTGCTTGTAACAGCCTGATTGCTCAATTAGTAAACTTACCCTGCACT
GTTCTAATTCTGAAAGCTCACAGTCTCTTCACTGTCCTTGTTGAAAGATGGTACTTTCAT
TCTTTGTACTACTTGCCCTTTATGATTTCTTTCTTATGTAAGTTAAGTAGAAAAAAAAAA
TTATACAGGTACATATTGTCCTTTCTTCTCTAGCAAGATTGTTTCCCTAATCTCAGCATT
CAGGACTCTCTCCTTTCTAATATGTTAGCCTTCCTTAGTTAATACAAAATTTACATTTAA
AATGTTGCTACATCTGCTTCAACATTTTGCAATTTACTCTTGCCAAAGACTTTCTCAGAA
AGGAGGCCAAACATTTTGCTACTCCTGCATATTTCAGAATCCAATTGAGTAGAACAGTTT
CTAAACAAAGGCAGTCTAATTTTTCCCTCATGCTTGAATTCTCTGCAGATATTTCTTCTT
CCAGTAATATAAGGAAAATTTTAATATTAGTACTAGTGTACATGTAAGAGATCTGAGTTA
GATGGGTAAAATTGTGTTTTCTTATTAACCAAAGGAAATGTGATCCAACCATCCTGGAAT
CTGGTTCTTGATTACTATTTGTTCCTTATTTTGTGTTTCTTTTCTTTATAGAAATTCAAA
ATAAATGTGATTTCTGCCATCTTTTTCTTCAGTATTACATATTCTCTTGAAAATCTTTAC
CTCAGAAGCTGATTTTAAAGTGTCCTTGAGCTGAATGTCATGCAAACTAAATGTCTTTGA
ACATCTTAAATCCTATTCTTTGACATAAATTTAGAAGAGGTATATCCAAAAGACTCTGTC
TGTACTTGTCATTGCTACTCTTACCAGGTGTGAGATCGCATAGCTATTTTAAAGACTCAC
TGTCCAAAGCAGTTAAGCTCTGTTTCCAACTTCTTATCCATTCTTATCTAGAACATAATG
TAGAGGGAGCACATTCAGTACTAGGTCAACAATATTTCTCATGGCTATTTTAAGCAGGCC
TATGTCATTTTGTCTGGTTCAATGCCAGGTACTTGTTTTTCAAGACTAATTATTTTAGTT
AGTTTTATGACATACCTTAGTATCTCTTTATAATCCTAATGTTCCCTTCTGAAAAGAACA
ATGTTACTGACTCTTCTATCCAGCATAGATTTTGAGTAATTTTATTGTTTCATTTTGGGT
AGGAGTTGTGATAGATTTCTGTCTGCTGATTGCTCTTCAAAGTTTATGAAATGGTTTTCA
TTATTTTATAAGTTTCCTACTGCTGCTATAACATATTACTATAAATTTAATGGTTTAAAA
CACCTTACAGTTCTTTAGCTTAGAATTCCAACATAGATAGGCCTTACTGGGCTAAAATTA
GGCATTGGCAGGGCTTTGTTCCTTTCTGTAGGCTCTAAAAGAGAACCTGTTTCCTTGCCT
TTTCCAACTTCTAGGGGCTACCCACACTGCTTGGCTTGTGTTCTCCTACCTCTGTCTTCA
AAGCTAGCAATGACAGATAGAGTCCTCCTCACGTCACAGTCACTACAACCTTCTCTCTTC
CCTCCCCTTCCATGGTTAAGGACCCTTGTCCTTAGGCCCACCTGGATAATGCAGAATTAT
CTCCCTGTTTTAAGGTCAGCTGGTTAGCGACCTTAATTCCATCACTTCTCAAATTCTCCT
TTGTCATGTAAGGTACTGTACTTACAGGTCCAGGGAGTTTTAGAACATGGACGTCTTTAG
GGACCCATTATTCTCCCTCCCACTATTATGGTTTCCATTCATGAAACAAAACAAACTTTA
GATCTTTATTAAGCCCATTATATTTACTGTTTTATTAATATAATTTATCCAAAGAACCTC
CCTTTGAGCTCTTAGTTGTGTAATCTCTAAACTGAGAGTCATTCAGTTCTCTTTTGTTAG
TATTGGTTATTTTTATAAATGTGGCAATTATGTAATACCAAATTACAGTAATCTGTCATG
ATTTTATCATAAGAAAGGATGGATAATCTATTGTATCAACACATTCTAAAAGTTTTAGAA
TTATCTGGCTGTATATAGAATTAGTCTGATCACCTCTTCAGAATAAGGAAATTTAGTATT
CTAAAACCATGTACTTTTCTTTATGTTTAACGAAATAAATTAATCCAAATCACTCAGACT
TTTCTGAGACTTTAATTTCAACTTATTTTTCTCCTTAGAGAAACTTAAAAATTTTTTTCT
TAATTCCTTTTTTTTTTAACTCCTTAAAACTACATAAGGAAAAGTATGTCTAAACTGCTG
TGATAACACCTTACCTCCACTTGGAAAAAACTAAAGGCTTACTTACTCAGTCTGGCCAAA
GCTGAAATAATGCTTTCATCAAACAGATGTCATAATGATAATTAAATTAATAGAATGAAA
ATAAAAAAGTTTATCCTTTAATACAGCTGAATAGAAAAATGTTTGAATATGATATGTTTA
CCTCTGTATCTTGGGGGCAAAACTTGTTCTTTTTTACATCCCTAATTCAGGATTATTTTA
CACCAGAACTGTCCAATTGATATATATAAAATGCAAAAGTTATATAGTAATGTAATTTTA
AATTTTCTAGTAACCACATTAAAGTAGGCAAGAAATATGTGAAAATTTTAAAAATATATT
TGATATAACCTGCTCTATCTAGTTATTATTTAATAATATGTGTAGGACCTCAAAAGTCTC
TTCTGATAATTCCTGGTAATTTGTTTTGAAGTTAGAGAGCATTTCTGTTGGCTCCAGTAG
ATCTAGACCATATTGGAGTTGAAACATATTTGACCTTCTGGATAAATGAGAATTGGTTTT
GTTTTTTTTTTGTTGTTGTTGTTGTTGTTTTTTCTACACAAACATAGCTTGAAATCTCCA
GAGTAATTTTCCATTGCATGTAAAGTGATTTGTGTAGAATTTGCCAAGTATAGTTCTTGT
TCTCTACAGTTTTATTACAGTAGGTTTTTTTTTACAATAGGTACTTTAAAATTTATCACT
TATTTAAAAGAAAAAATTTGTGTATTGTATAAAGTCATGTACACATATGACACATAGAAG
CTCTCCTCTTAGAAACAAGTGCGGTTGGTAAAGTGCATTGGTTTTGAAGTCCAGTATAAC
TAACAGGTGTTTAAGCTCAAGCTCTCAATGTATATCCAGGACATCAATGTGGCAACTTCT
AAGAGCTTCATGTCTATGCTATGTGCATTTTCCCTGCTGATACCATGTTTACTGGACATA
TTTTTCAAAAAAAAAAAAAAAAAACTGAGAAAACTGCATCAAAGAATTTTTGCAAGGATCT
TTGGAAATACCTTAAATTTATGGTATCTGCAATCACCAATACTTTGAGCACTGTGCAGGC
CTTGAAACTGTGGAATTCCCATGGCTTATTTGAAAAATTTCACCAGTGATACATTTATCA
TACCTAACCTTTGGAAGTTCTAAGTGTCTAGCCTTCTCCAGTATTAAGAGCCTAAGAGTG
AACTTTGATGTTGAACCTTTAGCAACACATTCTGAGTGTTTGAAATGCATTGATCATGTT
TTTGCTCTTTCTTGCTGTTCTTGGACAATAGCACTTCTCATGAGTTGCACAATTTGGTTT
TTATCCTTCAGAATTGTTCACTTGTCCAGCAGTTCATCCCGTGCTAACATACAAAAATCA
TTTTCTTACCACCTTCAATTATCTATACTTTTGTTTTCATCATAAATACCTATATCTTGC
CATCTTTTTCAATCTAATTACTATTTTTGCATGAATAAGAATTGGTGGGAGAAAACTTGG
AAAACCTCTCAAAATTAAAATACCAAATATACTTTGCCTAATGAAGAACAAAATGGTCAA
AACTAAATGCCCATGCAAACCCAAGTCCACTTATGGAGAGCATCATGGCTTTGATTTTTT
TAAGTTTTTTAAACCAAAGTTCTTAAGTGTGTAAATTTATGGGATTAGAATAGGCCCAGT
GAGCTTTTCACATATTAGTAGGAGAATGGGTAGAATAAGATCATGTTCTAGGGTTTATTA
GGAAATTTATCTGCTCTGAGAAACTTTCTTCAATCTCTAATCTAATGGGCAATTAGTCTT
CCTTTTGCTATTTCTTGAACTTAAAATTTGAGGGCTTTTTAAGCTATTAAAATCTTAGAG
ATAATCATTAATGCTTTGGGAAAAAATGCAGAAAAAGAAGGCTGGGGTCCATGAAGTTAA
TCATCTGTGGTAGAAGAGTTCTTTGTTATAATGAATTAAAATATCAGGTAGGGAAAAATC
CTTTTATGTACATAAACCTTTTTCATGAAGATGGTACTTGTTTTACCATCAGGTTAACAT
TCATATTAAGCATTTTTTTTTCTGATAGCGATGATAATTTTGCTCAAAGTAGAATAAAAT
GCTGACAAGGGAAATTCCTTGTCAGTATTTCGGTATGAATTTAACTACACTTTAGAAGTT
CATGAGCATTTTGGTATTAAATTACACTTAAATAAGCTAAGCATTGTAAACTGCCTACTT
ACACGTAAACCTGCCCACTTAACCTGTATCACATAAGCCTGATTCTAGTAACTGTCATAT
AAGCCTGATACTATTAACTTCATTTTAGTTTATAATTTCATATTATAAACAACTTGATAT
TATTGTCCTAATTTTAAACAACTGGACATTATTGTCCTTATATGGATTCTGTGAAGATTA
AAGTTATAAATGTAAGGATTTTATTTATTAAATATCTTTCAAGATCCCATTAATTTACTC
AGGAGTACCTTGCAGAAGCCTATTAATAAGTGTATCATGAACATCAAAAATAGAGTTTTT
ATAACTGCTTTTAATCTTTACCATGATCTTTAGCCCTTTCTAACCACTCATGTATACCTG
CCAAAGACAGATCAAGGGAACAGGAAAACTTGGCTCTTTTAAAATCTTAGGTCTTTCCTG
AATTCTATGTATATGAGCTGTCAAATGTATGTATCATTTTATTCATCCTTTGACATGTGT
ATTAGTAGATAGGAGAATTCACCAGCAGTTAATCTTAGAGTTAATTTTTATTACTTGGAA
TTTTGTCCCCATTTAACATTGTTTAATTTTAAAATGATGTTTAATTATTCTAAAAGGTAA
ACTTACATATTTCTTTTTTCAG**ATAGTCTTTATCTCTTACAAGGTTCAGTAGTGGCAGAT**
CATGACTACATTGGATTTCCTGAAATTCCAATTGGAGCATACCAAGCGAATATTCTGGTA
GAAGATGCAACTATTGGCGTAGTCGATAATGAATTACTGACATCAAGCAAAGATCGTGAA
CTATTAGAATTTAGAAATGCCAAAATCTCTCCATTGATAGAAGATCATAGTACTCTTGAA
AAGCAGACTTTCAGTCTGTTGGACTCTTCAAACCAAGTCCTTGAGTACTTAAGTTAGTAA
ATATCAAAATTCATTTAAAGTGCTTGTTTAGAAAGTATGACTCCTTGGATCTTTATCTTT
TGCATTCAAAAAAAAGTTGTGTAGAAGTGTTTTGATACGCTCAGAATGACTAAATCCTGA
AGGTTTATTTTATAACTCTTAATTTATCTAAACTTGTGTAATGTTTGAATGTCAGTATGT
ATGTGTGTGTGTGTGTGTGTGTGTGTGTATGTATATATGGAAAGGAAGCATATAAATGTATA
GAGCGTGACTGCTTCTTAGAAAGGAAACTAAATTCTAGCTCAAGACTCTCAGATCTTTCT
CTTGTATTGGTACCATATATTTTTTTTTGAATATTTTGCCCCTTTAAAATAAAAGTTTGA
ACTACCCAGAATGGGATTAATAAATATTTATTCAACTATAACAATGAATTAAGTGAAACT
TCTTTCTGTGTCATTGCAACTTTTAAAAATAATACTGTAGGAAAGAATTTGGAGTTGTTT
AATGGACTCTTACAGGATATTGATAATTTCACCTTCTAGGCTCATAATTTGTATCTGAAT
CTATATATTAGTTTGAAAACATTTGTGCATTTCCATAATATATATTAAAGTCAAAAG

===

Canis Gin-1

total length: 18477 bp (18477 bp excl N/X-runs)
GC level: 33.52 %
bases masked: 5266 bp (28.50 %)

	number of elements*	length occupied	percentage of sequence
SINEs:	11	1974 bp	10.68 %
Alu/B1	0	0 bp	0.00 %
MIRs	4	708 bp	3.83 %
LINEs:	3	560 bp	3.03 %
LINE1	2	445 bp	2.41 %
LINE2	1	115 bp	0.62 %
L3/CR1	0	0 bp	0.00 %
RTE	0	0 bp	0.00 %
LTR elements:	4	1146 bp	6.20 %
ERVL	1	271 bp	1.47 %
ERVL-MaLRs	3	875 bp	4.74 %
ERV_classI	0	0 bp	0.00 %
ERV_classII	0	0 bp	0.00 %
DNA elements:	5	1233 bp	6.67 %
hAT-Charlie	5	1233 bp	6.67 %
TcMar-Tigger	0	0 bp	0.00 %
Unclassified:	0	0 bp	0.00 %
Total interspersed repeats:		4913 bp	26.59 %
Small RNA:	2	147 bp	0.80 %
Satellites:	0	0 bp	0.00 %
Simple repeats:	5	206 bp	1.11 %
Low complexity:	0	0 bp	0.00 %

SW score	perc div.	perc del.	perc ins.	query sequence	position in query begin	end	(left)		matching repeat	repeat class/family	position in repeat begin	end	(left)	ID
989	13.3	1.3	0.0	UnnamedSequence	748	897	(17580)	+	SINEC_c1	SINE/tRNA-Lys	1	152	(57)	1
213	3.9	0.0	0.0	UnnamedSequence	932	957	(17520)	+	(T)n	Simple_repeat	1	26	(0)	2
383	31.2	1.7	2.6	UnnamedSequence	1333	1447	(17030)	C	L2a	LINE/L2	(3)	3423	3310	3
842	22.6	4.7	1.5	UnnamedSequence	2517	2709	(15768)	+	SINEC_c1	SINE/tRNA-Lys	1	199	(10)	4
237	29.3	21.4	2.0	UnnamedSequence	4510	4664	(13813)	+	MIR3	SINE/MIR	15	199	(9)	5
258	22.2	1.4	8.7	UnnamedSequence	4675	4748	(13729)	+	tRNA-Lys-AAG	tRNA	5	73	(3)	6
295	28.4	1.2	0.0	UnnamedSequence	4802	4882	(13595)	+	L1M5	LINE/L1	4487	4568	(1578)	7
284	8.7	2.1	5.3	UnnamedSequence	5123	5219	(13258)	+	(TA)n	Simple_repeat	2	95	(0)	8
1397	19.2	5.2	0.0	UnnamedSequence	5348	5618	(12859)	C	MER68	LTR/ERVL	(197)	366	82	9
860	18.8	4.5	1.5	UnnamedSequence	5619	5818	(12659)	+	SINEC_c2	SINE/tRNA-Lys	7	212	(0)	10
678	28.7	8.2	1.8	UnnamedSequence	5873	6026	(12451)	+	L1ME4c	LINE/L1	191	363	(467)	11
210	3.7	0.0	0.0	UnnamedSequence	6027	6053	(12424)	+	(TG)n	Simple_repeat	2	28	(0)	12
678	28.7	8.2	1.8	UnnamedSequence	6054	6263	(12214)	+	L1ME4c	LINE/L1	364	577	(253)	11
1094	10.2	7.2	0.0	UnnamedSequence	6302	6467	(12010)	+	SINEC_a1	SINE/tRNA-Lys	1	178	(1)	13
1235	7.5	1.1	0.6	UnnamedSequence	6481	6654	(11823)	C	SINEC_Cf3	SINE/tRNA-Lys	(4)	176	2	14
264	28.3	7.6	0.0	UnnamedSequence	7037	7128	(11349)	+	MLT1I	LTR/ERVL-MaLR	313	411	(0)	15
262	33.1	10.6	2.7	UnnamedSequence	7174	7381	(11096)	+	Charlie7	DNA/hAT-Charlie	72	295	(2317)	16
728	27.7	8.9	2.9	UnnamedSequence	7398	7724	(10753)	+	Charlie7	DNA/hAT-Charlie	348	693	(1919)	17
714	23.9	12.0	4.6	UnnamedSequence	7743	8067	(10410)	+	Charlie7	DNA/hAT-Charlie	759	1106	(1506)	18
363	27.2	12.1	2.5	UnnamedSequence	8085	8299	(10178)	+	Charlie7	DNA/hAT-Charlie	1174	1408	(1204)	19
349	24.7	0.0	0.0	UnnamedSequence	8432	8504	(9973)	C	tRNA-Lys-AAG	tRNA	(3)	73	1	20
1587	2.1	1.1	0.0	UnnamedSequence	8577	8763	(9714)	C	SINEC_Cf	SINE/tRNA-Lys	(0)	189	1	21
978	17.7	5.1	2.0	UnnamedSequence	8798	8993	(9484)	+	SINEC_c2	SINE/tRNA-Lys	10	211	(1)	22
340	37.1	5.2	4.9	UnnamedSequence	9130	9415	(9062)	C	MLT1J	LTR/ERVL-MaLR	(141)	371	85	23
522	27.8	9.0	0.5	UnnamedSequence	9666	9842	(8635)	C	MIRb	SINE/MIR	(0)	268	77	24
402	32.2	10.4	2.8	UnnamedSequence	9903	10103	(8374)	+	MIRb	SINE/MIR	50	265	(3)	25
437	32.9	4.6	1.1	UnnamedSequence	10893	11067	(7410)	+	MIRb	SINE/MIR	18	198	(70)	26
1645	21.3	6.6	5.6	UnnamedSequence	13747	14243	(4234)	C	MLT1D	LTR/ERVL-MaLR	(2)	503	2	27
435	18.5	6.3	7.7	UnnamedSequence	15004	15161	(3316)	C	MER33	DNA/hAT-Charlie	(0)	324	169	28
180	0.0	0.0	0.0	UnnamedSequence	15310	15329	(3148)	+	(TTG)n	Simple_repeat	2	21	(0)	29
261	8.3	0.0	0.0	UnnamedSequence	18055	18090	(387)	+	(TG)n	Simple_repeat	2	37	(0)	30

>Myotis (microbat) Gin-1 (scaffold:Myoluc2.0:GL429767:20303117:20321803:1)

TTTTTTCCTCAGTATGTGGAAAATTGTCAACATTTCTTACCACGTGGAACGAGACAAGAT
AATTTGTCATGATATATCTCAAAAATAACATTTGAATATTTTAGTTCATATACATCATAT
TGTTCCTTTTCTATACTACAAATTATCTCTCTCAGGTAATATTTTGTGAATATTCAGTTC
ATATGTAAAGCAGAAGTGTGATAGTCTGAACTGAAAAAGCAACTGAATTAGCTTATCATT
TAAATTGTATCTTTAATATATATATATAGATAGATAGATAGATAGATAGATAGATGATAG
ATTGATTTCAGAGAGAGGAAGGGAAAGGAAAAGAAGGATAGAAACATCAGTGATGAGAGA
GAATCCTTGATCTGCTGAAAGAAAGTCAAGAAAATTCTATGATCCTGTATAATAAAAGCC
TAATATGCTGAATGTCTGGTTGTCCTGCACACCCCCTACTAGGGATCCAGCTGCAACCCA
GGCATATGCCCTGACTGGGAATCCAACTGTGACTTCCTGGTTCATAGGTCTATGCTCAAC
CACAGAGCCATATCAGCCGGGCTAAATTATATCTTTAAATATCTTTTACCTAGGTTCACA
ATGGTACGTAGTGGAAAAAACGGTGAACTTCATCTTAAACAAATTGCGTATTATAAACGA
ACTGGTGAATATCATCCAACTACACTACCAAGTGAGAGAAGTGGAATAAGAAGAGCAGCA
AAAAAATTTGTCTTTAAAGGTAACATTAAGGCCCCAAATCTTGAATATAGAGTTAATATA
GATGCTTCTCCATATGTGTTCTTTATGGACTCCTGAAAGTTTCTGTAGTAATTGGTTGTT
GCTAAACAATTACTAGTTGAATTGATGGTGCCACTCAATCAAAAGCAAAATAAAAATCAC
ACAGAAGTTAACTGAAGGCCATGAAAAGGGCATTTAAATTTATAGACAGAAAAGATACCT
TGGGATAAAAAGCAATAACCTATACCTAAATTAACCTTGCCTGCTTTTTCCCTCAGGTGG
TTTCTGCCCTAATGGTGTATTTTGAAGACTCATGTTACTATCAATGAAAAACTTATTTGA
TGCTTAATATGAAAATGTTGACAAAGTTAACTTGTTTGTTTACTATACTTATTTCTGTGG
TTCCCTCCTTTCACTGCCATTAAATAATTTCTGAATGGGTTAAATATTAGTCAGTAGAAT
ATATGTATTGAAAGTTTATGATAATTTTTGCCTCTTTTTCTTCTTCCAAAGGGTAAGGGA
AGTTTGGAATGAGAAAAGTAAGAGGTATAGTCACAGATAACTGAATGTTGGTGCTATTAA
TGAGAATTTTGGCTAGAGGCACAGAATCAGGCTTTCCTCAAAGAAAGTCAAGAAAATTCT

ATGATCCTATATAATCTACACTAATAAAAGAGAAAGATGCAAATTGACCATACCTTCGCA
ACACCCACCAGCCAGTCAGGAGTGTGTATGCAAATTAACCCAACAAAGATGGCGGGTTAA
TTTGCATATGCAGGCGGCCGGGGGCGGGGCACTTGCGTTTTGCCATGGCAATGACGCAGG
CATTCCGCACCGCTCCAGGCCTCTGGGCAGCGTGGGAAGGCGGAAAGGCGGCTCCAGGAC
AGAGTGAAGGCGGAAAGGCGACTCCGGCCGGAGCAAAGGCAGTGCTGGCAGCCAGGGGAA
GGAAGGCCCATTCTTGCACTAATCTTCATGCATCGGGCCTCTAGTAAAAGCCTAATATGC
TAAGTGTCCAGTTATCCAGTCATCCATTCAACCAATCAAAGCGTAATATGCTAATGATAT
GCTAAGGCTGCTCAACTGCTCGCTATGACGTGCACTGACCACCAGGGGACAGACATCGAC
TGGTCACCAGTTGCTGTGATATGCACTGACCACCAGAGGGCAGATGCTCTGACTGGTAGG
TTAGCTTGCTGCTGGGGTCCGGCTGATTGGGACTGAGTGAGATGGGCCGGACACACCCTG
GAGCCCTCTCACAGTATCTTTCCGGCAGGTCAACCTCCCTTGTCCCTCCCCAACCCCAAT
CGTGCATTGGTGGGATCCCTCGGCCTGGCCTGCGCCCTCTTGCAATCCGGGACCTCTCGG
GGGATGTCGGAGAGCCGTTTTTGGCCCGACCCTGCAGGCCAGGCTTAGGGACCCCACTGG
GGCATGAATTCGTGCACCGGGCCTCTATAAATAAATAAATACCTATTCATTTCTCCCTGT
GTACATAACTCCCTTAAGCTTAAGAACAGACATACTTCTTAGACTAAAGTGAGGATTCAT
TGATTAATTTATTTAATCATTTATGTAAAAAATGTTTACAAAGTTCCTAATGAGCAGGTC
ACATCTCAGCCTTGGAGATACATTGACGAATAAGATAGTAAGGTTCCTGCCCTCAAGTTG
CTTAAATTCAAGTAAGGACTGTTCCACTGAGCATGTGCTTAATTCGTCATATTAGTAACT
TAGTGGTGTAATCTGGATTTCGAACAGTTACCTCATCTACACTGGAGAGGCGATGTTTCT
CTCCCTGCTAGATTCTTGTCAGGTATCCTCCAAAGGAAAACAAACTATCTTGAGGATTAA
TGTTCTCCGACTTTCTGGCTTTACCAACCCCACCTCCAACTCAAATCCAGAAAAATTTTA
AGAGAGGTGGTTGCTTACAAAAAATGGAGTTATGATGAAATACTATTTCAGAAAATTCTG
TTCCTGAAGTGGCTTATCTGTACATAGTTTGCTAATTCATGCCATTTTTAGAAGGTGTCT
GCAAGTATTTTTCTAGTTTGAGTATCCCATAGAAAGTTAATTTCATACTTCCATAGTTTT
ACATGCTTTGGTGAGCAAGCTAGTTCTACTTATATTGCTAGATACAAAGTTATAGCAGCT
AGTGCTCCTCATACATATCTAGTAAGTCTAACAGCTGAGAGCCTTTCAGATTGTATGTTA
ACATCATTACAAATATTCTGTCTTAGAGATAAAAAGGTTTTTAATTGTTTTTAAAAACCC
TCATGGTTTAAAGATAAGGCTGTGATCATCTTAAAATTATATAGATCTCTAAAAATTGCA
ATTATATTTTTGTCTCCTTTATTCTAG**ATAAAAAGTTGTTTTATGTTGGAAAAGACAGAA**
AACAAAATCGTTTAGTAATTATTTCAGAAGAAGAAAAAAAGAAAGTCCTAAGAGAATGCC
ATGAAAACAACACTGGAGCCCATCATGGCATATCCAGAACCCTCACTCTAGTGGAATCCA
ATTACTACTGGACTTCTGTGACCAATGATGTCAAACAGTGGGTATGGCTGTTATTTAGAA
TATTTTGAATAATGTATCTGTAATAGATATCCAAAATTCATTGCTCCAGATTTACTCTGG
ATAGCAGTTTTCATTTACCAGTTAACTGCCACGATATTTTGAATTTAATTAAAAAAGGCC
CGCCGCTTGTGACTTTAGATGCTTATGGTGTACAAATGGTTTAGTACCGTTTGTGTGGGT
AATTTAAAAAAAAAATTTTTATTGATTTTTTACAGAGAGGAAAGGAGAGGGATAGAGAGT
TAGAAACATCGATGAGCAAGAAACAACAATCAGCTACCTCCTGCACGCCCCCTACTGGGG
ATGTGCCCACTGCCAAGGTACATGCCCTTGACCGGAATTGAACCTGGGACCCTTGAGTCC
GCAGGCCGCCACTCTATCCACTGAGCCAAACCGGTTAGGGCTGTGTGGGTAATTTTTGTA
CAGAAAATTTTGTGTATTTTATTTTCCTTCAGAATTCTTCTTTAAAATCACTAAAGTATA
ATTTGAACTAGAACAAAGCATTATTTAAACATTATATTTAGACAGATTTTTTAGAACTAG
ACTTTCATCATTGATCTCTATATAGGCAGAATATAAATGGGGTTGAAATTCTTTTAACTA
AATGACCTCATTGATGGCAGTCATCCAATCTTGATGTGAATTCTTAAAGTGAATATGTTC
ATAAAAAGTCAGGCCCTGTGTGTGCTCTCATCTTTTGTGCCCATTTCATCTTGCCATAGA
AAATGTCAAATATGGCAGAAACCTAAAACCAGCTAAACGCAAAGTCAAGTTGTGAAGATA
ATGAAATTAGTCTGAGTTTTGTAACTTTGTAACCTACTAGGACATTCAACTTCTTACAAT
AAAAAGTTCACCCAGAAATTCTGGGATTCTTGCTGGTAGATGAACTGATTTCTACTCTTT
CTAGTACATTCAATTTGCAAATGGTAGGAATTCCTTGATATATATGTGAGATCCCTTTAA
TAATTATAATAGTGCATCTAAAGGATGGTCATTTTAGGCATAAATAATTAAACCAACACT
TAAAAAAGTATAATTTATGAGTTACCAGTTTATGAATCTATGCAGGTATTCCAAAGACCT
GGATATTTGTATTTGCCTTTAGAATCTGCCACAGTTCCTTTATAAACATTTGATGAAATC
TTTGTCAAATTTGAGGATGGATATGATTTTTGAAAATAACCAGGTTATTTGTATATAAGT
TGTAAAATAAAACTTTTGATAAAAAGTAATTATACATTGATATTTCTCTCTCTCTCTCTC
TCTCTCTCCCTCCCTCCCACCCTCCCTCCCTTCCTCCTTCTCCATCTCCCTCCCTTCCCC
TTCCTCTCTCTAAAAAATAATCTATAAAAAAGTGACTATAATTGAATAAGATTGTTTTAA
AATTTTGTTTAGGGGTTGGTTTAAAACTAGTTTCCAAAGTAGAATTCCAGAAATATGTTG
AACAATGACACCATCCATATGTTCATCCATTGATCTCATCTCATTCACATGAGAGCTCCA
TGATAGCATGGGTTTTGTTTTTATCCATTCTCTTTTCCCAGGACCTAAAGGAATTTTTGG
TATCTATAGATATTGTTTGAATAAAATTATGTGTTAATAAATATTTGTTGAAGTAGATGT
ATGAGTTAATTTAGAGTGATAATTTTTTTTTAAATGTTTTATTGATTTTTTTACAGAGAG
GAAGGGAGAGGGATAGAGAGTCAGAAACATTGATGAGAGAGAAACATCGATCAGCTGCCT
CCTGCACATCTCCTACTGGGGATGTGCCCGCAACCCAGGTACATGCCCTTGACCGGAATC
GAACCTGGGACCTTTCAGTCCGCAGGCTGACGCTCTATCCACTGAGCCAAACCGGTTTTG
GCTAGAGTGATAATTTTTTAAAAAACCTTTTGCTTATAGAAATTATTATATATTTACATA
CTGATTTCAAATTATAGCAAAGTTATCTTAAGATATATTCATTTCATAAATATTTATTGA
ATGTAAACCAGAATTATGTTCCATTTGAGTAAAACTCCTTATGATGGTCATTGAAGTCCT
AACCGGTTTGGCTCAGTGGATAGAGCGGCGGCCTGCAGACTCAAGGGTCCCAGGTTCGAT
TCTGGTCAAGGGCATGTACCTTGGTTGTGGGCACATCCCCAATAGGGAATGTGCAGGAGA
CAGCTGATCGATGTTTCTCGCTCATCGATGTTTCTAACTCTCTATCCCTCTCGCTTCCTC
TCTGTAAAAAATCAATAAAATATATATTAAAATAAAAAAAAGATGGTCATTGAATAGTAT
AGCAACTAATATTTACTTATTATCTTGAATAGGAGGTTTTATTTTATTCTAGAATAATCC
AAAAATTTCTTATTCAATACACAAAGTACCTATTAACTATTAAAGATTTATAAGACTGCT
TTTATTATGTTGAATATTTAAAATTTACATGTCTTTTCTTCACTGTGTGCCTGTTTTGCA
G**GTATATGCTTGTCATCATTGTCAGGTGGCAAAAAATACAGTTATTCTAGCACCTAAACA**
GCACCTCAAGGTGGAAAATCCATGGAGTATAGTTACTGTTGATCTAATGGGTCCATTTCA
TATAAGCAACAGACGTCATGTATATGCTATGATCATGACAGATTTGTTCACAAAATGGGT
TGTGATTTTGCCTCTGTGTGATGTTTCAGCATCAGAAGTTTCTAAAGCTATTATCAATAT
ATTTTTCTTATATGGACCTCCTCAGAAAATAATAATGGACCAAAGAGATGAGTTCATTCG
TCAGGTAAGACAGCTGAGGTCTAAGCTCTAAAATCAGTAAACATATTCTCGGGTGAGGAT
TATACAAACAAAAATCTGAGTAAAGAATTTTATTACATATATATTGCATATTCATTTACT
GACATAGATAATTATGTAAAAAATAGGTATTAAAACACTAAAATAAAAACCCATAAGGTT
AACCATGGAATTATTTTTAAGAGGAATAAGATTATAGATTTTTTCTTAATTTTTCAATTT
ATATGTATTATCAAATTGCTTTTATAATTTTAGTGATTAAAATATGCATAATTGTCTGTT
GCTTTTGTCATATAAATTACTAGTGCTCAGCAATAATTAGTTATCCTAATCTTCAAATAT
AGATGGCTCCTTCTAGACTCTTGCAGGTAGTGAAGCCCAAGTAACTAGATCTAGCCAAAG
AAATTGAGTGGATGGATGTGTGCCACTTTTACAGCCCGAGGTAGTGAAAAACTCGTGCTT
AATTCTTCAATCCCCCAATCTTACTCTTTTCTCTGGAGAACTGACAGAATATTCCAGATA
TTATACTGGAGAGTGATGGAATTTCTCTAATCCAGAATTGCTGGAGCACCTAGTGGAGAA
TAGTTGCTTTGGAAAGACATTTTGACCTACAACTGACTTTGAATGAGCAAACAGTAAAAC
TGTTGAATTAAGCCTCGGAAACTTGAATGTGTTTTGTAATTGCAACATAATCTTGACTAC
CTTGACTGACACAGCAGTGATCTGCAAAATAGGGTATGTAAGACCCTGTTTTGTTTGATG
ACAATATTAGAAATTCTGTTTATATTTCTTTTTATCTAATAAGAAAGAAATACAGCTTTA
CTAATAATGTGGACAGATTGATAATTGCATCCTGAGTCAATAAGGCAAATGATTGTGTGT
CACACCTACTGTGCCAAGATATCATAAGAGAAAAGTGAGAATCCTCAATATATAGGAATC
TTCAGTGGTTCCCTGTCTCTGGCTTTCAGCAATAATTGAATTTATGGCTATCGTGTAATT
TTCAGCAAAGTGAATCCTCACAGAATAGCCAGGTGGCTTAAATTTCTTGTAATAAAACAT
GATAATATTAATAATGCAGTCACCTATCAACCACAAGAAAATGATAGAGCTGACACTTCT
AATTAACTTGTGGTATTAGATCTTTCATAGACACATTAATGAAATGAAAATAAATATGAA
CTAATCCAATCTGATAGAAAATTGGCCACCAAAATTAAAAATTATAAAGTATACTATTTG
GAAATATTTGCTTTTACCTCTGTTAATGAGGAGCCTCACCTTAAGTGCATATACTGCTTC
AAGAAATTAAATAATGACAGCATAAAGCCAATATGAATCACCAGCAGTTTTTCCAGTCAT
GTTTAAAATCATGTGATAATCAATCCAGCACTTTGAAAATTTTACCAAAAGTAACAATAA
ATACTTATAAGTCTCTTTTAAGAGGTTAAATAATACAAAAAATTAAAAATCACACGATTG
GGAAAATGTGTTCTTTTTGCTGTGATTTAAATGGCTTAAATATTACAAAGAAAGCATAAT
GATTGCCAAATGTTTTATTTTGTCAGCAAATACAGAAAACACTGGTAAAGTGTTGTAATA
AGATCAGAACAGTGTACACAAAGTGGGAGATTTATCATATAGTTGGATGAAAATACACGT
TTCTATGGTAACTTGGCTTAGAATATTTGTTGAATTGTTATTCAATAAAATATAGATTTT
TTTTGTAAGCCTCTGAAGAAGAGAGAATAAACATTTTCAAATGTAAAATTACTTCTTTAA
TTTTAGAAATATGTCTTATGGAAAACCAAGATTGTAACCACTAGTGGGATGTCTGGTTTA
ATGGGAAGAAAAAGAGGCTATTGGGGTAAGGTTATCAAGGTAGCATCTCACAGATGGATT
CACTGGGTCATTCCTAGGAAATTTCTTGAAAGAAAGAAGTTGAAGCCATTCCAAAGGTGC
CACTTAAAACCATTGATTATGACCCCAGATTGAAGCTTACCCAAATATACCTATTCTCTG
GGATTTCTTTTTAGTTATTTTTTAATTAAAAGGAGATAGCTTACTACTACATAGTATACC
AGTATTAGCTAGAAATTGTATTCAGCTGCAAGTAACACAGAACACTTCTCTTCCTCCCAG
TATAAAAATTTAGGTAAAAGAAGGCCCAGGTTGGAAGGGTAACTCTGAATGTCATTGGGA
ACTGATACTTCTAGCTTTAAGAGGTGACTGTCAAGCTTGTGCTTCCAGTATGGCTGCCAG
AGCCCATGCCACAAAGTCTACATTTTAGAAAGAGGGGAAAGGTAATGGGCAAAAAACCCC
CCAAAAACTAGTACCAGCTGAGTCCACCCAACAACTACTAAAAGCCCAACAATTGCATTT
GTAACTTTTAAAAAAGATTTTTTTATTGATTTTCTTTTTAGAGAGAGAGGAAGGAAGAGA
GAGAGAAATATTGATGTTAGAGTGAAGCGTCAATCAGGTGCCTCCTGAATCAAACGGGCA
ACCTCTAGGTGCACAGGATGACGCCCAACCATCTCAGACACACTGGGCAGGGCTCTAAAC
TCATTTTGACTTCCTTTAGGTCGTGTACATTACAACAACAATAATAGTAACATTAAGAGT
TATAAATTGCCAAGAACTGTTTTAAGTCTTCACATTTACTCATTTAATTCTCAAAAGAAT

CCTGGGTGGGATTTTTCTGATTTTACAGATGAGGAAATTGAGTACACAGAGGTTTGGTGA
GTTGGCCAAGGTCATGTAGCTGATAATTGGATTATGTGACCTAGTGTTTTTTAGAAACTA
TTTACTGGATTAAAATGTTATTTGTACTCAGAGTTGATATTAAATTCTGTTCTTCCACTT
CCTTGATGTATGTGTGTCCCTGGCAAGGCACTATACTTAGAAAGCTCCAGCTTCTTATCT
TTAAAATGGGGTTAATATTACCTTATTAGATTGTCGTAATGTATAATGACAACAATATAA
AGCATTAGTCCATTGACCAGCACATAGTCAGGACTCAATAAATTATAATTATTATTTTAA
ATAAAAAATAGATGTTTTTATATAAAATGGCCATGGTCAATGCAAAAGGCAAGTTTCAAT
TTCTTTGAAACCTTTTAATCAAGGGAAAATAAAATCTTAAAGTCAGCTTTGTTTTATATA
TTTTTCAG**ATCAATATTGAACTGTGTGGATTGTTTGGCATGAAGCAAATTGTAATTTCTC**
ATGCCTCTCAAGCTGTTAACCAGACTGAAGGTATACCCAACACCATCAAAACATTTCTTT
CCAAACACTGTACTGACTACCCAAACAACTGGGATGATCACCTGTCAGCTGTTTCATTTG
CCTTCAATGTAACTCACTTGGTATGTGCCTTTTTGTAATTCTATACTTTTAAGTTATGAG
TATTTCTTGCCAAATAAGTGTTTTATTGTAAATTGTTGTTTATTTTCCTTAAAATAG**GAG**
CCTACTAAAAATACGCCATACTTTCAAATGTTTAATCGAAATCCTTATATGCCCGAGACT
TTAGATGTTCTCCACGAAGTGGATGGTGATGATACAAGTATGTTTGCCAAAATTCTAAGT
GCAATTAAAGAAGCTGATAAAATAATGGAGAATAAGACAACTTCAGTGGGCCAGGTGATT
CTATTTAATAGAAAACTCTATAACAGAAAAATCTATAAAGTGATATTTCAAAATTCTGTA
GCATATATACACACACGGATACAAACACACACACACACACACACACACACACATCTGATA
CATCCTTAAACCAAATTCTTTTTCCACCAAATTCTGCTTGTCTTGATTAAACATGGTGCT
TACTAGCTATGAAATTCTGAAAGACAGTTATAGGAAAAACATGCATTTTGGAATCAAGTA
GAGCAGAATTCAAATCCCTCATCTTACCGCTTTCCAGTTGTTTGACTTGGGCAAATTACT
TAATCTACTGCAGTTGACTTTGTTTGTAAAGTTGGGGTAATGCTGACTTTCAAATATTGT
TTTAAGGATTAAGTGGGACTCATACCTAACTTTGAAGTAGCATCTGAATAAATAATTATT
TATATTATTATCTGCATTTACCTTCAAGAAGCTATAGCTCTTTCATCCCCTTTGTTCTCT
CTCTCTTTTCCCCTGCCCCATACTTGTAG**ATGGAGAATAACACTGTTGATGAAACACATA**
AAAACAAAATCATTGTTAAAAAGAAACCAAAGCAGTTAAATCCATTTCAACTAAAAGTGG
GTCATGAAGTTTTAAGGCAAAGGAAAAACTGGTGGAAGGATGGTCGTTTCCAGTCGGAAT
GGGTTGGTCCTTGTGTCATAGACTATATTACAGAAAGTGGATGTGCTGTTCTGAGAGACA
ACACTGGGACTAGGCTTAAAAGACCTATCAAAATGTCTCACCTTAAGCCCTATGTAAGAG
AATCCAGTGAACAAGGTAAATATCTGTCACTTCTCTATACTTTTACTTTCTTTGAATGAG
GAGAGAAGTTAAAAATAAAACCCCACACATTTTCATTTTTATACAACTTGGTATAGTTTT
AGATCCCAGGTAGTCTGTTTTATTAGAATATCTCTTCTGGAACCATCAGGCTTAAAAGAG
ATTTTATTTTGATTGCTAGTGTGCTTCTCACACTTTCCCATAGTTCATGTGGGAGATACT
GAGCCGTTTCACAAAAGAATTAATGTGTTCTCTATGTTCATATTCAGTATTTTATGAGCA
ACTTTGTGTCTCATTTTAGCATGAAAAAACAGCAACTAAGATAATGCCAAAAACAAACAT
ACTGTCCAAAATCCTGTTGTGAGATAAATTTATTAGAGGACTTAAAAGAAATGTGATTAG
TTTTTAACATTGCATCATAGTCAGGTGGGTATGGGGGAGAGGGCAGAATGGTGTGTATTC
CAAGTGTTATAAGGTGCTATAAGAAGAGGTGATAACACCCTAAACAAAGTTATATATTCC
CCCAGATACGGTTCTGAGCAGTCAAGATATCCTGATTTCTTTAACCGTTTCTTTCATAGA
CTAGAGCTGAATGACATTTTGCTATGGCTTTCAGATTAAATCCCACTTTTGTGAGACTTT
TGTTTTGTCCGAGTGTATCTGCTCCCTAGAATTTCTCTGGGTTGGATAACTGAGATGGAG
TTCATTGGTACCTTAGCCATTCAACTTTGCCTTTTCCATAAAGAAAGATAAAGCATCAAT
ATTATTTGAATATGAATAACAATTTATGGAAGATACAATTTAGTATAGTTTTAAACAGTA
TAAATAGCTGAGTTCTTTAACAGTCTTAATAATAATTAGAAGAGCACCAGATAACCTACA
CTTTATAGGACACTAGAGGCCTAGTGCATGAATTCATGCACGGGTGGGGTCCAGCTGGCC
ACCTCCCCCGATTGGGGGGGGCAACCGGGGGTGGGGATGACTGGGGGGAGTGGCCACGGC
TGGCTGGGGGAAGGGGCCACAGGAGGTTGGCCAGCCAGCCCCGCCCCCGATTGGGGTGGA
GGGGCAGATCGGAGGCTGCCCCCCAATCGAGCTGGTTGGCTGACCACAGTGGGCGTCATA
GTGACTGGTCGTTCTAGTCGTTCTGGTGTTCTGGTCGCTTGGCTTTTATATATATACTAG
AGGCCTGGTGCACAAAATTCGTGTACGGGGGGGGGGTGTCCCTCAGCCCAGCCTGCACCC
TCTCCAATCTGGGACCCCTCGAGGGCAGTTGGACATCCCTCTCACAATCCAGGACTGCTG
GCTCCCAACCACTCACCTGCCTGCCTGCCTCGTCGCCCCCAACTGCCCTCCCTTGCAGGC
CTGGTCGCCCCTAACTGCCCTCCCCTGCAGGGCTGATCGCCCACAACTGCCCTCCCCTGT
TGGCCTGATCACCGCAACTTCCCTTCCCTGCAGGCTATCTTGTGGCGGCCATTGTGCCCA
CATGGTGGAGGCCATCTTTGACCACATGGGGGCAGCCATCTTGTGTTGGAGTGATGGTCA
ATTTGCTTATTACTATTTTATTAGATAGGATACTAGTGGCCCAGTGCACAAAATTCGTGC
ACGGGTAGGGTCACCATGCCTAGCCGGCGATCAGGGCTGATTGGGGCCTTCCTTCCCCAG
CTGCTGGCCGGGGCCTTCCTTCGTTTCACACCGCCTCCTGGTGGTCAGCACACGTTATAG
TGAGTGATCGAACTCCGATCTCCTGGTCGAACTCCCGAGGGGACACTTTGCATATTAGCC
TTTTATATATATAGATAGATGAAGCCCTTGGAGTCTGTTTGCTCAGTAATAGCTCAGTTG
TGGTTCTGATTTGGGTCACAGCTCTCATATTAGAAGCTCGTAGTGCCTTTCCATCTGCTT
GTGACAGATCCAGTTGCTCTATTAGTAATCTTATCTTGAACTATTTGAATTCAGAAAGCT
CATGGTACCTTCATTTTCTTTGTTAGAAGATAAGCACTTTATTTCTTGTGTTTCTTAATT
CTGAGCTATCCTTTATTATTTCTCCCTTCTGCAATTTAAGTAGAAAAAGCTTATGCGGAT
ACATTTCCTTTCTTCTTTAGCATGATTTTCTTCTTAACCTTTGACATTCAGCTTCTTTCT
AATGTGTTAACCTCATTAATACAAAATTTATATTTAAAGTATTCCTGTTTCAGGATCTGT
TCCAACGTTTTGCAATTTGCTCTTGCCAAAGAAAAAAGCCAAACATTTTGCTACTCCTGA
ATATTTTAGAATCCAGTTGTGTAGAACAGTTTCTAAACCGAGGCTGTATACTCTTTGCTG
CTTTCTTGAATTCTCTGCAGATACTTTTTCCAGTATATAAAGGAAGTTGCAATTTTAGTA
CTAGTGTACACATAAGGAACCTGAGATTGGTGGGTAAACTTGTTTTTCTTGTTAACCAAG
AAAGTGTTGATCTGGCCATCTTGGAATCTGCTTCGTGATTACTGTTTGGTCCTTTTTATG
TTTCTCTTATTTACAGAAATTCAAAATATAGTGGAACTTCAGTTCTCAAACTTAATTCAT
TTCAGAAGGCTGTTCAAGAAGCAGTTTGTTTGAAAACTGAATCCTTTTATTCCATTAGAA
ATAATGTAAATTGAATTAATCCGTTCCAGACCCCCAAATGATTCCCTGTTTTAAGCCTTC
TTATAGATAGTGCCTGTCTAATGTACTATTTAATCTATAAATAAACGCTTCTTAAATTCA
TTGAACCATCCCCCAATAGCCTTAAATGAATTTTTAAAAAAATCCTCACCCAGGTATATT
TTTTTCAATTGATTTTTAGACAGAGTGGAAGAGAGGGGGGCAGGGGAAGAGACAGAGAGA
GATACACACATTGATTGGACCCTTTGGTCCAAGGGCCCATTCACTAGCCAGGGCCTTAAA
TGAATATTTTAATATATCACTGCTTCTGAAATGCTATGACTGGCTAACTGCTTTTTGTTT
ATCCAGTCAGCAACAGTTTTTCTACCTGTTCAATTGCCTGTGATTGTGAGTACTTTCACA
CCCTTGGCAACATTAGCACTCTTTTTTTGTTTGTTTGTTTGTTTATCCTCACCAGAGGAT
ATTTTTCCACTGATTTTTAGAGAGAGTGGAAGGAAGGGACTAGAGACAGAGAGGGAGAGA
GAAACATCGATGTGAGAACATTGATTGGTTGCCTCCCACACCCGACCAGGCGGTATGTCC
CCTTGACCTGAATGGAACCAGGACCCTTCAGTCCTCTCACCAATGCTCTATCCACTGAAC
CAAACTGGCTAGGGCAACATTAGCGCTCTTGATGGTCTTTATGTTTTAAAATTGTGCTAA
TCATTTGATTTCCACAGCAACAAAAGCCTTCTGTCCTTTGTTGCCTGAGAGACACTTTTG
GACATGCTGAGGTATCAGCATGAAAGGGTTGTCAGGTCCAAAACTGAAGTTTTGGTTGAC
AACTGAGACATTTTTTTCAGTGAACTGTTTGATCGAGAACCGAATTGTTGGAGATGGGAG
ATGTTCCAGAAATGAGGTTTGACTGTAATGTGATATCGGCAGTCTTTTTTTCAATATCAC
ATATTTTCCTGAAAATCTTCACCTCAGAAGCTGATTTTAAAGAGTCTTGAACTAAATTTA
AAGCAAACAAAATGTCTTTGAATAAGCACCCCACTCTTTTATTTGACATACATTTGGATG
AGCTATAGCCAAAAGAACTATGAATTTGTCTGTACTTGTCATTGCTACTTTTACCAGCCT
TGAGATTTTGTAGTTGTATTAAAGGTTCATTGTCCAAAGCAGATATGCTTCTGATGCCAA
TTTCTTATCCATTCTTATCCAGAACATGGTGTAGAGAGAGCACATTCTGTACCATGTCTA
CAATGTTTCTCATTATTACTTTAAGCAATCCTATTTGTCATTTTGTCTCTCTAAATGACA
GGCATCTTTTCAAGATTAATTATTTTAACAAGAAATTTAATGATATACCATAGTGTCTCT
TTGTAATCATAATGTTCCCCTTTGAAATGAAAAATGTTGTTGAACCATTGCTTTATCCAT
TATAGATTTTAAATAGTTTCATTTGTTTGTTTTGTTTTGGATAGGAGTTATTGTGATAGA
TTGTGTCTGCCTCTCCCAAAAGATGTAGTGTGATGGCCCTTCACAATTTGTAAAATGGTT
TTCATTATATGTTGTTTCCTACTGCTGCTAAAGAAATTATCACACTTTTAGTGGCTTAAA
ACAATACCAGTTTATTATCTTTCAGTTCAGTAGCTTAGAAGGTCCAACATAGGTCTCACT
GGTAAAATTTTACTAGTAAAATCCAGTCATTGTCAGGGCTCTGTTCCCTTCTGTAGGCTC
TAGACAAGAATTTTTCCATGCCTTTTTTCACCTTCTAGAGCAGCGATTCTCAACCTGTGG
GTCGCGACCCCTTTGGCGGTCGAACGACCCTTTCACAGGGGTCGCCTAAGACCATCCTGC
ATATCAGACATTTACATTACGATTCATAACAGTAGCAACATTACAGTTATGAAGTAGCAA
CGAAAATAATTTTATGGTTGGGTCACAACATGAGGAACTGTATTTAAAGGGCCAGAAGGT
TGAGAACCACTGATCTGGAGGCTACCTGTACTGCTTAGCTTGTGGCCCTCTTCCTTTGTC
TTCAAAGCCAGCATCACATCACATCACTCCAACCTTCTATCTTCCACATTTAAGGACCGC
AGTTTTTGGGACCAACCAGATAATTCAGGATAAACTCCCCATATTAACATAGCTTGTTAG
TAACTTTAATTCCATCACTATCCTAGTTCATCTTTGCCATGTAAAGTAACATATTCACAC
ATTCCAGAGATTAGTACATGGAGATCTTTAGTGGCCCATTCTGCCCACCACAATAAGGTC
TACACCCATGAAACAAAACAAAGCTTAGTCTCTATATTATCAAGCCCATGATCTTGACTT
TATTATTTAATATAGTTTATGCAAAGAGCCTCCCTTTGACCTTTTAGTTATATAATCTGA
ACTCAGTCATTCAGTTCTCTGTTGTTAGTATTGGTTATTTATTATAACAACAACTATGTA
GAATCAAATTATGGTTATTGTTATTTTATCAGAGAAGATGAGTATGGCAATACATTCTAA
TGGTTTAGGATTATCAGGCTATATATAGGAATAGTCAAGTCATCTCTCTTCTGAATAAGA
AAATTAAGTATTCTGAAACCCTATACTTTTCTATTTGAAAAAATAAATTAATCCAAATCA
CTCAGACTTCTCTGAGGCTTTGATTTTAATTTATTTTTCTCCTTAGGGATATTTAAATTT
TTTTATTCTTTTTTTTTACAGAATAGTCATGGGGGTGTAAACTACAGCATAGTTAATATA
GTCAATAATATTTCATAAATAACTATGGTGTCAGATGGTACAAGGTTTATTGGGATGATC
ACTTAGTAAATTATATAATATCTAATCACTGGTGTACACCTGAAACTAATAGAATATTGT
ATGTCAACTATAACTGAAAAATAAATTAAAACACATAAACAAGCAAACAAAAAACTTTAG
AAAGGGTAATATGAACACAAACCTAAAAAAGTCTAAAACTAATTTGCTTCTTAAACTTAT

```
TTTTTACTCCTTAAAACTGCATAAACTGAAATACTGCTTTCATCAGACACAACTAAATCA
ATATATTGAGAAAAGAAAAATACTTAGTCTCTTGTGTAATGGGACTGAGTAGAAAAGTCC
TTTAAAAACGTTTGTTTAGCCCAGCCAGTGTCGCTCAGTGGTTGAGCACCGACCAGTGAA
TCAGGAGGTCAAGGCCCGGGTTGTGGGCTCAATCCCCAGTGGGGTACACAGCCAATCAAT
GATTCTCTCTCACTGATATTTCTATCTCTCCCTCTCCCATCCTCTCTGAAATCAATCAAA
ATATTTATCTCTCTATAATGAAGACAGAGCTTATCTTGTTCTTTTTTATATCCCTAACTC
AGAATTCTAGATCAGTACTATCCAATAGATATACAATACAAAAAATATGCAATATGTAAT
TTTAAATTTTCTAGTACATTTAAAAAGTACAAATAGGTGAAAATAATTTTAATAATATAT
TTTATATAACCTAGTGCAGTGGTTGGCAAACTGCGGCTGGCGAGCCACATGCGGCTCTTT
GGCCCCTTGAGTGTGGCCACGAAGTTTCAATCACACTGTACGTGCGTGCCTGCACGTGGT
ATTTTGTGGAAGAGCCACACTCAAGGGGCTGCAGTTTACCGACCACTGACCTAGTGTATC
TAAAATATTATTTCAATATGTATGTAAGGTCTCAAGAGTCACTGTTCTGGTAATTTTTTG
TAATCTTTTTTTGAAGTTAAAGAGCATTTCTATTGGTTCCAGTAGACTTAGACCATAGTA
GGAGTTAAACATATTTGACCTTTCTGAATGAATGAGAATATTGTGGGGTTTTTTTCTACA
CAAAAAAGGCTTGAAATCTCCAGAGTAGTTTTCTATTGCATTTGTGTAGAATTTGCTGAG
TATAATCCTTATTACAATACGTTACTTTTCTATATATATTAACGCCTAAGCGACCATTTG
ACCAGTAGCTATGATGCACACTGATCACCAGGGGGCAGACACTCAACGCACAGGCATGGA
AACATGAAACAGACTGATGAATCTCAGAGGGAAGTGGGGGGCAGGAAGAGATTAACCAAA
GATCTTATATGCATACTAGAGGTCTGGTGCATTTGTGCACTGGTGGGGTCCCTCGGCCTG
GCCAGCGGGGTTTGGGCCAAAACTGGCAGTCCAACATCCCCTGAGGGATCCCAGATTGTG
AGAGGGTGCAGACCAGGTCAAGGGATCACACCGGTGCACGAATCCATGCAAAAGGGCCTC
TAGTTTATAGTTTAAAAGAAAAAGTGTGTGTGTGTGTGTGTGTGTGTGTGTGTGTATCAC
ATATATAGAATGAGTATCACTTATATAGAACCTCTCTTCTGACAAATAAGTGTGGTAAAG
TCCATTTGTTACTAAGTCTAATATAACTAGTAAGTACTTAAGCTCAAGATCTATAAATGC
ATTTCTAGCATATCAAAGTGGTAACTTTATTTCTCTCTATATAAACTTTCAAATTCATAT
ATCATTTTATTCATTCTCTGAAATGTTTATTTAGTGGGTAGGAGAATCCAACGAAGTTAA
TCTTGCTAGTTAATTCTTATTACTTGGGATTTTGTTCTTCATTTAGCATTGTTTAATTTG
AAATGATGTTTAATTATAATTCTAAAAGGCAAACTTATGTTTCTTCTTTCAGACAGTCTT
TATCTCTTACAAAGTTCAGTAGTGGCAGATCATGACTACATTGGACTGCCTGAAATTCCA
GTTGGAGCATACCAAGCAAGTATTCTGGTAGAAGACGCAACTATTGGTGTAGCCGATAAT
GAACTATTGACATCAAGCAAGGATGGTGAACTATTAGGATATAGAAATACTAAAATCTCT
CCGTTAATAGAAGATAATAGTAATCTTGAAAAGCAGACATTCTGTTTGTTGGACTCCTCA
AACCAAGTCCTTGAGTATTTAAGTTAGTAAATATCAAAATTTATTTATAGTGCTTGTTGA
GAATATATAACCCCTTGAAACTTGATATTTTTTCCATTGAAAAAGGTTGTATAGAAGTGT
CTGGACACACTCTTAATAACTAAATTCTGAACATTTATTTTATAACTATTTATTGATCTA
AAGTTGTACCTTTTGAATGTTAGTGCATATATATAAGGGCAAAGAAAGCAGATAATATAT
AGAATTCACTTTTTCTTCTAAAAAAAATAGGCAATGCTTGTTTCTTAGGAAGTAAAGTAA
ATTTCAGCTCAAAACTTTCAGATCTTTTGATATGGTACCATGTTTTCAGTTTTAATTTTT
TAAAAAATATTTCTTTAAAAAATAAATTTGAACTATCTTGAATGGTATTAATAAATATTT
ATTCAATCATAACAATGAATTAAGTGAAATTTCTCTCTGTGTCACTGGAATGTCTAAAAA
CAATATTGTAGGGCTCTAGCCAGCATGGCTCAGTGGAAAGAGTGTCGGCCTATGGATTGA
AGGGTCTCCAGTTTAATTCCTGTCAAGGGTATGTACCTCTGTTGCAGGCTCGATCCGCGG
CTCCGATCCCCGGCTCTGGTCGAGGCG
```

==

Myotis Gin-1

total length: 18687 bp (18687 bp excl N/X-runs)
GC level: 36.80 %
bases masked: 6206 bp (33.21 %)

==

	number of elements*	length occupied	percentage of sequence
SINEs:	15	2389 bp	12.78 %
Alu/B1	0	0 bp	0.00 %
MIRs	3	552 bp	2.95 %
LINEs:	5	600 bp	3.21 %
LINE1	2	250 bp	1.34 %
LINE2	2	267 bp	1.43 %
L3/CR1	1	83 bp	0.44 %
RTE	0	0 bp	0.00 %
LTR elements:	2	568 bp	3.04 %
ERVL	0	0 bp	0.00 %
ERVL-MaLRs	2	568 bp	3.04 %
ERV_classI	0	0 bp	0.00 %
ERV_classII	0	0 bp	0.00 %
DNA elements:	10	1834 bp	9.81 %
hAT-Charlie	7	1564 bp	8.37 %
TcMar-Tigger	1	129 bp	0.69 %
Unclassified:	6	594 bp	3.18 %
Total interspersed repeats:		5985 bp	32.03 %
Small RNA:	12	1837 bp	9.83 %
Satellites:	0	0 bp	0.00 %
Simple repeats:	3	126 bp	0.67 %
Low complexity:	3	95 bp	0.51 %

==

SW score	perc div.	perc del.	perc ins.	query sequence	position in query begin	end	(left)		matching repeat	repeat class/family	position in repeat begin	end	(left)	ID	
762	21.2	3.3	9.2	UnnamedSequence	240	257	(18430)	C	Ves2_ML	SINE/tRNA	(9)	260	241	1	
302	6.1	2.0	0.0	UnnamedSequence	258	306	(18381)	+	(TAGA)n	Simple_repeat	4	53	(0)	2	
762	21.2	3.3	9.2	UnnamedSequence	307	562	(18125)	C	Ves2_ML	SINE/tRNA	(29)	240	1	1	
273	20.4	0.0	0.0	UnnamedSequence	1806	1854	(16833)	C	BAR1_ML	Unknown	(37)	272	224	3	
296	20.4	1.9	0.0	UnnamedSequence	1856	1909	(16778)	C	BAR1_ML	Unknown	(35)	274	220	3	
239	8.6	0.0	0.0	UnnamedSequence	2040	2074	(16613)	+	BAR1_ML	Unknown	17	51	(258)	4	*
752	9.9	0.9	0.0	UnnamedSequence	2073	2183	(16504)	C	BAR1_ML	Unknown	(197)	112	1	5	
335	33.9	4.1	0.0	UnnamedSequence	2297	2417	(16270)	C	L2b	LINE/L2	(2)	3373	3248	6	
1634	7.0	0.5	0.0	UnnamedSequence	3489	3701	(14986)	C	Ves	SINE/tRNA	(9)	214	1	7	
294	23.4	7.2	3.0	UnnamedSequence	4386	4482	(14205)	C	MARNA	DNA/TcMar-Mariner	(170)	416	316	8	

260	11.6	1.9	10.2	UnnamedSequence	4535	4640	(14047)	+	Ves2_ML	SINE/tRNA	151	248	(21)	9	
232	20.4	0.0	0.0	UnnamedSequence	4712	4755	(13932)	+	MARNA	DNA/TcMar-Mariner	410	453	(133)	10	
246	30.9	4.8	4.8	UnnamedSequence	4757	4902	(13785)	+	L2a	LINE/L2	3277	3422	(4)	11	
1775	4.6	1.8	0.5	UnnamedSequence	4943	5162	(13525)	C	Ves	SINE/tRNA	(0)	223	1	12	
1776	6.7	0.0	0.0	UnnamedSequence	5336	5558	(13129)	+	Ves	SINE/tRNA	1	223	(0)	13	
272	10.4	4.2	0.0	UnnamedSequence	6086	6133	(12554)	+	Ves2B_ML	SINE/tRNA	203	252	(0)	14	
238	22.8	4.9	2.4	UnnamedSequence	6438	6518	(12169)	+	MLT1J1	LTR/ERVL-MaLR	82	164	(280)	15	
482	30.0	7.9	0.9	UnnamedSequence	6837	7051	(11636)	+	Charlie7	DNA/hAT-Charlie	66	295	(2317)	16	
901	28.0	5.0	2.2	UnnamedSequence	7053	7410	(11277)	+	Charlie7	DNA/hAT-Charlie	330	697	(1915)	17	
691	23.3	6.7	0.8	UnnamedSequence	7429	7653	(11034)	+	Charlie7	DNA/hAT-Charlie	759	996	(1616)	18	
690	27.9	12.0	1.5	UnnamedSequence	7628	8028	(10659)	+	Charlie7	DNA/hAT-Charlie	971	1414	(1198)	19	*
334	17.5	3.9	5.0	UnnamedSequence	8466	8567	(10120)	C	Ves	SINE/tRNA	(4)	219	119	20	
570	20.7	9.3	0.7	UnnamedSequence	8675	8850	(9837)	C	MIRb	SINE/MIR	(0)	263	77	21	
470	28.5	7.7	2.3	UnnamedSequence	8909	9116	(9571)	+	MIRb	SINE/MIR	50	268	(0)	22	
297	8.9	0.0	0.0	UnnamedSequence	9789	9833	(8854)	+	(CA)n	Simple_repeat	2	46	(0)	23	
485	31.9	6.0	1.1	UnnamedSequence	9931	10098	(8589)	+	MIRb	SINE/MIR	22	197	(71)	24	
25	68.0	0.0	0.0	UnnamedSequence	10127	10151	(8536)	+	AT_rich	Low_complexity	1	25	(0)	25	
246	26.4	0.8	19.2	UnnamedSequence	12197	12313	(6374)	+	BAR1_ML	Unknown	211	309	(0)	26	
486	25.6	0.0	0.0	UnnamedSequence	13050	13178	(5509)	C	MarsTigger4	DNA/TcMar-Tigger	(1)	924	796	27	
395	21.0	5.2	8.4	UnnamedSequence	13301	13435	(5252)	C	Ves4_ML	SINE/tRNA	(8)	242	112	28	
1059	12.4	17.9	3.6	UnnamedSequence	13598	13815	(4872)	C	Ves4_ML	SINE/tRNA	(2)	248	1	29	
183	21.4	4.8	10.1	UnnamedSequence	14508	14590	(4097)	C	L3_Mars	LINE/CR1	(1251)	3171	3093	30	
1176	23.1	8.2	6.0	UnnamedSequence	14714	14920	(3767)	C	MLT1D	LTR/ERVL-MaLR	(7)	498	304	31	
1924	0.9	0.0	0.0	UnnamedSequence	14921	15132	(3555)	+	nhAT4a_ML	DNA/hAT-Charlie	1	212	(0)	32	
1176	23.1	8.2	6.0	UnnamedSequence	15133	15412	(3275)	C	MLT1D	LTR/ERVL-MaLR	(202)	303	1	31	
23	60.0	0.0	0.0	UnnamedSequence	15829	15858	(2829)	+	AT_rich	Low_complexity	1	30	(0)	33	
726	16.4	4.8	1.7	UnnamedSequence	15865	16032	(2655)	+	L1_Carn7	LINE/L1	6322	6494	(14)	34	
306	15.1	1.9	0.0	UnnamedSequence	16279	16331	(2356)	+	Ves2_ML	SINE/tRNA	1	54	(215)	35	
670	7.2	14.4	0.0	UnnamedSequence	16334	16444	(2243)	+	Ves3_ML	SINE/tRNA	83	209	(23)	36	
406	14.6	7.4	4.0	UnnamedSequence	16513	16633	(2054)	C	MER33	DNA/hAT-Charlie	(0)	324	200	37	
458	3.5	0.0	0.0	UnnamedSequence	16640	16697	(1990)	C	nhAT3_ML	DNA/hAT-Charlie	(0)	209	152	38	
463	12.5	20.0	0.0	UnnamedSequence	17071	17150	(1537)	C	BAR1_ML	Unknown	(0)	309	214	39	
455	7.5	11.0	2.2	UnnamedSequence	17153	17234	(1453)	+	HAL1-2_ML	LINE/L1	2404	2492	(141)	40	
636	15.7	3.7	0.0	UnnamedSequence	17241	17348	(1339)	+	BAR1_ML	Unknown	1	112	(197)	41	
291	13.9	0.0	0.0	UnnamedSequence	17348	17390	(1297)	C	BAR1_ML	Unknown	(259)	50	8	39	*
288	0.0	0.0	0.0	UnnamedSequence	17424	17455	(1232)	+	(TG)n	Simple_repeat	2	33	(0)	42	
33	67.5	0.0	0.0	UnnamedSequence	18410	18449	(238)	+	AT_rich	Low_complexity	1	40	(0)	43	
636	18.3	0.0	6.3	UnnamedSequence	18553	18686	(1)	+	Ves4_ML	SINE/tRNA	1	126	(124)	44	

>Horse Gin-1 (chromosome:EquCab2:14:67143758:67177474:1)
TCTCAACCCCAAGAAATGAAATAGGGAAGCAAGGGAGGAGCAGGAGGAGGGGAAATGAAG
AGGCGACAAGACGCTCCGGACTGTGCTGTCACTCCTCCACGCCCACACTGCCCTCCGCTC
CGGCGCTGGCGCTCAGAGGAGTGCGTGCCGAGGCGCCGCACAGCCGCCCGGAGACCAAAT
CACTGTCCAGCAAGGGCCGCCAACCTGGGGCGACCAGGTTCCGCATGTCAGGGCTCCTTC
GGGCTCGGGGCTCTCACCTGCCACGCTGCGGTCGCCACGCGAGCTGCTGGAGATGGCCGA
GCCCTTGCAGGTCTCGGTTTCTCCGTTAATGCCGGGGGCGGTAGGGAGGTGTGGGTCAAG
AGACTCCAGCTGTGAGCGTGGACGTGGGTCTTCCCTCTTCAGCAGCTGCTGAAGGACCAC
ACAGAGAGACCTCTGCACCGCACTACTACATCTAGCCAAGCTTGGGCACGCCTCTTTTTC
GCCCGCCTTGGGTCTCCCACGCAAACCTCACCGGGCGGAACAACTTCCGGCAGAAGCCAA
ACGATCCACTCTAATTTTCCGTGACCCAAGCGCTCCAGTCCCCATAAGTACCCACCTCTT
AACGGCACGGGTAGTTCCGCTTCCGGCAGCATCAGATAAGTCTTGAGAGGAAGCTTAAAT
TCTGTCGCTTGCATTTAGGACCACTTCGGTGAGTGGTCGTTCTGGTGTGCTTTGTCATAC
CTAATGATTTTTAAAGTGAGGCGTGACCTGACAATTTCAAAACCATTCGCGTCGCCTAGC
CTAAGGAAGGGTGGAATTGACTCCGGATATTTGCGCAGGCGCCCTTGGGGCGGGAGGGCC
TTGCTAGGGCGTTGCGGTGTCCCGAGGCAGCTGAGCAGAAAAGGGGCGAGTTCAGTTGTC
GGGCTTGGGGCGATGCCGACTCGGTGGAACCGGGTTTTACTTATTGGTCGTTTTCCTGCT
TGTGCTAAGTTTAACTGGACCTTGCCCTTAAGAATTAGTTAATGGGCCAACTAAACGCTC
TGTTAATGCTCCCTCGCAATTAATGTAGCGTCCGAGACTTTGAGCAAATTCAAAAGTTGA
TTGCGGCACTTGGCTGATACTAAAGGATTCTCCCCCTTACTTTATCACAGTTGTCATTTA
ATCTGAAATATTACGCTTTCCCCCAAATTAAAATCTGTGGAGGGGAGAAACGCGAGCTCA
GGGAGAATCTTGTCTCGTAGTTTTAAGGGAAAAGGACACTATCTCCGTGTGGGTTTTTAT
TGAATGAGAGTTTCTGAACCAAAGTAGACATAATTTTCAAAATGGAAATTCATTTATCAC
ATGGGGCTTTGTGTGTATGCGTATGGAGATGAAGGCATTTTAAACGATTTTGTACGTTGA
AGTGAAACATTGTAGAGAAATGTCTACTGTATTTAGATTTCCCAGTCTTTGGCCTTACGT
AAAATTGTGTTCTTCCATATTTCAGAGATTGAAATAGAATAATTGAGAAGTAAAAATTTT
GATCCCAAATTAACATGTTTATTGAAATATTAACAGATTTTAAGTTGGAAGAAATGCGTA
TTATAGCCTTTTAGAGGCTTAAATTGGTAGCCATGATGGGTTTTGCCCTTAGTGAAACAG
TTAAATCCTAATTTTAAAGTGGTGACTGTCTTTATTGCACATACAGGATAAAATTACTAA
GGGAATTAAATAATAAGTATTTACACGAGTATTTATTGATTTTATGTGATGCCGTACAGG
GACATTGTGTAAAAAGCTCAACCAGATATGATCCTGACATTTCCTCTTGCATAACTGGAA
GTTGCAACGTGCGACCTTCACTATCTTTCTTACTCCAGTGTGTTTTAATACACAAGCAAT
TATTATTTTAACAAAACTTATTGAAAATATGCGCATATCCAAAGAAGGCCTAATATAATC
TTCATTTTTATAATTCAAACCATTTAACTCTCCCAATCATTTAATAATATTTGGGTATAG
GAAGATAGTTTATTTATGTTTGTTCCAAATATTACAGCTTAAAACCATTTTCTCATATTA
AATACTTAAATAGACGGTTTCCAGGGAACAAATGGTGAATATACTTTGAAAAGAAAAGAC
TACTGGTTCCTTAAGTTATTACCGTACGTAAATAACAAATATTAGCAGCAAACCTTAGCC
ATCTACTTAAAAGCTAATTTTTTGTGAACTGATTTTTCTTTCTTACACAGTTTTTCACTT
GTCCTTGTCATCTGTCATTACTGCCAGAACTAGAAGCTTAGACTATAATCTCATTATAAA
GTATACAACTTTTGAGTCTTCTGGGTTTTGTTGTTACCCATAAAACATTATAAACATTGC
ATAAAACATAAGTTACAATTAATGAAAGGAATGAGATTTTTAAGGCTTTTTCTTTATACA
GATTTTGAGAAAAGAAAGGGTTCTGTGTTACTTTGAGCGGCTTCATATGGATGATAATAC
TTAAATATTTTAACAGTGATTATCTATTGCTTTACCATTTGCAAAGCACTTTTCAAATCC
ATTATCTTAGTGATCCACATAATTGTGAAAAAGGAGGGATAAGTATTGTCCTTTTTCCAA
ACGTGGATACTGAAGCCTAGGTATGTCACCCCCTAGAAATTCTGACTCCTAAAAGCCTTA
CTACCTTTTTAAGCACATCCTACTTGTAGTGCCATGTATATGCTAGAGAAGTTATGTTTT
CTGAAGCGAAGTAATAGTTACCTTTGGAACACGTGGTCTTTTTTAGTTCAGTGCTCCCCC
TCCTGTCAGGAAATGCAAGTAATTCTTTATTCATTCACAAGTAGTTGAATGTCCACAGCA
TCCCAGACACTGTGTGAAACAATGTAAACATTAAAAATTAATAAGACCTTCTCATTCCTC
ATTTATAATCTATTTATTACAAAAAACACTTTATAACTTTTCTAGCAGAGGCCTGAACCA
AGTATTATGAAACCTAAGGATGAGAAATAATTATTAGTGTAGCAGGGGGAAAAAACGTGA
AATTTGAAGCAGCAGAGACCAAGGTTCAGATGTCAGCACAGGCACTCTCTTAACTGTGTT
GCCTTGAGTAAACTGGATAATTTCTCTGAACCTCCATTTTTGTATCTACTCAGTGGATGT
AAGACTCGTACCTACTCCATAGGATGTGAGGATCATCGCATAAAGTACTGTATAAAGTTC
TCAATTAAAGAACTGCTTTTGTAGTTATTTAAAAGATTTCAAAAATTTTTTAAAAATTAT
TTCCTCTCCCTATAGTTTTGGCTTTTCGAGATGTCATATACATAGTAGCAAATAATATGT
TAGCCTTTCGTATCTGGCTTCTTTCACTTAGCATGATGATCTTGAGATTCATCCGTATTG
TTAGGTGTGTTAGTAGTTTGTTCCTTATCAATGCTGACTAGTATTCCACAGAGTGGATCT
ACCACAATTTGTTTATCCGTTCACTAGTTGATAAACATTTGGATTGTTTTCAGGTTTTGG
CTCTTATGAATAAAGCTTCCAAAAACATTCATATATAGATTTCTGTGTGGACCTATACTT
TCCTTTCTCTTGGGTTAAAACTGTTTTCCAACATATTTGCACTAGTTTGTATTTTCGCCA
GCAATGCTCTGCACCTTGGTAGTACTTAGTATTCTTAGATTTTCTTTTGTTTGTTTTTTT
TTTTATTTTTGATTGTTCTTTGCCATTTTGATGGATGTGTAGTGGTAGCTTATTGTGGTT
GTAATTTACATTTTTCATTGTCTAATGGTATTGAGCATCTTTTCATGTGTTTATTTGCTA
ACCATATCTTCTTTGATGAAGTGTGTGTTCAAATCTTTTTCCCATTCTTTGAATTGGATT
GTTTTCTTATTATTGAGCTTTGAGAGTTCTTTATAAATTCTGGACACAATTTATTATAAT
GTGTACTACAAATATTTTCTCAAACTGTGATTTCTCTTTTTATTTTCTTAACAGTATCTT
TATAAGAACAAAAGTTTTAATTTTTTCTTTTATGGATGGTTCTTTTTGTTTTCTGTCTAG
GAACCTAAGCTCACAAAGATTTTCTTTTTCGTTGTCTTCTAGAATTCTATAGTTTTAGAT
TTTATATTTAGGTCTATGCCCCATGATGAGTTAATTTTTACAGATGGTGCTCATTATCAA
TTGAAGTTCATCTTTTTTCATTTAGATAGATAGTTGTTCCAGCATCATTTGTTGTAAGTA
CTATCCTTTCTCTATTGAATTACCTTGGTACCTTTGTTGAAAATCAATTTGCCATTTCTA
TGTAGCTCTGTTTCTGGACATTCTATTTCTGTTCCATAGATCAATGGCTATCATTTTGCC
AATACCACACAGTTGTGATTACTATAGTGTTAAAGGAAGTCTTGAAACCAGGTACTGTGA
AATTCTTCAACTATGTTCTTGTTTTTCAAACTTTTTTAGCTATTCTAAGTCCTTTGGTTT
TCCATGTCAACTTTAGAATCAGCTTGTCAGTTTCTACAAAAGAGCTTGCTGGGGTTCCAA
CTGCGTTGCATTAATTCTGTAGATCAGTTTGCAGAGAACTCACATTTTAGTGAGTTTATC
CAGCTTTTTCTCTTTGTTAGGGTGGAAGTGACTGTTTTTCCAGCTTTCAGCATTCTAGAC
AGAAGATTATATTGGAATTCTTTTCATGCTATTTCAGAAAGGAATAATTTGCTTACATTG
TTGAGTATTATAATTTACCAATTTCAAGCTAAGTTAACCTTTTTTTTTTTTAATACTTTT
TGGTGAGGAAGATTGGCCCTGAGCTAACATCTGTTGCTGCTCTTCCTCTTTTTTTTTTTC
CCTCCTCAAAGCCCCAGCACATAGTTGTATATCCTAGTCATAAGTCATTCTAATTCTTCT

GTGTGGGTTGCTGCCACAGCATGGCTTGATGACCAGTGTGTAGGTCCGTGCCCAGGATCT
GAACCAGCAAATCTAGCCCACTGAAATAGAGTTCACGAATTTAACCACTCGGCCATGGGG
TGGCCCCTAAGTTAATCCTTTGAAGTCTTTTTCTTATCTGGAAAATGGGAGGTTGAATCA
CAATCTCCGGGCTTCCTTTCAGTTCTTTAGCCAAGATCTAATTTTTCTGTGCATGATGTG
ATATATTTAATTTTTCAAAAATCTGATCATATTTCTTTACTTCTTGAAAATTAAATATAT
TTGTATTATCCTTTGCTATGTTATATTTCTGTGAAAGATGCCAGGGAATGGAAATAATGA
AATAAACTGACCCTCCTGTTAATAATAATTAGTCTTTCACATTAGAAAGCCTTACTTCAT
GAACTTATTGGAAGCTAGAAATACATATCACCAAGCATGATGTATATGATTTAAGAAGTG
TAAAAATTAATTTTGCAAAGAGAAAAATCAAATAGGAAACCTCATTCTTTTGTGAAGTTA
CCTCTTGCCTTTTTTCTTTATTCAGGAAGTGCTTGGAAAACAGAAATGAGGAGTTGACCT
TGTAGGTCCTTTTGCGTTAGACTCTTAGAGAACTTGGTGAATGTTAAAAAAGTGAAACAC
CCATGTAGGGACTACCCAGATCCCACCTCCAGATAATGAAATAGATCATTAATCATACTC
CAGAAGCCCCCTTTCTTTCCTTTCCTAGTCACTACTCTTCCCTCATCCCCAAACCACAAG
TATGCTTTTTAAAACTTTATATTATAATCTGGTTTAAACCACAAATTAGGAAAGGAACAT
AATCCCCTAATTTCTCTCTGGGTATATGTGTTTGAAAATATAAAAATTAATTAAAATAAA
TGTATAGGTGTTTATACTAATGTGAAAATTAGATTGATTCTATAAAATATATTCATTATT
ATCTTCAAGACTGCTATGTTCATAGGGATATAAGGACGTATAAGTATGGTCCATTCCTAA
AGGATATTATGGTGAATATAACTCTAAACTTGACCTTTCCTTCCTCCTTTCCTCTTCTCT
TAGTGGAAGAGATCAAAAGACTAATGCCTCTGTTTGTGAGTTGGATCCTGTTCCCTACTG
CTGTGTCAGGAACTTTCTTCCATCAGTCATAATTTCTCTCCGTCTTGTTTCCAGTAGTCT
CTAGAAGCCCTTACCTTAGCATAAAGCTTTTCAGGTCATTCTCTTCTTGAAAGCAAAACA
ATCTCTCTTTAAAAATTTTTTTAATTGTAGTAAAATCACATAACATAAAATTTAGCATCT
TTTTTTTTTTATCATGAATGATATCAGGTAGGAATTCAACTTTTTATTCTAAATTGATAG
TCAATTTTTCATAAAATGCTATTTCTCTATTAGTTATACCATAAATTTTCATATATAAAT
GTTTGTTTCTAAACGCTCTATTCTTTCATGAAACTCTGCTTATATTTATATTAATAATAT
ACCATTTTATTACTATAACTTTATAGTATAACTTGATATCAAAAAGTTGCTTTTTTTAAT
CCCTGAAACATTTCCTTGGCTTCATTTCTCTATATATACTTACAGAGAAACTTTAGATTC
AACTTGTTAAATTTTATTTTCAAAAAAGACATTGGGATATTTGTTGATTCATCTTAACTA
TTTCTAAGTGTACAGTTCAGTGGCATTAAGTACATTCACATTGTTGTGCAGACATCAGCA
GAACTCTTTTCACCTTACATAACCGAAACTAGGTACTCTTTAAACAGTAACTCCTCCATT
ACTCCCAACCCTCTAGTGCCTGGCAACCACCATTCTACTGTCTGTCTCTATGAATTTATC
TACTCTAGGTACCTCATATAAGTGGAATTATACAGTCTTTGTCTTCTTGTGACTTGGTGA
CTTGGCTTATTTCACTTAACATAATGTCCTCAAGGTTCATCCATTTTGTAGTATTGTGTC
AGAATTTCCTTCCCCTTTAAGGCTGAATAATTACTCCGTTGTATGTATATACCACATTTT
GCTTAGCCATTCATCCATCTATGGAGGCTTAGGTTGCTTCTACCTTTTGGCTGTTGTGAA
TAATGCTGCTATGAACCTGAGTGTCAAAACAATCTTTTAACCCTACTAGAATGTAACTCC
ATAAGGGTTAAGAGGATATGTGTTTTGTTCGTTATTGCATTTCCAGTGTTGTCTGACAAG
TAGCAGGTAGCCAATAATATTTTTTAGATCAATGTATGGGCCTCATATTTGTCTCTAGCA
ACTGTCTTGTAGTTTCTTGCCTTCAAACTAAATTTAAGCTTACTAAATTCACTTTCTTGA
TGCCTCTTCATTCCTTACCTCATTGCAGACTAGTTTCCACCTGCCCTGCTCTACTATAAC
TATTGCCAAAACTCTAGACACATTTTACTTCTTATATCACTTAACCTCTCCTCTGCTTGG
CACATAAGAACTCTTTATTCCCTAGGCTTCTGTGGTTAGAACCTCTTCTAGCTTTTTTCC
TATCTCTCTAGCTATTCTCATGCCCATGAGGGATTTACAATATCTTTGGGAGAGAGACAT
ATAAATTAGTAGGCTCAATAAGATGTAGATTTTATAAAAGTTGGTACATGTTACTGTGAG
TTTACCAAGAAAGGTGTGCACAATTCTTCAGTTCTGCTTGAGAAGAAATAGATGTGGTCA
GGAGAGAATCATAGGTGAGTGAACTCATTAATGTGAATGTTAAAGGATAAGGATATGTAC
ACTGCTCTTCTTACTCAGTACACTTTCCATGAGCTCCTTCCTCCACTGCCTTTACTTCAT
CTACCGTCCACATGCTGAGTTCATCCGTGAGCTGAATACAACATGTTCAATGTCATCTTT
CTCCCTAAGCCAGAACTTCAGAAGTGATCCCAGACTCTTCTTTTCATACACTCCACACAT
CCAGTCAGTCACAGTTATCAGAAATATCTATCCATTTCCACTAACACTGCTTTGACTCAG
AATTTCATCATTTTTTCCTTACTTAGGCAGTAGTCCCTTAATTGACCTGTACTGGTCTTC
CTATCTCTTCTCCCCAGTCTATATTTCATATTGGTGAAGAATGGACTTTGTGGAATAGAA
ATATGATATATAACTCCTACTTATTCTTCAAAATTCAGGTTATCCCGGGTGGGGGTGGTC
AGAGAAGGCACTCTGTATGAAACAACACCTAACCAGGGTTTTGAAAGATGAGTAGGGTTG
GAGAGGTGGGGATGAGTTCTATTCCAGGCAAAAGTACCAATAAAAAGGTGTGAGCACACT
GCACAGAATGGTTGAAGAACTATGGAGATTTGTATAACTGGAGCTTAGAGTCTGGGTCAA
GAATGCCAAGAGATAAGTATGGAAGTATTGGTAAAAATCAAATCTTAAAAAATCTTGTAT
ACTGACTGAAGTGTTTAGATATCGTCAGGAATATCAGAAGGCCATTATTTCAAGTCAGGT
TAGTATTTTGATAAGACTTGATTTTAGGATGACCTCTGTGGCTGCAGTGAGGAGAGGGAC
AAAACTAGACAGGAGAAGCAAGGTCAGATACGAAGTGAGTCTGAACACAGTGGCCCTGGA
GTGAAGTCCAAGCTTCCACCTCATCCAAAATGCTTTGCCTCCCTCCCTAAGTGCATCTGG
GTGTCTGCACATCTGTTTTAATACTAGTTTTCTTTGCATATTCTGTCATAACACCATTCT
ATACTGTATAAATGCTGATTTACTCCTCTGTGCCTGTCCTTACACCATAAGCTCCTTGAG
GGCAGGGATTTCCTCTTTGATAGGAATACAATAACTTTGTATTTCTATCACCTAGTACAT
ATAACAATTTAAGGACTTGGTAAATGAATGAACAAACAAATAAAAAGACATATGGATGAA
TGGGCAAACAACTATAGTGGAGGGTAAAACACGGAAAGTTCTACAAAGGTGCAAAGAAGG
TCAGGGGAGGTCAAGATCACTTCAGATTCAGAAATCATGGAAGACTTGCTTCACTGAGGA
GCTAGAGCTTGATTTTGAGAGATGGGTAAGACTTAATCAGGTAGAATTGGATTGTTATGC
CACTCAAGGGAAAAGTGATGACATAAACCAAGGTACAGAGAATGGTTGGGATGTGGCCAA
AGGCGTATAAAGGAGACTGTTGAGAGAAGAAGCTGTAAGGCTTGTGTTGTGCCAGGTGGT
AGACACTAGGCTAAATAGTTTAGACTTAAGTCAGTAAGCAGTGGAGATGCCCAGGGGCAG
TCTCATAGTGTCTACCCTGTCAGGTAAATCATTTGGAAAATAAAAATGTGCTAAGGATGA
TTGATAATCTAAATAAGTTTCAAGGTAACTATGGCAAGCAAATAATCACCCCTTTAAGTG
TTCTCTTCTTTACTGAGCCAAGACAGCTGACCCTTATAAAAAATATAAGGTGAGAATAAC
ACACTTGTGAAAGGACTTGTGAAATAATGAAAATATGTAGAAGTTTTCTGACTTTTATGT
TTTCTGCCACTAGAAGAATCTCTCCCTTGATGATGACAAAAATAAGAAACCAAAAACCTC
AAAGAGCTGTCTTCAAAATTTTGTTTAGTGATTTCTTCTACTCCTTGACCTTTCGTACTA
TAGGCTTACTCTGAGAGGAATGAATTACTCTATTGTGATCCATTTTCAATTATTCAAAAA
TTAAGGGCTGGAAAGTGGAGAGATTAATTTCAGTAAATAGACTTTCTGAAGATGAATCTG
TGTGCATGTATTGTAAGTAGGAAAGCAAAGACTGTCCTAATGGCACAGATGCTTGAATGT
ACTGAGGGCTTCATTGTGTATTGAGTAATTAGATAAAGATTTTCTGGTATCCCCAGTGCT
ATAGACTGAATGTTTGTGTCCCCCTAAAATTCTTTTTTTTTTTTTTTTTTTTTTTTTATTA
TTTTTTTTTTTTTTTTTAATTTTTTTTTTTTTTTTTTTATTAATGTTATGATAGATTACAA
CCTTGTGAGATTTCAGTTGTACATTTTTGTTAGTCATGTTGTGGGTACACCACTTCCCCC
TCCGTACCCTCCCCCCACCCCCCCTTTTCCCTGGTAACCACCAATCAGATCTCCTTCTCA
ATATACTAATTTCCACCTATGAGTGGAGTCATATAGAGTTCGTCTTTCTCTGACTGACTT
ATTTCGCTTAACATAATACCCTCGAGGTCCATCCACATTGTTGTGAATGGGCCAATTTCG
TCTTTTTTTATGGCTGAGTAGTATTCCATTGTGTATATATACCACATCTTCTTTATCCAA
TCATTAGTTTCTGGGCATGTAGGCTGGTTCCACGTCTTGGCTATTGTAAATAATGCTGCA
ATGAACATAGGGGTGCAACGGACTCTTGAGATATCTGATATCAGGTTCTTAGGATAGATA
CCCAGTAATGGGATGGCTGGGTCATAGGGTATTTCTATTTTTAACTTTTTGAGAAATCTC
CATACTGTTTTCCATAGTGGCTGTACCAGTTTGCATTCCCACCAACAGTGTATGAGGGTT
CCTCTTTCTCCACAACCCCTCCAACATTTGTCGTTCTTGGTTTTGGATGTTTTTGCCAAT
CTAACGGGGGTAAGGTGATATCTTAGTGTAGTTTTGATTTGCATTTCCCTGATGATTAGC
GATGATGAACATCTTTTCATGTGTCTATTGGCCATATTCATATCTTCTTTTGAGAAATGT
CTGTTCATGTCCTCTGCCCATTTTTTGATCGGGTTGTTTGTTTTTTTGTTGTTAAGCAGT
GTGAGTTCTTTGTATATTATGGAGATTAACCCTTTGTCGGATAAGTGGCTTGTAAATATT
TTTTCCCAATTAGTGAGCTGTTTTTTTGTTTCAATCCTGTTTTCCCTTGCCTTGAAGAAG
CTCTTTAGTCTGATGAAGTCCCATTTGTTTATTCTTTCTATTGTTTCCCTCAACTGAGGA
GTTACAGTGTCTGAAAAGATTCTTTTGAAACTGATGTCAAAGAGTGTACTGCCTATATTC
TCTTCCAAAAGACTTATTGTCTCAGGCCTAATCTTTAGGTCTTTGATCCATTTTGAGTTT
ATTTTGGTGTGTGGAGAAAAAGAATGGTCAATTTTCAATCTTTTGCATGTGGCTGTCCAG
TTTTCCCAGCACCATTTGTTGAAGAGACTTTCTTTTCTCCATTGTAGGCCCTCTGCTCCT
TTGTCGAAGATTAGCTGTCCATAGTCCCCCTAAAATTCTTATGTTGAGATCCTAACCCCT
AATATGATGGTATTAGGAGATGGGACCTTTGATAGGTAATTAGGTCATGAGGATGGAGCC
CTCATGAATGGGATTAGTGCCCTTATGAAAGAGAGGGTAGGGAGTTCCCTAGCCTCTTCT
GCCATGTGAGGATGTAGCAAATGGGCCCTCTATGAACCAGAAAGCAAGCTCTCACCAGAC
ACTGAATCTGCTGGCTTGTTCTTGGGCTTCTCAGCCTCCAGAACTGAAAAATAAATGTTT
ATTGTTAATAAGCCACCCAGTCCATGGTATATTGTTATAGTGGCCTAAATGGACTAAGAC
ACCCCAGAGAAGGAAAATGGGGGGAGGGAGATTGTGGGAATTTAGAAGAGAGAGATTTTT
AAGTATAAGAGCAAAATATGAACATCACTGGTTTATGCAGACATTCATGTTTTCCAGTGG
CGAGTTTTTTTCTGTCAGTTGCAATTACCTACAACCCTCTGTGTTTTGGAACCTCTTCTT
CCTCTGAGGTATGAGACTTTGGTCTTTTGCTCCTGGAAGCTTTTTAGAGGCCTGAAAAGA
CTTCCCCTCCTCTTTTCTTTTTAAGGTCCTTCGGGAGATTTTGCAACCTTTACTGGTCTC
TGAAAGGAAGGCAATAAAGGATTTTATCTTTTTTTTTAATAAACCTCATCTTTCTACAGA
TATTATGCTCCCAGCTGGCTTGGATGGGATGTTCAAGGATTTCTTTTAGATGATTTAGAA
CATTGATGATACCAAAAGCCATTAAGGGAAAACAATAATTTGGAGTCATATATAGTGTGT
TATCATATTAAAGAATGTAGACCACAGTGATTTAATCTAAACTGAGAAAGGGCAGGTCTT
TCTGTGCCTTGCACTTGGGAGAGCTTGTGTCGCTATAGCCTAGAGATGAAAATAGGACAC
AGAGAAAAATGATTTTGGCTTGTTTGTTGGTTTGTTTTTTAACAGTTGAGGAGGATTAAT
ACATAGCTAGAATGACAGGCAGGCAAATGTAGACTGACGTAATCTATATCCCCTACTTGA
AACCACATGAGCCTTCCTGAATTGAAAATTGATGAATCTCTTCATCTCTTGGCAGAGGAA
AGGATGACATGAAAGTAAAGGCATCAAGGGGTCCTAAGTGACAGATTGGGAAGGAGGGGG

GTTAGTGTTTCACCACAGATAGTTTGAAAACACTTAAGATTTGGGGCAACATGAAACTGG
AAAACAATAAAAGCAGCTACAGGACAGGACTGAAGATACACATGGTAAAATCTCTTGATG
CCTTTACTATTTCCAATTAGGGGTTATTATGACTATAAAACGTCTGTGAACATTTTTATA
CTCACCTTTTGATAGTCATATGCTCATTTCACTGAGATGCAAACTTAAAAGTAGAATTAC
CACCCTTTGGGTAGTCATATGTTAGTTTTAGAGGAAACTGTTGCTTTCCCAAGATGGCTG
TAGCATTTTACACTCCCACGGACGATGTGTGAGTTTCATCTGTTACCTGTCCTCACGCTG
CCTCGTGTTGTTTTTCATTTTAGCCTTTCTGATAGTGTGTAGTTATAAGTCATTGTGGCT
TTAATTTGCATTGCCCTGAGGAGTAATGGTGTTGAGCACATTTTCACTTGCTTATTAACC
ATTTAAATATTCTCTTTTTTGAAGTGCCATGTAAGATTTCCCCCATTTTTTAAATTGGGT
TTTTATCCTTTCCTTTTTGATTTGTGGTAGTACTTTATATATGCTCCGTATGAGTGCTTT
GTTAGACATCTATATTAAAGATAGCTTCCCTCAGTCTGTGGATTACCCCTTTCCTTTCTT
AGTGGTATCTTTTGATGAACAGGTTTTAATTTTGATGAAGTCGAATTTGTTACAAATTTT
TTTCTTTTATGGATAGTGTTTTTTGTGTCCAGTTTAAGAAATCTTTGCTTTTCCCAAGAT
TTTGAAGATCTTTTCCTATATTTTCTTCCAGAAGCTCTATAGTTTACCTTTCACATTTAG
GTTTATGATTTGTCTGGAATTAATTTTGTGGAAGATGTAGGTAGATGATCAAGATTCATT
TTTTCCTATACAGATATACAATTGTTCCAACATTGTTTATTGAAAAGACCATTTCTTCCC
CCATCGTATTGATGCCTTTGTTGTAAATTAATTGACTATATACATGTGAATTTAATTATG
GACTCCCTGTTCTGTTTCATTGACCTATTTGTCTATCCTTATGCCAATACTTTGCTGTCT
TATTTACTGTAGTTTTATTGAAAGTCTTCAGTCTGATTCATTCATTTCTGAGATGTTTTA
GAAACTGTGAATAGCTACATTTGTTTGATTTGGGGTTACATCCATCTATTATAAAATGAA
AAAGTTCATATATTTAATATTTGATTGAAAGTGTATTAAACTTAGAACAAACTAATGGGT
TTTTTTAGGTTATGTTAGGACAGAATTAGCTTTGTAGAACCTTTGCTTTTAAAATGTCGT
ATACTTTATGCTAACCTAAGATTAAGGGATGTTAGAAATGTACTGATATTCTCACTGGAA
ATTATGAAAAGATCAGAAAGATTGTTATTGACTATGCCAAATGCTCTATGATAGGGGAAA
AGACTTAATTTAAGCCATAGTTCATAGGCACAGTCTATTCTGCATTATTCTAACATTCCC
TCTGCAGTGATGTGCTCTTTTAGCCTCATCGCTGCATCTTCTAGTCATCAGAAAACATCC
TTATTGTTTTTAGAAATTCAAAATTTTAGGCATTTATATTACTCAGTGTCCTAGCAGGAA
ATAGAAGATATACCTTGCTGGGATTTTAAAGAAGTTTAATAAAGCTCCTATTTACAGACA
TATGGACAGGGTCAAAGAAACCAACATGGCATCTGGAGACTAGCAACAGCAGGACATAGT
TACCACCTCTGGGGCTAGAGCAGCAGGAAGAAATGATGTTATAGACTTCCAGTGAGAAGT
AGAGACATGGAATATGGGCTACCTGACAGGAGCTATGGTTGTAGAAGGTTGCAGCTTAAA
CAAAGGAAGAACTCAGGAAATAAATATCCTGACCTCTTTTTTCTGCATTCTGATTGCCTG
CTGGTGCCTCCTTTTGGACAAGTCCAACCAGAAAACTATAAGTGCAATCTGTAAGAGTCA
GTCTCCTAGGGCATAGCAGCAGGGAGGAAAAGGCCAAAAATGGATCTGGATGGGTGGGGG
TGTTGGCAAACAGAATAACTAGCACAGAATAGTTCCTTTCAACTGAAAATATGAAAGAGG
GTTTTTTCTTCTTAAAGTAGTGGTGGCTTCCTATATTACTTAGGATCTAAAAATAGAGAA
GTTTTGCTTTGGCTAGTGCAGCCAGATTTTTCTTTATAACATACAGAAAGTACGCACAGG
ACCAAAAATCGCTTCTGTTCAAGAAAAAGCAGCTTATATGTATTTCAGAAGAGTGTGTGA
GATCTAGCATAGCTAAATATCTTCTCTGTCATTAATCATTTGAACATTTTTAAAGTATAT
CTTTCTCATAAATGATTTGAATAGTTTTTTTTTGCATTTTGCAATGACCATGAATATATT
TAGAAGTAATATAAAACTACATTTCTCCCCTTATAACTTATCATTTATTTAAATGTCACA
TCACAGAACCATTCTATGTAAGTAACATTTCAATTGATGATAATGAGAGACTTTAAGCAA
AATTTTTTTGAAGTGTTCTTTTTCATTTCCTTTTTTCCCCAGTATGTGGAAGTTTTTCAG
AATTTCTTATCACATGGAAGGAGACAAGATAATTTGTCATGATGTATCTCAAAAATAGCA
CTTGAATATTTTAGTTCATATGCAGCACACTGTTCCTTTTCTGTACTACAATTATCTCTG
TCACAGATAATATTCTGGGAATGTGCAACTCATTTGTCAGGAGAAATATGACAGAAGCCT
GAACTGAAATAGCAACTGAATTAGTTTATCATTTAAATTATATCTTTAAAT**ACCTTTTAC**
CTAGGTTCACGATGGTCCGTAGTGGAAAAAATGGTGACCTTCATCTTAAACAGATTGCTT
ATTATAAACGAACTGGTGAATATCATCCAACTACTCTGCCCAGTGAGAGAAGTGGCATAA
GAAGAGCAGCAAAAAAATTTGTCTTCAAAGGTAAAATTAATGCCCCAAATCATGAGTATA
GAGTTAATAGAGATGCTTCTCTATGTGTGTTCCATGTTTAATTCTGAGAATTTCTGTAGT
AGTTGGCTGTTGCTTAACAATTATCAGTTCAACTGATGATGCCACTCAGTCAAAAGCAAA
ATAAAAATCACACTGAAGCTAACTGAAGCCCAGTAAGCATGAAAGGGCATTTAAAAATCA
CAGACAAATGATACCATGAGAAAACCAAAACACTTCCCTAAATTAACCTCTCCTGCCGCT
GTCCTCAGATGGTTTCTACTCTAATGGTGCACTTTCAAGCACTCATGTCACTATCAGTGA
AGAACTTATCTGATGCTTAATAGAAAATGCTGGCAAAGTTAACTTGTTTGTTTACTATAT
TTATTTCTGTGGTCCCCCTCCCCCACTGCTATTAAACATTCTCAGAATTACATTTGTTTT
TGAACCCCTGAATGGATTAAATCTTTGAAAGGCACAGTGAAATATTTCTCTTGAAAGATT
ATGATAATTTTTCCCTCTCTCTCCTCCTCCTTCAAAGAGGAAGAAAAGTTTGGGATGAGA
TAAGCAGGAAATACAGTCACCAGATAACTAAATGAATGTTGGTGTTGTGAGTGGGAGTTT
TGCCTATAGGTACAGAATCAGGCTTCTTCAGAGCAGGTTAAGAAAATTCTAAGATAGTAA
GTAAATACCTGCTCATTTCTCCCTGTGTACACCCCTTCCAATAACTCCCTTATGCTTAAG
AACAGGCACACCTATTACCTCTTAAACTAAAATGAGCATTCATTCATTTATTTAATCATT
TACGTACCAAATGTTTACCGAGTTCCTACTGTGTGCCAGTCATACTTTAGGCCTTGGGGA
TTCACTGATGAATAATATAGATGAGATTCCTGCCCTCAAGTTGCTTACCTTGTAATTCAA
ATAAGGAATGTTCCACTCAGCATGTGCTTAATTAAGCATATTAGTAGCTTAAAGGTGTAT
TCTGGCATTTGGATAGTTCCCTCATCCGCACTGGAGAGCTGCTGGTTCTCTGTCTGCCCT
GGTTGCTTCCCAGCTGTCGGTTCTTCTCAGGTCTCCTCCAAAAGAAAGCAGAATTGCCGT
GAGGATTAAGGTTCTCCCACTTTCTGACTTTACCAACCCCACCTCCTACTCAAATTTAGA
AAAACTTAAGGGAGGTGGTGGCTTACACAAATGCATTTATGGTGAAATACTGTTTCGGAA
AATTTATTCCTGAAGTTGCTTGTCTGTGCATAATCCATTTGCTAATTCATGACATTTTTA
GGAGATGCCTGCAAGCATTTTTCTAGTTTGAGTACTCCCATTGAAAGATAATTTCATACT
CCTATAGTTATTATTCTGTGCTTTGATGAACAAGCTAGCTCTGCTTATATTGATAGCTAC
AAAATTATAGCAGTTAGTGCTCCTCCTACATATCTAGTAAGTAGCAGCTGAGGACTTGTC
AGATTGTATGGGAGCATGATTACAAATAAATATTCTGTCTTAGAGGGAAAAAAGTTTTTA
ATAAAAAAAAATCCTCTGTTTCGAAGATAAGGCTATCTTAAAACTATATAAATCTCTAAA
AATTGCAACTATATCTTTTTGGTATCGTTTATTCCAG**AAAAAAAGCTGTTTTATGTTGGA**
AAAGACAGAAAACAAAATCGTTTGGTAATTGTTTCAGAAGAAGAAAAAAAGAAAGTCCTA
AGAGAATGCCATGAAAATGACTCTGGAGCCCATCATGGCATATCGAGAACCCTTACTCTA
GTGGAATCCACTTACTATTGGACTTCTGTGACCAATGATGTCAAACAGTGGGTATGGCTT
ATGTATTTAGAATATTTTGAATAATGTCTTTGTAATAGGTAAACAAATTCATTATTGCTC
CTGATTTACTCTGGGTAGCACCTCTCAGTAAGTACTTTTTGTATGGGTTTTTAGAAGGAG
TTTTATTTCCCTTTAGAATTCTTCTTAAAATCACTAATGTGTAATTTGGACTAGAACAAA
GCATTATTTAAAGATTTTAAAATACCATATTTAGATTGGAGATTTTTTTGATCCAGATTT
TGATCACTGAACTCTTTTGTGCAGGCAGAATTTAAAGGGGAATGAAATTCACCTTTGACT
AAATCATTCCTTTTTGATGGCAGTCATCTTTTCTTGACAGGAGAATGTGTTCATAATAAG
TCAGGCCCAGGGCGTGCTCTCATCTTTTATGCCTATTTCATCTTGATATAGAAAATGTCA
AATATGACAGAAACTCAGAACCAATTAAATGCAAAATCAAGTTGTGAAGATGATGAAATC
AGTCTGAGATTTGTAACTTAATAATCCATTAGGACCTTCAGCTTCTTCTAATAGAAAATT
CACCTAGGAATTCTGCGATTCTTGCTAGTAGGTCAACTGATCTCCACTCTTTCTCATGTA
CTCTATATGGCACATGGTAGGAATTCCTTGGTATGTATGTCAGATCCCTTCAATAATTGT
AATAGTGCATCAAAAAGACTGTCATTTCAGGCATAAAGAATTAAACCAACTTTTAACAAG
GTATCTTTTATGAGTGCTCAGTTTATGAGTCTATGCAGGTATTCTAAAGACCTCGATATT
TATATTTGCCTTTAGAACGTGCCACACGTTCCTTTACAAGCATTCAGTGAAATCTTTGTC
ATTAAGAATGGGTATGATTTTTGGAAATAACCGGAGGTTATTTGTGCATAAGTTGGAAAA
TTAAAATTTTGATCAAAAGTAACTGTAATGCAATAAGATCATTTTTAAAATTTTCTTCAG
GAGTTGGTTTGAAAGTAGTTTCCAAAGGGGAATTCCAGAAATATGTTGAGCAATGGCAGC
ATCCATAAGTTGATCAGTTGATCTCTTCTCACGTAAATGAAAGCTCCCTGACAGCATGGG
GTTTGTTTTTGTTCATTCTCTTTTCCTGGGACCTAAAGGAATTTCTGGCATCTATAAATA
TTGTTTGAATAAAATTATGTTTTAATAAATATTGTTGGAATAGGTAAATGAATGAACTTA
CTACAGTGATAACTTTAAAAGAAAAAATCTTTTCATTATGGAAATTAGTGTATATTCATA
TACTGATTTAAAATTTATAGCAAACTTGTTTTAAGATCTATTCATTTAATAAATATTTAT
CGAATATAAACCGGAATTACATTCCACGTGAATAATACTCCTTAAGATGATCATTGAATA
GTAATAGCAAGTAATATTTGCTATTATTTTCTGAATAGAAGCTTTTATTCTATTCTGAGA
ATAGTCCAAAAGTTTCTCCTTCAAAACGCAAAGTACTGTTAACCATTTTAAAGTTTAGAT
TTATAAGACTGCTTTTATTATGTTGACTATATTAACATTTGTGTATCCTTTCTTTATTGT
GTGCCTGTTTTATAG**GTATATGCTTGTCAGCATTGTCAAGTGGCAAAAAATACAGTTATT**
CTAGCACCCAAACAGCACCTTCTCAAGGTGGAAAATCCATGGAGCATAGTTACTGTTGAT
CTAATGGGCCCATTTCATACAAGCAACAGAAGTCATGTGTATGCTATAATCATGACAGAT
TTGTTCACAAAATGGGTTGTGATTTTGCCTCTATGTGATGTTTCAGCATCAGAAATTTCT
AAAGCTATTATCAATATATTTTTCTTATATGGACCTCCTCAGAAAATAATAATGGACCAA
AGAGATGAGTTCATTCATCAGGTAAGACAAATAAACTACCTAGGTCTAGGAGCATATTTT
ACTCACCTTTCGGTGCCCAGCACACCTGTGCATTTTAGGGTGCTTAATAAGTTTCGATAA
ATTTCTACAGATATAGAGATTTAATATTGTATAGTACAAACGTAGTTGGACTGGAAATTA
AGGGACCAGAATTTTAGAACTTATTGTGCCACTAAGTATTTGTAAACCTATCTTCAGGTT
TCCTCAAACTAAAATAAGGAGGTTGGACCAACCAAATAATCTTTGTTTACTTCTAGCTTA
AAAATTCTATGATTGATATGTAGAGGGAGAATTGACTACATAGAGAAATGCATCATGTTC
ATGAATGAGAATATTCAATATTGAAAAGTATGAATTTTTTCCCAAATCAAAATTAATTTA
TGTAATTAATGAATTTCTAATCAGCATTAAAAAGGAGGGAGCTTTTTCTCTTGTCCTGGG
CTTTTTCCACATTAAAGTTAACCTGAAAAAATAAGCAGTCTAGAATATATACGAAAATTC
TAAAAAAGGAATATAATAACTACTACCCTTAAGGCATATTTAAAAATTTTTATAATTGAC
AGGGTACTGGTATAAGAAAAACTCAGAGGGAGCTGGACTCTCTTGGTTCACAGCACCTTT
GATATCTCAGTCCGAAAGAAATTCTAAAGTTATATTTATTAAGTAGTTAGGTCTTGTGTC

AGGGAGTCCCTAAGATTATCTCCAGGTTTTATGGTTTACTAAGAGTACTCAGCAAATGGT
TATACTCATGGCTAAGATTTAATATAATAAAAGGATGCAAAGAAAAGGCTCCTGGGGAGA
AGTCCAGGGGAAACCAGGAACAACTTTCCAAGATAGCATCTCCCAGTGGAATCACACAAG
ACTGGTTTAATTCTCTCAGCAAGAAGTTGTAGCAGCACATGTAAAATGTTATCTACCAGG
AAAGCTCATTAGAAGCTCAGTGCCTAGGATTTTTACTGGGGGCTGGTTACATACACACAC
TCTGCCTGGCATGTGGCAAAATTCTAGACTCCCAAAGAAAAGAAGGTGTTCAGCATAAAC
TGCTGGTTCGTATAAACAGTTTAGGCACAGTGAGCCACCCTTATCAGTTAGGGAATGGTG
GAAACCCTCCTGAAATCCAAGTTCCCGGACACTGGCCAAGGGCCAGCCTTGTAAGCAGGC
CTTTCAAAGGATAGCAGCCCCAGGTCTGCTATGTTAACTGTTTTGTGCACAGGTCCAAAC
AACTAAATAAGTATTTACATCCTAACTACTTAACCGTTAAAAAAAATAATACTCATAAAT
TGAAAGAAAAAATATTTTTATTTCATTCTTAGGTAAACACAATTACTTACTAATGGGATG
TGTGTATGTACCTGTTGACCACTACTCAATTTCTCAAACCTTGGGATGAAACTGAATGCT
CATTTCCTGTTTTACATTGATTTTTACAGAGTTGCTCTTTATCACAGCATCTACTGAAAA
ACCAGTTCTGTAAAGATAAGACATCAAAAGGAATGTAGCCTTATCTAATGTTGAAACTGT
AAACTACCTTTAGCTAGTAGTTTTCAAGGTGTCCAGTAGATGTTAAGGGGGCCCCTTGGC
AGAGTTTGGTAAGCCTGGTTAAAATGATAGAGCTGGCATCACAAATGAGTGGAGTAGGGA
AGGATTATTTAATAAATTTAGTTAACAATTGGCATAAGTAACAGCTTCCTAATTGACTTT
TTTTAATAATCTCTAGAGATGGAGGTAACATTCTAAGCTTAGGAACAGTGATATGAATTT
CAGTGGAAAAATCGCTAAATTTGGCTGCATATAAATTAGAAACTTACGTGTGGTAAAATA
GCCAAAACATATAAAGGCCAAGAAATAGACAAGCAAAAATAAAGCAAATCCATGCAGGAA
AGGGCTAATGTCTTTACTATAAAATATTCAAACAAATTAAAACATTTAGTCAATGGCAAA
AACATAATTTGCAAGAGAGAATAAAAATAATAAACATGGAAAAATGTTCAAATTCAATAG
TAATGAGTAATGCAACTTAAGACCATTTTTTGCTTATCTTATCAGCAAAAATAGTAATAA
TATCAAATAGACTCTAGGTAAATTATATAGCCTTCCATTACTGGTAACATTATAACTTGA
AGCTAACTCTTTTGTAAAGCAGTTTGCAAAAAGTCCGCTAAGAGCTATTTATATTTTTGT
CTTTTTGTTAGCAATCCTATTGCTAACAATTTTTTCTAAGGAACTTATCCAAAGAATAGA
GATAATTTTTTTATGATGCTATTTACCACAATGCTATTTATATTATTCAGAGACAGGAAA
CAACCTGAATATCCAAAAAAAGGAAGTATTTCAGAAAAATATGTCTAGCCACATGATGAA
ATATTTGTTGCCATTAAAAATGATGCTTACAAGGAAACAGAAGTAACACGAACAATGTTT
ACCACATTAAGTTAAAAATTTAGATAAAGAATTTTATTACATAATGTTATATATACATAT
ATTTACATAGATAACTGTGTAAAAGATATAAAAAAGTAAAATAAAAATCCTTAAGATTAC
CAAGGAGTTTTTTTAAAGAATGATGAGATTATAGATTTTTACTTAATTTTTGAGTTTAT
ATGTGTTATCATTATGTTCCTTTTATAGTTTTCAGTGATTAAAATATACATAATCATTTA
TTGCTTTGTGATATAAATTGCTAGTTCTTAGCAATAACTCATTCTCCTAGCCTTCCAAAC
GTAGAAGGCCAGCCTTCGAGCCTCCTGCAGGTAGACAAGGCCAAGGAACTAGATTTAGCC
AAAGAAATTAAGTGGATAGATGTGTGCCACTTTCCGCCTGAGGTAGTGAAAACCTCATGC
TCAGTTCTTCAGTCTCCGTGTCTTACTCTTTCTCTGGGGGGTTGACTGAATATTCCAGAT
ATTACGCTGTAAGGTGGTGGAATTTCTCTCATCCAGGATTGTTCACCTAAAGGGGAACAG
TTGCCTTGGAGAGACTGACCTCCAGCTGACTTGCAGGAGCAAGCAGTAAAACTGTTGCGT
TAAGCCACTAGGACTTGACTGTACTTGGTACCTGCAGCATAATCTTGCCTGCCTTGACTG
ACACAGCAGTGGTCTGCAAAATGGGACGTGTAGGACAATCCAGTTTGTGGGAATGGCAAT
ATTAAAACTTCTATTCATATTTCTTTTTTATCTAATAAGAAAGAAATGAAGATTTACTGA
TAATGTGTGCAGATTGATAATAGCATCCTGAATCAACAAGGCAGATTGTTGTGTGTCACA
CCGGTATGCGAAGGTATCCTGAGAGAAAAGTGAACATGTAGGCATCTGCGGTGGTTCCCT
CACTCATTCTTTTGTTTTCAACAGTAATTGAATTTATGGATATCACCTGGTTTTCACCAG
ACTACTCCCCCACAGAATAGCCAGGTAGCTTAATTTTCTTGCAGTGAAATACGATAATAA
CAATAATGCGATCGCCTGTCAACCACAAGAAAATGATAGAGCTGACACTTCTAAACTTCT
GATACTGGCTCTTTCATAGACACATTAATGAGATGAAAACAAATATGAACAAGTCAGATC
TGATGGGAAATTGGCCACCAAAATTCAAAATTATCAAGGAAACTATTTGAAATATTGATT
TGCTTCCACCTTTATTAATGAAGAGCGTCACCCTAAGTACGTAGAGTGCTTCAAGATAGT
AGATAAGGACAATATGAAGCCCTTATGAATTGCCAGTAGTTTTTGCAGTCTACTTAAAGT
CTTGTGATAATAAGCCCAAGACTTTGAAAATTTTACGAAAGGTAACAATAAAAAGCCTCT
GTATGAGGTTTAATGATATCAAAAAACTAAAAACCACATATGATTGAGACAGTTTGTTCT
ATTCGCTATGATTTAAATGGCTTATATATTACACAGAAAGCATAATGGTGACGAAATAAA
ATGTTTTCTTTTGTCAGCAAATACTGACAGAAAATGCTACTAAAGTGTTGAAATAAGATT
AGAATATTTTACACAATGTGGGAGATTTGTTACACAGTTGGATGAAAATACAGATGTTGC
CAACACAGCTTAGAGTATTTGTTAAAGTGTTTCAATAAAATGCACAAAGAACTTTTTGTA
AACCTCTCAAGGAAAGAGAATAAATGTTTTCAAAGGAAAAATTACTTCTTTAATTTAAAA
AATATGTCTTATGGAAAACCAAAATTGTAACCACTCATGGAATATCTGTTTTGATGGGGA
GGAAAAAGGCTGTTGGCGTCAAGATAGCAAGGTAGGATCTCACAAATGGACTCATTGGAT
CATTTCTAGGCAGTCTCTTGAAGCAAAGAAGTTGAAGGCTTTTCCAGAGGTCAGTAGTTT
AAAACCCTTGATTGTGACCTTGGATTAAGGCTGACCCAAATGACACCAATTTCTGGGATT
TCTTTTTAATTGCTTTTAAATTAAAAGGAGGCAGCCTAAATTGGCACATATATTAGCTAG
GAATTGGATTCAGCTGCAAGTAACAGAAAACTCCTGTCTTCCTCTCGATATATAAACTCC
AATAGGGGTTTATTTTATACTTAGATAAAAGAAGGCCAGGCTGTCCAAGGTTGGAAGGGT
AATGGAATGTCATTGGGAACCCATGCTTCCAGCTCTCTGCTTCTCCATCTTTGAGATGTG
ATTCTCAGTTTTGTGCTTACAATATGGCTGCCAGAGCCCATGCCACAAAGTCTACATTTC
AGAAAGAAAGAAGGGGGAGAGCAATGGGCAAAAAAAGCTTGCAACAGCTGAGTCTGCCCA
ACAACTTTTAAAAGCCCAACTTCCACTTTTTGGCTAGAACTTTATCATGACCACCACTAC
CTGCAAGGGAGCTTGGGAAATGTGATTTTGTTTTTATTCATTTTTTCCTAATTTATTAGC
TTTACCTTTTTAGTTACTCTGATACTACTTGTCTTACAATTAAGCAACAAGAAATATAAT
AGTCTTCCTTTTTTTTGTTTTTTGAGGAAGATTAGCCCTGAGCTACCTACTGTCAATCCT
CCTCTTTTTGCTGAGGAAGACTGGCCCTGAGCTAACATCCATGCCCATCTTCCTCTACTT
TATACGTGGGACGCCTACCACAGCATGGCTTTTTTGCCAAGCAGTGCCATGTCCGCACCC
AGGATCCGCACAGGCGAACCCCAGGCTGCCGAGAAGCGGAATGTGAGAACTTAACTTCTG
TGCCACTGGGCTGGCCCCTATAATAGTCTTCTTTTAAAAATCAAGTTGATCAGCCAAAAG
TTTACCCAGTTAAAACTAATAAGAAATAAGTCTGACGAATAAGGTAGGAAAAATAAGTTA
TTTTACATTTAACAGGCTTACTGTTGGAAGTTTTCTAAACTTGTTTTGACTTCTTTTAGG
CCTTGCACATACTAAATACAAAAACAACAATAATGGTAACATTAAACGTTATAAAGTGCT
TACTACTTGGCCAAGAATTGTTTTAAGTGCTTTACAAACATTTACTCAGTTAATCCTCGA
AAGAGTCCTGGGTAGAATTTTCCTCATTTGGCAGTTGAGGAAACTGGTTTAGTGACGTGG
CTATGGTCACATAGCTGTTAACTGGATTATGTGACTTAGTGTTTATTTGAGAAACTGTCT
ACTGAATTAAAATGTTATTTGAACTCAGAGTTGGTGTTAAATTCTGTTCTTCCATTTCCT
TGTTGTGTGTGTGTCTCTGGCAAGGCACTTAACCTTAAAGCTTCATTTAGCTTTTTATCT
TCAAAATTGGGTTAATAATGCCCCATAAGATCGTTGTAAGCTATAATGACAGTAATGTAA
AACATTGTGTGTTGACTAGCACATAATAAGGACTCAATAAATGGTGATCGCTGTTTTAAA
CAAAATATGTATGTTTTTATATAGAATGGCTATGGTCAATGAAAAAGGCAAGATTTAAAT
TCTTTTGAAAACTTAGAGTCAAGGGAAAAATAAAATACAAATTTTAAAGTCAGCTTTGTT
TTATATATTTTGCAG**ATCAATGTTGAACTTCGTGGATTGTTTGGCACAAAGCAAATTGTA**
ATTTCTCATGCCTCCCAAACTGTGAATCCAACTGAAAGTACACCCAGCACCATCAAAACG
TTTCTCTCCAAGTACTGTGCCGACCACCCAGACAACTGGGACGACCACCTGTCAGCCGTT
TCATTTGCCTTCAATGTGACTCATGTGGTATGTGCCTTTATACAGTTCTCTGCTTTTGAG
TTATGACTATTTCTTGGCTAGACAAATGTTTTATTAAAAATTGTTTATTTTCCTTAAAAC
AG**GAGTCTACTAAAAACACACCATATTTTCAAATGTTTAATCGAAATCCTTGTGTGCCTG**
AGACTTCAGATATTCTTCGAGAAGTGGATGGTGATAATACAAGTATGTTTGCCAGAATTC
TAGGTGCAATTAAAGAAGCTGATAAAATAATGGAGAATAAGACAACTTCAGCAGACCAGG
TGATTCTATTTAATAGAAAACTATAATCTAAAAATCTATACAGTGATTTTTTTTTAAATT
TCAGCATATATACACACACACAGATGCACATACACATACATGCACATACATATGTTGTTA
AACCAAATTTTTTTCTGTCAAATTCTGCTTGTCTTGATTAAACAGTATTCTTATTAGCTG
TGACATTCTGAGAGACAATAATGGGAAAGCCATGTATTTTGGAATCAAGTAGGGCTGAAT
TCAAATCCCTAGTCTTATTGCTTTCCAGTTGTTTGACTTGGGCAAATTACTTAACCTATC
TCAATTTACCTTCTTTGTAAAGTTGGGATAATAATACTGACTTTGGAATATCATTTTAAG
AATTAAATGGAATTCATGCCTACTTTTTATAGCATCTCAGTAAATAACCATTTTGTTATA
ATATTACTATCTGCATTTACCTTCAAGAAACTGTACCTCTTTCATCCTTTTGTTCTCTCT
CTTTTTTTTGTAG**ATGGAGGACAACAATTTTGATGAACTAAATAAAAGCAAAATCATTGT**
TAAAAAGAAACCAAAGCAATTAAATCCATTTCATCTAAAAGTGGGTCATGAAGTTTTAAG
ACAAAGGAAGAATTGGTGGAAGGATGGTCGTTTTCAGTCCGAATGGGTTGGTCCTTGTGT
CATAGATTATATTACAGAAAGTGGATGTGCAGTTCTGAGAGACAACACTGGGACTAGACT
TAAAAGACCTATCAAAATGTCCCACCTTAAGCCGTATGTGAGAGAATCCAGTGAACAAGG
TAAATATCTGTCACTTCTCCACATTTTTACTTTCTTTGACTGAGAAGTTAAAAATATAAG
AAATTCTCCCTCCTTTAGTTAGGTTTTCTTTTCCTTTCTCATTTTTATAAAATTTGGTAC
AGTTTTAGATACCAGGTGGTCTGATTTATTAGAATGTCTCTTCTGAACTGTCAGGCTTGT
AAGAGATTTTATTTTGATTTCTAATGCACTTTCCTTACACTTTTCCACAGTTCATGTGGG
AGATAATGAACCTGTTCCACAGAAAGAAGAATTACATCTTCTCTGTTTTCACTTTTGCAA
TATTTTATTAGTCACTTTGTGCCTCATTTTAGCATGAAAAACAGCAATTAAGAAAATTAA
TGCCCAAAACAAACATACTATCCAGAATCCTGTTGTGAGATAAATATATTAGGGAACTTA
AAAAATGTGATTAATTTTTAATAATCTATTATTGCATCATAGGTCATGGTGGGTACAGGG
GAGGGTACCGAGTGGGATGTGCTCAGAATGCTCGAAGGTGCTAAAAGAAGTGATCGCACC
CTAAATAAGGTTGTACTTGCCCCAAAATATGGTTTTGAACAGTCAAGATATTGTGATCTC
TCTAACCCTTTCTTTTCATAGACTGGAGCTAAATGACATTTTCCCATAGCTGTCATATTA
AATCCCACTTTACTGAGACTCTTTTATTTGGTCCTATTGTATCTGCTCCCTAAAATTTCT
CTGGGGTAGATAACTGAGAGGGAGTTAATTGGTACCTTAGCCAGTTAATTTTTCCTTTTC
TATAGAGAAAGATAAATCATGCATATTATTTGAATATGAGTAACAATTAATGGAAGATAC

AATTCAGTACATTTTTAAACAATATAAACAACTGAGTTATTCAACAGGCTTTTAAGTAAA
TAATAATTACAAAAGCAACAACAACAGATACCTACACATACCCTGTTTTTTAGGATATGA
AGCCCTTGGTGGCTGCTCAGTAACAGCTCAACTCTGCCTCTGATCTGTGTCACTACTGTT
GTCTTAAAAGCTTTTAGTGCCTTTCCTTATGCTGTGACAGACCCACTTGCTCTAGTGGTA
AACTTATCTTGGACCGTTCTAATTCTGAAAGCTCACAGTACCTTCATTCTCTTTGTTAGA
TGATGAGTGTTTTCATTCCCCAGGTGCCTCTTCTCAGTTCTGAGCTACCCTTTATAATTT
CTCCCTTCTGCAAGTTAAGTAGAAAAAAAAAAAGATTATACCTGTTGTCCTTTCTTCTTTA
GCCTGATTCTCCCCTTAATCTTTAGCATTCAGGATTTGCTCCCTTCTAATGTGTTAACCT
TCTTTAATTAATACAGAATTTACGTATAAAATGTTGCTGTATCAGGATCTGTTTCAACAT
TTTGCGATTTGCTCTTGCCAAAGACTTTCTTAGAAAAGAGGCCAAACATTTTGCTACTCC
TGCATATTTTGGAATCCAGTTGAGTAGAACAGTTTCTAAACAGAGACAATATAATTTTTG
CATCATGCTTGAATTCTCTGCAGATATTTCTTCCAGTAATAGGAAGGAAATTGCAATATT
AGTACTGGTTTACATATAAGGAACCTGAGATTGATAGGTCAAATTGTTTTTTCTTGTTAA
CCGAAGGAAGTACCGATCTGGCCTTCCTGCAATCTAGGTCGTGATTACTATTTGGTTCTT
TTGTGTTGCTCTTATTTGCAGAAATTCAAAACAAATGTGATTTCTGCCATCTTTTCTTCA
GTATCATATATTTTCCTGAAAATGTTCACCTCAGGAGCTGATTTTAAATAGTCCTTGAAC
TGAATTTAAGGCAAACAAAATGTGTTCCAATAAGCATCCCGTTTTTTTATTTGACGTAAA
TTTGGATGAACTTTAGCCAAAAGAACTATGAGTTTGTCTGTCTGTGCTTGTCAGTGCTAC
TCTTACCAGCCTTGAGATTTCGTAGCTGTATTAAAGACTCACTGTCCAAAGCAGATATGC
TTCTGTTGCCAACTTCTTATCCATTCTTATCTAGAACATGATGTGGAGAGAGCACATTCA
GTACTAGGTCTATAATTTTCTCATTGCTATGTTAGGCAGGCCTGTTTGTCATTTTGTCTA
GTTTAATGCCAGGTACCTTTTCAGGACGATTTTAACAAGAAATTTTATGGCATACCATAG
TGTCTCTTTGCAATCATAATGTTCCCCTTTGAAAGGAAAAATATTATTGACCCATTATTG
TATCTGACATAGATTTTGAGTAGTTTTATGTGTGTGTGTGTTTGTTTTGTTTTGGGTAGG
AGTTATTGTGATAGATTTGTGTCTGCCTCCTGTAAAGGTTTGAGTCTGATTTTCCTTCAC
AGAAAAACAAGTGAAATGTTTTTCATTATTTTGTAAGTTTTCTATCGCTGCCATAACATT
ACCACAAATGTAGTGACTTAAAACAACACAAGTTTATCATCTTACAGTTCTGCAGCTTGG
AAGTCCAACACAGGTCTCACTGGACTAAAATCAAGGCATTGGCAGGGCCGTGTTCCTTTT
GGAAGCTCTGGAAAAGAATCTGTTTCCTTGCCTTTTACACCTTCTAGAGGCTGCTCACGT
TGCTCGGCTTGTGGCCCTCTTCCTCTGTCCTCAAAGCCAGCAGTGGTGGATAGAGTCCTT
CTCACAGCGCGTCTCCCCAACTTCCTCTTCTTCTTCTCTCTTCCATATTTAAGGACCCTT
GTCCTTGGGCCTACCCAGATAATCTCCCTATTTTAAGGTCAGCTAGTTAGCAACCTTAAT
TCCATCACTACCCCAATTCTCCTTTGCCATGTAAGATGACTTATTCCCAGGTTCCAGGGG
TTAGTACATGGACGTCTTTAGGGGACCATTATGCTGCCTACCACAGTTATGGTCCACATG
CATGAAACAAAACAAAGCTTAGTCCTATATTATCCAGCCCATGATCTTGGCTTTATTATT
AATATAGTTTAGACAAAGAGCCTCCCTTTGGCCTCGTAGTTATCTAATCTCTAAACTAAT
AATTCAGTTCTCTGTTAGTATTGGTTACCTCTTATAATGACAATTATATAGTATCAAGTT
ATAGTTATTAGTTGTTATTTTATCATGAGAAAGGATGTACTAGTGTGGCAACACATTCTG
ATAGTTTTAGGATTATCAGGCCATACATAGGATTACTCTAGTGACCCGCCTGCAGAGTAA
GAAAACTAAGAATTCTGAAACTTTGTACTTTTCTTTCTATTTGAAGAAATAAATTAATCT
AAATCAGACTTTTCTGAAGCTTTGATTTTAACTTTTCTCCGTAGGGAAATTTAAAAATGT
ATTTCTCAATGTCATTATTTTACTCCTTAAAACTACAGAAGGAGAAGTATGTCAAAACTG
CTGAAAAAATTTCCTGTAATGTCTTGAAAATATCTCCATTTTTAGGAAAGTAAAAACTTA
ATTCATGCCTGAGTGGTTAAGTTCACATACTCCGCTTCAGAGTCCCAGGGTTTCGCCGGT
TCGGATCCTGGGTGTGCACCTAGCACCACTCCTCAAGCCATGCTGAGGCGGTGTCCCACA
TAGCAGAACCAGAAGGACCTACAAATAGAATATACAACCATGTACTGGTGGGCTTTGGGG
AGAAGAAGAAGAAAAATAAAAAGATTAGCAACAAATGTTAGCTCAGGGTCAATCTTTAAA
AAAAAAAATTATTCCATCTGGTCAAAACTGAAATAATACTTTCATCAAACAGATGTCATA
GTCATAACTAAATCAATAGAATGAGAAAAGAAAAATACTTCAGTAAAGTCTATAGTGTAA
TGTGACTGAGTGGAGAAGTCCTTTAATATGGTGTTGGTTTACCTCTGTCATAATCTGTCT
TGAGGGCAAAAATCTGTCTTGTTCTTTTCTATATGTCTAACTCAGAATTAATCTGAATCA
GTACTGTCCTATAAATGTATAATACAAAACTTATGTAATATATAATTTTAAATTTTCTAG
TATCCACACTAAAAAATTACAAATAGTTGAAAATAACTCTAATAATATATTTTATATAAC
CCAGTGTATCTAAAATATTATCTCAACATGTACATTAGGCCTCAAGAATCTTTTTTCTGG
TTTTCTTTGGTTTTTTGTGCTTTTTTGACGTTAAAGAGCATTTCTCTTTCTTCCAGCATT
TACTGGACCGTAGTAGGAGTTAAAACATATTTGACCTTTCTGAATGAATGAGAATTTTGT
TTTTTTCTACACAATCAATGCTTGAAATCTCCAGAGTAATTTTCCATTGCATATAAAGTA
ATTTGTGTAGAATTTGCCCTCTATAGTTTTATTATAATAAAGTACTTTAAAGTTTATCAC
TCATTTAAAAGAAAAAGTATGTGTGTGTATATACTCAGATATATATGTGTATATAAATAC
ACTTATATAGAAGCTCTCCTCTTACAAATGAGGTTGGTAAAGTCCATTTGTTTCTAAGTC
CCTTGTAACTAGAAGATTCTTAAGCTCCAGGTCTGTGAATGCATTTCCAGCAAATAAAAA
TGGTAACTTCTAAGAGCTTTACATATATGCATATTTTCCCAGTTGATATATGTTTCCTGG
ACATATTTTTCTAAAATAGACCTGCTTCATCTGTTTGGAAAAAAAATTTTGAGATAGAAA
ACTTCCTTCATCAAAAATCTCTGTAAGAATATTTAGAAATACCTTAAGTTCTGTGGTACG
TGCTGTCACGGATACTTTGCAGCATTGTTCAGGCCTTGAATCTATGGACTTCTCCATGGC
TTATTAGAAAAATTTGATCAGCAACACTTTGACCATACCTAACCTTTTTAAGTTCACTTA
TAAACTACTACCTAGCATTCTCCGGTATTAACAGGCCAACAGCAAACTTTGGTGTTAAAA
GTGTAGCAACACATTCGAAAAGTATTTCAATTTCATTGACCATTTCTCTTCCTTCTTGCT
GCTATTGCACATAGCACTTCTCATGGGCTACATGATTTTTCTTTATCTTTCATAATTATG
CCTCTTGTCCAGCAATTCATCCCATACTAACATAACAAAATTCATGGTCTTACCACTTTC
AATTATCCATATTTTTATTTCTGTCATAAATATGTGTATTTCTTAACAGCATTTTCAATG
TAGTTATTACTTTTGCATTCATAAGAATTGGTGGGATTAAACTTAGAAAACTTCTCAAAA
CTAAAATACCAAGAATACTTTGTCTGATGAAGAACCAAATAGGCAAAACTAAATGTCCTC
ACAAGCCCGAGTCCACTTATGCCATCTGTTGGTAGAGAGCATAGCTTGGATTCATCTAGT
GAAGTTTGTAAATTGAAAGTTCTTAAATGTATAAAATTCTGGGAGTGGAATAGGCCCAAG
TGAGCTTTTCACATAGTATGAGGATGGCTAGAGTTGGCTCATGTTCCAGAATATTTGTCT
GCTCTGAGAAACTTTCATTGATCTCCAATTTAAATGGGCAATTAATCTTCCTTTTGTTAA
TTCTCGAACTAAAATTTGAGGACTCTTTAAGCTATTAAAATCTTCAAGAAAATCTGTTAA
TGTTTTGGGAGAAAATCCAGAAAGAGAAGGCAGGATCCAGGAGGTTAATCAGCTGTGGTG
GAAGAGTTCCTTGTAATGAATTAAATGTCAGGTAGGGAGGAATCCTTTTATCTACATTAA
CCTTTATTTATAAAGATGCTACTGCTTTATCAGTTTAACATTCACTTTGAGCATTTTTTT
TCCTTGTAGTGATGATAAATTTGCTCAAAATAGAATAAAATGCTGACAAGAGAAATTTCT
TGTCAATATTTCAGTATGGAGTTAGCTACACCTTACAAGTTTATAAGTATTTTGTATTAA
GTTAGACTTAAATAAGCTGAGCTTTTAAAACTACTTTACTGCAGCCTACCTGCTTACACA
TTCCTATCATATAAGACTGATTCTAGAAACTTTATCTTCATTTTAGTTTATGACTTTATA
TTGTAACAATTTGACATTATTGTCCTAATATGGGTTCTATAAAGATTGAAAAATTATCTA
AAGTTGTAAATGTAAGGATTCAGTAATTGTTTTGTTAAAATATCTTTTAAGACCCCATTA
ATTCACCCAAAAATGCTTTGCAAAAGCATATTTAAGGATACCAACATTAAAAATATAGTT
TTATAACTTTGTTTTTAATCTATACCATAATCCTGAGCCCCTTCCTTGCCCACACCTACC
AAAGACAGGTCAAAGAAACAGGAAAACTTGACTCTTTTAAAATCCTTGGTCTTTCCTGAA
TTCTATATATATGTACTGTCAAGCTTATATATCATTTTACTTACTCTTTGGCATGTATAT
TTATTTAGTAGATAGGAGAACCCACCAGAAGTTAAACTTGATGGTTAATTTTTATTACTT
GGAATTTTGTTCCACATTTAACATTGTTTAATTCTTTTTAATGATGTTTGATTATAATTC
TAAAAGGCAAACTTATGATGTTTCTTCTTTCAG**ACAGTCTTTATCTCCTGCAAGGTTCAG**
TAGTGGCAGATCATGACTACATTGGATTGCCTGAAATTCCAGTTGGAGCATACCAAGCCA
GTATTCTGGTAGAAGATGCAACTATTGGTGTAGTCGAGAATGAATTACTGACATCAAGCA
AGGATCGTGAACTACTAGAATACAGAAATGCCAAAATCTCTCCATTGATAGAGGATCATA
GTACTCTTGAAAAGCAGACTTTCAGTCTTTTGGACTCTTCAAACCTTGAGTACTTAACTT
AGCAAATAACGAAATTCATTTAAAGTTCTTCTTTAGAATTACAACTCCTTGAATCTTGAT
ATCTTTTCCATTAAAAAAGGTTGTATAGAAGACGTATCTTTACACACTCTTAGTGACAGA
TCCTAAAGGTTTATTTTATAACTATTTATTTATGTAAAGTTGTGCCACGTTTGAATGTCA
GTGCATATATATAAAGGCAAAGGAAGTATATAATATATAGAATTTACTTTTTCTTCTAAA
AAGAGAACAGGCAAAGCATGCTTGCTTCTTAAGAAGTAAACTAAATTCCAGCTCAGAACT
CTCAAATCTTTGTGTGGTATTGATCCATGTTTTCAGTTTTAATTTTTTTTTAATATTCCC
CCCTTTTAAAAAATAAGTTTGAACTACCTAGAATGAGATTAATAAATATTTATTAAACCA
TAACAATGAATTAAGTGAAACTTCTTTGTATCATTGAAACTTCTAAACACAGTATTGTAG
GAAAATATTTAGAGTTGTTTAATGAGGTCTTACAGGATACTGGTACTTTTACCTTGTTGG
ACAGGCGTAGGATTAGAATCTGAAACTGTATGTTCGTTTGAAAACATTTGTGCATTTCCA
TAATATGTATTAAAGCCAAAAGCATCCATTAATAAGATTTTTCCTGCAATCATAAAAAAG
TAAATAACGATAAAATAATGTCAAAGATTTTCAACTATAATTCCTATGATATGTTTATCT
TCATTCAACAATATTTGTTATAAAGCACTTTGAAGCATACCGTGGGAAAATCAAAAGATG
AGTCAGATGTGTGCCTCATCCTCATGAGGGACTTACAGTCTGGTGAGGAATATAGAC

==

Horse Gin-1

total length: 33717 bp (33717 bp excl N/X-runs)
GC level: 35.57 %
bases masked: 13171 bp (39.06 %)

==

```
                 number of      length   percentage
                 elements*    occupied  of sequence
--------------------------------------------------
SINEs:                10         1822 bp    5.40 %
      Alu/B1           0            0 bp    0.00 %
      MIRs             7         1088 bp    3.23 %

LINEs:                17         5221 bp   15.48 %
      LINE1           10         3776 bp   11.20 %
      LINE2            7         1445 bp    4.29 %
      L3/CR1           0            0 bp    0.00 %
      RTE              0            0 bp    0.00 %

LTR elements:          8         2640 bp    7.83 %
      ERVL             2         1009 bp    2.99 %
      ERVL-MaLRs       5         1620 bp    4.80 %
      ERV_classI       0            0 bp    0.00 %
      ERV_classII      1           11 bp    0.03 %

DNA elements:          7         1981 bp    5.88 %
      hAT-Charlie      6         1767 bp    5.24 %
      TcMar-Tigger     0            0 bp    0.00 %

Unclassified:          0            0 bp    0.00 %

Total interspersed repeats:     11664 bp   34.59 %


Small RNA:             3          734 bp    2.18 %

Satellites:            4         1283 bp    3.81 %
Simple repeats:        2           97 bp    0.29 %
Low complexity:        5          127 bp    0.38 %
==================================================
```

SW score	perc div.	perc del.	perc ins.	query sequence	position in query begin	end	(left)		matching repeat repeat	class/family	position in repeat begin	end	(left)	ID	
351	28.3	9.1	0.8	UnnamedSequence	2480	2600	(31117)	C	MIRc	SINE/MIR	(21)	247	117	1	
422	34.7	3.9	2.0	UnnamedSequence	2976	3183	(30534)	+	MIR	SINE/MIR	3	217	(51)	2	
22	66.7	0.0	0.0	UnnamedSequence	3205	3240	(30477)	+	AT_rich	Low_complexity	1	36	(0)	3	
663	20.0	0.7	0.7	UnnamedSequence	3241	3386	(30331)	C	L1ME1	LINE/L1	(217)	5962	5817	4	
236	25.6	4.5	5.3	UnnamedSequence	3393	3524	(30193)	C	Sat-1_TS	Satellite	(318)	2918	2788	5	
1190	23.5	9.2	2.8	UnnamedSequence	3558	4278	(29439)	C	L1ME1	LINE/L1	(750)	5595	4821	4	
243	32.0	3.4	2.2	UnnamedSequence	4279	4454	(29263)	C	Sat-1_TS	Satellite	(1277)	1959	1782	6	
483	25.2	0.9	2.4	UnnamedSequence	4455	4609	(29108)	C	L1ME1	LINE/L1	(1503)	4643	4468	4	
1160	18.5	8.4	0.4	UnnamedSequence	4749	4987	(28730)	C	ERE1C	SINE/tRNA	(13)	258	1	7	
227	25.9	5.2	0.0	UnnamedSequence	5009	5066	(28651)	+	MIR3	SINE/MIR	125	185	(23)	8	
382	23.3	2.0	11.5	UnnamedSequence	5491	5664	(28053)	C	L1ME3A	LINE/L1	(73)	6169	5989	9	
27	59.3	0.0	0.0	UnnamedSequence	5735	5761	(27956)	+	AT_rich	Low_complexity	1	27	(0)	10	
279	6.5	2.2	0.0	UnnamedSequence	6136	6181	(27536)	C	L1MB2	LINE/L1	(0)	6171	6125	11	
294	25.0	9.4	0.0	UnnamedSequence	6280	6407	(27310)	C	L1MAB_EC	LINE/L1	(1475)	5419	5280	12	
2755	10.8	3.0	3.0	UnnamedSequence	6530	6988	(26729)	C	L1MB3	LINE/L1	(41)	6142	5684	13	
366	24.5	12.8	2.3	UnnamedSequence	7001	7117	(26600)	+	L2a	LINE/L2	3298	3426	(0)	14	
180	32.1	6.0	0.0	UnnamedSequence	7728	7811	(25906)	+	L2c	LINE/L2	2522	2610	(809)	15	
240	37.6	4.8	2.9	UnnamedSequence	7832	7998	(25719)	+	L2c	LINE/L2	2665	2834	(585)	15	
317	35.9	9.5	3.5	UnnamedSequence	8033	8423	(25294)	C	L2c	LINE/L2	(222)	3165	2756	16	
424	32.9	5.1	6.3	UnnamedSequence	8477	8732	(24985)	+	L2c	LINE/L2	3134	3386	(1)	15	
301	5.6	0.0	0.0	UnnamedSequence	9717	9752	(23965)	+	MLT1A	LTR/ERVL-MaLR	1	36	(338)	17	
339	4.4	0.0	0.0	UnnamedSequence	9753	9797	(23920)	+	(T)n	Simple_repeat	1	45	(0)	18	
2454	0.4	0.0	0.0	UnnamedSequence	9800	10079	(23638)	C	L1-1_EC	LINE/L1	(3)	6650	6371	19	
1243	32.8	1.8	4.2	UnnamedSequence	10080	11003	(22714)	C	Sat-1_TS	Satellite	(305)	2931	2029	20	
526	0.0	0.0	0.0	UnnamedSequence	11004	11063	(22654)	C	L1-1_EC	LINE/L1	(1207)	5446	5387	21	
2049	16.3	2.1	0.3	UnnamedSequence	11064	11401	(22316)	+	MLT1A0	LTR/ERVL-MaLR	22	365	(0)	22	
2890	22.5	2.8	1.1	UnnamedSequence	12368	13348	(20369)	C	L1M5	LINE/L1	(424)	5738	4735	23	
959	30.2	12.7	3.3	UnnamedSequence	13835	14306	(19411)	C	LTR33	LTR/ERVL	(0)	515	1	24	
259	29.7	1.0	2.9	UnnamedSequence	16028	16131	(17586)	C	L2B_ME	LINE/L2	(13)	3434	3333	25	
303	29.9	6.2	0.0	UnnamedSequence	17826	17922	(15795)	C	MARNA	DNA/TcMar-Mariner	(177)	409	307	26	
362	29.1	6.8	0.0	UnnamedSequence	17947	18063	(15654)	C	MARNA	DNA/TcMar-Mariner	(330)	256	132	26	
274	29.1	14.5	0.0	UnnamedSequence	18077	18193	(15524)	+	L2a	LINE/L2	3288	3421	(5)	27	
203	32.6	2.2	2.2	UnnamedSequence	18932	19022	(14695)	+	L2c	LINE/L2	3289	3379	(8)	28	
274	28.5	19.1	3.5	UnnamedSequence	19068	19214	(14503)	+	MIR3	SINE/MIR	30	199	(9)	29	
277	30.2	0.8	6.1	UnnamedSequence	19224	19343	(14374)	+	L1M5	LINE/L1	4487	4600	(1546)	30	
22	59.1	0.0	0.0	UnnamedSequence	19476	19497	(14220)	+	AT_rich	Low_complexity	1	22	(0)	31	
1996	16.7	4.9	4.2	UnnamedSequence	19547	19614	(14103)	C	MER119	DNA/hAT-Charlie	(54)	529	469	32	
2989	17.0	5.2	0.4	UnnamedSequence	19615	20151	(13566)	C	MER68	LTR/ERVL	(0)	563	1	33	
1996	16.7	4.9	4.2	UnnamedSequence	20152	20507	(13210)	C	MER119	DNA/hAT-Charlie	(115)	468	102	32	
706	29.6	9.8	0.5	UnnamedSequence	20892	21397	(12320)	+	L1ME4c	LINE/L1	100	652	(178)	34	
373	31.6	6.4	10.9	UnnamedSequence	21681	22025	(11692)	+	MLT1I	LTR/ERVL-MaLR	81	411	(0)	35	
615	25.7	11.1	2.4	UnnamedSequence	22049	22283	(11434)	+	Charlie7	DNA/hAT-Charlie	41	295	(2317)	36	
1180	26.4	4.2	1.4	UnnamedSequence	22285	22641	(11076)	+	Charlie7	DNA/hAT-Charlie	330	696	(1916)	37	
1030	24.1	10.2	0.3	UnnamedSequence	22674	23006	(10711)	+	Charlie7	DNA/hAT-Charlie	773	1138	(1474)	38	
640	26.2	8.7	3.2	UnnamedSequence	23008	23271	(10446)	+	Charlie7	DNA/hAT-Charlie	1142	1419	(1193)	39	
628	31.9	6.3	4.8	UnnamedSequence	23389	23799	(9918)	C	MLT1J	LTR/ERVL-MaLR	(0)	512	96	40	
1754	8.5	0.4	1.6	UnnamedSequence	23889	24138	(9579)	C	ERE2	SINE/tRNA	(1)	248	2	41	
239	30.6	11.1	3.6	UnnamedSequence	24326	24505	(9212)	C	Mon1f3	SINE/MIR	(1)	266	74	42	
528	29.0	7.0	3.4	UnnamedSequence	24566	24765	(8952)	+	MIRb	SINE/MIR	50	256	(12)	43	
226	27.4	0.0	0.0	UnnamedSequence	25438	25488	(8229)	+	Sat-1_TS	Satellite	1731	1781	(1455)	44	*
264	25.4	0.0	0.0	UnnamedSequence	25445	25499	(8218)	C	RNLTR2d	LTR/ERVK	(64)	460	406	45	
621	30.8	4.0	1.1	UnnamedSequence	25580	25753	(7964)	+	MIRb	SINE/MIR	20	198	(70)	46	
2009	21.5	5.1	3.2	UnnamedSequence	28536	29025	(4692)	C	MLT1D	LTR/ERVL-MaLR	(6)	499	1	47	
1668	10.6	5.7	0.0	UnnamedSequence	29584	29828	(3889)	+	ERE1C	SINE/tRNA	12	270	(1)	48	
555	16.8	5.2	3.2	UnnamedSequence	30059	30212	(3505)	C	MER33	DNA/hAT-Charlie	(0)	324	168	49	
208	26.9	0.0	0.0	UnnamedSequence	30498	30549	(3168)	+	(TA)n	Simple_repeat	1	52	(0)	50	
21	66.7	0.0	0.0	UnnamedSequence	33217	33237	(480)	+	AT_rich	Low_complexity	1	21	(0)	51	
21	52.4	0.0	0.0	UnnamedSequence	33277	33297	(420)	+	AT_rich	Low_complexity	1	21	(0)	52	
274	32.8	2.5	4.3	UnnamedSequence	33600	33717	(0)	C	L2c	LINE/L2	(5)	3382	3267	53	

Euarchontoglires

>Tupaia Gin-1 (genescaffold:TREESHREW:GeneScaffold_3028:264576:276530:-1)
TCTCCTCTTGGGATCTTGGACACGCACGCACACGCGCCCGGCGTGAGTTTTAAGCCACCG
GGTCCGGGACATGAACCGCGGTCGACTACGTGGGAGTCCAGCGACATAGCCATTACGCTA
ACTGCTGGTTCCCTTCCCTTTCCATTAATTTTTTGAAGACCTTGCATATTTGGAAATAAT
ATAAAACTTTATTTCTCTCCTCATAACGTAACATTTATTTAAATGTCTTGTAACAGAACT
ATCCTATGTCAGCAACCTTTCAATTGATAGCAGTGAGAGAACTTGAAGCAAACCTTTTTG
CAGAGTACTACTTTGTTTCCTGTTTTGTCAGTATATGGAAAATTTTCAGCATTTCTTATC
TCATGGAAAGAGACAGGATAATTTGTCATGATGTATCTCAAAAATAGCATTCAAATATTT
TAGTTTGCATACATCACTCTGTGTTCCTTTCTGTACTGTAATTATCTCCCTCACAGATAA
TATTCTGGGAATGCTCAATTCCGGTGTAAGCAGAAATGTGACAGAGCCTGAATTGAAATA
GCAACTGACATAGCTTATCAGTCCAGTTACATATTTTATTACCCTTTACCTAGGTTCACA
ATGGTCCGTAGTGGAAAAAATGGTGACCTTCATCTTAAACAGATTGCATATTATAAACGA
ACTGGCGAATATCATCCAACTACACTGCCAAGTGAGAGAAGTGGCATAAGAAGAGCAGCA
AAAAAATTTGTCTTCAAAGGTACAATTAACATCCCAGATCTTGAGTATGAGTTATAGATT
CTTTTCCACGTGTGTTCTGTGTGTAATCCTGAAAATCTCTGTACTAGTTGGTTGTTGCTA
AATCAGGTGAGCTGGTGATGACAATCAAAAACAAGATAAAAATTACACTGAAACGAATTG
AAGACTGGTAAAAATGAAAAGGGCAGTTAATTATATACAGAAAGTTATACCATGGAAGAA
AGCAAAACATTTACCTAGCTTTACTTCTGCTTTTTGCTCTCTGGTGGCTCCCATCCTAAT
GGTGCTCTTTCAAGCACTGTTGCCACTGTCAGTGGGGAATTGATTTGACGTTTCACATCA
AACTGTTGACAGAGTTAACTAGTTTGTTTACCAAAGTGAAAAATGGAAGAAACTAACGTT
TTTGTTTTTTTAATACTGACCAAGAGTTTTTCTTTATTTTTTTTTCATATGGTAATTCTA
TTTTTAATATTTTTATTAACTGCCATACTGTTTCCCATAGTGGCAGCACCATTTATGTAT
TACCATCAACAGTGCATAAGCATTCCTATTTCTCCACATTCTTGCCAACACTTGTTGTTT
TAGTTTTCCTTTTTTTCTTTTTTTCTTTTTTCTTTTTTTGATTTTTTATTTACTACCATC
ATTATCTTGTTTATATTACATTGAATCACATATAACATTACAGCGTATTACATTTCTTTT
TCCAGAAGGAATACCATTCATATCAGTCAAAACATAGACATTGAAATCCCCATTTCATTA
TCCAATAGCTTTTCATTATTTTTCCCAGTCATCATTATTATTATTCCAATTATTTTTTTA
AACAATCACTAAAACTATTTTTCTATTATTTCCCTCTAGCTACCAATACACATTCTTGGG
ATTATATTTATTTTGGGCTCAGATCTTCCTGAATGGATTAATATTTGAAAGGCAGTGAGA
TATTTATTTTGAAAAATCATTATTTTTTCATTCCGCCCCTCTTCTTTCAAAGGGAAAGAT
AATTTTGGGATAAGTAAAGCAACAAATAGTCTGTCATCAGGTAACCAAATGAATATTGGT
GTTTGCGATTAATGGGAGTTTTGCCTAAAGGTATAGCATTGTGCTTTTCCCCAAACAAGC
AAGAATATTCTAATACATAAATGTCTTCTCATGTCTCCCTAACAATAACTGCCATATACT
TAAGAACAGGCAGACTCATTACCTCATAGAGGAAAGGGAGAATTCATTCACTCATTTATT
TACCTAACAAAAGTTTACTGAGTTCCTGCTGTGTACTGAGCCTTGGAAATATATTGATAA
ATAAGATAGATGAGGTTTTCAGGGCCAGCAGGTGGCATGGTGGTTGGAGTGCTGGTCCCC
TACATGGCTGACCACGGTCTGAGTCCTGGACCCAGTGACTTGAGACTCCTGCTGGGTGTG
CATGTGTGTGCCTGGAATCCCGGGAGAATAGTGGAGAAGGGATTGCAGCATGGCCATCTC
CCATAGGGCAGGGGGTGATTTCTCACTTTCTCTCTCTCTCTGTCTCTCTGGTAAGCACAC
ATGCCCTATGGCAAAACCTAAAAAACAAAAGACATTTTTGCTCTCAAGTTGCTTACTTTA
TAACTTAATTAAGGAACGTTCCATTTGGCATGTGCTTAAGCACATTAGTAGCTTAATGAT
ATATTCTGAAGCTTGAGTAAGCTACCCCATCTGTACTGAAGAGGTGGTGATTCTCTCTGT
CCCCTGGTTGCTTTCCAGCTGCAAATTCTTCTCAGATCTCCCAAGGAAAATAGAAACCAT
TTTGAGCATTAAAGTTCTTCCTCTTTCTCACTTTATCATCCTTAATTTCTACTCAAATAT
AGAGAAACCAGAGGTGGTTGCTTATAAAAATGTAGTTATGATGAAGTGCTAGTGTATTTT
ACAAAATTCTGTTCCCAGAAGTTACTTATTTGTATGTAATTCACTTGCTAATTTATGCCA
TTTTCAGGAGAGTCTTACAAGTGTTTTCTTAGTGTGAGTATTCCCATAGAAAAATAATTT
TATACTCCCATGACTGTTACATGCTTTGATGAACAAATAACTGTACTTCTATTGATAGCT
ACAAAAGTATAACAGCTAGTGTTCCTCTTAGATGTCTAGTAAGTATAACAGCTGAAGGCC
TTTCAGATTGTATGGTGTTATTACAAATAAATTCTCTGTTTTAAATGGATAAAGATATTG
TAAAAATAGGACAATACTCATGGTTTTGATGCTAAGGCTGTGATTACCTTAAAACTAGAT
ATAGTTTTGCAATTCTATCTTTTTGATATTATTTATTCTAG**AAAAAAAGCTGTTTTATGT**
TGGAAAAGACAGAAAACAAAGTCGTTTGGTAATTGTTTCAGAAGAGGAAAAAAAGAAAGT
CCTAAGAGAATGCCATGAAAATGATACTGGGGCTCATCATGGCATATCCAGAACCCTCAC
CCTAGTGGAATCCAATTACTATTGGACTTCTGTGACCAATGATGTCAAACAGTGGGTATG
GCTTATGTATTTAGAATACTTTAAACAATATATTTCTAATAGATAATCAATATTCTAATA
GATAATCAGTATTTATTATTGTTCCAGGTTTATTCTAGGTATTACCTCTTATTAAGTAAC
TGTCATTTTGTGTGGTTAGTTTCTATTCATAAGACATTTAAAGGGATTTTATTTCCCTTC
CCAATTTCATATTACAAAATTATAATTTGAAGTAAAATGAAGCAGTATTTGAGCTACTGA
AAAAATGCCATGTTTAGATGAAAGATTATTTAGAGCCAGATTTTCATTATAGAACTACTT
TGTGTAGGCAGAATTTAAATGGGACCGAAATTCATTTGACTGAATCATTACCTCTTTAAT
AGCAGTAACACAAGTTTGTCTCTAAAGGAGAGTATGTTCAGTAATCTGATCCAATAATCA
GCCCAGATGTACCTCCATTTTTGAGGTAAAAATATCAAACACGGCAGAAACCTAAAAACC
AAACAAATGCAAAATCAAATTATGAACATAATGAAGTGTGACTTCTGTAACTTTAAAACT
CATTCTGACCTTCACCTTCTAATAGAAAATTCACATAGGAATTCTGAGGTTCTCGGAGTT
AGGACACCTGATCCCTACTCTTTCTGAACACCTGAGTATATCCTTTCTCTTCCTGATACA
CTGTTGGGTATATATCTACAGATATATCATGACTTTGCCAACGACTCAATAGATGCCACA
TAGCATGATTTCCTCAGTCTATGTGAGCTCTTTTTAACAATTGCAATAGTGCATCAGAAA
GACTAGCAGTTCAGCCATAAAAATTAAAATAACATTTAACTAAGTATCATTTGTGAGTTT
TCATTTCATGGGTCTGTGCTGGTATTCCAAAGACCTTGATATTTGTATTTGCCTTCAGAA
ACTGCCACACCTTCTTTTACAGACATTCAGTAAAATCTTTGTCAAATCTTCATTATTTGA
GAAGAGATTTGATTTTGGGAAATGATCAGAAGTTATTTGCAATGAAATAGCTTTAATTGG
AAAAAAAATTTTAATTCAGATTTGTAAAACTGCTTTCTAGTATGTTGACTATATTAACAT
TTATGTATATTTTCTTTATTGTGTGCCTGTTTTTAG**GTATATTCTTGTCAGCATTGCCAA**
GTGGCAAAAAATACAGTCATTCTAGCACCTAAACAGCACCTTCTCAAGGTAGAAAATCCG
TGGAGTATAGTTACTGTTGATCTCCTGGGACCTTTTCATACAAGCCACAGAAGTCATGTC
TATGCTATAATCATGACAGATTTGTTCACAAAATGGGTTGTAATTTTGCCTCTGTGTGAT
GTTTCAGCATCAGAAATTTCTAAAGCTATTATCAGTATATTTTTCTTATATGGACCTCCC
CAGAAAGTAATAATGGACCAAAGAGATGAATTCATTCAACAGGTAAGATAAATAAACTGC
TTAGGTCCAGAAGAATATCTTATCTTAATATAACACAGTCTCCTTTGTGTTATATTAAGG
TAGAGAGAATACTGTGTACCGTAAGAGTCGTTAATCTGGAAATTAGATCAGGATTTTAGT
ACTTAATTATGATACCAAGTCATTACAAACTTATCTTTGGGTCTCTTCAAACGAAAATGA
GGATGTTGGACCAAATAATCTTTGTTTATTTACAGCCTAAAGATTCTGTGATTGGTCAAG
GGAAACTTGACTAAACAGAAAAAATATCATATTCATGAGTGAGAATATTCATTANNNNNN
NNNNNNNNNNNNNNNNNNNNNNNNNNNNNNNNNNNNNNNNNNNNNNNNNNNNNNNNNNNN
NNNNNNNNNNNNNNNNNNNNNNNNNNNNNNNNNNNNNNNTCTCAATGTGGTGAGTGTGTGTACCC
TATAAAATTAAAAAGCAGGCAAGAATTTATTTGAAAGTTCTGAAAACGAAATAACTACTA
GACTTAAGGCATATTTTTAAAATTTTATAATTGACATAGTATTGATAAAAAAAAATCAGA
GGTGTCAAACTTTCTTGGTTCATAGCACCCTTGATGACCCAATTCAAAAGAAATACCTGG
GGAACCAGCAGTTAGCGTAATGGCTATGTCGCTGGACTCCCACGTAGTGGACTGCGGTTC
GCGTCCCAGACCCGGTGGCTTAGAACTCACGCCGGGTGTGCGTGCGTGCCCAAGATCCCG
AGAGGAGAATGGAGGAGAAGGAATTGCAGCGTGACCATCCCCCTGGGGGGTGATTTCTCA
CTTTCTGTCTCTATGTCTCTCTGTCTGTCTGGCGAGCGCACGCGCCCTATAGTGAAAATC
TCTCAAAAAAAAGTACCTGAAGTTATATTAGTAAGTAATTTGGAACTCTCAGGTGTGCCT
AATTGTCCCTGGGTTCCATTATTGGTCAGGAGAACTCACTCAGCGTGCAGTCACGTTTAT
GGCTAAGATTTAGTCATTAGTTATTCAACAAATATAATGCAAAAGCAGCCCAGAGAAAAT
GTGCATGGGGAGAGTCCAGAGGAAACCAGGTGTAAGTCTCCAAGATCTTCTGTCCCAGTG
GAGTCACACGGGACATGTTTAGTTCCCTTAATAATGCAGTGTGACAGCACCTAAGGAATG
TTACCTACCAGAGACGCTCATTAGGTATTAATGCCTAGGTTTTAATTTGGAGTTTGTCAC
ATAGGCACCCTCTACCTGGCATGTTACAAAATTTCAGACCCTCTGAAAGAAGGAAGGTGT
TTAATCAACCACATTGTTAGCATGAACTGTTTAGGCATGCCGAGCCACCCTTACCAGTTA
GGGAGTGATGGGAACCCTTTGCTGGTAACCAAAACCCAAGTTCCTAGACACTTGTCAAGA
GCCATCCTTGTATGGTTGTCACTTTCAGTCTGAGGTAATGAAAAGCTCCTGCTCAATTCC
TCCGTCTCCAGCCACGGGAAGTCACTGACTGTTACAGATGCTGCACTATAGGATGATGGA
ACTTCTCTCAGCCAGGCTTGCTGAGGAACCTAGTGAAGAACACCTGCCTTGGAGAGGCAC
TCAGACCAAAAGCTGACTTTACACAGCAAGAAGTAAAACTTCGTCGTGTTAAGCCACATA
CAAGTCTTGAATGTGTTTTAAATGCAAGCTAATCTTGCCTCCCTTTACTGACACAATCCA
GTTTTGGGGAAAACCGTTATTTATTTTTTAATAAATAAAAGTGATTTTAAAACTCCCATG
TTTTAAAAATACTTTTGTTATATTAAAATATATTTATTTCTTTTTTAATCAAAAAGTAAG
AAAGAAATGACGCTTTACTAATATTGATAGCATCCCAACTCACCCAACAAGGCAGATGAT
TGTGTTTTATAGATAACATGTGAACTTATCCTAAGAGAAAAGTGAGAACCCACAGCAAGA
AGAAGCAATCTCAGTGATCTCACTTTTTCTTTTCGTAGTTAATTAAATGTAAGGATATTA
TCTGATTTTCACCTCACTACTCCTCACAGAATGGCTGGGGTAGCTTTAATTTCTTGCAGT
GAAATAAGCTAATACTAAAACGCAAACACCTGTCAATCACAAGCATTGGCTAGAGATAAT
TATTCCATTTAACTTCTGGTATTAGGTGTTAGACATATTAATGCGATGAAAGTAAATGTT
AATTATTAATGTGTTCTTTTAAGAATTGGCCATCAAAATTCAAAATTATCAGGAAGATCT
GAAATAGTGATTGGTTTCTACCTTTAGTCATGAAGAGCCTTATCTTAGGTGAATAGAGTG

TTCCGCGTTACTAGCTATTGGAGACTAAAGCTATTTTGATTAATTCATCAGTTTTTCCAG
TAATGCTTAAAGTTGTGATAATCCAGAACTCTTTGAGATTTCACTATACTTGGGACCAGC
AGTTGGCGTGATGGCTATGTCACTAGACTCCCACGTAGTCAACCGCGGTTCGAGTCCAGG
ATCCCTTGGCTTAAACTCATGCCGGGCGCGTGCGTGCCCAAGATCCTGAGAGGAGAAAGG
AGAAGGGATTGCGGCGTGGCCATCCCCCCAGGGGGTGATTTCTCGCTTCCTGTCCTATGT
CTCTCTCTGTCTTTCTGGCGAGCACACGCACCCTAATTTCACTAAACTTAACAGTAAATG
TTTACATGCCTCTTTTGCGATGTTCATTAATAATAAATAAAAAGTCACATATTATTTAAA
AAGCTTGTTCTTTTTGCATAATGATAGCAAAATAAAATGTATTATTTTGTCAGCAAATAC
TGACTGCTAAATCATCAAAATAAACTTAGAAGATACTATACAATGTGGGGGGTATTATAC
ACTTGTATAAAAATGTGAACACTTCTAACCTTGGCTTAGCATATTTGCTAAATTGTTTAA
ATAAAATACTCAAGTACCTTTCTCTAAACCTCTGCAGGAAAGAGAATAAACATCCTCAAT
GGGAAAATTAATCTTCAAAAAATGCCTTGTGGAAAATTAAAATTTTAATGACCAATGAAC
TATCTAGTTGACTGGGCAGGGTAGGGGAGGCTCACCAGGCATGTATATCAAGATAGTATT
TCATGAACATATTCACAAGATTATTCCTAGGTAACCTGTTGAAAAAAAAACTATGAAGGG
TCATTTAAAAGCACTTATTAGGACCTCAGATTGAAGCTGACCCAAATAACAGCCTCAGGG
ATTTCTTTCTGTATTTTTGAATAAAAAGAGGCATCCTGCATGAGTACAGATATGTCAACG
AGGAACTGTGTTTAGCTACAACCAGCAGAGAGCACCCTTCCCTTCCCTTGTAAGTACACT
CCAGTAGGAGTTCATTTTGTACTCAGATAAGAGAAGGCCAGGCTGTCCGAGGCCAAGGAT
AGCTCTGAATATTACTGGAAACCTATGTCCCAACATCTTTGGGGTCTAACTCTCATCTCG
TGTTCACAGCATGGCTGCCCAAGCCCATCACGCAGTATCTACATTCCAGTTAGAAAGGCG
TAGGGTTGGAGGGGCAATGAGCAAAAAAATCTTGTACCACTTGAGTTCGCCCGTGAATTT
TCACATACATCTTTTTGGCCAAACCTGTGTCATACCACTATCCTCTGCTTGCAAGGGACC
TTGAGAAATATATTGGATTGTCTGAAAAGTCATGTGCCCTTTTTTGCATAGAAAAATGAA
AAAATACATCATCACTTTTTCAACAGCCCAATAGATGAGTTTTTGTTTCTCTTTTATTAG
CTTTACCATTTTAGTAATTGTCTCATTCTGAAATATTCTTCTTTCAAAATAAATTTATCA
GCCCAAATGTCTGCTTCAACTAATAACGAGGGGAACAGTCGGAAGGACNNNNNNNNNNNN
NNNNNNNNNNNNNNNNNNNNNNNNNNNNNNNNNNNNNNNNNNNNNNNNNNNNNNNNNNNN
NNNNNNNNNNNNNNNNNNNNNNNNNNNNNNNATTATTTTATTTAACTTTATTTATTTTATTTA
TTTTTACCCCGTTAGGGGAATTTGACCAGAGAGTGACAGAGTAAGAGATGTGGGGAAAGA
GAGAGAGAGAGAAAGTGAGAAGGCAAGCCGCCAGTTCTGAACTCCCTTGACTTATTTGAG
GCCCTGCACATCATATAACAGAAATAGAGCAACTAAGTGTCACCAAGTGCTTACTGCATG
CCAAGGACTGTTTTAAGTGCTTTACAAACATTAACTCAGTCCTTGAAAGATTCATGGATG
AGATTTTCCTCATTTCACAGGTGAGGAAACTGAGTACAGAGAGTTTCAGTGACTTGGCCA
AAATCAAATACCACTTAAATGGATTATGTGACCTACTATTAGAGAGACTTACTCCTGGAT
TTAAAAATTACTTGAACTTAGAGTCATTGTTAAATTCTATTCATCCGTATCCTTAATGTA
TGTGTGTATTTGCCAAGGCATTTAATCTTCAGCTTCTTATCCTCTGTTATTTTAATGGCT
TTGTGTTTGATTGTTTTTTTATTACCAGAGGGTGCTTGTGCTCTCCAGAGGGAAAGAGAG
AGAAATCACCTTCCCAGTAGGGCATGATCATACTGCAATCCTTTTTCCTCCATCCTATCA
GGATTGGGGCATGCAAGCCGCGCATGCGTGCGTGCACACACCCCTGGCATGTATTTCAAG
CCACCAGTCCAACCGTGTCTAGCTATATGTCCTCTAGCTCAGCAGACACAGGGCTTACCC
ACTGCTGTGCTAAGCCAGCCCATTTCTTATCTTAAAATGGGCTTTACAATACCTGATAAA
ATTGTCATAAGGTTTAATAACAATACTGTGAGGTATCTAGTGGGTGTTCTAACACATAGT
AAGGGTTCAATAAATAGCAACTACTATTTTAAACAAATTTGTATGTTTTAGTATGGAATG
GCTATGGTCAGTATAAAAGGTAGAGTCACATTTTTCCAGGNNNNNNNNNNNNNNNNNNNN
NNNNNNNNNNNNNNNNNNNNNNNNNNNNNNNNNNNNNNNNNNNNNNNNNNNNNNNNNNNNNNNN
NNNNNNNNNNNNNNNNNNNNNNNNNNNNNNNNNNNNNNNNNNNNNNNNNNNNNNNNNNNNNNNN
NNNNNNNNNNNNNNNNNNNNNNNNNNNNNNNNNNNNNNNNNNNNNNNNNNNNNNNNNNNNNNNN
NNNNNNNNNNNNNNNNNNNNNNNNNNNNNNNNNNNNNNNNNNNNNNNNNNNNNNNNNNNNNNNN
NNNNNNNNNNNNNNNNNNNNNNNNNNNNNNNNNNNNNNNNNNNNNNNNNNNNNNNNNNNNNNNN
NNNNNNNNNNNNNNNNNNNNNNNNNNNNNNNNNNNNNNNNNNNNNNNNNNNNNNNNNNNNNNNN
NNNNNNNNNNNNNNNNNNNNNNNNNNNNNNNNNNNNNNNNNNNNNNNNNNNNNNNNNNNNNNNN
NNNNNNNNNNNNNNNNNNNNNNNNNNNNNNNNNNN**NNNNNNNNNNNNNNNNNNNNNNNNNNNNN**
NNNNNNNNNNNNNNNNNNNNNNNNNNNNNNNNNNNNNNNNNNNNNNNNNNNNNNNNNNNNNNNN
NNNNNNNNNNNNNNNNNNNNNNNNNNNNNNNNNNNNNNNNNNNNNNNNNNNNNNNNNNNNNNNN
NNNNNNNNNNNNNNNNNNNNNNNNNNNNNNNNNNNNNNNNNNNNNNNNNNNNNNNNNNNNNNNN
NNNNNNNNNNNNNNNNNNNNNNNNNNNNNNNNNNNNNNNNNNNNNNNNNNNNNNNNNNNNNNNN
NNNNNNNNNNNNNNNNNNNNNNNNNNN**NNNNNNNNNNNNNNNNNNNNNNNNNNNNNNNNNNNNN**
NNNNNNNNNNNNNNNNNNNNNNNNNNNNNNNNNNNNNNNNNNNNNNNNNNNNNNNNNNNNNNNN
NNNNNNNNNNNNNNNNNNNNNNNNNNNNNNNNNNNNNNNNNNNNNNNNNNNNNNNNNNNNNNNN
NNNNNNNNNNNNNNNNNNNNNNNNNNNNNNNNNNNNNNNNNNNNNNNNNNNNNNNNNNNNNNNN
NNNNNNNNNNNNNNNNNNNNNNNNNNNNNNNNNNNNNNNNNNNNNNNNNNNNNNNNNNNNNNNN
NNNNNNNNNNNNNNNNNNNNNNNNNNNNNNNNNNNNNNNNNNNNNNNNNNNNNNNNNNNNNNNN
NNNNNNNNNNNNNNNNNNNNNNNNNNNNNNNNNNNNNNNNNNNNNNNNNNNNNNNNNNNNNNNN
NNNNNNNNNNNNNNNNNNNNNNNNNNNNNNNNNNNNNNNNNNNNNNNNNNNNNNNNNNNNNNNN
NNNNNNNNNNNNNNNNNNNNNNNNNNNNNNNNNNNNNNNNNNNNNNNNNNNNNNNNNNNNNNNN
NNNNNNNNNNNNNNNNNNNNNNNNNNNNNNNNNNNNNNNNNNNNNNNNNNNNNNNNNNNNNNNN
NNNNNNNNNNNNNNNNNNNNNNNNNNNNNNNNNNNNNNNNNNNNNNNNNNNNNNNNNNNNNNNN
NNNNNNNNNNNNNNNGAAATTACTAGCTATAATAATACTGACTTTGAAACGTTTTAAGAAT
TAAATAGGATTCCATGCCTACCTATTAGTGCCATCTCAATAAATGTCTATTTTGTTATAT
TATCATTATCTGCATGTACCTTCAAGTAATTATGGCTTTCTCGTCTTCTGTTCTGTTTTT
TTTTTTTTTTTTTGCATTTGCAG**ATGGAAAACAATCATTCGAATGAACTAAATAAAAGCA**
AGATCACCATTAAAAAGAAACCAAAGCAATTAAATCCGTTTCATTTAAAAGTGGGTCATG
AAGTTTTAAGACAAAGGAAAA**ACTGGTGGAAGGATGGTCGTTTCCAGTCCGAATGGGTTG**
GTCCTTGTGTTATAGACCACATTACAGAAAGTGGATGTGCTGTCCTGCGGGACAACACTG
GGGCAAGACTTAAAAGGCCGATCAAAATGTCCCACCTTAAGCCCTATGTAAGAGAATCTA
ACGAGCAAGGTAAATATGTTACTTCTCTACATTTTTACTTTCTTTAGTGAGAAGCTAAAA
ATACCAGAAACATTTCTTCCTCCCTTTGTTTAAGTTTTCTTTTTCTTTCTTTTTTTTTAA
TAAAATCTGTATAGTTTTAGATATCCTAGGTAGTCTGTTTTGGATTGTCAGAAAAGTCAT
GATGCATTTTTGCATAGAAAAACAGCAAAAAAAAAAAAAATACATCATGACTTTCCTGAC
AACCCAATAGAATGTCTCTTCTGCAACTGTCAGGCTTATGTGAGATGGTATTTTTATTTC
CAGTATGCTTTTCACAGCTCATGTGGAAGATAATGAACCTGTTTTGCTAAAAGAACACTT
GCATTTTCACTTTTACAATGCCCTGTTAATCACTTTGTGCCTTATTTTAGCATGAGAAAA
CAGCAGCTATGATCCTTAATGCCAAAGCAACATACTGTCCAGAATCCAGCTGTGAGATAA
ATATATTAGGGGATT

==

Tupaia Gin-1

total length: 11955 bp (10341 bp excl N/X-runs)
GC level: 36.64 %
bases masked: 4211 bp (35.22 %)

==

	number of elements*	length occupied	percentage of sequence
SINEs:	9	1605 bp	13.43 %
Alu/B1	0	0 bp	0.00 %
MIRs	3	382 bp	3.20 %
LINEs:	3	517 bp	4.32 %
LINE1	2	397 bp	3.32 %
LINE2	1	120 bp	1.00 %
L3/CR1	0	0 bp	0.00 %
RTE	0	0 bp	0.00 %
LTR elements:	3	824 bp	6.89 %
ERVL	1	504 bp	4.22 %
ERVL-MaLRs	2	320 bp	2.68 %
ERV_classI	0	0 bp	0.00 %

ERV_classII	0	0 bp	0.00 %
DNA elements:	10	1219 bp	10.20 %
hAT-Charlie	7	944 bp	7.90 %
TcMar-Tigger	0	0 bp	0.00 %
Unclassified:	0	0 bp	0.00 %
Total interspersed repeats:		4165 bp	34.84 %
Small RNA:	6	1223 bp	10.23 %
Satellites:	0	0 bp	0.00 %
Simple repeats:	0	0 bp	0.00 %
Low complexity:	2	46 bp	0.38 %

==

SW score	perc div.	perc del.	perc ins.	query sequence	position in query begin	end	(left)		matching repeat	repeat class/family	position in repeat begin	end	(left)	ID	
1091	2.3	0.8	0.0	UnnamedSequence	1	133	(11822)	C	TUS	SINE/tRNA	(142)	134	1	1	
793	14.7	0.0	0.7	UnnamedSequence	1185	1321	(10634)	C	L1M3	LINE/L1	(562)	5621	5486	2	
818	20.6	0.4	3.2	UnnamedSequence	1324	1583	(10372)	C	L1-1_Tbel	LINE/L1	(0)	6213	5961	3	
274	34.7	1.5	1.2	UnnamedSequence	1974	2061	(9894)	C	L2c	LINE/L2	(0)	3426	3313	4	
1053	22.2	3.6	2.4	UnnamedSequence	2062	2310	(9645)	+	Tu-III	SINE/tRNA	2	253	(5)	5	
190	35.1	9.9	8.1	UnnamedSequence	2311	2342	(9613)	C	L2c	LINE/L2	(92)	3334	3304	4	
390	27.4	0.0	5.5	UnnamedSequence	4122	4237	(7718)	C	MARNA	DNA/TcMar-Mariner	(170)	416	307	6	
247	27.6	9.2	3.9	UnnamedSequence	4816	4913	(7042)	+	MIR3	SINE/MIR	97	199	(9)	7	
22	45.5	0.0	0.0	UnnamedSequence	5171	5192	(6763)	+	AT_rich	Low_complexity	1	22	(0)	8	
231	23.4	2.1	0.0	UnnamedSequence	5217	5263	(6692)	C	MER119	DNA/hAT-Charlie	(0)	583	536	9	
1980	3.6	3.2	0.0	UnnamedSequence	5280	5532	(6423)	+	TUS	SINE/tRNA	1	261	(15)	10	
1963	24.9	2.8	3.4	UnnamedSequence	5569	6072	(5883)	C	MER68	LTR/ERVL	(4)	559	59	11	
226	28.4	2.2	3.3	UnnamedSequence	6265	6355	(5600)	+	MLT1I	LTR/ERVL-MaLR	322	411	(0)	12	
24	75.0	0.0	0.0	UnnamedSequence	6377	6400	(5555)	+	AT_rich	Low_complexity	1	24	(0)	13	
310	33.5	11.3	1.4	UnnamedSequence	6440	6633	(5322)	+	Charlie7	DNA/hAT-Charlie	73	285	(2327)	14	
394	32.7	7.0	3.0	UnnamedSequence	6636	6994	(4961)	+	Charlie7	DNA/hAT-Charlie	326	698	(1914)	15	
234	18.6	10.2	0.0	UnnamedSequence	7013	7071	(4884)	+	Charlie7	DNA/hAT-Charlie	759	823	(1789)	16	
1459	8.5	5.8	0.0	UnnamedSequence	7072	7295	(4660)	+	TUS	SINE/tRNA	2	238	(38)	17	
387	22.8	9.4	0.0	UnnamedSequence	7295	7421	(4534)	+	Charlie7	DNA/hAT-Charlie	809	947	(1665)	18	*
231	19.1	0.0	0.0	UnnamedSequence	7401	7442	(4513)	+	Charlie7	DNA/hAT-Charlie	957	998	(1614)	19	*
365	26.8	10.9	0.0	UnnamedSequence	7466	7603	(4352)	+	Charlie7	DNA/hAT-Charlie	1046	1198	(1414)	20	
227	37.6	7.5	4.6	UnnamedSequence	8066	8294	(3661)	C	MLT1J	LTR/ERVL-MaLR	(173)	339	104	21	
392	16.2	2.7	1.3	UnnamedSequence	8295	8369	(3586)	+	MariN1_Tbel	DNA/TcMar-Mariner	1	76	(0)	22	
492	12.9	0.8	17.7	UnnamedSequence	8613	8744	(3211)	C	TUB2B	SINE/tRNA	(2)	174	62	23	
410	24.9	16.5	3.4	UnnamedSequence	8805	8962	(2993)	C	Mon1a8	SINE/MIR	(26)	252	75	24	
521	23.1	10.5	11.2	UnnamedSequence	9136	9367	(2588)	C	TUBE1	SINE/tRNA	(0)	244	7	25	
331	29.8	7.9	1.5	UnnamedSequence	9383	9508	(2447)	+	MIRb	SINE/MIR	132	265	(3)	26	
482	10.6	0.0	10.5	UnnamedSequence	11622	11705	(250)	+	MariN1_Tbel	DNA/TcMar-Mariner	1	76	(0)	27	

>Rabbit Gin-1 (chromosome:oryCun2:11:22459094:22476069:-1)

GGCCCAAGTACTTGGGCCCCTACCTCCCATATGGGAGACCCTGGATGGAGTTCAGAGCTC
ATGGCTTTGGCTTGGCCAGTCCCAGCCTTTGCAGCCATTTGGGGACTAATCCAGCAGATG
GAAGATATCTCTCTCTCTCTGTCTCTGTAACTCTGCCTTTCAAATAAATAAGTAAATCTT
TATAAAGGAAAAAAAATTGTATTTTCCCACCATAATCTATTACCATTAATTTAAATATGG
CACAGTAAGACCATCTCATGTTAGTGACATTTCAACTGATGGCAATGAAATTTCTAAGCA
AATCTTTTGAAGTATACTTCCTTTTTTTTTTCCCAGTATATGGAAAATTTGCAGCATTTC
TTACTTCGTGGAAAGAGACAAGATAATTTGTCATGATGTGTCTCAAAAATAGCATCTGAA
TATTTTAGTTCATATTCATCTCTCTGTTCCTTTTCTGTCCTACAATTATCTCCATCACAG
ATAATATTCTGGGAATGCTCAATTCATACGTAAGCAGAAGTATGACAGAAGCCTGGATTG
AAATAGCAACAGCATTAGCTTAGCATTCAAGTTCTATCTTTAATTTTTACTAGGTTCGCA
ATGGTCCGTAGTGGAAAAAATGGTGACCTTCATCTTAAACAGATTGCGTATTATAAACGA
ACTGGTGAATATCATCCAACTACACTGCCAAGTGAAAGAAGTGGCATAAGAAGAGCAGCA
AAAAAATTTATCTTCAAAGGTAAAATTAATATCCAAAATCTTGAGTTTAGAGTTATAGGT
TCTCCACATGTGTTCTTTGTGGAAACCTAAGAGTTTCTGTAATAATAGGTTGTTGCTAGG
CAATTATCAGTCTAATTGATGATACTTTAGTCAAAAGTAAAATAAAAATCACACTGAAGC
TAACCGAAGGCCAATAAACATGAAAAGATGAATTTAATTATAGGCAGAAGATAATACCAT
GCAAAAAAATAAAACATTTACCTAAATTACCTTCTCCTGCTTTTGTCCTCCAGTAATTCC
TGCTCTGCTAGTGCCCGACACACACTGATGCCACTGTCAGTGAGGAACTCACTTGGTGCT
TAAAATGAATTGTTAACTAGCTTGTTTACTAAAATGAAAAGTTGATGGAATTGAGTGTAT
TTCCATGATTCCCCCCTCTGCTTCCTTTGTAAATATTCTCAGGGTTTATATTGGCTTTGG
AATCCTGAGTGGATTGAATACTGGAAAGGCAATGGCATATCTATATTGAAAGGTTAGGAT
AATTTTTCCTTCTTTTTCCCTCTCCTTCAAATAGTAAGATGGGTTTGGAATGAAATAAGT
GACAAATACCCAACTGAATGTTAGAGATGTTAATGGAAGTTTTGTCTGTAGGAATAGCGT
TAGGCTTTCTGCAAAGCAGGTAAGGAAAGTTCAAGTTCAGTAAGTGAATGCCTGCTCTTA
TCTCCCTGTGTGTATACCCCTACAAGTAACTCCCTTATGCTTAAGAATAGACCCATTATA
CTTAGACTAAAGTGAGAATTCATTCATTCATTTAATCATTTATCTTAATGATTTATCTAA
AAATGCTTACTGAGTTCCTATTAAGTGCTGATCTCATCCTAAGCCTTGGAGATGCAGTGA
TGGATAGGGCAAATATGATTCCCGCCCTGAGGTTGATTACCTTCTAATGTAAGTGAGCAG
TGTTCCACTCTGCATGTGCTTAAGCACGTTAGTAACGGGATGGTGTATTCTGGAGTGTAA
ATAGCCGCCATGTCCACACTGCAGAGATGGTGATTCACTCTACCCTGGTTGCTTCCCTTT
GCAGATTCTTCTCAGGTATCTTTCTATGAAAAACAGAAGTATCTTGAAGATTAAAGTTCT
CTCACATTCTCATTTTACCATCCCTACCTTCCACTCAAATCTAGAAAAACCTGAGAGATG
ATTGCTTACAAAACTAGTTATGATGAAATACTATTTTAACAGTCCCAAGAAACTGCTTAT
CTATACATACTTCCTTATAAGTTTATGCCATTTTTAGGAGCCACTTGCAAATATTTTCCT
AGTTTGAGTACTTCCATGGCAAGCTAACTTCCTACTGCCACAGTTAGGAGTGCGACATGC
TTTGATGAACAAGCCAGAGCTACTTGTGTTGGTAGCAAGAAAATTAGAGCAGCTAGTGGT
CTTTGTATGTATCTAGTAAGAATAACAACTGATCTTTCAGATCACACAAGTAAATGCTCT
GTTCTGAATGGAGGTTTTAGAAGAAGCAGGCAAATCCCTCATGTTTGAGATAAGGCTTTG
ATTACTTTAAAACTACAGAGGTCTCTAAAATTTGCAATTAATCTTTTGGCATCATTTATT
CCAG**AAAAAAAGCTATTTTATGTTGGAAAAGACCGAAAGCAAAATCGCTTGGTAATTGTT**
TCAGAAGAGGAAAAAAAGAAAGTCCTAAGAGAATGCCATGAAAATGACACTGGAGCTCAT
CACGGCATATCCAGAACCCTCACTTTAGTGGAAACCAGTTACTATTGGACTTCTGTGACC
AATGATGTCAAACAGTGGGTATGGCTTATGTATTTAGAATATTTCGAATACTATCTAATA
TTCAAAATTCAATATTGCTCCAAATTTACTCTGGGTATCACTTCTCATAAAATACTTTAT
GGGTAGTTTTTGTACAAAAGAGACATTTAAATGGAGTTCCATTTTCTTCCACAATTATTT
CAAATCACCAGAGTATGATTTAAACTAGAAAAAGGCATTATTTAAATGTTTGAAAATACC
ATATTTATACAGATTGTATAGAACCAGATTTTTAGTATTGAATTGCTTTGTGTAGCTAGA
ATTTAAATGGGACTGAAATTGATTTGAGTAAATCATTATCCTTCATTGTGGTCGTACAAG
ATTTTCTGTACTGAGGGGAATAATTTCAAAAGAAGTCAAGCCCAGAATGGGTTCTCACGA
GATAGAAAATGTCAGACACGGCAAAACCCAGAATCGAAATTGTGAAGATAATGAAATAAG
TCTGACTTCTGTATCTTTACAGCCTATTAGGACCTTCAAATCTTCTAATAGGAAATGCAT
CTGGGAAGTATGATGTTCTTGCTCAAAGGTCCTCTGAACTGTACTCCTTTTAGCGTACTG
TTTATCAAATGCTGGGAATTGCTTGGTATACGTGTAAGATCTCTTTAGTAGTTGTGATTA
CTAAATTACTGGTATCTGCCTTCAGAAGCTGCCATGCATTTCTTTACAGTTAGTGAAATC
TTTATCAAATCCTTGTCATTTGAGAATGGATATGCTTTTTTGAAACAGATGATCTGTAAT
AAATTCAAAAAGGGTAGGTAATTTTAATCAAAAGTAATTGTGACTATATGAGATGATTTT
TCAGTTTTTCTTCAGAGGTTAGTTTGAAAGTAGTTTCCAAAGTGCAGTTCCAAAGATACG
TTGAGCAATGGCAGTGTCCATAAACTGATCAACTGATCCCTTTTCATCTTACTCAAAAAC
ACCAAGACAGCAAAGGCTTTGTTTTTGTTTTTGCTTGTTTTTTCCTAGAACCTGGAGAAA

TTTGTGGCATCTATAAATACTGTTTGACTGAAATTATATTTCAATAAATGCTGTTGGAAT
AGATAATAAATGAACTTTTTAGAGTGATATCTTTAGAAGGAATCATCTTTTGCGTCTAGA
AATTACTGTATATTTACATGCTGATTTAAAATTATAGAATAAAATCTATTCCTTTAGTAA
ATATTTATTGAATATGAACCAGGATTGTGTTTCATGTGCATACAACTTCATAAGATGTTC
CCTTAATAATTGTAGTGACTAATATTTCCTTGATCTATTCTCAGTCCAGAACTTTCTCTT
CAAAATATAAAGTAGCTATTAATGATTCTAAAATTCAGATTTATAAAATTGTTTTTATAT
TATATTGATTGTGTTAACATTTATGTGTGTATATAAATATATATGTGCATTTTATTGT
GTGCCTCTTTGTAG**GTGTATGCTTGTCAGCATTGTCAAGTGGCAAAAAATACAGTTATTC**
TAGCACCTAAACAGCACCTTCTCAAGGTAGAAAATCCATGGAGTATAGTTACTGTTGATC
TGATGGGACCTTTTCATACAAGCAAGAGAAGTCATGTATATGCTATAATCATGACAGATT
TGTTCACAAAATGGGTGATGATTTTACCTCTGTGTGATGTTTCAGCATCAGAAATTTCCA
AAGCTATTATCAATGTATTTTTCTTATATGGACCTCCTCAGAAAATAATAATGGACCAGA
GAGATGAATTCATTCAACAGGTAAGATAAATAAACATAGGTGTGGGAGCATATCTTCCTC
CCTTTCAGGGTCCAGCATACAGTACCCTTTGCATTACATACAGAGGTTTAATATTGTGGA
CCACAGGTGGTTGAACTAGAAATTAGGAGACCAGAGTTTTACTGTTTACTATGCCACTAG
GAAATTGTAAAACTGTTTTCAGGTTTCTTCAAACTAAAACCAAAGAAGTAATCTAAATGT
TAAAGTGATCATAGATCATTAAAACCACAGTAATTCTTAAAACTACAGACATTCTTAACT
GTCGATTTGACAAAGATATAAAACAAAATTTTACAGAAATATTTTTGCAGCATATTTAAA
AAGACTTATTTATTTTCAAGATCAGAGTTAGAGAGAGAGAGAGAGAGAGAGAGAGAGAGA
GAGAGAGCGGGAGCGCACATGCACTAGAGACAGCTCTTCCACCCACCGGTTCACTTCCCT
GATGGCCACAATGGCCAGGGCTGGGCCAGGCAGGAGCCAGGAGCTTCAGCCAGGTCTCCC
ACATGTGTGGCAGGGGTGCAAACACTTGGGCCATCTTCTGTTTTCCTAGGCCATTTGCAA
GGAGCTGGATCAGAAGTGGAGCAGCTGGGACACAAACTAACACCAACTTGGTATCGTAGG
TGGCAACTTTACCCAATATGCCACAATACCATATTCAAACTGATGAGATTATAGATTTTT
TTTCTTAGTTTTTCAGTTTATATGTACTGTGATTTTGTTGCTTTAATGATTGCTTGAGTT
GAATGTATAAACAATCCATTGTGTTTTAATAAAAATTACTGGTGCTGGGACCCGTGTTGT
CATGCAGGAGGTTAAGCTGTTTTGCAACACCAGTCTCTCATGTGAACACCAGTTCATATC
CCAGCTGCTCCCCTTCTGATCCAGCTGCCTGGTAATGCAACTGGGAAAGAAGCAGATGGC
CCAGTGCATGGACCCCTGCCACCCATGTGGCAGGTGGAATTCCCAGATGGAATTCCTGCT
TCAACCTGGCCAAACCCCAGCCTTTCTAGCCATTTGAGGAGTGAAACAGTGGATTATCGC
TCCCTCTTATCTTGGTGTTTTTTTTTTTTTTTTTTTTTTTTTTTTGGACAGTGAAAGAGAGA
GAGACAGAGAGAAAGGTCTTCCTTCTGTTGGTTCACCCCCCAAATGGCCGCTCAGCTGGC
GCTGCGCCGATCCAAAGCCAGGAGCCAGGTGCCTCCTCCTGGTCTCTCATGTGGGTACAG
GGCCCAAGCACTTGGGCCATCCTCCACTGCCTTCCCAGGCCACAGCAGAGAGCTGGACTG
GAAGAGGGGCAACCAGGACAAGAACCTGGTGCCCATATGGGATGCCGGTGCCTCAGGTGG
AAGATTAACCAAGTGAGCCACGGTGCCGGCCCCTGACTTCTTATCTTTAAAATGGGATTA
ATCATATCTTATAAAATTGTTGCAAAGTTTAATGGTAATATAAAGCATCTACTGTGATGA
CCAGCGTGAGGACTCAGTACTAACTTCTGTTTTAAACAAAATATGCATCCTTTTTATATA
CAATGGCTTGGTCAGTGAAAAAATTTCAAATTCAAGTTTTTTTTTTTTTAAAATCATTTGA
AAAAATATTACATTAATTTTTAAAAGTTAGCTTTGTTTTATGTATGTTTCAG**ATCAATGT**
TGAGCTGTGTGGATTGTTTGGCACAAAGCAAATTGTGATTTCTCACACCTCTGGAACTAT
TAATCCAATTGAAAGTACACCTAGCACAATCAAAACATTTCTCGCCAAGCACTGTGTGGA
GCATCCAGACACCTGGGACGATCACCTGTCGGCTGTTTCATTTGCTTTCAATATCACTCA
CTTGGTGTGTTCCTATTTATAATCTTTTTTTTTTTGAAAGGCAAAGTCACACAGAGAGGC
AGAGGCAGAGAGAGAGAGAGAAACTTCCACCGGCTGGTTCACTCCCCAGATGGCTGCAAC
GGCTGGAGCGGGGTCTATCTGAAGCCAGGAGCCGGGTGCTTCCTCCTGGTCTCCCACTTG
GGCCATCTACTGCTTTCCCAGGCAGTAGCAGAGAGCAGTATCAGAAGTAGAGCAGCCAGA
TCTCCAACTGGTACCCACATGGGATGACAGCAGTGCAGATGGCAGCTACACCTGCTATGC
CACAGTGCCTGCCCTCCTTTTTATAATTCTGTATTTATGAGTTATGATTATCTCTTGCTA
GAAAAATGTTTTATTACAAATTTGCTATTTTCCTTAAAATAG**GAGCCTACTAAAAATACA**
CCATATTTTCAAATGTTTAATCGAAATGCTTACCTGCCTGAGACATCAGACTGCCTTAAT
GAAGTGGATGGTGACAATACAAGTATGTTTGCCAGAATTTTTGCTGCAGTTAAAGAAGCT
GATAAAACAACAGAGAATAAGACAACTTCAGTGGGCCAGGTGATTCTCTTTAATAGTAAA
ACTCTATGAAGTGATATTAAAAAAATAATTTCAGTATATACCAGTAAGTGTGCATATATA
CACAGACACATACACAAAAACATACAATATACCCTTAAGCCAAAATGTGTTATGTATCAT
GTTCTGCTTCTCTAGGTTAACTAGTAGTATGGTTTTTAGCTAAAAACCATGAATTTTGGA
ATCAAATTTTGGATTGAGATGGATGGTCTTGCTGCTTTCAAGTTGCTTGACTTTGGCAAA
TTCCTTAACCCATGTTAATGCTTTGAGATGTTATTTTAAGAATTCAGAAGGATTCGTGTT
TACCTTTTACTTATCATTATCTGCACTTAACTTTCAAGAAATTACAATTCTTTCATTCTT
TATTCTTTTTTTCCTTTTACATTTGCAG**ATGAAGAACAACAATGTGGATGAACTAAATAA**
AAGCAAGATCATTGTTAAAAAGAAACCAAAGCAATTAAATCCTTTTCATTTGAAAGTCGG
TCATGAAGTTTTGAGGCAAAGGAAAAATTGGTGGAAGGATGGTCGTTTCCAGTCTGAATG
GGTTGGTCCTTGTGTCATAGACTATATTACAGACAATGGATGTGCTGTTCTCAGAGACAA
CACTGGAGCTAGACTTAAAAGACCTATCAAAATGTCCCATCTTAAACCCTATGTAAGAGA
ATCCAGTGAACAAGGTAAGTGTCTGTCACTTCTCTATATTTTTAGTTTCTTCAAGTGAGG
CGAGAAATTAAAAATACAAGAAATCTTTCTTCCTCTCTATGAATAAGTTTCCTTTTCCCT
CTCATTTCTATGGTTGGTATAGTTTTAGATATCCTGGGTAGTCTCTTTTATTAGAATGCC
TCTTCTGGACCTGTCAGGCTTGTGAGCGATTTTACTTTGAAATCTATTTATTTCTCATAA
TACATAGGGGAGATACTGAACTTGTTTCACAGAGGAAGGATTACATCTGTTTTCACTTTC
ACAGTATCCTGTTAATCAATTTGTGCCTCATTTTCACATAGGAAAACAGAAACTAAGATC
ATTAGTGTATAGTCCAAGCATACTAGAATCTAGTTGTAAAAAGAATAAATTTGGATACTT
AAAAAATGTTACTAATTTTTTAAGATTTATTTTATTTATTTGAAAGGCAAATTTACATAG
AGAGAGAGAGGGAGAGAGAGAAATCTTCCATCCTCTGTTTCACTCCCCAAATGGCCACAA
CAGCTGTGGCTGGGCCAGGCCAGAGACAGGAATCAGGAACTTCATCCAGATCTCCCATGT
GGGTGCTATAGCTCAGGCACTTTGGCTGTCCTCCACTGCTTTCCCAAGCACATTAGCGGC
CAGCTGTGTCAGAAGTGGGACAGCTGGGACTCGAACTGGTGCTCAACTGGGATGCTGACA
TTGCAGGTGGTAGCTTAACCCAGTATGCCACAACACTGAGCCCTAGTCATGGATCTTTAA
TATTGCTTCATAGTGAGGTCATGGACGGAGGCAGATGAGGGTACCTGGTAGTGTGTATTC
AGAATGAAATATGATAGAAATACGGCTATATTTACCCTAAAATACGGCTGGTTCTTAACA
GGCTGTTCTAATCTCTGTAACCCTTCTTCCCCATAGATTAATTTAAGGACATTTTTGTCA
GGACTTTAAGCTCCCTCCTTTATCAAAACTTTCATTTTGTCCTGTTTTACCTGAGGTAGA
TAACTGAAGTGGAGTTAACTGGTACCTTAGTTAATTATGCTTTTGCTGTTAAGCAAGATA
AAGCATTAATATTATTTGAACATCATTAACAATGGAAGCTAAATTTATTATATTTTATAC
CTCATAAACAATTAAGTTATTTAACCAAGTAAATAAATAACAAGAGCAACAGCAACAAAT
AACCTGCACCTGCCCTATTCCTTTTTTAGATGTGAATTTCCCCAGGAGCTGCCTAGTAGC
TCAGCTCTGCTTCAGATCTGGCCACAACTCTTGTGAGAAGAGCTCTGTCCTTCTTTCTGT
GCCAGATCCAGTTGGTGTAGTCATAAACTTATCTTGGACTGTGTTCTAATTCTGAAAACT
CACAACTTCATTCTCGTGGCTAGAAGAAAAGCACTGTCATTTATTTTGCCTTTTCTCAGT
TCTGCGCTACTCTTTATGGTCATTCCCTTCCACAGTTAAGTATTGAAAAAAATATTCCAG
TGATGGGTCTGTTCCACCTCAGCATGATTTTCTTTTTACTCTTTGGCACTTGCTTCCTTC
TAAGGTGTTAATGTTCCTTAATTAATACAAAATTTACATTTAAAATGTTGCTAGACCGGA
TGTATTTCAACATTTTGCAATTTTGATATTGCCAAATACTTTCTTAGAAAGGAGAACAGA
TATTTTGCTGCTTCTGAATCTTTCAGAGCCCAGCTGAGAACAGTTTCTAAACAAAGGCTA
TGGAATTTATTGTCTCATGCTTGAATTTTCTGCAGCTGTTTTTTTCCAGTAATATAAAGA
AACTGTAATAGGACTAGTGATATACGTGTAAGGAACCTGAAATTAGTGAATTTGTTTTAG
TTTTTCTTGGAAACCAAAGGAAGCAGTCACTAATCTGACCATCCTAGACTCTAGTTCACT
TTTAAGTACTGTTTTTTGTGTTTCTCTCATTTGTAGAAATTCCAAATAAATGTAATTTCT
ACTATCTTTTTCTTCATTATCACATTTTCCTGAAAATCTTCACTTCACAAGCTAATTATA
TAATGTTTGAGCTGAATTTAAAGCAAACATATTATCTTTAAAGAATCATCCAATATTTTT
ATTTGATATAGATTTGATATAAATTTCATTTGATATGAATCCAAAGGAACTATAAGTTTG
TCTGTCTATACTTGTCATTGTTGCTTTTTTTTAACCACCCTTAAGACTTCATAGCTGTAT
TAAAGACTCTGTCTAAAGCCTGATATAATTGTATTGCCAGCTTCTTATCGCTGCTTATCT
ACAATATAATGTAGAAAATCACATTCAGTCTAAGTCTATAATATTTTTCATAGCTGTTTT
AAGGCCTAGTTGATATTTTATCTAGTACAATGCCAAATGGTTTTTCAGACTAGTTATTTA
ATAAAAAAAATTAAAATATAGTATCTTTGTAATCATGTTCCCCTTTGAAAGGAAAAATGA
TGTTATTGACTCATTTCCATCTGAGATGGATTTTTTACATAATCTAATAGTTAATTAAAA
TATATGGCTAGAACTTTCTCTTAAAGAGATTTTAAATATTTTATTTGAGAGGCAGAGTAA
CAGACAGAGAGGCAGAGACAGAGAAAAAAGTCTTCCATCCACTGTTTGTACCCCAAATGG
CTGCAATGGCTGGAGCAGGGCCAATCTGAAGCATGGAACCAGGAGCTTCCTCCAGGTCTC
CCATGTGAGTGCAGGGGCCCAAGCACTTGGGCCCTCTTTCATCTTTCACTGCTTCCCTAG
GCCATCAGCAGAGAGCTGGATCAGGAGCAGCCGGGACACCATCCAGTGCCCATGTGGGAT
GCCAGCACCGCAGGCAGAGGCTCAACCTACTGTGCCACAGTGCCAGCCCCTGAGATAGAT
TTTGAGCAGTAGTTTTTAATTTATTTCATTTTGTTTCTGATATATGTTGGTCTGCCAAAG
GTTTTAGTCTGATTTTCCTTCATAATTGAATATACCTAGTCTCCTTTTGTGTAATGATTG
TCATTATTATAAAATGTCCTATTGCTGCTATAACGAATTACTACAGATACAGTGGCTTAA
AACAACACAAACCTATTGTCTTACAGTTTTGCAGGTTAGAGGTCCAACGTAGGTGTCACT
GTAAGATGAAAGCCTTGGCAGGATTGGGTTCCTTTATGGAGGCAATAAAAGAGAAACCAT
TTCTTTGTTCTAGTTTCTAGAGGCTGTCTTCCTTGGCTTGTGGTCTTTTCTCTGTCTTCA
AAACCAGCGGTGGTGGGTTCAGTCCTTCACACATCGTATCACTCCAGCTTCTAGGATAGT
CTCCCTATTTTAAGATGAATCCCCTCACTGCCTTAATTCTCTTTAGCCTTGTAAGGTTGC
ATAGTCAGTTTCCAGGGAGTACTATGCAGAGACATCTCTGGGGCCTGTCTTCTGCCTTCC
ACACTTACAATCCGCATTCATGAAACAAAGCAAAACTTAGCCTCAGTGCTGTTAAGGTCC
TGATCTTGACATTCTTACTAACACAGTTTATGCAGAGTCTTCCACTGGCCTCTAGGTTGT

TTAATCTCTAAACCCAGGGTTGTTTCAGTTGTGTGTTGATAGAGTTAGTTATTATAATAA
CAATATGTAGTATTACATTATAGTTATTACTTGTTGTTTTATCCTGAAAAAGGATATAGA
GTATGACAGCACTTTCTAATAGTTTGAGGATTATCAGAGAATCTGTAGGAGTAGGTTAAT
CATCTCTCTTCAGAATAAGAAAATCAAGTCATCAAAGCCTTGTATTTTTCTAACTATTTG
AAGAAATAAATTAATGCAACTCCTTCAAACTTTTGTGAGGTTTTGATTTTAATTTATTTT
CTTCCTTTGGAGAAATATAAAACCAATTTTTTATTCTCATTTTTTAGTCCTTCCAACTGC
ATAGGAAAAAGTATGTTGAAACTGCTGATAACATATTGCTGACACATCTTTAAATTACCT
CAGTTTCAAAGAAGCTGTGGATGTAATTCAGTCTGATCAAGACTGAAAGGATGCCTTTAT
CCAACAGATATCATAATAATAATGAAATTAATAGAATGAGAAAAGAACATTGCTTGGTAA
AAATCTATATTATATAACTCAGCAGAAAATTCCTTTAATATGGTATGTTTGTTTCCTATC
CCTACTCTGCTGTTTTGAGGATAAAAACTTCATCATGTTCTTTTCTATATCCCTAACCCA
GGATTGTTCTAAATCAGCATTTCCAATAGAAATACATTGCAAAAACTGCACAATATATAA
TTCTAAATTTTCTAATAGCAATATTTCAAAAGTGTAAAGAAATAGGTAAAAAGAATTTTA
ATAATATATGTCCTATAACCCCAAATGTTATTAATTCAGCATATATGTAGGCTACAAGAG
TGTCTTTTCTGTTCATCTTGTTTAAAATTGAAGAGCATTTCAATTGGCTCCAATAGATTT
CAACCACAGTAGGAGTTGTAACATATGTGATCTTTGTGACTGAATTAGATTTTGTTTTTT
TCTACACAAACAGGCTCTTGACATCTTTGGAATAATTTTCTATTTTGTATAAAGTGATTT
GTGTAGATTTGCTGAAAATAATCCTTGTTCTCTACAGTTTTATTACAACAAAGTACTTTA
AAGGTTAACACTTACTGAAAACCAAAAGTGTGTGTATGTGTGCTACTTTCAGTACGGATC
AAGGGTTTCCTCTAACAAAGTCCCTCTGTTTCTAAAATCCAGTGTAACTAGGAGGCGTTT
AAGCTTATAGTTTCTGAATACATTTCCAGCAAATGAAACTGATGACTCCACAAGCTTTAT
ATCTAGGTATGTTTTTCCCAGTTGATAGGTTTCCCGGCCATGTTTTTCTAAACTAGAACT
GCTTCATCTGTATGGAAAAGCATTTTTCTTCTTCTTTTTTTTTTTTTTTAACAAAAATAC
AAGAGAACAAATCCATTTTTATTTATTTACTTTGTAGTGGTTTGAATCCTTGAGGGGTAC
AGCATCACACAGGTTATGTGTCTAATGGCCCTCTCAGGAAGGTTGCTTCAGAATTTGGCA
TGAACGATGCCACTGTCTTCATGAGCATGACTTACCTTTCCGCGGATTACTCTGATTTTG
TTAGTTTTGTGGCCAGGAGTCACTATGGTATTCTCTGCTTTGTACACATAAACACATCTC
TTGCCAGAATAGAATTCAGTTTCATTGCGAGCATAAACACCTCAATTTTAAGAAGTGCTG
TGTGCTCCCTTTGGTTCTGGAGACCTCACTTAGAGCCAGCAAAAATGGCACCTGGACATA
GTTACCTTTTAGACGTCCTATTCCCAACAGGCCTCCACAGGCTCCAAGATGGCAAAAAGG
GCAGGCATTTTTCTGTCAGCAGGTTAATAGTGAACTTCAATCTTGTAAAAATATATTCAG
TGGTTCATTGTCATTGGTCCTTTGTTCTTCCTTCTTGCTGCTGTCGGGCATAACATTTCT
CACAATTTAGTTCTTGTCTTGCAGAATTATTCCTTTTGTCCAGGAAATCATCCCATACCT
ATCATCTTAACATCTTCAGTTACCCGTACTTTTATTCTGTCATAAATATGTGTAACTTCT
TGTCATCTTTCAACCTATTCACTGTTTTTGCATTAATAAAATATGATGAGGTTAAATTCG
GAAAACCTTTCAAAACTGAAATACCAAATGTGCTTTGCCTAATGAAGAACTAAGTGGCCA
TACAAGCCCACTTATGCCATCTGTTGGTAGAGAGCATAGCATTTATGTGATTAACTATTA
AAGATTGTAAATAGTTCCTAAATTTACAAACTTCTGGGCTGCAATAGACTCAATGAGCTT
TTGTTTTCAAGATTTATTTATTTACTTGAAAGAGTTACACAGAGAGAGAAAGAGAGAGAG
AAAGAGAGAGAAAGGTCTTCCATCTGATGGTACACCCCCAGTTGGCTGCAACGGCCAGAG
CTGCGCCATTCCAAAGCCAGGAACCCGGAGCTTCTTCTGGGTCTCCCACATGGGTGCAGA
GGCCCAAGGGTTTGGGCCATCTTCTACTGCTTTCCCAGGCCATAGCAGAGAGCTGGATCG
GAAGTGAAGCAGCCAGGTCTCGAACCAGCACCCATATGGGATGCCACTGCTTCAGGCCAG
GGCATTAACCCACTGCACCACAGCCCTGGCTCCTCAGTGAGCTTTTTAAATATAATTACA
TTGGCCGGCGCTGTGGCTCAATAGGCTAATCCTCCACCTAGCGGCGCCGGCACACCGGGT
TCTAGTCCCGGTCGGGACGCCAGATTCTGTCCCGGTTGCCCCTCTTCCAGGCCAGCTCTC
TGCTGTGGCCCGGGAGTGCAGTGGAGGATGGCCCAAGTCCTTGGGCCCTGCACCCCATGG
GAGACCAGGAAAAGCACCTGGCTCCTGCCTTCGGATCAGCGCAGTGCGCCGGCCGCTGTG
CGCCAGCCGTGGCAGCCATTGGAGGGTGAACCAATGGCAAAAGGAAGACCTTTCTCTCTC
TCTCTCTCTCTCTCTCTCTCTCACTATCCACTCTGCCTGTCAAAAAAAAAAATATATA
TATATATATATAATTACATGGATGGGTTGAATAGATTGTTTTTCGAGATAGAATAGCAAA
TTTATCTGCTCTGAGAAACTTTCATTGATTACTAATTTAAAGGGGCAACAGATCTTCCTT
TCATCGGAAGTTAAAATTTAAAGACTCTTTGGGGCCGGAACTGTGGTGTGGCAGGTAAAG
CCACCGTGTGCAGTGCCAACACCCTTGTAGGCTCTGGTTCAAGTCCTGGCTGCTCCACTT
CCTATCCAGCTCTCTGCTATAGCCTGGAAAAGCAGTGGAAGATGATCCAAGTGCTTGGGC
CTCTGCACCTGTGTGAGAGACCCAGAAGCAGCTCCTGGCTCCTAGCTTCAGATTGGTGCA
GTTCAGGTGTTGCAGCCATTTGGGGAGTGAATCAGCAGATAGAAGATCGATCTCTCTCTC
TCTCTCTCTCTCTCTCTCCCTTTCTCTTTCTCTTTCTGCCTCTCTGTAACTCTGCCTTCC
AAATAATAAATAAATCTTTAAAAAATTTTAAAAACTCTAAGCTATCAAAATATTCAAGAG
AATCTGTTCATGTTTGGGCAGAAAATCTGGAAAGAAACCAGGTCAGGAATTAATGAGCTG
TGGTGGAAGACTTCCTTGTTGGAATGAATTAAAGTCTCAGATAGGGAGGGATCCCTTTAT
GCTGCTTTAGTATCAGCTTGACATTCATTTTAAGTATTTTTTCTTGATGTTATGATATAT
TTGCTCAAAATCGAATAAAAGGAAGATTCCTTGTAAATCTTTCTATCTGGATTAGGTAGG
CCTTAAAAGTTCCTATGAACTTTGGTAACAAATTAGGTTTAGGGGCCGGGGCTGGGGGCT
CATTTGGTTAATCCTCTGCCTGCAGCGCCGGCATCCCATATGGGCACCAGGTTCTAGTCC
CGGTTGCTCCTCTTCCAGTCCAGCTCTCTGCTGTGGCCCTGGAGGGCAATGGAGGATGGC
CCAAGTGCTTGGGCCCCTGCACCCGCATGGGAGACCAGGAAGAAGCACCTGGCTCCTGGC
TTCAGATTGGCGCAGCTCCAGCTGTAGTGGCCATTTGGGGAGTGAACCAATGGAAGGAAG
ACCTTTCTCTCTCTCTGTGTCTCTCTCACTGTCTGTAACTCTACCTGTCAAAAACAAAAA
CAAAAAACACATTCCTATGAACTTTGGTAACAAATTAGGTTTAAATAACTAAGCTTTTTA
AGCTAATCATTGCAGCCTACTTCCTTATACTTTCCTAACACATACACCTGATTCTAGTGA
CCTTTGTCTTCATTTACTGTACAATTTCATATTGTAGGAATTTAAACTAATTTTTAGTAT
GGGCTTTATAAAAATTGAAGCATTATCTAAAATTAATCATCTAAAGGCAAGGATTTAACA
ATTCTGCTTGTTAAAATACTTTGATTTCCGTATTAATGCATCCTGAAATGCTTTGTAGAG
GTGTATTGAGGATATCGTCATTATCAAAAGCAAAGTTTTATAATTTTTTTTCTAATCCAA
ACTACAATTCCTACTTTATTCCTGTCCCCCATACCTCCCCCATATATACCCAAGATGGAT
CAAGGGAACAGGAAATTTTGGCAGTTTTAAAATCCTTTATCTTTTCTGAAATCTTTTTCT
TTTTTAAAGAAGTATTTATTTATTATTTGAAAGGCAGAGTTAGAGTGGAAGAGGGAGAGA
AAGAGAGAGAGAGACAGAGATCTTCCATCTGCTAGTTCACTTCCTAAATGGCTGCAATGG
CTGGGGCTGAGCCAGGCCCGAGCCAGGAGCCAGGAGCTTCATCTGGAACCTCACACAGGT
GGCGGTGGCCCAGACACTTTGACCACCTTCCACCTTCCACTGTGCATTCCCAGGCACATT
AACAGGGAGCTGGATCAAAAGTGAAGCAGCCAGGACTCCAACTGGTACCCATATGGGATA
CTGGTGTTATAGGTGGCAGCTTAATCCATTGTGCTACAATGCTAGCCCCATTGCATTATT
TTCTATATGAACTGTCAAATGTGTATATTATTTTATTCATTCTTTAACTTGTGTAAATTA
GTACATAGGAGAACTGCCCAGAAGTTAATCTTCATAGTTAATTTTTATTATGTGGAATTT
TGTTCTTCATGTAACATTATGTAATTTTAAAATGTTTAATTATAGTTCAAGAAGGCCAAC
TTATGATATCTTTTCTTTTAG**ACAGTCTTTATCTCTTGCAAGGTTCAATAGTGGCAGATC**
ATGACTACATTGGATTGCCTGACCTTCCAGTTGGAGCATACCAAGCAAATATTCTTGTAG
AAGATGCAACTATTGGTGTAGTTGATAATGAATTACTGACATCAAGCAAGGATCGTGAAC
TCTTAGAATATAGAAGTGTCAAAATCTCTCCATTGATGGAAGATCATGGTACTCTTGAAA
AACAGACTTTCAGTCTATTGGACTCCTCAAATCAAGTCCTTGAGTACTTAAGTTAATAAA
TACTGACATTTATTTAAAATGTTTGTTTAGAATGTATAAATCCCTTAAATCTTGATATTT
TATGCAATAAAAAATATTGTAGAGGATAAATGTCTTGACATGCTTTTAATGACTAAATCC
TGAAGGTATATTTTCTAACTATTTATTTATGTAAATTTGTGCCACATTTGACTATCAGTA
TACATATAATGAATAAAGGCAAAGGAAGAATACAATACATAGAAATGAGTTTTTCTTCTA
AAAAGAGAACAGGCAAAGCACCTTTACTTTGATGAATATCAAAGATTTTAAACTATAATT
TCTATGATATCACTCATTCAACCAAATTTTAGTATAAAGCAGTATGGAAAATTACCTTTC
CTCACATGGCGCTTACTATCTGGTTAGAAATATAGACCATTATAAGCTGTGTATAACACA
AACTACAAGTTAGTAAATCCTAAATTCCAGGAAAAAAGTATAGAGAATGTACAATAGGGA
GGCAAAACAGAGCAGTCATTTCTGAACCTGAAACGCAGGTTATCCTCATCTGTGAGGGCT
CAGATGAGTTTGTCCTCGAGAAATGCAGAGGATTTGGAGAGGAAGAACCAGAGAAT

==

Rabbit Gin-1

```
total length:      16976 bp  (16976 bp excl N/X-runs)
GC level:         37.71 %
bases masked:      4911 bp ( 28.93 %)
==================================================
               number of      length   percentage
               elements*    occupied  of sequence
--------------------------------------------------
SINEs:                15        3818 bp   22.49 %
      Alu/B1           0           0 bp    0.00 %
      MIRs             2         141 bp    0.83 %
```

LINEs:	1	119 bp	0.70 %
LINE1	0	0 bp	0.00 %
LINE2	1	119 bp	0.70 %
L3/CR1	0	0 bp	0.00 %
RTE	0	0 bp	0.00 %
LTR elements:	1	407 bp	2.40 %
ERVL	0	0 bp	0.00 %
ERVL-MaLRs	1	407 bp	2.40 %
ERV_classI	0	0 bp	0.00 %
ERV_classII	0	0 bp	0.00 %
DNA elements:	3	294 bp	1.73 %
hAT-Charlie	1	151 bp	0.89 %
TcMar-Tigger	0	0 bp	0.00 %
Unclassified:	0	0 bp	0.00 %
Total interspersed repeats:		4638 bp	27.32 %
Small RNA:	1	8 bp	0.05 %
Satellites:	0	0 bp	0.00 %
Simple repeats:	4	123 bp	0.72 %
Low complexity:	6	150 bp	0.88 %

==

SW score	perc div.	perc del.	perc ins.	query sequence	position in query begin	end	(left)		matching repeat	repeat class/family	position in repeat begin	end	(left)	ID	
1138	12.5	2.1	1.1	UnnamedSequence	1	186	(16790)	+	CSINE2B	SINE/C	137	324	(3)	1	
242	13.4	3.0	9.7	UnnamedSequence	129	194	(16782)	+	SINEC1_AMe	SINE/tRNA	140	201	(0)	2	*
315	40.3	1.7	0.0	UnnamedSequence	1551	1669	(15307)	C	L2b	LINE/L2	(4)	3371	3251	3	
232	21.7	7.6	10.0	UnnamedSequence	3197	3288	(13688)	C	MARNA	DNA/TcMar-Mariner	(178)	408	319	4	
231	22.0	0.0	2.0	UnnamedSequence	3383	3433	(13543)	+	MARNA	DNA/TcMar-Mariner	399	448	(138)	5	
21	71.4	0.0	0.0	UnnamedSequence	3879	3906	(13070)	+	AT_rich	Low_complexity	1	28	(0)	6	
200	31.9	0.0	0.0	UnnamedSequence	4393	4439	(12537)	+	MIR3	SINE/MIR	30	76	(132)	7	
1335	21.2	7.1	0.6	UnnamedSequence	4620	4649	(12327)	C	CSINE2B	SINE/C	(8)	319	288	8	
342	0.0	0.0	0.0	UnnamedSequence	4650	4687	(12289)	+	(GA)n	Simple_repeat	2	39	(0)	9	
1335	21.2	7.1	0.6	UnnamedSequence	4688	4950	(12026)	C	CSINE2B	SINE/C	(40)	287	7	8	
1035	23.2	6.9	2.6	UnnamedSequence	5087	5347	(11629)	+	CSINE2B	SINE/C	1	272	(55)	10	
1504	17.5	4.8	0.3	UnnamedSequence	5359	5673	(11303)	C	CSINE2B_OP	SINE/C	(0)	329	1	11	
28	81.6	0.0	0.0	UnnamedSequence	5857	5905	(11071)	+	AT_rich	Low_complexity	1	49	(0)	12	
861	19.3	5.3	3.4	UnnamedSequence	6145	6434	(10542)	C	CSINE2	SINE/C	(20)	324	2	13	
234	35.9	3.2	2.1	UnnamedSequence	6877	6970	(10006)	+	MIR	SINE/MIR	15	109	(153)	14	
1007	26.4	7.2	0.9	UnnamedSequence	7816	7858	(9118)	C	CSINE2B_OP	SINE/C	(4)	325	282	15	
186	4.3	0.0	0.0	UnnamedSequence	7859	7881	(9095)	+	(GA)n	Simple_repeat	2	24	(0)	16	
1007	26.4	7.2	0.9	UnnamedSequence	7882	8143	(8833)	C	CSINE2B_OP	SINE/C	(48)	281	1	15	
22	40.9	0.0	0.0	UnnamedSequence	8498	8519	(8457)	+	AT_rich	Low_complexity	1	22	(0)	17	
22	45.5	0.0	0.0	UnnamedSequence	8500	8521	(8455)	+	AT_rich	Low_complexity	1	22	(0)	18	
27	44.4	0.0	0.0	UnnamedSequence	9774	9800	(7176)	+	AT_rich	Low_complexity	1	27	(0)	19	
1221	22.5	2.4	4.9	UnnamedSequence	9915	10250	(6726)	C	CSINE2B_OP	SINE/C	(1)	328	1	20	
1144	22.1	27.9	1.8	UnnamedSequence	10397	10803	(6173)	C	MLT1D	LTR/ERVL-MaLR	(10)	495	1	21	
418	19.1	7.3	3.2	UnnamedSequence	11595	11745	(5231)	C	MER33	DNA/hAT-Charlie	(0)	324	168	22	
180	11.1	0.0	0.0	UnnamedSequence	12263	12289	(4687)	+	(T)n	Simple_repeat	1	27	(0)	23	
1557	17.8	2.4	4.3	UnnamedSequence	13199	13533	(3443)	C	CSINE2B_OP	SINE/C	(0)	329	1	24	
2275	6.3	7.5	0.0	UnnamedSequence	13563	13849	(3127)	+	CSINE3A	SINE/C	1	287	(63)	25	
294	2.9	0.0	0.0	UnnamedSequence	13850	13884	(3092)	+	(TC)n	Simple_repeat	1	35	(0)	26	
2275	6.3	7.5	0.0	UnnamedSequence	13885	13914	(3062)	+	CSINE3A	SINE/C	288	341	(9)	25	
22	54.5	0.0	0.0	UnnamedSequence	13915	13936	(3040)	+	AT_rich	Low_complexity	1	22	(0)	27	
1899	15.3	1.4	5.9	UnnamedSequence	14071	14425	(2551)	+	CSINE2	SINE/C	1	340	(4)	28	
1453	17.2	0.6	4.5	UnnamedSequence	14742	15064	(1912)	+	CSINE2B_OP	SINE/C	1	311	(18)	29	
1273	22.1	0.6	7.9	UnnamedSequence	15537	15889	(1087)	C	CSINE2B_OP	SINE/C	(0)	329	1	30	

>Mouse Gin-1 (chromosome:NCBIM37:1:99666149:99690886:1)
GAACGTGGGTAGCTCCCTAACTAGCGGGACTGCAATTTCGGGCTTTTCAAACTCTAGAAA
TGAAAAGAGGAAGCGACTCGGGAAGCGGAGGAGAAATCAAGCCTGAAAGGGACCCTGGGG
ATGTTCTGTCACTCGTCCACAAGCACGCTGCCCTCCGCGACTGTCCGTGGCTCTTGCAAG
ACCACGTGTCCCGCATCCGCACCGTGGAGCAGAGACAAAGCAAACACCTGGAGGCGGCGG
CGAGCCTCGGGCTGCCGGAGATGTCGCCTCTCCCGGTCCTCACCTGCCGTGGCTGCGGGC
AGCAAGCGATCTGCGGGAGATGCTTGAGCCCATCCAGGTTTCGGTTCCTCCCCCTTTAAT
TGCGGGGGCGAGGGAAGGACGCTTGTGCCGAGGCGGCTCTCGGAGGCGTGGACCTGAAGC
TTCCCTCGCCGGCCACCGCGGAACCGCTGGCACTGGCACACCTCAGGAGCCTCCGACACG
CCTCTTCCCTGCCCGCCCTCCACAGGGCTCCCACGGAAACTGCACCGGCCGGAACAGTTT
CCGGCGGACCAACGACCCGCCTGAGCCTTCCTGTTCCACAAGCGCCACCAGAACTCCGAC
CCCCGGCAGTTCCGCTTCCGGTAGCACCAGATAAATCGCGAGAGGAGAGTTAAAAACCTG
CCGTTTGCACTTAGGACCACTTCGGTGAGTGGTCGTTCTAGTGTGTTCTGTCATACCTAT
TTTTCTTTATTTTTTTCTTCATTTTTTTTAAAAACGGAGACGTAGCCTTCCAGTAACTTT
AAAACGGTCCCCTTCGACTGCTCTAAGGACGGGTTTGACTAGCGGAGGTTTCGCGCAGGC
GCCGTTTGGGGCGGGAGGGCCTTGAACTTGCACAGCGCCGCGGATGGTGGCTGCAAAGAG
GCGAGGACTGTTGGGCTCAGGGAGGTGGCGACTCGGTATAATTGAGCTGTCAACGGTCCT
TTTCCTGCTTAG**GCAAGGATTAGCGGGACCTAGCCCTCAGGGGTCCGTTAACGAGGTGTC**
AAAAAGCTTGCGGAGCTCTCCCTTGCCGGCGGCGGCGAATGCTGGACTGCCTTCAAAACT
CGAGTGCAGCGGCGCTTGTCCGAAGCTGGAGGAGTTTCGTCCTGCATAGTAGTCACCATT
CAGTGTGAAAACGTACACCTGTGTAAAGGAACGAGCTCAAGGGGGGTGGGGTGGGGACCT
TAAAGCTCTAAAGGGAAAATACACTTTTTAAAAAAATCTCAATTTTCAAAATGGACCTGA
ATTTGTCCTGTGCGGCTTTATCTGTATGCATATGGAAATAAAGGGAAAAGAGGCAGGGTA
TGGTCTGAAGTATTGCCGCCTCAGGCTTATCCCTGCCTCCTCCCAGTTAATATATAATTG
TACGGGTTCAGTTTTCAGAGATTAAAGCAGAATAATTCAAAAGTCGGAACCCTAATCCCA
CAATCCTGTGTTTATTAAAACATTACAACTTTTGTGTTTTGAAGTTAGAATACAAACTTA
CTGTGTAACCTTTCATTGGCTTTAATAGTAGCTTCATGCTAAAAATAAAAAGAATAGTTA
ATTGTTTTCATTGGTTACGCAAGACAAAATTATAGATGTTAAGCAATAAGTATTGGTAAA
AATATTTGTTGTTCTTAACGTTGTACGAGGAACATGCTGTAAAGACCTAATCAAATATCC
TGATGAAATTGCAACACTTTGGTGCTCAGCTTTTAACGGAATGCATATGCCCAGCTGCCT
CACCCGAGCTGACCGGCCTCCCTATCATGGGACACAGGGCTGTCCAACTGTGACTCAGAA
TAAACCATTCTCCTTGGTGCCTTTCCTGCTCATTTTATAGTAACAGTGACACGGTAGCTA
ATGTGGGCAGCTCTCACAATTGAGAGGCTTCTGTACAGCAAAGGAAACATTTGAGTGAAA
AGATACCCTGCATAGTAGGGAAAAACCATTCCCACTAGTAGAAGACTGCTGCCTGGAATG
TGCAGAGAAATCAAAATACTAAGGAAAAAATACAGAATCCAGTTTTATAAGTGGGTAAAG
GACTGAGTGAAAAATTCTCAAAAGAGAAATACATGTCATCGAAACTATGTTTTAAAACTG
TTAAACATCTTTATCCACTAGGTAAATGCAGATTAAGACAAATTTGAAATTTCATCTTAG
GCCATTAAGGTCACCATCCTTAAGAGGTCAAATAACAAAATGACAAAAGCATGCAGAGGT
GGGAGGAAGGGAGCTCTTATTCAGTTCTGGTGGGGTATGAACGGGTGCGGCACTACATGG
GAGTAAGCTCGGGGCCTCTCACAAACTAAAACTAGAACTTCCACGTGACCCAACTCTCCG

GCTTCTGGGCATATCCCAGGGTATGTGAATATTTTCACAGAGACATTTACATACCTTTGC
TTACTGCTCTTGTATTCACATTAGCAAGGAAATGAGATCACTCCAGATGTCCATCACAGA
ATGTCTGTAGAATACATGTGCTATGGACTGGTACTCAGCCTTAGAGGGAAATGACATTAA
TAAACATTTCAAAAGCAAACCAAAAGGAATGACACTGGAAAATACTGTCCTAAGTAAGTT
AGCTCAGGCTGAAAAAGATACCTATGTTCTCCCGTGTTCTGACGTATTCTTCTTAGATCT
AATCTTAAGACATAAATGTGTTAATTTAGGGGTGGTTATGTGTAGGGGCCAGGAATGGAG
AAGAGAGTCCATGATGGGGCGGGGGGACCAGTTCTGAGGGAGGTAACGGAGACCAATTGA
ACACATATGATATGAACATGCAATAGAGAATACAGAGAGGGCGAAAATGTGAAAGGAAAA
GGGAGAGAGGAGTGAGTGAACGGTGGGGCCAACAGGAAATAAGTATGAATTCCATACAGA
ATCCTACTGCTTTTTACGCTATTGTAATATCATAATAACATAATAACGATATAATATTTT
AGATTGTAATAAAGAACTTATCCAATAGGGAGTTCTTCAGTGACAGGGTCTTAAGTGGGA
CGACCAGAGGATGAGAAGACAAAGAAGAAAGTAAAACCAGGATCAGGGATCTTGCCAAAG
CTGATGGCTTATGTCAAGCAAGTTTATTAAGAAGTACAGCATTGTAAAAAGAAGTTTGAG
GGAGAAGTGGGACCATGTCAGTGGAAGGCACTGTCACATAGTGAGACAAAGACAGCAGAA
ATGTTGCTAATCAAGTCATGGTGTGCAAAGGGAACACTGAGTATCTCTAGAGCACCAGGA
CTGAGTGTGCAGTGACCTTGGCACTCCTATCTATAGCAACATTCAGTGGCAAAGGTTAGG
TTCTCCAGACCTAAAGCTACAGCTACCCCCAAGGCCCTGGTCCCAAGCAGACTCCAGGTT
TTAGAGAAGGGACTGTTCCTTGCCACAGGGCCTCAGGGAAAAGCTCCCAAGTCATGTCCC
TAGGCTATTACTGGCTCTGCCAGGCAACACTAGATGGTAATTTATCTAACCCAGATACAC
TCGGTTCCTAGAAACCACCATTCTATTTTCTGTTGATATGAATTTGACAACTTTTGCCTT
TTAAAGAAATTACTTAGCATAATATCCTCAAGTTATATCCATGTTCTTTTTTTTTTTTTT
TTTTGGTTTTTCGAGACAGGGTTTCTCTGTATAGCTCTGGCTGTCCTGGAACTCACTTTG
TAGACCAGGCTGGCCTCGAACTAAGAAATCCGACTGCCTCTGCCTCCCAAGTGCTGGGAT
TAAAGGTGTGCACCACCACGCCCAGCTTATATCCATGTTATTACATGTGTTAGAATTAGC
TCTTTTTAAAAGGCCGAATAATATTTCATTATGTATATAAGCCATATTTTTTATATTTAT
TTGTCATTAGGCACTTGGGTCCCATCCTGTTTTTAACTACTATGAGTGAAGCAGCTTGAG
CTAACCTTTCAAAATAGCCTCTAACCCCACTATAAAGTAAACTTCACGAGGGTCAGGAGG
ATATGCGTTTTGTTGGTTATCATTGTATCTCCCCCCTTTTTTTTTTTTTTTTTTTTTTTG
GTTTTTCGAGGCAGGGTTTCTCTGTGTAGCCCTGGCTGTCCTGGAACTCACTCTGTAGAC
CAGGCTGGCCTTGAACTCAGAATCCGCCTGCCTCTGGTATCTCCAATTTTGTTTCTGGCA
TGTGCCAGATAATCGTGTATGTGTGTGTTGGGGGTAGTCTCTCTGTCTTTCTGTCTGTGT
ATAGGTATACTATGCATGTGTGCAGTTGGGATTTAAAGCCAGGGCTTTATACATCCTAGT
CAAGTACTGACTGACCCCCTAGGCTACATGCCTTGTCATGGTTCATCCTTTTAGAGATGC
TTGAGAAACCATGATAGCTACATTTGTCATTTGCTTGAGGTATAGCCATCTACTATAAAT
GGCTGATAGCTCCATTTGAAACTAGAGCATTCAAAGTATGGTAGTGCGTGCCTCTATTTC
TAGGACTTAGGGCTAAGGAAGAAGATTGACAAGTCTCAGGGCAAGCCTGAGCTTCATCAA
AACCCTGTAAACTAAAACTCATTGTTTAATGATGATACCTTATAGCTAAATTGACCTGTG
TAGGTTCTGATAGGACTATCTTAGCATTACAGAGGCTTATGTGGTGGTGTCTTATTTGAT
GGAGACAGTTGCACAGTTGAGGCTTCCTCTTCCCTGGTGCGTCAAGTTGACAGCCTAGAT
TAGTCATCACAATCCTTAGGTATTTTAATGAGCAGTAAAGACCCACATTGTTTTTAGAAT
TTTGAAATTGGTATGAGGGTTCCAGCAGGAAGCAGGGACTACTTTCTTGGATTGAGCAGT
GATGCTCCTATTTTCACCTGGCATGGAGTGAGAAAGAGCACGTGAGCATGTGAGCCAGGA
GTTAGCACAGAGGAAAATACTCTGACCTGTTCTGCATTCCGATTCTCCTTGCCATTGCCT
GATGCTACAAATATGGCTTGGCAGCATCAGTCTCCTAGAGCAGCCAGCCAGCAAAGAAGG
ACCAGAAATCAGTGTGGATGGTTAAGTTGATAATATACAAAATTGATAGTGTACAAAGGA
TAAACAGGCTGAGAAAGAAATTAGGGGAACAACACCCTTTACAATAGTCACAAATAATAT
AAAATACCTTGGTGTGACTCTAAGGAAGTGAAAGATCTGTATGATAAGAACTTCAGGTCT
CTGAAGAAAGAAATCGAAGAATATCTCAGAAGATGGAAAGATCTCCCATGCTCATGGATT
GGCAGGATTAATATAGTAAAAACTGGCTATCTTGCCAAAAGCAATCTACAAATTCAATGC
AATCCCCATCGAAATTCCAACTCAATTCTTCACTGAGTTAGAAAGGGCAATTTGCAAATT
CATCTGGAATAACAAAAACCTAGGATAGCAAAAACTATTCTCAACAATAAAAGAACCTCT
GGTGGAATCACCATCCATGACCTCAAGCTGTACTACAGAGCAATTGTGATAAAAACTGCA
TGGTACTGGTACAGTGACAGACAGGTAGATCAGTGGAATAGAATTGACCCAGAAATGACA
TTTCTTTTGATGCCAAGAGATTCCAAGCTAACTTTTGAAGTATACTCACTCACTTCCTTC
CCTTTCCTAGTTTTACAGGGTTGTTTTCTGTTTGTTAGTTTGTTTTAGCGTTTCTTATAT
CACATGAAGGAGACAAGATCATTTGTCGTGCTAGATCTTAAAAGTAGCGTTCAGATGCTT
TAGTTTGTAGACATCACTCTTGTCCCTGTTCTGCTCCGCAGTTATCTCCCTCACAGATGA
TATTCTGGGAATGCTCAATCCATGCATAAGCAGAAATGTGACAGAGGCCTGAACGGAAAT
AGCAACACAATTAGCTTGTCATTTAAGAGTAACATCTTTAAATAATCTTCCCTAG**GTTCG**
TGATGGTCCGTAGTGGGAAAAATGGTGACCTTCATCTTAAACAGATTGCATACTATAAGC
GCACAGGGGAATATCACCCCACTACACTGCCGAGTGAGAGAAGCGGCATAAGACGAGCAG
CTAAGAAATTTGTCTTCAAAGGTGAGACCAGTGTCTGAGTCTCAACTTATAGTTCTTCTC
CATGTGTTGTAGAACCCTTGCAAATTCTCTAGTAACTTGGTTTTTGCTAAGTGGTTGTCA
GTTAAATGAGAGATTATCTCAGAAGTAAAGTAAAACATGAAAGCTAACTGAAGGCTGGTA
AGCATGAAGAAGACATAGTAAGGGGCTGGAGAGATGGCTCAGCAGCTATGAGCATGTACT
GATCAAGTCCAGTTTCCAGTGCCCTGGGTGGGCAGCTCACAGCCACCTCTAACTCCAGTG
TCTAGTCTCAGTGTGTATCAGCACTCAACTGCACATACATTTACAACTGCGCGCGCTCGC
GCGCACACACACACACACACACACTCTCTCTCTCTCTCTCTCTCTCTCTCTCTCTCTCTC
TCGCACGCGCGCGCACGCGCGTGCAGGCGTGCATGCTCTCTCTCAATTTAAAATAAAAAA
TAAATCAAAAAAGAAAAGAAAATGACCTGTTTAATAGATCAAAAGGAATAGCCATGAAAC
AGATTACATAAATTAATTTATCTTGCTTTTGCCACCTAACCTTCAGACACTAATGCCACC
ATGCTGAGAAAATTATTGTATGCTTAGTAAGGCAACATGAACAGAGCTAACTAGTTTACT
TACTCCCCTCCCCTCACACTGTACCTTAAATTATTCTCAGGATTGCATTTGTTTTCTTGC
CTTGATGCTTCATCAGTACATTGAATATTTGACAGGCAGTAGGGCAATTTTGAAAGCCTT
AACAGCTTTGCCTCCTTTCTAGCTGCCTCCATGGGGAAAGTTCCCTGAGCGTGAGATATG
CAAAAGGTACAGTCAACAAACACCAAATGAGTGTTAGTGCTATTAGTGGCTGTTAAAGCA
TTAGGCTTTCTCCAAATCAGGGCAGAATCTAAGGATGTAAATTCTAAGGTCATAAGTAAA
CTCCTGCTCATATATCCCTATACAGATTCCTGCCAGGAACTCCTATTAACAATAGGTAGA
CCTCTTACACTAAAGTGGACGTGCATGCATTCATGTCAAAAATGCTTATTGAATTTCTGT
GCTGTGCCAATCATATCATAGGCCCTGGAGATGTATTGAGTAAGATAGTGTTTCTGTTTA
GTAATTGCCTTAGTATCTTGATGTGTTCTGAAGTCTGCATAGCACCCCACATGAACTGGA
GACATAATGGTTCTCCCTGTCCCCTGCTTGCTCCTCCAGTTAGGTATCTTCACAGGACAA
GAGAGCTGAGAATTGTGTCCCTCCCACTGTGCCATCCTACCTTTATTCAGATCAGAACAA
GGAGAGGAAGGCTGTTGCTTATAAAAACAAAACAACAATAACAACAACAACAACAAACCC
AGCTATTAATACTTTTACAGAAAAGTTGGTACCCAGAAGCATCTCATCAATACAGAACTC
ATTTTATAACCTACGCCATTTTTAGCTTCCACGAGTATTTCCCTAATTTGAGTACTTCCA
TAGAAAGGTTGTGTCATATTCCCATAGTTGGCACATGATTTGGTGAGCAAGCTCACTCCA
TTTCTGTTGATACCGACAGGATTATAGCAGATAGTGGTCCTTCCGGATCTTCAGAATGCA
TGGAAGTATTATTGAAAGGAAATAATTCTTTTTTAAAGAAAGATTTTTATGAGAATAAAA
TTAAAAATCTTCATAGCTTTAAGTTAAGGCTATAGAGCTTTGAAATTTATATTTTTTTAT
GTCATTTGTTTCAG**AAAAAAAGCTGTTTTATGTTGGAAAAGACAGAAAACAAAATCGTTT**
GGTAGTTGTTTCGGAAGAGGAGAAGAAGAAAGTACTGAGAGAGTGCCATGAGAATGGCCC
TGGTGTCCATCACGGCATTTCCAGAACCCTCACTCTAGTGGAGTCTGGCTACTACTGGAC
GTCCGTGACCAATGACGTCAAGCAGTGGGTATGGCTTCCACGCAGGGCTTAGGAGTGTCT
TACTGTCTTTCTGATAAATACAGAGCTCAGTGTTGCTTAAGAGGTACTGGCCATCTCACC
TGTGGAGTAAGTACGTTATAGGTTATTTCTGAAATAAGAAAATATTTCAATTAAAGGTAA
CTTTACTTCCCTTTATAATTACCTTACATCAGTAGAGTATAAATTAAACTAAGAAAATAC
AATATTTTGACAAGTTTATTGAGAATTACAGCTCCATTTTGCAGAATTCAGTTGAAGTTG
AAATTCTTTTGCTATTATATCCTTAGCACCAACGTGAATGATTTTTTTATATGATGAAAA
TACATCTCTTTATAATAAATCTGTTCCAGTGTGAACTCTCAGCTTTTGTCCCTATTCTGT
CTTAAGATAGAAAACTGTCAAATCAGCCAGGCTGTGGTGGTGCACATCTTTAATCCCATT
GCTTGGAAGGCAGAGGCAGGTGGATCTCTGAGTTTTAGGCCAGCATGGTCTCCAGAACAA
GTTCCTGCCGGGGCAGCCAGGACCACACAGAGAAGCCCTGTCTTGAAAAAACACAACAAG
CAAACAAACAAAAACCAAACAAAAAGAGAAAGAGAGAGAGAGAGAGAGAGAGAAAGGA
AGGAAAGAGGGAGGGAGGGAGGGAGGGAGGGAGGGAGGGAGGGAGGGAGGGAGGGAGGGA
GGGAGGGAGGAAAGAAGGAAGGGAGGGAGGAAGGAAAGAAAGGAAAGAGCTGAGCAGGGG
TGGCACACACCTTTAATCCCAGCACTCGGGAGGCAGAGGGAGGCAGATTTCTGAGTTCGA
GGCCAGCCTGGTCTACAGAGTGAGTTCCAGGACAGCCAGGGCTATACAATGAAACCCTGT
CTTGAAAAACCAAAAACAAACAAACAAAAAAAGGAGGAAAAGAAGAAAGGAAGGTGTCAA
ATCTAAATAGCAGGAACTTAAACCCAAACTGTAAGCATTCTAAATGTTCCTGTACAGCAT
TCAGTCAAACAGCCATCAAGTGGTTACAATTTGAGAAAAGATGTCCTTTTAAAAATCATG
CTGTTTATAGCTGGGGTGTGGGGAGCAGGTGTGGCGGCAGTCCCAAAAGTGCCAGGCACT
GCAGCTAAGTCTTATGACTTGCACCTGACTTCCTCATATAAGCCACAAACATCTTGAGAG
CTGCACAGGTGTACCAGGATACTGGTGAATCCATTTTGATGGAGATATGCCCCTGCTGCC
CTGATTAGCTGAAGCTGTGTGCCTGGTGAGGTGGCGTGGCCTGCTGTGCGTGGATGGGAA
CTGAGAGTATATAAGAGTGAGAGGCCCGGGTTAGAGGGAGGATTATTATTCGAGAGAGGA
TTACTATTAGTGGGGAGATATATAAACAGGGGAGATATAAAAACAAGGGAGATATAAACA
AGGGAGATATAAACAAGGGGAGATATAAAAACAGGGGAGATATAAACAGGGGAGATATGA
ACAAGGGAGATATAGAGAAAGAAGAAACAGGACTGAATAAACGTGTGCAGAAGGATCCTG
TAGCAGCGTCGTTCTTCCTGGCCTGTTGAGCGCGCGCAACAGTTGGTGCTGAAACCCGGG
AAGAAAAACTCGGGACGGGACGAGAAGACCTGGGACGAGAAACCCAGGAAAAGAAACATC
TTCAGGCAAGAGCGAAGACCCCCTGCTACAGGGAGGATTCAGAACTGCATCACGGGGAAG
GAGTGGTTAATAAAAGTTCCCGTAAAACAGACTGTTGAGAAGGATCCGGCGTGGATTCAG

AACTCTTCAGCTGGGGAACGGTACTGATGAAGAGAAAGAAGAAAGATGAGGACTGAATAA
ACTGCTGTTAGAAGGACTGGTGGTCGTGTCGTTCTTGCTGGTCGAGAGTGGACGCGACAA
TTGGTGGCCCGTACGGGGAACCGACTCCCCCACCGAGTTCAGAACTTTCAGCAGTCAGTG
GTTGCAGGCAGGGTAAGTTCACGGTGAGTGAAACTTGCGACCCCAGGAGTTTGGGAAGGA
CCTCGGATAAAATAGAGGTGAGCATAAAGTTGCCAGGAAGTAGGCACAAAGTAACCCAGG
AGTTTGGGAAGGACCTCGGATAAAATAGAGGGGAATATAAAGTTGCCAGGAAGCAGGCAC
AAGGTAACGAAAGGTTCCCGGCTTTGGGACAAGTTAAGGTTCCCGGCTTTGGGACAAGTT
AAGGTTCCCTGTTTGGGGACAAGTTAAGGAACTATGATAACCTCAGTGTAGTGATCAATA
GATCCTCGCTGTGTAGTTATGCTTTTTTCTCCCATTGACCCTTTGTGGGTAGGTCTGATA
GTTTTGGTCTTGTTTGTTCTGATATATGGACTCTGTTACTGTTTGAATCAAGAGGCAGTC
AAGACAGGTCAGAAAATCCTTACAGAGCAACAAGAAAGTATGTCGGAAAAGGAGAAGGGC
TTAAAAAGAAAAAGGAAAAAGAAAGACAGTGTTATCAGGTGGACAGAAAGGACAAAATAA
AAGAGCCGAGGTGGAGGAGGAAGGCGAATTAGCTTTTGTGCCGCCTCCCTATGCCTCCTC
AGCAGCCACCTGTAGGTGGACCTTCTGTCCAGAGATTTGGCTATTGACTCCCCAGATGGA
CACACAGGTGATTGAGGTTATGGTCTCATTACCACGAGGTATTGTGTCCATCTCCCCTGG
GATCGAGTAGATCAGGTGTTGACGTGGGCGAGAGGGTCTGTTTGTGTGTTTCCACAGGAC
CAGACTGAACCTCTTTGGGTGGTGGAGAGATTGGTGAGACGCTACAAGAATGAGGCTCCT
GATCCAGTTGCCCCGTGGATGTGGTGGATGATCCCACAAGCACAAACGATGGAGCCGAGA
TGGGAGATCCTTTCGGTATTCCAGAAGCCGATACCAGCTCGACATGACATTCAAATTTTT
CCACGCTTTTTGATCCCTGAATTCCCCCTAAAGAGATAGCCCCGCTGGCCATCTATCCCT
TGCTTCAGGGAAGATGAGCGGGGATGAGAGCCCTGGGATGTATGCTTGTTATAGTGTGTG
TGTGTTTTGTGTTTGGCACATGTGTTAGGTGCAGAGTGTGCGACCCGCACTTTCGCCATG
GTAGCGTAGGCTTTTGCTGCAGTGGAGGCGGGACAATCTCCTCAGATTCGGTTTGCCGCT
CTAAAAGAAATTATGCTGCGTTATGCCGTGGGGTGCGAGGCTAAGCACTGCACAGAGGAT
AGCTTGCTGTTGGCATCCTGTGGAAGGTATGTCTGATTGCATGAAGGTTCAGTGTCCTAG
TTCCCTTCCCTCAGGAAAAACGACACGGGAGCTGGCCAAGACCTCTCTGGGTGATGAGCC
TAAGGGATGGTTTTGTGTAGGGCCCCTATGCTTGCACACTGGGGATCAGACCTCTACCTT
CACCCATGAGGCTTGCTTGCAGCAATTAAGATCTGGCCATAGGTTAATTAATATCCTGGC
CTTTTGATGCACCTGCCGCAAGCAAAACACAATCTCCCCAGGAGTGGCTTGGCATGATAG
AGAGGTAATCAGTGATAAGACTCCCTGGGCATGTCACCAACCTAAGACAGGGATCAAACC
AATGCTGTTTGTCACCCAAGGACGGGTAAGGGGCATGGCTGCTGGGGGCTATCTACAGAC
ATTCTCTCTGCCAAAAAAGAAAAAAGGGGGAATTGTGGGGAGCAGGTGTGGCGGCAGTCC
CAAAAGTGCCAGGCACTGCAGCTAAGTCTTATGACTTGCACCTGACTTCCTCATATAAGC
CACAAACATCTTGAGAGCTGCACAGGTGTACCAGGATACTGGTGAATCCATTTTGATGGA
GATATGCCCCTGCTGCCCTGATTAGCTGAAGCTGTGTGCCTGGTGAGGTGGCGTGGCCTG
CTGTGCGTGGATGGGAACTGAGAGTATATAAGAGTGAGAGGCCCGGGTTAGAGGGAGGAT
TATTATTCGAGAGAGGATTACTATTAGTGGGGAGATATATAAACAGGGGAGATATAAAAA
CAAGGGAGATATAAACAAGGGAGATATAAACAAGGGGAGATATAAAAACAGGGGAGATAT
AAACAGGGGAGATATGAACAAGGGAGATATAGAGAAAGAAGAAACAGGACTGAATAAACG
TGTGCAGAAGGATCCTGTAGCAGCGTCGTTCTTCCTGGCCTGTTGAGCGCGCGCAACACT
GGGGAATGCATTTAAGCTGAGAAGTGAGATGATCAGGCATCAGATGAGGAGGACTGTCCA
GACCGGTCCACTCCCATTTCTTGTCCATGCTTCTGTGCCTTCACACCATCTGCCCTCCTA
CCTATCGCCGTTAAACAAAAGACTCTTACCATAATGAGGAAGTATTGTGGTTTTGTTCTA
CATTTTTTTTCTTAGAAGTTGGTTGAAAATACTTTCCTAACTGAGATTCTGGAAATGTTT
TAAAAATAATACCTTCTATAAATTAGTCAGCAGTGGTTCTCAACCTTCCTGATGCTGTGA
CGCTTATAGTTTCTCATGTTGTGGTGACCTCAACCATAAAATTATTTTATTACTTCATAG
CTGTCATTTTGCTACTGTTATAAATCGTACTATAGCTCTTTGATATGCAGGTTATATGAT
ATGCAACCCCCAAGGGGTCTCAGGCCACAGGTTGAGAAACACGGACATTGCATGAAGGTT
CATGGTACCTAGGCTTTCCCTAGGTAGTGGGAAGGTTTCTGCCATCCTTATTTTTTTTAT
TAAAATTGCAGTTTAGCAAATATTAGTGAAATAGACGAATGAATAAACTGTAGAATAAAA
ATTGTAGGAAGAAATATCATTTGTATGTATACTTTATTACATAGTTCCATGGTGGCTTAA
AATTATGGAAAAGGAGAATATTGCATGATAGTTCATTTGAGGTTAGTTTTTCCTTTTTGT
ATTCTTCCTTTTTTTTTAAAGATAAGCTCTTATTATATAACCCAACTTGAATTTATTTAT
TTATTTATTTATTTATTTATTTTGGGGCCATTGTTGTGCTGATTCTGTGACTATGGCAGC
TCTGCAGCGTCTAAACTAGCCACCACAGCTAGTTGTAGAAATTTTTTGTCAGCTAAAGAT
AAAATAATAGAAAACTTACTTGAGATCTTGATTCAGTATATATTTGCATATGAACCAGAA
TTCCATTTCATTTGCGTAAGAGCCTACTGAGACCGTCGTTTAGTCCAAGTAGCAGGGAAG
AGCCCCTGTGCTGCGGCTCTTTGGACTTACTAGCTGCACAGCTGCTGCCACTGGTAATTG
CCTGGTGTTCCAAATAAAAGGTATCATTTAAAGGAATTTCTAAGACACACAGTACCTGTT
GACCATGTTAAGATTCTGCTTTATAAAACTGATTTATGTTATAGTGAATATTTTAACATT
TATATGTATTTTCCTTATTGTGTGCCTCTCTGTAG**GTATATGCTTGTCAGCATTGCCAAG**
TAGCAAAAAATACAGTTATCGTAGCACCTCAGCAGCACCTTCCCATGGTGGGAAACCCGT
GGAGTGTAGTTACTGTTGATCTGATGGGACCTTTCCATACAAGCAACAGAAGTCATGTGT
ATGCTATAATCATGACAGATTTGTTCACAAAATGGGTTATGATTTTGCCTTTATGTGATG
TTTCAGCATCAGAAATTTCTAAAGCTATTATCAATATATTTTTTCTATATGGACCTCCTC
AGAAAATAATAATGGACCAAAGAGATGAATTCATTGAACAGGTAGGAAAAGTACTCACAT
TCGGGAGCATGTATCACTCTTTCAGTGTCTGGCACACCGTGCTTTGTGTTTTAGAGGGTG
TTTAATACACTGAAATAAATTTCTGTAAATATGGAGGTAGATAATGTATACTACTAGATT
TTGTTGTAAACCTATCTTTTGATCTCTTTAAACTAAAATGAGATGATTGGATCAAATAAT
ACTTTTTTACTTGAAGCTTAAGAACAACTAAAAATAAAAATATAATAAAATGATGTTAAC
GAGTGCTATAATTGAACATATTTATATCATGTTAAAATATTAAGAATAATTTTATATGTG
CATATTATATATTTACATAGGTAGCTGTGATAATATGTATACTAACTAAATATGTATATG
TATATATACATACTAATATGTAGACAAACGAAAAGTCTTCACTAATGTTGCACATTAACA
TAGCACACTAAGTCATTCATTAGGCACATATGGTATGTGAAGCACAGTAGGGGAAAAGTG
GAAAACAGCTAGTAGAAGTAGATTCCTCAGTCCTTCTTTCATTTCAACCATTAAGTGAAT
TTATGAATACAAAATCTAAATTTCACCAAGCTACCCATAGTGGTCACGTAACTCAAGAGT
CTTACAGCCTCCCACCTAAGAGTTGGTGAGGAGTCATACTCAGGTGCAAGCAATAAAGAA
TTCCTCTTCCTTTCCCAAGATCACAATTGAAATAGAAGTTTTGTACTCACATAAAAGAGA
AGGTTAGCGTGAAAGTTGAGCATCAGTGGGACCCAGGCTCCACACTACTCTTCTATCTTT
GGAGTGGAATTGTCAGTCTTGTGCTAACAGTAGAGCCACCAGAGCCCAGGTCATAATATT
TATATTACAGTTAACAAGAGAGGACAGGGAACAAAAAAGCTAGTGCTTGCCGAGTTTGTA
CAGCAATTTTAATTTAAATTTTTTTCTACTATAGCTTTCATGTGACCATGTCTACCCTCT
GAGGGTCTTGGGGAAAGTGTGTTTGGTTGGAGAGATAGATAGATAGATAGATAGATAGAT
AGATATACATATACATACATACACATACATACATATACAGAGAAACTTATCAGTGGAAGG
TTAGCTAAATTAAACTAAATAATAACACAGCAACAACAACTGGGATCATCAAGGCAGAAA
AATAGTTGTTTTACATTACTTACTTACAGTGTCTGAATTAAAAAGTGACATGATCACAGA
GTTAGTTTTGAATTCTGTTTTTCCTTAAGGTGCTTAGACTACCAAAGCTTAATTAATAAG
CTTCTGTCTTTAAAATGAAGTTAGTAATACATCATAAAATTGTCTTAGCATGTTATGACA
AAAATGTAAGACAAAACTAATATGTGGATCAGACTCAGTCTGTAGGACTTTACCCAAATG
CATGTGTTTTATCTAAATGCCTGTGGCCAATGGAAGACAAAATCTGGGTTTCTTGAAATT
TTTTTAAGCCAGAGGGAAAGTGTGAACTTTAAGCAGTCTGTTTTCTTTTCATATTTTTTA
G**ATCAATGTAGAACTATATAGATTATTTGGTGCAAAAGAGATTGTAATTTCTCGTGCTTC**
TGGAAGTGTTAATCCAGCTGAAAACACACCTAGCACCATCAAAACATTCCTCTCCAAACA
CTGTGCCGACCACCCCAACAGCTGGGATGAGCATCTGCCAGCCCTTTCCTTTGCCTTCAA
TGTCACTCACTTGGTAGGTGCCTTTTTTATTATTCATTGCTCATTCTAAATTCCAGGGAT
ATGAAAGGGTTTGGGGAGGGTTGTTTTATTTAATTACAAATGTCCTGGTTTTCTTTAAAA
TAG**GAGCCTAATAAAAACACACCGTATTTCCAAATGTTCAATCGGAATCCTTGCGTGTTG**
GAGTGTCCTCCTGAAGAGGGCAGTGAAGGTACAAGTGTGTTTGCCAGAATCGTAGCTGCG
ATTAGAGAGGCTGATGGCGTGGTGGAGAACAAGACACCAGCATCGAGCCAGGTGATTCTA
TCTACTAGGAAAGTTCCCTTTGTGGACCCCTACCAAAGAGGAATTGTGTTTAAAATCTCA
GCATGTGGACATGTTCACATATGTGGCCATGAAAAAAAGCAAGAAAAGGGGGAGGCGTTT
TTTATGCATGTCCTCATGTGTAAGCAAGTGTGTGGAGACTAGTAATGGACACGTAATATC
TTCCTTATATTTTGAGCCAAGGTTGCTCACTGAAACCAGAGCTCACCGACACAGCTGGAC
CAGCTGACCAGAGAGCCCCTGGATGCTTCAGTCCCAGTCCTTTCCACCTCCTTTCCCCCC
AGTTGCAGGGGTTACTGCCATGTGCTGCCATGTGCAAGGCATGCCGGGAATCTAACCCAG
GTCCTCATGAGTGTTTCAGGCATTTCCCACTGAACATGTTCTCCAAACCCTAAACAGTGT
GCTCATAGGCTATGAAATTCTGAGAGGCTTACAGGAAAGTCATGAACTGGGGAGACTGGG
TAGACCTGGTTTCTAACCTGTGAGAATTCCTGTTAGCAGTTGGAGAATTCTGTACTCAGT
CCATGTGAATCCAAGTATTCCAAGTCACTTGGCCTGCTTTCTGCCCTCTGTGTACAGAGT
GCCGATACTGACTTTAAAGTGTGCTTAGAACACATGAGGAGTCCATGCTTCAGCTCAGGA
GCAGTTCAATAATAGCATTGCCATAATCTAATCAGCGTGTGCCGTTATATTCAAGGCCTG
TCATGATTATTCTTTGCATTATAG**ATGGAGAACAACAATTCGGATGAACTGAGTAAAAGC**
AAAGTTGTTAAAAAGAAAGCAAAGCAGTTAAATCCCTTTCATTTGAAAGTGGGTCATGAG
GTTTTACGACAGAGGAAGAACTGGTGGAAGGATGGCCGCTTCCAGTCTGAGTGGGTCGGG
CCTTGTGTCATCGACTACATTACAGAAAGTGGATGTGCTGTCCTCAGAGATAACACGGGA
ACCAGACTCAAACGACCTATCAAAATGTCCCACCTTAGGCCCTATGTGAGAGAGTCCAGT
GAGCAGGGTAAGTATCTGCTACCTCTCTAGGGTGTTAGTTTCCACAAGAAAGATTAGACT
TTCAACCTACCTTCCTTCCTTCCTTCCTTCCTTCCTTCCTTCGGTTAAAATCCTCTCCAT
CTAAGTTTTATAAAACTTGATGGTCTTACTTCTCTCCAGGAGTCTGATTAGAATGTACTT
TCATAAACTCATTTTTCTTTTTAGTTTTTTGTTGAAAATAGATTTTTATACAATATATTC
TGATTATCTTTTTTTCATCCAATATATTCTGATTACCTTTTTCCCTTCTCTAACTACCTC
AGATCCTCCCCACCCCCCCCCCACCCTCATCCACTCTTTTTCTTTCTCTTTCATTAGGA
AACAAGCATGTAAGATTGATTAATGAGTGATTGATTGATTGATGTTGTTATATAATATAA

AGCAAACAATGCAGGATTTTTGTCTTGAATTCTACTGTGCTTTTCATAACTGCTATGAAA
AATGCTGAACCTGTTTCACATAAGGAACGATTCCATCTTGAGTTTTGCATACTATCCTCT
TACCTGCTGCCTCATTTTTATGTGAAAAAGTAGCAGCTCAGATCATTATCGCCAAAGAGA
AGCATGTTGTCCAGAATCTAGCTGTGAAATAACTAAGGGAACTTTGAAACTGTGTTTCCT
TTTAATGTTGCTTCATAAAGTCATAAATTAAGGTGGAGGATGGAGAATACTCAGAAAGCT
ATAGAATTATACCATTGCATTCCATTTATAGTCCCCCAAGCTATGCCAAAGAGCACCCTG
GCTATGCCAGAGAGCACCCTGACTATGCCAGAGAGCACCCTGACTATGCCAGAGAGCACC
TTGACTATGCCAGAGAGCACCCTATGCCAGAGAGCACCCTGACTATGCCAGAGAGCACCC
TGACTATGCCAGAGAGCACCTTGACTATGCCAGAGAGCACCCTGACTAAGCCAGAGAGCA
CCCTGACTATGCCAGAGAGCACCCTGACTATGCCAGAGAGCACCCTGACTATACCAGAGA
GCACCCTGACTATGCCAGAGAGCATCCTGATCTTCAGCTTTCTCCCTTGTAGGACTTAAA
TGACATTTTGTCCTGGTTTTCTCATTAGGCCCTCTACTTCAGCAAGATTATTTTATTTTA
TCCTAATGTATTTGCTCCTTGGAGCTTCTCGGGGTTAGACAAATGAGAGGAATTATTTGA
TACCTTTTATAATTATGCTTTTTCTATAAGATAATTTGTGTCATTTTCATGCATGCATAT
CATATACTTTGCTAATTTTGTACAAGGAATATATGCTAGACAGTAATATCTGTACAAATG
TTCGAATGAAAGCTAAATGTAATGGTGATGCAATATATTTGTAAATATCATTAACAAGTT
ACTTAATAGACATAATAACAAGCAATAAGGAATGACCTCTGCTTAACCCTAGTCCTGTGT
TGAGTATGAAGCCCTTCTCGGGCTCTTCAGTATATGAGTTCTTCTGAGTTCTTATCTACT
CTGGTCTGTACCTCGGCACTTCTGTTACAAGCTCTTGGTACTTTCATTTCTTGTGATGGA
ACCAGTTGCTCTAGTTACAGACTTATCTTAGACTGTTCTAACTCTGAACATGTGGATAAT
TTCAATCTCCTGGTTAGGACAGTACATTCATTTCTTTGTGTCGTCCCACTTCAGAGCTAT
TTATAGACCCTCCCTTCTACAAGCTAAGTAGGGGAAAAGGGATTCTATGAGTTCTTTAAT
TTCATGAAGACTTCATTCTCAGGCCTTCATCTTCAGAACTCATTTCCTTCTAATGTGTTA
AGCTACAGTTATATAAGATTTGCCTATAAAACATTACGGTGCCAGGGCCTTGTACAACAG
TCTTCTGTTTTTCTCTTATCAAAGACATTTAGAAAGGAAATCAAATTATTTTCTTTTTTG
GTGTTTCAGAAACAAGTTGAGAACACAAGCTTAAATCTTCTGGAAATGTTTCTTGTAGTA
ATACAAGACAATTGGAGTGTTACTATTAGTGTTACTATTAGTAGTGTGAACATAGGCATT
TGAAGGTGATAGGTGGATTTATTTTAAAATTTCATATCAACCATACTACAAATTAGTTCT
TCAGTCATACTTAATCCTTGTGTGTGTGTGTGTGTGTGTGTGTGTGTGTGTGTGTGTTGG
TTGGCAGATATACAAATTAAATAAAAATGGCCTTTTCCATCTTTTTCCGTGGCATCATGC
ATTTTCCTAAAAGCCTTTATTACTTCAGCAGCTGACTTTGAAGAATTCACAAAGTGAATT
TAATCAAAACAATATCTTTAACCATCAGACACTTTTTACTTGACATGAGAAATATCCAAA
AGGAGCTCAGGTATCTGTTGACATCTGTCATATCTGTCTTTACTACTGTTAATTTCTTGG
CTGTATCTAAAACCGATGGACTGACGTTGCCAAGTTCCTGTCATCTGTTTCTGGAACATA
ATACTGTGCTAACACATTCAGTAGTACTAGGTTAACAGTATTTGTCAATGCTATTTTAAG
CAGACCTGTTTGCTGTTTTGTCTAATTTGATGTTGAGCACTCTAAGCTACTTATTTTGAT
AAGAATTTCAAAAATAACTAGTGTGCCTTTGTAATCATAACCTATTGTAAAAGGGTAAGT
GTGTTGTTGTCCCGTCCGACACATTCTGCCTTTAAATGTGTTTGCTTTGTTGGTGGTAGA
TGCGGGTCGACCTTATCCAGAAGTCTCATTCTCTCCTTCATGATTAAAAACCCATTTGTG
AAATAATTTTTATTATGCTAGTTTTATATTACTGCCATAACAATATACAACAAATTTAGT
GCTTTAAGCAGCACTCATTTATTACCATAAATTTAGGTACTTTAGAATTTCTACATGAGT
ATTATCAGGCTAAAATTAAGTCATTCACAGACTCATGTTTCTAGACCATCCAGAAGACAA
GAGAATGGATTTCCTTGCCCTTTCTAGCTTCTAGATACTTCCTTTACTTCTTGATGTATT
CCTTGTCTTCTGTTCTCAAAGCCAGCAATGCCTCAAATGTCTCCCTCATCTGCATTTGAG
GACATTTGCTTTTAGATCCACCCAAATATTTTAAGATGATCTCCCTTATCATTGATTGGC
AGCTTTAATTTTATGACTACCCACATTCCTTTGTCGTGTAAAGCAACATATTCACAGGTT
GGTGAGGTTAATGGGTGGGCACTTTGGGAGACTATTATTATGATATAATAGTTAATGGTT
TCACTAATAAAACAAGCATTAGAACATGAAATTACACAAGCTACACATTATTAGTAATAC
AATGTATGCAGAGAAATTGTTCCTTTTTCCATGTTTCTATATACTCTCCAGACCCAGATT
CCATCCAGTTCATTAATAGGATACTATTAATATTATAACAGTATTAATATAGTATCAAAT
TATAGTTGTTTTATCATGGAAAGGATATACCACAAGAGGAATATAATGTAAAAGCTTGAA
AATAATCACATTTTTTTTTTGATGCCTCACACATCTGGTTTCCAACTAGGGTGCTGGGAA
TAGATGTAGTTTATAAGCTGGTAATGTTTTGGTATCTGTAGGGCCAGGCCTTGCCTGAAT
TCCCCAGACTTTTATATTCCTTTATCTCCAAGATCTTGAACATTGCCTCTCACTCAATAA
AGAAGCCATCCTTGGGCTGGAGAGATGGCTCAGTGGTTAAGAGCACTCACTGCTCTTCCA
ATGATCCTGAGTTCAATTCCCAGCAACCATATGGTGACTCACAACCATTGTAATGGGATC
TGATACCCTCTTCTGGTGTGTCTGAAGACAGTGATAGTGTACTCACATATATACATTTTA
TATATATATATATATATATAATGTATATATATATATATACATATATACATTTTAGGTTTA
TTATATATATATATATAATAAACCTAAAAATAAATCTTGAAAGAAAGGAAAGGAAAGGAAAG
GAAAGGAAAGGAAAGGAAAGGAAAGGAAAGGAAAGAAAAAAGAGGTCCTTATGAGAGACA
TCACTTGTACTTTACGAGAGTCTAGGTAACTTCTGTTATTTTAGTGCCGGGCCAATCCTG
ACTCCAACAGGCTATTATATATCTAGCTACCTTAGTAATTCTCCCTAAACTCTTTTTTTT
TTTTTTTTTTTTTTTTTTTTTGGTTTTTTGAGACAGGGTTTCTCTGTGCAGCCCTGGCTG
TCCTGGAACTCACTCTGTAGACCAGGCTGGCCTCGAACTCAGAAATCCGCCTCCCTCTGC
TTCCCGAGTGCTGGGATTAAAGGCGTGAGCCACCACGCCCGGCTCCTCCCTAAACTCTTA
TCTTAAGAGTTGTTTTGGAGTCAACAGTACTCATCTTTATGGAATAAAGGAAAGGCCATA
TGTACTTTGTAAAGAGTTTCAATATAAATGTGTTTTGGGTTTTGTTGTATGCATGGGATT
GAGTTAATTATCACCAAGAAACTTTTTTCCCAAAATTGTGCTGTGCTTAAATATGGCTAA
AATAAATTGCCTTGTTTAAGACTCCAGACATTTGAACCATTCTGGCTAAGTCATGTTGAA
GCAGATTTCCTTCCTGCCTCTTGCAGAGTGTTTCTCTGTTGGCCTTCATGTTTACAGACA
CTCCTACACATGCCCACTCATGCTGCCTGCTTATAGTGTGGATTTCCAAGAGCCACTAAT
GTTCATAAAATTTAAGTTTTTAAGCGTATGAGCTTCTGAAGAGGAAATACTTTTCTCAAA
CATGCTACTAAAGCTGATACGATTTCCACTGTTCTCTGATCTAAATGGGCGATTGGTCTT
CTGTGTTTAGACTTAGATTGGAGAGTTCTATAAGCTATTAAAATCTGCAAGAGAATCTGT
TAGTGTTTGGAAGGTCCAGAAAGAGAATGCAGGGATGATTCATCAATTATAGTAGATGAG
TTCCTTTTTGTAATGAATTAGGTATCAGACCTGATAGAAATGGTTTCTATTGTAGTCTTT
AGATTGAAGGGCAGTGTTACTTTCCTGTGAGTTTAACAACTTATTTTAAAGCTGTTTTCC
TGAGGGTTAATGATGACATTGCTAAAGTATAACAAAATACCTAGAAGGAAAGTTTCCCCA
TTAGTGTAGATTAGGTAGACCTTGAACATTCCTAAGGTTTTTTGCTCTCAGATACAACTT
AAATAAAAGTTTAGCTACTTCACTGCAGCTTGCCTTCTTGATATTCCTGTCACTTGAGTC
AGATTTTTGCTACTTTATCTTCATCTAGTTTATGGCTTTATATTATAACTGATAATGATA
TAATAATGATATAATAGTACTACTATGAATTCTTTACAAGTTGGATGACATCTGAAATTA
GAAATATATAGTGTAGCAATTCAGTTGGCTAAAAAGAGCTATATTAATTCACAAAAAATG
CACTGTAGAAGCGTATTAAGCATATCATCGGAATCCAAAATTATAGTTGTGTAATTTTGT
TTTAACCTGTCCTGCTGCTGCTCCTTGTCCCACTTAGATAATGTACACCACTGAGCAAGA
AATGGGAAGCTTTATTTCAATATATTTGGTGTTCCTACAGTCTTCAAGTGTGAACTATTG
GGGGGCGTATGTGTACACAATTTTGTTCACTTTTTGTGTTTGGTTATGTTTACTGGATAA
GAGGTTGGGTTTTGGGGGGCGGGCAGGGTGGGAGTTCTGTTTGACTCTACCTCCCAAGTG
GTAGGATTAAAGATGTGTGGTACAGTGCTCACTTTGATAAGAGAGCTTTTCAAGTTAATA
GTGATAACTTATTTTTATTGCTTTAACTTTTGTTCCTCACTTAATGTCTAGCTATAAAAA
TTGGTTTCCCGAGCCAGGCTTGGTGGCGCACGCCTTTAATCCCAGCACTTGGGAGGCAGA
GGCAGGCGGATTTCTGAGTTCGAGGCCAGCCTGGTCTACAAAGTGAGTTCCAGGACAGCC
AGAGCTACACAGAGAAACCCTGTCTCAAAAAAACCAAAAAAAAAAAAAAAAAAAAAAATTG
GTTTCCCTTCAATTCTAAAATGAAATGTTGATCTCTTTCTTTCAG**ACAGTCTTTATCTCT**
TGCAAGGTTCGATAGTGGCAGATCACGACTACATTGGATTACCTGAGATCCCAGTTGGAA
CATACCAGGCAAATATTCTGGTAGAAGATGCAACTATTGGGATAGCTGATAATGAACTAT
TGATATCAAGCAAGGATCATGAACTCTTAGAATATAGAAATTCCAAAATCTCTGCATTGG
TAGAAGACCACAGTTCTCTTGAGAAACAGACTTTTAGCCTACTGGATTCTTCAAATCAAG
TTCTCGAGTACTTAAGTTAGTAAATACCAAAATTAATGTAAAATGCTTGCACAGAATGTA
TATATTTCTAGTTTGTAAGTATTTTATCTATTAAAATAGGTTGTATAGAAGAGAGATCTT
GGTACTATGTTAATAACTAAATTCTGAAAGCTTATTTTAAACCTTCCTAAAGTTGTTACA
TGTTTGAATACAAATAAATATATGTAACAGAAGTATACCATGCGTAGAATTCAGAAATTT
CTCAGAAAACAGACAAAATACGTTGTTAGAAAATAAATTCTCGTGGAGAAGTCTCACCTT
TTTCTATATGGGTACTACATTTTCTCTTTTAACTTTTCAGCAATACATACCCCTTTTAAA
TAAATTTAAACTAATTATAGCTTAGTTAATAAATGTTGAGTGAACAATAACATAAATGAA
ACGAAACTTTATCTGTGACATTGGAACTTTTTAAATTTGTATTTGTGTGTGTCTGTGTGT
GTCTGTGTGTGCGCGCGCGCATGTGCATGTATACGTGTGTTTCTGAGTGAGGCTTTATGT
ATGTGAGTACAGTGCCCTAGTGTAAAGGCTAGGCAAGGGTATTGGATATGCTGAAGCTGA
AGTTACAGGTGATTGGGCACCAACCAGGTTGAGTACTGGGAACCACACTAGGATCCACTG
CACCAGCAGAGCACACTCCAGCTGAAGGACAAGTGACTGTCAGGGAAAGAATGAAGTCTC
TGACCTTGTACACAAACCAAAGACATTGCAGATAGAATTCTAGAAAGAAACTAAAACAGA
GTGGTGCTAGTAGGCTTTGCCTACTCTCCTCTACAGAGCAGAGTTCAGGAAGGGAGCCTA
CTGCAGTGCCTACATGGTCGATAAAGTGATTGTCTCTTTGTCACCTCAATGAAGTCACTT
TCTTCTAAGATTCTTTTCGAGATCATCTGACTTGAATTTGTCCTATTTTACTTGTTCTAT
TTAAGGAGGCCAATTAGCTTTCAGATTGATTTCTCGGTGGAAGATTCTAAATAGCTAAAA
ATAATGTTTTCCCTTCCTAAGTAATTTCCCATCAGATTTTTGCTTTGTGTGTATGTGAGT
CATATCAGGCTACATTATTCTTCTTGCAAATCATCCCCAGATCTTAGTAGCTTAAAACAA
TAAAGCTTTATTTCTTGACCACCTGCATGTCCTTCTTGATCATCAGTGACTGACTGCAGT
TTCATCTCAAATGTTGAGTGATGGAGCTTAATTCCTGAAATGGCAAAGGGACAATAACAT
TGAACTGAGGCACTGGATCAAAGGGACTCACCAAGACATTGTGCAAATCACTACAACGTT
TTAATGTCATGTTTTGCTTATGGGTATAGAATTTCATCTTGTTTTGTAATTAATGGTGAT
AATTTGTACTGTGAATATTTCAAACTCCACTGAAGTTTACCATCTCCAGGGGTGTTTCAG
GCTTCTCCTTACTACCTGTACCCACTGGTCCTTCCCTCCCTCCCACCCCCGGTTGATAAT

```
GATGACCCCTTGAAACTGATCCCCTGTGAACAACCATGTAAGGTAATTATCAGATAAACC
ACCCACAGACTCTGCTCAGACTAGTAAAGAGCTTTCCCTTCTGAATTACTGAAATAAGAG
CATTTAAGTGAGGTGTTGTCTAGATTAACTTAATCTATGGACATGTCTGTGAAAGACTGT
CTTGAGTCTGTGGCTGAGGTGGGAATATGCGCTCACTGTGTGGTGCTATCCCCTAGTGGC
TGGGAAGCCGATCTGTTT
```

Mouse Gin-1

```
==================================================
Mouse Gin-1
total length:      24738 bp  (24738 bp excl N/X-runs)
GC level:         41.04 %
bases masked:       9138 bp ( 36.94 %)
==================================================
               number of      length   percentage
               elements*    occupied  of sequence
--------------------------------------------------
SINEs:                12        1838 bp    7.43 %
      Alu/B1           7         932 bp    3.77 %
      B2-B4            5         827 bp    3.34 %
      IDs              0          79 bp    0.32 %
      MIRs             0           0 bp    0.00 %

LINEs:                 6        1762 bp    7.12 %
      LINE1            4        1610 bp    6.51 %
      LINE2            2         152 bp    0.61 %
      L3/CR1           0           0 bp    0.00 %

LTR elements:          9        4617 bp   18.66 %
      ERVL             0           0 bp    0.00 %
      ERVL-MaLRs       6        1011 bp    4.09 %
      ERV_classI       0           0 bp    0.00 %
      ERV_classII      3        3606 bp   14.58 %

DNA elements:          1         192bp    0.78 %
      hAT-Charlie      1         192 bp    0.78 %
      TcMar-Tigger     0           0 bp    0.00 %

Unclassified:          0           0 bp    0.00 %

Total interspersed repeats:     8409 bp   33.99 %


Small RNA:             0           0 bp    0.00 %

Satellites:            0           0 bp    0.00 %
Simple repeats:       13         598 bp    2.42 %
Low complexity:        6         131 bp    0.53 %
==================================================
```

```
SW   perc perc perc  query            position in query     matching   repeat            position in repeat
score   div. del. ins.  sequence         begin end    (left)   repeat     class/family    begin  end    (left)  ID

  279   29.2  9.3  0.1  UnnamedSequence   1719  1862 (22876) + MTE2b      LTR/ERVL-MaLR      223    376    (3)   1
 1103   32.6  6.3  2.9  UnnamedSequence   1878  2606 (22132) + L1_Mur3    LINE/L1           5119   5871    (9)   2
  511   28.4  8.9  2.4  UnnamedSequence   3480  3600 (21138) C L1MB3      LINE/L1          (193)   5990   5861   3
 1290    5.4  0.0  0.0  UnnamedSequence   3601  3747 (20991) C B1_Mm      SINE/Alu           (0)    147      1   4
  511   28.4  8.9  2.4  UnnamedSequence   3748  3896 (20842) C L1MB3      LINE/L1          (323)   5860   5703   3
  212   27.7  1.5  4.5  UnnamedSequence   3925  3992 (20746) + L2a        LINE/L2           3298   3363   (63)   5
  774   11.4  3.0  0.7  UnnamedSequence   4016  4147 (20591) C B1_Mus2    SINE/Alu           (0)    147     13   6
  448   23.5  4.9  5.6  UnnamedSequence   4148  4175 (20563) C ID_B1      SINE/B4            (0)    222     64   7
  235   30.3  2.5  3.9  UnnamedSequence   4219  4297 (20441) C ID         SINE/ID          (144)     80      3   7
  273   22.3 14.1  2.3  UnnamedSequence   4614  4691 (20047) C MTD        LTR/ERVL-MaLR    (332)     87      1   8
 4042    2.7  1.9  0.2  UnnamedSequence   5033  5518 (19220) + L1_Mus3    LINE/L1           4316   4809 (1838)   9
  380   28.9  9.1  5.5  UnnamedSequence   6202  6377 (18361) + B3A        SINE/B2              1    182   (16)  10
  351    0.0  0.0  0.0  UnnamedSequence   6384  6422 (18316) + (TC)n      Simple_repeat        1     39    (0)  11
   21   47.6  0.0  0.0  UnnamedSequence   6465  6485 (18253) + AT_rich    Low_complexity       1     21    (0)  12
  226   28.9  6.0  1.1  UnnamedSequence   7045  7128 (17610) C L2a        LINE/L2            (0)   3426   3339  13
  213    3.9  0.0  0.0  UnnamedSequence   7351  7376 (17362) + (CAA)n     Simple_repeat        2     27    (0)  14
  937   16.9  0.0  2.7  UnnamedSequence   8365  8516 (16222) + B1_Mus1    SINE/Alu             1    148    (0)  15
  277   10.8  0.0  0.0  UnnamedSequence   8518  8577 (16161) + (GA)n      Simple_repeat        1     60    (0)  16
  711   11.2  0.0  0.0  UnnamedSequence   8578  8684 (16054) + (GGGA)n    Simple_repeat        2    108    (0)  17
 1192    8.8  0.0  0.0  UnnamedSequence   8688  8835 (15903) + B1_Mus1    SINE/Alu             1    148    (0)  18
 2017    9.5  0.6  6.9  UnnamedSequence   9017  9354 (15384) + RLTR10     LTR/ERVK             1    318   (72)  19
  271    9.7  0.0 11.0  UnnamedSequence   9327  9407 (15331) + RLTR10     LTR/ERVK           259    331   (59)  19 *
  982    4.6  1.5  0.0  UnnamedSequence   9392  9521 (15217) + RLTR10     LTR/ERVK           259    390    (0)  19
 8102    2.6  1.3  2.6  UnnamedSequence   9556 10540 (14198) + RLTR10-int LTR/ERVK            16    988  (999)  19
 8885    1.8  0.1  0.0  UnnamedSequence  10620 11611 (13127) + RLTR10-int LTR/ERVK           992   1984    (3)  19
 2017    9.5  0.6  6.9  UnnamedSequence  11614 11951 (12787) + RLTR10     LTR/ERVK             1    318   (72)  19
  271    9.7  0.0 11.0  UnnamedSequence  11924 12004 (12734) + RLTR10     LTR/ERVK           259    331   (59)  19 *
  982    4.6  1.5  0.0  UnnamedSequence  11989 12118 (12620) + RLTR10     LTR/ERVK           259    390    (0)  19
 1057   14.6 16.7  0.0  UnnamedSequence  12391 12582 (12156) C URR1A      DNA/hAT-Charlie    (0)    226      3  20
   22   36.4  0.0  0.0  UnnamedSequence  12648 12669 (12069) + AT_rich    Low_complexity       1     22    (0)  21
  288    0.0  0.0  0.0  UnnamedSequence  12891 12922 (11816) + (TTTA)n    Simple_repeat        2     33    (0)  22
   23   47.8  0.0  0.0  UnnamedSequence  13889 13911 (10827) + AT_rich    Low_complexity       1     23    (0)  23
  235   39.7  6.4  2.8  UnnamedSequence  14421 14560 (10178) C MLT1J      LTR/ERVL-MaLR    (143)    369    225  24
   23   47.8  0.0  0.0  UnnamedSequence  14583 14605 (10133) + AT_rich    Low_complexity       1     23    (0)  25
  297    0.0  0.0  0.0  UnnamedSequence  14673 14705 (10033) + (TAGA)n    Simple_repeat        2     34    (0)  26
  180   17.6  0.0  0.0  UnnamedSequence  14706 14739  (9999) + (CATATA)n  Simple_repeat        5     38    (0)  27
  596   27.0 12.0  6.6  UnnamedSequence  15797 16070  (8668) C B4A        SINE/B4            (4)    288      1  28
  300    2.7  0.0  0.0  UnnamedSequence  16746 16782  (7956) + (TTCC)n    Simple_repeat        2     38    (0)  29
  454   19.5  4.0  1.6  UnnamedSequence  16930 17054  (7684) C Lx7        LINE/L1           (16)   7680   7553  30
  351    0.0  0.0  0.0  UnnamedSequence  18799 18837  (5901) + (TG)n      Simple_repeat        2     40    (0)  31
  679   31.1  4.5  4.0  UnnamedSequence  19454 19896  (4842) C MLT1D      LTR/ERVL-MaLR      (0)    505     13  32
  920   22.8  5.7  9.4  UnnamedSequence  20195 20353  (4385) + MLTR18C_MM LTR/ERVK            99    266  (434)  33
 1370    7.3  0.5  5.0  UnnamedSequence  20354 20518  (4220) + B2_Mm2     SINE/B2              2    163   (32)  34
  390    9.1  3.9  0.0  UnnamedSequence  20519 20595  (4143) + (TA)n      Simple_repeat        1     80    (0)  35
 1370    7.3  0.5  5.0  UnnamedSequence  20596 20616  (4122) + B2_Mm2     SINE/B2            164    180   (15)  34
  516    3.2  0.0  0.0  UnnamedSequence  20618 20679  (4059) + (GGAAA)n   Simple_repeat        4     65    (0)  36
```

920	22.8	5.7	9.4	UnnamedSequence	20680	20813	(3925)	+	MLTR18C_MM	LTR/ERVK	267	384	(316)	33
216	0.0	0.0	0.0	UnnamedSequence	20814	20837	(3901)	+	(T)n	Simple_repeat	1	24	(0)	37
1284	4.1	0.0	0.0	UnnamedSequence	20838	20984	(3754)	C	B1_Mus2	SINE/Alu	(0)	147	1	38
1317	25.0	2.5	3.1	UnnamedSequence	20985	21310	(3428)	+	RLTR18B	LTR/ERVK	189	512	(0)	39
269	30.5	0.0	0.0	UnnamedSequence	22352	22410	(2328)	C	B1_Mur3	SINE/Alu	(86)	63	5	40
1299	4.8	0.0	0.0	UnnamedSequence	22512	22658	(2080)	+	B1_Mus2	SINE/Alu	1	147	(0)	41
21	28.6	0.0	0.0	UnnamedSequence	22659	22679	(2059)	+	AT_rich	Low_complexity	1	21	(0)	42
21	66.7	0.0	0.0	UnnamedSequence	23330	23350	(1388)	+	AT_rich	Low_complexity	1	21	(0)	43
186	7.1	0.0	0.0	UnnamedSequence	23444	23471	(1267)	+	(TG)n	Simple_repeat	1	28	(0)	44
459	29.9	6.8	5.5	UnnamedSequence	23487	23649	(1089)	C	B3A	SINE/B2	(14)	202	38	45
379	26.0	2.9	0.0	UnnamedSequence	24052	24155	(583)	C	MLT1I	LTR/ERVL-MaLR	(0)	411	305	46
267	32.0	4.9	1.9	UnnamedSequence	24635	24736	(2)	+	MTE2a	LTR/ERVL-MaLR	60	164	(215)	47

>Bushbaby (Otolemur) Gin-1 (genescaffold:BUSHBABY1:GeneScaffold_2823:271513:287952:-1)
ACCGCAGGACACAGAAGAGATTCTGGTGGGATTAAAAAAAAATTTTCGTGAAGCTCCTGT
TTATTGGCAGGCAGGGACAAAGACACCAACAAGGCCCTGGGGCCTGGCAGCAGCAGGAAG
TAGTTACCATCTCTGCGGCTGGTGTGGCGAGAAGATGAGATGGTCAAGAGTATATTTAGG
AGTGATATAAGCTGTATTTCTCCTGTTGTAACCTGTCATTATTTATTTGGATGTCACGTA
ACGAGACCACACTGTGCGAGCCGTATTCCAGCTGGCGGGAGTAGGAGATTCGAAGCAGAC
CTTTTTGCAGTGTACTTGTTCATTCCCTTTCTTTCCCAGCATGTGGAAGTTTTTTAGCAT
TTCTTATCACATGGAAAGAGGCAAGATAATTTTTCGTGCTGTATCTCAAAAATAGCATTT
GAATATTTTCGTTCATTGACATCATTCTTCCTTTCCTGTACTGCAGTTATCTCCCTCACA
AATAATATTTTAGGAATGCTCAATTCATATGTAAATATAAGTATGACAGAGTCGGAACTG
AAATAGCAACTGAACTCACTTGTGATTTATTTCTTAAAATAACTTTCACCTAGGTTCATA
ATGGTCCGCAGTGGAAAAAATGGGGACCTCCACCTTAAACAGATCGCGTATTACAAACGG
ACCGGGGAGTATCACCCCACCACACTGCCAAGTGAGAGAAGTGGCATACGAAGAGCGGCG
AAAAAGTTTGTTTTCAAAGGTAAAATTGGTGTCCGCAATCTTGAGTGCAGAATACAGACT
TTTCTACGTGTGTTCTTTGAAGAATCCTAGGAATTNNNNNNNNNNNNNNNNNNNNNNNNN
NNNNNNNNNNNNNNNNNNNNNNNNNNNNNNNNNNNNNNNNNNNNNNNNNNNNNNNNNNNN
NNNNNNNNNNNNNNNNNNNNNNNNNNNNNNNNNNNNNNNNNNNNNNNNNNNNNNNNNNNN
NTTTTCCTAAATTTACAGCTTCTGCTTTTTGCCCTCTGATTCACCTGCTCCAGTGGGGCC
CGTTAAAGCCCTGATGCCACTTTAACATGCCTCCCTGTCAGTGAGGGACACCCCAAGCTT
AATGTGAAAAGGTGACAAAGTTAACTAGTCTGTATATTGAAATGGAGAACGGAATTAGCT
GTATATATTAATGTGGTTTCCCCCTCCTACTGCCATTTAAATTTTCTTAGGATCACATTT
ATTTTAAGACCCTGAATCTCGGCTCGGCGCCTGTGGCTCAAGCGGCTAAGGCGCCAGCCA
CATACACCTAAGGTGGCGGGTTCGAATCCCCAGCCCGGGCCCGCCAAACAACAATGATGG
CTGCAACCAAAAAATGGCCAGCGCTGTGGCAGGCACCTGTGGTCCCAGCTACTTGGGAGG
CTGAGGCAGGAGACTCGCTTGAGCCCAGGAGTTGGAGGTTGCTGTGAGCTGTGATGCCAG
GGCTCTCTACCCAGGGGGATGGCTTGAGTCTCCGTCTCAAAAAAGAAAAAAAAAAAAAAA
GACCCTGAATCTCTGAAGAGATTAACTATTTGAAAGGCAGTGGGATATTTATCCTGAAAG
TCATGATAATTTTTCCCTCTTTTCCTTCTTCCTTTGAAGGGGAAGAGAAGTTTGGGTAAT
TAAATAAATGCTGGTACTATTAATGGGAGTTTTGCCTGTAAGTCTAGCATTAGGCTTTCC
CCAAAGTAGGTGAAGGCGGTTCGTCTCTCTGTGTACAACCCTACCAGTAACGGGCAGGCT
GGTTACTCTTAGACTAAGATGAGGACTCATTCCTTTATTTAATACTCTGTGCAGCAAATG
TTTACTGACTTTCTATGTGCCAGTCACATTCTAGGCCATGGAGATATATTGATGAATAAG
AAAGATAAGATTTCTGCCTTCAGGTTACTTACCCTGTAAGTTAAGTAAGGACTGCTCCAC
TCAGCCTGTGTTCAGCACACGGGGAGGTTGATGGTGTATTCTGGAGTTTGAATTGCTGCC
CTGTCCATACTGGGGAGGTGGATGCTGGGTCTCTCCTAGTTGTCTCCCCACTGCAGGTTC
TTCTCAGGTGTCTTCCTAAGGAAAACAGAATTGTCTCGAGGCTGAAGGTTCCCTCACTTT
ACCATCCCCACCTTCTACTGAAATCTAGAAAAACACCAAAGAGGTGGTTGCTTACAAAAA
TGCAGCTGTGATAAAATAAATTTTAGAAAATTCTGTTCTAGGAAATTGCTCTTCTATACA
TAATTTATTTGCTAATTTAGACCATTTTTAGGAGGCACCTGCATGTATTTCCTTAGTTTG
AGAACTTCCATGGAATGGTACTTTTATATTTCCATAGTTGTTACATGCTTTGAAAAACAA
GCTAGCTCTGCTTGCCTTAAAAGCTACAAAATCATAGCGGCCTGTGCTGCTTGTGCAGAG
CAGGTACGTGCAGGCGCTGAGGACCTTCCCAGTTGTGCCGTAACACCTTTGCAAGTGACT
ACTCTGTTTTAAGTGGGATAAGAATTCCTAAAACAAAACCCCTCATGGTTTGCGGGTAAG
TCTATAATTATCTGAAAACAATGTAGATTTATAAAACCTGTACTTCTATCTCTTTGATAT
CATTTATTCCAG**AAAAAAAGCTGTTTTATGTTGGAAAAGACAGAAAACAAAATCGTTTGG**
TAATTGTTTCAGAGGAAGAGAAGAAGAAAGTGCTAAGAGAATGCCATGAAAATGACGCTG
GAGCTCACCATGGCATATCCCGAACCCTCACGCTGGTGGAGTCCAGTTACTATTGGACTT
CTGTGACCAATGATGTCAAACAGTGGGTATGGCTTACGTATTTAGAGTATGTTGAATTAT
GTCTAGTCGATACTCAAAAAGTCATGATGGCTCCCGGTTTACTCTGGGTATCACATCTCA
TTAAGTACTGTTTGGGGGTTGTAGTTTTTGTTCAAGAAAACACGTAAAGGGAGTTTTAGT
TCCCTTTTAATTATTTTAAATCACTAAACTATAAGTTGAGCAAGAAAGAAGCATTACTTG
AGCATTTGAAAATACCACATTGAGATGGGAGATCATTTAGAACCAGGTTTTTATCATTGA
ACTCCTTTGGCTAGGCAGAATTTAAACGCAGCTAGCATTTGTCTGACTAAGCTGTTACCT
CTTTAATGACGGTCAGACAAGCTCTTGTTGAGAGCCGAGGACATTCTAATAAGTTTGGCC
CACCGTGTGCTCTCATCTCCCAGGCCTAGCCCATCTTCAGGTAGAAAATGTCAAACGTGG
TAGAGACCCAAAACCAAATAAATGCAAAGTCAGATAATAAGTCTGATTTTGTGAAGACAA
ATTCACAAAACTGAAGATACTAAGTCTGACGTTTGTAACTGTGTCACCTGTTAGGCCCTT
CCACTTCTAACTGGTAGGTCACCTGATCCTGTTCTTCCTGGTGGACTGTATGCCAAATGC
TAAGAATTCCTCGGCATGGACATGAGAGCTCTTTAAAAATAGTAATGGTGCATTGAGAAG
ATGGGCCTATAAGTCATAAGGAACTAAACCAGCTGTTAATTGTCATTTTTGAGTTACTAG
TTTGTGAGTGTGTGAAGATATTCCAAAGACAAATATAAATTTGTATTTGCCTTTGGCACC
TGCCATGCATTTCTTTACCAGCATTCAGCGAAATCTTTGTAAAACCTTTATTATTTAAAA
ATGGATGATCTAATGATTTTTGGAAATAATTAGAGGTTATTTGCGACAAAATTTAGTGAA
TAAGTTTATATTTGAAAATTTTTTATTAAAAATAACTGTGACTATAATGTAATAAAATAT
TTTAAAAATTTTTCTTCAGTAGTTTGAAAGTCGTTTCCAAACTAGAATTCCAGAACTATA
TTGAGCAATGATATCACCCATAAATAGGTCAATTGATCCTTCTCACTTCACTCAACTTCC
ATGACAGATGACAGCAGAGATTAGTTTTGGGGTTTTTTTGGGGGGGAGGGAGGGGTAGTT
TAGAGTTTTCCTGTGACCTAGACTAGAGCACGGGTAGAGAGCTTCTTCATGTTGGGAGGC
TGCGTTATTTAGCTGTGACCAAAAACGGCCACATTCAAGAAAGTTCCTTCTAGATGATTT
AATAACCTGCATTATTCTGTAAAGTCTAACTATGTACCTACGATTTCAAAAAGTGAAAAC
TATTTGTTAATTTTAAAGTTGACCTTTTAACGACCTTGTGTCTGCTTTTTGTTGGAAAGA
TTTGAGTATCTGGTCCCAGCATGGAGGCCCCAGTGGTCAGACAGTCTCTGGATGGGCATC
AGTCATATCTGANNNNNNNNNNNNNNNNNNNNNNNNNNNNNNNNNNNNNNNNNNNNNNNN
NNNNNNNNNNNNNNNNNNNNNNNNNNNNNNNNNNNNNNNNNNNNNNNNNNNNNNNNNNNN
NNNNNNNNNNNNNNNNNNNNNNNNNNNNNNNNNNNNNNNNNNNNNNNNNNNNNNNNNNNN
NNNNNNNNNNNNNNNNNNNNNNNNNNNNNNNNNNNNNNNNNNNNNNNNNNNNNNNNNNNN
NNNNNNNNNNNNNNNNNNNNNNNNNNNNNNNNNNNNNNNNNNNNNNNNNNNNNNNNNNNN
NNNNNNNNNNNNNNNNNNNNNNNNNNNNNNNNNNNNNNNNNNNNNNNNNNNNNNNNNNNN
NNNNNNNNNNNNNNNNNNNNNNNNNNNNNNNNNNNNNNNNNNNNNNNNNNNNNNNNNNNN
NNNNNNNNNNNNNNNNNNNNNNNNNNNNNNNNNNNNNNNNNNNNNNNNNNNNNNNNNNNN
NNNNNNNNNNNNNNNNNNNNNNNNNNNNNNNNNNNNNNNNNNNNNNNNNNNNNNNNNNNN
NNNNNNNNNNNNNNNNNNNNNNNNNNNNNNNNNNNNNNNNNNNNNNNNNNNNNNNNNNNN
NNNNNNNNNNNNNNNNNNNNNNNNNNNNNNNNNNNNNNNNNNNNNNNNNNNNNNNNNNNN
NNNNNNNNGAGAATCTGGTACCCAGCATGGAGGCCCAATCTCAGAAAGTACTATGGATAG
GCATCAATCATATCTGAGGATCTGGTCCCAACATAGAGGCCCCCATCTCAGACAGTCCT
CTGGATGGGTATCAGTCAAATCTGAGGATATGGTCCCAGCATGGAGGACCAGTTCTCAGA
CAGTCCTCTGGATGGGCATCAGTCATATCTGAGGATCTGGTCCCAGCATGAAGGCCCCCA
TCTCAGACGGTCCTCTGGATGGGCATCAGTCATATCTGAGGATCTGGTCCCAGCATGGAG
GCCCAGGTCTCAGACAGTCCTCTGGATGGGCATCAGTCATATCTGAGGATCTGGTCCCAG
CATGGAGGCCCCCATCTCAGACGGTCCTCTGGATGGGCATCAGTCATATCTGAGGATCTG
GTCCCAGCATGGAGGCCCAGGTCTCAGACAGTCCTCTGGATGGGCATCAGTCATCTCTGA
GGATCTGGTCCCAGCATGGAGGCCCCCATCTCAGACGGTCCTCTGGATGGGCATCAGTCA
TATCTGAGGATCTGGTCCCAGCATGGAGGCCCAGATCTCAGACAGTCCCTCTGGATGTGC
ATCAGTCATATCTGAGGATCTGGTCCCAGCATGGAGGCCCCCATCTCAGACAGTCCTCTG
GATGGGCATCAGTCATATCTGAGGATCTGGTCCCAGCATGGAGGCCCCCATCTCAGACGG
TCCTCTGGATGGGCATCAGTCATATCTGAGGATCTGGTCCCAGCATGGAGGCCCCCATCT
CAGACGGTCCTCTGGATGGGCATCAGTCATATCTGAGGATCTGGTCCCAGCATGGAGGCC
CCCATCTCAGACAGTCCTCTGGATGGGCATCAGTCATATCTGAGGATCTGGTCCCAGCAT
GGAGGCCCCCATCTCAGACGGTCCTCTGGATGGGCATCAGTCATATCTGAGGATCTGGTC
CCAGCATGGAGGCCCCCATCTCAGACAGTCCTCTGGATGGGCATCAGTCATATCTGAGGA
TCTGGTCCCAGCATGGAGGCCCCCATCTCAGACGGTCCTCTGGATGGGCATCAGTCATAT
CTGAGGATCTGGTCCCAGCATGGAGGCCCCCATCTCAGACAGTCCTCTGGATGGGCATCA
GTCATATCTGAGGATCTGGTCCCAGCATGGAGGCCCCCATCTCAGACAGTCCTCTGGATG
GGCATCAGTCATATCTGAGGATCTGGTCCCAGCATGGAGGCCCCCATCTCAGACGGTCCT
CTGGATGGGCATCAGTCATATCTGAGGATCTGGTCCCAGCATGGAGGCCCCCATCTCAGA
CGGTCCTCTGGATGGGCATCAGTCATATCTGAGGATCTGGTCCCAGCATGGAGGCCCCCA
TCTCAGACAGTCCTCTGGATGGGCATCAGTCATATCTGAGGATCTGGTCCCAGCATGGAG
GCCCCCATCTCAGACGATCCTCTGGATGGGCATCAGTCATATCTGAGGATCTGGGTCTCA
GCATGGAGGCCCCCNNNNNNNNNNNNNNNNNNNNNNNNNNNNNNNNNNNNNNNNNNNNNN
NNNNNNNNNNNNNNNNNNNNNNNNNNNNNNNNNNNNNNNNNNNNNNNNNNNNNNNNNNNN

NNNNNNNNNNNNNNNNNNNNNNNNNNNNNNNNNNNNNNNNNNNNNNNNNNNNNNNNNNNN
NNNNNNNNNNNNNNNNNNNNNNNNNNNNNNNNNNNNNNNNNNNNNNNNNNNNNNNNNNNN
NNNNNNNNNNNNNNNNNNNNNNNNNNNNNNNNNNNNNNNNNNNNNNNNNNNNNNNNNNNN
NNNNNNNNNNNNNNNNNNNNNNNNNNNNNNNNNNNNNNNNNNNNNNNNNNNNNNNNNNNN
NNNNNNNNNNNNNNNNNNNNNNNNNNNNNNNNNNNNNNNNNNNNNNNNNNNNNNNNNNNN
NNNNNNNNNNNNNNNNNNNNNNNNNNNNNNNNNNNNNNNNNNNNNNNNNNNNNNNNNNNN
NNNNNNNNNNNNNNNNNNNNNNNNNNNNNNNNNNNNNNNNNNNNNNNNNNNNNNNNNNNN
NNNNNNNNNNNNNNNNNNNNNNNNNNNNNNNNNNNNNNNNNNNNNNNNNNNNNNNNNNNN
NNNNNNNNNNNNNNNNNNNNNNNNNNNNNNNNNNNNNNNNNNNNNNNNNNNNNNNNNNNN
NNNNNNNNNNNNNNNNNNNNNNNNNNNNNNNNNNNNNNNNNNNNNNNNNNNNNNNNNNNN
NNNNNNNNNNNNNNNNNNNNNNNNNNN**NNNNNNNNNNNNNNNNNNNNNNNNNNNNNNNNN**
NNNNNNNNNNNNNNNNNNNNNNNNNNNNNNNNNNNNNNNNNNNNNNNNNNNNNNNNNNNN
NNNNNNNNNNNNNNNNNNNNNNNNNNNNNNNNNNNNNNNNNNNNNNNNNNNNNNNNNNNN
NNNNNNNNNNNNNNNNNNNNNNNNNNNNNNNNNNNNNNNNNNNNNNNNNNNNNNNNNNNN
NNNNNNNNNNNNNNNNNNNNNNNNNNNNNNNNNNNNNNNNNNNNNNNNNNNNNNNNNNNN
NNNNNNNNNNNNNNNNNNNNNNNNNNNNNNNNNNNNNNNNNNNNNNNNNNNNNNNNNNNN
NNNNNNNNNNNNNNNNNNNNNNNNNNNNNNNNNNNNNNNNNNNNNNNNNNNNNNNNNNNN
NNNNNNNNNNNNNNNNNNNNNNNNNNNNNNNNNNNNNNNNNNNNNNNNNNNNNNNNNNNN
NNNNNNNNNNNNNNNNNNNNNNNNNNNNNNNNNNNNNNNNNNNNNNNNNNNNNNNNNNNN
NNNNNNNNNNNNNNNNNNNNNNNNNNNNNNNNNNNNNNNNNNNNNNNNNNNNNNNNNNNN
NNNNNNNNNNNNNNNNNNNNNNNNNNNNNNNNNNNNNNNNNNNNNNNNNNNNNNNNNNNN
NNNNNNNNNNNNNNNNNNNNNNNNNNNNNNNNNNNNNNNNNNNNNNNNNNNNNNNNNNNN
NNNNNNNNNNNNNNNNNNNNNNNNNNNNNNNNNNNNNNNNNNNNNNNNNNNNNNNNNNNN
NNNNNNNNNNNNNNNNNNNNNNNNNNNNNNNNNNNNNNNNNNNNNNNNNNNNNNNNNNNN
NNNNNNNNNNNNNNNNNNNNNNNNNNNNNNNNNNNNNNNNNNNNNNNNNNNNNNNNNNNN
NNNNNNNNNNNNNNNNNNNNNNNNNNNNNNNNNNNNNNNNNNNNNNNNNNNNNNNNNNNN
NNNNNNNNNNNNNNNNNNNNNNNNNNNNNNNNNNNNNNNNNNNNNNNNNNNNNNNNNNNN
NNNNNNNNNNNNNNNNNNNNNNNNNNNNNNNNNNNNNNNNNNNNNCAGATTTTTAATGAG
GATTGGAATTGCAATGTATCTTATATCTGCTAAGTCTGTAACAGCTGCATATTCTTCAAA
TGATTCACAGGTGTCATCCTGTTCAATGACCTTTACTGAATGGGGAAAATGTTTGTCTGA
AATTTCCTTTTGAAGAAGTGTTTTGGAAAAAAATAGCTTTTTTCAAAATTTTTGCCACTT
GTGACATATGGACCTGGTTTTACCTTGCAAATAGATATTCAGGGTGTTTCAATTTGACAT
GATACCACACAGAGACATTCCTAGAGACATCGTCTGTCAGTAACTCCTAGAGACATCTTC
TAGCTTCATGTCTCTACTATTCCTAGAGACATCTTCTTTCCATAATTCCCCATGGCTGAT
TCTGTTCTTCATAAATTTAATTATCTCTTCTCACAGGGATAAACTTTTGGCCAGCACCTG
TCCCTGGGATAGCCAGCACACTTGAGCATGGTCTGGCGGATGACACCACATACCTCCTCA
TTCAGCTATAGCATGTTGACCACTCCTGTGTTGCATTTGCAGGAACCTAGTCAGGAATGC
TCTCAAGTGGCTGCAAAGTGTCATTTAAAATAGCAGCTTTAGCAGAGATTAGGCTGATGC
AAGTTGTCATGAAAAGAAATGAGGACATCTGGATTCAGTAATACTTTTTTATCTGTGCAA
TAAACCCGTCATATTTTCCTGTACACTCACTAAATTTACTGAGTTCAGTCCAATCACCTT
CTGTTTACCTTGAAAGTTGTTGAAGGTGTCTATCTATTCTCTATGTTCTGTTCACAAGAG
CACCCAAAGCAAGTGACTGTAGCAAAGAAAATCCTCTATTATGACCCAAATGAAGTATAA
AACCCGCCCCTTGTCAGTAGTATCTGTTGATTCACCCATAGTGACTGCATAATATATATT
AGTTCTTAAGTAAGAGAAATCTGATTTCCTTAAGTGCTAAGTTTGACAGTGGGTATCTCT
GGCATTTCACTTTGACTCTTGTTTTATTGTTATTGTGAAGGTCCATCCTCATTCCTTCAA
ATGCACATTCTGGCTTGTGATTGATTATCTGACACATTTATTTTAGAGCAATTTTTGTGA
AACTACGAGTAAGTTAAAAATTTGTGTGTTACTTTTAAATAAGAGTTCCGCCAGTGCAGA
CAGCTAAAAATATCAAGGAAGTGTTTGGGAAGGATGTAGTTAATGAAGTCTCAGTATCTG
GATAGTTTGAAAAGTTCCATTCTGGTGATTTTAATATTGACAATGAGCCACATGGTCAAC
GTGAGACCAGGGTAGATAAGAAGCCAAAAGTTGCAGTGGGAGCTAATCTTTCAACCTACC
CATGAATTAGCAAAATTTGGCATTACTATTCCATAATATTGGACCATTTGAAACAAGTAG
ACAAGAGGAAGCTGGGTAGATGGGTTCCAGGAGGATTAAGTGAGCTTCAGAAGAGAAACT
GTGTTGAAGCTTGCCTTTCTTTGCTGTGACAACACAAAGGAGAACCTTTCTCTGTCACAT
TGTTACATGTGATGGAAACTGGATTCTTTCTGACAACCCTAAGAGTTTGGCACACTGGTT
GGATAAAGATGAAGCCCCGGAACACAGACCAAAAGCGAATATTTATCAAAAAAAAGCTAC
TGGTGTCTGTTGGTCTAGTGCTGGTATTATCCACCACAGCTACAGGGGCTAGAGTTGGAA
ACTCAGTCACCCACTGTATTCTCCAGACCTCACACCAGCTGACTGCCACTTCTCCCAGGC
TTTGGACCATTTACTGCAAGGAAAATAGTCAATTTTCAACCAGGTGTGGAAAACACCTTT
TGTAATTTCTTTGCCACTCACTCTCCAGGCTTCTTCAGTGCTGGCAGAAACAAGTTACAG
TTAAGATGGCAAAACTGTTATAGTTTAGATGCATATTGTGATTAATTGCACTGCTTCTAG
TTTGAGATATAATAAACTAAGCTTTTGATTTGAAATCAGACATTTCTTATTACCTAATAT
TTCACTTTTTAAGTATTGCATGAGGTTGTTCTGTTAAGTTGAAGCCTCGTTCATGCTGCT
GGTGAGTTATGGTTCTCTCTGAAATAGGCAGGTGTTTGCACTTTGGAGTGTTCTCAGGGT
CTAAGCATCCTACAACTTTCACAATTTCCACATCACTGAACGGTCTCCCTTCTTTCCTGA
GTATATCAGCTACTTTTTAAGTTGCTTCAAGGTCATTATTTCTAGGTCTTACTACTGCTT
CAAAGAATTGTCTTTGTTGGGATGCTGAGGCAGGAGTCAGAGGTTGTTGTGAGCTAGACT
GAGGCCATAACACTCTGGCCGAGGCGACAGAGCAAGATTCTCTCTCAAGGGAGGAAAAAA
ACAAGAATTAACTTTACTTTTGCTTTTCATCTCTTAATTTCTGCAATACAACCTTTTATG
TGACTTCCTCTAAATTAAAATGCGTGGGCCTTATGAGCGTTATAGTCCCAGTGCCCACCG
AATATCTTCACTGTTGGTCCTGCAGTATCACAGAGCAAGCAGATCATCTTATTCTTAGCA
GAAATAAGATAACATTGCAATTCCAAATCCTCATTAAAAAATCTGTTTTCTTCCTTCAGT
GTTCTCTTGGTCTTCTTTGACATGATGGGCTGTTAGCAGACAGAATTAAAAAGTATTGTA
AAATCATGCAACTATTCACAAATCCACTTCAGACCGACACACAGTGCACCAAAGTCAAAA
GTTGTACCTCATCCCCTGTAAACTCTCACCAGCCAGCCTCATGCGATTGTGGGGCCGTGG
GCAGGAGAAGTTAGAGCTGAATCATGAGCTTGGCAGGTGTCTGTTCAGGCTTTGATTTAC
TAATGTTCTGATTGTGCCACTTAATCTGGGAGGATTATTTCATTGAAACTTATTTTATTC
TTGGGTATTTTATTAATAAATATGTTCATTTGAAAAAATTAAATAAAAATAAAAAAGATG
AACATCGCACTGTGGGTGGATTCAGGCCAGTGCTGATGATGCCACTCAGTAATTGTGTGT
CTCATGTGTTTCTTCAGACTGAAATGAAAAGATTGCACTGAATAATCTGTGTTTAATTCT
AGCTTAAAAATCCTATAGTTGACAGGTAAAAGGAGATTTGACTAAATGGAAAAATACTTG
TTTCAATATTGAAAGGTATTTTTCCCATTGCCCTGGCCTTTTTCAGATTAGCATTAATCT
GAAAATAAGTAGGCAAAAATATATATGAAAAATATTTAACAGAAATAAAATAACTACTAG
GCATAAGGCAAACTTAAGGGGTATTTTAAAGTTTTTATAATTAACGGGGTACTGTATAAA
AATAAAAATCAGCGATAGCCAGACTTCCTTGGTTCAAAGCCGCCCCGGGGAAACTGCATA
CAGGCTTCTGAAGTCTCCTTCCCCAAGGAAGTTGCACAGGCTGTGTTTGATACCCACAGC
AGTGAGTTGTGACAGATGTGGATGTCGTCCACCAAGAAAGGTTACCAGAGGCTCAGCACC
CAGGGATTTAACTGGTCATGTAGGCACCCTCTGCTTGGAATGTACAGACATTCCAGATTC
TGTGAAGGAAAGAAGGTATTTGTCCTTCTCACCAAAACCTAAGTTCCTGGACACTAGCCC
AGGGCCAGCTCTGTAAGCAGCCCTTCAGGGACTGTAGCTGTCTCAGACCTGTGTTGTCCA
GTCCTCTGGAAGTCCCATGTTACATTAACTCTCCTCTACACAGGCCCATCCACACTTCGT
ATTTATGTCTTAACCACTTAGTAGCCATTTAAAAAATAATGCCCATAAATTGAAAGAAAA
AATATTTTTATTTCATTCTTAATAACCGGAATTGCTTGCTGATGGATTATGTATACCTGT
TGACCACTGCCTAGTATCTCAGACCTTGGAACCATATTTTTCCTGCCACCCTCATTTCCT
GCTTGACCTTGGTTTTCACATTGTCATTGATTTTTATCACAGCATTGCCTGAACAAAAAC
AGTTCTGTAAAGACACGATGTCGTTGTAAGGATTATAACCTACTCCAGCGTCCAGCTACC
TTAAGTTAAGTTATGGTTTCTGACAGGTGTTGAGGGGACAGCTTGGCACAGAGTTTGGTG
ACTCTGTGTAACATAGCTTACACTGTTTGCTGAATCGTTTCAGTAAAGTGGACAAAAAAC
TTTCTGCAAAACTCTGAAGGATAGAAAAGAAACATATTCAATGGGAAAATTAATTTTTTA
AGTGTCTTATGGGAAATTAAATTTTTAAGGACTGATGACTTCCCTGGTCTGAGTGGGGAG
GAGGCGGGGGGGTCAGAATGTCAGGCCAGCGTTTCACAAATGGATTTACTTTGTTTCAGG
AGGTTGCCAGGTTCTGGCCAACATTTTTATATTTTTAAAGTCAGAGTCTCATGCTGTCAC
CCTGGGTAGAGTGCCGTGGTGTCACAGCTCACAGCAACCTCCAACTCTCAGGCTCAAGTG
ATCCTCTTGCTTCAGCCTCCCGAGTAGCTGGGACTACAAGGCACCCACCACGGTGTCTGG
CTGGTTTTTCTGTTTTTAGTAGAGATGGATGGGCTCGTGCTCTTGCTCTTGCTCAGGCTC
GTCTTGAACTCCTGAGCTCAAGCAGTCCACCCGCCTTGGCCTCCCAGAGTGCAGGGATTG
TAGGAGTGAGCCATGGGCACCCAGCCTTCCTGGCAAACTTTTGAAACAAGGAAGTTGCAG
CCTTTTCCATTTCTCAGCACTTCATAATGATGCTGACCCAAATGACACCTTTTCTCTGGA
ATTTCTTTCTACTTCCTTTTGAATTAAAGGCAGGCAACCTGAATTCATACAGAAGTATAT
ATACACAAATTTGTACACACACGCATACATACAGAGGGTGCCAAATAATGTATACATATT
TTAAGAAAGGAAAAGACTATATTAAAATCATACTCAAGTCACATTTAATTTCTGCAATTA
CAAGAGATACAACCTAACATATGTTATTCTTGTATTTTCTTACTATATATGCCAATAATC
TCTCTCTGTATAAGCTAGGAATTGTATTCAGCTCAGGTAACAGAGGACTACTTCCCCTGC
CCCAGTATATAAATTCCAATAGGAGTTTGTATGTTGTACTCAGGAAAAGAAAGCCAGGCT
GTTGAAAGCTGGATGGGTAGCTCTGCCTGTCACTGGGAGCCCAGGAGCTTCTAGCTTTCT
GTCTTTGGGATGTGACTCTCAGTTTGTATGCACGATACAGCTGCCAGAGCCCTTGTCACA
ATGTCTGCCCTTCAGTTAGAGAGAAGGGGGAGAGCAAAGAGCAAACTCAGCTTGTACCAG
CAGACGGCATCCAACAACTTTCACTTAGATCTTTTTGGCCAGAACTGTGTCACGTGAACA
CTCTTACCTGCAAGGGAACTTGGGAAGTGTACTTTAGTTTTGATTTTCCCTCCTGTTATT
AGCTTTACGTGTTTAGTTATTCTGATGCTACTTATGCAAAATATTCCTTTAAATGATAAA
TTTATCAGGCAAAGATTTATCTATTACAAATAGTAAGAAAAAAAGTCCAAGGGGTAAGTT
AGGAAATTATTTTTCATTTAACAAGCTTACTGTTAGAAATTTTCTAAAATTATTTTTATT
TCTTTTAGATCCTGAATAGTATAAGATATAACAACAATTACAGCAGTTAAAAGTTATAAA
ATGCAAAAAAAAAAAAAAAGTTATAAAATGCTTGCTACCTGCCGAGAATTGTTTTAAGTG

```
CCTTATAAACACTAACTCACTTAATCGTCAGAAGAATCATGGATAGGATTTTGTTTCTTT
CACAGATGAGGAAAAGCTGAGTACACTGAAGTTCAGTGGGCTGGCCAAGGTCACACACCT
GTTAACTGGGTTGTGTGACTTAGTGTTCATTTGGCAGATTGTCTACTGAATTAAAAATTA
TTTAAACTTAAATTCTGTTCTTTCATTTCCTTGTTGTAAGTATTTGGCAAGGCACTTCAA
TCAGCATCTTACCTTTAAAATGAAATTAATATTACCTCATAAAAATTTTGTCAGGGGTCA
ATGATAATGACATAAAGCGTCTAGTGGGTTATCCAGGCAGATAATGACTCCAATTAACTG
GGAACTACTGCCTTTAAACAATGGGTGTTTTTTATGTAGAGCTTGAGTCAGTGAAGAAGC
CAGATGCCATTTCTTTGAAACCTTTTTTTTTTTTANNNNNNNNNNNNNNNNNNNNNNNNN
NNNNNNNNNNNNNNNNNNNNNNNNNNNNNNNNNNNNNNNNNNNNNNNNNNNNNNNNNNNN
NNNNNNNNNNNNNNNNNNNNNNNNNNNNNNNNNNNNNNNNNNNNNNNNNNNNNNNNNNNN
NNNNNNNNNNNNNNNNNNNNNNNNNNNNNNNNNNNNNNNNNNNNNNNNNNNNNNNNNNNN
NNNNNNNNNNNNNNNNNNNNNNNNNNNNNNNNNNNNNNNNNNNNNNNNNNNNNNNNNNNN
NNNNNNNNNNNNNNNNNNNNNNNNNNNNNNNNNNNNNNNNNNNNNNNNNNNNNNNNNNNN
NNNNNNNNNNNNNNNNNNNNNNNNNNNNNNNNNNNNNNNNNNNNNNNNNNNNNNNNNNNN
NNNNNNNNNNNNNNNNNNNNNNNNNNNNNNNNNNNNNNNNNNNNNNNNNNNNNNNNNNNN
NNNNNNNNNNNNNNNNNNNNNNNNNNNNNNNNNNNNNNNNNNNNNNNNNNNNNNNNNNNN
NNNNNNNNNNNNNNNNNNNNNNNNNNNNNNNNNNNNNNNNNNNNNNNNNNNNNNNNNNNN
NNNNNNNNNNNNNNNNNNNNNNNNNNNNNNNNNNNNNNNNNNNNNNNNNNNNNNNNNNNN
NNNNNCTGTTTCTTTAAAATAGGAGCCTACTAAAAATACACCATATTTCAAATGTTTAAT
CGAAATCCTTATATGGCGGAGATTTCAGATAGTCTTCAGGAAGTGGATGGTGGCAATACA
AGTATGTTCACCAGAATTCTAGAAGCTGTTAAAGAAGCTGATGAAACAGTGGCGAATAAG
ACACCATCAGTGGCCCAGGTGATTCTATTTAATAGAAAAACCCTAGAAATCTGTAAAGTG
ACATCTACTTTTAAAGTTTTGGCATATATGCATGTGTATGCATGCACACACATGAGATAC
CCTTACACCAGAATTTTTTATCTGTCAAATTTTGCTTGTCTTGTTATAGCATATACTTAT
TACCTATGAAATGCTGAGGCAATAATGAGAAAACTCATTTTCAAACTTGTTTCCGATTTT
GCAGTCAAGTAGAGCTGGATTCAGATGGAGTTGTGCTGCTCATCAGCTGTTTGGCATTAT
TAGCAAATTACCCTTCTTTAGTTTACTTTCTATGTAAGGACGAGTCTTTGAAACATTGTT
TTGAGAATTAAATGAGATTCATGCCTAACTTCTAGTAGCATTTCAGTAAATCGCCATAAT
ATGCATTTACTTTCAAGAAATTAGAGGCCCTTACATCCTTTGGTTCTTTTTTCTTTTTAT
TCTTACAGGTGGAGAACAACAATTTGGATGAACTAAATAAAAGCAAGATCATTATTAAGA
AGAAACCAAAGCAAATAAATCCATTTCACTTAAAAGTGGGGCATGAAGTTTTAAGACAAA
GGAAAAATTGGTGGAAGGATGGTCGTTTCCAGTCTGAGTGGGTCGGTCCCTGTGTCATAG
ACTACATCACAGACAGTGGCTGTGCCGTCCTGAGGGACGGCTCTGGGGCGAGGCTGAAGA
GGCCCATCAAGATGTCCCACCTGAAGCCCTACGTGAGAGAGGCCGGAGAGCACGGTGAAT
GGCGGTCACTTCTCTGCTTTTTACTTTCTTTGAGAAGAGAACTTAAAAATAATAGGAACC
TTTTTTCTCCTCTCTGGTTGGGTTTTCTTTTCCTTTCTCATTTTTATAAACTTGGGACAG
TTTAGGTGTCCCAGGTGACGGTCTTTTTGTTAGATGTCTCTTCAGGATTGTCGGGCTTGT
GAGCAGTTTCATTCTGATTTCTAGTGTGCACTCTCATGGTTCTCACAGGAAACTATGTGC
ACCTGTTTCCACTGAAGGAAAAAATAGCATCTTTTCAATTTTTGGTTTCTTAGTGCCCTA
TTAGTCGTTTTGTGCCTCATTTTATTATGAAAAACAACAGTAACAAAAATCACTAATGCC
AAAAACAAACATATTGTCCAGAATCCAGTTGTAAGATTGATGTATCAAAGGACTTAAAAT
ATCATTAATGTTGCTTTGTAGTGAGGTCATGGTAGGGCGCAGCGGGGGAGAGAGCGGGTG
```

```
==================================================
Bushbaby Gin-1
total length:      16440 bp  (13237 bp excl N/X-runs)
GC level:         41.64 %
bases masked:       3009 bp ( 18.30 %)
==================================================
               number of      length   percentage
               elements*    occupied  of sequence
--------------------------------------------------
SINEs:                7         1268 bp    7.71 %
      Alu/B1          2          413 bp    2.51 %
      MIRs            4          577 bp    3.51 %

LINEs:                1          101 bp    0.61 %
      LINE1           0            0 bp    0.00 %
      LINE2           1          101 bp    0.61 %
      L3/CR1          0            0 bp    0.00 %
      RTE             0            0 bp    0.00 %

LTR elements:         2          440 bp    2.68 %
      ERVL            1          219 bp    1.33 %
      ERVL-MaLRs      1          221 bp    1.34 %
      ERV_classI      0            0 bp    0.00 %
      ERV_classII     0            0 bp    0.00 %

DNA elements:         4         1091 bp    6.64 %
      hAT-Charlie     1          336 bp    2.04 %
      TcMar-Tigger    0            0 bp    0.00 %

Unclassified:         0            0 bp    0.00 %

Total interspersed repeats:     2900 bp   17.64 %


Small RNA:            0            0 bp    0.00 %

Satellites:           1           58 bp    0.35 %
Simple repeats:       0            0 bp    0.00 %
Low complexity:       1           51 bp    0.31 %
==================================================
```

SW score	perc div.	perc del.	perc ins.	query sequence	position in query begin	end	(left)		matching repeat repeat	class/family	position in repeat begin	end	(left)	ID
2192	3.2	0.4	0.4	UnnamedSequence	1221	1498	(14942)	+	GarnAlu1	SINE/B4	1	278	(0)	1
383	26.7	5.0	0.0	UnnamedSequence	1792	1892	(14548)	C	L2a	LINE/L2	(11)	3415	3310	2
267	30.8	10.4	8.1	UnnamedSequence	3580	3858	(12582)	C	MARNA	DNA/TcMar-Mariner	(170)	416	132	3
592	30.9	4.9	2.5	UnnamedSequence	9519	9881	(6559)	+	MMAR1	DNA/TcMar-Mariner	419	794	(493)	4
341	27.3	3.5	2.6	UnnamedSequence	9907	10019	(6421)	+	MMAR1	DNA/TcMar-Mariner	987	1100	(186)	5
450	23.7	3.1	0.1	UnnamedSequence	10457	10566	(5874)	+	AluJ_Mim	SINE/Alu	174	329	(1)	6
30	80.4	0.0	0.0	UnnamedSequence	11106	11156	(5284)	+	AT_rich	Low_complexity	1	51	(0)	7
195	30.9	4.7	4.7	UnnamedSequence	11216	11300	(5140)	+	MIR3	SINE/MIR	115	199	(9)	8
801	27.1	5.0	0.4	UnnamedSequence	11562	11780	(4660)	C	MER68	LTR/ERVL	(151)	412	184	9
1285	22.8	5.7	0.8	UnnamedSequence	11939	12274	(4166)	C	MER119	DNA/hAT-Charlie	(130)	453	102	10
1507	18.8	1.3	3.7	UnnamedSequence	12564	12866	(3574)	C	AluJo	SINE/Alu	(16)	296	1	11

238	19.0	1.7	0.0	UnnamedSequence	13049	13106	(3334)	C	Sat-1_TS	Satellite	(2767)	469	411	12
349	34.1	8.6	1.7	UnnamedSequence	13382	13602	(2838)	C	MLT1J	LTR/ERVL-MaLR	(183)	329	94	13
558	25.7	6.5	1.1	UnnamedSequence	13881	14049	(2391)	C	MIR	SINE/MIR	(15)	247	70	14
256	33.3	12.8	1.0	UnnamedSequence	14108	14286	(2154)	+	MIRb	SINE/MIR	61	260	(8)	15
277	32.3	15.3	1.2	UnnamedSequence	15415	15558	(882)	+	MIRb	SINE/MIR	35	198	(70)	16

>Macaque Gin-1 (chromosome:MMUL_1:6:99335946:99369350:-1)

CAATTTCTGGGGTCTCAAACCCAAGAAATGAAAAAGGGAAGCCATGGAGGAGCAGGAGGA
GAAATCAAGGCGCGAAAGGACCCTCAAAGCTATACTGTCACTCCACGCCCAGGCTGTCAT
TCTCTGCAGCACATGACCCTCGGTGGAGCGCGTGTTTAGGCTCTGCAGAGCACAGGAGAC
AAAGCAGAAGCCCAGCAGCCGCCTCAACCCTGGCATGATCAGGTTCCGCATCTCCGGTTC
TCCTCCAGTTTTGGGGCCCTCACCTGCCACGAATGCGGTCGTCACGCGAGCAGTTGTGAG
ATGGTCAAGCCCATTCAGGTCTAGGTTACCCACTTTCATCCCGGAGGCAGGGGTGAGGTG
TTCGCCAAGTTACTTCAGTTGTAGGCGTGGACGTAGGCTACTTCCTTCAGTAACAGCTAT
AGGGCCACATAGGGGCACCTCAGGCTACTACACCTCAGAAATCTCCGTCACGCCTCTTCC
CCGCCCTCCTCGGGTCTCACGCTGAAACCTCACCGGGCGGAACGATTTCCGGCAAGAGCC
AAACGATCCGTCCTAACCTTTCTGACCGTGGTTCCAAGGCTCCCAAAAGGACTGGCCTCT
AAAGGACCTGGGTAGTTCCGCTTCCGGCAGCGCCAGATAAATCACGAGAGGAAGCTTAAA
TCTGTCGTTTGAATTTAGGACCACCTCGGTGAGTGGTCGTTCTGGTGTGCTGTGTCATAC
CTACTTGTTTTTTAAAGTGAGGCGTAACCCGACAGTAATTTCAAAACCATTAGCCTCGAC
CGGCCTAAGGAAGGGTTTAATTGACTCTGTGGGGAGATTTTGCGCAGGCGCCGTTGAGAC
GGGAGGGCCTTGGGCGTTGCGATGTCCCGCGCTAGCTGAGCAGAAAGGGCGAGTGCCGTT
TCGGGCCTGGGGAGGTGCCTGCTGGGTGTAATTGAGTTTTACCAAATGGTCATTTTCCTG
CTGATTCTAAGTGTGGTTGGACCTTGCCCTTAAGATTCCGTTAGCAAGTCGCCCAAAGCT
TAGGAAATGCTCCCTCACAGCGTTTGAAACTCCGAGCACTTTTGGCCGGCATTGAAGAAT
TCTCCCCATTACTTTGATCAGTCATTTAATCAGAAATTTTGCCCCAAATTTAAATTAGTG
GCGGGGAGAAACGTGAGGTCAAGAGGGAATCCTGTCACGTAGTTTTAAAAGAAAAATATC
CACTTTATCTCAGTGTAGGTTTTTCTTAAATGGGCATTTCTGAACCAAAGTAGATACTTT
TTAAAGTGGAAATGCATTTATCCCATGTGGCTTCATGTGAATGCATATGGCGATGAAGGC
ATTTTTTTTTGTTAAATGAATTTGTACTCTGAAGTGAACCAGATTACAGAGAAGTGTATT
TAAATTTACAACTCCTTGACGTTGCATGAAATTGTGCGTGTCCATTTTTTGGAGCTTAAA
ATAGAATAATTCAAATGTCAGAATTCCGATCCAAGTTAACGTGTTGATTGAAAATTGTAG
ATTTTAAGATGGAACTAACGGGTATTTTAACCTTTTAGGGGCTTAAATTGGTAGCTTTGT
TTTTAACCCTTGGTGAAACAGTTAAGTCCTAATCTTGACGTAATACCTAACTGCCTTACT
GTATTGAAGACAAACATAAAATTATCAAAGGAATTAAGCAATGAGTTGATTTTATTTGAT
GTTATGGAGGGGACAAAGTGTGTAAAGCTTAACCAAATAAGACCCTGACATTCCTCATGC
AAAACTACAAGTTGTAATCAACCACTTTCATTTTTTCTTATTCCTACACGTTTAAGTACA
TAAGTAATGATTTTAAGAGAAATTCTTGAAAATATGAGCATATCCAAAGAAGTCCCAGGA
CAGTCTTTCTTTATGTAGTTGAAACCACTTAATAAGAAGATACTTATATTTGTCCCGAAT
ATTACAGTTTCAAGCCATTTTCTCATATTAACTACTTTTCAAATACGTTGTTCCTAGGGA
CCAAGTAGTGTATGTATGGGCTTTGAAAAAACAAAGACAAAGACTGTTTCTTTTATGTTA
CTTTTGTGTATGAATACCAATTATTAGCATTAAAACTTTAAACTACTTCAAGGGCTATGC
TTTCTGTGAACTGAGTTTTCTTTCTTACGCATTTTCTTTTTCTCTTGTCATCTGCCTCTC
TACCTCAAGAACCAGCAGTTTGTGAACTGCATTAGACTGTAATATCGTTATAAAAGAATA
CAACTCCTGAGTCTTCTGATTTTCTTCTTAGCACTAAAGCTTTATAAACATTGCTATAAA
GCATTAACACCATTAATGAAAGGAAAGATATTTTAAAATATTTTTTCATGTAGATGAAGA
TTTTGAGAAAAGAAAGGCTTCTCTGTTACTCTAAATAGCCTGATATGGATGATAATACTT
AGATATGATAATAATTATACATTACTTTACCATATACAAAGGACTTTTAAGTCCATTATT
TTCATCCTCATGATTGCAAAAAAGGAAAAGAGATTATGTCCTCTTCCAAACATAGATATT
AAGGCTTAAGTCTATACCTTCTTAGACACAGTTTACTTATAGTGCCATGTTCGTGAAAGT
GAACTATAATGTTTTCAAAAGTGAAATAAGTTACCTTTGGAATATTTGCTATAATTTTTA
ATTCTCCTTTCAGGAAATGCAAGTAATTCTTTATTCATTCACAAATAGTTGAGTGTCCAC
TGTGTTCTGAACATGATGTTAAACAATGTAAACATAAAAAATTAATAAGCTATTTCCATT
CCTCGTTTATAATCAAACTTATTGATTACAAAAATCACTTTAAAATTTGTGTAACAGAGG
CCTGAATCAAGTATTATAAAAGCTAAGGATGACAAGTTAATTGGCTTATTGGTATAGCAC
CAGGGAAAAGCATAAAATTTGAAGTAAGAGACTCCAAGTTACAAATGTCAGCGCGTGTAC
TCACTTTATTACCTTTAGTAAATTGGACAATCTCTGGACCCCCATTTTCTTACCTGTAAA
ATGGATATAAAACTAACACCAACTCCATGGAGTATTGTGAGGATTATCATAGTAAAATAC
TGCATAACTATTTGCAGCTGTAGTCGTGAGTCACTTAGCAGTGGGAATAAATTCTGAGAA
ATGCTTTGTTAGGCAGTTTCAGCAATGCACGAATACAGACTACCTACATGAACCTAGATG
GTGTGTGTGTGCGTGTGCATATATATATATATTTTTCTTCATATAAAAAAAAATGTCCCT
GCACATTATTGGGTATCATTGATTTCTCCTGCTTGATCTGCAGTGCTAATATCAAATGCC
CTATATCAGGTTCTATATATGCTCCCCTATCATCTTATCAGACCAGTAGGCAGTCCATCC
TTGACCAAAACATTCTTATGTGGTGCAATTGCATTGGCAATTGTTTTTAAAATTTTTAAT
TTTTTAAAATTATTTCCTCTTCCTATACTTTTGGCTTTTCCAGATGTCATATAAACAGAA
ATATATAATATATAGCCGTTTGTGTGAGGCTTATTTCACTTAGCATAATGATTTTGAGAT
TTATTCATATTGTTAGGTCTATTTGTAGTTGTTCCTTATCAATGCTTAATAGTATTCCGT
ATGTGGATCTACTATAATTTGTTTATCCATTCACCAGTTGATGAACATTTGTTTTCAGGT
TTTGGCTGTTAGGAATAAAACTTCTAGAAACATTCACTTACAGGTTTTTAGGTGTATATA
TTTTCTTTAGGGTTATTATCTAAGATTGGTATTGCTGGACCATATGGTAAATTGATGTTT
AATTTTATAAGAAATTGCCAAACTGTTTTCCAACATGTTTACATCATTTTCTATTATCAC
CAGCAAGAGTATCCTTGGTAGTACCTAGTATTGTTAGGTTTTGTTTTGTTTTTGTTGTCG
TTAGTGTGTAGTCATGTCTTATTGTGGTTTTAATTTACATTTTTTCATTGTCTATTGATG
TTGAGCATTTTTTCATGTGTTTATTTACTAACCATATCTCTGGTGAAGTGTCTGTATCTC
TAGGTTTTAAATTGGGTTGTTTTCTTGTTAAGCTTTGAGAGTTCTTTATAACTTCTAGAT
AAAAGTTATTTATCATATATATATGTATTGCAAATATTTTATCCCAGTGTTATTTTTCTC
TGTTTTTGGCAGTTCTTTAAAGAATAAAAATTTTAATTTTTTCTTTATGCATAGGTGCTT
TTTCTTTGCTATCTAGGAAGCCATTACCCAACCTAAGCTCACAAAGAATTTTTGTTGTTG
TTTTCTAGAAGTTGTATAGTTTTAAGTTGTACGTTTAGATCTGTGACCCATCATGAAGTA
ATTTTTACATAACATGTGAGGTATTAGTTGAGGTTCACCTTTTTTCAAAGGATGTTTAAT
TGTTACAGCATTTGTTGAAAAGATTATCCTTTCTTCATTGAATTACCTTGGCACCTTTGT
TAAAAATCAGTTTACCGTAACTGAGCGACTGTTTCTGGCACTATTCTGTTCCATAGATTT
AAGGCTGTCATTTTGCCATTATAACACAGTCTTCATTACCATAGCAGTATAGAAAGTCCT
GAAACCAAATAATGTGAAATTCTTCAACTATATTCATTTCTCAAAGTTGTTTTAATTATT
CTGAGTCCTTTAGCTTTCCATATCAATTTTAGAATCAGCTTGTCAGTTTCTGCAAAAGAG
CTTGAGGGTTCCTGATTGGGACTGCATTGAATCTATAGATCATTTGGATAAAATTGGCAT
CTTGTCCCACTTTTTCTCAGAGATAGAGGGGGAGTTACTCTAGCTTTCTGCATTCTAGTC
AGAAGCCTATATGGGAATTTTTAGTGGTATTTCAGAAAGAAATAATTTTGCTCCACGATT
ATATTCACTATAATATCTTTAGTGGAAGAGATCACGGACGAATCCCTGTTTGTGAATTGG
ATATCATCCCCTGCTGCCATCTCAGGAACGTCATACCATAATTTTTTTCCCTCTGTTTTC
AGTGGTCTTTCTAGAAGCCCGTCCGCTAGCATTAAACTTTTTGGGTTATTCCTTTGTTGA
AAGCAAAACAATCTTGTTTTTTAAAAAAAATTGTTGTAATTGTAGTAAATCACACATAAC
ATGAAATTTACCATCTTAACCATTTTTAAGTGTACATACAGTTCAGTGGCATTAAGTACA
TTCACATGGGTTTACAACTATTACCACCATCCATCTCAAGAAGTCTTTTCATTTTGTATA
ACTGAAACTCTTGACCAGTTAAACAATAACTCTTCCATTCTCTCCTCCCCACCCTCTGTT
CTATGCAACCATCATTGTTCTTTTTGTCTGTATGTATTTGACTACTCTGAGTACCTCATA
TAGGTGGAATCATACAGTATTTATCTTTTTGTGACTCATTCATTTCACTTAGCTTAACGT
CCTCAAGATTCATCCATATGGTAACGTGTGTCAGAATCTCCTTCCTTTTAAAGGTGTGTG
AATAAATACACCATGTTTTGTTTATCCATGCAGCCATTGATGGACACTTGAGTTGCTTCC
ATCATTTGGCTGTTGTGGGTGATGTGGCTAATAACACGGCTGTCAAAAATTTTAACCCCA
CTAGAATGTAAACTCAATGAAGGTCAGGAGAATCTACATCTTGTTTATTATCCTGTCTCC
GACGTTGTAACTGGCACATAGCAGTTACTCAGTAACGTTTTTTAGATCAAGGAATGAACC
TTACATCTGCCTCTAGCAACTATCTTGTAGCTCCTTGCCTTTATAGCTAAGCTCAAGTTT
CTAAATCCACTTTCTGACTTTCCACTCTTTCCTTGTGTCATTTCAGTCTGGTTGCCAACC
GCCCTGCTATACTGTAACTGTACTGTTACCAAAACCACTAGCTACATTATGTTGCCAAAA
CCACTAGCTACATTATGATTCTTATATCACTTAGCCTCTCTGTCTACTTCTAAGAACGGT
GTATTTGCCAAACTTCTATTACCAGAACCTCTTCCTGCTTTTTTCCTATGTCTCTGGCTA
TTCTTATGTCCCTAAAGGTTTACAATATATTTGGGAGAGACACATATAAACTAATATATT
CAATAGAGTGTTTCAGATTTTATGATAAATGTTGACACATATTACTGTGAATGTACCAAG
GAAAGTGTGCACAGTTCTCCCTGAGATTGGAGATGTGATCAGAAAGGATTCATGGATGAG
TGAACTCATTAATGTTGAAGGATAAGGATATGTCCACTGCACTTCCTACTCTGTACACTT
TCCATCTGATCCTTCTTCCATTGCCTTAACTTCACCTACTTTTTACATGCCGAGTTTATC
CATGAAGAATACAACCTGCTTAATATTGTCATCTTTCTCCCTAAGCCAGAGCTTCAGACA
GAAGTGATCCCAGATTCTTCTTTGCATTTACTCCACACATCTAGTCAGTCACAGTTATTA
TATAATGTATCTATCCATCTATACTGGCACTGCTTTGGCTCAGAATTTCCTCATTTCTTG
CTTTAATTGTACAGTAGTTTCCTAGCTGACTGGTACAGATATTCCTGTCTCTCCCCCAAT
CTCTATTCCATATTGGGGTACAATACACTTTATAGAATAGAAATCTAATCTTGTAACTCC
AAGTCATTCTTAAAATGTTAAGTTATTCCAGGTTGGAGTGGTTAGAAGACACTCTGTAGG
AAATGACACCTAGCCAGAGTTTTGAAAGATGATTAGGGGCTGGAGAAATAGCAATAAAGT
TCATTCCAGGCAAAAGTATCAATGAAAAGGTATGAACACACTGCATGGGATAGTTGAAGA
ACTGTGGTTATTTTTTGAATTGAGAATGCCAAGAGAAAAACGTGGAGAGACTGATAAAGA

ACCATATCTTATAAAGCTTTGCATAATGGCAGAGGTATTTAGATATCATTAGTGTCATGG
TCTTAATGGTTGTATTCTCCCAAAATTCATGTGTTGAAATCCTAACCACCAAGTTGATGG
TATTAAGAGACTGCACTTTGGGGCTGGGCGCAGTAGCTCACACCTGTAATCCTAGCACTT
TGGGAGGCTGAGATGGGCGGATCACTTGAGGTCAGGAGTTCGAGAGCAGCCTGGCCAACA
TAGTGAAACCCCATCTCTACTAAAAATATAAAAATTAGCTGGACATGGTGGCATGCGCCC
ATAGTCCCAGCTACTCGAGAGGCTGTGGAACGAAATTTGCTTGAGCCTGGGAGGCAGAGG
TTACAGTAAGCTGAGATCATGCCACTGCACTCCAGTCTGGGCGACAGAGCACAACTCTGT
CTAAAAAAAAAAAGAGGTAGAACTTTGGAGAGGTGACTTGATCAGGGGTCAGAGCTCTCA
TTAATGGGATTAGTGCCTATCTTTTAAAGAGTCTGAGAGAAACCCCTTCCCCCTTCCCTC
ATGTGAAGTTACAGTGAGAAGACAGCTGCCCATAAGAAAGTGAACCCTTAGTAGACACTA
AATTTGCTGGCATCTTGATCTTGTACTTCCTAGCCTCCAGAACTGTGAGAAAGAAATTTC
TGTTGATAAGCCATCCAGTTTATGGTATATTTGTTATAGCCTCCCAAGTGGACTAAGACA
ATCAGGAATGGCAAAAGGCGTATTTCGAGTTAAGTGAATATTTTGATAAGATTTGTGTTT
TAGTTTGACCTCTGTGACTGCAGTGAGGACAAGGACTATATTAGAGACAGAGGACACAAG
GTCAGATAGAAGGTGAGTGTGAAGTAAGGCAGTAGCAATGGAATAAAGTTCAAGGTTCTA
CCTCATCCACAATCAATGCTTTTCCTCCTTCTCCAGGTGGATCTGTGTCTCTGCACATCT
GTTTTGATAGTTCTCTTCGCATACATCTGTTATAACACCACTCTAAACTATATAAATGCT
GATATACTCCTCTTTTCCTCTCATACCATAAGCTCTGTGAGGACAGGGATTTCATCTTAT
TTAACTTTACATTAGTGTCATCTAGTATATATAAGGGTTTTAGAGCTCAGAACTGAATGA
GCAAATTCAAGGTTATATGGATGAACAGGCAAAAAACTATTAAAAAGGGTAAAACTTGGA
AGTTCTACAGTAGGCACAAAGAAGATTAGGAGAGGTCAAGATCACTTTAGATTCAGAGAT
CATGGAACGTTTGCTTCATTGAGGTGCTAGAGCTTGATTTCGAAAGATGGGTAAGGCTTA
ATCAGGTGGAAGGATATACCATTTAGGGGGAAATGATGTAAACCAAGGTATAGAGAATGG
TTGGGGATATGGCCATGGGTATACAAAGGAAACTAGTGAGAGAAGAAGCTGTGTTATGTC
CTGCCAGGTTGTAGAGGATAGGCTAAATAGTCTAGACCTTAGCCACTAAGTAGAAGATAA
TGCCCAGAGGCAGTCTCATAGTGTCCACTCTGTCAAGTAAATCATTTGGAAGATAAAAAT
ATGCTAGGAATAGTCTAAGTAAGTTTCAAGGTGACTATGTCAGGCAAATAACCACGCCTT
TAAGTCTTAGTTCTGTTGTCGAGTCAAGACAACTCACCCATATAAAAATGAAAATAATAT
ACTTGTGAAAGCATTTGTGAAATACGAAAATCTGTAGGAAATGTATGGTTTTTATGTCTT
CTCCCAGTGATTTTTATGTCCCTCTCCCTAACATAGATATGTGTTAGTCTTCTCCCACTA
GTTTGATAAGAAAAAAACAAACCAAAAACCTTAAAGAGCTCAGTCTCCAAAGATTTTTGT
AGTGGTTTCCTTTTCTCCTTGTCCCTGTGTACTATAGGTTTACTCTGAGGAGAGCAAATT
TCTCTATTTCGATCTATTTTCAGTTATTCAAAAGTTAAAGATTTCAAAGCAGAGAAGTTA
ATTTCAGTAAATAGACCTTTTCTGAGGAGTTTTTATATATTCGTTATAAATAGAAAAACC
AACAGTGTTCTAATGGCATAGACTTAATATACTGAAGACTTCATCTTTCATTAGATAGAA
AAATTTTCTGGTCTCTCCAGGAGGTAAATGGGTAGGGGGAGGAAGACAAGAGATGGTAGG
AACTTGGAACCCCAGAGGTTTTTAAATATAAGAGCAAAATACAGAAGCTACTGTGTATTA
CACAGACTCACATTTTTTTTTTTTTCCAATGGCTAGTTTGTTTTCTGCCAGTTCCAGTTA
CCCACATCCCTCTGTGTTTTGGGGCCTGTACTTCCTCTAAGGTGTGAGAGTTTGTTTTTT
TCCTTCTGTGGGCTTTTACAGGCCTGAAAAAGCTTCTCCCCTTTTCTTTTTAAGGCCCTT
CAGGAGTTTTTGCAATCTTTACTGGTCTCTAAAAAGGAAGGAAATACACAATTTTTATCT
TTCTTTTATAAACCTCATCTTCCTAGAGATACCATGCTCCCAGCTGGCTACGATGGGATA
TTTCTAGATTTTCTTTTAGATCGCTTAGATGATTTGGGACATTAATAGTTACAAAAACTA
TTAAGGGAAAACTATAACAGGAGGTTATATATAGTATATTATTAATTTTATAGTATGTAT
AGTCTGTATATTGATTTCATCTAAATCTCTAAATATATAGATTTCATCTATACATTTATA
GTATGTATAGTCTGTATATTGATTTCATCTAAACAGGTTTCTGTCCCTTAGACTTGAAAG
AACGTGTATCACTGTAGTATAGAGATGAAAATAAAGGACACAAACTAAAAATGACTTTGG
TTTCTTTTTTTTTTTTTGAGAGTTGAGGATGATTAATGCAGAGCTAGAATGACAGGCAGG
CAAATGGATGGGTAGACAAACGGGTTCTATATTCCCAACTTGAAATCACACAACGCTTCA
TGAATTGAAAATTGATTAGGAAGTAAAAGTTTGCCATGGAATCCTGGCAGAGGAGAGAGT
ATAGTGAGGATGACATGGAAAGTAAAGGTATCAAGAGGTCTTACGTGGCAGATTGGAGGG
CAGTAGGATACCACTAGTGTTTCACCACAGATGGTTTGAAAAGCACTGACCTAGAGCAAC
GTGAAACTTGGTAGCAATAGAAGTGGCTACACGACCAGAACAGAGTATATACGTGGTAAA
ACTTCTTGATACCTTTACTGTTTCCATGTAGTGGCTATTATGACTACAAAATATTTATAA
ACATTTTTGTACACAGATTTTGATAGTCATAGACTCATTGCACTGGAATATAAACTTAGA
ATTGCCAAGCCTTTAGGGTAAATTTGTGTTTAGCTTTAGAAGAAACTCAGTTTTCCAAAG
TGGTTGCAACATTTTTTCCTCCCACAATGTATGAGAGTTCCAGTTGCTACCTGTCTGCAC
CAACACTTGGTGTTGTCTTTAGTTTTTAATTTTAGCCGTTCAGTAGTGTGTAGTTCTTAG
TCATCGTCGTTTTATTTGCATTGCCCTGAGGAATAACGATGTTGAGCATATTTTCACTTG
CTTATTGACCATTGGATATTCTCTTTTATGAAGTGCTGTGTAAAAATTTCCCCCCATTTT
TTGAATTGGGTTTTGTCCTTTTCTTTTTGATTTTTAGAAGTTCCTTACATACACTGGGTC
TGACTCCTTTGTTAGATATATGTATTAAAGGTATCTTCTCCCAGTGTGTGTCCTGTGTTT
TCACTTTCTTAATGGTGTCTTTGATGAGTACACATTTTTATTTTGATAAAGCACTATTGT
TAATTTTCTTTCTTTTAAGGATAGTGTTTTTTGTGTCTTATTTAAGAAATCTTTGCTTAT
CCTAAGATTTGAAAGATCTTTTCCTATGTTTTCTTCCAGAATCATTGTCATTTATCTTTC
ACATTTAAGTTTATGATTTGCCTGGAATTAATTTTTGTGTAAGATGTGAGGTAGGTGGTT
AAGGTTCATTTTTCCCCCTAGTACAGTTATCTAGTTGTCTAGCATTATTTATTGAAAAGA
CCACCTCTTTCCTCATTGTATTGGTATCTTTGTTGTAAATGTATTGACTTTACATGTGTG
AATATAATTCTGGACTCCTTGTTCAATTACAGTGATGTGTCTGTCCGTCCTTACGCCAGT
TGGCTTGTCTTATTTACTTTATTTGTATAAAAAGTCTTAAATCTCATCCATTTATCTTTG
AGATGATTTAGAAACTGTGATAGGCTACATTTGTTTGGTTTGGGGTACCATCTATTAGAT
GAAAAAAATCATATATTTAATAATCAGTTGGAAATGAATTAAACTTAGAACAAACTAATT
GTCTTATAATACTTTATAGAGATAATTAACTTGTTTTTTAGGTCATGATGGGACTGTGTT
AACATTTCAGAACCTTTGTTTTTAAATTGACATACACTTTGTGTTAACTTAAGGTAAGAA
ATGTACTGTGACTCTCTCTGGAAGTTAAAAATAGATCAGAAAGATGGATTTACATTTGCA
AAGAGCTGTATTATAGGGGAAATGAATTGATAAGCTGTTTAAGCCATGGTGATAGGCACA
GTCTGTTCTGCTTTGTTCCAGCATTCTCTTTCCAGTAAATACTCTTTTAGTCTCATCCCT
AGATCCTCTAGTGGTCAGTAAACATCTTTTTTTTTAATGTAATTTCAAAATTGTAAGATA
CGTGTCAGAATCCTAGCAGGAGACAGAAGACATTCTGGTGGGATTTTAAAGAAAATTTAA
TGGAACTTCTTTCTATAGACAGACAGTAACAAAGTAAACAGCATGGCACCTGGCGACTAG
CAGCAGCAAACATAGTTACCACCTTAGGGCTGAGGTGCAAGAAGAAGAAATAACCATGAA
TATATTTAGAAATAATATAAAGCTGTATTTCTTCTCTTATAACTTATTTATTTAAATGTC
ACATGACAGGACCTTCCTGTGTTACCATCCCGTGAAAGTAACATTTCAGTTGATAGCAAA
TGAAATTTTAAGTAAACCTTTTTGAAGTGTACTGGTTCATTTCCTTTTTTCTCAGTATGT
GGAAAATTTTCAGCATTTCTTATCAAGTGGAAAGAGACAAGATAATTTGTCATGGGGTAT
ATCAAAAATAGCATTTGTGTATTTTCGTTCATATACATCACTGTTCCTTTCCTGTACTGC
AATTATCTCCCTCACAAATAAATTCTGGGAATTCTCAGTTCATATGTAAATAGAAATATG
ACAGAAGTCTTAACTTAAATAGCAATTCAGTTAGCTTAAAATTTAAGTTACCTTTTTAAA
TACCTTTTACCTAG**GTTCACAATGGTCCGTAGTGGAAAAAATGGTGACCTTCACCTTAAA**
CAGATTGCATATTACAAACGAACTGGTGAATATCATCCAACTACACTGCCAAGTGAGCGA
AGTGGTATAAGAAGAGCAGCAAAAAAATTTGTCTTCAAAGGTAAAATTCATGTTCAAAAT
TTTGATTATTGATTCTTCCCTACATTCTTCTTTGTGGAAATTTGAGAACTTCTGTAGTAA
TTGGTTGTTGCTAAATAATTACCAGTTGAATTGATGATGCCACTCAATCAAAAGCAAAAT
AAAAATTACACTCAGCTAACTGAAGGCCAGTAAGCATGAAAAGGGTGGTTTAAATTATAG
ACAGAATATGATACCATGAAAGAAAACAAAACATTTTTCTAAATTAATGACTTCTGCTTT
TACCCTTTCGTGGTCTCTAATGGTGTACTTTCCAGCACTGATGCCACTTTCAATGAGGAA
CTTTTTTGATGCTTAATAGGAAAATGTTGACAAAGTTAACTGGTTTGTTTACTAACATGA
AAATTGGAAGGAATTAACTATATTTCTGTGATCATGTCCCCCCAACCCCACCACTGCCAT
TAAATATTCTCAGGATCACATTCATTTTAGGACCCTTGGATCTTCCTGAATGGACTAAAT
ATTTGAAAGGCAGTGGGATATTTATCTTGGAAGGTCATGATAATTTTTCCCTCTTTTTCC
GCATATAAAGGGGAAGAGAAATTTGGGATGAGATAAGCAACAAGTAGTCACCAGATAATT
AAATGGATTTTGTGTTATAAATGGTAATTTTGTCTCTTAGGCTTACCCCAAAGCAGGTTA
ATAAAACTGTAAGATAATAAATAAATGCCTGCTTGCGTCTCCCTGTGTATCCCTACCAAT
AACTCCCTTATACTTAAGATTAGGCATACCCATTACCTCTTAAACTGAAGTGAGGATTCA
TTCATTCATATATTTGATAATTTATGTAACAAATATTAACTGGGTTTCTGCTATGTGCCA
GTCACATTCTAAGCCTTGGAGATATATTGAAGAATAACATAGATAAGGTTCCTGCTCTTA
GATTGCTTATCTTCTAATTCTAAAAAGTAAGGAATGTTCCACTCATCAATCTGCTTACTC
ATATTAGTAGCTGAATGGTGTATTTTGGAATTTGAATAGCTAGCCCATTCACACTAGAGA
GGTAGATGGTGATTTGCTCCATCCCCTGGTTACTTCCGAGCTGAAGATTCTTCTCAGTTG
TACTCCTAAGGAAACAGAATTGTCTTGAGGATTAAAGTTTTTTCCACTTCCCCACTTCAC
CATCCCTACCTTCTACTCAGATATAGAAAAAGCGGAGAGAGCTGTTTGCTTGCAAAAACG
CAGTGATGATAAAACATTATTTTAGAAAATTCTGTTCCAGGTAGGTGTTTCTCTATATGT
AATTTATTTGCTAATCTGTTCCATTTTTAGGATGTGCCTGCAAAATCTTCCTACTTTAAG
TACTCCCATAGAGAGATAATTTTGTATTTCTGTACTTATTACATGCTTTGGTGAACAAGC
TGGCTCTGTTTATATTGATAGGTACAAAATTATAGCAGCTACCGCCCTTCCTAAGTGTCT
AAGTAAGTGTAACAGCAAAGAACTTTTGTGTGGTAGCATCATTACAAATAAATATTTTGT
TTTAAATGCAGTAAGATTTTGTAAAACAAAACAAAAACCTTCATGGTTTAAACAGAAGGC
TATGATTCTCTTAGAACTACATACTTCTCTAAAAATTACAATTCTATGTTTTTGGTATCA
TTTATTCCAG**ATAAAAAGCTGTTTTATGTTGGAAAAGACAGAAAACAAAATCGTTTGGTA**
ATTGTTTCAGAAGAGGAAAAGAAGAAAGTCTTAAGAGAATGCCATGAAAATGACAGTGGA
GCTCATCATGGTATATCTAGGACCCTCACTCTGGTAGAATCCAATTATTATTGGACATCT
GTGACCAATGATGTCAAACAGTGGGTATGGCTTATGTATTTAGAATATTATGAATACTGT
CTTTCTGATAGATATTTACAATTTATTATTTCTCCAGGTTTATTCTGGGTATCACCTCTC

ATTAAGTATTTTTGGCATGGTTATTTTTATTTAAAAACCATTTAAAGGGAGTTGTATTTT
CCTTCACAGTTTTTTAAAATAACTTAAGTATAATTTGAAAAGGAAAGAAGCATCATTTAA
ACACTTGAAAATGCCATATTTAGATAAGAGATTATTTAGAATCAGATTTGTATCATTGAA
TTTTTTTGTGTAGGCAGAATTTAAATGGAGCTGGAATTCGTTTGACTAAATCATTACCTT
TTCAATGGCAATCATACAAGGTTTTTTTAGGAGGAGAATATGTTCATAGTAAGTCAGGCC
TAGTATGTGCTCTCATCTTTTGTGCTTGTTCCATCTTGAGTTAGAATGTCATGTATGACA
GAAACCCAAAACCAAATATAACCAAAATCAAATTTTGAGAATCATGAAATAAGTCTGAGT
TTTGTAACTTTATGACCCATTGGGACCTTCAACTTCTCCTAATAGAAAATTCACCTATAG
GAATTTTGGGGTTCTTGCTAGAAGTCAACTGATCCTACTCTTTCCAGTGTACTGTATGCC
AAATGGTAGGCATTCCTTGGTATATCTGTGAAATCTCCTTAATAATTGTAGTAGTGCATC
AAAAAGACTGGCATTTCAAGTGTAAAGAATTAAACCAACAGTTAAGTATCATTTGTGAGT
TATCAGTTTATGGGTCTGTTCAGGTATTCCAAAGACCTTCATAATTTATATATTTTTCCT
GCCATGCATTTTTTACAAACATTCAGTTAAATCTTTGTCGAATTGTTATCACTGCAGAAT
GAATATGATTTTTGGAAATAATCAGAAGTTATTTGTGATAAAATCCAACAAATAAGTTTA
AATTGGAAAATGAAAATTCTGATCAAAAGTAACTGTGACTATAAGGTGATTAAACAGCTT
TTTAATTTTTCTTCAGAAGCTAGTTTGAAAGTAGTTTCTAGAGTGGAATTCCAGAGATAT
ATTGAGAAATGGCACCCATCCATAAATTGAGCAGTTAATCTTGTCTCTCCTCACTTAAAT
GAAAGCTGCAGGACAGCATGGGTTTTGTTTATGTTTATTCTTTTCTCATGATTGAGATAA
ATTTCTCCATCTATACATATTTTATGAATGAAATTATATTTCAATAAATATGGTTGGAAT
AGATTAATGAATAGACTTCTTAACGTGATAACTTTAGGAGGAAACATTTTGTGCATATAG
AAATTATTGTAAACTTACACACTGATTTTAAATTATAGGAAACTTGTTTAAGATCTTTTA
AAGTATTTAATAAATATGTGTTCAGTGAACCAGATTCATGTTTCATGTGAATAAGACCCA
ATAAGATGGTCATTTAGTCGTATAGTCCTCCTTTAGTATAAGCAAGGGATTGGTTCCAGC
ACCACACTCATATAACCAAATCTACTCATACTGAAGTCTTTCAGTCGATCCTGTGAAACT
CGCATATGCAAGAAGTCAGCTTTCTGCATCCTTGAGTTTCTCATTCCTCCCTACCAATAC
TGTACTTTTTATTTGTGTTTAGTTAAAAAAAAAAAGCCATGTATAAGCTGACCTTCTCAGT
CCAAACCCATGTTGTTCGAGGGACAGCTGTAGTGGCAACTACAGTTGTTTGTTGTTGTTT
TATTTGTTTGTTGTTCTAAATAGGTTTAATCCAGTGATTCTCAGTCCAGAAGTTTCTTCT
TCAAAACAGGAAGTGTACCTATTAACCATTTTAAAATTCAGATTTATAAGACTGCTTTTA
TATTGTGTAGAGTGTTAACATTTGTATGTATTTTCTTTATTGTGTGCCTGTTTGTAG**GTA**
TATGCTTGTCAGCATTGCCAAGTGGCAAAAAATACAGTTATTGTAGCACCGAAACAGCAC
CTTCTCAAGGTGGAAAATCCATGGAGTTTAGTTACTGTTGATCTGATGGGACCTTTTCAT
ACAAGCAACAGAAGTCATGTATATGCTATAATCATGACAGATTTGTTCACCAAATGGGTT
GTGATTTTGCCTCTATGTGATGTTTCAGCATCAGAAGTTTCTAAAGCTATTATCAATATA
TTTTTCTTATATGGACCTCCTCAGAAAATAATAATGGACCAAAGAGATGAATTCATTCAA
CAGGTAAGACAAATAAACTACTTAGGTCTGGGAACATATCTTACTCCTTTTCAGTGTCCA
GAACCAGTGCCCTCTGCATTATATTACAGAGTGCTTAATATATTCAGATAAATTGCTATA
GCTATAGAGGTATGATATTAAATACTACAAGGATAATTGTACTGGAAATTAGGAGATTAA
GATTTTAATACTTACTGTGGGTTATTGTAAACCCATCTTCAGATATCCTCAAATTAAAAT
GAAGAGGTTGGACCAAATCTTTATTTACTTCTAGATTAAAAACCCGTGATTGATTGGACC
TGGTGGCTCACGCCTATAATCCCAACACTTTAGGAGGCTGAGGTGGGTGGATCACTTGAG
CTCAGGAGTTCAAGACCACCCTGGGCAACAGGGCAAAACCCTATCTCTACAAAAAATACA
AAAATTAGCTGGGCATGGTGGTAGGAACCAGTAATCACAGCTACTCAGGAAGCTGTGGCA
GGAGAATCGCTTGAACCTGGGAAGTAGAGGTTGCAGTGAGCCGAGATCGCACCATTGCAC
TCCAGCCTGAGTGACAGAGAGACTCCATCTCAAAAAGAAAAACAAAAAATTAGCCAGGTG
TGGTGGCATGCACCTGTGGTCCCAGGTACTTGGGAGGCTGAGATAGGAGGATCACTTGAG
CCCAGGAGGCAGAGGTTGCAATGAACTGAGATTGTGTCACTGCACTTCAACCTGGGCAAC
AGAGTAAGACCTTGTCTCAAATAATAATAATAATAATTAATAATAATAATTATGATTGAC
AGGTAAAGGGAGACTTAAATAAATGGAAAAATACATTGTGTTCATGAATAAGACTATTCA
GTATTGAAAGGTACTTTTTCCCTTGTCCTGGCCTTTTCCCAGTTAAAATTAACCTGAAAG
GATAAACAGGGGAGAATATATATGAAAACTTTTTATAAGAAATATAATAAGTACTAGACT
TAAGGCATATGTTAAAACTTTTTGTAATTGACAGGGTACATGTTTAAGAAAAATCAGAGA
TAGCCAGACTTGGTTCAAAGCATCTTTGATGGCTCAGTCCAAAAGAAATATCTAAAGTTA
TATTTATTAAGTAGATAGGACATATCATGGCACCCCAGGATTATTCCCAGTTTTCATAAT
TCTCTAGCAGGACTCAGCATATAGTCATATTCATGGCTAAGATTTATTCAGCAAAGACAA
AAGACACATTGGGAGAACCAGGTACAAGTTTCTAAGATCTCCTTCCCCAATAGAGTTACA
CAGGACATGTTTAATTCCTTTAGCAGAGTTGTGACAACACAGGGAAGCTCACTAGAGGCT
CAGTGCCTAGGGTTTTTATTGGGGGCTGGTGACATAGGCACCTTCTACCTGGCATGTACA
AAAATTTCAGACTCTTAGAAGGAAAGATTGTATTTGGTCTTATAACCAAAACCTAAGCTC
CTAGATGTTAGCCAAGGTGCAACCTTGTAAGCAGGCCTTTCAAAGAATAGCAGTCACAGA
TCCGCTGTGTTAATATGTTAAAGTTAACTGTCAGGTTAATTCTTTTCTTCACAGGTCCAT
CCAAACAACTTAATAAGTATTTATGTTGTAACCACTTAGTAGGAATTTAAAATACTCATA
AATTGAAAGAAATATATTTTTGTGTCATTCTTTTTTTTTGAGACAGGGTCTCATTCTGTC
ACCCAGCCTGGAGTGCTGTGGCACTGTCTTGGCTCAATGCAACCTCCGCTTTCCAGACTC
AAGTGATCCTGCCACCTTAGCCTCCCAGCTAATTTTTGTACCTTTTTTCTTTTAGAGACA
GGTTTCACCATGTTGCCCAGGCTGGTTTCGAACTCCTGAACTCACACGATCCACCTGCCT
TGACTTCCCAGAGTGCTGGAATTACTGGTGTGAGCCACCACACCCACCCCTTTCATTTCA
TTCTTAACCGTAATTACTTGCTAATGGGTAATGTGTACCTGTTGACCACTGCTTACCTTC
TCAGACCTTGGAATCATATTGTACACTGCCACCCTCATTTCCTGTTTCACACTGATTTTT
ACATAGTAACTGCTTTTTATCACAGCATTCGATGAAGAAAACAGTTCTGTAAAGATGTGA
TGTCATCAAATGAATTGTAACCTGATCTAATGTTGAAACTGAACTGCCTTAAACTGGTAG
TTTTCATGGTGTCCAACAGATGTTAAGGGGTAACTTAGCACAAAGTTTGGTAACAGTGCT
TTAAATGATAAAGCTAGTGTCACAAATGTGTCTAGTAGAGGAGGAACATGTAATTAATTA
ACAGGTAACAATTTGCTAATTGTTTAAAATTATTTCTGTAATATGATTTTAGAATCTAAT
ACCAAAAGAATTTTCAGGTAAATTAAAGAATTTTAGTTTTTAAAGTAGAAACAGAAAAAA
ATATATTTGACTTTTAATCTCTAGAAATGGAGGTCGCATTCTAAACCTAGGAACAGTGGT
ATAAACTTCAATGGAAAAAGATTAAATTTGGCTGCATAATAATAGGAGATTTATGTGGTC
ATTTTGTAAATGAAGGCAAGGTAGTCTTGCCTGCCTTGATTGACATAGCAGTGGTCTGTG
CAGTGTAGTACGTAAGGCAATCCAGTCTGGGGGAAGACTATTGGAACTTCTACTTATATT
TTTTTAAATCCAAAAAATAAGAAATGAAGCTTTATTAATAATGTATACAGATTGATAATA
GCATCCTGACTCAATCAACAAGACAGCTATGTCACACATACTATATGAAGGTATCCTGAA
AGAAAAGTGAGAATCCACATGTTTCCTCACTCATACTTTTGCTTTCAGCAGTTGAATGAA
AATATGGATATCATCTAGCATTCACCACACTACTCACAAAATGGCCAGGGAGCTTAGAGT
GTTGCAGTGAGAGCAGATAATACTAAAAGGCAAACACCCATCAACCACAAGAAAATGATA
GTGCTGGCACGTCTACGTAACTTCCAGCTCTTAGTTAGACACAGCAATGAGATGAATACA
AATATTAACTATAGTAGTCCTCATTTATCTGCAGTTTTACTTTCTGAGGTTTCAGTTCCT
ATAGTCAAGTGCAGTCCAAAAATATTAAATACAAAATTCCAGAAATAAACTATTTATATA
TTTTTTTAATTGCACACCATTCTGCGTATAGTGATGAAATCTCATGCTGTCCTGCTCTGT
CCAAATGGGATGTGAATCATCCCTTTGTCTAGCATATCCTCATTGTATATTCTACTCCCT
CCCCATCATTACTCACCTTGTAGCTATCTTGGTTATCAGATCAAATGTCCTGGTTAGTAG
TGTTTGTATCCAAGTTACCCTTATTTTACTTAAGAATAGCCCCAAAGTGTAGGAGTAGTG
ATGTTGGCATATTGTCAGTCATCCTATTTTTAGTTGTTAATCTCTTACTGTGCGTAATTT
ATAAATTAATCTCCATCATAAGTGTATATGTATAGGGGAAAACATAGTATAGATAGGGTA
TGGTACTATCTGCAGTATCAAACATTCACTAGGGGCCTTGGAACATATTCTCTGAAGATA
AAGGGAGACTGCAGTTATCAGATCTGATAGGAAATTAGCCATCAAAATTCAAAATTATTG
AGAATACTCTTTGAAATATTGGTTCACTTCCACTTTTATTAATGAAGAGCCTTACCCTAA
GTGCACATAATGTTTCAGGGTATTTGCTAATGATAATATGAAGTCATTATGATTAGCTAG
CAGGTTTTCCAGTCGTGTTTAAAGTCATCTGGTAGCCAAACCACGACTTGGAAAATTTTA
CTAAAGGTAACAATAGGGCCAAGTGTGGTGGCTCATACCTCAGCTTTTTGGGAGGCTGAG
GTGAGAGGATAACTTGAGACTATGGGTTCAAGACCAGCCCATGCAATATAGCAAGACTCC
ATCTCTACGAAAAAAAAAAAAAAAAATTAATTAGCTGGACATGATGTTGCACACCCATAGT
CCCAGCAACTCAGGAGGCTAAGGTGTAAGGATTGCTTAAGCCCAGGAGGTCAAGGCTGTA
GTGAGCTATGATTGTACCACTGCATTCCAGTTTGGGTAACAGTGAGACCCTGTCTCAAAA
AAAAAAAAAAAAAAAAAAAAAAGTAATAATAGGTTGTGCTTGGTGGCTCATGCTTGTTGT
AATCCTACCAGGCAGATTGCTTGAGCCTAGGAGTTCGAGACTAGCCTAAGCAACATGGCG
AAATGCCATCTCTACAAAAAATACAAAAATTAGCCAGGGGTGGTGGCTCACACTTGTAGT
CCAGCTACTTGGGAGGCTGAGATGGGAAGATCGCTTGAGCCCAGGAGGTTGAAGTTGCAG
TGAGCCAAGATCATGCACTCTAGCCTAGGTGACAGAGTGAGACCCAGTCTCAAAAAAAAA
AAAAGTAACAATAAATATTTGCAGGCCTATTTTATGAGATTGCTTAAACAAAAAAGGGAC
ATATGATTGGGAAAACTTGTTCTTTTTGCTGTGATTAAAATGGCTTCACTATTACACAGA
AAGTATAACGATGATAGTACAGAATGAACTCTTGTCAGCAAATACTGAAACAGAAAACAC
TGCTAAAGATTATAACATAATACACAATGTAGGAGGTTTATTGTATACTTGAATAAAAAT
ACAGATGTTTCTTAGAGTATTTGCTAAATTGTTTCAATAAAATGCACGAAGAACTTTTTG
TAAACCTCTGAAGGAAAGAAAATAAACATATTCAGTAGAAAAATTAATTTTTTTACAATG
TCTTACAGAAAACTAGAATTGTAAGAACTGATGGAATATCAGCTGGTTGGGGGGCTGATA
GGGTAAGATAGGATAAGATAGGATAAATGATATCATTTGTCAAGATACCATTTCACAAAT
GGATTCCCTGGATCATTTCTAGGCAACCTTTTGAAACAAAGAAGGTAAAGCCTTTTTCAA
GGGTCATTTAAAACCATTGATCAAGACTCAGATTGAAGCTGACCCAAATGACACCTATTC
TCTGGGATTTGTTTCTAATTACTTTTGAATTAAAAATAGGCATCCTGAATTAGTATGTAT
ATCAGCTAGGAAGTGTATTCAATCCAAGTAACAGACTGTCTTTCCCTATCCCAATATAAT
AATATGTGTTACATATATTAAACCAATTGGGAGTGATTAAGAGTCACTCCTATTGTATTC
ACAATATGGCTGCCAGAGCACATGCCACAATGCCTACATTTCAGTCAGAAAGAAGGGAGA
GAGTATTGGGCAAACAAAACTAGTACCAGCTGAGTTTGCCTAACAACTTTTTGGCTAGAA

TTATGTCACATGACCACCCCTACCTGCAAGGGGGTTTGGGACATGTAGTTTTCTTTTTGT
TGCTTTCCGCTCTTGTATTAACTTTGCTTATTTAGTTATTCTGATACTACTGGTCTGAAA
TACTCTTTTTTTAAAAGACAAATTTATGAGCCAAAGGTTTACTCAACTAAAAATAATAAG
GAAAAAAAGTCTGAAGGGTGAGGTAGGAAAAAAATATTATTTTATATTTAACAGGCTTAC
TGTTACAAGTTTTGTAAACTCATTTTGGTTTCTTTTAAGTGTTACACTTAATATGGTAAC
ACCAATAATAGTAGTTAAAAGTTATAAAATGCTTATTATTGCCAATAACTGTTTTTACAT
GCTTTTTAAACATTAACTTACGACCTAGTGGGTTGTTTTTGAGACACTGTCTACTGAATT
AAAAAGTTACTTGAGGGCTGGATGCAGTGGCTCATGCCTATAACCTCAAAACTTTGAAAG
ACTGAGGTGGGTAGATTGCTTGAGTCCAGGAGTTTGAGACCAGCCTGGGCAACATGGTGA
AACTCCATCTGTACAAAATATTTAAAAATTAGCTGGGCATGGTGACACATGCCTGTAGTT
CAAGCTGCTAGGCAAGCTGAGGTGAGAGGATTGCTTGAGTCCAGGTGGTGGAGGCTGCCC
TCCAGCCTGTGCAACAGAGCAAGACCCTGTCTCCCACTGCCTACCCCCACAAAAAAAGAA
AAAAATTAAACTCAAAGTTAGTGGTAAGTTCTCTTCTCCTGTTTCCTTGTTGTATGTGTG
TATTTGGCAAGGCATTTAATCTCTCAAAGCTTCAGCATCTTACCTTTAAAATGAAATTAA
TAATACCTCAAAAACTTGTTGTAAGGTTTAATGACAATAATGTAAAGTGTCTAGTGTGAT
GATCAGCATGTAGTAAAGACTCAATAAATGGTAACCACTATTTTATACATGAAATTTTCC
ATATTTTCATATAGAATGTTATGGTCAACAAAAAAGCAAGATTCAGCCAGGCATGGTGGC
TCACACCTGTAATCCCAGCACTTTGGGAGGACAAGGCAGGTGGATCACAAGGTCAGGAGT
TCAAGACTAGCCTGGCCAATATGGTGAAACCCCGTCTCTACTAAAAATAATGAAAATTAG
CCGGGCGTGGTGGCGCGTGCCTGTAGTCCCAGCTACTTAGGAGGCTGAGGCAGAAGAATC
GCTTGAACCTGGGAGGCAGAGGTTGTAGTGAGCTGAGATTGCGCCACTGCACTCCAGGCT
GGGCAACTGAGCGAGACTCCATCTCAAATAATAACAATAATAATAATAAAAATAAAACAC
AAGATTCTAATTCTTTTGAAGCTTTTTTTAAGTCAGTGGAAAAATAAAACACAAATTTTA
AAAAGCCAGCTTTGTTTTATATATTTTTCAG**ATCAATATTGAACTGTACAGATTGTTTGG**
CATAAAGCAGATTGTAATTTCTCACACCTCTGGAACTGTTAGTCCAATGGAAACTACACC
TAGCACAATCAAAGCATTTCTCTCCAAACACTGTGCTGACCACCCAAACAACTGGGATGA
TCACCTATCAGCTGTTTCATTTGCCTTCAATGTAACTCACTTGGTACGTGCCTTTTTATA
ATTCTGTACTTCTGAGTTATGACTATTTATTGGCCAGATAAATGTTTTATTATAAAATTG
ACATTTATTTTCTTTAAAATAG**GAACCTACTAAAAATACACCATATTTTCAAATGTTTAG**
TCGAAATCCTTATATGCCTGAGACTTCAGATAGTCTTCATGATGTGGATGGTGATAATAC
AAGTATGTTTGCCAAAATTCTAGATGCAATTAAAGAAGCTGATGAAATAATGGAGAATAA
GACAACTTCACTGGGCCAGGTGATTCTATTCAATAGAAAAACTATAAAACTTTATAAAGT
TATATTTATTTTTTAAATTTCAGCATATACATGTGTGCATGTACACAGATACGCATACAT
ACTTGCCATAATTTTTTATCCATCAGATTTTTATTGTCTTGATTAAATAGTTTACTAGCT
ATGACATGCTGAGAGGCAATAATGAGAAAGCTATGGATTTTGGAATCAAGTAGAGCCGGA
TTCAAATCTTTAGTCTTAACTGCTTTTGAGTTGTTTGACTTTGGCAAATTCCTTAACCTA
TCTTAGTTTACCTTCTATATAAAGTAGGGGTTAATAATACTGACTTGGAAATGTTGTTTT
AAGAATTAAATATAATTCATGCCTACCTTTTAGTAGCATTTCAAGTAACCATTTTGTTGT
AATATTCTCATTGTCTACATTTACCTTCAAGATATTATAGCTCTTCCATCTCTTCTTTTT
CTTTTTACACTTGTAG**ATGGAGAACAACAATTTGGATGAACTAAATAAAAGCAAGATCAT**
TGTTAAAAAGAAACCCAAACAATTAAATCCATTTCATTTAAAAGTGGGTCATGAAGTTTT
AAGACAAAGGAAAAATTGGTGGAAGGATGGTCGTTTTCAATCTGAATGGGTTGGTCCTTG
TGTCATAGACTATATTACAGAAAGTGGATGTGCTGTCCTGAGAGACAATACTGGGGTTAG
ACTGAAAAGACCTATCAAAATGTCCCACCTTAAGCCCTACATAAGAGAATCCAGTGAACA
AGGTAAATATTTGTCACTTCTCTACATTTTTATTGCTTTGGGTAAGGAGAAAAGTTAAAA
ATAACAGAAACCTTTCTCCCCTCTTTTGTTATGTTTTCTTTTCCCTTCTTCATAAAATTT
GTTAGCTATCCCAGGTAGTCTGTTTTATTACAGTGTCTCTTCTGGAACTGTCAGGCCTGT
GAGAATTTTTTTTTTTTTTTTTTTTTTTTGAGACGGCGTTTCCCCCTTTTTGCCCAAGCTG
GAGTGCAGTGGTGCGATCTCGGCTCACTGCACCCTCCGCTTCCCAGGTTCAAGCAGTTCT
CCTGCCTCAGCCTCTGAGTAGCCGGGATTACAGATGCGTGCCACCACGCCCGGCTAATTT
TTTGTATTTTTAGTAGAAACAGTATTTCACCATGTTAGCCAGGCTGGTCTTCAACTCCTG
ATCTCAGGTGATGCACCCGCCTCGACCTCCTGAAGTGGTTGGATTACAGGTAGGAACCAG
GGCGCCCAGGCATTTTTATTTTTTATTTCTAGTATATTTTTCATAGTTTATGTGGGAGAT
ACTGAACTTGTTTTCTTCTGAAGGAAAGATAACAGCTTAACATCTCTATTTTTGCTTTCT
CAATTTTTTTTTATTTTATTTTATTTTATTTTTTTGAGACAGAGTCTCACTCTGTCCCCC
AGGCTGGAGTGTGGTCGTGTAATCTCAGCTCTCTGCAACCTCTGCTTCCTGGGTTCAAGC
AATTCTCATGCGTCAGCCTCCCGAGTAGCTGGGATTATAGGCACATGCCACCATACCCAG
CTAATTTTTTGTATTTTTGGTAGAGATGAGGTTTCACCATGTTGGCCAGGCTGTTTTTTA
ACTCTTGGCCTCAAGTGGTTTGCCTGCCTCAGCCTCCCAAAGTGCTGGGATTGCATGCAT
GTGCCATTGCACCTGGCCTCACAAATATTGTTAGTCACTTCGTGCCTCTTTTTGGTGTGA
AAAACAGCACCTACGATCATTAATGCCAGAAGCAAACATACTGTCCAGAATCCAGTTGTG
AGATAAATATTTGGGTACTTTAAAAATGCCATTAATTTTTAATATTGCTTTGTAGTTAGT
TAGGTCATGGTTGGGGGCCAGGTGTGGGTGTGGAGTGGGGTATCCTCAGAATGCTGTAAG
GTGCTGTAAGAAGAGGTGATAGCACTCTGTAAACAAGTTATACTTGCCCCTAAATTTGAC
TGGTTCTGAACAGTCAAGATATCCTGTTCTCTGTAACTCTTTCTTTCATAGCCTAGTTAA
GTGATGTTTTTGCCATGGCTTTTAGATTATGTTCCCTGCTTTTATCAAGGCTTTTATTTT
GTCCTAATGCATCTGCTTCCTAGAGCTTCTCTGAAGTGGATAACTGACAGGGAGTTAATG
GGTAACTTAGCCAGATGAAGCATTAATGTTATTTGGATATAAGTAACATTTAATGGAAAA
TATATTTATAACAATATAAACAAGTTGTATGACAGGCTTTAGCAAATATTAATAACAAAA
AAAGTAACAACAACAAATAACCTACACTTACCCTATGCCTTTTCTTAAGATGTAAAACCC
TTGTGGGCTGCTTAGTAATGATAGCTCAACTCTGCTTCTAATCTGGGGTACAACTCTCAT
GTTAAAACCTCTTAGTACCTTCCCTTCTTGTGACAGACCCAGTTGTTTAGTCAGTAAACT
TACCTTAAACTATGTTCTAATCCTGAAAACTCACAAGTGCTTTCGTCTCTTTGTTAGAAG
ATAAAAGCTTGCATTGTTTGTGCCTCTTCTCAGTTCTGAGCTACCTTTAATGATCTCTCT
CTTCTGCAAGTTAAGTAGGGAAAAAAAGGTTATACAAGTACATGTTGTTCTGTCTTCTTT
AGCATGATTTTCCCCTTAATATTTGACATTCATGATTTGCTCACTTCTAATTTGTTAACC
TTCTTTAATACAAAATTTACATTTAAAATTTTGCTAAACCAGGATCCATCAACATTTTGT
AATTTGTTTTTAAGAAAAGAGTTTGTCAGAAAAGAAGTCAAATATTGTGCTTCTTCTGAA
CATTTCAGAACTCAGTGTTTCAGAACTCATCTTCTCTGTTTGAAAACAGTCTCTCAACAG
AGGCAGTATAATATTTGCCTCATACTTGAATTCTTTGCACAAGTTTCTTCCAGTAATATA
AGGATAAGTAATATTGCAATATGACTACTGGTGTATGCATAAGGAACTTAAGACTAATAA
ATGAATCTGTTTTAGTTTTTGTTAACCAAAGGAACTACTGATCCGGTCATCCTAGAATCT
GGTTCTTACTACTTGATCCTTTTTTTACATTTTTCTCATTTGTAGAAATTCAAAGTAAGT
GTGATTTCTACCATCTTTTCCCTCTATATTACATATTTTCTTGAAAATCTTTTCCATGAT
TTTCTTGAAAATGACCTCACAAATTGATTTTAAAGAGTCCTTGAGCTGAATTAAAGCAAA
CAAAATGATTTTGAATAATCATTGAAATTTTTATTTGACATAAGTTTCGGTATGCTATAG
CCAAAAGAACTATAAATTTATTTGTCCACATTTGTCATTGCTGCTCTTTGCTAGGCTTGA
AATTTCATAGCTGTATTAAAGACTGTCTAAAGTAGATATACTTCTGTTGCCAACTTCTTA
TCCATTCTTATCTAGAACTAAATGTGGAGTGAGCACATTCAGTACTAGTTATATAATATT
TGTCATTGCTATTTTAAGCAGGCCTGTTTGTTTTTTTGTGTAGTTCATTGCCAAGTGCTT
TCTCAGACTATTTGGACAAGAAATCTTAAAATACCGTAGTGTCTTTTTGTAATCATAATG
CTCCTCTTTGAAAGGAAAATTAATCTGACAGATTTTGCATAGTTTTGAATTTATTTTTTT
TGTGATATATTTGGGTCTGCCTCACCGAAGATTTTAGCCTGATTTCCCTTCACAGTTGAA
AGCCCCATTTGTGAAATGATTTTCATTATTGTATAAGTTTCTTCTTGTTGCCGTAACAAA
TTACTACAGATTTAATGGCTTTAAAACAACACACGTTTATTATCTTACAGTTCTGAAGGT
TAGAAGTTCAACATAGATCTTACTGGGCTAAAATCAAGGTATTGGCAGTGTTGTGTTCCT
ATCTGGACGCTCTAGAAAAGAATCCATTTTCTTGTCTTTTCCAGCTTCTAGAGGTTACCT
TCATTTCTTGGCTTGTGGTCCCTCTATCTTCAAAGCCAACAATAGTGGATTGAGTTTTTC
TTACATCACCTTACTCCAATCTCCTCTTCTGCCTCCCTCTTCTGTATTTGAGGACCCTTG
ACCTTTGGCCTGCCCAAAAAGTCCAGGATTATCACCCTATTTTAAGGTCATCTGATTAGC
AACCTTAATTCCATCGCTACCTAAATTCTCCTTCGCCATGTAAGGTTATATATTCACAGG
ATCCAAGGAGTAGTATGTTGACATCTTTAGGGGTCTATTATACTGCCTACTACAATTATG
ATCTGCATTCATGAAACAAAGCAAAGCACAGTCTCTATAGTATCAAGTCCATGATCTTGA
CGTTATTATTAATATAGTTTATGCAGAGAGCCTTTCTTTGGCCTCTTAGTTAATATCATT
TTTTCTTTCTTTCTCTCTTTTTTTTTTTTTTTTTTTGAGACAGGTTCTTACATGGTTGCC
CGTGCTGGAGTGCAGTTGCACAATAATAGCTCACTGTAGCCTTGACTTCCTGGGCTTAAG
CAATCTTCCCACATCAGCCTACCAAGTAGCTTGGAATACATGTATGCATCACCATGCCTG
GCTGATTTTTTTTATTTTTTTATAGAGACAGGATCTTACTATGTTGCCCAGGTTGGCCTCAA
ACTTCTGGGCTCAAGCAGTCCTCCCACCTAGACCTCCCAGAGTGTTGGAATTACAGGTGT
GAGCCATTGTGCCCAGTCCAGTTATATAATTTCTAAACCAACAGTTATTCAGTTCTCTAT
TGACAGTATTAGTTATTATAATGACAGTGATGTAGTATCAAGTTATAGTTATTATTTTGT
CTTGAGAAAGGATATACTAGTATGGCAGCACATTCTAATAGGTTGAGGATTATCAGACCA
TATATAGGAGTAGTTTAGTCACTTTAAAATAAGAAAAGTAGTCTGAAACCTTTATCTTTT
TTTCTGATTGAAGAAATAAATTAATCCAAATCAGACTTTTTTGAAGCTGTAATTTTTATT
TATTTTTCTCCTTAGGGAAATTTAAAAATTGATTTTTTTAATCTATTACTTTTACTCCTT
AAAACAACATTAAGGCTGGGCGCAGTGGCTCATGCCTGTAACCCCAGCATTTTGAGAGGC
CGAGGTGGGATTGCCTGAGCTCAGGAGTTTGAGACCAGCCTGGGCAATATACTAAAACCC
CGTCCGTACAAAAAAATACAAAAATTAGCTGGGCATGGTGATACGCACCTTTAATCTTAG
CTACTTAGGGGGCTGAGGCAGAGGATCACTTGAGCCTGGGAGATTGAGCTGCAGTGAGCC
AAGATTGCACCACCACTGCACTCCAGCCTGAGTGACAAAGTGAGACCCCATCTTTTTTTT
TTTTTTTTTTTTTTGAGATGGAGTTCAACTCTTGTTGCCCAGGCTGGAGTGCAGTGGTGC
GATCTTGGCTCACTGCAACCTCTGCCTCCTGGGTTCAAGTGATTCTCTTGCCTCGGCCTC
CCAAAGTGCTGGGATTATAGGCATGAGCCACCACGCCTGGCTGAGGCCCCCTCTCTTAAA

AAAAAAAACCCTACAATAAAAATGAGTATCAAAACTGCTGATTAACACCTTCCTGAAATA
TCTTGAAAATATTTTAATTTCAAAGAAACTAAAGACTTACTCATTCAGTCTTGTCAAAAC
TGTGATGATGCTTTCATCAAACAGATGTCATAATCATAATTAATAGAATGAGAAAAAAAT
ACTTAGTGAAATGTATTATGTTATGTAACTGACTGGAAAAGATCTTTAATGTGATATGTT
TGTTTACTCCCCACCTCTGTGTTTTGAGGGCAAAAAGTTTAATCATGTTCTTTTCTATAT
CCCTAACTCAGGATTGTTTTAGATTAGTGCTGTCCAATAGATTAATAATAATACAAGAGC
TATGTAATTTAAAATTTTCTAGTAGCCACATTTTGAAAGGTACAGAAATAAGTGAAAATA
ACCTTAATAATATATTTTATATAACCCAGTATCCAAAATATTATTTGTTCAACATGTAAG
GCCCCAAGAGTCTCTTTTCTGATTATCTTTATTGAAATTAAAGAGCATTTCTCTTAGCTC
CACTAGATTTAGACCATAGTAGGAGTTGAAACACATTTAACCTTGCAAATGAATGAGAAT
TTTTTTTCTAAACAAGCAGGCCTTTGACATATTCAGAGTAGTTTTCTATTGTGTATAAAG
TGATTTATGTAGAATTTGCTGCGTGTAGTCCTTGTTCTCTACAGTTTTATTACAATAAGT
TACTTTAAAGATTAATTTTTTTACAAGAAAAAGTGTGTGTGTGTGTGTGTGTGTCTTATT
TTACTTACATACAAAAGCTCTCCTCTTAAAATCAAGTGAGGTTGGTAATGTCCAATTGTT
TCTAACCAGTGTAACTAGGAGGTACTCAAGTTCAAGGTCTCTGAATCCATTTCAGCAAAT
TAAAATGATAATTTCTAAGAGCTTTACATCTAGGCATATTTTTTCCCTATTGACATATGT
TTACTAGACATATTTTTCTAAAATAGTCCTACTTCATCTGTATGGAAAAACTTTTACAGA
CAGAAAACCTCCCAGTTAATCCTCTGAAAGAGTCTTTAGACATTTCTTAAATGCCTATGG
TATCTGCCATCACTGATAACTTTGCAGGAGTGTACAGGCCTTGAACCTATGGAATTCTCC
ATGACTTAGTAGAAAATTTCATCAGCAACACTTTTACCCTACCTAACCTTTTAAAGTTCT
CTTATAAACCACTTCTTAGCCTTTTCTAGTATTAACAGTAGAGTAGTTGCAAACTTTGAT
GTTAAACCTCTAGCAACATACTCTGAAAGTGTTTCGAGTTTATTGATCATGTTTTCTTCC
CTCTTGCTGTTATTGGGTATAACACATCTCATGTGTTGTTTTGTTGTTGTTGAGAAAGAG
TCTTGCTCTGTCACCTAGGCTGGAGTGCAGTGCAATCTCACCTTACTGCCATCTCCACCT
CCTGGGTTGAAGCGATTCACCTAAGCCTCTCAAGTAACTGGGATTACAGGCACATGCCAC
CATGCCTGTTTTTGTATTTTTAGTAGAGACGGGGTTTCGCCGTGTTAGTCAGGTTGGTCT
CGAACTCCTGACCTCAAGTCATCTGCCCACATCGGCTTCCCAAAGTGCTGGGATTACAAG
CATGAGCCACTGCCCAGCCGCATGATTTTTTTTTTTTTTTGTCTTTCAGAATTATGGGCA
GAATAAATGACCATACGAGCCCAAGCCCACTTATGCCATCTTTTGGTAGAGAGAATAGCT
TTGTGTGATCTATTAAAGTTTGTAAATTGAAAGTTGTTAAATGCATGAACTTCTGGGATT
GAAATAGGTTCAGTGAGCTATTTGTAATAATGTTATGAGGATGAGTAGAGTTGGACTGAT
TTTTCAAAATATACTGGCAAATTCATCTGTTCTGAGACACTTAATATACTAGTAAATTCA
TCTGTTCTGAGACATTGGTCTCTAATCTAAACAAGCATTTAATCTTCCTTTCGCTTTTTT
TATCTTAAAATTTGAGGGCTCCTTAACCCATTAACATCTTCAGGAGACCAACCTAGGCAA
CACAGTTAGCCAGGCATGGCGCCATGTGCCTATAGTCTCAGCTACTCGGGAGGCTGAGGT
GAGAAGGATCACTTGAGCCCAGAAGGTCAACGATGCAGTGAGCTGTGATCATGCCATTGC
ACTCCAGCCTCGGTGACAGAGCAAGACTATGAATGAATGAATGAATGATAAGTAAATAAA
GATAAATATATCTATATCTATATTCTGATGTTTTGGGGGAACATTTAGAAAGAGAAGGCA
GGGGTCCAGGAAGTTAATCAGCTTTGGTAAAAGAGTTTCTTATAGTAATGGATTGAAATA
TCAGATAGGGAGGAATCCTTTTATACATATTAGTCTGTATATAGAAGATTTGCCATCAGT
TTAACATTCATTTTAAACATTTTTTTCTTGATGGTGATGATACATTTGCTCAACGTACAA
TAAAAGACTGACAAGGAAGACTCCCCGTCAGCACTTCAGTATGGCTTAGGTAGACCTTAA
AAGTTCCTAAGAGTTTTGGTGTCAAATTAGATTTAAATAAGCTAAGCTTTTTACGCTACT
CCATTGCAACCTACTTTCTTACACATTGTCACATAAGCCTGAGTTGGCATGTTGTTTTTA
ACTTCATACTGTAACAATTTGATATTATTTTTCCAATATGGGTTTTATAAAGATTAGAAA
ATTATGTCAACTTATAAATGCAAGGATTTAATAATTATATTTGTTAAAATAATTTTATGA
CCCTATTAATTCACCCAGAAATGCTTTACAGAAGCATATTAAAGATAACATTATCAAAAA
TAGTTTTATAATTTTTTTTTTTTTTTTTTTTTTTTTGAGATAGGGTCTTAGTCTGTCACCCA
TGCTGGAGTGCAGTGGTGCCATCATGGCTCACTGCAACCTCAATCTCCCAGGCTCAAGTG
ATCCTTCCACCTCAGCCTCCTAAGTAGCTAGGACCACAGACACCCTCAACCACACCTTGC
TAATTTTTTTGTATTTTTTGTAGAGACGAGTTTTTTTTTTTTCCGCCGTGTTAGCCAGGC
TGATCTCGAACTCCTGGGTTTAAGCAATCCACTGGCTTTGGCTTTGGAATGCTGAGATTA
CAGGCGTCAGGCACTGTGCACAGCCTTATAATTTTATTTTTAATCTATACCATAATTTTC
ACTCCTATCCTGTGTTCCTCATACATACATATCTACCAAAGACAGGTCGAGGGAATAGAA
AAATGGCTGTTTTAAAATCCTTGACCTTTCCTGCTTTTTTTATGTATGAACTGTCAAATG
TATATGTCATTTTTTTTTCTTCTTTGACATTCATATTTATTTAGTAGACCGTAGAACTCC
CTAGAAGTTAATCTTGATAGTTAATTTTTATTACTTGGGATTTTATTCCTCACTTAAAAT
TGTTTAATTAAAAAATATGCTTAATTATAATTCTAAAAGTCAAATGTATGATTTTTCTTG
TTTCAG**AAAGTCTTTATCTCTTACAAGGATCAGTAGTGGCAGATCATGACTACATTGGAT**
TACCTGAAATTCCGGTTGGAGCATATCAAGCAAATATTCTGGTGGAAGATGCAACTATTG
GTATAGTTGATAATGAATTACTGACATCAAGCAAGGATCGTGAACTATTAGAATATAGAA
ATACGAAAATCTCTCCATTGATAGACGATCATAGTACTCTTGAAAAGCAGACTTTCAGTC
TGTTGGACTCTTCAAACCAGGTTCTTGAATACTTAAGTTAGTAAATACCAAAATTTATTT
AAAGTGCTTGTTTAGAATGTATAAATTCTTAATGATATTCATTAAAAAAGGTTGTATAGA
AGAAGTATCTTGACACATTCTTAATGACTAATTTCTGAAGGTTTATTTTATAGTTATTAT
CTAAAATTGTGCCACATTTGAATATCAGTATACATATGTATGAAGGCAAAGGAACATGTA
ATATATAGAATTCACTTCTTCTTCTAAAAAGAGAACAGGCAAAGCACCCCTACTTCTTAG
GAAATAAATTCCAGCCCAAATCTCTCAAACCTTTGTATGGTTTTGGTACCGTTTCTCAGT
TTTAAATTTTCTGAAATTCTCTACCTTTTAAACAGTAAGTTTGAACTACCTTGAGTGGAA
TTTATAAATATTGAATCAACTGTAACAATGAATGAAATGAAGCTACTTTCTGTTTCATTA
GAACTTCTAAAAACAAGATTGTATGAAAAAATATCGGATTATTTAATGAGCTTTCACAGG
ATATTGGTAATTTCAAGTTATCTAATTGGTTAGATAGGGTCAGGATTAGGGTCTGAAACT
ACGTATTGGTTGGGAAGCATTTGTTTATTTTCATACTATATATTAAAGATAAAACCATCA
GCCAGGCATGGTGACCCACACCTGTAATCCCAGCACTTTGGGAAG

```
==================================================
Macaque Gin-1
total length:      33405 bp  (33405 bp excl N/X-runs)
GC level:         36.05 %
bases masked:      13797 bp ( 41.30 %)
==================================================
               number of      length   percentage
               elements*    occupied  of sequence
--------------------------------------------------
SINEs:                23        5064 bp   15.16 %
      Alu/B1          17        4315 bp   12.92 %
      MIRs             5         731 bp    2.19 %

LINEs:                10        4050 bp   12.12 %
      LINE1            4        2869 bp    8.59 %
      LINE2            6        1181 bp    3.54 %
      L3/CR1           0           0 bp    0.00 %
      RTE              0           0 bp    0.00 %

LTR elements:          4        1357 bp    4.06 %
      ERVL             1         382 bp    1.14 %
      ERVL-MaLRs       3         975 bp    2.92 %
      ERV_classI       0           0 bp    0.00 %
      ERV_classII      0           0 bp    0.00 %

DNA elements:         11        3042 bp    9.11 %
      hAT-Charlie      7        1731 bp    5.18 %
      TcMar-Tigger     3        1092 bp    3.27 %

Unclassified:          0           0 bp    0.00 %
```

Total interspersed repeats: 13513 bp 40.45 %

Small RNA: 0 0 bp 0.00 %

Satellites: 0 0 bp 0.00 %
Simple repeats: 5 131 bp 0.39 %
Low complexity: 5 153 bp 0.46 %

==

SW score	perc div.	perc del.	perc ins.	query sequence	position in query begin	end	(left)		matching repeat repeat	class/family	position in repeat begin	end	(left)	ID	
185	32.5	14.0	0.0	chromosome:MMUL_1:6:99335946:99369350:-1	2415	2528	(30877)	C	MIRc	SINE/MIR	(21)	247	118	1	
424	31.9	5.5	5.2	chromosome:MMUL_1:6:99335946:99369350:-1	2872	3065	(30340)	+	MIR	SINE/MIR	4	205	(63)	2	
1230	21.5	5.2	2.5	chromosome:MMUL_1:6:99335946:99369350:-1	3082	3387	(30018)	C	Tigger3d	DNA/TcMar-Tigger	(1)	320	7	3	
30	66.7	0.0	0.0	chromosome:MMUL_1:6:99335946:99369350:-1	3404	3433	(29972)	+	AT_rich	Low_complexity	1	30	(0)	4	
1986	24.0	8.3	0.5	chromosome:MMUL_1:6:99335946:99369350:-1	3434	4743	(28662)	C	L1ME1	LINE/L1	(723)	5983	4549	5	
216	34.6	3.9	0.0	chromosome:MMUL_1:6:99335946:99369350:-1	4897	4974	(28431)	+	L2c	LINE/L2	1643	1723	(1696)	6	
2483	15.8	3.6	3.0	chromosome:MMUL_1:6:99335946:99369350:-1	5077	5572	(27833)	C	L1MB3	LINE/L1	(6)	6177	5679	7	
359	32.2	8.3	1.6	chromosome:MMUL_1:6:99335946:99369350:-1	5576	5695	(27710)	+	L2b	LINE/L2	3248	3375	(0)	8	
269	36.3	9.4	1.2	chromosome:MMUL_1:6:99335946:99369350:-1	5698	5920	(27485)	+	L2c	LINE/L2	1825	2065	(1354)	6	
279	33.3	6.4	10.5	chromosome:MMUL_1:6:99335946:99369350:-1	6628	6815	(26590)	C	L2c	LINE/L2	(214)	3173	2993	9	
1699	19.5	2.6	6.0	chromosome:MMUL_1:6:99335946:99369350:-1	6893	6981	(26424)	+	MLT1A0	LTR/ERVL-MaLR	1	76	(289)	10	
2145	14.0	0.0	0.0	chromosome:MMUL_1:6:99335946:99369350:-1	6982	7273	(26132)	+	AluSz	SINE/Alu	1	292	(20)	11	
1699	19.5	2.6	6.0	chromosome:MMUL_1:6:99335946:99369350:-1	7274	7560	(25845)	+	MLT1A0	LTR/ERVL-MaLR	77	365	(0)	10	
264	38.8	4.3	2.6	chromosome:MMUL_1:6:99335946:99369350:-1	7727	7956	(25449)	+	L2c	LINE/L2	3123	3356	(31)	6	
198	32.3	12.0	2.6	chromosome:MMUL_1:6:99335946:99369350:-1	8000	8174	(25231)	C	L2c	LINE/L2	(114)	3273	3083	12	
21	75.0	0.0	0.0	chromosome:MMUL_1:6:99335946:99369350:-1	9445	9472	(23933)	+	AT_rich	Low_complexity	1	28	(0)	13	
252	18.7	1.4	5.8	chromosome:MMUL_1:6:99335946:99369350:-1	9864	9948	(23457)	+	MER5C	DNA/hAT-Charlie	185	313	(0)	14	
2370	23.5	3.5	1.6	chromosome:MMUL_1:6:99335946:99369350:-1	10039	11024	(22381)	C	L1M5	LINE/L1	(424)	5738	4728	15	
245	29.2	2.8	4.7	chromosome:MMUL_1:6:99335946:99369350:-1	13108	13215	(20190)	C	L2B_ME	LINE/L2	(11)	3436	3331	16	
376	34.4	4.6	0.4	chromosome:MMUL_1:6:99335946:99369350:-1	14976	15194	(18211)	C	MARNA	DNA/TcMar-Mariner	(222)	364	137	17	
224	24.1	1.7	1.7	chromosome:MMUL_1:6:99335946:99369350:-1	15222	15280	(18125)	+	L2a	LINE/L2	3291	3349	(77)	18	
962	26.5	0.0	3.4	chromosome:MMUL_1:6:99335946:99369350:-1	15564	15809	(17596)	C	MER8	DNA/TcMar-Tigger	(1)	238	1	19	
2058	14.3	0.7	0.0	chromosome:MMUL_1:6:99335946:99369350:-1	16616	16909	(16496)	+	AluSx1	SINE/Alu	5	300	(12)	20	
1040	18.7	0.0	0.0	chromosome:MMUL_1:6:99335946:99369350:-1	16910	17059	(16346)	+	AluSc8	SINE/Alu	133	282	(30)	21	
230	0.0	0.0	3.3	chromosome:MMUL_1:6:99335946:99369350:-1	17060	17090	(16315)	+	(TAA)n	Simple_repeat	2	31	(0)	22	
270	27.3	3.9	0.0	chromosome:MMUL_1:6:99335946:99369350:-1	17106	17182	(16223)	+	L1M5	LINE/L1	4486	4565	(1581)	23	
1172	15.7	15.5	0.5	chromosome:MMUL_1:6:99335946:99369350:-1	17433	17726	(15679)	C	MER68	LTR/ERVL	(12)	551	192	24	
517	20.4	0.0	0.0	chromosome:MMUL_1:6:99335946:99369350:-1	17746	17833	(15572)	C	MER68	LTR/ERVL	(461)	102	15	24	
1719	19.1	3.2	3.8	chromosome:MMUL_1:6:99335946:99369350:-1	17880	17971	(15434)	C	MER119	DNA/hAT-Charlie	(113)	470	385	25	
908	21.4	0.4	9.3	chromosome:MMUL_1:6:99335946:99369350:-1	17972	18229	(15176)	C	Alu2_OG	SINE/Alu	(0)	237	1	26	
1719	19.1	3.2	3.8	chromosome:MMUL_1:6:99335946:99369350:-1	18230	18507	(14898)	C	MER119	DNA/hAT-Charlie	(199)	384	102	25	
567	22.6	15.8	1.9	chromosome:MMUL_1:6:99335946:99369350:-1	18907	19130	(14275)	+	Charlie7	DNA/hAT-Charlie	37	295	(2317)	27	*
1017	25.5	6.7	3.1	chromosome:MMUL_1:6:99335946:99369350:-1	19130	19332	(14073)	+	Charlie7	DNA/hAT-Charlie	327	545	(2067)	28	
2656	19.4	3.0	1.6	chromosome:MMUL_1:6:99335946:99369350:-1	19333	19872	(13533)	+	MER44B	DNA/TcMar-Tigger	2	548	(2)	29	
1017	25.5	6.7	3.1	chromosome:MMUL_1:6:99335946:99369350:-1	19873	20040	(13365)	+	Charlie7	DNA/hAT-Charlie	546	711	(1901)	28	
643	26.4	12.1	6.0	chromosome:MMUL_1:6:99335946:99369350:-1	20045	20116	(13289)	+	Charlie7	DNA/hAT-Charlie	759	821	(1791)	30	
1720	18.9	2.9	2.3	chromosome:MMUL_1:6:99335946:99369350:-1	20117	20422	(12983)	+	AluJr	SINE/Alu	1	308	(4)	31	
1594	15.4	9.2	1.0	chromosome:MMUL_1:6:99335946:99369350:-1	20432	20704	(12701)	+	AluJb	SINE/Alu	1	295	(17)	32	
643	22.9	18.7	2.8	chromosome:MMUL_1:6:99335946:99369350:-1	20705	21104	(12301)	+	Charlie7	DNA/hAT-Charlie	822	1300	(1312)	30	
198	10.3	0.0	0.0	chromosome:MMUL_1:6:99335946:99369350:-1	21117	21145	(12260)	+	(ATAGG)n	Simple_repeat	3	31	(0)	33	
242	23.2	0.0	0.0	chromosome:MMUL_1:6:99335946:99369350:-1	21177	21232	(12173)	+	Charlie7	DNA/hAT-Charlie	1358	1413	(1199)	34	
342	25.1	0.0	1.1	chromosome:MMUL_1:6:99335946:99369350:-1	21566	21659	(11746)	C	MLT1J	LTR/ERVL-MaLR	(358)	252	94	35	
24	45.8	0.0	0.0	chromosome:MMUL_1:6:99335946:99369350:-1	21808	21831	(11574)	+	AT_rich	Low_complexity	1	24	(0)	36	
190	28.9	1.3	1.3	chromosome:MMUL_1:6:99335946:99369350:-1	21904	21980	(11425)	C	THER1_MD	SINE/MIR	(2)	272	196	37	
1460	19.4	9.3	0.0	chromosome:MMUL_1:6:99335946:99369350:-1	22036	22293	(11112)	+	AluJb	SINE/Alu	1	282	(30)	38	
552	28.1	6.0	0.6	chromosome:MMUL_1:6:99335946:99369350:-1	22375	22542	(10863)	+	MIRb	SINE/MIR	89	265	(3)	39	
2282	10.0	0.0	0.0	chromosome:MMUL_1:6:99335946:99369350:-1	22606	22886	(10519)	+	AluSx3	SINE/Alu	2	282	(30)	40	
207	6.9	0.0	0.0	chromosome:MMUL_1:6:99335946:99369350:-1	22887	22915	(10490)	+	(TAA)n	Simple_repeat	2	30	(0)	41	
24	84.2	0.0	0.0	chromosome:MMUL_1:6:99335946:99369350:-1	23503	23540	(9865)	+	AT_rich	Low_complexity	1	38	(0)	42	
545	27.1	5.6	2.7	chromosome:MMUL_1:6:99335946:99369350:-1	23664	23841	(9564)	+	MIRb	SINE/MIR	24	206	(62)	43	
2142	12.8	0.3	0.3	chromosome:MMUL_1:6:99335946:99369350:-1	24426	24729	(8676)	C	AluSq	SINE/Alu	(7)	306	3	44	
1968	16.1	0.0	0.3	chromosome:MMUL_1:6:99335946:99369350:-1	24843	25158	(8247)	C	AluSz6	SINE/Alu	(0)	312	1	45	
2062	19.5	4.8	4.8	chromosome:MMUL_1:6:99335946:99369350:-1	27210	27714	(5691)	C	MLT1D	LTR/ERVL-MaLR	(0)	505	1	46	
1870	20.5	0.0	0.0	chromosome:MMUL_1:6:99335946:99369350:-1	27847	28158	(5247)	C	AluJo	SINE/Alu	(0)	312	1	47	
309	23.2	15.6	5.2	chromosome:MMUL_1:6:99335946:99369350:-1	28496	28513	(4892)	+	ID_B1	SINE/B4	65	96	(128)	48	
1801	16.1	1.8	1.4	chromosome:MMUL_1:6:99335946:99369350:-1	28514	28793	(4612)	+	AluJb	SINE/Alu	1	281	(31)	49	
1252	12.5	0.0	0.0	chromosome:MMUL_1:6:99335946:99369350:-1	28795	28962	(4443)	C	AluSq	SINE/Alu	(10)	303	136	50	
677	13.9	3.9	1.9	chromosome:MMUL_1:6:99335946:99369350:-1	29306	29459	(3946)	C	MER33	DNA/hAT-Charlie	(1)	323	167	51	
198	0.0	0.0	0.0	chromosome:MMUL_1:6:99335946:99369350:-1	29733	29754	(3651)	+	(TG)n	Simple_repeat	2	23	(0)	52	
1827	14.6	5.0	0.0	chromosome:MMUL_1:6:99335946:99369350:-1	30339	30619	(2786)	C	AluSz	SINE/Alu	(17)	295	1	53	
907	18.2	0.2	1.1	chromosome:MMUL_1:6:99335946:99369350:-1	31006	31168	(2237)	+	AluJb	SINE/Alu	74	274	(28)	54	
180	0.0	0.0	0.0	chromosome:MMUL_1:6:99335946:99369350:-1	31169	31188	(2217)	+	(TGAA)n	Simple_repeat	4	23	(0)	55	
26	69.7	0.0	0.0	chromosome:MMUL_1:6:99335946:99369350:-1	31706	31738	(1667)	+	AT_rich	Low_complexity	1	33	(0)	56	
1764	17.8	0.6	3.6	chromosome:MMUL_1:6:99335946:99369350:-1	31812	32125	(1280)	C	AluJb	SINE/Alu	(7)	305	1	57	
314	18.8	0.0	0.0	chromosome:MMUL_1:6:99335946:99369350:-1	33341	33405	(0)	+	AluJb	SINE/Alu	2	46	(256)	58	

>Human Gin-1 (chromosome:GRCh37:5:102421104:102456455:-1)

TCAATTTCTGGCGTCTCGAACCCAAGAAATGAAAAAGGGAAGCCATGGGGGAGCAGGAGG
AGAAATCAAGGCGCGAAAGGACCCTCAAAGCTGTACTGTCACTCCACGCCCAGGCTGTCA
TTCTCTCCAGCACAAGCCCCTCGGTGGAGCATGTGTTTAGGGTCTGCAGAGCACAGGAGA
CAAAGCAGAAGCCCAGCAGCCGCCTCAACCCTGGCATGATCAGGTTCCGCATCTCCGGTT
CTCCTCCGGTTTTGGGCCCTCACCTGCCACGAATGCGGTCGTCACGCGAGCAGTTGTGAG
ATGGTCAAGCCCATTCAGGTCTAGGTTACCCCCTTTCATCCCGGAGGCAGGGGTGAGGTG
TTTGTCAAGAGACTTCAGTTGTAGGCGTGGACGTAGGCTACTTCCTTCAGTAACAGCTAT
AGGGCCACATAAGGGAACCTCAGGCTACTACACCTCAGGAATCTCCGTCACGCCTCTTCC
CCGCCCTCCTCGGGTCTCACGCCGAAACCTCACCGGGCGGAACGATTTCCGGCAAGAGCC
AAATGATCAGTCCTAACCTTTCTGACCGTGGTTCCAAGGTTCCCCAAAGGACTGCCCTCT
AAAGGACCCGGGTAGTTCCGCTTCCGGCAGCGCGAGATAAATCACGAGAGGAAGCTTAAA
TCTGTCGTTTGAATTTAGGACCACCTCGGTGAGTGGTCGTTCTGGTGTGCTGTGTCATAC
CTACTGTTTTTTAAAGTGAGGCGTAACCCGACAGTAATTTCAAAACCATTCGCCTCGACC
GGCCTAAGGAAGGGTTTAATTGAGTCTGTGGGGAGATTTTGCGCAAGCGCCTTTGAGATA
GGAGGGCCTTGGGCGTTGCGATGTCCCGCGCTAGCTGAGCAGAAAGGGCGAGTGCTGTTT
CGGGCCTGGGGAGGTGCCTACTGGGTGTAATTGAGTTTTACCAAACGGTCATTTTCCTGC
TGATTCTGAGTGTGGTTGGACCTTGCCCTTAAGATTCCGTTAACGAGTCGCCCAAAGCTT
AGGAAATGCTCCCTCACAGCGTTTGAGACTAAGCACTTTTGGCCGGCGTTGAAGAATTCT
CCCCATTACTTTGATCAGTCATTTAATCAGAAATTTTGCCCCAAATTTAAATACGTGGCG
GGGAGAAACGTGAGGTCAAGAGGGAATCCTGTCACGTAGTTTTAAAAGAAAAATGTCCAC
TTTATCTCAGTGTAGGTTTTTCTTAAATGGGCATTTCTGAACCAAAGTAGATACTTTTTA
AAGTGGAAATGCATTTATCCCATGTGGCTTCATGTGAATGCATATGGCGATGAAGGCATT
TTTTTGTTCAATGAATTTGTACTCTGAAGTGAACCAGATTACAGAGAAGTGTATTTAAAT
TTACAGGTCCTTGACGTTGCATAAAATTGTGCGTGTCCATTTTTTGGAGCTTAAAATAGA
ATAATTCAAATGTCAGAATTCCGATCCAAGTTAACATGTTGATTGAAAATTGCAGATTTT
AAGATGGAACCAGCGGGTATTATAACCTTTTAGGGGCTTAAATTGGTGGCTTTGTTTTTA
ACCCTTGGTGAAACAGTTAAGTCCTAATCTTGACATAATACCTAACTGCCTTACTTTATT
GAACACAAAATTATTAAAGGAATTAAGCAATGAGTTGATTTTATTTGCTTTTATGGAGGG
GACAAAGTGTATAAAGCTTAACCAAATGTGACCCTGACATTCCTCTTGCAAAACTACAAG
TTGTAATCAATCACTTTCATTTTTTCTTATTCCTACATGTTTAAGTACATTAAGTAATGA
TTTTAAGAGAAATTCTTGAAAATATGAGCATATCCAAAGAAGTCCCAGGACAGTCTTTCT
TTATGTAGTAGAAACCACTTAATCCCCCTTGTAAATGATACCTTTACTTAGCAATCAGAG
TATAAGAAGATATTTATATTTGTCTCGAATATTACAGTTTCAAGCCATTTTCTCATATGT
ATGGGCTTTGAAAAAAAAAAAAACAACTTTCATTTACGTTACTTTTGTATATGAATACCAA

TTATTAGCAGTAAAACTTTTAACTACTTCAAAGGCTATGCTTTTCGTGAACTGAGTTTTC
TTTCTTACGCATTTTCTATTTCTCCTTGTCATCTGTCTCTCTACCTCCAGAACCAGCAGT
TTGTGAACTGCATTAGACTGTTATATCGTTATAAAAGGATACAATTCGTGAGTCTTCTGA
TTATTTTCTTCTTAGCACTGAAGCTTTATAAACATTGCTATAAAACATTAACACCATTAA
TGAAAGGAAAGAGATTTTAAAATATTTTTTCATGTAGGTGAAGATTTTGGGAAAAGAAAG
GGTTTTATGTTACTTTAAACAGCCTGATATGGATGATAATACTTAGATATGATAATAATT
ATACATTACCATATACAAAGGACTTTTAATCATCCTCGTAATTGCAAAAAAGGGATAGAT
ATTATGTCCTTTTCCAAACATAGATACTAAGGCTTAAGTCTATACCTTCTTAGACACAGT
TTACTTATAGTGCCATGTATGTGAAAGTGAACTGTAATGTTTTCAAAAGTGAAATAATTT
ACCTCTGGAATATTTGCTATAATTTTTAATTCTCCTTTCAGGAAATGCAGGTAATTCTTT
ATTCATTCACAAATAGTTGAGTGTCCACTGTGTTCTGAACATTATGTTAAACAATGTAAA
CATAAAAAATTAATAAACTCTTTCCATTCCTCATTTATAATCTAAATTATTGATTACAAA
AATCACTTTATAATTTGTGTAACAGAGGCCTGAACCAAGTATTATAAAAGCTAAGGATAA
CAAGTAATTGACTTACTGGTATAGCAGGGGGGAAAAGCGTGAAATTTGAAGTAAGAGACA
CCAAGTTAAAAGTGTCAGCGCATGTACTCACTTTATTACCTTGAGTAAATTGGACAATCT
CTGAACCTCCATTTTCCTACCTGTAAAATGGATATAAAACTAATACCTACTTCATGAAGT
ACTGTGAGGATTATCATAGTAAAATACTGCATAACTATTTGCAGCTGTAGTCGTGAGTCA
CTTAGCAATGGAATAAATTCTGAGAAATGCTTTGTTAGGCAGTTTCAGCATTGCACGAAT
ACAGACTCTACCTACATGAACCTAGATGATATGTGTGTGTGTGTGTGTGTATATTTTTTT
CTTCATATAGAGAACCAAATGTCCCTGCACATTATTGGGTATCATTTATTTCTCCTACTT
GATCTGCAGTGCTAATATCAAATGACCTATATCAGGTTCTATATATGCTCTGCTATCATC
TTACGGGACCAATAGGCAGTCCATCCTTGACCAAAACATTCTTATGCAGTGCAATTGCGT
TGGCATTGTTTTTAAAATTTTTAATTTTTTAAAATTATTTCCTCTTCCTATACTTTTGGC
TTTTCCAGATGTCATATAAACAGAAATATAAAATATATAGCCATTTGTGTCTGGCTTATT
TCACTTAGCATAATGATTTTGAGATTTATTCATATTGTTAGGTCTATTTATAGTTTGTTC
CTTATCAATCCTTAATAGTATTCCAGTATGTGGATCTACTGTAATTTGTTTATCCATTCA
CCAGTTGATGAACATTTGTTTTCAGGTTTTGGCTGATATGAATAAAACTTCTAAAAACAT
ACACTTACAGGTTTTTAGGTGTACATATTTTCTTTTGGGTTAGTATCTAAGATTGGTATT
GCTGGACCATATGGTAAATTGATGTTTAACTTTATAAGAAATTGCCAAACTGTTTTCCAA
CATGTTTACATCATTTTCTATTATCACCAGTAAGAGCATCCTTGGTAGTACCTAGCATTG
TTAGGTTTTGTTTTATTTTTGTTGTTGTTAGTGTGTAGTCATATCTTATGGTAGTTTTAG
TTTACATTTTTTATTATCTATTGATGTTGAGCATTTTTTCATGTGTTTATTTGCTAATCA
TATCTCTTTAGTGAAGTGTATTTCCAGGTTTTAAATTGGGTTGTTTGCTTTCTTGTTAAG
CTTCGAGAGTTCTTTATAAATTCTAGAAAAAGTTATTTATCATATGTATGTATTGCAGAT
ATTTTATCCCAGTGTTATTTCTCTTTTTTTAGCAGTTCTTTAAGGAATAAAAATTTTAAT
TTTTTCTTTATGCATAGCTGCTTTTTCTTTGCTGTCTGGGAAGCCATTACCCAACCTGAG
CTCACAAAGATTTTTGTTGTTGTCTTCGAGAAGTTGTATAGTTTTAAGTTGTACATTTAG
ATCTGTGAGCCATCATGAATTAATTTTTACATATCATTTGAGGTATGAGTTGAGGTTCAT
CTTTTTTCAAAGGATGTTTAATTGTTACAGCATTTGTTGAAAAGATTATCCTTTCTTCAT
TGAATTACCTTGGCACCTTTGTTGAAAATCAGTTTACTATAACTGAGTGGCTATTTCTGT
CACTATTCTGTTCGATAGATTTAAGGCTGTCATTTTGCCATTATAACACAGTCTTGATTA
CCATAGTAGTATAGAAAGTCCTGAAACCAAATAATGTGAAATTCTTCAACTATATTCATT
TCTCAAAGTTGTTGTAATTATTCTGAGTCCTTTAGCTTTCCATATCAATTTTAGAATCAG
CCTGTCAGTTTCTGCAAAAGAGCTTGAGGGTTCCTGATTGGGACTGCATTGAATCTATAG
ATCAATTTGGAGAAAATTGGCATTTTGTCCCACTTTTTCTCAGAGGTAGAGTGGGAGTTA
CTCCAGCTTTCTGCATTCTAGTCAGAAGCCTATATGGGAATGTTTAGTGGCATTTCAGAA
AGAAATAATTTTCTTCCATGGTTATATTCACTATAATATCTTTATTGGAAGAGATCACAG
GACGAATCCCTCTATTTGTGAATTGGATGTCATCCCCTGCTGCCATCTCAGGACATCATA
CCATAAGTTTTTTCTCCTTTGTTTTCAGTGGTCTTTCTAGAAGCCCTTCCATTAGCATTA
AACTTTTTGGGTTACTCCCTTGTCGAAAGCAAAACAATCTTGTTCAAAAAAAAAAATTGTT
TTAATTGTAGTAAATCACACATAACATGAAATTTACCATCTTAACCATTTTTAAGTGTGC
AGTTCAGTGGCTTTAAGTACATTCACATTTGTTTTACAACTATTACCACCATCCATCCCA
AGAACTCTTTTCATTTTGTGTAACTGAAACTCTTGACCAGTTAAATAATAACTCTTACAT
TCTCTCCTCCTCACCCCCTGTTCTATGCAACCATCATTATTCTTTCTGTCTGTATGTATT
TGACTACTCTGACTACCTCATATAGGTGGAATCTTACAGTATTTATCTTTTTGTGACAGG
TTCATTTCACTAAGCTTAATGTCCTCAAGATTCATCCATGTGGTAACGTGTGTTAGAATC
TCCTTCCCTTTAAAGGTGTGTGAATATATACACCGTATTTTGTTTATCCATTCAGCCATT
GATGGACACTTGAGTTGCTTCCATCATTTGGCTGTTGTGGGTGATGTGGCTATGAACATG
ACTGTCAAAACTTTTAACCCCACTAGAATGTAAACTCAATGAAGGTCAAGAGAATCTACA
TCTTGTTTATTATCCTGTCTCCAACGTTGTAACTGGCACATAGCAGTTACTCAGTCACAT
TTTTTAGATCAATGAATGAACCTTATATCTGTCTCTAGCAACTATCTTGTAGCTCCTTGC
CTTTATAGCTAAGCCCAAGTTTCTAAATCCACTTTCTGAATTTCCATTCATTCCTTGTGT
CATTTCAGTCTGGTTGCCAACTGCCCTGCTATACTGTAACTATACTGTATACTGTTGCCA
AAACCACTAGCTACATTATGGTTCTTATATCACTTAGCCTCTCTGTGTACTTCTAAGAAG
TCTGTATTTCCCAGACTTCTATTACCAGAACCTCTTCTGGCTTTTTTCCTGTTTCTCTGG
CTAGTCTCATGTCCCTAAAGATTTACAATATATTTGGGAGAGAGACATATAAACTAATAT
ATTCAATAGAGTGTTTCAGATTTTATGATAAAGGTTGACACATATTACTGTGAATGTACC
AATGAGGGTGTGCACAATTCTCCCTGAGATTGGGGATGTGGTCAGAAAGGATTCATGGAT
GAATGAACTCATTAATGTGAACGTTGAAAGATAAGGATATGTTCACTGCACTTCCTACTG
TGTACACTTTCCATCTGATCCTTCTTCCATTGCCTTAACTTCACCTACTTTTTACATGCC
GAGTTCATCCATGAAGAATAGAACCTGCTTAATATTGTCATCTTTCTCCCTAAGCCAGAG
CTTCAGACAGAAGTGATCCCAGATTCTTCTTTGTGTTTACTCCACACATCTAGTCAGTTA
CAGTTCTTATATAATGTATCTATCCATTTATACTGACACTGCTTTGGCTCAGAATTTCCT
CATTTCTTGCTTTAATTATACAGTAGCTTCCTAGCTGACTGGTACAGGTATTCCTGTCTC
TACCCCAATCTCTATTCCATATTGGTGCACAATACACTTTATAGAATAGAAATCTAATCT
TGTAACTCCAACTCATTCTTAAAATTTTAAGTTATTCCAGGTTGGAGTGGTTAGAAGACA
CTCTGCAGGAAATTACACCTAGCCAGAGTTTTGAAATATGATGAGGGGCTGGAGAAATAG
CAATAAAAGTTCATTCCAGGCAAAAATATCAATGAAAAGGTGTGAGCACACTGCATGGGA
TGGTTGAAGAACTGTGGTTATGTTTTGAATTGAGAATGCCAAGAGAAAAAGATGGAGAGA
TTGATAAAAACCATATCTTATAAAGCCTTGTATACTGGCAGAGGTATTTAGATATCATTA
GTGCCATGGTCTTAATGGTTGTATTCTCCCAAAATTCATATGTTGAAATCCTAACCACCA
AGGTGATGGTATTAAGAGGTTGGACCTTGGGGCTGGGCGCGGTGGCTCACACCTGTAAGC
CCAGCACTTTGGGAGGCTAAGGTGGGTGGATCACTTGAGGTCAGGAGTTCGAGACCAGCC
TGGCCAACATCGTGAAACCTCATCTCTACTAAAAATATAAAAATTAGCTGGGCATGGTGG
CATGCACCCGTAGTCCCAGTTACTCGAGAGGCTGAGGAACAAAATTTGCTTGAGCCTGGG
AGGCAGAGGTTGCAGTAAGCTGAGATCATGCCACTGCACTCCAGCCTGGGCGACAGAGCG
CAACTCTGTCTCAAAAAAAAAAAAAAAAAAAGAGGTGGAACTTTGAGGAGGTGACTTGATC
ATAGGTCAGAGCTCTCATTAATGGGATTAGTGCCTGTATTTTAAAGAGTCTGAGAGAAAC
CCCTTTCCCCTTCCCTCATGTGAAGTTACAGTGAGAAGACAGCTGCCTATAAGAAAGTGA
ACCCTCAGTAGACACTAAATTTGCTGGCATCTTGATCTTGTATTTCCTAGCCTCCAGAAC
TGTGAGAAAGAAATTTCTGTTTATAAGCCATCCAGTTTATGGTATATTTGTTATAGCCGC
CCAAGTGGACTAAAACAATCAGGAATGGCAAAAGATGTATTTCGAGTTAAGTGAATATTT
TGATAAGATTTGTGTTTTAGTGTGACCTCTGTGACTGCAGTGAGGACAGGGACCATATTA
GAGACAGAGGAGGCAAGGTCAGATAGAAGGTGAGTGTGAGCTAAGGCAGTGGCAATGGAA
TAAAGTTCAAGGTTCTACCTCATCTACAATCAGTGCTTTTCCTCCTTTTCCAGGTGAATC
TGGGTCTCTACACATCTGTTTTGATAGTACTCTTTGCATACATCTGTTAAAACACCACTC
TAAACTGTATAAATGCTGATTTCCTCCTCTTTTCCTCTCATTTATACCATAAGCTCCGTG
AGGACAGGGATTTCATTTTATTTAACTTTGTATTAGTATCATCTAGTATATATAAGGGTT
TAAGAGCTCAGTAACTGAATGAGCAAATTCAAGGTTATATGGATGAACAGGCAAAAAACT
ATTAAAAAGGGTAAAGCATGGAAGTTCTACAGTAGGCACAAAGAAGATTAGGAGAGGTCA
AGATCACTTTAGATTCAGAGATCATGGAACACTTGCTTCATTGAGATGCTAGAGCTTGAT
TTTGAAAGATGGGTAAGGCTTAATCAGGTGGAAGGATATACCATTTAGGGGAAAATGATG
TAAACCAAGGTATAGAGAATGGTTGGGGATATGGCCATGGGTATAGAAAGGAAAGTAGTG
AGAGAAGAAGCTGTATTATGTTCTGCCAGGTTGTAGAGGGTAGGCTAAATAGTCTAGACC
TTAGCCACTAAGTGGAAGATAATGCCCAGAGGCAGTCTCATGGTGTCCACTCTGTCAAGT
AAATCATTTGTAAGATAAAAATATGCTAGGGATAGTCTAAATTAAAGTTTCAAGGTGACT
ATGTCAAATAACTACACCTTTAAGTCTTAGTTCTGTTGAGCCAAGACAACTCACCCATAT
AAAAATGAAAATAATACACTTGTGAAAGCATTTGTGAAATATGAAAATCTGTAGGAAATA
GATGGTTTTTATGTCTTCTCCCAATGATGTTTATGTCCCTCTCCCTAACATAGATATATG
TTAGTCTTCTCCCACTACTTTGATGAGAAAAAAACAAACCAAAAACCTTAAAGAGCTCAG
TCTCCAAAGATTTTTGTAGTGCTTTCCTTTTCTCCTTGTCCCTTGTACTATAGGTTTACT
CTGAGGAGAGCAAATTTCTCTATTTCGATCTACTTTCAATTATTCAAAAGTTAAAGATTT
CAAAGCAGAGAAGTTAATTTCAGTAAATAGACCTTTTCTGAGAAGTTTTTGTATATTTAT
TATAAATAGGAAAACCAACAGTGTCCTAATGGCACGTTAACTTAATATACTGAAGACTTC
ATCTTACATTAGATAGAAAAATTTTCTGGTCTCTCCAGGAGGTAAATGGGTAGGGGGAGG
AAGACAAGAGATGGTAGGAACTTGGAACCAAGAGGTTTTTAAATATAAAAGCAAAATACG
GAAGCTACTGTGGATTATGCAGACTCACATTTTTTTTCCAATGGCTGGTTTGTTTTCTAC
CAGTTCTGGTTACCCACATCCCTCTGTGTTTTGGGGCCTGTACTTCCTCTAAGGTGTGAG
AGTTTGGTCTTTTCCTTCTGTGGGCTTTTACAGGCCTGAAAAAGCTCCTCCCCTTTTCTT
TTTAAGGTCCTTCAGGAGTTTTTGCAACCTTTACTGGTCTCTAAAAGGAAGGAAATACAC
AATTTTTATCTTCCTTTTATAAACCTCGTCTTCCTACAGATACCATCCTCCCAACTGGCT
AGTATGGGATATTTCTAGATTTTCCTTTGGATTGCTTAGACGATTTGTGACATTGATAGT
TCCAAAAACTATTAAGGGAAAACTATAACAGGAGGTTATATGTAGTATATTATTAATTTT

ACAGTATGTATAGTCTATATATTGATTTCATCTAAACTGAGAAGGGACAGGTTTCTGTCC
CTTAGACTTGAAAGAATGTGTTATCATTGTAGTATAGAGATGAAAATAGAGGACACAAAG
AAAAAATGATTTTGGTTTCTTTTTTTTTTAAGAGTTGAGGACGATTAATGCAGAGCTAGA
ATGATAGGCAGGCAAATGGATGGGTAGACAAACAGGTTCTATATTCCCAACTTGAAACGA
CACAAGGCTTCATGAATTGAAAATTGATTAGGAAGTAAAAGTTTGCCATGGAGTCCTGGC
AGAGGAGAGAGTGTAGTGAGGATGACATGGAAAGTAAAGGCATCAGGAGGTCTTATGTGG
CAGATTGGAGGGCAGTAGGGTGCCACTAGTGTTTCACCACAGATGGTTTGAAAAGCACTG
ACCTGGAGCGACATGAAACTTGGTAACAATAGAAGTGGCTACAGGACCAGAACAGAGTAT
ACGCATGGTAAAACTTCTTGATACCTTTACTGTTTCCATTTAGTGGCTATTATGACTATA
AAATATCTATGAACATTTTTGTACACATATTTTGATAGTCATAGACTAATTTCACTGGAA
TATAAAATTAGAATTGCCAAACCTTTAGGGTAAATTTGTGCTCAGCTTTAGAAGAAACTC
AGTTTTCCTAGGTGGTTGCAACATTTTATCCTCCCACAATGTATGAGAGTTCCAGTTGCT
GCCTGTCTTCACCAACACTTGGTGTTGTCTTTGGTTTTTAATTTTAGCCTGTTCAGTAGT
GTGTAATTCTAAGTCATCGTGGTTTTATTTGCATTGCCCTGTGGAATAATGATGTTGAGC
ACATTTTCACGTGCTTATTGACCATTGGATATTCTCTTTTATGAAGTGCTGTGTAGAAAT
TTCCCCCCATTTTTTGAATTGGGTTTTTGTCCTTTTCTTTTTGATTTTTAGAAGTTCCTT
GCATACACTGGGTCTGAGTCCTTTGTTAGATATATGTATTACAGATATCTTCTCCCAATG
CGTGTCCTATGTTTTCACTTTCTTAATGGTGTCTTTCATGAATACACATTTTTACTTTGA
TAAAGCCCTATTGTTAATTTTTTTTTCTTTTAAGGATAGTGTTTTTTTTTTGTGTCTTAT
TTAAGAAATCTTTGCTTAGCCTAAGATTTGAAAGATCTTTTCCTATGTTTTCTTCCAGAA
TCATTGTTATTTATCTTTCACATTTAAGTTTATGATTTATCTGGAATTAATTTTTGTGTA
AGATGTGAGGTAGGTAGTTAAGGTTCATTTTTTCCCCTAATACAGTTATCTAATTGTCCA
ACATTATTTATTGAAAAGACCACCTCTTTCCTCATTGTGTTGATATCTTCATTGTAAACG
TATTGACTTTACATGTGTGGATGTAATTCTGTACTCCTTGTTCAATTACAGTGATGTATT
TGTCTGTCCTTATGCCAATTGGCTTGTCTTATTTACTTTATTTGTATAAAAAGTCTTAAA
TCTCATCCATTCATCTTTGAGATGATTCAGAAACTGTGATAGGCTGCATTTGTTTGGTTT
GGGATGCAGCCATCTATTAGAAGATGAAAAAAATCATGTATTTAATAATCAATTGGAAGT
GAATTAAACTTAGAACAAACTGATTGTCTTATAATACTTTATAGAGATAATTAACCTGTT
TTTTAGGTTATGATGGGACTGTGTTAACATTTCAGAACCTTTTCTTTTAAATTGACATAT
ACTTTATGTTAACCTAAGGTAAGAAATGTACTGTTACTCTCTCTGGAAGTTAAAAGTAGA
TCAGAAAGACGGTTTTACATTTGCAAAGAGCTGTGTCATAGGGAAAGTGAATTGGTAAGC
TGTTTAAGCCATGACAATAGGCACAGTCTGTTTCTGCTTTGTTCCAGCATTCTCTTTCCA
GTAAATACTCTTAGTCTCATCCCTAGATCTTCCAGTGGTCAGTAAACATCTTTTTTTAAA
TGTAATTTCAAAATTGTAAGATTTATGTCAGAATCCTAGCAGGAAACAGAAGACATTCTG
GTGGGATTTTAAAGAAAATTTAATGGAGCTCCTTTCTATAGACAGACAGTAGCAAAGTAA
ACAACATGGCACCTGGAGACTAGCAAAAGCAAAAATAGTTACCACCTTAGGGCTGAGGTG
ACAAGAAGAAGAAATAACCATGAATATATTTAGAAATAATATAAAGCTATATTTCTTCTC
TTATAACTTATTTATTTAAATGTCACATGACAGGATCCTCCTGTGTTACCATCCTGTGAA
TGTAACATTTCAGTTGATAGCAATGAAATTTTAAGCAAAACTTTTTGAAGTGTACTGGTT
CATTTCCTTTTTTCTCAGTATGTGGAAAATTTTCAGCATTTCTTATCAAATGGAAAGAGA
CAAGATAATTTGTCATGGGGTATATCAAAAATAGCATTTGAGTATTTTTGTTCATATACA
TCGCTGTTCCTTTCCTGTACTGCAATTATCTCCCTCACAAATAAATTCTGGGAATTCTCG
GTTCATATGTAAATAGAAATATGACAGAAGTCTTAACTGAAATAGCAATTCAGTTAGCTT
AAAATTTAAGTTATCTTTTTAAATACCTTTTACCTAG**GTTCACAATGGTCCGTAGTGGAA**
AAAATGGTGACCTTCATCTTAAACAGATTGCATATTACAAACGAACTGGTGAATATCATT
CAACTACACTGCCAAGTGAGAGAAGTGGCATAAGAAGAGCAGCAAAAAAATTTGTCTTCA
AAGGTAAAATTAATGTTCAAATTTTGATTATTGAGTTGTAGATTCTTCCCCACATTCTTC
CTTGTGGAAGTTTGGGAATTTCTGTAGTAGTTGGTTGTTGCTAAACAATTACCAGTTGAA
TTGATGATGCCACTCAATCAAAAGCAAAATAAAAATTACACTCAAGCTAACTGAAGGCCA
ATAAGCGTGAAAAGGGTTGTTTAAATTATAGACAGAATATGATACCATGAAAGAAAACAA
AACATTTTTCTAAATTAACAACTTCTGCTTTTACCCTCTCGTGGTCTCTAATGGTGCACT
TTCCAGCACTGTTGCCAATTTCAATGAGGAACTTTTTTGATGCTTAATATGAAAATGTTG
ACAAAGTTAACTGGTTTGTTTACTAAAATGAAAACTGGAAGGAATTAACTATATTTCTGT
GATCATGTCCCCTTGAACCCCACCACTGCCATTAAATATTCTCAGGATCACATTCATTTT
AGGACCCTTGGATCTTCCTGAATGGATTAAATATTTGAAAGGCAGTGGGATATTTATCTT
GGAAGGTCATGATAATTTTTGCCTCTTTTTCCCCTTATAAAGGGGAAGAGAAGTTTGGGA
TGAGATAAACAACAAGTAGTCACCAGATAATTGAATGAATGTTTGTGTTACAAATGGGAA
TTTTGCCTCTTAGGCTTACCCCAAAACAGGTTAATAAAACTCTAGGATAATAAATAAATG
TCTGCTTGCATCTCCCTGTGTATATCCCTACCAATAACTCCCTTATACTTAAGATTAGGC
AGACCTATTACCTCTTAAACTAAAGTGAGGATTCTTTCATTCATATATTTGATAATTTAT
GTAACAAATGTTAACTGGGTTTCTGCTGTGTGCCAGTCACATTCTAAGCCTTGGAGATAC
ATTGATGAATAACATAGATAAGGTTCCTGCTCTTAGGTTGCTTATCTTCTAATTCTAAAA
AATAAGGAGTGTTCCACTCATCAGTTGGCTTACTCATATTAGTAGCTGAATGGTGTATTT
TGGAGTTTGAATTGCTAGCCCATTCACATTGGAAAGGTAGATGGTGATTCGCTTCATACC
CTGGTTACTTCCTAGCTGCAGATTCTTCTCAGGTGTCCTCCTAAGGAAACAGAGAATTGT
CTTGAGGATTACAGTTTTTTCCACTTGCCCGCTTCACCATCCCTACCTTCTCAAATATAG
AAGAAGCTGAGAGAGCTGTTTGCTTGCAAAAATACAGTGATGATAAAACATTATTTTAGA
AAATTCTGTTCCAGGAAGTTGTTTTTCTGTACATAATTTATTTGCTAATCTATGCCATTT
TTAGGAGGTGCCTGCAAAATCTTCCTACTTTGAGTACTCCCAGAGAGAGATAATTTCATA
TTTCCGTACTTATTACGTGCTTTGGTGAACAAGCTTGCTCTACTTATATTGATAGCTACA
GAATTATAGCAGCTACTGCCCTTCCTAAGTATCTAGTAAGTGTAACAGCTAAGAACCTTT
GTGTTGTAGCATCATTAAAAATAAATATTCTGTTTTAAATGCAGTAAGATTTTACAAAAC
AAAACAAAAACCTTCATGGTTTAAAGAGAAGGCTATGATTCTCTTAAAACTACGTAGATC
TCTAAAAATTGCAATTCTATCTTTTGGTATCATTTATTCCAG**AAAAAAAGCTGTTTTATG**
TTGGAAAAGACAGAAAACAAAATCGTTTGGTAATTGTTTCAGAAGAGGAAAAAAAGAAAG
TCTTAAGAGAATGCCATGAAAATGACAGTGGAGCTCATCATGGTATATCCAGGACCCTCA
CTCTGGTAGAATCCAATTATTATTGGACATCTGTGACCAATGATGTCAAACAGTGGGTAT
GGCTTATGTATTTAGAGTATTATGAATAATGTCTTTCTGATAGATACTTAAAATTTATTA
TTTCTCCAGATTTATTCTGGGTATCACCTCTCATTAAGTATTTTTGGCAATCACTTAAAG
GGAGTTGTATTTCCCTTCACAGTTATTTTAAATCACTTAAGTATAATTTGAACAGGAAAG
AAGCATTATTTAAACACTTGAAAATACCATATTTAAATAAGAGATTATTTAAAAAATCAG
ATTTGTATCATTGAACTTTTTTGTGTAGGCAGAATTTAAATGGAGCTGGAATTCATTTGA
CTAAATCATTACTTCTTCAATGGCAGTCATACAAGGTTTTCTTAAGGGGAGAATATGTTC
ATAGTAAGTCAAGCCCAGTATGTGCTCTTATCTTTTGTGCCTGTTCCATTTTGAGGTAGA
ATGTCATACATGACAGAAACCCAAAACCAAATATAACCAAAATCAAATTTTGAGGATAAT
GAAATAAGTCTGAGTTTTGTAACTTTATGACCCATTGGGACCTTCAACTTCTAGTAGAAA
ATTCACCTATAGGAATTTTGGGGTTCTTGCTGGAAGTCAGCTGATTCTACTCTTTCCAGT
GTACTGTATGTCAAATGGTAGGAATTCCTTGGTATATCTGTGAGATCTCCTTAATAATTG
TAGTAGTACATCAAAAAGAGTGGCATTTCAAGCATAAAGAATTAAACCAAAATTTCATAA
GTATAATTTGTGAGTTATCAGTTTGTGGGTCTATTCAGGTATTCCAAAGACCTTCATAAT
TTATATATTTTTCCTGCTGTGCATTTTTTACAAACATTCAGTGAAATCTTTGTCAAATTG
TTATCACTGGAGAATGAATATGATTTTTGGAAATAATCAGAAGTTATTTGTGATAAAATC
CAACAAATAAGTTTAAATTGGAAAGTGAAAATTTTGATCAAAAGTAACTGTGACTATAAT
GGGATTAAATGGCTTTTAAATTTTTCTTCAGAAGCTAGTTTGAAAGTAGTTTCTAGAGTG
GAATTCCAGAGATACATTGAGAAATGGCACCCATCCATAAATTGATCAGTTGATCTTGTC
TCACCTCACTCAAATGAAAGCTGCAGGACAGCATGGGTTTTGTGTATGTTTATTCTTTTC
CCACGATTGAGAGAAATTGCTCCATCTATACATTTTATATGAATGAAATTATATTTCAAT
AAATATGGTTGGAATAGATTAATGAATAAGCTTCTTAATGTGATAACTTTAGAAGGAAAC
ATTTTATGTATATAGAAATTATTGTAAATTTACATACTGATTTAATAGGAAACTTAAGAT
CTTTTTAAGTATTTAATAAATATTTATTGAATGTGAACCAGATTTATGTTCCATGTGAAT
AAGACCCAATAAGATGGTCATTTGGTAGTATAGTCTTCCCTCAGTATATGCAAGGGATTG
GTTCCAGCACCACACTCCTGTCACCAAATCTACTCATACCGAAGTCCTGCAGTCAGCCCT
GTGAAACTCACATATACAAGAAGTCAGCTTTCTGTATACTTGAGTTTCTCATCCCTCCCT
ATCAATAGAGTACTTTTTATTTGTGTTTAGTTAAAAAAAAAAAGCCATGTATAAGCTGAC
CTTCACAGTCCAAACCCATGTTGTTTGAGGGTCAGCTGTAGTGGCTACTACAGTTGTTTG
TTGTTGTTTTATTTGTTTGTTATTCTAAATAGGTTTAATCCAGTGATTCTCAGTCCAGAA
GTTTCTCCTTCAAAACACAAAGTATACCTATTAACCATTTTAAAATTCAGATTTATAAGA
CTGCTTTTATATTGTGTTGAGTGTTAACATTTGTATGTGTTTTCTTTATTGTGTGCCTGT
TGTAG**GTATATGCTTGTCAGCATTGCCAAGTGGCAAAAAATACAGTTATTGTAGCACCGA**
AACAGCACCTTCTCAAGGTGGAAAATCCATGGAGTTTAGTTACTGTTGATCTGATGGGGC
CTTTTCATACAAGCAACAGAAGTCATGTATATGCTATAATCATGACAGATTTGTTCACCA
AATGGATTGTGATTTTGCCTCTATGTGATGTTTCAGCATCAGAAGTTTCTAAAGCTATTA
TCAATATATTTTTCTTATATGGACCTCCTCAGAAAATAATAATGGACCAAAGAGATGAAT
TCATTCAACAGGTAAGACAAATAAACTACTTAGGTCTGGGAGCATATCTTACTTCTTTTC
AGTGTCCAGAACCAGTGCCCTCTGCATTATATTACAGAGTGCTTAATAAGTTCAGATAAA
TTTCTGTAGGTATAGAGGTATGATATTAAATACTACAAGGATAGTTGTACTGGAAATTAG
GAGATTAGGATTTTAATACTTACTGTGGGTTATTGTAAACCTATCTTCAGATATCCTCAA
ATTAAAATGAGGAGGTTGGACCAAATAATCTTTATTTACTTCTAGATTAAAAACCCATGA
TTGGTTGGGCCTGGCTCATGCCTATAATCCCAACAGTTTAGGAGGCTGAGGTGGGTGGAT
CACTTGAGCTCAGGAGTTCGAGACCACCTGGGCAATAGGGCGAAACTCCATCTCTACAAA
AAATACAAAATTTAACTGGGCATGGTGGTGGGCGCCTGTAATCACAGCTACTCGGGAGGC
TGTGGCAGGAGAATCACTTGAACCTGGGAGGTAGAGGTTGCAGTGAGCCAAGATTGCACC
ACTGCACTCCAGCCTGAGCGACAGAGCGAGACTCCATCTCGAAAAGAAAAAAAAATTAGC

CAGGTGTGGTGGCACACACCTGTGGTCCCAGCTACTCGGGAGGTTGAGATAGGAGGATCA
CTTGAGCCCGGGAGGCGGAGGTTACAATGAACTGAGATTGTGCCACTGCACTTCAATCTG
GGCAACAGAGTGAGACCTTGTCTCAAAAAATAACAATAATAATAATAACACTATGATTGA
CAGGTAAAGGGAGACTTAAATAAATGGAAGAATACATTTTCTTCATGAATAAGAATAGTC
AGTATTGAAAGGTACTTTCTCCCTTGCTCTGGCCTTTTCCCAGTTAAAATTAACCTGAAA
GGATAAGCAGGGGAGAATATATATGAAAACTTTTTATAAGAAATACAACAACTGCTAGAC
TTAAGGCATATGTTAAAACTTTTTGTAATTGACAGGGTATATGTTTAAGAAAAATCAGAG
ATAGCCAGACTTGGTTGAAAGCATCTTTGATGGCTCAGTCCAAAAGAAATACCTAAAGTT
AATATGTATTAACTAGTAGATAGGACATGTGCCATGGCACCCCAGGATTATTCCCAGTTT
CCATAATTCTCTAGGAGGACTCAGCATATAGTCATACTCATGGCTAAGATTTATTCAGCA
AAGAGAAAAGACACATTGGGAGAACCAGGTACAAGTTTCCAAGATCTCCTTCCCCAGTGG
AGTTACACAGGACGTGTTTAATTCCTTTAGCAGAGTTGTGACAACATAAGGAAGCTCACT
AGAGTCTCAGTGCCTAGGGTTTTTACTGGGCGCTGGTCACATAAGCACCTTCTACCTGGC
ATGTACAAAAATTCCAGACTCTCAGAAGGAAAGATTGTGTTTGGTCTTATAACCAAAATC
TAAGCTCCTAGACATTAGCCAAGGTCCAACCTTGTAAGCATGCCTTTCAAAGAACAGCAG
TCACAGATCTGCTTTGTTAATATGTTAAAATTAACTGTCAGGTTAACTCTTTTCTTCACA
GGTCCATCCAAACAACTTAATAAGTATTTATGTTCTAACCACTTAGTAGCAATTTAAAAT
ACTCATAAATTGAAAGAAAATATATTTTTGTTCCATTCTTTTCTTTTTTTTTGAGACGGG
GTCTCATTCTGTCACCCAGCCTGGAGTGCTGTGGCACCGTCTTGGCTCACTGCAACCTCC
ACTTTCCAGGCTCAAGTGATACCCCCACCTTAGCCTTCTGGCTAATTTTTGTACCTTTTT
TCTTTTAG**AGACAGGTTTCACCATGTTGCCCAGGCTGGTTTTGAACTCCTGAACTCACAT**
GATCCACCTGCCTCGACCTCCCAGAGTGCTGGAATTACTGGCGTGAGCTACCGCACCCAG
CCCTTTCATTTCATTCTTAACCATAATTACTTGCTAATGGGTAATGTGTACCTGTTGACC
ACTGCTTAACTTCTCAGACCTTGGGATCATATTGTACACTGCCACCCTCATTTCCTGTTT
CACACTGATTTTTACATAGTAACTGCTTTTTATCACAGCATTCGATGAAGAAAACAGTTC
TGTAAAGATATGATGTCATCAAACAAATTGTAACCTGATCTAATGTTGAAACTGAACTAC
CTTAAACTGGTAGTTTTCATGGTGTCAAACAGATGTTAAGGGGGCAACTTGGCACAAAGT
TTGGTAACAATGGTTTAAATGATAAAGCTAGTGTCACAAATGTGTCTAGTAGAGGAGGAT
CATGTAATTAATTAACAGGTAACAATTTGCTAATTGTTTAAAATTATATCTGTAATATGA
TTTTAGAATCCAATACCAAAAGTATTTTCAGGTAAATTAAAAGAATTTTAATTTTTAAAG
TAGAAACAGAAAAAAATATATTTGACATTTAATCTCTAGAAATGGAGGTCACATTCTAAA
CCTAGGAATAGTGGTATAAACTTCAATGGAAAAACATTAAATTTGGCTGCATAATAATAG
GAGATTTATGTGGTAAAGTAATCAACATATAAAAGCCAATCTTGCAAATAAAGCAAGATA
ATTTTGTAAATGAAGGCAAGGTAGTCTTGCCTGCCTTGATTGACATAGCAGTGGTCTGAG
CAATGGAGTACGTAAAGGCCATCCAGTTTGGGGGAAGGCTATTGGAACTTCTATTTATAT
TTTTTAAATCCCAAAAATAAGAAATGAAGCTTTATTAATAATGTATACGATTGATAATAG
CATTCTGACTCAATCAACAAGACATGTATGTCACACATGCTATGTGAAGGTATCCTGAAA
GAAAAGTGAGAATCCACATGTTTCCTCACCCATACTTTTGCTTTCAGCAGTTGAATGAAA
GTATGGATATCATCTAGCATTCACCACACTACTCACAAAATGGCCAGGGAGCTTAGATTT
TTGCAGTGAAAGCAGATAATACTAAAAGGCAAACACCCATCATCAACCACAAGAAAGTGA
TAGCGCCGGCACGTCTATTTAACTTCCAGCTCTTTGTTAGACACATCAATGAGATGAATA
CAAATATTAACTACAATAGTCCCCATTTATCTGCAGTTTTACTTTCTGAGGTTTCAGTTC
CTATAGTCAACTGCAGTCCAAACATATTAAATACAAAATTTCAGAAATAAATGATTTAAA
AGTTTTTTAATTGCACACCATTCTGCGTATAGTGATAAAATCTCATGCTGTCCTGCTCTG
TCCAACTGGGACGTGAATCATCTCTTTGTCTAGCATATCTTCATTGTATATGCTACTCTC
TCCCCATCATTAGTCACTTCATAGCTGTCTTGGTTATCAGATCAAATGTAGTGGTTAGTA
GTGTGTATCCAAGTTACCCTTATTTTATTTAAGAATGCCCCCAAAGTGCAAGAGTAGTGA
TGTTGGCATATTGTTAATCGTCCTATTTTATTTTTAGTTGTTAACCTCTTACTGTGCCTA
ATTTAAAAATTAATCTCTATCATAGGTATGTATGTATAGGGGAAAACATAGTATAAATAG
GGTTAGGTACTACCTGCAGTATCAAACATTCACTAGGGGCCTTGAAACATATTCTCTGAA
GGTAAAGGGAGACTATAGTTATCAGATCTGATAGGAAATTAGGCATCAAAATTCAAAATT
ATCAAGAATACTCTTTGAAATATCAGTTCACTTCCACTTTTATTGATGGAGAGCCTTACC
CTAAGTGCATATAATGTTTCAAGGTATTTGCTAATGATAATATGAAGCCATTATGATTAG
CTAGCAGTTTTTCCAGTCGTGTTTAAAGTCATTTGATAACCAAACCAGGACTTTGAAAAT
TTTTCTAAAGGTAACAATAGGGCCAAGCATGGTGGCTCATACCTCAGCTTTTTGGGAGGC
TGAGGTGAGAGGATAACTTGAGACCAGGAGTTCAAGACCAGCCCATGCAATACAACAAGA
CTCCGTCTCTACAAAAAAAAAAAAAATTTTTAATTAACTGGACGTGGTGTTGCACACTCA
TAGTCCCAGCAACTCAGGAGGCTAACGTGTAAGGATTGCTTAAGCCCAGGAGGTGAAGGC
TGTAGTGAGCTATGGTTGCACCACTGCATTCCAGTTTGGGCAACAGTGAGACCCTGTCTC
AAAAAAATAATAATAATAATAATAATAGGCTGTGCTCAGTGGCTCATGCCTGTTGTAATC
CTACCAGCCAGATTGCTTGAGCCTAGGAGATCGAGACTAGCCTAAGCAACATGGTGAAAT
GCCATCTCTACAAAAAATGCAACAATTAGCCAGGAGTGGTAGGGCACACCTGTAGTCCAG
CTACTTGGGAGGCTGAGATGGGAAGATCGCTTGAGCCCAGGAGGTTGAGATTGCAGTGAG
CCAAGATCATGCACTCTAGCCTGGGTAACAGACTGAGACCCTGTCTCAAAAAAAAAAAAT
AAAAAATAAAGGGTAACAATAAATATTTGCAGGCCTATTTTGTGAGATTGCTTAAACAAA
AAACCAAAAAAGCGACATATGATTGGGAAAACTTGTTCTTTTTGCTGTGATTAAAATGGC
TTCACTATTACACAGAAAGCATAACGATGACAGTACAAAATGAACTCTTTTGTCAGCAAA
TACTGATGCAGAAAACACTGCTAAATATTATAACATAATACACAATGTGGGAGGTTTATT
GTATAGTTGAATAAAAATACAGATGTTTCTAATGGCTTAGCTTAGAGTATTTGCTAAATT
GTTCCAATAAAATGCACAAAGAACTTTTTGTAAACCTCTGAAGGAAAGAGAACAAACATA
TTCAGTAGGAAAATTAATTTTTTTAAATGTCTTACAGAAAACTAAAATTGTAAGAACTGA
CGGAATATCTGGTTTGATGGTTGGGGGGCTGACAGAGTAAGGATGTCAAGATACCATTTC
ACAAATGGATTCCCTGGATCATTCCTAGGCAAGCTTTTGAAACAAAGAAGTTAAAGCCTT
TTTCAAGGGTCATTTCAAACCATTAATCAAGACTCAGATTGAAGCTGACCCAAATGACAC
CTATTCTTTGGGATTTCTTTCTAATTTTGAATTAAAAATAGTCACCCTGAATTAGTATGT
ATATCAGCTAGGAAGTGTATTCAATCCAAGTAACAGACTTCCTTTCCCTATCTCAATATA
TTAATATATATTGTATATATATTATCCCAATATATATTGGGAGTGATTGAGAGTCACTCC
TATTGTATTCACAACGTGGCTGCTGGAGCACATGCCACAATGCCTACATTTCAGTCAGAA
AGAAGGGAGAGAGTGTTGGGCAAACAAAGCTAGTACCAGCTGAGTTTGCCTAACAACTTT
TACTTACAACTTTTTGGCTAGAATTATGTCACATGACCACCCCTACCTGCAAGGGAGTTT
GGGAAATGTAGTTTTCTTTTTATTGCTTTCCCCTCTTGTATTAACTTTGCCTATTTAGTT
ATTCTGATACTACTCGTCTGAAATATTCTTTTTTAAAAAGACAAATTTATGAGCCAAAGG
TTTACTCAACTAAAAATAATAAGGAAAAAAAAAGTCTGAAAGGTGAGGTAGGAAAAAAAT
ATTATTTTATATTTAATGGGTTTACTGTTAAAAGTTTTGTAAACTCATTTTAAGTGCTAC
ACATAATATAGTAACACCAATAATAGTAGTTATAAGTTAAAAGTTATAAAATGCTTATTG
CCAATAACTGTTTTACATGCTTTTTAAACATTAACTTATGACCTAGTGGGTTGTTTTTGA
GACACTGTCTACTGAATTAAAAAGTTACTTGAGGGCCGGGTACAGTGGCTCATGCCTGTA
ATCTCAAAACTTTGAAAGACTGAGGTGCATGGATTGCTTGAGTCCAGGAGTTTGAGACCA
GCCTGGGCAACATGGTGAAACTCCATCTGTACAAAATATTTAAAAATTAGGTGGGCATGG
TGACACATGCCTGTAGTCCAAGCTGCTAGGCAGGCTGAGGTGAGAGGATCGCTTGAACCC
AGGTAGTGCAGGCTGCCCTCCAGCCTGTGCGACAGAGCAAGACCCTGTCTCCCACCGCCC
CCCACCAAAAAAAAGAAAAAAATTAAACTCAAAGTTAACGTTAAGTTCTGTTCTTCCATT
TCCTTGTTGTATGTGTGTATTTGGCAAGGCACTTAATCTCTCAAAGCTTCAGTATCTTAC
CTTTAAAATGAAATTAATAATACCTCAAAAACTTGTAAGGTTTAATGACAATAATGTAAA
GTGTCTAGTGTGATGGGACCAGTGTGTAGTAAAGACTCAATAAATGGCAATCACTATTTT
ATACATAAAATTTTACATATTTTATGTATGTGTTCATATAGAATGTTATGGTCAATAAAA
AAGCAAGATTCAGCCAGGCGCAGTGGCTCATGCCTGTAATCCCAGCACTTTGGGAGGCCA
AGGCAGGTGGATCACAAGGTCAGGAGTTCAAGACTAGCCTGGCCAATATGGTGAAACCCC
GTCTCTACTAGAAATACGAAAATTAGCCGGGTGTGGTGGCGCACACCTGTAGTCCCAGCT
ACTCAGGAAGCCGAGGCAGAAGAATTGCTTGAACCTGGGAGGCAGAGGTTGCAGTGAGCC
GAGATTTTGCCACTGCACTCCAGCCTGGGTGACTGAGCAAGACTCCATCTCAAAAAAATA
AATAGATAAATAAAAGGCAAGATTCTAATTCTTTTGAAGCTTTTTTTTAAGTCAATGGAA
AAATAAAACACAAATTTTAAAAAGCCACCTTTGTTTTATATATTTTTCAG**ATCAATATTG**
AACTGTACAGATTGTTTGGCATAAAGCAAATTGTAATTTCTCACACCTCTGGAACTGTTA
ACCCAACGGAAAGTACACCTAACACAATCAAAGCATTTCTCTCCAAACACTGTGCTGACC
ACCCAAACAATTGGGATGATCACCTATCAGCTGTTTCATTTGCCTTCAATGTAACTCACT
TGGTATGTGCCTTTTTATAATTCTGTACTTCTGAGTTATGACTATTTCTTGGCCAGATAA
ATGTTTTATTACAAAATTGACGTTTATTTTCTTTAAAATAG**GAACCTACTAAAAATACAC**
CATATTTTCAAATGTTTAGTCGAAATCCTTATATGCCTGAGACTTCAGATAGTCTTCATG
AAGTGGATGGTGATAATACAAGTATGTTTGCCAAAATTCTAGATGCAATTAAAGAAGCTG
ATAAAATAATGGAGAATAAGACAACTTCACTGGGCCAGGTGATTCTATTCAATAGAAAAA
CTGTAATCTAAAACTTTATAAAAGTTATATTTATTTTTAAAATTTCAGCATATACATGTG
TGCATGCACACACACACACATACTTACTTGCCATAATTTTTTATCTATCAGATTTTTCTT
GTCTTGATTAAATAGTTGGTTTACTAGCTATGAAATGCTGAGAGGCAATAATGAGAAAGC
TATGGATTTTGGAATCAAGTAGAGCTGGATTCAAATCTGTAGTCTTAACTGCTTTTGAGT
TGTTTGACTTCGGCAAATTACTTAACCTATCTTAGTTTACCTTCTATATAAAGTAGGAGC
TAATAATACTGACTTTGAAATGTTTTAAGAATTAAATAGAATTCATACCTACCTTTTAGT
AGCATCTCAAGTACCATTTTGTTACAATATTCTCATTATTTGCATTTACCTTCAAGATAT
TATAGCTCTTTCATCTCTTTGTTCTTTTTCTTTTTACACTTGTAG**ATGGAGAACAACAAT**
TTGGATGAACTAAATAAAAGCAAGATCATTGTTAAAAAGAAACCCAAACAATTAAATCCA
TTTCATTTAAAAGTGGGTCATGAAGTTTTAAGACAAAGGAAAAATTGGTGGAAGGATGGT
CGTTTTCAGTCTGAATGGGTTGGTCCTTGTGTCATAGACTATATTACAGAAAGTGGATGT
GCTGTCCTGAGAGACAACACTGGGGTTAGACTGAAAAGACCTATCAAAATGTCCCACCTT

AAGCCCTACATAAGAGAATCCAGTGAACAAGGTAAATATCTGTCACTTCTCTACATTTTT
ATTGCTTTAGGTAAGGAGAATAGTTAAAAATAACAGAAACCTTTCTCCCCTCTTTTGTTA
GGTTTTCTTTTCCCTTCTTCATAAAATTTGTTACAGTTTTAGCTATCCCAGGTAGTCTGT
TTTATTACAGTGTCTCTTCTGGAACTGTCAGGCCTGTGAGATTTTTTTTTTGAGACAGAG
TTTCCCTCTTTCTGCCCAAGCTGGAGTGCAATGGCGCGATCTTGGCTCACTGCAACCTCT
GCCTCCCGGGTTCAAGCAATTCTGCCTCAGCCTCCCGAGTAGCTGGGACTACAGGTGGAT
ACCACCATGCCCAGCTAATTTTTTGTATTTTTAGTAGAAACGGGGTTTCACCATGTTAGC
TAGGCTGGTCTTGAACTCCTGACCTCCCGTGACCTGCCCGCCTTGACTTCCCAAAGTGCT
TGGATTACAGGCATGAAACAGGGTGCCTGGGCATTTTTATTTTTATTTCTAGTATGTTTT
TCATAGTTTATGTGGGAGATACTGAACTTGTTTTCCTCTGAAGGAAAGATAACATCTTAA
CATCATCTCTGTTTTTGCTTTCTCAATGTTTTGTTTGTTTGTTTGTTTGAGACAGAGTCT
CATTCTGTCCCCCAGGCTGGAGTGTAGTTGTGTAATCTCAGCTCACTGCAACTTCTGCTT
CCTGGGTTCAAGCAATTCTTGTGCCTCAGCCTCCCAAGTAGCTGGGATTATAGGCACATG
CCACCATGCCCAGCTAATTTTTTTGTATTTTTGGTAGAGATGAGGTTTCACCATGTTGGC
CAGGCTGGTTTTGAACTCTTGGCCTCTAGGGATTTGCCTGCCTTGGCCTCCCAAAGTGCT
GGGATTGCGTGCATGTGCCATTGCACCTGGCCTTACAAATGTATTAGTCACTTCGTGCCT
CTTTTTGGCATGAAAAACAGCAACTGTGATCATTAATGCCAAAAACAAACATACTGTCCA
GAATCTAGTTGTGAGATAAATATTAGGGTACTTTAAAAATGACATTAATTTTTAATATTG
CTTTATAGTTAGGTCATGGTTGGGGGCCAGGTGTGGGTGCAGAGTGGGGAATCCTCAGAA
TGCTATAAGGTGCTGTAAGAAGAGGTGACAGCACTCTAAACAAGTTATATTTGCCCCTAA
ATTTGACTCATTCTGAACAGTCAAGATACCCTAGTAAAGTGATGTTTTTGCCATGGCTTT
TAGATTATGTTCCCCACTTTATCAAGGCTTTTATTTTGTCCTAATGTGTCTGCTTCCTAG
ATCTTCTCTGAGGTGGATAACTGAGAGAGAGTTAATGAGTAACTTAGCCAGGTGAAACAT
TAATATTATTTGGATATAAGTAACATTTAATGGAAAATATATTTATAACAATATAAACAA
GTTATATGACAGGCTTTAGCAAATATTAATAACAAAAAGAGTAACAACAACAAATAACCT
ACACTTACCCTATGCCTTTTCTTGAGTTCTAAAGCCCATGTGGGCTGCTTAGTAATGATA
GCTCAACTCTGCTTCTAATCTGGGTTACAACACTCATGTTAAAACCTCTTAGTACCTTTC
CTTCTTGTGACAGACCGAGTTGTTTAGTCAGTAAACTTATCTTGAACTACATTCTAATCC
TGAAAACTCACAAGTGCTTTCGTTCTCTTTGTTAGAAGATAAAAGCTTGCATTGTTTGTA
CCTCTTCTCAGTTCTGAGCTACCTTTAATGATCTCTCTCTTCTGCAAGTTAAGTAGGGAA
AAAAAAGGTTATACAAGTATATGTTGTTCTAGCTTCTTTAGCATGATTTTCCCCTTAATC
TTTGACATTCATGATTTGCTCTCTTCTAAAGTGTTAAGCTTCTTTAATACAAAATTTAAT
TTAAAATTTTGCTAAACCAGGATCTATCAACATTTTGTAATTTGTTCTTGTCAAAGAGTT
TCTCAGAAAAGAAGTCAAATATTGTGCTTCTTCTGAACATTCAGAACTCAGTATTTCAGA
ACTCATCTTCTCTGTTTGAGAACAGTCTCTAAACAGAGGCAGTATAATATTTGCCTCATA
CTTGAATTCTTTGCACAAGATTCTTCCAGTAATATAAGGACAAATAATATTGCAATATGA
CTACTGGTGTATGCATAAGGAATTTAAGACTAATTAGATGAGTCTGTTTTAGTTTTTCTT
GTTAACTAAAGGAACTACTGATCTGGCCATCCTAGAATCTGGTTCTTATTGCTTGATCCT
TTTTTTACATTTTTCTCATTTGTAGAAATTAAAAGTAAATGTGATTTCTGCCATCTTTTT
CCTCAATATTACATACTTTCTTGAAAATCTTTTCCATGATTTTCTTGAAAATCACCTCAC
AAGTTGATTTGAAAGAGTCCTTGAGCTGAATTAAAGCAAACAAAATGATTTTGACTAATC
ATTGAAGTTTTTATTTGACATAAATTTTGACATGCTATAGCCAAAAGAACTATAAATTTA
TTTGTCCACATTTGTCATTGCTGCTCTTTGCTAGCCTTGAAATTTCATAGCTCTGTTAAA
AACTCACTGTCTAAAGTAGATATACTTCTATTGCCAACTTCTTATCCATTCTTATCTAGA
ACTAAATGTAGAGTGAGCACATTCAGTACTAGGTATACAATATTTTTCATTGCTATTTTA
AGCAGGCCTGTTTGTTTTTTTGTCTAGTTCATTGCCAAGCGCTTTTTCAGACTATTTAGA
CCAAGAAATCTTAAAATACCATAGTGTCTTTTTGTAATCATAATGCTCCTCTTTGAAAGG
AAAAAATAATCTGACATAGATTTTGCACAGTTTTGAATTTATTTTGTTTGTGATAGATTT
GGATCTGCCTCACCCAAGGTTTTAGCCTGATTTCCCTTCACAGTTGAAAGCCCCATTTGT
GAAACCATTTTCATTATTGTATAAGTTTCCTCTTGTTGCTGTAACAAATTACTACAGATT
TAATGGTTTAAAACAACACACATTTATTATATTACAATTCTGAAGGTTAGAAGTTCAACA
TAGATCTTACTGGGCTAAAATCAAGGTATTGGCACTGTTGTGTTCCTGTCTGGAAGCTCT
AGAAAATAATCCATTTTCTTGTCTTTTCCAGCTTCTAGAGGTTACCTTCATTTCTTGGCT
TGTGGTCCCTCTATCTTCAAAGCCAACAATAGTGGGTTGAGTCCTTCTTACATCACCTTA
CTCCAACCTCCTCTTCTGCCTCCCTCTTCTATATTTGAGGACCCTTGACCTTGGCCCTGC
CCAAAAAGTCCAGGATAATCCCCCTATTTTAAGGTCATCTGATTAGCAACCTTAATTCCA
TCACTACCTAAATTCTCCTTCACCATGTAAGATTACATATTCACAGGATCCAAGGAGTAG
TATGTTGACATCTTTAGGGGCCTATTATGCTGCCTACCACAATTATGGTCTGCATTCATG
AAACAAAGCAAAGCACAGTCCCTATAGTGTCAAGACCATGATCTTGACGTTAATATAGTT
TATGCAGAGAGCCTTTTTTTGGCCTCTTAGTTATATCTTTTTTTTTTCTTTCTCTTTTTT
CCTTTTTTTTTTTTTTTTTTTTTTTTGAGACAGGTTCTTACATGGTTGCCCGTGCTGGAG
TGCAGTTGCACAATAATAGCTCACTGCAGCCTTGACTTCCTGGGCTCAAGCAATCTTCCC
ACATCAGCCTCCCAAGTAGCTTGGAATGTAGGCATGCATCACCATGCCTGGCTGAGTTTT
TCGTTTTTTATAGAGACAGGGTCTTACTATGTTGCCCAGGTTGGTCTCAAACTCCTGGAC
TCAAGCAGTCCTCTCACCTAGACCTCCCAAGTGTTGGAATTACAAGTGTGAGCCATTGTG
CCCGTCCAGTTATATAATTTCTTAACCAATAGTTACTCAGTTATCTGTTGACAGTATTAG
TTATTATAATGACAGTGATTTAGTATCAAGTTATAGTTATTATTTTATCTTGAGAAAGGA
TATACTAGTATGGCAGCACATTCTAATAGTTTGAGGATTATCAAACCATATATAGGATTA
GTTTAGTCACTTCTCTTTAAAATAAGAAAATTAAGTAGTCTGAAACCTTTATCTTTTTTT
TTCTGATTGAAGAAATACATTAATCTAAATCAGACTTTTTTGAAGCTGTAATTTGTATTT
ATTTTTCTCAGGGAAATTTAAAAATTGATTTTTTAATTATTATTTTTATTCCTTAAAACA
ACATTAAGGCTGGGCACAGTGGCTTATGCCTGTAATCCCAGCATTTTGAGAGGCCGAGGT
AGGATTGCCTGAGCTCAGGAGTTCGAGACCAGCATGGGCAACATAGTGAAACCCCGTCCC
TACAAAAATTAGCCGGGCATGGTGATACGCACCTTTAGTTTTAGCTACTTAGCGGGCTGA
GGCAGAGGATCACTTGAGCCCGGGAGACTGAGCTGCAATGAGCCAACATTGCACCGCTGC
ACTCCAGCCTGAGTGACAGAGTGAGACCCCATCTTTTTTTTTTTTTGAGATGGAGTTCAA
CTATTATCACACAGGCTGGAGTGCAATGGTGTGATCTTGGCTGCCTGCAACCTCCGCCTC
CTGGGTTCAAGCAATTCTCCTACCTCGGCCTCCTGAGTAGCTGGGATTACAGGCGCCCAC
CTCCACACCCAGCTAATATTTGTATTTTTAGTAGAGACGGGGTTTCACCATGTTGGCCAG
GCTGGTCTCAAACTCCTGACATCAAGTGATCCACCCGCCTTGGCCTCCCAAAGTACTGGG
ATTACAGGCGTGAGCCACCATGCCTGGCTGAGACCCCTGGGATTATAGGCGTGAGCCACC
ATGCCTGGCTGAGACCCCATCTCTTTAAAAAAAAAACCTACATTAGAAATGGATATCAAA
ACTGCTGATTAATACCTTCCTGAAATGTCTTGAAAATATTTTAATTTCAAAGAAACTAAA
GACTTATTCAGTCTTGTCAAAACCAAAATGATGCTTTCATCAAACAGATGTCATAATCAT
AACTAAATTAATGGAATGAGAAAAGATAAATACTTAGTGAAATGTACTGTGTAATGTAGC
TGACTAGAAAAGACCTTTAATATGATATGTTTGCTTACTCCCCGCCTCTGTTTTGAGGGC
AGAAAGTTTAATCATATTCTTTTCTGTATCCCTAACTCATGATTGTTTTAGATTAGTGTT
GTCCAATAGATGAATAATAATACAAGAGCTATGTAATTTAAAATTTTCTAATAGCCACAT
TTTGAAAAGTACAGAAATAAATGAAAATAACCTTAATAATATATTTTATATAATCCAGTG
TATCCAAAATATTACTTGTTCAACATGTAAGGCCACAAGAGTCTCTTTTCTGATCTCTTT
ACTGAAATTAAAGAGCATTTCTCTTAGCTCCAGTAGATTTAGACCATAGTAGGAGTTGAA
ACATATTTGACCTTTGCAAATGAATGAGAATTTTTTTTGTAAACAAACAGGCCTTTGACA
TATTCAGAGTGGTTTTCTATGTGTATAAAGTGATTTATGTAGAATTTGCTGTGTGTAGTC
CTTGTTCTCTACAGTTTTATTACAATAAGTTACTTTAAAGGTTAACTTTTTTTAGAAGAA
AAAGAGTGTGTGTGTGTGTGTGTGTGTGTGTTCGTGTGTGACTTATTTTACTTACATACA
GAAGCTCTCTTAAAATCAAGTGAGGTTGGTATGTCCATTTGTTTCTAACCAGTGTAACTA
GGAGGTACTCAAGTTCAAGGTCTCTGAATCCATTTCAGCAAATTAAAATGAAAATTTCTA
AGAGCTTTACATCTAGGCATATTTTTCCCTATTGACATATGTTTACTAGACATATTTTTC
TAAAGTAGTCCTACTTCATCTGTATGAAAAAACTTTTACAGACAGAAAACCTCCTTCATT
AATCCTCTGAAAGAGTCTTTGGGCATTTCTTAAATGCCTATGGTAACTGCCATCACTGAT
AACTTTGTAGGAATGTACAGGCCTTGAACCTATGGAATTCTCCATGACTTAGTAGAAAAT
TTCATCAGCAACATGTTTACCCTATCTAACCTTTTAATGTTCTCTTATAAACTACCGCCT
AGCCTTCTCTCGTATTAACAGTAGGGTAGTTGCAAACTTTGATGTTAAACCTCTAGCAAC
ACATTCTGAAAGTGTTTCAATGTTATTGATCATGTTTTCTTCCCTCTTGCTGCTATTGGG
TATAACACATCTCATGTGTTGCATGATTTTCGTTGTTGTTGTTGAGACAGAGTCTCGCTC
TGTCACCTAGGCTGGAGTGCAATGGTGCGATCTCGCCTTACTGCAACCTCCACCTCCTGG
GTTCAAGCAATTCTCTCACCTAAGCCTCCCCAGTAGCTGGGATTACAGGCACATGCCACC
ATGCCTGACTAATTTATGTATTTTTAGTAGAGATGGGGTTTCACCGTGTTAGCCAGGTTG
GTCTCGAACTCCTAACCTTAAGTGATCTGCCCGCATTGGTTTCCCAAAGTGCTGGCATTA
CAGGCATGAGCCACTGCCTGGCTGCATGATTTTTATTTTATTTTATTTTTTTTTTGTCTTT
CAGAATTATGGGAGAATAAATGGCCATACAAGCCCAAGCCCACTTATGCCATCTTTTGGT
AGAGAGAATAGCTTTGTGTGATCTGTTAAAGTTTATAAATTGAAAGTTGTTAAATGCATG
AATTTCTGGGGTTGAAATAGGTTCAGTGAGCTATTTGTAATAATGTTATGAGGGTGAGTA
CAGTAGGATTGATTTTCAAAATATACTACCAAATTCATCTGTTCTGAGACACTTCCATTG
GTCTCTAATCTAAATAGGCAATTAATCTTCCTTTTGCTTTTTTGGTCTTAAAATTTGAGG
GCTCCTTAACCCATTAACATCTTCAGGAGACCAACCTAGGCAACACAATTAGCCAGGCAT
AGTGACATGTGTCTATGGTCCCAGCTATTTGGGAGGCTGAGGTGGGAGGATCACTTGAGC
CCAGAAGGTCAAGGATGCAGTCAGCTGTTATCACGCCATTGCACTCCAGCCTGGGTGACA
GAGCAAGACCATGAATGAATGAATGAGTGAATGAATGAATGAATGAATGAATGAAAAATA
AGTAAATAAAGATAAATAAATATATCTATATCTATATTCTGATGTTTTGGGGGAACATTT
AGAAAGAGAAGGTAGGGATCCAGGAAGTTAATCAGCTTTGGTAAAAGAGTTTCTTATTGT
AATGAATTCAAATATCAGAAAGGGAGGAATCCTTTTATACATATTAGTCTGTGTATAGAA
GATTTGCCATCAGTTTAACATTCATTTTAAACATTTTTTTCTTGATGGTGATGATAAATT

```
TGCTCAACATACAATAAAACACTGACCAGGAAGACTCCCTGTCAGTACTTCAGTATGGCT
TAGGTAGACCTTAAAAGTTCCTAAGAATTTTGGTATCAAATTAGATTTAAATAAGCTAAG
CTTTTTAAGCTACTCCATGGCAGCCTGCCTTCTTACACATTATCACATAAGCCTCAGTTG
GCATGTTGTTTTTAACTTCATACTGTAACAATTTGATGATATTATCTTTCCAATATGGGT
TTTATAAAGATTGGAAGATTATCTCAAGTTGTAAATGCAAGGATTTAATAATTCTATTTG
TTAAAATAATTATATGACCCTATTAATTCACCCAGAAATGCTTTACAGAAGCATATTAAG
GATAACATTATCAAAAATAGTTTTATAATTTTTTTTTTAAGACAGGGTTTTGCTCTGTCA
CCCATGCTGGAGTGCAGTGGTGCCATCATGGCTCACTGCAACCTCAATCTCCCAGGCTCA
AGTGATCCTTCCATCTCAGCCTCCTAAGTAGCTAGGACCATAGACACCCTTAACCACACC
TTGCTAATTGTTTGTATTTTTTGTAGAGAAGGGGTTTCGCCATGTTGGCCAAGCTGATCT
TGAACTCCTGGGCTTAAGCAATCTACCGGCTTTGGCTTTCCAAAGTGCTGAGATTACAGG
CATGAGGCACTGTGCACAGCCTTATTATTTTATTTTTAATCTATACCATAATTCTCACTC
CTTCTATCCCGTTGTCCGCATATATACATATCTACCAAAGACAGGTTGAGGGAACAGGAA
AATGGCTGTTTTAAAATCCTTGACCTTTCCTGCATTGTTTATGTATGAACTGTCAAATTT
ATATGTCATTTTTTTATTCTTTGACATTCATATTTAGTAGACAGTATAACTCCCTAGAAG
TTAATCTTGATAGTTAATTTTTATTACTTGGGATTTTGTTCCTCACTTAAAATTGTTTAA
TTAAAAAATATGTTTAATTATAATTCTAAAAGTCAAATGTATGATTTTTCTTTTTTCAGA
AAGTCTTTATCTCTTGCAAGGTTCAGTAGTGGCAGATCATGACTACATTGGATTGCCTGA
AATTCCGATTGGAGCATATCAAGCAAATATTCTGGTGGAAGATGCAACTATTGGTATAGT
CGATAATGAATTACTGACATCAAGCAAGGATCGTGAACTATTAGAATATAGAAATACGAA
AATCTCTCCATTGATAGACGATCATAGTTCTCTTGAAAAGCAGACTTTCAGTCTGTTGGA
CTCTTCAAACCAGGTTCTTGAATACTTAAGTTAGTAAATACCAAAATTTATTTAAATTGT
TTGTTTAGAATATAAATTCTTAATGATATCTTATTCATTAAAAACGTTGTATAGAAGAAG
TATCTTGACACATTCTTAATGACTATATTCTGAAGGTTTATTTTATAGTTCTTATCTAAA
ATTATGCCACATTTGAATATCAGTATACGTACATATGAAGGCAAAGGAAGCATGTAATAT
ATAGAATTCACTTTTTCTTCTAAAAAGAGAACAGGCAAAGCACCCCTACTTCTTGGGAAA
TAAATTCCAGCCCAAATCTCTCAAACCTTTGTATGGTTTTGGCACCGTTTCTAAGTTTTA
AATTTTCTGAAATTCTCTACCATTTAAACAGTAAGTTTGAACTACCTTGAATGGAATTGA
TAAATACTGAATCAACTGTAACAATGAATGAAATGAAGCTTCTTTCTGTTTCATTAGAAC
TTCTAAAAACAAGATTGTATGAAAAAATATAGGGTTATTTAATAGGCTTTCACAGAATAT
TGGTAATTTCAAATTATCTAATTGGTTAGATAGGGTCAGGATTAGGGTCTGAAACTATGT
ATTGGTTGGGAAACAATTTGTTTATTTTCATACTATATATTAAAGATAAAACCATCGACC
AGGCATGGTGGCCCACACCTGTAATCCCAGCGCTTTGGGAAGCCAAGGCAGGATGATCAC
TTGACCCCAGGAGTTCGAGACCAGCCTGGGCAACACAGGGAGACCCTATCTCTACAGAAA
AATGTTTAAAAATTAGCCAGGTGTGGTGGCACATGCCTGCGGTCCCAGCTACTCAGGAGG
CTGAAGTAGGAGCATTGCTTGACCCCAGGAGGTCGAGGCTGCAGTGAGCCGCGCTCATGC
CACAGCTCTCCAGCCTGGGCGACAGGGCAAGACCTGTCTCAACAACAACAACAAAAGTAT
CCGTTAAGAATTTTTTCTGCAATCATAAAATCATAAAATAACCAGTATCAAAGATTTTAA
ACTGTAATTTCTATGATATTCATAAATATAAAATAAATATTGTTCAATAAATATTTATTC
TAAAGCACTATGAAAAGAACTGTGGGAAAAGTAAAAGATGAGTCAAACGTGTACCTCATT
CTTATAAAGGACTTACTGTCTGGTAGGTAATAAACTATGTAAGCCATGTACAGCACAAGT
TAAGGCATGCTAAATTGCAGAAGACAGGTGTAGATAATGTACTTAGGGAGGCAAAAAGAG
AGAAGTTGTTTTTACATAGGAAATTTGTGTTTATCCTTATGGAAGAAGGATCATTTAAGC
TTGACCTTGAAGAATGTATAGGATTTGGTAAATGGATACAAAAGAATTAGGGGAGAAGTA
ATACACAGAAATGGAAAAGTATTGTAATGATTCATAAAAAGGCAGGGCTTCTTGTTGTCT
ACACTGCCTCGGGTATAAAGTGAAGAAGCAAGATAATGAGGGATAAAGCTGAAAAGGTAA
GTAGGAAACCTTATGGAGAGCTTTGAATACTATTAAGGATTGATATGTGCCCCTCCCAAA
ATTTGTGTTGAAGTCTTAACCCCCACCACCTCAAAATGTGACCTTATTTGGAAATGAGGT
TGTTGCAAGTGTAATTAGTTGAGATCATATTTCAATAGGGTGGGCTCTAATACAGTATTA
CTGGTGTTCTGATAAAAGGGGAAAATTTGAACACAGACTCAAGGAGAATGCCATGTGAAG
ATAAAGGCAGAGTTTAAGATTATGTTTCTATATGCCAAGAAATGACAGAGATTGCCAGAA
AACTACCAGAAGTTAGGGGAGAGGCATTGAATACATTTTTCCTCATTCCTCAGAAGACCA
ACCCTGTCAATACCTTATCTCAGACTTTTGCCTCCAGAACTGTGAGACAATAAAATTCTA
TTGTTTCAGCCATCCAGTTTGTGGGACTTTGTTACAGTAGCTCTAGCAATAATACAAATG
CCTGGCTAAATTATTTCTTTTCTGAGAGCAAAAACTTTGTATAGGAAACTGTCATAATCA
GAACTGTGTTTTTGGAAGTTTAATCTGGCAACTAGTAAAGAACAGATTAGAAGAGGAGAA
AACAAGGCATGGAGATTAGTTGAGAGGACTTCGCAGTGATGACGAATACTAAAAACCTAA
ACTGGGCCTGTAGCATTGGTAATGAATGGAGTCTGTCTTAAGATCAGTACAGAATATCGC
ACATATTATATTTCACAACCATACTGAACAGTGATGTGATATCAAAGATGGAGACCTGGA
GTATTCTGATTACTCATTTAAGAAGAAAGATTATGTTTAATATAGGCTAAAGGAAAAGTG
TTTAGGGTAGGGTGTGAATCTAGGACCTCATCTTATACCACAGACCAAATAAATTTCAAA
CAGAATTCCAGGAAGAAAAAAACAAAACTTTTTTTAAAAAAGCGTAATTGTATCAAACAG
TGCTACCTATCCTGCTTTCCTCAGAAGAGCAAAGTTTAGAAAAGAGTCATATTAGAATAT
CTTTTTGGTAAA
```

```
==================================================
Human Gin-1
total length:      35352 bp  (35352 bp excl N/X-runs)
GC level:         36.33 %
bases masked:      15498 bp ( 43.84 %)
==================================================
               number of      length   percentage
               elements*    occupied  of sequence
--------------------------------------------------
SINEs:                22        5261 bp   14.88 %
      ALUs            18        4632 bp   13.10 %
      MIRs             4         629 bp    1.78 %

LINEs:                11        4672 bp   13.22 %
      LINE1            4        2869 bp    8.12 %
      LINE2            7        1803 bp    5.10 %
      L3/CR1           0           0 bp    0.00 %

LTR elements:          7        2100 bp    5.94 %
      ERVL             3         590 bp    1.67 %
      ERVL-MaLRs       4        1510 bp    4.27 %
      ERV_classI       0           0 bp    0.00 %
      ERV_classII      0           0 bp    0.00 %

DNA elements:         11        3108 bp    8.79 %
     hAT-Charlie       7        1757 bp    4.97 %
     TcMar-Tigger      3        1094 bp    3.09 %

Unclassified:          0           0 bp    0.00 %

Total interspersed repeats:    15141 bp   42.83 %


Small RNA:             0           0 bp    0.00 %

Satellites:            0           0 bp    0.00 %
```

```
Simple repeats:        4           131 bp    0.37 %
Low complexity:        9           226 bp    0.64 %
==================================================
```

SW score	perc div.	perc del.	perc ins.	query sequence	position in query begin	end	(left)		matching repeat repeat	class/family	position in repeat begin	end	(left)	ID	
188	32.2	6.8	0.0	UnnamedSequence	2638	2696	(32656)	C	L2c	LINE/L2	(1)	3425	3363	1	
473	31.4	4.4	4.4	UnnamedSequence	2850	3032	(32320)	+	MIRb	SINE/MIR	23	205	(63)	2	
1349	21.9	4.3	1.3	UnnamedSequence	3049	3353	(31999)	C	Tigger3d	DNA/TcMar-Tigger	(1)	320	7	3	
30	66.7	0.0	0.0	UnnamedSequence	3369	3398	(31954)	+	AT_rich	Low_complexity	1	30	(0)	4	
1981	23.2	8.9	0.3	UnnamedSequence	3399	4706	(30646)	C	L1ME1	LINE/L1	(217)	5962	4549	5	
273	32.0	10.9	0.0	UnnamedSequence	4859	5014	(30338)	+	L2a	LINE/L2	1641	1813	(1606)	6	
2519	15.4	3.7	2.4	UnnamedSequence	5039	5529	(29823)	C	L1MB3	LINE/L1	(3)	6180	5684	7	
363	29.6	9.2	1.6	UnnamedSequence	5538	5657	(29695)	+	L2a	LINE/L2	3298	3426	(0)	6	
194	34.9	5.8	10.5	UnnamedSequence	6577	6765	(28587)	C	L2c	LINE/L2	(214)	3173	2993	1	
1771	17.7	2.4	5.8	UnnamedSequence	6842	6930	(28422)	+	MLT1A0	LTR/ERVL-MaLR	1	88	(277)	8	
2301	12.0	0.0	0.0	UnnamedSequence	6931	7230	(28122)	+	AluSz	SINE/Alu	1	300	(12)	9	
1771	17.7	2.4	5.8	UnnamedSequence	7231	7517	(27835)	+	MLT1A0	LTR/ERVL-MaLR	89	365	(0)	8	
395	36.2	4.6	3.0	UnnamedSequence	7684	7942	(27410)	+	L2c	LINE/L2	3123	3385	(2)	10	
215	32.1	11.4	2.1	UnnamedSequence	7962	8136	(27216)	C	L2c	LINE/L2	(114)	3273	3083	11	
252	13.9	0.0	0.0	UnnamedSequence	9805	9840	(25512)	+	MER5C1	DNA/hAT-Charlie	228	263	(0)	12	
2421	21.8	3.6	1.7	UnnamedSequence	9931	10923	(24429)	C	L1M5	LINE/L1	(424)	5738	4728	13	
297	31.7	12.6	0.0	UnnamedSequence	11427	11593	(23759)	C	LTR33	LTR/ERVL	(7)	508	321	14	
444	30.2	1.7	0.0	UnnamedSequence	13015	13133	(22219)	C	L2a	LINE/L2	(4)	3422	3302	15	
367	34.5	4.7	3.1	UnnamedSequence	14833	15089	(20263)	C	MARNA	DNA/TcMar-Mariner	(189)	397	137	16	
216	24.1	0.0	1.9	UnnamedSequence	15117	15171	(20181)	+	L2a	LINE/L2	3291	3344	(82)	17	
1002	24.2	0.8	4.6	UnnamedSequence	15452	15698	(19654)	C	MER8	DNA/TcMar-Tigger	(1)	238	1	18	
242	24.7	5.1	1.2	UnnamedSequence	16425	16502	(18850)	+	MIR3	SINE/MIR	119	199	(9)	19	
2102	12.3	0.3	0.0	UnnamedSequence	16512	16795	(18557)	+	AluSx1	SINE/Alu	13	297	(15)	20	
1137	16.1	0.0	0.0	UnnamedSequence	16796	16950	(18402)	+	AluSc8	SINE/Alu	133	287	(25)	21	
251	27.3	3.9	0.0	UnnamedSequence	16987	17063	(18289)	+	L1M5	LINE/L1	4486	4565	(1581)	22	
1194	15.2	10.7	0.5	UnnamedSequence	17308	17621	(17731)	C	MER68	LTR/ERVL	(0)	563	184	23	
524	19.3	0.0	0.0	UnnamedSequence	17633	17720	(17632)	C	MER68	LTR/ERVL	(461)	102	15	23	
1828	17.9	3.3	3.0	UnnamedSequence	17767	17855	(17497)	C	MER119	DNA/hAT-Charlie	(113)	470	385	24	
1432	16.5	13.9	1.7	UnnamedSequence	17856	18122	(17230)	C	AluJb	SINE/Alu	(13)	299	1	25	
1828	17.9	3.3	3.0	UnnamedSequence	18123	18400	(16952)	C	MER119	DNA/hAT-Charlie	(199)	384	102	24	
526	22.4	16.1	1.9	UnnamedSequence	18845	19069	(16283)	+	Charlie7	DNA/hAT-Charlie	35	295	(2317)	26	*
1074	25.5	5.6	2.9	UnnamedSequence	19069	19273	(16079)	+	Charlie7	DNA/hAT-Charlie	327	548	(2064)	27	
2823	18.2	2.4	1.5	UnnamedSequence	19274	19815	(15537)	+	MER44B	DNA/TcMar-Tigger	1	547	(3)	28	
1074	25.5	5.6	2.9	UnnamedSequence	19816	19984	(15368)	+	Charlie7	DNA/hAT-Charlie	549	711	(1901)	27	
717	23.7	5.8	6.2	UnnamedSequence	19989	20060	(15292)	+	Charlie7	DNA/hAT-Charlie	759	821	(1791)	29	
1576	19.6	3.2	2.4	UnnamedSequence	20061	20345	(15007)	+	AluJr	SINE/Alu	1	287	(25)	30	
198	0.0	0.0	0.0	UnnamedSequence	20346	20367	(14985)	+	(TAA)n	Simple_repeat	2	23	(0)	31	
1604	15.9	8.8	1.0	UnnamedSequence	20368	20650	(14702)	+	AluJb	SINE/Alu	1	305	(7)	32	
717	23.7	5.8	6.2	UnnamedSequence	20651	20849	(14503)	+	Charlie7	DNA/hAT-Charlie	822	1031	(1581)	29	
648	25.2	10.4	1.0	UnnamedSequence	20843	21178	(14174)	+	Charlie7	DNA/hAT-Charlie	1041	1413	(1199)	33	*
24	77.4	0.0	0.0	UnnamedSequence	21355	21385	(13967)	+	AT_rich	Low_complexity	1	31	(0)	34	
480	29.6	9.2	1.0	UnnamedSequence	21432	21615	(13737)	C	MLT1J	LTR/ERVL-MaLR	(213)	299	101	35	
280	4.1	3.6	13.7	UnnamedSequence	21581	21636	(13716)	C	LTR67B	LTR/ERVL	(499)	121	71	36	*
25	48.0	0.0	0.0	UnnamedSequence	21773	21797	(13555)	+	AT_rich	Low_complexity	1	25	(0)	37	*
26	42.3	0.0	0.0	UnnamedSequence	21775	21800	(13552)	+	AT_rich	Low_complexity	1	26	(0)	38	
1490	17.4	9.3	0.0	UnnamedSequence	21994	22251	(13101)	+	AluJb	SINE/Alu	1	282	(30)	39	
486	31.2	6.6	2.9	UnnamedSequence	22301	22498	(12854)	+	MIRb	SINE/MIR	61	265	(3)	40	
2337	9.7	0.3	0.0	UnnamedSequence	22573	22862	(12490)	+	AluSx4	SINE/Alu	2	292	(20)	41	
24	77.4	0.0	0.0	UnnamedSequence	23475	23505	(11847)	+	AT_rich	Low_complexity	1	31	(0)	42	
499	28.5	5.9	2.9	UnnamedSequence	23633	23802	(11550)	+	MIRb	SINE/MIR	24	198	(70)	43	
2108	11.4	1.0	1.0	UnnamedSequence	24402	24690	(10662)	C	AluSx3	SINE/Alu	(21)	291	3	44	
1963	15.9	0.0	0.7	UnnamedSequence	24810	25112	(10240)	C	AluSz6	SINE/Alu	(0)	312	1	45	
21	75.0	0.0	0.0	UnnamedSequence	26083	26110	(9242)	+	AT_rich	Low_complexity	1	28	(0)	46	
2112	19.2	5.4	5.2	UnnamedSequence	27138	27641	(7711)	C	MLT1D	LTR/ERVL-MaLR	(0)	505	1	47	
1904	19.0	0.3	0.0	UnnamedSequence	27777	28087	(7265)	C	AluJr	SINE/Alu	(0)	312	1	48	
30	86.5	0.0	0.0	UnnamedSequence	28394	28430	(6922)	+	AT_rich	Low_complexity	1	37	(0)	49	
26	38.5	0.0	0.0	UnnamedSequence	28407	28432	(6920)	+	AT_rich	Low_complexity	1	26	(0)	50	*
1715	16.5	5.3	0.0	UnnamedSequence	28448	28713	(6639)	+	AluJb	SINE/Alu	1	280	(32)	51	
2296	10.8	0.0	0.0	UnnamedSequence	28714	29008	(6344)	C	AluSq2	SINE/Alu	(16)	296	2	52	
272	12.1	0.0	0.0	UnnamedSequence	29017	29049	(6303)	C	AluYc	SINE/Alu	(265)	34	2	53	
634	17.0	2.6	1.9	UnnamedSequence	29395	29550	(5802)	C	MER33	DNA/hAT-Charlie	(1)	323	167	54	
261	5.7	0.0	0.0	UnnamedSequence	29826	29860	(5492)	+	(TG)n	Simple_repeat	1	35	(0)	55	
2082	13.0	0.0	0.0	UnnamedSequence	30462	30737	(4615)	C	AluSz	SINE/Alu	(28)	284	9	56	
186	10.3	0.0	0.0	UnnamedSequence	30751	30779	(4573)	+	(TTTTA)n	Simple_repeat	2	30	(0)	57	
1001	18.1	0.0	0.0	UnnamedSequence	31127	31270	(4082)	+	AluJb	SINE/Alu	132	275	(37)	58	
384	2.2	0.0	0.0	UnnamedSequence	31271	31315	(4037)	+	(TGAA)n	Simple_repeat	4	48	(0)	59	
1867	18.4	0.0	0.3	UnnamedSequence	31949	32241	(3111)	C	AluJb	SINE/Alu	(20)	292	1	60	
25	69.2	0.0	0.0	UnnamedSequence	32507	32545	(2807)	+	AT_rich	Low_complexity	1	39	(0)	61	
1976	15.3	0.3	1.0	UnnamedSequence	33477	33776	(1576)	+	AluJb	SINE/Alu	1	298	(14)	62	
324	37.1	8.0	5.1	UnnamedSequence	33883	34350	(1002)	C	L2c	LINE/L2	(9)	3378	2898	63	
2468	15.9	4.9	0.2	UnnamedSequence	34351	34796	(556)	+	MLT1C	LTR/ERVL-MaLR	1	467	(0)	64	
261	40.2	10.5	1.7	UnnamedSequence	34797	34999	(353)	C	L2c	LINE/L2	(522)	2897	2672	63	

Marsupials

>Monodelphis Gin-1 (chromosome:BROADO5:3:5052175:5085807:-1)
TGAAGTCAAACCCAACTTGACAGTTGTTAGATTCATGTCTTGTTTTATCTGCAGGTATAT
TTCAAAGGACATTTTTGGTCTGTCTTCAGTTTGAACCTTTGTATCGACTATTTGGATGAC
TATCATATTTTGACTTTCCTCTATTTTGCATGCCATTTCCAAAAGGTTTTGAACTTTTAA
TTCATAATTTTCTTATTAATATTATTCTCCTAATTCTAGCACAATGGAAAAAACAAAAAA
CTGTATTTACGTATCTGCCAAAGAGACCTTCAGGGCTGAGGGGGCACAGGCCTATCCATC
CATTGGTTATGTGCTAGCCTAACAAATAAATTGATATTTTTACCCAAAGATCAGTCTCTC
AAAAAATGAAAAAAGAAGCAAAAGGTTTCTTTTTATTTTACTTGAAATAAATTTATTGAA
GTATTTTTTAAATTGTTACTGTTTTTAATTGTTAAATTGTTACTATAAAAACCCTTTCAG
AAATATTTTTGAGGTCACATAAATATAAATCTATATTAGTGACATAATTTAGGACAGAAG
CAGGTCTAGGATAATAATCAAACTAACTAGATCTTTAAATCTTTTTCTCTCAATTTAAGA
ATGGTCCGTAGTGGCAAAAATGGTGGTCTTCATCTAAAGCAGATTGCATATTATAAGCGA
ACAGGTGAATATCATCCAACTACATTGCCAAGTGAGAGAAGCGGAATAAGAAGAGCAGCA
AAAAAGTTTGAATTCAAAGGTAAAGTTAACATCTACAATCTGTCCTCTCGATTTGTGTTA
AACCTCAGGAACTTAGAATCTCTGGTAAAAATTACTTAAATTGATTTTCTGCTAACGGAA
AAAAAGGTTTTCCTATTGTTTTAGTAATATCTATATAGTCCCTGTTTCCCTTTCCATCAC
CCTCTCCATTCTCTAGCCCAGAGTTCAATACAAAAATGTAATTTTTATTCAGTAAATTCT
TATTCCCATTTCTCTATTGATTCAATAAGTTCTTAAAAAGAGAAGCTTCTTTGGTGTCAC
TAATGTTGACTCCTCTCATGTGAGTTTTCTTGAATCAGAAAAACTGTAAATACTGTTCTT
CTTTCATGTAAAGAATGAAATGTTTGTTCTTGGTCTTCCCTCTGCTCAAACATGCATACA
CGTCAAGCACTAATGGGGATTGTCCTTAAAATTGCTGCAGGAATGTAGAAATAGGGTACA
AGCTGTTTGGGGAATGCAGAAAACCTGGGATCAAGGCCCATCGTAGACATTTCTGGGCTC
TGGGACCCTGGCTTAAGTTACGCTCTCAGGGCTCCCAAACAGCTCTTTAAGCCGATAAAT
TATAAACTGCAAAATCGATTCAGTTCTCATCGGTCCGGGAAGTTCTTCCTTGAAAAGTTC
TCTATTCCACGCAAGTCACCGGTTGGTCCACAGAAAAGAGCCTCCGGTTTCCCCGGAGGA
AAAAGGCCGCCTTGCTCATTTGCTCAAAGTTGGACGTGCTCGTTGGCTGGTCCTCTGATG
CCCTCATTTCCCTTCCGTGGAGACCGATGAGTGCCTTGAACAATAGAGTAAGGCAAGTCG
GTGCTGTGCATCCTAGCAGTCGGCTCTGAGTTTTCCTCGGGTACAGAGGGCCTGGCAGGT
CCGGAGCGCTCTGACCCTTCAGTGAGGCCCCGTTCATCCCTGGCCCCCTCCTTCCCAGCA
ATCCCTCCGCTGAGACAGTGAGATGGGCTGCATAGGAGGAGAACCAAGCAGGTTCCACGT
GCTCTGAGGTCTGGCAGTGCGGCTTAGACTACGCGCACACACACGCACACACACACACAC
ATGGACACACACTTGCACACATGCACACACTTGCACACACTTGCACACATGCACACACTT
GCACACATGCACACACACTCGCACACATGCACACACGCGCACGCACGCACACACACGCAC

GCAGACACGCACACACATGCCCACACACGCATGCACACACACGCACACACACACGGTATC
AGCCACCTCCCACAAAAGTAGGTAGTGGTGCTTCCTTTTCCCAGAGGACGCTCTGCTTCT
CGTACGTTTGCTCCAGAATGGCGCCCACAGAAGGACCACCGATCTTTACTTACCCCTGGG
AGTGGCAGAGGAGGGTAGGGGGCACACGAGCAGCACTCATCAAGCAATTCTCTGCCAGGG
TATTCTGCTAAGGGCTAAGGATGCAAAGACAGCCAAAAGAAGATGATGGGAATAATATTT
AACGGGATACAGAGGGGAAATTGGAGTGGCCTGAAGGAAGGCATTAAGATTTGGAAAGAC
TGGAGAGGCTTCAGGCCGAGCATAGCATTCATACGTCCCTTAAGGTTCACAGCGTGCTTT
CCAGCTGTCTTGTTTTATTCTTAGGAAAACTGACGCCGTCTTTCTGCATGTTAGAAGTGA
GGAATGGGAGCGGGTGGAAGTGAAATGATTTTCCCACAGTCACCCAGGTAGTAAGTGTCT
GAGGGCAGATTCGAGTTCAGGTCTTCCCACCTGTGACCAGTACTCTGGTCCCCTCAACGA
CCTGCCTGCCTCGTAGAAGGTGAGAGTTGGCCGGGAGCTAGAGATGAAGAGGGAGAGAAT
TCTGGGTGTTCGGAACAGCCAGTGAAAATGCGTGGAGCTAGAAAATATTTGCTTTTCGTT
AGAGTAACAGAATGATTCTGAGACTGCATTTAATGCCTTGACTTGAACTTAAATGGAATC
ATTCTGGAACAGAACTTATTTAAAGAACATTTTAAAAGCATTTTCCGTGCTGATTCTCAA
GATCATCCCCCCCGTTTACAAAGGCACTTCTTCAGGGTAGCAGCTTGGAGACCCAGTCTT
GGTGCCTGCTGAAAACAGCCATTCTGTGCAAAGTACTGCAAACCGAAGGTTCCTCTTAGG
CATCTGGTGTGTATAACGGCGAAGAACGTTCTAGATATATCTCGAAACCCGTCTCCTCCA
TCTGGTCTTTGGCAGTGGAAGAGTTGTAGAACTACTGAGTTTGGGAATACTGAGCAGGCC
ACTTTTTAAGCGACACATTGCAGCACAGAGGAAGCGTGCACATCTCACAATAACGTTCTG
TCTTTTGTAATTCTAG**AAAAAAAGCTGTTCTATGTGGGAAAGGACAAGAAACAAAGCCGA**
TTGGTAATTATCTCGGAAGAAGAGAAGAGCAAAGTCCTAAGAGAATGCCATGAAAATAAC
ACGGGGACGCACCATGGCATCTCCAGAACTCTTACTCTAGTAGAGTCTGGCTACTATTGG
ACATCTGTGACCAATGATGTCAAACACTGGGTACGCTCACAGGCTTAGAGTGTTGGCATC
GTTTTGTTGTCGCGCACATCCCAAAGGTGACTTGGCTCGGATTCACTCACTGTGAAATGG
TGACGCTGCCCTGCGCTGCCATTTGGGCAAAATCGCCGTTTTCCTTTTGAACGAGACACG
TGAAGTCAGTGAACAAGTCCCTGTTCTTGTAAGCTCGTATTAGGCACCAGGGACGGTCCT
TACGGCCAGGGTTACAAGACAGCCCCTTCCTTCAAGGACTGACACTTTGATGGGGGGAGA
AAGCACATAAAGATTGTACAGTGTACATGGAAGGTAATCTCAGAGGCCCCAAGAAAGGCT
GCAGCAAGTGGGGTTCAGCGGCGTCCTGAAGGAGGCCAGGAAAGCCAGCAGGGGGAAGTG
AGAGGAACAAAATTCTCCCTATGGGGTTCAGTTAGTGGAAAGGTCGAGTCAGGAGATAAA
GTGCCAAAGGCCAGTGACTAAGTGCGGGCTAGTAAAGAATAAGACTGGGAAGATAGGAAA
GGGCTACTCAAAATTGTGATGAAAGAGTTGGTTTTTGTTTCTTTGGCCAAATTCTCCAGA
AAAATCATTGTATACGTGGGCTATCTTTAGAATGCACTGAAGTACTTTGAATAATATGAT
TTAATTAGGGACAAAAATTAACCTGGGTAACAGACATCGTTTCAGTGTAAACATTACATT
CTTAGAGGAGGCAGGATTTTGATCCAGATTTTATGAATGAACTTCTGTGCGATGGCAGAG
ATCAAATAGAATTAGAATTCATTGTGTTTCAGATCTATTATCTCTATGAACCTTGGAGTT
CCAACTTGCCATTTAGGGATGGTCCTTTGCTCTCTCAGTACTTTTTTGTGCTGCTCTCTT
TCCATCGTTCCCCAGTAAAAGACAGATAACAACTGAACTCTAAAATGGAATTTAGGGAAA
CATGTCACCCTATGCAAAGTAAAATAATGAAACATTTTTCCCTGGTGTTGGAACTCTCTC
CTCTGGTATAGATTGTAATCTGTGTGACTTTGTCCCAAACTTCTCGTGGACCCCCTAACC
AAGGATCCTCCTGATCTGCCAGAGGCCCTCCTCGCGGCCTCGTGCTCAGTCTTCCCATTC
CTCCCGATGACTGTTTGTCCTACCTGCCTTCTCTTCCTTCCGTCCCCTTTTCTTGTCTGA
TGTCTTTGGTCCCTCTTCTTATTGGTGAGTCATCGCCCCATCACTGCACTGGAGCGCTCA
CACACACACTTCCTCTCAACTCCCTGAAGTTTCGCATTGCACAAAATACAATTCTGTGTT
CCTCGTAACCACCAGAAAACTATTGGTGCCTTTCTCTTTCCCGTTGGGTCTTTGTGCCCA
GGAGTATCTCGGGATCCTTAAGTCCCAGCCCAGCCTCCCTGATGAAAGTCACTGCGCTCT
CTCCCTCATCCCCTTGTCTGCCATCAGGACCGCCACGACAGCTCTGTACCCAGGGACTGA
TTCCGTGTCCTCTGCATTCACGTTCGCCTGAAAGACTGGACAGCGTTCCCTTCTCTGTGT
TCCACGGCGCGCTCCTTTTTCAGTGCTTGTGGCTCTCAATGGAAGCCCCTGTCGTTGTCT
AAGACTTCATGCCACGAGCATAGGGGTCCACAGATAGAACCCCCCTTTCACTCAGCGTCC
TGCACAACTCATCCTCTGTGACGTGTGTGGTGGATGTCTTTGGCAAGCGTGTCTTCCAGT
CCCATCGTGTGATAACTGTATTACACAACAGCGTTGACTTTGTCTGGGTTTCTAAATAAT
CTTTGTACCAGGTTAGCATATTCTTAGGAAATCAGAGAGCCTGGATTCGAATCCTGATTC
CAGGATTGATTGCTTTGTGACCTTAAGCAGGTCCTTCTCTAGGTCTAGTTATTCCTTCAT
TTATAAAATGAGGATATTGGACTAGACAGCCTCTGAGGGCACTTCCAGCTCTAGATCTAA
GATTCATGATCTCTCGAGTGTGTGGAAGTGCCTCATTGATGGAGAGACTTTCATTTTGTT
GTTGAGTCAAATACATTTTTGCAGTCAGGTAACATTGGGATAATAGCAGTGATTAAAAAT
GGTATTAATATAGGACTTTAAGGTTTGAAAGCATTATCCATTTATCTATCTATCATCTAT
CTATCCATCTATCCATCCAGCTATCATCTATCTATCTATCCATCTATCTATCCATCCATC
TATCAGCTATCTATCTATCTATCTATCTATCTATCTATCTATCCATGTATCATCTATCTA
TCTATCCATCTATCCATCCATCTATCATCTATCTATCTATCTATCTATCCATCTATCTAT
CTATCAGCTATCTATCTATCTATCTATCTATCTATCCATCTATCCATCCATCTATCTATC
TATCCCATTTTATTTTATACTTAAAATAATCCTGGGAGATAGAGATTATTATTCCTGTTT
TACAAATGAAGACACTGAGGCAGAGAGGAGTTAATTGATTTACCTAGGATCACATAGCTA
GTGTTAGGACTTGAACTCAGATCTTCCTAACTCTAAGGCTAGCACTCCATCCACTGCAAC
ATCTTAAAAAAACCCAACCTTAAAGAACATAGCCTATGAAAGTGTGTCATTTTCTTTTTC
ATGTTCTCATAGTGCCCATCTCCAAAAAAAGGTAACCAGAATAAGCTATATGGGTCAAAA
GTTGCCTCTCTGTGGCTCTTTTTGGTAACGTGTCGCCTGATTTTTCCCATTCCTTTCAGT
CTAGTCTCCTTTCACTGGTAACTTGAAAGCTGATTCCTGAAGGACTTGAACATTATGACG
CCTTCGGTGTGATGTCTTGAGTGTGCATTTGGTAACGTTTACTCCTTCTTGACTTCGTAT
TGAAAAATGCTCGCTCCCTGCTGTGATGTCAGTCCCCAGATAGTAAACTGCTGTGTTTAG
GGGCCATCAGTGAGGACACATAGGTGTTGGCTCTGTGTAAATCTGCTCCGTGTCGTTCAG
TTGTGTTCTTTCGAGCCACCTTTAAGTCTTTGGGGATCGTGCGGTTGATCGCGAGCCACT
TTTATTCAGACTTAATTTTCCGTCTAGAAGATGCTCAATTCCCAGAACCACCTACTGCTG
TCTATGGACACTTTGTAGATACGTTGTTAGGGTCCAGCGCGTCGCCCACCACCAAGGAAA
CCCCCAGACCCTGCAATCCAGCGGGAGGGTCCTTTTTTCTGGTCGGCGCCAGGGGAAGCT
CAGCCCAGGAGTGCCGCCTGCAGTTGTGAGTGCAGAGTCTTGTCTACCCGAAGCCTGACA
GCACGTCACTAGCCACCATAGCGCCCGCACGTCCTCCTGGACTCTACTTTGGGCGTCAGA
TGTTCTACAGTGATGCGTGCTGCACTGACCAAGCAGCTCCCTGCTTGGGGCGCCTTCGAC
GTTATAAAGCTCATCACTGTTCCCTTTCGCTGCCTGAAAGGAAATTGTTTGCTGTCTGAC
TGACTTCATTTGGTATCTTCATAGAGACAATTATTTTATCTCAATTTGACTTCTCTTCGT
AATGGTGATTCTCCGGGTTCGTGTCTTTTGTCTTTCTCATCTTCCCCCAACAGTGGATTT
AAATGAGGCAGAGTCTCTTGCTTTTACATGTTCGCAGTGTGTCCTCATTCTGATCCTTGT
GATCGGTGTGGATGTTGAACTTTACTCCAGGCAGTCAGTGCTATACTTAAGCATTGTGAC
AGTTCGTTTGAAAGGTTCCTGGTTCCAAGCAACAGCTCTTCCTGGAGGCCAGGACAGACG
ATTTCATTTTTTTCTTCTGGTGACGTTGCTTGGTATTCCCCGTGTCCGTGACCTTCCCCG
GCTCCAGATCTGTGATTTCGTTTTGAAGAAAATCCCAGTGCTGGAGAGAGAGGAAGCCTC
TGAGTAATCTGTAAGCACTTGCACAAATGTTTGAAGCCTGAACCCTATTTTATAACATTT
TTGCCTTATCGATGCCTTTTACTGGTGTCACATTCTCTGCTCACAGTCCCTCATCTCTTT
ATGAAGTGAAAGAAGCTCTGAGTCAGCATTTCTGTGACCCAGATTTATCGTTACAATCCG
AATTCGGCATCTTTTAAGCTCCGTTTCATTCGCATGATTTTAGTCATTGGATGCATTGTC
CTCTCGACTTCCCTAGGCTTTAATTCAGATCAGTCTTTCCCTATTTTTAGAAAATCCTCA
TCGTTGCAGCTTCAGTAGCATTTTACTCCCTTCTTGGGCTGCAGTTTGCACTCATGTGAC
AGACACGCCTTGGCTGACTGTCTCATTTTGCAGATGATGGACGCGGGGCCTGGAGAACGC
GGCAGAGGCAGGCTTCCCAGCCCCACCCTTCAGCCCCGGGCCCAGCCACCTTCCACTGTA
CGTCCCCGTGTCGTGGATCGGGTCCAGGGCCCAGAGCGATAGGGCATCTCCCCCAAGAGC
ACACGCACGGCCAGGATGGGACACCGGGTTCTTGACTCCGCTCGTCGTTCTTCCGCTCCG
CCAGGCTACCTCGGTCCCTCTTGGCTGTGCTCGGGCGTCTGCGGGACTTCCACCGCGGGG
TCTGAAGGGTCCAGACTTTCTCTGCTTCCTCCTGGGCACTAGACACTGGGGTGAGCCACG
ATGCCATTCAGGCCGATCTTGTGGCTAGCACTCCTGGACACCGGAGTGATCACTGAAACT
ACGTTCACGTGCTTCCTCCGTAACATCCGACCCCTTCTTTCATCGGTTTGCAGCTTCCGC
GTCTCTCGGTCTCCTCCACAGACACTAGTGGATTAGTCTATCGGTGCACCAGATACTCTG
TCATCAGAGGTTTCACATTTGATGCTTATCGACCGACTGAAAACTACTGGCGTCTTAGTG
TTCCCCAACCCCATGTCACCACCATTGTCGTCGAAGGGGGCCGTGTGGGACCTCAAGAAA
CTGCTTGTTTCATGTACTCAGCGGGCCTTTGGAGAAATGAGCCCTACGATTAAGCTCCTA
CCACATAGTCAGGCACTGTGCGAGGCACCGGAGATATAAAGCGAAAGTGAAACAATGCAT
CCTCCATTCAGCCACTAAAGGGATTTTCTGAAAGTGCACATCTGACATGCCAAACCCCAC
TTCCCTCCCTCCTGCCCACTCAGTAAACGTCCTTGGCTCCCTATTGCCTCTGGGGCAAAT
ACAAAATACAATCCCATTCAAAACCCTTTATAGCCTAGCCCCTCTTCCCTTCTAGTCTTC
TTAGACTTTTATTTCCCAACACATACTCTTTGACTGAATGGCATTGGTCTCCTGCTGTTC
CATGACCAAGACACTCCATCTCTTGACTCCAGGCATTTTCTCTGCTATCCCCCATGCAAG
GAATGCTCTACCTTCCCCTTCTCTGACTTCCCCAGCTTCCTTTAAGTTCCAACTAAAATC
CTACCTTTTACAGGAAGCCTTTCTTTATCCCTCTTAATTCCACCACTGTCCCTCTTGTTG
TTTTCCATTTATCCTGTATATATATATATACACATATATATGTATAATCCTGCATGTGTA
TATGCAAAATATAAGCAAAAACAAGTTTTGTTTTTCCATAAATACTTCTTAAAGCTAGTG
CTATCTTTAATGATCTTTGTTTTAATGTATAATGGAAAACTTGGCCAGTCAAATCCCACT
AAATATACAATTTTGTTTGAGAGGGAGAGATATTTAATGAGGATTTAGAAAACACATGCA
CTTTGCGAAAATTTTCAGTTTTATACAAATAAATCTATCAAAATGATGTCTATAAATGAA
AACTTGAGAGTCTTTAATAAAAATCCTGAAGATGTGAGACACAAATATTACTGTATTTGG
AGAGAATTGAGTAATTTCTACTAGGCACACTCTCCTATTAGAATAAACAAATTTCTTAAC
CCCCCAAACCAAGCAAAAGGAAGTTGGGTGGAAAGGAGGGAGCCAGAAAGAATTTGTATC
TGGATTCTCCTCTTCCCACCAGAGCACTACAAGCCAGGAGGGATCCAGAGAGAGATGGAG
AGAAAGAGAATAGAGTAAAACCTGTCAGAAGACCAGAGCCTTTAAATCTTTCTCACCACT

TAGCCTTGTCATATGTAAAAATAAATAATGACGAGTTATAAAAATATACTATTTAGATGG
AAAAACTGTTCCAATATAGCTTCAAAACTGTTTAACTCGCTAATCAGGTTCAAACTGTTC
AGATACTTTTGATAGAGGTAATCAAAAGTATCCTCAGTGATGAAGTGAAGTCAAATGTGA
ACTTCCCTTCAGGGCCAGACGCAAGGGTGCTAAAGAGATTTATGTAAACATAAAATGTTT
ATTGAAAAATATAAGGATAAATAAAGGATTTCTAATTCTAAGGGATTCTAAATCCACCCA
AACAAACTCCCAGTTTCCTCAAGGAACCACTTCTGCTGTGTTTCACAGGCTACACTCATT
CTCCCTGATCTAACTAATCCAAATTTATACTATCTAAATAAAAACTACTGCTAATATCCT
TATAAATCTTAATTTTGCCTTCAAATTATAAAATAGTAAAGGTTGGTTGGTTGAGTCTCC
ACAAGAATCCAATTAATGTTATGGGCAGGAAGGATGCCCTTGGGGTTCGGGAAGGATACC
TTTTGCAAGCATGCAAGACTCCAAAACTTAGCTTAAAAACAAAAAGAGATTTATTAATTT
AGAAAGTAATGTTGAGAATGGCCAGGAGGATAGCAAGGTGGGACAGCAAGATGGGGAGCA
GTTCCTGGGAAGACAGCATGAAAGGAAAGGCTGTTCTTCCATGGGGACAGCATGGGTGGA
GAGGCTTTCCCCCAGATGACCTCAGTTCTGGGGTTTATATACTCTTTAGATGCTATGTCC
TAGTGTGGACCTGACCTGGAGTATTAGTCCCTCGGGCACTTGGTGGGGTGGGGTGGTCAT
TTGACTGAGGCCTGCCTGGAGGCATAAAGATTCATCTCTTTGTCCATCTTAAATCTAGAG
GACAAAAGAGGGTAAACATCTCAGGACCCTGGGTGGGGCCATTGGGTGTTTCTAGGTTAA
GAGTCAGAGGATGCAAGGGCAAAGGAGTTTCCCTGGTAATAGTTCGCCTGGGTTTCTGGG
GGTACAGTGCCCATGTCATTAGGACTCTGAGTCTGTTACCAAACCAAAGACCAGTTTTTT
CTTTGGTCTTCTCAGAGTTCAACAATCTATAAAACCACAATAGATAGACAATAGTGTTTC
CCAAGTTGGGAAAGGAGAACTCTGAGTTCCTTCAGGTCTTTCCCTGAGACAGGGAGAGGC
AAAGATGTTACTTCCTCCAGCCCTGAGAGAGGTCTCTGGGTGTGTCTCTGCCAACTGCCA
GAGAGAGTAATTGACACAGAAAACGGTTCTAACTGTCACATCTGGAATTGACACGCTCTC
TCAGCCCTGCTTCCAACTCCGGCTCCTTCTCAAGCAGCCTCTTCACTTCTTATCCCTAAA
CGAATAAACCAAGATTTCTTTTCTAATGTCATCCTTACACATCTCACATGTTAGGTTTCT
AGTCAAATTTCTTTTTGCTAGAATCCATGTTCTCCCTGCATTGAGGGTTGGAAACAGCCT
TGTACATTCTAAGCTATAGACAACATTTTGGGGCTAGTGCAGGCATCCCTTTCCATTTGA
AGAGCAAATAAATCAGCCTTAAATCAAGGAAGACTCTTTTAACATCAGAAAGCTTCACTC
TCAGTTACATGAAAGTACTCCTTGCAGCTCACTTTACTGGGCCTTATTTTTCTAAGCCAA
GGAGATGAAGGCTTCCTTACTAGGAGGAGAGCAAAGGCAAGCTCTCCAGTCTTATACTTA
CTCCCCTCATGCACTCTTCGATCCAGAGCCTCTGGCCTCCTGGTTGTTCCATCTCTCAGC
TCTGACATTTTCTCTGGCTGCCTCATGCTTGGAATGCTTGGTCTCCTGGTCTCTGCCTGC
TCCCTTCCCTGGCTTCTTCCTTTAAGCCCTAAATAAATGACTTCTACAGAAAGCCCTTCT
CAGCCCCTCTTCATTTTACATGCTGGAGGGGATTGCGCCTCTCCCTCTCTCCATGTACCT
AAGGGCATTTCTCACATGACCCATCCTTCTGCCCAACAGCCCAGTAGAAGCACTTCCTCC
CTTCCTTGTCTGTGGTAAGGCCAGGCGACTCACACGTGGCATGAGGGTGCAGTTTGGTCG
CTCAGTCTCGAAAAGACTCGCCATCACTGATATAGATCCTTCCAACTACTCAATTTCTCT
TTTCAAGTTTCTTTGGATTCCTGTGTTTATACATCAAAAATTTTACACACTCACGTCTA
GTCTTTTATTCAGGAATGTTTAAAAAGTTCTCTATTTTACTAAAATTCCAGTAAAGTAGC
CTCTTATAGGTTTATATTTAGCTTTGGAACGTTGTCTTTGGTTGTAAACAGTATTCCTTT
TTTTACCTTTTGTAATAATGATTCCTGAAGTCTCCATTCTTTTGACCTTGATTTTTACCC
TATGATTCTAAAAGATCTGTGCTCTTTTAAGGTATGATTTCTTGAAATATACCAGCCTTT
TAAAAAATATTAAGATTTTTGGGAAATCCAGGGATTCCTAAGTTATTTCTCTTGATATAT
TATACATGTCAGTTTTCTTTAAAATCAACTACTCGTTTTCTTCCACTTTCTCTTCAAAGC
CGTTTATAAGCTTCCTTCCAATTTTATATTTCAGGACTTTTATTTAAATATTTATCTTTT
GCCTCATTTCTCCTATGTATTCTTGTAGTCCTTGTGGAAAATCCATTTTTCTCTTCTTTC
TGCTTGCAGTTATTATGAAGTTAGATTTGTAATCGTTTTCCTTGAGTCATTGATCTCTCC
AGCTTTACTTTCCAGATTGGGACTTTGTGCTAGAGCCAGAATTTTCCTGTAATACTTTTG
ACTGGGCTAATTTTCCCTGCTTGCTCTCATTCTGGGTTCATTGGTTCAAGCACTGACCTC
CATTTCCTAGCTTTTGGCTTTAGTCTTTGAGGTTGAAAGTACTGAATTTTCATTGTCTTT
TCTGGCATCTTGGAACAGTGTTAGGAACTTCAGAGTGCTCCTGTGCCACACCTGCCCTGA
TCTGAGTGTTTTTATACTTTCCTAGCCCAAGCCTCTCTTCAGATATTTCAAGGCCCCAGC
CCTTGCACCACTAAAACCCTCAGGTCTGTGACTTTTTCAGGGTGCTTCTATGCCTATGAT
GTTTTTATGTAATAACAACAAAATAGTATAATGATGATGATGATGATGAGTATAATATAT
AAAAGTATATTATAGAAGCATAAATTTTAATAACAATAATAAAGTATAACAAAAAATACT
TAAATATAAGGTAATATCTGAAAGGTAACCAGATCATTAATGGATTAATGTGGCTTTAGA
TTTGGAGACCAGAAGACCAGAGTTCAAATACTGCCTCATTGCTTACTATTGTACTATCAG
GGAATCAAGGAACCAAAGTTTTGACAGGTACTTGATAGGGAACAGAAAAAGAGTACACCA
GTTCTCCCTCCCCCCCCCCCCCCCAGGCAGAACTGTCTGTATGATAGAGTTAGATCTGA
CCAGAGCTGAAGGCTAAGAGAAAATCCCCTTCAATCTCTTTCTTCTTTAGTTGATTGTAT
TTGTAATCCCCTCTAGTACATTTAAGATGTCTGAGTTCTCAGTAAAGTAAAAATAGCTAT
TTATTGGATAAGGGGTTAGGGTATAAATTAGGAAAGAGGAACCAGGAAGTCCCTGAGCTA
TCTCCCTCCTATAGCTGTCTAGAGGGGTTTGAGGCACAAGTCTGGGACAAAGTCCCAGAC
GATCTTAATGTCTTTGTAAGTTCAAGATGATGGTATTTCTTGGCACAGGCTATTGCCTTC
ACCAAAGGGAAAAAGTCCCTATCCCACCACTGTAAAACAGATGGTTAGTCTGGATCTTTT
TCTACTCAACAAGGGATTTGTACATTTGTATCTAAGTAGGCTCAATGAGAATTTTGCTCC
TTCCCTCTTCAAGTCAAAGAGAAAAAAGACAGCAGCCTCTAACTGCTCCTCCTTTTCTTA
GCTGGCATGTTCCATTCCCATCCCCATTGGCAGCCAGAGTTCCATCTCATCTTTTCATGA
GATAATTGTACAAAATCCAGTCCTTGCATTCATTTTAGCATTTCTCTCTTCCACACTACG
GGCAAATCACTTAAACTCTGTGTTTTATCCCTTTATCTGTAGCATACCAGGCATATTAGC
ATCTTGGATGTATTGATACTGTATCTTATGTATTCATTTACCAGACTCATGGTCATGTTA
CTTATTGACTATTGTATTGCCAAATCTTCAATCCAAAGGACAAGAGAATTCGTGAGCAGG
ACATTACCAGCCACAGTGTCCTAGCTCACACCTTGTCAGACCACATCCCTTTCCAAGTCA
AGTTTTCCTCTTGGCTTTCCTCTTGATCCTCTTGCCGCTCTAGCTTCTCTTGCTCCTCAT
GGCCTCTTGTTTCTCACAATGCTGTTGCCTCTCAGACTTCTCATTACTCCTCTTGCTCTC
TTGCTCATGAGAAGTATTACCGGAGCTCGCTGTTCCCCACATGTTTTACCTAGTTGTCTC
TGAGTCTTTATTCTTGCATTTCTCCCTCTATCTCTATTATCTATTCATCCATTTTCCTTT
AGTCTCCATTCTCTTTCTTAGGTCACCACCCACAAGTGAGTTGTGTAGGAACTGCAGGCA
GCTCCTACATTATCTGTAAAATTAAATGGTTAGACACAGTGACCTCCAAGGCCCCTTCCA
GCTAACAATCTGTGTTTGTGAGGGCAGAAGCAGCTTAGGAGATGAGGAAAAGTTTTTTAT
AGAAGATAATTCTTGATCTGTGTCCGGAAGAAAGAGATTCTGTGATGCAGAGGTGAGAAG
GCATATATTCAAGGAAGATAGATAGGGTGGGGTGTCCATTGATTGGGGAATGGCTGAACA
AACTGTGGTGTCTGCTGGTGATAGAGTACTATTATGCTCAAAGGATTGATGAACTGGAGG
GATTCCATGTGAACTGGAATGACCTCCAGGAAGTGATGCAGAGCGAGAGGAGCAGAACCA
GGAGAACGTTGTACACAGAGACTGATCCATTGTGGCACAATTGAATGTAATGGACTTCTC
CATTAATGGCAATGCAGTGACTCAGGACAATCCAGAGGGATGTAGGAGAAAGAATGTTAT
CCACATCCACAGAAAGAACTGTGGGAGTAGAAATGCAGAAGAAAAACACATGATTGATTG
TGTTGTTCAATGGGGATGTGACTAGGGTTTTGAGGTTAAAAGATCTCTCTCCTGCAAATA
TGAGTAACATGGAAATGGGTTTTGAACAATGATACATGTAGAACCCAATGAAACTGCTTG
TCAGCTGAGGGAGGGGGGGGGAAGAGAGGAGGGAAAAATCATGTATCATGTAACCATGCA
AAACTATTCTAAATTAAAAAAAAATTAGTTAACTACTGACTAAAATTAAAAAAAAAAGAT
AAATAGTGTAAGGCAAAAAGACAGGAGAGGGAGTGTCATATGTGCGGAACAGGTAGAAGT
TTGGCTAGATTGCAGAGTATAGAATTGGGATAATGGTCAATAGGGCAGCAAGGCGGGACA
GTCGATAGAGTACCAGAAGATCTGATTTCAGAGCTAGCCCCAGACACCAAATATGTGAAA
CTGAGCAAGTCAATTTCTAGCTGTGTGACCCTAGGCCAGTCACTCTTCCCTGTTTGCCTC
AGTTCCATCATCTATAAAGTGAGCTGGAGAAGGAAAAAGGAAACCACTCCAGTGTTGTCA
AGAAAACCCCAATGTGCTCCCAGAGAGTTGAAAATGGCTGAATGACAGCAATGACTAGTA
AGCCTAACAAAGCTGACCACTTTACACTGATCATTGAAAATGAGAATTAAAATATCTTTC
CCTTATCATAGATAAACCGTTTTATCCATCGCTTATAAAATGCATTGGGCTTGACCACTG
GGCCTTCGCTGACTAACTGTCCTGACTTCGGAGCTCTCCGGGTCACCGTTTGACCTAGTA
ATCCAACCCTGAGAGCAGGAGAGGTCGGGTGAGAGTCACAGGCCTCGGATCGGGCTCCGG
GTGGTAGAGACGTGGAAGCCTCTCTCAGAACCTGAGTGCCCTTTGCTTGGTCCCTTCTGG
CTCAGCACCCGCCAGGGCATCTCTTCATCACGGTCATCAGACTAGAGCCAATTCCATCCA
CGGGAGAGGAAGAGCTTTCCTCGCCCGCTCCTCCCTGCCCTTCCCCCATTGCACGCGTAT
CTGTCGAGTGAAAGGAGTAGCCTAACCGTAGCCAGCTCTGATGGTGTCTCTCCGTGTTTC
GCTTTACACTTCTTTACTTTTCAG**GTATATTCGTGTCAGCATTGCCAAGTGGCGAAAAAC**
TCCGCCGTCTTGGCCCAGAGATATCAGCCCCTTCCAGTGGAAGAGCCCTGGGCGGCGGTC
ACCGCGGACTTGATGGGGCCCTTCCCGTCCAGCAGCCGGAGCCACGTGTACGCCGTCATT
GTGACCGACGCGTTCACCAAGTGGGTTGTGGCTTTGCCTCTCCGCGACGTGGCTGCATCG
GAAGTCTCTAGAGCCATCATTAGCCTGTTTTTCTTATATGGCCCCCCTCAGAGAGTAGTG
ATGGATCAGGGAGATGAATTTATTAATCAGGTAAGAGAAACGGCCTCCCAGATAGAGCTT
CCAGCGCGATGGCCTGCCCTCCGGAGCCCAGGACTGCGCCCCTTCCGTGGCCTTTGTGCT
GTGTGGCCGCCCTTGGAGGCTGGGTTTTACACAGAGCAGCCCCCGCTCTTGCTAAAACCT
CAGGGTAGGACTCACTTCCTTCTGCAGATGAGGAAACGGAGTCAGGGAGGCTGTGCTCTG
TCACACCGGGCAGTACACACAGGCTTCCTTCATTCAGCAGATACTTCCCTTACAAGCAGC
TGATTCTGCTCCCACCTATCACCAGCATTCTTTTTATTTGCTTTCTTCTTTCTGTTCGAC
TCCGAGAAACCAATTAGGATCCTGTGGCCAAAGTTCAGGAGGCCTAAAAAGCCCAATTTC
CTATCAAAACAAGGGAAAATGAGATAAAGAAATAGAAAGTCTGATAGAGTTCCTTCATTT
GAAGTCTGTGAACCTGGTTAAAGAAAATGGATCACTCTGTAAGCCCTTCATCACCTGCCC
CCCCCCCCCCAGCTCTTTACACCTCATTCCCTTGCCCTGCACTCTGCAAGACGGTGACAC
TACCCGTGGTGGGACACCTTATCTCTTGCTTTTTCTTTAGCTGCCTCCCATGCCTGAATT
CCCTCCCCTCAGATTCTACCTTCTACAGTCATCAGTCAGCACCCTCTAAGACCTTCAGTG
TTCTCTTTTGGTGTTTCAGATACACAGCGTCTTACATCTCCCATTTTATTGTGGATTGAT
CAAGGAAGGGACTGTCTTGCCTTTCGTTATATCCTCAGCACTTAGAACTGTACCTGGCAC

AGAGTACCGTCTGCCGGCTTGACTCAAACATTTTGAGGAGAGCATCTGACTCGCCAAAAA
GAGTGAGCTAGAAGTGAAAGAAAGTATATCTTTGGTTTCCTTCTCAG**ATCAATGGAGAAA**
TCTTGGGGCTCCTTGGCACAATGGCAAATGGGTCACGTTTCTACCCTTCTCAACCAGATG
ATGCAAGTGAAAGAACTGCCAACACAATTAAAATGTTTCTTTCAAAATACTGCCTCGACC
ATCCAAACGATTGGGATGATCATCTACTAGCTATTTCATATGCCTTCAATTTGACTCATT
TGGTATATAACTTTTCCTTTCTTTGCTTACAAGTTGTTTATCCATCGTTTTTAGTTTGGC
CTCGAAGCATTTCATACCACATTCGCCTCTTTGTTTTCTTTGAAACAG**GAGCCTTCCAAA**
AACATACCCTATTTCCAGATGTTTAATCGAAATCCAGAGGAGCTGAGAGCTTTGGATATG
TTTCATGAAGGGGAAGGCGATAGCACATGTATGTTTGCCAAAATTTTACGGGCAGTTAAA
GAAGCTGATAAGATAATGCTGAACAAGACAACACCAACATGCCAAGTAAATCTTATTAAA
ATGATGTATTAATTAAAAGACTAACTATCTTTTAAGTGTTCATTATTTAGGAAAGATGTT
TTGAGAGACAATACATGTATCACGCAGTGGAATTGATTGTCAACTCCAGGAGAGGGGAGG
GAAGAAGGGAGGGAGACAACATGAATCATGCAACCTTGGAAAACTTAGGTATACATTTGT
TACTGGAATAAAATGAAGATAACAATTTAAAAAGAAATAAAACAAAAATAAATAAATAAA
TGACCATTATTTATCTAGCAATAACATTAAGGAAAAATGTATTTGTTTGAAATTCTGACT
TTTCATTTTGGCAGAACGCTCATTAGTTATCATAAAAGTGTTTGATCTTTCAAGAAAAAT
AACTTGTTTGTTTTCTTTCTACAAAATGCAG**ATGGACAGCCACACTCAAGATGAAGCACA**
AAGTAAAAGCAAGATTATCCTTAAAAGGAGACCAAAGCAGCTCAATCCATTTCACCTTAA
AATTGGACATGAAGTTCTTCGACAAAGGAAAAATTGGTGGAAGGATGGACGATTCCATTC
CGAATGGGTTGGTCCCTGTGTCATAGACTACATTACAGAAAAGGGATGTGCTGTTCTGAG
AGACAAGACAGGTGCCAGACTCAAGAGACCTATCAAAATGTCGCACCTCAAACCTTACAT
AAGAGATTCTGGGGAACAAGGTAATTCTCACTTATGGCTCCTGATTTTGCTCATTTTTTT
TAAAAGAGGCTAAAGATTGAAGATAACTATGTGATCTTATTCCTTTTATTCACATTTATA
GGAATTTATCGGAATAGGGTCTAGACTTGGGATTTCATTGGTACGAAGAACTCTTAGATA
AGGAATCTTTCTCTACAACTAGGTAGTACTTGAACTCAGATCTCTCTGGCAATGAGGACA
TTCTCTTCTCTGCTACAATAGCATGCCTCTTTATGCCTCTAAGTATATTTAATATATATC
GGATATTCTAAATACTTTTTATTAAAGTTTCTTTTTGGAAACTATTGATTGGTGGGAAGG
TTATTTAGTTCATTTTTGTACTTCATAGGAAAGATAGGTGCTATAGCTGTAAGTTTCCAT
GAACATGCCAGATGTTTTTCTGAACTTGCTTTGGTGCTTAGGTTACTAGCAGCTTTTCCT
TAATGTTTCCCTCACATCTTTTCCCCCAGGGTCTTCTTTGCCAGTAAGCATATAGTTTTA
TTTGAAGAAATTAGCTTGTAATCATTTGCATTTAAAATGTTTCCATTTAAAGGGAATGCT
ATTGTTTGCTGTTCATGTCAGTTGGATTCATCAAAGAAACAAGTGGGTGTGTGCCTTCAT
TAGGTCAGAGTGACCTAAAGGGGTCGTGTGGGTCCCCTCGGTGACCAGAATTTTGGCCGT
CACTTTAGAAGGCGATACAATTCAAAGGTTTAGGCTATTCCTAGATATTGATGGATGACC
ATAAAAATATCATCAATTATAGCTAATGCTCATCTAGCACCTACTATGTGCCAGGCACTA
TTTTTTCCACACACATTCTCTCATTGGATCCTTACAGCAATCCTGGGAGGTTGAGGCTAT
TATTGTGCCCGTTTTTCAAATGTGGAAATGGGGGCACGTGATATGGTCACACAGCTAGTA
AGTGTCTGGGATTGGATGTGAACTTCTCGCTCCAAGATTGGCGATTGAGATAGCGTACGG
TAACACACAGTATCATTTCTTATACTTTCCCTGTAAAAACATAACTACTCAAATTCTTAA
CATTCACACAACAAATTCAACGTGTGACACATACAGAAAAATATGTTTGAAATATACACA
GCATTATTTAATATTTTGTTTACTAAAAATAACAAAATCTAGGAATTAGAAAAATTAGAG
AATATGTCCTTTGCTCCTGTAGTTATATACCTGTACAGGAATACTATTCCACAGTGTGCC
CAAGAAGTAGTCCAGTAAGCCTTTGTCTTTCCCATCAGAATGTATGGAAGCTCCTGGGGC
AGGGAATATTTCATTTTTGGTCTTTCTATCTTTAGCACTTGGGTAGGCATTCAATAAATG
TTTATTGATTGACTGATAAAACTGAACCCATTTGGAAACCCGTCTTTCCCAAAATGTTCT
CTTGGGATAGAGAGCCAGGCTCGGAGACAGGTGGTCTTGGGTTCAAATGTGGCCACAGAC
ACTGCCTAGCTGTGTGACTCTGGACAGGTCACTTACCTACAAGTGCCTCAGTCCTTATTA
CCTCACTTCTCTCATGGAGCCAATACTGAGTATCCATTCTTGACAGCGTTCTCTGTATAG
CGTAGATATCCAAGAAACTGCCCATAAAATGTCCTGCCCCCCCCCCCTTCTGCTTTTTCA
GGTCTTGCCCACAGGAGTTTATTTGTACAAGCATTTTTAAATTGAAAGTCGTCCGAGTAT
TCACTTGGCGGCTCACAATGCTTTCTGTGGGTTCTTCTCAGTTCTCTCCTACTCTGACAA
ACATCAGGTTTCCTTCCGGGTTGCGGATATCCCCTTCAGGTCACGCTGTTTCCGTTTTGA
TCTCTCCTCGGTCAATGGTGTACCTCCCAGTGTCAGCCTGGCTGCTTTCCTAATCATTCT
GACCAAGCAGTGAGCGGGGCACTGCGACACTTTGCGACAGTTGTGCGACTGTCCTGAGTC
ACTGGTACGCTTGTCTGCGGCTCAGTTCTTCCAGAGCGCTCCTGGTAGTGAAGAGAGTGC
CGGCTCCACGCACACTGTCTCACGAGCCCCGGGGGTACTCTTGGCCGCTTGTTCTTCCAA
ATGAACTTGCTATTCTTTTCTCTAATTCAGTAAAAGGACGTTTTGGTCATTGGATTGTGG
TCGCGGCCACAGTCACAAAGAGGTTCGGGATGAGAATGGAACTGCAAAAGGGCTTGTGGG
GGATGGGAAGGGCCGGGAAGAATCTGACCGGGCCGATTGCTGCTTTTGCTCCTTGACCTC
TCCGGCCGTCTGCGGCCCTGTGGCTCGCCCTCCTGCTCTCCCCCACCTTGTCCAGATTCC
AGCCTCGGGCCTGGGCTGGGCCTCTTCTCTCCTCCCTCTCTACTGCTTCCCTTGGGAGTA
CTGCCTTCCAGAGTGGGCGTCAGATGGATGCCCGGAGCCAAACGTGTCCAAAGCAGACCT
CGTTTGTCTCCTTAGCCCCAATCCCAAATAAAAGCAAAAATTAAATTATGTATAAATAAG
TACAGAAATAATAAAAAAAAATAAAAATCTTAGATGTATAAAATTCTGAGTAAAAAATAAA
ATAAACCTATAATAGGTTCTTGAATCAGTAACAAGATCTAATTAAAGTGACTTATGTCCC
ACAAGCCTGTAGGACAATAGCAGCAATACAGTACTTTACCCATTTGCAGCCAGGTCTACA
GAAAATTGCCAAACTATAAGAGAGAAAGGACTAGGTTTCAGCAGCAAGTGGGAAAAATCA
TTCTCTGGTCTGATTGTACCTTCACTGTCAGAGATAGGGGGTGCCAGGATGATAGCCAAA
CTGAGGTACTTGACTCAGAATATTGCACAAGGAGTGTGGTACAAGGAGTCCTTCATCTTA
ATGTATGTCAAACACCACAACCTAGATTCTCACACTAGATTCAAGTCCTTCCGTATTGGC
ATAGAAGTTCAACAATTCTCCCTCGATTGGTTTGGTCCTTTTTGACCTCATGGTATTTGG
CTCTGTATAGTGGCTATCCTGGAATCAGACACAATCATTAAAGAAAGTCCCATAACATTT
ATCAATAAATGGCAGGTTTCCATAGACTAAAGCTTTGGGGTATATATAGTTATCAGGGCT
AATAGATACTGGAAACTTATCTTGTAAGCCTTATCCTTCAAGTCAAAAGTATTAATACTG
ATTTCATGTACTTCAAGTATTACCAAGGATAGGAAAAAAAGAAGAAGAAAAAAATCAAAT
ATTCTATTGTGAATATAAAGTAAAAATAATTAAAAATCATAATTCATAGTTCACATTTCA
TGTGACAATGTTTTAGCCAAGCAGAGGCCCAACAGAAAAGGTGCTCAATCAAAAATGAGA
TTTATATCCCTCTTAACTTGGCTAGGATCCAGTGTGAGTGTACATGATTTTTTCACCAGG
TAAAAACCTTTTAAAAACAGTAGTACAACAACTAATACAGATTCTAAGAAGTACCAAAAT
CCCCCAAATTATAAGGACCCATTTCAGAATAACTGCAAACAAACCCATTCCTTGCAGCCA
TGAGAGGTTCTACCAGCTATTATTAGGATTAACATTAATCTCTTCAGCTACTTGCTGTGT
GTTATTAAAAAGCCTTTGAAGCTCATGGATATTTGTCACAAGTTTTCCAGACCTATTTCT
ATAAGAACAACAAGACTGATTAATAAATGCACAAGCACCTCCCCATGCGGCAGTGAACAT
GTTGAGGGCCAGTCAATTTTGTAAGACTGTTTTTGCTAGAGTCAATCTCCTCCTGTAGAT
AAGAGACGGCTTTAGTGGTTTGGGAGATGGCTTGAGCTGTGTTTTGAGCCAATCTTTCAA
CCTCAGCTTAGATATTCCTAACAGAGTCTATTACTGCCCTCATTCCAAAATTTGGCAGGA
GAGCCCAGAAAAACCACTGTCCTGCTGATGAAGAGGCTACTGAATTTGGTCTTGACCTCT
TGGGTCTAGAATGCCACTCAGAGCCTAGAACTCTGGCTGTTGATTTGTCTATCCAGCCAA
CATATCTTGATCATGCACAAATATAAATGGAATAACATAGGCCAAACCACAAGTACTATA
CCATCCTGGTGGAAGGGTAGAGTAAGCTGAGGAGTTACAAATATAGAACGGTCCAGGGGT
GACCAAACTTCATGGGAAATCCTCCTTCCTCTGGGATGAGATTGTAATTGCCATAAAAGG
CATGTATTTATACTTCCCATCCATTTCATTTTTAAGCCGTATTTCTAGCACAGATAAATG
TGGAGGTAGGGAATGCAATAGTATGTTTGCACCGTGCCTTGTCTCCTATGTACTTACTGC
TACTCACTCAATGAACCCTTACCTCTTCTTACACACAAGTCTGAGGTGGGCAAATGATCA
GCCATAGGTAGTGCGGCTCCTAGATATCTGGAAATTACTTCTGAAGACCCATTAAAATCA
ATTGAGATTTTTAGCTCATCAAATTTTTCAAGTCACTGATACAGGTCATAATAATTTTGA
TAGGGTCCACCCATCATCATTAATGTGACAATTTAACAAAAGCAAAAGGAGCCAAAGTCT
GTTTAGACAACATGTGACCTCCTTGTGTTGCAAATTGTGGTAACATCTCATTACTGTGGC
CTGGAAGTGTTGCCCATAACAAAACAGAGCTCCAATGGATCTGATGACACACCCAGCACC
CATGAGGACCAATGCCTTTTCCCTGGAAAAGAGTTTGTCCAACTTGTCTATGGAATTTTA
TAATGGTATTATCACCAGTCCAGTCATAGGGCAAAGGTACAAATAAAGACAATGTAGCAG
AGAATATAGCTAATAGCCATAATATAATAATTGAGGGTGACATAGTAAAATGGAACATGA
TAGCTTAGGTTAATATGTGAATTTAAAAATTAAAATTAAATCTTAAAACAATCAGTAAAT
ACAATAAATGTGACAGGATACTGCGATATTCAAATAAAGATAAATTTTAATTACAAGTTT
GGATTATGGAGGTATCAGGGAAGAGGGAAAAGGGGTTTCCCTAGATGAGGTTTGAGGCTC
TCTCGGCTTTGAGCTAAGGTCAGGCCTCAGAGGCTGGTGGAGGGTTTCTGTTATTCCTGT
CTATGCTAATCTGTGCTAGTTTTTTTAAGTTCAAAGTCTTAGCAGTAGACAGAGGAAGAA
AAGTTCTGACCTTCAGAGCTGGTTGGGCCTGTCTCTTTGGGCTGATGCCCCCAAAGTACA
GCCTTTAGTCATGTACCAGGCCTATCAGAACAAGCTCTCTCATAGATTTTAGTAATGCAT
TTCTAGCCTGGCATAGTTGTAAGTAATTTTGCTCTGAATTAGGAGTTCCATTTGGTATCT
TCATTTGGCCAACTAGGTGTTCCCTCTCCCTGTGCCTGATTCACAAGAGAAAGAATCTTA
TTTATTTCCCTTTCCATTAGCAAGGCCTGGAGCAAATCTTCCACATCAATCCATGTGGGA
TTATATGTGCGGGAAATGCTTTCAACCTTATTTATGAGTAGTATAGACTAAGATTCAAAG
GAAGGTAATTTTTCTTTGAATCCCTCACTGTCCTACGGAGAGAAAGGTGCATGATGTCTA
ATGTTTAGTAAATCTCCCAGTATATTATAAGTAGGGATGTCTCTTAAAGGAAATAGACCT
TTATTCAAATCATCTATGATAACTGCTGCTTGGCCTGTTTTCTGGAGTTCAATGGATTGG
GTTGTAGTGAGGCTAGTAGGGGAAGGTAGGGTGGGCAAATGGATAGGGAGGGACTGGTCA
GGGGATGGGAAGTCAGGGCAAAAAGATTAGGGGTAAGGCTGGGGGTGTGGTGGAGAGGAG
TAAGATGGGTGAGGTCTAGGGGAAGAGTCATAGTAAGGTGGGTGAGATCAGTCAAGGGGA
AGAGAGTAGGGAAAAGGATGACCAAGAGAATAAGGATAGGAATATAGGGAAAATGAGTGG
GCACTGGGTAGTGAGGAGAAAACTCTTGAGGGTATGAGTAGTGGGATGGTAAGGGAGGGT
AGGGATATAGATTGGGAGGAAAGAGGATGGGAGGGGTGGGGTATGAGAGAGGTCTGACAG
AGAGAGGAGCTGAAGGGTTCAAGTCCTGGTTTGGGTCAGTATGAAGAGAAACTTCCTCAC

AATCCAGGTAGGATGATAAGGTGTGTTTCTTGCTAGATCTAATCAAGAGATCAGGAGGAG
GAACTGAAGGAGTTAAGCCAGAATTGCCTGGTTCCAAAGAAAGAATGGTTTCCTCATTAG
AGGGGATGGCTGATTTATCACTATGATATGCCCACAGGAGCCAGTATTTACAATCTTTCA
AATCTTTGTTTTTAATGACTAACTTAAAATCTTTTAAGATCTTGATGTCAAAAGAGCCAT
ATTTGGCCATGTATAAGATATGTCCATCTAGGCTTTGCAAAATTTTCTAGCTTCCTTCTT
TGTCATCCAATTATCCTTAGAAAGATTAAGATCATCCCAAGAATTAAAAAGTGTATGCAG
TGGTGAATCTGGAGGAATATTTCCTGATTTCCCTTCATTTGTTGCAGTATTTCTCATAGT
GTCTAGTCTGTCTCTCTGTCTTAATCTGCTTATTTAAGCTAGTCTGAGTATTGCTAAATG
GTCAATTTAAACAATGAAAAGTTCCTAAAACATAAAAAAATGGAGACACAAATTTCTTCA
ATGTTACTGTGGAAGGCAATGAGAGTAGGTTAAAAAAAAAAGATAATATAAGGAACTAAA
TATTGTTGAATAGCAATAGTACTAATAATGAATGTGTATTTATTATAATAATTGTTATTG
ATCAATAATAGTAAATAATCATGAACAAATATCAATTATTAATGTTAATTAATAATAGTT
TCTAGTGACTAATAATAGTTATGGAAAATTAATAGTTATTGTTATTGATAGTATTATTAA
TACTATTGTGTTCTAAAATTATTCCTTAATGCATTTAATTCAATTTAATTTTGGCTTTGT
TTAATTTTAGAAATTTGGTTTTAATTTTAATTCAATAATATTAATATTTAACTATTACTA
ATCCTTATATTTAAATGTATTCAATTTAATATTATTATTTTGGATCACTAGGAGTAATAG
TAGTAGGAAAGGAATTTTTTTAAATTTCCTGCAGTTTTATTTCTCACCACAGGGTGGTGC
TATAACTGTGTGTAGAGTTTAACACACTTGAGGTTAGACCTGATCTAAACAAGTAGAGAG
AAAGGAATGTGTGAGGGGGGAAGGGAGAAACCACCACCAGCACCACCACCACCAATACTA
CCAACACTTCCTCTTAGCTCAGTCCTAAAGAATGTTTGATGGGGAAAGGGAGGAAAGAAA
ATTCAAAAGAGAACTTCCTTGAAAATATTTAGTCTTTTCCCCTCCCCCAGAGGTAGTCTA
GTGGCCATGTGTTTTCTATTTAAAGTTGGGGGCACTAATTACTGTGAGTAACTCACATTT
AGATTTTTCCTGTCTCTAGGTCTGGCTCCCAAACTACCGAGCATGCAAATTCCCAACACT
CTGCTACAGTGGGTTCTCGGAGGGGCAGGGGAAGTGTGCTGGGTCTTAGGGCCTCCTCTC
ACTAATCCCTAGGGGGCCACCCCTCCACGCTGTGCCTCCAGTCCCCAGGCTGCGGTCATG
GCAGCTAGGAGCATGAGTCAATGTCCCCTGGTGTGGCACACCTAGAAAAGTACAGACTGT
CAGGGGGCGCGTGATATCGCATCTGCACCAAAGCTTGGACTTCTTCTCTAATGCCCCTAC
TGCATCAGTTAAANNNNNNNNNNNNNNNNNNNNNNNNNNNNNNNNNNNNNNNNNNNNNNNN
NNNNNNNNNNNNNNNNNNNNNNNNNNNNNNNNNNNNNNNNNNNNNNNNNNNNNNNNNNNN
NNNNNNNNNNNNNNNNNNNNNNNNNNNNNNNNNNNNNNNNNNNNNNNNNNNNNNNNNNNN
NNNNNNNNNNNNNNNNNNNNNNNNNNNNNNNNNNNNNNNNNNNNNNNNNNNNNNNNNNNN
NNNNNNNNNNNNNNNNNNNNNNNNNNNNNNNNNNNNNNNNNNNNNNNNNNNNNNNNNNNN
NNNNNNNNNNNNNNNNNNNNNNNNNNNNNNNNNNNNNNNNNNNNNNNNNNNNNNNNNNNN
NNNNNNNNNNNNNNNNNNNNNNNNNNNNNNNNNNNNNNNNNNNNNNNNNNNNNNNNNNNN
NNNNNNNNNNNNNNNNNNNNNNNNNNNNNNNNNNNNNNNNNNNNNNNNNNNNNNNNNNNN
NNNNNNNNNNNNNNNNNNNNNNNNNNNNNNNNNNNNNNNNNNNNNNNNNNNNNNNNNNNN
NNNNNNNNNNNNNNNNNNNNNNNNNNNNNNNNNNNNNNNNNNNNNNNNNNNNNNNNNNNN
NNNNNNNNNNNNNNNNNNNNNNNNNNNNNNNNNNNNNNNNNNNNNNNNNNNNNNNNNNNN
NNNNNNNNNNNNNNNNNNNNNNNNNNNNNNNNNNNNNNNNNNNNNNNNNNNNNNNNNNNN
NNNNNNNNNNNNNNNNNNNNNNNNNNNNNNNNNNNNNNNNNNNNNNNNNNNNNNNNNNNN
NNNNNNNNNNNNNNNNNNNNNNNNNNNNNNNNNNNNNNNNNNNNNNNNNNNNNNNNNNNN
NNNNNNNNNNNNNNNNNNNNNNNNNNNNNNNNNNNNNNNNNNNNNNNNNNNNNNNNNNNN
NNNNNNNNNNNNNNNNNNNNNNNNNNNNNNNNNNNNNNNNNNNNNNNNNNNNNNNNNNNN
NNNNNNNNNNNNNNNNNNNNNNNNNNNNNNNNNNNNNNNNNNNNNNNNNNNNNNNNNNNN
NNNNNNNNNNNNNNNNNNNNNNNNNNNNNNNNNNNNNNNNNNNNNNNNNNNNNNNNNNNN
NNNNNNNNNNNNNNNNNNNNNNNNNNNNNNNNNNNNNNNNNNNNNNNNNNNNNNNNNNNN
NNNNNNNNNNNNNNNNNNNNNNNNNNNNNNNNNNNNNNNNNNNNNNNNNNNNNNNNNNNN
NNNNNNNNNNNNNNNNNNNNNNNNNNNNNNNNNNNNNNNNNNNNNNNNNNNNNNNNNNNN
NNNNNNNNNNNNNNNNNNNNNNNNNNNNNNNNNNNNNNNNNNNNNNNNNNNNNNNNNNNN
NNNNNNNNNNNNNNNNNNNNNNNNNNNNNNNNNNNNNNNNNNNNNNNNNNNNNNNNNNNN
NNNNNNNNNNNNNNNNNNNNNNNNNNNNNNNNNNNNNNNNNNNNNNNNNNNNNNNNNNNN
NNNNNNNNNNNNNNNNNNNNNNNNNNNNNNNNNNNNNNNNNNNNNNNNNNNNNNNNNNNN
NNNNNNNNNNNNNNNNNNNNNNNNNNNNNNNNNNNNNNNNNNNNNNNNNNNNNNNNNNNN
NNNNNNNNNNNNNNNNNNNNNNNNNNNNNNNNNNNNNNNNNNNNNNNNNNNNNNNNNNNN
NNNNNNNNNNNNNNNNNNNNNNNNNNNNNNNNNNNNNNNNNNNNNNNNNNNNNNNNNNNN
NNNNNNNNNNNNNNNNNNNNNNNNNNNNNNNNNNNNNNNNNNNNNNNNNNNNNNNNNNNN
NNNNNNNNNNNNNNNNNNNNNNNNNNNNNNNNNNNNNNNNNNNNNNNNNNNNNNNNNNNN
NNNNNNNNNNNNNNNNNNNNNNNNNNNNNNNNNNNNNNNNNNNNNNNNNNNNNNNNNNNN
NNNNNNNNNNNNNNNNNNNNNNNNNNNNNNNNNNNNCAAGGGAAAAAGGGTTGTTAGGAA
GTTTTTACTAGCAATGGACCGCTGCTCAGGATCTAGCTTTTAATGAGGCTGGTAAGGAGC
ATTTTTTACAGGCAATCCCCTGAAAACCCTGCAAGGAAGGATAATTTTAGTCTCCTCATT
CCCAATTTTTATACCATCCAGATTCATATTCTTCTCTGATTACTACTGCTAATGTTTCTA
GTACAATGTTAAATAATAGAGGTGATAATGGGCATCCTTGTTTCACTCCTGATCTTATTG
GGAATGCTTCTAAATTGTCCTCATTACAGGTGATGCTTGCTGATAGTTTTAAATATATAC
TGTCTATTATTTTTAGGAAAGGCCCTTCTATTCCTATACTTTCTAGTGTTTTCAATAGGA
ATGAGGGTTGTATTTTGTCAAAGGCTTTTTCTGCATCTATTGAGATAATCATGTGATTTT
TGTTGGTTTGGCTATTGATATGGTCAATTATGTGGATGGTTTTCCTAATATTGAACCATA
CTTGCATTCCTGGTATAAATCGTACCTGGTCATAATGAATAACCCTTGTGATGACTTGCT
GGAGTCTTTTTGCTAGTATCCTATTTAAGATTTTTGCATATATGTTCATTAAGGAGATTG
GTCTATAGTTTTCTTTCTCTGCTTTTGATCTGCCTCGCTTTGGAATCAGTACTATATTTG
TGTCATAAAAGGAATTTGGTAGAACCCCTTCTTTGCTTATTATATCAAATAGTTTGTATA
GTATTGGGATTAGTTGTTCTTTGAATGTTTGATAGAATTCATTTGTGAATTCATCAGGCC
CTGGGGATCTTTTCTTAGGGAGTTCTTTGATGGCTTGTTCAATTTCTTTGATATGGGATT
ATTTAAGGATTCTATTTCTTTTTCTGTTAATCTAGGCAATTTATATTTTTGTTAATAATT
ATCCATATCACCTAGAATGCCATATTTATTGCCATATAATTGGGCAAAATAGTTCTTAAT
AATTATTTTAATTTCCTCTTCATTAGAGGTGAGGTCACCCTTTTCATCTTTGATATTGTT
AATTTGGTTTTCTTTCCTTTTTAAAATTAGATTAACCAGTACTTTATCAATTTTATTTTT
TCAAAGTACCTACTTCTAGTCTTATTTATTAATTCAATATTTCTTTCACTTCTACTTGTA
TTAATTTCTCCTTTAATTTTTAGGATTTCTACTTTAGTTTTTATCTGGGGATTTTTAATT
TGTTCTGTTTTTTAATTTGCATGCCCAATTCATTGACCTTTTCCCTCCCTGATTTGTTAA
TATGTGCACTCAGGGATATAAATTTCCCCCGACTACTGCTTTGGCTACATCCCATAGATT
TTGATATGATGTCTCATCATTGTCATTCTCTTCAATGAAATTATTGTTTCTATGATTATT
TCTTTAACCAACTGAATTTGTAAAATCATATTTTTTAATTTCCAATTAATTTTTATTTGC
CTTTCCATGTACGCTTACTAATTATTACTTTTATTGCATTATGATCTTAAAAGTTTAAAT
TTTTTATTTCTGCTCTTTTGCTTTTGTTTGCCATGTTTTCATTCCCTAGTGCATGGTTAA
TCTTTGTGAATGTACCATTTCCTGCTGAAAAGGTATATTCCTTTTTGCCATTATTTTTCT
CCATATATCTATTAACTATAATTTTCTACAATTTCATTCTCTTCTCTTACCTCTTTCTTA
TTTGCTTTTGGGTTTGATTTGGCTAGATCTGATAGAGGAAGGTTCAGGTCTCCCACTTGT
CTAGTTTTACTATCTATCTCCTCCTTAAACTTCAGTAATTTGTCTTTAAAAATTTGGATG
ATATTCCATTTCATGCATACAAATTGAATACTAATATTTCCTCATTGTCTATACTGCCTT
TTATCAGGATGTAATTACCTTCCCTATCTCTTTTAATCGGATCTATTTTCACTTTGACTT
TGTCAGATATAATGATTGAGACTCCTGCCTTCTTTTTCTCAGTCGATGCCAAATAGATTT
TGTTCCATCCTCTTACTTTTACCCTCTGTGTGTGTCTGCCTACCTCCCTAATGTGTGTTT
CTTATAGAAAACTTATCATAGGATTTTGGTTTTAATCCACTCTGCTATTGCTTCTGTTTT
ATGGATGGGTTCATCCCATTCAGATTCAGAGTTATGATTACCACCTGTGTACTCCCCATC
ATTTTGATTTCCTTTCCTAATCGTCCCTTCCTTCATTCACTATGGCCTTCTACACTAGTT
TTTTGCTTTTAATCCATTACCCCTAATCCCCACCTGTGTTTTACTTCCCTTTCCACCCCC
TCCCTTCACATTCCCCTCTTATTTTTTTAGGGTCTATTAAATTCCCTCCCCGCGCTCTTT
TTTTCCCTTTTGGTACTCCCCACCCGACTCCTCCCCTTAGTTTTTCCCTCTCAACCTCTT
TGTAGGGTAAGATAGAATTTTGATACCCCAATGGATCTAGATATTCTTCCCTCCCAGAGC
TGATTCCACTGTGAATAAGGTTTGAGTATTACCTATTAGCACTGTCTTCTTCTCCTTCTT
ATAGTAGTATTCTTCCCTTTCCCATCCCATGTACCTCTTTGTCATATAATTTATCCTATT
CTTCTTATTCCTTCAAGTTTTCTTGGTGCCATCTTCAACCCCACCCTTTCTATTTTTACA
TATCATCATAAACAACTTAGTACCCCAACTACTACCTATGGATAATTCTTTTAACTACTA
TGATAGTTAATACAGTTTTTCAGAGTTACAAATATCATTTTTTCATAAAGGAATATAAAC
AATTTGAGTAAGTGATCCTTGGTTGTAGACCCAATTCTCTTGCCTTTTTGAATATCATAT
TCCAAGCCTTGTGGTCCTTTAGTATGGAGGTTCCCAGATCCTGTGTAATCCTGATTGGTA
GTCTTTGAGATCTTAATTGTCTCTTTCTGACTTCTTGCAGTATTTTCTCCTTAGCTTGGA
AGCTCTTGAATTTGGCAATTATATTCCTGGAGGTTGTCTTTTGAGGATTTATTGTAGAGG
GTGATCTGTGGACTCTTTCAATGTCTATTTTTGTCCTCTTGTTCAAGAATATTAGGGCAG
TTTTCTTGGATAATTCCTTGTAGTATAATATCAAGGTTTCTGTTTATTTCCATGTTTTCA
GGTAGGCAGAATTGTCTCTCCTGGACCTGCTTTCCATGTCAGCCATCTTCTCAATGAGAT
ATTTTTTGTTTCCTCCTAATCTGGCATTCTTTTGACTTTGCTTTATCAATTCTTGCTGTC
TTGTGAGATCGTTGGCTTCTACTTGCCCAATTCTAGTTTTTAAAGACTGGTTTTCAGCTA
TGATCTTTTGATTTTCCTTCTTAGTTTAGTGTATTCTGCTTTTCATGGCTTTCAGCAGGT
CATTTCTGGTCTTCAATTTGCTTATCATTTCATTTGATTTCTGGGCTTCTTTTTCCAAGT
GGAAGATTCTGTCTTTTAAACTGTTAGTTTAATTTTAAACTATTTCCCACTTTTCTTGAC
AAGTTTATTTGGTCTTCTTCATAATCTCAAATCTGAGCTCCTCAAGAGCTTGTGACCAAT
TTCCATTTTTTGGGAAGGTTTAGATATGTTTGCTTGTTTGTCATCCTCTGCTGTATCCTT
GGTAGTCTGGTCTTTTTCTCCATAGAAATTATGGATGGTCAAGGTTCATTTCTTGTTTTT
TTGTTTGTTTGTTTTTTGTAGTTGCAGATTGTATTTCCTGGGTGTTGGTGGCCATTTTTG
TACTCGTTCTTTCTTTTCTTCCTAGTCAGAAGTCTGAGTAAGGAGGGCAGGTTGGTCTTT
GTATGGAGTTATGGAGCCTTTTTTTCCCTGAGGCCTCTTTGCCGTCTCAGTTGAGTCTGC
AATTGCAGCCCCTCTCTCCCCTATCTCCGTGTTTCTCATGAGGCTTCCTGGGGTCCCAAG
TTTTGCTGCTTCTGGTGTAAATCTGCTGTGTCCTCAGACAGCCCCCCAAGAACCTAGAGA

TTGCCCCTCACTTGCTTTGACTCTGACACATCTTAAGTGGAGTGGGGAGGGGAAATGAGC
TCATGTTTTTGTAGGAGTACCCTTTTTTTACTCCCTTTATAGCATGGAAATGCCCAAACT
CTGCCCACCTTCAAGCTATGGAAGAGCCCCTTCACTTGTCTGGTCCCAGTTTTTGTTCTT
TTGAGAAGTTTTATGTCTGTTTGTGGTAAGGCGAGGAAGCCCCTTGCAGTATACTATGCT
ACGGCCATTTTAGCCTGGAAGTCTGCACTAGTTTTATGCATTTTAATATCTTTTTGTTTG
GTTGTTATATGTTCTTCCCCAAGTTAGTTGCTCAAGTACTATAAAGTTGATCATACTCTT
ATTCCAAGAAAGACATGCAAAAGTAGTCACGGGTATTTCTGATGTCCAATTATATTCTGA
GCCAAAATGGCTAATTCATATTTTCAAATAAACTCTAACTTCCTCCTTGCACACCCATAT
CTTATTTTCCATTTGCTAGTTAGATCTTAAGAGCAAACCTAAATAATCTTCAATTTCCAG
AGACATACACATTTCCAAGAACCTGGGCTTTGGGGATTTGGAGGACATTCAGTCTAGATA
CCAGAAAATACGATGTTTATGTCAGAAATTTAGTTCTTTCATAAGTTACCTGAAGAGTTT
TAAATTTCCTCAAAGAGTTCAACCAGGATTGTTAAGATATCTTTTAAGATTATATTAATT
CATCCGCTTATGCTTCCAGAAATGTGCTGAAGGCAGAAGTGTATTTTTAAGCACATTTTA
AAAAAAGTGGACCTTTGATGTCATTGATATAGGGAACTCCTGGTGAGGCAGTGCCCTTTC
CCAGTTTAGGTCTGCATCTGCTCTGTAAATGATGTCCGTAGAGAGTTGATTAAAGTCACA
GAAGCAGCAATTTATGTTAAAGAAGGGACTTGAACTTGGGTTTTTCTATTTATTAAGCCC
GTTGCTTTTCAGCAGACTCCTTGAGAGCAGCATCATTTCTAAAGTTTATAATTTATAGTT
TGTTACTTCTGAGGGAAAACGTAGAGCTCAGAAACCACAAATTATCCAGCAACCTGCAAT
GATTTTTAAAGAAATCTTACTTTTTAAGAACTGCTCGTTGTCCCATGTCTGACCTATAAT
GGCTAAATGTTCTTCTTCATATGCCTATTCATTCTGTAGACAATGAGAAAAAGAAACGGT
TGTGTAGCTCGGCATCCTTATGGACTCTCGCTTTGTTGGGGAGCCCTAAACGATCGCTTT
TCTGAAAGATAAACTCGTGATTTCTTCCTTCTCGTTTTAG**ACAGTCATTATCATTTACAT**
GACCCAGTCGTGACCGATCACGACTATGTTGGCTTAGCCGAAATTCCGATTGGGTCCTAC
CAGCCAGAGATTCTTGTAGAAGACGCGCCTCTCTGTGCAGCGGATGAGAACCTGCCTGCA
CCAAACACGGGCCAGGAGCTGTCAGAAAGCAACAGCTCTAAAGTGTCTCGGCTCCTAGAA
GATGGTGCCGTCCAGCCTGTCCACTTAGATCTTGGAAAGCCGGCCTTCAGTCTGTTGGAC
GCCTCTAGCCACGGCCTGGAATATCTAAGTTAGTTAAATAAATTCATGCCATGTTTGTTT
AGAATCCACAACCTCCTTTATAGCCTTTGGCTCCCTGGGCTCAGAAAATCTATGTTCACA
CGCTATTAAGTCCTGAGATGGTTCTAACTATTTATTTAGTAAAATATGTATATAGGTGAA
GGGTGTGTATGCACATATATGTGTACATGCACATGTATAGAAAACACAATTATACTATGT
ATATGTATATGATGGCAAATGTATCTGAAACATTTATTATGAAAAGTGTATTTTCCCCCA
GAAAAGTACCGGGTGTGTGTTTAAAGCACCATCATTTTAATAAAAGAAAATTAAATTCCA
GCTTAGTATTTACTGTTTTTAACCCATTTTATTCTGTTTTTTCCATTAAATCTTTTGTTT
TTATGAATGTGGCTGATCTGACTCTACTCCCTAAAATTGAATGAATAGCGAAATGGATTT
TTATGCTCTATGAGATAATATGATGTTAGAGGAGACAATGAAGGTTTATATCAAATGGAA
TCTTAGTAGATGTTAGCTGTATCCACTTTGGGGATGGCATCAAAAATGCCTCTTCATCCA
AGAGCATACAAACCCTTAACCCAACCCATTAAT

```
==================================================
```

Monodelphis Gin-1

```
total length:      33633 bp  (31870 bp excl N/X-runs)
GC level:        41.33 %
bases masked:      16270 bp ( 48.38 %)
==================================================
               number of      length   percentage
               elements*    occupied  of sequence
--------------------------------------------------
SINEs:                11         1605 bp    4.77 %
      Alu/B1           0            0 bp    0.00 %
      MIRs            11         1605 bp    4.77 %

LINEs:                12         6722 bp   19.99 %
      LINE1            5         5506 bp   16.37 %
      LINE2            7         1216 bp    3.62 %
      L3/CR1           0            0 bp    0.00 %
      RTE              0            0 bp    0.00 %

LTR elements:         10         7055 bp   20.98 %
      ERVL             0            0 bp    0.00 %
      ERVL-MaLRs       0            0 bp    0.00 %
      ERV_classI       9         6543 bp   19.45 %
      ERV_classII      1          512 bp    1.52 %

DNA elements:          1          189 bp    0.56 %
      hAT-Charlie      1          189 bp    0.56 %
      TcMar-Tigger     0            0 bp    0.00 %

Unclassified:          0            0 bp    0.00 %

Total interspersed repeats:     15571 bp   46.30 %


Small RNA:             0            0 bp    0.00 %

Satellites:            0            0 bp    0.00 %
Simple repeats:        5          582 bp    1.73 %
Low complexity:        2          117 bp    0.35 %
==================================================
```

SW score	perc div.	perc del.	perc ins.	query sequence	position in query begin	end	(left)		matching repeat	repeat class/family	position in repeat begin	end	(left)	ID	
293	32.0	3.2	3.8	UnnamedSequence	1219	1403	(32230)	+	WALLSI4	SINE/MIR	43	226	(23)	1	
690	21.6	0.0	0.0	UnnamedSequence	1776	1951	(31682)	+	(CA)n	Simple_repeat	1	176	(0)	2	
389	26.4	2.0	0.8	UnnamedSequence	2387	2532	(31101)	C	MAR1c_Mdo	SINE/MIR	(68)	147	2	3	
269	27.3	0.0	0.0	UnnamedSequence	2550	2626	(31007)	C	L2-2_ME	LINE/L2	(110)	2431	2355	4	
191	26.7	18.6	0.8	UnnamedSequence	3450	3551	(30082)	C	L2a	LINE/L2	(25)	3401	3282	5	
611	23.6	5.5	1.8	UnnamedSequence	5068	5231	(28402)	+	MIR3_MarsA	SINE/MIR	39	208	(6)	6	
1130	6.7	2.2	1.1	UnnamedSequence	5375	5644	(27989)	+	(TCTA)n	Simple_repeat	1	272	(0)	7	
199	26.5	0.0	0.0	UnnamedSequence	5647	5695	(27938)	C	MIRb	SINE/MIR	(70)	198	150	8	
571	22.4	8.0	0.0	UnnamedSequence	5698	5822	(27811)	C	MIR3_MarsB	SINE/MIR	(70)	145	11	9	
516	18.6	4.1	0.0	UnnamedSequence	6385	6529	(27104)	C	ERV15_MD_LTR	LTR/ERV1	(0)	398	248	10	
242	22.4	2.9	1.4	UnnamedSequence	8209	8276	(25357)	C	L2B_ME	LINE/L2	(22)	3425	3357	11	*
1763	17.7	4.7	4.7	UnnamedSequence	8273	8716	(24917)	+	L2_Mars	LINE/L2	2845	3288	(164)	12	
207	10.0	0.0	0.0	UnnamedSequence	8717	8746	(24887)	+	(TA)n	Simple_repeat	1	30	(0)	13	
4747	7.0	4.4	0.8	UnnamedSequence	9314	9796	(23837)	C	ERV13b_MD_LTR	LTR/ERV1	(0)	914	420	14	
4393	4.8	0.9	0.0	UnnamedSequence	9797	10338	(23295)	C	MdoLTR2	LTR/ERV1	(0)	547	1	15	
4747	8.6	3.3	0.5	UnnamedSequence	10339	10719	(22914)	C	ERV13b_MD_LTR	LTR/ERV1	(495)	419	1	14	

759	17.4	12.0	1.3	UnnamedSequence	11030	11238	(22395)	+	L2_Mars	LINE/L2	3021	3251	(201)	16	
979	19.2	1.6	1.1	UnnamedSequence	11241	11429	(22204)	+	OposCharlie3a	DNA/hAT-Charlie	27	216	(0)	17	
324	25.5	4.3	9.0	UnnamedSequence	11454	11686	(21947)	C	L1_Opos	LINE/L1	(4623)	1827	1605	18	
244	29.4	6.6	3.5	UnnamedSequence	11676	11872	(21761)	C	L1_Opos	LINE/L1	(5014)	1436	1234	18	*
23	88.6	0.0	0.0	UnnamedSequence	12471	12549	(21084)	+	AT_rich	Low_complexity	1	79	(0)	19	
416	25.4	5.4	2.9	UnnamedSequence	12593	12649	(20984)	+	MIR3_MarsA	SINE/MIR	28	84	(130)	20	
4183	11.4	6.9	2.4	UnnamedSequence	12650	13375	(20258)	C	LTR99_MD	LTR/ERV1	(0)	758	1	21	
416	25.4	5.4	2.9	UnnamedSequence	13376	13417	(20216)	+	MIR3_MarsA	SINE/MIR	85	131	(83)	20	
1737	17.3	11.3	4.0	UnnamedSequence	13418	13929	(19704)	+	ERV7b_MD_LTR	LTR/ERVK	1	548	(0)	22	
416	25.4	5.4	2.9	UnnamedSequence	13930	13994	(19639)	+	MIR3_MarsA	SINE/MIR	132	196	(18)	20	
256	30.4	4.3	0.0	UnnamedSequence	14000	14114	(19519)	C	L2B_ME	LINE/L2	(189)	3258	3139	11	
3111	15.7	0.3	2.3	UnnamedSequence	14129	14697	(18936)	+	L1_Mdo3b	LINE/L1	5972	6529	(4)	23	
906	17.0	10.8	10.4	UnnamedSequence	14802	15051	(18582)	+	MAR1b_Mdo	SINE/MIR	1	251	(3)	24	
342	24.4	3.3	0.0	UnnamedSequence	16597	16686	(16947)	+	L2_Mars	LINE/L2	3327	3419	(33)	25	
617	18.1	1.5	0.0	UnnamedSequence	17330	17462	(16171)	+	L1_Opos0	LINE/L1	6393	6527	(26)	26	
24	57.9	0.0	0.0	UnnamedSequence	17484	17521	(16112)	+	AT_rich	Low_complexity	1	38	(0)	27	
347	23.2	0.0	0.0	UnnamedSequence	18068	18136	(15497)	C	WALLSI4	SINE/MIR	(6)	243	175	28	
786	21.6	11.6	0.0	UnnamedSequence	18735	18924	(14709)	C	THER1_MD	SINE/MIR	(5)	269	58	29	
346	18.2	21.0	0.7	UnnamedSequence	19223	19337	(14296)	+	L2B_ME	LINE/L2	3322	3451	(1)	30	
628	22.5	0.0	2.6	UnnamedSequence	19385	19539	(14094)	+	SINE1_Mdo	SINE/MIR	23	173	(34)	31	
283	27.0	9.0	2.2	UnnamedSequence	19540	19706	(13927)	C	L1_Mdo3	LINE/L1	(930)	5517	5340	32	
320	22.9	1.2	0.0	UnnamedSequence	20013	20095	(13538)	C	L1_Mdo3	LINE/L1	(1521)	4912	4829	32	
4955	9.1	3.6	1.1	UnnamedSequence	20420	21469	(12164)	C	ERV18_MD_I-int	LTR/ERV1	(528)	5722	4642	33	
321	17.9	0.0	16.3	UnnamedSequence	21492	21591	(12042)	C	ERV18_MD_I-int	LTR/ERV1	(1602)	4648	4563	33	
563	16.2	3.6	0.0	UnnamedSequence	21764	21874	(11759)	C	ERV18_MD_I-int	LTR/ERV1	(1688)	4562	4448	33	
491	18.6	2.3	14.9	UnnamedSequence	22016	22214	(11419)	C	LTR54_MD_I	LTR/ERV1	(511)	1500	1280	34	
771	24.0	1.7	1.3	UnnamedSequence	22270	22505	(11128)	C	ERV18_MD_I-int	LTR/ERV1	(2100)	4150	3914	33	
532	20.1	0.1	1.5	UnnamedSequence	22654	22796	(10837)	C	LTR54_MD_I	LTR/ERV1	(801)	1210	1091	35	
262	11.5	6.6	0.0	UnnamedSequence	22819	22879	(10754)	C	LTR54_MD_I	LTR/ERV1	(917)	1094	1030	35	
2104	6.9	2.1	0.0	UnnamedSequence	22880	23170	(10463)	C	LTR4_MD	LTR/ERV1	(384)	594	298	36	
3463	16.1	3.7	1.9	UnnamedSequence	23171	23896	(9737)	C	ERV18_MD_I-int	LTR/ERV1	(3293)	2957	2219	33	
3403	13.9	1.3	4.1	UnnamedSequence	23907	25144	(8489)	C	ERV18_MD_I-int	LTR/ERV1	(3991)	2259	959	33	
201	13.2	0.0	0.0	UnnamedSequence	25170	25207	(8426)	+	(CCA)n	Simple_repeat	3	40	(0)	37	
327	19.6	3.6	3.6	UnnamedSequence	27458	27568	(6065)	+	LTR156B_MD	LTR/ERV1	99	209	(580)	38	
8367	14.1	1.8	0.7	UnnamedSequence	27569	30247	(3386)	C	L1_Mdo3	LINE/L1	(3195)	4729	1925	39	
3059	22.7	7.6	3.7	UnnamedSequence	30217	31703	(1930)	C	L1_Mdo3	LINE/L1	(4924)	1760	269	39	*
416	25.0	0.9	0.0	UnnamedSequence	32228	32335	(1298)	C	WALLSI4_Mar	SINE/MIR	(19)	231	123	40	
247	23.9	1.5	1.5	UnnamedSequence	33184	33251	(382)	+	(TATATG)n	Simple_repeat	1	68	(0)	41	

Genomic alignments of a few Gin-1 genes

```
Homo sapiens          TCAATTTCTGGCGTCTCGAACCCAAGAAATGAAAAAGGGAAGCCAT-----GGGGGAGCAGGAGGAGAAAT------CAAGGCGCGAAAGG-ACCCTCAAAGCTGTACTGTCACTCCACG
Dasypus novemcinctus  GAAAGATCTGGCGTCTCAAACACAATAAGCGAAAAAGGGAAACAATAGGGAGGGGGAAGAGAAAAAGCAATGAAAAGCAAGACACCAAGGCCGCTTTCAGGGTTACACAGTC-CTCTACA

Homo sapiens          CCCAGGCTGTCATTCTCTCCAGCACAAGCCCCTCGGTGGAGCATGTGTTTAGGGTCTGCAGAGCA-CAGGAGACAAAGCAGAAGCCCAGCAGCCGCCTCAACCCTGGCATGATCAGGTTC
Dasypus novemcinctus  CCCGCGCTGCGGTTCGCAAAGGCACATGCCCTTCCGAGGAACTCGCGTCTAGGATCCGCAGTCAAGCAGGAGA-AAAACAACTGCCTGACAGCGGCCGCAAGCTTCGGGAGACTTGATTC

Homo sapiens          CGCATCTCCGGTTCTCCTCCGGTTTTGG-GCCCTCACCTGCCACGAATGCGGTCGTCACGCGAGCAGTTGTGAGATGGTCAAGCCCATTCAGGTCTAGGTTACCCCCTTTCATCCCGGAG
Dasypus novemcinctus  CGCATCCCGGGTTCTCCTCCAGTCTCGGTACCCTCACCTGCCACGGCTGCGATCGTCACGCAAGCTGCTG-GAGGTGACCAAGTCCATTCAGGTCTCGGTTTCCCCTTT--ATTCCAGGG

Homo sapiens          ---GCAGGGGT--GAGGTGTTTGTCAAGAGACTTCAGTTGTAGGCGTGGACGTAGG------CTACTTCCTTCAGTAACAGCTATAGGGCCACATAAGGGAACCTCAGG--CTACTACAC
Dasypus novemcinctus  AGTGCAGGGAGCAAAAATGTTCGTCGAGAGACTGTAACTATTGGTGAGGACGTGGATCTTGTCTTCTCGCTTCAATAGCGGCTGAAGAGGCACACAGAGTTACCTCAAGTACCACTTTAT

Homo sapiens          CTCAGGAATCTCCGTCACGCCTCTTCCCCGCCCTCCTCGGGTCTCACGCCGAAACCTCACCGGGCGGAACGATTTCCGGCAAGAGCCAAATGATCAGTCCTAACCTTTCTGACCGTGGTT
Dasypus novemcinctus  CTCACCAACGTTGGTCACGCCTCTTCCCCGCCAGCCTCGGGTCTCACTCAGAAATGGCACCGGACTGAACGATTTCCGGCAAGAGCCAACT------------CTTCCTTGAATGAGGCC

Homo sapiens          CCAAGGTTCCCCAAAGGACTGCCCTCTAAAGGACCCGGGTAGTTCCGCTTCCGGCAGCGCGAGATAAATCACGAGAGGAAGCTTAAATC-TGTCGTTTGAATTTAGGACCACCTCGGTGA
Dasypus novemcinctus  ATGCCGTACCCGTAAGTAC-GCCGCCTTAGCGACACGGGTAGTTCCGCTTCCGGCAGCACCAGATAGGTCGCTAGAGGAAGGTTAAATCCTGTCGTTTGCGTTTAGGACCACCTCGGTGA

Homo sapiens          GTGGTCGTTCTGGTGTGCTGTGTCATACCTACTGTTTTTTAAAGTGAGGCGTAACCCGACAGTAATTTCAAAACCATTCGCCTCGACCGGCCTAAGGAAGGGTTTAATTGAGTCTGTGGG
Dasypus novemcinctus  GTGGTCGTTTTGGTGTGATTTGTCATACCTACTGACTTTTAACATAAGACGTAACTTGA---TCATTTCGAAACCTTTCCCTTCGACTGGACAAAG------TTTAATTGACGCTGTGA-

Homo sapiens          GAGATTTTGCGCAAGCGCCTTTGAGATAGGAGGGCCTTGGGCGTTGCGATGTCCCGCGCTAGCTGAGC--AGAAAGGGCGAGTGCTGTTTCGGGCCTGGGGAGGTGCCTACTGGGTGTAA
Dasypus novemcinctus  ---ACATTGCGCAGGCGCCCTTGGGGCGGAAGGGGTTTGGGCGTTGCGACGTCCTGCGCGAGCTGAGCTGGGAAAGGGCGAGTTTTATTGCAGTGCTTGGGAGGTGCCTTCTCGGTGTTA

Homo sapiens          TTGAGTTTTACCAAACGGTCATTTTCCTGCTGATTCTGAGTGTGGTTGGACCTTGCCCTTAAGATTCCGTTAACGAGTCGCCCAAA-GCTTAGGAAATGCT------------------
Dasypus novemcinctus  CTGAGTTTTACCAAATATTCTTTTTCCTGATTTTTCAAAGTGTAGCTGGACCTTGTGTTAAAGAATGAGTTAACGAGCCACTGAAGCGCTCCTGAAATGCTTTCTAATGGTTAAAATAGT

Homo sapiens          ------CCCTCACAGCGTTTGAG---ACTAAGCACTTTTGGCCGGCG------TTGAAGAATTCTCCCCATTACTTT----GATCAGTCATTTAATCAGAAA---------TTTTGCCCC
Dasypus novemcinctus  GTCCGATCCTCCCAGCGCTAGAGCAAGTTCAGAAGTTGAGAGCGGCGCTTGTCTTAAAGAATTAT-CCCGTTATTTTCTCATAGTTGTCATTTAGTCTGGAATATTACTTTTTTTCCCCC

Homo sapiens          AAATTTAAATACGTGGCGGGGAGAAACGTGAGGTCAAGAGGGAATCCTGTCACGTAGTTTTAAAAGAAAAATGTCCACTTTATCTCAGTGTAGGTTTTTCTTAAATGGGCATTTCTGAAC
Dasypus novemcinctus  AAATTAAAATTTCCTGTGAGGGAGA-----AGTTCAGGGGGGGAGCCTGTCACTTATTTTTAAAGGAA-----TTCACTTTATCACTGTGTGG--TTTCCTTAAATGGGCATTTTATAGC

Homo sapiens          CAAAGTAGA----TACTTTTTAAAGTGGAAATGCATTTATCCCATGTGGCTTCATGTGAATGCAT-ATGGC-GATGAAGGCATTTTTTTGTTCAATGAATTTGTACTCTGAAGTGAACCA
Dasypus novemcinctus  CAGGGTAGAAACGTAATTTTCAAAATAGAAATTCACATAGCACATGGGGTTTCATGTGTTTGCATAATGGTAGGTGAAGCCA--TTTTCGTTAAATGTACGTGC---------TGAAGCA

Homo sapiens          GATTACAGAGAAG-------TGTATTTAAATTTACAGGTCCTTGACGTTGCATAAAATTGTGCGTGTCCATTTTTTGGAGCTTAAAATAGAATAATTCAAATGTCAGAATTCCGATCCAA
Dasypus novemcinctus  GAGTCTAGAAAAGTGTTTGATGTGTTTAGATCCCCC-GTCTTTCACGTTACAAAAAAAT-----TGTCA--TTTTTAAAGATCAAACTCGAATGATTTAAAAGTCAG............

Homo sapiens          GTTAACATGTTGATTGAAAATTGCAGATTTTAAGATGGAACCAGCGGGTATTATAACCTTTTAGGGGCTTAAATTGGTGGCTTTGTTTTTAACCCTTGGTGAAACAGTTAAGTCCTAATC
Dasypus novemcinctus  ........................................................................................................................

Homo sapiens          TTGACATAATAC---CTAACTGCCTTACTTTATTGAACACA------AAATTATTAAAGGAATTAAGCAATGAG-----------------TTGATTTTATTTGCTTTTATGGAGGGGA
Dasypus novemcinctus  .....ATGATACGTTTTAACTGCCTTACTTTATTGCTCACACGAGTAAAAATACTAAAGCAGTTGGGCAATAAGCATTTATGCAGGCATTTGTTGATTTTATTT-----TGTACAGAAAC

Homo sapiens          CAAAGTGTATAAAGCTTAACCAAATGTGACCCTGACATTCCTCTTGCAAAACTACAAGTTGTAATCAATCACTTTCATTTTTTCTTATTCCTACATGTTTAAGTACATTAAGTAATGATT
Dasypus novemcinctus  ACTAGTGTAAAAAACTCAATCAAATATGACTCTGGCATTTCACTTCCAAAACCGGAGGCTGTAGTCAGCTG--TCTATCTTCTCTAATTTTTATGTATTTTGATACA-TACTAAATTATT

Homo sapiens          TTAAGAGAAATTCTTGAAAATATGAGC----ATATCCAAAGAAGTCCCAGGACAGTCTTTCTTTATGTAGTAGAAACCACTTAA-TCCCCCTTGTAAATGATACCTTTACTTAGCAATCA
Dasypus novemcinctus  TTAACAAATATTCATGAAAATATGAGCGTGTATGTCCAAAGAAGACGTAAAATAATCTTCATTTATACAACACAGATCACTTAATTCTTTCTTGGAAGTGATATCT-------------

Homo sapiens          GAGTATAAGAAGA-----TATTTATATTTGTCTCGAATATT---------------------------ACAGTTTCAAGCCATTTTCTCATAT--------GTATGGGC----TTTGAAA
Dasypus novemcinctus  -------AGAAAGTTTTTTTTTTATCATTGTTCTGAATATTGTAACTTAAATTCTAAGTTAAGCGTCAACAGCTGAAATCCATTTTCGCATATTAACTACCACATGGACATTTTTTAGGA

Homo sapiens          A-------------------AAAAAAAACAACTTTCATTTACGTTACTTTTGTATATGAATACCA---ATTATTAGCAGTAAAACTTTT----AACTACTTCAAAGGCTATGCTTTTCGT
Dasypus novemcinctus  ACCAACAGTGAATTCAGTTTAAAAAGAGCTGTTGGTCTTTAAGCTATACCTGTGCCTAAATAGCAAATACTATCAGCAGCAACATTTTTATCCATCTACCTAAAAGGTCATGGCTCTTGT

Homo sapiens          GAACTGAGTTTTCTTTCTTACGCATTTTCTATTTCTCCTTGTCATCTGTCTCTCTACCTCCAGAACCAGCAGTTTGTGAACTGCATTAGACTGT----TATATCGTTATAAAAGGATACA
Dasypus novemcinctus  GAACTG-----CTTTTCTGATGTATTTTGTATTTGTTATC----TCTCTCTCTCTACTGTCAGGGCCACAAACTTGTGAATTGTGCTAGACTATATAATATAAAATCCT-AAAGTATATA

Homo sapiens          ATTCGTGAGTCTTCTGATTATTTTCTTCTTAGCACTGAAGCTTTATA----AACATTGCTATAAAACATTAACACCATTAATGAAAGGAAAGAGATTTTAAAATATTTTTTCATGTAGGT
Dasypus novemcinctus  AATCCTGAGTCTACTGG--GTTTCCATGTTAGCATTAAAACATTATAAATGAACATAAGTATAAAGCATTGA-ACAGTTAATGAAAAAAAAAAGATTTAAAAGTGCTTTTTCTTGTAGTT

Homo sapiens          GAAGATTTTGGGAAAAGAAAGGGTTTTATGTTACTTTAAACAGCCTGATATGGATGATAATACTTAGATATGATAATAATTATACA-----TTACCATATACAAAGGACTTTTAA-----
Dasypus novemcinctus  AAAGGTTTTGAGGAAAGAG------TCCTGTTA-TTTAAACAACTTGATATGGATGATAAT-------TATAACAGTAAATATATATTGCTTTACTATTTGCAAAGACATTTCAAATCCA

Homo sapiens          ---------TCATCCTCGTAATTGCAAAAAAGGGA-TAGATATTATGTCCTTTTCCAAACATAGATACTAAGGCTT-----------------------------AAGTCTATACCTTCT
Dasypus novemcinctus  TTGTATCATTGGTTCTCATAATTGAAAAAGGAGGACTATAAATTATGTCCCTTTTCCAAAGTAGATACTGAGACTTAAGCATCATGTCAGAAATTCTGACTCCTAAAGTTCATGCCTTTT

Homo sapiens          TAGACACAGTTTACTTATAGTGCCATGTATGTGAAAGTGAACTGTAATGTTTTCAAAAGTGAAATA-ATTTACCTCTGGAATATTTGCTATAATTTTTAATTCTCCTTTCAGGAAATGCA
Dasypus novemcinctus  TTAATGCACCCTAATTGTAAAGCCATATATATTTTCATGTGAAATAATGTTTTCTGAAGTGAAATATGTTTATCTTTGGGATATTTGCTAT--TTTGTTATTC----------------

Homo sapiens          GGTAATTCTTTATTCATTCACAAATAGTTGAGTGTCCACTGTGTTCTGAACATTATGTTAAACAATGTAAACATAAAAAATTAATAAACTCTTTCCATTCCTCATTTATAATCTAAATTA
```

```
Dasypus novemcinctus  ---ATTACTACCTTTCCTCACATATAGTTGAATGTCCACTATGTGGAAAACACTATATTAAACATTGTAAACACAGAAAATGAA-AGACT-TTTCCATTCTTTAATTATAATCACAAGTG

Homo sapiens          TTGATTACAAAAATCACTTTATAATTTGTGTAACAGAG-GCCTGAACCAAGTATTATAAAAGCTAAGGATAACAAGTAATTGACTTACTGGTATAGCAGGGGGGAAAAGC----GTGAAA
Dasypus novemcinctus  TTTACTACAAAA-CCACTTTA-AGTTGTCTTAGCAGAGAGACCTGACGAACTATTATGAAAGCTGAGGATGACAAGTAATTCAGTTATTACTGTATCAGGGAAGAAAAACTAAAATTAAA

Homo sapiens          TTTGAAGTAAGAGACACCAAGTTAAAAGTGTCAGCGCATGTACTCACTTT----ATTACCTTGAGTAAATTGGACAATCTC--TGAACCTCCATTTTCCTACCTGTAAAATGGATATAAA
Dasypus novemcinctus  TTTGGAGCAGGACAGACCAAGTTTCAAGTATTAGCTCATGCACTCACTATTTGAATTTTCGTGAGTAATTTAGGTAAGCTCTGTGAACCTCTATTTTCTTACCTATAGATTGCATAC---

Homo sapiens          ACTAATACCTACTTCATGAAGT-ACTGTGAGGATTATCATAGTAAAATACTGCATAACTATTTGCAGCTGTAGTCGTGAGTCACTTAGCAATGGAATAAATTCTGAGAAATGCTTTGTTA
Dasypus novemcinctus  ACTAATATGTACTTAATAAGGTGGTTGTGAGAGTCATCACAATAAAGTGATGTATAAATGTTT----TTGCAGTT------------------------TTTTTTAATTGCTTTAAAA

Homo sapiens          GGCAGTTTCAGCATTGCACGAATACAGACTCTACCTACATGAACCTAGATGATATGTGTGTGTGTGTGTGTGTATATTTTTTTCTTCATAT----------AGAGAACCAAATGTCC---
Dasypus novemcinctus  GTCAAGTT---------------------------------------TTATATGTGTGGATTTTTTTAATCTTCCATTTTTTTTTAAGTCAGTCTTAGGAGAAACTTACATATACTAA

Homo sapiens          ---CTGCACATTATTGGGTAT-CATTT------ATTTCTCCTACTTGATCTGCAGTGCTAATATCAAATGACCTATATCAGGTTCTATATATGCTCTGCTATCATCTTACGGGACCAATA
Dasypus novemcinctus  AATTCAGACATT-TTAGATGTACATTTGAAGGCATTTTGACAAATACATATAT-GTGTGAACACCA------CTATATCAAGGTATAGA-ATGTTT--CCATTACCTG-------TGGTA

Homo sapiens          GGCAG---TCCATCCTTGACCAAAACATTCTTATGCAGTGCAATTGCGTTGGCATTGTTTTTAAAATTTTTAATTTTTTAAAATTATTTCCTCTTCCTATACTTTTGGCTTTTCCAGATG
Dasypus novemcinctus  GGTAGTATTCTCCTCTTACCTCCAAC---CTAAGGCAATGC--CTAATCTGATACCCTTTCTGGA--------------------------CCCCCTATCCTTTTGACTTTTCCAGATA

Homo sapiens          TCATATAAACAGAAATATAAAATATATAGCCATTTGTGTCTGGCTTATTTCACTTAGCATAATGATTTTGAGATTTATTCATATTGTTAGGTCTATTTATAGTTTGTTCCTTATCAATCC
Dasypus novemcinctus  T--------------------ATAGTCACCCTTTTGTGCCTGATTT------CTTAGGATAATTA-AGCGAGATCCATTCAT-TTGTTGAGTGTATTCCTTGTTTGTTCCATATCAATGC

Homo sapiens          TTAATAGTATTCCAGTATGTGGATCTACTGTAATTTGTTTATCCATTCACCAGTTGATGAACATT-----TGTTTTCAGGTTTTGGCTGATATGAATAAAACTTCTAAAAACATACACTT
Dasypus novemcinctus  TGACTAGTAATCCACTGTGTGAATCTGCCACAA----TTTATCCATTCACCAGTTGATGAACATTTGGACTGTTTTCAATC-----CAATTATGTATAAAGCTTCTAGAAACATTCACTT

Homo sapiens          ACAGGTTTTTAGGTGTACATATTTTCTTTTGGGTTAGTATCTAAGATTGGTATTGCTGGACCATATGGTAAATTGATGTTTAACTTTATAAGAAATTGCCAAACTGTTTTCCAACATGTT
Dasypus novemcinctus  TCAGGTTTTTTTGTGGGCATATATTTCAC-------ATATATA----TGGGATTGCTGGGCCACATGAAAATTATATATTTAATTTTATAAGATATTGCCAAAC--TTATCCAGTGTATT

Homo sapiens          TACATCATTTTCTATTATCACCAGTAAGA----GCATCCTTGGTAGTACCTAGCATTGTTA-----GGTTTTGTTTTATTTTTG--TTGTTGTTAGTGTGTAGTCATATCTTATGGTAGT
Dasypus novemcinctus  TACACCATTTTGCATTTTCTACAGTAGTGCTCTACATCCTTGGTAGTACCTGGTATTGTCAATTATAGTTCTGTATTGTTTTTTACTTTTTGCCA-TGTGCAGTGGTATC--ACTATAAT

Homo sapiens          TTTAGTTTACATTTT-TTATTATCTATTGATGTTGAGCATTTTTTCATGTGTTTATTTGCTAATCATAT---CTCTTTAGTGAAGTGTA----------------TTTCCAGGTTTTAA
Dasypus novemcinctus  TTTTATTGGCATTTTCCTAATCGTTAATGATGTTGAACATATTTTCAAGTATTTATTTGCCAGCAATATATCTTCTTTGGTTAGGGGTATACAAATCTGTTACTTGTTTTTTGTTTTCAA

Homo sapiens          ATTGGGTTGTTTGCTTTCTTGTTAAGCTTCGAGAGTTCTTTATAAATTCTAGAAAAAGTTATTTATCATATGTATGTATTGCAGATATTT-TATCCCAGTGTTATTTCTCTTTTTTTAGC
Dasypus novemcinctus  ATTGCGGTG-TTGTTTTCTTATTGAATTTTGAGGGTTCTGTATAAGAAT--AAAGAAATTCTGTATCAAATATGTGTACTTCACATTTTTGCCTCCCAGTCTGAGATTTCTTTTTTTCGG

Homo sapiens          AGTTCTTT-AAGGAATAAAAATTTTAATTTTTTCTTTATGCATAGCTGCTTTTTCTTTGCTGTCTGGGAAGCCATTACCCAACCTGAGCTCACAAAGATTTTTGTTGTTGTCTTCGAGAA
Dasypus novemcinctus  TTTTCTTTGAAAGAGCAGAGGTTTTAATTTTTTTTTT...................................................................................

Homo sapiens          GTTGTATAGTTTTAAGTTGTACATTTAGATCTGTGAGCCATCATGAATTAATTTTTACATATCATTTGAGGTATGAGTTGAGGTTCATCTTTTTTCAAAGGATGTTTAATTGTTACAGCA
Dasypus novemcinctus  ........................................................................................................................

Homo sapiens          TTTGTTGAAAAGATTATCCTTTCTTCATTGAATTACCTTGGCACCTTTGTTGAAAATCAGTTTACTATAACTGAGTGGCTATTTCTGTCACTATTCTGTTCGATAGATTTAAGGCTGTCA
Dasypus novemcinctus  ........................................................................................................................

Homo sapiens          TTTTGCCATTATAACACAGTCTTGATTACCATAGTAGTATAGAAAGTCCTGAAACCAAATAATGTGAAATTCTTCAACTATATTCATTTCTCAAAGTTGTTGTAATTATTCTGAGTCCTT
Dasypus novemcinctus  ........................................................................................................................

Homo sapiens          TAGCTTTCCATATCAATTTTAGAATCAGCCTGTCAGTTTCTGCAAAAGAGCTTGAGGGTTCCTGATTGGGACTGCATTGAATCTATAGATCAATTTGGAGAAAATTGGCATTTTGTCCCA
Dasypus novemcinctus  ........................................................................................................................

Homo sapiens          CTTTTTCTCAGAGGTAGAGTGGGAGTTACTCCAGCTTTCTGCATTCTAGTCAGAAGCCTATATGGGAATGTTTAGTGGCATTTCAGAAAGAAATAATTTTCTTCCATGGTTATATTCACT
Dasypus novemcinctus  ........................................................................................................................

Homo sapiens          ATAATATCTTTATTGGAAGAGATCACAGGACGAATCCCTCTATTTGTGAATTGGATGTCATCCCCTGCTGCCATCTCAGGACATCATACCATAAGTTTTTCTCCTTTGTTTTCAGTGGT
Dasypus novemcinctus  ........................................................................................................................

Homo sapiens          CTTTCTAGAAGCCCTTCCATTAGCATTAAACTTTTTGGGTTACTCCCTTGTCGAAAGCAAAACAATCTTGTTCAAAAAAAAAATTGTTTTAATTGTAGTAAATCACACATAACATGAAAT
Dasypus novemcinctus  ........................................................................................................................

Homo sapiens          TTACCATCTTAACCATTTTTAAGTGTGCAGTTCAGTGGCTTTAAGTACATTCACATTTGTTTTACAACTATTACCACCATCCATCCCAAGAACTCTTTTCATTTTGTGTAACTGAAACTC
Dasypus novemcinctus  ........................................................................................................................

Homo sapiens          TTGACCAGTTAAATAATAACTCTTACATTCTCTCCTCCTCACCCCCTGTTCTATGCAACCATCATTATTCTTTCTGTCTGTATGTATTTGACTACTCTGACTACCTCATATAGGTGGAAT
Dasypus novemcinctus  ........................................................................................................................

Homo sapiens          CTTACAGTATTTATCTTTTTGTGACAGGTTCATTTCACTAAGCTTAATGTCCTCAAGATTCATCCATGTGGTAACGTGTGTTAGAATCTCCTTCCCTTTAAAGGTGTGTGAATATATACA
Dasypus novemcinctus  ........................................................................................................................

Homo sapiens          CCGTATTTTGTTTATCCATTCAGCCATTGATGGACACTTGAGTTGCTTCCATCATTTGGCTGTTGTGGGTGATGTGGCTATGAACATGACTGTCAAAACTTTTAACCCCACTAGAATGTA
Dasypus novemcinctus  ........................................................................................................................

Homo sapiens          AACTCAATGAAGGTCAAGAGAATCTACATCTTGTTTATTATCCTGTCTCCAACGTTGTAACTGGCACATAGCAGTTACTCAGTCACATTTTTTAGATCAATGAATGAACCTTATATCTGT
Dasypus novemcinctus  ........................................................................................................................

Homo sapiens          CTCTAGCAACTATCTTGTAGCTCCTTGCCTTTATAGCTAAGCCCAAGTTTCTAAATCCACTTTCTGAATTTCCATTCATTCCTTGTGTCATTTCAGTCTGGTTGCCAACTGCCCTGCTAT
Dasypus novemcinctus  ........................................................................................................................

Homo sapiens          ACTGTAACTATACTGTATACTGTTGCCAAAACCACTAGCTACATTATGGTTCTTATATCACTTAGCCTCTCTGTGTACTTCTAAGAAGTCTGTATTTCCCAGACTTCTATTACCAGAACC
Dasypus novemcinctus  ........................................................................................................................

Homo sapiens          TCTTCTGGCTTTTTTCCTGTTTCTCTGGCTAGTCTCATGTCCCTAAAGATTTACAATATATTTGGGAGAGAGACATATAAACTAATATATTCAATAGAGTGTTTCAGATTTTATGATAAA
Dasypus novemcinctus  ........................................................................................................................

Homo sapiens          GGTTGACACATATTACTGTGAATGTACCAATGAGGGTGTGCACAATTCTCCCTGAGATTGGGGATGTGGTCAGAAAGGATTCATGGATGAATGAACTCATTAATGTGAACGTTGAAAGAT
Dasypus novemcinctus  ........................................................................................................................

Homo sapiens          AAGGATATGTTCACTGCACTTCCTACTGTGTACACTTTCCATCTGATCCTTCTTCCATTGCCTTAACTTCACCTACTTTTTACATGCCGAGTTCATCCATGAAGAATAGAACCTGCTTAA
Dasypus novemcinctus  ........................................................................................................................

Homo sapiens          TATTGTCATCTTTCTCCCTAAGCCAGAGCTTCAGACAGAAGTGATCCCAGATTCTTCTTTGTGTTTACTCCACACATCTAGTCAGTTACAGTTCTTATATAATGTATCTATCCATTTATA
Dasypus novemcinctus  ........................................................................................................................

Homo sapiens          CTGACACTGCTTTGGCTCAGAATTTCCTCATTTCTTGCTTTAATTATACAGTAGCTTCCTAGCTGACTGGTACAGGTATTCCTGTCTCTACCCCAATCTCTATTCCATATTGGTGCACAA
Dasypus novemcinctus  ........................................................................................................................

Homo sapiens          TACACTTTATAGAATAGAAATCTAATCTTGTAACTCCAACTCATTCTTAAAATTTTAAGTTATTCCAGGTTGGAGTGGTTAGAAGACACTCTGCAGGAAATTACACCTAGCCAGAGTTTT
Dasypus novemcinctus  ........................................................................................................................

Homo sapiens          GAAATATGATGAGGGGCTGGAGAAATAGCAATAAAAGTTCATTCCAGGCAAAAATATCAATGAAAAGGTGTGAGCACACTGCATGGGATGGTTGAAGAACTGTGGTTATGTTTTGAATTG
Dasypus novemcinctus  ........................................................................................................................

Homo sapiens          AGAATGCCAAGAGAAAAAGATGGAGAGATTGATAAAAACCATATCTTATAAAGCCTTGTATACTGGCAGAGGTATTTAGATATCATTAGTGCCATGGTCTTAATGGTTGTATTCTCCCAA
Dasypus novemcinctus  ........................................................................................................................

Homo sapiens          AATTCATATGTTGAAATCCTAACCACCAAGGTGATGGTATTAAGAGGTTGGACCTTGGGGCTGGGCGCGGTGGCTCACACCTGTAAGCCCAGCACTTTGGGAGGCTAAGGTGGGTGGATC
Dasypus novemcinctus  ........................................................................................................................

Homo sapiens          ACTTGAGGTCAGGAGTTCGAGACCAGCCTGGCCAACATCGTGAAACCTCATCTCTACTAAAAATATAAAAATTAGCTGGGCATGGTGGCATGCACCCGTAGTCCCAGTTACTCGAGAGGC
Dasypus novemcinctus  ........................................................................................................................

Homo sapiens          TGAGGAACAAAATTTGCTTGAGCCTGGGAGGCAGAGGTTGCAGTAAGCTGAGATCATGCCACTGCACTCCAGCCTGGGCGACAGAGCGCAACTCTGTCTCAAAAAAAAAAAAAAAAAAGA
Dasypus novemcinctus  ........................................................................................................................

Homo sapiens          GGTGGAACTTTGAGGAGGTGACTTGATCATAGGTCAGAGCTCTCATTAATGGGATTAGTGCCTGTATTTTAAAGAGTCTGAGAGAAACCCCTTTCCCCTTCCCTCATGTGAAGTTACAGT
Dasypus novemcinctus  ........................................................................................................................

Homo sapiens          GAGAAGACAGCTGCCTATAAGAAAGTGAACCCTCAGTAGACACTAAATTTGCTGGCATCTTGATCTTGTATTTCCTAGCCTCCAGAACTGTGAGAAAGAAATTTCTGTTTATAAGCCATC
Dasypus novemcinctus  ........................................................................................................................

Homo sapiens          CAGTTTATGGTATATTTGTTATAGCCGCCCAAGTGGACTAAAACAATCAGGAATGGCAAAAGATGTATTTCGAGTTAAGTGAATATTTTGATAAGATTTGTGTTTTAGTGTGACCTCTGT
```

```
Dasypus novemcinctus  .............................................ATCAGGAA-GGCAATAG-----CTTCAAGTTAGGTGAGTGTTGTGATAAGGTTGGTGTTTCATAATGACCTCTGT

Homo sapiens          GACTGCAGTGAGGACAGGGACCATATTAGAGACAGAGGAGGCAAGGTCAGATAGAAGGTGAGTGTGAGCTAAGGCAGTGGCAATGGAATAAAGTTCAAGGTTCTACCTCATCTACAATCA
Dasypus novemcinctus  AGCTACTGTAAGAAGAAGGACTATGCCAGAAACTGGGGAGGCAAGGTCGGATA-----TGAGTCTGAACTCAGGCAATGGCAATGAAATGAAGTTCAAGCTTCTACCTCATCCAAGA---

Homo sapiens          GTGCTTTTCCTCCTTTTCCAGGTGAATCTGGGTCTCTACACATCT-GTTTTGATAGTACTCTTTGCATACATCTGTTAAAACACCACTCTAAACTGTATAAATGCTGATTTCCTCCTCTT
Dasypus novemcinctus  -TACTTTACCTCCCTCCATAAGTGAATCTGAGTCTCTACAAATCTCCTTTTTATAGCATTCTTTGCATACTTTTGTCATAATACCTTTCTGTATGATATAAATGCTGATTTACTTGTCTC

Homo sapiens          TTCCTCTCATTTATACCATAAGCTCCGTGAGGACAGGGATTTCATTTTATTTAACTTTGTATTAGTATCATCTAGTATATATAAGGGTTTAAGAGCTCAGTAACTGAATGAGCAAATTCA
Dasypus novemcinctus  TCCCTGTCATTAGT-CCATAAGCTTCTTGAGGACAGGAATTTCATCATGTTTAACTTTTTATTACCATCACCTAGAATGTATAAAGGTTT--------GGTAAATGAATGAACAAACAAA

Homo sapiens          AGGTTATATGGATGAACAGGCAAAAAACTA----TTAAAAAGGGTAAAGCAT-GGAAGTTCTACAGTAGG--CACAAAGAAGATTAGGAGAGGTCAAGATCACTTTAGATTCAGAGATCA
Dasypus novemcinctus  AGCTCATATGGATGAATGGCCAAACAACTGACTCTGATAGAAGATAAAACATGGGAAGTTCCACAATGGGTTTATAAGGGATTTTAAGAGAAGTAAAGATCACTTGTAATTCAGAGATCA

Homo sapiens          TGGAACACTTGCTTCATTGAGATGCTAGAGCTTGATTTTGAAAGATGGGTAAGGCTTAATCAGGTGGAA------GGATATACCATT-TAGGGGAAAATGAT---GTAAACCAAGGTATA
Dasypus novemcinctus  TTGAAAGCTTACTTCATTGAG......GAGTTTTATTTAGAAGGATGAATAAGTCTTGATCAGGTAGAACTTGAAAAATAGGCCATCACAGGAAAAAATGATCATATAAACCAAGGTCTA

Homo sapiens          GAGAATGGTTGGGGATATGGCCATGGGTATAGAAAGGAAAGTAGTGAGAGAAGAAGCTGTAT------TATGTTCTGCCAGGTTGTAGAGGGTAGGCTAAATAGTC-TAGACCTTAGCCA
Dasypus novemcinctus  GAGAATGGTTGGA-ATGGGACCATAGTTATGTAGAGGAGTTTCTTGAGAGAAAAAGCTATAAGGCTTATGTGTTCTGCCAGCTTGTAGAGGCCAAGCTAGGCGTTAGTAGGCTGTGGC--

Homo sapiens          CTAAGTGGAAGATAATGCCCAGAGGCAGTCTCATGGTGTCCACTCTGTCAAGTAAATCATTTGTAAGATAAAAATATGCTAGG-------GATAGTCTAAATTAAAGTTTCAAGGTGACT
Dasypus novemcinctus  ------------AATGCCCAGAAGCAGTCTGATAGTGTCTATTCAATCAAGTAAATTATTTGGAAAAT-AAAATATGCTAGGGAAGATTGATAGTCTAAAT---AATTTCAAGGTGACT

Homo sapiens          ATGT----CAAATAACTACACCTTTAAGTCTTAGTTCTGTT-------GAGCCAAGACAACTCA---CCCATATAAAAATGAAAATAATACACTTGTGAAAGCATTTGTGAAATATGAAA
Dasypus novemcinctus  ATGTAAAGCAAATAATCATTTGTTTAAAT----GTTCTGTTCTTTACAGATCCATGAAAACTGACAGCCCTTATAAAAATAAGAGTAACACACTTGTGAAAGGAT...............

Homo sapiens          ATCTGTAGGAAATAGATGGTTTTTATGTCTTCTCCCAATGATGTTTATGTCCCTCTCCCTAACATAGATATATGTTAGTCTTCTCCCACTACTTTGATGAGAAAAAAACAAACCAAAAAC
Dasypus novemcinctus  ........................................................................................................................

Homo sapiens          CTTAAAGAGCTCAGTCTCCAAAGATTTTTGTAGTGCTTTCCTTTTCTCCTTGTCCCTT-GTACTATAGGTTTACTCTGAGGAGAGCAAATTTCTCTATTTCGATCTACTTTCAATTATTC
Dasypus novemcinctus  ......................................TCCTCTATCCTTTGACCCTTTGTACTATAGGCTTACTCTAAGGAGAGCAT-TTACTCCATTTTAATCTATTTTTAGTTATT-

Homo sapiens          AAAAGTTAAAGATTTCAAAGCAGAGAAGTTAATTTCAGTAAATAGACCTTTTCTGAGA-AGTTTTTGTATATTTATTATAAATAGGAAAACCAACAGTGTCCTAATGGCACGTTAACTTA
Dasypus novemcinctus  GAAAGTTAAAGATTTGTAAGTGGAAAAATTAATTTCAGTAAATAGAC--TTTCTGAGGTGGAGTCTGTGCATTTATTGAAGGTCAGAATGTCAAAATTATTCTAAAACTAAAATAGTTGA

Homo sapiens          ATATACTGAAGACTTCAT-------CTTACAT-------TAGATAGAAAAATTTTCTGGTCTCTCCAGGAGGTAAATGGGTAGGGGGAGGAAGACAAGAGATGGTAGGAACTTGGAACCA
Dasypus novemcinctus  ATGCACTCATATGTTCCTTATGTTCCTTATATTGAGGAATATGTAAAAAGATTTTCTGGTATCCC.......................................................

Homo sapiens          AGAGGTTTTTAAATATAAAAGCAAAATACGGAAGCTACTGTGGATTATGCAGACTCACATTTTTTTTCCAATGGCTGGTTTGTTTTCTACCAGTTCTGGTTACCCACA-TCCCTCTGTGT
Dasypus novemcinctus  .....TTTTTAACTA-------ATAATTTGGAGGTCACTACAGCTTAAGCAGACATTCATTTTT---CCAATGGCCAATTTTTTCCCATTCGTGTATAAATAGCCCTACTGCCTTCATGG

Homo sapiens          TTTGGGGCCTGTACTTCCTCTAAGGTGTGAGAGTTTGGTCTTTTCCTTCTGTGGGCTTTT-ACAGGCCTGAAAAAGCTCCTCCCCTTTTCTTTTTAAGGTCCTTCAGGAGTTTTTGCAAC
Dasypus novemcinctus  TCTGGGGCATTTACTTTATATAAGGTATGAAAATTTG-TCTTTTGCTCCTCTGAGCTTTTTACAGGACTGAGAAAGCCTCTCCCTTTTTCCTTTTAAGGCCCTTCTGAAAGTTTTGCAAC

Homo sapiens          CTTTACTGGTCTCTAAAAGGAAGGAAATACACAATTTTTATCTTCCTTTTATAAACCTCGTCTTCCTACAGATACCATCCTCCCAACTGGCTAGTATGGGATATTTCTAGATTTTCCTTT
Dasypus novemcinctus  TTTTCTTTGGCTCTGAAGGGGAG------------TTTAGCTTCCTTTTGTGAACATCATCTTCCCACAGATACTATGCTCCTATCTGAC---TATAAGATTTTTCCAGAATTTCTTTT

Homo sapiens          GGATTGCTTAGACGATTTGTGACATTGATAGTTCCAAAAACTATTAAGGGAAAACTATAACAGGAGGTTATATGTAGTATATTATT----AATTTTACAGTATGTATAGTCTATATATTG
Dasypus novemcinctus  AAATGGTTTAGA---------ACATTGATGATCCCAAGAGCCATTAAGGTAAAACTATTTGTGGA-ATTATACATATTATATTAATTCCAAACTTCACAGAATAGAGA----CGATAGTA

Homo sapiens          ATTTCATCTAAACTGAGAAGGGACAGGTTTCTGTCCCTTAGACTTGAAAGAATGTGTTATCATTGTAGTATAGAGATGAAAATAGAGGACACAAAGAAAAAATGATTTTGGTTTCTTTTT
Dasypus novemcinctus  ATTTAACTTAAACTGAGAAGG----TGTCCCTGTCTTTTGGGTTTGGAAGAGCCTAC--------------GAGATGAAACTAGA--ATGCAAAGTAAAAGGATTTTTG---------T

Homo sapiens          TTTTTAAGAGTTGAGGACGATTAATGCAGAGCTAGAATGATAGGCAGGCAAATGGATGGGTAGACAAACAGGTTCTATATTCCCAACTTGAAACGACACAAGGCTTCATGAATTGAAAAT
Dasypus novemcinctus  TTTTTAAGAGTTGAGAA-GGCAAATACATAGCTAGAAGGAT----TGACAAATGAATGTG-----------------GTTTCCCTCTTGAAACCACACAGGCCTT-----ATTGAAAAT

Homo sapiens          TGATTAGGAAGTAAAAGTTTGCCATGGAGTCC--TGGCAGAGGAGAGAGTGTAGTGAGGATGACATGGAAAGTAAAGGCATCAGGAGGTCTTATGTGGCAGATTGGAGGGCAGTAGGGTG
Dasypus novemcinctus  TTGTGAAG-GGTGGAAATTTGCCATAGAGTCCTTTGGCACAGGGGC---TATACTGAGGAAAACG-GAAAAGTAAAGGCATCCAGGGATCTTATGTGACAGATTGGAAGAGGGGAGGG--

Homo sapiens          CCACTAGTGTTTCACCACAGATGGTTTGAAAAGCACTGACCTGGAGCGACATGAAACTTGGTAACAATAGAAGTGGCTACAGGACCAGAACAGAGTATACGCATGGTAAAACTTCTTGAT
Dasypus novemcinctus  --ACTAGTACTT---CACAGATAGTTTGTAAAGCACTGATCTAGAGCTATGTGAACCTGAAGAACAACACAAGTGTCTACAGGATAAGGACTGAGTGTA-------TAAAACCTCATGAT

Homo sapiens          ACCTTTACTGTTTCCATTTAGTGGCTATTATGACTATAAAATATCTATGAACATTTTTGTACACATAT----------TTTGATAGTCATAGACTAATTTCACTGGAATATAAAATTAGA
Dasypus novemcinctus  TCCTTTACTCTTTGCAGTTTG-GGTTAATTTGACTATAAGACTGCTATGAACAAATTTGTACCATTATGTGCACCACCTTTGGTAGTCATATACTCTCTTCTCTAGGATAT.........

Homo sapiens          ATTGCCAAACCTTTAGGGTAAATTTGTGCTCAGCTTTAGAAGAAACTCAGTTTTCCTAGGTGGTTGCAACATTTTATCCTCCCACAATGTATGAGAGTTCCAGTTGCTGCCTGTCTTCAC
Dasypus novemcinctus  ............................................................................CCCACTAGCAGTGTATGAGA-TTGCAGCTGCTCCATGTTCTCAT

Homo sapiens          CAACACTTGGTGTTGTCTTTGGTTTTTAATTTTAGCCTGTTCAGTAGTGTGTAATTCTAAGTCATCGTGGTTTTATTTGCATTGCCCTGTGGAATAATGATGTT------GAGCA-----
Dasypus novemcinctus  CAATACTTG---------------------TGCGCTTTTCTGGTAGTATGTAGTTATAAATCATTATAGTTTTAATTGCATGGCCCAGAGTAACAATTATGTTCATGTGAAGCACTGGC

Homo sapiens          -------------------------CATTTTCACGTGCTTATTGACCATTGGATATTCTCTTTTATGAAGTGCTGTGT-AGAAATTTCCCCCATTTTTTGAATTGGGTTTTTGTCCTTT
Dasypus novemcinctus  TTTTTGGCTATTTGAATATCATGATCTTTTTTCCCTTCTTTTTTACTTTTGGCTATTCTCTTTTATGAAGTGCCATGTAAGATATTTTCTCA---TTTTAAAACATGGATTTCATCCTTT

Homo sapiens          TCTTTTTGATTTTTAGAAGTTCCTTGCATACACTGGGTCTGAGTCCTTTGTTAGATATATGTATTACAGATATCTTCTCCCAATGCGTGTCCTATGTTTTCACTTTCTTAATGGTGTCTT
Dasypus novemcinctus  TCTTTTTTATTTGTTGA----CTTTATATTTGCTGGATATGAGTCCTTTGTT--------------GGATATCTTCACTCAGTTTGTGATGAACTGCTTCACCTTCTTAATGGTGTCTT

Homo sapiens          TC-ATGAATA-CACATTTTTACTTTGATAAAGCCCTATT----------GTTAATTTTTTT--------------TCTTTTAAGGATAGTGTTTTTTTTTTGTGTCTTATTTAAGAAA
Dasypus novemcinctus  TTGATGAACAGAAGGTTTTGATTGTGATGAAGTCTGATTTATTTTTTTGGTTTACTTGTTTTTGGTGTTTTTTTTAGTCTTTTATGGATAGTG---TTTTTTTGTGTCCTGTTTAAAAAA

Homo sapiens          TCTTT-GCTTAGCCTAAGATTTGAAAGATCTTTTCCTATGTTTTCTTCCAGAATCATTGTTATTTATCTTTCACATTTAAGTTTATGATTTATCTGGAATTAATTTTTGTGTAAGATGTG
Dasypus novemcinctus  TCTTTGGCCTATTCCAAGGATTTGAGGACATTTTCT--TGTTTTCTTCCAGAGGCTTTATAGTTTACCTTTCACATTTAGATTAATGATTTATCTGTAGTTAATTTTTGTGGAAAATGTG

Homo sapiens          AGGTAGGTAGTTAAGGT--TCATTTTTTCCCCTAATACAGTTATCTAATTGTCCAACATTATTTATTGAAAAGACCACCTCTTTCCTCATTGTGTTGATATCTTCATTGTAAACGTATTG
Dasypus novemcinctus  AAATAGGTGGTTAAGGTGATTGTTTTTTTTCCT-ATGCAGATAGGCAATTGC---------------------------------------------TGTCTTTGCTGTAAATTCATTA

Homo sapiens          ACTT-TACATGTGTGGATGTAATTCTGTACTCCTTGTTCAATTACAGTGATGTATTTGTCTGTCCTTATGCCA--ATTGGCTTGTCTTATTTACTTTATTTGTATAAAAAGTCTTAAA-T
Dasypus novemcinctus  ACTGAAATATATGTGAATCTAATTCTGGACTCCCTGTTCTGTTCCAGTATTTTATTTTCCTTTCTTTGTGCTAGTACCACAATGTCATATTTACTATAGCTTTATACTAAGTCTTAAAGT

Homo sapiens          CTCATCCATTCATCTTTGAGATGATTCAGAAACTGTGATAGGCTGCATTTGTTTGGTTTGGGATGCAGCCATCTATTAGAAGATGAAAAAAATCATGTATTTAATAATCAATTGGAAGTG
Dasypus novemcinctus  CTGAACCATTCATTTTT-AGATGTTTTAGAAAC--TGATAAGCTACATTTGTTT--TTTGCAATATAGCCATGTATGATAAAATG--AATAATCATATATTTAATATTTAACTGAAAATG

Homo sapiens          AATTAAACTTAGAACAAACTGATTGTCTTATAATACTTTATAGAGATAATTAACCTGTTTTTTAGGTTATGATGGGACTGTGTTAACATTTCAGAACCTTTTCTTTTAAATTGACATATA
Dasypus novemcinctus  TATTTAAATTCGATCAAATTAATGGTTATATAATACTTTCTTAAAAATATTTATTTATTTCT....TTATCATAGGACTGTATCAGCACTGCAGAACCTTTGTTTTTAAA--GGTTTATG

Homo sapiens          CTTTATGTTAACCTAAGGTAA--------GAAATGTACTGTTACTCTCTCTGGAAGTTAAAAGTAGATCAGAAAGACGGTT-TTACATTTGCAAAGAGCTGTGTCATAGGGAAAGTGAAT
Dasypus novemcinctus  CTTTATGCTAATATAAGATTAAATGATTAGAAATACAATGGCATCCTCATTGACAGTTATGAACATATTAGAAAAATGGTTATTGGACATGCAAAAAGCTGTATCTCAGG--AAGAAACT

Homo sapiens          TGGTAAGCTG-TTTAAGCCATGAC-AATAGGCACAGTCTGTTTCTGCTTTGTTCCAGCATTCTCTTTCCAGTAA----ATACTCTTAGTCTCATCCCTAGATCTTCCAGTGGTCAGTAAA
Dasypus novemcinctus  TAATAGACTGACTTAAGCTATGGTTCATAGGCCCAGACTTTTTCT---TTGTGCTAACAATCTCTG--CAGTAATATAATATT-TTAGTCTCATTCCTAGATGTTTTAGTGGTAGGTAAA

Homo sapiens          CATCTTTTTTTAAATGT--AATTTCAAAATTGTAAG-ATTTATG----TCAGAATCCTAGCAGGAAACAGAAGACAT--TCTGGTGGGATTTTAAAGAAAATTTAATGGAGCTCCTTT--
Dasypus novemcinctus  TATCCGTAATTGATTTTAGAATTTCAAAATTTTAAGCATTTAAAACATCCAGAGCTCCCATAGGAAAAAGAAGACATACTTTGCTGTAATTTTAAAGAAAACTTAATGAAGAAACTGTTG

Homo sapiens          --------CTATAGACAGACAGTAGCAAAGTAAACAACATGGCACCTGGAGACTAGCAAAAGCA-AAAATAGTTACCACC-TTAGGGCTGAGGTGACAAGAAGAAGAAATAACCATGAAT
Dasypus novemcinctus  ACATTCAACTGGACACAGAAATCA--AAAGAAACCAGCATATCACCTGGAGATTAGTCACATCAGGAAATAGTTAGCACCACTCCAGCTGCAGGAGCAA-AAGAAGAAGTGACCATGAAT

Homo sapiens          ATATTTAGAAATAATATAAAGCTATATTTCTTCTCTTATAACTTATTTATTTAAATGTCACATGACAGGATCCTCCTGTGTTACCATCCTGTGAATGTAACATTTCAGTTGATAGCAAT-
Dasypus novemcinctus  GTATTTAAAAGTAATATAAAACTACATTT-------------------ATTTAAATGGCACATAACAGGACTATCCTAT-------TAATATTTAAGTAATATTGCAGTTGATGACAATA

Homo sapiens          ---GAAATTTTAAGCAAAACTTTTTGAAGTGTACTGGTTCATTTCCTTTTTTCTCAGTATGTGGAAAATTTTCAGCATTTCTTATCAAATGGAAAGAGACAAGATAATTTGTCATGGGGT
Dasypus novemcinctus  AGAGAGACTTTAAGCAAGACTTTTTGAAATATATTTAATCATTTCCTTTTTTTTCCGTTAGTGGAAAATTTTCAGCATTTCTTGTCACATGGAAAGAGACAAGATAATTTGTCATGATGT

Homo sapiens          ATATCAAAAATAGCATTTGAGTATTTTTGTTCATATACATCGCTGT-TCCTTTCCTGTACTGCAATTATCTCCCTCACAAATAA-ATTCTGGGAATTCTCGGTTCATATGTAAATAGAAA
Dasypus novemcinctus  ATCTCAAAA-CAGCATTTGAATATTTTAGTTCATATACATCACTCTATTCCTTTTTGTACTGCAGTTATCTTTCTCATAGATAATGTTCTGGGAAAGCTAAATTCAC--GTAAGCAGAAT

Homo sapiens          TATGACAGAAGTCTTAACTGAAATAGCAATTCAGTTAGCTTAAAATTTAAGTTATCTTTTTAAATACCTTTTACCTAGGTTCACAATGGTCCGTAGTGGAAAAAATGGTGACCTTCATCT
Dasypus novemcinctus  TATGACAGAAGCCTGAACTGAAGTAGCAACTAAATTAGCTTATCACTTAAATTATATCTTTAAATACCTTTC-CCTAGGTTAACAATGGTCCGTAGTGGAAAGAATGGTGACCTTCATCT

Homo sapiens          TAAACAGATTGCATATTACAAACGAACTGGTGAATATCATTCAACTACACTGCCAAGTGAGAGAAGTGGCATAAGAAGAGCAGCAAAAAAATTTGTCTTCAAAGGTAAAATTAATGTTCA
Dasypus novemcinctus  TAAGCAGATTGCATATTATAAACGAACTGGTGAATATCATCCCACTACATTGCCAAATGAGAGAAGTGGCATAAGAAGAGCAGCAAAAAAATTTGTCTTCAAAGGTAAAATTAATATGCT

Homo sapiens          -AATTTTGATTATTGAGT--------TGTAGATTCTTCCCCACATTCTTCCTTGTGGAAGTTTGGGAATTTCTGTAGTAGTTGGTTGTTGCTAAACAATTACCAGTTGAATTGATGATGC
```

```
Dasypus novemcinctus  TAACCTGGAGTATGGTATGGGGTTAATATAGATGCTTCTGCACATG-TTCTTTATGGAA-CCTGAAAATTTCTGTAGCAATTAGT-GTTGTTTAACAATTACTAGTTAA-----TGAAGC

Homo sapiens          CACTCAATCAAAAGCAAAATAAAAATTACACTCAAGCTAACTGAAGGCCAATAAGCGTGAAAAGGGTTGTTTAAATTATAGACAGAATA-----TGATACCATGAAAGAAA-ACAAAACA
Dasypus novemcinctus  CACCCAGTGAAAAACAAA-TAGAAATCTCACTGAAGCTATCTGAAGGCCAGTAAGCATTTAAAGTGCTATTGAAATGATAGGCAAAAAAAAAAATGACACCATAAAAGAAATATAACACA

Homo sapiens          TTTTTCTAAATTAACAACTTCTGCTTTTACCCTCTC--GTGGTCTCT-----AATGGTGCACTTTCCAGCACTGTTGCCAATTTCAATGAGGAACTTTTTGATGCTTAATATGAAAATG
Dasypus novemcinctus  TC-----------AAAATTCTGCTTTTGCTGACTCAGGTGGTTTTTCATATAATGGTGTACTTTCGAGCATTGATGCCACTTTTAATGAGGAACTGATTTTATGCTTCATATGAAAATG

Homo sapiens          TTGACAAAGTTAACTGGTTTGTTTACTAAAATGAAAACTGGAAGGAATTAACTATATTTCTGTGATCATGTCCCCTTGAACCCCACCACTGCCATTAAATATTCTCAGGATCACATTCAT
Dasypus novemcinctus  TTGAAAAA----ACTCATTGGTTTATTAAAGTGAAAAATGAAAGGAATTAACTATATTTCTATAGT----TTCCCT---ACTCC--CACTCCCATTAAATATTTTCCAGATTATATTTAT

Homo sapiens          TTTAGGACCCTTGGATCTTCCTGAATGGATTAAATATTTGAAAGGCAGTGGGATATTTATCTTGGAAGGTCATGATAATTTTTGCCTCTTTTTCCCC---TTATAAAGGGGAAGAGAAGT
Dasypus novemcinctus  TTT-GGACCCTAAG--TCTCCTGAATGAATTAAATATTTGTAAGGCAGTAGACTGTTTTTCTTCAAAGGTTATGATCATTTCCCCCCATTTCACCCCATCCTGCAAAGAAAAAAAGAAGT

Homo sapiens          TTGGGATGAGATAAACAACAAGTAGTCACCAGATAATTGAATGAATGTTTGTGTTACAAATGGGAATTTTGCCTCT-----------TAGGCTTACCCCAAAACAGGTTAATAAAACTCT
Dasypus novemcinctus  ----------ATAAACA-----------CTAGATAAGTACATGAACAGTTGTGATATTAATGGAAGTTTTACCTATAGGAACAGCATTAGGTTTTCCCCAAAGCAGGACAAGAAAATTGT

Homo sapiens          AGGATAATAAATAAATGTCTGCTTGCATCTCCCTGTGTATATCCCTACCAATAACTCCCTTATACTTAAGAT----TAGGCAGACCTATTACCTCTTAAACTAAAGTGAGGATTCTTTCA
Dasypus novemcinctus  AAGATAA---GTAAATGCCTCCT---------CTATGTATACTCATCCCAATAACTCCCACAGGTTGAAGATGGTTTGCCTAAATCCATTACTGCTTAGACTAAAGTGAGGAGTCACTCA

Homo sapiens          TTCATATATTTGATAATTTATGTAACAAATGTTAACTGGGTTTCTGCTGTGTGCCAGTCACATTCTAAGCCTTGGAGATACATTGATGAATAACATAGATAAGGTTCCTGCTCTTAGGTT
Dasypus novemcinctus  CTCATGTATTTATTCATTTATGCAACAAATAT-----GAGTCTCTACTATCTGCCAACCACATTTTAAGTCTTGGGGATACTTGGATAAATAAGATAGACAAGGTTCCTACCCTCAAGTT

Homo sapiens          GCTTATCTTCTAATTCTAAAAAATAAGGAGTGTTCCACTCATCAGTTGGCTTACTCATATTAGTAGCTGAATGGTGTATTTTGGAGTTTGAATTGCTAGCCCATTCACATTGGAAAGGTA
Dasypus novemcinctus  GTTTACCTCC----------AGGTCAGGAATGTTCCACTTAGCACGTC-CTTAGGATTATTAGTAGTTTAATGGCTTATTCTGGATTTTGAATAGTTGCCCCATCCACATTGGAGAAGT-

Homo sapiens          GATGGTGATTCGCTT-CATACCCTGGTTACTTCCTAGCTGCAGATTCTTCTCAGGTGTCCTCCTAAGGAAACAGAGAATTGTCTTGAGGATTACAGTTTTTTCCACTTGCCCGCTTCACC
Dasypus novemcinctus  ---GGCAATTCTTTTTCTTTCCTTGGTTTCTTCCTAGTTGCAGATTCTTCTCAGGTTTCCTCTGAGGAAAACAAAGAACTATTTTGAAAATTAAGGTCTC--CCACTTTCCCACCCTACC

Homo sapiens          ATCCCTACCTT---CTCAAATATAGAAGAAGCTGAGAGAGCTGTTTGCTTGCAAAAATACAGTGAT--GATAAAACATTATTTTAGAAAATTCTGTTCCAGGAAGTTGTTTTTCTGTACA
Dasypus novemcinctus  ATCACCACCTCTAACTCAA-TCTAGAAAATCCTGAGAGTATAGTT-ACTTACGAAAATGCAGCTATATGATGAAATACTATTTTA---AATTCTGTTCTAGGAAGTTGCTTATTTGTCTG

Homo sapiens          TAATTTATTTGCTAATCTATGCCATTTTTAGGAGGTGCCTGCAAA-ATCTTCCTACTTTGAGTACTCCCAGAGAGAGATAATTTCATATTTCCGTACTTATTACGTGCTTTGGTGAACAA
Dasypus novemcinctus  TAATTCATTTGCTAATTCATGCCATTTTTAGGAGACACCTGCAAGTATTTTTTCAGTTTGAATGCTC---AGTAGAGATAATTTCATATTCTGGCAAT-ATTGTGTGCTTTGGTGAACAA

Homo sapiens          GCTTGCTCTACTTATATTGATAGCTACAGAATTATAGCAGCTACTGCCCTTCCTAAGTATCTAGTAAGTGTAACAGCTAAGAACCTTT------GTGTTGTAGCATCATTAAAAATAAAT
Dasypus novemcinctus  ATCCACTCTACTAGTATTGATAACTATAAAATTATAGCAGCTAGTGCTCCTCCTAGACAGCTAGTACATATAATGGCCAAGGACCTTTCAAATTGTATGGTAGTATCATTAAAAATAAAT

Homo sapiens          ATTCTGTTTTAAATGCAGTAAGATTTTACAAAACAA----AACAAAAACCTTCATGGTTTAAAGAGAAGGCTATGATTCTCTTAAAACTACGTAGATCTCTAAAAA-TTGCAATTCTATC
Dasypus novemcinctus  ATTTCATTTTAGGAGGAGAAAGATTTTGTAAAAAAATGAAAACAAAAACCTT-ATAGTTTTAAGATGAAGCCATGATTTTCTTAAAACTACAGAGATCTCTAAAAAATTGTAATTCTATC

Homo sapiens          TTTT-GGTATCATTTATTCCAGAAAAAAAGCTGTTTTATGTTGGAAAAGACAGAAAACAAAATCGTTTGGTAATTGTTTCAGAAGAGGAAAAAAAGAAAGTCTTAAGAGAATGCCATGAA
Dasypus novemcinctus  TTTTTGGTATCATTTATTTCAGAAAAAAAGCTGTTTTATGTGGGAAAAGACAGAAAACAAAATCGTTTGGTAATTGTTTCAGAAGAAGATAAAAAGAAAGTCCTAAGAGAATGCCATGAA

Homo sapiens          AATGACAGTGGAGCTCATCATGGTATATCCAGGACCCTCACTCTGGTAGAATCCAATTATTATTGGACATCTGTGACCAATGATGTCAAACAGTGGGTATGGCTTATGTATTTAGAGTAT
Dasypus novemcinctus  AATGACACTGGAGCCCATCATGGCATTTCCAGAACACTTACTCTAGTGGAATCCAGTTACTATTGGACTTCTGTGACCAATGATGTCAAACAGTGGGTATGGC-TATGTATTTAAAGTAT

Homo sapiens          TATGAATAATGTCTTTCTGATAGATACTTAAAATTTATTATTTCTCCAGATTTATTCTGGGTATCACCTCTCAT---------------TAAGTATTTTTGGCAATCACTTAAAGGGAGT
Dasypus novemcinctus  TTTGAATAATGTCTTTATAATAAATATCTGAAATTCATCAATGCTCTAGATTTATTCTGGGTATCATCTCTCATATACTTTTGTATAGGAAGTTTTTATACAAAACTCTT----GAAGT

Homo sapiens          TGTATTTCCCTTCACAGTTAT---TTTAAATCACTTAAGTATAATTTGAACAGGAAAGAAGCATTATTTAAACA----CTTGAAAATACCATATTTAAATAAGAGATTATTTAAAAAATC
Dasypus novemcinctus  TTTATTTCCCTTTACAATTCTTTATTAAAATCATTGAAATATAATTTGAACAAAAAAGAATCACTAAACATACAGTCTTTTGAAAATACCTGATTTAGATACCTTTCTAAGTATAGAGCC

Homo sapiens          AGATTTGTATCATTGAACTTTTTTGTGTAGGCAGAATTTAAATGGAGCTGGAATTCATTTGACTAAATCATTACTTCTTCAATGGCAGTCATACAAGGTTTTCTTAAGGGGAGAATATGT
Dasypus novemcinctus  AGGTTTTCATCATT--ACTTCTTTATGTGGGTAGGACTTAAAGGGAACTGAAATTCTTTTGACTAAATTATTACCTCTTTGACTACAGTT-----GAGTTTT-----------------

Homo sapiens          TCATAGTAAGTCAAGCCCAGTATGTGCTCTTATCTTTTGTGCCTGTTCCATTTTGAGGTAG-AATGTCATACATGACAGAAACCCAAAACCAAATATAACCAAAATCAAATTTTGAGGAT
Dasypus novemcinctus  --------------------TGTGTGTACTCATCTTTTGTGCCTATTCCATCTTGGGTTAGAAATGTCAAATATGGCAGAAATCC-AAACCAACTAAATATACAATCAAGTTTTGTTCGT

Homo sapiens          AATGAAATAAGTCTGAGTTTTGTAACTTTATGACCCATTGGGACCTTCAAC---TTCTAGTAGAAAATTCACCTATAGGAATTTTGGGGTTCTTGCTGGAAG-TCAGCTGATTCTACTCT
Dasypus novemcinctus  AAGGAAAAGAGACTCATTTTTGTGACTTTATAGCCTATTAAGAGCTTTAACTTTTTCTGATAGAAAATTCCACTA-----------GGGTTTCTGCCAGTAGCTCAACTGAT----CCCT

Homo sapiens          TTCCAGTGTACTGTATGTCAAATGGTAGGAATTCCTTGGTATATCTGTGAGATCTCCTTAATAATTGTAGTAGTAC--ATCAAAAAGAGTGGCATTTCAAGCATAAAGAATTAAACCAAA
Dasypus novemcinctus  ATCCATTTTA----ATATTAAA-GGACCAAATTCCTTGTTATGTATGTGAAAGCTCTTTAATAATTATAGTAGTGCATATCAGAAAGCCTGCTATTTCATCCATAAAGGATTAAACCAAC

Homo sapiens          ATTTCA-TAAGTATAATTTGTGAGTTATCAGTTTGTGGGTCTATTCAGGTATTCCAAAGACCTTCATAATTTATATATTTTTCCTGCTGTGCA------TTTTTTACAAACATTCAGTGA
Dasypus novemcinctus  ATTCAACTAAATACTGTCTGTGAGATAGCAGTTTATGATTCTGTACAGATATTCCACAGACCTCTGTATTTGAAATTGCCTTGAAAATGTGCCACATATTCCTTTTCAAATATTCGGTGA

Homo sapiens          AATCTTTGTCAAATTGTTATCACTGGAGAATGAATATGATTTTTGGAAATAATCAGAAGTTATTTGTGATAAAATCCAACAAATAAGTTTAAATTGGAAAGTGAAAATTTTGATCAAAAG
Dasypus novemcinctus  AATCTTTGTCAAATCTTT-TCATTTGAAAATGAATATGGTTTTTGGAAATAATC--AAGTTATTTG-AATTAAAACTAATGAA------TACATTAAAAAATTAAAATTTTGATCAAAAG

Homo sapiens          TAACTGTGACTATAATGGGATTAAATGGCTTTTAAATTTTTCTTCAGAAGCTAG-TTTGAAAGTAGTTTCTAGAGTGGAATTCCAGAGATACATTGAGAAATGGCACCCATCCATAAATT
Dasypus novemcinctus  TA---------GTCATGTAATAAAATGGTTTTTAAAATTTTATTTATAAATTGGCTTTGAAAGTAGTTTCCAAAGT-GAATTCCAGAAATATATTGAGCCATAGTTACCATCAATAAGTT

Homo sapiens          GATCAGTTGATCTTGTCTCACCTCACTCAAATGAAAGCTGCAGGACAGCATGGGTTTTGTGTATGTTTATTCTTTTCCCACGATTGAGAGAAATTGCTC-CATCTATACATTTTATATGA
Dasypus novemcinctus  GAGCAACTGATTACTTCTCACCTCATTCAAATGAAAGCC-CATGGCAGCATGAGCTTTGTTTTTGTTTGTTTTTTTCCCAAGATATAGAGCAGTTTCTGGCATCTATAAATATTTTTTTA

Homo sapiens          ATGAAATTATATTTCAATAAATATGGTTGGAATAGATTAATGAATAAGCTTCTTAATGTGATAACTTTAGAAGGAAACATTTTATGTATATAGAAATTATTGTAAATTTACATACTGATT
Dasypus novemcinctus  ATAAAATGATCTTTTAATAACTTAAG--AGAATAGATAAATGAAT----TTCTCA--GTGACAGCTTTCAAAG-AATGATCTTTCATATATAAAAATTACTGTAT-TTCAAATACTGATT

Homo sapiens          TAATAGGAAACTTAAGATCTTTTTAAG--------TATTTAATAAATATTTATTGAATGTGAACCAGATTTATGTTCCATGTGAATAAGACCCA---ATAAGATGGTCATTTGGTAGTAT
Dasypus novemcinctus  TAAAATTATA--TCAAATCTGTTTAAGGATATATTCATTTATTAAGTATTTATTAGATGTGAACCAAAAGTATGTTCTGTGTGAATAAGACCTTCTTACAGTATGGTCATTTAATAGTA.

Homo sapiens          AGTCTTCCCTCAGTATATGCAAGGGATTGGTTCCAGCACCACACTCCTGTCACCAAATCTACTCATACCGAAGTCCTGCAGTCAGCCCTGTGAAACTCACATATACAAGAAGTCAGCTTT
Dasypus novemcinctus  ........................................................................................................................

Homo sapiens          CTGTATACTTGAGTTTCTCATCCCTCCCTATCAATAGAGTACTTTTTATTTGTGTTTAGTTAAAAAAAAAAAGCCATGTATAAGCTGACCTTCACAGTCCAAACCCATGTTGTTTGAGGG
Dasypus novemcinctus  ........................................................................................................................

Homo sapiens          TCAGCTGTAGTGGCTACTACAGTTGTTTGTTGTTGTTTTATTTGTTTGTTATTCTAAATAG--GTTTAATCCAGTG----ATTCTCAGTCCAGAAGTTTCTCCTTCAAAACACAAAGTAT
Dasypus novemcinctus  ......GTAATAACTAATACA---------------------TGCTTATCATTCTGAGTAGATGTTTAACTCAGTGGTCTATTCTCAGTCCTGAATCTTCTCCATCAAAACACAAG---T

Homo sapiens          ACCTATTAACCATTTTAAAATTCAGATTTATAAGACTGCTTTTATATTGTGTTGAGTGTTAACATTTGTATGTGTTTTCTTTATTGTGTGCCTGTTGTAGGTATATGCTTGTCAGCATTG
Dasypus novemcinctus  GCCTATAAATCATTTTAAAATTCAGATTCATAACATTGTTTTTATTTTGTATTTTATCTTGAT-----TATGTGTCTTTTTT------------TTTAGGTATATGCTTGTCAGCATTG

Homo sapiens          CCAAGTGGCAAAAAATACAGTTATTGTAGCACCGAAACAGCACCTTCTCAAGGTGGAAAATCCATGGAGTTTAGTTACTGTTGATCTGATGGGGCCTTTTCATACAAGCAACAGAAGTCA
Dasypus novemcinctus  CCAAGTGGCAAAAAATACAGTTATTTTAGCACCTAAACAACACCTTCTCAAGGTGGAAAATCCATGGAGTATAGTTACTGTTGATCTGATGGGACCTTTTCATACAAGTAACAAAAGTCA

Homo sapiens          TGTATATGCTATAATCATGACAGATTTGTTCACCAAATGGATTGTGATTTTGCCTCTATGTGATGTTTCAGCATCAGAAGTTTCTAAAGCTATTATCAATATATTTTTCTTATATGGACC
Dasypus novemcinctus  TGTATATGCTATAATCATGACAGATTTGTTTACAAAATGGGTTGTGATTTTGCCTCTAAGTGACGTTTCAGCATCAGAAATTACTAAAGCTATTAGCAATATATTTTTCTTATATGGACC

Homo sapiens          TCCTCAGAAAATAATAATGGACCAAAGAGATGAATTCATTCAACAGGTAAGACAAATAAACTACTTAGGTCTGGGAGCATATCTTACT-TCTTTTCAGTGTCCAGAACCAGTGCCCTCTG
Dasypus novemcinctus  TCCTCAGAAAATAATAATGGACCAAAGAGATGAGTTCATTCATCAGGTAAGAA----AAACTATTTATGTCTGTGAGTATATTTTAGTCTCCCTTCAGTGCCCAAAACAAGTGCCTTGTA

Homo sapiens          CATTATATTACAGAGTGCTTAATAAGTTCAGATAAATTTCTGTAGGTATAGAGGTATGATATTAAATACTACAAGGATAGTTGTACTGGAAATTAGGAGATTAGGATTTTAATACTTACT
Dasypus novemcinctus  CATAAT-----AAGGTACTTAATAAGTTTAGATAAA-TCCTGCAATTATAAAAG-ATG-TATTATATAGCACAAAG-TAGTCAGACTGGAAATTAGGAAGCCAGGATTATAGTACTTATT

Homo sapiens          G-------TGGGTTATTGTAAACCTATCTTCAGATATCCTCAAATTAAAATGAGGAGGTTGGACCAAATAATCTTTATTTACTTCTAGATTAAAAACCCATGATTGGTTGGGCCTGGCTC
Dasypus novemcinctus  TTTATCACTAAGTAATTGTAAAACTATTTTCCGGTTTCTTCAAACTAAAAT---GTGGTAAGATCAGATGATCTTTGTTTCCTTCCAGCTTAAAAA.......................

Homo sapiens          ATGCCTATAATCCCAACAGTTTAGGAGGCTGAGGTGGGTGGATCACTTGAGCTCAGGAGTTCGAGACCACCTGGGCAATAGGGCGAAACTCCATCTCTACAAAAAATACAAAATTTAACT
Dasypus novemcinctus  ........................................................................................................................

Homo sapiens          GGGCATGGTGGTGGGCGCCTGTAATCACAGCTACTCGGGAGGCTGTGGCAGGAGAATCACTTGAACCTGGGAGGTAGAGGTTGCAGTGAGCCAAGATTGCACCACTGCACTCCAGCCTGA
Dasypus novemcinctus  ........................................................................................................................

Homo sapiens          GCGACAGAGCGAGACTCCATCTCGAAAAGAAAAAAAAATTAGCCAGGTGTGGTGGCACACACCTGTGGTCCCAGCTACTCGGGAGGTTGAGATAGGAGGATCACTTGAGCCCGGGAGGCG
Dasypus novemcinctus  ........................................................................................................................

Homo sapiens          GAGGTTACAATGAACTGAGATTGTGCCACTGCACTTCAATCTGGGCAACAGAGTGAGACCTTGTCTCAAAAAATAACAATAATAATAATAACACTATGATTGACAGGTAAAGGGAGACTT
Dasypus novemcinctus  .................................................................................................TTCTATGATTTACATGTTAAAGG-GACTT

Homo sapiens          AAATAAATGGAAGAATACATTTTCTTCATGAATAAGAATAGTCAGTATTGAAAGGTACT--TTCTCCCT----------------------------TGCTCTGGCC------------
```

```
Dasypus novemcinctus  GACTAAATGGAAAAATATATCATATTCATACATGAAATTGCTTAATACTGAAAGATATTGATTCTTCCTAAATCAAAATTAATTTGTAAAAGTAATACCTTTCTAACTAGCATTTTAAAA

Homo sapiens          --------TTTTCCCAGTTAAAATTAACCTGAAAGGATAAGCAGGGGAGAATATATATGAAAACTTTTTATAAGAAATACAACAACTGCTAGACTTAAGGCATATGTTAAAACTTTTTGT
Dasypus novemcinctus  GAGGGAGATTTTCCAGTTTAAAATTAACTTTAAAGAATAAGTAAGCATGAATATATTTGAAAATTCTAAAAAAGAAATGTAGCAACTACTAGACTTAAGGCATATTTTTAAA-TTCTTAC

Homo sapiens          AATTGACAGGGTATATGTTTAAGAAAAATCAGAGATAGCCAGACTTGGTTGAAAGCATCTTTGATGGCTCAGTCCAAAAGAAATACCTAAAGTTAATATGTATTAACTAGTAGATAGGAC
Dasypus novemcinctus  AATTGACAAAGTATGAGGATAAAAATAATCAGAAATAGC-----------ATTGCACTTTTTGTGTCTTAATCCAAAAAAAAC-CCTAAAGTT-GTGTTTATTACAGAGT---TAAGTC

Homo sapiens          ATGTGCCATGGCACCCCAGGATTATTCCCAGTTTCCATAATTCTCTAGGAGGACTCA------GCATATAGTCATACTCATGGCTAAGATTTAT------------------------T
Dasypus novemcinctus  CTGTGTTTAAGGTCCCCAAGACCA-CCCCAGTTT---TGATTCTGTATGAGGACTCACAGGGCTCAGTGTGTTGTACTTATGGCTAA-ATTTATTATAGCCAAAGGATACAGAGCAAAAT

Homo sapiens          CAGCAAAGAGAAAAGACACATTG------------GGAGAACCAGGTACAAGTTTCCAAGATCTCCTTCCCCAGTGGAGTTACACAGGACGTGTTTAATTCCTTTAGC---AGAGTTGTG
Dasypus novemcinctus  CAGCAAACAGAAAAAGCACATGGGGTAAAGTCCAAGGGAAACCAGGCACCGGCTTTTAAGA-AACTTCCCTCAGTGGAGTCACGTGGGACACTCTTAATTACCTGGACCAACAAGTTGTG

Homo sapiens          ACAACATAAGGAAGCTCACTAGAGTCTCAGTGCCTAGGGTTTTTACTGGGCGCTGGTCACATAAGCACCTTCTACCTGGCATGTACAAAAATTCCAGACTCTCAGAAGGAAAGATTGTGT
Dasypus novemcinctus  ACAACAT.................................................................................................................

Homo sapiens          TTGGTCTTATAACCAAAATCTAAGCTCCTAGACATTAGCCAAGGTCCAACCTTGTAAGCATGCCTTTCAAAGAACAGCAGTCACAGATCTGCTTTGTTAATATGTTAAAATTAACTGTCA
Dasypus novemcinctus  ........................................................................................................................

Homo sapiens          GGTTAACTCTTTTCTTCACAGGTCCATCCAAACAACTTAATAAGTATTTATGTTCTAACCACTTAGTAGCAATTTAAAATACTCATAAATTGAAAGAAAATATATTTTTGTTCCATTCTT
Dasypus novemcinctus  ........................................................................................................................

Homo sapiens          TTCTTTTTTTTTGAGACGGGGTCTCATTCTGTCACCCAGCCTGGAGTGCTGTGGCACCGTCTTGGCTCACTGCAACCTCCACTTTCCAGGCTCAAGTGATACCCCCACCTTAGCCTTCTG
Dasypus novemcinctus  ........................................................................................................................

Homo sapiens          GCTAATTTTTGTACCTTTTTTCTTTTAGAGACAGGTTTCACCATGTTGCCCAGGCTGGTTTTGAACTCCTGAACTCACATGATCCACCTGCCTCGACCTCCCAGAGTGCTGGAATTACTG
Dasypus novemcinctus  ........................................................................................................................

Homo sapiens          GCGTGAGCTACCGCACCCAGCCCTTTCATTTCATTCTTAACCATAATTACTTGCTAATGGGTAATGTGTACCTGTTGACCACTGCTTAACTTCTCAGACCTTGGGATCATATTGTACACT
Dasypus novemcinctus  ..........................................TAAACAGTTAATAATGGGATGTGTGTACCTGTTGAGTACTGCTCAACTTCTCAAACCATGG-ATCAGATTGGTCATT

Homo sapiens          GCCACCCTCATTTCCTGTTTCACACTGATTTTTACATAGTAACTGCTTTTTATCACAGCATTCGATGAAGAAAACAGTTCTGTAAAGATATGATGTCATCAAACAAATTGTAACCTGATC
Dasypus novemcinctus  GCCACTCTTATTTCCC-TTCCACATTGATTTTTGCACAATAGTTGCTTGTTGTCACAACTTTCACTGGA-AAACCAGTTTTGCAAAGGTAGGACATCATCAAAAGGAATGTAGCTTGATC

Homo sapiens          TAATGTTGAAACTG--AACTACCTTAAACTGGTAGTTTTCATGGTGTCAAACAGATGTTAAGGGGGCAACTTGGCACAAAGTTTGGTAACAATGGTTTAAATGATAAAGCTAGTGTCACA
Dasypus novemcinctus  TATTGTTGACCCTGTGAGGTACATCAAGCTAGTAATGTTCACTGTGTCCAA-----GTAATGAGAACAACTTG--ACAGAGTGTGGTATCTGTGGTTATAATGATAAAGTCAGCATTACA

Homo sapiens          AATGTGTCTAGTAGAGGAGGATCATGTAATTAATTAAC--AGGTAACAATTTGCTAATTGTTTAAAATTATATCTGTAATATGATTTTAGAATCCAA------TACCAAAAGTATTTTCA
Dasypus novemcinctus  AGTAAGTGGAATAGGGAAAGAT----TAATTAGTAAACTTAATTCACAATTAAC--ATAGGTAACAATCAGA--CATAATATAATTTAGCATTTCAAACCATGTATCAAAATTGCATTCG

Homo sapiens          GGTAAATTAAAAGAATTTTAATTTTTAAAGTAGAAACAGAAAAAAATATATTTGACATTT-----AATCTCTAGAAATGGAGGTCACATTCTAAACCTAGGAATAGTGGTATAAACTTCA
Dasypus novemcinctus  GATAATTT-------TTTTAA--TATAACAGAAAATTAGTAGAAAATTCAGCTGACCTTTTTTTAAATCTCTAGTGTGAGAAATAA-----TAGCCTTAGGAA-AGTGATAAAAATTTCA

Homo sapiens          ATGGAAAAA---CATTAAATTTGGCTGCATAATAATAGGAGATTTA--TGTGGTAAAGTAATCAACATATAAAAGCCAATCTTGCAAATAAAGCAAGATAATTTTGTAAATGAAGGCAAG
Dasypus novemcinctus  AAGGAAAAAAAATCACTAAATTCGGCTGCATAAAAATTAAAAACCTAGGTGTGGTAAAATGATCAGAGCACAAAGACAGAT------AACAGAACAAGAAAA.................

Homo sapiens          GTAGTCTTGCCTGCCTTGATTGACATAGCAGTGGTCTGAGCAATGGAGTACGTAAAGGCCATCCAGTTTGGGGGAAGGCTATTGGAACTTCTATTTATATTTTTTAAATCCCAAAAATAA
Dasypus novemcinctus  ........................................................................................................................

Homo sapiens          GAAATGAAGCTTTATTAATAATGTATACGATTGATAATAGCATTCTGACTCAATCAACAAGACATGTATGTCACACATGCTATGTGAAGGTATCCTGAAAGAAAAGTGAGAATCCACATG
Dasypus novemcinctus  ........................................................................................................................

Homo sapiens          TTTCCTCACCCATACTTTTGCTTTCAGCAGTTGAATGAAAGTATGGATATCATCTAGCATTCACCACACTACTCACAAAATGGCCAGGGAGCTTAGATTTTTGCAGTGAAAGCAGATAAT
Dasypus novemcinctus  ........................................................................................................................

Homo sapiens          ACTAAAAGGCAAACACCCATCATCAACCACAAGAAAGTGATAGCGCCGGCACGTCTATTTAACTTCCAGCTCTTTGTTAGACACATCAATGAGATGAATACAAATATTAACTACAATAGT
Dasypus novemcinctus  ........................................................................................................................

Homo sapiens          CCCCATTTATCTGCAGTTTTACTTTCTGAGGTTTCAGTTCCTATAGTCAACTGCAGTCCAAACATATTAAATACAAAATTTCAGAAATAAATGATTTAAAAGTTTTTTAATTGCACACCA
Dasypus novemcinctus  ........................................................................................................................

Homo sapiens          TTCTGCGTATAGTGATAAAATCTCATGCTGTCCTGCTCTGTCCAACTGGGACGTGAATCATCTCTTTGTCTAGCATATCTTCATTGTATATGCTACTCTCTCCCCATCATTAGTCACTTC
Dasypus novemcinctus  ........................................................................................................................

Homo sapiens          ATAGCTGTCTTGGTTATCAGATCAAATGTAGTGGTTAGTAGTGTGTATCCAAGTTACCCTTATTTTATTTAAGAATGCCCCCAAAGTGCAAGAGTAGTGATGTTGGCATATTGTTAATCG
Dasypus novemcinctus  ........................................................................................................................

Homo sapiens          TCCTATTTTATTTTTAGTTGTTAACCTCTTACTGTGCCTAATTTAAAAATTAATCTCTATCATAGGTATGTATGTATAGGGGAAAACATAGTATAAATAGGGTTAGGTACTACCTGCAGT
Dasypus novemcinctus  ........................................................................................................................

Homo sapiens          ATCAAACATTCACTAGGGGCCTTGAAACATATTCTCTGAAGGTAAAGGGAGACTATAGTTATCAGATCTGATAGGAAATTAGGCATCAAAATTCAAAATTATCAAGAATACTCTTTGAAA
Dasypus novemcinctus  ........................................................................................................................

Homo sapiens          TATCAGTTCACTTCCACTTTTATTGATGGAGAGCCTTACCCTAAGTGCATATAATGTTTCAAGGTATTTGCTAATGATAATATGAAGCCATTATGATTAGCTAGCAGTTTTTCCAGTCGT
Dasypus novemcinctus  ........................................................................................................................

Homo sapiens          GTTTAAAGTCATTTGATAACCAAACCAGGACTTTGAAAATTTTTCTAAAGGTAACAATAGGGCCAAGCATGGTGGCTCATACCTCAGCTTTTTGGGAGGCTGAGGTGAGAGGATAACTTG
Dasypus novemcinctus  ........................................................................................................................

Homo sapiens          AGACCAGGAGTTCAAGACCAGCCCATGCAATACAACAAGACTCCGTCTCTACAAAAAAAAAAAAAAATTTTTAATTAACTGGACGTGGTGTTGCACACTCATAGTCCCAGCAACTCAGGAG
Dasypus novemcinctus  ........................................................................................................................

Homo sapiens          GCTAACGTGTAAGGATTGCTTAAGCCCAGGAGGTGAAGGCTGTAGTGAGCTATGGTTGCACCACTGCATTCCAGTTTGGGCAACAGTGAGACCCTGTCTCAAAAAAATAATAATAATAAT
Dasypus novemcinctus  ........................................................................................................................

Homo sapiens          AATAATAGGCTGTGCTCAGTGGCTCATGCCTGTTGTAATCCTACCAGCCAGATTGCTTGAGCCTAGGAGATCGAGACTAGCCTAAGCAACATGGTGAAATGCCATCTCTACAAAAAATGC
Dasypus novemcinctus  ........................................................................................................................

Homo sapiens          AACAATTAGCCAGGAGTGGTAGGGCACACCTGTAGTCCAGCTACTTGGGAGGCTGAGATGGGAAGATCGCTTGAGCCCAGGAGGTTGAGATTGCAGTGAGCCAAGATCATGCACTCTAGC
Dasypus novemcinctus  ........................................................................................................................

Homo sapiens          CTGGGTAACAGACTGAGACCCTGTCTCAAAAAAAAAAAATAAAAAATAAAGGGTAACAATAAATATTTGCAGGCCTATTTTGTGAGATTGCTTAAACAAAAAACCAAAAAAGCGACATAT
Dasypus novemcinctus  ........................................................................................................................

Homo sapiens          GATTGGGAAAACTTGTTCTTTTTGCTGTGATTAAAATGGCTTCACTATTACACAGAAAGCATAACGATGACAGTACAAAATGAACTCTTTTGTCAGCAAATACTGATGCAGAAAACACTG
Dasypus novemcinctus  ..........ACTTGTTATTTTTCCTGTGATTAAAATGTCTCAAATATTACTTAG----CATAATAATAACAGAATAAAATGTATCTTTTTATCAGCAAATACTAGCATAGAAAATACCA

Homo sapiens          CTAAA------------TATTATAACATAATACACAATGTGGGAGGTTTAT--------TGTATAGTTGAATAAAAATACAGATGTTTCTAATGGCTTAGCTTAGAGTATTTGCTAAATT
Dasypus novemcinctus  CTAAAATGTTAAAATAAGATTAGAACATAATATACAATGTGGGAAGTTAATAAACTTCCTGTACAGTTAGATGAAATTACAAGTGTTTCTAA---CATATCTTAGCATAT--GCTAAATT

Homo sapiens          GTTCCAATAAAATGCACAAAGAACTTTTTGTAAACCTCTGAAGGAAAGAGAACAAACATATTCAGTAGGAAAATTA--ATTTTTTTAA--ATGTCTTACAGAAAACTAAAATTGTAAGAA
Dasypus novemcinctus  GTTTCAATGACATGCACAAAGAATTTTTTATAAACCTCTGAAGGAAAGAG------CCTATTCAGTGGGAAAAATATGACTAATTAGACAATGTCCTCTAGAAAACTATAATTATAACCA

Homo sapiens          CTGACGGAATATCTGGTTTGATGGTTGGGGGGC-------TGACAGAGTAAGGATGTCAAGATACCATTTCACAAATGGATTCCCTGGATCATT-CCTAGGCAAGCTTTTGAAACAAAGA
Dasypus novemcinctus  CTGATGGAATATCTGCTTTGATAG---GGGAATAAAATAGTGATGGGGTAAGGATGTTAAGATAGCCTTTCATAAATGTAGTCACTGTGTCATAACTTAGTCATCCTCTTGAAACAAAGA

Homo sapiens          AGTTAAAGCCTTTTTCAAGGGT----CATTTCAAACCATTAATCAAGAC-TCAGATTGAAGCTGACCCAAATGACACCTATTCTTTGGGATTTCTT----TCTAATTTTGAATTAAAAAT
Dasypus novemcinctus  AGCTGAATCCTTTTCCAAAGGTTAGCCATTTAAAATTATGGATCAGGACCTCAGATTGAAGTTAATTTAAATGACACGTATTCCCTGGGATTTTTTAAAAATTATTTTTTAATTAAAAGA

Homo sapiens          AGTCACCCTGAATTAGTATGTATATC--------AGCTAGG----AAGTGTATTCAA-TCCAAGTAACAGA-----CTTCCTTTCCCTA---TCTCAATATATTAATATATATTGTATAT
Dasypus novemcinctus  AGGCATCCTGAATTAGTACATATATTGGGCTATAAGCTAGGACTTACTTGTATTAAGCTGCAAGTAACAGAAAACTCCTCCCCTCCCCACTCCCCCAGTATATAAA..............

Homo sapiens          ATATTATCCCAATATATATTGGGAGTGATTGAGAGTCACTCCTATTGTATTCACAACGTGGCTGCTGGAGCACATGCCACAATGCCTACATTTCAGTCAGAAAGAAGGGAGAGAGTGTTG
Dasypus novemcinctus  ........................................TTGTGCTCACAATATGGCTGCCAAAACCCATGTCACCTTTTTTACGTTTCAGGCAAAAAGAAGG--AAGAGCAACG

Homo sapiens          GGCAAACAAAGCTAGTACCAGCTGAGTTT--------------------GCCTAACAACTTTTACTTACAACTTTTTGGCTAGAATTATGTCACATGACCACCCCTACCTGCAAGGGAGT
Dasypus novemcinctus  GGCAGTAAAGGCTAACAGCAGATGAGTCTACGCCCACCAACTTTTAAGAGCCCCACAACTTCCATTTACATTTTTTTGGCCAGAACGTCATCCTATGTCCACCACTACCTACAAGGGAGC

Homo sapiens          TTGGGAAATGTAGTTTTCTTTTTATTGCTTTC-CCCTCTTGTATTAACTTTGCCTATTTAGTTATTCTGATACTACTCGTCTGAAATATTCTTTTTTAAAAAGACAAATTTATGAGCCAA
Dasypus novemcinctus  TTGGGAAATATAATTTTGTTTT-----CTTTCACCCTCAATTTTTAGCTTTATTTGATTACTCATTGTA-----ACTCGTT----ATAAGCTCTTCT----------------------

Homo sapiens          AGGTTTACTCAACTAAAAATAATAAGGAAAAAAAAAAGTCTGAAAGGTGAGGTAGGAAAAAAATATTATTTTATATTTAATGGGTTTACTGTTAAAAGTTTTGTAAACTCATTTT------
```

```
Dasypus novemcinctus  --GTTTAC---------AAT--TAAGCAAAAAGCAACTTCTAACAGTAAG-----------------------TCTAACAGGCTTACTGTTAGAAGTTTTCTAAATTCATTTTTCCTTT

Homo sapiens          ---------AAGTGCTACACATAAT-ATAGTAACACCAATAATAGTAGTTATAAGTTAAAAGTTATAAAATGCTTA----TTGCCAATAACTGTTTTACATGCTTTTTAAACATTAACTT
Dasypus novemcinctus  TTTTTGGCCATATCTTAAAGATAATGACAGCAACAATAACCAAAATA-TTAGCAGTTGAAAGTTATGAAGTGCTTACTACCTACCAAGAACTCTTTTAAATGCTATGCAGATCTTA....

Homo sapiens          ATGACCTAGTGGGTTGTTTTTGAGACACTGTCTACTGAATTAAAAAGTTACTTGAGGGCCGGGTACAGTGGCTCATGCCTGTAATCTCAAAACTTTGAAAGACTGAGGTGCATGGATTGC
Dasypus novemcinctus  ........................................................................................................................

Homo sapiens          TTGAGTCCAGGAGTTTGAGACCAGCCTGGGCAACATGGTGAAACTCCATCTGTACAAAATATTTAAAAATTAGGTGGGCATGGTGACACATGCCTGTAGTCCAAGCTGCTAGGCAGGCTG
Dasypus novemcinctus  ........................................................................................................................

Homo sapiens          AGGTGAGAGGATCGCTTGAACCCAGGTAGTGCAGGCTGCCCTCCAGCCTGTGCGACAGAGCAAGACCCTGTCTCCCACCGCCCCCCACCAAAAAAAAGAAAAAAATTAAACTCAAAGTTA
Dasypus novemcinctus  ........................................................................................................................

Homo sapiens          ACGTTAAGTTCTGTTCTTCCATTTCCTTGTTGTATGTGTGTATTTGGCAAGGCACTTAATCTCTCAAAGCTTCAGTATCTTACCTTTAAAATGAAATTAATAATACCTCAAAAACTTGTA
Dasypus novemcinctus  ........................................................................................................................

Homo sapiens          AGGTTTAATGACAATAATGTAAAGTGTCTAGTGTGATGGGACCAGTGTGTAGTAAAGACTCAATAAATGGCAATCACTATTTTATACATAAAATTTTACATATTTTATGTATGTGTTCAT
Dasypus novemcinctus  ........................................................................................................................

Homo sapiens          ATAGAATGTTATGGTCAATAAAAAAGCAAGATTCAGCCAGGCGCAGTGGCTCATGCCTGTAATCCCAGCACTTTGGGAGGCCAAGGCAGGTGGATCACAAGGTCAGGAGTTCAAGACTAG
Dasypus novemcinctus  ........................................................................................................................

Homo sapiens          CCTGGCCAATATGGTGAAACCCCGTCTCTACTAGAAATACGAAAATTAGCCGGGTGTGGTGGCGCACACCTGTAGTCCCAGCTACTCAGGAAGCCGAGGCAGAAGAATTGCTTGAACCTG
Dasypus novemcinctus  ........................................................................................................................

Homo sapiens          GGAGGCAGAGGTTGCAGTGAGCCGAGATTTTGCCACTGCACTCCAGCCTGGGTGACTGAGCAAGACTCCATCTCAAAAAAATAAATAGATAAATAAAAGGCAAGATTCTAATTCTTTTGA
Dasypus novemcinctus  ..............................................................................................AAAAGGCTAGATTCAAATTCTTTTTA

Homo sapiens          AGCTTTTTTTTAAGTCAATGGAAAAATAAAAC-ACAAATTTTAAAAAGCCACCTTTGTTTTA-TATATTTTTCAGATCAATATTGAACTGTACAGATTGTTTGGCATAAAGCAAATTGTA
Dasypus novemcinctus  AGCTTTGTA--AAATCAGTGGAAAAATAATATTACAGATTTTAAAAGTTAGCTTTGTTTTAATATATATTTTTCAGATCAATGTTGAACTGTATGGATTGTTTGGCACAAAGCATATTGTA

Homo sapiens          ATTTCTCACACCTCTGGAACTGTTAACCCAACGGAAAGTACACCTAACACAATCAAAGCATTTCTCTCCAAACACTGTGCTGACCACCCAAACAATTGGGATGATCACCTATCAGCTGTT
Dasypus novemcinctus  ATTTCTCATGCCTCTCAAACTGTTGATCCAACTGAGAGTACACCTAGCACAATCAAAACGTTCCTCTCCAAACACTGTGCTGACCACCCAAACAACTGGGATGATCACCTATCAGCTGTT

Homo sapiens          TCATTTGCCTTCAATGTAACTCACTTGGTATGTGCCTTTTTATAATTCTGTACTTCTGAGTTATGACT-ATTTCTTGGCCAGATAAATGTTTTATTACAAAATTGACGTTTATTTTCTTT
Dasypus novemcinctus  TCATTTGCCTTCAATGTAACTCACTTAGTATGTGCCGTTTTATAATTCTATACTTCTGAATTAAGACTCATTTCTTGCCTAGGTAAGTATTTTATTATAAACTTGCTGTTTATTTTCCTT

Homo sapiens          AAAATAGGAACCTACTAAAAATACACCATATTTTCAAATGTTTAGTCGAAATCCTTATATGCCTGAGACTTCAGATAGTCTTCATGAAGTGGATGGTGATAATACAAGTATGTTTGCCAA
Dasypus novemcinctus  AAAATAGGAGCCTGCTAAAAATACACCATATTTTCAGATGTTTAATCGAAATCATTATGTGCCTGAGACTTCAGATAGTCTTCATGGAGTGGATGCTGATAATACAAGTATGTTTGCAAA

Homo sapiens          AATTCTAGATGCAATTAAAGAAGCTGATAAAATAATGGAGAATAAGACAACTTCACTGGGCCAGGTGATTCTATTCAATAGAAAAACTGTAATCTAAAACTTTATAAAAGTTATATTTAT
Dasypus novemcinctus  AATTTTAGATGCAATTAAAGAAGCTGATAAAATAATGGAGAATAAGACAACTACAGTCAGCCAGGTAATTCTATTATTAAGAAAAACTATAATC--AATC-------AAGTGATCATTTT

Homo sapiens          TTTTAAAATTTCAGCATATACATGTGTGCATG------CACACACACACACATACTTACTTG---------------------------------CCATAAT-----TTTTTATCTATC
Dasypus novemcinctus  TTTTATATTTTCAGTATATATCTGTGTGTATATTTATACACATACATACATATACATACATATACATACATATACATACCCATTCATATAGTATACCCTTAATCAAAATATTTTCCCATT

Homo sapiens          AGATTTTTCTTGTCTTGATTAAATAGTTGGTTTACTAGCTATGAAATGCTGAGAGGCAATAATGAGAAAGCTATGGATTTTGGAATCAAGTAGAGCTGGATTCAAATCTGTAGTCTTAAC
Dasypus novemcinctus  AAATTCTACATGTCTTGATTTGATAGTATGCTAATTAGCGATGAAATTTTGC-AGACAACAGTGGGGAG...................................................

Homo sapiens          TGCTTTTGAGTTGTTTGACTTCGGCAAATTACTTAACCTATCTTAGTTTACCTTCTATATAAAGTAGGAGCTAATAATACTGACTTTGAAATGTT---TTAAGAATTAAATAGAATTCAT
Dasypus novemcinctus  ................................................CACCTTATATGTAAAGT--GAGGTCATAATACTGACTTGAAAGTGTTGTTTAAGAATTAAATGGGATTCAT

Homo sapiens          ACCTACCTTTTAGTAGCATCTCAAGTACCATTTTGTTACAATATTCTCATTATTTGCATTTACCTTCAAGATATTATAGCTCTTTCATCTCTTTGTTCTTTTTC------TTTTTACACT
Dasypus novemcinctus  GCCTACTTCTTAATAGC-------------TTTTGATA--------TTATTGCGTGTATTTGTCTTCAAGAGAGGATAGCTCATTTGTC-CTTTGTTCTTTTTGAGATTGTTTTTACATT

Homo sapiens          TGTAGATGGAGAACAACAATTTGGATGAACTAAATAAAAGCAAGATCATTGTTAAAAAGAAACCCAAACAATTAAATCCATTTCATTTAAAAGTGGGTCATGAAGTTTTAAGACAAAGGA
Dasypus novemcinctus  TGTAGGTGGAGAATGACAGTTGTGATGACCTAAATAAAGCCCAGATAATTGTTAAAAAGAAACCAAAGCAGTTAAATCCATTTAATCTAAAAGTGGGCCATGAAGTTTTAAGACAAAGGA

Homo sapiens          AAAATTGGTGGAAGGATGGTCGTTTTCAGTCTGAATGGGTTGGTCCTTGTGTCATAGACTATATTACAGAAAGTGGATGTGCTGTCCTGAGAGACAACACTGGGGTTAGACTGAAAAGAC
Dasypus novemcinctus  AAAATTGGTGGAAAGATGGTCGTTTCCCGTCTGAATGGGTTGGCCCTTGTGTCATAGACTATATTACAGAAAGTGGATGTGCTATTCTGAGAGACTACACTGGGACTAGACTTAAAAGAC

Homo sapiens          CTATCAAAATGTCCCACCTTAAGCCCTACATAAGAGAATCCAGTGAACAAGGTAAATATCTGTCACTTCTCTACATTTTTA-TTGCTTTAGGTAAGGAGAATAGTTAAAAATAACAGAAA
Dasypus novemcinctus  CCATCAAAATGTCCCACCTCAAGCCCTATATTAGGGAATCCAGTGAACAAGGTAAATATCAATTG-----TTACAATTTTACTTTTTCTGAATGAAGAGAGAAGCTAAAAATACACGAAT

Homo sapiens          CCTTT-----CTCCCCTCTTTTGTTAGGTTTTCTTTTCCCTTC-----------TTCATAAAATTTGTTACAGTTTTAGCTATCCCAGGTAGTCTGTTTTATTACAGTGTCTCTTCTGGA
Dasypus novemcinctus  CTCATTACCACCACCACCTTTGGTTAGGTTTGCTTTCTCCCTCTCAATTTTATATTTATAGAATTTGGTATTGTTTTAGATATCCCACATAGTCTATTTAAGTAGAGTGTCTGTTATGGA

Homo sapiens          ACTGTCAGGCCTGTGAGATTTTTTTTTTGAGACAGAGTTTCCCTCTTTCTGCCCAAGCTGGAGTGCAATGGCGCGATCTTGGCTCACTGCAACCTCTGCCTCCCGGGTTCAAGCAATTCT
Dasypus novemcinctus  ACTGTCAGGCTTGTAAGA......................................................................................................

Homo sapiens          GCCTCAGCCTCCCGAGTAGCTGGGACTACAGGTGGATACCACCATGCCCAGCTAATTTTTTGTATTTTTAGTAGAAACGGGGTTTCACCATGTTAGCTAGGCTGGTCTTGAACTCCTGAC
Dasypus novemcinctus  ........................................................................................................................

Homo sapiens          CTCCCGTGACCTGCCCGCCTTGACTTCCCAAAGTGCTTGGATTACAGGCATGAAACAGGGTGCCTGGGCATTTTTATTTTTATTTCTAGTATGTTTTTCATAGTTTATGTGGGAGATACT
Dasypus novemcinctus  .......................................................................TTATGTTCTGATTTCAGGTGTATTTT-CATAGTTCATGTGGGAGATACT

Homo sapiens          GAACTTGTTTTCCTCTGAAGGAAAGATAACATCTTAACATCATCTCTGTTTTTGCTTTCTCAATGTTTTGTTTGTTTGTTTGTTTGAGACAGAGTCTCATTCTGTCCCCCAGGCTGGAGT
Dasypus novemcinctus  GAACCTGTTTTA--CAGAAGAAAGGATTCTATCT--------TCTCTCATTTTACTTTTACA..........................................................

Homo sapiens          GTAGTTGTGTAATCTCAGCTCACTGCAACTTCTGCTTCCTGGGTTCAAGCAATTCTTGTGCCTCAGCCTCCCAAGTAGCTGGGATTATAGGCACATGCCACCATGCCCAGCTAATTTTTT
Dasypus novemcinctus  ........................................................................................................................

Homo sapiens          TGTATTTTTGGTAGAGATGAGGTTTCACCATGTTGGCCAGGCTGGTTTTGAACTCTTGGCCTCTAGGGATTTGCCTGCCTTGGCCTCCCAAAGTGCTGGGATTGCGTGCATGTGCCATTG
Dasypus novemcinctus  ........................................................................................................................

Homo sapiens          CACCTGGCCTTACAAATGTATTAGTCACTTCGTGCCTCTTTTTGGCATGAAAAA-CAGCAACTGTGATCATTAATGCCAAAAACAAACATACTGTCCAGAATCTAGTTGTGA---GATAA
Dasypus novemcinctus  ..................TATTAGTCATTTTGTGCCTCATTTGAGCATGAAAATTCAGCAGCTAAGGTCATTAATGCCAAAAACAAACATACTGTCCAGAATCCAATTTTGAAGAGAAAT

Homo sapiens          ATATTAGGGTACTTTAAAAATGACATTAATTTTTAATATTGCTTTATAGTTAGGTCATGGTTGGGGGCCAGGTGTGGGTGCAGAGTGGGGAATCCTCAGAATGCTATAAGGTGCTGTAAG
Dasypus novemcinctus  GTATCAGGGGACT--GAAAAT---ATTAATTTTTGGTATAACATTATAGGCAGGTTATGA--GAGGGGCAGGTGGGAGTATGGGGTGGAATTTACTCAGAATGCCATAAA----TATTAG

Homo sapiens          AAGAGGTGACAGCACTCTAAACAAGTTATATTTGCCCCTAAATTTGACTCATTCTGAACAGTCAAGATACCCTA-------------------------------GTAAAGTGATGTTTT
Dasypus novemcinctus  AAAAAGTGATAGCCCCCTAAAAAGGTTATACTTTCCCCAAAATATAAATGGTAATGAACAGTCAAGATATCCTAATCTTTCTAAGTTGTTCTTTCACAGATGAAAGCCAAATGACATTTT

Homo sapiens          TGCCATGGCTTTTAGATTATGTTCCCCACTTTATCAAGGCTTTTATTTTGTCCTAATGTGTCTGCTTCCTAGATCTTCTCTGAGGTGGATAACTGAGAGAGAGTTAATGAGTAACTTAGC
Dasypus novemcinctus  TGCCATAACTCTCAAATTAGGG--CTCACTCTACATAGACCTTTATTTTGTCCTACTATATCTATCCCCTAGAGTTTCTCTGAGATGGATAACTGAGGGGAAGTTAATTAACACCTTAAC

Homo sapiens          C---------------------------AGGTGAAACATTAATATTATTTGGATATAAGTAACATTTAATGGAA---------AATATATTTAT-AACAATATAAA------CAAGTTA
Dasypus novemcinctus  CAGTTAATTGTGCTTTTTCTAAAAAAGAAAGATAAAGCATCAATATTATTTGAAATTGAGTATAATTTATGGGAAATACAATATAGTATATTTGTAAATAATATGAACAACAGCAACTCA

Homo sapiens          TATGACAGGCTTTAGCAAATATTAATAACAAAAAGAGTAACAACAACAAATAACCTACACTTACCCTATGCCTTTTCTTGAGTTCTAAAGCCCATGTGGGCTGCTTAGTAATGATAGCTC
Dasypus novemcinctus  GTTAATAGGCGTATGTAAACAAGAATAATAGCAGG---AACAACAGGAAATAACTTTTCCTTACCCTGTACC................................................

Homo sapiens          AACTCTGCTTCTAATCTGGGTTACAACACTCATGTTAAAACCTCTTAGTACCTTTCCTTCTTGTGACAGACCGAGTTGTTTAGTCAGTAAACTTATCTTGAACTACATTCTAATCCTGAA
Dasypus novemcinctus  ........................................................................................................................

Homo sapiens          AACTCACAAGTGCTTTCGTTCTCTTTGTTAGAAGATAAAAGCTTGCATTGTTTGTACCTCTTCTCAGTTCTGAGCTACCTTTAATGATCTCTCTCTTCTGCAAGTTAAGTAGGGAAAAAA
Dasypus novemcinctus  ........................................................................................................................

Homo sapiens          AAGGTTATACAAGTATATGTTGTTCTAGCTTCTTTAGCATGATTTTCCCCTTAATCTTTGACATTCATGATTTGCTCTCTTCTAAAGTGTTAAGCTTCTTTAATACAAAATTTAATTTAA
Dasypus novemcinctus  ........................................................................................................................

Homo sapiens          AATTTTGCTAAACCAGGATCTATCAACATTTTGTAATTTGTTCTTGTCAAAGAGTTTCTCAGAAAAGAAGTCAAATATTGTGCTTCTTCTGAACATTCAGAACTCAGTATTTCAGAACTC
Dasypus novemcinctus  ........................................................................................................................

Homo sapiens          ATCTTCTCTGTTTGAGAACAGTCTCTAAACAGAGGCAGTATAATATTTGCCTCATACTTGAATTCTTTGCACAAGATTCTTCCAGTAATATAAGGACAAATAATATTGCAATATGACTAC
Dasypus novemcinctus  ........................................................................................................................

Homo sapiens          TGGTGTATGCATAAGGAATTTAAGACTAATTAGATGAGTCTGTTTTAGTTTTTCTTGTTAACTAAAGGAACTACTGATCTGGCCATCCTAGAATCTGGTTCTTATTGCTTGATCCTTTTT
Dasypus novemcinctus  ........................................................................................................................

Homo sapiens          TTACATTTTTCTCATTTGTAGAAATTAAAAGTAAATGTGATTTCTGCCATCTTTTTCCTCAATATTACATACTTTCTTGAAAATCTTTTCCATGATTTTCTTGAAAATCACCTCACAAGT
```

```
Dasypus novemcinctus    ...................................................................................................................................

Homo sapiens            TGATTTGAAAGAGTCCTTGAGCTGAATTAAAGCAAACAAAATGATTTTGACTAATCATTGAAGTTTTTATTTGACATAAATTTTGACATGCTATAGCCAAAAGAACTATAAATTTATTTG
Dasypus novemcinctus    ...................................................................................................................................

Homo sapiens            TCCACATTTGTCATTGCTGCTCTTTGCTAGCCTTGAAATTTCATAGCTCTGTTAAAAACTCACTGTCTAAAGTAGATATACTTCTATTGCCAACTTCTTATCCATTCTTATCTAGAACTA
Dasypus novemcinctus    ...................................................................................................................................

Homo sapiens            AATGTAGAGTGAGCACATTCAGTACTAGGTATACAATATTTTTCATTGCTATTTTAAGCAGGCCTGTTTGTTTTTTTGTCTAGTTCATTGCCAAGCGCTTTTTCAGACTATTTAGACCAA
Dasypus novemcinctus    ...................................................................................................................................

Homo sapiens            GAAATCTTAAAATACCATAGTGTCTTTTTGTAATCATAATGCTCCTCTTTGAAAGGAAAAAATAATCTGACATAGATTTTGCACAGTTTTGAATTTATTTTGTTTGTGATAGAT---TTG
Dasypus novemcinctus    ..........................................................................GGCAAAGATTTTGAGTAGTTTTA---TTGTTTTGTTT-TAGTGGATGTATTG

Homo sapiens            GATCTGCCTCACCCAAG-GTTTTAGCCTGATTTCCCTTCACAGTTGAAAGCCCCATTTGTGAAACCATTTTCATTATTGTATAAGTTTCCTCTTGTTGCTGTAACAAATTACTACAGATT
Dasypus novemcinctus    GGATAGCATCAACAAAAAGTTTTCATCTCATTGGCCTCTATAGTC-----CCCCATTTGTGAAATATTTTTCATTATTGTATTAATTTCCTATTGCTGCTTTAACAAATTATCATAGATT

Homo sapiens            TAATGGTTTAAAACAACACACATTTATTATATTACAATTCTGAAGGTTAGAAGTTCAACATAGATCTTACTGGGCTAAAATCAAGGTATTGGCACTGTTGTGTTCCTGTCTGGAAGCTCT
Dasypus novemcinctus    TA--GACTTGAAACACAAAAGCTTTA--ACCTTA-AATTCT-----TTAGAAGTCCAACATGGGTCTCAGTGGGCTAGAATCA-GGCTTTGGC----CTGTGTTCCC-TCTGGAGGCTCT

Homo sapiens            AGAAAATAATCCATTTTCTTGTCTTTTCCAGCTTCTAGAGGTTACCTTCATTTCTTGGCTTGTGGTCC-----CTCTATCTTCAAAGCCAACAATAGTGGGTTGAGTCCTTCTTACATCA
Dasypus novemcinctus    ACAGGAGAATCAGTTTTCTTGCCTGTTTCAGCTTTTTGAGGCCACCTGCATTCTTTGGCTTGTGGTCCTCTTCCTCTGTCTTCAAAGATAGCAATTACAGGTTGGGTCCTTCTGACATTG

Homo sapiens            CCTTACTCCAACCTCCTCTTCTGCCTCCCTCTTCTATATTTGAGGAC----CCTTGACCTTGGCCCTGCCCAAAAAGTCCAGGATAATCCCCCTATTTTAAGGTCATCTGATTAGCAACC
Dasypus novemcinctus    CATCACTCCAGCTTCCTCTTTTGCCTCCCTCTTCTACATTTAAGAATGGTGTGTTTACATTGGGCCCACTTGGAAA-TCCAGAATAAT...................................

Homo sapiens            TTAATTCCATCACTACCTAAATTCTCCTTCACCATGTAAGATTACATATTCACAGGATC----CAAGGAGTAGTATGTTGACATCTTTAGGGGCCTATTATGCTGCCTACCACAATTATG
Dasypus novemcinctus    ..................................ATGAGGTGGCATATTCAAAGATTCTAGGCAGGGATGAGTCTGTGGACATCTTGGTGGACC-ATTATTTTGCTTAC-ACAATTATA

Homo sapiens            GTCTGCATTCATGAAACAAAGCAAAGCACAGTCCCTATAGTGTCAAGACCATGATCTTGAC------GTTAATATAGTTTATGCAGAGAGCCTTTTTTTGGCCTCTTAGTTATATCTTTT
Dasypus novemcinctus    GTCTGCATTCATGA--CAAAGCAAAGCTTAGTCCCTATGGCATCAAGCCCATGGTCTTGACTTCATTATTAATATAGTTTATGTAAGAGCCTTCTTTTGATCTCTTACCTAATCGTTAT

Homo sapiens            TTTTTCTTTCTCTTTTTCCTTTTTTTTTTTTTTTTTTTTTTTGAGACAGGTTCTTACATGGTTGCCCGTGCTGGAGTGCAGTTGCACAATAATAGCTCACTGCAGCCTTGACTTCCT
Dasypus novemcinctus    TTAGTTTTCTCTATT....................................................................................................................

Homo sapiens            GGGCTCAAGCAATCTTCCCACATCAGCCTCCCAAGTAGCTTGGAATGTAGGCATGCATCACCATGCCTGGCTGAGTTTTTCGTTTTTTATAGAGACAGGGTCTTACTATGTTGCCCAGGT
Dasypus novemcinctus    ...................................................................................................................................

Homo sapiens            TGGTCTCAAACTCCTGGACTCAAGCAGTCCTCTCACCTAGACCTCCCAAGTGTTGGAATTACAAGTGTGAGCCATTGTGCCCGTCCAGTTATATAATTTCTTAACCAATAGTTACTCAGT
Dasypus novemcinctus    ...................................................................................................................................

Homo sapiens            TATCTGTTGACAGTATTAGTTATTATAATGACAGTGATTTAGTATCAAGTTATAGTTATT---ATTTTATCTTGAGAAAGGATATACTAGTATGGCAGCACATTCT-AATAGTTTGAGGA
Dasypus novemcinctus    ..............TTATTTATTATAAAGATAATGATGTATTATCAATTTTTTATTATTGCCATTTCATTATGAGGACGGATATACTAGTATGGTAAAACATTTTAAATAATTTTAGGA

Homo sapiens            TTATCAAACCATATATAGGA-TTAGTTTAGTCACTTCTCTTTAAAATAAGAAAATTAAGTAGTCTGAAACCTTTATCTTTTTTTTTCTGATTGAAGAAATACATTAATCTAAATCAGAC-
Dasypus novemcinctus    TTATCAAGCTATATATATCATTTAGACTAGTCA--TCTCTTGAGAA---------------------------ATCTTGTGCTTTTTAGTTGAAGAAATAAA-TAATCCAGATCACTCA

Homo sapiens            ---TTTTTTGAAGCTGTAATTTGTATTTATTTTTCTCAGGGAAATTTAAAAATTGATTTTTTAATTATTATTTTTATTCCTTAAAACAACATTAAGGCTGGGCACAGTGGCTTATGCCTG
Dasypus novemcinctus    ACGTTTTGTGAGAC-----TTTGTGTTTTAGTTTC................................................................................................

Homo sapiens            TAATCCCAGCATTTTGAGAGGCCGAGGTAGGATTGCCTGAGCTCAGGAGTTCGAGACCAGCATGGGCAACATAGTGAAACCCCGTCCCTACAAAAATTAGCCGGGCATGGTGATACGCAC
Dasypus novemcinctus    ...................................................................................................................................

Homo sapiens            CTTTAGTTTTAGCTACTTAGCGGGCTGAGGCAGAGGATCACTTGAGCCCGGGAGACTGAGCTGCAATGAGCCAACATTGCACCGCTGCACTCCAGCCTGAGTGACAGAGTGAGACCCCAT
Dasypus novemcinctus    ...................................................................................................................................

Homo sapiens            CTTTTTTTTTTTTTGAGATGGAGTTCAACTATTATCACACAGGCTGGAGTGCAATGGTGTGATCTTGGCTGCCTGCAACCTCCGCCTCCTGGGTTCAAGCAATTCTCCTACCTCGGCCTC
Dasypus novemcinctus    ...................................................................................................................................

Homo sapiens            CTGAGTAGCTGGGATTACAGGCGCCCACCTCCACACCCAGCTAATATTTGTATTTTTAGTAGAGACGGGGTTTCACCATGTTGGCCAGGCTGGTCTCAAACTCCTGACATCAAGTGATCC
Dasypus novemcinctus    ...................................................................................................................................

Homo sapiens            ACCCGCCTTGGCCTCCCAAAGTACTGGGATTACAGGCGTGAGCCACCATGCCTGGCTGAGACCCCTGGGATTATAGGCGTGAGCCACCATGCCTGGCTGAGACCCCATCTCTTTAAAAAA
Dasypus novemcinctus    ...................................................................................................................................

Homo sapiens            AAAACCTACATTAGAAATGGATATCAAAACTGCTGATTAATACCTTCCTGAAATGTCTTGAAAATATTTTAATTTCAAAGAAACTAAAGACTTATTCAGTCTTGTCAAAACCAAAATGAT
Dasypus novemcinctus    ...................................................................................................................................

Homo sapiens            GCTTTCATCAAACAGATGTCATAATCATAACTAAATTAATGGAATGAGAAAAGATAAATACTTAGTGAAATGTACTGTGTAATGTAGCTGACTAGAAAAGACCTTTAATATGATATGTTT
Dasypus novemcinctus    ...................................................................................................................................

Homo sapiens            GCTTACTCCCCGCCTCTGTTTTGAGGGCAGAAAGTTTAATCATATTCTTTTCTGTATCCCTAACTCATGATTGTTTTAGATTAGTGTTGTCCAATAGATGAATAATAATACAAGAGCTAT
Dasypus novemcinctus    ...................................................................................................................................

Homo sapiens            GTAATTTAAAATTTTCTAATAGCCACATTTTGAAAAGTACAGAAATAAATGAAAATAACCTTAATAATATATTTTATATAATCCAGTGTATCCAAAATATTACTTGTTCAACATGTAAGG
Dasypus novemcinctus    ...................................................................................................................................

Homo sapiens            CCACAAGAGTCTCTTTTCTGATCTCTTTACTGAAATTAAAGAGCATTTCTCTTAGCTCCAGTAGATTTAGACCATAGTAGGAGTTGAAACATATTTGACCTTTGCAAATGAATGAGAATT
Dasypus novemcinctus    ...................................................................................................................................

Homo sapiens            TTTTTGTAAACAAACAGGCCTTTGACATATTCAGAGTGGTTTTCTATGTGTATAAAGTGATTTATGTAGAATTTGCTGTGTGTAGTCCTTGTTCTCTACAGTTTATTACAATAAGTTA
Dasypus novemcinctus    ...................................................................................................................................

Homo sapiens            CTTTAAAGGTTAACTTTTTTTAGAAGAAAAAGAGTGTGTGTGTGTGTGTGTGTGTGTTCGTGTGTGACTTATTTTACTTACATACAGAAGCTCTCTTAAAATCAAGTGAGGTTGGTAT
Dasypus novemcinctus    ...................................................................................................................................

Homo sapiens            GTCCATTTGTTTCTAACCAGTGTAACTAGGAGGTACTCAAGTTCAAGGTCTCTGAATCCATTTCAGCAAATTAAAATGAAAATTTCTAAGAGCTTTACATCTAGGCATATTTTTCCCTAT
Dasypus novemcinctus    ...................................................................................................................................

Homo sapiens            TGACATATGTTTACTAGACATATTTTTCTAAAGTAGTCCTACTTCATCTGTATGAAAAAACTTTTACAGACAGAAAACCTCCTTCATTAATCCTCTGAAAGAGTCTTTGGGCATTTCTTA
Dasypus novemcinctus    ...................................................................................................................................

Homo sapiens            AATGCCTATGGTAACTGCCATCACTGATAACTTTGTAGGAATGTACAGGCCTTGAACCTATGGATTCTCCATGACTTAGTAGAAAATTTCATCAGCAACATGTTTACCCTATCTAACCT
Dasypus novemcinctus    ...................................................................................................................................

Homo sapiens            TTTAATGTTCTCTTATAAACTACCGCCTAGCCTTCTCTCGTATTAACAGTAGGGTAGTTGCAAACTTTGATGTTAAACCTCTAGCAACACATTCTGAAAGTGTTTCAATGTTATTGATCA
Dasypus novemcinctus    ...................................................................................................................................

Homo sapiens            TGTTTTCTTCCCTCTTGCTGCTATTGGGTATAACACATCTCATGTGTTGCATGATTTTCGTTGTTGTTGTTGAGACAGAGTCTCGCTCTGTCACCTAGGCTGGAGTGCAATGGTGCGATC
Dasypus novemcinctus    ...................................................................................................................................

Homo sapiens            TCGCCTTACTGCAACCTCCACCTCCTGGGTTCAAGCAATTCTCTCACCTAAGCCTCCCCAGTAGCTGGGATTACAGGCACATGCCACCATGCCTGACTAATTTATGTATTTTTAGTAGAG
Dasypus novemcinctus    ...................................................................................................................................

Homo sapiens            ATGGGGTTTCACCGTGTTAGCCAGGTTGGTCTCGAACTCCTAACCTTAAGTGATCTGCCCGCATTGGTTTCCCAAAGTGCTGGCATTACAGGCATGAGCCACTGCCTGGCTGCATGATTT
Dasypus novemcinctus    ...................................................................................................................................

Homo sapiens            TTATTTTATTTTATTTTTTTTTGTCTTTCAGAATTATGGGAGAATAAATGGCCATACAAGCCCAAGCCCACTTATGCCATCTTTTGGTAGAGAGAATAGCTTTGTGTGATCTGTTAAAGT
Dasypus novemcinctus    .............................AAATGTGCAAAACTAAAGGACCACTCAGATCCAAGCCCCCTTAGACCGCCTGTTGTTAGAAAGCATAGTTTTGTTTGCTCTATCAAAAT

Homo sapiens            TTATAAATTGAAAGTTGTTAAATGCATGAATTTCTGGGGTTGAAATAGGTTCAGTGAGCTATTTGTAATAATGTTATGAGGGTGAGTACAGTAGGATTGATTTTCAAAATATACTACCAA
Dasypus novemcinctus    TTGTAAGTTGGAAGTTCTTTAATACATGAACTTCTGATGTTGAAATATGCTTAATGAACT-TTTCAAATCTTAGTGCAAGGACAGATAGGGTGGGATCATGTTCTAGGATGTACTTTCAA

Homo sapiens            ATTCATCTGTTCTGAGACACTTCCATTGGTCTCTAATCTAAATAGGCAATTAATCTTCCTTTTGC--TTTTTGGTCTTAAAATTTGAGGGCTCCTTAACCCATTAACATCTTCAGGAGA
Dasypus novemcinctus    ATGTACCTGCTGTGAGAAACTTTCATTCATCTCTAAAGCAAATGGGCCATTAATCTTCCTTTTATTATTTCTTGAATGTAAAATTGGTGGCCTGTTTAAACTATTAAAATCTTTAAGAG.

Homo sapiens            CCAACCTAGGCAACACAATTAGCCAGGCATAGTGACATGTGTCTATGGTCCCAGCTATTTGGGAGGCTGAGGTGGGAGGATCACTTGAGCCCAGAAGGTCAAGGATGCAGTCAGCTGTTA
Dasypus novemcinctus    ...................................................................................................................................

Homo sapiens            TCACGCCATTGCACTCCAGCCTGGGTGACAGAGCAAGACCATGAATGAATGAATGAGTGAATGAATGAATGAATGAATGAATGAAAAATAAGTAAATAAAGATAAATAAATATATCTATA
Dasypus novemcinctus    ..........................................................................................................................TATCTAT-

Homo sapiens            TCTATATTCTGATGTTTTGGGGGAACATTTAGAAAGAGAAGGTAGGGATCCAGGAAGTTAATCAGCTTTGGTAAAAGAGTTTCTTATTGTAATGAATTCAAATATCAGAAAGGGAGGAAT
```

```
Dasypus novemcinctus  ---------TAATGTTTTTGG---------------------------CCCAGGAAGTGAATTG---TTGTAGGAAGAGTTAATTTTTGTAATGAATTAAAATATCAAGTAAAAAAGAAT

Homo sapiens          CCTTTTATACATATTAGTCTGTGTATA-GAAGAT-----TTGCCATCAGTTTAACATTCATTTTAAACA-TTTTTTTCTTGATGGTGATGATAAATTTGCTCAACATACAATAAAACACT
Dasypus novemcinctus  TCTTTTATATACATTAATCCTCATATATAAAGATGCTGCTTGATGTTAGTTTAACATTCACTTTGACCATTTTTTTTCCTGATGGTGATGATACATTTGCTCAAAGTAGAATAAAGTGCT

Homo sapiens          GACCAGGAAGACTCCCTGTCAGTACTTCAGTAT-GGCTTAGGTAGACC-TTAAAAGTTCCTAAGAATTTTGGTATCAAATTAGATTTAAATAAGCTAAGCTTTTTAAGCTACTCCATGGC
Dasypus novemcinctus  GATAAGGAAAATACCCTGTCAGACCTTTGGTTCAGATTTAATCTGACCTTTAAAATTTCCTGAAAATTTTGGTATTAAATCAGACTTAAATAAGCTGAG-TTTTTAAGCTTCTCC-TTAT

Homo sapiens          AGCCTGCCTTCTTACACAT---TATCACATAAGCCT--------CAGTTGGCATGTT----GTTTTTAACTTCATACTGTAACAATTTGATGATATTATCTTTCCAATATGGGTTTTATA
Dasypus novemcinctus  AACTTCTCTACTTACACATTCCTATCACATAAACCTGGTTCTAGGAACTGTCTTAGTTTTAGTTTATTACTTCATATTGTAGCAATTTGG------TATTTTCCTAGTTTGGGGTCTATG

Homo sapiens          AAGATTGGAAGATTATCTCAAGTTGTAAATGCAAGGATTTAATAATTCTATTTGTTAAAATAATTATATGACCCTATTAATTCACCCAGAAATGCTTTACAGAAGCATATTAAGGATAAC
Dasypus novemcinctus  AAGACTGGGGAATTATCTAAAGTTGTAAATATAAAGATTTAACAATCCTGTTTGTT................................................................

Homo sapiens          ATTATCAAAAATAGTTTTATAATTTTTTTTTTAAGACAGGGTTTTGCTCTGTCACCCATGCTGGAGTGCAGTGGTGCCATCATGGCTCACTGCAACCTCAATCTCCCAGGCTCAAGTGAT
Dasypus novemcinctus  ........................................................................................................................

Homo sapiens          CCTTCCATCTCAGCCTCCTAAGTAGCTAGGACCATAGACACCCTTAACCACACCTTGCTAATTGTTTGTATTTTTTGTAGAGAAGGGGTTTCGCCATGTTGGCCAAGCTGATCTTGAACT
Dasypus novemcinctus  ........................................................................................................................

Homo sapiens          CCTGGGCTTAAGCAATCTACCGGCTTTGGCTTTCCAAAGTGCTGAGATTACAGGCATGAGGCACTGTGCACAGCCTTATTATTTTATTTTTAATCTATACCATAATTCTCACTCCTTCTA
Dasypus novemcinctus  ........................................................................................................................

Homo sapiens          TCCCGTTGTCCGCATATATACATATCTACCAAAGACAGGTTGAGGGAACAGGAAAA--TGGCTGTTTTAAAATCCTTGACCTTTCCTGCATTGTTTATGTATGAACTGTCAAATTTATAT
Dasypus novemcinctus  ....................................AGGTAAAAAGAACAGGAAGACCTGGATGTTTTTAAATCCTTGATGCTTCCTTT-TTATAAGTATATAAATGGTCAAAATTATAT

Homo sapiens          GTCATTTT-TTTATTCTTTGACAT--TCAT--ATTTAGTAGACAGTATAA---------CTCCCTAGAAGTTAATCTTGATAGTTAATTTTTATTA--CTTGGGATTTTGTTCCTCACTT
Dasypus novemcinctus  GTTTTTGTATTCATTATTTGACATGTTTATTTATTTAGTAGACAAGGGAGTCTCCTCTCCCCTCCCAAAGTTAATCTTAGTAGGTAATTTTAATTATTCTTTGGCATTTGTTCCTCATTT

Homo sapiens          AAAATTGTTTAATTAAAAAATATGTTTAATTATAATTCTAAAAGTCAAATGTATGATTTTTCTTTTTTCAGAAAGTCTTTATCTCTTGCAAGGTTCAGTAGTGGCAGATCATGACTACAT
Dasypus novemcinctus  ACCATTGTTTAACTTAAAAAC--------TTATGAT-----------------GTTTCTTTTTTTTTCAGACAGTCTTTATCTTTTACAAGGTTCAGTAGTGGCAGATCATGACTACCT

Homo sapiens          TGGATTGCCTGAAATTCCGATTGGAGCATATCAAGCAAATATTCTGGTGGAAGATGCAACTATTGGTATAGTCGATAATGAATTACTGACATCAAGCAAGGATCGTGAACTATTAGAATA
Dasypus novemcinctus  CGGATTGCCTGAAATTCCAGTTGGAGCATACCAAGCAAATATTCTGGTAGAAGATGCAACTATTGGTGTAGTTAATAATGAATTACTGACATCAAGCAAAGATCATGAACTATTAGAATA

Homo sapiens          TAGAAATACGAAAATCTCTCCATTGATAGACGATCATAGTTCTCTTGAAAAGCAGACTTTCAGTCTGTTGGACTCTTCAAACCAGGTTCTTGAATACTTAAGTTAGTAAATACCAAAATT
Dasypus novemcinctus  CAGAAATACCAAAATCTCTCCATTGATAGAAGATCGTGTTGCTCTTGAAAAGCAGGCTTTTAGTCTCTTGGACTCTTCAAACCACGTTCTTGAGTACTTAAATTAGTAAATACCAGAATT

Homo sapiens          TATTTAAATTGTTTGTTTAGAATATAAATTCTTAA---TGATATCTTATTC-ATTAAAAACGTTGTATAGAAGAAGTATCTTGACACATTCTTAATGACTATATTCTGAAGGTTTATTTT
Dasypus novemcinctus  CATTAATGTTGTTTA--GAGAATATAACCCTTAAACCTTGATGTCTCAATCTATTAAAAAAGGTCTAGGGAAGAAGTGTCTTGACAC--CCTTGATGACTATATCTTGAAGTTTT-TTTT

Homo sapiens          ATAGTTCTTATCTAAAATTATGCCACATTTGAATATCAGT--ATACGTACATATGAAGGCAAAGGAAGCATGTAATATATAGAATTCACTTTTTCTTCTAAAAAGAGAACAGGCAAAGCA
Dasypus novemcinctus  A-AACAACTGTTTAAAGTTGTGTC-TGTTTCCATGCCAGTGCATATATATATATGAAGTCAAAGGAAACAT----TATATAGAATTCACTTT---TCCCAAAAAGAGAACAGGCAAAGTA

Homo sapiens          CCCCTACTTCTTGGGAAATAAA-----TTCCAGCCCAAATCTCTCAAAC-CTTTGTATGGTTTTGGCACCGT-TTCTAAGTTTTAAATTTTCTGAAATTCTCTACCATTTAAACAGTAAG
Dasypus novemcinctus  CTATTGCTTCTTAGAAAGTAAATTAATTTCCAGCTCAAAACTTTCAACCTCTTTTCTTGGTATTCATACTGTGTTTTCAGTTTTACTTTTTCTGAAA-TATTTCCCTTTTAAAAAATAAG

Homo sapiens          TTTGAACTACCTTGAATGGAATTGATAAATACTGAATCAACTGTAACAATGAATGAAATGAAGCTTCTTTCTGTTTCATTAGAACTTCTAAAAACAAGATTGTATGAAAAA-ATATAGGG
Dasypus novemcinctus  TGATAACTATCTGGCAATGGGTTAATAAATATTGAATCAACCATAACAATGAGTTAAATGAAACTTCTCTC--TTTTGTTGGACTGTATAAAAATAGTACTGTAGGAAAAATTTTTAGGG

Homo sapiens          TTATTTAATAGGCTTTCACAGAATATTGGTAATTTCAAATTATCTAATTGGTTAGATAGGGTCAGGATTAGGGTCTGAAACTATGTATTGGTTGGGAAACAATTTGTTTATTTTCATACT
Dasypus novemcinctus  TTGTCTAGTGAG............................................................................................................

Homo sapiens          ATATATTAAAGATAAAACCATCGACCAGGCATGGTGGCCCACACCTGTAATCCCAGCGCTTTGGGAAGCCAAGGCAGGATGATCACTTGACCCCAGGAGTTCGAGACCAGCCTGGGCAAC
Dasypus novemcinctus  ........................................................................................................................

Homo sapiens          ACAGGGAGACCCTATCTCTACAGAAAAATGTTTAAAAATTAGCCAGGTGTGGTGGCACATGCCTGCGGTCCCAGCTACTCAGGAGGCTGAAGTAGGAGCATTGCTTGACCCCAGGAGGTC
Dasypus novemcinctus  ........................................................................................................................

Homo sapiens          GAGGCTGCAGTGAGCCGCGCTCATGCCACAGCTCTCCAGCCTGGGCGACAGGGCAAGACCTGTCTCAACAACAACAACAAAAGTATCCGTTAAGAATTTTTTCTGCAATCATAAAATCAT
Dasypus novemcinctus  ........................................................................................................................

Homo sapiens          AAAATAACCAGTATCAAAGATTTTAAACTGTAATTTCTATGATATTCATAAATATAAAATAAATATTGTTCAATAAATATTTATTCTAAAGCACTATGAAAAGAACTGTGGGAAAAGTAA
Dasypus novemcinctus  ........................................................................................................................

Homo sapiens          AAGATGAGTCAAACGTGTACCTCATTCTTATAAAGGACTTACTGTCTGGTAGGTAATAAACTATGTAAGCCATGTACAGCACAAGTTAAGGCATGCTAAATTGCAGAAGACAGGTGTAGA
Dasypus novemcinctus  ........................................................................................................................

Homo sapiens          TAATGTACTTAGGGAGGCAAAAAGAGAGAAGTTGTTTTTACATAGGAAATTTGTGTTTATCCTTATGGAAGAAGGATCATTTAAGCTTGACCTTGAAGAATGTATAGGATTTGGTAAATG
Dasypus novemcinctus  ........................................................................................................................

Homo sapiens          GATACAAAAGAATTAGGGGAGAAGTAATACACAGAAATGGAAAAGTATTGTAATGATTCATAAAAAGGCAGGGCTTCTTGTTGTCTACACTGCCTCGGGTATAAAGTGAAGAAGCAAGAT
Dasypus novemcinctus  ........................................................................................................................

Homo sapiens          AATGAGGGATAAAGCTGAAAAGGTAAGTAGGAAACCTTATGGAGAGCTTTGAATACTATTAAGGATTGATATGTGCCCCTCCCAAAATTTGTGTTGAAGTCTTAACCCCCACCACCTCAA
Dasypus novemcinctus  ........................................................................................................................

Homo sapiens          AATGTGACCTTATTTGGAAATGAGGTTGTTGCAAGTGTAATTAGTTGAGATCATATTTCAATAGGGTGGGCTCTAATACAGTATTACTGGTGTTCTGATAAAAGGGGAAAATTTGAACAC
Dasypus novemcinctus  ........................................................................................................................

Homo sapiens          AGACTCAAGGAGAATGCCATGTGAAGATAAAGGCAGAGTTTAAGATTATGTTTCTATATGCCAAGAAATGACAGAGATTGCCAGAAAACTACCAGAAGTTAGGGGAGAGGCATTGAATAC
Dasypus novemcinctus  ........................................................................................................................

Homo sapiens          ATTTTTCCTCATTCCTCAGAAGACCAACCCTGTCAATACCTTATCTCAGACTTTTGCCTCCAGAACTGTGAGACAATAAAATTCTATTGTTTCAGCCATCCAGTTTGTGGGACTTTGTTA
Dasypus novemcinctus  ........................................................................................................................

Homo sapiens          CAGTAGCTCTAGCAATAATACAAATGCCTGGCTAAATTATTTCTTTTCTGAGAGCAAAAACTTTGTATAGGAAACTGTCATAATCAGAACTGTGTTTTTGGAAGTTTAATCTGGCAACTA
Dasypus novemcinctus  ........................................................................................................................

Homo sapiens          GTAAAGAACAGATTAGAAGAGGAGAAAACAAGGCATGGAGATTAGTTGAGAGGACTTCGCAGTGATGACGAATACTAAAAACCTAAACTGGGCCTGTAGCATTGGTAATGAATGGAGTCT
Dasypus novemcinctus  .............................................................................................CTGGTACATTTGTAATGGATAGAGCAT

Homo sapiens          GTCTTAAGATCAGTACAGAATATCGCACATATTATATTTCACAACCATACTGAACAGTGATGTGATAT-CAAAGATGGAGACCTGGAGTATTCTGATTACTCATTTAAG-AAGAAAGATT
Dasypus novemcinctus  TTCTTAAGATCATAGCAGAATATCTCACATATTTTATGCCTCAACCATTCTGAACAGTGATATGTGATACGAAAATGGAGACCTAAAATAGTCCTGTTACCAATTTAAATAAAAAAGATT

Homo sapiens          ATGT----TTAATATAGGCTAAAGGAAAAGTGTTTAGGGTAGGGTGTGAATCTAGGACCTCATCTTATACCACAGACCAAATAAATTTCAAACAGAATTCCAGGAAGAAAAAAACAAAAC
Dasypus novemcinctus  AAGCAGTGCTGGTACAGGCATAAGGAAAAGTGGCAGGAGTGATAAATCAACTTAGAACCTC-----ATACCGTAAACCAAGTGAATTGCAGGTAGAATTACAGAATGAAACTAAGAAAAC

Homo sapiens          TTTTTTTAAAAAAGCGTAATTGTATCAAACAGTGCTACCTATCCTGCTTTCCTCAGAAGAGCAAAGTTTAGAAAAGAGTCATATTAGAATATCTTTTTGGTAAA
Dasypus novemcinctus  T.......................................................................................................


Homo sapiens          TCAATTTCTGGCGTCTCGAACCCAAGAAATGAAAAAGGGAAGCCATGGGGGAGCAGGAGG-----------AGAAATCAAGGCGCGAAAGG-ACCCTCAAAGCTGTACTGTCA---CTCC
Choloepus hoffmanni   GAAAGCTTTGGCGTCTCAAAACCAATAAATGAAAAAGGGAAACAATGAGGAGGAAGGAAGGGGGAGGCAATGGAAATCAAGAGGCGAAAGGAACCCTCGAGGTTATACTGTCACTCCTCC

Homo sapiens          ACGCCCAGGCTGTCATTCTCTCCAGCACAAGCCCCTCGGTGGAGCATGTGTTTAGGGTCTGCAGAG---CACAGGAGACAAAGCAGAAGCCCAGCAGCCGCCTCAACCCTGGCATGATCA
Choloepus hoffmanni   ACGCCCAAGCAGCCATTCGCACCCGCACATGCCCT---GAGGACCGCGCGTCTAGGCTCCGCAGAATGCCGCTGGAGACGAAACTACTACCCAACAGCTG---CAACCTTCGGGCGACTG

Homo sapiens          GGTTCCGCATCTCCGGTTCTCCTCCGGTTTT-GGGCCCTCACCTGCCACGAATGCGGTCGTCACGCGAGCAGTTGTGAGATGGTCAAGCCCATTCAGGTCTAGGTTACCCCCTTTCATCC
Choloepus hoffmanni   GGTTCCGCATCCCGGATTCCCCTCCAGTCTCGGGGGCCTCACCTGCCACCGCTGCAGTAGTCACACGAGCTGCTG-GAGATGAGCAAGCCCAGTCATGTCTCGGTT-TCCCCTTTAGTCA

Homo sapiens          CGGAGGCAGGGGT-----GAGGTGTTTGTCAAGAGACTTCAGTTGTAGGCGTGGACGTAGGCTACTTCCTTCAGTAACAGCTATAGGGCCACATAAGGGAACCTCAGG---CTACTACAC
Choloepus hoffmanni   AGGGAGTGGGGGGTGCAAAAGGTGTTCATCAAGAAA--------------TGGGTCTATATT-CTCCCTTCAGCAGCAG-----------CATGGAGGAATCTCAAGGCACTGCTTCAC

Homo sapiens          CTCAGGAATCTCCGTCACGCCTCTTCCCCGCCCTCCTCGGGTCTCACGCCGAAACCTCACCGGGCGGAACGATTTCCGGCAAGAGCCAAATGATCAGTCCTAACCTTTCTGACCGTGGTT
Choloepus hoffmanni   CTCATGAATCTCGGTCACGCCTCTTCCCCGCCAGACTCGGGTCTCAATCAGAAATGTTACCGGGCGGAATGATTTCCGGCAAGAGCCCAA..............TTTTTGACAGAGCTT

Homo sapiens          CCAAGGTTCCCC-AAAGGACTGCCCTCTAAAGGACCCGGGTAGTTCCGCTT-CCGGCAGCGCGAGATAAATCACGAGAGGAAGCTTAAATC-TGTCGTTTGAATTTAGGACCACCTCGGT
Choloepus hoffmanni   CTCAGCCCCCTCTAGCACGCCACCACCTTAACGACATGGGTAGTTCCGCTTTCCGGCAGCACCAGAAAAGTCGCTAGAGGAAGCATAAATCGTGTGGTTTGCGTTTAGTACCACCTCGGT

Homo sapiens          GAGTGGTCGTTCTGGTGTGCTGTGTCATACCTACTGTTTTTTAAAGTGAGGCGTAACCCGACAGTAATTTCAAAACCATTCGCCTCGACCGGCCTAAGGAAGGGTTTAATTGAGTCTGTG
```

```
Choloepus hoffmanni  GAGTGGTCGTTCTAGTGTGCTTTGTCATACTTACCGACTTTTAACGTGAGACATGACTTG---GTAATTTCAAAACTATTCTCTTCGACTCGCCTAAG------TTTAATTGACTCTGTG

Homo sapiens         GGGAGATTTTGCGCAAGCGCCTTTGAGATAGGAGGGCCTTGGGCGTTGCGATGTCCCGCGCTAGCTGAGCAG--AAAGGGCGAGTGCTGTTTCGGGCCTGGGGAGGTGCCTACTGGGTGT
Choloepus hoffmanni  G----ACATTGCGCAGGCGCCCGTGGGGCGGGAGGGCTTTAGGTGTTGCGACGTCCTGCATGAGCTGAGTAGCGAAGGGGCGAGTTCTATTGCCAGGCCTGGGAGGTGCCTTCTCGGTGT

Homo sapiens         AATTGAGTTTTACCAAACGGTCATTTTCCTGCTGATTCTGAGTGTGGTTGGACCTTGCCCTTAAGATTCCGTTAACGAGTC-GCCCAAAGCTTAGGAAATGCTCCCTCACAGC-------
Choloepus hoffmanni  TACCGAGTTTTACTTAATGTTCTTTATCCTGCTTGTGCAACCCGTAACTGGACCTTGCTCTGAAAAATTAGTTAACGAGCCTGCGAAACGGTCCTAAAATGCTTCCTCACAGCTAAAATA

Homo sapiens         ---------------GTTTGAG---ACTAAGCACTTTTGGCCGGCG------TTGAAGAATTCTCCCCATTACTTTGATCA-----GTCATTTAATCAGAAAT--------TTTGCCCC
Choloepus hoffmanni  GTGCCCCATATTCCCAGCTAGAGTAAATTGAAAAGTTGAGTGCGGCACTTGTATTAAAGAATTATCCC-GTTATTTT-CTCACAGTTATTATTTATTCTGGAATATTTCTTTTTTGCCCC

Homo sapiens         AAATTTAAATACGTGGCGGGGAGAAACGTGAGGTCAAGAGGGAATCCTGTCACGTAGTTTTAAAAGAAAAATGTCCACTTTATCTCAGTGTAGGTTTTTCTTAAATGGGCATTTCTGAAC
Choloepus hoffmanni  AAAATAAAATCTGTGG-GAGGAGAAACTGGATCTAAAGGGGGAAGATCTTTATGTATTTTTAAAGAAA-----TCCACTTTATCACAATGT----TTTCCTTAAATGGGCATTTCTGAGC

Homo sapiens         CAAAGTAGA----TACTTTTTAAAGTGGAAATGCATTTATCCCATGTGGCTTCATGTGAATGCATA-TGGC-GATGAAGGCATTTTTTTGTTCAATGAATTTGTACTCTGAAGTGAACCA
Choloepus hoffmanni  CAAAATGGAAAAATAATTTTCAAAATAGAAATTCATATATCTCATTTGGTTTCATGTGTTTGTGTAATGGTAGATGAAGCCATTTTT--ATTAAACGAACGTAAATGCACAAGTGAAGCA

Homo sapiens         GATTACAGAGAAGT------GTATTTAAATTTACAGGTCCTTGACGTTGCATAAAATTGTGCGTGTCCATTTTTTGGAGCTTAAAATAGAATAATTCAAATGTCAGAATTCCGA-TCCAA
Choloepus hoffmanni  GATTACAGAGAAGTTCTGGTGTATTTAGATTTCCCGGTCTTTGATGTTACATAAAATTGTGCCCTTTCATTTTATAAAGATTAAAGTCGAATAATTTAAAAGTCAGAATTCTGACCCCAA

Homo sapiens         GTTAACATGTTGATTGAAA-ATTGC-------AGATTTTAAGATGGAACCAGCGGGTATT---ATAACCTTTT-----AGGGGCTTAAATTGGTGGCTTT----GTTTTTAACCCTTGGT
Choloepus hoffmanni  GTTAACGTGCTTATTGAAATATTACTGGTTTAATGTTTTAAGTTGGAAAACATGTATATTTCCATAACTTCTTAAAGAAGAGGCTTAAATTGGTAACGTTAATGGTTTTTGAC---TGGT

Homo sapiens         GAAACAGTTAAGTCCTAAT----CTTGACATAATACCTAACTGCCTTACTTTATTGAACAC------AAAATT--ATTAAAGGAATTAAGCAATG-----------AGTT------GATT
Choloepus hoffmanni  GAAACAGTCAGATCCTAACTTTATTTAAAATGATACTTAACTGCCTTACTTTATTGCACACACAGATAAAATTTAACTAAAGGAATTAAGCAATATGTATTTATGCAGTTATTGTAGATT

Homo sapiens         TTATTTGCTTTTATGGAGGGGACAAAGTGTATAAAGCTTAACCAAATGTGACCCTGACATTCCTCTTGCAAAACTACAAGTTGTAATCAATCACTTTCATT---TTTTCTTATTCCTACA
Choloepus hoffmanni  TTAT---CTTGTACAAAAAACATATGGTGTAAAAAGCTCAACCATATATTACCCTGGCATTTCTTTTCCAGAACCAGAAGGTAATTTCAGCTGTCTTCTCTGCCTTTTCTTATTCTTATG

Homo sapiens         TGTTTAAGTACATTAAGTAATGATTTTAAGAGAAATTCTTGAAAATATGAGC----ATATCCAAAGAAGTCCCAGGACAGTCTTTCTTTATGTAGTAGAAACCACTTAA-TCCCCCTTGT
Choloepus hoffmanni  TATTTTAATGCA-CAGTAAACTATTTTAACAAAAATTCTTGAAAATAAGAGCATGTATGTCCAAAGAAGACCTAAAATAA----TCTATATCCAACTCAAACCACTTAGTTTTCTCTCAG

Homo sapiens         AAATGATACCTTTACTTAGCAATC--AGAGTATAAGAAGA------TATTTATATTTGTCTCGAATATTACAGTTT----------------------CAAGCCATTTTCTCA------
Choloepus hoffmanni  AAGTGATACCTTTGCTTAATAATCTTTGGATCTAGAAAGATTTTTTTATTTATCCTTGTTCTGAATATTATAGCTTAAAATTCTGAATGTTAGCAGCTGAAAACCATTTTCTCATATTAA

Homo sapiens         ------------------------------TATGTATGGGCTTTGAAAAAAAAAAAACAACTTTCA----TTTACGTTACTTTTGTATATGAATACCA-----ATTATTAGCAGTAAAA
Choloepus hoffmanni  CTACAGACATGGACATTTTCTAGGAACCAACTATGAATACACTTTGAGAAGAAAAG---AACTTTTGGTGCTTTAAGCTATTCCTGTGCCTAAAGGCAAATATTATTATCAGCAGCAAAA

Homo sapiens         CTTTTAA----CTACTTCAAAGGCTATGCTTTTCGTGAACTGAGTTTTCTTTCTTACGCATTTTCTATTTCTCCTTGTCATCTGTCTCTCTACCTCCAGAACCAGCAGTTTGTGAACTGC
Choloepus hoffmanni  TTTTTAACCATCTACCTAAAAGACCATGGATTTTGTGAGAAGATTTTTCTTTCTGATG-ATTTTATGTTTC----TGTTACCTA--TCTCTACTGTCAGGGCCAGAAGCTTGTGAATTGC

Homo sapiens         ATTAGACT----GTTATATCGTTATA--------------AAAGGATACAATTCGTGAGTCTTCTGATTATTTTCTTCTTAGCACTGAAGCTTTATAAACATTGCTATAAAACATTAAC-
Choloepus hoffmanni  TCTAGACTACAAACTAGTGCATTATAAACTAGTATTTTATAAAGTATACAAATCTTGAGT--------------------------AAAACAAATAAAAGTTGCTATAAAGCATTATTA

Homo sapiens         ACCATTAATGAAAGGAAAGAGATTTTAAAATATTTTTTCATGTAGGTGAAGATTTTGGGAAAAGAAAGGGTTTTATGTTACTTTAAACAGCCTGATATGGATGATAATACTTAGATATGA
Choloepus hoffmanni  ATCAGTGATGAAAGAAAAGAAATTTTAAAATATTTTTCCTTGTAGATAAAGATTTAGAGAAAAGAAAGAGTTCTCTGATAC-TTGAACAGCCTT..........................

Homo sapiens         TAATAATTATACATTACCATATACAAAGGACTTTTAATCATCCTCGTAATTGCAAAAAAGGGA---TAGATATTATGTCC-TTTTCCAAACATAGATACTAAGG---------------
Choloepus hoffmanni  .....................................TGATTCTTGTAACTGAAAAAATGAAAAACTAGGTATTATGTCCTTTTTCCAAATATAGATACTGAGATCTTAAGTAAGTCTCA

Homo sapiens         -----------------------CTTAAGTCTATACCTTCTTAGACACAGTTTACTTATAGTGCCATGTATG--TGAAAGTGAACTGTAATGTTTTCAAAAGTGAA--ATAATTTACCT
Choloepus hoffmanni  TACCTGAAATTTCATAACTGACTCCTAAGGTCCTTACCTTCTTAGATGCACCCTAATTGTAAAGCTATATATATTTACAAGTGAA--ATCATGTTTTCCAAAGTGAAATATAAATTATCT

Homo sapiens         CTGGAATATTTGCTATAATTTTTAATTCTCCTTTCAGGAAATGCAGGTAATTCTTTATTCATTCACAAATAGTTGAGTGTCCACTGTGTTCTGAACATTATGTTAAACAATGTAAACATA
Choloepus hoffmanni  CTGGATAATTTGC...........................................................................................................

Homo sapiens         AAAAATTAATAAACTCTTTCCATTCCTCATTTATAATCTAAATTATTGATTACAAAAATCACTTTATAATTTGTGTAACAGAGGCCTGAACCAAGTATTATAAAAGCTAAGGATAACAAG
Choloepus hoffmanni  ........................................................................................................................

Homo sapiens         TAATTGACTTACTGGTATAGCAGGGGGGAAAAGCGTGAAATTTGAAGTAAGAGACACCAAGTTAAAAGTGTCAGCGCATGTACTCACTTTATTACCTTGAGTAAATTGGACAATCTCTGA
Choloepus hoffmanni  ........................................................................................................................

Homo sapiens         ACCTCCATTTTCCTACCTGTAAAATGGATATAAAACTAATACCTACTTCATGAAGTACTGTGAGGATTATCATAGTAAAATACTGCATAACTATTTGCAGCTGTAGTCGTGAGTCACTTA
Choloepus hoffmanni  ........................................................................................................................

Homo sapiens         GCAATGGAATAAATTCTGAGAAATGCTTTGTTAGGCAGTTTCAGCATTGCACGAATACAGACTCTACCTACATGAACCTAGATGATATGTGTGTGTGTGTGTGTGTATATTTTTTTCTTC
Choloepus hoffmanni  ........................................................................................................................

Homo sapiens         ATATAGAGAACCAAATGTCCCTGCACATTATTGGGTATCATTTATTTCTCCTACTTGATCTGCAGTGCTAATATCAAATGACCTATATCAGGTTCTATATATGCTCTGCTATCATCTTAC
Choloepus hoffmanni  ........................................................................................................................

Homo sapiens         GGGACCAATAGGCAGTCCATCCTTGACCAAAACATTCTTATGCAGTGCAATTGCGTTGGCATTGTTTTTAAAATTTTTAATTTTTTAAAATTATTTCCTCTTCCTATACTTTTGGCTTTT
Choloepus hoffmanni  ........................................................................................................................

Homo sapiens         CCAGATGTCATATAAACAGAAATATAAAATATATAGCCATTTGTGTCTGGCTTATTTCACTTAGCATAATGATTTTGAGATTTATTCATATTGTTAGGTCTATTTATAGTTTGTTCCTTA
Choloepus hoffmanni  ........................................................................................................................

Homo sapiens         TCAATCCTTAATAGTATTCCAGTATGTGGATCTACTGTAATTTGTTTATCCATTCACCAGTTGATGAACATTTGTTTTCAGGTTTTGGCTGATATGAATAAAACTTCTAAAAACATACAC
Choloepus hoffmanni  ........................................................................................................................

Homo sapiens         TTACAGGTTTTTAGGTGTACATATTTTCTTTTGGGTTAGTATCTAAGATTGGTATTGCTGGACCATATGGTAAATTGATGTTTAACTTTATAAGAAATTGCCAAACTGTTTTCCAACATG
Choloepus hoffmanni  ........................................................................................................................

Homo sapiens         TTTACATCATTTTCTATTATCACCAGTAAGAGCATCCTTGGTAGTACCTAGCATTGTTAGGTTTTGTTTTATTTTTGTTGTTGTTAGTGTGTAGTCATATCTTATGGTAGTTTTAGTTTA
Choloepus hoffmanni  ........................................................................................................................

Homo sapiens         CATTTTTTATTATCTATTGATGTTGAGCATTTTTTCATGTGTTTATTTGCTAATCATATCTCTTTAGTGAAGTGTATTTCCAGGTTTTAAATTGGGTTGTTTGCTTTCTTGTTAAGCTTC
Choloepus hoffmanni  ........................................................................................................................

Homo sapiens         GAGAGTTCTTTATAAATTCTAGAAAAAGTTATTTATCATATGTATGTATTGCAGATATTTTATCCCAGTGTTATTTCTCTTTTTTTAGCAGTTCTTTAAGGAATAAAAATTTTAATTTTT
Choloepus hoffmanni  ........................................................................................................................

Homo sapiens         TCTTTATGCATAGCTGCTTTTTCTTTGCTGTCTGGGAAGCCATTACCCAACCTGAGCTCACAAAGATTTTTGTTGTTGTCTTCGAGAAGTTGTATAGTTTTAAGTTGTACATTTAGATCT
Choloepus hoffmanni  ........................................................................................................................

Homo sapiens         GTGAGCCATCATGAATTAATTTTTACATATCATTTGAGGTATGAGTTGAGGTTCATCTTTTTTCAAAGGATGTTTAATTGTTACAGCATTTGTTGAAAAGATTATCCTTTCTTCATTGAA
Choloepus hoffmanni  ........................................................................................................................

Homo sapiens         TTACCTTGGCACCTTTGTTGAAAATCAGTTTACTATAACTGAGTGGCTATTTCTGTCACTATTCTGTTCGATAGATTTAAGGCTGTCATTTTGCCATTATAACACAGTCTTGATTACCAT
Choloepus hoffmanni  ........................................................................................................................

Homo sapiens         AGTAGTATAGAAAGTCCTGAAACCAAATAATGTGAAATTCTTCAACTATATTCATTTCTCAAAGTTGTTGTAATTATTCTGAGTCCTTTAGCTTTCCATATCAATTTTAGAATCAGCCTG
Choloepus hoffmanni  ........................................................................................................TTTTAGAATCAGCCTG

Homo sapiens         TCAGTTTCTGCAAAAGAGCTTGAGGGTTCCTGATTGGGACTGCATTGAATCTATAGATCAATTTGGAGAAAATTGGCATTTTG--------------TCCCACTTTTTCTCAGAGGTAG
Choloepus hoffmanni  TTGATTTCTACAAAAAAA-------------------GCTTACTTTGAACCTATAGATCAGTTTGGGGAGAATTGACGTCTTAGTGAGTTTATCTGGCTGCTGCTTGTTGTTAGAGTGAA

Homo sapiens         AGTGGGAGTTACTCCAGCTTTCTGCATTCTAGTCAGAAGCCTATATGGGAATGTTTAGTGGCATT------TCAGAAAGAAATAATTTTCTTCCATGGTTATATTCACTATAATATCTTT
Choloepus hoffmanni  AGTGACGCTTTTGCCAGCTTTTCACATTCTATGAAAAAGCTTGTGTGGGATTTTTTTCTTTTTTTTTTTTCTTTTAAAGACAGAATTTACTTCCATTGTT....................

Homo sapiens         ATTGGAAGAGATCACAGGACGAATCCCTCTATTTGTGAATTGGATGTCATCCCCTGCTGCCATCTCAGGACATCATACCATAAGTTTTTTCTCCTTTGTTTTCAGTGGTCTTTCTAGAAG
Choloepus hoffmanni  ........................................................................................................................

Homo sapiens         CCCTTCCATTAGCATTAAACTTTTTGGGTTACTCCCTTGTCGAAAGCAAAACAATCTTGTTCAAAAAAAAAATTGTTTTAATTGTAGTAAATCACACATAACATGAAATTTACCATCTTA
Choloepus hoffmanni  ........................................................................................................................

Homo sapiens         ACCATTTTTAAGTGTGCAGTTCAGTGGCTTTAAGTACATTCACATTTGTTTTACAACTATTACCACCATCCATCCCAAGAACTCTTTTCATTTTGTGTAACTGAAACTCTTGACCAGTTA
Choloepus hoffmanni  ........................................................................................................................

Homo sapiens         AATAATAACTCTTACATTCTCTCCTCCTCACCCCCTGTTCTATGCAACCATCATTATTCTTTCTGTCTGTATGTATTTGACTACTCTGACTACCTCATATAGGTGGAATCTTACAGTATT
Choloepus hoffmanni  ........................................................................................................................

Homo sapiens         TATCTTTTTGTGACAGGTTCATTTCACTAAGCTTAATGTCCTCAAGATTCATCCATGTGGTAACGTGTGTTAGAATCTCCTTCCCTTTAAAGGTGTGTGAATATATACACCGTATTTTGT
```

```
Choloepus hoffmanni  ........................................................................................................................

Homo sapiens         TTATCCATTCAGCCATTGATGGACACTTGAGTTGCTTCCATCATTTGGCTGTTGTGGGTGATGTGGCTATGAACATGACTGTCAAAACTTTTAACCCCACTAGAATGTAAACTCAATGAA
Choloepus hoffmanni  ........................................................................................................................

Homo sapiens         GGTCAAGAGAATCTACATCTTGTTTATTATCCTGTCTCCAACGTTGTAACTGGCACATAGCAGTTACTCAGTCACATTTTTTAGATCAATGAATGAACCTTATATCTGTCTCTAGCAACT
Choloepus hoffmanni  ........................................................................................................................

Homo sapiens         ATCTTGTAGCTCCTTGCCTTTATAGCTAAGCCCAAGTTTCTAAATCCACTTTCTGAATTTCCATTCATTCCTTGTGTCATTTCAGTCTGGTTGCCAACTGCCCTGCTATACTGTAACTAT
Choloepus hoffmanni  ........................................................................................................................

Homo sapiens         ACTGTATACTGTTGCCAAAACCACTAGCTACATTATGGTTCTTATATCACTTAGCCTCTCTGTGTACTTCTAAGAAGTCTGTATTTCCCAGACTTCTATTACCAGAACCTCTTCTGGCTT
Choloepus hoffmanni  ........................................................................................................................

Homo sapiens         TTTTCCTGTTTCTCTGGCTAGTCTCATGTCCCTAAAGATTTACAATATATTTGGGAGAGAGACATATAAACTAATATATTCAATAGAGTGTTTCAGATTTTATGATAAAGGTTGACACAT
Choloepus hoffmanni  ........................................................................................................................

Homo sapiens         ATTACTGTGAATGTACCAATGAGGGTGTGCACAATTCTCCCTGAGATTGGGGATGTGGTCAGAAAGGATTCATGGATGAATGAACTCATTAATGTGAACGTTGAAAGATAAGGATATGTT
Choloepus hoffmanni  ........................................................................................................................

Homo sapiens         CACTGCACTTCCTACTGTGTACACTTTCCATCTGATCCTTCTTCCATTGCCTTAACTTCACCTACTTTTTACATGCCGAGTTCATCCATGAA--GAATAGAACCTGCTTAATATTGTCAT
Choloepus hoffmanni  .......CTTCTTACTCTGTACATTT--AACTATATTTTTCTTCTGCTGCCTTAATTTCAGCTACTATTT-------GAGTTCATCCATCAGCTGACCAGAACATATTCAGTATT-CAGT

Homo sapiens         CTTTCTCCCTAAGCCAGAGCTTCAGACAGAAGTGATCCCAGATTCTTCTTTGTGTTTACTCCACACATCTAGTCAGTTACAGTTCTTATATAATGTATCTATCCATTTATACTGACACTG
Choloepus hoffmanni  GTTTCTCCCTAAGCCAGAACCTCAGA----AGTGATTCCAAACTTTTC-----ATTCATTCCATACCTCTAGTTAGTCGCA--TTTTAAA-AATTTATCTATCCATTTA------CACTG

Homo sapiens         CTTTGGCTCAGAATTTCCTCATTTCTTGCTTTAATTATACAGTAGCTTCCTAGCTGACTGGTACAGGTATTCCTGTCTCTAC--CCCAATCTCTATTCCATATTGGTGCACAATACACTT
Choloepus hoffmanni  CTTTGACTTGGAATTTCATCATTTCTTGCCTTAATTATACAGTAGTTCTCTAGGTGATCTGTACTGACCTCCCCACCTCTTCAGCTCAACCTCTCTTCCATACTACTGCAGAATGGACTT

Homo sapiens         TATAGAATAGAAATCTAATCTTGTAACTCCAACTCATTCTTAAAATTTTAAGTTATTCCAGGTTGGAGTGGTTAGAAGACACTCTGCAGGAAATTACACCTAGCCAGAGTTTTGAAATAT
Choloepus hoffmanni  TGTGGAATAGAAATCTAACCTTAAAACTCTTATTTGTTCTACAAA-----------------------------AACTCATGCTGTAGGAAATAGCATCTAGCCTGAGTTTTGAAAACT

Homo sapiens         GATGAGGGGCTGGAGAAATAGCAATAAAAGTTCATTCCAGGCAAAAATATCAATGAAAAGGTGTGAGCACACTGCATGGGATGGTTGAAGAACTGTGGTTATGTTTTG---AATTG----
Choloepus hoffmanni  TGAATGTGGTTTGAGTAGTTAGAGATGAAGTCTATTCCAGGCAAAAGT-TCAGTGAAAAGATGTGAACACACTGCATGGAATGCTTGAAAAACTATGGTGATTTGTTGTGTAACTGGACC

Homo sapiens         --------------AGAATGCCAAGAGAAA-------AAGATGGAGAGATTGATAAAAACCATATCTTATAAAGCCTTGTATACTGGCAGAGGTATTTAGATATCATTAGTGCCATGGTC
Choloepus hoffmanni  TTAGAGTTTGAAATAGCTTGCCAAGATTAAGCATATAAACATGAAGAGGTTAGTAAAGACCATATCTTGAGAATCCTTGTATGTTGACTGAGGAGTTTAAATACCAT............

Homo sapiens         TTAATGGTTGTATTCTCCCAAAATTCATATGTTGAAATCCTAACCACCAAGGTGATGGTATTAAGAGGTTGGACCTTGGGGCTGGGCGCGGTGGCTCACACCTGTAAGCCCAGCACTTTG
Choloepus hoffmanni  ........................................................................................................................

Homo sapiens         GGAGGCTAAGGTGGGTGGATCACTTGAGGTCAGGAGTTCGAGACCAGCCTGGCCAACATCGTGAAACCTCATCTCTACTAAAAATATAAAAATTAGCTGGGCATGGTGGCATGCACCCGT
Choloepus hoffmanni  ........................................................................................................................

Homo sapiens         AGTCCCAGTTACTCGAGAGGCTGAGGAACAAAATTTGCTTGAGCCTGGGAGGCAGAGGTTGCAGTAAGCTGAGATCATGCCACTGCACTCCAGCCTGGGCGACAGAGCGCAACTCTGTCT
Choloepus hoffmanni  ........................................................................................................................

Homo sapiens         CAAAAAAAAAAAAAAAAAAGAGGTGGAACTTTGAGGAGGTGACTTGATCATAGGTCAGAGCTCTCATTAATGGGATTAGTGCCTGTATTTTAAAGAGTCTGAGAGAAACCCCTTTCCCCT
Choloepus hoffmanni  ........................................................................................................................

Homo sapiens         TCCCTCATGTGAAGTTACAGTGAGAAGACAGCTGCCTATAAGAAAGTGAACCCTCAGTAGACACTAAATTTGCTGGCATCTTGATCTTGTATTTCCTAGCCTCCAGAACTGTGAGAAAGA
Choloepus hoffmanni  ........................................................................................................................

Homo sapiens         AATTTCTGTTTATAAGCCATCCAGTTTATGGTATATTTGTTATAGCCGCCCAAGTGGACTAAAACAATCAGGAATGGCAAAAGATGTATTTCGAGTTAAGTGAATATTTTGATAAGATTT
Choloepus hoffmanni  .....................................................................AGGGAAGGCAATAG-----CTTCAAGGTAGGTGAGTGTTGTGATAAGGTTT

Homo sapiens         GTGTTTTAGTGTGACCTCTGTGACTGCAGTGAGGACAGGGACCATATTAGAGACAGAGGAGGCAAGGTCAGATAGAAGGTGAGTGTGAGCTAAGGCAGTGGCAATGGAATAAAGTTCAAG
Choloepus hoffmanni  GTGTTTCAGGATGACCTCGGTAGCTATAATAAGGAGAGGGACTATACTGGTGGCAGAGGAAGCAAGGTCAGATA-----TGAGTCTGAATTAAGGCAGTGGCAGTGAAATGAGTTTCAAG

Homo sapiens         GTTCTACCTCATCTACAATCAGTGCTTTTCCTCCTTTTCCAGGTGAATCTGGGTCTCTACACATCTGTTTTGATAGTACTCTTTGCATACATCTGTTAAAACACCACTCTAAACTGTATA
Choloepus hoffmanni  TTTCTATGTCATCCAAGGTGCTTTCTTTATGCCTCTCCATAAGTGAATCTAGGTCTC-----------TTTTACTGCATTCTTTGCATGCTTTTGTCATAATGCCATTTTGTACTATATG

Homo sapiens         AATGCTGATTTCCTCCTCTTTTCCTCTCATTTATACCAT-AAGCTCCGTGAGGACAGGGATTTCATTTTATTTAACTTTGTATTAGTATCATCTAGTATATATAAGGGTTTAAGAGCTCA
Choloepus hoffmanni  AATGTTGATTTACTTCTCTGTTCCTGTCA-TTAGACCATAAAGTTCCTTGAAAATAGGAATTTCATCTTGTTTAGCTTTTTATTCCCATCACAAAGTACCTATAAAG--------GCTTG

Homo sapiens         GTAACTGAATG------AGCAAATTCAAGGTTATATGGATGAACAGGCAAAA----AACTATTAAAAAGGGTAAAGCAT-GGAAGTTCTACAGTAGG-CACA-AAGAAGATTAGGAGAGG
Choloepus hoffmanni  GTAAATGAATGAACAAAAACAAATGAAAGGTTATATAGATGAATGGGCAAACAAGTAACTACGATAAAAGCTAAAACATGGGAAGTTCCACAGTAAGTTACAGAGGGAGTTCAGGAGGAG

Homo sapiens         TCAAGATCACTTTAGATTCAGAGATCATGGAACACTTGCTTCATTGAGATGCTAGAGCTTGATTTTGAAAGATGGGTAAGGCTTAATCAGGTG------GAAGGATATACCATTTAGGGG
Choloepus hoffmanni  TAGAGAGCACTTATAAGTCAGAGATCATGGAATGCTTTCTTCATTGAGGAGGTAGAGCTTGATTTTGAAGGATGGATAAGACTTGAATGGGTAGAACTTGAAAAATAGACCGTTCAAGGG

Homo sapiens         AAA----ATGATGTAAACCAAGGTATAGAGAATGGTTGGGGATATGGCCATGGGTATAGAAAGGAAAGTAGTGAGAGAAGAAGCTGTA------TTATGTTCTGCCAGGTTGTAGAGGGT
Choloepus hoffmanni  GAAAATGATGATATAAACCAAGGTCCAGAGAATGGTT-GGGATGTGGCCATAGTTATATAAAGGAGACTATTGAGAGAAGAAGCTGTAAGGCCTATATGTTCTGCCAGGTTGTAGAAGCC

Homo sapiens         AGGCTAAATAGTCTAGACCTTAGCCACTAAGTGGAAGATAATGCCCAGAGGCAGTCTCATGGTGTCCACTCTGTCAAGTAAATCATTTGTAAGATAAAAATATGCTAGGG-------ATA
Choloepus hoffmanni  AAGCTAAA-----TAGACATTGGTCAGTAAACTG-TGGCAGTGTCCAGAAGGAGTCTCATAATGTCTATTCTGTCAAGTGAATCGTTTGGAAAATAAAAATGTGCTGAGGAAGATTAATA

Homo sapiens         GTCTAAATTAAAGTTTCAAGGTGACTATGT----CAAATAACTACACCTTTAAGTCTTAGTTCTGT-------TGAGCCAAGACAACTCACCCATATAAAAATGAAAATAATACACTTGT
Choloepus hoffmanni  GTCTAAAT--ATGTTTCAAGGTGACTATGTAAAGCAAATAATCATCTGTTTAAGT----GTTCTGTTCTTTACTCAGCCGAGAAA------------TAAGATGAGAATAACATACTTCT

Homo sapiens         GAAAGCATTTGTGAAATATGAAAATCTGTAGGAAATAGATGGTTTTTATGTCTTCTCCCAATGATGTTTATGTCCCTCTCCCTAACATAGATATATGTTAGTCTTCTCCCACTACTTTGA
Choloepus hoffmanni  GAAAGGAT................................................................................................................

Homo sapiens         TGAGAAAAAAACAAACCAAAAACCTTAAAGAGCTCAGTCTCCAAAGATTTTTGTAGTGCTTTCCTTTTCTCCTTGTCCCTTGTACTATAGGTTTACTCTGAGGAGAGCAAATTTCTCTAT
Choloepus hoffmanni  ....................................................................CTCTTGGCTGTTTGTAATCTTGGCTTACACTGAAGAAAGATAGTTACTCTAT

Homo sapiens         TTCGATCTACTTTCAATTATTCAAAAGTTAAAGATTTCAAAGCAGAGAAGTTAATTTCAGTAAATAGACCTTTTCTG---AGAAGTTTTTG-TATATTTATTATAAATAGGAAAACCAAC
Choloepus hoffmanni  TTTGATCTATTTCCAATTACTCAAAAGTTAAGGATTGGAAA-TGGAGGGGTTAATTTCAGTAAATAGACATTCTCTGGAAATAAATGTTTGTTCCATTTATGGTAGGTAGGGCAGCCACC

Homo sapiens         AGTGTCCTAATGGCACGTTAACTTAATATACTGAAGACTTCATCTTACATTAGATAGAAAAATTTTCTGGTCTCTCCAGGAGGTAAATGGGTAGGGGGAGGAAGACAAGAGATGGTAGGA
Choloepus hoffmanni  ACTG....................................................................................................................

Homo sapiens         ACTTGGAACCAAGAGGTTTTTAAATATAAAAGCAAAATACGGAAGCTACTGTGGATTATGCAGACTCACATTTTTTTTCCAATGGCTGGTTTGTTTTCTACCAGTTCTGGTTACCCACAT
Choloepus hoffmanni  ........................................................................................................................

Homo sapiens         CCCTCTGTGTTTTGGGGCCTGTACTTCCTCTAAGGTGTGAGAGTTTGGTCTTTTCCTTCTGTGGGCTTTTACAGGCCTGAAAAAGCTCCTCCCCTTTTCTTTTTAAGGTCCTTCAGGAGT
Choloepus hoffmanni  ........................................................................................................................

Homo sapiens         TTTTGCAACCTTTACTGGTCTCTAAAAGGAAGGAAATACACAATTTTTATCTTCCTTTTATAAACCTCGTCTTCCTACAGATACCATCCTCCCAACTGGCTAGTATGGGATATTTCTAGA
Choloepus hoffmanni  ............................................................TAAGCTTCATTTCCTCATGCATACCATTACTCCATCTGATTAGCATGGGTTTCCTCCAGG

Homo sapiens         TTTTCCTTTGGATTGCTTAGACGATTTGTGACATTGATAGTTCCAAAAACTATTAAGGGAAAACTATAACAGGAGGTTATATGTAGTATAT---TATTAATTTTACAGTATGTATAGTCT
Choloepus hoffmanni  TATTTCTTTAGATGTCTTAGGGTATC----------ACGGATCTAAGAACCATGAAGAAAAACTTACAAATTTGAATAATCTCCAGCATATCAATACAAAATTTACAGACTGGGAACATA

Homo sapiens         ATATATTGATTTCATCTAAACTGAGAAGGGACAGGT-TTCTGTCCCTTAGACTTGAAAGAATGTGTTATCATTGTAGTATA----------GAGATGAAAATAGAGGACACAAAGAAAAA
Choloepus hoffmanni  ACATTCCAATATTTTCTGAGTAGCAGTAGAATGCATCTTCTGTTC------CTTGAATAGGGGTATACTCATGTTAGACTAGCCTGAATTTGAGATAAAAACAAAGTACTTCA-------

Homo sapiens         ATGATTTTGGTTTCTTTTTTTTTTAAGAGTTGAGGACGATTAATGCAGAGCTAGAATGATAGGCAGGCAAATGGATGGGTAGACAAACAGGTTC---TATATTCCCAACTTGAAACGACA
Choloepus hoffmanni  -------TACTTTCATGGCATCTTA-----TAGGAAAGATGTA---AGAGGTATAGAGGTATG-------ATGCAGACGTAGACAA--AGATTCAGTTATGATACCTAACTAAAACAACA

Homo sapiens         CAAGGCTTCATGAATTGAAAATTGATTAGGAAGTAAAAGTTTGCCATGGAGTCCTGGCAGAGGAGAGAGTGTAGTGAGGATGACATGGAAAGTAAAGGCATCAGGAGGTCTTATGTGGCA
Choloepus hoffmanni  AAATCTTT--TGAAATAAAAAGAATTTAGGACCATAGATTGTATTCTCAGCCCCAGGCAGTGGAGAAGAGGCAAGAAAGACAA-ATCAAAAG-AAAAGTATCA................

Homo sapiens         GATTGGAGGGCAGTAGGGTGCCACTAGTGTTTCACCACAGATGGTTTGAAAAGCACTGACCTGGAGCGACATGAAACTTGGTAACAATAGAAGTGGCTACAGGACCAGAACAGAGTATAC
Choloepus hoffmanni  ........................................................................................................................

Homo sapiens         GCATGGTAAAACTTCTTGATACCTTTACTGTTTCCATTTAGTGGCTATTATGACTATAAAATATCTATGAACATTTTTGTACACATATTTTGATAGTCATAGACTAATTTCACTGGAATA
Choloepus hoffmanni  ........................................................................................................................

Homo sapiens         TAAAATTAGAATTGCCAAACCTTTAGGGTAAATTTGTGCTCAGCTTTAGAAGAAACTCAGTTTTCCTAGGTGGTTGCAACATTTTATCCTCCCACAATGTATGAGAGTTCCAGTTGCTGC
Choloepus hoffmanni  ........................................................................................................................

Homo sapiens         CTGTCTTCACCAACACTTGGTGTTGTCTTTGGTTTTTAATTTTAGCCTGTTCAGTAGTGTGTAATTCTAAGTCATCGTGGTTTTATTTGCATTGCCCTGTGGAATAATGATGTTGAGCAC
```

```
Choloepus hoffmanni  ........................................................................................................................

Homo sapiens         ATTTTCACGTGCTTATTGACCATTGGATATTCTCTTTTATGAAGTGCTGTGTAGAAATTTCCCCCCATTTTTTGAATTGGGTTTTTGTCCTTTTCTTTTTGATTTTTAGAAGTTCCTTGC
Choloepus hoffmanni  ........................................................................................................................

Homo sapiens         ATACACTGGGTCTG--------AGTCCTTTGTTAGATATATGTATTACAGATATCTTCTCCCAATGCGTGTCCTATGTTTTCACTTTCTTAATGGTGTCTTTC-ATGAATACACATTTTT
Choloepus hoffmanni  ATACAATGATTCTGTTAGTTATAGTCTCTAGTT---TACATTGATTGCAT---CCCTCCCCCAGTGCCTCCCCT---------------TAATGGTGTTTTTGATGAACAGAAGGTTTT

Homo sapiens         ACTT-TGATAAAGCCCTA-TTGTTAATTTTTTTTTCTTTTAAGGATAGTGTTTTTTTTTTGTGTCTTATTTAAGAAATCTTTGCTTAGCCTAAGATTTGAAAGATCTTTTCCTATGTTTT
Choloepus hoffmanni  GCTTGTCATGAAGTCCAAATTGTTA---TTTTTATCTTTTATAGA--GTGGTTTTTTTT---GTCCTGTTTAATAAATCTTTGCCTATCCCAAGGTTTTGAGGCTATTTTCTTGT---TT

Homo sapiens         CTTCCAGAATCATTGTTATTTATCTTTCACATTTAAGTTTATGATTTATCTGGAATTAATTTTTGTGTAAGATGTGAGGTAGGTAGTTAAGGTTCATTTTTTCCCCTAATACAGTTATCT
Choloepus hoffmanni  CTTTCAGAGGCTTTGTTTTTTACTTTTCACATTTAGGTTTATAATTTATCTGGAATTAATTTTTGTGGAAAATGTGAGATACGTGGTTAATGTTACTTTTTTTTCT--ATGCAGATCTCC

Homo sapiens         AATTG-TCCAACATTATTTATTGAAAAGACCACCTCTTTCCTC-ATTGTGTTGATATCTTCATTGTAAACGTATTGACTTTACATGTGTGGATGTAATTCTGTACTCCTTGTTCAATTAC
Choloepus hoffmanni  AATTGCTCCAGCATCATTTGTTGAGAAGAATGTCTCTTTCCCCTGTCGAACTGATGCCTTTGTTGTAAGTTCATTGACC----------GCATCCAATGCTGGATTCCCTATTCTGTTCT

Homo sapiens         AGTGATGTATTTGTCTGTCCTTATGCCAATTGGCT--TGTCTTATTTACTTTATTTGTATAAAAAGTCTTAAATCTCATCCATTCA-----------------TCTTTGAGATGATTCAG
Choloepus hoffmanni  AGTAATCTGTCATCCTCTCCTTATGCTAATAGCACAGTGTCTTATTTACTATAGCTTTATACTAGTTTTAAAATCTGATCCATTCACTTTTTTATCCATTCACTTTTTTAGATATTTTAA

Homo sapiens         AAACTGTGATAGGCTGCATTTGTTTGGTTTGGGATGCAGCCATCTATTAGAAGATGAAAAAAATCATGTATTTAATAATCAATTGGAAGTGAATTAAACTTAGAACAAACTGATTGTCTT
Choloepus hoffmanni  AAACTGTGATAAGCTACATTTGTTTT-TTTGCAATATAGCCATGTGTGATTAAGTGAATAAT-TCATGTATTTAACATTTAACTGAAAGTGAATATATATTAAAACAAATTAATGGTTTT

Homo sapiens         ATAATACTTTATAGAGATAATTAACCT-----------GTT-TTTTAGGTTATGATGGGACTGTGTTAACATTTCAGAACCTTTTCTTTTAAATTGACATATACTTTATGTTAACCTA--
Choloepus hoffmanni  ATAATACTTTATAATAGCAATGAACCTTAGATACTTAGGTTATCTTAGGTTATCATAGGGCTCTATTAGCACTGCAGAATCTTTTCTTTTTAA-TGGTTTATCTTCTATGCTAACCTAGA

Homo sapiens         -------AGGTAAGAAATGTACTGTTACTCTCTCTGGAAGTTAAAAGTAGATCAGAAAGACGGTT-TTACATTTGCAAAGAGCTGTGTCATAGGGAAAGTGAATTGGTAAGCTG-TTTAA
Choloepus hoffmanni  TTAACAGAGATTAGAAATACAATGGCAGCACCATTGGAAGTTATGAACATATTAGAAAGATGGTTATTCAACATGGAAAGAAGTATGTCATAAGAAAA--TACTTAATAGGCTGACTTAA

Homo sapiens         GCCATGA-CAATAGGCACAGTCTGTTTCTGCTTTGTTCCAGCATTCTCTTTCCAGTA--AATAC---TCTTAGTCTCATCCCTAGATCTTCCAGTGGTCAGTAAACATC-----TTTTTT
Choloepus hoffmanni  ACCATGGTTCATAGACATAATCT-TTTCTTCTTTGTTCTAACATTCT-----CAGCAATAATATAAATTTAGTGTCATTCCTAGATCTTTTAGTTGTATGTAAATATCCATAATTGTTT

Homo sapiens         TAAATGTAATTTCAAAATTGTAAG-ATTT---ATGT-CAGAATCCTAGCAGGAAACAGAAGACAT--TCTGGTGGGATTTTAAAGAAAATTTAATGGAGCTCCTTTCTATAGACAGACAG
Choloepus hoffmanni  TCAG---AATATCATGATTTTAAGCATTTAAAATGTCCAGAGTCCTCGCAGGAAACAGAAGACATACTCTGCTGGAATTTTAAGGAAAACTTAATGAAGAAACTGTTTACAAAGGTATGG

Homo sapiens         TAGCAAAGTAAA-------CAACATGGCACCTGGAGACTAGCAAAAGCA-AAAATAGTTACCACCTTAGGGCTGAGGTGACAAGAAGAAGAAATAACCATGAATATATTTAGAAATAATA
Choloepus hoffmanni  ACACTGAGTCAAAAGAATTCAGTATGGCACCTGGAGATTAGCAATAGCAGGAAATAG---CACCCCTAGAACTGAAGGAGCAAAGAGAAGAAATGA........................

Homo sapiens         TAAAGCTATATTTCTTCTCTTATAACTTATTTATTTAAATGTCACATGACAGGATCCTCCTGTGTTACCATCCTGTGAATGTAACATTTCAGTTGATAGCAATGAAATTTTAAGCAAAAC
Choloepus hoffmanni  ........................................................................................................................

Homo sapiens         TTTTTGAAGTGTACTGGTTCATTTCCTTTTTTCTCAGTATGTGGAAAATTTTCAGCATTTCTTATCAAATGGAAAGAGACAAGATAATTTGTCATGGGGTATATCAAAAATAGCATTTGA
Choloepus hoffmanni  ......................TTTCTTTTTCCCCAGTATATGAAAA--TTTCAGCATTCTTT--CACATG-AAAGAGACAAGATAATT-GTCATGAT-TGTTTCAAAA-TAACATT-GA

Homo sapiens         GTATTTTTGTTCATATACATCG--CTGTTCCTTTCCTGTACTGCAATTATCTCCCTCACAAATAA-ATTCTGGGAATTCTCGGTTCATATGTAAATAGAAATATGACAGAAGTCTTAACT
Choloepus hoffmanni  ATATT---AGTCAAAAACATCATTCTGTTCCTTTTTGTTACTGCAATT--TCTGCTCATAGATAATATTCTGGGAAAGCTAAATTCATTTGTAAGCAGAAATATGATAGAAGGCTAAACT

Homo sapiens         GAAATAGCAATTCAGTTAGCTTAAAATTTAAGTTATCTTTTTAAATACCTTTTACCTAGGTTCACAATGGTCCGTAGTGGAAAAAATGGTGACCTTCATCTTAAACAGATTGCATATTAC
Choloepus hoffmanni  GAAATAGCAACTGAATTAGCTTAGCACTTAAATTATATCTTTAAATACCTTTC-CCTAGGTTAACAATGGTCCGTAGTGGAAAAAATGGTGACCTTCATCTTAAACAGATTGCATATTAT

Homo sapiens         AAACGAACTGGTGAATATCATTCAACTACACTGCCAAGTGAGAGAAGTGGCATAAGAAGAGCAGCAAAAAAATTTGTCTTCAAAGGTAAAATTAATGTTCAAATTTTGATTATTGAGTTG
Choloepus hoffmanni  AAACGAACTGGTGAATATCATCCAACTACACTGCCAAGTGAGAGAAGTGGTATAAGAAGAGCAGCAAAAAAATTTGTCTTCAAAGGTAAAATTAATATGCAAACCTGGACTATATGGTTA

Homo sapiens         ---TAGATTCTTCCCCACATTCTTCCTTGTGGAAGTTTGGGAATTTCTGTAGTAGTTGGTTGTTGCTAAACAATTACCAGTTGAATTGATGATGCCACTCAATCAAAAGCAAAATAAAAA
Choloepus hoffmanni  ATACAGATGCTTCTCCACATGTTT---TGTGGAA-TCTGAGAATTTCTGCAGTGATTAGT-GTTCCTTAACAATTACTAGCTAAGTTGATGCTGCCACCCAGTCAGAAGCAAAATAGAAA

Homo sapiens         TTACACTCAAGCTAACTGAAGGCCAATAAGCGTGAAAAGGGTTGTTTAAATTATAGACAGAATATGATACCATGAAAGAAAACAAAACATTTTTCTAAATTAACAACTTCTGCTTTTACC
Choloepus hoffmanni  TCTCACTGAAGCTAACTAAAGGCCCGTAAGCATTTAAAGGGAAGTTTAAATCATAGGCAAAAAATGACACCATAAAAGAAAACAAAACAC----CAAAATTAAC---TTCTGCTTTTGCT

Homo sapiens         --CTCTCGTGGT-----CTCTAATGGTGCACTTTCCAGCACTGTTGCCAATTTCAATGAGGAACTTTTTTGATGCTTAATATGAAAATGTTGACAAAGTTAACTGGTTTGTTTACTAAAA
Choloepus hoffmanni  GACTCAAGTGGTTCCTACTCTAATGGTGCACTTTCAAGCACTGATGCCACTTTCAAAGAGGAACTAATTTAATGCTTCACATGAAAATGTTGACAAAACTA-----TGTGTTTATTAAAG

Homo sapiens         TGAAAACTGGAAGGAATTAACTATATTTCTGTGATCATGTCCCCTTGAACCCCACCACTGCCATTAAATATTCTCAGGATCACATTCATTTAGGACCCTTGGATCTTCCTGAATGGATT
Choloepus hoffmanni  TGAAAAATGAAAGGAATTAACTGTATTTTTATGGT--TTCCCCCTT--------CCACTGCCATTAAATATTCTCAGGATTATATTTATGTTTGA--TCTTAAGTCT-CCTGAATGTATT

Homo sapiens         AAATATTTGAAAGGCAGTGGGATATTTATCTTGGAAGGTCATGATAATTTTTGCCTCTTTTTCCCC--TTATAAAGGGGAAGAGAAGTTTGGGATGAGATAAACAACAAGTAGTCACCAG
Choloepus hoffmanni  AAATATTTGAAAAACAGTGGGATATTTACCTTAAAAGGTTGTGAAAATCGCCTCCCCCTTGTCCCCACTTACAAAGTAAGAGAGAGATTT--------------------AATTGCCAG

Homo sapiens         ATAATTGAATGAATGTTTGTGTTACAAATGGGAATTTTGCCT-----------CTTAGGCTTACCCCAAAACAGGTTAATAAAACTCTAGGATAATAAATAAATGTCTGCTTGCATCTCC
Choloepus hoffmanni  ATAAGTAAATGAATGATGGTGATATTAATGGGAGTTTTGCCTGTAGGTACAGAATTAGGTTTTCCCCAAAGCAGTTCAAGAAAATTATAAGATAA---ATAAATGCCTGCTCATTTCTTC

Homo sapiens         CTGTGTATATCCCTACCAATAACTCCCTTATACTTAAGATTAGGC----AGACCTATTACCTCTTAAACTAAAGTGAGGATTCTTTCATTCATATATTTGATAATTTATGTAACAAATGT
Choloepus hoffmanni  CTGTAAATA-CTCTCCCAATAACTTCCTTAAGCTAAAGATTATTTGCCTAAATCCATTATCACTTAGACTAAAATGAGAATTCACTCATTGATTTATTTACTCATTTATGCAACAATTGT

Homo sapiens         TAACTGGGTTTCTGCTGTG-TGCCAGTCACATTCTAAGCCTTGGAGATACATTGATGAATAACATAGATAAGGTTCCTGCTCTTAGGTTGCTTATCTTCTAATTCTAAAAAATAAGGAGT
Choloepus hoffmanni  TA-----GTCTCTACTCTTCTGCTAATCACATCCTAGGCCTTGGGGACACATTGATGAATAAGATAGATAAGATTTCTTCCTTCAAGTTGCTTACCT-CTGATCC----AAATAAGGAAT

Homo sapiens         GTTCCACTCATCAGTTGGCTTACTCATATTAGTAGCTGAATGGTGTATTTTGGAGTTTGAATTGCTAGCCCATTCACATTGGAAAGGTAGATGGTGATTCGCTTCATACCCTGGTTACTT
Choloepus hoffmanni  GTTCCACTCAGCACATG-CTTAGGCATATTAGTAGCTTAATG-CATATTCTGGAGTTTGAATAACTGCCCCA----CATTGGAGAG----ATGGTGATTTTTTCCGTTCCTTGATTGCTT

Homo sapiens         CCTAGCTGCAGATTCTTCTCAGGTGTCCTCCTAAGGAAACAGAGAATTGTCTTGAGGATTACAGTTTTTCCACTTGCCCGCTTCACCATCCCTACCTT---CTCAAATATAGAAGAAGC
Choloepus hoffmanni  CCCAGCTGCACATTCTTCTCAGGTATCCTCAGAGGAAAACAGA--ACTATCTTGAGGATCAAGATTCTCCCCACTTTCACACTCTACCATCCCCACCTCTAACTCAG-TCCAGAAAAACT

Homo sapiens         TGAGAGAGCTGTTTGCTTGCAAAAATACAGTGATGATAAAACATTATTTTAGAAAATTCTGTTCCAGGAAGTTGTTTTTCTGTACATAATTTATTTGCTAATCTATGCCATTTTTAGGAG
Choloepus hoffmanni  GGAGAGAAGTGGTTGCTTACAAAAATGCAGCTGTGATGAAATATTGTTTTGGA---TTTTGTTCCAGGAAGTTGCTTATCTGTCTGTGATTCATTTGCTGACTCATGCTATTTTTAGGAG

Homo sapiens         GTGCCTGCAAAATCTTCCTACTTTGAGTACTCCCAGAGAGAGATAATTTCATATTTCCGTACTTATTACGTGCTTTGGTGAACAAGCTTGCTCTACTTATATTGATAGCTACAGAATTAT
Choloepus hoffmanni  GCACCTGCAAA----------------------------------------TATTTT...............................................................

Homo sapiens         AGCAGCTACTGCCCTTCCTAAGTATCTAGTAAGTGTAACAGCTAAGAACCTTTGTGTTGTAGCATCATTAAAAATAAATATTCTGTTTTAAATGCAGTAAGATTTTACAAAACAAAACAA
Choloepus hoffmanni  ................................................................................TTCAGTTTT...............................

Homo sapiens         AAACCTTCATGGTTTAAAGAGAAGGCTATGATTCTCTTAAAACTACGTAGATCTCTAAAAATTGCAATTCTATCTTTT-GGTATCATTTATTCCAGAAAAAAAGCTGTTTTATGTTGGAA
Choloepus hoffmanni  .....................................................TCTAAAAATTGCAGTTCTATCTTTTTGGTATCATTTATTTCAGAAAAAAAGCTGTTTTATGTTGGAA

Homo sapiens         AAGACAGAAAACAAAATCGTTTGGTAATTGTTTCAGAAGAGGAAAAAAAGAAAGTCTTAAGAGAATGCCATGAAAATGACAGTGGAGCTCATCATGGTATATCCAGGACCCTCACTCTGG
Choloepus hoffmanni  AAGACAGAAAACAAAATCGTTTGGTAATTGTTTCAGAAGAAGAAAAAAACAAAGTCCTAAGAGAATGCCATGAAAATGACATTGGAGCCCATCATGGCATATCCAGAACCCTTACTCTAG

Homo sapiens         TAGAATCCAATTATTATTGGACATCTGTGACCAATGATGTCAAACAGTGGGTATGGCTTATGTATTTAGAGTATTATGAATAATGTCTTTCTGATAGATACTTAAAATTTATTATTTCTC
Choloepus hoffmanni  TGGAATCCAGTTATTATTGGACTTCTGTGACCAATGATGTCAAACAGTGGGTATGGCTTATGCATTTAGAGTATTTTGAATAATGTCTTT------AATATCCAAAATTCATCAATGCTC

Homo sapiens         CAGATTTATTCTGGGTATCACCTCTCATTAAGTATTTTTGGC----------AATCACTTAAAGGGAGTTGTATTTCCCTTCACAGTTAT---TTTAAATCACTTAAGTATAATTTGAAC
Choloepus hoffmanni  TAGATTTAGTCTCA--------------TAAGTACTTTTTGCCTCGGAAGTTAAACATTTAAAGGAAGCTTTATTCCCCTTCACAACTCTTCATTAAAATCATTAAAGTATAATTTGAAC

Homo sapiens         AGGAAAGAAGCATTATTTAAACA----------CTTGAAAATACCATATTTAAATAAGAGATTATTTAAAAAATCAGATTTGTATCATTGAACTTTTTTGTGTAGGCAGAATTTAAATGG
Choloepus hoffmanni  TAGAAAGAATCACTAATTAATCATATGAACAGTCTTGAAAATACCAGATTTAGATACCTTTCTAAGTATAGAACCAGATTTTCATCGTTAAACTCCTTTGTGTAGGTAGAATTTAAATGG

Homo sapiens         AGCTGGAATTCATTTGACTAAATCATTACTTCTTCAATGGCAGTCATACAAGGTTTTCTTAAGGGGAGAATATGTTCATAGTAAGTCAAGCCCAGTATGTGCTCTTATCTTTTGTGCCTG
Choloepus hoffmanni  GGCTGAAATTCATTTGACTAGATCATTACTTCTTTGATTACAGTCAGAAAAGCTTTTGCTTGAGGAAGAATATCTTCATAATAAGTCAAGCTCAGTGTGTGCTC--ATCTTTTGTGACTA

Homo sapiens         TTCCATTTTGAGGTAG-AATGTCATACATGACAGAAACCCAAAACCAAATATAACCAAAATCAAATTTTGAGGATAATGAAATAAGTCTGAGTTTTGTAACTTTATGACCCATTGGGACC
Choloepus hoffmanni  TTCCACCTTAAAGTAGAAATATCAAATATGTCAGAAATCCAAAACCAACTAAATGTAAAATCAAGTTGTGTTGATAAAGAAATGAGTCTGATTTTTGTAACTTTATAGCCCCGTGGGACC

Homo sapiens         TTCAA---CTTCTAGTAGAAAATTCACCTATAGGAATTTTGGGGTTCTTGCTGGAAG-TCAGCTGAT-TCTACTCTTTCCAGTGTA--CTGTATGTCAAATGGTAGGAATTCCTTGGTAT
Choloepus hoffmanni  TTCAATTTCTTCTAATAGAAAATTCACC--TAGGAATTCTAGGGTTCTTGCTGGTGGAAAAACTGATCCCTATTCTTTCTAGCATACTCTGTTTGCCAAA-GGAAGGAATTCCTTGGTAT

Homo sapiens         ATCTGTGAGATCTCCTTAATAATTGTAGTAGTACATCAAAAAGAGTGGCATTTCAAGCATAAAGAATTAAACCAAAATTTCA-TAAGTATAATTTGTGAGTTATCAGTTTGTGGGTCTAT
Choloepus hoffmanni  GTAAATGAGAGCTTTTTAATAATTTTAATAGTGCATGAAAAAGACTTCCATTTCATCCATAAAGGATTAAACCAACATTCAACTAAGTATCATTTATGAATTTGCAGTTCATGAATCTGT

Homo sapiens         TCAGGTATTCCAAAGACCTTCATAATTTATATATTTTTCC------TGCTGTGCATTTTTTACAAACATTCAGTGAAATCTTTGTCAAATTGTTATCACTGGAGAATGAATATGATTTTT
Choloepus hoffmanni  ATAGGTATTCCAAAGACCTCTAT-ATTTGGATTTGCCTCCAGATCATGCCACATACTCCTT---TACATTCAATGAAATCTTTGTCAAATCTTT-TTATTTTAGAATGAATACATTTTTT

Homo sapiens         GG-AAATAATCAGAAGTTATTTGTGATAAAATCCAACAAATAAGTTTAAATTGGAAAGTGAAAATTTTGATCAAAAGTAACTGTGACTATAATGGGATTAAATGGCTTTTAAATTTTTCT
```

```
Choloepus hoffmanni  GGAAAATAATCAGAAGTTATTT..................................................................................................

Homo sapiens         TCAGAAGCTAGTTTGAAAGTAGTTTCTAGAGTGGAATTCCAGAGATACATTGAGAAATGGCACCCATCCATAAATTGATCAGTTGATCTTGTCTCACCTCACTCAAATGAAAGCTGCAGG
Choloepus hoffmanni  ........................................................................................................................

Homo sapiens         ACAGCATGGGTTTTGTGTATGTTTATTCTTTTCCCACGATTGAGAGAAATTGCTCCATCTATACATTTTATATGAATGAAATTATATTTCAATAAATATGGTTGGAATAGATTAATGAAT
Choloepus hoffmanni  ........................................................................................................................

Homo sapiens         AAGCTTCTTAATGTGATAACTTTAGAAGGAAACATTTTATGTATATAGAAATTATTGTAAATTTACATACTGATTTAATAGGAAACTTAAGATCTTTTTAAGTATTTAATAAATATTTAT
Choloepus hoffmanni  ........................................................................................................................

Homo sapiens         TGAATGTGAACCAGATTTATGTTCCATGTGAATAAGACCCAATAAGATGGTCATTTGGTAGTATAGTCTTCCCTCAGTATATGCAAGGGATTGGTTCCAGCACCACACTCCTGTCACCAA
Choloepus hoffmanni  ........................................................................................................................

Homo sapiens         ATCTACTCATACCGAAGTCCTGCAGTCAGCCCTGTGAAACTCACATATACAAGAAGTCAGCTTTCTGTATACTTGAGTTTCTCATCCCTCCCTATCAATAGAGTACTTTTTATTTGTGTT
Choloepus hoffmanni  ........................................................................................................................

Homo sapiens         TAGTTAAAAAAAAAAAGCCATGTATAAGCTGACCTTCACAGTCCAAACCCATGTTGTTTGAGGGTCAGCTGTAGTGGCTACTACAGTTGTTTGTTGTTGTTTTATTTGTTTGTTATTCTA
Choloepus hoffmanni  ........................................................................................................................

Homo sapiens         AATAGGTTTAATCCAGTGATTCTCAGTCCAGAAGTTTCTCCTTCAAAACACAAAGTATACCTATTAACCATTTTAAAATTCAGATTTATAAGACTGCTTTTATATTGTGTTGAGTGTTAA
Choloepus hoffmanni  ........................................................................................................................

Homo sapiens         CATTTGTATGTGTTTTCTTTATTGTGTGCCTGTTGTAGGTATATGCTTGTCAGCATTGCCAAGTGGCAAAAAATACAGTTATTGTAGCACCGAAACAGCACCTTCTCAAGGTGGAAAATC
Choloepus hoffmanni  ..........................................ATGCTTGTCAGCATTGCCAAGTAGCAAAAAATACAGTTGTTCTAGCACCTAAACAACGCGTTCTCAAGGTGGAAAATC

Homo sapiens         CATGGAGTTTAGTTACTGTTGATCTGATGGGGCCTTTTCATACAAGCAACAGAAGTCATGTATATGCTATAATCATGACAGATTTGTTCACCAAATGGATTGTGATTTTGCCTCTATGTG
Choloepus hoffmanni  CGTGGAGTATAGTTACTGTTGATCTGATGGGACCTTTTCATACAAGCAACAGAAGTCATGTATATGCTATAATCATGACAGATTTGTTTACAAAATGGGTTGTGATTTTGCCTCTATGTG

Homo sapiens         ATGTTTCAGCATCAGAAGTTTCTAAAGCTATTATCAATATATTTTTCTTATATGGACCTCCTCAGAAAATAATAATGGACCAAAGAGATGAATTCATTCAACAGGTAAGACAAATAAACT
Choloepus hoffmanni  ATGTTTCAGCATCAGAAATTACTAAAGCCATTATCAATATATTTTTCTTATATGGACCTCCTCAGAAAATAATAATGGACCAAAGAGATGAATTCATTCATCAGGTAAGAC----AAACT

Homo sapiens         ACTTAGGTCTGGGAGCATATCTTACTT-CTTTTCAGTGTCCAGAACCAGTGCCCTCTGCATTATATTACAGAGTGCTTAATAAGTTCAGATAAATTTCTGTAGGTATAGAGGTATGATAT
Choloepus hoffmanni  ATTTATGTCTGAGAGCATATCTTACTCACCTTTCAGTGCCCAGAACAAGTGCCTTGTACATAAT------GGGTACTTAATAAGCTTAGATAAA-TGCTGCAGTTAT-GAGATTTAATAT

Homo sapiens         TAAATACTACAAGGATAGTTGTACTGGAAATTAGGAGATTAGGATTTTAATACTTACTG------TGGGTTATTGTAAACCTATCTTCAGATATCCTCAAATTAAAATGAGGAGGTTGGA
Choloepus hoffmanni  ---ATAGTACAAAAGTACTTGGACTGGAAATTAGGAAACCAGGATTTTAATACTTGTTTTATCACTAAGTAATTGTAAAACTATTGTCAAGTTTCCTCAAACTAGAATAAGGAGGTAAGA

Homo sapiens         CCAAATAATCTTTATTTACTT-CTAGATTAAAAACCCATGATTGGTTGGGCCTGGCTCATGCCTATAATCCCAACAGTTTAGGAGGCTGAGGTGGGTGGATCACTTGAGCTCAGGAGTTC
Choloepus hoffmanni  TCAGATAATCTTTGTTTCCTTAATAGCTT...........................................................................................

Homo sapiens         GAGACCACCTGGGCAATAGGGCGAAACTCCATCTCTACAAAAAATACAAAATTTAACTGGGCATGGTGGTGGGCGCCTGTAATCACAGCTACTCGGGAGGCTGTGGCAGGAGAATCACTT
Choloepus hoffmanni  ........................................................................................................................

Homo sapiens         GAACCTGGGAGGTAGAGGTTGCAGTGAGCCAAGATTGCACCACTGCACTCCAGCCTGAGCGACAGAGCGAGACTCCATCTCGAAAAGAAAAAAAAATTAGCCAGGTGTGGTGGCACACAC
Choloepus hoffmanni  ........................................................................................................................

Homo sapiens         CTGTGGTCCCAGCTACTCGGGAGGTTGAGATAGGAGGATCACTTGAGCCCGGGAGGCGGAGGTTACAATGAACTGAGATTGTGCCACTGCACTTCAATCTGGGCAACAGAGTGAGACCTT
Choloepus hoffmanni  ........................................................................................................................

Homo sapiens         GTCTCAAAAAATAACAATAATAATAATAACACTATGATTGACAGGTAAAGGGAGACTTAAATAAATGGAAGAATACATTTTCTTCATGAATAAGAATAGTCAGTATTGAAAGGTACT--T
Choloepus hoffmanni  ........................AAAAAATCTATGATTTACATATAAAGGGA-ACTTGTCAAAAAGGAAAAACAAATCACATTCATAAAAGAAAATAATC----CTGAAAGAAACTGAT

Homo sapiens         TCTCCCTTGCTC-----------------------------------------TGGCCTTTTCCCAGTTAAAATTAACCTGAAAGGATAAGCAGGGGAGAATATATATGAAAACTTTTTAT
Choloepus hoffmanni  TCTTCCTAAATCAAAATCAATTTATAAAAATAATTCTAATTAACATTTAAGAGAGCTTTTTCTTATTTAAAATTCACTTTGAAGAATAAGTAGTCATGAATGTATTTGAAAATTCTAAAA

Homo sapiens         AAGAAATACAACAACTGCTAGACTTAAGGCATATGTTAAAACTTTTTGTAATTGACAGGGTATATGTTTAAGAAAAATCAGAGATAGCCAGACTTGGTTGAAAGCATCTTTGATGGCTCA
Choloepus hoffmanni  AATAAATGTAATGACTACTAGAC--AAGATATATTTTTAAA-TTCTTATAATTGACAAGGTACTGGTATAAGAATAAACAGAGATAGC------------ATGGTACCCTTATTGTCTTC

Homo sapiens         GTCCAAAAGAAATACCTAAAGTTAATATGTATTAACT-AGTAGATAGGACATGTGCCATGGCACCCCAGGATTATTCCCAGTTTCCATAATTCTCTAGG---------AGGACTCAGCAT
Choloepus hoffmanni  ATCCAAAAGAA----------TTAAGTTGTGTTTATTAAGTAGTTAGGTCCTGTGTTGGAAGTCCCCAAGATCACCCCAGGTT----TGATTCTCTGGGGGGATTTACAGGACTCAGCAT

Homo sapiens         ATAGTCATACTCATGGCTAAGATTT---------------------------ATTCAGCAAAGAGAAAAGACACATTG-----------GGAGAACCAGGTACAAGTTTCCAAGATCTCCT
Choloepus hoffmanni  ATAGTCATACTTATGGCCAAGATTTTATTACAGTGAAAGGATACAGACCAAACTCAACAAAGAGAAAAAGCACATGGGGCAAAGTCCAGGGAAACGAGGCAATAGCTTCCTAGA-AACCT

Homo sapiens         TCCCCAGTGGAGTTACACAGGACGTGTTTAATTCCTTTAGCA--GAGTTGTGACAACATA-------------------AGGAAGCTCACTAGAGTCTCAGT-GCCTAGGGTTTTTACTG
Choloepus hoffmanni  CTTTCAGTGGAATCATACAGTGTACTCTTGATACCCCCAGCAATGAGTTGTGGCAACATGTGTGAAATGTTGTCGATCTGGGAAGTTCATTTAAGATTCAGTAACCCAGAGTTTTTATTA

Homo sapiens         GGCGCTGGTCACATAAGCACCTTCTACCTGG---CATGTACAAAAATTCCAGACTCTCAGAAGGAAAGATTGTGTTTGGTCTTATAACCAAAATCTAAGCTCCTAGACATTAGCCAAGGT
Choloepus hoffmanni  GAGGCTGGTCACATAGGCACCCTCTGCCTGGCATCATGTCCCAAAATTCAAGACTTCCAGAAGGAA.....................CCAAATTCCAAGTTCCTAGATGCCAAACAAGGG

Homo sapiens         CCAACCTTG-TAAGCATGCCTTTCAAAGAACAGCAGTCACAGATCTGCTTTGTTAATATGTTAAAATTAACTGTCAGGTTAACTCTTTTCTTCACAGGTCCATCCAAACAACTTAATAAG
Choloepus hoffmanni  CCAAACTTATTAAGCAAACTTTTCAAAGGATAGCAGTCCCAGGTCTGC--------------------------CATCTTAACTTTTTTCTTCACAGG----TCCAGACAACCTCATAAG

Homo sapiens         TATTTATGTTCTAACCACTTAGTAGCAATTTAAAATACTCATAAAT--TGAAAGAAAATATATTTTTGTTCCATTCTTTTCTTTTTTTTTGAGACGGGGTCTCATTCTGTCACCCAGCCT
Choloepus hoffmanni  CATTTATGTTTTAACT-TTTAGTAACCATTTTAAAAATTAATATATACTGAAAGAAAAAAATATT.......................................................

Homo sapiens         GGAGTGCTGTGGCACCGTCTTGGCTCACTGCAACCTCCACTTTCCAGGCTCAAGTGATACCCCCACCTTAGCCTTCTGGCTAATTTTTGTACCTTTTTTCTTTTAGAGACAGGTTTCACC
Choloepus hoffmanni  ........................................................................................................................

Homo sapiens         ATGTTGCCCAGGCTGGTTTTGAACTCCTGAACTCACATGATCCACCTGCCTCGACCTCCCAGAGTGCTGGAATTACTGGCGTGAGCTACCGCACCCAGCCCTTTCATTTCATTCTTAACC
Choloepus hoffmanni  .........................................................................................................ACTTCCTTCTTAAAC

Homo sapiens         ATAATTACTTGCTAATGGGTAATGTGTACCTGTTGACCACTGCTTAACTTCTCAGACCTTGGGATCATATTGTACACTGCCACCCTCATTTCCTGTTTCACACTGATTTTTACATAGTAA
Choloepus hoffmanni  AG------TTAATAACAAAATATGTAAACCTATTGAGCACTGCTCACCTTGTCAAACCTT-AGATCAGATTGGACGCTGCCACTCTTATTTCCTGCTCCACATTGATTTTTATACAATAG

Homo sapiens         CTGCTTTTTATCACAGCATTCGATGAAGAAAACAGTTCTGTAAAGATATGATGTCATCAAACAAATTGTAACCTGATCTAATGTTGAAACTGAACTACCTTAAACTGGTAGTTTTCATGG
Choloepus hoffmanni  TTAATTTTTATCACAACTTTCACTGAA-AAACCAGTTTTGCAAAGATACAACATCATCAAAAGGAATATAACCTTATCTAGTATTGACACTGAGCTACCTCAAGCTAGTAATTTTCACTG

Homo sapiens         TGTCAAACAGATGTTAAGGGGGCAACTTGGCACAAAGTTTGGTAACAATGGTTTAAATGATAAAGCTAGTGTCACAAATGTGTCTAGTAGAGGAGGATCATGTAATTAATTAACAGGTAA
Choloepus hoffmanni  TTTCCCTCCTATGTTAGGGGGACATGTTGGCACAGAATTTGGTAAC..........................................................................

Homo sapiens         CAATTTGCTAATTGTTTAAAATTATATCTGTAATATGATTTTAGAATCCAATACCAAAAGTATTTTCAGGTAAATTAAAAGAATTTTAATTTTTAAAGTAGAAACAGAAAAAAAATATATT
Choloepus hoffmanni  ........................................................................................................................

Homo sapiens         TGACATTTAATCTCTAGAAATGGAGGTCACATTCTAAACCTAGGAATAGTGGTATAAACTTCAATGGAAAAACATTAAATTTGGCTGCATAATAATAGGAGATTTATGTGGTAAAGTAAT
Choloepus hoffmanni  ........................................................................................................................

Homo sapiens         CAACATATAAAAGCCAATCTTGCAAATAAAGCAAGATAATTTTGTAAATGAAGGCAAGGTAGTCTTGCCTGCCTTGATTGACATAGCAGTGGTCTGAGCAATGGAGTACGTAAAGGCCAT
Choloepus hoffmanni  ........................................................................................................................

Homo sapiens         CCAGTTTGGGGGAAGGCTATTGGAACTTCTATTTATATTTTTTAAATCCCAAAAATAAGAAATGAAGCTTTATTAATAATGTATACGATTGATAATAGCATTCTGACTCAATCAACAAGA
Choloepus hoffmanni  ........................................................................................................................

Homo sapiens         CATGTATGTCACACATGCTATGTGAAGGTATCCTGAAAGAAAAGTGAGAATCCACATGTTTCCTCACCCATACTTTTGCTTTCAGCAGTTGAATGAAAGTATGGATATCATCTAGCATTC
Choloepus hoffmanni  ........................................................................................................................

Homo sapiens         ACCACACTACTCACAAAATGGCCAGGGAGCTTAGATTTTTGCAGTGAAAGCAGATAATACTAAAAGGCAAACACCCATCATCAACCACAAGAAAGTGATAGCGCCGGCACGTCTATTTAA
Choloepus hoffmanni  ........................................................................................................................

Homo sapiens         CTTCCAGCTCTTTGTTAGACACATCAATGAGATGAATACAAATATTAACTACAATAGTCCCCATTTATCTGCAGTTTTACTTTCTGAGGTTTCAGTTCCTATAGTCAACTGCAGTCCAAA
Choloepus hoffmanni  ........................................................................................................................

Homo sapiens         CATATTAAATACAAAATTTCAGAAATAAATGATTTAAAAGTTTTTTAATTGCACACCATTCTGCGTATAGTGATAAAATCTCATGCTGTCCTGCTCTGTCCAACTGGGACGTGAATCATC
Choloepus hoffmanni  ........................................................................................................................

Homo sapiens         TCTTTGTCTAGCATATCTTCATTGTATATGCTACTCTCTCCCCATCATTAGTCACTTCATAGCTGTCTTGGTTATCAGATCAAATGTAGTGGTTAGTAGTGTGTATCCAAGTTACCCTTA
Choloepus hoffmanni  ........................................................................................................................

Homo sapiens         TTTTATTTAAGAATGCCCCCAAAGTGCAAGAGTAGTGATGTTGGCATATTGTTAATCGTCCTATTTTATTTTTAGTTGTTAACCTCTTACTGTGCCTAATTTAAAAATTAATCTCTATCA
Choloepus hoffmanni  ........................................................................................................................

Homo sapiens         TAGGTATGTATGTATAGGGGAAAACATAGTATAAATAGGGTTAGGTACTACCTGCAGTATCAAACATTCACTAGGGGCCTTGAAACATATTCTCTGAAGGTAAAGGGAGACTATAGTTAT
```

```
Choloepus hoffmanni ........................................................................................................................

Homo sapiens        CAGATCTGATAGGAAATTAGGCATCAAAATTCAAAATTATCAAGAATACTCTTTGAAATATCAGTTCACTTCCACTTTTATTGATGGAGAGCCTTACCCTAAGTGCATATAATGTTTCAA
Choloepus hoffmanni ........................................................................................................................

Homo sapiens        GGTATTTGCTAATGATAATATGAAGCCATTATGATTAGCTAGCAGTTTTTCCAGTCGTGTTTAAAGTCATTTGATAACCAAACCAGGACTTTGAAAATTTTTCTAAAGGTAACAATAGGG
Choloepus hoffmanni ........................................................................................................................

Homo sapiens        CCAAGCATGGTGGCTCATACCTCAGCTTTTTGGGAGGCTGAGGTGAGAGGATAACTTGAGACCAGGAGTTCAAGACCAGCCCATGCAATACAACAAGACTCCGTCTCTACAAAAAAAAAA
Choloepus hoffmanni ........................................................................................................................

Homo sapiens        AAAATTTTTAATTAACTGGACGTGGTGTTGCACACTCATAGTCCCAGCAACTCAGGAGGCTAACGTGTAAGGATTGCTTAAGCCCAGGAGGTGAAGGCTGTAGTGAGCTATGGTTGCACC
Choloepus hoffmanni ........................................................................................................................

Homo sapiens        ACTGCATTCCAGTTTGGGCAACAGTGAGACCCTGTCTCAAAAAAATAATAATAATAATAATAATAGGCTGTGCTCAGTGGCTCATGCCTGTTGTAATCCTACCAGCCAGATTGCTTGAGC
Choloepus hoffmanni ........................................................................................................................

Homo sapiens        CTAGGAGATCGAGACTAGCCTAAGCAACATGGTGAAATGCCATCTCTACAAAAAATGCAACAATTAGCCAGGAGTGGTAGGGCACACCTGTAGTCCAGCTACTTGGGAGGCTGAGATGGG
Choloepus hoffmanni ........................................................................................................................

Homo sapiens        AAGATCGCTTGAGCCCAGGAGGTTGAGATTGCAGTGAGCCAAGATCATGCACTCTAGCCTGGGTAACAGACTGAGACCCTGTCTCAAAAAAAAAAAATAAAAAATAAAGGGTAACAATAA
Choloepus hoffmanni ........................................................................................................................

Homo sapiens        ATATTTGCAGGCCTATTTTGTGAGATTGCTTAAACAAAAAACCAAAAAAGCGACATATGATTGGGAAAACTTGTTCTTTTTGCTGTGATTAAAATGGCTTCACTATTACACAGAAAGCAT
Choloepus hoffmanni ........................................................................................................................

Homo sapiens        AACGATGACAGTACAAAATGAACTCTTTTGTCAGCAAATACTGATGCAGAAAACACTGCTAAATATTATAACATAATACACAATGTGGGAGGTTTATTGTATAGTTGAATAAAAATACAG
Choloepus hoffmanni ........................................................................................................................

Homo sapiens        ATGTTTCTAATGGCTTAGCTTAGAGTATTTGCTAAATTGTTCCAATAAAATGCACAAAGAACTTTTTGTAAACCTCTGAAGGAAAGAGAACAAACATATTCAGTAGGAAAATTAATTTTT
Choloepus hoffmanni ........................................................................................................................

Homo sapiens        TTAAATGTCTTACAGAAAACTAAAATTGTAAGAACTGACGGAATATCTGGTTTGATGGTTGGGGGGCTGACAGAGTAAGGATGTCAAGATACCATTTCACAAATGGATTCCCTGGATCAT
Choloepus hoffmanni ........................................................................................................................

Homo sapiens        TCCTAGGCAAGCTTTTGAAACAAAGAAGTTAAAGCCTTTTTCAAGGGTCATTTCAAACCATTAATCAAGACTCAGATTGAAGCTGACCCAAATGACACCTATTCTTTGGGATTTCTTTCT
Choloepus hoffmanni ........................................................................................................................

Homo sapiens        AATTTTGAATTAAAAATAGTCACCCTGAATTAGTATGTATATCAGCTAGGAAGTGTATTCAATCCAAGTAACAGACTTCCTTTCCCTATCTCAATATATTAATATATATATTGTATATATAT
Choloepus hoffmanni ........................................................................................................................

Homo sapiens        TATCCCAATATATATTGGGAGTGATTGAGAGTCACTCCTATTGTATTCACAACGTGGCTGCTGGAGCACATGCCACAATGCCTACATTTCAGTCAGAAAGAAGGGAGAGAGTGTTGGGCA
Choloepus hoffmanni ........................................................................................................................

Homo sapiens        AACAAAGCTAGTACCAGCTGAGTTTGCCTAACAACTTTTACTTACAACTTTTTGGCTAGAATTATGTCACATGACCACCCCTACCTGCAAGGGAGTTTGGGAAATGTAGTTTTCTTTTTA
Choloepus hoffmanni ........................................................................................................................

Homo sapiens        TTGCTTTCCCCTCTTGTATTAACTTTGCCTATTTAGTTATTCTGATACTACTCGTCTGAAATATTCTTTTTTAAAAAGACAAATTTATGAGCCAAAGGTTTACTCAACTAAAAATAATAA
Choloepus hoffmanni ........................................................................................................................

Homo sapiens        GGAAAAAAAAAGTCTGAAAGGTGAGGTAGGAAAAAAATATTATTTTATATTTAATGGGTTTACTGTTAAAAGTTTTGTAAACTCATTTTAAGTGCTACACATAATATAGTAACACCAATA
Choloepus hoffmanni ........................................................................................................................

Homo sapiens        ATAGTAGTTATAAGTTAAAAGTTATAAAATGCTTATTGCCAATAACTGTTTTACATGCTTTTTAAACATTAACTTATGACCTAGTGGGTTGTTTTTGAGACACTGTCTACTGAATTAAAA
Choloepus hoffmanni ........................................................................................................................

Homo sapiens        AGTTACTTGAGGGCCGGGTACAGTGGCTCATGCCTGTAATCTCAAAACTTTGAAAGACTGAGGTGCATGGATTGCTTGAGTCCAGGAGTTTGAGACCAGCCTGGGCAACATGGTGAAACT
Choloepus hoffmanni ........................................................................................................................

Homo sapiens        CCATCTGTACAAAATATTTAAAAATTAGGTGGGCATGGTGACACATGCCTGTAGTCCAAGCTGCTAGGCAGGCTGAGGTGAGAGGATCGCTTGAACCCAGGTAGTGCAGGCTGCCCTCCA
Choloepus hoffmanni ........................................................................................................................

Homo sapiens        GCCTGTGCGACAGAGCAAGACCCTGTCTCCCACCGCCCCCCACCAAAAAAAAGAAAAAAATTAAACTCAAAGTTAACGTTAAGTTCTGTTCTTCCATTTCCTTGTTGTATGTGTGTATTT
Choloepus hoffmanni ........................................................................................................................

Homo sapiens        GGCAAGGCACTTAATCTCTCAAAGCTTCAGTATCTTACCTTTAAAATGAAATTAATAATACCTCAAAAACTTGTAAGGTTTAATGACAATAATGTAAAGTGTCTAGTGTGATGGGACCAG
Choloepus hoffmanni ........................................................................................................................

Homo sapiens        TGTGTAGTAAAGACTCAATAAATGGCAATCACTATTTTATACATAAAATTTTACATATTTTATGTATGTGTTCATATAGAATGTTATGGTCAATAAAAAAGCAAGATTCAGCCAGGCGCA
Choloepus hoffmanni ........................................................................................................................

Homo sapiens        GTGGCTCATGCCTGTAATCCCAGCACTTTGGGAGGCCAAGGCAGGTGGATCACAAGGTCAGGAGTTCAAGACTAGCCTGGCCAATATGGTGAAACCCCGTCTCTACTAGAAATACGAAAA
Choloepus hoffmanni ........................................................................................................................

Homo sapiens        TTAGCCGGGTGTGGTGGCGCACACCTGTAGTCCCAGCTACTCAGGAAGCCGAGGCAGAAGAATTGCTTGAACCTGGGAGGCAGAGGTTGCAGTGAGCCGAGATTTTGCCACTGCACTCCA
Choloepus hoffmanni ........................................................................................................................

Homo sapiens        GCCTGGGTGACTGAGCAAGACTCCATCTCAAAAAAATAAATAGATAAATAAAAGGCAAGATTCTAATTCTTTTGAAGCTTTTTTTTAAGTCAATGGAAAAATAAAACACAAATTTTA---
Choloepus hoffmanni .................................................AAAAGACAAGATTCAAATTCTTTTTAAGCTTTTTTTTTAATCAATGGAAAAATAAAGTACAGATTTTTTTT

Homo sapiens        AAAAGCCACCTTTGTTTTAT-ATATTTTTCAGATCAATATTGAACTGTACAGATTGTTTGGCATAAAGCAAATTGTAATTTCTCACACCTCTGGAACTGTTAACCCAACGGAAAGTACAC
Choloepus hoffmanni AAAACTTAGCTTTGTTTTATTATATTTTTCAGATCAATGTTGAACTATATAGATTGTTTGGCACAAAGCATATTGTAATTTCTCATGCCTCTC-AACTGTTGATCCAACTGAAAGTACAC

Homo sapiens        CTAACACAATCAAAGCATTTCTCTCCAAACACTGTGCTGACCACCCAAACAATTGGGATGATCACCTATCAGCTGTTTCATTTGCCTTCAATGTAACTCACTTGGTATGTGCCTTTTTAT
Choloepus hoffmanni CTAGCACAATCAAAACCTTTCTTTCCAAACACTGTGCTGACTACCCAAACAACTGGGATGATCACCTATCAGCTGTTTCATTTGCCTTCAATGTAACTCACTTAGTATGTGCCTTTTTAT

Homo sapiens        AATTCTGTACTTCTGAGTTATGACT----ATTTCTTGGCCAGATAAATGTTTTATTACAAAATTGACGTTTATTTTCTTTAAAATAGGAACCTACTAAAAATACACCATATTTTCAAATG
Choloepus hoffmanni AATTTTATATTTCTGAATTATGACTGGTTATTTCTTGCCTAGATAAGTATTC-ATTACAAACTT----CCTGTTTTCCTTAAAATAGGAACCTACTAAAAATACACCATATTTTCAAATG

Homo sapiens        TTTAGTCGAAATCCTTATATGCCTGAGACTTCAGATAGTCTTCATGAAGTGGATGGTGATAATACAAGTATGTTTGCCAAAATTCTAGATGCAATTAAAGAAGCTGATAAAATAATGGAG
Choloepus hoffmanni TTCAATCGAAATCATTACGTTCCTGAGACTTCAGATAGTCTTCATGGAGTGGATGCTGATAATACAAGTATGTTTGCAAAAATTCTAGATGCAATTAAAGAAGCTGATAAAATAATGGAA

Homo sapiens        AATAAGACAACTTCACTGGGCCAGGTGATTCTATTCAATAGAAAAACTGTAATCTAAAACTTTATAAAAGTTATATTTATTTTTAAAATTTCAGCATATACATGTGTGCATGCACACACA
Choloepus hoffmanni AATAAGACAACTAGAGCCAGCCAGGTAATTCTATTATTAAGAAAAACTATA---TAA---TCAATATAGTGACCATTTATTTTTAAATTTTCATTTTATGTGTGCACATATACATACATA

Homo sapiens        CACACATACTTACTTGCCATA-----------ATTTTTTATCTATCAGATTTTTCTTGTCTTGATTAAATAGTTGGTTTACTAGCTATGAAATGCTGAGAGGCAATAATGAGAAAGCTAT
Choloepus hoffmanni CATACAGACACATATGATATACCCATAAGCAAACTATTTTTCTATTAAATTCTGCTTGTCTTGATTAGACAGTATTCCTGTTAGCTATGAAATTCAG-GATATAACAGTAAGA.......

Homo sapiens        GGATTTTGGAATCAAGTAGAGCTGGATTCAAATCTGTAGTCTTAACTGCTTTTGAGTTGTTTGACTTCGGCAAATTACTTAACCTATCTTAGTTTACCTTCTATATAAAGTAGGAGCTAA
Choloepus hoffmanni ...............................................................................TCACCCCAATCAGCTTA-----TATATAAAGT-GGAGC-AC

Homo sapiens        TAATACTGACTTTGAAAT---GTTTTAAGAATTAAATAGAATTCATACCTACCTTTTAGTAGCATCTCAAGTACCATTTTGTTACAATATTCTCATTATTTG------CATTTACCTTCA
Choloepus hoffmanni TAATAGTGACTTTGAAGTACTGTTTTAAGAATT----------CATGCCTACCTCTTAGCAGCATCTCAATAAATAATTGTTCTTAGTATT---ATTATTTGGTATATTGTTTGTCTTCA

Homo sapiens        AGATATTATAGCTCTTTCATCTCTTTGTTCTTTTTCTTTTTACACTTGT-AGATGGAGAACAACAATTTGGATGAACTAAATAAAAGCAAGATCATTGTTAAAAAGAAACCCAAACAATT
Choloepus hoffmanni AGAAATTATAACTAATTTATCCCTTTGTTCTTTCTCTTTT-GCATTTGTTAGATGGAGAACAACAATTTTGATGAACTAAATAAAAGCAAGATCGTTGTTAAAAAGAAACCAAAGCAGTT

Homo sapiens        AAATCCATTTCATTTAAAAGTGGGTCATGAAGTTTTAAGACAAAGGAAAAATTGGTGGAAGGATGGTCGTTTTCAGTCTGAATGGGTTGGTCCTTGTGTCATAGACTATATTACAGAAAG
Choloepus hoffmanni AAATCCATTTCATCTAAAAGTGGGTCATGAAGTTTTAAGACAAAGGAAAAATTGGTGGAAGGATGGTCGTTTCCAGTCCGAATGGGTTGGCCCTTGTGTCATAGACTATATTACAGAAAA

Homo sapiens        TGGATGTGCTGTCCTGAGAGACAACACTGGGGTTAGACTGAAAAGACCTATCAAAATGTCCCACCTTAAGCCCTACATAAGAGAATCCAGTGAACAAGGTAAATATCTGTCACTTCTCTA
Choloepus hoffmanni TGGATGTGCTGTCCTGAGAGACAACACTGGTTCCAGACTTAAAAGACCTATCAAAATGTCCCACCTCAAGCCCTATATACGAGAATCCAGTGAACAAGGTAAATGTCAGTTG-----CTA

Homo sapiens        CATTTTTA-TTGCTTTAGGTAAGGAGAATAGTTAAAAATAACAGAAACCTTTCTCC-------CCTCTTTTGTTAGGTTTTCTTTTCCCTTC-----------TTCATAAAATTTGTTAC
Choloepus hoffmanni CATTTTTACTTTCTTTAAATAAGGAGAAAGGCTAAAAATCCC----ACCCCCCACCACCACCACCTCTTTGGTTAGGTTTTCTTTCTCCCTCTCATTTTTATATTTATAAAATGGGGTAT

Homo sapiens        AGTTTTAGCTATCCCAGGTAGTCTGTTTTATTACAGTGTCTCTTCTGGAACTGTCAGGCCTGTGAGATTTTTTTTTGAGACAGAGTTTCCCTCTTTCTGCCCAAGCTGGAGTGCAATGG
Choloepus hoffmanni TGTTTTAGATATCCCAAGTAGTC---TTTAGTAGAATGTTTGTTCTGAAACTGTCGGGCTTGTGAGA.....................................................

Homo sapiens        CGCGATCTTGGCTCACTGCAACCTCTGCCTCCCGGGTTCAAGCAATTCTGCCTCAGCCTCCCGAGTAGCTGGGACTACAGGTGGATACCACCATGCCCAGCTAATTTTTTGTATTTTTAG
Choloepus hoffmanni ........................................................................................................................

Homo sapiens        TAGAAACGGGGTTTCACCATGTTAGCTAGGCTGGTCTTGAACTCCTGACCTCCCGTGACCTGCCCGCCTTGACTTCCCAAAGTGCTTGGATTACAGGCATGAAACAGGGTGCCTGGGCAT
```

```
Choloepus hoffmanni  ........................................................................................................................

Homo sapiens         TTTTATTTTTATTTCTAGTATGTTTTTCATAGTTTATGTGGGAGATACTGAACTTGTTTTCCTCTGAAGGAAAGATAACATCTTAACATCATCTCTGTTTTTGCTTTCTCAATGTTTTGT
Choloepus hoffmanni  TTGTATTCTGATTTCCACTGTATTTT-CATAGTTCATGTGGGAAATACTGAACCTGTTTTAC------AGAAGGATTGTATCT--------TCTCTATATTTACTTTCAGAAT.......

Homo sapiens         TTGTTTGTTTGTTTGAGACAGAGTCTCATTCTGTCCCCCAGGCTGGAGTGTAGTTGTGTAATCTCAGCTCACTGCAACTTCTGCTTCCTGGGTTCAAGCAATTCTTGTGCCTCAGCCTCC
Choloepus hoffmanni  ........................................................................................................................

Homo sapiens         CAAGTAGCTGGGATTATAGGCACATGCCACCATGCCCAGCTAATTTTTTTGTATTTTTGGTAGAGATGAGGTTTCACCATGTTGGCCAGGCTGGTTTTGAACTCTTGGCCTCTAGGGATT
Choloepus hoffmanni  ........................................................................................................................

Homo sapiens         TGCCTGCCTTGGCCTCCCAAAGTGCTGGGATTGCGTGCATGTGCCATTGCACCTGGCCTTACAAATGTATTAGTCACTTCGTGCCTCTTTTTGGCATGAAAAAC-AGCAACTGTGATCAT
Choloepus hoffmanni  ...................................................................TATTAGTCACTTTGTCTCTCATTTGAGCATGAGAAACCAGCAACTAAAATCAT

Homo sapiens         TAATGCCAAAAACAAACATACTGTCCAGAATCTAGTTGTGAGATAAATAT--TAGGGTACTTTAAAAATGACATTAATTTTTAATATTGCTTTATAGTTAGGTCATGGTTGGGGGCCAGG
Choloepus hoffmanni  TAATGCCAAAAATAAACATACTGTCCAGAATCTAGTTTTGAGACAAATATATTAGGGGACT----AAAAAATATTAATTTTTAAT-------TATAGGCAGGTCATGGAGTGGGGACAGA

Homo sapiens         TGTGGGTGCAGAGTGGGGAATCCTCAGAATGCTATAAGGTGCTGTAAGAAGAGGTGACAGCACTCTAAACAAGTTATATTTGCCCCTAAATTTGACTCATTCTGAACAGTCAAGATACCC
Choloepus hoffmanni  TGAGAGTGTAGGGTGGGATTTACTCAGAATGCTATAAAGTGCT-TATAAGATGGTGATAGCACCCTAAAGAGGTTATATTTGCCCCAAAATGTGAATGGCTCTGA-CAGTCAAGATATCC

Homo sapiens         T-------------------------AGTAAAGTGATGT----TTTTGCCATGGCTTTTAGATTATGTTCCCCACTTTATCAAGGCTTTTATTTTGTCCTAATGTGTCTGCTTCCTAGA
Choloepus hoffmanni  TGATCTTTGTAAGCCTTTCTTTCATAGAGTAGAGCTAAATGACATTGGGCCATGACTTTCA-ATTAG----CTCACTTTATAACGACTTTTATTT-GTCT--GTTTATCTATCCCCTAGA

Homo sapiens         TCTTCTCTGAGGTGGATAACTGAGAGAGAGTTAATGAGTAACTTAGCCAG---------------------------GTGAAACATTAATATTATTTGGATATAAGTAACATTTAATGG
Choloepus hoffmanni  GTTTCTCTGAGATGGATAACTTGGGG-GAGTTAACTGACACTTTAACCAGTTAATTGTACTTTTTCTAAAAAGCAAAGATAAAGCATCATTATTATTTGAAATTGAGTAACATTTA-TGG

Homo sapiens         AAAATATA------------TTTATAACAATATAAACAA----------GTTATAT-GACAGGCTTTAGCAAATATTAATAACAAAAAGAGTAACAACAACAAATAACCTACACTTACC
Choloepus hoffmanni  GAAATACAATATAGTAATTTTTTTTAAAGAATATGAACAGCAGCAACTAGGTTATTTTAACAGGCCTATGTAAATT---ATAACAGTAACGACAACAATAAGAAATAACTTTCACTTACC

Homo sapiens         CTATGCCTTTTCTTGAGTTCTAAAGCCCATGTGGGCTGCTTAGTAATGATAGCTCAACTCTGCTTCTAATCTGGGTTACAACACTCATGTTAAAACCTCTTAGTACCTTTCCTTCT---T
Choloepus hoffmanni  CTATACCTTTTTTTTAGGGTATGAACCCCTTGGGGCTACTCAGT---GATAGCTCAACTCTGCTTCTGATCTGGGCCATAATCATGGTGTTAAAAGCTCTTAGTATCTTTCCTTCTGCTT

Homo sapiens         GTGACAGACCGAGTTGTT-TAGTCAGTAAACTTATCTTGAACTACATTCTAATCCTGAAAACTCACAAGTGCTTTCGTTCTCTTTGTTAGAAGATAAAAGCTTGCATTGTTTGTACCTCT
Choloepus hoffmanni  GTGACAAAGTCAGCTGTTCTAGCCAGACAATTTATCTTCGATTGTGTTCTAATTCTGAAAACTCACAAGTACCTTCAGTTTCTTTGTTAGAAGATAAGCGCTTTCATTTCTTGTGCTGCT

Homo sapiens         TCTCAGTTCTGAGCTACCTTTAATGATCTCTCTCTTCTGCAAGTTAAGTAGGGAAAAAAAAAGGTTATACAAGTATATGTTGTTCTAGCTTCTTTAGCATGATTTTCCCCTTAATCTTTGA
Choloepus hoffmanni  TCTCAGCTCCAAGCTACTTTTTATAATCTCTCCCTTGTACAAGTTAAGTA--GAAGAAAATGATTGTACAGGTACATGTTTTCCTTTCTTCTTCAGCATGATTTTCTCCTTAATCTTTGG

Homo sapiens         CATTCATGATTTGCTCTCTTCTAAAGTGTTAAGCTTCT----TTAATACAAAATTTA-ATTTAAAATTTTGCTAAACCAGGATCTA--TCAACATTTTGTAATTTGTTCTTGTCAAAGAG
Choloepus hoffmanni  CATCCAGAATTCATTTCCTTCCAATTTGTTAACTTTCCCTAATTAATATAAAAGTTATATTTAAAGTGTTGCTGTATCAGGATCTGTTTCAACATTTTGCAATTTGCTCTTGCCGAAGAC

Homo sapiens         TTTCTCAGAAAAGAAGTCAAATATTGTGCTTCTTCTGAACATTCAGAACTCAGTATTTCAGAACTCATCTTCTCTGTTTGAGAACAGTCTCTAAACAGAGGCAGTATAATATTTGCCTCA
Choloepus hoffmanni  TTTCTCAGAAAGACGACCAAATATTTTGCTTTACCTG--------------AGTATTTC---------------------AGAACAGTTTCTAAACAAAGGCAGTATAATTCTTGCCCTA

Homo sapiens         TACTTGAATTCTTTGCACAAGATTCTTCCAGTAATATAAGGACAAATAATATTGCAATATGACTACTGGTGTATGCAT-AAGGAATTTAAGACTAATTAGATGAGTCTGTTTTAGTTTTT
Choloepus hoffmanni  TGTTTGAATT-TCTGCATATTTTTCTTTCTGTAATATAAGG------AATTTGTCAATATTAATACTGGTGTACACATAAAGGGACCTGGGATTTTACGGGTGAATTGGTTTTCTTTTTT

Homo sapiens         CTTGTTAACTAAAGGAACTACTGATCTGGCCATCCTAGAATCTGGTT----CTTATTGCTTGATCCTTTTTTTACATTTTTCTCATTTGTAGAAATTAAAAGTAAATGTGATTTCTGCCA
Choloepus hoffmanni  TCTTTTGACCAAGGGAAATGTTTATCTGTCCATCCTAGAACGTGGTTGCTGATTACCATTTGGTCCCTTTTTTGTGTTTCTCTTTTTGATAGAAATTCAAAATAAGTGTGATTTCTGCCA

Homo sapiens         TCTTTTTCCTCAATATTACATACTTTCTTGAAAATCTTTTCCATGATTTTCTTGAAAATCACCTCACAAGTTGATTTGAAAGAGTCCTTGAGCTGAAT-TAAAGCAAACAAAATGATTTT
Choloepus hoffmanni  TCTTTTTCCTCAGTATCATGTATTTTCCTGAAAATCTTC---------------------ACTCACAAGCTT------AAGAGTTTTTGAGCAGAATGTAAAGCAAACACATTGTCTTT

Homo sapiens         GACTAATCATTGA-AGTTTTTATTTGACATAAATTTTGACATGCTATAGCCAAAAGAACTATAAATTTATTTGTCCACATTTGTCATTGCTGCTCTTTGCTAGCCTTGAAATTTCATAGC
Choloepus hoffmanni  GAATAATCATGCACCTTTTTTATTTGGCATGAATTTAGATGAGCTGAATCCAAAAGAACTA-GAATTTGTGTGTGCACA--TGCCATTACAAC----TACCAACCTTGAGGTTTCATAAC

Homo sapiens         TCTGTTAAAAACTCACTGTCTAAAGTAGATATACTTCTATTGCCAACTTCTTATCCATTCTTATCTAGAACTAAATGTAGAGTGAGCACATTCAGTACTAGGTATACAATATTTTTCATT
Choloepus hoffmanni  TGTATTAAAGACTTCCTGTCTAAAGCAGATATGCTTCTGTTGCCAACTTCTTATCTATTCTTATCTAGAGTATATTGTAGAGGGAGCA----CAGTACTGTCTATA----------ATT

Homo sapiens         GCTATTTTAAGCAGGCCTGTTTGTTTTTTTGTCTAGTTCATTGCCAAGCGCTTTTTCAGACT-------------ATTTAGACCAAGAAATCTTA--AAATACCATAGTGTCTTTTTGT
Choloepus hoffmanni  ACTATTTTAAGCAGGCCTGTTTGTCATTTCATGTAGTTCAATGCCAAGAACCTTTGCAGACTCATTATTTTAATGAAATCACAACAAGAACTGTTATGACATACCATAATGTCTCTTAAT

Homo sapiens         AATCATAATGCTCCTCTTTGAAAGGAAAAAATAAT--------------------------CTGACATAGATTTTGCACAGTTTTGAATTTATTTTGT------------TTGTGATA
Choloepus hoffmanni  AATCATAATGTTCCCTTTTGAATGGAAAAACAATTCAGTGGCTCTAATTACATTCACAGTGCAGACATAGATTTTGAATAATTTT---TTTGTTTTGTTTTGGTGGAGGTATTATGATA

Homo sapiens         GATTTGGATCTGCCTCAC--CCAAGGTTTTAGCCTGATTTCCCTTCACAGTTGAAAGC-CCCATTTGTGAAACCATTTTCATTATTGTATAAGTTTCCTCTTGTTGCTGTAACAAATTAC
Choloepus hoffmanni  GATTTATGTCTGTCTCACAAAAAAAGTTTTTATCTGATTGTCCTTCACAGTCAAAATCTCCCATTCATGAAATATTTTTCATTATT-----AATTTCCTATTGCTGCTATAATAAACTAC

Homo sapiens         TACAGATTTAATGGTTTAAAACAACACACATTTATTATATTACAATTCTGAAGGTTAGAAGTTCAACATAGATCTTACTGGGCTAAAATCAAGGTATTGGCACTGTTGTGTTCCTGTCTG
Choloepus hoffmanni  CACAAATTTA--GATTTGAAAC-ACACGTATTT-TGATCTTA-GGTTCT-----TTAGAAGTCCAAATCAAATCTCAGTGAACTAAAACCAAGCT-TTGGCCAGACTGTGTTTCC-TCTG

Homo sapiens         GAAGCTCTAGAAAATAATCCATTTTCTTGTCTTTTCCAGCTTCTAGAGGTTACCTTCATTTCTTGGCTTGTGGTC-----CCTCTATCTTCAAAGCCAACAATAGTGGGTTGAGTCCTTC
Choloepus hoffmanni  GAGGCCCTAGGAGAGAATCAGTTTCCTTGCCTTTTTTAGCTTTTAGAGGCCACCCACATCCCTTGGTTTGTGGCCCTCTTCCTCCATCTTCAAAGCTAGCAATTACAGGTTGAGTTCTTC

Homo sapiens         TTACATCACCTTACTCCAACCTCCTCTTCTGCCTCCCTCTTCTATATTTGAGGACCCT----TGACCTTGGCCCTGCCCAAAAAGTCCAGGATAATCCCCCTATTTTAAGGTCATCTGAT
Choloepus hoffmanni  TCACACTGGGTCACTCCAACTTCCTCTTTTGCCTCCCTTTTCCACATTTAAGAGCACTGTGATTACATTGGGCCCACTGGGTAA-TCCAGGATA..........................

Homo sapiens         TAGCAACCTTAATTCCATCACTACCTAAATTCTCCTTCACCATGTAAGATTACATATTCACAGGATCCAAGGAGTAGTATGTTGACATCTTTAGGGGCCTATTATGCTGCCTACCACAAT
Choloepus hoffmanni  .........................................ATGTAAGGCAGCATATTCAAAGGTTCCAGGGATTAGTCCGTGGACATC-TTAGGGGACCATAATTCTACCTACCACAAT

Homo sapiens         TATGGTCTGCATTCATGAAACAAAGCAAAGCACAGTCCCTATAGTGTCAAGACCATGATCTTGAC------GTTAATATAGTTTATGCAGAGAGCCTTTTTTTGGCCTCTTAGTTATATC
Choloepus hoffmanni  TATGGTCTGCATTCATGATACAAAGCAAAGCTTAGTCCCTATGGCATCAAGCCCATGGTCTTGACTTTATGATTAATATAGTTTGTGTAAAGAGCCTTCTTTTGGCCTCTTAGTTATA..

Homo sapiens         TTTTTTTTTTCTTTCTCTTTTTTCCTTTTTTTTTTTTTTTTTTTTTTTTTGAGACAGGTTCTTACATGGTTGCCCGTGCTGGAGTGCAGTTGCACAATAATAGCTCACTGCAGCCTTGACT
Choloepus hoffmanni  ........................................................................................................................

Homo sapiens         TCCTGGGCTCAAGCAATCTTCCCACATCAGCCTCCCAAGTAGCTTGGAATGTAGGCATGCATCACCATGCCTGGCTGAGTTTTTCGTTTTTTATAGAGACAGGGTCTTACTATGTTGCCC
Choloepus hoffmanni  ........................................................................................................................

Homo sapiens         AGGTTGGTCTCAAACTCCTGGACTCAAGCAGTCCTCTCACCTAGACCTCCCAAGTGTTGGAATTACAAGTGTGAGCCATTGTGCCCGTCCAGTTATATAATTTCTTAACCAATAGTTACT
Choloepus hoffmanni  ..........................................................................................................AGCTAATAGTCATG

Homo sapiens         CAGTT--ATCTGTTGACAGTATTAGTTAT----TATAATGACAGTGATTTAGTATCAAGTTATAGTTATTATTTTATCTTGAGAAAGGATATACTAGTATGGCAGCACATTCTAA-TAGT
Choloepus hoffmanni  TCGTTTGCTCTATTGTTAATATCGGTTATCAATTATAATGATAATGATT----AT-------TAGTTGTCATTTTATCATGAAGAAGGATGTACTAGTATGGTAAAACTTTCTAAATAGT

Homo sapiens         TTGAGGATTATCAAACCATATATAGGATTAGTTTAGTCACTTCTCTTTAAAATAAGAAAATTAAGTAGTCTGAAACCTTTATCTTTTTTTTTCTGATTGAAGAAATACATTAATCTAAA-
Choloepus hoffmanni  TTTAGGATTATCAAGCTATATATAGGATTAGTCTGGTCA--TATCTTCAGAATAAGAAAATTAAGTATTTTGAAACCTTGTTCTTTTCTTTT-TATTAGAATAAATA-GGTAATCCAAAT

Homo sapiens         ---TCAGACTTTTTTGAAGCTGTAATTTGTATTTATTTTTCTCAGGGAAATTTAAAAATTGATTTTTTAATTATTATTTTTATTCCTTAAAACAACATTAAGGCTGGGCACAGTGGCTTA
Choloepus hoffmanni  CATTCAGACTTTTGTGAGACTTTATGTTTTAGTTTCTT..................................................................................

Homo sapiens         TGCCTGTAATCCCAGCATTTTGAGAGGCCGAGGTAGGATTGCCTGAGCTCAGGAGTTCGAGACCAGCATGGGCAACATAGTGAAACCCCGTCCCTACAAAAATTAGCCGGGCATGGTGAT
Choloepus hoffmanni  ........................................................................................................................

Homo sapiens         ACGCACCTTTAGTTTTAGCTACTTAGCGGGCTGAGGCAGAGGATCACTTGAGCCCGGGAGACTGAGCTGCAATGAGCCAACATTGCACCGCTGCACTCCAGCCTGAGTGACAGAGTGAGA
Choloepus hoffmanni  ........................................................................................................................

Homo sapiens         CCCCATCTTTTTTTTTTTTTGAGATGGAGTTCAACTATTATCACACAGGCTGGAGTGCAATGGTGTGATCTTGGCTGCCTGCAACCTCCGCCTCCTGGGTTCAAGCAATTCTCCTACCTC
Choloepus hoffmanni  ........................................................................................................................

Homo sapiens         GGCCTCCTGAGTAGCTGGGATTACAGGCGCCCACCTCCACACCCAGCTAATATTTGTATTTTTAGTAGAGACGGGGTTTCACCATGTTGGCCAGGCTGGTCTCAAACTCCTGACATCAAG
Choloepus hoffmanni  ........................................................................................................................

Homo sapiens         TGATCCACCCGCCTTGGCCTCCCAAAGTACTGGGATTACAGGCGTGAGCCACCATGCCTGGCTGAGACCCCTGGGATTATAGGCGTGAGCCACCATGCCTGGCTGAGACCCCATCTCTTT
Choloepus hoffmanni  ........................................................................................................................

Homo sapiens         AAAAAAAAAACCTACATTAGAAATGGATATCAAAACTGCTGATTAATACCTTCCTGAAATGTCTTGAAAATATTTTAATTTCAAAGAAACTAAAGACTTATTCAGTCTTGTCAAAACCAA
Choloepus hoffmanni  ........................................................................................................................

Homo sapiens         AATGATGCTTTCATCAAACAGATGTCATAATCATAACTAAATTAATGGAATGAGAAAAGATAAATACTTAGTGAAATGTACTGTGTAATGTAGCTGACTAGAAAAGACCTTTAATATGAT
Choloepus hoffmanni  ...............................................................................ATTGTGTAATGTAG--------AAGTGTTCCTTATAATGGT

Homo sapiens         ATGTTTGCTTACTCCCCGCCTCTGTTTTGAGGGCAGAAAGTTTAATCATATTCTTTTCTGTATCCCTAACTCATGATTGTTTTAGATTAGTGTTGTCCAATAGATGAATAAT---AATAC
```

```
Choloepus hoffmanni  ATGCTTGTTTATTTCAC-TCTGTGTCTTGAGGGTCAAGACTGTG-TCTTGTTCATTTCTATATCCCTGATTCAGTGTAGTTCTAGAGCAGTGCTGTCCAATAGAAATATAATGCAAAAAT

Homo sapiens         AAGAGCTATGTAATTTAAAATTTTCTAATAGCCACATTTTGAAAAGTACAGAAATAAATGAAAATAACCTTAATAATATATTTTATATAATCCAGTGTATCCAAAATATTACTTGTTCAA
Choloepus hoffmanni  AGGTAATATGTAATTTTAAGTATTCTAATTA------TTCAAAAAGCACAGAAAT----GAAAATGATTGTAATAATATATCTTATATAAACCAGTATAGCCAAAATATTATCATTTCAA

Homo sapiens         CATGTA---AGGCCACAAGAGTCTCTTTTCTGATCTCTTTACTGAAATTAAAGAGCATTTCTCTT--------AGCTCCAGTAGATTTAGACCATAGTAGGAGTTGAAACATATTTGACC
Choloepus hoffmanni  CATGTCTGTAGGCCCCAAG----TTTTTTTTGTTTTTTT--------------GTTTTTTTTTGGTAGATAAGCTTTATTGTAATTAAATAATATT--GTGTTGAGA--TGTTTG--T

Homo sapiens         TTTGCAAATGAATGAGAATTTTTTTTGTAAACAAACAGGCCTTTGACATATTCAGAGTGGTTTTCTAT-GTGTATAAAGTGATTT-ATGTAGAATTTGCTGTGTGTAGTCCTTGTTCTCT
Choloepus hoffmanni  TTTGAGAG-----GTTGGTTTTTTTCCTATGTAAATAGGCCTT-GACATCTTCAGAGTAATTTTCCATTGCATATAAAAAGATTTTATATAGAATTTCCTGAGTATAATCTGTTATTATT

Homo sapiens         ACAGTTTTATTACAATAAGTTACTTTAAAGGTTAACTTTT-TTTAGAAGAAAAAGAGTGTGTGTGTGTGTGTGTGTGTGTGTTCGTGTGTGACTTATTTTACTTACATACAGAAGCTCTC
Choloepus hoffmanni  ATAGTTTTAGTACAATAAGGTACTTTAAAGTTTATCAATTACTTAAAAGAAAAAGGGTGTATGTATATATGTATGTATGTGT----ATGTAATATATC------ACATACAGAAGCACTC

Homo sapiens         -TTAAAATCAAGTGAGGTTGGTATGTCCATTTGTTTCTAACCAGTGTAACTAGGAGGTACTCAAGTTCAAGGTCTCTGAATCCATTTC-AGCAAATTAAAATGAAAATTTCTAAGAGCTT
Choloepus hoffmanni  CTTAAAAACAGATGAAGTTGAAGT----------------CCACTGTAAC------------------AAGGTCTCTGAATGCATTTCCAACAAATAAAGTTGGTAGCTTCCAACAGCTT

Homo sapiens         TACATCTAGGCATATTTTTCCCTATTGACAT-ATGT-TTACTAGACATATTTTTCTAAAGTAGTCCTACTTCATCTGTATGAAAAAACT-TTTACAGACAGAAAACCTCCTTCATT--AA
Choloepus hoffmanni  TAAATTTAGGCAAGTTATTCCCTGTCAATGTTATGTCTTACTGGACATTTTTTTCTAAAATAGACCTTCAT--TCTGTATCAAAAAAATATTTTGGGATTGAAAGCT--CTTCATCTAAG

Homo sapiens         TCCTCTGAAAGAGTCTTTGGGCATTTCTTAAATGCCTATGGTAACTGCCATCACTGATAACTTTGTAGGAATGTACAGGCCTTGAACCTATGGAATTCTCCATGACTTAGTAGAAAATTT
Choloepus hoffmanni  ATATCTGCAGTTATCTTCAGAAATTCCTTAAGAGC-TATGGTGTCTGCCTTCAGTAATA-CTTTGCAGCATTATA-AGATCTTTAACCTATGGAACTATCCA---------GAAAAATTT

Homo sapiens         CATCAGCAACATGTTTACCCTATCTAACCTTTTAATGTTCTCTTATAAACTACCGCCTAGCCTTCTCTCGTATTAACAGTAGGGTAGTTGCAAACTTTGATGTTAAACCTCTAGCAACA-
Choloepus hoffmanni  CATCAGCAATACTTTAACCATACCTAACCTTTTTAAGTTCCCATATAAACTACTACTTTGCCTTTTCCAG--TTAACAGTAGGAGAGTAGCCAATTTTGATGCTGAATTTTTAACAACAC

Homo sapiens         --------------CATTCTGAAAGTGTTTCAATGTTATTGATCATGTTTTCTTCCCTCTTGCTGCTATTGGGTATAACACATCTCATGTGTTGCATGATTTTCGTTGTTGTTGTTGAGA
Choloepus hoffmanni  AACTTCTAGCTGCCCACTCTAAAAGC-TTTCAATTTCAAAGATTATTTTTTCTTCTTTCTTGATACTATTGGACAT............................................

Homo sapiens         CAGAGTCTCGCTCTGTCACCTAGGCTGGAGTGCAATGGTGCGATCTCGCCTTACTGCAACCTCCACCTCCTGGGTTCAAGCAATTCTCTCACCTAAGCCTCCCCAGTAGCTGGGATTACA
Choloepus hoffmanni  ........................................................................................................................

Homo sapiens         GGCACATGCCACCATGCCTGACTAATTTATGTATTTTTAGTAGAGATGGGGTTTCACCGTGTTAGCCAGGTTGGTCTCGAACTCCTAACCTTAAGTGATCTGCCCGCATTGGTTTCCCAA
Choloepus hoffmanni  ........................................................................................................................

Homo sapiens         AGTGCTGGCATTACAGGCATGAGCCACTGCCTGGCTGCATGATTTTTATTTTATTTTATTTTTTTTTGTCTTTCAGAATTATGGGAGAA--TAAATGGCCATACAAGCCCAAGCCCACTT
Choloepus hoffmanni  ...................................................................................ATGGGCAAAACTAAAGGGCCATATAAGTCTAAGCTTACCC

Homo sapiens         ATGCCATCTTTTGGTAGAGAGAATAGCTTTGTGTGATCTGTTAAAGTTTATAAATTGAAAGTTGTTAAATGCATGAATTTCTGGGGTTGAAATAGGTTCAGTGAGCTATTTGTAATAATG
Choloepus hoffmanni  AGGCCATCTTTTGGCAGAAAGCATAGCTTTGTTTGATTTATCAAAATTTGTAAGTTGGAAGTTTTTAAATACATGAACTTCTGATACTGAAATAGGCTTTGTGAGCTTTTTA-AATCTTA

Homo sapiens         TTATGAGGGTGAGTACAGTAG--GATTGATTTTCAAAATATACTACCAAATTCATCTGTTCTGAGACACTTCCATTGGTCTCTAATCTAAATAGGCAATTAATCTTCCTTTTGC--TTTT
Choloepus hoffmanni  GTGTGGGGATAGATAGGGTAGAAGGTTGTATTTGCAGATATAC------------CTGCTAGGAGAAACTTTCATCCAGCTCTAAAGTAAATGGGCAATTAATCTTCCTTTTGTTATTTC

Homo sapiens         TTGGTCTTAAAATTTGAGGGCTCCTTAACCCATTAACATCTTCAGGAGACCAACCTAGGCAACACAATTAGCCAGGCATAGTGACATGTGTCTATGGTCCCAGCTATTTGGGAGGCTGAG
Choloepus hoffmanni  TTGAACATAAAATGTGCGGACTGTTTAAGCTATTAACATCTTTAAGAG........................................................................

Homo sapiens         GTGGGAGGATCACTTGAGCCCAGAAGGTCAAGGATGCAGTCAGCTGTTATCACGCCATTGCACTCCAGCCTGGGTGACAGAGCAAGACCATGAATGAATGAATGAGTGAATGAATGAATG
Choloepus hoffmanni  ........................................................................................................................

Homo sapiens         AATGAATGAATGAAAAATAAGTAAATAAAGATAAATAAATATATCTATATCTATATTCTGATGTTTTGGGGGAACATTTAGAAAGAGAAGGTAGGGATCCAGGAAGTTAATCAGCTTTGG
Choloepus hoffmanni  ........................................CATCTAT----------TAATGTTTTGGGAGAAAATACAGAAAGAAAAGATATGTGTCCAGGAAGTTAATCAGCTATGG

Homo sapiens         TAAAAGAGTTTCTTATTGTAATGAATTCAAATATCAGAAAGGGAGGAATCCTTTTATACATATTAGTCTGTGTATAGAAGA----------TTTGCCATCAGTTTAACATTCATTTTA--
Choloepus hoffmanni  AGGAAAAGTTCATTGTTGTAATCAATTAAAATGTCAGCTAAAAAAGAATCTTTTTATGTACATTAACCTTCATATATAAAAATGCTGCTGCTTTGATATTAGTTTAACATTCATTTGAGC

Homo sapiens         AACATTTTTTTCTTGATGGTGATGATAAATTTGCTCAACATACAATAAAACACTGACCAGGAAGACTCCCTGTCAGTACTTCAGTAT-GGCTTAGGTAGACCTTAAAAGTTCCTAAGAAT
Choloepus hoffmanni  ATTCTTTTTCTCCTGATGGTGATGAAAAATTTTCTCAAAATAGAATAAAATGCTGATAAGGAAACTTCCCTATCAATACTTTGGTACAGATTTAACTAGACCTTAAAAGTTCCTGAGAAT

Homo sapiens         TTTGGTATCAAATTAGATTTAAATAAGCTAAGCTTTTTAAGCTACTCCATGGCAGCCTGCCTTCTT-ACACAT---TATCACATAAGCCTCAGTT---GGCATGTTGT-----TTTTAAC
Choloepus hoffmanni  TTTGGTATTAAATCAGAATTAAATAAACTAAAC-TTTTAAGCTACTTCATTGTAACTTCTCTGCTTAACACATTGCTATCGCATAAACCT-GGTTCTAGGAATTTTATCTTAGTTTTAGT

Homo sapiens         T----------TCATACTGTAACAATTTGATGATATTATCTTTCCAATATGGGTTTTATAAAGATTGGAAGATTATCTCAAGTTGTAAATGCAAGGATTTAATAATTCTATTTGTTAAAA
Choloepus hoffmanni  TTATTTCTTCATCATATTATAGCAATT---TGGTATTATTTTCCTATTTTGGATTCTATAAAGTTTGGGGAATTATCTGAAGTTGTAAATATAAGGATTTAACAATCCTATTTGTTA...

Homo sapiens         TAATTATATGACCCTATTAATTCACCCAGAAATGCTTTACAGAAGCATATTAAGGATAACATTATCAAAAATAGTTTTATAATTTTTTTTTTAAGACAGGGTTTTGCTCTGTCACCCATG
Choloepus hoffmanni  ........................................................................................................................

Homo sapiens         CTGGAGTGCAGTGGTGCCATCATGGCTCACTGCAACCTCAATCTCCCAGGCTCAAGTGATCCTTCCATCTCAGCCTCCTAAGTAGCTAGGACCATAGACACCCTTAACCACACCTTGCTA
Choloepus hoffmanni  ........................................................................................................................

Homo sapiens         ATTGTTTGTATTTTTTGTAGAGAAGGGGTTTCGCCATGTTGGCCAAGCTGATCTTGAACTCCTGGGCTTAAGCAATCTACCGGCTTTGGCTTTCCAAAGTGCTGAGATTACAGGCATGAG
Choloepus hoffmanni  ........................................................................................................................

Homo sapiens         GCACTGTGCACAGCCTTATTATTTTATTTTTAATCTATACCATAATTCTCACTCCTTCTATCCCGTTGTCCGCATATATACATATCTACCAAAGACAGGTTGAGGGAACAGGAAAA--TG
Choloepus hoffmanni  .....................................................................................................GGTCAAAAGAACAGGAAAGCCTG

Homo sapiens         GCTGTTTTAAAATCCTTGACCTTTCCTGCATTGTTTATGTATGAACTGTCAAATTTATATGTCATTTT-TTTATTCTTTGACATTCATATTTA----GTAGACAGTATAACT--------
Choloepus hoffmanni  CGTGTTTTAAAATCCTTGATCGTTCTTGTATTAGATGAATATAAACTGTCAAAATTATATGTTCTTGTATTCAATATTTGACATGTATATTTATTTAGTAGACA--AGAACTTCTCCCCC

Homo sapiens         --CCCTA--GAAGTTAATCTTGATAGTTAATTTTTATTACTTGGGATTTTGTTCCTCACTTAAAATTGTTTAA-TTAAAAAATATGTTTAATTATA--------ATTCTAAAAGTCAAAT
Choloepus hoffmanni  TCCCCTGCCAAAATTAATCTTAATGGGTAATTTTTATTAATTGGAATTTT-TTCTTTATTTACCATTGTTTAACTTAAAATTTATTTTTAATTATCTTTTAATTATTCTAATGGACAAAC

Homo sapiens         GTATGATTTTTCTTTTTTCAGAAAGTCTTTATCTCTTGCAAGGTTCAGTAGTGGCAGATCATGACTACATTGGATTGCCTGAAATTCCGATTGGAGCATATCAAGCAAATATTCTGGTGG
Choloepus hoffmanni  TTATGATGTTTCTTTTTTCAGACAGTCTTTATCTCTTACAAGGTTCAGTAGTGGCAGATCATGACTACATTGGATTGCCTGAAATTCCAGTTGGAGCATACCAAGCAAATATTCTGGTAG

Homo sapiens         AAGATGCAACTATTGGTATAGTCGATAATGAATTACTGACATCAAGCAAGGATCGTGAACTATTAGAATATAGAAATACGAAAATCTCTCCATTGATAGACGATCATAGTTCTCTTGAAA
Choloepus hoffmanni  AAGATGCAACAATTGGTGTAGTCGATAATGAATTACTGACATCAAGCAAAGATCATGAACTATTAGAATATAGACATGCCAAAATCTCTCCATTGATAGAAGATCATGGTACTCTTGAAA

Homo sapiens         AGCAGACTTTCAGTCTGTTGGACTCTTCAAACCAGGTTCTTGAATACTTAAGTTAGTAAATACCAAAATTTATTTAAATTGTTTGTTTAGAATATA-AATTCTTAA----TGATATCTTA
Choloepus hoffmanni  AGCAGACTTTCAGTCTTTTGGACTCTTCAAACCAAGTCCTTGAGTACTTAAGTTAGTAAATACCAAAATTCATGTCAAGCATTTGTTTAGAGTGTATAACCCTTGAATCTTAATGTCTCA

Homo sapiens         TTCATTAAAA-ACGTTGTATAGAAGAAGTATCTTGACACATTCTTAATGACTATATTCTGAAGGTTTATTTTATAGTT-----CTTATCTAAAATTATG-CCACATTTGAATATCAGTAT
Choloepus hoffmanni  TCTATCAAAATAGGTTGTATAGAAGAAGTATGTTGACATACCCT---TGACTAAATCTTGAAGTTTT-TTTTGTAACTATTTATTTATCTAAAATTGTGACCATGTTTGAATGTCAGTGT

Homo sapiens         ACGTACATATGAAGGCAAAGGAAGCATGTAATATATAGAATTCACTTTTTCTTCTAAAAAGAGAACAGGCAAAGCACCCCTACTTCTTGGGAAATAAA-----TTCCAGCCCAAATCTCT
Choloepus hoffmanni  ATATATGTATAAAGTCAGAGGAAACA--TAATATATAGAATTCACTTT---TTCTAAAAAGAGAACAGGCAAAACACAATTGCTTCTTAGAAAGTAAATTAATTTCCAGCTCAAAACTGT

Homo sapiens         CAAACCTTTGTATGGTTTTGGCACCGT-TTCTAAGTTTTAAATTTTCTGAAATTC-TCTACCATTTAAACAGTAAGTTTGAACTACCTTGAATGGAATTGATAAATACTGAATCAACTGT
Choloepus hoffmanni  CAACTCTTTCCCTGATATCCATACAATGTTTTCAGTTTTAATTTTTCTGGGATACTTCTCTTTTTTAAAGAATAGGTTCTAACTGCCTAGTAGTGAATTAATAAATATTGAATCAACCAT

Homo sapiens         AACAATGAATGAAATGAAGCTTCTTTCTGTTTCATTAGAACTTCTAAAAACAAGATTGTATGAAAAA-ATATAGGGTTATTTAATAGGCTTTCACAGAATATTGGTAATTTCAAATTATC
Choloepus hoffmanni  AACAACAAATTGAGTGAAACTTCTCTCTTTGTTGTTGCAGTGTGTAAAAATAATATTGTAGGAAAAATATTTAGGTTTGTCTAATGGACTCTTATAGGATATTGGTAATTTC--------

Homo sapiens         TAATTGGTTAGATAGGGTCAGGATTAGGGTCTGAAACTATGTATTGGTTGGGAAACAATTTGTTTATTTTCATACTATATATTAAAGATAAAACCATCGACCAGGCATGGTGGCCCACAC
Choloepus hoffmanni  -ACTGTGGTAGGTAGA--------TAAGGTCTGAAACTATATTATTGTTCTGGGACCATTTGTTCATTTCCATAATATATATTTAAGCCCAAA..........................

Homo sapiens         CTGTAATCCCAGCGCTTTGGGAAGCCAAGGCAGGATGATCACTTGACCCCAGGAGTTCGAGACCAGCCTGGGCAACACAGGGAGACCCTATCTCTACAGAAAAATGTTTAAAAATTAGCC
Choloepus hoffmanni  ........................................................................................................................

Homo sapiens         AGGTGTGGTGGCACATGCCTGCGGTCCCAGCTACTCAGGAGGCTGAAGTAGGAGCATTGCTTGACCCCAGGAGGTCGAGGCTGCAGTGAGCCGCGCTCATGCCACAGCTCTCCAGCCTGG
Choloepus hoffmanni  ........................................................................................................................

Homo sapiens         GCGACAGGGCAAGACCTGTCTCAACAACAACAACAAAAGTATCCGTTAA---GAATTTTTTCTGCAATCATAAA------------------ATCATAAAATAACCAGTATCAAAGATT
Choloepus hoffmanni  ....................................GCATCTGTTAATAAGACTTTTTTCTGCAATCTTAAAGAAAAAGAAGTAAATAACAATCATAAAATAACCAATATCAAAGATT

Homo sapiens         TTAAACTGTAATTTCTATGATATTCATAAAT-ATAAAATAAATATTGTTCAATAAATATTTATTC---TAAAGCACTATGAAAAGAACTGTGGGAAAAGTAAAAGATGAGTCAAACGTGT
Choloepus hoffmanni  TTAAACTATAATTTCTGCATTATTCATGACTCATTCAGCAAATTTTTTTTTTTTTTTTTTTTTTTGTAAAGCACAGAGAAAAGCACTATGGGAATATAAAAAGAT--GTCAGAAATGC

Homo sapiens         ACCTCATTCTTATAAAGGACTTACTGTCTGGTAGGTAATA---AACTATGTAAGCCATGTACAGCACAAG------TTAAGGCATGCTAAATTGCAGAAGACAGGTGTAGATAATGTACT
Choloepus hoffmanni  ACC--------ATAAGGGGCTTATAATCTGGTAAGGAATATAGAACT-TATAAGACATGTACAACAGAGGTTACAATTAGAAAGTGCTAAATTCCCTAAGAGAAGTAGAGATAGTGTACT

Homo sapiens         TAGGGAGGCAAAAAGAGAGAAGTTGTTTTTACATAGGAAATTTGTGTTTATCCTTATGGAAGAAGGATCATTTAAGCTTGACCTTGAAGAATGTATAGGATTTGGTAAATGGATACAAAA
```

```
Choloepus hoffmanni  TAAGGAGGCAAAAAAGGGG--GTTGTTTCTAAGTAG---------GTTTGTGCTTATGGAAGATGGGTCATTTGGGCTAGGCCTTGAAGAATGCATAGGATTTGGTGAGTGAGTACAAAA

Homo sapiens         GAATTAGGGGAGAAGTAATACACAGAAATGGAAAAGTATTGTAATGATTCATAAAAAGGC--AGGGCTTCTTGTTGTCTACACTGCCTCGGGTATAAAGTGAAGAAGCAAGATAATGAGG
Choloepus hoffmanni  GAAATAAGGGAGAAG--------AAAAATAAAAA------GTAATGATTCACAGAAGGGCTAAGGGCTTTTTTTTGTCTACACT-----GGGTTTAGAAAAAAATACAAAGTTAAGGGGA

Homo sapiens         GATAAAGCTGAAAAGGTAAGTAGGAAACCTTATGGAGAGCTTTGAATACTATTAAGGATTGATATGTGCCCCTCCCAAAATTTGTGTTGAAGTCTTAACCCCCACCACCTCAAAATGTGA
Choloepus hoffmanni  GATAAGGCTGACAAGGTGAGTAGGGAACCTTATGGGAGCCTTTGAAT.........................................................................

Homo sapiens         CCTTATTTGGAAATGAGGTTGTTGCAAGTGTAATTAGTTGAGATCATATTTCAATAGGGTGGGCTCTAATACAGTATTACTGGTGTTCTGATAAAAGGGGAAAATTTGAACACAGACTCA
Choloepus hoffmanni  ........................................................................................................................

Homo sapiens         AGGAGAATGCCATGTGAAGATAAAGGCAGAGTTTAAGATTATGTTTCTATATGCCAAGAAATGACAGAGATTGCCAGAAAACTACCAGAAGTTAGGGGAGAGGCATTGAATACATTTTTC
Choloepus hoffmanni  ........................................................................................................................

Homo sapiens         CTCATTCCTCAGAAGACCAACCCTGTCAATACCTTATCTCAGACTTTTGCCTCCAGAACTGTGAGACAATAAAATTCTATTGTTTCAGCCATCCAGTTTGTGGGACTTTGTTACAGTAGC
Choloepus hoffmanni  ........................................................................................................................

Homo sapiens         TCTAGCAATAATACAAATGCCTGGCTAAATTATTTCTTTTCTGAGAG-CAAAAACTTTGTATAGGAAACTGTCATAATCAGAACTGTGTTTTTGGAAGTTTAATCTGGCAACTAGTAAAG
Choloepus hoffmanni  ..................GACAAGCTAAATT-TTTATTTTCTGAAAATCACTGATTTTGTATAGGGAGTTGTCATGATCAGAACTAC---TTAAGTAGTTTAATCTGGCAGCTGATAAAA

Homo sapiens         AACAGATTAGAAGAGGAGAAAACA-AGGCATGGAGATTAGTTGAGAGGACTTCGCAGTGATGACGAATACTAAAAACCTAAACTGGGCCTGTAGCATTGGTAATGAATGGAGTCTGTCTT
Choloepus hoffmanni  AATAGATTAAAGAAGGAGAGAAAACAGACATGGAGATTGGTTGATAGAATTTTGCAATGATGATGGGTAATGAAAACCTAAACAAGGCTGGTAGCATTGGTAGGGGATAGAACATTTCTT

Homo sapiens         AAGATCAGTACAGAATATCGCACATATTATATTTCACAACCATACTGAACAGTGAT--GTGATATCAAAGATGGAGACCTGGAGTATTCTGATTACTCATTTAAG-AAGAAAGATT----
Choloepus hoffmanni  AAAATCATTACAAATTATCTCACATGTTACTTGCATCAACCATCCTGAATGGTAATATGTGATATTGGAAATGGAGACCTTAAGTATTCCAGTTACCAATTTAAATAAGAAAGATTTAAT

Homo sapiens         -ATGTTTAATATAGGCTAAAGGAAAAGTGTTTAGGGTAGGGTGTGAATCTAGGACCTCATCTTATACCACAGACCAAATAAATTTCAAACAGAATTCCAGGAAGAAAAAAACAAAACTTT
Choloepus hoffmanni  TATGTT-AATATCATATAAAGG-----TGTTT------GGGAGTTAACTTAGGACCACATCTTATACCACAAACCAAATGAATTACGGGTAGAATTCCAGAAAGAAA............

Homo sapiens         TTTTAAAAAAAGCGTAATTGTATCAAACAGTGCTACCTATCCTGCTTTCCTCAGAAGAGCAAAGTTTAGAAAAGAGTCATATTAGAATATCTTTTTGGTAAA
Choloepus hoffmanni  .......................................................................................................
```

```
Homo sapiens        TCAATTTCTGGCGTCTCGAACCCAAGAAATGAAAAAGGGAAGCCATGGGGGAGCAGGAGGA---GAAATCAAGGCGCGAAA-GGACCCTCAAAGCTGTACTGTCAC---TCCACGCCCAG
Loxodonta africana  GAAATCTTCCGTGTCACAAACCCAAGAAATGAAAAAGGGAGACAATGGAGCAGAAGGAGAAATGGAAATCAAGAAGCTAAAGGGACCCTCAGAACTGTGCAGTCACTCTTCCCCGACCAC

Homo sapiens        GCTGTCATTCTCTCCAGCACAAGCCCCTCGGTGGAGCATGTGTTTAGGGTCTGCAGAGCACAG---GAGACAAAGCAGAAGCCCAGCAGCCGCCTCAACCCTGGCATGATCAGGTTCCGC
Loxodonta africana  GCTGCCATTCACTTCCGCACATGTCCCTCGTAGGAGCGCGCGTCTAGGCTCAACAGAGTGCAGCAAGAGACAAAGAAACCACCCAGCGGCGGCCGCAACCCTCCAGCGACCA--------

Homo sapiens        ATCTCCGGTTCTCCTCCGGTTTT-GGGCCCTCACCTGCCACGAATGCGGTCGTCACGCGAGCAGTTGTGAGATGGTCAAGCCCATTCAGGTCTAGGTTACCCCCTTTCATCCCGGAGGCA
Loxodonta africana  ------GGTTGTCCTCTGGTCTTGGGGCTCTCACCTGCCACGACCGCGGTTGTCACGCGAGCTACTG-GAGATGGCCGAGCCCATTCAGGTCTCGGCT-TCCCCTTTAATCCCGGGGGCA

Homo sapiens        GGGGT------GAGGTGTTTGTCAAGAGACTTCAGTTGTAGGCGTGGACGTAGGCTACTTCCTTCAGTAACAGCTATAGGGCCACATAAGGGAACCTCAGG---CTACTACACCTCAGGA
Loxodonta africana  GGTGGAGGCAAAAGGTGTTCGTTACGAGACCGTCAATGCGGGCGAG---------TCCTCCTTACAGCAGTAGCTGTTCAGCCTCATAGAAGTACCTCAAGGAACTACTTCACTCCACGA

Homo sapiens        ATCTCCGTCACGCCTCTTCCCCGCCCTCCTCGGGTCTCACGCCGAAACCTCACCGGGCGGAACGATTTCCGGCAAGAGCCAAATGATCAGTCCTAACCTTT-CTGACCGTGGTTCCAAGG
Loxodonta africana  CGCTTGGTCACGCCTCTTCTCCGCCAG-CTCGGGTCTCTCTCAGAAACCTCACCGGGCAGAACGATTTCCGGCAAGAGACTAACGATCCGCCCTAACCTTTCCTCATCAAGGCGCCGCAG

Homo sapiens        TTCCCCAAAGGACTGCCCTCTAAAGGACCCGGGTAGTTCCGCTTCCGGCAGCGCGAGATAAATCACGAGAGGAAGCTTAAAT-CTGTCGTTTGAATTTAGGACCACCTCGGTGAGTGGTC
Loxodonta africana  TCCGCAGAGGCACGCTTCCCTTAACAATCCGGGTAGTTCCGTTTCCGGCAGCACCAGATAAGTCGCGAGAGGAAGCTTAAATCCTGTCGTTTGCGTAAAGGACCACCTTGGTGAGTGGTC

Homo sapiens        GTTCTGGTGTGCTGTGTCATACCTACTGTTTTTTAAAGTGAGGCGTAACCCGACAGTAATTTCAAAACCATTCGCCTCGACCGGCCTAAGGAA---GGGTTTAATTGAGTCTGTGGGGAG
Loxodonta africana  GTTCTAATGTGC--TGTCATACCTACTGATTTTTAACGTGAAGCGTGACTTGATAATAATTTCGAAACCGTTCGCTTCGACTGGCCTAAAAAAAATGGGTTTAATTGACTCAGGAGG---

Homo sapiens        ATTTTGCGCAAGCGCCTTTGAGATAGGAGGGCCTTGGGCGTTGCGATGTCCCGCGCTAGCTGAGCAG-AAAGGGCGAGTGCTGTT-TCGGGCCTGGGGAGGTGCCTACTGGGTGTAATTG
Loxodonta africana  ---TTGCGCAGGCGCGCTTGGAGCGGGAGGGCCTTGGGCGTTGCGACGTCCCACCTGAGCTGAGCAGAAAAGGGCAAGTTTTATTGTTAAGCCT-AGGAGGTG-CTGTTTGGTTCAATTG

Homo sapiens        AGTTTTACCAAACGGTCATTTTCCTGCTGATTCTGAGTGTGGTTGGACCTTGCCCTTAAGATTCCGTTAACGAGTCGCCCAAAGCTTAGGAAATGCTCCCTCAC---------AGCGTTT
Loxodonta africana  AGTTTTACTTAATGATCTTTTTCCTGCTTATGCT-----TAGTTGGACTTTGTCTTCAAGAACGCGTTAACCATCCGCTC-------AGGAAATAGTCCCTCACATCTAAAGTAGCGTCT

Homo sapiens        GAGACT--AAGCACT---------------------------TTTGGCCGGCGTTGAAGAATTCTCCCCATTACTTTGATCAGTCATTTAATCAGAAATTTTGCCCCAAATTTAAATAC
Loxodonta africana  GAGACTCCGAGCACTGGAGCAAATTGAAAAATTTAGCGTGGTGTTTGACCGACCTTATAGAATTCTCCCCGTTGT----AGTTGCCATTTAAAATGGAATATTAGCCTTTGTCCAAAT--

Homo sapiens        GTGGCGGGGAGAAACGTGAGGTCAAGAGGGAATCCTGTCACGTAGTTTTAAAAGAAAAATGTCCACTTTATC-TCAGTGTAGGTTTTTCTTAAATGGGCATTTCTGAACCAAAGTAGA--
Loxodonta africana  ---------GGAAAC------TCAA-AGGGTGTCTTGTCACGTATTTTTAAACGGAAAGTATAC-TTTTATCAGCAGTGTGGGTTTTCCTTAAACGGACTTTTTTGAGCCAAAGTGGAAA

Homo sapiens        --TACTTTTTAAAGTGGAAATGCATTTATCCCATGTGGCTTCATGTGAATGCAT-ATGGCGATGAAGGCATTTTT-TTGTTCAATGAATTTGTACTCTGAAGTGAACCAGATTACAGAGA
Loxodonta africana  AGTAATTTACAAAATGGAAATTTATTTATCCCACGTGGCTTTATGTGTGTGCGTAATGGTAGACAAGTCATTTTTATTGCAC----AAATTGTACGCTGAAATGAAGCAGATTTTAGGAA

Homo sapiens        AGTGT---ATTTAAATTT--ACAGGTCCTTGACGTTGCATAAAATTGTGCGTGTCCATTTTTTGGAGCTTAAAATAGAATAATTCAAATGTCAGAATTCCGATCCAAGTTAACATGTTGA
Loxodonta africana  TGTGTCTAGTGTAGATTTCCCCCAGGCTTTG--GTTACACGAAATTGTGCGTTTCCATTTTTCAGAATTTAAGATAGAAGAATTTAAATGTCAAAATTTGATCCCAAGTTAACGTGTGTG

Homo sapiens        TTGAAA-ATTGCAG------ATTTTAAGATGGAACCAGCGGGTATTATAACCTTTTAGGGGCTTAAATTGGTGGCTTT---GTTTTTAACCCTTGGTGAAACAGTTAAGTCCTAATCTTG
Loxodonta africana  TTAAAAGATTACAGTTTTATGTTTTAAGTTGAAACACATGCATACTGTAACTTTTTCGAGGCTTAAATTAGTAACCTTGACGGTTTTTGCTTTTGGTGAAACA------TCCTAATTTCA

Homo sapiens        AC-ATAATACCTAACTGCCTTACTTTATTGAACAC------AAAATTATTAAAGGAATTAAGCAATGAGT------------------TGATTTTATTTGCTTTTATGGAGGGGACAAAG
Loxodonta africana  CCTAAAATACTGAACTCCCTTATTTTATTGTACACAAAAATAAAATAATGAGAGAAATTAGGCAGTAAGTATTTATGCAGGTATCTGCTGATTTTATTTGGTGTTATGCAAGGGACATAG

Homo sapiens        TGTATAAAGCTTAACCAAATGTGACCCTGACATTCCTCTTGCAAAACTACAAGTTGTAATCAATCACTTTCATTTTTTCTTATTCCTACATGTTTAAGTACATTAAGTAATGATTTTAAG
Loxodonta africana  TGTAAAAAGTT----CAAATATGACCCTTACATACCCATTGCAAAACTAGAAGTTATAATCAGGTATCTCCACTTT----------TACATGTTTTAATACACGAA-TAGTTACTTTAAC

Homo sapiens        AGAAATTCTTGAAAATATGAGCATATCCAAAGAAGTCCCAGGACAGTCTTTCTTTATGTAGTAGAAACCACTTAATCCCCCTTGTAAATGATACCTTTACTTAGCAATCAGAGTATAAGA
Loxodonta africana  AAATATTCTGGAAAA-ATGAGGATGTCCAATG--------------------TTTGAGTA-TAGGAAGCTTTAAA----------AAATTTTACCTTTGT-------------------

Homo sapiens        AGATATTTATATTTGTCTCGAATATTACAGTTTCAAGCCATTTTCTCATAT--------------------------------------GTATGGGCTTTGAAAAAAAAAAAACAACT-T
Loxodonta africana  ---------------TCCTGAATATTACAGCTTAAAGCCATTTTCTCATATTAACTACTCAAATAGATGGTTTCTAGGGACCAACTTGCAAATATACTTTGAAAAGAAAAAAGCTGCTGG

Homo sapiens        TCATTTACGTTACTTTTGTATATGAATACCAATTATTAGCAGTAAAACTTTTAACTACTTCAAAGGC-TATGCTTTTCGTGAACTGAGTTTTCTTTCTTACGCATTTTCTATTTCTCC--
Loxodonta africana  TTCTTTAAGCTCTTCCTGTATGTAAATCGCAAATATTAGCAGCAAA--TTTTTGTTGATTAAACGACTTGTGCTTTGTGTGAACTGATTTTTCTTTCTGATTCATTTTCTATTTCTCTCA

Homo sapiens        ---------TTGTCATCTGTCTCTCTACCTCCAGAACCAGCAGTTTGTGAACTGCATTAGACTGTTATATCGTTATAAAAGGATACAATTCGTGAGTCTTCTGATTATTTTCTTCTTAGC
Loxodonta africana  TTTTCTCTACTGTCATT--TCTCTCTACTGGCAGGGCCAGCAGTTCGTGAATTGCACAGAACTATAAACTCATTAT-AAAGTATACAACT--TAAGTCATCTGG--GCTTTAGTGTTAGC

Homo sapiens        ACTGAAGCTTT----ATAAACATTGCTATAAAACATTAA-CACCATTAATGAAAGGAAAGAGATTTTAAAATATTTTTTCA------------TGTAGGTGAAGATTTTGGGAAAAGAAA
Loxodonta africana  AGTAAAACATTGTGAATAAACATGAGTATAAAACATTAACCACAGTCAATGAAAGGATAGAGATCTCAAAATTGTTATCTACAAGGAAATCTTTGTAGATAAACCTTTTGAGAAAAGAAA

Homo sapiens        GGGTTTTATGTTACTTTAAACAGCCTGATATGGATGATAATACTTAGATATGATAATAATTATACATTACCATATACAAAGGACTTTTAATCATCCTCGTAATTGCAAAAAAGGGATAGA
Loxodonta africana  GTGTTCTTTG..............................................................................................................

Homo sapiens        TATTATGTCCTTTTCCAAACATAGATACTAAGGCTTAAGTCTATACCTTCTTAGACACAGTTTACTTATAGTGCCATGTATGTGAAAGTGAACTGTAATGTTTTCAAAAGTGAAATAATT
Loxodonta africana  ........................................................................................................................

Homo sapiens        TACCTCTGGAATATTTGCTATAATTTTTAATTCTCCTTTCAGGAAATGCAGGTAATTCTTTATTCATTCACAAATAGTTGAGTGTCCACTGTGTTCTGAACATTATGTTAAACAATGTAA
Loxodonta africana  ........................................................................................................................

Homo sapiens        ACATAAAAAATTAATAAACTCTTTCCATTCCTCATTTATAATCTAAATTATTGATTACAAAAATCACTTTATAATTTGTGTAACAGAGGCCTGAACCAAGTATTATAAAAGCTAAGGATA
Loxodonta africana  ........................................................................................................................

Homo sapiens        ACAAGTAATTGACTTACTGGTATAGCAGGGGGGAAAAGCGTGAAATTTGAAGTAAGAGACACCAAGTTAAAAGTGTCAGCGCATGTACTCACTTTATTACCTTGAGTAAATTGGACAATC
Loxodonta africana  ........................................................................................................................

Homo sapiens        TCTGAACCTCCATTTTCCTACCTGTAAAATGGATATAAAACTAATACCTACTTCATGAAGTACTGTGAGGATTATCATAGTAAAATACTGCATAACTATTTGCAGCTGTAGTCGTGAGTC
Loxodonta africana  ........................................................................................................................

Homo sapiens        ACTTAGCAATGGAATAAATTCTGAGAAATGCTTTGTTAGGCAGTTTCAGCATTGCACGAATACAGACTCTACCTACATGAACCTAGATGATATGTGTGTGTGTGTGTGTGTATATTTTTT
Loxodonta africana  ........................................................................................................................

Homo sapiens        TCTTCATATAGAGAACCAAATGTCCCTGCACATTATTGGGTATCATTTATTTCTCCTACTTGATCTGCAGTGCTAATATCAAATGACCTATATCAGGTTCTATATATGCTCTGCTATCAT
Loxodonta africana  ........................................................................................................................
```

```
Homo sapiens        CTTACGGGACCAATAGGCAGTCCATCCTTGACCAAAACATTCTTATGCAGTGCAATTGCGTTGGCATTGTTTTTAAAATTTTTAATTTTTTAAAATTATTTCCTCTTCCTATACTTTTGG
Loxodonta africana  ........................................................................................................................

Homo sapiens        CTTTTCCAGATGTCATATAAACAGAAATATAAAATATATAGCCATTTGTGTCTGGCTTATTTCACTTAGCATAATGATTTTGAGATTTATTCATATTGTTAGGTCTATTTATAGTTTGTT
Loxodonta africana  ........................................................................................................................

Homo sapiens        CCTTATCAATCCTTAATAGTATTCCAGTATGTGGATCTACTGTAATTTGTTTATCCATTCACCAGTTGATGAACATTTGTTTTCAGGTTTTGGCTGATATGAATAAAACTTCTAAAAACA
Loxodonta africana  ........................................................................................................................

Homo sapiens        TACACTTACAGGTTTTTAGGTGTACATATTTTCTTTTGGGTTAGTATCTAAGATTGGTATTGCTGGACCATATGGTAAATTGATGTTTAACTTTATAAGAAATTGCCAAACTGTTTTCCA
Loxodonta africana  ........................................................................................................................

Homo sapiens        ACATGTTTACATCATTTTCTATTATCACCAGTAAGAGCATCCTTGGTAGTACCTAGCATTGTTAGGTTTTGTTTTATTTTTGTTGTTGTTAGTGTGTAGTCATATCTTATGGTAGTTTTA
Loxodonta africana  ........................................................................................................................

Homo sapiens        GTTTACATTTTTTATTATCTATTGATGTTGAGCATTTTTTCATGTGTTTATTTGCTAATCATATCTCTTTAGTGAAGTGTATTTCCAGGTTTTAAATTGGGTTGTTTGCTTTCTTGTTAA
Loxodonta africana  ........................................................................................................................

Homo sapiens        GCTTCGAGAGTTCTTTATAAATTCTAGAAAAAGTTATTTATCATATGTATGTATTGCAGATATTTTATCCCAGTGTTATTTCTCTTTTTTTAGCAGTTCTTTAAGGAATAAAAATTTTAA
Loxodonta africana  ........................................................................................................................

Homo sapiens        TTTTTTCTTTATGCATAGCTGCTTTTTCTTTGCTGTCTGGGAAGCCATTACCCAACCTGAGCTCACAAAGATTTTTGTTGTTGTCTTCGAGAAGTTGTATAGTTTTAAGTTGTACATTTA
Loxodonta africana  ........................................................................................................................

Homo sapiens        GATCTGTGAGCCATCATGAATTAATTTTTACATATCATTTGAGGTATGAGTTGAGGTTCATCTTTTTTCAAAGGATGTTTAATTGTTACAGCATTTGTTGAAAAGATTATCCTTTCTTCA
Loxodonta africana  ........................................................................................................................

Homo sapiens        TTGAATTACCTTGGCACCTTTGTTGAAAATCAGTTTACTATAACTGAGTGGCTATTTCTGTCACTATTCTGTTCGATAGATTTAAGGCTGTCATTTTGCCATTATAACACAGTCTTGATT
Loxodonta africana  ........................................................................................................................

Homo sapiens        ACCATAGTAGTATAGAAAGTCCTGAAACCAAATAATGTGAAATTCTTCAACTATATTCATTTCTCAAAGTTGTTGTAATTATTCTGAGTCCTTTAGCTTTCCATATCAATTTTAGAATCA
Loxodonta africana  ........................................................................................................................

Homo sapiens        GCCTGTCAGTTTCTGCAAAAGAGCTTGAGGGTTCCTGATTGGGACTGCATTGAATCTATAGATCAATTTGGAGAAAATTGGCATTTTGTCCCACTTTTTCTCAGAGGTAGAGTGGGAGTT
Loxodonta africana  ........................................................................................................................

Homo sapiens        ACTCCAGCTTTCTGCATTCTAGTCAGAAGCCTATATGGGAATGTTTAGTGGCATTTCAGAAAGAAATAATTTTCTTCCATGGTTATATTCACTATAATATCTTTATTGGAAGAGATCACA
Loxodonta africana  ........................................................................................................................

Homo sapiens        GGACGAATCCCTCTATTTGTGAATTGGATGTCATCCCCTGCTGCCATCTCAGGACATCATACCATAAGTTTTTTCTCCTTTGTTTTCAGTGGTCTTTCTAGAAGCCCTTCCATTAGCATT
Loxodonta africana  ........................................................................................................................

Homo sapiens        AAACTTTTTGGGTTACTCCCTTGTCGAAAGCAAAACAATCTTGTTCAAAAAAAAAATTGTTTTAATTGTAGTAAATCACACATAACATGAAATTTACCATCTTAACCATTTTTAAGTGTG
Loxodonta africana  ........................................................................................................................

Homo sapiens        CAGTTCAGTGGCTTTAAGTACATTCACATTTGTTTTACAACTATTACCACCATCCATCCCAAGAACTCTTTTCATTTTGTGTAACTGAAACTCTTGACCAGTTAAATAATAACTCTTACA
Loxodonta africana  ........................................................................................................................

Homo sapiens        TTCTCTCCTCCTCACCCCCTGTTCTATGCAACCATCATTATTCTTTCTGTCTGTATGTATTTGACTACTCTGACTACCTCATATAGGTGGAATCTTACAGTATTTATCTTTTTGTGACAG
Loxodonta africana  ........................................................................................................................

Homo sapiens        GTTCATTTCACTAAGCTTAATGTCCTCAAGATTCATCCATGTGGTAACGTGTGTTAGAATCTCCTTCCCTTTAAAGGTGTGTGAATATATACACCGTATTTTGTTTATCCATTCAGCCAT
Loxodonta africana  ........................................................................................................................

Homo sapiens        TGATGGACACTTGAGTTGCTTCCATCATTTGGCTGTTGTGGGTGATGTGGCTATGAACATGACTGTCAAAACTTTTAACCCCACTAGAATGTAAACTCAATGAAGGTCAAGAGAATCTAC
Loxodonta africana  ........................................................................................................................

Homo sapiens        ATCTTGTTTATTATCCTGTCTCCAACGTTGTAACTGGCACATAGCAGTTACTCAGTCACATTTTTTAGATCAATGAATGAACCTTATATCTGTCTCTAGCAACTATCTTGTAGCTCCTTG
Loxodonta africana  ....................................ACACATAGGTGTT---CAATATTATTTGTTAGATCAGT-AATGAATCC-ACAGCTTTCTCTAGCAGCTGTGCTGTAGCTCCTTG

Homo sapiens        CCTTTATAGCTAAGCCCAAGTTTCTAAATCCACTTTCTGAATTTCCATTCATTCCTTGTGTCATTTCAGTCTGGTTGCCAACTGCCCTGCTATACTGTAACTATACTGTATACTGTTGCC
Loxodonta africana  CCTTCACAGCTAAGCTAAATTTACTAAATCTGCTTTCTCAATGCCTGTTCATGCA-GATCCCATTGCAATCTGGTTTCTACCTGCCCTGCTGTACTGCAACTA-----------CTGTC

Homo sapiens        AAAACCACTAGCTACATTATGGTTCTTATATCACTTAGCCTCTCTGTGTACTTCTAAGAAGTCTGTATTTCCCAGACTTCTATTACCAGAACCT------CTTCTGGCTTTTTTCCTGTT
Loxodonta africana  GAAACCACCTGAGACATTTTAGTTCTTATATTATTAACCTTTCCT---TACTTCTAAGAACTCTTTATTACTTATGCTTCTA-TGCCAGAACCTTTATTGCTTTTTTTTTCTTCCTATT

Homo sapiens        TCTCTGGCTAGTCTCATGTCCCT-AAAGATTTACAATATATTTGGGAGAGAGACATATAAACTAATATATTCAATAGAGTGTTTCAGATTTTATGATAAAGGTTGACACATATTACTGTG
Loxodonta africana  TCT----CTATTCCCATACCCTTGAGGGATTTATAGTATATTTGG--AAAACACATATAAACTAGTA-AGTCAATAAAGTGTTGCAGATTTTATGGTAAAAGTCAGTACATGTTACTGT-

Homo sapiens        AATGTACCAATGAGGGTGTGCACAATTCTCCCTGAGATT---GGGGATGTGGTCAGAAAGGATTCATGGATGAATGAACTCATTAATGTGAACGTTGAAAGATAAGGATATGTTCACTGC
Loxodonta africana  AGTATACCAAGGAAGACATGCACAGT--GACCTGAGAGGGAAGGAGATGTGGTTAGAAAAGATTAGTAGATGAGTGAATTCATTAATGTGAATGTTGAAGGACAAGACTGTGGCCGCTTC

Homo sapiens        ACTTCCTACTGTGTACACTTTCCATCTGATCCTTCTTCCATTGCCTTAACTTCACCTACTTTTTACATGCCGAGTTCATCCATGAAGAATAGAACC----TGCTTAATATTGTCATCTTT
Loxodonta africana  TCTTCTTAC--TCTACGCTTTCCATGTGATCCTTCTTCCACTGCCTTAACTTCCTCTTCCATCTATGTACTGAGTTCATCAGT---GAGCTGAACCAGTGTGTTCAGTACTGGCTTC-TT

Homo sapiens        CTCCCTAAGCCAGAGCTTCAGACAGAAGTGATCCCAGATTCTTCTTTGTGTTTACTCCACACATCTAGT----CAGTTACAGTTCTTATATAATGTATCTATCCATTTATACTGACACTG
Loxodonta africana  CACCCAAAGGTAGAACTC----CAGAAGTAATCCCAGACTCTTC-----ATTCACTCCATAAGTCCAGTGGACTAGTCACAATTCTTTCAAA----ATTTATCCATTTCCATTGACACTC

Homo sapiens        CTTTGGCTCAGAATTTCCTCATTTCTTGCTTTAATTATACAGTAGCTTCCTAGCTGACTGGTACAGGTATTCCTG--TCTCTACCCCAATCTCTATTCCATATTGGTGCACAATACACTT
Loxodonta africana  CTTTGACTTAG-ATTTATCGTTTCTTCCCTTAATTATACAGTAGTCCTCTAGCTGGTCTGAACTGACTTTCCCACCTCTCCACCCCAATCTATCT----------TGCAGAACGGACTT

Homo sapiens        TATAGAATAGAAATCTAATCTTGTAACTCCAACTCATTCTTAAAATTTTAAGTTATTCCAGGTTGGAGTGGTTAGAAGACACTCTGCAGGAAATTACACCTAGCCAGAGTTTTGAAATAT
Loxodonta africana  TGTAGAATAAAAACCT........................................................................................................

Homo sapiens        GATGAGGGGCTGGAGAAATAGCAATAAAAGTTCATTCCAGGCAAAAATATCAATGAAAAGGTGTGAGCACACTGCATGGGATGGTTGAAGAACTGTGGTTATGTTTTGAATTGAGAATGC
Loxodonta africana  ........................................................................................................................

Homo sapiens        CAAGAGAAAAAGATGGAGAGATTGATAAAAACCATATCTTATAAAGCCTTGTATACTGGCAGAGGTATTTAGATATCATTAGTGCCATGGTCTTAATGGTTGTATTCTCCCAAAATTCAT
Loxodonta africana  ........................................................................................................................

Homo sapiens        ATGTTGAAATCCTAACCACCAAGGTGATGGTATTAAGAGGTTGGACCTTGGGGCTGGGCGCGGTGGCTCACACCTGTAAGCCCAGCACTTTGGGAGGCTAAGGTGGGTGGATCACTTGAG
Loxodonta africana  ........................................................................................................................

Homo sapiens        GTCAGGAGTTCGAGACCAGCCTGGCCAACATCGTGAAACCTCATCTCTACTAAAAATATAAAAATTAGCTGGGCATGGTGGCATGCACCCGTAGTCCCAGTTACTCGAGAGGCTGAGGAA
Loxodonta africana  ........................................................................................................................

Homo sapiens        CAAAATTTGCTTGAGCCTGGGAGGCAGAGGTTGCAGTAAGCTGAGATCATGCCACTGCACTCCAGCCTGGGCGACAGAGCGCAACTCTGTCTCAAAAAAAAAAAAAAAAAAAGAGGTGGAA
Loxodonta africana  ........................................................................................................................

Homo sapiens        CTTTGAGGAGGTGACTTGATCATAGGTCAGAGCTCTCATTAATGGGATTAGTGCCTGTATTTTAAAGAGTCTGAGAGAAACCCCTTTCCCCTTCCCTCATGTGAAGTTACAGTGAGAAGA
Loxodonta africana  ........................................................................................................................

Homo sapiens        CAGCTGCCTATAAGAAAGTGAACCCTCAGTAGACACTAAATTTGCTGGCATCTTGATCTTGTATTTCCTAGCCTCCAGAACTGTGAGAAAGAAATTTCTGTTTATAAGCCATCCAGTTTA
Loxodonta africana  ........................................................................................................................

Homo sapiens        TGGTATATTTGTTATAGCCGCCCAAGTGGACTAAAACAATCAGGAATGGCAAAAGATGTATTTCGAGTTAAGTGAATATTTTGATAAGATTTGTGTTTTAGTGTGACCTCTGTGACTGCA
Loxodonta africana  ........................................................................................................................

Homo sapiens        GTGAGGACAGGGACCATATTAGAGACAGAGGAGGCAAGGTCAGATAGAAGGTGAGTGTGAGCTAAGGCAGTGGCAATGGAATAAAGTTCAAGGTTCTACCTCATCTACAATCAGTGCTTT
Loxodonta africana  ........................................................................................................................

Homo sapiens        TCCTCCTTTTCCAGGTGAATCTGGGTCTCTACACATCTGTTTTGATAGTACTCTTTGCATACATCTGTTAAAACACCACTCTAAACTGTATAAATGCTGATTTCCTCCTCTTTTCCTCTC
Loxodonta africana  ........................................................................................................................

Homo sapiens        ATTTATACCATAAGCTCCGTGAGGACAGGGATTTCATTTTATTTAACTTTGTATTAGTATCATCTAGTATATATAAGGGTTTAAGAGCTCAGTAACTGAATGAGCAAATTCAAGGTTATA
Loxodonta africana  ........................................................................................................................

Homo sapiens        TGGATGAACAGGCAAAAAACTATTAAAAAGGGTAAAGCATGGAAGTTCTACAGTAGGCACAAAGAAGATTAGGAGAGGTCAAGATCACTTTAGATTCAGAGATCATGGAACACTTGCTTC
Loxodonta africana  ........................................................................................................................

Homo sapiens        ATTGAGATGCTAGAGCTTGATTTTGAAAGATGGGTAAGGCTTAATCAGGTGGAAGGATATACCATTTAGGGGAAAATGATGTAAACCAAGGTATAGAGAATGGTTGGGGATATGGCCATG
Loxodonta africana  ........................................................................................................................
```

```
Homo sapiens       GGTATAGAAAGGAAAGTAGTGAGAGAAGAAGCTGTATTATGTTCTGCCAGGTTGTAGAGGGTAGGCTAAATAGTCTAGACCTTAGCCACTAAGTGGAAGATAATGCCCAGAGGCAGTCTC
Loxodonta africana ........................................................................................................................

Homo sapiens       ATGGTGTCCACTCTGTCAAGTAAATCATTTGTAAGATAAAAATATGCTAGGGATAGTCTAAATTAAAGTTTCAAGGTGACTATGTCAAATAACTACACCTTTAAGTCTTAGTTCTGTTGA
Loxodonta africana ........................................................................................................................

Homo sapiens       GCCAAGACAACTCACCCATATAAAAATGAAAATAATACACTTGTGAAAGCATTTGTGAAATATGAAAATCTGTAGGAAATAGATGGTTTTTATGTCTTCTCCCAATGATGTTTATGTCCC
Loxodonta africana ........................................................................................................................

Homo sapiens       TCTCCCTAACATAGATATATGTTAGTCTTCTCCCACTACTTTGATGAGAAAAAAACAAACCAAAAACCTTAAAGAGCTCAGTCTCCAAAGATTTTTGTAGTGCTTTCCTTTTCTCCTTGT
Loxodonta africana ...........................................................................................TTATTTTAGTAATTTTTCTATCCCATTAC

Homo sapiens       CCCTTGTACTATAGGTTTACTCTGAGGAGAGCAAATTTCTCTATTTCGATCTACTTTCAATTATTCAAAAGTTAAAGATTTCAAAGCAGAGAAGTTAATTTCAGT-AAATAGACCTTTTC
Loxodonta africana CCTTTATAATCTAGGCTTACT-TGAGAAGA-CAGGTT-----ATTTTGACCTACTTCCAATTATTCACAAGTCAAAGATTGGACAGTGGAGGAATTAATTTTTATAAAATAGACTTCTTT

Homo sapiens       TGAGA---AGTTTTTG-TATATTTATTATAAATAGGAAAACCAACA--------------------GTG---TCCTAATGGCACGTTAACTTAATATA-------CTGAAGACTTCATCT
Loxodonta africana CGGGGATCAGTCTTTGTTCCATTTATTTTAAGTGGGGTGACCGTCATCAGCCATTATTTCCAGGATGTGACCTCCTCAGGGTTTAAAAATTTCATTTATAGTGTTTTGAATATTTAATCT

Homo sapiens       TACATT-AGATAG-------AAAAATTTTCTGGTCTCTCC--AGGAGGTAAATGGGTAGGGGGAGGAAGACAAGAGATGGTAGGAACTTGG-----AACCAAGAGGTT-----TTTAAAT
Loxodonta africana TATATTAAGGCAATTTGACTAAAAATTAACTGGGCACTCCCTAGAAAGGAAAT...........................TAAGAACTCAGCTACTAACCGAGAGGTTGGCAGTTCAAAT

Homo sapiens       ATAAAAGCAAAATACGGAAGCTACTGTGGATTATGCAGACTCACATTTTTTT--------TCCAATGGC--------TGGTTTGTTTTCTACCAGTTCTGGTTACCCACATCCCTCTGTG
Loxodonta africana .....................TACTCTGAAACACACAGGGTCACCATGCATTGGAGTTGACTCAACGGCAACAGGTTTGGTTTGGTTTTGGGATGTTCCCCTAACACAGTCTGGTGGAGG

Homo sapiens       TTTTGGGGCCTGTACTTCCTCTAAGGTGTGAGAGTTT---GGTCTTTTCCTTCTGTGGGCTTTTACAGG---CCTGAAAAAGCTCCTCCCC----------TTTTCTTTTTAAGGTCCTT
Loxodonta africana CACTTGGGCCTGTATCCCCTCTAAGGTATGAGAACTTACAGGAGTTTGCCTACCGGGGGCTTTTACAGATACTTTTAGAAGGTTTGTCCCCAAATTCCTTTCTCTCTCTTTCACTACTTA

Homo sapiens       CAGGAGTTTTTGCAACCTTTACTGGTCTCTAAAAGGAAGGAAATACACAATTTTTATCTTCCTTTTATAAACCTCGTCTTCCTACAGATACCATCCTCCCAACTGGCTAGTATGGGATAT
Loxodonta africana CTGAAGTTTTT-TAGCCTTTCCTGGGTATTTTAGGGGGATCAATGAAAA---TTAAACTTTCTTTTGT----TTCTTGTTCCCATCAATACTAGACT-CCATTTGGCCATGATGCGGTTT

Homo sapiens       TTCTAGATTTTCCTTTGGATTGCTTAGACGATTTGTGACATTGATAGTTCCAAAAACTATTAAGGGAAAACTATAACAGGAGGTTATATGTAGTATATTATTAATTTTACAGTATGTATA
Loxodonta africana TTCTAGGTTGTCTCTTAAATGGCTCCA---------GGTCTTGATGGTAGCAGAAATCATCAAGGGAAAAC.................................................

Homo sapiens       GTCTATATATTGATTTCATCTAAACTGAGAAGGGACAGGTTTCTGTCCCTTAGACTTGAAAGAATGTGTTATCATTGTAGTATAGAGATGAAAATAGAGGACACAAAGAAAAAAATGATTT
Loxodonta africana ........................................................................................................................

Homo sapiens       TGGTTTCTTTTTTTTTTAAGAGTTGAGGACGATTAATGCAGAGCTAGAATGATAGGCAGGCAAATGGATGGGTAGACAAACAGGTTCTATATTCCCAACTTGAAACGACACAAGGCTTCA
Loxodonta africana ........................................................................................................................

Homo sapiens       TGAATTGAAAATTGATTAGGAAGTAAAAGTTTGCCATGGAGTCCTGGCAGAGGAGAGAGTGTAGTGAGGATGACATGGAAAGTAAAGGCATCAGGAGGTCTTATGTGGCAGATTGGAGGG
Loxodonta africana ........................................................................................................................

Homo sapiens       CAGTAGGGTGCCACTAGTGTTTCACCACAGATGGTTTGAAAAGCACTGACCTGGAGCGACATGAAACTTGGTAACAATAGAAGTGGCTACAGGACCAGAACAGAGTATACGCATGGTAAA
Loxodonta africana ........................................................................................................................

Homo sapiens       ACTTCTTGATACCTTTACTGTTTCCATTTAGTGGCTATTATGACTATAAAATATCTATGAACATTTTTGTACACATATTTTGATAGTCATAGACTAATTTCACTGGAATATAAAATTAGA
Loxodonta africana ........................................................................................................................

Homo sapiens       ATTGCCAAACCTTTAGGGTAAATTTGTGCTCAGCTTTAGAAGAAACTCAGTTTTCCTAGGTGGTTGCAACATTTTATCCTCCCACAATGTATGAGAGTTCCAGTTGCTGCCTGTCTTCAC
Loxodonta africana ........................................................................................................................

Homo sapiens       CAACACTTGGTGTTGTCTTTGGTTTTTAATTTTAGCCTGTTCAGTAGTGTGTAATTCTAAGTCATCGTGGTTTTATTTGCATTGCCCTGTGGAATAATGATGTTGAGCACATTTTCACGT
Loxodonta africana ........................................................................................................................

Homo sapiens       GCTTATTGACCATTGGATATTCTCTTTTATGAAGTGCTGTGTAGAAATTTCCCCCCATTTTTTGAATTGGGTTTTTGTCCTTTTCTTTTTGATTTTTAGAAGTTCCTTGCATACACTGGG
Loxodonta africana ........................................................................................................................

Homo sapiens       TCTGAGTCCTTTGTTAGATATATGTATTACAGATATCTTCTCCCAATGCGTGTCCTATGTTTTCACTTTCTTAATGGTGTCTTTCATGAATACACATTTTTACTTTGATAAAGCCCTATT
Loxodonta africana ........................................................................................................................

Homo sapiens       GTTAATTTTTTTTTCTTTTAAGGATAGTGTTTTTTTTTTGTGTCTTATTTAAGAAATCTTTGCTTAGCCTAAGATTTGAAAGATCTTTTCCTATGTTTTCTTCCAGAATCATTGTTATTT
Loxodonta africana ........................................................................................................................

Homo sapiens       ATCTTTCACATTTAAGTTTATGATTTATCTGGAATTAATTTTTGTGTAAGATGTGAGGTAGGTAGTTAAGGTTCATTTTTTCCCCTAATACAGTTATCTAATTGTCCAACATTATTTATT
Loxodonta africana ........................................................................................................................

Homo sapiens       GAAAAGACCACCTCTTTCCTCATTGTGTTGATATCTTCATTGTAAACGTATTGACTTTACATGTGTGGATGTAATTCTGTACTCCTTGTTCAATTACAGTGATGTATTTGTCTGTCCTTA
Loxodonta africana ........................................................................................................................

Homo sapiens       TGCCAATTGGCTTGTCTTATTTACTTTATTTGTATAAAAAGTCTTAAATCTCATCCATTCATCTTTGAGATGATTCAGAAACTGTGATAGGCTGCATTTGTTTGGTTTGGGATGCAGCCA
Loxodonta africana ........................................................................................................................

Homo sapiens       TCTATTAGAAGATGAAAAAAATCATGTATTTAATAATCAATTGGAAGTGAATTAAACTTAGAACAAACTGATTGTCTTATAATACTTTATAGAGATAATTAACCTGTTTTTTAGGTTATG
Loxodonta africana ........................................................................................................................

Homo sapiens       ATGGGACTGTGTTAACATTTCAGAACCTTTTCTTTTAAATTGACATATACTTTATGTTAACCTAAGGTAAGAAATGTACTGTTACTCTCTCTGGAAGTTAAAAGTAGATCAGAAAGACGG
Loxodonta africana ........................................................................................................................

Homo sapiens       TTTTACATTTGCAAAGAGCTGTGTCATAGGGAAAGTGAATTGGTAAGCTGTTTAAGCCATGACAATAGGCACAGTCTGTTTCTGCTTTGTTCCAGCATTCTCTTTCCAGTAAATACTCTT
Loxodonta africana ........................................................................................................................

Homo sapiens       AGTCTCATCCCTAGATCTTCCAGTGGTCAGTAAACATCTTTTTTTAAATGTAATTTCAAAATTGTAAGATTTATGTCAGAATCCTAGCAGGAAACAGAAGACATTCTGGTGGGATTTTAA
Loxodonta africana ........................................................................................................................

Homo sapiens       AGAAAATTTAATGGAGCTCCTTTCTATAGACAGACAGTAGCAAAGTAAACAACATGGCACCTGGAGACTAGCAAAAGCAAAAATAGTTACCACCTTAGGGCTGAGGTGACAAGAAGAAGA
Loxodonta africana ........................................................................................................................

Homo sapiens       AATAACCATGAATATATTTAGAAATAATATAAAGCTATATTT-CTTCTCTTATAACTTATT------TATTTAAATGTCACATGACAGGATCCTCCTGTGTTACCATCCTGTGAATGTAA
Loxodonta africana AGTAACCATGAATATGGTTGGTAGTGATTTAAAATTATATTTTCTTGCCTTATCATTTATTGTCATTTATGTAAGTGTCACATAACAGGA------------CCATCCTATGTAAGTGA

Homo sapiens       CATTTCAGTTGATAGCAAT----GAAATTTTAAGCAAAACTTTTTGAAGTGTACTG---GTTCATTTCCTTTTTT----CTCAGTATGTGGAAAATTTTCAGCATTTCTTATCAAATGGA
Loxodonta africana CATTGCAGTTGATGGCAATAGGAGAGACTTTAA-CAAGATTTTTCAAAGTGTACTACTTGTTCATTTCCTTTTTTTCTCCCTGATATGTGGAAAATTTTCAGCATTTCTTTTCACATGGA

Homo sapiens       AAGAGACAAGATAATTTGTCATGGGGTATATCAAAAATAGCATTTGAGTATTTTTGTTCATATACATCGCT--GTTCCTTTCCTGTACTGCAATTATCT--CCCTCACAAATAA-ATTCT
Loxodonta africana AAGAGACAAGATAATTTGTCATGCTGTATCTCAAAAATAGCATTTGAATATTTTAGCTCATATGCATCACTCTATTCCTTTTCTGTACTGCAATTAACCAACCCCCTCAGATAATATTCT

Homo sapiens       GGGAATTCTCGGTTCATATGTAAATAGAAATATGACAGAAGTCTTAACTGAAATAGCAATTCAGTTAGCTTAAAATTTAAGTTATCTTTTTAAATACCTTTTACCTAGGTTCACAATGGT
Loxodonta africana GGGAAAGCTAATGTCATATGTAAGTGGAA-----------GCCTGAACTGAAATAGCAATG-AATTAGCT---AATTCAAATTCTATTTTTAAATACCGTT-ACCTAGGTTAACAATGGT

Homo sapiens       CCGTAGTGGAAAAAAATGGTGACCTTCATCTTAAACAGATTGCATATTACAAACGAACTGGTGAATATCATTCAACTACACTGCCAAGTGAGAGAAGTGGCATAAGAAGAGCAGCAAAAAA
Loxodonta africana CCGTAGTGGAAAAAAATGGTGACCTTCATCTTAAGCAGATTGCATATTATAAACGAACTGGTGAATATCATCCAACTACACTGCCAAGTGAGAGAAGTGGCATAAGAAGAGCAGCAAAAAA

Homo sapiens       ATTTGTCTTCAAAGGTAAAATTAATGTTCA-AATTTTGATTATTGAGTTG---TAGATTCTTCCCCACAT-TCTTCCTTGTGGAAGTTTGGGAATTTCTGTAGTAG--TTGGTTGTTGCT
Loxodonta africana ATTTGTCTTTAAAGGTAAAATTAATGTGCACAACCTTGGGTACAGAATTAATATAGATGTGTCTCCAAATGTGTTCTTTGTGAAATCCTGAGAATCTGTATTGTTGGATTGGTTATTGCT

Homo sapiens       AAACAATTACCAGTTGAATTGATGATGCCACTCAATCAAAAGCAAAATAAAAATTACACTCAAGCTAACTGAAGGCCAATAAGCGTGAAAAGGGTTGTTTAAATTATAGACAGAATATGA
Loxodonta africana TAACATTTAATGGTAAAATTGATGATGCCACTCAGTCAAAAGCAAAATAAAAATTACACTGAAGCTAAGTGAAGGCCAG-AAGTATGGAAAGGGCAGTTTAAATTA----CAAAAACTGG

Homo sapiens       TACCATGAAAGAAAACAAAACATTTTTCTAAATTAACAACTTCTGCTTTTAC--CCTCTCGTGGT-----CTCTAATGGTGCACTTTCCAGCACTGTTGCCAATTTCAATGAGGAACTTT
Loxodonta africana TACCATGAAAGGAAACAAAACAG----CTAAGTTAACTTCTCATGCTTTTACAACCTGACGTGGTTCCTACTCTAATGGTGCAGTTTCATGCACTGAGACTGCTATTAATGAGAAACGGA

Homo sapiens       TTTGATGCTTAATATGAAAATGTTGACAAAGTTAACTGGTTTGTTTACTAAAATGAAAACTGGAAGGAATTAACTATATTTCTGTG-ATCATGTCCCCTTGAACCCCACCACTGCCATTA
Loxodonta africana TTTGGTGGTTAATATGAAAATGTTGA-AAAGTTAACTAATTTGTTTACCGAAGTTAAAA-TAGAAGGAAT-GATTATATTTATGTGTATGATCTGTCTTC--------CCACTGCCATTA

Homo sapiens       AATATTCTCAGGATCACATTCATTTTAGGACCCTTGGATCTTCCTGAATGGATTAAATATTTGAAAGGCAGTGGGATATTTATCTTGGAAGGTCATGATAAT---TTTTGCCTCTTTTTC
Loxodonta africana AATATTCTTAGAATTGTATTTATTTTTGGACCCTAAG-TCCCCCTGAATGGA----ATGTTTGAAAGGTGGTAGAATGCGTATCTTGAAAGGTTATGATAATAGTTTATCCTTCTTATCC

Homo sapiens       CCCTTATAAAGGGGAAGAGAAGTTTGGGATGAGATAAACAACAAGTA--GTCACCAGATAATTGAATGAATGTTTGTGTTACAAATGGGAATTTTGCCT-----------CTTAGGCTTA
Loxodonta africana CTCCTTCAAAGGAGAAGAAAAGTTTGGGGTGAGATGAGCAGCAAGTACAATCAT-AGATAACTAAAGGAATGTTGGTGCTATTATTGGGAGTTTTGCCTGTAGGTTCAGAATGAAGATTT

Homo sapiens       CCCCAAAACAGGTTAATAAAACTCTAGGATAATAAATAAATGTCTGCTTGCATCTCCCTGTGTATATCCCTACCAATAACTCCCTTATACTTAAGATTAGGCAGACCTATTACCTCTTAA
Loxodonta africana CTCCAAAGGAGGTCAAGAAAATTGTAAGATGAGAAAT----GCCTATTCATTTCTCCCTGTGTACACTTGTCCCTATAACTTCCTTTTGCCAAAAAATTAGCA--TTTATTACCTCTTAG
```

Homo sapiens ACTAAAGTGAGGATTCTTTCATTCATATATTTGATAATTTATGTAACAAATGTTAACTGGGTTTCTGCTGTGTGCCAGTCACATTCTAAGCCTTGGAGATACATTGATGAATAACATAGA
Loxodonta africana ACTAAAGTGAGGAGTCATTCATTCATTTATTTACTCATTTATGCAACATATGTTTACCGAGTCCCTACTATGTGCCAATCACATTCTAGGCCTCAGGGATGCGTTGATGAGTAAAATAGG

Homo sapiens TAAGGTTCCTGCTCTTAGGTTGCTTATCTTCTAATTCTAAAAAATAAGGAGTGTTCCACTCATCAGTTGGCTTACTCATATTAGTAGCTGAATGGTGTATTTTGGAGTTTGAATTGCTAG
Loxodonta africana TAAGATT--------------------------------GAAGTAAACACTATTCCACTACACATGTG-TTTAGGCACTCTAGTAGCTTGGTAGTATATTCTGAAGTTTGAATAGCTGC

Homo sapiens CCCATTCACATTGGAAAGGTAGATGGTGATTCGCTTCATACCCTGGTTACTTCCTAGCTGCAGATTCTTCTCAGGTGTCCTCCTAA----GGAAACAGAGAATTGTCTTGAGGATTACAG
Loxodonta africana CCCATCTACATTGGAGGGGT----GGTGGTTCTTTCCATTTCCTAGTTGCCTCCCAGCTATAGATTCTTCTC-GGTGTCCTCCCAAAAAGGAAAGCAAAAAATTATCGTGAAGATTA-AG

Homo sapiens TTTTTTCCACTTGCCCGCTTCACCATCCCTACCT---TCTCAAATATAGAAGAAGCTGAGAGAGCTGTTTGCTT-GCAAAAATACAGTGATGATAAAACATTATTTTAGAAAATTCTGTT
Loxodonta africana GCTCTCCCACTTTCCCACTCTTCCTTCCACACCTCCATCTCAGATCCAGAAGAACCTGAGAAAGATGGTTGCTTTAAAAAAATGTAACTATGATGAAAC--TATTTGGGAAAATTCTGTT

Homo sapiens CCAGGAAGTTGTTTTTCTGTACATAATTTATTTGCTAATCTATGCCATTTTTAGGAGGTGCCTGCAAAA--TCTTCCTACTTTGAGTACTCCCAGAGAGAGATAATTTCATATTTCCGTA
Loxodonta africana CCAAGGTGTTGCTTATCTCTACGTAATTCATTTACTAATTCATACCATTTTTAGGAGGTACCTTCAAATATTACTTCTAATTCAAGTGCTC---AACAAAGGTGATTTCATACTCCCGTA

Homo sapiens CTTATTACGTGCTTTGGTGAACAAGCTTGCTCTACTTATATTGATAGCTA-CAGAATTATAGCAGCTACTGCCCTTCCTAAGTATCTAGTAAGTGTAACAGCTAAGAACCTTT---GTGT
Loxodonta africana GTTATTGCATGCTTTGGTGAACAACCCAGCTCTACTTGTATTCATAACTAGTAAAATTATAGCAGCTA-----------GATATCTAGTACTTATAACAGCTGAGAACCTTTCAGATTG

Homo sapiens TGTAGCATCATTAAAAATAAATATTCTGTTTTAAATGCAGTAAGATTTTACAAAACAAAACAAAAACCTTCATGGTTTAAAGAGAAGGCTATGATTCTCTTAAAACTACGTAGATCTCTA
Loxodonta africana TGAAAGGTCATTACAAATAGATATTCTGTCTTAGGGGAAGAAAAGCTTATTAAAGAACAAATGA-----CATGGTTAGAAGATAAGGGGATGATTATCTTAAAACTTTATCGATAGCTA

Homo sapiens AAAATTGCAATTCTATCTTTT-GGTATCATTTATTCCAGAAAAAAAGCTGTTTTATGTTGGAAAAGACAGAAAACAAAATCGTTTGGTAATTGTTTCAGAAGAGGAAAAAAAGAAAGTCT
Loxodonta africana AAAATTCCAATTCTATCTTTTTGGTATCATT-GTTCCAGAAAAAAAGCTATTTTATGTTGGAAAAGACAGAAAACAAAATCGTTTGGTAATTGTTTCAGAAGAGGAAAAAAAGAAAGTCC

Homo sapiens TAAGAGAATGCCATGAAAATGACAGTGGAGCTCATCATGGTATATCCAGGACCCTCACTCTGGTAGAATCCAATTATTATTGGACATCTGTGACCAATGATGTCAAACAGTGGGTATGGC
Loxodonta africana TGAGAGAATGCCATGAAAATGACACTGGAGCCATCATGGCATATCCAGAACTCTCACTCTAGTGGAATCCAGCTACTATTGGACTTCTGTGACCAATGATGTCAAACAGTGGGTATGGC

Homo sapiens TTATGTATTTAGAGTATTATGAATAATGTCTTTCTGATAGATACTTAAAATTTATTATTTCTCCAGATTTATTCTGGGTATCACCTCTCATTAAGTATTTTTGGCA-------------
Loxodonta africana TTATGTATTTAGAATATTTTGAATAATGTCTTTGTAATAGATATCCAAAACTCATTTTTGCTACAGATT-ACTCAGGCTATAACTTCTCATTAAGTACTTTTTGTATGGGTATTTTTTAT

Homo sapiens -----ATCACTTAAAGGGAGTTGTATTTCCCTTCACAGTTATT--TTAA-ATCACTTAAGTATAATTTGAACAGGAAAGAAGCATTATTTAAACACTTGAAAATACCATATTTAAATAAG
Loxodonta africana ACAAAAACCTTGAGAGGTAGTTTTATTTCACTTCACAATTTTTCATTAATATCTCTAATGCATAATTTGAACTAGAAAGAAGTATTATTTAAATATTTTAAAGTACCAGATTTAGATAGG

Homo sapiens AGATTATTTAAAAAATCAGATTTGTATCATTGAACTTTTTTG-TGTAGGCAGAATTTAAATGGAGCTGGAATTCAGTAAATCATTTGACTAAATCATTACTTCTTCAATGGCAGTCATCACAAGGTTTT
Loxodonta africana AAATTATTTAGAA--CTAGCTTTTCATTGTTGAACTCTTTTGGTGTAGGTAGAATTTGAATGAAGTTCAAATTCATTTGACTAAATCCTTATGTCTTTGATTGCAGTC-----------

Homo sapiens CTTAAGGGGAGAATATGTTCATAGTAAGTCAAGCCCAGTATGTGCTCTTATCTTTTGTGCCTGTTCCATTTTGAGGTAGAA--TGTCATACATGACAGAAACCCAAAACCAAATATAACC
Loxodonta africana -----------------------CAAGTCAGGCCTATTGTGTGCTCTCATCTTT-GCGTCAACTCTATCTTGTGGTAGAAAATGTCAGATATG-TAGAAATGCAAAACTAACTA-AATA

Homo sapiens AAAATCAAATTTTGAGGATAATGAAATAAGTCTGAGTTTTGTAACTTTATGACCCATTGGGACCTTCAA---CTTCTAGTAGAAAATTCACCTATAGGAATTTTGGGGTTCTTGCTGGAA
Loxodonta africana AAAATCAAGTTGTAATGACAAAGGAATTAGCCTGATTTTTGTAACTTTATAATCTATTTGAGCCTTCAATCTCTCCTAATAGAAAATTCACCTA------------GGGTTTTGC---AG

Homo sapiens GTCAGCTGATTCT-ACTCTTTCCAGTGTACTGTAT--GTCAAATGGTAGGAATTCCTTGGTATATCTGTGAGATCTCCTTAATAATTGTAGTAGTACATCAAAAAGAGTGGCATTTCAAG
Loxodonta africana GTCAGCTGATCCCCACTCTTTCTGGTGTACTTTCTTTGCCAAATCGTAGACATCCCTTGGTATGT-----------------ACATGTAATAGGGCATCACAAAGACTGATATTTTAGC

Homo sapiens CATAAAGAATTAAACCAAAATTTCAT-AAGTATAATTTGTGAGTTATCAGTTTGTGGGTCTATTCAGGTATTCCAAAGACCTTCATAATTTATATATTTTTCCTGCTGTGCATTTTTTAC
Loxodonta africana CGTAAAGGATTAAACCAACATTTAACCAAGTATCATTTGTGAGTTATCCATTTGTGAGT---------TATTCCAAATACCTGTATATTTGAA----TTTGCCTTCAGAACCCGC----C

Homo sapiens AAACATTCAGTGAAATCTTTGTCAAATTGTTATCACTGGAGAATGAATATGATTTTTGGAAATAATCAGAAGTTATTTGTGATAAAATCCAACAAATAAGTTTAAATTGGAAGTGAAAA
Loxodonta africana ACACATTCAGTGAAAATTTTACCAGATGTTTATCATTTGAAAATGGATATGATTTTTGGAAATAATCAGAAGTTATTTGTGTTGAAAACTAGTGAATGATTTT------GAAAGTAAAAA

Homo sapiens TTTTGATCAAAAGTAACTGTGACTATAATGGGATTAAATGGCTTTTAAATTTTTCTTCAGAAGCTAGTTTGAAAGTAGTTTCTAGAGTGGAATTCCAGAGATACATTGAGAAATGGCAC-
Loxodonta africana TTTTGATAAAAAGCAACTGTGGCTGTAATGTAATAAGATGGTTTTAAAAATTTTATACAGAAGTTGGTTGAGAGTAGTTTCCAAAG----ATTCCAGAAATATATTGAGCAGTGGCACA

Homo sapiens CCATCCATAAATTGATCAGTTGATCTTGTCTCACCTCACTCAAATGAAAGCTGCAGGACAGCATGGGTTTTGTGTATGTTTATTCTTTT--CCCACGATTGAGAGAAATTGCTC-CATCT
Loxodonta africana CCATCAATAAGTTGGTCAATGGGTGACTTCTCCCCTTACTCAAATGAAAGCTCCATGAC-----------TGTTTTTGTTCACTCTTTTTTCCCAGGACCTAGAGCAATTTCTGGCATCT

Homo sapiens ATACATTTTATATGAATGAAATTATATTTCAATAAATATGGTTGGAATAGATTAATGAATAAGCTTCTTAATGTGATAACTTTAGAAGGAAACATTTTATGTATATAGAAATTATTGTAA
Loxodonta africana ATAAATATTGTTGGAATAAAATTATATTTTAATAAATATTATTGGAATAGATGGATGAGTAAACTTCTTCGGGTGATAACTTGGAAAGAAAAAATCTCTTACATATAGTAATTTTTGTAT

Homo sapiens ATTTACATACTGATTTAA------TAGGAAACTAAGATCTTTTTAAG---TATTTAATAAATATTTATTGAATGTGAACCAGATTTATGTTCCATGTGAATAAGACCCAATAAGATGGT
Loxodonta africana TTTTACATACCAATTCAAAAATTATAGCAAACTTA------TTTCAAGATCTATTTATTTAATATGTATTGAACGTGAACCAGAATTATATTCCATGTGAATAAGACCCT-TAAAGTGGT

Homo sapiens CATTTGGTAGTATAGTCTTCCCTCAGTATATGCAAGGGATTGGTTCCAGCACCACACTCCTGTCACCAAATCTACTCATACCGAAGTCCTGCAGTCAGCCCTGTGAAACTCACATATACA
Loxodonta africana TATTTAATAGTA...

Homo sapiens AGAAGTCAGCTTTCTGTATACTTGAGTTTCTCATCCCTCCCTATCAATAGAGTACTTTTTATTTGTGTTTAGTTAAAAAAAAAAAGCCATGTATAAGCTGACCTTCACAGTCCAAACCCA
Loxodonta africana ....GTAGCAACTAGTATTTGCAGCCTAA-----

Homo sapiens TGTTGTTTGAGGGTCAGCTGTAGTGGCTACTACAGTTGTTTGTTGTTGTTTTATTTGTTTGTTATTCTAAATAGGTTTAATCCAGTGATTCTCAGTCCAGAAGTTTCTCCTTCAAAACAC
Loxodonta africana ----------------------------CTATAATGATCT--ATTTCAGTCCAGAAGCT-CTGGTTCTAAACA-

Homo sapiens AAAGTATACCTATTAACCATTTTAAAATTCAGATTTATAAGACTGCTTTTATATTGTGTTGA--GTGTTAACATTTGTATGTGTTTTCTTTATTGTGTGCCTGTT--GTAGGTATATGCT
Loxodonta africana ---GAGTACCTGTGAGCCATTTTAAAATTCAGATTTATAATATTGCTTT---ATTATGTTGACTATATTAACATTTATGTATTTTATTTTTATTATGTGCCTATTTTGCAGGTGTATGCT

Homo sapiens TGTCAGCATTGCCAAGTGGCAAAAAATACAGTTATTGTAGCACCGAAACAGCACCTTCTCAAGGTGGAAAATCCATGGAGTTTAGTTACTGTTGATCTGATGGGGCCTTTTCATACAAGC
Loxodonta africana TGTCAGCATTGCCAAGTGGCAAAAAATACAGTTACTCTAGCACCTAAACAGCACCTTCTCAAGGTGGAAAATCCATGGAGTATAGTTACTGTTGATCTGATGGGACCTTTCACACAAGC

Homo sapiens AACAGAAGTCATGTATATGCTATAATCATGACAGATTTGTTCACCAAATGGATTGTGATTTTGCCTCTATGTGATGTTTCAGCATCAGAAGTTTCTAAAGCTATTATCAATATATTTTTC
Loxodonta africana AACAGAAGTCATGTATATGCCATAATCATGACAGATTTGTTCACAAAATGGGTTGTGATTTTGCCTCTATGTGATGTTTCAGCATCTGAAATTTCTAAAGCTATTATCAATATATTTTTC

Homo sapiens TTATATGGACCTCCTCAGAAAATAATAATGGACCAAAGAGATGAATTCATTCAACAGGTAAGACAAATAAACTACTTAGGTCTGGGAGCATATCTTACTTCT-TTTCAGTGTCCAGAACC
Loxodonta africana TTATATGGACCTCCTCAGAAAATAATAATGGACCAAAGAGATGAGTTCATTAATCAGGTAAGA-----AAGCTGTTCAG-----------ATCTTACTTATCTTTCTTTGCCCATCGCC

Homo sapiens -AGTGCCCTCTGCATTATATTACAGAGTGCTTAATAAGTTCAGATAAATTTCTGTAGGTATAGAGGTATGATATTAAATACTACAAGGATAGTTGTACTGGAAATTAGGAGATTAGGATT
Loxodonta africana AAGTGCCCTACACGTTAT-----AGGATGCTTAATGAGTACAGATGTATTGCATTGGGTA...

Homo sapiens TTAATACTTACTGTGGGTTATTGTAAACCTATCTTCAGATATCCTCAAATTAAAATGAGGAGGTTGGACCAAATAATCTTTATTTACTTCTAGATTAAAAACCCATGATTGGTTGGGCCT
Loxodonta africana ....

Homo sapiens GGCTCATGCCTATAATCCCAACAGTTTAGGAGGCTGAGGTGGGTGGATCACTTGAGCTCAGGAGTTCGAGACCACCTGGGCAATAGGGCGAAACTCCATCTCTACAAAAAATACAAAATT
Loxodonta africana ....

Homo sapiens TAACTGGGCATGGTGGTGGGCGCCTGTAATCACAGCTACTCGGGAGGCTGTGGCAGGAGAATCACTTGAACCTGGGAGGTAGAGGTTGCAGTGAGCCAAGATTGCACCACTGCACTCCAG
Loxodonta africana ....

Homo sapiens CCTGAGCGACAGAGCGAGACTCCATCTCGAAAAGAAAAAAAAATTAGCCAGGTGTGGTGGCACACACCTGTGGTCCCAGCTACTCGGGAGGTTGAGATAGGAGGATCACTTGAGCCCGGG
Loxodonta africana ....

Homo sapiens AGGCGGAGGTTACAATGAACTGAGATTGTGCCACTGCACTTCAATCTGGGCAACAGAGTGAGACCTTGTCTCAAAAAATAACAATAATAATAATACACTATGATTGACAGGTAAAGGGA
Loxodonta africana ....TAAAAATTCTATGATTGACATATAAAGAGG

Homo sapiens GACTTAAATAAATGGAAGAATACATTTTCTTCATGAATAAGAATAGTCAGTATTGAAAGGTACTTTCTCCCTTGCTCTGGCCTTTTCCCAGTTAAAATTAACCTGAAAGGATAAGCAGGG
Loxodonta africana GACTTGACTAAATGGAGAAATACATTATGTTGATGAATGAGAATACTT--TATTGAAAGATGCTTTTTCTTTGCTCAAGTCTTTTTCCAG-----ATTACCCTGAAAGAACAAGCAGGG

Homo sapiens GAGAATATATATGAAAACTTTTTATAAGAAATACAACAACTGCTAGACTTAAGGCATATGTTAAAACTTTTTGTAATTGACAGGGTATATGTTTAAGAAAAATCAGAGATAGCCAGAC--
Loxodonta africana ATGGATGTATTTACAAATTCTGAAAAGAAATGTAAAGGTTACTAGACTTAAGGTGTAT-TTAAAATTTTGTATAATTAATAAAGCACTGGT-----AATAATCAGAGATAGCCAGACTA

Homo sapiens TTGGTTGAAAGCATCTTTGATGGCTCAGTCCAAAAGAAATACCTAAAGTTAATATGTATTAACTAGTAGATAGGACATGTGCCATGGCACCCCAGGATTATTCCCAGTTTCCATAATTCT
Loxodonta africana TTGGTTCACAGCACACTTAGTATCTCATTTCAAAAGAAATATCTGAAGTT------TATTA---AGTAGTTAGGTCCTGTGTCTCTGGTCCCCAGTACCA-CCCCAGGTTCAATGATTCA

Homo sapiens CTAGGAGGACTCAGCATATAGTCATACTCATGGCTAAGATTTAT-------------------------TCAGCAAAGAGAAAAGACACAT-----------TGGGAGAACCAGGTACAA
Loxodonta africana CTAGGAGTACTTGGCATATTGTCATACTCATGGCTAAAATGTATTATATACCAAAGTATACAAAGCAAAATCAGCAAAGGGAAAAGGTGCATGGGAAAAAAATCTGGGAAAATCAGATGCAA

Homo sapiens GTTTCCAAGATCTCCTTCCCCAGTGGAGTTACACAGGACGTGTTTAATTCCTTTAGCA--GAGTTGTGACAACATA------------------AGGAAGCTCACTAGAGTCTCAGTGCC
Loxodonta africana ACTTGCAAGAG-TCTTTTACCAGTAGAGACACACAGAGTGTACTTAATTCTTTTAGCAATGAATTTTGACAGCACACATGAAATGTTGTCTACCACGAAGTTCATTAGAGACTCAATGCT

Homo sapiens TAGGGTTTTTACTGGGCGCTGGTCACATAAGCACCTTCTACCTGGCATGTACAAAAATTCCAGACTCTCAGAAGGAAAGATTGTGTTTGGTCTTATAACCAAAATCTAAGCTCCTAGACA
Loxodonta africana CAAGGTTTTTCTTGGGAGCTGGTCACATAAGCAGTCTCTGCCTAACAGGTACCGAAATTCTGCACTCCCAGAAGGAAAGCAGGTGTT................................

Homo sapiens TTAGCCAAGGTCCAACCTTGTAAGCATGCCTTTCAAAGAACAGCAGTCACAGATCTGCTTTGTTAATATGTTAAAATTAACTGTCAGGTTAACTCTTTTCTTCACAGGTCCATCCAAACA
Loxodonta africana ....

```
Homo sapiens        ACTTAATAAGTATTTATGTTCTAACCACTTAGTAGCAATTTAAAATACTCATAAATTGAAAGAAAATATATTTTTGTTCCATTCTTTTCTTTTTTTTTGAGACGGGGTCTCATTCTGTCA
Loxodonta africana  ........................................................................................................................

Homo sapiens        CCCAGCCTGGAGTGCTGTGGCACCGTCTTGGCTCACTGCAACCTCCACTTTCCAGGCTCAAGTGATACCCCCACCTTAGCCTTCTGGCTAATTTTTGTACCTTTTTTCTTTTAGAGACAG
Loxodonta africana  ........................................................................................................................

Homo sapiens        GTTTCACCATGTTGCCCAGGCTGGTTTTGAACTCCTGAACTCACATGATCCACCTGCCTCGACCTCCCAGAGTGCTGGAATTACTGGCGTGAGCTACCGCACCCAGCCCTTTCATTTCAT
Loxodonta africana  ........................................................................................................................

Homo sapiens        TCTTAACCATAATTACTTGCTAATGGGTAATGTGTACCTGTTGACCACTGCTTAACTTCTCAGACCTTGGGATCATATTGTACACTGCCACCCTCATTTCCTGTTTCACACTGATTTTTA
Loxodonta africana  ...TAACTACAGTTACTTACTAATGGGATGTGTGTACCTGTTGAACATTGTTCAGCTTGTCAAACCGTGGAATGAGATTGAATACTGCCACCTTCATTTCTTGTTCCACATTGATTTTTA

Homo sapiens        CATAGTAACTGCTTTTTATCACAGCATTCGATGAAGAAAACA-GTTCTGTAAAGATATGATGTCATCAAACAAATTGTAACCTGATCTAATGTTGAAACTGAACTACCTTAAACTGGTAG
Loxodonta africana  CACAGTAGTTACTTTTTATCACAGTGTCTGCCAAAAAAAACCTGTTCTGCAAAGATATTATATCGTCAAAAGGAATGCAACTGGCTCTAATGTTGAAACTGAACTACCTCAAGTTAGTAG

Homo sapiens        TTTTCATGGTGTCAAACAGATGTTAAGGGGGCAACTTGGCACAAAGTTTGGTAACAATGGTTTAAATGATAAAGCTAGTGTCACAAATGTGTCTAGTAGAGGAGGATCATGTAATTA---
Loxodonta africana  TTTTTATGGTGTCCAACAGACATTAAGGGGGCCCCTTGGCACAGAGTTTGGTAACTGTGGTTAAAATGATAAAACTGGCATCACAAAGGAGTTGAATAGAGAAGAATTATTTAATTAACT

Homo sapiens        ---------ATTAACAGGTAACAATTTG--CTAATTGTTTAAAATTATATCTGTAATATGATTTTAGAATCCAATACCAAAAGTATTTTCAGGTAAATTAAAA--GAATTTTAATTTTTA
Loxodonta africana  TCATTAAGCATTAACAGGTAACAATCAGAATAATAATATTTTGGGATCGTAT-----------------ACCATATACTAAAATTACTTGAGGCCAATTATGACTTATTTTTAGTAATCA

Homo sapiens        AAGTAGAAACAGA-----AAAAAATATATTTGACATTT------AATCTCTAGAAATGGAGGTCACATTCTAAACCTAGGAATAGTGGTATAAACTTCAATGGAAAAACAT---TAAATT
Loxodonta africana  AAATATAAAATTAGTTTTAAAAAATGTAGTTGCCTTTTTAAAAAAATCTCTAGCAATGCAGATTACATTCTAAGCTTATTAACAGTGTTAAAAATTTCTAAAGAAAGACATCACCAATTT

Homo sapiens        TGGCTGCATAATAATAGGAGATTTA--TGTGGTAAAGTAATCAACATATAAAAGCCAATCTTGCAAATAAAGCAAGATAATTTTGTAA---------ATGAAGGCAAGGTAGTCTTGCCT
Loxodonta africana  TGGCTGTGTAAAAATTAGAAACTTATCTATAGTAAAATAACCAAAACATAAAGGCAGGT------AACAGACCAAGAAAAATACATAAAACAGATCCATGAGGAAAAGGGATTTTT....

Homo sapiens        GCCTTGATTGACATAGCAGTGGTCTGAGCAATGGAGTACGTAAAGGCCATCCAGTTTGGGGGAAGGCTATTGGAACTTCTATTTATATTTTTTAAATCCCAAAAATAAGAAATGAAGCTT
Loxodonta africana  ........................................................................................................................

Homo sapiens        TATTAATAATGTATACGATTGATAATAGCATTCTGACTCAATCAACAAGACATGTATGTCACACATGCTATGTGAAGGTATCCTGAAAGAAAAGTGAGAATCCACATGTTTCCTCACCCA
Loxodonta africana  ........................................................................................................................

Homo sapiens        TACTTTTGCTTTCAGCAGTTGAATGAAAGTATGGATATCATCTAGCATTCACCACACTACTCACAAAATGGCCAGGGAGCTTAGATTTTTGCAGTGAAAGCAGATAATACTAAAAGGCAA
Loxodonta africana  ........................................................................................................................

Homo sapiens        ACACCCATCATCAACCACAAGAAAGTGATAGCGCCGGCACGTCTATTTAACTTCCAGCTCTTTGTTAGACACATCAATGAGATGAATACAAATATTAACTACAATAGTCCCCATTTATCT
Loxodonta africana  ........................................................................................................................

Homo sapiens        GCAGTTTTACTTTCTGAGGTTTCAGTTCCTATAGTCAACTGCAGTCCAAACATATTAAATACAAAATTTCAGAAATAAATGATTTAAAAGTTTTTTAATTGCACACCATTCTGCGTATAG
Loxodonta africana  ........................................................................................................................

Homo sapiens        TGATAAAATCTCATGCTGTCCTGCTCTGTCCAACTGGGACGTGAATCATCTCTTTGTCTAGCATATCTTCATTGTATATGCTACTCTCTCCCCATCATTAGTCACTTCATAGCTGTCTTG
Loxodonta africana  ........................................................................................................................

Homo sapiens        GTTATCAGATCAAATGTAGTGGTTAGTAGTGTGTATCCAAGTTACCCTTATTTTATTTAAGAATGCCCCCAAAGTGCAAGAGTAGTGATGTTGGCATATTGTTAATCGTCCTATTTTATT
Loxodonta africana  ........................................................................................................................

Homo sapiens        TTTAGTTGTTAACCTCTTACTGTGCCTAATTTAAAAATTAATCTCTATCATAGGTATGTATGTATAGGGGAAAACATAGTATAAATAGGGTTAGGTACTACCTGCAGTATCAAACATTCA
Loxodonta africana  ........................................................................................................................

Homo sapiens        CTAGGGGCCTTGAAACATATTCTCTGAAGGTAAAGGGAGACTATAGTTATCAGATCTGATAGGAAATTAGGCATCAAAATTCAAAATTATCAAGAATACTCTTGAAATATCAGTTCACT
Loxodonta africana  ........................................................................................................................

Homo sapiens        TCCACTTTTATTGATGGAGAGCCTTACCCTAAGTGCATATAATGTTTCAAGGTATTTGCTAATGATAATATGAAGCCATTATGATTAGCTAGCAGTTTTTCCAGTCGTGTTTAAAGTCAT
Loxodonta africana  ........................................................................................................................

Homo sapiens        TTGATAACCAAACCAGGACTTTGAAAATTTTTCTAAAGGTAACAATAGGGCCAAGCATGGTGGCTCATACCTCAGCTTTTTGGGAGGCTGAGGTGAGAGGATAACTTGAGACCAGGAGTT
Loxodonta africana  ........................................................................................................................

Homo sapiens        CAAGACCAGCCCATGCAATACAACAAGACTCCGTCTCTACAAAAAAAAAAAAAATTTTTAATTAACTGGACGTGGTGTTGCACACTCATAGTCCCAGCAACTCAGGAGGCTAACGTGTAA
Loxodonta africana  ........................................................................................................................

Homo sapiens        GGATTGCTTAAGCCCAGGAGGTGAAGGCTGTAGTGAGCTATGGTTGCACCACTGCATTCCAGTTTGGGCAACAGTGAGACCCTGTCTCAAAAAAATAATAATAATAATAATAATAGGCTG
Loxodonta africana  ........................................................................................................................

Homo sapiens        TGCTCAGTGGCTCATGCCTGTTGTAATCCTACCAGCCAGATTGCTTGAGCCTAGGAGATCGAGACTAGCCTAAGCAACATGGTGAAATGCCATCTCTACAAAAAATGCAACAATTAGCCA
Loxodonta africana  ........................................................................................................................

Homo sapiens        GGAGTGGTAGGGCACACCTGTAGTCCAGCTACTTGGGAGGCTGAGATGGGAAGATCGCTTGAGCCCAGGAGGTTGAGATTGCAGTGAGCCAAGATCATGCACTCTAGCCTGGGTAACAGA
Loxodonta africana  ........................................................................................................................

Homo sapiens        CTGAGACCCTGTCTCAAAAAAAAAAATAAAAAATAAAGGGTAACAATAAATATTTGCAGGCCTATTTTGTGAGATTGCTTAAACAAAAAACCAAAAAAGCGACATATGATTGGGAAAAC
Loxodonta africana  ...............................................AATATTTATAAGTCT--CTTGTGAATTTTCTTAA-CAAAAAACAAAAA----GTCACACGATTGGGGAAAC

Homo sapiens        TTGTTCTTTTTGCTGTGATTAAAATGGCTTCACTATTACACAGAAAGCATAACGATGACAGTACAAAATGAACTCTTTTGTCAGCAAATACTGATGCAGAAAACACTGCTAAA-TATTAT
Loxodonta africana  TTGTTCTTTTTGCTGTGATTAAAATGGCTCAAATATTACATAGAAAGCATAATGGTGACA-----AAATACTTCCTTTTGTTAGCAAATAATAACATAGAAAGC--TGCTAAAGTACTG-

Homo sapiens        AACATAATACACAATGTGGGAGGTTTATTGTATAGTTGAATAAAAAATACAGATGTTTCTAATGG--CTTAGCTTAGAGTATTTGCTAAATTGTTCCAATAAAATGCACAAAGAACTTTTT
Loxodonta africana  ------ACATACAATGTGGGAGGTTTATTATACAGTTGGATGAAAATATAGATGTTTCTAATATATCTCAGCTTAGGGTCTTTGCTAAATTCTT-----GAAATACACAA----CTTTTT

Homo sapiens        GTAAACCTCTGAAGGAAAGAGAACAAACATATTCAGTAGGAAAATTAATTTTTTT------AAATGTCTTACAGAAAACTAAAATTGTAAGAACTGACGGAATATCTGGTTTGATGGTTG
Loxodonta africana  GTAAAGCTCTGAAGAAAGGAGAATAAACATTTGCAAT-GGAAAATTACTTTAACTTAAAAAAAAAAGTCTTATAAAAAACTACAGTTGTAACTACTGATAGAATGTCTGCTTTGATGGAGA

Homo sapiens        GGG-----GGCTGACAGAGTAAGGATGTCAAGATACCATTTCACAAATGGATTCCCTGGATCATTCCTAGGCAAGCTTTTGAAACAAAGAAGTTAAAGCCTTTTTCAAGGGTCA----TT
Loxodonta africana  AAAAAAAAGACTGGTGGGGTAACAGTATCAAGATAGCAACCTGCAAATGTATTCACTGCATCATTCCTAGGCCACCTCTTGAA-CACAGAAGATGAATCTCTTCCAAAAGTCAGCCATT

Homo sapiens        TCAAACCATTAATCAAGAC-TCAGATTGAAGCTGACCCAAATGACACCTATTCTTTGGGATTTCTTT----CTAATTTTGAATTAAAAATAGTCACCCTGAATTAGTATGTATATCAGCT
Loxodonta africana  TAAAATCATTGATCAGGACCTCAGATTGAAGCTGACCCAAATGATACCTATTCCCTGGGATTGTTTTTAAATTATTTTTTAATTAAAAGGAGGCAGCCTGAATTAGTACATGTATTAGCT

Homo sapiens        AGGAAGTGTATTCAA-TCCAAGTAACAG---ACTTCCTTTCCCTATCTCAATATATTAA-----------TATATATTGTATATATATTATCCCAATATATATTGGGAGT----GATTG
Loxodonta africana  AGGAGTTATATTAAGCTGCAAGGAACAGAGAACTTCCCTCACCCA---AAATATATGAACTCTATGAAGAGTTTATTTCGTATTTATGTAA----AAGAAGTCTGGGTGGTCTAAGGTTG

Homo sapiens        AGAGTCACTCC---TATTGTATTCACAACGTGGCTGCTGGAGCACATGCCACAATGCCTACATTTCAGTCAGAAAGAAGGGAGAGAGTGTTGGGC--AAACAAAGCTAGTACCAGCTGAG
Loxodonta africana  GAAGTCACCCTAAATCTTTTGCTCACAGTATGA--GCCAGAGCCCCT-------TTTCTACATTTTAGGCAGAGAGAAGAGGAAGGGCCATGTACAAAAAAAAAAAAAAGCATCAGCTGAG

Homo sapiens        TTTGCCTAACAACTTTTA-----CTTACAAC----------TTTTTGGCTAGAATTATGTCACATGACCACCCCTACCTGCAAGGGAGTTTGGGAAATGTAGTTTTCTTTTTATTGCTT
Loxodonta africana  CCTGCCCAACAATTTTGAAGAGCCCCACAACTTCTACTTCCTTTTTTTACCGGAACTGTGCTGTATGACCACCCCTACCTTCAAACAAGCTTGGGAAATACAGTTT--GGTTTGTTGTTT

Homo sapiens        TCCCCTCTTGTATTAACTTTGCCTATTTAGTTATTCTGATACTACTCGTC-------------------TGAAATATTCTTTTTTAAAAAGACAAATTTATGAGCCAAAGGTTTACTCA
Loxodonta africana  TCTCCTTATTTATTAGCTTTGCCTTTTTAGTTACCCTGATATTGTTTGCTTATAATTAAGCAACAAGAAATATAATATTCTTCTTTAAAAAA--TAGTATATCTTCCAATGGTTTACCTA

Homo sapiens        ACTAAAAATAATAAGGAAAAAAAAAGTCTGAAAGGTGAGGTAGGAAAAAAATATTATTTTATATTTAATGGGTTTACTGTTAAAAGTTTTGTAAAC------TCATTTTAAGTGCTACAC
Loxodonta africana  ATTAAAAA-AACAAGGGATAAA-----------------GTAGAAAAAATAAGTTATTTTACACTTATCAGGCCTATTGTTAGAAGTTTTCTGAACTATTGATTTCTTTTAGGCCCTGAAC

Homo sapiens        ATAATATAGTAACACCAATAATAGTAGTTATAAGTTAAAAGTTATAAAATGCTTA----TTGCCAATAACTGTTTTACATGCTTTTTAAACATTAACTTATGACCTAGTGGGTTGTTTTT
Loxodonta africana  ATAATATAA------CAATAATAATAGC----AATTAAGAGTTGTAAAGTGCTTACTGCCTGCCAAGAACTGCTTTTCATGCTTTATGAATATTAACTCAT..................

Homo sapiens        GAGACACTGTCTACTGAATTAAAAAGTTACTTGAGGGCCGGGTACAGTGGCTCATGCCTGTAATCTCAAAACTTTGAAAGACTGAGGTGCATGGATTGCTTGAGTCCAGGAGTTTGAGAC
Loxodonta africana  ........................................................................................................................

Homo sapiens        CAGCCTGGGCAACATGGTGAAACTCCATCTGTACAAAATATTTAAAAATTAGGTGGGCATGGTGACACATGCCTGTAGTCCAAGCTGCTAGGCAGGCTGAGGTGAGAGGATCGCTTGAAC
Loxodonta africana  ........................................................................................................................

Homo sapiens        CCAGGTAGTGCAGGCTGCCCTCCAGCCTGTGCGACAGAGCAAGACCCTGTCTCCCACCGCCCCCCACCAAAAAAAAGAAAAAAATTAAACTCAAAGTTAACGTTAAGTTCTGTTCTTCCA
Loxodonta africana  ........................................................................................................................

Homo sapiens        TTTCCTTGTTGTATGTGTGTATTTGGCAAGGCACTTAATCTCTCAAAGCTTCAGTATCTTACCTTTAAAATGAAATTAATAATACCTCAAAACTTGTAAGGTTTAATGACAATAATGTA
Loxodonta africana  ........................................................................................................................

Homo sapiens        AAGTGTCTAGTGTGATGGGACCAGTGTGTAGTAAAGACTCAATAAATGGCAATCACTATTTTATACATAAAATTTTACATATTTTATGTATGTGTTCATATAGAATGTTATGGTCAATAA
Loxodonta africana  ........................................................................................................................
```

```
Homo sapiens         AAAAGCAAGATTCAGCCAGGCGCAGTGGCTCATGCCTGTAATCCCAGCACTTTGGGAGGCCAAGGCAGGTGGATCACAAGGTCAGGAGTTCAAGACTAGCCTGGCCAATATGGTGAAACC
Loxodonta africana   ........................................................................................................................

Homo sapiens         CCGTCTCTACTAGAAATACGAAAATTAGCCGGGTGTGGTGGCGCACACCTGTAGTCCCAGCTACTCAGGAAGCCGAGGCAGAAGAATTGCTTGAACCTGGGAGGCAGAGGTTGCAGTGAG
Loxodonta africana   ........................................................................................................................

Homo sapiens         CCGAGATTTTGCCACTGCACTCCAGCCTGGGTGACTGAGCAAGACTCCATCTCAAAAAAATAAATAGATAAATAAAAGGCAAGATTCTAATTCTTTTGAAGCTTTTTTTT------AAGT
Loxodonta africana   .........................................................................AAAAGGCAAGATTCAAATTCTTTTGAAGCTTTTTTTTTTTTTTTAACA

Homo sapiens         CAATGGAAAAATAAAACACAAATTTTAAAAAGCCACCTTTGTTTTA-TATATTTTTCAGATCAATATTGAACTGTACAGATTGTTTGGCATAAAGCAAATTGTAATTTCTCACACCTCTG
Loxodonta africana   CGGTGGAAAAACTAAC-----ATTTTTAGAAGTCAACTTTGTTTTAATATGTTTTTTAGATCAATGTAGAACTGTATGGATTGTTTGGCACAAAGCAGATTGTAATTTCTCATGCCTCTC

Homo sapiens         GAACTGTTAACCCAACGGAAAGTACACCTAACACAATCAAAGCATTTCTCTCCAAACACTGTGCTGACCACCCAAACAATTGGGATGATCACCTATCAGCTGTTTCATTTGCCTTCAATG
Loxodonta africana   AAACTGTTGATCCTACTGTAAGTACACCTAGCACAATCAGAACGTTTCTCTCCAAACACTGTGCTGACCACCCGAACAACTGGGATGATCATCTGTCAGCTGTTTCGTTTGCCTTCAATA

Homo sapiens         TAACTCACTTGGTATGTGCCTTTTTATAATTCTGTACTTCTGAGTTATGACT----ATTTCTTGGCCAGATAAATGTTTTATTACAAAATTGACGTTTATTTTCTTTAAAATAGGAACCT
Loxodonta africana   TAGTTCACTTGGTATGTGCCTTTTTATAATGCTGTACTTTGAAGTTGTAACTGATTATTTCTTGGCTAGATAAATATTT------------GCTGTTTATTTTCCTTAAAATAGGAGCCT

Homo sapiens         ACTAAAAATACACCATATTTTCAAATGTTTAGTCGAAATCCTTATATGCCTGAGACTTCAGATAGTCTTCATGAAGTGGATGGTGATAATACAAGTATGTTTGCCAAAATTCTAGATGCA
Loxodonta africana   GCTAAAAATACACCATATTTTCAAATGTTTAATCGAAATCCTTACATGCCTGAGACTTCAGATAGTCTTCATGAAGTGGATGGTGATAATACAAGTATGTTTGCCAAAATTCTAGATGCA

Homo sapiens         ATTAAAGAAGCTGATAAAATAATGGAGAATAAGACAACTTCACTGGGCCAGGTGATTCTATTCAATAGAAAAACTGTAATCTAAAACTTTATAAAAGTTATA-------TTTATTTTTAA
Loxodonta africana   GTTAAAGAAGCTGATAAAATAATGGCAAATAAGACAACTTCAGCGGGCCAGGTGATTCTATTTAATAGAAAAA-----------AACTCTATAATCAATATAGTGATCATTTATTTTTAA

Homo sapiens         AATTTCAGCATATACATGTGTGCATGCACACACACAC-ACATACTTACTTGCCATAATTTTTTATCTATCAGATTTTTCTTGTCTTGATTAAATAGTTGGTTTACTAGCTATGAAATGCT
Loxodonta africana   GATTT-------------TGAGGATATACACACACGTTATATACT--CTTAAGATAAAAATTT-TCTACCAAATCTA-CTTGTCTTGATTAAACACTGTGTGTATTAGCTCTGAATT--T

Homo sapiens         GAGAGGCAATAATGAGAAAGCTATGGATTTTGGAATCAAGTAGAGCTGGATTCAAATCTGTAGTCTTAACTGCTTTTGAGTTGTTTGACTTCGGCAAATTACTTAACCTATCT-----TA
Loxodonta africana   GAGAGACAACAGTGGGAAAACAATGGATTTTGAAATCAGGTAGAGACAGATTTAAATCCCTAGTCTTG-CTGCTTTCTATTTGTTTGACTCAGACA--TTATTTAACCTATCTCTACCTC

Homo sapiens         GTTTACCTTCTATATAAAGTAGGAGCTAATAATACTGACTTTGAAAT---GTTTTAAGAATTAAATAGAATTCATACCTACCTTTTAGTAGCATCTCAAGTAC----CATTTTGTTACAA
Loxodonta africana   AGTTACCTTACATGTAAAGG------CAGTAATAACAACTTCGAAATACTGTTTTAAGAATTAAATGGGATTCATGCCTACCTTTTACTAGCATTTCAGTAACTAATCATTTTGTTATAA

Homo sapiens         TATTCTCATTATTT-------GCATTTACCTTCAAGATATTATAGCTCTTTCAT--------CTCTTTGTTCTTTTTCTTTTTACACTTGTAGATGGAGAACAACAATTTGGATGAACTA
Loxodonta africana   TATCCTAATCATTTTGTTACAATATCTGCAGTCAAGAAATTACAGCTCTTTTATACCCTGCTCTTTTTTTTTTTTTTTTTTGGTACTTGTAGCTGGAGAACAACAATTTTGATGACGTA

Homo sapiens         AATAAAAGCAAGATCATTGTTAAAAAGAAACCCAAACAATTAAATCCATTTCATTTAAAAGTGGGTCATGAAGTTTTAAGACAAAGGAAAAATTGGTGGAAGGATGGTCGTTTTCAGTCT
Loxodonta africana   AATAAAAGCAAGATTGTTGTTAAAAAGAAACCAAAGCAGTTAAATCCATTTCATCTAAAAGTGGGTCATGAAGTTTTAAGACAAAGGAAAAATTGGTGGAAGGATGGTCGTTTCCAGTCC

Homo sapiens         GAATGGGTTGGTCCTTGTGTCATAGACTATATTACAGAAAGTGGATGTGCTGTCCTGAGAGACAACACTGGGGTTAGACTGAAAAGACCTATCAAAATGTCCCACCTTAAGCCCTACATA
Loxodonta africana   GAATGGGTTGGCCCTTGTGTCATAGACTATATTACAGAAAGTGGTTGTGCTGTCCTGAGAGACAACACTGGGGCTAGACTTAAAAGACCGATCAAAATGTCTCACCTTAAGCCCTACGTG

Homo sapiens         AGAGAATCCAGTGAACAAGGTAAATATCTGTCACTTCTCTACATTTTTA-TTGCTTTAGGTAAGGAGAATAGTTAAAAATAACAGAAAC-CTTTCTCCCCTCTTTTGTTAGGTTTTCTTT
Loxodonta africana   AGAGAATCCACTGAACAAGGTAATTATCAATCACTTCTCTACATTTTTACTTTCTTTGAATGAGGAGAGAAGATTAAAATACAAGAGACTTTTTCTTTTCATTTTGGTTGGGTCTTCTTT

Homo sapiens         TCCCTTC-----TTCATAAAATTTGTTACAGTTTTAGCTATCCCAGGTAGTCTG------TTTTATTACAGTGTCTCTTCTGGAACTGTCAGGCCTGTGAGATTTTTTTTTGAGACAGA
Loxodonta africana   TCCTCTCTTATATTTAGAGATTTTGGTATAGTTTTAGAAATCCCAGGTAGTCTGGTTTTTTTTTTTTGGAATGTGTGTTCTGGAACTGTCAGGCTTGTGAGA.................

Homo sapiens         GTTTCCCTCTTTCTGCCCAAGCTGGAGTGCAATGGCGCGATCTTGGCTCACTGCAACCTCTGCCTCCCGGGTTCAAGCAATTCTGCCTCAGCCTCCCGAGTAGCTGGGACTACAGGTGGA
Loxodonta africana   ........................................................................................................................

Homo sapiens         TACCACCATGCCCAGCTAATTTTTTGTATTTTTAGTAGAAACGGGGTTTCACCATGTTAGCTAGGCTGGTCTTGAACTCCTGACCTCCCGTGACCTGCCCGCCTTGACTTCCCAAAGTGC
Loxodonta africana   ........................................................................................................................

Homo sapiens         TTGGATTACAGGCATGAAACAGGGTGCCTGGGCATTTTTATTTTTATTTCTAGTATGTTTTTCATAGTTTATGTGGGAGATACTGAACTTGTTTTCCTCTGAAGGAAAGATAACATCTTA
Loxodonta africana   ...................................TTTTATTTTGATTTCTAATACACTTT-CATAGTTCATATCGGAGGTGCTGAACCTGTGTCAC------AGAAGAATTATGTCT--

Homo sapiens         ACATCATCTCTGTTTTTGCTTTCTCAATGTTTTGTTTGTTTGTTTGTTTGAGACAGAGTCTCATTCTGTCCCCCAGGCTGGAGTGTAGTTGTGTAATCTCAGCTCACTGCAACTTCTGCT
Loxodonta africana   ------TCTCTG-TTTTGCTTTCATAATG...........................................................................................

Homo sapiens         TCCTGGGTTCAAGCAATTCTTGTGCCTCAGCCTCCCAAGTAGCTGGGATTATAGGCACATGCCACCATGCCCAGCTAATTTTTTTGTATTTTTGGTAGAGATGAGGTTTCACCATGTTGG
Loxodonta africana   ........................................................................................................................

Homo sapiens         CCAGGCTGGTTTTGAACTCTTGGCCTCTAGGGATTTGCCTGCCTTGGCCTCCCAAAGTGCTGGGATTGCGTGCATGTGCCATTGCACCTGGCCTTACAAATGTATTAGTCACTTCGTGCC
Loxodonta africana   ........................................................................................................TTAGTTACTTTGTGCC

Homo sapiens         TCTTTTTGG---CATGAAAAA--CAGCAACTGTGATCATTAATGCCAAAAACAAACATACTGTCCAGAATCTAGTTGTGAGATAAATATTAGGGTACTTTAAAAATGACATTAATTTTTA
Loxodonta africana   TCATTTTAGTAGCATGAAAAAAACAGCAACTAAGATATGTAATGCCAAGAATA----TACTGT--GGAATTCAGTT------------TAGGAAACTTAAAAAACGTGATTAATTTTTA

Homo sapiens         ATATTGCTTTATAGTTAGGTCATGGTTGGGGGCCAGGTGTGGGTGCAGAGTGGGGAATCCTCAGAATGCTATAAGGTGCTGTAAGAAGAGGTGACAGCACTCTAAACAAGTTATATTTGC
Loxodonta africana   ATGTAGTGTCACAGTCGGGCCACGGGTGGGC-TCATTTGAGGGTGTGGGGTGGCTTATGTTCAGAATGTTATAAGGTGATACAAG--GAGGTGATAGTTCCCTAAATGAGTTATACTTGC

Homo sapiens         CCCTAAATTTGACTCATTCTGAACAGTCAAGATACCCTAGT--------------------------AAAGTGA-TGTTTTTGCCATGGCTTTTAGATTATGTTCCCCACTTTATCAAG
Loxodonta africana   CCCCAAATGTGACTGATTCTGAACAGGCAAGATACCCTGATCTCTCTAATCGTTTCTGTCATCGACTAAAACTAAACATTTTTGCCATGGCTTTCAGATTAAGG--CCTTCCTTACTGGG

Homo sapiens         GCT--TTTATTTTGTCCTAATGTGTCTGCTTCCTAGATCTTCTCTGAGGTGGATAACTGAGAGAGAGTTAATGAGTAACTTAGCCAGGTGAAACATTAATATTATTTGGATATAAGTAAC
Loxodonta africana   ATTGTTTTATTTTGTCTT......................................................................................................

Homo sapiens         ATTTAATGGAAAATATATTTATAACAATATAAACAAGTTATATGACAGGCTTTAGCAAATA------TTAATAACAAAAAGAGTAACAA-------CAACAAATAACCTACACTTACCCT
Loxodonta africana   .............................TAACAAAAACATTTGATGGAAAGTACCATATACTGTAATTTAAAACAATATAAACAACTAAGTTATTCAACAAATAACCTTCACTTAGCCT

Homo sapiens         ATGCCTTTTCTTGAGTTCTAAAGCCCATGTGGGCTGCTTAGTAATGATAGCTCAACTCTGCTTCTAATCTGGGTTACAACACTCATGTTAAAACCTCTTAGTACCTTTCCTTCT---TGT
Loxodonta africana   ATGCCATTTTTTAGGGTATGATGACCTCAAGGGC-ACTAAGT---GATAGTGCAACTCTGCTTTTGCT-TGGGCTGCAGTTCTTGCGTTAAAAGCTCTTTGTACCTGTTCTTCTGCTTGT

Homo sapiens         GACAGACCGAGTTGTT-TAGTCAGTAAACTTATCTTGAACTACATTCTAATCCTGAAAACTCACAAGTGCTTTCG--TTCTCTTTGTTAGAAGATAAAAGCTTGCATTGTTTGTACCTCT
Loxodonta africana   GACAGACCCAATTGCTCTAGCCAGAAAACATGTCTTGAACAGTGTTCTAATTCTTAAAACTCACAAGTACCTTCACTTTTTTTTTGTTAAAAGACGGGCTTTTTCATTCCTTGTACCTCT

Homo sapiens         TCTCAGTTCTGAGCTACCTTTAATGATCTCTCTCTTCTGCAAGTTAAGTAGGGAAAAAAAAGGT-------------------TATACAAGTATATGTTGTTCTAGCTTCTTTAGCATG
Loxodonta africana   TCTCGATTCTGATCTACCCTTTATGGTCTCTCCCTTCTGCAACTTAAGTAGAAAAAAAAAAATGTATATATATATATATGCATATTACACAAGTACATGTTGTTTTGTGTTTTTCAACATG

Homo sapiens         ATTTTCCCCTTAATCTTTGACATTCATGATTTGCTCTCTTCTAAAGTGTTAAGCTTCTTTAAT----ACAAAATTTA-ATTTAAAATTTTGCTAAACCAGGATCTAT--CAACATTTTGT
Loxodonta africana   ATTTTCCTCGTAATCTTTGACAGT-AGGACTTGCTTCTTTCCAATATGTTACCCTTCCTTAATTAATACAAATTTTACATTTAAAATGTTTCTGTATCATGATGTGTTTCAACATTTTGC

Homo sapiens         AATTTGTTCTTGTCAAAGAGTTTCTCAGAAAAGAAGTCA-AATATTGTGCTTCTTCTGAACATTCAGAACTCAGTATTTCAGAACTCATCTTCTCTGTTTGA--GAACAGTCTCTAAACA
Loxodonta africana   AATTTGCTCTTGCCAAAAACTTGCTTAGAAAGGAGGTAATAACATTTTGCTTCTCCTGAA-------------TGTTTTAGAACCCA--------GTTGGATAGAACAGTTCCTAAACA

Homo sapiens         GAGGCAGTATAATATTTGCCTCATACTTGAATTCTTTGCACAAGATTCTTCCAGTAATATAAGGACAAATAATATTGCAATATGACTACTGGTGTATGCATAAGGAATTTAAGACTAATT
Loxodonta africana   GAGGCAGTATAATTTTTGCCCCATAGTTAAATT-----------------------------------------GGCAGCATTAGTACTGGTGTTCACATAAGAAATCTGAGATTAAT-

Homo sapiens         AGATGAGTCTGTTTTAGTTTTTCTTGTTAACTAAAGGAACTACTGATCTGGCCATCCTAGAATCTGGTTCTT----ATTGCTTGATCCTTTTTT---TACATTTTTCTC--ATTTGTAGA
Loxodonta africana   AGGTGAAACAGTTCTCTTTTTTCTTGTTAACCAAAGGAGGTA-TGACCAGGCCATCCTAGAATCTGGTTCTTGATTACTATTTGGTTCTTTTGTGTGTGTGTTTTTCTCTGACTTGCAGA

Homo sapiens         AATTAAAAGTAAATGTGATTTCTGCCATCTTTTTCCTCAATATTACATACTTTCTTGAAAATCTTTTCCATGATTTTCTTGAAAATCACCTCACAAGTTGATTTGAAAGAGTCCTTGAGC
Loxodonta africana   AATTCAAAATAAATACTATTTCTGCCACCTTTTCCCTCAGTATCACCTGCTTTCCTGAAAATCTT---------------------CACCTCA-------ATTTTAAAGAGTCTGTGAGC

Homo sapiens         TGAATT-AAAGCAAACAAAATGATTTTGACTAATCATTGAAGTTTTTATTTGACATAAATTTTGACATGCTATAGCCAAAAGAACTATAAATTTATTTGTCCACATTTGTCATTGCTGCT
Loxodonta africana   TGAATTTACAGCGAACAAAATGTCTTTGAATAGTCATCCAACTTTTTATTTGGTGTAAATTTGGATGAGCTCTATCCAAAAGAACTATGAATTTGTCTGTCTGTACTTGTTGTGTCTGCT

Homo sapiens         CTTTGCTAGCCTTGAAATTTCATAGCTCTGTTAAAAACTCACTGTCTAAAGTAGATATACTTCTATTGCCAACTTCTTATCCATTCTTATCTAGAACTAAATGTAGAGTGAGCACATTCA
Loxodonta africana   CTT-ACCAACCTTGAGATTTCATAGCTGTGTTAAAGTCTCACAGTCTAAAGCAGATATGCTTCCGTTGCCAACTTCTTACCCATTCTTATCTAGGACATAAAGTAGAAGGAGCACATTCA

Homo sapiens         GTACTAGGTATACAATATTTTTCATTGCTATTTTAAGCAGGCCTGTTTGTTTTTTTGTCTAGTT-CAT-TGCCAAGCGCTTTTTCAGACTATTT------------AGACCAAGAAATCT
Loxodonta africana   GTACTAGGTCTACAGTGTTTTTCAGTACTATTTTAAGAAGGCTTCTTTGTCATTTTGTCTAATTCCATGTGCCAAGCACCTTTTCTGAGTATTTTTTATTAAAATAATGACAAGAAATCT

Homo sapiens         TA--AAATACCATAGTGTCTTTTTGTAATCATAATGCTCCTCTTTGAAAGGAAAAA---------------ATAATCT-----GACATAGATTTTGCACAGT---TTTGAATTTATTTTG
Loxodonta africana   TATGACATACCACA--ATCTCTTGGTAATCGTGATGGCCCTCTTTGAATGGGAAAATGATGTTATTGACCCACAATTCAAGTAGACATAGATTTTGAGTAGTTTGTTTTGTTTCATTTTT

Homo sapiens         T------------TTGTGATAGATTTGGATCTGCCTCACC-CAAGGTTTTAGCCTGATTTCCCTTCACAGTTGAAAG-CCCCATTTGTGAAACCATTTTCATTATTGTATAAGTTTCCTC
Loxodonta africana   TGGATGAGGAATATTATGATAGGTTTGTGTCTGCCTCACCAAAAGGTTTTAGTCTGATTGTCCTTCACAGTTCAAAGTCCTCAATTGTAGAACAGTTTTTATTATT-----AGTTTCCTA

Homo sapiens         TTGTTGCTGTAACAAATTACTACAGATTTAATGGTTTAAAACAACACACATTTATTATATTACAATTCTGAAGGTTAGAAGTTCAACATAGATCTTACTGGGCTAAAATCAAGGTATTGG
Loxodonta africana   TTGCTGCCATAACAAATTGCCACAAATTCGATGGCTTAAAACAACATAGATTTATTATCTTATAGTTCTGTAGGTTAGAAGCCCAATACAGGTCCCACTGACCTAAAATCAA-GCATTGG
```

```
Homo sapiens        CACTGTTGTGTTCCTGTCTGGAAGCTCTAGAAAATAATCCATTTTCTTGTCTTTTCCAGCTTCTAGAGGTTACCTTCATTTCTTGGCTTGTGGTCC-----CTCTATCTTCAAAGCCAAC
Loxodonta africana  CAGAGCTGTGTTTCTTTTTAGTGGCTCTAGAGGAAAACCAGGTTCCTTGCCTTTTCCGGCTTCTAGAGGCCACCCACATTCTTTGACGCATGGCCCTGTTTCTCCACTTTCAAAGCCAGC

Homo sapiens        AATAGTGGGTTGAGTCCTTCTTACATCACCTTACTC-CAACCTCCTCTTCTGCCTCCCTCTTCTATATTTGAGGACCCTTG-----ACCTTGGCCCTGCCCAAAAAGTCCAGGATAATCC
Loxodonta africana  AATGGTGGATTAAGTGCTTCTCACACTGCATCACTCTTGACTTCCTCTTCTGCCTGCCTCTTCCACATTTAAGGACCATTGTGATAACATTGGGCCCACTAGGATAAACCAGAATAATCT

Homo sapiens        CCCTATTTTAAGGTCATCTGATTAGCAACCTTAATTCCATC-ACTACCTAAATTCTCCTTCACCATGTAAGATTACATATTCACAGGATCCAAGGAGTAGTATGTTGACATCTTTAGGGG
Loxodonta africana  CCCTATTTTAAGGTCAGCTGATTAGCAACCTTAATTCCATCTGTTACCTTAACTCCCCTTTCCCTTATAAGGTAACATATTCACAGGTTCCGGGAATTAGTGCGTGGACGTCTTTGAGGG

Homo sapiens        CCTATTATGCTGCCTACCACAATTATGGTCTGCATTCATGAAACAAAGCAAAGCACAGTCCCTATAGTGTCAAGACCATGATCTTGAC------GTTAATATAGTTTATGCAGAGAGCCT
Loxodonta africana  GCCATTGTACTGCCGACCACAAGTATAATCTACATTCATGAAATAAAGCAAAACTTAGTCCCTATGGCATCAAGCCCATGATCTTGACTTTATTATTAATATGGTCTATGCAAAAACCTT

Homo sapiens        TTTTTTGGCCTCTTAGTTATATCTTTTTTTTTCTTTCTCTTTTTTCCTTTTTTTTTTTTTTTTTTTTTTTTGAGACAGGTTCTTACATGGTTGCCCGTGCTGGAGTGCAGTTGCACAAT
Loxodonta africana  TCTTTTGGCCTCTTGGTTATAT..................................................................................................

Homo sapiens        AATAGCTCACTGCAGCCTTGACTTCCTGGGCTCAAGCAATCTTCCCACATCAGCCTCCCAAGTAGCTTGGAATGTAGGCATGCATCACCATGCCTGGCTGAGTTTTTCGTTTTTTATAGA
Loxodonta africana  ........................................................................................................................

Homo sapiens        GACAGGGTCTTACTATGTTGCCCAGGTTGGTCTCAAACTCCTGGACTCAAGCAGTCCTCTCACCTAGACCTCCCAAGTGTTGGAATTACAAGTGTGAGCCATTGTGCCCGTCCAGTTATA
Loxodonta africana  ........................................................................................................................

Homo sapiens        TAATTTCTTAACCAATAGTTACTCAGTTATCT--GTTGAC----AGTATTAGTT-----ATTATAATGACAGTGATTTAGTATCAAGTTATAGTTAT--------TATTTTATCTTGAGA
Loxodonta africana  .AATCCCTAAATTAAGACTCATCCAGTTCTCTCTGTTGTTCATTAGAATTGGTTTCTCTGTTGCAATGATAATAATACAGTATCAAATTTTAGTTATAAAATTGTCATTTTATCACAGGA

Homo sapiens        AAGGATATACTAGTATGGCAGCACATTCTAATAGTTTGAGGATTATCAAACCATATATAGGATTAGTTTAGTCACTTCTCTTTAAAATAAGAAAATTAAGTAGTCTGAAACCTTTATCTT
Loxodonta africana  AAGGATGTACTCGTATGGTGACACATTCTAATAGTTTTAGGATTATCAGGCCACTTATAGGATTAGTCTAGTTATCTCTCTTCAGAATAAGAAAATTAAGTATTTTGAAACC-TTGTGCT

Homo sapiens        TTTTTTTCTGATTGAAGAAATACATTAATCTAAATCAGACTTTTTTGAAGCTGTAATTTGTATTTATTTTTCTCA---GGGAAATTTAAAAATTGATTTTTTAATTATTATTTTTATTCC
Loxodonta africana  TTTCTTTGTATGTGAAGAAAGAAATCAG-ATCACTAAGACTTTTGAGATACTTCGATTTTAATTTATTTTTCTCCTTTGGGAAATTTTAAA.............................

Homo sapiens        TTAAAACAACATTAAGGCTGGGCACAGTGGCTTATGCCTGTAATCCCAGCATTTTGAGAGGCCGAGGTAGGATTGCCTGAGCTCAGGAGTTCGAGACCAGCATGGGCAACATAGTGAAAC
Loxodonta africana  ........................................................................................................................

Homo sapiens        CCCGTCCCTACAAAAATTAGCCGGGCATGGTGATACGCACCTTTAGTTTTAGCTACTTAGCGGGCTGAGGCAGAGGATCACTTGAGCCCGGGAGACTGAGCTGCAATGAGCCAACATTGC
Loxodonta africana  ........................................................................................................................

Homo sapiens        ACCGCTGCACTCCAGCCTGAGTGACAGAGTGAGACCCCATCTTTTTTTTTTTTTGAGATGGAGTTCAACTATTATCACACAGGCTGGAGTGCAATGGTGTGATCTTGGCTGCCTGCAACC
Loxodonta africana  ........................................................................................................................

Homo sapiens        TCCGCCTCCTGGGTTCAAGCAATTCTCCTACCTCGGCCTCCTGAGTAGCTGGGATTACAGGCGCCCACCTCCACACCCAGCTAATATTTGTATTTTTAGTAGAGACGGGGTTTCACCATG
Loxodonta africana  ........................................................................................................................

Homo sapiens        TTGGCCAGGCTGGTCTCAAACTCCTGACATCAAGTGATCCACCCGCCTTGGCCTCCCAAAGTACTGGGATTACAGGCGTGAGCCACCATGCCTGGCTGAGACCCCTGGGATTATAGGCGT
Loxodonta africana  ........................................................................................................................

Homo sapiens        GAGCCACCATGCCTGGCTGAGACCCCATCTCTTTAAAAAAAAAACCTACATTAG-AAATGGATATCAAAACTGCTGATTAATACCTTCCTGAAATGTCTTGAAAATATTTTAATTTCAAA
Loxodonta africana  .........................................AAAATTACATAAGGAAAAGTATGTCAAAGCTGCTGAT-AACGTCTTCCTGAAATATCTTGAAAATATCTCACTTTTAAA

Homo sapiens        GAAACTAAAGAC---TTATTCAGTCTTGTCAAAACCAAAATGATGCTTTCATCAAACAGATGTCATAATCATAACTAAATTAATGGAATGAGAAAAGATAAATACTTAGTGAAATGTACT
Loxodonta africana  GAAACCAAAGACAATTTATTCTGTCTGTTCAAAACTGAAACGATGCTTTCATC-AACAAATGTCCTAACTATAAC--AATTAGT....................................

Homo sapiens        GTGTAATGTAGCTGACTAGAAAAGACCTTTAATATGATATGTTTGCTTACTCCCCGCCTCTGTTTTGAGGGCAGAAAGTTTAATCATATTCTTTTCTGTATCCCTAACTCATGATTGTTT
Loxodonta africana  ........................................................................................................................

Homo sapiens        TAGATTAGTGTTGTCCAATAGATGAATAATAATACAAGAGCTATGTAATTTAAAATTTTCTAATAGCCACATTTTGAAAAGTACAGAAATAAATGAAAATAACCTTAATAATATATTTTA
Loxodonta africana  ........................................................................................................................

Homo sapiens        TATAATCCAGTGTATCCAAAATATTACTTGTTCAACATGTAAGGCCACAAGAGTCTCTTTTCTGATCTCTTTACTGAAATTAAAGAGCATTTCTCTTAGCTCCAGTAGATTTAGACCATA
Loxodonta africana  ........................................................................................................................

Homo sapiens        GTAGGAGTTGAAACATATTTGACCTTTGCAAATGAATGAGAATTTTTTTTGTAAACAAACAGGCCTTTGACATATTCAGAGTGGTTTTCTATGTGTATAAAGTGATTTATGTAGAATTTG
Loxodonta africana  ........................................................................................................................

Homo sapiens        CTGTGTGTAGTCCTTGTTCTCTACAGTTTTATTACAATAAGTTACTTTAAAGGTTAACTTTTTTTAGAAGAAAAAGAGTGTGTGTGTGTGTGTGTGTGTGTGTTCGTGTGTGACTTATTT
Loxodonta africana  ........................................................................................................................

Homo sapiens        TACTTACATACAGAAGCTCTCTTAAAATCAAGTGAGGTTGGTATGTCCATTTGTTTCTAACCAGTGTAACTAGGAGGTACTCAAGTTCAAGGTCTCTGAATCCATTTCAGCAAATTAAAA
Loxodonta africana  ........................................................................................................................

Homo sapiens        TGAAAATTTCTAAGAGCTTTACATCTAGGCATATTTTTCCCTATTGACATATGTTTACTAGACATATTTTTCTAAAGTAGTCCTACTTCATCTGTATGAAAAAACTTTTACAGACAGAAA
Loxodonta africana  ........................................................................................................................

Homo sapiens        ACCTCCTTCATTAATCCTCTGAAAGAGTCTTTGGGCATTTCTTAAATGCCTATGGTAACTGCCATCACTGATAACTTTGTAGGAATGTACAGGCCTTGAACCTATGGAATTCTCCATGAC
Loxodonta africana  ........................................................................................................................

Homo sapiens        TTAGTAGAAAATTTCATCAGCAACATGTTTACCCTATCTAACCTTTTAATGTTCTCTTATAAACTACCGCCTAGCCTTCTCTCGTATTAACAGTAGGGTAGTTGCAAACTTTGATGTTAA
Loxodonta africana  ........................................................................................................................

Homo sapiens        ACCTCTAGCAACACATTCTGAAAGTGTTTCAATGTTATTGATCATGTTTTCTTCCCTCTTGCTGCTATTGGGTATAACACATCTCATGTGTTGCATGATTTTCGTTGTTGTTGTTGAGAC
Loxodonta africana  ........................................................................................................................

Homo sapiens        AGAGTCTCGCTCTGTCACCTAGGCTGGAGTGCAATGGTGCGATCTCGCCTTACTGCAACCTCCACCTCCTGGGTTCAAGCAATTCTCTCACCTAAGCCTCCCCAGTAGCTGGGATTACAG
Loxodonta africana  ........................................................................................................................

Homo sapiens        GCACATGCCACCATGCCTGACTAATTTATGTATTTTTAGTAGAGATGGGGTTTCACCGTGTTAGCCAGGTTGGTCTCGAACTCCTAACCTTAAGTGATCTGCCCGCATTGGTTTCCCAAA
Loxodonta africana  ........................................................................................................................

Homo sapiens        GTGCTGGCATTACAGGCATGAGCCACTGCCTGGCTGCATGATTTTTATTTTATTTTATTTTTTTTTGTCTTTCAGAATTATGGGAGAATAAATGGCCATACAAGCCCAAGCCCACTTATG
Loxodonta africana  ........................................................................................................................

Homo sapiens        CCATCTTTTGGTAGAGAGAATAGCTTTGTGTGATCTGTTAAAGTTTATAAATTGAAAGTTGTTAAATGCATGAATTTCTGGGGTTGAAATAGGTTCAGTGAGCTATTTGTAATAATGTTA
Loxodonta africana  ........................................................................................................................

Homo sapiens        TGAGGGTGAGTACAGTAGGATTGATTTTCAAAATATACTACCAAATTCATCTGTTCTGAGACACTTCCATTGGTCTCTAATCTAAATAGGCAATTAATCTTCCTTTTGCTTTTTTGGTCT
Loxodonta africana  ........................................................................................................................

Homo sapiens        TAAAATTTGAGGGCTCCTTAACCCATTAACATCTTCAGGAGACCAACCTAGGCAACACAATTAGCCAGGCATAGTGACATGTGTCTATGGTCCCAGCTATTTGGGAGGCTGAGGTGGGAG
Loxodonta africana  ........................................................................................................................

Homo sapiens        GATCACTTGAGCCCAGAAGGTCAAGGATGCAGTCAGCTGTTATCACGCCATTGCACTCCAGCCTGGGTGACAGAGCAAGACCATGAATGAATGAATGAGTGAATGAATGAATGAATGAAT
Loxodonta africana  ........................................................................................................................

Homo sapiens        GAATGAAAAATAAGTAAATAAAGATAAATAAATATATCTATATCTATATTCTGATGTTTTGGGGGAACATTTAGAAAGAGAAGGTAGGGATCCAGGAAGTTAATCAGCTTTGGTAAAAGA
Loxodonta africana  ........................................................................................................................

Homo sapiens        GTTTCTTATTGTAATGAATTCAAATATCAGAAAGGGAGGAATCCTTTTATACATATTAGTCTGTGTATAGAA----------GATTTGCCATCAGTTTAACATTCAT-TTTAAACATTTT
Loxodonta africana  ..................................................ACACATTAACCTTCCTATATAAAGATGTCCCTGCTTAGCCATCAGCGTAACATTCATGCTTCAACATTTT

Homo sapiens        TTT--CTTGATGGTGATGATAAATTTGCTCAACATACAATAAAACACTGACCAGGAAGACTCCCTGTCAGTACTTCAGTATGG----CTTAGGTAGACCTTAA--AAGTTCCTAAGAATT
Loxodonta africana  CTTTCCCTAATGGAGAAGATAAATT-GCTGAAAATAAATTGCAGTGCTGGCAAGGATGATTCCTTGTGAGTACTTTGGTATGGATTTTTTAGTTAGACCTAAATTAAGTTCCTGAGAATT

Homo sapiens        TTGGTATCAAATTAGATTTAAATAAGCTAAGCTTTTTAAGCTACTCCATGGCAGCCTGCCTTCTTACACATT---ATCACATAAGCCTCAGTTGGCATGTTGTTTTTAACTTCATACTGT
Loxodonta africana  TTGGTATTAAGTCAGATTTACATGAGCTGAGCTTTG-AAGCTACTTCATTACAGCCTCGCTATTTACACATTCCTATCACATAAACCT--GATCACATA--------AGCCTGATTCTAG

Homo sapiens        AACAATTTGATGATATTATCTTTCCAATATGGGTTTTATAAAGATTGGAAGATTATCTCAAGTTGTAAATGCAAGGATTTAATAATTCTATTTGTTAAAATA-ATTATATGACCCTATTA
Loxodonta africana  AA----------GCCTTATCTTT---GTATTAGTTC---------------ATAACTTCATATTGTAT--------------CAATTCTGTTTGTTAAACTATCTTCCATGATCCCATTA

Homo sapiens        ATTCACCCAGAAATGCTTTACAGAAGCATATTAAGGAT------AACATTAT-------CAAAAATAGTTTTATAATTTTTTTTTTAAGACAGGGTTTTGCTCTGTCACCCATGCTGGAG
Loxodonta africana  ATTCATCCAGAAATTCTTTATACATGCATATTAAGGATATTATCAACATTTTTCAAAGACAAATATAGTTTTACAA............................................

Homo sapiens        TGCAGTGGTGCCATCATGGCTCACTGCAACCTCAATCTCCCAGGCTCAAGTGATCCTTCCATCTCAGCCTCCTAAGTAGCTAGGACCATAGACACCCTTAACCACACCTTGCTAATTGTT
Loxodonta africana  ........................................................................................................................
```

```
Homo sapiens        TGTATTTTTTGTAGAGAAGGGGTTTCGCCATGTTGGCCAAGCTGATCTTGAACTCCTGGGCTTAAGCAATCTACCGGCTTTGGCTTTCCAAAGTGCTGAGATTACAGGCATGAGGCACTG
Loxodonta africana  ........................................................................................................................

Homo sapiens        TGCACAGCCTTATTATTTTATTTTT----AATCTATACCATAATTCTCACTCCTTCTATCCCGTTGTCCGCATATATACATATCTACCAAAGACAGGTTGAGGGAACAGGAAAAT--GGC
Loxodonta africana  ....................TTTTTAAAAATACATACCATAATTGTC-CGACTTCCTTCC--TTGGCCCCAT----------CTACCAAAGACAGGTCAAGGAAACAGGAAAATCTAGC

Homo sapiens        TGTTTTAAAATCCTTGACCTTTCCTGCATTGTTTATGTATGAACTGTCAAATTTATATGTCATTTTTTTATTCTTTGACATTCATATTTAGTAGACAGTATAACTCCCTAGAAGTTAATC
Loxodonta africana  TGTTTTAAAATCCTTGGTCTTTCTTGCATTCACTGTGTAATAACTGTAAAAATTATATAT-------TCATTCTTTCATATGTGTATTTAGTAGACAAGAGAACTTCC-AAAAGTTAATC

Homo sapiens        TTGATAGTTAATTTTTATTACTTGGGATTTTGTTCCTCACTTAAAATTGTTTAATTAAAAAAT-ATGTTTAATTATAATTCTAAAAGTCAAATGTATGATTTTTCTTTTTTCAGAAAGTC
Loxodonta africana  TTGATAGTTAATTTTTATTACTTGAAATTTTGTTCCTCAATTAACATTTTTTAGTTTTAAAATGATGTTTAATTATCATTCTAAAAAGCAAACTTATGATGTTTCTTCTTTCAGACAGTC

Homo sapiens        TTTATCTCTTGCAAGGTTCAGTAGTGGCAGATCATGACTACATTGGATTGCCTGAAATTCCGATTGGAGCATATCAAGCAAATATTCTGGTGGAAGATGCAACTATTGGTATAGTCGATA
Loxodonta africana  TGTATCTCTTACAAGGTTCAGTAGTAGCAGATCATGACTACATCGGGTTGCCTGAAATTCCACTTGGAGCATACCAAGCAAATATTCTCGTAGAAGATGCAACTATTGCTGTAGTTGACA

Homo sapiens        ATGAATTACTGACATCAAGCAAGGATCGTGAACTATTAGAATATAGAAATACGAAAATCTCTCCATTGATAGACGATCATAGTTCTCTTGAAAAGCAGACTTTCAGTCTGTTGGACTCTT
Loxodonta africana  ATGAATTACTAACATCAACCAAGGACCGTGAACTATTAGAATATAGAAATGCTAAAGTCTCTCCACTGATAGAAGATCGTGGTACTCTTGACAAACAGACTTTCAGTCTGTTGGATTCTT

Homo sapiens        CAAACCAGGTTCTTGAATACTTAAGTTAGTAAATACCAAAATTTATTTAAATTGTTTGTTTAGAA--TATAAATTCTTAA----TGATATCTTATTCATTA-AAAACGTTGTATAGAAGA
Loxodonta africana  CAAATCAAGTCCTTGAGTACTTAAGTTAGTAAATACCAAAATTTATTTAAAATGTTTGTTTATAATGTGTAAACCGTTGAATCTTGATATCTCATCTATCAGAAAAAGTTGTGT---AGA

Homo sapiens        AGTATCTTGACACATTCTTAATGACTATATTCTGAAGGTTTATTTTATAGTT-----CTTATCTAAAATTATG-CCACATTTGAATATCAGTATACGTACATATGAAGGCAAAGGAAGCA
Loxodonta africana  AGTGACTTGACACACTCTTGATAACTAAATCCTG-ATGTTTATTTTATAACTATTTATTTATCTAAAGTTGTGACCATGTTTGAATGTCA--ATGCATAGATATACAGGCAGAGGAAGCA

Homo sapiens        TGTAATATATAGAATTCACTTTTTCTTCTAAAAAGAGAACAGGCAAAGCACCCCTACTTCTTGGGAAATAAATTCCAGCCCAAATCTCTCAAACCTTTGTATGGTTTTGGCACCGTTTCT
Loxodonta africana  TAACATAAATAGAATTCA---GTTCTTCTAAAAAGAGAATAGGCAAAGCGTACTTGCTTCTTAGGAAGTAAATTCTTGTTCAAAACTCCTAAATCTT----TGATATCTGTATTGTGTTT

Homo sapiens        AAGTTTTAAATTTTCTGAAATTCTCTACCA-----------------------------TTTA-----------------------------------------AACAGTAAGTTTG---
Loxodonta africana  CAGTTTTAATTTTTATGGAATGCTCTACCAGGCGCTTCTTGGAAATCGTATGGGGCAGTTCTACTCTGTCCTATAGGGTTGCTAAGAGTCGGAATTGACTTGACAGCAGTGGGCTTGTGT

Homo sapiens        -----------AACTACCTTGAATGGAATTGATAAATACTGAATCAACTGTAACAATGAATGAAATGAAGCTTCTTTCTGTTTCATTAGAACTTCTAAAAACAAGATTGTATGAAAAAAT
Loxodonta africana  AAGTAAATTCCAGCTCCCTACAATGGGATTAAT-----TTGAATCAGCCATAACAATGAATTAAATGAAACTCCCCTATGTGTCATTAAAGCTTGTAAAAATAATCTTGTAGGAAAAAAT

Homo sapiens        ATAGGGTTATTTAATAGGCTTTCACAGAATATTGGTAATTTCAAATTATCTAATTGGTTAGAT-AGGGTCAGGATT----AGGGTCTGAAACTATG-TATTGGTTGGGAAACAATTTGTT
Loxodonta africana  TCAGGGTTGTCTAAGGGAACCTTACAAGATATTGGTAATTTCACCTT---------TTTAGATAAGGATTAAGGTCTAAAAAGGTCTGGAGCTGTATTATTGGTT-TGAAACAG-GTGTT

Homo sapiens        TATTTTCATACTATATATTAAAGATAAAACCATCGACCAGGCATGGTGGCCCACACCTGTAATCCCAGCGCTTTGGGAAGCCAAGGCAGGATGATCACTTGACCCCAGGAGTTCGAGACC
Loxodonta africana  CATTTCCATAATATATACTAAAGCCAAAAGCATC......................................................................................

Homo sapiens        AGCCTGGGCAACACAGGGAGACCCTATCTCTACAGAAAAATGTTTAAAAATTAGCCAGGTGTGGTGGCACATGCCTGCGGTCCCAGCTACTCAGGAGGCTGAAGTAGGAGCATTGCTTGA
Loxodonta africana  ........................................................................................................................

Homo sapiens        CCCCAGGAGGTCGAGGCTGCAGTGAGCCGCGCTCATGCCACAGCTCTCCAGCCTGGGCGACAGGGCAAGACCTGTCTCAACAACAACAACAAAAGTATCCGTTAAGAATTTTTTCTGCAA
Loxodonta africana  ........................................................................................................................

Homo sapiens        TCATAAAATCATAAAATAACCAGTATCAAAGATTTTAAACTGTAATTTCTATGATATTCATAAATATAAAATAAATATTGTTCAATAAATATTTATTCTAAAGCACTATGAAAAGAACTG
Loxodonta africana  ........................................................................................................................

Homo sapiens        TGGGAAAAGTAAAAGATGAGTCAAACGTGTACCTCATTCTTATAAAGGACTTACTGTCTGGTAGGTAATAAACTATGTAAGCCATGTACAGCACAAGTTAAGGCATGCTAAATTGCAGAA
Loxodonta africana  ........................................................................................................................

Homo sapiens        GACAGGTGTAGATAATGTACTTAGGGAGGCAAAAAGAGAGAAGTTGTTTTTACATAGGAAATTTGTGTTTATCCTTATGGAAGAAGGATCATTTAAGCTTGACCTTGAAGAATGTATAGG
Loxodonta africana  ........................................................................................................................

Homo sapiens        ATTTGGTAAATGGATACAAAAGAATTAGGGGAGAAGTAATACACAGAAATGGAAAAGTATTGTAATGATTCATAAAAAGGCAGGGCTTCTTGTTGTCTACACTGCCTCGGGTATAAAGTG
Loxodonta africana  ........................................................................................................................

Homo sapiens        AAGAAGCAAGATAATGAGGGATAAAGCTGAAAAGGTAAGTAGGAAACCTTATGGAGAGCTTTGAATACTATTAAGGATTGATATGTGCCCCTCCCAAAATTTGTGTTGAAGTCTTAACCC
Loxodonta africana  ........................................................................................................................

Homo sapiens        CCACCACCTCAAAATGTGACCTTATTTGGAAATGAGGTTGTTGCAAGTGTAATTAGTTGAGATCATATTTCAATAGGGTGGGCTCTAATACAGTATTACTGGTGTTCTGATAAAAGGGGA
Loxodonta africana  ........................................................................................................................

Homo sapiens        AAATTTGAACACAGACTCAAGGAGAATGCCATGTGAAGATAAAGGCAGAGTTTAAGATTATGTTTCTATATGCCAAGAAATGACAGAGATTGCCAGAAAACTACCAGAAGTTAGGGGAGA
Loxodonta africana  ........................................................................................................................

Homo sapiens        GGCATTGAATACATTTTTCCTCATTCCTCAGAAGACCAACCCTGTCAATACCTTATCTCAGACTTTTGCCTCCAGAACTGTGAGACAATAAAATTCTATTGTTTCAGCCATCCAGTTTGT
Loxodonta africana  ........................................................................................................................

Homo sapiens        GGGACTTTGTTACAGTAGCTCTAGCAATAATACAAATGCCTGGCTAAATTATTTCTTTTCTGAGAGCAAAAACTTTGTATAGGAAACTGTCATAATCAGAACTGTGTTTTTGGAAGTTTA
Loxodonta africana  ........................................................................................................................

Homo sapiens        ATCTGGCAACTAGTAAAGAACAGATTAGAAGAGGAGAAAACAAGGCATGGAGATTAGTTGAGAGGACTTCGCAGTGATGACGAATACTAAAAACCTAAACTGGGCCTGTAGCATTGGTAA
Loxodonta africana  ........................................................................................................................

Homo sapiens        TGAATGGAGTCTGTCTTAAGATCAGTACAGAATATCGCACATATTATATTTCACAACCATACTGAACAGTGATGTGATATCAAAGATGGAGACCTGGAGTATTCTGATTACTCATTTAAG
Loxodonta africana  ........................................................................................................................

Homo sapiens        AAGAAAGATTATGTTTAATATAGGCTAAAGGAAAAGTGTTTAGGGTAGGGTGTGAATCTAGGACCTCATCTTATACCACAGACCAAATAAATTTCAAACAGAATTCCAGGAAGAAAAAAA
Loxodonta africana  ........................................................................................................................

Homo sapiens        CAAAACTTTTTTTAAAAAAGCGTAATTGTATCAAACAGTGCTACCTATCCTGCTTTCCTCAGAAGAGCAAAGTTTAGAAAAGAGTCATATTAGAATATCTTTTTGGTAAA
Loxodonta africana  ...............................................................................................................
```

```
Homo sapiens  TCAATTTCTGGCGTCTCGAA--CCCAAGAAATGAAAAAGGGAAGCCATGGGGGAGCAGGA---------GGAGAAATCAAGGCGCGAAAGGACCC--TCAAAGCTGT-ACTGTCACTCC-
Bos taurus    GAGACCTCCGTTATCTCAAAAGCCCAG-------AAGAGGGAAGGAATGGAGGAGGAGGAAGTCGCGGAGGAAAAATGAAGACGCCGAAGGATCCCTTCCAGGCTGTGACGGCCACTCCT

Homo sapiens  --ACGCCCAGGCTGTCATTCTCTCCAGC-ACAAGCCCCTCGGTGGAGCATGTGTTTAGGGTCTGCAGAGCACAGGAGACAAAGCAGAAGCCCAGCAGCCGCCTCAACCCTGGCATGATCA
Bos taurus    CCACACCCGGGCCGCCTTTCTCTCTGGCCACACGCCCCGCGGAGGAGC----------GGCAGCCAGAGCTTTG------AAACAGCTGCCCTGCGGCGGCCGCAAACCTCTGGGGACTA

Homo sapiens  GGTTCCGCATCTCCGGTTCTCCTCCGGTTTTG--------------GGCCCTCACCTGCCACGAATGCGGTCGTCACGCGAGCAGTTGTGAGATGGTCAAGCCCATTCAGG---TCTAG
Bos taurus    GGCTCCGCATCTC-GATTCTCCTCAGTTCTCGCCCCGCCCCCCCTGCTCCCCGCACCTGCCACGGCTGCGGTCATCACGCGAACTGC----AGATGTCCGAGTCCATTCAGGGGCTCCAT

Homo sapiens  GTTACCCCCTTTCATCCCGGAGG-------------------CAGGGGTGAGGTGTTTGTCAAG--AGACTTCAGTTGTAGGCGTGGACGTAGG-CTACTTCCTTCAGTAACAGCTATA
Bos taurus    GC--CCCCCTTTAATCCCGGGGGAGCGGGGGTTGGGGGGCGGTCAGGATTGAGGCGCCCGTCAAAGAAGACTCCAGCTGCTAGCGGGAACGTGGGTCTCCTCTCCTCAGCAGCAGCTGAA

Homo sapiens  GGGCC-ACATAAGGGAACCTCAGG--CTACTACACCTCAGGAATCTCCGTCACGCCTCTTCCCCGCCCTCCTCGGGTCTCACGCCGAAACCTCACCGGGCGGAACGATTTCCGGCAAGAG
Bos taurus    TGGCCCACAAAGAGGTACCGAAGGTACTACCGCACTTCGCGAAGCCCGGTCACGCCTCTTCCCCTCCCTCTCCGGGTCTCACACAGAAACCTCCCCGGGCGGAACGATTTCCGGCAAGAG

Homo sapiens  CCAAATGATCAGT-CCTAACC-TTTCTGACCGTGGTTCCAAGGTTCCC---------CAAAGGACTGCCCTCTAAAGG-----ACCCGGGTAGTTCCGCTTCCGGCAGCGCGAGATAAAT
Bos taurus    CCAAACGATCCGCGCCTAACCTTTTCTGACCGCGTCTCCGAGACACCCGCCCCCCGGCAGAAACACGCCCTTTGTAAACCACCACGCGGATAGTTCCGCTTCCGGCAGCACCACAAAAGT

Homo sapiens  CACGAGAGGAAGCTTAAATC-TGTCGTTTGAATTTAGGACCACCTCGGTGAGTGGTCGTTCTGGTGTGCTGTGTCATACCTACTGTT--TTTTAAAGTGAGGCGTAACCCGACAGTAATT
Bos taurus    CGCGAGAGGAAGCTTAAATCTTGACGGTAGCCTTTTGGACCACTTCGGTGAGTGGTCGTCTTAGTGTGC--TCTCACAGCTGCTGATCGTTTTAAAATGAGGCGAGACCTGAC--TAATT

Homo sapiens  TCAAAACCATTCGCCTCGACCGGCCTAAGGAAGGGTTTAATTGAGTCTGTGGGGAGATTTTGCGCAAGCGCCTTTGAGATAGGAGGGCCTT-----GGGCGTTGCGATGTCCCGCGCTAG
Bos taurus    TCAAAGCCGGGAGAGGCGACCAGTTTGAAGAAGGGTGTAATTGGCTTTATGGAGA----TTGCGCAGGCGCCCTTGGGGCGGGAGGGCCCTGTTTGGGGCGTTACGCTGCCGC-CGCCCG

Homo sapiens  CTGAGCAG--AAAGGGCGAGTGCTGTT-TCGGGCCTGGGGAGGTGCCTACTGGGTGTAA-------------------------------TTGAGTTTTACCAAAC-GGTCATTTTCCT
Bos taurus    CCGAGCGGCCAAGGGGCGAGTTCCGTTGTCGGGCTGAGGGAGGCGCCTACCAAGTGTAAAAAAAAGTATATATATATAACCAGATTGAACCCTGAGTTTTACTTAACGGGTCATTTTCTT

Homo sapiens  GCTGATTCTGAGTGTGGTTGGACCTTGCCCTTAAGATTCCGTTAACGAGTCGCCCAAA-GCTTAGGAAATGCTCCCTCACA-----GCGTTTGAGACT--AAGCA--------------
Bos taurus    GCTTACGCTAAATTTAGCTGGACCTTGCCCC--AGAGTTAGTTAACAAGTAACCAGAACGCTCTGGGAATG------CACAAAGTGGCATCTGAAACTGGGAGCAAACTCGGAAGTTGAG

Homo sapiens  -----CTTTTGGCCGGCGTTGAAGAATTCT------------CCCCATTACTTTGATCAGTCATTTAATCAGAAA--------TTTTGCCCCAAATTTAAATACGTGGCGGGGAGAAACG
Bos taurus    TGTGGCATTTGGCTGAAAGGAAAGGATTCTCTTCCCCCCAGCCCCCCTTATCCTGGTT-GTCGTTTCAGCAGATATTTCCCGGTTTTGCCGCAGATCAAAAATCTGCGGGGAGAGCAATG

Homo sapiens  TGAGGTCAAGAGGGAATCCTGTCACGTAGTTTTAAAAGAAAAATGTCCACTTTATCTCAGTGTAGGTTTT-TCTTAAATGGGCATTTCTGAACCAAAGTAG----ATACTTTTTAAAGTG
Bos taurus    CGAGCTAACAGGAGAATCTTGTCCTTTTGTTTTAAAAGAAAA---------TTACACCTCTGTGGGTTTTCTTTTAAATTACTGTGTCTGAACCAAAATGGAAAATAGTTTTTAAA---
```

```
Homo sapiens  GAAATGCATTATCCCATGTGGCTTCATGTGAATGCATATGGCGATGAAGGCATTTTTTTGTTCAATGAATTTGTACTCTGAAGTGAACCAGATTACAGAGAA-------GTGTATTTAA
Bos taurus    AAATTTCATTTTTCACACGTGGCTTCATT----TGCATACGGAGATGAAGGCGTTTTTAAGT----GAAATTTGCACGCTGAAGCGAAGCAGATTATAGGGAAGTGTCTTGTGTAGTTGG

Homo sapiens  ATTTACAGGTCCTTGACGTTGCATAAAATTGTGCGTGTCC-ATTTTTTGGAGCTTAAAATAGAATAATTCAAATGTCAGAATTCCGATCCAAGTTAACATGTTGATGAAA-ATTGCAGA
Bos taurus    ATTTCCCAGTCGTTTTCGTTACATAAAATTGTGGTCTTCCGATTTTTCAGAGATTAAAATAGAGTAATGCAGGAATAAAATTAATGATCCCAAGTAAAATGCTCACTGAAATATTACAGA

Homo sapiens  TTTT--------AAGATGGAACCAGCGGGTATTATAACCTTTTAGGGGCTTAAATT-GGTGGCTTTGTT---TTTAACCCTTGGTGAA--ACAGTTAAGTCCTAAT----CTTGACATAA
Bos taurus    TTTTAAGTTTTAAAGTTGAAACTCGTGCATATTATAACCTTTTAGAGGCTTAAATTAGGTGGTCCTGATGGTTTTGCCCTAGGTGAAACACAGTTAAATCCTAATTTTATTTAAAGTGG

Homo sapiens  TACCTAACTGCCT-TACTTTATTGAACACAAAATTATTAAAGGAATTAAGCAATGAGT------------------------TGATTTTATTTGCTTTTATGGAGGGGACAAAGTGTAT
Bos taurus    TACTTAACTGCCTGTGTTGCGCACAAGATAAAATTATTAAAGGAATTAAGTAATAAGTATTTGCACTTAAAAGAAGTATTCACTGGTTTCATTTAATGTTTTACCAGGGACAAAGTGTAA

Homo sapiens  AAAGCTTAACCAAATGTGACCCTGACA-TTCCTCTTGCAAAACTACAAGTTGTAATCAATCACTTTCATT---TTTTCTTATTCCTACATGTTTAAGTACATTAAGTAATGATTTTAAGA
Bos taurus    AAAA--TAATCAGATTTGACCCTGACAGTTCATC------AATTAGGAATTGTAATCAGACAGCTTTATTGCCTTTTCTTACTCCTAGGTATTTCAACACA-CAAGCAATTATTTGTCAA

Homo sapiens  GAAATTCTTGAAAATATGAGCATATCC---AAAGAAGTCCCAGGACAGTCTTTCTTTATGTAGTAGAAACCACTTAA-TCCCCCTTGTAAATGATACCTTTACTTAGCAATCAGAGTATA
Bos taurus    AGAATTCTTGAAAATATGAGCATATCCAAAAAAAAAGGTGTAATGTAATCTTCATCTTTATAACTCAAGCCACCTAATTCTCCCTTGTAAACGATTTCTTTCCTTAATAAT--ATTTGCA

Homo sapiens  AGAAGA-----TATTTATATTTGTCTCGAATATTACAGTTTCAAGCCATTTTCTCATA---------------------------------TGTATGGGCTTT-GAAAAAAAAAAAACA
Bos taurus    AACAGACATTTTGTTTATATTTGTTCTGAGTATTACAGCTAAAGCCATTTTCTCAAATTAACTGCTGGGATGGTTTCTAGGGACCAAGTAGTGAATGTACTTTCGAGAAGGGAAAAATT

Homo sapiens  ACT--TTCATTTACGTTACTTTTGTATATGAATACCAATTATTAGCAGTAAAACTTTTAACTACTTCAAAGGCTATGCTTTTCGTGAACTGAGTTTTCTTTCTTACGCATTTTCTATTTC
Bos taurus    ATTAGTTCTTTTAAGTTTCATCAATAAGTAAATAGCAAACACGCGCAGGAAACTTTTAGCCTTCCTCTTGGACT.............................................

Homo sapiens  TCCTTGTCATCTGTCTCTCTACCTCCAGAACCAGCAGTTTGTGAACTGCATTAGACTGTTATATCGTTATAAAAGGATACAATTCGTGAGTCTTCTGATTATTTTCTTCTTAGCACTGAA
Bos taurus    ........................................................................................................................

Homo sapiens  GCTTTATAAACATTGCTATAAAACATTAACACCATTAATGAAAGGAAAGAGATTTTAAAATATTTTTTCATGTAGGTGAAGATTTTGGGAAAAGAAAGGGTTTTATGTTACTTTAAACAG
Bos taurus    ........................................................................................................................

Homo sapiens  CCTGATATGGATGAT-AATACTTAGATA---TGATAATAATTATACAT-----TACCATATACAAAGGACTTTT--AATC------------ATCCTCGTAATTGCAAAAAG---GGA
Bos taurus    .....TATGGATGATAAATACTTAAACATTTTAACAGTAATTATATATTGCTGTACCGTATGCAAAGAGCATTTCAAATCCATTATCTCATTGGTCTTTATAATTGTGAAAAGAAAGGA

Homo sapiens  TAGATATTATGTC--CTTTTCCAAACATAGATACTAAGGCTTAAGTCTATACCTTCTTAGACACAGTTTACTTATAGTGCCATGTATGTGAAAGTGAACTGTAATGTTTTCAAAAGTGAA
Bos taurus    TAAGTAATATTTCTGTTTTGTCACACATGGATACTGAGACTTAGGT.........................................................CTTTCTGAAGTGAA

Homo sapiens  ATAAT--TTACCTCTGGAATATTTGC--------TATAATTTTTAATTCTCCTTTCAGGAAATGCAGGTAATTCTTTATTCATTCACAAATAGTTGAGTGTCCACTGTGTTCTGAACATT
Bos taurus    GTAATTGTCACCTTTGAAACCTGTGCTCTTTTTATATTAATGCTACTTCTCCTGTCAGGAAATGCAAGTAATTC-TTGTTCATTTACAAATAGTTGAATAGCCACTGTGTCC........

Homo sapiens  ATGTTAAACAATGTAAACATAAAAAATTAATAAACTCTTTCCATTCCTCATTTATAATCTAAATTATTGATTACAAAAATCACTTTATAATTTGTGTAACAGAGGCCTGAACCAAGTA--
Bos taurus    ..................................................................CTATAAAAGTCGCCTTGTAGCTTTCTAACAGAAGCCTGAACCAAGTATC

Homo sapiens  ----TTATAAAAGCTAAGGATAACAAGTAATTGACTTACTGGTATAGCAGGG-------GGGAAAAGCGTGAAATTTGAAGTAAGAGACACCAAGTTAAAAGTGTCAGCGCATGTACTCA
Bos taurus    AATGTTATGAAAGCTGAGGATGAGAAATAATTAAATTAGTGGGGGAGAGGGGTTGAGGTTGGAAAAACATGAAATTTGAAACAACAGAGACCAAGGTTAAATGTCAATGGATGGGCTCA

Homo sapiens  CTTTATTACCTTGAGTAAATTGGACAA--TCTCTGAACCTCCATTTTCCTACCTGTAAAATGGATATAAAACTAATACCTACTTCATGAAGTACTGTGAGGATTATCATAGTAAAATACT
Bos taurus    CTCTATTGCCTTGAGGAAAGTGGACAATCTCTCTGAAGCTACGTTTTTTATCTCTAAAATGTATGCAGAATTACTACCTACCCCTTAGGATGTTGTGAGGATCATCACAGTGTAGTACC

Homo sapiens  GCATAACTATTTGCAGCTGTAGTCGTGAGTCACTTAGCAATGGAATAAATTCTGAGAAATGCTTTGTTAGGCAGTTTCAGCATTGCACGAATACAGACTCTACCTACATGAACCTAGATG
Bos taurus    ATATACATGTTTGCAGCTGT----------------------------TTTTAAAATTGCTTT..........................................................

Homo sapiens  ATATGTGTGTGTGTGTGTGTATATTTTTTTCTTCATATAGAGAACCAAATGTCCCTGCACATTATTGGGTATCATTTATTTCTCCTACTTGATCTGCAGTGCTAATATCAAATGACCT
Bos taurus    ...TGTG.................................................................................................................

Homo sapiens  ATATCAGGTTCTATATATGCTCTGCTATCATCTTACGGGACCAATAGGCAGTCCATCCTTGACCAAAACATTCTTATGCAGTGCAATTGCGTTGGCATTGTTTTTAAAATTTTTAATTTT
Bos taurus    ..........................................................................................GTTATTTTTAAAA--ATTAATTTT

Homo sapiens  TTAAAATTATTTCCTCTTCCTATACTTTTGGCTTTTCCAGATGTCATATAAACAGAAATATAAAATA----TATAGCCATTTGTGTCTGGCTTATTTCACTTAGCATAATGATTTTGAGA
Bos taurus    AAAAAATTGTTTCCCATCTATATAGTTTTGG------------CATAAAAAGAGCAGCATGTAGTATGTTTGTAGCCTTTTGTGTCTGG---GTTTCACTTAGTCCAATAATTTTGAGG

Homo sapiens  TTTATTCATATTGTTAGGTCTATTTATAGTTGTTCCTTATCAATCCTTAATAGTATTCCAGTATGTGGATCTACTGTAATTTGTTTATCCATTCACCAGTTGATGAACAT-----TTGT
Bos taurus    TTCGTCCCTA---TTAAATATATTTGTAGTTCCTTTTTG-------CTGAGGAGTATTCCGCTACGTGGATCTACCACAATTTGTTTATCCATTCACTTGTTGAAGAGCATTTAGATTGT

Homo sapiens  TTTCAGGTTTTGGCTGATATGAATAAAACTTCTAAAAACATACACTTACAGGTTTTTAGGTGTACATAT-------TTTCTTTTGGGTTAGTATCTAAGATTGGTATTGCTGGACCATAT
Bos taurus    TTTTAGGTTTTGGCTGTTACGAATAAACATTCTAGAAATATTCACATACAGATTTTTGTGTGGACATGTACTTTCATTTCTCTTAAGTTA.............................

Homo sapiens  GGTAAATTGATGTTTAACTTTATAAGAAATTGCCAAACTGTTTTCCAACATGTTTACATCATTTTCTATTATCACCAGTAAGA----GCATCCTTGGTAGTACCTAGCATTGTTAGGTTT
Bos taurus    .................................AAGCTGTTTTCCAACATGTTTACACCATTTTTATTCTTACCAGCAGTGCTTTGCATGCTTGGTAGTACTTAGTATTGGCAGATTT

Homo sapiens  TGTTTTATTTTTGTTGTTGTTAGTGTGTAGTCATATCTTATGGTAGTTTTAGTTTACATTTTTATTATCTATTGATGTTGAGCATTTTTTCATGTGTTTATTTGCTAATCATATCTT
Bos taurus    TTTTTTTTTGCCATTTTGATGGATTTGTAGTGGTATCTTATTGTG...........................................................................

Homo sapiens  TAGTGAAGTGTATTTCCAGGTTTTAAATTGGGTTGTTTGCTTTCTTGTTAAGCTTCGAGAGTTCTTTATAAATTCTAGAAAAAGTTATTTATCATATGTATGTATTGCAGATATTTTATC
Bos taurus    ......................................................................................................................TC

Homo sapiens  CCAG-TGTTATTTCTC--TTTTTTTAGCAGTTCTTTAAGGAATAAAAATTTTAATTTTTTC-TTTATGCATAGCTGCTTTTTCTTTGCTGTCTGGGAAGCCATTACCCAACCTGAGCTCA
Bos taurus    CCAGCTGTGATTACTCTGTTTTCTTAACAGTATTTTAAAAGAACATAAGTTTAATTTTTCTTTTACGGGTGGTT-CTTTATGTTTGCTATCT--GAAGTCATTGTCTAACTTAAGCTTA

Homo sapiens  CAAAGATTTT---TGTTGTTGTCTTCGAGAAGTTGTATAGTTTTAAGTTGTACATTTAGATCTGTGAGCCATCATGAA-----TAATTTTTACATATCATTTGAGGTATGAGTTGAGGT
Bos taurus    CAAAAATTTTCTCTTTTACTGTCTTCTAGAAATTCTCAACCTTTAGTTTTTGCATTTAGATCTGTAAACCATCGTGAATAATTTTAATTTCTTTGTATGGTGCAAGGTGTTTGTTGAGAT

Homo sapiens  TCATCTTTTTTCA-AAGGATGTTTAATTGTT-------ACAGCATTTGTTGAAAAGATTATCCTTTCTTCATTGAATTACCTTGGCACCTTTGT-TGAAAATCAGT--TTACTATAACTG
Bos taurus    TCATCTTTTTTAATGTGGATGTTTAGTTAGTTGCTTCAGCATCATTTGTTGTAAACATTACTCTTTCTCTGTTGAGTTACCTTGGCACCTATGTAAAAAAATCAGTACATACCATTTCTC

Homo sapiens  AGTGGCTATTTCT---GTCACTATTCTGTTCGATAGATTTAAGGCTGTCATTTTGCCATTATAACACAGTCTTGATTACCATAGTAGTATAGAAAGTCCTGAAACCAAATAATGTGAAAT
Bos taurus    TGTAGCTGTATCTCTGGATACTATTCTGTTCCATAGATTGATGGCTATCATTTTGCCAGTACCACTGGGTCTTGATTACAATTGCAATA-AGTAAGTCTTGAAACAAGGTAATATACAAA

Homo sapiens  TCTTCAACTATATTC--ATTTCTCAAAGTTGTTGTAATTATTCTGAGTCCTTTAGCTTTCCATATCAATTTTAGAATCAGCCTGTCAGTTTCTGCAAAAGAGCTTG-AGGGTTCCTGATT
Bos taurus    TTTTCAACTATGTTCTTGTTTTCCAAACTTGTTTTAGCTATTGTGAGTCATCTGGTTTTCCATATCAATTTTAGAATCAGCTTGTCGATTTCTACAAAAGAGCTTGTTGGGTTTCTG-TT

Homo sapiens  GGGACTGCATTGAATCTATAGATCAATTTGGAGAAAATTGGCAT---------TTTGTCCCACTTTTTCTCAGAGGTAGAGTGGGAGTTACTCCAGCTTTCTGCATTCTAGTCAGAAGCC
Bos taurus    TGCATTGCATTGATTCTGTAAATCAATTTGGGGAGAATTGACATCTTAGTAAGTTTATACAGCTTTTT-TCATTGTTAGGGTGGGGAGTGATCTTTTTTTCCA----CCAGGCAGAAGCC

Homo sapiens  TATATGGGAATG-TTTAGTGGCATTTCAGAAAGAAATAATTTTCTTCCATGGTTATATTCACTATAATATCTTTATTGGAAGAGATCACAGGACGAATCCCTCTATTTGTGAATTGGATG
Bos taurus    TGTAGAAGAATTCTTTAATGCTATTTA----------------CTTCCATTGTTGAAT--ATTATAAT.....................................................

Homo sapiens  TCATCCCCTGCTGCCATCTCAGGACATCATACCATAAGTTTTTTCTCCTTTGTTTTCAGTGGTCTTTCTAGAAGCCCTTCCATTAGCATTAAACTTTTTGGGTTACTCCCTTGTCGAAAG
Bos taurus    ........................................................................................................................

Homo sapiens  CAAAACAATCTTGTTCAAAAAAAAAATTGTTTTAATTGTAGTAAATCACACATAACATGAAATTTACCATCTTAACCATTTTAAGTGTGCAGTTCAGTGGCTTTAAGTACATTCACATT
Bos taurus    ........................................................................................................................

Homo sapiens  TGTTTTACAACTATTACCACCATCCATCCCAAGAACTCTTTTCATTTTGTGTACTGAAACTCTTGACCAGTTAAATAATAACTCTTACATTCTCTCCTCCTCACCCCCTGTTCTATGCA
Bos taurus    ........................................................................................................................

Homo sapiens  ACCATCATTATTCTTTCTGTCTGTATGTATTTGACTACTCTGACTACCTCATATAGGTGGAATCTTACAGTATTTATCTTTTTGTGACAGGTTCATTTCACTAAGCTTAATGTCCTCAAG
Bos taurus    ........................................................................................................................

Homo sapiens  ATTCATCCATGTGGTAACGTGTGTTAGAATCTCCTTCCCTTTAAAGGTGTGTGAATATATACACCGTATTTTGTTTATTCCATTCAGCCATTGATGGACACTTGAGTTGCTTCCATCATTT
Bos taurus    ........................................................................................................................

Homo sapiens  GGCTGTTGTGGGTGATGTGGCTATGAACATGACTGTCAAAACTTTTAACCCCACTAGAATGTAAACTCAATGAAGGTCAAGAGAATCTACATCTTGTTTATTATCCTGTCTCCAACGTTG
Bos taurus    ........................................................................................................................

Homo sapiens  TAACTGGCACATAGCAGTTACTCAGTCACATTTTTTAGATCAATGAATGAACCTTATATCTGTCTCTAGCAACTATCTTGTAGCTCCTTGCCTTTATAGCTAAGCCCAAGTTTCTAAATC
Bos taurus    ........................................................................................................................

Homo sapiens  CACTTTCTGAATTTCCATTCATTCCTTGTGTCATTTCAGTCTGGTTGCCAACTGCCCTGCTATACTGTAACTATACTGTATACTGTTGCCAAAACCACTAGCTACATTATGGTTCTTATA
Bos taurus    ........................................................................................................................

Homo sapiens  TCACTTAGCCTCTCTGTGTACTTCTAAGAAGTCTGTATTTCCCAGACTTCTATTACCAGAACCTCTTCTGGCTTTTTTCCTGTTTCTCTGGCTAGTCTCATGTCCCTAAAGATTTACAAT
Bos taurus    ........................................................................................................................
```

```
Homo sapiens  ATATTTGGGAGAGAGACATATAAACTAATATATTCAATAGAGTGTTTCAGATTTTATGATAAAGGTTGACACATATTACTGTGAATGTACCAATGAGGGTGTGCACAATTCTCCCTGAGA
Bos taurus    ........................................................................................................................

Homo sapiens  TTGGGGATGTGGTCAGAAAGGATTCATGGATGAATGAACTCATTAATGTGAACGTTGAAAGATAAGGATATGTTCACTGCACTTCCTACTGTGTACACTTTCCATCTGATCCTTCTTCCA
Bos taurus    ........................................................................................................................

Homo sapiens  TTGCCTTAACTTCACCTACTTTTTACATGCCGAGTTCATCCATGAAGAATAGAACCTGCTTAATATTGTCATCTTTCTCCCTAAGCCAGAGCTTCAGACAGAAGTGATCCCAGATTCTTC
Bos taurus    ........................................................................................................................

Homo sapiens  TTTGTGTTTACTCCACACATCTAGTCAGTTACAGTTCTTATATAATGTATCTATCCATTTATACTGACACTGCTTTGGCTCAGAATTTCCTCATTTCTTGCTTTAATTATACAGTAGCTT
Bos taurus    ........................................................................................................................

Homo sapiens  CCTAGCTGACTGGTACAGGTATTCCTGTCTCTACCCCAATCTCTATTCCATATTGGTGCACAATACACTTTATAGAATAGAAATCTAATCTTGTAACTCCAACTCATTCTTAAAATTTTA
Bos taurus    ........................................................................................................................

Homo sapiens  AGTTATTCCAGGTTGGAGTGGTTAGAAGACACTCTGCAGGAAATTACACCTAGCCAGAGTTTTGAAATATGATGAGGGGCTGGAGAAATAGCAATAAAAGTTCATTCCAGGCAAAAATAT
Bos taurus    ........................................................................................................................

Homo sapiens  CAATGAAAAGGTGTGAGCACACTGCATGGGATGGTTGAAGAACTGTGGTTATGTTTTGAATTGAGAATGCCAAGAGAAAAAGATGGAGAGATTGATAAAAACCATATCTTATAAAGCCTT
Bos taurus    ........................................................................................................................

Homo sapiens  GTATACTGGCAGAGGTATTTAGATATCATTAGTGCCATGGTCTTAATGGTTGTATTCTCCCAAAATTCATATGTTGAAATCCTAACCACCAAGGTGATGGTATTAAGAGGTTGGACCTTG
Bos taurus    ........................................................................................................................

Homo sapiens  GGGCTGGGCGCGGTGGCTCACACCTGTAAGCCCAGCACTTTGGGAGGCTAAGGTGGGTGGATCACTTGAGGTCAGGAGTTCGAGACCAGCCTGGCCAACATCGTGAAACCTCATCTCTAC
Bos taurus    ........................................................................................................................

Homo sapiens  TAAAAATATAAAAATTAGCTGGGCATGGTGGCATGCACCCGTAGTCCCAGTTACTCGAGAGGCTGAGGAACAAAATTTGCTTGAGCCTGGGAGGCAGAGGTTGCAGTAAGCTGAGATCAT
Bos taurus    ........................................................................................................................

Homo sapiens  GCCACTGCACTCCAGCCTGGGCGACAGAGCGCAACTCTGTCTCAAAAAAAAAAAAAAAAAAGAGGTGGAACTTTGAGGAGGTGACTTGATCATAGGTCAGAGCTCTCATTAATGGGATTA
Bos taurus    ........................................................................................................................

Homo sapiens  GTGCCTGTATTTTAAAGAGTCTGAGAGAAACCCCTTTCCCCTTCCCTCATGTGAAGTTACAGTGAGAAGACAGCTGCCTATAAGAAAGTGAACCCTCAGTAGACACTAAATTTGCTGGCA
Bos taurus    ........................................................................................................................

Homo sapiens  TCTTGATCTTGTATTTCCTAGCCTCCAGAACTGTGAGAAAGAAATTTCTGTTTATAAGCCATCCAGTTTATGGTATATTTGTTATAGCCGCCCAAGTGGACTAAAACAATCAGGAATGGC
Bos taurus    ........................................................................................................................

Homo sapiens  AAAAGATGTATTTCGAGTTAAGTGAATATTTTGATAAGATTTGTGTTTTAGTGTGACCTCTGTGACTGCAGTGAGGACAGGGACCATATTAGAGACAGAGGAGGCAAGGTCAGATAGAAG
Bos taurus    ........................................................................................................................

Homo sapiens  GTGAGTGTGAGCTAAGGCAGTGGCAATGGAATAAAGTTCAAGGTTCTACCTCATCTACAATCAGTGCTTTTCCTCCTTTTCCAGGTGAATCTGGGTCTCTACACATCTGTTTTGATAGTA
Bos taurus    ........................................................................................................................

Homo sapiens  CTCTTTGCATACATCTGTTAAAACACCACTCTAAACTGTATAAATGCTGATTTCCTCCTCTTTTCCTCTCATTTATACCATAAGCTCCGTGAGGACAGGGATTTCATTTTATTTAACTTT
Bos taurus    ........................................................................................................................

Homo sapiens  GTATTAGTATCATCTAGTATATATAAGGGTTTAAGAGCTCAGTAACTGAATGAGCAAATTCAAGGTTATATGGATGAACAGGCAAAAAACTATTAAAAAGGGTAAAGCATGGAAGTTCTA
Bos taurus    ........................................................................................................................

Homo sapiens  CAGTAGGCACAAAGAAGATTAGGAGAGGTCAAGATCACTTTAGATTCAGAGATCATGGAACACTTGCTTCATTGAGATGCTAGAGCTTGATTTTGAAAGATGGGTAAGGCTTAATCAGGT
Bos taurus    ........................................................................................................................

Homo sapiens  GGAAGGATATACCATTTAGGGGAAAATGATGTAAACCAAGGTATAGAGAATGGTTGGGGATATGGCCATGGGTATAGAAAGGAAAGTAGTGAGAGAAGAAGCTGTATTATGTTCTGCCAG
Bos taurus    ........................................................................................................................

Homo sapiens  GTTGTAGAGGGTAGGCTAAATAGTCTAGACCTTAGCCACTAAGTGGAAGATAATGCCCAGAGGCAGTCTCATGGTGTCCACTCTGTCAAGTAAATCATTTGTAAGATAAAAATATGCTAG
Bos taurus    ........................................................................................................................

Homo sapiens  GGATAGTCTAAATTAAAGTTTCAAGGTGACTATGTCAAATAACTACACCTTTAAGTCTTAGTTCTGTTGAGCCAAGACAACTCACCCATATAAAAATGAAAATAATACACTTGTGAAAGC
Bos taurus    ........................................................................................................................

Homo sapiens  ATTTGTGAAATATGAAAATCTGTAGGAAATAGATGGTTTTTATGTCTTCTCCCAATGATGTTTATGTCCCTCTCCCTAACATAGATATATGTTAGTCTTCTCCCACTACTTTGATGAGAA
Bos taurus    ........................................................................................................................

Homo sapiens  AAAAACAAACCAAAAACCTTAAAGAGCTCAGTCTCCAAAGATTTTTGTAGTGCTTTCCTTTTCTCCTTGTCCCTTGTACTATAGGTTTACTCTGAGGAGAGCAAATTTCTCTATTTCGAT
Bos taurus    ..............................................................CTCTGGGCTCTTTGTAATCTAAGCCTACTGTGAAGAAAGAAAGTTATTCCATTTTAAT

Homo sapiens  CTACTTTCAATTATTCAAAAGTTAAAGATTTCAAAGCAGAGAAGTTAATTTCAGTAAATAGACCTTTTCTGAGAAGTTTTTGTA---TATTTATTATAAATAGGAAAA-----------
Bos taurus    GTATTTTCAATTGCTCAAAGGTTAAGGGT...AAAGTCGGGGAATTAATTTCAGTAAATAGATTTTTCTTGGAAGAAATCTATACCCCATTTACAGTAGATGGGCAGCTACCACTGGTCA

Homo sapiens  ------CCAACAGTGTCCTA---ATGGCACGTTAACTT-------AATATACTGAAGACTTCATCTTACATTA---------GATAGAAAAATTTTCTGGTCTCTCCAGGAGGTAAATGG
Bos taurus    TTATTTCCAGCAGAGTCCTGCTCAGGACTCAGAGACTTTAGAGACAATGCCCTGGGAGCTTAATCTGATACTGAGGCAGTCTGACACATGATTCCTCCCACCCC.............TGG

Homo sapiens  GTAGGGGGAGGAAGACAAGAGATGGTAGGAACTTGGAACCAAGAGGTTTTTAAATATAAAAGCAAAATACGGAAGCTACTGTGGATTATGCAGACTCACATTTTTTTTCCAATGGCTGGT
Bos taurus    AGAAGGGAAAGGGGGAGGGGTATTGTGGGAACTCAGA-----GAGGTTTTTAACTGTAAGGGCAAAATACAGACATCACTGTGCTTTTTGCAGACATTCGTGTTTCCTC--ATGGCCAGT

Homo sapiens  TTGTTTTCTACCAGTTCTGGTTACCCACATCCCTCTGTGTTTTGGGGCCTGTACTTCCTCTAAGGTGTGAGAGTTTGGTCTTTTCCTTCTGTGGGC-TTTTACAGGCCTGAAAAAGCTCC
Bos taurus    TT-TTTCCTA....................CCTCTGTGTTTTAAGGTCTGTATTTCCTTTGAGGTCTGAGAGTTTGGTCTTTTACTCCTGCAGGCTTTTTAGAGGCCTGATGAGACTTC

Homo sapiens  T--CCCCTTTTCTTTTTAAGGTCCTTCAGGAGTTTTTGCAACCTTTACTGGTCTCTAAAAGGAAGGAAATACACAATTTTTATCTTCCTTTT-------ATAAACCTCGTCTTCCTACAG
Bos taurus    TTCCCCCCATTTCTTTTAATGTCCTTCAGGAA-ATTTGCAACGTTTACTGGTCTCTGAAAAGAAGGCAACAGATAATTT-----TACATTTTAAACAAAATAAA---CATCTTTCTGCAG

Homo sapiens  ATACCATCCTCCCAACTGGCTAGTATGGGATATTT--CTAGATTTTCCTTTGGATTGCTTAGACGATTTGTGACATTGATAGTTCCAAAAACTATTAAGGGAAAAC-------TAT----
Bos taurus    GTACTGTGCTCTCAG-TGGCTAAACTGGGATGTTTTATTAAAGTTTTCTT--------TTAGATGATTTAGAATATTGGTGGTCCCAAAAGTCAACAAGGGAAAACATGGGGTCATTGGG

Homo sapiens  ---------------------------AACAGGAGGTTATATGTAGTATATTATTAATTTTACAGTATGTATAGTCTATATATTGATTTCATCTAAACTGAGAAGGGACAGGT--TTCTG
Bos taurus    GGAATGACCCCATGGGGTCACATGGGGAATGCGGGGTCATTTATAGTATATGATACATTTTACAGAATATAGA----CCATAATGATT-----TAAACTAAGAAAAAGCAGGTCCTTCTG

Homo sapiens  TCCCTTAGACTTGAAAGAATGTGTTATCATTGTAGTATAGAGATGAAAATAGAGGACACAAAGAAAAAATGATTTTGGTTTCTTT--------TTTTTTTAAGAGTTGAGGACGATTAAT
Bos taurus    GCTCTTAGACTTGGAAGAGCTTG-TGTCAGTGTATCTTAGAGA--GAAATGTAAGATA-----GGAAAATGATTTTTGCTTGTTTCTTGGTTGTTTTTTTAAGAGTGAAGGAGAATTAAT

Homo sapiens  GCAGAGCTAGAATGATAGGCAGGCAAATGGATGGGTAGACAAACAGGTTCTATATTCCCAACTTGAAACGACACAAGGCTTCATGAATTGAAAATTGATTAGGAAGTAAAAGTTTGCCAT
Bos taurus    ACAAAACTGGAATGA----CAGGCAAATAGATTGGTAGACTGACAG--TCTATACTCCCTACTTGAAACCACACACAGCTTCCTAAATTGAAAATTGATGAAGGA-TGGAAGTTTGGTAC

Homo sapiens  GGAGTCC--TGGCAGAGGAGAGAGTGTAGTGA----GGATGACATGGAAAGTAAAGGCATCAGGAGGTCTTATGTGGCAGATTGGAGGGCAGTAGGGTGC-CACTAGTGTTTCACCACAG
Bos taurus    AGAATCCTTTGGCAGAGGAAAGGCTGTAGACAGGATGGATGACAT-GAAAGTAAAAGCATCGAGGGGTCTCCTGTCGCAGATTG---------AGGTTGCGGAGCAGCGCTTCCCCACCG

Homo sapiens  ATGGTTTGAAAAGCACTGA--CCTGGAGCGACATGAAACTTGGTAACAATAGAAGTGGCTACAGGACCAGAACAGAGTATACGCATGGTAAAACTTCTTGATACCTTTACTGTTTCCATT
Bos taurus    TCAGCTGGAAAAGCACTGAGGTTTAGAGC-ACGCGGAACCGGAGAACACCAGACGTGTCTACAG--CAAGGGCTGAGTGCGCACGGGGCAGACCCTCTCGACACGCTTCCTGTTGCCAGC

Homo sapiens  TAGTGGCTATTATGACTATAAAATATCTATGAACATTTTTGTACACATATTTTGATAGTCATAGACTAATTTCACTGGAATATAAAAT------TAGAATTGCCAAACCTTTAGGGTAAA
Bos taurus    T---------------------TCATCTAGGAAAACTTCTGTACACATCTTTTGATGGTCATACACTCATTTCACTAGGATGTAAACTTAAAAATAGAATTGCCAAGTCCTTAGGGTA--

Homo sapiens  TTTGTGCTCAGCTTTAGAAGAAAC--TCAGTTTTCCTAGGTGGTTGCAACATTTTATCCTCCCACA----ATGTATGAGAGTTCCAGTTGCTGCCTGTCTTCACCAACACTTGGTGTTGT
Bos taurus    ----TGCTTAGCTTTAGAAGAAGCTGTCAGTTTTCCAAAGTGACTGTAGCCTTTTACACTTACACAAACCATGTGTGAGAG--------------TATCCTC-CCAACACC---TGCTGT

Homo sapiens  CTTTGGTTTTTAATTTTAGCCTGTTCAGTAGTGTGTAATTCTAAGTCATCGTGGTTTTA-TTTGCATTGCCCTGTGGAATAATGATG-TTGAGCACATTTTCACGTGCTTATTGACCATT
Bos taurus    CTAT-GTTTTTAAATTTAGCCTTTTTGGTAGTGTATAGTTGTAAATCATTTTGGCTTTAGTTTGCATTGCCCTG-GGAGTAATGATGTTTGAGGACATTTTCACTTGCTTATTATCCCTT

Homo sapiens  GGA-TATTCTC-TTTTATGAAGTGCTGTGTAGAAATTTCCCCCCATTTTTTGAATTGGGTTTTTGTCCTTTTCTTTTTGATTTT-TAGAAGTTCCTTGCATACACTGGGTCTGAGTCCTT
Bos taurus    TGAGCATTCACTTTTTTTGAAATCTCATGT-GAGGTATTTCCCTGTTTTT----TTATATTTTTATCCTTTGCCTTTTGATTTTGTCATAATTCATTGTATTTGCTGGGTATGAGTCCTT

Homo sapiens  TGTTAGATATATGTATTACAGATATCTTCTCCCAATGCGTGTCCTATGTTTTCACTTTCTTAATGGTGTCTTTC-ATGAATACACATTTTTACTTTGATAAAGCCCTA-TTGTTAATTTT
Bos taurus    TGTTAGATATATATATTAAAGTTATCCTCTCCCAGTCAGTGGATTATCTTTTCCCTTTCTTAATG--GTCTTTCGATGA--ACAGATTTTGATTTTAATAAAATCTAACTTGTTATAATT

Homo sapiens  TTTTTCTTTTAAGGATAGTGTTTTTTTTTTGTGTCTTATTTAAGAAATCTTTG-CTTAGCCTAAGATTTGAAAGATCTTTTCCTATGTTTTCTTCCAGAATCATTGTTATTTATCTTTCA
Bos taurus    TTTTTCTTTTATGAATAGGACTTTTTGT-----TCCTGTTTCAGAAATCTTTATCTTTTCCCAAGGTTTTGAAGATCTTTTTCTATGTTTTTGTCCAGAAGCTTTATACTTTACTTTTCA

Homo sapiens  CATTTAAGTTTATGATTTATCTGGAATTAATTTTTGTGTAAGATGTGAGGTAGGTAGTTAAGGTTCATTTTTTCCCCTAATACAGTTATCTAATTG-TCCAACATTATTTATTGAAAAGA
Bos taurus    TATTTAGGTTTGTGATTTATCTGGAA-----TTTTGT---AGATGTT-GGTAGATGGTTGATATTAATTTTTT--TCTGATACAAATATCCAATTGCTTCAGCATCATTTATTGAAAAGA
```

```
Homo sapiens  CCACCTCTTTCCTCATTGTGTTGATATCTTCATTGTAAACGTATTGACTTTACATGTGTGGATGTAATTCTGTACTCCTTGTTCAATTACAGTGATGTATTTGTCTGTCCTTATGCCAAT
Bos taurus    CCATCTCTTCTCCCATTGAATCAATGCCCTTGTTGTAAAGTAATTGACTGTATACATGTGGATTTAATTCTGGACTCTATATTCTATTTCCATGGCCTGTTTGT-----CTTATGCCAA-

Homo sapiens  TGGCT----TGTCTTATTTACTTTATTTGTATAAAAAGTCTTAAA-TCTCATCCATTCATCTTTGAGATGATTCAGAAACTGTGATAGGCTGCATTTGTTTGG-TTTGGGATGCAGCCAT
Bos taurus    -GACTTCACTGTTGTATTTACTGTAGTTTTATAAATGGTTTTAAAGTCTGATCCATTTATTTTTGAGATGTTTTAGAAATTGTAATAAGGTACATTTGTTTGATTTTTGTTTGTAACCAT

Homo sapiens  CTATTAGAAGATGAAAAAAATCATGTATTTAATAATCAATTGGAAGTGAATTAAACTTAGAACAAACTGATTGTCTTATAATACTTTATAGAGATAATTAACCTGTTTTTTAGGTTATGA
Bos taurus    TTATTACAAAATG-GAAAAATTATATATTAA----------------GAATTTAA-TTAGAATAAACTG--------------TTTTATAGAAATAATTAACCTGTCTTTTA-GTTATGA

Homo sapiens  TGGGACTGTGTTAACATTTCAGAACCTTTTCTTTTAAATTGACATATACTTTATGTTAACCTAAG----------GTAAGAAATGTACTGTTACTCTCTCTGGAAGTTAAAAGTAGATCA
Bos taurus    TAGGAGAGAATTAGTCTTACAGAACGTTTGCTTTTAAAATAGCCTACACTTTATACTAATCTAAGGTTAAGGGATGTTAGAAACATACTGATATTCC-----AGAGTTGTGAAAAGATCA

Homo sapiens  GAAAGACGGTT-TTACATTTGCAAAGAGCTGTGTCATAGGGAAAGTGAATTGGTAAGCTGTTTAAGCCATGA-CAATAGGCACAGTCTGTTTCTGCTTTGTTCCAGCATTCTCTTTCCAG
Bos taurus    GAAAGATGGTTATTAAATGTGCAAAGTGCTGTTTCATTGGCAAAGAGACTTAATAGGCTATTTAAGACCTAATTCATAGGCCTAGTCTG-TTCTGCTTTGTTCTGACCTTCTCTCTGCAG

Homo sapiens  TAAATA----CTCTTAGTCTCATCCCTAGATCTTCCAGTGGTCAGTAAACATCTTTTTTTA-AATGTAATTTCAAAATTGTAAG-ATTTATGTCAG-----AATCCTAGCAGGAAACAGA
Bos taurus    -AAATATCTTCTTTTATCCTTATCCTTAGATCTTCTGGTGGTCAGTAAACGTCCATATTAATTTTGAAAATTCAAAATGTTAAGTCTTTGCATCAGTTAACATTTCTAGTGGGAAACAGA

Homo sapiens  AGACAT--TCTGGTGGGATTTTAAAGAAAATTTAATGGAGCTCCTTTCTATA-----GACAGACAGTAGCAAAGTAAACAACATGGCACCTGGAGACTAGCAAAAGCA-AAAATAGTTAC
Bos taurus    AGATATACTCTGGTGGGATTTTAAAGAAAATTTAATAAAGCTCCTGTTTACACACCTGATGGGAAGGGTCAAAGAAGGCAACGTGGCTCCTGGAGACTAGCGACAGCAGAAAATAGCTAC

Homo sapiens  CACCT-TAGGGCTGAGGTGACAAGAAGAAGAAATAACCATGAATATATTTAGAAATAATATAAAGCTATATTTCTTCTCTTATAACTTAT---TTATTTAAATGTCACATGACAGGATCC
Bos taurus    CACCTCTAGGGCTGAAGTGTCAAG-AGAGGAAATGACCATGAATATATTTAGAAGTAACATAAAACTACATTTTTCCTTTTATCACCTATCATTTATTTAAATGTCACATAAGAGAA---

Homo sapiens  TCCTGTGTTACCATCCTGTGAATGTAACATTTCAGTTGATA-GCAAT--GAAATTTTAAGCAAAACTTTTTGAAGTGTACTGGTTCATTTCCTTTTTTCTCAGTATGTGGAAAATTTTCA
Bos taurus    ----------CCGTCCTATATAAGTAACTTTTCACGTGATGTGTAATAAGAGACTTGAAGCAAAACTTTTTGAAGTGTACTTGTTC-----CTTTTTTCCTAGTATGGGAAAATTTTTAA

Homo sapiens  GCATTTCTTATCAAATGGAAAGAGACAAGATAATTTGTCATGGGGTATATCAAAAATAGCATTTGAGTATTTTTGTTCATATACATCG--CTGTTCCTTTCCTGTACTGCAATTATCTCC
Bos taurus    GTATTTCTTTTCACTTGGAAAGAGACAAGATAATGTGTCATGTTGTATCTCAAAAACAGCATCTGGATATTCTAGTTCATATACATCATACTGTTCCTTTTCTGTACTGCAGTTATTTCT

Homo sapiens  C-TCACAAATAA-ATTCTGGGAATTCTCGGTTCATATGTAAATAGAAATATGACAGAAGTCTTAACTGAAATAGCAATTCAGTTAGCTTAAAATTTAAGTTATCTTTTTAAATACCTTTT
Bos taurus    TGTCACTGATAATATTCCAGGAAAGTTCAATTCATGGATAAGCACAAATATGACACAAGCCTGAACTGAAATAGCAACTGAATTAGCTTATCACTTAAATTGTATCTTTAAATACCTTTT

Homo sapiens  ACCTAGGTTCACAATGGTCCGTAGTGGAAAAAATGGTGACCTTCATCTTAAACAGATTGCATATTACAAACGAACTGGTGAATATCATTCAACTACACTGCCAAGTGAGAGAAGTGGCAT
Bos taurus    GCCTAGGTTCCTGATGGTTCGTAGTGGAAAAAATGGTGACCTTCATCTTAAACAGATTGCATATTATAAACGAACTGGTGAATATCATCCAACTACACTGCCTAGTGAGAGAAGTGGCAT

Homo sapiens  AAGAAGAGCAGCAAAAAAATTTGTCTTCAAAGGTAAAATTAATGTTCAAATTTTGATTATTGAGTT----GTAGATTCTTCCCCACATTC--TTCCTTGTGGAAGTTTGGGAATTTCTGT
Bos taurus    AAGAAGAGCAGCAAAAAAATTTGTCTTCAAAGGTAAAAT--AT-CCCCTGTTTTTAGTATAGACTTTTATATAGGTGCTTCCCCATGTGGAGTTCTTTGTGGAGTCCTGAGGATTTCTGT

Homo sapiens  AGTAGTTGGTTGTTGCTAAACAATTACCAGTTGAATTGATGATGCCACTCAATCAAAAGCAAAATAAAAATTACACTCAAGCTAACTGAAGGCCAATAAGCGTGAAAAGGGTTGTTTAAA
Bos taurus    AGCAATTGGTTGTTGCT--------------TAAATTGATAATGGCGTTCAGTCGAAAGCAAAATAAAAATCACGTTGAAGCTAACTGAAGGCTAGTAAGCATGAAAGGGGCATTTTAAA

Homo sapiens  TTATAGACAGAATATGATACCATGAAAGAAAACAAAACATTTTTCTAAATTAACAACTTCTGCTTTTACCCTCTCGTGGTCTCTA-----ATGGTGCACTTTCCAGCACTGTTGCCAATT
Bos taurus    TCATAGACAGAAAATGATAGCATGAAAAAACC---AGCACTTGCCTGAATTAACG----CCGCTCTTGTTCCTTGGCGAGGCCTACTTCGGTGGTGCACTTGCGGGCACTCACGTCACCC

Homo sapiens  TCAATGAGGAACTTTTTTGATGCTTAATATGAAAATGTTGACAAAGTTAACTGGTTTGTTTACTAAAATGAAAACTGGAAGGAATTAACTAT-ATTTCTGTGATCATGTCCCCTTGAACC
Bos taurus    TCAGTGAAGAACTGACTGGATGCCTAATGTGAAAATGTTGACAAAGTTAACT----TATTTACT------------------------CTATTACTTCCGTGATTCCTCCCTCT------

Homo sapiens  CCACCACTGCCATTAAATATTCTCAGGATCACATTCATTTTAGGACCCTTGGATCTTCCTGAATGGATTAAATATTTGAAAGGCAGTGGGATATTTATCTTGGAAGGTCATGATAATTTT
Bos taurus    ----CCCTGCTGTTAAATGGTTTCAGGATCATATTTATCTTTGAACTCCTA-ATCTCCCTGAGTGAATTAAATGTTTGAAAGGCAGTGGAATATTTATCTTGAAAGATTATGATCATTCT

Homo sapiens  TGCCTCTTT---TTCCCCTTATAAAGGGGAAGAGAAGTTTGGGATGAGATAAACAACAAGTA--GTCACCAGATAATTGAATGAATGTTTGTGTTACAAATGGGAATTTTGCCT------
Bos taurus    CCCCTCTTTGTCCTCCTCCTCCAAAAGGGAAGAGAAGTTTGAGAT-ATATAAGCAAAAAGTACAGTCACCAGATGGCTAAATGAATGTTGGTGTTATTAACGGGAATTTTGCCTGTAGGT

Homo sapiens  -----CTTAGGCTTACCCCAAAACAGGTTAATAAAACTCTAGGATAATAAATAAATGTCTGCTTGCATCTCCCTGTGTATATCCCTACCAATAACTCCCTTATACTTAAGATTAGGCAG-
Bos taurus    ACAGAATCAGGCTTTTCTCAAAGCAGGTCAAGAAAATTCTAAAATTATAAATAAATACCTGCTTATTTCTCCCTGTGTACATCCCTTCCAGTAGCTCCCTTTAATTGAAGAATAGGCAGT

Homo sapiens  ---------------------------------------------ACCTATTACCTCTTAAACTAAAGTGAGGATTCTTTCATTCATATATTTGATAATTTATGTAACAAATGTTAACTG
Bos taurus    CTTCCCACCCAGGGACTGAACCTGCATCTGTCATGTCTCTTACACACCTATTACATTTTACACTAAAG--AGTATTCGTTCATTCGTTCACTTAATGATTTATGCACCAATTGTTTACTG

Homo sapiens  GGTTTCTGCTGTGTGCCAGTCACATTCTAAGCCTTGGAGATACATTGATGAATAACATAGATAAGGTTCCTGCTCTTAGGTTGCTTATCTTCTAATTCTAAAAAATAAGGAGTGTTCCAC
Bos taurus    AGTTCCTACTATGTGCCAGTCACATTTTAGGCCTTGGGAATACACTGATGAATGAAATAGATTAGGCTCCTGGCCTTAAGCTGCTTACTTTCTAATTC----AAGTAAGGAATGTTCCAC

Homo sapiens  TCATCAGTTGGCTTACTCATATTAGTAGCTGAATGGTGTATTTTGGAGTTTGAATTGCTAGCCCATTCACATTGGAAAGGTAGATGGTGATTCGCTTCATACCCTGGTTACTTCCTAGCT
Bos taurus    TCAGCATATTCTTAGCTAAGCACACTAAC--AATGGCGTCTTTTTGAGTTTGAAT-----ATTCATCCACACTGGAGAAGT----GGCAGTTCTCTTCATGCCTTGATTACAT--TGCTC

Homo sapiens  GCAGATTCTTCTCAGGTGTCCTCCTAAGGAAACAGAGAATTGTCTTGAGGATTACAGTTTTTTCCACTTGCCCGCTTCACCATCCCTA---CCTTCTCAAATATAGAAGAAGCTGAGAGA
Bos taurus    ATAGACTCTTTTTAGGTGTC-TCCAAAGGAAA-GCAGAACTGTCCTGAAGATTAAAGTTCTGCTACTTTCTTGGCTTTACTAACCCTATTTCCTACTCAAATCCAGAAAAACTTAAGAGA

Homo sapiens  GCTGTTTGCTTGCAAAAATACAGTGATGATAAAACATTATTTTAGAAAATTCTGTTCCAGGAAGTTGTTTTTCTGTACATAATTTATTTGCTAATCTATGCCATTTTTAGGAGGTGCCTG
Bos taurus    GGTGGTGGCTTACAAAAGTACAGCTCTGATGAAATGCTCTTTTGGAAAATTCTATTCCAG-AAGTTGCTTATCTGTACATAATTTATTTGCTAATTCATGCCATTTTTAGGAGGTGCCTG

Homo sapiens  C----AAAATCTTCCTACTTTGAGTACTCCCAGAGAGAGATAATTTCATATTTCCGTACTTATTACGTGCTTTGGTGAACAAGCTTGCTCTACTTATATTGATAGCTACAGAATTATAGC
Bos taurus    CTGCAAGTGTTTTTCTAGTTTGATTACTCCCATAGGAAGATGATTTTATACTACCATA-TTACCATGTGCTTTGGTGAACAAGTTAGCTCTACTTGATTTGATAGCTACAAAACTATAGC

Homo sapiens  AGCTACTGCCCTTCCTAAGTATCTAGTAAGTGTAACAGCTAAGAACCTTT------GTGTTGTAGCATCATTAAAAATAAATATTCTGTTTTAAATGCAGTAAGATTTTACAAAACAAAA
Bos taurus    AGCTA------GATCTACATATCCAGTAAGTATAACAGTGGAAGATGTTTCAGATTGTATGGTAGCATCATCA----CAAATATTCTATCTTAGAGGGAGAAAGATTTAAC----CAAAA

Homo sapiens  CAAAAACCTTCATGGTTTAAAGAGAAGGCTATGATTCTCTTAAAACTACGTAGATCTCTAAAAATTGCAATTCTATC-TTTTGGTATCATTTATTCCAGAAAAAAAGCTGTTTTATGTTG
Bos taurus    CAAAAACCCTCATGGTTTGAAGATAAGGCTGTGATTATCTTAAAACTATATAGATGTCTAAAAATTGCAATGATATCATTTTGGTGTTTTTTATTCTAGAAAAGAAGCTGTTTTATGTCG

Homo sapiens  GAAAAGACAGAAAACAAAATCGTTTGGTAATTGTTTCAGAAGAGGAAAAAAAGAAAGTCTTAAGAGAATGCCATGAAAATGACAGTGGAGCTCATCATGGTATATCCAGGACCCTCACTC
Bos taurus    GAAAAGACAGAAAACAGGATCGCTTGGTAGTTGTTTCAGAAGAAGAAAAAAAGAAAGTCCTAAGAGAATGCCATGAAAATGACACTGGAGCCCATCATGGCATATCCAGAACCCTTACTC

Homo sapiens  TGGTAGAATCCAATTATTATTGGACATCTGTGACCAATGATGTCAAACAGTGGGTATGGCTTATGTATTTAGAGTATTATGAATAATGTCTTTCTGATAGATACTTAAAATTTATTATTT
Bos taurus    TAGTGGAATCCAGTTACTATTGGACTTCTGTGACCAATGATGTCAAACAGTGGGTATGGCTTATGTCTTTAGAATATTTTGAATAATGTCTTTATGGTATACATTCAAAATTCATTACTG

Homo sapiens  CTCCAGATTTATTCTGGGTATCACCTCTCATTAAGTATTTTTGGCA------------------------------------------ATCACTTAAAGGGAGTTGTATTTCCCTTC
Bos taurus    CTTTAGATTAACTCTAGATATCACTTCTCATTAAGTACTTTTTGTATGGTCACTTCTCAAGTACTTTTTGTATGGGTAGTTTTTTGTGCAGAACATTTGAAGAGAGTTTTATTTCCCTTC

Homo sapiens  ACAG---TTATTTTAAATCACTTAAGTATAATTTGAACAGGAAAGAAGCATTATTTAAACACTTGAAAATACCATATTTAAATAAGAGATTATTTAAAAAATCAGATTTGTATCATTGAA
Bos taurus    AAAAGTCTTCTTTAAAATCACTGAAATATAATTTGAACTAGAACAAAACATTATTTAAATATTTGAATTTACTACATTTA-------GATTATCTTGGGCA-CAGATTTTCATCATTGAA

Homo sapiens  CTTTTTTGTGTAGGCAGAATTTAAATGGAGCTGGAATTCATTTGACTAAA------TCATTACTTCTTCAATGGCAGTCATACAAGGTTTTCTTAAGGGGAGAATATGTTCATAGTAAGT
Bos taurus    CTATTTTGTGT-GGCAGAATTTAAATGGGGCTGAAGTTTCTTTGACTAAAAATAAGTCACTACATTTTTAATGACATTCATACAGTCTTTTCTTGAGAAGAGAATATGTTTGTGATAAAT

Homo sapiens  CAAGCCCAGTATGTGCTCTTATCTTTTGTGCCTGTT-CCATTTTGAGGTAGAA--TGTCATACATGACAGAAACCCAAAACCAAATATAACCAAAATCAAATTTTGAGGATAATGAAATA
Bos taurus    CAAGCCCAGTATGT-TTCTCATCTTTTGTGCCTGTTACTGTCTTGACATAGAAAATGTCAAGTATGACAGAAACCCCCAACCAAA--------AAATGAAGTTGTGAAGATAATGAAGTT

Homo sapiens  AGTCTGAGTTTTGTAACTTTATGACCCATTGGGACCTTCAACTTCT---AGTAGAAAATTCACCTATAGGAATTTTGGGGTTCTTGCTG-GAAGTCAGCTGATTC-TACTCTTT--CCAG
Bos taurus    CTTCTGAGTTTTGTAACGTTATAACCCATTAGGACCTTCAGCTTCTTCTGATAGAAAATTCA--------------GGGATTCTTGCTGCTAGGTCAGCTGATCCCTACTCGTGTGCTCA

Homo sapiens  TGTACTGTATGT--CAAATGGTAGGAATTCCTTGGTATATCTGTGAGATCTCCTTAATAATTGTAGTAGTACATCAAAAAGAGTGGCATTTCAAGCATAAAGAATTAAACCAAAATTTCA
Bos taurus    TGTGCTGTCTGTAGCAAATGGTAGAAATTCCTTGGTTTATGTGTGAGGATCCTTTAATAATTGTGATAGTGCATTAAAAGGC-TGTCATTTCAGGCATAAAAAATTAA----------CA

Homo sapiens  TAAGTATAATTTG-TGAGTTATCAGTTTGTGGGTCTATTCAGGTATTCCAAAGACCTTCATAATT-TATATATTTTT----CCTGCTGTGCATTT-TTTACAAACATTCAGTGAAATCTT
Bos taurus    TA--TTTAATTTAACCAAGTATCA-TTTGTGAGTCTGTGAAGGTATTCCAAAGACCTCAGTGTTTGTATTCACCTTTAGAACATGCCACACACTCCTTTTCCAATATTCAGTGAAGTCTT

Homo sapiens  TGTCAAATTGTTATCACTGGAGAATGAATATGATTTTTGGAAATAATCAGAAGTTATTTGTGATAAAATCCAACAAATAAGTTTAAATTGGAAAGTGAAAATTTTGATCAAAAGTAACTG
Bos taurus    TGTCAAATCTTTATCATTGAGAAATGAAT------TTTGGATGTAA-----GGTTATTTGTGATGAGAACTA--------GTTTAAGTTAGAAAATAAAAATTT-AATCAAAAGTAACT-

Homo sapiens  TGACTATAATGGGATTAAATGGCTTTTAAATTTTTCTTCAGAAGCTAGTTTGAAAGTAGTTTCTAGAGTGGAATTCCAGAGATACATTGAGAAATGGCACCCATCCATAAATTGATCAGT
Bos taurus    -----ATATTTCAGTAAAATCATTTTAAAATTTTTCTTCAGGCCTTGATTTGAAAGTAGTTTCCAAAATAGAGTTCCGGAAATATGTTGAACAATGGCATCT-TCCACAAGTCGGTCAAT

Homo sapiens  TGATCTTGTCTCACCTCACTCAAATGAAAGCTGCAGGACAGCATGGGTTTTGTGTATGTT--TATTCTTTTCCCACGATTGAGAGAAATTGCTC-CATCTATACATTTTATATGAATGAA
Bos taurus    TTATATCTTCTCATCTCACTACAGTGAAAACACCATGACAGCATGGGCTGGATTTTTGTTCGTACTCTTTTCCTAGGACCTAGAGGAATTTCTGGCATCTAAAAATATTGCTTGAATAAA

Homo sapiens  ATTATATTTCAATAAATATGGTTGGAATAGATTAATGAATAAGCTTCTTAATGTGATAACTTTAGAAGGAAA-CATTTTATGTATATAGAAATTATTGTAAATTTACATACTGATTTAA-
Bos taurus    GTTATATTTTAATAAATATTACTGCAGTAGATGAATGAA----CTTATTAGAGTGGTATCTTTAAAAGAAAAATATTTTTTGCTTATAGAAATTAT---ATACTCACATACTGATGTAAA

Homo sapiens  ----TAGGAAAC-----TTAAGATCTTTTTAAGTATTTAATAAATATTTATTGAATGTGAACCAGATTTATGTTCCATGTGAATAAGACCCAATAAGATGGTCATTTGGTAGTATAGTCT
Bos taurus    ATTGTAGCAAATATGTTTTGAGATCTATTCA----TTTAATAAGTATTTATTGACTGTAAACCAGAATTATGTTCCATGTGAATAAGACCCATTAACATGGTCATTGAATAGT-------
```

```
Homo sapiens  TCCCTCAGTATATGCAAGGGATTGGTTCCAGCACCACACTCCTGTCACCAAATCTACTCATACCGAAGTCCTGCAGTCAGCCCTGTGAAACTCACATATACAAGAAGTCAGCTTTCTGTA
Bos taurus    ----------------------TGATTTCA---------------------ATTAATTAATAGCAA......................................................

Homo sapiens  TACTTGAGTTTCTCATCCCTCCCTATCAATAGAGTACTTTTTATTTGTGTTTAGTTAAAAAAAAAAAGCCATGTATAAGCTGACCTTCACAGTCCAAACCCATGTTGTTTGAGGGTCAGC
Bos taurus    ..................................................TTAATTAATAGCAAA.......................................................

Homo sapiens  TGTAGTGGCTACTACAGTTGTTTGTTGTTGTTTTATTTGTTTGTTATTCTAAATAGGTTTAATCCAGTGATTCT-----CAGTCCAGAAGTTTCTCCTTCAAAACACAAAGTATACCTAT
Bos taurus    .................................TATTTG---ATTATTCTGAATAGGTTTCATT---TTATTCTGAGTACAGTCCAAAGTTTCTCCCTTAAAATATAAAGTACCTATAT

Homo sapiens  TAACCATTTTAAAATTCAGATTTATAAGACTGCTTTT---ATATTGTGTTGAGTGTTAACATTTGTATGTGTTTTCTTTATTGTGTGCCTGTT--GTAGGTATATGCTTGTCAGCATTGC
Bos taurus    CAACCGTTACAAAGTTCAGATTTATAATACTGCTTTTTTTATATTGGAT---ATATTAACACTTGTCTATCTTTTCTTTATTATATGCCTGTTTTATAGGTATATGCTTGTCAGCATTGC

Homo sapiens  CAAGTGGCAAAAAATACAGTTATTGTAGCACCGAAACAGCACCTTCTCAAGGTGGAAAATCCATGGAGTTTAGTTACTGTTGATCTGATGGGGCCTTTTCATACAAGCAACAGAAGTCAT
Bos taurus    CAAGTGGCAAAAAATACAGTTATTCTAGCACCTAAACAGCACCTTCTCAAAGTGGAGAATCCATGGAGTATAGTTACTGTTGATCTGATGGGCCCATTTCATACAAGCAACAGAAGTCAT

Homo sapiens  GTATATGCTATAATCATGACAGATTTGTTCACCAAATGGATTGTGATTTTGCCTCTATGTGATGTTTCAGCATCAGAAGTTTCTAAAGCTATTATCAATATATTTTTCTTATATGGACCT
Bos taurus    GTATATGCTATAATCATGACAGATTTGTTCACAAAATGGGTTGTGATTTTGCCTCTTTGTGATGTTTCAGCATCAGAAATTTCTAAAGCTATTATCAACATATTTTTCTTATATGGGCCT

Homo sapiens  CCTCAGAAAATAATAATGGACCAAAGAGATGAATTCATTCAACAGGTAAGACAAATAAACTACTTAGGTCTGGGAGCATATCTTACTT-CTTTTCAGTGTCCAGAACCAGTGCCCTCTGC
Bos taurus    CCTCAGAAAATAATAATGGACCAAAGAGATGAATTCATTCATCAGGTAAGACAAATAAACTACTTAGATCTAGGAGCATATTTTACTCACTTTTCAGTGTACAACA-------------C

Homo sapiens  ATTATATTACAGAGTGCTTAATAAGTTCAGATAAATTTCTGTAGGTATAGAGGTATGATATTAAATACTACAAGGATAGTTGTACTGGAAATTAGGAGATTAGGATTTT------AATAC
Bos taurus    AGCTTATTATATGATGCTTAATAAGTTTAGATAAATTCCTACAGGTATAAAGGTTTAATATTGTATAGTACAAAAGTAGTTGAACTGGAAATTAAGAGACCAGTATTTTAGTATCAGTAC

Homo sapiens  TTACTGTG------GGTTATTGTAAACCTATCTTCAGATATCCTCAAATTAAAATGAGGAGGTTGGACCAAATAATCTTTATTTACTTCTAGATTAAAAACCCATGATTGGTTGGGCCTG
Bos taurus    TTACTATGCCACTAAATAATTG-AAACTTATCTTTAGTTTTCTTCAAACTAAAAT---GAGATGGGACCAAATAGTCTTTTTTTGCTACTAGCTTAAAAA....................

Homo sapiens  GCTCATGCCTATAATCCCAACAGTTTAGGAGGCTGAGGTGGGTGGATCACTTGAGCTCAGGAGTTCGAGACCACCTGGGCAATAGGGCGAAACTCCATCTCTACAAAAAATACAAAATTT
Bos taurus    ........................................................................................................................

Homo sapiens  AACTGGGCATGGTGGTGGGCGCCTGTAATCACAGCTACTCGGGAGGCTGTGGCAGGAGAATCACTTGAACCTGGGAGGTAGAGGTTGCAGTGAGCCAAGATTGCACCACTGCACTCCAGC
Bos taurus    ........................................................................................................................

Homo sapiens  CTGAGCGACAGAGCGAGACTCCATCTCGAAAAGAAAAAAAAATTAGCCAGGTGTGGTGGCACACACCTGTGGTCCCAGCTACTCGGGAGGTTGAGATAGGAGGATCACTTGAGCCCGGGA
Bos taurus    ........................................................................................................................

Homo sapiens  GGCGGAGGTTACAATGAACTGAGATTGTGCCACTGCACTTCAATCTGGGCAACAGAGTGAGACCTTGTCTCAAAAAATAACAATAATAATAATAACACTATGATTGACAGGTAAAGGGAG
Bos taurus    ...............................................................................................TTCTGTGATTGACATGTAGAGGGAG

Homo sapiens  ACTTAAATAAATGGAAGA-----ATACATTTTCTTCATGAATAAGAATAGTCAGTATTGAAAGGTACTTTCTCCCTTGCTCTGGCCTTTT---CCCAGTTAAAATTAACCTGAAAGGATA
Bos taurus    AATTGACTAAATGGAGAAGTATTATACATCAT-TTCATGAATGAGAATATTCAATATTGGAGGAAGCATTTTCCCTTGCCCTGGCCTTTTTATCCAAATTAAAGTTAACCTTAAAGAATA

Homo sapiens  AGCAGGGGAGAATATATATGAAAACTTTTTATAAGAAATACAACAACTGCTAGACTTA-AGGCATATGTTAAAACTTTTTGTAATTGACAGGGTATATGTTTAAGAAAAATCAGAGATAG
Bos taurus    AGCAGACAGTAATATATACAAAAAATCTAACAAAGGAATATA----CTACTATATTTAGAGGTATATTATTAAA-TTTTTACAATTGACAAAAGACTGGTATAA------------ATAA

Homo sapiens  CCAGACTTGGTTGAAAGCATCTTTGATGGCTCAGTCCAAAAGAAATACCTAAAGTTAATATGTATTAACTAGTAGATAGGACATGTGCCATGGCA-CCCCAGGATTATTCCCAGTTTCC-
Bos taurus    CCAGACTAAGTTCACAGCACCTTTGATATCTCAATCCAGAAGAAATACCTAAAGTT-ATACTTATTAAATAGT---TAGGGCTTGGGTCAGGGGGTTCCTAAGGTGATCTCCAGGTTTTA

Homo sapiens  -----ATAATTCTCTAGGAGGACTCAGCATATAGTCATACTCATGGCTAAGATTTATTC---------------AGCAA-------AGAGAAAAGACACAT-------------TGGGAG
Bos taurus    TATTAATAATTCACTAGGAGGACTCTGCAGATGGACATACTCATGCCTAAGATTTATATAATTAAAGGATACAAAGCAAGATTAACAGAGAAAAGGCTCATGGGGATAAGTCCAGGGGAA

Homo sapiens  AACCAGGTACAAGTTTCCAAGATCTCCTTCCCCAGTGGAGTTACACAGGACGTGTTTAATTCCTTTAGC--AGAGTTGTGACAACATA-------------------AGGAAGCTCACTA
Bos taurus    AACCAGGAACAAACTTGTAAGACATTCTCTCCCAGTGGAGTCTCACAGGACATGGTTAATTCTCCCAGTGAAGAGTTGTAGCAACACATGTAAATTGTTGTCTACTTGGGAAGCTTATTA

Homo sapiens  GAGTCTCAGTGCCTAGGGTTTTTACTGGGCGCTGGTCACATAAGCACCTTCTACCTGGCATGTACAAAAATTCCAGACTCTCAGAAGGA---------------------------AAGA
Bos taurus    CAGACTTGGTGCCTGGGATTTTAAATGGAAGCCGATCACATTGGCACCC-----------------AAAATTCTAGAGTCTCTAAAGAAGTTTTCAGCATAAACCACCTTCTTTGTAAAA

Homo sapiens  TTGTGTTTGGTCTTATAA-------------------------------------CCAAAATCTAAGCTCCTAGACATTAGCCAAGGTCCAACCTTGTAAGCATGCCTTTCAAAGAACAG
Bos taurus    CAGT-TTAGGCACCATGAGCCATCCTTAATCCGTTAGGGAATGGTAGAAACCCTCACAGAATCCAGGTTCCTAGTCATCAGCCAAGGGCCAACTTTGTAAGCAGGACTTTCACAGGATAG

Homo sapiens  CAGTCACAGATCTGCTTTGTTAATATGTTAAAATTAACTGTCAGGTTAACTCTTTTCTTCACAGGTCCATCCAAACAACTTAATAAGTATTTATGTTCTAACCACTTAGTAGCAATTTAA
Bos taurus    CAGCCTTAGTTCTGCTGTGTTAA---------------------------CTCTTCCTACACAGG----TCCAAACAACTT----AGTGTTTATGTCCTAATCACT---TAGCCATTTAA

Homo sapiens  ------AATACTCATAAATTGAAAGAAAATATATTTTTGTTCCATTCTTTTCTTTTTTTTTGAGACGGGGTCTCATTCTGTCACCCAGCCTGGAGTGCTGTGGCACCGTCTTGGCTCACT
Bos taurus    GAGAATAATAGTCATAAATTGAAAGAAAAC-TAT......................................................................................

Homo sapiens  GCAACCTCCACTTTCCAGGCTCAAGTGATACCCCCACCTTAGCCTTCTGGCTAATTTTTGTACCTTTTTTCTTTTAGAGACAGGTTTCACCATGTTGCCCAGGCTGGTTTTGAACTCCTG
Bos taurus    ........................................................................................................................

Homo sapiens  AACTCACATGATCCACCTGCCTCGACCTCCCAGAGTGCTGGAATTACTGGCGTGAGCTACCGCACCCAGCCCTTTCATTTCATTCTTAA----CCATAATTACTTGCTAATGGGTAATGT
Bos taurus    ........................................................................TTTCATTTCATTCTTAAATAGCTTCAGTTAGTTACTAATGGGATATAT

Homo sapiens  GTACCTGTTGACCACTGCTTAACTTCTCAGACCTTGGGATCATATTGTACACTGCCACCCTCATTTCCTGTTTCACACTGATTTTTACATAGTAACTGCTTTTTATCACAG---------
Bos taurus    ATGCCTGTTGACCTCTGCTCAGTTTTTCAAACCTTGGAATCAGATTAAACACTGTAA---TCATTTCCTGTTTCACAATGATTTTTATACAATTGTTGCTTTTTATCATAGAGCGTAGAG

Homo sapiens  ------------------------------------CATTCGATGAAGAAAACAGTTCTGTAAAGATATGATGTCATCAAACAAATTGTA-----ACCTGATCTAATGTTGAAAC--TGA
Bos taurus    CAAAAGTCACTTAGTCGTGTCCAACTCTTTGTGACCCCTTGGACGAAAAGAACACTTCTATAGAGATACATCATTATCGAGAGAAATGTAGTCTTACCTAATCTAATGTTGAAACCATAA

Homo sapiens  ACTACCTTAAACTGGTAGTTTTCATGGTGTCAAACAGATGTTAAGGGGGCAACTTGGCACAAAGTTTGGTAACAATGGTTTAAATGATAAAGCTAGTGTCACAAATGTGTCTAGTAGAGG
Bos taurus    ACTACCTTAAGCTAGTAGTTTCCAAGGTGTCCAACAGATGCCAAGGGGGCCATTTGGCACAGAGTTCGGTAATTATGTTCAAAATGATAGAGCTGGTATCACAAGAGAGTGGAGTTGGGG

Homo sapiens  -AGGATCATGTAA----------TTAATTAACAG-----GTAACAATTTGCTAATTGTTTAAAATTATATCTGTAATATGATTTTAGAATCC---AATACCAAAAGTATTTTCAGGTAAA
Bos taurus    AAGGATTATTTAATACATTTAATTTAGTTAATAGCATAAATAAAAATTTTCTAATTATTTCTATTTATTTCTATAATATGATTTTAGGATTACTGCATACCAAAATTATTTTCAGATAAA

Homo sapiens  TTAAAAGAATTTTAATTTTTAAAGTAGAAACAGAAAAAAATATATTTGAC--------------ATTTAATCTCTAGAAATGGAGGTCACATTCTAAACCTA-GGAATAGTGGTATAAAC
Bos taurus    TTAAAATTTTTGTGTTTTTTAA--TTGAAACAAACAAAAATTCAATTAACTTAAAAAAAAAAAAAAAAAAACTCCAGAGACTGACTTGACATTCCAGGCTTAGGGAACAGAGGCATAAAT

Homo sapiens  TTCAATGGAAAAA-CATTAAATTT-GGCTGCATAATAATAGGAGATT--TATGTGGTAAAGTAATCAACATATAAAAGCCAATCTTGCAAATAAAGCAAGATAATTTTGTAAATGAAGGC
Bos taurus    TTGAATGGAAAAATCACCAAATTTGGGCTGTTTAAAAATTAGAAATTTGTATGTGGTAAAAT..........................................................

Homo sapiens  AAGGTAGTCTTGCCTGCCTTGATTGACATAGCAGTGGTCTGAGCAATGGAGTACGTAAAGGCCATCCAGTTTGGGGGAAGGCTATTGGAACTTCTATTTATATTTTTTAAATCCCAAAAA
Bos taurus    ........................................................................................................................

Homo sapiens  TAAGAAATGAAGCTTTATTAATAATGTATACGATTGATAATAGCATTCTGACTCAATCAACAAGACATGTATGTCACACATGCTATGTGAAGGTATCCTGAAAGAAAAGTGAGAATCCAC
Bos taurus    ........................................................................................................................

Homo sapiens  ATGTTTCCTCACCCATACTTTTGCTTTCAGCAGTTGAATGAAAGTATGGATATCATCTAGCATTCACCACACTACTCACAAAATGGCCAGGGAGCTTAGATTTTTGCAGTGAAAGCAGAT
Bos taurus    ........................................................................................................................

Homo sapiens  AATACTAAAAGGCAAACACCCATCATCAACCACAAGAAAGTGATAGCGCCGGCACGTCTATTTAACTTCCAGCTCTTTGTTAGACACATCAATGAGATGAATACAAATATTAACTACAAT
Bos taurus    ........................................................................................................................

Homo sapiens  AGTCCCCATTTATCTGCAGTTTTACTTTCTGAGGTTTCAGTTCCTATAGTCAACTGCAGTCCAAACATATTAAATACAAAATTTCAGAAATAAATGATTTAAAAGTTTTTTAATTGCACA
Bos taurus    ........................................................................................................................

Homo sapiens  CCATTCTGCGTATAGTGATAAAATCTCATGCTGTCCTGCTCTGTCCAACTGGGACGTGAATCATCTCTTTGTCTAGCATATCTTCATTGTATATGCTACTCTCTCCCCATCATTAGTCAC
Bos taurus    ........................................................................................................................

Homo sapiens  TTCATAGCTGTCTTGGTTATCAGATCAAATGTAGTGGTTAGTAGTGTGTATCCAAGTTACCCTTATTTTATTTAAGAATGCCCCCAAAGTGCAAGAGTAGTGATGTTGGCATATTGTTAA
Bos taurus    ........................................................................................................................

Homo sapiens  TCGTCCTATTTTATTTTTAGTTGTTAACCTCTTACTGTGCCTAATTTAAAAATTAATCTCTATCATAGGTATGTATGTATAGGGGAAAACATAGTATAAATAGGGTTAGGTACTACCTGC
Bos taurus    ........................................................................................................................

Homo sapiens  AGTATCAAACATTCACTAGGGGCCTTGAAACATATTCTCTGAAGGTAAAGGGAGACTATAGTTATCAGATCTGATAGGAAATTAGGCATCAAAATTCAAAATTATCAAGAATACTCTTTG
Bos taurus    ........................................................................................................................

Homo sapiens  AAATATCAGTTCACTTCCACTTTTATTGATGGAGAGCCTTACCCTAAGTGCATATAATGTTTCAAGGTATTTGCTAATGATAATATGAAGCCATTATGATTAGCTAGCAGTTTTTCCAGT
Bos taurus    ........................................................................................................................

Homo sapiens  CGTGTTTAAAGTCATTTGATAACCAAACCAGGACTTTGAAAATTTTTCTAAAGGTAACAATAGGGCCAAGCATGGTGGCTCATACCTCAGCTTTTTGGGAGGCTGAGGTGAGAGGATAAC
Bos taurus    ........................................................................................................................
```

```
Homo sapiens   TTGAGACCAGGAGTTCAAGACCAGCCCATGCAATACAACAAGACTCCGTCTCTACAAAAAAAAAAAAAAATTTTTAATTAACTGGACGTGGTGTTGCACACTCATAGTCCCAGCAACTCAG
Bos taurus     ........................................................................................................................

Homo sapiens   GAGGCTAACGTGTAAGGATTGCTTAAGCCCAGGAGGTGAAGGCTGTAGTGAGCTATGGTTGCACCACTGCATTCCAGTTTGGGCAACAGTGAGACCCTGTCTCAAAAAAATAATAATAAT
Bos taurus     ........................................................................................................................

Homo sapiens   AATAATAATAGGCTGTGCTCAGTGGCTCATGCCTGTTGTAATCCTACCAGCCAGATTGCTTGAGCCTAGGAGATCGAGACTAGCCTAAGCAACATGGTGAAATGCCATCTCTACAAAAAA
Bos taurus     ........................................................................................................................

Homo sapiens   TGCAACAATTAGCCAGGAGTGGTAGGGCACACCTGTAGTCCAGCTACTTGGGAGGCTGAGATGGGAAGATCGCTTGAGCCCAGGAGGTTGAGATTGCAGTGAGCCAAGATCATGCACTCT
Bos taurus     ........................................................................................................................

Homo sapiens   AGCCTGGGTAACAGACTGAGACCCTGTCTCAAAAAAAAAAAAATAAAAAATAAAGGGTAACAATAAATATTTGCAGGCCTATTTTGTGAGATTGCTTAAACAAAAAACCAAAAAAGCGACA
Bos taurus     ........................................................................................................................

Homo sapiens   TATGATTGGGAAAACTTGTTCTTTTTGCTGTGATTAAAATGGCTTCACTATTACACAGAAAGCATAACGATGACAGTACAAAATGAACTCTTTTGTCAGCAAATACTGATGCAGAAAACA
Bos taurus     ........................................................................................................................

Homo sapiens   CTGCTAAATATTATAACATAATACACAATGTGGGAGGTTTATTGTATAGTTGAATAAAAATACAGATGTTTCTAATGGCTTAGCTTAGAGTATTTGCTAAATTGTTCCAATAAAATGCAC
Bos taurus     ........................................................................................................................

Homo sapiens   AAAGAACTTTTTGTAAACCTCTGAAGGAAAGAGAACAAACATATTCAGTAGGAAAATTAATTTTTTTAAATGTCTTACAGAAAACTAAAATTGTAAGAACTGACGGAATATCTGGTTTGA
Bos taurus     ........................................................................................................................

Homo sapiens   TGGTTGGGGGGCTGACAGAGTAAGGATGTCAAGATACCATTTCACAAATGGATTCCCTGGATCATTCCTAGGCAAGCTTTTGAAACAAAGAAGTTAAAGCCTTTTTCAAGGGTCATTTCA
Bos taurus     ........................................................................................................................

Homo sapiens   AACCATTAATCAAGACTCAGATTGAAGCTGACCCAAATGACACCTATTCTTTGGGATTTCTTTCTAATTTTGAATTAAAAATAGTCACCCTGAATTAGTATGTATATCAGCTAGGAAGTG
Bos taurus     ........................................................................................................................

Homo sapiens   TATTCAATCCAAGTAACAGACTTCCTTTCCCTATCTCAATATATTAATATATATTGTATATATATTATCCCAATATATATTGGGAGTGATTGAGAGTCACTCCTATTGTATTCACAACGT
Bos taurus     ........................................................................................................................

Homo sapiens   GGCTGCTGGAGCACATGCCACAATGCCTACATTTCAGTCAGAAAGAAGGGAGAGAGTGTTGGGCAAACAAAGCTAGTACCAGCTGAGTTTGCCTAACAACTTTTACTTACAACTTTTTGG
Bos taurus     ........................................................................................................................

Homo sapiens   CTAGAATTATGTCACATGACCACCCCTACCTGCAAGGGAGTTTGGGAAATGTAGTTTTCTTTTTATTGCTTTCCCCTCTTGTATTAACTTTGCCTATTTAGTTATTCTGATACTACTCGT
Bos taurus     ........................................................................................................................

Homo sapiens   CTGAAATATTCTTTTTTAAAAAGACAAATTTATGAGCCAAAGGTTTACTCAACTAAAAATAATAAGGAAAAAAAAAAGTCTGAAAGGTGAGGTAGGAAAAAAATATTATTTTATATTTAAT
Bos taurus     ........................................................................................................................

Homo sapiens   GGGTTTACTGTTAAAAGTTTTGTAAACTCATTTTAAGTGCTACACATAATATAGTAACACCAATAATAGTAGTTATAAGTTAAAAGTTATAAAATGCTTATTGCCAATAACTGTTTTACA
Bos taurus     ........................................................................................................................

Homo sapiens   TGCTTTTTAAACATTAACTTATGACCTAGTGGGTTGTTTTTGAGACACTGTCTACTGAATTAAAAAGTTACTTGAGGGCCGGGTACAGTGGCTCATGCCTGTAATCTCAAAACTTTGAAA
Bos taurus     ........................................................................................................................

Homo sapiens   GACTGAGGTGCATGGATTGCTTGAGTCCAGGAGTTTGAGACCAGCCTGGGCAACATGGTGAAACTCCATCTGTACAAAATATTTAAAAATTAGGTGGGCATGGTGACACATGCCTGTAGT
Bos taurus     ........................................................................................................................

Homo sapiens   CCAAGCTGCTAGGCAGGCTGAGGTGAGAGGATCGCTTGAACCCAGGTAGTGCAGGCTGCCCTCCAGCCTGTGCGACAGAGCAAGACCCTGTCTCCCACCGCCCCCCACCAAAAAAAAGAA
Bos taurus     ........................................................................................................................

Homo sapiens   AAAAATTAAACTCAAAGTTAACGTTAAGTTCTGTTCTTCCATTTCCTTGTTGTATGTGTGTATTTGGCAAGGCACTTAATCTCTCAAAGCTTCAGTATCTTACCTTTAAAATGAAATTAA
Bos taurus     ........................................................................................................................

Homo sapiens   TAATACCTCAAAAACTTGTAAGGTTTAATGACAATAATGTAAAGTGTCTAGTGTGATGGGACCAGTGTGTAGTAAAGACTCAATAAATGGCAATCACTATTTTATACATAAAATTTTACA
Bos taurus     ........................................................................................................................

Homo sapiens   TATTTTATGTATGTGTTCATATAGAATGTTATGGTCAATAAAAAAGCAAGATTCAGCCAGGCGCAGTGGCTCATGCCTGTAATCCCAGCACTTTGGGAGGCCAAGGCAGGTGGATCACAA
Bos taurus     ........................................................................................................................

Homo sapiens   GGTCAGGAGTTCAAGACTAGCCTGGCCAATATGGTGAAACCCCGTCTCTACTAGAAATACGAAAATTAGCCGGGTGTGGTGGCGCACACCTGTAGTCCCAGCTACTCAGGAAGCCGAGGC
Bos taurus     ........................................................................................................................

Homo sapiens   AGAAGAATTGCTTGAACCTGGGAGGCAGAGGTTGCAGTGAGCCGAGATTTTGCCACTGCACTCCAGCCTGGGTGACTGAGCAAGACTCCATCTCAAAAAAATAAATAGATAAATAAAAGG
Bos taurus     ..........................................................................................................TGGTCAAGGAAAGG

Homo sapiens   CAAGATTCTAATTCTTTTGAAGCTTTTTTTTAAGTCAATGGAAAAATAAAACACAAATTTTAAAAAGCCACCTTTGTTTT-ATATATTTTTCAGATCAATATTGAACTGTACAGATTGTT
Bos taurus     CAAGATTTAAATTCTTTTGAAACCTTTCC----ATCAAGGGAAAAATAAAATACAGACTTTTAAA-GTCACTGTTGTTTTTATGTATTTTTCAGATCAATGTAGAGCTATGTGAATTGTT

Homo sapiens   TGGCATAAAGCAAATTGTAATTTCTCACACCTCTGGAACTGTTAACCCAACGGAAAGTACACCTAACACAATCAAAGCATTTCTCTCCAAACACTGTGCTGACCACCCAAACAATTGGGA
Bos taurus     TGGCACAAAGCAAATTGTTATTTCTCATGCCTCCCAAACAATTAATCCAGCTGAAAGTACACCTAGCACTATCAAAACCTTTCTTTCCAAACATTGTGTGGACTACCCAAACGACTGGGA

Homo sapiens   TGATCACCTATCAGCTGTTTCATTTGCCTTCAATGTAACTCACTTGGTATGTGCCTTTTTATAATTCTGTACTTCTGAGTTATGACTATTTCTTGGCCAGATAAATGTTTTATTACAAAA
Bos taurus     TGACCACCTGCCAGCTGTTTCATTTGCCTTCAACGTCACTCACTTGGTATGTATCTATTTATAATTCAGTACTTTTGAGTTATGATTATCTCTTGGC----TGCATGTTTTATTACAGAT

Homo sapiens   TTGACGTTTATTTTCTTTAAAATAGGAACCTACTAAAAATACACCATATTTTCAAATGTTTAGTCGAAATCCTTATATGCCTGAGACTTCAGATAGTCTTCATGAAGTGGATGGTGATAA
Bos taurus     T----GTTTATTTTTCTTGA--TAGGAACCTACTAAAAATACACCATATTTTCAAATGTTTAATCGAAATCCTTACATGCCTGAATCTTCAGATA---TTCGTGAAGTGGATGGTGATAA

Homo sapiens   TACAAGTATGTTTGCCAAAATTCTAGATGCAATTAAAGAAGCTGATAAAATAATGGAGAATAAGACAACTTCACTGGGCCAGGTGATTCTATTCAATAGAAAAACTGTAA----TCTAAA
Bos taurus     TACAAGTATGTTTGCCAAAATTCTAGATGCAATTAAAGAAGCTGATAAAATAATGGAGAATAAGACAACTTCAGTGGGCCAGGTGATTCTAATAGA-AAAAAAACTATAAAGTCTATAAA

Homo sapiens   ACTTTATAAAAGTTATATTTATTTTTAAAATTTCAGCATATACATGTGTGCATGCACACACACACAC-ATACTTACTTGCCATAATTTTTTATCTATCAGATTTTTCTTGTCTTGATTAA
Bos taurus     ATGATACAAAAA--------AGTTTT-----TTCAGTATATATA------CACACACACATACATATGATACACACTGAAACCAAATTTTT-TCTATTGAATTCTGCATGTCTTGATTAA

Homo sapiens   ATAGTTGGTTTACTAGCTATGAAATGCTGAGAGGCAATAATGAGAAAGCTATGGATTTTGGAATCAAG-TAGAGCTGGATTCAAATCTGTAGTCTTAACTGCTTTTGAGTTGTTTGACTT
Bos taurus     ACTGT--GTTTATTAGCTATGAAATTCTGAGAGACATTAATAGGA---CCATGTGTTTTGGAATGAAGGTGCAGCTGAATTCAAACCCCTGGTCTT----GCTTCCCAGTTGTTTAACTT

Homo sapiens   CGGCAAATTACTTAACCTATCTTAGTTTACCTTCTATATAAAGTAGGAGCTAATAATACTGACTTTGAAATG----TTTTAAGAATTAAATAGAATTCATACCTACCTTTTAGTAGCATC
Bos taurus     GGGCCTGTTACTT--CCTCTCATA------TTTCTTTATAAAGTTGGGG----TAATACTGATTTTGAAATACTATTTTAAAAAATTAAGTGGGATTCTTCCGTAGCTTTCAGTAGCATC

Homo sapiens   TCAAGTACCATTTTGTTACAATATTCTCATTATTTGCATTTACCTTCAAGATATTATAGCTCTTTCATCTCTTTGTTCTTTTTCTTTTTACACTTGTAGATGGAGAACAACAATTTGGAT
Bos taurus     TCAGTAATATTTTTATTATAATATT------ATCTGTATTTGCCTTCAAGAAACTATAGCTGTTTCATC--------CTTTTTTTTTAATACTTGCAGATGGAGAACAACAATTGTCAT

Homo sapiens   GAACTAAATAAAAGCAAGATCATTGTTAAAAAGAAACCCAAACAATTAAATCCATTTCATTTAAAAGTGGGTCATGAAGTTTTAAGACAAAGGAAAAATTGGTGGAAGGATGGTCGTTTT
Bos taurus     GAATTAAATAAAAGCAAAATCATTGTTAAAAAGAAACCGAAGCAACAAAATCCATTTCATCTGAAAGTGGGTCATGAAGTGTTAAGGCAGAGGAAAAACTGGTGGAAGGATGGTCGCTTC

Homo sapiens   CAGTCTGAATGGGTTGGTCCTTGTGTCATAGACTATATTACAGAAAGTGGATGTGCTGTCCTGAGAGACAACACTGGGGTTAGACTGAAAAGACCTATCAAAATGTCCCACCTTAAGCCC
Bos taurus     CGGTCTGAATGGGTCGGCCCTTGTGTCATAGACTACATTACGGAGAACGGGGGCGCTGTTCTGCGAGACAGCTCTGGGGCTAGACTTAAACGACCTATCAAGATGTCCCACCTGAAGCCC

Homo sapiens   TACATAAGAGAATCCAGTGAACAAGGTAAATATCTGTCACTTCTCTACATTTTTA-TTGCTTTAGGTAAGGAGAATAGTTAAAAATAACAGAAACCT----TTCTCCCC---------TC
Bos taurus     TATGTCAGAGAATCGGGTGAACAAGGTAAACATCTGTCACCTTTCTACGTTTGTACTTTCTTTGAATGAATAGAGAAGTT-AAATTATAAGAAATCTCTCCTTCTCCTCCCTGCTTTTCC

Homo sapiens   TTTTGTTAGGTTTTCTTTTCCCTTCT-----TCATAAAATTTGTTACAGTTTTAGCTATCCCAGGTAGT-CTGTTTT-ATTACAGTGTCTCTTCTGGAACTGTCAGGCCTGTGAGATTTT
Bos taurus     TTTGGTTAGGTTTTATTTTCCCTTTTCATTCTTATAAAATTTTGTACAGTGTTGG--AGCCCAAAGAGTGTTGTTTTAATTAACATATGGTTTCTGAAGCTGTCAGGTTTGTAAGA....

Homo sapiens   TTTTTTGAGACAGAGTTTCCCTCTTTCTGCCCAAGCTGGAGTGCAATGGCGCGATCTTGGCTCACTGCAACCTCTGCCTCCCGGGTTCAAGCAATTCTGCCTCAGCCTCCCGAGTAGCTG
Bos taurus     ........................................................................................................................

Homo sapiens   GGACTACAGGTGGATACCACCATGCCCAGCTAATTTTTTGTATTTTTAGTAGAAACGGGGTTTCACCATGTTAGCTAGGCTGGTCTTGAACTCCTGACCTCCCGTGACCTGCCCGCCTTG
Bos taurus     ........................................................................................................................

Homo sapiens   ACTTCCCAAAGTGCTTGGATTACAGGCATGAAACAGGGTGCCTGGGCATTTTTATTTTTATTTCTAGTATGTTTTTCATAGTTTATGTGGGAGATACTGAACTTGTTTTCCTCTGAAGGA
Bos taurus     ........................................................................................................................

Homo sapiens   AAGATAACATCTTAACATCATCTCTGTTTTTGCTTTCTCAATGTTTTGTTTGTTTGTTTGTTTGAGACAGAGTCTCATTCTGTCCCCCAGGCTGGAGTGTAGTTGTGTAATCTCAGCTCA
Bos taurus     ........................................................................................................................

Homo sapiens   CTGCAACTTCTGCTTCCTGGGTTCAAGCAATTCTTGTGCCTCAGCCTCCCAAGTAGCTGGGATTATAGGCACATGCCACCATGCCCAGCTAATTTTTTTGTATTTTTGGTAGAGATGAGG
Bos taurus     ........................................................................................................................
```

```
Homo sapiens  TTTCACCATGTTGGCCAGGCTGGTTTTGAACTCTTGGCCTCTAGGGATTTGCCTGCCTTGGCCTCCCAAAGTGCTGGGATTGCGTGCATGTGCCATTGCACCTGGCCTTACAAATGTATT
Bos taurus    ........................................................................................................................

Homo sapiens  AGTCACTTCGTGCCTCTTTTTGGCATGAAA-AACAGCAACTGTGATCATTAATGCCAAAAACAAACATACTGTCCAGAATCTAGTTGTGAGATAAATAT--TAGGGTAC-TTTAAAAATG
Bos taurus    .................TCTTAGCATGACATGATAGTAGCTAAGATAAATAATGCCAAAA-CAAACA--CTATCTGGAATTCTGTTGTGAGATAAATATATTAGGGGACATTTAAAAATG

Homo sapiens  ACATTAATTTTTAATATTGCTTTATAGTTAGGTCATGGTTGGGGGCCAGGTGTGGGTGCAGAGTGGGGAATCCTCAGAATGCTATAAGGTGCTGTAAGAAGAGGTGA-CAGCACTCTAAA
Bos taurus    TGATTGATTTTTAATGTTGCATCTTAAAACGGCCATGGTAAATG--CAGGGGAAGGTG---AGTGAGAGGTTCTGAGAATGAT-----GCGCTATTATAAGAGGTGAACAGCACCCTAGA

Homo sapiens  CAAGTTATATTTGCCCCTAAATTTGACTCATTCTGAACAGTCAAGATACCCTAGTAAAGTGATGTTTTTGCCATGGCTTTTAGATTATGTTCCCCACTTTATCAAGGCTTTTATTTTGTC
Bos taurus    TGA-TGCTACTTGCTCCACAATGTGAATC----TGAATAGTTAAGATATCCTGAT............TTGATATGGCTTTCAGTTTAAAT--CCTACTTTATTGAAGCTTTTATTTTGTC

Homo sapiens  CTAATGTGTCTGCTTCCTAGATCTTCTCTGAGGTGGATAACTGAGAGAGAGTTAATGAGTAACTTAGCCAG---------------------------GTGAAACA--TTAATATTATTT
Bos taurus    CTATAGTATCTGCTTTCTAGACTTTCTTTGAGGTAGGTAACTGAAAGGGAATAAATTGGTATCTTAGCTAGTTACTTTTGTTTTTTCTGTAAAGAAAGATAAAATAATTCAAAATTTTTT

Homo sapiens  GGATATAAGTAACATTTAATGGAAAATATA-----------TTTATAACAATATAAACAA----GTTATATGACAGGCTTTAGCAAATATTAA-TAACAA--AAAGAGTAACAACAACAA
Bos taurus    GAATCTGAGTACCAATTAATA-AAGATACAATTTAGTGCCTTTTAAAACAGTATAAACAACTGTGTTATTTAACAGTCTTAAGTAAATAATAAATAATAATTAAAAAATAATGATAATAG

Homo sapiens  ATAACCTACACTTACCCTATGCCTTTTCTTGAGTTCTAAAGCCCATGTGGGCTGCTTAGTAATGATAGCTCAACTCTGCTTCTAATCTGGGTTACAACACTCATGTTAAAACCTCTTAGT
Bos taurus    ATGACC---------------CTCTTTCTTAGGATATGAAGCCCTTG-AGGCTGCTCAA----------------CTGTTTCTGATCTGGGTCACAACTCTCATGTTAGAAGCTCTTAGT

Homo sapiens  ACCTTTCCTTCT---TGTGACAGACCGAGTTGTTTAGTCAGTAAACTTATCTTGAACTACATTCTAATCCTGAAAACTCACAAGTGCTTTCGTTCTCTTTGTTAGAAGATAAAAGCTTGC
Bos taurus    ACCTTTCTTTCTGCTTGTGACAGAACCAGTTGCTC---TAGTAAACTTATCTTGGAC--CATTCCAACTCAGAAAGCTCCCA-GCACCTTCATTCTCT--GTTAGAGGA--AGCACTTTT

Homo sapiens  ATTGTTTGTACCTCTTCTCAGTTCTGAGCTACCTTTAATGATCTCTCTCTTCTGCAAGTTAAGTAGGGAAAAAAAAAGGTTATACAAGTATATGTTGTTCTAGCTTCTTTAGCATGATTTT
Bos taurus    ATTCCTTGTGCCT--TCTCAGTTCTAAGCTACCCTTTATGATTTCTTCCTTCTGCCAGTTAAGTAGG--AACAAAAGATTATA-AGGTGCAGGTTGTCCTTTCTTCC-------GATTTT

Homo sapiens  CCCCTTAATCTTTGACATTCATGATTTGCTCTCTTCTAAAGTGTTAAGCTTCTT----TAATACAAAATTTA-ATTTAAAATTTTGCTAAACCAGGATCTATC--AACATTTTGTAATTT
Bos taurus    CCCTTCAATCCTTGGTGTTCAGGACTTGATTCCTTCTAATGTGTTAACCTTCCTAAATTAATGCAAAACCTGTGTTTAAAATGTTACTGCATCAGGATCTGTCTCACCATTTTGTAACTT

Homo sapiens  GTTCTTGTCAAAGAGTTTCTCAGAAAAGAAGTCAAATATTGTGCTTCTTCTGAACATTCAGAACTCAGTATTTCAGAACTCATCTTCTCTGTTTGAGAACAGTCTCTAAACAGAGGCAGT
Bos taurus    GCTCTTGCCAAAGCATTTCTTA---AGGAGACCTAACATTCTGCTAC--------------AACTGAATATTTCAGAATCCAGCTGAAT------AGAACAATTTCTAAACTA--GCAAT

Homo sapiens  ATAATATTTGCCTCATACTTGAATTCTTTGCACAAGATTCTTCCAGTAATATAAGGACAAATAATATTGCAATATGACTACTGGTGTATGCATAAGGAATTTAAGACTAATTAGATGAGT
Bos taurus    ACAATTTTTGCTTCATGCTTAAATTCTCTACAGATTCTTCTTCCAGTAATACAAGGGAAA------TTGCAAAATTAGTTCTAGTGTACATATAAGGAACCTGAGATTGATGGGTAAAAT

Homo sapiens  CTGTTTTAGTTTTTCTTGTTAACTAAAGGAACTACTGATCTGGCCATCCTAGAATCTGGTTCTTATTG--CTTGATCCTTTTTT-TACATTTTTCTCATTTGTAGAAATTAAAAGTAAAT
Bos taurus    T-------GTTTTTCTTATTAATCAAAAGAAGTACTAATCCAGC-----TGAAGTCTGGTTCTTGTTTACTCTGATCGGTTTTTGTGTATTTCTCTTATTTGTAGAAATTCAAAACAAGT

Homo sapiens  GTGATTTCTGCCATCTTTTTCCTCAATATTACATACTTTCTTGAAAATCTTTTCCATGATTTTCTTGAAAATCACCTCACAAGTTGATTTGAAAGAGTCCTTGAGCTGAATT-AAAGCAA
Bos taurus    GTGATTGCTATCACCTTTTCCTTTGGTAGCACATATTTTCCTGAAAATCTT---------------------CACCTCAAAAGCTGATTTTAAAG--TCTTTGAATTAAATTTAAAGTAA

Homo sapiens  ACAAAATGATTTTGACTAATCATTGAAGTTTTTATTTGACATAAATTTTGACATGCTATAGCCAAAAGAACTATAAATTTATTTGTCCACATTTGTCATTGCTGCTCTTTGCTAGCCTTG
Bos taurus    ATATAATGCCTCTGAGTAGTCATCCCATTCTTT---TGACGTAAATTTGAACATAGTGTAGCCAAAAGGACTATGAATTTGTCTG-----------CACTGTTGCTCTT-ACCAGCCTCG

Homo sapiens  AAATTTCATAGCTCTGTTAAAAACTCACTGTCTAAAGTAGATATACTTCTATTGCCAACTTCTTATCCATTCTTATCTAGAACTAAATGTAGAGTGAGCACATTCAGTACTAGGTATACA
Bos taurus    AGATGTCGTAACTGCAGTAAAG-CTCACCAGCCGGAACAGACATGCTTCTGTTGCCAACTTCTCATCCATACCGA----------------GAGGGGGCACATTCAGTACTAGGCTGGCA

Homo sapiens  ATATTTTTCATTGCTATTTTAAGCAGGCCTGTTTGTTTTTTTGTCTAGTTCATTGCCAAGCGCTTTTTC-AGACTATTTAGACCAAGAAATCTTAAA--ATACCATAGTGTCTTTTTGTA
Bos taurus    GTGTTTCTCATTGCTGTTTTAAGCAGGCCTGTGTGTCTGTCTGTCTAGTTCAGTTCCAGGCACCTTTTCAAGACTATTTTAAC----AAATTTTATGCCATACCATCGTGTCTGTTCATA

Homo sapiens  ATCATAATGCTCCTCTTTGAAAGGAAAAA---------------ATAATC-----TGACATAGATTTTGCACAGTTTTGAATTTATTTTG----TTTGTGATAGATTTGGATCTGCCTCA
Bos taurus    GTCATAATGTTTTCCTTTGAGTGGAAAAATGTTATTGACTCCCCATTATTGTATGTGACATAGATTTTGAGTAATTTTGTTTTGATTGGGAATTATCGTGATCAGTATGTGTCTGCCTCC

Homo sapiens  CCC-AAGGTTTTAGCCTGATTTCCCTTCACAGTTGAAAGCCCCATTTGTGAAACCATTTTCATTATTGTATAAGTTTCCTCTTGTTGCTGTAACAAATTACTACAGATTTAATGGTTTAA
Bos taurus    CCTAAAGGCCCTGGTCTGATTGCCCCTCACAG------------TTTGAGAAATGGTTTTTATTCTTTAATAAGTTTCCCATTGCTGCCATAACAATTTACCACAAATTTAGTGGCTTAA

Homo sapiens  AACAACACACATTTATTATATTACAATTCTGAAGGTTAGAAGTTCAACATAGATCTTACTGGGCTAAAATCAAGGTATTGGCACTGTTGTGTTCCTGTCTGGAAGCTCTAGAAAATAATC
Bos taurus    A-------ACACTTACTGTCTCACAGTTCTGTA-CTTAGAAATCTGGTATAGATCTCAGTGGGCTACAGT-AAGGCA-TGGCAGGGCTGTGTTCTTTTCTGGAGGCTCAAGAGGGTG---

Homo sapiens  CATTTTCTTGTCTTTTCCAGCTTCTAGAGGTTACCTTCATTTCTTGGCTTGTGGTCC---CTCTATCTTCAAAGCCAACAATAGTGGGTTGAGTCCTTCTTACATCACCTTACTCCAACC
Bos taurus    ---TTTTGTGCATTTTCCACCTTCTAGAGGCTGCTCAGATCACTTGACTTGTGGTCCTCTTCCTGTCTTAAAAGCCAGCAGTG--GGGTAGAGTTCTTCTCACATTATATCACTCCAGCC

Homo sapiens  TCCTCTTCTGCCTCCCTCTTCTATATTTGAGGACCCTTGACCTTGGCCCTGCCCAAAAAGTCCAGGATAATCCCCCTATTTTAA--------GGTCATCTGATTAGCAACCTTAATTCCA
Bos taurus    TCCTATTCTTCCTTCATCCTCTGTGTTTCAGGTCACCTGTGCTTGGGCCCATCCTGATAATCCAAGATATCCTCTCTACTTTAAGGTTGATTGGTCAGTTGGTTAGCAACCTTAATTCCA

Homo sapiens  TCACTACCTAAATTCTCCTTCACCATGTAAGATTACATATTCACAGGATCCAAGGAGTAGTATGTTGACATCTTTAGGGGCCTATTATGCTGCCTACCACAATTATGGTCTGCATTCATG
Bos taurus    TCACTACCCTAATCCTCCTTTACCATGTTAGGTAACGTAATCACAAGGTAAATGAATTAGTACATAGCTACCATTAGGGGTCCACTATTCTGCCTAACACAGTTATGGTCTGCACTCATA

Homo sapiens  AAACAAAGCAAAGCACAGTCCCTATAGTGTCAAGACCATGATCTTGACGTTAATATAGTTTATGCAGAGAGCCTTTTTTTGGCCTCTTAGTTATATCTTTTTTTTTTCTTTCTCTTTTTT
Bos taurus    ATAGAAAACAGAGCTTAGTCCCTATATTAAATAAAGTTTGACTTTATTGTTAATGCA...............................................................

Homo sapiens  CCTTTTTTTTTTTTTTTTTTTTTTTTGAGACAGGTTCTTACATGGTTGCCCGTGCTGGAGTGCAGTTGCACAATAATAGCTCACTGCAGCCTTGACTTCCTGGGCTCAAGCAATCTTCCC
Bos taurus    ........................................................................................................................

Homo sapiens  ACATCAGCCTCCCAAGTAGCTTGGAATGTAGGCATGCATCACCATGCCTGGCTGAGTTTTTCGTTTTTTATAGAGACAGGGTCTTACTATGTTGCCCAGGTTGGTCTCAAACTCCTGGAC
Bos taurus    ........................................................................................................................

Homo sapiens  TCAAGCAGTCCTCTCACCTAGACCTCCCAAGTGTTGGAATTACAAGTGTGAGCCATTGTGCCCGTCCAGTTATATAATTTCTTAACCAATAGTTACTCAGTTATCTGTTGACAGTATTAG
Bos taurus    ...................................................................AGTTATGTCATCTCA-AAGTAAGAATCATTCAGTTCTCTGTT---AGTACTGG

Homo sapiens  TTATTATAATGACAGTGATT---TAGTATCAAGTTATAGTTATT-ATTTTATCTTGAGAAAGGATATACTAGTATGGCAGCACATTCTAATAGTTTGAGGATTATCAAACCATATATAGG
Bos taurus    TTTATCTACT-ACAATAATTAAGTAGTATCAAACTACAGTTATTTATTTTATCATGAGAAAAGCC---CTAGTATGGCAACACATCATGCTAGTTTTAGGAT-ATCAGACCATGTATAGG

Homo sapiens  ATTAGTTTAGTCACTTCTCTTTAAAATAAGAAAATTAAGTAGTC-TGAAACCTTTATCTTTTTTTTTCTGATTGAAGAAATACATTAATCTAAATC----AGACTTTTTTGAAGCTGTAA
Bos taurus    ATTAGTCAAGACACTTCATTTCAGAATGAGAAAATT-----GTCATGAAACCTT----ATATTTTTTCTATTTGAAAAAATAAGTTAATCCAAATCACTGAGACTTTTCTGAGGCCTTGG

Homo sapiens  TTT---------GTATTTATTTTTCTC---AGGGAAATTTAAAAATTGATTTTTTAATTATTATTTTTATTCCTTAAAACAACATTAAGGCTGGGCACAGTGGCTTATGCCTGTAATCCC
Bos taurus    TTTTAATACGTGGTTTTAATTTTTCTCCTTAGGGAAATTTTAATATT-CTTACCTCACTTTGTTTTTTACTC--TAAGAC........................................

Homo sapiens  AGCATTTTGAGAGGCCGAGGTAGGATTGCCTGAGCTCAGGAGTTCGAGACCAGCATGGGCAACATAGTGAAACCCCGTCCCTACAAAAATTAGCCGGGCATGGTGATACGCACCTTTAGT
Bos taurus    ........................................................................................................................

Homo sapiens  TTTAGCTACTTAGCGGGCTGAGGCAGAGGATCACTTGAGCCCGGGAGACTGAGCTGCAATGAGCCAACATTGCACCGCTGCACTCCAGCCTGAGTGACAGAGTGAGACCCCATCTTTTTT
Bos taurus    ........................................................................................................................

Homo sapiens  TTTTTTTGAGATGGAGTTCAACTATTATCACACAGGCTGGAGTGCAATGGTGTGATCTTGGCTGCCTGCAACCTCCGCCTCCTGGGTTCAAGCAATTCTCCTACCTCGGCCTCCTGAGTA
Bos taurus    ........................................................................................................................

Homo sapiens  GCTGGGATTACAGGCGCCCACCTCCACACCCAGCTAATATTTGTATTTTTAGTAGAGACGGGGTTTCACCATGTTGGCCAGGCTGGTCTCAAACTCCTGACATCAAGTGATCCACCCGCC
Bos taurus    ........................................................................................................................

Homo sapiens  TTGGCCTCCCAAAGTACTGGGATTACAGGCGTGAGCCACCATGCCTGGCTGAGACCCCTGGGATTATAGGCGTGAGCCACCATGCCTGGCTGAGACCCCATCTCTTTAAAAAAAAAACCT
Bos taurus    .......................................................................................................................T

Homo sapiens  ACATTAGAAATGG-ATATCAAAACTGCTGATTAATACCTTCCTGAAATGTCTTGAAAATATTTTAATTTCAAAGAAACTAAAGACTT----ATTCAGTCTTGTCAAAACCAAAATGATGC
Bos taurus    AAATAAGAAAAAGTATGTCAAAACTGCTGAT-AATACCTTCTTGACATGTCTTGAAAATACCTCTCCTTCTGAGAAACTAAAGACTTAATCAGTCAGTCTGGTCAAAACTGAAATAATGC

Homo sapiens  TTTCATCAAACAGATGTCATAATCATAACTAAATTAATGGAATGAGAAAAGATAAATACTTAGTGAAATGTACTGTGTAATGTAGCTGACTAGAAAAGACCTTTAATATGATATGTTTGC
Bos taurus    TTTCATCAAGCA---TTTATATTAGTAACTAAATCAATAGAATGAGAAA--ATAAAAATTAGGTAAATTCTGTCATGTAATGTGACTGAGTAGACTAGTCCTTTAATATGGT---TTTGT

Homo sapiens  TTACTCCCCGCCTCTGTTT----TGAGGGCAGAAAGTTTAATCATATTCTTTTCTGTATCCCTAACTCATGATTGTTTTAGATTAGTGTTGTCCAATAGATGAATAATAATACAA----G
Bos taurus    TTA-------TCTCTGTTTATAATGGGGGCAAAAACTT--ATCTTGTTCTTTTCTTTATCTGTAACTCAGGATTATTCTAGATTAGTA----CTAATAGATATATAATGAAAAAACTATG

Homo sapiens  AGCTATGTAATTTAAAATTTTCTAATAGCCACATTTTGAAAAGTACAGAAATAAATGAAAATAACCTTAATAATATATTTTAT-ATAATCCAGTGTATCCAAAATATTACTTGTTCAA--
Bos taurus    TAATATATAATTTAAAATTTTCTAGTAACCATGTTAAAAAAAG--------TAGGTGAAAATAA--TTTGTTATATATTTTATAATAATCCAATATATCTAAAATAGTATTATTTCAGCA

Homo sapiens  --CATGTAAGGCCACAAGAGTCTCTTTTCTG---ATC--------TCTTTACTGAAATTAAAGAGCATTTCTCTTAGCTCCAGTAGATTTAGACCATAGTAGGAGTTGAAACATATTTGA
Bos taurus    TTCATGTAAGGCCTCAAAAATCTCTTTCTGGTAAATCTTTGGTAATCTTTTTTGAAGTTAAAGAACATTTCTGTTGGCTGTACTAGGTTTAGAACAAAGTAGGA-TGGAAACATATTTGA

Homo sapiens  CCTTTGCAAATGAATGAGA---ATTTTTTTTGTAAACAAACAGGCCTTTGACATATTCAGAGTGGTTTTCTATG-TGTATAAAGTGATTTATGTAGAATTTGCTGTGTGTAGTCCTTGTT
Bos taurus    CCTTTCCAAATGAATGAGAATTATTTTTTTCTGTACAAAGGCAA---CTTGAAATCTCCAGAGTAGTTTTCCATTCCATATAGAGTAACTTTTGTAGACTTTGCTGAATATAATCCTGATT
```

```
Homo sapiens  CTCTACAGTTTTATTACAATAAGTTACTTTAAAGGTTAACTTTTTTTAGAAGAAAAAGAGTGTGTGTGTGTGTGTGTGTGTGTTCGTGTGTGACTTATTTTACTTACATACAGAAGC-
Bos taurus    AGCTGCAGTTTTGTCTCACTAAGTTACTTTAAAGCTTACTT------------AAGAAAGTGTGTGTATACATCAG-ATGTAGATTGG-------------TTACTTACATTTGGAAGAT

Homo sapiens  ----TCTCTTAAAATCAAGTGAGGTTGGTATGTCCATTTGTTTCTAACCAGTGTAACTAGGAGGTACTCAAGTTCAAGGTCTCTGAATCCATTTCAGCAAATTAAAATGAAAATTTCTAA
Bos taurus    CTTGTCTTTTACAAACAAGTGAGGTTGGT...........................................................................................

Homo sapiens  GAGCTTTACATCTAGGCATATTTTTCCCTATTGACATATGTTTACTAGACATATTTTTCTAAAGTAGTCCTACTTCATCTGTATGAAAAAACTTTTACAGACAGAAAACCTCCTTCATTA
Bos taurus    ........................................................................................................................

Homo sapiens  ATCCTCTGAAAGAGTCTTTGGGCATTTCTTAA-ATGCCTATGGTAACTGCCATCACTGATAACTTTGTAGGAATGTACAGGCCTTGAACCTATGGAATTCTCCATGACTTAGTAGA-AAA
Bos taurus    .................TTAGGAAATACTTAACATGCTTATAGTAACCGCCCTCACTCGTA--------------------CCTGGTGGCTCAGAAACCCTCCAAGACTTATTAGACAGG

Homo sapiens  TTTCATCAGCAA----CATGTTTACCCTATCTAACCTTTTAATGTTCTCTTATAAACTACCGCCTAGCCTTCTCTCGTATTAACAGTAGGGTAGTTGCAAACTTTGATGTTAAACCTCTA
Bos taurus    TGTCATCAGCAGGTGTCATTCTTACCCCGCCTAACCTTCCAA-------------GTGACTACCTAGCTTTCTCTGATATTAACAG---ACTAAAAGCAAACTTTGATGTTGAACCTCTA

Homo sapiens  GCAACACATTCTGAAAGTGTTTCAATGTTATTGATCATGTTTTCTTCCCTCTTGCTGCTATTGGGTATAACACATCTCATGTGTTGCATGATTTTCGTTGTTGTTGTTGAGACAGAGTCT
Bos taurus    G-AACACATTCTAAAGGTGTTTCAATTTCACTGATCCTTTTTTCCTCTTTCCTGCTACTGTTGAGCATAGCACATATCCTGGGTTGCCTGATTTTTTTT.....................

Homo sapiens  CGCTCTGTCACCTAGGCTGGAGTGCAATGGTGCGATCTCGCCTTACTGCAACCTCCACCTCCTGGGTTCAAGCAATTCTCTCACCTAAGCCTCCCCAGTAGCTGGGATTACAGGCACATG
Bos taurus    ........................................................................................................................

Homo sapiens  CCACCATGCCTGACTAATTTATGTATTTTTAGTAGAGATGGGGTTTCACCGTGTTAGCCAGGTTGGTCTCGAACTCCTAACCTTAAGTGATCTGCCCGCATTGGTTTCCCAAAGTGCTGG
Bos taurus    ........................................................................................................................

Homo sapiens  CATTACAGGCATGAGCCACTGCCTGGCTGCATGATTTTTATTTTATTTTATTTTTTTTTGTCTTTCAGAATTATGGGAGAA--TAAATGGCCATACAAGCCCAAGCCCACTTATGCCATC
Bos taurus    ........................................................................ATGGGCAAAACTAAATGTCCATACAAGCCCAAGTCTACTTATGCCACC

Homo sapiens  TTTTGGTAGAGAGAATAGCTTTGTGTGATCTGTTAAAGTTTATAAATTGAAAGTTGTTAAATGCATGAATTTCTGGGGTTGAAATAGGTTCAGTGAGCTATTTGTAATAATGTTATGAGG
Bos taurus    TGTTAGGAAAGAGCATAGCTTTGTTTGATCTTTTGATGTTTGTAAATTTAAAGTTCATAAATGTGTGACTTAC-AGGGTTGGAATAGATTCAGTTAGCTTTTTCCAATATTAGTATGAGG

Homo sapiens  GTGAGTACAGTAGGATTGATTTTCAAAATATACTACCAAATTCATCTGTTCTGAGACACTTCCATTGGTCTCTAATCTAAATAGGCAATTAATCTTCCTTTTGCTTTTT--TGGTCTTAA
Bos taurus    ATAAGTATTGTGGAATCATGTTCTGGGATATATTAGCACATTTATCTGCTTTGAGAAACTTTCATTGATCTTTAGTCTAAATGAGCTATTAATGTTCCTTTTGCTCTTTCTTGAACTTAA

Homo sapiens  AATTTGAGGGCTCCTTAACCCATTAACATCTTCAGGAGACCAACCTAGGCAACACAATTAGCCAGGCATAGTGACATGTGTCTATGGTCCCAGCTATTTGGGAGGCTGAGGTGGGAGGAT
Bos taurus    AATTTGAGGGTTCTTTAAGCTATTAAAATCTTTAAGAGAT................................................................................

Homo sapiens  CACTTGAGCCCAGAAGGTCAAGGATGCAGTCAGCTGTTATCACGCCATTGCACTCCAGCCTGGGTGACAGAGCAAGACCATGAATGAATGAATGAGTGAATGAATGAATGAATGAATGAA
Bos taurus    ........................................................................................................................

Homo sapiens  TGAAAAATAAGTAAATAAAGATAAATAAATATATCTATATCTATATTCTGATGTTTTGGGGGAACATTTAGAAAGAGAAGGTAGGGATCCAGGAAGTTAATCAGCTTTGGTAAAAGAGTT
Bos taurus    .......................................TCTGT----TAATGTTTTGGGAGAAAATTCAAAAAGAGATGACAGGGGTCCAGGAAGCTAATCAGTGGTAGTAGAATAGTT

Homo sapiens  TCTTATTGTAATGAATTCAAATATCAGAAAGGGAGGAATCCTTTTATACATATTAGTCTGTGTATAGAA----------GATTTGCCATCAGTTTAACATTCATTTTAAACATTTTTTT-
Bos taurus    CCTTGTTGTAATGAATTAAAATACTATGTAGGGTGGAATTCTTTTATCTACATTAACCTTTGTTTATAAAGATTCTGCTGCTTTGCCATCTGTTTAACCCTCATTTTAAACATTTTTTTT

Homo sapiens  --CTTGATGGTGATGATAAATTTGCTCAACATACAATAAAACACTGACCAGGAAGACTCCCTGTCAGTACTTCAGTATGG-CTTAGGTAGACCTTAAAAGTTCCTAAGAATTTTGGTATC
Bos taurus    TCCCTTACAGTGAAGATAAATTTGCTGAAAATAGAATAAAATACTAAGAAGGAAAATTCCTTGTCAGTATTTTAGTATGGATTTAGCTATACCTCGAAAGTTCATGAGCATTTTAATATT

Homo sapiens  AAATTAGATTTAAATAAGCTAAGCTTTTTAAGCTACTCCATGGCAGCCTGCCTTCTTACACAT---TATCACATAAGCCTCAGTT--GGCATGTTGTTTTTA-------------ACTTC
Bos taurus    AAATTAGACCTAAGTAAGCTAA-----TTCACCTGATCCATTGTAGCCTGCCTACTTTCGTATACCTCTTATATAAACCTGATTCTAGGAACTTTATCTTCATTGTAGTTTAATCACTTC

Homo sapiens  ATACTGTAACAATTTGATGATATTATCTTTCCAATATGGGTTTTATAAAGATTGGAAGATTATCTCAAGTTGTAAATGCAAGGATTTAATAATTCTATTTGTTAAAATA-ATTATATGAC
Bos taurus    ATAGTGTGACAATTTGATGTTATTGTC---CTAATATGAATTCTATAAAGATTAAAGAATTACCTAAAGTTGTAAATATAAGGATTTAACAATTCCATTTGTTAGTGTATCTTTCAGGAC

Homo sapiens  CCTATTAATTCACCCAGAAATGCTTTACAGAAGCATATTAAGGATAACAT---TATCAAAAATAGTTTTATAATTTTTTTTTTAAGACAGGGTTTTGCTCTGTCACCCATGCTGGAGTGC
Bos taurus    CCCATAAATGCATCCAGAAATGCTTTG-AGATATATATTAAGGATATCATCAACATCAAAAATA........................................................

Homo sapiens  AGTGGTGCCATCATGGCTCACTGCAACCTCAATCTCCCAGGCTCAAGTGATCCTTCCATCTCAGCCTCCTAAGTAGCTAGGACCATAGACACCCTTAACCACACCTTGCTAATTGTTTGT
Bos taurus    ........................................................................................................................

Homo sapiens  ATTTTTTGTAGAGAAGGGGTTTCGCCATGTTGGCCAAGCTGATCTTGAACTCCTGGGCTTAAGCAATCTACCGGCTTTGGCTTTCCAAAGTGCTGAGATTACAGGCATGAGGCACTGTGC
Bos taurus    ........................................................................................................................

Homo sapiens  ACAGCCTTATTATTTTATTTTTAATCTATACCATAATTCTCACTCCTTCTATCCCGTTGTCCGCATATATACATATCTACCAAAGACAGGTTGAGGGAACAGGAAAATGGCTGTTTTAAA
Bos taurus    ............TTTTGTTTTTAACCTATACCATGATTCTCAGCCCTTTCCACCC-----CCTCACTTACAC----CTACCAAAGACATATCAAGAGAATA..AAAAAGTCTGGCTT---

Homo sapiens  ATCCTTGACCTTTCCTGCATTGTTTATGTATGAACTGTCAAATTTATATGTCATTTT-TTTATTCTTTGACATTCA--TATTTAGTAGACAGTATAACTCCCTAGAAGTTAATCTTGATA
Bos taurus    -TCCTTGATCTCTCCTGAATTCTGTATGTATGAGCCGTCAGATTCATATATCATTTTATTCATTCTTCGACGTGTACTTATTTAGGAGATACGAGAACCCATCAGAAATTAATCTTGATA

Homo sapiens  GTTAATTTTTATTACTTGGGATTTTGTTCCTCACTTAAAATTGTTTAATTAAAAAATATGTTTAATTATAATTCTAAAAGTCAAATGTATGATTTTTCTTTTTTCAGAAAGTCTTTATCT
Bos taurus    GTTAATTTGTATTACTTGGGATTTTATTCCTTATTTTATATTATTTCATTTGAATGTG-GTTTAATTATAGTTCTAAAAGGCAAACTTGTG---TTCCTTCTTTCAGATAGTCTCCATCT

Homo sapiens  CTTGCAAGGTTCAGTAGTGGCAGATCATGACTACATTGGATTGCCTGAAATTCCGATTGGAGCATATCAAGCAAATATTCTGGTGGAAGATGCAACTATTGGTATAGTCGATAATGAATT
Bos taurus    CTTACACGGTTCAGTGGTGGCGGATCATGACTATGTTGGAATGCCCGAGCTGCCAGTTGGAGCATACCAAGCAAGTATTCTGGTGGAAGATGCGGCTATTGGAGTAGACGACAGTGAGTT

Homo sapiens  ACTGACATCAAGCAAGGATCGTGAACTATTAGAATATAGAAATACGAAAATCTCTCCATTGATAGACGATCATAGTTCTCTTGAAAAGCAGACTTTCAGTCTGTTGGACTCTTCAAACCA
Bos taurus    ACTGACGTCAAGCAAGGATCGTGAACTATTAGAATACAGAAATGCCAAAATCTCTCCATTGATGGAAGATCATAATGCTCTTGAAAAGCAGACTTTTAGTCTCTTGGACTCTTCAAACCA

Homo sapiens  GGTTCTTGAATACTTAAGTTAGTAAATACCAAAATTTATTTAAATTGTTTGTTTAGAATATAAAT---TCTTAATGATATCTTATTCATT-AAAAACGTTGTATAGAAGAAGTATCTTGA
Bos taurus    AGTCCTTGAGTACTTAACTTAATAAACACAAAAATTCATTTAAAGTG-CTATTTAGAGAATACATCACCTTGAATCTTATCTTTTCCATTCAAAAAGATTACATAGAAAAAGTATCTTGA

Homo sapiens  CACATTCTTAATGACTATATTCTGAAGGTTTATTTTATAGTT-CTTATCTAAAATTATGCCACATTTGAATATCAGT----ATACGTACATATGAAGGCAAAGGAAGCATGTAATATATA
Bos taurus    CAAACTCTTAATGACTAAATCCTGAAGGTTTATTTTGTAATTATTTATCTGAAGTTGTTCTTCTTTTGTATATATATATGGATATATATATATTTAGGCAAAGGAAGCATGTAATATATG

Homo sapiens  GAATTCACTTTTTCTTCTAAAAAGAGAACAGGCAAAGCACCCCTACTTCTTGGGAAATAAATTCCAGCCCAAATCTCTCAAACCTTTGTATGGTTTTGGCACCGT-TTCTAAGTTTTAAA
Bos taurus    GATATTATTTTTTCTTCTACAAATACAACAGGCAAAGCATGCTTGCTTCTTAGGAAGTAAACTAAATTCCAA----CTCAAAACTT----TGGTATTGTTACCATGTTTTCAGTTTCAAT

Homo sapiens  TTTTCTGAAATTCT--CTACCATTTAAACAGTAAGTTTGAACTACCTTGAATGGAATTGATAAATACTGAATCAACTGTAACAATGAATGAAATGAAGCTTCTTTCTGTTTCATTAGAAC
Bos taurus    TTTTTTAAAAATATTCCCCCCTTTTAAAAACT--GTTTGAACTACCTAGAACCGCATTAATAAATATTTATT---------GAATGTGTTAAATGAAACCTATTCCTGTGTCATTGAAAC

Homo sapiens  TTCTAAAAACAAGATTGTATGAAAAAATATAGGGTTATTTAATAGGCTTTCACAGAATATTGGTAATTTCAAATTATCTAATTGGTTAGATAGGGTCAGGATTAGGGTCTGAAACTATGT
Bos taurus    ATCTAAAAAT---ATTGTA-------------GTTACTTAATGGGCTCCTACAGTGTATTGGTAAGTTCACCTTA----------TAGACAGGCTAAGGATTAGGATCTGAAACTATAT

Homo sapiens  ATTGGTTGGGAAACAATTTGTTTATTTTCATACTATATATTAAAGATAAAACCATCGACCAGGCATGGTGGCCCACACCTGTAATCCCAGCGCTTTGGGAAGCCAAGGCAGGATGATCAC
Bos taurus    ATTGGATTGAA---AATGTGTGCATTTCCATAGTATATATTAAAGTCAAAAGCATC................................................................

Homo sapiens  TTGACCCCAGGAGTTCGAGACCAGCCTGGGCAACACAGGGAGACCCTATCTCTACAGAAAAATGTTTAAAAATTAGCCAGGTGTGGTGGCACATGCCTGCGGTCCCAGCTACTCAGGAGG
Bos taurus    ........................................................................................................................

Homo sapiens  CTGAAGTAGGAGCATTGCTTGACCCCAGGAGGTCGAGGCTGCAGTGAGCCGCGCTCATGCCACAGCTCTCCAGCCTGGGCGACAGGGCAAGACCTGTCTCAACAACAACAACAAAAGTAT
Bos taurus    ........................................................................................................................

Homo sapiens  CCGTTAAGAATTTTTTCTGCAATCATAAAA-------TCATAAAATAACCAGTATCAAAGATTTTAAACTGTAATTTCTATGATATTCATAAATATAAAATAAATATTGTTCAATAAATA
Bos taurus    ......AAATTTTTTTCTGCAGTCTTAAAAAAGTAAATTATAAAATAACTGATATTAAAGATTTTAAACTATAATTTCTATAATA...................................

Homo sapiens  TTTATTCTAAAGCACTATGAAAAGAACTGTGGGAAAAGTAAAAGATGAGTCAAACGTGTACCTCATTCTTATAAAGGACTTACTGTCTGGTAGGTAATAAACTATGTAAGCCATGTACAG
Bos taurus    ........................................................................................................................

Homo sapiens  CACAAGTTAAGGCATGCTAAATTGCAGAAGACAGGTGTAGATAATGTACTTAGGGAGGCAAAAAGAGAGAAGTTGTTTTTACATAGGAAATTTGTGTTTATCCTTATGGAAGAAGGATCA
Bos taurus    ........................................................................................................................

Homo sapiens  TTTAAGCTTGACCTTGAAGAATGTATAGGATTTGGTAAATGGATACAAAAGAATTAGGGGAGAAGTAATACACAGAAATGGAAAAGTATTGTAATGATTCATAAAAAGGCAGGGCTTCTT
Bos taurus    ........................................................................................................................

Homo sapiens  GTTGTCTACACTGCCTCGGGTATAAAGTGAAGAAGCAAGATAATGAGGGATAAAGCTGAAAAGGTAAGTAGGAAACCTTATGGAGAGCTTTGAATACTATTAAGGATTGATATGTGCCCC
Bos taurus    ........................................................................................................................

Homo sapiens  TCCCAAAATTTGTGTTGAAGTCTTAACCCCCACCACCTCAAAATGTGACCTTATTTGGAAATGAGGTTGTTGCAAGTGTAATTAGTTGAGATCATATTTCAATAGGGTGGGCTCTAATAC
Bos taurus    ........................................................................................................................

Homo sapiens  AGTATTACTGGTGTTCTGATAAAAGGGGAAAATTTGAACACAGACTCAAGGAGAATGCCATGTGAAGATAAAGGCAGAGTTTAAGATTATGTTTCTATATGCCAAGAAATGACAGAGATT
Bos taurus    ........................................................................................................................
```

```
Homo sapiens  GCCAGAAAACTACCAGAAGTTAGGGGAGAGGCATTGAATACATTTTTCCTCATTCCTCAGAAGACCAACCCTGTCAATACCTTATCTCAGACTTTTGCCTCCAGAACTGTGAGACAATAA
Bos taurus    ........................................................................................................................

Homo sapiens  AATTCTATTGTTTCAGCCATCCAGTTTGTGGGACTTTGTTACAGTAGCTCTAGCAATAATACAAATGCCTGGCTAAATTATTTCTTTTCTGAGAGCAAAAACTTTGTATAGGAAACTGTC
Bos taurus    ........................................................................................................................

Homo sapiens  ATAATCAGAACTGTGTTTTTGGAAGTTTAATCTGGCAACTAGTAAAGAACAGATTAGAAGAGGAGAAAACAAGGCATGGAGATTAGTTGAGAGGACTTCGCAGTGATGACGAATACTAAA
Bos taurus    ........................................................................................................................

Homo sapiens  AACCTAAACTGGGCCTGTAGCATTGGTAATGAATGGAGTCTGTCTTAAGATCAGTACAGAATATCGCACATATTATATTTCACAACCATACTGAACAGTGATGTGATATCAAAGATGGAG
Bos taurus    ........................................................................................................................

Homo sapiens  ACCTGGAGTATTCTGATTACTCATTTAAGAAGAAAGATTATGTTTAATATAGGCTAAAGGAAAAGTGTTTAGGGTAGGGTGTGAATCTAGGACCTCATCTTATACCACAGACCAAATAAA
Bos taurus    .......................................................AAAGGAAAAATGATTAGAGGAAAG---AAATCAAAGACTTCATCTTATATTACCGACCAAATAGA

Homo sapiens  TTTCAAACAGAATTCCAGGAAGAAAAAAACAAAACTTTTTTAAAAAAGCGTAATTGTATCAAACAGTGCTACCTATCCTGCTTTCCTCAGAAGAGCAAAGTTTAGAAAAGAGTCATATT
Bos taurus    TTCCACATAGAATTCCAGAAAGAAG------------CCTTAAGAAAACATAGTTGTCTCAAGTAGTGATTCTTGTCCTTCTCCCATTAGCAGAACAAAGTTGATGATGGAGTCATACT

Homo sapiens  AGAATATCTTTTTGGTAAA
Bos taurus    GAA--CTCTGTTTGGTAAA
```

```
Homo sapiens  TCAATTTCTGGCGTCTCGAACCCAAGAAATGAAAAAGGGAAGCCATGGGGGAGCAGGAGGAGAAATCAAGGCGCGAAAGGACCCTCAAAGCTGTACTGTCACTC---CACGCCCAGGCTG
Mus musculus  GCAATTTCGGGCTTTTCAAACTCTAGAAATGAAAAGAGGAAGCGACTCGGGAAGCGGAGGAGAAATCAAGCCTGAAAGGGACCCT-GGGGATGTTCTGTCACTCGTCCACAAGCACGCTG

Homo sapiens  TCATTCTCTCCAGCACAAGCCCCTCGGTGGAGCATGTGTTTAGGGTCTGCA-----GAGCACAGGAGACAAAGCAGAAGCCCAGCAGCCGCCTCAACCCTGGCATGATC--AGGTTCCGC
Mus musculus  CCCTCCGCGACTGTCCGTGGCTCTTGCAAGACCACGTGTCCCGCATCCGCACCGTGGAGCA---GAGACAAAGCAAACACCTGGAGGCGGCGGCGAGCCTCGGGCTGCCGGAGATGTCGC

Homo sapiens  ATCTCCGGTTCTCCTCCGGTTTTGGGCCCTCACCTGCCACGAATGCGGTCGTCACGCGAGCAGTTGTGAGATGGTCAAGCCCATTCAGGTCTAGGTTAC--CCCCTTTCATCCCGGAGGC
Mus musculus  CTCTCCCG----------------GTCCTCACCTGCCGTGGCTGCGGGCAGCAAGCGATCTGCGG-GAGATGCTTGAGCCCATCCAGGTTTCGGTTCCTCCCCCTTTAATTGCGGGGGC

Homo sapiens  AGGGGTGAGGTGTTTGTCAAGAGACTTCAGTTGTAGGCGTGGACGT-AGGCTACTTCCTTCAGTAACAGCTATAGGGCCACATAAGGGAACCTCAGGC--TACTACACCTCAGGAATCTC
Mus musculus  GAGGGAAGGACGCTTGTGCCGAGGCGGCTCTCGGAGGCGTGGACCTGAAGCTTCCCTCGCCGGCCACCGC---------------GGAACCGCTGGCACTGGCACACCTCAGGAGCCTC

Homo sapiens  CGTCACGCCTCTTCCC----CGCCCTCCTCGGGTCTCACGCCGAAACCTCACCGGGCGGAACGATTTCCGGCAAGAGCCAAATGATCAGTCCTAACCTTTCTGACCGTGGTTCCAAGGTT
Mus musculus  CGACACGCCTCTTCCCTGCCCGCCCTCCACAGGGCTCCCACGGAAACTGCACCGGCCGGAACAGTTTCCGGCG---GACCAACGACCCGCCTGAGCCTTCCTG-------TTCCACAAGC

Homo sapiens  CCCCAAAGGACTGCCCTCTAAAGGACCCGGGTAGTTCCGCTTCCGGCAGCGCGAGATAAATCACGAGAGGAAGCTTAAA--TCTGTCGTTTGAATTTAGGACCACCTCGGTGAGTGGTCG
Mus musculus  GCCACCAGAACTCC--------GACCCCCGGCAGTTCCGCTTCCGGTAGCACCAGATAAATCGCGAGAGGAGAGTTAAAAACCTGCCGTTTGCACTTAGGACCACTTCGGTGAGTGGTCG

Homo sapiens  TTCTGGTGTGCTGTGTCATACCTACTGTTTTTT----------------------AAAGTGAGGCGTAACCCGACAGTAATTTCAAAACCATTCGCCTCGACCGGCCTAAGGAAGGGTT
Mus musculus  TTCTAGTGTGTTCTGTCATACCTATTTTTCTTTATTTTTTTCTTCATTTTTTTTAAAAACGGAGACGTAGCCTTCCAGTAACTTTAAAACGGTCCCCTTCGACTGCTCTAAGGACGGGTT

Homo sapiens  TAATTGAGTCTGTGGGGAGATTTTGCGCAAGCGCC-TTTGAGATAGGAGGGCCTTGGGCGTTGCGATGTCCCGCGCTAGCTGAGCAGAAAG-GGCGAGTGCTGTTTCGGGCCTGGGGAGG
Mus musculus  TGACTA-------GCGGAGGTTTCGCGCAGGCGCCGTTTGGGGCGGGAGGGCCTTGAAC-TTGCACAGCGCCGCGGATGGTGGCTGCAAAGAGGCGAGGACTGTT--GGGCTCAGGGAGG

Homo sapiens  TGCCTACTGGGTGTAATTGAGTTTTACCAAACGGTCATTTTCCTGCTGATTCTGAGTGTGGTTGGACCTTGCCCTTAAGATTCCGTTAACGAGTCGCC-CAAAGCTTAGGAAATGCTCCC
Mus musculus  TGGCGACTCGGTATAATTGAGCTGT---CAACGGTCCTTTTCCTGCTTAGGCAAGGATTAGCGGGACCTAGCCCTCAGGGGTCCGTTAACGAGGTGTCAAAAAGCTTGCGGAGCTCTCCC

Homo sapiens  TC--------------------ACAGCGTTTGAGACT-------AAGCACTTTTGGCCGGCGTTGAAGAATTCTC-CCCATTACTTTGATCAGTCATTTAATCAGAAATTTTGCCCCAA
Mus musculus  TTGCCGGCGGCGGCGAATGCTGGACTGCCTTCAAAACTCGAGTGCAGCGGCGCTTGTCCGAAGCTGGAGGAGTTTCGTCCTGCATAGTAGTCA-CCATTCAGTGTGAAAACGTACACC--

Homo sapiens  ATTTAAATACGTGGCGGGGAGAA---ACGTGAGGTCAAGAGGGAATCCTGTCACGTAGTTTTAAAAGAAAAATGTCCACTTTATCTCAGTGTAGGTTTTTCTTAAATGGGCATTTCTGAA
Mus musculus  -------TGTGTAAAGGAACGAGCTCAAGGGGGGTGGGGTGGGGACCTT-----AAAGCTCTAAAGGGAAAATA--CACTTT------------------TTAAA--AAAATCTC----

Homo sapiens  CCAAAGTAGATACTTTTTAAAGTGGAAATGCATTTATCCCATGTGGCTTCATGTGAATGCATATGGCGATGAAGGCATTTTTTTGTTCAATGAATTTGTACTCTGAAGTGAACCAGATTA
Mus musculus  -----------AATTTTCAAAATGGACCTGAATTTGTCCTGTGCGGCTTTATCTGTATGCATATGGAAATAAAGG----------------------------GAAAAGAGGCAGGGTA

Homo sapiens  CAG--AGAAGTGT-----ATTTAAATTTACAGGTCCTTGAC--------GTT-GCATAAAATTGTGCGTGTCCATTTTTTGGAGCTTAAAATAGAATAATTCAAATGTCAGAATTCCGAT
Mus musculus  TGGTCTGAAGTATTGCCGCCTCAGGCTTA----TCCCTGCCTCCTCCCAGTTAATATATAATTGTACGGGTTCAGTTTTCAGAGATTAAAGCAGAATAATTCAAAAGTCGGAACCCTAAT

Homo sapiens  -CCAAGTTAACATGTTGATTGAAA-ATTGCAGATTTT--------AAGATGGA--ACCAGCGGGTATTATAACCTTTTAGGGGCTTAAATTGGTGGCTTTGTTTTTAACCCTTGGTGAAA
Mus musculus  CCCACAATCCTGTGTTTATTAAAACATTACAACTTTTGTGTTTTGAAGTTAGAATACAAACTTACTGTGTAACCTTTCATTGGCTTTAA-TAGTAGCTTCATGCTAAA-----AATAAAA

Homo sapiens  CAGTTAAGTCCTAATCTTGACATAATACCTAACTGCCTTACTTTATTG---AACACAAAATTATTAAAGGAATTAAGCAATGAGT-----TGATTTTATTTGCTTTTATGGA--------
Mus musculus  --------------------AGAATAGTTAATTGTTTTCATTGGTTACGCAAGACAAAATTAT---AGATGTTAAGCAATAAGTATTGGTAAAAATATTTGTTGTTCTTAACGTTGTAC

Homo sapiens  -GGGGACAAAGTGTATAAAGCTTAACCAAATGTGACCCTGACATTCCTCTTGCAAAACTACAAGTTGTAATCAATCACTTTCATTTTTTCTTATTCCTACATGTTTAAGTACATTAAGTA
Mus musculus  GAGGAACATGCTGTA-AAGACCTAATCAAATAT.......................................................................................

Homo sapiens  ATGATTTTAAGAGAAATTCTTGAAAATATGAGCATATCCAAAGAAGTCCCAGGACAGTCTTTCTTTATGTAGTAGAAACCACTTAATCCCCCTTGTAAATGATACCTTTACTTAGCAATC
Mus musculus  ........................................................................................................................

Homo sapiens  AGAGTATAAGAAGATATTTATATTTGTCTCGAATATTACAGTTTCAAGCCATTTTCTCATATGTATGGGCTTTGAAAAAAAAAAAACAACTTTCATTTACGTTACTTTTGTATATGAATA
Mus musculus  ........................................................................................................................

Homo sapiens  CCAATTATTAGCAGTAAAACTTTTAACTACTTCAAAGGCTATGCTTTTCGTGAACTGAGTTTTCTTTCTTACGCATTTTCTATTTCTCCTTGTCATCTGTCTCTCTACCTCCAGAACCAG
Mus musculus  ........................................................................................................................

Homo sapiens  CAGTTTGTGAACTGCATTAGACTGTTATATCGTTATAAAAGGATACAATTCGTGAGTCTTCTGATTATTTTCTTCTTAGCACTGAAGCTTTATAAACATTGCTATAAAACATTAACACCA
Mus musculus  ........................................................................................................................

Homo sapiens  TTAATGAAAGGAAAGAGATTTTAAAATATTTTTTCATGTAGGTGAAGATTTTGGGAAAAGAAAGGGTTTTATGTTACTTTAAACAGCCTGATATGGATGATAATACTTAGATATGATAAT
Mus musculus  ........................................................................................................................

Homo sapiens  AATTATACATTACCATATACAAAGGACTTTTAATCATCCTCGTAATTGCAAAAAAGGGATAGATATTATGTCCTTTTCCAAACATAGATACTAAGGCTTAAGTCTATACCTTCTTAGACA
Mus musculus  ........................................................................................................................

Homo sapiens  CAGTTTACTTATAGTGCCATGTATGTGAAAGTGAACTGTAATGTTTTCAAAAGTGAAATAATTTACCTCTGGAATATTTGCTATAATTTTTAATTCTCCTTTCAGGAAATGCAGGTAATT
Mus musculus  ........................................................................................................................

Homo sapiens  CTTTATTCATTCACAAATAGTTGAGTGTCCACTGTGTTCTGAACATTATGTTAAACAATGTAAACATAAAAAATTAATAAACTCTTTCCATTCCTCATTTATAATCTAAATTATTGATTA
Mus musculus  ........................................................................................................................

Homo sapiens  CAAAAATCACTTTATAATTTGTGTAACAGAGGCCTGAACCAAGTATTATAAAAGCTAAGGATAACAAGTAATTGACTTACTGGTATAGCAGGGGGGAAAAGCGTGAAATTTGAAGTAAGA
Mus musculus  ........................................................................................................................

Homo sapiens  GACACCAAGTTAAAAGTGTCAGCGCATGTACTCACTTTATTACCTTGAGTAAATTGGACAATCTCTGAACCTCCATTTTCCTACCTGTAAAATGGATATAAAACTAATACCTACTTCATG
Mus musculus  ........................................................................................................................

Homo sapiens  AAGTACTGTGAGGATTATCATAGTAAAATACTGCATAACTATTTGCAGCTGTAGTCGTGAGTCACTTAGCAATGGAATAAATTCTGAGAAATGCTTTGTTAGGCAGTTTCAGCATTGCAC
Mus musculus  ........................................................................................................................

Homo sapiens  GAATACAGACTCTACCTACATGAACCTAGATGATATGTGTGTGTGTGTGTGTGTATATTTTTTTCTTCATATAGAGAACCAAATGTCCCTGCACATTATTGGGTATCATTTATTTCTCCT
Mus musculus  ........................................................................................................................

Homo sapiens  ACTTGATCTGCAGTGCTAATATCAAATGACCTATATCAGGTTCTATATATGCTCTGCTATCATCTTACGGGACCAATAGGCAGTCCATCCTTGACCAAAACATTCTTATGCAGTGCAATT
Mus musculus  ........................................................................................................................

Homo sapiens  GCGTTGGCATTGTTTTTAAAATTTTTAATTTTTTAAAATTATTTCCTCTTCCTATACTTTTGGCTTTTCCAGATGTCATATAAACAGAAATATAAAATATATAGCCATTTGTGTCTGGCT
Mus musculus  ........................................................................................................................

Homo sapiens  TATTTCACTTAGCATAATGATTTTGAGATTTATTCATATTGTTAGGTCTATTTATAGTTTGTTCCTTATCAATCCTTAATAGTATTCCAGTATGTGGATCTACTGTAATTTGTTTATCCA
Mus musculus  ........................................................................................................................

Homo sapiens  TTCACCAGTTGATGAACATTTGTTTTCAGGTTTTGGCTGATATGAATAAAACTTCTAAAAACATACACTTACAGGTTTTTAGGTGTACATATTTTCTTTTGGGTTAGTATCTAAGATTGG
Mus musculus  ........................................................................................................................

Homo sapiens  TATTGCTGGACCATATGGTAAATTGATGTTTAACTTTATAAGAAATTGCCAAACTGTTTTCCAACATGTTTACATCATTTTCTATTATCACCAGTAAGAGCATCCTTGGTAGTACCTAGC
Mus musculus  ........................................................................................................................
```

```
Homo sapiens  ATTGTTAGGTTTTGTTTTATTTTTGTTGTTGTTAGTGTGTAGTCATATCTTATGGTAGTTTTAGTTTACATTTTTTATTATCTATTGATGTTGAGCATTTTTTCATGTGTTTATTTGCTA
Mus musculus  ........................................................................................................................

Homo sapiens  ATCATATCTCTTTAGTGAAGTGTATTTCCAGGTTTTAAATTGGGTTGTTTGCTTTCTTGTTAAGCTTCGAGAGTTCTTTATAAATTCTAGAAAAAGTTATTTATCATATGTATGTATTGC
Mus musculus  ........................................................................................................................

Homo sapiens  AGATATTTTATCCCAGTGTTATTTCTCTTTTTTTAGCAGTTCTTTAAGGAATAAAAATTTTAATTTTTTCTTTATGCATAGCTGCTTTTTCTTTGCTGTCTGGGAAGCCATTACCCAACC
Mus musculus  ........................................................................................................................

Homo sapiens  TGAGCTCACAAAGATTTTTGTTGTTGTCTTCGAGAAGTTGTATAGTTTTAAGTTGTACATTTAGATCTGTGAGCCATCATGAATTAATTTTTACATATCATTTGAGGTATGAGTTGAGGT
Mus musculus  ........................................................................................................................

Homo sapiens  TCATCTTTTTTCAAAGGATGTTTAATTGTTACAGCATTTGTTGAAAAGATTATCCTTTCTTCATTGAATTACCTTGGCACCTTTGTTGAAAATCAGTTTACTATAACTGAGTGGCTATTT
Mus musculus  ........................................................................................................................

Homo sapiens  CTGTCACTATTCTGTTCGATAGATTTAAGGCTGTCATTTTGCCATTATAACACAGTCTTGATTACCATAGTAGTATAGAAAGTCCTGAAACCAAATAATGTGAAATTCTTCAACTATATT
Mus musculus  ........................................................................................................................

Homo sapiens  CATTTCTCAAAGTTGTTGTAATTATTCTGAGTCCTTTAGCTTTCCATATCAATTTTAGAATCAGCCTGTCAGTTTCTGCAAAAGAGCTTGAGGGTTCCTGATTGGGACTGCATTGAATCT
Mus musculus  ........................................................................................................................

Homo sapiens  ATAGATCAATTTGGAGAAAATTGGCATTTTGTCCCACTTTTTCTCAGAGGTAGAGTGGGAGTTACTCCAGCTTTCTGCATTCTAGTCAGAAGCCTATATGGGAATGTTTAGTGGCATTTC
Mus musculus  ........................................................................................................................

Homo sapiens  AGAAAGAAATAATTTTCTTCCATGGTTATATTCACTATAATATCTTTATTGGAAGAGATCACAGGACGAATCCCTCTATTTGTGAATTGGATGTCATCCCCTGCTGCCATCTCAGGACAT
Mus musculus  ........................................................................................................................

Homo sapiens  CATACCATAAGTTTTTTCTCCTTTGTTTTCAGTGGTCTTTCTAGAAGCCCTTCCATTAGCATTAAACTTTTTGGGTTACTCCCTTGTCGAAAGCAAAACAATCTTGTTCAAAAAAAAAAT
Mus musculus  ........................................................................................................................

Homo sapiens  TGTTTTAATTGTAGTAAATCACACATAACATGAAATTTACCATCTTAACCATTTTTAAGTGTGCAGTTCAGTGGCTTTAAGTACATTCACATTTGTTTTACAACTATTACCACCATCCAT
Mus musculus  ........................................................................................................................

Homo sapiens  CCCAAGAACTCTTTTCATTTTGTGTAACTGAAACTCTTGACCAGTTAAATAATAACTCTTACATTCTCTCCTCCTCACCCCCTGTTCTATGCAACCATCATTATTCTTTCTGTCTGTATG
Mus musculus  ........................................................................................................................

Homo sapiens  TATTTGACTACTCTGACTACCTCATATAGGTGGAATCTTACAGTATTTATCTTTTTGTGACAGGTTCATTTCACTAAGCTTAATGTCCTCAAGATTCATCCATGTGGTAACGTGTGTTAG
Mus musculus  ........................................................................................................................

Homo sapiens  AATCTCCTTCCCTTTAAAGGTGTGTGAATATATACACCGTATTTTGTTTATCCATTCAGCCATTGATGGACACTTGAGTTGCTTCCATCATTTGGCTGTTGTGGGTGATGTGGCTATGAA
Mus musculus  ........................................................................................................................

Homo sapiens  CATGACTGTCAAAACTTTTAACCCCACTAGAATGTAAACTCAATGAAGGTCAAGAGAATCTACATCTTGTTTATTATCCTGTCTCCAACGTTGTAACTGGCACATAGCAGTTACTCAGTC
Mus musculus  ........................................................................................................................

Homo sapiens  ACATTTTTTAGATCAATGAATGAACCTTATATCTGTCTCTAGCAACTATCTTGTAGCTCCTTGCCTTTATAGCTAAGCCCAAGTTTCTAAATCCACTTTCTGAATTTCCATTCATTCCTT
Mus musculus  ........................................................................................................................

Homo sapiens  GTGTCATTTCAGTCTGGTTGCCAACTGCCCTGCTATACTGTAACTATACTGTATACTGTTGCCAAAACCACTAGCTACATTATGGTTCTTATATCACTTAGCCTCTCTGTGTACTTCTAA
Mus musculus  ........................................................................................................................

Homo sapiens  GAAGTCTGTATTTCCCAGACTTCTATTACCAGAACCTCTTCTGGCTTTTTTCCTGTTTCTCTGGCTAGTCTCATGTCCCTAAAGATTTACAATATATTTGGGAGAGAGACATATAAACTA
Mus musculus  ........................................................................................................................

Homo sapiens  ATATATTCAATAGAGTGTTTCAGATTTTATGATAAAGGTTGACACATATTACTGTGAATGTACCAATGAGGGTGTGCACAATTCTCCCTGAGATTGGGGATGTGGTCAGAAAGGATTCAT
Mus musculus  ........................................................................................................................

Homo sapiens  GGATGAATGAACTCATTAATGTGAACGTTGAAAGATAAGGATATGTTCACTGCACTTCCTACTGTGTACACTTTCCATCTGATCCTTCTTCCATTGCCTTAACTTCACCTACTTTTTACA
Mus musculus  ........................................................................................................................

Homo sapiens  TGCCGAGTTCATCCATGAAGAATAGAACCTGCTTAATATTGTCATCTTTCTCCCTAAGCCAGAGCTTCAGACAGAAGTGATCCCAGATTCTTCTTTGTGTTTACTCCACACATCTAGTCA
Mus musculus  ........................................................................................................................

Homo sapiens  GTTACAGTTCTTATATAATGTATCTATCCATTTATACTGACACTGCTTTGGCTCAGAATTTCCTCATTTCTTGCTTTAATTATACAGTAGCTTCCTAGCTGACTGGTACAGGTATTCCTG
Mus musculus  ........................................................................................................................

Homo sapiens  TCTCTACCCCAATCTCTATTCCATATTGGTGCACAATACACTTTATAGAATAGAAATCTAATCTTGTAACTCCAACTCATTCTTAAAATTTTAAGTTATTCCAGGTTGGAGTGGTTAGAA
Mus musculus  ........................................................................................................................

Homo sapiens  GACACTCTGCAGGAAATTACACCTAGCCAGAGTTTTGAAATATGATGAGGGGCTGGAGAAATAGCAATAAAAGTTCATTCCAGGCAAAAATATCAATGAAAAGGTGTGAGCACACTGCAT
Mus musculus  ........................................................................................................................

Homo sapiens  GGGATGGTTGAAGAACTGTGGTTATGTTTTGAATTGAGAATGCCAAGAGAAAAAGATGGAGAGATTGATAAAAACCATATCTTATAAAGCCTTGTATACTGGCAGAGGTATTTAGATATC
Mus musculus  ........................................................................................................................

Homo sapiens  ATTAGTGCCATGGTCTTAATGGTTGTATTCTCCCAAAATTCATATGTTGAAATCCTAACCACCAAGGTGATGGTATTAAGAGGTTGGACCTTGGGGCTGGGCGCGGTGGCTCACACCTGT
Mus musculus  ........................................................................................................................

Homo sapiens  AAGCCCAGCACTTTGGGAGGCTAAGGTGGGTGGATCACTTGAGGTCAGGAGTTCGAGACCAGCCTGGCCAACATCGTGAAACCTCATCTCTACTAAAAATATAAAAATTAGCTGGGCATG
Mus musculus  ........................................................................................................................

Homo sapiens  GTGGCATGCACCCGTAGTCCCAGTTACTCGAGAGGCTGAGGAACAAAATTTGCTTGAGCCTGGGAGGCAGAGGTTGCAGTAAGCTGAGATCATGCCACTGCACTCCAGCCTGGGCGACAG
Mus musculus  ........................................................................................................................

Homo sapiens  AGCGCAACTCTGTCTCAAAAAAAAAAAAAAAAAAAGAGGTGGAACTTTGAGGAGGTGACTTGATCATAGGTCAGAGCTCTCATTAATGGGATTAGTGCCTGTATTTTAAAGAGTCTGAGAG
Mus musculus  ........................................................................................................................

Homo sapiens  AAACCCCTTTCCCCTTCCCTCATGTGAAGTTACAGTGAGAAGACAGCTGCCTATAAGAAAGTGAACCCTCAGTAGACACTAAATTTGCTGGCATCTTGATCTTGTATTTCCTAGCCTCCA
Mus musculus  ........................................................................................................................

Homo sapiens  GAACTGTGAGAAAGAAATTTCTGTTTATAAGCCATCCAGTTTATGGTATATTTGTTATAGCCGCCCAAGTGGACTAAAACAATCAGGAATGGCAAAAGATGTATTTCGAGTTAAGTGAAT
Mus musculus  ........................................................................................................................

Homo sapiens  ATTTTGATAAGATTTGTGTTTTAGTGTGACCTCTGTGACTGCAGTGAGGACAGGGACCATATTAGAGACAGAGGAGGCAAGGTCAGATAGAAGGTGAGTGTGAGCTAAGGCAGTGGCAAT
Mus musculus  ........................................................................................................................

Homo sapiens  GGAATAAAGTTCAAGGTTCTACCTCATCTACAATCAGTGCTTTTCCTCCTTTTCCAGGTGAATCTGGGTCTCTACACATCTGTTTTGATAGTACTCTTTGCATACATCTGTTAAAACACC
Mus musculus  ........................................................................................................................

Homo sapiens  ACTCTAAACTGTATAAATGCTGATTTCCTCCTCTTTTCCTCTCATTTATACCATAAGCTCCGTGAGGACAGGGATTTCATTTTATTTAACTTTGTATTAGTATCATCTAGTATATATAAG
Mus musculus  ........................................................................................................................

Homo sapiens  GGTTTAAGAGCTCAGTAACTGAATGAGCAAATTCAAGGTTATATGGATGAACAGGCAAAAAACTATTAAAAAGGGTAAAGCATGGAAGTTCTACAGTAGGCACAAAGAAGATTAGGAGAG
Mus musculus  ........................................................................................................................

Homo sapiens  GTCAAGATCACTTTAGATTCAGAGATCATGGAACACTTGCTTCATTGAGATGCTAGAGCTTGATTTTGAAAGATGGGTAAGGCTTAATCAGGTGGAAGGATATACCATTTAGGGGAAAAT
Mus musculus  ........................................................................................................................

Homo sapiens  GATGTAAACCAAGGTATAGAGAATGGTTGGGGATATGGCCATGGGTATAGAAAGGAAAGTAGTGAGAGAAGAAGCTGTATTATGTTCTGCCAGGTTGTAGAGGGTAGGCTAAATAGTCTA
Mus musculus  ........................................................................................................................

Homo sapiens  GACCTTAGCCACTAAGTGGAAGATAATGCCCAGAGGCAGTCTCATGGTGTCCACTCTGTCAAGTAAATCATTTGTAAGATAAAAATATGCTAGGGATAGTCTAAATTAAAGTTTCAAGGT
Mus musculus  ........................................................................................................................

Homo sapiens  GACTATGTCAAATAACTACACCTTTAAGTCTTAGTTCTGTTGAGCCAAGACAACTCACCCATATAAAAATGAAAATAATACACTTGTGAAAGCATTTGTGAAATATGAAAATCTGTAGGA
Mus musculus  ........................................................................................................................

Homo sapiens  AATAGATGGTTTTTATGTCTTCTCCCAATGATGTTTATGTCCCTCTCCCTAACATAGATATATGTTAGTCTTCTCCCACTACTTTGATGAGAAAAAAACAAACCAAAAACCTTAAAGAGC
Mus musculus  ........................................................................................................................

Homo sapiens  TCAGTCTCCAAAGATTTTTGTAGTGCTTTCCTTTTCTCCTTGTCCCTTGTACTATAGGTTTACTCTGAGGAGAGCAAATTTCTCTATTTCGATCTACTTTCAATTATTCAAAAGTTAAAG
Mus musculus  ........................................................................................................................
```

```
Homo sapiens  ATTTCAAAGCAGAGAAGTTAATTTCAGTAAATAGACCTTTTCTGAGAAGTTTTTGTATATTTATTATAAATAGGAAAACCAACAGTGTCCTAATGGCACGTTAACTTAATATACTGAAGA
Mus musculus  ........................................................................................................................

Homo sapiens  CTTCATCTTACATTAGATAGAAAAATTTTCTGGTCTCTCCAGGAGGTAAATGGGTAGGGGGAGGAAGACAAGAGATGGTAGGAACTTGGAACCAAGAGGTTTTTAAATATAAAAGCAAAA
Mus musculus  ........................................................................................................................

Homo sapiens  TACGGAAGCTACTGTGGATTATGCAGACTCACATTTTTTTTCCAATGGCTGGTTTGTTTTCTACCAGTTCTGGTTACCCACATCCCTCTGTGTTTTGGGGCCTGTACTTCCTCTAAGGTG
Mus musculus  ........................................................................................................................

Homo sapiens  TGAGAGTTTGGTCTTTTCCTTCTGTGGGCTTTTACAGGCCTGAAAAAGCTCCTCCCCTTTTCTTTTTAAGGTCCTTCAGGAGTTTTTGCAACCTTTACTGGTCTCTAAAAGGAAGGAAAT
Mus musculus  ........................................................................................................................

Homo sapiens  ACACAATTTTTATCTTCCTTTTATAAACCTCGTCTTCCTACAGATACCATCCTCCCAACTGGCTAGTATGGGATATTTCTAGATTTTCCTTTGGATTGCTTAGACGATTTGTGACATTGA
Mus musculus  ........................................................................................................................

Homo sapiens  TAGTTCCAAAAACTATTAAGGGAAAACTATAACAGGAGGTTATATGTAGTATATTATTAATTTTACAGTATGTATAGTCTATATATTGATTTCATCTAAACTGAGAAGGGACAGGTTTCT
Mus musculus  ........................................................................................................................

Homo sapiens  GTCCCTTAGACTTGAAAGAATGTGTTATCATTGTAGTATAGAGATGAAAATAGAGGACACAAAGAAAAAATGATTTTGGTTTCTTTTTTTTTTAAGAGTTGAGGACGATTAATGCAGAGC
Mus musculus  ........................................................................................................................

Homo sapiens  TAGAATGATAGGCAGGCAAATGGATGGGTAGACAAACAGGTTCTATATTCCCAACTTGAAACGACACAAGGCTTCATGAATTGAAAATTGATTAGGAAGTAAAAGTTTGCCATGGAGTCC
Mus musculus  ........................................................................................................................

Homo sapiens  TGGCAGAGGAGAGAGTGTAGTGAGGATGACATGGAAAGTAAAGGCATCAGGAGGTCTTATGTGGCAGATTGGAGGGCAGTAGGGTGCCACTAGTGTTTCACCACAGATGGTTTGAAAAGC
Mus musculus  ........................................................................................................................

Homo sapiens  ACTGACCTGGAGCGACATGAAACTTGGTAACAATAGAAGTGGCTACAGGACCAGAACAGAGTATACGCATGGTAAAACTTCTTGATACCTTTACTGTTTCCATTTAGTGGCTATTATGAC
Mus musculus  ........................................................................................................................

Homo sapiens  TATAAAATATCTATGAACATTTTTGTACACATATTTTGATAGTCATAGACTAATTTCACTGGAATATAAAATTAGAATTGCCAAACCTTTAGGGTAAATTTGTGCTCAGCTTTAGAAGAA
Mus musculus  ........................................................................................................................

Homo sapiens  ACTCAGTTTTCCTAGGTGGTTGCAACATTTTATCCTCCCACAATGTATGAGAGTTCCAGTTGCTGCCTGTCTTCACCAACACTTGGTGTTGTCTTTGGTTTTTAATTTTAGCCTGTTCAG
Mus musculus  ........................................................................................................................

Homo sapiens  TAGTGTGTAATTCTAAGTCATCGTGGTTTTATTTGCATTGCCCTGTGGAATAATGATGTTGAGCACATTTTCACGTGCTTATTGACCATTGGATATTCTCTTTTATGAAGTGCTGTGTAG
Mus musculus  ........................................................................................................................

Homo sapiens  AAATTTCCCCCCATTTTTTGAATTGGGTTTTTGTCCTTTTCTTTTTGATTTTTAGAAGTTCCTTGCATACACTGGGTCTGAGTCCTTTGTTAGATATATGTATTACAGATATCTTCTCCC
Mus musculus  ........................................................................................................................

Homo sapiens  AATGCGTGTCCTATGTTTTCACTTTCTTAATGGTGTCTTTCATGAATACACATTTTTACTTTGATAAAGCCCTATTGTTAATTTTTTTTTCTTTTAAGGATAGTGTTTTTTTTTTGTGTC
Mus musculus  ........................................................................................................................

Homo sapiens  TTATTTAAGAAATCTTTGCTTAGCCTAAGATTTGAAAGATCTTTTCCTATGTTTTCTTCCAGAATCATTGTTATTTATCTTTCACATTTAAGTTTATGATTTATCTGGAATTAATTTTTG
Mus musculus  ........................................................................................................................

Homo sapiens  TGTAAGATGTGAGGTAGGTAGTTAAGGTTCATTTTTTCCCCTAATACAGTTATCTAATTGTCCAACATTATTTATTGAAAAGACCACCTCTTTCCTCATTGTGTTGATATCTTCATTGTA
Mus musculus  ........................................................................................................................

Homo sapiens  AACGTATTGACTTTACATGTGTGGATGTAATTCTGTACTCCTTGTTCAATTACAGTGATGTATTTGTCTGTCCTTATGCCAATTGGCTTGTCTTATTTACTTTATTTGTATAAAAAGTCT
Mus musculus  ........................................................................................................................

Homo sapiens  TAAATCTCATCCATTCATCTTTGAGATGATTCAGAAACTGTGATAGGCTGCATTTGTTTGGTTTGGGATGCAGCCATCTATTAGAAGATGAAAAAAATCATGTATTTAATAATCAATTGG
Mus musculus  ........................................................................................................................

Homo sapiens  AAGTGAATTAAACTTAGAACAAACTGATTGTCTTATAATACTTTATAGAGATAATTAACCTGTTTTTAGGTTATGATGGGACTGTGTTAACATTTCAGAACCTTTTCTTTTAAATTGAC
Mus musculus  ........................................................................................................................

Homo sapiens  ATATACTTTATGTTAACCTAAGGTAAGAAATGTACTGTTACTCTCTCTGGAAGTTAAAAGTAGATCAGAAAGACGGTTTTACATTTGCAAAGAGCTGTGTCATAGGGAAAGTGAATTGGT
Mus musculus  ........................................................................................................................

Homo sapiens  AAGCTGTTTAAGCCATGACAATAGGCACAGTCTGTTTCTGCTTTGTTCCAGCATTCTCTTTCCAGTAAATACTCTTAGTCTCATCCCTAGATCTTCCAGTGGTCAGTAAACATCTTTTTT
Mus musculus  ........................................................................................................................

Homo sapiens  TAAATGTAATTTCAAAATTGTAAGATTTATGTCAGAATCCTAGCAGGAAACAGAAGACATTCTGGTGGGATTTTAAAGAAAATTTAATGGAGCTCCTTTCTATAGACAGACAGTAGCAAA
Mus musculus  ........................................................................................................................

Homo sapiens  GTAAACAACATGGCACCTGGAGACTAGCAAAAGCAAAAATAGTTACCACCTTAGGGCTGAGGTGACAAGAAGAAGAAATAACCATGAATATATTTAGAAATAATATAAAGCTATATTTCT
Mus musculus  ........................................................................................................................

Homo sapiens  TCTCTTATAACTTATTTATTTAAATGTCACATGACAGGATCCTCCTGTGTTACCATCCTGTGAATGTAACATTTCAGTTGATAGCAATGAAATTTTAAGCAAAACTTTTTGAAGTGTACT
Mus musculus  ..................................................................TGACATTTCTTTTGATGCCAA-GAGATTCCAAGCTAA--CTTTTGAAGTATACT

Homo sapiens  GGTTCATTTCCTT---------------------TTTTCTCAGTATGTGGAAAATTTTCAGCATTTCTTAT--CAAATGGAAAGAGACAAGATAATTTGTCATGGGGTATATCAAAAAT
Mus musculus  CACTCACTTCCTTCCCTTTCCTAGTTTTACAGGGTTGTTTTCTGTTTGTTAGTTTGTTTTAGCGTTTCTTATATCACATG-AAGGAGACAAGATCATTTGTCGTGCTAGATCTTAAAAGT

Homo sapiens  AGCATTTGAGTATTTTTGTTCATATACATCGCT---GTTCCTTTCCTGTACTGCAATTATCTCCCTCACAAATAA-ATTCTGGGAATTCTCGGTTCATATGTAAATAGAAATATGACAGA
Mus musculus  AGCGTTCAGATGCTTTAGTTTGTAGACATCACTCTTGTCCCTGTTCTGCTCCGCAGTTATCTCCCTCACAGATGATATTCTGGGAATGCTCAATCCATGCATAAGCAGAAATGTGACAGA

Homo sapiens  AGTCTTAACTGAAATAGCAATTCAGTTAGCTTAAAATTTAAG--TTATCTTTTTAAATACCTTTTACCTAGGTTCACAATGGTCCGTAGTGGAAAAAATGGTGACCTTCATCTTAAACAG
Mus musculus  GGCCTGAACGGAAATAGCAACACAATTAGCTTGTCATTTAAGAGTAACATCTTTAAATAA-TCTTCCCTAGGTTCGTGATGGTCCGTAGTGGGAAAAATGGTGACCTTCATCTTAAACAG

Homo sapiens  ATTGCATATTACAAACGAACTGGTGAATATCATTCAACTACACTGCCAAGTGAGAGAAGTGGCATAAGAAGAGCAGCAAAAAAATTTGTCTTCAAAGGTAAAATTAATGTTCAAATTTTG
Mus musculus  ATTGCATACTATAAGCGCACAGGGGAATATCACCCCACTACACTGCCGAGTGAGAGAAGCGGCATAAGACGAGCAGCTAAGAAATTTGTCTTCAAAGGTGAGACCAGTGTCTGAGTCTCA

Homo sapiens  ATTATTGAGTTGTAGATTCTTCCCCACATTCTTCCTTGTGGAAGTTTGGGAATTTCTGTAGTAG-TTGGTTGTTGCTAAACAATTACCAGTTGAATTGATGATGCCACTCAATCAAAAGC
Mus musculus  ACT-------TATAG-TTCTTCTCCATGTG-----TTGTAGAACCCTTGCAAATTCTCTAGTAACTTGGTTTTTGCTAAGTGGTTGTCAGTTAAATGAGAGAT-----TATCTCAGAAGT

Homo sapiens  AAAATAAAAATTACACTCAAGCTAACTGAAGGCCAATAAGCGTGAAAA------------GGGTTGTTTAAATTATAGACAGAATATGATACCATGAAAGAAAACAAAA-----------
Mus musculus  AAAGTAAAA----CATGAAAGCTAACTGAAGGCTGGTAAGCATGAAGAAGACATAGTAAGGGGCTGTCTCAATTTAAAATAAAAAATAA-ATCAAAAAAGAAAAGAAAATGACCTGTTTA

Homo sapiens  -------------------CAT--------TTTTCTAAATTAACAACTTCTGCTTTTACCCTCTCGTGGTCTCTAATGGTGCACTTTCCAGCACTGTTGCCAATTTCAATGAGGAACTTT
Mus musculus  ATAGATCAAAAGGAATAGCCATGAAACAGATTACATAAATTAATTTATCTTGCTTTTGCCACCTA-----------------ACCTTCAGACACTAATGCCACCAT-GCTGAGAAAATTA

Homo sapiens  TTTGATGCTTAATATGAAAATGTTGACAAAGTTAACTGGTTTGTTTACTAAAATGAAAACTGGAAGGAATTAACTATATTTCTGTGATCATGTCCCCTTGAACCCCACCACTG--CCATT
Mus musculus  TTGTATGCTTAGTAAGGCAACATGAACAGAGCTAACTAGTTTACTTACT--------------------------------------------CCCCTC---CCCTCACACTGTACCTTA

Homo sapiens  AAATATTCTCAGGATCACATTCATTTTAGGACCCTTGGATCTTCCTGAATGGATTAAATATTTGAAAGGCAGTGGGATATTTATCTTGGAAGGTCATGATAATTTTTGCCTCTTTTTCCC
Mus musculus  AATTATTCTCAGGATTGCATTTGTTTTCTTGCC-TTGATGCTTCATCAGTACATTGAATATTTGACAGGCAGTAGGGCAATT----TTGAAAGCCTTAACAGCTTTGCCTCCTTTCTAGC

Homo sapiens  CTTATAAAGGGGAAGAGAAGTTTGGG-ATGAGATAAACAACAAGTA--GTCACCAGATAATTGAATGAATGTTTGTGTTACAAATGGGAATTTTGCCTCTTAGGCTTACCCCAAAACAGG
Mus musculus  TGCCTCCATGGGGAAAGTTCCCTGAGCGTGAGATATGCAAAAGGTACAGTCAACAAACA-CCAAATGAGTGTTAGTGCTATTAGTGGCTGTTAAAGC-ATTAGGCTTTCTCCAAATCAGG

Homo sapiens  TTAATA-----------AAACTCTAGGATAATAAATAAATGTCTGCTTGCATCTCCCTGTGTATATCCCTACCAATAACTCCCTTATACTTAAGATTAGGCAGACCTATTACCTCTTAAA
Mus musculus  GCAGAATCTAAGGATGTAAATTCTAAGGTCATAAGTAAACTCCTGCTCATATATCCCTATACAGATTCCTGCCAGGAACTCCT-----ATTAACAATAGGTAGACCTCTTAC-------A

Homo sapiens  CTAAAGTGAGGATTCTTTCATTCATATATTTGATAATTTATGTAACAAATGTTAACTGGGTTTCTGCTGTGTGCCAGTCACATTCTAAGCCTTGGAGATACATTGATGAATAACATAGAT
Mus musculus  CTAAAGTGGACGTGCATGCATTCAT----------------GTCAAAAATGCTTATTGAATTTCTGTGCTGTGCCAATCATATCATAGGCCCTGGAGATGTATTGAGTAA-------GAT

Homo sapiens  AAGGTTCCTGCTCTTAGGTTGCTTATCTTCTAATTCTAAAAAATAAGGAGTGTTCCACTCATCAGTTGGCTTACTCATATTAGTAGCTGAATGGTGTATTTTGGAGTTTGAATTGCTAGC
Mus musculus  AGTGTTTCTGTTTAGTAATTGCCT........................................................TAGTATCT---TGATGTGTTCTGAAGTCTGCATAGC-ACC

Homo sapiens  CCATTCACATTGGAAAGGTAGATGGTGATTCGCTTCATACCCTGGTTACTTCCTAGCTGCAGATTCTTCTCAGGTGTCCTCCTAAGGAAACAGAGAATTGTCTTGAGGATTACAGTTTTT
Mus musculus  CCACATGAACTGGAGACATAA----TGGTTCTCCCTGTCCCCTGCTTGCTCC------------TCCAGTTAGGTATCTTCACAGGACAAGAGAG-------CTGAGAATTGTGTCCCT-

Homo sapiens  TCCACTTGCCCGCTTCACCATCCCTACCTTC--TCAAATATAGAAGAAGCTGAGAGAGCTGTTTGCTTGCAAAAATACAGTGATGATAA-------------------------AACAT
Mus musculus  --------CCCACTGTGCCATCC-TACCTTTATTCAGAT-CAGAACAAGGAGAGGAAGGCTGTTGCTTATAAAAACAAAACAACAATAACAACAACAACAACAAACCCAGCTATTAATAC

Homo sapiens  TATTTTAGAAAATTCTGTTCCAGGAAGTTGTTTTTCTGTACATAATTTATTTGCTAATCTATGCCATTTTTAGGAGGTGCCTGCAAAATCTTCCTACTTTGAGTACTCCCAGAGAGAGAT
Mus musculus  TTTTACAGAAAAGTTGGTACCCAGAAGCATCTCATCAATACAGAACTCATTTTATAACCTACGCCATTTTTAGC---TTCCACGAGTATTTCCCTAATTTGAGTACTTCCATAGAAAGGT
```

```
Homo sapiens  AATTTCATATTTCCGTACTTATTACGTGCTTTGGTGAACAAGCTTGCTCTACTTATATTGATAGCTACAGAATTATAGCAGCTACTGCCCTTCCTAAGTATCTAGTAAGTGTAACAGCTA
Mus musculus  TGTGTCATATTCCCATAGTTGGCACATGATTTGGTGAGCAAGCTCACTCCATTTCTGTTGATACCGACAGGATTATAGCAGATAGTGGTCCTTCCGGATCTTCAGAA-------------

Homo sapiens  AGAACCTTTGTGTTGTAGCATCATTAAAAATAAATA-TTCTGTTTTAAATGCAGTAAGATTTTAC--AAAACAAAAC-AAAAACCTTCATGGTTTAAAGAGAAGGCTATGATTCTCTTAA
Mus musculus  --------TGCATGGAAGTATTATTGAAAGGAAATAATTCTTTTTTAAA----GAAAGATTTTTATGAGAATAAAATTAAAAATCTTCATAGCTTTAAGTTAAGGCTAT-----------

Homo sapiens  AACTACGTAGATCTCTAAAAATTGCAATTCTATCTTTTGGTATCATTTATTCCAGAAAAAAAGCTGTTTTATGTTGGAAAAGACAGAAAACAAAATCGTTTGGTAATTGTTTCAGAAGAG
Mus musculus  --------AGAGCTTTGAAATTTA------TATTTTTTTATGTCATTTGTTTCAGAAAAAAAGCTGTTTTATGTTGGAAAAGACAGAAAACAAAATCGTTTGGTAGTTGTTTCGGAAGAG

Homo sapiens  GAAAAAAAGAAAGTCTTAAGAGAATGCCATGAAAATGACAGTGGAGCTCATCATGGTATATCCAGGACCCTCACTCTGGTAGAATCCAATTATTATTGGACATCTGTGACCAATGATGTC
Mus musculus  GAGAAGAAGAAAGTACTGAGAGAGTGCCATGAGAATGGCCCTGGTGTCCATCACGGCATTTCCAGAACCCTCACTCTAGTGGAGTCTGGCTACTACTGGACGTCCGTGACCAATGACGTC

Homo sapiens  AAACAGTGGGTATGGCTT------ATGTATTTAGAGTATTATGAATAATGTCTTTCTGATAGATACTTAAAATTTATTATTTCTCCAGATTTATTCTGGGTATCACCT------------
Mus musculus  AAGCAGTGGGTATGGCTTCCACGCAGGGCTTAGGAGTGTC----TTACTGTCTTTCTGATAAATAC--AGAGCTCAGTGTTGCTTAAGAGGTACTGGCCATCTCACCTGTGGAGTAAGTA

Homo sapiens  -CTCATTAAGTATTTTTGGCA-----------ATCACTTAAAGGGAGTTGTATTTCCCTTCACAGTTATTTTAAATCACTTAAGTATAATTTGAACAGGAAAGAAGCATTATTTAAACAC
Mus musculus  CGTTATAGGTTATTTCTGAAATAAGAAAATATTTCAATTAAAGGTAACTTTACTTCCCTTTATAATTACCTTACATCAGTAGAGTATAAATTAAAC-----------------TAA----

Homo sapiens  TTGAAAATACCATATTTAAATAAGAGATTATTTAAAAAATCAGATTTGTATCATTGAACTTTTTTGTGTAGGCAGAATTTAAATGGAGCTGGAATTCATTTGACTAAATCATTACTTCTT
Mus musculus  --GAAAATACAATATTTTGACA--AGTTTATTGAGAATTACAGCTCCATTTT-------------------GCAGAATTCAGTTGAAGTTGAAATTCTTTTG------CTATTATATCCT

Homo sapiens  CAATGGCAGTCATACAAGGTTTTCTTAAGGGGAGAATATG------TTCATAGTAAGTCAAGCCCAGTATGTGCTCTTATCTTTTGTGCCTGTTCCATTTTGAGGTAGAATGTCATACAT
Mus musculus  TAGCACCAA-CGTGAATGATTTTTTTATATGATGAAAATACATCTCTTTATAATAAATCTGTTCCAGTGTGAACTCTCAGCTTTTGTCCCTATTCTGTCTTAAGATAGAA.........

Homo sapiens  GACAGAAACCCAAAACCAAATATAACCAAAATCAAATTTTGAGGATAATGAAATAAGTCTGAGTTTTGTAACTTTATGACCCATTGGGACCTTCAACTTCTAGTAGAAAATTCACCTATA
Mus musculus  ........................................................................................................................

Homo sapiens  GGAATTTTGGGGTTCTTGCTGGAAGTCAGCTGATTCTACTCTTTCCAGTGTACTGTATGTCAAATGGTAGGAATTCCTTGGTATATCTGTGAGATCTCCTTAATAATTGTAGTAGTACAT
Mus musculus  ........................................................................................................................

Homo sapiens  CAAAAAGAGTGGCATTTCAAGCATAAAGAATTAAACCAAAATTTCATAAGTATAATTTGTGAGTTATCAGTTTGTGGGTCTATTCAGGTATTCCAAAGACCTTCATAATTTATATATTTT
Mus musculus  ........................................................................................................................

Homo sapiens  TCCTGCTGTGCATTTTTTACAAACATTCAGTGAAATCTTTGTCAAATTGTTATCACTGGAGAATGAATATGATTTTTGGAAATAATCAGAAGTTATTTGTGATAAAATCCAACAAATAAG
Mus musculus  ........................................................................................................................

Homo sapiens  TTTAAATTGGAAAGTGAAAATTTTGATCAAAAGTAACTGTGACTATAATGGGATTAAATGGCTTTTAAATTTTTCTTCAGAAGCTAGTTTGAAAGTAGTTTCTAGAGTGGAATTCCAGAG
Mus musculus  ........................................................................................................................

Homo sapiens  ATACATTGAGAAATGGCACCCATCCATAAATTGATCAGTTGATCTTGTCTCACCTCACTCAAATGAAAGCTGCAGGACAGCATGGGTTTTGTGTATGTTTATTCTTTTCCCACG-ATTGA
Mus musculus  ..........................................................................................................CTTTCCCTAGGTAGTGG

Homo sapiens  GAGAAATTGCTCCATCTATACATTTTATATGAATGAAATTATATTTCAATAAATATGGTTGGAATAGATTAATGAATAAGCTTCTTAATGTGATAACTTTAGAAGGAAACATTTTATGTA
Mus musculus  GAAGGTTTCTGCCATCCTTATTTTTTTTAT---TAAAATTGCAGTTTAGCAAATATTAGTGAAATAGACGAATGAATAAACT--GTAGAATAAAAATTGTAGGAAGAAATATCATTTGTA

Homo sapiens  TATAGAAATTATTGTAAATTTACATACTGATTTAATAGGAAACTTAAGATCTTTTTAAGTATTTAATAAATATTTATTGAATGTGAACCAGATTTATGTTCCATGTGAATAAGACCCAAT
Mus musculus  TGTATACTTTATTACATAGTTCCATGGTGGCTTAA.....................................................................................

Homo sapiens  AAGATGGTCATTTGGTAGTATAGTCTTCCCTCAGTATATGCAAGGGATTGGTTCCAGCACCACACTCCTGTCACCAAATCTACTCATACCGAAGTCCTGCAGTCAGCCCTGTGAAACTCA
Mus musculus  ........................................................................................................................

Homo sapiens  CATATACAAGAAGTCAGCTTTCTGTATACTTGAGTTTCTCATCCCTCCCTATCAATAGAGTACTTTTTATTTGTGTTTAGTTAAAAAAAAAAAGCCATGTATAAGCTGACCTTCACAGTC
Mus musculus  ........................................................................................................................

Homo sapiens  CAAACCCATGTTGTTTGAGGGTCAGCTGTAGTGGCTACTACAGTTGTTTGTTGTTGTTTTATTTGTTTGTTATTCTAAATA---GGTTTAATCCAGTGATTCTCAGTCCAGAAGTTTCTC
Mus musculus  ...........................................................TAATTGCCTGGTGTTCCAAATAAAAGGTATCATTTAA-------------AGGAATTTCT-

Homo sapiens  CTTCAAAACACAAAGTATACCTATTAACCATTTTAAAATTCAGATTTATAAGACTGCTTTTATATTGTGTTGAGTGTT--AACATTTGTATGTGTTTTCTTTATTGTGTGCCTGT-TGTA
Mus musculus  ----AAGACACACAGTA--CCTGTTGACCATGTTAAGATTCTGCTTTATAAAACTGATTT-ATGTTATAGTGAATATTTTAACATTTATATGTATTTTCCTTATTGTGTGCCTCTCTGTA

Homo sapiens  GGTATATGCTTGTCAGCATTGCCAAGTGGCAAAAAATACAGTTATTGTAGCACCGAAACAGCACCTTCTCAAGGTGGAAAATCCATGGAGTTTAGTTACTGTTGATCTGATGGGGCCTTT
Mus musculus  GGTATATGCTTGTCAGCATTGCCAAGTAGCAAAAAATACAGTTATCGTAGCACCTCAGCAGCACCTTCCCATGGTGGGAAACCCGTGGAGTGTAGTTACTGTTGATCTGATGGGACCTTT

Homo sapiens  TCATACAAGCAACAGAAGTCATGTATATGCTATAATCATGACAGATTTGTTCACCAAATGGATTGTGATTTTGCCTCTATGTGATGTTTCAGCATCAGAAGTTTCTAAAGCTATTATCAA
Mus musculus  CCATACAAGCAACAGAAGTCATGTGTATGCTATAATCATGACAGATTTGTTCACAAAATGGGTTATGATTTTGCCTTTATGTGATGTTTCAGCATCAGAAATTTCTAAAGCTATTATCAA

Homo sapiens  TATATTTTTCTTATATGGACCTCCTCAGAAAATAATAATGGACCAAAGAGATGAATTCATTCAACAGGTAAGACAAATAAACTACTTAGGTCTGGGAGCATATCTTACTTCTTTTCAGTG
Mus musculus  TATATTTTTTCTATATGGACCTCCTCAGAAAATAATAATGGACCAAAGAGATGAATTCATTGAACAGGTAGGA-----AAAGTACTCACATTCGGGAGCATGTATCACT--CTTTCAGTG

Homo sapiens  TCCAGAAC-CAGTGCCCTCTGCATTATATTACAGAGTGCTTAATAAGTTCAGATAAATTTCTGTAGGTATAGAGGTATGATATTAAATACTACAAGGATAGTTGTACTGGAAATTAGGAG
Mus musculus  TCTGGCACACCGTGCTTTGTG-----TTTTAGAGGGTGTTTAATACACTGAAATAAATTTCTGTAAATATGGAGGTA-GATAATGTATACTACTAGATT--------------------

Homo sapiens  ATTAGGATTTTAATACTTACTGTGGGTTATTGTAAACCTATCTTCAGATATCCTCAAATTAAAATGAGGAGGTTGGACCAAATAATCTTTATTTACTTCTAGATTAAAAACCCATGATTG
Mus musculus  --------------------------TTGTTGTAAACCTATCTTTTGATCTCTTTAAACTAAAATGAGATGATTGGATCAAATAATACTTTTTTACTTGAAGCTTAAGAAC.........

Homo sapiens  GTTGGGCCTGGCTCATGCCTATAATCCCAACAGTTTAGGAGGCTGAGGTGGGTGGATCACTTGAGCTCAGGAGTTCGAGACCACCTGGGCAATAGGGCGAAACTCCATCTCTACAAAAAA
Mus musculus  ........................................................................................................................

Homo sapiens  TACAAAATTTAACTGGGCATGGTGGTGGGCGCCTGTAATCACAGCTACTCGGGAGGCTGTGGCAGGAGAATCACTTGAACCTGGGAGGTAGAGGTTGCAGTGAGCCAAGATTGCACCACT
Mus musculus  ........................................................................................................................

Homo sapiens  GCACTCCAGCCTGAGCGACAGAGCGAGACTCCATCTCGAAAAGAAAAAAAAATTAGCCAGGTGTGGTGGCACACACCTGTGGTCCCAGCTACTCGGGAGGTTGAGATAGGAGGATCACTT
Mus musculus  ........................................................................................................................

Homo sapiens  GAGCCCGGGAGGCGGAGGTTACAATGAACTGAGATTGTGCCACTGCACTTCAATCTGGGCAACAGAGTGAGACCTTGTCTCAAAAAATAACAATAATAATAATAACACTATGATTGACAG
Mus musculus  ........................................................................................................................

Homo sapiens  GTAAAGGGAGACTTAAATAAATGGAAGAATACATTTTCTTCATGAATAAGAATAGTCAGTATTGAAAGGTACTTTCTCCCTTGCTCTGGCCTTTTCCCAGTTAAAATTAACCTGAAAGGA
Mus musculus  ........................................................................................................................

Homo sapiens  TAAGCAGGGGAGAATATATATGAAAACTTTTTATAAGAAATACAACAACTGCTAGACTTAAGGCATATGTTAAAACTTTTTGTAATTGACAGGGTATATGTTTAAGAAAAATCAGAGATA
Mus musculus  ........................................................................................................................

Homo sapiens  GCCAGACTTGGTTGAAAGCATCTTTGATGGCTCAGTCCAAAAGAAATACCTAAAGTTAATATGTATTAACTAGTAGATAGGACATGTGCCATGGCACCCCAGGATTATTCCCAGTTTCCA
Mus musculus  ........................................................................................................................

Homo sapiens  TAATTCTCTAGGAGGACTCAGCATATAGTCATACTCATGGCTAAGATTTATTCAGCAAAGAGAAAAGACACATTGGGAGAACCAGGTACAAGTTTCCAAGATCTCCTTCCCCAGTGGAGT
Mus musculus  ........................................................................................................................

Homo sapiens  TACACAGGACGTGTTTAATTCCTTTAGCAGAGTTGTGACAACATAAGGAAGCTCACTAGAGTCTCAGTGCCTAGGGTTTTTACTGGGCGCTGGTCACATAAGCACCTTCTACCTGGCATG
Mus musculus  ........................................................................................................................

Homo sapiens  TACAAAAATTCCAGACTCTCAGAAGGAAAGATTGTGTTTGGTCTTATAACCAAAATCTAAGCTCCTAGACATTAGCCAAGGTCCAACCTTGTAAGCATGCCTTTCAAAGAACAGCAGTCA
Mus musculus  ........................................................................................................................

Homo sapiens  CAGATCTGCTTTGTTAATATGTTAAAATTAACTGTCAGGTTAACTCTTTTCTTCACAGGTCCATCCAAACAACTTAATAAGTATTTATGTTCTAACCACTTAGTAGCAATTTAAAATACT
Mus musculus  ........................................................................................................................

Homo sapiens  CATAAATTGAAAGAAAATATATTTTTGTTCCATTCTTTTCTTTTTTTTTGAGACGGGGTCTCATTCTGTCACCCAGCCTGGAGTGCTGTGGCACCGTCTTGGCTCACTGCAACCTCCACT
Mus musculus  ........................................................................................................................

Homo sapiens  TTCCAGGCTCAAGTGATACCCCCACCTTAGCCTTCTGGCTAATTTTTGTACCTTTTTTCTTTTAGAGACAGGTTTCACCATGTTGCCCAGGCTGGTTTTGAACTCCTGAACTCACATGAT
Mus musculus  ........................................................................................................................

Homo sapiens  CCACCTGCCTCGACCTCCCAGAGTGCTGGAATTACTGGCGTGAGCTACCGCACCCAGCCCTTTCATTTCATTCTTAACCATAATTACTTGCTAATGGGTAATGTGTACCTGTTGACCACT
Mus musculus  ........................................................................................................................

Homo sapiens  GCTTAACTTCTCAGACCTTGGGATCATATTGTACACTGCCACCCTCATTTCCTGTTTCACACTGATTTTTACATAGTAACTGCTTTTTATCACAGCATTCGATGAAGAAAACAGTTCTGT
Mus musculus  ........................................................................................................................

Homo sapiens  AAAGATATGATGTCATCAAACAAATTGTAACCTGATCTAATGTTGAAACTGAACTACCTTAAACTGGTAGTTTTCATGGTGTCAAACAGATGTTAAGGGGGCAACTTGGCACAAAGTTTG
Mus musculus  ........................................................................................................................
```

```
Homo sapiens  GTAACAATGGTTTAAATGATAAAGCTAGTGTCACAAATGTGTCTAGTAGAGGAGGATCATGTAATTAATTAACAGGTAACAATTTGCTAATTGTTTAAAATTATATCTGTAATATGATTT
Mus musculus  ........................................................................................................................

Homo sapiens  TAGAATCCAATACCAAAAGTATTTTCAGGTAAATTAAAAGAATTTTAATTTTTAAAGTAGAAACAGAAAAAAATATATTTGACATTTAATCTCTAGAAATGGAGGTCACATTCTAAACCT
Mus musculus  ........................................................................................................................

Homo sapiens  AGGAATAGTGGTATAAACTTCAATGGAAAAACATTAAATTTGGCTGCATAATAATAGGAGATTTATGTGGTAAAGTAATCAACATATAAAAGCCAATCTTGCAAATAAAGCAAGATAATT
Mus musculus  ........................................................................................................................

Homo sapiens  TTGTAAATGAAGGCAAGGTAGTCTTGCCTGCCTTGATTGACATAGCAGTGGTCTGAGCAATGGAGTACGTAAAGGCCATCCAGTTTGGGGGAAGGCTATTGGAACTTCTATTTATATTTT
Mus musculus  ........................................................................................................................

Homo sapiens  TTAAATCCCAAAAATAAGAAATGAAGCTTTATTAATAATGTATACGATTGATAATAGCATTCTGACTCAATCAACAAGACATGTATGTCACACATGCTATGTGAAGGTATCCTGAAAGAA
Mus musculus  ........................................................................................................................

Homo sapiens  AAGTGAGAATCCACATGTTTCCTCACCCATACTTTTGCTTTCAGCAGTTGAATGAAAGTATGGATATCATCTAGCATTCACCACACTACTCACAAAATGGCCAGGGAGCTTAGATTTTTG
Mus musculus  ........................................................................................................................

Homo sapiens  CAGTGAAAGCAGATAATACTAAAAGGCAAACACCCATCATCAACCACAAGAAAGTGATAGCGCCGGCACGTCTATTTAACTTCCAGCTCTTTGTTAGACACATCAATGAGATGAATACAA
Mus musculus  ........................................................................................................................

Homo sapiens  ATATTAACTACAATAGTCCCCATTTATCTGCAGTTTTACTTTCTGAGGTTTCAGTTCCTATAGTCAACTGCAGTCCAAACATATTAAATACAAAATTTCAGAAATAAATGATTTAAAAGT
Mus musculus  ........................................................................................................................

Homo sapiens  TTTTTAATTGCACACCATTCTGCGTATAGTGATAAAATCTCATGCTGTCCTGCTCTGTCCAACTGGGACGTGAATCATCTCTTTGTCTAGCATATCTTCATTGTATATGCTACTCTCTCC
Mus musculus  ........................................................................................................................

Homo sapiens  CCATCATTAGTCACTTCATAGCTGTCTTGGTTATCAGATCAAATGTAGTGGTTAGTAGTGTGTATCCAAGTTACCCTTATTTTATTTAAGAATGCCCCCAAAGTGCAAGAGTAGTGATGT
Mus musculus  ........................................................................................................................

Homo sapiens  TGGCATATTGTTAATCGTCCTATTTTATTTTTAGTTGTTAACCTCTTACTGTGCCTAATTTAAAAATTAATCTCTATCATAGGTATGTATGTATAGGGGAAAACATAGTATAAATAGGGT
Mus musculus  ........................................................................................................................

Homo sapiens  TAGGTACTACCTGCAGTATCAAACATTCACTAGGGGCCTTGAAACATATTCTCTGAAGGTAAAGGGAGACTATAGTTATCAGATCTGATAGGAAATTAGGCATCAAAATTCAAAATTATC
Mus musculus  ........................................................................................................................

Homo sapiens  AAGAATACTCTTTGAAATATCAGTTCACTTCCACTTTTATTGATGGAGAGCCTTACCCTAAGTGCATATAATGTTTCAAGGTATTTGCTAATGATAATATGAAGCCATTATGATTAGCTA
Mus musculus  ........................................................................................................................

Homo sapiens  GCAGTTTTTCCAGTCGTGTTTAAAGTCATTTGATAACCAAACCAGGACTTTGAAAATTTTTCTAAAGGTAACAATAGGGCCAAGCATGGTGGCTCATACCTCAGCTTTTTGGGAGGCTGA
Mus musculus  ........................................................................................................................

Homo sapiens  GGTGAGAGGATAACTTGAGACCAGGAGTTCAAGACCAGCCCATGCAATACAACAAGACTCCGTCTCTACAAAAAAAAAAAAAAATTTTTAATTAACTGGACGTGGTGTTGCACACTCATAG
Mus musculus  ........................................................................................................................

Homo sapiens  TCCCAGCAACTCAGGAGGCTAACGTGTAAGGATTGCTTAAGCCCAGGAGGTGAAGGCTGTAGTGAGCTATGGTTGCACCACTGCATTCCAGTTTGGGCAACAGTGAGACCCTGTCTCAAA
Mus musculus  ........................................................................................................................

Homo sapiens  AAAATAATAATAATAATAATAATAGGCTGTGCTCAGTGGCTCATGCCTGTTGTAATCCTACCAGCCAGATTGCTTGAGCCTAGGAGATCGAGACTAGCCTAAGCAACATGGTGAAATGCC
Mus musculus  ........................................................................................................................

Homo sapiens  ATCTCTACAAAAAATGCAACAATTAGCCAGGAGTGGTAGGGCACACCTGTAGTCCAGCTACTTGGGAGGCTGAGATGGGAAGATCGCTTGAGCCCAGGAGGTTGAGATTGCAGTGAGCCA
Mus musculus  ........................................................................................................................

Homo sapiens  AGATCATGCACTCTAGCCTGGGTAACAGACTGAGACCCTGTCTCAAAAAAAAAAAAATAAAAAATAAAGGGTAACAATAAATATTTGCAGGCCTATTTTGTGAGATTGCTTAAACAAAAAA
Mus musculus  ........................................................................................................................

Homo sapiens  CCAAAAAAGCGACATATGATTGGGAAAACTTGTTCTTTTTGCTGTGATTAAAATGGCTTCACTATTACACAGAAAGCATAACGATGACAGTACAAAATGAACTCTTTTGTCAGCAAATAC
Mus musculus  ........................................................................................................................

Homo sapiens  TGATGCAGAAAACACTGCTAAATATTATAACATAATACACAATGTGGGAGGTTTATTGTATAGTTGAATAAAAATACAGATGTTTCTAATGGCTTAGCTTAGAGTATTTGCTAAATTGTT
Mus musculus  ........................................................................................................................

Homo sapiens  CCAATAAAATGCACAAAGAACTTTTTGTAAACCTCTGAAGGAAAGAGAACAAACATATTCAGTAGGAAAATTAATTTTTTTAAATGTCTTACAGAAAACTAAAATTGTAAGAACTGACGG
Mus musculus  ........................................................................................................................

Homo sapiens  AATATCTGGTTTGATGGTTGGGGGGCTGACAGAGTAAGGATGTCAAGATACCATTTCACAAATGGATTCCCTGGATCATTCCTAGGCAAGCTTTTGAAACAAAGAAGTTAAAGCCTTTTT
Mus musculus  ........................................................................................................................

Homo sapiens  CAAGGGTCATTTCAAACCATTAATCAAGACTCAGATTGAAGCTGACCCAAATGACACCTATTCTTTGGGATTTCTTTCTAATTTTGAATTAAAAATAGTCACCCTGAATTAGTATGTATA
Mus musculus  ........................................................................................................................

Homo sapiens  TCAGCTAGGAAGTGTATTCAATCCAAGTAACAGACTTCCTTTCCCTATCTCAATATATTAATATATATTGTATATATATTATCCCAATATATATTGGGAGTGATTGAGAGTCACTCCTAT
Mus musculus  ........................................................................................................................

Homo sapiens  TGTATTCACAACGTGGCTGCTGGAGCACATGCCACAATGCCTACATTTCAGTCAGAAAGAAGGGAGAGAGTGTTGGGCAAACAAAGCTAGTACCAGCTGAGTTTGCCTAACAACTTTTAC
Mus musculus  ........................................................................................................................

Homo sapiens  TTACAACTTTTTGGCTAGAATTATGTCACATGACCACCCCTACCTGCAAGGGAGTTTGGGAAATGTAGTTTTCTTTTTATTGCTTTCCCCTCTTGTATTAACTTTGCCTATTTAGTTATT
Mus musculus  ........................................................................................................................

Homo sapiens  CTGATACTACTCGTCTGAAATATTCTTTTTTAAAAAGACAAATTTATGAGCCAAAGGTTTACTCAACTAAAAATAATAAGGAAAAAAAAAGTCTGAAAGGTGAGGTAGGAAAAAAAATATT
Mus musculus  ........................................................................................................................

Homo sapiens  ATTTTATATTTAATGGGTTTACTGTTAAAAGTTTTGTAAACTCATTTTAAGTGCTACACATAATATAGTAACACCAATAATAGTAGTTATAAGTTAAAAGTTATAAAATGCTTATTGCCA
Mus musculus  ........................................................................................................................

Homo sapiens  ATAACTGTTTTACATGCTTTTTAAACATTAACTTATGACCTAGTGGGTTGTTTTTGAGACACTGTCTACTGAATTAAAAAGTTACTTGAGGGCCGGGTACAGTGGCTCATGCCTGTAATC
Mus musculus  ........................................................................................................................

Homo sapiens  TCAAAACTTTGAAAGACTGAGGTGCATGGATTGCTTGAGTCCAGGAGTTTGAGACCAGCCTGGGCAACATGGTGAAACTCCATCTGTACAAAATATTTAAAAATTAGGTGGGCATGGTGA
Mus musculus  ........................................................................................................................

Homo sapiens  CACATGCCTGTAGTCCAAGCTGCTAGGCAGGCTGAGGTGAGAGGATCGCTTGAACCCAGGTAGTGCAGGCTGCCCTCCAGCCTGTGCGACAGAGCAAGACCCTGTCTCCCACCGCCCCCC
Mus musculus  ........................................................................................................................

Homo sapiens  ACCAAAAAAAAGAAAAAAATTAAACTCAAAGTTAACGTTAAGTTCTGTTCTTCCATTTCCTTGTTGTATGTGTGTATTTGGCAAGGCACTTAATCTCTCAAAGCTTCAGTATCTTACCTT
Mus musculus  ........................................................................................................................

Homo sapiens  TAAAATGAAATTAATAATACCTCAAAAACTTGTAAGGTTTAATGACAATAATGTAAAGTGTCTAGTGTGATGGGACCAGTGTGTAGTAAAGACTCAATAAATGGCAATCACTATTTTATA
Mus musculus  ........................................................................................................................

Homo sapiens  CATAAAATTTTACATATTTTATGTATGTGTTCATATAGAATGTTATGGTCAATAAAAAAGCAAGATTCAGCCAGGCGCAGTGGCTCATGCCTGTAATCCCAGCACTTTGGGAGGCCAAGG
Mus musculus  ........................................................................................................................

Homo sapiens  CAGGTGGATCACAAGGTCAGGAGTTCAAGACTAGCCTGGCCAATATGGTGAAACCCCGTCTCTACTAGAAATACGAAAATTAGCCGGGTGTGGTGGCGCACACCTGTAGTCCCAGCTACT
Mus musculus  ........................................................................................................................

Homo sapiens  CAGGAAGCCGAGGCAGAAGAATTGCTTGAACCTGGGAGGCAGAGGTTGCAGTGAGCCGAGATTTTGCCACTGCACTCCAGCCTGGGTGACTGAGCAAGACTCCATCTCAAAAAAATAAAT
Mus musculus  ........................................................................................................................

Homo sapiens  AGATAAATAAAAGGCAAGATTCTAATTCTTTTGAAGCTTTTTTTTAAGTCAATGGAAAAATAAAACACAAATTTTAAAAAGCCACCTTTGTTTTATATATTTTTCAGATCAATATTGAAC
Mus musculus  .....AATGGAAGACAAAAT-CTGGGTTTCTTGAAA--TTTTTTTAAGCCAGAGGGAAAGTG-----TGAACTTTAAGCAGTCTGTTTTCTTTT-CATATTTTTTAGATCAATGTAGAAC

Homo sapiens  TGTACAGATTGTTTGGCATAAAGCAAATTGTAATTTCTCACACCTCTGGAACTGTTAACCCAACGGAAAGTACACCTAACACAATCAAAGCATTTCTCTCCAAACACTGTGCTGACCACC
Mus musculus  TATATAGATTATTTGGTGCAAAAGAGATTGTAATTTCTCGTGCTTCTGGAAGTGTTAATCCAGCTGAAAACACACCTAGCACCATCAAAACATTCCTCTCCAAACACTGTGCCGACCACC

Homo sapiens  CAAACAATTGGGATGATCACCTATCAGCTGTTTCATTTGCCTTCAATGTAACTCACTTGGTATGTGCCTTTTT-----------ATAATTCTGTACTTCTGAGTTATGACTATTTCTTG
Mus musculus  CCAACAGCTGGGATGAGCATCTGCCAGCCCTTTCCTTTGCCTTCAATGTCACTCACTTGGTAGGTGCCTTTTTTATTATTCATTGCTCATTCTAAATTCCAGGGATATGAAAGGGTTTGG

Homo sapiens  GCCAGATAAATGTTTTATTACAAAATTGACGT--TTATTTTCTTTAAAATAGGAACCTACTAAAAATACACCATATTTTCAAATGTTTAGTCGAAATCCTTATATGCCTGAGACTTCAGA
Mus musculus  G---GAGGGTTGTTTTATTTAATTACAAATGTCCTGGTTTTCTTTAAAATAGGAGCCTAATAAAAACACACCGTATTTCCAAATGTTCAATCGGAATCCTTGCGTGTTGGAGT-------
```

```
Homo sapiens  TAGTCTTCATGAAGTGGATGGTGATAATACAAGTATGTTTGCCAAAATTCTAGATGCAATTAAAGAAGCTGATAAAATAATGGAGAATAAGACAACTTCACTGGGCCAGGTGATTCTATT
Mus musculus  --GTCCTCCTGAAGAGGGCAGTGAAGGTACAAGTGTGTTTGCCAGAATCGTAGCTGCGATTAGAGAGGCTGATGGCGTGGTGGAGAACAAGACACCAGCATCGAGCCAGGTGATTCTATC

Homo sapiens  CAATAGAAAAACTGTAATC-TAAAACTTATAAAAGTTATATTTATTTTTAAAATTTCAGCATAT---------ACATGTGTGCATGCACACACACACACATACTTACTTGCCATAATT
Mus musculus  TACTAGGAAAGTTCCCTTTGTGGACCCCTACCAAAGAGGAATT-GTGTTTAAAATCTCAGCATGTGGACATGTTCACATATGTG...................................

Homo sapiens  TTTTATCTATCAGATTTTTCTTGTCTTGATTAAATAGTTGGTTTACTAGCTATGAAATGCTGAGAGGCAATAATGAGAAAGCTATGGATTTTGGA-ATCAAGTAGAGCTGGATTCAAATC
Mus musculus  .............................TAAACAGTGTGCTCATAGGCTATGAAATTCTGAGAGGC--TTACAGGAAAGTCATGAACTGGGGAGACTGGGTAGACCTGGTTTCTAACC

Homo sapiens  TGTAG-------TCTTAACTGCTTTTGAGTT------------GTTTGACTTCGG------CAAATTACTTAACCTATCTTTAGTTTACCTTCTATATAAAGTAGGAGCTAATAATACTG
Mus musculus  TGTGAGAATTCCTGTTAGCAGTTGGAGAATTCTGTACTCAGTCCATGTGAATCCAAGTATTCCAAGTCACTTGGCCTGCTTT---CTGCCCTCTGTGTACAGAG-----TGCCGATACTG

Homo sapiens  ACTTTGAAATGTTTTAAGAATTAAATAGAATTCATACCTACCTTTTAGTAGCATCTCAAGTACCATTTTGTTACAATATTCTCATTATTTGCATTTACCTTCAAGATATTATAGCTCTTT
Mus musculus  ACTTTAAAGTGTGCTTAGAACACATGAGGAGTCCATGCTTCAGCTCAGGAGCAGTTCAATAATAGCATTGCCATAATCTAATCAGCGTGTGCCGTTATATTCAAGG-------------

Homo sapiens  CATCTCTTTGTTCTTTTTCTTTTTACACTTGTAGATGGAGAACAACAATTTGGATGAACTAAATAAAAGCAAGATCATTGTTAAAAAGAAACCCAAACAATTAAATCCATTTCATTTAAA
Mus musculus  ---CCTGTCATGATTATTCTTTGCA---TTATAGATGGAGAACAACAATTCGGATGAACTGAGTAAAAGCAAAGT---TGTTAAAAAGAAAGCAAAGCAGTTAAATCCCTTTCATTTGAA

Homo sapiens  AGTGGGTCATGAAGTTTTAAGACAAAGGAAAAATTGGTGGAAGGATGGTCGTTTTCAGTCTGAATGGGTTGGTCCTTGTGTCATAGACTATATTACAGAAAGTGGATGTGCTGTCCTGAG
Mus musculus  AGTGGGTCATGAGGTTTTACGACAGAGGAGAACTGGTGGAAGGATGGCCGCTTCCAGTCTGAGTGGGTCGGGCCTTGTGTCATCGACTACATTACAGAAAGTGGATGTGCTGTCCTCAG

Homo sapiens  AGACAACACTGGGGTTAGACTGAAAAGACCTATCAAAATGTCCCACCTTAAGCCCTACATAAGAGAATCCAGTGAACAAGGTAAATATCTGTCACTTCTCTACATTTTTATTGCTTTAGG
Mus musculus  AGATAACACGGGAACCAGACTCAAACGACCTATCAAAATGTCCCACCTTAGGCCCTATGTGAGAGAGTCCAGTGAGCAGGGTAAGTATCTGCTACCTCTCTAGGGTGTTA..........

Homo sapiens  TAAGGAGAATAGTTAAAAATAACAGAAACCTTTCTCCCCTCTTTTGTAGGTT---TTCTTTTCCCTT----CTTCATAAAATTTGTTACAGTTTTAGCTATCCCAGGTAGTCTGTTTTA
Mus musculus  ..........................TCCTTCCTTCCTTCCTTCCTTCGGTTAAAATCCTCTCCATCTAAGTTTTATAAAACTTG--ATGGTCTTACTTCTCTCCAGGAGTCTG----A

Homo sapiens  TTACAGTGTCTCTTCTGGAACTGTCAGGCCTGTGAGATTTTTTTTTGAGACAGAGTTTCCCTCTTTCTGCCCAAGCTGGAGTGCAATGGCGCGATCTTGGCTCACTGCAACCTCTGCCT
Mus musculus  TTAGAATGTACTTTCATAAACT.................................................................................................

Homo sapiens  CCCGGGTTCAAGCAATTCTGCCTCAGCCTCCCGAGTAGCTGGGACTACAGGTGGATACCACCATGCCCAGCTAATTTTTTGTATTTTTAGTAGAAACGGGGTTTCACCATGTTAGCTAGG
Mus musculus  .......................................................................................................................

Homo sapiens  CTGGTCTTGAACTCCTGACCTCCCGTGACCTGCCCGCCTTGACTTCCCAAAGTGCTTGGATTACAGGCATGAAACAGGGTGCCTGGGCATTTTTATTTTTATTTCTAGTATGTTTTTCAT
Mus musculus  .......................................................................................ATTTTTGTCTTGAATTCTACTGTGCTTTTCAT

Homo sapiens  AGTTTATGTGGGAGATACTGAACTTGTTTTCCTCTGAAGGAAAGATAACATCTTAACATCATCTCTGTTTTTGCTTTCTCAATGTTTTGTTTGTTTGTTTGTTTGAGACAGAGTCTCATT
Mus musculus  AACTGCTATGAAAAATGCTGAACCTGTTT--CACATAAGGAACGATTCCATCTTGA...............................................................

Homo sapiens  CTGTCCCCCAGGCTGGAGTGTAGTTGTGTAATCTCAGCTCACTGCAACTTCTGCTTCCTGGGTTCAAGCAATTCTTGTGCCTCAGCCTCCCAAGTAGCTGGGATTATAGGCACATGCCAC
Mus musculus  .......................................................................................................................

Homo sapiens  CATGCCCAGCTAATTTTTTTGTATTTTTGGTAGAGATGAGGTTTCACCATGTTGGCCAGGCTGGTTTTGAACTCTTGGCCTCTAGGGATTTGCCTGCCTTGGCCTCCCAAAGTGCTGGGA
Mus musculus  .......................................................................................................................

Homo sapiens  TTGCGTGCATGTGCCATTGCACCTGGCCTTACAAATGTATTAGTCACTTCGTGCCTCTTTTTGGCATGAAAAA-CAGCAACTGTGATCATTAATGCCAAAAACAAACATACTGTCCAGAA
Mus musculus  ..........................................TTACCTGCTGCCTCATTTTTATGTGAAAAAGTAGCAGCTCAGATCATTATCGCCAAAGAGAAGCATGTTGTCCAGAA

Homo sapiens  TCTAGTTGTGAGATAAATATTAGGGTACTTTAAAAATGACATTAATTTTTAATATTGCTTTATAGTTAGGTCATGGTTGGGGGCCAGGTGTGGGTGCAGAGTGGGGAATCCTCAGAATGC
Mus musculus  TCTAGCTGTGAAATAACTA--AGGGAACTTTGAAACTG-TGTTTCCTTTTAATGTTGCTTCATA---AAGTCAT-----------AAATTAAGGTGGAGGATGGAGAATACTCAGAAAGC

Homo sapiens  TATAAGGTGCTGTAAGAAGAGGTGACAGCACTCTAAACAAGTTATATTTGCCCCTAAATTTGACTCATTCTGAACAGTCAAGATACCCTAGTAAAGTGATGTTTTTGCCATGGCTTTTAG
Mus musculus  TATA...................................................................................................TTTTGTCCTGGTTTTCTC

Homo sapiens  ATTATGTTCCCCACTTTATCAAGGCT--TTTATTTTGTCCTAATGTGTCTGCTTCCTAGATCTTCTCTGAGGTGGATAACTGAGAGAGAGTTAATGAGTAACTTAGCCAG----------
Mus musculus  ATTAGGCCCTCTACTTCAGCAAGATTATTTTATTTTATCCTAATGTATTTGCTCCTTGGAGCTTCTCGGGGTTAGACAAATGAGAG-GAATTATTTGATAACTTTGCTAATTTTGTACAA

Homo sapiens  ---------GTGAAACATTAATAT---------TATTTGGATATAAGTAACATTTAATGG----AAAATATATTTATAACAAT-ATAAACAAGTTATATGACAGGCTTTAGCAAATATTA
Mus musculus  GGAATATATGCTAGACAGTAATATCTGTACAAATGTTCGAATGAAAGCTAAATGTAATGGTGATGCAATATATTTGTAAATATCATTAACAAGTTACTTAATAGAC-------------

Homo sapiens  ATAACAAAAAGAGTAACAACAACAAATAACCTACACTTACCCTATGCCTTTTCTTGAGTTCTAAAGCCCATGTGGGCTGCTTAGTA-ATGAT--------AGCTCAACTCTGCTTCTAAT
Mus musculus  ATAATAACAAG-----CAATAAGGAATGACCTCTGCTTAACCCTAGTCCTGTGTTGAGTATGAAGCCCTTCTCGGGCTCTTCAGTATATGAGTTCTTCTGAGTTCTTATCTACT-CTGGT

Homo sapiens  CTGGGTTACAACACTCATGTTAAAACCTCTTAGTACCTTTCCTTCTTGTGACAGACCGAGTTGTT-TAGTCAGTAAACTTATCTTGAACTACATTCTAATCCTGAAAACTCACAAGTGCT
Mus musculus  CTGTACCTCGGCACTTCTGTTACAAGCTCTTGGTACTTTCATTTCTTGTGATGGAACCAGTTGCTCTAGTTA-CAGACTTATCTTAGACTG--TTCTAACTCTGAACAT--GTGGATAAT

Homo sapiens  TTCGTTCTCTTTGTTAGAAGATAAAAGCTTGCATTGTTTGTACCTCTTCTCAGTTCTGAGCTACCTTTAATGATCTCTCTCTTCTGCAAGTTAAGTAGGGAAAAAAAAGGTTATACAAGT
Mus musculus  TTCAATCTCCTGGTTAG--GACAGTACATTCATTTCTTTGTG--TCGTCCCACTTCAGAGCTA---TTTATAGACCCTCCCTTCTACAAGCTAAGTAGGGGAAAAGG-GATTCTATGAGT

Homo sapiens  ATATGTTGTTCTAGCTTCTTTAGCATGATTTTCCCCTTAATCTTTGACATTCATGATTTGCTCTCTTCTAAAGTGTTAAGCTTCTTTAATACAAAATTTAATT-TAAAATTTTGCTAAAC
Mus musculus  -------TCTTTAATTTCATGAA---GACTTCATTCTCAGGCCTTCATCTTCAGAACTCATTTCCTTCTAATGTGTTAAGCTACAGTTATATAAGATTTGCCTATAAAACATTACGGTGC

Homo sapiens  CAGGATCT-----ATCAACATTTTGTAATTTGTTTCTTGTCAAAGAGTTTCTCAGAAAAGAAGTCAAATATTGTGCTTCTTCTGAACATTCAGAACTCAGTATTTCAGAACTCATCTTCTC
Mus musculus  CAGGGCCTTGTACAACAGTCTTCTGT--TTTTCTCTTATCAAAGACATTT--AGAAAGGAAATCAAATTATTTTCTTTTTT----------------GGTGTTTCAGAAACAAG------

Homo sapiens  TGTTTGAGAACAGTCTCTAAACAGAGGCAGTATAATATTTGCCTCATACTTGAATTCTTTGCACAAGATTCTTCCAGTAATATAAGGACAAAT--AATATTGCAAT-------ATGACTA
Mus musculus  ---TTGAGAACA-------------------------------CAAGCTTAAATCTTCTGGAAATGTTTCTTGTAGTAATACAAG-ACAATTGGAGTGTTACTATTAGTGTTACTATTA

Homo sapiens  CTGGTGTATGCATAAGGAATTTAAGACTAATTAGATGAGTCTGTTTTAGTTTTTCTTGTTAACTAAAG---GAACTACTGATCTGGCCATCCTAGAATCTGGTTCTTAT---TGCTTGAT
Mus musculus  GTAGTGTGAACATAGGCATTTGAAGGTGA--TAGGTGGATTTATTTTAAAATTTCATATCAACCATACTACAAATTAGTTCTTCAGTCATACTTAATCCTTGTGTGTGTGTGTGTGTGTG

Homo sapiens  CCTTTTTTTACATTTTTTCTCATTTG-------TAGAAATTAAAAGTAAATGTGATTTCTGCCATCTTTTTCCTCAATATTACATACTTTCTTGAAAATCTTTTCCATGATTTTCTTGAAA
Mus musculus  TGTGTGTGTGTGTGTGTGTTGGTTGGCAGATATACAAATTAAA--TAAAAATGGCCTTTTCCATCTTTTTCCGTGGCATCATGCATTTTCCTAAAAGCCTTT-----------------

Homo sapiens  ATCACCTCACAAGTTGATTTGAAAGAGTCCTTGAGCTGAATTAAAGCAAACAAAATGATTTTGACTAATCATTGAAGTTTTTATTTGACATAAATTTTGACATGCTATAGCCAAAAGAAC
Mus musculus  ATTACTTCAGCAGCTGACTTTGAAGAATTCACAAAGTGAATTTAATCAAA-ACAATATCTTTAACCA-TCAG-ACACTTTTTACTTGACATGA----------GAAATATCCAAAAGGAG

Homo sapiens  TATAAATTTATTTGTCCACATTTGTCATTGCTGCTCTTTGCTAGCCTTGAAATTTCATAGCTCTGTTAAAAACTCACTGTCTAAAGTAGATATACTTCTATTGCCAACTTCTTATCCATT
Mus musculus  CTCAGGT--ATCTGTTGACATCTGTCATATCTG-TCTTTACTACTGTT--AATTTCTTGGCTGTAT-------------CTAAAACCGATGGACTGACGTTGCCAAGTTCCTGTC-ATC

Homo sapiens  CTTATCTAGAACTAAATGTAGAGTGAGCACATTCAGTA---CTAGGTATACAATATTTTTCATTGCTATTTTAAGCAGGCCTGTTTGTTTTTTGTCTAGTTCATTGCCAAGCGCTTTTT
Mus musculus  TGTTTCTGGAACATAATACTGTGCTAACACATTCAGTAGTACTAGGTTAACAGTATTTGTCAATGCTATTTTAAGCAGACCTGTTTGCTGTTTTGTCTAATTTGATGTTGAGCACTCT--

Homo sapiens  CAGACTATTTA---GACCAAGAAATCTTAAAATACCATAGTGTCTTTTTGTAATCATAATGCTCCTCTTTGAAAGGAAAAAATA-----------ATCTGACATAGATTTTGCACAGTT
Mus musculus  -AAGCTACTTATTTTGATAAGAATTTCAAAAATAAC-TAGTGTGCCTTTGTAATCATAA----CCTATTGTAAAAGGGTAAGTGTGTTGTTGTCCCGTCCGACAC--ATTCTGC----CT

Homo sapiens  TTGAA----TTTATTTTGTTTGTGATAGATTTGGATCTGCCTCACCCA-AGGTTTTAGCCTGATTTCCCTTCACAGTTGAAAGCCCCATTTGTGAAACCATTTTCATTATTGTATAAGTT
Mus musculus  TTAAATGTGTTTGCTTTGTTGGTGGTAGATGCGGGTCGACCTTATCCAGAAGTCTCATTC----TCTCCTTCATGATTAAAAA-CCCATTTGTGAAATAATTTTTATTATGCT---AGTT

Homo sapiens  TCCTCTTGTTGCTGTAACAAATTACTACAGATTTAATGGTTTAAAACAACACACATTTATTATATTACAATTCTGAAGGTTAGAAGTTCAACATAGATCTTACTGGGCTAAAATCAAGGT
Mus musculus  TTATATTACTGCCATAACAATATACAACAAATTTAGTGCTTT-AAGCAGCACTCATTTATTACCATAAATTTAGGTACTTTAGAATTTCTACATGAGTATTATCAGGCTAAAATTAAGTC

Homo sapiens  ATTGGCACTGTTGTGTTCCT-----GTCTGGAAGCTCTAGAAAATAATCCATTTTCTTGTCTTTTCCAGCTTCTAGAGGTTACCTTCATTTCTTGG-------CTTGTGGTCCCTCTATC
Mus musculus  ATTCACAGACTCATGTTTCTAGACCATCCAGAAG----ACAAGAGAATGGATTTCCTTGCCCTTTCTAGCTTCTAGATACTTCCTTTACTTCTTGATGTATTCCTTGT----CTTCTGTT

Homo sapiens  TTCAAAGCCAACAATAGTGGGTTGAGTCCTTCTTACATCACCTTACTCCAACCTCCTCTTCTGCCTCCCTCTTCTATATTTGAGGACCCTTGACCTTGGCCCTGCCCAAAAAGTCCAGGA
Mus musculus  CTCAAAGCCAGCAATG-------------------------------------CCTCAAATGTCTCCCTCATCTGCATTGAGGACATTTGCTTTTAGATCCACCCAAATATTTTAAGA

Homo sapiens  TAATCCCCCTATTTTAAGGTCATCTGATTAGCAACCTTAATTCCATCACTACCTAAATTCTCCTTCACCATGTAAGATTACATATTCACAGGATCCAAGGAGTAGTATGTTGACATCTTT
Mus musculus  TGATCTCCCT-------TATCAT-TGATTGGCAGCTTTAATTTTATGACTACCCACAT--TCCTTTGTCGTGTAAAGCAACATATTCACAGGTTGGTGAGGTTAATGGGTGGGCA-CTTT

Homo sapiens  AGGGGCCTATTATGCTGCCTACCACAATTATGGTCTGCATTCATGAAACAAAGCAAAGCACAGTCCCTATAGTGTCAAGACCATGATCTTGACGTTAATATAGTTTATGCAGAGAG----
Mus musculus  GGGAGACTATTATTATG-ATATAATAGTTAATGGTTTCACTAATAAAACAAGCATTAGAACA-----TGAAATTACACAAGCTACACATTATTAGTAATACAATGTATGCAGAGAAATTG

Homo sapiens  --CCTTTTTTTGGCCTCTTAGTTATATCTTTTTTTTTTCTTTCTCTTTTTTCCTTTTTTTTTTTTTTTTTTTTTTTTTTGAGACAGGTTCTTACATGGTTGCCCGTGCTGGAGTGCAGTTGC
Mus musculus  TTCCTTTTT..............................................................................................................

Homo sapiens  ACAATAATAGCTCACTGCAGCCTTGACTTCCTGGGCTCAAGCAATCTTCCCACATCAGCCTCCCAAGTAGCTTGGAATGTAGGCATGCATCACCATGCCTGGCTGAGTTTTTCGTTTTTT
Mus musculus  .......................................................................................................................

Homo sapiens  ATAGAGACAGGGTCTTACTATGTTGCCCAGGTTGGTCTCAAACTCCTGGACTCAAGCAGTCCTCTCACCTAGACCTCCCAAGTGTTGGAATTACAAGTGTGAGCCATTGTGCCCGTCCAG
Mus musculus  .......................................................................................................................
```

```
Homo sapiens  TTATATAATTTCTTAACCAATAGTT-ACTCAGTTATCTGTT--GACAGTATTAGTTATTATAATGACAGTGATTTAGTATCAAGTTATAGTTATTATTTTATCTTGAGAAAGGATATACT
Mus musculus  .TATATACTCTCCAGACCCAGATTCCATCCAGTTCATTAATAGGATACTATTAAT-ATTATAACAGTATTAATATAGTATCAAATTATAG---TTGTTTTATCATG-GAAAGGATATACC

Homo sapiens  AGTATGGCAGCACATTCTAATAGTTTGAGGATTATCAAACCATATATAGGATTAGTTTAGTCACTTCTCTTTAAAATAAGAAAATTAAGTAGTCTGAAACCTTTATCTTTTTTTTTCTGA
Mus musculus  ACAAGAGGAATATAATGTAAAAGCTTGAAAATAATCA...................................................................................

Homo sapiens  TTGAAGAAATACATTAATCTAAATCAGACTTTTTTGAAGCTGTAATTTGTATTTATTTTTCTCAGGGAAATTTAAAAATTGATTTTTTAATTATTATTTTTATTCCTTAAAACAACATTA
Mus musculus  ........................................................................................................................

Homo sapiens  AGGCTGGGCACAGTGGCTTATGCCTGTAATCCCAGCATTTTGAGAGGCCGAGGTAGGATTGCCTGAGCTCAGGAGTTCGAGACCAGCATGGGCAACATAGTGAAACCCCGTCCCTACAAA
Mus musculus  ........................................................................................................................

Homo sapiens  AATTAGCCGGGCATGGTGATACGCACCTTTAGTTTTAGCTACTTAGCGGGCTGAGGCAGAGGATCACTTGAGCCCGGGAGACTGAGCTGCAATGAGCCAACATTGCACCGCTGCACTCCA
Mus musculus  ........................................................................................................................

Homo sapiens  GCCTGAGTGACAGAGTGAGACCCCATCTTTTTTTTTTTTTGAGATGGAGTTCAACTATTATCACACAGGCTGGAGTGCAATGGTGTGATCTTGGCTGCCTGCAACCTCCGCCTCCTGGGT
Mus musculus  ........................................................................................................................

Homo sapiens  TCAAGCAATTCTCCTACCTCGGCCTCCTGAGTAGCTGGGATTACAGGCGCCCACCTCCACACCCAGCTAATATTTGTATTTTTAGTAGAGACGGGGTTTCACCATGTTGGCCAGGCTGGT
Mus musculus  ........................................................................................................................

Homo sapiens  CTCAAACTCCTGACATCAAGTGATCCACCCGCCTTGGCCTCCCAAAGTACTGGGATTACAGGCGTGAGCCACCATGCCTGGCTGAGACCCCTGGGATTATAGGCGTGAGCCACCATGCCT
Mus musculus  ........................................................................................................................

Homo sapiens  GGCTGAGACCCCATCTCTTTAAAAAAAAAACCTACATTAGAAATGGATATCAAAACTGCTGATTAATACCTTCCTGAAATGTCTTGAAAATATTTTAATTTCAAAGAAACTAAAGACTTA
Mus musculus  ........................................................................................................................

Homo sapiens  TTCAGTCTTGTCAAAACCAAAATGATGCTTTCATCAAACAGATGTCATAATCATAACTAAATTAATGGAATGAGAAAAGATAAATACTTAGTGAAATGTACTGTGTAATGTAGCTGACTA
Mus musculus  ........................................................................................................................

Homo sapiens  GAAAAGACCTTTAATATGATATGTTTGCTTACTCCCCGCCTCTGTTTTGAGGGCAGAAAGTTTAATCATATTCTTTTCTGTATCCCTAACTCATGATTGTTTTAGATTAGTGTTGTCCAA
Mus musculus  ........................................................................................................................

Homo sapiens  TAGATGAATAATAATACAAGAGCTATGTAATTTAAAATTTTCTAATAGCCACATTTTGAAAAGTACAGAAATAAATGAAAATAACCTTAATAATATATTTTATATAATCCAGTGTATCCA
Mus musculus  ........................................................................................................................

Homo sapiens  AAATATTACTTGTTCAACATGTAAGGCCACAAGAGTCTCTTTTCTGATCTCTTTACTGAAATTAAAGAGCATTTCTCTTAGCTCCAGTAGATTTAGACCATAGTAGGAGTTGAAACATAT
Mus musculus  ........................................................................................................................

Homo sapiens  TTGACCTTTGCAAATGAATGAGAATTTTTTTTGTAAACAAACAGGCCTTTGACATATTCAGAGTGGTTTTCTATGTGTATAAAGTGATTTATGTAGAATTTGCTGTGTGTAGTCCTTGTT
Mus musculus  ........................................................................................................................

Homo sapiens  CTCTACAGTTTTATTACAATAAGTTACTTTAAAGGTTAACTTTTTTTAGAAGAAAAAGAGTGTGTGTGTGTGTGTGTGTGTGTGTTCGTGTGTGACTTATTTTACTTACATACAGAAGCT
Mus musculus  ........................................................................................................................

Homo sapiens  CTCTTAAAATCAAGTGAGGTTGGTATGTCCATTTGTTTCTAACCAGTGTAACTAGGAGGTACTCAAGTTCAAGGTCTCTGAATCCATTTCAGCAAATTAAAATGAAAATTTCTAAGAGCT
Mus musculus  ........................................................................................................................

Homo sapiens  TTACATCTAGGCATATTTTTCCCTATTGACATATGTTTACTAGACATATTTTTCTAAAGTAGTCCTACTTCATCTGTATGAAAAAACTTTTACAGACAGAAAACCTCCTTCATTAATCCT
Mus musculus  ........................................................................................................................

Homo sapiens  CTGAAAGAGTCTTTGGGCATTTCTTAAATGCCTATGGTAACTGCCATCACTGATAACTTTGTAGGAATGTACAGGCCTTGAACCTATGGAATTCTCCATGACTTAGTAGAAAATTTCATC
Mus musculus  ........................................................................................................................

Homo sapiens  AGCAACATGTTTACCCTATCTAACCTTTTAATGTTCTCTTATAAACTACCGCCTAGCCTTCTCTCGTATTAACAGTAGGGTAGTTGCAAACTTTGATGTTAAACCTCTAGCAACACATTC
Mus musculus  ........................................................................................................................

Homo sapiens  TGAAAGTGTTTCAATGTTATTGATCATGTTTTCTTCCCTCTTGCTGCTATTGGGTATAACACATCTCATGTGTTGCATGATTTTCGTTGTTGTTGTTGAGACAGAGTCTCGCTCTGTCAC
Mus musculus  ........................................................................................................................

Homo sapiens  CTAGGCTGGAGTGCAATGGTGCGATCTCGCCTTACTGCAACCTCCACCTCCTGGGTTCAAGCAATTCTCTCACCTAAGCCTCCCCAGTAGCTGGGATTACAGGCACATGCCACCATGCCT
Mus musculus  ........................................................................................................................

Homo sapiens  GACTAATTTATGTATTTTTAGTAGAGATGGGGTTTCACCGTGTTAGCCAGGTTGGTCTCGAACTCCTAACCTTAAGTGATCTGCCCGCATTGGTTTCCCAAAGTGCTGGCATTACAGGCA
Mus musculus  ........................................................................................................................

Homo sapiens  TGAGCCACTGCCTGGCTGCATGATTTTTATTTTATTTTATTTTTTTTTGTCTTTCAGAATTATGGGAGAATAAATGGCCATACAAGCCCAAGCCCACTTATGCCATCTTTTGGTAGAGAG
Mus musculus  ........................................................................................CATGCCCACTCATGCTGCCTGCTTATAGTG--

Homo sapiens  AATAGCTTTGTGTGATCTGTTAAAGTTTATAAATTGAAAGTTGTTAAATGCATGAATTTCTGGGGTTGAAATAGGTTCAGTGAGCTATTTGTAATAATGTTATGAGGGTGAGTACAGTAG
Mus musculus  --TGGATTTCCAAGAGCCACTAATGTTCATAAAATTTAAGTTTTTAAGCGTATGAGCTTCTGAAGAGGAAATA-----------CT---------------------------------

Homo sapiens  GATTGATTTTCAAAATATACTACCAAATTCATCTGTTCTGAGAC-ACTTCCATTGGTCTCTAATCTAAATAGGCAATTAATCTTCCTTTTGCTTTTTTGGTCTTAAAATTTGAGGGCTCC
Mus musculus  ------TTTCTCAAACATGCTACTAAAG---------CTGATACGATTTCCACTGTTCTCTGATCTAAATGGGCGATTGGTCTTC------TGTGTTTAGACTT-AGATTGGAGAGTTCT

Homo sapiens  TTAACCCATTAACATCTTCAGGAGACCAACCTAGGCAACACAATTAGCCAGGCATAGTGACATGTGTCTATGGTCCCAGCTATTTGGGAGGCTGAGGTGGGAGGATCACTTGAGCCCAGA
Mus musculus  ATAAGCTATTAAAATCTGCAAGAGA...............................................................................................

Homo sapiens  AGGTCAAGGATGCAGTCAGCTGTTATCACGCCATTGCACTCCAGCCTGGGTGACAGAGCAAGACCATGAATGAATGAATGAGTGAATGAATGAATGAATGAATGAATGAAAAATAAGTAA
Mus musculus  ........................................................................................................................

Homo sapiens  ATAAAGATAAATAAATATATCTATATCTATATTCTGATGTTTTGGGGGAACATTTAGAAAGAGAAGGTAGGGATCCAGGAAGTTAATCAGCTTTGGTAAAAGAGTTTCTTATTGTAATGA
Mus musculus  ..................ATCTGT----------TAGTGTTT----GGAAGGTCCAGAAAGAGAATGCAGGGAT------GATTCATCAATTATAGTAGATGAGTTCCTTTTTGTAATGA

Homo sapiens  ATTCAAATATCAGAAAGGGAGGAATCCTTTTATACATATTAGTCTGTGTATAGAAGA----------TTTGCCATCAGTTTAAC-ATTCATTTTAAACATTTTTTTCTTGATGGTGATGA
Mus musculus  ATT-AGGTATCAGACCTGATAGAAATGGTTTCT--ATTGTAGTCTTTAGATTGAAGGGCAGTGTTACTTTCCTGTGAGTTTAACAACTTATTTTAAAGCTGTTTTCCTGAGGGTTAATGA

Homo sapiens  TAAATTTGCTCAACATACAATAAAACACTGACCAGGAAGACTCCCTGTCAGTACTTCAGTATGGCTTAGGTAGACCTTAAAAGTTCCTAAGAATTTTGG-TATCAAATTAGATTTAAATA
Mus musculus  TGACATTGCT-AAAGTATAACAAAATACCTAGAAGGAAAGTTTCCC-------CATTAGTGTAGATTAGGTAGACCTTGAACATTCCTAAGGTTTTTTGCTCTCAGATACAACTTAAATA

Homo sapiens  AGCTAAGCTTTTTAAGCTACTCCATGGCAGCCTGCCTTCTTACACAT---TATCACATAAGC-------------CTCAGTTGGCATGTTGTTTTTAACTTCATACTGTAACAATT----
Mus musculus  A---AAGTTT----AGCTACTTCACTGCAGCTTGCCTTCTTG-ATATTCCTGTCACTTGAGTCAGATTTTTGCTACTTTATCTTCATCTAGTTTATGGCTTTATATTATAACTGATAATG

Homo sapiens  ------TGATGATATTATCTTTCCAATATGGGTTTTATAAAGATTGGAAGATTATCTCAAGTTGTAAATGCAAGGATTTAATAATTCTATTTGTTAAAATAATTATATGACCCTATTAAT
Mus musculus  ATATAATAATGATATAATAGTACTACTATGAATTCTTTACAAGTTGGATGA-CATCTGAAATTAGAAATATATAG-TGTAGCAATTCAGTTGGCTAAAA-------AGAGCTATATTAAT

Homo sapiens  TCACCCAGAAATGCTTTACAGAAGCATATTAAGGATAACATT---ATCAAAA---ATAGTTTTATAATTTTTTTTTTAAGACAGGGTTTTGCTCTGTCACCCATGCTGGAGTGCAGTGGT
Mus musculus  TCA-CAAAAAATGCACTGTAGAAGCGTATTAAGCATATCATCGGAATCCAAAATTATAGTTGTGTAA.....................................................

Homo sapiens  GCCATCATGGCTCACTGCAACCTCAATCTCCCAGGCTCAAGTGATCCTTCCATCTCAGCCTCCTAAGTAGCTAGGACCATAGACACCCTTAACCACACCTTGCTAATTGTTTGTATTTTT
Mus musculus  ........................................................................................................................

Homo sapiens  TGTAGAGAAGGGGTTTCGCCATGTTGGCCAAGCTGATCTTGAACTCCTGGGCTTAAGCAATCTACCGGCTTTGGCTTTCCAAAGTGCTGAGATTACAGGCATGAGGCACTGTGCACAGCC
Mus musculus  ........................................................................................................................

Homo sapiens  TTATTATTTTATTTTTAATCTATACCATAATTCTCACTCCTTCTATCCCGTTGTCCGCATATATACATATCTACCAAAGACAGGTTGAGGGAACAGGAAAATGGCTGTTTTAAAATCCTT
Mus musculus  ........................................................................................................................

Homo sapiens  GACCTTTCCTGCATTGTTTATGTATGAACTGTCAAATTTATATGTCATTTTTTTATTCTTTGACATTCATATTTAGTAGACAGTATAACTCCCTAGAAGTTAATCTTGATAGTTAATTTT
Mus musculus  ................................................................................................AAGTTAATAGTGATAACTTATTTT

Homo sapiens  TATTACTTGGGATTTTGTTCCTCACTTAAAATTGTTTAATTAAAAAATATGTTTAATTATAATTCTAAAAGTCAAATGTATGATTTTTCTTTTTTCAGAAAGTCTTTATCTCTTGCAAGG
Mus musculus  TATTGCTTTAACTTTTGTTCCTCACTTAA---TGTCTAGCTAAAAAAATTGGTTTCCCTTCAATTCTAAAA-TGAAATGT-TGATCTCT-TTCTTTCAGACAGTCTTTATCTCTTGCAAGG

Homo sapiens  TTCAGTAGTGGCAGATCATGACTACATTGGATTGCCTGAAATTCCGATTGGAGCATATCAAGCAAATATTCTGGTGGAAGATGCAACTATTGGTATAGTCGATAATGAATTACTGACATC
Mus musculus  TTCGATAGTGGCAGATCACGACTACATTGGATTACCTGAGATCCCAGTTGGAACATACCAGGCAAATATTCTGGTAGAAGATGCAACTATTGGGATAGCTGATAATGAACTATTGATATC

Homo sapiens  AAGCAAGGATCGTGAACTATTAGAATATAGAAATACGAAAATCTCTCCATTGATAGACGATCATAGTTCTCTTGAAAAGCAGACTTTCAGTCTGTTGGACTCTTCAAACCAGGTTCTTGA
Mus musculus  AAGCAAGGATCATGAACTCTTAGAATATAGAAATTCCAAAATCTCTGCATTGGTAGAAGACCACAGTTCTCTTGAGAAACAGACTTTTAGCCTACTGGATTCTTCAAATCAAGTTCTCGA

Homo sapiens  ATACTTAAGTTAGTAAATACCAAAATTTATTTAAATTGTTTGTTTAGAA--TATAAATTCTTAAT-----GATATCTTATTCATTAAAA-ACGTTGTATAGAAGAAGTATCTTGACACAT
Mus musculus  GTACTTAAGTTAGTAAATACCAAAATTAATGTAAAATGCTTGCACAGAATGTATATATTTCTAGTTTGTAAGTATTTTATCTATTAAAATAGGTTGTATAGAAGAGAGATCTTGGTACTA
```

```
Homo sapiens  TCTTAATGACTATATTCTGAAGGTTTATTTTATAGTTCTTATCTAAAATTATGCCACATTTGAATATCAGTATACGTACATATGAAGGCAAAGGAAGCATGTAATATATAGAATTCACTT
Mus musculus  TGTTAATAACTAAATTCTGAAAGCTTATTTTAAA---CCTTCCTAAAGTTGTTACATGTTTGAATACAAATAAATATAT--------GTAACAGAAGTATACCATGCGTAGAATTCAGAA

Homo sapiens  TTTCTTCTAAAAAGAGAACAGGCAAAGCACCCCTACTTCTTGGGAAATAAATTCCAGCCCAAATCTCTCAAACCTTTGTATGGTTTTGGCACCGTTTCTAAGTTTTAAATTTTCTGAAAT
Mus musculus  ATTTCTC-----AGAAAACAGACAAAATAC-----GTTGTTAGAAAATAAATTCTCGTGGAGAAGTCTCACCTTTTTCTATATGGGTACTACATTTTCTC--TTTTAACTTTTCAGCAAT

Homo sapiens  TCTCTACCATTTAAACAGTAAGTTTGAACTACCTTGAATGGAATTGATAAATACTGAATCAACTGTAACAATGAATGAAATGAAGCTTCTTTCTGTTTCATTAGAACTTCTAAAAACAAG
Mus musculus  ACATACCCCTTTTA--AATAAATTTAAACTAATTATAGCTTAGTTAATAAATGTTGAGTGAACAATAAC-ATAAATGAAACGAAACTT-TATCTGTGACATTGGAACTTTTTAAA.....

Homo sapiens  ATTGTATGAAAAAATATAGGGTTATTTAATAGGCTTTCACAGAATATTGGTAATTTCAAATTATCTAATTGGTTAGATAGGGTCAGGATTAGGGTCTGAAACTATGTATTGGTTGGGAAA
Mus musculus  ........................................................................................................................

Homo sapiens  CAATTTGTTTATTTTCATACTATATATTAAAGATAAAACCATCGACCAGGCATGGTGGCCCACACCTGTAATCCCAGCGCTTTGGGAAGCCAAGGCAGGATGATCACTTGACCCCAGGAG
Mus musculus  ........................................................................................................................

Homo sapiens  TTCGAGACCAGCCTGGGCAACACAGGGAGACCCTATCTCTACAGAAAAATGTTTAAAAATTAGCCAGGTGTGGTGGCACATGCCTGCGGTCCCAGCTACTCAGGAGGCTGAAGTAGGAGC
Mus musculus  ........................................................................................................................

Homo sapiens  ATTGCTTGACCCCAGGAGGTCGAGGCTGCAGTGAGCCGCGCTCATGCCACAGCTCTCCAGCCTGGGCGACAGGGCAAGACCTGTCTCAACAACAACAACAAAAGTATCCGTTAAGAATTT
Mus musculus  ........................................................................................................................

Homo sapiens  TTTCTGCAATCATAAAATCATAAAATAACCAGTATCAAAGATTTTAAACTGTAATTTCTATGATATTCATAAATATAAAATAAATATTGTTCAATAAATATTTATTCTAAAGCACTATGA
Mus musculus  ........................................................................................................................

Homo sapiens  AAAGAACTGTGGGAAAAGTAAAAGATGAGTCAAACGTGTACCTCATTCTTATAAAGGACTTACTGTCTGGTAGGTAATAAACTATGTAAGCCATGTACAGCACAAGTTAAGGCATGCTAA
Mus musculus  ........................................................................................................................

Homo sapiens  ATTGCAGAAGACAGGTGTAGATAATGTACTTAGGGAGGCAAAAAGAGAGAAGTTGTTTTTACATAGGAAATTTGTGTTTATCCTTATGGAAGAAGGATCATTTAAGCTTGACCTTGAAGA
Mus musculus  ........................................................................................................................

Homo sapiens  ATGTATAGGATTTGGTAAATGGATACAAAAGAATTAGGGGAGAAGTAATACACAGAAATGGAAAAGTATTGTAATGATTCATAAAAAGGCAGGGCTTCTTGTTGTCTACACTGCCTCGGG
Mus musculus  ........................................................................................................................

Homo sapiens  TATAAAGTGAAGAAGCAAGATAATGAGGGATAAAGCTGAAAAGGTAAGTAGGAAACCTTATGGAGAGCTTTGAATACTATTAAGGATTGATATGTGCCCCTCCCAAAATTTGTGTTGAAG
Mus musculus  ........................................................................................................................

Homo sapiens  TCTTAACCCCCACCACCTCAAAATGTGACCTTATTTGGAAATGAGGTTGTTGCAAGTGTAATTAGTTGAGATCATATTTCAATAGGGTGGGCTCTAATACAGTATTACTGGTGTTCTGAT
Mus musculus  ........................................................................................................................

Homo sapiens  AAAAGGGGAAAATTTGAACACAGACTCAAGGAGAATGCCATGTGAAGATAAAGGCAGAGTTTAAGATTATGTTTCTATATGCCAAGAAATGACAGAGATTGCCAGAAAACTACCAGAAGT
Mus musculus  ........................................................................................................................

Homo sapiens  TAGGGGAGAGGCATTGAATACATTTTTCCTCATTCCTCAGAAGACCAACCCTGTCAATACCTTATCTCAGACTTTTGCCTCCAGAACTGTGAGACAATAAAATTCTATTGTTTCAGCCAT
Mus musculus  ........................................................................................................................

Homo sapiens  CCAGTTTGTGGGACTTTGTTACAGTAGCTCTAGCAATAATACAAATGCCTGGCTAAATTATTTCTTTTCTGAGAGCAAAAACTTTGTATAGGAAACTGTCATAATCAGAACTGTGTTTTT
Mus musculus  ........................................................................................................................

Homo sapiens  GGAAGTTTAATCTGGCAACTAGTAAAGAACAGATTAGAAGAGGAGAAAACAAGGCATGGAGATTAGTTGAGAGGACTTCGCAGTGATGACGAATACTAAAAACCTAAACTGGGCCTGTAG
Mus musculus  ........................................................................................................................

Homo sapiens  CATTGGTAATGAATGGAGTCTGTCTTAAGATCAGTACAGAATATCGCACATATTATATTTCACAACCATACTGAACAGTGATGTGATATCAAAGATGGAGACCTGGAGTATTCTGATTAC
Mus musculus  ........................................................................................................................

Homo sapiens  TCATTTAAGAAGAAAGATTATGTTTAATATAGGCTAAAGGAAAAGTGTTTAGGGTAGGGTGTGAATCTAGGACCTCATCTTATACCACAGACCAAATAAATTTCAAACAGAATTCCAGGA
Mus musculus  ................................GCTGAAGGACAAGTGACT--GTCAGGGAAAGAATGAAGTCTCTGACCTTGTAC-ACAAACCAAAGACATTGCAGATAGAATTCTAGAA

Homo sapiens  AGAAAAAAACAAAACTTTTTTTAAAAAAGCGTAATTGTATCAAACAGTGCTACCTATCCTGCTTTCCTCAGAAGAGCAAAGTTTAGAAAAGAGTCATATTAGAATATCTTTTTGGTAAA
Mus musculus  AGAAA---------------CTAAAACAGAGTGGTGCTAGTAGGCTTTG--------CCTACTCTCCTCTACAGAGCAGAGTTCAGGAAGGGAGCCTACTGCAGTGCCTACATGGTCGA
```

```
Homo sapiens        TCAATTTCTGGCGTCTCGAACCCAAGAAATGAAAAAGGGAAGCCATGGGGGAGCAGGAGGAGAAATCAAGGCGCGAAAGGACCCTCAAAGCTGTACTGTCACTCCACGCCCAGGCTGTCA
Microcebus murinus  GCAATTTCTGGCGTCTCAAACCCAAGAAACGAAAAGGGGAGGCAGTGGGGA---AAGAGGAGATACTAAGAGGCAAAAGGACCTTCGGGGCTGTACAGTCGCTCCACGCCCA--------

Homo sapiens        TTCTCTCCAGCACAAGCCCCTCGGTGGAGCATGTGTTTAGGGTCTGCAGAGCACAGGAGACAAAGCAGAAGCCCAGCAGCCGCCTCAACCCTGGCATGATCAGGTTCCGCATCTCCGGTT
Microcebus murinus  --CGCTCTGGCACATGCCCCTCGTAGGAGCACGTGTGCAGATTCCGCA--------GAGACAAAGCAACTGACCAGCAGCGGCCGCAACCCTCAGGGAATCAGGTTCCGCGTCTCCGGCT

Homo sapiens        CTCCTCCGGTTTT-GGGCCCTCACCTGCCACGAATGCGGTCGTCACGCGAGCAGTTGTGAGATGGTCAAGCCCATTCAGGTCTAGGTTACCCCCTTTC----ATCCCGG-----AGGCAG
Microcebus murinus  CTCCCTCGGTCTCGGGGCCCTCACCTGCCACGACTGCGGTCGTCACGCGAGCTGCTG-GAGATGGCCGAGCCCATTCGGGTCTAGGTTTTCTTCTTTTTATAATCCGGGGCAGCGGGCAG

Homo sapiens        GGGTGAGGTGTTTGTCAAGAGACTTCAGTTGTAGGCGTGGACGTAGGCTACTTCCTTCAGTAACAGCT-ATAGGGCCACATAAGGGAACCTCAG--GCTACTACACCTCAGGAATCTCCG
Microcebus murinus  GGGCGAGGAGCTCGTCAAGAGACTTCAGCTGTAGGCGTGGACGTGGGTTTCTTCCTTCTGTAGCAGCTGAGGGGGCCACATAGAGGTACCTCAAGCGCTACTACACCTTGCCCACATCCG

Homo sapiens        TCACGCCTCTTCCCC-GCCCTCCTCGGGTCTCACGCCGAAACCTCACCGGGCGGAACGATTTCCGGCAAGAGCCAAATGATCAGTCCTAACCTTT-CTGACCGTGGTTCCAAGGTTCCCC
Microcebus murinus  CCACGCCTCTCCTCCAGCCCTAGTCGGGTCTTACGCGGAAACCTCACCGGGCGGAACGATTTCCGGCGAGAGCCAGATGATCCGCCCTGACTTTTCCTGACTGAGGTTCC----------

Homo sapiens        AAAGGACTGCCCTCTAAAGGACCCGGGTAGTTCCGCTTCCGGCAGCGCGAGATAAATCACGAGAGGAAGCTTAAAT-CTGTCGTTTGAATTTAGGACCACCTCGGTGAGTGGTCGTTCTG
Microcebus murinus  -ACGGACTACCGTCTTAACGACTCGGGTAGTTCCGCTTCCGGCGGCACCAGATAAATTGCGAGAGGACCCTTAAATCCTGTCGT..................................

Homo sapiens        GTGTGCTGTGTCATACCTACTGTTTTTTAAAGTGAGGCGTAACCCGACAGTAATTTCAAAACCATTCGCCTCGACCGGCCTAAGGAAGGGTTTAATTGAGTCTGTGGGGAGATTTTGCGC
Microcebus murinus  ........................................................................................................................

Homo sapiens        AAGCGCCTTTGAGATAGGAGGGCCTTGGGCGTTGCGATGTCCCGCGCTAGCTGAGCAGAAAGGGCGAGTGCTGTTTCGGGCCTGGGGAGGTGCCTACTGGGTGTAATTGAGTTTTACCAA
Microcebus murinus  ........................................................................................................................

Homo sapiens        ACGGTCATTTTCCTGCTGATTCTGAGTGTGGTTGGACCTTGCCCTTAAGATTCCGTTAACGAGTCGCCCAAAGCTTAGGAAATGCTCCCTCACAGCGTTTGAGACTAAGCACTTTTGGCC
Microcebus murinus  ........................................................................................................................

Homo sapiens        GGCGTTGAAGAATTCTCCCCATTACTTTGATCAGTCATTTAATCAGAAATTTTGCCCCAAATTTAAATACGTGGCGGGGAGAAACGTGAGGTCAAGAGGGAATCCTGTCACGTAGTTTTA
Microcebus murinus  ..............................................AACTATGCCCCAAATTAAAATCCGTGGCGAGGGAAAACCTGAGCTAAAGGGGAAATGTTGCCA-ATAGTTTTA

Homo sapiens        AAAG--AAAAATGTCCACTTTATCTCAGTGTAGGTTTTTCTTAAATGGGCATTTCTGAACCAAAGTAGATACTTTTTAAAGTGGAAATGCATTTATCCCATGTGGCTTCATGTGAATGCA
Microcebus murinus  AAAGGAAAAAATGTACGCTTT------GTGTGAATTTTCCTAAAATGGGCATTTCCGAACCAAAGCAGATACTTTTAAAAA-GGAAATTCATTTATCCCATGTGGTTTCATGTGTCTGCA

Homo sapiens        TATGGCGATGAAGGCATTTTTTT-GTTCAATGAATTTGTACTCTGAAGTGAACCAGATTACAGAGAAGTGT-------ATTTAAATTTACAGGTCCTTGACGTTGCATAAAATTGTGCGT
Microcebus murinus  TATGAAGATGAAGGCATTTTTTTTGAAAAATGAATTTGTACGCTGAAGTGAAGCATATTATACAGAAGTGTCTGGTGTATTTAGATTTCCCAGTCCTTGACTTTGCATACAATTGTGGGT

Homo sapiens        GTCCATTTTTTGGAG-CTTAAAATAGAATAATTCAAATGTCAGAATTCCGATCCAAGTTAACATGTTGATTGAAA-ATTGCAGATTTTAAGATGGAACCAGCGGGTATTATAACCTTTTA
Microcebus murinus  TTTCATTTTTTTGAGGATTAAAATAGTATAATTTAAAAGTCATATTCTCACCCCAAGTTAATGTGTTTATTGAAATAGTACAGATTTTAAGTTGGAACACATGTGTATTATAATCCATTA

Homo sapiens        GGGGCTTAAATTGGTGG-----CTT--TGTTTTTAACCCTTGGTGAAACA--GTTAAGTCCTAATCTTG----ACATAATACCTAACTGCCTTACTTTATTGAACACAAA------ATTA
Microcebus murinus  AAGG-TTACATTGGTAGGTAGCCTTGATGGTTTTTACCCTTGGTGAAACACCGTTAAATCCTAATTTTATTTAAAGTAATACCTAATTGCCTT----TATTGGACACAAACATAAAATTA

Homo sapiens        TTAAAGGAATTAAGCAATGAGT------TGATTTTATTTGCTTTTATGGAGGGGACAAAGTGTATAAAGCTTAACCAAATGTGACCCTGACATTCCTCTTGCAAAACTACAAGTTGTAAT
Microcebus murinus  TCAAAGAAATTAAGCAATAAGTATTTGTTAATTTTATTTGATGTCATGCAAGGGACATAGTGCAAAAAGTTCAACCAGTTGTGACCCTGACATACCTCTTGCAAAACTAGAAGTTGTAAT

Homo sapiens        CAATCACTTTCATT--TTTTCTTATTCCTACATGTTTAAGTACATTAAGTAATGATTTTAAGAGAAATTCTTGAAAATATGAGCATATCCAAAGAAGTCCCAGGACAGTCTTTCTTTATG
Microcebus murinus  CAGCCACCTTCATTACTTTTTCTTTTCCTACATGTTTAAATACAC-AAGTAATTATTTTAATAGAATTTCTTGAATATATGAACATATCCAAGGAAGTCCTAAAGTACTATTTATTCA--

Homo sapiens        TAGTAGAAACCACTTAATCC-CCCTTGTAAATGATACCTTTACTTAGCAATCAGAGTATAAGAAGATATTTATATTTGTCTCGAATATTACAGTTTCAAGCCATTTTCTCATATGT----
Microcebus murinus  -------AACCACTTAATTCTCCCTTGTAAATGATACCTTTAGTAATC--TTTGAGTA-AGGAAGATATTTGTACTGAATATTATTAATACAGCTTAAAACCATTTTCTCATATTTTGAA

Homo sapiens        ----------------------------ATGGGCTTTGAAAAAAAAAAAACAACT--TTCATTTACGTTACTTTTGTATATGAATACCAATTATTAGCAGTAAAACTTTTAACTACTTC
Microcebus murinus  ATGGACTGTTTCTAGGACACAAGTTGTGAATAGGCTTTGAAAAAA------TTATTGGTTCTTTTAAATTGTTTTTGTATATAAATACCAAATATTAACAACAAAACTTTTAACGACTTC

Homo sapiens        AAAGGCTATGCTTTTCGTGAACTGAGTTTTCTTTCTTACGCATTTTCTATTTCTCCTTGTCATCTGTCTCTCTACCTCCAGAACCAGCAGTTTGTGAACTGCATTAGACTGTTATATCGT
Microcebus murinus  AAAGGCTATGCGTTTTGTAAACTGATTTTTCTTTCTTAGGCATTTTCCATTTCTTCTTATCA----TCTCTCAACCTCCAGAACC----------------------------------

Homo sapiens        TATAAAAGGATACAATTCGTGAGTCTTCTGATTATTTTCTTCTTAGCACTGAAGC-TTTATAAACATTGCTATAAAACATTAACACCATTAATGAAAGGAAAGAGATTTTAAAATATTTT
```

```
Microcebus murinus  -----AAGTATACAACACCTGACTCTTCTGG--GTTTTCTTGTTAGCATTAAAACATGAATAAACGTTGCTATAAAGCATAATCACTATTAGTGAAAGAAAAGAGATTTTAAAATCTTTT

Homo sapiens        TTCATGTAGGTGAAGATTTTGGGAAAAGAAAGGGTTTTATGTTACTTTAAACAGCCTGATATG---GATGATAATACTTAGATATGATAATAATTATACATTAC-----CATATACAAAG
Microcebus murinus  TCCATGTAGATAAAGATTTTGAG-AAAGAAAGAATTCTCTGTTACTTTAAACAGCCTGACATGAGTGATGATAATACATAAATATAACAATAATTATATATTGCTTTAGTATTTGCAAAG

Homo sapiens        GACTTTT--AATC-------------ATCCTCGTAATTGCAAAAAA---GGGATAGATATTATGTCC-TTTTCCAAACATAGATACTAAGGCTT-------------------------
Microcebus murinus  GACTTTTCAAATCCATTATCTCATTGATCTTCATACTTGTGAAAAAGGTGGAGTAGATATTATGTCCTTTTTCCAAACTTGAATACTGAGGCTTAGGTACATGTTACATGCCTGAAATTC

Homo sapiens        ---------AAGTCTATACCTTCTTAGACACAGTTTACTTATAGTGCCATGTATGTGAAAGTGAACTGTAATGTTTTCAAAAGTGAAATAATTTACCTCTGGAATATTTGCTATAATTTT
Microcebus murinus  TGACTCCCCAAGTCTATATCTTCTTAGACACACCCTACTTGTAGT-TCATACATGTGAAAATAGAGCGTACTGTTTTCTGAAGTGAAATAGGTAACCGTTGGAACTTTTGCTAT-TTTTT

Homo sapiens        TAAT----------TCTCCTTTCAGGAAATGCAGGTAATTCTTTATTCATTCACAAATAGTTGAGTGTCCACTGTGTTCTGAACATTATGTTAAACAATGTAAACAT-AAAAAATTAATA
Microcebus murinus  TAATTAATGTTACGTCTCCTGTCAGGAAATGCAGGTAATTCTTTCGTCATTCACAAATAGTTGAA-GCCCACTGTATTCCAAACACTATGTTAAAGAATGTAAACATAAAAAAATTAATA

Homo sapiens        AACTCTTTCCATTCCTCATTTATAATCTAAATTATTGATTACAAAAATCACTTTATAATTTGTGTAACAGAGGCCTGAACCAAGTATTATAAAAGCTAAGGATAACAAGTAATTGACTTA
Microcebus murinus  AGACATTCCTATTCCTTTTTCATAATCTAAATTACCTGTTACAATAATCACTTTATAACTTGTCTAACAGAGGCCTGAATCAAATTTTATAAAAGCTAAAGATAACAAGTAATTGAGTTA

Homo sapiens        CTGGTATAGCA--GGGGGGAAAAGCGTGAAATTTGAAGTAAGAGACACCAAGTTAAAAGTGTCAGCGCATGTACTCACTTTATTACCTTGAGTAAATTGGACAATCTCTGAACCTCCA-T
Microcebus murinus  CTAGTACAACATGGAGGGAAAAAACAGAAAATTTGAAGCAAGAGACACCAAGGTTCAAATGTCAGCTCATGCACTTACTGTGTTGCCTTGAGTAAACTGGACAATCTGTGAATCTCCATT

Homo sapiens        TTTCCTACCTGTAAAATGGATATAAAACTAATACCTACTTCATGAAGTACTGTGAGGATTATCATAGTAAAATACTGCATAACTATTTGCAGC-TGTAGTCGTGAGTCACTTAGCAATGG
Microcebus murinus  TTTCTTATCTATAAAAT-GATATAAAACTGA----TAGTTCATAGAGTATTCTGAGGATCATCATAGTAAAGTACTGTGTAAC-----ATAGCATATAGTCAT-AGTCACT---ACATGG

Homo sapiens        AATAAATTCTGAGAAATGCTTTGTTAGGCAGTTTCAGCATTGCACGAATACAGACTCTACCTACATGAACCTAGATGATATGTGTGTGTGTGTGTGTATATTTTTTTCTTCATATAGA
Microcebus murinus  CATAAGTTCTGAG-AATGCATCCTTAG.............................................................................................

Homo sapiens        GAACCAAATGTCCCTGCACATTATTGGGTATCATTTATTTCTCCTACTTGATCTGCAGTGCTAATATCAAATGACCTATATCAGGTTCTATATATGCTCTGCTATCATCTTACGGGACCA
Microcebus murinus  ........................................................................................................................

Homo sapiens        ATAGGCAGTCCATCCTTGACCAAAACATTCTTATGCAGTGCAATTGCGTTGGCATTGTTTTTAAAATTTTTAATTTTTTAAAATTATTTCCTCTTCCTATACTTTTGGCTTTTCCAGATG
Microcebus murinus  ........................................................................................................................

Homo sapiens        TCATATAAACAGAAATATAAAATATATAGCCATTTGTGTCTGGCTTATTTCACTTAGCATAATGATTTTGAGATTTATTCATATTGTTAGGTCTATTTATAGTTTGTTCCTTATCAATCC
Microcebus murinus  ........................................................................................................................

Homo sapiens        TTAATAGTATTCCAGTATGTGGATCTACTGTAATTTGTTTATCCATTCACCAGTTGATGAACATTTGTTTTCAGGTTTTGGCTGATATGAATAAAACTTCTAAAAACATACACTTACAGG
Microcebus murinus  ........................................................................................................................

Homo sapiens        TTTTTAGGTGTACATATTTTCTTTTGGGTTAGTATCTAAGATTGGTATTGCTGGACCATATGGTAAATTGATGTTTAACTTTATAAGAAATTGCCAAACTGTTTTCCAACATGTTTACAT
Microcebus murinus  ........................................................................................................................

Homo sapiens        CATTTTCTATTATCACCAGTAAGAGCATCCTTGGTAGTACCTAGCATTGTTAGGTTTTGTTTTATTTTTGTTGTTGTTAGTGTGTAGTCATATCTTATGGTAGTTTTAGTTTACATTTTT
Microcebus murinus  ........................................................................................................................

Homo sapiens        TATTATCTATTGATGTTGAGCATTTTTTCATGTGTTTATTTGCTAATCATATCTCTTTAGTGAAGTGTATTTCCAGGTTTTAAATTGGGTTGTTTGCTTTCTTGTTAAGCTTCGAGAGTT
Microcebus murinus  ........................................................................................................................

Homo sapiens        CTTTATAAATTCTAGAAAAAGTTATTTATCATATGTATGTATTGCAGATATTTTATCCCAGTGTTATTTCTCTTTTTTTAGCAGT-TCTTTAAG-GAATAAAA--------------AT
Microcebus murinus  ....................................................................TCTTATTTTTAAACAATATCTTTAAAAGAATAAAAGATAAAAATAAAGATAT

Homo sapiens        TTT----------AATTTTTTCTTTATGCATAGCTGCTTTTTCTTTGCTGTCTGGGAAGCCATTACCCAACCTGAGCTCACAAAGATTTTT-GTTGTTGTCTTCGAGAAGTTGTATAGTT
Microcebus murinus  TTTTTTATTAAAAAAATTTTTCTTTATGGATTGGTGTTTTTTGTTTGCTATCTAGGAAGTTGTTACCTAACCTAAGCTCACAAAGATTTTTTGTTGTTGTTTTCTAGAAGTTGTATAGTT

Homo sapiens        TTAAGTTGTACATTTAGATCTGTGAGCCATCATGAATTAATTTTTACATATCATTTGAG---------------------GTATGAGTTGAGGTTCATCTTTTTTCAAA-GGATGTTTA
Microcebus murinus  TTATGTTTTACATTTAGGTCTGTGACCCATCATAATTTAATTTTTACATATAATTTAGTTGGTCAATTAATTTCTTGCTAGTTTTAGTAGAGGTTCATCTTTTTTCATATGGATGTCCA

Homo sapiens        ATTGTTACAGCATTTGTTGAAAAGATTATCCTTTCTTCATTGAATTACCTTGGCACCTTTGTTGAAAATCAGTTTACTATAACTGAGTGGCTATTTCTGT---CACTATTCTGTTCGATA
Microcebus murinus  ATTGTTCCAACATTTGTTGAAAAGATTATTCTTTCTTCATTGAATTACCCTGACACCTTTGCTGAAAATCAGTTTACCATATCTGAGTGGCTGTTTCTGGATACTCTCTCCTGTTT-ATA

Homo sapiens        GATTTAAGGCTGTCATTTTGCCATTATAACACAGTCTTGATTACCATAGTAGTATAGAAAGTCCTGAAACCAAATAATGTGAAATTCTTCAACTATATTCATTTCTCAAAGTTGTTGTAA
Microcebus murinus  GCTCTATGGCTCTCATTTTGCCAGCGCCACACAGTCTTGATTACCATAGCATTATTGGAAGTCTTCAAATTGGATAGTGTGAAATTCTTCAACTGTGTTCATTTTTCAGAGTTGTTTTAA

Homo sapiens        TTATTCTGAGTCCTTTAGCTTTCCATATCAATTTTAGAATCAGCCTGTCAGTTTCTGCAAAAGAGCTTGAGGGTTCCTGATTGGGACTGCATTGAATCTATAGATCAATTTGGAGAAAAT
Microcebus murinus  CTATTCTAAGTCCTTTGGTTTTCCATATTAATTTTAGGATCAGCATGTTAGTTTCTACAAAAGAGATTGTGGATTTTTGACTGGGATTGCATTGAATCTATAGATAAATTTGTGGAGAAT

Homo sapiens        TGGCAT---------TTTGTCCCACTTTTTCTCAGAGGTAGAGTGGGAGTTACTC----CAGCTTTCTGCATTCTAGTCAGAAGCCTATATGGGAATGTTTAGT-GGCATTTCAGAAAGA
Microcebus murinus  TGGCATCTTAATCAGTTTGTTTAGCTGTTTCTCATTGTTAGGCTGAGAATGTCTCTTTCCAGCTTACTACATTCTAGACAGAAG--TATATGGGAATTTTTAATTAATATTTCAGAAAGA

Homo sapiens        AATAATTTTCTTCCATGGTTATATTCACTATAATATCTTTATTGGAAGAGATCACAGGACGAATCCCTCTATTTGTGAATTGGATGTCATCCCCTGCTGCCATCTC-AGGACATCATACC
Microcebus murinus  AATAATTTACTTCCATTGTTATGTTCACTGTGCTA-CCTTATTGGAAGCGATCACAAAACTAATCCC---ATTTGTGAATTTGATGTCATCCCTTACTGCCCTCTTAAGAACATTGTACA

Homo sapiens        A-----TAAGTTTTTTCTCCTTTGTTTTCAGTGGTCTTTCTAGAAGCCCTTCCATTAGCATTAAACTTTTTGGGTTACTCCCTTGTCGAAAGCAAAACAATCTTGTTCAAAAAAAAAATT
Microcebus murinus  ATCATGTAATTTTTTTCTCCTCTGTTTCCAATGGTCTTCCTAGGAGCTCTTCCATTAGCATTAAGCTTTCTGGGTTATTCCCTTCTTGAAAACAAAACAATC---TTTAAAAAATGAGTT

Homo sapiens        GTTTTAATTGTAGTAAATCACACATAACATGAAATTTACCATCTTAACCATTTTTAAGTGTGCAGTTCAGTGGCTTTAAGTACATTCACATTTGTTTTACAACTATTACCACCATCCATC
Microcebus murinus  TTTTTAATTGTAGTAAAATACATGTAACATGAAATTTAACATCTTAACCACTTTTAAATGTACAGTTCAGTGGCATTAAGTACATTCACATT--GTGTATAACCATTACTACCATGCATG

Homo sapiens        CCAAGAACTCTTTTCATTTTGTGTAACTGAAACTCTTGACCAGTTAAATAATAACTCTTACATTCTCTCCTCCTCACCCCCTGTTCTATGCAACCATCATTATTCTTTCTGTCTGTATGT
Microcebus murinus  TCCAGAACTCTTTTCATCTTGTACAACTGAAACTCTTTACTGATTAAATGATAACTCCCCCATTCCTCCCTCTCTACACCCAGTG...................................

Homo sapiens        ATTTGACTACTCTGACTACCTCATATAGGTGGAATCTTACAGTATTTATCTTTTTGTGACAGGTTCATTTCACTAAGCTTAATGTCCTCAAGATTCATCCATGTGGTAACGTGTGTTAGA
Microcebus murinus  ................TACCTCATATAAGTGGAATCAGACAGTATTCATCTTTTTATGACTGGTTTATTTCACTTAGCATAATGTCTTTAAGATTCATTGATGTCGTAGCATGTTGGAGA

Homo sapiens        ATCTCCTTCCCTTTAAAGGT--------------GTGTGAATATA--------------------------------TACACCGTATTTTGTTTATCCATTCAGCCATTGATGGACACTT
Microcebus murinus  ATCTCCTTCCTTTTTAAGATAGACACACACACACATATGCATATACATATATATATATATATATATACACACACACGCACACCATATTTTGTTTATTCATTCATCCACTGATGGACACTT

Homo sapiens        GAGTTGCTTCCATCATTTGGCTGTTGTGGGTGATGTGGCTATGAACATGACTGTCA----AAACTTTTAACCCCACTAGAATGTAAACTCAATGAAGGTCAAGAGAATCTACATCTTGTT
Microcebus murinus  GGGTTGCTTCTACCTTTCGGCTATTGTTGATTATGCTGCTATGAACATGGCTGTCAAAACAAACTTTTAACCCTGC............................................

Homo sapiens        TATTATCCTGTCTCCAACGTTGTAACTGGCACATAGCAGTTACTCAGTCACATTTTTTAGATCAATGAATGAACCTTATATCTGTCTCTAGCAACTATCTTGTAGCTCCTTGCCTTTATA
Microcebus murinus  ........................................................................................................................

Homo sapiens        GCTAAGCCCAAGTTTCTAAATCCACTTTCTGAATTTCCATTCATTCCTTGTGTCATTTCAGTCTGGTTGCCAACTGCCCTGCTATACTGTAACTATACTGTATACTGTTGCCAAAACCAC
Microcebus murinus  ........................................................................................................................

Homo sapiens        TAGCTACATTATGGTTCTTATATCACTTAGCCTCTCTGTGTACTTCTAAGAAGTCTGTATTTCCCAGACTTCTATTACCAGAACCTCTTCTGGCTTTTTTCCTGTTTCTCTGGCTAGTCT
Microcebus murinus  ........................................................................................................................

Homo sapiens        CATGTCCCTAAAGATTTACAATATATTTGGGAGAGAGACATATAAACTAATATATTCAATAGAGTGTTTCAGATTTTATGATAAAGGTTGACACATATTACTGTGAATGTACCAATGAGG
Microcebus murinus  ........................................................................................................................

Homo sapiens        GTGTGCACAATTCTCCCTGAGATTGGGGATGTGGTCAGAAAGGATTCATGGATGAATGAACTCATTAATGTGAACGTTGAAAGATAAGGATATGTTCACTGCACTTCCTACTGTGTACAC
Microcebus murinus  ........................................................................................................................

Homo sapiens        TTTCCATCTGATCCTTCTTCCATTGCCTTAACTTCACCTACTTTTTACATGCCGAGTTCATCCATGAAGAATAGAACCTGCTTAATATTGTCATCTTTCTCCCTAAGCCAGAGCTTCAGA
Microcebus murinus  ........................................................................................................................

Homo sapiens        CAGAAGTGATCCCAGATTCTTCTTTGTGTTTACTCCACACATCTAGTCAGTTACAGTTCTTATATAATGTATCTATCCATTTATACTGACACTGCTTTGGCTCAGAATTTCCTCATTTCT
Microcebus murinus  ........................................................................................................................

Homo sapiens        TGCTTTAATTATACAGTAGCTTCCTAGCTGACTGGTACAGGTATTCCTGTCTCTACCCCAATCTCTATTCCATATTGGTGCACAATACACTTTATAGAATAGAAATCTAATCTTGTAACT
Microcebus murinus  ........................................................................................................................

Homo sapiens        CCAACTCATTCTTAAAATTTTAAGTTATTCCAGGTTGGAGTGGTTAGAAGACACTCTGCAGGAAATTACACCTAGCCAGAGTTTTGAAATATGATGAGGGGCTGGAGAAATAGCAATAAA
Microcebus murinus  ........................................................................................................................

Homo sapiens        AGTTCATTCCAGGCAAAAATATCAATGAAAAGGTGTGAGCACACTGCATGGGATGGTTGAAGAACTGTGGTTATGTTTTGAATTGAGAATGCCAAGAGAAAAAGATGGAGAGATTGATAA
Microcebus murinus  ........................................................................................................................

Homo sapiens        AAACCATATCTTATAAAGCCTTGTATACTGGCAGAGGTATTTAGATATCATTAGTGCCATGGTCTTAATGGTTGTATTCTCCCAAAATTCATATGTTGAAATCCTAACCACCAAGGTGAT
Microcebus murinus  ........................................................................................................................

Homo sapiens        GGTATTAAGAGGTTGGACCTTGGGGCTGGGCGCGGTGGCTCACACCTGTAAGCCCAGCACTTTGGGAGGCTAAGGTGGGTGGATCACTTGAGGTCAGGAGTTCGAGACCAGCCTGGCCAA
```

```
Microcebus murinus  ........................................................................................................................

Homo sapiens        CATCGTGAAACCTCATCTCTACTAAAAATATAAAAATTAGCTGGGCATGGTGGCATGCACCCGTAGTCCCAGTTACTCGAGAGGCTGAGGAACAAAATTTGCTTGAGCCTGGGAGGCAGA
Microcebus murinus  ........................................................................................................................

Homo sapiens        GGTTGCAGTAAGCTGAGATCATGCCACTGCACTCCAGCCTGGGCGACAGAGCGCAACTCTGTCTCAAAAAAAAAAAAAAAAAAAGAGGTGGAACTTTGAGGAGGTGACTTGATCATAGGTC
Microcebus murinus  ........................................................................................................................

Homo sapiens        AGAGCTCTCATTAATGGGATTAGTGCCTGTATTTTAAAGAGTCTGAGAGAAACCCCTTTCCCCTTCCCTCATGTGAAGTTACAGTGAGAAGACAGCTGCCTATAAGAAAGTGAACCCTCA
Microcebus murinus  ........................................................................................................................

Homo sapiens        GTAGACACTAAATTTGCTGGCATCTTGATCTTGTATTTCCTAGCCTCCAGAACTGTGAGAAAGAAATTTCTGTTTATAAGCCATCCAGTTTATGGTATATTTGTTATAGCCGCCCAAGTG
Microcebus murinus  ........................................................................................................................

Homo sapiens        GACTAAAACAATCAGGAATGGCAAAAGATGTATTTCGAGTTAAGTGAATATTTTGATAAGATTTGTGTTTTAGTGTGACCTCTGTGACTGCAGTGAGGACAGGGACCATATTAGAGACAG
Microcebus murinus  ..............................TATTTCAAGTTAGGTGGGTATTTTGATATGGTTTGTGTTTTATGATAACCTCTGTGGCTGCAGGGAGGAGACGGACTATGT---AGACAG

Homo sapiens        AGGAGGCAAGGTCAGATAGAAGGTGAGTGTGAGCTAAGGCAGTGGCAATGGAATAAAGTTCAAGGTTCTACCTCATCTACAATCAGTGCTTTTCCTCCTTTTCCAGGTGAATCTGGGTCT
Microcebus murinus  GAGAGGCAAGGTCAGATACGAGGTGAGTCTGAACTAAGGCAGTGGTGGT-----AAAGTTCAAGCTTCTAATTTATCC----CAAGTGCTTTTCCTCCCTCTCCAAGT----CTGATTCT

Homo sapiens        CTACACATCTGTTTTGATAGTACTCTTTGCATACATCTGTTAAAACACCACTCTAAACTGTATAAATGCTGATTTCCTCCTCTTTTCCTCTCATTT-ATACCATAAGCTCCGTGAGGACA
Microcebus murinus  TTA------TGTTTTTATAATACACTTTGCATACTTCTGTTAAAACAACATTCTATAGTGTATAAATGCTGATTTATGCCTCTGTTCCTGTTATTTGACAGCATA-GCTTTATGAGGGTA

Homo sapiens        GGGATTTCATTTTATTTAACTTTGTATTAGTATCATCTAGTATATATAAGGGTTTAAGAGCTCAGTAACTGAATGAGCAAATTCAAGGTTATATGGATGAACAGGCAAAAAACTATTAAA
Microcebus murinus  AGGATTTCATCTTGTTTAGTTTTGCATTCCCATCATCTAATACATATAAGAGTTTAAGTTCTCAGCAAGTGAGCAAACAAATGTGAGGTTATATGGATAAACAAGCAATAAACTATAAAA

Homo sapiens        AAGGGTAAAGCATGG-AAGTTCTACAGTAGGCACAAAGAAGATTAGGAGAGGTCAAGATCACTTTAGATTCAGAGATCATGGAACACTTGCTTCATTGAGATGCTAGAGCTTGATTTTGA
Microcebus murinus  AAGGATAAAACATGGGAAGTGCTATAGTAGATACAAAGAAGGTCAGGACAAGTCAAGATCATTT------CAGAGGTGATAGAAGACTTGCTTCATTGAGGTGCTAGA-CATGATTTTGA

Homo sapiens        AAGATGGGTAAGGCTTAATCAGGTGGAA------GGATATACCATTTAGGGGAAA--ATGATGTAAACCAAGGTATAGAGAATGGTTGGGGATATGGCCATGGGTATAGAAAGGAAAGTA
Microcebus murinus  AAGATGAGTAAAACTTAATCATATAGAATTAGAAGGATGTACCACTCAGGGGAAACAATCATATAAACTAAAGTACAGAGAATGGTTGGG-ATATGGCAATAGGTAAACAAAGGAAACTA

Homo sapiens        GTGAGAGAAGAAGCTGTA----TTATGTTCTGCCAGGTTGTAGAGGGTAGGCTAAATAGTCTAGACCTTAGCCACTAAGTGGAAGATAATGCCCAGAGGCAGTCTCATGGTGTCCACTCT
Microcebus murinus  ACTAGAGAAGAAGCTCTAAGGCTTATGTTCTGCCAGGTTATAGAGGATAGGCTAAGT-GTTTAGACTTGAGTCAATAAGTAGTAGAGAA-GCCCAGAGGCAATCTAATAGTGTTCACTCT

Homo sapiens        GTCAA-GTAAATCATTTGTAAG----------------------ATAAAAATATGCTAGGGATAGTCTAAATTAAAGTTTCAAGGTGACTATGTCAA----ATAACTACACCTTTAAGT-
Microcebus murinus  GTCAAAGGAAATCATTTGTAAATGATTTTACAAAGTAAATCAAAATAAAAATATGCCAGGTATAGTCTAAAT--AAGTTGCAAAGTTCCTATGTCAAGCAAATAATCATGCTTCTAAGTG

Homo sapiens        --CTTAGTTCTGTTGAGCCAAGACAACTCACCCATATAAAAATGAAAATAATACACTTGTGAAAGCATTTGTGAAATATGAAAATCTGTAGGAAATAGATGGTTTTTATGTCTTCTCCCA
Microcebus murinus  TTCTGCTCTTTACTGAGCCAAGACAACTGACTCTTATAAAAATAAAAATAGTACACTTTTGAAAGGACTTATGAAATATGAAAATATATAGAAAATTTCTGATTTT-ATGTCTTCCACTG

Homo sapiens        ATGATGTTTATGTCCCTCTCCCTAACATAGATATATGTTAGTCTTCTCCCACTACTTTGATGAGAAAAAAACAAACCAAAAACCTTAAAGAGCTCAGTCTCCAAAGATTTTTGTAGTGCT
Microcebus murinus  A------------CTTCTCTCTTCCTTAATGATATG-----------------------AAAAACAAAGTAAGCAAAAAACCTCAAAGAGCTAATTCTTCAAAATTTTGTGTAGTGAT

Homo sapiens        TTCCTTTTCTCCTTGTCCCTT-GTACTATAGGTTTACTCTGAGGAGAGCAAATTTCTCTATTTCGATCTACTTTCAATTATTCAAAAGTTAAAGATTTCAAAGCAGAGAAGTTAATTTCA
Microcebus murinus  TTCCTTTCCTCCTTGACCCTTTGTACTATAGATTTACTTTGAGGATACCAAATTGCTCTATCTTGATCTATTTTCAATTATTCAAAAGTTAAAG-TTTGAAAGTAGAAAAATTAATTTCA

Homo sapiens        GTAAATAGACCTTTTCTGAGAAG---TTTTTGTATATTTATTATAAATAGGAAAACCAACAGTGTCCTAATGGCAC-GTTAACTTAA-TATACTGAAGACTTCATCTTA--------CAT
Microcebus murinus  GTAAATAGACCTTTTCTGAGAAGGATTTTTTGTGCATTTATTATAGGTAG-AAAGCCAACGGTGTCCTTACAGCACAGATAACTTAGGCATACTGAAGACTTCATCTTATATTGAGTCAT

Homo sapiens        TAGATAGAAAA-ATTTTCTGGTCTCTCCAGGAGGTAAATGGGTAGGGGGAGGAAGACAAGAGATGGTAGGAACTTGGAACCAAGAGGTTTTTAAATATAAAAGCAAAATACGGAAGCTAC
Microcebus murinus  TAGATAAAAGATATTCTTGTATATCCCCAGGAGGGAATTATGTAGAGAGAAGAGTACAGGAAATTGTGAGAACTTGGAACAGAGGGTTTTATAAGTATAAGAGCAAAATACAGGAGCCAC

Homo sapiens        TGTGGATTATGCAGACTCACATTTTTTTTCCAATGGCTGGTTTGTTTTCTACCAGTTCTGGTTACCCACATCCCTCTGTGTTTTGGGGCCTGTACTTCCTCTAAGGTGTGAGAGTTTGGT
Microcebus murinus  TGTGGTTTATGCAGACGC---TTGTTTTTCTGATGGCCAGCTT-TTTTCTACCAGTTACAATTACCCACACCCCTCTGTGTTTGGGGTCCTGTACTTCTCTTAAT---------TTTGAT

Homo sapiens        CTTTTCCTTCTGTGGGCTTTT-ACAGGCCTGAAAAAGCTCCTCCCCTTTTCTTTTTAAGGTCCTTCAGGAGTTTTTGCAA-CCTTTACTGGTCTCTAAAAGGAAGGAAATACACAATTTT
Microcebus murinus  CTTTTCCTCCTGTGAGCTTTTCATAGGCCTGAAAAAAACTTTCTCCTTTTCTTTTTAAGGTTCTTCAGGAATTTTTGCAAGCTTTTACTGGGCTCTAAAAGGGAGGAAATA-AGGATTTT

Homo sapiens        TATCTTCCTTTTATAAACCTCGTCTTCCTACAGATACCATCCTCCCAACTGGCTAGTATGGGATATTTCTAGATTTTCCTTTGGATTGCTTAGACGATTTGTGACATTGATAGTTCCAAA
Microcebus murinus  TGTCTGTCTTTTATAAGCTTCATCTTCCTACAGATTCCATGGTCTCAGCTAACTAGGATGGGATATTTCTAGATTTTCCTTTAGAT---------GATTTGGAACACTGATGGTCCCAAA

Homo sapiens        AACTATTAAGGGAAAA----CTATAACAGGAGGTTATATGTAGTATATTATTAATTTTACAGTATGTATAGTCTATATATTGATTTCATCTAAACTGAGAAGGGACAGGTTTCTGTCCCT
Microcebus murinus  AGCCATTAAAGGGAAAGAAACTGTAGCTTGAGGTTATATATAGTATATTATAAATTTTGTAGCATGTAAA--CTATGTAATGATT-------------GAAGGGACAGATCTCT--CCCT

Homo sapiens        TAGACTTGAAAGAATGTGTTATCATTGTAGTATAGAGATGAAAATAGAGGACACAAAGAAAAAATGATTTTGGTTTCTTTTTTTTTTAAGAGTTGAGGACGATTAATGCAGAGCTAGAAT
Microcebus murinus  TGCACTTAAAAGAGCTTGT-GTCAGTGTTGCCTAGAGATGAAAATAG--TACACAAAGAAAAAATGATTTGTGTTT----GTTTTTAAGAGTTGAGGAGGGTTAATGTAGAGCTAGTTT

Homo sapiens        GATAGGCAGGCAAATGGATGGGTAGACAAAC-AGGTTCTATATTCCCAACTTGAAACGACACAAGGCTTCATGAATTGAAAATTGATTAGGAAGTAAAAGTTTGCCATGGAGTCCTGGCA
Microcebus murinus  GAAAGGCAAGCAAATTGATAGCTAGACAGACTCAACTCTACATTCCCTACTTGAAACCACATTAGCCTTCCCGAAGTGAAAATTGATCAAGA-GTAGAAGTTTGGCATGG..........

Homo sapiens        GAGGAGAGAGTGTAGTGAGGATGACATGGAAAGTAAAGGCATCAGGAGGTCTTATGTGGCAGATTGGAGGGCAGTAGGGTGCCACTAGTGTTTCACCACAGATGGTTTGAAAAGCACTGA
Microcebus murinus  ......................................................................GCAGTAGGGAGGTACTAGTACTTCATCACAGTTACTTTGAAAAGTGCTAA

Homo sapiens        CCTGGAGCGACATGAAACTTGGTAACAATAGAAGTGGCTACAGGACCAGAACAGAGTATACGCATGGTAAAACTTCTTGATACCTTTACTGTTTCCATTTAGTGGCTATTATGACTATAA
Microcebus murinus  CCTAGAGCAACATGAAACTGGACAACAGTAGAAGTAGCTACAGGACAAGGGCTGAGCATACACATGGTAAAACCTCTTGATGCCTTTACTATTTCCAATTAGGGGTTATTATGACTATAA

Homo sapiens        AATATCTATGAACATTTTTGTACACATATTTTGATAGTCATAGACTAATTTCACTGGAATATAAAATTAGAA------TTGCCAAACCTTTAGGGTAAATTTGTGCTCAGCTTTAGAAGA
Microcebus murinus  AACATCTATGAACATTTTTATACACATCTTTTGATAGTCATACACTGATATTTTTGGGTTGTAAACTTAGAAGTAGAATTACCAAGTCTTTAGGGTAGATGTATGTTTAGCTTTAGAAGA

Homo sapiens        AACTCAGTTTTCCTAGGTGGTTGCAACATTTTATCCTCCCA----CAATGTATGAGAGTTCCAGTTGCTGCCTGTCTTCACCAACACTTGGTGTTGTCTTTGGTTTTTAATTTTAGCCTG
Microcebus murinus  AACTCAGTTTTCCAAAGTGGTTGCAGCATTTTACACTCCCATAGACAATGTATGAGAGTTCCAGTTGCTACTTGTCCTCACCAACACTTGGTGTTGTCCGTG-TTTTTAATTTTATCCTT

Homo sapiens        TTC--AGTAGTGTGTAATTCTAAGTCATCGTGGTTTTA-TTTGCATTGCCCTGTGGAATAATGATGTTGAGCACATTTTCACGTGCTTATTGACCATTGGATATTCTCTTTTATGAAGTG
Microcebus murinus  TTTTGGGCAGTGTATAGTTGTAAATCACTGTGGTTTTAATTTGCATTGCCGTG-AGAGTAATGATGTTGAGCACATTTTCCCTTGTATAGTCACCATTTGAATGTATCTTTTATGAAGTG

Homo sapiens        CTGTGTAGAAATTTCCCCCCATTTTTTGAATTGGGTTTTTGTCCTTTTCTTTTTGATTTTTAGAAGTTCCTTGC-ATACACTGGGTCTGAGTCCTTTGTTAGATATATGTATTACAGATA
Microcebus murinus  CCATGTAAGAGTTGTCCCCCATTTT--GAATTGGATTTTT------CATTTTTTGATTTGTAGAAGTTCCTTTCCATATTCTAGGTATGAGTCCTTTGTTGGATGTATGTATTAAAGATA

Homo sapiens        TCTTCTCCCAATGCGTGTCCTATGTTTTCACTTTCTTAATGGTGTCTTTC-ATGAATACACATTTTTACTTTGATAAAGCCCTATTGTTAATTTTTTTTTCTTTTAAGGATAGTGTTTTT
Microcebus murinus  TCTTTTTCCAGTCTGTGTCTTATCTTTTCACTTTCTTA-TGGTGTCTTTTGATGAACAGGAGTTTTGATTTTGATAAAGTTCTATT---------TTTTTATTTTATAGATAGTGTTTTT

Homo sapiens        TTTTTGTGTCTTATTTAAGAAATCTTTGCTTAGCCTAAGATTTGAAAGATCTTTTCCTATGTTTTCTTCCAGAATCATTGTTATTTATCTTTCACATTTAAGTTTATGATTTATCTGGAA
Microcebus murinus  T----GTGTCCTGTTTAAGAAATCTTTGCTTATTGCAAGATTTTAAAGATCTTTTCCTATATTTTCATCCAGAAGTGTTGTCCTTTATCTTTCACATTTAAGTTTATGATTTATCTGGAA

Homo sapiens        TTAATTTTTGTGTAAGATGTGAGGTAGGTAGTTAAGGTTCATTTTTTCCCCTAATACAGTTATCTAATTGTCCAACATTATTTATTGAAAAGACCACCTCTTTCCTCATTGTGTTGATAT
Microcebus murinus  TT----TTTGTGGAAGATTTGAGGTAAGTGGTTAAGGTTTATTTTTTTCCT--ATAGAAATATTTAGTTAT--AGCATCATTTATTGAAAAGACCACCTCTTCCTTCATTATACTGATGT

Homo sapiens        CTTCATTGTAAACGTATTGACTTTACATGTGTGGATGTAATTCTGTACTCCTTGTTCAATTACAGTGATGTATTTGTCTGTCCTTATGCCAAT--TGGCTTGTCTTATTTACTTTATTTG
Microcebus murinus  CTTCATTGTAAACG------CTTTATATATATGGATCTAATTCTAGACTCCCTCTTCCATTACAATGATGTATTTGTCTGTCCTTATGCCAGTACTGCACTGTCTTA----CTGTATTCA

Homo sapiens        TATAAAAAGTCTTAAATCTCATCCATTCATCTTTGAGATGATTCAGAAACTGTGATAGGCTGCATTTGTTTGGTTTGGGATGCAGCCATCTATTAGAAGATGAAAAAAATCATGTATTTA
Microcebus murinus  TATGGAAAGTCTAAAATCTGATTTATTCATTTTTGAGATGTTTTGGAAACTATGATAAGCTATATTTGTG------GGGGTATAGCCATCTACTATAAAGTGAAATAT-TCATATATTTA

Homo sapiens        ATAATCAATTGGAAGTGAATTAAACTTAGAACAAACTGATTGTCTTATAATACTTTATAGAGATAATTAACCTGTTTTTTAGGTTATGATGGGACTGTGTTAACATTTCAGAACCTTTTC
Microcebus murinus  GTATTTAATTGCAAATGAATTAAACTTAGAACAAACTAGTT----TATAGTACCTTGTAGAGATAATTAACCTGTTTTTTAGTTTATAATGGGATAGTGTTAGCATTGCAGAACCTTTGC

Homo sapiens        TTTTAAATTGACATATACTTTATGTTAACCTAAGGTAAGAAATGTACTGTTACTCTCTCTGGAAGTTAAAAGTAGATCAGAAAGACGGTTTTACATTTGCAAAGAGCTGTGTCATAGGGA
Microcebus murinus  TTTTAAAGTTGCTTACACTTTGTGCTAACCTAAGATTAGAAATGCACTGGTATTCTCTCTCAAAGTTATAAATAGATCACAAAGGTATTTTTTTCTGTGCAAGGAGCTATATCATGGGGA

Homo sapiens        AAGTGAATTGGTAAGCTGTTTAAGCCATGACAATAGGCACAGTCTGTTTCTGCTTTGTTCCAGCATTCTCTTTCCAGTAA---ATACTCTTAGTCTCATCCCTAGATCTTCCAGTGGTCA
Microcebus murinus  A-GAGACTTGATAAGCTGGTTAAGCCACCATGATAGGCATAGTCTGT--CTGTTTTGTTCCAACATTCTCTTTGTAGTAATCTACTCTTTTAGTCTCATACCTAGATCCTCTAGTGGTCA

Homo sapiens        GTAAACATC-----TTTTTTTAAATGTAATTTCAAAATTGTAAGATTTATGTCAGAATCCTAGCAGGAAACAGAAGACAT--TCTGGTGGGATTTTAAAGAAAATTTAATGGAGCTCCTT
Microcebus murinus  GTAAACATCCATATTGTTTTTAGA----ATTTCAACATTGTAAGCATTTTATCAGAGTCACAGCAGGAAACAGAAGCCATACTCTGGTGGGATTTTAAAGGAAATTTAATGAAGCTCCTA

Homo sapiens        TCTATAGACAGACAGTAGCAAAGTAAACAACATGGCACCTGGAGACTAGCAAAAGCA-AAAATAGTTACCACCTTAGGGCTGAGGTGACAAGAAGAAGAAATAACCATGAATATATTTAG
Microcebus murinus  TTTACAGATAGATAAGGACAGAGACACCAACGTGGCACTGGGAGACTAGCAACAGCAGAAAACAGTTACCACTTCTAGGGTGAAGTGGCAAGGAGAAGAAATGATCATGAATATATTTAG

Homo sapiens        AAATAATATAAAGCTATATTTCTTCTCTTATAACTTATT----------TATTTAAATGTCACATGACAGGATCCTCCTGTGTTACCATCCTGTGAATGTAACATTTCAGTTGATAGCAA
Microcebus murinus  AATTAACATAAAACTATATTTCTTCCCTTATAACCTATCATTTGTTAAATATTTAAATATCACATAATAGGA-------------CCATCCTGTATAAGTAACATTGCAGTTGATAGCAA

Homo sapiens        TGAAA--TTTTAAGCAAAACTTTTTGAAGTGTACTGGTTCATTTCCTTTTTT-CTCAGTATGTGGAAAATTTTCAGCATTTCTTATCAAATGGAAAGAGACAAGATAATTTGTCATGGGG
```

```
Microcebus murinus  TGAGAGATTTTAAGTAAACTTTTTTGAAGTGTACTTGTTCATTTCCTTTTTTTCCCAGTATGTGGAAAATTTTCAGCATTTATTATCACATGGAAAGAGACAAGATAATTTTTCATGCTG

Homo sapiens        TATATCAAAAATAGCATTTGAGTATTTTTGTTCATATACATCGCTGTTCCTTTCCTGTACTGCAATTATCTCCCTCACAAATAA-ATTCTGGGAATTCTCGGTTCATATGTAAATAGAAA
Microcebus murinus  CATCTCAAAAATAGCATTTGAATATTTTAGTTCATATACATCACTGTTCCTTTCCTGTACTGCAATTATCTCCCTCACAAATAATATTTGGGAATGCTCAATTCACATGTAAAAATAAA

Homo sapiens        TATGACAGAAGTCTTAACTGAAATAGCAATTCAGTTAGCTTAAAATTTAAGTTATCTTTTTAAATACCTTTTACCTAGGTTCACAATGGTCCGTAGTGGAAAAAATGGTGACCTTCATCT
Microcebus murinus  TATGACAGA-GCCTGAACTGAAATAGCAACTGAATTAGCTTA-----TAATTTATTTCTTTAAATAACTTTTACTTAGGTTCATGATGGTCCGTAGTGGAAAAAATGGTGACCTTCATCT

Homo sapiens        TAAACAGATTGCATATTACAAACGAACTGGTGAATATCATTCAACTACACTGCCAAGTGAGAGAAGTGGCATAAGAAGAGCAGCAAAAAAATTTGTCTTCAAAGGTAAAATTAATGTTCA
Microcebus murinus  TAAACAGATTGCATATTATAAACGAACTGGTGAATATCATCCAACAACACTGCCAAGTGAGAGAAGTGGCATACGAAGAGCAGCAAAAAAATTTGTCTTCAAAGGTAAAATTAGTGTCCA

Homo sapiens        AATTTTGAT-TATTGAGTTGTAGATTCTTCCCCACAT-TCTTCCTTGTGGAAGTTTGGGAATTTCTGTAGTAGTTGGTTGTTGCTAAACAATTACCAGTTGAATTGATGATGCCACTCAA
Microcebus murinus  GAATCTTATATATAGTATTATAGATTCTTCTACACATGTGTTCTTTGTGGAATCCTGAGAGTTTCTATGGCAGTTGGTTGTTGCCAAACTATTACCAGTTGAATTGATGATGCCACTCAA

Homo sapiens        TCAAAAGCAAAATAAAAATTACACTCAAGCTAACTGAAGGCCAATAAGCGTGAAAAGGGTTGTTTAAATTATAGACAGAATATGATACCATGAAAGAAAACA-AAACATTTTTCTAAATT
Microcebus murinus  TCAAAAGCAAAATAAAATTCACACTGAAGCTAACTGAAGGCCAGTTAGCATG-AAAGGGTAGTTTAAATTATATACAGAAAATGATACTGTGGGAGAAAACAGAAACATTTATCTGAATT

Homo sapiens        AACAACTTCTGCTTTTACCCTCTCGTG----GTCTCTAATGGTGCACTTTCCAGCACTGTTGCCAATTTCAATGAGGAACTTTTTTGATGCTTAATATGAAAATGTTGACAAAGTTAACT
Microcebus murinus  AACAGCTTCTACTTTTGCCCTCTGGTACACCTACTCTAATGGTGCACTTTTAAGCACTAATACCATAGTCAGTGAGGAACTTATTTGAGGCTTAATATGAAAATGTTGACAAAGTTAACT

Homo sapiens        GGTTTGTTTACTAAAATGAAAACTGGAAGGAATTAACTATATTTCTGTG-ATCATGTCCCCTTGAACCCCACCACTGCCA-TTAAATATTCTCAGGATCACATTCATTTTAGGACCCTTG
Microcebus murinus  AGTTTGTTTACTAAAATGAAAAAGGGAAGGAATTAGCTATTTTTATTTATATGATTTCCCCG------CTCTTACTGCCATTTAAATATTCTTAGGATTACATTTATTTTAGGCC-----

Homo sapiens        GATCTTCCTGAATGGATTAAATATTTGAAAGGCAGTGGGATATTTATCTTGGAAGGTCATGATAATTTTTGCCTCTTTTT---CCCCTTATAAAGGGGAAGAGAAGTTTGGGATGAGATA
Microcebus murinus  ------CTTGAGTGGATTAAATATTTGAAAGGCAGTGGGACATTTATCCTAAAAGGTCATAATAATTTTTCCCTCTTTTCCTGCCTCCTTCAAAGGGAAAGAGAAGTTTGGGTTGAGATA

Homo sapiens        AACAACAAGT--AGTCACCAGATAATTGAATGAATGTTTGTGTTACAAATGGGAATTTTGCC-----------TCTTAGGCTTACCCCAAAACAGGTTAATAAAACTCTAGGATAATAAA
Microcebus murinus  AGCAGAAAGTACAATCACCAGATAATTAAATGAATATTGGTACTATTAATGGGAGCTTTGCAGGTTGGTATAGCATTAGGCTTTCTCCAAAACAGGCCAAGAAAATTCTAAGGTAATAAA

Homo sapiens        TAAATGTCTGCTTGCATCTCCCTGTGTATATCCCTACCAATAACTCCCTTATACTTAAGATTAGGCAGACCTATTACCTCTTAAACTAAAGTGAGGATTCTTTCATTCATATATTTGATA
Microcebus murinus  TAAATAACTGCTCCTGTCTCCCTGTGTACAACCCTACCAATAA------------------TAGGCAGATCCATTACCTCTTAGACTAACATTAGAATTCATTCATTCATTTACTTAACA

Homo sapiens        ATTTATGTAACAAATGTTAACTGGGTTTCTGCTGTGTGCCAGTCACATTCTAAGCCTTGGAGATACATTGATGAATAACATAGATAAGGTTCCTGCTCTTAGGTTGCTTATCTTCTAATT
Microcebus murinus  ATTTGTGTAATAAGTGTGTACTGAGTTT---CTATGTGCCAGTCACATTTTAGGCCTTGGAGAAATATTGATGAATAAAATAGATAAGCTTCCTGCCCTCAGGTTACTTACCTTCTAATT

Homo sapiens        CTAAAAAATAAGGAGTGTTCCACTCATCAGTTGGCTTACTCATATTAGTAGCTGAATGGTGTATTTTGGAGTTTGAATTGCTAGCCCATTCACATTGGAAAGGTAGATGGTGATTCGCTT
Microcebus murinus  T----AAGTAAAGAATGTTCCACTCAGCA-TGTGCTTAAGCACATTAGTAGCTTAATGGTGTATTCTGGAGTTTGAATAGTTACCCTCT----ACTGGAGAGTTAGGTGGTGGTTCTCTG

Homo sapiens        CATACCCTGGTTACTTCCTAGCTGCAGATTCTTCTCAGGTGTCCTCCTAAGGAAACAGAGAATTGTCTTGAGGATTACAGTTTTTTCCACTTGCCCGCTTCACCATCCCTACCTT---CT
Microcebus murinus  CATCCTCTAGTTGCTTCCCAGCTGCAGATTCTTCTCAGGTGTCCTTGTAAGGAAA-AGAGTATTGTCGTGTGGTTTAAGGTTCT---------CCTACTTAATCAGCCCTACTTTCCACT

Homo sapiens        CAAATATAGAAGAAGCTGAGAGAGCTGTTTGCTTGCAAAAATACAGTGATGATAAAACATTATTTTAGAAAATTCTGTTCCAGGAAGTTGTTTTTCTGTACATAATTTATTTGCTAATCT
Microcebus murinus  CAAATCTAGAAAAACATGAAAAAGGTGGTTGCTTACAAAAATGCAGTTATGATAAAATGCTATTTTAGAAAATTCTGTTCTAGGAAATTGCTTATCTGTACATAATTTATTTGCTAATTT

Homo sapiens        ATGCCATTTTTAGGAGGTGCCTGCAAA-ATCTTCCTACTTTGAGTACTCCCAGAGAGAGATAATTTCATATTTCCGTACTTATTACGTGCTTTGGTGAACAAGCTTGCTCTACTTATAT-
Microcebus murinus  ATACCA-TTTTAGGAGGCACTTGCAGGTATTTTCTTAGTTTGAGTACTTCCGTAGAAAGTTAACTTTATGCTTCCATACTTATTATATGCTTTGATAAACAAGCTAGCTCTACTTATATA

Homo sapiens        -TGATAGCTACAGAATTATAGCAGCTACTGCCCTTCCTAAGTATCTAGTAAGTGTAACAGCTAAGAACCTTT------GTGTTGTAGCATCATTAAAAATAAATATTCTGTTTTAAATGC
Microcebus murinus  TTGATAGCTACAAAATTATAGCAGCTAGTGCTTCTTCTAGATA--------GTGTAACAGCTGAGGACCTTTCCAATTGTATGGTAGCACCATAACAAATAAATACTCTGTTTTAAGTAG

Homo sapiens        AGTAAGATTTTACAAAACAAAACAAAAACCTTCATGGTTTAAAGAGAAGGCTATGATTCTCTTAAAACTACGTAG---ATCTCTAAAAATTGCAATTCTATCTTTT-GGTATCATTTATT
Microcebus murinus  AGTAACGTTTAATAAAACG----------CCTCATGGTTTAAAGTTAAGGCTATGATTATATTAAAACTGTATAGGTAATTTCTAAAAACTGCACTTCTATTTTTTTGACATCACTTATT

Homo sapiens        CCAGAAAAAAAGCTGTTTTATGTTGGAAAAGACAGAAAACAAAATCGTTTGGTAATTGTTTCAGAAGAGG-AAAAAAAGAAAGTCTTAAGAGAATGCCATGAAAATGACAGTGGAGCTCA
Microcebus murinus  CCAGAAAAAAAGCTGTTTTATCTTGGAAAAGACAGAAAACAAAATCATTTGGTAATTGTTTCAGAAGAGGGAAAAAAAGAAAGTCCTAAGAAAATGCCATGAAAGTGACACTGGAGCCCA

Homo sapiens        TCATGGTATATCCAGGACCCTCACTCTGGTAGAATCCAATTATTATTGGACATCTGTGACCAATGATGTCAAACAGTGGGTATGGCTTATGTATTTAGAGTATTATGAATAATGTCTTTC
Microcebus murinus  TCAAGGTATATCTAGAACCCTCACACTAGTGGAATCCACTTACTATTGGACTTCTGTGACCAGTGATGTCAAACAGTGGGTATGGCTTATGTATTTAGAATATTTTTAATAATGTCTTAA

Homo sapiens        TGATAGATACTTAAAATTTATTATTTCTCCAGATTTATTCTGGGTATCACCTCTCATTAAGTATTTTTGGC------------------AATCACTTAAAGGGAGTTGTATTTCCCTTCA
Microcebus murinus  TG----------------------CCCCCAGTTTACTCTGGGTATTACCTCTCATTAAGTACTTTTGTATGGTAGTTTTGTGAAAAAACAATTAAAGGGGTTTTATTTCCTTTGA

Homo sapiens        CAGTTATTTTAAATCACTTAAGTATAATTTGAACAGGAAAGAAGCATTATTTAAACACTTGAAAATACCATATTTAAATAAGAGATTATTTAAAAAATCAGATTTGTATCATTGAACTT-
Microcebus murinus  ---TTCTTTTAAATCACTAAACTATAATTTGAGCAACAAAAAAGCATTACTTGAATATTTGAACATACCATATTTAGATAGGAGATTACTT--AGAACCAGATTTTTATTATAGAACTCG

Homo sapiens        TTTTGTGTAGGCAGAATTTAAATGGAGCTGGAATTCATTTGACTAAATCATTACTTCTTCAATGGCAGTCATACAAGGTTTTCTTAAGGGGAGAATATGTTCATAGTAAGTCAAGCCCAG
Microcebus murinus  TTTTGTGCAGGCAGAATTTAAATGGGGCTAGAATTTGTGTGACTAAATCATTACCTCTT.............................................................

Homo sapiens        TATGTGCTCTTATCTTTTGTGCCTGTTCCATTTTGAGGTAGAATGTCATACATGACAGAAACCCAAAACCAAATATAACCAAAATCAAATTTTGAGGATAATGAAATAAGTCTGAGTTTT
Microcebus murinus  ........................................................................................................................

Homo sapiens        GTAACTTTATGACCCATTGGGACCTTCAACTTCTAGTAGAAAATTCACCTATAGGAATTTTGGGGTTCTTGCTGGAAGTCAGCTGATTCTACTCTTTCCAGTGTACTGTATGTCAAATGG
Microcebus murinus  ........................................................................................................................

Homo sapiens        TAGGAATTCCTTGGTATATCTGTGAGATCTCCTTAATAATTGTAGTAGTACATCAAAAAGAGTGGCATTTCAAGCATAAAGAATTAAACCAAAATTTCATAAGTATAATTTGTGAGTTAT
Microcebus murinus  ........................................................................................................................

Homo sapiens        CAGTTTGTGGGTCTATTCAGGTATTCCAAAGACCTTCATAATTTATATATTTTTCCTGCTGTGCATTTTTTACAAACATTCAGTGAAATCTTTGTCAAATTGTTATCACTGGAGAATGAA
Microcebus murinus  ........................................................................................................................

Homo sapiens        TATGATTTTTGGAAATAATCAGAAGTTATTTGTGATAAAATCCAACAAATAAGTTTAAATTGGAAAGTGAAAATTTTGATCAAAAGTAACTGTGACTATAATGGGATTAAATGGCTTTTA
Microcebus murinus  ........................................................................................................................

Homo sapiens        AATTTTTCTTCAGAAGCTAGTTTGAAAGTAGTTTCTAGAGTGGAATTCCAGAGATACATTGAGAAATGGCACCCATCCATAAATTGATCAGTTGATCTTGTCTCACCTCACTCAAATGAA
Microcebus murinus  ........................................................................................................................

Homo sapiens        AGCTGCAGGACAGCATGGGTTTTGTGTATGTTTATTCTTTTCCCACGATTGAGAGAAATTGCTCCATCTATACATTTTATATGAATGAAATTATATTTCAATAAATATGGTTGGAATAGA
Microcebus murinus  ........................................................................................................................

Homo sapiens        TTAATGAATAAGCTTCTTAATGTGATAACTTTAGAAGGAAACATTTTATGTATATAGAAATTATTGTAAATTTACATACTGATTTAATAGGAAACTTAAGATCTTTTTAAGTATTTAATA
Microcebus murinus  ........................................................................................................................

Homo sapiens        AATATTTATTGAATGTGAACCAGATTTATGTTCCATGTGAATAAGACCCAATAAGATGGTCATTTGGTAGTATAGTCTTCCCTCAGTATATGCAAGGGATTGGTTCCAGCACCACACTCC
Microcebus murinus  ........................................................................................................................

Homo sapiens        TGTCACCAAATCTACTCATACCGAAGTCCTGCAGTCAGCCCTGTGAAACTCACATATACAAGAAGTCAGCTTTCTGTATACTTGAGTTTCTCATCCCTCCCTATCAATAGAGTACTTTTT
Microcebus murinus  ........................................................................................................................

Homo sapiens        ATTTGTGTTTAGTTAAAAAAAAAAAAGCCATGTATAAGCTGACCTTCACAGTCCAAACCCATGTTGTTTGAGGGTCAGCTGTAGTGGCTACTACAGTTGTTTGTTGTTGTTTTATTTGTTT
Microcebus murinus  ........................................................................................................................

Homo sapiens        GTTATTCTAAATAGGTTTAATCCAGTG---ATTCTCAGTCCAGAAGTTTCTCCTTCAAA-ACACAAAGTATACCTATTAACCATTTTAAAATTCAGATTTATAA-GACTGCTTTTATATT
Microcebus murinus  .......TAGAAAAGTTTAATACAGTGTCAAGTTCTAGTCCA-AAGTTTCTCTTTCAAAGACACCAAACTACCCTGTTAGCCATTTTAAAATTCAGATTTCTAAAGACGTCGTTTGTAAT

Homo sapiens        GT-GTTGAGTGT--TAACATTTGTATGTGTTTTCTTTATTGTGTGCCTGTT-GTAGGTATATGCTTGTCAGCATTGCCAAGTGGCAAAAAATACAGTTATTGTAGCACCGAAACAGCACC
Microcebus murinus  ATTGTTGAATATATTAACATCTGTGTGTATATTCTTTATCGTATGCCTGTTTGTAGGTGTATGCTTGCCAGCATTGCCAAGTGGCAAAAAATACAGTTATTCTAGCACCTAAACAGCATA

Homo sapiens        TTCTCAAGGTGGAAAATCCATGGAGTTTAGTTACTGTTGATCTGATGGGGCCTTTTCATACAAGCAACAGAAGTCATGTATATGCTATAATCATGACAGATTTGTTCACCAAATGGATTG
Microcebus murinus  TTCTCAAGGTGGAAAATCCATGGAGTATAGTTACTGTTGATCTGATGGGACCTTTTCATACAAGCAACAAAAGTCATGTATATGCTATAATCATGACAGATTTGTTCACAAAATGGGTTA

Homo sapiens        TGATTTTGCCTCTATGTGATGTTTCAGCATCAGAAGTTTCTAAAGCTATTATCAATATATTTTTCTTATATGGACCTCCTCAGAAAATAATAATGGACCAAAGAGATGAATTCATTCAAC
Microcebus murinus  TGATTTTGCCTCTGTGTGATGTTTCAGCATCAGAGATTTCTAAAGCTATTATCAATATATTTTTCTTATATGGACCTCCTCAGAAAATAATAATGGACCAAAGAGATGAATTCATTCAAC

Homo sapiens        AGGTAAGACAAATAAACTACTTAGGTCTGGGAGCATATCTTACTTCTTTTCAGTGTCCAGAAC-CAGTGCCCTCTGCATTATATTACAGAGTGCTTAATAAGTTCAGATAAATTTCTGTA
Microcebus murinus  AGGTAAGAC----AAACTACTTAGGTATGGGAGCATGTCTTATTCCCTTTCATTGTCCAGCACACAATGCCATTTGTGTTATATTACAGAGTGCTTAATAAATTCAGATAAATTGCTACA

Homo sapiens        GGTATAGAGGTATGATATTAAATACTACAAGGATAGTTGTACTGGAAATTAGGAGATTAGGATTTTAATACTTACTG------TGGGTTATTGTAAACCTATCTTCAGATATCCTCAAAT
Microcebus murinus  GGTATAGAGGTATGATATTGTATGCTACAGGGGTCATTGGATTGGAAATTAGTACATCAGGAATTTAGTACTTATAGAGCCACTAAGTAATTGTAAACCTATCTTCAGGTTTCTTCAAAC

Homo sapiens        TAAAATGAGGAGGTTGGACCAAATAATCTTTATTTACTTCTAGATTAAAAACCCATGATTGGTTGGGCCTGGCTCATGCCTATAATCCCAACAGTTTAGGAGGCTGAGGTGGGTGGATCA
Microcebus murinus  TAAAATGAAAAGAGTGTACCAAATAATATTTGTTTACTTGTAGCATAAAAATCC..................................................................

Homo sapiens        CTTGAGCTCAGGAGTTCGAGACCACCTGGGCAATAGGGCGAAACTCCATCTCTACAAAAAATACAAAATTTAACTGGGCATGGTGGTGGGCGCCTGTAATCACAGCTACTCGGGAGGCTG
```

```
Microcebus murinus  ....................................................................................................

Homo sapiens        TGGCAGGAGAATCACTTGAACCTGGGAGGTAGAGGTTGCAGTGAGCCAAGATTGCACCACTGCACTCCAGCCTGAGCGACAGAGCGAGACTCCATCTCGAAAAGAAAAAAAAATTAGCCA
Microcebus murinus  ....................................................................................................

Homo sapiens        GGTGTGGTGGCACACACCTGTGGTCCCAGCTACTCGGGAGGTTGAGATAGGAGGATCACTTGAGCCCGGGAGGCGGAGGTTACAATGAACTGAGATTGTGCCACTGCACTTCAATCTGGG
Microcebus murinus  ....................................................................................................

Homo sapiens        CAACAGAGTGAGACCTTGTCTCAAAAAATAACAATAATAATAATAACACTATGATTGACAGGTAAAGGGAGACTTAAATAAATGGAAGAATACATTTTCTTCATGAATAAGAATAGTCAG
Microcebus murinus  .........................................ATTGTTGACAGGTAAAGGGAGATTTGAGGAAATGGAAAAATACATCATGTT--------------TCAA

Homo sapiens        TATTGAAAGGTACTTTCTCCCTTGCTCTGG-CCTTTTCCCAGTTAAAATTAACCTGAAAGGATAAGCAGGGGAGAATATATATGAAAACTTTTTATAAGAAATACAACAACTGCTAGACT
Microcebus murinus  TATTGAAACTTTTTTTTCCCATTGCCCTGGACTTTTTTCTGATTAACATTAACCTGAAAAAATAAGTAGACAAAAATACATATGAAAATT-----TAAAAAATTATATAACTACTAGACT

Homo sapiens        TAAGGCATATGTTAAAACTTTTTGTAATTGACAGGGTATATGT-TTAAGAAAAATCAGAGATAGCCAGA----CTTGGTTGAAAGCATCTTTGATGGCTCAGTCCAAAAGAAATACCTAA
Microcebus murinus  TAACGCATATTTCAAAA-TTTTTATAATTGACAAGGTACTCATATAAAAAAAAATCAGAGATAGCCAGACTTTCTTGGTTCAAAACACCTTTGATGGCTCAGTTCAAAAGAAATACCTAA

Homo sapiens        AGTTAATATGTATTAACTAGTAGATAGGACATGTGCCATGGCACCCCAGGATTATTCCCAGTTTCCATAATTCTCTAGGAGGACTCAGCATATAGTCATACTCATGGCTAAGATTTAT--
Microcebus murinus  AGTT-ATATTTATTAATCAGT---TAGGACCGGTGTGAGGGGTCCTTAAGATTATTCTCAGGTTCCATGATTCGTCAGGAGGACTCAGTGTATAGAAATACTCTTGGCTAAGATTTATTC

Homo sapiens        ----------------------TCAGCAAAGAGAAAAGACACATTGGGA------------GAACCAGGTACAAGTTTCCAAGATCTCCTTCCCCAGTGGAGTTACACAGGACGTGTTTA
Microcebus murinus  AGTGAAATGATACAAAGCAAAATCAGCAAAGAGAAAAGGCTTATTGGGAAAAAACTAAGGGAAACCAGGTACAAGCTTCCATGATCTCCTTTCCCAGTGAAGTTACACAGGACATGTTTA

Homo sapiens        ATTCCTTTAGCA--GAGTTGTGACAACA-----------------TAAGGAAGCTCACTAGAGTCTCAGTGCCTAGGGTTTTTACTGGGCGCTGGTCACATAAGCACCTTCTACCTGGCA
Microcebus murinus  ATTCTCACAGCAGTGAGTTGTGACAATACATGAAATGTTGTCTACCAGGAAAGCTCATTAGAGGCTCAGTGCCTAGGGTTTTTATTGGAGGCTGGATATTTAAGCACTGTCCGCCTGGCA

Homo sapiens        TGTACAAAAATTCCAGACTCTCAGAAGGAAAGATTGTGTTTGGTCTTATAACCAAAATCTAAGCTCCTAGACATTAGCCAAGGTCCAACCTTGTAAGCATGCCTTTCAAAGA--ACAGCA
Microcebus murinus  TGTACCAAAATTCCAGACTCTGTGAAGGAAAGAAGGTGTTTGGCTTTCTAACCAAAACCTACATTCCTAGACACTAGCCAAAGGCCAACTTTGTAAGCAAGCCTGTCAAAGAATATAGCA

Homo sapiens        GTCACAGATCTGCTTTGTTAATATGTTAAAATTAACTGTCAGGTTAACTCTTTTCTTCACAGGTCCATCCAAACAACTTAATAAGTATTTATGTTCTAACCACTTAGTAGCAATTTAAAA
Microcebus murinus  GTCTCAAATCTGCTTTGTTAATCTGCTAGAGTTAAATG-------AACTCTTTTCTGCACAGGTTCATCCA---ACTTTAGTAAGTATTTATGTCCTGA....................

Homo sapiens        TACTCATAAATTGAAAGAAAATATATTTTTGTTCCATTCTTTTCTTTTTTTTTGAGACGGGGTCTCATTCTGTCACCCAGCCTGGAGTGCTGTGGCACCGTCTTGGCTCACTGCAACCTC
Microcebus murinus  ....................................................................................................

Homo sapiens        CACTTTCCAGGCTCAAGTGATACCCCCACCTTAGCCTTCTGGCTAATTTTTGTACCTTTTTTCTTTTAGAGACAGGTTTCACCATGTTGCCCAGGCTGGTTTTGAACTCCTGAACTCACA
Microcebus murinus  ....................................................................................................

Homo sapiens        TGATCCACCTGCCTCGACCTCCCAGAGTGCTGGAATTACTGGCGTGAGCTACCGCACCCAGCCCTTTCATTTCATTCTTAACCATAATTACTTGCTAATGGGTAATGTGTACCTGTTGAC
Microcebus murinus  ....................................................................................................

Homo sapiens        CACTGCTTAACTTCTCAGACCTTGGGATCATATTGTACACTGCCACCCTCATTTCCTGTTTCACACTGATTTTTACATAGTAACTGCTTTTTATCACAGCATTCGATGAAGAAAACAGTT
Microcebus murinus  ....................................................................................................

Homo sapiens        CTGTAAAGATATGATGTCATCAAACAAATTGTAACCTGATCTAATGTTGAAACTGAACTACCTTAAACTGGTAGTTTTCATGGTGTCAAACAGATGTTAAGGGGGCAACTTGGCACAAAG
Microcebus murinus  ....................................................................................................

Homo sapiens        TTTGGTAACAATGGTTTAAATGATAAAGCTAGTGTCACAAATGTGTCTAGTAGAGGAGGATCATGTAATTAATTAACAGGTAACAATTTGCTAATTGTTTAAAATTATATCTGTAATATG
Microcebus murinus  ....................................................................................................

Homo sapiens        ATTTTAGAATCCAATACCAAAAGTATTTTCAGGTAAATTAAAAGAATTTTAATTTTTAAAGTAGAAACAGAAAAAAATATATTTGACATTTAATCTCTAGAAATGGAGGTCACATTCTAA
Microcebus murinus  ....................................................................................................

Homo sapiens        ACCTAGGAATAGTGGTATAAACTTCAATGGAAAAACATTAAATTTGGCTGCATAATAATAGGAGATTTATGTGGTAAAGTAATCAACATATAAAAGCCAATCTTGCAAATAAAGCAAGAT
Microcebus murinus  ....................................................................................................

Homo sapiens        AATTTTGTAAATGAAGGCAAGGTAGTCTTGCCTGCCTTGATTGACATAGCAGTGGTCTGAGCAATGGAGTACGTAAAGGCCATCCAGTTTGGGGGAAGGCTATTGGAACTTCTATTTATA
Microcebus murinus  ....................................................................................................

Homo sapiens        TTTTTTAAATCCCAAAAATAAGAAATGAAGCTTTATTAATAATGTATACGATTGATAATAGCATTCTGACTCAATCAACAAGACATGTATGTCACACATGCTATGTGAAGGTATCCTGAA
Microcebus murinus  ....................................................................................................

Homo sapiens        AGAAAAGTGAGAATCCACATGTTTCCTCACCCATACTTTTGCTTTCAGCAGTTGAATGAAAGTATGGATATCATCTAGCATTCACCACACTACTCACAAAATGGCCAGGGAGCTTAGATT
Microcebus murinus  ....................................................................................................

Homo sapiens        TTTGCAGTGAAAGCAGATAATACTAAAAGGCAAACACCCATCATCAACCACAAGAAAGTGATAGCGCCGGCACGTCTATTTAACTTCCAGCTCTTTGTTAGACACATCAATGAGATGAAT
Microcebus murinus  ....................................................................................................

Homo sapiens        ACAAATATTAACTACAATAGTCCCCATTTATCTGCAGTTTTACTTTCTGAGGTTTCAGTTCCTATAGTCAACTGCAGTCCAAACATATTAAATACAAAATTTCAGAAATAAATGATTTAA
Microcebus murinus  ....................................................................................................

Homo sapiens        AAGTTTTTTAATTGCACACCATTCTGCGTATAGTGATAAAATCTCATGCTGTCCTGCTCTGTCCAACTGGGACGTGAATCATCTCTTTGTCTAGCATATCTTCATTGTATATGCTACTCT
Microcebus murinus  ....................................................................................................

Homo sapiens        CTCCCCATCATTAGTCACTTCATAGCTGTCTTGGTTATCAGATCAAATGTAGTGGTTAGTAGTGTGTATCCAAGTTACCCTTATTTTATTTAAGAATGCCCCCAAAGTGCAAGAGTAGTG
Microcebus murinus  ....................................................................................................

Homo sapiens        ATGTTGGCATATTGTTAATCGTCCTATTTTATTTTTAGTTGTTAACCTCTTACTGTGCCTAATTTAAAAATTAATCTCTATCATAGGTATGTATGTATAGGGGAAAACATAGTATAAATA
Microcebus murinus  ....................................................................................................

Homo sapiens        GGGTTAGGTACTACCTGCAGTATCAAACATTCACTAGGGGCCTTGAAACATATTCTCTGAAGGTAAAGGGAGACTATAGTTATCAGATCTGATAGGAAATTAGGCATCAAAATTCAAAAT
Microcebus murinus  ....................................................................................................

Homo sapiens        TATCAAGAATACTCTTTGAAATATCAGTTCACTTCCACTTTTATTGATGGAGAGCCTTACCCTAAGTGCATATAATGTTTCAAGGTATTTGCTAATGATAATATGAAGCCATTATGATTA
Microcebus murinus  ....................................................................................................

Homo sapiens        GCTAGCAGTTTTTCCAGTCGTGTTTAAAGTCATTTGATAACCAAACCAGGACTTTGAAAATTTTTCTAAAGGTAACAATAGGGCCAAGCATGGTGGCTCATACCTCAGCTTTTTGGGAGG
Microcebus murinus  ....................................................................................................

Homo sapiens        CTGAGGTGAGAGGATAACTTGAGACCAGGAGTTCAAGACCAGCCCATGCAATACAACAAGACTCCGTCTCTACAAAAAAAAAAAAAAATTTTTAATTAACTGGACGTGGTGTTGCACACTC
Microcebus murinus  ....................................................................................................

Homo sapiens        ATAGTCCCAGCAACTCAGGAGGCTAACGTGTAAGGATTGCTTAAGCCCAGGAGGTGAAGGCTGTAGTGAGCTATGGTTGCACCACTGCATTCCAGTTTGGGCAACAGTGAGACCCTGTCT
Microcebus murinus  ....................................................................................................

Homo sapiens        CAAAAAAATAATAATAATAATAATAATAGGCTGTGCTCAGTGGCTCATGCCTGTTGTAATCCTACCAGCCAGATTGCTTGAGCCTAGGAGATCGAGACTAGCCTAAGCAACATGGTGAAA
Microcebus murinus  ....................................................................................................

Homo sapiens        TGCCATCTCTACAAAAAATGCAACAATTAGCCAGGAGTGGTAGGGCACACCTGTAGTCCAGCTACTTGGGAGGCTGAGATGGGAAGATCGCTTGAGCCCAGGAGGTTGAGATTGCAGTGA
Microcebus murinus  ....................................................................................................

Homo sapiens        GCCAAGATCATGCACTCTAGCCTGGGTAACAGACTGAGACCCTGTCTCAAAAAAAAAAAATAAAAAATAAAGGGTAACAATAAATATTTGCAGGCCTATTTTGTGAGATTGCTTAAACAA
Microcebus murinus  .....................................................GTAACAATAAGTGTTTATAAGC-TCTTTTACGAGATTTCTTAATAAC

Homo sapiens        AAAACCAAAAAAGCGACATATGATTGGGAAAACTTGTTCTTTTTGCTGTGATTAAAAT----GGCTTCACTATTACACAGAAAGCATAACGATGACAGTACAAAATGAACTCTTTTGTCA
Microcebus murinus  AAAA---AAAAAGTCACATATGACTGGGAAAACTTGTTCTTTTTGCTGTGAGTGAAATAAATGGCTTAAATATTACACA--AAGCATAATGGTGACAAAATCAAATATATGCATTTGTCA

Homo sapiens        GCAAATACTGATGCAGAAAACACTGCTAAATATTATAACATAATACACAATGTGGGAGGTTTATTGTATAGTTGAATAAAAATACAGATGTTTCTAATGGCTTAGCTTAGAGTATTTGCT
Microcebus murinus  AGAAGTAC----ACAGAGAACACTGCTAAAGATTAGAGCATAATATACAATGTGAAAGGTTTATCATACAGTTGGATAAAAATACAGATATTTCTAA---CATAGCTTAGAGTATCGGCT

Homo sapiens        AAATTGTTCCAATAAAATGCACAAAGAACTTTTTGTAAACCTCTGAAGGAAAGAGAACAAACATATTCAGTAGGAAAATTAATTTTTTTAAATGTCTTACAGAAAACTAAAATTGTAAGA
Microcebus murinus  AATTTGTTTCAGTAAAGTGCACAAAGGCCTTTTTGTAATCCTCTGAAGGAAAGAAAAGAAACATATTCTGTGGGAAAATTACTTTTAAAAGATGTCTTATGGAAA..............

Homo sapiens        ACTGACGGAATATCTGGTTTGATGGTTGGGGGGCTGACAGAGTAAGGATGTCAAGATACCATTTCACAAATGGATTCCCTGGATCATTCCTAGGCAAGCTTTTGAAACAAAGAAGTTAAA
Microcebus murinus  ..........................................................................CA---TTTTGAAACAAAGAAGTTGAA

Homo sapiens        GCCTTTTTCAAGGGTCATTTCAAACCATTAATCAAGA-CTCAGATTGAAGCTGACCCAAATGACACCTATTCTTTGGGATTTCTTTCTAATT----TTGAATTAAAAATAGTCACCCTGA
Microcebus murinus  GTCTTTTCCAAGGGTTATTTAAAATCATTGATCAGCAGTTCATATTGAAGCTGACCCAAATGAC--CTTTTCTCCAGGATTTCTTTCTAATTCCTTTTGAATTAATGATAGGCAGCCTGA

Homo sapiens        ATTAGTATGTAT----------ATCAGCTAGGAAGTGTATTCAA-TCCAAGTAACAGACTTCCTTTCCCTATCTCAATATATTAATATATATTGTA-----TATAT-------------
Microcebus murinus  ATTTGTGTGTGTGTGTGTGTTCATCAGCTAGGAATTGTACTCAGCTACAAGTAACACAAGTCCTCTCCCTCCCCCAATATATAAACTCCAATTGGAATTTTTATGTTCTACTCAAGAAAA

Homo sapiens        ---------ATTATCCCAATATATATTGGGAG---------TGATTGAGAGTC---ACTCCTA------------TTGTATTCACAACGTG------------------------GCT
```

```
Microcebus murinus  GCAAGCCAGGTTGTCCAAAGCTGGAAGGGTAGCTCTAAATGTCGTTGGAAATCTATGCTCCTAGCTTTCTGTTCCACTGTCTTTATGATGTGACTCAATCTTGTGCCCAACTGTATAGCT

Homo sapiens        GCTGGAGCACATGCCACAATGCCTACATTTCAGTCAGAAAGAAGGGAGAGAGTGTTGGGCAAACAAAGCTAGTACCAGCTGAGTTTGCCTAACAACTTTTACTTACAACTTTTT------
Microcebus murinus  GCTAGCGCCCATGCCACAATGTATACATTTCAGTCAGAAAGAAGGGGAAGAGCAATGAGCAAACAAAGCTAGTACCAGCTAAGTATGTCCAACAACTTTCGTTTACTTTTTTTTTTTTT

Homo sapiens        -----GGCTAGAATTATGTCACATGACCACCCCTACCTGCAAGGGAGTTTGGGAAATGTAGTTTTCTTTTTATTGCTTTCCCCTCTTGTATTAACTTTGCCTATTTAGTTATTCTGATAC
Microcebus murinus  TTTTTGGCCAAAACTG---CACATGACCACCCTTACGTCCAAGGGAGCTTGGGAAATGTAGTTTTGTTTCT-----TATTTTCTCTCATATTACCTTCACCTGTTTAGTTATTCTGATAT

Homo sapiens        TACTCGTCTGAAATATTCTTTTTTAAAAAGACAAATTTATGAGCCAAAGGT---TTACTCAACTAAAAATAATAAGGAAAAAAAAAGTCTGAAAGGTGAGGTAGGAAAAAAATATTATTT
Microcebus murinus  TACTTGTCCT-AATATTCTTTT---AAAAGACAAATTTATCAGCCAAAAGTGTACTACTCTATTAAAAATAGTAAGAAAAAAA-----CTGCAGGTTGCGGTAGGAAAAA----------

Homo sapiens        TATATTTAATGGGTTTACTGTTAAAAGTTTTGTAAACTCA----------TTTTAAGTGCTACACAT-----AATATAGTAACACCAATAATAGTAGTTATAAGTTAAAAGTTATAAAAT
Microcebus murinus  TACATTTCACAGGCTTTCTATTAGAAGCTTTCTTAACTCATTTTGACTTCTTTTAGGTCCTGCATATTATAAGATATAAGAACAACAATAATACTAGC----AGTTAAAAGTTATAAAAC

Homo sapiens        GCTTATTGCCAATAACTGTTTTACATGCTTTTTAAACATTAACTTATGACCTAGTGGGTTGTTTTTGAGACACTGTCTACTGAATTAAAAAGTTACTTGAGGGCCGGGTACAGTGGCTCA
Microcebus murinus  ACTACTTGCCAAGAATTGTTTTAAGTGCTTTGTAAACACTAACTCATGATCTAGTG---TTTATTTGGAAGACTATCTACTAAGTTAAAAAGTTATTTGA....................

Homo sapiens        TGCCTGTAATCTCAAAACTTTGAAAGACTGAGGTGCATGGATTGCTTGAGTCCAGGAGTTTGAGACCAGCCTGGGCAACATGGTGAAACTCCATCTGTACAAAATATTTAAAAATTAGGT
Microcebus murinus  ........................................................................................................................

Homo sapiens        GGGCATGGTGACACATGCCTGTAGTCCAAGCTGCTAGGCAGGCTGAGGTGAGAGGATCGCTTGAACCCAGGTAGTGCAGGCTGCCCTCCAGCCTGTGCGACAGAGCAAGACCCTGTCTCC
Microcebus murinus  ........................................................................................................................

Homo sapiens        CACCGCCCCCCACCAAAAAAAAAGAAAAAAATTAAACTCAAAGTTAACGTTAAGTTCTGTTCTTCCATTTCCTTGTTGTATGTGTGTATTTGGCAAGGCACTTAATCTCTCAAAGCTTCAG
Microcebus murinus  ........................................................................................................................

Homo sapiens        TATCTTACCTTTAAAATGAAATTAATAATACCTCAAAAACTTGTAAGGTTTAATGACAATAATGTAAAGTGTCTAGTGTGATGGGACCAGTGTGTAGTAAAGACTCAATAAATGGCAATC
Microcebus murinus  ........................................................................................................................

Homo sapiens        ACTATTTTATACATAAAATTTTACATATTTTATGTATGTGTTCATATAGAATGTTATGGTCAATAAAAAAGCAAGATTCAGCCAGGCGCAGTGGCTCATGCCTGTAATCCCAGCACTTTG
Microcebus murinus  ........................................................................................................................

Homo sapiens        GGAGGCCAAGGCAGGTGGATCACAAGGTCAGGAGTTCAAGACTAGCCTGGCCAATATGGTGAAACCCCGTCTCTACTAGAAATACGAAAATTAGCCGGGTGTGGTGGCGCACACCTGTAG
Microcebus murinus  ........................................................................................................................

Homo sapiens        TCCCAGCTACTCAGGAAGCCGAGGCAGAAGAATTGCTTGAACCTGGGAGGCAGAGGTTGCAGTGAGCCGAGATTTTGCCACTGCACTCCAGCCTGGGTGACTGAGCAAGACTCCATCTCA
Microcebus murinus  ........................................................................................................................

Homo sapiens        AAAAAATAAATAGATAAATAAAAGGCAAGATTCTAATTCTTTTGAAGCTTTTTTTTAAGTCAATGGAAAAATAAAACACAAATTTTAAAAAGCCACCTTTGTTTTATATATTTTTCAGAT
Microcebus murinus  ........................GCAGGATTCCAATTCTTGTGAGGCCTTTTTTTAA-TCAGTGGAAAAATACAATGCAAGTTATAAAAAGCTAGCTTTATTTTATATTTTTTTCAGAT

Homo sapiens        CAATATTGAACTGTACAGATTGTTTGGCATAAAGCAAATTGTAATTTCTCACACCTCTGGAACTGTTAACCCAACGGAAAGTACACCTAACACAATCAAAGCATTTCTCTCCAAACACTG
Microcebus murinus  TAATGTTGAACTGTATGGATTGTTTGGCACAAAGCAAATTGTAGTTTCTCACACCTCTGGAACTGTTAAGCCAACTGAAAGTACACCTAGCACAGTCAAAACATTTCTCTCTAAACACTG

Homo sapiens        TGCTGACCACCCAAACAATTGGGATGATCACCTATCAGCTGTTTCATTTGCCTTCAATGTAACTCACTTGGTATGTGCCTTTTTATAATTCTGTACTTCTGAGTTATGACTATTTCTTGG
Microcebus murinus  TGCTGAATACCCAAACAATTGGGATGATCACCTATCAGCTGTTTCATTTGCCTTCAATGTAACTCACTTGGTATGTGCCTTTTACTAATTCTATCCTTCTGAGTTATGACTGTTTCTTGG

Homo sapiens        CCAGATAAATGTTTTATTACAAAATTGACGTTTATTTTCTTTAAAATAGGAACCTACTAAAAATACACCATATTTTCAAATGTTTAGTCGAAATCCTTATATGCCTGAGACTTCAGATAG
Microcebus murinus  CTAGATTACTGTCTTATTACAAAATTG----CTGTTTTCTTTAAAATAGGAGCCTACTAAAAATACACCATATTTTCAAATGTTTCATCGAAATCCTTATATGGCTGAGACTTCAGATAG

Homo sapiens        TCTTCATGAAGTGGATGGTGATAATACAAGTATGTTTGCCAAAATTCTAGATGCAATTAAAGAAGCTGATAAAATAATGGAGAATAAGACAACTTCACTGGGCCAGGTGATTCTATTCAA
Microcebus murinus  TCTTCATGAAGCTGATGGTGATAATACAAGTATGTTTACCAGAATTCTAGATGCAGTTAAAGAAGCTGATAAAATAATGGAAAGTAAGACAACTTCAGTGGGCCAGGTGATTCTATTTAA

Homo sapiens        TAGAAAAACTGTAATCTAAAACTTTATAAAAGTTATATTTATTTTTAAAATTTCAGCATATACATGTGTGCATGCACACACACAC---ACATACTTACTTGCCATAATTTTTTATCTATC
Microcebus murinus  CAGAAAAACTATAATCTAGAACTCTATAAA-GTGATACTTATTTTTAAAGTTTCAGCATATACACATGTGTATGCATGC-CACATGGTAGACCCTTAC--ACCAAAATTTTTTATCTATC

Homo sapiens        AGATTTTTCTTGTCTTGATTAAATAGTTGGTTTACTAGCTATGAAATGCTGAGAGGCAATAATGAGAAAGCTATGGATTTTGGAATCAAGTAGAGCTGGATTCAAATCTGTAGTCTTAAC
Microcebus murinus  ACATTTT----GTTTTGATTATGTAATATGCTTATTAGCAATAAAATTCTGAG--GCAACAATAAGAAAACCATTGATTTTGGAATCAAGTAGAGATGGATTCAGCTTGATAGTCTTGA-

Homo sapiens        TGCTTTTGAGTTGTTTGACTTCGGCAAATTACTTAACCTATCTTAGTTTACCTTCTATATAAAGTAGGAGCTAATAATACTGACTTTGAAA--TGTTTTAAGAATTAAATAGAATTCATA
Microcebus murinus  TGCTTATCCATAGTTTGACTTTAACAAATTACTTAACCTATGTTAGTTTGCCTTCTGTATATAGTAGGAG-TAATAATACTGACTTTGAATGTTGTTTTAAGAATTAAATGGGATTCATG

Homo sapiens        CCTACCTTTTAGTAGCATCTCA---AGTACCATTTTGTTACAATATTCTCATTATTTGCATTTACCTTCAAGATATTATAGCTCTTTCATCTCTTTGTTCTTTTTC-TTTTTACACTTGT
Microcebus murinus  CGTAACTTTTAGTAGCATTTCAGTAAATTACAATTATTTATAATATTATCATTATCTACATTTACCTTCAAGAAATTATAGCTCTTTCGTTCCTTTGTTTTTTTTTGTTTTTACACTTAT

Homo sapiens        AGATGGAGAACAACAATTTGGATGAACTAAATAAAAGCAAGATCATTGTTAAAAAGAAACCCAAACAATTAAATCCATTTCATTTAAAAGTGGGTCATGAAGTTTTAAGACAAAGGAAAA
Microcebus murinus  AGGTGGAAAACAACAATTTGGATGAACTAAATAAAAGCAAGATCATTGTTAAAAAGAAACCAAAGCAATTAAATCCTTTTCATTTAAAAGTGGGTCATGAAGTTTTAAGACAAAGGAAAA

Homo sapiens        ATTGGTGGAAGGATGGTCGTTTTCAGTCTGAATGGGTTGGTCCTTGTGTCATAGACTATATTACAGAAAGTGGATGTGCTGTCCTGAGAGACAACACTGGGGTTAGACTGAAAAGACCTA
Microcebus murinus  ATTGGTGGAAGGATGGTCGTTTCCAGTCTGAATGGGTTGGTCCTTGTGTCATAGACTATATTACAGAAAGTGGATGTGCTGTCCTGAGAGACAACACTGGGGCTAGACTTAAAAGACCTA

Homo sapiens        TCAAAATGTCCCACCTTAAGCCCTACATAAGAGAATCCAGTGAACAAGGTAAATATCTGTCACTTCTCTACATTTTTA-TTGCTTTAGGTAAGGAGAATAGTTAAAAATAACAGAAACCT
Microcebus murinus  TCAAAATGTCCCACCTTAAGCCCTATGTAAGAGAATCCAGTGAACAAGGTAAGTATCTGTCACTTCTCTACACTTTTACTTTCTTTGAATGAGTAGAGAACTTAAAAATAACAGAAACTT

Homo sapiens        TTCTCCCCTCTTTTGTTAGGTTTTCTTTTCCCTTCTTCATAAAATTTGTTACAGTTTTAGCTATCCCAGGTAGTCTGTTTTATTACAGTGTCTCTTCTGGAACTGTCAGGCCTGTGAGAT
Microcebus murinus  TCTTCTCCTCTTTGGTCAGATTTTCCTTTCTCATTTTTATAAAATTTGGTATAGTTTTAGATAACCCAGGTAGTCTTTTTT.......................................

Homo sapiens        TTTTTTTTTGAGACAGAGTTTCCCTCTTTCTGCCCAAGCTGGAGTGCAATGGCGCGATCTTGGCTCACTGCAACCTCTGCCTCCCGGGTTCAAGCAATTCTGCCTCAGCCTCCCGAGTAG
Microcebus murinus  ........................................................................................................................

Homo sapiens        CTGGGACTACAGGTGGATACCACCATGCCCAGCTAATTTTTTGTATTTTTAGTAGAAACGGGGTTTCACCATGTTAGCTAGGCTGGTCTTGAACTCCTGACCTCCCGTGACCTGCCCGCC
Microcebus murinus  ........................................................................................................................

Homo sapiens        TTGACTTCCCAAAGTGCTTGGATTACAGGCATGAAACAGGGTGCCTGGGCATTTTTATTTTTATTTCTAGTATG-TTTTTCATAGTTTATGTGGGAGATACTGAACTTGTTTTCCTCTGA
Microcebus murinus  ..................................................ATTTTTATTTTGATTTCTAGTGTGCTTTTTCATAGTTCACATGGGAAATACTGAACCTG-TATCTACTAA

Homo sapiens        AGGAAAGATAACATCTTAACATCATCTCTGTTTTTGCTTTCTCAATGTTTTGTTTGTTTGTTTGTTTGAGACAGAGTCTCATTCTGTCCCCCAGGCTGGAGTGTAGTTGTGTAATCTCAG
Microcebus murinus  AGGAAGGATTATGTCT--------TTTCTATTTTTGCTTTCACAATG.........................................................................

Homo sapiens        CTCACTGCAACTTCTGCTTCCTGGGTTCAAGCAATTCTTGTGCCTCAGCCTCCCAAGTAGCTGGGATTATAGGCACATGCCACCATGCCCAGCTAATTTTTTTGTATTTTTGGTAGAGAT
Microcebus murinus  ........................................................................................................................

Homo sapiens        GAGGTTTCACCATGTTGGCCAGGCTGGTTTTGAACTCTTGGCCTCTAGGGATTTGCCTGCCTTGGCCTCCCAAAGTGCTGGGATTGCGTGCATGTGCCATTGCACCTGGCCTTACAAATG
Microcebus murinus  ........................................................................................................................

Homo sapiens        TATTAGTCACTTCGTGCCTCTTTTTGGCATGAAAAACAGCAACTGTGATC-------ATTAATGCCAAAAACAAACATACTGTCCAGAATCTAGTTGTGAGATAAATAT--TAGGGTACT
Microcebus murinus  ..TTAGTCATTTTGTGCCTCATTTTAGCATGAAAGACAGCATCTAAGATCCCCTATCACTAATGCCAAAACCAAACATACTACCCAGAATCCAGTTGTGAGATAAATATATTAAGAAACT

Homo sapiens        TTAAAAATGACATTAATTTTTAATATTGCTTTATAGTTAGGTCATGGTTGGGGGCCAGGTGTGGGTGCAGAGTGGGGAATCCTCAGAATGCTATAAGGTGCTGTAAGAAGAGGTGACAGC
Microcebus murinus  T--AAAATATCATTAATTTTTGATGTTGCTTTATAGTTAGGTCATGGGTAGGGAATAGGGGAGGATACAGAGTAGTATATACTCAGAACGCTGTAAATTGCTTTAGAAGAA--------C

Homo sapiens        ACTCTAAACAAGTTATATTTGCCCCTAAATTTGACTCATTCTGAACAGTCAAGATACC----------------------CTAGTAAAGTGATGTTTTTGCCATGGCTTTTAGATTATGT
Microcebus murinus  ACTCTAGACAGGTTATACTTGCCCCAAAGTATGATTGGTTCTGACCGGTCAGGATCCTAATCTCTCTGCCCTTTCATAGGTTAGTTAAATGACATTTTTGCCATGGCTTTAAG------T

Homo sapiens        TCCCCACTTTATCAAGGCT--TTTATTTTGTCCTAATGTGTCTGCTTCCTAGATCTTCTCTGAGGTGGATAACTGAGAGAGAGTTAATGAGTAACTTAGCCAG----------------
Microcebus murinus  CCCCCACTCTATCAAGACTGTTTTATTTTATCCTAATGTATTTGCTCCCTAGAGCTTCCCTGAAGTGAATAACTAAGAGGGAGTTAATTGGTACCTCAGTTAATTATGCTTTTTCTATAA

Homo sapiens        -----GTGAAACATTAATATTATTTGGATATAAGTAACATTTAATGGAAAATA---TATTTATAA-CAATATAAACAAGTTATATGACAGGCTTTAGCAAATATTAATAACAAAAAGAGT
Microcebus murinus  AAAACATGAAACATTAATCTTATCTGAATATGAATAACATTTAATGGAAGATAGTGTATTTTTAAACAGCACAATTAAGTTATTTAACAGGCTTTAGTAAATA------ACAACAAGACC

Homo sapiens        AACAACAACAAATAACCTACACTTACCCTATGCCTTTTCTTGAGTTCTAAAGCCCATGTGGGCTGCTTAGTAATGATAGCTCAACTCTGCTTCTAATCTGGGTTACAACACTCATGTTAA
Microcebus murinus  AA-AACAACAAATAACCTACAGTTACCCTGTACTTTTTTTAGA--TATGAAGCCCTTGGGGGTTGCTCAGTAATAATAGCTCAACTCTGCTTCTGATCTGGACCACATCTCCTGTGTTAA

Homo sapiens        AACCTCTTAGTACCTTTCCTTCTTGTGACAGACCGAGTTGTT-TAGTCAGTAAACTTATCTTGAACTACATTCTAATCCTGAAAACTCACAAGTGCTTTCGTTCTCTTTGTTAGAAGATA
Microcebus murinus  AAGCTCTTAGTGCCTTTCCTTTTTGTGACAGACCCAATTGCTCTTATCAGTAAACTTATCTTGTATTATGTTCTAATTCTGAAAACTCACAAGTACTTTCATTCTCTTTGTTAGAAGATA

Homo sapiens        AAAGCTTGCATTGTTTGTACCTCTTCTCAGTTCTGAGCTACCTTTAATGATCTCTCTCTTCTGCAAGTTAAGTAGGGAAAAAAAAGGTTATACAAGTATATGTTGTTCTAGCTTCTTTAG
Microcebus murinus  AATGCTTTCATTCCTTGTGCCTCTTCTTATTTCTGAGTTACCTTTAAAGATCCTTCCCTTCCACAAATTAAGTAGG---AAAAAAGATTATACAAGTACAT------------------

Homo sapiens        CATGATTTTCCCCTTAATCTTTGACATTCATGATTTGCTCTCTTCTAAAGTGTTAAGCTTCTTTAATACAAAATTTA-ATTTAAAATTTTGCTAAACCAGGATCTAT--CAACATTTTGT
Microcebus murinus  ----------TCCTTAAT--------------------------------------------TAATACAAAATTTACATTTAAAATGTTGATATACTAAGATATGTTTCAACATTTC--

Homo sapiens        AATTTGTTCTTGTCAAAGAGTTTCTCAGAAAAGAAGTCAAATATTGTGCTTCTTCTGAACA-TTCAGAACTCAGTATTTCAGAACTCATCTTCTCTGTTTGAGAACAGTCTCTAAACAGA
```

```
Microcebus murinus  AGTTTGCTCTTGCCAAAGACTTTCTTAGAAAGGAGATCAGATATTTTGCTTCTTTTATATAGTTCAGAACCCAATT-----GAA---------------AAGGACAGTTTCTAAACAGA

Homo sapiens        GGCAGTATAATATTTGCCTCATACTTGAATTCTTTGCACAAGATTCTTCCAGTAATATAAGGACAAATAATATTGCAATATGACTACTGG-----------------------------
Microcebus murinus  GGAAGTATAATTTTTTCCTCATGCTTGAATTCTCTGCAGAAGTTTTTTTCAGTAATATAAGGAAA-------TTGCAGTATAACTAAAGGAAATATTCACTGAAGGAAGGATTATATCTT

Homo sapiens        ---------TGTATGC----ATAAGGAATTTAAGACTAATTAGATGAGTCTGTTTTAGTTTTTCTTGTTAACTAAAGGAACTACTGATCTGGCCATCCTAGAATCTGGTTCTT----ATT
Microcebus murinus  TTCTATTTTTGTTTTCTAAAATGAGGAACCTGAGATTGATCAG-TGAATTTGTTTTAGTTTTCCTTGTTAACCAAAGGAAGTATGGATCTGGCCATCCGAGAATCTAGTTCTTGATTACT

Homo sapiens        GCTTGATCCTTTTTTTACATTTTTCTCATTTGTAGAAATTAAAAGTAAATGTGATTTCTGCCATCTTTTTCCTCAATATTACATACTTTCTTGAAAATCTTTTCCATGATTTTCTTGAAA
Microcebus murinus  ATTTGGTCCTTTATT-GTGTTTCTCTCATTTGGATAAATTTAAAATAAGTGTGATTTCTGCCATCTTTTAGCTCAGTATCACATATTTTCCTAAAAATT-------------------

Homo sapiens        ATCACCTCACAAGTTGATTTGAAAGAGTCCTTGAGCTGAATTAA-AGCAAACAAAATGATTTTGACTAATCATTGAA-GTTTTTATTTGACATAAATTTTGACATGCTATAGCCAAAAGA
Microcebus murinus  GTCACCTAACAACCTGATTTTAAAGTGTCCTTGAGCTGAATTTATGGCAAACAAAGTGTCTTTGAATAATCATCCAACTTTTTTATTTGACACAAATTTGGATAAGCTGTATCCAA----

Homo sapiens        ACTATAAATTTATTTGTCCACATTTGTCATTGCTGCTCTTTGCTAGCCTTGAAATTTCATAGCTCTGTTAAAAACTCACTGTCTAAAGTAGATATACTTCTATTGCCAACTTCTTATCCA
Microcebus murinus  -CTATAAATGTATCTGTCAATACTTGTCATTGCTGCTCTTCACCAGCCCTGAGAATTCAGAACTATATTAAGGACTCACTATCTAAAGCAGATATACTTACGTTGCCAACTTCTTATTCA

Homo sapiens        TTCTTATCTAGAACTAAATGTAGAGTGAGCACATTCAGTACTAGGTATACAATATTTTTCATTGCTATTTTAAGCAGGCCTGTTTGTTTTTTTGTCTAGTTCATTGCCAAGCGCTTTTTC
Microcebus murinus  TTCTTATCTAGAATACAATGTACAGAGAGCACATTCAGTACGAAGTCTACAATATTTGTTATTGCTATTTTAAGCAGGCCTATTTGTTATTTTGTGTAGTTCAATGCTAGGCACTTTTTC

Homo sapiens        AGACTATTTAG---ACCAAGAAATCTTAAA--ATACCATAGTGTCTTTTTGTAATCATAATGCTCCTCTTTGAAAGGAAAAAATA--------------------ATCTGACATAGATT
Microcebus murinus  AGACCAGTTAGTTTAGTAGGAAATTTTAAGATATACCATAGTGTCTCTTTGTAATCATAATGTTCCCCCCTTAAAAATAAAAATGATGTTATTGACCCATTATTTTATCCAACGTAAATT

Homo sapiens        TTGCACAGTTTTGAATTTATTTTGTTTGTGATAGATTTGGATCTGCCTCAC-CCAAGGTTTTAGCCTGATTTCCCTTCACAGTTGAAAGCCC-CATTTGTGAAACCATTTTCATTATTGT
Microcebus murinus  GTGAGTAGTTTTTAATTTATTTTGTTT-TGATAGATGCGGGTCAGCCATACACAAAAGTTTTAGTCTGATTTCTCTTCCCAGTTGAAAGCCCACATTTGTGAAATGATTTGCATTATTGT

Homo sapiens        ATAAGTTTCCTCTTGTTGCTGTAACAAATTACTACAGATTTAATGGTTTAAAACAACACACATTTATTATATTACAATTCTGAAGGTTAGAAGTTCAACATAGATCTTACTG--------
Microcebus murinus  ATAAGTATCCTGTTGCTGCTATAA-AAATTGCTAAAAACTTAATGGCTTAAAATAACACAAATTTATTATCTTATAGTTATCTAGGTTAGCAGTCCAAAATAGATCTTACTGGGCTAAAA

Homo sapiens        -GGCTAAAATCAAGGTATTGGCACTGTTGTGTTCCTGTCTGGAAGCTCTAGAAAATAATCCATTTTCTTGTCTTTTCCAGCTTCTAGAGGTTACCTTCATTTCTTGGCTTGTGGTCCCTC
Microcebus murinus  TGGCTAAAATCAAGTCATTGGCAGGGTTGTATTCCTATCCAGGTGATTTTTTAAAT--CTAATTTTTTAATCCTT.............................................

Homo sapiens        TATCTTCAAAGCCAACAATAGTGGGTTGAGTCCTTCTTACATCACCTTACTCCAACCTCCTCTTCTGCCTCCCTCTTCTATATTTGAGGACCCTTGACCTTGGCCCTGCCCAAAAAGTCC
Microcebus murinus  ........................................................................................................................

Homo sapiens        AGGATAATCCCCCTATTTTAAGGTCATCTGATTAGCAACCTTAATTCCATCACTACCTAAATTCTCCTTCACCATGTAAGATTACATATTCACAGGATCCAAGGAGTAGTATGTTGACAT
Microcebus murinus  ........................................................................................................................

Homo sapiens        CTTTAGGGGCCTATTATGCTGCCTACCACAATTATGGTCTGCATTCATGAAACAAAGCAAAGCACAGTCCCTATAGTGTCAAGACCATGATCTTGACGTTAATATAGTTTATGCAGAGAG
Microcebus murinus  ........................................................................................................................

Homo sapiens        CCTTTTTTTGGCCTCTTAGTTATATCTTTTTTTTTTCTTTCTCTTTTTTCCTTTTTTTTTTTTTTTTTTTTTTTTGAGACAGGTTCTTACATGGTTGCCCGTGCTGGAGTGCAGTTGCAC
Microcebus murinus  ........................................................................................................................

Homo sapiens        AATAATAGCTCACTGCAGCCTTGACTTCCTGGGCTCAAGCAATCTTCCCACATCAGCCTCCCAAGTAGCTTGGAATGTAGGCATGCATCACCATGCCTGGCTGAGTTTTTCGTTTTTTAT
Microcebus murinus  ........................................................................................................................

Homo sapiens        AGAGACAGGGTCTTACTATGTTGCCCAGGTTGGTCTCAAACTCCTGGACTCAAGCAGTCCTCTCACCTAGACCTCCCAAGTGTTGGAATTACAAGTGTGAGCCATTGTGCCCGTCCAGTT
Microcebus murinus  ........................................................................................................................

Homo sapiens        ATATAATTTCTTAACCAATAGTTACTCAGTTATCTGTTGACAGTATTAGTTATTATAATGACAGTGATTTAGTATCAAGTTATAGTTATTATTTTATCTTGAGAAAGGATATACTAGTAT
Microcebus murinus  ........................................................................................................................

Homo sapiens        GGCAGCACATTCTAATAGTTTGAGGATTATCAAACCATATATAGGATTAGTTTAGTCACTTCTCTTTAAAATAAGAAAATTAAGTAGTCTGAAACCTTTATCTTTTTTTTTCTGATTGAA
Microcebus murinus  ........................................................................................................................

Homo sapiens        GAAATACATTAATCTAAATCAGACTTTTTTGAAGCTGTAATTTGTATTTATTTTTCTCAGGGAAATTTAAAAATTGATTTTTTAATTATTATTTTTATTCCTTAAAACAACATTAAGGCT
Microcebus murinus  ........................................................................................................................

Homo sapiens        GGGCACAGTGGCTTATGCCTGTAATCCCAGCATTTTGAGAGGCCGAGGTAGGATTGCCTGAGCTCAGGAGTTCGAGACCAGCATGGGCAACATAGTGAAACCCCGTCCCTACAAAAATTA
Microcebus murinus  ........................................................................................................................

Homo sapiens        GCCGGGCATGGTGATACGCACCTTTAGTTTTAGCTACTTAGCGGGCTGAGGCAGAGGATCACTTGAGCCCGGGAGACTGAGCTGCAATGAGCCAACATTGCACCGCTGCACTCCAGCCTG
Microcebus murinus  ........................................................................................................................

Homo sapiens        AGTGACAGAGTGAGACCCCATCTTTTTTTTTTTTTGAGATGGAGTTCAACTATTATCACACAGGCTGGAGTGCAATGGTGTGATCTTGGCTGCCTGCAACCTCCGCCTCCTGGGTTCAAG
Microcebus murinus  ........................................................................................................................

Homo sapiens        CAATTCTCCTACCTCGGCCTCCTGAGTAGCTGGGATTACAGGCGCCCACCTCCACACCCAGCTAATATTTGTATTTTTAGTAGAGACGGGGTTTCACCATGTTGGCCAGGCTGGTCTCAA
Microcebus murinus  ........................................................................................................................

Homo sapiens        ACTCCTGACATCAAGTGATCCACCCGCCTTGGCCTCCCAAAGTACTGGGATTACAGGCGTGAGCCACCATGCCTGGCTGAGACCCCTGGGATTATAGGCGTGAGCCACCATGCCTGGCTG
Microcebus murinus  ........................................................................................................................

Homo sapiens        AGACCCCATCTCTTTAAAAAAAAAACCTACATTAG-AAATGGATATCAAAACTGCTGATTAATACCTTCCTGAAATGTCTTGAAAATATTTAATTTCAAAGAAACTAAAGACT---TAT
Microcebus murinus  ......................AAAATTACATAAGGAAAAGTATAGCCAAACTGCTG-TTAACACCATGTTGAAATGTCTTGAAAATACCCCAGTTTGAAAGAAACTCAAGACTCGCTAT

Homo sapiens        TCAGTCTTGTCAAAACCAAAATGATGCTTTCATCAAACAGATGTCATAATCATAACTAAATTAATGGAATGAGAAAAGATAAATACTTAGTGAAATGTACTGTGTAATGTAGCTGACTAG
Microcebus murinus  TTAGTTTGGTCAAAACTAAAATGATGCTTTCATCAAACAGATGTCATA-TCATAACTGAATTAATAGAATGAGAAAAGAAAAATACTTATCAAAGTCTTTTGTATAATGTAACTGAGTAG

Homo sapiens        AAAAGACCTTTAATATGATATGTTTGCT---------------------TACTCCCCG---------CCTCTGTTTTGAGGGCAGAAAGTTTAATCATATTCTTTTCTGTATCCCTAA
Microcebus murinus  AAAAGTCCTTTAATGTGGTATGTTAGATATTCTCTCTCTTTCTGTCTCTCTCCTCCCCGACCCCCTCCTCCTTTGTTTTGAAGGCAAAAACTT----CATGTTTTTTT-TATATCCCTAA

Homo sapiens        CTCATGATTGTTTTAGATTAGTGTTGTCCAATAGATGAATAATA-------ATACAAGAGCTATGTAATTTAAAATTTTCTAATAGCCACATTTTGAAAA-GTACAGAAATAAATGAAAA
Microcebus murinus  CTCTGAATTGATCTAGATCGGTATTGTCCAATTGATAAGTATTACAAAACTATGTAA----TATGTAATTTTAAATTTTCTGGTAGCCACATTTTAAAAATACAAATAAATAGGTGAAAA

Homo sapiens        TAACCTTAATAATATATTTTATATAATCCAGTGTATCCAAAATATTACTTGTTCAACATGTA----AGGCCACAAGAGTCTCTTTTCTGATC-TCTTTACTGAAATTAAAGAGCATTTCT
Microcebus murinus  TGATTTTAATAATATATTTTCTATAACCTAATATATCCAAAATATTATTAGTTCAACATATATCTAAAGCCCTAAGAGTTTCTTTTCTGATAATCTTTATTGAAATTAAAGAGCATTTCT

Homo sapiens        CTTAGCTCCAGTAGATTTAGACCATAGTAGGAGTTGAAACATATTTGACCTTTGCAAATGAATGAGAATTTTTTTTGT---AAACAAACAGGCCTTTGACATATTCAGAGTGGTTTTCTA
Microcebus murinus  ATTGGCTTCAG---ATTTAGACCATAATAGGAATTGAAACATATTTGACCTTTGTGAATGAATCAGAATTTTTTTTTTTTCTGCACGAACGGGCCTTTGACAGCTTCAGAGTAGTTTTCCA

Homo sapiens        T-GTGTATAAAGTGATTTATGTAGAATTTGCTGTGTGTAGTCCTTGTTCTCTACAGTTTTATTACAATAAGTTACTTTAAAGGTTAACTTTT-TTTAGAAGAAAAAGAGTGTGTGTGTGT
Microcebus murinus  TTATGTGTAAAATAATTTGTGTGGAATTTGCCGAGTATAATCCTTGTTCACTATGGTTTGATTATAATAAGTTACTTTCAATGTTATCATTTATTTAAAAGAAAAA-------------

Homo sapiens        GTGTGTGTGTGTGTTCGTGTGTGACTTATTTTACTTACATACAGAAGCTCTC---TTAAAATCAAGTGAGGTTGGTAT-GTCCATTTGTTTCTAACCAGTGTAACTAGGAGGTACTCAAG
Microcebus murinus  ---TGTGTGTATATTT--------CTTATATTACTTACACATATAAGCTCTCCTTTTATAAGCAGGTGAGGTTGGTAAAGTACATTTGTTTCTAAGTCCAGTAACTAGGAGGTGCTTAAG

Homo sapiens        TTCAAGGTCTCTGAATCCATTTC-AGCAAATTAAAATGAAAATTTCTAAGAGCTTTACATCTAGGCATATTTTTCCCTATTGACATATGTTTACTAGACATATTTTTCTAAAGTAGTCCT
Microcebus murinus  TTCAAA-TATCTGAATCAATTTCCAGGAAGTCAAAGTGGTAACTTTTAAGAGCTATACATATAAGTATATTTTTCCCTTTTGG--TATGTTTACAGGACATATTTTTCTAAAATAGACCT

Homo sapiens        ACTTCATCTGTATGAAAAAACTTTTACAGACAGAAAACCTCCTTCATTA--ATCCTCTGAAAGAGTCTTTGGGCATTTCTTAAATGCCTATGGTAACTGCCATCACTGATAACTTTGTAG
Microcebus murinus  GCTTCATCTATATGGAAAAAAACTTTCCAACAAATAACCTACTTCATCACAGATCTCTGAAAGTGTCTTTGGAAATTCCTTAAACGC-TGTGGTATCTGCTGTCACTAATGACTTTGCAG

Homo sapiens        GAATGTACAGGCCTTGAACCTATGGAATTCTCCATGACTTAGTAGAAAATTTCATCAGCAACATGTTTACCCTATCTAACCTTTTAATGTTCTCTTATAAACTACCGCCTAGCCTTCTCT
Microcebus murinus  CAGTGTGTAGGCCTTAAAC-TATAGAATTCCCCATGGCTTATTATAAA-TTTCATCAGCAGCACTTTTACCATACCTGACATTTTAAGTTCTCTTGTAAAGTACTACCTAGCCTTCTCC

Homo sapiens        CGTATTAACAGTAGGGTAGTTGCAAACTTTGATGTTAAACCTCTAGCAACACATTCTGAAAGTGTTTCAATGTTATTGATCATGTTTTCTTCCCTCTTGCTGCTATTGGGTATAACACAT
Microcebus murinus  AGTATTAACAGTAGGCTAATAGCAAACTTTGATGTTAAACCTCTAGCAGCACACTCTGACAGTGTTTTAATATCATTGATGATTTTCTCTTCCCTCTTGCTGCTATTGGGTATAACGTTT

Homo sapiens        CTCATGTGTTGCATGATTTTCGTTGTTGTTGTTGAGACAGAGTCTCGCTCTGTCACCTAGGCTGGAGTGCAATGGTGCGATCTCGCCTTACTGCAACCTCCACCTCCTGGGTTCAAGCAA
Microcebus murinus  ATCATGGATTGCATGATTTATTTTTTTGT...........................................................................................

Homo sapiens        TTCTCTCACCTAAGCCTCCCCAGTAGCTGGGATTACAGGCACATGCCACCATGCCTGACTAATTTATGTATTTTTAGTAGAGATGGGGTTTCACCGTGTTAGCCAGGTTGGTCTCGAACT
Microcebus murinus  ........................................................................................................................

Homo sapiens        CCTAACCTTAAGTGATCTGCCCGCATTGGTTTCCCAAAGTGCTGGCATTACAGGCATGAGCCACTGCCTGGCTGCATGATTTTTATTTTATTTTATTTTTTTTTGTCTTTCAGAATTATG
Microcebus murinus  .....................................................................................................................ATG

Homo sapiens        GGAGAA--TAAATGGCCATACAAGCCCAAGCCCACTTATGCCATCTTTTGGTAGAGAGAATAGCTTTGTGTGATCTGTTAAAGTTTATAAATTGAAAGTTGTTAAATGCATGAATTTCTG
Microcebus murinus  GGCAAAACTAAATGGCCATA-AAGCCTAAGCCCACTTATGCCATCTTTTAATAGAGAGAATAGTTTTGTGTGATCTATTGAAGTTCACAAATTTAAAGTTGTTAAATAAATAAATTTCTG

Homo sapiens        GGGTTGAAATAGGTTCAGTGAGCTATTTGTAATAATGTTATGAGGGTGAGTACAGTAGGATTGATTTTCAAAATATACTACCAAATTCATCTGTTCTGAGACACTTCCATTGGTCTCTAA
```

```
Microcebus murinus  GGATTGAAATAGTCTCAATGAGCTATTCAT----ATGTTAATATAATGAATAAAGTGGGATCATTTTCCAGGATATACTAGCAAATTTATGAGCTCTGAAA-ACTTTCGTTGCTGTCTAA

Homo sapiens        TCTAAATAGGCAATTAATCTTCCTTTTGCTTTTTTGGTCTTAAAATTTGAGGGCTCCTTAACCCATTAACATCTTCAGGAGACCAACCTAGGCAACACAATTAGCCAGGCATAGTGACAT
Microcebus murinus  TCTAAATGGGCAATTTATCTTCCTTTTGCTT.........................................................................................

Homo sapiens        GTGTCTATGGTCCCAGCTATTTGGGAGGCTGAGGTGGGAGGATCACTTGAGCCCAGAAGGTCAAGGATGCAGTCAGCTGTTATCACGCCATTGCACTCCAGCCTGGGTGACAGAGCAAGA
Microcebus murinus  ........................................................................................................................

Homo sapiens        CCATGAATGAATGAATGAGTGAATGAATGAATGAATGAATGAATGAAAAATAAGTAAATAAAGATAAATAAATATATCTATATCTATATTCTGATGTTTTGGGGGAACATTTAGAAAGAG
Microcebus murinus  ........................................................................................................................

Homo sapiens        AAGGTAGGGATCCAGGAAGTTAATCAGCTTTGGTAAAAGAGTTTCTTATTGTAATGAATTCAAATATCAGAAAGGGAGGAATCCTTTTATACATATTAGTCTGTGTATAGAAGATTTGCC
Microcebus murinus  ........................................................................................................................

Homo sapiens        ATCAGTTTAACATTCATTTTAAACATTTTTTTCTTGATGGTGATGATAAATTTGCTCAACATACAATAAAACACTGACCAGGAAGACTCCCTGTCAGTACTTCAGTATGGCTTAGGTAGA
Microcebus murinus  ........................................................................................................................

Homo sapiens        CCTTAAAAGTTCCTAAGAATTTTGGTATCAAATTAGATTTAAATAAGCTAAGCTTTTTAAGCTACTCCATGGCAGCCTGCCTTCTTACACATTATCACATAAGCCTCAGTTGGCATGTTG
Microcebus murinus  ........................................................................................................................

Homo sapiens        TTTTTAACTTCATACTGTAACAATTTGATGATATTATCTTTCCAATATGGGTTTTATAAAGATTGGAAGATTATCTCAAGTTGTAAATGCAAGGATTTAATAATTCTATTTGTTAAAATA
Microcebus murinus  ........................................................................................................................

Homo sapiens        ATTATATGACCCTATTAATTCACCCAGAAATGCTTTACAGAAGCATATTAAGGATAACATTATCAAAAATAGTTTATAATTTTTTTTTTAAGACAGGGTTTTGCTCTGTCACCCATGCT
Microcebus murinus  ........................................................................................................................

Homo sapiens        GGAGTGCAGTGGTGCCATCATGGCTCACTGCAACCTCAATCTCCCAGGCTCAAGTGATCCTTCCATCTCAGCCTCCTAAGTAGCTAGGACCATAGACACCCTTAACCACACCTTGCTAAT
Microcebus murinus  ........................................................................................................................

Homo sapiens        TGTTTGTATTTTTTGTAGAGAAGGGGTTTCGCCATGTTGGCCAAGCTGATCTTGAACTCCTGGGCTTAAGCAATCTACCGGCTTTGGCTTTCCAAAGTGCTGAGATTACAGGCATGAGGC
Microcebus murinus  ........................................................................................................................

Homo sapiens        ACTGTGCACAGCCTTATTATTTTATTTTTAATCTATACCATAATTCTCACTCCTTCTATCCCGTTGTCCGCATATATACATATCTACCAAAGACAGGTTGAGGGAACAGGAAAATGGCTG
Microcebus murinus  ........................................................................................................................

Homo sapiens        TTTTAAAATCCTTGACCTTTCCTGCATTGTTTATGTATGAACTGTCAAATTTATATGTCATTTTTTTATTCTTTGACATTCATATTTAGTAGACAGTATAACTCCCTAGAAGTTAATCTT
Microcebus murinus  ........................................................................................................................

Homo sapiens        GATAGTTAATTTTTATTACTTGGGATTTTGTTCCTCACTTAAAATTGTTTAATTAAAAAATATGTTTAATTATAATTCTAAAAGTCAAATGTATGATTTTTCTTTTTTCAGAAAGTCTTT
Microcebus murinus  ...............................................................................................................ACAGTCTTT

Homo sapiens        ATCTCTTGCAAGGTTCAGTAGTGGCAGATCATGACTACATTGGATTGCCTGAAATTCCGATTGGAGCATATCAAGCAAATATTCTGGTGGAAGATGCAACTATTGGTATAGTCGATAATG
Microcebus murinus  ATCTGTTTCAAGGTTCAATAGTGGCAGATCGTGACTATATCGGATTGGCTGGCATTCCAGTTGGAGCGTACCAAGCAAATATTCTGGTGGAAGATGCAACTATTGTTATAGTTGATAATG

Homo sapiens        AATTACTGACATCAAGCAAGGATCGTGAACTATTAGAATATAGAAATACGAAAATCTCTCCATTGATAGACGATCATAGTTCTCTTGAAAAGCAGACTTTCAGTCTGTTGGACTCTTCAA
Microcebus murinus  AATTGCTGACATCAAGCAAAGATTGTGAACTATTAGAATATAGAAATGCCAGAATCTCTCCATTGATAGAATATCATGGTAGTCTTTAAAAACAGGTGTTCAGTCTGTTGGACTCTTCAA

Homo sapiens        ACCAGGTTCTTGAATACTTAAGTTAGTAAATACCAAAATTTATTTAAATTGTTTGTTTAGAA--TATAAATTCTTAA----TGATATCTTATTCATTAAAAAC-GTTGTATAGAAGAAGT
Microcebus murinus  ACCAAGTTCTTGAGTACTTAAGTTAGTAAATACCAAAATCCATTTAAAGTGCTTGTTTAGAATGTATAAACCCTTAAATCTTGATATATTATTCATTAAAAAAGGTTGTCTAGAA.....

Homo sapiens        ATCTTGACACATTCTTAATGACTATATTCTGAAGGTTTATTTTATAGTTCTTATCTAAAATTATGCCACATTTGAATATCAGTATACGTACATATGAAGGCAAAGGAAGCATGTAATATA
Microcebus murinus  ........................................................................................................................

Homo sapiens        TAGAATTCACTTTTTCTTCTAAAAAGAGAACAGGCAAAGCACCCCTACTTCTTGGGAAATAAATTCCAGCCCAAATCTCTCAAACCTTTGTATGGTTTTGGCACCGTTTCTAAGTTTTAA
Microcebus murinus  ........................................................................................................................

Homo sapiens        ATTTTCTGAAATTCTCTACCATTTAAACAGTAAGTTTGAACTACCTTGAATGGAATTGATAAATACTGAATCAACTGTAACAATGAATGAAATGAAGCTTCTTTCTGTTTCATTAGAACT
Microcebus murinus  ........................................................................................................................

Homo sapiens        TCTAAAAACAAGATTGTATGAAAAAATATAGGGTTATTTAATAGGCTTTCACAGAATATTGGTAATTTCAAATTATCTAATTGGTTAGATAGGGTCAGGATTAGGGTCTGAAACTATGTA
Microcebus murinus  ........................................................................................................................

Homo sapiens        TTGGTTGGGAAACAATTTGTTTATTTTCATACTATATATTAAAGATAAAACCATCGACCAGGCATGGTGGCCCACACCTGTAATCCCAGCGCTTTGGGAAGCCAAGGCAGGATGATCACT
Microcebus murinus  ........................................................................................................................

Homo sapiens        TGACCCCAGGAGTTCGAGACCAGCCTGGGCAACACAGGGAGACCCTATCTCTACAGAAAAATGTTTAAAAATTAGCCAGGTGTGGTGGCACATGCCTGCGGTCCCAGCTACTCAGGAGGC
Microcebus murinus  ........................................................................................................................

Homo sapiens        TGAAGTAGGAGCATTGCTTGACCCCAGGAGGTCGAGGCTGCAGTGAGCCGCGCTCATGCCACAGCTCTCCAGCCTGGGCGACAGGGCAAGACCTGTCTCAACAACAACAACAAAAGTATC
Microcebus murinus  ........................................................................................................................

Homo sapiens        CGTTAAGAATTTTTTCTGCAATCATAAAATCATAAAATAACCAGTATCAAAGATTTTAAACTGTAATTTCTATGATATTCATAAATATAAAATAAATATTGTTCAATAAATATTTATTCT
Microcebus murinus  ........................................................................................................................

Homo sapiens        AAAGCACTATGAAAAGAACTGTGGGAAAAGTAAAAGATGAGTCAAACGTGTACCTCATTCTTATAAAGGACTTACTGTCTGGTAGGTAATAAACTATGTAAGCCATGTACAGCACAAGTT
Microcebus murinus  ........................................................................................................................

Homo sapiens        AAGGCATGCTAAATTGCAGAAGACAGGTGTAGATAATGTACTTAGGGAGGCAAAAAGAGAGAAGTTGTTTTTACATAGGAAATTTGTGTTTATCCTTATGGAAGAAGGATCATTTAAGCT
Microcebus murinus  ........................................................................................................................

Homo sapiens        TGACCTTGAAGAATGTATAGGATTTGGTAAATGGATACAAAAGAATTAGGGGAGAAGTAATACACAGAAATGGAAAAGTATTGTAATGATTCATAAAAAGGCAGGGCTTCTTGTTGTCTA
Microcebus murinus  ........................................................................................................................

Homo sapiens        CACTGCCTCGGGTATAAAGTGAAGAAGCAAGATAATGAGGGATAAAGCTGAAAAGGTAAGTAGGAAACCTTATGGAGAGCTTTGAATACTATTAAGGATTGATATGTGCCCCTCCCAAAA
Microcebus murinus  ........................................................................................................................

Homo sapiens        TTTGTGTTGAAGTCTTAACCCCCACCACCTCAAAATGTGACCTTATTTGGAAATGAGGTTGTTGCAAGTGTAATTAGTTGAGATCATATTTCAATAGGGTGGGCTCTAATACAGTATTAC
Microcebus murinus  ........................................................................................................................

Homo sapiens        TGGTGTTCTGATAAAAGGGGAAAATTTGAACACAGACTCAAGGAGAATGCCATGTGAAGATAAAGGCAGAGTTTAAGATTATGTTTCTATATGCCAAGAAATGACAGAGATTGCCAGAAA
Microcebus murinus  ........................................................................................................................

Homo sapiens        ACTACCAGAAGTTAGGGGAGAGGCATTGAATACATTTTTCCTCATTCCTCAGAAGACCAACCCTGTCAATACCTTATCTCAGACTTTTGCCTCCAGAACTGTGAGACAATAAAATTCTAT
Microcebus murinus  ........................................................................................................................

Homo sapiens        TGTTTCAGCCATCCAGTTTGTGGGACTTTGTTACAGTAGCTCTAGCAATAATACAAATGCCTGGCTAAATTATTTCTTTTCTGAGAGCAAAAACTTTGTATAGGAAACTGTCATAATCAG
Microcebus murinus  ........................................................................................................................

Homo sapiens        AACTGTGTTTTTGGAAGTTTAATCTGGCAACTAGTAAAGAACAGATTAGAAGAGGAGAAAACAAGGCATGGAGATTAGTTGAGAGGACTTCGCAGTGATGACGAATACTAAAAACCTAAA
Microcebus murinus  ........................................................................................................................

Homo sapiens        CTGGGCCTGTAGCATTGGTAATGAATGGAGTCTGTCTTAAGATCAGTACAGAATATCGCACATATTATATTTCACAACCATACTGAACAGTGATGTGATATCAAAGATGGAGACCTGGAG
Microcebus murinus  ........................................................................................................................

Homo sapiens        TATTCTGATTACTCATTTAAGAAGAAAGATTATGTTTAATATAGGCTAAAGGAAAAGTGTTTAGGGTAGGGTGTGAATCTAGGACCTCATCTTATACCACAGACCAAATAAATTTCAAAC
Microcebus murinus  ........................................................................................................................

Homo sapiens        AGAATTCCAGGAAGAAAAAAACAAAACTTTTTTTAAAAAAGCGTAATTGTATCAAACAGTGCTACCTATCCTGCTTTCCTCAGAAGAGCAAAGTTTAGAAAAGAGTCATATTAGAATATC
Microcebus murinus  ........................................................................................................................

Homo sapiens        TTTTTGGTAAA
Microcebus murinus  ...........
```
