# Supplementary material for: Extensive intron gain in the ancestor of placental mammals
Source: Biol Direct. 2011 Nov 23;6:59. doi: 10.1186/1745-6150-6-59 (PMC3257199; doi:10.1186/1745-6150-6-59)
Supplement: Additional file 4 — Conservation of intron positions in domesticated genes. [file 1745-6150-6-59-S4.PDF]

Additional file 4 - Conservation of intron positions in domesticated genes

This large supplementary file contain genomic alignments for all placental-specific intron gains. This has the advantage over presentation of intron position conservation in a table or figure, since one can readily see the genes and their exons and introns compared at the nucleotide level over quite large evolutionary distances (at least 80 -100 million years) and, what is surprising, see the remarkable level of conservation at the nucleotide level. The human gene is always aligned with at least one representative of the placental superorders Afrotheria, Xenarthra, Laurasiatheria and Euarchontoglires. In this way a direct insight into the extent of conserved intron positions can be seen, as well as the several cases of highly conserved introns. It should be noted that most of the mammalian genomes are now available as low-coverage (~2x) assemblies. Sequencing errors in some of these genomes can still have surprising effects for apparent lineage-specific insertions of introns. A careful reinspection of such a data is always needed to exclude sequencing errors. Exons are in red.

PGBD1 gene

```
>chromosome:GRCh37:6:28248714:28270926:1
AGGATAGTTAAGTAAATTATGGAACACTCATACAGTATTACTTAGCTGTTAAAGAATAAG
CTGATCTGTGAAGATCTCCAAGTTGTCTTAACCGAAAAGAGCTAAGGTGCAAAATTACAT
TATGATACCATAAGTGTTTTAAAATGTATAAATACTTATTCACTAAACAAAATGTTCATT
AAGTGCTACTGTGTGGCAGGTAGTGTCCTAGATAAATATGAACAAAATACAATGGTTAAC
TCTAAAGAGGGGACACTTTTCGTTTTATGGTCCTTTTGCAACTTGACTTTTTTATTGTAC
ATATATTATGTATACTTAAAGAAAATAATTTGTACAGCTTACTATAATGGTCTTTTTATT
GATCTGTCTCTCCTACCAGCCAGTGATGACCCCGAAGACAGACATTTACCACTGTGTTGG
TTTTCAATCGAAATGATGTACATAAATTTGGGGCGTGCTTGCACCGTCCGAATGGGCCTG
GGGTCCGGCGGCGGAGAGTGACCTGGGCGGGGCAGGAGGTGGCAGCGGGGGTCCTCCCGG
CTCACCAGAGAGACGAGCGGCCGTGCTCCTAGAGAGGCAGGGAACCCGCCAGACTCCGCC
ACTCCGCTGCGGGCGCGGCGCAGGGAGAAGCTTTTGTACCCGCCCAGCTGCTGGAGGCGC
CGGCAGCGCCCGCCAGACCCGCCAGCCCAGCGGCCCGGGCTCTGGGGAAACCGGCGCTCC
CGACAGGGGAGCACCGGGCCTCTGAGCTCCCTCGGGAGCCTTTCACGAGGTCAGCTACGT
CTTTGTTGTGCGCGTTCCTGAACTTTTGGTCCATTAACCAACAATTAGACACGCCGGTAT
TCAGTGCCTGGCGCGGTGCTAGATGCTGGGTGTAATCTCAGAAAAATACATTCAGGGGCG
CGCCTGAGGGTGCTGGCTGCTGGCATCTCAGGTGCTTTACGTGCATTCGTGAAGAAGCCC
ATCAGTATTTCTTGAATACCAGGTACGTGCCGGGCATGTAGAGTCTATGCAGAGATAAAC
TCCTGTCCTCAAGAACCCGGGTGGGGATGGGGTTGGTGATCTGTAATACGAGATGGAGAT
GAGGGTTGGGTGCAGTTTTCTATGGGAATACCCCATCTCAACCTGTGTGGCTTACTTTTC
CTGTCTATATGTTAGGGTTAGGGGTTGCCAGGGAAGGTTTTACAATAGAACATTTACGCT
GGGCCTTGGGAATTTACCCTTAGAAGGGCGGTCCTGACTGTATAGAGTGGGTGGAAAAAA
ATTGAGTACTTAGCATGGCAGTGACATAAGAAATTTGGGAAGACCATGGCTTTCTTGAAA
GCAGGAACCAAGTCTTTTATTCCAGAGTTAACTTTATGCCTGGAAAATAATGAGATGCTC
CACAAATGTTGATTGAGTGACAAACAAATCAGCAAAACCCAGTAAAGATTAGATCCTGGG
GAGTTTGAATTTACTTCAACGAGTTTAATGAAAACTTTGTGTGTGTTTATGTGTGTGTGA
GGTATGTTGAATATGGTCACAACTGTGCTTTAGGGAATATTAATCCAGTGACCATAGAAG
CGGGGAGTTGGGCAGGAATACCGTAACAGAACATTATTGTAACAGTGAATGAAAATAATA
GTAAAATTATAAGAAGGAAAGAATGGCTGCAAGAGGCGTTTTTAAGAATTGACTCACTGA
TTGTGTATAGAGGCTGTTTAAAAGACTGAATGCGTGGAAGATGATGGTGATGCCAGTAGA
AATGGAGAAATACCGAGTGAACGCAGCTTTGAGGGACTCTGATTTCAGTTTGAGATCTGT
TGAAATTATAGTGCAGTAGAGGCCCACAGCTGCTGGCATTTCAAGATGTGGGTTTGGAGT
GCAAAAAGCAAGCTGGTGCCAAAGATTTGAGTCTTCTTGGAACAGAGATATATTGAAAGT
GTGACTAAGGAGATCACAAAGGAAAAAATGAAAAGAGGGAATAAAAGAACTTGATGCATG
TCTACTGTTTTTCAGTTATGTATTGTTGCATCAAAAATAAAACTCTAAACTTAGTGGCTG
AAAACAAGCTCAGCTTATTGTTCCTCAGGATTATGCGGGTTGACTAGGCGGTTCTACTGG
TCTCACCTGGAGTTACTAATAGAGCTGCATTCAGGTGGTGAATTATTTGAGCACTAGGCC
TGGCTGGGCTTTAACTTGGGCATTTTGGTTCTCATCCACATGACCTCTGTATAGCTAGCT
TGGGCTTCCTTACAGCATGGCAGTCTTATGGCAGTGAGCCAAGAGCTACAGCAGCACTCT
CTCTTACAATGTCATTTCTGCTGTATTCCACTGGTCAAAGCAAGTCATAGGCCAGAACAG
ATTCAAGGGAAGGAAAAAGAGACTCCACCCCTTGAATGACAACAGTGGCAAAATCTCATC
ACAAAAGGTATTTGAGAATGGGAGACTTTGGAAACATTCTGTTACACCTACATTTAAAGT
GGAATATTCAATGAGAGTTACACAGATATAGAAGACCTTTGAGACTGTATTATTTGTCCA
TGAAGGCAGCCATCTGCCTCCTCAATGGGTAAATTATCCCCTCTCCCCAGCTGAAGTGCA
AGCTACTCGAGCTTTCTTATTGCTAACACTTCTTCCCTCATGTCCTACATCCCTTACCTT
TGGACTCAGGCCCTTTAGTACTAGTTCAAAATATACTTCTGATTCATTTCACTGATAATA
GAAGCAAGCAGGCTCAGCTTTTGAGCATTACCTACAGTGGGGACAAAAATGTAAGTAGGT
TACATACAGTTTTGAAGCTCTTATAATTTGCATGTTGCTGATAAATTCTTATGCCTCAGA
TCTCTATTTCTGAATATAGACCCCAAGCTAAGTGAAGCTTTAGCCTCTAAGCTCAACATG
TATGAAGCTTTGCCAGGCCCTGCTCCTGAAAATGAAGATGGCCTTGTGAAAGTGAAGGAG
GAAGATCCCACCTGGGAGCAGGTGTGCAACTCACAGGAGGGCAGCTCCCACACTCAGGAG
ATTTGCCGCCTGCGCTTTCGGCACTTCTGCTACCAGGAGGCTCACGGACCCCAGGAAGCT
CTGGCCCAACTCCGAGAACTTTGTCATCAATGGCTGAGACCGGAGATGCACACCAAGGAA
CAGATAATGGAACTGCTGGTGCTGGAGCAGTTCCTGACCATCCTGCCCAAGGAGCTCCAG
CCCTGTGTGAAGACATATCCTCTGGAGAGTGGAGAGGAGGCAGTGACAGTGCTGGAGAAT
CTAGAGACAGGAAGTGGAGACACAGGACAACAGGTGGGAAGAGAATGTGTGTGGTGATGT
GGGAGAAGAAAAGGGGGACATGTATCGGTTTATTTTTAACGTTTTATTTTGAAATAACTC
CTAATTTATAGAAGTATTAGAAGAATAGAGAACTCCCGTAAAACTCTTCATCCAAATTTG
CCAGTTGTTAACATTTGTTTGATGAAGTCCCATTGTTGTGTACACACTCGTACAAGGAAG
TGCATTCCCCCCCCCCAAGATGCATATGTATATGTATACACACACATACATACACATATG
CAGTATTTTTTTTTCCTGAATCTATTTGAGAGTCAGTTGCAAACACTATATCCCTTTACC
TCACTGTTGACCAGGCTGGAGTGCAGTGGTACGATCATAGCATCATTGCAGCCTTGAACT
GCAATGGTGGAAGGCTCAAGCAATCCTCCCACCTCAGGCTCCTGAGTAGATGGGACTACA
GGTGCATGCCACCATGCCTGGCTAATTTATTTTTAATTTTTTGTAGCGGGGGTGGGGTCT
CACTATATTGCCCAGGCTGATCTTGAACTCCTGGCCTCAAGTGATCATGCTGCCTTGGCC
TCCCAAAGTGTTGGGATTACAGGCATGAGCCACTGCACCTGGCTATTCGGTGTGTATTTC
TGAAGAGTGAGGATATTCTCTTGTATCACAAGAATACAAAAATCAAGTTTAGGGAAATTA
AACTTACGATACTATCATATAACCTTAGTCCTTATTCATATTTTGCCATGTTATCCTAAT
AATGTTCTTTATGATAATTTTCCTGTGATTCAGGATACAAGCCAGCATCACATATTGCAC
TTAGTTGCCATATCTCTAGTCTGTGAATCTGGAGTAGCTGCTGTCTTTGTCATGACGTTG
TTATTTTTGAAGAGTACAGCTAGTTTTTTTGGAGACTATCCCTCAATTTGAGTTTGTCCT
ATATTTTCTCATGATTCAGGCTGCACATTTTGGCTAGAATACCACAGAGGTAATGTTGTG
TTCTCAGTGCGTTACTTCAGGAGGTGCGTAGTGATGATTTGTCCCATTACTGATGAGGTT
ATGGTGGTACCTGTCAATTTTCTCCACTAGAAAGTTACCATTTTCCCCTTTGTAATTACT
AAGTAATCTGTGGGAGACATTTTGAGGTGGTGTACATATCCTGTTCCTCATTAAATTTTC
ACCAGCTAGTTTAAACATCCACTTGTTTGTTGTTTTTGTTTTTCTGGGCTGGGATCCTTT
GCCTGTCTTTTCTGCCTTGTTTGCTGCTGTATCCCCAGCATGTAGAACAGTGCCAGGCTC
TTAGCATGCAGTCCATATATGCATGCCCTTTCAAAATAAATCTTTTGTTTCCAGGCCTCT
GTCTATATTCAGGGACAGGACATGCACCCAATGGTGGCAGAATATCAAGGAGTCTCTTTG
GAGTGTCAGAGCCTCCAGCTCCTGCCTGGGATAACCACCCTGAAGTGTGAACCTCCACAG
CGTCCTCAAGGGAACCCCCAAGAAGTGAGTGGTGAGTGCTCGAGTGAGTTTAGTGAGGAC
GCTGGGAGCTGGCCACTGACACTTGCACAGCCTTCTTGACCTCAAGTTTGTATACATTGT
GAAATGATTACCACAAGCTAACTTACATATCTCACAGTTACCTTTTATGTGTCTGTGGTA
```

AGAATTCCTAAGATCTACTCTCATGAAATTTCAAGTATACAATATATTATTATTACTAAC
TATAGTCAGCATTCTGTACGTAGGTCTTCAGAGCTTATTCATCTTATAACTGCAAGTTTA
TACCCTGTGACCAACATCAAGTTTTATTTTTTTGTCTAACCCTGTATCTGAACAAACTTG
TAAAGACCTGCTTTATTTTACACTGTGCATTTTAAGCTGGTTTATTTAGTATGTATGTGT
TGTGGACAAGTACATATAATAGTAAAAGTGAATCGGGGCAAGGCTGTTTTTTTCCAGCAA
ACTTTATTCCTTCTAGAAATGCAACAACAGCAATAAAAACATTCCATAGTGATCCATTAT
GGAAGCAAAGAAAAGCAGAGCAAAGAAAAGTGATGTTCTGTTTTATATTTTTGGGTTAGA
ATTACCACATGTGCTCTTCATTTGATAATTTTGTACTCTGCCATCTGCCAACTGCCAACT
TTGTGACCTAGGCAAGTCATTTAATAGGTTATTCAGTTAATCAACAATTATCTGTTTGAT
CTCTGTGTGTACTCTGCACTCAGCTGTGATTCTTCTGAATGTGCCTAGGATTTTGGAGGC
AATAATTTGGACCTTCTTGATTATTTTGAGGATTGGAAATAATCTTTTAAAGTAACAGCA
CAGTGGCTGGCCTTGTGCAGTCATCATCACCATTTTGATTATTGTTGGTTCTTTGGGATC
AGTACCTCCTAACAGTTTTTCTAGTTCATGGCTAACATCTCTTCTCTATTTTTTTAAATA
TCTTTATTTTCCTGTCTCTCTCTCCATCTTCCTTTCTTCTGCTTTCCTGTTTCCCCTCAG
TCTCATCTCTCTACTCTCTACATCCTTTCTTTTGCCTCTGTGTTCTCTCCTTGATCCTTC
CTTTATCACCAGCTTCTTCCTCCCCTTTATTTTTGTCTATGATTTTTATTTTTTCTTTAT
GTGAAAGTGCATGACTGTAAAGCACCTTATGAGTTCATAAATCCCACTCCATTTTTTGGG
TGAGAGACCCTAAAGTTGTAGAATTTTAGAATTTCTGACAGAATGAAAGCAGTTGTGATG
CTCTTTCTCACACTGTAAAATCTACGTCTTAGTTCTTGAATGTTTAACAAACATAACATT
TGGGGGAAAAGGCTGGGTCTCCAAAAAGGGTACCCTAAAGGCCATCATGTCTCATTTAGG
ACTCTTGCCCTCTCTCTCTGCAG**GGCCTGTTCCCCACGGATCAGCTCATCTCCAGGAAAA**
AAACCCCAGAGACAAGGCTGTAGTGCCTGTGTTTAACCCAGTCAGGTCCCAGGTAAGTAG
GATGCCCAAGCTTGTAGGAATATCAGTAGTGGTCTTTCTCCCTCATTCCATGTTCCTTGC
CCTTCTCTTGGCTCACACTCATCATCGTGAGAGCCATTCAGGTGGCATAGTCCCTCTGCC
TTCACCTTGTCACAGAATATCACAAAGTGTGTTGCATCATTCGAGGAGCTTGTATTGATT
ATATGCCATTGCCTCATTTCATTGTTCAGGAGGCACGTCCAAATCTCAAGGCATGGGAGT
GAGGGGTGGAGTGAGGCTCCTGGTTTCAGAAAGTGCTCCACTCATTTGATGTTGAGTAAC
TGATATTTTCTCCTTTGAAGTCTCTGGTTTATCTCCCCAGCTTTAGAATAAGGGCCCTTT
CTCACACTACTTTATATTCAGCATGTATGCAATTAATGCTTGGTGATAATAATGGAAATT
CTATATATCTAAGGTAATACTTAACATCTTCTTCACAAAATGGACTCTGGTTTTTTTTCT
TATTAGTGGTTCATTCATTTTCCTAGTCATTTATTTACAAAAAAAAAATAACTCTCTCAT
TTAAGTCCTCTTGCATACTGCTGCCACATCAGTTTCCTCAAGACACTTGCTCAGTCCTCT
TCTATGGCTTCTAATTAATTTTTAATTCTATCAGACCCAGTGCCTCCTGTTATTAACAAA
TACTTCCTGATAAGAAGTATCTAGAAAATAAAACCTACTCATGCACATTCTGAAAATCAA
CATAATTCCCTTAAATGTAATTTAATGGTATAATGTAGAAACAAAAAAGTTATTTATTTT
AAAAGTAGTGTTTCAAAATGCAAGTGCTTGATGTGATTATAACTAGGGAAATAACAACGT
AGTCAGGTGTTTGCACTGAAATATAATTAGTAAGTTCAAATTAAGGAGGAATAAGAAGAC
CAGAAGCCCAAGAAAATCAAAGAGCAATGGTATAGGTGGACAGTGAAATGAAACTAGTAT
TGAGTCAGATAATACAAGACTAGACAGAGAAAGTGGGGCCCTTAATTGAAGTAAGGCTTA
GATGTTGTAAATTGAATTATGTCCCTCCAAAAGATATATGAAAATCCTAACCCCACGTAC
CTGTGAATGTGACCTGATTTGGAAACAGGCTCTGTCTTAGTCCATTTTGTGTTGCTAATA
ACAGAATTCCTGAGACTGGGTAATTTATTTTAAAAAGAGGTTTATTTAGCTCAAAGTTCT
ACAACCTGGGAAGTCCAAGATCATCAGCCTATCTGGTTAGGATCTCATGCTGCTTTAGCT
CATGGCAGAAAGTGGAATGATGGATAATCAAGTGTGTGCAAGGAGACCACACATTAGAGG
CAGCCATCCTTTAAACAGTCTGCTCTTGGAGCTGAGCAAGAGCAAGAATAGAAAGCTTAT
TCAAAGAAATAACAATAGGCCGGGTGCAGTGGCTCTCAACTGTAATCCCAGAACTTTGGG
AGGCCATGGCAGGAGGATTGCTTGAGGCCAGGAGTTTGAGACCAGCCTGAGCAACACAGC
AAGACCCTGTATCTATTTAAAAAAAAAGAATAGGCCAGGCGTGGTGGCTCATGCCTGTAA
TCCCACACTTTGGGAGGCTGAGGTGGGTGGATCACGAGGTCAGGAGTTCGAGACCAGCCT
GGCCTACCTGGCAAAACCCCGTCTCTACTAAAAATACAAAAAAAAGGAGCCAGGTGTGGC
AGGCACCTGCAATGCCAGCTACTCGGGAGGCTGAGGCAGGAGAATCGCTTGAACCTGGGA
GATGGAGGTTGCAGTGAGCCGAGATCATGCCATTACACTCCAGCCTGGGCAACAAGAGCA
AGACTCCATCTCAAAAAAATTAAAATAAAATAAATTAAAAATAAATTTTAAAAATAGAGA
AATAATAACAAAAATCTTCACAAAACTTGTGAAAGATATAAATGTCCAGGTACAGGAAGA
TCTGAGAACACTGAACAGAATCATCCCAAATAAGACTACTCCAAGGCATATAATAATCAA
ACTCTCAACTGTCCAGGAAAAAGAGAGGATCTGAAAAGCAGCAAGAGACAAGAAACAAAT
AAGGAGCTCCAACTTGTCTGGCAATAGACTTCTCAATGGCAATCATACAGGCCAGGAGAA
AGTGGAATGACATTTTCAAAGTACTCCAAGAAAAAAACTGACATTCAAGAATATTGTATC
CAGCAGAATTATCTTTCATATATGAAGGAGAAAGTCTTTCCCAAACAAACAAGCTGAGAG
AGTACACCACCACCAGACACATCTTACAAGAAATGCTAAAGAATTCTTCAGTCTAAAAGG
AAAGAAGACTAATGTGCATAAAGAAAACTTTTGAAGGCATAAAAGTCACTGATAAAATTA
AGTACATGGACAAACCCAGAATACTTTATTACTGTAATAGTGACATACAGTATACTCACA
ACTGTGAAGCCCCAAGACATACCTATCAAAAACAATAATAGGACTGGACACAGTGGCTCA
TGCCTGTAAACCCAGTTCTTTGGGAGGCTGAAGTGGGAGGATCACTTGAGACAAGGAGTT
TGAGACCAGCCCAGGCAACATGGTAAGATCCTGATGCTATACCTGGAGACATGAGACAGA
GTGAGACCATATCTTTAAAACAAAACAAGACAAACAATAATGGCTAGCAACCTGTTAAGA
GATAGGTAATATAAAGATATAAATTGAGACAACGAAAAGTCAAAATCTAGAGGAATGGAA
TTAAAGCATATAATTTTTTTCTGTGTGTTTCTGCCTTTGTTTCTACTCTTGTATTTGGGA
TCTAAGATAAGTTGTTATCTCTTTAAAATAACTTATTATATCTGAAGGTCTTCTTTGTAA
GCCTCATGATAACTACAATGCAAAAACCTATAATAAATTCACTAAAAATAGCAACAAAGG
AAGGCATACTATAAGAGAAAATCACTTAGCCACAGAGGTTTTTTGTTGTTGTTGTTTGAG
ATAGGGTCTCACTCTTTCACTCTGGCTGGAGTGCAGTGGTATCATCTCAGCTCACTGCAA
CCTCTGCCTCCCGAGTTCAAGTGATCCTTCCACCTCAGCCTCCCAAGTAGCTGGGACCAC
AGGTGTGTGCCACCATGCCTGGGTAATTTTTTGTATTTTTTGTAGACCTGGGTTTTTGCC
ATGTTGCCCAGGCTGGCTTGAACTTCTGGCCTCAAGCAGTCTGCCCACCTTGACCTCCCA
AAGTTCTGGGATTACAGGCGTGAGACACCATGCCCGGCTGTATATAACAATTATAAATAC
CTATGCATGCAACACCAGAGCTCCCAGGTATATAAGGAAAACATTAATAGATCTAAAAGG
AGAGTTAGACTGCAATACAATAATAGTAGGAGATTTTAACACCCCACTCTCAGTAATTGA
CACATCATCCAGACAGAAAGTCAACAGAGAAACGATGGAATTAAACTACATGCTAGATCT
AATAAGCCTAACTGATATTTACTGAACATGTAACCCAACTGCTACAGAATGCACATTCTT
TTCATCAGCACGTGGGACATAAGAATAGACCATATCTTAGGCCACAAACAAATCTGAACA
AATTTTTAAAAAATATAAATCATATCAAGTATCTTTTCTAATCACAGTGGAATAAAACTA
GAAAGCAATAACAAGAAAAACCTTGGAAACTACACAAACGCGTGGAAATTAAATAGCATG
CTTCTGAACAACTAATTTGTCAGGGAAGAAATTAAGAAGGAAATGTAAAAATGTCTTGAA
ACTAATGAAAATAGAAAATGTGACATATCAAAATTTGTGGGATACTGCAAAAGCAGTACT
AAGAAGGAAATTTATAGCAATAAACACCTATATCAGAAAAGTAAAAAGACATCAAATGCA
CAACCTAATGATACACCTCAAGGAACTAGAAAAGCAAGAACAAACCAAACTGAAATTAGT
AGAAGGAAACAAATAAAGATTAGAACAGAAATAACTGAAATTGAGACCAAAAAAAAAAAA
AAAAAACCCAGAAGAACAATGAAACAAGAAGTTGGATTTTTTTTAAAAGATAAACAAAAT
CAAGTCTTTAGCTGGACAAACAAAAAAGGAGAAGACCCAAATGAATAAAATCAGAAATGA
AAAAGACATAACAGCTGAGACCTCAGAAATACAAAGAAACCATTAGAGACTTATGAACAA
CTATGACAATAAATTTGAAAACCTGGAAGAAATGGATAAATTCCTAGATGCATGCCAACT
ACCAAGATTGGACCATGAAAAAATAGAAAACCTCAGCAAACCAATAACAAGTAACGAGAT
AGAATCTGTAATAAAAAGTCTTCCATCAAAGAAAAGCCCAGGACCTGTTGGCTTCCCTGC
TGAATTCTACCAAACATTTAAAAAGAACTAATACCAATCCTATTCAAATTCTTCAAAAAA
AAAAAAAAAAAATTGAAGAGGAGGGACTACTTCCAAATTCATTCTAGGAGGCCAGCATTGC
CCTGATACCAAAACTAGACTAGGATACAAAATAAAAAGAAAACTACAGACCAACATCACT
GATGAGCATAGGTGCAAAAATTCTCAATGAAACACTAGCAAACTGAATTCAACAACACAT
TAAAAAGATCATTCACCATGATCAAGTGGGATTCACTCCAGGGATGCAAGGATGGTTCAA
CATATGCAAATCAATAAATGTGATATATCACATTAATATAATCAAGAACAAACACCATAT
GATTATTTCAATAGATGCTGAAAAGCATTTGATAAAATTCATTGCTTTTTGATAAAAACC
CTTGTCAAAATTGGTATAAAAGGAACATACCTCAAAATAAGGGCCATATATGACAAACCT
ACAGCTAACATCAAACTGAAGGCCTTTTCTCTAAGGACTGGAACAAGATAAGAATGCCCA
TCTCACCACTGTTGTTCAACATAATACCCGAAGTCCTGGCCAGAGCAATTATGCAAGAGA
AGGAAATAAATGGCATCCAAACCGGATAGCAAAAAGTCAGGTTAGTCTGTTCGCAGATGA
TATAATCTTATACCTCGAAAAACCTGAAGACTACACCAAAAAAACTGTTAGTGCTGATAA
ACAAACTAAGTAAAGTTGCAGGATAAAAAATCAACACTCAAAAATTAGTAGCATTTATAT
ACTTCAATAGTGAACAATCTGAGATAGAAATCAAGAAAATGATCGCATTTACAAAAGCTA
CAAAAAATATAAAATACTTAGGGAATTTAAATACTAAAAATTTAAACACCAAATTAAGTT
AAATTTAGTGTTTAAATTCCCTAGGCATTTTATATTTTTTAGTATTTTATTTATAATTTA
TAATTTAGCATTTAATTACTAAAGAAGTGAAAGACCTGTATAAGGAAAACTAAAACTCTC
ATGGAACAAATTGAAGAGGACACAAAAACACTGGAAAGATATTCCATTCTAATGGATTGG
AAGAATTTATATTATTAAAATGACAATACTTCCCAAAACAATTTACAGATTCAGTGTAAT
CACTATCAAAATACCAATGACATTCTTCACAAAAATCAGAAAAAAAAATTCATGTGGTGA
GCCCTTAATCTAATGACTCATATTCTTATAAGAAGTCCATATTTGCAAATTGACCAAGGG
GTTGAAAAAAAAGAAAGAAAAATGCCCAAGTGAAAACACACACAGGGAGAATGCCATGTG
ATCACTGAGGCAAAGATTTGTATAACACATCTGCAAGCCGAGGAACACCAAGAATTGCTG
ACAAACACTAAAAACTTGGAGAGAGGCAAGGAAAATTATCCCATGGAGCTTTTTTTCTTT
TCTTTTCTTTTCTTTTTCTTTCTTTTTGAGACAGTCTTGCTCTGTTGCCAGGCTGGAGTG
CAGTGGCGCGATCTCAGCTCACTGCAACCTCTGGCTCCCTGGTTCAAGTGATTCTCCTGC
CTCAGCCTCCCGAGTAGCTGAGATTACAGGCAAGCGCCATCACGCCCAGCTAATTTTTGT
ATTTTTAGTAGAGATGGGGTTTCACCATGTTGGCCAGGATGGTCTCGATCTCCTGACCTA
GTGATGTGCCCACCTTGGCCTCCCAAAGTGCTGGGATTACAGGCATGAGCCACCACGCCA

GGCCTCCCATAGAGCTTTCAAAGATAACATGGCCCTACTGATATCTTGATTTTGGATTTC
TAGCCTCCAGAGCCATGAGATAATACAATTCTGTTGTTTTCCACTATTGAGGTTGTGGTA
CTTTGTTACAAGAGCCATAGGAAAGCAATATAAGTTACAAAACAAATTATGGACTGATAA
AGGGAGAAAATAAGGAGGTATGATATTACAGATACACTTCCTTCATTGTGTTTCACTTTA
TTGTGCTTTGCAGTATTGAAAGTTTGTGATGACCCTGTGTGGAACAAGTCTGTCAGCACC
ATTTTTCTACAGCATGTGCTCACTTTGTGTCACATTTTGGTAATTCTTAAATTCTTAAAC
GTGACACTCTGTGTCACATTTTGGTAATTCTTAAAATGTTTCAAACTTTATTGTTATATC
TGTCATGGTGATCTGTGATCAGTTATTTTTGATGTTACTATTGAATTTGTTTTGGTGTGC
CACAAACCATGCCCGTTTAAAATGTCAGACCTAATCAATAAACATTGTATGTGTCCTGAC
TCCTCCATTGACCAGCTGTTCCCTCATCTCTTTTCCTTTCCTTGGTCCTTGCTATGTCCT
GAGACACAATAATATTGAAATGAAGCCATTTATAATACTACTACTACAGTGACCTCTATG
TGTTCAAGTGAAAGGAAGAGTCACATGCCTCTCAGGTTCAATAAAAAGCTAGAAGTGATT
AAGTATAGTAAGGAAGGCATGTTGAAAGCCGAGATAGGCCAAAAGCTAGGAGGCCTCTTG
TGCCAAACAGGCAGGTGGTGAATGCAAAGGAAATGTTCTTGAAGGAAATGAAAAGTGCTA
CTCTGGTGAACATATGAATGATAAGAAAGCAAAAAGCTTTTTGCTGATATGGAGAAAGTT
TGAGTGGTTTGGATAGAAGATCACACCAGCCACAGCATTGCCTTGAGCCAAAGGCTAATC
CAGAGCACATCTCTTACTCTCTTCAATTCTGTGAAGGCTGAGAGAGGTGAGGAAGCTACA
GAAGAATAATCGGAAGCTAGCAGAGGTTGGTTCGTGAGGTGTAAGGAAGAAGCCATCTCT
ATAAAATGCATCAGCAACAAGTGCTGATGCAGATGCTGCAGCAGGTTATCCAGATCTAGC
TAAAATCACTAATGAAGGTGGCTACACTAAACAACAGATTTTCAATGAAGATGAAACCAC
CTTTTATTGAAATAAGATGCCATCTAGGACTTTGATTGCTAGAGAGGAGAAGTCAGTGCC
TGGCTTCAAAGCTTCAAAGGACAGCCTGACTTGTTAGGGGCTTACGCAGTTGGTGATTTT
AAAGTTGAAGCCAGTGCTCATTTATCATTCTGAAAATCCTAGGGTCCATAAGAATTATGC
TAAACCTACTCTGCCTGTACTCTAGAAATGGAACAACAAAGCCTGGATGATAGCACATCT
GTTTACAGCATGGTTTACTGAATATTTTAAGCCTACTGTTGAGACATACTACTCAAAAAA
GAAAAAAAGATTTCTTTGAAAATATTACTGCTTATTCACAGAGCACCTAGTCACACAAGA
GCTCTGAAGGAGAGGTACAAGGAAATTAATGTTTTCAAGCCTGCCAATACAATACACATT
CTGTAGCCCATGGATCAAGGAGTAATTTTGACTTTCAAGTCTTATTATTTGAGAAATACA
TTTTTTAAGGCTATAGCTGCCATAGATAGTGATTCCATAGATAGTGATGGATCTGGGCAA
AGTAAATTAAAAACCTTCTGGAAAGGATTCATCATTCTAGATGCCATTAACATTTGTGAT
TCAAGGGAGGAGGTGAAAATACCAACATTAACAGGAGTTTGGAAGAAATTGGTTCTAACA
CTCATCATTGTCTTTGAGAGGTTTAGGACTTCAGTGGATAGTGTAGCTGCAGATGTGGTG
GAAAGGACTAGTATTAGAAGTGGAACCTGAAGATGTGACTGAATTGCTACAATCTCATGA
CAAAACTTGAACAGATAAGTTACTTCTTATGGGTGAGAAAAGAAAGTGGTTTGAGATTGA
ATCTACTCCTGGTGAAGCATTGTTGAAATGACAACAAAGGATTTAGAATATTAAATAAAC
GTAGTTGATAAAGCAGCAGCAGGTTTGAGATGATTGACTACAATTTTGAAAGAAGTTCTA
CTCTGGGTAAAATGCTATCAAACAACATTGCATGCTACACAGAAATCTTTTGTGAAAGGA
AGAGTCAATCAAGGTGGCCAACATTACTGTTGTCTTATTTTAAGCAACTGCCACAGCCAC
CGAAACCTTCAGCAACCACCACATTGATTAGTCAGCATCGTTCAACATCGCAGGAAGCCC
CTCTACCAGCAAAAAGATCACAACTCTCTGAAGGCTCAGATGATTATTAGCATGTATTAG
CAATAAAGTATTTTTAAATTAAGATATATACTTTTTTAGACACAATGCTTATGTACGATT
AATAGACTACATAGTATAGTGTAAACATGACTTTCACATGCACTCGGAAGCCAAGAAATT
TCTGTGACTTGCTTTATTGTGATATTTGCCTTATTGTGGTGGTCTGGAACTGAACCTGCA
ATATCTCTGAGGTAGGCCTATGTTTAAATGAGGTGGGCTTTGCCTTTTATTATCAAAATA
ATTATCTGTAGTTTGGAATAGGGTGATGATGAGTTAAAGAGAAAACATCTTCTGTCTTGA
AACCCCCTCCTTGTCAAAACAGATATTCATTCTCTAAAAGACTGGCACTGTGTCCTTTTT
CAGGCAACAAGAAAGAGAAAGTAGATTGGAAAATGAGAGAAAATCAAGAGGCTAATGAAA
CCATGTGGTAGCCTTTTGGGACAGTGTCAGAGAGATAGAAAATCTCAGACAGTTCCCCAA
ATAAATAAATTCAGTGTATGATTTTTCCAGCCTACATATCATTACTAGCTTTGAAATGTT
TTTAAACAATCTTTGTTGATGAAATTCAGTGAGATTTCTGCATTTACATTAGATTCCAAA
TCTGTTAAAAGGAGTCTCAAAACTCTGTTAGGTATTTGTAGTCAGTTTAATTGCATTTGT
TATAACTGCAATATTATATTGAAGTGCTGTTCAACCAAATACATAGAAAATGCTGTAAAA
TAGTGTGTAGAACAATCCAAGTGAGTCAACTTTTGTGACAATGTCTTTAATCGATGTAAG
CTTCCTTCTCTAACTTTCTTGAATTACTGACTTTTTTTCAGTATCATCCTATAAAAACTC
TTCAGGTTTCCTATGTTTCAGTGTAGCTGATTTATACTGATTGTAGGTTTGGTTCAGGAG
GACACTCAAAAGTCCTGCAGGATGTGGAAGAATTCTTTGTAGTGTGGGACTGTCCCACAT
ATTTCAAGATGCCTAGCATCTCTGGCCTCACCCTCTGAATGGCATCGGCTTGAGGTGGTG
GTGGACTTCATTGTTATAACCAGAAAGGTTCCCACAAGTTTACAAAATGCCCTGAGGGGC
TGGGACTACCGGTCTACAGCGTGGTATTCAACACCCTTCACAACTGGATTCCTTACCATG
TGAAAACAAAGAGGCTATTTTCTTTTTTTGTCTTTTTAG**ACATTGGTGAAGACTGAGGAA**
GAAACAGCCCAGGCCGTTGCTGCAGAGAAGTGGTCACATCTGAGTCTGACTCGGAGGAAC
CTCTGTGGGAACTCAGCTCAGGAGACAGTTATGAGCCTCAGTCCGATGAGTAAGGCCAGG
CCTCTGGCTGGACCCCTGTCTGGACTCTCATTTTCCTGTCACTCACCCTGGCTTGGCCCT
TGGGAGGCCACGTTTCCTACAGACCCACACCCTTCCTTTCCCTTCTTCTGGCCTCTGCCT
CCAGACTTCTAGCCCTTATCACTTTGCATCCTTCCCAATCACATTTTCTTCTGTTACTAA
TCAGTTTACTGATTGTTGTGGTTATCTTCTCTGATCATCTCTTCTAGTTCCCTGATTCCT
TCAGTGACTCCTTTGTATCCTTTTCCCCTTTTTTCTTCCCTGTCAAGGATTCCATATAAC
TTGTCTTTCAGATATCCCTTCATACTTGGAAGCCCCATGAACACCTCTGTCTGACCTTGT
CTTTACCTCAGAGGAGGTCATCTTCACTCTCTAAGAACACGACAGTTCATGTTTATTTTA
TTTTTTTTAGTTTTTTTAAAGATGGGGTCTCGCTTTGTTGCCCATGCTGGAGCATAGTGG
CACGATGATAGTTCACTGCAGCCTCAAACTCCTGGGCTCAACTGATCCTCCCACCTCAGC
CTTCTAAAAAGCTGGGGCTACACCCAGGTAATTTTTTGTAGACACGGAGTCTCGCTATGT
TGCCCAGGCTGGTCTTGAACTCCTGGCCTGAAGCAGTCCTTCCACCTTGGCCTCCCAAAG
CACCCAGCCTAATTTTCTTGAAATGTGCTCTGCCCTGTTGGGCCCTCTCTGCAGCTGGGC
TTTGGTGCCTTAATTAACCTTTCTTATCATTTTCCCATACCATGCCACATTTTGTAGGAT
TGCTGAAACTGTGCACCCTTGAAACACCAATATGATTTGTTTTTTGTTTGATTTGGAACA
TCTATTCCCTACCCTGGAAGTTTTTTTTTTTTTTTTTTTTTTTTTTTTTCAAAATTCACA
TAACAGATGACATTTAAATCATTAATGTTATATTTTAG**CTGAAGAAATTGTAACTAAAGA**
TAGATTGTTTAAAGCAAAGCAAGAAACTTCTGAAGAAATGGAACAAAGTGGAGAAGCCTC
AGGAAAGCCCAACAGGTGAGTGTCCATGGTCCTCAGTGAATCAAATCCTGCAGATATAAG
AAATTGGAATGGAATCAGAGAAACCAATTGGTCAAAGGCCTGCTTCTCTGGAATAAGATA
TGACAACTGCCACTCACTTCTGTGATCATAGGAGGTCCATGCCAAGAAAGTGACCTATTC
AAAATTTTCAATATGCAATAAATATACAAAACCTTCAGTTGATACTCAGTTTCCTCCAAG
AAGCCTATCCTGATCCAGTCTGTCCTGCTTGATTACCCTTCCCTGGGCTCCCTTACATTT
TTATGGAGCTGTGGAGAAGCTAGAAGGCAGATAATTTAACAAGAATGAGGGCTACAGATG
AAGCTGCCATGACAGTTAAGAAAGAAATGTCAGAGTGGAAGAGGACAAGCAGGGTACAGG
TTGAGCATTCCAAATCTGAAATGTTCCAAAATCTGAGCTGTTTGAGCACCAGCATGATGC
TCAAAGGAAATGCTCATTGGAGCATTTTGGATTTTGGATCTTTGGTTAGGGATGCCCAAC
TGGTATAATGCAAATATTCCAAAATCTGAAAAAAATCCAAAATTCAAAAATCTTCTGACC
CCAAGCATTTCAGATAAGGGAAAATTAACCTGTATTTTCAACCGCCATTGGGAGACCTGA
ACAAGGTATAGGTCAAAAGGAAAGTTAGCTGATGAAGTAGAAATGAATGACAGTGGTGTG
TGCTTCATAAGTGCTGGTTGTGGGAATGAATGAACTTGTAACAGATACTCCTATGCAAAG
CAAGAAACTTCTGAAGAAATGGAACAAGGTGGAGAAGCCTCGGGAATGCCCAACATTCCG
AGGCTTCTCAGGATTGCAGAAAGAGAGGGAACAAATCACAAGCAGAGGGTTGGCTTCTGG
TGAGGCCATTTGGTCCTCAGAGACTTGAGAAAAGAGAGAGTAAGCAAGGTGGGCTACTTG
GCCAAAGAGCTGAGAAGGGGGTGTGAATTTTCCTTATTGACATTTGTTTAACTTAGAGAA
ACTGGGAGCTAAATCTCAGCTAAAGTAGAAATGTCTTAAAGCTCAAGAGGAGAAGTGAAA
TTTTGCATTAAGATTAGTGAAGACTGAGCCAGGCTCCACTAAAGGAAGAGATATGATGTT
CAGTAACTTAGAGCAGAGTTATGCAACTGGAGAATAGGAATCTGCTATTGTGCCTGTAGG
TACATTTAGTATCAGGCAACTAACAGGTAATTCATAGTGCCTATAATAAAGATATTCATA
TAACAAAAAGTGTGAAGGTAGGCTATTTCAGGTTAGGTGCAATAGCTTAACCATGGTATC
AAAGGTCTAGCCCCCTGCCCTCCTTCTAGTCCCCCTCCCTCACCCCCACCCCACTTTCTT
AGCTCACTCTTTTTTCCCATTTCTCACTCCCATTTCCTTCCCTCACAATATTGGGCTTAA
TGCACTGTGGTTCTTAGAGTTTAGATGTCAACAGTAGAAAGACTCAGTGTGATCAAGGAA
TGTCACTGAAGTTATGGATGTCAGGGAACTTTAAGATGGATGTGTTGAATACATGCAGAC
ACTGAAGACACCCTGGATGATAAACACTTTACATAAAGAAATGTATGTAGTGTAAATTAT
ATTAAAATATACTCTTCATGCTTCTTAAATGTCTTTGAAAACTTGACTAGTTTACCAACA
GAACACTAATAAATTAAATGCACATGTCTAAAAATTAAAAACTTTGTGTAAAATAATACT
TTTCAGTTGGGTGCAGTGGCTCATGCTTGTAATCCCAGCACTTTGGGAGGCCGAGGTGAG
TGGATCACGAGGTCAGGAGTTTGAGACCAGCCTGGCCAACACAGTGAAACCCTGTCTCTA
CTAAAAATACAAAAATTAGTTGGGCATGGTGGCACGTGCCTGTAGTCCCAGCTACTCGGG
AGGCTGAGGCAGGAGAATTGCTTGAACCTGGGAGGCAGAGGTGGCAGTGAGCTGAAATCA
TGCCACTGCACTCCAGCCTGGGTGACAGAGCTAGACTCTGTCTCAAAAAAAAAAAAAAAC
AAAACAAAACTTTTCATCTTTGTAACTTTAGCTTACTTCCTTGTAAACCCCATAAAAATC
AGTCCAAGTGCAATGAGTTGAGTTTCATTTGTTTTTCCCCAGAGTAGTATGTCCATCAGA
TGAATGTAGAACTAGCAGGGGCTAAGTCCTTCCATGTTCAGAAATTCTGTGAGAGATGTC
AGAAGACCTGGTAAATAATAACCTTTTTTTCTTGTTCAGATTCAGAACCAATTGACAGGG
ATGCAGGAGTAAAATAGATGGTTCTGGAAACCTCAGTTTCTTACTGACAGAAGTTAGACT
GGATAAAAACGGCTTAAAGGCAGAGCTAAATAGGCTTGTACTGGCAGAGGAGGAGTGTAG
CAGATGTCCCTCTGCATAGGTCTTCTCAATGCAACTTGGGGCACCAAGGAGCATGGGATG
TCTGCATCCTGCTCCTAAAGAGGACAGAGGGAGGAGTGTACCAAGGATACTCAGTTATTT
CTCCCAGGCTCAACAGTTAGTTCCATCCTGAGGAGTGAGTGAGAGGTGGAGAGTGGCTAG
AAATGTTTAATCTAGCCAAAGTCCTCCCCATGGAACTTAAGAGAAATAAGTAAGTATCC

CCCCACACCCACCCCAAGCCCCAAAAGATTTAAGCTTCAGCAGATTTATCATGTGTGAGA
GAATATGATTGAGGATTTTTATAACATGTTCTTTTTTTCTTTCCCAG**AGAGTGTGCACCC**
CAGATTCCTTGTAGTACTCCTATTGCTACTGAAAGGACAGTTGCACATTTGAACACTCTG
AAGGACCGTCACCCAGGTGATTTGTGGGCCCGCATGCACATCTCATCCCTGGAATATGCT
GCAGGAGACATTACCCGAAAAGGGAGAAAAAAAGACAAAGCTCGAGTGAGTGAACTGCTC
CAAGGCCTCTCATTCTCTGGTGACTCAGATGTGGAAAAAGATAATGAGCCTGAGATCCAG
CCTGCTCAAAAGAAGTTAAAGGTATCATGTTTCCCAGAAAAGAGTTGGACCAAAAGAGAC
ATTAAACCCAATTTTCCAAGCTGGTCAGCACTGGATTCTGGACTTTTGAATCTCAAGAGC
GAAAAGTTGAACCCAGTAGAGCTTTTTGAATTATTTTTTGATGATGAAACATTCAACTTA
ATTGTCAATGAAACCAATAATTATGCTTCTCAGAAAAATGTCAGCTTGGAAGTCACAGTT
CAGGAAATGAGGTGTGTGTTTGGTGTCTTACTTTTGAGTGGATTTATGAGGCATCCTAGA
AGGGAAATGTATTGGGAAGTCTCTGACACCGATCAGAACCTGGTTAGAGATGCAATCAGA
AGGGACAGATTTGAATTGATTTTCTCAAACCTGCACTTTGCAGATAATGGCCACCTAGAT
CAAAAAGATAAGTTTACAAAGTTGAGACCTCTCATAAAACAAATGAATAAAAATTTCCTC
TTGTATGCTCCCCTGGAAGAATACTATTGCTTTGATAAGTCAATGTGTGAATGCTTTGAT
AGTGACCAATTCCTGAATGGAAAGCCTATTAGAATTGGCTATAAAATTTGGTGTGGTACA
ACCACACAGGGTTATCTGGTTTGGTTTGAACCCTATCAAGAAGAATCAACTATGAAGGTA
GATGAGGATCCTGATCTTGGGTTAGGTGGAAATCTAGTGATGAACTTCGCTGATGTTCTT
TTAGAGAGAGGTCAGTATCCCTATCACCTGTGTTTTGATAGCTTCTTTACAAGTGTCAAA
TTGTTGTCAGCCTTGAAAAAGAAGGGGGTGAGGGCAACAGGAACAATTCGTGAGAACAGG
ACCGAAAAATGTCCCCTTATGAATGTAGAACATATGAAAAAAATGAAGAGAGGGTATTTT
GATTTCCGAATAGAAGAAAACAATGAGATAATTTTGTGTCGTTGGTATGGGGATGGCATT
ATCAGTCTGTGCTCCAATGCTGTGGGCATAGAACCAGTCAATGAGGTAAGCTGTTGTGAT
GCTGATAACGAAGAAATCCCTCAGATAAGTCAACCATCCATAGTAAAAGTGTATGATGAA
TGCAAGGAAGGTGTAGCTAAAATGGATCAAATTATTTCGAAATACAGGGTGAGGATAAGA
AGCAAGAAATGGTACTCAATTTTGGTGAGCTACATGATTGATGTAGCCATGAACAATGCA
TGGCAACTACACAGAGCCTGTAACCCAGGTGCTTCTCTAGACCCCTTGGATTTTCGGAGA
TTTGTTGCACATTTCTACTTGGAACACAATGCTCATCTGTCAGATTAGGGTACATAAAAT
GGACATAGTGCAGACATTAATAAGACATAGAAAAATAATAATTATACATGCTGTTGTACC
CTCCCAAAGTAAATCTGATATATGTAATGAAGTTATTAAATAATACTTTTAAAAATCAGA
CATTTATATAGAGTTTCAAAGACTATTGTAACAAGTAATGTTAAAAATTGTCTGTGAGAA
TGTTGAACTGTAGTACCTTTCTCTATGTCAAGTTTTGTGTCAGACATGGGAAATCATGTA
TTTGTTCAATTGACTACTTTGTGCACTTATTTATTTATTTTTTGAGACACAGTCTCGCTC
TGCGGCCAAGCTGGAGTGCAGTGGCACGATCTTGGCTCACTGCAACCTCTGACACCCTAG
TTCAAGCGATTCTCCTGCCTCAGCCTCCCAAGTAGCTGGGATTACAGGCACGTGCTGCCA
TGCCTAGCTAATTTTTGTATTTTTAGTAGAGACAGGGTTTCACCATGTTGGCCAGGATGG
TCTCGATCTGACCTCGTGATCTGCCTGCCTTGGCCTCCCAAAGTGCTGGGATTACAGGCG
TGAGCCACCATGCCCAGCTATTTTGTGCATTTAAAGAAGGAAATCCTACCTCTTAAAAAA
AATTATCTGGAGAATGCCATTTTTAAGATGCAAGCAATGTTACAGAAACCATAGAATGGT
GCTGACTCAATAGTTCAAACTAGTGACACAGCCTATGAAGTAAGAATGATCTAAACAAAA
CATAGGTGGTAAGAGACTAAAAACCTTAGCATTGGTGTAAAACTGGATCGGATTGTGTGT
AAGTGAGAAGGGTCAGGCATGGATATTGAAGGAGAGTGCTATAAAGGAAAAACCAGAGGT
GAAGCAGTGCCTT

```
Loxodonta africana  TATTGGTGGAAATAAGTCATAATCCAGCACAGATTCAAGGGAAGGAGAAATAGACTC-ACCTTTTGA-TGTGAACAGTGGCAAAATCTCATTACAAAAGATATTTGGGGGTAGACGTTAT
Homo sapiens        CACTGGTCAAAGCAAGTCATAGGCCAGAACAGATTCAAGGGAAGGAAAAAGAGACTCCACCCCTTGAATGACAACAGTGGCAAAATCTCATCACAAAAGGTATTTGAGAATGGGAGA---

Loxodonta africana  AATTTCAGTCATCTTTGGAAATAGTCTACTGTACCTACATTTGAGGTGGAATATTCAGTGAGAGATACACAGAGATAGATGAC-TTTGAGAATATAGTATCTGTC-ATGCAGGTAGCCAC
Homo sapiens        ------------CTTTGGAAACATTCTGTTACACCTACATTTAAAGTGGAATATTCAATGAGAGTTACACAGATATAGAAGACCTTTGAGACTGTATTATTTGTCCATGAAGGCAGCCAT

Loxodonta africana  CTGGCTCCTTCATGAGTAAAGTTATCTCCCTTTCCTAGCTAAAATGTAAACTGCTCAAGCACTCTTAAAAGTCTAATAAGCCCTTCTTCCCTCATATTTCACATCCTTTAGCTCTTGGCT
Homo sapiens        CTGCCTCCTCAATGGGTAAA-TTATCCCCTCTCCCCAGCTGAAGTGCAAGCTACTCGAGCTTTCTTA------TTGCTAACACTTCTTCCCTCATGTCCTACATCCCTTACCTTTGGACT

Loxodonta africana  GGACCCCCTCAG----GGTTGAAGATGTACTTCTGATTAATTTCACTGATAAAGATAGGCAAACGGGCTCAGCTTTTGAACATGACCTACTGTGGGGACAAAAATATTACCATGTGGTGT
Homo sapiens        CAGGCCCTTTAGTACTAGTTCAAAATATACTTCTGATTCATTTCACTGATAA-TAGAAGCAAGCAGGCTCAGCTTTTGAGCATTACCTACAGTGGGGACAAAAATGTAAGTAGGTTACAT

Loxodonta africana  AGCGTTTTGAAGCTCTTGTAATTTTCATGTTACTGACACATTCTCATGCCTCAGATAGACATTTAGAAAAACATTTCTGAACTGTAAACCACA-GTCAAGTGATCCCTGTTCTAGCATCT
Homo sapiens        ACAGTTTTGAAGCTCTTATAATTTGCATGTTGCTGATAAATTCTTATGCCTCAGATC----TCTA--------TTTCTGAA-TATAGACCCCAAGCTAAGTGAA----GCTTTAGCCTCT

Loxodonta africana  AAGCTTAACATGTATGAAGCTT---CAGGCCCTGCTCCTGAAGATGAAGAAGGCCTTGTGAAAGTGAAGGAGGAAGATCCCACATGGGAACAGGCATGTGGCTTACAGGAGAGCAGCTCC
Homo sapiens        AAGCTCAACATGTATGAAGCTTTGCCAGGCCCTGCTCCTGAAAATGAAGATGGCCTTGTGAAAGTGAAGGAGGAAGATCCCACCTGGGAGCAGGTGTGCAACTCACAGGAGGGCAGCTCC

Loxodonta africana  CACACTTGGGAGGTTTGCCGCCTGCGCTTCCGGCAGTTCTGCTACCATGAGGCTTCTGGGCCCCGTGAGGTCCTGGCTCAACTCCGAGAACTTTGCCGTCGATGGCTAAAGCCAGAAACA
Homo sapiens        CACACTCAGGAGATTTGCCGCCTGCGCTTTCGGCACTTCTGCTACCAGGAGGCTCACGGACCCCAGGAAGCTCTGGCCCAACTCCGAGAACTTTGTCATCAATGGCTGAGACCGGAGATG

Loxodonta africana  CACACCAAAGAGCAGATCCTGGAGCTGTTGGTGCTAGAGCAGTTCTTGGCCATCCTGCCCAAGGAGCTTCAGGTCTGCGCGCAAGTGTATCATCTGGAGAGTGGAGAGGAGGTGGTGACA
Homo sapiens        CACACCAAGGAACAGATAATGGAACTGCTGGTGCTGGAGCAGTTCCTGACCATCCTGCCCAAGGAGCTCCAGCCCTGTGTGAAGACATATCCTCTGGAGAGTGGAGAGGAGGCAGTGACA

Loxodonta africana  GCGCTGGAAAATCTAGAGGCAGAACATGGAGACATGGGACAACAGGTGGGAAGAGAATGT-TGTGGTGCTATGGGAGAAGAAATGGGGGAACATTCCTCTTGTAATTTTTTAACTTTTTA
Homo sapiens        GTGCTGGAGAATCTAGAGACAGGAAGTGGAGACACAGGACAACAGGTGGGAAGAGAATGTGTGTGGTGATGTGGGAGAAGAAAAGGGGG-ACATGTATCGGTTTA-TTTTTAACGTTTTA

Loxodonta africana  TTTTGAAATAACT-TTAACTTATAGAAGCATTGGGAAAATAGTACAAAGAACTCCCA--------TTCACCTAGATTTGCTAGTTGTTAACATTTGTTTCGTGATGCCCCAT-------A
Homo sapiens        TTTTGAAATAACTCCTAATTTATAGAAGTATTAGAAGAATAG-----AGAACTCCCGTAAAACTCTTCATCCAAATTTGCCAGTTGTTAACATTTGTTTGATGAAGTCCCATTGTTGTGT

Loxodonta africana  ATGCACTTGTGCATGTACTTTTTTGTCTTTATATTTACATACACACACGTATGTGTATATACACACACACACAGGAGGCAGTCCCAGGGTTACGAACATCCCACTGGTTATGAACCAACC
Homo sapiens        ACACACTCGTACAAGGAAGTGCATTCCCCCCCCCCAAGATGCATATGTATATGTATACACACACATACATACA...............................................

Loxodonta africana  CCTATAAAGCCTCTTATATTAAAAATTCAAGGCATATACAATGGTTTGTAGTAACAGGCACTAATACTAGTTTGCAACCCACATCAAAACATTATTATTACTGTATTATTATGTTAAAGA
Homo sapiens        ........................................................................................................................

Loxodonta africana  TGTTTTAGTGTATCTGGAAGTGTTTCTTTAATTTTTTTAGGCATAGGAAGGTACACTATATACAAAGACAAACATTCAACTAACTGATGTGTGATACGAACCGTATGTAACCTAACTGTA
Homo sapiens        ........................................................................................................................

Loxodonta africana  TCAACTTAAATGTTTGGAACTCATTCTCGTAGTCCAGGAACTGCATATGTATATGAAGAAGCCCTGGTGGTGCAGTGGTTAACCTCTCAGCTGCAAACCAAAAGGTGGGTGGCTGTCTGC
Homo sapiens        ........................................................................................................................

Loxodonta africana  GGGAGGAAGATGTGGCAGCCTACTTCCATAAAGATTACGGCCTTGGAAACCCTATGGGGCAGTTCTACCCTGTCCTGTAGGGTAGCTGTAAGTTGGAATTGACTTGATGGCAATGGCTTT
Homo sapiens        ........................................................................................................................

Loxodonta africana  TTATATAGATGTCTATGTTGTTGGTGTTGTTTACTGCTGTTGAGCTGGCCCACATCTCATGGCGACTCCATGCACAGCGGGATCATTGTGGTCGATAAGGTTTTCGTTGGCTGGTTTTTC
Homo sapiens        ........................................................................................................................

Loxodonta africana  AGAAGCAGATCGTCAGGCTTTTTTCCTAGTCCATCTTAGTCTGGAAGCTCCGCTAAAACCTGTTCAGCATCATAGCAACACACAAGCCTCCACAGACGGACAGGTAGTGGCTGCACATTG
Homo sapiens        ........................................................................................................................

Loxodonta africana  GCCAGGAATCGAACCTGGGTGTCCTGCACAGAAGGCGAGAATTCTACTGCTGAACTGTATATGTATATACACGCGCACACACGTATTTTTTTCCTGAACCATGGGACAGTTAGTTGCAGA
Homo sapiens        ........................................................................................................................

Loxodonta africana  CAGCATACCCCTTTCCTTCTAAACATTTCAGTATGTATTTCCTAAGAACAAAAACATTCTCCTGAATAACCAGAATACAACTATCAAATTTAGGGAACTTAAAGTTGTTATGATGCTATC
Homo sapiens        ..........................TTCGGTGTGTATTTCTGAAGAGTGAGGATATTCTCTTGTATCACAAGAATACAAAAATCAAGTTTAGGGAAATTAAACTT---ACGATACTATC

Loxodonta africana  ATCTAAATTATAGTCCTTGTTCAAATTTGGCCATATTATGCCAGTAATGTTCTTTTTAGCAATCTCCCCCCACCCCCCCATGCCCCAAACCAGGGTACAATCTAGCGTCACATGATGCAG
Homo sapiens        ATATAACCT-TAGTCCTTATTCATATTTTGCCATGTTATCCTAATAATGTTCTTTATGATAATTTTCCTGTG---------------ATTCAGGATACAAGCCAGCATCACATATTGCAC

Loxodonta africana  TC-GATGTGAT-TCTTTAGTTTCCTTTAATGTGGAGTAATTCCTGTCTGT-CAATGACTTTGATGTTTTTGAAGAGCGTAGCTAGTTATTTTGTAGAATGCCCCTCAATTTGCGTTTGTC
Homo sapiens        TTAGTTGCCATATCTCTAGT--CTGTGAATCTGGAGTAGCTGCTGTCTTTGTCATGACGTTGTTATTTTTGAAGAGTACAGCTAGTTTTTTTGGAGACTATCCCTCAATTTGAGTTTGTC

Loxodonta africana  TTATATTTTCTCATGGTTAGATTCAAGTTGTGCATTTTTGCTAGAATGCTACTGAAGTA-TGTTGTGTTCGCCTCAGTGCATCACATCAGGAGACATGTGATGATGGTTTGTCCCATTAC
Homo sapiens        CTATATTTTCTCATG-----ATTCAGGCTGCACATTTTGGCTAGAATACCACAGAGGTAATGTTGTGTTC---TCAGTGCGTTACTTCAGGAGGTGCGTAGTGATGATTTGTCCCATTAC

Loxodonta africana  TGGTGACGTTAGGGTAGGATCTGTCAGATGTCTCCACTGTAAAATTTGTATTTTCCCCTTTGCA-TTAATAAATAATCTGTGGGAGATACGTTGAG--------TGTATCCTATTCCTCA
Homo sapiens        TGATGAGGTTATGGTGGTACCTGTCAATTTTCTCCACTAGAAAGTTACCATTTTCCCCTTTGTAATTACTAAGTAATCTGTGGGAGACATTTTGAGGTGGTGTACATATCCTGTTCCTCA

Loxodonta africana  ACGAACTTTCGCCCACTAGTTCAAGCAGCCATCTGTTT-TTGTTTTT----TTCCAAGCAGGGAGTCCTTTACCTGTCTTCTCTAACTGGTTCTCCATTGTAACCCCAGCACCTAGAACA
Homo sapiens        TTAAATTTTCACCAGCTAGTTTAAACATCCACTTGTTTGTTGTTTTTGTTTTTCTGGGCTGGGA-TCCTTTGCCTGTCTTTTCTGCCTTGTTTGCTGCTGTATCCCCAGCATGTAGAACA

Loxodonta africana  GGGCTTGCCTCTTAGTAGGTATGCAGTCAGTATTTGCCAAATGAATGCATGTCTCTCTCTTTCTACTCTGTCTTACACTCTGGTTCTTTAAACGATTAAATGGGTCTCGTCTTGTGTCCA
Homo sapiens        GTGCCAGGCTCTTAGCA----TGCAGTCCATATATGC...................................................................................
```

```
Loxodonta africana  CTTGGCAATTTCCCTAACTGCTGTTAGGTCCAGTTCCTACTAAGATACCCTTTCAAGGTGAGTCTTTTGTCCCCAGGCCTCAGTCTACATTCAAGGACAGGACATGCATCTAGCAGTGAC
Homo sapiens        ............................................ATGCCCTTTCAAAATAAATCTTTTGTTTCCAGGCCTCTGTCTATATTCAGGGACAGGACATGCACCCAATGGTGGC

Loxodonta africana  CGAATACCAAGGAGCCTCTTTGGAGTGTCAGCACCTCCAGCTCCTGCCTGAAGTAACCACCCTGAAGTATGAACCTCCAGAG-ATCC--------ACTTCCAAGAAGTGAGTGGTGAGTG
Homo sapiens        AGAATATCAAGGAGTCTCTTTGGAGTGTCAGAGCCTCCAGCTCCTGCCTGGGATAACCACCCTGAAGTGTGAACCTCCACAGCGTCCTCAAGGGAACCCCCAAGAAGTGAGTGGTGAGTG

Loxodonta africana  CCAGAGGGTGAGTTTAGTGGGGACATTGCAGTAAGACCACCGACTGGTTCATAGTCTTATCTGTACCCTCAAGTTGTTGTCTTTTTTATTTATCTATTTTTGCCTAGCCCTGTAGGTAAG
Homo sapiens        CTCGA--GTGAGTTTAGTGAGGACGCTGGGAGCTGGCCACTGACACTTGCACAGCCTTCT---TGACCTCAAGTT.............................................

Loxodonta africana  CAAGTTTTTAAAAACATACGGAATTGTAAAATGTTGGAATTCTAAGATCATCTTGTCCAACACCTTCAATTAATAGTAGGAAACCAAAACCCAGAGCTCATAAATGCTCTGCCCAGGCTC
Homo sapiens        ........................................................................................................................

Loxodonta africana  AACGGAGGCAGAGCTGCTTCTAGAATTTAAGTCCCCTGACCCTCTGACCAAAACTCTCTCTCTACTACCTCTCTCCATTCTACATTTTCAATTCTATATTTGAGGACATTTTAGAGATTT
Homo sapiens        ........................................................................................................................

Loxodonta africana  ACGTGGTAGCTTTCCACAAACTTACCATTTCTCCCATAGTAACATTTTCTTCATTCTCTTCCCTGCCTCTAATCTTTCCACCATTTTAAAGATGGTTATTTAGGCTTAAACAGGTTCTGC
Homo sapiens        ........................................................................................................................

Loxodonta africana  CCTTCTGTCCCTTTGAAGTACACCATCCTCTCTGCAGAGCTCTCCCCACATCCAGGCTTCATTTCTAGATAAACCTCCCCAAATACTGGCTACATTTCAGCTTTGTATAGGCCCCTGACA
Homo sapiens        ........................................................................................................................

Loxodonta africana  GTGGGATAAACCATACAAAGAATTCAAAGTCTGTAGTCTGCAGTCTGTTGGCTGACTGGGGAGAACAGACAGTACACAGTTCAAAGGGGAGATCTTAGAACCCCACAGTGAGTGTTTTTA
Homo sapiens        ........................................................................................................................

Loxodonta africana  ACCTTTGTGTGAGGAGCACTACCAATTCTTTCAAGGGAATTCTTTCTTCTCTGGCAGATGCATGTACATCCAAAATTCTGCATATAATTTCAGGAGGTTCATAGTTTAGAATATCTACGT
Homo sapiens        ........................................................................................................................

Loxodonta africana  TGGTGATCTATGAATATTGATGTTTTAATGCTAAGTGCAGAATAAGTGGCTCACATAATAAACGGTATGGAAACATTAGCGGGAGATAGAATGATACATACAGGTTTCAGATTATTTGTG
Homo sapiens        ........................................................................................................................

Loxodonta africana  ATATTTACCCATTGCAGCATAAATAGAGCTTGATATTTCCCTACTTACATAGCTGATGGTTCTGTCACATTTCTTCAGTTACTAAAAACCACTACCACATGGAGCCCCAAGTGTTTTTAT
Homo sapiens        ........................................................................................................................

Loxodonta africana  TTATGTGAAGAATTTAGAGGGAATAGAGACCCCTGTGTTCTTGGGGGTCAAGGAAAGTTTCTGTGGAGGGGTGGACCTGGCACTGAGCTTTGAAGGATGGCAGGATTTGGAATGGAGCAG
Homo sapiens        ........................................................................................................................

Loxodonta africana  AAATGGGAGTGGGTAAGCATTCCTTTTTACCTATGAGTTGCTAAACATATTTACTGAAAGGGATTTCATTTCATTTTGATTGGAGGAAAGGGGATAATGGAAAATGGAGGAGACTGGTTG
Homo sapiens        ........................................................................................................................

Loxodonta africana  TCACAAAGGTTGTGGTAATTGAGAGAAAGATAACAGCAGTTGGAAGATACAACATCCTTTAAATGAAGGCTTAAAAACTACATGAAATGGCTATGCAGAGCTGGACCCCAGTGGCATTAT
Homo sapiens        ........................................................................................................................

Loxodonta africana  CTCAGGAAGAGGGATGGCTTCCCTTAGCTCTCTAAGCCACCATGAGAATGCACCACTGCAGTGGTATGTGTGATGGGGGTGAGGATGCTCTCTGGTTCCCTCGGAAGCTCTCCTTTTCAT
Homo sapiens        ........................................................................................................................

Loxodonta africana  TTCCAAGTGCACTTTATATCCTCCTAGATTAAAAAAAAAAAAAAAAATTTAAAACAAAACAGAAAAACTAGTTACCAGCAAGTCTGTTTTTACTCACGGCAGCCCCGTGTGTGTCAGAGTA
Homo sapiens        ........................................................................................................................

Loxodonta africana  GAACTGTCCTCCATACCCTGATTTTTCAGAAGTAGATGGCAGGGCCTTTCTTCCAAGGCGCCTCTGGCTGGGTGGACTGAAACCTCCAACCTTTCAGTTAGCAGCCAAGCACATTAACCG
Homo sapiens        ........................................................................................................................

Loxodonta africana  TTTGCACCATACAGGAACTCCTTATGTCTTTTTCTTTTTTTTTAATTGTGTTTTTGGTGAGAGTTTATATAGCAAATTAGGTTCTCATTTAACAATTTATGTGCTTATTGTTCAGTGATG
Homo sapiens        ........................................................................................................................

Loxodonta africana  TCGGTTACATTTTTCACAGTGTGTCACCATTCTCATTATTTCCATTTTAGTTGTTCTGTTTCCATTAACCTAGCTTCCCTGTTCCCCCTTACCTTCTCATCTTTAGTCTAGGGTAAATGT
Homo sapiens        ........................................................................................................................

Loxodonta africana  TTACTGTTCGGTTTCATTTAGATGGTGATTTAGAGGAGCACAGTACTTACAGGTGATATTATTTATTTTATGAGCCAATCTGTCATTTGGCTGAAAGGTGACATCCAGGAGTGGTTTGAG
Homo sapiens        ........................................................................................................................

Loxodonta africana  TTCCAGTTTAAAGGATATCCCAGGGTGATAGTCTTGGGGGTTCCTTTAGTCTCTTCTGGTCTGGTAAGACTGGACCTTTTTTTTAGGAATTTGAGTTTTGTTCTACATTTTTCTCGCATT
Homo sapiens        ........................................................................................................................

Loxodonta africana  CTGTCCAGACCATCTATTGTGTCCCTGGGCGGAACGGTCAGTAGTGATAGCCAGGTATCATCTAGTTCTTCTGGTCTCCAGGTAGGTGAAGCTGTTGTTTGTGTAGACTGTTAGTCCTGT
Homo sapiens        ........................................................................................................................

Loxodonta africana  AGACTAGTTTCTTCTTTGAGTCTTTGGTTTCCTTCTTTCTCTTTTGCTCCAGACGAATATCCTTATATCTTCGTAACCCCTCCCAAGTATTCTGGAGACTCCGGCTAAGGGCCTGCTTGC
Homo sapiens        ........................................................................................................................

Loxodonta africana  CTATCAGGTACAAACAGCTAGTTATAACCCAGGCACGTTTCTTCGGTCACCTGGACACCAGATAAACTGAAGATCAATTTCCCAGGCATACGTGCATCCTTCCCTTCCAGGCTGTTTCCT
Homo sapiens        ........................................................................................................................

Loxodonta africana  ATGTGGCTAGAAGGTAGGGTTTGTTCAGGGAAGTGCTGGCTGGAGGGAATGGAATGATCAATTGGAGCCATATCTTTAAATTTGAACCAGGAAATATGGACTTAATCATTAAAAAGTTGT
Homo sapiens        ........................................................................................................................

Loxodonta africana  ATGCCATCGAAGTCTTTATTTTTTAATCAAAAACATCATTTATAGTGATTGTATTATTTTTGTGTGTGTGCTATTTCATTCTTACCTGTCGTGAAATACAAGATTTAAAAGGAAAACTTG
Homo sapiens        ........................................................................................................................

Loxodonta africana  AGTCCTTTATTTTCACTAATCTGTTAATGCTCAATTGTGATTGATTCTTAGTGTCAAACTTGTAAAGTGTTTAGGTATGTTGATGGAGAAGAAGGCACCTTGGGATATCATCTGGTTTAT
Homo sapiens        ........................................................................................................................

Loxodonta africana  TGCATTTTTCACCATTTTCTCCGGTTCTGTTTGGCAGAGGTGAGAGCAGTGGAGCAGCATGATGTTTAAAATGAGCTTTCATACATTTGAAAACATCCCTCTGCCTCTTTCTCAGGCTTA
Homo sapiens        ........................................................................................................................

Loxodonta africana  ACTCAGAAGGAGGTTAAGGAGCAAGCACAAGGTCTGATGTGGCCTTTGCTCTCTCACGTTTCCATTCTCTTTGACCTCTTTCCTCACTGCATTATGTCTTTGCCCCCTTCTATTTCTGTT
Homo sapiens        ........................................................................................................................

Loxodonta africana  GCCACCCTTTCCCTTCCCCAACTCTTTGCCCTTTTCTCTGCCAGAAGACTTGCTTTA-----CACTGTATTTTTTAAGCTGTTGGATTTAGTGTGTGTGCATTGTGGGGAGGT-----TA
Homo sapiens        ............................................AAGACCTGCTTTATTTTACACTGTGCATTTTAAGCTGGTTTATTTAGTATGTATGTGTTGTGGACAAGTACATATA

Loxodonta africana  GTAGTGACAGTCAGTTAGGGGCAAGGCTG-TTACTTCTAGCAAGCTTTATTCCAGTAGTGCAGTGCCTGGCACTGTGTGGGCATCGTCACTGTTTTGATTATTATTGGTTCCTTTGGGAT
Homo sapiens        ATAGTAAAAGTGAATC-GGGGCAAGGCTGTTTTTTTCCAGCAAACTTTATTC.AACAGCACAGTGGCTGGCCTTGTGCAGTCATCATCACCATTTTGATTATTGTTGGTTC-TTTGGGAT

Loxodonta africana  CAGCAGCTCCCAACATGGAAGATTCTTAAAGGTGCTCCTAGCTCATGCCTAACGTCTTTTTTTTCCCTCTCTC----TTTGTATTTCCTTTCCTTTCTTTTCACCTTTATCTTTTTTGCT
Homo sapiens        CAGTACCTCCTAACA----------------GTTTTTCTAGTTCATGGCTAACATCTCTTCTCTATTTTTTTAAATATCTTTATTTTCCTGTCTCTCTCTCCATCTTCCTTTCTTCTGCT

Loxodonta africana  TTCCTTTTCCCCCTTTGTCTCATCTTTCTGCTCTCTTCTACTTTTCTTCCATTTCTTTGTTCTCTGCTTGATCTTTTCTTTCTCATCAGCTTCTTCCTCCCCTCTCATTCTTTTCATGTT
Homo sapiens        TTCCTGTTTCCCCTCAGTCTCATCTCTCTACTCTCTACATCCTTTCTTTTGCCTCTGTGTTCTCTCCTTGATCCTTCCTTTATCACCAGCTTCTTCCTCCCCTTTA..............

Loxodonta africana  TTTTTTTTAATTGTGCTTATGTGAAAGTTTGCAGTGCAAATTAGTTTCCCATTCAAAAATTTGTACACACGTTGTTTTGTGATATTGGTTATAATCCCTGCAGTATGTCAGCACTTTTTG
Homo sapiens        ..................................................................................................................TTTTTG

Loxodonta africana  CCTTTCCCTTTCTACCGTAGGTTCTACATGTCCATTCATCTAGTTTTTCTGTCCCTTCCTGCCTTCTCATCTTTGCTTTTGGGCAACATGTTTTTTTATTTTTCTTCATGTATGTGAAAA
Homo sapiens        TCT....................................................................................ATGATTTTTATTTTTTCTT----TATGTGAAAG

Loxodonta africana  TGGACAGCTGTAAAGCACCTCATGAGTTCATAAACTCCACACAGTTTTGTGGGTGGGTATCCTTAAAGTT--------TTAGAGCTCCCGAGAGAATGAAAATAGTTGTGATGCTCTGTC
Homo sapiens        TGCATGACTGTAAAGCACCTTATGAGTTCATAAATCCCACTCCATTTTTTGGGTGAGAGACCCTAAAGTTGTAGAATTTTAGAATTTCTGACAGAATGAAAGCAGTTGTGATGCTCTTTC

Loxodonta africana  TCAAATGGTCTGAAGATCTGAGGGTGTAGAAACCTTGTCTTCTCTTCGTCTTAGTTCTTGTGTGTTTAACAAACATAAAAAAGTTTGGG-----AGGCTGAGTCCCCACAAAGGACACTC
Homo sapiens        TCACAC------------------TGTAAAA-----------TCTACGTCTTAGTTCTTGAATGTTTAACAAACATAACA---TTTGGGGGAAAAGGCTGGGTCTCCAAAAAGGGTAC-C

Loxodonta africana  CTAAAGGCCATTGTGTCTTACCCAGGACTTTTGTCTCCTTCGCCCACAGGGCCTGCTCCCCAGGGGCCAGCCCTTCTCCAGGAAGAAAACCTCAGAGACAAGGCAGCAGTGCCTGTGTTT
Homo sapiens        CTAAAGGCCATCATGTCTCATTTAGGACTCTTGCCCTCTCTCTCTGCAGGGCCTGTTCCCCACGGATCAGCTCATCTCCAGGAAAAAAACCCCAGAGACAAGGCTGTAGTGCCTGTGTTT

Loxodonta africana  AACTCAGCCAGGTCCCAGGTGAGCAGGGTGCCTGGGCTTGTGGGAGCTTCAGCAGCAATC-----------CTCCATCTCCCCAGTCACCTCCTTACCCC-------------TGAGAGC
Homo sapiens        AACCCAGTCAGGTCCCAGGTAAGTAGGATGCCCAAGCTTGTAGGAATATCAGTAGTGGTCTTTCTCCCTCATTCCATGTTCCTTGCCCTTCTCTTGGCTCACACTCATCATCGTGAGAGC

Loxodonta africana  CTCTCAGGTGGTATAGTCCTCCTCCTTT--GCTTGTCACAGGACATTGCAGACCATGTGCATGTCA-CCAAGGAGCTCTTATTGATTCCATGCTACTGCCTC----TGTGTTCCTGGGGG
Homo sapiens        CATTCAGGTGGCATAGTCCCTCTGCCTTCACCTTGTCACAGAATATCACAAAGTGTGT-TGCATCATTCGAGGAGCTTGTATTGATTATATGCCATTGCCTCATTTCATTGTTCAGGAGG

Loxodonta africana  AGTATCCACACCTCAAGGTGTAGGATTGAGGGGTAGAATCAGGCCTTCAGTTTCAGGAAGTGCCCCACTTGTTTGATGCTTAGTGACTGATGTTCG-GCTTCTGAGGTCTCTAATTTCTG
Homo sapiens        CACGTCCAAATCTCAAGGCATGGGAGTGAGGGGTGGAGTGAGGCTCCTGGTTTCAGAAAGTGCTCCACTCATTTGATGTTGAGTAACTGATATTTTCTCCTTTGAAGTCTCTGGTTTATC
```

Loxodonta africana TCTTCTGCCTGAGAATCAGGAACTTTTCTCAAATCTTTACAGTCTCCATGTTCAGCACATGTCCAATTACTATTAAGTGATGATAATGGTGAAATTCTGTATATCCAAGACGAAACTTTG
Homo sapiens TCCCCAGCTTTAGAATAAGGGCCCTTTCTCACACT-------ACTTTATATTCAGCATGTATGCAATTAATGCTTGGTGATAATAATG--GAAATTCTATATATCTAAGGTAATACTTAA

Loxodonta africana CATTTTCTTCACAAGATGAAATGTTTTTCCCCTTCTTATTCCCTCAGTAAAAAAAAAAAAACCAAACCCGCTGCCGTCGAGTCGATTCCGACTTATAGGTCGCTGTTCCTTCAGTAGGTC
Homo sapiens CATCTTCTTCACAAAATGGACTCTGGTTTTTTTTCTTATT..AGTGGTTC

Loxodonta africana GATCATTTTCCTAGTTATTTAGTTTTTTTAAGTTAAAT--CTCTCTGATATAAGCCCTCTTGCACACAGCTGCTAGATCATTTTTCTCAAG-----CTCTCA-----------------
Homo sapiens ATTCATTTTCCTAGTCATTTA-TTTACAAAAAAAAAAATAACTCTCTCATTTAAGTCCTCTTGCATACTGCTGCCACATCAGTTTCCTCAAGACACTTGCTCAGTCCTCTTCTATGGCTTC

Loxodonta africana -----AGTTTGTAATTCCGTCAAGCCCAGTGCCTCCTGTTGTTAACAAATACTTTACCTTCCTGAAAAGAAATACACAGAAAATAAAACCTACCAGTACATATTTTTTAAATTAACATTA
Homo sapiens TAATTAATTTTTAATTCTATCAGACCCAGTGCCTCCTGTTATTAACAAATA------CTTCCTGATAAGAAGTATCTAGAAAATAAAACCTACTCATGCACATTCTGAAAATCAACATAA

Loxodonta africana TTTTCTTAGCTACAA--------TATACTGGAGAAACAAAGGAAAAGTAATTTATTAAAAAAATAAAAGTATGGAGGCGGGGCCAAGATGGCGGACTAGGTGGACGCTACCGTGGAGCCC
Homo sapiens TTCCCTTAAATGTAATTTAATGGTATAATGTAGAAACAA---AAAAGTTATTTATTTTAAAAGTA...

Loxodonta africana TCTTGCAACAAAGACTCGGAAAAACAAGGGAATCGATCACATACATAACAATCTGTGAACTCTGAACAACAAGCACAGACTTAGAGATGGAAAACGAACAAATACGGGCAGACAGCGACC
Homo sapiens ....

Loxodonta africana GTTTTCAGAACTAGGAGCCAGCGTACCAGGCAGGTGACCTTCGGAGCCCGATCTGGGGCAGAGCCCAGGGGGGCAGACGGCACAGAAGGGGGGCCCCAGCCCTTCCCCCCCGAACCCATC
Homo sapiens ....

Loxodonta africana CCAGGAGGAAGTCTAGCTGGTTGGCGCGGGTGGCGTAGCGGCGCAGCCGGAGGGAGAAGCACCCGGGAGGCAGTGACTGATCTGGGAGTGGGGAGAACAGCGTCCCAGCCGGGAAGCCAT
Homo sapiens ....

Loxodonta africana CCCGCCAGGAGTTTGGCAGGAAGCCGGCGGGGTGTGGGGGGGTCAGCTATATTTCCCTAAAGCGACCCCCGGGGCGGGGACCACACGTTCGTGCGGGGGGACGCACACCCAGTCCGCGCG
Homo sapiens ....

Loxodonta africana TGTGGCACGGCGCACTGGAGGGAGAAGTCCCCGGGAGGAAGTGACTGGTCTCGGAGCGGGGAAAGCAGCGCCCCAGCAGGGGAGCCGTCCCGCTGGGATTTGGGCGCGCACGCGGGCGGG
Homo sapiens ....

Loxodonta africana GCGTGAGCGCGGGGTCCAATTATATTCCCCTGAACTGACCCAGGGGGCGGGCCCACCTGGTCGTGCAGGTGACGCCCACCCAATTCGCGCATGCAGTGCGGCGCTCCAGAAGGGGAAGTC
Homo sapiens ....

Loxodonta africana TCCGGGAGGAAGTGACTGGTCCCTGAGCGGGGTAAGCAGCGTCCCAGCCGGGAAGTCGTCCTGCTGGGATTTGGGCACGCGCGCGGCGGGGCGTGACCGCGGGGCCCAATTGTATTCGCC
Homo sapiens ....

Loxodonta africana TGAATAGACCCTGGGGGCGGGCCCACCCGTTCCTGCGGGAAACGCCCACCCAGTTCACGTGAGCAGTGCCGCGCACCGGAGGGAGAAGTTCCCGGGAGGAAGTGACTGGTCTCGGAGCAG
Homo sapiens ....

Loxodonta africana GGAAAGCCACGTCCCAGCCAGGGACCCGTCCCGCCCGGATTTTGGCGGACGGGGGCGGAGCGTGAACGCGGTGTTCAGCTCTATATTCTGTGGTGCTACACTCCTAGCTCTCTGATCCCT
Homo sapiens ....

Loxodonta africana CCCCCACCCTCCCCAGGCGGCTCCATTAACATCCGAATACCCGGAGCCAGAGGGAGAATTCAGATAGGGATCTGACTGCATTTTTTTTCGCTGATTACCTGGAAAATCTAGTTTGCCAGT
Homo sapiens ....

Loxodonta africana GATGGCTCGGAGACAGCAGTCCATATCAAACCACATAAAGAAACAGACCATGACAGCTTCTCCAACCCCCCAAACAAAAGAATCAAAATCTTTCCCAAATGAAGATACAATCCTGGAATT
Homo sapiens ....

Loxodonta africana ATCAGATACAGAATATAAAAAACTAATTTACAGAATGCTTAAAGATATCACAAATGAAATTAGGATAAATGCAGAAAAAGCCAAGGAACACACTGATAAAACTGTTGAAGAACTCAAAAA
Homo sapiens ....

Loxodonta africana GATTATTCAAGAACATAGTGGAAAAATTAATAAGTTGCAAGAATCCATAGAGAGACAGCATGTAGAAATCCAAAAGATTAACAATAAAATTACAGAATTTGACAACGCAATAGAAAGTCA
Homo sapiens ....

Loxodonta africana GAGGAGCAGACTCAAGCAATTAGAATGTAGACTGGGACTTCTGGAGGACCAGGGAAACAACACCAACATAGCTGAAAAAAAAAAAACAGATAAAAGAATTAAAAAAAATGAAGAAACCCT
Homo sapiens ....

Loxodonta africana AAGAATCATGTGGGACTCTATCAAGAAGGATAACTTGCGAGTGATTGGAGTCCCAGAACAGGGAGGGGGGACAGAAAACACAGAGAAAATAGTTGAGGAACTCCTGACAGAAAACTTCCC
Homo sapiens ....

Loxodonta africana TGACATCATGAAAGACGAAAGGATATCTATCCAAGATGCTCATCGAACCCCATTTAAGATTGATCCAAAAAGAAAAACACCAAGACATATTATCATCAAACTTCCCAAAACCAAAGACAA
Homo sapiens ....

Loxodonta africana ACAGAAAATTTTAAAAGCAGCCAGGGAGAAAAGAAAGGTTTCCTTCAAGGGAGAATCAATAAGAATAAGTTCAGACTACTCAGCAGAAACCATGCAGGCAAGAAGGGAATGGGACGACGT
Homo sapiens ....

Loxodonta africana ATACAGAGCACTGAAGGAGAAAAACTGCCAACCAAGGATCATATATCCAGCAAAACTCTCTCTGAAATATGAAGGAGAAATTAAGATATTTACAGATAAACACAACTTTAGAGAATTTGC
Homo sapiens ....

Loxodonta africana AAAAACTAAACAAAGACTGCAAGAAATGCTAAAGCAGATTGTTTGGCCGGATGACCAATAATATCAGGTACCAGCACAATACAAGGTCACAAAACAGAATGTCCTGATATCAATGCAACT
Homo sapiens ....

Loxodonta africana CAAATAGGGAAAGCACAAAAACAAAGAAATTAAGACATTCTAAAAAATAAATAAATAAACAAAATAATACACATAACAGAAAATCATGGAAATCAATAGATAAACGATCACAATAATCAA
Homo sapiens ....

Loxodonta africana AAAGAGGGACTAAATATAGGAGGCATTGAACTGCCAGATGGAGAGTGATACAAGGTGATATAGAAGGATACAAGTTAGGTTTTTACTTAGAAAAATAGGGGTAAATAATAAGGTAACCAC
Homo sapiens ....

Loxodonta africana AAAAAGGAATATCAATTCCATAACTCAAGAAAAAAGCCAAGAAAAACGTAACGACTCAATAAACACAAAGTTAAACGTTATGAAAATGAGGATCTCACAAGCTACTATGAAAAACGTCTC
Homo sapiens ....

Loxodonta africana AGCACAAAAAAGCATGCGGAAAAATGAAATGGCTAACAACACACATGAAAAGGCATCAAGATGACAGCACTAAAAACTTATTTATCTATAATTACGCTGAATGTAAATGGACTAAATGCA
Homo sapiens ....

Loxodonta africana CCAATAAAGAGACAGAGAGTCACGGACTGGTTAAAGAAACATGATCCATCTATATGCTGCCTACAAGAGACACACCTTAGACTTAGAGACACAAACAAACTAAAACTCAAAGGATGGAAA
Homo sapiens ....

Loxodonta africana AAAATATATCAAGCAAACAATAAACAAAAAAGAAGAGGAGTAGCAATATTAATTTCTGACAAAATAGACTTTAGACTTAAATCCACCACAAAGGATAAAGAAGGACACTATATAATGATA
Homo sapiens ....

Loxodonta africana AAAGGGACAATTGATCAGGAAGACATAACCATATTAAATATTTACGCACCTAATGACAGGGCTGCAAGATACATAAATCAAATTTTAACAGAACTGAAAAGTGAGATAGACACCTCCACA
Homo sapiens ....

Loxodonta africana TATATAGTAGGAGACTTCAACACACCACTTTCGGAGAAGGACAGGACATCCAGTAAGAAGCTCAATAGAGACACGGAAGACCTACTTACAACAATCAACCAACTTGACCTCATTGACTTA
Homo sapiens ....

Loxodonta africana TACAGAACTCTCCACCCAACTGCTGCAAAATATACTTTTTTTTCTAGCGCACATGGAACATTCTCTAGAATAGACCACATATTAGGTCATAAAACAAATCTTTGCAGAATCCAAAACATC
Homo sapiens ....

Loxodonta africana GAAATATTACAAAGCATCTTCTCAGACCACAAGGCAATGAAGCTAGAAATCAATAACAGAAAAACTAGGGAAAAGAAATCAAATACTTGGAAAATGAACAATACCCTCTGGAAAAAGACT
Homo sapiens ....

Loxodonta africana GGGTTATAGAAGACATCAAGGAGGGAATAAGGAAATTCTTAGAAAGCAACGAGAATGAAAATACTTCCTATCAAAACCTCTGGGACACAGCAAAAGCAGTGCTCAGAGGCCAATTTATAT
Homo sapiens ....

Loxodonta africana CGATAAATGCACACATACAAAAAGAAGAAAGAGCCAAAATCAGAGAACTGTCCCTACAACTTGAACAAATAGAAAGTGAGCAACAAAAGAATCCATCAGGCACCAGAAGAAAACAAATAA
Homo sapiens ....

Loxodonta africana TAAATATNNNNNNNNNNNNNNNNNNNNNNNNNNNNNNNNNNNNNNNNNNNNNNNNNNNNNNNNNNNNNNNNNNNNNNNNNNNNNNNNNNNNNNNNNNNNNNNNNNNNNNNNNNNNNNNNN
Homo sapiens ....

Loxodonta africana NNNNNNNNNNNNNNNNNNNNNNNNNNNNNNNNNNNNNNNNNNNNNNNNNNNNNNNNNNNNNNNNNNNNNNNNNNNNNNNNNNNNNNNNNNNNNNNNNNNNNNNNNNNNNNNNNNNNNNNN
Homo sapiens ....

Loxodonta africana NNNNNNNNNNNNNNNNNNNNNNNNNNNNNNNNNNNNNNNNNNNNNNNNNNNNNNNNNNNNNNNNNNNNNNNNNNNNNNNNNNNNNNNNNNNNNNNNNNNNNNNNNNNNNNNNNNNNNNNN
Homo sapiens ....

Loxodonta africana NNNNNNNNNNNNNNNNNNNNNNNNNNNNNNNNNNNNNNNNNNNNNNNNNNNNNNNNNNNNNNNNNNNNNNNNNNNNNNNNNNNNNNNNNNNNNNNNNNNNNNNNNNNNNNNNNNNNNNNN
Homo sapiens ....

Loxodonta africana NNNNNNNNNNNNNNNNNNNNNNNNNNNNNNNNNNNNNNNNNNNNNNNNNNNNNNNNNNNNNNNNNNNNNNNNNNNNNNNNNNNNNNNNNNNNNNNNNNNNNNNNNNNNNNNNNNNNNNNN
Homo sapiens ....

```
Loxodonta africana  NNNNNNNNNNNNNNNNNNNNNNNNNNNNNNNNNNNNNNNNNNNNNNNNNNNNNNNNNNNNNNNNNNNNNNNNNNNNNNNNNNNNNNNNNNNNNNNNNNNN
Homo sapiens        ....................................................................................................

Loxodonta africana  NNNNNNNNNNNNNNNNNNNNNNNNNNNNNNNNNNNNNNNNNNNNNNNNNNNNNNNNNNNNNNNNNNNNNNNNNNNNNNNNNNNNNNNNNNNNNNNNNNNN
Homo sapiens        ....................................................................................................

Loxodonta africana  NNNNNNNNNNNNNNNNNNNNNNNNNNNNNNNNNNNNNNNNNNNNNNNNNNNNNNNNNNNNNNNNNNNNNNNNNNNNNNNNNNNNNNNNNNNNNNNNNNNN
Homo sapiens        ....................................................................................................

Loxodonta africana  NNNNNNNNNNNNNNNNNNNNNNNNNNNNNNNNNNNNNNNNNNNNNNNNNNNNNNNNNNNNNNNNNNNNNNNNNNNNNNNNNNNNNNNNNNNNNNNNNNNN
Homo sapiens        ....................................................................................................

Loxodonta africana  NNNNNNNNNNNNNNNNNNNNNNNNNNNNNNNNNNNNNNNNNNNNNNNNNNNNNNNNNNNNNNNNNNNNNNNNNNNNNNNNNNNNNNNNNNNNNNNNNNNN
Homo sapiens        ....................................................................................................

Loxodonta africana  NNNNNNNNNNNNNNNNNNNNNNNNNNNNNNNNNNNNNNNNNNNNNNNNNNNNNNNNNNNNNNNNNNNNNNNNNNNNNNNNNNNNNNNNNNNNNNNNNNNN
Homo sapiens        ....................................................................................................

Loxodonta africana  NNNNNNNNNNNNNNNNNNNNNNNNNNNNNNNNNNNNNNNNNNNNNNNNNNNNNNNNNNNNNNNNNNNNNNNNNNNNNNNNNNNNNNNNNNNNNNNNNNNN
Homo sapiens        ....................................................................................................

Loxodonta africana  NNNNNNNNNNNNNNNNNNNNNNNNNNNNNNNNNNNNNNNNNNNNNNNNNNNNNNNNNNNNNNNNNNNNNNNNNNNNNNNNNNNNNNNNNNNNNNNNNNNN
Homo sapiens        ....................................................................................................

Loxodonta africana  NNNNNNNNNNNNNNNNNNNNNNNNNNNNNNNNNNNNNNNNNNNNNNNNNNNNNNNNNNNNNNNNNNNNNNNNNNNNNNNNNNNNNNNNNNNNNNNNNNNN
Homo sapiens        ....................................................................................................

Loxodonta africana  NNNNNNNNNNNNNNNNNNNNNNNNNNNNNNNNNNNNNNNNNNNNNNNNNNNNNNNNNNNNNNNNNNNNNNNNNNNNNNNNNNNNNNNNNNNNNNNNNNNN
Homo sapiens        ....................................................................................................

Loxodonta africana  NNNNNNNNNNNNNNNNNNNNNNAAGGATCATGGAAGAAAAATAGGATCAACCCTAGGTAGCCCTAATACAGGGCATAAACAGAATCCAAAACATTACCAA
Homo sapiens        ....................................................................................................

Loxodonta africana  AAATGATGAAGAGAAACCTGATAACTGGGAGCTCCTAAAAATCAAACACCTATGCTCATCTAAAGACTTCACCAAAAGAGTAAAAAGACCACCTACAGAATGGGAAAGAATTTTCAGCTA
Homo sapiens        ....................................................................................................

Loxodonta africana  TGACATCTCCGACCAGCGCCTGATCTCTAAAATCTACATGATTCTGTCAAAACTCAACCACAAAAAGACAAACAACCCAATCAAGAAGTGGGCAAAGGATATGAACACACATTTCACTAA
Homo sapiens        ....................................................................................................

Loxodonta africana  AGAAGATGTTCAGGCAGCCAACAGATACATGAGAAAATGCTCTCGATCATTAGCCATTAGAGAAATGCAAATTAAAACTACGATGAGATTCCCTCTCACACCAACTAGACTGGCATTAAT
Homo sapiens        ....................................................................................................

Loxodonta africana  CCAAAAAACACAAAATAATAAATGTTTGAGAGGCTGCGGAGAGATTGGAACTCTCATACACTGCTGGTGGGGTTGTAAAATGGTACAACCACTTTGGAAATCCATCTGGCGTTATCTTAA
Homo sapiens        ....................................................................................................

Loxodonta africana  ACAGTTAGAAATAGAACTACCATACAACCCAGAAATCCCACTCCTCGGAATATACCCTAAAGATACAAGAGCCTTCACACAAACAGATATATGCACACCCATGTTTATTGCAGCTCTGTT
Homo sapiens        ....................................................................................................

Loxodonta africana  TACAATAGCAAAAAGCTGGAAGCAACCAAGATGTCCGTCAACGGATGAATGGGTAAATAAATTGTGGTGTATTCACACAATGGAATACTACGCATCGATAAAGAACAGTGACGAATCTCT
Homo sapiens        ....................................................................................................

Loxodonta africana  GAAACATTTCATAACATGGAGGAACCTGGAAGGCATTATGCTGAGCGAAATGAGTCAGTTGCAAAAGGACAAATATTGTATAAGACCACTATTATAAGATCTTGAGAAATAGAAAAAACG
Homo sapiens        ....................................................................................................

Loxodonta africana  GAGAAGAACATATGCTTATGTGGTTACAAAGCGGGGAGGAAGGGAGGGAGGGAGAGGGCTTTTTATTGATCAGTCTGTAGATAAGAACTGCTTTGGGTGAAGGGAAAGACAACACTCAAT
Homo sapiens        ....................................................................................................

Loxodonta africana  ACAAGGAAGGTCAGCCTAATTGGACTGGATTAAAAGCAAAGAGGTTTCCGGGATAAAATGAAAGCTTCAAAGGTCAGCGGAGCAGGGGCTGGGGTCTGGGGAACTTGGTCTGAGGTGACT
Homo sapiens        ....................................................................................................

Loxodonta africana  TCTAAGTCAATGGGCAAAATGATTCTATTATGAAAACACTCTGCATCCCACTTTGAATTGTGGCGCCTGGGGTCCTAAATGCAAACAAGCGGCCATCTAAGATACATCAATTGGTCTCAA
Homo sapiens        ....................................................................................................

Loxodonta africana  CCCACCTGGAGCAAAGGCAAAGGAAGAACACCAAGGTCACACGACAACTAAGAACCCAAGAGACAGAAAGGGCCACATGAACCAGAGACCTACATTATCCTGAGACCAGAAGAACTAGTT
Homo sapiens        ....................................................................................................

Loxodonta africana  GGTGCCCGGCCACAATCGATGTCTGCCCTGTCAGGGAGAACAACAGACAACTCCTGAGGGAGCAGGAGACCAATGGGATACAGACCCCAAATTCTCATGAAAATACCATACCTAATGGTA
Homo sapiens        ....................................................................................................

Loxodonta africana  TGATTGCGACTAGAGGAATCCCAGAGACAATGCTCCCCAGATCTTCTGATGGCACAGGACAGGAACCATCCGCGAAGACAAATCATCAGGCATGAAAAGGACTGGTCAGTGAGGGGGAGA
Homo sapiens        ....................................................................................................

Loxodonta africana  GAGATACTGATGAAGAGTGAGCTAATTAACTCAGGTGGACACAGGAGAGTGTGTTGGCAACTCTTGACTGGAGGGGGGATGGGAAGATAGAGAGAGAGGGAAGATGGCAAAATTGGCACG
Homo sapiens        ....................................................................................................

Loxodonta africana  AAACGAGAGACTGTAAGGGCTGACTCAATAGGGGGAGAGCAAGTGGGAGAAGGGAGTAAGATGTATGTAAACCTACATGTGACAGACTGATTGGAATGGTAAATGTTCACTTGAAGCTTA
Homo sapiens        ....................................................................................................

Loxodonta africana  ATAAAAATTAAAAAAAAAAAGAAAGTCTGCTAAATAAAAAAATAAAAGTATGTATTTCAAAATGAAAATTTTTGTACATGATTACACCAGAAGAAATAACAAAGTAGTCATTCATTTGCA
Homo sapiens        ....................................................................................................

Loxodonta africana  TGACATATAATCAAGTTTGGATTAAAAGACCAGACAAAGAAAGGGGGAAGTAACCATAATTGAAGTAAGGCAATGATGTCAAGGCAAAGTATGGACTGACAAAGTGAAGAAAACAAGGAG
Homo sapiens        ....................................................................................................

Loxodonta africana  ATATTCTTTAAAAGGAGGTGGACTTT-----TTAGTGCCAAAATAATTCTGTTTACAGTTTGTGATTGGGCAGTGATGA--TAGAGAGGAAATATCATCAGTCTG--------------
Homo sapiens        .......TTTAAATGAGGTGGGCTTTGCCTTTTATTATCAAAATAATT--ATCTGTAGTTTGGAATAGGGTGATGATGAGTTAAAGAGAAAACATCTTCTGTCTTGAAACCCCCTCCTTG

Loxodonta africana  --------------------TAACAAACTGGGCACTGTATCCTATCTCAGGCAATGGGAATGAGAGGGCAGATT----------AGAAAATTAAAAGCTTAGTGAAAAGCCATGTGACC
Homo sapiens        TCAAAACAGATATTCATTCTCTAAAAGACTGG-CACTGTGTCCTTTTTCAGGCAACAAGAAAGAGAAAGTAGATTGGAAAATGAGAGAAAATCAAGAGGCTAATGAAA--CCATGTG...

Loxodonta africana  AACTGTCTTAATTATCTGGTGCTGCTATAACGGAAATAAGCACCAGTGGATGGCATTAACAAACCGAAATTTATTTTCTCACGGTTTAGGAGGCTGGAAGTCCAAATTCAGGCACCAGCC
Homo sapiens        ....................................................................................................

Loxodonta africana  CTGGGGGAATGCTTTCTCTCTCTCTCAGCTCTGGAGGAAGGTCCTTGTCTTTTCTGAGCTTCTGCTCTTGGGCAGTCTTCACGTGCTTGGCATCTCTCTTACCCCTTCTCTCCTTGCTAG
Homo sapiens        ....................................................................................................

Loxodonta africana  CTTGCATTTAATCTCTTTTGTATCTCAAAAGAGATTGACTCAAGATACACCCTACACTAATCCTGCCTCATTGACATAACAGAGACAACCCATTCCCAAATGGGATTAAAACCACAGGCA
Homo sapiens        ....................................................................................................

Loxodonta africana  TAGAGGTTAGGATTTATATTATAACACGTATTTTGGGGAGGACACAATTCAATCCATGACATTCCACCCTTTGGCTCCCAAAAATTCATGCCCTTACCACATGCAAAACACAGTCACCCC
Homo sapiens        ....................................................................................................

Loxodonta africana  ATCACATCATCATCAAAGTCTTAAGTCAACTGAAGTCCAAAATCTCATATTCTGACTCAAATATGGGTTAGACTTTAGGTGTAAATTTTTTTTTTTTTAAACTTTATTTGAGAGGTTTTA
Homo sapiens        ....................................................................................................

Loxodonta africana  CAATATTTTTAGTCTTTGTGGCTTTATATTATTAACCTATTTGACCCGTGGCATTTGGAAAAATATTAGGTTTACGATGGTGACAAATATGGTGTGAAAACACTCAAGTGATTTAAAAGA
Homo sapiens        ....................................................................................................

Loxodonta africana  TGACTTAAAAAAAACAAACACCAAACCCAGTGCCATTGAGTGGATTCTGATTCATCACGACCCAATACGATAGAGTAGAACTGCCGTATAGGATTTCCGGGGAGCAGCTGGTGGATTCGA
Homo sapiens        ....................................................................................................

Loxodonta africana  ACTGCCGACCTTTTGGTTAGTAGCCGTAGCTCTTAAGCACTACACCACCAGGGTTTCCAAAAGATGACTTAAGAGCTATAAAGTAAACAGCCTGAATCTGAATTATATTAAGAAAAAAAT
Homo sapiens        ....................................................................................................

Loxodonta africana  ATTTTTAAAAGGCAAAAGAGCTCCGCCTGGTGTTGGACATAACTAATGAGGATTTTTTTTTTTTTTGATGGAATTTTTTTTTTTTTTTTAAATTTTTATTAGCTTCAAGTGACATTTACC
Homo sapiens        ....................................................................................................

Loxodonta africana  ATTCCAATCAGTCTGTCACATGTAGGTTACATACATCTTACTCCCTTCTCCCACTTGCTNNNNNNNNNNNNNNNNNNNNNNNNNNNNNNNNNNNNNNNNN
Homo sapiens        ....................................................................................................

Loxodonta africana  NNNNNNNNNNNNNNNNNNNNNNNNNNNNNNNNNNNNNNNNNNNNNNNNNNNNNNNNNNNNNNNNNNNNNNNNNNNNNNNNNNNNNNNNNNNNNNNNNNNN
Homo sapiens        ....................................................................................................
```

```
Loxodonta africana  NNNNNNNNNNNNNNNNNNNNNNNNNNNNNNNNNNNNNNNNNNNNNNNNNNNNNNNNNNNNNNNNNNNNNNNNNNNNNNNNNNNNNNNNNNNNNNNNNNNNNNNNNNNNNNNNNNNNNN
Homo sapiens        ........................................................................................................................

Loxodonta africana  NNNNNNNNNNNNNNNNNNNNNNNNNNNNNNNNNNNNNNNNNNNNNNNNNNNNNNNNNNNNNNNNNNNNNNNNNNNNNNNNNNNNNNNNNNNNNNNNNNNNNNNNNNNNNNNNNNNNNN
Homo sapiens        ........................................................................................................................

Loxodonta africana  NNNNNNNNNNNNNNNNNNNNNNNNNNNNNNNNNNNNNNNNNNNNNNNNNNNNNNNNNNNNNNNNNNNNNNNNNNNNNNNNNNNNNNNNNNNNNNNNNNNNNNNNNNNNNNNNNNNNNN
Homo sapiens        ........................................................................................................................

Loxodonta africana  NNNNNNNNNNNNNNNNNNNNNNNNNNNNNNNNNNNNNNNNNNNNNNNNNNNNNNNNNNNNNNNNNNNNNNNNNNNNNNNNNNNNNNNNNNNNNNNNNNNNNNNNNNNNNNNNNNNNNN
Homo sapiens        ........................................................................................................................

Loxodonta africana  NNNNNNNNNNNNNNNNNNNNNNNNNNNNNNNNNNNNNNNNNNNNNNNNNNNNNNNNNNNNNNNNNNNNNNNNNNNNNNNNNNNNNNNNNNNNNNNNNNNNNNNNNNNNNNNNNNNNNN
Homo sapiens        ........................................................................................................................

Loxodonta africana  NNNNNNNNNNNNNNNNNNNNNNNNNNNNNNNNNNNNNNNNNNNNNNNNNNNNNNNNNNNNNNNNNNNNNNNNNNNNNNNNNNNNNNNNNNNNNNNNNNNNNNNNNNNNNNNNNNNNNN
Homo sapiens        ........................................................................................................................

Loxodonta africana  NNNNNNNNNNNNNNNNNNNNNNNNNNNNNNNNNNNNNNNNNNNNNNNNNNNNNNNNNNNNNNNNNNNNNNNNNNNNNNNNNNNNNNNNNNNNNNNNNNNNNNNNNNNNNNNNNNNNNN
Homo sapiens        ........................................................................................................................

Loxodonta africana  NNNNNNNNNNNNNNNNNNNNNNNNNNNNNNNNNNNNNNNNNNNNNNNNNNNNNNNNNNNNNNNNNNNNNNNNNNNNNNNNNNNNNNNNNNNNNNNNNNNNNNNNNNNNNNNNNNNNNN
Homo sapiens        ........................................................................................................................

Loxodonta africana  NNNNNNNNNNNNNNNNNNNNNNNNNNNNNNNNNNNNNNNNNNNNNNNNNNNNNNNNNNNNNNNNNNNNNNNNNNNNNNNNNNNNNNNNNNNNNNNNNNNNNNNNNNNNNNNNNNNNNN
Homo sapiens        ........................................................................................................................

Loxodonta africana  NNNNNNNNNNNNNNNNNNNNNNNNNNNNNNNNNNNNNNNNNNNNNNNNNNNNNNNNNNNNNNNNNNNNNNNNNNNNNNNNNNNNNNNNNNNNNNNNNNNNNNNNNNNNNNNNNNNNNN
Homo sapiens        ........................................................................................................................

Loxodonta africana  NNNNNNNNNNNNNNNNNNNNNNNNNNNNNNNNNNNNNNNNNNNNNNNNNNNNNNNNNNNNNNNNNNNNNNNNNNNNNNNNNNNNNNNNNNNNNNNNNNNNNNNNNNNNNNNNNNNNNN
Homo sapiens        ........................................................................................................................

Loxodonta africana  NNNNNNNNNNNNNNNNNNNNNNNNNNNNGACTGTGATCCGCCGCTGTGATAACCTTAGAGGTCCTATCACCGTCTTTCACTGGAGGCGGTGTCAGAGGTCTGGGAGTGTGAGTGTTCACA
Homo sapiens        ........................................................................................................................

Loxodonta africana  GAGACCTTACCATTTTAATGATCAGGCGCGGAGTCTGAGATGTCGTAGCTGAATATTCTTTCCCAGTACTGGGCGTGTCTTTTTACTCTTTTGGTGAAGTCTTTAGATGAGCATAGGTGT
Homo sapiens        ........................................................................................................................

Loxodonta africana  TTGATTTTTAGGAGCGCCCAGTTATCTGGTTTCTCTTCATCATTTTTGGTAATGTTTTGTATTCTGTTTATGCCCTGTATTAGGGCTCCTAGGGTAGATCCTATTTTTTCTTCCATGATT
Homo sapiens        ........................................................................................................................

Loxodonta africana  TTTATCGTTTTAGTCTTTATGTTTAGGTCTTTGATCCACTTGGAGTTAGTTTTTGTGCATGGTGTGAGGTATGGGTCCTGTTTCATTCTTTTGCAAATGGATATCCAGGTATGCCAGCAC
Homo sapiens        ........................................................................................................................

Loxodonta africana  CATTTGTTAAAAAGACTATTATTTTCCCAATTAACTGACACTTGTCCTTTGTCAAATATCAGCTGCTCATAAATGGATGGATTTATATCTGGATTCTCAATTCTGTTCCATTGGCCTATG
Homo sapiens        ........................................................................................................................

Loxodonta africana  TGCCTGTTGTTGTACCAGTACCAGGCTGTTTTGACTACTGTAGCTATATATAGGTCTGAAATCAGGTAGGGTGAGGCCTCCACTTTCTTCTCTTTTTCAGTAATGTTTTGCTTATCCGGG
Homo sapiens        ........................................................................................................................

Loxodonta africana  GGTTCTTTCCTTTCCATATGAAATTAGTGATTTGTTTCTCTATTCCCTTAAAGTATGACATTGGTATTTGGATTGGAAGTGCGTTGTATGTATAAATGGCTTTTGGTAGAATAGACATTT
Homo sapiens        ........................................................................................................................

Loxodonta africana  TTACTATGTTAAGTCTTCCTATCCATGAGCAGGGTATGTTTTTCCATTTAAGTGTGTCCTTTTGAATTTCTTGTAGCAGAGTTTTATAGTTTTCTTTGTATAGGTCTTTTACATCCTTGG
Homo sapiens        ........................................................................................................................

Loxodonta africana  TAAGATTTATTCCTAAGTATTTTATCTTCTTGGGGGCTACTGTGAATGGTATTGATTTGGTTATTTCCTCTTCGATGTTCTTTTTGTTGATGTAGAGGAATCCAAGTGATTTTTGTATGT
Homo sapiens        ........................................................................................................................

Loxodonta africana  TTATTTTATATCCTGAGACTCTTCCAAACTCTTCTATTAGTTTCAGTAGTTTTCCGGAGGATTCCTTAGGGTTTTCCATGTATACGATCATGTCATCTGCAAATAGTGATAGCTTTACTT
Homo sapiens        ........................................................................................................................

Loxodonta africana  CCTCCTTGCCAATCTGGATACCCTTTATTTCTTTGTCTAGCCTAATTGCTCTGGCTAGGACTTCAAGTACGATGTTGAATAAGAGTGGTGGTAAAGGGCATCCTTGTCTGGTTCCCGTTC
Homo sapiens        ........................................................................................................................

Loxodonta africana  TCAAGGGAAATGCTTTCAGGTTCTCTCCATTTAGAGTGATATTGGCCGTCGGCTTTGCATATATGCCCTTTATTATGTTGAGGAATTTTCCTTCAATTCCTATTTTGGTGAGAGTTTTTA
Homo sapiens        ........................................................................................................................

Loxodonta africana  TCATAAATGGGTGTTGGACTTTGTCAAATGCCTTTTCTGCATCAATTGATAAGATCATGTGGTTTTTGTCTTTTGTTTTATTTATGTGATGGATTACATTAATGGTTTTTCTGATATTAA
Homo sapiens        ........................................................................................................................

Loxodonta africana  ACCAGCCTTGCATACCTGGTATAAATCCCACTTGATCAGGGTGAATTATTTTTTTCATGCGTTGTTGGATTCTATTCGCTAGAATTTTATTAAGGATTTTTGCATCTATGTTCATGAGGG
Homo sapiens        ........................................................................................................................

Loxodonta africana  ATATAGGTCTAAAATTTTCTTTTTTTGTAATGTCTTTACCTGGTTTTGGTATCAGGGAGATGGTAGCTTCATAGAATGAGTTGGGTAGTATTCCGTCTTTTTCTATACTTTGAAATACCT
Homo sapiens        ........................................................................................................................

Loxodonta africana  TCAATAGTAATGGTGTTAAGTCTTCCCTGAAGGTTTGGTAGAACTCTGCAGTGAAGCCATCTGGGCCAGGGCTTTTTTTTGTTGGGAGTTTTTTGATTACCGTTTCAATCTCTTGTTTTG
Homo sapiens        ........................................................................................................................

Loxodonta africana  TTATGGGTCTATTTAGTTGTTCTACTTCTGAATGTGTTAGTTTAGGTAAGTAGTNNNNNNNNNNNNNNNNNNNNNNNNNNNNNNNNNNNNNNNNNNNNNNNNNNNNNNNNNNNNNNNNNN
Homo sapiens        ........................................................................................................................

Loxodonta africana  NNNNNNNNNNNNNNNNNNNNNNNNNNNNNNNNNNNNNNNNNNNNNNNNNNNNNNNNNNNNNNNNNNNNNNNNNNNNNNNNNNNNNNNNNNNNNNNNNNNNNNNNNNNNNNNNNNNNNN
Homo sapiens        ........................................................................................................................

Loxodonta africana  NNNNNNNNNNNNNNNNNNNNNNNNNNNNNNNNNNNNNNNNNNNNNNNNNNNNNNNNNNNNNNNNNNNNNNNNNNNNNNNNNNNNNNNNNNNNNNNNNNNNNNNNNNNNNNNNNNNNNN
Homo sapiens        ........................................................................................................................

Loxodonta africana  NNNNNNNNNNNNNNNNNNNNNNNNNNNNNNNNNNNNNNNNNNNNNNNNNNNNNNNNNNNNNNNNNNNNNNNNNNNNNNNNNNNNNNNNNNNNNNNNNNNNNNNNNNNNNNNNNNNNNN
Homo sapiens        ........................................................................................................................

Loxodonta africana  NNNNNNNNNNNNNNNNNNNNNNNNNNNNNNNNNNNNNNNNNNNNNNNNNNNNNNNNNNNNNNNNNNNNNNNNNNNNNNNNNNNNNNNNNNNNNNNNNNNNNNNNNNNNNNNNNNNNNN
Homo sapiens        ........................................................................................................................

Loxodonta africana  NNNNNNNNNNNNNNNNNNNNNNNNNNNNNNNNNNNNNNNNNNNNNNNNNNNNNNNNNNNNNNNNNNNNNNNNNNNNNNNNNNNNNNNNNNNNNNNNNNNNNNNNNNNNNNNNNNNNNN
Homo sapiens        ........................................................................................................................

Loxodonta africana  NNNNNNNNNNNNNNNNNNNNNNNNNNNNNNNNNNNNNNNNNNNNNNNNNNNNNNNNNNNNNNNNNNNNNNNNNNNNNNNNNNNNNNNNNNNNNNNNNNNNNNNNNNNNNNNNNNNNNN
Homo sapiens        ........................................................................................................................

Loxodonta africana  NNNNNNNNNNNNNNNNNNNNNNNNNNNNNNNNNNNNNNNNNNNNNNNNNNNNNNNNNNNNNNNNNNNNNNNNNNNNNNNNNNNNNNNNNNNNNNNNNNNNNNNNNNNNNNNNNNNNNN
Homo sapiens        ........................................................................................................................

Loxodonta africana  NNNTACATTCAGCGTAATTATAGATAAGTAAGTTTTTAGTGCTGTCATTTTGATGCCTTTTCATGTGTGTTGTTGGCCATTTCATTTTTCCACATGCTTTTTTGTGCTGAGACGTTTTTC
Homo sapiens        ........................................................................................................................

Loxodonta africana  TTAGTAGCTTGTGAGATCCTCATTTTCATAATGTTTAACTTTGTGTTTATTGAGTCGTTACGTTTTTCTTGGCTTTTTTCTTGAGTTATGGGATTGATATTTCTTTTTGTGGTTACCTTT
Homo sapiens        ........................................................................................................................

Loxodonta africana  TTATTTACCCCTATTTTTCTAAGTAAAAACCTAACTTGTATCCTTCTATTTCGCCTTGTATCACTCTCCATCTGGCAGTTCAATGCCTCCTATATTTAGTCCCTCTTTTTGATTATTTTG
Homo sapiens        ........................................................................................................................

Loxodonta africana  ATCGTTTATCTATTGATTTCCGTGATTTCCTGTTGTGTGTATTATTTTGTTTATTTATTTATTTTTTAGAATTAGTCTTAATTTGTTTGTTTTTGTGCTTTCCCTGTTTGAGTTGCGTTG
Homo sapiens        ........................................................................................................................

Loxodonta africana  ATATCAGGACGTTCTGTTTTGTGACCTTGTATTGTTCTGGTACCTGATATTATTGGTCATCCGGCCAAACAATCTCCTTTAGCATTTCTTGCAGTCTTGGTTTAGTTTTTGCAAATTCTC
Homo sapiens        ........................................................................................................................

Loxodonta africana  TAAACTTGTGTTTATCTGTAAATATCTTAATTTCTCCTTCATATTTCAGAGAGAGTTTTGCTGGATATATGATCCTTGGTTGGCAGTTTTTCTCGTTTAGTGCTCTGTATATGTCGTCCC
Homo sapiens        ........................................................................................................................
```

```
Loxodonta africana  ATTCCCTTCTTGCCTGCATGGTTTCTGCTGAGTAGTCTGAACTTATTCTTATTGATTCTCCCTTGAAGGAAACCTTTCTTTTCTCCCTGGCTGCTTTTAAAATTTTCTGTTTGTCTTTGG
Homo sapiens        ........................................................................................................................

Loxodonta africana  TTTTGGCAAGTTTGATGATGATATGTCTTGGTGTTTTTTCTTTTTGGATCAATCTTAAATGGGGTTCGATGAGCATCTTGGATAGATATCCTTTCTTCTTTCATGATGTCAGGGAAGTTT
Homo sapiens        ........................................................................................................................

Loxodonta africana  TGTGTCAGGAGTTCTTCAACTATTTTCTCTGTGTTTTCTGTCCCCCCTCCCTGTTCTGGGACTCCAATCACTCGCAAGTTATCCTTGTTGATAGAGTCCCACATGATTCTTAGGGTTTCT
Homo sapiens        ........................................................................................................................

Loxodonta africana  ACATATAATTAAATTCTTTTATCTGATTTTTTTTCAGCTATGTTGGTGTTGTTTCCCTGGTCCTCCAGAAGTCCCAGTCTACATTCTAATTGCTCGAGTCTGCTCCTCTGACTTTCTATT
Homo sapiens        ........................................................................................................................

Loxodonta africana  GCGTTGTCAAATTCTGTAATTTTATTGTTAATCTTTTGGATTTCTACATGCTGTCTCTCTATGGATTCTTGCAACTTGTTAATTTTTCCACTATGTTCTTGAATAATCTTTAAGAGTTCT
Homo sapiens        ........................................................................................................................

Loxodonta africana  TCAACAGTTTTATCAGTGTGTTCCTTGGCTTTTTCTGCATCTCCCCTAATTTCATTTGCTGTCCCTCTAATGGTGCATTCTGTAAATTAGTTTTAAATATTANNNNNNNNNNNNNNNNNNNN
Homo sapiens        ........................................................................................................................

Loxodonta africana  NNNNNNNNNNNNNNNNNNNNNNNNNNNNNNNNNNNNNNNNNNNNNNNNNNNNNNNNNNNNNNNNNNNNNNNNNNNNNNNNNNNNNNNNNNNNNNNNNNNNNNNNNNNNNNNNNNNNNNNN
Homo sapiens        ........................................................................................................................

Loxodonta africana  NNNNNNNNNNNNNNNNNNNNNNNNNNNNNNNNNNNNNNNNNNNNNNNNNGGGTTCGGGGGGGAAAGGGGTTGGGCCCCCTTTCTGTGCCGTTCTGCCCCCCTTGGCTCTGCCCCCGATCG
Homo sapiens        ........................................................................................................................

Loxodonta africana  AAGCTCCGAAGGTCACCTGCTGGTGCGCTGGCTCCTAGTTCTGAAAACAGTCGCTGTCTGCCCGTATTTGTTCGTTTTCCGTCTCTAAGTCTGTGCTTGTTGTTCAGAGTTCGTAGATTG
Homo sapiens        ........................................................................................................................

Loxodonta africana  TTATGTATGTGATCGATTCCCTTGTTTTTCCGAGTCTTTGTTGCAAGAGGGATCCGCGGTAGCGTCCACCTAGTCCGCCATCTTGGCCCCCGCCTTAGGTGTAAATTTTTATGGAGCAAA
Homo sapiens        ........................................................................................................................

Loxodonta africana  ATTCCCCTTTACCTGTGAACCTGTGAAACCTAGATCACAAGTTATCTGTTTCCAAAGTACAATGGTGGAACAGGCACATGGTAGATATTTCCATTACAAATGGGAGAAATTGGAGGGAAA
Homo sapiens        ........................................................................................................................

Loxodonta africana  GAAGGAATAATGGGCACCAAGCAAGTCCAAAACCCAGCAGAACAATTTATGTTCACTTTCAAGGTTTGAAAATAATCCTTTGTCCTCTGAGACCATCTAGGTAATGGTCCTGCCCTCTAG
Homo sapiens        ........................................................................................................................

Loxodonta africana  ACACTGGTCGTTTGCCATGCCCTCTGGGTTCTGAGTGTAGGCCCCTTGGCCCTGGGCTTCAGCTCAGCCTTCCAGGCCCTCTGGAATGGCAACTCTGCTTCCTTGGCTTTAGGCGGACCC
Homo sapiens        ........................................................................................................................

Loxodonta africana  ATCTTCCTAGTCCATCTGAATGTCAACGCCACCCCCTTGGCCCCATTCTTCTGCCCCTTGAACATGGCAGCCCCACCTCCTCAACTTTGGGCAGCTGCCCCATCCCCCGGCACAACTTGA
Homo sapiens        ........................................................................................................................

Loxodonta africana  TGGCAGCCCACGCTCCAGAACCAAGTTGGCAAAGATCCAACTCTTTGAAACCAAGAAGGCCCTGCCTCTCATGCTCCTTCCAACAGTTCTGCTAATCTCCAAGCTGGTCTTTTTCTTGGA
Homo sapiens        ........................................................................................................................

Loxodonta africana  AGACAACAGAAGTAGCTCCTTTGGTCTGTTTCCTGCCTGTAGAATTCTAAGAGTCATATGGCATTCTTTTCTTTCTTTGTCCCCTTCAGTCTAAGCTGATAGGTCTTCTGTTGTGGTAGT
Homo sapiens        ........................................................................................................................

Loxodonta africana  TGGTTAGATCCACCAGTCACAGGCTTAATCTCTTTAAGAAAAGATTGTGTAGCCACATTCTTGGTGAACATTCTAAGACATGTGTCCTTAGTTTGTACAACAGAATTTTCCAAATCTTTA
Homo sapiens        ........................................................................................................................

Loxodonta africana  AGTTCTGTTTTCATTTTACACAGTTTATTTTTAAGTCCTTCTCTTTCCTCTTGCATTTTACTGTAGGTGGCAAGGAGAAACCATGCTTCCCCTTTAAAATCTTGCTTAGAAATCTCTTCA
Homo sapiens        ........................................................................................................................

Loxodonta africana  GCCAGATATCCAAGTTCATCACTTACAACCTCTACCTTCCACCAAACATTTGAACATAATTCTTTGCCACTGCATAAAAAACATTACCTTTCCTCCACTGTCCAATCACATATTCACCAT
Homo sapiens        ........................................................................................................................

Loxodonta africana  TTTCTTTTAAAGCCTTTCTAGAGCACATTTAACATCCACAATTCTAGCAATATCCCTGTTGCTGGCACCATATGTGTTCTTCTGAGCCATCACCAGAATTGCCTTTGACGTCTGTCTTAG
Homo sapiens        ........................................................................................................................

Loxodonta africana  TCATCTAGTGTTGCTGTAACAGAAATACCATAAGTGGATGGCTTTAACAAAGAGAAATTTATTCTCTCACAGTCCAGTGGGTTAGATGGAATTCAGGGCACCAGCTCCAGGGGAAGACTT
Homo sapiens        ........................................................................................................................

Loxodonta africana  TCTCTCTCTATCGGCTCTGGAGGAGGGTCCTTGTCATCAGACTTGCCTTGATCTGGGAGAATCTCAGCACAGGAACCTCAGGTCCAAAGGATGCGCTTTGCTCCCAGCACTGCTTTCTTG
Homo sapiens        ........................................................................................................................

Loxodonta africana  ATGGTATGAGTTCCCCATGTCTCTCTGCTCACTTCTCTCTTTTATATCTCAAAAGAGATTGGCTTAAGACACTGCCTAACCTTGAAGACCTCATCAGTATAATTGCCGCTAATCCATCTT
Homo sapiens        ........................................................................................................................

Loxodonta africana  ATTACATCATAGTGATAGGATTTACAACATATAGGGAAATCATGTCAGAAGATAAAATGGTAAACGATCATACAATCATGCAAGGGAATCATGACCTAGCCAAATTGACAGATATTTTGG
Homo sapiens        ........................................................................................................................

Loxodonta africana  GAGGATACAATTTAATCCATGACAACATCCATAATTCTACCAACAGTCTCTTCAAGGCAATTTAGGCTTTTACTATTCGGCACATTAAAATTCTTTCAACCACTACCTGTTACCCAATTC
Homo sapiens        ........................................................................................................................

Loxodonta africana  CAAAACCGCTTCCACAATTTAGGTATCTGTTAGAGTGGCACGTCACTCTTTTGGTACCAAGTCCTGTCTTAGTTATCTAGTGCTGCAATAACAGAAATACCACAATTGGATGCTTTTAAC
Homo sapiens        ........................................................................................................................

Loxodonta africana  AAACATGAATTTATTTTCTCACAGTTCAGGCTGGAAATGCAGGTTCAGGGCGCCGGCTCTAGGGGAAGACTTTTTCTATCTGTCGGGTATGGAGTAAGGTTCTTGTCTCTTTTGAGCTTC
Homo sapiens        ........................................................................................................................

Loxodonta africana  TGCTCCTGGGCGATCTTCATGTATTTGGCATCTCTCTTCCCCTGTCTCTCTTTGCTAGCTTGACTTTTATGTCTCAAAAGAGATTAACTCAAGGTATACCCTATACTAATCCTGTCTTAT
Homo sapiens        ........................................................................................................................

Loxodonta africana  TAACATAACAAAGACATCCCATTCCCAAATGGGATTATAAACACACAAACAGGCATAGAGGTTAGGATTTACAGCACATATTTTGGGGTACACAGTTCAATCCAGACCTTTGGGGACAAT
Homo sapiens        ........................................................................................................................

Loxodonta africana  TTTAGAAAACCACAGGCAACACCCAAAATAAAGAAATCATTTTAGCATATAATTTTTCCAACCTAGTAGAACTGCCCCATATGGTTTCCAAGGAGCACCTAGTGGATTTGAACCACCACC
Homo sapiens        ........................................................................................................................

Loxodonta africana  CTTTTGGTTAACAGCTATAGCTCTTAACTACTACACCACCAGGGTTTCAGATATCTTATCACTCTCCTATATTTGAAATATTTT-AAACAATCTTTTTAAATGAAATTCAGTGAGATATC
Homo sapiens        ..................................................................CTAGCTTTGAAATGTTTTTAAACAATCTTTGTTGATGAAATTCAGTGAGATTTC

Loxodonta africana  TGTACGTATATTAGGCTCCAGGTCTGTTAAAAAGAGATCTCAAAACTCCTCCATCTTATATTTACACTCAGTTTAATTGCATTTATAGTAACTGCAGTACTGCATTGAATTCCCCTTCAA
Homo sapiens        TGCATTTACATTAGATTCCAAATCTGTTAAAAGGAG-TCTCAAAACTCTGTTAG---GTATTTGTAGTCAGTTTAATTGCATTTGTTATAACTGCAATATTATATTGAAGTGCTGTTCAA

Loxodonta africana  ATACTTAGTATGAAAATGCTAGAAAATAGTG----GAATGATCCAAGTGAACCCATTAATCAATACTTTTGAGACAATGTCTTTATTCCATGTAAGCTTATTTCTCCATAACCTCCTAAA
Homo sapiens        CCAAATACATAGAAAATGCTGTAAAATAGTGTGTAGAACAATCCAAGTGAGTCAA----------CTTTTGTGACAATGTCTTTAATCGATGTAAGCTTCCTTCTCTA-ACTTTCTTGAA

Loxodonta africana  TTACTGG--GTTTTTCAATATCATCCTATAAAAACTTTTCAGAATTCTTACATACTTAAGGGCAGCCAATTTAT------TTTAGGTTTAATCCAGCAGAACACTTGAAAGTCATGCAGG
Homo sapiens        TTACTGACTTTTTTTCAGTATCATCCTATAAAAACTCTTCAGGTTTCCTATGT--TTCAGTGTAGCTGATTTATACTGATTGTAGGTTTGGTTCAGGAGGACACTCAAAAGTCCTGCAGG

Loxodonta africana  ATGTGTAGCAGTACTTTGTAGTGTGGAACTGTCCCACACATTGCAATATGTCTGCCATCTCTGGCATCATCCTCTGAATGGCATTGGCAGGAGGTGATGGTAGGCAACATTATTATAACC
Homo sapiens        ATGTGGAAGAATTCTTTGTAGTGTGGGACTGTCCCACATATTTCAAGATGCCTAGCATCTCTGGCCTCACCCTCTGAATGGCATCGGCTTGAGGTGGTGGTGGACTTCATTGTTATAACC

Loxodonta africana  AAAAAGGTTTCCACAGATTTAAAGAATGCCCCTGAGTGGTTTGTACCACTGGTCTACAGTGTGGTA-----CACTCTTCATAACTGGATCCCTTATCCTTACCACAGTGAAAACAAAGAG
Homo sapiens        AGAAAGGTTCCCACAAGTTTACAAAATGCCC-TGAGGGGCTGGGACTACCGGTCTACAGCGTGGTATTCAACACCCTTCACAACTGGATTCCTTACCAT-------GTGAAAACAAAGAG

Loxodonta africana  ------CTCATGTGTTGTCTTTTCAGACGTTGGTGAAGACTGAGGAAGAAGCAGCCCAGGCCCTTGCCCCACAGGAGTGGCCTCATCTGGGTTTGGCTGAGAGGAACCTCTCTGGGAACT
Homo sapiens        GCTATTTTCTTTTTTTGTCTTTTTAGACATTGGTGAAGACTGAGGAAGAAACAGCCCAGGCCGTTGCTGCAGAGAAGTGGTCACATCTGAGTCTGACTCGGAGGAACCTCTGTGGGAACT

Loxodonta africana  CAGCTCAGGAAAAGGTTACAAACCTCAGTCTGATGAGTAAGGACAGACCTCTGGCCAGTCCCCTGTCTATGCTTTC----TCCCATCATTAACCTTGG-TTGTTCCCGGTGAACCCATGT
Homo sapiens        CAGCTCAGGAGACAGTTATGAGCCTCAGTCCGATGAGTAAGGCCAGGCCTCTGGCTGGACCCCTGTCTGGACTCTCATTTTCCTGTCACTCACCCTGGCTTGGCCCTTGGGAGGCCACGT

Loxodonta africana  TTCCTACAAA----------TGCTGACCCTTCTTCTG---CTAGTCTCCAGACTTCTCTCAGCCCCTATCCTTTTGTGTCCCTCTCCATCG-ATT-----------CACACAGCTTGTT
Homo sapiens        TTCCTACAGACCCACACCCTTCCTTTCCCTTCTTCTGGCCTCTGCCTCCAGACTTCT---AGCCCTTATCACTTTGCATCCTTCCCAATCACATTTTCTTCTGTTACTAATCAGTTTACT

Loxodonta africana  GATTCTTGTTGTTACCTTCTCTGATCATCCCTCT--GTATTCTGATTCCCTCAGCAATTTC------TCCTTTGCTCCTCTTTTTCTTCCCTATCAAGAGTTGTATATAACTTGTATTTG
Homo sapiens        GATTGTTGTGGTTATCTTCTCTGATCATCTCTTCTAGTTCCCTGATTCCTTCAGTGACTCCTTTGTATCCTTTTCCCCT-TTTTTCTTCCCTGTCAAGGATTCCATATAACTTGTCTTT-

Loxodonta africana  TAGACATCCCTTCATACCTGGAAGCCCC----ACATCCTTCTCTGCCC---TCTTCACTTCA---GAAGTTCTCTTCACTCTCTGAGAATGCTACCGTCCATGTATACTTTTCTCCAAAT
Homo sapiens        CAGATATCCCTTCATACTTGGAAGCCCCATGAACACCTCTGTCTGACCTTGTCTTTACCTCAGAGGAGGTCATCTTCACTCTCTAAGAACACGACAGTTCATGCCTAATTTTCTTGAAAT
```

```
Loxodonta africana  GTGCTTTTCCCTATTAGATCCTCTCCTTAGCTGAGCCCTGCTGCCCTACTTGACCTTTTTCAATATCTTTCCATGTTGTGCCACATTTTGCGGGCTTGATGAGACTGAGAACTCTTGGGA
Homo sapiens        GTGCTCTGCCCTGTTGGGCCCTCTCTGCAGCTGGGCTTTGGTGCCTTAATTAACCTTTCTTATCATTTTCCCATACCATGCCACATTTTGTAGGATTGCTGAAACTGTGCACCCTTGAAA

Loxodonta africana  TATCTGT-TGATTTGTGTAGTTTTTTTTTGTTTGTTTTTTAATTTGGAATATCCTGTGTTCTACCCTGGAGGGTGTTTTTTTT------------------GAAAATCAGGAAGCAGGT
Homo sapiens        CACCAATATGATTTG--------TTTTTTGTTTG-------ATTTGGAACAT-CTATTCCCTACCCTGGAAGTTTTTTTTTTTTTTTTTTTTTTTTTTTTCAAAATTCACATAACAGAT

Loxodonta africana  GACATTTAAATCCCTAATG-TATATTTCAGCTGAGGAAGTTGTGACTAAAGATGGATTGTTT-CATCAAAGCAGGAAACTTCTGAAGAAGTGGACCCAGGTGGGGAGACCTCAGGAATAC
Homo sapiens        GACATTTAAATCATTAATGTTATATTTTAGCTGAAGAAATTGTAACTAAAGATAGATTGTTTAAAGCAAAGCAAGAAACTTCTGAAGAAATGGAACAAAGTGGAGAAGCCTCAGGAAAGC

Loxodonta africana  CCAACAGGTAAGTGT-CATGGTCCTGAGTGGAATGAATCCTGCAAGTATAAGAAGAAACTAGAATTGAGTCAGGGAAACCAGATGGTCAGAAGGCTGCTTCCCTGGGATAATATAGAAAG
Homo sapiens        CCAACAGGTGAGTGTCCATGGTCCTCAGTGAATCAAATCCTGCAGATAT---AAGAAATTGGAATGGAATCAGAGAAACCAATTGGTCAAAGGCCTGCTTCTCTGGAATAAGATATGACA

Loxodonta africana  AGTGCCGCTCTTACCTCTGTGGTCATAGGAGGCCCCATGTCAAGAATGTGACCCG----------TCAATATACAACAATTATGCAAAAACATTCACTTGTAGCACAGACCTTTGTACCC
Homo sapiens        ACTGCCACTC--ACTTCTGTGATCATAGGAGG-TCCATGCCAAGAAAGTGACCTATTCAAAATTTTCAATATGCAATAAATATAC-AAAACCTTCAGTTGATACTCAG----TTTCCTCC

Loxodonta africana  AGGAATCCTATCCTGAACCAGTCTACTCTGTGTGATCACTCTTTGCTGGGTTCCCTTATGTTTT--------TGTAGAGAAGTCAGAAGGCAGAGAACCTAAACCAGAATGAAGGCTGCC
Homo sapiens        AAGAAGCCTATCCTGATCCAGTCTGTCCTGCTTGATTACCCTTCCCTGGGCTCCCTTACATTTTTATGGAGCTGTGGAGAAGCTAGAAGGCAGATAATTT-AACAAGAATGAGGGCTACA

Loxodonta africana  AGTGAGGCCGCCATGACAATTAAGAAAGAGATACCATCAGAGCTGGAAGAGGACTTAGGGAGGACAAGTAGA---------GTGTTTTCAGCCTTCATTCAAGAGAGTCCCGAATAAGGA
Homo sapiens        GATGAAGCTGCCATGACAGTTAAGAAAGAAAT---GTCAGAG-TGGAAGAGGACTTAGGGAAGATAAGGGAAAATTAACCTGTATTTTCAACCGCCATT---GGGAGACCTGAACAAGGT

Loxodonta africana  AAACATCAGAGGGAAAG-TAGCCAATGAAGAAGGAGAAATTAAAATTAGTAGGAGATGCTTAATAAGTGTCAGTTGCATGAATGA--GAATGTGTAATACGGATACTCCTAAAGTG----
Homo sapiens        ATAGGTCAAAAGGAAAGTTAGC---TGATGAAGTAGAAATGAATGACAGTGGTGTGTGCTTCATAAGTGCTGGTTGTGGGAATGAATGAACTTGTA--ACAGATACTCCTAAGGTGGAGA

Loxodonta africana  -------------------------------------------GAAAGGGAGGGACCACGTCACAAGCAGAGGATTGGTTTC---TGAGGCCATTAACTTCCCCAAGACTTGAGAAAAAA
Homo sapiens        AGCCTCGGGAATGCCCAACATTCCGAGGCTTCTCAGGATTGCAGAAAGAGAGGGAACAAATCACAAGCAGAGGGTTGGCTTCTGGTGAGGCCATTTGGTCCTCAGAGACTTGAGAAAA--

Loxodonta africana  GGAGAAAGGAAACAA--TGGAATAATAACCTGAAGAGC----AGGGCCATGGGAATT---CTTGTTGGCATTTGTTTGTTTTAGGGAAACAGGGAGATAAATTTCAGCTAAAATTAAAAT
Homo sapiens        -GAGAGAGTAAGCAAGGTGGGCTACTTGGCCAAAGAGCTGAGAAGGGGGTGTGAATTTTCCTTATTGACATTTGTTTAACTTAGAGAAACTGGGAGCTAAATCTCAGCTAAAGTAGAAAT

Loxodonta africana  GCCTTAAAGCTTAAGAGAAAA---GGAACTTTGCATAAACCTTTTTTTTAGTGAGGCTTGAGCC--------------AAGAGTGGTGATCTTCAGTGACTGAGTGGAGAGT--TGTAT
Homo sapiens        GTCTTAAAGCTCAAGAGGAGAAGTGAAATTTTGCATTAA------GATTAGTGAAGACTGAGCCAGGCTCCACTAAAGGAAGAGATATGATGTTCAGTAACTTAGAGCAGAGTTATGCAA

Loxodonta africana  CTGGAGAATAGGAATCTGTTACTGTGCCTCTTGGGTGTATTCAGCATTAGGTAACTGAAACCATGACTCACAGTGCTTTAAGAATAAAGATATTTTATTCTTTTATGTAACAAGAAGTCT
Homo sapiens        CTGGAGAATAGGAATCTGCTATTGTGCCT-GTAGGTACATTTAGTATCAGGCAACTAACA-GGTAATTCATAGTGCCT--ATAATAAAGATA---------TTCATATAACAAAAAGTGT

Loxodonta africana  TAAGATAGGCTGTTCCAGGTTTGGTACAGCAGCTCAACTACCATAATGGGTTTAACGTATGTCTGTGGTCAATATGTGACATGTAAAATAGCCACTTTTGTTTTTTGTGCATGTGTTTTA
Homo sapiens        GAAGGTAGGCTATTTCAGGTTAGGTGCAATAGCTTAACCA................................................................................

Loxodonta africana  AATTTATTTAAATCGTATTGTGATAGGTATTGTTCCGTTTCTTACTTTTTCAGTCAGCATAATGTTTTAAAATCCATCTATATTTCTATGTATATATTTATTCCTTTCCTTATAGCTGTT
Homo sapiens        ........................................................................................................................

Loxodonta africana  CCATAGGTACCCCATGGAATACATCCACCATGTTTTGCCTATCAGCTTTCCCCAGAGATGGTCACCCACATTGCCTCTAGCTCCCCATCACCATGATCCGTGCTACAGTAATCATCCTCA
Homo sapiens        ........................................................................................................................

Loxodonta africana  AACATGCCCCTTGGGTGAAAATTTCTTTCAGATAAATACCTAGGAACAGGATAACTGGATCATAGGATGTGCTTATATTAAATTTACCCACTGCCATCGAGTCAGTTCTGACTCATAGTG
Homo sapiens        ........................................................................................................................

Loxodonta africana  CCCCCATAGGGTTCCCAAGGCTGTAGATCTCTACAGAAGCAGACTGCTGCATCTTTCTCCCACAGATCGCTGGTCATTTCGAACCACCGACCTTTCAGTTAGCAGCCAATTACTTTAACC
Homo sapiens        ........................................................................................................................

Loxodonta africana  ACTGAGCAACCAGGGCTCCTAAATTTAATTTGGTTCTGCTAAATTGACTTCTAGAAAAGATACATGATCTCTACTCCCAGAACAGTAGATGACAGTTCATTTATTTCCATATCTCCTCCA
Homo sapiens        ........................................................................................................................

Loxodonta africana  ATACTTGGCATGATTCAGTTTTCATATTTGGGGATCCAGGCTCTTGGTCTCTTCCTGTTCTACCATCCTTAGTGCATTGGTTATTTGGCCTTTAACCACAAGATGGTGCTGCAGCTCTAG
Homo sapiens        ........................................................................................................................

Loxodonta africana  GTACTGCATCTTTGCTTAAAACCAGGAAGAAGGGTACCTCTAGCTTTATCTGTTCCTTTTATCAAGAAAGCAGAAACCTTACAGAAACCCCCAGCAGTCTTCTCCTTATTCTCATTGGTT
Homo sapiens        ........................................................................................................................

Loxodonta africana  GAAATTGGAGTCACATGATCATCCTAGCTACAAGAGAGACAGGGGAATTGTATATCTAGCCAAGGGGAATGGGATTACCACGACTGATTTAGACCAGTCATAAGTTGTAGACTGGACCTG
Homo sapiens        ........................................................................................................................

Loxodonta africana  GAAACATTGCTGTCCCAGGCACAATCAGGTTCATGGTTAGTTGGGAGGAAGAGGGCAGGAGATGTTGGCAGGAAAATGACAGTCTGCCACAGTCATGGAGCAGATTATTAGGGATCTGAA
Homo sapiens        ........................................................................................................................

Loxodonta africana  ACTTTCTTTCACAGGATGTCACAGGCTAGGATGAACTAGAGGGAGGTGTGGATCAAGAATTGGGTTTGGAGAATCAAGATGAACTAATAAAAGAGAAACCAAGGCATAGTTATATTGAAA
Homo sapiens        ........................................................................................................................

Loxodonta africana  AGACTTTTTCAGGCACACCTTTAGAAGTTTCTAACTTTTATTTTATTTCATATTCTTCTTTTCCTGTACTGATACATTCAGCAAACATTTTGTCAGTCATATTCAAAGTCATATATAATA
Homo sapiens        ........................................................................................................................

Loxodonta africana  CAGAGTCCCTACCCTCAGTAAAAGAGCCCTGGTAGCATAGTGGCTAAGTACTTGTCTGCTAACTAGAAGGTCTGCAGCCTCTCCAAGGGAAAAAGATGTGGCAGCCTGCTTCCGTACAGA
Homo sapiens        ........................................................................................................................

Loxodonta africana  TTACAACCTTGGAAACTCCATGGGGCAGTTCTGCTCTGTCCTATAGGGTGGCTATGAGTTGGAATTGACTGGACAGCAACAGGTTTGGTTTTTGGTTTTACTGTCAGTAAGTCAAATGGG
Homo sapiens        ........................................................................................................................

Loxodonta africana  AAAAGAGACACTGCAGATGAGGAAGCACACAACTACCACACACTATGATGAAGTGTCATGGTAGCTATGTTAGAATATGCAGGAGAGGCCCCTGAGATTTGAGTATCAGAGAAAGTGTCT
Homo sapiens        ........................................................................................................................

Loxodonta africana  CAGAGGAGGTGACATCTGGGACAAGACTTGATGAGTAAGTAGGAATTTGCTAGATAAAGTGGAAAAGAAGTAGAAAAATAGAATTGCAAATGCCTAGAGAGTATACGAGAAGAACTACCA
Homo sapiens        ........................................................................................................................

Loxodonta africana  GAGAGTGGAGTCATAGGAACCACAGAAGAAGACGGTTTCAAAGAGCAAATTTCTGCTTGAAAAATTCTGAGTCATCTTTGGAATAGGAAGAGAGGTCATATTTAGAAATACCAAAGACTG
Homo sapiens        ........................................................................................................................

Loxodonta africana  AATAGAAAATTTGAGAAGAGTGATGAAGATGTGAAATAGCTAATGTGGGGAACTGGGGAGAAAGGAAAAAGATAAAGTGAGAAGGATTTCTGGGCAATATTGAACTAGCTGAAGTTGAGG
Homo sapiens        ........................................................................................................................

Loxodonta africana  ACCATTATTTTTAGCAGTGTCTACTTAATTGTGTGATTTCTTTGGCAGCTCACTCTTGAATGAGGAACAGAGAAAGCCAACAGTAGTACTGATCTGATGCCGAAGTTTCACTGGGCAGAT
Homo sapiens        ........................................................................................................................

Loxodonta africana  GAGACAGATGGACACTGGGGCAGGGTGGTTGATGATCCTGGTAAGAGAGGGGCTAACGTGGTAGGTCATGGGGTCTGGACTGACTAGGAGGAAGGTGAAAACCAGAGAGAATGTAAGGTG
Homo sapiens        ........................................................................................................................

Loxodonta africana  AGAGTGAGATGGAAGAACAGGTCTCATGGAGTGAAGAGAATGAAGAGGCTAGAGCGGAAAGAAATTGGGACTAGAGAATGAGCTATTGGAGTTTAGATTTCAGCAATGAGTGAAAAGGGT
Homo sapiens        .................................................................................GTTCTTAGAGTTTAGATGTCAACA----GTAGAAAGACT

Loxodonta africana  CAGTGTAACGGCAGGATTCAAGGAATGTCCTTGAAGTTGAGGGTGTCAGAGAACTCTAAGATGGATGTGTTGAATGGATCATACATGTGGACATTGACAACACTCAGGATGATAAACTCT
Homo sapiens        CAGTGTGA---------TCAAGGAATGTCACTGAAGTTATGGATGTCAGGGAACTTTAAGATGGATGTGTTGA-------ATACATGCAGACACTGAAGACACCCTGGATGATAAACACT

Loxodonta africana  TTTAAGTAAATAAGTGAAGGTATGTAGTATAAAAAAAAAATTTTTTGTTTTTTTATTTTTTAAACATACTCTTTTCAGTGCTTTTTTCCCTTAGGTCTTTTAAAACTAGACTAGTTTACT
Homo sapiens        T-----TACATAAAGAAATGTATGTAGTGTAAA-------------------TTA-TATTAAAATATACTCTTC---ATGCTTCTTAA----ATGTCTTTGAAAACTTGACTAGTTTACC

Loxodonta africana  AATTAGACA-TAGTAAATGAAATGCAAATTTCTAGAGTTAAAAAAGGAGTTTGTGTAAAATAATACTTTTCATCTTAGTAACTTTAGCTTACTTCCTTGTAAACCCCATAAAAATCAGTT
Homo sapiens        AACAGAACACTAATAAATTAAATGCACATGTCTAAAAATTAAAA---ACTTTGTGTAAAATAATACTTTTCATCTTTGTAACTTTAGCTTACTTCCTTGTAAACCCCATAAAAATCAGTC

Loxodonta africana  CAAGTACAGTAAATCTGAGTTTCTCTTACATTTCTCCCTGGAGCAGCATGTCCACTGGATGGGTGTAGAACTAGGACAGGCTAAGTCCTCCCATATTCAGAGGTTCTGTGAGAGGTATGA
Homo sapiens        CAAGTGCAATGAGT-TGAGTTTCATTTG--TTTTTCCCCAGAGTAGTATGTCCATCAGATGAATGTAGAACTAGCAGGGGCTAAGTCCTTCCATGTTCAGAAATTCTGTGAGAGATGTCA

Loxodonta africana  GGGGACCCAGTAAACTCCAGC-TCTTTTCCTTGTCCAAGTTCAGAGCCAATTGACGGAGACACAGGAGTCAAACAGATGGTGCTGGAAACCTCAGCTTTATTACTGACAGAAGCTAGGCT
Homo sapiens        GAAGACCTGGTAAATAATAACCTTTTTTTCTTGTTCAGATTCAGAACCAATTGACAGGGATGCAGGAGTAAAATAGATGGTTCTGGAAACCTCAG-TTTCTTACTGACAGAAGTTAGACT

Loxodonta africana  AGATGAAG-CAGCTCAGAGGCAGAGCTAAGTGGGCTCCTACAGGCAGAGCTGGAGTATACCACACCTCCTCTGGCATAGGCCTGCCTGATAGAAGTTGAG-CAAAAAGGACCAACAAATG
Homo sapiens        GGATAAAAACGGCTTAAAGGCAGAGCTAAATAGGCTTGTACTGGCAGAGGAGGAGTGTAGCAGATGTCCCTCTGCATAGGTCTTCTCAATGCAACTTGGGGCACCAAGGAGCATGGGATG

Loxodonta africana  TCTGTATCCTGCTTCTAAAGAGGACAGAGAGAATC-TGCACTAAGGATGCCTAGTTCCTTCTCCCAGATTCAACAAGTAGTTGAGTCATGAGGAGTGACTGAGAGGTAAGGAGTGAAGCT
Homo sapiens        TCTGCATCCTGCTCCTAAAGAGGACAGAGGGAGGAGTGTACCAAGGATACTCAGTTATTTCTCCCAGGCTCAACAGTTAGTTCCATCCTGAGGAGTGAGTGAGAGGTGGAGAGTG--GCT

Loxodonta africana  AGAATTGTTAAGTGTAGCTGAAGTCCTCTCCATGGAAACACGAGAAGTTGTTGGTTGATGAATCATGGTGCCCTTATGATAACGAAACAAAATGTTGCCTGATCCTGTGCCATCTTCATG
Homo sapiens        AGAAATGTTTAATCTAGCCAAAGTCCTCCCCATGGAAACTTAAGA...........................................................................
```

```
Loxodonta africana  ATTGTTGTATGTTTGAACCCATTGTTGTTGCCAGTGTCTCAATCCAACTCATATATAATGTCATTTATCAAACATGAGAAGTAAGTAATCCCCTGCAAAAGAAGATTTAGGAGGCTTCAG
Homo sapiens        ...................................................................................................AAAAGATTTAAG---CTTCAG

Loxodonta africana  AGGGTTTATCATGTATGAAAGAATAAGACTGAGGATTT--ATAGCATGTGTTGTTTTTTCTTTCCCAGAGACTGTGTACCCCAGATCTCTTATAGCATGCCTGCCCCTCCTAAAAGGACA
Homo sapiens        CAGATTTATCATGTGTGAGAGAATATGATTGAGGATTTTTATAACATGTTCT-TTTTTTCTTTCCCAGAGAGTGTGCACCCCAGATTCCTTGTAGTACTCCTATTGCTACTGAAAGGACA

Loxodonta africana  GTTGCACATTTGAACACTCTGAAGGACCGTCACCTGGGTAACCTGTGGGCCCAGATGCACATTTCATCCTTGGAATATGGTGCAGGAGACATGACCCGTAAAGGCAAAAAAAAAGACAAA
Homo sapiens        GTTGCACATTTGAACACTCTGAAGGACCGTCACCCAGGTGATTTGTGGGCCCGCATGCACATCTCATCCCTGGAATATGCTGCAGGAGACATTACCCGAAAAGGGAGAAAAAAAGACAAA

Loxodonta africana  CCTCGAGTGAGTGAACTGCTCCAAGGCCTCGCATTTTCCGGTGACTCAGAGGTGGAGGAAGATAATGAGCCTGAGACTCAGCCTGCTCAAAAGAAGCAGAAGGTGTCAAGTGTGCCAGAG
Homo sapiens        GCTCGAGTGAGTGAACTGCTCCAAGGCCTCTCATTCTCTGGTGACTCAGATGTGGAAAAAGATAATGAGCCTGAGATCCAGCCTGCTCAAAAGAAGTTAAAGGTATCATGTTTCCCAGAA

Loxodonta africana  AAGAATTGGACCAAAAGAGACATTAAACCCAGCTTCCCAAGTTGGTCAGCACTGGATTCTGGACTTTTGAATCTCAAGAATGAAAAGTTGAACCCAGTCGAACTCTTTGAATTATTTTTT
Homo sapiens        AAGAGTTGGACCAAAAGAGACATTAAACCCAATTTTCCAAGCTGGTCAGCACTGGATTCTGGACTTTTGAATCTCAAGAGCGAAAAGTTGAACCCAGTAGAGCTTTTTGAATTATTTTTT

Loxodonta africana  GATGATGACACTTTCAACTTGATTGTAAATGAGACCAATAATTATGCTTCTCAGAAAAATGTCAACTTGGAAGTCACAGTTCAGGAACTGAAGTGTGTGTTTGGTGTCCTGCTTTTGAGT
Homo sapiens        GATGATGAAACATTCAACTTAATTGTCAATGAAACCAATAATTATGCTTCTCAGAAAAATGTCAGCTTGGAAGTCACAGTTCAGGAAATGAGGTGTGTGTTTGGTGTCTTACTTTTGAGT

Loxodonta africana  GGGTTTGTGAGGCGTCCTAGAAGGGGGATGTATTGGGAAATCTCTGATGCCGATAAGAATCTGGTTAGAGATGCAATTAGAAGGGACAGATTTGAATTGATTTTCTCATACCTACATTTT
Homo sapiens        GGATTTATGAGGCATCCTAGAAGGGAAATGTATTGGGAAGTCTCTGACACCGATCAGAACCTGGTTAGAGATGCAATCAGAAGGGACAGATTTGAATTGATTTTCTCAAACCTGCACTTT

Loxodonta africana  GCAGATAATAGCCACCTAGACCAAAAAGATAAGTTTACAAAATTAAGGCCTCTCATAAAGCAAATGAATAAAAAATTTCCTCTTGTATGCTCCCCTAGAAGAATACTATTGTTTTGATAAG
Homo sapiens        GCAGATAATGGCCACCTAGATCAAAAAGATAAGTTTACAAAGTTGAGACCTCTCATAAAACAAATGAATAAAAAATTTCCTCTTGTATGCTCCCCTGGAAGAATACTATTGCTTTGATAAG

Loxodonta africana  TCCATGTGTGAATGCTTTGATAGTGACCAGTACCTGAAGGGAAAGCTTACAAGAATTGGCTATAAAATTTGGTGTGGTACAACCACACAGGGTTATCTGGTTTGGTTTGAGCCCTATCAA
Homo sapiens        TCAATGTGTGAATGCTTTGATAGTGACCAATTCCTGAATGGAAAGCCTATTAGAATTGGCTATAAAATTTGGTGTGGTACAACCACACAGGGTTATCTGGTTTGGTTTGAACCCTATCAA

Loxodonta africana  GAAGAACCAACTGTGAAGGCAGATAAAGATCTTGATCTTGGTTTAGGTGGGAATCTAGTGATGAACTTTGCTGATGTTCTTTTAGAGAGAGGTCAATATCCCTATCACCTGTGTTTTGAT
Homo sapiens        GAAGAATCAACTATGAAGGTAGATGAGGATCCTGATCTTGGGTTAGGTGGAAATCTAGTGATGAACTTCGCTGATGTTCTTTTAGAGAGAGGTCAGTATCCCTATCACCTGTGTTTTGAT

Loxodonta africana  AGTTACTTTACAAGTGTCAAACTGATGTCAGCCTTGAAGAAGAAGGGACTGAGGGCAACAGGAGCAATTCGTGAAAGCAGGGCTGAAAAATGTCCCCTTATGAATGCAGAACACATGAAA
Homo sapiens        AGCTTCTTTACAAGTGTCAAATTGTTGTCAGCCTTGAAAAAGAAGGGGGTGAGGGCAACAGGAACAATTCGTGAGAACAGGACCGAAAAATGTCCCCTTATGAATGTAGAACATATGAAA

Loxodonta africana  AAAATGAAGAAGGGGCATTTCGATTTTCGAGTGGAGGAAAATGATGAGATAATGTTGTGTCGTTGGCATGGTGATGGCATTATCAGCCTGTGCTCCAATGCTGTAGGCATAGAACCAGTG
Homo sapiens        AAAATGAAGAGAGGGTATTTTGATTTCCGAATAGAAGAAAACAATGAGATAATTTTGTGTCGTTGGTATGGGGATGGCATTATCAGTCTGTGCTCCAATGCTGTGGGCATAGAACCAGTC

Loxodonta africana  AATGAAGTAAGTTGTTT---TGCTGATGATGAAGAGATCCTTCAGATAAGTCAGCCATCCATAGTAAAACTGTATGATGAATGCAGGGAAGGTGTAGCTAAAATGGATCAAATCATTTCT
Homo sapiens        AATGAGGTAAGCTGTTGTGATGCTGATAACGAAGAAATCCCTCAGATAAGTCAACCATCCATAGTAAAAGTGTATGATGAATGCAAGGAAGGTGTAGCTAAAATGGATCAAATTATTTCG

Loxodonta africana  AAATATAGGGTGAGGATAAGAAGCAAGAAATGGTACTCAATTTTGGTAAGCTACATGATTGATCTAGCCATGAACAATGCATGGCAGCTACACAGAGCCTGTAACCCCAGTGCTTCTCTA
Homo sapiens        AAATACAGGGTGAGGATAAGAAGCAAGAAATGGTACTCAATTTTGGTGAGCTACATGATTGATGTAGCCATGAACAATGCATGGCAACTACACAGAGCCTGTAACCCAGGTGCTTCTCTA

Loxodonta africana  GACCTCTTGGATTTTCGGAGATGTGTTGCACATTTCTACTTGGAACCCAATGCTAATCTGTCAGATTAAGGCAGATAAAATGGACACAGTGCAGACCTTAACAAGTTACAGGAAAGTCAT
Homo sapiens        GACCCCTTGGATTTTCGGAGATTTGTTGCACATTTCTACTTGGAACACAATGCTCATCTGTCAGATTAGGGTACATAAAATGGACATAGTGCAGACATTAATAAGACATAGAAAAATAAT

Loxodonta africana  AACTACACATTAGATTGTA-CATCCCAAAGCAAACCTGATGTATGTAATAAGATCATGAATTAAGA-TTTTAAAAATCAGACATTTATATAGAGTTTCAAAGACTATTATAACAAGTAAT
Homo sapiens        AATTATACATGCTGTTGTACCCTCCCAAAGTAAATCTGATATATGTAATGAAGTTATTAAATAATACTTTTAAAAATCAGACATTTATATAGAGTTTCAAAGACTATTGTAACAAGTAAT

Loxodonta africana  GTTAAAAATTATCTGTAAAAATATTGAATTCCAGTGGTACCTTTTTCTATGTCCAATTTTGATGTCAGATGTGGGAAATCATTTGTTTG---AATTGATTATTTTGTGCATTTAAAGAAT
Homo sapiens        GTTAAAAATTGTCTGTGAGAATGTTGAACT---GTAGTACCTTTCTCTATGTCAAGTTTTG-TGTCAGACATGGGAAATCATGTATTTGTTCAATTGACTATTTTGTGCATTTAAAGAAG

Loxodonta africana  GAA-TCCTGCCTCTTTTTTAAAAAATTGTTTCTGGAGAATGCTATTTTAAAAATGCAGGCAATGTTGTAGTAACCATAGAGTGGTGCCCAGTGTGTAGTCTAAA-----AATAGAGCCCA
Homo sapiens        GAAATCCTACCTCTT----AAAAAAAATTATCTGGAGAATGCCATTTTTAAGATGCAAGCAATGTTACAGAAACCATAGAATGGTGCTGACTCAATAGTTCAAACTAGTGACACAGCCTA

Loxodonta africana  TGAGGTAAGGATGATCTAAATAAAATGTAGGGGGTGAGCAGCCAAAGAACTCAGCATTGGTGTAAAACTGGATCACACTGTGTGTAGTGTGTAGATGAGAAGGGCCAGGCACCGCTGTTG
Homo sapiens        TGAAGTAAGAATGATCTAAACAAAACATAGGTGGTAAGAGACTAAAAACCTTAGCATTGGTGTAAAACTGGATCGGATT-------GTGTGTAAGTGAGAAGGGTCAGGCATGGATATTG

Loxodonta africana  CAGGAGAGCGCTGTAAAAGAAAAAGTCAAGAGATGAAGCAGTGTCTTTGAATATTGAAATGAAAAGCATGTCCCACTGTA
Homo sapiens        AAGGAGAGTGCTATAAAGGAAAAA—CCAGAGGTGAAGCAGTGCCTTTGAATATTGAAATGATAGGTGTATCCTATGGTG


Homo sapiens          AGGATAGTTAAGTAAATTATGGAACACTCATACAGTATTACTTAGCTGTTAAAGAATAAGCTGATCTGTGAAGATCTCCAAGTTGTCTTAACCGAAAAGAGCTAAGGTGCAAAATTACAT
Dasypus novemcinctus  ........................................................................................................................

Homo sapiens          TATGATACCATAAGTGTTTTAAAATGTATAAATACTTATTCACTAAACAAAATGTTCATTAAGTGCTACTGTGTGGCAGGTAGTGTCCTAGATAAATATGAACAAAATACAATGGTTAAC
Dasypus novemcinctus  ........................................................................................................................

Homo sapiens          TCTAAAGAGGGGACACTTTTCGTTTTATGGTCCTTTTGCAACTTGACTTTTTTATTGTACATATATTATGTATACTTAAAGAAAATAATTTGTACAGCTTACTATAATGGTCTTTTTATT
Dasypus novemcinctus  ........................................................................................................................

Homo sapiens          GATCTGTCTCTCCTACCAGCCAGTGATGACCCCGAAGACAGACATTTACCACTGTGTTGGTTTTCAATCGAAATGATGTACATAAATTTGGGGCGTGCTTGCACCGTCCGAATGGGCCTG
Dasypus novemcinctus  ........................................................................................................................

Homo sapiens          GGGTCCGGCGGCGGAGAGTGACCTGGGCGGGGCAGGAGGTGGCAGCGGGGGTCCTCCCGGCTCACCAGAGAGACGAGCGGCCGTGCTCCTAGAGAGGCAGGGAACCCGCCAGACTCCGCC
Dasypus novemcinctus  ........................................................................................................................

Homo sapiens          ACTCCGCTGCGGGCGCGGCGCAGGGAGAAGCTTTTGTACCCGCCCAGCTGCTGGAGGCGCCGGCAGCGCCCGCCAGACCCGCCAGCCCAGCGGCCCGGGCTCTGGGGAAACCGGCGCTCC
Dasypus novemcinctus  ........................................................................................................................

Homo sapiens          CGACAGGGGAGCACCGGGCCTCTGAGCTCCCTCGGGAGCCTTTCACGAGGTCAGCTACGTCTTTGTTGTGCGCGTTCCTGAACTTTTGGTCCATTAACCAACAATTAGACACGCCGGTAT
Dasypus novemcinctus  ........................................................................................................................

Homo sapiens          TCAGTGCCTGGCGCGGTGCTAGATGCTGGGTGTAATCTCAGAAAAATACATTCAGGGGCGCGCCTGAGGGTGCTGGCTGCTGGCATCTCAGGTGCTTTACGTGCATTCGTGAAGAAGCCC
Dasypus novemcinctus  ..............................................................................................CCCTGCGCGCAGACG---AGAGGCCC

Homo sapiens          ATCAGTATTTCTTGAATACCAGGTACGTGCCGGGCATGTAGAGTCTATGCAGAGATAAACTCCTGTCCTCAAGAACCC-------------GGGTGGGGATGGGGTTGGTGA-------
Dasypus novemcinctus  G---GGATGTGTTGAGTGCCGGGTGAGTACCAGGCA----------------GGAGAAGACCCTGTCCTCAGGGAGCTCGGCCCTGCGTAAGGGGAAAGGAAGGAGGCGAGGAGCGCCGC

Homo sapiens          -------------------------TCTGTAATACGAGATGGAGATGAGGGTTGGGTGCAGT------------------TTTCTATGGGAATACCCCATCTCAACCTGTGTGGCTTAC-
Dasypus novemcinctus  GAACAGCGCGCCCAGGGGGCGTCTGTCTGTAATTCCAGATGACGCAGGAGGCAGGAGGCCGTGCCCGGGCTGAGCTCTCCCTTCCATGGCAGCACCC-GCCTCCGCGTGTGTGGCTTGGA

Homo sapiens          ----------TTTTCCTGTCTATATGTTAGGGTTAGGGGTTGCCAGGGAAGGTTTTACAATAGAAC---ATTTACGCTGGGCCTTGGGAATTTACCCT--TAGAAGGGCGGTCCTGACT-
Dasypus novemcinctus  GGACGGACGGTTGCCCTGTCTGTTCATCAGGGCCGGGATTGGCCAGGGAAGGTTTTACCAGGAGACGGCACGGGGGCCGGGCCTGGGGGGTTTACCTTATCCCAAGGGCGACCCGAGCCC

Homo sapiens          ----GTATAGAGTGGGTGGAAAAAAATTGAGTACTTAGCATGGCAGTGACATAAGAAATTTGGGAAGACCATGGCTTTCTTGAAAGCAGGAACCAAGTCTTTTATTCCAGAGTTAACTTT
Dasypus novemcinctus  TCAGTTAAAATGCGGGTGGAGAAAA-CTGAACAGTCGGCGTGGCAGTGGCGTGAGGAGTCTGGGAATACCAAGGCTTTCAAGAAAGGAGGGTCTATGTCTTTTACTCCAGGTTTGGCTTT

Homo sapiens          ATGCCTGGAAAATAATGAGATGCTCCACAAATGTTGATTGAGTGACAAACAAATCAGCAAAACCCAGTAAAGATTAGATCCTGGGGA-GTTTGAATTTAC--TTCAACGAGTTTAATGAA
Dasypus novemcinctus  CTGCCTGGAAAATAGTGAAGCGCTCAATAAATATTGT--------CAAACAAACAAACAAAAAACACTAAAAATCAGGTCTTGGGGGTGCCTGAAATGACCCTCTAAACAATCTGACTAT

Homo sapiens          AACTTTGTGTGTGTTTA-------TGTGTGTGTGAGGT-ATGTTGAATATGGTCACAACTGTGCTTTAGGGAATATTAATCCAGTGACCATAGAAGCGGGGAGTTGGGCAGGAATACCGT
Dasypus novemcinctus  AGTTAAGTGAGCAATTGAATCTCTTTTGTTTTTGAGGGGATGTTCCGTGTGGTCGGAATTGTGCTTTAGGAAATATTAATCCAGT-----------CAGTGGGTGAGGTA---------T

Homo sapiens          AACAGAACATTATTGTAACAGTGAATGAAAATAATAGTAAAATTATAAGAAG-GAAAGAATGGCTGCAAGAGGCGTTTT----TAAGAATTGACT-------CACTGATTGTGTATAGAG
Dasypus novemcinctus  AACAGAAAATGATTGTGATCATGTACGTCAATGGTAATAAGGGAATAAAAAGAGCAGGAAGGGCTGCGGGAGGCGTTTTGGAGGAAGAATTGGCTGGACTTGGAATGACTGGATTTAGAG

Homo sapiens          GCTGTTTAAAAGACTGAATGCGTGGAAGATGATGGTGATGCCAGTAGAAATGGAGAAATACCGAGTGAACGCAGCTTTGAGGGACTCTGATTTCAGTTTGAGATCTGTTGAAATTATAGT
Dasypus novemcinctus  GCTGGTTTCGAGCCTGCGTGCTTGGAA.......................................................GACTCTAATTTCAGTTTGGGATGTGGTGAAATTACGGT

Homo sapiens          GCAGTAGAGGCCCACAG--------CTGCTGGCATTTCAAGATGTGGGTTTGGAGTGC-AAAAAGCAAGCTGGTGCCAAAGATTTGAGTCTTCTTGGAACAGAGATATATTGAAAGTGTG
Dasypus novemcinctus  G----AGAGTTCTGCAGCTGGAGGTCTGCTGGCATTCCCAGAGGTAGATCTGGAGTATGAAAAGGCAAGTTGGAGCCAGA------AGCGTCCTTAGAATGGAGATGTATTGAAA--CTG

Homo sapiens          ACTAAGGAGATCACAA-AGGAAAAAATGAAAAGAGGGAATAAAAGAACTTGATGCATGTCTACTGTTTTT--CAGTTATG-TATTGTTGCATCAAAAATAA-----AACTCTAAAC----
Dasypus novemcinctus  ATTGATGAGATCACAACAGGAGAAGTTG----GGGAGAGTAAAAGTACTTGCAGCATGCCTACTGTTTCTTACAATCATACTATTGTT--ATAAACAATAAGCAATAACCAAAAATACCA

Homo sapiens          -------TTAGTGGCTGAAAACAAGCTCAGCTTATTGTTCCTCAGGATTATGCGGGTTGACTAGG-CGGTT--CTACTGGTCTCACCTGGAGTTACTAATAGAGCTGCATTCAGGTGGTG
Dasypus novemcinctus  GATACCGCCACTGACTGAACA-----TCGGCTTATTATGTCTCATGATGCT-CGGGATGACTTGGTCGGTCTGCTGCTGGTCTCAC..................................

Homo sapiens          AATTATTTGAGCACTAGGCCTGGCTGGGCTTTAACTTGGGCATTTTGGTTCTCATCCACATGACCTCTGTATAGCTAGCTTGGGCTTCCTTACAGCATGGCAGTCTTATGGCAGTGAGCC
Dasypus novemcinctus  ........................................................................................................................

Homo sapiens          AAGAGCTACAGCAGCACTCTCTCTTACAATGTCATTTCTGCTGTATTCCACTGGTCAAAGCAAGTCATAGGCCAGAACAGATTCAAGGGAAGGAAAAAGAGACTCCACCCCTTGAATGAC
```

```
Dasypus novemcinctus  ........................................................................................................................

Homo sapiens          AACAGTGGCAAAATCTCATCACAAAAGGTATTTGAGAATGGGAGACTTTGGAAACATTCTGTTACACCTACATTTAAAGTGGAATATTCAATGAGAGTTACACAGATATAGAAGACCTTT
Dasypus novemcinctus  ........................................................................................................................

Homo sapiens          GAGACTGTATTATTTGTCCATGAAGGCAGCCATCTGCCTCCTCAATGGGTAAATTATCCCCTCTCCCCAGCTGAAGTGCAAGCTACTCGAGCTTTCTTATTGCTAACACTTCTTCCCTCA
Dasypus novemcinctus  ........................................................................................................................

Homo sapiens          TGTCCTACATCCCTTACCTTTGGACTCAGGCCCTTTAGTACTAGTTCAAAATATACTTCTGATTCATTTCACTGATAATAGAAGCAAGCAGGCTCAGCTTTTGAGCATTACCTACAGTGG
Dasypus novemcinctus  ........................................................................................................................

Homo sapiens          GGACAAAAATGTAAGTAGGTTACATACAGTTTTGAAGCTCTTATAATTTGCATGTTGCTGATAAATTCTTATGCCTCAGATCTCTATTTCTGAATATAGACCCCAAGCTAAGTGAAGCTT
Dasypus novemcinctus  ........................................................................................................................

Homo sapiens          TAGCCTCTAAGCTCAACATGTATGAAGCTTTGCCAGGCCCTGCTCCTGAAAATGAAGATGGCCTTGTGAAAGTGAAGGAGGAAGATCCCACCTGGGAGCAGGTGTGCAACTCACAGGAGG
Dasypus novemcinctus  ...............................................GAAGATTATGATGG-----TGGAGCCAAAGGAAGAGGAACACGCTTGGGAGCAGGAGACCAGATTACAGGGGA

Homo sapiens          GCAGCTCCCACACTCAGGAGATTTGCCGCCTGCGCTTTCGGCACTTCTGCTACCAGGAGGCTCACGGACCCCAGGAAGCTCTGGCCCAACTCCGAGAACTTTGTCATCAATGGCTGAGAC
Dasypus novemcinctus  ACCATTCTCCCAGTCAAGATATCTTCCGGCAGCGCTTCAGGCAACTCTGCTATCAGGAGACTCCTGGTCCCCGGGAGGCCCTTAGCCGACTCCGAGTGCTCTGTCGTGAGTGGCTGAGGC

Homo sapiens          CGGAGATGCACACCAAGGAACAGATAATGGAACTGCTGGTGCTGGAGCAGTTCCTGACCATCCTGCCCAAGGAGCTCCAGCCCTGTGTGAAGACATATCCTCTGGAGAGTGGAGAGGAGG
Dasypus novemcinctus  CAGAGAGGCACACGAAGGAGCAGATATTGGAATTGCTGGTGCTGGAACAATTCTTGACTATCCTGCCTGAGAAGTTCCAGTCCTGGGTGCGGAGACATCACCCTAAGAGTGGAGAGGAGG

Homo sapiens          CAGTGACAGTGCTGGAGAATCTAGAGACAGGAAGTGGAGACACAGGACAACAGGTGGGAA-GAGAATGTGTGTGGTGATGTGGGAGAAGAAAAGGGGGACATGTATCGGTTTATTTTTAA
Dasypus novemcinctus  CCGTGACTGTGCTGGAGAATTTAGAGAAAGGACTTGATGAACCAGGACTGCAGGTGGGAAGGGGAACATGTGATCTGTTGTGGGAG..................................

Homo sapiens          CGTTTTATTTTGAAATAACTCCTAATTTATAGAAGTATTAGAAGAATAGAGAACTCCCGTAAAACTCTTCATCCAAATTTGCCAGTTGTTAACATTTGTTTGATGAAGTCCCATTGTTGT
Dasypus novemcinctus  ........................................................................................................................

Homo sapiens          GTACACACTCGTACAAGGAAGTGCATTCCCCCCCCCCAAGATGCATATGTATATGTATACACACACATACATACACATATGCAGTATTTTTTTTTCCTGAATCTATTTGAGAGTCAGTTG
Dasypus novemcinctus  ........................................................................................................................

Homo sapiens          CAAACACTATATCCCTTTACCTCACTGTTGACCAGGCTGGAGTGCAGTGGTACGATCATAGCATCATTGCAGCCTTGAACTGCAATGGTGGAAGGCTCAAGCAATCCTCCCACCTCAGGC
Dasypus novemcinctus  ........................................................................................................................

Homo sapiens          TCCTGAGTAGATGGGACTACAGGTGCATGCCACCATGCCTGGCTAATTTATTTTTAATTTTTTGTAGCGGGGGTGGGGTCTCACTATATTGCCCAGGCTGATCTTGAACTCCTGGCCTCA
Dasypus novemcinctus  ........................................................................................................................

Homo sapiens          AGTGATCATGCTGCCTTGGCCTCCCAAAGTGTTGGGATTACAGGCATGAGCCACTGCACCTGGCTATTCGGTGTGTATTTCTGAAGAGTGAGGATATTCTCTTGTATCA---------CA
Dasypus novemcinctus  ...................................................................TCAGTGTATATTTCCTAAGA-TGAGTACATTCTCTTGAATAATCAGATAATCA

Homo sapiens          AGAATACAAAAATCAAGTTTAGGGAAATTAAACTT---ACGATACTATCATATA-ACCTTAGTCCTTATTCATATTTTGCCATGTTATCCTAATAATGTTCTTTATGATAATTTTCCTGT
Dasypus novemcinctus  GAAATATAGTTATGTAGTTTGGGGAACTAAAATTTGTTATGGTACTATCATCTATACTGTAGTCCTTGTTCCAGTTTTACCATATTACCCCAGTAAAGTTA---ATGGTCATTCCCCC-C

Homo sapiens          GATTCAGGATACAAGCCAGCATCACATATTGCACTTA------GTTGCCATATC--TCTAGTCT--GTGAATCTGGAGTAG------CTGCTGTCTTTGTCATGACGTTGTTATTTTTGA
Dasypus novemcinctus  ATTCCAGGATACAATTCAGTTTCATAGGTTGCATCTATTAATAGTTGCCAGACCACTTTAGTCTCCTTTAATCTGGAACTTTCCCTCCATCTTTCTTTGTCATGAC--TGACATTTTCAA

Homo sapiens          AGAGTACAG-CTAGTTTTTTTGGAGACTATCCCTCAATTTGAGTTTGTCCTATATTTTCTCAT-----GATTCAGGCTGCACATTTTGGCTAGAATACCACAGAGGTAATGTTGTGTTCT
Dasypus novemcinctus  GGGGCATAGGCCAGTTATTTTGTAGAATACCCCTCAGTTTGGGTTTGCCTCATATTTTTCCATAATTAGATCCAAGTTGTGCATTTTGGCTAGAATAAC-----------------TCCT

Homo sapiens          CAGTGCGTTACTTCAGGAGGTGCGTAGTGATGATTTGTCCCATTACTGATGAGGTTATGGTGGTACCTGTCAA--TTTTCTCCACTAGAAAGTTACCATTTTCCCCTTTGTAATTACTAA
Dasypus novemcinctus  CCGCGCATTCCATCAGAAGGCACATGGCGATGGTTTCTCCCATTACTGATGATGTTAATGTTGTGCCTGTCAGATTTCTCTCCACTATAAAGTTAC-----------TTGCAATTA----

Homo sapiens          GTAATCTGTGGGAGACATTTTGAGGTGGTGTACATATCCTGTTCCTCATTAAATTTTCACCAGCTAGTTTAAACATCCACTTGTTTGTTGTTTTTGTTTTTCTGGGCTGGGATCCTTTGC
Dasypus novemcinctus  ATAATCTGTGGGAGATAGTTTGAGACAGTGTACATATTCCATTCCTCATCAGAGTTCATCCATCTGGGTT-------TTCTTGTTTGTT--------TTCTCTGGCCAAGGAGTCTTCAT

Homo sapiens          CTGTCTTTTCTGCCTTGTTTGCTGCTGTATCCCCAGCATGTAGAACAGTGCCAGGCTCTTAGCA----TGCAGTCCATATATGCATGCCCTTTCAAAATAAATCTTTTGTTTCCAGGCCT
Dasypus novemcinctus  -TGTCTTGTCTGCCTTATTTGCTTCTGTATCCCCAGCATCTAGAACAGGGCGTGGCTCCTAGCAGGTGTACAACCATTGTGTGCGTGCCTTTTCAGGTTGAGTCTTTTGTCCCCAGGCTT

Homo sapiens          CTGTCTATATTCAGGGACAGGACATGCACCCAATGGTGGCAGAATATCAAGGAGTCTCTTTGGAGTGTCAGAGCCTCCAGCTCCTGCCTGGGATAACCACCCTGAAGTGTGAACCTCCAC
Dasypus novemcinctus  CAGACTATACTCAGGGACAGGAAATGGTTCCAGTGGTGACAGAATATCAAGGAGCCTCTTTGGAGTGTCAGAGCCTCCAGTTCCTGCCTGGGGGAACCGTCCTGAAATGTGAACCTCCAG

Homo sapiens          AGCGTCCTCAAGGGAACCCCCAAGAAGTGAGTGGTGAGTGCTCGA--GTGAGTTTAGTGAGGACGCTGGGAGCTGGCCACTGACACTTGCACAGCCTTCTTGACCTCAAGTTTGTATACA
Dasypus novemcinctus  AGCTCC---------AGCCCCAAGAAGTGAATGGTGAGTGCCAGAGGGTGAGTTTAGTGGGGGCATTGGGACAAGGCTACTGA.....................................

Homo sapiens          TTGTGAAATGATTACCACAAGCTAACTTACATATCTCACAGTTACCTTTTATGTGTCTGTGGTAAGAATTCCTAAGATCTACTCTCATGAAATTTCAAGTATACAATATATTATTATTAC
Dasypus novemcinctus  ........................................................................................................................

Homo sapiens          TAACTATAGTCAGCATTCTGTACGTAGGTCTTCAGAGCTTATTCATCTTATAACTGCAAGTTTATACCCTGTGACCAACATCAAGTTTTATTTTTTTGTCTAACCCTGTATCTGAACAAA
Dasypus novemcinctus  ........................................................................................................................

Homo sapiens          CTTGTAAAGACCTGCTTTATTTTACACTGTGCATTTTAAGCTGGTTTATTTAGTATGTATGTGTTGTGGACAAGTACATATAATAGTAAAAGTGAATCGGGGCAAGGCTGTTTTTTTCCA
Dasypus novemcinctus  ........................................................................................................................

Homo sapiens          GCAAACTTTATTCCTTCTAGAAATGCAACAACAGCAATAAAAACATTCCATAGTGATCCATTATGGAAGCAAAGAAAAGCAGAGCAAAGAAAAGTGATGTTCTGTTTTATATTTTTGGGT
Dasypus novemcinctus  ........................................................................................................................

Homo sapiens          TAGAATTACCACATGTGCTCTTCATTTGATAATTTTGTACTCTGCCATCTGCCAACTGCCAACTTTGTGACCTAGGCAAGTCATTTAATAGGTTATTCAGTTAATCAACAATTATCTGTT
Dasypus novemcinctus  ........................................................................................................................

Homo sapiens          TGATCTCTGTGTGTACTCTGCACTCAGCTGTGATTCTTCTGAATGTGCCTAGGATTTTGGAGGCAATAATTTGGACCTTCTTGATTATTTTGAGGATTGGAAATAATCTTTTAAAGTAAC
Dasypus novemcinctus  ........................................................................................................................

Homo sapiens          AGCACAGTGGCTGGCCTTGTGCAGTCATCATCACCATTTTGATTATTGTTGGTTCTTTGGGATCAGTACCTCCTAACAGTTTTTCTAGTTCATGGCTAACATCTCTTCTCTATTTTTTTA
Dasypus novemcinctus  ........................................................................................................................

Homo sapiens          AATATCTTTATTTTCCTGTCTCTCTCTCCATCTTCCTTTCTTCTGCTTTCCTGTTTCCCCTCAGTCTCATCTCTCTACTCTCTACATCCTTTCTTTTGCCTCTGTGTTCTCTCCTTGATC
Dasypus novemcinctus  ........................................................................................................................

Homo sapiens          CTTCCTTTATCACCAGCTTCTTCCTCCCCTTTATTTTTGTCTATGATTTTTATTTTTTCTTTATGTGAAAGTGCATGACTGTAAAGCACCTTATGAGTTCATAAATCCCACTCCATTTTT
Dasypus novemcinctus  ........................................................................................................................

Homo sapiens          TGGGTGAGAGACCCTAAAGTTGTAGAATTTTAGAATTTCTGACAGAATGAAAGCAGTTGTGATGCTCTTTCTCACACTGTAAAATCTACGTCTTAGTTCTTGAATGTTTAACAAACATAA
Dasypus novemcinctus  ........................................................................................................................

Homo sapiens          CATTTGGGGGAAAAGGCTGGGTCTCCAAAAAGGGTACCCTAAAGGCCATCATGTCTCATTTAGGACTCTTGCCCTCTCTCTCTGCAGGGCCTGTTCCCCACGGATCAGCTCATCTCCAGG
Dasypus novemcinctus  ........................................................................................................................

Homo sapiens          AAAAAAACCCCAGAGACAAGGCTGTAGTGCCTGTGTTTAACCCAGTCAGGTCCCAGGTAAGTAGGATGCCCAAGCTTGTAGGAATATCAGTAGTGGTCTTTCTCCCTCATTCCATGTTCC
Dasypus novemcinctus  ........................................................................................................................

Homo sapiens          TTGCCCTTCTCTTGGCTCACACTCATCATCGTGAGAGCCATTCAGGTGGCATAGTCCCTCTGCCTTCACCTTGTCACAGAATATCACAAAGTGTGTTGCATCATTCGAGGAGCTTGTATT
Dasypus novemcinctus  ........................................................................................................................

Homo sapiens          GATTATATGCCATTGCCTCATTTCATTGTTCAGGAGGCACGTCCAAATCTCAAGGCATGGGAGTGAGGGGTGGAGTGAGGCTCCTGGTTTCAGAAAGTGCTCCACTCATTTGATGTTGAG
Dasypus novemcinctus  ........................................................................................................................

Homo sapiens          TAACTGATATTTTCTCCTTTGAAGTCTCTGGTTTATCTCCCCAGCTTTAGAATAAGGGCCCTTTCTCACACTACTTTATATTCAGCATGTATGCAATTAATGCTTGGTGATAATAATGGA
Dasypus novemcinctus  ........................................................................................................................

Homo sapiens          AATTCTATATATCTAAGGTAATACTTAACATCTTCTTCACAAAATGGACTCTGGTTTTTTTTCTTATTAGTGGTTCATTCATTTTCCTAGTCATTTATTTACAAAAAAAAAATAACTCTC
Dasypus novemcinctus  ........................................................................................................................

Homo sapiens          TCATTTAAGTCCTCTTGCATACTGCTGCCACATCAGTTTCCTCAAGACACTTGCTCAGTCCTCTTCTATGGCTTCTAATTAATTTTTAATTCTATCAGACCCAGTGCCTCCTGTTATTAA
Dasypus novemcinctus  ........................................................................................................................

Homo sapiens          CAAATACTTCCTGATAAGAAGTATCTAGAAAATAAAACCTACTCATGCACATTCTGAAAATCAACATAATTCCCTTAAATGTAATTTAATGGTATAATGTAGAAACAAAAAAGTTATTTA
Dasypus novemcinctus  ........................................................................................................................

Homo sapiens          TTTTAAAAGTAGTGTTTCAAAATGCAAGTGCTTGATGTGATTATAACTAGGGAAATAACAACGTAGTCAGGTGTTTGCACTGAAATATAATTAGTAAGTTCAAATTAAGGAGGAATAAGA
Dasypus novemcinctus  ........................................................................................................................

Homo sapiens          AGACCAGAAGCCCAAGAAAATCAAAGAGCAATGGTATAGGTGGACAGTGAAATGAAACTAGTATTGAGTCAGATAATACAAGACTAGACAGAGAAAGTGGGGCCCTTAATTGAAGTAAGG
```

```
Dasypus novemcinctus  ........................................................................................................................

Homo sapiens          CTTAGATGTTGTAAATTGAATTATGTCCCTCCAAAAGATATATGAAAATCCTAACCCCACGTACCTGTGAATGTGACCTGATTTGGAAACAGGCTCTGTCTTAGTCCATTTTGTGTTGCT
Dasypus novemcinctus  ........................................................................................................................

Homo sapiens          AATAACAGAATTCCTGAGACTGGGTAATTTATTTTAAAAAGAGGTTTATTTAGCTCAAAGTTCTACAACCTGGGAAGTCCAAGATCATCAGCCTATCTGGTTAGGATCTCATGCTGCTTT
Dasypus novemcinctus  ........................................................................................................................

Homo sapiens          AGCTCATGGCAGAAAGTGGAATGATGGATAATCAAGTGTGTGCAAGGAGACCACACATTAGAGGCAGCCATCCTTTAAACAGTCTGCTCTTGGAGCTGAGCAAGAGCAAGAATAGAAAGC
Dasypus novemcinctus  ........................................................................................................................

Homo sapiens          TTATTCAAAGAAATAACAATAGGCCGGGTGCAGTGGCTCTCAACTGTAATCCCAGAACTTTGGGAGGCCATGGCAGGAGGATTGCTTGAGGCCAGGAGTTTGAGACCAGCCTGAGCAACA
Dasypus novemcinctus  ........................................................................................................................

Homo sapiens          CAGCAAGACCCTGTATCTATTTAAAAAAAAAGAATAGGCCAGGCGTGGTGGCTCATGCCTGTAATCCCACACTTTGGGAGGCTGAGGTGGGTGGATCACGAGGTCAGGAGTTCGAGACCA
Dasypus novemcinctus  ........................................................................................................................

Homo sapiens          GCCTGGCCTACCTGGCAAAACCCCGTCTCTACTAAAAATACAAAAAAAAGGAGCCAGGTGTGGCAGGCACCTGCAATGCCAGCTACTCGGGAGGCTGAGGCAGGAGAATCGCTTGAACCT
Dasypus novemcinctus  ........................................................................................................................

Homo sapiens          GGGAGATGGAGGTTGCAGTGAGCCGAGATCATGCCATTACACTCCAGCCTGGGCAACAAGAGCAAGACTCCATCTCAAAAAAATTAAAATAAAATAAATTAAAAATAAATTTTAAAAATA
Dasypus novemcinctus  ........................................................................................................................

Homo sapiens          GAGAAATAATAACAAAAATCTTCACAAAACTTGTGAAAGATATAAATGTCCAGGTACAGGAAGATCTGAGAACACTGAACAGAATCATCCCAAATAAGACTACTCCAAGGCATATAATAA
Dasypus novemcinctus  ........................................................................................................................

Homo sapiens          TCAAACTCTCAACTGTCCAGGAAAAAGAGAGGATCTGAAAAGCAGCAAGAGACAAGAAACAAATAAGGAGCTCCAACTTGTCTGGCAATAGACTTCTCAATGGCAATCATACAGGCCAGG
Dasypus novemcinctus  ........................................................................................................................

Homo sapiens          AGAAAGTGGAATGACATTTTCAAAGTACTCCAAGAAAAAAACTGACATTCAAGAATATTGTATCCAGCAGAATTATCTTTCATATATGAAGGAGAAAGTCTTTCCCAAACAAACAAGCTG
Dasypus novemcinctus  ........................................................................................................................

Homo sapiens          AGAGAGTACACCACCACCAGACACATCTTACAAGAAATGCTAAAGAATTCTTCAGTCTAAAAGGAAAGAAGACTAATGTGCATAAAGAAAACTTTTGAAGGCATAAAAGTCACTGATAAA
Dasypus novemcinctus  ........................................................................................................................

Homo sapiens          ATTAAGTACATGGACAAACCCAGAATACTTTATTACTGTAATAGTGACATACAGTATACTCACAACTGTGAAGCCCCAAGACATACCTATCAAAAACAATAATAGGACTGGACACAGTGG
Dasypus novemcinctus  ........................................................................................................................

Homo sapiens          CTCATGCCTGTAAACCCAGTTCTTTGGGAGGCTGAAGTGGGAGGATCACTTGAGACAAGGAGTTTGAGACCAGCCCAGGCAACATGGTAAGATCCTGATGCTATACCTGGAGACATGAGA
Dasypus novemcinctus  ........................................................................................................................

Homo sapiens          CAGAGTGAGACCATATCTTTAAAACAAAACAAGACAAACAATAATGGCTAGCAACCTGTTAAGAGATAGGTAATATAAAGATATAAATTGAGACAACGAAAAGTCAAAATCTAGAGGAAT
Dasypus novemcinctus  ........................................................................................................................

Homo sapiens          GGAATTAAAGCATATAATTTTTTTCTGTGTGTTTCTGCCTTTGTTTCTACTCTTGTATTTGGGATCTAAGATAAGTTGTTATCTCTTTAAAATAACTTATTATATCTGAAGGTCTTCTTT
Dasypus novemcinctus  ........................................................................................................................

Homo sapiens          GTAAGCCTCATGATAACTACAATGCAAAAACCTATAATAAATTCACTAAAAATAGCAACAAAGGAAGGCATACTATAAGAGAAAATCACTTAGCCACAGAGGTTTTTTGTTGTTGTTGTT
Dasypus novemcinctus  ........................................................................................................................

Homo sapiens          TGAGATAGGGTCTCACTCTTTCACTCTGGCTGGAGTGCAGTGGTATCATCTCAGCTCACTGCAACCTCTGCCTCCCGAGTTCAAGTGATCCTTCCACCTCAGCCTCCCAAGTAGCTGGGA
Dasypus novemcinctus  ........................................................................................................................

Homo sapiens          CCACAGGTGTGTGCCACCATGCCTGGGTAATTTTTTGTATTTTTTGTAGACCTGGGTTTTTGCCATGTTGCCCAGGCTGGCTTGAACTTCTGGCCTCAAGCAGTCTGCCCACCTTGACCT
Dasypus novemcinctus  ........................................................................................................................

Homo sapiens          CCCAAAGTTCTGGGATTACAGGCGTGAGACACCATGCCCGGCTGTATATAACAATTATAAATACCTATGCATGCAACACCAGAGCTCCCAGGTATATAAGGAAAACATTAATAGATCTAA
Dasypus novemcinctus  ........................................................................................................................

Homo sapiens          AAGGAGAGTTAGACTGCAATACAATAATAGTAGGAGATTTTAACACCCCACTCTCAGTAATTGACACATCATCCAGACAGAAAGTCAACAGAGAAACGATGGAATTAAACTACATGCTAG
Dasypus novemcinctus  ........................................................................................................................

Homo sapiens          ATCTAATAAGCCTAACTGATATTTACTGAACATGTAACCCAACTGCTACAGAATGCACATTCTTTTCATCAGCACGTGGGACATAAGAATAGACCATATCTTAGGCCACAAACAAATCTG
Dasypus novemcinctus  ........................................................................................................................

Homo sapiens          AACAAATTTTTAAAAAATATAAATCATATCAAGTATCTTTTCTAATCACAGTGGAATAAAACTAGAAAGCAATAACAAGAAAAACCTTGGAAACTACACAAACGCGTGGAAATTAAATAG
Dasypus novemcinctus  ........................................................................................................................

Homo sapiens          CATGCTTCTGAACAACTAATTTGTCAGGGAAGAAATTAAGAAGGAAATGTAAAAATGTCTTGAAACTAATGAAAATAGAAAATGTGACATATCAAAATTTGTGGGATACTGCAAAAGCAG
Dasypus novemcinctus  ........................................................................................................................

Homo sapiens          TACTAAGAAGGAAATTTATAGCAATAAACACCTATATCAGAAAAGTAAAAAGACATCAAATGCACAACCTAATGATACACCTCAAGGAACTAGAAAAGCAAGAACAAACCAAACTGAAAT
Dasypus novemcinctus  ........................................................................................................................

Homo sapiens          TAGTAGAAGGAAACAAATAAAGATTAGAACAGAAATAACTGAAATTGAGACCAAAAAAAAAAAAAAAAAAACCCAGAAGAACAATGAAACAAGAAGTTGGATTTTTTTTAAAAGATAAACA
Dasypus novemcinctus  ........................................................................................................................

Homo sapiens          AAATCAAGTCTTTAGCTGGACAAACAAAAAAGGAGAAGACCCAAATGAATAAAATCAGAAATGAAAAAGACATAACAGCTGAGACCTCAGAAATACAAAGAAACCATTAGAGACTTATGA
Dasypus novemcinctus  ........................................................................................................................

Homo sapiens          ACAACTATGACAATAAATTTGAAAACCTGGAAGAAATGGATAAATTCCTAGATGCATGCCAACTACCAAGATTGGACCATGAAAAAATAGAAAACCTCAGCAAACCAATAACAAGTAACG
Dasypus novemcinctus  ........................................................................................................................

Homo sapiens          AGATAGAATCTGTAATAAAAAGTCTTCCATCAAAGAAAAGCCCAGGACCTGTTGGCTTCCCTGCTGAATTCTACCAAACATTTAAAAAGAACTAATACCAATCCTATTCAAATTCTTCAA
Dasypus novemcinctus  ........................................................................................................................

Homo sapiens          AAAAAAAAAAAAAAATTGAAGAGGAGGGACTACTTCCAAATTCATTCTAGGAGGCCAGCATTGCCCTGATACCAAAACTAGACTAGGATACAAAATAAAAAGAAAACTACAGACCAACAT
Dasypus novemcinctus  ........................................................................................................................

Homo sapiens          CACTGATGAGCATAGGTGCAAAAATTCTCAATGAAACACTAGCAAACTGAATTCAACAACACATTAAAAAGATCATTCACCATGATCAAGTGGGATTCACTCCAGGGATGCAAGGATGGT
Dasypus novemcinctus  ........................................................................................................................

Homo sapiens          TCAACATATGCAAATCAATAAATGTGATATATCACATTAATATAATCAAGAACAAACACCATATGATTATTTCAATAGATGCTGAAAAGCATTTGATAAAATTCATTGCTTTTTGATAAA
Dasypus novemcinctus  ........................................................................................................................

Homo sapiens          AACCCTTGTCAAAATTGGTATAAAAGGAACATACCTCAAAATAAGGGCCATATATGACAAACCTACAGCTAACATCAAACTGAAGGCCTTTTCTCTAAGGACTGGAACAAGATAAGAATG
Dasypus novemcinctus  ........................................................................................................................

Homo sapiens          CCCATCTCACCACTGTTGTTCAACATAATACCCGAAGTCCTGGCCAGAGCAATTATGCAAGAGAAGGAAATAAATGGCATCCAAACCGGATAGCAAAAAGTCAGGTTAGTCTGTTCGCAG
Dasypus novemcinctus  ........................................................................................................................

Homo sapiens          ATGATATAATCTTATACCTCGAAAAACCTGAAGACTACACCAAAAAAACTGTTAGTGCTGATAAACAAACTAAGTAAAGTTGCAGGATAAAAAATCAACACTCAAAAATTAGTAGCATTT
Dasypus novemcinctus  ........................................................................................................................

Homo sapiens          ATATACTTCAATAGTGAACAATCTGAGATAGAAATCAAGAAAATGATCGCATTTACAAAAGCTACAAAAAATATAAAATACTTAGGGAATTTAAATACTAAAAATTTAAACACCAAATTA
Dasypus novemcinctus  ........................................................................................................................

Homo sapiens          AGTTAAATTTAGTGTTTAAATTCCCTAGGCATTTTATATTTTTTAGTATTTTATTTATAATTTATAATTTAGCATTTAATTACTAAAGAAGTGAAAGACCTGTATAAGGAAAACTAAAAC
Dasypus novemcinctus  ........................................................................................................................

Homo sapiens          TCTCATGGAACAAATTGAAGAGGACACAAAAACACTGGAAAGATATTCCATTCTAATGGATTGGAAGAATTTATATTATTAAAATGACAATACTTCCCAAAACAATTTACAGATTCAGTG
Dasypus novemcinctus  ........................................................................................................................

Homo sapiens          TAATCACTATCAAAATACCAATGACATTCTTCACAAAAATCAGAAAAAAAAAATTCATGTGGTGAGCCCTTAATCTAATGACTCATATTCTTATAAGAAGTCCATATTTGCAAATTGACCA
Dasypus novemcinctus  ........................................................................................................................

Homo sapiens          AGGGGTTGAAAAAAAAGAAAGAAAAATGCCCAAGTGAAAACACACACAGGGAGAATGCCATGTGATCACTGAGGCAAAGATTTGTATAACACATCTGCAAGCCGAGGAACACCAAGAATT
Dasypus novemcinctus  ........................................................................................................................

Homo sapiens          GCTGACAAACACTAAAAACTTGGAGAGAGGCAAGGAAAATTATCCCATGGAGCTTTTTTTCTTTTCTTTTCTTTTCTTTTTCTTTCTTTTTGAGACAGTCTTGCTCTGTTGCCAGGCTGG
Dasypus novemcinctus  ........................................................................................................................

Homo sapiens          AGTGCAGTGGCGCGATCTCAGCTCACTGCAACCTCTGGCTCCCTGGTTCAAGTGATTCTCCTGCCTCAGCCTCCCGAGTAGCTGAGATTACAGGCAAGCGCCATCACGCCCAGCTAATTT
```

```
Dasypus novemcinctus  ........................................................................................................................

Homo sapiens          TTGTATTTTTAGTAGAGATGGGGTTTCACCATGTTGGCCAGGATGGTCTCGATCTCCTGACCTAGTGATGTGCCCACCTTGGCCTCCCAAAGTGCTGGGATTACAGGCATGAGCCACCAC
Dasypus novemcinctus  ........................................................................................................................

Homo sapiens          GCCAGGCCTCCCATAGAGCTTTCAAAGATAACATGGCCCTACTGATATCTTGATTTTGGATTTCTAGCCTCCAGAGCCATGAGATAATACAATTCTGTTGTTTTCCACTATTGAGGTTGT
Dasypus novemcinctus  ........................................................................................................................

Homo sapiens          GGTACTTTGTTACAAGAGCCATAGGAAAGCAATATAAGTTACAAAACAAATTATGGACTGATAAAGGGAGAAAATAAGGAGGTATGATATTACAGATACACTTCCTTCATTGTGTTTCAC
Dasypus novemcinctus  ........................................................................................................................

Homo sapiens          TTTATTGTGCTTTGCAGTATTGAAAGTTTGTGATGACCCTGTGTGGAACAAGTCTGTCAGCACCATTTTTCTACAGCATGTGCTCACTTTGTGTCACATTTTGGTAATTCTTAAATTCTT
Dasypus novemcinctus  ........................................................................................................................

Homo sapiens          AAACGTGACACTCTGTGTCACATTTTGGTAATTCTTAAAATGTTTCAAACTTTATTGTTATATCTGTCATGGTGATCTGTGATCAGTTATTTTTGATGTTACTATTGAATTTGTTTTGGT
Dasypus novemcinctus  ........................................................................................................................

Homo sapiens          GTGCCACAAACCATGCCCGTTTAAAATGTCAGACCTAATCAATAAACATTGTATGTGTCCTGACTCCTCCATTGACCAGCTGTTCCCTCATCTCTTTTCCTTTCCTTGGTCCTTGCTATG
Dasypus novemcinctus  ........................................................................................................................

Homo sapiens          TCCTGAGACACAATAATATTGAAATGAAGCCATTTATAATACTACTACTACAGTGACCTCTATGTGTTCAAGTGAAAGGAAGAGTCACATGCCTCTCAGGTTCAATAAAAAGCTAGAAGT
Dasypus novemcinctus  ........................................................................................................................

Homo sapiens          GATTAAGTATAGTAAGGAAGGCATGTTGAAAGCCGAGATAGGCCAAAAGCTAGGAGGCCTCTTGTGCCAAACAGGCAGGTGGTGAATGCAAAGGAAATGTTCTTGAAGGAAATGAAAAGT
Dasypus novemcinctus  ........................................................................................................................

Homo sapiens          GCTACTCTGGTGAACATATGAATGATAAGAAAGCAAAAAGCTTTTTGCTGATATGGAGAAAGTTTGAGTGGTTTGGATAGAAGATCACACCAGCCACAGCATTGCCTTGAGCCAAAGGCT
Dasypus novemcinctus  ........................................................................................................................

Homo sapiens          AATCCAGAGCACATCTCTTACTCTCTTCAATTCTGTGAAGGCTGAGAGAGGTGAGGAAGCTACAGAAGAATAATCGGAAGCTAGCAGAGGTTGGTTCGTGAGGTGTAAGGAAGAAGCCAT
Dasypus novemcinctus  ........................................................................................................................

Homo sapiens          CTCTATAAAATGCATCAGCAACAAGTGCTGATGCAGATGCTGCAGCAGGTTATCCAGATCTAGCTAAAATCACTAATGAAGGTGGCTACACTAAACAACAGATTTTCAATGAAGATGAAA
Dasypus novemcinctus  ........................................................................................................................

Homo sapiens          CCACCTTTTATTGAAATAAGATGCCATCTAGGACTTTGATTGCTAGAGAGGAGAAGTCAGTGCCTGGCTTCAAAGCTTCAAAGGACAGCCTGACTTGTTAGGGGCTTACGCAGTTGGTGA
Dasypus novemcinctus  ........................................................................................................................

Homo sapiens          TTTTAAAGTTGAAGCCAGTGCTCATTTATCATTCTGAAAATCCTAGGGTCCATAAGAATTATGCTAAACCTACTCTGCCTGTACTCTAGAAATGGAACAACAAAGCCTGGATGATAGCAC
Dasypus novemcinctus  ........................................................................................................................

Homo sapiens          ATCTGTTTACAGCATGGTTTACTGAATATTTTAAGCCTACTGTTGAGACATACTACTCAAAAAAGAAAAAAAGATTTCTTTGAAAATATTACTGCTTATTCACAGAGCACCTAGTCACAC
Dasypus novemcinctus  ........................................................................................................................

Homo sapiens          AAGAGCTCTGAAGGAGAGGTACAAGGAAATTAATGTTTTCAAGCCTGCCAATACAATACACATTCTGTAGCCCATGGATCAAGGAGTAATTTTGACTTTCAAGTCTTATTATTTGAGAAA
Dasypus novemcinctus  ........................................................................................................................

Homo sapiens          TACATTTTTTAAGGCTATAGCTGCCATAGATAGTGATTCCATAGATAGTGATGGATCTGGGCAAAGTAAATTAAAAACCTTCTGGAAAGGATTCATCATTCTAGATGCCATTAACATTTG
Dasypus novemcinctus  ........................................................................................................................

Homo sapiens          TGATTCAAGGGAGGAGGTGAAAATACCAACATTAACAGGAGTTTGGAAGAAATTGGTTCTAACACTCATCATTGTCTTTGAGAGGTTTAGGACTTCAGTGGATAGTGTAGCTGCAGATGT
Dasypus novemcinctus  ........................................................................................................................

Homo sapiens          GGTGGAAAGGACTAGTATTAGAAGTGGAACCTGAAGATGTGACTGAATTGCTACAATCTCATGACAAAACTTGAACAGATAAGTTACTTCTTATGGGTGAGAAAAGAAAGTGGTTTGAGA
Dasypus novemcinctus  ........................................................................................................................

Homo sapiens          TTGAATCTACTCCTGGTGAAGCATTGTTGAAATGACAACAAAGGATTTAGAATATTAAATAAACGTAGTTGATAAAGCAGCAGCAGGTTTGAGATGATTGACTACAATTTTGAAAGAAGT
Dasypus novemcinctus  ........................................................................................................................

Homo sapiens          TCTACTCTGGGTAAAATGCTATCAAACAACATTGCATGCTACACAGAAATCTTTTGTGAAAGGAAGAGTCAATCAAGGTGGCCAACATTACTGTTGTCTTATTTTAAGCAACTGCCACAG
Dasypus novemcinctus  ........................................................................................................................

Homo sapiens          CCACCGAAACCTTCAGCAACCACCACATTGATTAGTCAGCATCGTTCAACATCGCAGGAAGCCCCTCTACCAGCAAAAAGATCACAACTCTCTGAAGGCTCAGATGATTATTAGCATGTA
Dasypus novemcinctus  ........................................................................................................................

Homo sapiens          TTAGCAATAAAGTATTTTTAAATTAAGATATATACTTTTTTAGACACAATGCTTATGTACGATTAATAGACTACATAGTATAGTGTAAACATGACTTTCACATGCACTCGGAAGCCAAGA
Dasypus novemcinctus  ........................................................................................................................

Homo sapiens          AATTTCTGTGACTTGCTTTATTGTGATATTTGCCTTATTGTGGTGGTCTGGAACTGAACCTGCAATATCTCTGAGGTAGGCCTATGTTTAAATGAGGTGGGCTTTGCCTTTTATTATCAA
Dasypus novemcinctus  ........................................................................................................................

Homo sapiens          AATAATTATCTGTAGTTTGGAATAGGGTGATGATGAGTTAAAGAGAAAACATCTTCTGTCTTGAAACCCCCTCCTTGTCAAAACAGATATTCATTCTCTAAAAGACTGGCACTGTGTCCT
Dasypus novemcinctus  ........................................................................................................................

Homo sapiens          TTTTCAGGCAACAAGAAAGAGAAAGTAGATTGGAAAATGAGAGAAAATCAAGAGGCTAATGAAACCATGTGGTAGCCTTTTGGGACAGTGTCAGAGAGATAGAAAATCTCAGACAGTTCC
Dasypus novemcinctus  ........................................................................................................................

Homo sapiens          CCAAATAAATAAATTCAGTGTATGATTTTTCCAGCCTACATATCATTACTAGCTTTGAAATGTTTTTAAACAATCTTTGTTGATGAAATTCAGTGAGATTTCTGCATTTACATTAGATTC
Dasypus novemcinctus  ........................................................................................................................

Homo sapiens          CAAATCTGTTAAAAGGAGTCTCAAAACTCTGTTAGGTATTTGTAGTCAGTTTAATTGCATTTGTTATAACTGCAATATTATATTGAAGTGCTGTTCAACCAAATACATAGAAAATGCTGT
Dasypus novemcinctus  ........................................................................................................................

Homo sapiens          AAAATAGTGTGTAGAACAATCCAAGTGAGTCAACTTTTGTGACAATGTCTTTAATCGATGTAAGCTTCCTTCTCTAACTTTCTTGAATTACTGACTTTTTTTCAGTATCATCCTATAAAA
Dasypus novemcinctus  ........................................................................................................................

Homo sapiens          ACTCTTCAGGTTTCCTATGTTTCAGTGTAGCTGATTTATACTGATTGTAGGTTTGGTTCAGGAGGACACTCAAAAGTCCTGCAGGATGTGGAAGAATTCTTTGTAGTGTGGGACTGTCCC
Dasypus novemcinctus  ........................................................................................................................

Homo sapiens          ACATATTTCAAGATGCCTAGCATCTCTGGCCTCACCCTCTGAATGGCATCGGCTTGAGGTGGTGGTGGACTTCATTGTTATAACCAGAAAGGTTCCCACAAGTTTACAAAATGCCCTGAG
Dasypus novemcinctus  ........................................................................................................................

Homo sapiens          GGGCTGGGACTACCGGTCTACAGCGTGGTATTCAACACCCTTCACAACTGGATTCCTTACCATGTGAAAACAAAGAGGCTATTTTCTTTTTTTGTCTTTTTAGACATTGGTGAAGACTGA
Dasypus novemcinctus  ........................................................................................................................

Homo sapiens          GGAAGAAACAGCCCAGGCCGTTGCTGCAGAGAAGTGGTCACATCTGAGTCTGACTCGGAGGAACCTCTGTGGGAACTCAGCTCAGGAGACAGTTATGAGCCTCAGTCCGATGAGTAAGGC
Dasypus novemcinctus  ........................................................................................................................

Homo sapiens          CAGGCCTCTGGCTGGACCCCTGTCTGGACTCTCATTTTCCTGTCACTCACCCTGGCTTGGCCCTTGGGAGGCCACGTTTCCTACAGACCCACACCCTTCCTTTCCCTTCTTCTGGCCTCT
Dasypus novemcinctus  ........................................................................................................................

Homo sapiens          GCCTCCAGACTTCTAGCCCTTATCACTTTGCATCCTTCCCAATCACATTTTCTTCTGTTACTAATCAGTTTACTGATTGTTGTGGTTATCTTCTCTGATCATCTCTTCTAGTTCCCTGAT
Dasypus novemcinctus  ........................................................................................................................

Homo sapiens          TCCTTCAGTGACTCCTTTGTATCCTTTTCCCCTTTTTTCTTCCCTGTCAAGGATTCCATATAACTTGTCTTTCAGATATCCCTTCATACTTGGAAGCCCCATGAACACCTCTGTCTGACC
Dasypus novemcinctus  ........................................................................................................................

Homo sapiens          TTGTCTTTACCTCAGAGGAGGTCATCTTCACTCTCTAAGAACACGACAGTTCATGTTTATTTATTTTTTTTAGTTTTTTTAAAGATGGGGTCTCGCTTTGTTGCCCATGCTGGAGCATA
Dasypus novemcinctus  ........................................................................................................................

Homo sapiens          GTGGCACGATGATAGTTCACTGCAGCCTCAAACTCCTGGGCTCAACTGATCCTCCCACCTCAGCCTTCTAAAAAGCTGGGGCTACACCCAGGTAATTTTTTGTAGACACGGAGTCTCGCT
Dasypus novemcinctus  ........................................................................................................................

Homo sapiens          ATGTTGCCCAGGCTGGTCTTGAACTCCTGGCCTGAAGCAGTCCTTCCACCTTGGCCTCCCAAAGCACCCAGCCTAATTTTCTTGAAATGTGCTCTGCCCTGTTGGGCCCTCTCTGCAGCT
Dasypus novemcinctus  ........................................................................................................................

Homo sapiens          GGGCTTTGGTGCCTTAATTAACCTTTCTTATCATTTTCCCATACCATGCCACATTTTGTAGGATTGCTGAAACTGTGCACCCTTGAAACACCAATATGATTTGTTTTTTGTTTGATTTGG
Dasypus novemcinctus  ........................................................................................................................

Homo sapiens          AACATCTATTCCCTACCCTGGAAGTTTTTTTTTTTTTTTTTTTTTTTTTTTCAAAATTCACATAACAGATGACATTTAAATCATTAATGTTATATTTTAGCTGAAGAAATTGTAACTA
Dasypus novemcinctus  ........................................................................................................................

Homo sapiens          AAGATAGATTGTTTAAAGCAAAGCAAGAAACTTCTGAAGAAATGGAACAAAGTGGAGAAGCCTCAGGAAAGCCCAACAGGTGAGTGTCCATGGTCCTCAGTGAATCAAATCCTGCAGATA
```

```
Dasypus novemcinctus  ........................................................................................................................

Homo sapiens          TAAGAAATTGGAATGGAATCAGAGAAACCAATTGGTCAAAGGCCTGCTTCTCTGGAATAAGATATGACAACTGCCACTCACTTCTGTGATCATAGGAGGTCCATGCCAAGAAAGTGACCT
Dasypus novemcinctus  ........................................................................................................................

Homo sapiens          ATTCAAAATTTTCAATATGCAATAAATATACAAAACCTTCAGTTGATACTCAGTTTCCTCCAAGAAGCCTATCCTGATCCAGTCTGTCCTGCTTGATTACCCTTCCCTGGGCTCCCTTAC
Dasypus novemcinctus  ........................................................................................................................

Homo sapiens          ATTTTTATGGAGCTGTGGAGAAGCTAGAAGGCAGATAATTTAACAAGAATGAGGGCTACAGATGAAGCTGCCATGACAGTTAAGAAAGAAATGTCAGAGTGGAAGAGGACAAGCAGGGTA
Dasypus novemcinctus  ........................................................................................................................

Homo sapiens          CAGGTTGAGCATTCCAAATCTGAAATGTTCCAAAATCTGAGCTGTTTGAGCACCAGCATGATGCTCAAAGGAAATGCTCATTGGAGCATTTTGGATTTTGGATCTTTGGTTAGGGATGCC
Dasypus novemcinctus  ........................................................................................................................

Homo sapiens          CAACTGGTATAATGCAAATATTCCAAAATCTGAAAAAAATCCAAAATTCAAAAATCTTCTGACCCCAAGCATTTCAGATAAGGGAAAATTAACCTGTATTTTCAACCGCCATT---GGGA
Dasypus novemcinctus  ..............................................................................AAGGGACAAGTAGC--GTATTTTCAACCTTTATTCAGGAAA

Homo sapiens          GACCTGAACAAGGTATAGGTCAAAAGGAAAGTTAGCTGATGAAGTAGAAA----TGAATGACAGTGGTGTGTGCTTCATAAGTGCTGGTTGTGGGAATGAATGAACTTGTAACAGATACT
Dasypus novemcinctus  TACCTATACAAGGAAAAGGTCCAAATGAAAGT-AGCCGATGAAGAAAAAGAAATTTAAAGATAGTAGTAGGTGCCGAATAGGTATTGGTTATGGCAATGGATTAATGCATAACATA----

Homo sapiens          CCTATGCAAAGCAAGAAACTTCTGAAGAAATGGAACAAGGTGGAGAAGCCTCGGGAATGCCCAACATTCCGAGGCTTCTCAGGATTGCAGAAAGAGAGGGAACAAATCACAAGCAGAGGG
Dasypus novemcinctus  --------------GATACTTCTGAAG-------------------------GAGAACG--------------------------------------GGGGGCAGATCTCAAGGAGAGGG

Homo sapiens          TTGGCTTCTGGTGAGGCCATTTGGTCCTCAGAGACTTGAGAAAA-GAGAGAGTAAGCAAGGTGGGCTACTT-GGCCAAAGAGCTGAGAAGGGGGTGTGAATTTTCCTTATTGACATTTGT
Dasypus novemcinctus  CTG-CTGCTTGTGAGAGCATTAACTCCTCAGAGAGTTGAGAAGACGGAAACGAGACAAGGTGGGATAATTAGGCTGAAGAGCAGGGCAGGCAG---GGATTCTCCTTATTTGCATTAAT

Homo sapiens          TTAACTTAGAGAAACTGGGAGCTAAATCTCAGCTAAAGTAGAAATGTCTTAAAGCTCAAGAGGAGAAGTGAAATTTTGCATTAAGATTAGTGAAGACTGAGCCAGGCTCCACTAAAGGAA
Dasypus novemcinctus  TTGTGTTAGGGAAAC--AGAGCTAAATCTCAGCTAAAATAGAG-TATCTTGAAGTTTAAGAG---AAGTGAAAGTTTTCAT-AAACCTACTGTAGCCTGAGTCAAGTTCCGCCAAAGGAA

Homo sapiens          GAGATATGATGTTCAGTAACTTAGAGCAGAGTTATGCAACTGGAGAATAGGAATCTGCTATTGTGCCTGTAG-GTACATTTAGTATCAGGCAACTAACA-GGTAATTCATAGTGCCT--A
Dasypus novemcinctus  GAGAAAGAACATTCAGTGACTTAGTGCAGAGTAAT--AACTGGAGAATAGGAACCTGTTATTTTACCTGTTGTATGCATTCAGCATTAGGCAAATGAAACCATAACTCACAGCGCCTCAA

Homo sapiens          TAATAAAGATA---------TTCATATAACAAAAAGTGTGAAGGTAGGCTATTTCAGGTTAGGTGCAATAGCTTAACCATGGTATCAAAGGTCTAGCCCCCTGCCCTCCTTCTAGTCCCC
Dasypus novemcinctus  AAGCAGAGACATGTTATTGTTTCACATAAAAACAATTCTGAAGGTAGTCTGTTT-ATGTTTGGTGCAGCCGCTCAACCTTG---CCAAAGATCCAGCCACCCACCC-------------

Homo sapiens          CTCCCTCACCCCCACCCCACTTTCTTAGCTCACTCTTTTTTCCCATTTCTCACTCCCATTTCCTTCCCTCACAATATTGGGCTTAATGCACTGTGGTTCTTAGAGTTTAGATGTCAACAG
Dasypus novemcinctus  ------CACACACA---CACTTCCTTGTATCACTC-TTCTACCCATTCCCCACTCCCATTCCCTTCCCCTAGCATGTTGGATTTAATG...............................

Homo sapiens          TAGAAAGACTCAGTGTGATCAAGGAATGTCACTGAAGTTATGGATGTCAGGGAACTTTAAGATGGATGTGTTGAATACATGCAGACACTGAAGACACCCTGGATGATAAACACTTTACAT
Dasypus novemcinctus  ........................................................................................................................

Homo sapiens          AAAGAAATGTATGTAGTGTAAATTATATTAAAATATACTCTTCATGCTTCTTAAATGTCTTTGAAAACTTGACTAGTTTACCAACAGAACACTAATAAATTAAATGCACATGTCTAAAAA
Dasypus novemcinctus  ........................................................................................................................

Homo sapiens          TTAAAAACTTTGTGTAAAATAATACTTTTCAGTTGGGTGCAGTGGCTCATGCTTGTAATCCCAGCACTTTGGGAGGCCGAGGTGAGTGGATCACGAGGTCAGGAGTTTGAGACCAGCCTG
Dasypus novemcinctus  ........................................................................................................................

Homo sapiens          GCCAACACAGTGAAACCCTGTCTCTACTAAAAATACAAAAATTAGTTGGGCATGGTGGCACGTGCCTGTAGTCCCAGCTACTCGGGAGGCTGAGGCAGGAGAATTGCTTGAACCTGGGAG
Dasypus novemcinctus  ........................................................................................................................

Homo sapiens          GCAGAGGTGGCAGTGAGCTGAAATCATGCCACTGCACTCCAGCCTGGGTGACAGAGCTAGACTCTGTCTCAAAAAAAAAAAAAAACAAAACAAAACTTTTCATCTTTGTAACTTTAGCTT
Dasypus novemcinctus  ................................................................................................................TTCAACTT

Homo sapiens          ACTTCCTTGTAAACCCCATAAAAATCAGTCCAAGTGCAATGAGTTGAGTTTCATTTGTTTTTCCCCAGAGTAGTATGTCCATCAGATGAATGTAGAACTAGCAGGGGCTAAGTCCTTCCA
Dasypus novemcinctus  CCTTA-TTATGAACCCCATAGAAATCAGTTCAAACAC------------------------------------CCTGTCCACCACAAAAGTGCACAACTAGGAGAGGCTAAATCCTTCTA

Homo sapiens          TGTTCAGAAATTCTGTGAGAGATGTCAGAAGACCTGGTAAATAATAACCTTTTTTTCTTGTTCAGATTCAGAACCAATTGACAGGGATGCAGGAGTAAAATAGATGGTTCTGGAAACCTC
Dasypus novemcinctus  TTTTCAGAAATTCTCTGAGGG---------GGCCCTGTACATAACAA-CTCTCTTCCTTGTCCAAGTTCAGAACCAATTGACAGACACACAAGCATCAAACAGTTGGTGCTG........

Homo sapiens          AGTTTCTTACTGACAGAAGTTAGACTGGATAAAAACGGCTTAAAGGCAGAGCTAAATAGGCTTGTACTGGCAGAGGAGGAGTGTAGCAGATGTCCCTCTGCATAGGTCTTCTCAATGCAA
Dasypus novemcinctus  ........................................................................................................................

Homo sapiens          CTTGGGGCACCAAGGAGCATGGGATGTCTGCATCCTGCTCCTAAAGAGGACAGAGGGAGGAGTGTACCAAGGATACTCAGTTATTTCTCCCAGGCTCAACAGTTAGTTCCATCCTGAGGA
Dasypus novemcinctus  ........................................................................................................................

Homo sapiens          GTGAGTGAGAGGTGGAGAGTGGCTAGAAATGTTTAATCTAGCCAAAGTCCTCCCCATGGAAACTTAAGAGAAATAAGTAAGTATCCCCCCACACCCACCCCAAGCCCCAAAAGATTTAAG
Dasypus novemcinctus  ........................................................................................................................

Homo sapiens          CTTCAGCAGATTTATCATGTGTGAGAGAATATGATTGAGGATTTTTATAACATGTTCTTTTTTTCTTTCCCAGAGAGTGTGCACCCCAGATTCCTTGTAGTACTCCTATTGCTACTGAAA
Dasypus novemcinctus  ........................................................................................................................

Homo sapiens          GGACAGTTGCACATTTGAACACTCTGAAGGACCGTCACCCAGGTGATTTGTGGGCCCGCATGCACATCTCATCCCTGGAATATGCTGCAGGAGACATTACCCGAAAAGGGAGAAAAAAAG
Dasypus novemcinctus  ........................................................................................................................

Homo sapiens          ACAAAGCTCGAGTGAGTGAACTGCTCCAAGGCCTCTCATTCTCTGGTGACTCAGATGTGGAAAAAGATAATGAGCCTGAGATCCAGCCTGCTCAAAAGAAGTTAAAG----GTATCATGT
Dasypus novemcinctus  ........................................TCTGAGGACTCAGGCACTGGGGAAGACATTGATCTGGAGCTGCAGCTAGTGAAGAAGAGGCAGAAGACAGGTGTCAAAT

Homo sapiens          TTCCCAGAAAAGAGTTGGACCAAAAGAGACATTAAACCCAATTTTC-CAAGCTGGTCAGCACTGGATTCTGGACTTTTGA--ATCTCAAGAG-CGAAAAGTTGAACCCAGTAGAGCTTTT
Dasypus novemcinctus  CTC----AGCAGGTATGGACCAAAAGAGATGTCTGTCCTGACTCTGGCCAGTTGGACAACATCAGAT-CTTCATATTTGAGTATCTCAAAAGTCAGGGAACTGAGTCCTGTACGCCTCTT

Homo sapiens          TGAA-TTATTTTTTGATGATGAAACATTCAACTTAATTGTCAATGAAACCAATAATTAT-GCTTCTCAGAAAAATGTCAGCTTGGAAGTCACAGTTCAGGAAATGAGGTGTGTGTTTGGT
Dasypus novemcinctus  TGAAATTTTTTTCTGATGAAGGAACTCTTAATTTAATTGTTAAGGAAACCAATCGTTATCGCTTGGCAGAAAAATTTCAACCTGGGTCTCACAACCCAGGAATTGAAGTGTGTTTTGGGC

Homo sapiens          GTCTTACTTTTGAGTGGATTTATGAGGCATCCTAGAAGGGAAATGTATTGGGAAGTCTCT---GACACCGATCAGAACCTGGTTAGAGATGCAATCAGAAGGGACAGATTTGAATTGATT
Dasypus novemcinctus  ATTCTGATTTTAAGCGGGTACATCTGCTATCCCAGGAGAAGGATGTTTTGGGAAACATCTCCTGATTCACATCACCACCTTGTGGCTGATGCAATTAGAAGAGATAGATTTGAACTGATC

Homo sapiens          TTCTCAAACCTGCACTTTGCAGATAATGGCCACCTAGATCAAAAAGATAAGTTTACAAAGTTGAGACCTCTCATAAAACAAATGAATAAAAATTTCCTCTTGTATGCTCCCCTGGAAGAA
Dasypus novemcinctus  TTCTCATACCTGCATTTTGCAGATAACAGTGAACTCGCTGAAACTGATAGATTTGCCAAAGTCAGGCCTCTCATTGTTCGGATGAACTGCAATTTCCAGAAGCATGCACCCTTGGAAGAG

Homo sapiens          TACTATTGCTTTGATAAGTCAATGTGTGAATGCTTTGATAGTG---------ACCAATTCCTGAATGGAAAGCCTATTAGAATTGGCTATAAAATTTGGTGTGGTACAACCACACAGGGT
Dasypus novemcinctus  TTCTATAGCTTTGGTGAGTCCAGGTGTGAGTACTTTGGGCATGGGGGGTCCAAGCAGCTGCACATGGGAAAATCTTCCCGGCTTGGCTATAAGATCTGGTGTGGGACAACCAGCAGAGGC

Homo sapiens          TATCTGGTTTGGTTTGAACCCTATCAAGAAGAATCAACTATGAAGGTAGATGAGGATCCTGATCTTGGGTTAGGTGGAAATCTAGTGATGAACTTCGCTGATGTTCTTTTAGAGAGAGGT
Dasypus novemcinctus  TACTTGGTGTGGTTTGAGCCCTCGCAGGGTACACTGTTCACCAAACCAGATGACGGTTTGGACCTGGGA------GGCAGTATGGTGATAAAATTTGTGGAGGCCCTTCAGCAGCGAGGC

Homo sapiens          CAGTATCCCTATCACCTGTGTTTTGATAGCTTCTTTACAAGTGTCAAATTGTTGTCAGCCTTGAAAAAGAAGGGGGTGAGGGCAACAGGAACAATTCGTGAGAACAGGACCGAAAAATGT
Dasypus novemcinctus  TGTCTGCCATACCACATATTTTTTGACAAGGTTTTTACAAGTGTCAAACTCATGTCCATTTTGAGGAAAAAGGGGGTGAAGGCCACGGGGACTCTCCGTGAGTACAGGACTGAGAGATGT

Homo sapiens          C-CCCTTATGAATGTAGAACATATGAAAAAAATGAAGAGAGGGTATTTTGATTTCCGAATAGAAGAAAACAATGAGATAATTTTGTGTCGTTGGTATGGGGATGGCATTATCAGTCTGTG
Dasypus novemcinctus  CTCCCTGAAAGATCCCAAAGAACTGAAGAAAATGAAGAGGGGCTCATTTGATTACAAAGTTGACGAGAGTGAGGAGATCATTGTATGCCGCTGGCATGACAGCAGCGTGGTCAACATCTG

Homo sapiens          CTCCAATGCTGTGGGCATAGAACCAGTCAATGAGGTAAGCTGTTGT---GATGCTGATAAC--GAAGAAATCCCTCAGATAAGTCAACCATCCATAGTAAAAGTGTATGATGAATGCAAG
Dasypus novemcinctus  CTCCAATGCGGTGGGCATAGAGCCAATGAAACTGATGAGTCGGTCTTCAGGAGCAGCCAACCTGCGGA-----CCCAGGTCCACCAGCCATCACTTGTAAGGCTGTACCAGGAGAAGGTG

Homo sapiens          GAAGGTGTAGCTAAAATGGATCAAATTATTTCGAAATACAGGGTGAGGATAAGAAGCAAGAAATGGTACTCAATTTTGGTGAGCTACATGATTGATGTAGCCATGAACAATGCATGGCAA
Dasypus novemcinctus  GGGGGCGTCAGCCGGATGGACCAGAACATTGCCAATTACAAGGTGAAGATTCGGGGCATGAAATGGTACTCGAGCTTCATGAACTACATGAT-GACACAGCCATGAACAATGCATGGCAG

Homo sapiens          CTACACAGAGCCTGTAACCCAGGTGCTTCTCTAGACCCCTTGGATTTTCGGAGATTTGTTGCACATTTCTACTTGGAACACAATGCTCATCTGTCAGATTAGGGTACATAAAATGGACAT
Dasypus novemcinctus  CTACACAGAGCCTGTAACCCAGGAGCT-CTTTAGACTCTT--GATTTTCGAAGATATGTTGCACATTTCTACTTGGAGCACAATGCTAATCTGTTAGATTAATGTAGATAAA---GGCAC

Homo sapiens          AGTGCAGACATTAATAAGACATAGAAAAATAATAATTATACATGCTGTTGTACCCTCCCAAAGTAAATCTGATATATGTAATGAAGTTATTAAATAATACTTTTAAAAATCAGACATTTA
Dasypus novemcinctus  AATTCAGACATTAACAAGACACAG-----------CTACACATTAGATTGTACTCATCCAAAGCAAACCTGATGTATGTAATGAAATAACTA------TTTTTTTAAAATGAAAC----A

Homo sapiens          TATAGAGTTTCAAAGACTATTGTAACAAGTAATGTTAAAAA-TTGTCTGTGAGAATGTTGAACT----GTAGTACCTTTCTCTATGTCAAGTTTT-GTGTCAGACA--TGGGAAATCATG
Dasypus novemcinctus  TATATAGTTTCAAAGACTATCGTAGCAAATAATGTTAAGAATTTACCCCTAAGAATGTTGAACTCAAGTTGGTACTTTTCTTGATGTCTAATTTTGGTGTCAGACACTTGGGTAATAAT-

Homo sapiens          TATTTGTTCAATTGACTACTTTGTGCACTTATTTATTTATTTTTTGAGACACAGTCTCGCTCTGCGGCCAAGCTGGAGTGCAGTGGCACGATCTTGGCTCACTGCAACCTCTGACACCCT
Dasypus novemcinctus  ------TTTAATTGTTTACA....................................................................................................

Homo sapiens          AGTTCAAGCGATTCTCCTGCCTCAGCCTCCCAAGTAGCTGGGATTACAGGCACGTGCTGCCATGCCTAGCTAATTTTTGTATTTTTAGTAGAGACAGGGTTTCACCATGTTGGCCAGGAT
Dasypus novemcinctus  ........................................................................................................................

Homo sapiens          GGTCTCGATCTGACCTCGTGATCTGCCTGCCTTGGCCTCCCAAAGTGCTGGGATTACAGGCGTGAGCCACCATGCCCAGCTATTTTGTGCATTTAAAGAAGGAAATCCTACCTCTTAA--
```

```
Dasypus novemcinctus  ....................................................................................TTGTGCATTTAAAGAATGAAATCTTGCTTTTTTTTT

Homo sapiens          -AAAAAATTATCTGGAGAATGCCATTTTTAAGATGCAAGCAATGTTACAGAAACCATAGAATGGTGCTGACTCAATAGTTCAAACTAGTGACACAG-CCTATGAAGTAAGAATGATCTAA
Dasypus novemcinctus  TGTAATTGTCTCTGGAGAATGCCGGTTTTAAAATGCAGGCAGTGTTATAATAGCCATAGAGT-GCACTGAATCCATAGTCCAAA--ACTGAAACAGATACATGAGGTAAGGATGATCTAA

Homo sapiens          ACAAAACATAGGTGGTAAGAGACTAAAAACCTTAGCATTGGTGTAAAACTGGATCGGATTGTGTGTAAGTGAGAAGGGTCAGGCATGGATATTGAAGGAGAGTGCTATAAAGGAAAAACC
Dasypus novemcinctus  ACA.....................................................................................................................

Homo sapiens          AGAGGTGAAGCAGTGCCTT
Dasypus novemcinctus  ...................


Homo sapiens  AGGATAGTTAAGTAAATTATGGAACACTCATACAGTATTACTTAGCTGTTAAAGAATAAGCTGATCTGTGAAGATCTCCAAGTTGTCTTAACCGAAAAGAGCTAAGGTGCAAAATTACAT
Mus musculus  ........................................................................................................................

Homo sapiens  TATGATACCATAAGTGTTTTAAAATGTATAAATACTTATTCACTAAACAAAATGTTCATTAAGTGCTACTGTGTGGCAGGTAGTGTCCTAGATAAATATGAACAAAATACAATGGTTAAC
Mus musculus  ........................................................................................................................

Homo sapiens  TCTAAAGAGGGGACACTTTTCGTTTTATGGTCCTTTTGCAACTTGACTTTTTTATTGTACATATATTATGTATACTTAAAGAAAATAATTTGTACAGCTTACTATAATGGTCTTTTTATT
Mus musculus  ........................................................................................................................

Homo sapiens  GATCTGTCTCTCCTACCAGCCAGTGATGACCCCGAAGACAGACATTTACCACTGTGTTGGTTTCAATCGAAATGATGTACATAAATTTGGGGCGTGCTTGCACCGTCCGAATGGGCCTG
Mus musculus  ......................................................TGTTGTTGTT--GTTGAAATGATTTACGGAAATG-AGCGCGTGTTTGGTTTCTTCGAAACTACC--

Homo sapiens  GGGTCCGGCGGCGGAGAGTGACCTGGGCGGGGCAGGAGGTGGCAGCGG--GGGTCCTCCCGGCTCACCAGAGAGACGAGCGGCCGTGCTCCTAGAGAGGCAGGGAACCCGCCAGACTCCG
Mus musculus  -----------AGGAGAG------GTGCGGGTCAGGAAA--ACCACGACCTGGCTCCACCGGTGCACCAGAGATT---GTCACCAGGCTTCTAAGGAG-CACAACGCTTGCCGGAAGTTG

Homo sapiens  CCACTCCGCTGCGGGCGCGG--CGCAGGGAGAAGCTTTT--GTACCCG--CCCAGCTGCTGGAGGCGCCGGCAGC------GCCCGCCAGAC------CCGCCAGCC--CAGCGGCCCGG
Mus musculus  CCAGTGAGTCTCCAGCTCCAACTACAGGAAGTGCCCCCTCGGTGCCCAGCTCCGTCTGCAGGAAGTGCCCCCTGTCCTTCATCCCTTTGGACTCCTTCCCGCCGACCTTCATCGTCGCGG

Homo sapiens  GCTCTGGGGAAACCGGCG-----CTCCCGACAGGGGAGCACCGGGCCTCTGAGCTCC---CTCGGGAGCCTTTCACGAGGTCAG--------CTACGTCTTTGTTGTGCGCGTTCCTGAA
Mus musculus  GCTCGGAGACTAACAGCAAGAACCGCCAGGCTGTGGCGC-TCTGGCCTCTTGGCTCTGGTCGCAGGCTTTTTTCATGAGCTTCGGCACATCCTCACGTCCTCGTAGCACACGTTCCCTAA

Homo sapiens  CTTTTGGTCCATTAACCAACAATTAGACACGCCGGTATTCAGTGCCTGGCGCGGTGCTAGATGCTGGGTGTAATCTCAGAA-AAATACATTCAGGGGCGCGCCTGAGGGTGCTGGCTGCT
Mus musculus  ACTTT-------TAAGCAGCAAATAGACACCC-----TTAGCTGCTGGGCGC---------TGGCGTATATAATTTTAGCGTAGATCTGGGCAGG--------TTAGAGCCCTGGC-GAA

Homo sapiens  GGCATCTCAGGTGCTTTACGTGCATTCGTGAAGAAGCCCATCAGTATTTCTTGAATACCAGGTACGTGCCGGGCATGTAGAGTCTATGCAGAGATAAACTCCTGTCCTCAAGAACCCGGG
Mus musculus  GGCACTACAGGTACATCTATTACGTTCGT-----------CCGGTCTTTGTTGGGTATCAGGTACGTGCTGCGATTCTAG--TTTATGTAGAGGT------CTGCC---AGGAGCAGGGG

Homo sapiens  TGGGGATGGGGTTGGTGATCTGTAATACGAGATGGAGATGAGGGTTGGGTGCAGTTTTCTATGGGAATACCCCATCTCAACCTGTGTGGCTTACTTTTCCTGTCTATATGTTAGGGTTAG
Mus musculus  TGGCTGTG----CAGTGATCTGCAATAGAAGCTGGAGA-ACGGAGTACGTGCA--CCCAGAGGTGCACACCCGGTTGCCACCTGTGTGGCTCAGTTTTCCCATTCCTATTTTAGGTTCGA

Homo sapiens  GGGTTGCCAGGGAAGGTTTTACA---ATAGAACA---TTTACGCTGGGCCTTGGGAATTTACCCTTAGAAGGGCGGTCCTGACTGTATAGAGTGGGTGGAAAAAAATTGAGTACTTAGCA
Mus musculus  GGGTAGCCACGATAGATTATGCAGGTACAGAGCAGGTCTTGTGTCAG----TAGGAATTTA-CATTGGAAGGATAGAC----CAATACAGAGCAG--GAAAAAAAACTG-----------

Homo sapiens  TGGCAGTGACATAAGAAATTTGGGAAGACCATGGCTTTCTT-GAAAGCAGGAACCAA-GTCTTTTATTCCAGAGTTAACTTTATGCCTGGAAAATAATGAGATGCTCCACAAATGTTGAT
Mus musculus  TCACCCTGAATTCGAGGAACTGGGAAGTCCATGGTTTTTTAGTAGGTAGGAACTAACTTCTTTTATTCTAGGATTAACTTTACTCATGGAAAATAATTAGGTGCTT----AATAT--AC

Homo sapiens  TGAGTGACAAACAAATCAGCAAAACCCAGTAAAGATTAGATCCTGGGGAGTTTGAATTTACTTCAACGAG-----TTTAATG------AAAACTTTGTGTGTGTTTATGTGTGTGTGAGG
Mus musculus  TTGGTGGTAAACTAAACGG-GAGACGCAGGTAAGATTGGATTCT-GGGAGTTTGAAGTTAGCCCAAGGAGAAGTTTCTGATGGTTGCCATGGTTTTGTACGTGCGTGTGCGTGTGCGAGA

Homo sapiens  TATGTT-GAATATGGTCACAACTGTGCTTTAGGGAATATTAATCCAGTGACCATAGAAGCGGGGAGTTGGGCAGGAATACCGTAACAGAACATTATTGTAACAGTGAATGAAAATAATAG
Mus musculus  TTTGTTAGAGTAAGAGCAAAATTGTGCTTTAGGAAATA-------------CACATCAATGATGTGATGTACAA---------AACAAAAC--TATTGCAATAGTGGATGAAAATGGTAA

Homo sapiens  -TAAAATTATAAGAAGGAAAGAATGGCTGCAAGAGGCGTTTTTAAGAATTGACT-------CACTGATTGTGTATAGAGGCTGTTTAAAAGACTGAATGCGTGGAAGATGATGGTGATGC
Mus musculus  CTAAGGAAATAGAAAGGAAGGAATTGTTGTCAGAGATATTTTATAGAAATAATTAGCCAAACACAGCGATCATATAGGGCCTGAGGTTAAGCCTAACTGCATGGGAAGTGGT--TGACCT

Homo sapiens  CAGTAGAAATGGAGAAATACCGAGTGAACGCAGCTTTGAGG-GACTCTGATTTCAGTTTGAGATCTGTTGAAATTATAGTGCAGTAGAGGCCCACAGCTGCTGGCATTTCAAGATGTGGG
Mus musculus  CAATAGAAGTGGAAAAATACATAAGGAACCCCAGTGTGAGGTAACCCTGATTTCCTTTCAGTATGTGTGGAAATGATAGCGCAGGAGAGACATCC---TGCTGGCACTGGAAAATGTGGG

Homo sapiens  TTTGGAGTGCAAAAAGCAAGCTGGTGCCAAA----GATTTGAGTCTTCTTGGAACAGAGATATATTGAAAGTGTGACTAAGGAGATCACAAAGGAAAAAATGAAAAGAGGGAATAAAAGA
Mus musculus  CCTGGAGT-CTAAAGGAAGCTTGGAACCAGAAAAGGATGCGGATGTCCTTGAAACAGA--TGTGCTGGAACTG---------AGATCACAAAAGGAAAAAGGAGATGAGGAAAAGAAGGG

Homo sapiens  ACTTGATGCATGTCTACTGTTTTTCAGTTATGTATTGTTGCATCAAAAATAAAACTCTAAACTTAGTGGCTGAAAACAAGCTCAGCTTATTGTTCCTCAGGATTATGCGGGTTGACTAGG
Mus musculus  ACCTGCTGAGCCTCCACTGCTTT--GGGTGTCTGTCGTTCTAT-AAGAATAAAAACAAAACCCGAATAA-TGAAAAGA-------------GTGTCTTGGAGTCCTATAGATTGATTGGC

Homo sapiens  CGGTTCTACTG---GTCTCACCTGGAGTTACTAATAGAGCTGCATTCAGGTGGTGAATTATTTGAGCACTAGGCCTGGCTGGGCTTTAACTTGGGCATTTTGGTTCTCATCCACATGACC
Mus musculus  CAATTCTACTGTGGGTCCTGCTTGGGGCTGCTCATACAGCTGTATTTAGCTTATAAATTATATAGGCTCTAGGCCCAGCTAGGGTTTAGCCTAGGAGGCCTGATTC-----CAAGTGACC

Homo sapiens  TCTGTATAGCTAGCTTGGGCTTCCTTACAGCATGGCAGTCTTATGGCAGTGAGCCAAGAGCTACAGCAGCACTCTCTCTTACAATGTCATTTCTGCTGTATTCCACTGGTCAAAGCAAGT
Mus musculus  TTTACATGACTAGCTTGGGTCTCCTCACAGCATGGTTGTCTTATGGCAATGTCCTAAGAGCTGCAG-AGAGTTTCCTTTTTCTATGTCATGTCTGTTATA--CTGTTTGTCAAAGCAAGC

Homo sapiens  CATAGGCCAGAACAGATTCAAGGGAAGGAAAAAGAGACTCCACCCCTTGAATGACAACAGTGGCAAAATCTCATCACAAAAGGTATTTGAGAATGGGAGACTTTGGAAACATTCT---GT
Mus musculus  CATAGGCCAGCACAGATTTTGAGACATAATTATGGGA---------------GGCA----TGGCAAGATCTCACTACAGAAGGCATTAGTGG-TGGGAAATTGTGGAAGAACAATAACGT

Homo sapiens  TACACCTACATTTAAAGTGGAATATTCAATGAGAGTTACACAGATATAGAAGACCTTTGAGACTGTATTATTTGTCCATGAAGGCAGCCATCTGCCTCCTCAATGGGT-AAATTATCCCC
Mus musculus  AAGATCTGCATAGAAAGTGAAATATTCAATG-------CATATCGATAAATGACCTTTGA-ATTGTACTGTGTTTCTGCCAAGATAGCTCTCTGGCTTCTCCATGTGTCAAATCATCCCT

Homo sapiens  -------------TCTCCCCAGCTGAAGTGCAAGCTA--CTCGAGCTTTCTTATTGCTAACACTTCTTCCCTCATGTCCTACATCCCTTACCTTTGGACTCAGGCCCTTTAGTACTAGTT
Mus musculus  AATTGAGACACAGTCTCTTTATTT--AGTCCAGGCTGACCTTGAACTTGCTTATTCTTAGCCTTTCTTCTCTCATGG--TGTATCCCTTATCTCTGGGCTCAAATCCTTTAGT----GTT

Homo sapiens  CAAAATATACTTCTGATTCATTTCACTGATAATAGAAGCAAGCAGGCTCAGCTTTTGAGCATTACCTACAGTGGGGACAAAAATGTAAGTAGGTTACATACAGTTTTGAA---GCTCTTA
Mus musculus  CAGGATATGACTTTGATGC---------------------GTAGGGTTAGCCTTTGAGCACAGC--ATAGTCGAGACAAAAATAAAAATATGTTTCATAAGATTTTAAGTTGACTTTTA

Homo sapiens  TAATTTGCATGTTGCTGATAAATTCTTATGCCTCAGATCTCTAT------------TTCTGAA-TATAGACCCCA---AGCTAAGTGAAGCTTTAGCCTCTAAGCTCAACATGTATGAAG
Mus musculus  T---------TTTCTGTTCAATTCTTATACTGTAGATGTTCATCTAGAAATGCATTTCTGAAACACAGACTTCAGATAGCTGAGCTGTTCTGCAGCTTCTAAGTTCAACATGTATGAAG

Homo sapiens  CTTTGCCAGGCCCTGCTCCTGAAAATGAAGATGGCCTTGTGAAAGTGAAGGAGGAAGATCCCACCTGGGAGCAGGTGTGCAACTCACAGGAGGGCAGCTCCCACACTCAGGAGATTTGCC
Mus musculus  TTTTGCTGGGTTCTGCTCCTGAGAATGAAGGCCGCCTTGTGACAGTGAAGGTGGAAGATCCCACCTGGGGACAGCCAGGCAACTCACAGGAGGGCAGGGCGCATGCTCAGGAACTTTACC

Homo sapiens  GCCTGCGCTTTCGGCACTTCTGCTACCAGGAGGCTCACGGACCCCAGGAAGCTCTGGCCCAACTCCGAGAACTTTGTCATCAATGGCTGAGACCGGAGATGCACACCAAGGAACAGATAA
Mus musculus  GCCTGCGATTTCGGCACTTCTGTTACCATGAGGCAGCAGGACCCCGCGATGCTCTCAGCCAGCTCCGGGTTCTCTGTTGTGAGTGGCTGAGGCCGGAGCTGCACACCAAAGAGCAGATCC

Homo sapiens  TGGAACTGCTGGTGCTGGAGCAGTTCCTGACCATCCTGCCCAAGGAGCTCCAGCCCTGTGTGAAGACATATCCTCTGGAGAGTGGAGAGGAGGCAGTGACAGTGCTGGAGAATCTAGAGA
Mus musculus  TGGAGCTGCTGGTGCTGGAGCAGTTCCTCACCATCCTGCCTGAAGAGTTTCAGGCCTGGGTGAAGACATATCCTCTGCAGAGAGGTGATGAAGCGGCGACATTACTGGAGAATCTAGCCT

Homo sapiens  CAGGAAGTGGAGACACAGGACAACAGGTGGGAAGAGAATGTGTGTGGTGATGTGGGAGAAGAAAAGG---GGGACATGTATCGGTTTATTTTTAACGTTTTATTTTGAAATAACTCCTAA
Mus musculus  CGGAACGTGGAGACACAGGGCAGCAGGTGGGGAAATAATGAGCACGCTTGTGTGGGAAGGGAGTGGGGTAGGAATGTGTATCTGCTAATGCGCACCCTCTTATTCTGAAATAATTCAGAA

Homo sapiens  TTTATAGAAGTATTAGAAGAATAGAGAACTCCCGTAAAACTCTTCATCCAAATTTGCCAGTTGTTAACATTTGTTTGATGAAGTCCCATTGTTGTGTACACACTCGTACAAGGAAGTGCA
Mus musculus  TTTAGAGAAGGACCATCAAAACAG-----------CATTCTCTTCACCTAGATTTAGCTCTTGGTAACACTGGTTCCATGATGTGCC.................................

Homo sapiens  TTCCCCCCCCCCAAGATGCATATGTATATGTATACACACACATACATACACATATGCAGTATTTTTTTTTCCTGAATCTATTTGAGAGTCAGTTGCAAACACTATATCCCTTTACCTCAC
Mus musculus  ................TGCATGTGTCTATGTATACACACAGATGCATGCTGATTTGTTTTTCTTTTTTTTTT................................................

Homo sapiens  TGTTGACCAGGCTGGAGTGCAGTGGTACGATCATAGCATCATTGCAGCCTTGAACTGCAATGGTGGAAGGCTCAAGCAATCCTCCCACCTCAGGCTCCTGAGTAGATGGGACTACAGGTG
Mus musculus  ........................................................................................................................

Homo sapiens  CATGCCACCATGCCTGGCTAATTTATTTTTAATTTTTTGTAGCGGGGGTGGGGTCTCACTATATTGCCCAGGCTGATCTTGAACTCCTGGCCTCAAGTGATCATGCTGCCTTGGCCTCCC
Mus musculus  ........................................................................................................................

Homo sapiens  AAAGTGTTGGGATTACAGGCATGAGCCACTGCACCTGGCTATTCGGTGTGTATTTCTGAAGAGTGAGGATATTCTCTTGTATCACAAGAATACAAAAATCAAGTTTAGGGAAATTAAACT
Mus musculus  ........................................................................................................................

Homo sapiens  TACGATACTATCATATAACCTTAGTCCTTATTCATATTTTGCCATGTTATCCTAATAATGTTCTTTATGATAATTTTCCTGTGATTCAGGATACAAGCCAGCATCACATATTGCACTTAG
Mus musculus  ........................................................................................................................

Homo sapiens  TTGCCATATCTCTAGTCTGTGAATCTGGAGTAGCTGCTGTCTTTGTCATGACGTTGTTATTTTTGAAGAGTACAGCTAGTTTTTTTGGAGACTATCCCTCAATTTGAGTTTGTCCTATAT
Mus musculus  ........................................................................................................................

Homo sapiens  TTTCTCATGATTCAGGCTGCACATTTTGGCTAGA---ATACCACAGAGGTAATGTTGTGTTCTCAGTGCGTTACTTCAGGAGGTGCGTAGTGATGATTTGTCCCATTACTGATGAGGTTA
Mus musculus  .........ACCCAAGCTGTACACTCGGGCTAGATGCCTACCACATAAGGCAGCGTGCCCTCTGGCTG-GTCCTCCCAGGAAGCACACAGCGATGCTTTTGTCCTTCAC-----AGTTCA
```

```
Homo sapiens  TGGTGGTACCTGTCA-ATTTTCTCCACTAGAAAGTTACCATTTTCCCCTTTGTAATTACTAAGTAATCTGTGGGAGACATTTTGAGGTGGTGTACATATCCTGTTCCTCATTAAATTTTC
Mus musculus  TGCTAGTGCTTGTCAGAGTTTCTTCTCTAGGGAATTG---TTTTACCCTTTGTAGTTGTTGTGTAGTCTGTGGAAGACATTTGGCGAGAGTATACAGACCCTAGGCCTCATTACATTTTT

Homo sapiens  ACCAGCTAGTTTAAACATCCACTTGTTTGTTGTTTTTGTTTTTCTGGGCTGGGATCCTTTGCCTGTCTTTTCTGCCTTGTTTGCTGCTGTATCCCCAGCATGTAGAACAGTGCCAGGCTC
Mus musculus  TACTGCTAGCTCAGACATGC-------TGTCATTGTGTATTTCCGGGGATAGATCCTTTCTCTTGTTTTTCCTGCCTTG---ACTTCTGAGTCCCCAGATTCTGAGGGGGACCTGCGTGG

Homo sapiens  TTAGCATGCAGTCCATATATGCATGCCCTTTCAAAATAAATCTTTTGTTTCCAGGCCTCTGTCTATATTCAGGGACAGGACATGCACCCAATGGTGGCAGAATATCAAGGAGTCTCTTT-
Mus musculus  TGAGTTTGCAGCGCTAATATGAATGCTTTTTCAAAGTGACTCCTTTGTCTCTAGGCTACTCTCTGTGCTCAGGAACAGGACATGCACCTAGTGGTGACAGAGTATCCAGGAGCCTCTATG

Homo sapiens  --GGAGTGTCAGAGCCTCCAGCTCCTGC---CTGGGATAACCACCCTGAAGTGTGAACCTCCACAGCGTCCTCAAGGGAACCCCCAAGAAGTGAGTGGTGAGTGCTCG--AGTGAGTTTA
Mus musculus  GAGGAGTATCAGAGCCTCCATCTCCTGCAGTCTTCGGTCACTCCCTGGACAAGAGACTTTCCAGAGCTC-----------CCCCTGGGATGTGAGAGGTGAGTGCTAGGAAATGAGTTTA

Homo sapiens  GTGAGGACGCTGGGAGCTGGCCACTGACACTTGCACAGCCTTCTTGACCTCAAGTTTGTATACATTGTGAAATGATTACCACAAGCTAACTTACATATCTCACAGTTACCTTTTATGTGT
Mus musculus  ATGAGGACCCT-GAAGGAGGCTGCTGA.............................................................................................

Homo sapiens  CTGTGGTAAGAATTCCTAAGATCTACTCTCATGAAATTTCAAGTATACAATATATTATTATTACTAACTATAGTCAGCATTCTGTACGTAGGTCTTCAGAGCTTATTCATCTTATAACTG
Mus musculus  ........................................................................................................................

Homo sapiens  CAAGTTTATACCCTGTGACCAACATCAAGTTTTATTTTTTTGTCTAACCCTGTATCTGAACAAACTTGTAAAGACCTGCTTTATTTTACACTGTGCATTTTAAGCTGGTTTATTTAGTAT
Mus musculus  ........................................................................................................................

Homo sapiens  GTATGTGTTGTGGACAAGTACATATAATAGTAAAAGTGAATCGGGGCAAGGCTGTTTTTTTCCAGCAAACTTTATTCCTTCTAGAAATGCAACAACAGCAATAAAAACATTCCATAGTGA
Mus musculus  ........................................................................................................................

Homo sapiens  TCCATTATGGAAGCAAAGAAAAGCAGAGCAAAGAAAAGTGATGTTCTGTTTTATATTTTTGGGTTAGAATTACCACATGTGCTCTTCATTTGATAATTTTGTACTCTGCCATCTGCCAAC
Mus musculus  ........................................................................................................................

Homo sapiens  TGCCAACTTTGTGACCTAGGCAAGTCATTTAATAGGTTATTCAGTTAATCAACAATTATCTGTTTGATCTCTGTGTGTACTCTGCACTCAGCTGTGATTCTTCTGAATGTGCCTAGGATT
Mus musculus  ........................................................................................................................

Homo sapiens  TTGGAGGCAATAATTTGGACCTTCTTGATTATTTTGAGGATTGGAAATAATCTTTTAAAGTAACAGCACAGTGGCTGGCCTTGTGCAGTCATCATCACCATTTTGATTATTGTTGGTTCT
Mus musculus  ........................................................................................................................

Homo sapiens  TTGGGATCAGTACCTCCTAACAGTTTTTCTAGTTCATGGCTAACATCTCTTCTCTATTTTTTTAAATATCTTTATTTTCCTGTCTCTCTCTCCATCTTCCTTTCTTCTGCTTTCCTGTTT
Mus musculus  ........................................................................................................................

Homo sapiens  CCCCTCAGTCTCATCTCTCTACTCTCTACATCCTTTCTTTTGCCTCTGTGTTCTCTCCTTGATCCTTCCTTTATCACCAGCTTCTTCCTCCCCTTTATTTTTGTCTATGATTTTTATT--
Mus musculus  ..........................................................................CAGCAGCTTCTTCCTCCCTTCT-CTCTTACCGGTGGCTTTAACTCA

Homo sapiens  TTTTCTTTATGTGAAAGTGCATGACTGTAAAGCACCTTATGAGTTCATAAATCCCACTCCATTTTTTGGGTGAGAGACCCTAAAGTTGTAGAATTTTAGAATTTCTGACAGAATGAAAGC
Mus musculus  TATCACATATGTGA------------GTAAAGTACCTACCAAGTCTCTCACTCCTGCTCAGTCTTGTGGGTGAGCAACAGCTGAGCTTCAGACTCTTAGAAAC-----------------

Homo sapiens  AGTTGTGATGCTCTT-TCTCACACTGTAAAATCTACGTCTTAGTTCTTGAATGTTTAACAAACATAACATTTGGGGGAAAAGGCTGGGTCTCCAAAAAGGGTACC-CTAAAGGCCATCAT
Mus musculus  -GTTGCAGTGCTTTTAGCTCAGTCCATGAAATCTGTTTCTTGG-----------------------------------GAGAGTGGATCCATACAGAGGGAGCTTCCCAAGGCAATTTT

Homo sapiens  GTCTCATTTAGGACTCTTGCCCTCTCTCTCTGCAGGGCCTGTTCCCCACGGATCAGCTCATCTCCAGGAAAAAAACCCCAGAGACAAGGCTGTAGTGCCTGTGTTTAACCCAGTCAGGTC
Mus musculus  GTCTCAGGAAGGACTCTTGTCTTCTCCCTTTATAAGATGTGCTCTTCAAGGAGTAGCCTGTCTCCAGGAAGAAAGTTCCAAAGACAGTGCCGTGGTGCC-GAGTCTAGCTCAGCTAGGTT

Homo sapiens  CCAGGTAAGTAGGATGCCCAAGCTTGTAGGAATATCAGTAGTGGTCTTTCTCCCTCATTCCATGTTCCTTGCCCTTCTCTTGGCTCACACTCATCATCGTGAGAGCCATTCAGGTGGCAT
Mus musculus  CCAGGTGAGC-TGCCGTCCAGGTTTATGGAAAC----TTAGTGGCAATCCTCCCT--TCCCTTG----TTGCCTTTCTCTTGGCCCACTCTC-TCAGTCCAAAAGCCTTTAAGGAG----

Homo sapiens  AGTCCCTCTGCCTTCACCTTGTCACAGAATATCACAAAGTGTGT---TGCATCATTCGAGGAGCTTGTATTGATTATATGCCATTGCCTCATTTCAT----TGTTCAGGAGGCACGTCCA
Mus musculus  -GTGCCTTCACGTTCACTTTGTCTCAGACTATCACAGACTGTGTATGTTCATCATGCAGAGAGCCTATGATGGT----GGCTTCAGTTTTATTTTGTGCCATGTTTTGGAGGCACATTTA

Homo sapiens  AATCTCAAGGCATGGGAGTGA--GGGGTGGAGTGAGGCTCCTGGTTTCAGAAAGTGCTCCACTCATTTGATGTTGAGTAACTGATATTTTCTCCTTTGAAGTCTCTGGTTTATCTCCCCA
Mus musculus  GATCTCAAGGTATCAGGGTAAAGAGGGTGGTATAAGACT.................................................................................

Homo sapiens  GCTTTAGAATAAGGGCCCTTTCTCACACTACTTTATATTCAGCATGTATGCAATTAATGCTTGGTGATAATAATGGAAATTCTATATATCTAAGGTAATACTTAACATCTTCTTCACAAA
Mus musculus  ........................................................................................................................

Homo sapiens  ATGGACTCTGGTTTTTTTTCTTATTAGTGGTTCATTCATTTTCCTAGTCATTTATTTACAAAAAAAAAATAACTC--TCTCATTTAAGTCCTCTTGCATACTGCTGCCACATCAGTTTCC
Mus musculus  ............................................................AGGACAGAGTGGCTCTTTCCCATATAAGTCTTTTT-----------CTCCATTTGTTCTC

Homo sapiens  TCAAGACAC------------TTGC--TCAGTCCTCTTCTATGGCTTCTAATTAATTTTTAATTCTATCAGACCCAGTGCCTCCTGTTATTAACAAATACTTCCTGATAAGAAGTATCT
Mus musculus  TCAAGGCACTGTCTTTATGATATTGCCTTCTCTGCTTTTCCATGACTTC-------CTGTTAATCACATCAGACCTCAGACCTCATGTTGTTATCAACTACTT------------TATCT

Homo sapiens  A---GAAAATAAAACCTACTCATGCACATTCTGAAAATCAACATAATTCCCTTAAATGTAATTTAATGGTATAATGTAGAAACAAAAA----AGTTATTTATTTTAAAAGTAGTGTTT--
Mus musculus  GTCTGTAAATGCAACCTACTTAAACATACTTTAAATAAAAACCTAATTGCTGTAAATGTA--------GTATAACATGGATACAAAGAAAACAGTTATATATTACATATATATTATTTAT

Homo sapiens  --CAAA--ATGCAAGTGCTTGATGTGATTATAACTAGGGAAATAAC----AACGTAGTCAGGTGTTTGCACTGAAATATAATTAGTAAGTTCAAATTAAGGAGGAATAAGAAGACCAGAA
Mus musculus  TACAAGCTATAAAAGTCTTCAATATGATTATATATGAGGAAATGACAAAGAAAGTAGTTAGATGTTTGCACCAATGTATAATCAATGACTGAAAATTAAGAAGGAATAATA----TAGGA

Homo sapiens  GCCCAAGAAAATCAAAGAGCAATGGTATAGGTGGACAGTGAAATGAAACTAGTATTGAGTCAGATAATACAAGACTAGACAGAGAAAGTGGGGCCCTTAATTGAAGTAAGGCTTAGATGT
Mus musculus  GTCCAAGAAAGTC-TAGGGTAGAGGTGTGTGTGCACAGAAGATGAAAGCTAATATTAAATCAAATTATAAAAGACCAAACAGACCAAA------ATTTAACTGAAGCTGAGCAAACA---

Homo sapiens  TGTAAATTGAATTATGTCCCTCCAAAAGATATATGAAAATCCTAACCCCACGTACCTGTGAATGTGACCTGATTTGGAAACAGGCTCTGTCTTAGTCCATTTTGTGTTGCTAATAACAGA
Mus musculus  TATGAGTTAAATTTTGTCCCCTCAAAAGATATG-GTAAGTCCTAATTGTAGGTGCCCATGAAGATGGCCTGACTTG............................................

Homo sapiens  ATTCCTGAGACTGGGTAATTTATTTTAAAAAGAGGTTTATTTAGCTCAAAGTTCTACAACCTGGGAAGTCCAAGATCATCAGCCTATCTGGTTAGGATCTCATGCTGCTTTAGCTCATGG
Mus musculus  ........................................................................................................................

Homo sapiens  CAGAAAGTGGAATGATGGATAATCAAGTGTGTGCAAGGAGACCACACATTAGAGGCAGCCATCCTTTAAACAGTCTGCTCTTGGAGCTGAGCAAGAGCAAGAATAGAAAGCTTATTCAAA
Mus musculus  ........................................................................................................................

Homo sapiens  GAAATAACAATAGGCCGGGTGCAGTGGCTCTCAACTGTAATCCCAGAACTTTGGGAGGCCATGGCAGGAGGATTGCTTGAGGCCAGGAGTTTGAGACCAGCCTGAGCAACACAGCAAGAC
Mus musculus  ........................................................................................................................

Homo sapiens  CCTGTATCTATTTAAAAAAAAAGAATAGGCCAGGCGTGGTGGCTCATGCCTGTAATCCCACACTTTGGGAGGCTGAGGTGGGTGGATCACGAGGTCAGGAGTTCGAGACCAGCCTGGCCT
Mus musculus  ........................................................................................................................

Homo sapiens  ACCTGGCAAAACCCCGTCTCTACTAAAAATACAAAAAAAAGGAGCCAGGTGTGGCAGGCACCTGCAATGCCAGCTACTCGGGAGGCTGAGGCAGGAGAATCGCTTGAACCTGGGAGATGG
Mus musculus  ........................................................................................................................

Homo sapiens  AGGTTGCAGTGAGCCGAGATCATGCCATTACACTCCAGCCTGGGCAACAAGAGCAAGACTCCATCTCAAAAAAATTAAAATAAAATAAATTAAAAATAAATTTTAAAAATAGAGAAATAA
Mus musculus  ........................................................................................................................

Homo sapiens  TAACAAAAATCTTCACAAAACTTGTGAAAGATATAAATGTCCAGGTACAGGAAGATCTGAGAACACTGAACAGAATCATCCCAAATAAGACTACTCCAAGGCATATAATAATCAAACTCT
Mus musculus  ........................................................................................................................

Homo sapiens  CAACTGTCCAGGAAAAAGAGAGGATCTGAAAAGCAGCAAGAGACAAGAAACAAATAAGGAGCTCCAACTTGTCTGGCAATAGACTTCTCAATGGCAATCATACAGGCCAGGAGAAAGTGG
Mus musculus  ........................................................................................................................

Homo sapiens  AATGACATTTTCAAAGTACTCCAAGAAAAAAACTGACATTCAAGAATATTGTATCCAGCAGAATTATCTTTCATATATGAAGGAGAAAGTCTTTCCCAAACAAACAAGCTGAGAGAGTAC
Mus musculus  ........................................................................................................................

Homo sapiens  ACCACCACCAGACACATCTTACAAGAAATGCTAAAGAATTCTTCAGTCTAAAAGGAAAGAAGACTAATGTGCATAAAGAAAACTTTTGAAGGCATAAAAGTCACTGATAAAATTAAGTAC
Mus musculus  ........................................................................................................................

Homo sapiens  ATGGACAAACCCAGAATACTTTATTACTGTAATAGTGACATACAGTATACTCACAACTGTGAAGCCCCAAGACATACCTATCAAAAACAATAATAGGACTGGACACAGTGGCTCATGCCT
Mus musculus  ........................................................................................................................

Homo sapiens  GTAAACCCAGTTCTTTGGGAGGCTGAAGTGGGAGGATCACTTGAGACAAGGAGTTTGAGACCAGCCCAGGCAACATGGTAAGATCCTGATGCTATACCTGGAGACATGAGACAGAGTGAG
Mus musculus  ........................................................................................................................

Homo sapiens  ACCATATCTTTAAAACAAAACAAGACAAACAATAATGGCTAGCAACCTGTTAAGAGATAGGTAATATAAAGATATAAATTGAGACAACGAAAAGTCAAAATCTAGAGGAATGGAATTAAA
Mus musculus  ........................................................................................................................

Homo sapiens  GCATATAATTTTTTTCTGTGTGTTTCTGCCTTTGTTTCTACTCTTGTATTTGGGATCTAAGATAAGTTGTTATCTCTTTAAAATAACTTATTATATCTGAAGGTCTTCTTTGTAAGCCTC
Mus musculus  ........................................................................................................................

Homo sapiens  ATGATAACTACAATGCAAAAACCTATAATAAATTCACTAAAAATAGCAACAAAGGAAGGCATACTATAAGAGAAAATCACTTAGCCACAGAGGTTTTTTGTTGTTGTTGTTTGAGATAGG
Mus musculus  ........................................................................................................................
```

```
Homo sapiens  GTCTCACTCTTTCACTCTGGCTGGAGTGCAGTGGTATCATCTCAGCTCACTGCAACCTCTGCCTCCCGAGTTCAAGTGATCCTTCCACCTCAGCCTCCCAAGTAGCTGGGACCACAGGTG
Mus musculus  ........................................................................................................................

Homo sapiens  TGTGCCACCATGCCTGGGTAATTTTTTGTATTTTTTGTAGACCTGGGTTTTTGCCATGTTGCCCAGGCTGGCTTGAACTTCTGGCCTCAAGCAGTCTGCCCACCTTGACCTCCCAAAGTT
Mus musculus  ........................................................................................................................

Homo sapiens  CTGGGATTACAGGCGTGAGACACCATGCCCGGCTGTATATAACAATTATAAATACCTATGCATGCAACACCAGAGCTCCCAGGTATATAAGGAAAACATTAATAGATCTAAAAGGAGAGT
Mus musculus  ........................................................................................................................

Homo sapiens  TAGACTGCAATACAATAATAGTAGGAGATTTTAACACCCCACTCTCAGTAATTGACACATCATCCAGACAGAAAGTCAACAGAGAAACGATGGAATTAAACTACATGCTAGATCTAATAA
Mus musculus  ........................................................................................................................

Homo sapiens  GCCTAACTGATATTTACTGAACATGTAACCCAACTGCTACAGAATGCACATTCTTTTCATCAGCACGTGGGACATAAGAATAGACCATATCTTAGGCCACAAACAAATCTGAACAAATTT
Mus musculus  ........................................................................................................................

Homo sapiens  TTAAAAAATATAAATCATATCAAGTATCTTTTCTAATCACAGTGGAATAAAACTAGAAAGCAATAACAAGAAAAACCTTGGAAACTACACAAACGCGTGGAAATTAAATAGCATGCTTCT
Mus musculus  ........................................................................................................................

Homo sapiens  GAACAACTAATTTGTCAGGGAAGAAATTAAGAAGGAAATGTAAAAATGTCTTGAAACTAATGAAAATAGAAAATGTGACATATCAAAATTTGTGGGATACTGCAAAAGCAGTACTAAGAA
Mus musculus  ........................................................................................................................

Homo sapiens  GGAAATTTATAGCAATAAACACCTATATCAGAAAAGTAAAAAGACATCAAATGCACAACCTAATGATACACCTCAAGGAACTAGAAAAGCAAGAACAAACCAAACTGAAATTAGTAGAAG
Mus musculus  ........................................................................................................................

Homo sapiens  GAAACAAATAAAGATTAGAACAGAAATAACTGAAATTGAGACCAAAAAAAAAAAAAAAAAACCCAGAAGAACAATGAAACAAGAAGTTGGATTTTTTTTAAAAGATAAACAAAATCAAGT
Mus musculus  ........................................................................................................................

Homo sapiens  CTTTAGCTGGACAAACAAAAAAGGAGAAGACCCAAATGAATAAAATCAGAAATGAAAAAGACATAACAGCTGAGACCTCAGAAATACAAAGAAACCATTAGAGACTTATGAACAACTATG
Mus musculus  ........................................................................................................................

Homo sapiens  ACAATAAATTTGAAAACCTGGAAGAAATGGATAAATTCCTAGATGCATGCCAACTACCAAGATTGGACCATGAAAAAATAGAAAACCTCAGCAAACCAATAACAAGTAACGAGATAGAAT
Mus musculus  ........................................................................................................................

Homo sapiens  CTGTAATAAAAAGTCTTCCATCAAAGAAAAGCCCAGGACCTGTTGGCTTCCCTGCTGAATTCTACCAAACATTTAAAAAGAACTAATACCAATCCTATTCAAATTCTTCAAAAAAAAAAA
Mus musculus  ........................................................................................................................

Homo sapiens  AAAAAATTGAAGAGGAGGGACTACTTCCAAATTCATTCTAGGAGGCCAGCATTGCCCTGATACCAAAACTAGACTAGGATACAAAATAAAAAGAAAACTACAGACCAACATCACTGATGA
Mus musculus  ........................................................................................................................

Homo sapiens  GCATAGGTGCAAAAATTCTCAATGAAACACTAGCAAACTGAATTCAACAACACATTAAAAAGATCATTCACCATGATCAAGTGGGATTCACTCCAGGGATGCAAGGATGGTTCAACATAT
Mus musculus  ........................................................................................................................

Homo sapiens  GCAAATCAATAAATGTGATATATCACATTAATATAATCAAGAACAAACACCATATGATTATTTCAATAGATGCTGAAAAGCATTTGATAAAATTCATTGCTTTTTGATAAAAACCCTTGT
Mus musculus  ........................................................................................................................

Homo sapiens  CAAAATTGGTATAAAAGGAACATACCTCAAAATAAGGGCCATATATGACAAACCTACAGCTAACATCAAACTGAAGGCCTTTTCTCTAAGGACTGGAACAAGATAAGAATGCCCATCTCA
Mus musculus  ........................................................................................................................

Homo sapiens  CCACTGTTGTTCAACATAATACCCGAAGTCCTGGCCAGAGCAATTATGCAAGAGAAGGAAATAAATGGCATCCAAACCGGATAGCAAAAAGTCAGGTTAGTCTGTTCGCAGATGATATAA
Mus musculus  ........................................................................................................................

Homo sapiens  TCTTATACCTCGAAAAACCTGAAGACTACACCAAAAAAACTGTTAGTGCTGATAAACAAACTAAGTAAAGTTGCAGGATAAAAAATCAACACTCAAAAATTAGTAGCATTTATATACTTC
Mus musculus  ........................................................................................................................

Homo sapiens  AATAGTGAACAATCTGAGATAGAAATCAAGAAAATGATCGCATTTACAAAAGCTACAAAAAATATAAAATACTTAGGGAATTTAAATACTAAAAATTTAAACACCAAATTAAGTTAAATT
Mus musculus  ........................................................................................................................

Homo sapiens  TAGTGTTTAAATTCCCTAGGCATTTTATATTTTTTAGTATTTTATTTATAATTTATAATTTAGCATTTAATTACTAAAGAAGTGAAAGACCTGTATAAGGAAAACTAAAACTCTCATGGA
Mus musculus  ........................................................................................................................

Homo sapiens  ACAAATTGAAGAGGACACAAAAACACTGGAAAGATATTCCATTCTAATGGATTGGAAGAATTTATATTATTAAAATGACAATACTTCCCAAAACAATTTACAGATTCAGTGTAATCACTA
Mus musculus  ........................................................................................................................

Homo sapiens  TCAAAATACCAATGACATTCTTCACAAAAATCAGAAAAAAAAAATTCATGTGGTGAGCCCTTAATCTAATGACTCATATTCTTATAAGAAGTCCATATTTGCAAATTGACCAAGGGGTTGA
Mus musculus  ........................................................................................................................

Homo sapiens  AAAAAAAGAAAGAAAAATGCCCAAGTGAAAACACACACAGGGAGAATGCCATGTGATCACTGAGGCAAAGATTTGTATAACACATCTGCAAGCCGAGGAACACCAAGAATTGCTGACAAA
Mus musculus  ........................................................................................................................

Homo sapiens  CACTAAAAACTTGGAGAGAGGCAAGGAAAATTATCCCATGGAGCTTTTTTTCTTTTCTTTTCTTTTCTTTTTCTTTCTTTTTGAGACAGTCTTGCTCTGTTGCCAGGCTGGAGTGCAGTG
Mus musculus  ........................................................................................................................

Homo sapiens  GCGCGATCTCAGCTCACTGCAACCTCTGGCTCCCTGGTTCAAGTGATTCTCCTGCCTCAGCCTCCCGAGTAGCTGAGATTACAGGCAAGCGCCATCACGCCCAGCTAATTTTTGTATTTT
Mus musculus  ........................................................................................................................

Homo sapiens  TAGTAGAGATGGGGTTTCACCATGTTGGCCAGGATGGTCTCGATCTCCTGACCTAGTGATGTGCCCACCTTGGCCTCCCAAAGTGCTGGGATTACAGGCATGAGCCACCACGCCAGGCCT
Mus musculus  ........................................................................................................................

Homo sapiens  CCCATAGAGCTTTCAAAGATAACATGGCCCTACTGATATCTTGATTTTGGATTTCTAGCCTCCAGAGCCATGAGATAATACAATTCTGTTGTTTTCCACTATTGAGGTTGTGGTACTTTG
Mus musculus  ........................................................................................................................

Homo sapiens  TTACAAGAGCCATAGGAAAGCAATATAAGTTACAAAACAAATTATGGACTGATAAAGGGAGAAAATAAGGAGGTATGATATTACAGATACACTTCCTTCATTGTGTTTCACTTTATTGTG
Mus musculus  ........................................................................................................................

Homo sapiens  CTTTGCAGTATTGAAAGTTTGTGATGACCCTGTGTGGAACAAGTCTGTCAGCACCATTTTTCTACAGCATGTGCTCACTTTGTGTCACATTTTGGTAATTCTTAAATTCTTAAACGTGAC
Mus musculus  ........................................................................................................................

Homo sapiens  ACTCTGTGTCACATTTTGGTAATTCTTAAAATGTTTCAAACTTTATTGTTATATCTGTCATGGTGATCTGTGATCAGTTATTTTTGATGTTACTATTGAATTTGTTTTGGTGTGCCACAA
Mus musculus  ........................................................................................................................

Homo sapiens  ACCATGCCCGTTTAAAATGTCAGACCTAATCAATAAACATTGTATGTGTCCTGACTCCTCCATTGACCAGCTGTTCCCTCATCTCTTTTCCTTTCCTTGGTCCTTGCTATGTCCTGAGAC
Mus musculus  ........................................................................................................................

Homo sapiens  ACAATAATATTGAAATGAAGCCATTTATAATACTACTACTACAGTGACCTCTATGTGTTCAAGTGAAAGGAAGAGTCACATGCCTCTCAGGTTCAATAAAAAGCTAGAAGTGATTAAGTA
Mus musculus  ........................................................................................................................

Homo sapiens  TAGTAAGGAAGGCATGTTGAAAGCCGAGATAGGCCAAAAGCTAGGAGGCCTCTTGTGCCAAACAGGCAGGTGGTGAATGCAAAGGAAATGTTCTTGAAGGAAATGAAAAGTGCTACTCTG
Mus musculus  ........................................................................................................................

Homo sapiens  GTGAACATATGAATGATAAGAAAGCAAAAAGCTTTTTGCTGATATGGAGAAAGTTTGAGTGGTTTGGATAGAAGATCACACCAGCCACAGCATTGCCTTGAGCCAAAGGCTAATCCAGAG
Mus musculus  ........................................................................................................................

Homo sapiens  CACATCTCTTACTCTCTTCAATTCTGTGAAGGCTGAGAGAGGTGAGGAAGCTACAGAAGAATAATCGGAAGCTAGCAGAGGTTGGTTCGTGAGGTGTAAGGAAGAAGCCATCTCTATAAA
Mus musculus  ........................................................................................................................

Homo sapiens  ATGCATCAGCAACAAGTGCTGATGCAGATGCTGCAGCAGGTTATCCAGATCTAGCTAAAATCACTAATGAAGGTGGCTACACTAAACAACAGATTTTCAATGAAGATGAAACCACCTTTT
Mus musculus  ........................................................................................................................

Homo sapiens  ATTGAAATAAGATGCCATCTAGGACTTTGATTGCTAGAGAGGAGAAGTCAGTGCCTGGCTTCAAAGCTTCAAAGGACAGCCTGACTTGTTAGGGGCTTACGCAGTTGGTGATTTTAAAGT
Mus musculus  ........................................................................................................................

Homo sapiens  TGAAGCCAGTGCTCATTTATCATTCTGAAAATCCTAGGGTCCATAAGAATTATGCTAAACCTACTCTGCCTGTACTCTAGAAATGGAACAACAAAGCCTGGATGATAGCACATCTGTTTA
Mus musculus  ........................................................................................................................

Homo sapiens  CAGCATGGTTTACTGAATATTTTAAGCCTACTGTTGAGACATACTACTCAAAAAAGAAAAAAAGATTTCTTTGAAAATATTACTGCTTATTCACAGAGCACCTAGTCACACAAGAGCTCT
Mus musculus  ........................................................................................................................

Homo sapiens  GAAGGAGAGGTACAAGGAAATTAATGTTTTCAAGCCTGCCAATACAATACACATTCTGTAGCCCATGGATCAAGGAGTAATTTTGACTTTCAAGTCTTATTATTTGAGAAATACATTTTT
Mus musculus  ........................................................................................................................

Homo sapiens  TAAGGCTATAGCTGCCATAGATAGTGATTCCATAGATAGTGATGGATCTGGGCAAAGTAAATTAAAAACCTTCTGGAAAGGATTCATCATTCTAGATGCCATTAACATTTGTGATTCAAG
Mus musculus  ........................................................................................................................
```

```
Homo sapiens  GGAGGAGGTGAAAATACCAACATTAACAGGAGTTTGGAAGAAATTGGTTCTAACACTCATCATTGTCTTTGAGAGGTTTAGGACTTCAGTGGATAGTGTAGCTGCAGATGTGGTGGAAAG
Mus musculus  ........................................................................................................................

Homo sapiens  GACTAGTATTAGAAGTGGAACCTGAAGATGTGACTGAATTGCTACAATCTCATGACAAAACTTGAACAGATAAGTTACTTCTTATGGGTGAGAAAAGAAAGTGGTTTGAGATTGAATCTA
Mus musculus  ........................................................................................................................

Homo sapiens  CTCCTGGTGAAGCATTGTTGAAATGACAACAAAGGATTTAGAATATTAAATAAACGTAGTTGATAAAGCAGCAGCAGGTTTGAGATGATTGACTACAATTTTGAAAGAAGTTCTACTCTG
Mus musculus  ........................................................................................................................

Homo sapiens  GGTAAAATGCTATCAAACAACATTGCATGCTACACAGAAATCTTTTGTGAAAGGAAGAGTCAATCAAGGTGGCCAACATTACTGTTGTCTTATTTTAAGCAACTGCCACAGCCACCGAAA
Mus musculus  ........................................................................................................................

Homo sapiens  CCTTCAGCAACCACCACATTGATTAGTCAGCATCGTTCAACATCGCAGGAAGCCCCTCTACCAGCAAAAAGATCACAACTCTCTGAAGGCTCAGATGATTATTAGCATGTATTAGCAATA
Mus musculus  ........................................................................................................................

Homo sapiens  AAGTATTTTTAAATTAAGATATATACTTTTTTAGACACAATGCTTATGTACGATTAATAGACTACATAGTATAGTGTAAACATGACTTTCACATGCACTCGGAAGCCAAGAAATTTCTGT
Mus musculus  ........................................................................................................................

Homo sapiens  GACTTGCTTTATTGTGATATTTGCCTTATTGTGGTGGTCTGGAACTGAACCTGCAATATCTCTGAGGTAGGCCTATGTTTAAATGAGGTGGGCTTTGCCTTTTATTATCAAAATAAT-TA
Mus musculus  ..............................................................................................GCCTTATAGTATCAGAATAGTCTG

Homo sapiens  TCTGTAGTTTGGAATAGGGTGATGATGAGTTAAAGAGAAAACATCTTCTGTCTTGAAACCCCCT-----CCTTGTCAAAACAGATATTCATTCTCTAAAAGACTGG-CACTGTGTCCTTT
Mus musculus  TGTGCCCATTGGGACTGTGTAGTAACAAGGTAAACAGAAATGATC--CAATCTTGAGAGCCACTAGACCTTTTATCAGAGCCTACATCCAGTCTGGGAAGGACTGGATACTGTATCCTTT

Homo sapiens  TTCAGGCAACAAGAAAGAGAAAGTAGATTGGAAAATGAGAGAAAA-TCAAGAGGCTAATGAAACCAT--GTGGTAGCCTTTTGG-GACAGTGTCAGA--GAGATAGAAAATCTCAGACAG
Mus musculus  T-CAGGCAATGGGAAAT-GAGTGTAAATAAGAACATGAAACAAAAGTTAAGAGATTAATGAAAATATATGTGGTAGACTTTTGGAGACATTATCAAATAAAGACAAAGAACTATAAGCGA

Homo sapiens  TTCCCCAAATAAATAAATTCAGTGTATGATTTTTCCAGCCTACATATCATTACTAGCTTTGAAATGTTTTTAAACAATCTTTGTTGATGAAATTCA-GTGAGATTTCTGCATTTACATTA
Mus musculus  CCACCAAAATAATTAT-TATAGCATATAATTTTT-------AAATAGTAT----AGATATGCAATATTTT-AAATAATTTTTATTAATAAAATTTTTGCAAGATCTCTACATTTACA--G

Homo sapiens  GATTCCAAATCTGTTAAAAGGAGTCTCAAAACTCTGTTAGGTATTTGTAGTCAGTTTAATTGCATTTGTTATAACTGCAATATTATATTGAAGTGCTGTTCAACCAAATACATAGAAAAT
Mus musculus  AGATCCAAATCTGCTAAAAAGAGCCTCAAAACTCC------------------------------TTGTTACATCTTCGATGTTACATC---------CTCAACCAC---TGCAGGAAAT

Homo sapiens  GCTGTAAAATAGTGTGTAGAACAATCCAAGTGAGTCAACTTTTGTGACAATGTCTTTAATCGATGTAAGCTTCCTTCTC--TAACTTTCTTGAATTACTGACTTTTTTTCAGTATCATCC
Mus musculus  G-------ATACTGAACTGAACAGGT-GAGTGAGTCCATGTTTGTAACAATGTCTTTATTCTGTGCAAGCTTTCTTCTAGGTAACATTCTTAAGTTGTTTTCCTTGCTTTGGTATTATTT

Homo sapiens  TATAAAAACTCTTCAGGTTTCCTATGTTTCAGTGTAGCTGA--TTTATACTGATTGTAGGTTTGGTTCAGGAGGACACTCAAAAGTCCTGCAGGATGTGGAAGAAT-TCTTTGTAGTGTG
Mus musculus  CGTAACAA--TCTCAGACATCTTAT-TATAGGTGCAGTAGGCCTGTGTATTGG-TGCAGAGTTAGTCTACCAGGGCACTCCAATGCCCTGTACCATGTGGGACAGCGTTGCTGTTGTGTG

Homo sapiens  GGACTGTCCCACATATTTCAAGATGCCTAGCATCTCTGGCCTCACCCTCTGAATGGCATCGGCTTGAGGTGGTGGTGGACTTCATTGTTATAACCAGAAAGGTTCCCACAAGTTTACAAA
Mus musculus  GG-------CAGTCATTGTCAGGTATCCAG----------------------TGGCAGTGGATTGTGCTGGTGCTGAAC---------ATAAGTA-----GTTC--------------

Homo sapiens  ATGCCCTGAGGGGCTGGGACTACCGGTCTACAGCGTGGTATTCAACACCCTTCACAACTGGATTCCTTACCATGTGAAAACAAAGAGGCTATTTTCTTTTTTTGTCTTTTTAGACATTGG
Mus musculus  ATCCCCAGAGTGGCTGGCACCACAGGTTTACAGTG-----TTCAACATCTTTAATA................................................................

Homo sapiens  TGAAGACTGAGGAAGAAACAGCCCAGGCCGTTGCTGCAGAGAAGTGGTCACATCTGAGTCTGACTCGGAGGAACCTCTGTGGGAACTCAGCTCAGGAGACAGTTATGAGCCTCAGTCCGA
Mus musculus  ........................................................................................................................

Homo sapiens  TGAGTAAGGCCAGGCCTCTGGCTGGACCCCTGTCTGGACTCTCATTTTCCTGTCACTCACCCTGGCTTGGCCCTTGGGAGGCCACGTTTCCTACAGACCCACACCCTTCCTTTCCCTTCT
Mus musculus  ........................................................................................................................

Homo sapiens  TCTGGCCTCTGCCTCCAGACTTCTAGCCCTTATCACTTTGCATCCTTCCCAATCACATTTTCTTCTGTTACTAATCAGTTTACTGATTGTTGTGGTTATCTTCTCTGATCATCTCTTCTA
Mus musculus  ........................................................................................................................

Homo sapiens  GTTCCCTGATTCCTTCAGTGACTCCTTTGTATCCTTTTCCCCTTTTTTCTTCCCTGTCAAGGATTCCATATAACTTGTCTTTCAGATATCCCTTCATACTTGGAAGCCCCATGAACACCT
Mus musculus  ........................................................................................................................

Homo sapiens  CTGTCTGACCTTGTCTTTACCTCAGAGGAGGTCATCTTCACTCTCTAAGAACACGACAGTTCATGTTTATTTTATTTTTTTTAGTTTTTTTAAAGATGGGGTCTCGCTTTGTTGCCCATG
Mus musculus  ........................................................................................................................

Homo sapiens  CTGGAGCATAGTGGCACGATGATAGTTCACTGCAGCCTCAAACTCCTGGGCTCAACTGATCCTCCCACCTCAGCCTTCTAAAAAGCTGGGGCTACACCCAGGTAATTTTTTGTAGACACG
Mus musculus  ........................................................................................................................

Homo sapiens  GAGTCTCGCTATGTTGCCCAGGCTGGTCTTGAACTCCTGGCCTGAAGCAGTCCTTCCACCTTGGCCTCCCAAAGCACCCAGCCTAATTTTCTTGAAATGTGCTCTGCCCTGTTGGGCCCT
Mus musculus  ........................................................................................................................

Homo sapiens  CTCTGCAGCTGGGCTTTGGTGCCTTAATTAACCTTTCTTATCATTTTCCCATACCATGCCACATTTTGTAGGATTGCTGAAACTGTGCACCCTTGAAACACCAATATGATTTGTTTTTTG
Mus musculus  ........................................................................................................................

Homo sapiens  TTTGATTTGGAACATCTATTCCCTACCCTGGAAGTTTTTTTTTTTTTTTTTTTTTTTTTTTTTCAAAATTCACATAACAGATGACATTTAAATCATTAATGTTATATTTTAGCTGAAGAA
Mus musculus  ........................................................................................................................

Homo sapiens  ATTGTAACTAAAGATAGATTGTTTAAAGCAAAGCAAGAAACTTCTGAAGAAATGGAACAAAGTGGAGAAGCCTCAGGAAAGCCCAACAGGTGAGTGTCCATGGTCCTCAGTGAATCAAAT
Mus musculus  ........................................................................................................................

Homo sapiens  CCTGCAGATATAAGAAATTGGAATGGAATCAGAGAAACCAATTGGTCAAAGGCCTGCTTCTCTGGAATAAGATATGACAACTGCCACTCACTTCTGTGATCATAGGAGGTCCATGCCAAG
Mus musculus  ........................................................................................................................

Homo sapiens  AAAGTGACCTATTCAAAATTTTCAATATGCAATAAATATACAAAACCTTCAGTTGATACTCAGTTTCCTCCAAGAAGCCTATCCTGATCCAGTCTGTCCTGCTTGATTACCCTTCCCTGG
Mus musculus  ........................................................................................................................

Homo sapiens  GCTCCCTTACATTTTTATGGAGCTGTGGAGAAGCTAGAAGGCAGATAATTTAACAAGAATGAGGGCTACAGATGAAGCTGCCATGACAGTTAAGAAAGAAATGTCAGAGTGGAAGAGGAC
Mus musculus  ........................................................................................................................

Homo sapiens  AAGCAGGGTACAGGTTGAGCATTCCAAATCTGAAATGTTCCAAAATCTGAGCTGTTTGAGCACCAGCATGATGCTCAAAGGAAATGCTCATTGGAGCATTTTGGATTTTGGATCTTTGGT
Mus musculus  ........................................................................................................................

Homo sapiens  TAGGGATGCCCAACTGGTATAATGCAAATATTCCAAAATCTGAAAAAAATCCAAAATTCAAAAATCTTCTGACCCCAAGCATTTCAGATAAGGGAAAATTAACCTGTATTTTCAACCGCC
Mus musculus  ........................................................................................................................

Homo sapiens  ATTGGGAGACCTGAACAAGGTATAGGTCAAAAGGAAAGTTAGCTGATGAAGTAGAAATGAATGACAGTGGTGTGTGCTTCATAAGTGCTGGTTGTGGGAATGAATGAACTTGTAACAGAT
Mus musculus  ........................................................................................................................

Homo sapiens  ACTCCTATGCAAAGCAAGAAACTTCTGAAGAAATGGAACAAGGTGGAGAAGCCTCGGGAATGCCCAACATTCCGAGGCTTCTCAGGATTGCAGAAAGAGAGGGAACAAATCACAAGCAGA
Mus musculus  ........................................................................................................................

Homo sapiens  GGGTTGGCTTCTGGTGAGGCCATTTGGTCCTCAGAGACTTGAGAAAAGAGAGAGTAAGCAAGGTGGGCTACTTGGCCAAAGAGCTGAGAAGGGGGTGTGAATTTTCCTTATTGACATTTG
Mus musculus  ........................................................................................................................

Homo sapiens  TTTAACTTAGAGAAACTGGGAGCTAAATCTCAGCT---AAAGTAGAAATGTCTTAAAGCTCAAGAGGAGAAGTG-AAATTTTGCATTAAGATTAGTGAAGACTGAGCCAGGCTCCACTAA
Mus musculus  ....................AGCTATACCACAACCCCCAAAATAGAAGTATTTTGAAGCTAAAAGGAAGAAGAGTGGCTCTTTTATTTAACCTGGTAAAGACAGAGCTAAGTTCCACTAT

Homo sapiens  AGGAAGAGATATGATGTTCAGTAACTTAGAGCAGAGTTATGCAACTGGAGAATAGGAATCTGCTATTGTGCCTG-TAGGTACATTTAGTATCAGGCAACTAACAG-GTAATTCATAGTGC
Mus musculus  AGGACAGGATATG-------GCCACTCA----------------CAGGAGCATGGGAAGCTGCCATTTTGACTGCTGGGTATATGTC-TGTCAGATGCTTGAAACCATGATTCATAGCAC

Homo sapiens  CTATAA---TAAAGATATT---------CATATAACAAAAAGTGTGAAGGTAGGCTATTTCAGGTTAGGTGCAATAGCTTAACCATGGTATCAAAGGTCTAGCC----------CCCTGC
Mus musculus  CGATTAGATTAAAAATGTTTTATTGTTTCATGTAGCAAACC-TATGA-GGTAG----CTACAGTTTG-------TAGACCTGCTATGATTCCTGAGCTCGAGCCTCTGGCAGACCCATGG

Homo sapiens  CCTCCTTCTAGTCCCCCTCCCTCACCCCCACCCCACTTTCTTAGCTCACTCTTTTTTCCCATTTCTCACTCCCATTTCCTTCCCTCACAATATTGGGCTTAATGCACTGTGGTTCTTAGA
Mus musculus  TCCTCTTCCAGTCCCACATCCTAACCCTCTCTT---TCCCTTGCCTTACTCTTC---CCCATTTACTACTCCCAGAGCCTGATCTCACCATATTGGTCTTAATGCACCACAGGTCTTGGA

Homo sapiens  GTTTAGATGTCAACAGTAGAAAGACTCAGTGTGATCAAGGAATGTCACTGAAGTTATGGATGTCAGGGAACTTTAAGATGGATGTGTTGAATACATGCAGACACTGAAGACACCCTGGAT
Mus musculus  GTTTAGATCTCAGCATTAAAAGGTTTCAATGTCATGATGGAA..............................................................................

Homo sapiens  GATAAACACTTTACATAAAGAAATGTATGTAGTGTAAATTATATTAAAATATACTCTTCATGCTTCTTAAATGTCTTTGAAAACTTGACTAGTTTACCAACAGAACACTAATAAATTAAA
Mus musculus  ........................................................................................................................

Homo sapiens  TGCACATGTCTAAAAATTAAAAACTTTGTGTAAAATAATACTTTTCAGTTGGGTGCAGTGGCTCATGCTTGTAATCCCAGCACTTTGGGAGGCCGAGGTGAGTGGATCACGAGGTCAGGA
Mus musculus  ........................................................................................................................

Homo sapiens  GTTTGAGACCAGCCTGGCCAACACAGTGAAACCCTGTCTCTACTAAAAATACAAAAATTAGTTGGGCATGGTGGCACGTGCCTGTAGTCCCAGCTACTCGGGAGGCTGAGGCAGGAGAAT
Mus musculus  ........................................................................................................................
```

```
Homo sapiens  TGCTTGAACCTGGGAGGCAGAGGTGGCAGTGAGCTGAAATCATGCCACTGCACTCCAGCCTGGGTGACAGAGCTAGACTCTGTCTCAAAAAAAAAAAAAAACAAAACAAAACTTTTCATC
Mus musculus  .......................................................................................................CTTTTCATC

Homo sapiens  TTTGTAACTTTAGCTTACTTCCTTGTAAACCCCATAAAAATCAGTCCAAGTGCAATGAGTT-GAGTTTC--ATTTGTTTTTCCCCAGAGTAGTATGTCCATCAGATGAATGTAGAACTAG
Mus musculus  TT---AATGTTGCCTTGCTTCCTTGTAGATCCCACACAAACTAGTCCAACTGCAATAAATTTGAGTTTCTTCTTTGTTTCTCCCCAAAGAAATTTATCTACCAGATGAATGTAAAGCTAA

Homo sapiens  CAGGGGCTAAGTCCTTCCATGTTCAGAAATTCTGTGAGAGATGTCAGAAGACCTGGTAAATAATAACCTTTTTTTCTTGTTCAGATTCAGAACCAATTGACAGGGATGCAGGAGTAAAAT
Mus musculus  GGGATGCTCAGTCCTTCCTTGCTCAGAAATCCTG--AGAGTTGTGAGAGGACCCTGTAAATGATCAACT............................GACAGGGACACA--AGTGGGAC

Homo sapiens  AGATGGTTCTGGAAACCTCAGTTTCTTACTGACAGAAGTTAGACTGGATAAAAACGGCTTAAAGGCAGAGCTAAATAGGCTTGTACTGGCAGAGGAGGAGTGTAGCAGATGTCCCTCTGC
Mus musculus  AGATAGTTCTGGAAATCTCAAGA-CTGGCTGACAGAAGCTAGCCTTGATAAGGA--GCTTAAACACAGAGCCAACTAGGCT--------CAGAATAAAAAAGTG--------CCTCCCCC

Homo sapiens  ATAGGTCTTCTCAATGCAACTTGGGGCACCAAGGAGCATGGGATGTCTGCATCCTGCTCCTAAAGAGG--------------------ACAGAGGGAGG-AGTGTACCAAGGATACTCA
Mus musculus  AGAGGCCTGCCCAGCAAGACACAGGACAA-AAGCAGCATAGAAGGACAGAG--------CGAGGGAGGGAGGGAGGGAGGGAGGGAGGGAGGGAGGGAGGGAGGGAGGGAGGGAGGGCCA

Homo sapiens  GTTATTTCTCCCAGGC---TCAACAGTTAGTTCCATCCTGAGGAGTGAGTGAGAGGTGGAGAGTGGCTAGAAATGTTTAATCTAGCCAAAGTCCTCCCCATGGAAACTTAAGAGAAATAA
Mus musculus  GCT---TCTCATGGGCATCTCCACCATTAGTTAGGACTTGAAGAGTGAGTGCCAGATGGGGAGAAGTGAGAA-TGCTAAGTGCAGC--AAGCCTTTCCCAGCAAAACCAGAGTGAAA--A

Homo sapiens  GTAAGTATCCCCCCACACCCACCCCAAGCCCCAAAAGATTTAAGCTTCAGCAGATTTATCATGTGTGAGAGAATATGATTGAGGATTTTTATAACATGTTCTTTTTTTCTTTCCCAGAGA
Mus musculus  GTCCATCTCCTTT-GTACTTACCCCGTGT---AGAAGAAAGAGATTTGGGGTGTTTTGTTTTGT-TAGTAGCATATGTTTT----TTTTT--------TTTTTTTTTTTTTCCTCAGAGA

Homo sapiens  GTGTGCACCCCAGATTCCTTGTAGTACTCCTATTGCTACTGAAAGGACAGTTGCACATTTGAACACTCTGAAGGACCGTCACCCAG---GTGATTTGTGGGCCCGCATGCACATCTCATC
Mus musculus  TT-----------------------CTCCTTCCCCCACTGAAAAGGCAGTCTCCACTTTGAACACTGTCGGGAGCTGCCACCCACCCCATGACTCCTGGGCCAAGATGCACATTGCGTC

Homo sapiens  CCTGGAATATGCTGCAGGAGACATTACCCGAAAAGGGAGAAAAAAAGACAAAGCTCGAGTGAGTGAACTGCTCCAAGGCCTCTCATTCTCTGGTGACTCAGATGTGGAAAAAGATAATGA
Mus musculus  CTTGCAGTA------------CATTACTCAGAAAGGGAGGGAAAAAGCCAAATTTAGAGTGATCGAGCTGCTCCAAGGCCATGCGTTTTCTGATGAGTCAGAGACAGAGGAAGACAATGA

Homo sapiens  GCCTGAGATCCAGCCTGCTCAAAAGAAGTTAAAGGTATCATGTTTCCCAGAAAAGAGTTGGACCAAAAGAGACATTAAACCCAATTTTCCAAGCTGGTCAGCACTGGATTCTGGACTTTT
Mus musculus  GCCTGAGGTACAGCCTGAGAGAAAGAAGATAAAGGCATCCAGCATCCCTGAGAAGAGCTGGACCAAACGAGACAGTAAGCCTAACTTTCCAAGCTGGTCAGCCCTGGATTCTGGACTTTT

Homo sapiens  GAATCTCAAGAGCGAAAAGTTGAACCCAGTAGAGCTTTTTGAATTATTTTTTGATGATGAAACATTCAACTTAATTGTCAATGAAACCAATAATTATGCTTCTCAGAAAAATGTCAGCTT
Mus musculus  GAATCTTAAGAGTGAGAAGTTGAACCCAGTAGAGCTCTTTGAATTATTTTTTGATGGTGAAACATTCAACTTGATTGTCAATGAAACCAATAATTATGCTTCTCAGAAAAATGTCAGCCT

Homo sapiens  GGAAGTCACAGTTCAGGAAATGAGGTGTGTGTTTGGTGTCTTACTTTTGAGTGGATTTATGAGGCATCCTAGAAGGGAAATGTATTGGGAAGTCTCTGACACCGATCAGAACCTGGTTAG
Mus musculus  AGAAGTCACAGTGCAGGAAATGAGGTGTGTGTTTGGTGTTCTGCTCTGCAGTGGATTTGTAAGGCATCCCAGAATGGGGATGTATTGGGAAATCTCTGATTCTGATCAGACCCTGGTTAG

Homo sapiens  AGATGCAATCAGAAGGGACAGATTTGAATTGATTTTCTCAAACCTGCACTTTGCAGATAATGGCCACCTAGATCAAAAAGATAAGTTTACAAAGTTGAGACCTCTCATAAAACAAATGAA
Mus musculus  AAATGAAATTAGAAGGGACAGATTTGAATTAATTTTCTCATGCCTGCATTTTGCAGATAATAAGCACCTCGATCAAAAAGATAAGTTTTCAAATTTGAGGCCTCTCATAAAGCAAATGA-

Homo sapiens  TAAAAATTTCCTCTTGTATGCTCCCCTGGAAGAATACTATTGCTTTGATAAGTCAATGTGTGAATGCTTTGATAGTGACCAATTCCTGAATGGAAAGCCTATTAGAATTGGCTATAAAAT
Mus musculus  -AAAATTTTCCTCTTGTATGCCCCCCT-GAGGAATACTATTGTTTTGACAAGTCAATGTGTGAATGCTTTGATTGTGACCAATTTCTGAATGGGAAGCCTCTTCAAATTGGCTATAAAAT

Homo sapiens  TTGGTGTGGTAC----AACCACACAGGGTTATCTGGTTTGGTTTGAACCCTATCAAGAAGAATCAACTATGAAGGTAGATGAGGATCCTGATCTTGGGTTAGGTGGAAATCTAGTGATGA
Mus musculus  TTGGTGTGGTACAACTAACTACGCAAGGCTATCTGGTTTGGTTTGAGCCTTACCAAGAAGACTC------GGGTGTGGATAAGGAGCTGGACCTTGGGCCAGGTGGAAATCTAATAATGA

Homo sapiens  ACTTCGCTGATGTTCTTTTAGAGAGAGGTCAGTATCCCTATCACCTGTGTTTTGATAGCTTCTTTACAAGTGTCAAATTGTTGTCAGCCTTGAAAAAGAAGGGGGTGAGGGCAACAGGAA
Mus musculus  GTTTTGCTGATGTTCTTCTGGAGAAAGGTCACCATCCCTATCACTTGAGTTTTGATAGCTTCTTTACACCTGTTAAACTGATGTCAGCTTTGAAGAAGAAGGGAGTGAAGGCTACAGGAT

Homo sapiens  CAATTCGTGAGAACAGGACCGAAAAATGTCCCCTTATGAATGTAGAACATATGAAAAAAATGAAGAGAGGGTATTTTGATTTCCGAATAGAAGAAAACAATGAGATAATTTTGTGTCGTT
Mus musculus  CGCTTTGTGAAAACAAAAC--AAAAATGTCCACTTATGAATGTGGAACTTATGAAAAAGATGAACCGGGGGTATTTTGATTTCATAGTGGAAGAAAAGGAGGAGATATTTTTATGCCGTT

Homo sapiens  GGTATGGGGATGGCATTATCAGTCTGTGCTCCAATGCTGTGGGCATAGAACCAGTCAATGAGGTAAGCTGTTGTGATGCTGATAACGAAGAAATCCCTCAGATAAGTCAACCATCCATAG
Mus musculus  GGCATGGTGATGGCTTTATTAGTCTGTGTTCAAATGCTGTAGGCATAGAACCAGTCAGCAAAGCAAGC---TGTGTAGCTGACGATAAGGAGTTCCTTCCTTCAGGT--AACATCCATTG

Homo sapiens  TAAAAGTGTATGATGAATGCAAGGAAGGTGTAGCTAAAATGGATCAAATTATTTCGAAATACAGGGTGAGGATAAGAAGCAAGAAATGGTACTCAATTTTGGTGAGCTACATGATTGATG
Mus musculus  TGAAGTTATGTGAGGAATGCAGGAAAGGTGTAGCTAAAATGGATCAAATTATTTCCAAATACAGGGTGGGATTAAGAAGTAAGAAGCGGCTCTGGGTTTTGGTGAGTTACATGGTCGATG

Homo sapiens  TAGCCATGAACAATGCATGGCAACTACACAGAGCCTGTAACCCAGGTGCTTCTCTAGACCCCTTGGATTTTCGGAGATTTGTTGCACATTTCTACTTGGAACACAATGCTCATCTGTCAG
Mus musculus  TAGCCATGAGCAATGCGTGGCAGTTGCACAGAATCTGTAACCCAGGCTCCCCTTTAGATCTCCCAGGTTTCCAGAAATGTGTGGCATGTTTTCGCTTGGGACATGATGCTAATCTGTCTG

Homo sapiens  ATTAGGGTACATAAAATGGACATAGTGCAGACATTAATAAGACATAGAAAAATAATAATTATACATGCTGTTGTACCCTCCCAAAGTAAATCTGATATATGTAATGAAGTTAT-TAAATA
Mus musculus  ATTAGGACGCGTAAA--GAACACAGTAACAACAC-------ACACACACAAATTATAAGTGTA---GTAAATCTACATTACCAAAGCAAATGTAATGGAGAAGGTGTGATTATGTCACTG

Homo sapiens  ATACTTTTAAAAATCAGACATTTATATAGAGTTTCAAAGACTATTGTAACAAGTAATG--TTAAAAATTG---TCTGTGAGAATGTTGAACTGTAGTACCTTTCTCTATGTCAAGTTTTG
Mus musculus  GTATTGTTTACA--------------------TCAAGGATTGTTACAGCGAACAATGCTTTAAAAATAACATTCTGTGAG--TGTTGGATTTGTGTTGCTTT---TATGTCTAGTTTTG

Homo sapiens  TGTCAGACATGGGAAATCATGTATTTGTTCAATTGACTACTTTGTGCACTTATTTATTTATTTTTTGAGACACAGTCTCGCTCTGCGGCCAAGCTGGAGTGCAGTGGCACGATCTTGGCT
Mus musculus  GGTCAGATATATGAAATGACTTACTTGTTAAAT.......................................................................................

Homo sapiens  CACTGCAACCTCTGACACCCTAGTTCAAGCGATTCTCCTGCCTCAGCCTCCCAAGTAGCTGGGATTACAGGCACGTGCTGCCATGCCTAGCTAATTTTTGTATTTTTAGTAGAGACAGGG
Mus musculus  ........................................................................................................................

Homo sapiens  TTTCACCATGTTGGCCAGGATGGTCTCGATCTGACCTCGTGATCTGCCTGCCTTGGCCTCCCAAAGTGCTGGGATTACAGGCGTGAGCCACCATGCCCAGCTATTTTGTGCATTTAAAGA
Mus musculus  .......................................................................................................TTTTATATATTAAAAGA

Homo sapiens  AGGAAATCCTACCTCTTAAAAAAAATTA-------TCTGGAGAATGCCATTTTTAAGATGCAAGCAATGTTACAGAAACCATAGAATGGTGCTGACTCAATAGTTCAAACTAGTGACA--
Mus musculus  GCAAAAACCTACCATTTCCTCACTTTCAGTTTTGTTTTAGAGATTGACATTTTGAAAATGCAGGCAATATCACA--CTCTATAGAATGACACTGAATTCATAGTCCAGAATAGATACAAA

Homo sapiens  ---------CAGCCTATGAAGTAAGAATGATCTAAACAAAACATAGGTGGTAAGAGACTAAAAACCTTAGCATTGGTGTAAAACTGGATCGGATTGTGTGTA----AGTGAGA-AGGGTC
Mus musculus  ACAGATATACAACTCATGCGG-GAGGATATGCTAAC--GGATATAGGTA-TAAGGGAGTAAATATTTCAGC-------------TGGATCACACTATATGTATATAGTTGAGACAGGGCC

Homo sapiens  AGGCATGGATATTGAAGGAGAGTGCTATAAAGGAAAAACCAGAGGTGAAGCAGTGCCTT
Mus musculus  AGGCATGAAAATTGC.......................TGTGTGTGTAGCTGTGCCTT
```

```
Homo sapiens           AGGATAGTTAAGTAAATTATGGAACACTCATACAGTATTACTTAGCTGTTAAAGAATAAGCTGATCTGTGAAGATCTCCAAGTTGTCTTAACCGAAAAGAGCTAAGGTGCAAAATTACAT
Oryctolagus cuniculus  .......................................................................................................TGGGCTGCAAAATTAAAT

Homo sapiens           TATGATACCAT-AAGTGTTTTAAAATGTATA------AATACTTATTCACTAAACAAAATGTTCATTAAGTGCTACTGTGTGGCAGGTAGTGTCCTAGATAAATATGAACAAAATA----
Oryctolagus cuniculus  TGTGATCCCGTTAAGCGTTTTAAAATACATATACACGAGTACTTTTTCACCAAACAAGAGGTTTATTAAATGCTA-----------------------------TACACAAAACATTGA

Homo sapiens           CAATGGTTAACTCTAAAGAG-GGGACACTTTTCGTTTTATGGTCCTTTTGCAACTTGACTTTTTTATTGTACATATATTATGTATACTTAAAGAAAATAATTTGTACAGCTTACTATAAT
Oryctolagus cuniculus  CAGTAGTTAGCTCTGGGGAATGAGGCACTTTTGATTTTACCATCTTTGCACAAATTGATTCTTT------ACATATATAATCTGCAT------AAAATAATCTGTACAGTTTGCTGACCT

Homo sapiens           GGTCTTTTTATTGATCTGTCTCTCCTACCAGCCAGTGATGAC-CCCGAAGACAGACATTTACCACTGTGTTGGTT-----------TTCAATCGAAATGATGTACATAAATTTGGGGCG
Oryctolagus cuniculus  GAACTATTGGTTGATCTTCCATTTCT--------GTGAAGACTCCCGAAGAGTGAGATTCGTCTTTGTGTTTGCTATTCTGTCGCGTTTTAACTGAAAGCCCCTCCGTAAACGTGTGACG

Homo sapiens           TGCTTGCACCGTCCGAATGGGCCTGGGGTCCGGCGGCGGAGAGTGACCTGGGCGGGGCAGGAGGTGGCAGCGGGGGTCCTCCCGGCTCACCAGAGAGACGAGCGGCCGTGCTCCTAGAGA
Oryctolagus cuniculus  CGCTCGGGCCGTGCAGGTGCCCCGGGCATCGCGCGTTGGCCCGAGGCCCGCGCGGG--CGGGCGTGG-GGCGGCGCTCCGCCCGACGCACCACAGAGAGGAGCGGCCGCGCTCCTAGAGA

Homo sapiens           GGCAGGGAACCCGCCAGACTCCGCCACTCCGCTGCGGGCGCGGCGCAGGGAGA------------------AGCTTTTG--------TACCCGCCCAGCTGCTGGA---GGCGCCGGCA
Oryctolagus cuniculus  GGCGCG--AGCCGCCGGAAGCTGCG----CGTTGAGCGC-CGGCACCGGGAGACCGGAGACCGTTGACTCTTAGCTCTGGGTGTGAGACGCGCTCTCGGCCCTTGTCCTCGTCGCAGGGA

Homo sapiens           GCGCCCGCCAGACCCGCCA-GCCCAGCG----------GCCCGGGCTCTGGGGAAACCGGC--GCTCCCGACAGGGGAGCACCGGGCCTCTGAGCTCCCTCGGGA----GCCTTTCACGA
Oryctolagus cuniculus  GCCCCGGGGAGACTCACCCCGCCCAGCGAGACGCCGCGGCCCGGCCTCTGGGGAGACTGGCCGGCACCT-----------CCCAGGCCTCCGCGTTCCGGTGGGAACGAGCCTTTCGCGA

Homo sapiens           GGTCAGCTACGTCTTTGTTGTGCGC-GTTCCTGAACTTT--TGGTCCATTAACCAACAATTAGACACGCCGG--TATTCAGTGCCT-----------GGCGCGGTGCTAGATGC-TGGGT
Oryctolagus cuniculus  GGTCGGCTACATCTTTGTTGTGTACAGTTCCTAAACTTTAATGGTCTTTTAACCAGCAGTGAAACACACACGCTTATTGAGTGTCTACCGTGTGCCAGGCGCTATGCGAGGTGCAGGGGT

Homo sapiens           GTAATCTCAGAAAAAATACATTCAGGGGCG--CGCCTGAGGGTGC--TGGCTGCTGGCATCTCAGGTGC-TTTACGTGCATTCGTGAAGAAGCCCATCAGTATTTCTTGAATACCAGGTAC
Oryctolagus cuniculus  GCAATCTTAGA----TACAGCCCGCCCCCTCCGCCCGAGGGTGCCCTGGCTACTGGCATCTCGGGTACATCTGCGTGCCTTCGTCAAGAAGTCCAGCAGTGTTTGTTGACTACCAGGTAC

Homo sapiens           GTGCCGGGCATGTAGAGTCTATGCAGAGATAAACTCCTGTCCTCAAGAACCCGGGTGGGGATGGGGTTGGTGATCTGTAATACGAGATGGAGATGAGGGT-TGGGTGCAGTTTTCTATGG
Oryctolagus cuniculus  GTGCCGGGCTCTTGGAGT--------ATATAAGCTCCTGTGTTTAGGAG--CATGTCGGGGTGAAGT----------------GAAATGGAGAGAGGGGCGTGGCTTCAGGTTTGTATGG

Homo sapiens           GAATACCCCATCTCAACCTGTGTGGCTTA------------CTTTTCCTGTCTATATGTTAGGGTTAGGGGTTGCCAGGGAAGGTTTTACAATAGAACATTTACGCTGGGCCTTGGGAAT
Oryctolagus cuniculus  T-ACACCCCACCTCCACCTGCGTGGCCTAGAGACAGAATAGTTTTCCCGGTCTGTACTT----------AGTTG---------------------GCATTTGCCCTGGGCGTTGGGAGT

Homo sapiens           TTACCCTTAGAAGGGCGGTCCTGACTGTATAG---AGTGGGTGGAAAAAAATTGAGTACTTAGCATGGCAGTGACATAAGAAATTTGGGAAGACCATGGCTTTCTTGAAAGCAGGAACCA
Oryctolagus cuniculus  TTACCATTGGAAGCACAGTGGTGG-TGTAAAGGACAGAAGATGGAGACTTGTTGAGTAGCCAGCATGGCAGTGA------------------------CTTTCGTGAAGGTAGAGGTGA
```

```
Homo sapiens           AGTCTTTTATTCCAGAGTTAACTTTATGCCTGGAAAATAATGAGATGCTCCACAAATGTTGATTGAGTGACAAACAAATCAGCAA---AACCCAGTAAAGATTAGATCCTGGGGAGTTTG
Oryctolagus cuniculus  A---TTTAAATGAGGACTTAGCTGTATGTCT-GAAAATAATGAGGCACTCGAAAAGTTGAGATAAATTGACAAACCAACCAGTCACTTAAACCAATAGTGATCAGCCCCTGAAGAAGCAG

Homo sapiens           AATTTACTTCAACGAGTTTAATGAAAACTTTGTGTGTGTTTATGTGTGTGTGAGGTATGTTGAATATGGTCACAACTGTGCTTTAGGGAATATTAATCCAGTGACCATAGAAGCGGGGAG
Oryctolagus cuniculus  AATTTAGTCCAAGGAG........................................................................................................

Homo sapiens           TTGGGCAGGAATACCGTAACAGAACATTATTGTAACAGTGAATGAAAATAATAGTAAAATTATAAGAAGGAAAGAATGGCTGCAAGAGGCGTTTTTAAGAATTGACTCACTGATTGTGTA
Oryctolagus cuniculus  ........................................................................................................................

Homo sapiens           TAGAGGCTGTTTAAAAGACTGAATGCGTGGAAGATGATGGTGATGCCAGTAGAAATGGAGAAATACCGAGTGAACGCAGCTTTGAGGGACTCTGATTTCAGTTTGAGATCTGTTGAAATT
Oryctolagus cuniculus  ........................................................................................................................

Homo sapiens           ATAGTGCAGTAGAGGCCCACAGCTGCTGGCATTTCAAGATGTGGGTTTGGAGTGCAAAAAGCAAGCTGGTGCCAAAGATTTGAGTCTTCTTGGAACAGAGATATATTGAAAGTGTGACTA
Oryctolagus cuniculus  ........................................................................................................................

Homo sapiens           AGGAGATCACAAAGGAAAAAATGAAAAGAGGGAATAAAAGAACTTGATGCATGTCTACTGTTTTTCAGTTATGTATTGTTGCATCAAAAATAAAACTCTAAACTTAGTGGCTGAAAACAA
Oryctolagus cuniculus  ........................................................................................................................

Homo sapiens           GCTCAGCTTATTGTTCCTCAGGATTATGCGGGTTGACTAGGCGGTTCTACTGGTCTCACCTGGAGTTACTAATAGAGCTGCATTCAGGTGGTGAATTATTTGAGCACTAGGCCTGGCTGG
Oryctolagus cuniculus  ....................................................................CTCATATAGCTGCATTCAGCTGGTGAATCATCTGGGCACTAGGCCTAGGTGG

Homo sapiens           GCTTTAACTTGGGCATTTTGGTTCTCATCCACATGA--CCTCTGTATAGCTAGCTTGGGCTTCCTTACAGCATGGCAGTCTTATGGCAGTGAGCCAAGAGCTACAGCAGCACT--CTCTC
Oryctolagus cuniculus  GCTTTAGCTTGGAAGCCTTGGTTTTGATCCACATGAACTCTCTATATGGCCAGCTTGGGCTTCCTCACAGCATGGCAGT-----------GTGCCAAGAGCTATAGGGGCATTTTCTCTC

Homo sapiens           TTACAATGTCATTTCTGCTGTATTCCACTGGTCAAAGCAAGTCATAGGCCAGAACAGATTCAAGGGAAGGAAAAAGAGACT-CCACCCCTTGAATGACAACAGTGGCAAAATCTCATCAC
Oryctolagus cuniculus  TTTCAGTGTCATTTCTACCATTCTTCATCGGTTAAAACACATCA-------------------GGGAAGGAGAAATAGACTGCCTCCTACTGGAGGAGTG-AGTGGAAAA--CTGATTGC

Homo sapiens           AAAAGGTATTTGAGAATGGGAGACTTTGGAAACATTCTGTTACACCTACATTTAAAGTGGAATATTCAATGAGAGTTACACAGATATAGAAGACCTTTGAGACTGTATTATTTGTCCATG
Oryctolagus cuniculus  AAAAGGTGTTTGGGGATAGGAGACTTAG-----AGTCTACCACACTAACATTTAAACTAAAATACTCAAT-------ATGCAGAGATAAGTGAACTTCAGGACTGTATTATCCATCTACA

Homo sapiens           AAGGCAGCCATCTG---CCTCCTCAATGGGTAAATTATCCCCTCTCCCCAGCTGAAGTGCAAGCTACTCGAGCTTTCTTATTGCTAACACTTCTTCCCTCATGTCCTACATCCCTTACCT
Oryctolagus cuniculus  AAGACACCTATGTAGGTCTTCCCCA----GTAAAGTGACCCCTTTCCTCTGCTGAAGTGTAAGGGGCTCAAGCATTCTT---GTTAACCCTTCTTCCCTCAT--TCTGTATCCCTTTTCT

Homo sapiens           TTGGACTCAGGCCCTTTAGTACTAGTTCAAAATATACTTCTGATTCATTTCACTGATAATAGAAGCAAGCAGGCTCAGCTTTTGAGCATTACCTACAGTGGGGACAAAAATGTAAGT-AG
Oryctolagus cuniculus  CTGGGTTCAA-CCCCTTAGT----GTTCAAGAAATACTTAGGGTTCGCTTCTCCAATAGTAGAGGCAAGCAGTCTCAGCTTTTGAGTGCGTCCTACAGTGGAGACAAAGATATAAGTTAA

Homo sapiens           GTTACATACAGTTTTGAAGCTCTTATAATTTGCATGTTGCTGATAAATTCTTATGCCTCAGATCTCTATTTCTGAA--------------TATAGACCCCAAGCTAAGTGAAGCTTT---
Oryctolagus cuniculus  GTTGCA--CAGCTTTGAAGCTTTTGTAATGTACATGTTATTGATCAGTTCCTATGCCTTAGATGTGTATTTGGGAAAACACTTCTGGAAGCATAGACCTTA-GATAAGTGAACCTTTTTC

Homo sapiens           -AGCCTCTAAGCTCAACATGTATGAAGCTTTGCCAGGCCCTGCTCCTGAAAATGAAGATGGCCTTGTGAAAGTGAAGGAGGAAGATCCCACCTGGGAGCAGGTGTGCAACTCACAGGAGG
Oryctolagus cuniculus  TAGCCTCTAAACTCAACATGTATGAAGCTTTGCCAGGCTCTGCTCCTGAAAATGAAGACGGCCTTGTGAAAGTGAAGGTGGAAGACCCCACCTGGGAGCAGGGGTACAGCTCCCAAGAGG

Homo sapiens           GCAGCTCCCACACTCAGGAGATTTGCCGCCTGCGCTTTCGGCACTTCTGCTACCAGGAGGCTCACGGACCCCAGGAAGCTCTGGCCCAACTCCGAGAACTTTGTCATCAATGGCTGAGAC
Oryctolagus cuniculus  GCAGCCCCCACACTCAGGAGCTTTGCCGCCTGCGTTTCCGGCAGTTCTGCTACCAGGAGGCACCTGGTCCCCGAGAAGCTATGGCACAGCTCCGAGAACTTTGCCGTCAGTGGTTGAGAC

Homo sapiens           CGGAGATGCACACCAAGGAACAGATAATGGAACTGCTGGTGCTGGAGCAGTTCCTGACCATCCTGCCCAAGGAGCTCCAGCCCTGTGTGAAGACATATCCTCTGGAGAGTGGAGAGGAGG
Oryctolagus cuniculus  CAGAGATGCACACGAAAGAGCAGATCCTGGAGCTGCTGGTGCTGGAGCAGTTCCTGACCATCCTGCCAGAGGAGCTGCGGGCCTGGATGCAGATGTATCCTCTGCAGAGTGGGGAGGAGG

Homo sapiens           CAGTGACAGTGCTGGAGAATCTAGAGACAGGAAGTGGAGACACAGGACAACAGGTGGGAAGAGAATGTGTGTGGTGATGTGGGAGAAGAAAAGGGGGACATGTATCGGTTTATTTTTAAC
Oryctolagus cuniculus  CAGTGACCGTGCTGGAGAGTCTTGCGACAGGACGTGCAGACGCAGGACAGCAGGTGGGAAGGGAGTGTGTGAGATGGTGTGGAAGAAG-AAGTGGAAACGTGTGTCTGTTCATTTTTAAC

Homo sapiens           GTTTTATTTTGAAATAACTCCTAATTTATAGAAGTATTAGAAGAATAG-AGAACTCCCGTAAAACTCTTCATCCAAATTTGCCAGTTGTTAACATTTGTTTGATGA------AGTCCCAT
Oryctolagus cuniculus  TTTTTATTTAGAAATAATTTATAACCTATAGAAGCATTGGGAGAACAATACAAATAACGT-TCACTCCTCACCCAGATTCACCGGTTGTTAACATTTGTTTCATGCTGTGTTTGTTCCCC

Homo sapiens           TGTTGTGTACACACTCGTACAAGGAAGTGCATTCCCCCCCCCCAAGATGCATATGTATATGTATACACACACATACATACACATATGCAGTATTTTTTTTTCCTGAATCTATTTGAGAGT
Oryctolagus cuniculus  TGTCCTGCACATACTC..........................CAATAAACATTTGCACATGTATACACACACATATATATA.......................................

Homo sapiens           CAGTTGCAAACACTATATCCCTTTACCTCACTGTTGACCAGGCTGGAGTGCAGTGGTACGATCATAGCATCATTGCAGCCTTGAACTGCAATGGTGGAAGGCTCAAGCAATCCTCCCACC
Oryctolagus cuniculus  ........................................................................................................................

Homo sapiens           TCAGGCTCCTGAGTAGATGGGACTACAGGTGCATGCCACCATGCCTGGCTAATTTATTTTTAATTTTTTGTAGCGGGGGTGGGGTCTCACTATATTGCCCAGGCTGATCTTGAACTCCTG
Oryctolagus cuniculus  ........................................................................................................................

Homo sapiens           GCCTCAAGTGATCATGCTGCCTTGGCCTCCCAAAGTGTTGGGATTACAGGCATGAGCCACTGCACCTGGCTATTCGGTGTGTATTTCTGAAGAGTGAGGATATTCTCTTGTATCACAAGA
Oryctolagus cuniculus  ........................................................................................................................

Homo sapiens           ATACAAAAATCAAGTTTAGGGAAATTAAACTTACGATACTATCATATAACCTTAGTCCTTATTCATATTTTGCCATGTTATCCTAATAATGTTCTTTATG--ATAATTTTCCTGTGATTC
Oryctolagus cuniculus  ...........................................................................TGTTGTCCCAGTAGTGTTGTTTGTAGAATGAATTATAT-TGATCC

Homo sapiens           AGGATACAAGCCAGCATCACATATTGCACTTAGTTGCCATATCTCTAGTCTGTGAATCTGG--AGTAGCTGCTGTCTTTGTCATGACGTTGTTATTTTTGAAGAGTACA-GCTAGTTTTT
Oryctolagus cuniculus  AGCATATAAGCTAGCATTACATGTTGCATTTAGTTGC--------------ATGAATTTGCTCAGT-----CTTTCTCTGTCATGACATTGATGTTTTTGAAAGATGTACTCCTGTTTTA

Homo sapiens           TTGGAGACTATCCCTCAATTTGAGTTTGTCCTATATTTTCTCATGATT-----CAGGCTGCACATTTTGGCTAGAATACCACAGAGGTAATGTTGTGTTCTCAGTGCGTTACTTCAGGAG
Oryctolagus cuniculus  TTGGAGT-----------TTTTGATTTGTTTTATATTTTCTCATGATTAGTTCTAGCCTGTACATTTTGGCTAGCATGTCATAGAGCTGATGTATTCTCCTTAGTTTAGCACTTCAGGAA

Homo sapiens           GTGCGTAGTGATGATTTGTCCCATTACTGATGAGGTTATGGTGGTACCTGTCAATTTTCTCCACTAGAAAGTTACCATTTTCCCCTTTGTAATTACTAAGTAATCTGTGGGAGACATTTT
Oryctolagus cuniculus  GCAAACA-TAATGGTTTATCCCACTACTGGTGAGTTTACAGTGGAATCTGACAGATTTCCCCATGAGAAAATTACCATCTTCCC------------TAAGTAATTTGTGGGAGACCTTTT

Homo sapiens           GAGGTGGTGTACATATCCTGTTCCTCATTAAATTTTCACCAGCTAGTTTAAACATCCACTTGTTTGTTGTTTTTGTTTTTCTGGGCTGGGATCCTTTGCCTGTCTTTTCTGCCTTGTTTG
Oryctolagus cuniculus  GAGACAGTGTACTTACCCA---------------TTCACACACTGGTTTAT-CATCCGTCTGTTTTTTGTGTCTCTTTTTCTGGGCAAGGATCCTTTGTCTGTCTTACCTGCCATGTTTG

Homo sapiens           CTGCTGTATCCCCAGCATGTAGAACAGTGCCAGGCTCTT----AGCATGCAGTCCATATATGCATGCCCTTTCAAAATAAATCTTTTGTTTCCAGGCCTCTGTCTATATTCAGGGACAGG
Oryctolagus cuniculus  CTGCTGCATCCCCAGCACATAGAACAGGGCCTGGCTCTTTGTGGGTATACAGGCCATATTTGCATGCTTTTTCAAGGTGAATCGTTTGTCTCCAGGCCTCTTTCTACATTCAGGGACAAG

Homo sapiens           ACATGCACCCAATGGTGGCAGAATATCAAGGAGTCTCTTTGGAGTGTCAGAGCCTCCAGCTCCTGCCTGGGATAACCACCCTGAAGTGTGAACCTCCACAGCGTCCTCAAGGGAACCCCC
Oryctolagus cuniculus  ACATGCACCTAGTGGTAACAGAATATCAAGGAACCTCTTTGGAGTGTCAGGGTCTCCAGCTGTTGCCTGAGGTAACCACCCTGAAGTGTGAACCTCCACAGCTT------------CCCC

Homo sapiens           AAGAAGTGAGTGGTGAGTGCTCGA--GTGAGTTTAGTGAGGACGCTGGGAGCTGGCCACTGACACTTGCACAGCCTTCTTGA-----------------CCTCAAG--------------
Oryctolagus cuniculus  AAGAAGTGAGTGGTGAGTGCCAGAGGGTGAGTTTAGTGAGGACAGAGGGACCAAGCCACCAATAGCTGCAT-GTCTTCTTAGTAGAAACTTGTGTGTATCTTCAAGTTTTTGTTTCTGTT

Homo sapiens           TTTGTATACATTGTGAAATGATTACCACAAGCTAAC----TTACATATCTCACAGTT-ACCTTTTATGTGTCTGTGGTAAGAATTCCTAAGATCTACTCTCATGAAATTTCAAGTATACA
Oryctolagus cuniculus  TTTATATGCATCGTGAAATGATTACCAGAAGTTGTCATTGTCTGTTACCTCACAGTTAACCTCATGAGTGTGTGTAAGAATATTACATAAGAC---CTCCCAGCAAATTTCAAATATGCA

Homo sapiens           ATATATTATTATTACTAACTATAGTCAGCATTCTGTACGTAGGTCTTCAGAGCTTATTCATCTTATAACTGCAAGTTTATACCCTGTGACCAACATCAAGTTTTATTTTTTTGTCTAACC
Oryctolagus cuniculus  ATATGTTGTTAT---TTGCTATAACCA-TATTATGTGT-TACATCTGCAGAACTT------------ACTACAATTTTGTACCCTTTGACCAACATCA---TTCTGTTTTCTGTCTCATC

Homo sapiens           CTGTATCTGAACAAACTTGTAAAGACCTGCTTTATTTTACACTGTGCATTTTAAGCTGGTTTATTTAGTATGTATGTGTTGTGGACAAGTACATATAATAGTAAAAGTGAATCGGGGCAA
Oryctolagus cuniculus  CTATATCTAAGCAGGCTTTTAAA.................................................................................................

Homo sapiens           GGCTGTTTTTTTCCAGCAAACTTTATTCCTTCTAGAAATGCAACAACAGCAATAAAAACATTCCATAGTGATCCATTATGGAAGCAAAGAAAAGCAGAGCAAAGAAAAGTGATGTTCTGT
Oryctolagus cuniculus  ........................................................................................................................

Homo sapiens           TTTATATTTTTGGGTTAGAATTACCACATGTGCTCTTCATTTGATAATTTTGTACTCTGCCATCTGCCAACTGCCAACTTTGTGACCTAGGCAAGTCATTTAATAGGTTATTCAGTTAAT
Oryctolagus cuniculus  ........................................................................................................................

Homo sapiens           CAACAATTATCTGTTTGATCTCTGTGTGTACTCTGCACTCAGCTGTGATTCTTCTGAATGTGCCTAGGATTTTGGAGGCAATAATTTGGACCTTCTTGATTATTTTGAGGATTGGAAATA
Oryctolagus cuniculus  ........................................................................................................................

Homo sapiens           ATCTTTTAAAGTAACAGCACAGTGGCTGGCCTTGTGCAGTCATCATCACCATTTTGATTATTGTTGGTTCTTTGGGATCAGTACCTCCTAACAGTTTTTCTAGTTCATGGCTAACATCTC
Oryctolagus cuniculus  ........................................................................................................................

Homo sapiens           TTCTCTATTTTTTTAAATATCTTTATTTTCCTGTCTCTCTCTCCATCTTCCTTTCTTCTGCTTTCCTGTTTCCCCTCAGTCTCATCTCTCTACTCTCTACATCCTTTCTTTTGCCTCTGT
Oryctolagus cuniculus  .........................................................................CCTACATCTGCTTTGCCTTGTCTCTGCTCCCTGTCTTTCCATTCTGT

Homo sapiens           GTTCTCTCCTTGATCCTTCCTTTATCACCAGCTTCTTCCTCCCCTTTATTTTTGTCTATGATT-TTTATTTTTTCTTTATGT----GAAAGTGCATGACTGTAAAGCACCT--TATGAGT
Oryctolagus cuniculus  GTTATCTGCTTGCTCTTAGCTTTTTTACC-----TTTCTTCCCCTCTTTATTTAATCTTGATTGTTTAGTTTCTCTTTATGTATGTGAAAATGCATC---------CACTTACTACGAGT

Homo sapiens           TCATAAATCCCACTCCATTTTTTGGGTGAGAGACCCTAAAGTTGTAGAATTTTAGAATTTCTGACAGAATGAAAGCAGTTGTGATGCTCTTTCTCACACTGTAAAATCTACGTCTTAGTT
Oryctolagus cuniculus  TTATAAACTCCACTCCGTTTTGTGGGTGGGACTTTATAAAGTTGTAGAATTTCAGG-CCCCTGACACAGTGAAAATAGCTGCGGTGCTCTATTTGAGACAGTAAGGTCCACATCTCAGTT

Homo sapiens           CTTGAATGTTTAACAAACATAACATTTGGGGGAAAAGGCTGGGTCTCCAAAAAGGGTAC-CCTAAAGGCCATCATGTCTCATTTAGGACTCTTGCCCTCTCTCTCTGCAGGGCCTGTTCC
Oryctolagus cuniculus  --TGGATATTTAGCAAATGTGAGACTCGGGG--------TGGG---CAACAAAGGGTACTCCTAAAGGTCATCTTGTCTCACTTAGGACTCTTGTCTCCTTGCTCTCCAGGGTTTGCTCC
```

```
Homo sapiens            CCACGGATCAGCTCATCTCCAGGAAAAAAACCCCAGAGACAAGGCTGTAGTGCCTGTGTTTAACCCAGTCAGGTCCCAGGTAAGTAGGATGCCCAAGCTTGTAGGAATATCAGTAGTGGT
Oryctolagus cuniculus   TCAGGGACCAGCCATTCTCCAGGAAGAAAGCTCCAGAGACAAGACAGCAGTGCCTGAGCTAAACCCATCCAGGTTCCAGGTGAGCAGAGTGCCCAGGCTTGTGGAAACCTCAGTAGCAGC

Homo sapiens            CTTTCTCCCTCATTCCATGTTCCTTGCCCTTCTCTTGGCTCACACTCATCATCGTGAGAGCCATTCAGGTGGCATAGTCCCTCTGCCTTCACCTTGTCACAGAATATCACAAAGTGTGT-
Oryctolagus cuniculus   CCTCCTCC----TAACTGGTTCTTTGCTCTTCTGTCGGCTCACGCTCCTTACCCCGAAAACATTTCAGGTGGCACAGTGCATCTGCTTTCACTGTGTCACGGAGTGTCACAGATCGTGTG

Homo sapiens            TGCATCATTCGAGGAGCTTGTATTGATTATATGCCATTGCCTCATTTCAT----TGTTCAGGAGGCACGTCCAAATCTCAAGGCATGGGAGTGAGGGGTGGAGTGAGGCTCCTGGTTTCA
Oryctolagus cuniculus   TATGTCATCCCAGG-GCTTTTGTTGATTACATGCTTTTACCTCGCTTCATACCATGCTTAGAGGGTGCATCCAGACCTCAAGGTATGGCAGGCAAGGGTGGAGTGAGGCCCCTGGTTTCC

Homo sapiens            GAAAGTG-CTCCACTCATTTGATGTTGAGTAACTGATATTTTCTCCTTTGAAGTCTCTGGTTTATCTCCCCAGCTTTAGAATAAGGGCCCTTTCTCACACTACTTTATAT---------T
Oryctolagus cuniculus   AAAATTACCTCCTCTGATTTGATGACAAG----TGATATTTTATCTTTAGGGGTCTCTGGTTTCTCTCTCTAGCTTAAGAAGCAGGACATTTTCTCACATTGCTTTATACTGTCAGTGGT

Homo sapiens            CAGCATGTATGCAATTAATGCTTGGTGATAATAATGGAAATTCTATATATCTAAGGTAATACTTAACATCTTCTTCACAAAATGGACTCTGGTTTTTTTTCTTATTAGTGGTTCATTCAT
Oryctolagus cuniculus   CAGCACATATCTAACTA.......................................................................................................

Homo sapiens            TTTCCTAGTCATTTATTTACAAAAAAAAAATAACTCTCTCATTTAAGTCCTCTTGCATACTGCTGCCACATCAGTTTCCTCAAGACACTTGCTCAGTCCTCTTCTATGGCTTCTAATTAA
Oryctolagus cuniculus   ........................................................................................................................

Homo sapiens            TTTTTAATTCTATCAGACCCAGTGCCTCCTGTTATTAACAAATACTTCCTGATAAGAAGTATCTAGAAAATAAAACCTACTCATGCACATTCTGAAAATCAACATAATTCCCTTAAATGT
Oryctolagus cuniculus   ........................................................................................................................

Homo sapiens            AATTTAATGGTATAATGTAGAAACAAAAAAGTTATTTATTTTAAAAGTA-GTGTTTCAAAATGCAAGTGCTTG-ATGTGATTATAACTAGGGAAATAACAACGTAGTCAGGTGTTTGCAC
Oryctolagus cuniculus   .................................ATTCACCCCAGAATTATGTATTTCAAAATGAAAATTCTTGGACATGATAATACCTGAGCAAATAACAAAGTAATTAGATGTTTACCC

Homo sapiens            TGAAATATAATTAGTAAGTTCAAATTAAGGAGGAATAAGAAGACCAGAAGCCCAAGAAAATCAAAGAGCAATGGTATAGGTGGACAGTGAAATGAAACTAGTATTGAGTCAGATAATACA
Oryctolagus cuniculus   TAATATATAAACAGTAAATCTAAATTAAGGAGGAATAAAAGAATCAGAAGTCTCCAAAAATTGAAGGGCCCTGGTATAGGTGAACAGTAGAATGAAATTAGTATTGAG............

Homo sapiens            AGACTAGACAGAGAAAGTGGGGCCCTTAATTGAAGTAAGGCTTAGATGTTGTAAATTGAATTATGTCCCTCCAAAAGATATATGAAAATCCTAACCCCACGTACCTGTGAATGTGACCTG
Oryctolagus cuniculus   ........................................................................................................................

Homo sapiens            ATTTGGAAACAGGCTCTGTCTTAGTCCATTTTGTGTTGCTAATAACAGAATTCCTGAGACTGGGTAATTTATTTTAAAAAGAGGTTTATTTAGCTCAAAGTTCTACAACCTGGGAAGTCC
Oryctolagus cuniculus   ........................................................................................................................

Homo sapiens            AAGATCATCAGCCTATCTGGTTAGGATCTCATGCTGCTTTAGCTCATGGCAGAAAGTGGAATGATGGATAATCAAGTGTGTGCAAGGAGACCACACATTAGAGGCAGCCATCCTTTAAAC
Oryctolagus cuniculus   ........................................................................................................................

Homo sapiens            AGTCTGCTCTTGGAGCTGAGCAAGAGCAAGAATAGAAAGCTTATTCAAAGAAATAACAATAGGCCGGGTGCAGTGGCTCTCAACTGTAATCCCAGAACTTTGGGAGGCCATGGCAGGAGG
Oryctolagus cuniculus   ........................................................................................................................

Homo sapiens            ATTGCTTGAGGCCAGGAGTTTGAGACCAGCCTGAGCAACACAGCAAGACCCTGTATCTATTTAAAAAAAAAGAATAGGCCAGGCGTGGTGGCTCATGCCTGTAATCCCACACTTTGGGAG
Oryctolagus cuniculus   ........................................................................................................................

Homo sapiens            GCTGAGGTGGGTGGATCACGAGGTCAGGAGTTCGAGACCAGCCTGGCCTACCTGGCAAAACCCCGTCTCTACTAAAAATACAAAAAAAAGGAGCCAGGTGTGGCAGGCACCTGCAATGCC
Oryctolagus cuniculus   ........................................................................................................................

Homo sapiens            AGCTACTCGGGAGGCTGAGGCAGGAGAATCGCTTGAACCTGGGAGATGGAGGTTGCAGTGAGCCGAGATCATGCCATTACACTCCAGCCTGGGCAACAAGAGCAAGACTCCATCTCAAAA
Oryctolagus cuniculus   ........................................................................................................................

Homo sapiens            AAATTAAAATAAAATAAATTAAAAATAAATTTTAAAAATAGAGAAATAATAACAAAAATCTTCACAAAACTTGTGAAAGATATAAATGTCCAGGTACAGGAAGATCTGAGAACACTGAAC
Oryctolagus cuniculus   ........................................................................................................................

Homo sapiens            AGAATCATCCCAAATAAGACTACTCCAAGGCATATAATAATCAAACTCTCAACTGTCCAGGAAAAAGAGAGGATCTGAAAAGCAGCAAGAGACAAGAAACAAATAAGGAGCTCCAACTTG
Oryctolagus cuniculus   ........................................................................................................................

Homo sapiens            TCTGGCAATAGACTTCTCAATGGCAATCATACAGGCCAGGAGAAAGTGGAATGACATTTTCAAAGTACTCCAAGAAAAAAACTGACATTCAAGAATATTGTATCCAGCAGAATTATCTTT
Oryctolagus cuniculus   ........................................................................................................................

Homo sapiens            CATATATGAAGGAGAAAGTCTTTCCCAAACAAACAAGCTGAGAGAGTACACCACCACCAGACACATCTTACAAGAAATGCTAAAGAATTCTTCAGTCTAAAAGGAAAGAAGACTAATGTG
Oryctolagus cuniculus   ........................................................................................................................

Homo sapiens            CATAAAGAAAACTTTTGAAGGCATAAAAGTCACTGATAAAATTAAGTACATGGACAAACCCAGAATACTTTATTACTGTAATAGTGACATACAGTATACTCACAACTGTGAAGCCCCAAG
Oryctolagus cuniculus   ........................................................................................................................

Homo sapiens            ACATACCTATCAAAAACAATAATAGGACTGGACACAGTGGCTCATGCCTGTAAACCCAGTTCTTTGGGAGGCTGAAGTGGGAGGATCACTTGAGACAAGGAGTTTGAGACCAGCCCAGGC
Oryctolagus cuniculus   ........................................................................................................................

Homo sapiens            AACATGGTAAGATCCTGATGCTATACCTGGAGACATGAGACAGAGTGAGACCATATCTTTAAAACAAAACAAGACAAACAATAATGGCTAGCAACCTGTTAAGAGATAGGTAATATAAAG
Oryctolagus cuniculus   ........................................................................................................................

Homo sapiens            ATATAAATTGAGACAACGAAAAGTCAAAATCTAGAGGAATGGAATTAAAGCATATAATTTTTTTCTGTGTGTTTCTGCCTTTGTTTCTACTCTTGTATTTGGGATCTAAGATAAGTTGTT
Oryctolagus cuniculus   ........................................................................................................................

Homo sapiens            ATCTCTTTAAAATAACTTATTATATCTGAAGGTCTTCTTTGTAAGCCTCATGATAACTACAATGCAAAAACCTATAATAAATTCACTAAAAATAGCAACAAAGGAAGGCATACTATAAGA
Oryctolagus cuniculus   ........................................................................................................................

Homo sapiens            GAAAATCACTTAGCCACAGAGGTTTTTTGTTGTTGTTGTTTGAGATAGGGTCTCACTCTTTCACTCTGGCTGGAGTGCAGTGGTATCATCTCAGCTCACTGCAACCTCTGCCTCCCGAGT
Oryctolagus cuniculus   ........................................................................................................................

Homo sapiens            TCAAGTGATCCTTCCACCTCAGCCTCCCAAGTAGCTGGGACCACAGGTGTGTGCCACCATGCCTGGGTAATTTTTTGTATTTTTTGTAGACCTGGGTTTTTGCCATGTTGCCCAGGCTGG
Oryctolagus cuniculus   ........................................................................................................................

Homo sapiens            CTTGAACTTCTGGCCTCAAGCAGTCTGCCCACCTTGACCTCCCAAAGTTCTGGGATTACAGGCGTGAGACACCATGCCCGGCTGTATATAACAATTATAAATACCTATGCATGCAACACC
Oryctolagus cuniculus   ........................................................................................................................

Homo sapiens            AGAGCTCCCAGGTATATAAGGAAAACATTAATAGATCTAAAAGGAGAGTTAGACTGCAATACAATAATAGTAGGAGATTTTAACACCCCACTCTCAGTAATTGACACATCATCCAGACAG
Oryctolagus cuniculus   ........................................................................................................................

Homo sapiens            AAAGTCAACAGAGAAACGATGGAATTAAACTACATGCTAGATCTAATAAGCCTAACTGATATTTACTGAACATGTAACCCAACTGCTACAGAATGCACATTCTTTTCATCAGCACGTGGG
Oryctolagus cuniculus   ........................................................................................................................

Homo sapiens            ACATAAGAATAGACCATATCTTAGGCCACAAACAAATCTGAACAAATTTTTAAAAAATATAAATCATATCAAGTATCTTTTCTAATCACAGTGGAATAAAACTAGAAAGCAATAACAAGA
Oryctolagus cuniculus   ........................................................................................................................

Homo sapiens            AAAACCTTGGAAACTACACAAACGCGTGGAAATTAAATAGCATGCTTCTGAACAACTAATTTGTCAGGGAAGAAATTAAGAAGGAAATGTAAAAATGTCTTGAAACTAATGAAAATAGAA
Oryctolagus cuniculus   ........................................................................................................................

Homo sapiens            AATGTGACATATCAAAATTTGTGGGATACTGCAAAAGCAGTACTAAGAAGGAAATTTATAGCAATAAACACCTATATCAGAAAAGTAAAAAGACATCAAATGCACAACCTAATGATACAC
Oryctolagus cuniculus   ........................................................................................................................

Homo sapiens            CTCAAGGAACTAGAAAAGCAAGAACAAACCAAACTGAAATTAGTAGAAGGAAACAAATAAAGATTAGAACAGAAATAACTGAAATTGAGACCAAAAAAAAAAAAAAAAAAACCCAGAAGAA
Oryctolagus cuniculus   ........................................................................................................................

Homo sapiens            CAATGAAACAAGAAGTTGGATTTTTTTTAAAAGATAAACAAAATCAAGTCTTTAGCTGGACAAACAAAAAAGGAGAAGACCCAAATGAATAAAATCAGAAATGAAAAAGACATAACAGCT
Oryctolagus cuniculus   ........................................................................................................................

Homo sapiens            GAGACCTCAGAAATACAAAGAAACCATTAGAGACTTATGAACAACTATGACAATAAATTTGAAAACCTGGAAGAAATGGATAAATTCCTAGATGCATGCCAACTACCAAGATTGGACCAT
Oryctolagus cuniculus   ........................................................................................................................

Homo sapiens            GAAAAAATAGAAAACCTCAGCAAACCAATAACAAGTAACGAGATAGAATCTGTAATAAAAAGTCTTCCATCAAAGAAAAGCCCAGGACCTGTTGGCTTCCCTGCTGAATTCTACCAAACA
Oryctolagus cuniculus   ........................................................................................................................

Homo sapiens            TTTAAAAAGAACTAATACCAATCCTATTCAAATTCTTCAAAAAAAAAAAAAAAAAATTGAAGAGGAGGGACTACTTCCAAATTCATTCTAGGAGGCCAGCATTGCCCTGATACCAAAACTA
Oryctolagus cuniculus   ........................................................................................................................

Homo sapiens            GACTAGGATACAAAATAAAAAGAAAACTACAGACCAACATCACTGATGAGCATAGGTGCAAAAATTCTCAATGAAACACTAGCAAACTGAATTCAACAACACATTAAAAAGATCATTCAC
Oryctolagus cuniculus   ........................................................................................................................

Homo sapiens            CATGATCAAGTGGGATTCACTCCAGGGATGCAAGGATGGTTCAACATATGCAAATCAATAAATGTGATATATCACATTAATATAATCAAGAACAAACACCATATGATTATTTCAATAGAT
Oryctolagus cuniculus   ........................................................................................................................

Homo sapiens            GCTGAAAAGCATTTGATAAAATTCATTGCTTTTTGATAAAAACCCTTGTCAAAATTGGTATAAAAGGAACATACCTCAAAATAAGGGCCATATATGACAAACCTACAGCTAACATCAAAC
Oryctolagus cuniculus   ........................................................................................................................
```

```
Homo sapiens           TGAAGGCCTTTTCTCTAAGGACTGGAACAAGATAAGAATGCCCATCTCACCACTGTTGTTCAACATAATACCCGAAGTCCTGGCCAGAGCAATTATGCAAGAGAAGGAAATAAATGGCAT
Oryctolagus cuniculus  ........................................................................................................................

Homo sapiens           CCAAACCGGATAGCAAAAAGTCAGGTTAGTCTGTTCGCAGATGATATAATCTTATACCTCGAAAAACCTGAAGACTACACCAAAAAAACTGTTAGTGCTGATAAACAAACTAAGTAAAGT
Oryctolagus cuniculus  ........................................................................................................................

Homo sapiens           TGCAGGATAAAAAATCAACACTCAAAAATTAGTAGCATTTATATACTTCAATAGTGAACAATCTGAGATAGAAATCAAGAAAATGATCGCATTTACAAAAGCTACAAAAAATATAAAATA
Oryctolagus cuniculus  ........................................................................................................................

Homo sapiens           CTTAGGGAATTTAAATACTAAAAATTTAAACACCAAATTAAGTTAAATTTAGTGTTTAAATTCCCTAGGCATTTTATATTTTTTAGTATTTTATTTATAATTTATAATTTAGCATTTAAT
Oryctolagus cuniculus  ........................................................................................................................

Homo sapiens           TACTAAAGAAGTGAAAGACCTGTATAAGGAAAACTAAAACTCTCATGGAACAAATTGAAGAGGACACAAAAACACTGGAAAGATATTCCATTCTAATGGATTGGAAGAATTTATATTATT
Oryctolagus cuniculus  ........................................................................................................................

Homo sapiens           AAAATGACAATACTTCCCAAAACAATTTACAGATTCAGTGTAATCACTATCAAAATACCAATGACATTCTTCACAAAAATCAGAAAAAAAAATTCATGTGGTGAGCCCTTAATCTAATGA
Oryctolagus cuniculus  ........................................................................................................................

Homo sapiens           CTCATATTCTTATAAGAAGTCCATATTTGCAAATTGACCAAGGGGTTGAAAAAAAAGAAAGAAAAATGCCCAAGTGAAAACACACACAGGGAGAATGCCATGTGATCACTGAGGCAAAGA
Oryctolagus cuniculus  ........................................................................................................................

Homo sapiens           TTTGTATAACACATCTGCAAGCCGAGGAACACCAAGAATTGCTGACAAACACTAAAAACTTGGAGAGAGGCAAGGAAAATTATCCCATGGAGCTTTTTTTCTTTTCTTTTCTTTTCTTTT
Oryctolagus cuniculus  ........................................................................................................................

Homo sapiens           TCTTTCTTTTTGAGACAGTCTTGCTCTGTTGCCAGGCTGGAGTGCAGTGGCGCGATCTCAGCTCACTGCAACCTCTGGCTCCCTGGTTCAAGTGATTCTCCTGCCTCAGCCTCCCGAGTA
Oryctolagus cuniculus  ........................................................................................................................

Homo sapiens           GCTGAGATTACAGGCAAGCGCCATCACGCCCAGCTAATTTTTGTATTTTTAGTAGAGATGGGGTTTCACCATGTTGGCCAGGATGGTCTCGATCTCCTGACCTAGTGATGTGCCCACCTT
Oryctolagus cuniculus  ........................................................................................................................

Homo sapiens           GGCCTCCCAAAGTGCTGGGATTACAGGCATGAGCCACCACGCCAGGCCTCCCATAGAGCTTTCAAAGATAACATGGCCCTACTGATATCTTGATTTTGGATTTCTAGCCTCCAGAGCCAT
Oryctolagus cuniculus  ........................................................................................................................

Homo sapiens           GAGATAATACAATTCTGTTGTTTTCCACTATTGAGGTTGTGGTACTTTGTTACAAGAGCCATAGGAAAGCAATATAAGTTACAAAACAAATTATGGACTGATAAAGGGAGAAAATAAGGA
Oryctolagus cuniculus  ........................................................................................................................

Homo sapiens           GGTATGATATTACAGATACACTTCCTTCATTGTGTTTCACTTTATTGTGCTTTGCAGTATTGAAAGTTTGTGATGACCCTGTGTGGAACAAGTCTGTCAGCACCATTTTTCTACAGCATG
Oryctolagus cuniculus  ........................................................................................................................

Homo sapiens           TGCTCACTTTGTGTCACATTTTGGTAATTCTTAAATTCTTAAACGTGACACTCTGTGTCACATTTTGGTAATTCTTAAAATGTTTCAAACTTTATTGTTATATCTGTCATGGTGATCTGT
Oryctolagus cuniculus  ........................................................................................................................

Homo sapiens           GATCAGTTATTTTTGATGTTACTATTGAATTTGTTTTGGTGTGCCACAAACCATGCCCGTTTAAAATGTCAGACCTAATCAATAAACATTGTATGTGTCCTGACTCCTCCATTGACCAGC
Oryctolagus cuniculus  ........................................................................................................................

Homo sapiens           TGTTCCCTCATCTCTTTTCCTTTCCTTGGTCCTTGCTATGTCCTGAGACACAATAATATTGAAATGAAGCCATTTATAATACTACTACTACAGTGACCTCTATGTGTTCAAGTGAAAGGA
Oryctolagus cuniculus  ........................................................................................................................

Homo sapiens           AGAGTCACATGCCTCTCAGGTTCAATAAAAAGCTAGAAGTGATTAAGTATAGTAAGGAAGGCATGTTGAAAGCCGAGATAGGCCAAAAGCTAGGAGGCCTCTTGTGCCAAACAGGCAGGT
Oryctolagus cuniculus  ........................................................................................................................

Homo sapiens           GGTGAATGCAAAGGAAATGTTCTTGAAGGAAATGAAAAGTGCTACTCTGGTGAACATATGAATGATAAGAAAGCAAAAAGCTTTTTGCTGATATGGAGAAAGTTTGAGTGGTTTGGATAG
Oryctolagus cuniculus  ........................................................................................................................

Homo sapiens           AAGATCACACCAGCCACAGCATTGCCTTGAGCCAAAGGCTAATCCAGAGCACATCTCTTACTCTCTTCAATTCTGTGAAGGCTGAGAGAGGTGAGGAAGCTACAGAAGAATAATCGGAAG
Oryctolagus cuniculus  ........................................................................................................................

Homo sapiens           CTAGCAGAGGTTGGTTCGTGAGGTGTAAGGAAGAAGCCATCTCTATAAAATGCATCAGCAACAAGTGCTGATGCAGATGCTGCAGCAGGTTATCCAGATCTAGCTAAAATCACTAATGAA
Oryctolagus cuniculus  ........................................................................................................................

Homo sapiens           GGTGGCTACACTAAACAACAGATTTTCAATGAAGATGAAACCACCTTTTATTGAAATAAGATGCCATCTAGGACTTTGATTGCTAGAGAGGAGAAGTCAGTGCCTGGCTTCAAAGCTTCA
Oryctolagus cuniculus  ........................................................................................................................

Homo sapiens           AAGGACAGCCTGACTTGTTAGGGGCTTACGCAGTTGGTGATTTTAAAGTTGAAGCCAGTGCTCATTTATCATTCTGAAAATCCTAGGGTCCATAAGAATTATGCTAAACCTACTCTGCCT
Oryctolagus cuniculus  ........................................................................................................................

Homo sapiens           GTACTCTAGAAATGGAACAACAAAGCCTGGATGATAGCACATCTGTTTACAGCATGGTTTACTGAATATTTTAAGCCTACTGTTGAGACATACTACTCAAAAAAGAAAAAAAGATTTCTT
Oryctolagus cuniculus  ........................................................................................................................

Homo sapiens           TGAAAATATTACTGCTTATTCACAGAGCACCTAGTCACACAAGAGCTCTGAAGGAGAGGTACAAGGAAATTAATGTTTTCAAGCCTGCCAATACAATACACATTCTGTAGCCCATGGATC
Oryctolagus cuniculus  ........................................................................................................................

Homo sapiens           AAGGAGTAATTTTGACTTTCAAGTCTTATTATTTGAGAAATACATTTTTTAAGGCTATAGCTGCCATAGATAGTGATTCCATAGATAGTGATGGATCTGGGCAAAGTAAATTAAAAACCT
Oryctolagus cuniculus  ........................................................................................................................

Homo sapiens           TCTGGAAAGGATTCATCATTCTAGATGCCATTAACATTTGTGATTCAAGGGAGGAGGTGAAAATACCAACATTAACAGGAGTTTGGAAGAAATTGGTTCTAACACTCATCATTGTCTTTG
Oryctolagus cuniculus  ........................................................................................................................

Homo sapiens           AGAGGTTTAGGACTTCAGTGGATAGTGTAGCTGCAGATGTGGTGGAAAGGACTAGTATTAGAAGTGGAACCTGAAGATGTGACTGAATTGCTACAATCTCATGACAAAACTTGAACAGAT
Oryctolagus cuniculus  ........................................................................................................................

Homo sapiens           AAGTTACTTCTTATGGGTGAGAAAAGAAAGTGGTTTGAGATTGAATCTACTCCTGGTGAAGCATTGTTGAAATGACAACAAAGGATTTAGAATATTAAATAAACGTAGTTGATAAAGCAG
Oryctolagus cuniculus  ........................................................................................................................

Homo sapiens           CAGCAGGTTTGAGATGATTGACTACAATTTTGAAAGAAGTTCTACTCTGGGTAAAATGCTATCAAACAACATTGCATGCTACACAGAAATCTTTTGTGAAAGGAAGAGTCAATCAAGGTG
Oryctolagus cuniculus  ........................................................................................................................

Homo sapiens           GCCAACATTACTGTTGTCTTATTTTAAGCAACTGCCACAGCCACCGAAACCTTCAGCAACCACCACATTGATTAGTCAGCATCGTTCAACATCGCAGGAAGCCCCTCTACCAGCAAAAAG
Oryctolagus cuniculus  ........................................................................................................................

Homo sapiens           ATCACAACTCTCTGAAGGCTCAGATGATTATTAGCATGTATTAGCAATAAAGTATTTTTAAATTAAGATATATACTTTTTTAGACACAATGCTTATGTACGATTAATAGACTACATAGTA
Oryctolagus cuniculus  ........................................................................................................................

Homo sapiens           TAGTGTAAACATGACTTTCACATGCACTCGGAAGCCAAGAAATTTCTGTGACTTGCTTTATTGTGATATTTGCCTTATTGTGGTGGTCTGGAACTGAACCTGCAATATCTCTGAGGTAGG
Oryctolagus cuniculus  ........................................................................................................................

Homo sapiens           CCTATGTTTAAATGAGGTGGGCTTTGCCTTTTATTATCAAAATAATTATCTGTAGTTTG------GAATAGGGTGATGATGAGTTAAAGAGAAAACATCTTCTGTCTTGAAACCCCCTCC
Oryctolagus cuniculus  ......TTTAAAGGAGGTGGGCTTT-----TTAGTATCAGAATAATT--CTGTACTTTGACTTAGGACTGGTGGAGTGATAAGGAAAAGAAAACACATCTTCTGTCTTAAGCCCTCCCCC

Homo sapiens           -------TTGTCAAAACAGATATTCATTCTCTAAAAGACT-GGCACTGTGTCCTTTTTCAGGCAACAAGAAAGAGAAAGTAGATTGGAAAATGAGAGAAAATCAAGAGGCTAATGAAACC
Oryctolagus cuniculus  ACTCCCGGTAGCAAAACAGACATTCAATCTGTAAAATTCTGGGCACTGTGGCC-CTGGCAGACAACAGGGGAGAGGATAAATGCTGGAAAAT...AAAAAATTAAAAAATAAA--AAGCC

Homo sapiens           ATGTGGTAGCCTTTTGGGACAGTGTCAGA--GAGATAGAAAATCTCAGACAGTTCCCCAAATAAATAAATTCAGTGTATGATTTTTCCAGCCTACATATC--ATTACTAGCTTTGAAATG
Oryctolagus cuniculus  AAGAGATAGGTCTTGGGAACAGAGTCAGATAAAGGTAGAAAACCATAGAT-GCCGCCCAAATAAATA----------------TTTCTAACCTAGATATCTTATGACTGCTTTTGAAATA

Homo sapiens           TTTTTAAACAATCTTTGTTGATGAAATTCAGTGAGATTTCTGCATTTACATTAGATTCCAAATCTGTTAAAAGGAGTCTCAAAA---CTCTGTTAGGTATTTGTAGTCAGTTTAATTGCA
Oryctolagus cuniculus  TTTTAAAATGATGTTTATTAATGAGACTCAGTGAGAGCTCTGCATTTATATCAAGTTCCA--------------AGTCTCAGAACTTCTCAATTATATATTTGTAATCAGTTCAGGTACA

Homo sapiens           TTTGTTATAACTGCAATATTATATTGAAGTGCTGTTCAACCAAATACATAGAAAATGCTGTAAAATAGTGTGTAGAACAATCCAAGTGAGTCAACTTTTGTGACAATGTCTTTAATCGAT
Oryctolagus cuniculus  TTTCTAGTAACTACAATATTGTGT.....................................AAAAAAGTGAGTAGAACACATTAAATGAATCAAGTTTGGTGA---TGTCTTTATTCCAT

Homo sapiens           GTAAGCTTCCTTCTCT--AACTTTCTTGAATTACTGACTTTTTTTCAGTATCATCCTATAAAAACTCTTCAGGTTTCCTATGTTTCAGTGTAGCTGATTTATACTGATTGTAGGTTTGGT
Oryctolagus cuniculus  GTAAGCCTCCTTCACTGTAACATGATTAAATTACT---TGTTCCCCAGTTTTCTGCTGTAAAAAAATTTCAAGATTCTTAGAATTAAGTGTGTCTACTTGATATTGATTATGGGTTTA-T

Homo sapiens           TCAGGAGGACACTCAAAAGTCCTGCAGGATGTGGAAGAATTCTTTGTAGTGTGGGACTGTCCCACA------TATTTCAAGATGCCTAGCATCTCTGGCCTCACCCTCTGAATGGCATCG
Oryctolagus cuniculus  TCAGCAGTACTCCTAGAAGTCCTGCAGGATGCAGGACAGTGTGCTGTAGCGTGGGACCATGGGACAGGGGACCATGGCAGGCTGTCTAGCATTTCTGGCCTCACCCTCTGAATGGCACTG

Homo sapiens           GCTTGAGGTGGTGGTGGA-CTTCATTGTTATAACCAGAAAGGTTCCCACAAGTTT---ACAAAATGCCCTGAGGGGCTGGGACTACCGGTCTACAGCGTGGTATTCAACACCCTTCACAA
Oryctolagus cuniculus  GCATGAAGTGGTGATGGGGCATCATTGCTATACCCAGAAAGGTTCCCGCAGAATTCCTACAGAATACACTGAGGGC-----ACCATTGGGCTGCCTTGTGGTGTTCAGCACA--------

Homo sapiens           CTGGATTCCTTACCATGTGAAAACAAAGAGGCTATTTTCTTTTTTTGTCTTTTTAGACATTGGTGAAGACTGAGGAAGAAACAGCCCAGGCCGTTGCTGCAGAGAAGTGGTCACATCTGA
Oryctolagus cuniculus  ----------------GTGAAAGTTAGGAGACCATTGTTTG-------CCTTTTAGATGTTGGTGAAGACCGAGGGCGAGGGAAGTCCAGCTTTTGCCCCAGAGCGGTGGTCACATCTGG
```

```
Homo sapiens             GTCTGACTCGGAGGAACCTCTGTGGGAACTCAGCTCAGGAGACAGTTATGAGCCTCAGTCCGATGAGTAAGGCCAGGCCTCTGGCTGGACCCCTGTCTGGACTCTCATTTTCCTGTCACT
Oryctolagus cuniculus    CTCTGGCGAAGAGGAACCCGCGTGAGAACTCGGCCCAGAAGA---TGACCTGCGTCAGTCTGATGAGTAAGGACGGGC-TCTGGCTGCTCCCCTGTGCTTGCTGTCCTTCTCCCATCACT

Homo sapiens             CACCCTGGCTTGGCCCTTGGGAGGCCACGTTTCCTACAGACCCACACCCTTCCTTTCCCTTCTTCTGGCCTCTGCCTCCAGACTT---CTAGCCCTTATCACTTTGCATCCTTCCCAATC
Oryctolagus cuniculus    ------GACGTGGCCC----------ACGCTTCCCGCAGACCCTCACCCTTCCTTTCC-----TCTGCTCTCTACTCCTGGACTTGGCTCAGCCTTTGCCACCTGGTGTCCCTCCCAATC

Homo sapiens             ACATTTTCTTCTGTT-----ACTAATCAGTTTACTGATTGTTGTGGTTATCTTCTCTGATCATCTCTTCTAGTTCCCTGATTCCTTCAGTGACTCCTTTGTATCCTTTTCCCCTTTTTTC
Oryctolagus cuniculus    ACGTTTCCTTCTGTAGTGGAACCACCCTGCTTACTA----------TGAACGTCTCTGAGCCTCC-------TTCCCCAAGTCCCACAGTGATTCCTCCTCGGCTT-----CCTTTTTAC

Homo sapiens             TTCCCTGTCAAGGATTCCATATAACTTGTC--TTTCAGATATCCCTTCATACTTGGAAGCCCCATGAACACCTCTGTCTGACCTTGTCTTTACCTCAGAGGAGGTCATCTTCACTCTCTA
Oryctolagus cuniculus    TTCGCTATCGAGGGGTCCGTATAACTTGTCGTTTTCCGATATCCTTTTACATGTGGGAAGCCCATGGATGCTTCTGCCTGTCC----CTTCACCACAGGGAAGTTC-TCTTC----CCTG

Homo sapiens             AGAACACGACAGTTCATGTTTATTTTATTTTTTTTAGTTTTTTTAAAGATGGGGTCTCGCTTTGTTGCCCATGCTGGAGCATAGTGGCACGATGATAGTTCACTGCAGCCTCAAACTCCT
Oryctolagus cuniculus    AGAGCAGTTTTGGCCATG......................................................................................................

Homo sapiens             GGGCTCAACTGATCCTCCCACCTCAGCCTTCTAAAAAGCTGGGGCTACACCCAGGTAATTTTTTGTAGACACGGAGTCTCGCTATGTTGCCCAGGCTGGTCTTGAACTCCTGGCCTGAAG
Oryctolagus cuniculus    ........................................................................................................................

Homo sapiens             CAGTCCTTCCACCTTGGCCTCCCAAAGCACCCAGCCTAATTTTCTTGAAATGTGCTCTGCCCTGTTGGGCCCTCTCTGCAGCTGGGCTTTGGTGCCTTAATTAACCTTTCTTATCATTTT
Oryctolagus cuniculus    ..................................TTTAATTTTCCTTGAATGTGCTGTGCCCCGCTGGGCCCCCTCCCCAGCAGGACTTTGGTGCCCTAAATGATTTTTCTTATTTCCCT

Homo sapiens             CCCATACCATGCCACATTTTGTAGGATTGCTGAAACTGTGCACCCTTGAAACACCAAT--ATGATTTGTTTTTTGTTTGATTTGGAACATCTATTCCCTACCCTGGAAGTTTTTTTTTTT
Oryctolagus cuniculus    CCCATACTCTACCTTATTTTGTAGGCTTCCTGAAACCATG-ACCCCTGAGATACCTGTCAATGTTTTGTTTT------AATTTGACGTATCTATTCCTTACCCTGGAGGGTTTTTTCCT-

Homo sapiens             TTTTTTTTTTTTTTTTTTCAAAATTCACATAACAGATGACATTTAAATCATTAATGTTATATTTTAGCTGAAGAAATTGTAACTAAAGATAGATTGTTTAAAGCAAAGCAAGAAACTTCT
Oryctolagus cuniculus    --------------------AAATTCGTGTAACAAGTGACATTTCAATCACTAATGTACTATTACAGATGAAGAAATTGTGACTAAAGATGGATTGACTAATGTAGAGCAAGAAACTTCC

Homo sapiens             GAAGAAATGGAACAAAGTGGAGAAGCCTCAGGAAAGCCCAACAGGTGAGTGTCCATGGTCCTCAGTGAATCAAATCCTGCAGATATAAGA---AATTGGAATGGAATCAGAGAAACCAAT
Oryctolagus cuniculus    AAAGAAATGGAACAGGGTGGAAAGACTTCAGCAGAACCCAACAGGTGAGTGTTC-TGGCTCT-GGTAGATCAAATCAT--AGATCTAAGACTGAATTGGAATGAAACAAAAGAAACTAAT

Homo sapiens             TGGTCAAAGGCCTGCTTCTCTGGAATAAGATATGACAACTGCCAC--TCACTTCTGTGATCATAGGAGGTCCATGCCAAGAAAGTGACCTATTCAAAATTTTCAATATGCAATAAATATA
Oryctolagus cuniculus    CAGTGAAAAGCCTGCTTCCCC-GAAGAGTACAGGACAGATGCCACCATCACTTCTGTGATCA----AGACGGATGCCAAGAGGGTGACCTG----------TCAATATAC-GTAATTGTA

Homo sapiens             CAAAACCTTC-AGTTGATACTCAGTTTCCTCCAAGAAGCCTATCCTGATCCAGTCTGTCCTGCTTGATTACCCTTCCCTGGGCTCCCTTACATTTTTATGGAGCTGTGGAGAAGCTAGAA
Oryctolagus cuniculus    CAAAACCTTCAAGGTAGCACACACTTGGCTCCAAGAACCCTGACCTGACCCAGTCTGTCCTGCTTGATAATTCTTCCTCGGGCTTCCTTATGCTTTCATGGAGCTGTAGATAAACAAACA

Homo sapiens             GGCAGATAATTTAACAAGAATGAGGG-----CTACAGATGAAGCTGCCATGACAGTTAAGAAAGAAATGTCAGAGTGGAAGAGGACAAGCAGGGTACAGGTTGAGCATTCCAAATCTGAA
Oryctolagus cuniculus    TAAAGAGAATTTAGATGG---GAGGGAATATCTACAAGCGAGGCTGCCATGACAGTTAAGAAAGAAATACTAGA----------ACAAAGAGG...........................

Homo sapiens             ATGTTCCAAAATCTGAGCTGTTTGAGCACCAGCATGATGCTCAAAGGAAATGCTCATTGGAGCATTTTGGATTTTGGATCTTTGGTTAGGGATGCCCAACTGGTATAATGCAAATATTCC
Oryctolagus cuniculus    ........................................................................................................................

Homo sapiens             AAAATCTGAAAAAAATCCAAAATTCAAAAATCTTCTGACCCCAAGCATTTCAGATAAGGGAAAATTAACCTGTATTTTCAACCGCCATTGGGAGACCTGAACAAGGTATAGGTCAAAAGG
Oryctolagus cuniculus    .......................................................................GTGTTTTCAACCTCTGTTAGGAGAGCTGAACAAGAGATGGATCAAAAGG

Homo sapiens             AAAGTTA-GCTGATGAAGTAG--AAATGAATGACAGTGGTGTGTGCTTCATAAGTGCTGGTTGTGGGAATGAATGAACTTGTAACAGATACTCCTATGCAAAGCAAGAAACTTCTGAAGA
Oryctolagus cuniculus    AAAGTCACTCTGATAAAGAAGGAAAATTAATGATAGGAGCAGGTGTTTCATGAATGTTAGTTGTGGGAATGAGTCAG--TGTAATACATACTCCTA-----------AAACT........

Homo sapiens             AATGGAACAAGGTGGAGAAGCCTCGGGAATGCCCAACATTCCGAGGCTTCTCAGGATTGCAGAAAGAGAGGGAACAAATCACAAGCAGAGGGTTGGCTTCTGGTGAGGCCATTTGGTCCT
Oryctolagus cuniculus    .............................................................GAAAGGTAGGGAGCAAATCCCAGGCAGAGGGTTGGCTTCTGGTGAGGCCATTAACTCCT

Homo sapiens             CAGAGACTTGAGAAAAGAGAGAGTAAGCA---AGGTGGGCTACTT-GGCCAAAGAGCTGAGAAGGGGGTGTGAATTTTCCTTATTGACATTTGTTTAACTTAGAGAAACTGGGAGCTAAA
Oryctolagus cuniculus    TAGAGACTTGAGAAAAAAG-GAGAAAGCAAACAGGTGGGATGCTTAGGCTAAGGAACAGGGCAG-----------------TACTGATGTTCAGTCATTTAAGGAAGCAGGGAACTAAC

Homo sapiens             TCTCAGCTAAAGTAGAAATGTCTTAAAGCTCAAGAGGAGAAGTGAAATTTTGCATTAAGATTAGTGAAGACTGAGCCAGGCTCCACTAAAGGAAGAGATATGATGTTCAGTAACTTAGAG
Oryctolagus cuniculus    TCTCAGCTAAAGTTGAAATGACTTGAAGCTTAA---GAGATGTAAAATTTT-CATTAAG-CTAGTGAGGACTGAGCCACGCTGCACTAAA--AAGAAATCTAATATTCAGGGGCTTGGTG

Homo sapiens             CAGAGTTATGCAACTGGAGAATAGGAATCTGCTATTGTGCCTGT-AGGTACATTTAGTATCAGGCAACTAACAGGTAATTCATAGTGCCTATAATAAAGATATTCATATAACAAAAAGTG
Oryctolagus cuniculus    CAGAGTTATT--ACTGGAGAATAGGAGTGTGCTACTGTGCCTGTCATGTGCATGCAGCATCAGTAACTGAAGCCGTGGCTCACGATGTCTGTAAC--------------------AGTC

Homo sapiens             TGAAGGTAGGCTATTTCAGGTTAGGTGCAATAGCTTAACCATGGTATCAAAGGTCTAGCC--CCCTGCCCTCCTTCTAGTCCCCCTCCCTCACCCCCACCCCACTTTCTTAGCTCACTCT
Oryctolagus cuniculus    TGAAGGTGAGCTGTTCCACATTTAGTGCAGCAGCTTAACCATGATGTCGAAGCTGCAGCCTACTCAGATCTGCACCT-----CCCTGCTCGTCTCCCACCCTACTTCCTTGCCTCACTC-

Homo sapiens             TTTTTCCCATTTCTCACTCCCATTTCCTTCCCTCACAATATTGGGCTTAATGCAC-TGTGGTTCTTAGAGTTTAGATGTCAACAGTAGAAAGACTCAGTGTGA---------TCAAGGAA
Oryctolagus cuniculus    GTTTTCCCAT-----------------TTCCCTCACCAGATTGGGCTTAATGCACTTGTAGCTCTGAGAGTCTAGAGTTCTGC-TTGGAAAGGCTCTGTGCCATGACAGAATTTAAAGAC

Homo sapiens             TGTCACTGAAGTTATGGATGTCAGGGAACTTTAAGATGGATGTGTTGAA-------TACATGCAGACACTGAAGACACCCTGGATGATAAACACTTTACATAAAGAAATGTATGTAGTGT
Oryctolagus cuniculus    TGCTGCCAAAGTGGAGAGTGGCAGGGGCCTCTGGGATGAATGTGTTGAAAGCGATTTGCAGGTAGACACTGAAGACACCCAGAACAGTAAATGCATTA-GTAAATACATACATGTAGTGC

Homo sapiens             AAATTATATTAAAATATACTCTTCAT---GCTTCTTAAA-----TGTCTTTGAAAACTTGACTAGTTTACCAACAGAACACTAATAAATTAAATGCACATGTCTAAAAATTAAAAACTTT
Oryctolagus cuniculus    GAGTTAAATTAAAACACACTCTTCTTACAACTTCTTCCCCCTTTTGTCTTCTAAAAGTAGACTAGTTTACAGATAAGACACTAGTAAAT...............................

Homo sapiens             GTGTAAAATAATACTTTTCAGTTGGGTGCAGTGGCTCATGCTTGTAATCCCAGCACTTTGGGAGGCCGAGGTGAGTGGATCACGAGGTCAGGAGTTTGAGACCAGCCTGGCCAACACAGT
Oryctolagus cuniculus    ........................................................................................................................

Homo sapiens             GAAACCCTGTCTCTACTAAAAATACAAAAATTAGTTGGGCATGGTGGCACGTGCCTGTAGTCCCAGCTACTCGGGAGGCTGAGGCAGGAGAATTGCTTGAACCTGGGAGGCAGAGGTGGC
Oryctolagus cuniculus    ........................................................................................................................

Homo sapiens             AGTGAGCTGAAATCATGCCACTGCACTCCAGCCTGGGTGACAGAGCTAGACTCTGTCTCAAAAAAAAAAAAAAAACAAAACAAAACTTTTCATCTTTGTAACTTTAGCTTACTTCCTTGTA
Oryctolagus cuniculus    ............................................................................................CTCTAAAAATTTAATTTACTTCCTTGAA

Homo sapiens             AACCCCATAAAAATCAGTCCAAGTGCAATGAGTT-GAGTTTCATTT------------GTTTTTCCCCAGAGTAGTATGTCCATCAGATGAATGTAGAACTAGCAGGGGCTAAGTCCTTC
Oryctolagus cuniculus    AACCCCATACAAATCCATTAATTTGCAATGAATTTGAGTTCCTTTTTTGTTTTGTTTTGTTTTTCCCAAGAGAATTATGTC-ACCAGATGACTGTAGAGCTAGGAGAGGCTAATTCCTCC

Homo sapiens             CATGTTCAGAAATTCTGTGAGAGATGTCAGAAGACCTGGTAAATAATAACCTTTTTTTCTTGTTCAGATTCAGAACCAATTGACAGGGATGCAGGAGTAAAATAGATGGTTCTGGAAACC
Oryctolagus cuniculus    TGTGTTCACAAATTTTCTGAGAGGTGTGAGGAGACCTGGTAAATTACCACCTCCTTC-CTTGTCCAAGTTCAAGAACAATTGACAGGGACATGGGAGTACAGCAGATGCTTCTGGAAGCC

Homo sapiens             TCAG-TTTCTTACTGACAGAAGTTAGACTGGATAAAAACGGCTTAAAGGCAGAGCTAAATAGGCTTGTACTGGCAGAGGAGGAGTGTAGCAGATGTCCCTCTGCATAGGTCTTCTCAATG
Oryctolagus cuniculus    TCAACTTTACCACTGACAGAAGGCAGACTGGA-GGAAATGGCTCAGAGACAGAGCTAAACAGGCTTGCACTAGCAGAGAAGGAGTAGAGCGGGTGTCTTCCTGAACAGG-----------

Homo sapiens             CAACTTGGGGCACCAAGGAGCATGGGATGTCTGCATCCTGCTCCTAAAGAGGACAGAGGGAGGAG-TGTACCAAGGATACTCAG--TTATTTCTCCCAGGCTCAACAGTTAGTTCCATCC
Oryctolagus cuniculus    ---------GCAGAAAGGAAAGTGGAATGTCTGTA--------------------GAGGGAGGGGCTGCACAAAGGATG-TCAGATTTGCTTCTCCTGGAATTCACGGTTAGTTATGACC

Homo sapiens             TGAGGAGTGAGTGAGAGGTGGAGAGTGGCTAGAAATGTTTAATCTAGCCAAAGTCCTCCCCATGGAAACTTAAG-AGAAATAAGTAAGTATCCCCCCACAC-----------CCACCCCA
Oryctolagus cuniculus    TGAGGAGTGAGTGAGAGGTGG-GAGAGGCCAGAAAATTTAATTCTACCTGAAATCCTCCCTACAGAATTCTGAGCAGTGACAAGCAGCCAACCCCCCAACCTTCTGTCTGTCCTGCCCTG

Homo sapiens             AGCCCCAAAAGATTTAAGCTTCAGCAGATTTATCATGTGTGAGAGAATATGATTGAGGATTTTTATAACATGTTCTTTTTTT-CTTTCCCAGAGAGTGTGCACCCCAGATTCCTTGTAGT
Oryctolagus cuniculus    GGTTACAAAAGATTTCTGCTTCAG---ATTTGTCATGTGTGAGAGGCTGAGAC-GAGGACTTG-GTAGCATGTACTCTTTTTACTTTCCCAGAGATTGCGCGCCTCACATCCCTGGTAGT

Homo sapiens             ACTCCTATTGCTACTGAAAGGACAGTTGCACATTTGAACACTCTGAAGGACCGTCACCCAGGTGATTTGTGGGCCCGCATGCACATCTCATCCCTGGAATATGCTGCAGGAGACATTACC
Oryctolagus cuniculus    ACTGCTGTTCCCACTGAAAGGACGGTCTCCCATTTGAATACGCTGAAGGCCCGTCTCCCACGGGATTCGTGGGCCCGGATGCACATCGCATCCTTGCAGTATGCCGCAGGAGACATTACC

Homo sapiens             CGAAAAGGGAGAAAAAAAGACAAAGCTCGAGTGAGTGAACTGCTCCAAGGCCTCTCATTCTCTGGTGACTCAGATGTGGAAAAAGATAATGAGCCTGAGATCCAGCCTGCTCAAAAGAAG
Oryctolagus cuniculus    CGGAAAGGGAGAAAAAAGGACAAAGCTCGAGTGAGTGAATTGCTCCAAGGACTCGCGTTTTCTGATGACTCAGATGTGGAGGAAGATAACGAGTCCGAGACCCAGCCTGCTCAGAAGAAG

Homo sapiens             TTAAAGGTATCATGTTTCCCAGAAAAGAGTTGGACCAAAAGAGACATTAAACCCAATTTTCCAAGCTGGTCAGCACTGGATTCTGGACTTTTGAATCTCAAGAGCGAAAAGTTGAACCCA
Oryctolagus cuniculus    CCGAAGGTGTCTGGTGTCCCGGAGAGGAACTGGACTAAACGAGACATTCAGCCCAACTTTCCAAGCTGGTCGGCATTGGATTCTGGACTTTTAAATCTCAAGAGTGAAAAGTTGAACCCA

Homo sapiens             GTAGAGCTTTTTGAATTATTTTTTGATGATGAAACATTCAACTTAATTGTCAATGAAACCAATAATTATGCTTCTCAGAAAAATGTCAGCTTGGAAGTCACAGTTCAGGAAATGAGGTGT
Oryctolagus cuniculus    GTAGAGCTCTTTGAGTTATTTTTTGATGATGAAACATTCAATTTGATTGTCAATGAAACCAATAATTATGCTTCTCAAAAAAATGTCAACTTGGAAGTCACAGTTCAGGAAATGAGGTGT

Homo sapiens             GTGTTTGGTGTCTTACTTTTGAGTGGATTTATGAGGCATCCTAGAAGGGAAATGTATTGGGAAGTCTCTGACACCGATCAGAACCTGGTTAGAGATGCAATCAGAAGGGACAGATTTGAA
Oryctolagus cuniculus    GTGTTTGGTGTCCTGCTTTGGAGTGGATTTGTAAGGCGTCCCAGGAGGGAAATGTATTGGGAAGTCTCTGATGCTGATCAGAACCTGGTCAGAAATGCCATTAGAAGGGATAGATTTGAA

Homo sapiens             TTGATTTTCTCAAACCTGCACTTTGCAGATAATGGCCACCTAGATCAAAAAGATAAGTTTACAAAGTTGAGACCTCTCATAAAACAAATGAATAAAAATTTCCTCTTGTATGCTCCCCTG
Oryctolagus cuniculus    TTGATTTTCTCATACTTGCATTTTGCAGATAACAGCCACCTAGATCAAAAGGATAAGTTTTCAAAATTGAGGCCTCTCATAAAGCAAATGAATAAAAATTTCCTCTTGTATGCTCCCCTA

Homo sapiens             GAAGAATACTATTGCTTTGATAAGTCAATGTGTGAATGCTTTGATAGTGACCAATTCCTGAATGGAAAGCCTATTAGAATTGGCTATAAAATTTGGTGTGGTACAACCACACAGGGTTAT
Oryctolagus cuniculus    GAAGAATATTACAGCTTTGATAAATCAATGTGTGAATGCTTTGATAGTGACCAATTCCTGAACGGAAAGCCTATTAGAATCGGCTATAAAATTTGGTGTGGCACAACCACACAGGGTTAT

Homo sapiens             CTGGTTTGGTTTGAACCCTATCAAGAAGAATCAACTATGAAGGTAGATGAGGATCCTGATCTTGGGTTAGGTGGAAATCTAGTGATGAACTTCGCTGATGTTCTTTTAGAGAGAGGTCAG
Oryctolagus cuniculus    CTGGTTTGGTTTGAGCCCTATCAAGAAGAATCAACCATGGAGGCAGATAAGGATCTTGATCTTGGATTAGGTGGAAATCTAGTGATGAATTTTGCTGATGTCCTTTTAGAGAGAGGTCAG
```

```
Homo sapiens           TATCCCTATCACCTGTGTTTTGATAGCTTCTTTACAAGTGTCAAATTGTTGTCAGCCTTGAAAAAGAAGGGGGTGAGGGCAACAGGAACAATTCGTGAGAACAGGACCGAAAAATGTCCC
Oryctolagus cuniculus  TATCCCTATCACCTGTGTTTTGATAGCTTCTTTACAAGTGTCAAATTAGTGTCAGCTTTGAAGAAAAAGGGCGTGAAGGCAACAGGAACAATACGTGAGAATAGGACTGAAAAATGTCCA

Homo sapiens           CTTATGAATGTAGAACATATGAAAAAAATGAAGAGAGGGTATTTTGATTTCCGAATAGAAGAAAACAATGAGATAATTTTGTGTCGTTGGTATGGGGATGGCATTATCAGTCTGTGCTCC
Oryctolagus cuniculus  CTGATGAACTCAGAACATATGAGAAAAATGAAGAAGGGGCATTTTGATTTTCGAGTGGAAGAAAATGATGAGATAATTTTGTGCCGTTGGCATGGCGATGACATTATCAGTCTGTGCTCC

Homo sapiens           AATGCTGTGGGCATAGAACCAGTCAATGAGGTAAGCTGTTGTGATGCTGATAACGAAGAAATCCCTCAGATAAGTCAACCATCCATAGTAAAAGTGTATGATGAATGCAAGGAAGGTGTA
Oryctolagus cuniculus  AATGCTGTAGGCATAGAACCAGTCAGTGAGATAAGTTGTTT---TGCTGACGATCAAGAGATCCCTCAGGTAAGTCAACCGTCCATTGTGAAATTGTTTGATGAATGCAGGAAAGGTGTA

Homo sapiens           GCTAAAATGGATCAAATTATTTCGAAATACAGGGTGAGGATAAGAAGCAAGAAATGGTACTCAATTTTGGTGAGCTACATGATTGATGTAGCCATGAACAATGCATGGCAACTACACAGA
Oryctolagus cuniculus  GCTAAAATGGATCAAATTATTTCCAAATATAGGGTAAGAATAAGGAGCAAGAAGTGGTACTCAATTTTGGTGAGCTACATGATTGATGTAGCTGTGAACAATGCATGGCAGTTACACAGA

Homo sapiens           GCCTGTAACCCAGGTGCTTCTCTAGACCCCTTGGATTTTCGGAGATTTGTTGCACATTTCTACTTGGAACACAATGCTCATCTGTCAGATTAGGGTACATAAAATGGACATAGTGCAGAC
Oryctolagus cuniculus  GCCTGCAACCCAGGTGCCTCTCTAGACCTACTGGATTTTCGAAGATGCATTGCCCATTTCTACTTGGGACATGATACTAATCTGCCAGATTAAGGCAAATCAAATGGAAGTAGAATGGAC

Homo sapiens           ATTAATAAGACATAGAAAAATAATAATTATACATGCTGTTGTACCCTCCCAAAGTAAATCTGATATATGTAATG-----AAGTTATTAAATAATACTTTTAAAAATCAGACATTTATATA
Oryctolagus cuniculus  ---CAAAAGACACAG-AAAATTCTAACTACACATTAGATTGTATCCTACCAAAGCAAATTTGATGTATATAATGAAACTAAGTAATTAAGTAGTATTTTT--AAATCAGATGTTTATATA

Homo sapiens           GAGTTTCAAAGACTATTGTAACAAGTAATGTTAAAAATTGTCTGTGAGAATGTTGAACT---GTAGTACCTTTCTCTATGTCAAGTTTTGTGTCAGACATGGGAAATCATGTATTTGTTC
Oryctolagus cuniculus  GAATTTCAAATACTATTA------------TAAAAATTACCTGTGAAAATGTTGACCTCCAGTGGTACCTTTCTC--TGTCTAATTTTGTGTCAGGTGTGGGAAATCACTTATTTGC--

Homo sapiens           AATTGACTACTTTGTGCACTTATTTATTTATTTTTTGAGACACAGTCTCGCTCTGCGGCCAAGCTGGAGTGCAGTGGCACGATCTTGGCTCACTGCAACCTCTGACACCCTAGTTCAAGC
Oryctolagus cuniculus  --TGGATT................................................................................................................

Homo sapiens           GATTCTCCTGCCTCAGCCTCCCAAGTAGCTGGGATTACAGGCACGTGCTGCCATGCCTAGCTAATTTTTGTATTTTTAGTAGAGACAGGGTTTCACCATGTTGGCCAGGATGGTCTCGAT
Oryctolagus cuniculus  ........................................................................................................................

Homo sapiens           CTGACCTCGTGATCTGCCTGCCTTGGCCTCCCAAAGTGCTGGGATTACAGGCGTGAGCCACCATGCCCAGCTATTTTGTGCATTTAAA--GAAGGAAATCCTACCTCTTAAAAAAAATTA
Oryctolagus cuniculus  ........................................................................ATTTTGTGCAGTTAAAAAGAATGAAATCCTGCCTCTTTAAAAACATTT

Homo sapiens           TCT--GGAGAATGCCATTTTTAAGATGCAAGCAATGTTACAGAAACCATAGAATGGTGCTGACTCAATAGTTCAAACTAGTGACACAGCCTATGAAGTAAGAATGATCTAAACAAAACAT
Oryctolagus cuniculus  TCTTTAGAGAATGCCAATTTAAAAATGCAGGCAATGTCA---GTACCATAGAATGGCACTAAATCCACAGTCCAAAATAGAAGTAGAGCCCAT-AAGTAGTGATGATCTAAATAAAACAT

Homo sapiens           AGGTGGTAAGAGACTAAAAACCTTAGCATTGGTG-TAAAACTGGATCGGATTGTGTGTAAGTGAGAAGGGTCAGGCATGGATATTGA---AGGAGAGTGCTATAAAGGAAAAACCAGAGG
Oryctolagus cuniculus  GGGTAATAAGAGGCTGAAGACTGCAGCATTGGTGAGAAAACTGGCTCAGGTTGTGTGTAGGTGAGGAGGGCT--GCCTGGATGTTCAGAGAGGAGAGAACCCTGAAGAAAAGTCCAAAGG

Homo sapiens           TGAAGCAGTGCCTT
Oryctolagus cuniculus  TAGATCAATGCCTG
```

```
Homo sapiens  AGGATAGTTAAGTAAATTATGGAACACTCATACA-----GTATTACTTAGCTGTTAAAGAATAAGCTGATCTGTGAAGATCTCCAAGTTGTCTTAACCGAAAAGAGCTAAGGTGCAAAAT
Bos taurus    GGGATAGTTAAGTAAATTA------GCCCATACACTGGAATACTAATTAGCTCTTAAAAGATGAACTGGTGTGGGAAGATCGCCAAGTT-----AACTGAAAA-AGATAAGATGTAAAAT

Homo sapiens  TACATTATGATACCATAAGTGTTTTAAAATGTATAAATACTTATTCACTAAACAAAATGTTCATTAAGTGCTACTGTGTGGCAGGTAGTGTCCTAGATAAATATGAACAAAATAC----A
Bos taurus    AATATCATGATGCCAGGGGTGGTTTAAAATACATA------------CTAAACAAA-TGTTTGTTGAGTGCTATTGTGTGGCTGGTGGTGTCCTGGGAGGGTATACACAAAATACTAACA

Homo sapiens  ATGGTTAACTCTAAAGAG--GGGACACTTTTCGTTTTATGGTCCTTTTGCAACTTGA---------------------CTTTTTTATTGTAC----ATATATTATGTATACTTAAAGAA
Bos taurus    GTGGTTAACTGTGGGGAGTTGGGATACTTTTCATTTCA--GTCTCTGTGCAACATGAGGGTGTTTTTGTTTACTATGGGACTTTTTAATATATTTTGATATATTGTGTATATTTAAATTT

Homo sapiens  AATAATTTGTACAGCTTACTATAATGGTCTTTTTATTGATCTGTCTCTCCTACCAGCCAGTGATGACCCCGAAGACAGACA------------TTTACCACTGTGTTGGTTTTCAATCGA
Bos taurus    TTAAATGTGTGCTGCTTGCTCTATTGCAATGTTTATTCATCTGTCTCCCC-AGCAGCCTGTGAGGACTCTAAAGACAGAAACTCAGTTTTGTCTTCATCATCTATTTGGTTTTTAATCGA

Homo sapiens  AATGATGTACATAAATTTGGGGCGTGCTTGCACCGTCCGAATGGGCCTGGGGTCCGGCGGCGGAGAGTGACCTGGGCGGGGCAGGAGGTGGCAGCGGGGGTCCTCCCGGCTCACCAGAGA
Bos taurus    AATAAT-TGCAAAAATGAGGGGTAGTTTGGGGATATCCGGATGG--------TCTGTCGGAGACTATGCTACTGAGCCTGTTGGGCTGTGGCAGCTGGAGTCTTCCAGGCGCACCAGAGA

Homo sapiens  GACGAGCGGCCGTGCTCCTAGAGAGGCAGGGAACCCGCCAGACTCCGCCACTCCGCTGCGGGCGCGGCGCAGGGAGAAGCTTTTGTACCCGCCCAGCTGCTGGAGGCGCCGGCAGCGCCC
Bos taurus    GATGTGCGGCCGCGTTCCTAGAGAGACGCAGAGCCGGCT-------------------CGGCCGCAGCCCGGAA----------------TCCTGACGGCAGGAAGTGGGGGCA-CGCCC

Homo sapiens  GCCAGACCCGCCAGCCCAGCGGCCCGGGCTCTGGGGAAACCGG-CGCTCCCGACAGGGGAGCACCGGGCCTCTGAGCTCCCTCGGGAGCCTTTCACGAGGTCAGCTACGTCTTTGTTGTG
Bos taurus    ACCAGGC-------------------------GAGGTAGCTGGATTCTCTGGACTGGAGAGC--TGCGCCTCTCATCTCCGATGGGA-TTTTTCACTGGGTCAGCTACGTCTTTGTTGTG

Homo sapiens  CGCGTTCCT-GAACTTTTGGTCCATTAACCA-----ACAATTAGACACG--------CCGGTATTC--AGTGCCTGGCGCGGTGCTAGATGCTGGG-TGTAATCTCAGAAAAATACATTC
Bos taurus    CTTGATCCTTAAACTTTTGGTCTGTTAGCAGTTAAGACACTCATTCACCGAGTGCCCCCTGTATTCCAAGTGCCCCCTGTATT-CCAAGTGCTGGGATGCGATCGCAGTAAGATACGTTC

Homo sapiens  AGGGGCGCGCCTGAGGGTGCTGGCTGCTGGCATCTCAGGTGCTTTACGTGCATTCGTGAAGAAGCCCATCAGTATTTCTTGAATACCAGGTACGTGCCGGGCATGTAGAGTCTATGCAGA
Bos taurus    TGGG-----CCCGCGGG----ACATCCTGGCATCCCAGTACATCTCCGTGCATTCAGCAGGACATCCACCAGTATTTGCTGAATACCAGGTA--GGCCGG----------------CAAA

Homo sapiens  GATAAACTCCTGTCCTCAAGAACCCGGGTGGG--------GATGGGGTTGGTGATCTGTAATACGAGATGGAGA-TGAGGG-----------------TTGGGTGCAGTTTTCTATGGGA
Bos taurus    GATAAGACCCTGTCCTCAAGGAGCAAGGGGTGATGGTCGTGATGAGGGCTGTGACCTGT-----GAGTTGGAGAATGAAGGGAGTTGGTGAGGCAGGCTTGGGCTCACTTTTCCATGGGA

Homo sapiens  ATACCCCATCTCAACCTGTGTGGCTTACTTTTC----CTGTCTATATGTTAGGGTTAGGGGTTGCCAGGGAAGGTTTTACAATAGAACATTTACGCTGGGCCTTGGGAATTTACCCTTAG
Bos taurus    ACGCCC----TCCGCCTGAGTGGCTCAGAGGACAAGATTGTCTGTACGTTAGGTTTGGGGGTGGC----------------------ATTTGCGCTGGACCTTGGGGATTCACGGTAAG

Homo sapiens  AAGGGCGGTCCTGACT-----GTATAGAGTGGGTGGAAAAAAATTGAGTACTTAGCATGGCAGTGACATAAGAAATTTGGGAAGACCATGGCTTTCTTGAAAGCAGGAACCAAGTCTTTT
Bos taurus    AAGACTAATCGTGGCCATAAAGTACAAGGTGGGTGGAACAAATTTGAGTA-TTAGC-TGGCAGTGACACGAGGTGTTTGAGAAGACGATGGCTTTCTTGCAAGTAGGGTCTGTGTCTTTT

Homo sapiens  ATTCCAGAGTTAACTTTATGCCTGGAAAATAATGAGATGCTCCACAAATGTTGATTGAGTGACAAACAAATCAGCAAAACCCAGTA--AAGATTAGATCCTGGGGAGTTTGAATTTACTT
Bos taurus    ATTCTGGGGATAGCT-----CCTGGAAAATAATGATGTGCTCGATAAATGTTTATCGAGTGGCAAACACGTAAGTGAAACTCAGTTCTAAGATCGAATCCTGGGGAATTTGAATTTAGTT

Homo sapiens  CAACGAGTTTAATGAAAACTTTGTGTGTGTTTATGTGTGTGTGAGGTATGTTGAATATGGTCACAACTGTGCTTTAGGGAATATTAATCCAGTGACCATAGAAGCGGGGAGTTGGGCAGG
Bos taurus    CAACCAG------GAGCAATTGAAATTAGCATTTGGGGGTGGGGGGT-TGTCAGATATGGTCCGAATTGTGCTTTAAGAAATACCAATCCAGTGATGA-GGAAGTGGTG----GAGCAGG

Homo sapiens  AATACCGTA-ACAGAACATTATTGTAACAGTGAATGAAAATAATAGTAAAATTATAAGAAGGA---AAGAATGGCTGCAAGAGGCGTTTTTAAGAATTGACTCACTG-----------AT
Bos taurus    AATACCACACAGATAATATTGTGATAATAGTGACTGTCAATCATAATGGTGTAAAAAGGAAGATGAAGGAGTGGATGCAAGAGGCCTTTTGGAGGAAGAACTGACTGGACCCAGGGTGAT

Homo sapiens  TGTGTATA-------GAGGCTGTTTAAAAGACTGAATGCGTGGAAGATGATGGTGATGCCAGTAGAAATGGAGAAATACCGAGTGAACGCAGCTTTGAGGGACTCTGATTTCAGTTTGAG
Bos taurus    TATGTACACTATATGTAGGCTGGCTTTGAGCCTGAATACATGGAGGATGGTGGTGATGCCAATAGAAATAGAAAAATACAGAGTGAGTGCAGCTTTGAGG-AGGCTGATTTCAGTTGGGG

Homo sapiens  ATCTGTTGAAATTATAGTGCAGTAGAGGCCCACAGCTGCTGGCAT----------TTCAAGATGTGGGTTTGGAGTGCAAAAAGCAAGCTGGTGCCAAA--GATTTGAGTCTTCTTGGAA
Bos taurus    ATGTGTTGAAATGACAGGGAAGGA-AGGTCCAAGGCTGGAGGCATCCTGTGGCTTTTCAAGATGTGAGTCTGGAATGTGAAAAGCAAGTCAGAACCAGATAGATAATAGTGTTCTTAGAA

Homo sapiens  CAGAGATATATTGAAAGTGTGACTAAGGAGATCACAAAGGAAAAAATGAAAAGAGGGAATAAAAGAACTTGATGCATGTCTACTGTTTTTCAGTTATGTATTGTTGCATCAAAAATAAAA
Bos taurus    CAGAGATATATTGAA-TTTTGAT---GAAGATAACAAAAGGAAAAATGA---GAGGGAGAAACAGAAGTTGAAATATTTCTGCTGTTTATC-------TATTGTTTCCTTAAAAAGAATG

Homo sapiens  CTCTAAACTTAGTGGCTGAAAACAAGCTCAGCTTATTGTTCCTCAGGATTATGCGGGTTGACTAGGCGGTTCTACTGGTCTCACCTGGAGTTACTAATAGAGCTGCATTCAGGTGGTGAA
Bos taurus    CCCCAAACTTAGTGGTGTAAGACAACTTCAGCTTATTATTTCTTATGATTCTGTAGGTTGACTGGGCAGTGTTACTGGACTCAC------------------------TCATGTGGC---

Homo sapiens  TTATTTGAGCACTAGGCCTGGCTGGGCTTTAACTTGGGCATTTTGGTTCTCATCCACATGACCTCTGTATAGCTAGCTTGGGCTTCCTTACAGCATGGCAGTCTTATGGCAGTGAGCCAA
Bos taurus    -----TGGGGACTAGGCTCAGCTCCGCTTCAGCTGGGGCTCTGTGGTTCTCATCCACGTGACCTCTGCATGGCTAGCTTGGGCCTTCTCACAGCAAGGCAGTCTCATGGCTGTGTGCCAG

Homo sapiens  GAGCTACAGCAGCACTCTCTCTTACAATGTCATTTCTGCTGTATTCCACTGGTCAAAGCAAGTCATAGGCCAGAACAGATTCAAGGGAAGGAAAAAGAGACTCCACCCCTTGAATGACAA
Bos taurus    GCACTACA-CAGCACTTCCACTTACAGTGTCATATCTGCCATATTTTATCGGTCAGAGCAAGTCACAGACCAGCACAGATTCAAGGGAAGGAAAAAGA--CTCCACCCCAT-AATGGGAA

Homo sapiens  CAGTGGCAAAATCTCATCACAAAAGGTATT-------TGAGAATGGGAGACTTTGGAAACATTCTGTTACACCTACATTTAAAGTGGAATATTCAATGAGAGTTACACAGATATAGAAGA
Bos taurus    AAGTAACAAAATCTCATCACAACAGATCTTAGGCGATGGAGACTTGGAGACTTTTGAAA--GTCTGCTTCACCTA------AGGTAAAGTATTCTGTGAGAAAGACACAGAGATAGATGA

Homo sapiens  CCTTTGAGACTGTATTATTTGTCCATGAAGGCAGCCATCTGCCTCCTCAATGGGTAAATTATCCCCTCTCCCCAGCTGAAGTGCAAGCTACTCGAGCTTTCTTATTGCTAACACTTCTTC
Bos taurus    CC...................................................................CTCCATCTAAAGTATAAGCTGCTCAGGCATTCTTATT---AACCCTTCTTC

Homo sapiens  CCTCATGTCCTACATCCCTTACCTTTGGACTCAGGCCCTTTAGTACTAGTTCAAAATATACTTCTGATTCATTTCACTGATAATAGAAGCAAGCAGGCTCAGCTTTTGAGCATTACCTAC
Bos taurus    TCTTAT-------ATCCCCTCCCTTTGGGCTCTACTCCCTTAGGA----GTCAAGGTATACTTT-----------ATTGATAAAACAGGCAAGCAGGCCCAGTTTTTGAGCATCACTTAG

Homo sapiens  AGTGGGGACAAAAATGTAAGTAGGTTACATACAGTTTTGAAGCTCTTATAATTTGCATGTTGCTGATAAATTCTTATGCCTCAGATCTCTATTTCTGAATATAGACCCCAAGCTAAGTGA
Bos taurus    AGTGGGGACAAAAAGATAAATGTGTTACATAGATTTTTGAAGCTCTTGTTATTCTCATGTTACTGATCAATACTTACGCCTCAGGTGTACATTTGGAAA-----------AGCTAAGTGA

Homo sapiens  A--GCTTT---AGCCTCTAAGCTCAACATGTATGAAGCTTTGCCAGGCCCTGCTCCTGAAAATGAAGATGGCCTTGTGAAAGTGAAGGAGGAAGATCCCACCTGGGAGCAGGTGTGCAAC
Bos taurus    ACTGTTTTCGAAGCTTTTAAGATCACCATGGATGAAGTTTTGCAGGACTCCGCTCCTGAAAATGGAGACGGCCTTGTGAAAGTGAAGGAGGAAGACCCCACATGGGAACAGACATGCAGC

Homo sapiens  TCACAGGAGGGCAGCTCCCACACTCAGGAGATTTGCCGCCTGCGCTTTCGGCACTTCTGCTACCAGGAGGCTCACGGACCCCAGGAAGCTCTGGCCCAACTCCGAGAACTTTGTCATCAA
Bos taurus    TCACAGCAGAGTGTCCCCAATACTCGGGAGCTTTGCCGCCTGCGCTTCCGGCACTTCTGCTACCAGGAGGTACCTGGGCCCCGGGAGGCTCTGGCCCAGCTCCGAGAACTTTGCCATCAG

Homo sapiens  TGGCTGAGACCGGAGATGCACACCAAGGAACAGATAATGGAACTGCTGGTGCTGGAGCAGTTCCTGACCATCCTGCCCAAGGAGCTCCAGCCCTGTGTGAAGACATATCCTCTGGAGAGT
```

```
Bos taurus     TGGTTGAGCCCAGAGACCCACAGCAAGGAGCGGATCTTGGAGCTGCTGGTGCTGGAGCAGTTCCTGACCATCCTACCCGAGGAGCTCCAGGCCAGGGTGCAGGCGTGTTCTTTGGAGAGT

Homo sapiens   GGAGAGGAGGCAGTGACAGTGCTGGAGAATCTAGAGACAGGAAGTGGAGACACAGGACAACAGGTGGGAAGAGAATGTGTGTGGTGATGTGGGAGAAGAAAAGGGGGACATGTATCGGTT
Bos taurus     GGGGAGGATGCAGTGATAGCGCTGGAGAGTCTAGAGAGAGACACAGGAGACTCAGGAGAACAGGTGGGAAGAGAATGTGTGTGGTGGTGTGGGAGATGTAATGGCGAACATTTGTCTGTT

Homo sapiens   TATTTTTAACGTTTTATTTTGAAATAACTCCTAATTTATAGAAGTATTAGAAGAATAGAGAACT-CCCGTAAAACTCTTCATCCAAATTTGCCAGTTGTTAACATTTGTTTGATGAAGTC
Bos taurus     AATTTTTAAC-------TCTGAAATAATTTATAACTTGGGAGAGT------AGTGCAGAGAACTGTGTGTATGATTC-ACACACATGTTAACGAAATGTTAACATTTGTTACAGGAA---

Homo sapiens   CCATTGTTGTGTACAC-ACTCGTACAAGGAAGTGCATTCCCCCCCCCCAAGATGCATA-----TGTATATGTATACACACACATACATACA-CATATGCAGTATTTTTTTTTCCTGAATC
Bos taurus     -------TGTGCACCCTTCTTGTGTGAGCAAGTGC---TCCCTCTCTCTGTATGCATACATATTGTGTGTGCACGCACACACCCCCATACAGCTTATGCAG---TTTTTTTTCTTGAA-C

Homo sapiens   TATTTGA-----GAGTCAGTTGCAAACACTATATCCCTTT----ACCTCACTGTTGACCAGGCTGGAGTGCAGTGGTACGATCATAGCATCATTGCAGCCTTGAACTGCAATGGTGGAAG
Bos taurus     CATTTGAAAATGGAATTAGTTGGAGATACCTTTTATCTCTAAACACTTCA......................................................................

Homo sapiens   GCTCAAGCAATCCTCCCACCTCAGGCTCCTGAGTAGATGGGACTACAGGTGCATGCCACCATGCCTGGCTAATTTATTTTTAATTTTTTGTAGCGGGGGTGGGGTCTCACTATATTGCCC
Bos taurus     ........................................................................................................................

Homo sapiens   AGGCTGATCTTGAACTCCTGGCCTCAAGTGATCATGCTGCCTTGGCCTCCCAAAGTGTTGGGATTACAGGCATGAGCCACTGCACCTGGCTATTCGGTGTGTATTTCTGAAGAGTGAGGA
Bos taurus     ..................................................................................................GTGTGTTTCTGAAGAAGAAAGA

Homo sapiens   TATTCTCTTGTATCACAAGAATACAAA-AATCAAGTTTAGGGAAATTAAA---CTTACGATACTATCATAT-AACCTTAGTCCTTATTCATATTTTGCCATGTTATCCTAATAATGTTCT
Bos taurus     CGTTCTTTTGTATAACTAGATTATAGATTATCATGTTTTGGGAAATTAAAATTTTTATGATAGTATCACCTCAGTAGTAGTCCTTGTTCAGATGTATCCGTGATAGCCCAATAGTGTTCC

Homo sapiens   TTATGATAATTTTCCTGTGATTCAGGATACAAGCCAGCATCACATATTGCACTTAGTTGCCATATCTCTAGTCT--GTGAATCTGGAGTAGCTGCT--GTCTTTGTCATGACGTTGTTAT
Bos taurus     TTATGACAGTTTTGCCCCGATT-----------CCATCATCACACATTT--TTTAGTTGCTGTCTCTTCAGTCTTCTTAGATCTGGAACAGTTGTTCAGTCTCTGTCATGACAATGACAT

Homo sapiens   TTTTGAAGAGTACAGCTA-GTTTTTTTGGAGACTATCCCTCAATTTGAGTTTGTCCTATATTTTCTCATGAT-----TCAGGCTGCACATTTTGGCTAGAATACCACAGAGGTAATGTTG
Bos taurus     TTTTGAAGAGTACAGATCTGTTACTTTTTGGAATGCCCCTCAATTTGGGTTTGTCTTGTATTTTCTCACGATAAGACCCAGGTAGTACATTTTGGCTGGAATACCACAGCAGTGACATTG

Homo sapiens   TG---TTCTCAGTGCGTTACTTCAGGAGGTGCGTAGTGATGATTTGTCCCATTACTGATGAGGTTATGGTGGTACCTGTCAATTTTCTCCACTAGAAAGTTACCATTTTCCCCTTTGTAA
Bos taurus     TGGTCTCCTCAGTGCATCACTTCAGGAGGCACACGGTGGTGGATCATCCTGTTGCTGATGAGGTTAAGGTGGTATATGTCAGATTTCTCCACTATCAAGTTACCATTTTCCCCTTTGTAA

Homo sapiens   TTACTAAGTAATCTGTGGGAGACATTTTGAGGTGGTGTACATATCCTGTTCCTCATTAAATTTTCACCAGCTAGTTTAAACATCCACTTGTTTGTTGTTTTTGTTTTTCTGGGCTGGGAT
Bos taurus     TT----AGTAATCTGTGGGAGACATTTGGAGATGGTATACATATCCTATTTCTCATTAAATTTCCACACACTGGTTTAAGCATCAATCTG-GGGTTTTGTTTTGTTTTCCAGGTAGGGGT

Homo sapiens   CCTTTGCCTGTCTTTTCTGCCTTGTTTGCTGCTGTATCCCCAGCATGTAGAACAGTGCCAGGCTC---TTAGCATGCAGTCCATATATGCATGCCCTTTCAAAATAAATCTTTTGTTTCC
Bos taurus     TCTTTGCCTGTATTGTGTCTCTTCTTTGCT----------TAGCATGTAGAACAGAATCTGGCTTTAGTAGGCATTCAGTCCATATTTGCACGCCTTTTCAAAGTGAATCTTTTGTCCGC

Homo sapiens   AGGCCTCTGTCTATATTCAGGGACAGGACATGCACCCAATGGTGGCAGAATATCAAGGAGTCTCTTTGGAGTGTCAGAGCCTCCAGCTCCTGCCTGGGATAACCACCCTGAAGTGTGAAC
Bos taurus     AGGCTTCTGTCTACACTGTAGGACAGGACCTGCACCTCCTGGTGACAGAATATCAAGGAACCTCTTCAGAGTGTCAGAGCGCCCAGCTCCTGTTGGGGGCAACCACCCTGAAGAGTGAGC

Homo sapiens   CTCCACAGCGTCCTCAAGGGAACCCCCAAGAAGTGAGTGGTGAGTGCTCGA--GTGAGTTTAGTGAGGACGCTGGGAGCTGGCCACTGACACTTGCACAGCCTTCTTGACCTCAAGTTTG
Bos taurus     CTCCAGAGTCCCTCCAGGAGACCTCCCAAGAAGTGAGTGGTGAGTGCCAGAGGGCGAAGTTAGTAAAGATACTGGGAACAGGCCACTGACAGGTGTGTGGCCTTCT..............

Homo sapiens   TATACATTGTGAAATGATTACCACAAGCTAACTTACATATCTCACAGTTACCTTTTATGTGTCTGTGGTAAGAATTCCTAAGATCTACTCTCATGAAATTTCAAGTATACAATATATTAT
Bos taurus     ........................................................................................................................

Homo sapiens   TATTACTAACTATAGTCAGCATTCTGTACGTAGGTCTTCAGAGCTTATTCATCTTATAACTGCAAGTTTATACCCTGTGACCAACATCAAGTTTTATTTTTTTGTCTAACCCTGTATCTG
Bos taurus     .................................................................................................TCCTTAGCCTAGCCCTTTCTCTG

Homo sapiens   AACAAACTTGTAAAGACCTGCTTTATTTTACACTGTGCATTTTAAGCTG--GTTTATTTAGTATGTATGTGTTGTGGACAAGTACATATAATAGTAAAAGTGAATCG-GGGCAAGGCTGT
Bos taurus     AC--AGC------AGACCGGCTTCA-----CACTGTGCTTTTTAAGCTGTTGGTTTTCTGGCATATATGCATTGCGG----GGAGATTAGGTGGTAATAGTGAATCATGGGCTAGGCTAT

Homo sapiens   TTT---TTTCCAGCAAACTTTATTCCTTCTAGAAATGCAACAACAGCAATAAAAACATTCCATAGTGATCCATTATGGAAGCAAAGAAAAGCAGAGCAAAGAAAAGTGATGTTCTGTTTT
Bos taurus     TTTATTTTCCTAGCAAGCTTTATTCTCTCTAGTA-TGAAGGAACAGCACCAAGAAGATTCCACAGTGATTCACTGTTGAAGCAAAGAAAAGTA--------------ATGCTATGTTCT

Homo sapiens   ATATTTTTGGGTTAGAATTACCACATGTGCTCTTCATTTGATAATTTTGTACTCTGCCATCTGCCAACTGCCAACTTTGTGACCTAGGCAAGTCATTTAATAGGTTATTCAGTTAATCAA
Bos taurus     --------GGGTTAGAAT-ATCCCCTGTGCACTTCGTTTAGTAATTCTGGACTCCCTCTATTGTTA......................................................

Homo sapiens   CAATTATCTGTTTGATCTCTGTGTGTACTCTGCACTCAGCTGTGATTCTTCTGAATGTGCCTAGGATTTTGGAGGCAATAATTTGGACCTTCTTGATTATTTTGAGGATTGGAAATAATC
Bos taurus     .......................................................................................CCCCCTTATACAGATAAGGAATGGGAAAGAATC

Homo sapiens   TTTTAAAGTAACAGCACAGTGGCTGGCCTTGTGCAGTCATCATCACCATTTTGATTATTGTTGGTTCTTTGGGATCAGTACCTCCTAACAGTTTTTCTAGTTCATGGCTAACATCTCTTC
Bos taurus     TTTCTAAGTAACAGTGCAGTGAC-------GTGCAGTCATTATCACTGTTTGGATTATGGTT---TCCAAGGAAGCAGATTCTC--AAAAGTTCTCCTAGTTTGCAGGTAGCATCTCTTC

Homo sapiens   TCTATTTTTTTAAATATCTTTATTTTCCTG--TCTCTCTCTCCATCTTCCTTTCTTCTGCTTTCCTGTTTCCCCTCAGTCTCATCTCTCTACTCTCTACATCCTTTCTTTTGCCTCTGTG
Bos taurus     TATGTTTTTTTAGAGATTTTTTTTATCCTCACTTTCTCTTTTCATCTTTCTCTTACCTGCTTTCCTGTTGCCCCTTGGCTTTGTCTCTGT--TCTCCACTTCTTACCTTT--CTTCTTTG

Homo sapiens   TTCTCTCCTTGATCCTTCCTTTATCACCAGCTTCTTCCTCCC---CTTTATTTTT-GTCTATGATTTTTATTTTTTTCTTT-----ATGTGAAAGTGCATGACTGTAAAGCACCTTATGAG
Bos taurus     TTCTCTGGTTAATGTTTCTTTAATCACCAACTTCTTCTTCCCTCTCCTTATTTTTCATCTGTGATTTCTGATTTTTCTTTTATGTCTGTGAAAGTGCATCACTGGAAAGCATCTCATGAG

Homo sapiens   TTCATAAATCCCACTCCATTTTTTGGGTGAGAGACCCTAAAGTTGTAGAATTTTAGAATTTCTGACAGAATGAAAGCAGTTGTGATGCTCTTTCTCACACTGTAAAATCTACGTCTTAGT
Bos taurus     TTCATAAACACCACTT-ATTCTGTGGGTGGGGGATGCTAAAGCT--------CTAGAATTCCTGACA--ATGAAAACAGACGTGATGTTCTAGCT-------TAAGATCTGTATCTTAGT

Homo sapiens   TCTTGAATGTTTAACAAACATAACATTTGGGGGAAAAGGCTGGGTCTCCAA-AAAGGGTACCC-TAAAGGCCATCATGTCTCATTTAGGACTCTTGCCCTCTCTCTCTGCAGGGCCTGTT
Bos taurus     TCTTGGATGTTTAAAAAACATAAGATTTAGGGT------CTGGGTCCCCCACAAAGGGCTCTTGTAAAAGCTACTATTTCTC-TTCAGGACTCTTGTCTGCTCCGTCCACAAGGCCAGCT

Homo sapiens   CCCCACGGATCAGCTCATCTCCAGGAAAAAAACCCCAGAGACAAGGCTGTAGTGCCTGTGTTTAACCCAGTCAGGTCCCAGGTAAGTAGGATGCCCAAGCTTGTAGGAATATCAGTAGTG
Bos taurus     CCCCAGAGACCAGACCCTCTCCAGGAAGAGAATCCCAGAGACCAGGCTGCAGTACTGGGGTTGAACTCAGCCAGGTCCCAGGTGAGCAGGGTGCCCAGGC-TGTGGGAACCTCAGTAGTA

Homo sapiens   GTCTTTCTCCCTCATTCCA--TGTTCCTTGCCCTTCTCTTGGCTCACACTCATCATCGTGAGAGCCATTCAGGTGGCATAGTCCCTCTGCCTTCACCTTGTCACAGAATATCACAAAGTG
Bos taurus     GCCCTCCTTCCCCCGCCCACCCATCCGTGGCCCTTCTCTTGGCTCACACTGGTCACTCTGAGAGCCTTTCAGGTGGCACAATCCCTCTGCCTTCACCTTGTCACCGAATACCACACACTA

Homo sapiens   TGT-TGCATCATTCGAGGAGCTTGTATTGATTATATGCCATTGCCTCATTTCAT----TGTTCAGGAGGCACGTCCAAATCTCAAGGCATGGGAGTGAGGGGTGGAGTGAGGCTCCTGGT
Bos taurus     TGTGCGCCTCATCCG-GGAGCTCACAC---ATACACGCTGTTGCCTCATTCCGCACCATATGCAGAAGGCACATCCAAATCTCA-------GGTATAAGGGATAGAGTGAGGCCCTTGGT

Homo sapiens   TTCAGAAAGTG-CTCCACTCATTTGATGTTGAGTAACTGATATTTTCTCCTTTGAAG--TCTCTGGTTTATCTCCCCAGCTTTAGAATAAGGGCCCTTTCTCACACTACTTTATATTCA-
Bos taurus     TTCCGAAAGTGCCTCCACTCATTTGATGCCCAGTGACAGATGTTTTCTCTTTTGGGGAACCTCTGGTTTCTCTCCGCAGTCTGAGAACCA-ACCTCTTTCTCATATTCCTTTATATTCCC

Homo sapiens   --------GCATGTATGCAATTAATGCTTGGTGATAATAATGGAAATTCTATATATCTAAGGTAATACTTAACATCTTCTTCACAAAATGGACTCTGGTTTTTT-------TTCTTATTA
Bos taurus     AGTGTTCTGCACATGCCCAATTCATACTTGGTGATGGTAATGGCAGTACTGTGT------------------ATCTTCTTCACAGAGTGGACTCTTGTTCATTCTTCCCCTTATTTTTA

Homo sapiens   GTGGTTCATTCATTTTCCTAGTCATTTATTTACAAAAAAAAAATAACTCTCTCATTTAAGTCCTCTTGCATACTGCTGCCACATCAGTTTCCTCAAGACA----CTTGCTCAGTCCTCTT
Bos taurus     GTGGTCCAGTCATTTTCCGAGTTGTTTAGTT--TGTAAGTGTTTAACTCTCCCATTGAAGCCCTTT---------CCTCTGCGTTCATTTCCTCCAGACAGGTTTTCATGCAGTCCTCTC

Homo sapiens   CTATGGCTTCTAAT---TAATTTTTAAT----TCTATCAGACCCAGTGCCTCCTGTTATTAACAAAT--ACTTCCTGATAA----------------GAAGTATCTAGAAAATAAAACCT
Bos taurus     CTCTCTGTTTTCACCTGTGATTTCTAATTGGCTCTGTCAGACCCTGGGCCTCCTAGTCTTAATACTTTACCTTCCTGAAAACCAGTGTATAGCAAGTGAAATGTATAGCAAGTAAAACTT

Homo sapiens   -ACTCATGCACATTCTGAAAATCAACATAATT-CCCTTAAATGTAATTTAATGG--------TATAATGT----AGAAACAAAAAAGTTATTTATTTTAAAAGTAGTGTTT---------
Bos taurus     CCCTTATGTATATTT---AAATCAATGTAATTATCCTAAAATGTAACATAATGGAGAAACAAAATAATTTATTAAGAAAAGAAAAAATTCCCTATCCCATAAGCCGTGTGTGTATGAATT

Homo sapiens   --CAAAATGCAAGTGCTTGATGTGATTATAACT-AGGGAAATAACAACGTAGTCAGGTGTTTGCACTGAAATATAATTAGTAAGTTCAAATTAAGGAGGAATAAGAAGACCAGAAGCCCA
Bos taurus     TAAAAAATGCAAACATTTGA--ACATAATAACTCCAAGAAATAA--------TTAAATG-TTGCACCATAAT--------CAAGTTTGAATTAAGTTGGAGTAAGAAGATCAACAGTCT-

Homo sapiens   AGAAAATCAAAGAGCAATGGTATAGGTGGACAGTGAAATGAAACTAGTATTGAGTCAGATAATACAAGACTAGACAGAGAAAGTGGGGCCC----TTAATTGAAGTAAGGCTTAGAT---
Bos taurus     GGGCAATGAAAGGGCAGAGATATGGGTGGTCAGTAGAATGAAAGTAGTGTTGAGTCAAATAATGAAAGACCAGCCAGAGAAAGAGGAAATTCATATTAACTGAAGTAGGGCTAAGATGAA

Homo sapiens   GTTGTAAATTGAATTATGTCCCTCCAAAAGATATATGAAAATCCTAACCCCACGTACCTGTGAATGTGACCTGATTTGGAAACAGGCTCTGTCTTAGTCCATTTTGTGTTGCTAATAACA
Bos taurus     GTTATGGGCTGTATTGTGTCCGG--AAAAGATATATTAAAGTCCTAA-CCCAGGTGCCTGTGAC--TGATCTGATTTGGATATAGACT................................

Homo sapiens   GAATTCCTGAGACTGGGTAATTTATTTTAAAAAGAGGTTTATTTAGCTCAAAGTTCTACAACCTGGGAAGTCCAAGATCATCAGCCTATCTGGTTAGGATCTCATGCTGCTTTAGCTCAT
Bos taurus     ........................................................................................................................

Homo sapiens   GGCAGAAAGTGGAATGATGGATAATCAAGTGTGTGCAAGGAGACCACACATTAGAGGCAGCCATCCTTTAAACAGTCTGCTCTTGGAGCTGAGCAAGAGCAAGAATAGAAAGCTTATTCA
Bos taurus     ........................................................................................................................

Homo sapiens   AAGAAATAACAATAGGCCGGGTGCAGTGGCTCTCAACTGTAATCCCAGAACTTTGGGAGGCCATGGCAGGAGGATTGCTTGAGGCCAGGAGTTTGAGACCAGCCTGAGCAACACAGCAAG
Bos taurus     ........................................................................................................................

Homo sapiens   ACCCTGTATCTATTTAAAAAAAAAGAATAGGCCAGGCGTGGTGGCTCATGCCTGTAATCCCACACTTTGGGAGGCTGAGGTGGGTGGATCACGAGGTCAGGAGTTCGAGACCAGCCTGGC
Bos taurus     ........................................................................................................................

Homo sapiens   CTACCTGGCAAAACCCCGTCTCTACTAAAAATACAAAAAAAAGGAGCCAGGTGTGGCAGGCACCTGCAATGCCAGCTACTCGGGAGGCTGAGGCAGGAGAATCGCTTGAACCTGGGAGAT
```

```
Bos taurus    ........................................................................................................................

Homo sapiens  GGAGGTTGCAGTGAGCCGAGATCATGCCATTACACTCCAGCCTGGGCAACAAGAGCAAGACTCCATCTCAAAAAAATTAAAATAAAATAAATTAAAAATAAATTTTAAAAATAGAGAAAT
Bos taurus    ........................................................................................................................

Homo sapiens  AATAACAAAAATCTTCACAAAACTTGTGAAAGATATAAATGTCCAGGTACAGGAAGATCTGAGAACACTGAACAGAATCATCCCAAATAAGACTACTCCAAGGCATATAATAATCAAACT
Bos taurus    ........................................................................................................................

Homo sapiens  CTCAACTGTCCAGGAAAAAGAGAGGATCTGAAAAGCAGCAAGAGACAAGAAACAAATAAGGAGCTCCAACTTGTCTGGCAATAGACTTCTCAATGGCAATCATACAGGCCAGGAGAAAGT
Bos taurus    ........................................................................................................................

Homo sapiens  GGAATGACATTTTCAAAGTACTCCAAGAAAAAAACTGACATTCAAGAATATTGTATCCAGCAGAATTATCTTTCATATATGAAGGAGAAAGTCTTTCCCAAACAAACAAGCTGAGAGAGT
Bos taurus    ........................................................................................................................

Homo sapiens  ACACCACCACCAGACACATCTTACAAGAAATGCTAAAGAATTCTTCAGTCTAAAAGGAAAGAAGACTAATGTGCATAAAGAAAACTTTTGAAGGCATAAAAGTCACTGATAAAATTAAGT
Bos taurus    ........................................................................................................................

Homo sapiens  ACATGGACAAACCCAGAATACTTTATTACTGTAATAGTGACATACAGTATACTCACAACTGTGAAGCCCCAAGACATACCTATCAAAAACAATAATAGGACTGGACACAGTGGCTCATGC
Bos taurus    ........................................................................................................................

Homo sapiens  CTGTAAACCCAGTTCTTTGGGAGGCTGAAGTGGGAGGATCACTTGAGACAAGGAGTTTGAGACCAGCCCAGGCAACATGGTAAGATCCTGATGCTATACCTGGAGACATGAGACAGAGTG
Bos taurus    ........................................................................................................................

Homo sapiens  AGACCATATCTTTAAAACAAAACAAGACAAACAATAATGGCTAGCAACCTGTTAAGAGATAGGTAATATAAAGATATAAATTGAGACAACGAAAAGTCAAAATCTAGAGGAATGGAATTA
Bos taurus    ........................................................................................................................

Homo sapiens  AAGCATATAATTTTTTTCTGTGTGTTTCTGCCTTTGTTTCTACTCTTGTATTTGGGATCTAAGATAAGTTGTTATCTCTTTAAAATAACTTATTATATCTGAAGGTCTTCTTTGTAAGCC
Bos taurus    ........................................................................................................................

Homo sapiens  TCATGATAACTACAATGCAAAAACCTATAATAAATTCACTAAAAATAGCAACAAAGGAAGGCATACTATAAGAGAAAATCACTTAGCCACAGAGGTTTTTTGTTGTTGTTGTTTGAGATA
Bos taurus    ........................................................................................................................

Homo sapiens  GGGTCTCACTCTTTCACTCTGGCTGGAGTGCAGTGGTATCATCTCAGCTCACTGCAACCTCTGCCTCCCGAGTTCAAGTGATCCTTCCACCTCAGCCTCCCAAGTAGCTGGGACCACAGG
Bos taurus    ........................................................................................................................

Homo sapiens  TGTGTGCCACCATGCCTGGGTAATTTTTTGTATTTTTTGTAGACCTGGGTTTTTGCCATGTTGCCCAGGCTGGCTTGAACTTCTGGCCTCAAGCAGTCTGCCCACCTTGACCTCCCAAAG
Bos taurus    ........................................................................................................................

Homo sapiens  TTCTGGGATTACAGGCGTGAGACACCATGCCCGGCTGTATATAACAATTATAAATACCTATGCATGCAACACCAGAGCTCCCAGGTATATAAGGAAAACATTAATAGATCTAAAAGGAGA
Bos taurus    ........................................................................................................................

Homo sapiens  GTTAGACTGCAATACAATAATAGTAGGAGATTTTAACACCCCACTCTCAGTAATTGACACATCATCCAGACAGAAAGTCAACAGAGAAACGATGGAATTAAACTACATGCTAGATCTAAT
Bos taurus    ........................................................................................................................

Homo sapiens  AAGCCTAACTGATATTTACTGAACATGTAACCCAACTGCTACAGAATGCACATTCTTTTCATCAGCACGTGGGACATAAGAATAGACCATATCTTAGGCCACAAACAAATCTGAACAAAT
Bos taurus    ........................................................................................................................

Homo sapiens  TTTTAAAAAATATAAATCATATCAAGTATCTTTTCTAATCACAGTGGAATAAAACTAGAAAGCAATAACAAGAAAAACCTTGGAAACTACACAAACGCGTGGAAATTAAATAGCATGCTT
Bos taurus    ........................................................................................................................

Homo sapiens  CTGAACAACTAATTTGTCAGGGAAGAAATTAAGAAGGAAATGTAAAAATGTCTTGAAACTAATGAAAATAGAAAATGTGACATATCAAAATTTGTGGGATACTGCAAAAGCAGTACTAAG
Bos taurus    ........................................................................................................................

Homo sapiens  AAGGAAATTTATAGCAATAAACACCTATATCAGAAAAGTAAAAAGACATCAAATGCACAACCTAATGATACACCTCAAGGAACTAGAAAAGCAAGAACAAACCAAACTGAAATTAGTAGA
Bos taurus    ........................................................................................................................

Homo sapiens  AGGAAACAAATAAAGATTAGAACAGAAATAACTGAAATTGAGACCAAAAAAAAAAAAAAAAAACCCAGAAGAACAATGAAACAAGAAGTTGGATTTTTTTTAAAAGATAAACAAAATCAA
Bos taurus    ........................................................................................................................

Homo sapiens  GTCTTTAGCTGGACAAACAAAAAAGGAGAAGACCCAAATGAATAAAATCAGAAATGAAAAAGACATAACAGCTGAGACCTCAGAAATACAAAGAAACCATTAGAGACTTATGAACAACTA
Bos taurus    ........................................................................................................................

Homo sapiens  TGACAATAAATTTGAAAACCTGGAAGAAATGGATAAATTCCTAGATGCATGCCAACTACCAAGATTGGACCATGAAAAAATAGAAAACCTCAGCAAACCAATAACAAGTAACGAGATAGA
Bos taurus    ........................................................................................................................

Homo sapiens  ATCTGTAATAAAAAGTCTTCCATCAAAGAAAAGCCCAGGACCTGTTGGCTTCCCTGCTGAATTCTACCAAACATTTAAAAAGAACTAATACCAATCCTATTCAAATTCTTCAAAAAAAAA
Bos taurus    ........................................................................................................................

Homo sapiens  AAAAAAAATTGAAGAGGAGGGACTACTTCCAAATTCATTCTAGGAGGCCAGCATTGCCCTGATACCAAAACTAGACTAGGATACAAAATAAAAAGAAAACTACAGACCAACATCACTGAT
Bos taurus    ........................................................................................................................

Homo sapiens  GAGCATAGGTGCAAAAATTCTCAATGAAACACTAGCAAACTGAATTCAACAACACATTAAAAAGATCATTCACCATGATCAAGTGGGATTCACTCCAGGGATGCAAGGATGGTTCAACAT
Bos taurus    ........................................................................................................................

Homo sapiens  ATGCAAATCAATAAATGTGATATATCACATTAATATAATCAAGAACAAACACCATATGATTATTTCAATAGATGCTGAAAAGCATTTGATAAAATTCATTGCTTTTTGATAAAAACCCTT
Bos taurus    ........................................................................................................................

Homo sapiens  GTCAAAATTGGTATAAAAGGAACATACCTCAAAATAAGGGCCATATATGACAAACCTACAGCTAACATCAAACTGAAGGCCTTTTCTCTAAGGACTGGAACAAGATAAGAATGCCCATCT
Bos taurus    ........................................................................................................................

Homo sapiens  CACCACTGTTGTTCAACATAATACCCGAAGTCCTGGCCAGAGCAATTATGCAAGAGAAGGAAATAAATGGCATCCAAACCGGATAGCAAAAAGTCAGGTTAGTCTGTTCGCAGATGATAT
Bos taurus    ........................................................................................................................

Homo sapiens  AATCTTATACCTCGAAAAACCTGAAGACTACACCAAAAAAACTGTTAGTGCTGATAAACAAACTAAGTAAAGTTGCAGGATAAAAAATCAACACTCAAAAATTAGTAGCATTTATATACT
Bos taurus    ........................................................................................................................

Homo sapiens  TCAATAGTGAACAATCTGAGATAGAAATCAAGAAAATGATCGCATTTACAAAAGCTACAAAAAATATAAAATACTTAGGGAATTTAAATACTAAAAATTTAAACACCAAATTAAGTTAAA
Bos taurus    ........................................................................................................................

Homo sapiens  TTTAGTGTTTAAATTCCCTAGGCATTTTATATTTTTTAGTATTTTATTTATAATTTATAATTTAGCATTTAATTACTAAAGAAGTGAAAGACCTGTATAAGGAAAACTAAAACTCTCATG
Bos taurus    ........................................................................................................................

Homo sapiens  GAACAAATTGAAGAGGACACAAAAACACTGGAAAGATATTCCATTCTAATGGATTGGAAGAATTTATATTATTAAAATGACAATACTTCCCAAAACAATTTACAGATTCAGTGTAATCAC
Bos taurus    ........................................................................................................................

Homo sapiens  TATCAAAATACCAATGACATTCTTCACAAAAATCAGAAAAAAAAAATTCATGTGGTGAGCCCTTAATCTAATGACTCATATTCTTATAAGAAGTCCATATTTGCAAATTGACCAAGGGGTT
Bos taurus    ........................................................................................................................

Homo sapiens  GAAAAAAAAGAAAGAAAAATGCCCAAGTGAAAACACACACAGGGAGAATGCCATGTGATCACTGAGGCAAAGATTTGTATAACACATCTGCAAGCCGAGGAACACCAAGAATTGCTGACA
Bos taurus    ........................................................................................................................

Homo sapiens  AACACTAAAAACTTGGAGAGAGGCAAGGAAAATTATCCCATGGAGCTTTTTTTCTTTTCTTTTCTTTTCTTTTTCTTTCTTTTTGAGACAGTCTTGCTCTGTTGCCAGGCTGGAGTGCAG
Bos taurus    ........................................................................................................................

Homo sapiens  TGGCGCGATCTCAGCTCACTGCAACCTCTGGCTCCCTGGTTCAAGTGATTCTCCTGCCTCAGCCTCCCGAGTAGCTGAGATTACAGGCAAGCGCCATCACGCCCAGCTAATTTTTGTATT
Bos taurus    ........................................................................................................................

Homo sapiens  TTTAGTAGAGATGGGGTTTCACCATGTTGGCCAGGATGGTCTCGATCTCCTGACCTAGTGATGTGCCCACCTTGGCCTCCCAAAGTGCTGGGATTACAGGCATGAGCCACCACGCCAGGC
Bos taurus    ........................................................................................................................

Homo sapiens  CTCCCATAGAGCTTTCAAAGATAACATGGCCCTACTGATATCTTGATTTTGGATTTCTAGCCTCCAGAGCCATGAGATAATACAATTCTGTTGTTTTCCACTATTGAGGTTGTGGTACTT
Bos taurus    ........................................................................................................................

Homo sapiens  TGTTACAAGAGCCATAGGAAAGCAATATAAGTTACAAAACAAATTATGGACTGATAAAGGGAGAAAATAAGGAGGTATGATATTACAGATACACTTCCTTCATTGTGTTTCACTTTATTG
Bos taurus    ........................................................................................................................

Homo sapiens  TGCTTTGCAGTATTGAAAGTTTGTGATGACCCTGTGTGGAACAAGTCTGTCAGCACCATTTTTCTACAGCATGTGCTCACTTTGTGTCACATTTTGGTAATTCTTAAATTCTTAAACGTG
Bos taurus    ........................................................................................................................

Homo sapiens  ACACTCTGTGTCACATTTTGGTAATTCTTAAAATGTTTCAAACTTTATTGTTATATCTGTCATGGTGATCTGTGATCAGTTATTTTTGATGTTACTATTGAATTTGTTTTGGTGTGCCAC
Bos taurus    ........................................................................................................................

Homo sapiens  AAACCATGCCCGTTTAAAATGTCAGACCTAATCAATAAACATTGTATGTGTCCTGACTCCTCCATTGACCAGCTGTTCCCTCATCTCTTTTCCTTTCCTTGGTCCTTGCTATGTCCTGAG
```

```
Bos taurus     ........................................................................................................................

Homo sapiens   ACACAATAATATTGAAATGAAGCCATTTATAATACTACTACTACAGTGACCTCTATGTGTTCAAGTGAAAGGAAGAGTCACATGCCTCTCAGGTTCAATAAAAAGCTAGAAGTGATTAAG
Bos taurus     ........................................................................................................................

Homo sapiens   TATAGTAAGGAAGGCATGTTGAAAGCCGAGATAGGCCAAAAGCTAGGAGGCCTCTTGTGCCAAACAGGCAGGTGGTGAATGCAAAGGAAATGTTCTTGAAGGAAATGAAAAGTGCTACTC
Bos taurus     ........................................................................................................................

Homo sapiens   TGGTGAACATATGAATGATAAGAAAGCAAAAAGCTTTTTGCTGATATGGAGAAAGTTTGAGTGGTTTGGATAGAAGATCACACCAGCCACAGCATTGCCTTGAGCCAAAGGCTAATCCAG
Bos taurus     ........................................................................................................................

Homo sapiens   AGCACATCTCTTACTCTCTTCAATTCTGTGAAGGCTGAGAGAGGTGAGGAAGCTACAGAAGAATAATCGGAAGCTAGCAGAGGTTGGTTCGTGAGGTGTAAGGAAGAAGCCATCTCTATA
Bos taurus     ........................................................................................................................

Homo sapiens   AAATGCATCAGCAACAAGTGCTGATGCAGATGCTGCAGCAGGTTATCCAGATCTAGCTAAAATCACTAATGAAGGTGGCTACACTAAACAACAGATTTTCAATGAAGATGAAACCACCTT
Bos taurus     ........................................................................................................................

Homo sapiens   TTATTGAAATAAGATGCCATCTAGGACTTTGATTGCTAGAGAGGAGAAGTCAGTGCCTGGCTTCAAAGCTTCAAAGGACAGCCTGACTTGTTAGGGGCTTACGCAGTTGGTGATTTTAAA
Bos taurus     ........................................................................................................................

Homo sapiens   GTTGAAGCCAGTGCTCATTTATCATTCTGAAAATCCTAGGGTCCATAAGAATTATGCTAAACCTACTCTGCCTGTACTCTAGAAATGGAACAACAAAGCCTGGATGATAGCACATCTGTT
Bos taurus     ........................................................................................................................

Homo sapiens   TACAGCATGGTTTACTGAATATTTTAAGCCTACTGTTGAGACATACTACTCAAAAAAGAAAAAAAGATTTCTTTGAAAATATTACTGCTTATTCACAGAGCACCTAGTCACACAAGAGCT
Bos taurus     ........................................................................................................................

Homo sapiens   CTGAAGGAGAGGTACAAGGAAATTAATGTTTTCAAGCCTGCCAATACAATACACATTCTGTAGCCCATGGATCAAGGAGTAATTTTGACTTTCAAGTCTTATTATTTGAGAAATACATTT
Bos taurus     ........................................................................................................................

Homo sapiens   TTTAAGGCTATAGCTGCCATAGATAGTGATTCCATAGATAGTGATGGATCTGGGCAAAGTAAATTAAAAACCTTCTGGAAAGGATTCATCATTCTAGATGCCATTAACATTTGTGATTCA
Bos taurus     ........................................................................................................................

Homo sapiens   AGGGAGGAGGTGAAAATACCAACATTAACAGGAGTTTGGAAGAAATTGGTTCTAACACTCATCATTGTCTTTGAGAGGTTTAGGACTTCAGTGGATAGTGTAGCTGCAGATGTGGTGGAA
Bos taurus     ........................................................................................................................

Homo sapiens   AGGACTAGTATTAGAAGTGGAACCTGAAGATGTGACTGAATTGCTACAATCTCATGACAAAACTTGAACAGATAAGTTACTTCTTATGGGTGAGAAAAGAAAGTGGTTTGAGATTGAATC
Bos taurus     ........................................................................................................................

Homo sapiens   TACTCCTGGTGAAGCATTGTTGAAATGACAACAAAGGATTTAGAATATTAAATAAACGTAGTTGATAAAGCAGCAGCAGGTTTGAGATGATTGACTACAATTTTGAAAGAAGTTCTACTC
Bos taurus     ........................................................................................................................

Homo sapiens   TGGGTAAAATGCTATCAAACAACATTGCATGCTACACAGAAATCTTTTGTGAAAGGAAGAGTCAATCAAGGTGGCCAACATTACTGTTGTCTTATTTTAAGCAACTGCCACAGCCACCGA
Bos taurus     ........................................................................................................................

Homo sapiens   AACCTTCAGCAACCACCACATTGATTAGTCAGCATCGTTCAACATCGCAGGAAGCCCCTCTACCAGCAAAAAGATCACAACTCTCTGAAGGCTCAGATGATTATTAGCATGTATTAGCAA
Bos taurus     ........................................................................................................................

Homo sapiens   TAAAGTATTTTTAAATTAAGATATATACTTTTTTAGACACAATGCTTATGTACGATTAATAGACTACATAGTATAGTGTAAACATGACTTTCACATGCACTCGGAAGCCAAGAAATTTCT
Bos taurus     ........................................................................................................................

Homo sapiens   GTGACTTGCTTTATTGTGATATTTGCCTTATTGTGGTGGTCTGGAACTGAACCTGCAATATCTCTGAGGTAGGCCTATGTTTAAATGAGGTGGGCTTTGCCTTTTATTATCAAAATAATT
Bos taurus     ........................................................................................................................

Homo sapiens   ATCTGTAGTTTGGAATAGGGTGATGATGAGTTAAAGAGAAAACATCTTCTGTCTTGAAACCCCCTCCTTGTCAAAACAGATATTCATTCTCTAAAAGACTGGCACTGTGTCCTTTTTCAG
Bos taurus     ........................................................................................................................

Homo sapiens   GCAACAAGAAAGAGAAAGTAGATTGGAAAATGAGAGAAAATCAAGAGGCTAATGAAACCATGTGGTAGCCTTTTGGGACAGTGTCAGAGAGATAGAAAATCTCAGACAGTTCCCCAAATA
Bos taurus     ........................................................................................................................

Homo sapiens   AATAAATTCAGTGTATGATTTTTCCAGCCTACATATCATTACTAGCTTTGAAATGTTTTTAAACAATCTTTGTTGATGAAATTCAGTGAGATTTCTGCATTTACATTAGATTCCAAATCT
Bos taurus     ........................................................................................................................

Homo sapiens   GTTAAAAGGAGTCTCAAAACTCTGTTAGGTATTTGTAGTCAGTTTAATTGCATTTGTTATAACTGCAATATTATATTGAAGTGCTGTTCAACCAAATACATAGAAAATGCTGTAAAATAG
Bos taurus     ........................................................................................................................

Homo sapiens   TGTGTAGAACAATCCAAGTGAGTCAACTTTTGTGACAATGTCTTTAATCGATGTAAGCTTCCTTCTCTAACTTTCTTGAATTACTGACTTTTTTTCAGTATCATCCTATAAAAACTCTTC
Bos taurus     ........................................................................................................................

Homo sapiens   AGGTTTCCTATGTTTCAGTGTAGCTGATTTATACTGATTGTAGGTTTGGTTCAGGAGGACACTCAAAAGTCCTGCAGGATGTGGAAGAATTCTTTGTAGTGTGGGACTGTCCCACATATT
Bos taurus     ........................................................................................................................

Homo sapiens   TCAAGATGCCTAGCATCTCTGGCCTCACCCTCTGAATGGCATCGGCTTGAGGTGGTGGTGGACTTCATTGTTATAACCAGAAAGGTTCCCACAAGTTTACAAAATGCCCTGAGGGGCTGG
Bos taurus     ..................................................................................................................GGTTGG

Homo sapiens   GACTACCGGTCTACAGCGTGGTATTCAACACCCTTCACAACTGGATTCCTTACCAT-------GTGAAAACAAAGAGGCTATTTTCTTTTTTTGTCTTTTTAGACATTGGTGAAGACTGA
Bos taurus     TCCTACTGGTGTGCAGTGTGGTAGTCAGCACCCTTCACAACCAGTCCCTTTATCTTTACCACAGTGAAGACTAAGAGGCCATTTG------TTGTCCTTGTAGACATCAGTGAAGACTGA

Homo sapiens   GGAAGAAACAGCCCAGGCCGTTGCTGCAGAGAAGTGGTCACATCTGAGTCTGACTCGGAGGAACCTCTGTGGGAACTCAGCTCAGGAGACAGTTATGAGCCTCAGTCCGATGAGTAAGGC
Bos taurus     GGAAG---CAGCGCCGCTCCTTGTCTCAGAGGAATGGCCAGTTCCGAGTCTG---------------------------GCTCAGGAGGAGGCTACAGACGTCAGTCTCATGGGTGAGGA

Homo sapiens   CAGGCCTCTGGCTGGACCCCTGTCTGGACTCTCATTTTCCTGTCACTCACCCTGGCTTGGCCCTTGGGAGGCCACGTTTCCTACAGACCCACACCCTTCCTTTCCCTTCTTCTGGCCTCT
Bos taurus     CAGGCCTCTGACTGG-----TGTCCA-------CCTTTCCCGTCATTAACCCTGACTCAGCCATCATGAACCCACATTTCCTACA------CACACTTCTTTCCCCTTCTTCTGGGCTCG

Homo sapiens   GCCTCCAGACTTCT---AGCCCTTATCACTTTGCATCCTTCCCAATCACATTTTCTTCTGTTACTAA----TCAGTTTACTGATTGTTGTGGTTATCTTCTCTGATCATCTCTTCTAGTT
Bos taurus     ATCTCCACACTTCTCTCAGCC--TGTCATTTTGTATCCCTCACAATCACAGTACCTTCTATTACTAGACACTCAGCCTACTGATTCTTGCTGTT-----CTCTGATCATCCGTCTGTGTT

Homo sapiens   CCCTGATTCCTTCAGTGACTCCTTTGTATCCTTTTCCCCTTTTTTCTTCCCTGT-CAAGGATTCCATATAACTTGTC--TTTCAGATATCCCTTCATACTTGGAAGCCCCATGAACACCT
Bos taurus     CCCTGATTCCCTCAATGTCTCCT------CCTCTGCTCCTTTTTTTCCCCCTTTGCAAGGGTCCCATATAATTTGTCATTTTCACACATCCCTTAATATCTGGAAATTCCACAAACACCT

Homo sapiens   CTGTCTGACCTTGTCTTTACCTCAGAGGAGGTCATCTTCACTCTCTAAGAACACGACAGTTCATGTTTATTTTATTTTTTTTAGTTTTTTTAAAGATGGGGTCTCGCTTTGTTGCCCATG
Bos taurus     CTGCCTTATTCCTTCTTCACATCAGAGGAAGTTCTCTTTACTCT--GAGAACACTAAAAGTTATGTG.....................................................

Homo sapiens   CTGGAGCATAGTGGCACGATGATAGTTCACTGCAGCCTCAAACTCCTGGGCTCAACTGATCCTCCCACCTCAGCCTTCTAAAAAGCTGGGGCTACACCCAGGTAATTTTTTGTAGACACG
Bos taurus     ........................................................................................................................

Homo sapiens   GAGTCTCGCTATGTTGCCCAGGCTGGTCTTGAACTCCTGGCCTGAAGCAGTCCTTCCACCTTGGCCTCCCAAAGCACCCAGCCTAATTTTCTTGAAATGTGCTCTGCCCTGTTGGGCCCT
Bos taurus     ....................................................................................TAATTTCTTTAAATAGTCTTTGTCCCATTAGGCCTT

Homo sapiens   CTCTGCAGCTGGGCTTTGGTGCCTTAATTAACCTTTCTTATCATTTTCCCATACCATGCCACATTTTGTAGGATTGCTGAAACTGTGCACCCTTGAAACACCAATATGATTTGTTTTTTG
Bos taurus     CTC----------CCCTGGA-----AACTGACCTTTCTTACTATCTTCCCACACTCCACCACGTTTT-TAGGATTGCTATGAGTATGTTCC--TGGGACACCTGT------TGATTTTTG

Homo sapiens   --TTTGATTTGGAACATC-TATTCCCTACCCTGGAAGTTTTTTTTTTTTTTTTTTTTTTTTTTTTCAAAATTCACATAACAGATGACATTTAAATCATTAATGTTATATTTTAGCTGAA
Bos taurus     GTTTTAATTTGAAACATCCTATTCCCTACCCTGGAGATATTTTCT----------------------AGATTCAGGTGGCA-ATGACATTTGAATTATCAATGTGTTATTTTAGCTGAA

Homo sapiens   GAAATTGTAACTAAAGATAGATTGTTTAAAGCAAAGCAAGAAACTTCTGAAGAAATGGAACAAAGTGGAGAAGCCTCAGGAAAGCCCAACAGGTGAGTGTCCATGGTCCTCAGTGAATCA
Bos taurus     GAAGTTGTAACTAAAGATGGATTTTTTAATGCAAAGCAAGAAACTTCTGAAGAAATGGAACGAGGTGCTGAGGCCTCAGGAATAGTGAACAGGTGAGTGTCCAC--------GTAGAATA

Homo sapiens   AATCCTGCAGATATAAGAAATTGGAATGGAATCAGAGAAACCAATTGGTCAAAGGCCTGCTTCTCTGGAATAAGATATGACAACTGCCACTCACTTCTGTGATCATAGGAGG-TCCATGC
Bos taurus     GCTCCTACAAAAGTAAAAAATTGGAATGAAACCAGAGAAACCAGTTGGT-AAACGTCTGCTTCCCTGGAATAATACAGAAAAAGTAACACTCACTTCTATGATCAGAGGAGGCCCCACGT

Homo sapiens   CAAGAAAGTGACCTATTCAAAATTTTCAATATGCAATAAATATACAAAACCTTCAGTTGATACTCAGTTTCCTCCAAGAAGCCTATCCTGATCCAGTCTGTCCTGCTTGATTACCCTTCC
Bos taurus     CAAGACACTGACCTG----------TCGGTATATCATAATTACACAAAACCTTCAGTTGGCACACGGTTGCCTTCAAGAATCCTCT-------CAGTT--CCTTGCCTGATCACTGTTCC

Homo sapiens   CTGGGCTCCCTTACATTTTTATGGAGCTGTGGA----GAAGCT---------------AGAAGGCAGATAATTTA---ACAAGAATGAGGGCTACAGATGAAGC---TGCCATGACAGTT
Bos taurus     CCTGGCTCCCTTATGTTTTTGTGGAGCTATAGATACTGAAGCCAGAAAAAGAAGATAGAGAAGGCAGAGGATTGAGATAGGAGAGTGAGGGCTGCAGATGAGGCTGTTGTCATGATGGTT

Homo sapiens   AAGAAAGAAATGTCAGAG-TGGAAGAGGAC---AAGCAGG-GTACAGGTTGAGCATTCCAAATCTGAAATGTTCCAAAATCTGAGCTGTTTGAGCACCAGCATGATGCTCAAAGGAAATG
Bos taurus     ---AAAGAAATATCAGATCTGGAAAAGGACTTAAGACAGGAAGATAGGTAGGGTATTTTCAGCCT.......................................................

Homo sapiens   CTCATTGGAGCATTTTGGATTTTGGATCTTTGGTTAGGGATGCCCAACTGGTATAATGCAAATATTCCAAAATCTGAAAAAAATCCAAAATTCAAAAATCTTCTGACCCCAAGCATTTCA
Bos taurus     ........................................................................................................................

Homo sapiens   GATAAGGGAAAATTAACCTGTATTTTCAACCGCCATTGGGAGACCTGAACAAGGTATAGGTCAAAAGGAAAGTTAGCTGATGAAGTAGAAATGAATGACAGTGGTGTGTGCTTCATAAGT
```

```
Bos taurus     .....................................................................................TAGATTCAGGTGGCAATGACATTTGAATTATCAAT

Homo sapiens   GCTGGTTGTGGGAATGAATGAACTTGTAAC-------AGATACTCCTATGCAAAGCAAGAAACTTCTGAAGAAATGGAACAAGGTGGAGAAGCCTCGGGAATGCCCAACATTCCGAGGCT
Bos taurus     GT--GTTATTTTAGCTGAAGAAGTTGTAACTAAAGATGGATTTTTTAATGCAAAGCAAGAAACTTCTGAAGAAATGGAACGAGGTGCTGAGGCCTCAGGAATAGTGAACA..........

Homo sapiens   TCTCAGGATTGCAGAAAGAGAGGGAACAAATCACAAGCAGAGGGTTGGCTTCTGGTGAGGCCATTTGGTCCTCAGAGACTTGAG---AAAAGAGAGAGTAAGCAAGGTGGGCTACTTG-G
Bos taurus     .............GAAGCAGAAAGACCAGATCATGAGCAGAAGGTGGGCTTCTGGTGAGACCACCAACTCCTTGGAGACTTGAGTAAAGAAGAAAAAGGAAACAGCTTGGGATAATTATA

Homo sapiens   CCAAAGAGCTGAGAAGGGGGTGTGAATTTTCCTTATTGACATTTGTTTAACTTAGAGAAACTGGGAGCTAAATCTCAGCTAAAGTAGAAATGTCTTAAAGCTCAAGAGGAGAAGTGAAAT
Bos taurus     TTGAAGAGCAGGGCAGGGGATGAGAATT---CTTCTTGACATTTGTTCATCTTAGGCAAACAGGGACCACAATCTCAGTTAAAAGAGAAACGTCTTAAAGCTT-----GAGAAGTGAAAT

Homo sapiens   TTTGCATTAAGATTAGTGAAGACTGAGCCAGGCTCCACTAAAGGAAGAGATATGATGTTCAGTAACTTAGAGCAGAGTTATGCAACTGGAGAATAGGAATCTGCTATTGTGCCTGT-AGG
Bos taurus     CTTTCATAAAGCTT-GTGAAGTCTGAGCCAAGCTGTACTAAACGAGAAGTAACA-----CTGTGACTTAGCTCAGGACTAT--AATTAGATAATACAAATCTGCTCATGTGTCTGTCAGG

Homo sapiens   TACATTTAGTATCAGGCAACTAACA-GGTAATTCATAGTGCCT---ATAATAAAGATA---------TTCATATAACAAAAAGTGTGAA--GGTAGGCTATTTCAGGTTAGGTGCAATAG
Bos taurus     TACATTTA-CATCAGATAACTGAAACCATGACTCACAGTGCCTCTGAGAATCAAAGTATCTTACTGTTTGATGTGACAAGAAGTCTGAAGGGGTAGGCTGTTCTAGGTTTGA------GG

Homo sapiens   CTTAACCATGGTATCAAAGGTCTAGC--CCCCTGCCCTCCTTCTAGTCCCCCTCCCTCACCCCCACCCCACTTTCTTAGCTCACTCTTTTTTCCCATTTCTCACTCCCATTTCCTTCCCT
Bos taurus     CTCCATCATGATATCAGAGATTGAGTGACCCCCAACATCCT...............................................................................

Homo sapiens   CACAATATTGGGCTTAATGCACTGTGGTTCTTAGAGTTTAGATGTCAACAGTAGAAAGACTCAGTGTGAT--------------CAAGGAATGTCACTGAAGTTATGGATGTCAGGGAAC
Bos taurus     ..........................GCTTTTGGAGTTT-----TCAGCAATGGAAAG-TTCAGTGTAGTGAAGTGACAGGATTCAAGGAGGATCCCTGAAGTTGAGGATGTCAGGGAAC

Homo sapiens   T-TTAAGATGGATGTGTTGAATACATGCAGACACTGA-----AGACACCCTGGATGATAAACACTTTACATAAAGAAATGTATGTAGTGTAAATTATATTAAAATATAC--TCTTCA-TG
Bos taurus     TCTTAAGATGGATGTGTTGAATGGATGGGGTCACTGAGAGTCAGACACGACTGAGCATAAATGCTCTAAGCAGATAAACATATGTACTGTAAATTATATTACACCATACGTTTTTCACTG

Homo sapiens   CTTCTTAA------ATGTCTTTGAAAACTTGACTAGTTTACCAACAGAACACTAATAAATTAAATGCACATGTCTAAAAATTAAAAACTTTGTGTAAAATAATACTTTTCAGTTGGGTGC
Bos taurus     CTTTTTTCTCCCTTATGTCTTCTTAAATTAGACCA-TTTACCAACAGGACATTAGTAAATAAAACGCAGATTTCTAGAATTTAAGAAGCTTGTATGAAGTAATAG...............

Homo sapiens   AGTGGCTCATGCTTGTAATCCCAGCACTTTGGGAGGCCGAGGTGAGTGGATCACGAGGTCAGGAGTTTGAGACCAGCCTGGCCAACACAGTGAAACCCTGTCTCTACTAAAAATACAAAA
Bos taurus     ........................................................................................................................

Homo sapiens   ATTAGTTGGGCATGGTGGCACGTGCCTGTAGTCCCAGCTACTCGGGAGGCTGAGGCAGGAGAATTGCTTGAACCTGGGAGGCAGAGGTGGCAGTGAGCTGAAATCATGCCACTGCACTCC
Bos taurus     ........................................................................................................................

Homo sapiens   AGCCTGGGTGACAGAGCTAGACTCTGTCTCAAAAAAAAAAAAAAAACAAAACAAAACTTTTCATCTTTGTAACTTTAGCTTACTTCCTTGTAAACCCCATAAAAATCAGTCCAAGTGCAAT
Bos taurus     ........................................................TTTTCATCTTAGTAACTTTAGCACACTTCTTTGAGAAACCCATAAAAATCAGTTCAAGGGCAAT

Homo sapiens   GAGT-TGAGTTTCA--TTTGTTTTTCCCCAGAGTAGTATGTCCATCAGATGAATGTAGAACTAGCAGGGGCTAAGTCCTTCCATGTTCAGAAATTCTGTGAGAGATGTCAGAAGACCTGG
Bos taurus     CAGTCTGAGTTTCTCTTTTGTTTCTCCCCAAAACAGTGTATCCACCAGATGAATGCAGAACTAGAGGAGGCTAAATCCTTCTTTGTTCAGAAAGTCTGTTAGAGATG--AGGGGGCTCAG

Homo sapiens   TAAATAATAACCTTTTTTTCTTGTTCAGATTCAGAACCAATTGACAGGGATGCAGGAGTAAAATAGATGGTTCTGGAAACCTCAG-TTTCTTACTGACAGAAGTTAGACTGGATAAAAAC
Bos taurus     TAAATAATCACCCATTTCT-TTGTCTAAATTCAGAACCAATTGATAGAGACATAGGAGTAAAACAGATAGT-CTAGAAACCTCAGCTTTATGACTGAGAGAAGCTAGACC--AATAAAGC

Homo sapiens   GGCTTAAAGGCAGAGCTAAATAGGCTTGTACTGGCAGAGGAGGAGTGTAGCAGATGTCCCTCTGCATAGGTCTTCTCAATGCAACTTGG-GGCACCAAGGAGCATGGGATGTCTGCATCC
Bos taurus     CGCTCAGAGGCAGAGCTAAATGGATGCATAGTGGC----------TGCATC------CTTTCCGCATAGGCCTTCCTGATCCATCTTGGCTGGGGGAAGGAACATGGAATGTCTGCAGCC

Homo sapiens   TGCTCCTAAAGAGGACAGAGGGAGGAGTGTACCAAGGATACTCAGTTATTTCTCCCAGGCTCAACAGTTAGTTCCATCCTGAGGAGTGAGTGAGAGGTGGAGAGTGGCTAGAAATGTTTA
Bos taurus     TGCTCAGAAAGAGGACAG----------------------CCCAGTTCCTTCTCCCAGATTCAACAGTTAA----GTCATGAGGAATGAGTTGGAAGAAGGGAG--GCCAGAAATGCTCA

Homo sapiens   ATCTAGCCAAAGTCCTCCCCATGGAAACTTAAGAGAAATAAGTAAGTATCCCCCCACACCCACCCCAAGCCCCAAAAGA---TTTAAGCTTCAGCAGATTTATCATGTGTGAGAGAATAT
Bos taurus     GTCTGGCCAGAGCCCTCCCCACAGAAACACGAGTGAGA------AGTATTCCCCA-----------------CAGAAGAATCTTTAGGTGTCAGTGGATTTATCCTGTATCAGTAAATAA

Homo sapiens   GATTGAGGATTTTTATAACATGTTCTTTTTTTCTTTCCCAGAGAGTGTGCACCCCAGATTCCTTGTAGTACTCCTATTGCTACTGAAAGGACAGTTGCACATTTGAACACTCTGAAGGAC
Bos taurus     GACTGAGAATTTTTACAGCCTATG--TTTTTTCTTTCTCAGAGACTGTGTACCCCAGACCCCTTGTGATACCCCTGTTCCTGCTGAAAGGACACTTGTGCATTTGAACACTCTCAAGGAC

Homo sapiens   CGTCACCCAGGTGATTTGTGGGCCCGCATGCACATCTCATCCCTGGAATATGCTGCAGGAGACATTACCCGAAAAGGGAGAAAAAAAGACAAAGCTCGAGTGAGTGAACTGCTCCAAGGC
Bos taurus     CGTCATCCGGGGGACTTGTGGGCTCGGATGCACATCTCCTCCCTGGAATATGCTGCAGGAGACATCACCCGGAAAGGGAGGAAGAAAGACAAAGCTCGAGTGAGTGAACTGCTCCAAGGG

Homo sapiens   CTCTCATTCTCTGGTGACTCAGATGTGGAAAAAGATAATGAGCCTGAGATCCAGCCTGCTCAAAAGAAGTTAAAGGTATCATGTTTCCCAGAAAAGAGTTGGACCAAAAGAGACATTAAA
Bos taurus     CTTGCATTTTCTGGTGATTCAGATGTGGAGGAGGATAACGGGCCTGAGACCCAGCCTGCTCCAAAACGGCCAAAAGGGCCAGGTATCCCAGAGAAGCACTGGACCAAAAGAGATATTAAA

Homo sapiens   CCCAATTTTCCAAGCTGGTCAGCACTGGATTCTGGACTTTTGAATCTCAAGAGCGAAAAGTTGAACCCAGTAGAGCTTTTTGAATTATTTTTTGATGATGAAACATTCAACTTAATTGTC
Bos taurus     CCCAGCTTTCCATGCTGGTCAGCACTGGATTCTGGACTTTTGAATCTCAAGAGTGAAAAGTTGAACCCAGTAGAGTTCTTTGAGTTATTTTTTGATGATGAAACATTCAACCTGATTGTC

Homo sapiens   AATGAAACCAATAATTATGCTTCTCAGAAAAATGTCAGCTTGGAAGTCACAGTTCAGGAAATGAGGTGTGTGTTTGGTGTCTTACTTTTGAGTGGATTTATGAGGCATCCTAGAAGGGAA
Bos taurus     AGTGAAACCAATAATTATGCTTCTCAGAAAAATGTCAACTTGGAAGTCACAGTTCAGGAAGTGAGGTGTGTGTTTGGTGTCCTGCTTTTGAGTGGACTCGTGCGGCGTCCCAGAAGGGGA

Homo sapiens   ATGTATTGGGAAGTCTCTGACACCGATCAGAACCTGGTTAGAGATGCAATCAGAAGGGACAGATTTGAATTGATTTTCTCAAACCTGCACTTTGCAGATAATGGCCACCTAGATCAAAAA
Bos taurus     ATGTACTGGGAAACGTCTGATGCTGACCAGAACCTGGTTAGAGATGCAATCAGAAGGGATAGATTTGAATTGATTTTCTCATACCTGCATTTTGCAGATAATAGCCATCTAGATCAGAAT

Homo sapiens   GATAAGTTTACAAAGTTGAGACCTCTCATAAAACAAATGAATAAAAATTTCCTCTTGTATGCTCCCCTGGAAGAATACTATTGCTTTGATAAGTCAATGTGTGAATGCTTTGATAGTGAC
Bos taurus     GATAAGTTCACAAAATTGAGGCCTCTCATAAAGCAAATGAATAAAAATTTTCTCCTATATGCTCCCCTCGAAGAATACTACTGTTTTGATAAGACAATGTGTGAGTGCTTTGATAGTGAC

Homo sapiens   CAATTCCTGAATGGAAAGCCTATTAGAATTGGCTATAAAATTTGGTGTGGTACAACCACACAGGGTTATCTGGTTTGGTTTGAACCCTATCAAGAAGAATCAACTATGAAGGTAGATGAG
Bos taurus     CAATTTCTGAATGGTAAATCTATTAGAATTGGCTATAAAATCTGGTGTGGTGCTACCACAGAGGGTTATCTGGTTTGGTTTGAGCCTCATCAAGAAGAAGCAACTCTGATGGCAGGTAAG

Homo sapiens   GATCCTGATCTTGGGTTAGGTGGAAATCTAGTGATGAACTTCGCTGATGTTCTTTTAGAGAGAGGTCAGTATCCCTATCACCTGTGTTTTGATAGCTTCTTTACAAGTGTCAAATTGTTG
Bos taurus     GATCCTGATCTTGGTTTCGGTGGAAACCTCGTGATGAATTTTGCTGATGTTCTTTTAGCAAGGGGTCAGTATCCATATCACCTGTGCTTCGATGGCTTCTTTACAAGTGTCACATTGGTG

Homo sapiens   TCAGCCTTGAAAAAGAAGGGGGTGAGGGCAACAGGAACAATTCGTGAGAACAGGACCGAAAAATGTCCCCTTATGAATGTAGAACATATGAAAAAAATGAAGAGAGGGTATTTTGATTTC
Bos taurus     TCAGCCTTGAAGAAGAAAGGGGTGAGGGCCACAGGGACAATTCGTGAGAGCAGGACTGAAAAATGCCCCCTTATGAGTGCAGAACATATGAAAACAATGAAGAAGGGGTACTGTGATTTC

Homo sapiens   CGAATAGAAGAAAACAATGAGATAATTTTGTGTCGTTGGTATGGGGATGGCATTATCAGTCTGTGCTCCAATGCTGTGGGCATAGAACCAGTCAATGAGGTAAGCTGTTGTGATGCTGAT
Bos taurus     CGAGTAGAAGAAAATGATGAGATAATTTTGTGTCGTTGGCATGGTGATGGTATTATCAGTCTGTGTTCCAATGCTGTTGGCATAGAGCCAGTCGATGAGATACACTGTTT---TGCCAGT

Homo sapiens   AACGAAGAAATCCCTCAGATAAGTCAACCATCCATAGTAAAAGTGTATGATGAATGCAAGGAAGGTGTAGCTAAAATGGATCAAATTATTTCGAAATACAGGGTGAGGATAAGAAGCAAG
Bos taurus     GACAAGGAGATCCCTCAGATAAGTCAACCATCCATAGTAAAACTGTATGATGAATGCAGGGAAGGTGTAGCTAAAATGGAGCAGATCACTTCCAAATACAGGGTAAGAATAAGAAGCAAG

Homo sapiens   AAATGGTACTCAATTTTGGTGAGCTACATGATTGATGTAGCCATGAACAATGCATGGCAACTACACAGAGCCTGTAACCCAGGTGCTTCTCTAGACCCCTTGGATTTTCGGAGATTTGTT
Bos taurus     AAATGGTACTCAGTTCTGGTGAGCTATATGATTGACATAGCCATGAGCAACGCATGGCAGCTACACAGAGCCTGTAACCCAGGCGCCTCTCTAGACCTCGGGGATTTTCGGAGATACGTT

Homo sapiens   GCACATTTCTACTTGGAACACAATGCTCATCTGTCAGATTAGGGTACATAAAATGGACATAGTGCAGACATTAATAAGACATAGAAAAATAATAATTATACATGCTGTTGTACCCTCCCA
Bos taurus     GCACATTTCTACTTGGAGCATAATGCGAATATGTCAGATTAAGGCAGGTGAAACAGACACAATGAAGACATTAATAAAACACAGAAAAGTCACAGCTATACAGCAGATTGTA----CCCA

Homo sapiens   AAGTAAATCTGATATATGTAATGAAGTTATTAAATAATACTT---TTAAAAATCAGACATTTATATAGAGTTTCAAAGACTATTGTAACAAGTAATGTTAAAAATTGTCTGTGAGAATGT
Bos taurus     AAGCAAACATGATGTATGTATACAATTAAATAACTAATATTTCTGTTAAAA-----------ATGTAGAGTTTCAAAAAC--TTAGAACAAATGA------------TCTGTAAAAATGT

Homo sapiens   TGAA---CTGTAGTACCTTTCTCTATGTCAAGTTTTGTGTCAGAC--ATGGGAAATCATGTATTTGTTCAATTGACTACTTTGTGCACTTATTTATTTATTTTTTGAGACACAGTCTCGC
Bos taurus     TGAAATCCATTGGTACCTTG-TGTATGTCTAATTTTGATATAAACATATGGGAAACCCTTTATTAGTTGCATTGAATTGATTATCCATTTA.............................

Homo sapiens   TCTGCGGCCAAGCTGGAGTGCAGTGGCACGATCTTGGCTCACTGCAACCTCTGACACCCTAGTTCAAGCGATTCTCCTGCCTCAGCCTCCCAAGTAGCTGGGATTACAGGCACGTGCTGC
Bos taurus     ........................................................................................................................

Homo sapiens   CATGCCTAGCTAATTTTTGTATTTTTAGTAGAGACAGGGTTTCACCATGTTGGCCAGGATGGTCTCGATCTGACCTCGTGATCTGCCTGCCTTGGCCTCCCAAAGTGCTGGGATTACAGG
Bos taurus     ........................................................................................................................

Homo sapiens   CGTGAGCCACCATGCCCAGCTATTTTGTGCATTTAAAGAAGGAAATCCTACCTCTTAAAAAAAATTATCTGGAGAATGCCATTTTTAAGATGCAAGCAATGTTACAGAAACCATAGAATG
Bos taurus     .................................................................................................AACATAAAAATAACCACAGGATG

Homo sapiens   GTGCTGACTCAATAGTTCAAACTAGTGACACAGCCTATGAAGTAAGAATGATCTAAACAAAACATAGGTGGTAAGAGACTAAAAACCTTAGCATTGGTGTAAAACTGGATCGGATTGTGT
Bos taurus     GCGCTGAATCCATAGCTGAAAATCAAGACAGAGCCCATGAGGTCAGGAAGATCTAAATAAAACA--GGTG-TCAGAGGCTGATGACCTGAGCATTGGTATAC--CCACTTCCGATTGTGT

Homo sapiens   GTAAGTGAGAAGGGTCAGGCATGGATATTGAAGGAGAGTGCTATAAAGGAAAAACCAGAGGTGAAGCAGTGCCTT
Bos taurus     GTA--------------GGCAGGGATGTTGAAGGAGTGTTCTGTAAAGGGAAAACTGGAGGTGAAGCAGTGTCTT
```

PNMA2 gene

>chromosome:GRCh37:8:26361617:26372208:-1
CCCCCACACCGACCTGGTCGTTGATGTTCTGCAGGGCTTCTTTGCCCGAGGCCCTCCGGA
TCTCCACCTTCCGGCCCGATTCAACAGATGGCTCTCCGCCGGCGCGCCGGCCCTGCCGCG
AGTACGGCGGCTGCGGGTCCGGGCCCAAGTGGGCGACGTCCCGCTGCTGAGCCACCACCT
GCCCCGAGCCCCGGCCCACCGACAGCGAGTCGAAGTCGATGGTCTTGTTCTCCGAGGACC
CTTCCACCGGGCAGAACATGCCACAGAATTTCTGCCGGGGCTTGGGGCTGCCGGAGCCCA
GGTTTTTAAAAAAAGCAGGGACCTCTTCGTCCTCTGCCGCCTTCCCGGATTGCTTTCTCT
CGGCCATGGCTCGGGGGCTCGGGTAGGGTCCTCGCCGTCCGTCCGTCCGTCTGCCAGTCC
CTAAGTCTCCCTCGCTGTGTGCACAAGCCCCCTAGCTGCTGCCGCGGCTGCCGTTCGCCG
CCCACCGCAAGGTGCACAGAAAGGAAGGAGGGCTGGCGAGCCGGCGACCCACCCTCCCGG
TCCCTCCCCCTGACCCCGCTTCTCCCCTCCCTGCCCACGCCCCCCGCGGCTACAGCGCGA
GCTCGGGGAAGCGGCAAAGTTGGATCTGGGAGGGCGTCCGCTGCGGCTGCGGGAGCCGAA
AGGCTGCCAGTTCCCGGCAGCGCTGGGAGGAGGGAGGCGCGGGCGGGGCGCGGCGCCGGG
GAGGGGAGCGGTGCTCAGGGGAGGGCTGGAGGGGAGGGAAGGAGAGAGAGAGGGGAGGGC
GGCACCGCCCCTAGCCCCGCGCTCCGGAAGTGAAGCGGCCAGACCACCAGCTAATGGATG
CGGAGCGGAGGGCCCGCTGACCGCTCTCCGCGCCTGGGTAAGTATCGGGCAGGGCACGCG
GAGTGAGGACCGGAGGACGCAGCCCCCAGCCCCAGTGCCCCTCTCCTCCACCTGCCTGGC
CCAGGCGCCTCTGCCCCGCCGCATGGCGGAGGTGGGCCGGGGGCGAGGGCGCACTGTGGA
TAGGGGTCCCGCCCCGTTACCCTGGGTGTGAGCTGATGGATGGGAAAAGGGGACAAGCCG
GGCGCCAGGCTACTCAGGGTGTGGGAAGCGGGATGGCACTGAAGATGAGCAAGGATAGCA
CAGGCAGGGTGGACAGGAGGGGGGCAGCCAAGACGCTGGACACCCCAGCTTCAGTTTGTC
CACTCGCCTTGGCAGGCTGCAGGGAGCAGAGACTGAAACGAGAAGGTTCCCGCTTCGTGC
ATCCACTTTGGTGTGGTTGGGGTGACGGGGGAGTGGGGGGGGGCACACAACATGGCGGG
AAGAAATTCAGAGGGGCGTAGCAGATGGGAGAGGAATATAGAGAGAGACGCAGGTCTGCG
GTCTATGGGGGTGTAGCAAAGAGTGCAGTCGGGAATTCCAGCCCAGGGCCACGACTTAAA
GACGGGGTGCAGACCGACTCTATTGCGCAAGGCGCCATGGACCGTGTCTGCGCCACTGCG
CCAAGAAGGGCGCAGGAACGTGTCGCGATTTGGTTTATTCTGCTTCCTCCACCAAAAAAA
AAAAAAAAAAAAAAAAAAGAGAGAAAAAACAAACTGAAACCCCCTTCCCAAATCTCCAGC
TTTCCCCTGCGCCCCAGAGCCCCGCCTGGTTCCCCCGCCCCTGTTGCGCACTGCGCC**ACA**
GTCGCAGCGTTTCCCGGGTTACCAGATGCCACGTGTTTGGGGAGTAAGTGCGGGTGCGCA
TCTGGTGCGGAGTAGGAGGTAGGATGTGGGCTGTGAGTGCCATGAATTGTGTGGGGCCAA
GCAGGGGAAAGGTGTGTAGGAACTTGGTGTGTGTGTGCAGTAGCTGAGCCACGGCCACCG
GGTTGGTAAGTAACCAGGGGGCAATTAGATTTGTGTGCCTAACACGGTCAATGCTGGTCC
TTCATTGCTTATTCCCATCTCCACGTCCTTTCCGCGTATCAGTCCCTGCCTCTGCGCGAT
GACAGCTCCGCCTCTCTTTCCACAGTGGCAAAATCCTGCAGAAATGGTGCCCCTCCTTTG
GTGGCCCGGGAGGGAGTTCTGGGCATGCTCAGTAACCAGGGTGGGTTTGGGCTGAGAGGC
CGTTTACCGCAGGGCCACCGCGTAGGGCCGTGTAACCAGGGATCCAGTCCCATTGTCAGC
CCAGGATTAAATGCGGTTACCAACCACCCCCACAGTCCAATTGTGAGAGATAGTTTGGTG
AATATAGTTACATCTGCTGGACAATCAGTCGCAATTTGAAGATCAGCCCATCTTGATAGG
TGGAAAGGCGTCTCCGGATGAGAAACAGACTGGGGAAGAAGAATATAGGAGTGGAGGGGC
TGAGAGAACCGGAGGGGGCATTTTGGACTTAGCGGCAGATGTGCTTTGCATTTGAATAAA
GCATTTGAAAAAAGCGCTTCCAACTGAATTACATCATTTCCTACTCACAGCCCCACTGTG
AGATGCGTGCCTTATCAGCACTGCTTTCCAGGTTAGGAAACAGATTTAGAGAGCTTGGGG
CGCATAGCCAAGGTTGGGTATTTAGTTAAATGATCCAGCAATTCCAGTGGGAGTCTTCCA
CCTCAAAGTCTGGCTTCTTTTCCAGCTTGTCCTGTGACCCCCTTTATGTGAGAAAATACA
AGGTTCTTCTATTTCTACCGTATCCAGATATTTCCATCCATGGCCCTATTGTATATCAAG
GGTGTTTCTTGCCATGTTTTTATTTGAAGGTAACACTGCAGGTCACACTGGGAGGAAGAG
TCATCTGAAGATAGCCTAACTTCAGCATAGCAGTCATTCTGTAGACCATAGAGATGCTTT
TTCCTGGGCAGTGTCTTTTTACATTCCCTGTTTCCTCTAAATATCCCAGTGGGGAGGAAG
GTTGCCCCCAGCTGGTTTTTTTATTTTTGTTTTTGTTTTCAG**ACAGAGTCTCACACTGTC**
ACCCAGGCTGGAATGCAATGGTGCAAACGCAACTCACTGTAGCCTTGACCCACTCGGCTC
AAGCAGTCCTCCCTCCTCAGCCTCCCAAGTAGTTGGGACTACAGGCGAGTGCCACACGCC
CAGCTAATTTTTTTATTTTTCCTTTTTGTAGAGACAGGGTCGCACTATGTTGCTGGTCTC
AAACTCCTGGGCTCAAGTGATCCTCCCGCTTTGGCCTCCCAAAGTGCTGGGTTTACAGGT
GTAAGCCACTGCACCTGGCCCTCCCCCACAGTCCCCACATTTTAGAAAGGAAAAGTCAGG
CATGGAGAAGTCAGCAGTCTCAGGTCTCTGGGGCTCCTGGGCACAGGGGTGAAGCAGCAT
TTTGCCCTCCTGGCTCTCTGGCACCCCTGGTGCCTTCAGCACTTCCCAGAAGACTCAGGA
GATCTTCCCACAGCAGGAGCACTGCTGATAGAGTTACAGGACAGCACTGTGGCGTATGGC
CCCCATTCGAATTGATGAAGATGTTTAGGCCAAGGGGGAAATGGTTGTAGCCCCTTAACA
CACACTCCGCAGCAGTAACTGGGAGGTATTAGGAACTCTAACTTTTTTTTTTTTTTTTTT
TGAGACAGAGTCTTGCTCTGTTACTCAGGTTAGAGTGCAGTGGTGCAATCGTGGCTCACT
GCAACTTCCGTCTCCCAGGTTTAAGCGATTCTCCTGCCTCAGCCTCCCAAGTAGCTGGGA
TTACAGGCACGTGCCACCACACCCAGATAATTTTTGTATTTTTGGTAGAGACGGGGTTTG
GCCATGTTGGCCAGGCTGGTTTCGAATTCCTGACCTCAGTGATCTACCCGCCTTGGCTTC
CCAAAGTGCTGGGATTACAGGCATGAGTCACCACGCCTGGCCAACTCTAACTTTTTTACG
TTCTCTAATCCTCACAGGTTCTCCAGAACAAAATCCTTGATAGGTGTGCTTCCTCCTTTT
TTCTTTTTTCTCAAATGATGGGTTAAAGTTCAAATGTTAGGAAAGGCTGGGTGTGGTGAT
TCAAGTGTGTAATTCAGCACTGTGAGAGGCTGTGGCGGGAGGATTGCTTGAGCCCAGGAG
TTTGAGACCAGGCTGAACAACGTAAGGAGACTGCGTCTCTACACAAAATTTAAAAAACTA
GCCAGGCATGGTGGCATGTGCCTGTGGTCCCAGGTACTCAGGAGGTCGAGGTGGGAGGAT
TGCTTGAGCCCAGGAGAGATCAAGGCTACTACAGTGAGCCGTGTTTACACCACTGCATTC
CAGCCTCGGCAACAGAGTGAGACCCTGTCTCAAAAAACAGAAACAGAAACAAATGCTTGG
AGAGTGTTGTGTTAGTAACTTATTGTCTTAGTCTATTTTGTGTTGCTATAAAGGAATACC
TGAGGCTGGATAATTTATAAAGAAAAGAGGTTTATTTGGGTCACAGTTCTGCAGGCTGTA
CAAGAAGTATGGTGCTGACATCTGCATCTGGTGACCCCTCAGGAAGCTTCCAGTCATAGC
AGAAAGCAAAGAGGGAGCAGATGCGGCAAGAAAGGGAGCAAGAGTGAGGGGAGGGAGGTG
GCAGTCTGTTTTTAACAATCAGATCTCGAGGAAGCTAATAGAACAAGAACCCACTCATTA
CCCCAAGAATGGCACCAAGCCATTCATAAGGGATCCACTCTCATGACCCAAACACATCCC
ACCAGGCCCCACCTCCAACACTGGGGATCACATTTCAGCATGAGATTTGGAGGGGACAAA
CCTTCAAACTATTTCACTTACCATTCATAGCAAAAGCAAAGCCCAAATGTGTGTTTGCAA
AGCCTTCCAGACACAACAAAAGAGAATTAATTCATAATTCCCGATTTAACTTAGTCGGCC
CTGAGATTTTTTTCCCTGAATTGTTATACCTAGATTAAACTATTTTTAAACGTTTGAGTG
ATTCCTCATTAGTTTTGTTAATCAAAGAGGTAAAGCAACTTAATATTGAGTAGTATTGTG
AAGGATTATCATATTTTTGAATAAGTGCATTAACGGTCTTCCTACTGAATATTAATGAAG
CATAAATACCTGTTCTTATTGTTTATGTGTAACAG**AGCAGCTTGGCTTGGCTGGAGCTAA**
GAGCCAGACACACCACTGTGTGGAGGTGGGTGATGTCTTCCTGTGCTAAAAGGTGAATAA
ATAAGCTCCTCACCTCTCGCGGAACACTCGGGAACACATCAACAGGTATTTAGCCACATC
TCCTGATTTCATCTTTCAATGGCAAAGACCTTGAATCCTGTCCTTAACTGGGGCTCAGAC
ATTAATCAACAGGTCTGATGAGCATATGGTGGGGACACCAGATTTCATTGGAGTCCCCAA
GAGAAAAAAAAGTCACAGTGAGCAAAGAAGGGGTCTGACAGAGTGGGACAAATGCAAGCG
CAGTGTAAAGCAGAACCGCCATCTGCCCCCAGTTCCATTCCAGATTAGAGTATGGATGCT
TCCAAGGAAAGAGAGTGCTGTGTAGTAAAACTGAACATTGTTTCACCAGGGGTCCAAGCC
GCCCTGCTGGGAGGCTTCTCTTCAAGAGTTCTGGGTCCCAGAGTGGAAGGCATTTTCCCA
TCAACTGGAGAGAGACGAAACATCAGAGACCAGGAGGCTGTGGAGAAAGCAGCTGTCCCA
GGTGCCTCAACTATCAGAGAAGGGTCAGCGTCACGTGGCTGCCAGCATCTTTGAGAAAAT
CACTGGCAATCGGACTTCAGAGCTGCGGGCACAGGTGTGGTTAGAACTGAGATACGACCT
GCCCACCTGGGTCAGGCCTAAAGACAAGAAGTCCTGAGTTCTTGCCACTGAGTAGGCCAG
GGTCATTTGTCCAGAAAACTTTGTGACTGTCTTTGAGTGACCTAGTCTGGGACCCATTCA
TTGGTGGGTTCTAAGGTTAGAAGCTCATCCAGGATATTTTCAATATTAAGTCAGTGCATA
GCTGCACCACTAACAAATTGGTGCCTGTAGAGTCAGAGTGGGTCAATTCTTAGGACAATG
GCGCTGGCACTGTTAGAGGACTGGTGCAGGATAATGAGTGTGGATGAGCAGAAGTCACTG
ATGGTTACGGGGATACCGGCGGACTTTGAGGAGGCTGAGATTCAGGAGGTCCTTCAGGAG
ACTTTAAAGTCTCTGGGCAGGTATAGACTGCTTGGCAAGATATTCCGGAAGCAGGAGAAT
GCCAATGCTGTCTTACTAGAGCTTCTGGAAGATACTGATGTCTCGGCCATTCCCAGTGAG
GTCCAGGGAAAGGGGGGTGTCTGGAAGGTGATCTTTAAGACCCCTAATCAGGACACTGAG
TTTCTTGAAAGATTGAACCTGTTTCTAGAAAAAGAGGGGCAGACGGTCTCGGGTATGTTT
CGAGCCCTGGGGCAGGAGGGCGTGTCTCCAGCCACAGTGCCCTGCATCTCACCAGAATTA
CTGGCCCATTTGTTGGGACAGGCAATGGCACATGCGCCTCAGCCCCTGCTACCCATGAGA
TACCGGAAACTGCGAGTATTCTCAGGGAGTGCTGTCCCAGCCCCAGAGGAAGAGTCCTTT
GAGGTCTGGTTGGAACAGGCCACGGAGATAGTCAAAGAGTGGCCAGTAACAGAGGCAGAA
AAGAAAAGGTGGCTGGCGGAAAGCCTGCGGGGCCCTGCCCTGGACCTCATGCACATAGTG
CAGGCAGACAACCCGTCCATCAGTGTAGAAGAGTGTTTGGAGGCCTTTAAGCAAGTGTTT
GGGAGCCTAGAGAGCCGCAGGACAGCCCAGGTGAGGTATCTGAAGACCTATCAGGAGGAA
GGAGAGAAGGTCTCAGCCTATGTGTTACGGCTAGAAACCCTGCTCCGGAGAGCGGTGGAG
AAACGCGCCATCCCTCGGCGTATTGCGGACCAGGTCCGCCTGGAGCAGGTCATGGCTGGG
GCCACTCTTAACCAGATGCTGTGGTGCCGGCTTAGGGAGCTGAAGGATCAGGGCCCGCCC
CCCAGCTTCCTTGAGCTAATGAAGGTAATACGGGAAGAAGAGGAGGAAGAGGCCTCCTTT
GAGAATGAGAGTATCGAAGAGCCAGAGGAACGAGATGGCTATGGCCGCTGGAATCATGAG
GGAGACGACTGAAAACCACCTGGGGGCAGGACCCACAGCCAGTGGGCTAAGACCTTTAAA
AAATTTTTTTCTTTAATGTATGGACTGAAATCAAACCATGAAAGCCAATTATTGACCTT
CCTTCCTTCCTTCCTTCCCTCCCTTCCTCCTTCTCTCCTTCTCTCCTCCTCTCTCCTCTC

```
CTCTCCTCTCTTTCCTTCCTTCCTTCCTTTTTTCTTTTTCTCTTTCTTCTTTATTTCTTG
GGTCTCACTCTCATCACCCAGGCTAGAGTGCAGTGGCACAAAAATCTCGGCTCACTGCAG
CCTTGACTTCCCAGGCTCAGGCTCAGGTGATCCTCACACCTTAGCCTCCCAAGTACCTGG
GACTACAGGCACGCACCACCATGCCTAGCTATTCTTTTGTATTTTTGGTAGAGACAGGGT
TTTGCTGTGTTGCTCAGGCTGGTCTGGAACCCCTAGGCTCAAATGATGTGCCCAACTCGG
CCTCCCAAAGTGCTGGGATTACAGGCATGAACCGCCATGCCTGGCCCTTGATTTTTCTTT
TTAAGAAAAAAATATCTAGGAGTTTCTTAGACCCTATGTAGATTATTAATGAACAAAAGA
TTAAACTCCAAATATTAAATAGTAAGCCTGAAGGAATCTGAAACACTTGTACTTCCAATT
TTCTTTAAATAATCCCAAATAGACCAGAATTGGCCCATACCATAGAAGAAAGAATTGGCA
GTCAAAAAAAAAAAATACCTTTTGTAATGTTTGAAAAATAAAGCTGTTTGACTTGTCAGGT
GTTTTCCTTTCTCAAATCAGCAAATTCTCTCTGAGTGCCTGGCTTTGTGAGACACTGTAC
AAGGAGTTACAAGACTACAGCTATAACCTGCAGTTGAGCAGTTATAAACCTACAAAATGG
GCCCTGCCCTCAGAGAGGTTCCAGTCTAGATGAGGAGCTGATCTAGACAGGTAAAAGGCT
AACTAACCCTTTGTGTAAATAAGTTCATCACCCCAGTAAAAGTGTCATCACCCAGTGAAT
AGGACCACCTCTGCCTGCAGATTTTTGTTGTTGTTGTTGTCATTGTTGTTGTTGTTTTAA
CCTGGGAAGTGTTCTTCCTGCCTTTCTGCTAGGTGTCAGATAGATGGTCCCAGAGCTAGG
TGCTGTGTCAGGCCCTGAAGACACAGATGACTCAACCTAAGCTTTACTTTCCAGAGGTCC
ACAGCCTGAGAGGTGTCCCCAAAGAAAGGGGGACATGAGGGGACTGCATGCTTGAGAGCA
GGGTTGTTTAGGGCAGGTTTGGATTTAGTGAGCAGGCTGGTTTGCTTAGAGAAGGCTTTT
AGTGGCAACAAAGGATGAAGAGGAGAGAAAAGGAACTCACATTTATTGAGGGCCTACTGT
GTGCAAAGTGTTTCATGTATATCTCATTGAATGTATACAGCCACCCTGTTGTGGTATAAT
TTTGCTCTTTATAAAGAGAAAGACCGAAGCTCAGATGAGTTAAGTGGTCTCCTCAACACC
AAAATGCCAAGAAGTGATGGAGCCTAGACAGAAGCCCAGAACTTTCTGACTCACACTAGT
CCATCCTCTACCATCACGATGACTTTCAAATTGTGCTCTGCAGTTCTGCAGATTTTCTAG
CAGTGCCATCTCCAAAATGTGTTTTAAACTCTTTATTTTTTTAATTATTATTAGTATTAT
TTTGAGACTGAGTCTTGCTCTATCACCCAGGCTGGAGTGCAGTGGTGCAATCTCAGCTCA
CTGCAACCTCCGCCTCCCAGGTTCAAGCGATTTCGTGCCTCAGCCTCCCGAGTAGCTGGG
ATTACAGGCACCCACCACCACGCCCAGCTAATTTTTGTATTTTTAGTAGAAATGGGGTTT
CACCATGTTGGCCAGGCTGGTCTCGAACTCCTGACCTCAAGTGATCCACTCACCTCGGCC
TCCCAAAGTGCTGGGATTACAGGTGTGAGCCACCATGCCTGGGCTAAACTCTTTAAGTCT
CTAGTAAATGCAGCTAGATTCAAATGGGCTGATAACCAAATTTTAACACATCAGCATTCA
CCACCAGGTTTACTTTTATTTTCAGATTGGCTCATTTTGTGCAGACCTTAGAGCAAAGTT
TCCTTTATGGTATCTGTGTACGTATCCAAACTTCTTTTAATTGTTCACAGATTTTAAAAG
CGGTAGCACCACATGGTTGTGTAGATCAGACCTGTGTATTTAGATCAGACCTGTGTATCA
CGTAAGTGTGTGAGTGCAGTGCAGATGAGCACCATTTAGTTATATGTGCTAGGCAAATCT
CCAACACAGTTGATGTGTAGTCTTGTGGTAGATTTGTGCATACTGTAAGCAAATTGCTTA
GCTTCTCTAGACATCAGTTTCCACATCTGAAAAATAAGAAGATGAGAGTACACGGTTGTT
ATGAACAAATGACTTAATGCTTTTTAAGCACGTTGCATGACATCTGGAACACAGAAAGCC
CTCAATACATTGAAGCTCTTAGGATTTTCACGATGTTCCTGTCTGCTCAATGCATGCTTT
CTTTATTGTTCTGACAGTTGTGTGGTAACAAGCTAATATGCTTCCAGTTGACTTCCAGTC
TACCCTGGTGTTAGAAACCGTTTCATCTCTTATTGTAAATTTGAGTGCTTGTTGTTTTTT
ATATTTGTGATGACTCTTCCAGCAGTTGTTGACAATTGTTAGAGGTTTGACTTTTAAATA
ATTACTTATTTTTTCTGATTGTGGTTCAGTTTAACTGAAGAATATCCTGAGATTGTAAGA
AAAGCATTTTTTAAAAGGTATCACTTGTGATCATTTATCTTTCTAAATTCTATTTTTAAT
ACTGTTCCACCAAAGTGATGCAGTGGTTACCATGACACCCTAATTTCATGTGTTTTTGTA
TTTATGAAAATAGTTTCATTGTCATTTATTGGCGGTATACAAAGTAAAATGTTATAAATG
TGAAGTTATAAAATAAATATATGCTAATAAAATCCTGAGTTTTTCTGTTTCCTATATCGT
GGTCTCTATAAAGTACTGCTTGAAGAAGGAGCTTTGTTACTGAAAAATTTTGAGAGATAA
GTTGAGAAAATCAAGCTAAAGCCAAAAGGTCTTAGAATCCTGTAGGCAAGAAAACCTGAA
AGCAAAAAAATGCTTGAACAAGAATACTTGAACTATGTCTCTGGGGACAAGGGAGAGCTC
GGTCCACCCATTATCGATTTCTCTTTTAAAATATACAGAAACAACAGATGGAGGTCAGTT
GCCTCAGAGACCCCCCAGCTCATTTGCTTAGGAAAGTAACTGTACAGATAAATTCCCAGA
CTCTATTCTGCCAAGGGTGAATGACCCTGAAGGTCGAATATTGCTTTTCTGGGCTGAAGT
CACCCTTTTTCTTCTTCTTCTTCTTCTTTTTTTTGAGACAAGGTGTCACCCAGGCTGGAG
TATAGTGGCACAATCTCGGCTCACTGCAAACTCTGCCTACCAGCTTCAAGTGATTCTCAT
GCCTCAGCTTCCCGAGTAGCTGGGATTACAGGGGTGCACCACCACGTTCCAGCTAAGTTT
TTGTATTTTTAGTAGAGACAGGGTTTCACTAT
```

```
Mus musculus  CGACATCGCGCTGCTGAGCCACCACTTGCCCCGAGCCCCGGCCCACTGAAAGCGAGTCGAAGTCGATGGTCTTGTTCTCCGAGGACCCTTCCACAGGGCAGAACATGCCGCAGAATTTCT
Homo sapiens  CGACGTCCCGCTGCTGAGCCACCACCTGCCCCGAGCCCCGGCCCACCGACAGCGAGTCGAAGTCGATGGTCTTGTTCTCCGAGGACCCTTCCACCGGGCAGAACATGCCACAGAATTTCT

Mus musculus  GCCGCGGCTTGGGACTGCCGGAGCCCAGGTTTTTAAAAAAGGCAGGGACCTCTTCGTCCTCTGCTGCCTTTCCAGATTGCTTTCTCTCGGCCATGGCTCAGGGGGCGCTGGGGAAGGTCC
Homo sapiens  GCCGGGGCTTGGGGCTGCCGGAGCCCAGGTTTTTAAAAAAAGCAGGGACCTCTTCGTCCTCTGCCGCCTTCCCGGATTGCTTTCTCTCGGCCATGGCTC-GGGGGCTC-GGGTAGGGTCC

Mus musculus  TGTCTGTTCGTCCGTCCGTCCGTGGTTTCCTAAGTCTCCCTAGCTGGGTGTGCACTAGCCTCTT-GCTGCCGCCGCGGCTGCCTCTCCCTGCCTACCGCAAGGTGCACAGAAAGGATGGA
Homo sapiens  TCGCCGTCCGTCCGTCCGTCTGCCAGTCCCTAAGTCTCCCTCGCT--GTGTGCACAAGCCCCCTAGCTGCTGCCGCGGCTGCCGTTCGCCGCCCACCGCAAGGTGCACAGAAAGGAAGGA

Mus musculus  GAGCTGGCGAACCGGCGACCCACCCCTCGCGGTCCCTCCTCCTGACCCCGCTTTCTCCCTCCCTGCCCACGCCCCCCGGCGCCGCCACGCCACCTCCG------AGCAAAGTTGGATCTG
Homo sapiens  GGGCTGGCGAGCCGGCGACCCA-CCCTCCCGGTCCCTCCCCCTGACCCCGCTTCTCCCCTCCCTGCCCACGCCCCCCGCGGCTACAGCGCGAGCTCGGGGAAGCGGCAAAGTTGGATCTG

Mus musculus  AGCGGGCGGTCGCTGCGGC-----------AGAGGATGCCAGTTCCCGGCAACGCGGGGCGGGGCGGAGTG-GGGCGGGGC---------AGGAGGAACGCAG-GATCAGGAGGGAGGGG
Homo sapiens  GGAGGGCGTCCGCTGCGGCTGCGGGAGCCGAAAGGCTGCCAGTTCCCGGCAGCGCTGGGAGGAGGGAGGCGCGGGCGGGGCGCGGCGCCGGGGAGGGGAGCGGTGCTCAGGGGAGGGCTG

Mus musculus  ACAAGGAGGGAGAGGACCCAGAAGGAGGAGACGGAGAAGGGAGGGTACGAGGGGGTGGGGGGCGCCCGGAG-CCTGCGCCCCGGAAGTAAAGCGGACACTCCACCCGCTAATGGACGCGC
Homo sapiens  GAGGGGAGGGA--------------AGGAGAGAGAGAGGGGAGGGC-----------GGCACCGCCCCTAGCCCCGCGCTCCGGAAGTGAAGCGGCCAGACCACCAGCTAATGGATGCGG

Mus musculus  GGCAGCCGCCCGACAGACCTCTCTCGGCGCCAGGGTGAGTGTCGGATAGCTATAGCCGGGCAAGGACCCGAAGGCTCAGACCCTAG-TCCAGAGCCCCTCTCCCCGACCTGCCT------
Homo sapiens  AGCGGAGGGCCCGCTGACCGCTCTCCGCGCCTGGGTAAGTATCGGGCAGGGCACGCGGAGTGAGGACCGGAGGACGCAGCCCCCAGCCCCAGTGCCCCTCTCCTCCACCTGCCTGGCCCA

Mus musculus  -------------CACTGCATTGCTAAGGTGGCCCTGGGGC---------------------------TCCCCTGGCCTCGGGTGAGAGCTGAGGGTTGAGGGAGCCAAGGTTCAAGCCT
Homo sapiens  GGCGCCTCTGCCCCGCCGCATGGCGGAGGTGGGCCGGGGGCGAGGGCGCACTGTGGATAGGGGTCCCGCCCCGTTACCCTGGGTGTGAGCTGATGGATGGGAAA---AGGGGACAAGCCG

Mus musculus  GA----AGGCT-CTTGGAAACTGGGCA--GGGGTGACCCTGGAAAC-AGCAAAGTCTACTCC------------------GGGATAACCAAGTATCCGG-CACCAATGCTTCAGTTTACC
Homo sapiens  GGCGCCAGGCTACTCAGGGTGTGGGAAGCGGGATGGCACTGAAGATGAGCAAGGATAGCACAGGCAGGGTGGACAGGAGGGGGGCAGCCAAGACGCTGGACACCCCAGCTTCAGTTTGTC

Mus musculus  CACGACCTGGGCGCCAGGGCGCGCACTGCAAGGAGCAGAAG--GAAAGGAGAATGTTCTCCCTGCTTGCAGCCCC-----TGTGGTTG---------------GGAGGTGGGGCTCACAA
Homo sapiens  CAC-------TCGCCTTGGCAGG--CTGCAGGGAGCAGAGACTGAAACGAGAAGGTTCCCGCTTCGTGCATCCACTTTGGTGTGGTTGGGGTGACGGGGGAGTGGGGGGGGGGCACACAA

Mus musculus  CATGGCGGGGGCGAAAAAGCAGAGGAGCATAGCAAATGCAAGCGGTGACCGGGGGAGAAAGCAGCAAGCCTGCGGTGCGGGGGGGGGGGGGGGGGCATAGTAAAGAATGCAGACTGAGG
Homo sapiens  CATGGCGGGA--AGAAATTCAGAGGGGCGTAGCAGATGGGAGAGGAA---TATAGAGAGAGACGCAGGTCTGCGGTCTATGGGGGTG-----------TAGCAAAGAGTGCAGTC-GGGA

Mus musculus  ATT--------GCGCTTCAGCATAGAAACTGGG----GACCCGTAACATCATCTGAAAAGGCCATGCCCTGCACATCAGCCATTGCCTCAGAAAAGGCTCAGAAGAGTCCTG-GATTTGA
Homo sapiens  ATTCCAGCCCAGGGCCACGACTTAAAGACGGGGTGCAGACCGACTCTATTG-CGCAAGGCGCCATGGACCGTGTCTGCGCCACTGCGCCAAGAAGGGCGCAGGAACGTGTCGCGATTTGG

Mus musculus  TATCTCCAA-----TCCCTCAAAACACACACACACACACACACACACACACACACACACACACACACACACCCAAAACGGAAAATGGTTGGGGACTAAGCAGACTGAAGCACACT-----
Homo sapiens  TTTATTCTGCTTCCTCCACCAAAAAAAAAA----------------------------------------AAAAAAAAAAAAAAGAGAGAAAAAACAAACTGAAACCCCCTTCCCA

Mus musculus  ------CATCTTGCTTTCAAGTC-----GCCCCGCCTAGCTTCCCCGCCCCTGTTGCACGCTGCGCAGGAG----------GCCCGGGAGTTTGCCAGATGTTCCTTCTCTAGGGCCGCA
Homo sapiens  AATCTCCAGCTTTCCCCTGCGCCCCAGAGCCCCGCCTGGTTCCCCCGCCCCTGTTGCGCACTGCGCCACAGTCGCAGCGTTTCCCGGG---TTACCAGATGCCACGTGTTTGGGGAGTAA

Mus musculus  GC---GGTTTGCATGGAGGGTAGAGGTAGATTGCTGTTCAGGGGAGGGTTACATGCTGTG-------TGAGTTGTGGGCAGCCAAA------------GTGTGGGGATTTGGAGTGTGTG
Homo sapiens  GTGCGGGTGCGCATCTGGTGCGGAG------------TAGGAGGTAGGATGTGGGCTGTGAGTGCCATGAATTGTGTGGGGCCAAGCAGGGGAAAGGTGTGTAGGAACTTGGTGTGTGTG

Mus musculus  CGCGCGTGCACACGGTTCAGCCACGACCGCCAGGTTCG----------------------------------------------AGGCTTTTCCTTGCTTAGTCCCACCTCCTGGTCC
Homo sapiens  TGCA-------GTAGCTGAGCCACGGCCACCGGGTTGGTAAGTAACCAGGGGGCAATTAGATTTGTGTGCCTAACACGGTCAATGCTGGTCCTTCATTGCTTATTCCCATCTCCACGTCC

Mus musculus  TGGCTTGGT---------TGCATTTA---------AGCCCCTCCTGTTTTTCCAGAGTGGCTGAAAGCTGCAAAAGTGTTGCCCATCCTTTAGGGGTTCTGGAGCGAGATCTGAGCATGC
Homo sapiens  TTTCCGCGTATCAGTCCCTGCCTCTGCGCGATGACAGCTCCGCCTCTCTTTCCACAGTGGCAAAATCCTGCAGAAATGGTGCCCCTCCTTTGGTGGCCCGGGAGGGAGTTCTGGGCATGC

Mus musculus  TCACTAAGATGGATGGGTTTGGTCTAGGAGAGGCAGTTTACTGCAGCGTGTTCTCACAGGGCGTTGTGACCAAGAAATCAGTCCCATTGTCAGCCTTTGCTTAGGTAACGATTGCTTGCA
Homo sapiens  TCAGTAACCAGGGTGGGTTTGGGCT--GAGAGGCCGTTTACCGCAGGGCCACCGCGTAGGGCCGTGTAACCAGGGATCCAGTCCCATTGTCAGCCC-----------AGGATTAAATGCG

Mus musculus  GATATCAACGCCC--------CCAGTTGCCCGAGATAGCTTGATTGATGGAGTCATAATTTGTGCGCACACACACACAATCCTGTAGGAGCCGAAAG-------GAGGGCCTATGGACCT
Homo sapiens  GTTACCAACCACCCCCACAGTCCAATTGTGAGAGATAGTTTGGTGAATATAGTTACATCTGCTGGACAATCAGTCGCAATTTTGGAGGGGCTGAGAGAACCGGAGGGGGCATTTTGGACT

Mus musculus  TAGCACCAGAAATGCTTGGAATTT----------TCTTATAAA---GTGTCCATTGGAATT--GTCAATGCCACACTAAAGCCC-----TGAG----GTCTTTCATCCTCCCTGCCTTCC
Homo sapiens  TAGCGGCAGATGTGCTTTGCATTTGAATAAAGCATTTGAAAAAAGCGCTTCCAACTGAATTACATCATTTCCTACTCACAGCCCCACTGTGAGATGCGTGCCTTATCAGCACTGCTTTCC

Mus musculus  AAGTTAGGACACCAGTTTATGAGTCATTACGC------CTAAAGTCATGTGTCTAGTT-----GTCCAG-------------GCCTTTGCCCCAAGAGCCTGGTTCCTTCAACAGGTTA
Homo sapiens  AGGTTAGGAAACAGATTTAGAGAGCTTGGGGCGCATAGCCAAGGTTGGGTATTTAGTTAAATGATCCAGCAATTCCAGTGGGAGTCTTCCACCTCAAAGTCTGGCTTCTTTTCCAGCTTG

Mus musculus  TCCTATAGGCCTCTTTA-GTGAGAAAAGTACAAAATCCTTGCA-TTCTTCCATACCCAAGCATTTCCCT-GGTGGTTCTTTTGTACATCAAGGGTGTTTCATTTACAGTCTTAAAATCTT
```

```
Homo sapiens  TCCTGTGACCCCCTTTATGTGAGAAAA-TACAAGGTTCTTCTATTTCTACCGTATCCAGATATTTCCATCCATGGCCCTATTGTATATCAAGGGTGTTTC...................

Mus musculus  TTTTGGTTAACCTTCATTTATGTATATGGATGTTTTGCTTGAATGTTTGTACATGCACTGTGTACAGGCCTGGGGCCATTGGCGGCCAGACAAAAGTGCCAGATTCCCCTGGAATTGAAG
Homo sapiens  ........................................................................................................................

Mus musculus  TTACAGAGTAAGCCAACTTGTGGGTGCTAGTAACCAAACCTGTGCATTTCACCACTAAGCAATCCCTCCAGATGTGTGAGGCCTTAGTTAATTGAAGAGCAACCCTTGTAGGTATGGTTA
Homo sapiens  ........................................................................................................................

Mus musculus  CCATTGGGTTAAAGGTCTCAAAGGCTAGCAGATTTCAGCATGACAATTTTTCTGTGAATCTGGACATTTGGTGTTTTTTGTTCATTTGTTTGTATTTGTCTTTTTTGTTTGTTCGTTCGT
Homo sapiens  ........................................................................................................................

Mus musculus  TTTCTGTTTGTTTCGGTTTTTCAAGAGAGGGTTTCTCCGTGTGACAGCTCTGGCTGTCCTAGAACATACTCTGTAGACCAGGCTGGCCTGGAACTCAGAGTTCTGCCTGCCTCTGCTTCC
Homo sapiens  ........................................................................................................................

Mus musculus  CAAGTGCTAGGATTACAGGCATGCGCCACCACTACCCAGCATGTCGCTTGTGTTTGTTTTTGCTCTTTCTGAGACAAGGTCCTGTTTGTAAGCCCAGGCTGACCTCAAACTCCCGATGTA
Homo sapiens  ........................................................................................................................

Mus musculus  GCCAAGGGAGTTAAACTCCTGGCTCACTTACCTTCACCACTTAAGTGCTGGGGTAATGGGTATGTGCCACGGCGCCTGGCTTATGCAGTGCTTGGGATCTAACTCAAGGCTTTGTACATA
Homo sapiens  ........................................................................................................................

Mus musculus  GTGACAGCACTCTGCCAACTAGGCTACAGTCCTAGCCCCTCCAGAGCTCTTCACATCTCATTCCTCGAAACAACTCCAAGCAGAGGAAGTTCACTCCCATATTTTACAAGGGAATC-TCA
Homo sapiens  ..................................................................................................CCCACATTTTAGAAAGGAAAAGTCA

Mus musculus  GGCCTAGATTGGTCTGAAGTTCCGGG-CTCCCATGCTGCCAAGCACAGGG-TGAGACAGCATCTTGCTCTCCTGGTTCT--GACACAGTGGAACAGCCTTCAACACCTTCCAGGAAGACC
Homo sapiens  GGCATGGAGAAGTCAGCAGTCTCAGGTCTCTGGGGCTCCTGGGCACAGGGGTGAAGCAGCATTTTGCCCTCCTGGCTCTCTGGCACCCCTGGT--GCCTTCAGCACTTCCCAG-AAGACT

Mus musculus  ----GGATCTGCTCCTG---------CTGCTGCCATAGTTA-AGACCAGCATCTGTGGCATACAACCTCC-TTCAGAGTGATGGGAGATGTTCAAACCAAGGAGGAAGTGGATATAGCAC
Homo sapiens  CAGGAGATCTTCCCACAGCAGGAGCACTGCTGATAGAGTTACAGGACAGCA-CTGTGGCGTATGGCCCCCATTCGAATTGATGA-AGATGTTTAGGCCAAGGGGGAAATGGTTGTAGCCC

Mus musculus  CCTAACACACCCTCTACA--AGTCTCTGAGAGGCACAGTCAGGCACCAAATTACAACATTTCAGTTGACATAACACATGTGCCCTATAAGAATGCATGGTCTAGTGATGGCACTCTGACA
Homo sapiens  CTTAACACACACTCCGCAGCAGTAACTGGGAGGTA.....................................................................................

Mus musculus  TTCAGCCAATTAGGAAGCGGCTTCTCTGGAGGCATCCTCATGACTAAGTGACCACACTGTCACTGGGAGCACTCCCTTTTACATTCTCCAGTCTCCCCAGCCCTGCAGAGCAAGCACGCT
Homo sapiens  ........................................................................................................................

Mus musculus  TGGTGCACACATCACTTTGCAGTGGATATGTGTTCTGTCTTTGCCCTTTTCTCAACGGCCCAGTTAAATTTAAATGACAATGTCAAGTTTAAATGTCTTAGTTACTTTTCTATTTCTCTG
Homo sapiens  ........................................................................................................................

Mus musculus  GCAGAGCACCATGACCAAGGCAGCTTATAGAAGAAAGCATTTGATGGGGGCTTACCGTCTCAGAGGATGAGTTCATAACCAGTGTGGGCAGGAATATGGCAGCAGGCAGGGAGGCATGGA
Homo sapiens  ........................................................................................................................

Mus musculus  GGTGGAGCAGAGAGCTGATATCCGATCCACAAGCACAGGACAGAGACTGACAGTGAGAAGGGCATGGCCTTCTGAAATCTTAAAGCCCACCCCCAGTGGCACACCTCCTCCAAAATCCTT
Homo sapiens  ........................................................................................................................

Mus musculus  CTCAAACAGTTCCACCAGCTAAGGACCAAGCATTTGAACACATGAGCTTATGGGGCCCGCTCTCCTTCAAAGCACCACAGGAAGAGCCGGGTTGGTAACTTACCATGCATGGCCCTTGAA
Homo sapiens  ........................................................................................................................

Mus musculus  AAGCCTGGCTAGACACAGCCACAGAAGACTGACTCCTAACTCCAAAGTGTACTTCTTTTTCTGAGATTTGAATTTTCTCCGTTCTACCCATTTTAAGTACTCGTAAGTGTTTGAATGATT
Homo sapiens  ........................................................................................................................

Mus musculus  CTGTTTAGTTTTGTTTGTGTCTTCTTTTGGAGACATGATTTCTCTATATAACCCAGATTGACTTCAAACTTGCAGTCCTCCTGCCTCAACTTCACAAATGCTGGGAGTGCTACCAAATAC
Homo sapiens  ........................................................................................................................

Mus musculus  AATTCAGTTGGATTTGTTTTTTAAAGATGGTTCTCATGTAGCCCAGGCTGGCCTCCCACTCCCTATGAGGTTAAAAAGGCTCTTAAACCCCTGATTCTCCTGTCTCCAGCTTTCAGGGGC
Homo sapiens  ........................................................................................................................

Mus musculus  TGGTGTTACAGGGAGGGAGCACCATACCCAGTTTATACGGTACTGGAAATTGAATGCCAGGCTTTCTAAAGGCTTCTTCTAGGCACGCATTCTGCAAAGTGAGGTGCGTTCCCCGAACTA
Homo sapiens  ........................................................................................................................

Mus musculus  CCTGGGCTTTTTCTTCTAAGAAGTATGGTGACTCTGTCCTGTTTAGGATGCTTTATTAAATTCTGAGGATTGTCATTCCTTTCTTTCTTTACTAAACTGTTACGTTTTTAATAAGTGCAT
Homo sapiens  .......................................................................................................TTTTGAATAAGTGCAT

Mus musculus  TAATA--TTTGCTACTAGGTTTTAG----ACCTAAAAAGATGCTATTCATGTTTGTGTGTAACAGGGCAGCTTGACTCGACTGAAATTGAGACACATACATCCTATTGTATAGAGATGTG
Homo sapiens  TAACGGTCTTCCTACTGAATATTAATGAAGCATAAATACCTGTTCTTATTGTTTATGTGTAACAGAGCAGCTTGGCTTGGCTGGAGCTAAGAGCCAGACACACCACTGTGTGGAGGTGGG

Mus musculus  TGACATCTTCCTGATCTGGAAGCTGAGT-ACTTGGCTCCTTGGTTCTTGAAAAACATTCTGGAACACATGAACAGGTATTTAACTACATCTCCTAATTCCATCTTTTGAATGGTGAAGGT
Homo sapiens  TGATGTCTTCCTGTGCTAAAAGGTGAATAAATAAGCTCCTCACCTCTCGCGGAACACTCGGGAACACATCAACAGGTATTTAGCCACATCTCCTGATTTCATCTTTC-AATGGCAAAGAC

Mus musculus  CTTGAATCCTGCTCACTGGTAGGGCTT---------TCAG-----------------GGTGGAAAGACTGGATTTTAGCTGGTTCTCACCGGGGAAAAGG----ACAGTTGGCAAAGAA
Homo sapiens  CTTGAATCCTGTCCTTAACTGGGGCTCAGACATTAATCAACAGGTCTGATGAGCATATGGTGGGGACACCAGATTTCATTGGAGTCCCCAAGAGAAAAAAAAGTCACAGTGAGCAAAG--

Mus musculus  AAGGGATTCACCAGAGTGGGGTAGACACAGGCACAGTGGGAGTCAGAGCCCTTGGTTCACCCCTAGTCCCA-CCTAGTGTGTGTGTGTGTGTGTGTGTGTGTGTGTGTGTGTGTGTGTGT
Homo sapiens  AAGGGGTCTGACAGAGTGGGACAAATGCAAGCGCAGTGTAAAGCAGAACCGCC-ATCTGCCCCCAGTTCCATTCCAG------------------------------ATTAGAGTATGG

Mus musculus  GTGTCCCCTGGGAAGTAGAGGGCTGTGTAGTCAAAATGAGCCTCTGTTTCCCTAGGAGTCCTTGCCATCTCACTGTGTGACTCCCC----AGAGT--TGGGTGCTAGAGTGGAAGCCACC
Homo sapiens  ATGCTTCCAAGGAAAGAGAGTGCTGTGTAGTAAAACTGAACAT-TGTTTCACCAGGGGTCCAAGCCGCCCTGCTGGGAGGCTTCTCTTCAAGAGTTCTGGGTCCCAGAGTGGAAGGCA-T

Mus musculus  TCCCCCATTGACTAGAGAGAGGCGAAGTGACAGAAACCAGGAGATGGTGGGAAAAGCGATTGTTCCAGGTGCCTC-CCGAT--GACAAGAGTCAGCACCACGCAGTTACAACAACCAGCC
Homo sapiens  TTTCCCATCAACTGGAGAGAGACGAAACATCAGAGACCAGGAGGCTGTGGAGAAAGCAGCTGTCCCAGGTGCCTCAACTATCAGAGAAGGGTCAGCGTCACGTGGCT---GCCAGCATCT

Mus musculus  CTCGGAAG--CAGCAGCGACCCCACTGCAGAGCAACAGCCCACCCTACGTACGCT----------GCAGGACCCGTCAGCCTTGCTGTGGGTCTGAAGACAGACAGGGAGTGTCTGGTTC
Homo sapiens  TTGAGAAAATCACTGGCAATCGGACTTCAGAGCTGCGG----GCACAGGTGTGGTTAGAACTGAGATACGACCTGCCCACCTGGGT-CAGGCCTAA----AGACAAGAAGTCCTGAGTTC

Mus musculus  TTGTGACTGGGTAAAGGGACAGAGCATTTGGCCCAAGAGGCTTTGAGAC---CTCTTGGAGACCAAGTC------------------------------CAGAAGCATATCCTGGGACAA
Homo sapiens  TTGCCACTGAGTA---GGCCAGGGTCATTTGTCCAGAAAACTTTGTGACTGTCTTTGAGTGACCTAGTCTGGGACCCATTCATTGGTGGGTTCTAAGGTTAGAAGCTCATCCTAGGACAA

Mus musculus  TGGCGGTGGCCCTGCTGGAGGAGTGGTGTAAGATCATGGGTGTAGACGTCCAGAAGTCACTGCTGGTGGTTGACATTCCGGTGGACTGTGGTGAACCTGAGATCCAGACAGTCCTCCAGG
Homo sapiens  TGGCGCTGGCACTGTTAGAGGACTGGTGCAGGATAATGAGTGTGGATGAGCAGAAGTCACTGATGGTTACGGGGATACCGGCGGACTTTGAGGAGGCTGAGATTCAGGAGGTCCTTCAGG

Mus musculus  AGGCTCTGAAGTGTGTCGGCAGCTACCGGCTACTAGGCAAGATATTCCAGAAGCAGGACAACACCAGTGTTGTCTTAGTGGAACTTATGGAGGACACAGATATGTCTGTGGTCCCCAGTG
Homo sapiens  AGACTTTAAAGTCTCTGGGCAGGTATAGACTGCTTGGCAAGATATTCCGGAAGCAGGAGAATGCCAATGCTGTCTTACTAGAGCTTCTGGAAGATACTGATGTCTCGGCCATTCCCAGTG

Mus musculus  AGGTACAGGGCAAGGGAGGTGTCTGGAAAGTGATCTTCAAGACCCCTAATCAGGACACTGAGTTTCTCCAAAGACTGAACCTCTTTCTAGAAAAAGAAGGGCAGACGGTCGCAGGGATGT
Homo sapiens  AGGTCCAGGGAAAGGGGGGTGTCTGGAAGGTGATCTTTAAGACCCCTAATCAGGACACTGAGTTTCTTGAAAGATTGAACCTGTTTCTAGAAAAAGAGGGGCAGACGGTCTCGGGTATGT

Mus musculus  TCCGAGCCCTTAAGCATGAGGGAGTGTCTCCAGCCACCCCACCCTGCACGTCCCCTGAGTTATTGGCCCATTTGACGGGGCAGGCAATGGTTCACGGCCAGAGGCCTCTGTTGCCTGTGA
Homo sapiens  TTCGAGCCCTGGGGCAGGAGGGCGTGTCTCCAGCCACAGTGCCCTGCATCTCACCAGAATTACTGGCCCATTTGTTGGGACAGGCAATGGCACATGCGCCTCAGCCCCTGCTACCCATGA

Mus musculus  AGTACTGTAAGATGAGAATATTCTCTGGGAGCACAGCAGCTGCCCCAGAGGAGGAGCCCTTTGAGGTCTGGTTGGAACAGGCCACTGAGATAGCCAAGGAATGGCCCATCCCTGAAGCAG
Homo sapiens  GATACCGGAAACTGCGAGTATTCTCAGGGAGTGCTGTCCCAGCCCCAGAGGAAGAGTCCTTTGAGGTCTGGTTGGAACAGGCCACGGAGATAGTCAAAGAGTGGCCAGTAACAGAGGCAG

Mus musculus  AAAAGAAAAGGTGGGTGGCAGAGAGCCTCCGTGGCCCTGCCCTGGACCTCATGCACATAGTACAGGCAGACAACCCTTCCATCAGTGTGGGGGAATGTCTGGAGGCCTTTAAGCAGGTGT
Homo sapiens  AAAAGAAAAGGTGGCTGGCGGAAAGCCTGCGGGGCCCTGCCCTGGACCTCATGCACATAGTGCAGGCAGACAACCCGTCCATCAGTGTAGAAGAGTGTTTGGAGGCCTTTAAGCAAGTGT

Mus musculus  TTGGGAGCACGGAGAGCCGTAGAACCTCACAGGTAAAATACCTAAGAACCTATCAGCAAGAAGGGGAGAAAATCTCAGCCTACGTGCTCCGGCTGGAAACTCTGCTCCGGAGAGCTGTGG
Homo sapiens  TTGGGAGCCTAGAGAGCCGCAGGACAGCCCAGGTGAGGTATCTGAAGACCTATCAGGAGGAAGGAGAGAAGGTCTCAGCCTATGTGTTACGGCTAGAAACCCTGCTCCGGAGAGCGGTGG

Mus musculus  AAAAAAGGGCCATCCCCCGGAACATTGCTGACCAGGTACGCCTGGAGCAGGTCATGGCTGGTGCCAACCTCGGCAACGTTCTGTGGTGCAGGCTACAGGAGCTGAAAGATCAGGGCCCAC
Homo sapiens  AGAAACGCGCCATCCCTCGGCGTATTGCGGACCAGGTCCGCCTGGAGCAGGTCATGGCTGGGGCCACTCTTAACCAGATGCTGTGGTGCCGGCTTAGGGAGCTGAAGGATCAGGGCCCGC

Mus musculus  TCCCCACCTTTTTGCAGCTGATGAAGGTGATACGTGAGGAAGAAGAAGAAGAAGACGCCTACTTTGAACAAGAGAGCAGGGAGGAGCCGGGGGAAAGGGAGGGCTCTGGCTGCTGGAACA
Homo sapiens  CCCCCAGCTTCCTTGAGCTAATGAAGGTAATACG---GGAAGAAGAGGAGGAAGAGGCCTCCTTTGAGAATGAGAGTATCGAAGAGCCAGAGGAACGAGATGGCTATGGCCGCTGGAATC

Mus musculus  ATTCAAGAAACAACTGAGAGATATCCCCACTCCGTCCCTGATCTGGTGGGCGATGCTTGACAAGAGACGATGCTTGACAAGGCTAACCCCATTACAGTGGATTCTTCTTAAGTGCA--GG
Homo sapiens  ATGAGGGAGACGACTGAAAA------CCAC------------CTGG-GGGCAGGAC----CCACAGCCAGTG--------GGCTAAGACCTTTAAAAAATTTTTTTCTTTAATGTATGGG

Mus musculus  ACTGAAGCTAAACATTAGGACTGAAGGTTTTTGTCTTTTCTTTCTTTTCTTTTGTTTTGATAGGGTCTCACCTTATAGCCCAAGTTGGCCTTGAACTGACATTCTTCCTGCCTCAACCTC
Homo sapiens  ACTGAAATCAAACCATGAAAGCCAA--TTATTGACCTTCCTTCCTTCCTTCCT...................................................................

Mus musculus  CCAAATTGAGAGTTCTGGGATTAGAGGAACATCCCACCACTAGTTCCCCATCTCGGTTTTTCTTTCTGAGAAAAAAAAAAAAATACAAGACTAAGTTCCAATACCTAAAACTTCTGAACTT
Homo sapiens  ........................................................................................................................

Mus musculus  TAGTTTCCTTTAAGTACACCAGGGTTGGCCAGACAGCTCGGTTGATCAAGTTACTTACCATTCTCTTCAGAGGGAGCAATAATGTGAACAGGAGATAGGAATTGGCCAGGCAGGGGCTGG
```

```
Homo sapiens   ........................................................................................................................

Mus musculus   AGGGATGCTCAGCCATTAAGCACATTTGCTATTCTTTTGGAGGAGTTCAGATCGCAGCACCCATGTAGTGGTTCACAATTCTCTATAACGGGGATCCAACACCTTCTTCTGGTCTCTTTG
Homo sapiens   ........................................................................................................................

Mus musculus   GGTACTTTGCATATGTGGTACACATGCATACATGCAGACCATACACATAAAATAAAAACAAATAAATCTTTAAGAAGAAAAGAACCTGGCTAGGCAGTTTCCAGGCATTCCAAAAGTGTC
Homo sapiens   ........................................................................................................................

Mus musculus   CCTGGTCTTCTGGTGCATGAACCTCACAAGGAGCTCTGATTTCCCATTGTCCCCTTCCTGAAGGTTACACTTGAGGGTGGGACCTTCAGTCAGACACTAATCCATTTGTGGATAGTGTTA
Homo sapiens   ........................................................................................................................

Mus musculus   GAATTAGGTCCATAGATTTTTCATGATAAAATGGCTTATATCTGTTCCCCAGTTTTAGCCTAATCTCCTATATGATTTTAGGGGTAGCTCATCAGAGGGGGAAATTTACAGTAGCCTTGA
Homo sapiens   ........................................................................................................................

Mus musculus   TCAGGACTTTTCTGAGTCTCATTAAGAGAAAACCATTGCCTTCTGGTCAGAAAAATGGAATCTACCTCTTGCCTAGGCCGAGACCTACCATTGCAAGCTAACCAAAACATAAGCGTTAAT
Homo sapiens   ........................................................................................................................

Mus musculus   CAAGTCGGGCAGGTCCATCACCAGGCTAATGGACTGCCCATTAGGAAGGCAGATTCACCTAACTCTATGCTAATGAGATGGAAGGAATAATTATCCCTTTAATGAAGTAATGTCCTTTTC
Homo sapiens   ........................................................................................................................

Mus musculus   CTTCCCAGAGTCCCTGCTATCTGTACAGTCACTACAGAACTACCATGAAAAGTAATTTTCACTTTTTCTTTTTCTTTTTGCTGGCCCAGGCCTAAGCGCACATTCTTTAGTACTTTGAGC
Homo sapiens   ........................................................................................................................

Mus musculus   TGTTTCACTCTCTCCATGTGGCTGCATGCTAACCCTCTACCCACTCAAAAGAAAGCGTGGCTTTCTCTCTTCTTTTTCTCTATATCCCTCTTCCAGGTATGTTTGATCCCCATAAGCCAT
Homo sapiens   ........................................................................................................................

Mus musculus   AGGGTAGAAGGAGAGAACCAATTCCTGCAAGCTGTCATTTTACACACACACACATGTACAAATAATGTAATTTTTAAAACCACAACAAGTAGACCAATGTTTACTCATACCTTTGAAAAA
Homo sapiens   ........................................................................................................................

Mus musculus   TGGACTGACCTTATTTAAAAAAAAAATTATACAAAACACATATATCCTACTTAATGTGTGCAACAACCTACTCTTTATAGGGTGTGTGTGTGTGTGCGCTTGCATGCATGTATGTGTGTG
Homo sapiens   ........................................................................................................................

Mus musculus   TGGAGGAGGGGTGGGGCTTGTGGTACACTTCATAAAGGGAAAGATGCAAAGGTCAACTAAGTTAAATGGCCTCAGCACCAAAATGCTCAGAAGTGAAACCAGAGCAGAGGCCCAGAACAA
Homo sapiens   ........................................................................................................................

Mus musculus   GACTTTAGGGATCCACCCTCTGTGCGTCGCTCAAATGTGTTTTCTTAGTTCTGTAGAGGACTGATTTTAAGCACTAGTGTTTTAAACTCCTTACATCTCAGACACCCAGCAGGTAGTATC
Homo sapiens   ..................................................................................TAAACTCTTTAAGTCTCTAGTAAAT-GCAGCTAGATTC

Mus musculus   AAGTGTGCTAATGACCAAGTCTCAGTATCTCCGTGTTCACCA---AGCTTGCTTTTGTTTCCAAACTGACCCA--------GGACTGCAGAACAAAGCTCCCTGC-TGGCAATGGTGAGC
Homo sapiens   AAATGGGCTGATAACCAAATTTTAACACATCAGCATTCACCACCAGGTTTACTTTTATTTTCAGATTGGCTCATTTTGTGCAGACCTTAGAGCAAAGTTTCCTTTATGGTATCTGTGTAC

Mus musculus   CTATTCCAAAAGCCCTGTAATTGTGCACAGACTTGAAAAGCCATAGCGCCTTGTGAGTATGTGTGCCAGGTC------------------------------AAGAGTACTAGGGCACC
Homo sapiens   GTAT-CCAAACTTCTTTTAATTGTTCACAGATTTTAAAAGCGGTAGCACCACATGGTTGTGTAGATCAGACCTGTGTATTTAGATCAGACCTGTGTATCACGTAAGTGTGTGAGTGCAGT

Mus musculus   ATAAACAAACACTGCTTAGTTGCCTG--CCAGGCAGAGCTCGGGCACAGTTAGTGTGCAGCCT-GTGGTAGATCCCTGTGTG--GTTAGCAAGCTGCTTGGTTTCTCTGAACATCCGTTT
Homo sapiens   GCAGATGAGCACCATTTAGTTATATGTGCTAGGCAAATCTCCAACACAGTTGATGTGTAGTCTTGTGGTAGATTTGTGCATACTGTAAGCAAATTGCTTAGCTTCTCTAGACATCAGTTT

Mus musculus   CCTTATCTAAAAA---------------TGGGCTGCCATTAGGATCAAGTGGCACAATGCTTTTTAAGGGCTTGGAGTAACGTTTGGAACTTAGAAGCCCCTCCATATACTGAGGATCTT
Homo sapiens   CCACATCTGAAAAATAAGAAGATGAGAGTACACGGTTGTTATGAACAAATGACTTAATGCTTTTTAAGCACGTTGCATGACATCTGGAACACAGAAAGCCCTCAATACATTGAAGCTCTT

Mus musculus   TGTATTTTTGTGATAGT--TGTCAGCTCAATGCATGTTCTCTTGATTGTTT---CCATTGTATGGAAACAAGTCACAAAGTAACAGACTTACA------TTCCAGTCGGGATGCTCCTGC
Homo sapiens   AGGATTTTCACGATGTTCCTGTCTGCTCAATGCATGCTTTCTTTATTGTTCTGACAGTTGTGTGGTAACAAGC-------TAATATGCTTCCAGTTGACTTCCAGTCTA-----CCCTGG

Mus musculus   AGTGAGGAAGAACTTTGGCT-----TGGAAGTAAGTGTGCACTAGCTGGCCGTCCACATTTGTGGTAAGTATTCCAGAAGTAGTTGGTGACTGTTAATCGACTTGACTTTGGAATGATGA
Homo sapiens   TGTTAGAAACCGTTTCATCTCTTATTGTAAATTTGAGTGC--TTGTTGTTT-TTTATATTTGTGATGACTCTTCCAGCAGTTGTTGACAATTGTTAG-AGGTTTGACTTTTAAATAATTA

Mus musculus   CCTGTTCTTCCGGGTTGTGGGTTAGTTTAACTGAAGAATAA---GAAGTTCTAAGAAAGGTGGTTTGTTTGTTTATTTAAGTCATCATCTGTGATGGTTTATCTTTCGGAAATATATTTC
Homo sapiens   CTTATTTTTTCTGATTGTGGTTCAGTTTAACTGAAGAATATCCTGAGATTGTAAGAAAAGCATTTT------TTAA--AAGGTATCACTTGTGATCATTTATCTTTCTAAATTCTATTTT

Mus musculus   TCAACACTGTCCAGCCACAGTGATGTCAGTGATGGGATTGTCCTTGACCACTCTAGCCTGACTCCACGTGGCATTATGTTCCTGGGAATAGCTTTGCCGTTGTACTTACCAACAGAATAC
Homo sapiens   T-AATACTGTTCCACCAAAGTGATG-CAGTGGTT------------ACCATGACACCCTAATTTCATGTGTTTTTGTATTTATGAAAATAGTTTCATTGTCAT--TTATTGGCGGTATAC

Mus musculus   AGAGTTAAATGTTACCACTGCGAAGTTACCAAATAAATCTGAGCTAGTGAAATCGTGAGTTGTTTTCTGCTTCCTATA--------------------TGCCTGAGTTTTAAGGAGCTTC
Homo sapiens   AAAGTAAAATGTTATAAATGTGAAGTTATAAAATAAATATATGCTAATAAAATCCTGAGT--TTTTCTGTTTCCTATATCGTGGTCTCTATAAAGTACTGCTTGAA---GAAGGAGCTTT

Mus musculus   ATTACTAAAA------GCAAAAC--------AAAATCAAGCTAAAGGGGCTGGAGAAATGGCTCCGTGGTTAAGAGCACTTGCTGATCTTCCAGAGGTCCTGAGCCCAGTTTCTAGCACC
Homo sapiens   GTTACTGAAAAATTTTGAGAGATAAGTTGAGAAAATCAAGCTAAAG..........................................................................

Mus musculus   TACATCTGGTGGCTCCTAAGTATCTGTAACTCCAATTCCAAGGCCTTTGGAGGCCTTAAAGCTGCTTCAACTC------TCCGAAGTGCATTTGGCGAGCATCTCATATTGTGGTGCTGG
Homo sapiens   ..........................................GCCTTTGGCCTTCTTGAAATTGCTTCTCCTTCTTTTTTCTAAAGTGCAATGGGGGAGTATCTTTCACTGGG---CCAT

Mus musculus   ACACACTGT-GAGCAGTCCATGAGAGGTGAGTCCCCACCCCCTCAAGGGGTTCT-----CTGTTCCCA-TGGTTTGAACAAACTCCTCCCAGGAC--TCTCACCTCAAGTCTTAT----C
Homo sapiens   ACATCCTGCAGACCAGCCCATTAAAGAGGAATGTCC-------CAAGGGGGCCCAACGCCATCTCCCACTCACCCAAGCAAACTCTCCAAGGGGTGGTCTCATCCTATACTTCATACTTC

Mus musculus   TCCACTCCTAACCCTTAAGGATGGAACAATCCAAGATTCCAGAACCATTGATTTTGAAGATCATTTTCCTTTTGAGTAGGTGTTCCTGGTGCAAGCTGCAGGCCAGTCCATCAGCTTCCT
Homo sapiens   TCCAGTCCTGACTCTCAAATACAGGCC---CCAAGATTCTTGAATCACTG---CTGAGGATAACTTTCC-----ACCAGTGCTGTCTGGTGAAGACTG------AGGCCATCAGCCTCCT

Mus musculus   GCATGTTTCAGAACACTGATGTCATGTTGCCCACCACTGCATCAGCCTTGTGCA------------TCCTGCAGGCGTCTGCTTCCCAAACTACACGCAAG-------------GCACTC
Homo sapiens   GCCT-TTTTAGGCCTTTGGTGTCACCTGCCATGCACCTGCACCCACCATGTCCACTAGGCCACCCCTCCTCTTGGGTTCTGCTGCCCATCCTAAATGCAAGACTTTGCTCAGCAGCTCTC

Mus musculus   ACTTTTA
Homo sapiens   GGCTTTG


Bos taurus     CCCAAGTGCGCGACGTCCCGCTGCTGGGCCACCACCTGCCCCGAGCCCCGGCCCACCGTCAGCGAGTCGAAGTCGATGGTCTTGTTCTCCGAGGACCCCTCCACCGGGCAGAACATGCCA
Homo sapiens   CCCAAGTGGGCGACGTCCCGCTGCTGAGCCACCACCTGCCCCGAGCCCCGGCCCACCGACAGCGAGTCGAAGTCGATGGTCTTGTTCTCCGAGGACCCTTCCACCGGGCAGAACATGCCA

Bos taurus     CAGAATTTCTGCCGAGGCTTGGGGCTGCCTGAGCCCAGGTTTTTAAAAAAGGCAGGGACCTCTTCGTCCTCTGCCGCCTTCCCGGATTGCTTCCTCTCAGCCATGGCGTGGGGGCTCGGG
Homo sapiens   CAGAATTTCTGCCGGGGCTTGGGGCTGCCGGAGCCCAGGTTTTTAAAAAAAGCAGGGACCTCTTCGTCCTCTGCCGCCTTCCCGGATTGCTTTCTCTCGGCCATGGCTCGGGGGCTCGGG

Bos taurus     GAGGGTCCTTGCCGTTCGTCCGTCCGTCTGCTGGTCCCTACGTCTGCCTGTCTCTAGGCACAAACCCCCTGCCCCGGCTGCAGCCGCGGCTGCCGCTCTCTGCCTACCGCAAGGTGTACA
Homo sapiens   TAGGGTCCTCGCCGTCCGTCCGTCCGTCTGCCAGTCCCTAAGTCTCCCTCGCTGTGTGCACAAGCCCCCT-----AGCTGCTGCCGCGGCTGCCGTTCGCCGCCCACCGCAAGGTGCACA

Bos taurus     GAAAGGAAGGAGGGCTGGCGAACGAGCGACCCACCCCTCTCGGTCCCTCCCCCTGACCCCGCTTTCTCCCCTCCCTGCCCACGCCCCC-----------CGCCAGCTCCCGCGAGCAGCA
Homo sapiens   GAAAGGAAGGAGGGCTGGCGAGCCGGCGACCCA-CCCTCCCGGTCCCTCCCCCTGACCCCGC-TTCTCCCCTCCCTGCCCACGCCCCCCGCGGCTACAGCGCGAGCTCGGGGAAGCGGCA

Bos taurus     AAGTTGGGTCTGGGCGGGTGGCCGCTGCGGCAGCGAGAGCGGAGAGGCTGCCAGTTCCCGGCAGCTGCGGGAGGAGCGAGGCGCGGGCGGGGCG-------GAGGCGGGGCGCGGCACGC
Homo sapiens   AAGTTGGATCTGGGAGGGCGTCCGCTGCGGCTGCGGGAGCCGAAAGGCTGCCAGTTCCCGGCAGCGCTGGGAGGAGGGAGGCGCGGGCGGGGCGCGGCGCCGGGGAGGGGAGCGGTGCTC

Bos taurus     CGGGGAGGG-----GAGCGAGGAGCCGGGGAGGCCAGGGAGGG-----------------CCTCGCTCCGGAAGTGAAGCTGCCAGACCACCGGCTAATGGA-GCGCAGCGGAGGGCCCG
Homo sapiens   AGGGGAGGGCTGGAGGGGAGGGAAGGAGAGAGAGAGGGGAGGGCGGCACCGCCCCTAGCCCCGCGCTCCGGAAGTGAAGCGGCCAGACCACCAGCTAATGGATGCGGAGCGGAGGGCCCG

Bos taurus     CTGACCGCTCTAGGCGCCACGGTGAGTACCCGGCAGGGCACGCGGGGGCGAAGATCGTAGGGCGCAGTCCCTGGTCCCCGCGCCCCTCTCCCGGACCTGCCCGTCCCAGGCGCCTCTTCC
Homo sapiens   CTGACCGCTCTCCGCGCCTGGGTAAGTATCGGGCAGGGCACGC-GGAGTGAGGACCGGAGGACGCAGCCCCCAGCCCCAGTGCCCCTCTCCTCCACCTGCCTGGCCCAGGCGCCTCTGCC

Bos taurus     TCACCGCATTGCGGAGGTGGGCCCACGACGAGTGCCCACCGTGGGGAGGGCTCCCTCCCC-CTGCCCCGGGTGTGAGCTGATGGGTGGGGAAAGTGGCACACTCTGGCGGGCTCCAGGAT
Homo sapiens   CCGCCGCATGGCGGAGGTGGGCCGGGGGCGAGGGCGCACTGTGGATAGGGGTCCCGCCCCGTTACCCTGGGTGTGAGCTGATGGATGGGAAAAGGGG-ACAAGC---CGGGCGCCAGGCT

Bos taurus     ACCCATGGTGGAGGGGCGGGGGAGAGAAGGAGGGGCGGCACTGGGGATGGGGAAGAGATGGCGCAGGCCGCGCGGAAAAGGGGGGACCATAAGAGGCGCGGGGACACTAGAGCTTCGGTC
Homo sapiens   ACTCAGGGTGTGGG-------------AAGCGGGATGGCACTGAAGATGAGCAAG-GATAGCACAGGCAGGGTGGACAGGAGGGGGGCAGCCAAGACGC-TGGACACCCCAGCTTCAGTT

Bos taurus     TAACCACCGGCCGTGGTGGAATACTGGGAGCAGAGATTGAAACGAGA---TTCTCACTTACTGCTCCCAC---------------------AGTAGTGATGGTGGGGCGCAT--TATGG
Homo sapiens   TGTCCACTCGCCTTGGCAGGCTGCAGGGAGCAGAGACTGAAACGAGAAGGTTCCCGCTTCGTGCATCCACTTTGGTGTGGTTGGGGTGACGGGGGAGTGGGGGGGGGCACACAACATGG

Bos taurus     CCGGGAGAGACACAGAGGGGCGTAGCAAATACGAAGTGGGTGGGTGTAGAGACAGAAAATAGATCTACAGTCTGTGCAAGTGTAGCAAGGCGAGGAGTTGGCAGGTACAGCCCAGCGCGG
Homo sapiens   CGGGAAGAAATTCAGAGGGGCGTAGCAGATGGGA----GAGGAATATAGAGAGAG-ACGCAGGTCTGCGGTCTATGGGGGTGTAGCAAAGAGTGCAGTCGGGAATTCCAGCCCAGGGCCA

Bos taurus     CAACTTAAGGATATAGTGAGAGGGGACACCGGTGCGCGGTATTGGCCGAGGGCGCCGTGTACCGTCGCTGTGCCACTGCGCCAAGAAGGGTCCGGCAGAAGGTCTTCATCTGATATCTTC
Homo sapiens   CGACTTAAAGACGGGGTGCAGACCGACTCTATTGCGC-----------AAGGCGCCATGGACCGTGTCTGCGCCACTGCGCCAAGAAGGGCGCAGGAACGTGTCGCGATTTGGTTTATTC

Bos taurus     CGCCCCTCCCTCC-----------------TGCCAAAGGGGGAAAGATACACATGGAAGCCCACCCGGCCAAATCCCCAGCCCTACCCGGGGCCACTGGCGCCCCGCCTCATTCTACAGC
Homo sapiens   TGCTTCCTCCACCAAAAAAAAAAAAAAAAAAAAAAAAAGAGAGAAAAAACAAACTGAAACCC-CCTTCCCAAATCTCCAGCTTTCCCCTGCGCC-CCAGAGCCCCGCCTGGTTCCCCCGC
```

```
Bos taurus     CCATGTAGCGCACTGCGCCAGATCCGCGGGGTTTCCTAGGCTGCCAGAAGCCGCGTGCCTGGGCGCTA---GTGCGCGCGAGTCCAGTACAGGATCAGCATGTGGGGCGC-GGCTGTGAT
Homo sapiens   CCCTGTTGCGCACTGCGCCACAGTCGCAGCGTTTCCCGGGTTACCAGATGCCACGTGTTTGGGGAGTAAGTGCGGGTGCGCATCTGGTGC-GGAGTAGGAGGTAGGATGTGGGCTGTGA-

Bos taurus     GGTTGCCTTGAATTGTGTGAGGCCAAGCAGCGGAAAGGTGTATGGGGACGTGGTGTGTATGCACGAGTAGATCAGCCTCAGCCATGGAATTAGTGGACATCCAGGGGGCATTTTGATGTA
Homo sapiens   --GTGCCATGAATTGTGTGGGGCCAAGCAGGGGAAAGGTGTGTAGGAACTTGGTGTGTGTGTGC-AGTAGCTGAGCCACGGCCACCGGGTTGGTAAGTAACCAGGGGGCAATTAGATTT-

Bos taurus     ACGCCAGTACATCAGGCCAGTAACACGGCCTCCAAGGACCCTTCATTGCTTACTTCTATTCCCCGTGTCTTTTCCACGTCTCAGTCCCTGCCCAGGGTTGATTACAGCCCCGCCCCTCTC
Homo sapiens   ------GTGTGCC-------TAACACGGTCAATGCTGGTCCTTCATTGCTTATTCCCA-TCTCCACGTCCTTTCCGCGTATCAGTCCCTGCCTCTGCGCGATGACAGCTCCGCCTCTCTT

Bos taurus     TCCACAGTGGCAAAACCCTGCAGAAACGATGCCCATCCTTTGGCAGCTCGAGAAGGAGCTCTGGGCATGCTCAGTGATCAGGGTGGGTTTAGACTGGGAAACTGTTAACCACAGGAGCGT
Homo sapiens   TCCACAGTGGCAAAATCCTGCAGAAATGGTGCCCCTCCTTTGGTGGCCCGGGAGGGAGTTCTGGGCATGCTCAGTAACCAGGGTGGGTTTGGGCTGAGAGGCCGTTTACCGCAGGGCCAC

Bos taurus     CGTGCCAGGCTGAGCAACCAAAGATTCGGTCCCATTGTCAGCCCTTGCCCTTGGAAGGACTAAATGCGGATACCCAGCCCTCCTCCA---CAAATGCCAGGGAAA----GGTGAATGGAG
Homo sapiens   CGCGTAGGGCCGTGTAACCAGGGATCCAGTCCCATTGTCAGCCC-----------AGGATTAAATGCGGTTACCAACCACCCCCACAGTCCAATTGTGAGAGATAGTTTGGTGAATATAG

Bos taurus     TTAAACTTAGAGGACAACCAATCCCAGTTGGAAGATAAGCCCACCATGATAGG-----------------------------ATGAGGAGGAAGGACAGAGGAACGTACCTGTGCAGATG
Homo sapiens   TTACATCTGCTGGACAATCAGTCGCAATTTGAAGATCAGCCCATCTTGATAGGTGGAAAGGCGTCTCCGGATGAGAAACAGACTGGGGAAGAAGAATATAGGAGTG-----GAGGGGCTG

Bos taurus     AGAGAGCAGAGACGGACG-TTTGGACCCAGCACCAGATGTGCTTTGCATTTGAAT-------------AAAGTGCCTCCACACGAATT---CCATTTCCTACTCCTAGCAGACCCCTGGG
Homo sapiens   AGAGAACCGGAGGGGGCATTTTGGACTTAGCGGCAGATGTGCTTTGCATTTGAATAAAGCATTTGAAAAAAGCGCTTCCAACTGAATTACATCATTTCCTACTCACAGCCCCACTGTGAG

Bos taurus     ATGAGTGCTTCCTTATCACCACTTTCCAGGGTAGGAAACAGAGTTAGA----------TGCTTTGCCCAAGGTGACTATATGGTTAAATGATCCAACAAATCCAGTGGG-GTCTTCCACC
Homo sapiens   ATGCGTGCCTTATCAGCACTGCTTTCCAGGTTAGGAAACAGATTTAGAGAGCTTGGGGCGCATAGCCAAGGTTGGGTATTTAGTTAAATGATCCAGCAATTCCAGTGGGAGTCTTCCACC

Bos taurus     TGAAAGTCTAGTTCTTTTTCCACTTTATCCTAT------CTTTATGTGAGA---------TTTTCACATTTTTTCCCCTATCCAAATATTTCCAGTCTTGGTCTTATCCTGAAGCACCCA
Homo sapiens   TCAAAGTCTGGCTTCTTTTCCAGCTTGTCCTGTGACCCCCTTTATGTGAGAAAATACAAGGTTCTTCTATTTCTACCGTATCCAGATATTTCCATCCATGGCCCTAT.............

Bos taurus     GGTGGCTCAGACCATAAAGAATCAGCCTGCAATGCAAGAGACCCAGGTTCCATCCCTGGGTGGGGAAGATACCCTGGAGAAGGAAATTGCAACCCATTCCAGTATTCTTGCCTCAGAAAT
Homo sapiens   ........................................................................................................................

Bos taurus     CCCATGGACAGAGGAGCCTGGAGGGCTGTAGTCCATGAGGTCACAAAGAGTGGGACATGACCAGTGACTAACACTTTCACTGGCCTTACGGTGTACCAGGGATGTTTCATTTGCCATATT
Homo sapiens   ..........................................................................................GTATATCAAGGGTGTTTC--TTGCCATGTT

Bos taurus     TCTATTTGGAAGGTTGCATTACAGATTGAGATAGCCTTTGGAGGAAGAAGCCACTCAAAGGTAGCCTAACCTGAGCAGGACAGTTA---TGTAAGACTTTAAGAGGTGTTTCTTCCTGGG
Homo sapiens   TTTATTTG-AAGGTAACACTGCAGGTCA------CACTGGGAGGAAGA-GTCATCTGAAGATAGCCTAACTTCAGCATAGCAGTCATTCTGTAGACCAT--AGAGATGCTTTTTCCTGG-

Bos taurus     TATGCAATGTC-TTCAACGTCTCTTGTTTCCTCTAAATATTATAACCGGGAAGGTCACCCCTATATCTTAGAAGAGGGTCATGAGGCATGAGGGAGGAGTCAATGTACTCAGATCCCTGG
Homo sapiens   ---GCAGTGTCTTTTTACATTCCCTGTTTCCTCTAAATATCCCAGTGGGGAGG.....CCCCACATTTAGAAAGGAAAAGTCAGGCATG---GAGAAGTCAGCAGTCTCAGGTCTCTGG

Bos taurus     GGCTGCTGGGCAAGGACTCAATCCAGCATTTTGCCCTCCCTGCCCTCTGGCACACGCACCTCCTTTCAGCACCTTCTAGA--ACTCAGGATATGTGCCTACAGCAGGAGCAGCTCCCCCG
Homo sapiens   GGCTCCTGGGCACAGGGGTGAAGCAGCATTTTGCCCTCCTGGCTCTCTGGCACCC-CTGGTGCCTTCAGCACTTCCCAGAAGACTCAGGAGATCTTCCCACAGCAGGAGCA---CTGCTG

Bos taurus     CTTTAGCTGCAAGTTGGTGTCTGTGGCATATGGACCCCATTTGGACTGATGGAAAATTTTAGGGCAAG-----AATGATCGTATCCACTTA----ATACTCCATGACAATAACTCAAGAT
Homo sapiens   ATAGAGTTACAGGACAGCA-CTGTGGCGTATGGCCCCCATTCGAATTGATGAAGATGTTTAGGCCAAGGGGGAAATGGTTGTAGCCCCTTAACACACACTCCGCAGCAGTAACT......

Bos taurus     AGAAGCTCTTCCCTTTTTAAATCAGCCGGTCTCCACCGCTTCTTCACAGATGTGCCTCCTGTCTTTTTCTTTGCTTTTCAAATGACCCATTCAAGTTCAGATGCTAGGAAGAAAGCATTG
Homo sapiens   ........................................................................................................................

Bos taurus     TGTTACTATCTTAACATTTATAGCAAAAGAAAACCCCTAGTTGTACATTTGCAAAGCATAACCAGGCATAACCAAAGAGAATTAATTCATAACTTCAAGTTTAATTTATTTGGCTCTGAG
Homo sapiens   .........CTTACCATTCATAGCAAAAGCAAAGCCCAAAT-GTGTGTTTGCAAAGCCTT-CCAGACACAACAAAAGAGAATTAATTCATAATTCCCGATTTAACTTAGTCGGCCCTGAG

Bos taurus     TTTTTAGTAGATAGTAAAACCTATATTAAGTTGTATTTATGTTAGTATATTAAAGCATATTATGCAAGTTATATTGCTAAACCCATATTAAGTTGATTTTAAATGTTTGAATGATTC-TA
Homo sapiens   ATTTT...................................................................ATTGTTATACCTAGATTAAACTATTTTTAAACGTTTGAGTGATTCCTC

Bos taurus     ATTGGATTT-TTAATCAAAGGAATGAACTAACTCAATATTGTCTAGTAGCATTAATTAGATTATGAAGGATTTGGGGGGATTTTTTTTTTTTGGCTTCATGGCAATGCTTGTGGGCCTGG
Homo sapiens   ATTAGTTTTGTTAATCAAAGAGGTAAAGCAACTTAATATTGAGTAGTA-----------TTGTGAAGGATT.................................................

Bos taurus     CAGTGAAAGCACCAAGTCTCAACCACTGGACCGCAAGAAAATTTCCAAGAATTCCTGTATTTTTCAATAAGTGTATTAACAGTGTTCACCCTGGGTATTGTCATTGTTGAGTCTATAAGT
Homo sapiens   .....................ATCA--------------------------------TATTTTTGAATAAGTGCATTAACGGTCTTCCTACTGAATATTA....................

Bos taurus     CATGTCTGCTTCCCCATGGACTGTAACCTGCCAGGGTCCTTTGTCCACAGGATTTCCCAGGCAAGAATACTGGAGTGAGTTGCCATTTCCTCCTCCAGGGACATTGGATATTATCGTCAT
Homo sapiens   ........................................................................................................................

Bos taurus     AAAAATACACTATTTACTATATTTATACAGGGCAGCTTGGCTTGGCTGGAGCAGAGACACCCAGACACACCATTGTGCTGAG-TGGGTGACATCTTCTTGTACTAGAAGCTGAATACATA
Homo sapiens   .................TATGTGTA-ACAGAGCAGCTTGGCTTGGCTGGAGCTAAGAG--CCAGACACACCACTGTGTGGAGGTGGGTGATGTCTTCCTGTGCTAAAAGGTGAATAAATA

Bos taurus     AGCGCCTTGGCTGTCACAGAACATTCTGGAACA----AACAGGTACCTAACAAACAGCCTCCCGATTTCACTTTTCATGTGAGAAAGATCTTGAGTCCTGTCCATCACTGGAGCTC---C
Homo sapiens   AGCTCCTCACCTCTCGCGGAACACTCGGGAACACATCAACAGGTATTTAGCCAC--ATCTCCTGATTTCATCTTTCA-ATGGCAAAGACCTTGAATCCTGTCCTTAACTGGGGCTCAGAC

Bos taurus     ATTAACTGGCATGCCTGATGAGTATATGGTGGGGAAGCCCAATTGCAGGAGGGTCCCCAAGAGAATAAAAAGTCACTGTAAGCAAAGAAGAGCGATGA--------------TTCAACTA
Homo sapiens   ATTAATCAACAGGTCTGATGAGCATATGGTGGGGACACCAGATTTCATTGGAGTCCCCAAGAGAAAAAAAAGTCACAGTGAGCAAAGAAGGGGTCTGACAGAGTGGGACAAATGCAAGCG

Bos taurus     TAATATAAGGCAGTCTCCCTGTTCCCCCCTTCCGCTTCCATCCCTCACCAG-GTACCCATGCTTCCCAGAAATGAGAGAGCTGTGTGGTAAAACTGAGCCTTGTTTCCCTAGGAGCCCAA
Homo sapiens   CAGTGTAAAGCAGAACCGCCATCTGCCCC---CAGTTCCATTCCAGATTAGAGTATGGATGCTTCCAAGGAAAGAGAGTGCTGTGTAGTAAAACTGAACATTGTTTCACCAGGGGTCCAA

Bos taurus     GCCACTCTGCTGGGAAACTTCCCTCCAAGAGTTCTGGCTGCCAGAGTGG-AGGCATCTCCCCACTGGCTAGAGAGAGGTGAGGAGGAAGAGACCCAGAGA--GGGGTGAAAGGAACTA--
Homo sapiens   GCCGCCCTGCTGGGAGGCTTCTCTTCAAGAGTTCTGGGTCCCAGAGTGGAAGGCATTTTCCCATCAACTGGAGAGAGACGAAACATCAGAGACCAGGAGGCTGTGGAGAAAGCAGCTGTC

Bos taurus     CCACGTGTTTC---------CGGTGGGGCAGGTCCCCAC-----CCGGCCTCCT---------------CAACGAGACTGCAGAGCCTCTGGCCATGGTCCTGTCATAACTGAGATACTA
Homo sapiens   CCAGGTGCCTCAACTATCAGAGAAGGGTCAGCGTCACGTGGCTGCCAGCATCTTTGAGAAAATCACTGGCAATCGGACTTCAGAGCTGCGGGCACAGGTGTGGTTAGAACTGAGATACGA

Bos taurus     -------ACCTGGGGCAGGTCTGGAGACCTCACGGAAAGGGCCAAGTTCTTGCCACTGAGCA-GCAAGGGTGATTGGTCCA------------------------------------GGA
Homo sapiens   CCTGCCCACCTGGGTCAGGCCTAAAG-----ACAAGAAGTCCTGAGTTCTTGCCACTGAGTAGGCCAGGGTCATTTGTCCAGAAAACTTTGTGACTGTCTTTGAGTGACCTAGTCTGGGA

Bos taurus     CCTGTTAACTGGTGAGTTAGAAGTCTAGAAGCTCATCTGGGATATTCTCAATTTGAACTCAGCGCCAGTCTGTCCCATAAGCTAATCAGTAGCCATAGAGTTCCAGCCAGTCAGTATCCA
Homo sapiens   CCCATTCATTGGTGGGTTCTAAGGTTAGAAGCTCATCCAGGATATTTTCAATATTAAGTCAGTGCATAGCTGCACCACTAACAAATTGGTGCCTGTAGAGTCAGAGTGGGTCAATTCTTA

Bos taurus     G--CAATGGCGCTGGCCCTGTTGGAGGACTGGTGTAGGATTCTGAGTGTGGATGATCAGAAATCACTGATGGTTATGGGGATCCCAGTGGACTGCAGTGAGGATGAGATTCAGGAGGTCC
Homo sapiens   GGACAATGGCGCTGGCACTGTTAGAGGACTGGTGCAGGATAATGAGTGTGGATGAGCAGAAGTCACTGATGGTTACGGGGATACCGGCGGACTTTGAGGAGGCTGAGATTCAGGAGGTCC

Bos taurus     TGCAGGAGACTTTAAAGCCTTTGGGCAGGTATAAACTCCTTGGCAAAATATTCCGGAAGCAGGAGAATGCCAACGCGGTGCTGTTAGAGTTGCTGGAGGACCCTGAGGTCTCTGTGATCC
Homo sapiens   TTCAGGAGACTTTAAAGTCTCTGGGCAGGTATAGACTGCTTGGCAAGATATTCCGGAAGCAGGAGAATGCCAATGCTGTCTTACTAGAGCTTCTGGAAGATACTGATGTCTCGGCCATTC

Bos taurus     CCAGCAAGGTTCAGGGCAAGGGGGGCATCTGGAAAGTCATCTTCAAGACCCCTAACCAGGACACAGAATTTCTGGAAAGACTGAACCTCTTTCTAGAAAAAGAGGGACAGACGGTCTCGG
Homo sapiens   CCAGTGAGGTCCAGGGAAAGGGGGGTGTCTGGAAGGTGATCTTTAAGACCCCTAATCAGGACACTGAGTTTCTTGAAAGATTGAACCTGTTTCTAGAAAAAGAGGGGCAGACGGTCTCGG

Bos taurus     GAATGTTCCGGGCACTTGGGCACCAGGGGTTGTCTCCAGCAGCCATGCCCTGCATCTCACCAGAGTTACTGGCCCACGTGTTGGGACAGGTGATAGCACATCCCCCTCAGCCTCTGCTCC
Homo sapiens   GTATGTTTCGAGCCCTGGGGCAGGAGGGCGTGTCTCCAGCCACAGTGCCCTGCATCTCACCAGAATTACTGGCCCATTTGTTGGGACAGGCAATGGCACATGCGCCTCAGCCCCTGCTAC

Bos taurus     CCATGAGGTACCGGAAGCTGAGAGTGTTCTCAGGGAGCGCAGTGCCTGCCCCGGAGGAAGAGCCCTTTGAAGTCTGGTTGGAACAGGCCACTGAGATAGTCAAAGAGTGGCCGGTAGCAG
Homo sapiens   CCATGAGATACCGGAAACTGCGAGTATTCTCAGGGAGTGCTGTCCCAGCCCCAGAGGAAGAGTCCTTTGAGGTCTGGTTGGAACAGGCCACGGAGATAGTCAAAGAGTGGCCAGTAACAG

Bos taurus     AGGCAGAAAAGAAGAGATGGTTGATGGAAAGCCTTCGCGGGCCAGCCCTGGACCTCATGCACATAGTCCAGGCAGACAATCCATCCATCAGCGTGGAGGAGTGTTTGGAAGCATTTAAGC
Homo sapiens   AGGCAGAAAAGAAAAGGTGGCTGGCGGAAAGCCTGCGGGGCCCTGCCCTGGACCTCATGCACATAGTGCAGGCAGACAACCCGTCCATCAGTGTAGAAGAGTGTTTGGAGGCCTTTAAGC

Bos taurus     AAGTGTTTGGGAACCTGGAGAGCCGCCGGACCTCCCAGGTGAAGTACCTGAAAACCTATCAGGAGGAAGGAGAGAAAGTCTCAGCCTACGTGTTACGGTTAGAAACCCTGCTGCGGAGAG
Homo sapiens   AAGTGTTTGGGAGCCTAGAGAGCCGCAGGACAGCCCAGGTGAGGTATCTGAAGACCTATCAGGAGGAAGGAGAGAAGGTCTCAGCCTATGTGTTACGGCTAGAAACCCTGCTCCGGAGAG

Bos taurus     CGGTGGAGAAGCGGGCGATCCCCAGGAACATCGCTGATCAAATCCGCCTGGAGCAGGTCATGGCCGGGGCAAGCCTCAGTGAGGTACTCTGGTGTCGACTTAGGGAGCTGAAAGACCAGG
Homo sapiens   CGGTGGAGAAACGCGCCATCCCTCGGCGTATTGCGGACCAGGTCCGCCTGGAGCAGGTCATGGCTGGGGCCACTCTTAACCAGATGCTGTGGTGCCGGCTTAGGGAGCTGAAGGATCAGG

Bos taurus     GCCGACCTCCCAGCTTCCTGCAGTTAATGAAGGTCATCCGGGAAGAAGAGGAGGAAGAGGCCACTTTCGAAAATGAGAATACTGAAGAGCCGGAGGGAGGGGATGGCTATGGCCACTGGG
Homo sapiens   GCCCGCCCCCCAGCTTCCTTGAGCTAATGAAGGTAATACGGGAAGAAGAGGAGGAAGAGGCCTCCTTTGAGAATGAGAGTATCGAAGAGCCAGAGGAACGAGATGGCTATGGCCGCTGGA

Bos taurus     GTAACGAGGCCAATGACTAAAACCCAACTCCGAGGGCGACCCACAGATGGTGGTTGGAGGGACAGCCACGTTATTAGGCTAAGACCTTTTCAGTCTTTTTTTCTCTATAATTTGCACAGT
Homo sapiens   ATCATGAGGGAGACGACTGAAAACCACCTGGGGGCAGGACCCACAGCCAGTG------------------------GGCTAAGACCTTTAAAAAATTTTTTTCTT--TAATGTATGGGAC

Bos taurus     TGAAATCAAAGCATTCAAGCCAGGTTTTATTTTGTTTTTCTTTCATCTTTTTGGCTGCATCGTGGGGTATGTGGGATCTTAGTCCCCCAACCAGGGATTGAATTCATGTGCCCTGCATTG
Homo sapiens   TGAAATCAAACCATGAAAGCCAATTATTGACCTTCCTTCCTTCCTTCCTT......................................................................

Bos taurus     AAAGTGGAGCCTTAACCTCTGAGTGGCCAGGGAAGTCCCTCAAGCAAGGAAGATCTTGATTCTTCTTCATAAGGAAAAAAAAAAAATATGAGAGCTTCTTGGACTCTACATAGATAATTG
Homo sapiens   ........................................................................................................................
```

```
Bos taurus    CTAAGTCTCTTCAGTCGTGTTTGATTCTGTGCAACCCTGACCTAATGGACTATAGCCTACCGGGCTCCTCTGTCCATGGGATTCTCCAGGTAAGAATACTGGAGTGGGTTGCCATGCCCT
Homo sapiens  .....................................................................................................................

Bos taurus    CCTTCAGGGGATCTTCCCGATCCAGGGACCAACCCTGCGATTCACAGATGATTATTAATAAACAAAAACTTAAATTCCAAATAGGAGGTCAGAAGCCTAAAGGAATCTGAACCAGGTG--
Homo sapiens  .............................................GATTATTAATGAACAAAAGATTAAACTCCAAATATTAAATAGTAAGCCTGAAGGAATCTGAAACACTTGTA

Bos taurus    ---------TCCTTTAAATCCCTCCCAATAGACC-----------------AAAAGAAAGAAATGATGTGAGAAAAAAAAAAATCTCTTTTGTCATCTATTTAAAAAATCCCAGCTGTTT
Homo sapiens  CTTCCAATTTCTTTAAATAATCCCAAATAGACCAGAATTGGCCCATACCATAGAAGAAAGAATTGGCAGTCAAAAAAAAAAAATACCTTTTGTAA--TGTTTGAAAAAT-AAAGCTGTTT

Bos taurus    GAATTA--ATTTGTTTTCCTTTCTCAAATCAGCAAGCTGTGTCTCTGAGTACC-----ATATAAGACAGTGTTCAGAGAGCT---------------------------AACCATCATA
Homo sapiens  GACTTGTCAGGTGTTTTCCTTTCTCAAATCAGCAAAT--TCTCTCTGAGTGCCTGGCTTTGTGAGACACTGTACAAGGAGTTACAAGACTACAGCTATAACCTGCAGTTGAGCAGTTATA

Bos taurus    AACCTACCAGCCTGGCCCTGCTCATGG-------CCTCTGAATATGAAAAGCTAA-----------------ATAAATAACCTTGTGTGTAAAGAAG-GAAGCACACACTGGAAGCCAGT
Homo sapiens  AACCTACAAAATGGGCCCTGCCCTCAGAGAGGTTCCAGTCTAGATGAGGAGCTGATCTAGACAGGTAAAAGGCTAACTAACCCTTTGTGTAAATAAGTTCATCACC-------------

Bos taurus    AGAAGCCAAGAGAAGTGTCGTTGCTGAATGAGTATGACTCCTCCATCCTG--GATTCCTCTCTTTCCTTT----------------------CTTGGGAAGTACTCTCCACGTC--TCT
Homo sapiens  -----CCAGTAAAAGTGTCATCACCCAGTGAATAGGACCACCTCTGCCTGCAGATTTTTGTTGTTGTTGTTGTCATTGTTGTTGTTGTTTTAACCTGGGAAGTGTTCTTCCTGCCTTTCT

Bos taurus    GCTGGGTGTCAGATAGATGGTTCCAG--------GCAGCATGTTACCCCCAAGGC--------------------------CACAGAAGTCCATGGTGTGAGGGACATTCTCAAGAAAG
Homo sapiens  GCTAGGTGTCAGATAGATGGTCCCAGAGCTAGGTGCTGTGTCAGGCCCTGAAGACACAGATGACTCAACCTAAGCTTTACTTTCCAGAGGTCCACAGCCTGAGAGGTGTCCCCAAAGAAA

Bos taurus    GGTAGACATCACAGGAGGGCCTG--GAGGAGCAGGGACGCACTGACCATGTTTTGATT-------CAGGCCAG--TGGTTAGAGAAGAAGATTTTTAGTGACGACAAAAGACAAAGAAGA
Homo sapiens  GGGGGACATGAGGGGACTGCATGCTTGAGAGCAGGGTTGTTTAGGGCAGGTTTGGATTTAGTGAGCAGGCTGGTTTGCTTAG---AGAAGGCTTTTAGTGGCAACAAAGGATGAAGAGGA

Bos taurus    GAGGAAGTGAGCTGATAGTTACTGAGAAAATGCTCTGCGCCAGGCATCTTACATGTATTTCCTTTCATGTGTACAGCATCCCCAGTGTGGGATAATTATGCTCATTTTAAAAGAGAGGGG
Homo sapiens  GAGAAAAGGAACTCACATTTATTGAGGGCCTACTGTGTGCAAAGTGTTTCATGTATATCTCATTGAATGTATACAGCCACCCTGTTGTGGTATAATTTTGCTCTTTATAA..........

Bos taurus    GGACTTCCCTGATGTTCCAGTGGTTAGGACTCCACAGTTCCAAAAATAAAAAAGCAGGGGTTGAAGATTTGATCCCTGGTTGGAGAGCTAAGATCCCACATTGCCAAATGGCACAGCCAA
Homo sapiens  .....................................................................................................................

Bos taurus    AACTAAACAAATTATAATTTTTAAAAATATCTCCCAAATAAAAATAACTAGATAAGTGAGAAAGAAAAAAAAGTCCTCATGGGTTACATGGTCTGCTCAACATCAAAACGCTAACAGGTG
Homo sapiens  .......................................................AGAGAAAGACCGAAGCTCAGATGAGTTAAGTGGTCTCCTCAACACCAAAATGCCAAGAAGTG

Bos taurus    GCAGAGCACA-ACGGAAGTCGGGACCTCTGTGACTCTCCCTAATCCGCTCTCCACCGTGATCGCAGTTTTCAAATCGTGCTTTGTAGTTCTGCAGGGACATTTCAGCAATTCCATGTTGA
Homo sapiens  ATGGAGCCTAGACAGAAGCCCAGAACTTTCTGACTCACACTAGTCCATCCTCTACCATCACGATGACTTTCAAATTGTGCTCTGCAGTTCTGCA--GATTTTCTAGCAGTGCCATCTCCA

Bos taurus    AATTGTATTTTACACTCTTTAAATCTCTATTAGATAACAGGTAGACAGACACAAGTGTGCTGATATCCAGATTTTAACATACTGGCATTTGCCAGCAGGCTCACTGTTGTTTCCAGATTG
Homo sapiens  AAATGTGTTTTAAACTCTTTAAGTCTCTAGTAAAT-----GCAGCTAGATTCAAATGGGCTGATAACCAAATTTTAACACATCAGCATTCACCACCAGGTTTACTTTTATTTTCAGATTG

Bos taurus    ACTCATTTTGTGCAGACCTTGGAAAAAAGATTCCCTGGTGAGATCCAAGCACACAGCTAAACTTCTACTCATTCTTCGTAGATTAAGGAAGTCATAGCACTTCAGAGGTGTTTCATTCAG
Homo sapiens  GCTCATTTTGTGCAGACCTTAGAGCAAAGTTTCCTTTATGGTATCTGTGTACGTATCCAAACTTCTTTTAATTGTTCACAGATTTTAAAAGCGGTAGCACCACATGGTTGTGTAGATCAG

Bos taurus    GCTTATGCAT--------GTTCTGTG----ATGCCAA-GTGTGCTTCAAGCATAAATGAGCATCATTCAGT--TAAGCACTACCCAAGGCTCCAACACAATCGATGTCTCACATCCTGGC
Homo sapiens  ACCTGTGTATTTAGATCAGACCTGTGTATCACGTAAGTGTGTGAGTGCAGTGCAGATGAGCACCATTTAGTTATATGTGCTAGGCAAATCTCCAACACAGTTGATGTGTAGTCTTGTGGT

Bos taurus    AGCTTTGTATATACAACAAACAATTGGGCTAGCTTCTCTAAGTATCAGTTTTCCTCATCAGAAAAATGGGAGGCCTATAGAATAGGGTTGTTGTTAGGATCAAATGACATAATGCCTTTT
Homo sapiens  AGATTTGTGCATACTGTAAGCAAATTGCTTAGCTTCTCTAGACATCAG-TTTCCACATCTGAAAAATAAGAAGATGAGAGTACACGGTTGTTAT---GAACAAATGACTTAATGCTTTTT

Bos taurus    AAGCACTTAACGTGGTGTCCGGAACACAGAAAGCCCTCAATACGTGGAGGCTCTTTCTCTTATCTTTCCCATGCTTATTGTCTGATCAGTGCATGTTCTCTTC-TTTTTCTA--AGTCCT
Homo sapiens  AAGCACGTTGCATGACATCTGGAACACAGAAAGCCCTCAATACATTGAAGCTCT---TAGGATTTTCACGATG-TTCCTGTCTGCTCAATGCATGCTTTCTTTATTGTTCTGACAGTTGT

Bos taurus    GCAATAATGAGTAAATAGGTAATCTGTCCGGTTGCGA--CAACATTGCATTAGAAACCA-TTAAGTACTTGTGGTAAGTTGGCGCGCTTATTG--CATTATCTTTGTGATCAGTTTTCCA
Homo sapiens  GTGGTAACAAGCTAATATGCTTCCAGTTGACTTCCAGTCTACCCTGGTGTTAGAAACCGTTTCATCTCTTATTGTAAATTTGAGTGCTTGTTGTTTTTATATTTGTGATGACTCTTCCA

Bos taurus    GCTATTGTTGACTATTGTTAGTGA------TTATAAGAAATAAATTATTCTTTCTGATGGTAAATAATTTTAACTGAAGGAATATCTTGAGATTGTAAGAAAAGGCTTTTATTTAAAAAA
Homo sapiens  GCAGTTGTTGACAATTGTTAGAGGTTTGACTTTTAAATAATTACTTATTTTTTCTGATTGTGGTTCAGTTTAACTGAA-GAATATCCTGAGATTGTAAGAAAAGCATTTTTT--------

Bos taurus    AAAAATGTACCACTTGTGAGTACTTACCTTTACAAATAATAGTTCAATTTTTCAATACTG-TCAAACAAAGTGAT--------------ACACCCGGATTTCATGTGTTTTTTGTATTT
Homo sapiens  -AAAAGGTATCACTTGTGATCATTTATCTTTCTAAATTCTA-------TTTTTAATACTGTTCCACCAAAGTGATGCAGTGGTTACCATGACACCCTAATTTCATGTG-TTTTTGTATTT

Bos taurus    GTAAAAATGGCCTCATTGTTCTCATGTATTCTCAGT------AGTAAAGTGTTATAAATGTGAAGTTATAAAATAAATCCATTCTGGTAAGATTCTGAATTTTTTGTGCTTTGTGTATTA
Homo sapiens  ATGAAAATAGTTTCATTG---TCATTTATTGGCGGTATACAAAGTAAAATGTTATAAATGTGAAGTTATAAAATAAATATATGCTAATAAAATCCTGAGTTTTTC-TGTTTCCTATATCG

Bos taurus    TGGTTCTGTATAAACTTTTGCTTAACAAAGGGATTTAATTACTAAAAAAAAAATTTTTTTTGAGAGATAGAGATTAGAAAATCAAGCTAAAACCACAAAAGATCTTGAATTCTTCTAGGAA
Homo sapiens  TGG-TCTCTATAAAGTACTGCTTGAAGAAGGAGCTTTGTTACTGAAAAA-------TTTTGAGAGATA-AGTTGAGAAAATCAAGCTAAAGC--CAAAAGGTCTTAGAATCCTGTAGG--

Bos taurus    AAAAAAAAAACCGAGAGC---AAAATGCTGGGATGGAAATACTTGGACTTGGTCTCCAAAGATGAGAGGGAGCTTAGTTCACCCATCACTGATTTCCCTTTTAACCTACA-AGAAATGAC
Homo sapiens  -CAAGAAAACCTGAAAGCAAAAAAATGCTTGAACAAGAATACTTGAACTATGTCTCTGGGGACAAGGGAGAGCTCGGTCCACCCATTATCGATTTCTCTTTTAAATATACAGAAACAAC

Bos taurus    AGATGGAGGCCGGGTCCCTTGGAGA-CCTCCAGCCCATTTGCTTAGACAAGTAACTGTACCTTGACATTCACCAGCCTCTATTCTGCCAAAGAGGAAGTGACCCAAAGGTGAAATACTGC
Homo sapiens  AGATGGAGGTCAGTTGCCTCAGAGACCCCCCAGCTCATTTGCTTAGGAAAGTAACTGTACAGATAAATTC-CCAGACTCTATTCTGCCAAGGGTGAATGACCCTGAAGGTCGAATATTGC

Bos taurus    ATTTCTTGGATGAAGTCATTTTTTGCTATATAGATATATTGTCCTGGTGCTCCAGTCATGTCTGACTTTTTGCAACCTCATGGGCTAGCCCACCGGGCTTCTCTGTCCATGGGATTTTCC
Homo sapiens  TTTTCTGGGCTGAAGTCA.......................................................................................................

Bos taurus    AGGTAAGAATACTGGAGTGGATTGCTTTTCCTTCTCCAGGGGATCTTCTCGACCCAAGAATTGAACCTGAGTCCCCTATGTCGGCAAGCAGATGTTTTACCG
Homo sapiens  .....................................................................................................


Homo sapiens        CCCCCACACCGACCTGGTCGTTGATGTTCTGCAGGGCTTCTTTGCCCGAGGCCCTCCGGATCTCCACCTTCCGGCCCGATTCAACAGATGGCTCTCCGCCGGCGCGCCGGCCCTGCCGCG
Loxodonta africana  CTCCCATACCAACCTGGTCGTTGATGTTCTGCAGGGCTTCCTTGCCCGAGGCCCGACGGATCTCCACCTTCCGGCTCGATTCAACAGATGGCTCCCCGCCGGCACG-CGGCCCTGCCGGG

Homo sapiens        AGTACGGCGGCTGCGGGTCCGGGCCCAAGTGGGCGACGTCCCGCTGCTGAGCCACCACCTGCCCCGAGCCCCGGCCCACCGACAGCGAGTCGAAGTCGATGGTCTTGTTCTCCGAGGACC
Loxodonta africana  AGTACGGCGGCTGCGGGT-CGGGCCCAAGT-GGCGACGTCCCGCTGCTGAGC--ACAACTG-CCCGAGCCC--GCCCA-CGACAGCGAGTCGAAATCGATG--CCTGGTCT-CGAAGACC

Homo sapiens        CTTCCACCGGGCAGAACATGCCACAGAATTTCTGCCGGGGCTTGGGGCTGCCGGAGC---CCAGGTTTTTAAAAAAAGCAGGGACCTCTTCGTCCTCTGCCGCCTTCCCGGATTGCTTTC
Loxodonta africana  CTTCATC--GGCAG-ACATG-CACAG-ATTTCTGTCGGGCCT--GGGCTGCTGGGTCGCGCCAGGT-----ACACCGGCAGG-------TCGTCCTCCGTGTTCCACGCGCGTCGC---C

Homo sapiens        TCTCGGCCATGGCTCGGGGGCTCGGGTAGGGTCCTCGCCGTCCGTCCGTCCGTCTGCCAGTCCCTAAGTCTCCCTCGCTGTG---TGCACAAGCCCCCTAGCTG-----CTGCCGCGGCT
Loxodonta africana  GGTCCGCCATGCCC-----GTCCAACTGCGGCCGC--CCGCCCGCCTGCGCACCCGCCCGACCCT-----TGCCTTGGTGCGCTCCGCACTCGGCGCCTTGGTGCGGGCCCGCCGCGGCC

Homo sapiens        GCCGTTCGCCGCCCACCGCAAGGTGCACAGAAAGGAAGG---AGGGCTG---GCGAGCCGGCGACCCACCCTCCCGGTCCCTCCCCCTGACCCCGCTTCTCCCCTCCCTGCCCACGCCCC
Loxodonta africana  CCGGCGCG------ACTGCG----GCGCGGGAGGGGCGGGCCGGGGCGGGGCGCGAGGCGG...........................................................

Homo sapiens        CCGCGGCTACAGCGCGAGCTCGGGGAAGCGGCAAAGTTGGATCTGGGAGGGCGTCCGCTGCGGCTGC-GGGAGCCGAAAGGCTGCCAGTTCCCGGC-AGCGCTGGGAGGA-GGGAGGCGC
Loxodonta africana  .GGCGGAGCCGGGGCGGGCCGGGGCGGGGCCTACGGGAGGCTGTGGGTAGGCAAGCGCT--GGCTACAAAGGATCGAAAGGCGG---GTGCCGGACTAGGGCTGCGAGGGCCGGTGACGC

Homo sapiens        GGGCGGGGCGCGGCGCCGGGGAGGGG---------AGCGGTGCTCAGG---------------------------GGAGGGCTGGAGGGGAGGGAAGGAGAGAGAGAGGGGAGGGCGGC
Loxodonta africana  GGCTGAGCCGCAGCGCGGAGGGCGGGCTGCAGAGCCGCAGTGCCCCAGCTGCACCCTCACTTCCCGCCCCCCAGGCGGAGCGCAGGGTGCGAG-------GAGAGAGAGGGAAGGGCGGG

Homo sapiens        ACCGCCCCTAGCCCCGCGCTCCGGAAGTGAAGCGGCCAGACCACCAGCTAATGGATGCGGAGCGGAGGGCCCGCTGACCGCTCTCCGCGCCTGGGTAAGTATCGGGCAGGGCACGCGGAG
Loxodonta africana  A--GCCGCGGGCACGGCGC.....................................................................................................

Homo sapiens        TGAGGACCGGAGGACGCAGCCCCCAGCCCCAGTGCCCCTCTCCTCCACCTGCCTGGCCCAGGCGCCTCTGCCCCGCCGCATGGCGGAGGTGGGCCGGGGGCGAGGGCGCACTGTGGATAG
Loxodonta africana  ........................................................................................................................

Homo sapiens        GGGTCCCGCCCCGTTACCCTGGGTGTGAGCTGATGGATGGGAAAAGGGGACAAGCCGGGCGCCAGGCTACTCAGGGTGTGGGAAGCGGGATGGCACTGAAGATGAGCAAGGATAGCACAG
Loxodonta africana  .....................................................................................................................CAG

Homo sapiens        GCAGGGTGGACAGGAGGGGGGCAGCCAAGACGCTGGACACCCCAG--CTTCAGTTTGTC-CACTCGCCTTGG-CAGGCTGCAGGGAGCAGAGACTGAAACGAGAAGGTTCCCGCTTCGTG
Loxodonta africana  GCGGTGCCGAGAATAGGGTTCCATCCGAGGCGCCGGAAAACCAAAGCCTCCAGTTCACCTCACGGGCCCTGGGTGGGCTGCGTGGAGCAAAGGATGACGCGAGAACATTCTTGCTTGGTG

Homo sapiens        CATCCACTTTGGTGTGGTTGGGGTGACGGGGGAGTGGGGGGGGGGCACACAACATGGCGGGAAGAAATTCAGAGGGGCGTAGCAGATGGGAGAGGAATATAGAGAGAGACGCAGGTCTGC
Loxodonta africana  TATCCCC------------------------AGGGGGTGGGGGGTGCACAACGTGTTCAGAAGAAATGCAGAGGGGCGTAGCAAATGAGGAGG---TATAAAGAGAGACTTAAGCCTGT

Homo sapiens        GGTCTATGGGGGTGT-AGCAAAGAGTGCAGTCGG-GAATTCCAGCCCAGGGCCACGACTTAA---AGACGGGGTGCAGACCGACTCTATTGCGCAAGG-CGCCATGGACCGTGTCTGCGC
Loxodonta africana  GGTTTATGGGAGTTTTAGCAAGGAGTGCAGTCTGCGGATTCCAGTGCAG--CCACTTGTAAAGACAGAGAGGGTGTAGACCCGCTACATTGCCTGAGGGCCCCATGTAATATTTCTGCGC

Homo sapiens        CACTGCGCCAAGAAGGGCGCAGGAACGTGTCGCGATTTGGTTTATTCTGCTTCCTCCACCAAAAAAAAAAAAAAAAAAAAAAAAAGAGAGAAAAAACAAACTGAAACCCCCTTCCCAAAT
Loxodonta africana  CCCTGCGCCAAGAAGGGCCAAGGAGAGTGTCTTGATTTGATATCTTCCACCGCCCCTCTCCAAAGGGGAAAATACGTAGAATAAAG--------------------CCCACTCCCTAAAT
```

```
Homo sapiens        CTCCAGCTTTCCCCTGCGCCCCAGAGCCCCGCCTGGT-TCCCCCGCCCCTGTTGCGCACTGCGCCACAGTCGCAGCGTTTCCCGGGTTACCAGATGCCACGTGTTTGGGGAGTAAGTGCG
Loxodonta africana  CTCCAGGCCTTCCTCACGCCCAAGCACCCCGCCTAGTCTCCCCCCCCCCTGTTGCGCACTGCGCCAGAG------------CAGGG---CCAGACGCCACGTGCCTGTGGTGTTAGTGTG

Homo sapiens        GGTGCGCATCTGGTGCGGAGTAGGAGGTAGGATGTGGGCTGTGA----GTGCCATGAATT--GTGTGGGGCCAAGCAGGGGAAAGGTGTGTAGGAACTTGGTGTGTGTGTGCAG-TAGCT
Loxodonta africana  --TGCGCATCCGCTGGGGACTAGGAGGCAGGGTGCGCACTGTGATAGAATGCAGAGAATTACGTTTGGGGACAAGAGGGGCAACTGTGTGGAGG--TGTGGTGTTTCTGTGGGGGTGTTT

Homo sapiens        GAGCCACGGCCACCGGGTTGGTAAGTAACCAGGGGGCAATTAGATTTGTGTGCCTAACACGGTCAATGCTGGTCCTTCATTGCTTATTCCCATCTCCACGTCCTTTCCGCGTATCAGTCC
Loxodonta africana  CAGCCATGACCACCGGGTCGGTAGGCATTCAGGTGTCATTTTGATTTGTGTTAGTAACGCCGTCGATGCTTACTCTTC-----TTGTCCTC---------TTCTTACCGTATATCAATCT

Homo sapiens        CTGCCTCTGCGCGATGACAGCTCCGCCTCTCTTTCCACAGTGGCAAAATCCTGCAGAAATGGTGCCCCTCCTTTGGTGGCCCGGGAGGGAGTTCTGGGCATGCTCAGTAACCAGGGTGGG
Loxodonta africana  CTGCTTCTGATCGAGTGCAGCCCC-CCTCTTTTCCTACAGTGGCAAAATTTTGCGAAAATGATGCCCACCCATCCGAGGCTCAGAAGGGAGATCTGGCCTTGCTCTGTGACCAGGGCGGG

Homo sapiens        TTTGGGCTGAGAGGCCGTTTACCGCAGGGCCACCGCGTAGGGCCGTGT-AACCAGGGATCCAGTCCCATTGTCAGCCC-----------AGGATTAAATGCGGTTACCAACCACCCCCAC
Loxodonta africana  TTTGGGCTAAGAGGCGATTTCCCGCAGCATCACCACGCCAGGCATTACGGGCCAAGGATTCAGTACCGTTGTCAACCCTTGTCCCTGGAAGGATTAAATGTGGATCCC--CCACCCCT--

Homo sapiens        AGTCCAATTGTGAGAGATAGTTTGGTGAATATAGTTACATCTGCTGGACAATCAGTCGCAATTTGAAGATCAGCCCATCTTGATAGGTGGAAAGGCGTCTCCGGATGAGAA--ACAGACT
Loxodonta africana  ------ATTGTCAGTGATGGTTTGGTGAATGGGGTTAAATTTGGAGGACGACTAGTCACAGTGTGAAGATGAGCCCATC---ATAGGTGGGAATGAACCTCAAAATGAGGAGGGGAGAAT

Homo sapiens        GGGGAAGAAGAATATAGGAGTGGAGGGGCTGAGAGAACCGGAGGG---GGCATTTTGGACTTAGCGGCAGATGTGCTTTGCATTTGAATAAAGCATTTGAAAAAAGCGCTTCCAACTGAA
Loxodonta africana  GAAGAAGAAGAATACACATATGGAGCAGGCGAGAGACCCCCATAGGCAGGTATTCCGGATTTAGCATCAGATAGACTT---------------CTTTTGAACTAAATGCTTTCACGTGAA

Homo sapiens        TTACATCATTTCCTACTCACAGCCCCACTGTGAGATGCGT------GCCTTATCAGCACTGCTTTCCAGGTTAGGAAAC--AGATTTAGAGAGCTTG-GGGCGCATAGCCAAGGTTGGGT
Loxodonta africana  TTACGTTATTACCTACTCACGGCAGCCCTGTTAGATAAATGGGACAGGTTTATTGTCACTATTTTCCAGGTTAGGAAACTGTGATTTAAAAGGATTGAGGGACCTTGGCCAGGGTGACCT

Homo sapiens        ATTTAGTTAAATGATCCAGCAATTCCAGTGGGAGTCTTCCACCTCAAAGTCTGGCTTCTTTTCCAGCTTGTCCTG-TGACCCCCTTTATGTGAGAAAATACAAGGTT-----CTTCTATT
Loxodonta africana  ATCTAGTTAACTGATGCAACAGTTCCACTGGGGGTCTTCCATCTGAAAATCT-GTTCCTTTTCCAGTTTATCCTATTGGTTCTGTTGATGTGAGAAAATATAAGGTTTTCACTTTTTTTT

Homo sapiens        TCTACCGTATCCAGATATTTCCATCCATGGCCCTATTGTATATCAAGGGTGTTTCTTGCCATGTTTTTATTTGAAGGTAACACTGCAGGTCACACTGGGAGGAAGAGTCATCTGAAGATA
Loxodonta africana  TTTTCCATATCTAAGTACTTCCATCCATGGCCCTATTGGATACCAAGGGTGTTTCACTTGCTTTTTTTATTTGAAAGTCACATTGCAGGT..............................

Homo sapiens        GCCTAACTTCAGCATAGCAGTCATTCTGTAGACCATAGAGATGCTTTTTCCTGGGCAGTGTCTTTTTACATTCCCTGTTTCCTCTAAATATCCCAGTGGGGAGGAAGGTTGCCCCCAGCT
Loxodonta africana  .........................................................................................................................

Homo sapiens        GGTTTTTTTATTTTTGTTTTTGTTTTCAGACAGAGTCTCACACTGTCACCCAGGCTGGAATGCAATGGTGCAAACGCAACTCACTGTAGCCTTGACCCACTCGGCTCAAGCAGTCCTCCC
Loxodonta africana  .........................................................................................................................

Homo sapiens        TCCTCAGCCTCCCAAGTAGTTGGGACTACAGGCGAGTGCCACACGCCCAGCTAATTTTTTTATTTTTCCTTTTTGTAGAGACAGGGTCGCACTATGTTGCTGGTCTCAAACTCCTGGGCT
Loxodonta africana  .........................................................................................................................

Homo sapiens        CAAGTGATCCTCCCGCTTTGGCCTCCCAAAGTGCTGGGTTTACAGGTGTAAGCCACTGCACCTGGCCCTCCCCCACAGTCCCCACATTTTAGAAAGGAAAAGTCAGGCATGGAGAAGTCA
Loxodonta africana  .........................................................................................................................

Homo sapiens        GCAGTCTCAGGTCTCTGGGGCTCCTGGGCACAGGGGTGAAGCAGCATTTTGCCCTCCTGGCTCTCTGGCACCCCTGGTGCCTTCAGCACTTCCCAGAA-GACTCAGGAGATCTTCCCACA
Loxodonta africana  .....CTCATGTCCTTGGGGCTCCTGGGGATAGGAGTAAAATAGCATTTTGTTTTCCTGGCCTTTTGGCACTTATGGTGCCTTCAGCACCTCCTAGAAGGACTCAGGAGATGTACCTGCA

Homo sapiens        GCAGGAGCA-CTGCTGATAGAGTTACAGGACAGCACTGTGGCGTATGGCCCCCATTCGAATTGATGAAGATGTTTAGGCCAAGGGGGAAATGGTTGTAGCCCCTTAACACACACTCC-GC
Loxodonta africana  ACAGGAGCAGCTGCTGTTCTAGCTACAGGTTGGTATCATAGCATATGGACCTTCCTCAGATTGATGGGGATTTTCATGCCAAGGGGAGAACGATTGTAGCACCCTAACGTGTACTCCAGC

Homo sapiens        AGCAGTAACTGGGAGGTATTAGGAACTCTAACTTTTTTTTTTTTTTTTTTTGAGACAGAGTCTTGCTCTGTTACTCAGGTTAGAGTGCAGTGGTGCAATCGTGGCTCACTGCAACTTCCG
Loxodonta africana  AACGCTGACTGGGAGGCATCAGACACTCT............................................................................................

Homo sapiens        TCTCCCAGGTTTAAGCGATTCTCCTGCCTCAGCCTCCCAAGTAGCTGGGATTACAGGCACGTGCCACCACACCCAGATAATTTTTGTATTTTTGGTAGAGACGGGGTTTGGCCATGTTGG
Loxodonta africana  .........................................................................................................................

Homo sapiens        CCAGGCTGGTTTCGAATTCCTGACCTCAGTGATCTACCCGCCTTGGCTTCCCAAAGTGCTGGGATTACAGGCATGAGTCACCACGCCTGGCCAACTCTAACTTTTTTACGTTCTCTAATC
Loxodonta africana  .........................................................................................................................

Homo sapiens        CTCACAGGTTCTCCAGAACAAAATCCTTGATAGGTGTGCTTCCTCCTTTTTTCTTTTTTCTCAAATGATGGGTTAAAGTTCAAATGTTAGGAAAGGCTGGGTGTGGTGATTCAAGTGTGT
Loxodonta africana  .........................................................................................................................

Homo sapiens        AATTCAGCACTGTGAGAGGCTGTGGCGGGAGGATTGCTTGAGCCCAGGAGTTTGAGACCAGGCTGAACAACGTAAGGAGACTGCGTCTCTACACAAAATTTAAAAAACTAGCCAGGCATG
Loxodonta africana  .........................................................................................................................

Homo sapiens        GTGGCATGTGCCTGTGGTCCCAGGTACTCAGGAGGTCGAGGTGGGAGGATTGCTTGAGCCCAGGAGAGATCAAGGCTACTACAGTGAGCCGTGTTTACACCACTGCATTCCAGCCTCGGC
Loxodonta africana  .........................................................................................................................

Homo sapiens        AACAGAGTGAGACCCTGTCTCAAAAAACAGAAACAGAAACAAATGCTTGGAGAGTGTTGTGTTAGTAACTTATTGTCTTAGTCTATTTTGTGTTGCTATAAAGGAATACCTGAGGCTGGA
Loxodonta africana  .........................................................................................................................

Homo sapiens        TAATTTATAAAGAAAAGAGGTTTATTTGGGTCACAGTTCTGCAGGCTGTACAAGAAGTATGGTGCTGACATCTGCATCTGGTGACCCCTCAGGAAGCTTCCAGTCATAGCAGAAAGCAAA
Loxodonta africana  .........................................................................................................................

Homo sapiens        GAGGGAGCAGATGCGGCAAGAAAGGGAGCAAGAGTGAGGGGAGGGAGGTGGCAGTCTGTTTTTAACAATCAGATCTCGAGGAAGCTAATAGAACAAGAACCCACTCATTACCCCAAGAAT
Loxodonta africana  .........................................................................................................................

Homo sapiens        GGCACCAAGCCATTCATAAGGGATCCACTCTCATGACCCAAACACATCCCACCAGGCCCCACCTCCAACACTGGGGATCACATTTCAGCATGAGATTTGGAGGGGACAAACCTTCAAACT
Loxodonta africana  .........................................................................................................................

Homo sapiens        ATTTCACTTACCATTCATAGCAAAAGCAAAGCCCAAATGTGTGTTTGCAAAGCCTT-CCAGACACAACAAAAGAGAATTAATTCATAATTCCCGATTTAACTTAGTCGGCCCTGAGATTT
Loxodonta africana  .........ACCATTCATAACAAAAGAAATGCC---------------AAAGACTAACCAGACGTAGCAAAAAAGAATTAGTCCATGATTCCAAGTTTAGTGTATTTGGCCCTAAGATTT

Homo sapiens        TTTTCCCTGAATTGTTATACCTAGATTAAACTATTTTTAAACGTTTGAGTGATTCCTCATTAGTT--TTGTTAATCAAAGAGGTAAAGCAACTTAATA----TTGAGTAGT---------
Loxodonta africana  TCTTTTTCTAATTGCTATCTATATATTAAATTATTTTTAAATGTTTGACCGATTCCTAACTGGGTATTTTTTAATCAAAGAAAGGAAGCAGTTCAATAGCTGTTTAGTAGAGCTAGCTAA

Homo sapiens        ATTGTGAAGGATTATCATATTTTTGAATAAGTGCATTAACGGTCTTCCT-ACTGAATATTAATGAAGCATAAATACCTGTTCTTATTGTTTATGTGTAACAGAGCAGCTTGGCTTGGCTG
Loxodonta africana  ATCGTGAAGGATTGTCATATTTTT-AATAAGTGTAGAAACAGTCTTTATGACTGCATATTATTGAGCTATAAAACCACATGATT----TTTATGTGTAGCAGGACATCTTGACTTGGCTG

Homo sapiens        GAGCTAAGAGCCAGACACACCACTGTGTGGAGGTGGGTGATGTCTTCCTGTGCTAAAAGGTGAATAAATAAGCTCCTCACCTCTCGCGGAACACTCGGGAACACATCAACAGGTATTTAG
Loxodonta africana  GAGCTAAGATTCAGAGACACCATCGTGTGGAGTTGGGTGACATCATCCTATACTAGAAGCTGAATAA----GCTGTTCAC-TCTCAAGGAACATCCTGGAACATATAAACAGGTATTTAG

Homo sapiens        CCACATCTCCTGATTTCATCTTTCA-ATGGCAAAGACCTTGAATCCTGTCCTTAACTGGGGCTCAGACATTAATCAACAGGTCTGATGAGCATATGGTGGGGACACCAGATTTCATTGGA
Loxodonta africana  CCACATCTTTTGATTTCATCCCTTATGTGGGAAAAATCTTGGATCTTGTCCAAAACTGGGGTTTAGACATTGATTGACATGTCTGATGAGCATATGCTGGGGAAGCTGTATTTCAGAGG-

Homo sapiens        GTCCCCAAGAGAAAA---AAAAGTCACAGTGAGCAAAGAAGGGGTCT-GACAGAGTGGGACAAATGCAAGCGCAGTGTAAAGCAGAACCGCCA-TCTGCCCCCAGTTCCATTCCAGATTA
Loxodonta africana  GTCCCCAAGAGAAAATAAAAAAGTCACTATAATCAAAGAAGAATTCTTGACAGAGTGGGCTAGACACAGGTACAATGTAAGACAAAACCCCTGGTCTACCCCCACTTCCTTCCCAAACTA

Homo sapiens        GAGTATGGATGCTTCCAAGGAAAGAGAGTGCTGT-GTAGTAAAACTGAACATTGTTTCACCAGGGGTCCAAGCCGCCCTGCTGGGAGGCTTCTCTTCAAGAGTTCTGGGTCCCAGAGTGG
Loxodonta africana  GAATATGTATGCTTCCTGGTA-------TGCTTCAGTGGTAAAACTGAAAATTGTTTCACTAGGAGTCCAAGTCACGCTACTGTGAGACTTCTCTTCAAGAGTTCAGGGTGCCAGAGTGG

Homo sapiens        AAGGCATTTTCCCATCAACTGGAGAGAGACGAAACATCAGAGACCAGGAGGCTGTGGAGAAAGCAGCTGTCCCAGGTGCCTCAACTATCAGAGAAGGGTCAG---CGTCACGTGGCTGCC
Loxodonta africana  AAAGTATTTCACCGCTGACTAGAGAAAG------TATCAGAGACCGGGAGACAGTGGTCAAAGCAGCTGTTCCAGGTGCCTCGGTGATTGAAGAAAGGTCAGGACCACCACGTGGCTGCC

Homo sapiens        AGCATCTTTGAGAAAATCACTGGCAATCGGACTTCAGAGCTGCGGGCACAGGTGTGGTTAGAACTGAGATACGACCTGCCCA-CCTGGGTCAGGCCTAAAG-----ACAAGAAGTCCTGA
Loxodonta africana  AGCATCGCTGAGAGAAACAGCACCAAGAGGACTGCAGAGCCGCAGGTAGTGGCTCTGTTATAGGTGAGACACAACCTACCCGTCCTGGATCAGTTCTAGAGACATTACAGGAAGTGCTGA

Homo sapiens        GTTCTTGCCACTGAGTAGGCCAGGGTCATTTGTCCAGAAAACTTTGTGACTG-TCTTTGAGTGACCTAGTCTGGGACCCATTCATTGGTGGGTTCTAAGGTTAGAAGCTCATCCAGGATA
Loxodonta africana  GTTGTTGCCGCTGGGCAAGTAAGGGTTAACAGTCCAGGAGATTTTGAGACCACTCTTAGAGTGACCTGACCTAGGATCCACCCCCT-------------------AGGTCATCCAGGATT

Homo sapiens        TTTTCAATAT-TAAGTCAGTGCATAGCTGCACCACTAACAAATTGGTGCCTGTA-GAGTCAGAGTGGGTCAATTCTTAGGACAATGGCGCTGGCACTGTTAGAGGACTGGTGCAGGATAA
Loxodonta africana  TTTTTTTAATCTAAGGCAGTGCATATCTGCCCCACAAACTAACTGGTGCCCCTACGAGTCAGGGCAAGTGGATACTTGGAGCGATGGCACTGGCACTGCTAGAGGACTGGTGTGGGATAA

Homo sapiens        TGAGTGTGGATGAGCAGAAGTCACTGATGGTTACGGGGATACCGGCGGACTTTGAGGAGGCTGAGATTCAGGAGGTCCTTCAGGAGACTTTAAAGTCTCTGGGCAGGTATAGACTGCTTG
Loxodonta africana  TGAGTGTGGATACTCAGAAATCACTGTTGGTTATGGGGATACCAGTGGAGTGTGATGAGCCTGAGATTCAGGAGGTTCTTCAGGAGAATTTGAAGTCCCTGGGCAGGTATAGACTGCTTG

Homo sapiens        GCAAGATATTCCGGAAGCAGGAGAATGCCAATGCTGTCTTACTAGAGCTTCTGGAAGATACTGATGTCTCGGCCATTCCCAGTGAGGTCCAGGGAAAGGGGGGTGTCTGGAAGGTGATCT
Loxodonta africana  GCAAAATATTCAGGAAGCAGGATAATGCCAATGCTGTCTTATTAGAGCTTATGGAGGATATTGATATCTCCGTGATCCCCAGTGAGGTGCAGGGAAGGGGCGGTGTCTGGAAAGTGGTCT

Homo sapiens        TTAAGACCCCTAATCAGGACACTGAGTTTCTTGAAAGATTGAACCTGTTTCTAGAAAAAGAGGGGCAGACGGTCTCGGGTATGTTTCGAGCCCTGGGGCAGGAGGGCGTGTCTCCAGCCA
Loxodonta africana  TTAAGACCCCTAATCAGGACACTGAATTTCTTGAAAGACTGAACCTCTTCTTAGAAAAAGAGGAACAGACATTAGCGGGTATGTTCCGAGCCCTTGGGCATGAGGGAGTGTCCACAGCTG

Homo sapiens        CAGTGCCCTGCATCTCACCAGAATTACTGGCCCATTTGTTGGGACAGGCAATGGCACATGCGCCTCAGCCCCTGCTACCCATGAGATACCGGAAACTGCGAGTATTCTCAGGGAGTGCTG
Loxodonta africana  CTGTCCCCTCCATATCACCAGAGTTACTGGCCAATTTGGTGGGACAGGCAATAGCGCATGCACCTCAGCCTCTGCTACCCATGAGATACCGAAAACTGAGAATATTCTCAGGCAGTGCTG
```

```
Homo sapiens        TCCCAGCCCCAGAGGAAGAGTCCTTTGAGGTCTGGTTGGAACAGGCCACGGAGATAGTCAAAGAGTGGCCAGTAACAGAGGCAGAAAAGAAAAGGTGGCTGGCGGAAAGCCTGCGGGGCC
Loxodonta africana  TTCCAGCCCCAGAGGAAGAGCCCTTTGAAATCTGGTTGGAACAGGCCACTGAGATAGTCAAAGAGTGGCCAGTAGCAGAGGCAGAAAAGAAAAGGTGGTTGATGGAAAGCCTGCGTGGCC

Homo sapiens        CTGCCCTGGACCTCATGCACATAGTGCAGGCAGACAACCCGTCCATCAGTGTAGAAGAGTGTTTGGAGGCCTTTAAGCAAGTGTTTGGGAGCCTAGAGAGCCGCAGGACAGCCCAGGTGA
Loxodonta africana  CTGCTCTGGACCTCATGCATATAGTGCAGGCAGACAACCAGTCCATAAGCGTGGAAGAGTGTTTGGAGGCATTTAAACAAGTGTTTGGAAGCCTGGAGAGCCGCAGGACCTCACAGGTTA

Homo sapiens        GGTATCTGAAGACCTATCAGGAGGAAGGAGAGAAGGTCTCAGCCTATGTGTTACGGCTAGAAACCCTGCTCCGGAGAGCGGTGGAGAAACGCGCCATCCCTCGGCGTATTGCGGACCAGG
Loxodonta africana  GGTATCTGAAGACCTATCAGGAGGAGGGCGAGAAAGTCTCTGCCTATGTGTTACGGTTAGAAACCTTGCTTCGGAAAGCTGTTGAGAAGCGTGCCATTCCTAGAAATATTGCAGATCAGG

Homo sapiens        TCCGCCTGGAGCAGGTCATGGCTGGGGCCACTCTTAACCAGATGCTGTGGTGCCGGCTTAGGGAGCTGAAGGATCAGGGCCCGCCCCCCAGCTTCCTTGAGCTAATGAAGGTAATACGGG
Loxodonta africana  TCCGTTTGGAACAAATCATGGCTGGGGCGAGCCTTAGTGAAATCCTTTGGTGCAGGCTTAGGGAGCTGAAGGATCAAGGACCGCCTCCCAGCTTCCTTGAGTTGATGAAGGTAATACGGG

Homo sapiens        AAGAAGAGGAGGAAGAGGCCTCCTTTGAGAATGAGAGTATCGAAGAGCCAGAGGAACGAGATGGCTATGGCCGCTGGAATCATGAGGGAGACGACTGAAAACCACCTGGGGGCAGGACCC
Loxodonta africana  AAGAAGAAGAGGAAGAGGCCTCTTTTGAACATGAGAATCTTGAAGATCCAGATGAGGCGGAAACCTATGGCCAGTGGGTTAACCAGGCCAGTGCGTAAGGTCGAAACTGGAATAGGA-TC

Homo sapiens        ACAGCCAGTG--------------GGCTAAGACCTTTAAA-AAATTTTTTTCTTTAATGTATGGGACTGAAATCAAACCATGAAAGCCAATTATTGACCTTCCTTCCTTCCTTCCTTCCC
Loxodonta africana  ACAG-TAGTGGCCTCACTCTCCTAGGCTAAGACCTTTCAATATTTTTTTTTCCTTCATGCACTGGGTTAAAATCAAGACATTCAATTTAAGTCTTAACTCTTCT...............

Homo sapiens        TCCCTTCCTCCTTCTCTCCTTCTCTCCTCCTCTCTCCTCTCCTCTCCTCTCTTTCCTTCCTTCCTTCCTTTTTTCTTTTTCTCTTTCTTCTTTATTTCTTGGGTCTCACTCTCATCACCC
Loxodonta africana  ........................................................................................................................

Homo sapiens        AGGCTAGAGTGCAGTGGCACAAAAATCTCGGCTCACTGCAGCCTTGACTTCCCAGGCTCAGGCTCAGGTGATCCTCACACCTTAGCCTCCCAAGTACCTGGGACTACAGGCACGCACCAC
Loxodonta africana  ........................................................................................................................

Homo sapiens        CATGCCTAGCTATTCTTTTGTATTTTTGGTAGAGACAGGGTTTTGCTGTGTTGCTCAGGCTGGTCTGGAACCCCTAGGCTCAAATGATGTGCCCAACTCGGCCTCCCAAAGTGCTGGGAT
Loxodonta africana  ........................................................................................................................

Homo sapiens        TACAGGCATGAACCGCCATGCCTGGCCCTTGATTTTTCTTTTTAAGAAAAAAAATATCTAGGAGTTTCTTAGACCCTATGTAGATTATTAATGAACAAAAGATTAAACTCCAAATATTAAA
Loxodonta africana  .............................................AAGAAAAAATATCTGAGAGTTTCTTGGACCCTACATAGATTAATAT----AAAAGATTACATACCAACTGTGAGA

Homo sapiens        TAGTAAGCCTGAAGGAATCTGAAACACTTGTACTTCCAATTTTCTTTAAATAATCCCAAATAGACCAGAATTGGCCCATACCATAGAAGAAAGAATTGGCAGTCAAAAAAAAAAATACCT
Loxodonta africana  TCATAAGCTGAAAGGAATCTGAAACACCAGTACTTAAAGCTTCCTTAAAATA--CCCAAATACACTAAAATTTATCCATAA-ATAAAAGAAATAATTGACAT----GAGAAAAAATATTT

Homo sapiens        TTTGTAA--TGTTTGAAAAATAAAGCTGTTTGACTTGTCAGGTGTTTTCCTTTCTCAAATCAGCAAATTCTCTCTGAGTGCCTGGCTTTGTGAGACACTGTACAAGGAGTTACAAGACTA
Loxodonta africana  ATTGTAAAATATTTGAAAACCTAAGCTGTTTTACTTATTAAGTCTTTATCTTTCTCCTATCAGCAAATTCTTTCTGAGTCCCTAGCATCGTGAGGCACTTTACAGAGAAGTGCGAG----

Homo sapiens        CAGCTATAACCTGCAGTTGAGCAGTTATAAACCTACAAAATG-GGCCCTGCCCTCAGAGAGGTTCCAGTCTAGATGAGGAG-CTGATCTAGACAGGTAAAAGGCTAACTAACCCTTTGTG
Loxodonta africana  -----------------------TCATGAACTTACCAAACATGGCCATGCCCTCAGGGAGTTTACAGTCTAGTGGAGGAGGCGGGCCTACAAAAGTGAAAAGTTAAATAACCATATGTG

Homo sapiens        TAAATAAGTTCATCACCCCAGTAAAAGTGTCATCACCCAGTGAATAGGACCACCTCTGCCTGCAGATTTTTGTTGTTGTTGTTGTCATTGTTGTTGTTGTTTTAACCTGGGAAGTGTTCT
Loxodonta africana  TAAATAAGTTCACATCACCAATAAAAGTGTTGTTGCCAAATGAATACTACCATCTCGGCCTTTAGGTTTCT---------------------TTCTTCTCCTTACTTGGGAAGCGCTCA

Homo sapiens        TCCTGCCTTTCTGCTAGGTGTC-AGATAGATGGTCCCAGAGCTAGGTGC-TGTGTCAGGCCCTGAAGACACAGATGACTCAACCTAAGCTTTA-CTTTCCAGAGGTCCACAGCCTGAGAG
Loxodonta africana  TCTCGCTTCTCTGTGAGGTGTCTGGACAGTTGGTCCCAGGACTAGGCACTTGTGTTAGGACCTGAAGAAACAGGTGACTCCACCTAAATGCCAGCTTTCAAGAGTTCCAGCAGCTGAGTG

Homo sapiens        GTGTCCCCAAAGAAAGG-GGGACATGAGGGGACTGCATGCTTGAGAGCAGGGTTGTTTAGGGCAGGTTTGGATTTAGTGAGCAGGCTGGTTTGCTTAGAGAAGGCTTTTAGTGGCAACAA
Loxodonta africana  GCGTCCTCAAAGAATGGTTGGAAATTCAAGGAGAAAAAGCAGGACAGTGAAAATGTGCAGGGCACGTTTGGATTTAGTGAGCAGGTTAG------AGAAGAAGGCTTTTAGTGGTA--AA

Homo sapiens        AGGATGAAGAGGAGAGAAAAGGAACTCACATTTATTGAGGGCCTACTGTGTGCAAAGTGTTTCATGTATATCTCATTGAATGTATACAGC--------CACCCTGTTGTGGTATAA-TTT
Loxodonta africana  AGGGCAAAGAGGAGAGGAAGGAAACTCACGTTTATTGAGGGCCTGCTAGGTGCCAGATGTTT-ACACATATCTCATTTAATGTGTACGGCAACTCTAACACCCTAATGTGGTATCGTTTT

Homo sapiens        TGCTCTTTATAAAGAGAAAGACCGAAGCTCAGATGAGTTAAGTGGTCTCCTCAACACCAAAATGCCAAGAAGTGATGGAGCCTAGACAGAAGCCCAGAACTTTCTGACTCACACTAGTCC
Loxodonta africana  TGCTCTTT-TGGAAAGAGAAAC--AAGCTCAGATGA-TTAAGTGGTCTTCTCAATGTCAAAATGCTGAGAAGTAACAGAGGCTAGATA------------TGTCTAACTCATGTTAATCT

Homo sapiens        ATCCTCTACCAT--------------CACGAT---GACTTTCAAATTGTGCTCTGC---AGTTCTGCAGATTTTCTAGCAGTGCCATCTCCAAAATGTGTTTTAAACTCTTTATTTTTTT
Loxodonta africana  GCTCTCTACCACGATAAAAAAAAAAAACACGATAGAGCTTTTCAAATTGTGTTTGGCAGAAGTTTT-----TTTTCCACCAGTTCCATCTGTAAAATGCATTTTAAAGTATTTA.......

Homo sapiens        AATTATTATTAGTATTATTTTGAGACTGAGTCTTGCTCTATCACCCAGGCTGGAGTGCAGTGGTGCAATCTCAGCTCACTGCAACCTCCGCCTCCCAGGTTCAAGCGATTTCGTGCCTCA
Loxodonta africana  ........................................................................................................................

Homo sapiens        GCCTCCCGAGTAGCTGGGATTACAGGCACCCACCACCACGCCCAGCTAATTTTTGTATTTTTAGTAGAAATGGGGTTTCACCATGTTGGCCAGGCTGGTCTCGAACTCCTGACCTCAAGT
Loxodonta africana  ........................................................................................................................

Homo sapiens        GATCCACTCACCTCGGCCTCCCAAAGTGCTGGGATTACAGGTGTGAGCCACCATGCCTGGGCTAAACTCTTTAAGTCTCTAGTAAATG-CAGCTAGATTCAAATGGGCTGATAACCAAAT
Loxodonta africana  .........................................................................AACCCATAATGAATAACAGGTAGACTCAAATGTGTTTATAGCCAAAT

Homo sapiens        TTTAACACATCAGCATTCACCACCAGGTTTACTTTTATTTTCAGATTGGCTCATTTT-GTGCAGACCTTAGAGCAAAGTTTCCTTTATGGTATCTGTGTACGTATCCAAACTTCTTTTAA
Loxodonta africana  TTTAGCACGTTGGCATTCACCAACAGGTTCGCTTT-ATTTCCAGATTGACTCATTTTTGTGCAGACCTTGGAACAAAGCTTCTTTTGCAGTATCTAAGCACATATTCCAACTTTTTTTCA

Homo sapiens        TTGTTCACAGATTTTAAAAGCGGTAGCACCACATGGTTGTGTAGATCAGACCTGTGTATTTAGATCAGACCTGTGTATCACGT-AAGTGTGTGAGTGCAGTGCAGATGAGCACCATTTAG
Loxodonta africana  TT-------GAGTAAGAAAACTGTAGTACTGCATGGGTGTTTAAGTCAGGCCCATGTGTGT-----------CTTGTCATGTAAAGTGTGCTAGTACA-TTTGTAGGAACACCATTCAG

Homo sapiens        TTATATGTGCTAGGCAAATCTCCAACACAGTTGATGTGTAGTCTTGTGGTAGATTTGTGCATACTGTAAGCAAATTGCTTAGCTTCTCTAGACATCAGTTTCCACATCTGAAAAATAAGA
Loxodonta africana  CTAAA--TGCCAGACACAGTTCCAACACCGTTGATATATAGCATCACAGTAGCTTTGTGTATACAAAAACCAAATTACTTAACTTCTGGAAACTTCCGTTTCCTCATCTGAAAAATGCAT

Homo sapiens        AGATGAGAGTACACGGTTGTTATGAACAAATGACTTAATGCTTTTTAAGCACGTTGCATGACATCTGGAACACAGAAAGCCCTCAATACATTGAAGCTC-TTAGGATTTTCACGATG-TT
Loxodonta africana  AGG---------GTGGTTGTTAGGATCAAATGACATAATGCCTTTTAAGCACTTAGCATGATGTCTGGAACGTAGAAAGCCCTCAATAAGTTGAGACACATTATTATTTTTATGATGCTT

Homo sapiens        CCTGTCTGCTCAATGCATGCTTTCTTTATTGTTCTGACAGTTGTGTGGTAACAAGCTAATATG----CTTCCAGTTGACTTCCAGTCTACCCTGGTGTTAGAAACCGTTTCATCTCTTAT
Loxodonta africana  ATTGTCTGTTCAGTGCATGCTTTCTTTATTGTTACAACAGTTGTGCAGTAAGAAA--AATATGTAACCTATCAGTCGACTTGTGA--TAACGTTGCATTGGAAACCGTTTTAGCTCTTAC

Homo sapiens        TGTAAATTTGAGTGCTTGTTGTTTTTTATATTTGTGATGACTCTTCCAGCAGTTGTTGACAATTGTTAGAGGTTTGACTTTTAAATAATTACTTATTTTTTCTGATTGTGGTTCAGTTTA
Loxodonta africana  TCTAAGTTTGAGTGCTTGTTGTCTTTCATATTTGTGATGAGGATTCACACA---GTTGACAGTTGCTAGTGATTTG------AAACAGTAATTTACTCTTTCTGATCTTGGATCATTTTC

Homo sapiens        ACTGAAGAATATCCTGAGATTGTAAGAAAAGCA--TTTTTTAAAAGGTATCACTTGTGATCATTTATCTTTCTAAATTCTA----TTTTTAATACTGTTCCACCAAAGTGATGCAGTGGT
Loxodonta africana  ACTAAAGAATATCCTGAGATTGTAAGAAAAACATTTTTTTTTAAAGGTATCATCTGTGA----TTATTTCTCTAAGTTACACATTTTCTCAGTACTCTTCAACCAAAGGCATATAATTGT

Homo sapiens        TA-CCATGACACCCTAATTTCATGTGTTTTTGTATTTATGAAAATAGTTTCATTG---TCATTTATTGGCGGTATACAAAGTAAAATGTTATAAATGTGAAGTTATAAAATAAATATATG
Loxodonta africana  CCTCCGTTGTACCCCAATTTCATGTGTTTTTGTATTAATGAAAATGGCCTCATTGTTCTCATTTACTGAAAGTATGTAAAGT-GAATGTTACAAATGTGAAGTTATAAAATAAATATATG

Homo sapiens        CTAATAAAATCCTGAG-TTTTTCTGTTTCCTATATCGTGGTCTCTATAAAGTACTG------CTTGAAGAAGGAGCTTTGTTACTGAAAAA-TTTTGAGAGATAAGTTGAGAAAATCAAG
Loxodonta africana  CTAATAAAATTCTGCGTTTTTTCTGTTTCATGTATTGTACT-TCTGTGTACTACTGATATCTCTCGAAGAAGGAGTTTCATTCTAAAAAAATTTTGAGAGACAAGGTTAGAAAATCAAG

Homo sapiens        CTAAAGCCAAAAGGTCTTAGAATCCTGTAGGCAAGAAAACCTGAAAGCAAAAAAATGCTTGAACAAGAATACTTGAACTATGTCTCTGGGGACAAGGGAGAGCTCGGTCCACCCATTATC
Loxodonta africana  CTGAAGCCAAAAGGTCTTGAGCTCCTCTAGGCAAGAAAATTTGAGAGCAAA-------------AAGAATACTTGGGCTGTGTCTCCAGAGATGACAGAAAGTTCAGTTCACCCATCGTT

Homo sapiens        GATTTCTCTTTTAAAATATAC--AGAAACAACAGATGGAGGTCAGTTGCCTCAGAGACCCCCCAGCTCATTTGCTTAGGAAAGTAACTGTACAGATAAATTC-CCAGACTCTATTCTGCC
Loxodonta africana  GATTTCCCTCTTAAAACACACAGAGAAACCACAGATGGAGGCCAGTCCCCTCAGAGA-CCTACAACTCATTTGCTTAGGAAAAAAATTGTTCAGATAAAGTCATCAGCCTCT--------

Homo sapiens        AAGGGTGAATGACCCTGAAGGTCGAATATTGCTTTTCTGGGCTGAAGTCACCCTTTTTCTTCTTCTTCTTCTTCTTTTTTTTGAGACAAGGTGTCACCCAGGCTGGAGTATAGTGGCACA
Loxodonta africana  --GATGAAATTACC--GAAGAT-GGTTGTTTGTGTTCTGGGCTATGTTCACC....................................................................

Homo sapiens        ATCTCGGCTCACTGCAAACTCTGCCTACCAGCTTCAAGTGATTCTCATGCCTCAGCTTCCCGAGTAGCTGGGATTACAGGGGTGCACCACCACGTTCCAGCTAAGTTTTTGTATTTTTAG
Loxodonta africana  ........................................................................................................................

Homo sapiens        TAGAGACAGGGTTTCACTAT
Loxodonta africana  ....................
```

```
Homo sapiens          CCCCCACACCGACCTGGTCGTTGATGTTCTGCAGGGCTTCTTTGCCCGAGGCCCTCCGGATCTCCACC-TTCCGGCCCGATTCAACAGATGGCTCTCCGCCGGCGCGCCGGCCCTGCCGC
Dasypus novemcinctus  CCCCTCCACCAACCTGGTCGTAGATGTTCTGCAGGGCTTCCTTGCCCGA-GCCCGCAGGATCTCCACCTTTCCGGCTCGATTCAACAGATGGCTCCCCGCTGGCGCG-CGGCCCTGCCAG

Homo sapiens          GAGTACGGCGGCTGCGGGTCCGGGCCCAAGTGGGCGACGTCCCGCTGCTGAGCCACCACCTGCCCCGAGCCCCGGC---CCACCGACAGCGAGTCGAAGTCGATGGTCTTGTTCTCCGAG
Dasypus novemcinctus  GAGTA-----GCGCTGGGGTCGGGTCCAAGTGGGCGACGTCCCGCTGCTGA......GTGCGCGCCGAGCCGCGGCGGCCCACGCTGTACGCGTCGAAGTCGATGGTCTTGTTCTCGTAG

Homo sapiens          GACCCTTCCACCGGGCAGAACATGCCACAGAATTTCTGCCGGGGCTTGGGGCTGCCGGAGCCCAGGTTTTTAAAAAAAGCAGGGACCTCTTCGTCCTCTGCCGCCTTCCCGGATTGCTTT
Dasypus novemcinctus  GCGCCCTCCACCGCGGCGAACATGCCGCCGTACTTC..........TGGGGCTGCCTGA-CCCAGGTTTTTAAAAAG-GCAGGGACCTCTTCGTCCTCGGCCTGTTTCCCGGACTGCTTC

Homo sapiens          CTCTCGGCCATGGCTCGGGGGCTCGGGT---------AGGGTCCTCGCCGTC----CGTCCGTCCGTCTGCCAGTCCCTAAGTCTCCCTCGCTGTGTGCACAAGCCCCCTAGCTGCTGCC
Dasypus novemcinctus  CTCTCGGCCATGGCGCGGGGGCTCGGGGGCTCGGGGGAGGGAGCTCTCCGTCAGTCCGTCCGTCCGTCTACCGGTCGCTAAATCTCCCTCTCTGTG--CAAAAATCCCCTGGTTGCTACA
```

```
Homo sapiens           GCGGCTGCCGTTCGCCGCCCACCGCAAGGTGCACAGAAAGGAAGGAGGGCTGGCGAGCCGGCGACCCACCC-TCCCGGTCCCTCCCCCTGACCCCGCTTCT--CCCCTCCCTGCCCACGC
Dasypus novemcinctus   GCGACTGTCGCTTTCTGCCCACCGCAAGGTGTACAGAAAGGAAGGAGGGCTCTCGAGCGGGCGTCCCACCCCTCGCAGTCCCTCCCCCTGACCCGGCTTTCTCCCCCTCTCTGCCCACGC

Homo sapiens           CCCCCGCGGCTACAGCGCGAGCTCGGGGAAGCGGCAAAGTTGGATCTGGGAGGGCGTCCGCTGCGGCTGCGGGAGCCGAAAGGCTGCCAGTTCCCGGCAGCGCTGGGAGGAGGG-AGGCG
Dasypus novemcinctus   CCCCCGCAGCC---GCGCGAGCTCCGACGAGCCGCGAAGTTCGGCCCGGGAGG-CGGCCGCTGCGGCAGCGGGAGCCGAGTGGCTGCCAGTTCCCGAAAGCGGGGGTACGAGGGGAGGCG

Homo sapiens           CGGGCGGGGCGCGGCGCCGGGGAGGGGAGCGGTGCTCAGGGGAGGGCTGGAGGGGAGGGAAGGAGAGAGAGAGGGGAGGGCGGCACCGCCCCTAGCCCCGCGCTCCGGAAGTGAAGCGGC
Dasypus novemcinctus   CGGGCGGGGCGCGGGGCCGGGGCGGGGAGGCG---------------------------------------GGGGAGGGCCGCGCCGCGCACAGCCCCGCGCTCCGGAAGTGAAGCGGC

Homo sapiens           CAGACCACCAGCTAATGGATGCGGAGCGGAGGGCCCGCTGACCGCTCTCCG-CGCCTGGGTAAGTATCGGG-CAGGGCACGCGGAG-TGAGGACCGGAGGACGCAGCCCCCAGCCCCAGT
Dasypus novemcinctus   CAGACCCCCGGCTAATGGATGCGGAGCGGAGGACCCGCTGGCCGCTCGCGGGCGCCTGGGTAAGTAGGGGGGCAGGGCCCGCGGAGGCGAGGGCCCGAGAGTGCAGCCCCTGGCCCCCTC

Homo sapiens           GCCCCTCTCCTCCACCTGCCTGGCCCAGGCGCCTCTGCCCC-GCCGCATGGCGGAGGTGGGCCGGGGGCGAGGGCGCACTGTGGATAGGGGTCCCGCCCCGTTACCCTGGGTGTGAGCTG
Dasypus novemcinctus   GCCCCTCTCCCCCACCTGCCTGGCCCTGGCGCCTCTTCCCCCTTCACTTGGCGGGGCTGGGCCGGAGGCGAGTGCGCCTCGTGGATAGGGCTCCCACCCTTGTGCCCGGGGTGGGAGCTG

Homo sapiens           ATGGATGGGAAAAGGGGACA-AGCCGG--GCGCCAGGCTACTCAGGGTGTGGGAAGCGGGATGGCACTGAAGATGAGCAAGGATAGCACAGGCAGGGTGGACAGGAGGGGGGCAGCCAAG
Dasypus novemcinctus   AGGGGTGTGGAAAGATGAAGCAGCTGGAGGCGCCAGGACTCCCGGGGGAAGGGCAGCG--------CTGGCAGTGCGGAAAGGTGGCCCGGGCAGCGCAGAGAGGAGAGGTCCAGCCGAG

Homo sapiens           ACGCTGGACACCCCAGCTTCAGTTTGTCCACTCGCCTTGGCAGGCTGCAGGGAGCAGAGACTGAAACGAGAAGGTTCCCGCTTCGTGCATCCACTTTGGTGTGGTTGGGGTGACGGGGGA
Dasypus novemcinctus   GCACGGCAAGCCGAAGCCTCGGTCTACCCACGGGCCTCGGTGGGCTGC-GGAAGCAAAGAC--AAGCGAGAAGGTTCTCGTTTGGTGCCTCCACCGCGGTG------------------

Homo sapiens           GTGGGGGGGGGGCACACAACATGGCGGGAAGAAATTCAGAGGGGCGTAGCAGATGGGAGAGGAATATAGAGAGAGACGCAGGTCTGCGGTCTATGGGGGTGTAGCAAAGAGTGCAGTCGG
Dasypus novemcinctus   -------GGGGCCGCACAACATGGCGGGAGGAA-TGCAGAGGGGAGTAGCAATGGGGGTTGTAAT-------GAGAAACATCTCTATCGTCTGTGGGAGGAGAGCAGAGAGCGCAGTCTG

Homo sapiens           GAATTCCAGCCCAGGGCCACGACT--TAAAG----ACGGGGTGCAGACCGACTCTATTGCGCAAGG-CGCCATGGACCGTGTCTGCGCCACTGCGCCAAGAAGGGC--GCAGGAACGTGT
Dasypus novemcinctus   CAAATTCGCAC----GCTGCAACTCGTAAAGCGGTAGAGGGTGCGGACCCGCTGTGTTGCCCGAGGGCGCCATATACCATATCTGAGCCCCTGTGCCAGGAAGGGCCAACAGGA--GTGC

Homo sapiens           CGCGATTTGGTTTATTC----TGCTTCCTCCACCAAAAAAAAAAAAAAAAAAAAAAAAAAGAGAGAAAAAACAAAC--TGAAACCCCCTTCCCAAATCTCCAGCTTTCCCCTGCGCCCCAG
Dasypus novemcinctus   CTCGATTTGGCCACTTCCACCTGTATCCTGGCCCAAA-----------------------GGGGGAAAATGAGCCTTGAAGCCCACTCCTTAAGTCCCCAGCCGGTCTCCGCGCCCTGG

Homo sapiens           A-GCCCCGCCTGGT-TCCCCCGCCCCTGTTGCGCACTGCGCCACAGTCGCAGCGTTTCCCGGGTTACCAGATGCCACGTGTTTGGGGAGTAAGTGCGGGTGCGCATCTGGTGCGGAGTAG
Dasypus novemcinctus   GCGCGCCGCCTCATCTCCCCCGCCCCTGTTGCGCACTGCGCCACCGCCGCTGGGTTTCTCTGGCTGCCAGACGCCGCTC--CTGGGGCGTAAGTGCTCGCGCGCGTC-GGCGGGGAGGGG

Homo sapiens           GAGGTAG--GATGTGGGCTGTGA----GTGCCATGAATTGTGTGGGGCCAAGCAGGGGAAAGGTGTGTAGGAACTTGGTGTGTGTGTGCAGTAGCTG-AGCCACGGCCACCGGGTTGGTA
Dasypus novemcinctus   AGCGGAGCGGGTGTGGGGCGTGAAAGGGTGTCTTGACTTGTGTGGGGCCAGCAGTGGAGAGGGTGTGTGGAGATGTGGTGTGCATGTGCGGATGTTCCAGCAACGGCCACCAGGTTGGGG

Homo sapiens           AGTAACCAGGGGGCAATTAGATTTGTGTGCCTAACACGGTCAATGCTGGTCCTTCATTGCTTATTCCCATCTCCACGTCCTTTCCGCGTATCAGTCCCTGCCTCTGCGCGATGACAGCTC
Dasypus novemcinctus   AGCATCCAGTGGGCATTTAGATTTGTGTGGGTAATGTGGTCTCTGCGGGCTCTTCACTGCTGACTCCTG-------------------GTCCGTTCCTGCCTCTGGTCGAGTGCCACCC

Homo sapiens           CGCCTCTCTTTCCACAGTGGCAAAATCCTGCAGAAATGGTGCCCCTCCTTTGGTGGCCCGGGAGGGAGTTCTGGGCATGCTCAGTAACCAGGGTGGGTTTGGG--CTGAGAGGCCGTTTA
Dasypus novemcinctus   CGCCCCTCCTTCCACAGTGGCCAAATCTTACCGAAATGGTGCCCATCCTTGGGCGGCCCGGAAGGAAGACCTGAGCATGCTGGGTAACCAGGGTAGGTTTAGGGTCCAAGAGG-------

Homo sapiens           CCGCAGGGCCACCGCGTAGGGCCGTGTAACCAGGGATCCAGTCCCATTGTCAGCCC-----AGGATTAAATGCGGTTACCAACCACCCCCACAGTCCAATTGTGAGAGATAGTTTGGTGA
Dasypus novemcinctus   ----------------------GAGTAGACCGGGATTCAGTCTCATTGTCAGCACTTGGAAAGGTTAAAGGCGGAT--------TCTCCGCTCTCC-ATTGTAAGAGATA----GGTCA

Homo sapiens           ATATAGTTACATCTGCTGGACAA-TCAGTCGCAATTTGAAGATCAGCCCATCTTGATAGGTGGAAAGGCGTCTCCGGATGAGAAACAGACTGGGGAAGAAGAATATAGGAGTGGAGGGGC
Dasypus novemcinctus   ATAGGATGAAATCTGGAGGACAACTCAGTCACAGTAAGAAGAGGAGCTC-TCCTGATACTTGGGAAGGAGTCT...............................................

Homo sapiens           TGAGAGAACCGGAGGGGGCATTTTGGACTTAGCGGCAGATGTGCTTTGCATTTGAATAAAGCATTTGAAAAAAGCGCTTCCAACTGAATTACATCATTTCCTACTCACAGCCCCACTGTG
Dasypus novemcinctus   .......................................................................AAGTGGTTTTTCATGAATTACACTGTTTCCTACTCCCAGCAAGCCTGTG

Homo sapiens           AGATGCGT------GCCTTATCAGCACTGCTTTCCAGGTTAGGAAAC---AGATTTAGAGAGCTTGGGGCGCATAGCCAAGGTTGGGTATTTAGTTAAATGATCCAGCAATTCCAGTGGG
Dasypus novemcinctus   AGGTGAGTGGGACAGGTTTATCCTGACCGTCTTCCAGGAGAAGAGACTATAAATTTAGAAAG------------------------------GAGAAACAGTACAGCAGTTCCATTGAG

Homo sapiens           AGTCTTCCACCTCAAAGTCTGGCTTCTTTTCCAGCTTGTCCTGTGAC--------CCCCTTTATGTGAGAAAATACAAGGTTCTTCTATTTCTACCGTATCCAGATATTTCCATCCATGG
Dasypus novemcinctus   TGTTGTCCACTGAAAAGTCTAGTTCGTTTTCTACTTTATTATGATATCAAGCTTGCCCCGTTATGCGAGAAAATACAAGGCCTTAAATTTTTTCCTATATACAGACATTTCCATCTGTGG

Homo sapiens           CCCTATTGTATATCAAGGGTGTTT--------CTTGCCATGTTTTTATTTGAAGGTAACACTGCAGGTCACA-------CTGGGAGGAAGAGTCATCTGAAGATAGCCTAACTTCAGCAT
Dasypus novemcinctus   CCATACTGTGCATCAAGGGTATTTTTTTTTCACTTGCCATCTTTTCA-TTGAAAGTCGCATTGCAGGTCGGAATTGCTTTTGCAAGGAGGCGCCATCCCAAGAGAGCCTAACCTCAGCAT

Homo sapiens           AGCAGTCATTCTGTAGACCATAGAGATGCTTTTTCCTGGG----CAGTGTCTTTTTACATTCCCTGTTTCCTCTAAATATCCCAGTGGGGAGGAAGGTTGCCCCCAGCTGGTTTTTTTAT
Dasypus novemcinctus   GGTGGATATTATGTAGACCTTACAGATATCTCTTCCTGGGTATTCAGTGTCTTTTT................................................................

Homo sapiens           TTTTGTTTTTGTTTTCAGACAGAGTCTCACACTGTCACCCAGGCTGGAATGCAATGGTGCAAACGCAACTCACTGTAGCCTTGACCCACTCGGCTCAAGCAGTCCTCCCTCCTCAGCCTC
Dasypus novemcinctus   ........................................................................................................................

Homo sapiens           CCAAGTAGTTGGGACTACAGGCGAGTGCCACACGCCCAGCTAATTTTTTTATTTTTCCTTTTTGTAGAGACAGGGTCGCACTATGTTGCTGGTCTCAAACTCCTGGGCTCAAGTGATCCT
Dasypus novemcinctus   ........................................................................................................................

Homo sapiens           CCCGCTTTGGCCTCCCAAAGTGCTGGGTTTACAGGTGTAAGCCACTGCACCTGGCCCTCCCCCACAGTCCCCACATTTAGAAAGGAAAAGTCAGGCATGGAGA-----AGTCAGCAGT-
Dasypus novemcinctus   ....................................................................CTCCATATTTTCAAAGGGGAAAGTGAGGCATGGAGAGAGAGAGAGAGCAGAG

Homo sapiens           ---CTCAGGTCTCTGGGGCTCCTGGGCACAGGGGTGAAGCAGCATTTTGCCCTCCTGGCTCTCTGGCACCCCTGGTGCCTTCAGCACTTCCCAGAAG-ACTCAGGAGATCTTCCCACAGC
Dasypus novemcinctus   GGACTCAGATCTCTAGGCTGCTGAGGAATAGGGGCAG-----------------------------------GAAGTCTTCAGTACTTCCTAGAAGTACTCTGGAGATATGCCTGGTAT

Homo sapiens           AGGAGCA--CTGCTGATAGAGTTACAGGACAGCA-CTGTGGCGTATGGCCCCCATTCGAATTGATGAAGATGTTTAGGCCAAGGGGGAAATGGTTGTAGCCCCTTAACACACACTCCGCA
Dasypus novemcinctus   GGGAGCAGCTTGCTACTGTAGCTATAGGTTGGCATCTGGGGCATATGGACCCTGCACAGATTGATGGAGATTTTCATACCAAGGAGAAAATGGTTTAGCACCTTAGCACACATGCCACA

Homo sapiens           GCAGTAACTGGGAGGTATTAGGAACTCTAACTTTTTTTTTTTTTTTTTTTGAGACAGAGTCTTGCTCTGTTACTCAGGTTAGAGTGCAGTGGTGCAATCGTGGCTCACTGCAACTTCCGT
Dasypus novemcinctus   GCAATAACTAGGAGGCATTAGGAACTCT............................................................................................

Homo sapiens           CTCCCAGGTTTAAGCGATTCTCCTGCCTCAGCCTCCCAAGTAGCTGGGATTACAGGCACGTGCCACCACACCCAGATAATTTTTGTATTTTTGGTAGAGACGGGGTTTGGCCATGTTGGC
Dasypus novemcinctus   ........................................................................................................................

Homo sapiens           CAGGCTGGTTTCGAATTCCTGACCTCAGTGATCTACCCGCCTTGGCTTCCCAAAGTGCTGGGATTACAGGCATGAGTCACCACGCCTGGCCAACTCTAACTTTTTTACGTTCTCTAATCC
Dasypus novemcinctus   ........................................................................................................................

Homo sapiens           TCACAGGTTCTCCAGAACAAAATCCTTGATAGGTGTGCTTCCTCCTTTTTTCTTTTTTCTCAAATGATGGGTTAAAGTTCAAATGTTAGGAAAGGCTGGGTGTGGTGATTCAAGTGTGTA
Dasypus novemcinctus   ........................................................................................................................

Homo sapiens           ATTCAGCACTGTGAGAGGCTGTGGCGGGAGGATTGCTTGAGCCCAGGAGTTTGAGACCAGGCTGAACAACGTAAGGAGACTGCGTCTCTACACAAAATTTAAAAAACTAGCCAGGCATGG
Dasypus novemcinctus   ........................................................................................................................

Homo sapiens           TGGCATGTGCCTGTGGTCCCAGGTACTCAGGAGGTCGAGGTGGGAGGATTGCTTGAGCCCAGGAGAGATCAAGGCTACTACAGTGAGCCGTGTTTACACCACTGCATTCCAGCCTCGGCA
Dasypus novemcinctus   ........................................................................................................................

Homo sapiens           ACAGAGTGAGACCCTGTCTCAAAAAACAGAAACAGAAACAAATGCTTGGAGAGTGTTGTGTTAGTAACTTATTGTCTTAGTCTATTTTGTGTTGCTATAAAGGAATACCTGAGGCTGGAT
Dasypus novemcinctus   ........................................................................................................................

Homo sapiens           AATTTATAAAGAAAAGAGGTTTATTTGGGTCACAGTTCTGCAGGCTGTACAAGAAGTATGGTGCTGACATCTGCATCTGGTGACCCCTCAGGAAGCTTCCAGTCATAGCAGAAAGCAAAG
Dasypus novemcinctus   ........................................................................................................................

Homo sapiens           AGGGAGCAGATGCGGCAAGAAAGGGAGCAAGAGTGAGGGGAGGGAGGTGGCAGTCTGTTTTTAACAATCAGATCTCGAGGAAGCTAATAGAACAAGAACCCACTCATTACCCCAAGAATG
Dasypus novemcinctus   ........................................................................................................................

Homo sapiens           GCACCAAGCCATTCATAAGGGATCCACTCTCATGACCCAAACACATCCCACCAGGCCCCACCTCCAACACTGGGGATCACATTTCAGCATGAGATTTGGAGGGGACAAACCTTCAAACTA
Dasypus novemcinctus   ........................................................................................................................

Homo sapiens           TTTCACTTACCATTCATAGCAAAAGCAAAGCCCAAATGTGTG--TTTGCAAAGCCTTCC-AGACACAACAAAAGAGAATTAATTCATAATTCCCGATTTAACTTAGTCGGCCCTGAGATT
Dasypus novemcinctus   ........ACCATTTATAGCAAAAGAAACACCTAGGTTTATGCATTTGCAAAGCCTGAGGAGACAAAACAAAAGACAATCTATTCTTAATTCCAAGTTTAACATCTCTAGCCTTGAGTTT

Homo sapiens           T---TTTTCCCTGAATTGTTATACCTAGATTAAACTATTTTTAAACGTTTGAGTGATTCCTCATTAGTT-TTGTTAATCAAAGAGGTAAAGCAACTTAATATTGAGTAGTATTGTGAAGG
Dasypus novemcinctus   TGTTTTTTTTTCTAATTGTTATTTCTATATTGAATTGTTGTTAAATGTTTGAACAATTCCTTACTGGGTATTTTTTACTAAAGAAGTGAAGCAGTTCAATA------GATGTTGCAAAGG

Homo sapiens           ATTATCATATTTTTGAATAAGTGCATTAACGGTCTTCCT-ACTGAATATTAATGAAGCATAAATACCTGTTCTTATTGTTTATGTGTAACAGAGCAGCTTGGCTTGGCTGGAGCTAAGAG
Dasypus novemcinctus   GTTGTCATACTTTTTAATAAGTGCATTAATAGTCTTCATCATCGGAAATTATCGAGCCATAAAAATCCATTGCTATTCTTTATATGAAACAGG------CAGCGTGGTTGGAGCTAAGAC

Homo sapiens           CCAGACACACCACTGTGTGGAGGTGGGTGATGTCTTCCTGTGCTAAAAGGTGAATAAATAAGCTCCTCACCTCTCGCGGAACACTCGGGAACACATCAACAGGTATTTAGCCACATCTCC
Dasypus novemcinctus   TAAGACACACCATCGT-TGGAGATGAGTGACACCTTCCTGTGCTAGAAGCAGGATAAATAAGCTTTTTGACTC--ATGGAACATTCTGGGACACAGCAACAGGTATCTAGCCACATCAAC

Homo sapiens           TGATTTCATCTTTCA-ATGGCAAAGACCTTGAATCCTGTCCTTAACTGGGGCTCAGACATTAATCAACAGGTCTGATGAGCATATGGTGGGGACACCAGATTTCATTGGAGTCCCCAAGA
Dasypus novemcinctus   TGATTTTATCTTTCATGTGGGAAAGATCTTGAGTCCTGTCCATAACTGGGGCTCAGACAGTAAT-AGCA--TATGGTGAGCATATGGTGGGGAAGCCAAATTGCAGAAAGGTCCCCAAGG
```

```
Homo sapiens          GAAAAAAAAGTCACAGTGAGCAAAGAAGGGGTCTGACAGAGTGGGACAAATGCAAGCGCAGTGTAAAGCAGAACCGCCATCTGCCCCCAGTTCCATTCCAGATTAGAGTATGGATGCTTC
Dasypus novemcinctus  GAAAAGAA--TCACTGTTAGCAAAGAGGAGGTCTGATGGAGGGGGATAGACCAAGGTACGATGGGAGGCAGATCCCTGGTCAAACCCCACTTCCACCCTAAACTAGGGTATGTATACGTC

Homo sapiens          CAAGGAAAGAGAGTGCTGTGTAGTAAAACTGAACATTGTTTCACCAGGGGTCCAAGCCGCCCTGCTGGGAGGCTTCTCTTCAAGAGTTCTGGGTCCCAGAGTGGAAGGCATTTTCCCATC
Dasypus novemcinctus  TTGGGATGGAAGAGGCCATGTGGTAAAACTGAAAATTGTTTTGCTAGGAGTCCAAGCCACTCTGCTATAAGACTTCTGTCCAAGAGTTCTCGGCATCGGAGCAGAAGGTGTTTCACCATT

Homo sapiens          AACTGGAGAGAGACGAAACATCAGAGACCAGGAGGCTGTGGAGAAAGCAGCTGTCCCAGGTGCCTCAACTATCAGAGAAGGGTCAG---CGTCACGTGGCTGCCAGCATCTTTGAGAAAA
Dasypus novemcinctus  TAGTAGAAAAAGGCAAAGCATCAGAGATGAGGAGACGATGGTGAAAGTTAGTGT-TCAAGTGCATCGGCAATCA-AGAAGGATCAGCACCACCACGCAGCTGCCAGCATCAGTGAAAACA

Homo sapiens          TCACTGGCAATCGGACTTCAGAGCTGCGGGCACAGGTGTGGTTAGAACTGAGATACGACCTGCCCACC-TGGGTCAGGCCTAAAG-----ACAAGAAGTCCTGAGTTCTTGCCACTGAGT
Dasypus novemcinctus  ACA---ACAACC-GACTGCAGAGCCACAGA-ACATG-ACACTTATAACTGAGATACAACCCACCCACCATGAGTCTGTTCTAGAGACATAACAGGAAGTACTGCACTTTTGCCACTGGGC

Homo sapiens          AGGCCAGGGTCATTTGTCCAGAAAACTTTGTGACTGTCTTTGAGTGACCTAGTCTGGGACCCATTCATTGGTGGGTTCTAAGGTTAGAAGCTCATCCAGGATATTTTCAATATTAAGTCA
Dasypus novemcinctus  TAGTAAGAGTTATTGGTCCCGAAGA------------CATTGAGGAATC---TCTTAGACCCATTTGCTGGTGGGTTATAAGTTTAGGAGTTCATTCAGGATCTTTAAAAT-CAAAGGCT

Homo sapiens          GTGCATAGCTGCACCACTAACAAATTGGTGCCTGTAGAGTCAGAGTGGGTCAATTCTTAGGACAATGGCGCTGGCACTGTTAGAGGACTGGTGCAGGATAATGAGTGTGGATGAGCAGAA
Dasypus novemcinctus  GTGCTTTTCTGCACCACAAACTAATTGGTA---------------------AATATTTGGGACAATGGCACTGGCACTGTTAGAGGACTGGTGCAGGATAATGAGTCTGGATGCTCAGAA

Homo sapiens          GTCACTGATGGTTACGGGGATACCGGCGGACTTTGAGGAGGCTGAGATTCAGGAGGTCCTTCAGGAGACTTTAAAGTCTCTGGGCAGGTATAGACTGCTTGGCAAGATATTCCGGAAGCA
Dasypus novemcinctus  ATCACTGCTGGTTATGGGGATACCAGTGGACTGTGATGAGGCTGAGATTCAGGAGGTTCTTCAGGAGACTTTAAAGCCTCTGGGCAGGTACAGACTGCTTGGAAAAATATTCAGGAGGGA

Homo sapiens          GGAGAATGCCAATGCTGTCTTACTAGAGCTTCTGGAAGATACTGATGTCTCGGCCATTCCCAGTGAGGTCCAGGGAAAGGGGGGTGTCTGGAAGGTGATCTTTAAGACCCCTAATCAGGA
Dasypus novemcinctus  GGATAATGCCAGTGCTGTCTTATTAGAGCTTATGGAGGATGTTGACAGCTCGGTGATCCCTACTGAGATTCAGGGAAAGGGAGGTATCTGGAAAGTGATCTTTAAGACCCCTAACCAGGA

Homo sapiens          CACTGAGTTTCTTGAAAGATTGAACCTGTTTCTAGAAAAAGAGGGGCAGACGGTCTCGGGTATGTTTCGAGCCCTGGGGCAGGAGGGCGTGTCTCCAGCCACAGTGCCCTGCATCTCACC
Dasypus novemcinctus  TGCTGAATTTCTCGAAAGACTGTACCTCTTTCTAGAAAAAGAAGGCCAGACAGTATCAGGTATGTTCCAAGCCCTTGGTCATGAGGGGGTACCTACAGCCGCAGCCCCCTGCATATCACC

Homo sapiens          AGAATTACTGGCCCATTTGTTGGGACAGGCAATGGCACATGCGCCTCAGCCCCTGCTACCCATGAGATACCGGAAACTGCGAGTATTCTCAGGGAGTGCTGTCCCAGCCCCAGAGGAAGA
Dasypus novemcinctus  AGAGTTATTGGCCCAACTAGTGGGACAGGCAATGGTGCATGCATGTCAGCCTCTGCTATCCAGGAGATACCGGAAACCGAGAGTATTCTCTGGGAGTGCTGTTCCAGCCCCAGAGGAAGA

Homo sapiens          GTCCTTTGAGGTCTGGTTGGAACAGGCCACGGAGATAGTCAAAGAGTGGCCAGTAACAGAGGCAGAAAAGAAAAGGTGGCTGGCGGAAAGCCTGCGGGGCCCTGCCCTGGACCTCATGCA
Dasypus novemcinctus  GCCCTTTGAAATCTGGTTGGAACAGGTCACTGAGATTGTCAAGGAGTGGCCAGTACCGGAGGCAGAAAAGAAGAGGTGGTTGATGGAAAGTCTGTGCGGCCCTGCCCTGGACCTCATGCG

Homo sapiens          CATAGTGCAGGCAGACAACCCGTCCATCAGTGTAGAAGAGTGTTTGGAGGCCTTTAAGCAAGTGTTTGGGAGCCTAGAGAGCCGCAGGACAGCCCAGGTGAGGTATCTGAAGACCTATCA
Dasypus novemcinctus  TGTATTGCAGGCAGACAATCCATCCATAAGCATGGGAGAGTGTTTGGAGGCATTTAAACAAGTTTTGGGGAGCCTGGAGAGCCGCAGGACCTCACAGGTTAAATTTCTGAAAACCTATCA

Homo sapiens          GGAGGAAGGAGAGAAGGTCTCAGCCTATGTGTTACGGCTAGAAACCCTGCTCCGGAGAGCGGTGGAGAAACGCGCCATCCCTCGGCGTATTGCGGACCAGGTCCGCCTGGAGCAGGTCAT
Dasypus novemcinctus  GGAGGAAGGAGAAAAAGTCTCAGCCTATGTGTTACGGTTAGAAGCCTTGCTCCGGAGAGCTGTGGAGAAACATGCCATTCCTAGAAATATAGCAGACCAGGTCCGTTTGGAGCAAGTCAT

Homo sapiens          GGCTGGGGCCACTCTTAACCAGATGCTGTGGTGCCGGCTTAGGGAGCTGAAGGATCAGGGCCCGCCCCCCAGCTTCCTTGAGCTAATGAAGGTAATACGGGAAGAAGAGGAGGAAGAGGC
Dasypus novemcinctus  GGCTGGGGCAAGTCTCAGTGAAATACTCTGGTGTAGGCTTAGGGAGCTGAGAGATCAGGGACCACCTCCCAGCTTCCTTGAGTTAATGAAGGT......................NAGGC

Homo sapiens          CTCCTTTGAGAATGAGAGTATCGAAGAGCCAGAGGAACGAG------ATGGCTATGGCCGCTGGAATCATGAGGGAGACGACTGAAAACCACCTGGGGGCAGGACCCACAGCCAGTGG--
Dasypus novemcinctus  AACTTTTGAAAATGAGAACCAGGAGGAGCCAGACGAGTGGGCCAGCCATGGCCATGGCTATTAGGATAATG-GGGCCACGACTGAGGAGCAACT-GGAGCAGGTCCTACAGCCAGGGGCC

Homo sapiens          ----------------------GCTAAGACCTTTAAAAAATTTTTTTCTTTAATGTATGGGACTGAAATCAAACCATGAAAGCCAATTATTGACCTTCCTTCCTTCCTTCCTTCCCTCCC
Dasypus novemcinctus  CTGGGGACATGCACTTAACTAAGCTAAGGCCTTCTCATTATTTTTTTCTTTAATGTACCAGATTGAAATCAAGGCACTCAAGGCAAGT................................

Homo sapiens          TTCCTCCTTCTCTCCTTCTCTCCTCCTCTCTCCTCTCCTCTCCTCTCTTTCCTTCCTTCCTTCCTTTTTTCTTTTTCTCTTTCTTCTTTATTTCTTGGGTCTCACTCTCATCACCCAGGC
Dasypus novemcinctus  ........................................................................................................................

Homo sapiens          TAGAGTGCAGTGGCACAAAAATCTCGGCTCACTGCAGCCTTGACTTCCCAGGCTCAGGCTCAGGTGATCCTCACACCTTAGCCTCCCAAGTACCTGGGACTACAGGCACGCACCACCATG
Dasypus novemcinctus  ........................................................................................................................

Homo sapiens          CCTAGCTATTCTTTTGTATTTTTGGTAGAGACAGGGTTTTGCTGTGTTGCTCAGGCTGGTCTGGAACCCCTAGGCTCAAATGATGTGCCCAACTCGGCCTCCCAAAGTGCTGGGATTACA
Dasypus novemcinctus  ........................................................................................................................

Homo sapiens          GGCATGAACCGCCATGCCTGGCCCTTGATTTTTCTTTTTAAGAAAAAAATATCTAGGAGTTTCTTAGACCCTATGTAGATTATTAATGAACAAAAGATTAAACT-CCAAATATTAAATAG
Dasypus novemcinctus  .......................CTTGATTCTTCTTTCTAAGACAAAAATGCCCAAGAGCTTCTT-GACCCTATGTAAATTATTAAT-AACAAAAGAGTAAATTTCCAAATATTAGAGAA

Homo sapiens          TAAGCCTGAAGGAATCTGAAACACTTGTACTTCCAATTTTCTTTAAATAATCCCAAATAGACCAGAATTGGCCCATACCATAGAAGAA-AGAATTGGCAGTCAAAAAAAAAAATACCTTT
Dasypus novemcinctus  TAAGCCTAAAGGAATCTG-AATACCAGTAATTCAGGTTTACTTTAAAT-ACCCTAAATACACCAGAATTAACCCATACCATAGAAAAAGAAAATTGACTTT------AGAAAATACCTTT

Homo sapiens          TGTAATGTTTGAAAAATAA--AGCTGTTTGACTTGTCAGGTGTTTTCCTTTCTCAAATCAGCAAATTCTCTCTGAGTGCCTGGCTTTGTGAGACACTGTACAAGGAGTTACAAGACTACA
Dasypus novemcinctus  GGT---------AAAATAGTTTACTATTT-ATTTGTGATGTATTTTTCTTTCTCAAATGAGCAGGTGCT---TAAGTCCCTGGTATTGTGAGGCAGTGTATAAGCATATACAG-------

Homo sapiens          GCTATAACCTGCAGTTGAGCAGTTATAAACCTA--CAAAATGGGCCCTGCCCTCAGAGAGGT-TCCAGTCTAGATGAGGAGCTGATCTAGACAGGTAAAAGGCTAACTAACCCTTTGTGT
Dasypus novemcinctus  --------------------AGCCATAAACCTGCCCAAAGTGTGCTGTGCTCTCGAGGAGTTGTATAATCTAGTGGAGAAGA-GGCCTAGACATGTGAAAAAATAGGTAATTA-TAGTGT

Homo sapiens          AAATAAGTTCATCACCCCAGTAAAAGTGTCATCACCCAGTGAATAGGACCACCTCTGCCTGCAGATTTTTGTTGTTGTTGTTGTCATTGTTGTTGTTGTTTTAACCTGGGAAGTG---TT
Dasypus novemcinctus  AAATAAGTTCATGGGGCCAATA------------------GAATATGACCATCGCTGCCCTTAGGTTTC-----------------------------TTCTCCTTTGGGAAGGAAGCTT

Homo sapiens          CTTCCTGCCTTTCTGCTAGGTGTCAG-ATAGATGGTCCCAGAGCTAGGTGCTGTGTCAGGCCCTGAAGACACAGATGACTCAACCTAAGCTTTACTTTCCAGAGGTCCACAGCCTGAGAG
Dasypus novemcinctus  TCCTCTGTTTCTCTGCCAGGTGTCTGAATGGATTGTTCAAGGCCTAGGGGACATGTTAAG-CATGAACACGCAAACGATTTGCCCTAATGCCTGTTCTCAGAACATCTGCAGTCTGAGAG

Homo sapiens          GTGTCCCCAAAGAAAGGG--GGACATGAGGGGACTGCATGCTTGAGAGCAGGGTTGTTTAGGGCAGGTTTGGATTTAGTGAGCAGGCTGGTTTGCTTAGA---GAAGGCTTTTAGTGGCA
Dasypus novemcinctus  GCATTCTCAAACAATGGGTAGGAGTTAAAGCAAGTTCCTCC--AAGAGTGAGCATTCACAGGGCATGTTTGGGTTTAGTGAGCAGGCCAGTCTGGTTACAGAGGAAGGTTTTTAGTGGCG

Homo sapiens          ACAAAGGATGAAGAGGAGAGAAAAGGAACTCACATTTATTGAGGGCCTACTGTGTGCAAAGTGTTTCATGTATATCTCATTGAATGTATACAGCCACCCTGTTGTGGTATAATTTTGCTC
Dasypus novemcinctus  GCAAA----------GAGAGGGAGAGAGCTCATATTTGTAGAGGACCTACTGTGTGCCAGATATTTGATGCACATCTCATTAAATGTATACAGCAACCCTAATA-GGTATTATTTTGCTC

Homo sapiens          TTTATAAAGAGAAAGACCGAAGCTCAGATGAGTTAAGTGGTCTCCTCAACACCAAAATGCCAAGAAGTGATGGAGCCTAGACAGAAGCCCAGAACTTTCTGACTCACACTAGTCCATCCT
Dasypus novemcinctus  TTTTTGAA-AGAGAAACTGAAGCTCACATGGATTAAGCAGTCTTTTCAATATCCAAATGCTAGGAAGTGATAGATGATAG-----AGCCCAAAACAGCCTCTCTCATGCCAGTACACCCT

Homo sapiens          CTACCATCACGATGACTTTCAAATTGTGCTCTGCAGTTCTGCAGATTTTCTAGCAGTGCCATCTCCAAAATGTGTTTTAAACTCTTTATTTTTTTAATTATTATTAGTATTATTTTGAGA
Dasypus novemcinctus  CCACCATGATGGTGGTTTCCAAGTTGTGCTCCCCAG------AGATTCTTCAGCAGGTCCATCTTTAAAATACATTCCAAA.......................................

Homo sapiens          CTGAGTCTTGCTCTATCACCCAGGCTGGAGTGCAGTGGTGCAATCTCAGCTCACTGCAACCTCCGCCTCCCAGGTTCAAGCGATTTCGTGCCTCAGCCTCCCGAGTAGCTGGGATTACAG
Dasypus novemcinctus  ........................................................................................................................

Homo sapiens          GCACCCACCACCACGCCCAGCTAATTTTTGTATTTTTAGTAGAAATGGGGTTTCACCATGTTGGCCAGGCTGGTCTCGAACTCCTGACCTCAAGTGATCCACTCACCTCGGCCTCCCAAA
Dasypus novemcinctus  ........................................................................................................................

Homo sapiens          GTGCTGGGATTACAGGTGTGAGCCACCATGCCTGGGCTAAACTCTTTAAGTCTCTAGTAAAT-GCAGCTAGATTCAAATGGGCTGATAACCAAATTTTAACACATCAGCATTCACCACCA
Dasypus novemcinctus  ......................................AAACTCTTTAAATTCACCCTAAATAGCAGGTAGGCTCAAA----CTAA-AACCAAATTTTAAAGCATTTGCAGTCACCAACA

Homo sapiens          GGTTTACTTTTATTTTCAGATTGGCTCATTTTGTGCAGACCTTAGAGCAAAGTTTCCTTTATGGTATCTGTGTACGTATCCAAACTTCTTTTAATTGTTCACAGATTTTAAAAGCGGTAG
Dasypus novemcinctus  GGTTTGGTTTTATTCCCAAGCTAACTCATTTTGTGCAGACCTGAGAACAAAGCTTCTCTTTCAGTGCCCGAGTGCATGTTCAAACTTCTTTTACTTATTTGCTAGGTAAGGACAGTGTAG

Homo sapiens          CACCACATGGTTGTGTAGATCAGACCTGTGTATTTAGATCAGACCTGTGTATCACGT-AAGTGTGTGAGTGCAGTGCAGATGAGCACCATTTAGTTATATGTGCTAGGCAAATCTCCAAC
Dasypus novemcinctus  TACTGCATGGGTGTTTAAATCAGACCTATTTGT--------GTCCTG----TCGTGTAAAGCGTGGAAGTTCAGCATTCGCGA-TGCCATTCA--CGTAGGTGCCAGGCAAAGATCTGAC

Homo sapiens          ACAGTTGATGTGTAGTCTTGTGGTAGATTTGTGCATACTGTAAGCAAATTGCTTAGCTTCTCTAGACATCAGTTTCCACATCTGAAAAATAAGAAGATGAGAGTACACGGTTGTTAT---
Dasypus novemcinctus  ACAGTTGATGTATAGCATCATGCTAGCTTTGTGTGTAC---AAGAAAGTTAGTTAACATCTCTAAACATCAGTTTCCTCATCTTAAAAATGGAAAAATAATAATATAAGGTTGTTGTTAG

Homo sapiens          GAACAAATGACTTAATGCTTTTTAAGCACGTTGCATGACATCTGGAACACAGAAAGCCCTCAATACATTGAAGCTCTTAGGATTTTCACGATGTTC-CTGTCTGCTCAATGCATGCTTTC
Dasypus novemcinctus  GATCAAATGACTTAA----------------GGCATGATGTCTGGAATATATG----CCCCAATAGATTGAATTCTTCACGGTTTT-ATGATGTTTATTGTCTGTTCAATGCATGCTTTC

Homo sapiens          TTTATTGTTCTGACAGTTGTGTGGTAACAAGCTAATATG----CTTCCAGTTGACTTCCAGTCTACCCTGGTGTTAGAAACCGTTTCATCTCTTATTGTAAATTTGAGTGCTTGTTGTTT
Dasypus novemcinctus  TTGATTG-TATAATAGTTGTGCAGTAACAAGTAAATACATAATCTGTCAACAGAGTTGTGATGAAACT--GCATT-GAGACAATGCTAACTCTTATTGTAAACTTGAGTGCTTACTGACT

Homo sapiens          TTTATATTTGTGATGACTCTTCCAGCAGTTGTTGACAATTGTTAGAGGTTTGACTTTTAAATAATTACTTATTTTTTCTGATTGTGGTTCAGTTTAACTGAAGAATATCCTGAGATTGTA
Dasypus novemcinctus  TTTATATTTGTTATGAGCATCCAAACAGGTGTTGAC---TGTTGATGATTTGACTTCAGAACAGTTATTTACTCATTCTGATTGTGAACCATTTTTAGAGGAGACTATCCTAAAATTGTA

Homo sapiens          AGAAAAGCATTTTTTAAAAGGTATCACTTGTGATCATTTATCTTTCTAAATTCTA----TTTTTAATACTGTTCCACCAAAGTGATGCAGTGGTTA-CCATGACACCCTAATTTCATGTG
Dasypus novemcinctus  AGAAAATC-CTTTTTAAAAAGTACCATTTAGGGTCA------TTTCTAAGTTATACATTTTCTCAGTACTCTTAAACAAAAGTGATAGAATTGTCATCCATTACACCCTGATTTCATGTA

Homo sapiens          TTTTTGTATTTATGAAAATAGTT---TCATTGTCATTTATTGGCGGTATACAAAGTAAAATGTTATAAATGTGAAGTTATAAAATAAATATATGCTAATAAAATCCTGAGTTTTTCTGTT
Dasypus novemcinctus  TTTTTGTATTTAAGAAAATGGCCTCATCATTCTCATTTACTCACAGTATCCAGTGTAAAC--TTACAAATGCAAAGTTATCAAATAAATATGCGTTAATAAAATTCTGTGTTTT--CATT

Homo sapiens          TCCTATATCGTGG-TCTCTATAAAGTACTGCTTGAAGAAGGAGCTTTGTTACT----GAAAAATTTTGAGAGATAAGTTGAGAAAATCAAGCTAAAGCCAAAAGGTCTTAGAATCCTGTA
Dasypus novemcinctus  TCGTATACCGTGGTTCTGGATGAACTATTGCTTGAGGAAGAAGTTTCATTACTTGAAAAAAAAATTTGAGAGAGAAGATTAG-AAACCAAGCCAAAGCAAAAAGGTCTTTAAATC---TA
```

```
Homo sapiens          GGCAAGAAAACCTGAAAGCAAAAAAATGCTTGAACAAGAATACTTGAACTATGTCTCTGGGGACAAGGGAGAGCTCGGTCCACCCATTATCGATTTCTCTTTTAAA--ATATACAGAAAC
Dasypus novemcinctus  GGCAAGAAAATCTAAGAAC---AACATGCTAGAATAAGAATATTTGGCCTGTGTCTCTAGGGATGAAAGAGAGCTCAGTTCACCCATCATTGATTTTGCTTTTAAGACACACACAGAAAT

Homo sapiens          AACAGATGGAGGTCAGTTGCCTCAGAGACCCCCCAGCTCATTTGCTTAGGAAAGTAACTGTACAGATAAATTCCCAGACTC-TATTCTGCCAAGGGTGAATGACCCTGAAGGTCGAATAT
Dasypus novemcinctus  AACAGATGGAGGCAAGTTCCCTTCGAGACCTCCAAGA-CATTTGCGTAGGGAAGTAATTGTACGTATAAATGCACCCACCCTTATTCAGCCAAGGATAAGTAA-CCTAAAGGTGGAAACT

Homo sapiens          TGCTTTTCTGGGCTGAAGTCACCCTTTTTCTTCTTCTTCTTCTTCTTTTTTTTGAGACAAGGTGTCACCCAGGCTGGAGTATAGTGGCACAATCTCGGCTCACTGCAAACTCTGCCTACC
Dasypus novemcinctus  TGTGTTTCTGGGCTGAATTCCCC.................................................................................................

Homo sapiens          AGCTTCAAGTGATTCTCATGCCTCAGCTTCCCGAGTAGCTGGGATTACAGGGGTGCACCACCACGTTCCAGCTAAGTTTTTGTATTTTTAGTAGAGACAGGGTTTCACTAT
Dasypus novemcinctus  .........................................................................................................
```

PNMA3 gene

```
>chromosome:GRCh37:X:152224166:152229427:1
AGGTCCCAGCAGCGGGATGGACAGCGGGGAGATGGCTGACCCTCCTTGGGGTGGTGTGCG
TGACCTGCATGAGTGCCTGGGGCATCTCCTGCACGGAATGGCACCCGCACCAGGTGCCCC
TGGCCTCGCCAGCCAGAGGACCAGTCCTGGACGGGGAGGGGGAGGACAAGAGCAGGTGAA
CAGGCCCCAGAACCCGGCTCCCTGACCCTGGCCATAATGCAGAGCTGCCTGGCGCACCTC
AGACCACTCCCTGGCCCTCTGGTGGGCACGCCGGGGTGCAGACCCCTCGCCCCGCTGGCA
CCTCCGTGGCACGTGCGGGAGGGGCAGGAGCCGGGACCAGGAACCGCTGTGGTGAGAGCC
AAAGGCACGGGGACTGGCCGCCACAGACCTTTGGCCACCGCAGTGGGGATGGGGGTGGGC
GGGCGCTTGTGTGCCCGGGGGCCCGGGGGCCCCCGTGGCGGGGGGGCTAGGGGAGAGGAG
CAGGTGACGTCACCCGGGGACCTGGGGCCCCCAGGGGCGGCGGCGAGAGCCCGACCTCGT
GACCCGTCCGCGCCAATGGCCCTGCGGTCTCCCGGACGACGCGGAGGGGTCCGCGCTGCA
CCGCCGCCGCGCCCCCGCCCCGTGTCCGCACTGCAGAGAGCCGCGGCGAGCAGGCGCCGG
CGCACTGGCCCACGTGCTGCGCGAGCGAGGGAGAGCCACAGTCTGAGCGAACGTCCGCGC
TGGGAGCCAGGGGTGCCCGACCCCCGTCCGCCGCCGCCGCCGCCGCCGCGCATAGCCCCC
GGAGAGCCCTCTGGGGACCCCGACCAGAAGGGACCTTGCCCTGGGAGAAGGTAACAGACC
CACCTGGGGAGGGAGACGCTGGGCCCCGGGAGACTCCGCCGCCCCTCGCCTGTCCGCCCT
ATCAGAACTGAAGCAGCCTGCAGGGCAGGGGTCCAGGGTCCTGGGGCAGCGCGGGGAGGG
GGCTGAGGCCCGCAGAGACCCCACGGGGGGTGGGGGTGGGGGGCTGGGCATGGGCCGGGC
CTGGCTGCAGAGGCGCCGCACGGGGAGGAGGGCGGCTGGGGGTGGGGGTGGGGGTGGGCG
TGGATGTGGGCGTGCGGAGAGGTGGGCATTAACCTCGCTCTCGCCCTGCTCGCATTCACA
GGCTGTGGAGACCTGGGCCTTCTGCGATCACCCTAGGAGTTGATCCAGATATGTGCCTCA
CGCCCTGATCACTCCCCCCAAATTAGTATCCGCAGAGATTCGAGGACATGCCGTTGACCT
TGTTACAGGACTGGTGTCGGGGGGAACACCTGAACACCCGGAGGTGCATGCTCATCCTGG
GGATCCCCGAGGACTGTGGCGAGGATGAGTTTGAGGAGACACTCCAGGAGGCTTGCAGGC
ACCTGGGCAGATACAGGGTGATTGGCAGGATGTTTAGGAGGGAGGAGAACGCCCAGGCGA
TTCTACTGGAGCTGGCACAAGATATCGACTATGCTTTGCTCCCAAGGGAAATACCAGGAA
AGGGGGGGCCCTGGGAAGTGATTGTAAAACCCCGTAACTCAGATGGGGAATTTCTCAACA
GACTGAACCGCTTCTTAGAGGAGGAGAGGCGGACCGTGTCAGATATGAACCGAGTCCTCG
GGTCGGACACCAATTGTTCGGCTCCAAGAGTGACTATATCACCAGAGTTCTGGACCTGGG
CCCAGACTCTGGGGGCAGCAGTGCAGCCTCTGCTAGAACAAATGTTGTACCGAGAACTAA
GAGTGTTTTCTGGGAACACCATATCCATCCCAGGTGCACTGGCCTTTGATGCCTGGCTTG
AGCACACCACTGAGATGCTACAGATGTGGCAGGTGCCCGAGGGGGAAAAGAGGCGGAGGC
TGATGGAATGCTTACGGGGCCCTGCTCTCCAGGTGGTCAGTGGGCTCCGGGCCAGCAATG
CTTCCATAACTGTGGAGGAGTGCCTGGCTGCCTTGCAGCAGGTGTTCGGACCTGTGGAGA
GCCATAAAATTGCCCAGGTGAAGTTGTGTAAAGCCTATCAGGAGGCAGGAGAGAAAGTAT
CTAGCTTTGTGTTACGTTTGGAACCCCTGCTCCAAAGAGCTGTAGAAAACAATGTGGTAT
CACGTAGAAACGTGAATCAGACTCGCCTGAAACGAGTCTTAAGTGGGGCCACCCTTCCTG
ACAAACTCCGAGATAAGCTTAAGCTGATGAAACAGCGAAGGAAGCCTCCTGGTTTCCTGG
CCCTGGTGAAGCTCCTGCGTGAGGAGGAGGAATGGGAGGCCACTTTAGGTCCAGATAGGG
AGAGTCTGGAGGGGCTGGAAGTAGCCCCAAGGCCACCTGCCAGGATCACTGGGGTTGGGG
CAGTACCTCTCCCTGCCTCTGGCAACAGTTTTGATGTGAGGCCTTCCCAGGGCTACCGGC
GCCGGAGGGGCAGAGGCCAACACCGAAGGGGTGGTGTGGCAAGGGCTGGCTCTCGAGGCT
CAAGAAAACGGAAACGCCACACATTCTGCTATAGCTGTGGGGAAGACGGCCACATCAGGG
TACAGTGCATCAACCCCTCCAACCTGCTCTTGGTAAAGCAGAAGAAACAGGCTGCAGTTG
AGTCGGGAAACGGGAACTGGGCTTGGGACAAGAGCCATCCCAAGTCCAAGGCCAAGTAGG
CTCGGGAGAACAGGGCAACATTTCCTACCACAGGCCAAGGAGACAAAAGAGATATTGGAA
GGAGGGGAAAGAGAAGCCCAGACAAACAGCAGATGAGTTGAGTGGGGCAGAGGGACAGGG
CAGCCAGACCAAGGCCAAGCCTTCTCACCCTTGGCCAGCTGGAAGGGACTTCAGCAACCA
AGACCACCTGGCAACAGGCTCAGTGGGGGTCAGGTCCAGGTCCCCGAAGAGGTGCTGGAG
AGGAAAGCAGGGAGCCACTGCATCCAGCACATGGGGTGCCTGGGCCTCAGATGGGGACCC
CAAAGAAGCAGAAGCTGAAGAAGGTACGGCTGGGGGTTCTGTCCTGCTCATCCAACCACC
CCTAAATACCCACCCTGTGGACTTTGAGCTGAACATGCCCACTGGCCCCCAGGCCACATG
GGACCTGGAGGAGCCTACCTGGGGCCTGCCCCTGCCAGCAGGTGCCAGGGCTGGTGAGGA
AGAGCTGGGGGGCAGAGGTAAAGCCCTGCAGGGGAGGCCACAGGGTCCATCCCGTCTTCA
GGATCATCTACACTGCACTAGGGGAGCCCCAGGAAGGCAGCACCCTGGAGGCCCTGTGCC
AGTGAGGACAGGAGACCCTAAGGCCCCGGGAGCCCAGTGCCAGCCAGAGGTTGTGCAGGC
AAGGAGACCAAAGATTGATGAGAAGACCCCCAGCAGGGGTACTGGGTACCCGGCAGGCCA
GTGCCCTCACAGTTGACTTGGACCAGGGTGGCTGTGAAGGGAAGTCTTTGTTGCAAAGGA
GGAGGAGGAAAAGGGAGGACTTGGTAGGGTTTTGTTTCTTCTGCTTGTTTCTGTACAGGG
CCACCAGACTCCTGGAGAGATCAAGCAAGGAGAACCTGGGGCTGCCATGGCCAAAGCAAC
TCAACAGATGCCAATGCCAATTCCAAGGCCAGCCACAACCCTGCCACCTTGGGGAATCCA
GCCTGGAGGCATCCCCTAAGCAGCCAGCCATGGCCTGGGTGGAGGCACCTGAAGACGTCT
GTCCCAAACTCCCCCAGCCCTGAGCTGGGAGATGACAGGGGGAAAGAGGCCCTCTCAAGG
GTGCCAGATGCCTGGGTCTCCCAAGAGGGGTCCCCCAACTCACCGTTCCCGGGACAGGCT
GCCCCCTGTTCCAGGAAGCTCATCCTCACCTGTGTAGGCCCCTGTAGTGACCCACGCGTC
CAGCAGACGCCCACCCACCGCTAGCCGTTGTTCCTGTGCAAAGTAGTGTGCTATGCACCC
ACCCAGGTGGCCGCCTCTGGGCCCAAGGCACATGCTGTGAGCTTCCTGTGAGCCCAGGCT
CTGCTCACTGCTGTCCCGCGTCATGAGCACCACCTCTGCTTTCCCTGTGTAGATCTAGGC
CAGTGGCTGCTTGTTCTTGTGGAGCTGTGTGTGTTCTTCTCTGAGCAGCTCCTCCCCGGA
GTCCCCCAGCACAGTCCCAGGAGATGACAGGAAGGAAGCACCAGGGCAAGGCGGACGCTC
ACCCTGTGACCACGATGGTGACCGTGACTGTGGGAGGAAGAACTGGACCCAGGACGGAGC
GGGGCTGCCCTGCCTGAGGCTCCCGAGGAGCTTTGTGCTTTGGTGTTCCACCCCTGTTGT
TACTCATGACTCAGTTTCCTTGACCTGGTAGGGTGTTCCCTGCTGTGTTTTCCAGTGTCC
TGTGACTGTCCTGTGCGGGCCATAGGGCAGGGCCCTGTCCCAGCAGATGGGCTTGGGAGG
GGGCTCCCTAAAGCCAGTGGACACTGCCAGAGTCTACCTTCCTGGCAAGAGGCAGACCCC
GGGGCCCTCAGGAAGGAGGGAGTTGGCAGCGGGGGCTGCAGCAGGAGTAGGAGCAGATGA
GGCGTCTTGCCAGGAACCTCAGGAGGAGGGGGCCCGGGACCTGTGTGGGACCTGTGTCCT
GTGGTGGCCGTTTGCAGTTTCTCTCTGTGTTGTGATTCCCTTCTCTTCAACGTTTTCAGT
ACGTGTTTCTCTTCAATAAACTTCATTCAGTGTTCCAGCCGGCCTCGCCTCTGCTGTGGG
AAACTGGGGATGAGGTTGGCTGGGGATGGGGTTGGGGTGGAGTGAGGGCCACAGTGGCTT
TGTTAACACTGGTGTATACTTGGGGCTCGGTCTGCAAGGCATTCTAGAATGCCCTATCTG
CGCATGTTGGTCATGAGCTAGGGTTCACGGCCCACGGACCCAGGCAAAGAAACCCAGCTG
AGCAGGCTGGATAGTCCCAGTAGACAGGACACTGGTAGGCCCAGGCAGTGGCTGGTTGGG
TCCTCAGGGCACTGGTTGAGCTTCTCAGAAATGAATAACATATTTAAGGAAGGGTGAGGG
ACAGCAGGCTGGACCAGTGGCCCCAGGATCCCTTGTATCACTATTGGGCTTCCACACCTG
GAGCATCAAAACCAGGCCCATCTCTGTGAGTGTGTGTGAGATGGTGAATATGTGTGAGTA
TGCGTACTAGTGATTGTGTGAGAGTGAGTGTAGGAGAGTTTGTGTGTGTCATGTGTCAGC
GAATGTGTGAATATGTGAGTGTGTGAGATGGTGTGTGAATATGTTAATGTGTGTTTGTAT
TTGAGTGTGAGTATGCATCAGTTTTTGTGTGTGAGTGAATGT
```

```
Homo sapiens        AGGTCCCAGCAGCGGGATGGACAGCGGGGAGATGGCTGACCCTCCTTGGGGTGGTGTGCGTGACCTGCATGAGTGCCTGGGGCATCTCCTGCACGGAATGGCACCCGCACCAGGTGCCCC
Loxodonta africana  TGCCCACACGGGCGTGGCAGACA-CGCCCGCGTAGCACACACTCTGTGGG----TGTATCTGGTGTGCGTGCGCAGGTGGGTTGTCTCC---ACAGAATCCCACCTNNNNCAGG------

Homo sapiens        TGGCCTCGCCAGCCAGAGGACCAGTCCTGGACGGGGAGGGGGAGGACAAGAGCAGGTGAACAGGCCCCAGAACCCGGCTCCCTGACCCTGGCCATAATGCAGAGCTGCCTGGCGCACCTC
Loxodonta africana  ---------------GAAGGCCAG-----------GGGAAGGAGACCCAGAGC-----------CCCCCAGATCCG-----CCGCCGCCGCCCAGAGCACAGTGTTAGCAGGCGC-CCTC

Homo sapiens        AGACCACTCCCTGGCCCTCTGGTGGGCACGCCGGGGTGCAGACCCCTCGCCCCGCTGGCACCTCCGTGGCACGT-GCGGGAGGGGCAGGAGCCGGGACCAGGAACCGCTGTGGTGAGAGC
Loxodonta africana  ACA--------------------GAGCACCCCGAGATGAGGCCCCCTGGCCACGCCTGGGCGTAAGGAG-ACGTCTCGGGCAGCTCGAGGTCCGAAGCCAGGTG----------------

Homo sapiens        CAAAGGCACGGGGACTGGCCGCCACAGACCTTTGGCCACCGCAGTGGGGATGGGGGTGGGCGGGCGCTTGTGTGCCCGGGGGCCCGGGGGCCCCCGTGGCGGGGGGGCTAGGGGAGAGGA
Loxodonta africana  ---AGGCAGAGTGACTGCCC.....................................................CGCCTGGG---CCAGTGCCCCTTGGGG------------GAGAGGGAG

Homo sapiens        GCAGGTGACGTCACCCGGGGACCTGGG-------GCCCCCAGGGGCGGCGGCGAGAGCCCGACCTCGTGACCCGTCCGCGCCAATGGCCCTGCGGTCTCCCGGACGACGCGGAGGGGTCC
Loxodonta africana  GCCCGTGACGTCACCCGCGGTCCGGAGCTCTTGAGGCTGCGGAGGCAGCGGCTGCAGCCCCGCCGCGTGACCCGCGTGCGCCAATGGCCCAGCGGTCTCCGGGGCGACGCG--GGGGTCC
```

```
Homo sapiens         GCGCT------------GCACCGCCGCCGCGCCCCCGCCCCGTGTCCGCACTGCAGAGAGCCGCGGCGAGCAGGCGCCGGCGCACTGGCCCACGTGCTGCGCGAGCGAGGGAGAGCCACA
Loxodonta africana   GCGCTCCTCCACCACCACCGCCGCCGCCGCGCCCCCGCCCCGCGTCCGCACTGCAGAGA-CGGCGGCGAGCAGGCGC-------GCGGGCCACGTGCCACGC-----------------

Homo sapiens         GTCTGAGCGAACGTCCGCGCTGGGAGCCAGGGGTGCCCGACCCCCGTCCGCCGCCGCCGCCGCCGCGCATAGCCCCCGGAGAGCCCTCTGGGGACCCCGACCAGAAGGGACCTTGCC
Loxodonta africana   ---------CACGGGCGGGCTGGACTCGAGAGGGGCGCAG-------------------------GGTGAACAGACC------------------CGCCGACCAAGCGAGACCGTGCC

Homo sapiens         CTGGGAGAAGGTAACAGACCCACCTGGGGAGGGAGACGCTGGGCCCCGGGAGACTCCGCCGCCCCTCGCCTGTCCGCCCTATCAGAACTGAAGCAGCCTGCAGGGCAGGGGTCCAGGGTC
Loxodonta africana   ..................................................GCCCCCCCCGCCCCGCCCCT-----CCTTTTCGGGAAGGTGGCGGTC--CAGAGAAGGTATGAGGAG--

Homo sapiens         CTGGGGCAGCGCGGGGAGGGGGCTGAGGCCCGCAGAGACCCCACGGGGGGTGGGGGTGGGGGGCTGGGCATGGGCCGGGCCTGGCTGCAGAGGC-GCCGCACGGGGAGGAGGGCGGCTGG
Loxodonta africana   ------------GAGGAGGGGGCTCCGG-CCGCGATGGCCCT-------------------------GCACCCTCCGCCCCCAGGGGACGCGGCGGCTGCGCAGGGCGGAAGG----GGG

Homo sapiens         GGGTGGGGGTGGGGGTGGGCGTGGATGTGGGCGTGCGGAGAGGTGGGCATTAACCTCGCTCTCGCCCTGCTCGCATTCACAGGCTGTGGAGACCTGGGCCTTCTGCGATCACCCTAGGAG
Loxodonta africana   GGGCGGGGGAGGGGCTGGGGGC----------------------GGCACTGACC-CGCTC---CCCTGGTCGGGTTCCCAGGCCGTGGAG--CCGGGC------------CCCTGGGAG

Homo sapiens         TTGATCCAGA-TATGTGCCTCACGCCCTGATCACTCCCCCCAAATTAGTATCCGCAGAGATTCGAGGACATGCCGTTGACCTTGTTACAGGACTGGTGTCGGGGGGAACACCTGAACACC
Loxodonta africana   TTGGCCCAGATTAAGCGCATCCCGCACTGATCACCGGAGGCCAAT-----TCCGGGGAAGGT-GGCGACATGCCGGTGGCCATGCTGCAGGACTGGTGCCGTGGGGAGCACTTGGACATC

Homo sapiens         CGGAGGTGCATGCTCATCCTGGGGATCCCCGAGGACTGTGGCGAGGATGAGTTTGAGGAGACACTCCAGGAGGCTTGCAGGCACCTGGGCAGATACAGGGTGATTGGCAGGATGTTTAGG
Loxodonta africana   CAGAGGGCCCTGCTCATCCTGGGCATCCCTGAGGACTGTAGCGAGGTGGAGCTGGAGGAGACGCTGCAGGCGGCACTCCGGCGCCTGGGCAAGTATCGGGTCGTCGGCAGGATGTTTAGG

Homo sapiens         AGGGAGGAGAACGCCCAGGCGATTCTACTGGAGCTGGCACAAGATATCGACTATGCTTTGCTCCCAAGGGAAATACCAGGAAAGGGGGGGCCCTGGGAAGTGATTGTAAAACCCCGTAAC
Loxodonta africana   AGGGAGGAGAATGCCCAGGCTTTTCTCCTGGAGCTGGCGCGAGACATTGACTATGCTCTGGTCCCCAGGGCAATACCAGCAAAGGGGGGCCCCTGGGAAGTGATTGTAAAGCCCCGCAAC

Homo sapiens         TCAGATGGGGAATTTCTCAACAGACTGAACCGCTTCTTAGAGGAGGAGAGGCGGACCGTGTCAGATATGAACCGAGTCCTCGGGTCGGACACCAATTGTTCGGCTCCAAGAGTGACTATA
Loxodonta africana   TCAGACGGCGAATTTCTCAACCGACTGAACCGCTTCCTAGAGGAGGAGAGGCGCTCAGTGGCTGACATGAACCGTGTACTTGGGTCTGGCCCCGCCTACCCGGCCCCTGGAATGGCCATC

Homo sapiens         TCACCAGAGTTCTGGACCTGGGCCCAGACTCTGGGGGCAGCAGTGCAGCCTCTGCTAGAACAAATGTTGTACCGAGAACTAAGAGTGTTTTCTGGGAACACCATATCCATCCCAGGTGCA
Loxodonta africana   TCCCCTGAATTCTGGGCCTGGGCACAAACCCTGGGGGCGGCGGTGCAGCCTCTGCTAGAACAAACGCTGTACCGAGAGCTCCGCGTGTTCTCTGGGCACACCGTGTCCATCCCTGGGGCG

Homo sapiens         CTGGCCTTTGATGCCTGGCTTGAGCACACCACTGAGATGCTACAGATGTGGCAGGTGCCCGAGGGGGAAAAGAGGCGGAGGCTGATGGAATGCTTACGGGGCCCTGCTCTCCAGGTGGTC
Loxodonta africana   CTGGCCTTTGACGCCTGGCTGGAGCACACCACCGACGTGCTTCAGATGTGGCAGGTGCCGGAGGGGGAAAAGAGGCGGCGGCTGATGGAATGCTTGCGGGGCCCCGCGCTGCAGGTGGTC

Homo sapiens         AGTGGGCTCCGGGCCAGCAATGCTTCCATAACTGTGGAGGAGTGCCTGGCTGCCTTGCAGCAGGTGTTCGGACCTGTGGAGAGCCATAAAATTGCCCAGGTGAAGTTGTGTAAAGCCTAT
Loxodonta africana   AGCGGGCTGAGAGCGAACAACTCGGCAGTCACGGTGGCGGAGTGCCTGGCGGCACTGCAGCAGGTATTTGGGCCTGTGGAGAGCCGCAAAATCACCCAGGTGAAGGTTTGCAAGGCCCGC

Homo sapiens         CAGGAGGCAGGAGAGAAAGTATCTAGCTTTGTGTTACGTTT-GGAACCCCTGCTCCAAAGAGCTGTAGAAAACAATGTGGTAT-CACGTAGAAACGTGAA-TCAGACTCGCCTGAAACGA
Loxodonta africana   CAGGAGATAGGAGAGAAGGTGTCTAGCTTTGTGGTCCGTTTAGAAACCCCTGCTCCAAAGAGCTGTAGAGAAAAATGCCATCTCCCCGAAGGAATGTCAACCCAGGCTCGGCTGAAGCAC

Homo sapiens         GTCTTAAGTGGGGCCACCCTTCCTGACAAACTCCGAGATAAGCTTAAGCTGATGAAACAGCGAAGGAAGCCTCCTGGTTTCCTGGCCCTGGTGAAGCTCCTGCGTGAGGAGGAGGAATGG
Loxodonta africana   ATCCTGGCCCGGGCCTCCATGACTCCCTCACTCCGTGGCAAGCTGGAGATCCTCGACCAACGAGGGTGTCCCCCCACTTTTCTGGAGTTGATGAAGCTCATTCGCGATGAAGAGGAGTGG

Homo sapiens         GAGGCCACTTTAG------GTCCAGATAGGGAGAGTCTGGAGGGGCTGGAAGTAGCCCCAAGGCCACCTGCCAGGATCACTGGGGTTGGGGCAGTACCTCTCCCTGCCTCTGGCAACAGT
Loxodonta africana   GAAACAACCATGGCAGTGAGCCAGGAGAAGCAGAGGC-----AAGTAGGAAAGGGCCGGAGGGGCTCCAGCAG-----ACAGGTGATGGTGGAGG-----TCCATGCCCCTG--------

Homo sapiens         TTTGATGTGAGGCCTTCCCAGGGCTACCGGCGCCGGAGGGGCA-------GAGGCCAACACCGAAGGGGTGGTGTGGCAAGGGCTGGCTCTCGAGGCTCAAGAAAACGGAAACGCCACAC
Loxodonta africana   --------------CACCCCAGGCT-CCGATGCCGGCAGGATCCTTCATCGAGAACAGCACCCAGACGGCA------CAGGGAGGGGCTCCCCTG--TCACTGAAGCGCAAGCGCCTGCC

Homo sapiens         ATTCTGCTATAGCTGTGGGGAAGACGGCCACATCAGGGTACAGTGCATCAACCCCTCCAACCTGCTCTTGGTAAAGCAGAAGAAACAGGCTGCAGTTGAGTCGGGAAACGGGAACTGGGC
Loxodonta africana   GTGCTCCTATGGCAGTGGGGATGATGACCAAGTCATG------TGTCCCGGGGCTGAGAACCTGCCCCTG-TCAAGCAGAAGAGACAGGCTACCGTGGAGTCGG--AACGGGAGCTGGGC

Homo sapiens         TTGGGACAAGAGCCATCCCAAGTCCAAGGCCAAGTAGGCTCGGGAGAACAGGGCAACATTTCCTACCACAGGCCAAGGAGACAAAAGAGATATTGGAAGGAGGGGAAAGAGAAGCCCAGA
Loxodonta africana   TTGGGACCAGGGCCAGCCCAAGCCCGAGCCCAAGTAGGCTCAGGAGACCAGGTCGACTCCCCCCTACACAGGCCAAGGAGACAAAAGAGACACTGGAAGAAGGGGAAGGGGAGCCCCAGG

Homo sapiens         CAAACAGCAGATGAGTTGAGTGGGGCAGAGGGACAGGGCAGCCAGACCAAGGCCAAGCCTTCTCACCCTTGGCCAGCTGGAAGGGACTTCAGCAACCAAGACCACCTGGCAACAGGCTCA
Loxodonta africana   CAGACAGCAG-----------GGGGCA----------ACAGTGGAACCAGGGCTGAGCCCCCTCCCCCAGTGCCAGTGGGAAGGGACTCTGCTGACCAA-ACCACTTGT-----GGTTCC

Homo sapiens         GTGGGGGTCAGGTCCAGGTCCCCGAAGAGGTGCTGGAGAGGAAAGCAGGGAGCCACTGCATCCAGCACATGGGGTGCCTGGGCCTCAGATGGGGACCCCAAAGAAGCAGAAGCTGAAGAA
Loxodonta africana   AGGAAGCCCAGGAGCAAGGCACGGAGGAGGAG--GAGGAGGAGGGGCCGGAGAC----CACCCAGCAGGTGGGGGTTCAGTGTGATGGGTGGGGAGTCC---GATTCTGGGACTGAAGAG

Homo sapiens         GGTACGGCTGGGGGTTCTGTCCTG---CTCATCC---------AACCACCCCTAAATACCCACCCTGTGGACTTTGAGCTGAACATGCCCACTGGCCCCCAGGCCACATGGGACCTGGAG
Loxodonta africana   GTCGCAGCTGGGGGCTCAGACCTGGGACTCAGCCCCTTCTTTGGGCACCCCCCTGACACCCACCTTGTGGACTCTGAGCTGAGGATGACCACTGGTCCCCAGGCCATGGGGGGCCTGGAA

Homo sapiens         GAGCCTACCT---------------------GGGGCCTGCCCCTGCCAGCAGGTGCCAGGGCTGGTGAGGAAGAGCTGGGGGG--CAGAGGTAAAGCCCTGCAGGGGAGGCCACAGGGTC
Loxodonta africana   GAGCTTTCCTCAGGGCCACCACAGTTGGGGGTGGGACCACCCCTGCCACGAGGAGCCAGCGCTGGAGAGGGAGAGCAGCGTGGCCCAGAGGTAATGCCCTGGAGAGGAGACCACAGGGTG

Homo sapiens         CATCCCGTCTTCAGGATCATCTACACTGCACTAGGGGAGCCCCAGGAAGGCAGCACCCTGGAGGCCCTGTGCCAGTGAGGACAGGAGACCCTAAGGCCCCGGGAGCCCAGTGCCAGCCA-
Loxodonta africana   AACCCCGTCTTCGGGAT-ATCTACACTGCCCTAGGAGAGCCCCGGGTAGGCAGCACCCTGGAGCTCCTGCGCCCCTGAGGGCAGCAGACACCCAGGTCC--AGAGCCCAGTGCAGGCCAA

Homo sapiens         ----------------------GAGGTTGTGCAGGCAAGGAGACCAAAGATTGATGAGAAGACC------------CCCAGC---------AGGGGTACTGGGTACCCGGCAGGCCAGTG
Loxodonta africana   CCCTCTCCTGGCCTGCAGCCTGGAGGGTGT---GGTGAGGAGCACAGACTGTCAGGAGAGGACCATGAGGCCAGAGCCCGGCTCAGGTGTAGGGGGCTCTGGAGGCCTGGTGGACCAGTG

Homo sapiens         CCCTCACAGTTGACTTGGACCAG--GGTGGCTGTGAAGGGAAGTCTTTGTTGCAAAGGAGGAGGAGGAAAAGGGAGGACTTGGTAGGGTTTTGTTTCTTCTGCTTGTTTCTGTACAGGGC
Loxodonta africana   ACCCCACAGCTGGCTT---CCTGTCAGTGGGCGTGAGAGCAG--CTTTGGGCAGGAGGAAGAGGAGGAGGAAGGGGGCTTGGGGGGGGTGTCTTTTGTTCTGCTTGTT--TGTGCAGGAC

Homo sapiens         CACCAGACTCCTGGAGAGATCAAGCAAGGAGAACCTGGGGCTGCCATGGCCAAAGCAACTCAACAGATGCCAATGCCAATTCCAAGGCCAGCCACAACCCTGCCACCTTGGGGAATCCAG
Loxodonta africana   CATGAAGAT--GGGAGCAAACAGGCGAGGAGAACCTGAGACTTTCCTGGCCAA----------------------------CCAGACCTCCAGCATTTTTGCCACCCAGG.........

Homo sapiens         CCTGGAGGCATCCCCTAAGCAGCCAGCCATGGCCTGGGTGGAGGCACCTGAAGACGTCTGTCCCAAACTCCCCCAGCCCTGAGCTGGGAGATGACAGGGGGAAAGAGGCCCTCTCAAGGG
Loxodonta africana   .....................................................................CCACTGCCCTGGTCTGGGAGATGATAGGGGG-AGGCAGTCATTTCTAGGG

Homo sapiens         TGCCAGATGCCTGGGTCTCCCAAGAGGGGTCCCCCAACTCACCGTTCC-CGGGACAGGCTGCCCCCTGTTCCAGGAAGCTCATCCTCACCTGTGTAGGCCCCTGTAGTGACCCACGCGTC
Loxodonta africana   GGCCAGCTGCCT-GGTCTCCCAAGAAGGGGACCCC-CTTCACCACCCCTTGGGGTGGGCTGCCCCCTGTTCTGGGAAGCCCCCCTGTATCTCTGTAGGCCTC-GTAGTTACCCACGTGTC

Homo sapiens         CAGCAGACGCCCACCCACCGCTAGCCGTTGTTCCTGTGCAAAGTAGTGTGCTATGCACCCACCCAGGTGGCCGCCTCTGGGCCCAAGGCACATGCTGTGAGCTTCCTGTGAGCCCAGGCT
Loxodonta africana   AGGCGAATACCACCCC.........................................TACCCAGGT..................................................

Homo sapiens         CTGCTCACTGCTGTCCCGCGTCATGAGCACCACCTCTGCTTTCCCTGTGTAGATCTAGGCCAGTGGCTGCTTGTTCTTGTGGAGCTGTGTGTGTTCTTCTCTGAGCAGCTCCTCCCCGGA
Loxodonta africana   ..................................................ATCATGGTCAACTGATGCTTGTTCTTGTGGAGGTGTGTGTGTTCTTACCTGAGCAGTTTCTCTCTGGA

Homo sapiens         GTCCCCCAGCACAGTCCCAGGAGATGACAGGAAGGAAGCACCAGGGCAAGGCGGACGCTCACCCTGTGACCACGATGGTGACCGTGACTGTGGGAGGAAGAACTGGACCCAGGACGGAGC
Loxodonta africana   G-ACCCCAGCCCAATCTCTGAAGACAGCAGCAAGGACGGATGCGGGAGTGGC-CACCCTCACCCCATGA--------------GCCACTGAGGGAAGGAAGAAGGGACC--GGGTGGGTT

Homo sapiens         GGGGCTGCCCTGCCTGAGGCTCCCGAGGAGCTTTGTGCTTTGGTGTTCCACCCCTGTTGTTACTCATGACTCAGTTTCCTTGACCTGGTAGGGTGTTCCCTGCTGTGTTTTCCAGTGTC-
Loxodonta africana   GTGG-----------GGAGCCCCTTGGGAACTGTG---CTTGAAGCTGCACCCCAGCTGTTGTTTGTGACTCAGTTTCCTTGACATGGTGACGTGTGCTCTGCTGTGTTTTCCAGTGTCC

Homo sapiens         --CTGTGACTGTCCTGTGCGGGCCATAGGGCAGGGCCCTGTCCCAGCAGATGGGCTTGGGAGGGGGCTCCCTAAAGCCAGTGGACACTGCCAGAGTCTACCTTCCTGGCAAGAGGCAGAC
Loxodonta africana   TACTGTGTCTGTCCC---TGGGCCACA--GCAGGGACCACGCCCAGTGGGTGGTTCCAGGGAAGGATCCCCTGAAGTGAGTGGACGTCGCTAGAGTCCATTGTCCTGACAG---------

Homo sapiens         CCCGGGGCCCTCAGGAAGGAGGGAGTTGGCAGCGGGGGCTGCAGCAGGAGTAGGAGCAGATGAGGCGTCTTGCCAGGAACCTCAGGAGGAGGGGGCCCGGGACCTGTGTGGGACCTGTGT
Loxodonta africana   ------------AGGAGAGTGGGAG--GGCTGCAGTGGGCG----GGGAGGGGGGGCGGCTGAGGCTCCTCATTAGGGACCCTGGGAGGAGGG----------------TGGCCCCGTGG

Homo sapiens         CCTGTGGTGGCCGTTTGCAGTTTCTCTCTGTGTTGTGATTCCCTTCTCTTCAACGTTTTCAG-TACGTG-TTTCTCTTCAATAAACTTCATTCAGTGTTCCAGCCGGCCTCGCCTCTGCT
Loxodonta africana   CCTCTGGTGGTGGTTATCCATTAC---CTCTGTTGTGATTGCCTTCCCTTCAATCATTCCAGTTAAGTGTTTTCTCTTCAATAAATCTTATCAAGTGTTTTGCTGAGTCTGGCCTCTGCT

Homo sapiens         GTGGGAAACTGGGGATGAGGTTGGCTGGGGATGGGGTTGGGGTGG-AGTGA--------GGGCCACAGTGGCTTT---GTTAACACTG-------GTGTATACTTGGGGCTCGGTCTGCA
Loxodonta africana   GTGGGCAGTGGAGGGGAGGGCCTGCGGGGCGGGGGGCAGGTGGGGCAGTGGCCTGTGTTCAGCCACAGCTGCTGCTGAGGGACCACTGGGGACCTGTGTGTGTGTGATGCTCGGTCTGTG

Homo sapiens         AGGCATTCTAGAATGCCCTATCTGCGCATGTTGGTCATGAGCTAGGGTTCACGGCCCACGGACCCAGGCAAAGAAACCCAGCTGAGCAGGCTGGATAGTCCCAGTAGACAGGACACTGGT
Loxodonta africana   AGGGGTTGTGTGGTCTCTTA--------------------------------------------GGCCAAAGGAACTTCAT------------------------GACAGGATGCTGAT

Homo sapiens         AGGCCCAGGCAGTGGCTGGTTGGGTCCTCAGGGCACTGGTTGAGCTTCTCA----GAAATGAATAACATATTTAAGGAAGGGTGAGGGACAGCAGGCTGGACCAGTGGCCCCAGGATCCC
Loxodonta africana   TGGCATGGGAAGTGAGTGGTTGTGACCCTTGAACGCTTGGGGAGCCCCCCAAAACGAAATAGGGAAGATCTGCGGGGCGTGGTGAGGGAGGAGGGGCTGGAAGGGCAGCC----------

Homo sapiens         TTGTATCACTATTGGGCTTCCACACCTGGAGCATCAAAACCAGGCCCATCTCTGTGAGTGTGTGTGAGATGGTGAATATGTGTGAGTATGCGTACTAGTGATTGTGTGAGAGTGAGTGTA
Loxodonta africana   ---------------GGCTCCATAGTCAGAG-ACCTACGCTAGGGTTAGCT------GTATGTGTGAGTGAGTGCATGAGAGTGAGTGTGTGTGT--GTGAGTGACTGAGGGT--GTGCA

Homo sapiens         GGAGAGTTTGTGTGTGTCATGTGTCAGCGAATGTGTGAATATGTGAGTGTGTGAGATGGTGTGTGAATATGTTAATGTGTGTTTGTATTTGAGTGTGAGTATGCATCAGTTTTTGTGTGT
Loxodonta africana   CGAGAGTGAGTGCGTGTGA-GTCTGAGTGCATGAGTGAA----TGAATGTGCA----GGTGTGTGAATCTGAGTGTGTGTATGTGTATATGAGTGAGTGGAAGTGGAAATGTAAGTGTGT

Homo sapiens         GAGTGAATGT
Loxodonta africana   GCACGTGTGT
```

Genomska poravnava (BLASTZ) PNMA3 gena med človekom in Cavio (1 intron v 5'UTR), sprememba pri rodents !!

```
Homo sapiens     AGGTCCCAG--CAGCGGG-----------ATGGACAG--------CGGGGAGATGGCTGACCCTCCTTGGGGTGGTGTGCGTGACCTGCATGAGTGCCTGGGGCATCTCCTGCACGGAAT
Cavia porcellus  AGGCTCCAGGACAGCTGGCTCCCTCCAGCATGGACAGGCAGCAACCAGCCAGATGGGGGCCCCTCAGTAGGG------GTCTCACTTAGGGGAGCCACCAGGGT-TCCTAGGTACAGCGT

Homo sapiens     GGCACCCGCACCAGGTGCCCCT-GGCCTCGCCAGCCAGAGGACCA-----GTCCTGGACGGGGAGGGGGAGGACAAGAGCAGGTGAACAGGCCCCAGAACCCGGCTCCCTGACCCTGGCC
Cavia porcellus  CTCACCTGTAGTAGGTGCACCTCAGCTAGGCTGTCCCCTGGACCAGCCCTGTCCTGGATGAGGATGGGCAGAGATATTGCAGAGAAAGAAGCCCTACAG---GTATCCTTG--CCTGGCC

Homo sapiens     ATAATGCAGAGCTGCCTGGCGCACCTCAGACCACTCCCTGGCCCTCTGGTGGGCACGCCGGGGTGCAGACCCCTCGCCCCGCTGGCACCTCCGTGGCACGTGCGGGAGGGGCAGGAGCCG
Cavia porcellus  AGAGCACATCCTGATTTGCTGCAGATCTGGTCACT--CTGGCA--CTGGTGTCAAGGCAGGGG.....................................

Homo sapiens     GGACCAGGAACCGCTGTGGTGAGAGCCAAAGGCACGGGGACTGGCCGCCACAGACCTTTGGCCACCGCAGTGGGGATGGGGGTGGGCGGGCGCTTGTGTGCCCGGGGGCCCGGGGGCCCC
Cavia porcellus  ....................................................................................................

Homo sapiens     CGTGGCGGGGGGGCTAGGGGAGAGGAGCAGGTGACGTCACCCGGGGACCTGGGGCCCCCAGGGGCGGCGGCGAGAGCCCGACCTCGTGACCCGTCCGCGCCAATGGCCCTGCGGTCTCCC
Cavia porcellus  ....................................................................................................

Homo sapiens     GGACGACGCGGAGGGGTCCGCGCTGCACCGCCGCCGCGCCCCCGCCCCGTGTCCGCACTGCAGAGAGCCGCGGCGAGCAGGCGCCGGCGCACTGGCCCACGTGCTGCGCGAGCGAGGGAG
Cavia porcellus  ....................................................................................................

Homo sapiens     AGCCACAGTCTGAGCGAACGTCCGCGCTGGGAGCCAGGGGTGCCCGACCCCCGTCCGCCGCCGCCGCCGCCGCGCATAGCCCCCGGAGAGCCCTCTGGGGACCCCGACCAGAAGGGA
Cavia porcellus  ....................................................................................................

Homo sapiens     CCTTGCCCTGGGAGAAGGTAACAGACCCACCTGGGGAGGGAGACGCTGGGCCCCGGGAGACTCCGCCGCCCCTCGCCTGTCCGCCCTATCAGAACTGAAGCAGCCTGCAGGGCAGGGGTC
Cavia porcellus  ....................................................................................................

Homo sapiens     CAGGGTCCTGGGGCAGCGCGGGGAGGGGGCTGAGGCCCGCAGAGACCCCACGGGGGGTGGGGGTGGGGGGCTGGGCATGGGCCGGGCCTGGCTGCAGAGGCGCCGCACGGGGAGGAGGGC
Cavia porcellus  .......................................................................................CGGGGAGCTTTGC

Homo sapiens     GGCTGGGGGTGGGGGTGGGGGTGGGCGTGGATGTGGGCGTGCGGAGAGGTGGG-----CATTAACCTCGCTCTCGCCCTGCTCGCATTCACAGGCTGTGGAGACCTGGGCC--TTCTGCG
Cavia porcellus  CATGGGAAGCAGGGGCGGGGCGGGGCG-----GTGGG-GCGCAGAGGGGCGGGGCAAGCACTCAGGGCACTCTCACCCCTTTTGCACTGACAGGCTGTGGAACCCTGAGACACTTCTGCC

Homo sapiens     ATCACCCTAGGAGTTGATCCAGA--TATGTGCCTC-----ACGCCCTGATC--ACTCCCCCCAAATTAGTAT---------CCGCAGAGATTCGAGGACA-TGCCGTTGACCTTGTTACA
Cavia porcellus  TTCTTACTTCGCACCGGCCCGGAATTCTTTGCGTCGTGGTACACTCTGAGCTAACTGCCGC----TTAGCGTTAGATCAGGCTGGAGAAA---GAGGACAATGGCTGTGGTTCTGTTAGA

Homo sapiens     GGACTGGTGTCGGGGGGAACACCTGAACACCCGGAGGTGCATGCTCATCCTGGGGATCCCCGAGGACTGTGGCGAGGATGAGTTTGAGGAGACACTCCAGGA-GGCTTGCAGGCACCTGG
Cavia porcellus  GGACTGGTGCAAGGGAATGGACCTGGACCCCAGGAAGGCCCTGCTGATCGTGGGCATTCCTGTGGAGTGCAGTGAGGCTGAAATCAAGGAGACCGTGCAGGAAGGCCTGTAC-CCCCTGA

Homo sapiens     GCAGATACAGGGTGATTGGCAGGATGTTTAGGAGGGAGGAGAACGCCCAGGCGATTCTACTGGAGCTGGCACAAGATATCGACTATGCTTTGCTCCCAAGGGAAATACCAGGAAAGGGGG
Cavia porcellus  GCATGTACAGGATAGTGGGCAGAATGTTCAGGAGGGAGGACAACGCTAAGGCTGTCCTCATCGAGCTGGCCGACACTGTTAATTATGCTACGATACCCAGTTGTATACCAGGAAAGGGGG

Homo sapiens     GGCCCTGGGAAGTGATTGTAAAACCCCGTAACTCAGATGGGGAATTTCTCAACAGACTGAACCGCTTCTTAGAGGAGGAGAGGCGGACCGTGTCAGATATGAACCGAGTCCTCGGGTCGG
Cavia porcellus  GTTCCTGGGAAGTAGTGGTTAAACCCCGTACCGAAGATGAAGAGTTTCTCAGTAGGCTGAACTATTTCCTGAAAGATGAGGGCCGGAAAATTGAAGATGTGGCCAGAGTGCTAGGGTGTC

Homo sapiens     ACACCAATTGTTCGGCTCCAAGAGTGACTATATCACCAGAGTTCTGGACCT-GGGCCCAGACTCTGGGGGC-AGCAGTGCAGCCTCTGCTAGAACAAATGTTGTACCGAGAACTAAGAGT
Cavia porcellus  GGGGCTTTTCTGTGG-----AGGATGAGGATGTAG----------GGACTTCGGGTCCAG--TCAGTTCGCTACCTGTGCATCCTGTGAAGGAAAGTCTGTGGTACCGAAAGCTGAAAGT

Homo sapiens     GTTTTCTGGGAACACCATATCCATCCCAGGTGCACTGGCCTTTGATGCCTGGCTTGAGCACACCACTGAGATGCTACAGATGTGGCAGGTGCCCGAGGGGGAAAAGAGGCGGAGGCTGAT
Cavia porcellus  GTTTTCAGGGAGCCCTTCCCCAGGCTCAGGCGAAGAGCCCTTCGAGGCCTGGCTGGAGCATGTCACGGAGCTGATGCAGGTGTGGCAGGTGTCAGAGGCAGAGAAGCGCAGGCGCCTGCT

Homo sapiens     GGAATGCTTACGGGGCCCTGCTCT-----CCAGGTGGTCAGTGGGCTCCGGGCCAGCAATGCTTCCATAACTGTGGAGGAGTGCCTGGCTGCCTTGCAGCAGGTGTTCGGACCTGTGGAG
Cavia porcellus  AGAGAGCCTGCGGGGCTCAGCGCTGGCCACCATGCGG-----GTGCTCCGGGCCAACGATGACTCCATCACGGTGGTGCAGTGCCTAGACGCCCTGAGGGAGATGTTTGGCAGCAAAGAG

Homo sapiens     AGCCATAAAATTGCCCAGGTGAAGTTGTGTAAAGCCTATCAGGAGGCAGGAGAGAAAGTATCTAGCTTTGTGTTACGTTTGGAACCCCTGCTCCAAAGAG-----CTGTAGAAAACAATG
Cavia porcellus  GATGCTAAGACCGAGCAGCACTTGTTTCTCCAGATGCAGCACAAGTCTGAAGAAAAGGTCTCTGCTTTCTTGCTGCGCCTAGAGCCACTGCTGCAGAAAGTGGCCCGGCAGAGCCCATT-

Homo sapiens     TGGTATCACGTAGAAACGTGAATCAGACTCGCCTGAAACGAGTCTTAAGTGGGGCCACCCTTCCTGACAAACTCCGAGATAAGCTTAAGCTGATGAAACAGCGAAGGAAGCCTCCTGGTT
Cavia porcellus  ---TAGAGTGCAGGG-CACGGACATGGTTCGCCTCAAACATGTCCTAGCTCAGGCCTCTGTGACCGGGGCCCTGCGGGGCAAGCTGGAGCTCCTAGAACAGCAAGGGTGCCCCCCGAGCT

Homo sapiens     TCCTGGCCCTGGTGAAGCTCCTGCGTGAGGAGGAGGAATGGGAGGCCACTTTAGGTCCAGATAGGGAGAGTCTGGAGGGGCTGGAAGTAGCCCCAAGGCCACCTGCCAGGATCACTGGGG
Cavia porcellus  TCCTGGAGTTAGTGAAGCTTGTCCGAGAAGAAGAGGCGTGGGAGCATGCGGTGACGGTGACTAAGGAGAAGCAGACAGGGCAGA................

Homo sapiens     TTGGGGCAGTACCTCTCCCTGCCTCTGGCAACAGTTTTGATGTGAGGCCTTCCCAGGGCTACCGGCGCCGGAGGGGCAGAGGCCAACACCGAAGGGGTGGTGTGGCAAGGGCTGGCTCTC
Cavia porcellus  ....................................................................................................

Homo sapiens     GAGGCTCAAGAAAACGGAAACGCCACACATTCTGCTATAGCTGTGGGGAAGACGGCCACATCAGGGTACAGTGCATCAACCCCTCCAACCTGCTCTTGGTAAAGCAGAAGAAACAGGCTG
Cavia porcellus  .......................................................................................AGGAAACAGG---

Homo sapiens     CAGTTGAGTCGGGAAACGGGAACTGGGCTTGGGACAAGAGCCATCCCAAGTCCAAGGCCAAGTAGGCTCGGGAGAACAGGGCAACATTTCCTACCACAGGCCAAGGAGACAAAAGAGATA
Cavia porcellus  --GAGGAATCGGGAAACGGGAAGGGGGCTGGGGCTCTGAGCCACCCCAAGCCCTAGGAAGTACAGGCTCAGCACACCCAGGCG----TGCCTCACCCTGGCGCGCAGGACACAGGGCTCC

Homo sapiens     TTGGAAGGAGGGGAAAGAGAAGCCCAGACAAACAGCAGATGAGTTGAGTGGGGCAGAGGGACAGGGCAGCCAGACCAAGGCCAAGCCTTCTCACCCTTGGCCAGCTGGAAGGGACTTCAG
Cavia porcellus  TCC-----AGGGGAGGGAGGAGCTCAGACTAGCAGCAGGAGACCCGAGCAGGGTGGAGGGGCAGGGCAGCTGGGCCCAACCCCAGCCCCCTCACTCCCCACCAGCCAGGAGGGCCTCCCC

Homo sapiens     CAACCAAGACCACCTGGCAACAGGCTCAGTGGGGGTCAGGTCCAGGTCCCCGAAGAGGTGCTGGAGA-------GGAAAGCAGGGAGCCACTGCATCCAG--------------CACAT
Cavia porcellus  ACCCCAGGTACAAACCGAACCTGGCACGGGGAGGCCTGCGTTCGGGGAACCCAGGAGGCGCCAGAGGCGTGGTTGGGGAACCGGCAGCTGCAGCACTCAGGAAGCAGCAGATACCCTTAT

Homo sapiens     GGGGTGCCTGGGCCTCAGATGGGGACCCCAAAGAAGCAGAAGCTGAAGAAGGTACGGCTGGGGGTTCTGTCCTG---CTCATCCAACCACCCCTAAATACCCACCCTGTGGACTTTGAGC
Cavia porcellus  GCGGCTCTTGAGCTTT-------GAGTCCATCAAGGTAGAGGCTGATGATAGTGAGGATGGGATCTCTGGCCAGGATTCCACCAGGTTACCTCCAGATGCCCACCCTGTGGAGTCTGAGC

Homo sapiens     TGAACATGCCCACTGGCCCCCAGGCCACATGGGACCTGGAGGAGCCTACCTGGGGCCTGCCCCTGCCAGCAGGTGCCAGGGCTGGTGAGGAAGAGCTGGGGGGCAGA--GGTAAAGCCCT
Cavia porcellus  ACAAGACACCCACTGGCCCCAAGGCCACATTGAGCAAAGAGGTGCCTGTCCCAGGGCCACCCTTGCCAGAAGAATCCCAGACCAGTACATCAGAGGCTGGAGACAGACAGGCCACACTCC

Homo sapiens     GCAGGGGAGGCCACAGGGTCCATCCCGTCTTCAGGATCATCTACACTGCACTAGGGGAGCCCCAGGAAGGCAGCACCCTGGAGGCCCTGTGCCAGTGAGGACAGGAGAC------CCTAA
Cavia porcellus  GGAGGGGAAGTCGCAGGGTGCAGCCCATCTTCAGGATCATTTATACCGCTCTAGGGGAGCCCCACGAATGCAGCATCCTAGACCCCCTGCGTGATTAAGGGCCTGGGGCTCAGCACCCAA

Homo sapiens     GGCCCCGGGAGCCCAGTGCCAGCCAGAGGTTGTGCAGGCAAGGAGACCAAAGATTGATGAGAAGACCCCCAGCAGGGGTACTGGGTACCCGGCAGGCCAGTGCCCTCACAGTTGACTTGG
Cavia porcellus  GGCCCTTGGGGCC---------CCAAACGGTGTGGAG--AAGGAA----------GAGGAGGA-------AGAGGAGGGAGTGGGT----GAGAGGACAACTTCTTTCCA----------

Homo sapiens     ACCAGGGTGGCTGTGAAGGGAAGTCTTTGTTGCAAAGGAGGAGGAGGAAAAGGGAGGACTTGGTAGGGTTTTGTTTCTTCTGCTTGTTTCTGTACAGGGCCAC-CAGACTCCTGGAGAGA
Cavia porcellus  --------------GACGGGAGGC------------GATGGAAGAGGAGCAAGGAGGAATAGATGTGG---GGAGTGTTCTGTTGGATTCC-TGCAGGGCCCTGCAGAATC----AGAAA

Homo sapiens     TCAAGCAAGGAGAACCTGGGGCTGCCATGGCCAAAGCAACTCAACAGATGCCAATGCCAATTCCAAGGCCAGCCAC-AACCCTGCCACCTTGGGGAATCCAGCCTGGAGGCATCCCCTAA
Cavia porcellus  GCAAGCAGGGAGAACC--GGGCTG--------------------CAGCTGCCAATGCCAA-------GCCAGCACCGAACCCTGCTG-ATTCGGGCACCCAGCCTAGAGGCA--CCCTGA

Homo sapiens     GCAGCCAGCCATGGCCTGGGTGGAGGCACCTGAAGACGTCTGTCCCAAACTCCCCCAGCCCTGAGCTGGGAGATGACAGGGGGAAAGAGGCCCTCTCAAGGGTGCCAGATGCCTGGGTCT
Cavia porcellus  G-GGCCAGCTGCAGTCTGCATGAGGATGCCCGAAGGCATCTG-CTCAGTCTCTCCTGA---------AGGAGATGTTTGAGAG-AAGAGGCCATCTCACGTGTGCCAGTTGCCTGGCTCT

Homo sapiens     CCCAAGAG---GGGTCCCCCA--ACTC--------------ACCGTTCCCGGGACAGGCTGCCCCCTGTTCCAGGAAGCTCATCCTCACCTGTGTAGGCCCCTGTAGTGACCCACGCGTC
Cavia porcellus  CTCACTGGTGCGAGTGCCCCATTACTCCTCCATTTATTGTTCCTGTCCCTGGCATAAATTGCTTCCTG-TCCGGGAAGCCCA--CGCATGTTGGTAAG-CCCTGTAGTGACCCATGGTGC

Homo sapiens     CAGCAGACGCCCACCCACCGCTAGCCGTTGTTCCTGTGCAAAGTAGTGTGCTATGCACC-------------------------------------CACCCAGGTGGCCGCCTCTGGGCC
Cavia porcellus  AGGTAAACGCTCACCCCAACCTGGCCGCTGTTCTTGGGCAAAGCTGTGAGGTGGGCATCAACCCTGCACGGAGGAGAGGGCCTGGCCCTACCGGGGCACCCAGCCATCCACCTGCAGGCC

Homo sapiens     CAAGGCACATGCTGTGAGCTTCCTG-TGAGCCC----AGGCTCTGCTCACTGCTGTCCCGCGTCATGAGCACCACCTCTGCTTTCCCTGTGTAGATCTAGGCCAG----TGGCTGCTTGT
Cavia porcellus  T-GGGCATGTGTGGTGAGCTTCCTTCTGAGCCTGCCGAGCCTCTGACCGCTCCTGTCCGGTGCTGTGAGGTCAACCTCTGCTTTCTATGACTGTATCCGGGCCTGCTACCTGCCCCTTGT

Homo sapiens     TCTTGTGGAGCTGTGTGTGTTCTTCTCTGAGCAGCTCCTCCCCGGAGTCCCCCAGCACAGTCCCAGGAGATGACAGGAAGGAAGCACCAGGGCAAGGCGGACGCTCACCCTGTGACCACG
Cavia porcellus  CCTCGAGGAGTTGTTTGTGTTCTCATTGGAACAGGTCCTGTCTGGAGACCCCAAGCTTCGTCCCAGTGGGAGA---GAAGGATGGACGACGATGGAG-------TCACCTTGTGACTGTG

Homo sapiens     ATGGTGACCGTGACTGTGGG---------------AGGAAGAACTGGACCCAGGACGGAG---CGGGGCTGCCCTGCCTGAGGCTCCCGAGGAGCTTTGTGCTTTGGTGTTCCACCCCTG
Cavia porcellus  ACTGTGACCATGA--GTGGGATGACAACTTTGGACAAGAAGAACTGGACGAGGGATGGAGGGACAAGGCTG------------------GGGACTT--TGCTTTGACATTCTTCCCATG

Homo sapiens     TTGTTACTCATGACTCAGTTTCCTTGACCTGGTAGGGTGTTCCCTGCTGTGTTTTCCAGTGTCCTGTGACTGTCCTGTGCGGGCCATAGGGCAGGGCCCTGTCCCAGCAGATGGGCTTGG
Cavia porcellus  TTGTTATTTGTGATTTAA-TTCTTTGGTGTGGTGGGGCATTTTTTGTTGCACTTTCTGGTGTCTTGGGACTGTCTAAAGCTGGGCATAGGGCAGGGCCA---CCCAGAAGTT--------

Homo sapiens     GAGGGGGCTCCCTAAAGCCAGTGGACACTGCCAGAGTCTACCTTCCTGGCAAGAGGCAGACCCCGGGGCCCTCAGGAAGGAGGGAGTTGGCAGCGGGGGCTGCAGCAGGAGTAGGAGCAG
Cavia porcellus  ------TCAACCTAAAGTGAGTGGGCATTGCTAGGTGTCACCATCCTGGCGAGAGGCAGA-TCTGGAGCCCTCAGGAAGGAG------------------GCAGATGAGGTCGGAGCTG
```

```
Homo sapiens      ATGAGGCGTCTTGCCAGGAACCTCAGGAGGAGGGGCCCGGGACCTGTGTGGGACCTGTGTCCTGTGGTGGCCGTTTGCAGTTTCTCTCTGTGTTGTGATTCCCTTCTCTTCAACGTTTT
Cavia porcellus   TGGAG-------------------GGTGAAGGGTGACC------------AGACCTGTGGCCC-TCATGGCCCCCAGGAG-TCCTTTCTGTTTTGTTATG--TTTCTTCCCAATG-GTT

Homo sapiens      CAGTACGTGTTTCTCTTCAATAAACTTCATTCAGTGTTCCAGCCG-GCCTCGCCTCTGCTGTGGGAAACTGGGGATGAGGTTGGCTGGGGATGGGGTTGGGGTGGAGTGAGGGCCACAGT
Cavia porcellus   CATCAAATGTTTTTCTTCAATAAACTTTACTGGATGTTCCAACTGCATCCCATCTCAGCTGTGGGTTAGTTTAGAGAAAG--GGCAGCAGCTGGGGTGGGGGTGCAGTGGGGGGC-----

Homo sapiens      GGCTTTGTTAACACTGGTGTATACTTGGGGCTCGGTCTGCAAGGCATTCTAGAATGCCCTATCTGCGCATGTTGGTCATGAGCTAGGGTTCACGGCCCACGGACCCAGGCAAAGAAACCC
Cavia porcellus   -------TTAGCAGTGGGG----CTGGGGGGCAGGTTCACGAGGGCCTGTGGAGTCCTCT---TGC.............................................

Homo sapiens      AGCTGAGCAGGCTGGATAGTCCCAGTAGACAGGACACTGGTAGGCCCAGGCAGTGGCTGGTTGGGTCCTCAGGGCACTGGTTGAGCTTCTCAGAAATGAATAACATATTTAAGGAAGGGT
Cavia porcellus   ...........CTGGCTGGCTCGGGTTAATGGGATACTGGTGGGTGTGGGT----GCCACTGAGGAACTTGGGGAGCTG-----------CAGGAATGAGTTAGACAT-----------

Homo sapiens      GAGGGACAGCAGGCTGGACCAGTGGCCCCAGGATCCCTTGTATCACTATTGGGCTTCCACACCTGGAGCATCAAAACCAGGCCCATCTCTGTGAGTGTGTGTGAGATGGTGAATATGTGT
Cavia porcellus   --GGGACAGT-GGCAGAACCGGAGACCCCAGGGTCCTTGGCAGTACTCAGAGGGCTCAGCATTTGCAG.................................................

Homo sapiens      GAGTATGCGTACTAGTGATTGTGTGAGAGTGAGTGTAGGAGAGTTTGTGTGTGTCATGTGTCAGCGAATGTGTGAATATGTGAGTGTGTGAGATGGTGTGTGAATATGTTAATGTGTGTT
Cavia porcellus   ........................................................................................................................

Homo sapiens      TGTATTTGAGTGTGAGTATGCATCAGTTTTTGTGTGTGAGTGAATGT
Cavia porcellus   ...............................................


Homo sapiens             AGGTCCCAGCAGCGGGATGGAC---------AGCGGGGAGATGGCTGACCCTCC-TTGGGGTGGTGTGCGTGACCT-GCATGAGTGCCTGGGGCATCTCCTGCACGGAATGGCACCCGCA
Canis lupus familiaris   GTGCACCCACAGCGGCCTCGGCCCCTGCGCAAGCTTGGGGACTGCCGACCCTCCATTGGGGTGGAGTGTGTGAATGAGCCTGTGCGCATGTGGCAGTTACCGCACGTGACCGCACCTGGA

Homo sapiens             CCAGGTGCCCCTGGCCTCGCCAGCCAGAGGACCAGTCCTGGACGGGGAGGGGGAGGACAAGAGCAGGTGAACAGGCCCCAG---------------------AACCCGGCTCCCTGACC
Canis lupus familiaris   CGAGGTGCTTCTCGCCTAGCTGGG-ACAGGACCAGTTCTGGA---GGTGGGGGAGGAGGAAAACAAGCGAAGAAGCCCCCATAACGCACCCCCCACACGCACACACCCTGTTCCCC-ACC

Homo sapiens             CTGGCCATAATGCAGAGCTGCCTGGCGCACCTCAGACCACTCCC-TGGCCCTCTGGTGGGCACGCCGGGGTGCAGACCCCTCGCCCCGCTGGCACCTCCGTGGCACGTGCGG-GAGGGGC
Canis lupus familiaris   CATGCCAGAGCGCAACGCGGGCAGGCGCGTCTCAGGCCTGTCCAGCCGCTCTCCCGTGGGCACCCGGGGATGCGGACCCCTCGCCAGGCTGATGCCCTGAGGTCACGTCCCGCGAGGGGC

Homo sapiens             AGGAGCCGGGACCAGGAACCGCTGTGGTGAGAGCCAAAGGCACGGGGACTGGCCGCCACAGACCTTTGGCCACCGCAGTGGGGATGGGGGTGGGCGGGCGCTTGTGTGCCCGGGGGCCCG
Canis lupus familiaris   CGGCGGCGGT----GGACGGGCTTTGG-----GTCTAGGAC---GGGACGCCGAGTCGCGGACCCCCAGGGTCCCCA----------------------CCTGTG----------CCA

Homo sapiens             GGGGCCCCCGTGGCGGGGGGGCTAGGGGAGAGGAGCAGGTGACGTCACCCGGGGACCTGGGGCCCCCAGGGGCGGCGGCGAGAGCCCGACCTCGTGACCCGTCCGCGCCAATGGCCCTGC
Canis lupus familiaris   GCGGCCCCT----CGGGGG----CGGGGGAAGGAGCGGGTGACGTCACGCGCGGTCCCGGGGTGCCC-GCGGCGGTGGCCACAGCCCGGCCGCGTGACCCGCGCGCGCCAATGGCCGGGC

Homo sapiens             GGTCTCCCGGACGACGCGGAGGGGTCCGCGCTGCACCGCCGCCGCGCCCCCGCCCCGTGTCCGCACTGCAGAGAGCCGCGGCGAGCAGGCGCCGGCGCACTGGCCCACGTGCTGCGC-GA
Canis lupus familiaris   GATCTCCGGGGCGACGCGGAGGG-TCCGCGCTCTGCCGCCACTGCGCCCTCGCCCCGAGTCCGCACTGCAGAGAGTGGTGGCGAGCAGGCGCCGGCGCGCCGGCCCACGTGCCGCGCAGA

Homo sapiens             GCGAGGGAGAGCCACAGTC----------TGAGCGAACGTCCGCGCTGGGAGCCAGGGGTGCCCGACCCCCGTCCGCCGCCGCCGCCGCCGCCGCGCATAGCCCCCG-GAGAGCCCTCTG
Canis lupus familiaris   GAGAGGGACGGGCAGAGGAAGGAAAAGAGGGAGTGAGAGAGCGCCCCGGGCAAGAGGCGCGCACGACC--------------CGGCCGCCGCTCCGCGCAGCCCCTGCGCGCCTCCCGCG

Homo sapiens             GGGACCCCGACCAGAAGGGACCTTGCCCTGGGAGAAGGTAACAGACCCACCTGGGGAGGGAGACG--CTGGGCCCCGGGAGACTCCGCCGCCCCTCGCCTGTCCGCCCTATCAGAACTGA
Canis lupus familiaris   GGGACCTCCACCAGGGAGGACCCCCACCCGAG-GAAGGTACCAGACCC----GGGGAGGGTGCGGGCTTGGCCCCCGGGAAACCCCGCCGCCCTCCGC----CCGCCCCAGCGGGACTGC

Homo sapiens             AGCAGCCTGC-AGGGCAGGGGTCCAGGGTCCTGGGGCAGCGCGGGGAGGGGGCTGAGGCCCGCAGAGACCCCACGGGGGGTGGGGGTGGGGGGCTGGGCATGGGCCGGGCCT-GGCTGCA
Canis lupus familiaris   GGCAGCCCGCGGGGGCAGGGGGACGGGCT-------------GGGGAGGGGGCCGTGGCCCCTGCGGGC----CGGTGGAAGGCGGGGCGGGGCGGGGCGGGGCTCGAGCCTGGGCTGCA

Homo sapiens             GAGGCGCCGCAC-GGGGAGGAGGG-CGGCTGGGGGTGGGGGTGGGGGTGGGCGTGGATGTGGGCGTGCGGAGAGGTGGGCATTAACCTCGCTCTCGCCCTGCTCGCATTCACAGGCTGTG
Canis lupus familiaris   GAGGCGCCGCCCGGGGGAGGAGGGTCACCTCGGGGTGGGAGGTGGGGT-------------------------GTCAGCCTTAACCTCGCTCTCGCCCGGTTCGTATTCACAGGCTGTG

Homo sapiens             GAGACCTGGGC---CTTCTGCGATCACCCTAGGAGTTGATCCAGATATGTGCCTCACGCCCTGATCACTCCC-----------------------------CCCAAATTAGTATCC----
Canis lupus familiaris   GAGACCTGGGCAGTTCTCTCTGA--GTCCTAGGCTCCCGCCCCAATTCATTCTCCCCCACCCGCCCAGTACCATCCTGGTCTGCTACGGAACCGTTTCCTGCCCCTAGTATTAGCCCCGC

Homo sapiens             ----GCAGAGATTCGAGGACATGCCGTTGACCTTGTTACAGGACTGGTGTCGGGGGGAACACCTGAACACCCGGAGGTGCATGCTCATCCTGGGGATCCCCGAGGACTGTGGCGAGGATG
Canis lupus familiaris   GCAGGCGGAAATTGCGCGCGATGGCGGTGACGATGCTGCAGGACTGGTGCAGGTGGATGGGCGTCAGCGCTCGAAGGGGTCTGCTCATTCTGGGCATCCCAGAGGACTGCGATGAAGCCG

Homo sapiens             AGTTTGAGGAGACACTCCAGGAGGCTTGCAGGCACCTGGGCAGATACAGGGTGATTGGCAGGATGTTTAGGAGGGAGGAGAACGCCCAGGCGATTCTACTGGAGCTGGCACAAGATATCG
Canis lupus familiaris   AACTCCAAGAGTCCCTGGAGGCCGCCCTCTGGCCCATGGGCCACTTTACTGTGCTCGGCAAAGTGTTCCGGGAGGAGGATAATGCCACCGCGGCCCTAGTCGAGCTTGACCGGGAAGTCA

Homo sapiens             ACTATGCTTTGCTCCCAAGGGAAATACCAGGAAAGGGGGGGCCCTGGGAAGTGATTGTAAAACCCCGTAACTCAGATGGGGAATTTCTCAACAGACTGAACCGCTTCTTAGAGGAGGAGA
Canis lupus familiaris   ACTATGCGTTGGTCCCCAGGGAAATCCCGGGCACTGGGGGTCCCTGGAACGTGGTTTTTGTGCCCCGTTGCTCAGGCGACGAGTTCCTCAGTCGCGTGTTCCACTTCCTGGAGCAACAGG

Homo sapiens             GGCGGACCGTGTCAGATATGAACCGAGTCCTCGGGTCGGACACCAATTGTTCGGCTCCAAGAGTGACTATATCACCAGAGTTCTGGACCTGGGCCCAGACTCTGGGGGCAGCAGTGCAGC
Canis lupus familiaris   GGCAGACGGTGGAGAGTGTGGCTGGGGCCCTGGGGCTGG------------------------GACTGCG----CAGGGT----GTGCTGGCTCCGATCGGTCAGTCAGGCAGTCCAGC

Homo sapiens             CTCTGCTAGAACAAATGTTGTACCGAGAACTAAGAGTGTTTTCTGGGAACACCATATCCATCCCAGGTGCACTGGCCTTTGATGCCTGGCTTGAGCACACCACTGAGATGCTACAGATGT
Canis lupus familiaris   CCTGGGTGGAGACCATGCGGTACCAGCGCCTGGGCGTGTTTTCCGGGAGGGATCAGCCCGCTCTGGGGGAGGAGTCCTTTGAGGCTTGGCTACACCACACTGCCGACATGCTACATGTGT

Homo sapiens             GGCAGGTG---CCCGAGGGGGAAAAGAGGCGGAGGCTGATGGAATGCTTACGGGGCCCTGCTCTCCAGGTGGTCAGTGGGCTCCGGGCCAGCAATGCTTCCATAACTGTGGAGGAGTGCC
Canis lupus familiaris   GGCAGGGGGTCTCGGAAAGGGAGAAGAGGAGGCGGCTGATGGAAGGCCTTCGAGGGACGGCCCTGCAGCTCGTGCACGGACTCCTGGCCGAGAACCCTGCCAGGACCGCGCAGGACTGCC

Homo sapiens             TGGCTGCCTTGCAGCAGGTGTTCGGACCTGTGGAGAGCCATAAAATTGCCCAGGTGAAGTTGTGTAAAGCCTATCAGGAGGCAGGAGAGAAAGTATCTAGCTTTGTGTTACGTTTGGAAC
Canis lupus familiaris   TGGCGGCCCTGATCCAGGTGTTTGGAGACAATGAGTCCCAGGCCACACTCCGGGTGAAGTGCTTAACTGCTCAGCAGCACTCAGGAGAGCGGCTCTCTGCTTTCGTGTTGAGGCTGGAAG

Homo sapiens             CCCTGCTCCAAAGAGCTGTAGAAAACAATGTGGTATCACGTAGAAAC---GTGAATCAGACTCGCCTGAAACGAGTCTTAAGTGGGGCCACCCTTCCTGACAAACTCCGAGATAAGCTTA
Canis lupus familiaris   TCCTGCTGCAGAAAGCCATTGAG---AAGGGGGCCCTGGCCAGAGCCTCAGCTGACCACGTGCGCCTGAGGCAGGTGCTCACCAGGGCCAACCTCACGGAGCCACT--GGATGAAGCT--

Homo sapiens             AGCTGATGAAACAGCGAA------GGAAGCCTCCTGGTTTCCTGGCCCTGGTGAAGCTCCTGCGTGAGGAGGAGGAATGGGAGGCCACTTTAGGTCCAGATAGGGAGAGTCTGG--AGGG
Canis lupus familiaris   --CTGAGGAAGCTGAGAATGGTTGGGAGGTCTCCAAGTTTCCTAGAGATGCTGGGGCTTGTTCGGGAGTCTGAGGCATGGGAGGCCAGTCTA-GCCAGGAGTGAGAGAGCCCAGGCGGAG

Homo sapiens             GCTGGAAGTAGCCCCAAGGCCACCTGCCAGGATCACTGGGGTTGGGGCAGTACCTCTCCCTGCCTCTGGCAACAGTTTTGATGTGAGGCCTTCCCAGGGCTACCGGCGCCGGAGGGGCAG
Canis lupus familiaris   GAAAGGGCTGGCGCCCAGACCAATGCCCAGGCTGA.....................................................................................

Homo sapiens             AGGCCAACACCGAAGGGGTGGTGTGGCAAGGGCTGGCTCTCGAGGCTCAAGAAAACGGAAACGCCACACATTCTGCTATAGCTGTGGGGAAGACGGCCACA-----TCAGGGTACAGTGC
Canis lupus familiaris   ...........................................................................CTGGCTATGAGCCAGAGGGCCTCACCCGGTCAGGAGACAGGGA

Homo sapiens             ATC-----AACCCCTCCAACCTGCTCTTGGTAAAGCAGAAGAAACAGGCTGCAGTTGAGTCGGAAACGGAACTGGGCTTGGACAAGAGCCATCCCAAGTCC----AAGGCCAAGTAG
Canis lupus familiaris   GGCTGGGGAGCCCCTC-------------GAGGAGGGGCTCAAGCCCATCCCAGACGAGTCAGGAAACGAGGATGGGGCTGGGGGGCTGGGCCTCCCCAAGTCCTCCTCAGG-CAAATAG

Homo sapiens             GCTCGGGAGAAC--AGGGCAACATTTCCTACCACAGGCCAAGGAGACAAAAGAGATATTGGAAGGAGGGGAAAGAGAAGCC------CAGACAAACAGCAGATGAGTTGAGTGGGGCAGA
Canis lupus familiaris   GCTCGGAAGGCCCAAGGGC-CCCCCTCTCCTCTCAGGCAGCCGAG----------TTCTGGAGGCAGGGGGAGGCCATGCCAGGGCGCAGCCTCACCCCACATGAG--GAGCAGGGCCGA

Homo sapiens             GGGACAGGGCAGCCAGACCAAGGCCAAGCCTTCTCACCCTTGGCCAGCTGGAAGGGACTTCAGCAACCAAGACCACCTGGCAACAGGCTCAGTGGGGGTCAGGTCCAGGTCCCCGAAGAG
Canis lupus familiaris   GGGAAGGGCCCACCCGGCCCAAGCCAAGCCCCCTCCCTCTACATTAGCTGGAAAGGACTCCAACCACAAAGCCAAATTGGC-------------------------------TCAAAGAG

Homo sapiens             GTGCTGGAGAGGAAAGCAGGGAGCCACTGCATCCAGCACATGGGGTGCCTGGGCCTCAGATGGGGACCCCAAAGAAGCAGAAGCTGAAGAAGGTACGGCTGGGGGTTCTGTC------CT
Canis lupus familiaris   GTGCCAGCAATGCAAGCAAGAGACCATCACACCCAGCACAAGGGGTACCTTACCCTCAGACAAGGACCCCAACACAGCAGAGACTGAAGGAGGTGCAACTGGGAACTCAGGCATCAATCC

Homo sapiens             GCTCATCCAACCACCCCTAAATACCCACCCTGTGGACTTTGAGCTGAACATGCCCACTGG-CCCCCAGGCCACATGGACCTGGAGGAGCCTACCTGGGGCCTGCCCCTGCCAGCAGGTG
Canis lupus familiaris   CATTGTCTGGCCACTCCCAAATGCCCACCCCATGGGCTCTGAGTTAAAGATGCCCACTGGTCCTCTGGGCCACATG...........................................

Homo sapiens             CCAGGGCTGGTGAGGAAGAGCTGGGGGGCAGAGGTAAAGCCCTGCAGGGGAGGCCACAGGGTCCATCCCGTCTTCAGGATCATCTACACTGCACTAGGGGAGCCCCAGGAAGGCAGCACC
Canis lupus familiaris   ........................................................................................................................

Homo sapiens             CTGGAGGCCCTGTGCCAGTGAGGACAGGAGACCCTAAGGCCCCGGGAGCCCAGTGCCAGCCAGAGGTTGTGCAGGCAAGGAGACCAAAGATTGATGAGAAGACCCCCAGCAGGGGTACTG
Canis lupus familiaris   ..................................................................TGCAGACGAAGAGACCAAAAGCCAAAGAGAAGACCCCAAGCTGGAGGACTG

Homo sapiens             GGTACCCGGCAGGCCAGTGCCCTCACAGTTGACTTGGACCAGGGTGGCTGTGAAGGGAAGTCTTTGTTGCAAAGGAGGAGGAGGAAAAGGGAGGACTTGGTAGGGTTTTGTTTCTTCTGC
Canis lupus familiaris   TGCCCCCGGTGG............................................................................................................

Homo sapiens             TTGTTTCTGTACAGGGCCACCAGACTCCTGGAGAGATCAAGCAAGGAGAACCTGGGGCTGCCATGGCCAAAGCAACTCAACAGATGCCAATGCCAATTCCAAGGCCAGCCACAACCCTGC
Canis lupus familiaris   ........................................................CTCCCATGGC---AGCACCACACCCAGCCCCAGT-CCAATC---AGGCACACCCAATCCCTGC

Homo sapiens             CACCTTGGGGAATCCAGCCTGGAGGCATCCCCTAAGCAGCCAGCCATGGCCTGGGTG-GAGGCACCTGAAGACGTCTGTCCCAAACTCCCCCAGCCCTGAGCTGGGAGATGACAGGGGGA
Canis lupus familiaris   CAACTCAGGCAG-CCAGCCTGGGAAGCACCCCTCAGTGGCCAGCCCCAGCCTAGGTGAGGGGCACCTGAAGCCATCTG---------CCACCCACGCTGGACTGGAAGATGTTAGGGA--

Homo sapiens             AAGAGGCCCTCTCAAGGGTGCCAGATGCCTGGGTCTCCCAAGAGGGGTCCCCCAACTCACCG-TTCCCGGGACAGGCTGCCCCCTGTTCCAGGAAGCTCATCCTCACCTGTGTAGGCCCC
Canis lupus familiaris   ------CCATCTCAAGGGTGCCAGCCTCTTGGGCCTCCCAAAACGGGGACCCTTGCCCACTGACCCCCGGGGCACGTTGCTCCATGTTCTGGGAAGCCCACCTGTGTCTCTGTAAGCTCC

Homo sapiens             TGTAGTGACCCACGCGTCCAGC-----------------------------AGACGCCCACC-----CAC-CGCTAGCCGTTGTTCCTGTGCAAAGTAGTGTGCTATGCA----------
```

```
Canis lupus familiaris  T-TAGTGACCCACGCGTCAAGCTATCCCTCCTCCCCACACCCTCCCCCCCCAAACACACACCACACACACACACTAGCCATCATTCCCATGCAGAGCCTTGAGGTGTGCAGGAACCTGCC

Homo sapiens            -------------CCCACCCAGGTGGCCGCCTCTGGGCCCAAGGCACATGCTGTGAGCTTCC-TGTGAGCCCAGGCTCTGCTCACTGCTGTCCCGC----------------------GT
Canis lupus familiaris  ATGGGCGTCTTGGCCTCCCGGGGCACCCACCTGTCAGCACAGG-----TGTCATGAGCTTCAGTGTCCACCAAGGCTCTGCTCGCTGCTGCGCCACCCAGGGATCCCTCTCCTCCACTGT

Homo sapiens            CATGAGCACCACCTCTGCTTTCCCTGTGTAGATCTAGGCCAGTGGCTGCTTGTTCTTGTGGAGC--------------------------------TGTGTGTGTTCTTCTCTGAGCAGC
Canis lupus familiaris  TGTGAGCTCAACCTCCGCTTTCTCAGTGTAGATTTAGGCCGAATGCTGCTTGTTCTTGTGGAGACGTGTGTGTGTGTGTGTGTGTGTGTGTGTGTGTGTGTGTGTGTTCTTATCTGAGCAGC

Homo sapiens            T---------CCTCCCCGGAGTCCCCCAGCACAGTCCCAGGAGATGACAGGAAGGAAGCACCAGGGCAAGGCGGACGCTCACCCTGTGACCACGATGGTGACCGTGACTGTGGGAGGAAG
Canis lupus familiaris  TGCCCTCACCCCACCCCGGAGACCCTGAGCCCAGTTCCAGGAGACGGCGGGAAGGATGGAGGAGGGCACGGCCGGTGCTCACCCCGCG----------TGACC-----TCCGGAAGGAAG

Homo sapiens            AACTGGACCCAGGACGGAGCGGGGCTGCCCTGCCTGAGGCTCCCGAGGAGCTTTGTGCTTTGGTGTTCCACCCCTGTTGTTACTCATGACTCAGTTTCCTTGACCTGGTAGGGTGTTCCC
Canis lupus familiaris  AACTGGCCCAAAGG----ACGGAGGTGGCCTGCGGCTGTCTCCACAGGAACT--GTGCTCTGATGTGCCGCC--TGTTGTCACTTGTGACTCAGTTTCCTTGGCTTGGCCAAGGGTCCCC

Homo sapiens            TGCTGTGTTTTCCAGTGTCCTGTGACTGTCCTGTGCGGGCCATAGGGCAGGGCCCTGTCCCAGCAGATGGGCTTGGGAGGGGGCTCCCTAAAGCCAGTGGACACTGCCAGAGTCTACCTT
Canis lupus familiaris  TGTTGCCTTTTC-AGCATCCTGGGACTGTCCCGTGCCGGCCATAGGGCAGGACCAGGCCCCAGCATGTCTGCCAGAGGGGGGGTGTCCTGGCGTGAGGGGACATGGCCAGTGTCCGTCGT

Homo sapiens            CCTGGCAAGAGGCAGACCCCGGGGCCCTCAGGAAGGAGGGAGTTGGCAGCGGGGGCTGCAGCAGGAGTAGGAGCAGATGAGGCGTCTTGCCAGGAACCTCAGGAGGAGGGGGCCCGGGAC
Canis lupus familiaris  CCTGGCCAGAGGTTGA--TCGTGGCCCTCGGGAAGGGAGACTATGGCAG-GAGGGCCACGGTGGTGGGGGATGCA---GAGGCGCCCCGTTTGGGACCCTGAGAGGAGGGAGGCTGTGAC

Homo sapiens            CTGTGTGGGACCTGTGTCCTGTGGTGGCCGTTTGCAGTTTCTCTCTGTGTTGTGATTCCCTTCTCTTCAACGTTTTCAGTACGTGTTTCTCTTCAATAAACTTCATTCAGTGTTCCAGCC
Canis lupus familiaris  CTATGGG-----------CTCTACTGACCGTTTTTAATTCCATTCTCTGTTGTCATTCCCTG-TCTTCAATGGTTGCAGTATAT-TTTCTCTTCAATAAATTTCATTGAATGTTTCAGCC

Homo sapiens            G-GCCTCGCCTCTGCTGTG------GGAA-------------ACTGGGGATGAGGTTGGCTGGGGATGGGGTTGGGGTGGAGTGAGGGCCACAGTGGCTTTGTTAACACTGGTGTATACT
Canis lupus familiaris  ATGTCTTGCCTCTGCTCTGGGCTCTGGAAGGAACAACTTTCTACTGGAGCTG----CAGGAGGGGATGGGGTTG----TGAGTGGCAGCATTAGTG-------TGGTACTGGAGTATTTC

Homo sapiens            TGGGGCTCGGTCTGCAAGGCATTCTAGAATGCCCTATCTGCGCATGTTGGTCATGAGCTAGGGTTCACGGCCCACGGACCCAGGCAAAGAAACCCAGCTGAGCAGGCTGGATAGTCCCAG
Canis lupus familiaris  AGGGG...................................................................................................GGCTGGATGGAGCCAA

Homo sapiens            TAGACAGGACACTGGTAGGCCCAGGCAGTGGCTGGTTGGGTCCTCAGGGCACTGGTTGAGCTTCTCAGAAATGAATA----ACATATTTAAGGAAGGGTGAGGGACAGCAGGCTGGACCA
Canis lupus familiaris  GTGACTGGAACTT--TGGGCACAAGAGATATCTGGTTGGGTCCACATGAAACTCAGGGAGC--CCCAGGTATAAGTAGGTTAGACATTCAAGGGAGGGGGCCAGATGGCAGGCAGGGCCT

Homo sapiens            GTGGCCCCAGGATCCCTTGTATCACTATTGGGCTTCCACACCTGGAGCATCAAAACCAGGCCCATCTCTGTGAGTGTGTGTGAGATGGTGAATATGTGTGAGTATGCGTACTAGTGATTG
Canis lupus familiaris  GATGGCCCAGGATC..........................................................................................................

Homo sapiens            TGTGAGAGTGAGTGTAGGAGAGTTTGTGTGTGTCATGTGTCAGCGAATGTGTGAATATGTGAGTGTGTGAGATGGTGTGTGAATATGTTAATGTGTGTTTGTATTTGAGTGTGAGTATGC
Canis lupus familiaris  ........................................................................................................................

Homo sapiens            ATCAGTTTTTGTGTGTGAGTGAATGT
Canis lupus familiaris  ..........................
```

PNMA3 missing 5' end pri Xenarthra (low coverage genome)

PNMA5 gene

>chromosome:GRCh37:X:152156768:152163271:-1
GTGCCGCACCCACACCCTTTTCTGGAACAGTACTTTCGGGCCGCTGCAAGCCGCAGGACC
TTCCCTCTGTGGAAGAAACCTGCTTTGGGAAACGACAGGAAGCAGTTTGGGTACTAGGTG
CGTAATGTTTGGTCGTGAAATCAGGAGAATAATGGGAGAATGGAATATTTTCAGTCATCT
GGCTCTCAGTGAGCAGCGAAAAAAAACTAATGCCCTTGTGAAATGCAGGCAGTAGCTGTA
TTTTTTGCCCTCCTCCCCTAGAACAGTCCAATAAAAAATAAACGAGGGCTATGACTCGTT
TATAAGGGCGCTCACGTGGCCTATACGTTGCACTGGGTGATTTGCACCCGGCCCTTCACC
CATGGGCTTGGGACGCCACAGCTCCCCTCCAATGAGGAAGAGCCACAGTGGCTGCTGGGC
CAAATGGCACGTGGCGAGCCTGGCTGCCACCGTCGTCGGCACGGGGGTGGGTGGGGTGAG
GAACCACCTTGCAGGAAGTAGGAGGGTTTATGTCACCAGTGCGCAACCGTGAGCGGGCCG
TGTGCAGTTCCGCTCATGGGGTAGGGGGGCGGTCGTCACCAGACAACCTCTTCCCGCCAC
AGCCTCGTGGCGCAGTCAGCACGGAGGCGGCAGCCGCCATAGACGTGTGGTGGTCGCGCT
GCGCGGGAACTCCGGCTTGGAGGCACCTCCGGCAGGTACGGAGCTGGACCCCGGGCTCGG
ACAGCAACCCAGATTACGGGGGAACCCCCCAGTAACTCTTGCTATTTTCCACGGGGTCTA
GGGGCTGGTGGCCATAGGTTTCCCTGGAGGCGGGATCCCACTAGATCACACCCAGCACGC
CAAGGAATCCCTGCCATTTCCAGGCAGAACTTCAGGGAGACCTGGCCGGGCGCGGTGGCT
CACACCTGTAATCCCAGCACTTTGGGAGGCCGAGGCGGGTGGATCACCTGAGGTCAGGAG
TTGGAGACCAGCCTGGCCAACATGGGGAAACCCCGTTTCTACTAAAAATACAAAAAGTAG
CCGGGCGTGGTGGTGTACGCCTGTAATCCCAGCTACTCAGGAGGCTGAGGCCGGAGAATC
ACTTGAACCCAGGGGGCGGAGGTTGCAGTGAGCCGAGATACGCTACTGCACTCCAGCCTG
GGTGACAAAGCCAGACTCCGTTTCAGAGAAAAACAAACAAACTTCAGGGAGACCTATTCC
ACCCTTGACCTGCCCGGAGACACTGCAGTCAGGATAGTAGCTCTTGGCCCTTGCCCTTGA
GGGAAAGTGGCTTCGAACCTTCCCCTCCTAGATTCTGCCTGCTTCCACTGATGTCGGTGG
TGTGGGTGTATGTAGATAACCGGGTGGGGATTGGGGGAGCAGGGGAATGGAGGTAGGGG
ATTTGGGGATATCAGTTAGACTTGCCTCGGGGTAGGAGTTGGACTTGGTGAGGGGGCAGG
GCTTGGGGTAGGAGTTGGACTGTGGGTGGGGGCAGGGCTTGAGGCACGAGTTAGACTTGG
GTGGTGGAAGGGGTTAGACTTGGTGGGGGGCTAAGCTTGGGGTAGAGGTGCAACTTAGTT
ATAGGCAGAGTTTGGCAGCAGGGGTGCAGGTTTTGAGGTGGGGCTTAGGAAGGGTTGCAA
CATGGCTGTAGGGGCGGAGCTTGGGAGGGAATGGAGTTTGCAGTGGGGGTAGGGCAGCAA
CCTTACCTTAGGATCCCTTCTGCCCTACCAGCAGCCCCCCTGCCCCCACCCCCTACCTCC
CTCCTCACCTCCCACCCCTCTCACCATTCGTCCCCCGACTCCTCCCCCCGCTCCGCTCCC
TCTGTCAGTTGAGCAGTTTCTTCTATCCCTGCACCAGGGTCCTAGTGAACACCTATGCAG
AGTCTGAATTGGCATGGCCTGGGCAGGGACATGTGTGTGGGACAGAATCACAGCAAAATA
CCTCTGCTTTGCCTTGGACAG**ATCCAGTGAAGCAGGACCCATCACGCCTGAGTCAGGAAC**
TGCCTAACTGGATAGAGTTGAAAAGCAGAGCGAGTCTGAAGTGGCTGTGTTATTGCAGCA
TCCGCGCTGCCAGCAGGGCCAACCCAGCAAGGTGAGCAGCTGTGAGTTGGGCCCTGTTGG
GAGGGGCTCTGGAACCCTGGCAAAGAGTGGGGAGAGGGGACTGCCTGCCAGGGTTCACAG
ACTCTGCAGGGTAGGGCCATCTCTAGGGCAAGAGTTGTTGGTTTCCTGCAGTCTGGCAGT
GGGGACCTTATCTGGGTCCCTTTTCTGCCTGATTTCAGCTTCCACACAATTGAAACCATG
AGCTCCTGTCCTTTCAGCACTCACTCCTCATGCCCATCTCGCCTTCCCTCTCCCACCCTC
CTTTTTGCTAGTACGCTTTATTGGTTGGAGGCCAATGACATCACCTGTTGCTTTTCAAGG
ACCTTGGCCTTACCACATGACACTCCCCTGCAATAGCAACCCATGGTCCATGGTATTCTC
TGTATCCATGGCAACACTGCAGCTTGTCACACAATCTCCACACCCACCAC**TCTCATTGCA**
TTGCGCAGAGCTCAGCCATCTTTCTCATCCGTCAGGCATTCCTGAGAAAGAGGGCCACTC
TACCTCTCTTGCTGCCAGCCTTCACTCCAGCAAGGAGGGTGCTGGGTGACCTGAGCCCAC
ATAGCCCCGAGTGAGCAGTGAGGCTGTCTCCTGCCCTCTTCTGCCTGGAGGGCTCGTCAG
TGTCCCCAGGTGTCAGGCCCTGCCTCCTGACGTTGGCCTTTTCACTACAGGCACCCGAAG
CACACACAGCAGAGTCCTCTGGACTTTGAGGAAGAACCCACAGCAGGAGGAAGTCAGCAG
GGAGTGGTGAGATGGGGAGATCTTTGCCTGGGGAGAACCTCCACACTCTACCCTCCCCAA
AATGACTTGGGGAACCCTGGGAAGAAAGGACTCCATCCCTGCTTGGGGGTGGGGGCAGAG
GAGGGGTGCTTGGGGGCGTAGGTTGGGGTGGGCATTAACAATGCTCTCATCCCATTTGCT
TTGCAG**GCTGTGTGAAACCTGGGACCACTTCTGCCTTCCTACGTGGCAGTGGCTCAGAGT**
TATTTGAGTGCTGTCAAACTGAGCTGATTGCTGCCCTAGTATTAGATCAGTCCATAGAAA
GTGGGAGCAATGGCACTGACACTGCTAGAGGATTGGTGCAAGGGGATGGACATGGACCCC
AGAAAGGCCCTGCTGATTGTAGGCATCCCCATGGAGTGTAGTGAGGTGGAAATTCAGGAC
ACTGTGAAGGCAGGCTTACAGCCCCTGTGCGCATACAGGGTCCTAGGGAGAATGTTCAGG
AGGGAAGACAATGCCAAGGCAGTCTTCATTGAACTGGCTGACACTGTCAATTACACTACT
CTGCCCAGTCACATACCAGGAAAGGGTGGCTCCTGGGAAGTGGTGGTAAAACCCCGTAAC
CCAGATGATGAGTTTCTCAGTAGACTGAACTACTTCCTGAAAGATGAGGGCCGAAGTATG
ACAGATGTGGCCAGAGCCCTGGGATGTTGCAGCCTCCCTGCCGAGAGCCTGGATGCAGAG
GTCATGCCCCAAGTTAGATCCCCACCTTTAGAGCCTCCGAAAGAAAGTATGTGGTACAGG
AAACTGAAAGTGTTTTCGGGAACTGCTTCCCCTAGCCCAGGCGAAGAGACCTTTGAAGAC
TGGCTAGAGCAGGTCACTGAGATAATGCCCATATGGCAAGTGTCTGAGGTGGAGAAGAGG
CGGCGTTTGCTGGAGAGCTTACGTGGGCCTGCTCTGTCAATCATGCGGGTGCTCCAGGCC
AACAATGACTCCATAACTGTGGAGCAGTGCCTTGACGCCCTAAAGCAGATCTTTGGGGAT
AAAGAGGACTTTAGAGCCTCTCAGTTTAGGTTTCTGCAGACCTCTCCGAAGATTGGAGAG
AAAGTCTCCACTTTCCTGCTGCGCTTAGAGCCCCTGCTGCAGAAAGCCGTGCACAAGAGC
CCCTTGTCAGTGCGCAGCACAGACATGATTCGTCTGAAACATCTCTTAGCTCGGGTCGCC
ATGACCCCCGCCCTCAGGGGCAAGCTGGAGCTCTTGGATCAGCGAGGGTGTCCTCCCAAT
TTTCTGGAGTTAATGAAGCTCATTCGAGATGAAGAAGAGTGGGAGAACACTGAGGCAGTG
ATGAAGAATAAGGAGAAGCCATCAGGGAGAGGCCGGGGGGCCTCCGGTAGACAGGCAAGA
GCAGAAGCCAGTGTCTCTGCACCTCAGGCCACCGTGCAGGCAAGGTCTTTTAGCGACAGC

```
AGCCCCCAGACCATACAGGGAGGCTTACCCCCATTAGTGAAACGCAGGCGGCTGTTAGGC
AGTGAAAGCACTAGGGGAGAAGACCATGGCCAGGCCACGTATCCCAAGGCTGAAAACCAG
ACTCCAGGCAGGGAGGGGCCACAGGCTGCAGGAGAGGAGTTGGGAAACGAGGCAGGGGCT
GGGGCCATGAGCCATCCCAAGCCCTGGGAAACATAGGCTCAGAAGACCGAAGCACTCCTT
CCCCTGGCAGGCAGCAGGGACATTTGAACAAGGGGAAGGGGCAGCTTACACTAACAGGGA
ACTTGACTGGGGCAGAGGGACAGGGAAGCCAGGTCGAGATCAAGCCCCCTTACTTTCCAC
CAGCTGGAAGGGACTTCACCAACCAAGACCAAATAGCATCTGGTTCAGTGGGGGCCGGGC
CCATAGCCCCTAACAGGTACAGGAGACACAAGCGGGGAACCACTACACTTAGCACATGGG
GTGCCTGGGCTTCAGATGGGGACCCCAGCGAAGCAGGGGCTGAAGAAGGTGTGGCTGGGG
GCTCTGGCCTGGTCCTCTGGACACCCCCAAATATCTACCCTGTGGACTTTGAGCTGAGCA
TGCCCACTGGCACCCAGGCCACGTGGGACCTGGAGGAGCCTGCCTGGGGCCTGCCTCTGC
CAGAAGGAGCCAGGGCTGCTGAGGAAGAGCTGTGGGGCAGAAGTGAAGCCCCGCAGGGGA
GGCCACAGGGTCCAGCCTGTCTTTAGGATCATCTACACTGCATGAGGGGAGCCCCAGGAA
GGCAGCATTCATAAGCCCCTGCACCAGTGAGGAAGAGAAAGAGGAGGAGAATTAAGAAGA
GGATGATCGGGAGGAGGAAGAGGAGAATAAGGAGGAGCAGGAGGAAGAGGGGGAGGAGAA
GGAGGAAGAGGGGGAGGAGGAAGAGGGGAAGGAGGAGGAAGAAGTGTAAGAAGAGAAGAG
AGGATGTGTTGGGGGTGGGTAGTTGTTTTTGGTTTGCGTCATCATCAGGCCCGGGCTCTG
CTCACTGCTGTTCCGTGTCAAGAACTCCACCTCTGCTTTGCCGGTGTAGATCTCGGCCAG
TGGCTGCTTGTTCTCTTGGGGATGTGTGTGTTTTTATCTGAGCAGCTCCTTTCCAGAGTA
CCCCAGCGCAGTTCCAGGAGACGGTGGGAAGGAGGCATGGCAGACGCTCACCTTGTGACT
GTGACCTCGACTGCAGGAGGACCAACCAGACACGGGAAGGAGCAGGGCTGCCTGAGGCTC
CCCAGGAAGCTTTGTGCTTTGGCGTTCCACCCCTGCTGTTACTCGTGACTCAGTTTCCTC
AACTTGGTGGGGTGTTCCCTGCTATGTTTTCCAGCATCCTGTGACTGTCCTGTGCAGTCT
ATAGGGCAGGGCCCTGCCCCAGCGGGTGGGCTCAGGAGGGGGTTCCCTGAAGCGAGTACA
CATTGCCAGAGCCCACCATCCTGGCAAGAGGTGGATCCTGGGGCCCTATGGAAGGAGGGA
TGTGGTGGGGGGGGCCACACTGAGGGAGGGGCCGGGGACTTGTGACCTGTAGTGGCTGTT
TGCAGTTTCTCTCTGTGTTAGATTCCCTTTTCTTCAGCAGTTTCAGTTCATGTTTCTCTT
CAGTAAATTTTGTTCAGTGTTCCAGTGGTGTCTTGCCTCTGCTGTGTGAAACTGGGGTGG
GAGGTGGTCTGCTGGGGATGGGGCCACAGTGGCCAGGCTGACATCGAGGTGTGTGTGGGG
CAGGGTCTATGAGGGCCTGTGGGGTGCCTTGGGCTGCATGGAGCCTGGCAACAGGATGCT
GGTGGTCATGAGCAGTGACTGGCTGGGTACCAAAGGCACTCCAGGTGTCCTCAGGAATAA
GTGACTTGGACATTGAAGGCTGGGCAAGGGGCAGCAGGTGGGTCCCAGGCCCCTGATCCC
TGGCAGCACTGTAGGGGTGTGGCTCAAGGCTTCCGGAAGGGTCAGAGAGGCCACGTGTGC
AACCAAGGCCAGCAACCGAGGTCAGAGAAGAGCCCCAGGAGGGCCTGGACAGGCAGTCAG
AGGCCCTGTGAAGAGCTGTGTGCAGGACTCGGGGTGGGGTAGGGGCTGGTGTGTGCCCAT
CCTCCCAGATCAGCCCGCAGGTGGCCAGCCTTGGCCTAGCCACTGAGGGATGTTTGGGGA
CAGTGTGTGTGTGATGGCCAAGGGGACAGAGTGTGGGCGAGAAAGGATCCCAAGTGCTC
ATGTCTTGGGGAGGGGGAGAGGAA
```

```
Homo sapiens           GTGCCGCACCCACACCCTTTTCTGGAACAGTACTTTCGGGCCGCTGCAAGCCGCAGGACCTTCCCTCTGTGGAAGAAACCTGCTTTGGGAAACGACAGGAAGCAGTTTGGGTACTAGGTG
Dasypus novemcinctus   ........................................................................................................................

Homo sapiens           CGTAATGTTTGGTCGTGAAATCAGGAGAATAATGGGAGAATGGAATATTTTCAGTCATCTGGCTCTCAGTGAGCAGCGAAAAAAAACTAATGCCCTTGTGAAATGCAGGCAGTAGCTGTA
Dasypus novemcinctus   ........................................................................................................................

Homo sapiens           TTTTTTGCCCTCCTCCCCTAGAACAGTCCAATAAAAAATAAACGAGGGCTATGACTCGTTTATAAGGGCGCTCACGTGGCCTATACGTTGCACTGGGTGATTTGCACCCGGCCCTTCACC
Dasypus novemcinctus   ........................................................................................................................

Homo sapiens           CATGGGCTTGGGACGCCACAGCTCCCCTCCAATGAGGAAGAGCCACAGTGGCTGCTGGGCCAAATGGCACGTGGCGAGCCTGGCTGCCACCGTCGTCGGCACGGGGGTGGGTGGGGTGAG
Dasypus novemcinctus   ...................................................................................................CAAGGGGGCCAGGGGGGCGGG

Homo sapiens           GAACCACCTTGCAGGAAGTAGGAGGGTTTATGTCACCAGTGCGCAACCGTGAGCGGGCCGTGTGCAGTTCCGCTCATGGGGTAGGGGGGCGGTCGTCACCAGACAACCTCTTCCCGCCAC
Dasypus novemcinctus   G-------TTCCCTGGGGCGCGTGGGCT-------CCAGGGCGCAGCCGCGAGCGGGCCGTGTGCAGCCCAGC----------GGGGGGCAGTC---ACTAGACC-CCTCTTCCCGCCAT

Homo sapiens           AGCCTCGTGGCGCAGTCAGCACGGAGGCGGCAGCCGCCATAGACGTGTGGTGGTCGCGCTGCGCGGGAACTCCGGCTTGGAGGCACCTCCGGCAGGTACGGAGCTGGACCCCGGGCTCGG
Dasypus novemcinctus   CACC-AGCGGCCCACACTGCACTGCAGCAGCGGCTG------ACGCGAGGTGGCTGCGGCGTGCGAGAGCTCCGGTCCAGAGGCATCCCCCGCGGGTAAGGCGCGGGA-CCCGGGCCCAG

Homo sapiens           ACAGCAACC-----------CAGATTACGGGGGAACCCCCCAGTAACTCTTGCTATTTTCCACGGGGTCTAGGGGCTGGTGGCCATAGGTTTCCCTGGAGGCGGGATCCCACTAGATCAC
Dasypus novemcinctus   GCAGCCCCCACTGCCTCCCGCTGAGTCCCAGGCGGCCCCCAACGTCCCCCCGCGACGTCCCAGGGG----CGGGTCTGCCG------GGCTCCCAGGGATGCGGATTCCCATCAGGT--C

Homo sapiens           ACCCAGCACGCCAAGGAATCCCTGCCATTTCCAGGCAGAACTTCAGGGAGACCTGGCCGGGCGCGGTGGCTCACACCTGTAATCCCAGCACTTTGGGAGGCCGAGGCGGGTGGATCACCT
Dasypus novemcinctus   AGCCGGCACGCGGAGGGCTCCCTGCCAT............................................................................................

Homo sapiens           GAGGTCAGGAGTTGGAGACCAGCCTGGCCAACATGGGGAAACCCCGTTTCTACTAAAAATACAAAAAGTAGCCGGGCGTGGTGGTGTACGCCTGTAATCCCAGCTACTCAGGAGGCTGAG
Dasypus novemcinctus   ........................................................................................................................

Homo sapiens           GCCGGAGAATCACTTGAACCCAGGGGGCGGAGGTTGCAGTGAGCCGAGATACGCTACTGCACTCCAGCCTGGGTGACAAAGCCAGACTCCGTTTCAGAGAAAAACAAACAAACTTCAGGG
Dasypus novemcinctus   ........................................................................................................................

Homo sapiens           AGACCTATTCCACCCTTGACCTGCCCGGAGACACTGCAGTCAGGATAGTAGCTCTTGGCCCTTGCCCTTGAGGGAAAGTGGCTTCGAACCTTCCCCTCCTAGATTCTGCCTGCTTCCACT
Dasypus novemcinctus   ........................................................................................................................

Homo sapiens           GATGTCGGTGGTGTGGGTGTATGTAGATAACCGGGTGGGGATTGGGGGGAGCAGGGGAATGGAGGTAGGGGATTTGGGGATATCAGTTAGACTTGCCTCGGGGTAGGAGTTGGACTTGGT
Dasypus novemcinctus   ........................................................................................................................

Homo sapiens           GAGGGGGCAGGGCTTGGGGTAGGAGTTGGACTGTGGGTGGGGGCAGGGCTTGAGGCACGAGTTAGACTTGGGTGGTGGAAGGGGTTAGACTTGGTGGGGGGCTAAGCTTGGGGTAGAGGT
Dasypus novemcinctus   ........................................................................................................................

Homo sapiens           GCAACTTAGTTATAGGCAGAGTTTGGCAGCAGGGGTGCAGGTTTTGAGGTGGGGCTTAGGAAGGGTTGCAACATGGCTGTAGGGGCGGAGCTTGGGAGGGAATGGAGTTTGCAGTGGGGG
Dasypus novemcinctus   ........................................................................................................................

Homo sapiens           TAGGGCAGCAACCTTACCTTAGGATCCCTTCTGCCCTACCAGCAGCCCCCCTGCCCCCACCCCCTACCTCCCTCCTCACCTCCCACCCCTCTCACCATTCGTCCCCCGACTCCTCCCCCC
Dasypus novemcinctus   ........................................................................................................................

Homo sapiens           GCTCCGCTCCCTCTGTCAGTTGAGCAGTTTCTTCTATCCCTGCACCAGGGTCCTAGTGAACACCTATGCAGAGTCTGAATTGGCATGGCCTGGGCAGGGACATGTGTGTGGGACAGAATC
Dasypus novemcinctus   ........................................................TGGCCACCTTTGCAGGCCCAGCGCGAGCAGGGCCTGAGCAAGGGCACAAG----GGGCAGACCC

Homo sapiens           ACAGCAAAATACCTCTGCTTTGCCTTGGACAGATCCAGTGAAGCAGGACCCATCACGCCTGAGTCAGGAACTGCCTAACTGGATAGAGTTGAAAAGCAGAGCGAGT-CTGAAGTGGCTGT
Dasypus novemcinctus   GGAGCAAAACACCTCTGCCTGGCCTTCCACAGCTCTGGTGCAGAGGGACCCACCACCCCTG-GCCAGCAGGTGGCTGATTGGACAAAACTAGAGGGCAGGCCATGTGCTG--GGGGCTGG

Homo sapiens           GTTATTGCAGCATCCGCGCTGCCAGCAGGGCCAACCC----AGCAAGGTGAGCAGCTGTGAGTTGGGCCCTGTTGGGAGGGGCTCT-GGAACCCTGGCAAAGAGTGGGGAGAGGG-GACT
Dasypus novemcinctus   CAGATCCCCGTGTCCCAACTGCTGG--GGGCCCTTTCTGGGAGCAAG------ACCTGGGATGGGGCCTCTATTGGGCAGGGCTTTTGGAACCCTGGTGAAGAGAAGGAGTTGGGAGGCT

Homo sapiens           GCCTGCCAGGGTTCACAGACTCTGCAGGGTAGGGCCATCTCTAGGGCAAGAGTTGTTGGTTTCCTGCAGTCTGGCAGTGGGGACCTTATCTGGGTCCCTTTTCTGCCTGATTTCAGCTTC
Dasypus novemcinctus   GCCAGCCAGGCTTGATATAGTCCATGGGCTCTGGTCCAAGCTAGGATGAGGGCTG.................................................................

Homo sapiens           CACACAATTGAAACCATGAGCTCCTGTCCTTTCAGCACTCACTCCTCATGCCCATCTCGCCTTCCCTCTCCCACCCTCCTTTTTGCTAGTACGCTTTATTGGTTGGAGGCCAATGACATC
Dasypus novemcinctus   ........................................................................................................................

Homo sapiens           ACCTGTTGCTTTTCAAGGACCTTGGCCTTACCACATGACACTCCCCTGCAATAGCAACCCATGGTCCATGGTATTCTCTGTATCCATGGCAACACTGCAGCTTGTCACACAATCTCCACA
Dasypus novemcinctus   .CCTGTTGCCCATTGAAGGCCTTGACCTT-------------CCTCTGCAATGGCAACCCATGGTCTA-GCCATCCTCTGTGTCCATGGCAACACTGCTGCTTCTCACCTGCCCTCCACA

Homo sapiens           CCCACCACTCTCATTGCATTGCGCAGAGCTC-AGCCATCTTTCTCATCCGTCAGGCATTCCTGAGAAAGAGGGCCACTCTACCTCTCTTGCTGCCAGCCTTCACTCCAGCAAGGAGGGTG
Dasypus novemcinctus   CCCCCTGGCCACGTTGCCTTGTGCACAGCTCCAGCTGTCTCTTT-------------TCTCTGAGTGTGGCAGTGGGGCTGTGTTTCCTTCCGCCAGCCTCTGCCCAGGCCAGGAGGGCA

Homo sapiens           CTGGGTGACCTGAGCCCACATAGCCCCGAGTG-AGCAGTGAGGCTGTCTCCTGCCCTCTTCTGCCTGGAGGGCTC-GTCAGTGTCCCCAGGTGTCAGGCCCTGCCTCCTGACGTTGGCCT
Dasypus novemcinctus   -TAGGTGACCTGGGCCTGAGGGGTTCCAAGCCCAGGAGAGAGGCATCTTCCTGCCCTCAGCTGCTTGGAAGACTTTGTCGGTGGCCTTGGGTGGCAGGCCTGGTCTCCTCACGTTGCCCT

Homo sapiens           TTTCACTACAGGCACCCGAAGCACACACAGCAGAGTCCTCTGGACTTTGAGGAAGAACCCACAGCAGGAGGAAGTC---AGCAGGGAGTGGTGAGATGGGGAGATCTTTGCCTGGGGAGA
Dasypus novemcinctus   TTTCCCCACAGGCTCCTGCGG-------------GTTCCTTGGACGTGGAGGCAGAGCTCACAGCAGCAGTAGGTCTGTAGTAAGGGGATGGGGTATGGGAAGATCT--GCCTGGGGGG-

Homo sapiens           ACCTCCACACTCTACCCTCCC-CAAAATGACTTGGGGAACCCTGGGAAGAAAGGACTCCA--TCCCTGCTTGGGG--GTGGGGGCAGAGGAGGGGTGCTTGGGGGCGTAGGTTGGGGTGG
Dasypus novemcinctus   -CCCCTGCACTCCACCCTCCCACACAGTGACTGTAGGAGCCCTGGGGCT---GGGCTCTGAGTCCCTGCACGGGGCAGTAGGGGTAGAGGAGGGGCCCTTGGC--CAGAGTGGGGGGTGG

Homo sapiens           GCATT-AACAATGCTCTCATCCCATTTGCTTTGCAGGCTGTGTGAAACCTGGGACCACTTCTGCCTTCCTACGTGGCAGTGGCTCAGAGTTATTTGAGTGCTGTCAAACTGAGCTGATTG
Dasypus novemcinctus   GAGTTTCATGGTGCTCTCACCCCTTCGTATCCACAGGCTGTG--GAACCCTGGGCCGATTCTGCCTTCGTATGTGGCCTTGGCCTTAAATCCTTTGAGCCCTGGTG---TGGACTGATTC

Homo sapiens           CTG-CCCTAGTATTAGATCAGTCCATAGAAAGTGGGAGCAATGGCACTGACACTGCTAGAGGATTGGTGCAAGGGGATGGACATGGACCCCAGAAAGGCCCTGCTGATTGTAGGCATCCC
Dasypus novemcinctus   CTGTCCCTCGGGTCAGATTGATCCCTAGAGTGCGGGAACAATGGCAGTGGCACTGCTAGAGGACTGGTGCAAGGGGATGGACCTGGATCCCAGGAAGGCACTGCTGGTTGTGGGGATCCC
```

```
Homo sapiens          CATGGAGTGTAGTGAGGTGGAAATTCAGGACACTGTGAAGGCAGGCTTACAGCCCCTGTGCGCATACAGGGTCCTAGGGAGAATGTTCAGGAGGGAAGACAATGCCAAGGCAGTCTTCAT
Dasypus novemcinctus  CCTGGACTGTAGTGAGGCCGAGATTACAGAGACCCTGCAGACAGGCTTAGAATCGCTGTGCTCATACAGAGTGCTGGGGAGAATATTCCGCAGGGAAGACAACGCTAAGGCAGTCTTCAT

Homo sapiens          TGAACTGGCTGACACTGTCAATTACACTACTCTGCCCAGTCACATACCAGGAAAGGGTGGCTCCTGGGAAGTGGTGGTAAAACCCCGTAACCCAGATGATGAGTTTCTCAGTAGACTGAA
Dasypus novemcinctus  CGAATTGGCTGACAGCATTGATTATGCCCTGATACCCGATCAGATACCGGGAAAGGGGGGCGCCTGGGAAGTGGTAGTGAAGCCCCGTAACCCGGATGAAGAGTTCATCAGCAGACTGAA

Homo sapiens          CTACTTCCTGAAAGATGAGGGCCGAAGTATGACAGATGTGGCCAGAGCCCTGGGATGTTGCAGCCTCCCTGCCGAGAGCCTGGATGCAGAGGTCATGCCCCAAGTTAGATCCCCACCTTT
Dasypus novemcinctus  CTGCTTCCTGAAGGACGAGGGCCGGAGAATGGTGGATGTGGCCAAAACCTTTGGGTACAGCAACCTCCCTGCAGGCTCCCCGCA---AGACGCCTTAGCCCAGGT--------------

Homo sapiens          AGAGCCTCCGAAAGAAAGTATGTGGTACAGGAAACTGAAAGTGTTTTCGGGAACTGCTTCCCCTAGCCCAGGCGAAGAGACCTTTGAAGACTGGCTAGAGCAGGTCACTGAGATAATGC-
Dasypus novemcinctus  GCAGCCCCTGAAAGAGAATATGTGGTACCGAAAGCTGAAAGTATTTTCAGGCAATGCTTTGCCAGGCCCGGGGGAGGAGCCCTTTGAGGCCTGGCTGGAGCATGCCACTGAGATCATGCA

Homo sapiens          -CCATATGGCAAGTGTCTGAGGTGGAGAAGAGGCGGCG-TTTGCTGGAGAGCTTACGTGGGCCTGCTCTGTCAATCATGCGGGTGCTCCAGGCCAACAATGACTCCATAACT-GTGGAGC
Dasypus novemcinctus  ACCTTGTGGCAGTTGACCGAGGCAGAGAAACGCTACCGCTTTGCTGGACACTTGCTGCGCTCCATCCTTGTCCAGCTGGCGCGTGCTTCAGGGCAACTACTATTCCATGACTGGTAGAGC

Homo sapiens          AGTGCCTTGACGCCCTAAAGCAGATCTTTGGGGATAAAGAGGACTTTAGAGCCTCTCAGTTTAGGTTTCTGCAGACCTCTCCGAAGATTGGAGAGAAAGTCTCCACTTTCCTGCTGCGCT
Dasypus novemcinctus  AGTGCCTGGCGGCGCTGCAGCAGGTGTTCGGACCTGTGGAGAGCCGCAAAATTGCCCAGGTGAAATTTTGTCAGGCCCATCAGGAGGTAGGAGAGAAAGTCTCTAGCTTCGTGTTGCGTT

Homo sapiens          TAGAGCCCCTGCTGCAGAAAGCCGTGCACAAGAGCCCCTTGTCAGTGCGCAGCACAGACATGATTCGTCTGAAACATCTCTTAGCTCGGGTCGCCATGACCCCCGCCCTCAGGGGCAAGC
Dasypus novemcinctus  TGGAACCCCTGCTCCAAAGAGCGGTGGAGAAGAACGCGGTGTCGCGGAGGAACGTCAACCAGGCACGCCTGAAGCAAGTCCTGGGAGGTGCCACGCTGACTGACAAACTTCGAGACAGGC

Homo sapiens          TGGAGCTCTTGGATCAGCGAGGGTGTCCTCCCAATTTTCTGGAGTTAATGAAGCTCATTCGAGATGAAGAAGAGTGGGAGAACACTGAGG---CAGTGATGAAGA------ATAAGGAG-
Dasypus novemcinctus  TGAAGCTGATGAGGCAGCGCAGAAAGCCTCCCGGGTTCTTGGCCCTGGTGAAGCTCCTGCGGGAGGAGGAGGAGTGGGAGGCTACTTTAGGCCCAGAGAGGGAGATACCTGACCCGGAGC

Homo sapiens          --AAGCCATCAGGGAGAGGCCGGGGGGCCTCCGGTAGACAGGCAAGAGCAGAAGCCAGTGTCTCTGCACCTCAGGCCACCGTGCAGGCAAGGTCTTTTAGCGACAGCAGCCCCCAGACCA
Dasypus novemcinctus  CCAAGCCTCCTGCTACA-GCCGGCGGGCT---------CAGG--------GCGGTCCCTGGCCTGGTCCTTGGGAGCAGTGTCCAGGCGAGGCCCTCC-----CAGCGGTCCCGGCGCCG

Homo sapiens          T-------------------ACAGGG-----------AGGCTTACCCCCA----TTAGTGAAACGCAGGCGGCTGTTAGGCAGTGAAAGCACTAGGGGAGAAGACCATGGCCAGGCCAC
Dasypus novemcinctus  TTGAGGCCGGGGCCAGCACCGGCGGGGGGGCGTGCTGCGGGCTGGCCCCCGGGGCCCAGGGCGGCGCAAACGCCACACGTTCTGCTACGGCTGTGGGGAGGAAGGCCACATCAGGGCACT

Homo sapiens          GTATCCCAAGGCTGAAAACCAGACTCCAGGCAGGGAGGGGCCACAGGCTGCAGGAGAGGAGTTGGGAAACGAGGCAGGGGCTGGGGCCATGAGCCATCCCAAGCCCTGGGAAACATAGGC
Dasypus novemcinctus  GTGTTTCAATCCCCCCAACCTGACCCTGGTGAAACAGAAGAGACAGGCCGCAAGC---GAGTCGGGAAACGGGAGCCGGGCTTGAGACGCGAGCCATCCCAAGCCCAAGCCCAGGCAGGC

Homo sapiens          TCAGAAGACCGA---AGCACTCCTTCCCCTGGCAGGCAGCAGGGACATTTGAACAAGGGGAAGGGGCAGCTTACA--CTAACAGGGAACTTGACTGGGGCAGAGGGACAGGGAAGCCAGG
Dasypus novemcinctus  TCAGGAGAGCGGGACGACACTTCTTCCC----AAGGGAGCAGGA----------AAGGCGAGGAGGCAGCCAAGAAGACAGCAGGGGGTGGAGGCAAGGCGCAGGCTCAGCGCCGCCGCA

Homo sapiens          TCGAGATCAAGCCCCCTTACTTTCCACCAGCTGGAAGGGACTTCACCAACCAAG--ACCAAATAGCATCTGGTTCAGTGGGGGCCGGGCCCATAGCCCCTAACAGGTACAGGAGACACAA
Dasypus novemcinctus  TCAAAACTGAGTCCCCCAGCCCTCCCCCAGCCGGGAGGGTTTCCACTGACGAAACTACCAG--------TGCGTCAGTGACAGCCAGGGGCAGGGCACCGATAAGGGGCCAAGGCCGCAA

Homo sapiens          GCGGGGAACCACTACACTTAGCACATGGGGTGCCTGGGCTTCAGATGGGGACCCCAGCGAAGCAGGGGCTGAAGAAGGTGTGGCTGGGGGCTCTGGCCTG-----------GTCCTCTG
Dasypus novemcinctus  GGAGGGGCCTGCC-------GCGCATG-------CAGGCTCGAGTAGGCTGCC----CGACTCTGAGGGTAAAGGAAGTGTGGCCCGAGATTCCGGCCTGGGAATCGGACCCGTAATTGG

Homo sapiens          GACACCCCCAAATATCTACCCTGTGGACTTTGAGCTGAGCATGCCCACTGGCACCCAGGCCACGTGGACCTGGAGGAGCCTGCCTGGGGCCTGCCTCTGCCAGAAGGAGCCAGGGCTGC
Dasypus novemcinctus  GCCGCCCCTGAACGCCCACCCCGTGGGCGCTGAGTTCCGGATAAGGATCGGCCCCAGATTCACCTGGGGCCTGGAGGAGCCAGCCCTGAGGCCACCGCTGC.................

Homo sapiens          TGAGGAAGAGCTGTGGGGCAGAAGTGAAGCCCCGCAGGGGAGGCCACAGGGTCCAGCCTGTCTTTAGGATCATCTACACTGCATGAGGGGAGCCCCAGGAAGGCAGCATTCATAAGCCCC
Dasypus novemcinctus  ....................................................................................................

Homo sapiens          TGCACCAGTGAGGAAGAGAAAGAGGAGGAGAATTAAGAAGAGGATGATCGGGAGGAGGAAGAGGAGAATAAGGAGGAGCAGGAGGAAGAGGGGGAGGAGAAGGAGGAAGAGGGGGAGGAG
Dasypus novemcinctus  ....................................................................................................

Homo sapiens          GAAGAGGGGAAGGAGGAGGAAGAAGTGTAAGAAGAGAAGAGAGGATGTGTTGGGGGTGGGTAGTTGTTTTTGGTTTGCGTCATCATCAGGCCCGGGCTCTGCTCACTGCTGTTCCGTGTC
Dasypus novemcinctus  ....................................................................................................

Homo sapiens          AAGAACTCCACCTCTGCTTTGCCGGTGTAGATCTCGGCCAGTGGCTGCTTGTTCTCTTGGGGATGTGTGTGTTTTTATCTGAGCAGCTCCTTTCCAGAGTACCCCAGCGCAGTTCCAGGA
Dasypus novemcinctus  ....................................................................................................

Homo sapiens          GACGGTGGGAAGGAGGCATGGCAGACGCTCACCTTGTGACTGTGACCTCGACTGCAGGAGGACCAACCAGACACGGGAAGGAGCAGGGCTGCCTGAGGCTCCCCAGGAAGCTTTGTGCTT
Dasypus novemcinctus  ....................................................................................................

Homo sapiens          TGGCGTTCCACCCCTGCTGTTACTCGTGACTCAGTTTCCTCAACTTGGTGGGGTGTTCCCTGCTATGTTTTCCAGCATCCTGTGACTGTCCTGTGCAGTCTATAGGGCAGGGCCCTGCCC
Dasypus novemcinctus  ....................................................................................................

Homo sapiens          CAGCGGGTGGGCTCAGGAGGGGGTTCCCTGAAGCGAGTACACATTGCCAGAGCCCACCATCCTGGCAAGAGGTGGATCCTGGGGCCCTATGGAAGGAGGGATGTGGTGGGGGGGGCCACA
Dasypus novemcinctus  ....................................................................................................

Homo sapiens          CTGAGGGAGGGGCCGGGGACTTGTGACCTGTAGTGGCTGTTTGCAGTTTCTCTCTGTGTTAGATTCCCTTTTCTTCAGCAGTTTCAGTTCATGTTTCTCTTCAGTAAATTTTGTTCAGTG
Dasypus novemcinctus  ....................................................................................................

Homo sapiens          TTCCAGTGGTGTCTTGCCTCTGCTGTGTGAAACTGGGGTGGGAGGTGGTCTGCTGGGGATGGGGCCACAGTGGCCAGGCTGACATCGAGGTGTGTGTGGGGCAGGGTCTATGAGGGCCTG
Dasypus novemcinctus  ....................................................................................................

Homo sapiens          TGGGGTGCCTTGGGCTGCATGGAGCCTGGCAACAGGATGCTGGTGGTCATGAGCAGTGACTGGCTGGGTACCAAAGGCACTCCAGGTGTCCTCAGGAATAAGTGACTTGGACATTGAAGG
Dasypus novemcinctus  ....................................................................................................

Homo sapiens          CTGGGCAAGGGGCAGCAGGTGGGTCCCAGGCCCCTGATCCCTGGCAGCACTGTAGGGGTGTGGCTCAAGGCTTCCGGAAGGGTCAGAGAGGCCACGTGTGCAACCAAGGCCAGCAACCGA
Dasypus novemcinctus  ....................................................................................................

Homo sapiens          GGTCAGAGAAGAGCCCCAGGAGGGCCTGGACAGGCAGTCAGAGGCCCTGTGAAGAGCTGTGTGCAGGACTCGGGGTGGGGTAGGGGCTGGTGTGTGCCCATCCTCCCAGATCAGCCCGCA
Dasypus novemcinctus  ....................................................................................................

Homo sapiens          GGTGGCCAGCCTTGGCCTAGCCACTGAGGGATGTTTGGGGACAGTGTGTGTGTGATGGCCAAGGGGACAGAGTGTGGGCGAGAAAGGGATCCCAAGTGCTCATGTCTTGGGGAGGGGGAG
Dasypus novemcinctus  ....................................................................................................

Homo sapiens          AGGAA
Dasypus novemcinctus  .....
```

```
Homo sapiens        GTGCCGCACCCACACCCTTTTCTGGAACAGTACTTTCGGGCCGCTGCAAGCCGCAGGACCTTCCCTCTGTGGAAGAAACCTGCTTTGGGAAACGACAGGAAGCAGTTTGGGTACTAGGTG
Loxodonta africana  ....................................................................................................

Homo sapiens        CGTAATGTTTGGTCGTGAAATCAGGAGAATAATGGGAGAATGGAATATTTTCAGTCATCTGGCTCTCAGTGAGCAGCGAAAAAAAACTAATGCCCTTGTGAAATGCAGGCAGTAGCTGTA
Loxodonta africana  ....................................................................................................

Homo sapiens        TTTTTTGCCCTCCTCCCCTAGAACAGTCCAATAAAAAATAAACGAGGGCTATGACTCGTTTATAAGGGCGCTCACGTGGCCTATACGTTGCACTGGGTGATTTGCACCCGGCCCTTCACC
Loxodonta africana  ....................................................................................................

Homo sapiens        CATGGGCTTGGGACGCCACAGCTCCCCTCCAATGAGGAAGAGCCACAGTGGCTGCTGGGCCAAATGGCACGTGGCGAGCCTGGCTGCCACCGTCGTCGGCACGGGGGTGGGTGGGGTGAG
Loxodonta africana  ....................................................................................................

Homo sapiens        GAACCACCTTGCAGGAAGTAGGAGGGTTTATGTCACCAGTGCGCAACCGTGAGCGGGCCGTGTGCAGTTCCGCTCATGGGGTAGGGGGGCGGTCGTCACCAGACAACCTCTTCCCGCCAC
Loxodonta africana  ....................................................................................................

Homo sapiens        AGCCTCGTGGCGCAGTCAGCACGGAGGCGGCAGCCGCCATAGACGTGTGGTGGTCGCGCTGCGCGGGAACTCCGGCTTGGAGGCACCTCCGGCAGGTACGGAGCTGGACCCCGGGCTCGG
Loxodonta africana  ....................................................................................................

Homo sapiens        ACAGCAACCCAGATTACGGGGGAACCCCCCAGTAACTCTTGCTATTTTCCACGGGGTCTAGGGGCTGGTGGCCATAGGTTTCCCTGGAGGCGGGATCCCACTAGATCACACCCAGCACGC
Loxodonta africana  ....................................................................................................

Homo sapiens        CAAGGAATCCCTGCCATTTCCAGGCAGAACTTCAGGGAGACCTGGCCGGGCGCGGTGGCTCACACCTGTAATCCCAGCACTTTGGGAGGCCGAGGCGGGTGGATCACCTGAGGTCAGGAG
Loxodonta africana  ....................................................................................................

Homo sapiens        TTGGAGACCAGCCTGGCCAACATGGGGAAACCCCGTTTCTACTAAAAATACAAAAAGTAGCCGGGCGTGGTGGTGTACGCCTGTAATCCCAGCTACTCAGGAGGCTGAGGCCGGAGAATC
Loxodonta africana  ....................................................................................................

Homo sapiens        ACTTGAACCCAGGGGGCGGAGGTTGCAGTGAGCCGAGATACGCTACTGCACTCCAGCCTGGGTGACAAAGCCAGACTCCGTTTCAGAGAAAAACAAACAAACTTCAGGGAGACCTATTCC
Loxodonta africana  ....................................................................................................

Homo sapiens        ACCCTTGACCTGCCCGGAGACACTGCAGTCAGGATAGTAGCTCTTGGCCCTTGCCCTTGAGGGAAAGTGGCTTCGAACCTTCCCCTCCTAGATTCTGCCTGCTTCCA------------
Loxodonta africana  ................................................GGAAGAGAGGGGGCTCTGGCCTGCCTCTCCTGGCCGCGGCCCACTTCCATCCGTGGGGTGTA

Homo sapiens        ------CTGATGTCGGTGGTGTGGGTGTATGTAGA-------------TAACCGGG-TGGGGATTGGGGGGAGCAGGGGAATGGAGGTAGGG--------GATTTGGGGATATCAGTTAG
```

```
Loxodonta africana  CTTCTGCTCAAGATGGGGATGTGGGGGCGTGGGGGGCACGGTGGAGGGCAACGGGGACGGGGGTGAGAAGGTGTGGGAGACTGGGGGCAGGGTGGGGCGGGGTGTGAGGAGGTTTGGGAG

Homo sapiens        ACTTGCCTCGGGGTAGGAGTTG-GACTTGGTGAGGGGGCAGGGCTTGGGGTAGGAGTTGGACTGTGGG--------TGGGGGCAG----------------GGCTTGAGGCACGAGT---
Loxodonta africana  CGTGGGGTGTGGATGTGGGGTGCAACCAGGATAGGGGGTGGG--TAAGGGTGTGAGCGGGA-TATGGGGGGCAGGGTGGGGGCAGATGTGAGGAGGTTAGGGGTTTGGAGTGTGGATGTG

Homo sapiens        ----------TAGACTTGGGTGGTGGAAGGGGTTAGACTTGGTGGGGGGCTAAGCTTGGGGTAGAGGTGCAACTTAGTTATAGGCAGAGTTTGGCAGCAGGGGTGCAGGTTTTGAGGTGG
Loxodonta africana  GGCGTCAACGTAGATTGGGGAGCGGGGAGATGTGGG----GGCTGGGGTGTGAG---GGGGTAGGGGCATGAAGTAGGCGGGGGCACCGGGTAGAGGGTAAGGGGAAGGGTGTAAGGAGG

Homo sapiens        GGC-TTAGGAAGGGTTGCAACATGGCTGTAGGGGCGGAGCTTG--GGAGGGAATGGAGTTTGCAGTGGGGG------------TAGGGCAGCAACCTTACCTTAGGATCCCTTCTGCCCT
Loxodonta africana  GGAGCTGTGGAGGGTGGAGGCACAGAGTGGGGGATGGGGTGTGAAGAAGGGGGCACAGGGTGCGGTGGGGTATGAGTGGGAGCTGGGGTAGCAACCTTACCTTAGGACTCTCTCTGCCGC

Homo sapiens        ACCAGCAGCCCCCC----------------TGCCCCCACCCCCTACCTCCCTCCTCAC-CTCCCACCCCTCTCACCATTCGTCCCCCGACTCCTCCCCCCGCTCCGCTCCCTCTGTCAGT
Loxodonta africana  AGCAGCAGCCCCCTAGTTATGCAGTTTTTATATCCCAACACTCGGGGTCCTTCTGGATACTTCCACC..........................................TCTGTCAGC

Homo sapiens        TGAGCAGTTTCTTCTATCC--CTGCACCAGGGTCCTAGTGAACACCTATGCAGAGTCTGAATTGGCATGGCCTGGGCAGGGAC-------ATGTGTGT----GGGACAGAATCACAGCAA
Loxodonta africana  --AGCAGGGTACCTTAACCATTTGCACTA-----TTCAAGGACTCC-ACGAAGAGTTTGAATTTGAGCGACCTGAGTAGGGGCTCAAAGTGTGTGTGTAGGGGGGGTAGGACCATCACAC

Homo sapiens        AATACCTCTGCTTTGCCTTGGACAGATCCAGTGAAGCAGGACCCATCACGCCTGAGTCAGGAACTGCCTAACTGGATAGAGTTGAAAAGCAGAGCGAGTCTGAAGTGGCTGTGTTATTGC
Loxodonta africana  AGCTGTTCTCCT--GCCTTGGACAGATCTAGTGCAGCAGGACTCA-CACCCTTGGGCCAGAAACGGCCTGACT--------------------------CAGACTCACTGTGGTGCAGC

Homo sapiens        AGCATCCGCGCTGCCAGCAGGGCCAACCCAG--CAAGGTGAGCAGCTGTGAGTTGGGCCC-TGTTGGGAGGGGCTCTGGAACCCTGGCAAAGAGTGGGGAGAGGGGACTGCCTGCCAGGG
Loxodonta africana  AGCACCCACCCTGGTGTCTACCCCAACTCAGAGCCAGGTGAGCAGTTGCACTGTGGGCTTGTGGCGGGAGGGGCCGTGTCACTCTGGCACAGAGCGGGAGAGGCTGCCTGCCTGC-----

Homo sapiens        TTCACAGACTCTGCAGGGTAGGGCCATCTCTAGGGCAAGAGTTGTTGGTTTCCTGCAGTCTGGCAGTGGGGACCTTATCTGGGTCCCTTTTCTGCCT-GATTTCAGCTTCCACACAATT-
Loxodonta africana  TTGCCCTACTCACCAGGGTGGGGTCTCAGCTAGGGTGCCAGCTGTCTATTCC---------AGCACTGTGGACCCTTTCTGGGTCCTTTTAATGCCCCGATCTCAGTTTCCACACAATTT

Homo sapiens        GAAACCATGAGCTCCTGTCCTTT-CAGCACTCACTCCTCATG----------CCCATCTCGCCTTCCCTCTCCCACCCTCCTTT-TTGCTAGTACGCTTTATTGGTTGGAGGCCAATGAC
Loxodonta africana  GAAACAATAAGGTCCTGTTTTTTTCAGAACTTACTCCTCACCAGCCACCTCCCCCAGCTC-CCTCTCGTCCCTCATCCCCTTGTGTTGCTAGGATGCTTCATTGGTCCAAGGCCAGTGAC

Homo sapiens        ATCACCTGTTGCTTTTCAAGGACCTTGGCCTTACCACATGACACTCCCCTGCAATAGCAACCCATGGTCCATGGTATTCTCTGTATCCATGGCAACACTGCAGCTTGTCACACAATCTCC
Loxodonta africana  ATCACCTATTGCTCCTCAAGGGCCTTAACCTCACCGCATGACACCCCTCTGCAGTCGAAGCCCCTAGTCCACGCCGTTCTCCGTATCCATGGCAACACCGCAGCTTGTCA-GTGCTCTCC

Homo sapiens        ACACCCACCACTCTCATTGCATTGCGCAGAGCTC-AGCCATCTTTCTCATCCGTCAGGCATTC-CTGAGAAAGAGGGCCACTCTACCTCTCTTGCTGCCAGCCTTCACTCCAGCAAGGAG
Loxodonta africana  ACACCCACTGGCCTCATTGCATTGTGCAGAGCTCCAGACATCTTTCTCAGTTGTCAGGCACTGGCTGAGA--------------------------------------------GGGG

Homo sapiens        GGTGCTGGGTGACCTGAGCCCACATAGCCCCGAGTGAGCAGTGAGGCTGTCTCCTGCCCTCTTCTGCCTGGAGGGCTCGT-CAGTGTCCCCAGGTGTCAGGCCCTGCCTCCTGACGTTGG
Loxodonta africana  AGCCCCAGGTGACCTGGGCCA-----------------------------TTCTGCTCTC--CTGCCTGGAGGGCTTGGGCAGTGGCCTTAGGAGTCAG-CCTTGCCTTCTGACCTCAG

Homo sapiens        CCTTTTCACTACAGGCACCCGAAGCACACA-CAGCAGAGTCCTCTGGACTTTGAGGAAGAACCCACAGCAGGAGGAAGTCAGCAGGG-AGTGGTGAGATGGGGAGATCTTTGCCTGGGGA
Loxodonta africana  CTTTTCCACCACAGGTACGTGCATCTCGCAGCAGCA----TCTGTGGACTTTGAGGAGGAACCCATAGCGGGAGCAGGTCAGCGGGTCACGGGTGCAGCAGGGGGAGCTCTACCTGGGGA

Homo sapiens        GAACCTCCACACTCTACCCTCCCCAAAATGACTTGGGGAACCCTGGGAAGAAAGGACTCCATCCCTGCTTGGGGGTGGGGGCAGAGGAGGGGTGCTTGGGGGCGTAGGTTGGGGTGGGCA
Loxodonta africana  GACCCCTCACACCCAAATCTCTCCAGAACGACTGCAGGAGGCCCGGGAGGCAGGGAGTGAAACCCTG-----------GCACAGAGG--------------------------------

Homo sapiens        TTAACAATGCTCTCATCCCATTTGCTTTGC-AGGCTGTGTGAAACCTGGGACCACTTCTGCCTTCCTACGTGGCAGTGGCTCAGAGTTATTTGAGTGCTGTCAAACTGAGCTGATTGCTG
Loxodonta africana  TTGATGAGGCTCTCA-CCCATTTGCATTCCTAGGCTGTGGAGACCGTGGGGAAGGTTCTGCCTTCACAC--------------------CTGCGT-CGGCCTGACTCAGCCGA-----G

Homo sapiens        CCCTAGTATTAGATCAGTCCATAGAAAGTGGGAGCAATGGCACTGACACTGCTAGAGGATTGGTGCAAGGGGATGGACATGGACCCCAGAAAGGCCCTGCTGATTGTAGGCATCCCCATG
Loxodonta africana  TCTCGGCCTTAGACCAGTCCCTAAACAGCGGCAGCCATGGCCGTGAACCTGTTAGAAGATTGGTGCAAGGGGATGGACCTGGACCCCAGGAAGGCCCTGCTGATTGTGGGGATCCCCATA

Homo sapiens        GAGTGTAGTGAGGTGGAAATTCAGGACACTGTGAAGGCAGGCTTACAGCCCCTGTGCGCATACAGGGTCCTAGGGAGAATGTTCAGGAGGGAAGACAATGCCAAGGCAGTCTTCATTGAA
Loxodonta africana  GAGTGCAGTGAGGCTGAGATCAAGGAGACCGTGAAAGTGGGCTTTCAGCCTTTGTTTTCCTATAGAGTGCTGGGCAGAATGTTCATGAGGGAAAACAACGCCAAGGCCGTCTTTATCGAG

Homo sapiens        CTGGCTGACACTGTCAATTACACTACTCTGCCCAGTCACATACCAGGAAAGGGTGGCTCCTGGGAAGTGGTGGTAAAACCCCGTAACCCAGATGATGAGTTTCTCAGTAGACTGAACTAC
Loxodonta africana  CTGGCAGAGCCCGTCAACTACTCTCTGATGCCTAGTCACATCCTGGGCAGGGGGGGCACCTGGGAGGTGGTGGTGAATCCCCGCAACCCAGATGATGAGTTTATCAGTAGACTGAACTAC

Homo sapiens        TTCCTGAAAGATGAGGGCCGAAGTATGACAGATGTGGCCAGAGCCCTGGGATGTTGCAGCCTCCCTGCC---GAGAGCCTGGATGCAGAGGTC--ATGCCCCAAGTTAGATCCCCACCTT
Loxodonta africana  TTCCTGAAAGACGAGGGCCGGCGAATGGTGGATGTGGCCAAAACCCTGGGGTATAGCAGCCCAGGTGCCACTGAGGGCAGGGAGCACGAGGTCTTAGGCCCAGAGGCAG--CACCGGGTT

Homo sapiens        TAGAGCCTCCGAAAGAAAGTATGTGGTACAGGAAACTGAAAGTGTTTTCGGGAACTGCTTCCCCTAGCCCAGGCGAAGAGACCTTTGAAGACTGGCTAGAGCAGGTCACTGAGATAATGC
Loxodonta africana  TGCAGCCCCTGAAGGAAAGCATGTGGTACCGGAAATTGAAAGTGTTTTCGGGAAATGCTTTCCCAGTACCCGGCGAGGAGACCTTTGAGGTCTGGCTAGAGCAGGCGACCGAGATGATGC

Homo sapiens        CCATATGGCAAGTGTCTGAGGTGGAGAAGAGGCGGCGTTTGCTGGAGAGCTTACGTGGGCCTGCTCTGTCAATCATGCGGGTGCTCCAGGCCAACAATGACTCCATAACTGTGGAGCAGT
Loxodonta africana  AGCTGTGGCAGGTATCTGAGGTGGAGAAGCGGCGGCGGCTGATGGAGAGCCTGCGCGGCTCTGCGCTGTCCATCATGCGGGTGCTGCGGGCCAACAACGATGCCATGACGGTGGAGGAGT

Homo sapiens        GCCTTGACGCCCTAAAGCAGATCTTTGGGGATAAAGAGGACTTTAGAGCCTCTCAGTTTAGGTTTCTGCAGACCTCTCCGAAGATTGGAGAGAAAGTCTCCACTTTCCTGCTGCGCTTAG
Loxodonta africana  GCCTTGACGCGCTGACGCAGATATTCGGCAACAAGGAGGACTATCGCACCTCTCAGTTCCACTTCCTTCAGGCCTTCCAGAAGTCTGGGGAGAAAATCTCCACTTTCCTGCTTCACTTGG

Homo sapiens        AGCCCCTGCTGCAGAAAGCCGTGCACAAGAGCCCCTTGTCAGTGCGCAGCACAGACATGATTCGTCTGAAACATCTCTTAGCTCGGGTCGCCATGACCCCCGCCCTCAGGGGCAAGCTGG
Loxodonta africana  AACCCCTGCTTCACAAGGCGGTGCAACACAGCCCCATCACCACTCGCAGTGCAGACATGATCCGTCTGAAGCACATCCTGGCCCGGGCCTCCATGACTCCCTCACTCCGTGGCAAGCTGG

Homo sapiens        AGCTCTTGGATCAGCGAGGGTGTCCTCCCAATTTTCTGGAGTTAATGAAGCTCATTCGAGATGAAGAAGAGTGGGAGAACACTGAGGCAGTGATGAAGAATAAGGAGAAGCCATCAGGGA
Loxodonta africana  AGATCCTCGACCAACGAGGGTGTCCCCCCACTTTTCTGGAGTTGATGAAGCTCATTCGCGATGAAGAGGAGTGGGAAACAACCATGGCAGTGAGCCAGGAGAAGCAGAGGCAAGTAGGAA

Homo sapiens        GAGGCCGGGGGGCCTCCGGTAGACAGGCAAGAGCAGAAGCCAGTGTCTCTGCACCTCAGGCCACCGTGCAGGCAAGGTCTTTTAGCGACAGCAGCCCCCAGACCATACAGGGAGGCTTAC
Loxodonta africana  AGGGCCGGAGGGGCTCCAGCAGACAGGTGATGGTGGAGGTCCATGCCCCTGCACCCCAGGCTCCGATGCCGGCAGGATCCTTCATCGAGAACAGCACCCAGACGGCACAGGGAGGGGCTC

Homo sapiens        CCCCATTAGTGAAACGCAGGCGGCTGTTAGGCAGTGAAAGCACTAGGGGAGAAGACCATGGCCAGGCCACGTATCCCAAGGCTGAAAACCAGACTCCAGGCAGGGAGGGGCCACAGGCTG
Loxodonta africana  CCCTGTCACTGAAGCGCAAGCGCCTGCCGTGCTCCTATGGCAGTGGGGATG------ATGACCAAGTCATGTGTCCCGGGGCTGAGAACCAGCCCTCAGCCAAGAAGCAGCAGCCAGTTG

Homo sapiens        CAGGAGAGGAGTTGGGAAACGAGGCAGGGGCTGGGGCCATGAGCCATCCCAAGCCCTGGGAAACATAGGCTCAGAAGACCGAAGCACTCCTTCCCCTGGCAGGCAGCAGGGACATTTGAA
Loxodonta africana  CAGTGGAAGAGTCGGGAAACGGGGTAGGGGCTGGGGCTTTGAGCCACCCCGAGCCCTAAGCAGCATAGGCTCAGGAGACCCTGGCACCCCTTCTCCTAGCACTCAACGGAGACACTGGAA

Homo sapiens        CAAGGGGAAGGGGCAGCTTACACTAACAGGGAACTTGACTGGGGCAGAGG--GACAGGGAAGCCAGGTCGAGATCAAGCCCCCTTACTTTCCACCAGCTGGAAGGGACTTCACCAACCAA
Loxodonta africana  GAAGGAGGAGGGGAGCCCCAGGCAGACA---------------GCAGAGGGCGACAGTGTAGCCTGGGC-----CGAGCCCCCTCCCCCAGTGCCAGCAGGAGGGGACTCTGCT------

Homo sapiens        GACCAAATAGCATCTGGTTCAGTGGGGGCCGGGCCCATAGCCCCTAACAGGTACAGGAGACACAAGCGGGGAACCACTACACTTAGCACATGGGGTGCCTGGGCTTCAGATGGGGACCCC
Loxodonta africana  GACCACATCACCCATGGTTCTGGGAGGCCCAGGAGCAGGGCATGGAGGAGG---AGGAG----GGGCGGGAGAC-----CACTCAGCAGGTGGGTAGCCAGAGCAGTGGGTGGGGAGTCC

Homo sapiens        AGCGAAGCAGGGGCTGAAGAAGGTGTGGCTGGGGGCTCTGGCCTGG-----------TCCTCTGGACACCCC-CAAATATCTACCCTGTGGACTTTGAGCTGAGCATGCCCACTGGCACC
Loxodonta africana  AAC---TCTGGGACTGAAGAAGTTACAACTGGGGGCTCAGGCCTGGGAATTGGCCCCTTGTTTGGGCCCCCCGCTGACATGCACCTTGTGGACTCTAAGCTCAGGATGACCAACGGTCTC

Homo sapiens        CAGGCCACGTGGGACCTGGAGGAGCCTGCCT---------------------GGGGCCTGCCTCTGCCAGAAGGAGCCAGGGCTGCTGAGGAAGAGCTGTGGGGC--AGAAGTGAAGCCC
Loxodonta africana  CAGGCTGTAGGGGGCCTGGAACAGCTTTCCTCAGGGCCACGACAGTCGGGGGTGGACCACCCCTGCCACGAGGAGCCAGGGCTGGAGAGGGAGAGCAGCATGGCCAAGAGGTAGTGCCC

Homo sapiens        CGCAGGGGAGGCCACAGGGTCCAGCCTGTCTTTAGGATCATCTACACTGCATGAGGGGAGCCCCAGGAAGGCAGCATTCATAAGCCCCTGCACCAGTGAGGAAGAGAAAGAGGAGGAGAA
Loxodonta africana  CGGAGGGGAGACCGCAGGGTCCACCCTGTCTTCAGGATCATCTACACAGCCCTAGGGGAGCCCCAAGAAGGCAGCACCTTAGAGCCCCTGCGCCCCTGAGGAGGAGAAGGAGGAGGCGAA

Homo sapiens        TTAAGAAGAGGATGATCGGGAG--GAGGAAGAGGAGAATAAGGAGGAGCAGGAGGAAGAGGGGGAGGA-GAAGGAGGAAGAGGGGGAGGAGGAAGAGGGGAAGGAGGAGGAAGAAGTGTA
Loxodonta africana  GCAGGAGCAGGAGAACAAAGAAACGAGGAGGAGGAGGAGAAGCAGGGGCAGGAGGAGGAGGAGGAGGACAAAGAAACAGGAGGAGGAGGAGAAGCAGGGGCAGGAGGAGGAGGG......

Homo sapiens        AGAAGAGAAGAGAGGATGTGTTGGGGGTGGGTAGTTGTTTTTGGTTTGCGTCATCATCAGGCCCGGGCTCTGCTCACTGCTGTTCCGTGTCAAGAACTCCACCTCTGCTTTGCCGGTGTA
Loxodonta africana  .......................................................................................................................

Homo sapiens        GATCTCGGCCAGTGGCTGCTTGTTCTCTTGGGGATGTGTGTGTTTTTATCTGAGCAGCTCCTTTCCAGAGTACCCCAGCGCAGTTCCAGGAGACGGTGGGAAGGAGGCATGGCAG-----
Loxodonta africana  .ATCATGGTCAACTGATGCTTGTTCTTGTGGAGGTGTGTGTGTTCTTACCTGAGCAGTTTCTCTCTGGAG-ACCCCAGCCCAATCTCTGAAGACAGCAGCAAGGACGGATGCGGGAGTGG

Homo sapiens        --ACGCTCACCTTGTGACTGTGACCTCGACTGCAGGAGGACCAACCAGACACGGGAAGGAGCAGGGCTGCCTGAGGCTCCCCAGGAAGCTTTGTGCTTTGGCGTTCCACCCCTGCTGTTA
Loxodonta africana  CCACCCTCACCCCATGA--------GCCACTGAGGGAAG-------GAAGAAGGGACCGGGT-GGGTTGTGGGGAGCCCCTTGGGAA---CTGTGC-TTGAAGCTGCACCCCAGCTGTTG

Homo sapiens        CTCGTGACTCAGTTTCCTCAACTTGGTGGGGTGTTCCCTGCTATGTTTTCCAGCATC---CTGTGACTGT-CCTGTGCAGTCTATAGGGCAGGGCCCTGCCCCAGCGGGTGGGCTCAGGA
Loxodonta africana  TTTGTGACTCAGTTTCCTTGACATGGTGACGTGTGCTCTGCTGTGTTTTCCAGTGTCCTACTGTGTCTGTCCCTGGGC------CACAGCAGGGACCACGCCCAGTGGGTGGTTCCAGGG

Homo sapiens        GGGGGTTCCCTGAAGCGAGTACACATTGCCAGAGCCCACCATCCTGGCAAGAGGTGGATCCTGGGGCC-CTATGGAAGGAGGGATGTGGTGGGGGGGGCC-----------ACACTGAGG
Loxodonta africana  AAGGATCCCCTGAAGTGAGTGGACGTCGCTAGAGTCCATTGTCCTGAC-AGAGGAGAGTGGGAGGGCTGCAGTGGGCGGGGAGGGGGGGCGGCTGAGGCTCCTCATTAGGGACCCTGGGA

Homo sapiens        GAGGGGCCGGGGACTTGTGACCTGTAGTGGCTGTTTGCAGTTTCTCTCTGTGTTAGATTCCCTTTTCTTCAGCAGTTTCAGTTCA-TG-TTTCTCTTCAGTAAATTTTGTTCAGTGTTCC
Loxodonta africana  GGAGGGT---GGCCCCGTGGCCTCTGGTGGTGGTTATCCATTAC-CTCTGTTGT-GATTGCCTTCCCTTCAATCATTCCAGTTAAGTGTTTTCTCTTCAATAAATCTTATCAAGTGTTTT

Homo sapiens        AGTGGTGTCTTGCCTCTGCTGTGTGAAACTGGGGTGGAGGTGGTCTGCTGGGGATGGGGCC------ACAGTGGCCAGGCTGACATCGA--GGTGTGTG---TGGGGCAGGGTCTATGA
Loxodonta africana  GCTGA-GTCTGGCCTCTGCTGTGGGCAGTGGAGGGGAG----GGCCTGCGGGGCGGGGGGCAGGTGGGGCAGTGGCCGG---TACCCCGACCAGTGTCTGTCCTGGCATGCGGTCTGTGA

Homo sapiens        GGGCCTGTGGGGTGCCTTGGGCTGCATGGAGCCTGGCAACAGGATGCTGGTGGTCATGAGCAGTGACTGGCTGGGTACCAAAGGCACTCCAGGTGTCCTCAGGAAT-AAGTGACTTGGAC
```

```
Loxodonta africana  GGGGTTGTGTGGTCTCTTAGGCCAAA-GGAACTTCATGACAGGATGCTGATTGGCATGGGAAGTGAGTGGTTGTGACCCTTGAACGCTTGGGGAGCCCCCCAAAACGAAATAGGGAAGAT

Homo sapiens        ATTGAAGGCTGGGCAAGGGGCAGCAGGTGGGTCCCAGGCCCCTGA--TCCCTGGCAGCACTGTAGGGGTGTGGCTCAAGGCTTCCGGAAGGGTCAGAGAGGCCACGTGTGCAACCAAGGC
Loxodonta africana  TTTGGAGAATGGGAGAAGAACAGCAGGCAGGAGCCAAGACCCTAATGTTACTCACTGGGCTGTG-----GTGCCTCAGGGCCTCC--------CAGAGAGG------------------

Homo sapiens        CAGCAACCGAGGTCAGAGAAGAGCCCCAGGAGGGCCTGGACAGGCAGTCAGAGGCCCTGTGAAGAG----CTGTGTGCAGGACTCGGGGTGGGGTAGGGGCTGG------TGTGTGCCCA
Loxodonta africana  --ATCACCGAGATCACACGTAAGGTCCTGG------TGGTCGTGAGGGCAGACACCCCTTGCCGAGGCTCCCCTGAGCCT--CCCAGGGCGGGGGGTGTGCTTGCCAGCCTGCCTGCCCG

Homo sapiens        TCCTCC------CAG---------ATCAGCCCGCAGGTGGCCAGCCTTGGCCTAGCCACTGAGGGATGTTTGGGGACA-GTGTGTGTGTGATGGCCAAGGGGACAGAGTGTGGGCGAGAA
Loxodonta africana  CTCACCTGTGCGCAGTCTACACCTGCCAGACCATCGGTGACAAGCATCACTCTATCCACCAAGGGACACTTGGGGACCTGGGGGTGTGTTATGGTGACTGACACAGGG-ATGGGACAGGC

Homo sapiens        AGGGATCCCA-AGTGCTCATGTCTT-----GGGGAGGGGGAGAGGAA
Loxodonta africana  TGGGCTAGTATGGTGCTCATGCCCTGTCCGGGTGAGGGGAGATAGAA


Homo sapiens        GTGCCGCACCCACACCCTTTTCTGGAACAGTACTTTCGGGCCGCTGCAAGCCGCAGGACCTTCCCTCTGTGGAAGAAACCTGCTTTGGGAAACGACAGGAAGCAGTTTGGGTACTAGGTG
Procavia capensis   ........................................................................................................................

Homo sapiens        CGTAATGTTTGGTCGTGAAATCAGGAGAATAATGGGAGAATGGAATATTTTCAGTCATCTGGCTCTCAGTGAGCAGCGAAAAAAAACTAATGCCCTTGTGAAATGCAGGCAGTAGCTGTA
Procavia capensis   ........................................................................................................................

Homo sapiens        TTTTTTGCCCTCCTCCCCTAGAACAGTCCAATAAAAAATAAACGAGGGCTATGACTCGTTTATAAGGGCGCTCACGTGGCCTATACGTTGCACTGGGTGATTTGCACCCGGCCCTTCACC
Procavia capensis   ........................................................................................................................

Homo sapiens        CATGGGCTTGGGACGCCACAGCTCCCCTCCAATGAGGAAGAGCCACAGTGGCTGCTGGGCCAAATGGCACGTGGCGAGCCTGGCTGCCACCGTCGTCGGCACGGGGGTGGGTGGGGTGAG
Procavia capensis   ........................................................................................................................

Homo sapiens        GAACCACCTTGCAGGAAGTAGGAGGGTTTATGTCACCAGTGCGCAACCGTGAGCGGGCCGTGTGCAGTTCCGCTCATGGGGTAGGGGGGCGGTCGTCACCAGACAACCTCTTCCCGCCAC
Procavia capensis   ........................................................................................................................

Homo sapiens        AGCCTCGTGGCGCAGTCAGCACGGAGGCGGCAGCCGCCATAGACGTGTGGTGGTCGCGCTGCGCGGGAACTCCGGCTTGGAGGCACCTCCGGCAGGTACGGAGCTGGACCCCGGGCTCGG
Procavia capensis   ........................................................................................................................

Homo sapiens        ACAGCAACCCAGATTACGGGGGAACCCCCCAGTAACTCTTGCTATTTTCCACGGGGTCTAGGGGCTGGTGGCCATAGGTTTCCCTGGAGGCGGGATCCCACTAGATCACACCCAGCACGC
Procavia capensis   ........................................................................................................................

Homo sapiens        CAAGGAATCCCTGCCATTTCCAGGCAGAACTTCAGGGAGACCTGGCCGGGCGCGGTGGCTCACACCTGTAATCCCAGCACTTTGGGAGGCCGAGGCGGGTGGATCACCTGAGGTCAGGAG
Procavia capensis   ........................................................................................................................

Homo sapiens        TTGGAGACCAGCCTGGCCAACATGGGGAAACCCCGTTTCTACTAAAAATACAAAAAGTAGCCGGGCGTGGTGGTGTACGCCTGTAATCCCAGCTACTCAGGAGGCTGAGGCCGGAGAATC
Procavia capensis   ........................................................................................................................

Homo sapiens        ACTTGAACCCAGGGGGCGGAGGTTGCAGTGAGCCGAGATACGCTACTGCACTCCAGCCTGGGTGACAAAGCCAGACTCCGTTTCAGAGAAAAACAAACAAACTTCAGGGAGACCTATTCC
Procavia capensis   ........................................................................................................................

Homo sapiens        ACCCTTGACCTGCCCGGAGACACTGCAGTCAGGATAGTAGCTCTTGGCCCTTGCCCTTGAGGGAAAGTGGCTTCGAACCTTCCCCTCCTAGATTCTGCCTGCTTCCACTGATGTCGGTGG
Procavia capensis   ........................................................................................................................

Homo sapiens        TGTGGGTGTATGTAGATAACCGGGTGGGGATTGGGGGGAGCAGGGGAATGGAGGTAGGGGATTTGGGGATATCAGTTAGACTTGCCTCGGGGTAGGAGTTGGACTTGGTGAGGGGGCAGG
Procavia capensis   ........................................................................................................................

Homo sapiens        GCTTGGGGTAGGAGTTGGACTGTGGGTGGGGGCAGGGCTTGAGGCACGAGTTAGACTTGGGTGGTGGAAGGGGTTAGACTTGGTGGGGGGCTAAGCTTGGGGTAGAGGTGCAACTTAGTT
Procavia capensis   ........................................................................................................................

Homo sapiens        ATAGGCAGAGTTTGGCAGCAGGGGTGCAGGTTTTGAGGTGGGGCTTAGGAAGGGTTGCAACATGGCTGTAGGGGCGGAGCTTGGGAGGGAATGGAGTTTGCAGTGGGGTAGGGCAGCAA
Procavia capensis   ........................................................................................................................

Homo sapiens        CCTTACCTTAGGATCCCTTCTGCCCTACCAGCAGCCCCCCTGCCCCCACCCCCTACCTCCCTCCTCACCTCCCACCCCTCTCACCATTCGTCCCCCGACTCCTCCCCCCGCTCCGCTCCC
Procavia capensis   ........................................................................................................................

Homo sapiens        TCTGTCAGTTGAGCAGTTTCTTCTATCCCTGCACCAGGGTCCTAGTGAACACCTATGCAGAGTCTGAATTGGCATGGCCTGGGCAGGGACATGTG---TGTGGGACAGAATCA-----CA
Procavia capensis   ..........................................................AGATTTTGAATTTGTGTGACCTGAACAGGGTTTCGGGGGCTGGGGGCCAGGACCATCACTCA

Homo sapiens        GCAAAATACCTCTGCTTTGCCTTGGACAGATCCAGTGAAGCAGGACCCATCACGCCTGAGTCAGGAACTGCCTAACTGGATAGAGTTGAAAAGCAGAGCGAGTCTGAAGTGGCTGTGTTA
Procavia capensis   GCCACACTCTTC-------CTGTGAACAGGTTCGGTGCAGCAGGACGCATC-----CAGGCCAGGAGCTGCCTGCCTGA----------------------------------------

Homo sapiens        TTGCAGCATCCGCGCTGCCAGCAGGGCCAACCCAGCAAGGTGAGCAGCTGTGAGTTGGGCCCTGTTGGGAGGGGCTCTGGAACCCTGGCAAAGAGTGGGGAGAGGGGACTGCCTGCCAGG
Procavia capensis   ---CACCAACTCGGTGTCCACCAGGAC--ACGGAGCCAGGTGAGCAGCGATGCTTTGGGT--TGTGGGAGGAGCCGTTAGCACCCTGACATAC----GACAGGAGAGGCTGCCTGCC--T

Homo sapiens        GTTCACAG-ACTCTGCAGGGTAGGGCCATCTCTAGGGCAAGAGTTGTTGGTTTCCTGCAGTCTGGCAGTGGGGACCTTATCTGGGTCCCTTTTCTG-CCTGATTTCAGCTTCCACACAAT
Procavia capensis   ACTCACCTTACTTGCCAGGGTGGGGTCTCAGCTAGAGTGATAGTTGTCTGTC---------CTAGTACCACAGACCGCCTGTGGGTCCCTTTTCTGGCCCGCTGTCCGTTTCCACATAAT

Homo sapiens        TG-AAACCATGAGCTCCTGTCCTTTCAGCACTCACTCCTCAT-GCCCATCT--------CGCCTTCCCTCTCCCACCCTC-CTTTTTGCTAGTACGCTTTATTGGTTGGAGGCCAATGAC
Procavia capensis   TGGAAACAACGAGTTCCTGTTGTCTCAGAATTCACTCCTCATTGGCCACCTTCCGGAGACCCCGCCCCTCCGTCATCCCCACGGGTGGCTGGGATGCTTTGGTGGTCCAAAGTCAGTGGT

Homo sapiens        ATCACCTGTTGCTTTTCAAGGACCTTGGCCTTACCACATGACACTCCCCTGCAATAGCAACCCATGGTCCATGGTATTCTCTGTATCCATGGCAACACTGCAGCTTGTCACACAATCTCC
Procavia capensis   ATCCGCTGCTGTTCATCCAGAGCCTTAAGCATGCCGCGCAACACCCCTCTGTGGTTGAAGCCCCTG-TCCGCCCTGTTCTTTGTATTCATGGCGACACTGCTGCTTGTCG-GCACTCCCC

Homo sapiens        ACACCCACCACTCTCATTGCATTGCGCAGAGCTC-AGCCATCTTTCTCATCCGTCAGGCATTCCTGAGAAAGAGGGCCACTCTACCTCTCTTGCTGCCAGCCTTCACTCCAGCAAGGAGG
Procavia capensis   A-GCCCGCTGGCCTCCCTGCGCTGTGCAGAGCTCCAGGTGTCTTTCTCAGCGGTCAAGT----------------------------TCTGGCTGACAG------------------

Homo sapiens        GTGCTGGGTGACCTGAGCCCACATAGCCCCGAGTGAGCAGTGAGGCTGTCTC--CTGCCCTCTTCTGCCTGGAGGGCTC-GTCAGTGTCCCCAGGTGTCAGGCCCTGCCTCCTGACGTTG
Procavia capensis   -----GGG----------------AGCCTCAGGCGA----TTGGGCCGTTTCTGCTGTGCTCTT--GCCTAGAGGGCTCTGACAGTGGCCTCGGGCTTC-GGCCCTGCCTCTTACCTCAG

Homo sapiens        GCCTTTTCACTACAGGCACCCGAAGCACACACAGCAGAGTCCTCTGGACTTTGAGGAAGAACCCAC-AGCAGGAGGAAGTCAGCAGG--GAGTGGTGAGATGGGGAGATCTTTGCCTGGG
Procavia capensis   TTCCTTCCACCACAGGTGCCTG---------CAGCAGCATCCCTAAGACTCTGAAGAGGAACCCCCAAGTGGGAGCAGGTCAGCAGGTTGGGAGGTTTGGAGCGGAG--CTCTGCTTGGG

Homo sapiens        GAGAACCTCCACACTCTACCCTCCCCAAAATGACTTGGGGAACCCTGGGAAGAAAGGACTCCATCCCTGCTTGGGGGTGGGGGCAGAGGAGGGGTGCTTGGGGGCGTAGGTTGGGGTGGG
Procavia capensis   GGGACCCCTCCCAC----------------TGACTACAGGAGCCCTGGGGAGCAGG....................................................GGTTGAGG----

Homo sapiens        CATTAACAATGCTCTCATCCCATTTGCTTTGCAGGCTGTGTGAAACCTGGGACCACTTCTGCCTTCCTACGTGGCAGTGGCTCAGAGTTATTTGAGTGCTGTCAAACTGAGCTGATTGCT
Procavia capensis   ---------TACTTTCCTTGGTTTGTATTCGCAGGCAGTGAAGACCCTTGGA-AGCTTACGCCTTAAGGCCCGCC----------------------GCATCCTAACTCCACGGA-----

Homo sapiens        GCCCTAGTATTAGATCAGTCCATAGAAAGTGGGAGCAATGGCACTGACACTGCTAGAGGATTGGTGCAAGGGGATGGACATGGACCCCAGAAAGGCCCTGCTGATTGTAGGCATCCCCAT
Procavia capensis   GTCTTAGTTGTCGATCAGTCCCTAGACCGTGGGCGCCATGGCCATGTCCTTGTTAGAAGATTGGTGCAAGGGCATGGACCTGGAACCC.....................GGGATCCCCAT

Homo sapiens        GGAGTGTAGTGAGGTGGAAATTCAGGACACTGTGAAGGCAGGCTTACAGCCCCTGTGCGCATACAGGGTCCTAGGGAGAATGTTCAGGAGGGAAGACAATGCCAAGGCAGTCTTCATTGA
Procavia capensis   GGAGTGCTATGAGGCTGAAATCAATGAGACC-TGAAAGCAAGCCTGGAGCCTCTGTGCTCCTACAGAGTCCTGGGCAGAATGTTC-TGAAGGAAGAAAATGCTAAGGCTGTCTTAATCCA

Homo sapiens        ACTGGCT-GACACTGTCAATTACACTACTCTGCCCAGTCACATACCAGGAAAGGGTGGCTCCTGGGAAGTGGTGGTAAAACCCCGTAACCCAGATGATGAGTTTCTCAGTAGACTGAACT
Procavia capensis   GCTGGCCCGAGCCTGTCAACTACGCTGTGATGCTCAGTCACATTCCAGGCAGAGGCGGCACCTGGGAAGTGGTGATGAAACCCCGAAACCCAGATGATGCCTTTTTCAGTAAACTCAACA

Homo sapiens        ACTTCCTGAAAGATGAGGGCCGAAGTATGACAGATGTGGCCAGAGCCCTGGGATGTTGCAGCCTCCCTGCCGAGAGCCTGGATGCAGAGGTCATGCCCCAAGTTAGATCCCCACCTTTAG
Procavia capensis   GCTTTCTTAAAGGTGAGGGGAGGCGAGTAGCAGATGTGGCCCAAAGCCTGGGGTATCAAGGCC-------CAGGGGCCC-----CCAAGGGCGG---CCAGCTTGAGGCCTCGGCTGTAG

Homo sapiens        AG----CCTCCG-----AAAGAAAGTATGTGGTACAGGAAACTGAAAGTGTTTTCGGGAACTGCTTCCCCTAGCCCAGGCGAAGAGACCTTTGAAGACTGGCTAGAGCAGGTCACTGAGA
Procavia capensis   AGGTAGCAGCCGGTGTCAGAGAAAGCATGTGGTACCGAAAACTGAAAATGTTTTCGGGAAGTGCTGTCCCAGCGCCTGATGAGGAGACCTTCGAGGTCTGGCTGGAGCACGTGACCGAGA

Homo sapiens        TAATGCCCATATGGCAAGTGTCTGAGGTGGAGAAGAGGCGGCGTTTGCTGGAGAGCTTACGTGGGCCTGCTCTGTCAATCATGCGGGTGCTCCAGGCCAACAATGACTCCATAACTGTGG
Procavia capensis   TGATGAAGGTGTGGCAGGTGTCAGAGGCCGAGAAGCGGCGGCGGCTGCTGGAGAGCCTGCGTGGCACGGCGCTGTCCATCATGCGGGTGCTGCGGGACAAGAACGAGGCCATGACGGTGC

Homo sapiens        AGCAGTGCCTTGACGCCCTAAAGCAGATCTTTGGGGATAAAGAGGACTTTAGAGCCTCTCAGTTTAGGTTTCTGCAGACCTCTCCGAAGATTGGAGAGAAAGTCTCCACTTTCCTGCTGC
Procavia capensis   AGGAGTGCCTGGATGCGCTGACTGAGATATTTGGCAGCAAGGAAGACTTCCGCATCTCTGAGTTTCGCTTCCTTCAGATCTTCCAGAAGTCTGAGGAGAAACTCTCCGCCTTCCTGCTCC

Homo sapiens        GCTTAGAGCCCCTGCTGCAGAAAGCCGTGCACAAGAGCCCCTTGTCAGTGCGCAGCACAGACATGATTCGTCTGAAACATCTCTTAGCTCGGGTCGCCATGACCCCCGCCCTCAGGGGCA
Procavia capensis   GCCTGGAACCCCTGCTCCAGAAAGTGGTGCAGAAAAATCCCATCTCCACCCTCAGTGCAGACATGATCCGCCTGAAGCATCTTCTGTCTCGGGCTTCCATGACCTCCTCACTCCGGGGCA

Homo sapiens        AGCTGGAGCTCTTGGATCAGCGAGGGTGTCCTCCCAATTTTCTGGAGTTAATGAAGCTCATTCGAGATGAAGAAGAGTGGGAGAACACTGAGGCAGTGATGAAGAATAAGGAGAAGCCAT
Procavia capensis   AGCTGCAGATCCTGGACCGGCGAGGCTGTCCGCCCACTTTCCTGGAGCTGGTGAAGCTCATTCGCGATGAAGAAGAGTGGGAGACGGCCGTGGCGGTGATGAGG---GGGCCGAGGCAAG

Homo sapiens        CAGGGAGAGGCCGGGGGGCCTCCGGTAGACAGGCAAGAGCAGAAGCCAGTGTCTCTGCACCTCAGGCCACCGTGCAGGCAAGGTCTTTTAGCGACAGCAGCCCCCAGACCATACAGGGAG
Procavia capensis   TAGACAACAGCCAGAGGATCTTCAACAGGAAGACGCCTATGGAGCTGCACGCTCCTGAGCCCCAGGTCCCTATGCCAGCAGGACTGTTCACGGAGAACAGCACCCAGACCACGCAGGAAG
```

```
Homo sapiens        GCTTACCCCCATTAGTGAAACGCAGGCGGCTGTTAGGCAGTGAAAGCACTAGGGGAGAAGACCATGGCCAGGCCACGTATCCCAAGGCTGAAAACCAGACTCCAGGCAGGGAGGGGCCAC
Procavia capensis   GGATCCCCGCCCCACTGAAGCGCAAGCGCTCTCCGCACAGCCATGG--CCATGGGAATAG----TGAACAGGCCACATGTTCCTGGCGTGTGGGCCAGCCACCAGAGGACCGGAAGCCGT

Homo sapiens        AGGCTGCAGGAGAGGAGTTGGGAAACGAGGCAGGGGCTGGGGCCATGAGCCATCCCAAGCCCTGGGAAACATAGGCTCAGAAGACCGAAGCACTCCTTCCCCTGGCAGGCAGCAGGGACA
Procavia capensis   CCGGTGCAGGAGAAGAGTCGGGAAATGGGGATGGGGCTGGGGCCATGAGCCACCCCGAGCCCTAGGAGCCATAGGCTCACAAGACCCTGGCACCCCTT---CTAGCAAGCAGCGAGCACA

Homo sapiens        TTTGAACAAGGGGAAGGGGCAGCTTACACTAACAGGGAACTTGACTGGGGCAGAGGGACAGGGAAGCCAGGTCGAGATCAAGCCCCCTTACTTTCCACCAGCTGGAAGGGACTTCACCAA
Procavia capensis   CCAGAAGAAGCGAAAGGGGAGCCCCAGGCAGACAGCGA-------------GGGGTGACGGTGAAGCCAGGGTCACATCA--------TACCTTTCTCCAGC-----------------

Homo sapiens        CCAAGACCAAATAGCATCTGGTTCAGTGGGGGCCGGGCCCATAGCCCCTAACAGGTACAGGAGACACAAGCGGGGAACCACTACACTTAGCACATGGGGTGCCTGGGCTTCAGATGGGGA
Procavia capensis   ---------------------GCCAGTGGGT----------------------------AGAGGCTCAGGTTGGGA--------ACGTAACCCTTG-----------------------

Homo sapiens        CCCCAGCGAAGCAGGGGCTGAAGAAGGTGTGGCTGGGGGCTCTGGCCTGGTCCTCT-----GGACACCCCCAAATATCTACCCTGTGGACTTTGAGCTGAGCATGCCCACTGGCACCCAG
Procavia capensis   ---TAACGAAAAGGGGGC-----AACTTGGGACTCAGGACTGGGCCTTGGCCCTTTCTCGGGGCCACTCCCTCATGCCTGTCTTGTGGATTCGGAGCTCAGGAGGAGCCCTGGTCCCCAG

Homo sapiens        GCCACGTGGGACCTGGAGGAGCCTGCCT---GGGGCCTGCCTCTGCCAGAAGGAGCCAGGGCTGCTGAGGAAGAGCTGTGGGGC--AGAAGTGAAGCCCCGCAGGGGAGGCCACAGGGTC
Procavia capensis   GCCATGGAGGACCTGCAGAAGCCTTCCTCAGGGGGCCT---CCTGCCCCGAGGGTCCATGACTCCGGAAGGGGAGCAGTGTGGCCGAGAGGTGACACCCCGGAAGGGAGACCGGAGGGTC

Homo sapiens        CAGCCTGTCTTTAGGATCATCTACACTGCATGAGGGGAGCCCCAGGAAGGCAGCATTCATAAGCCCCTGCACCAGTGAGGAAGAGAAAG--AGGAGGAGAATTAAGA---AGAGGATGAT
Procavia capensis   CACCCCGTCTTCAGGATCATTTACATGGCTCCAGGGGAGCCCCAGGAAGGCAGCACCCTGGAGTCCCTGTGCCCCTAAGGGAGAGACTGTCAGGAGAGGACTCCAGGACCAGAGCCCAGC

Homo sapiens        CGGGAGGAGGAAG---------------------------------------------AGGAGAATAAGGAGGAGCAGGAGGAAGAGGG---GGAGGAGAAGGAGGAAGAGGGGGAGGAG
Procavia capensis   CAGGTGCAGGGGGCTCTGGGCACCTGGTGAGCTGGTGGTTGTGAGGGCAGCTTTGGGCAGGAGGTAGAGGAGGAGGGGGACTGAGCGAGACTGGAGGCGGAGGACAAGCAGGGGCAGGAG

Homo sapiens        GAAGAGGGGA-----------AGGAGGAGGAAGAAGTGTAAG-AAGAGAAGAGAGGATGTGTTGGGGGTGGGTAGTTGTTTTTGGTTT---------------------------------
Procavia capensis   GAAAAGCAGAATGCTAGAATCAGGAGGACAAAGAAGCAAGAGCAGGCGAAGTAACAAGGGAGTTGGGAGATGCTTTCATCTTTGGTTTGTGGAAGGCCGCTGGGGACAGGAGCAGACAGG

Homo sapiens        ---GCGTCATCATCAGGCC---CGGGCTCTGCTCACTGCTGTTCCGTGTCAAGAACTCCAC--CTCTGCTTTG-------------------------------------CCGGTGTAGA
Procavia capensis   GGAGCGTACTCACCTGGCCAGTCAGACCCCAGGTACC-TTGTTCAATGCCAGG----CCACTGCCCTGGTCTGGGAGACCGCACAGGAGGTGGTTATCGTCAGCAGGCTTTCTGGGCAGA

Homo sapiens        TCT-------------------------CGGCCAGTG---------------------------------------------GCTGCTTGTTCTCTTGGGGATGTGTGTGTTTTTATCTG
Procavia capensis   TCTGTCTGTGGTAGCCCTTAGGTTGGGCCGCTCACTGTTCTGAGAAGGTCTCCTGTATTTCTGTAAGCACCACAGTTACCAAGCTCCTTGTCCTTGTAAAGGTGTGTGTGTTACTGCCTG

Homo sapiens        AGCAGCTCCTTTCCAGAGTACCCCAGCGCAGTTCCAGGAGACGGTGGGAAGGAGGCATGGCAG-----ACGCTCACCTTGTGACTGTGACCTCGACTGCAGGAGGACCAACCAGACACGG
Procavia capensis   AGCAGTTTCTCCCCAAG--ACGCCAGCCCAGTCTCAGAGGCCAGTGGGAAGCACACATGTCAGTGGTCACCCTCACCCTGCGA--GCGGCCCTG------GGAAGGAAGAGCGGACCAGG

Homo sapiens        GAAGGAGCAGGGCTGCCTGAGGCTCCCCAGGAAGCTTTGTGCTTTGGCGTTCCACCCCTGCTGTTACTCGTGACTCAGTTTCCTCAACTTGGTGGGGTGTTCCCTGCTATGTTTTCCAGC
Procavia capensis   GACCACGCA-GGCTGCAGGACCCCCTCTGGGA----CTGTGATCAGAGCT--CACTCCCGCTGTTGTTTGTAGCTGAGTTCCTTCAGCATGGTGCCCTGTACTCTGCCGTGTTTTCCAGT

Homo sapiens        A---TCCTGTGACTGTCCTGTGCAGTCTATAGGGCAGGGCCCTGCCCCAGCGGGTGGGCTCAGGAGGGGGTTCCCTGAAGCGAGTACACATTGCCAGAGCCCACCATCCTGGCAAGAGGT
Procavia capensis   GTTCTTCTGTCACTGTCCCAGGGGGCCC-TGTGGCAGGACCACGCCCCAGCGGGTAGGCT-GGGAAGGGGTCCCCTGTGG-------ACATCGCCAGAGTCCGCCATCCTCGCAGAAGGC

Homo sapiens        GGATCCTGGGGCCCTATGGAAGGAGGGATGTGGTGGGGGGGGCCACACTGAGGGAGGGGCC-------------------------------GGGGACTTGTGACCTGTAGTGGCTGTTT
Procavia capensis   AGAGCCTGAGTCCTCCAGGCAGGCAGA----GATGGGTAGGGCCGTGCCGGGGTGGGGGCCAAGGCTCCTCGTCAGGGCCCCCGGGAGCAGGGTGGACCCGCAGCCTGGGCGGGCTCTCC

Homo sapiens        GCAGTTTCTCTCTGTGTTAG-ATTCCCTTTTCTTCAGCAGTTTCAGTT----------CATGTTTCTCTTCAGTAAATTTTGTTCAGTGTTCCAGTGGTGTCTTGCCTCTGCTGTGTGAA
Procavia capensis   A------TCCCCTTTGCTGGTTTTCCCTTCCCTTAAATAGTTCCAGTTAAATGTTTCAAATGTTGCTCTTCAATAAACT-------ACATTTCTGTTGAGTCTGGTCTCTGCTGTGGG--

Homo sapiens        ACTGGGGTGGGAGGTGGTCTGCTGGGGATGGGGCCACAGTGGCCAG-GCTGACATCGAGGTGTGTGTGGGGCAGGGTCTATGAGGGCCTGTGGGGTGCCTTGGGCTGCATGGAGCCTGGC
Procavia capensis   -CTGTG--GAGAGGGAGCCTGCGGGGTACGGG---ACAGTGGCCAGTAGTCCCAACAAAGTCAGCATTG--------TTGTAGGGGGCCATGTGGTCCTCTAGGCCAAA-GGCGCTTCAT

Homo sapiens        AACAGGATGCTGGTGGTCATGAGCAGTGACTGGCTGGGTACCAAAGGCACTCCAGGTG-TCCTCAGGAATAAGTGACTTGGACATTGAAGGCTGGGCAAGGGGCAGCAGGTGGGTCCCAG
Procavia capensis   TACAGGATTCTGATTGGCACTGAAAGGAACTCCTTAATGACCCTTGGAGTTTGGGGAGCTCCCCCCCAAAGAATAGCAAAGACA---GAGGGTGGGCAGGGAACAGCTGGTAG.......

Homo sapiens        GCCCCTGATCCCTGGCAGCACTGTAGGGGTGTGGCTCAAGGCTTCCGGAAGGGTCAGAGAGGCCACGTGTGCAACCAAGGCCAGCAACCGAGGTCAGAGAAGAGCCCCAGGAGGGCCTGG
Procavia capensis   ........................................................................................................................

Homo sapiens        ACAGGCAGTCAGAGGCCCTGTGAAGAGCTGTGTGCAGGACTCGGGGTGGGGTAGGGGCTGGTGTGTGCCCATCCTCCCAGATCAGCCCGCAGGTGGCCAGCCTTGGCCTAGCCACTGAGG
Procavia capensis   ........................................................................................................................

Homo sapiens        GATGTTTGGGGACAGTGTGTGTGTGATGGCCAAGGGGACAGAGTGTGGGCGAGAAAGGGATCCCAAGTGCTCATGTCTTGGGGAGGGGGAGAGGAA
Procavia capensis   ................................................................................................
```

```
Homo sapiens            GTGCCGCACCCACACCCTTTTCTGGAACAGTACTTTCGGGCCGCTGCAAGCCGCAGGAC-----------CTTCCCTCTGTGGAAGAAA-CCTGCTTTGGGAAACGACAGGAAGCAGTTT
Canis lupus familiaris  GTGCCCTACCCATGTTCTTTTCCCAAATCGTGTTTTAGGATC-CTGTGGGTCTCAGGGCGAATATTCCGTCTTCCCTATAAGGAAAAAAGCGTACTTCATAAAAGAGCCAGAAGGAGTTT

Homo sapiens            GGGTACTAGGTGCGTAATGTTTGGTCGTGAAATCAGGAGAATAATGGGAGAATGGAATATTTT--CAGTCATCTGGCTCTCAGTGAGCAGCGAAAAAAAACTAATGCCCTTGTGAAATGC
Canis lupus familiaris  GGGTACCAGGTGAGTAATCCTTGGTCAAGAAGTCAAGGCAATAGTGGCAAAATGCAATCTTTTGATAGTTTTCTGTCTCTTGGTGAAGAGGGCATGAA---------CCTTGTGAAATAT

Homo sapiens            AGGCAGTAGCTGTATTTTTTGCCCTCCTCCCCTAGA--ACAGTCCAATAAAAAATAAACGAGGGCTATGACTCGTTTATAAGGGCGCTCACGTGGCCTATACGTTGCACTGGGTGATTTG
Canis lupus familiaris  AGGAAGCAGCTGTA-TTTTTGCCATCATCCCCTAGAATACAGCACAATAAAAAATAAAC-AGGGCTAAGA..................................................

Homo sapiens            CACCCGGCCCTTCACCCATGGGCTTGGGAC-GCCACA-----GCTCCCCTCCAATGAGG-----------------AAGAGCCACAGTGGCTGCTGGGCC--AAATGGCACGTGGCGAGC
Canis lupus familiaris  ......................CTGGTGACTGTCACAGCATGGCTTCTGTCCAGTGGGAGAAGAGATACATGCAACAGGAACTGCAATGGCTGCTAGGCCTGGCATGGCACATGATGGGC

Homo sapiens            CTGGCTGCC--------------ACCGTCGTC-------------GGCACGGGGGTGGGTGGGGTGAGGAA---CCACCTTGCAGGAAGTAGGAGGGTTTATGTCA------CCAGTGCG
Canis lupus familiaris  CAAGCTGCCCTCAGTTTGAATCAACAGTCATTTCCAGGCATAACAGGGACAGGGACAGGAGGCAGGAAGAAAAGCCACCTT---TGAGGGGGGTAGGTATGTGCCATACTTCTTAGAGAG

Homo sapiens            CAACCGTGAGCGGGCCGTGTGCAGTTCCGCTCATGGGGTAGGGGGGCGGTCGTCACCAGACAACCTCTTCCCGCCACAGC---CTCGTGGCGCAGTCAGCACGGAGGCGGCAGCCGCCAT
Canis lupus familiaris  CAGCTGGGAGCAGGCCATGCACAGCTCC---------ATAGGGG---GTTTGTCACCCAAGAACGTCTTCCTGCCACCACCAGCTCCCAGCCCAGTCTGCACTATGGCAGAAGTGAGGAG

Homo sapiens            AGACGTGTGGTGGTCG----CGCTGCGCGGGAACTCCGGCTTGGAGGCACCTCCGGCAGGTACGGAGCTGGACCCCGGGCTCGGACAGCAACCCAGATTACGGGGGAACCCCCCAGTAAC
Canis lupus familiaris  AGACACATGGTGGCTGTGGTTGGTGCTCAGGTTCTCTGTCTTGGACACATCTCCATCAGGTATGGCATAGGACCTGGGGCACAGAGAGCAACC-------AGAGGATACCCACCAATGAC

Homo sapiens            TCTTGCTATTTTCCACGGGGT-CTAGGGGCTGGTGGCCATAGGTTT-CCCTGGAGGCGGGATCCCACTAGATCACACCCAGCACGCCAAGGAATCCCTGCCATTTCCAGGCAGAACTTCA
Canis lupus familiaris  CCTTGCCATCTTCCCTGGCATCCCAGGGACTGGTGGCTATAAGTTTCCCCTGGAAGTGGGATCCCATTAGATCAGGTCTGGTACCCCA................................

Homo sapiens            GGGAGACCTGGCCGGGCGCGGTGGCTCACACCTGTAATCCCAGCACTTTGGGAGGCCGAGGCGGGTGGATCACCTGAGGTCAGGAGTTGGAGACCAGCCTGGCCAACATGGGGAAACCCC
Canis lupus familiaris  ........................................................................................................................

Homo sapiens            GTTTCTACTAAAAATACAAAAAGTAGCCGGGCGTGGTGGTGTACGCCTGTAATCCCAGCTACTCAGGAGGCTGAGGCCGGAGAATCACTTGAACCCAGGGGGCGGAGGTTGCAGTGAGCC
Canis lupus familiaris  ........................................................................................................................

Homo sapiens            GAGATACGCTACTGCACTCCAGCCTGGGTGACAAAGCCAGACTCCGTTTCAGAGAAAAACAAACAAACTTCAGGGAGACCTATTCCACCCTTGACCTGCCCGGAGACACTGCAGTCAGGA
Canis lupus familiaris  ........................................................................................................................

Homo sapiens            TAGTAGCTCTTGGCCCTTGCCCTTGAGGGAAAGTGGCTTCGAACCTTCCCCTCCTAGATTCTGCCTGCTTCCACTGATGTCGGTGGTGTGGGTGTATGTAGATAACCGGGTGGGGATTGG
Canis lupus familiaris  ........................................................................................................................

Homo sapiens            GGGGAGCAGGGGAATGGAGGTAGGGGATTTGGGGATATCAGTTAGACTTGCCTCGGGGTAGGAGTTGGACTTGGTGAGGGGGCAGGGCTTGGGGTAGGAGTTGGACTGTGGGTGGGGGCA
Canis lupus familiaris  ........................................................................................................................

Homo sapiens            GGGCTTGAGGCACGAGTTAGACTTGGGTGGTGGAAGGGGTTAGACTTGGTGGGG--GGCTAAGCTTGGGGTAGAGGTGCAACTTAGTTATAGGCAGAGTTTGGCAGCAGGGGTGCAGGTT
Canis lupus familiaris  ..........CAGAACTTGGACTTAGGTGG-GGATGGGGTCTGGGTGGGTGCAAACGACTTTACATAGGGCAGGG------CTTGTAAAGTGGTGGTGCTTGGTAG--GGGCCCTTGGGC

Homo sapiens            TTGAGGTGGGGCTTAGGAAGGGTTGCAACATGGCTGTAGGGGCGGAGCTTGGGAGGGAATG-GAGTTTGCAGTGGGGGTAG-------------GGCAGCAACCTTACCTTAGGATCCCT
Canis lupus familiaris  CAGAAGTTGAGTTTCAGTGGGGGTGGGACTTGAAGGCAGGGGTGAGGCATGGAAAGGGGTGAGAATTGGCAGTGGGGTCAGTTTTGTGGTGTGTGGCAATAGTCTTACTGCAGGATTGCT

Homo sapiens            TCTGCCCTACCAGCAGCCCCCCTGCCCCCACCCCCTACCTCCCTCCTCACCTCCCACCCCTCTCACCATTCGTCCCCCGACTCCTCCCCCCGCTCCGCTCCCTCTGTCAGTTGAGCAGTT
Canis lupus familiaris  TCTCATCTAGCAGTGGATCCTT..........................................................................................GTTTAGCAGTT

Homo sapiens            TCTTCTATCCCTGCACCAGGGTCCTAGTGAACACCTATGCAGAGTCTGAATTGGCATGGCCTGGGCAGGGACATGTGTGTGGGACAGAATCACAGCAAAATACCTCTGCTTTGCCTTGGA
Canis lupus familiaris  TCTTGTATCCATGCACCAGAGTAGTTGTGGACACCTCTGAAGACTCAGAATTGGTATAGCCTGAGCAGGGGCATGTG----AGGCACAACATTAGCAAGATATTTCTGCCTTACTTTTGA

Homo sapiens            CAGATCCAGTGAAGCAGGACCCATCACGCCTGAGTCAGGAACTGCCTAACTG--GATAGAGTTGAAAAGCAGAGCGAGTCTGAAGTGGCTGTGTTATTGCAGCATCCGCGCTGCCAGCAG
Canis lupus familiaris  CAGATCCAGTGGATTAGTAC---TCACTCCAAGGCCAGAAACTGCCTTCTTGAGGATAGAACTAAAGGGCAGATTACATGTTGAGTGCTGGTGGAATCCCAGAATCTGCACTGCCAGTGG

Homo sapiens            GGCCAACCCAGCAAGGTGAGCAGCTGTGAGTTGGGCCC----TGTTGGG----AGGGGCTCTGGAACCCTGGCAAAGAGTGGGGAGAGGGGACTGCCTGCCAGGGTTCACAGACTCTGCA
Canis lupus familiaris  GGCC---TTAGAAATGAGA----CACTGAAGTGAGCCTATGTTGTTGAAGCACAGGGGCTTCTGTGCTCTGGCAAAG----GAGGGGAGGGGCTGCGTGACAAGATTTACATTCTCTGCA
```

```
Homo sapiens              GGGTAGGGCCATCTCTAGGGCAAGAGTTGTTGGTTTCCTGCAGTCTGGCAGTGGGGACCTTATCTGGGTCCCTTTTCTGCCTGATTTCAGCTTCCACACAATTGAAACCATGAGCTCCTG
Canis lupus familiaris    GGGCAGGTCACCCCCTAGAGTAAGAATTGTCTATCTCTTGCAGTGTGACACTTTAGACCCTTTCTAGGTCCTTTTTCTGCCT-----------TCACACAATTGAA-CCATAAGCTCTTG

Homo sapiens              TCCTTTCAGCACTCACTCCTCA-TGCCCATCTCGCCTTCCCTCTCCCACCCTC-----CTTTTTGCTAGTACGCTTTATTGGTTGGAGGCCAATGACATCACCTGTTGCTTTTCAAGGAC
Canis lupus familiaris    TTTTTTCAGCACTAGATCCTTGCTGCCCATTCCCCATCTCTCCTCCCTCCCTCACAACCCAGTTACTAGGACACTTCACTGGTTAGAGGCCAGTGACATCACCTGTTGCTTCTCAATGGT

Homo sapiens              CTTGGCCTTACCACATGACACTCCCCTGCAATAGCAACCCATGGTCCATGGTATTCTCTGTATCCATGGCAACACTGCAGCTTGTCACACAATCTCCACACCCACCACTCTCATTGCATT
Canis lupus familiaris    CTTAACTTCACCATATGACACCACTTTGTGATAGAAATCCACGGTCCAT-GTGTTCTCAGTATCCATGGCAACACTGCACCTTGTCACACGCTTTCCACACC---------CATTGCATT

Homo sapiens              GCGCAGAGCT-CAGCCATCTTTCTCATCCGTCAGGCATTCC-TGAGAAAGAGGGCCACTCTACCTCTCTTGCTGCCAGCCTTCACTCCAGCAAGGAGGGTGCTGGGTGACCTGAGCCCAC
Canis lupus familiaris    GTGGAGAGCTCCAGCCATCTTTATCATCTGTCAGGCATTTTATGAGGAAGGGGA--ACTCCACATTTCCTTAATTTAGCCTTTGCCCTGGCAAAGGAGGTGCCAGGTGCCCTGGGCCTGT

Homo sapiens              ATAGCCCCGAGTGAGCAGTGAGGCTGTCTCCTGCCCTCTTCTGCCTGGAGGGCTCGTCAGTGTCCCCAGGTGTCAGGCCCTGCCTCCTGACGTTGGCCTTTTCACTACAGGCACCCGAAG
Canis lupus familiaris    ATAGCAC---GTGAG-AATGCAATAGTCTCATGTTCTCTCCTGCTCAGAGGGCTTTAAGGTGGCCCCCAGAGTC--GCCCTGCAGCCTGTAGGTGG---TTCCACGAC-----------

Homo sapiens              CACACACAGCAGAGTCCTCTGGACTTTGAGGAAGAACCCACAGCAGGAGGAAGTCAGCAGGGAGTGGTGAGATGGGGAGATCTTTGCCTGGGGAGAACCTCCACACTCTACCCTCCCCAA
Canis lupus familiaris    ----TCCTGCAGAGTC-----------------------------AGAGACAGTGAGCAG--------GAGACCGGGGA--CTTTGCTCAGATGGGACCT-----------------TAG

Homo sapiens              AATGACTTGGGGAACCCTGGGAAGAAAGGACTCCATCCCTGCTTGGG-----GGTGGGGGCAGAGGAGGGGTGCTTGGGGGCGTAGGTTGGGGTG--GGCATTAACAATGCTCTCATCCC
Canis lupus familiaris    AATTACC--GGGCACCCTGG---GTAAGGGCTGGATCACTGCCAGGGCAGTCAGTGAGGGGGGAGGAGGGTCACCTCGGGGTGGGAGGTGGGGTGTCAGCCTTAACCTCGCTCTCGCCCG

Homo sapiens              ATTTGC-TTTGCAGGCTGTGTGAAACCTGGGACCACTTCTGCCT---TCCTACGTGGCAGTGGCTCAGAGTTATTTG-------------AGTGCTGTCAA--------ACTGAGCTGAT
Canis lupus familiaris    GTTCGTATTCACAGGCTGTG-GAGACCTGGG--CAGTTCTCTCTGAGTCCTA---GGCTCCCGCCCCAATTCATTCTCCCCCACCCGCCCAGTACCATCCTGGTCTGCTACGGAACCGTT

Homo sapiens              TGCTGCCC-TAGTATTAGATCAGTCCA--TAGAAAGTGGGAGCAATGGCACTGACACTGCTAGAGGATTGGTGCAAGGGGATGGACATGGACCCCAGAAAGGCCCTGCTGATTGTAGGCA
Canis lupus familiaris    TCCTGCCCCTAGTATTAGCCCCGCGCAGGCGGAAATTGCGCGCGATGGCGGTGACGATGCTGCAGGACTGGTGCAGGTGGATGGGCGTCAGCGCTCGAAGGGGTCTGCTCATTCTGGGCA

Homo sapiens              TCCCCATGGAGTGTAGTGAGGTGGAAATTCAGGACACTGTGAAGGCAGGCTTACAGCCCCTGTGCGCATACAGGGTCCTAGGGAGAATGTTCAGGAGGGAAGACAATGCCAAGGCAGTCT
Canis lupus familiaris    TCCCAGAGGACTGCGATGAAGCCGAACTCCAAGAGTCCCTGGAGGCCGCCCTCTGGCCCATGGGCCACTTTACTGTGCTCGGCAAAGTGTTCAGGAAGGAGGAAAATGCTAAGTCAGTCT

Homo sapiens              TCATTGAACTGGCTGACACTGTCAATTACACTACTCTGCCCAGTCACATACCAGGAAAGGGTGGCTCCTGGGAAGTGGTGGTAAAACCCCGTAACCCAGATGATGAGTTTCTCAGTAGAC
Canis lupus familiaris    TCATTGAATTTGCTGACTCT--CAATTACACTATGATGC----TCAAATACCAGGAAATGGAGGTGCCAGGGAAGAGGAAGTAAAACCCTGGAACCCAGATGACGAATTTATCAATAGAC

Homo sapiens              TGAACTACTTCCTGAAAGATGAGGGCCGAAGTATGACAGATGTGGCCAGAGCCCTGGGATGTTGCAGCCTCCCTGCCGAGAGCCTGGATGCAGAGGTCATGCCCCAAGTTAGATCCCCAC
Canis lupus familiaris    TGCACTGCTTCCTGAAAGACAACAGCCAGAGAATGGCAGATGTCAGCAAAACACTGGGGTATAGAAACC-----ACTGAGGACATGGAGCCAGAGGGTTTGGCCCAAGTCAAACCACCAG

Homo sapiens              CTTTAGAGCCTCCGAAAGAAAGTATGTGGTACAGGAAACTGAAAGTG-TTTTCGGGAACTGCTTCCCCTAGCCCAGGCGAAGAGACCTTTGAAGACTGGCTAGAGCAGGTCACTGAGATA
Canis lupus familiaris    TTTTGCAGCACTTGAAAGAAAGCATGTGGTACTGAAAACTAAAAGTGTTTTTCAGGTAGCACTTTCCC-AGCCCAGCCAAAGAAAAGTTTGAAATCTGG----AGCAGGTCA--GAGATG

Homo sapiens              ATGCCCATATGGCAAGTGTCTGAGGTGGAGAAGAGGCGGCGTTTGCTGGAGAGCTTACGTGGGCCTGCTCTGTCAATCATGCGGGTGCTCCAGGCCAACAATGACTCCATAACTGTGGAG
Canis lupus familiaris    ATGTAGATGTGGCAAGTGTCTCAGGTAGAGAAGAGGTGACATCTGCTGCAAAGCATACATGGCTCTGCACTATCAATCACATGAGTGCTT--GGGCAGCAATGACTCTATGATTGTGGAG

Homo sapiens              CAGTGCCTTGACGCCCTAAAGCAGATCTTTGGGGATAAAGAGGACTTTAGAGCCTCTCAGTTTAGGTTTCTGCAGACCTCTCCGAAGATTGGAGAGAAAGTCTCCACTTTCCTGCTGCGC
Canis lupus familiaris    CAGTGCCTGGATGCCCTGAAGCAGATCGTTGGGAATAGAGAGGACTGTAGAACCTTACAGTTTAGCTCCCTTCAGACCATTCAGAAATTTGGAGAGAAAATCTCTGCTTTTTTGCTATGA

Homo sapiens              TTAGAGCCC-----CTGCTGCAGAAAGCCGTGCACAAGAGCCCCTTGTCAGTGCGCAGCACAGACATGATTCGTCTGAAACATCTCTTAGCTCGGGTCGCCATGACCCCCGCCCTCAGGG
Canis lupus familiaris    TAAGAGCCCCCCCGCCGATACAGAAAGGCTTGCAGTCCAGCCCCTTGTCAGTGCATAGCACCCACATGATTCACCTGAAACACATCCTAGTTCAAGCCCATATGACCACTACACTATGGG

Homo sapiens              GCAAGCTGGAGCTCTTGGATCAGCGAGGGTGTCCTCCCAATTTTCTGGAGTTAATGAAGCTCATTCGAGATGAAGAAGAGTGGGAGAACACTGAGGCA---GTGATGAAGAATAAGGAGA
Canis lupus familiaris    GTAAGCTAGACCTCCTGGATCACTGAGGCTGTGCTCCCATTTTTCTGGAGTTAATGCTGCTCATCTGAGATGAAGAGGACTGAGAGGCCACCATCTCATCTGAGATGAAGAGGACTGGGA

Homo sapiens              AGCCATCAGGGAGAGGCCGGGGGGCCTCCGGTAGACAGGCAAGAGCAGAAGCCAGTGTCTCTGCACCTCAGGCCACCGTGCAGGCAAGGTCTTTTAGCGACAGCAGCCCCCAGACCATAC
Canis lupus familiaris    GACCACCATGAAA-----------------------AGGCAAGTGTGAAGG------------------GGCCAC-------------------------------------------

Homo sapiens              AGGGAGGCTTAC-CCCCATTAGTGAAACGCAGGCGGCTGTTAGGCAGTGAAAGCACTAGGGGAGAAGACCATGGCCAGGCCACGTATCCCAAGGCTGAAAACCAGACTCCAGGCAGGGAG
Canis lupus familiaris    --AGAAGATTATGGCCTATCGTTGAAGCACAGGTCAATGCCATGCTGCTACAGTAGTGGGGAGGAAGGCCACAGCCAGGCACCATGTCCTCGGAACAAGAACCAACCTCTACTAAAGCAG

Homo sapiens              GGGCCACAGGCTGCAGGAGAGGAGTTGGGAAACGAGGCAGGGGCTGGGGCCATGAGCCATCCCAAGCCCTGGGAAACATAGGCTCAGAAGACCGAAGCACTCCTTCCCCTGGCAGGCAGC
Canis lupus familiaris    AAGTCTCATCTTCCAGCAGATGAGTCAGGAAACAACAAAGAGGCTGGAGCTATG----------AGCCCTAGGAAACATAGGCTCAAGAGATCCAGGCACCTCTTCCCCTAGCAGGGAGC

Homo sapiens              AGGGACATTTGAACAAGGGGAAGGGGCAGCTTACACTAAC---AGGGAACTTGACTGGGGCAGAGGGACAGGGAAGCCAGGTCGAGATCAAGCCCCCTTACTTTCCACCAGCTGGAAGGG
Canis lupus familiaris    AGAAAGGTTTTAACAAGGGGGA-AGGCACTTCACACAAACAGAAGGGGACTTGAGTGAGGCACAGGAGCAGGACAACTGGGTCCATGTCAAGAACTCTCCCTCTACATTAGCTGGAAAGG

Homo sapiens              ACTTCACCAACCAAGACCAAATAGCATCTGGTTCAGTGGGGGCCGGGCCCATAGCCCCTAACAGGTACAGGAGACACAAGCGGGGAACCACTACACTTAGCACATGGGGTGCCTGGGCTT
Canis lupus familiaris    ACTCCAACCACAAAG-CCAAAT------TGGCTCA-----------------------AAGAGGTGCCAGCAATGCAAGCAAGAGACCATCACACCCAGCACAAGGGGTACCTTACCCT

Homo sapiens              CAGATGGGGACCCCAGCGAAGCAGGGGCTGAAGAAGGTGTGGCTGGGGGCTCTGGC------CTGGTCCTCTGGACACCCCCAAATATCTACCCTGTGGACTTTGAGCTGAGCATGCCCA
Canis lupus familiaris    CAGACAAGGACCCCAACACAGCAGAGACTGAAGGAGGTGCAACTGGGAACTCAGGCATCAATCCCATTGTCTGGCCACTCCCAAATGCCCACCCCATGGGCTCTGAGTTAAAGATGCCCA

Homo sapiens              CTGG-CACCCAGGCCACGTGGGACCTGGAGGAGCCTGCCTGGGGCCTGCCTCTGCCAGAAGGAGCCAGGGCTGCTGAGGAAGAGCTGTGGGGCAGAAGTGAAGCCCCGCAGGGGAGGCCA
Canis lupus familiaris    CTGGTCCTCTGGGCCACATGGGATCTGG-GAAGCCCACCTG-----TGTCTCTGTAAG.............................................................

Homo sapiens              CAGGGTCCAGCCTGTCTTTAGGATCATCTACACTGCATGAGGGGAGCCCCAGGAAGGCAGCATTCATAAGCCCCTGCACCAGTGAGGAAGAGAAAGAGGAGGAGAATTAAGAAGAGGATG
Canis lupus familiaris    ........................................................................................................................

Homo sapiens              ATCGGGAGGAGGAAGAGGAGAATAAGGAGGAGCAGGAGGAAGAGGGGGAGGAGAAGGAGGAAGAGGGGGAGGAGGAAGAGGGGAAGGAGGAGGAAGAAGTGTAAGAAGAGAAGAGAGGAT
Canis lupus familiaris    ...............................................GAGAGAGAGAGAGAGAGAGAG-AGGCAGAGACACAGGCAGAGGGAGAAGC-----------AGGCTCCAT

Homo sapiens              GTGTTGGGGGTGGGTAGTTGTTTTTGGTTTGCGTCATCATCAGGCCCGGGCTCTGCTCACTGCTGTTCCGTGTCAAGAACTCCACCTCTGCTTTGCCGGTGTAGATCTCGGCCAGTGGCT
Canis lupus familiaris    GCACCGGGAGCCCGATGTGGGATTCGATCCCGG--GTCTCCAGGTTCAGGCTTTGCTCTCTCTCCTCCACTGTTGTGAGCTCAACTTCTGCTTTCTTGGTGAAGATTTAGGCTGGTTTCT

Homo sapiens              GCTTGTTCTCTTGGGGATGTG----TGTGTTTTTATCTGAGCAGCT---------CCTTTCCAGAGTACCCCAGCGCAGTTCCAGGAGACGGTGGGAAGGA--------GGCATGGCAGA
Canis lupus familiaris    GATTGTTCTTGTGGAGACGTGTGTATGTGTTCTTATCTGAGCAGCTGCCCCCACCCCACCCCGGAGACCCTGAGCCTAGTTCCAGGAGATGGCAGGAAGAATGGACAAGGGCATGGCTGG

Homo sapiens              CGCTCACCTTG--TGACTGTGACCTCGACTGCAGGAGGACCAACCAGAC-ACGGGAAGGAGCAGGGCTGCCTGAGGCTCCCCAGGAAGCTTTGTGCTTTGGCGTTCCACCCCTGCTGTTA
Canis lupus familiaris    TGCTCATCCTGCATGACTCT--------TTGAAGAAGAACCAGCCGGCCCAAAGGACGGAGGTGGCCTGCGGCTGTCTCCACAGGAA---CTGTGCTCTGATGTGCCGCC--TGTTGTCA

Homo sapiens              CTCGTGACTCAGTTTCCTCAACTTGGTGGGGTGTTCCCTGCTATGTTTTCCAGCATCCTGTGACTGTCCTGTGCAGTCTATAGGGCAGGGCCCTGCCCCAGCGGGTGGGCTCAGGAGGGG
Canis lupus familiaris    CTTGTGACTCAGTTTCCTTGGCTTGGCCAAGGGTCCCCTGTTGCCTTTTC-AGCATCCTGGGACTGTCCCGTGCCGGCCATAGGGCAGGACCAGGCCCCAGCATGTCTGCCAGAGGGGGG

Homo sapiens              GTTCCCTGAAGCGAGTACACATTGCCAGAGCCCACCATCCTGGCAAGAGGTGGATCCT------------GGGGCCCTATGGAAGGAG----GGATGTGGTGGG----GGGGGCCACACT
Canis lupus familiaris    GTGTCCTGGCGTGAGGGGACATGGCCAGTGTCCGTCGTCCTGGCCAGAGGTTGATCGTGGCCCTCGGGAAGGGAGACTATGAAAGGAGCTACAGGTGTGGCAGGAGCAGGAGG------T

Homo sapiens              GAGGGAGGGGCCGGG----GACTTGTGACCTGTAGTGGCTGTTTGCAGTTTCTCTCTGTGTTAGATTCCCTTTTCTTCAGCAGTTTCAGTTCATGTTTCTCTTCAGTAAATTTTGTTCAG
Canis lupus familiaris    GTGGCAGGAGCGGGGCCGTGACCTATGGGTTCTAGTGGCCATTAGATGTTCCTCTCTCTGTTGTCATTCTGTCCTTCAAATGGTTGCAGTAAATGTTTCTCTTCAATAAATGTCATTGAG

Homo sapiens              TGTTCCAGTGGTGTCTTGCCTCTGCTGTGTG-----AAACTGGGGT--------GGGAGGTGGTCTGCTGGGGATGGGGCCACAGTG---------GCCAGGCTGACATCGAGGTGTGTG
Canis lupus familiaris    TGTTTCAGCTGCATCGTGTCTCTGTTGTGCGCCATCAAAGGAAAGTTCTCTTGGGGGAGGTGGT------GGGA---GGCAACAGTAGCTTGGTTCGCCCAGTTGGCATCGGGGTATGGT

Homo sapiens              TGGGGCAGGGTCTATGAGGGCCTGTGGGGTGCCTTGGGCTGCATGGAGCCTGGCAACAGGATGCTGGTGGTCATGAGCAGTGACTGGCTGGGTACCAAAGGCACTCCAGGTGTCCTCAGG
Canis lupus familiaris    CAGGGCAGG------GAGGTGCTGTGCGGTCCTCTGGGCTGGATGGAGCCAAGTGACTGGAACTT--TGGGCACAAGAGATATCTGGTTGGGT-CCACATGAAACTCAGGGAGCCCCAGG

Homo sapiens              AATAAGTGACTTGGACATTGAAGGCTGGGCAAGGGGCAGCAGGTGGGTCCCA--GGCCCCTGATCCCTGGCAGCACTGTAGGG--GTGTGGCTCAAGGCTTCCGGAAGGGTCAGAGAGGC
Canis lupus familiaris    TATAAGTAGGTTAGACATTCAAGGGAGGGGGCCAGATGGCAGGCAGGGCCTGATGGCCCAGGAT--CTGG-------GTGGGGCTGAGTGGTTCAGGGCATTCAGACAGGTCAGAGAGGT

Homo sapiens              CACGTGTGCAA--CCAAGGCCAGCAACCGAGGTCAGAGAAGAGCCCCAGGAGGGCCTGGACAGGCAGTCAGA-GGCCCTGTGAAGAGCTGTGTGCAGGACTCGGGGTGGGGTAGGGGCTG
Canis lupus familiaris    CCCATGTGGAATGCCAGGGAC--CAGCTGGG-------------CACAGAAAGGTCTGGGCATGAGGTCAGACTCCCCTGTCGGGGGCTGCATGCAGGACTC----CGAGGTGGGGGATA

Homo sapiens              --------GTGTGTGCCCATCCT-CCCAGATCAGCCCGCAGGTGGCCAGCCTTGGCCTAGCCACTGAGGGATGTTTGGGGACAGTGTGTG-TGTGATGGCCAAGGGGACAGAGTGTGGGC
Canis lupus familiaris    CCCCATCTGTTTGTGATCAGCCTACCCCTGCCAGCTGACAAGTGGCCAGTCACAGTCTGGCCACTGAGGGACATTTGGGTCCCTGGGGGGCTGTGATGTC--AAGGGACAGAGGGCAGGC

Homo sapiens              GAGAAAGGGA-TCCCAAGTGCTCATGTCTTGGGGAGGGGGAGAGGAA
Canis lupus familiaris    TGGCTGGGGCTTTCTCAGTGCTTCTGCCTTGCCAGGAAGGAG-GAAA


Homo sapiens  GTGCCGCACCCACACCCTTTTCTGGAACAGTACTTTCGGGCCGCTGCAAGCCGCAGGACCTTCCCTCTGTGGAAGAAACCTGCTTTGGGAAACGACAGGAAGCAGTTTGGGTACTAGGTG
Mus musculus  ........................................................................................................................

Homo sapiens  CGTAATGTTTGGTCGTGAAATCAGGAGAATAATGGGAGAATGGAATATTTTCAGTCATCTGGCTCTCAGTGAGCAGCGAAAAAAAACTAATGCCCTTGTGAAATGCAGGCAGTAGCTGTA
Mus musculus  ........................................................................................................................

Homo sapiens  TTTTTTGCCCTCCTCCCCTAGAACAGTCCAATAAAAAATAAACGAGGGCTATGACTCGTTTATAAGGGCGCTCACGTGGCCTATACGTTGCACTGGGTGATTTGCACCCGGCCCTTCACC
Mus musculus  ........................................................................................................................
```

```
Homo sapiens   CATGGGCTTGGGACGCCACAGCTCCCCTCCAATGAGGAAGAGCCACAGTGGCTGCTGGGCCAAATGGCACGTGGCGAGCCTGGCTGCCACCGTCGTCGGCACGGGGGTGGGTGGGGTGAG
Mus musculus   ........................................................................................................................

Homo sapiens   GAACCACCTTGCAGGAAGTAGGAGGGTTTATGTCACCAGTGCGCAACCGTGAGCGGGCCGTGTGCAGTTCCGCTCATGGGGTAGGGGGGCGGTCGTCACCAGACAACCTCTTCCCGCCAC
Mus musculus   ........................................................................................................................

Homo sapiens   AGCCTCGTGGCGCAGTCAGCACGGAGGCGGCAGCCGCCATAGACGTGTGGTGGTCGCGCTGCGCGGGAACTCCGGCTTGGAGGCACCTCCGGCAGGTACGGAGCTGGACCCCGGGCTCGG
Mus musculus   ........................................................................................................................

Homo sapiens   ACAGCAACCCAGATTACGGGGGAACCCCCCAGTAACTCTTGCTATTTTCCACGGGGTCTAGGGGCTGGTGGCCATAGGTTTCCCTGGAGGCGGGATCCCACTAGATCACACCCAGCACGC
Mus musculus   ........................................................................................................................

Homo sapiens   CAAGGAATCCCTGCCATTTCCAGGCAGAACTTCAGGGAGACCTGGCCGGGCGCGGTGGCTCACACCTGTAATCCCAGCACTTTGGGAGGCCGAGGCGGGTGGATCACCTGAGGTCAGGAG
Mus musculus   ........................................................................................................................

Homo sapiens   TTGGAGACCAGCCTGGCCAACATGGGGAAACCCCGTTTCTACTAAAAATACAAAAAGTAGCCGGGCGTGGTGGTGTACGCCTGTAATCCCAGCTACTCAGGAGGCTGAGGCCGGAGAATC
Mus musculus   ........................................................................................................................

Homo sapiens   ACTTGAACCCAGGGGGCGGAGGTTGCAGTGAGCCGAGATACGCTACTGCACTCCAGCCTGGGTGACAAAGCCAGACTCCGTTTCAGAGAAAAACAAACAAACTTCAGGGAGACCTATTCC
Mus musculus   ........................................................................................................................

Homo sapiens   ACCCTTGACCTGCCCGGAGACACTGCAGTCAGGATAGTAGCTCTTGGCCCTTGCCCTTGAGGGAAAGTGGCTTCGAACCTTCCCCTCCTAGATTCTGCCTGCTTCCACTGATGTCGGTGG
Mus musculus   ........................................................................................................................

Homo sapiens   TGTGGGTGTATGTAGATAACCGGGTGGGGATTGGGGGGAGCAGGGGAATGGAGGTAGGGGATTTGGGGATATCAGTTAGACTTGCCTCGGGGTAGGAGTTGGACTTGGTGAGGGGGCAGG
Mus musculus   ........................................................................................................................

Homo sapiens   GCTTGGGGTAGGAGTTGGACTGTGGGTGGGGGCAGGGCTTGAGGCACGAGTTAGACTTGGGTGGTGGAAGGGGTTAGACTTGGTGGGGGGCTAAGCTTGGGGTAGAGGTGCAACTTAGTT
Mus musculus   ........................................................................................................................

Homo sapiens   ATAGGCAGAGTTTGGCAGCAGGGGTGCAGGTTTTGAGGTGGGGCTTAGGAAGGGTTGCAACATGGCTGTAGGGGCGGAGCTTGGGAGGGAATGGAGTTTGCAGTGGGGGTAGGGCAGCAA
Mus musculus   ........................................................................................................................

Homo sapiens   CCTTACCTTAGGATCCCTTCTGCCCTACCAGCAGCCCCCCTGCCCCCACCCCCTACCTCCCTCCTCACCTCCCACCCCTCTCACCATTCGTCCCCCGACTCCTCCCCCCGCTCCGCTCCC
Mus musculus   ........................................................................................................................

Homo sapiens   TCTGTCAGTTGAGCAGTTTCTTCTATCCCTGCACCAGGGTCCTAGTGAACACCTATGCAGAGTCTGAATTGGCATGGCCTG----GGCAGGGACATGTGTGTGGGACAGAATCACAGCAA
Mus musculus   ...................................................CTTGAGCAGACTCAGAATTAGCACAGCCTGCGTGGGCAGGGACATTCAGGATAGACCATGTTA----AA

Homo sapiens   AATACCTCTGCTTTGCCTTGGACAGATCCAGTGAAGCAGGACCCATCACGCCTGAGTCAGGAACTGCCTAACTGGATAGAGTTGAAAAGCAGAGCGAGTCTGAAGTGGCTGT--------
Mus musculus   TATTCCTTTGCCTTGCCTTGAACAGATC-AGTGG-GCAGGAACCAGCAC---------------TGCTTGTCTGGATAGAGCCAGAAGACTG--CCAGGATCAAGCACCAGCACAGGAAA

Homo sapiens   GTTATTGCAGCATCCGCGCTGCCAGCAGGGCC--------AACCCAGCAAGGTGAGCAGCTGTGAGTTGGGCCCTGTTGGGAGGGGCTCTGGAACCCTGGCAAAGAGTGGGGAGAGGGGA
Mus musculus   GTCACCATAGC---CACACT-TCAGTGGGGCACTCTACAGAAGACAGCAGGGTGAGCATCTATT------------TTGGGAATAGC-CTCTAACTGTAGCAGA-AGACTGGTGAAGGGG

Homo sapiens   CTGCCTGCCAGGGTTCACAGACTCTGCAGGGTAGGGCCATCTCTAGGGCAAGAGTTGTTGGTTTCCTGCAGTCTGGCAGTGGGGACCTTATCTGGGTCCCTTTTCTGCCT--GATTTCAG
Mus musculus   TTTCTTGCCAAGATTCACAGACT--GCAGGGTAGGGCCATACCTG-----AGAGTTGTCTTTTACTTTC----------TGGGGTCTTTTTTT------TTTTTTGCCTCTGATTCCAG

Homo sapiens   CTTCCACACAATTG--AAACCATGAGCTCCTGTCCTTTCAGCACTCACTCCTCATGCCCATCTCGCCTTCCCTCTCCCACCCTCC----TTTTTGCTAGTACGCTTTATTGGTTGGAGGC
Mus musculus   ATTCCATACTATTGTAAACCCATATGCT--TAATTTATCAGCACTCGCTCCTCA-----GTCTC---TGCCCTATCCTACTCTCCACACCTGTTGCTAAGATGCTTCATTAGTTGGAGAA

Homo sapiens   CAATGACATCACCTGTTGCTTTTCAAGGACCTTGGCCTTACCACATGACACTCCCCTGCAATAGCAACCCATGGTCCATGGTATTCTCTGTATCCATGGCAACACTGCAGCTTGTCACAC
Mus musculus   CCATGGCATCACCTGTTGTCCATAAAAGGCCTTAACCT----ATGTGGTAA---CTTATGGTAGAAATCTA-GATTCACACTGTTCT-TGTGTCCATGTCAATACTTTAGCTTGTCGTAC

Homo sapiens   AATCTCCACACCCACCACTCTCATTGCATTGCGCAGAGCTC-AGCCATCTTTCTCATCCGTCAGGCATTCCTGAGAAAGAGGGCCACTCTACCTCTCTTGCTGCCAGCCTTCACTCCAGC
Mus musculus   GTTCTTTGCACTCAGCACTGTGATCACATC-TGCTGAGTTCTAGTCATCTTTCTTATCTGTTGAGCATTTCTTCAGGTGGAGGAGAGTTTAT-----GTGTTGCCAATCTTGAACCTAAC

Homo sapiens   AAGGAGGGTGCTGGGTGACCTGAGCCCACATAGCCCCGAGTGAGCAGTGAGGCTGTCTCCTGCCCTCTTCTGCCTGGAGGGCTCGTCAGTGTCCCCAGGTGTCAGGCCCTGCCTCCTGAC
Mus musculus   AAGGAATGGGTTAAAT--------CCAATATAGCACTGAGTGGGTGGCAGAGTGCTCTCCTGTTCTTTCCT--------------------CCACTGGCATTGGTCTTTGCCTCCTGAC

Homo sapiens   GTTGGCCTTTTCACTACAGGCACCCGAAGCACACACAGCAGAGTCCTCTGGACTTTGAGGAAGAACCCACAGCAGGAGGAAGTCAGCAGGGAGTGGTGAGATGGGGAGATCTTTGCCTGG
Mus musculus   ATTGGCATTTTCCCCACAGGTACTTGCAGAATGCACAGTAGTGTCC-CTGGACTTGCAAGGAGG--TTGCTGCAACCAAAGGTCAGCAGGGTTCAGTGAGGTTTGGGGAGCTTT--CTG-

Homo sapiens   GGAGAACCTCCACACTCTACCCTCCCCAAAATGACTTGGGGAACCCTGGGAAGAAAGGACTCCATCCCTGCTTGGGGGTGGGGGCAGAGGAGGGGTGCTTGGGGGCGTAGGTTGGGGTGG
Mus musculus   -------------------------------TGATT----GAAC-----------AAGACTGAATGTCTG------------GGAAAAGGAAAGGTGATGGGAATCAT------------

Homo sapiens   GCATTAACAATGCTCTCATCCCATTTGCTTTGCAGGCTGTGTGAAACCTGGGACCACTTCTGCCTTCCTACGTGGCAGTGGCTCAGAGTTATTTGAGTGCTGTCAAACTGAGCTGATTGC
Mus musculus   ---TAAGTAACATCTTAACTTCATTTGCATTTCAGGTTGTG-GAAACCAGGA--CATCACTGCCTTTCTATATGGCATTGGTTTAGAATC-TTTGAGT--------------------

Homo sapiens   TGCCCTAGTATTAGATCAGTCCATAGAAAGTGGGAGCAATGGCACTGACACTGCTAGAGGATTGGTGCAAGGGGATGGACATGGACCCCAGAAAGGCCCTGCTGATTGTAGGCATCCCCA
Mus musculus   ---CCTAGTGTTAGGTCTGCTGATAGAGGGAGGGAACAATGGCCGTGGCTCTATTAGATGACTGGTGTAAGGGGATGGATCTGGATCCCAAGAAGGCTGTGCTCATTGTGGGCATCCCCG

Homo sapiens   TGGAGTGTAGTGAGGTGGAAATTCAGGACACTGTGAAGGCAGGCTTACAGCCCCTGTGCGCATACAGGGTCCTAGGGAGAATGTTCAGGAGGGAAGACAATGCCAAGGCAGTCTTCATTG
Mus musculus   TGCAGTACACTGAGGCTGCGATAAATGATGCTCTGAAAGAAGGCTTACCTCCCTTGTGTGCATACAAGGTGATAGGCAGGATGTTCAGGAGGGAAGATGAAGCTAAGGCAGTCTTAATTG

Homo sapiens   AACTGGCTGACACTGTCAATTACACTACTCTGCCCAGTCACATACCAGGAAAGGGTGGCTCCTGGGAAGTGGTGGTAAAACCCCGTAACCCAGATGATGAGTTTCTCAGTAGACTGAACT
Mus musculus   AACTGCCTGAAGTTGTTGATTATACTATGATGCCTACTCATATACCAGCAGAGGGTGGGGCCTGGGAAGTGGTTGTCAAACCCCGTAGCCCAGATGATGAGTTTATGAATAAGTTGATCT

Homo sapiens   ACTTCCTGAAAGATGAGGGCCGAAGTATGACAGATGTGGCCAGAGCCCTGGGATGTTGCAGCCTCCCTGCCGAGAGCCTGGATGCAGAGGTCATGCCCCAAGTTAGATCCCCACCTTTAG
Mus musculus   ACTTTCTAAGAGATGAAGGACGGAGAATAGTAGATGTGGCCAAAGCCCTGGGGTTTAGCACTGTTCCTACAGGGAAAATAGAGTTAAAGAATTTAGACCAAGACAAACCAAAAGGCTTGA

Homo sapiens   AGCCTCCG---AAAGAAAGTATGTGGTACAGGAAACTGAAAGTGTTTTCGGGAACTGCTTCCCCTAGCCCAGGCGAAGAGACCTTTGAAGACTGGCTAGAGCAGGTCACTGAGATAATGC
Mus musculus   AGTCTCTGTGTAATAATAGCACATGTTACAAAAAACTGAAAGTATTTTCTGGCAGTCCCTTTCCAGGCCCAGGAGAAGAAAGCTTTGAAACCTGGTTAGAGGAGGTCACTGAGCTGATGC

Homo sapiens   CCATATGGCAAGTGTCTGAGGTGGAGAAGAGGCGGCGTTTGCTGGAGAGCTTACGTGGGCCTGCTCTGTCAATCATGCGGGTGCTCCAGGCCAACAATGACTCCATAACTGTGGAGCAGT
Mus musculus   AGTTATGGCAGGTGTCCGAGAGGGAAAAGAAGCAGTGCCTTCTAGAGAGCTTGCGTGGCTCTGCCCTGTCAATCATGCAGGCACTGTGGACTAGCAATGACTCCCTAACCGTGGAGCAGT

Homo sapiens   GCCTTGACGCCCTAAAGCAGATCTTTGGGGATAAAGAGGACTTTAGAGCCTCTCAGTTTAGGTTTCTGCAGACCTCTCCGAAGATTGGAGAGAAAGTCTCCACTTTCCTGCTGCGCTTAG
Mus musculus   GCTTGAAAGCCCTAAAGCACATCTTTGGAAATAAAGAGGACTCTAAGGTCTTACAGTTCAGGTTCCTTCAGTCTTCTCAGAAACCTGCAGAGAAGGTATCTGACTACTTGCTACGTTTAG

Homo sapiens   AGCCCCTGCTGCAGAAAGCCGTGCACAAGAGCCCCTTGTCAGTGCGCAGCACAGACATGATTCGTCTGAAACATCTCTTAGCTCGGGTCGCCATGACCCCCGCCCTCAGGGGCAAGCTGG
Mus musculus   AGCCCCTGCTGCAAAAAGCAGTGCAGCAGAGCCCACTGTCAGCACACAGTGCAGATTCAATTCGTCTCAAGCACGTCTTATCTCAGGTCTCCATGACCACTGGCCTTCGGGGAAAGCTCT

Homo sapiens   AGCTCTTGGATCAGCGAGGGTGTCCTCCCAATTTTCTGGAGTTAATGAAGCTCATTCGAGATGAAGAAGAGTGGGAGAACACTGAGGCAGTGATGAAGAATAAGGAGAAGCCATCAGGGA
Mus musculus   CCCTCTTAGATCAGCAAGGGTGCCCACCTACTTTTCTGGAATTAATGAAACTTACTCGAGACGAGGAAGAGTGGGAAAGCACTGTGGTAGTGGAGGAGC------AGGAGCAGGTACGAA

Homo sapiens   GAGGCCGGGGGGCCTCCGGTAGACAGGCAAGAGCAGAAGCCAGTGTCTCTGCACCTCAGGCCACCGTGCAGGCAAGGTCTTTTAGCGACAGCAGCCCCCAGACCATA-CAGGGAGGCTTA
Mus musculus   GGGACAGCAGCGCATGTG------AGGCGGCAAATGAAGTT------------------GTCACC---CAGGCAGAGGATTCTAGGGAGGTCAGCACCCAGACCACAGGAGAGGAGATGA

Homo sapiens   CCCCCATTAGTGAAACGCAGGCGGCTGTTAGGCAGTGAAAGCACTAGGGGAGAAGACCATGGCCAGGCCACGTATCCCAAGGCTGAAAACCAGACTCCAGGCAGGGAGGGGCCACAGGCT
Mus musculus   ----CATCAGTGAAACGGAGGCGACTGCTCTGGAGACACAGTGCTGGGGAAGAGGGACAGAGGAAGGAATCAGGTTTCTGGGCTGAAAGTGAA---CCCGATGAGCAGAAACCTTATGTT

Homo sapiens   GCAGGAGAGGAGTTGGGAAACGAGGCAGGGGCTGGGGCCATGAGCCATCCCAAGCCCTGGGAAACATAGGCTCAGAAGACCGAAGCACTCCTTCCCCTGGCAGGCAGCAGGGACATTTGA
Mus musculus   AGAGCACAGGAGTCGGGGAACGAGAGAGGGGCTTGGGCAGTGAGCCACCCTAACCCCAAGGAAATAGAGGCTCAGGACAGCCAGGAATTCCTTCCTGTGGCAGGCAACAGAGATACTTTG

Homo sapiens   ACAAGGGGAAGGGGCAGCTTACAC----TAACAGGGAACTTGACTGGGGCAGAGGGACAGGGAAGCCAGGTCGAGATCAAGCCCCCTTACTTTCCACCAGCTGGAAGGGACTTCACCAAC
Mus musculus   ACAAAATCATGGGGCAGCCCAGACAAAGGAACAGGGGACATGAGTGTTGCAGAAGGGCAA------CAGGGCCAGGGTAAGGCACCCAACTTCTTACTAGCTAGAAATGATCCCAACAAG

Homo sapiens   CAAGACCAAATAGCATCTGGTTCAGTGGGGGCCGGGCCCATAGCCCCTAACAGGTACAGGAGACACAAGCGGGGAACCACT---ACACTTAGCACATGGGGT-GCCTGGGCTTCAGATGG
Mus musculus   CAAGAACAAATTCCACACAGCTCAGTGACTACCAAATGGCAGGACCGTGGGGAGTGTCAGAGACTGAAATGGGGAGCTAGTATGATAACTAGACCACAGGGTAATCCAGACCGCAG-TTG

Homo sapiens   GGACCCCAGCGAAGCAGGGGCTGAAGAAGGTGTGGCTGGGGGCTCTGGCCTGGTCCTCTGGACACCCCCAAATATCTACCCTGTGGACTTTGAGCTGAGCATGCCCACTGGCACCCAGGC
Mus musculus   GGATACTAGT------GGGTCACAAGATGGTGAGGATGGGTGTTC---------------------------------------------TGAGCTAAGAATGCCCACTGGCACTGAAGC

Homo sapiens   CACGTGGGACCTGGAGGAGCCTGCCTGGGGCCTGCCTCTGCCAGAAGGAGCCAGGGCTGCTGAGGAAGAGCTGTGGGGCAGAAGTGAAG--CCCCGCAGGGGAGGCCACAGGGTCCAGCC
Mus musculus   AGCACAGGGTGTGGAGGAGCCTGCCACAGGGTTGTCTTGGGCAGAGGACACCAGTGCCTGGGAACAAGCACGGCTAGGCAGGGAGACAGTACCCAGGAGAGGAGGCCGCAGGATCCAGCC

Homo sapiens   TGTCTTTAGGATCATCTACACTGCATGAGGGGAGCCCCAGGAAGGCAGCATTCATAAGCCCCTGCACCAGTGAGGAAGAGAAAGAGGAGGAGAATTAAGAAGAGGATGATCGGGAGGAGG
Mus musculus   TGTCTTCAGGATCATCTACACAGCTCTAGGGGAGCCCCATGAGGGCAGCACCCTTGAGTCCTTTCGTGAGTGA...............................................

Homo sapiens   AAGAGGAGAATAAGGAGGAGCAGGAGGAAGAGGGGGAGGAGAAGGAGGAAGAGGGGGAGGAGGAAGAGGGGAAGGAGGAGGAAGAAGTGTAAGAAGAGAAGAGAGGATGTGTTGGGGGTG
Mus musculus   ........................................................................................................................
```

```
Homo sapiens  GGTAGTTGTTTTTGGTTTGCGTCATCATCAGGCCCGGGCTCTGCTCACTGCTGTTCC---------GTGTCAAGAACTCCACCTCTGCTTTGCCGGTGTAGATCTCGGCCAGTGGC----
Mus musculus  ...............................GCCTAGGCTACGCTAGCTGCTCTCTTACCCACCTGGTGTTGTGAACTCCTTGTCAGCTTTCCTAGTGTACATCTAGGCCAGCTGCCTGC

Homo sapiens  TGCTTGTTCTCTTGGGGATGTGTGTGTTTTTATCTGAGCAGCTCCTTTCCAGAGTACCCCAGCGCAGTTCCAGGAGACGGTGGGAAGGAGGCATGGCAGACG--CTCACCTTGTGACTGT
Mus musculus  TGCTTGTTCTCTTGCAGATGTGTGTG------------------CTAACCTGAGTTCCTGAGCCTGGTTCTGGAA--CCATGG-ATGTTGACAAGGCTGATGACTTCAACTTGGGACTAT

Homo sapiens  GACCTCGACTGCAGGAGGACCAACCAGACACGGGAAGGAGCAGGGCTGCCTGAGGCTCCCCAGGAAGCTTTGTGCTTTGGCGTTCCACCCCTGCTGTTACTCGTGACTCAGTTTCCTCAA
Mus musculus  G..........................................................................TGTGATATTCCTCTCCTGTTATTACTTATAATTTTACTTTCTAGG

Homo sapiens  CTTGGTGGGGTGTTCCCTGCTATGTTTT--CCAGCATCCTGTGACTGTCCTGTGCAGTCTATAGGGCAGGGCCCTGCCCCAGCGGGTGGGCTCAGGAGGGGGTTCCCTGAAGCGAGTACA
Mus musculus  GTTGGTGGGGTGTTGCATGCTATGGTTCCACCAATTCCCTGTGCCTGGACTGTGTATGCTATAAGGGAGAACTACACTGTAGTGAGTGATCTCTTGAGA--------------------

Homo sapiens  CATTGCCAGAGCCCACCATCCTGGCAAGAGGTGGATCCTGGGGCCCTATGGAAGGAGGGATGTGGTGGGGGGGGCCACACTGAGGGAGGGGCCGGGGACTTGTGACCTGTAGTGGCTGTT
Mus musculus  ---------------------TAGCAGAA------------GGCCCTGTGTGAGCAGAG---------------------------------------CCTATGGCTTCTAGTGACCACT

Homo sapiens  TGCAGTTTCTCTCTGTGTTAGATTCCCTTTTCTTCAGCAGTTTCAGTTCATGTTTCTCTTCAGTAAATTT------TGTTCAG--------TGTTCCAGTGGTGTCTTGCCTCTGCTGTG
Mus musculus  CAAAGTTTC------TGTTA--------TGTTTTTAATGGTTTTAATACA-ATTTTTGTTGAATAAATCTCATGAATGTTCTGGCTATGTTCTTTCTGGCGATGTTCTGCCTC-GCTGTG

Homo sapiens  TGAAACTGGGGTGGGAGGTGGTCTGCTGGGGATGGGGCCACAGTGGCCAGGCTGACATCGAGGTGTGTGTGGGGCAGGGTCTATGAGGGCCTGTGGG-GTGCCTTGGGCTGCATGGAGCC
Mus musculus  AG----TGAGTTGGGCAGCAGT-------------------AGTGGCTGAGTTGACACTGGCATATATGCATGTCAGAGACCATGAGGGGCTATAGTCGTCTCCTGGGTGGGAAGGAACC

Homo sapiens  TGGCAACAGGATGCTGGTGGTCATGAG---CAGTGACTGGCTGGGTACCAAAGGCACTCCAGGTGTCCTCAGGAATAAGTGACTTGGACATTGAAGGCTGGGCAAGGGGCAGCAGGTGGG
Mus musculus  AGGCTACTGGTAGCCAATGGATATGGGGTACAATAGCTATTTGTCTTCCTAGGGCACTT--GGCAAGAACAGGGATAAAGGAGCTATGTGCTGAAGGGAGGATGCAGTATATAAGGCAGG

Homo sapiens  TCCCAGGCCCCTGATCCCTG--GCAGCACTGTAGGGGTGTGGCTCAAGGCTTCCGGAAGGGTCAGAGAGGCCACGTGTGCAACCAAGGCCAGCAACCGAGGTCAGAGAAGAGCCCCAGGA
Mus musculus  CCCCAG------GATTCCTGGTGCAGCAGTGTA.......................................................................................

Homo sapiens  GGGCCTGGACAGGCAGTCAGAGGCCCTGTGAAGAGCTGTGTGCAGGACTCGGGGTGGGGTAGGGGCTGGTGTGTGCCCATCCTCCCAGATCAGCCCGCAGGTGGCCAGCCTTGGCCTAGC
Mus musculus  ........................................................................................................................

Homo sapiens  CACTGAGGGATGTTTGGGGACAGTGTGTGTGTGATGGCCAAGGGGACAGAGTGTGGGCGAGAAAGGGATCCCAAGTGCTCATGTCTTGGGGAGGGGGAGAGGAA
Mus musculus  .........................................................................................................
```

MOAP1 gene

```
>chromosome:GRCh37:14:93647941:93651873:-1
AAGTCAAGCCCCCAAGTTAAGACAGTAACTCCTAAGCACTTACAATGGTCCCGAAAATCT
TCCTGTTCCCACGGCAAGCGTTAAAGAGTCACTCAGCTCTGAATAATCGGGTGGCTTTGG
CATTTCAGAGAAAGCCAACATAAAATAAATCCCCTGGGTCACCCGGCTAGGAGAAAGGTG
AGCGCCGCCAGCCTGGAGCACCAGCTACTAGTGATTAAGAACGGCGTGACTGCGGCGGGT
CACGCGCGGCCCGGCCCCCGTCGCCGCCGCCGGAGACCCGCTCTGAGAACCTGACCCGGC
AGGGAGGCGTCCCCGCCACTTGGGCCTCCGGGCGGCCGGCCCCGCCCCCTGCCGCGCTGC
AGCGCCGGCACGCCGCGACCAAGGCTGCGCAGGCGGCGCCAGGCCCGGGCCTCGCCGCCT
TGCAGCGCCGCGAGCCCAGCGCCCGTCAGCGGTCGCCGGCGGAGCCTGCTTGCAAAGCTG
AGGTCCCGGATCTCACCTTCCTGTCCCGTGGTGGCCAGCCCGCCCGCCGTCCCGGATGCC
GCCGCAGTCGGAGCTTCCGGCTCGGGCCGGAAGCGCGCGCGCCCCGCCCCCGAGGCCCGC
CCCTCATGACGTCAGATCGCCCCCGCCCGCCGCGCAGCTTCCCCGAGCGAGACCAAAACA
GGTGGAATCCGGGCTGGAGCCGGAGCTCCGGCGGCGCGGGTGGCGGCACGTCCCTCCAGG
TGGGTGGCTCTCTGGGTCTGGCGACTCAGCGCTCCTCAGCCTTGCCCCCTGGTGCCCGCT
CGCGACTGTCATCGCCCCATCACTTCTCGTTGCAGACAGTACCACAGGCACCTGGAGTAC
CGGCATCGGTCGCTGTGGCCCCCGAGTGTCCGTCAGAGCCTAGGGGAGCCTGCCCTCCCG
CGCCTCGTCGGGGCCCGGCCAGGCACCTTGGCCGCCGGCGCACGGACGCGGGCACGAGCA
CTAGATCACGGTGAGTGGAAGCGGGCCCGACGCGGCCCTGCAGGCCCGACATCCGCCAGG
CACCAGGGCTGGGAGGGGTGAGGGGCGCGCGCCGCAGTGCGGCTGCCCTGGCGTCGCCGC
TGGGGGCAGGGCGCGCGGGGCTTGGGGCCTGCACGCTTGCCCTGGACGTTGGCACCTTTA
GCTAACCTGTCTGCTCTGTCTTGCAGGCTGCTGGACCTCGGCACGTTGACAAGATTTCTC
TGGGGTACCGCGGAGGATTACTTTGAATTTCGGTGGTCGCCTGTGGTCTGGCATATTTAG
AACTTAAGTCTATTATTTCGGGCACCATGACTTTGAGGCTTTTAGAAGACTGGTGCAGGG
GGATGGACATGAACCCTCGGAAAGCGCTATTGATTGCCGGCATCTCCCAGAGCTGCAGTG
TGGCAGAAATCGAGGAGGCTCTGCAGGCTGGTTTAGCTCCCTTGGGGGAGTACAGACTGC
TTGGAAGGATGTTCAGGAGGGATGAGAACAGGAAAGTAGCCTTAGTAGGGCTTACTGCGG
AGACTAGTCACGCCCTGGTCCCTAAGGAGATACCGGGAAAAGGGGGTATCTGGAGAGTGA
TCTTTAAGCCCCCTGACCCAGATAATACATTTTTAAGCAGATTAAATGAATTTTTAGCGG
GAGAGGGCATGACAGTGGGTGAGTTGAGCAGAGCTCTTGGACATGAAAATGGCTCCTTAG
ACCCAGAGCAGGGCATGATCCCGGAAATGTGGGCCCCTATGTTGGCACAGGCATTAGAGG
CTCTTCAGCCTGCCCTGCAATGCTTGAAGTATAAAAAGCTGAGAGTGTTCTCGGGCAGGG
AGTCTCCAGAACCAGGAGAAGAAGAATTTGGACGCTGGATGTTTCATACTACTCAGATGA
TAAAGGCGTGGCAGGTGCCAGATGTAGAGAAGAGAAGGCGATTGCTAGAGAGCCTTCGAG
GCCCAGCACTTGATGTTATTCGTGTCCTCAAGATAAACAATCCTTTAATTACTGTCGATG
AATGTCTGCAGGCTCTTGAGGAGGTATTTGGGGTTACAGATAATCCTAGGGAGTTGCAGG
TCAAATATCTAACCACTTACCAGAAGGATGAGGAAAAGTTGTCGGCTTATGTACTAAGGC
TGGAGCCTTTGTTACAGAAGCTGGTACAGAGAGGAGCAATTGAGAGAGATGCTGTGAATC
AGGCCCGCCTAGACCAAGTCATTGCTGGGGCAGTCCACAAAACAATTCGCAGAGAGCTTA
ATCTGCCAGAGGATGGCCCAGCCCCTGGTTTCTTGCAGTTATTGGTACTAATAAAGGATT
ATGAGGCAGCTGAGGAGGAGGAGGCCCTTCTCCAGGCAATATTGGAAGGTAATTTCACCT
GAGTCTCAGGGAACCACGAAGGGATATGGCAATGAGTAGAGCATGAAGGTAGAACAGTCT
ATATACTCTTGTGACACATACAATCCCTACCTTGTGCTGCCAAGTAACTCATTTTTGTGC
AATTCTCAGTATAAGCCCTTTGTCGTTTCTGTGCCTATTTAAAGTCTCCTAAAGGTGTAA
TTGACTAGGAAGGATGTAGTTCTACACTGCCATTTACCTATTTAAATTCATCCTTGTGAA
TATCTTTGTTGTTGTTGTTGAGACAGAGTCTCGCTCTGTCACCCAGGCTGGAGTGCAGTG
GCGTGATCTTGGCTCACTGCATCCTCCGCCTTCCAGGTTTAAGCTATTCTCCTGCCTCAG
TTGCCCGAGTAGCTGGGACTACAGGCATGTGCCACCACGCCCAGCTAAGTTTTGCATTAT
CAGTAGAGACGGGGTTTCACCATGTTGGCCAGGCTAGTTTCGAACTCCTGACCTCAAGTG
ATCCACCTGCTTCGGCCTCTGAAAGTGCTGGGATTACAGGCGTGAGACACTGCGCCCAGC
CTCATCCCTGTGAATTTCTTATTGTACACAAGTTCTTTCACTATCTGTGTGCAGTGCTCT
GAGGGGACAGACAAGGCTTGGGTGTATATGCCAACCAGATCTCATTGAAGTATCAGCTTG
TTTGGTACTAGGTGCAAGTGTAGCATGTCACATGTGACCATGTTGGATCATTGACAATTT
TTAGGTATGTACTGACCTACATTTATGATGAAGATCCTGAGCGGAGGTTAAGATATTAAG
TTATTTTCCATATGAATCAGAATTATATTGATTCTGTGCAATCAAAACAAAAGGCAGAAT
AGAATGCTGAGATTGGTTAAGTTTGCAATGACCATCTTGAACCACAGATTTCTGCTATGT
GTCATCAAAACATCTAGTTCTGAGTAACATTTTCACGATTGTTATAAAATTATAGGTGTG
AACTTCTAAAATAAAGGAATGCTAATAAAATGACTTTGTCTGTTTTGTTTATATCACGGT
TCCAAGAGAGATTTCATTTGAATAAAGAGTTCCCAAAGCATGAATCTGCAAACCACTGAA
CCTACACAGACTTAGTTCCTTGGACTCTTTCCTCTTGGTGCTCAGGGGTAGGAAATTGTT
TTCTAAGTGAAGGGACCTTCTACCGAAGCAGTAAAACAATGGGATGAAACAAGACAGTGC
ACAAATTAGTGCGAGTAGTGCAGATGTATCAGTGTGTCAAATTTGGAAGGAGAAGGAAAG
AAATGAACATTGCATGCTGCTGCTTGGAAGATTGGACTAAAAGTTTCTCCATGTACTTTC
CCATTTAATCCTCTTGAAAAAAGGTTGAGCAGTATAATCTTAGCACTTTGAGAGGCTGAG
GCAGGAAGATCGCTTGAACCCAGGAGTTCGAGACCAGCCTGGGCAACATAGTAAGTACAA
AACATTTTTAAAAACTAGCCAGGCATGGTGGTGTGCGCCTGCAGACCCTGCTACTCAGGA
GGCTGAAGCTGGAGGACTGCCTGAGGCCAGGAAGTCAAGGCTGCAGCAAGCCATGATCAT
ACCACTGCACTCCAACCGGGTGACAGAGCAAGA
```

```
Homo sapiens          AAGTCAAGCCCCCAAGTTAAGACAGTAACTCCTAAGCACTTACAATGGTCCCGAAAATCTTCCTGTTCCCACGGCAAGCGTTAAAGAGTCACTCAGCTCTGAATAATCGGGTGGCTTTGG
Dasypus novemcinctus  ........................................................................................................................

Homo sapiens          CATTTCAGAGAAAGCCAACATAAAATAAATCCCCTGGGTCACCCGGCTAGGAGAAAGGTGAGCGCCGCCAGCC---TGGAGCACCAGCTACTAGTGATTAAGAACGGCGTGACTGCGGCG
Dasypus novemcinctus  ....................................GGTTCCCCGGCTCGGAGAA-GGTGCGCGCGGCCCGTCGATTAGAGCACAAGCTTATAGTGATTAAGCAGAGCGTGACGGCTGCC

Homo sapiens          GGTCACGCG-CGGCCCGGCCCCCGTCGCCGCCGCCGGAGACCCGCTCTGAG--AACCTGACCCGGCAGGGAGGCGTCCCCGCCACTTGGGCC-TCCGGGCGGCCGGCCCCGCCCCCTGCC
Dasypus novemcinctus  GGTCACGCGGCGGCCCGGCCCCCGTCGCGGCTGGCGGAACCTCAGTCAGTTTCGGCCCG-CCCGGCCCGAAAGCACCAGTGACACCCGGGCCGCCCCTGCAGCCCGCCCCGCCCCCTGCC

Homo sapiens          GCGCTGCAGCGCCGGCACGCCGCGACCAAGGCTGCGCAGGCGGCGCCAGGCCCGGGCCTCGCCGCCTTGCAGCGCCGCGAGCCCAGC-GCCCGTCAGCGGTCGCCGGCGGAGCCTGCTTG
Dasypus novemcinctus  GCGC--------CGGCCGCCCGCAACCCGGGCTGCGCAGGCAGAGCCAGGCCGGGGCCTCGCCTCCCTGCAGCGCGGCGAGCCCCGCTGCGGGGCGGAGGCAACCCACGGAGCCAGCGC-

Homo sapiens          CAAAGCTG---AGGTCCCGGATCTCACCTTCCTGTCCCGTGGTGGCCAGCCCGCCCGCCGTCCCGGATGCCGCCGCAGTCGGAGCTTCCGGCTCGGGCCGGAAGCGCGCGCGCCCCGCCC
```

```
Dasypus novemcinctus  ---AGCTGGGCAGGTGCTGGATCTCACCTTCCTGTCCCG-AGTGGCCCTCCCGCCCGCCGTCCCGGATGCCGCCGCAGTCGAGGCTTCCGGCTCGGACCGGAAACGCGCGCTCCCTCTCC

Homo sapiens          CCGAGGCCCGCCCCTCATGACGTCAGATCGCCCCCGCCCGCCGCGCAGCTTCCCCGAGCGAGACCAAAACAGGTGGAATCCGGGCTGGAGCCGGAGCTCCGGCGGCGCGGGTGGCGGCAC
Dasypus novemcinctus  GACGGGCCTGCCCCCTCTGACGTCAGATCGT----------------------------------------GTTAGAGTCAGGG--AGAGGTTGGGC-----CGGCGCCGGTGGCTGTCA

Homo sapiens          GTCCCTCCAGGTGGGTGGCTCTCTGGGTCTGGCGACTCAGCGCTCCTCA--GCCTTGCCCCCTGGTGCCCGCTCGCGACTGTCATCGCCCCATCACTTCTCGTTGCAGACAGTACCACAG
Dasypus novemcinctus  GTTTCCGGAGGTGG---------------------------GCTCCCCAAGGCCTCGCCCTATG--------------CTCTGGTCGCCCCGTCCGTCTTCTCCGCCGAGAGCAGCACAG

Homo sapiens          GCACCTGGAGTACCGGCA-TCGGTCGCTGTGGCCCC-------------------------CGAGTGTCCGTCAGAGCCTAGGGGAGCCTGCCCTCCCGCGCCT---------------
Dasypus novemcinctus  CTACTAGGCGGCCCTGCACCCACTTGCCGTCGCCTCTCAGCAGAGCCCTTGATAGCCCTGTCGAGTTTGCATCTGAGTCGAGGACAGGCCGCGCTTTCGCTCCTCCGGCCTGGCAGTCCC

Homo sapiens          -----CGTCGGGGCCCGGCCAGG--CACCTTGGCCGCCGGCGCACGGACGCGGGCACGAGCACTAGATCACGGTGAGTGGA-AGCGGGCCCGAC--GCGGCCCTGCAGGCC-CGACATCC
Dasypus novemcinctus  GGAAGCGTCGGAGCCGGGCTTGGGTCTCCTCGGCCCGGGGCAGATGGACATCGACA-----ACTAGATGAGGGTGAGGGGGCAGGGGGTCCTGCGAGCTGCTCCGCGGGCCGCGACGTCC

Homo sapiens          GCCAGGCA----CCAGGGCTGGGAGGGGTGAGGGGCGCGCGCCGCAGTGCGGCTGCCCTGGCGTCGCCGCTGGGGGCAGGGCGCGCGGGGCTT---GGGGCCTGCACGCTTGCCCTGGAC
Dasypus novemcinctus  GCCGGGGGGCGCCCGAGGCTGGGAGAGGCGGTGTG-------------ATGGCTGCC--GCCATCGCCCGCGGGGTGGGGAGATGCGGGGCTTCGCGGGTCCTGTTCGAGCGGCCTAGCA

Homo sapiens          GTTGGCACCTTTAGCTAACCTGTCTGCTCTGT-CTTGCAGGCTGCTGGACCTCGGCACGTTGACAAGATTTCTCTGGGGTACCGCGGAGGATTACTTTGAATTTCGGTGGTCGCCTGTGG
Dasypus novemcinctus  --CGACACCTTTAACTAACCTGACTGCTTTGTCCTCACAGGCTGTCG----TTGAGATAGGTACTCGGCCTATCTCGGGTACCGCAGAGGATTGCTGTAAGTTGCGGTGGGCATCAATGC

Homo sapiens          TCTGGCATATTTAGAACTTAAGTCTATTATTTCGGGCACCATGACTTTGAGGCTTTTAGAAGACTGGTGCAGGGGGATGGACATGAACCCTCGGAAAGCGCTATTGATTGCCGGCATCTC
Dasypus novemcinctus  CGCGGATAAACTAGAATTTAAGGCTATTATCTTGGGCACCATGACGCTGAGGCTCTTAGAAGACTGGTGTAGGGGGATGGATATGAACCCTCGGAAAGCGCTGTTGATTGCTGGCATCTC

Homo sapiens          CCAGAGCTGCAGTGTGGCAGAAATCGAGGAGGCTCTGCAGGCTGGTTTAGCTCCCTTGGGGGAGTACAGACTGCTTGGAAGGATGTTCAGGAGGGATGAGAACAGGAAAGTAGCCTTAGT
Dasypus novemcinctus  CCCGACCTGTCATGAGACAGACATCCAGGAGGCTCTGCAGGCTGGTTTAGCTCCCTTGGGCGAGTACAGACTGCTTGGGAGGATGTTCAGGAGGGAAGAGAACAGGAATGTAGCCTTAAT

Homo sapiens          AGGGCTTACTGCGGAGACTAGTCACGCCCTGGTCCCTAAGGAGATACCGGGAAAAGGGGGTATCTGGAGAGTGATCTTTAAGCCCCCTGACCCAGATAATACATTTTTAAGCAGATTAAA
Dasypus novemcinctus  TGGGCTCACTGAGGAGACTAGTCATGCTTTGGTCCCTAGGGAGATAGATGGCAAAGGGGAGCCCTGGAGAGTGATCTTTAAGCCCCCTGACCCAGATGATGCATTTTTAAACAGGTTAAA

Homo sapiens          TGAATTTTTAGCGGGAGAGGGCATGACAGTGGGTGAGTTGAGCAGAGCTCTTGGACATGAAAATGGCTCCTTAGACCCAGAGCAGGGCATGATCCCGGAAATGTGGGCCCCTATGTTGGC
Dasypus novemcinctus  TGAATTCTTAAAGGGAGAGGGCATGACAGTGGGTGAGTTCACCAGAGCTCTTGGGTATGGAAAC-TCTCCTTTGACCCAGACCAGGACGT..............................

Homo sapiens          ACAGGCATTAGAGGCTCTTCAGCCTGCCCTGCAATGCTTGAAGTATAAAAAGCTGAGAGTGTTCTCGGGCAGGGAGTCTCCAGAACCAGGAGAAGAAGAATTTGGACGCTGGATGTTTCA
Dasypus novemcinctus  ........................................................................................................................

Homo sapiens          TACTACTCAGATGATAAAGGCGTGGCAGGTGCCAGATGTAGAGAAGAGAAGGCGATTGCTAGAGAGCCTTCGAGGCCCAGCACTTGATGTTATTCGTGTCCTCAAGATAAACAATCCTTT
Dasypus novemcinctus  ........................................................................................................................

Homo sapiens          AATTACTGTCGATGAATGTCTGCAGGCTCTTGAGGAGGTATTTGGGGTTACAGATAATCCTAGGGAGTTGCAGGTCAAATATCTAACCACTTACCAGAAGGATGAGGAAAAGTTGTCGGC
Dasypus novemcinctus  ........................................................................................................................

Homo sapiens          TTATGTACTAAGGCTGGAGCCTTTGTTACAGAAGCTGGTACAGAGAGGAGCAATTGAGAGAGATGCTGTGAATCAGGCCCGCCTAGACCAAGTCATTGCTGGGGCAGTCCACAAAACAAT
Dasypus novemcinctus  ........................................................................................................................

Homo sapiens          TCGCAGAGAGCTTAATCTGCCAGAGGATGGCCCAGCCCCTGGTTTCTTGCAGTTATTGGTACTAATAAAGGATTATGAGGCAGCTGAGGAGGAGGAGGCCCTTCTCCAGGCAATATTGGA
Dasypus novemcinctus  ........................................................................................................................

Homo sapiens          AGGTAATTTCACCTGAGTCTCAGGGAACCACGAAGGGATATGGCAATGAGTAGAGCATGAAGGTAGAACAGTCTATATACTCTTGTGACACATACAATCCCTACCTTGTGCTGCCAAGTA
Dasypus novemcinctus  ........................................................................................................................

Homo sapiens          ACTCATTTTTGTGCAATTCTCAGTATAAGCCCTTTGTCGTTTCTGTGCCTATTTAAAGTCTCCTAAAGGTGTAATTGACTAGGAAGGATGTAGTTCTACACTGCCATTTACCTATTTAAA
Dasypus novemcinctus  ........................................................................................................................

Homo sapiens          TTCATCCTTGTGAATATCTTTGTTGTTGTTGTTGAGACAGAGTCTCGCTCTGTCACCCAGGCTGGAGTGCAGTGGCGTGATCTTGGCTCACTGCATCCTCCGCCTTCCAGGTTTAAGCTA
Dasypus novemcinctus  ........................................................................................................................

Homo sapiens          TTCTCCTGCCTCAGTTGCCCGAGTAGCTGGGACTACAGGCATGTGCCACCACGCCCAGCTAAGTTTTGCATTATCAGTAGAGACGGGGTTTCACCATGTTGGCCAGGCTAGTTTCGAACT
Dasypus novemcinctus  ........................................................................................................................

Homo sapiens          CCTGACCTCAAGTGATCCACCTGCTTCGGCCTCTGAAAGTGCTGGGATTACAGGCGTGAGACACTGCGCCCAGCCTCATCCCTGTGAATTTCTTATTGTACACAAGTTCTTTCACTATCT
Dasypus novemcinctus  ........................................................................................................................

Homo sapiens          GTGTGCAGTGCTCTGAGGGGACAGACAAGGCTTGGGTGTATATGCCAACCAGATCTCATTGAAGTATCAGCTTGTTTGGTACTAGGTGCAAGTGTAGCATGTCACATGTGACCATGTTGG
Dasypus novemcinctus  ........................................................................................................................

Homo sapiens          ATCATTGACAATTTTTAGGTATGTACTGACCTACATTTATGATGAAGATCCTGAGCGGAGGTTAAGATATTAAGTTATTTTCCATATGAATCAGAATTATATTGATTCTGTGCAATCAAA
Dasypus novemcinctus  ........................................................................................................................

Homo sapiens          ACAAAAGGCAGAATAGAATGCTGAGATTGGTTAAGTTTGCAATGACCATCTTGAACCACAGATTTCTGCTATGTGTCATCAAAACATCTAGTTCTGAGTAACATTTTCACGATTGTTATA
Dasypus novemcinctus  ........................................................................................................................

Homo sapiens          AAATTATAGGTGTGAACTTCTAAAATAAAGGAATGCTAATAAAATGACTTTGTCTGTTTTGTTTATATCACGGTTCCAAGAGAGATTTCATTTGAATAAAGAGTTCCCAAAGCATGAATC
Dasypus novemcinctus  ...........................................................TATTTATTTCAGAGTTTTGAGAGAAATTTCATTTGAACAAGAAGTTCCAAAAGCTTAAATC

Homo sapiens          TGCAAACCACTGAACCTACACAGACTTAGTTCC-TTGGACTCTTTCCTCTTGGTGCTCAGGGGTAGGAAATTGTTTTCTAAGTGAAG-----GGA-----CCTTCTACCGAAGCAGTAAA
Dasypus novemcinctus  TGAAGACCACTGAACTTACAGAATCTTAGTTCCCTTGGACTCTG--CTTTTGGTGCCCATGGGTAAGAAGTACTTTTTAAAGTGAGATTGCTGGAGCATCCCTGCTATGGAAGCCATAAA

Homo sapiens          ACAATGGGATGAAACAAGACAGTGCACAAATTAGTGCGAGTAGTG-----CAGATGTATCAGTGTGTCAAATTTGGAAG-GAGAAGGAAAGAAATGAACATTGCATGCTGCTGCTTGGAA
Dasypus novemcinctus  ACAGCAGAAGGAAACAAGACAGTATGCAAATTAGTGCAAGTTTTGTGGGCCAAATGCATCACTGTATTGAATTTGGAGGAAAAAAGGAAAGAAAGGAACATTGCATGCATTTGCTAGGAG

Homo sapiens          GATTGGACTAAAAGTTTCTCCATGTACTTTCCCATTTAATCCTCTTGAAAAAAGGTTGAGCAGTATAATCTTAGCACTTTGAGAGGCTGAGGCAGGAAGATCGCTTGAACCCAGGAGTTC
Dasypus novemcinctus  GACTGGACTAAATGTTTTCCCACATACTTTCTCACTTAATCCTCTTACAAA.....................................................................

Homo sapiens          GAGACCAGCCTGGGCAACATAGTAAGTACAAAACATTTTTAAAAACTAGCCAGGCATGGTGGTGTGCGCCTGCAGACCCTGCTACTCAGGAGGCTGAAGCTGGAGGACTGCCTGAGGCCA
Dasypus novemcinctus  ........................................................................................................................

Homo sapiens          GGAAGTCAAGGCTGCAGCAAGCCATGATCATACCACTGCACTCCAACCGGGTGACAGAGCAAGA
Dasypus novemcinctus  ................................................................


Homo sapiens        AAGTCAA------------GCCCCCAAGTTAAGACAGTAACTCCTAAGCACTTACAATGGTCCCGAAAATCTTCCTGTTCCCACGGCAAGCGTTAAAGAGTCACTCAGCTCTGAATAATC
Choloepus hoffmanni AAGTCAAACCAATGTTGGAGCCCCCAAGTTAAGACAGTAACTCTTAAACACTTACAATGGTCCCGAAAATCTTCCTGTTCCCACGGCAAGCGTTAAAGAGTCACTCAGTTCTGAATAATC

Homo sapiens        GGGTGGCTTTGGCATTTCAGAGAAAGCCAACATAAAATAAATCCCCTGGGTCACCCGGCTAGGAGAAAGGTGAGCGCCGCCAGCCTGGAGCACCAGCTACTAGTGATTAAGAACGGCGTG
Choloepus hoffmanni GGGTGGCTTTGGCATTTCAGAGAAAGCAACCATAAAAGAAATTCCCCGGTTC-CCTGGCTTGGAGAAGGGTGAGCGCGGCCAGCCTAGAGCACAAGCTAATAGTGATTAAGCACAGCGTG

Homo sapiens        ACTGCGGCGGGTCACGCG-CGGCCCGGCCCCCGTCGCCGCCGCCGGAGACCCGCTCTG-AGAACCTGACCCGGCAGGGAGGCGTCCCCGCCACTTGGGCCT-CCGGGCGGCCGGCCCCGC
Choloepus hoffmanni ACTGCAGCCGGTCACGCGACCGTCCGGCCCCTGTCGCCGTTGCCGGAGCCTCAGTCTGTTTCGGCCGCCCGGGCCGGGAGGCGCCGGAGACGCCGGGGCCTCCCCTGCAGCCCGCCCCGC

Homo sapiens        CCCCTGCCGCGCTGCAGCGCCGGCACGCCGCGACCAAGGCTGCGCAGGCGGCGCCAGGCCCGGGCCTCGCCGCCTTGCAGCGCCGCGAGCCCAGCGCCCGTCAGCGGTCGCCGGCGGAGC
Choloepus hoffmanni CCCCTGCCGCGCTGCAGGGCCGGCCTGCCCCGACC-AGGCTGCGAAGGCAGCGCCAGGCCCAGGCTTCGCCTCCCTGCAGCGCCGCGAGTCCGGCGCCGGGCAGTAGCTAACCGCACAGC

Homo sapiens        CTGC-TTGCAAAGCTGAGGTCCCGGATCTCACCTTCCTGTCCCGTGGTGGCCAGCCCGCCCGCCGTCCCGGATGCCGCCGCAGTCGGAGCTTCCGGCTCGGGCCGGAAGCGCGCGCGCCC
Choloepus hoffmanni CGGCACAGCAGGAC--AGGTTCCCGATCTCACCTTCCTGTCCCG-AGTGGCCCTCCCGCCTGCCGTCCCGGATGCCGCCGCAGTCAGGGCTTCCGGCTCGGACCGGAAGCGCGCGAAACA

Homo sapiens        CGCCCC-CGAGGCCCGCCCCTCATGACGTCAGATCGCCCCCGCCCGCCGCGCAGCTTCCCCGAGCGAGACCAAAACAGGTGGAATCCGGGCTGGAGCCGGAGCTCCGGCGGCGCGGGTGG
Choloepus hoffmanni CGCTCCGAAAGGCCCGCCCCTTCTGACGTCAGGGCGCCTTGACCCGCAG-----------CGAGCGCGCTGGGGATAGAGCCTATTCCCGCCCGGGTAGAGGTTGTGGCGGCGCACGGAG

Homo sapiens        CGGCACGTCCCTCCAGGTGGGTGGCTCTCTGGGTCTGGCGACTCAGCGCTCCTCAGCCTTGCCCCCTGGTGCCCGCTCGCGACTGTCATCGCCCCATCACTTCTCGTTGCAGACAGTACC
Choloepus hoffmanni CTGACAGTTCCCTGAGGTGG-------------------------GCGCCGCTCGGCCTTGCTCCTTGCTCCTTGCTC-------CGATCGCTCCGTCCCTCTTCGCCGCCGAAAGCAGC

Homo sapiens        ACAGGCACCTGGAGTACCGGCATCGGT------------------------CGCTGTGGCCCCCGAGTGTCCGTCA-----------GAGCCTAGGGGAGCCTGCCCTCCCGCGCCTCGT
Choloepus hoffmanni GCAGATACTGGGCGGTCCTGCACCGGTTTGCCGGCGCTCGGCACGCCTCTCTGCAGAGCCCTCCCTATCCCTGTCGAGCCTGCACGTGAGTCGAGGAGAGGCCGCCCTCCCGCTCCTC--

Homo sapiens        CGGGGCCCGG-----CCAGGCACCTTGGCCGCCGGCGCACGGACGCGGGCACGAGCACTAGATCACGGTGAGTGGAAGCGGG-CCCGACGCGGCCCTGCAGGCCCGACATCCGCCAGGCA
Choloepus hoffmanni --AGGCCTGGCTGCCCTGGGCACCTCGGCCCGGAGCAGATGGCCGTCAACA-----ACCAGATGAGGGTGAGGGGCCCCGGGCCCCGACG-------------TCGACGTCTCCC-----

Homo sapiens        CCAGGGCTGGGAGGGGTGAGGGGCGCGCGCCGCAGTGCGGCTGCCCTGGCGTCGCCGCTGGGGGCAGGGCGCGCGGGGCTTGGGGCCTGCACGCTTGCCCTGGACGTTGGCACCTTTAGC
Choloepus hoffmanni ----------GCGGGGTGGGGGATGCGGGTCCT-----------------------------------GCGTGAGCGGCCTGGGG-------------------CAGTGACACCTGTAAC

Homo sapiens        TAACCTGTCTGCTCTGT-CTTGCAGGCTG-CTGGACCTCGGCACGTTGACAAGATTTCTCTGGGGTACCGCGGAGGATTACTTTGAATTTCGGTGGTCGCCTGTGGTCTGGCATATTTAG
```

```
Choloepus hoffmanni  TGACCCGACTGCTTTGTCCTCACAGGCTTCCTGAAACAAGGGATTTTGACTCGACCTATCTCGGGTACCGCGGAGGATTACCGTGAATTGCAGTGGGGATCTATGCCACGGACAAATTAG

Homo sapiens         AACTTAAGTCTATTATTTCGGGCACCATGACTTTGAGGCTTTTAGAAGACTGGTGCAGGGGGATGGACATGAACCCTCGGAAAGCGCTATTGATTGCCGGCATCTCCCAGAGCTGCAGTG
Choloepus hoffmanni  AG-TTAAAACTATTCTTTTGGAAACCATGACGCTAAGGCTCTTAGAGGACTGGTGTAGGGGGATGGATATGAACCCTTGGAAAGCGCCGTTGATTGCCAGCATCCCCACGACCTGTAACG

Homo sapiens         TGGCAGAAATCGAGGAGGCTCTGCAGGCTGGTTTAGCTCCCTTGGGGGAGTACAGACTGCTTGGAAGGATGTTCAGGAGGGATGAGAACAGGAAAGTAGCC-TTAGTAGGGCTTACTGCG
Choloepus hoffmanni  AGGCGGACATCGAGGAAGCTCTTCATGCTGGTTTAGCTCCTTTGGGCGAGTACAGACTGCT-----GGATGTTCAGGAGAGAAAGAGACAGGATTGTAGCCTTTAATAGGGCTTACTGGG

Homo sapiens         GAGACTAGTCACGCCCTGGTCCCTAAGGAGATACCGGGAAAAGGGGGTATCTGGAGAGTGATCTTTAAGCCCCCTGACCCAGATAATACATTTTTAAGCAGATTAAATGAATTT-TTAGC
Choloepus hoffmanni  GAGACTAGTCGTGCGT--GTCCCTAGAGAGATAGCTGGAAAAGAGGGGCCCTGAAGAGTGATCTTTAAGTCCCCTGACCCAGATAATGAATTTTTAAACAGATTAAATGAGTTTCTTGGA

Homo sapiens         GGGAGAGGGCATGACAGTGGGTGAGTTGAGCAGAGCTCTTGGACATGAAAATGGCTCCTTAGACCCAGAGCAGGGCATGATCCCGGAAATGTGGGCCCCTATGTTGGCACAGGCATTAGA
Choloepus hoffmanni  GGGGGAGGGCATGACAGTGGGTGAGTTCACCAGAGCCCTTGGGTATGGGAACTGCCCTTTTGACCTTGACTGGGACATAATCCCAGAAATGCAGGCCCTTTTGTTGGCAGAGGCATTTGA

Homo sapiens         ---GGCTCTTCAGCCTGCCCTGCAATGCTTGAAGTATAAAAAGCTGAGAGTGTTCTCGGGCAGGGAGTCTCCAGAACCAGGAGAAGAAGAATTTGGACGCTGGATGTTTCATACTACTCA
Choloepus hoffmanni  TGCAGCTCTTCAGCCTACCCTGCAATACCTGAAATACAAAAAGCTGACAGTATTCTCCGGCAGTGATTCTCCAGAACCAGAAGAAGAAGAATTTGAACCCTGGCTGTTTCATACTACTCG

Homo sapiens         GATGATAAAGGCGTGGCAGGTGCCAGATGTAGAGAAGAGAAGGCGATTGCTAGAGAGCCTTCGAGGCCCAGCACTTGATGTTATTCGTGTCCTCAAGATAAACAATCCTTTAATTACTGT
Choloepus hoffmanni  AATGATAAAGACATGGCAGGTATCAGCTGCAGAAAAGAGAAGACAATAGCTAGAAAGCCTTAGAGGTCCAGCATTTGATATCATTCGTGTCCTCAAGACAGACAACCCTTTCATTACGGC

Homo sapiens         CGATGAATGTCTGCAGGCTCTTGAGGAGGTATTTGGGGTTACAGATAATCCTAGGGAGTTGCAGGTCAAATATCTAACCACTTACCAGAAGGATGAGGAAAAGTTGTCGGCTTATGTACT
Choloepus hoffmanni  CCTTGAATGTCTGCAGGCTCTTGAGCAAGTATTTTGGCTTACTGATAACCCTAGGGAGTTGCAGGTGAAGTTTCTAACCACTTATCAGCAGCCTGAAGAAACATTATCTGCTTATGTGCT

Homo sapiens         AAGGCTGGAGCCTTTGTTACAGAAGCTGGTACAGAGAGGAGCAATTGAGAGAGATGCTGTGAATCAGGCCCGCCTAGACCAAGTCATTGCTGGGGCAGTCCA---CAAAACAATTCGCAG
Choloepus hoffmanni  AAGGCTGGAGCCTTTATTACAGAAACTGGTAGAGAGAGGAGCAGTCGAGAGAGGACCTGTGAACCAGGCCCGCCTAGACCAAATCACTGCTGGGGCAGTCCACAGCAGAACACATCACAG

Homo sapiens         AGAGCTTAATCTGCCAGAGGATGGCCCAGCCCCTGGTTTCTTGCAGTTATTGGTACTAATAAAGGATTATGAGGCAGCT---------GAGGAGGAGGAGGCCCTTCTCCAGGCAATATT
Choloepus hoffmanni  AAAACTTTCTCTGCCAGAAGACAGCCCAGCCCCAGGCTTATTAGAGTTACTGACACTGATAAAGGATGGAGAGGCAGCTTAGGAGGAGGAGGAGGCGGAGGCTCTTCTCCAGGTAGGCTT

Homo sapiens         GGAAGGTAATTTCACCTGAGTCTCAGGGAACCACGAAGGGATAT--GGCAATGAGTAGAGCATGAAGGTAGAACAGTCTATATACTCTTGTGACACATACAA------TCCCTACCTTGT
Choloepus hoffmanni  AGAGGGGCATTTCACCTGAGTTTTAGGAAACCAGGAGGGGATGTTGGGCAGTGAGCAGAGCATGAAGGTCAAACAGTCCTTATACTTCAGTGACAGACCTTAACCAGCTCCCTACCTTGT

Homo sapiens         GCTGCCAAGTAACTCATTTTTGTGCAATTCTCAGTAT-AAGCCCTTTGTCGTTTCTGTGCCTATTTAAAGTCTCCTAAAGGTGTAATTGACTAGGAAGGATGTAGTTCTACACTGCCATT
Choloepus hoffmanni  GCTGC---------CACTTTTGTACACTTCTCAGTATAAAACCCTTTATCATTTCTGTGTCTATTTGACGTCTCCTAAATGTGTCATTTACTAAGAAAGATGTAGTTCCTCACTGTTATT

Homo sapiens         TACCTATTTAAATTCATCCTTGTGAATATCTTTGTTGTTGTTGTTGAGACAGAGTCTCGCTCTGTCACCCAGGCTGGAGTGCAGTGGCGTGATCTTGGCTCACTGCATCCTCCGCCTTCC
Choloepus hoffmanni  TATATATTTCAGTTCACAC......................................................................................................

Homo sapiens         AGGTTTAAGCTATTCTCCTGCCTCAGTTGCCCGAGTAGCTGGGACTACAGGCATGTGCCACCACGCCCAGCTAAGTTTTGCATTATCAGTAGAGACGGGGTTTCACCATGTTGGCCAGGC
Choloepus hoffmanni  .........................................................................................................................

Homo sapiens         TAGTTTCGAACTCCTGACCTCAAGTGATCCACCTGCTTCGGCCTCTGAAAGTGCTGGGATTACAGGCGTGAGACACTGCGCCCAGCCTCATCCCTGTGAATTTCTT-------------
Choloepus hoffmanni  ......................................................................................CTGTGATTTTTTTTTTTTTTTGTCATAC

Homo sapiens         ---ATTGTACACAAGTTCT-------TTCACTATCTGTGTGCAGTGCTCTGAGGGGACAGACAAGGCTTGG----GTGTATATGCCAACCAGATCTCATTGAAGTA---TCAGCTTGTTT
Choloepus hoffmanni  AAAATTGTACAAAAGGTATAAAATTGTACACTACATATATGCAGTGCTCTGAGATGACAGCCAAGGTCAGGCCAGATGCTTGTGCTGTCTAGAAGATACTGGTGCACTTTTGGCTTGTGT

Homo sapiens         GGTACTAGGTGCAAGTGTAGCATGTC-ACATGTGACCATGTTGGATCATTGACAATTTTTAGGTATGTACTGACCTACATTTATGATGAAGATCCTGAGCGGAGGTTAAGATATTAAGTT
Choloepus hoffmanni  GGGAACAGTTGCAAGTGTAGCATGTCCAGATGTGA---TATTGTATCA--GATAGTTTTTACATGTGTATTGTCTTACATTTATGAGGAAGATCATGAGCAGTAGTTAAGACCTTAAG-T

Homo sapiens         ATTTTCCATATGAATCAGAATTATATTGATTCTGTGCAATCAAAACAAAAGGCAGAATAGAATGCTGAGATTGGTTAAGTTTGCAATGACCATCTTGAACCACAGATTTCTGCTATGTGT
Choloepus hoffmanni  ATTTTCTGGGTTTCGCAGAATTACATTGTTTCTGTTCAATCACAAAAAAAGGCAGAATTGGATCCTGAGACTGA--AGGTCTGCATCTACCATTTTTAACCCCAGATTTCTGATGTGTGA

Homo sapiens         CATCAAAACATCTA---GTTCTGAGTAACATTTTCACGATTGTTATAAAATTATAGGTGTGAACTTCTAAAATAAAGGAATGCTAATAAAATGA--CTTTGTCTGTTTTGTTTATATCAC
Choloepus hoffmanni  GATCAAAGCATCTGGTTGTTCAGAATATCATTGTAGTAGGTGTTATAAA--------TGTGAACTTATAAAATAAAGGAATGCTAATCAAATGATCTTTTACCTATTGTATTTATATTGG

Homo sapiens         GGTTCCAAGAGAGATTTCATTTGAATAAAGAGTTCCCAAAGCATGAATCTGCAAACCACTGAACCTACACAGACTTAGTT-CCTTGGACTCTTTCCTCTTGGTGCTCA-GGGGTAGGAAA
Choloepus hoffmanni  CGTTTCAAGAGGGATTTCATTTGAACAAGCAGTTCCAAAAGCGTCAGTCTAAAAACCATTGAACT----CAATTTTATTTCCCTTGGGCTCATTCCTCTTTGTGCCCATGGGGTA-----

Homo sapiens         TTGTTTTCTAAGTGAAGGGACCTTCTACCGAAGCAGTAAAACAATGGGATGAAAC-AAGACAGTGCACAAATTAGTGCGAGTAGTG-----CAGATGTATCAGTGTGTCAAATTTGGAAG
Choloepus hoffmanni  --------TTACTGGAGCAACCTGCTATTGTAGCTGTAAAACAGTGGAATGAAACAAAAACAGTGTGCAATTCTGTGCAAGTTGTACGGTCCAGATGCATCAGTGTATTGAATTTATAGG

Homo sapiens         GAG-AAGGAAAGAAATGAACATTGCATGCTGCTGCTTGGAAGATTGGACTAAAAGTTTCTCCATGTACTTTCCCATTTAATCCTCTTGAAAAAAGGTTGAGCAGTATAATCTTAGCACTT
Choloepus hoffmanni  AAGAAAGGAAAGAAATGAAGATTGCATACTTCTGTT---AGGACTGGAAT--GTGTTTTCCCACATATTTTTTCATTTAATCCTCTTAAAAA...........................

Homo sapiens         TGAGAGGCTGAGGCAGGAAGATCGCTTGAACCCAGGAGTTCGAGACCAGCCTGGGCAACATAGTAAGTACAAAACATTTTTAAAAACTAGCCAGGCATGGTGGTGTGCGCCTGCAGACCC
Choloepus hoffmanni  .........................................................................................................................

Homo sapiens         TGCTACTCAGGAGGCTGAAGCTGGAGGACTGCCTGAGGCCAGGAAGTCAAGGCTGCAGCAAGCCATGATCATACCACTGCACTCCAACCGGGTGACAGAGCAAGA
Choloepus hoffmanni  ..........................................................................................................


Homo sapiens       AAGTCAA------------GCCCCCAAGTTAAGACAGTAACTCCTAAGCACTTACAATGGTCCCGAAAATCTTCCTGTTCCCACGGCAAGCGTTAAAGAGTCACTCAGCTCTGAATAATC
Procavia capensis  AATTCAAATCAATGCCAGAGCCCCCAACTTTGAAAAGTCACTCTTAAACACTTACAACGGTCCTGAAAATCTTCCTGTTCCCACGGCAAGCGTTAAAGAGTCACTCAGTTCTGAATAATC

Homo sapiens       GGGTGGCTTTGGCATTTCAGAGAAAGCCAACATAAAATAAATCCCCTGGGTCACCCGGCTAGGAGAAAGGTGAGCGCCGCCAGCCTGGAGCACCAGCTACTAGTGATTAAGAACGGCGTG
Procavia capensis  AGGTGGCTTTGGCATTTCAGAGAAAGCCACCATAAAAGAAATCCCATGGGTAACCTGGCTTGAAGAAAGGTGAGCTCGGCCAGCCT-GAGAGCAAGCTAACTGTGATTAAGTCCAGCGTG

Homo sapiens       ACTGCGGCGGGTCACGCGCGGCCCGGCCCCCGTCGCCGCCGCCGGAGACCCGCTCTGAGAACCTGACCCGGCAGGGAGGCGTCCCCGCCACTTGGGCCT-CCGGGCGGCCGGCCCCGCCC
Procavia capensis  ACT---------------------------GTCACCGCAGCCGGAGTCTCAAGCTG-TCACCCGGCCTGGCCGATAGGCGCCGGTGCTACGAGGGCCTCCCCAGCAGCCCGCCCCGCCC

Homo sapiens       CCTGCCGCGCTGCAGCGCCGGCACGCCGCGACCAAGGCTGCGCAGGCGGCGCCAGGCCCGGGCCTCGCCGCCTTGCAGCGCCGCGAGCCCAGCGCCCGTCAGCGGTCGCCGGCGGAGCCT
Procavia capensis  CCTGCCGTGCTGTGGCGCCAGCCAGCCGCGACCA---------AGGCGGCGCCAGGCCCAGGCCTCGCCACCACACAGCGCCAGGAGCTCCGCGCCGAGCAGTGGCCACCGGCAGAGCA-

Homo sapiens       GCTTGCAAAGCTG---AGGTCCCGGATCTCACCTTCCTGTCCCGTGGTGGCCAGCCCGCCCGCCGTCCCGGATGCCGCCGCAGTCGGAGCTTCCGGCTCGGGCCGGAAGCGCGCGCGCCC
Procavia capensis  ---AGCAAAGCCAGGCAGGTCCGGGATCTCACCTTCCTGTCCCG-GCTGGCCAGCCCGCCCGCCTTACCGAGGGCCGCTGCGGTCGGGGCTTCCGGCTCGGGCCGGAAGTGCTGGCGCCC

Homo sapiens       CGCCCC-CGAGGCCCGCCCCTCAT-GACGTCAGATCGCCCCCGCCCGCCGCGCAGCTTCCCCGAGCGAGACCAAAACAGGTGGAATCCGGGCTGGAGCCGGAGCT-CCGGCGGCGCGGGT
Procavia capensis  CGCCCCGAAAGGCCCGCCTCCTGCGGACGTCAGAGGGCCTCCTACCGCGGCGCCGCTGGTCTGAGAGC-ATCCCCCCAGGT----CTCATGGTGGGGCGGGGGCTGGTTGTGTCGCCGGT

Homo sapiens       GGCGGCACGTCCCTCCAGGTGGGTGGCTCTCTGGGTCTGGCGACTCAGCGCTCCTCAGCCTTGCCCCCTGGTGCCCGCTCGCGACTGTCATCGCCCCATCACTTCTCGTTGCAGACAGTA
Procavia capensis  GACGGTCTGTCCTTTCAGACGGGCGTCCTTCTGGGGCTGGCG-------------GGCCT-------------CGCTGGCGTCTCTGATCGCCTGAT--CCTCCCGCAGCGGAGAGCA

Homo sapiens       CCACAGGCACCTGG--AGTACCGGCATCGGTCGCTGTGGCCCCCGAGTG----------------------------------TCCGTCAGAGCCTAGGGGAGCCTGCCCTCCCGCGCC
Procavia capensis  CCGCAGA-GCCTAGATAGCATC-GCATCACTCGC----GTCCCTGGGCGCGCCTGTCTTCAGAGTCTAGCTACCCTGTCGCTTCTCCATCCGAACCGAGTAGAGGTCGTCTTCCCGCTCC

Homo sapiens       T------------CGTCGGGGCCCGGCC--AGGCACCTTGGCCGCCGGCGCACGGACGCGGGCA-CGAGCACTAGATCACGGTGAGTGGAA-GCGGGCCC--GACGCGGCCCTGCAGGCC
Procavia capensis  TCGGGCCTGGACACGTCGAGAGCCAGCCCTGGGCACCCCGAGCCCCGGCAGGTGGACGCGGGCATCGACTACTAGGTAACGGTGAGGGGGATGCGGGCCCTGAGAGCGGCTTCCCGGGCC

Homo sapiens       CGA-CATCCGCCAGGCAC---CAGGGCTGGGAG----GGGTGAGGGGCGCGCGCCGCAGTGCGGC--TGCCCTGGCGTCGCCGCTGG------------GGGCAGGGCGCGCGGGGCTT-
Procavia capensis  CCAGTGTCCGCTGGGGGCGCTCGAGGCCGTGAGACGCAGTCAAAAGGCGGCTGTCGCAGAGCGCCAAAGCCCCGGCACCGCTGCCGGCATTTCAGGGGTGTGGATGATCCGCAGGGCTTT

Homo sapiens       --GGGGCCTGCACGCTTGCCCTGGACGTTGGCACCTTTAGCTAACCTGTCTGCTCTGT-CTTGCAGGCTGCTGGACCTCGGCACGTTGACAAGATTTCTCTGGGGTACCGCGGAGGATTA
Procavia capensis  TCAGGTCCTGCTGTGAGGCGTGGGACGTTGACTCCGTTAACTAACTCAACGACTTTGTCCTTACAGGCAGCGGAAACTCAGGACGTTGAAATGACACAGCTGGAGGACCGCGGAGGATTA

Homo sapiens       CTTTGAATTTCGGTGGTCGCCTG----------------TGGTCTGGCATATTTAGAACTTAAGTCTATTATTTCGGGCACCATGACTTTGAGGCTTTTAGAAGACTGGTGCAGGGGGAT
Procavia capensis  CTGTGAATCGCAGGGGGCATTTACCACTCCAGCCCACCCCACCCTTGTCAAATCAGAATTTCAGTCGCCGCATCTTGGCACCATGACGCTGAGACTCTTAGAAGACTGGTGCAGGGGGAT

Homo sapiens       GGACATGAACCCTCGGAAAGCGCTATTGATTGCCGGCATCTCCCAGAGCTGCAGTGTGGCAGAAATCGAGGAGGCTCTGCAGGCTGGTTTAGCTCCCTTGGGGGAGTACAGACTGCTTGG
Procavia capensis  GGACATGAACCCTCGGAAAGCTCTCCTGATTGCCGGCATCCCCCTGGCTTGTAACGAGGCAGAAATCCAGGAGGCTCTGCAGGCGGGGTTGGCGTCCTTGGGCCAGCACCGACTGCTTGG

Homo sapiens       AAGGATGTTCAGGAGGGATGAGAACAGGAAAGTAGCCTTAGTAGGGCTTACTGCGGAGACTAGTCACGCCCTGGTCCCTAAGGAGATACCGGGAAAAGGGGGTATCTGGAGAGTGATCTT
Procavia capensis  GAGGATGTTCCGGAGGGAAGAGAACAGAGTCGTAGCCCTGGTAGGACTTTCTGAGGAGACTAGCCTTGCTGTGGTCCCTAAGGAGATACCAGGGAAGGGAGGTGCCTGGAGAGTGATCTT

Homo sapiens       TAAGCCCCCTGACCCAGATAATACATTTTTAAGCAGATTAAATGAATTTTTAGCGGGAGAGGGCATGACAGTGGGTGAGTTGAGCAGAGCTCTTGGACATGAAAATGGCTCCTTAGACCC
Procavia capensis  TAAACCCCCAGACCCAGATAATGAATTTTTAAACAGATTAAATGAATTCTTAGAGGGAGAGGGTTTGACAGTGGCTGAGTTTACTAGCGCTCTTGGGTATGGAAACGACCCTTTTGACCT

Homo sapiens       AGAGCAGGGCATGATCCCGGAAATGTGGGCCCCTATGTTGGCACAGGCATTA---GAGGCTCTTCAGCCTGCCCTGCAATGCTTGAAGTATAAAAAGCTGAGAGTGTTCTCGGGCAGGGA
Procavia capensis  AGACCAGAACTTAGTCCCACAAGTCGGGGCCCCTATATTGGCAGAGGCGTTAGATGAGGCTCTTCAGCCTGCCCTGCAGTACGTGAAATACAGAAAGCTGAAAGTCTTCTCTGGTAGCGA

Homo sapiens       GTCTCCAGAACCAGGAGAAGAAGAATTTGGACGCTGGATGTTTCATACTACTCAGATGATAAAGGCGTGGCAGGTGCCAGATGTAGAGAAGAGAAGGCGATTGCTAGAGAGCCTTCGAGG
Procavia capensis  TCCTCCAGAACCTGGAGAAGAAGAATTTGATTCCTGGCTGTTCCACACCACTCACATGATGAAGACATGGCAGGTGTCAGATGCAGAGAAAAGACGGCGACTGCTAGAGTGCCTTAGAGG
```

```
Homo sapiens        CCCAGCACTTGATGTTATTCGTGTCCTCAAGATAAACAATCCTTTAATTACTGTCGATGAATGTCTGCAGGCTCTTGAGGAGGTATTTGGGGTTACAGATAATCCTAGGGAGTTGCAGGT
Procavia capensis   CCCAGCACTGGATGTCCTTCGTGTTCTCAAGATAAACAGTCCCTTCCTTACGGTCCCTGAATGCCTACAGGCACTTGAGCAGGTGTTCGGGGTTATCGATAATCCTAGGGAGCTGCAGGT

Homo sapiens        CAAATATCTAACCACTTACCAGAAGGATGAGGAAAAGTTGTCGGCTTATGTACTAAGGCTGGAGCCTTTGTTACAGAAGCTGGTACAGAGAGGAGCAATTGAGAGAGATGCTGTGAATCA
Procavia capensis   CAAATTTCTAACCACTTATCAGAAGGCTGAAGAAAAATTATCTGCTTATATCCTAAGGCTGGAGCCTTTATTACAGAAACTAGTAGAGAGGGGAGCAATCGAGAAAGAAGTTGTGAATCA

Homo sapiens        GGCCCGCCTAGACCAAGTCATTGCTGGGGCAGTCCACAAAACAATTCGCAGAGAGCTTAATCTGCCAGAGGATGGCCCAGCCCCTGGTTTCTTGCAGTTATTGGTACTAATAAAGGATTA
Procavia capensis   GGCCCGCCTAGACCAAATCCTTGCTGGGGCAGAGGACAGAACACTTCGCAGGAAGCTTGATCTTCCAGAGGATGGCCCGGCACCTGGCTTGCTGCAGTTACTCATGCTGATAAAGGAAGA

Homo sapiens        TGAGGCAGCT---GAGGAGGAGGAGGCCCTTCTCCAGGCAATATTGGAAGGTAATTTCACCTGAGTCTCAGGGAACCACGAAGGG--ATATGGCAA----TGAGTAGAGCATGAAGGTAG
Procavia capensis   AGAGGCAGCTGAAGAGGAAGAAGAGGCCCTTTTCCAGGCAGGATTAGAGGGGAATTTCACCTGAAGCTTAAGAAACCAGGAGGGGCTATCTGACAATGAGTGAGTAGAGGGTGAAGGTAT

Homo sapiens        AACAGTCTATATACTCTTGTGACA-CATACAA-----TCCCTACCTTGTGCTGCCAAGTAACTCATTTTTGTGCAATTCTCAGTATAAGCCCTTTG---------TCGTTTCTGTGCCTA
Procavia capensis   GGCAGGCCATACACGCCAGTGATAGTCCATAACCACCTCCCTGCTTTATGCTGCCCAGTA----------GCACAGTGTCCAGTAGAAACCCTTTGCATGTGCCCCTTTGTCTGTGCCTC

Homo sapiens        TTTAAAGTCTCCTAAAGGTGTAATTGACTAGGAAGGATGTAGTTCTACACTGCCATTTACCTATTTAAATTCATCCTTGTGAATATCTTTGTTGTTGTTGTTGAGACAGAGTCTCGCTCT
Procavia capensis   TTTGACATCTCCTGAATGTGTCATTGACCAGGACAGATGGAATTCTGCACTGGTGTTTATATATTTAAAGTCACATCTGTAAA.....................................

Homo sapiens        GTCACCCAGGCTGGAGTGCAGTGGCGTGATCTTGGCTCACTGCATCCTCCGCCTTCCAGGTTTAAGCTATTCTCCTGCCTCAGTTGCCCGAGTAGCTGGGACTACAGGCATGTGCCACCA
Procavia capensis   ........................................................................................................................

Homo sapiens        CGCCCAGCTAAGTTTTGCATTATCAGTAGAGACGGGGTTTCACCATGTTGGCCAGGCTAGTTTCGAACTCCTGACCTCAAGTGATCCACCTGCTTCGGCCTCTGAAAGTGCTGGGATTAC
Procavia capensis   ........................................................................................................................

Homo sapiens        AGGCGTGAGACACTGCGCCCAGCCTCATCCCTGTGAATTTCTTATTGTAC-ACAAGTTCTTTCACTATCTGTGTGCAGTGCTCTGAGGGGACAGACAAGGCTTGGGTGTATATGCCAACC
Procavia capensis   ...........................................ATTGTACCAAAGGGTGTGTCACTGTATACATGCCATGGGCCCTGGTCACAGGCA-----------CCCAGGCCGACC

Homo sapiens        AGAT---CTCATTGAAGTATCAGCTTGTTTGGTACTAGGTGCAAGTGTAGCATGTCACATGTGACCATGTTGGATCATTGACAATTTTTAGGTATGTACTGACCTACATTTATGATGAAG
Procavia capensis   AGATGATCTCATTGAGTTGTCATTTTGTGTGATGACAGGAGCAAATGTAGCATGTCAGATATGATAATG-----TCATAGATACTTTTTAGATGTGTGTTGTCTTATATTTATAATGATA

Homo sapiens        ATCCTGAGCGGAGGTTAAGATATTAAGTTATTTTCCATATGAATCAGAATTATATTGATTCTGTGCAATCAAAACAAAAGGCAGAATAGAATGCTGAGATTGGTTAAGTTTGCAATGACC
Procavia capensis   A---TGAGCAGTGGT-----TATTAAA-TATTTTCTGTGTGACTCAGAATGTTACTGATTCTGTGCCAC-----CAAAAGGGGGAATTGCATGCCAGGAGTAA-AAAGTTTGCAAATGCC

Homo sapiens        ATCTTGA-ACCACAGATTTCTGCTATGTGTCATCAAAACATCTA---GTTCTGAGTAACATTTTCACGATTGTTATAAAATTATAGGTGTGAACTTCTAAAATAAAGGAATGCTAATAAA
Procavia capensis   GTCTCTATCCCCCAGGTGTCTGATGTGTG---TTAAACCGTGTAGATGTTCTGAATATCATTTTAATA--------GAAATCATAAATCTGAGTTTATAAAATAAAGGAATGCTAATGAA

Homo sapiens        ATGACTTTGTCTGTTTTGTTTATATCACGGTTCCAAGAGAGATTTCATTTGAATAAAGAGTTCCCAAAGCATGAATCTGCAAACCACTGAACCTACACAGACTTAGTTCCT-TGGACTCT
Procavia capensis   ATGACTTAGGC-GTTTTGCTTACATCAGAGTTTCAAGAGAGACTTCATTTGAGCAAATGGTTCTAAAAGCTTAAATGTGGAAACCACTTAGGTT--ACAATCTTAGTTCCTCTGGAC---

Homo sapiens        TTCCTCTTGGTGCTCAGGGG-TAGGAAATTGTTTTCTAAGTGA---------AGGGACCTTCTACCGAAGCAGTAAAACAATGGGATGAAACAAGACAGTGCACAAATTAGTGCGAGTAG
Procavia capensis   --CCTCCTGCTGTCTAGGGGCTAAGAAGTGCTTTTCCTAGTGAGATTCCCAGAGTGACCTGCCATTGAAACA................................................

Homo sapiens        TGCAGATGTATCAGTGTGTCAAATTTGGAAGGAGAAGGAAAGAAATGAACATTGCATGCTGCTGCTTGGAAGATTGGACTAAAAGTTTCTCCATGTACTTTCCCATTTAATCCTCTTGAA
Procavia capensis   ..............................................GAGGAGTGCATGCCTCTGCTAGAGAAA-----CTAAGTATTTTTCTACATACTTTCTTATTTAATCCTCTTAAG

Homo sapiens        AAAAGGTTGAGCAGTATAATCTTAGCACTTTGAGAGGCTGAGGCAGGAAGATCGCTTGAACCCAGGAGTTCGAGACCAGCCTGGGCAACATAGTAAGTACAAAACATTTTTAAAAACTAG
Procavia capensis   AAATGTTTG...............................................................................................................

Homo sapiens        CCAGGCATGGTGGTGTGCGCCTGCAGACCCTGCTACTCAGGAGGCTGAAGCTGGAGGACTGCCTGAGGCCAGGAAGTCAAGGCTGCAGCAAGCCATGATCATACCACTGCACTCCAACCG
Procavia capensis   ........................................................................................................................

Homo sapiens        GGTGACAGAGCAAGA
Procavia capensis   ...............

Homo sapiens         AAGTCAA------------GCCCCCAAGTTAAGACAGTAACTCCTAAGCACTTACAATGGTCCCGAAAATCTTCCTGTTCCCACGGCAAGCGTTAAAGAGTCACTCAGCTCTGAATAATC
Loxodonta africana   GAGTCAAACCAAAGCCGGAGCCCCCAACTAAGAACAGTCACTCTGAAACACTTACAGTGGTCCTGAAAATCTTCCTGTCCCCACGGCAAGCGTTAAAGAGTCACTCAGCTCTGAGTAGGC

Homo sapiens         GGGTGGCTTTGGCATTTCAGAGAAAGCCAACATAAAATAAATCCCCTGGGTCACCCGGCTAGGAGAAAGGTGAGCGC-CGCCAGCCTGGAGCACCAGCTACTAGTGATTAAGAACGGCGT
Loxodonta africana   AGGCGGCTTGGGCATTTCAGGGAGAGCCACCATAAGAGAACTCCCATGGGTCACCCGCCTCACAGAAGGGTGAGCTCGGGCCAGCCTGGAGAACAAGCTAACAGTGACTAAGTCCAGCGT

Homo sapiens         GACTGCGGCGGGTCACGCGCGGCCCGGCC---CCCGTCGCCGCCGCCGGAGACCCGCTCTGAGAACCTGACCCGGCAGGGAGGCGTCCCCGCCACTTGGGCCTCCGGGCGGCCGGCCCCG
Loxodonta africana   GACTGGGGCCGCTCGCGCGGGGACGGGGTCAGCCTGTC-------------------------ACCCGGCCCGGCA--GCAGCGCCGGCGCCACCATGGACTCCCAGCAGCCGG-CCCG

Homo sapiens         CCCCCTGCCGCGCTGCAGCGCCGGCACGCCGCGACCAAGGCTGCGCAGGCGGCGCCAGGCCCGGGCCTCGCCGCCTTGCAGCGCCGCGAGCCCAGCGCCCGTCAGCGGTCGCCGGCGGAG
Loxodonta africana   ACCCCTG-CGTGCAGCAGCGTCAGTCGGCCGCGACAAGGGC...............................................................................

Homo sapiens         CCTGCTTGCAAAGCTGAGGTCCCGGATCTCACCTTCCTGTCCCGTGGTGGCCAGCCCGCCCGCCGTCCCGGATGCCGCCGCAGTCGGAGCTTCCGGCTCGGGCCGGAAGCGCGCGCGCCC
Loxodonta africana   ........................................................................................................................

Homo sapiens         CGCCCCCGAGGCCCGCCCCTCATGACGTCAGATCGCCCCCGCCCGCCGCGCAGCTTCCCCGAGCGAGACCAAAACAGGTGGAATCCGGGCTGGAGCCGGAGCTCCGGCGGCGCGGGTGGC
Loxodonta africana   ........................................................................................................................

Homo sapiens         GGCACGTCCCTCCAGGTGGGTGGCTCTCTGGGTCTGGCGACTCAGCGCTCCTCAGCCTTGCCCCCTGGTGCCCGCTCGCGACTGTCATCGCCCCATCACTTCTCGTTGCAGACAGTACCA
Loxodonta africana   ........................................................................................................................

Homo sapiens         CAGGCACCTGGAGTACCGGCATCGGTCGCTGTGGCCCCCGAGTGTCCGTCAGAGCCTAGGGGAGCCTGCCCTCCCGCGCCTCGTCGGGGCCCGGCCAGGCACCTTGGCCGCCGGCGCACG
Loxodonta africana   .......................................................................................................................G

Homo sapiens         GACGCGGGCACGAGCACTAGATCACGGTGAGTGG-AAGCGGGCCCGACG--CGGCCCTGCAGGCCC-GACATCCGCCAGGCAC---CAGGGCTGGGAGGGG-----------------T
Loxodonta africana   GACGCAGGCGTCGGCACTAGATAAAGGTGGGCGGGAAGCGGGCCCTGCGAGCGGCTCCGCGGGCCCCGGGGTCCGCTGGGGGCGCCCGAGGCCGGGAGGCGAGGTCACATGGCTGCCGCT

Homo sapiens         GAGGGGCGCGCGCCGCAGTGCGGCTGCCCT-GGCGTCGCCGCTGGGGGCAGGGCGCGCGGGGCTT---GGGGCCTGCACGCTTGCCCTGGACGTTGGCACCTTTAGCTAACCTGTCTGCT
Loxodonta africana   GCGGAGCGCGGAGGCCTGGGTGGCCGCTCTCGGCGTCGCCGGGGGTGG-AGGATCCGCGGGGCCTTTCGCGTCCTGCTGTGCGGCCTGGGACCTTGACCCAGTTAGCTAACTCAACTGCT

Homo sapiens         CTGTCTT-GCAGGCTGCTGGACCTCGGCACGTTGACAAGATTTCTCTGGGGTACCGCGGAGGATTACTTTGAATTTCGGTGGTCGCCTGTGGTCTG---GCATATTTAGAACTTAAGTCT
Loxodonta africana   TTGTCCTCACAGGCTGCTGGAACTCGGGACGTTGACGTGACATAGCTGGGGGACCGCGGAGGACTACTGCGAATCGCGGTGGGCATCTGCGCCCCGCCGGTCCAATTAGAATTTAAACCT

Homo sapiens         AT-TATTTCGGGCACCATGACTTTGAGGCTTTTAGAAGACTGGTGCAGGGGGATGGACATGAACCCTCGGAAAGCGCTATTGATTGCCGGCATCTCCCAGAGCTGCAGTGTGGCAGAAAT
Loxodonta africana   CTGTATCTTGGGCACCATGACACTGAGGCTCTTGGAAGACTGGTGCAGGGGAATGGACATGAACCCTCGGAAAGCTCTGTTGATTGCCGGCATCCCCCTGACCTGTAACGAGGCGGAAAT

Homo sapiens         CGAGGAGGCTCTGCAGGCTGGTTTAGCTCCCTTGGGGGAGTACAGACTGCTTGGAAGGATGTTCAGGAGGGATGAGAACAGGAAAGTAGCCTTAGTAGGGCTTACTGCGGAGACTAGTCA
Loxodonta africana   CCAGGAGGCTCTGCAGGCAGGGTTAGCGTCCCTGGGCCAGTACCGACTACTTGGGAGAATGTTCAGGAGGGAAGAGAACAGGGTTGTAGCCCTTGTAGGACTTACTGAGGAGACTAGTCT

Homo sapiens         CGCCCTGGTCCCTAAGGAGATACCGGGAAAAGGGGGTATCTGGAGAGTGATCTTTAAGCCCCCTGACCCAGATAATACATTTTTAAGCAGATTAAATGAATTTTTAGCGGGAGAGGGCAT
Loxodonta africana   TGCTGTGGTCCCTAAGGAGATACCAGGGAAGGGCGGTGCCTGGCGAGTGATCTTTAAGCCCCCTGACCCAGATAATGAATTTTTAAACAGATTAAATGAATTCTTAGAGGGAGAGGGGCT

Homo sapiens         GACAGTGGGTGAGTTGAGCAGAGCTCTTGGACATGAAAATGGCTCCTTAGACCCAGAGCAGGGCATGATCCCGGAAATGTGGGCCCCTATGTTGGCACAGGCATTAGA---GGCTCTTCA
Loxodonta africana   GACAGTGGCTGAGTTTACCAGCGCTCTTGGGTATGGAAACGACCCTTTTGACCTAGACCAAAACCTAGTCCCACACGTGGGGGCCCCTATGTTGGCAGAGGCATTAGATGAGGCCCTTCA

Homo sapiens         GCCTGCCCTGCAATGCTTGAAGTATAAAAAGCTGAGAGTGTTCTCGGGCAGGGAGTCTCCAGAACCAGGAGAAGAAGAATTTGGACGCTGGATGTTTCATACTACTCAGATGATAAAGGC
Loxodonta africana   GCCGGCCCTGCAATATCTGCAATACAGAAAGCTGAAAGTGTTCTCAGGCAGCGATCCTCCAGAACCTGGAGAAGAAGGATTTGATTCCTGGCTGTTTCATACTACTCATATGATGAAGAC

Homo sapiens         GTGGCAGGTGCCAGATGTAGAGAAGAGAAGGCGATTGCTAGAGAGCCTTCGAGGCCCAGCACTTGATGTTATTCGTGTCCTCAAGATAAACAATCCTTTAATTACTGTCGATGAATGTCT
Loxodonta africana   ATGGCAGGTGTCAGATGCAGAGAAAAGAAGACGATTGCTAGAGTGCCTTAGAGGCCCAGCATTTGATGTCATTCGTGTCCTCAAGATAAACAATCCTTTCATTACGGTCCCTGAATGCCT

Homo sapiens         GCAGGCTCTTGAGGAGGTATTTGGGGTTACAGATAATCCTAGGGAGTTGCAGGTCAAATATCTAACCACTTACCAGAAGGATGAGGAAAAGTTGTCGGCTTATGTACTAAGGCTGGAGCC
Loxodonta africana   ACAGGCACTTGAGCAGGTATTTGGGGTTATCGATAATCCTAGGGAGCTGCAGGTCAAATTTCTAACCACTTATCAGAAGGCTGAAGAAAAGTTATCTGCCTATATCCTAAGGCTGGAGCC

Homo sapiens         TTTGTTACAGAAGCTGGTACAGAGAGGAGCAATTGAGAGAGATGCTGTGAATCAGGCCCGCCTAGACCAAGTCATTGCTGGGGCAGTCCACAAAACAATTCGCAGAGAGCTTAATCTGCC
Loxodonta africana   TTTATTACAGAAACTGGTAGAGAGGGGAACAATCGAGAAAGAAGTTGCGAATCAGGCCCGCCTAGACCAAATCCTTGCCGGGGCAGACGACAGAACCCTTCGGAGGAGGCTTGACCTGCC

Homo sapiens         AGAGGATGGCCCAGCCCCTGGTTTCTTGCAGTTATTGGTACTAATAAAGGATTATGAGGCAGCTGAGGAGGAGGAGG---CCCTTCTCCAGGCAATATTGGAAGGTAATTTCACCTGAGT
Loxodonta africana   GGAGGATGGCCCGGCACCTGGCTTATTGCAGTTACTGACACTGATAAAGGAAGAAGCAGCAGCTGAGGAGGAGGAGGAGGCCCTTCTTCAGGCAGGACTAGAGGGGAATTTCACCTGAGT

Homo sapiens         CTCAGGGAACCACGAAGGGATAT--GGCAATGAGTAGAGCATGAAGGTAGAACAGTCTATATACTCTTGTGACACATACAA------TCCCTACCTTGTGCTGCCAAGTAACTCATTTTT
Loxodonta africana   CTTAGGAAACCAGAAGGGGATATCTGACAGTGAGCAGAGCATGAAGGTACAGCAGCCCGTACTCACCAGAGTTAGATCTGAACCAACCCCCTGCCTTGTGCCGCCAAGTAACTCATCGTT

Homo sapiens         GTGCAATTCTCAGTATAAGCCCTTTGTCGTTTCTGTGCCTATTTAAAGTCTCCTAAAGGTGTAATTGACTAGGAAGGATGTAGTTCTACACTGCCATTTACCTATTTAAATTCATCCTTG
Loxodonta africana   GCACAGTGCTCAGTATAAAACCTTTGTCATTTCTGTGCCTACTTAATGTCTCCTAAGTGTGTCCTTGACTAGAAAAAATGGAATTTTGCACTGGTATTTATATATTTAAGTTCACACCTG

Homo sapiens         TGAATATCTTTGTTGTTGTTGTTGAGACAGAGTCTCGCTCTGTCACCCAGGCTGGAGTGCAGTGGCGTGATCTTGGCTCACTGCATCCTCCGCCTTCCAGGTTTAAGCTATTCTCCTGCC
Loxodonta africana   TGA.....................................................................................................................
```

```
Homo sapiens         TCAGTTGCCCGAGTAGCTGGGACTACAGGCATGTGCCACCACGCCCAGCTAAGTTTTGCATTATCAGTAGAGACGGGGTTTCACCATGTTGGCCAGGCTAGTTTCGAACTCCTGACCTCA
Loxodonta africana   ........................................................................................................................

Homo sapiens         AGTGATCCACCTGCTTCGGCCTCTGAAAGTGCTGGGATTACAGGCGTGAGACACTGCGCCCAGCCTCATCCCTGTGAATTTCTTATTGTACACAAGT-TCTTTCACTATCTGTGTGCAGT
Loxodonta africana   ......................................................................................CGTACAGAAGGGTGCATCACTACATGTATGCAGT

Homo sapiens         GCTCTGAGGGGACAGACAAGGCTTGGGTGTATATGCCAACCAGA---TCTCATTGAAGTATCAGCTTGTTTGGTACTAGGTGCAAGTGTAGCATGTCACATGTGACCATGTTGGATCATT
Loxodonta africana   GCTCTGAGGTTGCAGGCACAGCTCAGGGACATGGGCTGAGCAGAAGAGCTTGCTGAGATGTCAGTTTGTGTAATGACGGGAGCAAACGTAGCATGTCAGATGGGATAATATGGTATCGTA

Homo sapiens         GACAATTTTTAGGTATGTACTGACC--TACATTTATGATGAAGATCCTGAGCGGAGGTTAAGATATTAAGTTATTTTCCATATGAATCAGAATTATATTGATTCTGTGCAATCAAAACAA
Loxodonta africana   GGTCGTTTTTAGATGTGTATTGTCTCTTATATTTACCATGAA-ATCATGAACAGTGGTTAAGATATTAAAT-ATTTTCTGCATGAATCAGAATTTTATTGATTCTGTGCAATCAAAA---

Homo sapiens         AAGGCAGAATAGAATGCTGAGATTGGTTAAGTTTGCAATGACCATCTTGAACCACAGATTTCTGCTATGTGTCATCAAAACATCTA---GTTCTGAGTAACATTTTCACGATTGTTATAA
Loxodonta africana   --AGCGGAATTACATGCTGAGGCTGAA-AAGTTTGCAGCTGCCACCTCTAGCCCTAGGTGTCTGATATGCGTTG---AACGGTGTACATGTCCTGAATGTCGTTTTAATAGATATTATAC

Homo sapiens         AATTATAGGTGTGAACTTCTAAAATAAAGGAATGCTAATAAAATGACTT----TGTCTGTTTTGTTTATATCACGGTTCCAAGAGAGATTTCATTTGAATAAAGAGTTCCCAAAGCATGA
Loxodonta africana   A--------TGTGAATTTATAAAATAAAGGAATGCTAATGAAGTGACTTCTTTTATCTGTTCTACTTACATTAGGGTTTCAAGAGAGGTTTCATTTGAACAAATGGTCCCAAAAGCTTAA

Homo sapiens         ATCTGCAAACCACTGAACCTACACAGACTTAGTTCCT-TGGACTCTTTCCTCTTGGTGCTCA-GGGGTAGGAAATTGTTTTCTAAGTGAA---------GGGACCTTCTACCGAAGCAGT
Loxodonta africana   ATATGAAAACCACTGAGGTTATACAGTCTTCGTCCCTCTGGACTCTCTTCTCTTGGTTCCCACAGGATAGGAAGTGCTTTACTAAGTGAAATTGCTGGAGGGACCTGCTACTGAAGCAGT

Homo sapiens         AAAACAATGGGATGAAACAAGACAGTGCACAAATTAGTGCGAGTAGTGCAGATGTATCAGTGTGTCAAATTTGGAAGGAGAAGGAAAGAAATGAACATTGCATGCTGCTGCTTGGAAGAT
Loxodonta africana   GAAACAATGGAATGAAACAAAAC--TACACAAATT----------GTCCTTGTGTGCCATTGAGTCAGTCCTGG..............................................

Homo sapiens         TGGACTAAAAGTTTCTCCATGTACTTTCCCATTTAATCCTCTTGAAAAAAGGTTGAGCAGTATAATCTTAGCACTTTGAGAGGCTGAGGCAGGAAGATCGCTTGAACCCAGGAGTTCGAG
Loxodonta africana   ........................................................................................................................

Homo sapiens         ACCAGCCTGGGCAACATAGTAAGTACAAAACATTTTTAAAAACTAGCCAGGCATGGTGGTGTGCGCCTGCAGACCCTGCTACTCAGGAGGCTGAAGCTGGAGGACTGCCTGAGGCCAGGA
Loxodonta africana   ........................................................................................................................

Homo sapiens         AGTCAAGGCTGCAGCAAGCCATGATCATACCACTGCACTCCAACCGGGTGACAGAGCAAGA
Loxodonta africana   .............................................................
```

```
Homo sapiens  AAGTCAA------------GCCCCCAAGTTAAGACAGTAACTCCTAAGCACTTACAATGGTCCCGAAAATCTTCCTGTTCCCACGGCAAGCGTTAAAGAGTCACTCAGCTCTGAATAATC
Bos taurus    AAGTCAAACCTATGTCGGAGCCCCCAAGTTAAGACAGTAACTCTTAAACACTTACAGTGGTCCCGAAAATCTTCCTGTTCCCACGGCAAGCGTTAAAGAGTCACTCAGTTCTGAATAATC

Homo sapiens  GGGTGGCTTTGGCATTTCAGAG--AAAGCCAACATAAAATAAATCCCCTGGGTCACCCGGCTAGGAGAAAGGTGAGCGCCGCCAGCCTGGAGCACCAGCTACTAGTGATTAAGAACGGCG
Bos taurus    GGGTGGCTTTGGCATTTCAGAGAAAAAGCCACCATAAAAGAAATCCCCCGGGGTCACCGGCTGGGAGAATGGTGAGCACGGCCAGCCTGGAGCAAAGACTAAGTGTGATTAAGCACAGCG

Homo sapiens  TGACTGCGGCGGGTCACGC-GCGGCCCGGCCCCCGTCGCCGCCGCCGGAGACCCGCTCTGAG-AACCTGACCCGGCAGGGAGGCGT-CCCCGCCACTTGGGCC-TCCGGGCGGCCGGCCC
Bos taurus    TGACTGCAGCCGGTCACGCGGGGGGCTGGCCCCCGTCGCTGCTGCCCGAGCCCCAGCCTGTGTCGGTCGGCCCAGCCGGGAGGCTTCCCCCGACACCTGGGCCTTCCCTGCAGCCGGCCC

Homo sapiens  CGCCCCCTGCCGCGCTGCAGCGCCGGCACGCCGCGACCAAGGCTGCGCAGGCGGCGCCAGGCCCGGGCCTCGCCGCCTTGCAGCGCCGCGAGCCCAGCGCCCGTCAGCGGTCGCCGGCGG
Bos taurus    CGCCCCCTGTCGCGCAGCAGGGGCCGCCGGCCGCGACCAGGGCTGCGCAGGCGGCGCCAGACCCGGGCCTCTCCGCCTTGCAGCGCCGAGAGCCCAGTGCCGGGCAGCGGCCACCGGCCG

Homo sapiens  AGCCTGCTTGCAAAGCTG---AGGTCCCGGATCTCACCTTCCTGTCCCGTGGTGGCCAGCCCGCCCGCCGTCCCGGATGCCGCCGCAGTCGGAGCTTCCGGCTCGGGCCGGAAGCGCGCG
Bos taurus    AGGC----TGCAGAGCTGGGCAGGTCCCGGGTCTCACC-TCCTGT-CCGGGTTGGCCCGCCTGCCCGCCGTCCCGGATGCTGCCACACTCGTGGCTTCCGGCTCGGGCCGGAAGCGCGCA

Homo sapiens  CGCCCCGCCCCCG--AGGCCCGCCCCT---CATGACGTCAGATCGCCCCCGCCCGCCGCGCAGCTTCCCCGAGCGAGACCAAAACAGGTGGAAT--CCGGGCTGGAGCCGGAGCTCCGGC
Bos taurus    TGCCCCGCCCCCGAAAGACCCGGCCCTCCCCGTGACGTCAGG----------CCGCGGCACCGCCTCCTCAAGCGCTCTCCAATCGGCTCCAGCTGTCTGACTG--GCTAAAGTTGCCGA

Homo sapiens  GGCGCGGGTGGCGGCA-----CGTCCCTCCAGGTGGGTGGCTCTCTGGGTCTGG-CGACTCAGCGCTCCTCAGCCTTGCCCCCTGGTGC-CCGCTCGCGACTGTCATCGCCCCATCACTT
Bos taurus    GGCGCGGGTGGTGGCAAAGATCCCCACTTCTGGTGGGTGGCCGTCCGAGCCTGGCCGCTTCAGTCCCCCTCGAGGCTTCACCCTGGTGCTTCTCTCGTGGCCCTGGTCTCCCAGCTGTTC

Homo sapiens  CTCGTTGCAGACAGTACCACA----------GGCACCTGGAGTACCGGCATCGGTC-GCTGTGGCCCCCGAGTGTCCGTCAGAGCCTAGGGGAGCCTG-CCCTCCCGCGCCTCG----TC
Bos taurus    CGCCTCGCGGAAAGCCCCGCAGCGTCGGGCGGTCAGCTGTTTCCCCGGCGCGCCTCTGCTATCCCCTTTGGGTGTGCATC-GAGTGGAGAAGAGGCCGCCCCTCGCGTTCCTCGGGCCTG

Homo sapiens  GGGGCCCGG-----------------CCAGGCACCTTGGCCGCCGGCGCACGGACGCGGGCACGAGCA-CTAGATCACGGTGAGTGGAAGCGGGCCCGACGCGGCCCTGCAGGCCCGAC
Bos taurus    GCGGTCCGGGAAGCGTCGCGAGCAGGCCCTGGTGCCTCGGCTGCCGGCG-ATGGACTCAGACACCGACAGCTAGATC----------------------CGAGGCTCCGGTGAC-----

Homo sapiens  ATCCGCCAGGCACCAGGGCTGGGAGGGGTGAGGGGCGCGCGCCGCAGTGCGGCTGCCCT--GGCGTCGCCGCTGGGGGCAGGGCGCGCGGGGCTTGGGGCCTGCACGCTTGC-CCTGGA-
Bos taurus    -----------------------------------TGCTTGCCGCAGTGGGGCTGGCCTCAGGCGC--------------GGGTGCGCGGGCCTT-----CTCCGGGCTCGAGTCTAGGG

Homo sapiens  -----CGTTGGCACCTTT-AGCTAACCTGTCTGCTCTGT-CTTGCAGGCTGCTGGACCTCGGCACGTTGACAAGATTTCTCTGGGGTACCGCGGAGGATTACTTTGAATTTCGGTGGTCG
Bos taurus    TCATTCATTGACACCTTCAAACTGACCTGACTGCCTCGTCCTCGCAGCTCGTCGGAACTCCGGACGTTAACCAGACTTAACTGTAGTACCGCGGAGGAATACTGTGAATTGCGGCGGT-G

Homo sapiens  CCTGTGGTCT-GGCATATTTAGAACTTAAGTCTATTATTTCGGGCACCATGACTTTGAGGCTTTTAGAAGACTGGTGCAGGGGGATGGACATGAACCCTCGGAAAGCGCTATTGATTGCC
Bos taurus    TCAATGGTGTGGGAAAAATTGGAATTTAAATCTGTTGTTTGGGGCACCATGACGCTGAGGCTCTTAGGAGACTGGTGTAGGGGGATGGATGTGAACTCGCGGAAAGCGCCGTTGGTTGCC

Homo sapiens  GGCATCTCCCAGAGCTGCAGTGTGGCAGAAATCGAGGAGGCTCTGCAGGCTGGTTTAGCTCCCTTGGGGGAGTACAGACTGCTTGGAAGGATGTTCAGGAGGGATGAGAACAGGAAAGTA
Bos taurus    GGCATCGCCCAGACCTGTGATGTGGCAGAAA----------TCTGTGAGCTGGTGTAGCCCCCTTGGGGGAGTACAGACTCCTCGGGAGAATGTCCCGGAGGGACGAGATCAGGAATGTA

Homo sapiens  GCCTTAGTAGGGCTTACTGCGGAGACTAGTCACGCCCTGGTCCCTAAGGAGATACCGGGAAAAGGGGGTATCTGGAGAGTGATCTTTAAGCCCCCTGACCCAGATAATACATTTTTAAGC
Bos taurus    GCCTTAATAGGACTTACTGAGGAGACAAGTCATGTTCTGATCCCTAAGGAGATACCCGGAGGCAGGGGTGCCTGTAGAGTGATCTTTAAGTCCCCTGACCCAGATAGTTAATTTTTAAGC

Homo sapiens  AGATTAAATGAATTTTTAGCGGGAGAGGGCATGACAGTGGGTGAGTTGAGCAGAGCTCTTGGACATGAAAATGGCTCCTTAGACCCAGAGCAGGGCATGATCCCGGAAATGTGGGCCCCT
Bos taurus    AGATTAAATGAACTCTTAGAGGGGGAGGGCGTGACATTGGGTAAGTTTACCAGAGCTCTTACGTATGGAAACGAC-CCTTTGATCTAGACTAGGATGTGATTCTAGGAATGCGGACCCCT

Homo sapiens  ATGTTGGCACAGGCATTA---GAGGCTCTTCAGCCTGCCCTGCAATGCTTGAAGTATAAAAAGCTGAGAGTGTTCTCGGGCAGGGAGTCTCCAGAACCAGGAGAAGAAGAATTTGGACGC
Bos taurus    TTGTTGGCACAGGCAGTCCATAAGGCTCTTCAGCCTGCTCTGTAACACCTGAAATACAAAAAACTGAAAGTTTTCTCAGGCAGTGATCCTCCAGAGCC---AGAAGAAGAGTTTGAATCC

Homo sapiens  TGGATGTTTCATACTACTCAGATGATAAAGGCGTGGCAGGTGCCAGATGTAGAGAAGAGAAGGCGATTGCTAGAGAGCCTTCGAGGCCCAGCACTTGATGTTATTCGTGTCCTCAAGATA
Bos taurus    TGGCTGTTTCATACTACTCAAAAGATGAGGATATGAAAGTTGTAAGATGTCGAAAAAACAAGGCGATTGCTAGAGAGCTTCAGAGACTCAGCATCCGGTATCTTTCGTGTCCTCCAGATA

Homo sapiens  AACAATCCTTTAATTACTGTCGATGAATGTCTGCAGGCTCTTGAGGAGGTATTTGGGGTTACAGATAATCCTAGGGAGTTGCAGGTCAAATATCTAACCACTTACCAGAAGGATGAGGAA
Bos taurus    AACAATCCTTTAATCACAACTCCTGAATGCCTGCAGGCTCT--------------------------------GGGAGTTGCAGGTCAAATACCTGACCACTTGCCAGAAGGAGGGAGAA

Homo sapiens  AAGTTGTCGGCTTATGTACTAAGGCTGGAGCCTTTGTTACAGAAGCTGGTACAGAGAGGAGCAATTGAGAGAGATGCTGTGAATCAGGCCCGCCTAGACCAAGTCATTGCTGGGGCAGTC
Bos taurus    AATT-----GCTTAGGAACTAAGGCTGGAGCCTTTACTACAGAAACTGGTAGAGAGAGGAGACTTGGAGTGA-------------------GCCTGGAGCAGGTCCTTGCTGGAGCAGTC

Homo sapiens  CACAAAACAATTCGCAGAGAGCTTAATCTGCCAGAGGATGGCCCAGCCCCTGGTTTCTTGCAGTTATTGGTACTAATAAAGGATTATGAGGCAGCTGAGGAGGAGGAG------GCCCTT
Bos taurus    CACAGAACA------------------CCGGCGCAGG------------CAGGCTTCTTAGAGTTACTGACACTAATAAAGGATGAAGAGGCAGCTGAAGAGGAGGAAGAGGGCGCCCTC

Homo sapiens  CTCCAGGCAATATTGGAAGGTAATTTCACCTGAGTCTCAGGGAACCACGAAGGGATAT-GGCAATGAGTAGAGCATGAAGGTAGAACAGTCTATATACTCTTGTGACACATACAATCCCT
Bos taurus    CTCCAGGCAGGACTTGAGAGACATTTCATCTGAATCTTGGGAAATCAGGAAGGGCTATCTGCAGCAAGCAGAACACGGAGGCTGAACAGTCTGTATGCTC-AGTGATAGACATTA-ACCT

Homo sapiens  ACCTTGTGCTGCCAAGTAACTCATTTTTGTGCAATTCTCAGTATAAGCCCTTTGTCGTTTCTGTGCCTATTTAAAGTCTCCTAAAGGT-------GTAATTGACTAGGAAGGATGTAGTT
Bos taurus    ATCTTGTGCTGCCAAGTTACTCATGTTTGTGCAGTTCTTAGTATAA-TCCTTTGTCATTTCTGTGCCCATGTAATGTCTCCTAAATGTCTCTCTAGTCATTGACTA-GAAGGATGTAGTT

Homo sapiens  CTACACTGCCATTTACCTATTTAAATTCATCCTTGTGAATATCTTTGTTGTTGTTGTTGAGACAGAGTCTCGCTCTGTCACCCAGGCTGGAGTGCAGTGGCGTGATCTTGGCTCACTGCA
Bos taurus    CAGCACTGGTATTTATATATTTATATTCACCCATG.....................................................................................

Homo sapiens  TCCTCCGCCTTCCAGGTTTAAGCTATTCTCCTGCCTCAGTTGCCCGAGTAGCTGGGACTACAGGCATGTGCCACCACGCCCAGCTAAGTTTTGCATTATCAGTAGAGACGGGGTTTCACC
Bos taurus    ........................................................................................................................

Homo sapiens  ATGTTGGCCAGGCTAGTTTCGAACTCCTGACCTCAAGTGATCCACCTGCTTCGGCCTCTGAAAGTGCTGGGATTACAGGCGTGAGACACTGCGCCCAGCCTCATCCCTGTGAATTTCTTA
Bos taurus    ........................................................................................................................

Homo sapiens  TTGTACACAAGTTCTTTCACTATCTGTGTGCA----GTGCTCTGAGGGGA----CAGACAAGGCTTGGGTGTATATGCCAACCAGATCTCATTGAAGTATCAGCTTGTTTGGTACTAGGT
Bos taurus    TCGTACAGAAGTTTCATCACTACACACATACATGTGATACTCTGAGAGGATGTCCAGACATGGCTCAGGTGCACATGCTGATCAGATTTCGTTCAAATGTCGGCTTGTGTGATAATAGGG

Homo sapiens  G--CAAGTGTAGCATGTCACATGTGACCATGTTGGATCATTGACAATTTTTAGGTATGTACTGACCTACATTTATGATGAAGATCCTGAGCGGAGGTTAAGATATTAAGTTATTTTCCAT
Bos taurus    GGACCAGAGTAGC-TGTCCCACGTGA------TGCGTCATAGGTGGTTTTTAGGTATGTCTGGCCCTATATTAATCATGAAGATCATGAGCAGCGGTT----------TTTATTTTCTGT

Homo sapiens  ATGAATCAGAATTATATTGATTCTGTGCAATCAAAACAAAAGGCAGAATAGAATGCTGAGATTGGTTAAGTTTGCAATGACCATCTTGAACCACAGATTTCTGCTATGTGTCATCAAAAC
Bos taurus    GTGAATAGAAATTTTATTGATTCTATGCCATCAAAACAAGAGGCAGAATTCAACGCTGTGGTTGG-TAAGTTTGCAGCTGCCATCTCTAACCCCAGATTTCTCATGTGTGTCGTCAAAGC

Homo sapiens  ATCTAGTTCTGA---GTAACATT-----------TTCA-----CGATTGTTATAA----AATTATAGGTGTGAACTTCTAAAATAAAGGAATGCTAATAAAATGACTTTGT---CTGTTT
Bos taurus    ATCTATTTATTAAACGTCATATTAATAGGTTGCATTCAATTATAGATTGAATTAAATTCAATTATAGATGTGAATTTATAAAATA----AATGTGAATAAAATGACCTTTTAACCTATTT
```

```
Homo sapiens  TGTTTATATCACGGTTCCAAGAGAGATTTCATTTGAATAAAGAGTTCCCAAAGCATGAATCTGCAAACCACTGAACCTACACAG---------------ACTTAGTTCCTTGGACTCTT
Bos taurus    TGTTTATACCAAGTTTCCAAGAGCGATTTCACTTGAACAAAGTGTTTCAAAAGCATGAATCTGAAAGCCACTGAATTTGCACAGTCTTAGTCCTTTGGACACTTAGTCCCTTGGACACTT

Homo sapiens  TCCTCTTGGTGCTCAGGGGTAGGAAATTGTTTTCTAAGTGAA---------GGGACCTTCTACCGAAGCAGTAAAACAATGGGATGAAACAAGACAGTGCACAAATTAGTGCGAGTA---
Bos taurus    TCCTGCTGGTGCCCAGGGGTAGGGAGTAGTTTTCGAACTGAGAATGCTGGTGGGACCTTCTCCTGAGGC------ACAATGGAGTGAAATGAGAC--TGCGCAAATGAGTGCAAGCAGCA

Homo sapiens  -GTGCAGATGTATCAGTGTGTCAAATTTGGAAGGAGAA-GGAAAGAAATGAACATTGCATGCTGCTGCTTGGAAGATTGGACTAAAAGTTTCTCCATGTACTTTCCCATTTAATCCTCTT
Bos taurus    GGTCCCAAGGTATCAATGTGTTGAATCTGGAGGGTGAAGGGAAAGACAGGAGCATTGCATACCTCTGGGTGGCAGGCTAGACTAAATGGTCC----AGTACTTCCTCACTTAATCCTTGT

Homo sapiens  GAAAAAAGGTTGAGCAGTATAATCTTAGCACTTTGAGAGGCTGAGGCAGGAAGATCGCTTGAACCCAGGAGTTCGAGACCAGCCTGGGCAACATAGTAAGTACAAAACATTTTTAAAAAC
Bos taurus    AAAAA...................................................................................................................

Homo sapiens  TAGCCAGGCATGGTGGTGTGCGCCTGCAGACCCTGCTACTCAGGAGGCTGAAGCTGGAGGACTGCCTGAGGCCAGGAAGTCAAGGCTGCAGCAAGCCATGATCATACCACTGCACTCCAA
Bos taurus    ........................................................................................................................

Homo sapiens  CCGGGTGACAGAGCAAGA
Bos taurus    ..................
```

```
Homo sapiens  AAGTCAAGCC------------CCCAAGTTAAGACAGTAACTCCT--AAGCACTTACAATGGTCCCGAAAATCTTCCTGTTCCCACGGCAAGCGTTAAAGAGTCACTCAGCTCTGAATAA
Mus musculus  TCGTTAAACCAACGTCGGAGTCCCCAAGTTAAGACGGTAACTCTTTCAAACACTTACAGTGGTCCCGAAAATCTTCCTGTTCCCCCTGCAAGCGAGCAAGAGTCACTCAGTTCTGAACAG

Homo sapiens  TCGGGTGGCTTTGGCATTTCAGAGAAAGCCAACATAAAATAAATCCCCTGGGTCACCCGGCTAGGAGAAAGGTGAGCGCCGCCAGCCTGGAGCACCAGCTACTAGTGATTAAGAACGGCG
Mus musculus  TCAGGTGCCTTTGGCATTTCAGAACAAGCCACCTTAAGAGAAACCCTCTGGGTCGCGCAGCT--GAAGAAGGTGAGCGCGGC--GGCGGGAGCACGAGCTAGTAGTGATTAAGCTCAGCG

Homo sapiens  TGACTGCGGCGGGTCACGC-GCGGC-CCGGCCCCCGTCGCCGCCGCCGGAGACCCGCTCTGAGAACCTGACCCGGCAGGGAGGCGTCCCCGCCACTTGGGCCTCCGGGCGGCCGGCCCCG
Mus musculus  TGACTACGGCTGGTCACGCGGCGGCGGCGGGCCCCGTCGC--CCTCCGGAGCCCCA-----------------GGCGGGGGA-------ACCACCTG-------GAGATGCCGGGCCCG

Homo sapiens  CCCCCTGCCGCGCTGCAGCGCCGGCACGCCGCGACCAAGGCTGCGCAGGCGGCGCCAGGCCCGGGCCTCGCCGCCTTGCAGCGCCGCGAGCCCAGCGCCCGTCAGCGGTCGCCGGCGGAG
Mus musculus  CCCCCCACTGCACC----CGCCGCCGAGCTG-------AGCTGGGCA-GTGGCGCCAGGCTTGG----------CCTGCAGAGGCCCGAGCTCGGCTCCCGGC--CGGACGCCGGGCCAG

Homo sapiens  CCTGCTTGCAAAGCTGAG--GTCCCGGATCTCACCTTCCTGTCCCGTGGTGGCCAGCCCGCCCGCCGTCCCGGATGCCGCCGCAGTCGGAGCTTCCGGCTCGGGCCGGAAGCGCGCGCGC
Mus musculus  CCTGACAGCCAAGCCGAGCAGCCCCGGGTCTCACCTTCCTGTCCTG-AGTGGCCCGCCCGCTCGCAGTCCCGGATACCGCCGCATTCAGAGCTTCCGTCTAGGGCCGGAAACGCGC----

Homo sapiens  CCCGCCCCCGAGGCCCGCCCCTCATGACGTCAGATCGCCCCCGCCCGCCGCGCAGCTTCCCCGAGCGAGACCAAAACAGGTGGAATCCGGGCTGGAGCCGGAGCTCCGGCGGCGCGGGTG
Mus musculus  ------------GCCGGCCTCTCATGACGTCAGGCTGT--------------CAGCTTC-----------------------------------GCGCCG-------------------

Homo sapiens  GCGGCACGTCCCTCCAGGTGGGTGGCTCTCTGGGTCTGGCGACTCAGCGCTCCTCAGCCTTGCCCCCTGGTGCCCGCTCGCGACTGTCATCGCCCCATCACTTCTCGTTGCAGACAGTAC
Mus musculus  ----------CCTCCAG--AGGCGGTTGTC------------------------------TGTTCCCTGAGG--CGTTTGCTGCGGTCGTTCTTCTGCC----CTCG-------------

Homo sapiens  CACAGGCACCTGGAGTACCGGCATCGGTCGCTGTGGCCCCCGAGTGTCCGTCAGAGCCTAGGGGAGCCTG---CCCTCCCGCGCCTCGTCGGGGCCCGGCCAGGCACCTTGGCCGCCGGC
Mus musculus  -------GCCGGGAG--------GCGGGCGC-----------------------------GGGGAGCCCGGGTCTCTCCTAAACCCCGCAAA-----GGTCAGACGTCCTCTCCG-----

Homo sapiens  GCACGGACGCGGGCACGAGCACTAGATCACGGTGAGTGGAAGCGGGCCCGACGCGGCCCTGCAGGCCCGACATCCG---CCAGGCACCAGGGCTGGGAGGGGTGAGGGGCGCGCGCCGCA
Mus musculus  ----------GGACCCAAGCGATGTATC----TACGGGCAGGTGATCCTGA----------------TGGCGTTCGTTTCTAAGCGCCTGTGGCGGGTGGGCGGAGG------------

Homo sapiens  GTGCGGCTGCCCTGGCGTCGCCGCTGGGGGCAGGGCGCGCGGGGCTTGGGGCCTGCACGCTTGCCCTGGACGTTGGCACCTTTAGC-TAACCTGTCTGCTCTGTCTT-GCAGGCTGCTGG
Mus musculus  ----AACTGCTCGGGAGTCA.....................................................GACACGGTTAACTTAACGTGGATGCTCTGTTTTCTCAGGCTCCCGG

Homo sapiens  ACCTCGGCACGTTGACAAGATTTCTCTGGGGTACCGCGGAGGATTACTTTGAATTTCGGTGGT--CGCCTGTGGTCTGGCATATTTAGAACTTAAGTCTATTATTTCGGGCACCATGACT
Mus musculus  ACCTCTGCGTGTTAAAGAGAC-------GAGCAC----GCACATCACTGTAAGCGGCGGCGGCGGCGGCGGCGCCCTGGTCGAATTAGAATTTAAA-----TACTCTGAGCACCATGACA

Homo sapiens  TTGAGGCTTTTAGAAGACTGGTGCAGGGGGATGGACATGAACCCTCGGAAAGCGCTATTGATTGCCGGCATCTCCCAGAGCTGCAGTGTGGCAGAAATCGAGGAGGCTCTGCAGGCTGGT
Mus musculus  CTGAGACTTCTAGAAGACTGGTGCAGAGGGATGGATATGAATCCTCGGAAAGCACTATTGGTTGCCGGCATCCCTCCGACCTGCGGAGTGGCAGACATAGAGGAGGCCCTGCAGGCTGGC

Homo sapiens  TTAGCTCCCTTGGGGGAGTACAGACTGCTTGGAAGGATGTTCAGGAGGGATGAGAACAGGAAAGTAGCCTTAGTAGGGCTTACTGCGGAGACTAGTCACGCCCTGGTCCCTAAGGAGATA
Mus musculus  CTTGCTCCCTTAGGGGAACACAGACTGCTTGGGAGGATGTTCAGGAGGGATGAGAACAAGAATGTAGCCCTGATTGGGCTTACAGTAGAGACTGGCAGTGCCCTGGTCCCCAAGGAAATA

Homo sapiens  CCGGGAAAAGGGGGTATCTGGAGAGTGATCTTTAAGCCCCCTGACCCAGATAATACATTTTTAAGCAGATTAAATGAATTTTTAGCGGGAGAGGGCATGACAGTGGGTGAGTTGAGCAGA
Mus musculus  CCTGCAAAAGGAGGTGTCTGGAGAGTGATCTTTAAGCCTCCTGATACTGATAGTGACTTTTTGTGCAGATTAAATGAGTTTTTAAAGGGGGAGGGCATGACGATGGGTGAATTAACCAGA

Homo sapiens  GCTCTTGGACATGAAAATGGCTCCTTAGACCCAGAGCAGGGC---ATGATCCCGGAAATGTGGGCCCCTATGTTGGCACAGGCATTA---GAGGCTCTTCAGCCTGCCCTGCAATGCTTG
Mus musculus  GTTCTTGGGAATCGGAATGACCCTCTCGGCCTAGACCCGGGCATAATGATCCCTGAAATTCGAGCACCCATGTTAGCACAGGCATTAAATGAGGCCCTTAAGCCTACCCTGCAGTATCTG

Homo sapiens  AAGTATAAAAAGCTGAGAGTGTTCTCGGGCAGGGAGTCTCCAGAACCAGGAGAAGAAGAATTTGGACGCTGGATGTTTCATACTACTCAGATGATAAAGGCGTGGCAGGTGCCAGATGTA
Mus musculus  AGGTACAAAAAGCTGAGTGTGTTCTCAGGCAGGGATCCTCCAGGACCAGGTGAGGAAGAATTTGAATCTTGGATGTTTCATACTTCCCAAGTAATGAAAACATGGCAGGTGTCAGATGTA

Homo sapiens  GAGAAGAGAAGGCGATTGCTAGAGAGCCTTCGAGGCCCAGCACTTGATGTTATTCGTGTCCTCAAGATAAACAATCCTTTAATTACTGTCGATGAATGTCTGCAGGCTCTTGAGGAGGTA
Mus musculus  GAGAAAAGAAGGCGGTTGATAGAGAGCCTTAGAGGTCCAGCATTTGAAATTATTCGAGTCCTCAAGATAAACAACCCCTTCATTACAGTTGCAGAATGCCTGAAGACGCTTGAGACAATA

Homo sapiens  TTTGGGGTTACAGATAATCCTAGGGAGTTGCAGGTCAAATATCTAACCACTTACCAGAAGGATGAGGAAAAGTTGTCGGCTTATGTACTAAGGCTGGAGCCTTTGTTACAGAAGCTGGTA
Mus musculus  TTTGGGATTATTGATAATCCTAGAGCATTGCAGGTCAAATACCTTACTACTTATCAGAAGACTGATGAAAAGCTGTCTGCCTATGTTCTAAGGTTGGAGCCTTTATTGCAGAAACTGGTT

Homo sapiens  CAGAGAGGAGCAATTGAGAGAGATGCTGTGAATCAGGCCCGCCTAGACCAAGTCATTGCTGGGGCAGTCCACAAAACAATTCGCAGAGAGCTTAATCTGCCAGAGGATGGCCCAGCCCCT
Mus musculus  CAGAAAGGAGCAATTGAGAAAGAAGTTGTGAATCAGGCCCGTCTAGACCAAGTCATTGCTGGGGCAGTCCACAAATCAGTTCGAAGAGAGCTTGGGCTGCCGGAGGGTAGCCCAGCCCCA

Homo sapiens  GGTTTCTTGCAGTTATTGGTACTAATAAAGGATTATGAGGCAGCTGAGGAGGAGGAGGCCCTTCTCCAGGCAATATTGGAAGGTAATTTCACCTGAGTCTCAGGGAACCACGAAGGGATA
Mus musculus  GGCTTACTGCAGTTGCTGACACTGATAAAAGATAAGGAG---GCCGAAGAAGAAGAGGTCCTCCTTCAGGCCGAATTAGAAGGCTATTGCACTTGA--CCCAAG-----ACCCAAGGCT-

Homo sapiens  TGGCAATGAGTAGAGCATGAAGGTAGAACAGTCTATATACTCTTGTGACA-----CATACAATCCCTACCTTGTGCTGCC-AAGTAACTCATTTTTGTGCAATTCTCAGTATAAGCCCTT
Mus musculus  --GTCATGAGCAGAACAAGATGGAAGAG-------------CTCATTGCAGAGATCAGCCCATCCACTCTTTATGCTGCCAAAGTAACTC------------------------GCCCTT

Homo sapiens  TGTCGTTTCTGTGCCTATTTAAAGTCTCCTAAAGGTGTAATTGACTAGGAAGGATGTAGTTCTACACTGCCATTTACCTATTTAAATTCATCCTTGTGAATATCTTTGTTGTTGTTGTTG
Mus musculus  TGTCGTGT---TGTTTACTGAATGTCTCCCCAATGTTTTACTGA-TAAAAATGATGTCATTCTACACTGA----TAATTATTTAAATCTATCCCCATGGA...................

Homo sapiens  AGACAGAGTCTCGCTCTGTCACCCAGGCTGGAGTGCAGTGGCGTGATCTTGGCTCACTGCATCCTCCGCCTTCCAGGTTTAAGCTATTCTCCTGCCTCAGTTGCCCGAGTAGCTGGGACT
Mus musculus  ........................................................................................................................

Homo sapiens  ACAGGCATGTGCCACCACGCCCAGCTAAGTTTTGCATTATCAGTAGAGACGGGGTTTCACCATGTTGGCCAGGCTAGTTTCGAACTCCTGACCTCAAGTGATCCACCTGCTTCGGCCTCT
Mus musculus  ........................................................................................................................

Homo sapiens  GAAAGTGCTGGGATTACAGGCGTGAGACACTGCGCCCAGCCTCATCCCTGTGAATTTCTTATTGTACACAAGTTCTTTCACTATCTGTGTGCAGTGCTCTGAGGGGACAGACAAGGCTTG
Mus musculus  ............................................................ATTGTACAAAAGTTTTTGTGCTTCATGCACACAGGCTTTCAAAAGAACAG----------

Homo sapiens  GGTGTATATGCCAACCAGATCTCATTGAAGTATCAGCTTGTTTGGTACTAGGTGCAAGTGTAGCATGTCACATGTGACCATGTTG--GATCATTGACAATTTTTAGGTATGTACTGACCT
Mus musculus  -GTGCATGTGCTGCCCAGAGCTCACTGAGGTGTCAGCCAGTTTGGT-TTATATGCATGTTTAATA-GTCATATGTGATGGTGCTATCAGTTATCAATAGTTTTCAGGTGTT-----GCCC

Homo sapiens  ACATTTATGATGAAGATCCTGAGCGGA----GGTTAAGATATTAAGTTATTTTCCATATGAATCAGAATTATATTGATTCTGTGCAATCAAAACAAAAGGCAGAATAGAATGCTGAGATT
Mus musculus  ACATTTATGCTGAAGACCCTGAGCCAAGGCTGGTTAAGATATTTAGTT-TCTTTTATTAAAATCAG----TTATCGATTCTGTGCAAT-----CAAAAAGCACAACTGAATGCTGTGGTC

Homo sapiens  GG--------------TTAAGTTTGCAATGACCATCTTGAACCACAGATTTCTGCTATGTGTCATCAAAACATCTAGTTCTGAGTAACATTTTCACG-------------ATTGTTATAA
Mus musculus  AGTTCTACAACTAGTTTTAGGTTTG---TGACTATCT------------------GATATGTTGCCAAAACGTCTGCTTCTAAATATCATTTTCTTGGAGTAATAAAGAAATGATAATAA

Homo sapiens  AATTATAGGTGTGAACTTCTAAAATAAAGGAATGCT--------AATAAAATGACTTTGTCTGTTTTGTTTATATCACGGTT--------------------------------------
Mus musculus  AAT-----ATTTGATCTTTGTATACATTGTAATACTACATTTGAAGGAAAATAATTTT-TTGGCTTTGTTTTTAAGACAGTTTCTCTGTGCAGCTTTAGCTGTGCTGGAACCCACTCTAT

Homo sapiens  ---CCAAGAGAGATTTCATTTGAATAAAGAGTTCCCAAAGCATGAATCTGCAAACCACTGAACCTACACAGACTTAGTTCCTTGGACTCTTTCCTCTTGGTGCTCAGGGGTAGGAAATTG
Mus musculus  AGACCCAGGGTAGTCTCAGCTGAACAAA-AGTTTTTATAGTGTGAATCTGAAAGCCACTGAAATT--ACAGACTTAGTTCTTTGAACTCTTGGCACCTAG------GGGATAGGAACTTG

Homo sapiens  TTTTCTAAGTGAAGGGACCTTCTACCGAAGCAGTAAAACAATGGGATGAAACAAGACAGTGCACAAATTAGTGCGAGTAGTGCAGATGTATCAGTGTGTCAAATTTGGAAGGAGAAGGAA
Mus musculus  CTTGTT---TGACAGG---------------------------------AACCAGACTATAAACAGATTGTGGCGAGTAGT--AGGCCCATCA--ATATTGAATTTTGAGGGAGAAAGGG

Homo sapiens  AGAAATGAACATTGCATGCTGCTGCTTGGAAGATTGGACTAAAAGTTTCTCCATGTACTTTCCCATTTAATCCTCTTGAAAAAAGGTTGAGCAGTATAATCTTAGCACTTTGAGAGGCTG
Mus musculus  A-AGATGAAGGTT...........................................................................................................

Homo sapiens  AGGCAGGAAGATCGCTTGAACCCAGGAGTTCGAGACCAGCCTGGGCAACATAGTAAGTACAAAACATTTTTAAAAACTAGCCAGGCATGGTGGTGTGCGCCTGCAGACCCTGCTACTCAG
Mus musculus  ........................................................................................................................

Homo sapiens  GAGGCTGAAGCTGGAGGACTGCCTGAGGCCAGGAAGTCAAGGCTGCAGCAAGCCATGATCATACCACTGCACTCCAACCGGGTGACAGAGCAAGA
Mus musculus  ...............................................................................................
```

RGAG1

>chromosome:GRCh37:X:109601444:109700162:1
AAACCTCAAAGGGAGTGTAAAATGCAGAGTGTTGTCAACTTATCACACCAAGAATATTTT
TTTGCACCTTTTTAGTTTGGAAGGGTCAAATTGAGATGACAGAGGAATAGTGGATAAGAG
CTATAGTGCTATTAAATTACATTCTAGAGTTGGGAAAATCGAGACCCATAATCCTAGGAA
TTATTAATGATAAGAGCTAACACTTTCTGAGTGCTTACCACATGCCAGGCCCTATACTGA
ATGCATCATGTCCATTTTCTCATTTGATCCTGACAACCACCTGTGAGGCCTCATTTTGAA
GACAAGCAAGCTGAGGCTCAGACCTTGGATTTGTCCAAGGTCACTCAGTTGGTCATTTAC
AGAGCCCTGATTCAAACTCTCTTTTGCTTGATTACAAAGCCATTGCTCTAAAAGATTAGA
TTATACTGTCTGTATTTGCTTCTGGGAGCTTATGGAATTCACTCCTATCTGTTCTCACAA
GGAACTCTATTGGGTTTTGAAAAAGGCTAAAGAATTGAAAAGAAACTTTGTAGGCTGGGA
AACATAGTGTCCTTTGAGCAGTGAGATAGGCTAAGCTCAGAGGACTGGGCTCTGGAATGT
GGAAAGGAGACGTGCCCACTGGAGAGCAAGTCAGAACTTTCGTGAGCCCTGCTCCAGGTC
CCAGGAGAAAGTGCCAGCCATGAGGACTGGAGTATTGCCTGGTAAGGAGAAGATTATTAT
TTTATCTAACACGTTCTTCTATAACGCTCTATATGCTAGGCACTGGTCTGCACTGCTCTA
AGTACTTTATAAGTATTAACTCCTTTACATCTTATAACACTTCCCTGGAAATAGGCATTA
TTATTGCCCCCATAATACATATGAGTAAACTGAAGTTCAGAGATGTTAAATAACTTGTTC
AAGGTCACATAGCTGGTTAAGTGCCAGAGCCAGGCTTTGAACCTAGGCAGTCTGGATCTA
GTGGCTGTGTTATACTGCTTCGCAACAGAAACTTGATTTTTCTTTCTTCTGTGTTTATCA
TCCTTTCTCAATCACTCCTGGGCAAGTCTACTTTGCTAGCAAATTTCCCTTACCCCTTTG
GCTTTTTTTTCACCCATGAACCCTCTAATGTTTTCTGCAGCTTCTTTGAGCTCATTCTTT
ATCTTCCACACTGCCTCTTTTGGCATTACTCATGAACGACATGCTCTTTTCATCTGCCAA
ATATTTTTTCCATAAATTATCCCATCTCAACTTGGGAAGTCACAATGGGAAGTATTGTTC
CTATTTTGTGAATGAGGCAACTAAGGCAAGCAACTTTCCTGAAGTCTCACACAACCATCT
AGTGGCAAAGTGCACTAGAATCCAGATTCTCTGATTCTGAATCTAATGTTCTTGTCCATT
GCACCATCTGATGGAATTTAACTATCTATTCATTAATTAATTTATTCATTAATATTGTAA
AAATATACTGAGTGTCTTACTGTATGGTAAGGCCCTGGGTGCAAAAGTCGGTATAAAAAT
TGTTGCTCTTAAGCAACAGACAACACAGAGAGATAAGGTCTCACCATAAAAAGTTAAATA
GCAGCACATATACTAAGTGCCAAATGAGTGGTCTGGTCACATCATACAAGAATTTTGAGG
TTGGATATGTAGCATCAAAGACTGCTTTATCAGAAAGGTGAGACTTGAACTTGGCATAGA
AGGGTACTTAGAACTTAGAGAAGAGAATGGAGCAGCACTTTCCAAACTCTGGGAATGGTG
TGCATTAAAGCAGTCCTTCACAACCTTTATTGTGTCATGGCATGCAGAGAAAAGCATAGT
ATTTGTAAAGCACACTAGGCTGAATGGAGGAAGCAGTTTGACATCTAGAGGCAAGCATTC
TAGGAGTTTCAACAGCACTGTCTCTGGGCTCAGTTGAGGACAGAGAAGAAATCTCATAGC
CTCAGACACTTGTAATATGTTTGGGAAGGTCTGCACTAGGTATTGGGGGCACACTGTGGT
CATGGTGGGTCTAAAGACCACTGACAAGCCCACAGGGATGAAATGAGGGGAATTTCCAGA
GAAGCCATAGTAGATACCATTGGAAAGAAAAGTTATGGGCAAATGATGAAGGGCTTTTGA
ATTTTATTCTGTGGACCTACAGAATAGGTAGGCAAAATATGCATAGCACTGTTTTAGAAA
GATTCCACTGGTTTTCATATGGGAGATTAACTAGAGGGAAGAAGAGTAGTCAAGAGGCTA
TTACCATTGTCCAGGTGGGAGGTGATGTTATCTTGAACTAGATTGATAGTAATAGAGACA
GAGAATGGTGGACAGAATTGAGTTATATTTTGGAGGTAAAAATGTCAGGGTTTGGTAATA
GAATTGCATAATGGAGAGTAGAAGAAAGGGAGGTGACAAGGAGGATTTCAAATTTTCAAG
TTTAAGCAAATGCTTATGAATAGTGATGCCATTCTTTAAGATAGAGAGCCCAGGAGAAGT
AGAAAGATTTTTTTGGAGGGGAAGGACAAATGAGATGATGAGTTTCAAGTTGACATTTGG
AGGCAAGGAAACCAGTTAGGAGGCTATTGTAAAAGCCAAGTAAGACAAGGTGAGATCCTG
GACTACAATGGTAGCAGTGGGAATGGAAGGAAAATGGTGAATTGAGAGACATTATACAGG
AAGAATTGATAGGATTTTGTCACGGACTGACTTCTGGAGTTGAAAGGTAGAGGAAGGCTT
GAAGCCTAAGATATCTTCTGTCCTTTATCTTCTTAGTGATGCTCCGATCTTCGTGGCTTA
ATATACTATCTATTTGCTGATGAGTCACAAATTTGTATCTCCATACTTTTATGCCCAATT
GCCTGCTGGACATATCAACTTGCATGCCTAATAGGTATCTCCAACTTACCATGTTCAAAA
CTGCCCCTGCCCACAACCCCCAACAAAACCTGCTGCACTCACAAAACCTGCTCCTCACAG
TCTTCCCCGTCTCCCTAAATACCAATTTCATTCTTCCGGTTGCTCAGGCGGAAGACCTAG
GAATCAATCTTGATTTTTCCCTCTTTCATATCCTACATTTATTTGATCAGCAAATCTTAT
TGGTTCTACCTTCAGAATATATCTAGAATCTGACCACTTTTTCATTGCTACTATCCTGGT
TTATGTCACAATGATCTGCTTCCTGGATTATATTGCACTAGCCTCTCAACTGTTCTCCTT
GCTTCTGTTCTTGCCACCATCACTCAAGCCTATTATTCTCTCACTGTTTTTTGGAACCAG
GATAATTTTATTATGTGAATGTCAGATCATGTCACTCCTCTGCTCAAAACTCCTCCAATG
GCTTCCCATCTGACACTCAGTAACAACAAAACCCCTTATAATGGCCCATAATGCTGTATG
CAACCCAGATATTGACATACTCCTGTCCTTCAGGTGATGGATCAAATATCCCACTTGGTA
AAATCTTCTCTGATTACCATATTTAGTATTGCAAGCGCCTTCTCGCCATTCTCTATCTGC
CTTCCATGTTTTATTTCTTTATACTTTGAATACTAGCAGATGTTATATGTTTATCTATTT
ATTTGTTTATCATCTATCTCCTTCTGCTAGAACAGGAGCTGGAAAACTTTTTCTGTAAAG
GTCCAGATAATAAATATTTTAGGCTTTGTGAGCCACGTAATCTTTAACACAACTACCTAC
CTCTATCATTGTTATACAAAAGCTGCCATAGATGAGCATGGCTGTGTTCCAATAAAACTG
TATTTACCAAAACAGGGAGCGGGCTAGATTTAGCTAATGGGCCATAGTGTACTGACCCCT
GCTAGAATGTAAATTCCAGGAGGGCAAGGACTTTTGTTTACTGCTGTATCTCCAACATCT
AGAATAGTACCTGGCATATAGTAGGCACATGATTTATTTTACTGAATGAATGCATCAGTG
GGAGATTGGAAAGCTGTGGAATAAAAAATATATATATATAACAGAGCCATTGATGGTGCA
GAATCCTGAACAAAATCTAGCTTTGGATGGGTTTGCAGCATATCAACTCTCCTTCCTTCT
TTTTCCCCTCATCCCTACACTAAAACTTAAGCTTGTTATATAAAGTAAAAGTTGCTTTCA
TTGCCTGTGCAATTCTCCCAATGTGTGAAATTTCATCTGACTTCCTTTAATGTTGTCATT
TGTCTATTTAACCCTCCTGACTCAGACTCCCTCTAATAACAATAATAACTCAAATACTCT
GTACTAAAACCAGAACGCTGAAATCAGTATTTTTAATTAATTTGAAACAACTGCTAGCAA
TAGCTTCTATTGACAACTCTACGCTTTTCAACACTTAATCATATTCTGAAGTACTCCCAG
AAGCTGGGCGACTGTTACTCTTATGATATGAGATGATATTTTCCTTCCATGCATTCAATG
TGAATGGTTTGTTCTGAAAAACTTCAGCTTTCATGTACTCCTAATGGTAAGAATCACATG
GTAGGAAATATGATAAATGAAGTATTTACCAGTTCTTACTGACTGTTCTTTCATACTTCC
TTGGATCTCTTACAGGCACTAAAGATATCCTGTAGTTATATGACTTCCTATTGAAAATGC
ACTTTCACTCATTCATTATTTTATATAGTCTTCCACACATTGCTAACATTTACCAAACAT
TCACTACGTATGTGGTATTGTGCTGGGGGATTTATATATATTAATTCATTTAATTCTTAT
AAAACACCTAAGTGATATATACTATCATAATTTTATAGATGAAGGAACTGAAGTACTAAA
AGCTGAAGTAATTTGCTCAAGGCCCCACAACCAGCAGAAAAGGAAGCTAGCATTTCAACT
CAGGTCTGCCCAACTCCAGAGCCAAGATTTATCTATTGTTTTTTGTCACCTCAACAAATA
TTTACCATATGCCATGCAGCACACTAGGCAGTACCTACAAAGATGAATAGGGCATGATCT
CCTTGGCCTTCCAGAGGTAACACTAGTTGGGGCTATATGGGTGGGAATGGAGTGTCAGAT
GCAGAAGCTAAGGAATTTTCTCTTTACAAACAGGTGGGTGAGAAAGTAGACACAGCACTG
TGAATTTGTGGAGTTTTGAAAAATGGACAGAGGGCTACAGACTGAGAGGGAATAGGGCCA
CCAACTCTAGAATTAAGGTCAACTGATAGGGTAGAAACATGCCATTCTCAGGGGCTTAAG
CTGACAAAAGAAATTGCCCACTGTAGAAATGAGATTGGCTGCCCACAGAAAAGTATTTAC
TGATAGATAAGGGACCTCCCTATGTGTCATTAAGGAGTAATGCTCTAAAATGTAATCAGT
GGAAAACACAACTGTAGCCTTGGAAATGCTATGGCTGGGAAAGTCCATATAACTAAGACG
GGGTTCATGATATTGTGACATGTACAAACAAGTTATTTGACCATTATAGAGAGAAAAATA
AAAATAATTAGAAAGAGAAGTTGCTTTTATTACTCTGTGCCCTGGCCTACTGACACATTT
ATTGACTTTCTGATAGGGTGATGCATGGATGCATCAGACTGAATAAACTACATGACTCCA
TCTCTTCAGATCATCCCACACCATATACTATCTCTTCACTCACCAGTAATAACAGTCACC
ATATAACAAGTGCCTACAGAGTCAAACACTTTACATAAATTATCTTATTTAATCCTCAAG
GCAACTCTATGATATCATAGAATTCTATTATTAGTTCCATTTTCAAATGACGGAACTGAG
GCTCAGAGAGGTTATGTGACTTAAGCCCATAAAACGGAATGAGTGGTATATTAGTTTTCT
GTGACTACCATAACAAATTGCCACAAACTTGGTGGCTTAAAACAGCAGATATTTATACTT
TCACAGTTCTGGAGGCCAGAAGTTCAAAATCAAGGTGTTGGCAGGGCCATGCTCCCTACA
AAGGCTCTAGGGGAGATTCTGTTCCTTGCCTCTTCCTAGCGGTTCCAGGTGTTGCTTGGC
TTGTGGTAGCATCATTCCAATCCCTGCCTCTGTCTTCAGATGGGCTTCTCCCTTGTCTCT
GTGCCCTGTCACTTATAAGGACACTTATCATTGGATTTACAGCCCACCCTAATCCAGGAT
GATCTCATGTAGACATCCTTATCTTACATACATCAGCAAAGAGTCATATTCTAAATAAGT
TCACATTTTGCAGTTCCAGTTGGATGTAACTTTTGGGGGCGGTGACACAATTCAACCCAC
TATGTATAGCCTAGCTGGGACCCTAGCCTATCTGACTCCAAAACTCTGGCTCTTTCCACT
AACTAAAGCAGGCTGCTCCCACCAAACAAACAGCCTTTTGTCTTTATTTGGTTTCCTAAG
TAGATTTCCTACTGGGATTTATCAAGATGAATTAATAAATTGACATTTTGGCTTCCAAAT
TATTGCCTTATAAGTTTAGGTTGTGTGAAATTTGTCCCCTAGAGCCTGATCAATCCCTGG
CTAAAATGGAAAAAATTGAGTTTTGGAACCTGTGAAGCTGATGAATGGATCCTGAATAGG
ACCAAGGTCCATTTAGAGTATAAGCATCCCACAGCCTGCCAGCCATACTAATTTCTTTAC
TATATATGTGACCATAATATAATGGGATAGCATAATATTCTAAGTCTACCGAAGAATGTC
TAATCATCTAAGAGCTTACAGGGGGAATTTTTGTGTACCTTAATATTATATCCAGAAATA
TTCCCTGTAGTGCCAACTATATTTGTTCCTGAGCCTCAGCTCCCTGCATCAGGTCACAAG
GAAGATGTCTCTAGGAGTTCATTATCACATTAAGACAAGGATTCTGTAGGAAAATGGGAA
TCTAGAAACAGTAGGGACTTCCTGTACCTCTACATCAGTGTCTTTGCAGGGGAATTGCCC
TAGCTGACCAGTTGACACTGGCCTGCTCACACCTTAATTATGAATAGAATATTCCCAGGG
ATGTCTTTCTCAGATTGTGGTCAGTTTGTGGGCCGCCTATTGTGGTGGTCCACTGAGTGA

CACCTGGGGAGCTAGTTAAAAATGTCAGTTTCCTGGTCCTTTATAGCAGACTTACTGAGT
TAGTATATCTGAAGATAGGGTCAGGAGCCTGCATTTTAAAACAAGATTCTGGAATAATTC
TGATACATATTCAAGTTTGAGAACCATTGCCTTCTGGTGAAATCAGGCTTCCATGACGTT
GAAATACAGAATAAACTCCAAATGTTCATTTACAATTCCAGCCAATAATCACTTACCTTA
TTGCATTCTTGTAGCGTGATCTATATGCTTTAGTTCATGTGACAATATCATTTCATATGG
CTTAATTTAAATGAAAGCAAAGGCAGTTATGGCAAGATTTCTTAATCCTGCTCTCCTGAG
ACAATTTACATAATAACTTTTGGGGAGAATGCAATAATCTCTCTCTAATATCTTAAACTG
ATCTCTAAGGTTCCTTTTGGCTCCAACATTCTGATTCTATCAGTGTTCCTAGGGCTTCAA
ATCTGTCTGTGCAAAACACATTGCTTGTTACAGACTAAACCTATTTTCCTTCTTCCAGGC
AACAAGTTTCTGAAGAAAACTGGTTGCTCATGATCTTTCTGGGTGTTTTCTAATAATATA
TACCTTCTCACAATATTATTGCAAAGAGTGAGCGAGGTGATCTATGCAAAGTGCCAAGCA
TAGTGCCTGACACAAGGAAGTGGTTAGTAAATATGCATGCTGCTTCCAGTGCATTGTGAC
AATACTATCTCTTGAATTATTGAAAATATAACATCAGGAAACTTCAGCTGAATGAAAACT
AGGGAATTAGCTGTATTGACAATATTAGGTTTATTTGGCTTTAGGGGAACTAGATAGAAC
CGAAATCTCTGCAGACTGGCTCAGCTGCAGAATAGAGAGATGGCCCGGGGAGGACATGTG
TTGGTTCCTGCCATGCATATTGTCTTGGAGAAAAGCTGCTATGACTCTGGCCCAACAAAG
GTTTTATCCTAAAAGTCAGCTTCCAATCCCCAACATGATTCCTAAGCTAGCATTTCTCAA
AATGTATTCCATGGAGTACTAGTTTCTGAGGATTGATGTTGATAGTATTACATAAAGAGC
TCCATGAACAAGCAATGTTACAAACCACTGGATTAGTCAAATGTGGTTTCCTGGCTGCGA
GAATATTCAGAGTCCTTAATAGGTCAACAGGTAATGTGAATCTCCAAAAACTAATGGGGT
ATACCGGATTTGAAAATTTTTGTGACCTCACTCACAGAGATGGTGGTGAGGGACTAGTGG
TTCTGAAAAATGCAATTATGAGAACACTACTCCAAAATGACTAGTGACTTGCTAACAAGA
ATGGACACTTTTCAGCCCTTACATGATAAAAGTTAAGAGAACAATTACGTGCTAAACTAT
AGAGTACTGTCCCTCAGCCCCCTAGTGTCTCCATTTTCTTCTTTGATCAGCTTAGATTCT
ATAACCATCCTTAAAGTCACTGTTTAGCATACACCCTAGATTCCCTAGGCTCTCTCTGCT
TTCATGTGATTTGAATAACAGACATCCAATCGTTGTTAAATCCTACTCTCTTTATTCCCA
ACCTGCCCCAGCAGCCGAATGAGATTGGATAAAAGCTTATAACCGTGCTAACCAGTCTCG
CTTTAGATTCAGGACCACTAACTTCAAATGGGCCCTTAGTACTATCTGCTAACTTCACTA
CATTCATTTCATCCATTCATTCTTGAATGCTCCTGGGTGGCTTTTTTACATCTTTCCTTT
CCTCAAACTTCCGACACTTGTTTCTACATTTTCCCTTTCAGCTGCTACCTTGTTTCCTAT
TTCACTGAGAAAATAGAAGTTGTCAGAAAAGAACTCCCACCAATCTATTTGTATTTGTAT
CCATAAACTCTGCCTTCCCTCTTGTCACTGTGAGCGAACCATCTGGGATTCCACTGAATG
CTGACTCCTCTACTTATGCACTAAATTTTATTTCTTTTTGCCTAATCAAGGTCATTACTC
AAGTCATTCACTCTTCCCTCATGGCATCAATTTTTCTTTCATTGAACAACATCTTCAATG
TTTAAAAAATCTGTTCTCTTCACCCAAGTCATAGCCCCATTTCTCTCCTCCTCAGAGCAA
AAATTCTGGAATGAGTTGTCTCTGCTCATTCGTTCCAATTTCTCTCCTCTCATTTTCTCT
TGAATCCTCTCCAGTCAGGCTTTTGCCATCATCACTCCACCAAAATGATTCATATCAAGG
TCAACAATATCTCCTCAGTATTAAATTCAATGGTCATTTTACTGTTCTCCTTTTTTTTTC
GCCCAACAGCGGCATTTGACACAGTTTATCACTCTCTCCTTGAAACACTTTTTTCTTCCA
CTTGGCTTCCAGGACACCATGTAATTCTTGGCTCTTTCTACTTCACTAGCTACTATTTCT
TAATATCCTTTACAGGTATGTCTTCATTTTCATGATAACTAGGTTGTCTGATTGGCATCT
CAACTTGAAATTATCCAAGGTCAAAGTGACTATTTTCCTTATACCTGCTTCTCCCACAGC
CTTCTTTATCCATTCTTCTGGTTATTTAGGCCAAAGCCTTGGAGTCTGACTTGACTCCCT
TTTTCTCTCACACTCCAGATCCTATCTATCAGCAATACTTTCAGCTGAATAGTTAAAAAT
TTATTTGTTTAGAAACAGCATCTCACACTGTCACCCAGGCTGGAATGGAGTGGCACAATC
ATAGCTCACTATAACTTCAGACTCCTGGGCTCAAGGGATCCTCCCACCTCAGATTTCCCA
GTAGTTGGAACTACAGGTACTCACCACAGTGCTGGCTAATTATTATTATTATTATTATTA
TTATTATTATTATTATTATTATTTTGTAGAAACAGATGTCTTGCTTTGTTGCCCAGGCTG
GTCTTGAACTTCTGGCCTAAAGTTATTTTCCCACCATGGCCTCTCAAATTGTTGGGATTA
CAGGTGTGAGCCACTGCACCTGGCCTAAAAGTATATTTAGAATCTGAACACTTCTCTACT
GCTATAACCTTAATCCAAGCCAGCATCATTTCTTACTTGGATCATTACATAATCCATTAA
ACATGTAAATCAGATCATGTCATGCTCCTGCTCAAAACCGTCCGTGGCCTTTTATATTGT
TCACAATAAAAGCCAAAGTCTTTAGAGTGACCTACAAGATTCTACATGATCTACCTTCAT
TCCCATTACCTCTCTGACCTCATGCCCTGTCACTCTCCTCTTTGATTACTGAGATCAGCC
ATATGGCTTACTTGCTGTTTCTCAGACATACCAAGAAAACTCCCAGAGGTCCATTGCTTT
TCTCTCTAACTAGGATGTTTCTTCCTCATAGAGTGTCATGCTTATTCACTTGCTTCTGTC
AAATCTCTGTGCAAATGACACTTTATCAGTGATACTTTCTTTATCATCTTATTTAAAAAT
TGCCACTCTCTGCATGCTTCCTACAACTTTACTCTGCTTAATTTTTTTCCATAGTACTTG
TCATTTTCTGATATATTAATATTGATTTGTTTGTTTACTGTCTATCTTCACCACTAGAAT
ATAAGCTTCAGGAAGGCAGGGATTTTATTTTGTTGCTGCTGTATCCCTAGTGTGTAATGT
GGTAGCTGGCACATAATAGGTGCTCAATAAATTTCAAATTATTATAATAAAGAATGATTC
CAAGTGCAATAGGAATTCCAAGATTCCTGCCATCTTCAGATGGACCCAGATATCCTTCAT
CATCTGCAGCAATCCCCCACCCAGTGCCAAACTCTGTGACACTGCATGTCTGGTAGTGAG
TTTAGTCTTTACATGAGGACTTCCAGGAAGTTCCATAGATTCTTAAGCAAAGTGCTCCAT
TATTGAAGCTATTTAACTCCTAGAAAGATCTTTGTGGCTGTGTGCGGTGGCTCACGCCTG
TAATCCCAGCACTTTGGGAGGCTGAGGTGGGCAGATCAGGAAGTCAGGGAATTGAGACCA
TCCTGGCTAACACAGTGAAACCCCGTCTCTACTAAAAATAGAAAAAATTAACCGGGTGTG
GTGGCACGCTCCTGTGGTCCCAGCTACTCGGTAGGCTGAGACAGGAGAATTGCTTGAACC
CGGGGGGTGGAGGTTGCAGTGAGCAGAGATGGCACCACTGCACTACAGCCTGGGCGACAG
AGCAAGATTCTGTCTCAGTAAAAGAAAAAAAGAAAAAAAGAAAAAGAAAGATTTTTGCCA
AGGGTCCTTTTCTGATACATAACATTTTTTGAATAGGTATTAGGTGTACACGATACAAAG
TGCCAAATGTTCAAAGAGCAAGTAGTGAAAAGTATATTCTACCACTCCCACTGCTACCAA
CTTATTCCAAGCCACCCACATTTCTAAACTGGAATTATAGCACAATTTTTTTAGAAACAG
AAGTTAGATGATGTCGCTCCTCTGCTAACATCAGCCTGGGGCATGTCATCACACTCAAGG
TAAAAGCTAGAGTCTCCCTCTGCCCCCAGCCACCAAGATCCCTTCCTGGAGGCAAACACT
CTTACTAGTTTATTGTGAATTTTTTAGAGATAGCATGTATATTATACACACATATCTCCA
TGTGTATAATTCATTTTTAAACTGTACACACTATTTTTTATATTTCTTTTTTTTCTCACTT
TATTCTACCTCATTCTTTTTAATAGCTACAGTTGTATCAGTATGGCTACTGTATTCTCCA
GAGATTCTTAGTCTGGAGTCCATATATGGGTTTCAATGGGTCTATGAATCTCCTGAAAGT
GCATGTTGACACTTGTGTGCGTGTGTACATGTATATCTTTTAGCAGAGCAGGCCCATATC
TTTGATCAGATTGAAAGGAATCTGAGACCCAAAATGAGTAAGAAACCACTATATTGAGTC
TGAATCTGCCTTCCTGAAACACCTACCCTTTAGTCCTAATTCCACTGTTTGGAGCAGTTC
TGTCAGGACCTGGTACATAGAGGACATACAACAGGTAACTTGTGAAATGGATGAAGGGAC
AACATAAAATGAATCTATTCTGTCTCTCTCTTTTTAAAATTTTTAAATTTTTATTTATTA
TTATTTTTTAAAGACAGAGTATCACTCTTGTCACCCAGGCTGGAGTGCAATGGCACAATA
TTGGTTCACTGAAAACTTCCACTCCCAGGTTCAAGCAATTGTCCTGCCTCAGCCTCCCCA
GTAGCTAGGATTACAGGCACCTGCCACCATGCCCAGCTAATTTTTGTATTTTTAGTAGAG
ACATGGTTTCACCATGTTGGCCAGGCTGGTCACAAACTCCTGACCTCATGTGATCCACCC
GCCTAGGCCTCCCAAAGTGCTGGGATTACAAGCGTGAGCCACTGCGCCCGGCCTATTCTC
TCTTCTTTATGTTAGCTTTTGGTTAGCCTGAGGAAACTATCATATTCTAAGTCATCCCTT
TTCTAAAGTTAATTACTTAATTTTCTTAACTTTTCCTAATTTGACATGGTTTCCAAACCT
GTAGGCTTTCCCTACATGCTGTAATGTGGGTTTCTTCCTCAAATGCCATCGGGGCAAGCT
GGTAATTTAAAAAAGTGAAGTGGATCAGATGTGAGACAACGGCAAGTGGTGGGACCTGTG
GAGTCCACATGGCTAAGATGTTCACATTCAAACTCACATATAACACATCCATAACCAAAC
AAAAAATATTTTCAGGCCAGTTTTGGACTATGGGCAGGCAGCTTGCAAATATAAAAATTT
GGACCTCGTACAGTTTGTTGATACTCTTTTCACATCTTCTGCACTCTATCAATTCACTTT
TGTCCTCCCCATAGGCCTGACAATTTAATCAAACTAAGGTAGTGCCTGCATCTCAAATAC
CCCTTAAGATTCCTTGCTATTATTCCTTTGCCAATATGGCTCTGTCTGTATAAAATGCCT
TTGTGGAATATTCTCTACTTGCCCCTCAAGATCCACTTTTAAAAATAGACTATTTTTTAG
AGCAGTTTTAGGTTAACAGCAAAATTGAGAGGAACATACAGAGAGTTCCCGTTAGCCCCT
AAAGATCCAGTTTTCACCCTTCTTCATCCTCTTTGTGTACCCCCCGAGACTGACCTTTAT
AGACTGCATTTAAAAACATATTTTTAGTTATTGTGGGTACATAGTAGGTGTATATATTTT
TGGGGTACATGAGATGTTCTGATACAGGCATGCAATGTGAAATAAACACATCATGAAGAA
TGGGGTATCCATACCCTCAAGCATTTACTCACTGAGTCGCAAACAATCCAATTACAGTCC
TTAAATTATTTTTAAATGTACAGTTATCACTGGCTATAGTCACCCTGTTGTGCTATCAAA
TAGCAGATCTTATTCATTCTATTTTTTGTACCCATTGACCATCCCCAACTCCCCCCAGCC
CCCCACTACCTTCCCAGCCTCTGGTAACCATATGAATTGGCTTCCTTGTCCTCTGAATTC
TTCTTGGATATGGCCAATGTAAGGCACTAACAGGGGACTGGAGATCAGAGACAGGTTAAA
GTCTGCGTATTTATTCTCCTGGCTCCCTCTTTATGGAGTGGGTGCAGCTTGGCTGAATCC
TGTTAGAAGGATCTGCTGTGAGCCACAGACTTTTTTTTGGCAGTCATCTTCTAAAGCTAT
GGCTACTCTGTCTAGGTTCTGGTTAACTGCTTTCTCCCCTTGGCTTTTCAGGTCTCAGGG
TGGTAAGTGCTCCCTGGGGTGCTTCACCATCCTTTTTGCTTTCCCTTAACAATGCTCACA
CTGTTATAAACAGTCCGTTCATTAAACTCCACTCACTTCCCTGTTGAATATGCTCTCAAT
ATCCTACTAGATCCTTTACTGTTACCAATGCCTTCAACTCCCTTGTGTACTTGGTGTACT
CCCCATTGTTAAGGTCCCAGCTCCTAGGACAATGTGGTGCATTCCTAGATATCACTCCCT
TTGAGAAAGTTAGCTATTCCCCCACCTCTTTGAGAACATTTCTTGCAACACTCTGTGTTT
AGATCTTGGGCTTCTTATGATTGGGAATGAAGCCTATTCGTTATTGTTTCCTGGCCAAGT
AGATGTTTGGTGAGTATTGGTTATCTTTTTTGCTTTGTGAGGAGGATATAAAATCAAGGA
AGGAGGATATTTGCTTTTTATTTCTTTCAAATTATTTGATTTTAAAAGAAATGCCTACAC
ATTATTTTTTAAAAATTAAAATGAAAAGAAAAAAGTAGTAAAAGTGGCACCGTTCTTCAC
ATCCGTGCTGGAAGTAACAGTGGTAATGGTTTTTGTTGTGCATCCTTCCAGTATCTTTTT
ATGCACATAAAGGCTTAAGTTTGTGTACATAAAATGTAGACTCACATATTTCTCTGCTCC
TTGCTTTTTTTTTAACTTAACAACACACCTGTCTTTCTATATCAGCCAATGTAAGTATAC

AGCCCTACTTCATTATGTTTTATGGCTGCATTGTATTTCATTTGATGAATTTACCATAAT
TTATTTAACAAGCCTCTATTATTAGCTAGTTTATCTAATATTTTCACTGTTAAAAGCAAC
ACAACTTAAAACACAGAGACATACTGGATCAAATTTAAGCCCAAAAATATATGGCTGAGC
TCCAGAAGCAAGAAAGGAAAACTGTCAGATTCCAGAGACAAGAGAAAACTCACAGTCACA
GCAATGAGCATAAATTGACATTTCACCACTCACAGGAAAAATGGGACCAGGTATATTCCT
TAAAGGATGAGATTATAAGTCCTCTGTAGGGCTTAGAATTGAGTTAGAATTGTGCTTCCC
TCATAAAACAAAGAGTAGAAGGATGCTGCCCCATTTATTATTTAAAAGCAGATTTCCCAA
ATAGTCAGGGGTACAATAATAACAAATCTTATTTAGTACTTACTATGTGCCAAGCATTGT
TCTACATGATTTACATGTATTTATTTATTTAACCATCACAGAAACCCTATGATATTAGGT
ACTATTATAAACCCCATTTTATAAGTAGGGAGTAAAACGTTTAAGTAACTTGCGCAGCTA
GGAGGTGGCAGCTCTAAAATTCAGACTCAGGCATCCTGACTCCAGGACCATGCCTGTAAG
TAAATGTGCTATATTGCTTCTCTACAAACAGGATGTAGGTCATCTTCCTTAGGATGAGGT
GGAAATAAACTCACCCATAAGAAGTTTAAAACTTTAAACCTGAGCTGCACACTAGTATGA
AACCAAAATTCACAGTACCCACATAATGCAGAAACCCTGGGGTGAGAACTTAACATAAAA
CCTGGCTCAGAGCAGGAACACAATGAAACTCTAGCAGAGGCAAATGAAAAACCGCCTGTA
GACCCACCTCCATAACTCAGAGTCCAATTCTTACGGAAGGGTTCTAACAGAAAACAGTTC
CTGCAGAAGATAAGCTCACATGCAAAAAAATGATAAAGCACATGAGGAAATTGACTGGAA
TGAGTTAGTTGGTATATACAAGAAATGGGAGAATTATGTCCCAAGAAACTACAGATAATA
CAGTAATCTGAAAGGACTTTATTTTTTGGGTTTTTCTCATCTTTTTGTAGCCCTCATGTA
TGAAAGAACTTTAAAATGTTGTATGTTTGAAGGTTTCAAGATGTAAAAAAGGAACATAAT
ACATAAAGCAAGAGCACAACTTTCCTCGGTGTGTGTACACCAAATAAATAGGCCAGAAAT
GAAGAATATAGAAAATATGGGATGAGCATTGTAAGATTTTAGTTCTAGCCCCAATGAAAT
ATTTAAAAAGTCAAATGACAGTTTAAACTACAAATAAGTTATGAGTGAAGAGAGAACAAA
TGGAATAACAAATAGAGCCGAGATAATCATAGAATATAGCATGGCACTATAAAAATGCTA
AATATGAAAGGGAAAATAGAAGACACACATGATATAACAAGAAACTTCACATTCATCTAA
TAAAGCTCCAGAAAAAAAATAGGAAGAGATAATATTCAAAGAGAAATAGCTGATAATTTT
CTAAAACTGAACAGGACATGAGTCCTGAGATTAAAACCATGTACCTAGTTCTAAGCAGAA
TAAAGACAAATTGATACCCTGTAGATGCAACAGGGTGAAACTGTAGAACATCAAAGATAA
AATGAAAATCTTGAAACCTACTATAGACAAACAATGTTGCAAAATATTTCTGTAGAATAG
ATTCCTAAAATGGAATTTCTGGGCCAGGGTATATGCACATTAAAAAAATGAATATGTTTT
AAATGCATGAGTGAGTGAATTAATGAGTCTAGTAGTATTTCTCTCAATCTGGACATATAT
TTATTTTAGTACAGCCTGAGATTGGATCCCCCAGCTATCTATTTATTTATATATTTGTGA
CTGCATCACCACTATTGATTTCTATTGAGCATAAGACCAATTAAAATCCCCAGGACTTTT
TCAAATGAAAGGCTGTCAAATGAGGTCCTCTTTAACATGTACTTAGGCAATTAATCTTTA
AAACTTAGAAGTAGGGCTATTAAGACATTGGTGAGATCACTGAGAGCTAGAGCTGTTAAA
GAAGGTTTCATAAAGGAAGTAGGACTTAGGCTGCATAGAATTTATTTTCGGTGGAGGGTG
CAAAGGACAGCTTCTATGTTTGTGGGATGGAGGTAAGAAGAGTGATAAGGTGCATGAATA
GACCATAGGATAGGAAATGAGCTTGGGGTGGATATGTGGAGGGTGGGTGAGTAAAGAGAT
AAGGTTCAAGGGAAGGGGTGGGGCAAGATTATGGAGGGCCTAAGAAGCCAGGGAGAGTGT
CTGCATATGATATTATACATGGGCAATTGGAAGTCCCTTCAGCCTTTTTAAAGGAAATTA
TATGTTTTCTGACTGGCCAATGAAAGGCTAACTAACCCAGGAAAGAGCAAGAAGGCTGGG
TGAAGTAATGCAGGCCCCTAGTTCTCCTGTTTCTGGGTTACTGGATAGGTTATATAGCAA
GTAGGCAAGCTTAGCAAACAGCTCAGGGACACTAGGGTGACTTGATGTTAAACTGCCTGA
CTAGTGAGAATTAAATGTAATAAAACTGGAATTGCAAGACTAAAAATGGTATTTAGGCAG
AATAATCTACTAGCAAATTCAGGATTCTTTAGAGTTGAGTGGGAAATATAAGAAAGAAAG
AAAGATGCTGGATGCAGGAGAACAAGAGATGGAATGAGAAAGTGGAATATACACTGGAAT
GAGCAACAGGAAACTTTAGTTCTAGTCTCAGCTCTACCCCTACTAACCGCACAACTTTGT
GCAGATTACTTAAACATTGAGGCTAGTTTCCTCCTCTGCAAACAACCTAGAATACAAACT
CTAAAGGGTGGTAACCAGATCTGCCATGTTCATCATTGTATCTCTAGCCACTGGTACAGG
GCCTAGAACGTAGTAGGGGCTCAGTAAATATTGGATGAATAGATGAATGAATGAATGAAT
GAATGAATCAATCAATCAATCAATTAGAGCCTGCCCTGCCTGCCTCACAGAGTTATTATA
AACTTCAAATGAAATCAGGGATGGGGAAATATTACATGAACTTAAAACACTGAGCAATCC
GATGGTAAAATACTACAGCTACTACTCACAATTATTATTTTAAAAATTAAATTAGAATGC
TATTTGTAGTACTTTAAGGGTGAAATCATAAGTGGGCTAATGATTTCAAGTTTTCTTAGC
ATGGAAAGTAAGAAGAAAAGGAAAAGTATAAGACATTTTGAGTGAGGTAAATAAAACATC
AGAATCTGTGTTCTGAACCATGTTCTGTCATTTGCATTCAATGTGCTTATGGTAGGGCTA
ATATTGATGTCAGTTATAGTTAGCATTCATTGACTACATACAGTAATGCTATGTCCTGTC
TGTCCTTAAAAAAATAGAACACCCTTCAAAGAAGGTAGTCACATTCTCATTTTAGTGATG
TGGAAACTGAGATTCAGAAAGGTTAAATGACTTGCTTTTAAAACCATGGTAAGGTGGTTT
CAGAACCAGACATTTTCTCCCAGATATTTTTGTCTCTGAAGCCAGTGGGAAAAAAACTGC
AGATAAATAGATTTCTTTACACCATAAGGATGCCTTTGCTAAGAGACAGCAATTCAAAGA
AGGAATGGGCTGCCTCCTGAGCTAATGAGTTTCTCATCACTAGAAGCAGGATCGCTATGT
AATTTGCAGGGCCCAGTGCAAAATGAAATGGCCTTATGTCCAGGAAGCTGGTCTTGGCTG
GTAGTTTGCAAGCAGAAGTTAGCCTGCCTCCAGACAGGGATGTCAGAGTGAGGCGTCAAC
ATGAGCTGATGCACAGGACCAGATGGCCTTAATCTGAGACACCATGGCTCTATTCTATTA
GCCCAAGTGAGTTAAGGGAGCATCCCAAGGTAGCTGACATGACACCTTAGAGGCTTCAGA
TGAGAGAGAATTCTGCAAGAGGATTTGGAGAGAGCCATGGTGCCATGATCATCTGGGGCA
CTGAAAACATGCTCTGCCCTATGAGGCAGCACCAACACTCAGAAGAAAACAAAAAAGAAA
CTCTTTTAGTGGAGGTGAAGTGTAATGAAAAGAATTAGACAGATGTGGGTTCAAATCCTA
GCTCTGGCAATTACTAGCTAGCTATTTGCCTTTGAACAAATCACTTAAACCTCCCTGCGA
CTGTTCTGTCATCTAGAATATGATCACAGTACCTGCCTTGCAGAGGTGTGGGGAAGGAGG
CAAGTCAAATGTATCTGGCACTGGGTAAGCCCTCCATAAATGTTAACTGTTGGATGTGTT
TATAAACCACTGGGAGTGGGGACGGTATGACGTTGGGTCATTTGCCTTTCCTTGAGCACT
TCATGGACGAATAGCCAAATCTGCCGGACCCACCTAGGGGCATGGAAACCACTTCCCGCT
CTAGTGCTACTAACTACAGCACTGCAGTAGTGGTAGGCAGTGGGTGAAAGTGGAGTGGGG
CCACTAGTAGACAGTAATAACAATAAACTCTCCAGGAGAACAGGGCCCTCAAGCTTTTCC
CCTAGCCTTGAAGATACTTACAGGAGATAACTGCCCAAGTCCAATCTTGGAATCCTGCTA
GTTGAGTGTTTTACCCACAAGGACATTTCATGAGATCAGGACTAAGAATAAAATTCACTT
CTGCCCAGTAGCGCTATATGCATTAGTCACTTGGAATAGCAACAATCTTAATATCTTTCT
ATTTGTCCGCAAACCCCACCATCCCACCTCTTTAAAATTCCGTGAAATGGCCAGAATAGC
GAAACACCGATACCTACAGTCTTCCAGGAACTTCATTGTCCCTTATGCAACATAGAAGGA
ATGGTATGAGAACTAAGAGAACATTGCAGTAAATCACTGAATTTCTTGGCTTTCTTTTTT
TCTTTTAATAATAAAAAGCACTCCAATCAAACTATTAATTAACTTGTAAAAGGCTCGAAC
TATTATGTACTATTCCCCGCCCATGTCCATGTGCTGCACAGTATTTTCTTACTCAGGACT
GGTGGAACATTTCTTTTGCTTTTGTTTGAAAAATTACTTTTTATTTGGCCTTGGGCTGCT
ATCATAACTTCTGTTTTCACTAGATTGCATAAGCCATACATCTGCACACATTTTTGATTT
GTACAGGGAGTTTGAATTTTAATATCTACCTCCATCTCAATCCCAGCACTTTGGGAGGCC
GAGGCGGGCGGATCACGAGGTCAGGAGATCGAGACCATCCTGGCTAACACGGTGAAACCC
CGTCTCTACTAAAAATACAAAAAATTAGCCGGGCGTGGTAGCGGGCCCCTGTAGTCCCAG
CTACTCGGGAGGCTGAGGCAGGAGAATGGCGTGAACCCGGGAGGCGGAGCTTGCAGTGAG
CCGAGATCGCGCCACTGCACTCCAGCCTGGGCGACAGAGCGAGACTCCGTCTCAAAAAAA
AAAAAAAAAAAAAAAAATATCTACCTCCATCTCAAAAACCTCTCATCTTTTGTCCTTTTC
TTCAAGATTCTGATTTCCTTGTGGACATTCATTTTGATACTTACATAAATCTTGCGTTAT
TCTGCCCATTTTTTTGGCTACAAGGCTCAGCTTTGGGGCTGTAAATGTGAAATATTTCCA
TCATAACCTATCTTTGGGTATGGGAAGGTCCTTAATGCTGGAAGTTCATTGAATAATTGC
TTATTTTAATAGATGTTTATTTTCATTCACATTTCTCCCTCCACTCCATCCCTTGGGGCA
GTACCCTAGTTCAGTTCTTCATCTACTCTCTCCTTGACTGTTGCGATAGCCTCCTAAGCA
GCCACTCCACATCCAATCTCCCCATATCCAAAGTATCCTAAACATAGCTACCAGTCATCT
CTCTAAACCACAGCTTTTGGCAAAAGATCCTTGAAGTTTCCCTTTTGCCTAATGAGTAAC
GTACTAGCTCCTCATCCTGGCATTCGAAGCTCTCTATGTTTATATGTTTGCCTATCTTTC
TTTTCTAGCTTCTTTCCCATTACTTGTCTTCATATAGCTTTAGCTTTTGCCAAACTACCA
TTCTTTGTTTTCAGAGCACACCCTATACTTTCCAGCATCAGTGTTTCTTTCACCTGGAAT
TAACTGCCTGCCACAGAGATCTCCGTTCATCAGCCTATTCATTCTTTAATGCCCAAGACA
CATGCCACCAATTTCATTTGAATGGTCCTTAGAGACACTGAAATGGCGCCTGCTGTACAC
CTGTCCACCCCGACCCCCGCTGCCCACTGCTGGCTCTTCCAGTCTTGTGAAACATTAACC
TCTCCTAACAGCAAGCACAGCTTCTCCATGGAGAAGACACTGAAATGGCACCTGCTGGAC
ACCCGTCCACCCCCCACCCCTGCTGCCCACTGCTGCGTCTGCCAGTCTCGTGAAACATTA
ACCTCTCCTAACAGCAAGCACAGCTTCTCCATGGGGCCTGCTCACTCTGGCCCTCTGGTC
CCACCATTTTTCTCACTACTCAAATAAAGTTTGGAGACCTTTCCCTGATTTTTTAAGAAC
ATGCTAGCTCTTTCATGATACTCGTTATCATACCACCCCCATCTGCTTTTCAGTGTACCT
TTCAATTGCTCTCTAGTGCTCCATTCTTGATTGTTTTCCATTTCTATTATAGCTTTCAAG
GAGAGTCTTTACTTGGCCTACTTTCTTAGTAAATGGTTTGGTGAGATTCTGATTTTCATC
TGATATTAGTTCTTTCTCCAAGAGGGACAGAGAAAAATGAAAAAAATATCTTGCCTTCAG
TTATTCTTTTCATATTCTTTGAAATTCTAAGCCACAGAAGTCTTCTGTTAGGCTGCTACC
TTCATTTTAAGAGACTCTTCCCTAAATGACAAGTTGTGACTTCAGAACTAGAGAACATGG
CAGCCTCTCATGGCTTTCTGCTCAGTCCCATTTTTCTTTTCTGGATCCACTCTGGGCCTT
CTTTAAAGTGACTCAACTTCCCAAGCATGTGATTTTACGTATTTTAAAAACAAGTCTGGT
TTTTTCAACTCATTCTGAAATGTGGCTGCCAAAGTAAATACCCAACCCTCTCTAACACAT
ACATACCCTCTTTCACACACACATGCACATGCACATGTAAACATACTCCTGAAGGATGAG
GAAAGCAATGGTAAATGGGGAAGCAAAAAAATACCATTTTTCATTTAGACAGACTTCCCA
ATACCGATGAACTAAAATTCAGGGATTTGTGATTTGAAATGCATGGATGGCCTTTGAGTG
GTCTTTTCAAATCCCCTAAATATATCAATTTAATAACTTATTGAGCATCTACCATTTGAT
GAGTGCTGTGTAGATGTATCTATATATATCATAAAATTTATTTCTTGCTACAACTTTTGG

AAGAGATTATTATAATCCTCATTTACAGATGAGGAAACTGAGTCTCTGTGAGAGGAAATG
ATTTGCCCAAGATCCCTCAGGCAATAAGTAGTAAATCTGACTTTGAAACACAGGTCTGAT
TCCTTATCCCCATCAGATTTCCCAGGCATTCTGGTACAGGTGGTGTTTCAACAGATGGGA
ATGGGGCAGGGTGAGAAAGATAGTATGAATAAAGGTACAGACATGGGAAAATGTAGCTAC
ACATAGGACAGTAGTAGGAATTAAGGTTGCAAAATTGTGTTAGGGACAGAACACAAAAGG
ATTAGAAACTTGAACTTCCTTCCATTGTCCTGAAGGGACAATAAAAGATTTTGTAGTAGG
AAAACAATATAATCAGAGAGCCAAATCAAGAATGCAATCCAATTTACAATAGCCATACAA
AAAAAATTAAATACCTAAGAATACATCTAACCAAAGAGGTGAAAGATCTCTACAGGGAGA
ATTAACATACACTGCTGAGAGAAATCAGAGGCAACACAGACAAATGGAAAAACATTCCAC
ACTCATTAATTAGAAGAATCAATATTGTTAAAATGGCCATACTGCTCAAAGCAATCTACA
GATTCAACGCTATTCCTATCAAATAACCAATGTCACTTTTCACAGATTTAAAAAAAGATT
TTAAAATTCATATGGAATTTAAAACGAGCCCAAATAGTCAAAGTAAACCTAAGCCAAAAG
AACAAAGCTGGAGGCATCACATTACCCAACTTCAAACTATACTACAAGGCTACAGGAACC
AAAACAGCATGTTGCTGGTACAGAAAAAGACATATAGACCAATGCAACAGGATAGAGAAT
GCAGAAATAAAGCCACACACCTACAACCAACTAGTCTTTGACAAAGTTGATAAAAAATAA
GCAACAGGGACAGGACTTTCTATTCAATAAATGGTGCTGGGATAACTGGCATATGCAGAA
GATTGAAACTGGACCTCTGGCTATCACCATATATACAAAAATTAACTCAAGATTGATTAA
AGACTTAAATGTAAGACTTCAAACTATAAAAACTCCAGAAGGAAACCTGGGAAATATCCT
TTGGACATCAGCCTAGGCAAAGAATTTATGACTGAGTCATCAAAAGCAATTGAAACAAAA
CCAAAAATTGACAGGTGGGATCTAATTAAACCAAAGAGCTTCTGCACAGCAAAAGAAAAT
ATCGACAGGCACCCTACAGAACTGGATAAAATATTCATAAACTATGCATCCAACAAAGGA
CTAATATCCAGCATCTATAAGGAACTTAAATCAACAAGCAAAAAACAACCCCATTGAAAA
GAGGGCAAAGGACATGAACAGACGCTTTTCAAAAGAAGACATACAAGTGGCCAAAAGACA
TGAAAAAATGCTCATCATTACTAATCATCAGAGAAATGTGAATCAAAACCACAGTGAGAT
ACCATCTCACACCAGTCAGAATGGCTATTATTAAAACGTCAAAAACAGCAGATGTTGAAG
AGGTTGCAGTGAAAAGGGAATGTGTATACATTGCTGGTGGCAATGTAAGTTAGTTCAGCC
CCTGTGGAAAGCAGTTAAAGATTTCTCAAAGATCTAAAAATAGAAATACCATTTGATCCA
GCAGTCCTCTTACTGGGTATATATCCAAAGGAAAATAAACCATTCTACCAAAAAGACACC
TGCACTTGTATGTTCATCGCAGCACTATTCACAACAGTAAAGACATGGAATCAACATAGG
TGCCCATTGATAGTGGATCGGATAAAGAAAATGTACACGTACACCATGGAATACTACCCA
GCCATAAAAATGAACAAAATCATGTCCTTTATGGGAACATGGATGCAGCTGAAGGCCATT
ATCCTAAGCAAATTAATGCAAAAAAAAATCACATATTCTCACTTATAAGTGGGAGCTATA
CCTTGGGTACACATGGACATAAAGATGAGAACAATAGACATTAAGGACTCCAAAAACTAT
CTGGGTGATGGAATCAATAGAAGCCCAAACCTCAGCATCATGCAGTACACCCTTGTAATA
AACCTGTACATGTACCCCCTGAATCTAAAATAAAAATTAAAAAAAATTTAAACAGCTGCA
TTTTAGACAGATTAATCTAATTCAAGTCTGAATCTATGCTGTTGACAGTATTTTTAAAAA
TTCTTTCCTAAGAATTCTTTATTCACTCCAGGCCTATGCTTTGGTCAGAATTCTCTCTTT
GTTTTTCAATACTTTAGTGAAAATTCAGAAGGCCATGTGGAAAAAAACAGAGGAAACAAA
CCACACACAAACACAAGATAATTATATACAAGTCTGGGAAGAAAGAGCACAATGGGTCAT
GACGAACTTTATTGAACAGAAGGCGAATAAGCAACGTTCCAATTGTACAGGAAAAAAAAA
ACCCAGCAACCCAAACACCACCACCAACAATAAGAAACAAAGGCAGCCAGGATCTGGGAG
ACATGGATGAAGCTTGTGAAAATGACACTTTCTTAAAAGAGCAGAAGAGAAACTAAAAAG
GATGAAGGGGAGAACCATGGATTTGAGGGTTAAACTAAAAGGAAAAATAGAAAGGAAATA
GGAAGGAAGGGTGTAACTCATAGCTGGTTTTGATATATCATCCCAGTCTGACAACCAAGG
CAGTCTCCCTGCATTAGAAGGCCATGCTAAAAGTAGATGAGTTGTAAACAAGCCTCCCAC
TGGTCATTAGTTTGGCAAATTAGTGGGTTATCTCTTAGAGTCCTCTCTTTTTCTCCTTTC
CCTTTCCTCTGCATTCAATTTGTCACTCATTCCTACAAGTCTACCCCAGAAGGGTTGCCT
GAATCAAAGCCCTTCTCCCCATTTCCACTCACGGGGCCACTGTGTTAATTAAATTCCATG
TCGTTTCTCAGCTGGACCAGGCTTCCTGCCTCTAATTCGTTTCTTCCTCCCTCTAATTGA
GGCTTAGATCTGCCCCAGTATTGAGCTTCCTATACTCTCTTGCTTATAAACCTTCTTTGG
TGGCCCATGGCCTATAAGGTACAGTAGAACTCACTCAGTTTTACATGATGCAGTGGGAGT
GGAATCAAAGGGTTCCCCTAGACCCATGGATGAAGAGACGACTAAGGAGCTAAGTCTGGG
ATGAGAAACTCTTGCAAAAAGCCAACTAATAGCTTTTTCCTCCTCATCTCAGAACCACCC
CTCTTCCCAGAGTGTGTGTATACAAGATGAGTTACTTAAGATTGCACCCAACCTGATGGT
AGATTGGAGAAGGCTATCTGTCCCTTTCTCAGTTGAATATATGAGGGTCTTCTTCCCAGG
AGGCTTGCCCACACCAGTCTTCTTTCCCACCATGGCCCATCCCCTTTCCAAAGAGTATAA
ACTTCATACCCTCCTCCCTTCAAGAACTCCTATCTTTGCTTAGCAAAATTTGTTTCCCAA
GCTTGGAGAGAGGAAAGGGGCCTTTTTCCAAATCAGGGGTAAATAAAAAGTGGCAGAATC
TTACAGTAGACATCATATTAGAGCTCATTTTTATGAATTCTTCTACTGTATATAAGGTCC
TCACTTTCTTATTACTCAGCCTACCACTCCAGTTTCATCTTCCACTTTTACTGCTTCTCC
CCACCCCTTCACAATCCTGACATACCCTAAACTCCAGTCATACTGAACTTTCCAACATTC
CTCAGATAAATCAAGCCTTTGCAGAAATTCATGCACAAGCTGTTCCTTCTGCTTAGAACA
TCCTTACTCTTCTTATCTCGCCAGTAAATTCCTACTCTCCTTTAAGACCCTGCTCAACTA
TTGCCTCTTCTGGGAACTCTTTCCAGACTGTTCCAGATAGAATTAATTAATCGTCCTTTG
AGCTCTCATAGCATTTTTCCATATCCCTTTGATAGCACCATCCTTATCATGGCAAAACAA
TATAACAACTAAAGGGATGGGCTCTGGAGGCAGACTGCCTGGGTTTGAGTTCTTACTCTG
CCACTTATAAGCTATGTGATGTTGAGCCAATTACTTCGCCTTTCTTTGCTTCAGCTTCGT
CATGTATAAAACAAAATAAATAAGATGATACTTGTAAAACCTTTAGAACAGAACCTGACA
CATTGCAAATGTTAGTAAATATTATACATCATCATTTCTTCTATTGTTTTATTGTTATCG
TATATATAACCTACTTGTATATGTAATATACATGTGTTATTATATAGCACTATTATAGCA
TTACTTAACTTTTGCTGTGTCTGTGTCTTGTCTTCCAAGCTGGTTTATAGTGTTTTGAGG
GCAGAAACCTTGTATTACTTTCTCCCCCATAGTGCCTGGTGTTAAGTATGGGCTCATTAA
GTATTTGTTGGGGTCAGTATATTATACTGAAAAAAGCACTGAAATCTGAGAGATTTGGGT
TTGGATCTTGATTCTGCTCATAATTGTTGTATGATCTCAATCTTCCTGAGCCTCAGTTTC
CTCATCTATAAAATGAGGATGATAATAAGAGTACCTACCTCACAGGGATATTTTAAGAAT
TAAATAACTTAGTACATGTAAAACCTAGCACATAATAACATAATAAGAACTCACTAAATG
GTTTTTATTATCAGCTTTGTATCCCTTTGCATTTAACTTAATAAATACTTTTACTTGAAT
GAATGAACCCAGCCATTTTCTGGAACTTGAGGATCAAGGGAATCCAGGCAGATACTCTAT
TTGTTCCTAAGAATCCAGAGGAAAATGTGAGTCATCTCCATTCTGTATCATGGCTCACTC
TGAACTGGGTCACCATCCTCCAGAAGGTGACAAATGTGGCTGGCTGAGTCACTATAAGAG
CTTTTCTGTAAGGATTTGCCCGAACCCCCCACCATAGCTCATTGGACATAGTATCTTTCA
TATTAGGTGCGAGGTGCATACCCTTCCTGAAATGGGCAGAATAAAGAGGTGCAAAACTCC
TGGGGAATCAGAGTGGCTATGAATTCACTCCTCTTTCTTATTCTGTTGCCTCTTCCCTTT
GTTTGGTTTATTTGCTAATCAACTTTGCCCACACTCACATCTAGTGTAGCCCAAGTCTGT
GCAGAGAAGACACTTTGAAAGCCTAAGACTATCATAGTTCCAGCAGAATAATAGAAAAAT
AACAAGTGTTGACTATTCAGTTCTGATGTAGCACTGTATTGAAGAACATAACATTCTAGA
TCCAGATGGCCTGAATTTGAATCCCAGCTCCACCACCAACTAGCTGTGAGACCTCAGGCA
AATCACTTAACGTTTGCATCCTTTCATTTCTCCATCTATCAAATAGAAACAATAAAAGTA
CTTACCTACCTCTTAGGGTTTTGTGAGGGTTAAATGAGTTAATACATACAGAACACTTAA
AAGAATGTCTGGCACATAGTAGGTGCTATATAAATATTAGCTTTTAAAAACAGCTTTATT
GAGATATAATTCATCTATGTGAATTATATCTCAATAAATTCACCTATTTAAAGTGTACAG
TTCAGTCATTTTTAGTATTTCACAGAGTTGTGCAACCATCACCACAATCTAATTTTTAGA
AAATATTCATTACCCCAAAAAGGAATCTCATACCCACTGGTAGCCGTTTCCATCCCTGAA
GCTCCCTCTCCCCCTTCCCAGCTCCAGGTAATCACTAACCTACTTTCTGTCTGTATAGAT
TTGCCTCTTCTGGACATTTTATATAAATGAACTCATACAATAGGTGGTCTTCTGTGACTA
GCTTCTCTCCCTTAGCATAACTGTCTTTTAAAAAATTGCAACTTCCATTTTAGATTCAGG
GTGTACATGTCCAGGTTTGCTACATGGGTATATTGCATGATGCTGAGGTTTGGGGTACAG
CTGATCCCATCACCCAGACAGTGAGCATCCAATAGGTAGTTTTTCGGGCCTTGTCCACCT
CCCTTCCTCCCCTGTCTAGTAGTCTCCAGTGTCTATCGTTCCCATATTTATGTCCATGTG
TACCCAGTGTTTAGCTCCCACTTACAAGTGAGAACATAGCATAATGTTTTTAAGGTTCAT
CCATGTTGTATATATATCAGAGCTTACTTCCTTTTTAAGGCTGAATAATAGTTCATTATA
TGAATATATCATATTTTGTTTTTCCATTTATCAGTTGATTGACATTTTGGATTGTTTCTA
TCTTTTGGCTAGTATGAATAACACTGCTATCAATATTCATTTACAAGTTTTTGTGTAAAG
ATATGTTTTTAAATCTCTTGGGTATATATCTGGGAGTGGAATTGCTGGATCATATGGTAA
CACTATGTTTAATGGTTTGAGGAACCACCAAACTATTTTCCAAAGAGCCTGCACTATTTA
AAATTCCCTCCAGTAGTGGGAATGATGGTTTCAATTTTTCCACATCTTCATCAACACTTG
TTATTTTATTTTATTTTTATTTTATTAAATTATTATTTTTATTATATTTATTTTATTTTT
TATTTATTTTTTATTATAGCCATCCTACTGTGTGTAAAGTAGTATCTCATTGTGGTTTTG
ATTTGCATTTCTCTGATGGTTAATGAGGTTGAGTATCTTTTCATGTGTTTATTGACCATT
CTTATGTCTACTTTGGAGAAATGTCCCTACAAATTCTTTGCCCATTTTTAAATTGGGTTG
TCATTTTATTACTGAGTTGTAACAGTTCTTATATATTCTGCATGCAAATCTCTTGTCAGA
TATATGTTTGTGAATATTCTGTCATTCTGTGTAATTTTTTTTCATTTTCTTGAAGCTATT
ATTATTAAATAGAAATTTGTAAGTTCGCATATAATAAAGATGACAGATTGTATTAGAAAG
AATGAGATCTAAAATCACCTGATGCACCCTATGGTACACTACCCTGCTCCTGCACTCCTT
TGAAATTAAATTCAAAACAGATCAAGCTTCTCCTCTGTGCCAGGCACTGTGGGGGAATAA
GACCTAACCTCTATTCTCAAAGAGCTTCATAACACAGAAGAGAAGATGCAAACATGTGGA
AACTTAAATAATTATAACATGTTTAAATAGCAGTTCAGGACCACAACAGAAAAGATGCCA
CAGAGCACTATATGATTAATTTCCAAATGAATTGCATGGAAAAGAATTGCTATGGGAACT
CAAGGGAGGGAGAAAGCACTTGGGGCTGAGGTGGTCAGGGGAAGCTTTAAGGAGAAGGGA
TCTAAGCAGTCTGTAATTAACTACCGCCATGCCTGTGTAATACTAAACAAAGACTTGGAG
AGAAAAAAATATTGACTGTTTTTGAGGTGAGGCATCTTAGTGATTAAATTGATACCACCA
TTTCTTTTCAACCTCTGCAGGAATCCCCACAGTCCCACCTTTCCAATGAAGACTTACGTG
GCCTCTGTCTAGTCCAGAGGTTACCAATTAACAAAGTTAACAAGCCACATCTGGCTTACA

GACACGTTTGGTTTGGTCCAATTAAAATATTAGAACTAGCTGCTAAAACATTTAACTGGA
CAAATTTCACATAAAATATTAATTTCCAGTTTCTCTAGAAAAATCAGAAGACCTGGCTAC
CCTGCATTGGAACATGCATTCTCTGGGACCATGGCCAATGGCAACCCTGCTTGCACATGC
AGAATTCAGTTTGAGACTTCCTAGAATCATAGTTCTTAGACTAAAAGGTATCTTAAGAGT
GCATCTGTTTCTATTCCCTGACTTTGGGAATGTATGGTATTATAATATTACCCTCAATTT
ACAACTTGATTTAGAATATCAACTAAAACCCACAGAGAATTTGACTTGCTGCAGGCCACA
GGGCTATTAACTGGAAGAGGGAGGGCTAAAACCCATGTTCTCTACTTCTCAACGGAATGT
TCTTTCAACAATCTGTCTTTACAGTCTGGATGCATATTGTTTAGTAGCATTCTTCTGGCA
CTTCCTAATCACTGCTTTGTGTTGTTGGACGATTTTTGATATGATCGTTATCTCCCTCTC
TCTTTTTTTTTTTTTTTTTTTTTTGAGACGGTGTCTCACTCTGTCGCCCAGGCTGGAGTG
CGGTGGTGCGATCTTGGCTCACTGCAAGCTCCGCCTCCCGGGTTCACGCCATTCTCCTGC
CTCAGCCTCCCAAGTAGCTGGGACAACAGGCGCCCGCCATCACGCCCAGCTAATTTTCTT
TTTTTGTATTTTTAGTAGAGACGGGGTTTCATCATGTTAGCCAGGATGGTCTCGATCTCC
TGACCTCATGATCTGCCCGCCTCAGCCTCCCAAAGTGTTGGGATTACAGGCGTGAGCCAC
CGCCCAGCCTGATCATTATCTCTCTTAAATGACATTGTAACATCCCAAACTGCAAAGGGG
CCCACATCAAGTTTTTCTATGAAAGCCCATGACGTGGAGCACAATGCTTTGTACTAAATA
GGCACTCAGGTGGTATTTGTTATTGATTGATAGGATTCTGAAGAACTCAGTATGTAACCA
TCCTTCCAAACCTGGGTGTGTTAAAATGTGTTCATTGTTCAGAGACAAAGGTCTGGTACC
TTTAAGAAGTCTCAGCTTTGCAATGAGTTTGTGGGGCAGCTGCTTGGCATAGATACTGGG
GAGGGACAGGGAGGAGATCATTATGTAGGCACATGGCGCTAGGATGTACCTTACTTTGTC
TTGGCTGTTTTCTGAATGTTAAGAGGAAGGATGAAATAATAGCAGATATAGGAAAAGTTA
TTTAAAGCCTTGCCTTAGATCAGTGGTTCTCAGGCGTGAGGATGCATCAAAAGCCCCTGG
AGGGCTTCTTAAAACACAGATTGCTATATCCCACCCCTGAGTTTCTGGTTCAGTAGGTCT
GAAGTGAGTGGGGTCCACAAATATGCATTTCTAACAAGTTCTCAGGTGATGCTGATGCTC
CTGGTCCAGGGACCACACTTTAAGAACCATTGCTCTGCCAAGTAGTTGGCTCAAGAGTAG
CTAATTAGAGAAATCTGAGCACAGTTAGCAACTGGAAGCAAAAAGTCCATGAGGAAACTG
TGTGATGTACGTAGTGGGGAGAGTGTGAACTGGCTAGGAAACCAGAATGGTCAGATTCCA
GTACCAGGGGTATCATATCTTTTTAAGCAGTGTCCAAAGAATAAGTATATTTTAATTCAC
ATATTCCTGATTCATTTTACTAGTGTTTCCCAAAATGTGCACTGTGGAACCCTAGAAGAT
ATTAATAGGTAGGCAATGAGATTAAAAAGGATCCATAGAGAAAGATGTTGGAACATTTTG
AATTAAAGAAAATTGAACAGGATTCTTTACGGCTTCTAAGAGCCTTTAAAATGTGAATGA
ATGAATGCACTGCAAATCTCCAAGGAAATATAGAATATGCAGTATTTATTTTATTAATTT
ATGCAGAATATTCAGAATTAGTTGACCAAAGATCCTTCGTTAACACTTGGCTGTTTTTTT
GTAAAATGTTGTTTAGGAAACCTTCATTGGTTTACACCTGAAATGCCACAATATTTTATC
AATAGATGAGGTTGCTTGCAACAGTGGAAACAGCACTGCACTGGGAGTCAAAACATCTGG
GATTTTGTTCTGTCTCTGCCACTTAGGTCTGCCACAAGTTGGCTTGCTCCCATGAAAAAG
ATGGGTATGATTGAAATTTATCATCCCTTTGCAAATGGTAGCAGCCAGTAGCTCCACCCA
AAATAAGTAAATAAAATCTCACACAATCAATTTTTTTAAATCTAATTTACCATACCCTTA
AGGTTATTCAATCTTTTTGTTTTTTTTTTCTCATGTTACCTTGTGTAACAGGTATTCAAC
CTTCAAAGCCAGAAATATGAGAAGCCTTATGCTGAGAGTTTGATAGAAAGGAAGGGAGGA
AGGGAGAAAGTAGAAAGGGAGAAAAGGAAGAAGAGAGGAAGGGATGAAAGGAGGAAAGGC
GGGGCAGGGAGGAAAGGAAGAACAGCAGAAAGAAGGAAGGAAGGAAAAATAAGAAGTCAG
ACAGTCAGGTCATTGCAGACCATGATTCCATCTGAAAAGAAAAGGCTGAGAAGCATAGAC
ACATCCTGTGTTTATTTGATTCCTGGCTCTAGGAGAAAGTTTTATTTCACCAAGGACAGA
GTCAGCTCGCTTGTTTACCACAGAGTATTGGAAGTGTGTGAAGTGTGTTTGTCCCCCAAT
TAACCATTAGTTGTTTTTTTTTCACTTGAACAAGATACAAACTTGACCCAATGGAGAAAC
ATTGCAAGTTGTATGTGGGAGGATGCCATAAAATATCTACATTGATATTTAAATAAAATA
CCTGCCTACATTAAACGATCTTTGAAAAGAAACTGATGGGATTCAAGGCTGAATTATATT
TACTGAATTCCTACAAGGTACATAAATATATGCAACATGGAGTCCCTTGTAGATCCATGA
TGAAATCATATTTCTTTTAAACACAAGGGTCAATTCAAGTTAAAAAATAAAATAAATGTT
TACAAGCTTTCTGCCTTTTTTAAATTTCAAAGTGAAAATAATATTTAATTGCATTTAAAT
ATATACATATATTTTAATAACTCTGATTACCTATTTGATTGCATAAAAGAATGACGAGAA
CATCAACACTGCTTTTAGCCACAATATTTGAGAAGTAGCAGCCAGAAGTGTTTTTTTAAA
TATTTTATCTTGAAATCAGATCTCATGTACCTAGGTGAACTGAATATGCCCAGAGGATAT
CAGGGGACTCTCCACTACTGTAGATCCAAAAAACAGAGGGCTAAATAGAAATTCCACTTG
CTGTCATCCAGCAAAATAGAGATGGCTTTGCTGAGTCCTTAGATCATGTTTAGTTTCAGG
AGGGGTTGAGCTGTAATGACTAAATTATCTGGTCACTCTAACCAAGCAGCTAATTAATCC
AGTCAAACAAACTTATCAGTCATGAAGGTGAAATTGTTTTTTCATCTAGAATTTGATCAT
AAGGGCTTTCAAGAAGGAAGCTCCTTGAGTCTCCCTTGTAAACCCTTTCTGCAGTATGCC
TTTCTTTCCCACGTCTGTTGAAAATTATTTATTCTTGAACCAGCTTCAGTGGTGAACAGC
AGCTGGTTGCTACCCTCTCATACCCATTTTTTGTCATCTGGAACATGGTTATTCAGCGTA
GCTTCATCTTCAAAAGGTGCCTGATTTACTCTGGGATTGGGAATGGAGAGAATAATTTTG
AAGCTCTTTCTCTTTGCCTGTTTCCTTCCTTCCATGACTTTGGGTTATTTTCTAGTGATC
TTCAGTATTAAAAGGCATACTGACAGCTCTTTTCTCCCAGGCTGATTTTCAGCTTGTCTA
CTGAGCTTTTTTGGGTTTGGTTTTATGGAATGAAATCCCCTTGTTTGGTTTTCCCCACAT
TCACACTGAGGCTGTGAATGTCCACCAACTCAATTCTGAGCTACAATTGAGTAGTGTATT
AAGAATACATGTGACTGCCACATGGGCCCTGGACTGCTGAATTCATGGTCAAATCATTGT
TTTCCCAACCATCATGCTTGGGGGCACCACATCAGCCTACAGTGAACATGGATTAATAAG
TGCTGATTTAGAGTAAATAGTTTTCTGGTAAGTGGTACATTGAAGATTTCTCTTCCCCTT
TCTATGATTTGTATAGTAGCCACAATTTTCTATATCATTATTGCAGCTTCCCAGTACTAC
TTTATGTAGAAATCTTGCACATTCCTACCTCTGGCTTTTGCTCAGGCTACGACTCTATGG
CAGTGGCAGAACCGTTTTAGGAATGTCTTCTGCTGTAATTTTCTCTTTGTCCCACCACTT
CCACTTTAAACTTCTGGTGTATTCCCTAATCAAAGAGGCATCTAAGTTGTCTTTAGTGAG
AAACCATCTATATATACAAAGCCATAACATGTGGATGAAAAGGACATACTGTCAACTCTC
TTGTGCCAGACACCCAGATGTTGGCAAGTAGTACTTGGACCGAACAGCATAGTCGTTGAA
CAGCATCATCTTCCCCTTTTTTAATTCATCCAGATGTTTTGGCATCACAGACACATAGCC
AGATTTCATCCCTGATTTTGTCAAAGGCTTAAAAAAATGTGAAAAAATAATATGCTTCTC
CTCTGATGAACAATTACCAATGAACTCTGTTCAAAAGGGAAAACATAATGTTTCATTTAA
AAGACCAATAATATTGAATTAAGATAAAGTTCCCATGAGAGTATATTTTACATTCTCATG
AATCACGGATGAGTTTATTATGATCCATGCTTGTATGAAAGACTGACAAATAAATTCATA
TTGATTTGACATTTAAAATAGCATGTCTAAAAATGTACAGCATCACATTAAGTATCAAGT
AATTATTCAACTATCAGCTTCAAATGTACATGGGAGAAAATGGGTCCAGAACCCAAAATG
TAGTGAAAGATTTAAATGCTCAAATTTCTACCACAGGTTTTTTTTTTTTTTTTTTTTTTG
AGACAAGGTCTCACTCTATCACCCAGTCTGTGTGCAATGGTGTAATCATGGCTCACTGCA
GCCTCAAACTCCTGGGCTCGAGTGATCCTCCCACCTCAGCCTCCCAAGTAGTTAGGACTT
ACAGTCATGTGCCGTCATGCCTGGCTAATTTATTTTATTTTTATTTTTGTAGAGACAAGG
CCTTGCTTTGTTGTCCAGGTTGGTCTTGAACTCCTGGCCTCAAGCAGTCCTCTCACTTCG
GCCTCCCAAAGGACTAGGATTATAGGCGTGAGCCACTGTGCCCAGCCTTCTACCACAATT
TTTAAAGAAGCCAATACCATACATTAATCCATTTACAAGGGCTCACGCAAGAGTGCCTTT
TGAGCCTGTGTTTGACAGGAAGTTTCTATTCAGATCATTGTGCCTAGAGTATATAGAGGG
GTTACAGCAAAGTGCTGTACTTTGGGTTACAGTAAAGTGACTCAATTGATGTACCAAGAT
ATTGATCATTTACACCCTTTAGGCCTGAAAAATATACTTTTCTTTGTGTTCCATGAAACT
AAAAAGGCAGGGAAGCATTAAAGAGACAGCAATAAAAAGATTTGATGGTATGTATATGTG
AGTTTGAGTGTTTGAGCTGGCGTTGTGAGTTTGTGGGTGGATTAGAAAGCATATGGCTAA
AGACATGTGCAACAGATGCAAGGGCCTGGGCTTTGGCCTTCCCTTTGAAACTTCTTTATG
GGGACATGTGCTGCCTTGCTTCCTGGATTAAGACCACATTTGCACAATTTGCACATTGTC
CCTCTTGGGCCTGCTTCCCACCGGAGACTGCATCTTCCACAAAAACAAGGCAAGAAGTAA
AGAACTTAAGAGAGCTGATTGTGAAGTCCAACTACATGGGTTCAAATCCTGGTACCATCA
TTAATTAGCTGGGTCATTTTGGACCACTTAACTTCCAGGACCCTCATTTTCCTCATCTAC
ACAAAGGGCTAATAACAGTATTTACCTCATAGTTGTTATGTAAAGTGCTTATCCCAGAGC
CTGGCAGAGAGCAGACACTAGTTGATCATTATGATTATTGAGCAATTACTATGTACCAGG
TGCTTTATTTTCATCTCTGATCCTCACAACAGCCCTACAAGGCAGATACTATTATCTTCA
GTTTACAAGTGAAGAGGCTGAAGCTCAGTGAGATAAAATACACTGCTCAAGGTCACACAA
CCAGGAAGAGCAGAAACCAGGCTCCAAGTCCCTCTGTCCCTCACTCCTAAGGCCTGTGCT
ATTTGCCATATCATCATCCCACTGGTGCCTCTAAAAGTTTCTTCTTCTAGGGTTCATCCC
TCTTCTCTCTTCATCCTCCTCTTGGCCTACCCTGGCCTGAGAACTGCTTGGCACAATCCT
ATACAGCAGGTCCTTGAATAACATTGTTTTGTTCAAAGCCATTTAGTTATCACGTTAATG
GGGAAAATTTGGTTTTCCCCAGGGCCACTGTCCATGGACAGGATGGCGTCCTGACCAGGG
TGGTTCCCACCTTTTGTGCTCCTTGTGCTGCCAGGATGGGCTCCGGCCACCTGTGACCCT
GAACTGGAATCAGCGGGTTAGAAAATAACTGAATGAGTGAATACAAATTATTGGAAAATG
AAAACTCGTAAAGTACACAATGACCATACGAATCCATGATAATAAATGACACGATAGGAA
AGCGCTCAATGAGCGCTTTTGTGATTCTTCTGGAACTGCTGGGTGGCAGGAGGTGCTCCT
TATGATTTTGCTTTGCAAATGTTTATTCCTTGACCTGACCCGCCATTGCTACAACCGCTG
TCACTCATTGATTCACCAAAAATTAGGCAAATAATTCTCTTACTTGTTTTTATTAATCTT
TCCTAAATGTATGTGTAGCTCACACTTATTGTTAATGTTTAATTAACTTATTAATGTTTA
ATATTAGAAGTGTTTGGGTGGTTTTTGTTTGTTTGTTTTTTTGTTTGTTTTTGAGACAGG
GTCTCACTCCTCTCGCCCAGGCTGGAGTGCAGTGGCGCAATCACGGCTCACTGCAGCCTC
AACCTCCCAGGATTCAGGTGATCCTCCCACCTCAGCCTCCTGAGTAGCTGTGACTACAGA
CGTGTGCCACCATGCCTGGCTAAATTTTTGTATTTTTAGTAGAGACAGGGTTTCACCATG
TTGCCCAGGCTGGTATTTGGATCTTTATTTAGAAGTTTGGTGATATTTTTGTGACCAGAA
ATATGCTGTGGGTACTTAACTCTTGTTTATATCAATTAGCCTACAGTAAAATTAGTTTTG
TTATTCGTTGTTTCACTTAAAGTCCAGTTCCCAAGAACCTGTCAAGGACGTTCAGTGAGG
ACTTACTGTACTCAGAGACAGCAGAGAGCAAAGGGCAAGAAGGCTGGTTGGAGGACGAGG

AGGCCTAGAGCCCCTTGCCTGCTTTTGCACATGCATCTGCTCCTGGTGGATGACTCACTT
AACCTCTCCGTGCTTCTGCCTCTTCAACTGGAAAATGGAATCTGTAAGTTGCAGCACTAG
CTTCTGTGACATTCTACTAGGCCTCCAGGAAGAGGAGGCCATCCCTCTATATGTCCAGTC
AGCATATGGGGAACAGTGGGGCTGTGTGTGACTCACCCTGTGGTGTGCCAGCCTCAGGCT
TATACTAGCCTAGGCTTATACTCCCAGATCCTGAGCTAGCAGCTGGAGCTTTGGGATCTG
GAACTGTTACAGGGCCAAAGAACCTGCTTAGCTTCAGGGCTGCTTCTAGATAAACTGCCA
GGATGTGGGCAAAGCATTTGTCATGGAAGGTGGGGAGTGTATGTAGGTTGTTTCAGGGGC
TGCTTTGTGGGAAGCCAAACCTTTTGGCAATGGGCTCAGAACTGGCTTTCCTTTTTGGGT
GTCCTCTAAATTCCGAGGGTGCCCCCACACCAGAGGGTCTCCACTTCTGCCATCCCTAAC
CTAGTGGGGAGATGAATCTGATCTTTTGTCTCAAAGCCCACATTAAAGAGGACCATTGCA
TGCTTCATCACCAGGCTGTGACTCCCAGACCTCAACTCATGTCTTTTTCCCTCTTCACCA
GGGCAGAGGCTAGACTGTTTCCTCTCCTCTCCCTGAACAATAGCAGTGCCCAGATTTTGG
AGGGCATACCGCCTACCTGGCACTGTGCTAAGTACTTTGCCTGCATTTCCTTGGTAATCC
TCATGTAATCCTACAAGGGAAGTACCATTTTGTCACGATTTAACAGAAGAGGATACAAAA
GCTCAGAGCCTGGAGGTGACTTGCTCAAAGTCACATTGATAGTAAGTGGTAAAGCCGGGA
CTGAAATACAGGTCTAACTTTTGAGACAACCTTTTTTCTTTTTCTTTTTTTTTCTATTTCA
ATTACATAATCCTAATCCTGTGCGTGTTTGAAGGGGAGAGACAGGGGAGGGAGTGAGATG
GCAATTTGCTTGTGACCTAAGGAGGCCCTGAAATCTCCTGGGAATGAGGATGGGGGACCT
ACATGAGTTGCCCAGGGCACTGGTAATGCAGCCCACACCAGCAGTCAGTGATCTCCAACA
TGGAGGGCTCCAGGCCTGCCTAGCTGCTGGCTGCTACTGGAGGCCAGAAAAAGACAGCTC
CAGTGTCCTTCTAGGCTGGGGGTGGTTCTTGCCTCCAGGGCTTATGGAAGAACTTATCAG
ACAAAACTCTGCCCTTTCAAGCAGAAAAGTACTTTACAAAGAACATAGAGAGGTGAAGTG
GGTACTTTTCAATTTTTGCCTGATTATCTAATATCTAAATGCTTTTTCTTCACTTTGGAA
TCCTGACCTGACCTGTTTTGTGCCCTGTCCACCCACCACCACTGAGTCAAAGCTCAGGGC
CTGGCATCCAGTATTAGTCATACCTATTTGTTGCCCGAATGAATGGGGCTGAACCCTGGT
CTGACTCTAAAATACATGCTTGAAAAAAATTATTATGGAAATTTTCAAACACATACACAA
AAGTAAAAAATATAGTATAATGAACCCCCAAGTATCCATCACCCAGATTCAACATCTATC
ATGATTTTCCATGTTAGCTTTACCTATCTCTCTTTCTTTTTCCCTCTTATGCTTTACCCC
ACATGTTTCATTATGCAACTCAAAAATAGGATATTTTTATATACAATGACGAAATTATT
ATCCCCCCCATCGCCAAACTAACAATAATTCCCTTCAAACCATGCTCTTGAGCACTACAC
TGGAGTTCCTCCATTCTACTTTCTTTCCCAGTCCCCAACAGGAAGCATTTCTCAGGCATC
TGCTCTTGCACCTCCTCAGAGCTGGAATTACAGTTTGATGTGTATTGGGTACTCACAATG
TGCCCTTTTAGGTAAGCAGGGGGCCAGATTCCCCTCTCCCTGTTTTACAAAGAAGAATGG
CGACACACAGCTATTAAGTGCCTTGTACAAGGTCACACAGTCAATTAGTGGCTGATAAAT
GGGACTCAAGCACTGTGGCTGGGTCTATACAGCCCCAAATACAGTTTGCAAGGAAATTAA
ATGAAGGGACTCGTGTCCTGGGGACAGTACCGTGTGTTTTCTGTTGCTGTTGAAGTCATT
GCACTTAGCCCATCAGGACAGCCTGATTGATGGGATCAGTTGGACTCCTATGTTTGTTTT
CCTCATCTGACTGGATTGCAACAATCACCTACAGGCAGGCTGGCCACACCCGTGAGTTTT
TCTCTTCTTTTTTTCCATGTTCCTGGAGCTGACTCCGGAGCCCACATTTCTCCTGTCTTT
CTAGGCTTGGACTTGCTCTCTGCCAAAGCAGATGACTCCCAGAGCAGCCACAGTCTGGGA
CAGGGCAAATGTGAGAAGCAGAAGGACTAGCTTAACTGCTTTTCTGTTCCTGCCTCCTTC
TTGTTCCAAAGACTTACTCTTAGATGCTTGGCTCTCCTGAGCTTGATCTCCATGTCTAAT
TCTCTAGACCTCTTCCACTCCCCTGATATCCCACCCACATTTTCCAAGTAAGTCTCTCAG
AGCCTTGTATTCCAAAATACAAACTGGCTAGTGGAAAGAAGGAATCATTGCTGTTGGCTA
GTGGATGACTGATCAGTCTGAGTCCAACCAGGAAAATAGAAATCACTGAGTATTTATTTA
AGGGAAAAGGGATTCAGTGCAGGGAATTGATCACACAGGTGATGGAAAAGCTGTGATGCC
AAGCAGAGGACAATGAAGCAACCTAGAGATTAGTCACAGCAGGGAGCTGCTACCATTCCT
TGGCTAGAACCTCAGGGCTGGGACACTTAGTAGAAACTTGAGCCACAGTAACCTGGTCAG
CAGTGCCAGAGCCACAAAGAGACACAGTCACTGCTGGGGACACCACATGAAGCAGAGGGG
GAGGGAGAGAAATACCCTGGTTTCTCTCTCCCTTTTGCTTTCTAATATCCTAACAGTGCC
TTCCATTGGCCAAACCCATCAGAGCCTGGGGAACAGCCTGCAAAGGCCAACCTGCCCCAT
CAGCTTCCCTGCTACAAGAGCAGAGATGTGAAAAGGAAGTAATGGACTTGGGGGGGCAAA
CAGGCCTGGGGACAGCACAGATCTCCTCCTCCCTGCCACCCCCAACTAACTTATTTGGAT
ATGTCAGGGTCTATTTGGAAACAGTGCATTTACTCAACAAAATGATTCTAAAGCTGACAA
GAAAGACCCTTAAGGAAAAGGGTGTGGGTGGAGCACGAACCCGCTTCCTTGACTACCTGC
AAGTCTTAGCATCCAGATTCTAGCCCAGTCCTCTTATTTCACTTCCTCCTGCATGCTCCA
TCCCTCCTCCTCCCACGCATAGGCTGACTCCAACAAAGGCAGGCCGGCCATTTTGCTTTC
TGGCAATGTGTGATGCATTCATTGGCTCCCAGGACTGTGTAAACCCAACCTTCTTGCCTG
CAGGGTTTCAGCTTGGCAGCCAGGCTCTGAACTGCAGTGCTGTCAGGCAGGTGAACCCGC
TGGCACCACAATCCAGAAAACATATCTCCTGATTCCTCAGGTGTTCTACACGTTTGAATG
TGTAGATGAAGGGAGATGGCTGGGAGGGGGAAAAGAAAGACATCAAACTATTTATGTGCT
TTCCCACACCTTATTTCATTTACTCTCCACAACTCCTCAAGGCAGATGCCCTTCTTCCCA
TTAAAAAATAAATTTTTAAATCTTAGAATACTTTTAGATTTACAGGACAGTTGAAAAGAG
AGTACAGAGAATCCCTATGCATCCTTCACTCAGTTTCTCCTGATGTTAACATCTTACAAA
ATCATCTTTTGTCTGTTTTTACAAATGATGAAACTGGGGCTCAGAGAGGTTAGTGACTTG
CCCAAGGTCATAGAACTAGGACACAGCAGAAGAAACAGGCATAAATCCAGTTAGATCTTA
ACTGTATCACACCACCAAGTTGAATTATTTGTTTCCGACTCATTATATTGGAAGTCACTC
TCTGAACCTTGAACCCTTCACTTCACCTCATAGTTGGGTTGTAAACATTATCAGAGAGGA
TTTCTTCCATAGATCCATAGTATGTTAACACTAAAAGATACATCTAAGACCATTTAATCT
AGGGAAGGCAAATAAGTTTCATTTCATTTGCCGACTTTAATTGAGCAGTAGAGGCTGTTT
GGAATTCCGTGCTGAGAATGATTCTAAATCTATGTTTTTGCTCATTAGGAAAAAAATGCT
ATGACTGATTATTGATACCTGCCATATATGTTGACTAGGAGAGTGGTGGAACTCATGTTA
CCTATTAGCCGTTTCTCAGTGAATGTCTCTGCCTCATTAGTGGTACAGCTAAGAACAGAA
CTCAGGCTCCTGACTCATGGTCTCTTGTTCTTTCTGGTTTACAATGGACTCACAATAACA
ACAATAATATAATACAGTGTATCACATAATGAGTGTCTACTCACTGCCAGGCACTCTCCT
AAGCACATTCCACTTTCTGTACCTTACTTTTAATTCTCAGAACAACCCTTCAAGGTAGGC
ACATTCAACCCCAAACTTAGAAATGCAGAAGCCTAGGCTCAGTGAATTTTAAAAACTTAC
CTAAGGTCCACAGTGAGCAAGTGGTAAAACTGGAATTTAAACCTACGCTGGTCTGGCTCC
AAAGCCTAAACCCTGTGGTCTCACTTCCTGCAAACACAATGTTGAATTTGCGCTCACAGC
AGGTGGAGATGACCCTGGTCCCACTGCTTTGATTAAGACTAAACTGGAGTGAAAAAATCA
TTTTTGTTCTTACTAATTTTCTTAAAAAGATTTGTTCACAGTTCCTTACTTAGATGGAAC
TGATTTTTTCGGGCTGTCTCATATTAGATGTGATACAAATTGACATGTTAGGAGAGCTGA
TGTGAAAGGCGACCTGGTTCACTTACAAATAAATGATTAATATAATCTCATTGTGCGGCT
AGCCTGCATTACTGATTCTCTCACTTGGGAGGCTCAGAAAAACAACAGGTCAGACAATAA
AACCCATGCTAGCATAAGACTATAGTGTGGGATGACCATGGATGAGTCAGGGAGAATAAG
AAGATAATTAACTAAGAGGGAGTTCTATGGAGTTCGAAGATAATTAAAAAATGTTCCTTC
TCTATCCTAAGAATATCTGACATTATTAGCGAAGGCAATGAATAAGATAAGGGGAAGAGG
TGAGTTAATTAGAATTATGAGATACAACATTTTAAAGAAATTGACAGGGATCACATTTCA
ACGGAAAGACATTCAATTCAACCGACTTCATTATAGCAATTCAACATTGATTAGGGACAT
GTTATGTGTGTGGCTGTGCTCTGTGTGCTTAGAGGACTACTGAGATGCTGTAATCTTCCT
CCCTGACATTCTACTGAATAGATACCCAAGTTTTGGCAGCGCAGTAAATAATCTCTTACA
CACACATTTGGGTGCAATTTCTAAAATTGTTTCTGCAAAATAGTATTATGTGCCACTGAG
TAGATATCTTGTATGCCAGATATTACTGTTCCTGCCAGGCTAAATGTTCAAACAACATGG
AAGCCTCAGTGATAGCCAATCCATTATTTATCTACCTATATTCACTTCAGTTGCCAAAAC
TATTCTAAATATAAGGAATCCCTTCCTTAGAGGCCCCAATATGGCTTTCCGTATTGTTCT
GAGTCCTTTCTAACTGGTTAATGCCCATGCCATATTGGTTATCATAAATTTTGAATAGCA
TCTTTTCTCTGCTCAGAAGTGTCGCTCAGTAATACCCTACCTCTTCTGACTCCTCCTACA
GTGTAATCTCTCACCAGTCCTCACTACATTCTGAACTGATGTTCCAGCTCCACTTAAATC
TGTCATGTTTTGTTCCTTAACATATGCTATTCCCTTTACTTGGAATACACTCTCTTTCCT
TGTCTGCCTAACAATTGCTGCTTGTCTCGCAAATATTTTCTCTGTGAAGCTTTCTCAGTG
CCCTTAGGTACCCTCATGGCCCTCCTGAATTTATCTCTATTATAATGTTTTATATTTTAT
GGCAAGAGTTTGTTGGATCTTTGCCCACTTGAATGTGAGCCCCTTGAGAACAGGGACTAT
TTTATTTATGTCTGAGTCTCTAGGGAACAAAAGAGGGATTCAGGGCTTATGTGGTAAATG
AATCAATGAATGCTCCCAGTTCTTCGTCATCTTTAGAGGCTACTTCCAGGGCTTTTAAAT
TTGGACTCCCTTTTCTTCCAAGAGGTGGTGAGAGCAGGGTAGGTGGACATGATATTTAAT
GAATTTCTTTTAAGTGCCAGACATTGTGCTGAACCATTTACACAAACAATCTCACGTCTC
AAGTCGATGTTTTTATTTTCATCCTCGTTTTACAGATGAAAGTGGAAAATTCAGACCAGT
GACTTGCCACATTCAGAGGAGTAACAAAGATACATAGCAAGTAGGCTGCAAAGCTAGGAT
TTGAACGTAGGCTCCAATACCTGCATCAGCTCCCTTGCACTGTGCACTCTTCCCTCGCTT
GTGTGTATGGTGAGCAGAGTCAGCTCTGGGCAAAATGGTGAAATAAGCCAAGTTTGTCTT
TAGTGTCTTCAGACTCCCAGTGTGTCTCGGGGACTCAGTACTACAGTGCCACCTACATGC
TGCAACACCTACTATGGCTCCTACAGGCAGCCCATCCTGCTGAGAGCTTCTCCTTCCTAA
CTCCTCTAAGACCTTGACCTCTGGTTTGTGTTTTCTGAAGGTAAACTGCCTTAACCTAAC
CTTTTCCAGAGCACATATTATATTTATTGGTGCCTTCCCAGAAAGCAATCTTTAGCTTAG
GAGCAGGTAGCTCCCACGGGAGGCATGTAGGTGTTTCTGTGTTGTGGTAAGGGGAAGGGG
TTGTCTGGGGGCCTCTATCAAGATACACCATGGAGGCTTCTCCTACAACCAAAGGGTGTT
TGGCCCCTTTCTCAGATGGCTGAGGGCCCTGGAGTCAGATCAATAAGCATTTGAATCTTG
GCTCTGCCATTCACTGATTGCATGATTTCCCACAGTTAATCTCAGTGAGCCTTCGTTTCC
TCCCCTGTAAAATGGATCTAATAATAGTTCTCATCTCCTAGACGTGTTGTGAGGATGACA
TGAGATAACACACTTACGGTGTCCAGTACAAAATAAGCATGTAAAAATGTTAACTGTTCC
CTTAAATCAATTTTTAGTAGTCTTCTGCCTCACCCCCATAACCATCCAGGTCTCTCTTCG
CTTCCTTGTTTCTCTTTTCCTCTTCTCACCAACAAGTCTCTTTCTTCAAAGTCTCCCTTT
CCTTTCCCAACTCCCTCCCACCAGTACGCTCAACCTTTGGCAACCCTCTGTCCCACACTT

TCCAGGTCAATCCCAATTTCAGATAATCTCTCTTGATATCAAACTGCATGACCTGATTTG
AATCGGAAAACAGAGTGAAACAGAGTCCCTTGCTCTGAGGAATGTTGCTATGGTCTATCT
TAGATCAAGAAGACAAGAGCTGCCTTGGCAGCCCTACATCATAATCTGATGAGCATGAAG
AAGGCTCACCTTGCCTATAAATATTTGTTTTTTGCCTATGAGTGATGCCAGAAACCGTCA
TGGTCGGCATACAATTCCTTTGAGTCAAATTGCAAGGTTTGTCTGTCAGGGTTGCCTTGG
TAAGGCAGGGTTATTATTTATATCTGAGTGCAAATTCCTTGATCAGAGCCTTTCCTGTCA
GGGAGAGGAGACGGAAGGGTTCCAGGGAGCCTACTCCCCACGGAAGAGGAAGGCTGTGCT
AGCATGGATGGGGTATGTGAGTGTGGGAGCTCAGCTGACTGAGAGCTGTGTCCCCGGCTC
CATTTTGCCTAGGGGCGAGCCCTGTGTGTTGCAACAGATGTGTGCTTTGGAATCACCAAG
GTTGGGAAGCCAGGCCTCAGAGGGAGTGGCTGGAGGCTGATCTCATCCTGAAATGATGTG
GGCTTGGATTCCCAGAGGGTGGAGAGGAGGACCAGTCCAGAGAGAAGGACATGCTTTTGG
AGGGAGGTCTAAAGACAAAAAGAGGGCTTTAGTTATTTTTTGAGCTTAGGTGTAGGTGAT
GGTTGGGGGTAAGTGACAAAAGAAAATAGGTCCTGAAAGGGGGGTGTCTCCTTTCATGAT
TAGGAACAGAAGAATGAACTCAAGGACTGCCCCCTTTTCTTCAGGAAGCAAACAACTTCT
GAGGGTGTGACAGGGTTCACTGGGTGTTCCCCTTTAAGGGAGGTTAGCCCCTACGTGAAG
CTGCTTGGCTGGTAGAGGATAATTGGGTGTTCCCCAATCCAGATAAGCAGAAGCTCAGGT
GCCCCTCCCTGTAGGCTTTTCAGCTTTCTTTATCACTTGCTAGGCAGGCCCTGCCATCCT
TGGGAGTAGGTATGAGAGAGGGAGTTGAGAGGCCAGGCTTTCCCTGCCCCTGTTCTTCAC
AGAACACAGCTGAAAAAGGAAGGCCAGAGATGTGACACCCCCCAGCCCCAGGGATTCTAC
CACACATTGCAAACCTCACAGAGGATGCTTATTCTACAAAATTTGTGGGCATATTCATAG
GTACTATGGAGCTAAATAATTTGAAAACAATAACAAAACCCATTTACTGATTATTTGGTT
AATAAAGACCTCATTCCTTCAAAGTATGTTGCTAGGAGCCACTGAAAGTGGACTTGACCT
GAGGAGCAGCAAGGAGCTTCCTCCTGCTGTATACACAGACACACACAGACAGACAGACAG
ACACACACACACGCATGCGCGCACACACATGCACATGCCCGGATCCTTCTCCAAACACGT
CCATGATACCAGGGCAGCAGAATTGGGGCACATTAGAGACACAGGCTCTGCCCACATTCT
GAAGCTCCCTGTCATTCCCAGGTCTTGGTGCTTGGCCAGACTCCCCTTACCCAGACTCAT
AGCCTCATAGCTATCATTCCAAAACCCCAAAAGCTCTGGAAACTGAGAGCCTTTGTACGT
TTATGACAAACTCATTTGGCAGCGCAACTTGAACTGACATGAGGTTATTTATAGTCTTTA
TTTTTCACACCCTGTGTGAATATTCATGTGTTTTTGCTCCAGAAATATCAATGTACTTGA
TTACTGGGTACTGCCTCAGACCCCACTGGAGGTGATATATATGTGCTGTATACACCCAAT
CGCCTTTTAAAAGTCCAAAGCATTCTGAATTCCTTCAATTATACTAAGCCTCCCACTGTC
TTATAATGATCTGTTTGGCATGTCTCCCTCATTAGCCTGTGAACTTCTTCTCCAGTGCTC
AGCACACAGTCCAGCATGGGGTGGAAGCTCGACTAATGTTTTAGGAATGAAGAACGAACA
GTCTTTAGTGATTCCGGAGACTTGTTGTCATGTTCTTTCTTACATCTAAACTAAAGTACG
TCATGCTACCATTCGTTTATTCTGTGCCATCAATAGCGAGGAAGAACAGTTGGTCAGGCT
CCTTCATTTTTTTTCCTCTTGAGGCTAAATCGTCCTTATAATCCTAATACCTTTAACCTT
TTCCCCTAGATCCTCTTTTCCAATCCCTTAATCGTCTTCATTGCTGTCTTCTGAAGTCTT
TACAGGTTCTTTCCTTCCGAAATTGTGGCCTAGTGCTTAGGATAGGGCTGCAAAGAGCCC
AGCTATTACACATAATAGGCCTCTGGTTTCTATGGTGCTTTTCCGATCAGTTTCATTTGG
CTGGGCTAAGAAGCTTTGCCTTTAAAAATACACTCGCAGGACAAACTCCAGGGGCATGAA
CAACTTTCACCTGAGCATGCTGGTTGGTAATAGGAAGGGAAAATAGCACTGTCTGATCAT
AAGAAACTGTAATTACGAAAGCTGAAAGCAACAGGTAACACATGCAGTAGGCACCTGTAT
ACAAAAACCACTGTTGATAATTACGGTACCTCAGCCTATTATTTTTACCTTGGGAGAACC
GGCCTTGTTTGTGCGCATCATGTGCCTTTTCTCTCATAAGGTTGCTAAAGGCCATTTCTG
ACCACTTCCCATTAAATTCAGGGAGTGAACCTGACTCTGTCAGGGTTGCTGAAAATCACC
GGATTAAGTTAAACTCACTAATTAAGCCTGTTAGACTTCCTGTAACTCACGATTTTTCTT
GGCATAAGTAAAAACATTTCACTCAGAGGCAAAATGTGTTATATTAATAACCAGGAAACA
AGAAAATACACATAGTAGCATTTTCGGAAGCAAATTCTGTCATCCGCATTCTGTTGAAAC
GGCCATTGTCATTTCTCCTAATAGCCTAAAGTGTTTACTTTTGGGGTGGAAACTATACAT
ACTGTGATGGAGATTCAGAGATACGCTTTTGTCAGTTAAATTCGAAAAATATATTCAGGT
GTGCTTGTTGTGTCCCCCCCCCCCCCAAGCATGAGTCAGAGCCTTTCATGGGGTTTTGTG
TAAATGTATTTTCTAAGAGGTGCTGTTGGGATTTTGCATACAGGTCATTCTTTTTAAAAA
GGTCACGTTGCAATTTTTCTATCCTTTCTTGTGTTGTTATCCCTCAGGGAAGAAGATGTA
GAATGCCAAATTGCAAAGGGAGCTTGTAAAGAGGGGAACATTAAAATTGGATAACCAAGA
GCTCCCAGAGAGGGCCTCAATGGCTGAAAGGAGCCCCTTCTGAGCAAATGGGTAGCAGCG
TAAAAGCCAAATTAACCTGGGAGGAGAAGGTATGGAACTTTTGGAGAAACACTTTGTTTA
CCTCACCATCTTACCATATCCAGCTGGCTGTTTCTCCAAGAATGTTAGTGCTCCAGAAGG
GAGGGAAACCTGTCCCCACTGCTAATAGAAGAGGCTGAAATTTTCTTTCAGAGTTTCCAG
ATGTCCTTGGCAAATGCTAGCAATAGTCATTCTCTCTCTTTTAATAATTATAATAAGCTG
CTTTTTAAGGGAACATTAACTGTGTGGTAGGCTCTATGCTAAGTGTTTTATATAAGGTAT
CTCTATTAATCTTCACTATGATCCTTCCAAAAAGGTATTGCTATTACCCTCAATTTCCAT
AGGAGGAAACGGGCCCAAAGAAGTTAGACAACCTGGGCAAGGTCACCCAGCTAGTAAGTG
GAGGAGCCAGAACTTAAACGCAGATCCACCTGACACCAACACCTGGTTCTGAACCATGGT
GTGCAAGTCCTTGTAAAGTCAAACAGTCTCTTTAATAAGCTTGACTATATTTTCTCTTTT
ACCAAATCACAGAACATCTGGTCTGCTTAATTTTATGAGACTGTAATTACAGCAATGAAA
AGGATGTTAGCCTACTCTGAAAACCCTAGCTGAAGGGCTTTCTATTGCTTTGTTTAGAGA
TTTGTTCTTTTCTCTCCACCTCCACCCCCGACTCCCCAAATTGGATAGTAAGTTCTTCAA
GGAAGGTTCTGGAAAGTGGTTTTTTTTTTTTCTTCAAGTTCTGTGTTACATGTGCAGAAC
TTGCAGTTTGGTTACTTAGGTATACAAGTGCCATGGTGGTTTGCTGCACCCATCTACCCG
TCACCTACATTAGGTATTTCTCCTAATGCTATCCCTCCCCTAGCCCCCCACGCCCCACAG
GCCCCGGTGTGTGATGTTCCCCTCTCTGCTTCCATGTGTTCTCATTGTTCAACTCCCACT
TATGAGTGAGGACATGCGGTGTTTGGTTTTCTGATCTTGCGATAGTTTGCTGAGAATGAT
GGTTTCCAGCTTCATCCATGTCCCTGCAAAGGACGTGAACTCATTCTTTTTTATGGCTGC
ATAATATTCCATGGTGTATGTGTGCCACATTTTCTTTATCCAGTCTATCATTGATGGACA
TTTGGGTTGGTTCCAAGTCTTTGCTATTGTGAATAGTGCCGCAATAAACATAGGTGGGCA
TGTGTCTTTATAGCAGCATGATTTGTAATCCTTTGTGTATATGCCCAGTAATGGGATCGC
TGGGTCAAATGGTATTTCTAGTTCTAGATCCTTGAAGAATCTCCAGTGTCTTCCACGATA
TTTGAACTAATTTACACACCCACCAACAGTGTAAAAGCATTCCTCTTTTTGGTTCTGGAA
AGTCTTATGCTCCTCTGCTTTCTATAGTTCTCATAGTGCCTGGCATGTAGTGTGTGCTCC
ATAAATATTGACTGATTGATGAATGTGTGGATGTTTCATGCAATAGTTTTTGGGTTGTTC
AACACCATTTCTTAATGCTAAGCAAACAGGTTAGAACCTTCAAGATGTCTGTTGTCTTAA
TCTAGTTGCTTGCACTGAGGTAACTTTTTAATATCATCTCTGCACAGGAGCTGAATGCCT
TCTTTGGCACCTGGATGATTGATTGTGTTTTCTCCTTTTCCTCGGAAAGCTCTGGTATTG
CGCAAACGCTGCTTTAGGTGATTAACAATGCCGCCAACTTCACCAGTCACCTTTGCAGAC
AATTCTTGAATTTTATAGGAAAGTGGGAACCTGTTTAAGTATAGTTTCTAGTTAATTCTG
CTTCCCCATCCTGGTTTGCTCAATGCTACATAAAAGATTTTCTTTGTGGAATGAGACAGA
ATAGAAAAAATAAAAATACATTCTCAATGGCTGGGTGAAGAGAGTCCTTGAGATTTTACC
AGCTTGGTATTTCTCACTGTTGGAACTTCAACCCAGGATGCAGATTCATCCTAGTGAGCC
TCACAGGAAACTCTGCCTTGGTCTAGAAGATACAGTGCAAATATATAGGATAGGACAGGT
AAGGTCAGCAGAAGCCACTTTTCAGCACATACCATGTGATTGCTAGAATAATACAAATAA
CAACCATTTACTAGCAGATTACTATGTGGCAGGTACTGTGCTAAGCATTTTCCAGACATT
CATTATTGTTGCCATTTTTCCAGGTGAGCAAACTGCAGCTCAGAGAGTCTAAGTGAATTT
CTTTGTCTTTAAGCAGTATGAGATAGACCCTGGACTTGAACTCAGGTCCATCTGATTCCA
GAGTACATAGTCCTTTTGCTACAGTGCTTTGCAGGGAGCTGTGCTGGCTTCTCAAATGGT
TTGGGGCTATCTGTGTGAGGCTGACAAGAAAACTATTTAAAGGCTGGTAGAAGAGGGCCC
TAACAGAAGGTCTGACTCTCATTTTCTTTCTATTTGCAGAAGATAATTCTTTCCTGTTTC
AATATCAGAAGGGGATCTAGCTGAAGGCAAAAGGCCACAGGGTTCTGGTGAGGCACAAGT
TCAAACCTTGCTGTAGTTGGACCTGTTCTACTGACTATCAACGGCCTGGGTCTGTGGGTG
ATCATAGTGTCAGGGGGCTGGAGATTGGAAGTTCAGAGTGGAAGAGCTGGCATATGCCGC
AAAATGCACCCTGTTGGGATGGAGCAGGAAACTCAACTGCAGCTGAGTTTAGTGGGGCAC
CCAAAGAGAGTGGCAACAACAGGTATCAATTTAGAGAAGCACTTGGCTTTGGTGAAAGCA
GAAGTCCTGCCCAGCCTAGCTGAACTGAGCAGGAGCAGGTGAGGCTTAGGAGTCCTGCCA
GGTGCTAGTAGCAAGGATGGGACTCTAGTCCTTGGAGGCAAGGGTGAAGGAGTTGGGAGC
CTGGGAAGAACCAGACAAAATCCCAGTTCCACAGAAACTGAATGACTAGACTGGTGGCTC
TCAACTGAGACAATACTGCCCCCTAGGGGACTTTTGGAAAATGCAAGAGGGTATTCAGAG
GACTGGAAATGGGGATGCTAGATGTTCTCCAACGCCCACGACAGACTTGCAAAATGAAAA
ATAGTCTAGCATCCCGAATAATTTTTGAATGTTCTGCTGGACTTCCATGGAGCTGAAAAC
TGCTTTTCTAATTATCTAAACTTTTACTATAAATGTTTTATACCTAAGCATAAAGGGTTT
TGCTTCCCCTGCCCCGACCCCACAGTTTTAATCAACTATTCTGTAAAGATATGGAAGATT
ACATGGTTACAGAGGATCACACAGACTCATTCACCATTTCAGGAAATCATGTCAACAATT
GTGATGTTGCTTTTGGAATTAATCAATATAACATATCAGTTTGTGTCCATAACTGTTGCA
TTCATGGGGATTCTATATAAAGGCACAAGCTTCTGACTACTTCATTTAATCATTCAATGT
AGTCCTGTGTGAGCATTTACAGAGTTAAATACATAGTGTTTTATTATGTATTGCTTTATT
TTCTCTTTATGTGGCAGGACATATAGCTATCTTTTTGAACTTATGCGTGTAGGTAGGTTA
TAGTACCTAGAAATTTAATTTCAGGATAGTAAAGGGGTGAAGCAAAAGCAGATTTATTAT
AAAAGAGAGGATGTTAAGTCTGATAAGGTTGAACACCACTGTGCTAGCCTGATGGCTTCG
TGAGGGCCAGGACCATGTGTTTTGCTTCCGTTTGGCCTAATTAACTGAGGTGCTTATAAA
TAATGAATAATGAATGTGGATGCAAGCACACAGCTGGAGTCAGGCATGTAGGTCAGCATA
GTCGGGGTATATAAAACTGGCAGGAGATGGGGACTTTTTTTTTTGAGACGGGGACTTTTG
ATCACCAAAAGAAGGCAGGATCCCAGTTATCAGAAGAGAAACCATAGTTCAGAGTTTGCC
TCAAAGCTAGAGCGTCCTAATGTCAAGGCCCAGGGCTTCCTACAGTCCTCTTATCTTGAG
ACATGCTGGAGCCAAGGGACTGACGTCAGATGGTCCCAGTGGTGAAGAGAGTTGTGTTTA
CATTCTCACTTGCTGGTATCAATCGTAGGGGCCCAAAGACCTCACTACCTAAAAAGAGGG

CTGTTTTCCAGCTTAGCTGTACCTCTTTGCATTGTTTTGTCTGGCCAAGACAGAGAGGAT
ACCAGGGAAAATGAGGCTCGAGTATTGTACGTATAATCTGTCAATGCCCTCCATGCCCCC
ATTAGGATCTCAGGCTGTCTTGGCCTTCAGGCAGATCCAGCTCCCCCATTTTTCTGTTGT
TGCTGCTGCTGTTTTTTTTTTTTTCACTCCCCTGATCCCATCAATAATACGTGTACTCCTT
CCTTCTGGGTCCTGTCCTCAAAGGTAACTGCTAAATATCTGACAAAGGAATGGCTAAAGC
CTTCAGGGCTCCTGATAAGAGAATGGGCTCCCACTTAGCAAGAAGGGCAGGGGGCAGCAC
CTCACTTAGAGATATGGTACATAAGTACAGAGGGAGGTATTGAACCACCTGTTGTCAGGA
GCAGAAGGGACCCTGAGGTCATCTGGTTCACTAGATCCCAAACCTGGCTGTACAATGGAA
TTACCAGGAATGCTGTTGAAATACAGATGCAGTGTGGCTCCTAATCCTCAGAGTCTGATT
CAGTAAGTCCGAGATGAGGACTTGAATTATCTGCATATTATTAACAGTATCACTGGTGAT
TTTGATGAGGAACCCAGTTAGGGAAACCACTCAGACTGACTTTCTCGTTTTATGGGTAGC
CAGCTGAGTCACCGACAGGGATGATGACTCGCCTCGGGTCACAGAGCAAGTCATGGCAGA
GTTGGAAATGGAACTCAGGGTTTTTCCTCCCCTGTATTGGTGGTGGGACGATCTGACATT
ATTAGTGTGGGAAGAAGGACCTTAAATTTCACTGTACAAGCTGCTGCTCACTCTGCCTAG
AGGTAGAGGACAGAGGGCATCTTAAAGGTTGTTTTTCGTTATAATAAGCAGTACATTTAC
TTCAGAGTTTGGAAGTGAATCAACAAAATACCCATCAAGATTAGTAGGACAAGAGGAACT
CGCCAATATCTTTGACGATGTCTATCCATAGAAAGCCAAGTGAAATAATGGAGGTAATTA
TCCACCTATTTTCTTTGAACACTTTGACCGAAAATCTCCTGGTAACTGAGGGAAAATAGG
AGCTTTTAAAAATTGTTGTCTCAAGTAGCCTGAATGACTTCAACTCCCACTTTACTTTTC
TTTTTCTGTGGAGATGCTCTCCCAGATGCTTGTCTCCTGGGGGTAGCAGCTTTCCACAGA
CAAAATGTCCACAGCTTTGGGAAGCATGGAGGGAGAAGGGAAACATTAAGTGAAATGGGA
TGTCAGGTCAAGCTGCGTTGGTGGTGATGTAGTCACCTGTCGTTTCAAGATAACCTTTTG
GATCCTAGATATGTCGTAGCTGGGGCCTTGGGACAAGTCCCAGCAGCCTCCAAGCCACAA
GCTTATCATTTTGGCGCAATTCAAGGCAGCCTCCTTGAAAAGCTCCACGATGGCAGCTGC
TCCATCAGCAACTGGATGAAAACAATGCCAAATCTCTTTATTTAAAATTAGGCACTTCCT
ATTTTGGTTCCTAAATGGCAAATCTATCTGCTCTCTGCCTCCAGTCTTCTGTGTCCAAAG
CTGCCTTAATGTGAGGTATTTCAGCAGCGATCAGCAAGTGTCTTCCTATTTATAAGCAGC
AGAGCTGCCATGGGTCACTGCACTTGTATCTCTGAGCAAGAGAAGTCAGGGGGCCTATTG
GAAAGGAAAAATACTGTGGCCTCCGACAGTGAACCTTGGCGTGGGTCAGGGAAGACTGTT
CAAGTGCACCAGAGAATGGAGCTGTGATGAGTTCAGTTTTGTATCACTGGCATGGAAGTC
AGTAGCCTCAAGATCTGGGAAGAGTTTGGTCACCACTAATTTGCTACATGACCCTGGGCA
AGAGTCGTCCCTTCTCTGGGCTTCAGCCTCACCATTTCTGGGGAAAAGGATGCACCATTT
AATACATGGTGCCATTTACTGTGACAACCGGCCTGCCACCAAAATATAAAGCTGAATCTT
TATCTCACTCCTTATTCCAAAATATAATCCAATTAGGTCAGATTTAGATGTTAAAAAGAG
AAACCATTAAAAGATTAGAAGAAGGCATAAGAGAATATTGAGGTAGGGAGTCTTTGCAAA
GCAACGAGAAACTGTAGAATCTATGAAGAAAAAGAATGATAGATTTGACTATGCAAAAAT
TTAACAACCTGAGTATGGTGAAAGATATCTCATGCAAAGTGGAAAGGCAACATATAAACC
AGGATAAAGTCACAACATAGAAGGAAGACAATGGTTTATTATTCAGAATACTCCACCAGC
TCCAGTGTAAGAAGTGCATTATTTTCTTTACAGCACTTACCATGATTTACAATGACATGA
TTTAGCTACTTGTTTTTCTATTATTTCCTCTGCTTACATGTTAGCTTCATGAAAGCTCAG
GTTTTGCTTACTCACTATTATATTTCTAGTGCTAGACTGGTGCCTGGCATACAGTAGGTA
CTCAATAAATACTTGTTGAAGGAAGGAATAAACGAGCTTCTGTAGACCAATGAGAAGATA
ATGAACAATTCACTAGAAAAATGCTCAAAGAATAATGAACAGGCAATTCACAGAGGAAAG
GCAAAGGGCCTATGCCCTATTAAAAGGAACACCAATCAGTAATAATTAAGAAATGCAAAT
TAAAACAATATTGAGATGATTCAGATGTCAGCTTTTACCTCTCAAATTGGCAAAAATTTA
AAAGGTCACTAAAACCCAGGGTTTGTGAGAATCTGGTGAAACAGCACACTCATTCACTCT
TGATGCACATCTGGAGGGCAACTGGAAATGTTATTTGAGTCAGCAATTCCTGTCTTAGGA
GTTTACCTTTGCAAAATGCTCCATGTATAAGGATGTTCATTTAAACATTATTTGTAAATA
GGAGCAAATTGAAAAAACTAAATGACCTCGGTGAAGGACTGGTTAAATCATTTATAATAC
ATCCCAATAATGACATACTAAGCATTACTATGCTAATGATTAAGAGTTTGAGCCTTGGTT
AGACTGCCTAGGTTTGACTTCCTCCTCTAGCTTTTATGAGCTGTGTGACTTCGACCAAGT
TTCTAACCTCTCTGTGCCGTTTCTCATCTGTAAAATGGGAAAAGTAACAATTCATGAAGT
TGTGAAGAGTAAATGAGATAATTCATATGAAATATTCAGATAGGACCTGGCACAGAGCAT
TTAATAATTGCTACACATTACTATTCCAACTACTAGTATAATGATGACAATGACGATGTA
GCCACAAATAAAGACAAGCTAGATCTTTAAATACTGATGTGCAATGATATCTCCAATATA
GTATTGTGATAAAAGCAAGTTGCAGAAGAGTAAGTATAGTAGGATCTGATTTTTGTTAAG
GGTATTATATGTTACACATACAGGAAAAGACTGGAAGGATAGACAGACTGTCAACACTGG
GGAGTAGAATAGGAGAGGTAGATTTTCCCTTGAGCAATAATTAGAGTTGTTTGAAACTAG
CATGTGCTACTTTTATAATAAACAAAAGATGTAAAGGGAAAAAAAAGCTCCGTTTGGAAT
TCCGAGGACAGTTAATCCCACCAGGCTTCACATCCCCCAGTTCTTGGCCACAGCCCAAAC
GTTAAGCCTCCCTGCTCGTCATCTGTCCTCTCCCATACCATCCTCATACTGCTCAGTGCT
CCCATCCCTGCCACCCCGACTCATCCACCTCCCCACTACCGCCCTCTCTCACTCTTCTCA
GTACCCTCTGGAAGCCCCCACCTTGCAGTAAACAATTCCCCCTACACCCTGGATAAATTT
TACAGAGCACTATTGTCTACCTCCGGGCCTGGAAGTGCTGAAACCTGAGTCTCCACGGCG
GATCTCACGGCTTTGCCACCAGCGCCCTCTAGTGGACACTGTCCACACTCCCACACTACA
GGCACCCTAGGGCTGGGAGGTGGGGTCTGTGCTCTAGCTTCCCCTGTTTGCTTCCAGGCT
GTTAGTCCTCCGTCCTCAGCCAAGAACTCCTCCTTCTCATGCCATCTGGCTACTTCTCTC
CTCGTTGACGCTTCTTTATAACCATCCCCTCCTGGTCAACCCCCCCCACCCCCACATTC
AACGAAGAGTTCGACATCTGGCTCATTGTCTTCTTCTCCACCCCAAGTCCTGACATCATT
CTGGGTGAGTTGGACATCCGTGATCCATGTACAGGACACACTCTCATCCTAGCCTTACCA
TGCTTCATCATCAGTCAGAACCCCCTACCATCTACACCTTGCTGGCTGGCCTCAACGCTC
TTCTTTTTACCTCAGGAGGCTCTCCAGTTTTTTGAGCCCCCTCCATCCATTTCTTCCATC
CCATCAGCCCCTTTCACCTTACTACCTTCCCTCTCCATCCTGGAACATGATGATGATGAT
GATAAGAATAACAACTAATATGTATGTGCCAGGCATATACTACCCATCTTATGACATCAT
TTCAGTAATCTCATGTTATCCTCACAATAACTTTATGAGGAAGATACTATTGTGGTTTTC
CTTTTAATGAGGTGACTGAGGCTCAGAGAGGTTGAGTGACTTACCCAAAGTCCCACAGCA
GAGATGAGATTTGAAGTCAGGTCTGTTTGCTTTCAGAATCTTGCGTATATCCCCAATTCC
CTTGCCCTGTGTCTTTCTGCTGCATCCATGAGGCAAAATCCTAAGTCTGGATAAATTTAA
CTGACAGTTTCCTCCTCTGTGCTTATGCCCAGATGGAGGAACATTGCTGGAGAAAATCCC
ATCACTTTGTTGGTTAGTCCCACCAGAAAGTCATAGTTTCCAACCTCAGCGGGGCCCTCA
ACTCAGCTAAACAATCCTTCTAAGTAACTGTCCTGACAATAGCACTTGCAAACTTTCTCC
ACTGCCCTCTGGAGTCAGACTGTCTGGGTTAGAGTCTGCTTTGGCCACTTCCATGCTCCA
TACCATGCCAGCCTTTAAGAAAGGGTGAAGTGGAGGAAAAAGGATGAGGGGAAAGGGGAA
GTGATATGTATCGAGTGTCTTCTAGGTACCAGCCACTGTGCCAGGTGCTTTGCATGCAGT
GTTTCATTTAATCCTGCCAGCAGCCCTGCAAGGGCAATACTCTTGTTATTTTTGCTTTAC
AGAGGAGGAAGCTGAGGCACAGACAGGTTAAGTCACTCACTAACGTGACACAATTTGTAT
GTGGAGAAGCCATCTCATTCGTTTAGACCTTCCTTATCTTGATGAATTATTGTGACAAGC
TGATAAATGGGTCCCCTGTTACCTATCTCATGCCCTACAATCTACCTTCCATTCTGTTGC
TGGGACAATCTTTCTAAAATGCACATCTGACAGTGCTACTACTCTGCTTAAAGTCCTTCC
AGAGCTTCCCCTCAGTCTTCAGAATGAAACAGAAACCTTTGATCTTAATGATTTGGCCCT
GTCCTTCCTTTCTCACTGCATCCACTACCACTTGCCCATGCAGTCACACAGTACTATTTT
TGGCCACCTGATGACACCTTGCTTTCTTCTGAAGAAAGAAGAAAGCATATATATATATAT
TCTCTGCCTCCCCATCCTCCACACCCTTTCCTGCCCACTTTAAATGCTCCTCTTCTGTGC
TTTCACAGCATTCATGTTTATCCCAGTGTGTTATAATTTCCTGCTGTCTTCTCTGTTTCC
CCCATTAAACTGGAAGCATCTTGAGAACTGGGATTGTGCCTTAGTCATCTCTGTACTCCC
CAGGGCCGTAGCACCAGAGCCATGGTTAAATCTATGTTTGTTGAATGACTGAATTAATGA
ATGAATAGAGTGAATGAGAGAAGAAAAAGCTCTCACTTGGAAAGTTTAGTGACATGAGCT
GTATGGTATCTGATTCTTGGCTGAAGCTGCTGCAGTTTTTCCCTATGACTACAGTGCTAT
AAAAATATGTCTGCACATGGATAAAGACTGGAAGAGAACTTTCCACACAAAACTAAGAGT
TGTGTTAGAGTGGTGAGTGATGGATGCTTTTTTCCCCATTTTCAAAATGTTCTTTAGTGC
TGCTTTTGGATTGTTTTGGCAATGTAGCAGACATTGTAGAAATGGAATTTGGGGCTCCCA
TGGAGGAATCGACAACCAATATGTATTGATGCCCAGTGTACTCTGCTGGGTTCTGGGTGC
TGTGGGGAGATGGGTGGGTAAAGGCGACTCATTCTGACTCAGGTTTGCATGGTCAGGAAT
CTTGCCAATTACGTTGGATTTGCTGGAATAAAAAGATAACTTTCAAGAATTTTCCAAGCT
CCTGGGTTGAGGTATTTCTTTTCTCCCAAAGCTGGGCCGCAAACAAAGATGGCTTTTGTT
TGCAGTAAAATACTCATTCCTTCAATTTTGTCCTGCAAAGTGACTTCTATAGCTTTGGGA
GTCAGGTTTGCTGAGTGGGGAAACTGATTGTCCATGCTTCATTGATTTAGAAATGACCAA
AAATGTTTGCCTTTTCTGGCTCTTGAATAAAATGTTTAGGAATTTCATTGGCAAAATGAT
GAAATTGTTCCCTTTACTGCTGCAAGACTTCCAAAACTATATATCGTGGGTTAACTTAAC
CCAGAAGAGGCAAAAAAAAAAAAAAAATGGGAAGCAGACTCAGATGATTAACACTCTGCC
TTAATTGGGTGCTTACTCACTCTGCCGTGGCAAGGCACTGTGTTCTGGACTTTGGAGATA
TCTCATTTAAAACTCTCAACAGCCTGTGGGTAAGGGACTGTTATTGTTCTCATGGTAGAG
ATGGTCAAACTGAGTTTCATAGAGGCTCAGCTCTGAAATCATGAGACCAGGATTTGAAGC
CGAGTTCAGCTGGTTCCATGGGCACACACTAACCATTTGGCAACAGTGCCTCTAGACTAG
TCTGGCTACAAAGGGACAGAGGTATGTATTTATTTTGATTTAACGAACCGTTGTTTAATG
TTTACCATGTCCCGGGCACATGGTAACTGCTTTAAAAATGTTAGATTATTTACTTCTCAT
AGACAACCCTGTGAGGAGGTACTATTATTAATATCATATGACAGGTGGGAAAATGGAGGC
ACAGGGGTGTTGAATCACTTGCCCGGGGTCACTCAGGCTAGCATGTGACAGAGCTAGAGT
TACAATCCAAGTGGCCATGTTCAGGTGTGTGGCCCATTACCTTGACTCTGTGCTGGTTAG
CATGCTACCCCCTGTGCCCTGTCTGCATAGCTCTGGCTCAGAAACACTGAAGACATGGGC
CTCTTTTTGTAGTTTTGTAACAGATTTGTACTTTTTAAAGTATTTTCCACCATTAAAGCC
ATACGCTCTGCTTCTAAGCGTCCTGTGGGGTTGGTGGGAAGGGTGAAAACATACTCCTGT
CCTTCTATAGCAAGACTAGCTTAGAGGCAAGGTGGCCCAGTGGTTAGAGCATGCCCATGG

TGCCAGTGAACTCAGCTTCAAATCCTGGCCTTATGATTTCAGAGCTGAACCTCTCTGAAT
GTCTGTATCACTATCTTTGCTAAAGGAAGAGGAAGCTTTCTCCCGATATCTTCATTTACT
TCCAATTTTCGGTTTTTGGGGGAGAGCTGGGTTTCCTCCACAGCTAGGCGCTGGGTGGCG
AAGGGCTTTTGGACTGTCACTACTTTTCATTCGACAGAGACTTCAGGATAGCCCCCTTCA
AAGGCAGTCTGTTTGGGGTCATCAGGAACAGACAGAGTGCTCTTCCCTACCGCACTCATC
CCACCTAGTGCCATCTATCCTTACCCACTCTGAATCCCTCCTCTCGGCAGGCACTGCCTC
ATTCCCCAGAGCCAGGCCTAGACAATGGCGGCTGCTCAGCTTGGAGGGAGGGGGAGTGAA
GAGGCAGCAGGCCCAACCTGCCAGCTTGAAAGCTTCATTTGACAGGGGAGGAGGCACATC
TGGTTTGGGTTTTCAATTCAATTCAACAATGATTTATTGTGGGTCTGCCAGCTGCAGAGG
TTCTGGGCTAGGCTAGGCAGCAAGAGATGCAAAGATAAGTAAAATATTGTCTGTGCCCTT
AAAGGAGTTTTCTAGGTTGTAGGGGTGTGCAGACGCAAATGAGCATTTTTTTTTTATGCG
ATAAGCCCTGTGCTTAGCAGTTTACCTTGCCTACATTATTATGTCTTTGCATCTTCCCAG
CAGGTTTTGTGATGGAGGTGGGTAAACTATTATCACTTCATTTTATACACATGAGGAAAC
AGAGCCTCAGAGTGGAAGAGACTGGCCCAAGGTCACATTTTTAAGAATGGACCAAACCAG
TTCTGGAACTATTACATTCTTCTGCTCCCCAGAGGTGATGAAGATGATGATGATGACAAA
AAACACTTACTGAGCTCTGGAGGCCAAGTCTTGTGCTATGCAGGCTGCAGATATTATTTT
ACTGGCAACTTCCAACATCCCATTGAGGCTGATGTTGTTATCCCTTTTTCTAGAAGAGAA
AACGGAGGTAGCCTTGGGAAGTGATGGAGTTGGAATTTGAAACCAGGTCATTCCAGTCCA
CCAGCCTGTACCATAAAAGAGGTAGACACAGGGTGCCATGTTCAGGGGCAAGAGCAATGA
ATTGTGCCTGCAAAAGCTTCATGCCAGAGATGGCATTTACATTTGGCCTTGAAGGGTGAG
ATGGGGTTATGATTGGTAGAGTTGGGAGAGAGGGCATCCCAGTCAGAAGGGATATTCTGA
GCAAAGAACCCAAAGAGATGACTTGTGGAGCATTTTGATTGAGAGGCTGTAGGTTCATCC
GGCATGCAGGCCGGGGACTCTGGGTGTAGAGGAACAATGTGAAGGAAACCAGAGATACGC
CTGATGGTCTAACACATTTTTGTCAAGTTGAGGTTCTTATTTTTTTAATTAAGAGCAGGT
ACTCTGCCCCAAAGGCTTTTCAAATGATACTATCTTGCAACCCATGAGCAAACACACTCG
AGTCTCTCACTGTGTAAATTCTACTGTGCTGAGAGCTGCGGTGAATACAAAAGAAAACAA
CTCAATTCCTCAAGTGTGTGCCGATGGCCCCCGTGTGGTTGAGGAGGCAAGATACACACA
ATCAGGAAAAGCAGCAGCTAGCATAGGCAGTTAGTGATCAGGGCCAAATGAGTTTTTCCA
GCCGTCAGATTTCAAGGAACATGAACGAGAGGAAGGCCGGCAAGCTTAAGGCCGGGTGGG
GCAATCAGTGTAGCTAGCATTAAGGGCTCTGGCCAGGCCTCCCTTCCCCAAACTGCCACG
GTCTCCAACCATGTCTTGACCTCCCAGAGTTTGTTAGGGAAGATGGGACCTTATGGGATC
AGAGACAGCGCATGGGAGCTGGGAAACGGAGCCGAATACCTGATATTCCTGATCCTCCAA
TTTTCTACACTTATTCTCATTTCCTGGCAGCTCCTCCAGCTGCTGAATTGTATGTGTATG
TGTGAGCCGTATTTTAAATTAAGCAGTGAGTTCTTTCAGCTCATCATAAACATGCTAAAA
AGCTTCAGCCGCGGCCTGAGCCCACCCCCGCCCTTTACACACACAGGCAGCAGCTGGGGC
CACTCGACCCCACACTCTAATGTGCATTGCCTATGACTCACCAACAAAAACGACTGTATG
TTTCAGTCTCCCTGGGTAGCTGATTCCTAGTTGTGGTGGGAAATCTCAGTCCGATTTGGA
A**GGCAAAGGGCCTCAGCCCCGCCCTACTGACTGTGAATTCTACAACATATGGAGCGCCTA**
CCTTGTGCAAGGCATTGTGGGTAAGTCCATTCATCTCTCTGGGCTTTCCATTTCCTCAGC
TTCAAAATGCATGTACTAATCCTGCCGGCTTTACCTCCCTCCTAGGGCTCTGTGTCTCAG
CCACACTGGTCTTTCAATTTCTGCAACATTCTCGGTCTTTTCTCGCCCCAGGGCCTTTGT
TCGTGCTCCTGCCTGGACTGCCCTTCCTCTTGCTTTTGGCATGGAAGGCTCCTTCCTAAC
CTTTCAGTCTCAGCTTAACGTTACCTCCTGAGAAGAGCCTTCTCTGACCACCCACAGTTA
GATGCCTCCTGTTCCTCTCTTACATTTCTTTTATTAGCAATGCTTCCTTAACCCCATTTA
CTGCTCTGGGTAATTATGTCTATTTATATTTGTCTATTTATGGTATGTCTCTCCCACTGG
AATGTAAGCTCCAAGAGGACAGGGACCTTGTCTGTGTCACTCAGCAATGTGTCCCTCTCC
TCTAGAGTGGTGCTTGGCACATAGTAGGCTGACATAAGCAAAACCATCTGGAAAATATTT
TCTTCCTGAGGTTCTTGCCAGACATATGGTTGCTGAGGGAAAAATTATGGCAGAACTGTG
TCATTCCATAGGAAAAAAAATCAGGCTAAAAGGGAAGAGTTTGTTGAAAACAGTTCTGTT
TTAGAAACCAGTCCTTGGAGTCTCATCGCCAGACACAGTCTTATGAAATTAAACAAAAGA
AATAAAAAGCATAAAAAGCTAAGTTTTTATTGGGTATTTACTATGTGCCAGGAACTGTTC
TAAGTGTTCTACACAGATTATCTCATTTAATCCTCATCATAACCCAATGACGTCAACTCT
ATTATTAGCTCCATTTAACAGATGAAGGAATTGAGGCTCTGGGATGTTAAGAAAAGTGCC
CAAGGTCACACAGCTAGCAAGAGACAGAACCAAAAGTTGAACTCAAGGCAGTCTGAGTCC
AAGTCCACAAACTAAATCATCTATGCCACCTGCAGCCCCCACCTCATGTTACCCTACACC
TCCCACGCCACCCCCAGGCACGATTCTACTACAGCACTGGCCACATTGCAGAACAGGGGT
TTGTTTACTTGTCTGTCCTTCCTACTAGACGAGGAGCTTCTCAAGTGCAGAGACTGGATC
TTATTCATTTTTGCTTCCCCTGTGCCTGGAAGAGAGCTTGTTCTGGAATAGCTAACCAAG
GAATATTCGTTGAATGAGCAAATGAGTGAGGGAGTAAGTGAATCCATATTCCTTAATATT
TTCAGGCTTCAGGCTTATGGGCTCATGTGTATTTCCCTGGTCAAGCATAGCACTGGGCAC
AAGGGTATTCTGGTGCAAGAGGTGCCTCTCACCACAGGGTACAAGAAGGACTAGCTCTGG
CTGGGTAAACCAAAGCAGTGTTCCACAGAGCAAGGGGCTTGATCCAGAAGGTTTCATTTT
GTCATCGGTCTAAAAAGTAGCTGGACCTGTTTAGGACTTAAACAAACTTTCAGTGTTCCA
ATCCCATCTCAAATCCTTTGCAGAATGAGGCAGGGTGTGGGGAGGTGGTGGTGGGGCAGT
TATAAATAAACAAATACCCACAAACCGGCTGGTTTTTTAAACAGAATTTGCTTCAGTCAG
TGCTGCACCACATTTTCCCAACTGGCCTCCGTCGTGAGAACAAGTGGGAACTTTCATACT
GAATGGCTTTATTTTTATTCTGACCAGATGAATGGCAGGAGTTTTGGCTCGGGAGGCAGG
TTTCCGATCCTCAGCACACAGTTTGCTGCCCTACTGTGCCCCGTCCATCACACACAGCGC
CCTGCACAACATACCCAGTCATTGCATCTACTGCCTCAGCCCTTGAAAATAATTGGGTCA
TTGTTTTATTACACCAGCGAGAGAGCTGCAAGCCTGTTAGGATTATGTCATTGATATTTG
CGTGAAAAGGCAATCATGATATGCTGGTCATTTAACACAGCTGGGAATGCAACTGTGTTG
TCATTGTTTAAGGTTCTCATCCAGCCCCTCTGACTTTTTTGCACAGCCCAGCCCGTGAAA
ATGGATCACTCTGATGTATGTCACCTTTAAAATGCTTTCTAGCCCTTCAAGGGACCAAGC
AGGATTTAACTCTTTTCATGCACTTGGACTTTGCTGCTTGTAGGCACAGCAGTCTGAAGG
TGAGAGATGCTTGCTTTTCATACTTGTGGGAGTTGTACACCCTAGTGGCAGGATGCTGCC
ATACAGTGTCATTTGTTCACTAACTGCTGATCAGAGCCAAGGACAATTTTGGCTCAAGAA
CAAATGAGACCTAATGTCTGTGAGGGAAGACTATGTAGATAAGTCACGGGTCAGAGTTGT
GTTGCGACTTGTATTGTGTGCTAGTTTCAGTAGGACTTGTGCTTTCATTGCAATTGTCAG
TATGCTTAAGCAGGGGTCTCTGCTAATGAAATTCGAGCATATTTGCATTTATCGACAGGT
ATTACTGATGGTAGAACATCTTATTTCCACACAAGGCAATGAGTAACATTGATTATAGAA
GTGTAAACAAAGCTCAAATTCATGTGCCAATTTGTCTATTTCAACTCATCGATCCTCTTC
TTCCACTGGCATGCTAGCCTGAGTTTTATGGCATAAGGCCATTAAAGTATTCTTCAAAGG
AATCTGTTGCAGACACCTGTTCATCCCTCTTCTGGTCCCGGTCCTTGCCAAACCAGCTCT
GCCTTTGCCAACCACCTTGAGGTGTGACAGGAAAAGCTGACCCAATAAGGGAGTCAACTG
AAGGGATGGAGTGGCTTAGATGCAAATGGCCAAATCCCAATGGCCATGGCTATAAGGAAC
ACAGGTTCCTGAAGTTTTCTTGGCAACTAGCTCTAACTGCTTTGCCTTCTGAGTGCTTTG
GTATTTGTTTTATTCTAAGTTCCCAGTTTTGATCTATTTTTGCCTTATGGCTGCAGATTC
CCTTTCTGGAAAATGGGGCTCTGGCTGGGATCAAATCAGAGAACACACCCAGACTTGGCA
TAGTGCCTGGCACAGAGTAAGTGCTCAACAAATGGTAGCTGTTGTCTCTTTTTCCCTGTC
CAGGTTTCTTCTGGTTCTGTTCTCTGTACCTCCAGTATTGGCCCAGAACCAGGAACTGTT
CCCTCCCCTGACTGGCAAGTCAGATTACCTGGCCCTCAGACAAGCTGGGCTCCCTTTTCT
GGCAGCCTTTTGTTGTCCTTACCTGTTGCCATGTGCCACTCAGTGTAACCACAGGCAGAC
CTCTGCCAATTGCTGACATTAACCTATTGAATCCTGGCAGAGAAGAATAAGTTTGCAGAC
TGGAATTTGCATGTCTGGGCCAGATACGAGACCAGGAGGCAAAGCCATATTTACCTGTTC
TCCCCAGTTGTGGCAAATCACCCACATTCTGGTTAAAACCAGATGTTTCACACCCATGAC
TGCAATGCCACACATCAAACAGTTGCTTACAAAGAACACAAAGTGGTTTTCACATCGCAA
ATGTAACTTGCAGGTCACTTCTCAAATTTCCTCGCAGCAGCTACCATGCCTGGGTATTTT
TCCCTTAAAAATGACAACCTTGTTGGGTCTCTTGTAGGGACTTTCATTTGGAAGCCCTGG
GAGAACTTATATGCTCTTTCCAAGAGGGTCTCTGATGGCTTTAATTCACATGGAGTTAAT
ATATTGGAAAAGAAAAGAGATCTTCCCCAAACTCACCTGCTCCTTGAAAATGCTATTCTG
TTGCCTTGGCGAACAGCCAATCCAAACATTAAAAAAGAGATAGAGAATGCAGGAATCCTA
AATGCTATTCTCAGTGCATGTCCAAACAAGAACTGGGGGCTGAGGGGCAAAGAACAGAAA
GAGGAGAAAAGAGTTTTTGTAGCCTAGGCCGTGAGTTGATCCCACCCCGTCATGTCCTGT
ACCCTTCACGCTCATAAAGAAGACAGAGGAGGCCGGGCGCGGTGTCTCATGCCTGTAATC
CCAGCACTTTGGGTGGCCGAGGCAGGCGGATCACCTGAGGTCAGGTGTTGGAGACCAGCC
TGGCCAACATGGCGAAACCCTGTCCCTACTAAAAATACAAAAATTAGCCGGGGGTGGTGG
AACATGCCTGTAATCCCAGCTACTCGGGAGTCTGAGACAGGAGAATTGCTTGAACCTGGG
AGGTTGAGGTTGCAGTGAGCCAAGATTGTGCCATTGCACTCCAACCTGGGCAACAAGAGT
GAAACTCGGTCTCAAAAAAAAAAAAAAGAAAAAGAAAAAGAAAGAGGAAGGAGAAGGAGAA
GAAGGAGAAGGAGAAGGAGAGAAGAAGAAGGAGAAGGAGAACGAGAAGATGTGGTGGCAT
CATGACTTCTCTGCTAGGGTTAATTGCTCAGCCTGAGAGGCTCTCCAGGCTGGCAGGTCA
CACTAGAGCCAAGAGTGAAGGGTCCATTCAGTAAGGTCACTGCCATGATCAGTTCTCAAC
CACTTTAACTCAGTTTTTCTGTTGCTGCTTTGTTGGTTAGTGGTTTGTTTGTTTCTACCC
ATTTGGGAGAAGGGTAGGAAAGGAACTAAGGTGATCAACTATTCCATATGTGTCAGGCCC
TGTACTTGTCTTTTCAAACACTTATTCACACTCTGAGGGACTCTCTCAGGTCCAGCAACT
GTGTAGCTATGATGTGAAATAGCAGCTTCTCTCTATGCATTCACTGGGAATGGTTTGTTC
TGATCACACAATTTTTTCCAGTTCTTCCGACGTTAGGTGTTGCATGGTAAGAAACAGGTG
GTTAATTATTGAGACAGTTCTTATTTGGAATTGTCTCTTCCTCCTTCTCCTCTCAGGCAG
ATGCTAAAGGATGTCTGTAGTTTTTTCATGGCATTTTGCTAAAGATGCACATTCATTAAT
TCATCAAATAGCTTTTTTTGAGACATTATTATGAGTGAGGTGTTGTGCTATGATATGCTT
TATATACCATATCTTATTTCATCCTCACCACAAAGCTATGAGGTAGGTACTATCATTATC
TTAATTTTACAATGAAGAGACTGAGGCTCAGGAAACATAATGCAAGGTCACACTCAGTTA
ATAAGTTGAGAGCTAGGATTCAAACCCAGGTTTATCTGACTCAGTTACACTACACTTCCT

CCACATAGCACATTGTGGGCCCGTCACAGCTCTTGAGGACAAAATTCATCCTTAGAAAAG
TTTTCTTACTCCACTTTTTGCTGATACTACTGCTGCTGTTGCTGCTGCTACTACTATTAC
TACTACTGCTACTGTTTCTCCTCCTCTATTTATCCAGAACTGCATTTTTCCAGCACTCCT
AGGTGCTTTGAACCCAGCTACCAGTTGTAGAGTTCAAACCCAGGCCTGCATGAGTCCCTT
GCACCCATTCTACTTTTCTGCACTGCTTGTAATGTAACACAGGAGGTATGGGACATCCAT
AACTCAGGTAGGTAATTGACTTCAAGCTTAACATTCACTCACCAATCATTTATTGAGCAC
ATATTAGATAAGCAACCTATGGCTCCTGCCCTGGAGTTGTTCATAAGACCTTCCAGGCAG
ACAGCTATGGGACGAATACACTTGAATTTATTAAATCTACCTCAGGCAAAAAACCAAGCA
TGTAATTATAAGTAGTATATCAGAGTATGTGTAAATGGGGTTCTGTAAAATGTTTGAAAG
AGGCAGGCCTGGGTTAGTGGTGAGAACAAGGATCAAGAAGGCTCTGCCACCAAATAACAA
CATGAGTGTTGTCCCGTTAATCTCTCTGGGCTTCTGTTTCCAGATCAAGGACCTCAAAAT
AATAGTCATAGTCACCATTTATTGAGTGCTTTACTGTTAACCAGGCAGTATGCTAGGTGG
TTTTCATTTAGTCTTGACAACAGTATTCTCGCTTTCCCTTTTCTACAGCTGAGACTCAGA
GACATTAGGTAATGTCTAGGTCACACAGCTCTTCAGTGACCAGTTGTTTCTATGTCAGAA
TCCTCTTATAATCACTGAGTTGTATCGCTTCCCTTGCCTCCTGCTCCCAAGTCAAGTATT
CTCTACTATGTCGCCTGAAAATTAAAATTAAAATCAAGGTTAATCTTGTTCAACTGAGCC
AAGAGGATAAACATTAGAAATAGCAATTAGTAGGTAGAAATGTTGCCAGGAAGAGCTATA
CAAAAGCATAGAAAGGCAACCAAGGGAGTTAATGATGGGGGTGTGGCAGGGGTTGCATGT
TGATGGGTAATTGGATCCTGGCCAGCTCTTACCTTCTTCCTTCACCTTCCCACTCCGCAA
TTTCTCAGGCTTCTCTAGAGAGCACAACCCACCTAGAGACAGCCTGTGTTGCCACAGGAG
GGCACCAGGCCAGGAATGGCAGGCCTAGGGCAGTGGGGCTCTGGGCTGGGAACCATAAAT
AACCACTTTAATGAAAACCAGCTTTGGCCAAAAGCCTTATCATTTCTTAAGAGGTTCTGT
GCTTCTTCTAATGACCTAGTTACTTGAGAAAGGCCTAGCCTAACATATACAACAACAATA
ATAGTTGCCCTATAGTGAGTACTTACTATGGGCCAGGGCCTGTGCTAAACACTCACTAAT
TCATTTAATCCTCACACAGCCCTACAGGAGCAAGTACTATTATTTCCCCCACGTTACAGA
GCAGGCATCAGGCTGAGAGAGGCTAAGTCATTTGCTCAAGATCATATAGCTGGTTGGAGC
TGGAATTTGAAGCCTGAAGCATCTAGTTCCAGGGCCTGAGCTCTTTAATTCCTATATTTA
ATGAATGTCTGTCGAAGGAATAAACAGATGAATGAATAGACAAATGTATTCATTTCATTT
CACTTTAACAAACATTTATTGAGTGCTCTGTGTCAGCTCCTGTGCTAGGCATTGAGGATT
TATAAGTTTAGTGCGTGCGTGCGCGCACACACACACACACACAAACGCACACACACACAC
ACACACATCCTGCATGGCCCTTGCTCTCCAGGGGTTCACAAAAGCATTCTCTATTGTGGC
TTGATTTCTTCTGGAGAGAAGTAATTTGAATGTCTTGAACAGCAGTTTTTAATGATGCTA
TTAAATTTAACCTAAAGCGGGGGTTTTGCACCGGTGAGCAAAGTGTTTCAAATGAGAATG
TGTCTTTGATTAATCATCGATTCAGCCAGAGAATAGTTACTACCAGAATGGAGCTGCAAC
TCCTATTATATTAGCTAACTCTTCTTCCATCTATCATGTCTCTATCTTGTCGAACCCTCC
CACGATACAAAAGTTATCTGTACCCTAATGGACAATTATCCCATCCATCATTCCTGCTGG
TCTTGTTGATTGCTGGTAGTCTGCTTGCCCACGGAGGAGACTTACAAATAGTCATGACCA
GTGACGCACTGGAAAACTGCTCATTTTTAGACATTTTAAATCTATGTAGAATGAATTATT
TTTCAGGCTGTACTTAAGCTGCATTTGCTTTGTTGGTTATGTAAGAAGAGATATGAATTC
CTTACCTATGCGTGAGACATGGCGTCTGAATATTTTTAAGTTCCTGTGGGCAGCAGCCCT
ATACATATTTGTTAAAATGATGAAAGAATAAAGTAAACATACCTAGAGTAATTCCAGAGG
AGCAGGCTTCATACCTGGAGCTCGGGTGGGTGGCCATCATGTCAGGGTAGTCACCGTCTT
CCTTTTCCTTTCTAAGTACTTTGAGGAGAATCCCAAGTTGATTAGGGTCAAGGTGAGGAC
TCATGGTGCTGAAAATAGGGATTGAGTGGACAATGAGAGATGCCTGTTGAATTCCCTGGG
ACTATTGCTATGAGATTTGAGGGCAGAAATGCCCCCCTCTCCCATGTCCTACCCTAAGCC
TGGTAACACATTTAACTTTTTGTTACAGTAACTACCATGCCTAGTAAAACTGGAGTCACT
GGTTGCCCAGGGCAAAAACCCAAGAGTTGTAGTTCACTCATCTTTATTTCTCACCTCTAC
ATTCAGCCAGTCACTAAGCCCTGCCAAGTATATTTCCTAAGCATTCCTTGCATGTCACCT
CTTTTCTCCATTCCTACTGCCAGGGCTTTAGTTCAGGACTCCTCCCACCTCCAGACATCT
CTTGCCTTGATTCCTACAATACCTTCTGGTTTTCTCACCTTCTCTCCTTAGTCCTCTGTG
ACCATTCTCCCCAAAGTGGCCTGAGTGATCTTTCAAACATGCAAATCAAATCATTTCACA
CCTCCATCCCCTCCACTTAACACTTGTAAATAGCCTCACAACACCTGAGACTTGAGGCTC
TCAAAATGTGGGTCCTGGGGCCAGCAACACCAGCATCTCTGGCAACTTCTTAAAGTTAGA
GTACGTACTCATTAGAGTACCTACTGAATCAGAAACTCTGAGAGTGCTCACCTGACTCTG
TGTTTAACAGCCCTACAGGTGATTCTGATGAGCACTCAAATTTGAGAACTGGCACCCTAG
AGCCATGGTTCTCAATCCTGGCTGCACATTCAAATAAATGGAGGGAGCTTTTAAAAGTTC
TAATGCCTCTCCTGCAGAGTAATTAAATCAGTTTCTAGGGGTGGGATCCAGTCATAAGTA
TTGTTTTTAAAGTTCCCCAGGTGCTTCTAATATACAGTCAAGGCTGAGAACTACTGCTCT
ATAGGATGAAGTCCAAACTCCTTAGCTTGGCATTTAAGGTCTATCTCTTTGGTCATAACC
ACCCCCAACACTATGCTCTTGCTTGTTCATTCTAGAATTCAATCACAGGGAGTCATTTGC
AATTCTCTTAGAGCCACACATGGTCTCTTACCTCTGTGCATTTGCACACATCATTCCCAC
TGCTGTGCCCATCCCTTCCTCCCTCCCTTCACCCAGCTAACACCATCTCATCTACAACAC
ATAGCTCAGAAGACACCTCTAAGGAACAGTTTTCCCCGCCTGTCCAGTCTGGGTTAGATG
CCCCTCCTCTGTACTCCCACAGCGTCCACAACATAGCACTGGATACATTCTCTTGTTCTT
CTCTGGTTAGTTGTCTACTTTCTCTACCAGTCTGAGAGCTAGGATCCCCACCTGGCACAC
AGTAGATGTTCCATGATTCTGTACATCCTGTTCCCTCTTTTTGGAGTGACTTTCTGTGCC
TTGTCTGTGGCCTTCAGGATGCAGCTCAGATATCACTTCTTGGGAATCTCAGAATTCTCT
GTCTGGACCCTTTCTCTAGGCTCCCGCAACAATCTAAATGTAATCTGATCCTAGCCCTTA
GCACACAGGGCTATCAGGCTGCCATAGTCTGTTTACTTATCTGCCTCCCTCACTGAACTG
TAAGTAACTTGAGGTTAGGGACAGAGCCTTGTACATTCCTGTATCCCAGCTCCTAGCACA
AAGACTCACACATTATAGGTGCTCAGTAGCCATAACCATCTGCAGAACGAATAACTGAAT
CAATGATGACCTTTTCCTCTTCTCCATATCATCAGCCACTATTTCTGTCCAGGCCCCCGT
TGCTTTATTGCAATAGCCTTCTGACTATTTCCCATCTCTCCTTTCTCTGATGCCATCTGA
ACACTCCCCCCCTCTCCAAGATGTGGCTTTCTAAAACCACAGTCACCCTATCCCTCCCCT
GCTCAAACACTTTCAGTCATTCCTCATTGTCTGCAGGGCAAATATCAAAGTGGAATTCCA
GATCCTTCCTTCCCAAGAGTGGTCTGCCTTCCCAACCATACCTCCCCCCATTCCCCTATG
GAATACCAGCCACATCATCTGAGGGTCCTGGTGCAAAACGAAAATGTGGAACCCCGTGTT
AAAATATAATCAAGGATTTCAAGATAGCAGCAAAGGAGAGCATTAAACCAAGCATAGGGC
TCTTCTGACTTCAGGGTCCGATGCGACGGCACAGGTCTGCCCCTACGTGTATCCAGCGCA
CTGGCCAAATGCACTTGCTCCTCCTTCAACTGCTCTGTAGGTTCACTTTCTCCACGGCTT
TTGTCAGGCTGTTCCATCTCTTCTACCCCACTAGCCAATTCAAAGCTTGTCTTCCCTGTA
GATCCACCCTGTTCTTCAAGAATTGCTCAATTCTCATCTCCTACTGTTCCAGACCACAGT
AGTTTCTACCACACAGCCTGATATTAATAATTATTTTTATATGTGTGTAGTCTTGCCTGT
CTACTGAATTTTAAGCTTCTTGAGGGCAAGGACTCTATTTTGTTCATCTTTTTTTTTTTAT
AGACTTAGCATAGTACTTTGTAATCATCTCACTTGTTGAATGACTCAGGGAAGAAATAAT
ACAACTCCAGTCACTGAGTTTGCCAAAAAACAGTAGACACTGCAAACCTGATTTAAATCC
CTCATTATTAGCTATGCAACATTGAAGGAGTCACCTCACTTCTCTTTGCCATCTATAAAA
CGATGATACTTAGAGAAAAAGTGTTTTTTTTTTTTTTTGTTTTGTTTTTTTGGTTTTTTT
GTTTTTACAGAGGCTTGCTCTCTTGCCCAGGCTGGAGTACAGTGGCATGCAATCTCGGCT
CACTGCAACCTCCGCCTCCTGGGTTCAAGTGATTCTCCTGCCTCAGCCTCCCAAGTAGCT
GGGATTACGGGCCCATGCCACCATGCCCTGCTAATTTTGTATTTTTAGTAGAGATGGGGT
TTCACCATGTTGGCCAGGCTGGTCTTGAACTCCTGACCTCAAGTGTTCCACCTGTCTCGG
CCTCCCAAAGTGCTGGGATTACAGGCGTTGAGCCACCATGCCTGGCTAGAAAAAGTAATT
TTTTGTTAGTATCTTGTTCTTTGGCAATGTTTTCAAAATCTTTTGTTTTTTAATACATGT
AAAACTTATTTTATATTTTATATCACCTAATTGCAATACCTGAGGTCTTTGAAGACCTGA
TTCTGTTTTCATTGTTTCTGGCTTCATCGTGTTCTTTGTAATTTTGGATTATGAGTTCAT
GTTCAACCTGACTTTATCCTTGGTAATCCTATGAAAACTGGGTGGTGGGCATGTTCTTCC
AGGGAGGATTTGTGTTTGGTTCTTCCAAGTGCATCAGGGCACTATTAATCCAGGAGTTCT
TCCAGTGAATTGTTTAGTCCCAAAGGCAAGGGCCATGTCTTCTTTGGCTATATGATCCCA
GGGCTTAACTCAGGGACTTGTAGATGCTCAACATATATTTGCTGATTGAGTGGATGAATG
AATGTGGTACTTTACCGCTTCTTTTGGAAGCCCAATAAGAATTGTGACCTCTGGTATTAA
CAAAGAACAGTAGGCACATCAGGAAAATAATAATTACTAACATTTATTGAGCAATGATTA
TGTGTCAGGTATCATTCTAAGTGCTTTGTGTGTATTAACTCATTTAATTCTCACTATGAT
GCTGTGAGGTAGGTTACTATTATTATCTTCACATTACAGAGGAGGCACCAGACTCAGACA
GGCTGAATAACTTGGCCAAGGTCACATAGCTAGTGAATGGCGGAACACAGATATGAACCC
AGGCACCCTAGAGACACATCTACTTACCACCTTGTATTGGAGAAGAGGTCAACAGTAGTG
ACAGAAGCAATAGAGTGGAAATTTTCAGTACTGCCAAAACTATGAGAGCCATATAGTCCA
CACCAGGCATGAAAAGGAAGCTGGCATGGTCTTGTGTTGTCAGTTAACCCTTGTATATAG
GGTTTTCATTTCCTAACTAGAGAAGGAGGAGTTTGAAAGCACGAACCATGAATTACAGCT
CTTTGAAATGCTCAACATAGATTTGCTGAGTGAATAAATGACTGTTTTGAAATTAAACAC
AGGAGTGCTTTGAATAAATTACTTTGCATCTTGTATGTCAAACTGAAATTGGTGAGACAT
CTTTCACCTTGTGTTAAAAACTAAAAATCCCTAGATGTGGTCATAACATGGCTGGTTATA
ATGACACCTTGCATTTGTACTTGTGTATGATACTCAGGACTCCCTGAGAAAAGGAATGAA
GAAACTTGGATTTTGCCTTCATAGGCCCTGAGGCTGTCAGGAACCAAGGGCCACTGTGTT
TGTAATCCATTCCTTTGGCTGCTCTCCAAATCTAGGAAGACCTGGAGGAAAGGGGGGCTT
TATTTTCTATCTTTGCCAAGATCAGGAAATAATTTGTGAACTCTCAGTAAACATTCTGGG
GGCCTTCCCTCTAACACTGCTTTGGTGGAATAATATCTCCAGTTTTCACCAATCTTGGTT
CAATATTTACCTGGGGGTTTGCAGTGATCATGGGGACAGGGCTCCAACATGAAGTGAAGG
TTCATGAGAAGGAAAATGAGGGGAAGGCTGTGTGTGTGTGTGTGTGTGTGTGTGTGTGTG
CTGGCTGAGGGATGAAGGTGGGAGGAATACCAGGATTCTGAAGCAATGAGAGTGGGATTG
GGAGAGAGAGCTCATGTTGAAGAAGAAATAACTCACCAGTTCACATGGGCTCCTTTTCAA
GGGTTTTTCTTTATATAAACAATTTGCCTGGTTCCAATTAGAAGCCCACATTAGGCAGTT
AGCTCCAATTTGGCAGTTCATTCTTAATTTGGCTTTCCTTATGGGTGGAGGGAGGGCTG

ACAGTTAAAAGGTTGATGGGAAAAACCTTGGTATCCTGGTGTGAAGACAGACCCTGGGAT
CAGAAGGTTGCCCTGATGACTTTGCCAATGTGTTCATTGCCCCTGTCCTGCTGGGGCGCA
TACAGCAGGCCCTTCTTTCTCATACGCCCTTGACAGACCAGGACTCACTGCTAGAAAACC
CCTGGGGAAGTCATTGCTGAAGACTCTAGTGTGGGGCACATTAGGTTTGAGGAGCAGGGG
AAACATTCAGGCAGCTGGCAAGGTCTGGGCTACCAGGAGAGATTTGGGAGTTAGAAGCAT
ATTAGGTGAGAGTTGACACTGTCCAAAGGGAGTATATAGAAAGAAAAGCAGAGGAAAGGG
CTCATGGAGTCATTACAGCCTGTGAGGTGGGAACAGGGTTTGGGAGTACTAAGGGTTTGG
GGAGGGTTTATGTGTTTATTCACCCATTCATTTATTCAACATTTCTTGAGCTTTATGGCA
GATGCTGAGTTGAAGAGGAAGCTGGGGATTTAAGAGGGAGAAGAGTTAAGAGGGGATCCC
TGGGTGGATCCAGATGAAGGAGAAGGGAGGGGCTAAGTGCCTAGGCTCGCTCTTGCCAGG
AGAGCACATCTGTTAACACAAAGCTGCATCTGTATCTCAGCCAGCTCTCTTCCATTTCAT
CTCCTCCTCTCTACCCACACTTGCAAGACGGGGAGCCACATGGCGGCTTCCTACTCCCTT
GTACCACTACACCCCCTGCCGGGTTCTTTCTAGGCATTTAGCTGTGGCATCAGCAGCACC
CCTTCTGAGAGATCAGGCAATCCCCAAGGGTGAGTGGTAGTGGTAGTGGTGGGGAGGGTT
GATAGCTTCGAGAGAAGAGCTGGAGAAGGCGCTGTCTGGCTGTAATTAGCCCCAGCTGCT
GTTGTGAGGCAGCGTTGGGGGCCGTCAGGAGTGTACATGCATGTGTATGAACCTGTGAGC
GTGGCACCTCATTGCTATCACTCTATTTTTCACTGTATTGTGTGCTGGCTGCAGCCTCGC
TGCTCTGAAACCCCTCCTGTCCCTGAGGATCCCGAAAGACAGAGTGTTCCACAGGCAGCA
CCAGCTTGAATCACTAATGCTATTAAGCAGGAGGACAACAAATATAGGCCCCCATATGTC
CTGGATATGCAAGTCCCTGCTCTCTCTTTCCCCACCCCTGCTTCCTGAATGTTTCCAAGT
GAGTGAAGTCCCAGCACTTGAAATTAGCAACACCTCACTTCTGCACAGCCTTTTGGCAAA
GAGCTTTCACAGCTGCCATGTCATGCATCCTCAGACGCTTCTAGGAGAAGCATTTTATAG
GTGAGGACCCTGTGGTTCAGGGAGGTGGAGAGATTTGCACAGGCTAACCCAGACAGTCAG
TGGCAGACTTGTGTCCAGGAAGCAAGTCCTCGACCTCCTGGCCTGGTGGCCCATCCCCAG
CAGAGCACTGCCAAGAGGGCTGCTCCCGGGTGGCTCCAGCTGCTGCTGTCTGTGAGCTCG
TGAGAACCTGTCAGCTGTGGCTCCAAGACCTGCCTCAGTGATTGCTGAAAACGCTGAGAG
TGCTGAAAACCAGGACCCTCAAGCCGAGCCTGACGTCACATAGTTGATTGAGTACCTGCT
ATGTGCCCACCACTGTGCTGGTGCTTATGGGAGACAAAACAGGGGTAGTGCATAGCCCGT
ATCCTCAAAGAAGTGATAACTTGAATGGGGAAACAACTCTTAGATACCAAGTCCAAAGGC
AATTCCAAGCACTTCTCTCTCTTCCACTAAGCAGTCTGTTGTCAGTGACAATGACACAGG
AACCCCTTCTGAATTCATCTGTCTTATCGCAGGCCTGGGGTAGGAGAGGATGGGAGGATG
ACTAGGAAGGGTAATGAGGAAGGGCAGGGAGATGGGGGAAGGAAGGGAAAAACTGGAGCT
CCTAATAAACACCAGGCACATTGTGAAGTTTATATGTTATACATAGGTCATTTGTATTAT
GCTTATCATACATGTACATGAATATATACACCCAAACACATACACAAACACATACACAAT
CACTTTCCAGGGATTGACTCATCTATTTGGCTCACTCTCAATCATGAAAAGAGCTTAGGA
CAGTGACACTTTTAGAAAGCCCTGAATGAGTGTTAACTAGCCTTTCTTCTTATCATGATC
ATCTTCATTGTCATTATCGGCATATTATATAAATTATCTCATTTATTTCTTCTAACTACA
GGACTTAGCAACTATTATTTTTATTTCCAGTTTACAAATGAGGAAACCAGTATCTTATTT
TATCCAAGCTTGTGGGTACTGAGAATATAAAGAGGAATTCAATATGGTTCTAGGCAAACT
TTGGGGTCTTCCCTATCTAGTGGGAGGGACCGAGGCACACATAAGCCCTCATGGTCCAAT
AGGTGCTGTGGTAGAGGAACGTATCTGGGGAAGTTGAGGAAGTCTCCACGAATGAAGGAG
GCTTGAGCAGCATCTTGAGGGATGGCAGGTTATGTCAGGAGGAACAGTGGAGGCAGGGGA
AGACATTCCAGGCAAAGTGAGCAGTATTTGTGTGACAGGGCCTAGTGCATGTTTCAAAGG
GCAGGACAATTGTAGCTGGGTGCACCACTGGCAGCAGCAGGAGGGGAGTGGACGAGTATT
TGGGGTCAGCTATTCAGAGCCCTATTGGCCAAGCTGAAGGTTTTGTTCTGTGAGCAAGAG
GGAGCCACTGGAGCTTTTTATTAAGGCAGATTCGGGACAGACTAGGAAGACAGTCCTTAC
CAGGGTGTTGAAAGATTGGAGGGAAGAGAGGCCAGCTGCATGCGGACCAATTGGGAGACC
ATCTCCAGGGTCTGGGCAAGAAGTGGAACAGGCTCTGAACCACGACCGTGGCAGTGAGAG
CCGAGAGGAGGGGATGACTCAAGGCACATGTCAACAGGTAGAAACCTCAGGGCTGGCTGT
CATGGGGTCAAGGAGAGGGAAAAGGTGACAAGGACCCTGAGGTTCCAGCTTGGGTCCGTG
GTGATACTGTCAACTGAGGCAGGGAACACAAGACTGTTAGTAGAGAGGGAGAGTGAGGGG
AGACAGATGACAAGGTCTGTTTGGACTTGCTGGGTTTGGAGTGCTGGATAGCGGACATGT
CCAGCAAGAAACTGGGAATCTTGGCTTTGAGCCCAGCTGAGACATGGGGGCTGGAAAGCC
ACCACAGTCAAGGGATATTAGTTGACCTCAGGGGACTGGATGGGGTTTCTCCTCTCCCAT
TTTACTCATCCAGGGCAAGGGCTAGGGACAGAGCCAGGTGAATACCAGTATTTAATGAGG
GAGGTGGTGGGGGACACCAGCCCGGAGTGAGAAGACACTCTTCTTTGGACCTTCCCAGAA
ACATCATCCCTTTCCCCTCATGATCATGGTCCTTGTTCCAGGGCTGCACTCAGGCCAGTG
ACAGAAGGAAGGGAGGGACAGAGGGAGAAAGGGAGGAAAGAAGGGAGGGAAGGAGGGAGG
GGGGAGGAAAGAAGGGAAGGAAGGAGGGAGGGGGGAGGAGGAAGGAGGGAGGGAGGGGGA
AGAAGGAGGGAGTGAGGGAGGAAGGAAAGGGAGGAAGGAGGGAAGGAGGGAGGGAGGAGC
TCTGTGCACAGAACTCATGCCTGCACAGGGGCCATGAAGCAAGGACAGGACAGTCACTTC
AGCTCTGGAGTTCTTTAGTGTGGGAGTATCTTCTAACAAAACCTAATAAATTAACAGCTG
TGGGTTTGGGGCAGCAAGGACAAAGACCACAACTCTACAGTGACCCCTGGAGCACTCAGT
GCCACTGACTGCGTCTCCACTGCTGCAGAGCTGGGAGCACATTCATGACAGCATTTTTCA
AGCTCCTGTCTGTGCAAACCACAGCTGGTCTCCTAGGAGTCCTAACACTTTAATTAGACC
TAGCAGGTCCCAGAACAAAATCCCATAATCCCCAAATCTATAGCAACAAGTTAGGCATGA
GAGTTTTAGTTACATTCTGGATGTAAGAGAACCCTAACTGCCACTTTCTTTCTGAGTCTC
CAAATGACCTTTGACCAATCTCTTTACCAGAAATATTTATCTAGAATGGCCTTGGTCTCT
GACACCTTGAGGACTTCTACTGATTTCTCTGTTAAATCCCTGGAACCACACCAATAATAG
TTCTGAACATTTTACACTCATCATCTCATTTATTCCTCACAAATATTATAAACCCTGTTT
ATATATATGTGTAATATTTATAGTAACAAGAGTAGCAGATATTTTGCTGAGGGGAGGCAT
GGTTCTACACAGGTTACATGAAGTAATTCATAATTCCACATGAACAGCACTATGAGGTAT
TATTACTGTCTCCATTTCACAGGCAAAGAGCAAATCACAGAAAGAATGAGTAAGTTGTTC
AAGGTCACACAGCCTATAAGAGGCAAAGCCTGCCGGTGTGGCTCCACTGTGCTATTAACC
AGTAAGGTCTTCTATAGCCCCAACTAGCTCAAAATGGTTCTAAATGAAGAATCGCTGTGC
GTTTTGCACCTCAGACTGCTAGCATCGCTGATTTAGGTCTGGAGTTTAGATAAGTGAAAC
TACTCAGGGTCTTTGTGAAGTATCTTTAGAGCTGCTGCTTGGACAAGACTGGGTCAGGAC
TTCGAAGGCATTTCCATGTGCTCCTGGAGAGTCAGGATGTAGGCTGGGATTCATTATGCG
ACCCAGCTGTGTGACTTTCCTCCCCACCCCTTACCCCCATTCACCAAGATAGAATGCACT
GTTTCTTAAGGTGTGGCTTAAGGAGAGCTCTTTAAACATGCAGATTCTAGGGCTGAAGCA
CAAACCCACTCTGTATCTTTATAGAGATAACTGCTGGAATATGCATTTTAGTAAACTCTC
AGGCTGAATTACTTTGAGAATCATCAGAGTCTACTTTGACAGGATATAGGCTTGTCTTCT
GATTCTAGAAGTACCATAAGCCTAACTAGGGATGCTTCCCACTCCTCTTTCTTGCTGAGC
AGAGAAGCAAGGGGGACTGGAATGCAGAGAACATGCTGAGGGCATGCCTGCACAGAAAGC
TGAGCTCCTTGTCACCACATACCTTGAAAGAGTGGACTCTGTATCCCCAGGAGAGGATGG
GGAGGGACAGAGAGAGAAAGCTATGGAAAGCCTCTGGAAGCCTTAGTACCCTGGGGCCCA
GACAAGGGAGTCCCCTGAGGGAGGTAGAGAGTGGGGCTGTTGGAGGAGGAGAGCAGCTAT
GGGCCAGCAGCCTGAGGAGCTGAGAGTCACAGTGGGAGTGACTGTGGCTTCAGAGTGATG
CCATAGCTGCAGTGAGCTGTACAGGAACCAGAGACCCAAAGTTTTCTGTAGGGCTTCTTT
TGCTTTGTTAGTGCTTCAGGAGTTGGATCCTTCTGGACCCTGATCTCCCAGAAGTGATTC
CATTTCGCCTCTAGGAGGTGGGTAGCAAAGCTCAGTCTGTGGCTGGGGAAAGCCAAGGAA
GTGATTTCACGAATCTTTAGCAGTGAATCCACCTATTTAGTTAGCAGGGGATGGAGCAGA
TCAGAACTAGCGAGAGGAAAGGCTTAGGAATGCTCTCTAGTGGGACTGAGAAGAAAAGGT
GAGAGGGTGACATTAACTGGAGGAGATGGTGAGCCCCTGACCCCTGCAGTTGTGAGACTT
GCACTGAGTGGGATGATCTGCTGCTATAACCTATCCTCAGGCAATGAAGCATAAGCAGAG
AGCCAGGAGTGTTAATCATTGCTGATTTTTATTAAGCATTTCCTTTGTACTGGACACTGT
ACTGGGCTGTACATGCATCATCACATTCAGTCTTCACAACATTATATGATAGCTCCTATT
ACTGTGCCCATTTTACAGATGAGGTTTAGATAGGTTATACCACTTGCCCAAGGCCACACA
ACTAGTAAGTAGCAGAGCCAGAATTTGCACCCAAGTGGTTTTAATCCAAACTGTGTCCAC
TAGGCCTTAGGAAGGAGCCCACATAGAGCTTTGGGGATGCTGCATTCTTGACCTGTGGCT
GTAGGTGGGCAACTCCTCTTCATACCTTATAGCTCTTGGTAAATGAAGTGAATCACTGTA
TCTACCATGAATCTCCTTGGGAATGAGTTCAATGCGGGTATCAGAGGAAGCAAAGGGTGG
GAAGGGGGTCTCAAAAGTCTAAATCTTAGTGTGGTCCCCTCCTTTGACCTACTCTCTCTG
GGCTGAAGAAATGCCAGGATTGAGGGGGCAGCAGATTGTTAGATCTGTTTGTTCCATCTA
CTGAGGCCTCATAACATTTTTGGAGCCCTTGTTCAGTGACTCAGGTAATTCAGCCTGAGG
GTACCTTTGCAGGGAGTTCCCCTATCTCTTATCCTGTGTTAGTACATGAGATTATTCCTC
CTTCTTGTACCCCAGTTACATTAAGTGGGTCTTATTCCCCAAATCCGTAGTCTCTGCCCC
AATAAGTTGCCTGTACCCCATTTTAGCAAGAATAGAATCTTCCCTTTCTGCCAACAGAGC
TTCTTGGCACTTTGGCCTTGCCTGGTTCCTGCTTGCAGACTCCTGTTCTGAGGCTCATGT
TTGTACCTAGAATGATTTCATGTTCCTCCTCTCTCTTTGTCCCTCTATCAGAACCTGCTT
TCACGATGGGAGATTTAAGAGGCTGATCCTCCCAAGAGGTTGGCATTTTAACTGATGCCA
AAGCCCTGAGTCAAAAGAATTATATACTTAAAAATTTTCAAGCCCTTGGGAGCGGATGGA
GAGATTGGAAAGGCCAATGGAACATTCTTAATGCTCCTGGAATGCCACAAGGTGGACATA
AAATTCAGGCTTCTGCCAAGACAGGACTTGGATTATTTTTGTTATAATCAGAATCCGTAA
TCTGCCCCCACCCCAGCACTCAGGGCTTTGATCAAAAGTCTTCCAGACAATGAACCTCTA
TGATCCAAGTATCATTTTCTCAGAGCTCCTTAGAAGGAGGAGAGCTAAGAAACTTCATGA
ATTACCAAGGACCAAGCAAACAGGCGGTAGTGAAGTCAGAAACAAATTCCTCCCGAGACT
AGTTTTGTCCTATAGCCTGAAAACAAATCCGGTCTCCACCAGACATAAGGTGGAATTCCT
GTCTTGATTTTTTTTTAGCACTGATATTAACATTTTTCTTCTCAAATCCTCTTCAGAAC
CAGCCCTTTTAGAACACAAATTAGAAGCTGTTTATTTTGTCAATCAGAGAAACTGAAGGC
AGCCAGCCTTGGCCAAAGGAACATCTTATCCTTTGCCTTTGCTAAGGACAATGTTCCTTC
TATTGTGCTCTGTGGAAATAAAAGGGCAAAATATGCTTGCCTTCTTTTATTCTTATTCAT
GGCTGAGGGTCATATGAAAGTTCAGAGAAGAGCACAGTTTTTAGTATGGGTCGTGGCAGG

TTTTCCAGATGGCTTCCTAGCCAAGATATTTCTCCCGCTCACCTCCAAACAAGTTATGTA
CCCCTAAGAATGATTTGGTCTCAGTGTTAGGGGAAGAGGGTAGGTAGCAGGGCCTGGCTT
TGTCATCCTGGGCTATTTTGAGGGGACTTCAGCAGTCCCCGACAGAAGGACAAGAGGGGC
TGATTCTCCCTTTGTGAGCAGCCATTGGAGCTGCTTCAGGGACTGACAAGCCGGCCAGAG
ACAATGTCCTCTCTCAGGAAAGCAGTAAAAACATCAGAAATGCTCCTTTCCTGGGGAGCC
CTTAGTTATTTTGGCCTCTCTTTCCATTAAAATAGGAAAGCATTGAAAGGGACCATTCGT
GGGATAGCGTGGAGCCTCCCCATTGTGGCAGCATTTCTAACTGCGTTGAGAATTAACCAT
ATTGCTCTTCCTTTTTACCATGGAGGAGTTTCTAGATTTTTATTTATACCCTACCGCTTA
AAACATCTTTCACTGTTTCAGCAAATTGCAGCATAAAAGATGGAGAAGCCAGCAGGGGCT
GTGCTCACAATAATAGCTGTGGTTGCATTTAAGACATTCAAATGCCAAGGGTGCACTTCT
CCCATTAAGTCCAACATAAGAAATCAGGTGTGCACAACATTGATGAACAGAAGACCCCAA
AGGACAGGTGGAGTTTCTGGTATGCTGTGGTGGGGGCGGTGGGGTGGTGAGCTTGGGAAA
GAACGAACAGGCATTTTCATTTTCCAAACTAAGAAGCAGTTTCGGGCGAAGGGGAGCCGT
GGGGATGTCAGAAATTCTTCTAACGCCATGGACCTGCCCACACCTGAGTCAGATAAAGAT
GACCTTCCCTAGGCCCTGCTTTCCTCTCCCCCTCCCCAAAAGACAGCATGAGCATGAGTA
AGCCGGGTACTTACAGGTGCAGAAGAATTTGGAACAGGAAAATGGAGCTTACAGGCTCCC
GAATGCCTGCCGAGTCAGTTGCATCAGATTAGGAAGTGATGATCCTAAGAATAAGATGTT
TGGAAAATCTTGTCCCCCGAAAGCCCCCTGCTTTGTCTTCTCAGCCCACATCCAAACTCA
GACCACCCCCAGGATGTCTCTCATCCTGCCAGACTGCCTGGTGCCTCCGTGCCCCTTACC
GTGAGGGTGCTGGCAGCCACACTGCTTCTTTGAGCCTGTGGCCCGGCAAGGTAGTCAGGC
TCATGGGATTAGGTTGGCAGGAGCGACTGTCCTCTAAACGATTAGCCCAGAGAAAGAGAG
AGAGAGAGAGCAAGCAAACCCGAAAACGGGTCCAGGGTAGAGAGCTCCAGGTCCTTAGCA
TGTTCCTGGCGTATTCCAAAAGTGGCATCCACAGCAGAGAAAGTGTCCCAAAATAGCTCA
GGCTGCCAATGCCTTGCTGAAACCTGAGAAAGAATTGCTGAATTTAAAGATCTGGGTCGC
TGTGGGTGTGTGATCAAAGTGCAGATTTGTTGGTAGGAGGAACTCAAGCCATGAGCTTAG
GAGTGACATGAGGTCACTTGGGTCACGGCCTGCCTTCTTGTTTCCAACTTGCTGAAGTTG
GTGGCAGTGTCAGAGAGCTCCTCTGTGGTGAGGCAGGGGTTTGGAAAGAGGTCCTAAGGA
TGCTGTTTTCATGCCCAGGTCTCTGATGCGCTAACAATATGAAATAGCAGTGCCCTGTTT
TCTGCATTTCAAACAAAGGAACTGATACTATGCAAAGGTTTCTTTAGGTTATTGCTCCAT
GTCTGATGAAATTCCCTTTTCAGTAGATTTTTCATTTCTTTTGGCCTTTGGCATAAGTAA
ATGTCAGCTTCTTAAAAATCAACAGAGAAGAGCAAACAGTAGGGTCTCTGTGATTTTCTT
GGGATTTGAGAAATCTTCCCTCCCTAGCTTCTAACTGGATCATGTCTGTACATTGCTGCC
TGTGGTCCTGGAAAGGACGAGGTCCACCCAGACATGCTGTCTCATTTTTATATTTGCTGA
GATCTTGGGACACTCTCCTTTCATCAGGTATGTCAAAAACCTTAGGATTGTCTGCAGAGA
GTGTGGTTCAGTATGTGGAAAGGTCAGCATATGTCATTCTTGGACTGGCCCTGGGGTTTA
AGAAGAAAAAGCCCAGAATTTGGTTTCCAATTCCAGCTCTGCCTGTCTTTTGCTGTATGA
ATTTGGGCAAGTCACTTCTCTTCTCTGGACCTCAGTTTCCTCTTCTGTAAACTGAAGACA
ATAATCTTCATTCTAATTCTAACCCAAATGTTGTAAAGAGTAACTCAGAGAGTTGAAAGA
ATGCTATAAAGGGCAGGGTTTGGCACTCTCAGTAAGTGGTGACATTTTTCAGTTCTTCAA
AGGTCCCTGATTGGTAGCCACTACCTGCATCCACATGCCTATGGCGAGCCCAAGACATCC
CACAGTACTAGGCACTATGAGCTTCATGTCTAGGAGTCTAAATAGTTTCCACTTTCAGTT
GTGGGAATTTTGGCAAGTAGCTCAATGCTTAGTAGCTCAGTTTTCCCATCTGTCAAGTGG
GTATACAAACTAATAATAATACAGGTCCACAATCCCTTATCTGAAACTGTTGGAGCTAGA
CATGTTTTACAGTTCAAAATATTTGGATTTTAGAAAAGCAATGTGGTAGATATAACATAT
ATATCACACCTTAATGAGATCCAAGATAATACCCAGTGATCAAACTCACTAATATTTCTA
AATGGGATAAATGAATAGCCTCATGTCAATTCAGGCCCAGTTTTGCCACCAAATGAGCTT
GCCACAGAATTTCAGTTTTCAGAAGCTTTGGGATATCAGAGAGGCACATAGGAAATTGTG
GACAATGAAGGAAGTTAATACTTATTTCGTGCATGTAATGTACCAGGCATTGTTCTAAGT
ATACATTAACTCATTTAAGTGGCCCAATAAGCCTGTGAGGTAAGCAGTATTATTATTATC
ATCCCCATTTCATAGATGAGAAAACTGAGATTCAGAAGTTAAATCACTTGCCCAGGGTCC
CAAAGCCAGTTAGGAGCAATGCTGGGATTCCAACCCACACAGTTCAGCTCTTATCCACCT
TCTCTGCCTAACCCATGGGGTTAAATTGAGGCTTTAAATGCCTACAGGGACTGAGCAGGT
CGCTGAAAAAGTAAAGGTGGCCTTATGGGTACTGAGGCAAACTGGTGAGCACGTGCCTCA
CCTACAGGGAGCAGGTTGTTGAGCTCTGGCTGATTGTTGCTATATTGGAATTTGGATCGA
AGTCTTTCTCTCTCTCTCTCTCTCTCTCTCTCTGTGTGTGTGTGTGTGTGTGTGTGTGTG
TGTGTGTGTGTGTTTCAGAAAAGCCAGAAATCCAGCTTTCTATCTGAAATTTCCTGACTT
TTAAATGTTGGCAATTAATTTTAAAAAATGTTTAAAACGTTGTACGGGTCAAATAAACTC
TGTCTGCAGTCTGAATTTAGCCTGTGGCTGCCAGTTTGCAAGCTCTGCAATGAGTAAATA
GATGTGAAAGTGCTTTGGAAACTTAAAAACGTGCTCTAGAAACAGAAGAGTCTAATTTTA
GGGTTATATCTTGTTTTTTCCTTGACTGTGGTCTAGTTTTTTAAAAACTTGTATTCCCGA
CCTGATGGAGATGAAAAGTCAGAATTAAATAGAGACTTCAGTGAACTTGAGTACTTCATT
CCTTTGGCAGGCTGGATAAATGAGAAGCCTAGGTGTACTGGATGGACTAAGCCTCAGAAT
TCTTGCCACACCCACAAGCCCCTTCTAAGGAGTTTCCATCCACAGACCCTAGCTGAGGGA
AGTAGCAGTTTCTTATGAATAGGTGACTATTCTTGGTACCACGTGACCCTGCTTCCCCCA
GCAATAGCCACTTGAGCCAGGAGAGGGCATCTGATCCATGAGCAGTCACTCTATAGGCTG
GCAAACAGCCTATGAAGGAACCTGGGATGAGAACTTTGCCCAGCAAGTACAGTTACTGAA
TTTGGACCAATGAGAGTTTCTCTCTTGGGGAGTTTGAATACCAGACCTAGAAAGAGTGAG
CAATTGGCAACAGGGGCAGAAGCTGAACACAGAGAAGGCAGGCAGCAGGGACCCCGGATA
AGCAGGACCAGGAGAGATTACTGAACTTCAACTATGTGCCAGATTCTTTACACGTTGTTT
TGTTTAAAACATTCAAAGAACATGCGAGGTAGGTACTGTTATTATGCCTGTTTTACAGAT
GAGGAAACTGAGGTGAACTAACTTGAAGTATTGAGGTTCAAGCCCAGGCCTGTTTGAAGT
TAAAATGAGAGTGTAGGAACCCCAAAGCCAGTGGTTCTGAAACTCTAGTGTTTCAGAATC
ACTTGGGTTAAATGCAGGTTCCTTGGCGTCACCCCAGACCTATATTGAATCTGACTCTTT
GTGGGTAGAGTCTCCCTCCCCCTACAAATCTGTGTTTTAGCAGTTTTCCCAGGGAGCACT
TGATGCAGGGTTTCCCCACCACACTTTTATTTTGAGAAATGCAGTTTCAGAGGGTATGGC
AAGTACTGTGTACTGTGCTACCTTTACCTACACAACCAAGGTCAATGTCAACAGATGTGG
TCAAATTCCAATTTAAAGTCTTTTGGAAATGGGGCCAATTATGTAAAGAAGAGGGCATTA
GTTTGTTCTCTTCTCTCATCAAGGAAGTCTACATTATTACCAGTTATTTGAGAGAACAGA
AGATTGGGATGAATGCTTAGTAGTTGTAAACACGATCGGATCTGCACTTCAGGGTGGGTG
CAAAGCTAGCATATGTTCACTAAGCATTTTCCATGAGGTGCGTAAACTGTAGAAACTCAG
TGCTCTGGCAACAAAGAACATGAGAGAAAATTACCAAATGCAGATAGACATATTGTAAAC
CGTGGAGCTTAAAATGTAAACCTACTTGCTAGGCTGATCATAGAGGTTCTTCTCAGCCCC
AGAGAATTTGTGTAAGAATCCTGTCCATGCCTTTATTTACACCATTGATTAAAAATGCTA
ATCAGGAGAGGATCAAGGCTATGGACCTCCTTCTAGGTTGTTGCAGAACTCCCACTCTGG
CAATGGTATTCAGCTGGTTTTGAATTTTTCTGCTTTAAGAACTATCCCTGGGAAGACTAG
TGGTCTGAAGTTTGCTTGGAGAGTCTCAAGCCGGAAACTGATTTGTGGTCCAAATGCTTA
ATGGCATCAGACACTTATTCCTACAAGAGAGGCATGTGGTCCAAGGGGTGGCACAGGTCA
AATGTGATGGCCAAGTCCCGCCTCAGCAGGGCTTGAATCCGCACTTGGCAACTTTTATTT
CTCTCTGTCCATTCCTGTGGATACTTTATCTGTGTTAATGTCCTCATTTAGGCTAAGGTC
AAACTCTGTTCTTCTCTCTTCTGGCCAGAAAGACTTCCTGTTTTACAGGTATATGATCTT
ATCTGTGTTGATACCTTAACTGTGAGTGATTCCCTAAAGCAGGTCCTTGGACAAGTGCCC
ATCCTCCAGGAAAATTGCAGCAGTCCACCGTGCAATGAGAAAGCCTGAGGACTAGGTAGT
AAATGTTTCGCAGAGAAGAGAAATCTATTTCATGTATTATTCTTTATTCTGAGGTTATTT
CCTTCTCTTTTGGTGTTGAAAGGAAGCCCCTTCTTTTTAGAAAATGATGATTTGTGGTTG
TTCTGTTTCTTAATGATTTTACTTGGGAAAATAAAAATCTGGAAGTTCAATATCAGTCCC
TGAAATTTTGGGTTGCATTAGCTGCAAGTCTGGGAACCTGGTCTAATTTTGAGTGTTCTG
CTAGTACCTCTAGGTACCTCCTATTGCCACTTGTGTTCTAAGTGCTTATAAATTACCTGC
TTCGCAATCTGCAGCAGAATCTCTCCGAGGATTGACATCAAGCTTACAGCTTTGGAGTTT
ACGGTATACTGGTCTGTAGTTTCCTTATTTAAGAAAATTGGGACACTGCCCATTTGCAGT
CCTCTGGTATCCCTCCTGTTCTCTCTGATTCCACAGATCCTTCAGACCGGCACCTAATGC
AAGGCAGTTCCGTTTGCTCTTCTTATAATGTTCTTTTCCCTATAGAGTTGGACATGCATA
TACATGTTGTTTGGGGAATTTGCAGCACATCCAATGAAAGCATAATTCCCAACTAACAGT
CAAATCCTTTTCTTTGGAGCTACCCAGAACACACCTACCCTCTCTTCCATATGACAG**CCC**
TTCAGAGACTTGAAGACAGTGTGTGACTCTCCTGTGAGTTAAGAGCACTGGCTGAGTCAG
ACAGACCCGAGTGACAAGCCCAGCTTCGCTCCCTCTCAGCGGTGAGAGCCAAGGTAAGTT
ACTTCATTTTAATTTCTCTCTAGTTCAGTTCTTTAGCTGATTTAGAAAATGGGAGTAAAA
AAGCCACTTTGTTTGTTTATCGTTCAATAAATAAGATGAGGTATGTAAAGTGTTTAACAT
AGTGCCTGGAATAGTAAGCACTCAGTAAATGCTAGCTATTTCCCCCTCTTTATTTATAAA
GAGCCGGTGCGCATTTTAAGTGTCAGGGCTGTGGGGGAGGAAGGAAGTGGGGAGGGCTTC
TACTTCCCTGAATGATCAAGGAAGGAAGAAGATCCTAAAGTAGACCAGGTATTTCTTTAG
CCTTAGGTTGTCTGTTTTCAGTTATCTGGATGCCTATTTGCTAGGACTTGGGGAAAGAAT
ATTGTTGAGAATCTAGGGAAAGGCTTATTTTCAACAGCTGGAAAGCTATTTGTTGACATA
TTGAACCGTCTCTTGTTTATTCTTATTTCCCTGAGCAGCTAGCCATGCAATCCCCTAAAC
TCAATTACCTTTTGTGAGGCGGCAGGAGCATGACACCTCCTAGCCGTATTGATCAGTGCA
GACTGGGTGGGACGTGAGGGACCTGAGAGATGCTAAGCGGTAAATTCTGGGCTTTTCCTT
CCTTAGAAGGCCTCTCTTCTCTGTGTTCCTAGAGACTCTGGCTTTAATAGAACGGAAGTC
TATGCCTCACATGGGCTGAGCATGCAAGAACCCTCTCTCAGTAGCCTCCTTAGAAGCAGG
CTGATGAAAATAGTCACAATGGGTCAGAACTTGGAACCCTGGGGTGACCAGTGCTAGCAT
GGTTTTCTAACTGCAGCTCTTTGCACACACTGGGTTTTGCAAGCAATTGAATGATATGAA
CTCTGAATGCCATTGCAGCTAGAGAAAGTTTGGAAGGAGACACACCCAACTGTGAACAGA
CAGAGGAATGGGACTTGCGGGGAATGGGGGGTGGGGCATTTTCAGGAGTTTAGCTTTGTC
TGTACGGAGGATTTCAAGTCTTTATCCTAAGAATATATTCCTAGATTACTTATGGAATTA
CTAAACATAAATAAAAGTTAGAGAAAGAAGAAATTATTTTTAAAAACAAACCAAAAATAA

AACACAAAATAAAGTGGTACAACCATTAAGCTTGTCGGGGTAGAAAGATTGCTTGGGTCG
GGGGGTGGATGTCCGGGATTCCTAGAGAGAGCTTGCAAGGAGAGGAGTGAAAAGGGCTTG
AGAAAGAACCCTAGGGAAACATATCTTTAAAAAAAATAAAATTAACTTTTATTAGTTTTT
TTCTTTTCGTATTATGGGTCCATTATAGACCAATGAGAAAATACAGATAAGGAAAATGAA
AATTAAAAATCCCCTCTAATCCCATCATTCAACTGTGGTCACTGTTGGCGTTTTGGTATA
TATCCTTCCAGATTTTTTTTCTATGCAGAAATCTACATATTTATCTTTATGATAATCAGA
TCCTCATGTACACATTAGAATATATTTTCAATGGCTACAATAGTGGCTGCTACAATTGCA
CCATGATTCGTTTAATCATTAAACAAGTTGTGTTCTAACATTAGACACTTAGGTTATTTC
CAAAATTGTTGCATTAGAAATAAACAACAGTGCCCTGTAGCTGTGTGACATTGGGAAAGC
TAGTTAACCTCTCTGTTCCTCTGTTCTCATCTGGAAAATGAAGGTGCTAATAGAACCTAC
CTCGTAGTATGTTGTGAGTGTCAAATGAGATCATATAGGTAAAGCACTTAGCACAGGGCC
TGGCACAGTAAACGTTAGCTTTAATTGTTAGGTGTGTTAATAATAATAATAGTAGTTGTT
GTGAGGCCATTGTTAATAGAATGGCTATTCTGTGACTTTTTTTTTTTTTTTTTTTTTTTT
GCTTTGAAGGGAGAGGAGAGCGACTGGAATTTTTCTTTTGAGTAGTATGCTATAGTGAGC
TCTGGAGTCACACAGAGCTGTGTGCCTTTGGTCATGTCAATTCATCTCATGGAGGCTCAG
TTTCCTTACCTGTATACTGAGAATTTAAAGGACCTAGCTCACAGGGTAATTGTCAAGATC
AAATGAGATAATGCTGGTGAAGCCCTCAGCCCCATTCTTGGCATATGGGAAGAGTTCAAG
CAATATCAGTTGTTGTTGTTATTATTACTACAAGGATTCAGCCATTTGTAGTAAGAAAGT
TTCCTTGCTTTGTGGTGTGGGGATGTAGGGGAGATGGCAGTGAGAAGAGTACTCCGAGTT
TCTAATCACTTGTCTGATTACAAAGAGATAAAAATATTTTGGAAACAGAATGGCCAGGGC
TGAAATCTGGGATTTAAAAAAAATTATTATTATTATTTTATGTGACCGTGGTCACATAGA
CTTCTCCAAGCCCATTTCCTCATGTATAAAATGGGAATGATCTCAGTGCCTACCTTACAG
GTGTTGCTGGGAGGTCGGAATTATATAATGCATATGTAGTGCTTAGCCCAAGTGCTCAGT
TGTTGTTGGTCGTTAATTATGAGGTTTATAACGACCTGAATGCCTCCCTTGTCAAACTCT
ACATCCCTATACCTAGGCTACTTGAACTTCACCTGGAGGCTTTGCCCTATCCAAGGCTCA
GGGACCAATTCTCCCCTTAGACTGAGGGTTTCTCCCCTTAGACTGAGTGCACCAGTTCAG
GAAAACTGGAGTACTCTGGTAAACCTTTCTGCTAGCTCTACTCTGACCTTGTGCCCCAGT
TCTAGAAAGCAGGCTGTAGCCATGTTTGTATGAACAAGCCCTCAGCTGGTATGCTCAAGG
CTGGTGTGCTTTTCTTGCTAGCTGTCTGAAATCCATGGAGCCTTTCTGTTGAATCCTAGC
CCTATGGCAGGGATACCGGTCGCTGGATTTTTCTCCGTGTCTGTGACCCTGGGGTAAATT
GTCTTCGTTATGATCACCATTACAGGGCTCTATTAAGGTGTTTGCTTGCCAGCCCCCACT
GTCCATCCTCCCAAACCCTCTTTCCCTGCACCTGAACCTCTTTGAACGTTTCTGATGCTG
CTCCACTCAATCTCCAGCCTCAGTTTCTTTCCCTCGAGCCCTCTGCCTGTGCTTCCATGT
CATATCAGTTGCAGTGCCTGGGTCCCAGGACTCCTGTGTCTGTTCAACTGCAGAGGGCAC
GTATCTTGTTTCCTGAGCCAGGACCCTTTTCATCTCATCTTTTTAATTGTGTCAATCTCC
TCTTTTCTTTGGGCAGAGTGGGGTGGGGCATAGGCTGGTGGGACTGTTGGGGGCGACAGG
GAAGAGGGGTGGAGTTGCATAGGCCAAGTGCTAAGGCGGAGCTTCTGGAGGACTGTCAAG
TGAAAGGCGGCCAGAGGCAAATGTCAGAGCCTATATAGAGGCAGGAATCTGGGACCAGAG
AGACAGAGCAAGCAGTAGCAAGTCAGGAGCAGTGGGGCGGGTGGGCGGGCAGGCTGGCTG
GCGGGCAGGGGGGCGTGGCAGGGGATAGCAAAGGGAGATTGCAGAGAACAGCTGTTGGTA
CTGGGTCTTACTTTAATGAACTGGCTGGGCACGAGGTATAAGTTAATTGGCCAGAGTGAA
AGAGCTAGGGGCGCAAAGGATACTCTCTCCCTCTTCTCCGTCACTGGCAAGGTAAGATCT
TTCTGGGTTTTGCCTGGTGGGAAACTGTGGCATGTTCAGATATTTTTCTGAAGATGGGAG
GTGGAGGTCTATGGCTGCTTTTTTGACTCATCTTTCCCATTGCACCAATTATTACAATTA
TTACAATAACCAGGGTGTCAGCAGGAGGCCAGCATTTCTTTTATGACAACCCATCTTGGG
GTTGTCCAGGCTGGAGACAGAGGCAGGGGTCTCGGTAGTGTGACTGAGTAAGGGGGAGAG
GTGGATCTTTATAGCACCTTGGTGAAGAGTAGGGGGCTCTGGAACTGGATGGACCCAGGA
ATCTATGTGACTGCAGGCAAACTATTAAGCTTCTCTGGGTCTGAATTTTCTCATTTGTAA
GATAAAGGAAGAGTACAACCGAAATCTCTTAGTGTTGCTATGGGAACCACATGAGATGAT
ACATGTAAAGTGCCTAGCAGAGTGTTTGCCAGTCAGTGCATGCTAAAGAAATGTTCACTG
TTAGCGTTTCTGTTGCAGGTTTTCTCATTATTATGGAATCACAATTCCATGAGGTTGATG
ACTTTTGTGACATTGTCCCAAAGTAGCAAGTTTTCTGCTTTTGGCATGGGCAGGCTGTGG
GGACATGTCAACATTGTGTAATATTTGCATTGGCTTCCAAGCCGTGTGTCCAAAAGCCCC
TTCTGGGCTGCTTATTGGAGCAGGCTGGAGGGGTGAGGCTCTGGATTGCCCACCCCCATG
GCATCAGAGAAACTTGAAACTTGGACATGCCAGACACTGGGCTTTAAGTGACCCATGATC
TGGGATGTCAGAGAAATAATTGAATGCAAGCTCAGGGATGCCAAACACTAGGCAACCCCC
AGGAGGGGGTCAGAGTCAATGCTATACAGTTTGTTACTGTTGCAGTGACCCTATTTTAGC
CTTGACCCTGGCTAATCTGAGAAAGCTTGTAAAATGGGGATAATAATATCTATTGCCCAG
GAGATTGTAAGACCCAAATGGGATAATGCTTACAAAGGCCCTCACGTTATAGGAAGTTGT
GACAGTTGTCTAAAGTACAGTGCCTGGCACATACTACGTGAAGCAATAAATAAATAGTAG
TAATGGGGTGGTGGTTGCTGTTATTAATAAGAATAATGAAAATAATAAGCAGAATTTTCA
GTTCATGGAATGTTGCTCTCAGTCATTGATTAGATAAATGTCTCCCTTTTCCTGAGAACA
TTCAGGGCCATCAGAGTAGTAAAATGAGGATTAAACCACTGGCAGAATTCAATGAGCCCC
AGGAACTAATAATTATCCTGGGGCAAAGGACTTTCCCATTCAGGCTTGCCAGTACCTTGG
CTGGCTTGTGAGTACTGCCGTGGGATAGAATACAGGCCAGGAATCCGCATTTCTCACATT
TGGCCAGAAGGTGGTGCTGCTGCTTTATTTGAGTCAGGACTGCCCAGGCTCCTGGCTGCG
GAGGCTGCTGGCGTTTATAGCATTTACAAACTGAGAGGCTGGGTTGGTTTGAAAAGGATA
GAGGCAGAAGGGACATTTGCTCCAGCTCCAAAGTTGTTCATTGCTTCACAGCTTCTTTAA
TCTGATAGCCTCACCTTCAATGCCATATACCTGACAACGCATAAAAACAGGTCTCCACCA
TGAACAATTTGTCTGTTTCCAAATCTTCTTGATACTGGCTTTTGACCTCTAAACTTGTTT
CTTGTTTAC**AGATTTTCTTGCCTCCTGGGCTCCCTCCACACTATCCTGTGGCTGTGGCAG**
ATATGTCAATACCCTTACATTCACTGCGATTCAACAATACAATGAGGGAGGAAAATGTTG
AGCCCCAAAACAAGCAGATGGCCTTCTGTAGACCAATGACAGAGACCAGAGCAGACGTAC
AGATTCTGCATTCTCATGTACAGTTGCCTATAGTCTCAACTTCAGCCTCAGACCCTGGAG
GGACATCCACACAGTTGATGACATCTCCAGTCTTTGACACCATGTCTGCACCTCTAATGG
GAGTACCAAACTCTGGAGCATTGTCCCCACCCCTAATGCCAGCCTCAGACTCTGGGGCAC
TTTCCCCATTGCTAATGCCAGCCTCAGACTCTGGGGCACTGTCCCCATTGCTAATGCCAG
CCTTAGATTCTGGAACATTGTCCCCATTGCTGTCCACTTCAGAGTATGGGGTAATGTCCC
CAGGGATGATGACAATTCCTGATTTTGGAACGATGTCCGCAACGCTAATGGTAGCACCAG
ATTCTGCAGAGATATCACCATTGGCAATGCCAGCTCCATCCTCTGGAGTGGTGTGTACAC
CTATAATGAGCACTTCATCCTCTGAGGCAATGTCCACACCATTAATGCTAGCCCCAGATT
CTGGAGAGTTATCCCCAATTCTAATGCAAGATATGAATCCTGGAGTGATGTCTACACAGC
CAGTGCCAGCTCCCAGCTCTGAGGCAATGTCCCCATTGCAAATTACAGATGAAGACACCG
AAGCAATGTCCAAAGTGCTAATGACTGCTCTAGCCTCTGGAGAGATATCTTCGCTGCTAA
TGTCAGGCACGGACTCTGAAGCAATATCCTCACTGATAATGTCAGCTGTAGCTTCTGGAG
GTACATCACCCCAGCCAACAAGCACCCAAAACTCTGGGGGAATACCTACCCCGCTCATGT
CAGATCTAGACTCTGGAATAATGTCATCGCTTTTAATGTCATCTCCAGGCTCTGAAGTAA
TGTCCACACCGCTACTGTCAGTCCCAGATGCTGGAGAAATGTCCACATTACCAAAGCCAG
CTCCAGATGCTGAAGCAATGTCCCCAGCACTAATGACGGCCCTACCCTCTGGAGTGATGC
CCACCCAAACGATGCCAGCCCCAGGCTCTGGGGCGATGTCCCCATGGTCAACACAAAATG
TAGACTCTGAAATGATGTCTAATCCGCCAGTGAGAGCAACAGCCTCTGGGGTGATGTCTG
CACCACCAGTAAGAGCTTTAGATTCTGGAGCAATGTCCACACCGCTAATGGGAGCCCCAG
CCTCTGGAAATATGTCTACATTGCAAAAGACAGTTCCAGCCTCTGGAGCCATGACCACCT
CACTGATGACAGTCCCAAGCTCTGGAGTGATGTCCACAGAGCAAATGTCAGCCACAGCCT
CTAGAGTAATGTCCGCACAGTTAACAATGGCCAAAACTTCTGGAGCAATGCCCACAGGCT
CTATGAAAGCCGTGGCAAAACAATACAAGAGAGCCACAGCCTCTGGAAAGATGTCCACGC
CACTGAGGAGAGCTCCAACTTCTGGAGCAATGTCCACCCAACCAGTTACGGCAACAGCCT
CTGAAACAATGTCCATGCCACAATTGACAGTCCCAGCCTCTGGATCAATGTCCATGCTGC
AAATGAGAGCCCCTGTCTCTGAAGCAATGTCCATGCCACAAATGAGAACCATGGCCTCAG
GATTGACATCTGCAGCACAGATGAAAGCCATGACTTCTGGAGCAATGTCCACCCCACTAA
TGACAGCCCAAACCTCTGGATCAACATCCACGCTGTTAATGAGAGACACAGCCTCAGGAG
TGATGTCCTGTCCACAAATGAGATCTCTGGCCTCTGGAGCATTGTCCAAGCCACTAATGA
CACCCAAAGCCTCAGGAACGATGTTCACGGAGAAAATGACAACCACAGCTTCTGAAGCAA
TGCCCACACTGCTAATGAGAGACACAGTTTCTGGAGCTCTGTCCATGCCGCAAATGACAG
ACACAGCCTCTGGAGGGTTGTCTGCATCGCTAATGAGAGACACAGCTTCTGGAGCAATGT
CCACATCACAAATGACAGCCACAGTCTCTGGAGGGATGTCCATGCCACTAATGAGAGCTC
AAGACCCAGGAGTAATGCCTGCCTCACTAATGAGAGCCAAAGTGTCTGGAAAGATGCTCA
GTCAGCCAATGAGCACCCAAGATCCTGGAGGGATGTCCATGTCGCCCATGAAGTCCATGA
CCGCTGGAGGGATGCAGATGAATTCCCCAACCTCTGATGTGATGTCCACACCAACAGTGA
GAGCCTGGACCTCTGAAACAATGTCCACACCACTAATGAGAACCTCAGACCCTGGAGAGA
GGCCCTCACTGCTCACAAGAGCTTCATCCTCTGGAGAGATGTCCCTACCACTAATGAGAG
CTCCAGCTTCTGGAGAGATAGCCACGCCTCTGAGATCCCCAGCTTATGGAGCCATGTCTG
CTCCACAAATGACAGCCACAGCCTCTGGAATGATGTCATCCATGCCACAAGTGAAGGCTC
CCATCTCTGGAGCAATGTCCATGCCACTAACAAGATCCACAGCCTCTGGAGGGATGTCCA
TGCCACTGATGAGAGCCCCAGACTCTAGAGTGACATCCACATCACAAATGATGCCCACAG
CTTCTGGAGACATGTGCACACTACCAGTGCGAGCCCCAGCCTCTGGAGGGGTGTCCTCAC
CACTAGTAAGAGCTCCAGCCTCTGGAACTATGTCCACACCACTAAGGAGACCCTCAGCCT
GTGAGACTGTGTCCACAGAGTTAATGAGAGCTTCAGCCTCTGGACATATGTCCACTGCAC
AAACAACAGCCATGGTCTCTGGAGGGATGTCCAAGCCATTAATGAGAGCCCCGGCCTCTG
GAACAATGCCCATGCCTTTAATGTCAGCCATGGCTTCTGGAGAGATGTCTATGCCGCTAA
TGGAAACCATGGCCTCTGGAGCAACGTCCACATTGCAAACCAGTGTTGCGAACTCTAGAT
CTATGTCCTTGTCACAAACAACATATACCGTGTCTGGAAGGATGGCCACAGCGCCAATTA
GAGCCTCTGCTTCTGGAGCAAGGTCCACATCATTTATGAGAGCCTCAGTTTCTGGATCGA

TGCCCATGCCACTACCAAGAGCCACAGCCTCTGGATGTGGCATGGGTATGTCCATGCCAC
AAATGACAGCCACAGACTCTAGAGGGATGTCCACACCACTAATGAGGGCCTCAGGCCCTG
GAACAATGTCCACACCACAGACAGCCTTTGGAGTGATGTCCACTCCGGAAATCAAAGCCA
CAGACTCTGGAGAGGCATCCACCTCTCACATTAACATCACAGCCTCTGGATCAAAGCCCA
CATCGCACATGACTGCCACAACTCCTGAAACTGCGAAACCACCACCAAAGGAAGTGCCAT
CCTTCGGAATGTTGACCCCAGCACTCTGCTACCTCTTAGAAGAGCAGGAAGCAGCCCGGG
GCTCATGCTCTGTGGAGGAAGAGATGGAGATTGATGAGGAGAAGCAAATGAAGGGGTTTT
TGGACGATTCAGAGAGAATGGCATTTCTGGTATCTCTTCATCTGGGAGCAGCAGAGAGGT
GGTTCATCTTGCAAATGGAGGTAGGAGAACCTCTCTCACATGAAAATAAATCTTTCCTGA
GAAGATCCCAGGGCATATATGACTCCCTATCTGAGATAGACATCCTCAGTGCTGTTCTTT
GCCATCCCAAACAGGGCCAAAAGTCAGTCAGGCAGTATGCCACTGACTTCCTGCTGCTGG
CCCGACATTTGTCTTGGTCTGATGCCATTCTACGGACCAGGTTTCTGGAAGGACTCTCAG
AAGCTGTTACCACCAAAATGGGTCGGATCTTCCTGAAGGTGGCTGGCAGCCTAAAGGAGC
TGATAGACAGGTCTCTGTACACCGAGTGCCAGCTGGCTGAAGAGAAGGATTCCCCAGGCA
ACTCAAGCCAGGTTCTGCCAACAGCCTGTAAGCGGAATAATGAGGAGGCCATGGGGAATG
AACTGAGCTCTCAGCAGCAGACTGAGGAGGTAATGAGGGGGAAATCCGCTGAAGGTTTTT
GAGCAGAGAAATGAGGAGGTCACGACAGGGAATACATCCTGCTTCCTTGAGTAGAATTGG
GAAGAGGCAAGGGGGAGGCATGGAGCTCCTAGACCATTAAACCCCAGCAGTTTCATCAAG
TGAGCATGGCTCTGCAGTTATTTTGGGTATATTAACTACTTTGAGGAGCAATGTCCTGCC
CAAGTTACTGAGAACGTGGGATAGTTGAAGTACTAATTCTTCTCATTATGAGGGAACTGG
ATGGGCTCAGTTCCAATTCTCCCCTTGTTCCAAGTCCAATTCTAATAAATGGGTCTTTTG
GGAGCTGGGGCCCATCTACAGCAACATGTGTTGTGGAGTCAGGTCACTAGGGAATTAGAA
TAGCCTACTCCTTGGTCACACTGTATGCAAGTGAGATAAATTAGCAAAGTGTCTTGATCT
GCTAAGTCACAGAACTTATCCAGGCAGAACACCCGTTGCCATAGTAAGTGTGGCTCTTAC
CTCTGTTGTCTTTTTCTCACTCCCAG**CACCAGCATGTTTCCAAACGCTGTTACTACCTGA**
AAGAGCATGGAGACCCCCAAGAAGGTCTTCATGATCACCTTGGACAGAGCACAGGCCATC
ATCAGAAGGCCCATACCAACAAGTAAAACTCCATGGAATCTTCTCCTGTGATATCTGACT
CGACCGCTAACTGGAGGACTGGTTCCTGGACCCATTCCATTAAACTACATTATAAACCTT
GTTGCCTTCACCCTTCAGCCTCCCGCTCCAGGCACATCCCACCTTACCTCTCACTTGGCT
GATTTGACCTTCTCTTTCACCCACATGCCTAAGACCTGCCCTGACAATCAGAGTAGGGAC
TACAAGAGTGTATGGTGTATGAGGTTCTGAACTCCCATCACCACCGAGGCCAGTCCTAGT
TCTTGGTCCAGGGAGAAGTCTAGGCAGGATAAATCATATCTTACCTCAAAAAACCCTTAG
CGTATCTTGCCATGTGTCAGCAGGCAAACTTGAGGAAGCCCTGGTTCTATTTCACCTGGC
AGGGGAGCCTATAAGGAGAGCGATGCCCACTCCTATACCACCCTTGTAACCCAGTAGACT
TGCCCCATATGCCCCAACTCATAGCACCTAAAATGGGTCTCCAGGGTCCTTGCCCTGTTT
ATAGTATCCATCCTGAACCATTCGTTTTGACCCATAATAATAGTGATAGTATAATGGCTA
CCAGTTATTGATAATGTATCATGTGACAGGCATTGGGCTAAACTCTTGACAAGCACCATC
TCACTACATTCTTACAGTAACACAACTTGTTCTTGTGTTGTCAGCTTCTGACCTCTTGAT
CTGTTGTTTATGGCCTTTGTCTTGTCCCTCAGTGGGGGTTCTCTAGCTCTGACCTCTGGG
TGACCCCCTCAGTTGTTGATTGCTCTCACTCCCCTTGGCTGTTGTGCTTCATGGCCAGCA
CTCTTGATGAGGGTCTTGGTCTATAAACAGGCATAGATAGCTAGAACATAATGTGAGCAG
TGTTTGCCTTCAGGTGGTGGGGTTATGGGTAGTTTTTATTTTCTTCCTTCTACTTCTCTA
TACATTTCAGATTCTCTACAGTAAGCATGTGTTACATTTGTAATTATAAAAGTAAGTAAA
CTTCATTTATGAAAAAAGAGTTCATTGTGGCTCCTAGAATTTGATTTACTGAGTCCTTTA
TTCAACCAATATTAGTTATGTAACTTATGTCAGCTACTCTTTGGGGGACAGACGGATAAG
AATCCTGCTGATATGAACATTCCATTCCAATGGAGTGGGGAGAACAAGAACACACTGAAC
TTAGATTGGAATTGTGAAGGCTGTGAAGAGAAGAAATCAGGACAATGGGGAATTTGATGC
AAATAAGACCAATTTATGTATTCAATAGTAGTCCATATAATTGAGTTACTCAAAGATCTT
TCAACAATAGTAGGAGAGACATGATTAGAAACTTATTTTATATAGTTAAATTGGCAACAG
TTTGGAGACAGGATGGAGGAAGGGATCAGAGAGCAGCTGAGCCCTCTACTGCAATAGTCC
AGGTAACTGACTGGCTAGGTGACTCAATCTAGGGTGGTGGCAGCAGAGATGATAATTCAG
GAACAGAGACCTAACCTTCTCTGAGGACTCAAGTCCACTGCTCCAGGACTCAAGTCCACT
GCTCCATTACCCTAAACTTTGTGTCTGATTTCAGGAAGAGGAAAATGATACTCAGGAAAG
ACAGATTGGCAGATAAATC

```
Homo sapiens          AAACCTCAAAGGGAGTGTAAAATGCAGAGTGTTGTCAACTTATCACACCAAGAATATTTTTTTGCACCTTTTTAGTTTGGAAGGGTCAAATTGAGATGACAGAGGAATAGTGGATAAGAG
Dasypus novemcinctus  ........................................................................................................................

Homo sapiens          CTATAGTGCTATTAAATTACATTCTAGAGTTGGGAAAATCGAGACCCATAATCCTAGGAATTATTAATGATAAGAGCTAACACTTTCTGAGTGCTTACCACATGCCAGGCCCTATACTGA
Dasypus novemcinctus  ........................................................................................................................

Homo sapiens          ATGCATCATGTCCATTTTCTCATTTGATCCTGACAACCACCTGTGAGGCCTCATTTTGAAGACAAGCAAGCTGAGGCTCAGACCTTGGATTTGTCCAAGGTCACTCAGTTGGTCATTTAC
Dasypus novemcinctus  ........................................................................................................................

Homo sapiens          AGAGCCCTGATTCAAACTCTCTTTTGCTTGATTACAAAGCCATTGCTCTAAAAGATTAGATTATACTGTCTGTATTTGCTTCTGGGAGCTTATGGAATTCACTCCTATCTGTTCTCACAA
Dasypus novemcinctus  ........................................................................................................................

Homo sapiens          GGAACTCTATTGGGTTTTGAAAAAGGCTAAAGAATTGAAAAGAAACTTTGTAGGCTGGGAAACATAGTGTCCTTTGAGCAGTGAGATAGGCTAAGCTCAGAGGACTGGGCTCTGGAATGT
Dasypus novemcinctus  ........................................................................................................................

Homo sapiens          GGAAAGGAGACGTGCCCACTGGAGAGCAAGTCAGAACTTTCGTGAGCCCTGCTCCAGGTCCCAGGAGAAAGTGCCAGCCATGAGGACTGGAGTATTGCCTGGTAAGGAGAAGATTATTAT
Dasypus novemcinctus  ........................................................................................................................

Homo sapiens          TTTATCTAACACGTTCTTCTATAACGCTCTATATGCTAGGCACTGGTCTGCACTGCTCTAAGTACTTTATAAGTATTAACTCCTTTACATCTTATAACACTTCCCTGGAAATAGGCATTA
Dasypus novemcinctus  ........................................................................................................................

Homo sapiens          TTATTGCCCCCATAATACATATGAGTAAACTGAAGTTCAGAGATGTTAAATAACTTGTTCAAGGTCACATAGCTGGTTAAGTGCCAGAGCCAGGCTTTGAACCTAGGCAGTCTGGATCTA
Dasypus novemcinctus  ........................................................................................................................

Homo sapiens          GTGGCTGTGTTATACTGCTTCGCAACAGAAACTTGATTTTTCTTTCTTCTGTGTTTATCATCCTTTCTCAATCACTCCTGGGCAAGTCTACTTTGCTAGCAAATTTCCCTTACCCCTTTG
Dasypus novemcinctus  ........................................................................................................................

Homo sapiens          GCTTTTTTTTCACCCATGAACCCTCTAATGTTTTCTGCAGCTTCTTTGAGCTCATTCTTTATCTTCCACACTGCCTCTTTTGGCATTACTCATGAACGACATGCTCTTTTCATCTGCCAA
Dasypus novemcinctus  ........................................................................................................................

Homo sapiens          ATATTTTTTCCATAAATTATCCCATCTCAACTTGGGAAGTCACAATGGGAAGTATTGTTCCTATTTTGTGAATGAGGCAACTAAGGCAAGCAACTTTCCTGAAGTCTCACACAACCATCT
Dasypus novemcinctus  ........................................................................................................................

Homo sapiens          AGTGGCAAAGTGCACTAGAATCCAGATTCTCTGATTCTGAATCTAATGTTCTTGTCCATTGCACCATCTGATGGAATTTAACTATCTATTCATTAATTAATTTATTCATTAATATTGTAA
Dasypus novemcinctus  ........................................................................................................................

Homo sapiens          AAATATACTGAGTGTCTTACTGTATGGTAAGGCCCTGGGTGCAAAAGTCGGTATAAAAATTGTTGCTCTTAAGCAACAGACAACACAGAGAGATAAGGTCTCACCATAAAAAGTTAAATA
Dasypus novemcinctus  ........................................................................................................................

Homo sapiens          GCAGCACATATACTAAGTGCCAAATGAGTGGTCTGGTCACATCATACAAGAATTTTGAGGTTGGATATGTAGCATCAAAGACTGCTTTATCAGAAAGGTGAGACTTGAACTTGGCATAGA
Dasypus novemcinctus  ........................................................................................................................

Homo sapiens          AGGGTACTTAGAACTTAGAGAAGAGAATGGAGCAGCACTTTCCAAACTCTGGGAATGGTGTGCATTAAAGCAGTCCTTCACAACCTTTATTGTGTCATGGCATGCAGAGAAAAGCATAGT
Dasypus novemcinctus  ........................................................................................................................

Homo sapiens          ATTTGTAAAGCACACTAGGCTGAATGGAGGAAGCAGTTTGACATCTAGAGGCAAGCATTCTAGGAGTTTCAACAGCACTGTCTCTGGGCTCAGTTGAGGACAGAGAAGAAATCTCATAGC
Dasypus novemcinctus  ........................................................................................................................

Homo sapiens          CTCAGACACTTGTAATATGTTTGGGAAGGTCTGCACTAGGTATTGGGGGCACACTGTGGTCATGGTGGGTCTAAAGACCACTGACAAGCCCACAGGGATGAAATGAGGGGAATTTCCAGA
Dasypus novemcinctus  ........................................................................................................................

Homo sapiens          GAAGCCATAGTAGATACCATTGGAAAGAAAAGTTATGGGCAAATGATGAAGGGCTTTTGAATTTTATTCTGTGGACCTACAGAATAGGTAGGCAAAATATGCATAGCACTGTTTTAGAAA
Dasypus novemcinctus  ........................................................................................................................

Homo sapiens          GATTCCACTGGTTTTCATATGGGAGATTAACTAGAGGGAAGAAGAGTAGTCAAGAGGCTATTACCATTGTCCAGGTGGGAGGTGATGTTATCTTGAACTAGATTGATAGTAATAGAGACA
Dasypus novemcinctus  ........................................................................................................................

Homo sapiens          GAGAATGGTGGACAGAATTGAGTTATATTTTGGAGGTAAAAATGTCAGGGTTTGGTAATAGAATTGCATAATGGAGAGTAGAAGAAAGGGAGGTGACAAGGAGGATTTCAAATTTTCAAG
Dasypus novemcinctus  ........................................................................................................................

Homo sapiens          TTTAAGCAAATGCTTATGAATAGTGATGCCATTCTTTAAGATAGAGAGCCCAGGAGAAGTAGAAAGATTTTTTTGGAGGGGAAGGACAAATGAGATGATGAGTTTCAAGTTGACATTTGG
Dasypus novemcinctus  ........................................................................................................................

Homo sapiens          AGGCAAGGAAACCAGTTAGGAGGCTATTGTAAAAGCCAAGTAAGACAAGGTGAGATCCTGGACTACAATGGTAGCAGTGGGAATGGAAGGAAAATGGTGAATTGAGAGACATTATACAGG
Dasypus novemcinctus  ........................................................................................................................
```

```
Homo sapiens            AAGAATTGATAGGATTTTGTCACGGACTGACTTCTGGAGTTGAAAGGTAGAGGAAGGCTTGAAGCCTAAGATATCTTCTGTCCTTTATCTTCTTAGTGATGCTCCGATCTTCGTGGCTTA
Dasypus novemcinctus    ........................................................................................................................

Homo sapiens            ATATACTATCTATTTGCTGATGAGTCACAAATTTGTATCTCCATACTTTTATGCCCAATTGCCTGCTGGACATATCAACTTGCATGCCTAATAGGTATCTCCAACTTACCATGTTCAAAA
Dasypus novemcinctus    ........................................................................................................................

Homo sapiens            CTGCCCCTGCCCACAACCCCCAACAAAACCTGCTGCACTCACAAAACCTGCTCCTCACAGTCTTCCCCGTCTCCCTAAATACCAATTTCATTCTTCCGGTTGCTCAGGCGGAAGACCTAG
Dasypus novemcinctus    ........................................................................................................................

Homo sapiens            GAATCAATCTTGATTTTTCCCTCTTTCATATCCTACATTTATTTGATCAGCAAATCTTATTGGTTCTACCTTCAGAATATATCTAGAATCTGACCACTTTTTCATTGCTACTATCCTGGT
Dasypus novemcinctus    ........................................................................................................................

Homo sapiens            TTATGTCACAATGATCTGCTTCCTGGATTATATTGCACTAGCCTCTCAACTGTTCTCCTTGCTTCTGTTCTTGCCACCATCACTCAAGCCTATTATTCTCTCACTGTTTTTTGGAACCAG
Dasypus novemcinctus    ........................................................................................................................

Homo sapiens            GATAATTTTATTATGTGAATGTCAGATCATGTCACTCCTCTGCTCAAAACTCCTCCAATGGCTTCCCATCTGACACTCAGTAACAACAAAACCCCTTATAATGGCCCATAATGCTGTATG
Dasypus novemcinctus    ........................................................................................................................

Homo sapiens            CAACCCAGATATTGACATACTCCTGTCCTTCAGGTGATGGATCAAATATCCCACTTGGTAAAATCTTCTCTGATTACCATATTTAGTATTGCAAGCGCCTTCTCGCCATTCTCTATCTGC
Dasypus novemcinctus    ........................................................................................................................

Homo sapiens            CTTCCATGTTTTATTTCTTTATACTTTGAATACTAGCAGATGTTATATGTTTATCTATTTATTTGTTTATCATCTATCTCCTTCTGCTAGAACAGGAGCTGGAAAACTTTTTCTGTAAAG
Dasypus novemcinctus    ........................................................................................................................

Homo sapiens            GTCCAGATAATAAATATTTTAGGCTTTGTGAGCCACGTAATCTTTAACACAACTACCTACCTCTATCATTGTTATACAAAAGCTGCCATAGATGAGCATGGCTGTGTTCCAATAAAACTG
Dasypus novemcinctus    ........................................................................................................................

Homo sapiens            TATTTACCAAAACAGGGAGCGGGCTAGATTTAGCTAATGGGCCATAGTGTACTGACCCCTGCTAGAATGTAAATTCCAGGAGGGCAAGGACTTTTGTTTACTGCTGTATCTCCAACATCT
Dasypus novemcinctus    ........................................................................................................................

Homo sapiens            AGAATAGTACCTGGCATATAGTAGGCACATGATTTATTTTACTGAATGAATGCATCAGTGGGAGATTGGAAAGCTGTGGAATAAAAAATATATATATATAACAGAGCCATTGATGGTGCA
Dasypus novemcinctus    ........................................................................................................................

Homo sapiens            GAATCCTGAACAAAATCTAGCTTTGGATGGGTTTGCAGCATATCAACTCTCCTTCCTTCTTTTTCCCCTCATCCCTACACTAAAACTTAAGCTTGTTATATAAAGTAAAAGTTGCTTTCA
Dasypus novemcinctus    ........................................................................................................................

Homo sapiens            TTGCCTGTGCAATTCTCCCAATGTGTGAAATTTCATCTGACTTCCTTTAATGTTGTCATTTGTCTATTTAACCCTCCTGACTCAGACTCCCTCTAATAACAATAATAACTCAAATACTCT
Dasypus novemcinctus    ........................................................................................................................

Homo sapiens            GTACTAAAACCAGAACGCTGAAATCAGTATTTTTAATTAATTTGAAACAACTGCTAGCAATAGCTTCTATTGACAACTCTACGCTTTTCAACACTTAATCATATTCTGAAGTACTCCCAG
Dasypus novemcinctus    ........................................................................................................................

Homo sapiens            AAGCTGGGCGACTGTTACTCTTATGATATGAGATGATATTTTCCTTCCATGCATTCAATGTGAATGGTTTGTTCTGAAAAACTTCAGCTTTCATGTACTCCTAATGGTAAGAATCACATG
Dasypus novemcinctus    ........................................................................................................................

Homo sapiens            GTAGGAAATATGATAAATGAAGTATTTACCAGTTCTTACTGACTGTTCTTTCATACTTCCTTGGATCTCTTACAGGCACTAAAGATATCCTGTAGTTATATGACTTCCTATTGAAAATGC
Dasypus novemcinctus    ........................................................................................................................

Homo sapiens            ACTTTCACTCATTCATTATTTTATATAGTCTTCCACACATTGCTAACATTTACCAAACATTCACTACGTATGTGGTATTGTGCTGGGGGATTTATATATATTAATTCATTTAATTCTTAT
Dasypus novemcinctus    ........................................................................................................................

Homo sapiens            AAAACACCTAAGTGATATATACTATCATAATTTTATAGATGAAGGAACTGAAGTACTAAAAGCTGAAGTAATTTGCTCAAGGCCCCACAACCAGCAGAAAAGGAAGCTAGCATTTCAACT
Dasypus novemcinctus    ........................................................................................................................

Homo sapiens            CAGGTCTGCCCAACTCCAGAGCCAAGATTTATCTATTGTTTTTTGTCACCTCAACAAATATTTACCATATGCCATGCAGCACACTAGGCAGTACCTACAAAGATGAATAGGGCATGATCT
Dasypus novemcinctus    ........................................................................................................................

Homo sapiens            CCTTGGCCTTCCAGAGGTAACACTAGTTGGGGCTATATGGGTGGGAATGGAGTGTCAGATGCAGAAGCTAAGGAATTTTCTCTTTACAAACAGGTGGGTGAGAAAGTAGACACAGCACTG
Dasypus novemcinctus    ........................................................................................................................

Homo sapiens            TGAATTTGTGGAGTTTTGAAAAATGGACAGAGGGCTACAGACTGAGAGGGAATAGGGCCACCAACTCTAGAATTAAGGTCAACTGATAGGGTAGAAACATGCCATTCTCAGGGGCTTAAG
Dasypus novemcinctus    ........................................................................................................................

Homo sapiens            CTGACAAAAGAAATTGCCCACTGTAGAAATGAGATTGGCTGCCCACAGAAAAGTATTTACTGATAGATAAGGGACCTCCCTATGTGTCATTAAGGAGTAATGCTCTAAAATGTAATCAGT
Dasypus novemcinctus    ........................................................................................................................

Homo sapiens            GGAAAACACAACTGTAGCCTTGGAAATGCTATGGCTGGGAAAGTCCATATAACTAAGACGGGGTTCATGATATTGTGACATGTACAAACAAGTTATTTGACCATTATAGAGAGAAAAATA
Dasypus novemcinctus    ........................................................................................................................

Homo sapiens            AAAATAATTAGAAAGAGAAGTTGCTTTTATTACTCTGTGCCCTGGCCTACTGACACATTTATTGACTTTCTGATAGGGTGATGCATGGATGCATCAGACTGAATAAACTACATGACTCCA
Dasypus novemcinctus    ........................................................................................................................

Homo sapiens            TCTCTTCAGATCATCCCACACCATATACTATCTCTTCACTCACCAGTAATAACAGTCACCATATAACAAGTGCCTACAGAGTCAAACACTTTACATAAATTATCTTATTTAATCCTCAAG
Dasypus novemcinctus    ........................................................................................................................

Homo sapiens            GCAACTCTATGATATCATAGAATTCTATTATTAGTTCCATTTTCAAATGACGGAACTGAGGCTCAGAGAGGTTATGTGACTTAAGCCCATAAAACGGAATGAGTGGTATATTAGTTTTCT
Dasypus novemcinctus    ........................................................................................................................

Homo sapiens            GTGACTACCATAACAAATTGCCACAAACTTGGTGGCTTAAAACAGCAGATATTTATACTTTCACAGTTCTGGAGGCCAGAAGTTCAAAATCAAGGTGTTGGCAGGGCCATGCTCCCTACA
Dasypus novemcinctus    ........................................................................................................................

Homo sapiens            AAGGCTCTAGGGGAGATTCTGTTCCTTGCCTCTTCCTAGCGGTTCCAGGTGTTGCTTGGCTTGTGGTAGCATCATTCCAATCCCTGCCTCTGTCTTCAGATGGGCTTCTCCCTTGTCTCT
Dasypus novemcinctus    ........................................................................................................................

Homo sapiens            GTGCCCTGTCACTTATAAGGACACTTATCATTGGATTTACAGCCCACCCTAATCCAGGATGATCTCATGTAGACATCCTTATCTTACATACATCAGCAAAGAGTCATATTCTAAATAAGT
Dasypus novemcinctus    ........................................................................................................................

Homo sapiens            TCACATTTTGCAGTTCCAGTTGGATGTAACTTTTGGGGGCGGTGACACAATTCAACCCACTATGTATAGCCTAGCTGGGACCCTAGCCTATCTGACTCCAAAACTCTGGCTCTTTCCACT
Dasypus novemcinctus    ........................................................................................................................

Homo sapiens            AACTAAAGCAGGCTGCTCCCACCAAACAAACAGCCTTTTGTCTTTATTTGGTTTCCTAAGTAGATTTCCTACTGGGATTTATCAAGATGAATTAATAAATTGACATTTTGGCTTCCAAAT
Dasypus novemcinctus    ........................................................................................................................

Homo sapiens            TATTGCCTTATAAGTTTAGGTTGTGTGAAATTTGTCCCCTAGAGCCTGATCAATCCCTGGCTAAAATGGAAAAAATTGAGTTTTGGAACCTGTGAAGCTGATGAATGGATCCTGAATAGG
Dasypus novemcinctus    ........................................................................................................................

Homo sapiens            ACCAAGGTCCATTTAGAGTATAAGCATCCCACAGCCTGCCAGCCATACTAATTTCTTTACTATATATGTGACCATAATATAATGGGATAGCATAATATTCTAAGTCTACCGAAGAATGTC
Dasypus novemcinctus    ........................................................................................................................

Homo sapiens            TAATCATCTAAGAGCTTACAGGGGGAATTTTTGTGTACCTTAATATTATATCCAGAAATATTCCCTGTAGTGCCAACTATATTTGTTCCTGAGCCTCAGCTCCCTGCATCAGGTCACAAG
Dasypus novemcinctus    ........................................................................................................................

Homo sapiens            GAAGATGTCTCTAGGAGTTCATTATCACATTAAGACAAGGATTCTGTAGGAAAATGGGAATCTAGAAACAGTAGGGACTTCCTGTACCTCTACATCAGTGTCTTTGCAGGGGAATTGCCC
Dasypus novemcinctus    ........................................................................................................................

Homo sapiens            TAGCTGACCAGTTGACACTGGCCTGCTCACACCTTAATTATGAATAGAATATTCCCAGGGATGTCTTTCTCAGATTGTGGTCAGTTTGTGGGCCGCCTATTGTGGTGGTCCACTGAGTGA
Dasypus novemcinctus    ........................................................................................................................

Homo sapiens            CACCTGGGGAGCTAGTTAAAAATGTCAGTTTCCTGGTCCTTTATAGCAGACTTACTGAGTTAGTATATCTGAAGATAGGGTCAGGAGCCTGCATTTTAAAACAAGATTCTGGAATAATTC
Dasypus novemcinctus    ........................................................................................................................

Homo sapiens            TGATACATATTCAAGTTTGAGAACCATTGCCTTCTGGTGAAATCAGGCTTCCATGACGTTGAAATACAGAATAAACTCCAAATGTTCATTTACAATTCCAGCCAATAATCACTTACCTTA
Dasypus novemcinctus    ........................................................................................................................

Homo sapiens            TTGCATTCTTGTAGCGTGATCTATATGCTTTAGTTCATGTGACAATATCATTTCATATGGCTTAATTTAAATGAAAGCAAAGGCAGTTATGGCAAGATTTCTTAATCCTGCTCTCCTGAG
Dasypus novemcinctus    ........................................................................................................................

Homo sapiens            ACAATTTACATAATAACTTTTGGGGAGAATGCAATAATCTCTCTCTAATATCTTAAACTGATCTCTAAGGTTCCTTTTGGCTCCAACATTCTGATTCTATCAGTGTTCCTAGGGCTTCAA
Dasypus novemcinctus    ........................................................................................................................

Homo sapiens            ATCTGTCTGTGCAAAACACATTGCTTGTTACAGACTAAACCTATTTTCCTTCTTCCAGGCAACAAGTTTCTGAAGAAAACTGGTTGCTCATGATCTTTCTGGGTGTTTTCTAATAATATA
Dasypus novemcinctus    ........................................................................................................................
```

```
Homo sapiens          TACCTTCTCACAATATTATTGCAAAGAGTGAGCGAGGTGATCTATGCAAAGTGCCAAGCATAGTGCCTGACACAAGGAAGTGGTTAGTAAATATGCATGCTGCTTCCAGTGCATTGTGAC
Dasypus novemcinctus  ........................................................................................................................

Homo sapiens          AATACTATCTCTTGAATTATTGAAAATATAACATCAGGAAACTTCAGCTGAATGAAAACTAGGGAATTAGCTGTATTGACAATATTAGGTTTATTTGGCTTTAGGGGAACTAGATAGAAC
Dasypus novemcinctus  ........................................................................................................................

Homo sapiens          CGAAATCTCTGCAGACTGGCTCAGCTGCAGAATAGAGAGATGGCCCGGGGAGGACATGTGTTGGTTCCTGCCATGCATATTGTCTTGGAGAAAAGCTGCTATGACTCTGGCCCAACAAAG
Dasypus novemcinctus  ........................................................................................................................

Homo sapiens          GTTTTATCCTAAAAGTCAGCTTCCAATCCCCAACATGATTCCTAAGCTAGCATTTCTCAAAATGTATTCCATGGAGTACTAGTTTCTGAGGATTGATGTTGATAGTATTACATAAAGAGC
Dasypus novemcinctus  ........................................CCTACATCAGCATTTCCCAAAATGTATTCCACAGAATACTGGTCTCTGGAAAT...........................

Homo sapiens          TCCATGAACAAGCAATGTTACAAACCACTGGATTAGTCAAATGTGGTTTCCTGGCTGCGAGAATATTCAGAGTCCTTAATAGGTCAACAGGTAATGTGAATCTCCAAAAACTAATGGGGT
Dasypus novemcinctus  ........................................................................................................................

Homo sapiens          ATACCGGATTTGAAAATTTTTGTGACCTCACTCACAGAGATGGTGGTGAGGGACTAGTGGTTCTGAAAAATGCAATTATGAGAACACTACTCCAAAATGACTAGTGACTTGCTAACAAGA
Dasypus novemcinctus  ........................................................................................................................

Homo sapiens          ATGGACACTTTTCAGCCCTTACATGATAAAAGTTAAGAGAACAATTACGTGCTAAACTATAGAGTACTGTCCCTCAGCCCCCTAGTGTCTCCATTTTCTTCTTTGATCAGCTTAGATTCT
Dasypus novemcinctus  ........................................................................................................................

Homo sapiens          ATAACCATCCTTAAAGTCACTGTTTAGCATACACCCTAGATTCCCTAGGCTCTCTCTGCTTTCATGTGATTTGAATAACAGACATCCAATCGTTGTTAAATCCTACTCTCTTTATTCCCA
Dasypus novemcinctus  ........................................................................................................................

Homo sapiens          ACCTGCCCCAGCAGCCGAATGAGATTGGATAAAAGCTTATAACCGTGCTAACCAGTCTCGCTTTAGATTCAGGACCACTAACTTCAAATGGGCCCTTAGTACTATCTGCTAACTTCACTA
Dasypus novemcinctus  ........................................................................................................................

Homo sapiens          CATTCATTTCATCCATTCATTCTTGAATGCTCCTGGGTGGCTTTTTTACATCTTTCCTTTCCTCAAACTTCCGACACTTGTTTCTACATTTTCCCTTTCAGCTGCTACCTTGTTTCCTAT
Dasypus novemcinctus  ........................................................................................................................

Homo sapiens          TTCACTGAGAAAATAGAAGTTGTCAGAAAAGAACTCCCACCAATCTATTTGTATTTGTATCCATAAACTCTGCCTTCCCTCTTGTCACTGTGAGCGAACCATCTGGGATTCCACTGAATG
Dasypus novemcinctus  ........................................................................................................................

Homo sapiens          CTGACTCCTCTACTTATGCACTAAATTTTATTTCTTTTTGCCTAATCAAGGTCATTACTCAAGTCATTCACTCTTCCCTCATGGCATCAATTTTTCTTTCATTGAACAACATCTTCAATG
Dasypus novemcinctus  ........................................................................................................................

Homo sapiens          TTTAAAAAATCTGTTCTCTTCACCCAAGTCATAGCCCCATTTCTCTCCTCCTCAGAGCAAAAATTCTGGAATGAGTTGTCTCTGCTCATTCGTTCCAATTTCTCTCCTCTCATTTTCTCT
Dasypus novemcinctus  ........................................................................................................................

Homo sapiens          TGAATCCTCTCCAGTCAGGCTTTTGCCATCATCACTCCACCAAAATGATTCATATCAAGGTCAACAATATCTCCTCAGTATTAAATTCAATGGTCATTTTACTGTTCTCCTTTTTTTTTC
Dasypus novemcinctus  ........................................................................................................................

Homo sapiens          GCCCAACAGCGGCATTTGACACAGTTTATCACTCTCTCCTTGAAACACTTTTTTCTTCCACTTGGCTTCCAGGACACCATGTAATTCTTGGCTCTTTCTACTTCACTAGCTACTATTTCT
Dasypus novemcinctus  ........................................................................................................................

Homo sapiens          TAATATCCTTTACAGGTATGTCTTCATTTTCATGATAACTAGGTTGTCTGATTGGCATCTCAACTTGAAATTATCCAAGGTCAAAGTGACTATTTTCCTTATACCTGCTTCTCCCACAGC
Dasypus novemcinctus  ........................................................................................................................

Homo sapiens          CTTCTTTATCCATTCTTCTGGTTATTTAGGCCAAAGCCTTGGAGTCTGACTTGACTCCCTTTTTCTCTCACACTCCAGATCCTATCTATCAGCAATACTTTCAGCTGAATAGTTAAAAAT
Dasypus novemcinctus  ........................................................................................................................

Homo sapiens          TTATTTGTTTAGAAACAGCATCTCACACTGTCACCCAGGCTGGAATGGAGTGGCACAATCATAGCTCACTATAACTTCAGACTCCTGGGCTCAAGGGATCCTCCCACCTCAGATTTCCCA
Dasypus novemcinctus  ........................................................................................................................

Homo sapiens          GTAGTTGGAACTACAGGTACTCACCACAGTGCTGGCTAATTATTATTATTATTATTATTATTATTATTATTATTATTATTTTGTAGAAACAGATGTCTTGCTTTGTTGCCCAGGCTG
Dasypus novemcinctus  ........................................................................................................................

Homo sapiens          GTCTTGAACTTCTGGCCTAAAGTTATTTTCCCACCATGGCCTCTCAAATTGTTGGGATTACAGGTGTGAGCCACTGCACCTGGCCTAAAAGTATATTTAGAATCTGAACACTTCTCTACT
Dasypus novemcinctus  ........................................................................................................................

Homo sapiens          GCTATAACCTTAATCCAAGCCAGCATCATTTCTTACTTGGATCATTACATAATCCATTAAACATGTAAATCAGATCATGTCATGCTCCTGCTCAAAACCGTCCGTGGCCTTTTATATTGT
Dasypus novemcinctus  ........................................................................................................................

Homo sapiens          TCACAATAAAAGCCAAAGTCTTTAGAGTGACCTACAAGATTCTACATGATCTACCTTCATTCCCATTACCTCTCTGACCTCATGCCCTGTCACTCTCCTCTTTGATTACTGAGATCAGCC
Dasypus novemcinctus  ........................................................................................................................

Homo sapiens          ATATGGCTTACTTGCTGTTTCTCAGACATACCAAGAAAACTCCCAGAGGTCCATTGCTTTTCTCTCTAACTAGGATGTTTCTTCCTCATAGAGTGTCATGCTTATTCACTTGCTTCTGTC
Dasypus novemcinctus  ........................................................................................................................

Homo sapiens          AAATCTCTGTGCAAATGACACTTTATCAGTGATACTTTCTTTATCATCTTATTTAAAAATTGCCACTCTCTGCATGCTTCCTACAACTTTACTCTGCTTAATTTTTTTCCATAGTACTTG
Dasypus novemcinctus  ........................................................................................................................

Homo sapiens          TCATTTTCTGATATATTAATATTGATTTGTTTGTTTACTGTCTATCTTCACCACTAGAATATAAGCTTCAGGAAGGCAGGGATTTTATTTTGTTGCTGCTGTATCCCTAGTGTGTAATGT
Dasypus novemcinctus  ........................................................................................................................

Homo sapiens          GGTAGCTGGCACATAATAGGTGCTCAATAAATTTCAAATTATTATAATAAAGAATGATTCCAAGTGCAATAGGAATTCCAAGATTCCTGCCATCTTCAGATGGACCCAGATATCCTTCAT
Dasypus novemcinctus  ........................................................................................................................

Homo sapiens          CATCTGCAGCAATCCCCCACCCAGTGCCAAACTCTGTGACACTGCATGTCTGGTAGTGAGTTTAGTCTTTACATGAGGACTTCCAGGAAGTTCCATAGATTCTTAAGCAAAGTGCTCCAT
Dasypus novemcinctus  ........................................................................................................................

Homo sapiens          TATTGAAGCTATTTAACTCCTAGAAAGATCTTTGTGGCTGTGTGCGGTGGCTCACGCCTGTAATCCCAGCACTTTGGGAGGCTGAGGTGGGCAGATCAGGAAGTCAGGGAATTGAGACCA
Dasypus novemcinctus  ........................................................................................................................

Homo sapiens          TCCTGGCTAACACAGTGAAACCCCGTCTCTACTAAAAATAGAAAAAATTAACCGGGTGTGGTGGCACGCTCCTGTGGTCCCAGCTACTCGGTAGGCTGAGACAGGAGAATTGCTTGAACC
Dasypus novemcinctus  ........................................................................................................................

Homo sapiens          CGGGGGGTGGAGGTTGCAGTGAGCAGAGATGGCACCACTGCACTACAGCCTGGGCGACAGAGCAAGATTCTGTCTCAGTAAAAGAAAAAAAGAAAAAAAGAAAAAGAAAGATTTTTGCCA
Dasypus novemcinctus  ........................................................................................................................

Homo sapiens          AGGGTCCTTTTCTGATACATAACATTTTTTGAATAGGTATTAGGTGTACACGATACAAAGTGCCAAATGTTCAAAGAGCAAGTAGTGAAAAGTATATTCTACCACTCCCACTGCTACCAA
Dasypus novemcinctus  ........................................................................................................................

Homo sapiens          CTTATTCCAAGCCACCCACATTTCTAAACTGGAATTATAGCACAATTTTTTTAGAAACAGAAGTTAGATGATGTCGCTCCTCTGCTAACATCAGCCTGGGGCATGTCATCACACTCAAGG
Dasypus novemcinctus  ........................................................................................................................

Homo sapiens          TAAAAGCTAGAGTCTCCCTCTGCCCCCAGCCACCAAGATCC-CTTCCTGGAGGCAAACACTCTTACTAGTTTATTGTGAATTTTTTAGAGATAGCATGTATATTATACACACATATCTCC
Dasypus novemcinctus  .......................CCCTAGTCCCCAAGGGCCTCTTCCCATAAGCCACCACATTTACTAATTTAATGTGTATTTTTCAGAGATAGTCTATGTA-CATACACACATAT....

Homo sapiens          ATGTGTATAATTCATTTTTAAACTGTACACACTATTTTTTATATTTCTTTTTTTCTCACTTTATTCTACCTCATTCTTTTTAATAGCTACAGTTGTATCAGTATGGCTACTGTATTCTCC
Dasypus novemcinctus  ........................................................................................................................

Homo sapiens          AGAGATTCTTAGTCTGGAGTCCATATATGGGTTTCAATGGGTCTATGAATCTCCTGAAAGTGCATGTTGACACTTGTGTGCGTGTGTACATGTATATCTTTTAGCAGAGCAGGCCCATAT
Dasypus novemcinctus  ........................................................................................................................

Homo sapiens          CTTTGATCAGATTGAAAGGAATCTGAGACCCAAAATGAGTAAGAAACCACTATATTGAGTCTGAATCTGCCTTCCTGAAACACCTACCCTTTAGTCCTAATTCCACTGTTTGGAGCAGTT
Dasypus novemcinctus  ........................................................................................................................

Homo sapiens          CTGTCAGGACCTGGTACATAGAGGACATACAACAGGTAACTTGTGAAATGGATGAAGGGACAACATAAAATGAATCTATTCTGTCTCTCTCTTTTTAAAATTTTTAAATTTTTATTTATT
Dasypus novemcinctus  ........................................................................................................................

Homo sapiens          ATTATTTTTTAAAGACAGAGTATCACTCTTGTCACCCAGGCTGGAGTGCAATGGCACAATATTGGTTCACTGAAAACTTCCACTCCCAGGTTCAAGCAATTGTCCTGCCTCAGCCTCCCC
Dasypus novemcinctus  ........................................................................................................................

Homo sapiens          AGTAGCTAGGATTACAGGCACCTGCCACCATGCCCAGCTAATTTTTGTATTTTTAGTAGAGACATGGTTTCACCATGTTGGCCAGGCTGGTCACAAACTCCTGACCTCATGTGATCCACC
Dasypus novemcinctus  ........................................................................................................................

Homo sapiens          CGCCTAGGCCTCCCAAAGTGCTGGGATTACAAGCGTGAGCCACTGCGCCCGGCCTATTCTCTCTTCTTTATGTTAGCTTTTGGTTAGCCTGAGGAAACTATCATATTCTAAGTCATCCCT
Dasypus novemcinctus  ........................................................................................................................

Homo sapiens          TTTCTAAAGTTAATTACTTAATTTTCTTAACTTTTCCTAATTTGACATGGTTTCCAAACCTGTAGGCTTTCCCTACATGCTGTAATGTGGGTTTCTTCCTCAAATGCCATCGGGGCAAGC
Dasypus novemcinctus  ........................................................................................................................
```

```
Homo sapiens          TGGTAATTTAAAAAAGTGAAGTGGATCAGATGTGAGACAACGGCAAGTGGTGGGACCTGTGGAGTCCACATGGCTAAGATGTTCACATTCAAACTCACATATAACACATCCATAACCAAA
Dasypus novemcinctus  ....................................................................................................

Homo sapiens          CAAAAAATATTTTCAGGCCAGTTTTGGACTATGGGCAGGCAGCTTGCAAATATAAAAATTTGGACCTCGTACAGTTTGTTGATACTCTTTTCACATCTTCTGCACTCTATCAATTCACTT
Dasypus novemcinctus  ....................................................................................................

Homo sapiens          TTGTCCTCCCCATAGGCCTGACAATTTAATCAAACTAAGGTAGTGCCTGCATCTCAAATACCCCTTAAGATTCCTTGCTATTATTCCTTTGCCAATATGGCTCTGTCTGTATAAAATGCC
Dasypus novemcinctus  ....................................................................................................

Homo sapiens          TTTGTGGAATATTCTCTACTTGCCCCTCAAGATCCACTTTTAAAAATAGACTATTTTTTAGAGCAGTTTTAGGTTAACAGCAAAATTGAGAGGAACATACAGAGAGTTCCCGTTAGCCCC
Dasypus novemcinctus  ....................................................................................................

Homo sapiens          TAAAGATCCAGTTTTCACCCTTCTTCATCCTCTTTGTGTACCCCCCGAGACTGACCTTTATAGACTGCATTTAAAAACATATTTTTAGTTATTGTGGGTACATAGTAGGTGTATATATTT
Dasypus novemcinctus  ....................................................................................................

Homo sapiens          TTGGGGTACATGAGATGTTCTGATACAGGCATGCAATGTGAAATAAACACATCATGAAGAATGGGGTATCCATACCCTCAAGCATTTACTCACTGAGTCGCAAACAATCCAATTACAGTC
Dasypus novemcinctus  ....................................................................................................

Homo sapiens          CTTAAATTATTTTTAAATGTACAGTTATCACTGGCTATAGTCACCCTGTTGTGCTATCAAATAGCAGATCTTATTCATTCTATTTTTTGTACCCATTGACCATCCCCAACTCCCCCCAGC
Dasypus novemcinctus  ....................................................................................................

Homo sapiens          CCCCCACTACCTTCCCAGCCTCTGGTAACCATATGAATTGGCTTCCTTGTCCTCTGAATTCTTCTTGGATATGGCCAATGTAAGGCACTAACAGGGGACTGGAGATCAGAGACAGGTTAA
Dasypus novemcinctus  ....................................................................................................

Homo sapiens          AGTCTGCGTATTTATTCTCCTGGCTCCCTCTTTATGGAGTGGGTGCAGCTTGGCTGAATCCTGTTAGAAGGATCTGCTGTGAGCCACAGACTTTTTTTTGGCAGTCATCTTCTAAAGCTA
Dasypus novemcinctus  ....................................................................................................

Homo sapiens          TGGCTACTCTGTCTAGGTTCTGGTTAACTGCTTTCTCCCCTTGGCTTTTCAGGTCTCAGGGTGGTAAGTGCTCCCTGGGGTGCTTCACCATCCTTTTTGCTTTCCCTTAACAATGCTCAC
Dasypus novemcinctus  ....................................................................................................

Homo sapiens          ACTGTTATAAACAGTCCGTTCATTAAACTCCACTCACTTCCCTGTTGAATATGCTCTCAATATCCTACTAGATCCTTTACTGTTACCAATGCCTTCAACTCCCTTGTGTACTTGGTGTAC
Dasypus novemcinctus  ....................................................................................................

Homo sapiens          TCCCCATTGTTAAGGTCCCAGCTCCTAGGACAATGTGGTGCATTCCTAGATATCACTCCCTTTGAGAAAGTTAGCTATTCCCCCACCTCTTTGAGAACATTTCTTGCAACACTCTGTGTT
Dasypus novemcinctus  ....................................................................................................

Homo sapiens          TAGATCTTGGGCTTCTTATGATTGGGAATGAAGCCTATTCGTTATTGTTTCCTGGCCAAGTAGATGTTTGGTGAGTATTGGTTATCTTTTTTGCTTTGTGAGGAGGATATAAAATCAAGG
Dasypus novemcinctus  ....................................................................................................

Homo sapiens          AAGGAGGATATTTGCTTTTTATTTCTTTCAAATTATTTGATTTTAAAAGAAATGCCTACACATTATTTTTTAAAAATTAAAATGAAAAGAAAAAAGTAGTAAAAGTGGCACCGTTCTTCA
Dasypus novemcinctus  ....................................................................................................

Homo sapiens          CATCCGTGCTGGAAGTAACAGTGGTAATGGTTTTTGTTGTGCATCCTTCCAGTATCTTTTTATGCACATAAAGGCTTAAGTTTGTGTACATAAAATGTAGACTCACATATTTCTCTGCTC
Dasypus novemcinctus  ....................................................................................................

Homo sapiens          CTTGCTTTTTTTTTAACTTAACAACACACCTGTCTTTCTATATCAGCCAATGTAAGTATACAGCCCTACTTCATTATGTTTTATGGCTGCATTGTATTTCATTTGATGAATTTACCATAA
Dasypus novemcinctus  ....................................................................................................

Homo sapiens          TTTATTTAACAAGCCTCTATTATTAGCTAGTTTATCTAATATTTTCACTGTTAAAAGCAACACAACTTAAAACACAGAGACATACTGGATCAAATTTAAGCCCAAAAATATATGGCTGAG
Dasypus novemcinctus  ....................................................................................................

Homo sapiens          CTCCAGAAGCAAGAAAGGAAAACTGTCAGATTCCAGAGACAAGAGAAAACTCACAGTCACAGCAATGAGCATAAATTGACATTTCACCACTCACAGGAAAAATGGGACCAGGTATATTCC
Dasypus novemcinctus  ....................................................................................................

Homo sapiens          TTAAAGGATGAGATTATAAGTCCTCTGTAGGGCTTAGAATTGAGTTAGAATTGTGCTTCCCTCATAAAACAAAGAGTAGAAGGATGCTGCCCCATTTATTATTTAAAAGCAGATTTCCCA
Dasypus novemcinctus  ....................................................................................................

Homo sapiens          AATAGTCAGGGGTACAATAATAACAAATCTTATTTAGTACTTACTATGTGCCAAGCATTGTTCTACATGATTTACATGTATTTATTTATTTAACCATCACAGAAACCCTATGATATTAGG
Dasypus novemcinctus  ....................................................................................................

Homo sapiens          TACTATTATAAACCCCATTTTATAAGTAGGGAGTAAAACGTTTAAGTAACTTGCGCAGCTAGGAGGTGGCAGCTCTAAAATTCAGACTCAGGCATCCTGACTCCAGGACCATGCCTGTAA
Dasypus novemcinctus  ....................................................................................................

Homo sapiens          GTAAATGTGCTATATTGCTTCTCTACAAACAGGATGTAGGTCATCTTCCTTAGGATGAGGTGGAAATAAACTCACCCATAAGAAGTTTAAAACTTTAAACCTGAGCTGCACACTAGTATG
Dasypus novemcinctus  ....................................................................................................

Homo sapiens          AAACCAAAATTCACAGTACCCACATAATGCAGAAACCCTGGGGTGAGAACTTAACATAAAACCTGGCTCAGAGCAGGAACACAATGAAACTCTAGCAGAGGCAAATGAAAAACCGCCTGT
Dasypus novemcinctus  ....................................................................................................

Homo sapiens          AGACCCACCTCCATAACTCAGAGTCCAATTCTTACGGAAGGGTTCTAACAGAAAACAGTTCCTGCAGAAGATAAGCTCACATGCAAAAAAATGATAAAGCACATGAGGAAATTGACTGGA
Dasypus novemcinctus  ....................................................................................................

Homo sapiens          ATGAGTTAGTTGGTATATACAAGAAATGGGAGAATTATGTCCCAAGAAACTACAGATAATACAGTAATCTGAAAGGACTTTATTTTTTGGGTTTTTCTCATCTTTTTGTAGCCCTCATGT
Dasypus novemcinctus  ....................................................................................................

Homo sapiens          ATGAAAGAACTTTAAAATGTTGTATGTTTGAAGGTTTCAAGATGTAAAAAAGGAACATAATACATAAAGCAAGAGCACAACTTTCCTCGGTGTGTGTACACCAAATAAATAGGCCAGAAA
Dasypus novemcinctus  ....................................................................................................

Homo sapiens          TGAAGAATATAGAAAATATGGGATGAGCATTGTAAGATTTTAGTTCTAGCCCCAATGAAATATTTAAAAAGTCAAATGACAGTTTAAACTACAAATAAGTTATGAGTGAAGAGAGAACAA
Dasypus novemcinctus  ....................................................................................................

Homo sapiens          ATGGAATAACAAATAGAGCCGAGATAATCATAGAATATAGCATGGCACTATAAAAATGCTAAATATGAAAGGGAAAATAGAAGACACACATGATATAACAAGAAACTTCACATTCATCTA
Dasypus novemcinctus  ....................................................................................................

Homo sapiens          ATAAAGCTCCAGAAAAAAAATAGGAAGAGATAATATTCAAAGAGAAATAGCTGATAATTTTCTAAAACTGAACAGGACATGAGTCCTGAGATTAAAACCATGTACCTAGTTCTAAGCAGA
Dasypus novemcinctus  ....................................................................................................

Homo sapiens          ATAAAGACAAATTGATACCCTGTAGATGCAACAGGGTGAAACTGTAGAACATCAAAGATAAAATGAAAATCTTGAAACCTACTATAGACAAACAATGTTGCAAAATATTTCTGTAGAATA
Dasypus novemcinctus  ....................................................................................................

Homo sapiens          GATTCCTAAAATGGAATTTCTGGGCCAGGGTATATGCACATTAAAAAAATGAATATGTTTTAAATGCATGAGTGAGTGAATTAATGAGTCTAGTAGTATTTCTCTCAATCTGGACATATA
Dasypus novemcinctus  ....................................................................................................

Homo sapiens          TTTATTTTAGTACAGCCTGAGATTGGATCCCCCAGCTATCTATTTATTTATATATTTGTGACTGCATCACCACTATTGATTTCTATTGAGCATAAGACCAATTAAAATCCCCAGGACTTT
Dasypus novemcinctus  ....................................................................................................

Homo sapiens          TTCAAATGAAAGGCTGTCAAATGAGGTCCTCTTTAACATGTACTTAGGCAATTAATCTTTAAAACTTAGAAGTAGGGCTATTAAGACATTGGTGAGATCACTGAGAGCTAGAGCTGTTAA
Dasypus novemcinctus  ....................................................................................................

Homo sapiens          AGAAGGTTTCATAAAGGAAGTAGGACTTAGGCTGCATAGAATTTATTTTCGGTGGAGGGTGCAAAGGACAGCTTCTATGTTTGTGGGATGGAGGTAAGAAGAGTGATAAGGTGCATGAAT
Dasypus novemcinctus  ....................................................................................................

Homo sapiens          AGACCATAGGATAGGAAATGAGCTTGGGGTGGATATGTGGAGGGTGGGTGAGTAAAGAGATAAGGTTCAAGGGAAGGGGTGGGGCAAGATTATGGAGGGCCTAAGAAGCCAGGGAGAGTG
Dasypus novemcinctus  ....................................................................................................

Homo sapiens          TCTGCATATGATATTATACATGGGCAATTGGAAGTCCCTTCAGCCTTTTTAAAGGAAATTATATGTTTTCTGACTGGCCAATGAAAGGCTAACTAACCCAGGAAAGAGCAAGAAGGCTGG
Dasypus novemcinctus  ....................................................................................................

Homo sapiens          GTGAAGTAATGCAGGCCCCTAGTTCTCCTGTTTCTGGGTTACTGGATAGGTTATATAGCAAGTAGGCAAGCTTAGCAAACAGCTCAGGGACACTAGGGTGACTTGATGTTAAACTGCCTG
Dasypus novemcinctus  ....................................................................................................

Homo sapiens          ACTAGTGAGAATTAAATGTAATAAAACTGGAATTGCAAGACTAAAAATGGTATTTAGGCAGAATAATCTACTAGCAAATTCAGGATTCTTTAGAGTTGAGTGGGAAATATAAGAAAGAAA
Dasypus novemcinctus  ....................................................................................................

Homo sapiens          GAAAGATGCTGGATGCAGGAGAACAAGAGATGGAATGAGAAAGTGGAATATACACTGGAATGAGCAACAGGAAACTTTAGTTCTAGTCTCAGCTCTACCCCTACTAACCGCACAACTTTG
Dasypus novemcinctus  ....................................................................................................

Homo sapiens          TGCAGATTACTTAAACATTGAGGCTAGTTTCCTCCTCTGCAAACAACCTAGAATACAAACTCTAAAGGGTGGTAACCAGATCTGCCATGTTCATCATTGTATCTCTAGCCACTGGTACAG
Dasypus novemcinctus  ....................................................................................................

Homo sapiens          GGCCTAGAACGTAGTAGGGGCTCAGTAAATATTGGATGAATAGATGAATGAATGAATGAATGAATGAATCAATCAATCAATCAATTAGAGCCTGCCCTGCCTGCCTCACAGAGTTATTAT
Dasypus novemcinctus  ....................................................................................................
```

```
Homo sapiens         AAACTTCAAATGAAATCAGGGATGGGGAAATATTACATGAACTTAAAACACTGAGCAATCCGATGGTAAAATACTACAGCTACTACTCACAATTATTATTTAAAAATTAAATTAGAATG
Dasypus novemcinctus ....................................................................................................

Homo sapiens         CTATTTGTAGTACTTTAAGGGTGAAATCATAAGTGGGCTAATGATTTCAAGTTTTCTTAGCATGGAAAGTAAGAAGAAAAGGAAAAGTATAAGACATTTTGAGTGAGGTAAATAAAACAT
Dasypus novemcinctus ....................................................................................................

Homo sapiens         CAGAATCTGTGTTCTGAACCATGTTCTGTCATTTGCATTCAATGTGCTTATGGTAGGGCTAATATTGATGTCAGTTATAGTTAGCATTCATTGACTACATACAGTAATGCTATGTCCTGT
Dasypus novemcinctus ....................................................................................................

Homo sapiens         CTGTCCTTAAAAAAATAGAACACCCTTCAAAGAAGGTAGTCACATTCTCATTTTAGTGATGTGGAAACTGAGATTCAGAAAGGTTAAATGACTTGCTTTTAAAACCATGGTAAGGTGGTT
Dasypus novemcinctus ....................................................................................................

Homo sapiens         TCAGAACCAGACATTTTCTCCCAGATATTTTTGTCTCTGAAGCCAGTGGGAAAAAAACTGCAGATAAATAGATTTCTTTACACCATAAGGATGCCTTTGCTAAGAGACAGCAATTCAAAG
Dasypus novemcinctus ....................................................................................................

Homo sapiens         AAGGAATGGGCTGCCTCCTGAGCTAATGAGTTTCTCATCACTAGAAGCAGGATCGCTATGTAATTTGCAGGGCCCAGTGCAAAATGAAATGGCCTTATGTCCAGGAAGCTGGTCTTGGCT
Dasypus novemcinctus ....................................................................................................

Homo sapiens         GGTAGTTTGCAAGCAGAAGTTAGCCTGCCTCCAGACAGGGATGTCAGAGTGAGGCGTCAACATGAGCTGATGCACAGGACCAGATGGCCTTAATCTGAGACACCATGGCTCTATTCTATT
Dasypus novemcinctus ........................................................................ATCTGAGATTCTGTGGCTCTATTCTATT

Homo sapiens         AGCCCAAGTGAGTTAAGGGAGCATCCCAAGGTAGCTGACATGACACCTTAGAGGCTT-CAGATGAGAGAGAATTCTGCAAGAGGATTTGGAGAGAGCCATGGTGCCATGATCATCTGGGG
Dasypus novemcinctus AGCTTATAAGGGTTAGCATTGGAGCCCAAGGTGGCT-ACCTGACAGCTCAGAGACTTTCATTTGAGGGAAAATGCTGCAAGACAACTCAGAGAT-TTTATGGTCCTATTAGCATCTGGGA

Homo sapiens         CACTGAAAACATGCTCTGCCCTATGAGGCAGCACCAACACTCAGAAGAAAACAAAA-AAGAAACTCTTTTAGTGGAGGTGAAGTGTAATGAAAAGAATT--------------AGACAGA
Dasypus novemcinctus CACTGAAAACATACTCTGCCTTGTAAGGCAACACAAATACTCAGCTGAAAACAAAATAATTAGCTTTTTCAGTAATGGTTAGGTATAATGAAAAGAACTGGGGCTTTGAACTCAGACAGA

Homo sapiens         TGTGGGTTCAAATCCTAGCTCTGGCAATTACTAGCTAGCTATTTGCCTTTGAACAAATCACTTAAACCTCCCTGCGACTGTTCTGTCATCTAGAATATGATCACAGTACCTGCCTTGCAG
Dasypus novemcinctus TGTGGGTTCAGATGCTGGCTCTGTCA-----TATCTTGGTATGTGCCTTTGAACAAGTCACTTAAACCTCTCTGAGACTGTTCTTTCATTTGGAA-ATGATCATAGCACCTGTCTTAGAG

Homo sapiens         AGGTGTGGGGA------AGGAG-----GCAAGTCAAATGTATCTGGCACTGGGTAAGCCCTCCATAAATGTTAACTGTTGGATGTGTTTATAAACCACTGGGAGTGGGGACGGTA---TG
Dasypus novemcinctus ATTTCTGGTAAGATTCAAGGAGATTTTGCAAGGCAAATGCATCTGATTCTTGGCACGCTCTCAATAAATGTTGG-TGTTGT-----TTTATTATTTGATGGTGCTGGGGAGGGGAGTCTG

Homo sapiens         ACGTTGGGTCATTTGCCTTTCCTTGAGCACTTCATGGACGAATAGCCAAATCTGCCGGACCCACCTAGGGGCATGGAAACCACTTCCCGCTCTAGTGCTACTAACTACAGCACTGCAGTA
Dasypus novemcinctus ACAGTAGGTCATTTGCCACTCTTTGAGCTCTTAATGACCCAGTAGACAAGAAAGCCAGATCCACATAGGCACAGAGAAGCCCCTTCCCACTCTAATCTTCCTAACTACAGTGGGGCGATA

Homo sapiens         GTGGTAG--GCAGTGGG-------TGAAAGTGGAGTGGGG-CCACTAGTAGACAGTAATAACAATAAACTCTCCAGGAGAACAGGGCCCTCAAGCTTTTCCCCTAGCCTTGAAGATA---
Dasypus novemcinctus GTGGTGGTGGTGGTGGGCTAAGAATGAAGGTGGACTGGGGGCCTCTAGTAGACAGTAATAATAATGAACCTCCAAGGAGAATGGGACTCACAAAGTTTGTCCCTAGCCCTGCAGTCAACC

Homo sapiens         -CTTACAGGAGATAACTGCCCAAGTCCAATCTTGGAATCCTGCTAGTTGAGTGTTTTACCCACAAGGACATTTCATGAGATCAGGACTAAGAATAAAATTCACTTCTGCCCAGTAGCGCT
Dasypus novemcinctus CCTCACAGGATAGGG-TGTTTAAAGTCAATCTTAGAAGCATGCCAGGGAAGTATTTTACCTACATTGACTTTTCACAGGATCAGAACTAAAAATAAAATTCACTCCTGCTCAGCAAAGTT

Homo sapiens         ATATGCATTAGTCACTTGGAATAGCAACAATCTTAATATCTTTCTATTTGTCCGCAAACCCCACCATCCCACCTCTTTAAAATTCCGTGAAATGGC----CAGAATAGCGAAACACCGAT
Dasypus novemcinctus ATATGCATTAGTCACATGAACTAACAAGAATCTCAAAATCTCTCTACTTGTCTGTGAGACCC-CTGTCTTACCTCCTCACATTTTTGTGAAATGCCTCTATAGCAGAGCAGGTCACATAT

Homo sapiens         ACCTACAGTCTTCCAGGAACTTCATTGTCCCTTATGCAACATAGAAGGAATGGTATGAGAACTAAGAGAACATTGCAGTAAATCACTGAA-----TTTCTTGGCTTTCTTTTTTTCTTTT
Dasypus novemcinctus ACCCACAGTCTTCCGGGAACTTCATTATCCCTTACGTGGTACAGAAAGAATGGTATGAAAACTAAGGGAACATTACAATAAATCACAGAATAGTTTCTCTCTGCTCTCTTTCTCTCTCTT

Homo sapiens         AATAATAAAAAGCACTCCAATCAAACTATTAATTAACTT-GTAAAAGGCTCGAACTATTATGTACTATTCCCCG----------------CCCATGTCCATGTGCTGCACAGTATTTTCT
Dasypus novemcinctus AAGAA-AAACAACACCCAAATCAAACCATTAATTAGCTCGGTAAAAGACTGGAATTATTGTGTTCTAATCCCCACCCCCATGCCCCACGCCCCAGGTCCATGTGCTGCTCAGCATTTTCT

Homo sapiens         TACTCAGGACTGGTGGAACATTTCTTTTGCTTTTGTTTG--AAAAATTACTTTTTATTTGGCCTTGGGCTGC-TATCATAACTTCTGTTTTCACTAGATTGCATAAGCCATACATCTGCA
Dasypus novemcinctus TAGTCATGACTGGTAGAATATTTCTTTTGCTTTTGTGTGGAAAAAAACTCTTTTTAGTTGGCCTTGGCTCATACATCATAACTTCTCTTTCCACTAGATTGCATAAGCCATACA......

Homo sapiens         CACATTTTTGATTTGTACAGGGAGTTTGAATTTTAATATCTACCTCCATCTCAATCCCAGCACTTTGGGAGGCCGAGGCGGGCGGATCACGAGGTCAGGAGATCGAGACCATCCTGGCTA
Dasypus novemcinctus ....................................................................................................

Homo sapiens         ACACGGTGAAACCCCGTCTCTACTAAAAATACAAAAAATTAGCCGGGCGTGGTAGCGGGCCCCTGTAGTCCCAGCTACTCGGGAGGCTGAGGCAGGAGAATGGCGTGAACCCGGGAGGCG
Dasypus novemcinctus ....................................................................................................

Homo sapiens         GAGCTTGCAGTGAGCCGAGATCGCGCCACTGCACTCCAGCCTGGGCGACAGAGCGAGACTCCGTCTCAAAAAAAAAAAAAAAAAAAAAAAAATATCTACCTCCATCTCAAAAACCTCTCAT
Dasypus novemcinctus ....................................................................................................

Homo sapiens         CTTTTGTCCTTTTCTTCAAGATTCTGATTTCCTTGTGGACATTCATTTTGATACTTACATAAATCTTGCGTTATTCTGCCCATTTTTTTGGCTACAAGGCTCAGCTTTGGGGCTGTAAAT
Dasypus novemcinctus ....................................................................................................

Homo sapiens         GTGAAATATTTCCATCATAACCTATCTTTGGGTATGGGAAGGTCCTTAATGCTGGAAGTTCATTGAATAATTGCTTATTTTAATAGATGTTTATTTTCATTCACATTTCTCCCTCCACTC
Dasypus novemcinctus ....................................................................................................

Homo sapiens         CATCCCTTGGGGCAGTACCCTAGTTCAGTTCTTCATCTACTCTCTCCTTGACTGTTGCGATAGCCTCCTAAGCAGCCACTCCACATCCAATCTCCCCATATCCAAAGTATCCTAAACATA
Dasypus novemcinctus ....................................................................................................

Homo sapiens         GCTACCAGTCATCTCTCTAAACCACAGCTTTTGGCAAAAGATCCTTGAAGTTTCCCTTTTGCCTAATGAGTAACGTACTAGCTCCTCATCCTGGCATTCGAAGCTCTCTATGTTTATATG
Dasypus novemcinctus ....................................................................................................

Homo sapiens         TTTGCCTATCTTTCTTTTCTAGCTTCTTTCCCATTACTTGTCTTCATATAGCTTTAGCTTTTGCCAAACTACCATTCTTTGTTTTCAGAGCACACCCTATACTTTCCAGCATCAGTGTTT
Dasypus novemcinctus ....................................................................................................

Homo sapiens         CTTTCACCTGGAATTAACTGCCTGCCACAGAGATCTCCGTTCATCAGCCTATTCATTCTTTAATGCCCAAGACACATGCCACCAATTTCATTTGAATGGTCCTTAGAGACACTGAAATGG
Dasypus novemcinctus ....................................................................................................

Homo sapiens         CGCCTGCTGTACACCTGTCCACCCCGACCCCCGCTGCCCACTGCTGGCTCTTCCAGTCTTGTGAAACATTAACCTCTCCTAACAGCAAGCACAGCTTCTCCATGGAGAAGACACTGAAAT
Dasypus novemcinctus ....................................................................................................

Homo sapiens         GGCACCTGCTGGACACCCGTCCACCCCCCACCCCTGCTGCCCACTGCTGCGTCTGCCAGTCTCGTGAAACATTAACCTCTCCTAACAGCAAGCACAGCTTCTCCATGGGGCCTGCTCACT
Dasypus novemcinctus ....................................................................................................

Homo sapiens         CTGGCCCTCTGGTCCCACCATTTTTCTCACTACTCAAATAAAGTTTGGAGACCTTTCCCTGATTTTTTAAGAACATGCTAGCTCTTTCATGATACTCGTTATCATACCACCCCCATCTGC
Dasypus novemcinctus ....................................................................................................

Homo sapiens         TTTTCAGTGTACCTTTCAATTGCTCTCTAGTGCTCCATTCTTGATTGTTTTCCATTTCTATTATAGCTTTCAAGGAGAGTCTTTACTTGGCCTACTTTCTTAGTAAATGGTTTGGTGAGA
Dasypus novemcinctus ....................................................................................................

Homo sapiens         TTCTGATTTTCATCTGATATTAGTTCTTTCTCCAAGAGGGACAGAGAAAAATGAAAAAAATATCTTGCCTTCAGTTATTCTTTTCATATTCTTTGAAATTCTAAGCCACAGAAGTCTTCT
Dasypus novemcinctus ....................................................................................................

Homo sapiens         GTTAGGCTGCTACCTTCATTTTAAGAGACTCTTCCCTAAATGACAAGTTGTGACTTCAGAACTAGAGAACATGGCAGCCTCTCATGGCTTTCTGCTCAGTCCCATTTTTCTTTTCTGGAT
Dasypus novemcinctus ....................................................................................................

Homo sapiens         CCACTCTGGGCCTTCTTTAAAGTGACTCAACTTCCCAAGCATGTGATTTTACGTATTTTAAAAACAAGTCTGGTTTTTTCAACTCATTCTGAAATGTGGCTGCCAAAGTAAATACCCAAC
Dasypus novemcinctus ....................................................................................................

Homo sapiens         CCTCTCTAACACATACATACCCTCTTTCACACACACATGCACATGCACATGTAAACATACTCCTGAAGGATGAGGAAAGCAATGGTAAATGGGGAAGCAAAAAAATACCATTTTTCATTT
Dasypus novemcinctus ....................................................................................................

Homo sapiens         AGACAGACTTCCCAATACCGATGAACTAAAATTCAGGGATTTGTGATTTGAAATGCATGGATGGCCTTTGAGTGGTCTTTTCAAATCCCCTAAATATATCAATTTAATAACTTATTGAGC
Dasypus novemcinctus ....................................................................................................

Homo sapiens         ATCTACCATTTGATGAGTGCTGTGTAGATGTATCTATATATATCATAAAATTTATTTCTTGCTACAACTTTTGGAAGAGATTATTATAATCCTCATTTACAGATGAGGAAACTGAGTCTC
Dasypus novemcinctus ....................................................................................................

Homo sapiens         TGTGAGAGGAAATGATTTGCCCAAGATCCCTCAGGCAATAAGTAGTAAATCTGACTTTGAAACACAGGTCTGATTCCTTATCCCCATCAGATTTCCCAGGCATTCTGGTACAGGTGGTGT
Dasypus novemcinctus ....................................................................................................

Homo sapiens         TTCAACAGATGGGAATGGGGCAGGGTGAGAAAGATAGTATGAATAAAGGTACAGACATGGGAAAATGTAGCTACACATAGGACAGTAGTAGGAATTAAGGTTGCAAAATTGTGTTAGGGA
Dasypus novemcinctus ....................................................................................................

Homo sapiens         CAGAACACAAAAGGATTAGAAACTTGAACTTCCTTCCATTGTCCTGAAGGGACAATAAAAGATTTTGTAGTAGGAAAACAATATAATCAGAGAGCCAAATCAAGAATGCAATCCAATTTA
Dasypus novemcinctus ....................................................................................................

Homo sapiens         CAATAGCCATACAAAAAAAATTAAATACCTAAGAATACATCTAACCAAAGAGGTGAAAGATCTCTACAGGGAGAATTAACATACACTGCTGAGAGAAATCAGAGGCAACACAGACAAATG
Dasypus novemcinctus ....................................................................................................
```

```
Homo sapiens          GAAAAACATTCCACACTCATTAATTAGAAGAATCAATATTGTTAAAATGGCCATACTGCTCAAAGCAATCTACAGATTCAACGCTATTCCTATCAAATAACCAATGTCACTTTTCACAGA
Dasypus novemcinctus  ........................................................................................................................

Homo sapiens          TTTAAAAAAAGATTTTAAAATTCATATGGAATTTAAAACGAGCCCAAATAGTCAAAGTAAACCTAAGCCAAAAGAACAAAGCTGGAGGCATCACATTACCCAACTTCAAACTATACTACA
Dasypus novemcinctus  ........................................................................................................................

Homo sapiens          AGGCTACAGGAACCAAAACAGCATGTTGCTGGTACAGAAAAAGACATATAGACCAATGCAACAGGATAGAGAATGCAGAAATAAAGCCACACACCTACAACCAACTAGTCTTTGACAAAG
Dasypus novemcinctus  ........................................................................................................................

Homo sapiens          TTGATAAAAAATAAGCAACAGGGACAGGACTTTCTATTCAATAAATGGTGCTGGGATAACTGGCATATGCAGAAGATTGAAACTGGACCTCTGGCTATCACCATATATACAAAAATTAAC
Dasypus novemcinctus  ........................................................................................................................

Homo sapiens          TCAAGATTGATTAAAGACTTAAATGTAAGACTTCAAACTATAAAAACTCCAGAAGGAAACCTGGGAAATATCCTTTGGACATCAGCCTAGGCAAAGAATTTATGACTGAGTCATCAAAAG
Dasypus novemcinctus  ........................................................................................................................

Homo sapiens          CAATTGAAACAAAACCAAAAATTGACAGGTGGGATCTAATTAAACCAAAGAGCTTCTGCACAGCAAAAGAAAATATCGACAGGCACCCTACAGAACTGGATAAAATATTCATAAACTATG
Dasypus novemcinctus  ........................................................................................................................

Homo sapiens          CATCCAACAAAGGACTAATATCCAGCATCTATAAGGAACTTAAATCAACAAGCAAAAAACAACCCCATTGAAAAGAGGGCAAAGGACATGAACAGACGCTTTTCAAAAGAAGACATACAA
Dasypus novemcinctus  ........................................................................................................................

Homo sapiens          GTGGCCAAAAGACATGAAAAAATGCTCATCATTACTAATCATCAGAGAAATGTGAATCAAAACCACAGTGAGATACCATCTCACACCAGTCAGAATGGCTATTATTAAAACGTCAAAAAC
Dasypus novemcinctus  ........................................................................................................................

Homo sapiens          AGCAGATGTTGAAGAGGTTGCAGTGAAAAGGGAATGTGTATACATTGCTGGTGGCAATGTAAGTTAGTTCAGCCCCTGTGGAAAGCAGTTAAAGATTTCTCAAAGATCTAAAAATAGAAA
Dasypus novemcinctus  ........................................................................................................................

Homo sapiens          TACCATTTGATCCAGCAGTCCTCTTACTGGGTATATATCCAAAGGAAAATAAACCATTCTACCAAAAAGACACCTGCACTTGTATGTTCATCGCAGCACTATTCACAACAGTAAAGACAT
Dasypus novemcinctus  ........................................................................................................................

Homo sapiens          GGAATCAACATAGGTGCCCATTGATAGTGGATCGGATAAAGAAAATGTACACGTACACCATGGAATACTACCCAGCCATAAAAATGAACAAAATCATGTCCTTTATGGGAACATGGATGC
Dasypus novemcinctus  ........................................................................................................................

Homo sapiens          AGCTGAAGGCCATTATCCTAAGCAAATTAATGCAAAAAAAAATCACATATTCTCACTTATAAGTGGGAGCTATACCTTGGGTACACATGGACATAAAGATGAGAACAATAGACATTAAGG
Dasypus novemcinctus  ........................................................................................................................

Homo sapiens          ACTCCAAAAACTATCTGGGTGATGGAATCAATAGAAGCCCAAACCTCAGCATCATGCAGTACACCCTTGTAATAAACCTGTACATGTACCCCCTGAATCTAAAATAAAAATTAAAAAAAA
Dasypus novemcinctus  ........................................................................................................................

Homo sapiens          TTTAAACAGCTGCATTTTAGACAGATTAATCTAATTCAAGTCTGAATCTATGCTGTTGACAGTATTTTTAAAAATTCTTTCCTAAGAATTCTTTA----------------TTCACTCCA
Dasypus novemcinctus  ................TTAGGAAGATTATTCAGATTCAGGTGTGACTC---ACTGGCTCCTCTTTTTCATTTATTTCTTTCTTTTTAATTTTTTAGCCTTGCTGAGAACTCTTTGGTCTA

Homo sapiens          GGCCTATGCTTTGGTCAGAATTCTCTCTTTGTTTTTCAATACTTTAGTGAAAATTCAGAAGGCCATGTGGAAAAAAAC-----AGAGGAAACAAACCACACACAAACACAAGATAATTAT
Dasypus novemcinctus  GACTTTTACTTTGGTTGGAATTCTTTTTTTTTTTAGTAACA--TCCGTGCAAACT--GAAGACACTGTGGGAAAAAATTATGGAAAAGAAACAAAACATGCACAAATACCAGATAATTAT

Homo sapiens          ATACAAGTCTGGGAAGAAAGAGCACAATGGGTCATGACGAACTTTATTGAACAGAAGGCGAATAAGCAACGTTCCAATTGTACAGGAAAAAAAAAAACCCAGCAACCCAAACACCACCACC
Dasypus novemcinctus  GCATAAGTCTATGAAGAAAGAG-----TTGGTCATGATGAACTTTATTGAACTAAACATGAGGTAGCAATATCCCAACTGTTAAAGGAAAAAGA------GCCACCAAAACAATACCACT

Homo sapiens          AACAATAAGAAACAAAGGCAGCCAGGATCTGGGAGACATGGATGAAG-CTTGTGAAAATGACA--------------------------------------------CTTTCTTAAAAG
Dasypus novemcinctus  GACAGCAAAACAGAAAGGTAGCCAAAATCTGAGAGAAATGGATAAGGATTTGTGGAAATGACAGATCACTGTTAGATGTCAGATAAGTATCAGGTAAGTTCTGGTTCCCTTTCTTAAAAG

Homo sapiens          AGCAGAAGAGAAACTAAAAAGGATGAAGGGGAGAACCATGGATTTGAGGGTTAAACTAAAAGGAAAAATAGAAAGGAAATAGGAAGGAAGGGTGTAACTCATAGCTGGTTTTGATATATC
Dasypus novemcinctus  GATGGCAGAGAAACTAAAAAGGGGGCAGGGAAGACCCATGGAATTGAGCAACAAACTAAAAAAAAAGAAAGAGAGAACATAGAAAGAACAGATTACACTCATAGCTGCTTGTGGTGTAT-

Homo sapiens          ATCCCAGTC--TGACAACCAAGGCAGTCTCCCTG-CATTAGAAGGCCATGCTAAAAGTAGATGAGTTGTAAACAAGCCTCCCACTGGTCATTAGTTTGGCAAATTAGTGGGTTATCTCTT
Dasypus novemcinctus  ATCAAAACCACTGA-AACCAAGACAAGTTCACTGGCATTAGAAGGCCACACTGAAGGTAGAT-----------AAACCTTTTACATGTAATTAGTTT--------------CTTCCTCTT

Homo sapiens          AGAGTCCTCTCTTTTTCTCCTTTCCCTTTCCTCTGCATTCAATTTGTCACTCATTCCTACAAGTCTACCCCAGAAGG-GTTGCCTGAATCAAAGCCCTTCTCCCCATTTCCACTCACGGG
Dasypus novemcinctus  AGAGTC--------TCCCTCTCTCTCTTCCCACTACGTCTGATTAGTTACCAAGTCCTGTGAGTATTCATCACAGGAAGTCTCCAGAATCAAGACCCTTCTCTTCACTCTCA-TGACATG

Homo sapiens          GCCACTGTGTTAATTAAATT-CCATGTCGTTTCTCAGCTGGACC--------------------AGGCTTCCTGCCTCTAATTCGTTTCTTCCTCCCTCTAATTGAGGCTTAGATCTGCC
Dasypus novemcinctus  GCCACTATGGAAACCCAGTTTCCATTTC--CTCTCACCTGGACTGTAGCATCAGCTTCCTGATTAGGTTCCCTGCCTTTG-TCAGTCTCTTCCCCTCTCTAATCCAGCCTCCCTTCTACC

Homo sapiens          CCAGTATTGAGCTTCCTA-TACTCTCTTGCTTATAAACCTTCTTTGGTGGCCCATGGCCTATAAGGTACAGTAGAACTCACTCAGTTTTACATGATGCAGTGGGAGTGGAATCAAAGGGT
Dasypus novemcinctus  TCAAGACTGAGCTTCCTAATACTGTTGTGCTCATAAACTTCCTTTGGTGTACCAATGAGTATAGAATAAAGCAAAACTTTCTTCATTTTGAGTAAAATAGTGACAAAGGAGTCAAAAGAA

Homo sapiens          TCCCCTA--GACCCATGGATGAAGAGACGACTAAGGAGCTAAGTCTGGGATGAGAAACTCTTGCAAAAAGCCAACTAATAGCTTTTTCCTCCTCATCTCAGAACCACCCCTCTTCCCAGA
Dasypus novemcinctus  TCCCCTGATAACC-----ATGAAGA-------AAGGAGCTATATCTGGGATGAGTTACTCTTGGAATAAAGCCACCAATAGCTTTTCCTACTAGATCTCAGCACCG--CTGCTTCCCA--

Homo sapiens          GTGTGTGT-----ATACAAGATGAGTTACTTAAGATTGCACCCAACCTGATGGTAGATTGGAGAAGGCTATCTGTCCCTTTCTCAGTTGAATATATGAGGGTCTTCTTCCCAGGAGGCTT
Dasypus novemcinctus  -TGTGTGTACAACATGCACAATGGGTAATTTATGTTTGCACTCAACTAATGGGTGGA----------------------------CTGAATATCTGTGGGTCTTGCTCCTAGGAGTTCT

Homo sapiens          GCCCACACCAGTCTTCTTTCCCACCATGGCCCATCCCCTTTCCAAAGAGTATAAACTTCATAC--CCTCCTCCCTTCAAGAACTCCTATCTTTGCTTAGCAAAATTTGTTTCCCAAGCTT
Dasypus novemcinctus  CCCCGAAACAATATTCTCT-TTGCCATAGCCTATCTAC--TCTAGAGAGGATACACTT-ATGCTTCATCTCCACTCCAAGAATG--GATCCTTTCTTAGCCAAGCTCATTCCCCTAGACT

Homo sapiens          GGAGAGAGGAAAGG--GGCCTTTTTCCAAATCAGGGGTAAATAAAAAGTGGCAGAATCTTACAGTAGACATCATATTAGAGCTCATTTTTATGAATTCTTCTACTGTATATAAGGTCCTC
Dasypus novemcinctus  GAAGAAAGGAGGGGTATACTTTATTTTAATTCAGAGGGATATAAAAA----CAGAGG----------------------------------------TTGCGTTGTACACAAGGCATGC

Homo sapiens          ACTTTCTTATT-ACTCAGCCTACCACTCCAGTTTCATCTTCCACTTTTACTGCTTCTCCCCACCCCT-TCACAATCCTGACATACCCTAAACTCCAGTCATACTGAACTTTCCAACATTC
Dasypus novemcinctus  ACCCTCCAATTAAATCAACCCACTTCTCCAGTCT-ATCTCC--CTACCACCACTTCTTCCCAGCCCTGCCAGAATCCCAAATTACCTTATATACCAGTCATACTGAACTTTTCAACCTTC

Homo sapiens          CTCAGATAAATCAAGCCTTTGCAGAAATTCATGCACAAGCTGTTCCTTCTGCTTAGAACATCCTTACTCTTCTTATCTCGCCAGTAAATTCCTACTCTCCTTTAAGACCCTGCTCAACTA
Dasypus novemcinctus  CTTAGATAAACCATGCCTTTGCAGAAGT............................................................................................

Homo sapiens          TTGCCTCTTCTGGGAACTCTTTCCAGACTGTTCCAGATAGAATTAATTAATCGTCCTTTGAGCTCTCATAGCATTTT-TCCATATCCCTTTGATAGCACCATCCTTATCATGGCAAAACA
Dasypus novemcinctus  ..................................................TTGTTCTTTGGGTCACCAAAACATTTCATTTATACCTTTATTATAGT-CTACCCTTATTATGGTAATGGA

Homo sapiens          ATATAACAACTAAAGGGATGGGCTCTGGAGGCAGACTGCCTGGGTTTGAGTTCTTACTCTGCCACTTATAAGCTATGTG-----------ATGTTGAGCCAATT--ACTTCGCCTTTCT
Dasypus novemcinctus  GTATAGCAGCTAAGAGCCTGGACTCT-GAGCCAAATTGCCTGGTTTAAACCTTGAGGTCTTCCACTTACTAGATGTGTGATATATATATATATATTAAGTTATATAAACTTAAACTCTCT

Homo sapiens          TTGCTTCAGCTTCGTCATGTATAAAACAAAATAAATAAGATGATACTTGTAAAACCTTTAGAACAGAACCTGACACATTGCAAATGTTAG-TAAATATTATACATCATCATTTCTTCTAT
Dasypus novemcinctus  TTTCCTCTGTTTCTCCACCTATAAAATGGAATAAATGCTATAATACTAGTAAAGATCTTAGAACAGAAGCTGTCACATTCTAAATGTTAAATAAGCATTAGCCATTATCATCTCTTCTAT

Homo sapiens          TGTTTTATTGTTATCGTATATATAACCTACTTGTATATGTAATATACATGTGTTATTATATAGCACTATTATAGCATTACTTAACTTTTGCTGTGTCTGTGTCTTGTCTTCCAAGCTGGT
Dasypus novemcinctus  TACTGTATTGTTAA-------ATA--CTACTTGTAT--------------------------TATTATTATAGTAGTATCTAACTTCTCCCATGTTTATGTCTAGTGTCCCCAGCTGGT

Homo sapiens          TTATA-GTGTTTTGAGGGCAGAAACCTTGTATTACTTTCT-CCCCCATAGTGCCTGGTGTTAAGTATGGGCTCATTAAGTATTTGTTGGGGTCAGTATATTATACTGAAAAAAGCACTGA
Dasypus novemcinctus  TTAAAAGTGTTTTGAGGACAGAACAGTTGTTGCACTCTCTACCTCCATAGTGCCTTGTAT--AGTATGGGTTCACTGAATATTTGTTGAAGGCAGTATATTATAATGAAAATGGCACTTG

Homo sapiens          AATCTGAGAGATTTGGGTTTGGATCTTGATTCTGCTCATAATTGTTGTATGATCTCAATCTTCCTGAGCCTCAGTTTCCTCATCTATAAAATGAGGATGATAATAAGAGTACCTACCTCA
Dasypus novemcinctus  GGTCTGAGAGATTTGGGTTTAAATCCTTACTCTGTTGTTAGTTGATGTGGGATCTTAATCTCCCT------CAGTTTCTTCATCTGCAAGATG-GAATGGTAATA---GCACCTACCTCA

Homo sapiens          CAGGGATATTTTAAGAATTAAATAACTTAGTACATGTAAAACCTAGCACATAATAACATAATAAGAACTCACTAAATGGTTTTTATTATCAGCTTTGTATCCCTTTGCATTTAACTTAAT
Dasypus novemcinctus  TAAGGTTGCTGTAAGAATTAAATAGGTTA---TATGTAAAGCCTAGCACATGGGGA--------GCACTCACTAAATAATA---GTTATCAATTTTGCATCCCTTTGTATTTAGCTTAAC

Homo sapiens          AAATACTTTTACTTGAATGAAT------------GAACCCAGCCATTTTC---TGGAACTTGAGGATCAAGGGAATCCAGGCAGATACTCTATTTGTTCCTAAGAATCCAGAGGAAAATG
Dasypus novemcinctus  AAATACTTGTGAATGAATGAATGAATGAATGAAAGAATCCAGCTATTTTCCTTTGAAACTTGGGCATCAAGAGGATCCAGGCAAA...................................

Homo sapiens          TGAGTCATCTCCATTCTGTATCATGGCTCACTCTGAACTGGGTCACCATCCTCCAGAAGGTGACAAATGTGGCTGGCTGAGTCACTATAAGAGCTTTTCTGTAAGGATTTGCCCGAACCC
Dasypus novemcinctus  ........................................................................................................................

Homo sapiens          CCCACCATAGCTCATTGGACATAGTATCTTTCATATTAGGTGCGAGGTGCATACCCTTCCTGAAATGGGCAGAATAAAGAGGTGCAAAACTCCTGGGGAATCAGAGTGGCTATGAATTCA
Dasypus novemcinctus  ........................................................................................................................

Homo sapiens          CTCCTCTTTCTTATTCTGTTGCCTCTTCCCTTTGTTTGGTTTATTTGCTAATCAACTTTGCCCACACTCACATCTAGTGTAGCCCAAGTCTGTGCAGAGAAGACACTTTGAAAGCCTAAG
Dasypus novemcinctus  ........................................................................................................................

Homo sapiens          ACTATCATAGTTCCAGCAGAATAATAGAAAAATAACAAGTGTTGACTATTCAGTTCTGATGTAGCACTGTATTGAAGAACATAACATTCTAGATCCAGATGGCCTGAATTTGAATCCCAG
Dasypus novemcinctus  ........................................................................................................................

Homo sapiens          CTCCACCACCAACTAGCTGTGAGACCTCAGGCAAATCACTTAACGTTTGCATCCTTTCATTTCTCCATCTATCAAATAGAAACAATAAAAGTACTTACCTACCTCTTAGGGTTTTGTGAG
Dasypus novemcinctus  ........................................................................................................................
```

```
Homo sapiens          GGTTAAATGAGTTAATACATACAGAACACTTAAAAGAATGTCTGGCACATAGTAGGTGCTATATAAATATTAGCTTTTAAAAACAGCTTTATTGAGATATAATTCATCTATGTGAATTAT
Dasypus novemcinctus  ........................................................................................................................

Homo sapiens          ATCTCAATAAATTCACCTATTTAAAGTGTACAGTTCAGTCATTTTTAGTATTTCACAGAGTTGTGCAACCATCACCACAATCTAATTTTTAGAAAATATTCATTACCCCAAAAAGGAATC
Dasypus novemcinctus  ........................................................................................................................

Homo sapiens          TCATACCCACTGGTAGCCGTTTCCATCCCTGAAGCTCCCTCTCCCCCTTCCCAGCTCCAGGTAATCACTAACCTACTTTCTGTCTGTATAGATTTGCCTCTTCTGGACATTTTATATAAA
Dasypus novemcinctus  ........................................................................................................................

Homo sapiens          TGAACTCATACAATAGGTGGTCTTCTGTGACTAGCTTCTCTCCCTTAGCATAACTGTCTTTTAAAAAATTGCAACTTCCATTTTAGATTCAGGGTGTACATGTCCAGGTTTGCTACATGG
Dasypus novemcinctus  ........................................................................................................................

Homo sapiens          GTATATTGCATGATGCTGAGGTTTGGGGTACAGCTGATCCCATCACCCAGACAGTGAGCATCCAATAGGTAGTTTTTCGGGCCTTGTCCACCTCCCTTCCTCCCCTGTCTAGTAGTCTCC
Dasypus novemcinctus  ........................................................................................................................

Homo sapiens          AGTGTCTATCGTTCCCATATTTATGTCCATGTGTACCCAGTGTTTAGCTCCCACTTACAAGTGAGAACATAGCATAATGTTTTTAAGGTTCATCCATGTTGTATATATATCAGAGCTTAC
Dasypus novemcinctus  ........................................................................................................................

Homo sapiens          TTCCTTTTTAAGGCTGAATAATAGTTCATTATATGAATATATCATATTTTGTTTTTCCATTTATCAGTTGATTGACATTTTGGATTGTTTCTATCTTTTGGCTAGTATGAATAACACTGC
Dasypus novemcinctus  ........................................................................................................................

Homo sapiens          TATCAATATTCATTTACAAGTTTTTGTGTAAAGATATGTTTTTAAATCTCTTGGGTATATATCTGGGAGTGGAATTGCTGGATCATATGGTAACACTATGTTTAATGGTTTGAGGAACCA
Dasypus novemcinctus  ........................................................................................................................

Homo sapiens          CCAAACTATTTTCCAAAGAGCCTGCACTATTTAAAATTCCCTCCAGTAGTGGGAATGATGGTTTCAATTTTTCCACATCTTCATCAACACTTGTTATTTTATTTTATTTTTATTTTATTA
Dasypus novemcinctus  ........................................................................................................................

Homo sapiens          AATTATTATTTTTATTATATTTATTTTATTTTTTATTTATTTTTTATTATAGCCATCCTACTGTGTGTAAAGTAGTATCTCATTGTGGTTTTGATTTGCATTTCTCTGATGGTTAATGAG
Dasypus novemcinctus  ........................................................................................................................

Homo sapiens          GTTGAGTATCTTTTCATGTGTTTATTGACCATTCTTATGTCTACTTTGGAGAAATGTCCCTACAAATTCTTTGCCCATTTTTAAATTGGGTTGTCATTTTATTACTGAGTTGTAACAGTT
Dasypus novemcinctus  ........................................................................................................................

Homo sapiens          CTTATATATTCTGCATGCAAATCTCTTGTCAGATATATGTTTGTGAATATTCTGTCATTCTGTGTAATTTTTTTTCATTTTCTTGAAGCTATTATTATTAAATAGAAATTTGTAAGTTCG
Dasypus novemcinctus  .........................................................................................................AAATTTACAAATTCA

Homo sapiens          CATATAATAAA-GATGACAGATTGTATTAGAAAGAATGAGATCTA-AAATCACCTGATGCACCCTATGGTACACTACCCTGCTCC--TGCACTCCTTTGAAATTAAATTCAAAACAGATC
Dasypus novemcinctus  CATATGATAAAAGAACACAGACTGCATGAGAAGGC-TGAGATGTAGAAATCAGCTGAGATACCCTTCAGTGCACTATCCTGCTCCACTACTCTTCTTTGAAATTAAATCCAAAACAGATC

Homo sapiens          AAGCTTCTCCTCTGTGCCAGGCACTGTGGGGGAATAAGACCTAACCTCTATTCTCAAAGAGCTTCATAACACAGA----AGAGAAGATGCAAACATGTGGAAACTTAAA-TAATTATAAC
Dasypus novemcinctus  AAGCTTCTACACTGTGCCAGGCACTGTGGGG-AATAAGATTTGGC-TCTATCCTCAAAGAGCTTACTAAGTCAGAGGGAAGAGACGATGCAAACATGTGGAAACTTAAAATAATTATAAC

Homo sapiens          ATGTTTAAATAGCAGTTCAGGACCACAACAGAAAAGATGCCACAGAGCACTATATGATTAATTTCCAAATGAATTGCATGGAAAAGAATTGCTATGGGAACTCAAGGGAGGGAGAAAGCA
Dasypus novemcinctus  ATGTTTAAATAGCAGCTCAAGACCATAATAGAAAAGATGCCACAATGCA--GTGTGATTAATTGCCAAATGAATTGCATGGAAAATAATTGCTATGGGAACTCAT..............

Homo sapiens          CTTGGGGCTGAGGTGGTCAGGGGAAGCTTTAAGGAGAAGGGATCTAAGCAGTCTGTAATTAACTACCGCCATGCCTGTGTAATACTAAACAAAGACTTGGAGAGAAAAAAATATTGACTG
Dasypus novemcinctus  ........................................................................................................................

Homo sapiens          TTTTTGAGGTGAGGCATCTTAGTGATTAAATTGATACCACCATTTCTTTTCAACCTCTGCAGGAATCCCCACAGTCCCACCTTTCCAATGAAGACTTACGTGGCCTCTGTCTAGTCCAGA
Dasypus novemcinctus  .......................................................................................ATGAAGACTTATGTAGTGTCTGTCTAGCCCAGA

Homo sapiens          GGTTACCAATTAACAAAGTTAACAAGCCACATCTGGCTTACAGACACGTTTGGTTTGGTCCAATTAAAATATTAGAACTAGCTGCTAAAACATTTAACTGGACAAATTTCACATAAAATA
Dasypus novemcinctus  GGTCACCAATTAGCAA--TTTACAGGGGACATTTGGCTCACAGACACACTTTATTTGGCTCAATTTTAAAAATGGAATTA-TTGCCAAAA--TTTTAATGGACAAATTTTGCGCAAAGTG

Homo sapiens          TTAATTTCCAGTTTCTCTAGAAAAATCAGAAGACCTGGCTACCCTGCATTGGAACATGCATTCTCTGGGACCATGGCCAATGGCAACCCTGCTTGCACATG----CAGAATTCAGTTTGA
Dasypus novemcinctus  TGTATTTGCACTTTCCTTAGAAAAATCAGAAGACCTGGAATCCTTAGGTTGGAGCATGCACTTTC------CATG-----TTTCATCCCTGCTTGCATGTGTACACAGAA--AAGTTTGA

Homo sapiens          GACTTCCTAGAATCATAGTTCTTAGACTAAAAGGTATCTTAAGAGTGCATCTGTTTCTATTCCCTGACTTTGGGA-ATGTATGGTATTATAATATTACCCTCAATTTACAACTTGATTTA
Dasypus novemcinctus  GACTTCCTGAAATCATCAATCTTAGACTGAAAGGTATCTCAAGGATGCATCTGTTTTGGTTCCTTGCCTTTTGGATATTTATGATATTTT-----TACCCTTAATTTACA----------

Homo sapiens          GAATATCAACTAAAACCCACAGAGAATTTGACTTGCTGCAGGCCACAGGGCTATTAACTGGAAGAGGGAGGGCTAAAACCCATGTTCTCTACTTCTCAACGGAATGTTCTTTCAACAATC
Dasypus novemcinctus  GAATATTAACTAAAACCCATAGAGGCTGTGACTTCTCCAAGGTCACAGAGCTAGCAAGTAGAAGAGAGGGAACTAAAACCCACGTTTTCTGCTTCTCAACTCAAAGTTCTTTCAACAATA

Homo sapiens          TGTCTTTACAGTCTGGATGCATATTGTTTAGTAGCATTCTTCTGGCACTTCCTAATCACTGCTTTGTGTTGTTGGACGATTTTTGATATGATCGTTATCTCCCTCTCTCTTTTTTTTTTT
Dasypus novemcinctus  TATCTTTCCAGTCAAGATGCATAGTGTTTAGTAGTATTCTTCTGGCACTTTCTAACCACTACTCTATGTTGTTGGACAGTTTTTGATATGAAAGTCTTATCTCTC..............

Homo sapiens          TTTTTTTTTTGAGACGGTGTCTCACTCTGTCGCCCAGGCTGGAGTGCGGTGGTGCGATCTTGGCTCACTGCAAGCTCCGCCTCCCGGGTTCACGCCATTCTCCTGCCTCAGCCTCCCAA
Dasypus novemcinctus  ........................................................................................................................

Homo sapiens          GTAGCTGGGACAACAGGCGCCCGCCATCACGCCCAGCTAATTTTCTTTTTTTGTATTTTTAGTAGAGACGGGGTTTCATCATGTTAGCCAGGATGGTCTCGATCTCCTGACCTCATGATC
Dasypus novemcinctus  ........................................................................................................................

Homo sapiens          TGCCCGCCTCAGCCTCCCAAAGTGTTGGGATTACAGGCGTGAGCCACCGCCCAGCCTGATCATTATCTCTCTTAAATGACATTGTAACATCCCAAACTGCAAAGGGGCCCACATCAAGTT
Dasypus novemcinctus  .........................................................................AAACTATATTGTAACATTCCAGAGTGCAA-GGAACTCACATCGCACT

Homo sapiens          TTTCTATGAAAGCCC-ATGACGTGGAGCACAATGCTTTGTACTAAATAGGCACTCAGGTGGTATTTGTT-----ATTGATTGATAGGATTCTGAAGAACTCAGTATGTAACCATCCTTCC
Dasypus novemcinctus  TTTCTATGAAAGCCCCATAGCACGGAGGAAAGTACTTTGAATTTAACAGGTACTCAGTATATATTTGTTGGCTTATTGATCAATAGGTTTGTGAAAAAATTACTATATAACCCTTTTTAA

Homo sapiens          AAACCTGGGTGTGTTAAAATGTGTTCATTGTTCAGAGACAAAG--GTCTGGTACCTTTAAGAAGTCTCAGCTTTGCAATGAGTTTGTGGGGC-AGCTGCTTGGCATAGATACTGGGGAGG
Dasypus novemcinctus  AAACTTAGGTATGTTAAAATGTGCTTATTATTAAAAGACAAATACGTCTGTTACCTTTAAGTAGCCTCGGCTT-GCAACTACTTGTTGGGGCCAGCTGCTTGGATTTGATATTAGGGAAG

Homo sapiens          GACAGGGAGGAGA-TCATTATGTAGGCACATGGCGCTAGGATGTACCTTACTTTGT--CTTGGCTGTTTTCTGAATGTTAAGAGGAAGGATGAAATAATAGCAGATATAGGAAAAGTTAT
Dasypus novemcinctus  GGCAGAGAAGGGAAGTATTGTGCAGGCACATGCCCCCGGCATGTACCTTACTTTATACCCTGACTGTTTTCTGAGTGATGAGAGGAAAGG---AGTAACAGCAGGTGTTGGGAAAGTGGT

Homo sapiens          TTAAAGCCTTGCCTT---AGATCAGTGGTTCTCAGGCGTGAGGATGCATCAAAAGCCCCTGGAGGGCTTCTTAAAACACAGATTGCTATATCCCACCCCTGAGTTTCTGGTTCAGTAGGT
Dasypus novemcinctus  TTAAAGCCTTGCCTTTGAAGATCAAAGCTTCTGAAACTTGAGGGTACATCACATTGTCCTGGAGGGCTTGTTAAAACACAGATTGCAGGGCCTGTCCTGAAAGTTTCTGGTTTAGTAAAT

Homo sapiens          CTGAAGTGAGTGGGGTCCACAAATATGCATTTCTAACAAGTTCTCAGGT----------------------------------GATGCTGATGCTCCT--GGTCCAGGGACC--------
Dasypus novemcinctus  GTGAAGTTAGTGGGGCCCAAGAATTTGCATTTCTAACAAGTTTCCAAGTTTCCACACTTTAAGAACCATTGCCTTAGGTCAGGGATTCTTAAACTTTTTTGTTCCATGGGCCCTTTTGCC

Homo sapiens          -----------------------------------ACACT--------------TTAAGAACCAT------------------TGCTCTGCCAAG-----------------TAGTTGGC
Dasypus novemcinctus  AATCAGGTAAAAACCACAGACCCCTTACTAAATCCACACTATACTGTGTATTATTTAATAAATATATCACGCCCACACCAAAACGTCCTCACAAGAATAATAAAAAGGTAATTAATTGTC

Homo sapiens          TCA-AGAGTAGCTAATTAGAGAAATCTGAGCA-CAGTTAGCAACTGGAAGCAAAAAGTCCATGAGGAAACTGTGTGATGTACGTAGTGGGGAGAGTGTGAACTGGCTAGGAAACCAGAAT
Dasypus novemcinctus  TTAGAGAATAATTAATTAGAGAGATCTGAGCAACAGTAAGCAACAAGAAGAACAAA------------AGTGTGTGATGTATGCAGTGGGGAGAACATGAACTAGCCAGAAATCCAGAAG

Homo sapiens          GGTCAGATTCCAGTACCAGGGGTATCATATCTTTTTAAGCAGTGTCCAAAGAATA---AGTATATTTTAATTCACATATTCCTGATTCATTTTA-CTAGTGTTTCCCAAAATGTGCACTG
Dasypus novemcinctus  ACCTGGATTCCAGTAAGAGAGGTATT-----CTCTTAAGCACTGTCCAAAGGATATTAAATACAGTTTAATCGACACAATCCTGATTTATTTTAACCAGTGTCTCCCAAATTATGCACCA

Homo sapiens          TGGAACCCTAGAAGATATTAATAGGTAGGCAATGAGATTAAAAAGGATCCATAGAGAAA-GATGTTGGAACATTTTGAATTAAAGAAAATTGAACAGGATTCTTTACGGC-----TTCTA
Dasypus novemcinctus  CAGAACACTACAAGATGTTAACAGGCAGTCAAAGGGAT-AAAATGGATCCATAGTCAAATAAGGTTGAAATTTTTTGAGTTAAATAAAATTGAACAGGATTCTTTACTACAAGAATTCTT

Homo sapiens          AGAGCCTTTAAAATGTGAATGAATGAATGCACTGCAAATCTCCAAGGAAATATAGAATATGCAGTATTTATTTTATTAATTTATGCAGAATATTCAGAATTAGTTGACCAAAGATCCTTC
Dasypus novemcinctus  GGAGCCTTTAAAGTACTAATG------TGCACTACAAATCACCAAGGGGGTATAGAATATGCTGCATTT----------------------CTCAGACTTAATTGACAAATGATCCTTT

Homo sapiens          GTTAACACTTGGCTGTTTTTTTGTAAAATGTTGTTTAGGAAACCTTCATTGGTTTACACCTGAAATGCCACAATATTTTATCAATAGATGAGGTTGCTTGCAACAGTGGAAACAGCACTG
Dasypus novemcinctus  ATTAATAGCTGCCTAGTTTTCTGAAGAACATGGCTTGGGAAACATTCATTGTTTCACCTCCAGACTCCCAAAATAGTTTGTCAATGGGCAAGGCTGTTTATAACAGTGGAAAAAGCATTG

Homo sapiens          CACTGGGAGTCAAAACATCTGGGATTTTGTTCTGTCTCTGCCACTTAGGTCTGCCACAAGTTGGCTTGCTCCCATGAAAAAGATGGGTATGATTGAAATTTATCATCCCTTTGCAAATGG
Dasypus novemcinctus  GAATGGGGGTCAAAAG---TGGAGTTTTG----------------------------------ACTTGCTTCCATGAA---GATGGGTGTGACTGAAATTTATCATTTTTTTGCAAATGG

Homo sapiens          TAGCAGCCAGTAGCTCCACCCAAAATAAGTAAATAAAATCTCACACAATCAATTTTTTTAAATCTAATTTACCATACCCTTAAGGTTATTCAATCTTTTTGTTTTTTTTTTCTCATGTTA
Dasypus novemcinctus  TGGCAGCCAGTAGTTCCAGCCACCAAAACTG----------------------TTTTTAATCTATAATACACTGTGCCCTCAGGGATATTTAATCTT----------------------

Homo sapiens          CCTTGTGTAACAGGTATTCAACCTTCAAAGCCAGAAATATGAGAAGCCTTATGCTGAGAGTTTGATAGAAAGGAAGGGAGGAAGGGAGAAAGTAGAAAGGGAGAAAAGGAAGAAGAGAGG
Dasypus novemcinctus  ------------------------CAAAGCCAGAAGGGTGAGAAGCCTTATGTGGAGAATCTG.........................................................

Homo sapiens          AAGGGATGAAAGGAGGAAAGGCGGGGCAGGGAGGAAAGGAAGAACAGCAGAAAGAAGGAAGGAAGGAAAAATAAGAAGTCAGACAGTCAGGTCATTGCAGACCATGATTCCATCTGAAAA
Dasypus novemcinctus  ......................GGGGGAGGGAGG----------------------GAGGGAGGGAAAA---------------TTAGGTTGTTGTGGACCATGATTCCAAATGGATT

Homo sapiens          GAAAAGGCTGAGAAGCATAGACACATCCTGTGTTTATTTGATTCCTGGCTCTAGGAGAAAGTTTTATTTCACCAAGGACA--GAGTCAGCTCGCTTGTTTACCACAGAGTATTGGAAGTG
Dasypus novemcinctus  GAACAGGCTTAGAAGCATAGGCACATTCTATGTTTATTTGATTTCTGGCTTTCAGTGAAAGTTTTGTTTCACCAAGGACACTGAGCCAGCTT-TTTGCTTGCCACAGATCGTTG------
```

Homo sapiens TGTGAAGTGTGTTTGTCCCCCAATTAACCATTAGTTGTTTTTTTTTCACTTGAACAAGATACAAACTTGACCCAATGGAGAAACATTGCAAGTTGTATGTGGGAGGATGCCATAAAATAT
Dasypus novemcinctus ---GAAGTGTGTTTGTTCCCCAATTAATC------------TTTTTTGCTTAAAGAAAATACAAA--TGACTCAATGGAGGAACAGTGCAAATTGTATGTGGTGAGATGCCGCAAAATAT

Homo sapiens CTACATTGATATTTAAATAAAATACCTGCCTACATTAAACGATCTTTGAAAAGAAACTGATGGGATTCAAGGCTGAATTATATTTACTGAATTCCTACAAGGTACATAAATATATGCAAC
Dasypus novemcinctus CTACACTGATGT------------------------------------AGAAGAAACTGATGGGGTTTAAGATTCAATTATACTTACTGAATTCCTATAAGGTACAGAAATACATGCAA-

Homo sapiens ATGGAGTCCCTTGTAGATCCATGATGAAATCATATTTCTTTTAAACACAAGGGTCAATTCAAGTTAAAAAATAAAATAAATGTTTACAAGCTTTC-TGCCTTTTTTAAATTTCAAAGTGA
Dasypus novemcinctus ATAAGGTACCTTGCAATCCCATGGTGAAACA----TTTTTTAAAACACATTGGTAAATTCAGGT--AAAAATAATA-AAATCTTTACAAACTTTCCTAGCTTTTTAAAATTTCAAAGTGA

Homo sapiens AAATAATATTTAATTGCATTTAAATATATACATATATTTTAATAACTCTGATTACCTATTTGATTGCATAAAAGAATGAC---GAGAACATCAACACTGCTTTTAGCCACA---------
Dasypus novemcinctus AAATAATATTTAGTTGAATGGAAAAATATA-----------ATAACT----TTACCTGTTT---TGCATAGGAAAATGGCAATGAGAACATCAGCACTGCTTTTAACTAAATGTTCATTC

Homo sapiens ------ATATTTGAGAAGTAGCAGC-CAGAAGTGTTTTTTTAAATATTTTATCTTGAAATCAGATCTCATGTAC---------CTAGGTGAACTGAATATGCCCAGAGGATATCAGGGGA
Dasypus novemcinctus ATGGGCATATTTGAGAAGTAGCAACTCAGAAGTAGGATTTTAA-TCTTTTATCTTGAAATCAGATCTCATGTATATCTTGAAGCTAGGTGAACTGAATGTGCACAAAGGGTCTCATGGGA

Homo sapiens CTCTCCACTACTGTAGATCC-AAAAAACAGAGGGCTAAATAGAAATTCCACTTGCTGTCATCCAGCAAAATAGAGATGGCTTTGCT----GAGTCCTTAGATCATGTTTAGTTTCAGGAG
Dasypus novemcinctus CTCTCCACTGCTGTAGACCCCAAAAAACACAGTGTTAAATCAAAATAGCAGTTGGTGTCATACAGAAAAATAGAGATGGTTCTGTCTGTCGGGTTCTCAAACTATGTCTAGCCTCAGGAG

Homo sapiens GGGTTGAGCTGTAATGACTAAATTATCTGGTCACTCTAACCAAGCAGCTAATTAATCCAGTCAAACAAACTTATC----AGTCATGAAGGTGAAATTGTTTTTTCATCTAGAATTTGATC
Dasypus novemcinctus GGGTTGAGCCATATTAATGAAATTGTCT-------CCAGCCAAGCAACTGATTAATCCTGCCAAATAACCCTATAGTCAAGTCAAGAAGGTAAATTTGTTTTTTCATATAGTCTTTGACC

Homo sapiens ATAAGGGCTTTCAAGAAGGAAGCTCCTTGAGTCTCCCTTGTAAACCCTTTCTGCAGTATGCCTTTCTTTCCCACGTCTGTTGAAAATTATTTATTCTTGAACCAGCTTCAGTGGTGAACA
Dasypus novemcinctus TTAAGGGTTTTCAAGAAAGAAGCCTCTGGAGGCTCCCTGGTAAGTCTCTTCTGCTATATAACTCTCTTTCCCATGACTATTGAAAATTATTTACTCTTGAACCAGACCCAGTGGTGAACA

Homo sapiens GCAGCTGGTTGCTACCCTCTCATACCCATTTTTTGTCATCTGGAACATGGTTATTCAGCGTAGCTTCATCTTCAAAAGGTGCCTGATTTACTCTGGGATTGGGAATGGAGAGAATAATTT
Dasypus novemcinctus GCAGCTGATTGCCATTC-CGTACACTCATTTTTT-TCAACTGGATCTTCATTATTCAGAGTAGCTTTAATGTCAAAAGAAGCCTAATTCATCCAAGGAT---GGATG------------

Homo sapiens TGAAGCTCTTTCTCTTTGCCTGTTTCCTTCCTTCCATGACTTTGGGTTATTTTCTAGTGATCTTCAGTATTAAAAGGCATACTGACAGCTCTTTTCTCCCAGGCTGATTTTCAGCTTGTC
Dasypus novemcinctus -------TCTTCTCTTTGCCTGTTTCCTTCCTTCTACAATTTTGGGTTCCTTT-TATAGCTCCGTGGTTTAAAAATGCATTCTGAAAGCTCTGCTCTTCCAGGTGGCCTTTCAGTATTTC

Homo sapiens TACTGAGCTTTTTTGGGTTTGGTTTTATGGAATGAAATCCCCTTGTTTGGTTTTCCCCACATTCACACTGAGGCTGTGAATGTCCACCAACTCAATTCTGAGCTACAATTGAGTAGTGTA
Dasypus novemcinctus TACTGAGCATTTCTGGGTTTGTTTTAATGGAATGAAATCCATTTGTTTGGTTTTCCCCATATTCATATTGAGGCTGTGAATGTCAACTAACTCAATTCAGAGCTACAATTGAGTAGTGTA

Homo sapiens TTAAGAATACATGTGACTGCCACATGGGCCCTGGACTGCTGAATTCATGGTCAAATCATTGTTTTCCCAACCATCATGCTTGGGGGCACCACATCAGCCTACAGTGAACATGGATTAATA
Dasypus novemcinctus TTAAGAGTGCATGTGACTGCCACGTGGGCCCTGGGCTGCTAAATTCATGGGCAAGT---TTTTTCCCTAGCCATCATGCTCAGGAGCACCACCTGAAAGTGAAG--AACATGAATTAATA

Homo sapiens AGTGCTGATTTAGAGTAAATAGTTTTCTGGTAAGTG-GTAC-ATTGAAGATTTCTCTTCC-CCTTTCTATGATTTGT-ATAGTAGCCACAATTTTCTATATCATTATTGCAGCTTCCCAG
Dasypus novemcinctus AGCACTGGCTTAGGCTAAATATTTTTATGATAATTGTGTATTACTGATCATTTCTTTTTCATCTTTCTGTGTTTTTTTAAAGTAGTCATAATTTTCCTTATCATTATTGCATCGTAACAG

Homo sapiens TACTACTTTA-TGTAGAAATCTTGCACATTCCTACCTCTGGCTTTTGCTCAGGCTACGACTCTATGGCAGTGGCAGAACCGTTTTAGGAATGTCTTCTGCTGTAATTTTCTCTTTGTCCC
Dasypus novemcinctus TACCGCTTTAATATAGAAAATTTGCAAATTCCTGCTTCTAGCCTTTGTTCAGACTACAACTCCATGGCAGTGACAGAACCATTTTAGGACTGTCTTCTGTCATAATTTTCCCTT------

Homo sapiens ACCACTTCCACTTTAAACTTCTGGT---------GTATTCCCTAATCAAAGAGGCATCTAAGTTGT----CTTTAGTGAGAAACCATCTATATATACAAAGCCATAACATGTGGATGAAA
Dasypus novemcinctus -------CCATTTTTATCTTTTGGTTGCTTCCATATGTTCCTTTGTAATAGAGGCATCCAAGTAGTTGATCTTTAATGAAAAGCCATCTACATACACAAAGCCGGAACTTGTAGATGAAA

Homo sapiens AGGACATACTGTCAACTCTCTTGTGCCAGACACCCAGATGTTGGCAAGTAGTACTTGGACCGAACAGCATAGTCGTTGAACAGCATCATCTTCCCCTTTTTAATTCATCCAGATGTTTT
Dasypus novemcinctus AGGAAACATTGTTTAACCTCTCACGCCAAATACCCAGATGTTGGCCAATAGTACATGGACTAAACAGTCTAGTCATTGAACAATATCACCTCCCATTTTT---ATTCATCCAGATGTTTT

Homo sapiens GGCATCACAGACACATAGCCAGATTTCATCCCTGATTTTGTCAAAGGCTTAAAAAAATGTGAAAAAATAATATGCTTCTCCTCTGATGAACAATTACCAATGA--ACTCTGTTCAAAAGG
Dasypus novemcinctus AGCATGATGCAGATGTGACCAG-TTTTACCCATGATTTTGTCAAAGACTGAAAAAAGTATGAAAAA-TATCATACT-CTATTCTGATGAACAATTATCAATGCCCATTCTGTTCAGAAGA

Homo sapiens GAAAACATAATGTTTCA--TTTAAAAGACCAATAATATTGAATTAAGATAAAGTTCCCATGAGAGTATATTTT---ACATTCTCATGAATCACGGATGAGTTTATTATGATCCATGCTTG
Dasypus novemcinctus GAAAACA-GATATTTCACTTTTAAAAGACCAATAATACTGAATTAAGATAAAATTCC-ATGGGGGCATTTTTTTTTACATTCTCCTGAATCACAGATGAGCTTATTATGATCCATAATTG

Homo sapiens TATGAAAGACTGACAAATAAATTCATATTGATTTGACATTTAAAATAGCATGTCTAAAAATGTACAGCATCACATTAAGTATCAAGTAATTATTCAACTATCAGCTTCAAATGTACATGG
Dasypus novemcinctus TAGGAAAGAATGACAAATGAATTCATATTGATTAGACATTTAAAATGACATGTCTAAAAATGTATAGCACCACATTAAATATCAGGAAATTCTTCTTCTACCAGCTTTGAATGTAAGTGG

Homo sapiens GAG-AAAATGGGTCCAGAACCCAAAATGTAGTGAAAGATTTAAATGCTCAAATTTCTACCACAGGTTTTTTTTTTTTTTTTTTTTGAGACAAGGTCTCACTCTATCACCCAGTCTGTG
Dasypus novemcinctus AAGAAAAAAGGGACCAGTGCCCAAAACGTAATGAATTGTTTAAGTATTTAACT...

Homo sapiens TGCAATGGTGTAATCATGGCTCACTGCAGCCTCAAACTCCTGGGCTCGAGTGATCCTCCCACCTCAGCCTCCCAAGTAGTTAGGACTTACAGTCATGTGCCGTCATGCCTGGCTAATTTA
Dasypus novemcinctus ....

Homo sapiens TTTTATTTTTATTTTTGTAGAGACAAGGCCTTGCTTTGTTGTCCAGGTTGGTCTTGAACTCCTGGCCTCAAGCAGTCCTCTCACTTCGGCCTCCCAAAGGACTAGGATTATAGGCGTGAG
Dasypus novemcinctus ....

Homo sapiens CCACTGTGCCCAGCCTTCTACCACAATTTTTAAAGAAGCCAATACCATACATTAATCCATTTACAAGGGCTCACG-CAAGAGTGCCTTTTGAGCCTGTGTTTGACAGGAAGTTTCTATTC
Dasypus novemcinctus ...............TTCAGTCACAAATTTTTAAGAAGCTAAAACCATACATTTATCTATTTGTAAGTACTGATGTTAAGATTGCCTTTTTAGTCTA-ATTTGATTGGAAATTTATGGCC

Homo sapiens AGATCATTGTGCCTAGAGTATATAGAGGGGTTACAGCAAAGTGCTGTACTTTGGGTTACAGTAAAGTGACTCAATTGATGTACCAAGATATTGATCATTTACACCCTTT-----------
Dasypus novemcinctus AGATCACCGTGCCTGGGCTATATAG------------AAAGTAT----CCTTGAGTTATGGCAAAGTGATTCAATTGATATACCAAGGAATTGATTGTTTAAATCCTTGCAGGGGCTGGG

Homo sapiens --AGGCCTGA------------------------AAAATATACTTTTCTTTGTGTTCCATGAAACTAAAAAGGCAGGGAAGCATTAAAGAGACAGCAATAAAAAGATTTGATGGTATGTA
Dasypus novemcinctus AGGGGCCTGGGACAGCAAGGGTCCTTGGGTATTCAAAATATAATTTTTTATGTGTTC----------AGAAGGTTGGGAAGCACTAAGGAAGCATAAATTAAAAGATCTGATTATATGTT

Homo sapiens TATGTGAGTTTGAGTGTTTGAGCTGGCGTTGTGAGTTTGTGGGTGGATTAGAAAGCATATGGCTAAAGACATGTGCAACAGATGCAAGGGCCTGGGCTTTGGCCTTCCCTTTGAAACTTC
Dasypus novemcinctus ----------------------------------GTCTGTGTGAGGAT-----------------GAGATATGTACAACAGATGCAATCGTCTGG-------CCCCCCCTCTGAAACTTC

Homo sapiens TTTATGGGGACATGTGCT-------GCCTTGCTTCCTGGATTAAGACCACATTTGCACAATTTGCACATTGTCCCTCT-----TGGGCCTGCTTCCCACCGGAGACTGCATCTTCCACAA
Dasypus novemcinctus TTTCCTGGAACATGTGCTTGCCTGAGCCTTGCTTCCTGGATTAAGACCACATTTGTACCACTTCTATACTACTTCTCTAATCCTGGGCCTGCCCACTACCAAAGCCTGCATCTTCCATGA

Homo sapiens AAACAAGGCAAGAAGTAAAGAACTTAAGAGAGCTGATTGTGAAGTCCAACTACATGGGTTCAAATCCTGGTACCATCATTAATTAGCTGGGTCATTTTGGACCACTTAACTTCCAGGACC
Dasypus novemcinctus AA--CAGGGAGGCAGCAGAGAATTTAAGAGCTCTG-TTGTGAATTCCAACTGCCTGGGTTCAAATACAAGTACCATCACTGATTATCTGGGTCATCTCGGGCCAGTTAACTTCCTTGAAC

Homo sapiens CTCATTTTCCTCATCTACACAAAGGGCTAATAACAGTATTTACCTCATAGTT----------------------GTTATGTAAAGTGCTTATCCCAGAGCCTGGCAGAGAGCAGACACT
Dasypus novemcinctus CTCTTTTTCCTCATCTATAAAGAGGCATTATAACAGTACTTGCCTCATACTTTTTGTGAAGAATAAATTAACTAAGCTATACAAAGTGCTTATCCCAGAGTCTAGCATAGAGTAGGCAAC

Homo sapiens AGTTGATCATTATGATTATTGAGCAATTACTATGTACCAGGTGCTTTATTTTCATCTCTGATCCTCACAACAGCCCTACAAGGCAGATACTATTATCTTCAGTTTACAAGTGAAGAGGCT
Dasypus novemcinctus ATTTGGCTATTTTGATTATTGGG------------CCAGTTGCTTTATCTTTTTTTTT..

Homo sapiens GAAGCTCAGTGAGATAAAATACACTGCTCAAGGTCACACAACCAGGAAGAGCAGAAACCAGGCTCCAAGTCCCTCTGTCCCTCACTCCTAAGGCCTGTGCTATTTGCCATATCATCATCC
Dasypus novemcinctus ....GTGCTATCTTCCCGGAAGTCACAC

Homo sapiens CACTGGTGCCTCTAAAAGT----TTCTTCTTCTAGGGTTCATCCCTCTTCTCTCTTCATCCTCCTCTTGGCCTACCCTGGCCTGAGAACTGCTTGGCACAATCCTATACAGCAGGTCCTT
Dasypus novemcinctus TGCT-----TTTTAAAAGGGGGATTCTTCTTCTAGGAATCATCCCTCTTTTCTC---ATCCTGCCCTTGACCTACCCTGGTTTGAGAACTTCTTGGCACAAAGCTA..............

Homo sapiens GAATAACATTGTTTTGTTCAAAGCCATTTAGTTATCACGTTAATGGGGAAAATTTGGTTTTCCCCAGGGCCACTGTCCATGGACAGGATGGCGTCCTGACCAGGGTGGTTCCCACCTTTT
Dasypus novemcinctus ....

Homo sapiens GTGCTCCTTGTGCTGCCAGGATGGGCTCCGGCCACCTGTGACCCTGAACTGGAATCAGCGGGTTAGAAAATAACTGAATGAGTGAATACAAATTATTGGAAAATGAAAACTCGTAAAGTA
Dasypus novemcinctus ....

Homo sapiens CACAATGACCATACGAATCCATGATAATAAATGACACGATAGGAAAGCGCTCAATGAGCGCTTTTGTGATTCTTCTGGAACTGCTGGGTGGCAGGAGGTGCTCCTTATGATTTTGCTTTG
Dasypus novemcinctus ....

Homo sapiens CAAATGTTTATTCCTTGACCTGACCCGCCATTGCTACAACCGCTGTCACTCATTGATTCACCAAAAATTAGGCAAATAATTCTCTTACTTGTTTTTATTAATCTTTCCTAAATGTATGTG
Dasypus novemcinctus ....

Homo sapiens TAGCTCACACTTATTGTTAATGTTTAATTAACTTATTAATGTTTAATATTAGAAGTGTTTGGGTGGTTTTTGTTTGTTTGTTTTTTTGTTTGTTTTTGAGACAGGGTCTCACTCCTCTCG
Dasypus novemcinctus ....

Homo sapiens CCCAGGCTGGAGTGCAGTGGCGCAATCACGGCTCACTGCAGCCTCAACCTCCCAGGATTCAGGTGATCCTCCCACCTCAGCCTCCTGAGTAGCTGTGACTACAGACGTGTGCCACCATGC
Dasypus novemcinctus ....

Homo sapiens CTGGCTAAATTTTTGTATTTTTAGTAGAGACAGGGTTTCACCATGTTGCCCAGGCTGGTATTTGGATCTTTATTTAGAAGTTTGGTGATATTTTTGTGACCAGAAATATGCTGTGGGTAC
Dasypus novemcinctus ....

Homo sapiens TTAACTCTTGTTTATATCAATTAGCCTACAGTAAAATTAGTTTTGTTATTCGTTGTTTCACTTAAAGTCCAGTTCCCAAGAACCTGTCAAGGACGTTCAGTGAGGACTTACTGTACTCAG
Dasypus novemcinctus ....TACCCAG

Homo sapiens AGACAGCAGAGAGCAAAGGGCAAGAAGGCTGGTT-GGAGGACGAGGAGGCCTAGAGCCCCTTGCCTGCTTTTGCACATGCATCTGCTCCTGGTGGATGACTCACTTAACCTCTCCGTGCT
Dasypus novemcinctus AGACAGCAGGAAGCAAAGGGCATGAACACTGGTTTGGAGGGCGAGGGAACCTGTAGTCCCTTGCCAGCTCTCACACATGCTTTGGATCATAGTGGATGAGCCACTTAACATCTCTGTGCT

```
Homo sapiens           TCTGCCTCTTCAACTGGAAAATGGAATCTGTAAGTTGCAGCACTAG-----CTTCTGTGACATTCTACTAGGCCTCCAGGAAGAGGAGGCCATCCCTCTATATGTCCAGTCAGCATAT--
Dasypus novemcinctus   TCTATTTCTTGAACTGGAAAATGGAATCTGCAAACTGTAACACAAGAGTTGCTTCTGGGACATTTCACCTGGCCCAGAGGGAGAGCAGGCCATTCCTCTTTATCTCCAGTCAGCATAAAC

Homo sapiens           -GGGGAACAGTGGGGCTGTGTGTGACTCACCCTGTGGTGTGCCAGCCTCAGGCTTATACTAGCCTAGGCTTATACTCCCAGATCCTGAGCTAGC-----------------AGCTGGAGC
Dasypus novemcinctus   AGGGCAGTTGTGGAGTTATGTGTGATTCAGCTGGTG------------CATGCCTGGGCTCTCACAGACTTGTACTCCCAGATCCTGAATTAGTTGCAGACAACCTGGAATGGCTGGGCT

Homo sapiens           TTTGGGATCTGGAACTGTTACAGGGCCAAAGAACCTGCTTAGCTTCAGGGCTGCTTCTAGATAAACTGCCAGGA-TGTGGGCAAAGCATTTGTCATGGAAGGTGGGGAGTGTATGTAGGT
Dasypus novemcinctus   TTCTGGATTTAGAACAGTGA-AGTGTCAAAGAACCTACTCAATTCAAGGTCAGCTTCAGGATAA-CTGCTGGGAATGTAGATAAACCATTTGCCATGGAAGGCAGGGAGTGCGTGTGGGT

Homo sapiens           TGTTTCAGGGGCTGCTTTGTGGGAAGCCAAACCTTTTGGCAATGGGCTCAGAACTGGCTTTCCTTTTTGGGTGTCCTCTAAATTCCGAGGGTGCCCCCACACCAGAGGGT-CTCCACTTC
Dasypus novemcinctus   TGCTTTAG-------CTTG---------AAACCTTT--GTCATAGGCTCAAAACTGGCCTTCATTCTTGGGTATCCTCTAAAATCCTAGGGACCCTCCAGGTCAGAGGGGGCTCCACCTC

Homo sapiens           --------------TGCCATCCCTAACCTAGTGGGGAGATGAATCTGATCTTTTGTCTCAAAGCCCACATTAAAGAGGACCATTGCATGCTTCATCACCAGGCTGTGACTCCCAGA--CC
Dasypus novemcinctus   ATCTGGAGCCTCTGGGCTATACCTAGCCTAG----------------AACTTTTGCCTCAGGGCCTCCTTTACAGAGAATCATAG---------------GGTTATGACTTAGAGAGACC

Homo sapiens           TCAACTCATGTCTTTTTCCCTCTTCACCAGGGCAGAGGCTAG-ACTGTTTCCTCTCCTCTCCCTGAACAATAGCAGTGCCCAGATTTTGGAGGGCATACCGCCTACCTGGCACTGTGCTA
Dasypus novemcinctus   TCATCTAGCATCCTTTTCACCCTTCACCAAGGCCAAGGGTGGGACTATTTCCTCTCC-CTCCCTCAGCAATAACTGTACCCAGCTGTTGGATGGCTTAACTTATACCTGGCACTGGGCTA

Homo sapiens           AGTACTTTGCCTGCATTTCCTTGGTAATCCTCATGTAATCCTACAAGGGAAGTACCATTTTGTCACGATTTAACAGAAGAGGATACAAAAGCTCAGAGCCTGGAGGTGACTTGCTCAAAG
Dasypus novemcinctus   AGCCCATTGCATGCATTTCCTTGTTAACTCTCACATAGCCCTGTGAGGGAGGTGCCATTTTGTTGCTATTTTACACTAGAGAAAATGGAAGCTCATAGCATGAAGGTGACTTGCTCAGAG

Homo sapiens           TCACATTGATAGTAAGTGGTAAAGCCGGGACTGAAATACAGGTCTAACTTTTG----AGACAACCTTTTTTCTTTTTCTTTTTTTTCTATTTCAATTACATAATCCTAATCCTGTGCGTG
Dasypus novemcinctus   TCATGCTGAGAGTAAATGGTGGAGCCAGAATTCAGATGCAGGTCTATCTCATGTCAGAGACTGCCTATTTCCCACCT----------GATTTCAGTTACACACTCCT------GTACATG

Homo sapiens           TTTGAAGGGGA-------GAGACAGGGGAGGGAGTGAGATGGCAATTTGCTTG---TGACCTAAGGAGGCCCTGAAATCTCCTGGGAATGAGGATGG--GGGACCTACATGA--------
Dasypus novemcinctus   TTTGAGGGGGATACAGGTGATAGAGGGAAAGGGGCAAGTTGGTTATTGCACTGGAGTGTCCTAAGAAAGCTCTGGAATCTCCTGGGAATGAGAATTGAAGGGACATATATGACAGGCCTG

Homo sapiens           --GTTGCCCAGGGCACTGGTAATGCAGCCCACACCAGCAGTCAGTGATCTCCAACATGGAGGGCTCCAGGCCTGCCTAGCTGCTGGCTGCTACTG--GAGGCCAGAAAAAGACAGCTCCA
Dasypus novemcinctus   AATTTGCCCAGGGCATTTGAAATGCAGCCCATGCCAGTTTGTAGTGGTCCACCCTGTGGAAGGCT----GCCTGAATAGCCATTGGCTTTTGCAGCAGAAGCTGCAAAAAGAGAGCTCAG

Homo sapiens           GTGTCCTT---CTAGGCTGGGGGTGGTTCTTGCCTCCAGGGCTTATGGA-AGAACTTATCAGACAAAACTCTGCCCTTTCAAGCAGAAAAGTACTTTACAAAGAACATAGAGAGGTGAAG
Dasypus novemcinctus   GTGTCCCTAATCTAGGCTGGGA-TGGCTTCTGCCTCCAGG-CTCATGGGGAGAATTCAGCAGTAAACATTCTGGCCTTTTAAACAGAAAGGTGCTTTAGAATGAGCAAAGACAGGTAGAG

Homo sapiens           TGGGTACTT--TTCAATTTTTGCCTGATTATCTAATATCTAAATGCTTTTTCTTCACTTTGGAATCCTGACCTGACCTGTTTTGTGCCCTGTCCACCCACCACCACTGAGTCAAAGCTCA
Dasypus novemcinctus   TGGACATTTGGCTTTTTTTTTTCCTGGCCGCCTAATATCTAAACTCTCTCCCTAGGTTTGGGAATCCTGACCTGGTTTGTTTTGTGCCC------CAAACCACTCTTGAGTCAAAGCACA

Homo sapiens           GGGCCTGGCATC--CAGTATTAGTC-ATACCTATTT-GTTGCCCGAATGAATGGGGCTGAACCCTGGTCTGACTCTAAAATACATGCTT---GAAAAAAATTATTATGGAAATTTTCAAA
Dasypus novemcinctus   GTACCTGGCATAAATAGTATTACTCCATATGTATTTTGTTCCCCGAATGAATGGGATTGAACCCAAGTCTGACTCTAAAACCCACGCTTAAAGGAAAAAGTTATTTTGGACATTTCTGAA

Homo sapiens           CACATACACAAAAGTAAAAAATATAGTATAATGAACCCCCAAGTATCCATCACCCAGATTCAACATCTATCATGATTTTCCATGTTAGCTTTACCTATCTCTCTTTCTTTTTCCCTCT--
Dasypus novemcinctus   CACA--CACAAAAGTAGATAGAATAGTATAATAAACCCC-ATGTACCAATCACCCAGATTCAACAGCTATCAAGATTTTC-ACATTTGCTTTATCTATCTTCCTTTCTTTTTTCCTCTCT

Homo sapiens           --TATGCTTTACCCCA------------------------------------------CATGTTTCATTATGCAACTCAAAAA-TAGGGATATTTTTATATACAATGACGAAATTATTA
Dasypus novemcinctus   TTTATGCTTTGCTTTAATATTTTGAAGGAAATCTCAGATATTATAACATTTTGCCAAAACATAATTCAGTATGCATCTCAAAAAATGAAAATATTTTCATATACAATGATGATATTATGA

Homo sapiens           TCCCCCCCATCGCCAAACTAACAATAATTCCCTTCAAACCATGCTCTTGAGCACTACACTGGAGTTCCTCCATTCTACTTTCTTTCCCAGTCCCCAACAGGAAGCATTTCTCAGGCATCT
Dasypus novemcinctus   TTACACCTA--GTCAAACTACCAG--ATTTCTTTAAAACCGTGCTCTTGAACACTACACTATAATTCCTTCATTCTACTTTCTTTCCCAGTCCCCAGTAGAAAGCATTTCTCAGGCATCT

Homo sapiens           GCTCTTGCACCTCCTCAGAGCTGGAATTACAGTTTGATGTGTATTGGGTACTCACAATGTGCCCTTTTAGGTAAGCAGGGGGCCAGATTCCCCTCTCCCTGTTTTACAAAGAAGAATGGC
Dasypus novemcinctus   GCTCTTGCACCTCCACAGAGCTGGAATTGAAATTTGATTTGTACTGAGTACTCACAATGTGCCCTTTTAGGTAAGCAGGGGACCAGACTCCCCTCTCCCTGTTTTAAAAAGAGGAATGGA

Homo sapiens           GACACACAGCTATTAAGTGCCTTGTACAAGGTCACACAGTCAATTAGTGGCTGATAAATGGGACTCAAGCACTGTGGCTGGGTCTATACAGCCCCAAATACAGTTTGCAAGGAAATTAAA
Dasypus novemcinctus   GACACAGGGCTTTTAAGTGCCTTGAACAAGGTCACACAGTCAATTAGTAGCTGATGAATGGGACTCAAGCACCGTGGCTGGTTCTATGTAGCCCCAAACATAGTTAGAAAGGAAATTAAG

Homo sapiens           TGAAGGGACTCGTGTCCTGGGGACAGTACCGTGTGTTTTCTGTTGCTGTTGAAGTCATTGCACTT---------------------------------------AGCCCATCAGGACAG
Dasypus novemcinctus   TGAAGTGGCTCCTGTGCTGAGGATAGTACTGTGTGGTTTCTGTTGCAGTTGAAGTCATTGCCATTCAGTCTCAGACAAATCCTTTCCTTAGGACAGTTTGATAACAGCCCATTAAGACAG

Homo sapiens           CCTGATTGATGGGATCAGTTGGACTCCTATGTTTGTTTTCCTCATCTGACTGGATTGCAACAATCACCTACAGGCAGGCTGGCCACACCCGTGAGTTTTTCTCTTCTTTTTTTCCATGTT
Dasypus novemcinctus   CCTAATTGATGGGATCAGTTGGACTCCCATGTTTTTTCTCC-----------------------------------------CCACAGCTCCATGCTTTTCTGCTC-ATCACTCCATGTT

Homo sapiens           CCTGGAGCTGACTCCGGAGCCCACATTTCTCCTGTCTTTCTAGGCTTGGACTTGCTCTCTGCCAAAGCAGATGACTCCCAGAGCAGCCACAGTCTGGGACAGGGCAAATGTGAGAAGCAG
Dasypus novemcinctus   CTTGGGGCAGACGCTGAGGCCCTCATTGCTCCTCTCTTTCTAGAGTTAGACTTACTTTCTGCCAAAGCAGAAGACTCCCAGGGCACCCATAG------------------------GTAT

Homo sapiens           AAGGACTAGCTTAACTGCTTTTCTGTTCCTGCCTCCTTCTTGTTCCAAAGACTTACTCTTAGATGCTT-GGCTCTCCTGAGCTTGATCTCCATGTCTAATTCTCTAGACCTCTTCCACTC
Dasypus novemcinctus   CAGGGCTAGCTCAGC----------------------------------------CATTTGGGTGCCCAAGCTCACCTGGGCTTGACCTCTATGTCTGATTTCCCAGACCTCTTCCACTT

Homo sapiens           CCCTGATATCCCACCCACATTTTCCAAGTAAGTCTCTCAGAGCCTTGTATTCCAAAATACAAACTGGCTAGTGGAAAGAAGGAATCATTGCTGTTGGCTAGTGGATGACTGATCAGTCT-
Dasypus novemcinctus   TCTTGATCTCCTGCCCAAATTTTCC-----AGTATCTCATAATCTCTCATTCCAAAATGAAAATAGAC----------AAGTAATCCATTCTGTTGGCTAATGGAGAATCTGTCAGTCTA

Homo sapiens           GAGTCCAACCAGGAAAATAGAAATC-ACTGAGTATTTATTTAAGGGAAAAGGGATTCAGTGCAGGGAATTGATCACACAGGTGATGGAAAAGCTGTGATGCCAAGCAGAGGACAATGAAG
Dasypus novemcinctus   GAATCCAACTAGGAAAAAAGAAATCTACTGAATATTTAT-----GGAAGAAGAATTCAGTGCTGGGCATTGGTTACAGAGGTGATGGAGGAGCTGCAATG....................

Homo sapiens           CAACCTAGAGATTAGTCACAGCAGGGAGCTGCTACCATTCCTTGGCTAGAACCTCAGGGCTGGGACACTTAGTAGAAACTTGAGCCACAGTAACCTGGTCAGCAGTGCCAGAGCCACAAA
Dasypus novemcinctus   ........................................................................................................................

Homo sapiens           GAGACACAGTCACTGCTGGGGACACCACATGAAGCAGAGGGGGAGGGAGAGAAATACCCTGGTTTCTCTCTCCCTTTTGCTTTCTAATATCCTAACAGTGCCTTCCATTGGCCAAACCCA
Dasypus novemcinctus   ........................................................................................................................

Homo sapiens           TCAGAGCCTGGGGAACAGCCTGCAAAGGCCAACCTGCCCCATCAGCTTCCCTGCTACAAGAGCAGAGATGTGAAAAGGAAGTAATGGACTTGGGGGGGCAAACAGGCCTGGGGACAGCAC
Dasypus novemcinctus   ........................................................................................................................

Homo sapiens           AGATCTCCTCCTCCCTGCCACCCCCAACTAACTTATTTGGATATGTCAGGGTCTATTTGGAAACAGTGCATTTACTCAACAAAATGATTCTAAAGCTGACAAGAAAGACCCTTAAGGAAA
Dasypus novemcinctus   ........................................................................................................................

Homo sapiens           AGGGTGTGGGTGGAGCACGAACCCGCTTCCTTGACTACCTGCAAGTCTTAGCATCCAGATTCTAGCCCAGTCCTCTTATTTCACTTCCTCCTGCATGCTCCATCCCTCCTCCTCCCACGC
Dasypus novemcinctus   ........................................................................................................................

Homo sapiens           ATAGGCTGACTCCAACAAAGGCAGGCCGGCCATTTTGCTTTCTGGCAATGTGTGATGCATTCATTGGCTCCCAGGACTGTGTAAACCCAACCTTCTTGCCTGCAGGGTTTCAGCTTGGCA
Dasypus novemcinctus   ........................................................................................................................

Homo sapiens           GCCAGGCTCTGAACTGCAGTGCTGTCAGGCAGGTGAACCCGCTGGCACCACAATCCAGAAAACATATCTCCTGATTCCTCAGGTGTTCTACACGTTTGAATGTGTAGATGAAGGGAGATG
Dasypus novemcinctus   ........................................................................................................................

Homo sapiens           GCTGGGAGGGGGAAAAGAAAGACATCAAACTATTTATGTGCTTTCCCACACCTTATTTCATTTACTCTCCACAACTCCTCAAGGCAGATGCCCTTCTTCCCATTAAAAAATAAATTTTTA
Dasypus novemcinctus   ........................................................................................................................

Homo sapiens           AATCTTAGAATACTTTTAGATTTACAGGACAGTTGAAAAGAGAGTACAGAGAATCCCTATGCATCCTTCACTCAGTTTCTCCTGATGTTAACATCTTACAAAATCATCTTTTGTCTGTTT
Dasypus novemcinctus   ........................................................................................................................

Homo sapiens           TTACAAATGATGAAACTGGGGCTCAGAGAGGTTAGTGACTTGCCCAAGGTCATAGAACTAGGACACAGCAGAAGAAACAGGCATAAATCCAGTTAGATCTTAACTGTATCACACCACCAA
Dasypus novemcinctus   ........................................................................................................................

Homo sapiens           GTTGAATTATTTGTTTCCGACTCATTATATTGGAAGTCACTCTCTGAACCTTGAACCCTTCACTTCACCTCATAGTTGGGTTGTAAACATTATCAGAGAGGATTTCTTCCATAGATCCAT
Dasypus novemcinctus   ........................................................................................................................

Homo sapiens           AGTATGTTAACACTAAAAGATACATCTAAGACCATTTAATCTAGGGAAGGCAAATAAGTTTCATTTCATTTGCCGACTTTAATTGAGCAGTAGAGGCTGTTTGGAATTCCGTGCTGAGAA
Dasypus novemcinctus   ........................................................................................................................

Homo sapiens           TGATTCTAAATCTATGTTTTTGCTCATTAGGAAAAAAATGCTATGACTGATTATTGATACCTGCCATATATGTTGACTAGGAGAGTGGTGGAACTCATGTTACCTATTAGCCGTTTCTCA
Dasypus novemcinctus   ........................................................................................................................

Homo sapiens           GTGAATGTCTCTGCCTCATTAGTGGTACAGCTAAGAACAGAACTCAGGCTCCTGACTCATGGTCTCTTGTTCTTTCTGGTTTACAATGGACTCACAATAACAACAATAATATAATACAGT
Dasypus novemcinctus   ........................................................................................................................

Homo sapiens           GTATCACATAATGAGTGTCTACTCACTGCCAGGCACTCTCCTAAGCACATTCCACTTTCTGTACCTTACTTTTAATTCTCAGAACAACCCTTCAAGGTAGGCACATTCAACCCCAAACTT
Dasypus novemcinctus   ........................................................................................................................

Homo sapiens           AGAAATGCAGAAGCCTAGGCTCAGTGAATTTTAAAAACTTACCTAAGGTCCACAGTGAGCAAGTGGTAAAACTGGAATTTAAACCTACGCTGGTCTGGCTCCAAAGCCTAAACCCTGTGG
Dasypus novemcinctus   ........................................................................................................................

Homo sapiens           TCTCACTTCCTGCAAACACAATGTTGAATTTGCGCTCACAGCAGGTGGAGATGACCCTGGTCCCACTGCTTTGATTAAGACTAAACTGGAGTGAAAAAATCATTTTTGTTCTTACTAATT
Dasypus novemcinctus   ........................................................................................................................
```

```
Homo sapiens          TTCTTAAAAAGATTTGTTCACAGTTCCTTACTTAGATGGAACTGATTTTTTCGGGCTGTCTCATATTAGATGTGATACAAATTGACATGTTAGGAGAGCTGATGTGAAAGGCGACCTGGT
Dasypus novemcinctus  ........................................................................................................................

Homo sapiens          TCACTTACAAATAAATGATTAATATAATCTCATTGTGCGGCTAGCCTGCATTACTGATTCTCTCACTTGGGAGGCTCAGAAAAACAACAGGTCAGACAATAAAACCCATGCTAGCATAAG
Dasypus novemcinctus  ........................................................................................................................

Homo sapiens          ACTATAGTGTGGGATGACCATGGATGAGTCAGGGAGAATAAGAAGATAATTAACTAAGAGGGAGTTCTATGGAGTTCGAAGATAATTAAAAAATGTTCCTTCTCTATCCTAAGAATATCT
Dasypus novemcinctus  ........................................................................................................................

Homo sapiens          GACATTATTAGCGAAGGCAATGAATAAGATAAGGGGAAGAGGTGAGTTAATTAGAATTATGAGATACAACATTTTAAAGAAATTGACAGGGATCACATTTCAACGGAAAGACATTCAATT
Dasypus novemcinctus  ........................................................................................................................

Homo sapiens          CAACCGACTTCATTATAGCAATTCAACATTGATTAGGGACATGTTATGTGTGTGGCTGTGCTCTGTGTGCTTAGAGGACTACTGAGATGCTGTAATCTTCCTCCCTGACATTCTACTGAA
Dasypus novemcinctus  ........................................................................................................................

Homo sapiens          TAGATACCCAAGTTTTGGCAGCGCAGTAAATAATCTCTTACACACACATTTGGGTGCAATTTCTAAAATTGTTTCTGCAAAATAGTATTATGTGCCACTGAGTAGATATCTTGTATGCCA
Dasypus novemcinctus  ..............................................................................AAAGTAATTCTATGTACCACT--------ATCTTATATGC--

Homo sapiens          GATATTACTGTTCCTGCCAGGCTAAATGTTCAAACAACATGGAAGCCTCAGTGATAGCCAATCCATTATTTATCTACCTATATTCACTTCAGTTGCCAAAACTATTCTAAATATAAGGAA
Dasypus novemcinctus  ----------------------TAAATGCCCAA-CAGCAC---AGCCTTAGTAATAGCAAACCCATTAATCTTCTACCTATGTTCAATTTAATGGCCCAAATTATTCTACATATAAATAA

Homo sapiens          TCCCTTCCTTAGAGGCCCCAATATGGCTTTCCGTATTGTTC---------TGAGTCCTTTCTAACTGGTTAATGCCCATGCCATATTGGTTATCATAAATTTTGAATAGCATCTTTTCTC
Dasypus novemcinctus  TCCCATCCTTAGAGGCCCTGATATGACTTCACTTATTGTTTATGGCCAGGTGACTCCTTTCTGATTGGTCAATGCCCATGCCATACTGGTTAGTATAA---TTAAATCTTATC-TCTCTC

Homo sapiens          TGCTCAGAAGTGTCGCTCAGTAATACCCTACCTC-TTCTGACTCCTCCTACAGTGTAATCTCTCACCAGTCCTCACTACATTCTGAACTG-ATGTTCCAGCTCCACTTAAATCTGTCATG
Dasypus novemcinctus  TGCTTAGACGTGGCACTAAATAATACTTTATCTTATTCTGACTCCTCGTCTATTGTAATATCTCACCAGTCC-CACTGCATCCTCAACTGTACATTTTAGCACCACCCAAACCTGTCATG

Homo sapiens          TTTTGTTCCTTAACATATGCTATTCCCTTTACTTGGAATACACTCTCTTTCCTTGTCTGCCTAACAATTGCTGCTTGTCTCGCAAATATTTTCTCTGTGAAGCTTTCTCAGTGCCCTTAG
Dasypus novemcinctus  TTTTGTTCCCTAATATATACTATTCCCTTTACCTGGAGTCCACTTTCTTTTCTCTTCTGCCTAAAAATTGCTGCTTATCCTTCAA--ATTCTCTCTCAAAAATCTTCTCTGTGT-----G

Homo sapiens          GTACCCTCATGGCCCTCCTGAATTTATCTCTATTATAATGTTTTATATTTTATGGCAAGAGTTTGTTGGATCTTTGCCCACTTGAATGTGAGCCCCTTGAGAACAGGGACTATTTTATTT
Dasypus novemcinctus  GCCTTCCCATAGCCCTCTCAGATCTTCCTCTATTATAATGTATTGTACTGTATGGCAA-AGCTTGATCAGTCTT----CACTAGTCTGTGAATTCCTTGAGAACA-GGACTGTTCCGT--

Homo sapiens          ATGTCTGAGTCTCTAGGGAACAAAAGAGGGATTCAGGGCTTATGTGGTAAATGAATCAATGAATGCTCCCAGTTCTTCGTCATCTTTAGAGGCTACTTCCAGGGCTTTTAAATTTGGACT
Dasypus novemcinctus  -GGTCTGACTCTC...........................................................................................................

Homo sapiens          CCCTTTTCTTCCAAGAGGTGGTGAGAGCAGGGTAGGTGGACATGATATTTAATGAATTTCTTTTAAGTGCCAGACATTGTGCTGAACCATTTACACAAACAATCTCACGTCTCAAGTCGA
Dasypus novemcinctus  ........................................................................................................................

Homo sapiens          TGTTTTTATTTTCATCCTCGTTTTACAGATGAAAGTGGAAAATTCAGACCAGTGACTTGCCACATTCAGAGGAGTAACAAAGATACATAGCAAGTAGGCTGCAAAGCTAGGATTTGAACG
Dasypus novemcinctus  ........................................................................................................................

Homo sapiens          TAGGCTCCAATACCTGCATCAGCTCCCTTGCACTGTGCACTCTTCCCTCGCTTGTGTGTATGGTGAGCAGAGTCAGCTCTGGGCAAAATGGTGAAATAAGCCAAGTTTGTCTTTAGTGTC
Dasypus novemcinctus  ..............................................................GTAGCCAGAGTCAGCTCTGGGCAAAATGGTCAGGAAGGCAGAGTTTGTCTTCAG....

Homo sapiens          TTCAGACTCCCAGTGTGTCTCGGGGACTCAGTACTACAGTGCCACCTACATGCTGCAACACCTACTATGGCTCCTACAGGCAGCCCATCCTGCTGAGAGCTTCTCCTTCCTAACTCCTCT
Dasypus novemcinctus  ........................................................................................................................

Homo sapiens          AAGACCTTGACCTCTGGTTTGTGTTTTCTGAAGGTAAACTGCCTTAACCTAACCTTTTCCAGAGCACATATTATATTTATTGGTGCCTTCCCAGAAAGCAATCTTTAGCTTAGGAGCAGG
Dasypus novemcinctus  ........................................................................................................................

Homo sapiens          TAGCTCCCACGGGAGGCATGTAGGTGTTTCTGTGTTGTGGTAAGGGGAAGGGGTTGTCTGGGGGCCTCTATCAAGATACACCATGGAGGCTTCTCCTACAACCAAAGGGTGTTTGGCCCC
Dasypus novemcinctus  ........................................................................................................................

Homo sapiens          TTTCTCAGATGGCTGAGGGCCCTGGAGTCAGATCAATAAGCATTTGAATCTTGGCTCTGCCATTCACTGATTGCATGATTTCCCACAGTTAATCTCAGTGAGCCTTCGTTTCCTCCCCTG
Dasypus novemcinctus  ........................................................................................................................

Homo sapiens          TAAAATGGATCTAATAATAGTTCTCATCTCCTAGACGTGTTGTGAGGATGACATGAGATAACACACTTACGGTGTCCAGTACAAAATAAGCATGTAAAAATGTTAACTGTTCCCTTAAAT
Dasypus novemcinctus  ........................................................................................................................

Homo sapiens          CAATTTTTAGTAGTCTTCTGCCTCACCCCCATAACCATCCAGGTCTCTCTTCGCTTCCTTGTTTCTCTTTTCCTCTTCTCACCAACAAGTCTCTTTCTTCAAAGTCTCCCTTTCCTTTCC
Dasypus novemcinctus  .........................................................................................................CTGTCTTTTCATTGC

Homo sapiens          CAACTCCCTCCCACCAGTACGCTCAACCTTTGGCAACCCTCTGTCCCACACTTTCCAGGTCAATCCCAATTTCAGATAATCTCTCTTGATATCAAACTGCATGACCTGATTTGAATCGGA
Dasypus novemcinctus  CCACAGCCTCCCTATGGTTCTTCATTGCTATGGCAACCACATGTCCTGGATTTTTCAGGACATTTTTATTTCAGATATTCTGTCTTGTGGTCAAACCATGTG................

Homo sapiens          AAACAGAGTGAAACAGAGTCCCTTGCTCTGAGGAATGTTGCTATGGTCTATCTTAGATCAAGAAGACAAGAGCTGCCTTGGCAGCCCTACATCATAATCTGATGAGCATGAAGAAGGCTC
Dasypus novemcinctus  ...................CCATTCCTCAGAATAGTGT-GCCACAGTCCATCTTATATAAATAAGGTGAAAACAGACTTGTTGGCCC-ACATTATAGTCTAATGGGCACTGAGAAT-CTC

Homo sapiens          A------------------------------------CCTTGCCTATAAA------------------------------------------------TATTTGTTTTTTGCCTATGAGT
Dasypus novemcinctus  AGATCCTTCATTTAGAAAATGAGAGAAACAATTGTCCCTTAGCCCATGAAGTTGTGAGTATATTTTGTGAGATTTGAGGGTGAAAGGTTTGGCACAGATACCTTCTTTATGCCTGTGAGT

Homo sapiens          GATGCCAGAAACCGTCATGGTCGGCATACAATTCCTTTGAGTCAAATTGCAAGGTTT-GTCTGTCAGGGTTGCCTTGGTAAGGCAGGGTTATTATTTATATCTGAGTGCAAATTCCTTGA
Dasypus novemcinctus  GAAGCCATTAACT-TCACAACCAGTGCAGGATTCCTTTGAGTCAAATTGTAAGGTGTAGTCTAGGAAGGGTGCCTCCTTAAGGCAGGGTTATTATTTATATCTGAGTACAAATTCTTTCA

Homo sapiens          TCAGAGCCTTTCCTGTCAGGGAGAGGAGACGGAAGGGTTCCAGGGAGCCTACTCCCCACGGAAGAGGAAGGCTGTGCTAGCATGGATGGGGTATGTGAGTGTG--GGAGCTCAGCTGACT
Dasypus novemcinctus  TGAGATCCTTTCCTGTCAGAGAGAGGAATGGGAAGAGTTAGAGGGAGAAAACTCCCTAGGGAAGAGGAAGGCTATGTAAGCAGGGGTGGGGCCTGGGGGTGTGTGGGAAATCATCAGACA

Homo sapiens          GAGAGCTGTGTCCCCGGCTCCATTTTGCCTAGGGGCGAGCCCTGTGTGTTGCAACAGATGTGTGCTTTGGAATCACCAAGGTTGGGAAGCCAGGCCTCAGAGGGAGTGGCTGGAGGCTGA
Dasypus novemcinctus  GAAGATTGTGTCCCAGGCTCAATTTGGCCTAGGG-TGGACCCTGTGTGTTGCAACAGATTTGTGCTTTGGAATCATCAAGGTTGG-AAGCCAGGCCTCAGAGGGAGTGGTTGTAGACTGA

Homo sapiens          TCTCATCCTGAAATGATGTGGGCTTGGATTCCCAGAGGGTGGAGAGGAGGACCAGTCCAGAGAGAAGGACATGCTTTTGGAGGGAGGTCTAAAGACAAAAAGAGGGCTTTAGTTATTTTT
Dasypus novemcinctus  GCTCATCCTGAAATGCTGTGGGCTTGGGTTCCCAGAGGGCAGAGAGGAGGATGAGGGCAGAGAGAATGATATGTTTTTTGGAGTA----TAATGGAGAAAAGAGGGGATTGGTT--TCTT

Homo sapiens          TGAGCTTA-GGTGTAGGTGATGGTTGGGGGTAAGTGACAAAAGAAAATAGGTCCTGAAAGGGGGGTGTCTCCTTT----------CATGATTAGGAACAGAAGA--ATGAACTCAAGGAC
Dasypus novemcinctus  TAAGCTTTTAGTGTAGATGATAGCTGGGGATAGGTCCTAGAAGAAAAGGGTCCCTGAA---GGGGAGTTTCCTTTATCTGATTGGCAACATTTGAAACAGAAGAGAAAGAGCTTAAGGAC

Homo sapiens          TGCCCCCTTTTCTTCAGGAAGCAAACAACTTCTGAGGGTGTGACAGG-GTTCACTGGGTGTTCCCCTTTAAGGGAGGTTAGCCCCTACGTGAAGCTGCTTGGCTGGTAGAGGATAATTGG
Dasypus novemcinctus  TACTCCCTTTTCTTCAGGAAGCAAATAACTTCTGAGGGTGTGGCAAGTGCTCAGTGGGTGTTCCCC-CAGAGGAGGGTTAACCCCTCACTGATCCCGCTCGGGTGATAAAAGGTAATTGG

Homo sapiens          GTGTTCCCCAATCCAGATAAGCAGAAGCTCAGGTGCCCCTCCCTGTAGGCTTTTCAGCTTTCTTTATCACTTGCTAGGCAGGCCCTGCCATCCTTGGGAGTAGGTATGAGAGAGGGAGTT
Dasypus novemcinctus  TAGTTACCCAATCCAGATAACCTGAAACTCAGATGCCCCTCCCTATA-GCTTTTCTGCTTCCCTTATGTATTGC----CAGGGTCAGATATCCACTAGGGTAGGTGTGAGAGAGGAAGCA

Homo sapiens          GAGAGGCCAGGCTTTCCCTGC---------------CCCTGTTCTTCACAGAACACAGCTGAAAAAGGAAGGCCAGAGATGTGACACCCCCCAGCCCCAGGGATTCTACCACACATTGCA
Dasypus novemcinctus  GGCCTGCCACTCTCTCTCTGTCCCCCTGAACTTGAGCCCTGCTCTTAGC--AAGTCAGCTTACAAAAGGTGGCCAGAGATGTGA---CCCTCA----CAGTGATTCTACCACACTTTTCA

Homo sapiens          AACCTCACA-GAGGATGCTTATTCTACAAAATTTGTGGGCATATTCATAGGTACTATGGAGCTAAATAATT-------TGAAAACAATAACAAAACCCATT-TACTGATTATTTGGTTAA
Dasypus novemcinctus  AACCCCACATGAGGATATTTATTCTG-AAAATTTGCAAGTGTACTCATAGGGGCTATGGGCCCAAATATTTGCAATTATTATAACAATAACAGAAAAAGTTGTTTTGAACATTTGGTTAA

Homo sapiens          TAAAGACCTCATTCCTTCAAAGTATGTTGCTAGGAGCCACT----GAAAGTGGACTTGACCTGAGGAGCAGCAAGGAGCTTCCTCCTGCTGTATACACAGACACACACAGACAGACAGAC
Dasypus novemcinctus  GAAAGGCCCCATTCCTCTGAGTCATATTGCCAGAGACCACTCCACTAACATGGACTTACCCAGAGGAACAACAAGGAAGTTCATTCTGCTGTATACACACACACACACACACACACACAC

Homo sapiens          AGACACACACACACGCATGCGCGCACACACATGCACATGCCCGGATCCTTCTCCAAACACGTCCATGATACCAGGGCAGCAGAATTGGGGCACATTAGAGACACAGGCTCTGCCCACATT
Dasypus novemcinctus  ACACACACA...............................................................................................................

Homo sapiens          CTGAAGCTCCCTGTCATTCCCAGGTCTTGGTGCTTGGCCAGACTCCCCTTACCCAGACTCATAGCCTCATAGCTATCATTCCAAAACCCCAAAAGCTCTGGAAACTGAGAGCCTTTGTAC
Dasypus novemcinctus  ........................................................................................................................

Homo sapiens          GTTTATGACAAACTCATTTGGCAGCGCAACTTGAACTGACATGAGGTTATTTATAGTCTTTATTTTTCACACCCTGTGTGAATATTCATGTGTTTTTGCTCCAGAAATATCAATGTACTT
Dasypus novemcinctus  ........................................................................................................................

Homo sapiens          GATTACTGGGTACTGCCTCAGACCCCACTGGAGGTGATATATATGTGCTGTATACACCCAATCGCCTTTTAAAAGTCCAAAGCATTCTGAATTCCTTCAATTATACTAAGCCTCCCACTG
Dasypus novemcinctus  ........................................................................................................................

Homo sapiens          TCTTATAATGATCTGTTTGGCATGTCTCCCTCATTAGCCTGTGAACTTCTTCTCCAGTGCTCAGCACACAGTCCAGCATGGGGTGGAAGCTCGACTAATGTTTTAGGAATGAAGAACGAA
Dasypus novemcinctus  ........................................................................................................................

Homo sapiens          CAGTCTTTAGTGATTCCGGAGACTTGTTGTCATGTTCTTTCTTACATCTAAACTAAAGTACGTCATGCTACCATTCGTTTATTCTGTGCCATCAATAGCGAGGAAGAACAGTTGGTCAGG
Dasypus novemcinctus  ........................................................................................................................

Homo sapiens          CTCCTTCATTTTTTTTCCTCTTGAGGCTAAATCGTCCTTATAATCCTAATACCTTTAACCTTTTCCCCTAGATCCTCTTTTCCAATCCCTTAATCGTCTTCATTGCTGTCTTCTGAAGTC
Dasypus novemcinctus  ........................................................................................................................
```

```
Homo sapiens          TTTACAGGTTCTTTCCTTCCGAAATTGTGGCCTAGTGCTTAGGATAGGGCTGCAAAGAGCCCAGCTATTACACATAATAGGCCTCTGGTTTCTATGGTGCTTTTCCGATCAGTTTCATTT
Dasypus novemcinctus  ........................................................................................................................

Homo sapiens          GGCTGGGCTAAGAAGCTTTGCCTTTAAAAATACACTCGCAGGACAAACTCCAGGGGCATGAACAACTTTCACCTGAGCATGCTGGTTGGTAATAGGAAGGGAAAATAGCACTGTCTGATC
Dasypus novemcinctus  ........................................................................................................................

Homo sapiens          ATAAGAAACTGTAATTACGAAAGCTGAAAGCAACAGGTAACACATGCAGTAGGCACCTGTATACAAAAACCACTGTTGATAATTACGGTACCTCAGCCTATTATTTTTACCTTGGGAGAA
Dasypus novemcinctus  ........................................................................................................................

Homo sapiens          CCGGCCTTGTTTGTGCGCATCATGTGCCTTTTCTCTCATAAGGTTGCTAAAGGCCATTTCTGACCACTTCCCATTAAATTCAGGGAGTGAACCTGACTCTGTCAGGGTTGCTGAAAATCA
Dasypus novemcinctus  ........................................................................................................................

Homo sapiens          CCGGATTAAGTTAAACTCACTAATTAAGCCTGTTAGACTTCCTGTAACTCACGATTTTTCTTGGCATAAGTAAAAACATTTCACTCAGAGGCAAAATGTGTTATATTAATAACCAGGAAA
Dasypus novemcinctus  ........................................................................................................................

Homo sapiens          CAAGAAAATACACATAGTAGCATTTTCGGAAGCAAATTCTGTCATCCGCATTCTGTTGAAACGGCCATTGTCATTTCTCCTAATAGCCTAAAGTGTTTACTTTTGGGGTGGAAACTATAC
Dasypus novemcinctus  ........................................................................................................................

Homo sapiens          ATACTGTGATGGAGATTCAGAGATACGCTTTTGTCAGTTAAATTCGAAAAATATATTCAGGTGTGCTTGTTGTGTCCCCCCCCCCCCCAAGCATGAGTCAGAGCCTTTCATGGGGTTTTG
Dasypus novemcinctus  ........................................................................................................................

Homo sapiens          TGTAAATGTATTTTCTAAGAGGTGCTGTTGGGATTTTGCATACAGGTCATTCTTTTTAAAAAGGTCACGTTGCAATTTTTCTATCCTTTCTTGTGTTGTTATCCCTCAGGGAAGAAGATG
Dasypus novemcinctus  ........................................................................................................................

Homo sapiens          TAGAATGCCAAATTGCAAAGGGAGCTTGTAAAGAGGGGAACATTAAAATTGGATAACCAAGAGCTCCCAGAGAGGGCCTCAATGGCTGAAAGGAGCCCCTTCTGAGCAAATGGGTAGCAG
Dasypus novemcinctus  ........................................................................................................................

Homo sapiens          CGTAAAAGCCAAATTAACCTGGGAGGAGAAGGTATGGAACTTTTGGAGAAACACTTTGTTTACCTCACCATCTTACCATATCCAGCTGGCTGTTTCTCCAAGAATGTTAGTGCTCCAGAA
Dasypus novemcinctus  ........................................................................................................................

Homo sapiens          GGGAGGGAAACCTGTCCCCACTGCTAATAGAAGAGGCTGAAATTTTCTTTCAGAGTTTCCAGATGTCCTTGGCAAATGCTAGCAATAGTCATTCTCTCTCTTTTAATAATTATAATAAGC
Dasypus novemcinctus  ........................................................................................................................

Homo sapiens          TGCTTTTTAAGGGAACATTAACTGTGTGGTAGGCTCTATGCTAAGTGTTTTATATAAGGTATCTCTATTAATCTTCACTATGATCCTTCCAAAAAGGTATTGCTATTACCCTCAATTTCC
Dasypus novemcinctus  ........................................................................................................................

Homo sapiens          ATAGGAGGAAACGGGCCCAAAGAAGTTAGACAACCTGGGCAAGGTCACCCAGCTAGTAAGTGGAGGAGCCAGAACTTAAACGCAGATCCACCTGACACCAACACCTGGTTCTGAACCATG
Dasypus novemcinctus  ........................................................................................................................

Homo sapiens          GTGTGCAAGTCCTTGTAAAGTCAAACAGTCTCTTTAATAAGCTTGACTATATTTTCTCTTTTACCAAATCACAGAACATCTG-GTCTGCTTAATTTTATGAGACTGTAATTACAGCAATG
Dasypus novemcinctus  ......................AAACAGTCACCTCAATAAGCTCAACTGTATATTTTCTTTTACCAACTAGCAGAACATCTGAGTCTACTTGATTTTACGAGACTATAATTACAACAACT

Homo sapiens          AAAAGGATGTTAGCCTACTCTGAAAACCCTAGCT---GAAGGGCTTTCTATTGCTTTGTTTAGAGATTTGTTCTTTTCT--CTCCACCTCCACCCCCGACTCCCCAAATTGGATAGTAAG
Dasypus novemcinctus  GAAAGGATGTTAGTCTACTCTGAAAGCCCTAGATGAAGAAGAGCTTTCTATTGCTTTGTGTAGAGATTTGTTTTTCCCCCACACCACCTCCAC--------CCTCCAATTAGATGGCAAG

Homo sapiens          TTCTTCAAG-GAAGGTTCTGGAAAGTGGTTTTTTTTTTTTTCTTCAAGTTCTGTGTTACATGTGCAGAACTTGCAGTTTGGTTACTTAGGTATACAAGTGCCATGGTGGTTTGCTGCACCC
Dasypus novemcinctus  TTCTTCAAGAGAA...........................................................................................................

Homo sapiens          ATCTACCCGTCACCTACATTAGGTATTTCTCCTAATGCTATCCCTCCCCTAGCCCCCCACGCCCCACAGGCCCCGGTGTGTGATGTTCCCCTCTCTGCTTCCATGTGTTCTCATTGTTCA
Dasypus novemcinctus  ........................................................................................................................

Homo sapiens          ACTCCCACTTATGAGTGAGGACATGCGGTGTTTGGTTTTCTGATCTTGCGATAGTTTGCTGAGAATGATGGTTTCCAGCTTCATCCATGTCCCTGCAAAGGACGTGAACTCATTCTTTTT
Dasypus novemcinctus  ........................................................................................................................

Homo sapiens          TATGGCTGCATAATATTCCATGGTGTATGTGTGCCACATTTTCTTTATCCAGTCTATCATTGATGGACATTTGGGTTGGTTCCAAGTCTTTGCTATTGTGAATAGTGCCGCAATAAACAT
Dasypus novemcinctus  ........................................................................................................................

Homo sapiens          AGGTGGGCATGTGTCTTTATAGCAGCATGATTTGTAATCCTTTGTGTATATGCCCAGTAATGGGATCGCTGGGTCAAATGGTATTTCTAGTTCTAGATCCTTGAAGAATCTCCAGTGTCT
Dasypus novemcinctus  ........................................................................................................................

Homo sapiens          TCCACGATATTTGAACTAATTTACACACCCACCAACAGTGTAAAAGCATTCCTCTTTTTGGTTCTGGAAAGTCTTATGCTCCTCTGCTTTCTATAGTTCTCATAGTGCCTGGCATGTAGT
Dasypus novemcinctus  ...........................................................GATTCTGGACTGAGTCATACTCCTGTCCTTTCTGAAGCCCTCAGAATGCC-AGCATATAGT

Homo sapiens          GTGTGCTCCATAAATATTGACTGATTGATGAATGTGTGGATGTTTCATGCAATAGTTTTTGGGTTGTTCAACACCATTTCTTAATGCTAAGCAAACAGGTTAGAACCTTCAAGATGTCTG
Dasypus novemcinctus  AGGTGCTTGATCAATATTGACTGATTGATAAATGCTTGGATGTTTCATTCAATTGTTTCAGGGTTTTTCATGACACTTTCTTAATGCCAAACAACCAGGTTAGAACCTTCAGGTTGTCTC

Homo sapiens          TTGTCTTAATCTAGTTGCTTGCACTGAGGTAACTTTTTAATATCATCTCTGCACAGGAGCTGAATGCCTTCTTTGGCACCTGGATGATTGATTGTGTTTTCTCCTTTTCCTCGGAAAGCT
Dasypus novemcinctus  TTGCCTTAATCTAGTTTTTTGCTCCAAGATGACTTTTTAATATCGTCTCTGCACAGGAGCTAAATGATTTCTTTGGCATCTGGATGATGGATTTTGTTTTCCCATTTTCCTCCAAAAGCT

Homo sapiens          CTGGTATTGCGCAAACGCTGCTTTAGGTGATTAACAATGCCGCCAACTTCA--CCAGTCACCTTTGCAGACAATTCTTGAATTTTATAGGAAAGT--GGGAACCTGTTTAAGTATAGTTT
Dasypus novemcinctus  TCTGCATTTTGCAGATACTGCTTTAGGTGATTAACAATGCAGTCAGCTTTCACCCAGTCACCTTTTCAGACAATCCTTGAATTTTCTAGGAAAGTAGGGGGGCTTGTTTAAGTGTAGTTT

Homo sapiens          CTAGTTAATTCTGCTTCCCCATCCTGGTTTGCTCAATG-CTACATAAAAGATTTTCTTTGTGGAATGAGACAGAATAGA--------------------------------------AAAA
Dasypus novemcinctus  CCAGGT-ATTCTGCTCCCCAATCCTGATCGACTCAATGACTATCCCTTGGATTTCCTTTGTGGAATGAAACAGGATAAACTAAAAATTACTTTAAATTTACTACAACTTCTCTAATAAAA

Homo sapiens          ATAAAAAT------------ACATTCTCAATGGCTGGGTGAAGAGAGTCCTTGAGATTTTACCAGCTTGGTATTTCTCACTGT-TGGAACTTCAACCCAGGATG-CAGAT-TCATCCTA
Dasypus novemcinctus  AAAGAAATTAATAAAAAACAAACATAATTGATAGCTGGGTGAAAAGAGTCCTGAAGATTTTTCTAGATTGGTATTTCTTGTTGGATGGACCTCCAACTCAGCAGGGCAGATCTCATCCTG

Homo sapiens          GTGAGCCTCACAGGAAACTCTGCCTTGGTCTAGAAGATACAGTGCAAATATATAGGATAGGACAGGTAAGGTCAGCAGAAGCCACTTTTCAGCACATACCATGTGATTGCTAGAATAATA
Dasypus novemcinctus  GACAGCCTCAGAGAAACTTCTGCTTTGGTTAGGAAGATAAAGTAGAAACACACAGG------------------TGGAAGCCAGCTTTTAGCTCACACCAGGTGATTCCCAGAATAGTA

Homo sapiens          CAAATAACAACCATTTACTAGCAGATTACTATGTGGCAGGTACTGTGCTAAGCATTTTCCAGACATTCA-------------------------------------------TTATTGTTG
Dasypus novemcinctus  ACAATACCAGGGGATTACTATC-GATTACTATGGGCCAGGCACTTTGCTAAGAATTTTGTAGTCATACACTTACTTTCATCCCCAGAGGAACACTATAAAGTTAGGTAGTATTATTATCT

Homo sapiens          CCATTTTTCCAGGTGAGCAAACTGCAGCTCAGAGAGTCTAA-GTGAATTTCTTT--GTCTTTAAGCAGTATGAGATAGACCCTGGACTTGAACTCAGGTCCATCTGATTCCAGAGTACAT
Dasypus novemcinctus  CCATTTT-CCAGATGAGGAAACTACAGCTCAAAGAGTCTAAAGTGAACCTCCTAAAGTCCCTCAGCATTATGGGATAGACCTTGTAGTTGAACCCAGGTGTGTCTGACTCCAGAGGACCT

Homo sapiens          AGTCCTTTTGCTACAGTGCTTTGCAGGGAGCTGTGCTGGCTTCTCAAATGGTTTGGGGCTATCT-GTGTGAGGCTGACAAGAAAACTATTTAAAGGCTGGTAGAAG-AGGGCCCTAACAG
Dasypus novemcinctus  ACTC--TTGGCCACACTGCTCCGTAGGGAGTTAGGCCAACTTCTCAAATGGTTTAGGGCTGTCTAGTGTGATGCTGATAAAAGAGGTGTTTAAAGG-TGATAGAAGGAAGGCCTTGG--G

Homo sapiens          AAGGTCTGACTCTCATTTTCTTTCTATTTGCAGAAGATAATTCTTTCCTGTTTCAATATCAGAAGGGGATCTAGCTGAAGGCAAAAGGCCACAGGGTTCTGGTGAGGCACAAGTTCAAAC
Dasypus novemcinctus  AATGGCCAACTCTCTTTGTCTTCCTATTTGTGGAAGAGAGTTCTTTCCTGTTTCCGTATCCTATGGGAATCTAGCTGAGGGCCAAAAACTACAGGGATCTGTTGAGGCCCAAGTTTAAAC

Homo sapiens          CTTGCTGTAGTTGGACCTGTTCTACTGACTATCAACGGCCTGGGTCTGTGGGTGATCATAGTGTCAGGGGGCTGGAGATTGGAAGTTCAGAGTGGAAGAGCTGGCATATGC--CGCAAAA
Dasypus novemcinctus  TTAGCCATAGTTAGACTTGTCCTACAGATTGTCAACAGCCCAG----------------------AGGGGCTAAGGGTTGT-GGTGCTGAGTGACAGGGTTAGAGTTTACAACCCTAGA

Homo sapiens          TGCACCCTGTTGGGATGGAGCAGGAAACTCAACTGCAGCTGAGTTTAGTGGGGCACCCAAAGAGAGTGGCAACAACAGGTATCAATTTAGAGAAGCACTTGGCTTTGGTGAAAGCAGAAG
Dasypus novemcinctus  AGAATCCCA-TGGGGTGGGACAGGAAACTCAGCTTC--------CCAGAGTGGCACCCTAGGCGGGTGGCAACAACAGGTTGCAATGTAAGCAAGCAGTTAGCTTTTGTG---------G

Homo sapiens          TCCTGCCCAGCCTAGCTGAACTGAGCAGGAGCAGGTGAGGCTTAGGAGTCCTGCCAGGTGCTAGTAGCAAGGATGGGACTCTAGTCCTTGGAGGCAAGGGTGAAGGAGTTGGGAGCCTGG
Dasypus novemcinctus  TCCTGCCCA-TATAACTGAACTGAGCA-GAGGGGATGAAACTT.............................................................................

Homo sapiens          GAAGAACCAGACAAAATCCCAGTTCCACAGAAACTGAATGACTAGACTGGTGGCTCTCAACTGAGACAATACTGCCCCCTAGGGGACTTTTGGAAAATGCAAGAGGGTATTCAGAGGACT
Dasypus novemcinctus  ........................................................................................................................

Homo sapiens          GGAAATGGGGATGCTAGATGTTCTCCAACGCCCACGACAGACTTGCAAAATGAAAAATAGTCTAGCATCCCGAATAATTTTTGAATGTTCTGCTGGACTTCCATGGAGCTGAAAACTGCT
Dasypus novemcinctus  ........................................................................................................................

Homo sapiens          TTTCTAATTATCTAAACTTTTACTATAAATGTTTTATACCTAAGCATAAAGGGTTTTGCTTCCCCTGCCCCGACCCCACAGTTTTAATCAACTATTCTGTAAAGATATGGAAGATTACAT
Dasypus novemcinctus  ........................................................................................................................

Homo sapiens          GGTTACAGAGGATCACACAGACTCATTCACCATTTCAGGAAATCATGTCAACAATTGTGATGTTGCTTTTGGAATTAATCAATATAACATATCAGTTTGTGTCCATAACTGTTGCATTCA
Dasypus novemcinctus  ........................................................................................................................

Homo sapiens          TGGGGATTCTATATAAAGGCACAAGCTTCTGACTACTTCATTTAATCATTCAATGTAGTCCTGTGTGAGCATTTACAGAGTTAAATACATAGTGTTTTATTATGTATTGCTTTATTTTCT
Dasypus novemcinctus  ........................................................................................................................

Homo sapiens          CTTTATGTGGCAGGACATATAGCTATCTTTTTGAACTTATGCGTGTAGGTAGGTTATAGTACCTAGAAATTTAATTTCAGGATAGTAAAGGGGTGAAGCAAAAGCAGATTTATTATAAAA
Dasypus novemcinctus  ........................................................................................................................

Homo sapiens          GAGAGGATGTTAAGTCTGATAAGGTTGAACACCACTGTGCTAGCCTGATGGCTTCGTGAGGGCCAGGACCATGTGTTTTGCTTCCGTTTGGCCTAATTAACTGAGGTGCTTATAAATAAT
Dasypus novemcinctus  ........................................................................................................................
```

```
Homo sapiens          GAATAATGAATGTGGATGCAAGCACACAGCTGGAGTCAGGCATGTAGGTCAGCATAGTCGGGGTATATAAAACTGGCAGGAGATGGGGACTTTTTTTTTGAGACGGGGACTTTTGATCA
Dasypus novemcinctus  ........................................................................................................................

Homo sapiens          CCAAAAGAAGGCAGGATCCCAGTTATCAGAAGAGAAACCATAGTTCAGAGTTTGCCTCAAAGCTAGAGCGTCCTAATGTCAAGGCCCAGGGCTTCCTACAGTCCTCTTATCTTGAGACAT
Dasypus novemcinctus  ........................................................................................................................

Homo sapiens          GCTGGAGCCAAGGGACTGACGTCAGATGGTCCCAGTGGTGAAGAGAGTTGTGTTTACATTCTCACTTGCTGGTATCAATCGTAGGGGCCCAAAGACCTCACTACCTAAAAAGAGGGCTGT
Dasypus novemcinctus  .............................................................................................GACCTTGCCAACTAAAAGGAGAGCTAT

Homo sapiens          TTTCCAGCTTAG-CTGTACCTCTTTGCATTGTTTTGTCTGGCCA-AGACAGAGAGGATACCAGGGAAAATGAGGCTCGAGTATTGTACGTATAATCTGTCAATGCCCTCCATGCCCCCAT
Dasypus novemcinctus  TTCTCAGCTTAGACTGTGCCTTTTTGCATTGCTTTTCCTGGGTAGAGACAGGGATGCTCTTAGGAAAAATGAGCCTCAGTTACTGTCCTTATAAGCTGTCAATGCCCTGCATGCTCCCAG

Homo sapiens          TAGGATCTCAGGCTGTCTTG-GCCTTCAGGCAG-ATCCAGCTCCCCCATTTTTCTGTTGTTGCTGCTGCTGTTTTTT--TTTTTTCACTCCCCTGATCCCATCAATAATACGTGTACTCC
Dasypus novemcinctus  TGTTGTCTCAGGCAACTGTACGCCTGAAAGAAATATCCAG-TCCTCCATTTTTCTGTTGTTGCTGTTGCTGTTTCTCACTCCCCTTACTCCCTT--CCCCTTCAGTAAT--GTGCATCTT

Homo sapiens          TTCCTTCTGGGTCCTGTCCTCAAAGGTAACTGCTAAATATCTGACAAAGGAATGGCTAAAGCCTTCAGGGCTCCTGATAAGAGAATGGGCTCCCACTTAGCAAGAAGGGCAGGGGGCAGC
Dasypus novemcinctus  TTCCTTTTGTGTTCCGTCCTCAGAGGTGACTGCTAAATAGCTGACGTATGCATGTATAAAGCTTTCAGGGTTCCTGATGAAGGAATAGATATCCCGTTATCTGG---GGTAGGGG----T

Homo sapiens          ACCTCACTTAGAGATATGGTACATAAG--TACAGAGGGAGGTATTGAACCACCTGTTGTCAGGAGCAGAAGGGACCCTGAGGTCATCTGGTTCACTAGATCCCAAACCTGGCTGTACAAT
Dasypus novemcinctus  ATGTCTCTTAGAGAAACAATGTATGAACTTTCCCAGGAAGGTATTGGACCATCTAATATCAAAA-------------------CTTCTGGTTCACTGGATCCCTAACCTGACTACACAAC

Homo sapiens          GGAATTACCAGGAATGCTGTTGAAATACAGATGCAGTGTGGCTCCTAATCCTCAGAGT--CTGATTCAGTAAGTCCGAGATGAGGACTTGAATTATCTGCATATTATTAACAGTATCACT
Dasypus novemcinctus  AGAATTACTTGAAGGTTTGTTGAAATACAGATG---TGTGGGTCCTAATCCTCAGAGTGTCTGATTCAATAGGTCAGAG-TGGGGCCCTGGG--ATCTTCATGTTA--AAAAGTACCCCT

Homo sapiens          GGTGATTTTGATGAGGAACCCAGTTAGGGA-------AACCACTCAGACTGACTTTCTCGTTTTATGGGTAGCCAGCTGAGTCACCGACAGGGATGATGACTCGCCTCGGGTCACAGAGC
Dasypus novemcinctus  GGTGATTTTGCTAAAGAGCCTCGATGGAGAGCTCCTAATCCATAAAGACCAACCTTATCATTTTACTGGTAGCCAGGT-----------AGGGGTAGTGACTT-CCTCAGGTCACGCAAC

Homo sapiens          AAGTCATGGCAGAGTTGGAAATGGAACTCAGGGTTTTTCCTCCCCTGTATTGGTGGTGGGACGATCTGACATTATTA----------GTGTGGGAAGAAGGACCTTAAATTTCACTGTAC
Dasypus novemcinctus  AAGTCATGGCAGAG--GAAACTGGAATGCATGTCTCTT--TCCACTCTTTTGATGGTTGGAATATCTGGGGTGATTACGGCTGATTTGGGTGGGATGGACTACCTTAAATATTGTCATGG

Homo sapiens          AAGCTGCTGCTCACTCTGCCTAGAGGTAGAGGACAGAGGGCAT-CTTAAAGGTTGTTTTTCGTTATAATAAGCAGTACATTTACTTCAGAGTTTGGAAGTGAATCAACAAAATACCCATC
Dasypus novemcinctus  AGGATGCTGCTCACTCTGCCTGTTGGTAGATGATAGAGGAGATGCTAGGAGATTGCTTTACTTTATTAGAAGCAGTAAATTTACCATGGGCTTTGGGAAGGAAGCAACAAAATACCCATC

Homo sapiens          AAGATTAGTAGGACAAGAGGAACTCGCCAATATCTTTGACGATGTCTATCCATAGAAAGCCAAGTGAAATAATGGAGGTAATTATCCACCTATTTTCTTTGAACACTTTGACCGAAAATC
Dasypus novemcinctus  GAAGTTAGTAGGAAAATAAGAACCCGCCGATACCTTCGGCTGGGTCTACCTGTGGAAAGCAGAGCAAAACAATGGAGGTAATTATCCACCGATTTTGTTTGAACACTTTAAACAAAAATA

Homo sapiens          TCCTGGTAACTGAGGGAAAATAGGAGCTTTTAAAAATTGTTGTCTCAAGTAGCCTGAATGACTTCAACTCCCACTTTACTTTTCTTTTTCTGTGGAGATGCTCTCCCAGATGCTTGTCTC
Dasypus novemcinctus  TCCTCATAACTTTGGGAAAATATGAATTTTTAAAAA-TGTTGTCTCAAGTAGCCTGAATGAGTTCAGCTCCCACTTCACTGTTCTTTTTCCCTGGAAACACCCTCCTGGATACCCATCTC

Homo sapiens          CTGGGGGTAGCAGCTTTCCACAGACAAAATGTCCACAGCTTTGGGAAGCATGGAGGGAGAAG--------------GGAAACATTAAGTGAAATGGGATGTCAGGTCAAGCTGCGTTGGT
Dasypus novemcinctus  CTTGGGGT--------------ATGAAAAGCCCACAGTCCTGAGATATGTGTAGAGAAGGGTAAAACCCCACCTAGGAAACAACAAATGAAATGTAATATTA-ACCAAGCTCCCTTGGT

Homo sapiens          GGTGATGTAGTCACCTGTCGTTTCAAGATAACCTTTTGGATCCTAGATATGTCGTAGCTGGGGCCTTGGGACAAGTCCCAGCAGCCTCCAAGCCACAAGCTTATCATTTTGGCGCAATTC
Dasypus novemcinctus  GGTGGAGCAAGCACCTACCCTTT---GATAACCTTTTGGATTCTGGGCCTGCTCTAACTGGGACCTTGGGACAAGTCCCAGCAGCCTCTAAGCTTTCAGCTTATCACTTGGACATAATTT

Homo sapiens          AAGGCAGCCTCCTTGAAAAGCT-CCACGATGGCAGCTGCTCCATCAGCAACTGGATGAAAACAATGCCAAATCTCTTTATTTAAAATTAGGCACTTCCTATTTTGGTTCCTAAATGGCAA
Dasypus novemcinctus  GAGGCAGCCCCTTTGGACGGCTCCCGAGATGGCAGCTGCTCTGTTAGCAAATGAATGAAGACAATGCCAAATATCTTTATTCAGAA-AAAACACTTCCTATTTTGGTTCCTAAATGGCAA

Homo sapiens          ATCTATCTGCTCTCTGCCTCCAGTCTTCTGTGTCCAAAGCTGCCTTAATGTGAGGTATTTCAGCAGCGATCAGCAAGTGTCTTCCTATTTATAAGCAGCAGAGCTGCCATGGGTCACTGC
Dasypus novemcinctus  AACTGTCTGCCCTCTGCCCCCAGCCCTTTGCAGCCAAAGCTGCCTCAATCCAAAGTCTTCTAGCAGAGTTCAGC-AGCTTCTGCCTATTTATAATTAGCAGGGCTTCCATGGGTCACTGC

Homo sapiens          ACTTGTATCTCTGAGCAAGAGAAGTCAGGGGGC-CTATTGGAAAGGAAAAAT-------ACTGTGGCCT-CCGACAGTGAACCTTGGCGTGGGTCAGGGAAGACTGTTCAAGTGCACCAG
Dasypus novemcinctus  ATTCCTATCTCTGAGCAAGACAGGTCAGGGAACTTTTTTGGAAAGGGAAAATACAGGTCACTGTGGTCTGAAGACAGTCAGTCTTGGTGCAGGTCAGGGAAGGTGGTTCAAGTGCAGCAG

Homo sapiens          AGAATGGAGCTGTGATGAGTTCAGTTTTGTATCACTGGCATGGAAGTCAGTAGCCTCAAGATCTGGGAAGAGTTTGGTCACCACTAATTTGCTACATGACCCTGGGCAAGAGTCGTCCCT
Dasypus novemcinctus  GGGATGAAGCTTTGGGGAGCTCTGTCGTTTATCACTGTCACCAGAGTCATGCAACCCAGGATCTAGGCCCAAGTCTGCCACCACTAATTTGCTACATGATCATGGGCAAGCCTCTTCCCA

Homo sapiens          TCTCTGGGCTTCAGCCTCACCATTT-----CTGGGGAAAAGGATGCACCATTTAATACATGGTGCCATTTACTGTGACAACCGGCCTGCCACC--AAAATATAAAGCTGAATCTTTATCT
Dasypus novemcinctus  TCTCTGACCATCAGTTTCACCATTTGGGGGTGGGGGGGTGGATGCACCATTTAATACATGGTGCCATTGGCTGTGACAATTGGCCTGCCATTGGAAAAAAATAAAG..............

Homo sapiens          CACTCCTTATTCCAAAATATAATCCAATTAGGTCAGATTTAGATGTTAAAAAGAGAAACCATTAAAAGATTAGAAGAAGGCATAAGAGAATATTGAGGTAGGGAGTCTTTGCAAAGCAAC
Dasypus novemcinctus  ........................................................................................................................

Homo sapiens          GAGAAACTGTAGAATCTATGAAGAAAAAGAATGATAGATTTGACTATGCAAAAATTTAACAACCTGAGTATGGTGAAAGATATCTCATGCAAAGTGGAAAGGCAACATATAAACCAGGAT
Dasypus novemcinctus  ........................................................................................................................

Homo sapiens          AAAGTCACAACATAGAAGGAAGACAATGGTTTATTATTCAGAATACTCCACCAGCTCCAGTGTAAGAAGTGCATTATTTTCTTTACAGCACTTACCATGATTTACAATGACATGATTTAG
Dasypus novemcinctus  ........................................................................................................................

Homo sapiens          CTACTTGTTTTTCTATTATTTCCTCTGCTTACATGTTAGCTTCATGAAAGCTCAGGTTTTGCTTACTCACTATTATATTTCTAGTGCTAGACTGGTGCCTGGCATACAGTAGGTACTCAA
Dasypus novemcinctus  ........................................................................................................................

Homo sapiens          TAAATACTTGTTGAAGGAAGGAATAAACGAGCTTCTGTAGACCAATGAGAAGATAATGAACAATTCACTAGAAAAATGCTCAAAGAATAATGAACAGGCAATTCACAGAGGAAAGGCAAA
Dasypus novemcinctus  ........................................................................................................................

Homo sapiens          GGGCCTATGCCCTATTAAAAGGAACACCAATCAGTAATAATTAAGAAATGCAAATTAAAACAATATTGAGATGATTCAGATGTCAGCTTTTACCTCTCAAATTGGCAAAAATTTAAAAGG
Dasypus novemcinctus  ........................................................................................................................

Homo sapiens          TCACTAAAACCCAGGGTTTGTGAGAATCTGGTGAAACAGCACACTCATTCACTCTTGATGCACATCTGGAGGGCAACTGGAAATGTTATTTGAGTCAGCAATTCCTGTCTTAGGAGTTTA
Dasypus novemcinctus  ........................................................................................................................

Homo sapiens          CCTTTGCAAAATGCTCCATGTATAAGGATGTTCATTTAAACATTATTTGTAAATAGGAGCAAATTGAAAAAACTAAATGACCTCGGTGAAGGACTGGTTAAATCATTTATAATACATCCC
Dasypus novemcinctus  ........................................................................................................................

Homo sapiens          AATAATGACATACTAAGCATTACTATGCTAATGATTAAGAGTTTGAGCCTTGGTTAGACTGCCTAGGTTTGACTTCCTCCTCTAGCTTTTATGAGCTGTGTGACTTCGACCAAGTTTCTA
Dasypus novemcinctus  ........................................................................................................................

Homo sapiens          ACCTCTCTGTGCCGTTTCTCATCTGTAAAATGGGAAAAGTAACAATTCATGAAGTTGTGAAGAGTAAATGAGATAATTCATATGAAATATTCAGATAGGACCTGGCACAGAGCATTTAAT
Dasypus novemcinctus  ........................................................................................................................

Homo sapiens          AATTGCTACACATTACTATTCCAACTACTAGTATAATGATGACAATGACGATGTAGCCACAAATAAAGACAAGCTAGATCTTTAAATACTGATGTGCAATGATATCTCCAATATAGTATT
Dasypus novemcinctus  ........................................................................................................................

Homo sapiens          GTGATAAAAGCAAGTTGCAGAAGAGTAAGTATAGTAGGATCTGATTTTTGTTAAGGGTATTATATGTTACACATACAGGAAAAGACTGGAAGGATAGACAGACTGTCAACACTGGGGAGT
Dasypus novemcinctus  ........................................................................................................................

Homo sapiens          AGAATAGGAGAGGTAGATTTTCCCTTGAGCAATAATTAGAGTTGTTTGAAACTAGCATGTGCTACTTTTATAATAAACAAAAGATGTAAAGGGAAAAAAAAGCTCCGTTTGGAATTCCGA
Dasypus novemcinctus  ........................................................................................................................

Homo sapiens          GGACAGTTAATCCCACCAGGCTTCACATCCCCCAGTTCTTGGCCACAGCCCAAACGTTAAGCCTCCCTGCTCGTCATCTGTCCTCTCCCATACCATCCTCATACTGCTCAGTGCTCCCAT
Dasypus novemcinctus  ........................................................................................................................

Homo sapiens          CCCTGCCACCCCGACTCATCCACCTCCCCACTACCGCCCTCTCTCACTCTTCTCAGTACCCTCTGGAAGCCCCCACCTTGCAGTAAACAATTCCCCCTACACCCTGGATAAATTTTACAG
Dasypus novemcinctus  ........................................................................................................................

Homo sapiens          AGCACTATTGTCTACCTCCGGGCCTGGAAGTGCTGAAACCTGAGTCTCCACGGCGGATCTCACGGCTTTGCCACCAGCGCCCTCTAGTGGACACTGTCCACACTCCCACACTACAGGCAC
Dasypus novemcinctus  ........................................................................................................................

Homo sapiens          CCTAGGGCTGGGAGGTGGGGTCTGTGCTCTAGCTTCCCCTGTTTGCTTCCAGGCTGTTAGTCCTCCGTCCTCAGCCAAGAACTCCTCCTTCTCATGCCATCTGGCTACTTCTCTCCTCGT
Dasypus novemcinctus  ........................................................................................................................

Homo sapiens          TGACGCTTCTTTATAACCATCCCCTCCTGGTCAACCCCCCCCCACCCCCACATTCAACGAAGAGTTCGACATCTGGCTCATTGTCTTCTTCTCCACCCCAAGTCCTGACATCATTCTGGG
Dasypus novemcinctus  ........................................................................................................................

Homo sapiens          TGAGTTGGACATCCGTGATCCATGTACAGGACACACTCTCATCCTAGCCTTACCATGCTTCATCATCAGTCAGAACCCCCTACCATCTACACCTTGCTGGCTGGCCTCAACGCTCTTCTT
Dasypus novemcinctus  ........................................................................................................................

Homo sapiens          TTTACCTCAGGAGGCTCTCCAGTTTTTTGAGCCCCCTCCATCCATTTCTTCCATCCCATCAGCCCCTTTCACCTTACTACCTTCCCTCTCCATCCTGGAACATGATGATGATGATGATAA
Dasypus novemcinctus  ........................................................................................................................

Homo sapiens          GAATAACAACTAATATGTATGTGCCAGGCATATACTACCCATCTTATGACATCATTTCAGTAATCTCATGTTATCCTCACAATAACTTTATGAGGAAGATACTATTGTGGTTTTCCTTTT
Dasypus novemcinctus  ........................................................................................................................
```

```
Homo sapiens          AATGAGGTGACTGAGGCTCAGAGAGGTTGAGTGACTTACCCAAAGTCCCACAGCAGAGATGAGATTTGAAGTCAGGTCTGTTTGCTTTCAGAATCTTGCGTATATCCCCAATTCCCTTGC
Dasypus novemcinctus  ........................................................................................................................

Homo sapiens          CCTGTGTCTTTCTGCTGCATCCATGAGGCAAAATCCTAAGTCTGGATAAATTTAACTGACAGTTTCCTCCTCTGTGCTTATGCCCAGATGGAGGAACATTGCTGGAGAAAATCCCATCAC
Dasypus novemcinctus  ........................................................................................................................

Homo sapiens          TTTGTTGGTTAGTCCCACCAGAAAGTCATAGTTTCCAACCTCAGCGGGGCCCTCAACTCAGCTAAACAATCCTTCTAAGTAACTGTCCTGACAATAGCACTTGCAAACTTTCTCCACTGC
Dasypus novemcinctus  ........................................................................................................................

Homo sapiens          CCTCTGGAGTCAGACTGTCTGGGTTAGAGTCTGCTTTGGCCACTTCCATGCTCCATACCATGCCAGCCTTTAAGAAAGGGTGAAGTGGAGGAAAAAGGATGAGGGGAAAGGGGAAGTGAT
Dasypus novemcinctus  ........................................................................................................................

Homo sapiens          ATGTATCGAGTGTCTTCTAGGTACCAGCCACTGTGCCAGGTGCTTTGCATGCAGTGTTTCATTTAATCCTGCCAGCAGCCCTGCAAGGGCAATACTCTTGTTATTTTTGCTTTACAGAGG
Dasypus novemcinctus  ........................................................................................................................

Homo sapiens          AGGAAGCTGAGGCACAGACAGGTTAAGTCACTCACTAACGTGACACAATTTGTATGTGGAGAAGCCATCTCATTCGTTTAGACCTTCCTTATCTTGATGAATTATTGTGACAAGCTGATA
Dasypus novemcinctus  ........................................................................................................................

Homo sapiens          AATGGGTCCCCTGTTACCTATCTCATGCCCTACAATCTACCTTCCATTCTGTTGCTGGGACAATCTTTCTAAAATGCACATCTGACAGTGCTACTACTCTGCTTAAAGTCCTTCCAGAGC
Dasypus novemcinctus  ........................................................................................................................

Homo sapiens          TTCCCCTCAGTCTTCAGAATGAAACAGAAACCTTTGATCTTAATGATTTGGCCCTGTCCTTCCTTTCTCACTGCATCCACTACCACTTGCCCATGCAGTCACACAGTACTATTTTTGGCC
Dasypus novemcinctus  ........................................................................................................................

Homo sapiens          ACCTGATGACACCTTGCTTTCTTCTGAAGAAAGAAGAAAGCATATATATATATATTCTCTGCCTCCCCATCCTCCACACCCTTTCCTGCCCACTTTAAATGCTCCTCTTCTGTGCTTTCA
Dasypus novemcinctus  ........................................................................................................................

Homo sapiens          CAGCATTCATGTTTATCCCAGTGTGTTATAATTTCCTGCTGTCTTCTCTGTTTCCCCCATTAAACTGGAAGCATCTTGAGAACTGGGATTGTGCCTTAGTCATCTCTGTACTCCCCAGGG
Dasypus novemcinctus  ........................................................................................................................

Homo sapiens          CCGTAGCACCAGAGCCATGGTTAAATCTATGTTTGTTGAATGACTGAATTAATGAATGAATAGAGTGAATGAGAGAAGAAAAAGCTCTCACTTGGAAAGTTTAGTGACATGAGCTGTATG
Dasypus novemcinctus  ........................................................................................................................

Homo sapiens          GTATCTGATTCTTGGCTGAAGCTGCTGCAGTTTTTCCCTATGACTACAGTGCTATAAAAATATGTCTGCACATGGATAAAGACTGGAAGAGAACTTTCCACACAAAACTAAGAGTTGTGT
Dasypus novemcinctus  .......................................ATGGCTACAGTCCTGTAAAATTATGTCACCAGGTGAATGAGGACTGAAAGAAAACT.........................

Homo sapiens          TAGAGTGGTGAGTGATGGATGCTTTTTTCCCCATTTTCAAAATGTTCTTTAGTGCTGCTTTTGGATTGTTTTGGCAATGTAGCAGACATTGTAGAAATGGAATTTGGGGCTCCCATGGAG
Dasypus novemcinctus  ........................................................................................................................

Homo sapiens          GAATCGACAACCAATATGTATTGATGCCCAGTGTACTCTGCTGGGTTCTGGGTGCTGTGGGGAGATGGGTGGGTAAAGGCGACTCATTCTGACTCAGGTTTGCATGGTCAGGAATCTTGC
Dasypus novemcinctus  ........................................................................................................................

Homo sapiens          CAATTACGTTGGATTTGCTGGAATAAAAAGATAACTTTCAAGAATTTTCCAAGCTCCTGGGTTGAGGTATTTCTTTTCTCCCAAAGCTGGGCCGCAAACAAAGATGGCTTTTGTTTGCAG
Dasypus novemcinctus  ........................................................................................................................

Homo sapiens          TAAAATACTCATTCCTTCAATTTTGTCCTGCAAAGTGACTTCTATAGCTTTGGGAGTCAGGTTTGCTGAGTGGGGAAACTGATTGTCCATGCTTCATTGATTTAGAAATGACCAAAAATG
Dasypus novemcinctus  ........................................................................................................................

Homo sapiens          TTTGCCTTTTCTGGCTCTTGAATAAAATGTTTAGGAATTTCATTGGCAAAATGATGAAATTGTTCCCTTTACTGCTGCAAGACTTCCAAAACTATATATCGTGGGTTAACTTAACCCAGA
Dasypus novemcinctus  ........................................................................................................................

Homo sapiens          AGAGGCAAAAAAAAAAAAAAAAATGGGAAGCAGACTCAGATGATTAACACTCTGCCTTAATTGGGTGCTTACTCACTCTGCCGTGGCAAGGCACTGTGTTCTGGACTTTGGAGATATCTCA
Dasypus novemcinctus  ........................................................................................................................

Homo sapiens          TTTAAAACTCTCAACAGCCTGTGGGTAAGGGACTGTTATTGTTCTCATGGTAGAGATGGTCAAACTGAGTTTCATAGAGGCTCAGCTCTGAAATCATGAGACCAGGATTTGAAGCCGAGT
Dasypus novemcinctus  ........................................................................................................................

Homo sapiens          TCAGCTGGTTCCATGGGCACACACTAACCATTTGGCAACAGTGCCTCTAGACTAGTCTGGCTACAAAGGGACAGAGGTATGTATTTATTTTGATTTAACGAACCGTTGTTTAATGTTTAC
Dasypus novemcinctus  ........................................................................................................................

Homo sapiens          CATGTCCCGGGCACATGGTAACTGCTTTAAAAATGTTAGATTATTTACTTCTCATAGACAACCCTGTGAGGAGGTACTATTATTAATATCATATGACAGGTGGGAAAATGGAGGCACAGG
Dasypus novemcinctus  ........................................................................................................................

Homo sapiens          GGTGTTGAATCACTTGCCCGGGGTCACTCAGGCTAGCATGTGACAGAGCTAGAGTTACAATCCAAGTGGCCATGTTCAGGTGTGTGGCCCATTACCTTGACTCTGTGCTGGTTAGCATGC
Dasypus novemcinctus  ........................................................................................................................

Homo sapiens          TACCCCCTGTGCCCTGTCTGCATAGCTCTGGCTCAGAAACACTGAAGACATGGGCCTCTTTTTGTAGTTTTGTAACAGATTTGTACTTTTTAAAGTATTTTCCACCATTAAAGCCATACG
Dasypus novemcinctus  ........................................................................................................................

Homo sapiens          CTCTGCTTCTAAGCGTCCTGTGGGGTTGGTGGGAAGGGTGAAAACATACTCCTGTCCTTCTATAGCAAGACTAGCTTAGAGGCAAGGTGGCCCAGTGGTTAGAGCATGCCCATGGTGCCA
Dasypus novemcinctus  ........................................................................................................................

Homo sapiens          GTGAACTCAGCTTCAAATCCTGGCCTTATGATTTCAGAGCTGAACCTCTCTGAATGTCTGTATCACTATCTTTGCTAAAGGAAGAGGAAGCTTTCTCCCGATATCTTCATTTACTTCCAA
Dasypus novemcinctus  ........................................................................................................................

Homo sapiens          TTTTCGGTTTTTGGGGGAGAGCTGGGTTTCCTCCACAGCTAGGCGCTGGGTGGCGAAGGGCTTTTGGACTGTCACTACTTTTCATTCGACAGAGACTTCAGGATAGCCCCCTTCAAAGGC
Dasypus novemcinctus  ........................................................................................................................

Homo sapiens          AGTCTGTTTGGGGTCATCAGGAACAGACAGAGTGCTCTTCCCTACCGCACTCATCCCACCTAGTGCCATCTATCCTTACCCACTCTGAATCCCTCCTCTCGGCAGGCACTGCCTCATTCC
Dasypus novemcinctus  ........................................................................................................................

Homo sapiens          CCAGAGCCAGGCCTAGACAATGGCGGCTGCTCAGCTTGGAGGGAGGGGGAGTGAAGAGGCAGCAGGCCCAACCTGCCAGCTTGAAAGCTTCATTTGACAGGGGAGGAGGCACATCTGGTT
Dasypus novemcinctus  ........................................................................................................................

Homo sapiens          TGGGTTTTCAATTCAATTCAACAATGATTTATTGTGGGTCTGCCAGCTGCAGAGGTTCTGGGCTAGGCTAGGCAGCAAGAGATGCAAAGATAAGTAAAATATTGTCTGTGCCCTTAAAGG
Dasypus novemcinctus  ........................................................................................................................

Homo sapiens          AGTTTTCTAGGTTGTAGGGGTGTGCAGACGCAAATGAGCATTTTTTTTTTATGCGATAAGCCCTGTGCTTAGCAGTTTACCTTGCCTACATTATTATGTCTTTGCATCTTCCCAGCAGGT
Dasypus novemcinctus  ........................................................................................................................

Homo sapiens          TTTGTGATGGAGGTGGGTAAACTATTATCACTTCATTTTATACACATGAGGAAACAGAGCCTCAGAGTGGAAGAGACTGGCCCAAGGTCACATTTTTAAGAATGGACCAAACCAGTTCTG
Dasypus novemcinctus  ........................................................................................................................

Homo sapiens          GAACTATTACATTCTTCTGCTCCCCAGAGGTGATGAAGATGATGATGATGACAAAAAACACTTACTGAGCTCTGGAGGCCAAGTCTTGTGCTATGCAGGCTGCAGATATTATTTTACTGG
Dasypus novemcinctus  ........................................................................................................................

Homo sapiens          CAACTTCCAACATCCCATTGAGGCTGATGTTGTTATCCCTTTTTCTAGAAGAGAAAACGGAGGTAGCCTTGGGAAGTGATGGAGTTGGAATTTGAAACCAGGTCATTCCAGTCCACCAGC
Dasypus novemcinctus  ........................................................................................................................

Homo sapiens          CTGTACCATAAAAGAGGTAGACACAGGGTGCCATGTTCAGGGGCAAGAGCAATGAATTGTGCCTGCAAAAGCTTCATGCCAGAGATGGCATTTACATTTGGCCTTGAAGGGTGAGATGGG
Dasypus novemcinctus  ........................................................................................................................

Homo sapiens          GTTATGATTGGTAGAGTTGGGAGAGAGGGCATCCCAGTCAGAAGGGATATTCTGAGCAAAGAACCCAAAGAGATGACTTGTGGAGCATTTTGATTGAGAGGCTGTAGGTTCATCCGGCAT
Dasypus novemcinctus  ..............................................................................................TGAGAGGCTGCAGGTTCATCCAGC-T

Homo sapiens          GCAGGCCGGGGACTCTGGGTGTAGAGGAACAATGTGAAGGAAACCAGAGATACGCCTGATGGTCTAACACATTTTTGTCAAGTTGAGGTTCTTATTTTTTTAATTAAGAGCAGGTACTCT
Dasypus novemcinctus  CTGGGCAGGGTAACCTTGGTGTC-AGGGAGAATGGGAATGAAACTGGA--TACCCCTAAGGGTTTT-CACATTTTTGTCAAGTTGAAATTCTTGTGTTTC-AATTGAGAGCAGGTTCTCC

Homo sapiens          GCCCCAAAGGCTTTTCAAATGATACTATCTTGCAACCCATGAGCAAACACACTCGAGTCTCTCACTGTGTAAATTCTACTGTGCTGAGAGCTGCGGTGAATACAAAAGAAAACAACTCAA
Dasypus novemcinctus  ATCCAAAAGACTTTTAAAACGATACTGTCATGCAACCCATCAACAAATGCACATGGGACTCTTACCGTTTACATACTACCGGGCTGAGAAATGTGGAGAATACAAAAGAAAGCAACTTAT

Homo sapiens          TTCCTCAAGTGTGTGCCGATGGCCCC-CGTGTGGTTGAGGAGGCAAGATACACACAATCAGGAAAAGCAGCAGCTAGCATAGGCAGTTAGTGATCAGGGCCAAATGAGTTTTTCCAGCCG
Dasypus novemcinctus  TTCCTTATGCATTTGCTGAGAGCCCCATTTGTTGTTGGGGAGACAAGATACATAT-ACCTGAAAAAGCAGCAGCCAGCCCAGGCAGTTTGTGACCATGGCCAAATGAGTTTTCCCAACTG

Homo sapiens          TCAGATTTCAAGGAACATGAACGAGAGGAAGGCCGGCAAGCTTAAGGCCGGGTGGGGCAATCAGTGTAGCTAGCATTAAGGGCTCTGGCCAGGCCTCCCTTCCCCAAACTGCCACGGTCT
Dasypus novemcinctus  TCAGATGTCAAGGAACATGAAGGAGAGGAAAACAGGCAAGCTTAAGACCAGGTGGGGCTACTAGTGTGGCTGGCTCCAAGGGCTCTGAGGAGGCTTCCCTTCCCCAAATCACTGCTGTCC

Homo sapiens          CCAACCATGTCTTGACCTCCCAGAGTTTGTTAGGGAAGATGGGACCTTATGGGATCAGAGACAGCGCATGGGAGCTGGGAAACGGAGCCGAATACCTGATATTCCTGATCCTCCAATTTT
Dasypus novemcinctus  CTAGCCATGTCTTGATTTCTGGGGATTT-TCAGGGAAGGTGGGATTGTATGGGATCAGAGACAGCCCGTGGGAGCTGAAAAATAAGACTGGGTACCAGTTATTCCCGATCCTCCAATTTT

Homo sapiens          CTACACTTATTCTCATTTCCTGGCAGCTCCTCCAGCTGCTGAATTGTATGTGTATGTGTGAGCCGTATTTTAAATTAAGCAGTGAGTTCTTTCAGCTCATCATAAACATGCTAAAAAGCT
Dasypus novemcinctus  CTACACTTAGCTTCAATTCCTGGCAACTCC-ACAGCTGCTAGATTGTATGTGTATATGTGGAAGCATTTTAAATTAAGTGGTGACTGCTTTCAGTTCATCATAAACATGCTAAAAAGCT
```

```
Homo sapiens           TCAGCCGCGGCCTGAGCCCACCCCCGCCCTTTACACACACAGGCAGCAGCTGGGGCCACTCGACCCCACACTCTAATGTGCATTGCCTATGACTCACCAACA-AAAACGACTGTATGTTT
Dasypus novemcinctus   TCAGCCACAGGCAGAGCCCACTCCCACCCCTT--ACACTCAGGCAGCAGCTGGGGCCACTGGGCCCCATACTCAACTGTGCATTGCCTATGACTCATCAACACAAAAGGAC--CATGTTT

Homo sapiens           CAGTCTCCCTGGGTAGCTGATTCCTAGTTGTGGTGGGAAATCTCAGTCCGA-TTTGGAAGGCAAAGGGCCTCAGCCCCGCCCTACTGACTGTGAATTCTACAACATATGGAGCGCCTACC
Dasypus novemcinctus   CAGTCTCCCTGGGTAGCTGATTCCTAGATGTGGTGGGAAACCTCAGTGCAATTTTGGAAGGCAGAGGCGCTCAGTCCCGCCTTACTGATTGAGCAGTCTACAACATCTGGAGTGCCTACT

Homo sapiens           TTGTGCAAGGCATTGTGGGTAAGTCCATTCATCTCTCTGGGCTTTCCATTTCCTCAGCTTCAAAATGCATGTACTAATCCTGCCGGCTTTACCTCCCTCCTAGGGCTCTGTGTCTCAGCC
Dasypus novemcinctus   GTGTGCAAGGCATTGTGGGTAAGTCCCTTAATCTCTCT-GACTATCCATTTCCTTATCTTCAAAATGCATATAATAATCCTGCCTGCTTTACTGTCCTACCAGGGCTGTATGTCCCAATC

Homo sapiens           ACACTGGTCTTTCAATTTCTGCAACATTCT--CGGTCTTTTCTCGCCCCAGGGCCTTTGTTCGTG------CTCCTGCCTGGACTGCCCTTCCTCTTGCTTTTGG-CATGGAAGGCTCCT
Dasypus novemcinctus   ATGCTGGTCTTTCTATCTCTGGAACATTCCAAAAGACTTTTCCTGCCCCAGGGCATTTGCACATGCTGTTCCTTCCGCCTTGACTGACTTTGCTCTTGCCAAAGGTCCCAGGAGGTTTCT

Homo sapiens           TCCTAACCTTTCAGTCTCAGCTT-----------AACGTTACCTCCTGAGAAGAGCCTTCTCTGACCACCCACAGTTAG-ATGCCTCCTGTTCCTCTCTTACAT-TTCTTTTATTAGCAA
Dasypus novemcinctus   TGTTCAAGTTTCACTCTCAGCTTAAATGAGCTTAAATCTCACCTCCTGAGATGTGCCTTCTCTGACCA-CTATAGTTAGTCTCCCTCCTGCCCCTCTCCAATATCCTTTTTTTTTGGCAA

Homo sapiens           TGCTTCCTTAACCCCATTTACTGCTCTGGGTAATTATGTCTATTTATATTTGTCTATTTATGGTATGTCTCTCCCACTGGAATGTAAGCTCCAAGAGGACAGGGACCTTGTCTGTGTCAC
Dasypus novemcinctus   TGCTTCTTTCATCATGCTTACCAC-CTGAGTAATCACATCTCTTTATATTTACCTCCATATGGTCTGTCTCTCTTCCTGGAATGTAAGCTCCAAGAGGATGGGGACCTTGTCTGCATTGC

Homo sapiens           TCAGCAATGTGTCCCTCTCCTCTAGAGTGGTGCTTGGCACATAGTAGGCTGACATAAGCAAAACCATCTGGAAAATATTTTCTTCCTGAGGTTCTTGCCAGACATATGGTTGCTGAGGGA
Dasypus novemcinctus   TCAGTGCTACTTCCCTATCACCCAGAATGGTGCTTAGCACATAGTAGGCTAATGTAAGCAAAACCATCTGGGAAATATTTCCTCCCTGAGGTTTTTGCTGGACATATGGTTGCTGAGGGA

Homo sapiens           AAAATTATGGCAGAACTGTGTCATTCCATAGGAAAAAAAATCAGGCTAAAAGGGAAGAGT-----------------TTGTTGAAAACAGTTCTGTTTTAGAAACCAGTCCTTGGAGTCT
Dasypus novemcinctus   GAAATCACGGCAAAACTGTGTCATCTCATATG-AAAAAAATCAGGCTAAGAGAAAAAAATATCAGGCTAGAAAGGAGTTGTTGAAATCAGTTCTGTTTTAGAAA-----CCTTGGAGTCT

Homo sapiens           CATCGCCAGACACAGTCTTATGAAATTAAACAAAAGAAATAAAAAGCATAAAAAGCTAAGTTTTTATTGGGTATTTACTATGTGCCAGGAACTGTTCTAAGTGTTCTACACAGATTATCT
Dasypus novemcinctus   CATCACCAGACACAAGGTCTTAAAATCAAACAAAAAGAATAAGAAACATAAAAAGCTAAGTTTGTCTTAGGTGTGTACTATATGCCAGGAA-----CTAAGTGTTCTATAGAGATTATCT

Homo sapiens           CATTTAATCCTCATCATAA--CCCAATGACGTCAACTCTATTA-TTAGCTCCATTTAACAGATGAAGGAATTGAGGCTCTGGGATGTTAAGAAAAGTGCCCAAGGTCACACAGCTAGCAA
Dasypus novemcinctus   TGTTTAATCCTTATAATTATCCCCAAT------AATCCTCCTATTTAGGCCCATTTTACAGATGAGGCTATTGAGGCTCTGAGATGTTAAGCTAAGTGCCCATGATTGCACAGCTAGCGA

Homo sapiens           GAGACAGAACCAAAAGTTGAACTCAAGGCAGTCTGAGTC-CAAGTCCACAAACTAAATCATCTATGCCACCTGCAGCCCCCACCT--CATGTTACCCTACACCTCCCACGCCACCCCCAG
Dasypus novemcinctus   AAGGCAGAATC-AAAGTTGAACCC-AGGCTGTTTGAGTCTCTAGTTCATGCACTAAATCGTCTTTGC------CAACCTCCAGCTGACATGTTGCCCTG-----------CGGTCTGTG

Homo sapiens           GCACGATTCTACTACAGCACTGGCCACATTGCAGAACAGGGGTTTGTTTACTTGTCTGTCCTTCCTACTAGACGAGGAGCTTCTCAAGTGCAGAGACTGGATCTTATTCATTTTTGCTTC
Dasypus novemcinctus   GCATGCCTCCACCATAG---------CATTACAGTGCAGGGGCATCTTTATTTCTCTATCCTTCCCACTAGAC--CGAGCTTTTAAAGTGCAGGGTCTGGGTCTTCTTCCGTTTAGCTTT

Homo sapiens           CCCTGTGCCTGGAAGAGAGCTTGTTCTGGAATAGCTAACCAAGGAATATTCGTTGAATGAGCAAATGAGTGAGGGAGTAAGTGAATCCATATTCCTTAATATTTTCAGGCTTCAGGCTTA
Dasypus novemcinctus   CCCTGTTCCTAGAATAGAGCTTGTCCCAGAGTA-CATACCTGTCAATATCTGTTAAACAACTGAATG-----------AATAAATCCATGTTCC........................

Homo sapiens           TGGGCTCATGTGTATTTCCCTGGTCAAGCATAGCACTGGGCACAAGGGTATTCTGGTGCAAGAGGTGCCTCTCACCACAGGGTACAAGAAGGACTAGCTCTGGCTGGGTAAACCAAAGCA
Dasypus novemcinctus   ..................................................................................................................................

Homo sapiens           GTGTTCCACAGAGCAAGGGGCTTGATCCAGAAGGTTTCATTTTGTCATCGGTCTAAAAAGTAGCTGGACCTGTTTAGGACTTAAACAAACTTTCAGTGTTCCAATCCCATCTCAAATCCT
Dasypus novemcinctus   ..................................................................................................................................

Homo sapiens           TTGCAGAATGAGGCAGGGTGTGGGGAGGTGGTGGTGGGGCAGTTATAAATAAACAAATACCCACAAACCGGCTGGTTTTTTAAACAGAATTTGCTTCAGTCAGTGCTGCACCACATTTTC
Dasypus novemcinctus   ..................................................................................................................................

Homo sapiens           CCAACTGGCCTCCGTCGTGAGAACAAGTGGGAACTTTCATACTGAATGGCTTTATTTTTATTCTGACCAGATGAATGGCAGGAGTTTTGGCTCGGGAGGCAGGTTTCCGATCCTCAGCAC
Dasypus novemcinctus   ..................................................................................................................................

Homo sapiens           ACAGTTTGCTGCCCTACTGTGCCCCGTCCATCACACACAGCGCCCTGCACAACATACCCAGTCATTGCATCTACTGCCTCAGCCCTTGAAAATAATTGGGTCATTGTTTTATTACACCAG
Dasypus novemcinctus   ...............................................................................................................................CAG

Homo sapiens           CGAGAGAGCTGCAAGCCTGTTAGGATTATGTCATTGATATTTGCGTGAAAAGGCAATCATGATATGCTGGTCATTTAACACAGCTGGGAATGCAACTGTGTTGTCATTGTTTAAGGTTCT
Dasypus novemcinctus   TGAGAGAGCTGCAAGCCTGTTAGGATTATGTCATTGACATTTGCGTGAAAAGGCAATCATGACATGCTGGTCATTTAACACAGCTGGGAATGCAACTATGTTGTCATTGTTGGGGGTTCT

Homo sapiens           CATCCAG-CCCCTCTGACTTTTTTGCACAGCCCAGCCCGTGAAAATGGATCACTCTGATGTATGTCACCTTTAAAATGCTTTCTAGCCCTTCAAGGGACCAAGCAGGATTTAACTCTTTT
Dasypus novemcinctus   CATCCAAACCCTTCCCACTTCATTTCACAGCCCAGCCAATGTAAATGGATCACTCTGGTGTATGTAACCTTTAAAATGTTTTTGAGCCCAT-----------GCAGTATCTAATGCTTTC

Homo sapiens           CATGCACTTGGACTTTGCTGCTTGTAGGCACAGCAGTCTGAAGGTGAGAGATGCTTGCTTTTCATACTTGTGGGAGTTGTACACCCTAGTGGCAGGATGCTGCCATACAGTGTCATTTGT
Dasypus novemcinctus   CACACACTTGGACCTTTCTGTTTGTAGGCACAGCAGTCTGTAGGTGAGAGATGCTTACCTTTCACGAGTGTGGGACTTGTACACCCTAGTGGCAGGATGCTGTAATATAGTGTCATTTGT

Homo sapiens           TCACTAACTGCTGATCAGAGCCAAGGACAATTTTGGCTCAAGAACAAATGAGACCTAA---TGTCTGTGAGGGAAGACTATGTAGATAAGTCACGGGTCAGAGTTGTGTTGCGACTTGTA
Dasypus novemcinctus   TCACTCATTGCTAATCAGAGTCAAGGACAGTTTTCGCTCAAGAACAAAAAAGGGCTAATATTGTCTGTGAGGGAAGACTATTTAGACAGGTCATGATTCAGAGTT---------CTT---

Homo sapiens           TTGTGTGCTAGTTTCAGTAGGACTTGTGCTTTCATTGCAATTGTCAGTATGCTTAAGCAGGGGTCTCTGCTAATGAAATTCGAGCATATTTGCATTTATCGACAGGTATTACTGATGGTA
Dasypus novemcinctus   TTGTGTGTTAA----AGTTGGACCTGTGTTTTGATTGCAGTTGCCAGCCTGCTTAAGCAAGGGTCCCTACTAATGAAATTCAAGCATATTTGCATTTATCAACAGGTATTAATGATGGAA

Homo sapiens           GAACATCTTATTTCCACACAAGGCAATGAGTAACATTGATTATAGAAGTGTAAACAAAGCTCAAATTCATGTGCCAATTTGTCTATTTCAACTCATCGATCCTCTTCTTCCAC-TGGCAT
Dasypus novemcinctus   GAAAAGCTTAGTTCCAAACTAGGAAACAATTAGCATTGATTATAGAAGTTTAAATAGAACTCAAATTCATGTGCTAATTTTTCTATCGCAACCCTTCCATCCTCTTCTCCCACTTTGCAT

Homo sapiens           GCTAGCCTGAGTTTTATGGCATAAGGCCATTAAAGTATTCTTCAAAGGAATCTGTTGCAGACACCTGTTCATCCCTCTTCTGGTCCCGGTCCTTGCCA---AACCAGCTC--TGCCTTTG
Dasypus novemcinctus   GCTAGCAT-AGTTTTATAACATGTGGCCATTAATGTATTCTTTGAAGGAATCTATTGCAGACACCTGTTCTTCCTTGTTCTGGTCCTAGTCCCTGCTGAACAACCAGCTCTATGCCCTTG

Homo sapiens           CCAACCACCTTGAGGTGTGACAGGAAAAGCTGACCCAATAAGGGAGTCAACTGAAGGGATGGAGTGGCTTAGATGCAAATGGCCAAATCCCAATGGCCATGGCTATAAGGAACACAGGTT
Dasypus novemcinctus   CCAGCTATCTAGAG--GTGACAGACAGAGCTGGCCCAATAAGGGAGTTAGCTGAAGAGATAGAGTGGGGTAGACATGAATGTCCAAATCCCAATCTCCATAGATGTAAAGAACTCAGGCT

Homo sapiens           CCTGAAGTTTTCTTGGCAACTAGCTCTAACTGCTTTGCCTTCTGAGTGCTTTGGTATTTGTTTTATTCTAAGTTCCCAGTTTTGATCTATTTTTGCCTTATGGCTGCAGATTCCCTTTCT
Dasypus novemcinctus   CTTAAATTTCTCTTGGCAACTGATTCCACCTGCCTTGCCTTCTGGGGGCTTTGGTATTTG-----TTCTCGGTTCCTGGTTTTGATCTGCATTTTCAGTATGGCTGCAGTTTCCCTATCA

Homo sapiens           GGAAAATGGGGCTCTGGCTGGGATCAAATCAGAGAACACACCC---AGACTTGGCATAGTGCCTGGCACAGAGTAAGTGCTCAACAAATGGTAGCTGTTGTCTCTTTTTCCCTGTCCAGG
Dasypus novemcinctus   GTAAAATGAGGCTCTTGCTGGGATCAAATGAAAGAACATACCCAGAAGAACTGGCTCAGTGCCTGGCACAGAGTAAGTGCTCAACAAATGGTA---ATTGTCTACTTTTCCCTGCGGAAG

Homo sapiens           TTTCTTCTGGTTCTGTTCTCTGTACCTCCAGTATTGGCCCAGAACCAGGAACTGT--TCCCTCCCCTGACTGGCAAGTCAGATTACCTGGCCCTCAGACAAGCTGGGCTCCCTTTTCTGG
Dasypus novemcinctus   TTTCTTCTCCTCTCATTCTCTGCACCTT-------------GGACCAGGGCGTGTCCCCCCTCCCCTTATTGGCAAGCCAGGTCACCTGCCCCTCAGACAAGCTGAGCTCCCCTCCCTGG

Homo sapiens           CAGCCTTTTGTTGTCCTTACCTGTTGCCATGTGCCACTCAGTGTAACCACAGGCAGACCTCTGCCAATTGCTGACATTAACCTATTGAATCCTGGCAGAGAAGAATAAGTTTGCAGACTG
Dasypus novemcinctus   CAGCCTTCTGTTGTCCTTACCTGTTGCCATGTGACACACA--GTAACCACAGGCA-ATCTCTGCCAGATGCTGACATTAACCTACTAAATTCTGGCAGAGAAGAATAACGTTGCAGACAG

Homo sapiens           GAATTTGCATGTCTGGGCCAGATACGAGACCAGGAGGCAAAGCCATATTTACCTGTTCTCCCCAGTTGTG-GCAAATCACCCACATTCTGGTTAAAACCAGATGTTTCACACCCATGACT
Dasypus novemcinctus   GAATTTGCGTGTCTGGGCCAGATGGAAGACCAGGAGGCAAAGGCATATTTACCTGCCCTCCCCAATTGTGAGCAAGTCACCTGCATTCTGGTTAGAACCAGCTGTTTCACATCCATGGCT

Homo sapiens           GCAATGCCACACATCAAACAGTTGCTTACAAAGAACACAAAGTGGTTTTCACATCGCAAATGTAACTTGCAGGTCACTTCTCAAATTTCCTCGCAGCAGCTACCATGCCTGGGTATTTTT
Dasypus novemcinctus   GAAATGCCACACAT----TAGTCACTTACAAGGAACATAGAGTTGTTTTCACATCAGCAACATAACCTTCAGGTTACATCTCAGATTGCTT--AAACAGCTATTACACCTGGGTATTTTT

Homo sapiens           CCCTTAAAAATGACAACCTTGTTGGGTCTCTTGTAGGGACTTTCATTTGGAAGCCCTGGGAGAACTTATATGCTCTTTCCAAGAGGGTCTCTGATGGCTTTAATTCACATGGAGTTAATA
Dasypus novemcinctus   CTCTAAAAGA---------TGTTGGGTTTCTCATAGGCACTTTAATTTGGAAGCCCTGGTG----TTATCTGCAATTTCCAAGGGGGTCTCTGATAGCTTTAATTTGCAGGGAGTTAAAA

Homo sapiens           TATTGGAAAAGAAAAGAGATCTT-CCCCAAACTCACCTGCTCCTTGAAAATGCTATTCTGTTGCCTTGGCGAACAGCCAATCCAAACATTAAAAAAGAGATAGAGAATGCAGGAATCCTA
Dasypus novemcinctus   TATTAGAGAAGATAAAAGATCTTCCCCCAAACTCACCTGCTCTTTGAAAATGCTATGCAATTGCCTTGGCAAACAGCCAATCCAAACGTGAAAAAAGAGATAGAGAATGCAGGAATCTTG

Homo sapiens           AATGCTATTCTCAGTGCATGTCCAAACAAGAACTG-GGGGCTGAGGGGCAAAGAACAG-------AAAGAGGAGAAAAGAGTTTTTGTAGCCTAGGCCGTGAGTTGATCCCACCCCGTCA
Dasypus novemcinctus   AATGCTATTCCCAGTGCATGTCCAAACAAGAACAACGGGGGTGGGGGGTAGAAAGGAGCAACAGAAAAGAGGAAAAAAGA-------TAG---AGGCTGTGCATTCATCCCACCCCATCG

Homo sapiens           TGTCCTGTACCCTTCACGCTCATAAAGAAGACAGAGGAGGCCGGGCGCGGTGTCTCATGCCTGTAATCCCAGCACTTTGGGTGGCCGAGGCAGGCGGATCACCTGAGGTCAGGTGTTGGA
Dasypus novemcinctus   TGTCC-ATCCCCCATGCACTCATAAAGAGAAAAGAGGA...................................................................................

Homo sapiens           GACCAGCCTGGCCAACATGGCGAAACCCTGTCCCTACTAAAAATACAAAAATTAGCCGGGGGTGGTGGAACATGCCTGTAATCCCAGCTACTCGGGAGTCTGAGACAGGAGAATTGCTTG
Dasypus novemcinctus   ..................................................................................................................................

Homo sapiens           AACCTGGGAGGTTGAGGTTGCAGTGAGCCAAGATTGTGCCATTGCACTCCAACCTGGGCAACAAGAGTGAAACTCGGTCTCAAAAAAAAAAAAAGAAAAAGAAAAAGAAAGAGGAAGGAG
Dasypus novemcinctus   ..................................................................................................................................

Homo sapiens           AAGGAGAAGAAGGAGAAGGAGAAGGAGAGAAGAAGAAGGAGAAGGAGAACGAGAAGATGTGGTGGCATCATGACTTCTCTGCTAGGGTTAATTGCTCAGCCTGAGAGGCTCTCCAGGCTG
Dasypus novemcinctus   ..............................................................TGTCATCA-GACTTCCCTGCCAGGGTAAATTGCTCAGACTGAGAATTTCTCCAGTCTG

Homo sapiens           GCAGGTCACACTAGAGCCAAGAGTGAAGGGTCCATTCAGTAAGGTCACTGCCATGATCAGTTCTCAACCACTTTAACTCAGTTTTTCTGTTGCTGCTTTGTTGGTTAGTGGTTTGTTTGT
Dasypus novemcinctus   GCAGGCCTCACCAGGGCCAAGAGTGAAGGTTCAAGTCAGTGAGGTCACTCGGATCATCAGTTCTCAACTGCTTTGACTCAAGTTTTCCAATTGGGGTTCTTTGATTATTGGTGCATTTGT

Homo sapiens           TTCTACCCATTTGGGAGAAGGGTAGGAAAGGAACTAAGGTGATCAACTATTCCATATGTGTCAGGCCCTGTACTTGTCTTTTCAAACACTTATTCACACTCTGAGGGACTCTCTCAGGTC
Dasypus novemcinctus   TTCTTCCCATTTAGGA...........................................................................................................
```

```
Homo sapiens          CAGCAACTGTGTAGCTATGATGTGAAATAGCAGCTTCTCTCTATGCATTCACTGGGAATGGTTTGTTCTGATCACACAATTTTTTCCAGTTCTTCCGACGTTAGGTGTTGCATGGTAAGA
Dasypus novemcinctus  ........................................................................................................................

Homo sapiens          AACAGGTGGTTAATTATTGAGACAGTTCTTATTTGGAATTGTCTCTTCCTCCTTCTCCTCTCAGGCAGATGCTAAAGGATGTCTGTAGTTTTTTCATGGCATTTTGCTAAAGATGCACAT
Dasypus novemcinctus  ........................................................................................................................

Homo sapiens          TCATTAATTCATCAAATAGCTTTTTTTGAGACATTATTATGAGTGAGGTGTTGTGCTATGATATGCTTTATATACCATATCTTATTTCATCCTCACCACAAAGCTATGAGGTAGGTACTA
Dasypus novemcinctus  ........................................................................................................................

Homo sapiens          TCATTATCTTAATTTTACAATGAAGAGACTGAGGCTCAGGAAACATAATGCAAGGTCACACTCAGTTAATAAGTTGAGAGCTAGGATTCAAACCCAGGTTTATCTGACTCAGTTACACTA
Dasypus novemcinctus  ........................................................................................................................

Homo sapiens          CACTTCCTCCACATAGCACATTGTGGGCCCGTCACAGCTCTTGAGGACAAAATTCATCCTTAGAAAAGTTTTCTTACTCCACTTTTTGCTGATACTACTGCTGCTGTTGCTGCTGCTACT
Dasypus novemcinctus  ........................................................................................................................

Homo sapiens          ACTATTACTACTACTGCTACTGTTTCTCCTCCTCTATTTATCCAGAACTGCATTTTTCCAGCACTCCTAGGTGCTTTGAACCCAGCTACCAGTTGTAGAGTTCAAACCCAGGCCTGCATG
Dasypus novemcinctus  ........................................................................................................................

Homo sapiens          AGTCCCTTGCACCCATTCTACTTTTCTGCACTGCTTGTAATGTAACACAGGAGGTATGGGACATCCATAACTCAGGTAGGTAATTGACTTCAAGCTTAACATTCACTCACCAATCATTTA
Dasypus novemcinctus  ....................................................................................................................TTTA

Homo sapiens          TTGAGCACATATTAGATAAGCAACCTATGGCTCCTGCCCTGGAGTTGTTCATAAGACCTTCCAGGCAGACAGCTATGGGACGAATACACTTGAATTTATTAAATCTACCTCAGGCAAAAA
Dasypus novemcinctus  CTGGGCATATACTAGATGAGCA--CTGCAGGTC-TGCTTTGAG--TGCTCATA-GAC------GTCAAGCAAGTAGGGGACAAATGCTTTTAAATTTATTAAATTTAACTGATGTAAAAA

Homo sapiens          ACCAAGCATGTAATTATAAGTAGTATATCAGAGTATGTGTAAATGGGGTTCTGTAAAATGTTTGAAAGAGGCAGGCCTGGGTTAGTGGTGAGAACAAGGATCAAGAAGGCTCTGCCACCA
Dasypus novemcinctus  AACAAGTCCA-AACTGTGATGAGTATATCAGAGTATCTGTAAATGGGTATCTATAAAG-GCTCAAAAGGGGCAGGACTAATTTAGTAGTGAGAACAAG----AAGA-----------TA

Homo sapiens          AATAACAACATGAGTGTTGTC----CCGTTAATCTCTCTGGGCTTCTGTTTCCAGATCAAGGACCTCAAAATAATAGTCATAGTCACCATTTATTGAGTGCTTTACTGTTAACCAGGCAG
Dasypus novemcinctus  AATAGCTACATGAATATAGGCAAGTCCTTTAACCTAACTGGACTTCTGTTTCTGCGTGTAAGACCTCAGAAT----GTCATAGCCACCATTTATTGAGCA-------------------

Homo sapiens          TATGCTAGGTGGTTTTCATTTAGTCTTGACAAC-AGTATTCTCGCTTTCCCTTTTCTACAGCTGAGACTCAGAGACATTAGGTAATGT-------CTAGGTCACACAGCTCTTCAGTGAC
Dasypus novemcinctus  --------GTGGTTTCCATTTACTCCTCATAACCACTGTTGTCACTTTTCCTTTTCTATAGCTGAGGCTCTGAGAAGTGAAGTAATGTGCCTTCCTTAAGTCAAACAGCTTATCAGTGGT

Homo sapiens          CAGTTGTTTCTATGTCAGAATCCTCTT----------ATAATCACTGAGTTGTATCGCTTCCCTTGCCTCCTGCTC-----CCAAGTCAAGTATTCTCTACTATGTCGCCTGAAAATTAA
Dasypus novemcinctus  CAGTTGTTTCTATCTCCAAATTCTTTCTCCAGAATTCATAACCTCTAAGTTGTATTGTCTCCTTTGCCTGCTGACTGAACTCCAGATCAAGTATTTTCTACAACATTGTCTTAAAATTAA

Homo sapiens          AAT------TAAAATCAAGGTTAATCTTGTTCAACTGAGCCAAGAGGATAAACATTAGAAATAGCAATTAGTAGGTAGAAATGTTGCCAGGAAGAGCTATACAAAAGCATAGAAAGGCAA
Dasypus novemcinctus  AATCAAAATTAAAATCAAGGACAATCTTGTTCAACTGAGCCAAGAAGATAAGCATTAGAAATAGCAATTAGTAGGTAAAAACGTTGCCAGGAGGAGCTATACAATAGCGTGGAAAG---A

Homo sapiens          CCAAGGGAGTTA--ATGATGGGGGTGTGGCAGGGGTTGCATGTTGATGGGTAATTGGATCCTGGCCAGCTCTTACCTTCTTCCTTCACCTTCCCACTCCGCAATTTCTCAGGCTTCTCTA
Dasypus novemcinctus  TGCAGAGAGTTACAGGGATGAGGGTACGGCCTGGGCTGCATGTTGACAATTAATTGGATGCCCACCAGCCCTTACCTTCCTCCTTCACTTTCCCACTCAGCAGCTTCTTAGGCTTCTCTA

Homo sapiens          GAGAGCACAACCCACCTAGAGACAGCCTGTGTTGCCACAGGAGGGCACCAGGCCAGGAATGGCAGGCCTAGGGCAGTGGGGCTCTGGGCTGGGAACCATAAATAACCACTTT--AATGAA
Dasypus novemcinctus  GAGTACATAGCCCAGGAAGAGACAGCCCTAGATGCCACAGGAGGGCACCAGGTCAGGAATGGTTGGCCTAGGGCAGTAGGGCTCTAAGCAGGAAACCATAAATAACCACTTGAAAATGGA

Homo sapiens          AACCAGCTTTGGCCAAAAGCCTTATCATTTCTTAAGAGGTTCTGTGCTTCTTCTAATGACCTAGTTACTTGAGAAAGGCCTAGCCTAACATATAC------AACAACAATAAT----AGT
Dasypus novemcinctus  AACCAGCTTTTGCCAATAGCCATGTCATTTCTTAAGTGATTCCATG---CTTCCAATATGATAGTTACTTGAGAAAGGTCTAGCCTAGTATAGGCAATAATAACAACAATAATAATAAGT

Homo sapiens          TGCCCTATAGTGAGTACTTACTATGGGCCAGGGCCTGTGCTAA-------ACACTCACTAATTCATTTAATCCTCACACAGCCCTACAGGAGCAAGTACTATTATTTCCCCCACGTTACA
Dasypus novemcinctus  TGCCCTTTAGTACATACTGTCTATGAGCCC-TACCGTTGCTAAACGGTTTACATGCACGAACTCATTTAATCCTCACGCAG-CCTATTAGGGCAAGTACCATTATTTTCCTCATTTTACA

Homo sapiens          GAGCAGGCATCAGGCTGAGAGAGGCTAAGTCATTTGCTCAAGATCATATAGCTGGTTGGAGCTGGAATTTGAAGCCTGAAGCATCTAGTTCCAGGGCCTGAGCTCTTTAATTCCTATATT
Dasypus novemcinctus  TGGAAGGCTTCAGACCCAGAGAAGCCA------------AAGGTCATACGGTTGATTGGAGCTGGAATTTGAAGCCTGAAACATCTGGTTCCAGGACCAGAGCTC-TTAATTCCTGTATT

Homo sapiens          TAATGAATGTCTGTCGAAGGAATAAACAGATGAATGAATAGACAAATGTATTCATTTCATTTCACTTTAACAAACATTTATTGAGTGCTCTGTGTCAGCTCCTGTGCTAGGCATTGAGGA
Dasypus novemcinctus  TAATGAACAACTGTTAAAGGAATTAACAGATGAATGAACAGACAGATGTAT.....................................................................

Homo sapiens          TTTATAAGTTTAGTGCGTGCGTGCGCGCACACACACACACACACAAACGCACACACACACACACACACATCCTGCATGGCCCTTGCTCTCCAGGGGTTCACAAAAGCATTCTCTATTGTG
Dasypus novemcinctus  ........................................................................................................................

Homo sapiens          GCTTGATTTCTTCTGGAGAGAAGTAATTTGAATGTCTTGAACAGCAGTTTTTAATGATGCTATTAAATTTAACCTAAAGCGGGGGTTTTGCACCGGTGAGCAAAGTGTTTCAAATGAGAA
Dasypus novemcinctus  ........................................................................................................................

Homo sapiens          TGTGTCTTTGATTAATCATCGATTCAGCCAGAGAATAGTTACTACCAGAATGGAGCTGCAACTCCTATTATATTAGCTAACTCTTCTTCCATCTATCATGTCTCTATCTTGTCGAACCCT
Dasypus novemcinctus  ........................................................................................................................

Homo sapiens          CCCACGATACAAAAGTTATCTGTACCCTAATGGACAATTATCCCATCCATCATTCCTGCTGGTCTTGTTGATTGCTGGTAGTCTGCTTGCCCACGGAGGAGACTTACAAATAGTCATGAC
Dasypus novemcinctus  ........................................................................................................................

Homo sapiens          CAGTGACGCACTGGAAAACTGCTCATTTTTAGACATTTTAAATCTATGTAGAATGAATTATTTTTCAGGCTGTACTTAAGCTGCATTTGCTTTGTTGGTTATGTAAGAAGAGATATGAAT
Dasypus novemcinctus  ........................................................................................................................

Homo sapiens          TCCTTACCTATGCGTGAGACATGGCGTCTGAATATTTTTAAGTTCCTGTGGGCAGCAGCCCTATACATATTTGTTAAAATGATGAAAGAATAAAGTAAACATACCTAGAGTAATTCCAGA
Dasypus novemcinctus  ........................................................................................................................

Homo sapiens          GGAGCAGGCTTCATACCTGGAGCTCGGGTGGGTGGCCATCATGTCAGGGTAGTCACCGTCTTCCTTTTCCTTTCTAAGTACTTTGAGGAGAATCCCAAGTTGATTAGGGTCAAGGTGAGG
Dasypus novemcinctus  ........................................................................................................................

Homo sapiens          ACTCATGGTGCTGAAAATAGGGATTGAGTGGACAATGAGAGATGCCTGTTGAATTCCCTGGGACTATTGCTATGAGATTTGAGGGCAGAAATGCCCCCCTCTCCCATGTCCTACCCTAAG
Dasypus novemcinctus  ........................................................................................................................

Homo sapiens          CCTGGTAACACATTTAACTTTTTGTTACAGTAACTACCATGCCTAGTAAAACTGGAGTCACTGGTTGCCCAGGGCAAAAACCCAAGAGTTGTAGTTCACTCATCTTTATTTCTCACCTCT
Dasypus novemcinctus  ........................................................................................................................

Homo sapiens          ACATTCAGCCAGTCACTAAGCCCTGCCAAGTATATTTCCTAAGCATTCCTTGCATGTCACCTCTTTTCTCCATTCCTACTGCCAGGGCTTTAGTTCAGGACTCCTCCCACCTCCAGACAT
Dasypus novemcinctus  ........................................................................................................................

Homo sapiens          CTCTTGCCTTGATTCCTACAATACCTTCTGGTTTTCTCACCTTCTCTCCTTAGTCCTCTGTGACCATTCTCCCCAAAGTGGCCTGAGTGATCTTTCAAACATGCAAATCAAATCATTTCA
Dasypus novemcinctus  ........................................................................................................................

Homo sapiens          CACCTCCATCCCCTCCACTTAACACTTGTAAATAGCCTCACAACACCTGAGACTTGAGGCTCTCAAAATGTGGGTCCTGGGGCCAGCAACACCAGCATCTCTGGCAACTTCTTAAAGTTA
Dasypus novemcinctus  ........................................................................................................................

Homo sapiens          GAGTACGTACTCATTAGAGTACCTACTGAATCAGAAACTCTGAGAGTGCTCACCTGACTCTGTGTTTAACAGCCCTACAGGTGATTCTGATGAGCACTCAAATTTGAGAACTGGCACCCT
Dasypus novemcinctus  ........................................................................................................................

Homo sapiens          AGAGCCATGGTTCTCAATCCTGGCTGCACATTCAAATAAATGGAGGGAGCTTTTAAAAGTTCTAATGCCTCTCCTGCAGAGTAATTAAATCAGTTTCTAGGGGTGGGATCCAGTCATAAG
Dasypus novemcinctus  ........................................................................................................................

Homo sapiens          TATTGTTTTTAAAGTTCCCCAGGTGCTTCTAATATACAGTCAAGGCTGAGAACTACTGCTCTATAGGATGAAGTCCAAACTCCTTAGCTTGGCATTTAAGGTCTATCTCTTTGGTCATAA
Dasypus novemcinctus  ........................................................................................................................

Homo sapiens          CCACCCCCAACACTATGCTCTTGCTTGTTCATTCTAGAATTCAATCACAGGGAGTCATTTGCAATTCTCTTAGAGCCACACATGGTCTCTTACCTCTGTGCATTTGCACACATCATTCCC
Dasypus novemcinctus  ........................................................................................................................

Homo sapiens          ACTGCTGTGCCCATCCCTTCCTCCCTCCCTTCACCCAGCTAACACCATCTCATCTACAACACATAGCTCAGAAGACACCTCTAAGGAACAGTTTTCCCCGCCTGTCCAGTCTGGGTTAGA
Dasypus novemcinctus  ........................................................................................................................

Homo sapiens          TGCCCCTCCTCTGTACTCCCACAGCGTCCACAACATAGCACTGGATACATTCTCTTGTTCTTCTCTGGTTAGTTGTCTACTTTCTCTACCAGTCTGAGAGCTAGGATCCCCACCTGGCAC
Dasypus novemcinctus  ........................................................................................................................

Homo sapiens          ACAGTAGATGTTCCATGATTCTGTACATCCTGTTCCCTCTTTTTGGAGTGACTTTCTGTGCCTTGTCTGTGGCCTTCAGGATGCAGCTCAGATATCACTTCTTGGGAATCTCAGAATTCT
Dasypus novemcinctus  ........................................................................................................................

Homo sapiens          CTGTCTGGACCCTTTCTCTAGGCTCCCGCAACAATCTAAATGTAATCTGATCCTAGCCCTTAGCACACAGGGCTATCAGGCTGCCATAGTCTGTTTACTTATCTGCCTCCCTCACTGAAC
Dasypus novemcinctus  ........................................................................................................................

Homo sapiens          TGTAAGTAACTTGAGGTTAGGGACAGAGCCTTGTACATTCCTGTATCCCAGCTCCTAGCACAAAGACTCACACATTATAGGTGCTCAGTAGCCATAACCATCTGCAGAACGAATAACTGA
Dasypus novemcinctus  ........................................................................................................................

Homo sapiens          ATCAATGATGACCTTTTCCTCTTCTCCATATCATCAGCCACTATTTCTGTCCAGGCCCCCGTTGCTTTATTGCAATAGCCTTCTGACTATTTCCCATCTCTCCTTTCTCTGATGCCATCT
Dasypus novemcinctus  ........................................................................................................................
```

```
Homo sapiens          GAACACTCCCCCCCTCTCCAAGATGTGGCTTTCTAAAACCACAGTCACCCTATCCCTCCCCTGCTCAAACACTTTCAGTCATTCCTCATTGTCTGCAGGGCAAATATCAAAGTGGAATTC
Dasypus novemcinctus  ........................................................................................................................

Homo sapiens          CAGATCCTTCCTTCCCAAGAGTGGTCTGCCTTCCCAACCATACCTCCCCCCATTCCCCTATGGAATACCAGCCACATCATCTGAGGGTCCTGGTGCAAAACGAAAATGTGGAACCCCGTG
Dasypus novemcinctus  ........................................................................................................................

Homo sapiens          TTAAAATATAATCAAGGATTTCAAGATAGCAGCAAAGGAGAGCATTAAACCAAGCATAGGGCTCTTCTGACTTCAGGGTCCGATGCGACGGCACAGGTCTGCCCCTACGTGTATCCAGCG
Dasypus novemcinctus  ........................................................................................................................

Homo sapiens          CACTGGCCAAATGCACTTGCTCCTCCTTCAACTGCTCTGTAGGTTCACTTTCTCCACGGCTTTTGTCAGGCTGTTCCATCTCTTCTACCCCACTAGCCAATTCAAAGCTTGTCTTCCCTG
Dasypus novemcinctus  ........................................................................................................................

Homo sapiens          TAGATCCACCCTGTTCTTCAAGAATTGCTCAATTCTCATCTCCTACTGTTCCAGACCACAGTAGTTTCTACCACACAGCCTGATATTAATAATTATTTTTATATGTGTGTAGTCTTGCCT
Dasypus novemcinctus  ........................................................................................................................

Homo sapiens          GTCTACTGAATTTTAAGCTTCTTGAGGGCAAGGACTCTATTTTGTTCATCTTTTTTTTTTATAGACTTAGCATAGTACTTTGTAATCATCTCACTTGTTGAATGACTCAGGGAAGAAATA
Dasypus novemcinctus  ........................................................................................................................

Homo sapiens          ATACAACTCCAGTCACTGAGTTTGCCAAAAAACAGTAGACACTGCAAACCTGATTTAAATCCCTCATTATTAGCTATGCAACATTGAAGGAGTCACCTCACTTCTCTTTGCCATCTATAA
Dasypus novemcinctus  ........................................................................................................................

Homo sapiens          AACGATGATACTTAGAGAAAAAGTGTTTTTTTTTTTTTTTGTTTTGTTTTTTTGGTTTTTTTGTTTTTACAGAGGCTTGCTCTCTTGCCCAGGCTGGAGTACAGTGGCATGCAATCTCGG
Dasypus novemcinctus  ........................................................................................................................

Homo sapiens          CTCACTGCAACCTCCGCCTCCTGGGTTCAAGTGATTCTCCTGCCTCAGCCTCCCAAGTAGCTGGGATTACGGGCCCATGCCACCATGCCCTGCTAATTTTGTATTTTTAGTAGAGATGGG
Dasypus novemcinctus  ........................................................................................................................

Homo sapiens          GTTTCACCATGTTGGCCAGGCTGGTCTTGAACTCCTGACCTCAAGTGTTCCACCTGTCTCGGCCTCCCAAAGTGCTGGGATTACAGGCGTTGAGCCACCATGCCTGGCTAGAAAAAGTAA
Dasypus novemcinctus  ........................................................................................................................

Homo sapiens          TTTTTGTTAGTATCTTGTTCTTTGGCAATGTTTTCAAAATCTTTTGTTTTTTAATACATGTAAAACTTATTTTATATTTTATATCACCTAATTGCAATACCTGAGGTCTTTGAAGACCT
Dasypus novemcinctus  ........................................................................................................................

Homo sapiens          GATTCTGTTTTCATTGTTTCTGGCTTCATCGTGTTCTTTGTAATTTTGGATTATGAGTTCATGTTCAACCTGACTTTATCCTTGGTAATCCTATGAAAACTGGGTGGTGGGCATGTTCTT
Dasypus novemcinctus  ........................................................................................................................

Homo sapiens          CCAGGGAGGATTTGTGTTTGGTTCTTCCAAGTGCATCAGGGCACTATTAATCCAGGAGTTCTTCCAGTGAATTGTTTAGTCCCAAAGGCAAGGGCCATGTCTTCTTTGGCTATATGATCC
Dasypus novemcinctus  ........................................................................................................................

Homo sapiens          CAGGGCTTAACTCAGGGACTTGTAGATGCTCAACATATATTTGCTGATTGAGTGGATGAATGAATGTGGTACTTTACCGCTTCTTTTGGAAGCCCAATAAGAATTGTGACCTCTGGTATT
Dasypus novemcinctus  ........................................................................................................................

Homo sapiens          AACAAAGAACAGTAGGCACATCAGGAAAATAATAATTACTAACATTTATTGAGCAATGATTATGTGTCAGGTATCATTCTAAGTGCTTTGTGTGTATTAACTCATTTAATTCTCACTATG
Dasypus novemcinctus  ........................................................................................................................

Homo sapiens          ATGCTGTGAGGTAGGTTACTATTATTATCTTCACATTACAGAGGAGGCACCAGACTCAGACAGGCTGAATAACTTGGCCAAGGTCACATAGCTAGTGAATGGCGGAACACAGATATGAAC
Dasypus novemcinctus  ........................................................................................................................

Homo sapiens          CCAGGCACCCTAGAGACACATCTACTTACCACCTTGTATTGGAGAAGAGGTCAACAGTAGTGACAGAAGCAATAGAGTGGAAATTTTCAGTACTGCCAAAACTATGAGAGCCATATAGTC
Dasypus novemcinctus  ........................................................................................................................

Homo sapiens          CACACCAGGCATGAAAAGGAAGCTGGCATGGTCTTGTGTTGTCAGTTAACCCTTGTATATAGGGTTTTCATTTCCTAACTAGAGAAGGAGGAGTTTGAAAGCACGAACCATGAATTACAG
Dasypus novemcinctus  ........................................................................................................................

Homo sapiens          CTCTTTGAAATGCTCAACATAGATTTGCTGAGTGAATAAATGACTGTTTTGAAATTAAACACAGGAGTGCTTTGAATAAATTACTTTGCATCTTGTATGTCAAACTGAAATTGGTGAGAC
Dasypus novemcinctus  ........................................................................................................................

Homo sapiens          ATCTTTCACCTTGTGTTAAAAACTAAAAATCCCTAGATGTGGTCATAACATGGCTGGTTATAATGACACCTTGCATTTGTACTTGTGTATGATACTCAGGACTCCCTGAGAAAAGGAATG
Dasypus novemcinctus  ........................................................................................................................

Homo sapiens          AAGAAACTTGGATTTTGCCTTCATAGGCCCTGAGGCTGTCAGGAACCAAGGGCCACTGTGTTTGTAATCCATTCCTTTGGCTGCTCTCCAAATCTAGGAAGACCTGGAGGAAAGGGGGGC
Dasypus novemcinctus  ........................................................................................................................

Homo sapiens          TTTATTTTCTATCTTTGCCAAGATCAGGAAATAATTTGTGAACTCTCAGTAAACATTCTGGGGGCCTTCCCTCTAACACTGCTTTGGTGGAATAATATCTCCAGTTTTCACCAATCTTGG
Dasypus novemcinctus  ........................................................................................................................

Homo sapiens          TTCAATATTTACCTGGGGGTTTGCAGTGATCATGGGGACAGGGCTCCAACATGAAGTGAAGGTTCATGAGAAGGAAAATGAGGGGAAGGCTGTGTGTGTGTGTGTGTGTGTGTGTGTGTG
Dasypus novemcinctus  ........................................................................................................................

Homo sapiens          TGCTGGCTGAGGGATGAAGGTGGGAGGAATACCAGGATTCTGAAGCAATGAGAGTGGGATTGGGAGAGAGAGCTCATGTTGAAGAAGAAATAACTCACCAGTTCACATGGGCTCCTTTTC
Dasypus novemcinctus  ........................................................................................................................

Homo sapiens          AAGGGTTTTTCTTTATATAAACAATTTGCCTGGTTCCAATTAGAAGCCCACATTAGGCAGTTAGCTCCAATTTGGCAGTTCATTCTTAATTTGGCTTTCCTTATGGGTGGAGGGAGGGC
Dasypus novemcinctus  ........................................................................................................................

Homo sapiens          TGACAGTTAAAAGGTTGATGGGAAAAACCTTGGTATCCTGGTGTGAAGACAGACCCTGGGATCAGAAGGTTGCCCTGATGACTTTGCCAATGTGTTCATTGCCCCTGTCCTGCTGGGGCG
Dasypus novemcinctus  ........................................................................................................................

Homo sapiens          CATACAGCAGGCCCTTCTTTCTCATACGCCCTTGACAGACCAGGACTCACTGCTAGAAAACCCCTGGGGAAGTCATTGCTGAAGACTCTAGTGTGGGGCACATTAGGTTTGAGGAGCAGG
Dasypus novemcinctus  ........................................................................................................................

Homo sapiens          GGAAACATTCAGGCAGCTGGCAAGGTCTGGGCTACCAGGAGAGATTTGGGAGTTAGAAGCATATTAGGTGAGAGTTGACACTGTCCAAAGGGAGTATATAGAAAGAAAAGCAGAGGAAAG
Dasypus novemcinctus  ........................................................................................................................

Homo sapiens          GGCTCATGGAGTCATTACAGCCTGTGAGGTGGGAACAGGGTTTGGGAGTACTAAGGGTTTGGGGAGGGTTTATGTGTTTATTCACCCATTCATTTATTCAACATTTCTTGAGCTTTATGG
Dasypus novemcinctus  .......................................................................ATGAATACATATATCCACTCGTTCAACAAATATCTATTGAGTTTTGTCC

Homo sapiens          CAGATGCTGAGTTGAAGAGGAAGCTGGGGATTTAAGAGGGAGAAGAGTTAAGAGGGGATCCCTGGGTGGATCCAGATGAAGGAGAAGGGAGGGGCTAAGTGCCTAGGCTCGCTCTTGCCA
Dasypus novemcinctus  TAAGCACAAAGTTAAAGGAGAACTTGGAAATTTAAAAG--AGAAGAATTAAGAGAGAATCCCAGACTGGAGCTTGGATAAGGAGAAAGGAGGGGCTGAATATGTAGGCTTCTTCTTGCCT

Homo sapiens          GGAGAGCACATCTGTTAACACAAAGCTGCATCTGTATCTCAGCCAGCTCTCTTCCATTTCATCTCCTCCTCTCTACCCACACTTGCAAGACGGGGAGCCACATGGCGGCTTCCTACTCCC
Dasypus novemcinctus  GGAAAGCTCATTCATTTATTCA..................................................................................................

Homo sapiens          TTGTACCACTACACCCCCTGCCGGGTTCTTTCTAGGCATTTAGCTGTGGCATCAGCAGCACCCCTTCTGAGAGATCAGGCAATCCCCAAGGGTGAGTGGTAGTGGTAGTGGTGGGGAGGG
Dasypus novemcinctus  ........................................................................................................................

Homo sapiens          TTGATAGCTTCGAGAGAAGAGCTGGAGAAGGCGCTGTCTGGCTGTAATTAGCCCCAGCTGCTGTTGTGAGGCAGCGTTGGGGGCCGTCAGGAGTGTACATGCATGTGTATGAACCTGTGA
Dasypus novemcinctus  ........................................................................................................................

Homo sapiens          GCGTGGCACCTCATTGCTATCACTCTATTTTTCACTGTATTGTGTGCTGGCTGCAGCCTCGCTGCTCTGAAACCCCTCCTGTCCCTGAGGATCCCGAAAGACAGAGTGTTCCACAGGCAG
Dasypus novemcinctus  .................................................................................................................CAGGCAG

Homo sapiens          CACCAGCTTGAATCACTAATGCTATTAAGCAGGAGGACAACAAATATAGGCCCCCATATGTCCTGGATATGCAAGT-CCCTGCTCTCTCTTTCCCCACCCCTGCTTCCTGAATGTTTCCA
Dasypus novemcinctus  CACCCGCTTGAATCGCTAATGCTGCTAAGCAGAAAGACAACAAATATAGGCTCCTATATGTCCTGGGTACACAAGTCCCCTGCTTCTTCCCCCCATTCCCCCACTTCCTGAATGTTTCCT

Homo sapiens          AGT-GAGTGAAGTCCCAGCACTTGAAATTAGCAACACCTCACTTCTGCACAGCCTTTTGGCAAAGAGCTTTCACAGCTGCCATGTCATGCATCCTCAGACGCTTCTAGGAGAAGCATTTT
Dasypus novemcinctus  TGTCGAGTGAAGCCCTAGCCCTTGAAATTATCAACACCTCACATCTGCACAGCTTTTT..............................................................

Homo sapiens          ATAGGTGAGGACCCTGTGGTTCAGGGAGGTGGAGAGATTTGCACAGGCTAACCCAGACAGTCAGTGGCAGACTTGTGTCCAGGAAGCAAGTCCTCGACCTCCTGGCCTGGTGGCCCATCC
Dasypus novemcinctus  ........................................................................................................................

Homo sapiens          CCAGCAGAGCACTGCCAAGAGGGCTGCTCCCGGGTGGCTCCAGCTGCTGCTGTCTGTGAGCTCGTGAGAACCTGTCAGCTGTGGCTCCAAGACCTGCCTCAGTGATTGCTGAAAACGCTG
Dasypus novemcinctus  ........................................................................................................................

Homo sapiens          AGAGTGCTGAAAACCAGGACCCTCAAGCCGAGCCTGACGTCACATAGTTGATTGAGTACCTGCTATGTGCCCACCACTGTGCTGGTGCTTATGGGAGACAAAACAGGGGTAGTGCATAGC
Dasypus novemcinctus  ........................................................................................................................

Homo sapiens          CCGTATCCTCAAAGAAGTGATAACTTGAATGGGGAAACAACTCTTAGATACCAAGTCCAAAGGCAATTCCAAGCACTTCTCTCTCTTCCACTAAGCAGTCTGTTGTCAGTGACAATGACA
Dasypus novemcinctus  ........................................................................................................................

Homo sapiens          CAGGAACCCCTTCTGAATTCATCTGTCTTATCGCAGGCCTGGGGTAGGAGAGGATGGGAGGATGACTAGGAAGGGTAATGAGGAAGGGCAGGGAGATGGGGGAAGGAAGGGAAAAACTGG
Dasypus novemcinctus  ........................................................................................................................
```

```
Homo sapiens          AGCTCCTAATAAACACCAGGCACATTGTGAAGTTTATATGTTATACATAGGTCATTTGTATTATGCTTATCATACATGTACATGAATATATACACCCAAACACATACACAAACACATACA
Dasypus novemcinctus  ........................................................................................................................

Homo sapiens          CAATCACTTTCCAGGGATTGACTCATCTATTTGGCTCACTCTCAATCATGAAAAGAGCTTAGGACAGTGACACTTTTAGAAAGCCCTGAATGAGTGTTAACTAGCCTTTCTTCTTATCAT
Dasypus novemcinctus  ........................................................................................................................

Homo sapiens          GATCATCTTCATTGTCATTATCGGCATATTATATAAATTATCTCATTTATTTCTTCTAACTACAGGACTTAGCAACTATTATTTTTATTTCCAGTTTACAAATGAGGAAACCAGTATCTT
Dasypus novemcinctus  ........................................................................................................................

Homo sapiens          ATTTTATCCAAGCTTGTGGGTACTGAGAATATAAAGAGGAATTCAATATGGTTCTAGGCAAACTTTGGGGTCTTCCCTATCTAGTGGGAGGGACCGAGGCACACATAAGCCCTCATGGTC
Dasypus novemcinctus  ........................................................................................................................

Homo sapiens          CAATAGGTGCTGTGGTAGAGGAACGTATCTGGGGAAGTTGAGGAAGTCTCCACGAATGAAGGAGGCTTGAGCAGCATCTTGAGGGATGGCAGGTTATGTCAGGAGGAACAGTGGAGGCAG
Dasypus novemcinctus  ........................................................................................................................

Homo sapiens          GGGAAGACATTCCAGGCAAAGTGAGCAGTATTTGTGTGACAGGGCCTAGTGCATGTTTCAAAGGGCAGGACAATTGTAGCTGGGTGCACCACTGGCAGCAGCAGGAGGGGAGTGGACGAG
Dasypus novemcinctus  ........................................................................................................................

Homo sapiens          TATTTGGGGTCAGCTATTCAGAGCCCTATTGGCCAAGCTGAAGGTTTTGTTCTGTGAGCAAGAGGGAGCCACTGGAGCTTTTTATTAAGGCAGATTCGGGACAGACTAGGAAGACAGTCC
Dasypus novemcinctus  ........................................................................................................................

Homo sapiens          TTACCAGGGTGTTGAAAGATTGGAGGGAAGAGAGGCCAGCTGCATGCGGACCAATTGGGAGACCATCTCCAGGGTCTGGGCAAGAAGTGGAACAGGCTCTGAACCACGACCGTGGCAGTG
Dasypus novemcinctus  ........................................................................................................................

Homo sapiens          AGAGCCGAGAGGAGGGGATGACTCAAGGCACATGTCAACAGGTAGAAACCTCAGGGCTGGCTGTCATGGGGTCAAGGAGAGGGAAAAGGTGACAAGGACCCTGAGGTTCCAGCTTGGGTC
Dasypus novemcinctus  ........................................................................................................................

Homo sapiens          CGTGGTGATACTGTCAACTGAGGCAGGGAACACAAGACTGTTAGTAGAGAGGGAGAGTGAGGGGAGACAGATGACAAGGTCTGTTTGGACTTGCTGGGTTTGGAGTGCTGGATAGCGGAC
Dasypus novemcinctus  ........................................................................................................................

Homo sapiens          ATGTCCAGCAAGAAACTGGGAATCTTGGCTTTGAGCCCAGCTGAGACATGGGGGCTGGAAAGCCACCACAGTCAAGG-GATATTAGTTGACCTCAGGGGACTGGATGGGGTTT-CTCCTC
Dasypus novemcinctus  ..GTCCAGCAGGAAACTGGG--------CTTAGTGCTCAGGTGAGAGATGGGG-CTGGGAAGCTACTAGGGTCCATATGGTATTAGGTGACCCAATGGGACTAGATGGAGTTTTCTCCTC

Homo sapiens          TCCCATTTTACTCATCCAGGGCAAGGGCTAGGGACAGAGCCAGGTGAATACCAGTATTTAATGAGGGAGGTGGTGGGGGACACCAGCCCGGAGTGAGAAGACACTCTTCTT-TGGACCTT
Dasypus novemcinctus  TCCCATTATACTCCTCCAGTGCAAGGGCTGGGGACAGAGCCAGGTGAGCCATAGCACTTAAGCGGCAAGGTGGTGGGAG-CACTGGCCCAG-GTGAGAAATCACCCTTCTTCTGTTGCTT

Homo sapiens          CCCAGAAACATCATCCCTTTCCCCTCATGA----------TCATGGTCCTTGTTCCAGGGCTGCACTCAGGCCAGTGACAGAAGGAAGGGAGGGACAGAGGGAGAAAGGGAGGAAAGAAG
Dasypus novemcinctus  CTGAGAAATCTTATCCCCATCTTCTCTGGAGAAATCTTATTCCCATCCCCCTTTTCAGGGCTGTGCTCTGTTCAGCAA..........................................

Homo sapiens          GGAGGGAAGGAGGGAGGGGGGAGGAAAGAAGGGAAGGAAGGAGGGAGGGGGGAGGAGGAAGGAGGGAGGGAGGGGGAAGAAGGAGGGAGTGAGGGAGGAAGGAAAGGGAGGAAGGAGGGA
Dasypus novemcinctus  ........................................................................................................................

Homo sapiens          AGGAGGGAGGGAGGAGCTCTGTGCACAGAACTCATGCCTGCACAGGGGCCATGAAGCAAGGACAGGACAGTCACTTCAGCTCTGGAGTTCTTTAGTGTGGGAGTATCTTCTAACAAAACC
Dasypus novemcinctus  .......AGGATTGGGCTCCATGTGCAGAACTCA--------CAGGGACTCTGATATAAGGAAAGGACAAACACTTTTGCCTTTGAGTTCT------------GTCTTCTATTGAAATC

Homo sapiens          TAATAAATTAACAGCTGTGGGTTTGGGGCAGCAAGGACAAAGACCACAACTCTACAGTGACCCCTGGAGCACTCAGTGCCACTGACTGCGTCTCCACTGCTGCAGAGCTGGGAGCACATT
Dasypus novemcinctus  CAACAAATTGATGACTGGGGGTTTGGGACAAAAAGGACGAGAACCACAGCTATACAATGACTGCTGAAGCATCCAGTGCGAGTAACTGAGTCGTCTGCGCTGCAGAACTGTGATTACATT

Homo sapiens          CATGACAGCATTTTT---CAAGCTCCTGTCTGTGCAAACCACAGCTGGTCTCCTAGGAGTCCTAACACTTTAATTAGACCTAGCAGGTCCCAGAACAAAATCCCATAATC-CCCAAATCT
Dasypus novemcinctus  CACAATGGGATTTTTAAAAAAGATCTTGTGTGTGCAAACCATAGCCAGTCTCCTAGGAGTCCTAAAACTTTAATTAGACTTAGCAGGTTCTATAACCAAATCCCATAATCGCATAAATCT

Homo sapiens          ATAGCAACAAGTTAGGCATGAGAGTTTTAGTTACATTCTGGATGTAAGAGAAC-CCTAACTGC------------CACTTTCTTTCTGAGTCTCCAAATGACCTTTGACCAATCTCTTT
Dasypus novemcinctus  ATAGCAACAAGTTGGGCCTGAGACTTTTAGTTGCACTTTGAAAGTAAGAGAGCTCCAAATTGCCGTTTTATCTATTCATCTTCTTTCTGAGCCTCTGAATGACCCTTGACGAGTCTCATC

Homo sapiens          ACCAGAAATATTTATCTAGAATGGCCTTGGTCT---CTGACACCTTGAGGACTTCTACTGATTTCTCTGTTAAATCCCTGGAACCACACCAATAATAGTTCTGAACATTTTACACTCATC
Dasypus novemcinctus  CTCCAAACTATTTTTCCAGTATGGCCTTGGTCTCCCCTGACACCATGAGGACTTCTACTGATTTATCTGTTAAGTCCCCGAGTCCACGCCAATAATAGTTCTGAACACTTTACATGTATT

Homo sapiens          ATCTCATTTATTCCTCACAAATATTATAAACCCTGTTTATATATATGTGTAATATTTATAGTAACAAGAGTAGCAGATA--TTTTGCTGAGGG-----------GAGGCATGGTTCTAC
Dasypus novemcinctus  TCCTCAAATATTCTTCATGGATATTACAGACCCTGTTTATATGAA---ATAGTATTTATAGTAACAATAATAATAGGTAATATTTACTGAGTGCTTAATCTAGGCCAGGCCTAGTTCTAC

Homo sapiens          ACAGGTTACATGAAGTAATTCATAATTCCACATGAACAGCACTATGAGG----TATTATTACTGTCTCCATTTCACAGGCAAAG-AGCAAATCACAGAAAGAATGAGTAAGTTGTTCAAG
Dasypus novemcinctus  ACACTTCACATGAAATAACTCAT----------AACCACCCTCTGAGGTGGTTATTATCACTATCCAAATTGTACAGACAAAGAAACAAATCACAGAGCAATTGAATATATTGTTTTAA

Homo sapiens          GTCACACAGCCTATA-------------AGAGGCAAAGCCT-GCCGGTGTGGCTCCACTGTGCTATTAACCAGTAAGGTCTTCTATAGCCCCAACTAGCTCAAAATGGTTCTAAATGAA
Dasypus novemcinctus  GTCAGACAGCATATAAAGGGCCTGAGCTGAGAGTCAAACCCTGGACAGTGTGGCTCCAGTGTGCAGTTGACCACTGAGGTCATCCTTAGCCCCAACAAGCTCAAAATGCCTCTAGATGAG

Homo sapiens          GAATCGCTGTGCGTTTTGCACCTCAGACTGCTAGCATCGCTGATTTAGGTCTGGAGTTTAGATAAGTGAAA-CTACTCAGGGTCTTTGTGAAGTATCTTTAGAGCTGCTGCTTGGACAAG
Dasypus novemcinctus  GGCTCTCTGTCCATTTGGCCATGCAGATTGGCAGCATCCCAGATTTAAGTCTGTAGTCCAGAGGATTTAAATCTGCTCAGGAT.....................................

Homo sapiens          ACTGGGTCAGGACTTCGAAGGCATTTCCATGTGCTCCTGGAGAGTCAGGATGTAGGCTGGGATTCATTATGCGACCCAGCTGTGTGACTTTCCTCCCCACCCCTTACCCCCATTCACCAA
Dasypus novemcinctus  ........................................................................................................................

Homo sapiens          GATAGAATGCACTGTTTCTTAAGGTGTGGCTTAAGGAGAGCTCTTTAAACATGCAGATTCTAGGGCTGAAGCACAAACCCACTCTGTATCTTTATAGAGATAACTGCTGGAATATGCATT
Dasypus novemcinctus  ........................................................................................................................

Homo sapiens          TTAGTAAACTCTCAGGCTGAATTACTTTGAGAATCATCAGAGTCTACTTTGACAGGATATAGGCTTGTCTTCTGATTCTAGAAGTACCATAAGCCTAACTAGGGATGCTTCCCACTCCTC
Dasypus novemcinctus  ........................................................................................................................

Homo sapiens          TTTCTTGCTGAGCAGAGAAGCAAGGGGGACTGGAATGCAGAGAACATGCTGAGGGCATGCCTGCACAGAAAGCTGAGCTCCTTGTCACCACATACCTTGAAAGAGTGGACTCTGTATCCC
Dasypus novemcinctus  ........................................................................................................................

Homo sapiens          CAGGAGAGGATGGGGAGGGACAGAGAGAGAAAGCTATGGAAAGCCTCTGGAAGCCTTAGTACCCTGGGGCCCAGACAAGGGAGTCCCCTGAGGGAGGTAGAGAGTGGGGCTGTTGGAGGA
Dasypus novemcinctus  ........................................................................................................................

Homo sapiens          GGAGAGCAGCTATGGGCCAGCAGCCTGAGGAGCTGAGAGTCACAGTGGGAGTGACTGTGGCTTCAGAGTGATGCCATAGCTGCAGTGAGCTGTACAGGAACCAGAGACCCAAAGTTTTCT
Dasypus novemcinctus  ........................................................................................................................

Homo sapiens          GTAGGGCTTCTTTTGCTTTGTTAGTGCTTCAGGAGTTGGATCCTTCTGGACCCTGATCTCCCAGAAGTGATTCCATTTCGCCTCTAGGAGGTGGGTAGCAAAGCTCAGTCTGTGGCTGGG
Dasypus novemcinctus  ........................................................................................................................

Homo sapiens          GAAAGCCAAGGAAGTGATTTCACGAATCTTTAGCAGTGAATCCACCTATTTAGTTAGCAGGGGATGGAGCAGATCAGAACTAGCGAGAGGAAAGGCTTAGGAATGCTCTCTAGTGGGACT
Dasypus novemcinctus  ........................................................................................................................

Homo sapiens          GAGAAGAAAAGGTGAGAGGGTGACATTAACTGGAGGAGATGGTGAGCCCCTGACCCCTGCAGTTGTGAGACTTGCACTGAGTGGGATGATCTGCTGCTATAACCTATCCTCAGGCAATGA
Dasypus novemcinctus  ........................................................................................................................

Homo sapiens          AGCATAAGCAGAGAGCCAGGAGTGTTAATCATTGCTGATTTTTATTAAGCATTTCCTTTGTACTGGACACTGTACTGGGCTGTACATGCATCATCACATTCAGTCTTCACAACATTATAT
Dasypus novemcinctus  ........................................................................................................................

Homo sapiens          GATAGCTCCTATTACTGTGCCCATTTTACAGATGAGGTTTAGATAGGTTATACCACTTGCCCAAGGCCACACAACTAGTAAGTAGCAGAGCCAGAATTTGCACCCAAGTGGTTTTAATCC
Dasypus novemcinctus  ........................................................................................................................

Homo sapiens          AAACTGTGTCCACTAGGCCTTAGGAAGGAGCCCACATAGAGCTTTGGGGATGCTGCATTCTTGACCTGTGGCTGTAGGTGGGCAACTCCTCTTCATACCTTATAGCTCTTGGTAAATGAA
Dasypus novemcinctus  ........................................................................................................................

Homo sapiens          GTGAATCACTGTATCTACCATGAATCTCCTTGGGAATGAGTTCAATGCGGGTATCAGAGGAAGCAAAGGGTGGGAAGGGGGTCTCAAAAGTCTAAATCTTAGTGTGGTCCCCTCCTTTGA
Dasypus novemcinctus  ........................................................................................................................

Homo sapiens          CCTACTCTCTCTGGGCTGAAGAAATGCCAGGATTGAGGGGGCAGCAGATTGTTAGATCTGTTTGTTCCATCTACTGAGGCCTCATAACATTTTTGGAGCCCTTGTTCAGTGACTCAGGTA
Dasypus novemcinctus  ........................................................................................................................

Homo sapiens          ATTCAGCCTGAGGGTACCTTTGCAGGGAGTTCCCCTATCTCTTATCCTGTGTTAGTACATGAGATTATTCCTCCTTCTTGTACCCCAGTTACATTAAGTGGGTCTTATTCCCCAAATCCG
Dasypus novemcinctus  ........................................................................................................................

Homo sapiens          TAGTCTCTGCCCCAATAAGTTGCCTGTACCCCATTTTAGCAAGAATAGAATCTTCCCTTTCTGCCAACAGAGCTTCTTGGCACTTTGGCCTTGCCTGGTTCCTGCTTGCAGACTCCTGTT
Dasypus novemcinctus  ........................................................................................................................

Homo sapiens          CTGAGGCTCATGTTTGTACCTAGAATGATTTCATGTTCCTCCTCTCTCTTTGTCCCTCTATCAGAACCTGCTTTCACGATGGGAGATTTAAGAGGCTGATCCTCCCAAGAGGTTGGCATT
Dasypus novemcinctus  ........................................................................................................................

Homo sapiens          TTAACTGATGCCAAAGCCCTGAGTCAAAAGAATTATATACTTAAAAATTTTCAAGCCCTTGGGAGCGGATGGAGAGATTGGAAAGGCCAATGGAACATTCTTAATGCTCCTGGAATGCCA
Dasypus novemcinctus  ........................................................................................................................
```

```
Homo sapiens          CAAGGTGGACATAAAATTCAGGCTTCTGCCAAGACAGGACTTGGATTATTTTTGTTATAATCAGAATCCGTAATCTGCCCCCACCCCAGCACTCAGGGCTTTGATCAAAAGTCTTCCAGA
Dasypus novemcinctus  ........................................................................................................................

Homo sapiens          CAATGAACCTCTATGATCCAAGTATCATTTTCTCAGAGCTCCTTAGAAGGAGGAGAGCTAAGAAACTTCATGAATTACCAAGGACCAAGCAAACAGGCGGTAGTGAAGTCAGAAACAAAT
Dasypus novemcinctus  ........................................................................................................................

Homo sapiens          TCCTCCCGAGACTAGTTTTGTCCTATAGCCTGAAAACAAATCCGGTCTCCACCAGACATAAGGTGGAATTCCTGTCTTGATTTTTTTTTTAGCACTGATATTAACATTTTTCTTCTCAAA
Dasypus novemcinctus  ........................................................................................................................

Homo sapiens          TCCTCTTCAGAACCAGCCCTTTTAGAACACAAATTAGAAGCTGTTTATTTTGTCAATCAGAGAAACTGAAGGCAGCCAGCCTTGGCCAAAGGAACATCTTATCCTTTGCCTTTGCTAAGG
Dasypus novemcinctus  ........................................................................................................................

Homo sapiens          ACAATGTTCCTTCTATTGTGCTCTGTGGAAATAAAAGGGCAAAATATGCTTGCCTTCTTTTATTCTTATTCATGGCTGAGGGTCATATGAAAGTTCAGAGAAGAGCACAGTTTTTAGTAT
Dasypus novemcinctus  ........................................................................................................................

Homo sapiens          GGGTCGTGGCAGGTTTTCCAGATGGCTTCCTAGCCAAGATATTTCTCCCGCTCACCTCCAAACAAGTTATGTACCCCTAAGAATGATTTGGTCTCAGTGTTAGGGGAAGAGGGTAGGTAG
Dasypus novemcinctus  ........................................................................................................................

Homo sapiens          CAGGGCCTGGCTTTGTCATCCTGGGCTATTTTGAGGGGACTTCAGCAGTCCCCGACAGAAGGACAAGAGGGGCTGATTCTCCCTTTGTGAGCAGCCATTGGAGCTGCTTCAGGGACTGAC
Dasypus novemcinctus  ........................................................................................................................

Homo sapiens          AAGCCGGCCAGAGACAATGTCCTCTCTCAGGAAAGCAGTAAAAACATCAGAAATGCTCCTTTCCTGGGGAGCCCTTAGTTATTTTGGCCTCTCTTTCCATTAAAATAGGAAAGCATTGAA
Dasypus novemcinctus  ........................................................................................................................

Homo sapiens          AGGGACCATTCGTGGGATAGCGTGGAGCCTCCCCATTGTGGCAGCATTTCTAACTGCGTTGAGAATTAACCATATTGCTCTTCCTTTTTACCATGGAGGAGTTTCTAGATTTTTATTTAT
Dasypus novemcinctus  ........................................................................................................................

Homo sapiens          ACCCTACCGCTTAAAACATCTTTCACTGTTTCAGCAAATTGCAGCATAAAAGATGGAGAAGCCAGCAGGGGCTGTGCTCACAATAATAGCTGTGGTTGCATTTAAGACATTCAAATGCCA
Dasypus novemcinctus  ........................................................................................................................

Homo sapiens          AGGGTGCACTTCTCCCATTAAGTCCAACATAAGAAATCAGGTGTGCACAACATTGATGAACAGAAGACCCCAAAGGACAGGTGGAGTTTCTGGTATGCTGTGGTGGGGGCGGTGGGGTGG
Dasypus novemcinctus  ........................................................................................................................

Homo sapiens          TGAGCTTGGGAAAGAACGAACAGGCATTTTCATTTTCCAAACTAAGAAGCAGTTTCGGGCGAAGGGGAGCCGTGGGGATGTCAGAAATTCTTCTAACGCCATGGACCTGCCCACACCTGA
Dasypus novemcinctus  ........................................................................................................................

Homo sapiens          GTCAGATAAAGATGACCTTCCCTAGGCCCTGCTTTCCTCTCCCCCTCCCCAAAAGACAGCATGAGCATGAGTAAGCCGGGTACTTACAGGTGCAGAAGAATTTGGAACAGGAAAATGGAG
Dasypus novemcinctus  ........................................................................................................................

Homo sapiens          CTTACAGGCTCCCGAATGCCTGCCGAGTCAGTTGCATCAGATTAGGAAGTGATGATCCTAAGAATAAGATGTTTGGAAAATCTTGTCCCCCGAAAGCCCCCTGCTTTGTCTTCTCAGCCC
Dasypus novemcinctus  ........................................................................................................................

Homo sapiens          ACATCCAAACTCAGACCACCCCCAGGATGTCTCTCATCCTGCCAGACTGCCTGGTGCCTCCGTGCCCCTTACCGTGAGGGTGCTGGCAGCCACACTGCTTCTTTGAGCCTGTGGCCCGGC
Dasypus novemcinctus  .............................................................................................ACTGCCTCTTTGAGCCTATG------C

Homo sapiens          AAGGTAGTCAGGCTCATGGGATTAGGTTGGCAGGAGCGACTGTCCTCTAAACGATTAGCCCAGAGAAAGAGAGAGAGAGAGAGCAAGCAAACCCGAAAACGGGTCCAGGGTAGAGAGCTC
Dasypus novemcinctus  AAGGTAGTCAAGGTCTTGGGATTAGGTTGGCAAGAGCAACTGTCCTCCAAGCAATCCGCCCAGAGAAAGAGAGAGAG------CAAGCAAACCCAAAAACTGGTCCAGGATGGAGAGCTC

Homo sapiens          CAGGTCCTTAGCATGTTCCTGGCGTATTCCAAAAGTGGCATCCACAGCAGAGAAAGTGTCCCAAAATAGCTCAGGCTGCCAATGCCTTGCTGAAACCTGAGAAAGAATTGCTGAATTTAA
Dasypus novemcinctus  CAGGTCCTTGGCTTGTTCTAGGCGTATTCCAAAAGTGGCATCTGCAGCAGAGAAAGTGTCCCAAAATAGCTCAGGCTGCCAATGCCTCGCTAAAACCTGAAAAAGGACTGCTGAATTCAA

Homo sapiens          AGATCTGGGTCGCTGTGGGTGTGTGATCAAAGTGCAGATTTGTTGGTAGGAGGAAC------TCAAGCCATGAGCTTAGGAGTGACATGAGGTCACT-TGGGTCACGGCCTGCCTTCTTG
Dasypus novemcinctus  AGATCTGGGTTTCTGTGGGTGTGTGATCAAAGTGCGGATTTGCTGGTAGGAGTAACCTAGGCTCAAGCCATGAGCTTTGGAGCAACATGAGGTCATTCTGGGTTATGACCTGCCTTCTCG

Homo sapiens          TTTCCAACTTGCTGAAGTTGGTGGCAGTGTCAGAGAGCTCCTCTGTGGTGAGGCAGGGGTTTGGAAAGAGGTCCTAAGGATGCTGTTTTCATGCCCAGGTCTCTGATGCGCTAACAATAT
Dasypus novemcinctus  TTTCCAACTTGCTTAACTTCGGGGCATTATCAGAAAGCTCCCTTGTACCAAGGTACGGGGCTGGAAAGAGGTTCTGGAAATGCTGTTTTCATTTCCAGGTCTCTGATGTGATAACAATAT

Homo sapiens          GAAATAGCAGTGCCCTGTTTTCTGCATTTCAAACAAAGGAACTGATACTATGCAAAGGTTTCTTTAGGTTATTGCTCCATGTCTGATGAAATTCCCTTTTCAGTAGATTTTTCATTTCTT
Dasypus novemcinctus  GAAATAGCAGTGTTCTATATTCTGCATTTCAAACAAAGGAACTGATACTGTGCAAAGGTTTCTTTAGGATATTGCTTCATAACTGATGACATTCCCTTTTCAGTAGATTTTTCATTTCTT

Homo sapiens          TTGGCCTTTGGCATAAGTAAATGTCAGCTTCTTAAAAATCAACAGAGAAGAGCAAACAGTAGGGTCTCTGTGATTTTCTTGGGATTTGAGAAATCTTCCCTCCCTAGCTTCTAACTGGAT
Dasypus novemcinctus  TTGACCTTTGGCATCAGTAAATATCAGCTTCTT-AATATCAACAGAGAAGAGCAAACAGTAGGGTCTCTGTGATTTTCTTGGGATTTGAGAATTCTCTTCTCCCTGTCTTCTGACTCGAT

Homo sapiens          CATGTCTGTACATTGCTGCCTGTGGTCCTGGAAAGGACGAGGTCCACCCAGACATGCTGTCTCATTTTTATATTTGCTGAGATCTTGGGACACTCTCCTTTCATCAGGTATGTCAAAAAC
Dasypus novemcinctus  GGTGTCTGGGCACTGTTGCCTGTAGTACAGGCAAGGATGAAGTCCACCCAGGTACTCTGTCTCA---TTGTATTTGTTGGGATCTTAGGATATTCTCCTTTCATCCTTTCCAT-GAAAAG

Homo sapiens          CTTAGGATTGTCTGCAGAGAGTGTGGTTCAGTATGTGGAAAGGTCAGCATATGTCATTCTTGGACTGGCCCTGGGGTTTAA--GAAGAAAAAGCCCAGAATTTGGTTTCCAA-TTCCAGC
Dasypus novemcinctus  TTTAGAGTTGGCTGTAGAGAGTGGGGGTCAGGATGTGGAAAGGTCAGGATATA-CTTCCTTGGACTAGTCCTTGTGTTTAAGGGAAAAAAAAATAGAAAATCGAGTTTCTAAGTTCCATA

Homo sapiens          TCTGCCTGTCTTTTGCTGTATGAATTTGGGCAAGTCACTTCTCTTCTCTGGAC--CTCAGTTTCCTCTTCTGTAAACTGAAGACAATAATCTTCAT-TCTAATTCTAACCCAAATGTTGT
Dasypus novemcinctus  TCTGCCTCTCCTTTGCTGTGTGAATTTGGGCAAGTCACTTTCCTAAGGTGGACCACACAGTTTCCTCGTATGTAAA--ACAGCCTTTTATGTCCCCATCTACATCAAACCC-AATGTTGT

Homo sapiens          AAAGAGTAACTCAGAGAGTTGAAAGAATGCTATAAAGGGCAGGGTTTGGCACTCTCAGTAAGTGGTGACATTTTTCAGTTCTTCAAAGGTCCCTGATTGGTAGCCACTACCTGCATCCAC
Dasypus novemcinctus  A.......................................................................................................................

Homo sapiens          ATGCCTATGGCGAGCCCAAGACATCCCACAGTACTAGGCACTATGAGCTTCATGTCTAGGAGTCTAAATAGTTTCCACTTTCA-GTTGTGGGAATTTTGGCAAGTAGCTCAATGCTTAGT
Dasypus novemcinctus  .......................................................................TTTCTCCTTTCAAGCAGTGGGACTTTTGGCAAATTGCTTAATGCTCAGT

Homo sapiens          AGCTCAGTTTTCCCATCTGTCAAGTGGGTATACAAACTAATAATAATACAGGTCCACAATCCCTTATCTGAAACTGTTGGAGCTAGACATGTTTTACAGTTCAAAATATTTGGATTTTAG
Dasypus novemcinctus  ACCTCCATTTTCCCATCTGTCAAATGGGTATGCAAATGAATAATAAT-CAGATCCCAAATCTCTCATCTAAAATCTTTAGAGCTAGGTGTATTTTGGAGTTCAGAATTTTTGGATTTTAG

Homo sapiens          AAAAGCAATGTGGTAGATATAACATATAT-----ATCACACCTTAATGAGATCCAAGATAATACCCAGTGATCAAACTCACTAATATTTCTAAATGGGATAAATGAATAGCCTCATGTCA
Dasypus novemcinctus  AAAGGTAACATGGTTCTTATGACATATATTGTGTAACCCACCTACA-AAGTTCTGGGGCAGTACATTGTGATCAAATGCATTCCTATTTCTATATGGGATAAAAGTAGAGCCTCATGCCA

Homo sapiens          ATTCAGGCCCAGTTTTGCCACCAAATGAGCTTGCCACAGAATTTCAGTTTTCAGAAGCTTTGGGATATCAGAGAGGCACATAGGAAATTGTGGACAATGAAGGAAGTTAATACTTATTTC
Dasypus novemcinctus  ATTCAGATCCAGTTTTACTACCAAATGACCTTACCATAAAACTTCATTTTTCAGAATCTTTCAGATACCAGAACGATAGATAGGAG-TTGTGGACAACGAAAGAAGCGAATA--TATTTT

Homo sapiens          GTGCATGTAATGTACCAGGCATTGTTCTAAG-------TATACATTAACTCATTTAAGTGGCCCAATAAGCCTGTGAGGTAAGCAGTATTATTATTATCATCCCCATTTCATAGATGAGA
Dasypus novemcinctus  GTGC-----------CCAGCTTTGTTCTAAGAAGTTTATATATATTAACTCATTTAGTTGGTCCCTTAACCCTGTGAGGTAGG---TACTCTTGTTACCATCCCCATTTTATAGTTGAGG

Homo sapiens          AAACTGAGATTCAGAAG--TTAAATCACTTGCCCAGGGTCCCAAAGCCAGTTAGGAGCAATGCTGGGATTCCAACCCACACAGTTCAGCTC------------TTATCCACCTTCTCTGC
Dasypus novemcinctus  AAGTTGAGGTTCAGAAGGGTTAAATCACTTGCCCAAGGTCCCACAGCTAGGAAGGGACTGAACTGGGGTTCCAACTCAGACAATCCAGCTCAAGAATCTGTTTTAACCACCTTTTCTAC

Homo sapiens          CTAACCCATGGGGTTAAATTGAGGCTTTAAATGCCTACAGGGACTGAGCAGGTCGC--TGAAAAAGTAAAGGTGGCCTTATGGG--TACTGAGGCAAACTGGTGAGCACGTGCCTCACCT
Dasypus novemcinctus  CTATTCCATGGGATTAAGGTGAGGCTACAAATGCTCACAAGAGCCA---AGGTCACTGTGAAAAAGTAAAGGTGGCCACGGGGGGTGACTGTGGCAAACTTGTGGACACATGCCCCATCT

Homo sapiens          ACAGGGAGCAGGTTGTTGAGCTCTGGCTGATTGTTGCTATATTGGAATTTGGATCGAAGTCTTTCTCTCTCTCTCTCTCTCTCTCTCTCTGTGTGTGTGTGTGTGTGTGTGTGTGTGTGT
Dasypus novemcinctus  AAAGAGAGAGGCTTATTCAGTTCCAGTTGACTGTTGCTATGTCAGAGTAAGGATCTAAGTCTATTTCTC...................................................

Homo sapiens          GTGTGTGTGTTTCAGAAAAGCCAGAAATCCAGCTTTCTATCTGAAATTTCCTGACTTTTAAATGTTGGCAATTAATTTTAAAAAATGTTTAAAACGTTGTACGGGTCAAATAAACTCTGT
Dasypus novemcinctus  ...........CCAAAGAATCCAGAAATCCAGCTTTTTATGTGAAATCTCCTTACTCTTAAAGGTTGGTAACTAATTTTAAAA--TGTTAAAAACATTGTGTGGGTCAATTAAAATACAC

Homo sapiens          CTGCAGTCTGAATTTAGCCTGTGGCTGCCAGTTTGCAAGCTCTGCAATGAGTAAATAGATGTGAAAGTGCTTTGGAAACTTAAAAACGTGCTCTAGAAACAGAAGAGTCTAATTTTAGGG
Dasypus novemcinctus  CTGCAGTCTGCATCCAGCCTGTGGCTGCCAGTTTTCAAGCTCTGCAATGAGGAAATAGATGTGAAATTGCCTTGGAAAT--------GTGTTATCGAAACCGAAG-GTCTGGTTTTAGGG

Homo sapiens          TTATATCTTGTTTTTT------CCTTGACTGTGGTCTAGTTTTTTAAAAACTTGTATTCCCGACCTGATGGAGATGAAAAGTCAGAATTAAATAGAGACTTCAGTGAACTTGAGTACTTC
Dasypus novemcinctus  TTAAGTTTCTTTTTTTTTGCCCCCTTGACTGAGGTCTCGTTTTTGAAAACTGGGTGTTCCTGGTCTGATGGAGATGAAAAGTCAGAACGAAACAAAGGTATCAGTGAACTTGAACACTTC

Homo sapiens          ATTCCTTTGGCAGGCTGGAT-AAATGAGAAGCCTAGGTGTACTGGATGGACTAAGCCTCAGAATTCTTGCCACACCCACAAGCCCCTTCTAAGGAGTTTCCATCCACAGACCCTAGCTGA
Dasypus novemcinctus  ATTTCTTTGGCAGACTGGATGAGAAGAGAAGCCTAGGTGTACTGAAATGGCTAAGCCTCAGAATTCCTACCACACCCACGGACCCCTTCTAAGGCATTTCCATTCACAGACTCTAGCTGA

Homo sapiens          GGGAAGTAGCAGTTTCTTATGAATAGGTGACTATTCTTGGTACCACGTGACCCTGCTTCCC-CCAGCAATAGCCACTTGAGCCAGGAGAGGGCATCTGATCCATGAGCAGTCACTCTATA
Dasypus novemcinctus  GGGAAGTAGCAGTTTCTTATGAA----TGGCTGTGTTTGGGACCACATGGTCCTGCCTCCCACTGGCAGTAGTCGATTGAGCCAGGAGAGGGCATCTGACCCAAAAGCAGTTGCTCTATA

Homo sapiens          GGCTGGCAAACAGCCTATGAAGGAACCTGGGATGAGAACTTTGCCCAGCAAGTACAG---TTACTGAATTTGGACCAATGAGAGTTTCTCTCTTGGGGAGTTTGAATACCAGACCTAGAA
Dasypus novemcinctus  GGCTGGCAAGCAGCCTATGAGGGAATCCCATGCAAGCACTTTGACCAGTGAGCACAGTGATGACTAAATTTGGATCAATGAGAGTTTCTCTCTTGGGGAGTTTGAATACCAGAACTAGAA

Homo sapiens          AGAGTGAGCAATTGGCAACAGGGGCAGAAGCTGAACACAGAGAAGGCAGGCAGCAGGGACCCCGGATAAGCAGGACCAGGAGAGATTACTGAACTTCAACTATGTGCCAGATTCTTTACA
Dasypus novemcinctus  AGAGTGAGCAGTTGGCAACAGGACCAGAAGCTGAATACAGAGATGACAGGCAGCAGGGTCCATGAATAAGCAGGACCAGGAGAGGTTACTGAGCATCAGCTAAGTGTCAGACACTTTACA

Homo sapiens          CGT-TGTTTTGTTTAAAACATTCAAAGAACATGCGAGGTAGGTACTGTTATTATGCCTGTTTTACAGATGAGGAAACTGAGGTGAACTAACTTGAAGTATTGAGGTTCAAGCCCAGGCCT
Dasypus novemcinctus  CACATTGTTTGTTTAAAATATTCAAAGAGCCTGTGTGGTAGATACTGTTATTATCACTGTTTTACAGATGAAGAAACTGAGGTGAAATAACCTGTCATGCTAAGGTTTCAGCCCAGGCTT
```

Homo sapiens GTTTGAAGTTAAAATGAGAGTGTAGGAACCCCAAAGCCAGTGGTTCTGAAACTCTA--GTGTTTCAGAATCACTTGGGTTAAATGCAGGTTCCTTGGCGTCACCCCAGACCTATATTGAA
Dasypus novemcinctus ATTTAATGTTAAAATCTGGGTGTATGAATCCATAAGCCAGTGGCTCTTAAACTTTATTGTGTCTTACAATCATTTGGGTTAAATGCTGATTCCTTGGTCCCACCCCC-ACACATACTGAT

Homo sapiens TCTGACTCTTTGTGGGTAGAG-TCTCCCTCCCCCTACAAATCTGTGTTTTAGCAGTTTTCCCAGGGAGCACTTGATGCAGGGTTTCCCCACCACACTTTTATTTTGAGAAATGCAGTTTC
Dasypus novemcinctus TGTGGCTCTGTAAAAGTGAAGATCTC-----------AAATCTGCATTTTAACAGTCCTCCCAGGGGTCAGCTGATGTAGGGTGTTCCC----CAACTACACTTTGAGAAGTGCAGTTCT

Homo sapiens AGAGGGTATGGCAAGTACTGTGTACTGTGCTACCTTTACCTACACAACCAAGGTCAATGTCAACAGATGTGGTCAAATTCCAATTTAAAGTCTTTTGGAAATGGGGCCAATTATGTAAAG
Dasypus novemcinctus AGAGGCTACTGCAAGTACTGAACATTGTATTACTTCACATTTGGAAATGAGGTCTAGTTCAACAGATGTGGCCAAACTCCAATTTAAACTCTTTTGGCAATGGGGTCAATTCTGTGAAT

Homo sapiens AAGAGGGCATTAGTTTGTTCTCTTCTCTCATCAAGGAAGTCT---ACATTATTACCAGTTATTTGAGAGAAC---AGAAGATTGGGATGAATGCTTAGTAGTT-GTAAACACGATCGGAT
Dasypus novemcinctus AAAAGGACATGACTTTGCT-TCTTTTCTCATCAACCAAGGATGATACATTGGCTCCAGTTATCTGAGGGAGGGTGACAGGATTCAGATGCACACTTGGCAGTTTATATGAAGGACCCTAT

Homo sapiens CTGCACTTCAGGGTG---GGTGCAAAGCTAGCATATGTTCACTAAGCATTTTCCATGAGGTGCGTAAACTGTAGAAACTCAGTGCTCTGGCAACAAAGAACATGAGAGA--AAATTACCA
Dasypus novemcinctus GTGCTCTTCAGGGTGCTGAGCTCATAGCTACCATATGTGCACTGAACATTTTCCATGAAGAGCACCAATTGCAGGAACTTAGTGA-CTGACAACAAATAACATGGGAGGGTAACTTCTCA

Homo sapiens AATGCAGATAGACATATTGTAAACCGTGGAGCTTAAAATGTAAACCTACTTGCTAGGCTGATCATAGAGGTTCTTCTCAGCCCCAGAGAATTTGTGTAAGAATCCTGTCCATGCCTTTAT
Dasypus novemcinctus AAAACAGAAAGACGGATTTGAAACCAATGGATTTAAAATGAAATCCTACTTTCTAGCCTGATCATGCATGTTCTTATCAACGTTAGAGTAATTGTGTAAGATTCGCCTCCATGCCCTTAT

Homo sapiens TTACACCATTGATTAAAAATGCTAATCAGGAGAGGATCAAGGCT------------ATG--------GACCTCCTTCTAGGTTGTTGCAGAACTCCCACTCTGGCAATGGTATTCAGCT
Dasypus novemcinctus TTATGCTATTGATGAAAGG-GTTGACCTGGATAATATCAAGGCTGGAAAGGTGTGGCATGCCCTTAGTGACCTTCTTC-AGGTTGTTTCAG-----------GACAATGGTGCTCAGTC

Homo sapiens GGTTTTGAATTTTTCTGCTT-TAAGAACTATCCCTGGGAAGAC---TAGTGGTCTGAAGTTTGCTTGGAGAGTCTCAAGCCGGAAACTGATTTGTGGT-CCAAATGCTTAATGGCATCAG
Dasypus novemcinctus AGTTTTGAATTCCTTTACCTCTAGGAAATCTCCCTGGGAAGACAAATAGTAGTTAGAATTTTGCTTGGAGAGTCTCAAATAAGAACCCGGCTTGTGGTTCCAAATGCTTAATACTATCAG

Homo sapiens ACACTTATTCCTACAAGAGAGGCATGTGGTCCAAGGGGTGGCACAGGTCAAATGTGATGGCCAAGTCCCGCCTCAGCAGGGCTTGAATCCGCACTTGGCAACTTTTATTTCTCTC-TGTC
Dasypus novemcinctus GCATTTATTTCTACAAGAGGGCTGTGTGAACCAAGGGTTGGTCGAGGTCAAATGTGTTGACCAAATCCCGTCACAGCAGTGCTTGAATCCACATGCGGCAACTTTTATTTCTCTCATGCC

Homo sapiens CATTCCTGTGGATACTTT----ATCTGTGTTAATGTCCTCATTTAGGCTAAGGTCAAACTCTGTTCTTCTCTCTTCTGGCCAGAAAGACTTCCTGTTTTACAGGTATATGATCTTATCTG
Dasypus novemcinctus CACACCTATGGATACTTCTTTACCTATATTATTGTCCTCACTTAGGCTAAGGTCAAACTCTATTCTTCTCT-TTCCGACCAGAAAGACTTCCTGTCTTACAGGTATCTGATCTTATCTG

Homo sapiens TGTTGATACCTTAACTGTGAGTGATTCCCTAAAGCAGGTCCTTGGACAAGTGCCCATCCTCCAGGAAAATTGCAGCAGTCCACCGTGCAATGAGAAAGCCTGAGGACTAGGTAGTAAATG
Dasypus novemcinctus TGTTGATATTTTAACTCTGAGTGATTTCCTAAAGCAAGTCCTTGGACCAGTGCCCATCTTCGAGGAAAACTGTAGCAGTCCCTGGTACAATCCAAAAGCCTGAAGACTATGTAGTACATT

Homo sapiens TTTCGCAGAGAAGAGAAATCTATTTCAT-----GTATTATTCTTTATTCTGAGGTTATTTCCTTCTCTTTTGGTGTTGAAAGGAAGCCCCTTCTTTTTAGAAAATGATGATTTGTGGTTG
Dasypus novemcinctus TTTTT--GTAAAGTAAAATCTATTTAACTTAAAGGACAGTCCTTTATTCTGAGGTTATTAACTTCTCTTCTGGTGTTTAAAGAAA-TCCTTCCTTTTTATGAAATGATGATTTGGGGTTG

Homo sapiens TTCTGT-----TTCTTAATGATTTTACTTGGGAAAATAAAAATCTGGAAGTTCAATATCAGTCCCTGAAATTTTGGGTTGCATTAGCTGCAAGTCTGGGAACCTGGTCTAATTTTGAGTG
Dasypus novemcinctus TTTTGTCTTGATTCTTTAAGTCTTTACTTGGAAAAATAAAAACCTGGTGATCCAATGTCAGTTCCCAATATCTGGGTTTGCAAGAGTTTCAAGTCTGGGAACGTGGTCCTATTTTCAGAG

Homo sapiens TTCTGCTAGTACCTCTAGGTACCTCCTATTGCCACTTGTGTTCTAAGTGCTTATAAATTACCTGCTTCGCAATCTGCAGCAGAATCTCTCCGAGGATTGACATCAAGCTTACAGCTTTGG
Dasypus novemcinctus TACTGGGCATAC-TCTAGGTACCTCCCATCACTACTTATTTCCTAAGAGCTTATACATTACCTGCTTCATAAT-TGTGGCAGAATCTCTGAGGATTGATACCAAGCTCACAGCTCTGA

Homo sapiens AGTTTACGGTATACTGGTCTGTAGTTTCCTTATTTAAGAAAATTGGGACACTGCCCATTTGCAGTCCTCTGGTATCCCTCCTGTTCTCTCTGATTCCACAGATCCTTCAGACCGGCACCT
Dasypus novemcinctus AGCTTATGGTATAC-AGTCTGTAGTTTCCCTATTTAAGAAAATTGGGACGCTACCCATTTGCAGTCTTTTGGTATCCCTCCTGTTCTCTCTGATTCCACAAATACTTCAGGACAGCACCT

Homo sapiens AATGCAAGGCAGTTCCGTTTGCTCTTCTTATAATGTTCTTTTCCCTATAGAGTTGGACATGCATATACATGTTGTTTGGGGAATTTGCAGCACATCCAATGAAAGCATAATTCCCAACTA
Dasypus novemcinctus AATGCAAGGCAGATTCGTTCGCTCTT---ATAATGTTCTTTTCTCATCTGTAGAGTTGGACATGCATATACATGTTGTTTGGGGAATTTGCAGCACATCCGATGAAAGCATAATTCCCAGCTA

Homo sapiens ACAGTCAAATCCTTTTCTTTGGAGCTACCCAGAACACACCTACCCTCTCTTCCATATGACAGCCCTTCAGAGACTTGAAGACAGTGT--GTGACTCTCCTGTGAGTTAAGAGCACTGGCT
Dasypus novemcinctus ACAGTGAAATCTTTCCCTTTGTAGCGACTCAGA-CATTCCTACCTTCTCTTCCACATGACAGCTCTTTAGAGACTTGAAGACAATATTGGTGACTCTTCCAAGAGTTAAGAGCACTGGCT

Homo sapiens GAGTCAGACAGACCCGAGTGACAAGCCCAGCTTCGCTCCCTCTCAGCGGTGAGAGCCAAGGTAAGTTACTTCATTTTAATTTCTCTCTAGTTCAGTTCTTTAGCTGATTTAGAAAATGGG
Dasypus novemcinctus GAGTCAGACAGTCTTGAGTGACAAGCCCAGCTTCACTCCCTCTCAGCTGTGTGAGCCAAGGTAAGTTACTTAATTTTAATTTCTCTCTACTTCACTTCTTCAGCTGATTTGTGAAATGAG

Homo sapiens AGTAAAAAAGCC--ACTTTGTTTGTTTATCGTTCAATAAATAAGATGAGGTATGTAAAGTGTTTAACATAGTGCCTGGAATAGTAAGCACTCAGTAAATGCTAGCTATTTCCCCCTCTTT
Dasypus novemcinctus GGTAAAAAAAACCCCACTTCATTGGTTTATTGTACAGTAAAAAACAT----TATGTAAAGTGCTTAACACAGTGCCTGGAATAGTAAGTACTCAATAAATGTTAGCTATTTCCTCCTCTTT

Homo sapiens ATTTATAAAGAGCCG-GTGCGCATTTTAAGTGTCAGGGCTGTGGGGGAGGAAGGAAGTGGGGAGGGCTTCTACTTCCCTGAATGATCAAGGAAGGAAGAAGATCCTAAAGTAGACCAGGT
Dasypus novemcinctus ATTTATAAAGAGCTGAGTGGGAATATCAAGTGTTGGGAATGTTGGGGATCTGGG----GGAGAAGGTATCTCCCTCCTTGAATGGTCAGGGAAAGCAGAGAACACTGATGTAGACCAAAC

Homo sapiens ATTTCTTTAGCCTTAGGTTGTCTGTTTTCAGTTATCTGGATGCCTATTTGCTAGGACTTGGGGAAAGAATATTGTTGAGAATCTAGGGAAAGGCTTATTTTCAACAGCTGGAAAGCT--A
Dasypus novemcinctus ATTTCTTTATTCTTAGGTTGTCTATTTTCAGTTATCTGAGTACCTGATTGCTGGGAATGGGGGAGAACATATTGTTGAGAACCTAGAGAGAGATTTATTTTCAAGAGATGAAAAGCTCAA

Homo sapiens TTTGTTGACATATTGAACCGTCTCTTGTTTATTCTTATTTCCCTGAGCAGCTAGCCATGCAATCCCCTAAACTCAATTACCTTTTGTGAGGCGGCAGGAGCATGACACCTCCTAGCCGTA
Dasypus novemcinctus TTTGTTGACAAATTGAACAATCTTTTGTTTATTCTTATTTCTTGAGCAGGTAGTCATGCAATCCCCCAAACTCAATTAGCTTTTGTGAGGTTGCAGAAGCATGACACTTTCTAGCCTTA

Homo sapiens TTGATCAGTGCAGACTGGGTGGGACGTGAG------GGACCTGAGAGATGCTAAGCGGTAAATTCTGGGCTTTTCCTTCCTTAGAAGGCCTCTCTTCTCTGTGTTCCTAGAGACTCTGGC
Dasypus novemcinctus TTGATCAGTCCAGACTAGGCGGGACTTGAGCGACCTGGACCTGAGAGATGCTAAACAGAAATTTCTGGGCCTTTCCTTCCTGAGAAGGCCTTTCCTTACTGTGTTAATAGAGACTCTGAC

Homo sapiens TTTAATAGAACGGAAGTCTATGCCTCACATGGGCTGAGCATGCAAGAACCCTCTCTCAGTAGCCTCCTTAGAAGCAGGCTGATGA-AAATAGTCACAATGGGTCAGAACTTGGAACCCTG
Dasypus novemcinctus CTTAATAAAACAGAAGCATGTGCCCTAACTGGGCTAACTATGCAAGAGCCCTTTCTCAGCAGACTCCTTAGAAACAGGCTGATGATAAATGGTCACACTAAACCAGAACTTAAAGCCCTG

Homo sapiens GGGTGACCAGTGCTAGCATGGTTTTCTAACTGCAGCTCTTTGCACACACTGGGTTTTGCAAGCAATTGAATGATATGAACTCTGAATGCCATTGCAGCTAGAGAAAGTTTGGAAGGAGAC
Dasypus novemcinctus GG-TAACCAGTGCAAGAAGAGTTTTCTAACTGTAGCTCTTTGCACACACTGGGTTTGCAAGCAATTGCATGATATGAATTCTAAATGTAGCAGCAGTTAGGAAAAGTTTGGTAGGAGAC

Homo sapiens ACACCCAACTGTGAACAGACAGAGGAATGGGACTTGCGGGGAATGGGGGGTGGGGCATTTTCAGGAGTTTAGCTTTGTCTGTACGGAGGATTTCAAGTCTTATCCTAAGAATATATTCC
Dasypus novemcinctus ACACCCAATAGCTAACCGAGAGAGGAATGGGACTTTGCGGGTGGGAGGAGTAGGGTAGTTTCAGGATTTAGCTTTATCTGTCT----TATTTAAAAAATTTATACTGAGAATGTA-GCC

Homo sapiens TAGATTACTTATGGAATTACTAAACATAAATAAAAGTTAGAGAAAGAAGAAATTATTTTTAAAAACAAAC--CAAAAATAAA---------------------------------ACACA
Dasypus novemcinctus TAGATTACTTGGGTAATTTTTAAGCATAAATATAAGTTGAAAGTTAAAA----ATGCTTAAAAATAAACAACTAAAAGAAAGCCCTTGGAGACATTTTTTGGAGGTCACTGCTGACCCA

Homo sapiens AAATA-AAGTGGTACAACCATTAAGCTTGTCGGGGTAGAAAGATTGCTTGGGTCGGGGGGTGGATGTCCGGGATTCCTAGAGAGAGCTTGCAAGGAGAGGAGTGAAAAGGGCTTGAGAAA
Dasypus novemcinctus ACATACAAGTGGCACAACCATGAAGCTTGTTGGGGGAGAATGGTTGCTTGGGT-----GGGGGATGTTGAGGGTCCCTAGAGAGAGTATGCAGGAAAAAGAGTGGAGAAAGCTAAAGACA

Homo sapiens GAACCCTAGGGAAACATATCTTTAAAAAAAATAAAATTAACTTTTATTAGTTTTTTTCTTTTC------GTATTATGGGTCCATTATAGACCAATGAGAAAATACAGATAAGGAAAATGA
Dasypus novemcinctus GAACCCTAGGGAAATACAATTT--AAAAAATTAAAGATAGCTTAAT--GTTGTTTACTTTTTATAAAAGTAATATGGCTTCATTATAGACAAATGAGAAAATGCAGATAAGGAAAATGA

Homo sapiens AAATTAAAAATCCCCTCTAATCCCATCATTCAACTGTGGTCACTGTTGGCGTTTTGGTATATATCCTTCCAGATT---TTTTTCTATGCAGAAATCTACATATTTATCTTTATGATAAT
Dasypus novemcinctus AAA-TAAAAATCCCCTTTAATCCCACCACTTGGGTATGGTCAC------ATTTTTCATATATATCCTTCTAGACTTTATTTGTTCCATGTA-AAATATGCACATTTAT-TTGAGAAAAAT

Homo sapiens CA--GATCCTCATGTACACATTAGAATA-TATTTTCAATGGCTACAATAGTGGCTGCTACAATTGCACCATGATTCGTTTAATC-ATTAAACAAGTTGTGTTCTAACATTAGACATTAG
Dasypus novemcinctus CAATAATCATTAAGTACACACTGGAACAGCATTTTCAATAGTTACATAAATGGCAGCTACTGATGCATGCTGATTCAGTTGCTCAATTAAACGAACTATAGTCTAATATTGGATGTTTAC

Homo sapiens GTTATTTCCAAAATTGTTGCATTAGAAATAAACAACAGTGCCCTGTAGCTGTGTGACATTGGGAAAGCTAGTTAACCTCTCTGTTCCTCTG--TTCTCATCTGGAAAATGAAGGTGCTAA
Dasypus novemcinctus ATTATTTTCAATATTTTATTATCAT-----AACGACAGTGCTTTCTAGATGTGTGACCTTGGGAATATTAGGTAAACTCTCTGTGTCTTTAGTTTCTCATCTAGAAAATGAAGGATATAA

Homo sapiens TAGAACCTACCTCGTAGTATGTTGTGAGTGTCAAATGAGATCATATAGGTAAAGCACTTAGCACAGGGCCTGGCACAGTAAAC--------------------GTTAGCTTTAATTGTTA
Dasypus novemcinctus TAGTACATAAC----------TTGTGAGTATCAAATGAGATCATGTAAGCCAAGTGCTTAGCACCATGCCTGGGACAGTAAATGTAAGGTTTTTTTTTTTTTTTTGGTAAGTTTTTATTATTA

Homo sapiens GGTGTGTTAATAATAATAATAGTAGTTGTTGTGA-GGCCATTGTT---------AATA----GAATGGCTATTCTGTGACTTTTTTTTTTTTTTTTTTTTTTTGCTTGAAGGGAGAGG
Dasypus novemcinctus GATATAATAGTAATAATAATTGTTTCTGTTGCTATTGCTGTTGTTATACTAGTGAATAGAGTGAGTGGGTTTCCTGTGAC-----------------TTTTTTGGTTCAGGAGAGAGGGG

Homo sapiens AGAGCGACTGGAATTTTTC--TTTTGAGTAGTATGCTATAGTGAGCTCTGGAGTCACACAGAGCTGTGTGCCTTTGGTCATGTCAATTCATCTCATGGAGGCTCAGTTTCCTTACCTGTA
Dasypus novemcinctus AGAATGATTGGAGTTTTTCTTTTTTGAGTAGTATACTACAGTGAGCTCTGGAGCCAGAGAGAGCTGTGTGCTTTTGGTCATGTCAATTCACCT--GTGAGGCTCAGTTTCCTTATCTGTA

Homo sapiens TACTGAGAATTTAAAGGACCTAGCTCACAGGGTAATTGTCAAGATCAAATGAGATAATGCTGGTGAAGCCCTCAGCCCCATTCTTGGCATATGGGAAGAGTTCAAGCAATATCAGTTGTT
Dasypus novemcinctus AACTG-GGATTAAAAAGACCTAACTCACAGGGTAGCTGTTAAGACTAAGTGAGATAACGTGTGTAAGGCCCTCAGCATAGTTCTTGGCTTAT-CGAAAAGTTCAATCAGTGTCAGTTGCT

Homo sapiens GTTGTTATTATT---ACTACAAGGATTCAGCCATTTGTAGTAAGAAAGTTTCCTTGCTTTGTGGTGTGGGGATGTAGGGGAG------ATGGCAGTGAGAAGAGTACTCCGAGTTTCTAA
Dasypus novemcinctus ATTATTATAATTAACACTGTAGTGATTCAGGAATTGGCAGT---AAAGTTTCCTTGCTTGGCAGAGTGTGGGTAGTGGGGAGGAAGGAATAGCAATGGATAGGGCACTTAGAG-------

Homo sapiens TCACTTGTCTGATTACAAAGAGATAAAAATATTTTGGAAACAGAATGGCCAGGGCTGAAATCTGGGATTTAAAAAAAATTATTATTATTATTTTATGTGACCGTGGTCACATAGACTTCT
Dasypus novemcinctus -----------------AGGAAAT-ATAATAGTTAGGGACCAGGCTGGACTGGGCTCAAACCTGAGTTTTGCCAA--------TTTACTAGCTC-TGTGACCTTGGTCATAGA-ACTTCT

Homo sapiens CCAAGCCCATTTCCTCATGTATAAAATGGGAATGATCTCAGTGCCTACCTTACAGGTGTTGCTGGGAGGTCGGAATTATATAATGCATATGTAGTGCTTAGCCCAAGTGCTCAGTTGTTG
Dasypus novemcinctus CCAAGCCTCTTTCTGTATTTGGAAATCAGGAATGATGACAGTGCCTCCTTTCCATGGATTTCTGTAAGGCCCAAATCAT.......................................

Homo sapiens TTGGTCGTTAATTATGAGGTTTATAACGACCTGAATGCCTCCCTTGTCAAACTCTACATCCCTATACCTAGGCTACTTGAACTTCACCTGGAGGCTTTGCCCTATCCAAGGCTCAGGGAC
Dasypus novemcinctus ....CTAAGTCATTTAGACCTCACCTGAAGACACAACCCCATCCCAGTCTCAGGGAC

Homo sapiens CAATTCTCCCCTTAGACTGAGGGTTTCTCCCCTTAGACTGAGTGCACCAGTTCAGGAAAACTGGAGTACTCTGGTAAACCTTTCTGCTAGCTCTACTCTGACCTTGTGCCCCAGTTCTAG
Dasypus novemcinctus CCATGGTGTTCTTGGAC...

```
Homo sapiens          AAAGCAGGCTGTAGCCATGTTTGTATGAACAAGCCCTCAGCTGGTATGCTCAAGGCTGGTGTGCTTTTCTTGCTAGCTGTCTGAAATCCATGGAGCCTTTCTGTTGAATCCTAGCCCTAT
Dasypus novemcinctus  ........................................................................................................................

Homo sapiens          GGCAGGGATACCGGTCGCTGGATTTTTCTCCGTGTCTGTGACCCTGGGGTAAATTGTCTTCGTTATGATCACCATTACAGGGCTCTATTAAGGTGTTTGCTTGCCAGCCCCCACTGTCCA
Dasypus novemcinctus  ........................................................................................................................

Homo sapiens          TCCTCCCAAACCCTCTTTCCCTGCACCTGAACCTCTTTGAACGTTTCTGATGCTGCTCCACTCAATCTCCAGCCTCAGTTTCTTTCCCTCGAGCCCTCTGCCTGTGCTTCCATGTCATAT
Dasypus novemcinctus  ........................................................................................................................

Homo sapiens          CAGTTGCAGTGCCTGGGTCCCAGGACTCCTGTGTCTGTTCAACTGCAGAGGGCACGTATCTTGTTTCCTGAGCCAGGACCCTTTTCATCTCATCTTTTTAATTGTGTCAATCTCCTCTTT
Dasypus novemcinctus  ........................................................................................................................

Homo sapiens          TCTTTGGGCAGAGTGGGGTGGGGCATAGGCTGGTGGGACTGTTGGGGGCGACAGGGAAGAGGGGTGGAGTTGCATAGGCCAAGTGCTAAGGCGGAGCTTCTGGAGGACTGTCAAGTGAAA
Dasypus novemcinctus  ........................................................................................................................

Homo sapiens          GGCGGCCAGAGGCAAATGTCAGAGCCTATATAGAGGCAGGAATCTGGGACCAGAGAGACAGAGCAAGCAGTAGCAAGTCAGGAGCAGTGGGGCGGGTGGGCGGGCAGGCTGGCTGGCGGG
Dasypus novemcinctus  ........................................................................................................................

Homo sapiens          CAGGGGGGCGTGGCAGGGGATAGCAAAGGGAGATTGCAGAGAACAGCTGTTGGTACTGGGTCTTACTTTAATGAACTGGCTGGGCACGAGGTATAAGTTAATTGGCCAGAGTGAAAGAGC
Dasypus novemcinctus  ........................................................................................................................

Homo sapiens          TAGGGGCGCAAAGGATACTCTCTCCCTCTTCTCCGTCACTGGCAAGGTAAGATCTTTCTGGGTTTTGCCTGGTGGGAAACTGTGGCATGTTCAGATATTTTTCTGAAGATGGGAGGTGGA
Dasypus novemcinctus  ...............................CTCCCTGACTGGCAAGGTAAGATTTCCTGAGTTATACCTAGTGAGAAACTG--GCATCTTCAGGGATTTCTCTGAAGAAGAG-TATGGA

Homo sapiens          GGTCTATGGCTGCTTTTTTGACTCATCTTTCCCATTGCACCAATTATTACAATTATTACAATAACCAGGGTGTCAGCAGGAGGCCAGCATTTCTTTTATGACAACCC-ATCTTGGGGTTG
Dasypus novemcinctus  CTTCCACGACTGCTCTTGACTCTTGTCTTTCCCATTGTACCAAGTCTTAGAGT---------GACCAAGGTGTCAT---GAGGTCAATATCTCTTTTACCATACTCTTAACTCAGGGCTG

Homo sapiens          TCCAGGCTGGAGACAGAGGCAGGGGT-CTCGGTAGTGTGACTGAGTAAGGGGGAGA-------------GGTGGATCTTTATAGCACCTTGGTGAAGAGTAGGGGGCTCTGGAACTGGAT
Dasypus novemcinctus  TCCAGGCTGAAAAGAGAGGGTGGGATTCTTGGTACTCGCACTAAGTAAAGGGGAAAAGAGGGCAGACAAGGTGGATCATTATAACACAGTCATGAAGAGTAGGAG-CTCTGGAGTTAGA-

Homo sapiens          GGACCCAGGAATCTATGTGACTGCAGGCAAACTATTAAGCTTCTCTGGGTCTGAATTTTCTCATTTGTAAGATAAAGGAAGAGTACAACCGAAATCTCTTAGTGTTGCTATGGGAACC--
Dasypus novemcinctus  -GTCCTGGGAATCT--GTGATTGCATGCAAATTACTAAACTTCTCTGTGCCTTAGTTTTCTCATTTGCAAGATGAAGAAATAATACTACCAAGCTCTCTTAGTTTTGCTGTGGGGGGGCG

Homo sapiens          -------------ACATGAGATGATACATGTAAAGTGCCTAGCAGAGTGTTTGCCAGTCAGTGCATGCTAAAGAAATGTTCACTGTTAGCGTTTCTGTTGCAGGTTTTCTCATT-ATTAT
Dasypus novemcinctus  GGGGTGGGGTGGGGAATGAAATGATACATATAAAGTGCTTAACACAGTGCTTGAGAATCAGGACAATCTCAAGAAATGATAGCCATTGGTATTTCTGTTGCTAGTATTATCATTTATTAT

Homo sapiens          GGAATCACAATTCCATGAGGTTGATGACTTTTGTGACATTGTCCCAAAGTAGCAAGTTTTCTGCTTTTGGCATGGGCAGGCTGTGGGGACATGTCAACATTGTGTAATATTTGCATTGGC
Dasypus novemcinctus  GGAAGTAGAAAGCTATGAAACTGATGACTTTTGTGACATTGTCTCAAAGTAGCAAGTCTTCCATTTTTGGAATGGGCTGGCTGT-GGGATGTGCCAACATTGTGTAATGTGTGCATTGGC

Homo sapiens          TTCCAAGCCGTGTGTCCAAAAGCCCCTTCTGG-GCTGCTTATTGGAGCAGGCTGGAGGGGTGAGGCTCT-GGATTGCCCACCCCCATGGCATCAGAGAAACTTGAAACTTGGACATGCCA
Dasypus novemcinctus  TTCTAAGCTGTGTGCCAAGAAGCCCCTTCTGGAACTACTTATGAGGGTGGGCCAGAGGGGTGAGACCCCAAGATTGCCCACCCCTAAGTCATCATAGAAGTTCAAAATATGTATCCTTTA

Homo sapiens          GACACTGGGCTTTAAGTGACCCATGATCTGGGATGTCAGAGAAATAATTGAATGCAAGCTCAGGGATGCCAAACACTAGGCAACCCCCAGGAGGGGGTCAGAGTCAATGCTATACAGTTT
Dasypus novemcinctus  -----TAGACTTCAAGCGAGCCACAGTCTAGGATAGTATAGACATAACTGAATGCAACTTTAGGTATG-AAAAGGCTAGCAAACTCTGAAAAGGGGGTGAGAGTTAAAGACATACAGTTT

Homo sapiens          GTTACTGTTGCAGTGACCCTATTTTAGCCTTGACCCTGGCTAATCTGAGAAAGCTTGTAAAATGGGGATAATAATAT---CTATTGCCCAGGAGATTGTAAGACCCAAATGGGATAATGC
Dasypus novemcinctus  GTTACTGTCAGAGTGA--CTGTTTTAACCCCAACTCAGGCTAATCTGAGAAAGCTGGAGTAATGGAATTAATAATATTACTTGTTGCACAGGGTACTATGAGGTCCAAATGGAATAATGC

Homo sapiens          TTACAAAGGCCCTCACGTTATAGGAAGTTGTGACAGTTGTCTAAAGTACAGTGCCTGGCACATACTACGTGAAGCAATAAATAAATAGTAGTAATGGGGTGGTGGTTGCTGTTATTAATA
Dasypus novemcinctus  TTGT-AGGGCCCTTGC------------------------------TACAGTGCCTGACACACACTAAATGAG-----CAATAAATGGTGGTA-----GTAGTGATTGTTGTTAT--ATA

Homo sapiens          AGAATAATGAAAATAATAAGCAGAATTTTCAGTTCATGG-AATGTTGCTCTCAGTCATTGATTAGATAAATGTCTCCCTTTTCC---TGAGAACATTCAGGGCCATCAGAGTAGTAAAAT
Dasypus novemcinctus  ATAATAATAATAATAATAA-TAGGATTTTCTGTTCATGGAAATGCTGCTCTCAGTATTTGATCAGACAAATGCCTTCCTTTTCCACAGGAGGCCATTCAAGGCTATCAGAGTAAT-----

Homo sapiens          GAGGATTAAACCACTGGCAGAATTCAATGAGCCCCAGGAACTAATAATTATCCTGGGGCAAAGGACTTTCCCATTCAGGCTTGCCAGTACCTTGGCTGGCTTGTGAGTACTGCCGTGGGA
Dasypus novemcinctus  ------------ACTGGCAGGAATCAATGAGCCCAAGGACCTTACGATTAGGCTGGGGTAAGAGGCT-----------------------------AGGCTTGTGAGTGCTGGTATATGA

Homo sapiens          TAGAATACAGGCCAGGAATCCGCATTTCTCACATTTGGCCAGAAGGTGGTGCTGCTGCTTTATTTGAGTCAGGACTGCCCAGGCTCCTGGCTGCGGAGGCTGCTGGCGTTTATAGCATTT
Dasypus novemcinctus  TAGAAGATAACCCAAGAAACTGCACTACTCATAGTTGGCCAGTATGTGGTGCTGCTGCATCGTTTGCTACAGGACTAATCTCGCTCCTGGCTGCTGA.......................

Homo sapiens          ACAAACTGAGAGGCTGGGTTGGTTTGAAAAGGATAGAGGCAGAAGGGACATTTGCTCCAGCTCCAAAGTTGTTCATTGCTTCACAGCTTCTTTAATCTGATAGCCTCACCTTCAATGCCA
Dasypus novemcinctus  ........................................................................................................................

Homo sapiens          TATACCTGACAACGCATAAAAACAGGTCTCCACCATGAACAATTTGTCTGTTTCCAAATCTTCTTGATACTGGCTTTTGACCTCTAAACTTGTTTCTTGTTTACAGATTTTCTTGCCTCC
Dasypus novemcinctus  ........................................................................................................................

Homo sapiens          TGGGCTCCCTCCACACTATCCTGTGGCTGTGGCAGATATGTCAATACCCTTACATTCACTGCGATTCAACAATACAATGAGGGAGGAAAATGTTGAGCCCCAAAACAAGCAGATGGCCTT
Dasypus novemcinctus  ........................................................................................................................

Homo sapiens          CTGTAGACCAATGACAGAGACCAGAGCAGACGTACAGATTCTGCATTCTCATGTACAGTTGCCTATAGTCTCAACTTCAGCCTCAGACCCTGGAGGGACATCCACACAGTTGATGACATC
Dasypus novemcinctus  ........................................................................................................................

Homo sapiens          TCCAGTCTTTGACACCATGTCTGCACCTCTAATGGGAGTACCAAACTCTGGAGCATTGTCCCCACCCCTAATGCCAGCCTCAGACTCTGGGGCACTTTCCCCATTGCTAATGCCAGCCTC
Dasypus novemcinctus  ............................................ATTCTGGAGCAATATCCACAGGGCTTATGAAACCAATGACCAATGGAGCAATGTCCACACAGAGAGTGAGACCTCC

Homo sapiens          AGACTCTGGGGCACTGTCCCCATTGCTAATGCCAGCCTTAGATTCTGGAACATTGTCCCCATTGCTGTCCACTTCAGAGTATGGGGTAATGTCCCCAGGGATGATGACAATTCCTGATTT
Dasypus novemcinctus  AGACT...................................................................TGCAGCCTCTGAAACAGTGTCTATGCCACAGTTGATAGCCCCAGAATC

Homo sapiens          TGGAACGATGTCCGCAACGCTAATGGTAGCACCAGATTCTGCAGAGATATCACCATTGGCAATGCCAGCTCCATCCTCTGGAGTGGTGTGTACACCTATAATGAGCACTTCATCCTCTGA
Dasypus novemcinctus  TGAATCAATGTCCACACTGCAAATAAGAACCCCAGTGTCTGGAGCAATATCCATGTCACAAATGAGGGCCACAGCCTCAGGAGTGATGTCTACACCACAAATGAGAGCCACGGCCTCTGG

Homo sapiens          GGCAATGTCCACACCATTAATGCTAGCCCCAGATTCTGGAGAGTTATCCCCAATTCTAATGCAAGATATGAATCCTGGAGTGATGTCTACACAGCCAGTGCCAGCTCCCAGCTCTGAGGC
Dasypus novemcinctus  AGCAACATCCACCCCACTAATGACAGCCAAA...........................................CTGAAGTAATGTCTACACAGAGAGTGAGACCTCC------------

Homo sapiens          AATGTCCCCATTGCAAATTACAGATGAAGACACCGAAGCAATGTCCAAAGTGCTAATGACTGCTCTAGCCTCTGGAGAGATATCTTCGCTGCTAATGTCAGGCACGGACTCTGAAGCAAT
Dasypus novemcinctus  ---------------------------AGACTCTGAAGTAATGTCTACACCACAAATGACAACCACCACTTCTGGTGAGCTAGCCACAATACTAATGAGAGACATTTCTTCTGGAACTAT

Homo sapiens          ATCCTCACTGATAATGTCAGCTGTAGCTTCTGGAGGTACATCACCCCAGCCAACAAGCACCCAAAACTCTGGGGGAATACCTACCCCGCTCATGTCAGATCTAGACTCTGGAATAATGTC
Dasypus novemcinctus  GGCCATGCCACAAATGACAGCCACAGCTTCTGGAGGGATGTCTGTGCAACCATGGAGAGCTCCAGCTTCTGGAGCAAGGTCAACACAGCTAATTACAGATGCAGCCTCTGAAACAGTGTC

Homo sapiens          ATCGCTTTTAATGTCATCTCCAGGCTCTGAAGTAATGTCCACACCGCTACTGTCAGTCCCAGATGCTGGAGAAATGTCCACATTACCAAAGCCAGCTCCAGATGCTGAAGCAATGTCCCC
Dasypus novemcinctus  TATGCCACAGTTGATAGCCCCAGAATCTGAATCAATGTCCACACTGCAAATAAGAACCCCAGTGTCTGGAGCAATATCCATGTCACCATGGAGAGCTCCAGCTTCTGGAGCAAGGTCAAC

Homo sapiens          AGCACTAATGACGGCCCTACCCTCTGGAGTGATGCCCACCCAAACGATGCCAGCCCCAGGCTCTGGGGCGATGTCCCCATGGTCAACACAAAATGTAGACTCTGAAATGATGTCTAATCC
Dasypus novemcinctus  ACAGCTAATTACAGATGCAGCCTCTGAAACAGTGTCTATGCCACAGTTGATAGCCCCAGAATCTGAATCAATGTCCACACTGCAAATAAGAACCCCAGTGTCTGGAGCAATATCCATGTC

Homo sapiens          GCCAGTGAGAGCAACAGCCTCTGGGGTGATGTCTGCACCACCAGTAAGAGCTTTAGATTCTGGAGCAATGTCCACACCGCTAATGGGAGCCCCAGCCTCTGGAAATATGTCTACATTGCA
Dasypus novemcinctus  ACAAATGAGGGCCACAGCCTCAGGAGTGATGTCTACACCACAAATGAGAGCCACGGCCTCTGGAGCAACATCCACCCCACTAATGACAGCCAAAGCCTCTGGATCAATGTCAACATGGTC

Homo sapiens          AAAGACAGTTCCAGCCTCTGGAGCCATGACCACCTCACTGATGACAGTCCCAAGCTCTGGAGTGATGTCCACAGAGCAAATGTCAGCCACAGCCTCTAGAGTAATGTCCGCACAGTTAAC
Dasypus novemcinctus  AATGAGAGACACAAACTTGGGAGCATTGTCCATGCCACAAATGAGAGCTACTGTATCTGGAACAGTCTCCAAGCCACTAGTGACGTCTAAACCTTCTCGAGCAACATTCACACCACAAAT

Homo sapiens          AATGGCCAAAACTTCTGGAGCAATGCCCACAGGCTCTATGAAAG---------------CCGTGGCAAAACAATACAAGAGAGCCACAGCCTCTGGAAAGATGTCCACGCCACTGAGGAG
Dasypus novemcinctus  GACAACCACCACTTCTGGTGAGCTAGCCACAATACTAATGAGAGACATTTCTTCTGGAACTATGGCCATGCCACAAATGACAGCCACAGCTTCTGGAGGGATGTCTGTGCAGCTAAGGAG

Homo sapiens          AGCTCCAACTTCTGGAGCAATGTCCACCCAACCAGTTACGGCAACAGCCTCTGAAACAATGTCCATGCCACAATTGACAGTCCCAGCCTCTGGATCAATGTCCATGCTGCAAATGAGAGC
Dasypus novemcinctus  AGCTCCAGCTTCTGGAGCAAGGTCAACACAGCTAATTACAGATGCAGCCTCTGAAACAGTGTCTATGCCACAGTTGATAGCCCCAGAATCTGAATCAATGTCCACACTGCAAATAAGAAC

Homo sapiens          CCCTGTCTCTGAAGCAATGTCCATGCCACAAATGAGAACCATGGCCTCAGGATTGACATCTGCAGCACAGATGAAAGCCATGACTTCTGGAGCAATGTCCACCCCACTAATGACAGCCCA
Dasypus novemcinctus  CCCAGTGTCTGGAGCAATATCCATGTCACAAATGAGGGCCACAGCCTCAGGAGTGATGTCTACACCACAAATGAGAGCCACGGCCTCTGGAGCAACATCCACCCCACTAATGACAGCCAA

Homo sapiens          AACCTCTGGATCAACATCCACGCTGTTAATGAGAGACACAGCCTCAGGAGTGATGTCCTGTCCACAAATGAGATCTCTGGCCTCTGGAGCATTGTCCAAGCCACTAATGACACCCAAAGC
Dasypus novemcinctus  AGCCTCTGGATCAATGTCAACATGGTCAATGAGAGACACAAACTTGGGAGCATTGTCCATGCCACAAATGAGAGCTACTGTATCTGGAACAGTCTCCAAGCCACTAGTGACGTCTAAACC

Homo sapiens          CTCAGGAACGATGTTCACGGAGAAAATGACAACCACAGCTTCTGAAGCAATGCCCACACTGCTAATGAGAGACACAGTTTCTGGAGCTCTGTCCATGCCGCAAATGACAGACACAGCCTC
Dasypus novemcinctus  TTCTCGAGCAACATTCACACCACAAATGACAACCACCACTTCTGGTGAGCTAGCCACAATACTAATGAGAGACATTTCTTCTGGAACTATGGCCATGCCACAAATGACAGCCACAGCTTC

Homo sapiens          TGGAGGGTTGTCTGCATCGCTAATGAGAGACACAGCTTCTGGAGCAATGTCCACATCACAAATGACAGCCACAGTCTCTGGAGGGATGTCCATGCCACTAATGAGAGCTCAAGACCCAGG
Dasypus novemcinctus  TGGAGGGATGTCTGTGCAGCTAATGAGGGCCCAAGCCTCTGGAGCAATGTCCACTTCACAAATGACAGCCACAGCCTCTG------------------------GAACTGCAATCCCTGG

Homo sapiens          AGTAATGCCTGCCTCACTAATGAGAGCCAAAGTGTCTGGAAAGATGCTCAGTCAGCCAATGAGCACCCAAGATCCTGGAGGGATGTCCATGTCGCCCATGAAGTCCATGACCGCTGGAGG
Dasypus novemcinctus  AGCAAAGCCCACATCGCTCATCAGAGCCACAGCCTCTGGAAAGATGTTCAGTCAGCCAATGAGCACCCAATACTCTGGAGGGATGTCCACATCACTCAAGAGATCCTTGACTTCTGTAGG
```

```
Homo sapiens          GATG---------CAGATGAATTCCCCAACCTCTGATGTGATGTCCACACCAACAGTGAGAGCCTGGACCTCTGAAACAATGTCCACACCACTAATGAGAACCTCAGACCCTGGAGAGAG
Dasypus novemcinctus  GATGTCCTCACTACAGATGAAAGCCCCAGCATCTGAGGTGATGTCCACATCAAAAATCATAGCCCCAGCTTCTGGATCAATGTCTACACCACTAATGAGAGCCTCCAA------------

Homo sapiens          GCCCTCACTGCTCACAAGAGCTTCATCCTCTGGAGAGATGTCCCTACCACTAATGAGAGCTCCAGCTTCTGGAGAGATAGCCACGCCTCTGAGATCCCCAGCTTATGGAGCCATGTCTGC
Dasypus novemcinctus  ---------------------------CTCGGGAGAGATGTCTACACCACTAATGAGAGCCCCAGTTTCTGGAGAAATAGCCACACCTCTGAGAGCCCCAGCTTATGAAGCACTGCCCAC

Homo sapiens          TCCACAAATGACAGCCACAGCCTCTGGAATGATGTCATCCATGCCACAAGTGAAGGCTCCCATCTCTGGAGCAATGTCCATGCCACTAACAAGATCCACAGCCTCT--------------
Dasypus novemcinctus  TCCACAAATGACAGCCACAGGCGCTGGAACAATG---TCCATGCTACAGATGAGAGCTCCAGTCTCTGGAGCAATGTCCTCATTGCTAATGAGATCCACAACCTCTGGAGCTATGTCTGT

Homo sapiens          -------------------------GGAGGGATGTCCATGCCACTGATGAGAGCCCCAGACTCTAGAGTGACATCCACATCACAAATGATGCCCACAGC-TTCTGGAGACATGTGCACAC
Dasypus novemcinctus  GCCTCAGATGTCAGCCATAGCCTCAGGAGGGATGTCCATGCCACTGATGCAAACCCCAGCTGCTGGAGCAAAGTCTATGCCACAGGAGACACCCGCAGCTTTCTGGAAGGATGTGTGCAC

Homo sapiens          TACCAGTGCGAGCCCCAGCCTCTGGAGGGGTGTCCTCACCACTAGTAAGAGCTCCAGCCTCTGGAACTATGTCCACACCACTAAGGAGACCCTCAGCCTGTGAGACTG------------
Dasypus novemcinctus  TGTCAGTGCAG--CCCAG-ATCTGAAGTGATGTCCTCACCACTAGGAAGA--CCCATCTTCTGGAACTATGGCCATGCCACAAATGACAGCCACAGCCTCTGGAACTGCAATCCCTGGAG

Homo sapiens          ---TGTCCACAGAGTTAATGAGAGCTTCAGCCTCTGGACATATGTCCACTGCACAAACAACAGCCATGGTCTCTGGAGGGATGTCCAAGCCATTAATGAGAGCCCCGGCCTCTGGAACAA
Dasypus novemcinctus  CAAAGCCCACATCGCTCATCAGAGCCACAGCCTCTGGAAAGATGTTCAGTCCACAAATGACAGCCACAGCTTCTGGAGGGATGTCTGTGCAGCTAATGAGGGCCCAAGCCTCTGGAGCAA

Homo sapiens          TGCCCATGCCTTTAATGTCAGCCATGGCTTCTGGAGAGATGTCTATGCCGCTAATGGAAACCATGGCCTCTGGAGCAACGTCCACATTGCAAACCAGTGTTGCGAACTCTAGATCTATGT
Dasypus novemcinctus  TGTCCACTTCACAAATGACAGCCACAGCCTCTGAGGTGATGTCCACATCAAAAATCATAGCCCCAGCTTCTGGATCAATGTCTACACCACTAATGAGAGCCTCCAACTCGGGAGAGATGT

Homo sapiens          CCTTGTCACAAACAACATATACCGTGTCTGGAAGGATGGCCACAGCGCCAATTAGAGCCTCTGCTTCTGGAGCAAGGTCCACATCATTTATGAGAGCCTCAGTTTCTGGATCGATGCCCA
Dasypus novemcinctus  CTACACCACTAATGACAGCCACAGCTTCTGGAGGGATGTCTGTGCAGCTAATGAGGGCCCAAGCCTCTGGAGCAATGTCCACTTCACAAATGACAGCCACAG......GCTCTGAGCATG

Homo sapiens          TGCCACTACCAAGAGCCACAGCCTCTGGATGTGGCATGGGTATGTCCATGCCACAAATGACAGCCACAGACTCTAGAGGGATGTCCACACCACTAATGAGGGCCTCAGGCCCTGGAACAA
Dasypus novemcinctus  TTCCACAACCATGAGACCCAG-CTCTGAA................................AGCCACAGCCTCTGGAAAGATGTTCAGTCAGCCAATGAGCACCCAATACTCTGGAGGGA

Homo sapiens          TGTCCACACCAC---AGACAGCCTTTGGAGTGATGTCCACTCCGGAAATCAAAGCCACAGACTCTGGAGAGGCATCCACCTCTCACATTAACATCACAGCCTCTGGATCAAAGCCCACAT
Dasypus novemcinctus  TGTCCACATCACTCAAGAGATCCTT...............................................................................................

Homo sapiens          CGCACATGACTGCCACAACTCCTGAAACTGCGAAACCACCACCAAAGGAAGTGCCATCCTTCGGAATGTTGACCCCAGCACTCTGCTACCTCTTAGAAGAGCAGGAAGCAGCCCGGGGCT
Dasypus novemcinctus  ........................................................................................................................

Homo sapiens          CATGCTCTGTGGAGGAAGAGATGGAGATTGATGAGGAGAAGCAAATGAAGGGGTTTTTGGACGATTCAGAGAGAATGGCATTTCTGGTATCTCTTCATCTGGGAGCAGCAGAGAGGTGGT
Dasypus novemcinctus  ........................................................................................................................

Homo sapiens          TCATCTTGCAAATGGAGGTAGGAGAACCTCTCTCACATGAAAATAAATCTTTCCTGAGAAGATCCCAGGGCATATATGACTCCCTATCTGAGATAGACATCCTCAGTGCTGTTCTTTGCC
Dasypus novemcinctus  ........................................................................................................................

Homo sapiens          ATCCCAAACAGGGCCAAAAGTCAGTCAGGCAGTATGCCACTGACTTCCTGCTGCTGGCCCGACATTTGTCTTGGTCTGATGCCATTCTACGGACCAGGTTTCTGGAAGGACTCTCAGAAG
Dasypus novemcinctus  ........................................................................................................................

Homo sapiens          CTGTTACCACCAAAATGGGTCGGATCTTCCTGAAGGTGGCTGGCAGCCTAAAGGAGCTGATAGACAGGTCTCTGTACACCGAGTGCCAGCTGGCTGAAGAGAAGGATTCCCCAGGCAACT
Dasypus novemcinctus  ........................................................................................................................

Homo sapiens          CAAGCCAGGTTCTGCCAACAGCCTGTAAGCGGAATAATGAGGAGGCCATGGGGAATGAACTGAGCTCTCAGCAGCAGACTGAGGAGGTAATGAGGGGGAAATCCGCTGAAGGTTTTTGAG
Dasypus novemcinctus  ........................................................................................................................

Homo sapiens          CAGAGAAATGAGGAGGTCACGACAGGGAATACATCCTGCTTCCTTGAGTAGAATTGGGAAGAGGCAAGGGGGAGGCATGGAGCTCCTAGACCATTAAACCCCAGCAGTTTCATCAAGTGA
Dasypus novemcinctus  ........................................................................................................................

Homo sapiens          GCATGGCTCTGCAGTTATTTTGGGTATATTAACTACTTTGAGGAGCAATGTCCTGCCCAAGTTACTGAGAACGTGGGATAGTTGAAGTACTAATTCTTCTCATTATGAGGGAACTGGATG
Dasypus novemcinctus  ........................................................................................................................

Homo sapiens          GGCTCAGTTCCAATTCTCCCCTTGTTCCAAGTCCAATTCTAATAAATGGGTCTTTTGGGAGCTGGGGCCCATCTACAGCAACATGTGTTGTGGAGTCAGGTCACTAGGGAATTAGAATAG
Dasypus novemcinctus  ........................................................................................................................

Homo sapiens          CCTACTCCTTGGTCACACTGTATGCAAGTGAGATAAATTAGCAAAGTGTCTTGATCTGCTAAGTCACAGAACTTATCCAGGCAGAACACCCGTTGCCATAGTAAGTGTGGCTCTTACCTC
Dasypus novemcinctus  ........................................................................................................................

Homo sapiens          TGTTGTCTTTTTCTCACTCCCAGCACCAGCATGTTTCCAAACGCTGTTACTACCTGAAAGAGCATGGAGACCCCCAAGAAGGTCTTCATGATCACCTTGGACAGAGCACAGGCCATCATC
Dasypus novemcinctus  ..................CTAGCTCCAGCATGATCCTAAACGTTGTTGCTACCTGAAAGAGCACGGAGACCCTCGAGAAGATCTTCACAATCATCTTCGACAGAGCACAGGCCACCG--

Homo sapiens          AGAAGGCCCATACCAACAAGTAAAACTCCATGGAATCTTCTCCTGTGATATCTGACTCGACCGCTAACTGGAGGACTGGTTCCTGGACCCATTCCATTAAACTACATTATAAACCTTGTT
Dasypus novemcinctus  GAAAGACCCTCACTAACTAGTAATGCTTGCTGAACTCTTCT---GTGATATCTGACTCTGCTGCTAAATTGAGAACTGGTTTCTGAACTCATTCCATTCAATTATATCATAAACCTTGTT

Homo sapiens          GCCTTCACCCTTCAGCCTCCCGCTCCAGGCACATCCCACCTTACCTCTCACTTGGCTGATTTGACCTTCTCTTTCACC-CACATGCCTAAGACCTGCCCTGACAATCAGAGTAGGGACTA
Dasypus novemcinctus  GCCTCCTCCCTCCAGCTTCCTGCTCCAAGCAGCTCCCACCCCATATTCTATTCAGCTGACTTGACATTATCCTTCGCCTCTCATGTCTGTGATGTGTCCTGAAAACCAAACGATGGACTT

Homo sapiens          CAAGAGTGTATGGTGTATGAGGTTCTGAACTCCCATCACCACCGAGGCCAGTCCTAGTTCTTGGTCCAGGGAGAAGTCTAGGCAGGATAAATCATATCTTACCTCAAAAAACCCTTAGCG
Dasypus novemcinctus  TGAGCATATATGGTTTGTGAGATTCTACACTCCCATCACCATCAAGACCAGTCTTAAATCTTGGCCTGGGGAACAGTCTAGGTAGGA-AAATCATGCCTTACTTAAGA-----------G

Homo sapiens          TATCTTGCCATGTGTCAGCAGGCAAACTTGAGGAAGCCCTGGTTCTATTTCACCTGGCAGGGGAGCCTATAAGGAGAGCGATGCCCACTCCTATACCACCCTTGTAACCCAGTAGACTTG
Dasypus novemcinctus  TGTCTCACCACATGTCACCGAGCAAACTTGATGAAGCTCTGCTCCAGCTTCACCTGGAAGGGGATCTTATAAGGAGAGTGATGCCCATTCTTATACTACCCTTTTAACCCAGTAACCTTG

Homo sapiens          CCCC-ATATGCCCCAACTCATAGCACCTAAAA-TGGGTCTCCAGGGTCCTTGCCCTGTTTATAGTATCCATCCTGAACCATTCGTTTTGACCCATAATAATAGTGATAGTA-TAATGGCT
Dasypus novemcinctus  TCCCTAAGTGCCACAAC-CATAGCACCTAAAAATGGGTCTCTGTGACCCTTGCCCTGTTTGTAGTCTCCACCCAGAATCATTCATTTTGACCCATAATAATAACACCACTAGTAGTGGTT

Homo sapiens          ACCAGTTATTGATAATGTATCATGTGACAGGCATTGGGCTAAACTCTTGACAAGCACCATCTCACTACATTCTTACAGTAACACAACTTGTTCTTGTGTTGTCAGCTTCTGACCTCTTGA
Dasypus novemcinctus  AACAGTTATTGATTGCGTGCCCTGTGAGAAGTACTGGGCTAAACTTTTGACAAGCATCATTTCATTGCATTCTTATAATAAGACAAGTTGTTCTTGTGCTGTCAGTTTCTGACCTCTTGA

Homo sapiens          TCTGTTGTTTATGGCCTTTGTCTTGTCCCTCAGTGGGGGTTCTCTAGCTCTGACCTCTGGGTGACCCCCTCAGTTGTTGATTGCTCTCACTCCCCTTGGCTGTTGTGCTTCATGGCCAGC
Dasypus novemcinctus  TCTGTT---TATGACTCTTGTCTTGTCCCTCAGTATTGAATCTCTAGCTCTGACTTCTGGGTGACTGCTTCAGTTATTAATTGCCCTCAGTCCCCTTGGCTGTTATGCTTCATGGCCAGC

Homo sapiens          ACTCTTGATGAGGGTCTTGGTCTATAAACAGGCATAGATAGCTAGAACATAATGTGAGCAGTGTTTGCCTTCAGGTGGTGGGGTTA-TGGGT----AGTTTTTATTTTCTTCCTTCTACT
Dasypus novemcinctus  ACTCTTGATAAGGGTCTTTGTCTCTAAACAAGTATAGATAATCAGAACATACTATGAACAGTGTTTGCCTTCAGATAGTGGGGTTAGTGGGGTTTAAAATTTTTTTTTCTTCTTTATACT

Homo sapiens          TCTCTATACATTTCAGATTCTCTACAGTAAGCATGTGTTACATTTGTAATTATAAAAGTAAGTAAACTTCATTTATGAAAAAAGAGTTCATT-GTGGCTCCTAGAATTTGATTTACTGAG
Dasypus novemcinctus  TCTCTGTACTTTCTAGATACTTTGCAGTAAGTGTGTACCACATTTGTAATCATAAAAGCAAATAAAGTTCACTTATGAAAAAAGGAATAATTAGTGGCTAATAGAATTTG..........

Homo sapiens          TCCTTTATTCAACCAATATTAGTTATGTAACTTATGTCAGCTACTCTTTGGGGGACAGACGGATAAGAATCCTGCTGATATGAACATTCCATTCCAATGGAGTGGGGAGAACAAGAACAC
Dasypus novemcinctus  ........................................................................................................................

Homo sapiens          ACTGAACTTAGATTGGAATTGTGAAGGCTGTGAAGAGAAGAAATCAGGACAATGGGGAATTTGATGCAAATAAGACCAATTTATGTATTCAATAGTAGTCCATATAATTGAGTTACTCAA
Dasypus novemcinctus  ........................................................................................................................

Homo sapiens          AGATCTTTCAACAATAGTAGGAGAGACATGATTAGAAACTTATTTTATATAGTTAAATTGGCAACAGTTTGGAGACAGGATGGAGGAAGGGATCAGAGAGCAGCTGAGCCCTCTACTGCA
Dasypus novemcinctus  ........................................................................................................................

Homo sapiens          ATAGTCCAGGTAACTGACTGGCTAGGTGACTCAATCTAGGGTGGTGGCAGCAGAGATGATAATTCAGGAACAGAGACCTAACCTTCTCTGAGGACTCAAGTCCACTGCTCCAGGACTCAA
Dasypus novemcinctus  ........................................................................................................................

Homo sapiens          GTCCACTGCTCCATTACCCTAAACTTTGTGTCTGATTTCAGGAAGAGGAAAATGATACTCAGGAAAGACAGATTGGCAGATAAATC
Dasypus novemcinctus  ......................................................................................
```

```
Homo sapiens         AAACCTCAAAGGGAGTGTAAAATGC----AGAGTGTTGTCAACTTATCA-CACCAAGAA-----------------------------TATTTTT-TTGCACCTTTTTAGTTTGGAAGG
Loxodonta africana   AAACCTCAAATGGAGGATAAAACATGGAGAGAGCATTCTCAGTTTATTGGCTCCAAGAAAAAAATATATATATATAAAATATGTGTATATATTTTAATTTACCTTTTTAAGTTGGAAGG

Homo sapiens         GTCAAATTGAGATGACAGAGGAATAGTGGATAAGAGCTATAGTGCTATTAAATTACATTCTAGAGTTGGGAAAATCGAGACCCATAATCCTAGGAATTATTAAT-GATAAGAGCTAACAC
Loxodonta africana   GACAAGTTGAGATGACAGC--AACAGTGGATGGGAGCTATAGCGCTATTACATTACATTCTAGAGTTGGGAAAATTGAGACCCATAGTTCTAGAGATTATTAACAGGTAAGGGCTAATAC

Homo sapiens         TTTCTGAGTGCTTACCACATGCCAGGCCCTATACTGAATGCATCATGTCCATTTTCTCATTTGATCCTGACAACCACCTGTGAGGCCTCATTTTGAAGACAAGCAAGCTGAGGCTCAGAC
Loxodonta africana   ATTTTGAGTGCTCACTACATGCCAGGCCCTGTGTTAAATGCTTCATGTCTATTTTCTCATTTGATCCTGACAACCACTTTTGAGTCTTCATTTTACAGACAAGCAAACTGAGGCTCAGA-

Homo sapiens         CTTGGATTTGTCCAAGGT------CACTCAGTTGGTCATTTACAGAGCCCTGATTCAAACTCTCTTTTGCTTGATTACAAAGCCATTGCTCTAAAAGATTAGATTATACTGTCTGTATTT
Loxodonta africana   -------------AAGGTTGATTTCACCCAGTTAGACATTTGCAGAGCCC-------AACTCACTTTTGCCTGATTGCAAAGTC--TGCTATG----------------TGCCTCTATTT

Homo sapiens         GCTTCTGGGAGCTTATGGAATTCACTCCTATCTGTTCTCACAAGGAACTCTATTGGGTTTTGAAAAAGGCTAAAGAATTGAAAAGAAACTTTGTAGGCTGGGAA-------ACAT-----
Loxodonta africana   GCTTTGTGGAGCTCAGGGAATCCACCCTTTGCTGTCCTCACGATAAAATCTAGTGGGCTTTGAAAAAGGCTGAAGGGTCAGGAAGAAACTTTTTAGGCTGGAAACCTAGTAACACTGGCA

Homo sapiens         AGTGTCCTTTGAGCAGTGAGATAGGCTAAGCTCAGAGGACTGGGCTCTGGAATGTGGAAAGGAGACGTGCCCACTGGAGAGCAAGTCAGAACTTTCGTGAGCCCTGCTCCAGGTCCCAGG
```

```
Loxodonta africana  AGTGTCCTATGAGCAGTGAGATAGGCCAGGCTCAGAGGACTGGGGTTTGGAACCTGGGAAGGAGACATGTCCACTGGGATGCAACTCAGAGCTTGTGTAGGCACTGCTTTGGGCACAGGG

Homo sapiens        AGAAAGTGCCAGCCATGAGGACTGGAGTATTGCCTGGTAAGGAG----AAGATTATTATTTTATCTAACACGTTCTTCTATAACGCTCTATATGCTAGGCACTGGTCTGCACTGCTCTAA
Loxodonta africana  AGGACGTTCCAGCCATTGTAGTTGAAGAACCACTTGAC-AGGAGCAGTAAAGCCGTTGTCTTATTTAACAAGCCCTTCTTTAATGCTCTACATGCCAGGCACTGAACTGTACTTATCTAA

Homo sapiens        GTACTTTATAAGTATTAACTCCTTTACATCTTATAACACTTCCCTGGAAATAGGCATTATTATTGCCCCCATAATACATATGAGTAAACTGAAGTTCAGAGATGTTAAATAACTTGTTCA
Loxodonta africana  GTACT------------------------TTACAACAACTCTCTGAAAAAGGGTACTCTTATTATCCCCATGTTACATGTCAATACACTGAAGCTCAGAGATGTTAA-----TTGTCCA

Homo sapiens        AGGTCACATAGCTGGTTAAGTGCCAGAGCCAGGCTTTGAACCTAGGCAGTCTGGATCTAGTGGCTGTGTTATACTGCTTCGCAACAGAAA-CTTGATTTTTCTTTCTTCTGTGTTTATCA
Loxodonta africana  AGGTTGCACAGATGG-GAAATGCCAGAACCAGGCTTTGAACCCAGGCAGTCTGGCTCTAGTGTCCATGTTAAGCTGCCTCTCAACAGAAAGAATGATTTTTCTTTCTCTTGTGGTTGCCA

Homo sapiens        TCC-TTTCTCAATCACTCCTGGGCAAGTCTACTTTGCTAGCAAATTTCCCTTACCCCTTTGGCTTTTTTTTCACCCATGAACCCTCTAATGTTTTCTGCAGCTTCTTTGAGCTCATTCTT
Loxodonta africana  TCCTTTTTTCAACCATTCCTGGGCAAGTCTCCTTTGGCAGCAAATTTCCTCTACCTCATTGTTTTTTTCTCCACCAGTG-ACTTACCAGAGTTTTCCTCAGAGTCCTTGTGCTCCTTCTT

Homo sapiens        TATCTTCCACACTGCCTCTTTTGGCATTACTCATGAACGACATGCTCTTTTCATCTGCCAAATATTTTTTCCATAAATTATCCCATCTCAACTTGGGAAGTCACAATGGGAAGTATTGTT
Loxodonta africana  CATCCTCCACACTGCTTAGATTGGCGTTACTCTTGAACAACATGCTC--TTCACGTGCCAAATAGCTTTTCTATAAATTGTCTCATCTCAACTTGGGAAGTGAGGGTGGAAAGTATTGTT

Homo sapiens        CCTATTTTGTGAATGAGGCAACTAAGGCAAGCAACTTTCCTGAAGTCTCACACAACCATCTAGTGGCAAAGTGCACTAGAATCCAGATTCTCTGATTCTGAATCTAATGTTCTTGTCCAT
Loxodonta africana  CCTCTCTTACCAATGAGGAAACAAAGACAAGCAATTTGCCTAATGTCTCACACAACCATCT-GTGGCAAATTGGACTAGAAACCACATTCTCTGACTCTTAATCCAATGTTCTTTTCCAT

Homo sapiens        TGCACCATCTGATGGAATTTAACTATCTATTC----ATTAATTAATTTATTCATTAATATTGTAAAAATATACTGAGTGTCTTAC-TGTATGGTAAGGCCCTGGGTGC----AAAAGTCG
Loxodonta africana  TGTACCATCTGATGGAATTTACAAATCCATTCATCTATTCGTTCGTTCATTCATTCATATCATAACAATGTTTT--ATATTGTACACGTATGCTAAAGCACTGGGTGAGAAGGAAGGCAG

Homo sapiens        GTATAAAAATTGTTGCTCTTAAGCAACAGACAACA-CAGAGAGATAAGGTCTCACCATAAAAAGTTAAATAGCAG--------CACATATACTAAGTGCCAAATGAGTGGTCTGGTCACA
Loxodonta africana  GTATAAAAATCCTTGCCCTTAAGGAGCTGACAGCATTACTGAGATAGGATCTCACCAC-AAAAGTTGAATAACAACACAGAGTCACATATACTAACTATCACTTGAGGGGTCTAGTCAGG

Homo sapiens        TCATACAAGAATTTTGAGGTTGGATATGTAGCATCAAAGACTGCTTTATCAGAAAGGTGAGACTTGAACTTGGCATAGAAGGGTACTTAGAACTTAGAGAAGAGAATGGAGCAGCACTTT
Loxodonta africana  TCATACAGGAATTTAGAGGCTGGATATGCAGGGTCAAAGACAGCTTTATCAAGAAGGTAGGTCTTGAGTTTGGCATACAAGGACACTTAGAGCTTAGAGACTAGGATAGAGCAGCACATC

Homo sapiens        CCAAACTCTGGGAATGGTGTGCATTAAAGCAGTCCTTCACAACCTTTATTGTGTCATGGCATGCAG-AGAAAAGCATAGTATTTGTAAAGCACACTAGGCTGAATGGAGGAAGCAGTTTG
Loxodonta africana  CCAGGCCGGAGGAATGGTGTGCACCAAGGCACTGCTTCCCAACCTTTATCATGTCATGGTACACAGAAAAAAAGCATAGTATTTGTAAAGCACA-----CTGGATGGAGGAAGCAGATGG

Homo sapiens        ACATCTAGAGGCAAGCATTCTAGGAGTTTCAACAGCACTGTCTCTGGGCTCAGTTGAGGACAGAGAAGAAATCTCATAGCCTCAGACACTTGTAATATGTTTGGGAAGGTCTGCACTAGG
Loxodonta africana  CAGCCCAGAGGCAAGCAACCTATAAGTTC------CACTGTCCCTGGGCTCAGCT---GACTGGTAAG----CTCATAGCCTCGTATATTTGTAAC-CACTTAGGAAGGTCTGTACTAAG

Homo sapiens        TATTGGGGGCACACTGTGGTCATGGTGGGTCTAAAGACCACTGACAAGCCCACAGGG-ATGAAATGAGGGGAATTTCCAGAGAAGCCATAGTAGATACCATTGGAAAGAAAAGTTATGGG
Loxodonta africana  GTATCGGTGCACACTGCAGTCATGGTGGGCTTTAAGACCATTGACAAAACTGCAGTGCATGAAATGGAGGGAATTTTTGGAAGAGCCATGGCAGATACCATTGGAGAGAAAAGTTATGGC

Homo sapiens        CAAATGATGAAGGGCTTTTGAATTTTATTCTGTGGACCTACAGAATAGGTAGGCAAAATATGCATAGCACTGTTTTAGAAAGATTCCACTGGTTTTCATATGGGAGATTAACTAGAGGGA
Loxodonta africana  CAAATAATGAAGAGTCTTTGAATTTTATTCTGTAGACTTACAAAACAGGTAGTTGAAGTGCACATAGCTTTGTTTTAGAAAGATACCACTGACATCAGTAAAGAGGGTAGACTGGAGGGA

Homo sapiens        AGAAGAGTAGTCAAGAGGCTATTACCATTGTCCAGGTGGGAGGTGATGTTATCTTGAACTAGATTGATAGTAATAGAGACAGAGAATGGTGGACAGAATTGAGTTATATTTTGGAGGTAA
Loxodonta africana  AGGAAAGTAGTCAGGAGGCTATTACATTTTT------------------------------GAGTGATGGCAATGAAGACAGAGAAAAACGGGCAGAATCGAGTTGTATTTTGGACGTAA

Homo sapiens        AAATGTCAGGGTTTGGTAATAGAATTGCATAATGGAGAGTAGAAGAAAGG----GAGGTGACAAGGAGGATTTCAAATTTTCAAGTTTAAGCAAATGCTTATGAATAGTGATGCCATTCT
Loxodonta africana  AATTGACAGGGTTTGGTAAC-GGATTGCAT-ACGGAAAGTAGGAGGAAAAAATTGAGGAGATAAAGATGATTTCCAGGTTTCAAACTTGAGCACATGGGTGTGAATAGTGATGCCATTCT

Homo sapiens        TTAAGATAGAGAGCCCAGGAGAAGTAGAAAGATTTTTTTGGAGGGGAAGGACAAATGAGATGATGAGTTTCAAGTTGA--CATTTGGAGGCAAGGAAACCAGTTAGGAGGCTATTGTAAA
Loxodonta africana  CTAAGATGAAAAACCCATGGGAAGGAGCACGTTTTTTGGAGAGAAGAGGGACATATAAGATGACGAATTTTGAATTGACTCATTGAGAGTCAAGGGAAGCAGTTAAGAAG----------

Homo sapiens        AGCCAAGTAAGACAAGGTGAGATCCTGGACTACAATGGTAGCAGTGGGAATGGAAGGAAAATGGTGAATTGAGAGACATTATACAGGAAGAATTGATAGGATTTTGTCACGGACTGACTT
Loxodonta africana  -GCCAGGTGAGACAGGATG--------------------CGCAGTGGGAATGAATAGAAAATATTGGACTGGGAGGCATTATACAGGAAGACTTGACAGGATTTAGTTATGGACTAAATC

Homo sapiens        CTGGAGTTGAAAGGTAGAGGAAGGCTTGAAGCCTAAGATATCTTCTGTCCTTTATCTTCTTAGTGATGCTCCGATCTTCGTGGCTTAATATACTATCTATTTGCTGATGAGTCACAAATT
Loxodonta africana  CAGG-GTTGAAAGGTAGAAGAAGGCTTCAAGCCTTAGATCTCTTCTGCTATCTATTTCCTTGGTGATTCTTCCACTGTCATGGCTTAAAGTACTATCTATGTGC-AATGACTC-CAAAGT

Homo sapiens        TGTATCTCCA---------------TACTTTTATGCCCAATTGCCTGCTGGACATATCAACTTGCATGCCTAATAGGTATCTCCAACTTACCATGTTCAAAACTG-----------CCCC
Loxodonta africana  TGTATCTCCAGCCTGGACCTTTACATACTTTTATATCCAACTGCCTACTTAACATATTAACTTGGATGCCTAATAAGCATCTCAAATTTAGCATGTCCAAAACTGAACTCTTGATTTCCC

Homo sapiens        TGCCCACAACCCCCAACAAAACCTGCTGCACTCACAAAACCTGCTCCTCACAGTCTTCCCCGTCTCCCTAAATACCAATTTCATTCTTCCGGTTGCTCAGGCGGAAGACCTAGGAATCAA
Loxodonta africana  CACCCCAGACCCCCACCCAAGCCAGCTCCACTTGCCAAACCTGCTCCTTGCAGTATTCCTCATCTCACTAAATAGTAATGTTATTCTTTCAGTTACTTAGGC---AGACCTTGGAATCAT

Homo sapiens        TCTTGATTTTTCCCTCTTTCATATCCTACATTTATTTGATCAGCAAATCTTATTGGTTCTACCTTCAGAATATATCTAGAATCTGACCACTTTTTC--------ATTGCTACTATCCTGG
Loxodonta africana  CTTTGATCCTTTT-TCTCTCACATCCTA-----------TCGGCAAATCTTGCTGGTTCTACCATTAAAATATATCTAGAATCTGACCAATTTTCCTCACCTCCACTGCTAGTACCCTTG

Homo sapiens        TTTATGTCACAATGATCTGCTTCCTGGATTATATTGCACTAGCCTCTCAACTGTTCTCCTTGCTTCTGTTCTTGCCACCATCACTCAAGCCTATTATTCTCTCACTGTTTTTTGGAACCA
Loxodonta africana  TTCAAGTCATAACAATCTATCTCCTGAATTATA--GTACTAGCCTACCAAGTGTTCTACTTGCTTCT--TCCTTTCTCCAACACCCCAGACTATCGTCTACTGGTTG------AAGGCCA

Homo sapiens        GGATAATTTTATTA-TGTGAATGTCAGATCATGTCACTCCTCTGCTCAAAACTCCTCCAATGGCTTCCCATCTGACACTCAGTAACAACAAAACCCCTTATAATGGCCCATAA-TGCTGT
Loxodonta africana  GACTAATCTTACTAATACAAAAATCAGATCATGTCACTCTTCTGCTCAAAACCCCTTCAATGGCTTACTATCTTACACATAGTAAAAAGAAAAATTCTTACAACGGCCCGCAAATGCTGT

Homo sapiens        ATGCAACCCAGATATTGACATA---------CTCCTGTCCTTCAGGTGATGGATCAAATATCCC--ACTTGGTAAAATCTTCTCTGATTACCATATTTAGTATTGCAAGCGCCTTCTCGC
Loxodonta africana  ATACAACCCAGATATTCACATAACTTACTCCCTCACCTCCTTTAGGTATTGGACCAAATGTCCCCTACTCATTGAGATCTTCCCTGACTGCCAAGTTTGAAAAAGGAACCCCCCTC---C

Homo sapiens        CATTCTCTATCTGCCTTCCATGTTTTATTTC--TTTATAC-TTTGAATACTAGCAGATGTTATATGTTTATCTATTTATTTGTTTATCATCTATCTCCTTCTGCTAGAACAGGAGCTGGA
Loxodonta africana  CATTCCCTATCTGCCATCTTTGTTTTATTTTAATTGATGCGTTTTAACACCACTTGACATGATATACTTATCTATTTATTTGTTTATTGTTTGTCTCCTCCTATTAGAACAGGGGTCAAC

Homo sapiens        AAACTTTTTCTGTAAAGGTCCAGATAATAAATATTTTAGGCTTTGTGAGCCACGTAATCTTTAACACAACTACCTACCTCTATCATTGTTATACAAAAGCTGCCATAGATGA--------
Loxodonta africana  AAATTTTTTCTGTAAAGTGCCCGAGAGTAAATATTTTAGGCTTTGCAAGCCAGATGGTTTCTATTGCAACTATTCAACTCTGTTGTTGTTACACAAAAGCGGCCATAAATGATACATAAA

Homo sapiens        -------GCATGGCTGTGTTCCAATAAAACTGTATTTACCAAAACAGGGAGCGGGCTAGATTTAGCTAATGGGCCATAGTGTACTGACCCCTGCTAGAATGTAAATTCCAGGAGGGCAAG
Loxodonta africana  TGAATGAACATGGCTCTGTTCCAATAAAACTGTATTTGCCACAACAGATAGCAGGCCAGATTTGGCCCGTGGGCCAGAGTTTGCTGAATCCTACTAAACTGTAAGTTTCAGGAGGGCGAG

Homo sapiens        GACTTTTGTTTACTGCTGTATCTCC-AACATCTAGAATAGTACCTGGCATATAGTAGGCA---CATGATTTATTTTACTGAATGAATGCATCAGTGGGAGATTGGAAAGCTGTGGAATAA
Loxodonta africana  GATTTTTATTTACTACTATATCTCCCAACACGTAGAACAGTGCCTGGCACATAGTAGACAGTGCAT------TTTTGTCGAATGAATGAACCAGTGGAGGACTGG--------GGAATAA

Homo sapiens        AAAATATATATATATAACAGAGCCATTGATGGTGCAGAATCCTGAACAAAATCTAGCTTTGGATGGGTTTGCAGCATATCAACTCTCCTTCCTTCTTTTTCCCCTCATCCCTACACTAAA
Loxodonta africana  GGAGAAT-----TATAGTAAAACCATTGAGAGAGCAGAATCTTGAGCAAGAGCTGATTTTGGCTAGGTTTGCAGCGTATCAATTCTCCTTCCTCCCCTC-CCCCTCATCCCTACACTGTC

Homo sapiens        ACTTAAGCTTGTTATATAAAGTAAAAGTTGCTTTCATTGCCTGTGCAATTCTCCCAATGTGTGAAATTTCATCTGACTTCCTTTAATGTT--GTCATTTGTCTATTTAACCCTCCTGACT
Loxodonta africana  GCTTAAGCGTGTCTTATGAAGTAAAA-TTGCTTTCATTGTCTGTGCAGCTCTCCTGATGTGTGAAATTTCATCAGACTTCCTTTAACGTTCAGACATTTGTCTATTCAATCCTCCTGACT

Homo sapiens        CAGACTCCCTCTAATAACAATAATAACTCAAATACTCTGTACTAAAACCAGAACGCTGAAATCAGTATTTTTAATTAATTTGAAACAACTGCTAGCAATAGCTTCTATTGACAACTCTAC
Loxodonta africana  CAGACTC--TCTAATAACAATAATAACTCAA-TACTCTGCACTAAAACCAGAACGCCAAAATCTGAATTTTTAATTAATTTAAAACAACTGTTAGCAATAGCTTCGATGGACAGCTCTCC

Homo sapiens        GCTTTTCAACACTTAATCATATTCTGAAGTACTCCCAGAAGCTGGGCGACTGTTACTCTTATGATATGAGATGATATTTTCCTTCCATGCATTCAATGTGAATGGTTTGTTCTGAAAAAC
Loxodonta africana  ACTTCTCAACACTTCATCATGTTTTGAAGCACTCTCTGAAGTTGAGCAACTGTTACTCTTATGATATGGAACATTATTTTTCTTCCATGCATTTGCTGCGAATGGCTTGTTCTGATAAAC

Homo sapiens        TTCAGCTTTCATGTACTCCTAATGGTAAGAATCACATGGTAGGAAATATGATAAATGAAGTATTTACCAGTTCTTACTGACTGTTCTTTCATACTTCCTTGGATCTCTTACAGGCACTAA
Loxodonta africana  TTCAGCTTTCGAGTACTCCCAACAGCAGGAATCACATGGTAAGAGATATAGTAAATGCAGTATTCATCAGTACTTAGGGACTGTTCTCTCATCCTCCCCTGGATCTTTTATGGTCATTAA

Homo sapiens        AGATATCCTGTAGTTATATGACTTCCTATTGAAAATGCACTTTCACTCATTCATTATTTTATATAGTCTTCCACACATTGC-TAACATTTACCAAACATTCACTACGTATGTGGTATTGT
Loxodonta africana  AAGTTCCCTGGAGTTATATACCTTTCTATTGAAAATGCA----CATGAATTTATTATTTCATGTGATTCTTCAAATAATTCGTAACGTTCGCTGAGCATTCACTATGTATGTGGTACTGT

Homo sapiens        GCTGGGGGA-TTTATATATATTAATTCATTTAATTCTTATAAAACACCTAAGTGATATATACTAT------CATAATTTTATAGATGAAGGAACTGAAGTACTAAAAGCTGAAGTAATTT
Loxodonta africana  GCTAGGAAACTTTAAATATATTAACTCATTTAATTCTTATAAAACACCTGGGACATAGGCACTATTATTACCATCATTTTACAGATGAGGGAACTAAGGTACAGAAAGCTGACATAATTT

Homo sapiens        GCTCAAGGCCCCACAACCAGCAGAAAAGGAAGCTAGCATTTCAACTCAGGTCTGCCCAACTCCAGAGCCAAGATTTATCTATTGTTTTTTGTCACCTCAACAAATATTTACCATATGCCA
Loxodonta africana  GCTCAAGGCCCCACAGCTAGCAGAAAGGGGAGCTAGGGTTTGAGTCTAGGTCTGCCTGACTCCAGAGTCAAGATTTATTCACTGCTTTCTACCACTTCAACAAATATTTACCATATTCTA

Homo sapiens        TGCAGCACACTAGGCAGTACCTACAAAGATGAATAGGGCATGATCTCCTTGGCCTTCCAGAGGTAACACTAGTTGGGGCTATATGGGTGGGAATGGAG--TGTCAGATGCAGAAGCTAAG
Loxodonta africana  TGCAGTGTGCCAGGCACTACCTGAAAAATTGAATAAGGTGCTATCTTCCTGACCTCCCAGAGGTGACACTGATTGGGGCTGTGTGGGT-GGAGTGGAGTCTGTCAGATGTAGAAACCAAG

Homo sapiens        GAATTTTCTCTTTACAAACAGGTGGGTGAGAAAGTAGACACAGCACTGTGAATTTGTGGAGTTTTGAAAAAT--GGACAGAGGGCTACAGACTGAGAGGG----AATAGGGCCACCAACT
Loxodonta africana  GAATTCTTTGTTTAAGGACAGGTTGACGATTAAGTGCATATAACACTGTGAATTCACAGAGTTCTGAAAAATAGGGACCAAAGGCCATAGACTGAAAGGGAATAAATAGGGCCTCCAACT

Homo sapiens        CTAGAATTAAGGTCAACTGATAGGGTAGAAACATGCCATTCTCAGGGGCTTAAGCTGACAAAAGAAATTGCCCACTGTAGAAATGAGATTGGCTGCCCACAGAAAAGTATTTACTGATAG
Loxodonta africana  TTAGAGACAAGGAGAACCAATAGGCTGGAAAAATCCCACTCTCAGGAACTTAAACTGACAAGA-AAATTACCCACTGTAGAAATGAGATTGACTGTCCACACAAAATTGCTTACTTACAA

Homo sapiens        A--TAAGGGACCTCCCTATGTGTCATTAAGGAGTAATGCTCTAAAATGTAATCAGTGGAAAACACAACTGTAGCCTTGGAAATGCTATGGCTGGGAAAGTCCA-TATAACTAAGACGGGG
Loxodonta africana  GATTAAGGGACCTCCCTACATGTCATCAAGGAACAATGCTCTGAAGTGTAACCAGTAGAAAACAATACTATAGACTTGAAAATTCTATGGTTGGGAAAGTCCATTGAAATTAAGGAGGAG

Homo sapiens        TTCATGATATTGTGACATGTACAAACAAGTTATTTGACCATT-ATAGAGAGAAAAATAAAAATAATTAGAAAGAGAAGTTGCTTTTATTACTCTGTGCCCTGGCCTACTGACACATTTAT
```

```
Loxodonta africana  TTCATGATATTGTGATATGTGCA---AAGTTATTTGACCATTAAGAGAAACAAAAATAAAATTAATTAGAAAGAAAATTGGTTTCTCTCGCTCTGTGTCCTGCCCTGC-------TTCAT

Homo sapiens        TGACTTTCTGATAGGGTGATGCATGGATGCATCAGACTGAATAAACTACATGACTCCATCTCTTCAGATCATCCCACACCATATACTATCTCTTCACTCACCAGTAATAACAGTCACCAT
Loxodonta africana  CGTCTTTTTGTCAGAG-GATGGGTGGTTGCATCGGGCTCTATAAATTACATGACTTCATCTCTTCAGATCATCACATACCTTGTACTACCTCTTCATTCACCAGTAACAACGGCCACCAT

Homo sapiens        ATAACAAGTGCCTACAGAGTCAAACACTTTACATAAATTATCTTATTTAATCCTCAAGGCAACTCTATGATATCATAGAATTCTATTATTAGTTCCATTTTCAAATGACGGAACTGAGGC
Loxodonta africana  ATAAT------------GCCAAGTACTTTGCATAAATTATCTCATTTACATCTCACAACAATGCTATGAGAT----AGGTTCTACTATTAGCCCCATCTTCAGATGAGGGAACTGAGGC

Homo sapiens        TCAGAGAGGTTATGTGACTT----AAGCCCATAAAACGGAATGAGTGGTATATTAGTTTTCTGTGACTACCATAACAAATTGCCACAAACTTGGTGGCTTAAAACAGCAGATATTTATAC
Loxodonta africana  TCAGTGAGGCTATGTGACTTACTGAAGCCTAAAAAACTAAATGGGTGTTGTATTCAGTTTCTAAGGCTGCCATAATAAATAACTACAAGTTTGGTGGCTTAAAACAACAGAAATTTATTC

Homo sapiens        TTTCACAGTTCTGGAGGCCAGAAGTTCAAAATCAAGGTGTTGGCAGGGCCATGCTCCCTACAAAGGCTCTAGGGGAGATTCTGTTCCTTGCCTCTTC------CTAGCGGTTCCAGGTGT
Loxodonta africana  TCTCACAGTTCTGGAGGCCAGAAGTCTGAAATCAAGGTGTTGGTAAGGCCACACTCTCT--GAAGGCTCTAGGGGAGAATGT-TTCCTTGTCTCCTCCAGCTTCCTGTAGCTCCAAACAT

Homo sapiens        TGCTTGGCTTGTGGTAGCATCATTCCAATCCCTGCCTCTGTCTTCAGATGG-GCTTCTCCCTTGTCTCTGTGCC----CTGTCACTTATAAGGACACTTATCATTGGATTTACAGCCCAC
Loxodonta africana  TGCTTGGCTTGTGGCAGCATAACTCCAGTCTCTGCCT-TCTCTGCACATGGCTCTTTTCTTGTGTGTATGTGTCTTTTCTGTCCCTTATAAGGATACTTGTCACTGAATTTAGGGGCCAC

Homo sapiens        CCTAATCCAGGATGATCTCATGTAGACATCCTTATCTTACATACATCAGCAAAGAGTCATATTCTAAATAAGTTCACATTTTGCAGTTCCAGTTGGATGTAACTTTTGGGGGCGGTGACA
Loxodonta africana  GCTAATCCAGGATGATCTCAAG-----ATCCTTACCTTAATTACATCTGCAAAGACCCTTATTCCAAATAAGGTCATATTTTGAGCTTTCAGGTGGACATATCTTTTGGGAG----GACA

Homo sapiens        CAATTCAACCCACTATGTATAGCCTAGCTGGGAC---CCTAG-CCTATCTGACTCCAAAACTCTGGCTCTTTCCACTAACTAAAGCAGGCTGCTCCCACCAAACAAACAGCCTTTTGTCT
Loxodonta africana  CCATTTAACCCACTATAAGTGGCCTAGCTAAGACAAACCTAGATCTACCTGATTCCAAAACTCTGGCTCTTTCTGTTTAATAA----TGCTGCCTCTACCAAACAA----CATTTTGTCT

Homo sapiens        TTATTTGGTTTCCTA---AGTAGATTTCCTACTGGG---------------------ATTTATCAAGATGAATTAATAAATTGACATTTTGGCTTCCAAATTATTGCCTTATAAGTTTA
Loxodonta africana  TTATTTGATTTCCCACTGAGAAGAGGGTGTACTGATTAACATGGTTTACTTTGTAGCTAATTATCAGAATGAATTAATAAATTGACATCCTGGTTTCTAAAACATTGCCGTAAGAGTTTA

Homo sapiens        GGTTGTGTGAAATTTGTCCCCTAGAGCCTGATCAATCCCTGGCTAAAATGGAAAAAATTGAGTTTTGGAACCTGTGAAGCTGATGAATGGATCCTGAATA-GGACCAAGGTCCATTTAGA
Loxodonta africana  GATTGTGTGGAATTTATCCCCCAGAGCCTAATCAGTCTGTGGTTAAAACTGACAAAATTGAGTTTTGGAACCTGCAAAGCTGAGGATTGGATCCTGGATAAGGACCAAGGTCCCTTTAGG

Homo sapiens        GTATAAGCATCCCACAGCCTGCCAGCCATACTAATTTCTTTACTATATATGTGACCATAATATAATGGGATAGCATAATATTCTAAGTCTACCGAAGAATGTCTAATCATCTAAGAGCTT
Loxodonta africana  GTATAGCCATCCCACAGCCTACCAGCCCTACTTGCTTCTTTACTTTATAAGTGTCCCTAATATAATGGCATACCGCAAACTGCTGAGTGTACAGAAGAAATTCTAATCACTTGAGAGCTT

Homo sapiens        ACA------GGGGGAATTTTTGTGTACCTTAATATTATATCC-AGAAATATTCCCTGTAGTGCCAACTATATTTGTTCCTGAGCCTCAGCTCCCTGCATCAGGTCACAAGGAA---GATG
Loxodonta africana  ACCCAACCTGAGGGATTTTGTGTCCACCTTGATGTTATGTCCCAGAAAGATTCCTCATAACATCTATTATGTTTGCATCTGAGCCTCATTCCCCACCATCAAGTCACAGGGAATGAGAGG

Homo sapiens        TCTCTAGGAGTTCATTATCACATTAAGACAAGGATTCTGTAGGAAAATGGGAATCTAGAAACAGTAGGGACTTCCTGTACCTCTACATCAGTGTCTTTGCAGGGGAATTGCCCTAGCTGA
Loxodonta africana  TCTCTAAGAGCTCATTATCACATTAGCATGAGGATTTTG-AAGAAAATGGAAGTCTAGAAACAGTAGGGACTTCCTGTGCCTCTAAATCAGTCTCTTTGCTGGGCAACTGCTCCAGCTGA

Homo sapiens        CCAGTTGACACTGGCCTGCTCACACCTTAATTATGAATAGAATATTCCCAGGGATGTCTTTCTCAGATTGTGGTCAGTTTGTGGGCCGCCTATTGTGGTGGTCCACTGAGTGACACCTGG
Loxodonta africana  CCAGTTCACACTGGCCTGCTCACACTTTGATTATGAAGAGAATATCCCCAGGGATGGGTTTCTCAGATTGTGGTCAGCCTATGGACTGCCCATAGTGGTGGACGACTGAGTGCCACCCAA

Homo sapiens        GGAGCTAGTTAAAAATGTCAGTTTCCTGGTCCTTTATAGCAGACTTACTGAGTTAGTATATCTGAAGATAGGGT-CAGGAGCCTGCA--TTTTAAAACAAGATTCTGGAATAATTCTGAT
Loxodonta africana  GGAGCTTGTTAAAAATGTCCATTTCCTTGTCCTTATGCAAGGCCCTACTGAACTAGAATCTTTCAAGCTGGGGTCCAGAAACCCGCATTTTTTAAAACAAGTACCTGGAGTGGTTATAAT

Homo sapiens        ACATATTCAAGTTTGAGAACCATTGCCTTCTGGTGAAATCAGGCTTCCATGACGTTGAAATACAGAATAAACTCCAAATGTTCATTTACAATTCCAGCCAATAATCACTTACCTTATTGC
Loxodonta africana  ATACATTCAAGTTTGAGAGC--TTGCATTCTGATGAAATTAGGCTTCCACAATATTGAGATACAGATTAAACTCCAAATGTTCATTTGCAATTTCAGCCAATAACCAATTACGGTATTGC

Homo sapiens        ATTCTTGTAGCGTGATCTATATGCTTTAGTTCATGTGACAATATCATTTCATATGGCTTAATTTAAATGAAAGCAAAGGCAGTTATGGCAAG---ATTTCTTAATCCTGCTCTCCTGAGA
Loxodonta africana  ATTCTGGTAGCACGATCTGCAGGCTTTAGTTCATGTGACAATATCATTTTATATGGCTTGATTTAAATCAAAGCAAAGGCAGTTATGGCAAGAAAATTTCTTAATCCCTGTCTCCTGAGA

Homo sapiens        CAATTTACATAATAACTTTTGGGGAGAATGCAATAATCTCTCTCTAATATCTTAAACTGATCTCTAAGGTTCCTTTTGGCTCCAACATTCTGATTCTATCAGTGTTCCTAGGGCTTCAAA
Loxodonta africana  CAATTTATGTAATAACTTTTGAGGAGACTGCAATAACCTCTTCCTAATATCTTAAATTGATCTCTAAGGTCCCTTTC-ACTGCAACATTCTGACTCTATGAATGTTCCTAGGGCTTCAAA

Homo sapiens        TCTGTCTGTGCA-AAACACATTGCTTGTTACAGACTAAACCTATTTTCCTTCTTCCAGGCAACAAGTTTCTGAAGAAAACTGGTTGCTCATGATCTTTCTGGGTGTTTTCTAATAATATA
Loxodonta africana  TCTGTTTTTTAAGAAACACACTGCTTGTTAAAGACTAAACCTATTTCCTTTCTTCCAGGCAAAGAGTTTCAGGAGAACACTAGTTGCTGGTGATCTTCCTTGATGTTTTCTAACAACGTG

Homo sapiens        TACCTTCTCACAATATTATTGCAAAGAGTGAGCGAGGTGATCTATGCAAAGTGCCAAGCATAGTGCCTGACACA-AGGAAGTGGTTAGTAAATATGCATGCTG-----CTTCCAGTGCAT
Loxodonta africana  TACCTTCTCACAGTAGTGTTGTGAAGAGT----AGGATAATATATGCAAAGTGCTAAGCATAGTGCAGGATGTACAGAAAATTGTTAATAAATGTTCATGATGTTTATCTTCCAGTACAT

Homo sapiens        TGTGACAATACTATCTCTTGAATTATTGAAAATATAACATCAGGAAACTTCAGCTGAATGAAAACTAGGGAATTAGCTGTATTGACAATATTAGGTTTATTTGGCTTTAGGGGAACTAGA
Loxodonta africana  TGTCACAATAATCTCCCTCGAATTATTGAAAATATAACATCAGAG------AGCTGAATGAAAATTAGGGAATCAGCTCTATTGGCAATATTGGGTTTATTTGGCTTTAGGGGAAATGGA

Homo sapiens        TAGAACCGAAATCTCTGCAGACTGGCTCAGCTGCAGAATAGAGAGATGGCCCGGGGAGGACATGTGTTGGTTCCTGCCATGCATATTGTCTTGGAGAAAAGCTGCTATGACTCTGGCCCA
Loxodonta africana  TACAACCAAAATCTCTGAGGGTTGGCTCAGCTGCAGCATACTGAGATTATCCTGGGAAGG---GGGTGAGTTCCTGCCACTCACATTGCCTTGGAGAAAGGCTGCAGTGACTCTGACCTA

Homo sapiens        ACAAAGGTTTTATCCTAAAAGTCAGCTTCCAATCCCCAACATGATTCCTAAGCTAGCATTTCTCAAAATGTATTCCATGGAGTACTAGTTTCTGAGGATTGATGTTGATA-GTATTACAT
Loxodonta africana  GTAGAGA-TTCGGCTTAAAACTCAGCTTCTACTTCTCAACATGCTTATTAAGCTAGTACTTCTCCAAGTATGTTCCATGGAGTACTAATCCTTGAGGAAGGGTGTTGATAGGTATTATAC

Homo sapiens        ----AAAGAGCTCCATGAACAAGCAATGTTACAAACCACTGGATTAGTCA-----AATGTGGTTTCCTGGCTGCGAGAATATTCAGAGTCCTTAATAGGTCAACAGGT-AATGTGAATCT
Loxodonta africana  AAAAAAAGAGCTCCATGAACAAGCAAAGTTCTGAACCACTGGATTAATCAAAGTGAACAAGGTTTCCCAACTGCAGGAATATTC--AGTCCTTAATATGCTAACAGGCAAACGTGAATCT

Homo sapiens        CCAAAA----ACTAATG------GGGTATACCGGATTTGAAAATTTTTGTGACCTCACTCACAGAGATGGTGGTGAGGGACTAGTGGTTCTGAAAAATGCAATTATGAGAACACTACTCC
Loxodonta africana  CCAAGAGAGGGCTAGTGTACACAGGGTTTTCTAGACTTGAAAGGCCTGGTGATCTTATTCAGAACGATGGTGGTGAGGGGCTAGTGGCTCTGAGGAACACAATTATGAGAAGATT-----

Homo sapiens        AAAATGACTAGTGACTTGCTAACAAGAATGGACACTTTTCAGCCCTTACATGATAAAAGT--TAAGAGAACAATTACGTGCTAAACTATAGAGTACTGTCCCTCAGCCCCCTAGTGTCTC
Loxodonta africana  ------------GCTTTCTGAATTGAATGAACATCCTTTATCCCTTACATGACAAAAGTAACAAGAGAACAATTATGTGCTAAACTGTGGAGTACCGT--TTCAGTTCTCTCATGTCCT

Homo sapiens        CATTTTCTTCTTTGATCAGCTTAGATTCTATAACCATCCTTAAAGTCACTGTTTAGCATACACCCTAGATTCCCTAGGCTCTCTCTGCTTTCATGTGATTTGAATAACAGACATCCAATC
Loxodonta africana  CATTTTCTTCCTTGCCCAGCTTAGATTCCATATCCATTATTACAGTCACCCTTTTGCGTACACCCTAGCCTCACTTGCCTCTCTCTGGCTTCATCGTTCTTGAAT----GACACCCAACC

Homo sapiens        GTTGTTAAATCCTACTCTCTTTATTCCCAACCTGCCCCAGCAGCCGAATGAGATTGGATAAAAGCTTATAACCGTGCTAACCAGTCTCGCTTTAGATTCAGGACCACTAACTTCAAATGG
Loxodonta africana  CTGGTTAAATCCAACCCTGTTTATTCCAATCCTGCCTCAGTAGCTGAACAAGACTGGATAAAAACTTATAACCATGCCGACTAGTCTCCCTTTAGATTCATGACCACTAAACTCAAGTGG

Homo sapiens        GCCCTTAGTACTATCTGCTAACTTCACTACATTCATTTCATCCATTCATTCTTGAATGCTCCTGGGTGGCTTTTTTACATCTTTCCTTTCCTCAAACTTCCGACACTTGTTTCTACATTT
Loxodonta africana  GCTGCTAGTGCCACCTTGCAGTTTCACTACATTTCCTTCATCCATTCATTCTCATGCTCTCCTGGGTGATTGTCTTACACCCTCCCTCTTCCCAAACTTCCAATACCTCTTCCCCCATCG

Homo sapiens        TCCCTTTCAGCTGCT-ACCTTGTTTCCTATTTCACTGAGAAAATAGAAGTTGTCAGAAAAGA----------ACTCCCA-----------CCAATCTATTT-GTATTTGTATCCATAAA
Loxodonta africana  TTCCTCTCAGCTGCTGACCTTGCTTCCTATTTCATTGACAAAAGAGAAGCAGTCAGAAGAGAATGTCTACAGACTCCCACCATCATATCTTCCAACCTATTTGGCATATGTGTCCA----

Homo sapiens        CTCTGCCTTCCCTCTTGTCACTGTGAGCGAACCATCTGGGATTCCACTGAATGCTGACTCCTCTACTTATGCACTAAATTTTATTTCTTTTTGCCTAATCAAGGTCATTACTCAAGTCAT
Loxodonta africana  -----CCTTCCCTCTTGTCTCTATGAATGAAC........................................................................................

Homo sapiens        TCACTCTTCCCTCATGGCATCAATTTTTCTTTCATTGAACAACATCTTCAATGTTTAAAAAATCTGTTCTCTTCACCCAAGTCATAGCCCCATTTCTCTCCTCCTCAGAGCAAAAATTCT
Loxodonta africana  ........................................................................................................................

Homo sapiens        GGAATGAGTTGTCTCTGCTCATTCGTTCCAATTTCTCTCCTCTCATTTTCTCTTGAATCCTCTCCAGTCAGGCTTTTGCCATCATCACTCCACCAAAATGATTCATATCAAGGTCAACAA
Loxodonta africana  ........................................................................................................................

Homo sapiens        TATCTCCTCAGTATTAAATTCAATGGTCATTTTACTGTTCTCCTTTTTTTTTCGCCCAACAGCGGCATTTGACACAGTTTATCACTCTCTCCTTGAAACACTTTTTTCTTCCACTTGGCT
Loxodonta africana  ........................................................................................................................

Homo sapiens        TCCAGGACACCATGTAATTCTTGGCTCTTTCTACTTCACTAGCTACTATTTCTTAATATCCTTTACAGGTATGTCTTCATTTTCATGATAACTAGGTTGTCTGATTGGCATCTCAACTTG
Loxodonta africana  ........................................................................................................................

Homo sapiens        AAATTATCCAAGGTCAAAGTGACTATTTTCCTTATACCTGCTTCTCCCACAGCCTTCTTTATCCATTCTTCTGGTTATTTAGGCCAAAGCCTTGGAGTCTGACTTGACTCCCTTTTTCTC
Loxodonta africana  ........................................................................................................................

Homo sapiens        TCACACTCCAGATCCTATCTATCAGCAATACTTTCAGCTGAATAGTTAAAAATTTATTTGTTTAGAAACAGCATCTCACACTGTCACCCAGGCTGGAATGGAGTGGCACAATCATAGCTC
Loxodonta africana  ........................................................................................................................

Homo sapiens        ACTATAACTTCAGACTCCTGGGCTCAAGGGATCCTCCCACCTCAGATTTCCCAGTAGTTGGAACTACAGGTACTCACCACAGTGCTGGCTAATTATTATTATTATTATTATTATTATTAT
Loxodonta africana  ........................................................................................................................

Homo sapiens        TATTATTATTATTATTTTGTAGAAACAGATGTCTTGCTTTGTTGCCCAGGCTGGTCTTGAACTTCTGGCCTAAAGTTATTTTCCCACCATGGCCTCTCAAATTGTTGGGATTACAGGTGT
Loxodonta africana  ........................................................................................................................

Homo sapiens        GAGCCACTGCACCTGGCCTAAAAGTATATTTAGAATCTGAACACTTCTCTACTGCTATAACCTTAATCCAAGCCAGCATCATTTCTTACTTGGATCATTACATAATCCATTAAACATGTA
Loxodonta africana  ........................................................................................................................

Homo sapiens        AATCAGATCATGTCATGCTCCTGCTCAAAACCGTCCGTGGCCTTTTATATTGTTCACAATAAAAGCCAAAGTCTTTAGAGTGACCTACAAGATTCTACATGATCTACCTTCATTCCCATT
Loxodonta africana  ........................................................................................................................

Homo sapiens        ACCTCTCTGACCTCATGCCCTGTCACTCTCCTCTTTGATTACTGAGATCAGCCATATGGCTTACTTGCTGTTTCTCAGACATACCAAGAAAACTCCCAGAGGTCCATTGCTTTTCTCTCT
```

```
Loxodonta africana  ........................................................................................................................

Homo sapiens        AACTAGGATGTTTCTTCCTCATAGAGTGTCATGCTTATTCACTTGCTTCTGTCAAATCTCTGTGCAAATGACACTTTATCAGTGATACTTTCTTTATCATCTTATTTAAAAATTGCCACT
Loxodonta africana  ........................................................................................................................

Homo sapiens        CTCTGCATGCTTCCTACAACTTTACTCTGCTTAATTTTTTTCCATAGTACTTGTCATTTTCTGATATATTAATATTGATTTGTTTGTTTACTGTCTATCTTCACCACTAGAATATAAGCT
Loxodonta africana  ........................................................................................................................

Homo sapiens        TCAGGAAGGCAGGGATTTTATTTTGTTGCTGCTGTATCCCTAGTGTGTAATGTGGTAGCTGGCACATAATAGGTGCTCAATAAATTTCAAATTATTATAATAAAGAATGATTCCAAGTGC
Loxodonta africana  ........................................................................................................................

Homo sapiens        AATAGGAATTCCAAGATTCCTGCCATCTTCAGATGGACCCAGATATCCTTCATCATCTGCAGCAATCCCCCACCCAGTGCCAAACTCTGTGACACTGCATGTCTGGTAGTGAGTTTAGTC
Loxodonta africana  ........................................................................................................................

Homo sapiens        TTTACATGAGGACTTCCAGGAAGTTCCATAGATTCTTAAGCAAAGTGCTCCATTATTGAAGCTATTTAACTCCTAGAAAGATCTTTGTGGCTGTGTGCGGTGGCTCACGCCTGTAATCCC
Loxodonta africana  ........................................................................................................................

Homo sapiens        AGCACTTTGGGAGGCTGAGGTGGGCAGATCAGGAAGTCAGGGAATTGAGACCATCCTGGCTAACACAGTGAAACCCCGTCTCTACTAAAAATAGAAAAAATTAACCGGGTGTGGTGGCAC
Loxodonta africana  ........................................................................................................................

Homo sapiens        GCTCCTGTGGTCCCAGCTACTCGGTAGGCTGAGACAGGAGAATTGCTTGAACCCGGGGGGTGGAGGTTGCAGTGAGCAGAGATGGCACCACTGCACTACAGCCTGGGCGACAGAGCAAGA
Loxodonta africana  ........................................................................................................................

Homo sapiens        TTCTGTCTCAGTAAAAGAAAAAAAGAAAAAAAGAAAAAGAAAGATTTTTGCCAAGGGTCCTTTTCTGATACATAACATTTTTTGAATAGGTATTAGGTGTACACGATACAAAGTGCCAAA
Loxodonta africana  ......................................GAAATATGTTTGCCAAGGGTCTGTTT-TGATATGTAATATTTTT--AATAGGTATTAGATGTACTTGATACAACATGACAAA

Homo sapiens        TGTTCAAAGAGCAAGTAGTGAAAAGTATATTCTACCACTCCCACTGCTACCAACTTATTCCAAGCCACCCACATTTCTAAACTGGAATTATAGCACAATTTTTTAGAAACAGAAGTTAG
Loxodonta africana  TGCTCAAAGGAGAAGGAGTAAAAAGTATATTTCCCCACCTCCACTGCTACCACCCTGTTCTAAGCCACCCTTATTTTT---CTAGGATGATTGTGCAAATCCTTTAGAAACATAGGTCAG

Homo sapiens        ATGATGTCGCTCCTCTGCTAACATCAGCCTGGGGCATGTCATCACACTCAAGGTAAAAGCTAGAGTCTC-CCTCTGCC-CCCAGCCACCAAGATCCCTTCCTGGAGGCAAACACTCTTAC
Loxodonta africana  GTCATGTCACTTCTCTGCTAAAACCTGCCAGAGGTATCTCATCTCATTCAAAGTAAAAGCCAAAGTCTCATCTCTGTTGCCTGGCCACTAAGCTCCTTTCCTGGAGGCAACCTCTTTTGG

Homo sapiens        TAGTTTATTGTGAATTTTT-TAGAGATAGCATGTATATTATACACACATAT------CTCCATGTGTATAATTCATTTTTAAACTGTACACACTATTTTTTATA-TTTCTTTTTTTCTCA
Loxodonta africana  CAGTTTCTTGTGTATTTTTCTAGAGATAGCTTCAATATTATGCATATATATTTGTGGCTCTGTGTGTATAATTC-TTTTTAAACAATAAACATTATTTTTTATACCTTCCTTTCTTATAA

Homo sapiens        CTTTAT-TCTACCTCATTCTTTTTAATAGCTACAGTTGTATCA---GTATGGCTACTGTATTCTCCAGAGATTCTTAGTCTGGAGTCCATATATGGGTTTCAATGGGTCTATGAATCTCC
Loxodonta africana  CTCTATATCTACCTCGTTCTTTTTAATGGCTGCATAGTTATCCATTGTATGGCTGTTGTATTCTCTAAGGATTCCTAGACTGGAGTCCATGTACGGACTTCACTGGGTCTGTGAGCCTCC

Homo sapiens        TGAAAGTGCATGTTGACACTTGTGTGCGTGTGTACATGTATATCTTTTAGCAGAGCAGGCCCATATCTTTGATCAGATTGAAAGGAATCTGAGACCCAAA-ATGAGTAAGAAACCACTAT
Loxodonta africana  TGAAATTGCATGTGGATATTTGTATGCATGGGCATAGGCACATCTTTCTGGGGAGAGGGTCCGTACCTTTTATCAGATTTAAAGGGGTCTGAGACCCAAATGTGGTTAAGAAGCCACTAT

Homo sapiens        ATTGAGTCTGAATCTGCCTTCCTGAAACACCTACCCTTTAGTCCTAATTCCACTGTTTGGAGCAGTTCTGTCAGGACCTGGTACATAGAGGACATACAACAGGTAACTTGTGAAATGGAT
Loxodonta africana  ACTGAGTCTAAGCCTACCTCCCTGAAATTTTTACCCCTTTGTCCTAATTCTGACATTTGGAACAGTTCTATCAGAGCCTGGCACCTAGAGGATTTACTCAGTACATATTTGCAAATAAAT

Homo sapiens        GAAGGGACAACATAAAATGAATCTATTCTGTCTCTCTCTTTTTAAAATTTTTAAATTTTTATTTATTATTATTTTTTAAAGACAGAGTATCACTCTTGTCACCCAGGCTGGAGTGCAATG
Loxodonta africana  GAACGGACAACACAAAATAAATCTACTCCATCTCTGTGTT................................................................................

Homo sapiens        GCACAATATTGGTTCACTGAAAACTTCCACTCCCAGGTTCAAGCAATTGTCCTGCCTCAGCCTCCCCAGTAGCTAGGATTACAGGCACCTGCCACCATGCCCAGCTAATTTTTGTATTTT
Loxodonta africana  ........................................................................................................................

Homo sapiens        TAGTAGAGACATGGTTTCACCATGTTGGCCAGGCTGGTCACAAACTCCTGACCTCATGTGATCCACCCGCCTAGGCCTCCCAAAGTGCTGGGATTACAAGCGTGAGCCACTGCGCCCGGC
Loxodonta africana  ........................................................................................................................

Homo sapiens        CTATTCTCTCTTCTTTATGTTAGCTTTTGGTTAGCCTGAGGAAACTATCATATTCTAAGTCATCCCTTTTCTAAAGTTAATTACTTAATTTTCTTAACTTTTCCTAATTTGACATGGTTT
Loxodonta africana  ........................................................................................................................

Homo sapiens        CCAAACCTGTAGGCTTTCCCTACATGCTGTAATGTGGGTTTCTTCCTCAAATGCCATCGGGGCAAGCTGGTAATTTAAAAAAGTGAAGTGGATCAGATGTGAGACAACGGCAAGTGGTGG
Loxodonta africana  ................................................................................AGGTGAGGTAGACCAGGTGTGAGACAATAGCTAATGGTGA

Homo sapiens        GACCTGTG--GAGTCCACATGGCT-----AAGATGTTCACATTCAAACTCACATATAACACATCCATAACCA---------AACAAAAAATATTTTCAGGCCAGTTTTGGACTATGGGCA
Loxodonta africana  GACCTGTGAGGAGTCCATGTGGGTGTCTAAAGATGATCACATTCAAC--------TAATATCTCTATCACTATGTATGTATGATAAATGAATTTTGTATGCCAATTTGGGCTCATGGGCT

Homo sapiens        GGCAGCTTGCAAATATAAAAATTTGGACCTCGTACAGTTTGTTGATACTCTTTTCACATCTTCTGCACTCTATCAATTCACTTTTGTCCTCCCCATAGGCCTGACAATTTAATCAAACTA
Loxodonta africana  GCCAATCTGCAAATCTAATAATTTGGAATT------GTTTGTCAATACCCCTTTCACATCTTCTGCACTCCATCACTCCATCCTTATCTTTCTCATGCACCCTACAATTTAGTCAGACCA

Homo sapiens        AGGTAGTGCCTGCATCTCAAATACCCCTTAAGATTCCTTGCTATTATTCCTTTGCCAATATGGCTCTGTCTGTATAAAATGCCTTTGTGGAATATTCTCTACTTGCCCCTCAAGATCCAC
Loxodonta africana  AACT----TCTGATTCTT--------------ATTTCTTGCACATATTCCTTTGCCAATATAGCTC--TCTGCATAAAATGCTTATGCTGAGTGTTCTCTATGTGCCCCTTCAGATTCAC

Homo sapiens        TTTTAAAAATAGACTATTTTTTAGAGCAGTTTTAGGTTAACAGCAAAATTGAGAGGAACATACAGAGAGTTCCCGTTAGCCCCTAAAGATCCAGTTTTCACCCTTCTTCATCCTCTTTGT
Loxodonta africana  TTTT....................................................................................................................

Homo sapiens        GTACCCCCCGAGACTGACCTTTATAGACTGCATTTAAAAACATATTTTTAGTTATTGTGGGTACATAGTAGGTGTATATATTTTTGGGGTACATGAGATGTTCTGATACAGGCATGCAAT
Loxodonta africana  ........................................................................................................................

Homo sapiens        GTGAAATAAACACATCATGAAGAATGGGGTATCCATACCCTCAAGCATTTACTCACTGAGTCGCAAACAATCCAATTACAGTCCTTAAATTATTTTTAAATGTACAGTTATCACTGGCTA
Loxodonta africana  ........................................................................................................................

Homo sapiens        TAGTCACCCTGTTGTGCTATCAAATAGCAGATCTTATTCATTCTATTTTTTGTACCCATTGACCATCCCCAACTCCCCCCAGCCCCCCACTACCTTCCCAGCCTCTGGTAACCATATGAA
Loxodonta africana  ...................................................................................................................ATTAT

Homo sapiens        TTGGCTTCCTTGTCCTCTGAATTCTTCTTGGATATGGCCAATGTAAGGCACTAACAGGGGACTGGAGATCAGAGACAGGTTAAAGTCTGCGTATTTATTCTCCTGGCTCCCTCTTTATGG
Loxodonta africana  CTGGCTCCCTTGCCCTCTGGCTTCTTTTTGGATATGGCCAATGGAAGGCACTACCAGGAAATTGAAGATCAGAAGCAGACTGAAGTCTGGGTATTTATTCTCTCGGCTTCCTTTCTGTGG

Homo sapiens        AGTGGGTGCAGCTTGGCTGAATCCTGTTAGAAGGATCTGCTGTGAGCCACAGACTTTTTTTTGGCAGTCATCTTCTAAAGCTATGGCTACTCTGTCTAGGTTCTGGTTAACTGCTTTCTC
Loxodonta africana  GGTTTCTGCAGGTTGGTTGAGTCCTTCTAGAAGGATGTATTGCAAGCCACAGACCCTTTTG-GGTGGCCATCTTCTATAGCTACACCTACTCTGTCTGGGTTCTAGT-AACTACTTCCTC

Homo sapiens        CCCTTGGCTTTTCAGGTCTCAGGGTGGTAAGTGCTCCCTGGGGTGCTTCACCATCCTTTTTGCTTTCCCTTAACAATGCTCACACTGTTATAAACAGTCCGTTCATTAAACTCC----AC
Loxodonta africana  CCCTTGGCCCTTCAGTTCTAAGGACGGTAAAGACTCCCTGGGGGTC-----CATCCTTGTTGGTTTTCCTTAACGCTGCTCACCCTGTTATAAACAGTCC-CTCAATAAACTCCGAGCAC

Homo sapiens        TCACTT-CCCTGTT-GAATATGCTCTCAATATCCTACTAGATCCTTTACTGTTACCAATGCCTTCAACTCCCTTGTGTACTTGGTGTACTCCCCATTGTTAAGGTCCCAGCTCCTAGGAC
Loxodonta africana  TTACTTACCCTGTTTGAGTGTGCCATCTGGCCCCTACTGGATCCCCGATGGCTACAGAGGCCTTCC-TTCCCTTGTCTATTGGGTGAATTCCATCTTGTTCAGACCCAAGCTCCTT----

Homo sapiens        AATGTGGTGCATTCCTAGATATCACTCCCTTTGAGAAAGTTAGCTATTCCCCCACCTCTTTGAGAACATTTCTTGCAACACTCTGTGTTTA---------------------GATCTTG
Loxodonta africana  --TGTGAAGCTTTTTCAAGTGCCGTTCTCACTGCAAAAGTTAGCTATTCCCCTACCTCCTTAAGAACATTTCTTGCAGTATTCTGTGTTTATTTATATATGTTTCTCCCATGAGATCTTG

Homo sapiens        GGCTTCTTATGATTGGGAATGAAGCCTATTCGTTATTGTT--------TCCTGGCCAAGTAGATGTTTGGTGAGTATTGGTTATCTTTTTTGCTTT-------GTGAGGAGGATATAAAA
Loxodonta africana  AGTTTCCTAAGGCAAGGAATGAGGCTTATTCATCATTATTGTCTCAGTTTCTGGACAAATAGATGATCATTACATGTTTGTTATCTTTTTTTTTTTTTTTGCTGGGGAGTGGTGGTGAAG

Homo sapiens        TCAAGGAAGGAGGATATTTGCTTTT-TATTTCTTTCAAATTATTTGATTTT-AAAAGAAATGCCTACACATTATTTTTTAAAAATTAAAA----TGAAAAGAAAAAAGTA--GTAAAAGT
Loxodonta africana  TAAAGG---GAAGGTATTTGTTCTTATATTTATTTGAAATCATTTGATTTTGAAAAGAAATGCCTGCAC-----TTTGTAAAAAAAAAAAAAACTAAGAGAAAAAGAGCAAAGTAAAAAT

Homo sapiens        GGCA--CCGTTCTTCACATCCGTGCTGGAAGTAACAGTGGTAATGGTTTTTGTTGTGCATCCTTCCAGTATCTTTT-TATGCACATAAAGGCTTAAGTTTGTGTACATAAAATGTAGACT
Loxodonta africana  GAAAGTCTATCCCTCATATCCACGCTAGGGATAACCATGCTAATAGTTTGGGGTGTGCACCTTTCCAGTACTTTCCCTGTGCACTTAAAGGCATCCATTTGTATAAACACAACATAGACA

Homo sapiens        CACATATTTCTCTGCTCCTTGCTTTTTTTTTAACTTAACAA-----CACACCTGTCTTTCTATATCAGCCAATGTAAGTATACAGCCCTACTTCATTATGTTTTATGGCTGCATTGTATT
Loxodonta africana  CACATATTTCTCTGCCCCTTGCTTTTTTC---ACTTAACAATATATCACAACTATCTTTCTACGTCAGCCCATATAGGCATACAGACACACTTCATT--TTTTTATGGCTACACTGTATT

Homo sapiens        TCATTTGATGAATTTACCATAATTTATTTAACAAG-CCTCTATTATTAGCTA--------------------------------------GTTTATCTAATATTTTCACTGTTAAAAGCA
Loxodonta africana  CCATTGCAGGAATGTACCATAATTTATTTAACAAATCCCCTATTATTAGCTATAGGTCTGAACCACCGCACCACCAGGGTTCCACTGGATGTATATCTAATATTTTTCATG-------CA

Homo sapiens        ACACAACTTAAAACACAGAGACATACTGGATCAAATTTAAGCCCAAAAATATATGGCTGAGCTCCAGAAGCAAGAAAGGAAAACTGTCAGATTCCAGAGACAAGA-GAAAACTCACAGTC
Loxodonta africana  GTGCAACTCAAAACACAGAGACATGCTGAATAGGATACAACCACAAAAATATGCAG-TGGACCTGAAAAGTAAGAAAGGAAAACTCCCAGTTTCCAGAAACAAAAAGTGAACTCAAAATC

Homo sapiens        ACAGCAATGAGCATAAATTGACATTTCACCACTCACAGGAAAAATGGGACCAGGTATATTCCTTAAAGGATGAGATTATAAGTCCTCTGTAGGGCTTAGAATTGAGTTAGAATTGTGCTT
Loxodonta africana  ACAGCAATGAGCGTGAATTGAAGTTTCATAACTCACAGGGATAATGGATCCAGGTATACACCTTAGAGGCTGAGGTTTTAAGTCCTTTCTAGTACTTGGAATTGAGTTAGAACTGTGCTT

Homo sapiens        CCCTCATAAAACAAAGAGTAGAAGGATGCTGCCCCATTTATTATTTAAAAGCAGATTTCCCAAATAGTCAGGGGTACAATAATAACAAATCTTATTTAGTACTTACTATGTGCCAAGCAT
Loxodonta africana  CCTACACAAAACAAGGAGCAGAAGAGAGCTGCCCCATGAATGAATAGTGACCAGATTTACCAAATAGTCAGAAGCATAATAATAACACATCTTATTTAGTACTTACTATGTGT----CAC

Homo sapiens        TGTTCTACATGATTTAC---ATGTA----------------TTTATTTATTTAACCATCACAGAAACCCTATGATATTAGGTACTATTATAAACCCCATTTTATAAGTAGGGAGTAAAAC
Loxodonta africana  TAATCTACATGTTTTACATTATGTACTTATTTAATTTTTTTTTACTTATTTAATTATT-CAAAAACTTCATGA-GGTAGGTACTATTGTAATCCCCATTTTAGAAATGGTAAATAGAAA

Homo sapiens        GTTTAAGTAACTTGC----------GCAGCTAGGAGGTGGCAGCTCTAAAATTCAGACTCAGGCATCCTGACTCCAGGA-CCATGCCTGTAAGTA--AATGTGCTATATTGCTTCTCTAC
```

```
Loxodonta africana  GCTTAAGTAACTTGCTTAAGGTCAAACAGGTAGAGGCTGGCAAATCTAGGATTCAGACTCAGGTAGCCTGATTCCAGGATCCATGCTCTTAACTACTACTATACCATACTGCTTCTCCAC

Homo sapiens        AAACAGGATGTAGGTCATCTTCCTTAGGATGAGGTGGAAATAAACTCACCCATAAGAAGTTTAAAACTTTAAACCTGAGCTGCACACTAGTATGAAACCAAAATTCACAGTACCCACATA
Loxodonta africana  AAACAAGATTTAGGCTATCTGCCCTAGGATGGAATGGAAATAAGCTCATCCATAGAAAGTTTAGAACTCTAAACCTGAGCTGTACACTAGTAGGAGACCCAGATTCATACTATCAACATA

Homo sapiens        ATGCAGAAACCCTGGGGTGAGAACTTAACATAAAACCTGGCTCAGAGC-AGGAACACAATGAAACTCTAGCAGAGGCAAATGAAAAACCGCCTGTAGACCCACCTCCATAACTCAGAGTC
Loxodonta africana  CTGCAAAAACCCTAAGGTAAATACCTAACATA----------TAGAACTAGAAATAATATAGGACTCTGTCAGAGGCAAATAAAAAATTGCGCATACAGCCACCTCTGCAACTCAGAATC

Homo sapiens        CAATTCTTACGGAAGGGTTCTAACAGAAAACAGTTCCTGCAGAAGATAAGCTCACATGCAAAAAAATGATAAAGCACATGAGGAAATTGACTGGAATGAGTTAGTTGGTATATACAAGAA
Loxodonta africana  AAATTCTCACAGAACAGTTCTCACAGAAGACAACTCCTGCAGAAGATAAATGCACATGCAAAAAA--TATAAAGCAAAAGAGGAAACCCATTGCAATAAGTGAGTAAGTAGGTA-AAGAA

Homo sapiens        ATGGGAGAATTATGTCCCAAGAAACTACAGATAATACAGTAATCTGAAAGGACTTTATTTTTTGGGTTTTTCTCATCTTTTTGTAGCCCTCATGTATGAAAGAACTTTAAAATGTTGTAT
Loxodonta africana  ATGAGAGAATTCAGTGCCAAGGTACTACAGGTAATACCGATTTCTGAAAG...................................................AGACTTTAAAATAGTATAT

Homo sapiens        GTTTGAAGGTTTCAAGATGTAAAAAAGGAACATAATACATAAAGCAAGAGCACAACTTTCCTCGGTGTGTGTACACCAAATAAATAGGCCAGAAATGAAGAATATAGAAAATA-------
Loxodonta africana  GATTAAAATGTTCAAATGGATAAAAAAGAACAGAATACATAAAGCAAGA---------------ATGTGTATATATCAAAGAGA-AGTTCTAGAATGAAGACTATAGAAAAGATGGAAAA

Homo sapiens        -----TGGGATGAGCATTGTAAGATTTTAGTTCTAGCCCCAATGAAATATTTAAAAAGTCAAATGACAGTTTAAACTACAAATAAGTTATGAGTGAAGAGAGAACAAATGGAATAACAAA
Loxodonta africana  AGCATTGGGATGAGCATTGGAAGATATTAGTTCTAGTTCTGCTGTAATGTTC-AAAAGTCAATTGAGAGGTTAAAGTAAAAATGAGACTTAAGTGAAGACAGGATTAGTGAAATAACAAA

Homo sapiens        TAGAGCCGAGATAATCATAGAATATAGCATGGCACTATAAAAATGCTAAATATGAAAGGGAAAATAGAAGACACACATGATATAACAAGAAACTTCACATTCATCTAATA-AAGCTCCAG
Loxodonta africana  TAGAACCG----------AGAATATAGGACAGCAATATAAAAATGCTAAATATGAAAGAAAAATTAGGAGACACAGATGGCAGAATAAGAAGCTCCACTTATATCTAATAGAATTTCCAG

Homo sapiens        AA--AAAAAATAGGAAGAGATAATATTCAAAGAGAAATAGCTGATAA-TTTTCTAAAACTGAACAGGACATGAGTCCTGAGATTAAAACCATGTACCTAGTTCTAAGCAG----AATAAA
Loxodonta africana  AAGGAAAAAATAG---------------AAAGATAAATGGCTGAGAATTTTTCCATAACTGAATAAGATGTGAGTCCTCATGTTGAAATCATGGACCTAGTACTAAGCTGAATAAATAAA

Homo sapiens        GACAAATTGATACCCTGTAGATGCAACAGGGTGAAACTGTAGAACATCAAAGATAAAATGAAAATCTTGAAACCTACTATAGACAAACAATGTTGCAAAATATTTCTGTAGAATAGATTC
Loxodonta africana  GACAAATCGACATCCGCTAGACGCATCATGATAAAAATGCAGA-TGTCAAAGATAAAGGGAAAACCTTAAAACCTACTAGAGACAAATAATGTTACAAAGTATTTCTGTAGGATAGATTC

Homo sapiens        CTAAAA-TGGAATTTCTGGGCCAGGGTATATGCACATTAAAAAAA-TGAATATGTTTTAAATGCATGAGTGAGTGAATTAATGAGTCTAGTAG------TATTTCTCTCAATCTGGACAT
Loxodonta africana  TTAAAAGTGGAATTTCTGAATCAAGGGATATACACATTAAAAAAAATGAATGTATTTTAAATGAATGAGTGAGTGAATTAATGA-TCCAGTAGAGCCATTATTTCTCTCAGTCTGGACTT

Homo sapiens        A-TATTTATTTTAGTACAGCCTGAGATTGGATCCCCCAGCTATCTATTTATTTATATATTTGTGACTGCATCACCACTATTGATTTCTATTGAGCATAAGACCAATTAAAATCCCCAGGA
Loxodonta africana  GCTATTTCTTTTAGTACAGCCTCAGATTCCACTCCGCATCTAGCTATTTATTTATATATTTGCAACTGCATCACTACTATTGATTCATATTGAGCTTGAGGCCAATTAAAATCCCCAGAA

Homo sapiens        CTTTTTCAAATGAAAGGCTGTCAAATGAGGTCCTCTTTAACATGTACTTAGGCAATTAATCTTTAAAACTTAGAAGTAGGGCTATTAAGACATTGGTGAGATCACTGAGAGCTAGAGCTG
Loxodonta africana  CTTTCGCAAATGAAGGGCTGTCAAATGAGGTCCTCTCTAGCGTGTTCTTGGGCAATTAATTTTTAAAACTCCGATGCAAGACTATTCAGATATTAGACAGATCATTCAGAGCTGGAGCCA

Homo sapiens        TTAAAGAAGGTTTCATAAAGGAAGTAGGACTTAGGCTGCATAGAATTTATTTTCGGTGGAGGGTGCAAAGGACAGCTTCTATGTTTGTGGGATGGAGGTAAGAAGAGTGATAAGGTGCAT
Loxodonta africana  TTAAAGAAGGTTTCATAAAGTAAGTAGGACTGAGGCTGATTAGCATTTACGTTAGGTGGAGGAGACAGAGGAGGGCTTCTAGGTTTGTGGGATGGAGGTAAAAAGAGTGATGAGGTGCAT

Homo sapiens        GAATAGACCATAGGATAGGAAA--TGAGCTTGGGGTGGATATGTGGAGGGTGGGTGAGTAAAGAGATAAGGTTCAAGGGAAGGGGTGGG----GCAAGATTATGGAGGGCCTAAGAAGCC
Loxodonta africana  GAACAGGTCATAAGATAGGAAAAGTGAGTTTGGGGTGGAC-TGTGGAGGGTGGGAGAATAAAGAGATCAATTTCAATAGCCAGGCAGGAAGATACCAGACTATGGAGGCCCTAGAAAGCC

Homo sapiens        AGGGAG-AGTGTCTGCATATGATATTATACATGGGCAATTGGAAGTCCCTTCAGCCTTTTTA-AAGGAAATTATATGTTTTCTGACTGGCCAATGAAAGGCTAACTAACCCAGGAAAGAG
Loxodonta africana  AGGCAGAAGTGTTTGGATATGATACCATACATGGGCAATTGGGAGCCATTTCATCCTTTTAAGAAAGAAGTTATGTGTTATCTGACCAACCAAAGGAATGTTAACTAACCCAGGAAGTAA

Homo sapiens        CAAGAAGGCTGGGTGAAGTAATGCAGGCCCCTAGTTCTCCTGTTTCTGGGTTACTGGATAGGTTAT-ATAGCAAGTAGGCAAGCTTAGCAAACAGCTCAGGGACACTAGGGTGACTTGAT
Loxodonta africana  CAGGGAGACTGGCTAAATTAATACATGCCCCTAGTTCTTCTGTTTCTGAGTTACTGAGCAAATTATCCCAACAAGCTGTCAAGCTCCGCACACAGCTCAGGAACACTAGGGTGACATGAT

Homo sapiens        GTTAAACTGCCTGACTAGTGAGAATTAAATGTAATAAAACTGGAATTGCAAGACTAAAAATGGTATTTAGGCAGAATAATCTACTAGCAAATTCAGGATTCTTTAGAGTTGAGTGGGAAA
Loxodonta africana  GTTGAACCATCCGGCTAGTGGAAGCTATGTGTAATAAAACTGCTGTGGCAAGACTAAGAACGGTGGTTAGGAAGAACAGTGTAATAGCAAATTCAAGACTCTTTGCAGCTCAGTGGAAAT

Homo sapiens        TATAAGAAAGAAAGAAAGATGCTGGATGCAGGAGAACAAGAGATGGAATGAGAAAGTGGAATATACACTGGAATGAGCAACAGGAAACTTTAGTTCTAGTCTCAGCTCTACC-CCTACTA
Loxodonta africana  TATAGGAAAGTATGACA----CTGGGTGCAGGGAAAAAAGAGACAGAATGGGAGAATAGAAAACACACTGGAATAGGCACCAGAAAGCCTTAATTCTAGTCCCAGCTCCACCTCCCACTA

Homo sapiens        ACCGCACAACTTTGTGCAGATTACTTAAACATTGAGGCT-AGTTTCCTCCTCTGCAAACAACCTAGAATACAAACTCTA-AAGGGTGGTAACCAGATCTGCCATGTTCATCATTGTATCT
Loxodonta africana  ACCATGCGACCTTGTGCAGATGACATAGACAACGAGGCTCAGTTTCCTCATCTACAAACCTACTAGAATATAAACTCTACAAGGGTAGTAACCAGATCTACTTTGTTTACCTCTGTATCC

Homo sapiens        CTAGCCACTGGTACAGGGCCTAGAACGTAGTAGGGGCTCAGTAAATATTGGATGAATAGATGAATGAATGAATGAATGAATGAATCAATCAATCAATCAATTAGAGCCTGCCCTGCCTGC
Loxodonta africana  CTAGCCCTTAGCACAGGGTTTGGCACATAGTAGGGGCCCAGTAAATATTGGCTAAGTAGATAAACCAATGACTGAAGG--------------------TACTAACACCTGTCCTGCCTAT

Homo sapiens        CTCACAGAGTTATTATAAACTTCAAATGAAATCAGGGA------TGGGGAAATATTACATGAACTTAAAACACTGAGCAATCCGATGGTAAAATACTACAGCTACTACTCACAATTATTA
Loxodonta africana  CTCATAGAGTTATTGTGAGATTCAGATGAGATCAAAAAACAAACCAAGAAAGGGCCACGTGAACTTAAAACACTAAGCAATTCTGTGGTAAAATACTACAGCTAT--------------

Homo sapiens        TTTTAAAAATTAAATTAGAATGCTATTTGTAGTACTTTAAGGGTGAAATCATAAGTGGGCTAATGATTTCAAGTTTTCTTAGCATGGAAAGTAAGAAGAAAAGGAAAAGTATAAGACATT
Loxodonta africana  -----------------GAGGACTGGTTGCAGTACTTTAAGGGTGAAACGGTAAGTGGGCTAAAAATTTCAGGTTTTCTTAGCATGGAAAGTAAGAAAAATAGTGA-----CAAGGCACT

Homo sapiens        TTGAGTGA---------GGTAAATAAAACATCAGAATCTGTGTTCTGAACCATGTTCTGTCATTTGCATTCAATGTGCTTATGGTAGGGCTAATATTGATGTCAGTTATAGTTAGCATTC
Loxodonta africana  TCAAATGACTTACCCACTTTAAGAAATATATCA-----TGTGTTCTGTATATTGTTCTGTTGCTTGCATTCAATGTGTTCATGGTAGAGATAATAATGATGTTAATTATAGTTATCATTC

Homo sapiens        ATTGACTACATACAGTAATGCTATGTCCTGTC--TGTCCTTAAAAAAATAGAACACCCTTCAAAGAAGGTAGTCACATTCTCATTTTAGTGATGTGGAAACTGAGATTCAGAAAGGTTAA
Loxodonta africana  ATTGATTACATACAATG-TACTTTGTCCTATCCCTCTCCTTAGA---------TACCCTGTGAAGACAGTAATAACATTTCCATTTTACAAACGAGGAAACTGAGGTTCAGAAAGGTTAG

Homo sapiens        ATGACTTGCTTTTAAAACCATGGTAAGGTGGTTTCAGAACCAGACATTTTCTCCCAGATATTTTTGTCTCTGAAGCCAGTGGGAAAAAAACTGCAGATAAATAGATTTCTTTACACCATA
Loxodonta africana  ATGACTTGCTTT-AAAATCATGGTGAGGAAGTTATAGAACTGGAAATTTTATCTTAATTATCTTGCACTCTAAAGCCAGTGGGAGGAAA-CCACAGATAAACAGATTTCTTTACAGCATA

Homo sapiens        AGGATGCCTTTGCTAAGAGACAG--CAATTCAAAGAAGGAATGGGCTGCCTCCTGAGCTAATGAGTTTCTCATCACTAGAAGCAGGATCGCTATGTAATTTGCAGGGCCCAGTGCAAAAT
Loxodonta africana  AGGATGACCTTGCTAAGAGACAGAGCAGTCCAAACATGGAATGGGCTGCCTCCAGAGCTAATGAGATGCTCATCACTGGCGGAGGGAACACTACATAATTGGCAGGGCACAGTGCAAAAT

Homo sapiens        GAAATGGCCTTATGTCCAGGAAGCTGGTCTTGGCTGGTAGTTTGCAAGCAGAAGTTAGCCTGCCTCCAGACAGGGATGTCAGAGTGAGGCGTCAACATGAGCTGATGCACAGGACCAGAT
Loxodonta africana  GAAGTGTTTGCACTCCCATTAAGCTGGCCCTGACTGGAAGCTTTCAAGCAGAAGTTAGCCAGCCTCTAGGCAAGGATGATAGAGCTAGGAATCAGTGTTAGCTGAGGCACCAGACCAGAT

Homo sapiens        GGCCTTAATCTGAGACACCATGGCTCTATTCTATTAGCCCAAGTGAGTTAAGGGAGCATCCCAAGGTAGCTGACATGACACCTTAGAGGC-TTCAGATGAGAGAGAATTCTGCAAGAGGA
Loxodonta africana  GACCTTAATCTGAG................................GTTAGGGCAGCTTTCCAAGATGGCTGACATGACAGTTAAGAGGCCTTCAGATGAGGGAGAATTCTGTAAGATAA

Homo sapiens        TTTGGAGAGAGCCATGGTGCCATGATCATCTGGGG-CACTGAAAACATGCTCTGCCCTATGAGGCAGCACCAACACTCAGAAGAAAACAAAAAAG-AAACTCTTTTAGTGGAGGTGAAGT
Loxodonta africana  CTCAGTGAGATCCATGGTCCTATGATCATCTGGGGGCACTAAACACATGCTGTGCCCTGTGAGGCAGCACAAGCACTCAGGAGAAAACAAAAAATTAAACTGTTTTCGTGGTGGTGAGGC

Homo sapiens        GTAATGAAAAGAATTAGA----------CAGATGTGGGTTCAAA-TCCTAGCTCTGGCAATTACTAGCTAGCTATTTGCCTTTGAACAAATCACTTAAACCTCCCTGCGACTGTTCTGTC
Loxodonta africana  ACAATGGAAAGAACCAGGGCTTTGGGGTCAGATATGGGTTCAAAATCCTGACCATACCACTTACTAACCAGGTGTGTGCCTTTGAATAAGCCCTTTAAACCTCTCTGAGATTGTTCTCTA

Homo sapiens        ATCTAGAATATGATCACAGTACCTGCCTTGCAGAGGTGTGGGGAAG-------------GAGGCAAGTCAAATGTATCTGGCACTGGGTAAGCCCTCCATAAATGTTAACTGTTGGATGT
Loxodonta africana  ATCTGAAAAATAATAATAGTACCTATCTTGCAGGGTCGTGGTGAGGTTTCAGATGAGATGATGCAAGTTAAGTGGCTCTAGTATTGA-CAAGCCTTCAGTAAATCTTAGCTTTTT----T

Homo sapiens        GTTTATAAACCACTGGGAGTGGGGACGGTATGACGTTGGGTCATTTGCCTTTCCTTGAGCACTTCATGGACGAATAGCCAAATCTGCCGGACCCACCTAGGGGCATGGAAACCACTTCCC
Loxodonta africana  TTTTTTAATTTTATGGGAG------CAATATGACATTTGGTCATCGGCCACCCCTTGAGCACTGAAAGTTCCAGTAGACAAGTCTGCCAGATCCACCTAGGGACACAGAAACCCCTTCTC

Homo sapiens        GCTCTAGTGCTACTAACTACAGCACTGCAGTAGTGGTAGGCAGTGGGTGAAAGTGGAGTGGGGCCACTAGTAGACAGTAATAACAATAAACTCTCCAGGAGAACAGGGCCCTCAAGCTTT
Loxodonta africana  ATTCTAGTCCCCCTAACCGCAGTGGAGTGGTGGTGGTGGGCTGTGGGTGAAGGTGG-GCAGGGCCACTAGTAGACAGAAATAATAATAAACCTTCAAAGAGTATAGGACTCACAAGGTTT

Homo sapiens        TCCCCTAGCCTTGAAGATA----CTTACAGGAGATAACTGCCCAAGTCCAATCTTGGAATCCTGCTAGTTGAGTGTTTTACCCACAAGGACATTTCATGAGATCAGGACTAAGAATAAAA
Loxodonta africana  ACCCC-AGTCTTCCAGACACTCCCTCACAGAAGAGGACTGTTTAAATCCAATCTTGGAACCCTTCCAGGGGAGTGTTTTACCTAGTCTGACTTTTCACAGGATCAGAACTAAGAATAAAA

Homo sapiens        TTCACTTCTG----CCCAGTAGCGCTATATGCATTAGTCACT--TGGAATAGCAACAATCTTAATATCTTTCTATTTGTCCGCAAACCCCACCATCCCACCTCTTTAAAATTCCGTGAAA
Loxodonta africana  TTCACTTCTGACCCACCAGCAAAGATACATGCATTAGCTACTTGTGAAATAGCAAGAA-CTTAACACCTGTCTATTTG-----AAAGACTCCTGTCCCACCTCTTCAAAATTCTGTGAAA

Homo sapiens        TGGCCAGAATAGCGAAACACCGATACCTACAGTCTTCCAGGAACTTCATTGTCCCTTATGCAACATAGAAGGAATGGTATGAGAACTAAGAGAACATTGCAGTAAATCACTGAATTTCTT
Loxodonta africana  TGGCCTGCATAGCCAAGCTCAGATGCCCACGGTTTTCCAGGAACTTCATTGTCCTTTACACAGTACAGAAAGAATGGTATGAGAACTAAGGGAACATTGCAGTAAATCACTGAATGGCTT

Homo sapiens        GGCTTTC-------------------TTTTTTTCTTTTAATAATAAAAAGCACTCCAATCAAACTATTAATTAACT-TGTAAAAGGCTCGAACTATTATGTACTATTCCCCGCCCATGT
Loxodonta africana  GGCTTTCTTTCTTCTTCTTCTTCTTTTTTTTTTTTTTTAAATAAAAAGCAACACCCAAACCAAACCATCAATTAACTGGGCAAAAGGCTGGAACTGTTATGTACTAATTCCCAGCCACGT

Homo sapiens        CCATGTGCTGCACAGTATTTTCTTACTCAGGACTGGTGGAACATTTCTTTTGCTTTTGTTTGAAAAATTACTTTTTATTTGGCCTTGGGCTGCTATCATAACTTCTGTTTTCACTAGATT
Loxodonta africana  CCATGTGCTG---------------CCCAGGATGGGTGAAACATTTCTTTTGCTTTTGTTTGGGGGA-------GGGGGGGGGCTTGGGCTCATCTTGTAATTTTCCTTTCCACTAGATT

Homo sapiens        GCATAAGCCATACATCTGCACACATTTTTGATTTGTACAGGGAGTTTGAA-TTTTAATATCTACCTCCATCTCAATCCCAGCACTTTGGGAGGCCGAGGCGGGCGGATCACGAGGTCAGG
Loxodonta africana  GCGTGAGCCATACATATGCACACATTTTTGATTTTTACGGGGAGTCTGGATTTTTAATATCTGCTTCTACCCC...............................................

Homo sapiens        AGATCGAGACCATCCTGGCTAACACGGTGAAACCCCGTCTCTACTAAAAATACAAAAAATTAGCCGGGCGTGGTAGCGGGCCCCTGTAGTCCCAGCTACTCGGGAGGCTGAGGCAGGAGA
Loxodonta africana  ........................................................................................................................

Homo sapiens        ATGGCGTGAACCCGGGAGGCGGAGCTTGCAGTGAGCCGAGATCGCGCCACTGCACTCCAGCCTGGGCGACAGAGCGAGACTCCGTCTCAAAAAAAAAAAAAAAAAAAAAAAAATATCTACC
```

```
Loxodonta africana  ........................................................................................................................

Homo sapiens        TCCATCTCAAAAACCTCTCATCTTTTGTCCTTTTCTTCAAGATTCTGATTTCCTTGTGGACATTCATTTTGATACTTACATAAATCTTGCGT---TATTCTGCCCA-TTTTTTGGCTAC
Loxodonta africana  .......................................GGACCCTAATTTCCTCGTGGACATTCATTTTGA----------AATCTTGTGTACTTATTCTGCCCAATTTCTTTGGTTAC

Homo sapiens        AAGGCTCAGCTTTGGGGCTGTAAATGTGAAATATTTCCATCATAACCTATCTTTGG------GTATGGGAAGGTCCTTAATGC-----------------------TGGAAGTTCATTG
Loxodonta africana  GAGACACAGCTTTAGGGCCATAAATATGAAATATTTCCATCCTGACCTATTTTAGGGGGCAAGGGTGAGGAGGTCCTTAATGTCAGAGGGAGAAACAGAGTAGGTCGTGGAAGTTCATTG

Homo sapiens        AATAATTGCTTATTTTAATAGATGTTTATTTTCATTCACATTTC-TCCCTCCACTCCATCCCTTGGGGCAGTACCCTAGTTCAGTTCTTCATCTACTCTCTCCTTGACTGTT-GCGATAG
Loxodonta africana  AATAATTACTCATTTTAATAGATGTTCATTTTCTTTCACAGCTCTTCCCTTCCTTCCATCCCCGAGGCCTCTACCCTAGTTCAACTCTTTGTCTGCTCTCTCCTGGCCTTTCAGCAATAG

Homo sapiens        CCTCCTAAGCAGCCACTCCACATCCAATCTCCCCATATCCAAAGTATCCTAAACATAGCTACCAGTCATCTCTCTAAACCACAGCTTTTGGCAAAAGATCCTTGAAGTTTCCCTTTTGCC
Loxodonta africana  CTTCCTAACCAGCTGCACCATATCCAATCTCTTCCTTTCCAAACTATCTTAAACATAGCTACCACTCATCCCTCTAAACTACAGCTCTTGGTTAAAGATCCTTAGTGCTTCTGCCTTGCC

Homo sapiens        TAATGAGTAACGTACTAGCTCCTCATCCTGGCATTCGAAGCTCTCTATGTTTATATGTTTGCCTATCTTTCTTTTCTAGCTTCTTTCCCATTACTTGTCTTCATATAGCTTTAGCTTTTG
Loxodonta africana  TAATGAGTAAGATACAAACTTCCCATCACGGCATTCAAGTCTCTCCATAATTTTGCTCTCATCTACCTTTCTTTCCAGTCTTCTCTCATGTTAGTCATCTGCATGTAACTTTGGCTTCAG

Homo sapiens        CCAAACTACCATTCTTTGTTTTCAGAGCACACCCTATACTTTCCAGCATCAGTGTTTCTTTCACCTGGAATTAACTGCCTGCCACAGAGATCTCCGTTCATCAGCCTATTCATTCTTTAA
Loxodonta africana  CCATAC------------TTGACTGAACACACCCTATATTTTGCTGCTTGAGTTTTTCTTCCACCTGGAATCAACTCCTCTTCATAGAG--CTCTGTTCACCATCCTACTCATTATTTAA

Homo sapiens        TGCCCAAGACACATGCCACCAATTTCATTTGAATGGTCCTTAGAGACACTGAAATGGCGCCTGCTGTACACCTGTCCACCCCGACCCCCGCTGCCCACTGCTGGCTCTTCCAGTCTTGTG
Loxodonta africana  AGCCCATGTCAAATGCCATCAACTTCA.............................................................................................

Homo sapiens        AAACATTAACCTCTCCTAACAGCAAGCACAGCTTCTCCATGGAGAAGACACTGAAATGGCACCTGCTGGACACCCGTCCACCCCCCACCCCTGCTGCCCACTGCTGCGTCTGCCAGTCTC
Loxodonta africana  ..............................................AGACACTGAAATGATAGCTGCTG-------------------------CCCCCACGGTTGGCTCTGCCAGTCTG

Homo sapiens        GTGAAACATTAACCTCTCCTAACAGCAAGCACAGCTTCTCCATGGGGCC-TGCTCACTCTGGCCCTCTGGTCCCACCATTTTTCTCACTACTCAAATAAAGTTTGGAGACCTTTCCCTGA
Loxodonta africana  GTGGAACGGGGACCCCTCCTGACAACAAGTACAGCTTCTCTGTTGCTCCCTGCTCACTCTAGCCCTCTGTTCCCATCCTTTTATCCATCACCCAGATGAAGTTTTGAGGCCTCTGTCCAG

Homo sapiens        TTTT---TTAAGAACATGCTAGCTCTTTCATGATACTCGTTATCATACCACCCCCATCTGCTTTTCAGTGTACCTTTCAATTGCTCTCTAGTGCTCCATTCTTGATTGTTTTCCATTTCT
Loxodonta africana  TTTTCTCTTAACAAGAGGTTTGCTCTTTCATGATATTCTGTATCATACCACCTCCCCC--CTTTTCATTGCAGCTTTGAAGTGCTCTCTAGTGTTCCATTCTTGATTGCTTTCCATTTCT

Homo sapiens        ATTATAGCTTTCAAGGAGAGTCTTTACTTGGCCTACTTTCTTAGTAAATGGTTTGGTGAGATTCTGATTTTCATCTGATATTAGTTCTTTCTCCAAGAGGGACAGAGAAAAATGAAAAAA
Loxodonta africana  ATGACAGCTTTTAGGGACAGCCTTTACTTGGCCTATTTCTTTAATAAATGGTCTGATGAGATTCTGATTTTCATCTAATTTTAATTCTTTCTCCATGAGGGACAG----------AAAAA

Homo sapiens        ATATCTTGCCTTCAGTTATTCTTTTCATATTCTTTGAAATTCTAAGCCACAGAAGTCTTCTGTTAGGCTGCTACCTTCATTTTAAGAGACTC------TTCCCTAAATGACAAGTTGTGA
Loxodonta africana  GGACCTTGCTTTCAGCTGTTCTTGTT----------AAATGGTAAGTCACAGAAGTCTTCTTTCAGGCTGCTGCCTTCATGTCAAGAGACTCCTCACTTTCCCTCAGTGACGTCTTGTGA

Homo sapiens        CTTCAGAACTAGAGAACATGGCAGCCTCTCATGGCTTTCTGCTCAGTCCCATTTTTCTTTTCTGGATCCACTCTGGGCCTTCTTTAAAGTGACTCAACTTCCCAAGCATGTGATTTTACG
Loxodonta africana  CTTCAAAACTTGAAAACAT-GCAGCCCATCACGGCTATCTGCACAGTCTTCTTTTTCTCCTCTGGGTCCATTCTGGGCCTTCCTCAAAGTGACTCAACTTCCCTGACATTTGATTTTACC

Homo sapiens        TATTTTAAAAACAAGTCTGGTTTTTTCAACTCATTCTGAAATGTGGCTGCCAAAGTAAATACCCAACCCTCTCTAACACATACATACCCTCTTTCACACACACATGCACATGCACATGT-
Loxodonta africana  TATTTTGAAAACTAGTCTTGTTTTCTCAACTCCTTCTGAAATATAGTTGCTGAAGTAAATACCCACTCCTCTCCAATACACACATACTCTCTCTCACACACACATATATGTGCACATGTG

Homo sapiens        -------AAACATACTCCTGAAGGATGAGGAAAGCAATGGTAAATGGGGAAGCAAAAAAATACCATTTTTCATTTAGACAGACTTCCCAATACCGATGAACTAAAATTCAGGGATTTGTG
Loxodonta africana  CACACACAAACATACTCTCGAGGG-------------------GTGGAGAGGTAAAAAGAT--TATTTTTCATTTACGTAGAC-TCCCAATACTGATGAATTTAAAAATCAGGTTTCTTA

Homo sapiens        ATTTGAAATGCATGGATGGCCTTTGAGTGGTCTTTTCAAATCCCCT-AAATATATCAATTTAATAACTTATTGAGCATCTACCATTTGATGAGTGCTGTGTA-----------GATGTAT
Loxodonta africana  ACTTGAAATGCATGAGTGGCCTTCTGATGGTCTAT--GAACCACCTGAAATTTATCCATTCAATAATTTATTGAGCATCTACTATGTGCTGGGTACTATGTGCTAGGCACTGTGGTAGGC

Homo sapiens        CTATATATATCATAAAATTTATTTCTTGCTACAACTTTTGGAAGAGATTATTATAATCCTCATTTACAGATGAGGAAACTGAGTCTCTGTGAGAGGAAATGATTTGCCCAAGATCCCTCA
Loxodonta africana  CTGTCTATGTCATAAAATTTAATCCTCATCATAACTTTT----GAGATTATTATGATCCTCATTTACTTGTAAGAAAACTGAGTCTCAGCGAGGTGAAATGACTTGCCTAAGAACACAGG

Homo sapiens        GGCAATAAGTAGTAAATCTGACTTTGAAACACAGGTCTGATTCCTTATCCCCATCAGATTTCCCAGGCATTCTGGTACAGGTGGTGTTTCAACAGATGGGAATGGGGCAGGGTGAGAAAG
Loxodonta africana  ATCACTAATCATCAAAGCTCAGTTTGAAAGCAAAGTCTGATTCCTTATCACCATCAAGCCTCCCAGGCCGAAAGGAGCAGTTGGCATCCCAACAGATGGGAATGGGGCAGGGTGGGAAAG

Homo sapiens        -------------ATAGTATGAATAAAGGTACAGACATGGGAAAATGTAGCTACACATAGGACAGTAGTAGGAATTAAGGTTGCAAAATTGTGTTAGGGACAGAACACAAA---------
Loxodonta africana  CCAGAATGAATAAATAGTATGAACAAAGGTACAAACATGAGAAAATGTAGCTACAGATAGGAGGGTAGTAGGAGTTACATTTGCAAAGTGATGTTAGGGACAGAACGTACAAGGCCTTGA

Homo sapiens        -----AGGATTAGAAACTTGAACTTCCTTCCATTGTCCTGAAGGGACAATAAAAGATTTTGTAGTAGGAAAACAATATAATCAGAGAGCCAAATCAAGAATGCAATCCAATTTACAATAG
Loxodonta africana  AAAGCAGGCTTAGAAATTTAAACTTCTTTCCATTGGCCTGAGAGAACAATGAGAGATTTTGAAATATGACAATGACATAATTA.....................................

Homo sapiens        CCATACAAAAAAAATTAAATACCTAAGAATACATCTAACCAAAGAGGTGAAAGATCTCTACAGGGAGAATTAACATACACTGCTGAGAGAAATCAGAGGCAACACAGACAAATGGAAAAA
Loxodonta africana  ........................................................................................................................

Homo sapiens        CATTCCACACTCATTAATTAGAAGAATCAATATTGTTAAAATGGCCATACTGCTCAAAGCAATCTACAGATTCAACGCTATTCCTATCAAATAACCAATGTCACTTTTCACAGATTTAAA
Loxodonta africana  ........................................................................................................................

Homo sapiens        AAAAGATTTTAAAATTCATATGGAATTTAAAACGAGCCCAAATAGTCAAAGTAAACCTAAGCCAAAAGAACAAAGCTGGAGGCATCACATTACCCAACTTCAAACTATACTACAAGGCTA
Loxodonta africana  ........................................................................................................................

Homo sapiens        CAGGAACCAAAACAGCATGTTGCTGGTACAGAAAAAGACATATAGACCAATGCAACAGGATAGAGAATGCAGAAATAAAGCCACACACCTACAACCAACTAGTCTTTGACAAAGTTGATA
Loxodonta africana  ........................................................................................................................

Homo sapiens        AAAAATAAGCAACAGGGACAGGACTTTCTATTCAATAAATGGTGCTGGGATAACTGGCATATGCAGAAGATTGAAACTGGACCTCTGGCTATCACCATATATACAAAAATTAACTCAAGA
Loxodonta africana  ........................................................................................................................

Homo sapiens        TTGATTAAAGACTTAAATGTAAGACTTCAAACTATAAAAACTCCAGAAGGAAACCTGGGAAATATCCTTTGGACATCAGCCTAGGCAAAGAATTTATGACTGAGTCATCAAAAGCAATTG
Loxodonta africana  ........................................................................................................................

Homo sapiens        AAACAAAACCAAAAATTGACAGGTGGGATCTAATTAAACCAAAGAGCTTCTGCACAGCAAAAGAAAATATCGACAGGCACCCTACAGAACTGGATAAAATATTCATAAACTATGCATCCA
Loxodonta africana  ........................................................................................................................

Homo sapiens        ACAAAGGACTAATATCCAGCATCTATAAGGAACTTAAATCAACAAGCAAAAAACAACCCCATTGAAAAGAGGGCAAAGGACATGAACAGACGCTTTTCAAAAGAAGACATACAAGTGGCC
Loxodonta africana  ........................................................................................................................

Homo sapiens        AAAAGACATGAAAAAATGCTCATCATTACTAATCATCAGAGAAATGTGAATCAAAACCACAGTGAGATACCATCTCACACCAGTCAGAATGGCTATTATTAAAACGTCAAAAACAGCAGA
Loxodonta africana  ........................................................................................................................

Homo sapiens        TGTTGAAGAGGTTGCAGTGAAAAGGGAATGTGTATACATTGCTGGTGGCAATGTAAGTTAGTTCAGCCCCTGTGGAAAGCAGTTAAAGATTTCTCAAAGATCTAAAAATAGAAATACCAT
Loxodonta africana  ........................................................................................................................

Homo sapiens        TTGATCCAGCAGTCCTCTTACTGGGTATATATCCAAAGGAAAATAAACCATTCTACCAAAAAGACACCTGCACTTGTATGTTCATCGCAGCACTATTCACAACAGTAAAGACATGGAATC
Loxodonta africana  ........................................................................................................................

Homo sapiens        AACATAGGTGCCCATTGATAGTGGATCGGATAAAGAAAATGTACACGTACACCATGGAATACTACCCAGCCATAAAAATGAACAAAATCATGTCCTTTATGGGAACATGGATGCAGCTGA
Loxodonta africana  ........................................................................................................................

Homo sapiens        AGGCCATTATCCTAAGCAAATTAATGCAAAAAAAAATCACATATTCTCACTTATAAGTGGGAGCTATACCTTGGGTACACATGGACATAAAGATGAGAACAATAGACATTAAGGACTCCA
Loxodonta africana  ........................................................................................................................

Homo sapiens        AAAACTATCTGGGTGATGGAATCAATAGAAGCCCAAACCTCAGCATCATGCAGTACACCCTTGTAATAAACCTGTACATGTACCCCCTGAATCTAAAATAAAAATTAAAAAAAATTTAAA
Loxodonta africana  ........................................................................................................................

Homo sapiens        CAGCTGCATTTTAGACAGATTAATCTAATTCAAGTCTGAATC-----------TATGCTGTTGACAGTATTTTTA-AAAATTCTTTCCTAAGAATTCTTTATTCACTCCAGGCCTATGCT
Loxodonta africana  ....TGTGTTTTAGGAAGATTAATCTGATTCAGGTCTGAATCCCTGGCTCCTTTGTACTGTTGATAGTATTCAAACAAAGTTCTTTGCTCAGAATTCTTTATTCACTCCAAGCCTATGCT

Homo sapiens        TTGGTCAGAATTCTCTCTTTGTTTTTCAAT-ACTTTAGTGAAAATTCAGAAGGCCATGTGGAAAAA------AACAGAGGAAACAAACCACACACAAACACAAGATAATTATATACAAGT
Loxodonta africana  TTGGTCAGAATTCTCTCTCTGTTTTTAAATAACTTTAGTGCAAATTCAGAAGGCCATGTGGAAAAAGCATAGAAGAGAAGAAGCAAACCACACACGAACACAAGATAATTATGTACAAGT

Homo sapiens        CTGGGAAG-AAAGAGCACAATGGGTCATGACGAACTTTATTGAACAGAAGGCGAATAAGCAACGTTCCAATTGTACA---GGAAAAAAAAAACCCAGCAACCCAAACACCACCACCAACA
Loxodonta africana  CTGGGAAGAAAAGAGCACAGTGGGTCATGATGAACTTTATTGAACAGAGTATGAGTAAGCAATGTCCCAATTGTAAAAAGAAAAAAAAAAAAAGAAGCAACAAAAACCCCATCATCAAC-

Homo sapiens        ATAAGAAACAAAGGCAGCCAGGATCTGGGAGACATGGATGAAGCTTGTGAAAATGACACTTTCTTAAAAGAGCAGAAGAGAAACTAAAAAGGATGAAGGGGAGAACCATGGATTTGAGGG
Loxodonta africana  --ATGAAGCACAGGCGGCCAGGATCTGAGAGACATGAATGAGGGTTATGGAAATGGCACCTTCTTAAAAGGACAGAAACGAAACTACAAAAGGGGCAAGGGAGAGCCATGTTGTTTAACA

Homo sapiens        TTAAACTAAAAGGAAAAATAGAAAGGAAATAGGAAGGAAGGGTGTAACT---CATAGCTGGTTTTGATATATCATCCCAGTCTGACAACCAAGGCAGTCTCCCTG-CATTAGAAGGCCAT
Loxodonta africana  CTAGACTAAATGAGGAAA----AAGAAAATAAGAAGGGAGGGTAAACCCATACATAGCTGGTTTTGGTCTATCAACCCAGTCAGACAACAAAGGCAAGCTAGCTGTCCTAAGAAGGCCAC

Homo sapiens        GCTAAAAGTAGATGAG--TTGTAAACAAGCCTCCCACTGGTCATTAGTTTGGCAAATTAGTGGGTTATCTCTTAGAGTCCTCTCTTTTTCTCCTTTCCCTTTCCTCTGCATTCAATTTGT
Loxodonta africana  ACTGAGAGTAGATAAGCATTGTAAACAAACCTCCTACTTGTACTTAGTTTATCAAATTAGTTGGCTATCTTCTACAGTTTTCTTTTCTTCTCCTTCC----TCCTTTCCATTAAATTGGT

Homo sapiens        CACTCATT-CCTACAAGTCTACCCCAGAAGGGTTGCCTGAATCAAAGCCCTTCTCCCCATTTCCACTCACGGGGCCACTGTGTTAATTAAATTCCATGTCGTTTCTCAGCTGGACCAGGC
```

```
Loxodonta africana  CTTCAAGTGCCTGTGAGTCTACCTTAGAAGGGTCTCCTGAATCAAGGCTCTTCTCCCCATTCCCACTGACTGGGCCATTATGCTAATTAAGTTCCATGTCCTCTCTCACCTGGACTAGGC

Homo sapiens        TTCCTGCCTCTAATTCGTTTCTTCCTCCCTCTAATTGAGGCTTAGATCTGCCCCAGTATTGAGCTTCCTATA-CTCTCTTGCTTATAAACCTTCTTTGGTGGCCCATGGCCTATAAGGTA
Loxodonta africana  TTCCTGCTTCTAGCTAGTCTCTCCCTCTCTCTAGTTCAGGCTGCAGGCTGTCTCAAGAGCGAGTGTTCTAAAACTCCCCTGCCTATAAACCTCCTTTGGTGGTCCACTGACTATAGGGCA

Homo sapiens        CAGTAGAACTCACTCAGTTTTACATGATGCAGTGGGAGTGGAATCAAAGGGTTCCCCTAGACCCATGGATGAAGAGACGACTAAGGAGCTAAGTCTGGGATGAGAAACTCTTGCAAAAAG
Loxodonta africana  CAGTAGACCTCCCTTAGTTTTCCATAATGCAGTGGCAAAGGAACCAAAGGATTCCCTCAGGCCCATGGATGATGAGATGATTAAGGAGCTATGTCTGGAATGAGAAACTCTTGAAACGAG

Homo sapiens        CCAACTAATAGCTTTTTCCTCCTCATCTCAGAACCACCCCTCTTCCCA----GAGTGTGTGTATACAAGA-------------TGAGTTACTTAAGATTGCACCCAACCTGATGGTAGAT
Loxodonta africana  CCAGCTAAAGCCTTCTTCCTCCTAGTCTCAAAACCACCCCGTTCCCAAATGCGTATGTGTGTATACGGGGGGTGAGCGAGTATTGGGTAACTTA...TTGCATCCAACCTAAGAATAGAT

Homo sapiens        TGGAGAAGGCTATCTGTCCCTTTCTCAGTTGAATATATGAGGGTCTTCTTCCCAGGAGGCTTGCCCACACCAGTCTTCTTTCCCACCATGGCCCATCCCCTTTCCAAAGAGTATAAACTT
Loxodonta africana  TGAAGAGGGTTGTCTGTCCCTGTCTCAATTGAATATATGAGGGGTCTGCTCCTGGGAGGCTTCCCAACTATCATCTCCTTTCCCACCCGGGCCCATCTCCTTCCCAAAGAGTACATCCTT

Homo sapiens        CATACCCTCCTCCCTTCAAGAACTCCTATCTTTGCTTAGCAAAATTTGTTTCCCAAGCTTGGAGAGAGGAAAGGGGCCTTTTTCCAAATCAGGGGTAAATAAAAAGT-GGCAGAATCTTA
Loxodonta africana  CA-ACCCTCCTACC-CCAAGAACTCCTATCCTTACTTAGAAAAACTCATTTCCCAAGCTTAGGGAAAGGAAAGAACCTTTTTTAAAAATCAGCACTAGATAAAAAGGAGGGAGACTCTCA

Homo sapiens        CAGTAGACATCATATTAGAGCTCATTTTTATGAATTCTTCTACTGTATATAAGGTCC-TCACTTTCTTATTACTCAGCCTACCACTCCAGTTTCATCTTCCACTTTTACTGCTTCTCCCC
Loxodonta africana  CAGCAGACATTAGATGAGAGCTCATCTTTATTACTTTTTCTACTGGACAGAAAGCCCTTCACTTTCTGATTACCCAACTTACTTCTGCAGTCTCATCTTCCACTCTCACTGCTTCTCCCC

Homo sapiens        ACCCCTTCACAATCCTGACATACCCTAAACTCCAGTCATACTGAACTTTCCAACATTCCTCAGATAAATCAAGCCTTTGCAGAAATTCATGCACAAGCTGTTCCTTCTGCTTAGAACATC
Loxodonta africana  A-CCCTTCACAATCCCAACATACCCTAGACTTCAGTCATACTGAGCTTTTCCACATTCCTCAGATAAACC-------------AGTTCATGTACATACTGTTCC-------------CC

Homo sapiens        CTTACTCTTCTTATCTCGCCAGTAAATTCCTACTCTCCTTTAAGACCCTGCTCAACTATTGCCTCTTCTGGGAACTCTTTCCAGACTGTTCCAGATAGAATTAATTA-ATCGTCCTTTGA
Loxodonta africana  CTTGCTCCTCTTATCTCATCAGTGAATTCCTACCCTCCTTTGAGACCCTGCACAACTGTTGCTTCCTCTGTGAAACCTTTCCTGTCTGCCCCAGATAGAATTAACGGCCTCCTCCTTTGG

Homo sapiens        GCTCTCATAGCATTTT-TCCATATCCCTTTGATAGCACCATCCTTATCATGGCAAAACAATATAACAACTAAAGGGATGGGCTCTGGAGGCAGACTGCCTGGGTTTGAGTTCTTACTCTG
Loxodonta africana  GTTCCCATAGCATTTTATTCACACCCTTATTATAGCATCATCCATATCAGGGCAAAACAATGTAAAAACTAAAAGGATGGACTCTGGCGGCAAACTGCTGGGGTTTGCATCCTGACTGTA

Homo sapiens        CCACTTATAAGCTATGTGATGTTGAGCCAATTACTTCGCCTTTC-TTTGCTTCAGCTTCGTCATGTATAAAACAAAATAAATAAGATGATACTTGTAAAACCTTTAGAACAGAACCTGAC
Loxodonta africana  CCACTTCTAAGCTATAAGATGTTGGGCCAATTATTTCACCTCTCTTTTGCCTCAGTTTCTTCACCTGTCAAACAGAATAAACGAGAAAATACTTGTAAA---TTTA-------------

Homo sapiens        ACATTGCAAATGTT-AGTAAATATTATACATCATCATTTCTT-----CTATTGTTTTATTGTTATCGTATATATAACCTA---------------------------------------
Loxodonta africana  CCATTGTAAATGTTAAATAATTATTGT-TGTCATTATTTTTTTGGTGCCATTGCCTTTTCGTTAGCAGCCACAGCACTTAACCACTCTGCCACCAGGGCTCCTTTAGCTTTGTTCATCTC

Homo sapiens        ----CTTGTATATGTAA--TATACATGTGTTATTATATAGCACTATTATAGCATTACTTAACTTTTGCTGTGTCTGTGTCTTGTCTTCCAAGCTGGTTTAT-AGTGTTTTGAGGGCAGAA
Loxodonta africana  TTCTATTGTATTGCTAAGTTCCACTTGTATTATTGT-------TATTGTAGTATTATTTAATTTTTCCTGTGTTTATATTTTATTCCCCAAATCAGTTTTTAAGTGTTTTGAGGACAGGA

Homo sapiens        ACCTTGTATTACTTTC--TCCCCCATAGTGCCTGGTGTTAAGTATGGGCTCATTAAGTATTTGTTGGGGTCAGTATATTATACTGAAAAAAGCACTGAAATCTGAGAGATTTGGGTTTGG
Loxodonta africana  CACTTGTACTACCCCCAACCCCCTGTAATGCCTTGTATGA--TATGGGCTCATTAAATATTTATTGGAAGCAGTATATTATAATGGAATAAGCACCGGAGTCTGAGAGGTTTAAGTTTAA

Homo sapiens        ATCTTGATTCTGCTCATAATTGTTGTATGATCTCAATCTTCCTGAGCCTCAGTTTCCTCATCTATAAAATGAGGATGATAATAAGAGTACCTACCTCACAGGGATATTTTAAGAATTAAA
Loxodonta africana  ATCCTGGCTCTGTTCTTGGTTGTTGCATGATCTTAATCTCTCTGA-----AGTTCCCACATCTGTGAAATGGGGATGATAATAATAGTACCTTCCTCATAGGGATGTTGTAAGGATTAAA

Homo sapiens        TAACTTAGTACATGTAAAACCTAGCACATAATAACATAATAAGAACTCACTAAATGGTTTTTATTATCAGCTTTGTATCCCTTTGCATTTAACTTAATAAATACTTTTAC----TTGAAT
Loxodonta africana  CAAGTTGGTACTTATAAAGCCTAGC--------ACCTGGTAAGTGCTCACTATATTGTTGTTATTATCAGCTTTGTATCCCCTTGCATTTAGCTTAACAAATCCTTGTACATGAATGAAT

Homo sapiens        GAATGAACCCAGCCATTTTC---TGGAACTTGAGGATCAAGGGAATCCAGGCAGAT------ACTCTATTTGTTCCTAAGAATCCAGAGGAAAATGTGAGTCATCTCCATTCTGTATCAT
Loxodonta africana  GAATGAACCCAGTTATCTTCCTTTCAAATTTGGGGATTAAGGGGATCTGGGCAGATATCAAAACTCCACTTACTTCTAGAAATCAAGAGAAAAATGTGAGTCATTTCCATTCTGTTTCAT

Homo sapiens        GGCTCACTCTGAACTGGGTCACCATCCTCCAGAAGGTGACAAATGTGGCTGGCTGAGTCACTATAAGAGCTTTTCTGTAAGGATTTGCCCGAACCCCCCACCATAGCTCATTGGACATAG
Loxodonta africana  GGGTCACTTTGAACTGGGGCACCATTCTCCAGAAGGCAACAGACATGGCCAGCTGAGTCACTATAATATCCTTTCTGTACGAATTGACCTCCACTCCTTACCGTAGCTCATTGGACACAG

Homo sapiens        TATCTTTCA-----TATTAGGTGCGAGGTGCATACCCTTCCTGAAATGGGCAGAATAAAGAGGTGCAAAACTCCTGGGGAATCAGAGTGGCTATGAATTCACTCCTCTTTCTTATTCTGT
Loxodonta africana  TCCCTCACATACTGTACTAGGTGCAAGGTGCATATTATTCCTGCCATGGGCAGAAGAAAATGCTGCAAATTTACTACTGAGCCAAAATGTCTATGAATTCACTGCTCTTGCTTCTTTTGT

Homo sapiens        TGCCTCTTCCCTTTGTTTGGTTTATTTGCTAATCAACTTTGCCCACACTCACATCTAGTGTAGCCCAAGTCTGTGCAGAGAAGACACTTTGAAAGCCTAAGA--CTATCATAGTTCCAGC
Loxodonta africana  AGCCTCTTCCCT--------TTTATTTGTTAATCAACTTTCCCTACTTCCGCATCTACCATAGCCCAAATCTGCACAGAGGAGACACTTTCAAAGTGTGAGACTCTACCATGGCTACAGC

Homo sapiens        AGAATAATAGAAAAATAACAAGTGTTGACTATTCAGTTCTGATGTAGCACTGTATTGAAGAACATAACATTCTAGATCCAGATGGCCTGAATTTGAATCCCAGCTCCACCACCAACTAGC
Loxodonta africana  AGAATAATGGAGAAATGACAAGTGTGGGCTACTTAGGTCCAATATAGCACTGTGTTAAAGAATATAACACTCTAGAGCCAGACGGCCTGGATTTGAATCCCAGCTCCACCACTTAC----

Homo sapiens        TGTGAGACCTCAGGCAAATCACTTAACGTTTGCATCCTTTCATTTCTCCATCTATCAAATAGAAACAATAAAAGTACTTACCTACCTCTTAGGGTTTTGTGAGGGTTAAATGAGTTAATA
Loxodonta africana  ---GAGACTTTTGGTAAATCGCTTAACGTTTGTGTCCTTCAATTTCTCTATCTGTCAAATAGGGATAACAACAGTACCTGCCT---TCTAGGGTTTTTGTGAAGACTAAATGTGTTAATA

Homo sapiens        CATACAGAACACTTAAAAGAATGTCTGGCACATAGTAGGTGCTATATAAATATTAGCTTTTA-------------AAAACAGCTTTATTGAGATATAATTCATCTATGTGAATTATAT-
Loxodonta africana  GTTCTATAACATGTCGAAGAGTGTCTGGCA-ATAGTAGGTGCTATACAAGTATAAGCTATTATTATTATTGTTATTTTAGCATCTTTATTG-------ATTTATTGAGGTAATTTACATA

Homo sapiens        -------CTCAATAAATTCACCTATTTAAAGTGTACAGTTCAGTCATTTTTAGTA-TTTCACAGAGTTGTGCAACCATCACCACAATCTAATTTTTAGAAAATATTCATTACCCCAAAAA
Loxodonta africana  CCATAAAATCATGAAATACACCCATTTAAATTGCACAGCTCAGTT-TTTTTAGTATTTTCACAGAGTTGTGCAACCATCGCCACAATC-AAATTTTAAAATATTTTTAACACCCCAAAAA

Homo sapiens        GGAATCTCATACCCACTGGTAGCCGTT-TCCATCCCTGAAGCTCCCTCTCCCCCTTCCCAGCTCCAGGTAATCACTAACCTACTTTCTGTCTGTATAGATTTGCCTCTTCTGGACATTTT
Loxodonta africana  GGAACCCCGTACCCACTACCAGCCATTCCCCAACCCTGCACCTCCAAC--------CTCAGCCCCAGGCAGCCACTAATACACTTTCTGTC--TCTAGATTTGCCTATTCTAGACATTTC

Homo sapiens        ATATAAATGAACTCATACAATAGGTGGTCTTCTGTGACTAGCTTCTCTCCCTTAGCATAACTGTCTTTTAAAAAATTGCAACTTCCATTTTAGATTCAGGGTGTACATGTCCAGGTTTGC
Loxodonta africana  ATATAAATAGAATCATATAACATGTGGTCTCTTATGACTGGCTTTTTTACTTAGCATAAC............................................................

Homo sapiens        TACATGGGTATATTGCATGATGCTGAGGTTTGGGGTACAGCTGATCCCATCACCCAGACAGTGAGCATCCAATAGGTAGTTTTTCGGGCCTTGTCCACCTCCCTTCCTCCCCTGTCTAGT
Loxodonta africana  ........................................................................................................................

Homo sapiens        AGTCTCCAGTGTCTATCGTTCCCATATTTATGTCCATGTGTACCCAGTGTTTAGCTCCCACTTACAAGTGAGAACATAGCATAATGTTTTTAAGGTTCATCCATGTTGT-ATATATATCA
Loxodonta africana  .....................................................................................GTTTTCAGGGCTCATCCATGTTGTAACATATATTA

Homo sapiens        GAGCTTACTTCCTTTTTAAGGCTGAATAATAGTTCATTATATGAATATATCATATTTTGTTTTTCCATTTATCAGTTGATTGACATTTTGGATTGTTTCTATCTTTTGGCTAGTATGAAT
Loxodonta africana  GAACTTTATTCCTTTTTGTGGCTGAATAATAGTTCATT-TACAGATATACCACTTGTTGTTTATTCATTAATCTACTGATAGACA-TTTGTGTTGTTTCCATCCTTTGGCTATTATGAAT

Homo sapiens        AACACTGCTATCAATATTCATTTACAAGTTTTTGTGTAAAGATATGTTTTTAAATCTCTTGGGTATATATCTGGGAGTGGAATTGCTGGATCATATGGTAACACTATGTTTAATGGTTTG
Loxodonta africana  AATGCTGCCATAAACATTCATGTGCAAGTTTTTGTGTGGACATATGCTATCAATTCTCTTGGATATGTGTCTAGGAGTGG-ATTCCTGGGTCACATGGTAAATCTATGTTTAACTTTTTG

Homo sapiens        AGGAACCACCAAACTATTTTCCAAAGAGCCTGCACTATTTAAAATTCCCTCCAGTAGTGGGAATGATGGTTTCAATTTTTCCACATCTTCATCAACACTTGTTATTTTATTTTATTTTTA
Loxodonta africana  AGGAACCACCAAACTGCTTTGCACAGCAGCTGCACCCTTTTTCATTCTCACCAGCAGTGT--ATGAGGGTTACAATTTCTCCATGTCCTCACTAACACTTGTTATTGTCCGTC.......

Homo sapiens        TTTTATTAAATTATTATTTTTATTATATTTATTTTATTTTTTATTTATTTTTTATTATAGCCATCCTACTGTGTGTAAAGTAGTATCTCATTGTGGTTTTGATTTGCATTTCTCTGATGG
Loxodonta africana  ...............................................TTTTTGATTATAGCCATCCTAGAATGTGAGAAGTGGTATCTCATTGTGGTTTTGATTTGCATTTCCCTGATGA

Homo sapiens        TTAATGAGGTTGAGTATCTTTTCATGTGTTTATTGACCATTCTTATGTCTACTTTGGAGAAATGTCCCTACAAATTCTTTGCCCATTTTTAAATTGGGTTGTC---ATTTTATTACTGAG
Loxodonta africana  CTAATGATATTGAGCATCCTTTCACGTGCTTACTGGCCATTTGTATATCTTCTTTGGTAAAATGTCAATTGAAATCCTTTGCTCATTTTTAAATTGGGTTATTTGTCTTTTATTATCGAG

Homo sapiens        TTGTAACAGTTCTT-ATATATTCTGCATGCAAATCTCTTGTCAGATATATGTTTGTGAATATTCTGTC-ATTCTGTGTAATTTTTTTTCATTTTCTTGAAGCTATTATTATTAAATAGAA
Loxodonta africana  TTGTAATAGTTCTTTATGTATTCTGGATACAAGTCCCTTGTCAGATATAAGTTTGCAAATATTTTTCCCATCCTGTAGGTTGTCTTTTCACCTGCTTCTAGCTACTACTATTAAATATAA

Homo sapiens        ATTTGTAAGTTCGCATATAATAAAGATGACAGATTGTATTAGAAAGAATGAGATCTA-AAATCACCT--GATGCACCCTATGGTACACTACCCTGCTC--CTGCACTCCTTTGAAATTAA
Loxodonta africana  CTTTATAAGTTCACACGTGGTAAAGAGTACAGACTATATTAGAGAGGCAGAGATCTAGAAATCAGCTCAGAGATAGTCTTAGGTACAATACTCTGCTCTACAGCTCTCCTTTTAAATTAA

Homo sapiens        ATTCAAAACAGATCAAGCTTCTCCTCTGTGCCAGGCACTGTGGGGGAATAAGACCTAACCTCTATTCTCAAAGAGCTTCATAACACAGA----AGAGAAGATGCAAACATGTGGAAACTT
Loxodonta africana  ATCCAAAAGAGTTCAAGCATCTACTCTGTGCCAGGCACTGTGGGG-AGTAAGATCTGGCCTCTAGCCTCCAAGAGCTTACTAACTCAGAGGTAAGGGAAGATGCAAACATGTGGAAACTT

Homo sapiens        AAATAATTATAACATGTTTAAATAGCAGTTCAGGACCACAACAGAAAAGATGCCACAGAGCACTATATGATTAATTTCCAAATGAATTGCATGGAAAAGAATTGCTATGGGAACTCAAGG
Loxodonta africana  AAATAATTATAACATGTTTAAATAGCAGTTGAACACCACAATAGAAAAGATGCCACAAGGCGCTGTATGATTAATTGCCAAATGAATTGCACAGAAAATAATTGCTATGGGAACTCAAAA

Homo sapiens        GAGGGAGAAAGCACTTGGGGCTGAGGTGGTCAGGGGAAGCTTTAAGGAGAAGGGAT-CTAAGCAGTCTGTAATTAACTACCGCCATGCCTGTGTAATACTAAACAAAGACTTGGAGAGAA
Loxodonta africana  GAGGGAGAGAGCACTTGGGGCTGGGGTGGTCAGGGAAAGCTTTAAGAAGGGGGATTTCTAAGTGGTCTATAATTGACTGCAGCAATGCCTGTGTAATACTGAAGAAAGGCTTGAAGAGAA

Homo sapiens        AAAAATATTGACTGTTTTTGAGGTGAGGCATCTTAGTGATTAAATTGATACCACCATTTCTTTTCAACCTCTGCAGGAATCCCCACAGTCCCACCTTTCCAATGAAGACTTACGTGGCCT
Loxodonta africana  AAAAATATTGACTCAATTTGAGGTGGAGCATCTTAATGATTAAACTGATTACGCCATTTCATTTCAGCCTCTGCAGAAATCCCCATATTCTCATCTTTCCTATGTAGACTTAAGTGGCTT

Homo sapiens        CTGTCTAGTCCAGAGGTTACCAATTAACAAAGTTAACAAGCCACATCTGGCTTACAGACACGTTTGGTTTGGTCCAA-TTAAAAT----ATTAGAACTAGCTGCTAAAACATTTAACTGG
Loxodonta africana  CTCTCTAGTCCAAAGGTTAACAACAAACAACAACAACATGCCACATCTGACTCACAGAAACACTTTGTTTGGCCCCGTTTAAAATAAAAAATGGAATTAGTTGCCCAAAATTTTTAACGT

Homo sapiens        ACAAATTTCACATAAAATATTAATTTCCAGTTTCTCTAGAAAAATCAGAAGACCTGGCTACCCTGCATTGGAACATGCATTCTCTGGGACCATGGCCAATGGCAACCCTGCTTGCACATG
Loxodonta africana  GCAAATTTTACATAAAATATGTATTCCCAGTTTTTCTAGAAAAATCAAAAGACCTGGCAACTCTGGGTTGGAGCATGTACTCTCCAGGATGATGGCCTTTTTCACCTTCGCTTGTACCTG

Homo sapiens        CAGAAT--TCAGTTTGAGACTTCCTAGAATCATAGTTCTTAGACTAAAAGGTATCTTAAGAGTGCATCTGTTTCTATTCCCTGACTTTGGGAATGTATGGTATTATAATATTACCCTCAA
```

```
Loxodonta africana  TAGACTTCTCAGTTTGAGACTTCCTGGAATCATAGATCTTAGATC--ATGGTATCTCAAGAGTGCATCTCTTTCGATTCCCTGCCTTTGGGGG--------------------------

Homo sapiens        TTTACAACTTGATTTAGAATATCAACTAAAACCCACAGAGAATTTGACTTGCTGCAGGCCACAGGGCTATTAACTGGAAGAGGGAGGGCTAAAACCCATGTTCTCTACTTCTCAACGGAA
Loxodonta africana  --------------AAGAATATCAACTAAAACCCATAGAGGTTGTGACCTGCC-CAGGTCACAGATCTATTAAGCGGAAGAGGGAGAACTAGAATCCATGTTTTATATTCCTCAACT---

Homo sapiens        TGTTCTTTCAACAATCTGTCTTTACAGTCTGGATGCATATTGTTTAGTAGCATTCTTCTGGCACTTCCTAATCACTGCTTTGTGTTGTTGGACGATTTTTGATATGATCGTTATCTCCCT
Loxodonta africana  CGTTGTTTCAACACTCTGTCTTTGCAGTCTAGGTGCATAGTGTTTAGTAGTATTCTTCTGGCAGTTTCCAATCATTGCTTTG---TACTGGACAGCTTTTGCCATGATGGTCGTATCTCT

Homo sapiens        CTCTCTTTTTTTTTTTTTTTTTTTTTTGAGACGGTGTCTCACTCTGTCGCCCAGGCTGGAGTGCGGTGGTGCGATCTTGGCTCACTGCAAGCTCCGCCTCCCGGGTTCACGCCATTCTCC
Loxodonta africana  C...................................................................................................

Homo sapiens        TGCCTCAGCCTCCCAAGTAGCTGGGACAACAGGCGCCCGCCATCACGCCCAGCTAATTTTCTTTTTTTGTATTTTTAGTAGAGACGGGGTTTCATCATGTTAGCCAGGATGGTCTCGATC
Loxodonta africana  ....................................................................................................

Homo sapiens        TCCTGACCTCATGATCTGCCCGCCTCAGCCTCCCAAAGTGTTGGGATTACAGGCGTGAGCCACCGCCCAGCCTGATCATTATCTCTCTTAAATGACATTGTAACATCCCAAACTGCAAAG
Loxodonta africana  ......................................................................AAACTACATTTAACGTCCCAGAGTGCAGGG

Homo sapiens        GGGCCCACATCAAGTTTTTCTATGAAAGCCCATGACGTGGAGCACAATGCTTTGTACTAAATAGGCACTCAGGTGGTATTTGTTA-----TTGATTGATAGGATTCTGAAGAACTCAGTA
Loxodonta africana  AGGCCCACATCATGTTCTTCTACGGAAGCCCATAACATGGAGTAGGATGTTTTGCATTTAATAGGCATGTAATAAGTATTTGTTAACTTATTGATCAATACAAATGTGAAGACATCCTTG

Homo sapiens        TGTAACCATCCTTCCAAACCTGGGTGTGTTAAAATGTGTTCATTGTTCAGAGACAAAGGT--CTGGTACCTTTAAGAAGTCTCAGCTTTGCAATGAGTTTGTGGGGCA-GCTGCTTGGCA
Loxodonta africana  TGTGACCTTCCTTAAAACCTTGACTGTATTAAAATGTGCTTATTGTTCAGAGACAAATATGACTGATACCGTTCAGAAGTCTTACCTTTGCAATGACTTGGTGGGGCAAGCTGCTTGGAG

Homo sapiens        TAGATACTGGGGAGGGACAGGGAGGAGATCATTATGTAGGCACATGGCGCTAGGATGTACCTTACTTTGTCTTGGCTGTTTTCTGAATGTTAAGAGGAAGGATGAAATAATAGCAGATAT
Loxodonta africana  TTGATACTGGGAAGGGACAGGGAGGGAACTATTAGGCAAGCTCATGTCCCTGGGATGTACCTTACTTTATCTTGGCTGTTTTCTGAGTGATAAGAGGAAAGATGAAGTAATAGCAG-TAT

Homo sapiens        AGGAAAAGTTATTTAAAGCCTTGCCTTAGA---TCAGTGGTTCTCAGGCGTGAGGATGCATCAAAAGCCCCTGGAGGGCTTCTTAAAACACAGATTGCTATATCCCA-CCCCTGAGTTTC
Loxodonta africana  AGGGAAAGTGATTTAAAGTCTTACCTTAGAAGACCAAAGATTCTCAAACTTGAGAGGGCATCAGTATCACCTGCAGGGCTTGTTAAAA-ACGGATTGCTGGGGCCTGTCCCCAGAGTTTC

Homo sapiens        TGGTTC-AGTAGGTCTGAAGTGAGTGGGGTCCACAAATATGCATTTCTA-ACAAGTTCTCAGGTGATGCTGATGCTCCTGGTCCAGGGACCACACTTTAAGAACC-ATTGCTCTGCCAAG
Loxodonta africana  TGATTCGATTAGGTCTAAAGTGAGTGGGGTCCAAAACT--GCATTTCTATGAAAGTTCCCAGGTGATGCAGATGTCACTGGTCGAGGGACCACACTTCTAGAACCCATTGTCTT------

Homo sapiens        TAGTTGGCTCAAGAGTAGCTAATTAGAGAAATCTGAGCA-CAGTTAGCAACTGGAAGCAAAAAGTCCATGAGG-----------AAACTGTGTGATGTACGTAGTGGGGAGAGTGTGA-A
Loxodonta africana  --------TAGAGAGTATTTAG--------ATCTGAGCAACAATCAGCAACTAGAAGAAAAAAGGCAATGAGGGACCAAGGGTGAAAGCATGTGATGTATACAGTGTGGAGAGCATGGGA

Homo sapiens        CTGGCTAGGAAACCAGAATGGTCAGATTCCAGTACCAGGGGTATCATATCTTTTTAAGCAGTGTCCAAAG---AATAAGTATATTTTAATTCACATATTCCTGATTCATTTTACTAGTGT
Loxodonta africana  CTGGCCACGGAGCCAGAAGAGCTGGATTCCAGTACCTGAGGTGTC-----CTCTTAAGCAGCGTTCAAAGCATAATAAGTACGCTTTAACCCATATGTCCCTGATTTATTTTACAAAGCC

Homo sapiens        TTCCCAAAATGTGCACTGTGGAACCCTAGAAGATATTAATAGGTAGGCAATGAGATTAAAAAGGATCCATAGAGAAA-GATGTTGGAACATTTTGAATTAAAGAAAATTGAACAGGATTC
Loxodonta africana  TTCCCAAATTATGCACCATGGAGCACTACAAGATGTTAATAGGCATTTGATGGGGGAGAGAGGAATTTAGAGTCAAATAAGGTTGGAAAATTTTGAGTTAAACCAGATAGAACAGGGTTC

Homo sapiens        TTTAC-----GGCTTCTAAGAGCCTTTAAAATGTGAATGAATGAATGCACTGCAAATCTCCAAGGAAATATAGAATATGCAGTATTTATTTTATTAATTTATGCAGAATATTCAGAATTA
Loxodonta africana  TTTACTGCAAGACTTCTCAGAACCTTTAAAATGGTAAT------ATGCACTACAAATTTCCAAGGG--CAGAGAACAGGCAGCATTT----------------------CTCATACTTA

Homo sapiens        GTTGACCAAAGATCCTTCGTTAACACTTGGCTGTTTTTTTGTAAAATGTTGTTTAGGAAACCTTCATTGGTTTACACCTGAAATGCCACAATATTTTATCAATAGATGAGGTTGCTTGCA
Loxodonta africana  ATTAACCAAGGATCTTTTAGTAACACCTGGCTGCTTTTCTGTAGAACCTGGCTTAAAAAACATTTATTGTTTTATATCCAGGCTGCCAAAATATTTTATCAGTGGGTGAGGCTGCTTGTA

Homo sapiens        ACAGTGGAAACAGCACTGCACTGGGAGTCAAAACATCTGGGATTTTGTTCTGTCTCTGCCACTTAGGTCTGCCACAAGTTGGCTTGCTCCCATGAAAAAGATGGGTATGATTGAAATTTA
Loxodonta africana  ACAATAGAAATAGCACTGAACTGGGAGTCAAAAGGCCTGGGGTTTTGTCCTGCTTCTGACACTTAATTTTGTCTCAAGTTGACTTGCTCCCATGAA---GATAGGTGTAACTGAAATTTA

Homo sapiens        TCATCCCTTTGCAAATGGTAGCAGCCAGTAGCTCCACCCAAAATAAGTAAATAAAATCTCACACAATCAATTTTTTTAAATCTAATTTACCATACCCTTAAGGTTATTCAATCTTTTTGT
Loxodonta africana  TCATTCCTTTGCAAATGGTGGCAGCCAGTAGCCTCAGCCAAAA-----------------ACACA------TGTTTTATCTAGAATTCACTGTACCCTCAGGG----------------

Homo sapiens        TTTTTTTTTCTCATGTTACCTTGTGTAACAGGTATTCAACCTTCAAAGCCAGAAATATGAGAAGCCTTATGCTGAGAGTTTGATAGAAAGGAAGGGAGGAAGGGAGAAAGTAGAAAGGGA
Loxodonta africana  ------------------------------GTATTCAATCTTCAAAGCCAGAAGGGTGAGAAGCTTTATGCTGATGGTTTGATAGGAAAGAAGGGAGGGAGGGAGGAAGGAATAAGG--

Homo sapiens        GAAAAGGAAGAAGAGAGGAAGGGATGAAAGGAGGAAAGGCGGGGCAGGGAGGAAAGGAAGAACAGCAGAAAGAAGGAAGGAAGGAAAAATAAGAAGTCAGACAGTCAGGTCATTGCAGAC
Loxodonta africana  ----------AAAGGAGGGAAAGATGAAAGGAG.............................................AGAAAGTCAGATCTTTGCAGAC

Homo sapiens        CATGATTCCATCTGAAAAGAAAAGGCTGAGAAGCATAGACACATCCTGTGTTTATTTGATTCCTGGCTCTAGGAGAAAGTTTTATTTCACCAAGGACA--GAGTCAGCTCGCTTGTTTAC
Loxodonta africana  CATGATTCCAACTGGAATGAACAGGCTGAGAAGCACAGACACATCCTGTGTTTATTTGATTCCTGGCTCCCAGATAAAGTTTTGTTTCACCAAGGACACTGAGTCAACTTGTTTGTTTAC

Homo sapiens        CACAGAGTATTGGAAGTGTGTGAAGTGTGTTTGTCCCCCAATTAACCATTAGTTGTTTTTTTTT---CACTTGAACAAGATACAAACTTGACCCAATGGAGAAACATTGCAAGTTGTATG
Loxodonta africana  CACAGAGCATTGGAAGTGCGT---------CTGTCCCCCAATTAATCAGTAGTTGCTTTTTTTTTTCCACTTGAAGAA-ATACAAACTTG--------GAGAAACATTGCAAGTTATACG

Homo sapiens        TGGGAGGATGCCATAAAATATCTACATTGATATTTAAATAAAATACCTGCCTACATTAAACGATC-TTTGAAAAGAAACTGATGGGATTCAAGGCTGAATTA-TATTTACTGAATTCCTA
Loxodonta africana  TAACAGGATGCCACAAAATATCTGTATTGATATATAGATAAAATACATGCCCACATTAAACAGTCATTTGAAAAGAAACTGATGGGATTTAAGGTTCAATTAATACTGGCTGAATTTGTA

Homo sapiens        CAAGGTACATAAATATATGCAACATGGAGTCCCTTGTAGATCCATGATGAAATCATATTTCTTTTAAACACAAGGGTCAATTCAAGTTAAAAAATAAAATAAATGTTTACAAGCTTTCTG
Loxodonta africana  TAAGGTACATAAACGTATGTAACATGGAGTCCCTTGAAGTCCTGTGGTGAAAACAT----CTTCTGAACACAAGGGTTAATTCAGGTAAAAAAAAAAA--AATCTTTACCAGCTTTCTG

Homo sapiens        CCTTTTTTA---AATTTCAAAGTGAAAATAATATTTAATTGCATTTAAATATATACATATATTTTAATAACTCTGATTACCTATTTGATTGCATAAAAGAATGACGAGAACATCAAC---
Loxodonta africana  CCTTTTTTCTTTAATTTCAAAGTGAAAATA---TATAATGGCATAGAAAAATATA----TATTCTAATAACT--GTTTACATACTTT-TTGCATAGAGGAATGGCAA-AATGTTAACTAA

Homo sapiens        ACTGCTTTTAGCCACAATATTTGAGAAGTAGCAGC-CAGAAGTGTTTTTTTAAATATTTTATCTTGAAATCAGATCT-CATGTACCTA-------GGTGAACTGAATATGCCCAGAGGAT
Loxodonta africana  AATGCCAATGGCTGGGCATACTGAGAAATAGCAACTCAGAAGTGCTTTT--GAATCTTTTATCTTGAAATCAGATCTGTGTATATCTCAAAGTTTGGTGAACTGAATGTGCCCAAAGGGT

Homo sapiens        ATCAGGGGACTCTCCACTACTGTAGATCCAAAAAACAGAGGGCTAAATAGAAATTCCACTTGCTGTCATCCAGCAAAATAGAGATGGCTTTGCTGAGTCCTTAGATCATGTTTAGTTTCA
Loxodonta africana  CTC-GGGGACTCCTCACTGCTGTAGACCCAAAAAGCACAGGGCTCAATAGAAATTGCACTTGCCATCATACAGCAGAATAGAGATAACTCTGTCAGGTTTTCAGACCATGTCTAGCTTCA

Homo sapiens        GGAGGGGTTGAGCTGTAATGACTAAATTATCTGGTCACTCTAACCAAGCAGCTAATTAATCCAGTCAAACAAACTTATCAGTCAT-----GAAGGTGAAATTGTTTTT-TCATCTAGAAT
Loxodonta africana  GGAGATCTGGAGCTGCATTGATTGAATTGCCTGATCACTCTAGCCAAGCAACCGATTAATCCAGTCAAACAAACTTATCAGTCACATCAAGAAGGTGAAACTGTTTTTCTCAGCTAGATT

Homo sapiens        TTGATCATAAGGGCTTTCA-----------AGAAGGAAGCTCCTTG----AGTCTCCCTTGTAAACCCTTTCTGCAGTATGCCTTTCTTTCCCACGTCTGTTGAAAATTATTTATTCTTG
Loxodonta africana  TTGACCTTAAGGGTTTTCACCCCCTGCCCAAGAAAGAATCTTCCTGCCTGAGACTCTCTTGTATACCCCTTCTGCAGTATGCCTCTCTTTCTCATGACTACTGAGCATGATTTCCTCTTG

Homo sapiens        AACCAGCTTCAGTGGTGAACAGCAGCTGGTTGCTACCCTCTCATACCCATTTTTTGTCATCTGGAACATGGTTATTCAGCGTAGCTTCATCTTCAAAAGGTGCCTGATTTACTCTGGGAT
Loxodonta africana  AACCAGCTCCAGTGGTGAACAGCAGCCGGTTGTCACTCT-GCATACTCACGTTTTGTCTACTGAATCATGGCTATTCAGTGTAGCCTCAACTTCAGAAGGAACCTGGCTCACTCTGCAGT

Homo sapiens        TGGGAATGGAGAGAATAATTTTGAAGCTCTTTCTCTTTGCCTGTTTCCTTCCTTCCATGACTTTGGGTTATTTTCTAGTGATCTTCAGTATTAAAAGGCATACTGACAGCTCTTTTCTCC
Loxodonta africana  GAAGAGTAGGGAGAATCACTTTGAAGCTCTTTCTCCTTGCCTATTTCCTCCCTTCTATAATTTTCAGTTGCTTTCTATTGATTTGCAGTTTAAAATTGTGTGCTAAAAACTCTGCTCTTC

Homo sapiens        CAGGCTGATTTTCAGCTTGTCTACTGAGCTTTTTTGGGTTTGGTTTTATGGAATGAAATCCCCTTGTTTGGTTTTCCCCACATTCACACTGAGGCTGTGAATGTCCACCAACTCAATTCT
Loxodonta africana  CATGCTGCCTTTCAGTGTTTCTATTGAGCTTTCCTAAGTTTGCTTTTATGAAACGAAATTCCCTTGTTTGGTTTTCCCCACATTCACAGT------------------AATTTAATTCA

Homo sapiens        GAGCTACAATTGAGTAGTGTATTAAGAATACATGTGACTGCCACATGGGCCCTGGACTGCTGAATTCATGGTCAAATCATTGTTTTCCCAACCATCATGCTTGGGGGCACCACATCAGCC
Loxodonta africana  GAGCTACAACTGAATACTGTATTAAGAGTACATGTGATGGCCACTTGGGTTCTGGCCTGCTGAATTCATGGGTGAGTCATTGTTTTCCCAACCATCGTGCTCAGGGGCACCA-----GTC

Homo sapiens        TACAGTGAACATGGATTAATAAGTGCTGATTTAGAGTAAATAGTTTTCTGGTAAGTGGTACATTGAAGATTTCTCTTCCCCTTTCTATGATTTGTATAGTAGCCACAATTTTCTATATCA
Loxodonta africana  TGGGGTGAACATGAATTAATAAGTGCTGATTTAGATGAACTATTTTTCTGATAAGTGGT--------GATTTCCC--CCCCTTTCTGTGTTTTTTATGGTAACCGCAGTTTTCTACATTA

Homo sapiens        TTATTGCAGCTTCCCAGTACTACTTTA-TGTAGAAATCTTGCACATTCCTACCTCTGGCT-TTTGCTCAGGCTACGACTCTATGGCAGTGGCAGAACCGTTTTAGGAATGTCTTCTGCTG
Loxodonta africana  TCACTGCGGTCTCACAGTACTGCTTCAATGTAGAAAATCTACAGATTTCTACCCTTGGTTCTTTGCTCAGGCTACAACTCCATGGTAG-GGCAGAACCATTTTAAGA-CATCTTCTGCCA

Homo sapiens        TAATTTTCTCTTTGTCCCACCACTTCCACTTTAAACTTCTGGT---------GTATTCCCTAATCAAAGAGGCATCTAAGTTGT----CTTTAGTGAGAAACCATCTATATATACAAAGC
Loxodonta africana  TAGCTTCCTCTT-------------CCATTTTTAATTTCTGGTTACTGACATATATTCCCTCATAACAGAAGCATTCAAGTTGTTTGTCTTTAGTGAGAAGCCATCTACATACACGAAGC

Homo sapiens        CATAACATGTGGATGAAAAGGACATACTGTCAACTCTCTTGTGCCAGACACCCAGATGTTGGCAAGTAGTACTTGGACCGAACAGCATAGTCGTTGAACAGCATCATCTTCCCCTTTTT
Loxodonta africana  CATAACATGTAGATGGAAAGGAAACACCGTCAACCCTCTCATGCCAGACATCCAAATGTTGGCAAGTAGTACATGGTCCAAAGAGCCTAGTCATTGAACAATATCACCTCTCCCATTTTT

Homo sapiens        AATTCATCCAGATGTTTTGGCATCACAGACACATAGCCAGATTTCATCCCTGATTTTGTCAAAGGCTTAAAAAAATGTGAAAAAATAATATGCTTCTCCTCTGATGAACAATTACCAATG
Loxodonta africana  TATCCATCCAGATGTTTTGGCATCATGCAGACATAGACACTTTTTACCCATGATTTTGTCAAAGACTGAAAAAAGTATG-AAAAATATTATGCTTCTTCTCTGATGAACAACTGTCAATG

Homo sapiens        AA--CTCTGTTCAAAAGGGAAAAC-----ATAATGTTTCATTT-AAAAGACCAATAATATTGAATTAAGATAAAGTTCCCATGAGAGTATATTTTACATTCTCATGAATCACGGATGAGT
Loxodonta africana  AACTCTCTGTTCAGAATAGAAAAAAAAAGATAATGTTTCATGGAAAAAGACCAATAATACTGAATTAACATAAAATTCCCATGGGAGAATTTTTTACATTCTCGTGAATCACGGATGAGT

Homo sapiens        TTATTATGATCCATGCTTGTATGAAAGACTGACAAATAAATTCATATTGATTTGACATTTAAAATAGCATGTCTAAAAATGTACAGCATCACATTAAGTATCAAGTAATTATTCAACTAT
Loxodonta africana  TTATTATGATCCATGCTTGTA-GGAAGAGTGACAAATGAATTCATATTGATTAGACATTTAAAATACCATGTCTAAAAATGTACAGCATTGCATTAAATATCACACAATTATTCATCTAC

Homo sapiens        CAGCTTCAAATGTACATGGGAGAA--AATGGGTCCAGAACCCAAAATGTAGTGAAAGATTTAAATGCTCAAATTTCTACCACAGGTTTTTTTTTTTTTTTTTTTTGAGACAAGGTCTC
Loxodonta africana  CGGTTTCAAGTATAAGCGGAAGGACCAATGGA---------CAAAATGTAGTGAAAGATTTAAGCGCTCAACT...........................

Homo sapiens        ACTCTATCACCCAGTCTGTGTGCAATGGTGTAATCATGGCTCACTGCAGCCTCAAACTCCTGGGCTCGAGTGATCCTCCCACCTCAGCCTCCCAAGTAGTTAGGACTTACAGTCATGTGC
```

```
Loxodonta africana  ........................................................................................................................

Homo sapiens        CGTCATGCCTGGCTAATTTATTTTATTTTTATTTTTGTAGAGACAAGGCCTTGCTTTGTTGTCCAGGTTGGTCTTGAACTCCTGGCCTCAAGCAGTCCTCTCACTTCGGCCTCCCAAAGG
Loxodonta africana  ........................................................................................................................

Homo sapiens        ACTAGGATTATAGGCGTGAGCCACTGTGCCCAGCCTTCTACCACAATTTTTAAAGAAGCCAATACCATACATTAATCCATTTACAAGGGCTCACGCAAGAGTGCCTTTTGAGCCTGTGTT
Loxodonta africana  ...................................TTCTGCCATAAATTTTTAAGATGTTAAGAGAGTGTATTAATCCATCCATAAGGACTTATGTAAGTGTGCCTTTTTAGTCTGAATC

Homo sapiens        TGACAGGAAGTTTCTATTCAGATCATTGTGCCTAGAGTATATAGAGGGGTTACAGCAAAGTGCTGTACTTTGGGTTACAGTAAAGTGACTCAATTGATGTACCAAGATATTGATCATTTA
Loxodonta africana  TGATTGGAAATTTATAGTTAGATCACTGTGCCTGGAACATATCAA------------AAATAC----CCATGGGTTACAGCAAAGTGCCTCAACTGATATACTAGAATGTTGATCGCTTC

Homo sapiens        CACCCT-------------------------------------TTAGGCCTGAAAAATATACTTTTCTTTGTGTTCCATGAAACTAAAAAGGCAGGGAAGCATTAAAGAGACAGCAATAA
Loxodonta africana  AACCTTCAGAAGAGCTGGGAGGGGCCTGGGGCAACAAGGGCTCTTGGCCTTAAAAATATAATCTTCT--GTGTTCTAAGACATTAAAAAGGCTAGGAAGCACTAAGGAAGCACAAATAA

Homo sapiens        AAAGATTTGATGGTATGTATATGTGAGTTTGAGTGTTTGAGCTGGCGTTGTGAGTTTGTGGGTGGATTAGAAAGCATATGGCTAAAGACATGTGCAACAGATGCAAGGGCCTGGGCTTTG
Loxodonta africana  AAAGATTAGATGGTATACATGTATG----TGAGTGTGTGAACTGAGGTTAGGGGGATTGGACTGGATTAGAAAGCATATGGTTGTAGACATGTCCAACAGATGTAAGGGCTGGGGCTTTA

Homo sapiens        GCCT-TCCCTTTGAAACTTCTTTATGGGGACATGTGCT-------GCCTTGCTTCCTGGATTAAGACCACATTTGCACAATTTGCACATTGTCCCTCT-----TGGGCCTGCTTCCCACC
Loxodonta africana  GCCTCCCCCTTTAAAACTTCTTTATGGGGGCATGTGTTTACATGAACCTTGCTTCCTGGACTAAGACCACATTTGCACCATTTGCACATCACCCCTCTATTCCTGGGCCTGACTTCCATC

Homo sapiens        GGAGACTGCATCTTCCACAAAAACAAGGCAAGAAGTAAAGAACTTAAGAGAGCTGATTGTGAAGTCCAACTACATGGGTTCAAATCCTGGTACCATCATTAATTAGCTGGGTCATTTTGG
Loxodonta africana  AAAGACAACATCGTCCACGAAAACATGG-AGGCAGTGGACAATTTAAGAGCTCTGGCTATGAAGTCCAACTGCCTGGATTCAAATGCTGTTACCATCACTAATTAACTGGGTCATCCTGG

Homo sapiens        ACCACTTAACTTCCAGGACCCTCATTTTCCTCATCTACACAAAGGGCTAATAACAGTATTTACCTCATAGTTGTTATGTAAAGTGCTTATCCCAGAGCCTGGCAGAGAGCAGACACTAGT
Loxodonta africana  GCTAGTTAATATCTGTGACCCTTGCCTTCCTCGTCTATA.................................................................................

Homo sapiens        TGATCATTATGATTATTGAGCAATTACTATGTACCAGGTGCTTTATTTTCATCTCTGATCCTCACAACAGCCCTACAAGGCAGATACTATTATCTTCAGTTTACAAGTGAAGAGGCTGAA
Loxodonta africana  ..........................................................................................TTATCTCCATTTACAGATGAGGAGGCTAAA

Homo sapiens        GCTCAGTGAGATAAAATACACTGCTCAAGGTCACACAACCAGGAAGAGCAGAAACCAGGCTCCAAGTCCCTCTGTCCCTCACTCCTAAGGCCTGTGCTATTTGCCATATCATCATCCCAC
Loxodonta africana  GCTCAGAAAAAGAAAGTAACCTGCCCAAAGTCACAT---TGGAAAGAGCAAAATGCAGAATTCAAGTCCTTCCCTCTTTTACTCCTCAGCCTTGTGCTTTTGGCACTTTCATCGCCCCAC

Homo sapiens        ---------TGGTGCCTCTAAAAGTTTCTTCTTCTAGGGTTCATCCCTCTTCTCTCTTCATCCTCCTCTTGGCCTACCCTGGCCTGAGAACTGCTTGGCACAATCCTATACAGCAGGTCC
Loxodonta africana  CTCCACCCCTTATGCTTCTAGAGTTTCCTTCTTCTAGGAGTCATCCCTCTTCTCTTCTCACCCTCCCCTTGACCCACCCTGGCCCGAGAACTTCTTGGTACAAAGCTA............

Homo sapiens        TTGAATAACATTGTTTTGTTCAAAGCCATTTAGTTATCACGTTAATGGGGAAAATTTGGTTTTCCCCAGGGCCACTGTCCATGGACAGGATGGCGTCCTGACCAGGGTGGTTCCCACCTT
Loxodonta africana  ........................................................................................................................

Homo sapiens        TTGTGCTCCTTGTGCTGCCAGGATGGGCTCCGGCCACCTGTGACCCTGAACTGGAATCAGCGGGTTAGAAAATAACTGAATGAGTGAATACAAATTATTGGAAAATGAAAACTCGTAAAG
Loxodonta africana  ........................................................................................................................

Homo sapiens        TACACAATGACCATACGAATCCATGATAATAAATGACACGATAGGAAAGCGCTCAATGAGCGCTTTTGTGATTCTTCTGGAACTGCTGGGTGGCAGGAGGTGCTCCTTATGATTTTGCTT
Loxodonta africana  ........................................................................................................................

Homo sapiens        TGCAAATGTTTATTCCTTGACCTGACCCGCCATTGCTACAACCGCTGTCACTCATTGATTCACCAAAAATTAGGCAAATAATTCTCTTACTTGTTTTTATTAATCTTTCCTAAATGTATG
Loxodonta africana  ........................................................................................................................

Homo sapiens        TGTAGCTCACACTTATTGTTAATGTTTAATTAACTTATTAATGTTTAATATTAGAAGTGTTTGGGTGGTTTTTGTTTGTTTGTTTTTTTGTTTGTTTTTGAGACAGGGTCTCACTCCTCT
Loxodonta africana  ........................................................................................................................

Homo sapiens        CGCCCAGGCTGGAGTGCAGTGGCGCAATCACGGCTCACTGCAGCCTCAACCTCCCAGGATTCAGGTGATCCTCCCACCTCAGCCTCCTGAGTAGCTGTGACTACAGACGTGTGCCACCAT
Loxodonta africana  ........................................................................................................................

Homo sapiens        GCCTGGCTAAATTTTTGTATTTTTAGTAGAGACAGGGTTTCACCATGTTGCCCAGGCTGGTATTTGGATCTTTATTTAGAAGTTTGGTGATATTTTTGTGACCAGAAATATGCTGTGGGT
Loxodonta africana  ........................................................................................................................

Homo sapiens        ACTTAACTCTTGTTTATATCAATTAGCCTACAGTAAAATTAGTTTTGTTATTCGTTGTTTCACTTAAAGTCCAGTTCCCAAGAACCTGTCAAGGACGTTCAGTGAGGACTTACTGTACTC
Loxodonta africana  ...................................................................................................................TACTC

Homo sapiens        AGAGACAGCAGAGAGCAAAGGGCAAGAAGGCTGGTTGGAGGACGAGGAGGCCTAGAGCCCCTTGCCTGCTTTTGCACATGCATCTGCTCCTGGTGGATGACTCACTTAACCTCTCCGTGC
Loxodonta africana  AGGGACAGCAAACAGCAAAGGACAAGAGCACTGGTTGGAGGAGGAGGGTGCCT----TTCCATATCTG-----GTGCATGCACTAGGTCATGGTGGATGACCCACTTAACCTCTCCGTGC

Homo sapiens        TTCTGCCTCTTCAACTGGAAAATGGAATCTGTAAGTTGCAGCACTAG-----CTTCTGTGACATTCTACTAGGCCTCCAGGAAGAGGAGGCCATCCCTCTATATGTCCAGTCAGCATA--
Loxodonta africana  TCCTGTCTCTTCAACTGGAAAATGGAATCTGTAAACTGTAACACAAGGGTTGCTTCTGTGACGTTTTGCTTGGCCTTGATGGAGAGCAGGCCACCCCTCTATATGCCCAGTCAGCATAAG

Homo sapiens        TGGGGAACAGTGGGGCTGTGTGTGACTCACCCTGTGGTGTGCCAGCCTCAGGCTTATACTAGCCTAGGCTTATACTCCCAGATCCTGAGC---------------TAGCAGCTGGAGCT
Loxodonta africana  TGTGGCCCTTGGGGGCTGTGTGTGATTCAGCTGGT-GCATGCCAGCTGTGG------ACTATCTCAGCCTTGTACTCCCAGACTCTAAGTTATTCCAGACTGCCTGTAGTGACTGCGGCT

Homo sapiens        TTGGGATCTGGAACTGTTACAGGGCCAAAGAACCTGCTTAGCTTCAGGGCTGCTTCTAGATAAACTGCCAGGATGTGGGCAAAGCATTTGTCATGGAAGGTGGGGAGTGTATGTAGGTTG
Loxodonta africana  TCTGGATCTGGAACAG-TACATGGCCCAAGAACCTGATCAATTCAAGGGCAACTTCTGGAT-AACCATAGGGGTCTAGGCAAACCATATGGCATAG-----------------GGGTCG

Homo sapiens        TTTCAGGGGCTGCTTTGTGGGAAG----CCAAACCTTTTGGCAATGGGCTCAGAACTGGCTTTCCTTTTTGGGTGTCCTCTAAATTCCGAGGGTGCCCCCACACCAGAGGG---TCTCC-
Loxodonta africana  CTTTAAGGGCTGCTTTGTTGGAATCAGCCTGAAACTTTTGAT-GTGAGCTCAGAACTGGCCTTCCTTCCTGGGTCTCCTCTAAAATCCTAGGGTGCCTCCAGGTCAGAGGGGACTCTCCC

Homo sapiens        ---------ACTTC--TGCCATCCCTAACCTAGTGGGGAGATGAATCTGATCTTTTGTCTCAAAGCCCACATTAAAGAGGACCATTGCATGCTTCATCACCAGGCTGTGACTC--CCAGA
Loxodonta africana  TCATGTGGGGCCTCTGTGCTAATTGCAGCCTAGGAGGGTGGTGAAAC--ATCTTTTGCCTCAGGGCTCATGTTAAAGAGG---------------ATCACAAGGTTGTGACTCAGAGAGA

Homo sapiens        CCTCAACTCATG---TCTTTTTCCCTCTTCACCAGGGCAGAGGCT-AGACTGTTTCCTCTCCTCTCCCTGAACAATAGCAGTGCCCAGATTTTGGAGGGCATACCGCCTACCTGGCACTG
Loxodonta africana  GCTCAACTCCTGGTTTTTTTTTTCCCATTCACTAGAGCAAAGGATGGGGCTGCTCTTTCTCCTCTTCTTCAACAATAGCAGCTTCCACCTTTCAGAAGGCTTACTTTGTACCTGGTTCTG

Homo sapiens        TGCTAAGTACTTTGCCTGCATTTCCTTGGTAATCCTCATGTAATCCTACAAGGGAAGTACCATTTTGTCACGATTTAACAGAAGAGGATACAAAAGCTCAGAGCCTGGAGGTGACTTGCT
Loxodonta africana  TGCTAAGCCCTTTGCATGCATTCCGTCACTAATCTTCCCACACTCCTGTGAAGGAAATACCATTTTGTCACTATTTTACAGAAGAGGAAGCAGAAGCTCAGAGGATGGAGATGACTTGCT

Homo sapiens        CAAAGTCACATTGATAGTAAGTGGTAAAGCCGGGACTGAAATACAGGTCTAACTTTTGAGACAACCTTTTTTCTTTTTCTTTTTTTTTCTATTTCAATTACATAATCCTAATCCTGTGCGT
Loxodonta africana  CAGAATCACGCTGACAGTAAGTGGTGGAGCCAGGATTCAAATGCAGGTCTAATTTATGAGTCTGCCTCTTTCC---------CTACCTGATTTCAGTCACA------CAATCCTGTGTTT

Homo sapiens        GTTTGAAGGGGAGAGACAGGGGAGGGAGTGAGATGGCAATTTGCTTGTGACCTAAGGAGGCCCTGAAATCTCCTGGGAATGAGGATGGGGGACCTACATGAGTTGC----------CCAG
Loxodonta africana  GTTTGAATGGCAGACACAGGTGAG...................................................................ACCTATATGAGTTGCATGAATTTGCCCAG

Homo sapiens        GGCACTGGTAATGCAGCCCACACCAGCAGTCAGTGATCTCCAACATGGAGGGCTCCAGGCCTGCCTAGCTGCTGGCTGC--TACTGGAGGCCAGAAAAAGACAGCTCCAGTGTCCTTCTA
Loxodonta africana  GGCATTCAAAATGCAGATTGCA----------GTGGTCTGCCCTGTGGAGGGTTTCCGGCTGGCTTAGCCATTGACTACAGTAGCAAAGGCTATAAAAACAGAGCTCCAGTGTCCCTCTA

Homo sapiens        ----GGCTGGGGGTGGTTCTTGCCTCCAGGGCTTATGGAAGAACTTATCAGACAAAACTCTGCCCTTTCAAGCAGAAAAGTACTTTACAAAGAACATAGAGAGGTGAAGTGGGTACTTTT
Loxodonta africana  CATGAGGCAGTGGTGGTTC...CCTCTAGGGCCCATGGGAGAATCCATCAGAGAGCACTCTAGCCTTCCAAGTAGAAAGATGCTTTATAAAGAGCACAGACAGGTGAAATGGA-------

Homo sapiens        CAATTTTTGCCTGATTATCTAATATCTAAATGCTTTTTCTTCACTTTGGAATCCTGACCTGACCTGTTTTGTGCCCTGTCCACCCACCACCACTGAGTCAAAGCTCAGGGCCTGGCATCC
Loxodonta africana  -GATTTTTTTCTACCCACCTAATATC-AAGCCCCCTTCCTACGTTTGGGAATCCTGGCCTGGTCTGTTTTGTGCCCCCTTCCCCCATCACTCCTGAGTCAAAACATAGTGCTTAGCTAAC

Homo sapiens        AGTATTAGT-CATACCTATTTGTTGCCCGAATGAATGGGGCTGAACC----CTGGTCTGACTCTAAAATACATGCTTGAAAAAAATTATTATGGAAATTTTCAAACACATACACAAAAGT
Loxodonta africana  AG-ATTACTCCATATATATTTGTTGCCGGAATGAATGGGGTCAAACCCAAGCCTGTCTAACTGTAAAATCCATGCTTAAATAACATTTACTACGAAATTTTTGAACAC--ACACAAAAGT

Homo sapiens        AAAAAATATAGTATAATGAACCCCCAAGTATCCATCACCCAGATTCAACATCTATCATGATTTTCCATGTTAGCTTTACCTATCTCTCTTTCTTTTT-CCCTCTTATGC-----------
Loxodonta africana  AGATAGAATAGTATAGTGAACCCATATGTACCCATTACCCAGCTTGAACGGCTATCAGGATTTTTCATGTTTGCTTTACCTATTTCTGTTTCTTTTTATTCTTGTATGCTTTGCCTTAAT

Homo sapiens        -------------------------------TTTACCCCACATGTTTCATTATGCAACTCAAAAA--TAGGGATATTTTTATATACAATGACGAAATTATTATCCCCCCCATCGCCAAAC
Loxodonta africana  ATTTTGCAGGGAATCCCAGGCATTATATCATTTTACCCTATATACTTCAGTATGCATCTCAAAAACTTTGGGGTATTTTCAT-------------ATTATTATCACCCCTA--ACCAAAC

Homo sapiens        TAACAATAATTCCCTTCAAACCATGCTCTTGAGCACTACACTGGAGTTCCTCCATTCTACTTTCTTTCCCAGTCCCCAACAGGAAGCATTTCTCAGGCATCTGCTCTTGCACCTCCTCAG
Loxodonta africana  TAACAATAATTT-------------------AAAACTACGCTGCTGTTCCTCCATTCTACTTTCTTTCCCAGTTCCTGAGAAA-------------------------GCACCCCCACTG

Homo sapiens        AGCTGGAATTACAGTTTGATGTGTATTGGGTACTCACAATGTGCCCTTTTAGGTAAGCAGGGGGCCAGATTCCCCTCTCCCTGTTTTACAAAGAAGAATGGCGACACACAGCTATTAAGT
Loxodonta africana  AGCTGGAATTGCAGTTTGATGTGTTTTGGGTACTCACAATGTGCTCTTTTAGGTAAG--------CAGACTCTCTTCTCCCTGTTTTACAAAATGGAATAGAGACACAGAGCTATTAAGT

Homo sapiens        GCCTTGTACAAGGTCACACAGTCAATTAGTGGCTGATAAATGGGACTCAAGCACTGTGGCTGGGTCTATACAGCCCCAAATACAGTTTGCAAGGAAATTAAATGAAGGGACTCGTGTCCT
Loxodonta africana  GCCTTGCACAAGGTCACACAGTGAATTAGTGGCTGATGAATGGGACTCAAGCACTGTGGCTGTGTCTATACAGCCCCAAACACAGTTTGAAAGAAAATTAAATGAAGGGGCTTCTGTGTT

Homo sapiens        GGGGACAGTACCGTGTGTTTTCTGTTGCTGTTGAAGTCATTG-CACTTAG---------------------------------------CCCATCAGGACAGCCTGATTGATGGGATCAG
Loxodonta africana  GGGGACAGTACTGTGTGGTTTCTGTTGCTGTTGAAGTCATGGCCACTCAGTCTGGAGCAAATCCTTTCCCTGGGACAGCCTGATAATGACCCATTAATATAGCCTAATTGATGGGATCAG

Homo sapiens        TTGGACTCCTATGTTTGTTTTCCTCATCTGACTGGATTGCAACAATCACCTACAGGCAGGCTGGCCACACCCGTGAGTTTTTCTCTTCTTTTTTTCCATGTTCCTGGAGCTGACTCCGGA
Loxodonta africana  ATGATTTCT----TTGATCTTTTCCATCTGATTGAATTGCAGTCGCCACCTAGAGGCAGGCCAGCCACATCCCCATG-------CTGTTTTCTTTCCATGTTCCTGGAGCAGGCCCTGGG

Homo sapiens        GCCCACATTTCTCCTGTCTTTCTAGGCTTGGACTTGCTCTCTGCCAAAGCAGATGACTCCCAGAGCAGCCACAGTCTGGGACAGGGCAAATGTGAGAAGCAGAAGGACTAGCTTAACTGC
```

```
Loxodonta africana  GCCCTCATCTCTCCTCTCTTTCTAGTGTTGGACTTGCTGTTGGTCAAAGCAGAAGACTCCCAGCACACCCACAGGCATATCCAGGGCAAACACAAGAAGAAGCAGAACTAGCTCAGCTGC

Homo sapiens        TTTTCTGTTCCTGCCTCCTTCTTGTTCCAAAGACTTACTCTTAGATGCT-TGGCTCTCCTGAGCTTGATCTCCATGTCTAATTCTCTAGACCTCTTCCACTCCCCTGATATCCCACCCAC
Loxodonta africana  TTTTCTGTCCTTGTTCCCTTC-CGTTTCTAAGACTTCTCCTTAGGTGTGAAGGCTGTCCTAGATTTAACCTCTATGTCTAATTCCCCAGACCTCTTCCAC-CCCTTGATCTCCCACCCAA

Homo sapiens        ATTTTCCAAGTAAGTCTCTCAGAGCCTTGTATTCCAAAATACAAACTGGCTAGTGGAAAGAAGGAATCATTGCTGTTGGCTAGTGGATGACTGATCAGTCTGAGTCCAACCAGGAAAATA
Loxodonta africana  ATTTTCCAGATAAGTGTCTCAGAGT------TTGCAAAGTAAAAACTGGCTCATGGAAAGAAGGAA-CTTCCATGTTGGCTAATGGAGGGTTTATCAGTTAGAGTCCAACCAGAAAAACA

Homo sapiens        GAAATCACTGAGTATTTATTTAAGGGAAAAGGGATTCAGTGCAGGGAATTGATCACACAGGTGATGGAAAAGCTGTGATGCCAAGCAGAGGACAATGAAGCAACCTAGAGATTAGTCACA
Loxodonta africana  GGAACCACTGAG----TATTTAAGGAAGAAGGAATTCAATGCAGGGACTTGGTTACACAGATGATGTAAGAGCTGTGATGC-----------------------------------CATA

Homo sapiens        GCAGGGAGCTGCTACCATTCCTTGGCTAGAACCTCAGGGCTGGGACACTTAGTAGAAACTTGAGCCACAGT-AACCTGGTCAGCAGTGCCAGAGCCACA-AAGAGACACAGTCACTGCTG
Loxodonta africana  GCAAGGAACTGCTACCATTCCTGGGCTAGAACTTAGGAACTGAGTCACTTAATGGAAACTGGAGCCACGGTGAACCTGGTCAGCAGTGATGGAGCCATAGAAGCGACACAGTTACTACTA

Homo sapiens        GGGACACCACATGAAGCAGAGGGGGAGGGAGAGAAATACCCTG-GTTTCTCTCTCCCTTTTGCTTTCTAATATCCTAACAGTGCCTTCCATTGGCCAAACCCATCAGAGCCTGGGGAAC-
Loxodonta africana  GAGACATCACTAGCGGCACAGGGGATGGGAAAGAAATACCTTGAATTTCTCCCTTCCTCCCACTTTTCAATGTCCTGCCAGTGCCTTCCAGTGGCCAAGCCCACGGGAGCCTGCAAAACA

Homo sapiens        -AGCCTGCAAAGGCCAACCTGCCCCATCAGCTTCCCTGCTACA----AGAGCAGAGATGTGAAAAGGAAGTAATGGACTTGGGGGGGCAAACAGGCCTGGGGACAGCACAGATCT-----
Loxodonta africana  TAGTCTGCAAGGGTCAACC--------------CCCTG-TACAATATAGACCAGAGAAGGGAAAAAGAATGAGTGGATTT--GGGGGCAAACAGGACCAGGATCAGCACAGAGCTAATGG

Homo sapiens        ------------CCTCCTCCCTGCCACCCCCAACTAACTTATTTGGATATGTCAGGGTCTATTTGGAAACAGTGCATTTACTCAACAAAATGATTCTAAAGCTGAC-----------AAG
Loxodonta africana  TTCAACATATACTCCTCTCCCTGTGTCCTCCAACTAACTTATTTGGTTATACCAGGGTCTATCTGGAAACAATTTTTTCACCCAAAGGAATGATTCTAAAGCTGATGGAGAAAAAAAAAA

Homo sapiens        AAAGACCCTTAAGGAAAAGGGTGTGGGTGGAGCACGAACCCGCTTCCTTGA----CTACCTGCAAGTCTTAGCATCCAGATTCTAGCCCAGTCCTCTTATTTCACTTCCTCCTGCATGCT
Loxodonta africana  AAAGATTTTTAGGCATGAGGGTGGGGATGGAGCATGAACATGCCTCTTTGAGCATATACCTGAAAGTTGT--CATCTAGATTCTACCCCAGGTCTTCTATTTCATCTGTCTCTGCCT---

Homo sapiens        CCATCCCTCCTCCTCCCACGCATAGGCTGACTCCAACAAAGGCAGGCCGGCCATTTTGCTTTCTGGCAATGTGTGATGCATTCATTGGCTCCCAGGACTGTGTAAACCCAACCTTCTTGC
Loxodonta africana  ----CCCACCTTAGCACATGCACAG------TCTAACGGTGATGCTCGGGCCAATTTGCTTTCTGGCAACGTGTGATGCATTCATTGACTCTCAGAACTATCTAATCTCGGCCTTCTTGC

Homo sapiens        CTGCAGGGTTTCAGCTTGGCAGCCAGGCTCTGAACTGCAGTGCTGTCAGGCAGGTGAACCCGCTGGCACCACAATCCAGAAAACATATCTCCTGATTCCTCAGGTGTTCTACACGTTTGA
Loxodonta africana  CTTTAGGACCTCAACTTGGCAGCAAGGCTCTGAACTGCAGCATTGTCAGGCAGGTGAACCTGCTGACATCACAGTCCAGAGAACATACCTACTGATTCCTCAGCTGTTTGACATGTTCGA

Homo sapiens        ATGTGTAGATGAAGGGAGATGGCTGGGAGGGGGA------------------------------------AAAGAAAGACATCAAACTATTTATGTGCTTTCCCACACCTTATTTCATTT
Loxodonta africana  ATGGGGGGTAGAGCGGGGGCGGA-GGGAGGGGAAGGACAGTTTTCATTCCAAAGTGAAAACTGGCTAATGGAAAAAAGGAATCAAACCACTTACATGCTTTCCCACATCTTATCTCACTT

Homo sapiens        ACTCTCCACAACTCCTCAAGGCAGATGCCCTTCTTCCCATTAAAAAATAAATTTTTAAATCTTAGAATACTTTTAGATTTACAGGACAGTTGAAAAGAGAGTACAGAGAATCCCTATGCA
Loxodonta africana  ---------AACTCCTCAAGGCAGTTGCCCTTATGACCATTTAAAAAT-TTTTGTTTTATTTTGAAATAATTTTAGATTTACAGAAAAGAAGCAGAGATAGTACAGAGAGTTCTTGTATA

Homo sapiens        TCCTTCACTCAGTTTCTCCTGATGTTAACATCTTACAAAATCATCTTTTGTCTGTTTTTACAAATGATGAAACTGGGGCTCAGAGAGGTT-AGTGACTTGCCCAAGGTCATAGAACTAGG
Loxodonta africana  TCCTTCACGCAGTTTC-CCTAATGTTACCACCTTGCATAACCATCTTATGTCCGTTTTTACAAATGAGAAAACTGGGGCTCAGAGAAGTTAAGTGACTTGCCCAAGGTTACAGAGCTAGG

Homo sapiens        ACACAGCAGAAGAAACAGGCATAAATCCAGTTAGATCTTAACTGTATCACACCACCAAGTTGAATTATTTGTTTCCGACTCATTATATTGGAAGTCACTCTCTGAACCTTGAACCCTTCA
Loxodonta africana  ACATGGCTGAAGAAATAGCTATAGATCCAGGGTGGCCTTATC-GCATTATATCACTATGCCGAATTATCTGTTTCCATCTTACTAGGTGAGAAGTCCCTCTCTGAACCAGAAACCCTTCA

Homo sapiens        CTTCACCTCATAGTTGGGTTGTAAACATTATCAGAGAGGATTTCTTCCATAGATCCATAGTATGTTAACACTAAAAGATACATCTAAGACCATTTAATCTAGGGAAGGCAAATAAGTTTC
Loxodonta africana  TTTCACCTCATAGTTAAGTTCCAACCTTTATCAGAGAGCA-CTCTTCCATAGAGCTATAGAATGTTAGCACTGAAAGGGACATCTGTGGCCATTCGATCCAGGGGAT---AATTGGCTTC

Homo sapiens        ATTTCATTTGCCGACTTTAATTGAGCAGTAGAGGCTGTTTGGAATT----------------------------------CCGTGCTGAGAATGATTCTAAATCTATGTTTTTGCTCATT
Loxodonta africana  ATTTTATTTGCCAACTTTAATTGAGCATTAGAGGTTACTAGGAGCTGTGTCACTTGATGGACCTTGGAGCCACAGTGAACCAGTGTTGAGAATATTT---GGTCTATTTCTTTGCTTATC

Homo sapiens        AGGAAAAAAATGCTATGACTGATTAT--TGATACCTGCCATATATGTTGACTAGGAGAGTGGTGGAACTCATGTTACCTATTAGCCGTTTCTCAGTGA---------------------
Loxodonta africana  A-GCAAAGAGTACTGTGATTGATTATGATTATGCCTGCCATCAATGTTGAAGAGGAGAGTGGCAGAACCCATGTTACACACTAGCCACCCCTCAGCAAATCTCATTTTATAGACAAGGTA

Homo sapiens        ------------------------ATGTCTCTGCCTCATTAGTGGTACAGCTAAGAACAGAACTCAGGCTCCTGACTCATGGTCTCTTGTTCTTTCTGGTTTACAATGGACTCACAATAA
Loxodonta africana  TCTGAGGTCCAGAGAGGGGAAGTGATTTCTCTGCCTCATTAGTGGTACAGCTAAGAACAGAACTGGGGTACCTCACTCATGGTCTCATGTTCTTTATACTGTATAATGAACTTGCAATAA

Homo sapiens        CAACAATAATATAATACAGTGTATCACATAATGAGTGTCTACTCACTGCCAGGCACTCTCCTAAGCACATTCCACTTTCTGTACCTTACTTTTAATTCTCAGAACAACCCTTCAAGGTAG
Loxodonta africana  TAATAATAGTAGC--------TACCATTTATTGAGTGCCTACTTGCTGCCAGGCACCGTGTTAAGAACTTTCTACTTTCTACATCTTACTTTTAATCCTTACAACAACCCTTTAAGGTAG

Homo sapiens        GCACATTCAACCCCAAACTTAGAAATGCAGAAGCC---TAGGCTCAGTGAATTTTAAAAA--------------CTTACCTAAGGTCCACAGTGAGCAAGTGGTAAAACTGGAATTTAAA
Loxodonta africana  GTGTTTTTTTTTCAAATTTACAAATGCAGAAACCACCTAGGCTCA-TGAAGTTTAAAAAAACAAACAAACAAACCTGCCTAAGGGCCACAGTGTGCAGGTGGTAAAGCTGGAATTTAAA

Homo sapiens        CCTACGCTGGTCTGGCTCCAAAGCCTAAA----CCCTGTGGTCTCACTTCCTGCAAACACAATGTTGAATTTGCGCTCA-----------CAGCAGGTGGAGATGACCCTGGTCCCACTG
Loxodonta africana  CTGAGGCTGGTCTGGTTCCAAAGACTAAATAAACCCTGGGCTCTCTTTTCCCACAAACACAATGCTGAATTTGCCCTCATAGCTAGCCTACAGCAGGTAGAGATGACCCTGGGCGCATGG

Homo sapiens        CTTTGATTAAGACTAAACTGGAGTGAAAAAATCATTTTTGTTCTTACTAATTTTCTTAAAAAGATTTGTTCACAGTTCCTTACTTAGATGGAACTGATTTTTTCGGGCTGTCTCATATTA
Loxodonta africana  ATTTGGTTAGAGCTAAACTGTGGTGAAAAA-TCATTTTTGTTCTTGCTAATTTTTTAAAACAAATTTGCTTGCAGGCCCTTACCTAGATGGAACTGATTTCTTTGGGCCGTTTCATGTCA

Homo sapiens        GATGTGATACAAATTGACATGTTAGGAGAGCTGATGTGAAAGGCGACCTGGTTCACTTACAAATAAATGATTAATATAATCTCATTGTGCGGCTAGCCTGCATTACTGATTCTCTCACTT
Loxodonta africana  GATGTGTTAAAAATTGACATGTTAGGAGAGCTGACGTGAAAGGTGACCTGGTTCACTTACAA-TAAATCATTAATACAACCTCATTGTGCAGCCCGTCTGCATTACTGATTCTCTCACTT

Homo sapiens        GGGAGGCTCAGAAAAACAACAGGTCAGACAATAAAACCCATGCTAGCATAAGACTATAGTGTGGGATGACCATGGATGAGTCAGGGAGAATAAGAAGATAATTAACTAAGAGGGAGTTCT
Loxodonta africana  GGGAGGCTTAGAAAAACAACAGGTCAGACAACAAAATCCATGCTAGCATAAGACCATAGTGTGTGATGACCATGGATAAGTCATGGGGAATCAGAAGATAATTAACTAAGTTGGAGCTGT

Homo sapiens        ATGGAGTTCGAAGATAATT---AAAAAATGTTCCTTCTCTATCCTAAGAATATCTGACATTATTAGCGAAGGCAATGAATAAGATAAGGGGAAGAGGTGAGTTAATTAGAATTATGAGAT
Loxodonta africana  ATGGAGTCCAAAGATAATTCAAAAAAAAAATTCCTTCTCTAGCCTGAGAATATCTGACGTTATTAGCAAAGACGATGAATAGAATAAGGGGAAGGGGCGAATTAATTAGAATTATTACAG

Homo sapiens        ACAACATTTTAAAGAAATTGACAGGGATCACATTTCAACGGAAAGACATTCAATTCAACCGACTTCATTATAGCAATTCAACATTGATTAGGGACATGTTATG------TGTGTG-----
Loxodonta africana  AGAACATTTTAAAGAAATGGGCAGGGATCAAATTTCAAAGGGAAGTCATTCAATTCAGCTGAGTTCATTATAGCAATTCAACATTGATTAGGGACATATTATGCTCTTTTGTGTGCATTG

Homo sapiens        ------GCTGTGCTCTGTGTGCTTAGAGGA-CTACTGAGATGCTGTAATCTTCCTCCCTGACATTCTACTGAATAGATACCCAAGTTTTGGCAGCGCAGTAAATAATCTCTTACACACAC
Loxodonta africana  ATTAAGGACATATTATGTGTGCTTAGGGGAGCTATAAAGGTATGGTACTGTCTCTTCCTGTCAATCTACTGGACAAGTACCCAAGTTTTGGCAGGGTAGTAAAGAATCTCTCATATACAG

Homo sapiens        ATTTGGGTGCAATTTCTAAAATTGTTTCTGCAAAATAGTATTATGTGCCACTGAGTAGATATCTTGTATGCCAGATATTACTGTTCCTGCCAGGCTAAATGTTCAAACAACATGGAAGCC
Loxodonta africana  ATATGTGTGCAAATTCCAAATTTCCTTTTGCAAAATAGTATTATCTGCCACTGTATACGTATCTTGTGCGTCAGATACTACTGTTACTGTCAGGCTAAATGTCCAAACAGCATGGAAGTA

Homo sapiens        TCAGTGATAGCCAATCCATTATTTATCTACCTATATTCACTTCAGTTGCCAAAACTATTCTAAATATAAGGAATCCCTTCCTTAGAGGCCCCAATATGG-CTTTCCGTATTGTT------
Loxodonta africana  CCAGTAATAGCAAATTCATTAATTATCTACTTAGGTTTACTGGAATTGCCAAAATTATTCTTAATGGAAGTAATCCCTTCCTTAGAAACCCCAATATGGCCTTTATTTTTGTTTCTGGC

Homo sapiens        ---CTGAGTCCTTTCTAACTGGTTAATGCCCATGCCATATTGGTTATCATAAATTTTGAATAGCATCTTTTCTCTGCTCAGAAGTGTCGCTCAGTAATACCCTA-CCTCTTCTGACTCCT
Loxodonta africana  CAGCTGGGTCCTTTCTGATTGGTCAATGCCCATACTATACCGGTTATTTTAAATTTTAAATATCATC-TCTCTCTGCTCAGAGATGCCACTAGAGACTACCCTACCCTCTTCTGATACCT

Homo sapiens        CCTACAGTGTAATCTCTCACCAGTCCTCACTACATTCTGAACTGATGTTCCAGCTCCACTTAAATCTGTCATG-TTTTGTTCCTTAACATATGCTATTCCCTTTACTTGGAATACACTCT
Loxodonta africana  CCTCTTGTGTAATCTCTCACAAATCCTCATCATATCCTGAACACATTTTCCAGCACCACCTAAACCTGTCATGTTTTTGTTCCTTGACATATATTATTCCCTTTACCTGGAATACACTTT

Homo sapiens        CTTTCCTTGTCTGCCTAACAATT------------GCTGCTTGTC------------TCGCAAATATTTTCTCTGTGAAG-CTTTCTCAGTGCCCTTAGGTACCCTCATGGCCCTC-CTG
Loxodonta africana  CTTTTCTCGTCTGCCTAACAAGTTAAAAAAAAAAAAAAACTTGTCCTTTAAGTTCTATCTGAAATAACTTCTCCTTAAAGTCTTCCTCAAAGCCCTCAGGTGCCC-CATGACCCTCTCTA

Homo sapiens        AATTTATCTCTATTATAATGTTTTA---TATTTTATGGCA------------------------AGAGTTTGTTG--------------GATCTTTGCCCACTTGAATGTGAGCCCCTTG
Loxodonta africana  GAAATCCCCCTGCTATAGTATGATACGGTATGGCATGGCATGGCATGGCATGGTATGGTATGGTATGGTATGTTGCATGGTTGTGGTTTGATCTGGCCCCACCAGAATGTGCGTTCCTTG

Homo sapiens        AGAACAGGGACTATTTTATTTATGTCTGAGTCTCTAGGGAACAAAAGAGGGATTCAGGGCTTATGTGGTAAATGAATCAATGAATGCTCCCAGTTCTTCGTCATCTTTAGAGGCTACTTC
Loxodonta africana  AGAACAGGGACTGTTTGATTTAT-TGTGACTCTCCAGGGCATAAAGGAGGGGTTCAGGGCTTATGTGTTGAATAAATCAATGAATG-TGCTACTCCTTAGTCATCTTCAGAGGCTATTTT

Homo sapiens        CAGGGCTTTTAAATTTGGACTCCCTTTTCTTCCAAGAGGTGGTGAGAGCAGGGTAGGTGGACATGATATTTAATGAATTTCTTTTAAGTGCCAGACATTGTGCTGAACCATTTACACAA-
Loxodonta africana  CAGAGTTTCAGGAATTGGCCTCTCTTTTCCATCAAGAGGTGTTGAGGGTAGGGTAGATGGACATGATACTTAATGATTGTTTACTAAGTACCAGGCATTGTGCAGAACATCTTACCTGGC

Homo sapiens        ACAATCTCACGTCTCAAGTCGATGTTTTTATTTTCATCCTCGTTTTACAGATGA--AAGTGGAAAATTCAGACCAGTGACTTGCCACATTCAGAGGAGTAACAAAGATACATAGCAAGTA
Loxodonta africana  ATGCTCTCATGTATTCAGTAGGTGTTTCTATTTCTATCCATATTTTACAGATGAGGAAGCAG-AAATTCAGAGCAGCGACCTTCC----------------CAAAGTCGCATAGCTAGTA

Homo sapiens        GGCTGCAAAGCTAGGATTTGAACGTAGGCT--------CCAATACCTGCATCAGC---TCCCTT-----------GCACTGTGCACTCTTCCCTCGCTTGTGTGTATGGTGAGCAGAGTC
Loxodonta africana  GGCTGCAGATGCAGGATTTGAACCTAGGCTTTTTGTCGCCAATGCCTGTACCCCCCCATCCCTTCACCCCCCCACACACTGGGCACTTCTTCT-------TGTCTGTGGAAGGCAGGGTC

Homo sapiens        AGCTCTGGGCAAAATGGTGAAATAAGCCAAGTTTGTCTTTAGTGTCTTCAGACTCCCAGTGTGTCTCGGGGACTCAGT------ACTACAGTGCCACCTACATGCTGCAACACCTACTAT
Loxodonta africana  AGCTCTAGGCAAAAGGGTGAAGTAGGTCAA-TTTTTCTTTGGTGTCTTCAGCCTCCCACTGTATCTCTGGGACTCAGTGTCCTGGCAACCCCACCCCCCACCTCCTGCAGCCCCTCCTTT

Homo sapiens        GGCTCCTACAGGCAGCCCATCCTGCTGAG-AGCTTCTCCTTCCTAACTCCT--CTAAGA----------------CCTTGACCTCTGGTTTGTGTTTTCTGAAGGTAAACTGCCTTAACC
Loxodonta africana  GGCTCCTACAGGCAGCCCACCCTGCTGGGCAACTTTGTTTTTCAATATTCTGCCTAAGGTCTGTTTCAGCTTTTCCCCTTACGTCTGGGCTGTGCTTTCTGAAGGTACACCACCTTAACT

Homo sapiens        TAACCTTTTCCAGAGCACATA-TTATA-TTTATTGGTGCCTTCCCAGAAAGCAATCTTTAGCTTAGGAGCAGGTAGCTCCCACGGGAGGCATGTAGGTGTTTCTGTGTTGTGGTAAGGGG
Loxodonta africana  TGACCTTTTCCAGAACACATATTTACATTTTAATGATGCTTTCCCAGAACGAATTTTTTAATCCGGGATGAGGTAGCCCCTGGAAGAGGTGTGTGGGTGTTTTTGTGTTGTGGTGAGGAG

Homo sapiens        AAGGGGTTGTCTG-GGGGCCTCTATCAAGATACACCATGGAGGCTTCTCCTACAACCAAAGGGTGTTTGGCCCCTTTCTCAGATGGCTGAGGGCCCTGGAGTCAGATCAATAAGCATTTG
```

Loxodonta africana AAGGGGTTGTCTGTGGAGCCTTTCTCCAGATACACCAAGAAAGCTCCTCCTCCAACCAAAGAGTGCTTGTCTCCTTCCTTACATGGCTGAGGGCTCTGGAGTCAGATAAACAAGTGTTTG

Homo sapiens AATCTTGGCTCTGCCATTCACTGATTGCATGATTTCCCACAGTTAATCTCAGTGAGCCTTCGTTTCCTCCCCTGTAAAATGGATCTAATAATAGTTCTCATCTCCTAGACGTGTTGTGAG
Loxodonta africana AATCCCAGCCCCACCATTTACTGATTGTGTGATTT...

Homo sapiens GATGACATGAGATAACACACTTACGGTGTCCAGTACAAAATAAGCATGTAAAAATGTTAACTGTTCCCTTAAATCAATTTTTAGTAGTCTTCTGC-----CTCACCCCCATAACCATCCA
Loxodonta africana ....AAAAACTTTAGCTGTTTCCTTAAAACCATTTTTACTAGTCCTCAGCTCACCCCCACCCCAATTGCCATTCA

Homo sapiens GGTCTCTCTTCGC---------TTCCTTGTTTCTCTTTTCCTCTTCTCACCAACAAGTCTCTTTCTTCAAAGTCTCCCTTTCCTTTCCCAACTCCCTCC---CACCAGTACGCTCAACCT
Loxodonta africana AGTTCCTCTCCTCCCACTCTCTTCCCTCTCTTCTCTTTTCCTCTTCTCACCAACACGTGTCTTTTTTCAAAGCCTCCCCTTCCTTTCCCACCTCCCTCCTTCCTCCAGCACACTCAACCT

Homo sapiens TTGGCAACCCTCTGTCCCACACTTTCCAGGTCAATCCCAATTTCAGATAATCTCTCTTGATATCAAACTGCATGACCTGATTTGAATCGGAAAACAGAGTGAAACAGAGTCCCTTGCTCT
Loxodonta africana TTGGCAACCCTGCCTCCCAGAATTTCCAGGTTGATCCCAATTCCAGATATTCTCTCCTGATGTCAGACTGCACGACCTGACTTGAATT------CAGA---AAACAGAGTCCATTCCTTT

Homo sapiens GAGGAATGTTGCTATGGTCTATCTTAGATCAAGAAGACAAGAGCTGCCTTGGCAGCCCTACATCATAATCTGATGAGCATGAAGAAGGCTCACCTTGCCTATAAATATTTGTTTTTTGCC
Loxodonta africana GAGGAAAGTTGCCACAGTCCAACTTCTATAAAGAAGGTGAGAATTGACTTGGTGGCCCCACATCACAATCTGG---------GAAAGTTCACCCTGCCCCAAGATATCTCCTTTTTGCC

Homo sapiens TATGAGTGATGCCAGAAACCGTCATGGTCGGCATACAATTCCTTTGAGTCAAATTGC-AAGGTTTGTCTGTCAGGGTTGCCTTGGTAAGGCAGGGTTATTATTTATATCTGAGTGCAAAT
Loxodonta africana T--GAGTGATGCCAGAA-TGTTCACCATCAGTGCACAATTCATTTGAGTCAAATTGCCAGGGGTAGGCTGGGAGGGGTACCTCGTTAAGGCAGGGTTATTAATTACATCTGAATGCAAAT

Homo sapiens TCCTTGATCAGAGCCTTTCCTGTCAGGGAGAGGAGACGGAAGGGTTCCAGGGAGCCTACTCCCCACGGAA-GAGGAAGGCTGTGCTAGCATGGATGGGGTATGTGAG-TGTGGAGCTCA
Loxodonta africana TCCTTTATCAGACCCTTTTCTGTCACTGAGAGGAGAAGTAAGGGTTACAGGGAGACAACTCCTTGGGGAAAGGGGAAAGCTGTGTAAGCATGGGCGGGGCATGGGGGGTGTGGGAAATCA

Homo sapiens GCTGACTGAGAGCTGTGTCCCCGGCTCCATTTTGCCTAGGGGCGAGCCCTGTGTGTTGCAACAGATGTGTGCTTTGGAATCACCAAGGTTGGGAAGCCAGGCCTCAGAGGGAGTGGCTGG
Loxodonta africana GTGGACTGAAGTCTGTGGCCCCAGCTCAATTTTGTCT-GGGGTGGGCCCTGTGTGTTGCAACAGATGTGTGCTT-GGAATCACCAAGGTTGGGAAGCCAGGCCTCAGAGGGAGTGGCTGT

Homo sapiens AGGCTGATCTCATCCTGAAATGATGTGGGCTTGGATTCCCAGAGGGTGGAGAGGAGGACCAGTCCAGAGAGAAGGACATGCTTTTGGAGGGAGGTCTAAAGACAAAAAGAGGGCTTTAGT
Loxodonta africana AGGCTGAGCTCATCCTGAAATGATGTGAGCTTGGGTTTCTGGGGAGGGGAGAGGAGAATGAGGACAGAGAGGAGGCTATGCTTTTTGGGG----TGTCAAGATGAAAAGATGGCCTTAGC

Homo sapiens TATTTTTTGAGCTTA-GGTGTAGGTGATGGTTGGGGGTAAGTGACAAAAGAAAATAGGTCCTGAAAGGGGGGTGTCTCCTTTCATGATTAGGAACAGAAG---AATGAACTCAAGGACTG
Loxodonta africana T--TCTTTGAGTTTTTGGTTTAGGAAACGGTTGGGGGTAGGTGGTAAAAGAAAATGGCCCCT-------------CCTTTTCTGATTGGGAACAGAAGGAAAAAGAGCTCAAGGACTG

Homo sapiens CCCCCTTTTCTTCAGGAAGCAAACAACTTCTGAGGGTGTGACAGGGTTCACTGGGTGTTCCCCTTTAAGGGAGGTTAGCCCCTACGTGAAGCTGCTTGGCTGGTAGAGGATAATTGGGTG
Loxodonta africana CACCTTTTTCTTCAGGAAGCAATCAACTTCTGAGGGTGTGGCAGGGCTCACTGGGTGTTCCCCCTCAAGGGAGGTTAGCCCCTAACTGAAGCTGCTTGGCTGCTAAAGG-TAACTGGGTG

Homo sapiens TTCCCCAATCCAGATAAGCAGAAGCTCAGGTGCCCCTCCCTGTAGGCTTTTCAGCTTTCTTTATCACTTGCTAGGCAGGCCCTGCCATCCTTGGGAGTAGGTATGAGAGAGGGAGTTGAG
Loxodonta africana TGCCCCAATTCAGATAACTAGAAGCTCAGGTGCCCCTCCCCATAGGCTTTTCAGCTTTCTTGATCACTTGC----CAAACTCTGCCATCACT-GAAGTAGGTGTGAGA---GGAAGCAGG

Homo sapiens AGGCCAGGCTTTCCCTGC----------------CCCTGTTCTTCACAGAACACAGCTGAAAAAGGAAGGCCAGAGATGTGACACCCCCCAGCCCCAGGGATTCTACCACACATTGCAAA
Loxodonta africana CGGCCAGTCCCTCCCTGCCCCCTCTGAGCCTGAGCCCTGCTCTTTGCAGGACATAGCTTAAAATGGAAGACCAGAGATGGGA---CCCCCA----CAGGGATCCCACCATACCTTTCAAA

Homo sapiens CCTCACA-GAGGATGCTTATTCTACAAAATTTGTGGGCATATTCATAGGTACTATGGAGCTAAATAATT-------TGAAAACAATAACAAAACCCATTTACTGATTATTTGGTTAATAA
Loxodonta africana CCCCGCCTGAGGATGCTTATTCTG-AAAATCAGTAGGCAAGGTCACAGGGACTATGGGGCCAAATATTTGAGATCATTACAACAATAACAAAA-CCATTTACT................

Homo sapiens AGACCTCATTCCTTCAAAGTATGTTGCTAGGAGCCACT--------------GAAAGTGGACTTGACCTGAGGAGCAGCAAGGAGCTTCCTCCTGCTGTATACACAGACACACACAGAC
Loxodonta africana ......CATTCCTTTGAGGTATGTTGCCTGGGACCACTTTTGTATAGATCTTGAAAGTGAACTTGACCTAAGGGGCAGTGAGGAAGTTCCCTTTGCTTT------AAACACACACAGAC

Homo sapiens AGACAGACAGACACACACACACGCATGCGCGCACACACATGCACATGCCCGGATCCTTCTCCAAACACGTCCATGATACCAGGGCAGCAGAATTGGGGCACATTAGAGACACAGGCTCTG
Loxodonta africana ACACAG..................TACACACACACACTCACACAGCC--ATCCTCATCAGAACATACCCATGGTACCAGGACAGTGGAGTTGGGGCACAGT--------GTGCTCTG

Homo sapiens CCCACATTCTGAAGCTCCCTGTCATTCCCAGGTCTTGGTGCTTGGCCAGACTCCCCTTACCCAGACTCATAGCCTCATAGCTATCATTCCAAAACCCCAAAAGCTCTGGAAACTGAGAGC
Loxodonta africana CTTGCAGCCAG----TCCCTCTCATTCCCAAGTTCTAGTGCCAGGCTAGACTCCCCTTCCACAGGAGCGCAGGCCCATATCTACTGTTCTGAAATGCAAAAGGCTCTAAACACTGAAAGC

Homo sapiens CTTT-GTACGTTTATGACAAACTCATTTGGCAGCGCAACTTGAACTGACATGAGGTTATTTATAGTCTTTATTTTTCACACCCTGTGTGAATATTCATGTG-TTTTTGCTCCAGAAATAT
Loxodonta africana CTTTCGTAAGCTTATGGTAAACTTATTTGACAACAAAACTTGAACTGATGTGAAGCTATTTATAGTCTTTATTTACCACA-GCTGTGTGAATATGCATGTATTTTTTGCTCCAGAAATAT

Homo sapiens CAATGTACTTGATTACTGGGTACTGCCTCAGACCCCACTGGAGGTGATATATA-TGTGCTGTATACACCCAATCGCCTTTTAAAAGTCCAAAGCATTCTGAATTCCTTCAATTATACTAA
Loxodonta africana AATTGTACTTGATTTTTGGGTGCTGACTTAGATGCCTCTAGAGGTGATCCACACTGTACTATATGCATCATATTGCCTTTTAAAAATCTGAAACATTCCAAA----------------A

Homo sapiens GCCTCCCACTGTCTTATAATGATCTGTTTGGCATGTCTCCCTCATTAGCCTGTGAACTTCTTCTCCAGTGCTCAGCACACAGTCCAGCATGGGGTGGAAGCTCGACTAATGTTTTAGGAA
Loxodonta africana GCTTTCCACTGTCTTGTAATGACTTATTTGGCGTATCTCCCTCCTTAGCCTGTGAACTTCTTCTCCAGTGCTTAGCATGCAGCCTGACATGTGGTTGAGGCTCAACCAGTGTTTCAGGAA

Homo sapiens TGAAGAACGAACAGTCTTTAGTGATTCGGAGACTTGTTGTCATGTTCTTTCTTACATCTAAACTAAAGTACGTCATGCTACCATT------CGTTTATTCTGTGCCATCAATAGCGAGG
Loxodonta africana TAAAGAATGAATGGCCTTTAGTGTTTAAGGAGACTTGTTAAGAAGTTGTTTCTAACGTCTCAATGAAAGTGTCTCATACCGCCATTTACATTCATTTATTCTGTGCCATCAGCAGTGAGG

Homo sapiens AAGAACAGTTGGTCAGGCTCCTTCATTTTTTTT-----CCTCTTGAGGCTAAATCGTCCTTATAATCCTAATACCTTTAACCTTTTCCCCTAGATCCTCTTTTCCAATCCCTTAATCGTC
Loxodonta africana AAGAACAGCTGGTCATCCTCCCTTCATTTTTTTTTTTCCCCTCCTGGGGCTAAATCGTCCTTGTAATCCTAATACCTTTAACCTTTCCCCCTAGATCCTCTTTTCCAATCCCTTAATCACC

Homo sapiens TTCATTGCTGTCTTCTGAAGTCTTTACAGGTTCTTTCCTTCCGAAATTGTGGCCTAGTGCTTAGGATAGGGCTGCAAAGAGCCCAGCTATTACACATAATAGGCCTCTGGTTTCTATGGT
Loxodonta africana TTCATTGCTGTCTTCTAAAGCCGTTAAAGGTCCTTTGCTTCCGAAATTGTGGTCTAGTGCTTAGGATCTGGCTGCAAAGAGCTCGGCTATTACAAGTAATAGGTCTCTCGTTTCTATGGT

Homo sapiens G--CTTTTCCGATCAGTTTCATTTGGCTGGGCTAAGAAGCTTTGCCTTTAAAAATACACTCGCAGGACAAACTCCAGGGGC-ATGAACAACTTTCACCTGAGCATGCTGGTTGGTAATAG
Loxodonta africana GAACTTTCCCAGTCAGTTCCATTTGGCTGGATTGAGAAGCTTTGCCTTTTAAAAGACACTTACAGGGCAAACCTGAGGGGCCATGAATGACTTTCTCCTGAGCATGCTGGTTGGTAATAG

Homo sapiens GAAGGGAAAATAGCACTGTCTGATCATAAGAAACTGTAATTAC-GAAAGCTGAAAGCAACAGGTAACACATGCAGTAGGCACCTGTATACAAAAACCACTGTTGATAATTACGGT-ACCT
Loxodonta africana GAAGGGAAAAGAGCGCTGTCTGATCAGAGGAAACTGTAATTACAGCAAGTTGAAAGCAACAGGTAATGCAAGTAATAGAGACCTGCATGCAAAAACACTGTTGATAATTACAGTGACCT

Homo sapiens CAGCCTATTATTTTACCTTGGGAGAACCGGCCTTGTTTGTGCGCATCATGTGCCTTTTCTCTCATAAGGTTGCTAAAGGCCATTTCTGACCACTT----CCCATTAAATTCAGGGAGTG
Loxodonta africana CAGCCTGTTATT---GCCTCGGGAGAACCAGCCTTGTTTGTGCGCATCATGTGGCTTTTCTCTAACAAGGTTGCTAAAGGCCATTTCTGACCACTTACTTCCCATTACATGCAGGGAGTA

Homo sapiens AACCTGACTCTGTCAGGGTTGCTG-AAAATCACCGGATTAAGTTAAACTC---ACTAATTAAGCCTGTTGAGACTTCCTGTAACTCACGATTTTTCTTGGCATAAGTAAAAACATTTCACT
Loxodonta africana AACC-GACTGTGGCTGCGTTGTTGCAAAACCACTGGATTAAGTTAAACTCCTCACTAATTAAGCCTGTTAGACTTCCTGTAACTCACGCTTTTTCTTGGCATAAGTAAAAACTTTAGACT

Homo sapiens CAGAGGCAAAATGTGTTATATTAATAACCAGGAAACAAGAAAATACACATAGTAGCATTTTCGGAAGCAAATTCTGTCATCCGCATTCTGTTGAAACGGCCATTGTCATTTCTTCCTAATA
Loxodonta africana CAGAGTAAAAAA----TGTATTAATAACCAGGAAACAACAAAGTACACACGGTAGCATTTTCAGAGGCAATTTCTGTCATCCGCATTCTGTTGGAACTGCCATTATGATTTCTCATAATA

Homo sapiens GCCTAAAGTGTTTACTTTTGGGGTGGAAACTATACATACTGTGATGGAGATTCAGAGATACGCTTTTGTCAGTTAAATTCGAAAAATATATTCAGGTGTGCTTGTTGTGTCCCCCCCCCC
Loxodonta africana GTCCAGAGTGTTTCCTTTTGAGGTGGACACTCGACACACTGTGATGGAGATTCAAAGATACATTTTTGCCAGTTAAATTTGAGCACTCTATTCAGTTGTGCTTATGG--------CTTCT

Homo sapiens CCCAAGCATGAGTCAGAGCCTTTCATGGGGTTTTGTGTAAATGTATTTTCTAAGAGGTGCTGTTGGGATTTTGCATACAGGTCATTCTTTTTAAAAAGGTCACGTTGCAATTTTTCTATC
Loxodonta africana CCCAACCACGAGACAGAACCTTTTGTGGGGTTTTGTATACATGTATTTTCCTAGAGGCCCTGTTGGGTTTTCACATAGAGGTCATTCTTTTTTAAAAGGCCACATTACAATTTTTCTATC

Homo sapiens CTTTCTTGTGTTGTTATCCCTCAGGGAAGAAGATGTAGAATGCCAAATTGCAAAGGGAGCTTGTAAAGAGGGGAACATTAAAATTGGATAACCAAGAGCTCCCAGAGAGGGCCTCAATGG
Loxodonta africana CTTTCTCGTGTTGTTGTACCTCAGGGAAGGAGATGTTGGATGCCAAATTGTAAAGGCAGTTTGTCAAGAGAGGAATATTGAAATTGCATGACCAAGAGCTCCCAGAGAGGGGCATCAGTG

Homo sapiens CTGAAA-GGAGCCCCTTCTGAGCAAATGGGTAGCAGCGTAAAAGCCAAATTAACCT-GGGAGGAGAAGGTATGGAACTTTTGGAGAAACACTTTGTTTACCTCACCATCTTACCATATCC
Loxodonta africana CTGAAATGTGGCCCCTTCTGGGTAAGTAGGCAGCAGCATGAAAGCCAAATTAACCTGGGGAGAGGAAGAACATGAAACTGTTACAAGAATACTTCTTTCCCTCACCAGCTTACCA-AGCC

Homo sapiens AGCTGGCTGTTTCTCCAAGAATGTTAGTGCTCC--AGAAGGGAGGGAAACCTGTCCCCACTGCTAATAGAAGAGGCTGAAATTTTCTTTCAGAGTTTCCAGATGTCCTTGGCAAATGCTA
Loxodonta africana TGATGGACTCTTACACAAGAATGCTAGTGCTCCCAGGATGGGAGGAGAACCTGCCTTGTTCGCTAACAAGAAAGGTTGGGGTTTTCTTACACAGTTTCTAGATGTGTTTCGCCAACATTA

Homo sapiens GCAATAG-----------------------TCATTCTCTCTCTTTTAATAATTATAATAAGCTGCTTTTTAAGGGAACATTAACTGTGTGGTAGGCTCTATGCTAAGTGTTTTATATAA
Loxodonta africana GCAATAGTTTTTTGGGGGGATTTAGTGAGGATCATTTTCTCTCTTTTAATAATTATAGTAAGCTATCTTTT-AGGGAACATTA----TGTGGTAGGCTCTATGCTAAGTGCTTTACATGA

Homo sapiens GGTATCTCTATTAATCTTCACTATGATCCTTCCAAAAAGGTATTGCTATTACCCTCAATTTCCATAGGAGGAAACGGGCCCAAAGAAGTTAGACAACCTGGGCAAGGTCACCCAGCTAGT
Loxodonta africana GGTATTGC---------TCATAACAATCTTCAAAGTAGGTACTGTTATTGCCCTCAACTTCCACAGGGGGGAACTGGCTCAGAGAGGTTAGGCAATGTAGGCAAGATCACACAGCTAGT

Homo sapiens AAGTGGAGGAGCCAG-AACTAAACGCAGATCCACCTGACACC-AACACCTGGTTCTGAACCATGGTGTGCAAGTCCTTGTAAAGTCAAACAGTCTCTTTAATAAGCTTGACTATATTTT
Loxodonta africana AAGTGGCAGAACCAGAAACCTAAGGCCAGATCTGTCTGACACTGAAGACATGGTTCTTAACCTCCCTGCATAAGTCCTTGTAAAGTCAAACAGCCACCTCAGTGAGACTGACTATATTTT

Homo sapiens -CTCTTTTACCAAATCACAGAACATCTGGTCTGCTTAATTTTATGAGACTGTAATTACAGCAATGAAAAGGATGTTAGCCTACTCTGAAAACCCTAGCTGAAGGGCTTTCTATTGCTTTG
Loxodonta africana GTTCTTTTACCAAATAACAGAACAGCTCGTCTGCTTGATTTTACAAAACCACAATTACAGCAACTAAAAGAGTGTTAATCTACTCTGAAAGTCCTAGCTGAAGGACCTTCTATTGCTTTG

Homo sapiens TTTAGAGATTTGTTCTTTTCTCTCCACCTCCACCCCCGACTCCCCAAATTGGATAGTAAGTTCTTC--AAGGAAGGTTCTGGAAAGTGGTTTTTTTTTTTTCTTCAAGTTCTGTGTTACA
Loxodonta africana TGAAGGGATTTGTTCTTTCCCCTA-----ACACCCCCTTCTGCC--AATTTGGTAGCAAGGTCCTTCAAAGGAAAGTTCTGGA..................................

Homo sapiens TGTGCAGAACTTGCAGTTTGGTTACTTAGGTATACAAGTGCCATGGTGGTTTGCTGCACCCATCTACCCGTCACCTACATTAGGTATTTCTCCTAATGCTATCCCTCCCCTAGCCCCCCA
Loxodonta africana ....

Homo sapiens CGCCCCACAGGCCCCGGTGTGTGATGTTCCCCTCTCTGCTTCCATGTGTTCTCATTGTTCAACTCCCACTTATGAGTGAGGACATGCGGTGTTTGGTTTCTGATCTTGCGATAGTTTGC
Loxodonta africana ....

Homo sapiens TGAGAATGATGGTTTCCAGCTTCATCCATGTCCCTGCAAAGGACGTGAACTCATTCTTTTTTATGGCTGCATAAATATTCCATGGTGTATGTGTGCCACATTTTCTTTATCCAGTCTATCA

```
Loxodonta africana  ........................................................................................................................

Homo sapiens        TTGATGGACATTTGGGTTGGTTCCAAGTCTTTGCTATTGTGAATAGTGCCGCAATAAACATAGGTGGGCATGTGTCTTTATAGCAGCATGATTTGTAATCCTTTGTGTATATGCCCAGTA
Loxodonta africana  ........................................................................................................................

Homo sapiens        ATGGGATCGCTGGGTCAAATGGTATTTCTAGTTCTAGATCCTTGAAGAATCTCCAGTGTCTTCCACGATATTTGAACTAATTTACACACCCACCAACAGTGTAAAAGCATTCCTCTTTTT
Loxodonta africana  ........................................................................................................................

Homo sapiens        GGTTCTGGAAAGTCTTATGCTCCTCTGCTTTCTATAGTTCTCATAGTGCCTGGCATGTAGTGTGTGCTCCATAAATATTGACTGATTGATGAATGTGTGGATGTTTCATGCAATAGTTTT
Loxodonta africana  ................................................CCTGACACATAGTAGGTGCTTGATAAATATTGACTGATTGATAAATGATCGGATATTCCTTTCAATGGTTTC

Homo sapiens        TGGGTTGTTCAACACCATTTCTTAATGCTAAGCAAACAGGTTAGAACCTTCAAGATGTCTGTTGTCTTAATCTAGTTGCTTGCACTGAGGTAACTTTTTAATATCATCTCTGCACAGGAG
Loxodonta africana  TGGGTTGTTCAAGACGCTTTCTTAATGCTAAACAAGCAGGTTAGACCCTTCAAGGTGTCCGTTGCCTTAATCTGGCTGCTTGTACCAAGATGGCTTTTTAATATCGTCCCTGCACAGGAG

Homo sapiens        CTGAATGCCTTCTTTGGCACCTGGATGATTGATTGTGTTTTCTCCTTTTCCTCGGAAAGCTCTGGTATTGCGCAAACGCTGCTTTAGGTGATTAACAATGCCGCCAACTTCACC-AGTCA
Loxodonta africana  CTGAATGCTTTCTTTGGCACCTGGATGATTGATGGTGTTTTTCCCTTTTCCTCCAAAAGCACTGGCATCGTGCAGACACTGCGTTACATGATTAACAACGCAGCCAACTTTGCCCAGCCA

Homo sapiens        CCTTTGCAGACAATTCTTGAATTTTATAGGAAAGTGGGAA-----CCTGTTTAAGTATAGTTTCTAGTTAATTCTGCTTCCCCATCCTGGTTTGCTCAATG-CTACATAAAAGATTTTCT
Loxodonta africana  CCTTTTCAGACAACTCTTGAATTCTCTAGGAAAGTGGGGGAGAGCCCTATTGAAGTATAGTTCTTAGTTCCTTCTGTTTCCCAGTCCTGGTCTGCTCAGTAACTACACATCAGCTTTCCT

Homo sapiens        TTGTGGAATGAGACAGAATAGAAAAAATAAAAATACATTCTCAATGGCTGGGTGAAGAGAGTCCTTGAGATTTTACCAGCTTGGTATTTCTCACTGT-TGGAACTTCAACCCAGGATG-C
Loxodonta africana  TTGTGGAATGAGACAGGGTCAAAAAAATAAAGATACATTCCTGATGCCTGGGTGAAGAGAGTCTTTAGGATTTTACCAACTTGATATTTCTCCTTGGATGGAACTTGAACCCAGCATGGC

Homo sapiens        AGATTC-ATCCTAGTGAGCCTCACAGGAAACTCTGCCTTGGTCTAGAAGATACAGTGCAAATATATAGGATAGGACAGGTAAGGTCAGCAGAAGCCACTTTTCAGCACATACCATGTGAT
Loxodonta africana  AGATCCCATCCTGGTCAGCCTCACAGAAACCTCTGCTTTGTTCAGAAAGATGAAGTGTAAATATGCAGGATGGAATGAGTAAAGTTAGTAAAA-CCAGGTTTTAGCTCATACCGTGTGAT

Homo sapiens        TGCTAGAATAATACAAATAACAACCATTTACTAGCAGATTACTATGTGGCAGGTACTGTGCTAAGCATTTTCCAGACATTCATT------------------------------------
Loxodonta africana  TCCCAGAACAGTA-GCATAACTACCATTTACG........ACTATATATCAGGTACTGGGCTAAGCATTTCCTAAGCACTCATTTCATTTCATTCTTAGAAGAGCACTGTGATGGTAGGT

Homo sapiens        ------ATTGTTGCCATTTTTCCAGGTGAGCAAACTGCAGCTCAGAGAGTCTAAGTGAATTTCTTT--GTCTTTAAGCAGTATGAGATAGACCCTGGACTTGAACTCAGGTCCATCTGAT
Loxodonta africana  AGTATTATTATCTCCATTCT-CCAGATGAGGAAACTGCAGCTCAGAGAGTCTAAGTGAAACTTCTAAAGTCTCCTAGTAGTATGAGGTAGACCTTGGACTTGAACCCAAGTCTGTCTGAT

Homo sapiens        TCCAGAGTACATAGTCCTTTTGCTACAGTGCTTTGCAGGGAGCTGTGCTGGCTTCTCAAATGGTTTGGGGCTATCTGT-GTGAGGCTGACAAGAAAACTATTTAAAGGCTGGTAGAAGAG
Loxodonta africana  TCCAGAGTACACA--CCCTTGACTACAATGCTCTGTAGGGGGCCAGGCCAGTTTCTCAAAAGGTTTGAGGCTGTCTGGAGTGATGCTGACAAGATCGCTGTTTAAAGGCAGGTAGAAGAA

Homo sapiens        GGCCCTAACAGAAGGTCTGACTCTCATTTTCTTTCTATTTGCAGAAGATAATTCTTTCCTGTTTCAATATCAGAAGGGGATCTAGCTGAAGGCAAAAGGCCACAGGGTTCTGGTGAGGCA
Loxodonta africana  AGCCCTGAGAGTAG--CCAACTCT----GTCTTCCTATTTGCAGAAGATAGTTCTCTCCTGTTTAAATATCCTAAGG-----------------------------------TGAGGCC

Homo sapiens        CAAGTTCAAACCTTGCTGTAGTTGGACCTGTTCTACTG-ACTATCAACGGCCTGGGTCTGTGGGTGATCATAGTGTCAGGGGGCTGGAGATTGGAAGTTCAGAGTGGAAGAGCTGGCATA
Loxodonta africana  CAAGTTCAAATCTGGCTGTAGTTGGACTTGTCCTACTGGATTATCAACAGCCCAG---------------------AGTGGCCTGGACGT--GAAGTGCACAGTGGAAGGGCTGGCACC

Homo sapiens        TGCCGCAAAATGCACCCTGTTGGGATGGAGCAGGAAACTCAACTGCAGCTGAGTTTAGTGGGGCACCCAAA------------------GAGAGTGGCAACAACAGGTATCAATTTAGA
Loxodonta africana  TGCAAGTAGATGCGCCCC-TTGGGATGGGGCA------------CGGCTGAGTTCAATGTGGCACCCAAAAAAAAAAAAAAAACCCAAAGAGGGTGGGAATAATAGGTTGCAATCTAAG

Homo sapiens        GAAGCACTTGGCTTTGGTGAAAGCAGAAGTCCTGCCCAGCCTAGCTGAACTGAGCAGGAGCAGGTGAGGCTTAGGAGTCCTGCCAGGTGCTAGTAGCAAGGATGGGACTCTAGTCCTTGG
Loxodonta africana  CAAGCATTTGGCTTTGATGGAGGCATAGGTCCTGCCCAGCCTCGTTGAACTGATCAAGAGGGAGTAAGACTTAGGAATCCAGTCACACGCGGGTAGCAAGGATGGGACTCCATTCCTTGG

Homo sapiens        AGGCAAGGGTGAAGGAGTTGGGAGCCTGGGAAGAACCAGACAAAATCCCAGTTCCACAGAAACTGAATGAC----TAGACTGGTGGCTCTCAACTGAGACAATACTGCCCCCTAGGGGAC
Loxodonta africana  GGGCAGAAGTGAAGAGGGTGGGAGCCTGCTGAGAACCAGG-AACGCCCCAGTCCTACAGGATCTGAAAGACTGGATAGACCAGTGGCCCTCAGTTGAGGCGATACTGCCCCCTAGAGGA-

Homo sapiens        TTTTGGAAAATGCAAGAGGGTATTCAGAGGACTGGAAATGGGGATGCTAGATGTTCTCCAACGCCCACGACAGACTTGCAAAATGAAAAATAGTCTAGCATCCCGAATAATTTTTGAATG
Loxodonta africana  TTTTGGAAAATTTGTGAGGGCATTTAGTGGGTCAGCAACAGAGATGCC------------CACACAGGACAGTCTTGCAAAATGAAAAACAGTCCAGCATCTTGTATGGCTTTTGAGCA

Homo sapiens        TTCTGCTGGACTTCCATGGAGCTGAAAACTGCTTTTCTAATTATCTAAACTTTTACTATAAATGTTTTATACCTAAGCATAAAGGGTTTTGCTTCCCCTGCCCCGACCCCACAGTTTTAA
Loxodonta africana  TCGCGCTGGCTATTCATGGAGGTGAAAACC-CATGTATAATTGCC------------TGAATATTTTACACATAAACACAAAGGATTTG--TTGCCCTTCTCC.................

Homo sapiens        TCAACTATTCTGTAAAGATATGGAAGATTACATGGTTACAGAGGATCACACAGACTCATTCACCATTTCAGGAAATCATGTCAACAATTGTGATGTTGCTTTTGGAATTAATCAATATAA
Loxodonta africana  ........................................................................................................................

Homo sapiens        CATATCAGTTTGTGTCCATAACTGTTGCATTCATGGGGATTCTATATAAAGGCACAAGCTTCTGACTACTTCATTTAATCATTCAATGTAGTCCTGTGTGAGCATTTACAGAGTTAAATA
Loxodonta africana  ........................................................................................................................

Homo sapiens        CATAGTGTTTTATTATGTATTGCTTTATTTTCTCTTTATGTGGCAGGACATATAGCTATCTTTTTGAACTTATGCGTGTAGGTAGGTTATAGTACCTAGAAATTTAATTTCAGGATAGTA
Loxodonta africana  ........................................................................................................................

Homo sapiens        AAGGGGTGAAGCAAAAGCAGATTTATTATAAAAGAGAGGATGTTAAGTCTGATAAGGTTGAACACCACTGTGCTAGCCTGATGGCTTCGTGAGGGCCAGGACCATGTGTTTTGCTTCCGT
Loxodonta africana  ........................................................................................................................

Homo sapiens        TTGGCCTAATTAACTGAGGTGCTTATAAATAATGAATAATGAATGTGGATGCAAGCACACAGCTGGAGTCAGGCATGTAGGTCAGCATAGTCGGGGTATATAAAACTGGCAGGAGATGGG
Loxodonta africana  ........................................................................................................................

Homo sapiens        GACTTTTTTTTTGAGACGGGGACTTTTGATCACCAAAAGAAGGCAGGATCCCAGTTATCAGAAGAGAAACCATAGTTCAGAGTTTGCCTCAAAGCTAGAGCGTCCTAATGTCAAGGCCC
Loxodonta africana  ........................................................................................................................

Homo sapiens        AGGGCTTCCTACAGTCCTCTTATCTTGAGACATGCTGGAGCCAAGGGACTGACGTCAGATGGTCCCAGTGGTGAAGAGAGTTGTGTTTACATTCTCACTTGCTGGTATCAATCGTAGGGG
Loxodonta africana  ........................................................................................................................

Homo sapiens        CCCAAAGACCTCACTACCTAAAAAGAGGGCTGTTTTCCAGCTTAGCTGTACCTCTTTGCATTGTTTTGTCTGGCCAAGACAGAGAGGATACCAGGGAAAATGAGGCTCGAGTATTGTACG
Loxodonta africana  ........................................................................................................................

Homo sapiens        TATAATCTGTCAATGCCCTCCATGCCCCCATTAGGATCTCAGGCTGTCTTGGCCTTCAGGCAGATCCAGCTCCCCCATTTTTCTGTTGTTGCTGCTGCTGTTTTTTTTTTTTCACTCCCC
Loxodonta africana  ........................................................................................................................

Homo sapiens        TGATCCCATCAATAATACGTGTACTCCTTCCTTCTGGGTCCTGTCCTCAAAGGTAACTGCTAAATATCTGACAAAGGAATGGCTAAAGCCTTCAGGGCTCCTGATAAGAGAATGGGCTCC
Loxodonta africana  ........................................................................................................................

Homo sapiens        CACTTAGCAAGAAGGGCAGGGGGCAGCACCTCACTTAGAGATATGGTACATAAGTACAGAGGGAGGTATTGAACCACCTGTTGTCAGGAGCAGAAGGGACCCTGAGGTCATCTGGTTCAC
Loxodonta africana  ............................................................GGAAGACATCCAACCACCTTGTATCAGAGCCAGGAGGGATCCTAAAGTCATCTGGTTCAC

Homo sapiens        TAGATCCCAAACCTGGCTGTACAATGGAATTACCAGGAATGCTGTTGAAATACAGATGCAGTGTGGCTCCTAATCCTCAGAG--TCTGATTCAGTAAGTCCGAGATGAGGACTTGAATTA
Loxodonta africana  TGGATGCCAAAGGAGGCTGCACAAAAGAATCACTGGGAGGTTTGTTGAAATACAGATATAGTAGG--TCCTAACCCTCAGAGAGTCTGATTCAGAAGGCCTGA-ATCAGGGCCTGGA--A

Homo sapiens        TCTGCATATTATTAACAGTATCACTGGTGATTTTGATGAGGAACCCAGTTAGGGAAACCACTCAGACTGACTTTCTCGTTTTATGGGTAGCCAGCTGAGTCACCGACAGGGATGATGACT
Loxodonta africana  TCTGGATATTAATGT--GCACCATTGCTGATTTTGATGAGGAGCCTGGTTAGGGAAACCACTCAGACCAACCACCTCATCTTACTGGTAGCCAGCTGGACCCCAGAGAGGGGTGGTGACT

Homo sapiens        CGCCTCGGGTCACAGAGCAAGTCATGGCAGAGTTGGAAATGGAACTCAGGGTTTTCCTCCCCTGTATTGGTGGTGGGACGATCTGACATTATTA----------------------GT
Loxodonta africana  TGCCCCAGCTCACA-----AGTCATGGCAGAGTTGGAACTGGAACCCAGGTCTCTTCC--CACTGTATCAATGGTGGAAATATCTGACATCATTGTGGCTGGTTGGAGAGATCATTAGGG

Homo sapiens        GTGGGAAGAAGGACCTTAAATTTCACTGTACAAGCTGCTGCTCACTCTGCCTAGAGGTAGAGGACAGAGGGCATCTTAAAGG-TTGTTTTTCGTTATAATAAGCAGTACATTTACTTCAG
Loxodonta africana  GTGGGAAAGAAGATCTTAAATTTCATCCCAGGGGCTGCTGCCCATGCTGCCTATAGATAGGGGACAGAGAGGATGTGAAAAGATGGCTTTGTGTTATTATGAGCAATAAATTTTCTGCAG

Homo sapiens        AGTTTGGAAGTGAATCAACAAAATACCCATCAAGATTAGTAGGACAAGAGGAACTCGCCAATATCTTTGACGATGTCTATCCATAGAAAGCCAAGTGAAATAATGGAGGTAATTATCCAC
Loxodonta africana  GCTTTGGGAGAGAAGCAACAAAGTATCTACCAAGGT-AGTAGGACAATAAGAACTCGCTGATGTCTTTGGCTATGTCTGTCCATGGAAAGCCAAGTGAAATAATAGAGGCAATTATCCAC

Homo sapiens        CTATTTTCTTTGAACACTTTGACCGAAAATCTCCTGGTAACTGAGGGAAAATAGGAGCTTTTAAAAATTGTTGTCTCAAGTAGCCTGAATGACTTCAACTCCCACTTTACTTTTCTTTTT
Loxodonta africana  CTACTTTGTTCAAACACTATGACCAAAAATCTC-TCACAACTGAGGGAAAATATGAGCTTAAGAAAAT-GTTGTCTCAAGTAGCCTGAATGACTTCCGTTCCCACTTCACTATTCTCTTT

Homo sapiens        CTGTGGAGATGCTCTCCCAGATGCTTGTCTCCTGGGGGTAGCAGCTTTCCACAGACAAAATGTCCACAGCTTTGGGAAGCATGGAGGGAGAAGG--------------GAAACATTAAGT
Loxodonta africana  CCCTAGAGACACCCGCCTGGATGCCTGTCTCCTGGGGGT--------------GTGAAAATGTCCACAGCCCCAGGCGATGTGTAGAGAGAGGGAAAACCCTGCCAAGGAAACAACAAAT

Homo sapiens        GAAATGGGATGTCAGGTCAAGCTGCGTTGGTGGTGATGTAGTCACCTGTCGTTTCAAGATAACCTTTTGGATCCTAGATATGTCGTAGCTGGGGCCTTGGGACAAGTCCCAGCAGCCTCC
Loxodonta africana  GAAATGTAATATTAGGCCAAGCTGCATTGGAGGTGGTGCAAGCACCTGTCCTTTCAAGACAACCTTTTGGATCTTGGATCTGCACTAGCTGGGGCCGTGGGACAAGTCCTGGCAGCCTCC

Homo sapiens        AAGCCACAAGCTTATCATTTTGGCGCAATTCAAGGCAG-CCTCCTTGAAAAGCTCCACGATGGCAGCTGCTCCATCAGCAACTGGATGAAAACAATGCCAAATCTCTTTATTTAAAATTA
Loxodonta africana  AAGCTGTCAGCTTATTGCTTTGACATAATTTAAGGCAGCCCCCCTTGGAAGACTCCGGGATGGCAGCTGTTCTGTTAGCAACTGAATGAAAACAATGCCAAATTTCTTTATTTAGAA-TA

Homo sapiens        GGCACTTCCTATTTTGGTTCCTAAATGGCAAATCTATCTGCTCTCTGCCTCCAGTCTTCTGTGTCCAAAGCTGCCTTAATGTGAGGTATTTCAGCAGCGATCAGCAAGTGTCTTCCTATT
Loxodonta africana  GACACTTCCTATTTTGTTTCCTCAAAGGCAAATCTGTCTGTCCTCTGCC-CCGGCCCTATGCAGCCAAAGCTGCTTCAATCCGAGGTATTTCAGCAGCGTTCAGCAGGTGTCTGCCTATT

Homo sapiens        TATAAGCAGCAGAGCTGCCATGGGTCACTGCACTTGTATCTCTGAGCAAGAGAAGTCAGGGGGC-CTATTGGAAAGGAAAAAT-------ACTGTGGCCTCCGACAGTGAACCTTGGCGT
Loxodonta africana  TATAGTTAGTAG-GCCGCCATGGATCATGGCATTCTTATCTCTGAGCAAGAGAGGTCGAGGGACTTTATTGGAAAGGGAAAATACAGGCCACTGGGGCCC---ACAGTCAACCCTAGTGT

Homo sapiens        GGGTCAGGGAAGACTGTTCAAGTGCACCAGAGAATGGAGCTGTGATGAGTTCAGTTTTGTATCACTGGCATGGAAGTCAGTAGCCTCAAGATCTGGGAAGAGTTTGGTCACCACTAATTT
```

```
Loxodonta africana  AGGTCACAGAGGGAGGTTCAAGTGCAACAGGGAATGGAACTGTAGTGAACTCAGTCATGTGTCACTGTCATGGGTGTCAAGAAACCCAGGATTTAGGCCCA-TTCTGCCACCACTAATTT

Homo sapiens        GCTACATGACCCTGGGCAAGAGTCGTCCCTTCTCTGGGCTTCAGCCTCACCATTTCT-GGGGAAAAGGATGCACCATTTAATACATGGTGCCATTTACTGTGACAACCGGCCTGCCACC-
Loxodonta africana  GCTACGTGACCCTGAGCAAGGTGCTTCCCTTCTCTGGGCCTCAGCTTGACCATTTCTGGGGGGAAATGATGCGCCAATTAGCATATGGTGCCATTTCCTGTGACAACTGGCTTGTCATCA

Homo sapiens        --AAAATATAAAGCTGAATCTTTATCTCACTCCTTATTCCAAAATATAATCCAATTAGGTC--AGATTTAGATGTTAAA-AAGAGAAACCATTAAAAGATTAGAAGAAGGCATAAGAGAA
Loxodonta africana  GAAAAAAATAAAGCTGAATC----TCTCACTCCTTATTCCAAGATATATTCCAGATAAGTTGAAGATTTAAATGTTAAACAAAAGATACCATAAAAGGACTAGAATGGAGCATACGAGAA

Homo sapiens        -----------TATTGAGGTAGGGAGTC----------------------------------TTTGCAAAGCAACGAGAAACTGTAGAATCTA-TGAAGAAA------AAGAATGATAGA
Loxodonta africana  TATTATTAAGGTATTGAGGTAGGTGGTTAAAACAGAAAATTTTAAAAATTGTGGTGAGTATATATACATATAAAACAAAAACTGTAGAATTCAGTGAAGAAACTCAGTGAAAATGGCAAA

Homo sapiens        TTT-----------------------------------------------GACTATGCAAAAATTTAACAACCTGAGTATGGTGAAAGATATCTCATGCAAAGTGGAAAGGCAACATATA
Loxodonta africana  TTTTTTGTTATATATAATTTACCACAATGAAATTTATATTTAGTAGGAGAATCTTTGCAAAAATTTACCAACTTTGGTATGGCAAAAGATAACTCACACAGAGTTGAAAGACAAAGGATG

Homo sapiens        AACCAGGATA-AAGTCACAACATAGAAGGAAGACAATGGTTTATTATTCAGAATACTCCACCAGCTCCAGTGTAAGAAGTGCATTATTTTCTTTACAGCACTTACCATGATTTACAATGA
Loxodonta africana  AGCTAAGATAGAAGTCACAACATAGAAGGCAGAGAAAGGTTTGTTATTC---ATATTTCAAGGGCTCCTTTAAAACAACTGTAT-----------------TTTCCATAATGTGCAATCA

Homo sapiens        CA--------TGATTTAGCTACTTGTTTTTCTAT-TATTTCCTCTGCTTACATGTTAGCTTCATGAAAGCTCAGGTTTT-GCTTACTCACTATTATATTTCTAGTGCTAGACTGGTGCCT
Loxodonta africana  CAGTCTTTAGTTGTTTATTTACTTGTTTTTTTATCTGCTTCCTTTACTTACACAGCAGCCCCTGGAGAGCAGAGGCTTTGGCTTAATCACCACCGTATTCCTAGTGGTAGAACAGTGCCA

Homo sapiens        GGCATACAGTAGGTACTCAATAAATACTTGTTGAAGGAAGGAATAAACGAGCTTCTGTAGACCAATGAGAAGATAATG-AACAATTCACTAGAAAAATGCTCAAAGAATAATGAACAGGC
Loxodonta africana  GGCACACAGTAGGAGTTCAATAAATACTTGCGGAAGGAATGAATGAGTGAGCGTCTATAGATCAGTGAGAACAGGATGAAAAAATCCATTAGAAAACTGATCCAAGAAGAATGACTAGGT

Homo sapiens        AATTCACAGAGGAAAGGCAAAGGGCCTATGCCCTATTAAAAGGAACACCA-ATCAGTAATAATTAAGAAATGCAAATTAAAACAATATTGAGATGATTCAGATGTCAGCTTTTACCTCTC
Loxodonta africana  AACTCACAGAGGAAACACAAAGGGTCCATGCTACATAAAAACATGCAGCATCTCAGTTA----TAAGAAATGCAAATTAAAGCCGCATGGAGATGATTGAGATGCCATTTTTCAC--CTC

Homo sapiens        AAATTGGCAAAAATTTAAAAGGTCACTAAAA----CCCAGGGTTTGTGAGAATCTGGTGAAACA-GCACACTCATTCACTCTTGATGCACA-----------------TCTGGAGGGCAA
Loxodonta africana  AGATTGGAAAACATCTAAAAGG---CTGAAATAATCCCAGTGTTTGTGAGGGTAAGGTGAGACAGGCACCATCATTCATCCTTGGTACATGTCTAAATCAGCACAATTTCTGGAGGGCAA

Homo sapiens        CTGGAAATGTTATTTGAGTCAGCAATTCCTGTCTTAGGA--GTTTACCTTTGCAAAATGCTC-CATGTATAAGGATGTTCATTTAAACATTATTTGTAAATAGGAGCAAATTG--AAAAA
Loxodonta africana  CTTGAAATATCCTTTGACCCAGGAATTCTTCTTCTAGGAGTGTTCATCCTTTTGCAATCCTCACATGTATAAGGATGTTCATGTCAGTATTATTCGTAAATAGCAAAGAATTGTAAATAA

Homo sapiens        ACTAAATGACCTCGGTGAAGGACTGGTTAAATCATTTATAATACATCCCAATAATGACATACTAAGCATTACTATGCTAATGATTAAGAGTTTGAGCCTTG--GTTAGACTGCCTAGGTT
Loxodonta africana  CCTAAATGTCCTTAGTGGAGGACTGATTAAATAAATT-------ACTCAAATAATGGCATA...AACAACACTATGCTAATGACGCAGAATTTGGGCTCTGTGATCAGACTTCCTAGGGT

Homo sapiens        TGACTTCCTCCTCTAGCTTTTATGAGCTGTGTGACTTCGACCAAGTTTCTAA-CCTCTCTGTGCCG----TTTCTCATCTGTAAAATGGGAAAAGTAACAAT-------TCATGAAGTTG
Loxodonta africana  TGACTTCCTCTTCTAGGCCTTATGAACTGTGTGACCTTGATCAAGTTTCTAAACCTCTCTGTGCCTCAATTTTCAGGTCTGAAAAATGGAAAAAATAATAATACCTCCTTCATAGAGTTG

Homo sapiens        TGAAGAGTAAATGAGATAATTCATATGAAATATTCAGA-----------TAGGACCT----GGCACAGAGCATTTAATAATTGCTACACATTA-------------------CTATTCCA
Loxodonta africana  TGAAGAGTAAATGAAATAATCCATATGAAATGCTTAGAATATTTGAACATTGAACCTACCTGGCACAAAGCGTTCAATAACTGCTACCTATTAAAAAAAAATTTTTTTATTACTATTTCT

Homo sapiens        ACTACTAGTATAATGATGACAATGACGATGTAGCCACAAATAAAGACAAGCTAGATCTTTAAATACTGATGTGCAATGATATCTCCAATATAGTATTGTGATAAAAGCAAGTTGCAGAAG
Loxodonta africana  ACTACTACCATGAAGATGACAGTGAC-----------AAAAAAGGGCAAGCTAGATCTTTATATACTAACATGCAAT---AGCTACAGTATAGTATTGTGATAAATG-------------

Homo sapiens        AGTAAGTATAGTAGGATCTGATTTTT--GTTAAGGGTATT---ATATGTTACACATACAGGAAAAGACTGGAAGGATAGACAGACTGTCAACA----------CTGGGGAGTAGAATAGG
Loxodonta africana  --TATATATAGTAAGATGTGATTTTTTTATGAATGATACTGTAATAGATTATATATAGGGGAAAAGTCTGGAAGGATAGACTGACTGTCAACAGCGGTTATCTCTGGGCAACAGGATGGA

Homo sapiens        AGAGGTAGATTTTCCCTTGAGCAATAATTAGAGTTGTTTGAAACTAGCATGTGCTACTTTTATAATAAACAAAAGATGTAAAGGGAAAAAAAAGCTCCGTTTGGAATTCCGAGGACAGTT
Loxodonta africana  AGAGGTGGATTTTCACTTCGGTAATGATTAGAACTGTTTTAAACAAACATGTGTTAGTTTTATCATAAACAAAAGATGTAAAGAAAAAAAAAAAGCTTCATTTGGAACTCTGAGGACAGTT

Homo sapiens        AATCCCACCAGGCTTCACATCCCCCAGTTCTTGGCCACAGCCCAAA----CGTTAAGCCTCCCTGCTC-GTCATCTGTCCTCTCCC-ATACCATCCTCATACTGCTCAGTGCTCCCATCC
Loxodonta africana  AATCCCACCAGGCCTCACATCCCC-AGTCCTTGGCCACAGTCCAAATACACGTTAAGCCTTCCTGCCCCGTCATCTGTCCTCTCCCCATACTATCCCCATACTGTTCTGTGCTTCCATCC

Homo sapiens        CTGCCACCCC-GACTCATCCACCTCCCCACTACCGCCCTCTCTCACTCTTCTCAGTACCCTCTGGAAGCCCCCACCTTGCAGTAAACAATTCCCCCTACACC--CTGGATAAATTTTACA
Loxodonta africana  CCACCGCCCCTGACTCATCCGTCTCCCCATTACTGCCCTCTCCCACTCTTCTCAGTATCCTCTAGAAACCCC-ATCTCACGGTAAAAAATTCCCCCTACACTGAAAAAAAAAACTTCACA

Homo sapiens        GAGCACTATTGTCTACCTCCGGGCCTGGAAGTGCTGAAACCTGAGTCTCCACGGCGGATCTCACGGCTTTGCCACCAGCGCCCTCTAGTGGACACTGTCCACACTCCCACACTACAGGCA
Loxodonta africana  GAACACTGTTCTCTAGCTCCACGCTTAGGAGTGCTGAAACCCGAGTCGCGACAGATGAGTTCACGGCTTTGCCAGCAGCGCCCTCTAGTGGACACCGTTCACCCTCCCACAGCACAAGCA

Homo sapiens        CCCTAGGGCTGGGAGGTGGGGTCTGTGCTCTAGCTTCCCCTGTTTGCTTCCAGGCTGTTAGTCCTCCGTCCTCAGCCAAGAACTCCTCCTTCTCATGCCATCTGGCTAC---------TT
Loxodonta africana  CCCTAGGGCTTGGAGGTGGGGTCTGTGCTCTATCGTGCCCTGTTTGCTTCCAGACCGTTAGAGCTCGGTC---AGCCAACAACTCCTCCTTCTCATGCCATCTGGCTACAGCACCCTCTT

Homo sapiens        CTCTCCT-----CGTTGACGCTTCTTTATAACCATCCCCTCCTGGTCAACCCCCCCCCACCCCCACATTCAACGAAGAGTTCGACATCTGGCTCATTGTCTTCTTCTCCACCCCAAGTCC
Loxodonta africana  CTCTCCTATCTTCGTGGACTCTTCTTTTTAATCA-CCCCTCGTGGTCACCCCCC----------AGATTCAACGAAGAGTTGAACATCTGGCTCACTGTCTTCTTCTCCACCCCAAGTCC

Homo sapiens        TGACATCATTCTGGGTGAGTTGGACATCCGTGATCCATGTACAGGACACACTCTCATCCTAGCCTT----ACCAT--------GCTTCATCATCAGTCAGAACCCCC-TACCATCTACAC
Loxodonta africana  TGACATCATTCTGGGTGAGTTCAACATCCATGATCCACGTGCAGGACACACCCCCATCCTAGCCTTCATCACCATTCAGAACCGCTCCATC-----TCAGAACCCCGGCACCATCCACAT

Homo sapiens        CTTGCTGGCTGGCCTCAACGCTCTTCTTTTTACCTCAGGAGGCTCTCCAGTTTTTTGAGC--CCCCTCCATCCAT----TTCTTCCATCCCATCAGCCCCTTTCACCTTACTACCTTCCC
Loxodonta africana  CCTTCCTGCTGATCCCAGTGCTC-------------------CTCTCCAGGCTCTTGAGCTACCCCCCCGCCCCCGCCATTCTCCCA-CCTGGCAGCTCCTCCCACCTTACTTCCTTCCC

Homo sapiens        TCTCCATCCTGGAACATGATGATGATGATGATAAGAATAACAACTAATATGTATGTGCCAGGCATATACTACCCATCTTATGACATCATTTCAGTAATCTCATGTTATCCTCACAATAAC
Loxodonta africana  TCTCCATCCTTGAA---AACAATGAGGATGATAAGGATAACAATGAATATGTATGTGCCAGGCACATGCTAGGGATCTTACAACAGCATTTCATTCATCTCATGTCATCCTCACAGTAAT

Homo sapiens        TTTATGAGGAAGATACTATTGTGGTTTTCCTTTT-----AATGAGGTGACTGAGGCTCAGAGAGGTTGAGTGACTTACCCAAAGTCCCACAGCAGA-----------GATGAGATTTGAA
Loxodonta africana  TCTATGAGACAGTTACTGTTGTCTTTTTTTTTTTTTTAAGATGAGTGGACTGAGGCTCAGAGAGGTTGGGTGACTTACCCAAAGTCCCGCAGCGGGTTCAGGGCACGGTAGAGATTTGAA

Homo sapiens        GTCAGGTCTGTTTGC-------------------TTTCAGAATCTTGCGTATATCCCCAAT----TCCCTTGCCCTGTGTCTTTCTGCTGCATCCATGAGGCAAAA-TCCTAAGTCTGGA
Loxodonta africana  ACCAAGTCTGTTTGTCTAGCTCCTGAAAAGTAGATTTCAGAATCTTGCCTTTACCCTCAATCGACTCCCTTGCCCTATATCTTTCTGCCACATCCACCAGGCAAAAATCCCAAGCCTGGA

Homo sapiens        TAAATTTAACTGACAGTTTCCTCCTCTGTGCTTATGCCCAGATGGAGGAACATTGCTGGAGAAAATCCCATCACTTTGTTGGTTAGTCCCACCAGAAAGTCATAGTTTCCAACCTCAGCG
Loxodonta africana  GAAATTTAACTGACAGCCTCCTCCTCTGCTCTTACACCCAGATGGAGGAACACTGCTGGAGAAAATTCCATAACTTTGTTGATTAGTCCCACTAGAAAGTCATAGTTTCCAACCTCAGCT

Homo sapiens        GGGCCCTCAACTCAGCTAAACAATCCTTCTAAGTAACTGTCCTGACAATAGCACTTGCAAACTTTCTCCACTGCCCTCTGGAGTCAGACTGTCTGGGTTAGAGTC-TGCTTTGGCCACTT
Loxodonta africana  GGGCCCTTGACTCGGCTAAACAAACCTTCTAAGTAACTGTCCTTACAAGAGCACTTTCAAACTTTCTCCACTGCCCTCTGGAGTCAGACTGTCTGGGTTAGAATCCTGCCTCGGCCACTT

Homo sapiens        CCATGCTCCATACCATGCCAGCCTTTAAGAAAGGGTGAAGTGGAGGAAAAAGGATGAGGGGAAAGGGGAAGTGATATGTATCGAGTGTCTTCTAGGTACCAGCCACTGTGCCAGGTGCTT
Loxodonta africana  CCATGCTCCATACCGTGCAAGCCTTTAAGGAAGGGTGAGGTGGAAGAAAAGGGAATGAGGGGAAGGGGAAGTAACATGTACTGAGTGCCTTCTGTGTACCAGCCACTGTGCCAGGCGCTT

Homo sapiens        TGCATGCAGTGTTTCATTTAATCCTGCCAGCAGCCCTGCAAGGGCAATACTCTTGTTATTTTTGCTTTACAGAGGAGGAAGCTGAGGCACAGACAGGTTAAGTCACTC----ACTAACGT
Loxodonta africana  TGCATGCAGTAGCTCATGTAATCCTCCCAGCAACTCCGCAAGG------------------------------------------------GAGAGGTTAAGTTAGTCTATGGTCAGGGT

Homo sapiens        GACACAATTTGTATGTGGAGAAGCCATCTCATTCGTTTAGACCTTCCTTATCTTGATGAATTATTGTGACAAGCTGATAAATGGGTCCCCTGTTACCTATCTCATGCCCTACAATCTACC
Loxodonta africana  GACACAGCCTGTAAGTGGAGAAGCTATTTCATTAGTTTGTACCTTCCTTCTCTTGATGAATTACTCAGGCAGCCTGCTAAATAGTCTCCCTGTTACCTATCTCATGCCCTCCAGCCTATC

Homo sapiens        TTCCATTCTGTTGCTGGGACAATCTTTCTAAAATGCACATCTGACAGTGCTACTACTCTGCTTAAAGTCCTTCCAGAGCTTCCCCTCAGTCTTCAGAATGAAACAGAAACCTTTGATCTT
Loxodonta africana  CTCCATTCTCGTGCTGGGGTAATCTTTCTAAAATGTGGATCTGACTGTGCTTTTGCCCTGCTTAAAACCCTTCCAAAGC-ACCCCACAGTCTTC-GAATGAAACTGACACCTTTAACCTA

Homo sapiens        AATGATTTGGCCCTGTCCTTCCTTTCTCACTGCATCCACTACCACTTGCCCATGCAGTCACACAGTACTATTTTTGGCCACCTGATGACACCTTGCTTTCTTCTGAAGAAAGAAGAAAGC
Loxodonta africana  AA......................................................................................................................

Homo sapiens        ATATATATATATATTCTCTGCCTCCCCATCCTCCACACCCTTTCCTGCCCACTTTAAATGCTCCTCTTCTGTGCTTTCACAGCATTCATGTTTATCCCAGTGTGTTATAATTTCCTGCTG
Loxodonta africana  ........................................................................................................................

Homo sapiens        TCTTCTCTGTTTCCCCCATTAAACTGGAAGCATCTTGAGAACTGGGATTGTGCCTTAGTCATCTCTGTACTCCCCAGGGCCGTAGCACCAGAGCCATGGTTAAATCTATGTTTGTTGAAT
Loxodonta africana  ........................................................................................................................

Homo sapiens        GACTGAATTAATGAATGAATAGAGTGAATGAGAGAAGAAAAAGCTCTCACTTGGAAAGTTTAGTGACATGAGCTGTATGGTATCTGATTCTTGGCTGAAGCTGCTGCAGTTTTTCCCTAT
Loxodonta africana  ........................................................................................................................

Homo sapiens        GACTACAGTGCTATAAAAATATGTCTGCACATGGATAAAGACTGGAAGAGAACTTTCCACACAAAACTAAGAGTTGTGTTAGAGTGGTGAGTGATGGATGCTTTTTTCCCCATTTTCAAA
Loxodonta africana  ........................................................................................................................

Homo sapiens        ATGTTCTTTAGTGCTGCTTTTGGATTGTTTTGGCAATGTAGCAGACATTGTAGAAATGGAATTTGGGGCTCCCATGGAGGAATCGACAACCAATATGTATTGATGCCCAGTGTACTCTGC
Loxodonta africana  ................................................................................................GTATTGATGCCCCGTGTAC-CTGC

Homo sapiens        TGGGTTCTGGGTGCTGTGGGGAGATGGGTGGGTAAAGGCGACTCATTCTGACTCAGGTTTGCATGGTCAGGAATCTTGCCAATTACGTTGGATTTGCTGGAATAAAAAGATAACTTTCAA
Loxodonta africana  TGAGATTCTGGTCCTGTG---AGACGAGTGCGGAGAGA-GACTA--TCTGACTCAGGTTCTCATGGCCAGGAATCTTGCCAGTTCTGTTGGATTTGCTGGAATAGAATGAGAACTTTCAA

Homo sapiens        GAATTTTCCAAGCTCCTGGGTTGAGGTATTTCTTTTCTCCCAAAGCTGGGCCGCAAACAAAGATGGCTTTTGTTTGCAGTAAAATACTCATTCCTTCAATTTT-GTCCTGCAAAGTGACT
Loxodonta africana  GAATTTTCCAAGCTCCTGGATTGAGGTATTT--TTTCTCCCAAAGCTGGGCAGCAAACAAAGATGGCTTTTGTTTGCAGTGAAATGCTCACTCCTTCGATTTTTGCCCTTCAAAGTGTCC

Homo sapiens        TCTA---------TAGCTTTGGGAGTCAGGTTTGCTGAGTGGGGAAACTGATTGTCCATGCTTCATTGATTTAGAAATGACCAAAAATGTTTGCCTTTT---CTGGCTCTTGAATAAAAT
```

```
Loxodonta africana  TTCGAACCTGACCCAGCTTTGTGAGTCAGGTTTGCTGAGTGGGAAAATTGATTGCCTGTGCTTGGTGGGTTTCGAAATTACCAAAAAAACTTCTCTTTTTGTCTGGCTCTCAAATGAAAT

Homo sapiens        GTTTAGGAATTTCATTGGCAAAATGATGAAATTGTTCCCTTTACTGCTGCAAGACTTCCAAAACTATATATCGTG--GGTTAACTTAACCCAGAAGAGGCAAAAAAAAAAAAAAAAATGGG
Loxodonta africana  GTTTAGGAATTTCACTGGCAAAACGGTGAACTTGTTCC-TTTACAGAGGCAGGATTTCCAAAAGTACATATTGTGTTATATAACTTAACTCAGAAGAGGCAAAAAT----------TGGA

Homo sapiens        AAGC-----------AGACTCAGATGATTAACACTCTGCCTTAATTGGGTGCTTACTCACTCTGCCGTGGCAAGGCACTGTGTTCTGGACTTTGGAGATATCTCATTTAAAACTCTCAAC
Loxodonta africana  AAGCTGTACTCTCCAAGATTCAGAGAATGAATATGATGCCTTTATTGAATACTTACTT-------TGTGGCA-GTCACCGTGTTCAGGACTTTGTAGATATCTCATTTAAAACTCTCAAC

Homo sapiens        AGCCTGTGGGTAAGGGACTGTTATTGTTCTCATGGTAGAGATGGTCAAACTGAGTTTCATAGAGGCTCAGCTCTGAAATCATGAGACCAGGATTTGAAGCCGAGTTCAGCTGGTTCCATG
Loxodonta africana  AGCCTTTAGAGTAGGGACTGTTATTATCCCCATGTTAGAGATAGTAGAACCAAGCTTCA----------GCTCCTAAACCATAAAACTGGGGTTTGAAACAGAGTTCAGCTGGCTCCAAA

Homo sapiens        GGCACACACTAACCATTTGGC---------------------------------------AACAGTGCCTCTAGACTA-------------------GTCTGGCTACAAAGGGACAG---
Loxodonta africana  GGCAGCCACCACCCATCTGTCGGTGGAGGCTCGTGTGTTGCTCTGATGCTGAACAGGTCGAGCAGAGCATCCAGACTAAGACAGATTAGGAAGAAAGGCCTGGCGACATAGGGATGGCGC

Homo sapiens        ------AGGTATGTATTTATTTTGATT-------------TAACGA----------------ACCGTTGTTTA--ATGTTTACCATGTCCCGGGCACATGGTAACTGCTTTAAAAATGTT
Loxodonta africana  AGGACCAGGCA-GTGTTTTGTTCCATTGTGCATGGGGTTGCCACGAGTCGGGGGCCGATTAGACAGCAGTTCACAACATTTATTATGTGCCAGGTACA----------------AACATT

Homo sapiens        AGATTATTTACTTCTCATAGACAACCCTGTGAGGAGGTACTATTATTAATATCATATGACAGGTGGGAAAATGGAGGCACAGGG---------GTGT-TGAATCACTTGCCCGGGGTCAC
Loxodonta africana  AGCTCATTTAATCTGCATAAACAATTCTGTGAGGAGGTACTATTATCAACACCATGTGACAGATGAGGAAACAGTGGCATGGGGTGAGGGAGGGTGTGTAAGTCATTTGCCCAGGATCAG

Homo sapiens        TCAGGCTAGCATGTGACAGAGCTAGAGTTACAATCCAAGTGGCCATGTTCAGGTGTGTGGCCCATTACCTTGACTCTGTGCTGGTTAGCATGCTACCCCCTGTGCCCTGTCTGCATAGCT
Loxodonta africana  GCAGGCTAG----------------------------GCAGCTGTGTTCTGGTGTCTGGCTCTTAGCCTTAACCCTGTACTGGATAGCAAACTAGTCCCTGTGCCCTGTCTACACAGCT

Homo sapiens        CTGGCTCAGAAACACTGAAGACATGGGCCTCTTTTTGTAGTTTTGTAACAGATTTGTACTTTTTAAAGTATTTTCCACCATTAAAGCCATACGCTCTGCTTCTAAGCGTCCTGTGGGGTT
Loxodonta africana  CTGGCTCAGAAGCACCGACGACATGGGCCCCTAGTTGTAGTTTTAAAACAGATTTGTGCTTTCGGGGGTATTTTTC-CCAGTAAAGCTCCGTGTTCTGCTCCCAGAGGTCTTTGGGGG--

Homo sapiens        GGTGGGAAGGGTGAAAACATACTCCTGTCCTTCTATAGCAAGACTAGCTTAGAGGCAAGGTGGCCCAGTGGTTAGAGCATGCCCATGGTGCCAGTGAACTCAGCTTCAAATCCTGGCCTT
Loxodonta africana  ---AGGGAGGGTAA------------------------------------------TGGTCC..................................................

Homo sapiens        ATGATTTCAGAGCTGAACCTCTCTGAATGTCTGTATCACTATCTTTGCTAAAGGAAGAGGAAGCTTTCTCCCGATATCTTCATTTACTTCCAATTTCGGTTTTTGGGGGAGAGCTGGGT
Loxodonta africana  ...............................................CTAAAGGAGGAGGGAGCATCCTCCTCACACTTTCATTT----------TTTGGTGTCT----------TAGGT

Homo sapiens        TTCCTCCACAGCTAGGCGCTGGGTGGC-GAAGGGCTTTTGGACTGTCACTAC------------------TTTTCATTCGACAGAGACTTCAGGATAGCCCCCTTCAAAGGCAGTCTGTT
Loxodonta africana  TTCCTTGGTAGGTAGGCAATGGATGGTTGAAGGGTTTTGGGTGTGTCACCTCAGGAATCAAGCCCACCTCTTTTCATTCACTGGAGACTTCAAGATAGCCCCCTTCAAAGGCAATTTGTT

Homo sapiens        TGGGGTCATCAGGAACAGACAGAGTGCTCTTCCCTACCGCACTCATCCCACCTAGTGCCATCTATCCTTACCCACTCTGAATCCCTCCTCTCGGCAGGCACTGCCTCATTCCCCAGAGCC
Loxodonta africana  TGGAGTCACCAGGAGCAGACACCTGGCCTTGCCC--CCGC------------------CTATCTCTCTGCACTAAC-------------------------CTGCCTCAC-CCCCAGAGCC

Homo sapiens        AGGCCTAGACAATGGCGGCTGCT---CAGCTTGGAGGGAGGGGGAGT-GAAGAGGCAGCAGGCCCAACCTGCCAGCTTGAAAGCTTCATTTGACAGGGGAGGAGGCACATCTGGTTTGGG
Loxodonta africana  AGGCCTAGACAATGGCGGCTGCTGCTCAGCTCAGAGGGAGGGAAAGCCAAAGGAGCAGCAGGCCCAACCCACCAGCTTGAAAGCTTCATTTGACAGGGGAGGAGGCACATCTGGTTTGGG

Homo sapiens        TTTTCAATTCAATTCAACAATGATTTATTGTGGGTCTGCCAGCTGCAGAGGTTCTGGGCTAGGCTAGGCAGCAAGAGATGCAAAGATAAGTAAAATATTGTCTGTGCCCTTAAAGGAGTT
Loxodonta africana  TTTTCAATTCAATTCAACAATGATTTATTGTGGGCCTGCCAGCTGCAGAGGTTCTGGGCTAGGCTAGGCAGTAAGAGATGCAAAGATAAGTAAAATATTGTCCGTATCCTTTAAAGAGTT

Homo sapiens        TTCTAGGTTGTAGGGGTGTGCAGACGCAAATGAGCATTTTTTTTTTATGCGATAAGCCCTGTGCTTAGCAGTTTACCTTGCCTACATTATTATGTCTTTGCATCTTCCCAGCAGGTTTTG
Loxodonta africana  TAATAGGTATTAGGGGTGTTTAGACCTGAATGAGCAATTTTT----ATACAACAAGCCTTGTGCTTAGTGGTTTGCCTTGCATACATTACC---TCTTTGAACCTTTCCAGCAGGTTCTG

Homo sapiens        TGATGGAGGTGGGTAAACTATTATCACTTCATTTTATACACATGAGGAAACAGAGCCTCAGAGTGGAAGAGACTGGCCCAAGGTCACATTTTTAAGAATGGACCAAACCAGTTCTGGAAC
Loxodonta africana  TGATGGAGGCAGGGGAACTATTATTACTTCATTTTA--CACGTGTGGAAACTGGACCTTGGGGAGGAAGTGACTGGCCCAAGGTCACACAGTTAGGAAGTGACTAAGCCAAGCCTGGAAC

Homo sapiens        T-----------------------------------ATTACATTCTTCTGCTCCCCAGAGGTGATGAAGATGATGATGATGACAAAAAACACTTACTGAGCTCTGGAGGCCAAGTCTTG
Loxodonta africana  CTAGATATGCCTGGCTCCAAAGTCCAGACTCTTAACCATTACATTACCCTGCTTTCCATGACTGATGAAAATGATTATGA---CAAGTAACAGT----GAGCTCCATACGCCAAGCTTTG

Homo sapiens        TGCTATGCAGGCTGCAGATATTATTTTACTGGCAACTTCCAACATCCCATTGAGGCTGATGTTGTTATCCCTTTTTCTAGAAGAGAAAACGGAGGTAGCCTTGGGAAGTGATGGAGTTGG
Loxodonta africana  TGCTAAGTGGGCTGCAGACATTATTTCATGGGTAACTTCCAACATCCCGGTGGGGAAAGTATTGCTATGCCCTTTTCCAGAAGAGAGAACTGAGGTTGCCTTGGGGAGTGATGGGATTGG

Homo sapiens        AATTTGAAACCAGGTCATTCCAGTCCACCAGCCTGTACCATAAAAGAGGTAGACACAGGGTGCCATGT----TCAGGGGCAAGAGCAATGAATTGTGCCTGCAAAAGCTTCATGCCAGAG
Loxodonta africana  GATATGAACCTAGGTTATTCTAGCACACCAACCTGTACCCTAAAA-AGGTACACACAGG-TGCCATGAGGAATCAGGGAGAAGAGCAATGAATTGTGCCTGGAAAAGTTTCATGGCAGAG

Homo sapiens        ATGGCATTTACATTTGGCCTT-GAAGGGTGAGATGGGGTTATGATTGGTAGAGTTGGGAGAGAGGGCATCCCAGTCAGAAGGGATATTCTGAGCAAAGAACCCAAAGAGATGACTTGTGG
Loxodonta africana  ATGGTATTGGGATTTGGCCTTTGAAGGGTGAGTTGGGGTTTTGATAGGTGGAAGTGAGAAAGAGGACCTCCCAGTCAGAGAGGAAATTATAAGCAAAGGGCCCAAATAGAGGAATTATGG

Homo sapiens        AGCATTTT--GATTGAGAGGCTGTAGGTTCATCCGGCATGCAGGCCGGGGACTCTGGGTGTAGAGGAACAATGTGAAGGAAACCAGAGATACGCCTGATGGTCTAACACATTTTT-----
Loxodonta africana  GGTATTTTTTGGTTGAGAGGCTGCTGGTTCATTCAGCTTGTCAGCAGGGTACCCTGAGGGTAGGGGAGCAGTGGGAAGGAAACTGGATAT---TCTAAGGGTTTT-CACATTTTTTTCTT

Homo sapiens        -GTCAAGTTGAGGTTCTTATTTTTTTAATTAAGAGCAGGTACTCTGCCCCAAAGGCTTTTCAAATGATACTATCTTGCAACCCATGAGCAAACACACTCGAGTCTCTCACTGTGTAAATT
Loxodonta africana  TGTTAAATTGAAAGTTCTTGTTTTTCAATTAAGAGCAAGTTCTCTACCCAAATGGTTTTTCAAATGATACTATCATGCGAGGCATCAGCAAGCGTACACGGGGCTCTCACTGTGTACACA

Homo sapiens        CTACTGTGCTGAGAGCTGCGGTGAATACAAAAGAAAACAACTCAATTCCTCAAGTGTGTGCCGAT----GGCCCCCGTGTGGTTGAGGAGGCAAGATACACACA-ATCAGGAAAAGCAGC
Loxodonta africana  ATACTGTGCTGAGAGCTGCGGAGACTACAAAACAAAG-AACTCCATTCCTCAAGTGTGTGCTGAGTGAGGGCCCCCATGTGGTTTGGGAGACAAGATATACACACACCTGAAAAAGGACC

Homo sapiens        AGCTAGCATAGGCAGTTAGTGATCAGGGCCAAATGAGTTTTTCCAGCCGTCAGATTTCAAGGAACATGAACGAGAGGAAGGCCGGCAAGCTTAAGGCCGGGTGGGGCAATCAGTGTAGCT
Loxodonta africana  AGCCGGCCAGGGCAGTTTGTGATCAGGGCCAAAGGAATTTTTCCAATTGTCAAATTTCAAGGAACACGAAAGAAAGGGAGGCAAGCAAGCCTAAGACCTGCTGGGGCTACGAGTGTGGGT

Homo sapiens        AGCATTAAGGGCTCTGGCCAGGCCTCCCTTCCCCAAACTGCCACGGTCTCCAACCATGTCTTGACCTCCCAGAGTTTGTTAGGGAAGATGGGACCTTATGGGAT-CAGAGACAGCGCATG
Loxodonta africana  GCAGCGAAGGGCTCTGAGGAGCCCGCCCCTCCTGAAACCACTGCTGTCCCCATGCGTATCTTAACCTTTGGGGGTTTGTCAGGGAAGATAGGACTGCATGAGATTCAGAGACAGCCAGTG

Homo sapiens        GGAGCTGGGAAACGGAGCCGAATACCTGATATTCCTGATCCTCCAATTTTCTACACTTATTCTCATTTCCTGGCAGCTCCTCCAGCTGCTGAATTGTATGTGTATGTGTGAGCCGTATTT
Loxodonta africana  GAAGCAGAGAAACAAGGCAAGGTACCCGATATTTCTGATCCTCCAATTTTCTACACTTAGCCTCAATTCCTGGCAGCTCCCCCAGCTGCTGGATTGTATGTGTATGTGTGAGCGGTATTT

Homo sapiens        TAAATTAAGCAGTGAGTTCTTTCAGCTCATCATAAACATGCTAAAAAGCTTCAGCCGCGGCCTGAGCCCACCCCCGCCCTTTACACACACAGGCAGCAGCTGGGGCCACTCGACCCCACA
Loxodonta africana  TAAATTAAGCAGTGAGTGCTTTCAGCTCATCATAAACATGCTAAAAAGCTTCAGCCACAGCCTGAGCCCACTCCCTCCCCTTACGCTCA--GGCGGCAGCTGGGGCCACTCGACCCCACA

Homo sapiens        CTCTAATGTGCATTGCCTATGACTCACCAACAAAAACGACTGTATGTTTCAGTCTCCCTGGGTAGCTGATTCCTAGTTGTGGTGGGAAATCTCAGTCCGA-TTTGGAAGGCAAAGGGCCT
Loxodonta africana  CTCTAATGCGTGTTGCCTATGACTCACCAACAAAAAAGACTGTATGTTTCAGTCTCTCTGGGTAGCTGATTCCTAGTTGTGGTGGGAAATCTCAGTCCGAATTTGGAAGGCAGAGGGCCT

Homo sapiens        CAGCCCCGCCCTACTGACTGTGAATTCTACAACATATGGAGCGCCTACCTTGTGCAAGGCATTGTGGGTAAGTCCATTCATCTCTCTGGGCTTTCCATTTCCTCAGCTTCAAAATGCATG
Loxodonta africana  CAGCCCCGCCCTACTGACTGAGCATTCTACAACATATGGAGCGCCTACTATGTGCAAGGCATTTTGGGTAAGTCTCTTAATCTCTGTGA-CTTTCCATTTCCTCATCTTCAAAATACATG

Homo sapiens        TACTAATCCTGCCGGCTTTACCTCCCTCCTAGGGCTCTGTGTCTCAGCCACACTGGTCTTTCAATTTCTGCAACATTCTCG--GTCTTTTCTCGCCCCAGGGCCTTTGTTC-----GTGC
Loxodonta africana  CCTTAATTCTGCCTGCTTTACTCACCTACCAGGGCTGTATGTCTCTGCCATGCTATTCTTTCAATTTCTGGGACATTCCAAAAGTCTTTTCCTGCCTTGGGTCCTTTGCACATACTGTTC

Homo sapiens        TCCTGCCTGGACTGCCCTTCCTCTTGCTTTTGGCAT---GGAAGGCTCCTTCCTAACCTTTCAGTCTCAGCTTAA-CGTTACCTCCTGAGAAGAGCCTTCTCTGACCACCCACAGTTAGA
Loxodonta africana  TTCTGCCTAGACTGT------TCTTGCTTTTGTCATCAGAGGAGGTTTCTTCTCTCCCTTTCAGTCTCAGCTTAAACGTTACCTCCTGCGAAGTGCCTTCTCTGACCGCCTATAGTTAAT

Homo sapiens        TGCCTCCTGTTCCTCTCTTACATTTCTTTTATTAGCAATGCTTCCTTAACCCCATTTACTGCTCTGGGTAATTATGTCTATTTATATTTGTCTATTTATGGTATGTCTCTCCCACTGGAA
Loxodonta africana  TCCCTCCTGCCCCTCCCTAATACTCCTTTTATTGGAAAGGCTTCCTTCATCACAATTACCAC-CTGGGTAATTGTATCTGTTTATATTTACAGACATAT--------TCT-----TGAAG

Homo sapiens        TGTAAGCTCCAAGAGGACAGGGACCTTGTCTGTGTCACTCAGCAATGTGTCCCTCTCCTCTAGAGTGGTGCTTGGCACATAGTAGGCTGACATAAGCAAAACCATCTGGAAAATATTTTC
Loxodonta africana  TATAAGCTCCAAGAGGACAGGGACCTTGTCTGTATTGCTCAGCA-----------CCATCTGGAGTGGTGCTTGGCACATAGTAGGCTGACACA-GCAAAACCATCTGGGATGTATTTCC

Homo sapiens        TTCCTGAGGTTCTTGCCAGACATATGGTTGCTGAGGGAAAAATTATGGCAGAACTGTGTCATTCCATA-------GGAAAAAAAATCAGGCTAAAAGGGAAGAGTTTGTTGAAAACAGTT
Loxodonta africana  TCCCTGAGGTTTTTGCCAGACATTTGTTTGCTGAGGGAGAAATCATGGCAGAACTGTGTCATTCCATACTGAAAAAAAAAAAAAATTCAGGCTAAAAGGGAGGAGTTGCTTGAAAACAGTT

Homo sapiens        CTGTTTTAGAAACCAGTCCTTGGAGTCTCATCGCCAGACACAG--TCTTATGAAATTAAACAAAAGAAATAAAAAGCATAAAAAGCTAAGTTTTTATTGGGTATTTACTATGTGCCAGGA
Loxodonta africana  CTGTTGTAGAAACCAGCCTTTGGAGTCTACTCTCCAGACACAACTTCTTAGGAAATCAAACAAAAAGAATAAAACGCCTAAGAGGTTAACTTTTTACTGAGTGCTTACTATGTGCCAAGA

Homo sapiens        ACTGTTCTAAGTGTTCTACACAGATTATCTCATTTAATCCTCATCATAACCCAATGACGTCAACTCTATTATTAGCTCCATTTAACAGATGAAGGAATTGAGGCTCTGGGATGTTAAGAA
Loxodonta africana  ACTGTTCTAAGTGTTCTACACTGATTATCTCATTTAATTTTCATAATGGCCCAATGATGTAGATTCTATTTTTAGCTCCATTTTATAGATGAGGGAATTGAGGCTCTGAGATGTGAAGCA

Homo sapiens        AAGTGCCCAAGGTCACACAGCTAGCAAGAGACAGAACCAAAAGTTGAACTCAAGGCAGTCTGAGTCCA-AGTCCACAAACTAAATCATCTATGCCAC-CTGCAGCCCCCACCTCATGTTA
Loxodonta africana  AGGGTCCCAAGATCACCCAGCTTGTGGGAGGCAGAACCAAAAGTTGAACCCAAGGCAGTCTGAGTCCCTAGTCCATGCAATACATCACGGATGCCTCTCTGCAG-----ACAGGATGCTT

Homo sapiens        CCCTACACCTCCCACGCCACCCCCAGGCACGATTCTACTACAGCACTGGCCACATTGCAGAACAGGGGTTTGTTTACTT--GTCTGTCCTTCCTACTAGACGAGGAGCTTCTCAAGTGCA
Loxodonta africana  CCTTGCAGC------------CCCTGGCACACCTTGCCCAGAATACTCATCACATTGTAGTACAGGGGTTTGTTTACTCTCATCTGTCCTTCCCACTAGACTGAGAGCCTCTAAAGTGTA

Homo sapiens        GAGACTGGATCTTATTCATTTTTGCTTCCCCTGTGCCTGGAAGAGAGCTTGTTCTGGAATAGCTAACCAAGGAATATTCGTTGAATGAGCAAATGAGTGAGGGAGTAAGTGAATCCATAT
Loxodonta africana  GGGAC-----CATCTTCATTTTTGCTTCCCCTGCG--TAGAGTAGAGCTTGTTCCAGAGTA-CGGACCGATCAATATTTGTTGACTGAATGAATGAATGAG--------TGAATCCATGT

Homo sapiens        TCCTTAATATTTTCAGGCTTCAGGCTTATGGGCTCATGTGTATTTCCCTGGTC-------AAGCATAGCACTGGGCACAAGGGTATTCTGGTGCAAGAGGTGCCTCTCACCACAGGGTAC
Loxodonta africana  TCCTTTTTTCTTTTAGGCTTCAAGCTTATGGGCTCGTGTTCATTTCCCTGGTTTATATTAACGTGTAGCACTGGGGACAAGGGTGTGCTGGTGCAAGAGATCCCTTGCACCATGGGGTAA

Homo sapiens        AAGAAGGACTAGCTCTGGCTGGGTAAACCAAAGCAGTGTTCCACAGAGCAAGGGGCTTGATCCAGAAGGTTTCATTTTGTCATCGGTCTAAAAAGTAGCTGGACCTGTTTAGGACTTAAA
```

```
Loxodonta africana  AAAGAGGACCAGCTCTGACTGGTAAAACCAAAGCAATGTTCTATAGAGCAAGGCGCTTGATTCAAAGGGTTTCATTTTGTCGTCGGTTTAGAAAGTAGCTGGACCTGTTTAGGACTTAAA

Homo sapiens        CAAACTTTCAGTGTTCCAATCCCATCTCAAATCCTTTGCAGAATGAGGCAGGGTGTGGGGAGGTGGT---GGTGGGGCAGTTATAAATAAACAAATACCCACAAACCGGCTGGTTTTTTA
Loxodonta africana  CAAACTTTCAATGTTCAAATCCCATCTCAAATCCTTCTCAGAATGAGGCGGGTTGTGGGGGGAGAGGCAAGAGGAGGGAGTTATAAATAAACAAATACCCACAAACCGCCTGGTTTTTTA

Homo sapiens        AACAGAATTTGCTTCAGTCAGTGCTGCACCACATTTTCCCAACTGGCCTCCGTCGTGAGAACAAGTGGGAACTTTCATACTGAATGGCTTTATTTTTATT---CTGACCAGATGAATGGC
Loxodonta africana  AACAGAATTTACCTCAATCAGCGCTGCGCTACATCTTCCCAACTGGCCTCTGTCATGAGAACAAGTGGGAACGTTCATACTGAATGGCTTTATTTTTTTTTTCCGACCAGATGAATGGC

Homo sapiens        AGGAGTTTTGGCTCGGGAGGCAGGTTTCCGATCCTCAGCACACAGTTTGCTGCCCTACTGTGCCCCGTCCATCACACACAGCGCCCTGCACAACATACCCAGTCATTGCATCTACTGCCT
Loxodonta africana  AGGAGTTTTGGCTCGAGAGGCAGGTTTCAAATCCTTGACACACAGTTTGCTGCCCTACTGTGCCCCATCCGTCACACACAGTGCACTGCACAACATACCCAGTCATCACATCTGCTGCTT

Homo sapiens        CAGCCCTTGAAAATAATTGGGTCATTGTTTTATTACACCAGCGAGAGAGCTGCAAGCCTGTTAGGATTATGTCATTGATATTTGCGTGAAAAGGCAATCATGATATGCTGGTCATTTAAC
Loxodonta africana  CAGTCCTCAAAAA-AACTGGGTCATTGTTTTATTACACCAGCGAGAGAGCTGCAAGCCTGCTACGATTATGTCATTGACATTTGCGTGAAAAGGCAATCATGATATGCTGGTCATTTAAC

Homo sapiens        ACAGCTGGGAATGCAACTGTGTTGTCATTGTTTAAGGTTCTCATCCAG-CCCCTCTGACTTTTTTGCACAGCCCAGCCCGTGAAAATGGATCACTCTGATGTATGTCACCTTTAAAA-TG
Loxodonta africana  ACAGCTGGGAATGCAACTGTGTTGTCATTGTTCAAGGTTCTCATCCAAACTCTTCTGACTTCGTTTTCCAGCCCAACCAGTGTAAGTGGATCGCTCTGATGTATGTAACCTTTGAAAGTG

Homo sapiens        CTTTCTAGCCC-TTCAAGGGACCAAGCAGGATTTAACTCTTTTCATGCACTTGGACTTTGCTGCTTGTAGGCACAGCAGTCTGAAGGTGAGAGATGCTTGCTTTTCATACTTGTGGGAGT
Loxodonta africana  TTTTTTAGCCCCTTCAAAAGACCAAGCAGGATCTACCTCTTTCCATGCACTTGGACTTTGCT---TGTAGGCACAGCCGTCTGAAGGTGAGAGATGCTTGCTTT-CATGCGTGTGGGAGT

Homo sapiens        TGTACACCCTAGTGGCAGGATGCTGCCATACAGTGTCATTTGTTCACTAACTGCTGATCAGAGCCAAGGACAATTTTGGCTCAAGAACAAATGAGACCTAATGT---CTGTGAGGGAAGA
Loxodonta africana  TGTACACCCTAGTGGCAGGATGCTGCAATACAGTGTCATTTGTTCACTAAGTGCTAATCAGAGCCAAGGACAGTTTTGGCTCAAAAACAAATGAGGGCTAATGTTGTCTGTGGGGGAAGA

Homo sapiens        CTATGTAGATAAGTCACGGGTCAGAGTTGTGTTGCGACTTGTATTGTGTGCTAGTTTCAGTAGGACTTGTGCTTTCATTGCAATTGTCAGTATGCTTAAGCAGGGGTCTCTGCTAATGAA
Loxodonta africana  TTATTTAGACAATTCATGAATCAGAATTCTGTTGCGACTTCTATTGTGTGTTAGTTTAAGTAGGACTTGCACTTGGATTGCAGTTGTCAGTGTGCTTATGCAGGGGTCTCTGCTAATGAA

Homo sapiens        ATTCGAGCATATTTGCATTTATCGACAGGTATTACTGATGGTAGAACATCTTATTTCCACACAAGGCAATGAGTAACATTGATTATAGAAGTGTAAACAAAGCTCAAATTCATGTGCCAA
Loxodonta africana  ATTCAAGCATATTTGCATTTATCAACAGGTGTTAATGTTGGGAGAAAAGCTTATTTTCAAACAAGGAAATGATTAGCGTTGGCTATAGAA-TATAAACGGAGCTCAAAGTCATGTGCTAA

Homo sapiens        TTTGTCTATTTCAACTCATCGATCCTCTTCTTCCACTGGCATGC----TAGCCTGAGTTTTATGGCATAAGGCCATTAAAGTATTCTTCAAAGGAATCTGTTGCAGACACCTGTTCATCC
Loxodonta africana  TTTTTCTGTTGCAACTC-----TTCTCTTCTCCTACCGGCAGGCACGTTAGCTGGAGTTTTATGGCATGTGACCATTAATTTATTCTTCGAAGGAATCTATTGCAGGCACCTGGCCATCC

Homo sapiens        CTCTTCTGGTCCCGGTCCTTGCCAAACCAGCTCTG--CCTTTGCCAACCACCTTGAGGTGTGACAGGAAAAGCTGACCCAATAAGGGAGTCAACTGAAGGGATGGAGTGGCTTAGATGCA
Loxodonta africana  ATTTTCTAGTCCCAGTCCCTGGCAAACCAGCTCTGTGCCCTTGCCAGCCACCTTGAGGTGTGACAGGGAGAGTTGGCCCAATAAAGGAGTCAGCTGAAGAGATGGTCTGGGGTAGATGCC

Homo sapiens        AATGGCCAAATCCCAATGGCCATGGCTATAAGGAACACAGGTTCCTGAAGTTTTCTTGGCAACTAGCTCTAACTGCTTTGCCTTCTGAGTG-CTTTGGTATTTGTTT-TATTCTAAGTTC
Loxodonta africana  AACCTCCAAGTCCCAATCGCCCTGGCTATAACAAACTCAGGTTCCTAAATTTTTCTTGGCAACTGATTCCAACTGTCTTGTCTTTTGGGGAACTTTGGTATTTGTTTATTCTCTCTGTTC

Homo sapiens        CCAGTTTTGATCTATTTTTGCCTTATGGCTGCAGATTCCCTTTCTGGAAAATGGGGCTCTGGCTGGGATCAAATCAGAGAACACACCCAGA---CTTGGCATAGTGCCTGGCACAGAGTA
Loxodonta africana  CTGGTTTTGATCTGTCTTTTCCTTCTGGCTGTGGTTTTCCTTTCTGTAAAATGGGGCTTTTGCTGGAATCAAAT---AGAACGTACCCAGAAGACCCGGCACAGTGCCTGACACAGAGTA

Homo sapiens        AGTGCTCAACAAATGGTAGCTGTTGTCTCTTTTTCCCTGTCCAGGTTTCTTCTGGTTCTGTTCTCTGTACCTCCAGTATTGGCCCAGAACCAGGAACTGTTCCCTCCCCTGACTGGCAAG
Loxodonta africana  AGTGCTCCACAAATGGTAGTTGTTGTCTTCTTTTCCCTGTACAGGTTTCTTCTGGGTCAGTTCTCTGCACCTCCAATATTGGCCCTGGACTGGGGCCAGTTCCTTCCC-TGGCTGGGAAG

Homo sapiens        TCAGATTACCTGGCCCTCAGACAAGCTGGGCTCCCTTTTCTGGCAGCCTTTTGTTGTCCTTACCTGTTGCCATGTGCCACTCAGTGTAACCACAGGCAGACCTCTGCCAATTGCTGACAT
Loxodonta africana  CCAGGTCACTTGGCCCTCAAACAAGCTGAGCTCCCCTTTCTGGCAGCCTTCTGTTGTCCTTACCTGTCACCGTGTGGCACTCACAGTAACCACAGGCAGACCTCTGCCAGCTGCTAACAT

Homo sapiens        TAACCTATTGAATCCTGGCAGAGAAGAATAAGTTTGCAGACTGGAATTTGCATGTCTGGGCCAGATACGAGACCAGGAGGCAAAGCCATATTT-ACCTGTTCTCCCCAGTTGTG-GCAAA
Loxodonta africana  TAACCTGTTGAATTCTAGCAGAGAAGAACAACTTTGCAGACAGGAATTTGCATGTCTGGGCCAGATACAAGACCAAGAGGCAAGGCCGTATTTTATCTGCCCTCCCCAATTGTGAGCGAA

Homo sapiens        TCACCCACATTCTGGTTAAAACCAGATGTTTCACACCCATGACTGCAATGCCACACATCAAACAGTTGCTTACAAAGAACACAAAGTGGTTTTCACATCGCAAATGTAACTTGCAGGTCA
Loxodonta africana  TCACACACCTTCTGGTTCAAACCAGATGTTTCACACCCATGACTGCAATGCTACACATCAAACAGTTGCTTACAAGGAACATGGAATGGTTTTCACATCAGAAATGTAACTTGTAGGTCA

Homo sapiens        CTTCTCAAATTTCCTCGCAGCAGCTACCATGCCTGGGTATTTTTCCCTTAAAAATGACAACCTTGTTGGGTCTCTTGTAGGGACTTTCATTTGGAAGCCCTGGGAGAACTTATATGCTCT
Loxodonta africana  CTTCTCAAATTTCTTAAAAGTAGCTACCATGTCTGGATATTTTTCCTTTGGAGATGACAACTCTGTTGGGTCTCTTGAAGGGGTTTTAATTTGGAAGCCCTGGGAACAATTATCTGCTTT

Homo sapiens        TTCCAAGAGGGTCTCTGATGGCTTTAATTCACATGGAGTTAATATATTGGAAAAGAAAAGAGATCTTCCCCAAACTCACCTGCTCCTTGAAAATGCTATTCTGTTGCCTTGGCGAACAGC
Loxodonta africana  TTCCAAGGGGGTCTCTGAATGCTTTAATTTGTAGGGAGTTAAAGTGTTGGAGAAGATAAGAGATCTTCCCCAAACTCACCTGCTCTTTGAAAGTGCTATTCAGTTGCCTTGGCTAGCAGC

Homo sapiens        CAATCCAAACATTAAAAAAGAGATAGAGAATGCAGGAATCCTAAATGCTATTCTCAGTGCATGTCCAAACAAGAAC--TGGGGGCTGAGGGGCAAAGAACAGAAAGAGGAGAAAAG--AG
Loxodonta africana  CAATCCAAACACTAAAAAAGAGACAGAGAATGCAGGAATCCTGAATGTTATTCTCATCGCATGTCCAAACAAGAACAACGGGGGGGGGGGGGGGGACAAGAAAAAGAGGAGAAAAGGCGG

Homo sapiens        TTTTTGTAGCCTAGGCCGTGAGTTGATCCCACCCCGTCATGTCCTGTACCCTTCACGCTCATAAAGAAGACAGAGGAGGCCGGGCGCGGTGTCTCATGCCTGTAATCCCAGCACTTTGGG
Loxodonta africana  TTTTTGCAGCCCAGGCTGTGAGCCCATCCCATCTCATCGTGTCCCATTCTACGCACACTCCTAAAGAGGAGA................................................

Homo sapiens        TGGCCGAGGCAGGCGGATCACCTGAGGTCAGGTGTTGGAGACCAGCCTGGCCAACATGGCGAAACCCTGTCCCTACTAAAAATACAAAAATTAGCCGGGGGTGGTGGAACATGCCTGTAA
Loxodonta africana  ........................................................................................................................

Homo sapiens        TCCCAGCTACTCGGGAGTCTGAGACAGGAGAATTGCTTGAACCTGGGAGGTTGAGGTTGCAGTGAGCCAAGATTGTGCCATTGCACTCCAACCTGGGCAACAAGAGTGAAACTCGGTCTC
Loxodonta africana  ........................................................................................................................

Homo sapiens        AAAAAAAAAAAAAGAAAAAGAAAAAGAAAGAGGAAGGAGAAGGAGAAGAAGGAGAAGGAGAAGGAGAGAAGAAGAAGGAGAAGGAGAACGAGAAGATGTGGTGGCATCATGACTTCTCTG
Loxodonta africana  .........................................................................................GATAAAGTGTGACAGCATCCT--------TG

Homo sapiens        CTAGGGTTAATTGCTCAGCCTGAGAGGCTCTCCAGGCTGGCAGGTCACACTAGAGCCAAGAGTGAAGGGTCCATTCAGTAAGGTCACTGCCATGATCAGTTCTCAACCACTTTAACTCAG
Loxodonta africana  CCAGGGTCAAGTGCGTAGGCTGAGAGGTTCTCTAATTTGGCAGGCCACACCAAGGCCAAGAGCGAAGGTGCAGTCCAGTGAGGCCAGTATAATGATCAGTTCTCAACTGCTTTGACTTGG

Homo sapiens        TTTTTCTGTTGCTGCTTTGTTGGTTAGTGGTTTGTTTGTTTCTACCCATTTGGGAGAAGGGTAGGAAAGGAACTAAGGTGATCAACTATTCCATATGTGTCAGGCCCTGTACTTGTCTTT
Loxodonta africana  TTTTTCTGTTTTGAATTCTTTGGTTCTTGGTGTATTTGTTTCAACCCATTTGGGAAGAGGGTAGGGAAGAAGCTAACATGATTGACTATTTCGTATGTGCCAGGCTCAGTGCTTGTACTT

Homo sapiens        TCAAACACTTATTCACACTCTGAGGGACTCTCTCAGGTCCAGCAACTGTGTAGCTATGATGTGAAATAGCAGCTTCTCTCTATGCATTCACTGGGAATGGTTTGTTCTGATCACACAATT
Loxodonta africana  TTCAACACTCAAGCATA--CTGAAGGA-TTTTTGACCTTCAGCAACTGTGTGGTTATAATGTGAAACTGTGG----TTTCTATATATTCCCTGGGAAAGGTTTGTTCTGATGACTCTAAT

Homo sapiens        TTTTCCAGTTCTTCCGACGTTAGGTGTTGCATGGTAAGAAACAGGTGGTTAATTATTGAGACAGTTCTTATTTGGAATTGTCTCTTCCTCCTTCTCCTCTCAGGCAGATGCTAAAGGATG
Loxodonta africana  TTTCCC-GTGTTTTGAACATTGAGTAGTAGATGGTGAGAGACAGGTTGTTAAGTGTTTA-CCAGTACTTATTGGGAGTTACCTCTTCTTCTTTCAGATTTCATGCAAGTGGTCAACAGTG

Homo sapiens        TCTGTAGTTT-TTTCATGGCATTTTGCTAAAGATGCACATTCATTAATTCATCAAATAGCTTTTTTTGAGACATTATTATGA-GTGAGGTGTTGTGCTATGATATGCTTTATATACCATA
Loxodonta africana  TCTGGAATTTCTTTTATGCTATTTTATTGAAAATGCATATTCACTAATTCATAAAATCGC-CTCTTTGAGTCATTATGATGATATGCTACGTTTTGTGATTA-ATGCTTTACATATCATA

Homo sapiens        TCTTATTTCATCCTCACCACAAAGCTATGAGGTAGGTACTATCATTATCTTAATTTTAC-AATGAAGAGACTGAGGCTCAGGAAACATAATGCAAGGTCACACTCAGTTAATAAGTTGAG
Loxodonta africana  TCTTATTTTATCTTCACCACCAAGCTGTCAGGTAGGTACTGTCATTAGCTTCATTTTCCAAATGAGGAGATCGAAGCTCAGAGATTATAACGCAAGACCACA--CAGTCAATAAGTGGGG

Homo sapiens        AGCTAGGATTCAAACCCAGGTTTATCTGACTCAGTTACACTACACTTCCTCCACATAGCACATTGTGGCCCGTCACAGCTCTTGAGGACAAAATTCATCCTTAGAAAAGTTTTCTTACT
Loxodonta africana  AACTGGGATTCAAAC----AGCTGTTAGATGCAACTGCATTATACTTCCTCCACGTACTGTATTTTGGGCCCCTCACAGGCCTTGAGAACAAAATTTCTCCTTAGGGAAA-TTTCCTACT

Homo sapiens        CCACTTTTTGCTGAT-------ACTACTGCTGCTGTTGCTGCTGCTACTACTATTACTACTACTGCTACTGTTTCTCCTCCTCTATTTATCCAGAACTGCATTTTTCCAGCACT-CCTAG
Loxodonta africana  CTACATTTTGCTAATAGTACGAACAACCGCTGCCTCTACTACTACTACTATTGTTACTGCTACTACTGCTAT...........................TATTTATCCATTACTGGGTAG

Homo sapiens        GTGCTTTGAACCCAGCTACCAGTTGTAGAGTTCAAACCCAGGCCTGCATGAGTCC--CTTGCACCCATTCTACTTTTCTGCACTGCTTGTAATG-TAACACAGGAGGTATGGGACATCCA
Loxodonta africana  GTGCTTTATATTCAGCTGCTAATTATAATGCTGAAACCAAGACCTATATGAGTCCAGTGTGCTCCCATGCCATTTTTCTATATCGCTTGTATTGTTAACACAGGAGTCATGGGATATATA

Homo sapiens        TAACTCAGGTAGGTAATTGACTTCAAGCTTAACATT---CACTCACCAATCATTTATTGAGCACATATTAGATAAGCAACCTATGGCTCCTGCCCTGGAGTTGTTCATAAGACCTTCCAG
Loxodonta africana  CAATTTGGGGAGATTGTTGACTGCTAGCTCAACTACCAACATTCATCAAATGTTTACTGAGCATATACTAGATGAACAAGCTATAGCTCCTGCCTTCCAGCTGCTCATAAGACCTTCAAA

Homo sapiens        GCAGACAGCTATGGGACGAATACACTTGAA-----TTTATTAAATCTACCTCAGGCAAAAAACCAAGCATGTAATTATAAGTAGTATATCAGAGTATGTGTAAATGGGGTTCTGTAAAAT
Loxodonta africana  AGAGACAGATATGGAAAGAATAAATTAAAAAAAACTTTTTTTAATTTGCCTGATGT-AAAAACTAGGTACG-ACCTATAATCAGTATATCAGAGAATCTGAAAACAG------ACAAAGG

Homo sapiens        GTTTGAAAGAGGCAGGCCTGGGTTAGTGGTGAGAACAAGGATCAAGAAGGCTCTGCCACCAAATAACAACATGAGTGT-----TGTCCCGTTAATCTCTCTGGGCTTCTGTTTCCAGATC
Loxodonta africana  GCTTGAAAGAGGCAGGCCTACTTTAGTAGTGAGAATAAGAAGAAAG-AGGCCCTGCCGTTAAATAGCTACGTAAGCATAGGTAAGTCCC-TTAAACTCTCTGGGCTCCTGTTTCCACACC

Homo sapiens        AAGGACCTCAAAATAATAGTCATAGTCACCATTTATTGAGTGCTTTACTGTTAACCAGGCAGTATGCTAGGTGGTTTTCATTTAGTCTTGACAACAGTATTCTCGCTTTCCCTTTTCTAC
Loxodonta africana  TGGAACCTCAAAAGAATAGTAACAGCCACTGTTTATTGAGCAC-TTACTGTGCACTAGGCACCAAAC-AATTGGCTTCCGTTCAGTCCTGACAACCGCTTTGTCGCTTTCCCTTTTCTAC

Homo sapiens        AGCTGAGACTCAGAGACATTAGGTAATG---TCTAGGTCACACAGCTCTTCAGTGACCAGTTGTTTCTATGTC-AGAATCCTCTTATAATCACTGAGTTGTATCGCTTCCCTTGCCTCCT
Loxodonta africana  AGCGCAGGCTCAGAGAGGATAGGTAAAGTGCTCTAGGTCACACAGCTCATCAGCGGCCAATTGTTTCTATCTCTAGAATCCTCTCATAACCATTAAGTTGTATTGCCTCTCTTATCTCCT

Homo sapiens        G-----CTCCCAAGTCAAGTATTCTCTACTATGTCGCCTGAAAATTAAAATTAAAATCAAGGTTAATCTTGTTCAACTGAGCCAAGAGGATAAACATTAGAAATAGCAATTAGTAGGTAG
Loxodonta africana  GACTGAACTCCAAGTCCAGTATTCTCTGCTATGTTGCCTTAAAGTTAAAATTAAAATCAAGGGTAATCTTGTTCAACTGAGCCAAGAGGATAAACATTAGAAATAGTAATTAGTAGGTAG

Homo sapiens        AAATGTTGCCAGGAAGAGCTATACAAAAGCATAGAAAGGCAACCAAGGGAGTT---AATGATGGGGGTGTGGCAGGGGTTGCATGTTGATGGGTAATTGGATCCTGGCCAGCTCTTACCT
Loxodonta africana  AAATTTTGCCAGGAGGAGCTATACAAGAGTGCGGAAAGACACCCAAGTCAGTTAACAATGATGGGGGTGCAGC-GGGGTTGCATGTTGAAGGTTAATTGGTTCCCTGCCAGCCCGTACCT

Homo sapiens        TCTTCCTTCACCTTCCCACTCCGCAATTTCTCAGGCTTCTCTAGAGAGCACAACCCACCTAGAGACAGCCTGTGTTGCCACAGGAGGGCACCAGGCCAGGAATGGCAGGCCTAGGGCAGT
```

```
Loxodonta africana  TCCTCCTTCACCTTCCTGCTACG--------AAGCCTGCTCTAAAGAACAAGGCCCACCAAGAGACAGCCGGAGAT-CCACAGAGGGG---------------------CTCAGGCAGT

Homo sapiens        GGGGCTCTGGGCTGGGAACCATAAATAACCACTTTAATGAAAACCAGCTTTGGC-CAAAAGCCTTATCATTTCTTAAGAGGTTCTGTGCTTCTTCTAAT---GACCTAGTTACTTGAGAA
Loxodonta africana  GGGGCTCTGGGCTGGGAACCATAAATAACCGCCTTAATGAAAGCCAGCTTTTGCTTAAGAGCCTTGTCATTTCTTAAGAAATTTTTTGC--CTTCTAATATGGACTTAATTACTTGAGAA

Homo sapiens        AGGCCTAGCCTAACATATACAACAACAATAAT------------AGTTGCCCTATAGTGAGTACTTACTATGGGCCAGGGCCTGTGCTAAACACTCACTAATTCATTTAATCCTCACACA
Loxodonta africana  A-GCCTAGCCTACTCTATACAACAACAATAATAATAATAATGGCAGTTGATCTTTGGTGAGCACCTACTATGGGCTAATACTTG------ACATACACTAACTCATTTAATTCTCCTGCA

Homo sapiens        GCCCTACAGGAGCAAGTACTATTATTTCCCCCACGTTACAGAGCAGGCATCAGGCTGAGAGAGGCTAAGTCATTTGCTCAAGATCATATAGCTGGTTGGAGCTGGAATTTGAAGCCTGAA
Loxodonta africana  GCCCTTTGAGGGCACATATTATTATTTCTCCCGTTTTACACAGGAGGCATCAGGCTCAGAGAGGCTAAGTAACTTGCTCAAGGTCATACAGCTGGTTGGAGCTGGAATTTGAAGCTTGAA

Homo sapiens        GCATCTAGTTCCAGGGCCTGAGCTCTTTAATTCCTATATTTAATGAATGTCTGTCGAAGGAATAAACAGATGAATGAATAGACAAATGT----ATTCATTTCATTTCACTTTAACAAACA
Loxodonta africana  GCATCTGGTTCCAGGGCCAGAGTTC-TTAATTCCTGTATTTAATAAATGTCTGTTGGAGTAATAAACAGATGAATGAATGGACAAATGTATTAATTCATTACAGTTTACTTCAGCAAACA

Homo sapiens        TTTATTGAGTGCTCTG----TGTCAGCTCCTGTGCTAGGCATTGAGGATTTATAAGTTTAGTGCGTGCGTGCGCGCACACACACACACACACAAACGCACACACACACACACACACATCC
Loxodonta africana  TTTATTGAGTGCTCTGTATATGTCAGCTTCTGTGCTAGGCATTGAGGATTTAAGTGTTTA----------------------------ACACACACACACTCACACACACACACACCCCC

Homo sapiens        TGCATGGCCCTTGCTCTCCAGGGGTTCACAAAAGCA----TTCTCTATTGTGGCTTGATTTCTTCTGGAGAGAAGTAATTTGAATGTCTTGAACAGCAGTTTTTAATGATGCTATTAAAT
Loxodonta africana  CACATGGCCCCTGTCTTTGAGAAGCTTACAAAAAAACATTCTCTCTAATGTAGCATGATTTCTTTGGGGGAAAAGTAATTTGCGTTTCTTGAATAGTAG-TCTTAATGATGCTATTAAAT

Homo sapiens        TTAACCTAAAGCGGGGGTTTTGCACCGGTGAGCAAAGTGTTTCAAATGAGAATGTGTCTTTGATTAATCATCGATTCAGCCAGAGAATAGTTACTACCAGAATG-GAGCTGCAACTCCTA
Loxodonta africana  TTAACTCAAAGTGGGGGTTGTGCACCGGTGAGCAAAGAGTTTCAAAACAGAACATGTCTTTGATTAATCATTGATTCAGGCAGAAAATGGTTACTACCAGAAAGAGGGCTGCAACTCCTA

Homo sapiens        TTATATTAGCTAACTCTTCTTCCATCTATCATGTCTCTATCTTGTCGAACCCTCCCACGATACAAAAGTTATCTGTACCCTAATGGACAATTATCCCATCCATCATTCCTGCTGGTCTTG
Loxodonta africana  TAACATTAGCTAACTCTTCTTCCATCTATTATGTCTGTAT----TCTAACCCTCCCACGGCACAGTAGTTATCTGTACTCTAATGGACAATTATCCTATCCATCATTTGTGCTGGCCCTG

Homo sapiens        TTGATTGCTGGTAGTCTGCTTGCCCACGGAGGAGACTTACAAATAGTCATGACCAGTGACGCACTGGAAAACTGCTCATTTTTAGACATTTTAAATCTATGTAGAATGAATTATTTTTCA
Loxodonta africana  CTGATTGCTGGCAATCTACTCGCCCGCAGAGGAGACTTAC-AATAGTCACGACCGATGATGCAGTGGAAAATTGCTGATTTTTACACATTTTAAATCTGTGCCGAATGAATTCTTTCGCA

Homo sapiens        GGCTGTAC-TTAAGCTGCATTTGCTTTGTTGGTTATGTAAGAAGAGA-TATGAATTCCTTACCTATGCGTGAGACATGGCGTC--TGAATATTTTTAAGT--------TCCTGTGGGCAG
Loxodonta africana  GGCTGCCCTTTAAGCTGCATTTGTTTTTCTGGTTATATAAGAGCAGGTTATGAATTTTTTACCTGCATGTGAAATGTGGACTCTGTGAATATTTCAAAGGGAAAAAAACCCTCTAGGCAG

Homo sapiens        CAGCCCTATACATATTTGTTAAAATGATGAAAGAATAAAGTAAACATACCTAGAGTAATTCCAGAGGAGCAGGCTTCATACCTGGAGCTCGGGTGGGTGGCCATCATGTCAGGGTAGTCA
Loxodonta africana  CAGCCTCATAAATATTTGTTAAAATGCGGGCTGATCGAAATAAACACA--TGGGGTAATAAAAGAGGAGCAGGTCCCAAGATTAGGGTTCAGGGGGCTGGTCATCACATCAGAGTGACCA

Homo sapiens        CC-GTCTTCCTTTTCCTTTCTAAGTACTTTGAGGAGAATCCCAAGTTGATTAGGGTCAAGGTGAGGACTCATGGTGCTGAAAATAGGGATTGAGTGGACAATGAGAGATGCCTGTTGAAT
Loxodonta africana  CCAGTCTTCCTTTTCCTTTCCAGGTATTTTGAAGAGAAACCCAAGTTGATGAGGATCCAGGTGAGGACTCTGGGTACTGAAAGTAGTGGTTGAGTGGGGAGTGAGAGATGCCTGTTGAAT

Homo sapiens        TCCCTGGGACTATTGCTATGAGATTTGAGGGCAGAAATGCCCCCCTCTC----------CCATGTCCTACCCTAAGCCTGGTAACACATTTAACTTTTTGTTACAGTA--ACTACCATGC
Loxodonta africana  TCCCTGGGAGTATTGCTATGAGATTTGAGGACAGAAATGCTCTCCCTCCATGTCCCACTCCATGTCCCATCCCAGGCATGGTAACACACTTAACTTTTGTGTATGGTATTGCCAACATAC

Homo sapiens        CTAGTAAAACTGGAGTCA--------------------CTGGTTGCCCAGGGCAAAAACCCAAGAGTTGTAGTTCACTCATCTTTATT-----TCTCACCTCTACATTCAGCCAGTCACT
Loxodonta africana  CCAGTCCAATGGGAGTCATATTTCCTCCCCACACTCTCCTGGTTGCTCAAGGTAAAAACCCAAAGATTGTCTTTCACTCATCCCTATTCCTTACCTCACCTCTACATCCCGCTAGTCCCT

Homo sapiens        AAGCCCTGCCAAGTATATTTCCTAAGCATTCCTTGCATGTCACCTCTTTTCTCCATTCCTACTGCCAGGGCTTTAGTTCAGGACTCCTCCCACCTCCAGACATCTCTTGCCTTGATTCCT
Loxodonta africana  AAGCCCTGCCAAGCCTCCTTCCTAAATATTACTCAAATTTGACCCATTTTTTCCATTCCT---------TCTTAAGTTCAG--------CTATCTCTTG---CCTCTCACCTTAACACTT

Homo sapiens        ACAATACCTTCTGGTTTTCTCACCTTCTCTCCTTAGTCCTCTGTGACCATTCTCCCCAAAGTGGCCTGAGTGATCTTTCAAACATGCAAATCAAATCATTTCACACCTCCATCCCCTCCA
Loxodonta africana  GTAA....................................................................................................................

Homo sapiens        CTTAACACTTGTAAATAGCCTCACAACACCTGAGACTTGAGGCTCTCAAAATGTGGGTCCTGGGGCCAGCAACACCAGCATCTCTGGCAACTTCTTAAAGTTAGAGTACGTACTCATTAG
Loxodonta africana  ........................................................................................................................

Homo sapiens        AGTACCTACTGAATCAGAAACTCTGAGAGTGCTCACCTGACTCTGTGTTTAACAGCCCTACAGGTGATTCTGATGAGCACTCAAATTTGAGAACTGGCACCCTAGAGCCATGGTTCTCAA
Loxodonta africana  ........................................................................................................................

Homo sapiens        TCCTGGCTGCACATTCAAATAAATGGAGGGAGCTTTTAAAAGTTCTAATGCCTCTCCTGCAGAGTAATTAAATCAGTTTCTAGGGGTGGGATCCAGTCATAAGTATTGTTTTTAAAGTTC
Loxodonta africana  ........................................................................................................................

Homo sapiens        CCCAGGTGCTTCTAATATACAGTCAAGGCTGAGAACTACTGCTCTATAGGATGAAGTCCAAACTCCTTAGCTTGGCATTTAAGGTCTATCTCTTTGGTCATAACCACCCCCAACACTATG
Loxodonta africana  ........................................................................................................................

Homo sapiens        CTCTTGCTTGTTCATTCTAGAATTCAATCACAGGGAGTCATTTGCAATTCTCTTAGAGCCACACATGGTCTCTTACCTCTGTGCATTTGCACACATCATTCCCACTGCTGTGCCCATCCC
Loxodonta africana  ........................................................................................................................

Homo sapiens        TTCCTCCCTCCCTTCACCCAGCTAACACCATCTCATCTACAACACATAGCTCAGAAGACACCTCTAAGGAACAGTTTTCCCCGCCTGTCCAGTCTGGGTTAGATGCCCCTCCTCTGTACT
Loxodonta africana  ........................................................................................................................

Homo sapiens        CCCACAGCGTCCACAACATAGCACTGGATACATTCTCTTGTTCTTCTCTGGTTAGTTGTCTACTTTCTCTACCAGTCTGAGAGCTAGGATCCCCACCTGGCACACAGTAGATGTTCCATG
Loxodonta africana  ........................................................................................................................

Homo sapiens        ATTCTGTACATCCTGTTCCCTCTTTTTGGAGTGACTTTCTGTGCCTTGTCTGTGGCCTTCAGGATGCAGCTCAGATATCACTTCTTGGGAATCTCAGAATTCTCTGTCTGGACCCTTTCT
Loxodonta africana  ........................................................................................................................

Homo sapiens        CTAGGCTCCCGCAACAATCTAAATGTAATCTGATCCTAGCCCTTAGCACACAGGGCTATCAGGCTGCCATAGTCTGTTTACTTATCTGCCTCCCTCACTGAACTGTAAGTAACTTGAGGT
Loxodonta africana  ........................................................................................................................

Homo sapiens        TAGGGACAGAGCCTTGTACATTCCTGTATCCCAGCTCCTAGCACAAAGACTCACACATTATAGGTGCTCAGTAGCCATAACCATCTGCAGAACGAATAACTGAATCAATGATGACCTTTT
Loxodonta africana  ........................................................................................................................

Homo sapiens        CCTCTTCTCCATATCATCAGCCACTATTTCTGTCCAGGCCCCCGTTGCTTTATTGCAATAGCCTTCTGACTATTTCCCATCTCTCCTTTCTCTGATGCCATCTGAACACTCCCCCCCTCT
Loxodonta africana  ........................................................................................................................

Homo sapiens        CCAAGATGTGGCTTTCTAAAACCACAGTCACCCTATCCCTCCCCTGCTCAAACACTTTCAGTCATTCCTCATTGTCTGCAGGGCAAATATCAAAGTGGAATTCCAGATCCTTCCTTCCCA
Loxodonta africana  ........................................................................................................................

Homo sapiens        AGAGTGGTCTGCCTTCCCAACCATACCTCCCCCCATTCCCCTATGGAATACCAGCCACATCATCTGAGGGTCCTGGTGCAAAACGAAAATGTGGAACCCCGTGTTAAAATATAATCAAGG
Loxodonta africana  ........................................................................................................................

Homo sapiens        ATTTCAAGATAGCAGCAAAGGAGAGCATTAAACCAAGCATAGGGCTCTTCTGACTTCAGGGTCCGATGCGACGGCACAGGTCTGCCCCTACGTGTATCCAGCGCACTGGCCAAATGCACT
Loxodonta africana  ........................................................................................................................

Homo sapiens        TGCTCCTCCTTCAACTGCTCTGTAGGTTCACTTTCTCCACGGCTTTTGTCAGGCTGTTCCATCTCTTCTACCCCACTAGCCAATTCAAAGCTTGTCTTCCCTGTAGATCCACCCTGTTCT
Loxodonta africana  ........................................................................................................................

Homo sapiens        TCAAGAATTGCTCAATTCTCATCTCCTACTGTTCCAGACCACAGTAGTTTCTACCACACAGCCTGATATTAATAATTATTTTTATATGTGTGTAGTCTTGCCTGTCTACTGAATTTTAAG
Loxodonta africana  ........................................................................................................................

Homo sapiens        CTTCTTGAGGGCAAGGACTCTATTTTGTTCATCTTTTTTTTTTATAGACTTAGCATAGTACTTTGTAATCATCTCACTTGTTGAATGACTCAGGGAAGAAATAATACAACTCCAGTCACT
Loxodonta africana  ........................................................................................................................

Homo sapiens        GAGTTTGCCAAAAAACAGTAGACACTGCAAACCTGATTTAAATCCCTCATTATTAGCTATGCAACATTGAAGGAGTCACCTCACTTCTCTTTGCCATCTATAAAACGATGATACTTAGAG
Loxodonta africana  ........................................................................................................................

Homo sapiens        AAAAAGTGTTTTTTTTTTTTTTTGTTTTGTTTTTTTGGTTTTTTTGTTTTTACAGAGGCTTGCTCTCTTGCCCAGGCTGGAGTACAGTGGCATGCAATCTCGGCTCACTGCAACCTCCGC
Loxodonta africana  ........................................................................................................................

Homo sapiens        CTCCTGGGTTCAAGTGATTCTCCTGCCTCAGCCTCCCAAGTAGCTGGGATTACGGGCCCATGCCACCATGCCCTGCTAATTTTGTATTTTTAGTAGAGATGGGGTTTCACCATGTTGGCC
Loxodonta africana  ........................................................................................................................

Homo sapiens        AGGCTGGTCTTGAACTCCTGACCTCAAGTGTTCCACCTGTCTCGGCCTCCCAAAGTGCTGGGATTACAGGCGTTGAGCCACCATGCCTGGCTAGAAAAAGTAAT--TTTTTGTTAGTATC
Loxodonta africana  ..............................................................................................AAAATATAATGTTTTCTATTAGTGTG

Homo sapiens        TTGTTCTTTGGCAATGTTTTCAAAATCTTTTGTTTTTTAATACATGTAAAACTTATTTTATATTTTATATCACCTAATTGCAATACCTGAGGTCTTTGAAGACCTGATTCTG-TTTTCAT
Loxodonta africana  TTGTTCCTTGCTAATGTTTTCAAAAATTTTT-----TTAATACATGTAGAACTTATTTTATATTATATA-------ATTGTAATGTCTGAAGTCTTTGAGGATCTAATTCTGCTTTTTGT

Homo sapiens        TGTTTCTGGCTTCATCGTGTTCTTTGTAATTTTGGATTATGAGTTCATGTTCAACCTGACTTTATCCTTGGTAATCCTATGAAAACTGGGTGGTGGGCATGTTCTTCCAGGGAGGATTTG
Loxodonta africana  TGTTTCTGGCTTCATTAT---TTTTGTAATTTTAGATTATGAGTTCATGTTCAGCCAGACTTCATCCATGGCAATTGTATGAGAACTGGATTGAGAGTGTGTTCCTCCAGAGAGAATTTT

Homo sapiens        TGTTTGGTTCTTCCAAGTGCATCAGGGCACTATTAATCCAGGAGTTCTTCCAGTGAATTGTTTAGTCCCAAAGGCAAGGGCCATGTCTTCTTTGGCTATATGATCCCAGGGCTTAACTCA
Loxodonta africana  TGTTTGCTTCTTCCAGGTGT-TCCAGGCACTATTAACCAAGGACTACTTAGAGTGAATTGTTTAGCCCCAAAGGCAGGGGTCATGGCTTACTTGTCTATAGGACCCCAGCGCTTAGCTCA

Homo sapiens        GGGACTTGT-------AGATGCTCAACATATATTTGCTGATTGAGTGGATGAATGAATGTGGTACTTTACC-GCTTCTTTTGGAAGCCCAATAAGAATTGTGACCTCTGGTATTAACAAA
```

```
Loxodonta africana  GGGACTGGCCCCTTTCAGGTCCGCAACAAATATTTGCTGATTGAATGGTTGAATGGATAAGGCATTCTACCAGCTTCGTTTGGAAGCCAAATAAGAATTGTAACCTCTGGTGATAGCATA

Homo sapiens        GAACAGTAGGCACATCAGGAAAATAATAATTACTAACATTTATTGAGCAATGATTATGTGTCAGGTATCATTCTAAGTGCTTTGTGTGTATTAACTCATTTAATTCTCACTATGATGCTG
Loxodonta africana  GGGTAGTAGATGAATAAGGAAAATAAT...TACTAACATTTATTGAACATTTACTATGTGCCAGGTACCATTCTAAGTACTTTGTGTGTATTAATTCATTTAATTCTCAGTATACTTC-A

Homo sapiens        TGAGGTAGGTTACTATTATTATCTTCACATTACAGAGGAGGCACCAGACTCAGACAGGCTGAATAACTTGGCCAAGGTCACATAGCTAGTGAATGGCGGAACACAGATATGAACCCAGGC
Loxodonta africana  TGAGGTAGGT-ACCATTATTGTCCCAATTTTAGAGAGGTGGCACCAGGCTCAGACAGGCTAAGTAACTTGGCCAAGGTCACACAGCTAATAAATGGTAGAACTTGGATATGAACCTAGGC

Homo sapiens        ACCCTAGAGACACATCTACTTACCACCTTGTATTGGAGAAGAGGTCAACAGTAGTGACAGAAGCAATAGAGTGGAAATTTTCAGTACTGCCAAAA---CTATGAGAGCCATATAGTCCAC
Loxodonta africana  AACCTGGAGCCACATTTAACTGCCATCCTGTATTGAAGAAGAGAGCAACAGTAGTGACAGCAGCAATTGAGTGGACAATTTCAGTATTACCGAAGGGGCAGCGAGAGCCACACAGGCCAC

Homo sapiens        ACCAGGCATGAAAAGGAAGCTGGCATGGTCTTGTGTTGTCAGTTAACCCTTGTATATAGGGTTTTCATTTCCTAACTAGAGAAGGAGGAGTTTGAAAGCACGAACCATGAATTACAGCTC
Loxodonta africana  ACCAGGCATGGGAAGGGACTTGGCATGATCTTATATTGGTAGTTAACCCTTGTATGTAGTTTTTTTTTCCTCCAAATAGAATAGGAGCAGTTTGAAAGTAGGAACCATAGATTACAGCTC

Homo sapiens        TTTGAAATGCTCAACATAGATTTGCTGAGTGAATAAATGACTGTTTTGAAATTAAACACAGGAGTGCTTTGAATAAATTACTTTGCATCTTGTATGTCAAACTGAAATTGGTGAGACATC
Loxodonta africana  TTTGAAACGCTCAATATAGATTTGCTGAGTGAATAAATGAGTCTTTTGAAATTAAACACATGAGTACTTTGAGTAAGTTACTTCACATCTTGTGTGTCAAATTGAAATTGGTGACAAAGC

Homo sapiens        TTT-CACCTTGTGTTAAAAACTAAAAATCCCTAGATGTGGTCATAACATGGCTGGTTATAATGACACCTTGCATTTGTACTTGTGTATGATACTCAGGACTCCCTGAGAAAAGGAATGAA
Loxodonta africana  TTTTCACTTTGTTTTAAAACCTGAAGACACCTAGATATGGCCATAACATGTCTGGGTATGATGACACCTTGCGTTTGTACCAGTGTATGG-----------------GAATAGGACAGAA

Homo sapiens        GAAACTTGGATTTTGCCTTCATAGGCCCTGAGGCTGTCAGGAACCAAGGGCCACTGTGTTTGTAATCCATTCCTTTGGCTGCTCTCCAAATCTAGGAAGACCTGGAGGAAAGGGGGGCTT
Loxodonta africana  GAAAGTTAGATTCTGCCTTCATAGGCCCTGGGGCTGTCAGGAACCTAAGGTCACTAGGTTTGTAATCCATTCTTTGGCCTTATGTCCAAATCTAGGAGGACCTAGAGAAAAGGAGGACTT

Homo sapiens        TATTTTCT---ATCTTTGCCAAGATCAGGAAATAATTTG----TGAACTCTCAGTAA-----ACATTCTGGGGGCCTTCCCTCTAACACTG-----CTTTGG------TGGAATAATATC
Loxodonta africana  CATTTGCTCTTATCCTTGCCAA-ACTAGGATCGAATTTGAGTGTGGACTCTCTGTTGGAATGAAATCCTAG----TTTCTATCTATCTGTGGATATCATTGAGACACCTCCAAAAACATA

Homo sapiens        TCCAGTTTTCACC----AATCTTG--GTTCAATATTTACCTGGGGGTTTGCAGTGATCA-------TGGGGACAGGGCTCCAACATGAAGTGAAGGTTCATGAGAAGGAAAATGAGGGGA
Loxodonta africana  CCCTGAAATTACCTTTAGATCCTGAAGTTCAACCTTTACTTGGGGGTTCGCAGTGAGCAACTAGCTTGGGGGCAGGGCTCCAGAAGGAAG---AGGAATGGAGGAATGGAATTG--GGGA

Homo sapiens        AGGCTGTGTGTGTGTGTGTGTGTGTGTGTGTGTGTGTGCTGGCTGAGGGATGAAGGTGGGAGGAATACCAGGATTCTGAAGCAATGAGAGTGGGATTGGGAGAGAGAGCTCATGT-TGAAGA
Loxodonta africana  GGGCCGT---TGTCTATATGTGTGTGTGTATGTGTACTGGGTGAGGAGTGG----GGGGTGGGTGCCCAGATAATGAAGCAATGAGAGTGGGATTGGGATGGAGTGGGAGGGTACCATGA

Homo sapiens        AGAAATAACTCACCAGTTCACATGGGCTCCTTTTCAAGGGTTTTTCTTTATATAAACAATTTGCCTGGTTCCAAT--TAGAAGCCCACATTAGGCAGTTAGCTCCAATTTGGCAGTTCAT
Loxodonta africana  AGAAATAACTTCCCGGTTCAT-TTGGTTCATTTTCAAGGGCTTCTCTTTGCTGAAACAACTTGCCTGGTTCTAGTCCTAGAAGCCCAGATTGGGCAGTTAGTCCCTA-----AGGTTCAT

Homo sapiens        TCTTAATTTGGCTTTCCTTATGGGTGGGAGGGAGGGCTGACAGTTAAAAGGTTGATGGGAAAAACCTTGGTATCCTGGTGTGAAGACAGA-----------------------------
Loxodonta africana  ----AATTTG-CTTGCTTTATATGTGGGGGTGAGGTT----AATTAAGAGGGTGAAGGGAAGGATTTTGGGATCCTGGTATGAAGAAAGGGTTTGATTTCTCAGACCAATGTTGGCATGT

Homo sapiens        CCCTGGGATCAGAAGGTTGCCCTGATGACTTTGCCAATGTGTTCATTGCCCCTGTCCTGCTGGGGCGCATACAGCAGGCCCTTCTTTCTCATACGCCCTTGACAGACCAGGACTCACTGC
Loxodonta africana  CCCTTGGATCAAGAATTTGCCCTGATAACTGTGCTATTAC-TTCATTGAGCTC--CCCACCGAG----------------------------------TCCATAGACCAGGACTAACTCT

Homo sapiens        TAGAAAACCCCTGGGG-AAGTCATTGCTGAAGACTCTAGTGTGGGGCACATTAGGTTTGAGGAGCAGGGGAAACATTCAGGCAGCTGGCAAGGTCTGGGCTACCAGGAGAGATTTGGGAG
Loxodonta africana  AAGGAAATCTCTGGGGGAAGAGATTGTTGAAGACTTTAATGTGGGACACATTGGGTTTGAGGAGCATGGGGACCACTTAGGCAGCTGGGGAGGTCTGGG-TGGAAAGAGAGACATGGGAG

Homo sapiens        TTAGAAGCATATTAGGTGAGAGTTGACACTGTCCAAAGGGAGTATATAGAAAGAAAAGCAGAGGAAAGGGCTCATGGAGTCATTACAGCCTGTGAGGTGGGAACAGGGTTTGGGAGTACT
Loxodonta africana  CCAGGAGCATAT-AGGGGAGAGTTGAAACTGCTTAAAGGAAGTATAGAGAAAGAAAAGCAGAGGAAAGGGCTTATGGGGTTATTGTAGTCTGAGAAGTGGAAACAGGGCTTGGGAGTAGT

Homo sapiens        AAGGGTTTGGGGAGGGTTTATGTGTTTATTCACCCATTCATTTATTCAACATTTCTTGAGCTTTATGGCAGATGCTGAGTTGAAGAGGAAGCTGGGGATTTAAGAGGGAGAAGAGTTAAG
Loxodonta africana  AAGGGTTTGTGGAGGGTCCGT-TGTTCATCCATCCAACTATTCATTCATCCTTTATTGAGCTTTAGGCCAGATACAGAGTTGAAGAGGAAGTTAAGGATTTAAGAAGGAGAAGGGTTAAG

Homo sapiens        AGGGGATCCCTGGGTGGATCCAGATGAAGGAGAAGGGAGGGGCTAAGTGCCTAGGCTCGCTCTTGCCAGGAGAGCACATCTGTTAACACAAAGCTGCATCTGTATCTCAGCCAGCTCTCT
Loxodonta africana  AGGGGATCCCTGGGTGGATCCAGATGAAGGAGAAGGGAGGGGCCGAGTGCCTGGGCTCCCTCTTGCCAGGAGAACACATCTGTTAGCACAAAGCTGCATCTGTATCCCAGCCAGCTCTCC

Homo sapiens        TCCATTTCATCTCCTCCTCTCTACCCACACTTGCAAGACGGGGAGCCACATGGCGGCTTCCTACTCCCTTGTACCACTACACCCCCTGCCGGGTTCTTTCTAGGCATTTAGCTGTGGCAT
Loxodonta africana  TTCCCTTCATCCCCTCCTCTCTACCCACCCCCACAAGACAGGGAGCCACATGGCGGCTTCCTACTCCCCTGTTCC------CCCCCCCCAATGTTCTTTCTAGGCACTTAGCTGTGGCGC

Homo sapiens        CAGCAGCACCCCTTCTGAGAGATCAGGCAATCCCCAAGGGTGAGTGGTAGTGGTAGTGGTGGGGAGGGTTGATAGCTTCGAGAGAAGAGCTGGAGAAGGCG----CTGTCTGGCTGTAAT
Loxodonta africana  CGGCACCACCCCTTCTGAGAGCTCAGGCAATCCCCAGGGGCGGGTGAT-------GAGGGGGGCGGGGTTGACGG-TCTGAGAGAAAAGCCGGAGTAGGAGCTGTCTGTCTGTCTGTAAT

Homo sapiens        TAGCCCCAGCTGCTGTTGTGAGGCAGCGTTGGGGGCCGTCAGGAGTGTACATGCATGTGTATGAACCTGTGAGCGTGGCACCTCATTGCTATCACTCTATTTTTCACTGTATTGTGTGCT
Loxodonta africana  TAGCTTCAGCTGCTGTTGCCA-GCAGCGTTGGGGGCCATCAGGAGTGTGAATGCATGTATATGAACCTGAGTGCGTGGCGTCTCATCACTATCACTCTATTTTTCTCCGTATTGTGCGCT

Homo sapiens        GGCTGCAGCCTCGCTGCTCTGAAACCCCTCCTGTCCCTGAGGATCCCGAAAGACAGAGTGTTCCACAGGCAGCACCAGCTTGAATCACTAATGCTATTAAGCAGGAGGACAACAAATATA
Loxodonta africana  GGCTGATGCCTCGCTGCTGTGCAACCTCTCCTGTCCCTGAGGATCCCAGAGGACAGAGTGTTCCACAAGCAGCACCAGCTTGAATCACTAATGCTGCTAAGCAGAAAGACAACAAATATC

Homo sapiens        GGCCCCCATATGTCCTGGATATGCAAGTCCCTGCTCTCTCTTTCCCCACCCCTGCTTCCTGAATGTTTCCAAGTGAGTGAAGTCCCAGCACTTGAAATTAGCAACACCTC--ACTTCTGC
Loxodonta africana  GGCCCCCATACGCCTTGAATAC-CAAGCCCCTGTTC-CCCCCTCCACTCCCCCACTTCCTGAATATTTCCTAGTGAGTGAAGCCCCAGTCCTTGAAATTAGCAACACCTCATATCTCTGC

Homo sapiens        ACAGCCTTTTGGCAAAGAGCTTTCACAGCTGCCATGTCATGCATCCTCAGACGCTTCTAGGAGA-AGCATTTTATAGGTGAGGACCCTGTGGTTCAGGGAGGTGGAGAGATTTGCACAGG
Loxodonta africana  ACAGACTTTTGGCAAAGAGCTTTCAGAGATGTCATGCCAGGCATCCTCAGACAGCTCTGGGGGGCAGCATTCTACAGGTGAGGACCCTGAGGGTCAGAGAAGCAGAGAGATTTGCTCAGG

Homo sapiens        CTAACCCAGACAGTCAGTGGCAGACTTGTGTCCAGGAAGCAAGTCCTCGACCTCCTGGCCTGGTGGCCCATCCCCAGCAGAGCACTGCCAAGAGGGCTGCTCCCGGGTGGCTCCAGCTGC
Loxodonta africana  C---------------------------ATCCAGGATGCAGGCCCTTGGTCTCCTGAGGTTGTGCCCTGTCCCTAGCAGAGCACTGCCTCAATGAGCGCTC-----------------

Homo sapiens        TGCTGTCTGTGAGCTCGTGAGAACCTGTCAGCTGTGGCTCCAAGACCTGCCTCAGTGATTGCTGAAAACGCTGAGAGTGCTGAAAACCAGGA-CCCTCAAGCCGAGCCTGACGTCACATA
Loxodonta africana  -----TCTCTGTGCTTGTGAATACCTATGAGCCCTGACTCCAAGACTTGCCTCAGCAATCACTGAGAAAGCTGATACTGCTAATAACTAGGACCCCTGAAGTCAAGCCCAGCCTAAAAGA

Homo sapiens        GTTGATTGAGTACCTGCTATGTGCCCACCACTGTGCTGGTGCTTATGGGAGACA-AAACAGGGGTAGTGCATAGCCCGTATCCTCAAAGAAGTGATAACTTGAATGGGGAAACAACTCTT
Loxodonta africana  GCTTCTTTA------------------------GCCAGTGCTTATGGGGACACAAAAAGGAGTGGAATA-AGCCCCTGTCCTCAAAGAAATGATAACTTGAGTGGGAAGACAAACCCT

Homo sapiens        AGATACCAAGTCCAAAGGCAATTCCAAGCACTTCTCTCTCTTCCACTAAGCAGTCTGTTGTCAGTGACAATGACACAGGAACCCCTTCTGAATTCA--TCTGTCTTATCGCAGGCCTG--
Loxodonta africana  AGACAACAAGTCCAAAGGCAATTCCAAGAGCATCTCTTTCTTC-----------------CAGAGACAATGACTCAGAGACCACTTCTGCATTCATTTCTGTCCTATCACAGCCCTGTG

Homo sapiens        -GGGTAGGAGAGGATGGGAGGATGACTAGGAAGGGTAATGAGGAAGGGCAGGGAGATGGGGGAAGGAAGGGAAAAACTGGAGCTCCTAATAAACACCAGGCACATTGTGAAGTTTATATG
Loxodonta africana  TGGGCAGGGGTAGGTAAAGGGA-----------AGTAATAGTGAAGAG--GGAAGATGAAAGGAAGAAAGGAAGAACTAGAGTTCCTACTACATGCCAGGCACAGTGTGGAGTATATGTC

Homo sapiens        TTATACATAGGTCATTTGTAT-TATG-CTTATCATACATGTACATGAATATATACACCCAAACACATACACAAACACATACACAATCACTTTCCAGGGATTGACTCATCTATTTGGCTCA
Loxodonta africana  ATATATATATTTATATATATATATATTTTAATATATATG.................................ATGTACTTCATTACTTTCCATGGATTAACCCATCTA---------

Homo sapiens        CTCTCAATCATGAAAAGAGCTTAGGACAG----TGACACTTTTAGAAAGCCCTG-----------------AATGAGTGTTAACTAGCCTTTCTTCTTATCATGATCATCTTCATTGTCA
Loxodonta africana  -CCTCATCCATGTAAAGAGCTTAGAATGGTGCTTGGCACTTTTTGGAAGCCCTGCCTGGCACTTAGAATCTAATAAATGTTAACTTTTTTTTCTTCGTATC---ATCGTCATCATTATTA

Homo sapiens        TTATCGGC----ATATTATATAAATTATCTCATTTATTTCTTCTAACTACAG-----------GACT--------TAGCAACTATTATTTTTATTTCCAGTTTACAAATGAGGAAACCAG
Loxodonta africana  TTATCTGCATGAATATTATATACATTATCTCAATTAATCCTTGTAACTCCAGAAAAAAAAAAAAAACTCCAGAAGGTGGCATTTATTATTTTCATCCCCAGTTTATAAATGAGGAAACCAA

Homo sapiens        TATCTTATTTTATCCAAGCTTGTGGGTACTGAGAATATAAAGAGGAATTCAATATGGTTCTAGGCAAACTTTGGGGTCTTCCCTATCTAGTGGGAGGGACCGAGGCACACATAAGCCCTC
Loxodonta africana  TATCTTGTTGTATCTAAGCCTGAGGGTACTGAGAATACAAAGGAGAACCCAATATGGTCCCAGGCCACTTTTGGGGATCTCCTCTTCTAGTGGGAGGGACCAAGGCACCCATAAATACTC

Homo sapiens        ATGGTCCAATAGGTGCTGTGGTAGAGGA------------------------------ACGTATCTGGGGAAGTTGAGGAAGTCTCCACGAATGAAGG------AGGCTTGAGCAGCAT
Loxodonta africana  A-GGTCCAATGGGTGCTGTAACAGAGGACTGTACCAGTGCAACTGAGGACTGGATGACCACGTATCTGGGGAAGTAG-GGAAGGCTTTGCAGGTGAAAGACATACATATTTGAGCTGAAT

Homo sapiens        CTTGAGGGATGGCAGGTTATGTCAGGAGGAACAGTGGA-----GGCAGGGGAAGACATTCCAGGCAAAGTGAGCAGTATTTGTGTGACAGGGCCTAGTGCATGTTTCAAAGGGCAGGA--
Loxodonta africana  CTAATGGG--TGCAGGGCTTGCAAGGAGGAGCAGTGGAGGAGGGGCAGGGTAGGGCATTCCAGTCAGGCGGAAGTGCA-TTGCGTGACAGTGCACGGTGCTGGCTCTTCAGGGAAGGATG

Homo sapiens        CAATTGTAGCTGGGTGCACCACTGGCAGCAGCAGGAGGGGAGTGGACGAGTATTTGGGGTCAGC-TATTCAGAGCCCTATTGGCCAAGCTGAAGGTT-----TTGTTCTGTGAGCAAGAG
Loxodonta africana  CATCTTTGGTGGGCTGTGTGATTGGCGGCACCAGAAGGGAAGTGGAAAAGGTGGTCAGGTCAGCTTATTCAGGGCTTTGCTGGCCAAGCTGAGAGTTTACACTTGTTCTGTGAGCAAAAG

Homo sapiens        GGAGCCACTGGAGCTTTTT---ATTAAGGCAGATTCGGGACAGACT------------AGGAAGACAGTCCTT-----ACCAGGGTGTTGAAAGATTGGAGGGAAGAGAGGCCAGCTGCA
Loxodonta africana  GGAGTCACCCAAGGTTTTTAAAATTAAAACAGAGGAGTGACAGACCAGGTTTAGGTTGAGGAAGACAATTCCTACAGCACCAGGATAAGGAAGGGCTGGAGGGGAAAGAGGCTAAAAGCA

Homo sapiens        TGCGGACCAATTGGGAGACCATCTCCAGGGTCTGGGCAAGAAGTGGAACAGGCTCTGAACCACGACCGTGGCAGTGAGAGCCGAGAGGAGGGGATGACTCAAGGCACATGTCAACAGGTA
Loxodonta africana  GGGAGATGAGTTAGAAGGCCATCTCAAGTGTCCAGGCAAGTAGTG----------------------------CTAAGTGCAGGGAG--GGGGGTGACTCCAGGTACATGTCAATAGGTA

Homo sapiens        GAAACCTCAGGGCTGGCTGTCAT----GGGGTCAAGGAGAGGGAAAAGGTGACAAGGACCCTGAGGTTCCAGCTTGGGTCCGTGGTGATACTGTCAACTGAGGCA----------GGGAA
Loxodonta africana  GAAGCCATAGGGCTGTGTGTCATGGGGGGGGTCAAAGAGAAGGAAAAGGTGACGATGACTCTGAGGCTTTGGCTTGGGTAAGTGATGATACTGGTAACCAAGACATTGAGGAGAGGGGGC

Homo sapiens        CACAAGACTGTTAGTAGAGAGGGAGAGTGAGGGGAGACAGATGACAAGGTCTGTTTGGACTTGCTGGGTTTGGAGTGCT----GGAT-----AGCGGACATGTCCAGCAAGAAACTGGGA
Loxodonta africana  CGTAAGACTATTAGTAGGGAGGGGGAATGAGTGCAGACAGGTGACAAGTTCAGCCAGGACATGTTGGGTTTGGGGTGCCAGTGGGACATCCAAGTGGACATGTCCAGCAGGAAGCTGGAA

Homo sapiens        ATCTTGGCTTTGAGCCCAGCTGAGACATGGGGGCTGGAAAGCCACCACAGTC-AAGGGATATTAGTTGACCTCAGGGGACTGGATGGGGTTTCTCCTCTCCCATTTTACTCATCCAGGGC
Loxodonta africana  ATCTGGGCTTAGAGGCCAGATGAGAGATGGGGTCTGGGAAGCCATGAGGGCCCAAGTGGTATTAGCTGTCCCCATGGAACTGGGTGAGGTTTTTCTT-TCCCGTTGTACTCCTCCAGGGC

Homo sapiens        AAGGGCTAGGGACAGAGCCAGGTGAATACCAGTATTTAATGAGGGAGGTGGTGGGGGACACCAGCCCGGAGT----GAGAAGACACTCTTCTT-TGGACCTTCCCAGAAACATCATCCCT
```

Loxodonta africana AACGGCTGGGGACAGGGCCAAGCGAGCCCCAGTACTTAA-GAGGTGAGTGGTGGGGGACACCTGTTCAGGGGTGGGGGAAAATGCCCTTCTTCTGGCGCCTCTCAAAAACCTCACCCCC

Homo sapiens TTCCCCTCATGATCATGGTCCTTGTTCCAGGGCTGCACTCAGGCCAGTGACAGAAGGAAGGGAGGGACAGAGGGAGAAAGGGAGGAAAGAAGGGAGGGAAGGAGGGAGGGGGGAGGAAAG
Loxodonta africana TTCCCCTTAGGATCA...

Homo sapiens AAGGGAAGGAAGGAGGGAGGGGGGAGGAGGAAGGAGGGAGGGAGGGGGAAGAAGGAGGGAGTGAGGGAGGAAGGAAAGGGAGGAAGGAGGGAAGGAGGGAGGGAGGAGCTCTGTGCACAG
Loxodonta africana ....GGGGTGTGCTCTGAGTGTGG

Homo sapiens AACTCATGCCTGCACAGGGGCCATGAAGCAAGGACAGGACAGTCACTTCAGCTCTGGAGTTCTTTAGTGTGGGAGTATCTTCTAACAAAACCTAATAAATTAACAGCTGTGGGTTTGGGG
Loxodonta africana TACCCAGGCCCACACAGGGGCCAT-AAGGAAGGAAAGGACAAGGACTTCAGCTTTTGCGTTCTTAAGGG-AGGAATGTGTTCTAATGAAAGTCACTAAGTTAACCACTGGGGATTTGGGA

Homo sapiens CAGCAAGGACAAAGACCACAACTCTACAGTGACCCCTGGAGCACTCAGTGCCACTGACTGCGTCTCCACTGCTGCAGAGCTGGGAGCACATTCATGACAGCATTTTTCAAGCTCCTGTCT
Loxodonta africana CAAAAAGGACAAATACCAGAGCT--------------GGCTCATTCAGTGCAAGTAACTGAGTTTTCAGTGTAACAGAGCTGTGATTATATTCACGATGGGACTTTGAAAGATACTGT--

Homo sapiens GTGCAAACCACAGCTGGTCTCCTAGGAGTCCTAACACTTTAATTAGACCTAGCAGGTCCCAGAACAAAATCCCAT-AATCCCCAAATCTATAGCAACAAGTTAGGCATGAGAGTTTTAGT
Loxodonta africana GTTCAAAACACAGCCAGTCTTCTAGGTGTCCTAAAACTTTCATTAGACCTAGCAGTTCCCACATCCAAATCCTATAAATACATACATCTACAGCAATAAGTT..................

Homo sapiens TACATTCTGGATGTAAGAGAACCCTAACTGCCACTTTCTTTCTGAGTCTCCAAATGACCTTTGACCAATCTCTTTACCAGAAATATTTATCTAGAATGGCCTTGGTCTCTGACACCTTGA
Loxodonta africana ....

Homo sapiens GGACTTCTACTGATTTCTCTGTTAAATCCCTGGAACCACACCAATAATAGTTCTGAACATTTTACACTCATCATCTCATTTATTCCTCACAAATATTATAAACCCTGTTTATATATATGT
Loxodonta africana ....

Homo sapiens GTAATATTTATAGTAACAAGAGTAGCAGATATTTTGCTGAGGGGAGGCATGGTTCTACACAGGTTACATGAAGTAATTCATAATTCCACATGAACAGCACTATGAGGTATTATTACTGTC
Loxodonta africana ....

Homo sapiens TCCATTTCACAGGCAAAGAGCAAATCACAGAAAGAATGAGTAAGTTGTTCAAGGTCACACAGCCTATAAGAGGCAAAGCCTGCCGGTGTGGCTCCACTGTGCTATTAACCAGTAAGGTCT
Loxodonta africana ....

Homo sapiens TCTATAGCCCCAACTAGCTCAAAATGGTTCTAAATGAAGAATCGCTGTGCGTTTTGCACCTCAGACTGCTAGCATCGCTGATT--------TAGGTCTGGAGTTTAGATAAGTGAAACTA
Loxodonta africana ..................TCATCATGGCTCTAGATGAATGCTCTCTGTCCATTTTGCACCTTAGACTAGTAGTATCACTGATTCCCTGATTTAGATCTGCAGTCCAGATATTGAAATCTG

Homo sapiens CTCAGGGTCTTTGTGAAGTATCTTTAGAGCTGCTGCTTGGACAAGACTGGGTCAGGACTTCGAAGGCATTTCCATGTGCTCCTGGAGAGTCAGGATGTAGGCTGGGATTCATTATGCGAC
Loxodonta africana CTTAGGGTCTTTGTGAAGCAGTTTAGGAACTCCTACCTGGACAAGAGAGGGTCAGGATTTAGAAGG----------TGCACCTGATGAGTTGGGATGTAGAGTGAGGCTCATTATAGGAA

Homo sapiens CCAG--CTGTGTGACTTTCCTCCCCACCCCTTACCCCCATTCACCAAGATAGAATGCACTGTTTCTTAAGGTGTGGCTTAAGGA---------------------GAGCTCTTTAAACAT
Loxodonta africana TCAGAGCTGTGAAATTTTCCTTCCTACTCCTTGCCCTCATTCCGCAAGATGGGGTGCACTCTTTCTTAAGGTGTGGTCCAAGGACCTCTGTTTCAGTCACCTTGAGAGCTTTTTAAGAAT

Homo sapiens GCAGATTCTAGGGCTGAAGCACAAACCCA----CTCTGTATCTTTATAGAGATAACTGCTGGAATATGCATTTTAGTAAACTCTC----------------AGGCTGAATTACTTTGAGA
Loxodonta africana ACAGATTCCAGGGCTAAACCCCAAACCCAGAGACTCTCTATCTCTATGGGGATCATCCCTGAAAAATGGATTTTAACAAACTCCCTGAGTGATTCCAACCCAGATTAAATTAGTTCAAGA

Homo sapiens ATCATCAGAGTCTA--CTTTGACAGGATATAGGCTTGTCTTCTGATTCTAGAAGTACCATAAGCCTAACTAGGGATGCTTCCCACTCCTCTTTCTTGCTGAGCAGAGAAGCAAGGGGGAC
Loxodonta africana ACCACTCTCATTTAGTCATTGAGAGGATGCATGCAGGTCTTCTGATTCTAGAGGTACCATGAACATGCCTAGGGGTGCTTCT---TCCACTTCCACCCCCAGCAGAGAATCGGGGAGGGC

Homo sapiens TGGAATGCAGAGAACA--TGCTGAGGGCATGCCTGCACAGAAAGCTGAGCTCCTTGTC----ACCACATACCTTGAAAGA--GTGGACTCTGTATCCCCAGGAGAGGATGGGGAGG-GAC
Loxodonta africana TAGAATGAGGAGAGCACGTGCTGAGGGCATGCCTGCACAGAAAGCTCAGCTACCTTTCTGCAGCCACACCCCCAGAGAGACAGTGAACTCTGTATCCCTGAGAGAGGACTGGGAGGAGGA

Homo sapiens AGAGAGAGAAAGCTATGGAAAGCCTCTGGAAGCCTTAGTACCCTGGGGCCCAGACAAGGGAGTCCCCTGAGGGAGGTAG--AGAGTGGGGCTGTTGGAGGAG----GAGAGCAGCTATGG
Loxodonta africana AGGTAGGGGAAGCAAGGGAAAGCCTCTGGAAGCTGCAGAATGCTGGGGCCCAGAAGAGGGAGTCCCCGAAAGGTGAGAGTGAAAGGGGGGC-GCTGGAAGAGAGCAGCAGGCAGCAGTGG

Homo sapiens GCCAGCAGCCTGAGGAGCTGAGAGTCACAGTGGGAGTGACTGTGGCTTCAGAGTGATGCCATAGCTGCAGTGAGCTGTACAGGAACCAGAGACCCAAAGTTTTCTGTAGGGCTTCTTTTG
Loxodonta africana GCCAGCAGCCACAGGGTCAGAGAGCTACAGTGGGAGAGGTTGTGGCTGAAGAGTGCTGCCACAGCTGCAGGGAGCTTTCCAGGAACCAGAGACTCAAAGTTTTAGGTGGGATTAATTTCT

Homo sapiens CTTTGTTAGTGCTTCAGGAGTTGGATCCTTCTGGACCCTGATCTCCCAGAA--------------------GTGATTCCATTTCGCCTCTAGGAGGTGGGTAGCAAAGCTCAGTCTGTGG
Loxodonta africana TTTTGTTTGTGCTTTAGATGCTGCACCCTTCTGAACCCCACCCTCCCAGAAAAACAAAAACAAAAAACATTGCCATCGAATTTCACTGCTAGGGAATGGGGAGCAAAGCTCACTTTGTGG

Homo sapiens CTGGGGAAAGCCAAGGAAGTGATTTCACGAATCTTTAGCAGTGAATCCACCTATTTAGTTAGCAGGGGATGGAG-------------------CAGATCAGAACTAGCGAGAGGAAAGGC
Loxodonta africana TTGGGGAAAGCCAAGCGAAGAATTTCATGAATCTTTAGCAGTGAATCCATCTATTTAGTTAGCTGGGGGTGGGGGTGGTGTGGGGAATGGAGTCAATGCGAAACTGGTGAGAG-AAAGGC

Homo sapiens TTAGGAATGCTCTC---TAGTGGGACTGAGAAGAAAAGGTGA-GAGGGTGACATTAACTGGAGGAGATGGTGAGCCCCTGACCCCTGCAGTTGTGAGACTTGCACTGAGTGGGATGATCT
Loxodonta africana TTGGGAACACTCTCATCTAGTAAGACTGGGAGGAGGAGGTGAAGAGAGCCACTTTTAATGAGCGAGATGGGGAGTCCCCAAACCCTG--GTTGTTAGATCCGTACTGAGCAGGGCCATCT

Homo sapiens GCTGCTATAACCTATCCTCAGGCAATGAAGCATA-AGCAGAGAGCCAGGAGTGTTAATCATTGCTGATTTTTATTAAGCATTTCCTTTGTACTGGACACTGTACTGGGCTGT-ACATGCA
Loxodonta africana GCTGCTATAACCCATTCTCAGATGATGAGGCATACAGAAGAGAGCTGGGTATCTTAGTCATTGC---------------------------------ACTGTGCCGGGTTGTTACATACA

Homo sapiens TCATCACATTCAGTCTTCACAACATTATATGATAGCTCCTATTACT---GTGCCCATTTTACAGATGAG------------GTTTAGATAGGTTATACCACTTGCCCAAGGCCACACAAC
Loxodonta africana TCATCTCATTTAATCTTAACAACAT--------AGATCCTACTATTCATGTGTCCATTTTACAGATGAGACTCAGACACATGTCTATATAGATCAAATGACTTGCCCAAAGCCACACAAC

Homo sapiens TAGTAAGTAGCAGAGCCAGAATTTGCACCCAAGTGGTTTTAATCC-AAACTGTGTCCACTAGGCCTTAGGAAGGAGCCCACATAGAGCTTTGGGGATGCTGCATTCTTGACCTGTGGCTG
Loxodonta africana TAGTAAGTGTCAGAGCCAGGATTTGAACTCAGGTGGCCTTACTCCCAAAACCTGCCCATTAGGCCCTAAGAAGGAGCCCACATAGAGACGTGGGGGTGCCTCACCCTTGGCCGGTGCCTC

Homo sapiens TAGGTGGGCAACTCCTCTTCATACCTTATAGCTCTTGGTAAATGAAGTGAATCACTGTATCTACCATGAATCTCCTTGGGAATGAGTTCAATGCGGGTATCAGAGGAAGCAAAGGGTGGG
Loxodonta africana TGCATGGGCAACCACCCTTCAAATCTTACAGCTCTTGGTGAACGAAGTGAGTCACTGTATCTATCGTGCTCCTCATCGGGTATGAGTTCAGTGCTGGCCTCAGAGGAAGGAAAGGGTTGG

Homo sapiens AAGGGGGTCTCAAAAGTC-TAAATCTTAGTGTGGTC-CCCTCCTTTGACCTACTCTCTCTGGGCTGAAGAAATGCCAGGATTGAGGGGGCAGCAGATTGTTAGATCTGTT----------
Loxodonta africana AGGAGGGTCTC--AAGTCTTAAGTTCTTCTGTATTCTCCTTTCTTTGACCTA--CTCCCTAAACTGGTGAAATGTGAGGATTGA-GGCGCGGCAGAGTAGAAGATCTCTTTGATTCCTTG

Homo sapiens ------TGTTCCATCT---------ACTGAGGCCTCATAACATTTTTGGAGCCCTTGTTCAGTGACTCAGGTAATTCAGCCTGAGGGTA-CCTTTGCAGGGAGTTCCCCTATCTCTTATC
Loxodonta africana GCCCAGTGGTCTACCTCTTTTCCTAACTGAGGCCTCTTAACATTTTTGG----------------CTCAGGTGACTCAGCCCTAGGGTAGCCTTAATAGAAAATTCTCTTATCTCTTGTC

Homo sapiens CTGTGTTAGTACATGAGATTATTCCTCCTTCTTGTACCCCAGTTACATTAAGTGGG------TCTTATTCCCCAAATCCGTAGTCTCTGCCCCAATAAGTTGCCT-GTACCCCATTTTAG
Loxodonta africana CCACTCTAGTAGGTGGGATTCTTCCTCCTCCCTGTGCCTCAGTCTCATTAAGTGGGTCTTTTTCTTATTCCCCAAATCCTGAATCTTGGCCTCAATAAGTTGCCTCTTAGTCCATTTTAG

Homo sapiens CAAGAATAGAATCTTCCCTTTCTGCCAACAGAGCTTCTTGGCAC-TTTGGCCTTG-CCTGGTTCCTGCTT-GCAGACTCCTGTTCTGAGGCTCATGTTTGTACCTAGAATGATTTCATGT
Loxodonta africana CAAGAACGGAAACTTACC-TTCTGCCAACAAAGTTTCTTGGTACTTTTGGCCTTGAACTGGGTCTTGCTTCCCAGACTCCTGCTCCAAGGCTCATCTTTGCACCTACAATGAGCTCATGT

Homo sapiens TCCTCCTCTCTCTTTGTCCCTCTATCAGAACCTGCTTTCACGATGG-------------GAGATTTAAGAGGCTGATCCTCCCAAGAGGTTGGCATTTTAACTGATGCCAAAGCCCTGAG
Loxodonta africana TCTTTCTTTCTGTTCTCCCCGCTATCAGAATCTGCTGTCAGGATGGGATAAGTCCAGATGAGGCTTAATATGTTGACCCTCCCATGAAGATTGCATTTTAACTGACGCCAAAGCCCTGAG

Homo sapiens TCAAAAGAATTATATACTTAAAAATTTTCAAGCCCTTGGGAGCGGAT--GGAGAGATTGGAAAGGCCAATGGAACATTCTTAATGCTCCTGGAATGCCACAAGGTGGACATAAAATTCAG
Loxodonta africana TCAAAGGAATTATGTACTTAAGAATTTTCAAGCCCTTGGGAGTGGATAGGAAAAGATTTGGAAGGCCAAGGGAACCTTCTTAATGCTCTTGGAATGCCACAAGGCCGGCATAAAATTCAG

Homo sapiens GCTTCTGCCAAGACAGGACTTGGATTATTTTTGTTATAATCAGAATCCGTAATCTG-------CCCCCACCCCAGCACTCAGGGCTTTGATCAAA-AGTCTTCCAGACAATGAACCTCTA
Loxodonta africana GCTACTGCCAAGCCAGAACCTGGATTATTTTGGTTATACTCAGAATTCATAACATAAGGCAACCCCCAACCCCAACACTCAAGGCTCTGATAAAACAGTCTTCCAGATAATTAACATCTA

Homo sapiens TGATCCAAGTATCATTTTCTCAGAGCTCCTTAGAAGGAGGAGAGCTAAGAAACTTCATGAATTACCAAGGACCAAGCAAACAGGCGGTAGTGAAGTCAGAAACAAATTCCTCCCGAGACT
Loxodonta africana TGACCCGAACATCGTTTTCTCAAAGCTTCTCAGAAGGAGAAGGGCCGAGAAACTTCGTGAGTTACCAAAGA-TATACAAACAAGCAATAGTCAAGGTAAAACTAAATTCTTCCCAAGACC

Homo sapiens AGTTTTGTCCTATAGCCTGAAAACAAATCCGGTCTCCACCAGACATAAGGTGGAATTC-CTGTCTTGATTTTTTTTTTAGCACTGATATTAACATTTTTCTTCTCAAATCCTCTTCAGAA
Loxodonta africana AGTTTCTTTCTATGGCCTGAGAACTAATCCAGCCTTCCTCAGACACAAGGTGGATTTTACTTTCTTGA---TTTCTCCAGCACTGACATTAACACTGTTCTTCTCGAACCCTCCTCAGGC

Homo sapiens CCAGCCCTTTT---AGAACACAAATTAGAAGCTGTTTATTTTGTCAATCAGAGAAACTGAAGGCAGCCAGCCTTGGCCAAAGGAACATCTTATCCTTTGCCTTTGCTAAGGACAATGTTC
Loxodonta africana TCAGTACTCTTAGCAGAATACAAATATGAAGCTACTTATTTCTCCAATCAGAAAAACTGAAGGTAGCTAGCCTTGGTCTAAGGAATATCTTATACCTTTTCTTTCTTAAGGGCAATGATC

Homo sapiens CTTCTATTGTGCTCTGTGGAAATAAAAGGGCAAAATATGCTTGCCTTCTTTTATTCTTATTCATGGCTGAG--GGTCA-TATGAAAGTTCAGAGAAGAGCACAGTTTTT--------AGT
Loxodonta africana CTTCCATGATACTACATAGAAATACAAGGGCAAGACATTCTTGCCTTGTCCCGTTCTTCATCATGGCTGAGAAAGTCATTAGGAAAGTGC--AGAAGGATACCTTTTTTTTTTCTTCAGT

Homo sapiens ATGGGTCGTGGCAGGTTTTCCAGATGGCTTCCTAGCCAAGATATTTCTCCCGCTCACCTCCAAACAAGTTATGTACCCCTAAGAATGATTTGGTCTCAGTGTTAGGGGAAGAGGGTAGGT
Loxodonta africana ATGGATTGTGGCAGATTTTCCAAATAGCTTCCTATCAAAGACATTCCCATC-CTTGCCTCCAAACAAACTCTGTA-CCCTAAGAATGAATTGGTCTTGATGTTGGAGGAAGAGGTTTGGT

Homo sapiens AGCAGGGCCTGGCTTTGTCATCCTGGGCTATTTTGAGGGGACTTCAGCAGTCCCCGACAGAAGGAC---AAGAGGGGCTGATTCTCCCTTTGTGAGCAGCCATTGGAGCTGCTTCAGGGA
Loxodonta africana AGCAGGGCTTGGCTCTGTTATCTTGGGCTATTCTGAGGGAACTTCAGCAGCCCCCAAGAGAAGGAAGAGAAGAAGGGCTGAATGTCCCCTTCGGAGCAGCTGTT-------------GGA

Homo sapiens CTGACAAGCCGGCCAGAGACAATGTCC--TCTCTCAGGAAAGCAGTAAAAACATCAGAAATGCTCCTTTCCTGGGGAGCCCTTAGTTATTTTGGCCTCTCTTTCCATTAAAATAGGAAAG
Loxodonta africana CTGACAAGCTGACCAGAGACCATGTCCAGTCTTTCAGCAAGGCAGTAAAAACATCAGAAATGTTCCTTTCCTGGGGAGCTTGTATCTATTTTGGCCTCTCTTTCCATTAAAAGAGGGAGG

Homo sapiens CATTGAAAGGGACCA-TTCGTGGGATAGCGTGGAGCCTCCCCATTGTGGCAGCATTTCTAACTGCGTTGAGAATTAACCATATTGCTCTTCCTTTTTACCATGGAGGAGTTTCTAGATTT
Loxodonta africana CATTGAAAAGGGCCAGCTCACCGCATAGCATGGAGCCTCCCTATTGTAGCAGCGTTTCTAACTGCATTGCGAATCAACAAAA---------------GCCATGGAGGAGTTTCTAGATTC

Homo sapiens TTATTTATACCCTACCGCTTAAAACATCTTTCACTGTTTCAGCAAATTGCAGCATAAAAGATGGAGAAGCCAGCAGGGGCTGTGCTCACAATAATAGCTGTG-GTTGCATTTAAGACATT
Loxodonta africana TCATTAATACCCTCCTGCTTACAATATT--------TTCCAGCAAATTGCTGCATAAAAGATGGAGAAGCCAGCA-GGGCTGTGCCCACAATAGTAGCTGTGAGTTGCATTTAAGAAATT

Homo sapiens CAAATGCCAAGGGTGCACTTCTCCCATTAAGTCCAACATAAGAAATCAGGTGTGCACAACATTGATGAACAGAAGACCCCAAAGGACAGGTGGAGTTTCTGGTATGCTGTGGTGGGGGCG

```
Loxodonta africana  CAAATGCCAAGGGTGCACTTTCC.................................................................................................

Homo sapiens        GTGGGGTGGTGAGCTTGGGAAAGAACGAACAGGCATTTTCATTTTCCAAACTAAGAAGCAGTTTCGGGCGAAGGGGAGCCGTGGGGATGTCAGAAATTCTTCTAACGCCATGGACCTGCC
Loxodonta africana  ........................................................................................................................

Homo sapiens        CACACCTGAGTCAGATAAAGATGACCTTCCCTAGGCCCTGCTTTCCTCTCCCCCTCCCCAAAAGACAGCATGAGCATGAGTAAGCCGGGTACTTACAGGTGCAGAAGAATTTGGAACAGG
Loxodonta africana  ........................................................................................................................

Homo sapiens        AAAATGGAGCTTACAGGCTCCCGAATGCCTGCCGAGTCAGTTGCATCAGATTAGGAAGTGATGATCCTAAGAATAAGATGTTTGGAAAATCTTGTCCCCCGAAAGCCCCCTGCTTTGTCT
Loxodonta africana  ....................CTGAATGCCTGCCGAGTCAGTTGCATCAGATTAGGAAGTGATGATTGTAAGAACAAGATGTTTGAAAAAGCTTG-----------CCCCATCGTTTCTCT

Homo sapiens        TCTCAGCCCACATCCAAACTCAGACCACCCCC-AGGATGTCTCTCATCCTGCCAGACTGCCTGGTGCCTCCGTGCCCCTTACCGTGAGGGTGCTGGCAGCCACACTGCTTCTTTGAGCCT
Loxodonta africana  TCTCAGCCCGCATCCAAACTCAGACCACCTCCTAGGACATCTTCCATCCTGCCAGGCTGCTTGGTGCCTCCATGCCCCTTCCCATGAGGGTGCTGGCAGCAGCATTGC--CTTTGAGCCT

Homo sapiens        GTGGCCCGGCAAGGTAGTCAGGCTCATGGGATTAGGTTGGCAGGAGCGACTGTCCTCTAAACGATTAGCCCAGAGAAAGAGAGAGAGAGAGAGCAAGCAAACCCGAAAACGGGTCCAGGG
Loxodonta africana  GTGGCCCAGCAAGGTAGCCAGGCTCCTGGGATTAGGTTGGCAAGAGTGCCTGTCCTCCAGATGATCAGCCCGGAGAAAGAGAGAGAG------CACGCAAACCCAAAAACCGGTCCAGGA

Homo sapiens        TAGAGAGCTCCAGGTCCTTAGCATGTTCCTGGCGTATTCCAAAAGTGGCATCCACAGCAGAGAAAGTGTCCCAAAATAGCTCAGGCTGCCAATGCCTTGCTGAAACCTGAGAAAGAATTG
Loxodonta africana  TGGAGAGCTCCAGGTCCTTAGCATGTTCCTGGCGTATTCCGAAAGTGGCATCCACAGCAGAGAAAGTGTCCCAAAATAGCTCAGGCTGCCAATGCCTCGCTGAAACCTGAGAAAGAATTG

Homo sapiens        CTGAATTTAAAGATCTGGGTCGCTGTGGGTGTGTGATCAAAGTGCAGATTTGTTGGTAGGAGGAA------CTCAAGCCATGAGCTTAGGAGTGACATGAGGTCACTTGGGTCACGGCCT
Loxodonta africana  CTGAATTCAAAGATCTGGGTCGCTGTGGGTGTGTGATCAAAGCACAGAATTGTTGGTAGGAGGAAACTAGACTCAAACCAAGAGCTTAGGAGTGACGTGAGGTCACTTGGGTCACAACCT

Homo sapiens        GCCTTCTTGTTTCCAACTTGCTGAAGTTGGTGGCAGTGTCAGAGAGCTCCTCTGTGGTGAGGCAGGGGTTTGGAAAGAGGTCCTAAGGATGCTGTTTTCATGCCCAGGTCTCTGATGCGC
Loxodonta africana  GCCTTCTCCTTTTCAACTTGCTTAACTTGCTGGTGCTGTCAGAAAGCTCCCCTGTGCTGAGGCAGGGCCCTCAAAGAGGTTGTGGGGGATGCTGCTTTCATCCCTAGGTCTCTGATGCCA

Homo sapiens        TAACAATATGAAATAGCAGTGCCCTGTTTTCTGCATTTCAAACAAAGGAACTGATACTATGCAAAGGTTTCTTTAGGTTATTGCTCCATGTCTGATGAAATTCCCTTTTCAGTAGATTTT
Loxodonta africana  TCACAATATGAAATAGCAGTGCTCGGTTTTCTGCATTTCAAACAAGGGAACTGATACCATGCAAAGGCTTATTTATGATATTGCTTCATAACTAATGAAATTCTCTTTCCAGTAGATTTT

Homo sapiens        TCATTTCTTTTGGCCTTTGGCATAAGTAAATGTCAGCTTCTTAAAAATCAACAGAGAAGAGCAAACAGTAGGGTCTCTGTGATTTTCTTGGGATTTGAGAAATCTTCCCTCCCTAGCTTC
Loxodonta africana  TCATTTCTTTTGGCCTTTGGCATCAGCAAATGTCAGCTTCTTAAAA-TCAACAGTGAAGAGCAAACAGTAGGGTCTCTCTAATTTTCTTGGCATTTGAGAAATCTTCCCACCCTGGCTTT

Homo sapiens        TAACTGGATCATGTCTGTACATTGCTGCCTGTGGTCCTGGAAAGGACGAGGTCCACCCAGACATGCTGTCTCATTTTTATATTTGCTGAGATCTTGGGACACTCTCCTTTCATCAGGTAT
Loxodonta africana  TAACTGGATAGTGTCTGCACGTTGCTACCTATGGTA-TGGGAAGGACTAGGTCTACCCAGACAAGCTGTCTCATTTTTATATTTGCTGAGATCTTCAGATGCTCTCTTTTCATCAAGTAT

Homo sapiens        GTCAAAAACCTTAGGATTGTCTGCAGAGAGTGTGGTTCAGTATGTGGAAAGGTCAGCATATGTCATTCTTGGACTGGCCCTGGGGTTTAAGAAGAAAAAGCCCAGAATTTGGTTTCCAAT
Loxodonta africana  GTTGAAAATTTTAGGATTGTCTGCAGAGAGTGGGGGTTAGTATGTGAACAGGTCAGCATAAATCTTCCTTGAGCTGGTCTTGGGATTTAAGAAGAGAAGGCACAGAATTTGTCTTCTAGT

Homo sapiens        TCCAGCTCTGCCTGTCTTTTGCTGTATGAATTTGGGCAAGTCACTTCTCTTCTCTGGACCTCAGTTTCCTCTTCTGTAAACTGAAGACAATAATCTTCATTCTAATTCTAACCCAAATGT
Loxodonta africana  TCCAGCTCTGCATCTCCTTTGCTGTGTAAATTTGGGCAAGTC-----CCTTTTCTGGACCTCAGTTTCCTCCTCTGTCAAATGGAGACAATAATCCCCATCCTAATTCTAACCCAA-TGC

Homo sapiens        TGTAAAGAGTAACTCAGAGAGTTGAAAGAATGCTATAAAGGGCAGGGTTTGGCACTCTCAGTAAGTGGTGACATTTTTCAGTTCTTCAAAGGTCCCTGATTGGTAGCCACTA----CCTG
Loxodonta africana  TGTAAAGATTAATGCAAAGAGCCAAAAGACTGGTATTATGGAAAGGGCTGGACACTCTCAGTAAACAGTGACATTCTTCAG---------GGGCCCTGACTGGTAGTGCCTAGAGTTCTG

Homo sapiens        CATCCACATGCCTATGGCGAGCCCAAGACATCCCACAGTACTAGGCACTATGAGCTTCATGTCTAGGAGTCTAAATAGTTTCCACTTTCA-GTTGTGGGAATTTTGGCAAGTAGCTCAAT
Loxodonta africana  CATCAGCATGCCTAGAGCAAGCCTGATACAGACTACAATACTAGGCCCTATAGACTTCATGCCTAAGGGTTGATATTATTTCTGCTTTTAAGCTGTGGGACTTTTGACAAGTTGCTTAAT

Homo sapiens        GCTTAGTAGCTCAGTTTTCCCATCTGTCAAGTGGGTATACAAACTAATAATAATACAGGTCCACAATCCCTTATCTGAAACTGTTGGAGCTAGACATGTTTTACAGTTCAAAATATTTGG
Loxodonta africana  GCTCAGTACCTCAGTTTTCCCATCTGTCAAGTGGGTATGCAAAC--ATAATAATACAGATCCACAATCCCTTAT---------TGGGAGTTAGACATGTTTCGGAGTTCAGAATTTTTGA

Homo sapiens        ATTTTAGAAAAGCAATGTGGTAGATATAACATATATATCACACCTTAATGAGATCCAAGATAATACCCAGTGATCAAACTCACTAATATTTCTAAATGGGATAAATGAATAGCCTCATGT
Loxodonta africana  ATTTTAGAAAGGTAATATGGTGCATAT.............................................................................................

Homo sapiens        CAATTCAGGCCCAGTTTTGCCACCAAATGAGCTTGCCACAGAATTTCAGTTTTCAGAAGCTTTGGGATATCAGAGAGGCACATAGGAAATTGTGGACAATGAAGGAAGTTAATACTTATT
Loxodonta africana  .....................ACCAAACGAGCTTGCCACAAAATGTTGGTTTCCATAATCTTTTGCATGTCAGAATGGCAGATATGAAATTATAGACAAAGAAAAAAACAAACACTTATT

Homo sapiens        TCGTGCATGTAATGTACCAGGCATTGTTCTAAGTA-------TACATTAACTCATTTAAGTGGCCCAATAAGCCTGTGAGGTAAGCAGTATTATTATTATCATCCCCATTTCATAGATGA
Loxodonta africana  TAGTGCTTGCCATATGCAAAACATTGTTCTACGTAGTTTATATACATTAGCTCATTTAGTCAGCCCAACAGCCCTATGAAGTAGGCA---CTGTTATTATCATCCTCATTTTACAGATGA

Homo sapiens        GAAAACTGAGATTCAGAA--GTTAAATCACTTGCCCAGGGTCCCAAAGCCAGTTAGGAGCAATGCTGGGATTCCAACCCACACAGTTCAGCTC--------------------TTATCCA
Loxodonta africana  GGAAATTGAGGTTCAGAGAGGTTAAATCTCTTGCCCAGGGTCCCACAGCCAGTAAAGGGCAGACCTGGTATTCCAATCCAGACAGTCCAGCTCCAGAATCTGTGCTCTTAATCTTAACTA

Homo sapiens        CCTTCTCTGCCTAACCCATGGGGTTAAATTGAGGCTTTAAATGCCTACAGGGACTGAGCAGGTCGCTG--AAAAAGTAAAGGTGGCCTTATGGGTACTGAGGCAAACTGGTGAGCACGTG
Loxodonta africana  CCTTCTCTACCTATCCCACAAAGTTAAGGTGAGGCCCGAAGTGCTTACATGGGGCAAGCAGGTCACTGTGAAAAAGTGAGGGTGGCCGGGTAGAGACTGTGGCAAACTGGTGAGCACATA

Homo sapiens        CCTCACCTACAGGGAGCAGGTTGTTGAGCTCTGGCTGATTGTTGCTATATTGGAATTTGGATCGAAGTCTTTCTCTCTCTCTCTCTCTCTCTCTCTGTGTGTGTGTGTGTGTGTGTGT
Loxodonta africana  ACCTTTCTAAAGGGAGCAGATTACTCAACTCCAGCTGACTGTTGCCATGTTAGAACGAGGATCCAGGCCTTTCTCTTTTCCCC.....................................

Homo sapiens        GTGTGTGTGTGTGTGTGTGTTTCAGAAAAGCCAGAAATCCAGCTTTCTATCTGAAATTTCCTGACTTTTAAATGTTGGCAATTAATTTTAAAAAATGTTTAAAACGTTGTACGGGTCAAATA
Loxodonta africana  .....................AGAGAAGCCAGAAATCTGGCTTTTTATGTGAAATCTCTTGACTTTTAAATGTTGACAACTAATTTTAAAAAATGTTAAAAATATTGCATGGGTCAAATA

Homo sapiens        AACTCTGTCTGCAGTCTGAATTTAGCCTGTGGCTGCCAGTTTGCAAGCTCTGCAATGAGTAAATAGATGTGAAAGTGCTTTGGAAACTTAAAAACGT--GCTCTAGAAACAGAAGAGTCT
Loxodonta africana  AAATACATCTGCAGTCTGGATCCAGACTGTGGCTGCCAGTTTGCAAGGTCTGCATTGAGGAAATAGATGTGAAAGTGGTTTGGAAACTTAAAAATATATGTTATAGAAACAGAAG-TTCT

Homo sapiens        AATTTTAGGGTTA-----TATCTTGTTTTTTCCTTGACTGTGGTCTAGTTTTTTAAAAACTTGTATTCCCGACCTGATGGAGATGAAAAGTCAGAATTAAATAGAGACTTCAGTGAACTT
Loxodonta africana  CATTTTAGGGTTAAGTTTTTTTTTTTTTTTCCTTGACTGTGGTCTCGTTTTTTGAAAAC--GTGTTCCTGGCCTGATGGAGATGAAAAGTCGGAATTAAATAGAGACTCTAGTGAACTT

Homo sapiens        GAGTACTTCATTCCTTTGGCAGGCTGGATAAATGAGAAGCCTAGGTGTACTGGATGGACTAAGCCTCAGAATTCTTGCCACACCCACAAGCCCCTTCTAAGGAGTTTCCATCCACAGACC
Loxodonta africana  GAATGCTTCATTCCTCTGGCAGGCTGGATAAATGAGAAGCCTAGGTGTACTGAATGGGCTAAGCCTCGAAATTCCTGCCACACCCACGGGCCCCTTCTGAGGAGTTTCCATCCACAGACC

Homo sapiens        CTAGCTGAGGGAAGTAGCAGTTTCTTATGAATAGGTGACTATTCTTGGTACCACGTGACCCTGCTTCCCCC-AGCAATAGCCACTTGAGCCAGGAGAGGGCATCTGATCCATGAGCAGTC
Loxodonta africana  CTAGCTGAAGGAAGTAGCAATTTCTTATGAATAGGTAGCTATGTTTGGTACCATGTGACTCTGCTTCCCCCTGGCAGTAGCTGATTGAGCCAGAAGAGGGCATCTGATCCGAGACCAGTC

Homo sapiens        ACTCTATAGGCTGGCAAACAGCCTATGAAGGAACCTGGGATGAGAACTTTGCCCAGCAAGTACAGTTA---CTGAATTTGGACCAATGAGAGTTTCTCTCTTGGGGAGTTTGAATACCAG
Loxodonta africana  ACTCTATAGGCTGGCAAGCGGCCTATAAGGGAATCTGGTGCGAAAACTTTGCCCAACAAGCACAGTGATGACTAAATTTGGACCAATGAGAGTCTCTCTCTTGGGGAGTTTGAATACCAG

Homo sapiens        ACCTAGAAAGAGTGAGCAATTGGCAACAGGGGCAGAAGCTGAACACAGAGAAGGCAGGCAGCAGGGACCCCGGATAAGCAGGACCAGGAGAGATTACTGAACTTCAACTATGTGCCAGAT
Loxodonta africana  ACCTAGAAAGAGTGAGCAGTTGGCAACAGGGCCAGAAGCTGAATGCAGAGAAGGCAGGCAGCAAGGCCCCTGAATAAGCAGGACCAGGAGAGATTACTGAGCATCAACTATGTGCCAGAC

Homo sapiens        TCTTTACAC--GTTGTTTTGTTTAAAACATTCAAAGAACATGCGAGGTAGGTACTGTTATTATGCCTGTTTTACAGATGAGGAAACTGAGGTGAACTAACTTGAAGTATTGAGGTTCAAG
Loxodonta africana  ACTTTACACATGTTGTTTTGTTTAAAACATTCAAAGAGCCTACGAGGTAGGTACTGTTGTTATCCCTGTTTTACAGATGAGGAAACTGAAGTGAAGGAATTTGCAGTGCTGGGGTTTGAG

Homo sapiens        CCCAGGCCTGTTTGAAGTTAAAATGAGAGTGTAGGAACCCCAAAGCCAGTGGTTCTGAAACTCTAGTGT--TTCAGAATCACTTGGGTTA--AATGCAGGTTCCTTGGCGTCACCCCAGA
Loxodonta africana  CCCAGGCCTGTTTGATATTAAAATCAGGGTGTATGACCCCCTCAACTAGTGGTTCTTTAACTCTAGTGTATCTCAGAATCACTTGGGTTACAAATGCAGATTCCTTGGCCCCACCCCAGA

Homo sapiens        CCT------------------------------ATATTGAATCTGACTCTTTG-----------------------------------TGGGTAGAGTCTCCCTCCC----------CCT
Loxodonta africana  CCTGACTCTTTGAAAGTTTTGTTGTTGTTAGTTATAGTCAATGTGACTTTTTGGAGCAGATCGCCAGGCCTGTGTTCTGAGGTGCCTCTGGATAGGGTCTCCGTTTTGAAAGTAGAGCCC

Homo sapiens        ACAAATCTGTGTTTTAGCAGTTTTCCCAGGGAGCACTTGATGCAGG--GTTTCCCCA-CCACACTTTTATTTTGAGAA--ATGCAGTTTCAGAGGGTATGGCAAGTACTGTGTACTGTGC
Loxodonta africana  CCAAATCTGCATTTTAACAGTCCTCCCAGGGGGTGGCTGAAGCAGGAAGTTCCCCCAACCACAC----GTGGTGAAACGCACGCAGTTCTAGAGGGTATTGCAAGTGTTGTACATTGTAT

Homo sapiens        TACCTTTACCTACACAACCAAGGTCAATGTCAACAGATGTGGTCAAATTCCAATTTAAAGTCTTTTGGAAATGGGGCCAATTATGTAAAGAAGAGGGCATTAGTTTGTTCTCTTCTCTCA
Loxodonta africana  TGCCTTCGTCGTCTGAAACGAGGTCTATGCTAACAGATGTGGTCAAACTCCAATTTAAATTCTTTTGGAAATGGGGTCAGTTCTGCAAATGAAAGGACATCAGTTTGTCTTCTCTTCTCC

Homo sapiens        TCAAGGAAGTCTACATTATTACCAGTTATTTGAGAGAAC---AGAAGATTGGGATGAATGCTTAGTAGTT-GTAAACACGATCGGATCTGCACTTCAGGGTG---GGTGCAAAGCTAGCA
Loxodonta africana  TCAAGGAAAGAT-CACTGGCACCAGTTATCTGAGGAAGGCTGATGGGATTCAGATGCATGCTCAGCAGTTTGTATAGAGGACCTTATCTTGTCTTCAGAGTGCAGAGCTCATAGCTAGCA

Homo sapiens        TATGTTCACTAAGCATTTTCCATGAGGTGCGTAAACTGTAGAAACTCAGTGCTCTGGCAACAAAGAACATG---AGAGAAAATTACCAAATGCAGATAGACATATTGTAAACCGTGGAGC
Loxodonta africana  TACGTTCACTAAGCATTTTCCAAGAAAAGCACAAATTGCATGAACTTAGTG-TATTGCAACAAAGAATATGGGGAGAGAAAATTACCAAATGCAGAAAGACATATTGTAAACCATGGGAT

Homo sapiens        TTAAAATGTAAACCTACTTGCTAGGCTGATCATAGAGGTTCTTCTCAGCCCCAGAGAATTTGTGTAAGAATCCTGTCCATGCCTTTATTTACACCATTGATTAAAAATGCTAATCAGGAG
Loxodonta africana  TTAAAATGTAAACTAACT---------GATCATAGCGGTTCTTCTCAACTTTAGAGTAATTGTGTAAGAATCCCAGCCATGCCCTTATTTACGCCCTTGATTCAAA-TGTTACACAGGAC

Homo sapiens        AGGATCAAGGCTA--------TGG-------------ACCTCCTTCTAGGTTGTTGCAGAACTCCCACTCTGGCAATGGTATTCAG----------------------------------
Loxodonta africana  AGGATCAAGGCTAGAACTGCGTGGCATGCCATTAGAAACATGCTTCTAGATTGTTGCAGAAC-CCTACTCTGGCAAATGTGTTCAGTTCATTTGAGTTCGCCTGGTGGTGCAATGGTTAA

Homo sapiens        ---------CTGGT-----------------TTTGAATTTTTCTGCTTTAAGAACTATCCCTGGGAAGAC-------TAGTGGTCTGAAGTTTGCTTGGAGAGTCTCAAGCCGGAAACTG
Loxodonta africana  AGTGCTCGGCTGGTAACTGAAAGGTCGGTGGTTTGAACCCTTCTACTTTATGAAATCTCCCCAGGAAAACAAGTAAGTAATAGTGGGAAGGCTGCTTGAAGAGCCTCAAGTGAGAAGCTG

Homo sapiens        ATTTGTGGTCCAAATGCTTAATGGCATCAGACACTTATTCCTACAAGAGAGGCATGTGGTCCAAGGGGTGGCACAGGTCAAATGTGATGGCCAAGTCCCGCCTCAGCAGGGCTTGAATCC
Loxodonta africana  GTTTGTGGTCCAAATGCATAATGGCATAGGGAACCTATTCCTACAAGAGGGCCATATGGTCCAAGGGGTGGTACAGGTCAAATGAGTTGGCCAAGTACTGCCTTGGCAGTGCTTGGGTCC

Homo sapiens        GCACTTGGCAACTTTTATTTCTCTCTGTCCATTCCTGTGGATACTT--TATCTGTGTTAATGTCCTCATTTAGGCTAAGGTCAAACTCTGTTCTTCTCTCTTCTGGCCAGAAAGACTTCC
```

```
Loxodonta africana  ACACTTGGCAACTTTTGTTTCTCTCTCTT-GTTCCTGTGGACACTTCTTATTTATGTTAATGCTCTCACTTAGGCTAAGATCAGACTCTGTTCTTCCCGATTCTGGCCAGAAAGACTTCC

Homo sapiens        TGTTTTACAGGTATATGATCTTATCTGTGTTGATACCTTAACTGTGAGTGATTCCCTAAAGCAGGTCCTTGGACAAGTGCCCATCCTCCAGGAAAATTGCAGCAGTCCACCGTGCAATGA
Loxodonta africana  TGTCTTACAGGTATCTGATCTTATCTGTGTTGATATTTTAACTCTGAGTGATTCCCTAAAGCAGGTCCTTGGATAAGTGCCCATCCTCCAGGAAAATTGCAGTGGTCTGCAGGGCAAGGA

Homo sapiens        GAAAGCCTGAGGACTAGGTAGTAAATGTTTCGCAGAGAAGAGAAATCTATTTCAT-----GTATTATTCTTTATTCTGAGGTTATTTCCTTCTCTTTTGGTGTTGAAAGGAAGCCCCTTC
Loxodonta africana  GAAAGCTCGAGGACCATGTAGTGAATTTTTTGTAAAACTGAAAAAGTTATTTAATCAAAAGGATTGTCCTTT-TTCTGAGGTTATTTCCTTCTCTTTTGGCATTTAAAGAAAGCCCTCTC

Homo sapiens        TTTTT-AGAAAATGATGATTTGTGGT-----TGTTCTGTTTCTTAATGATTTTACTTGGGAAAATAAAAATCTGGAAGTTCAATATCAGTCCCT--GAAATTTTGGGTTGCATTAGCTGC
Loxodonta africana  TTTTTGATGACATGATGATCAGGGATGGTTTTGTTTTGTTTCTTTATGTCTTTCCTTGGAAAAATAAAAATTTGGCCACCCA---TCAGTCCCCCAGTGTTTTTGGTTTGCAATATCTGC

Homo sapiens        AAGTCTGGGAACCTGGTCTAATTT---------TGAG--TGTTCTGCTAGTACCTCTAGGTACCTCCTATTGCCACTTGTGTTCTAAGTGCTTATAAATTACCTGCTTCGCAATCTGCAG
Loxodonta africana  AAGTCTGGGAACCTGGTCTAATTTGTGGAGATCTGGGCATGCTTTGCCAGTACCTCTAGGTACCTCCTATCACCACTTATTTCCTAAGTGCTTATAAATGACCTGCTTCATA-TCTGCGG

Homo sapiens        CAGAATCTCTCCGAGGATTGACATCAAGCTTACAGCTTTGGAGTTTACGGTATACTGGTCTGTAGTTTCCTTATTTAAGAAAATTGGGACACTGCCCATTTGCAGTCCTCTGGTATCCCT
Loxodonta africana  CAGCATCTCTGTGAGGATTGACATCAAGCTTACAGCTCTGGAGTTTACAGTATACTAGTCTGTAGTTTCCTTATTTCAGAAAATTGGGACACTGCCCATTTGCAGTCCTTCGATATCCCT

Homo sapiens        CCTGTTCTCTCTGATTCCACAGATCCTTCAGACCGGCACCTAATGCAAGGCAGTTCCGTTTGCTCTTCTTATAATGTTCTTTTCCCTATAGAGTTGGACATGCATATACATGTTGTTTGG
Loxodonta africana  CCTGTTCTCTCTGATTCCACAAATACTTCAGGACGGCACCTAATGCAAGGCAGTTCCGTTTGCTCTT---ATAATGTTCTTTTCCCTATAGAGTTGGACGTGCATATACATGTTGTTGGG

Homo sapiens        GGAATTTGCAGCACATCCAATGAAAGCATAATTCCCAACTAACAGTCAAATCCTTTTCTTTGGAGCTACCCAGAACACACCTACCCTCTCTTCCATATGACAGCCCTTCAGAGACTTGAA
Loxodonta africana  GGAATTTGCAGCACATCCAATGAAAGTGTAATTCCCAACTAACAGTCAGATCCTTCCCTTTGGAGCTACCCAGAACACACCTACCCTCTCTTCCATATGACAGCCCTTCAGAGACTTGAA

Homo sapiens        GACAGTGTGTGACTCTCCTGTGAGTTAAGAGCACTGGCTGAGTCAGACAGACCCGAGTGACAAGCCCAGCTTCGCTCCCTCTCAGCGGTGAGAGCCAAGGTAAGTTACTTCATTTTAATT
Loxodonta africana  GACAGTGTGTGACTCTCCCGTGAGTTAAGAGCACCGGCTGAGTCAGGCAGACCCGAGTGACAAGCCCTGCTTCACTCGCTCTCAGCT-TAAGACTCAAGGTAAGTTACTTAATTTTCATT

Homo sapiens        TCTCTCTAGTTCAGTTCTTTAGCTGATTTAGAAAATGGGAGTAAAAAAG------CCACTTTGTTTGTTTATCGTTCAATAAATAAGATGAGGTATGTAAAGTGTTTAACATAGTGCCTG
Loxodonta africana  TCTCTCTGTTTCAGTTCTTCAGCTGATTTAGAAAATGGGGGTAAAAAAAAAAAAACCACTTCATTAGTTGATCGTGCAGTAAATAATATGATGTATGTAAAGAGCTTAACACAGTGCCTG

Homo sapiens        GAATAGTAAGCACTCAGTAAATGCTAGCTATTTCC-CCCTCTTTATTTATAAAGAGCCGG-TGCGCATTTTA-AGTGTCAGGGCTGT-GGGGGAGGAAGGAAGTGGGGAGGGCTTCTACT
Loxodonta africana  GCATAGTAAGTACTCAATGAACGCTAGCTATTTTCTCCCTTTTTATTTATAAAGAGCCGGGTGGGCATTTTTAGTGTAAAGGCTGTTGGGGGAGTGGAGGGTTGAGGAGGGTTTCTTCC

Homo sapiens        TCCCTGAATGATCAAGGAAGGAAGAAGATCCTAAAGTAGACCAGGTATTTCTTTAGCCTTAGGTTGTCTGTTTTCAGTTATCTGGATGCCTATTT------GCTAGGACTTGGGGAAAGA
Loxodonta africana  TCCTGGAATGGTCAGGAGAGGCAGAGGACCCTAA-TTAGACCAGATATTTCTTTATCCTTAGGTTGTCTCTTTTCAGTTATCTGGGTGCCCATTTCAGTTATCTGGGGCCAGGAGAGAGC

Homo sapiens        ATATTGTTGAGAATCTAGGGAAAGGCTTATTTTCAACAGCTGGAAAGCTATTTGTTGACATATTGAACCGTCTCTTGTTTATTCTTATTTCCCTGAGCAGCTAGCCATGCAATCCCCTAA
Loxodonta africana  ATATTGTTGAGAATCTAGGGAAAGGTTTATTTTCAAGGGCTGGAAAGCTATTTGTTGACATATTGAACCGTCTCTTGTTTATGCTTATTTCCCCGAGCAGCTAGCCATGCAATCCCCCAA

Homo sapiens        ACTCAATTACCTTTTGTGAGGCGGCAGGAGCATGACACCTCCTAGCCGTATTGATCAGTGCAGACTGGGTGGGACGTGAGGGACCTGAGAGATGCTAAGCGGTAAATTCTGGGCTTTTCC
Loxodonta africana  ACTCAATTACTTTCTGTGAGGCGGCAGGAGCATGACAACTCCTAGCCTTATTGATCTGTGCAAGTTGGGTGGGATGTGAGGGACCTTAGAGATGCTAAGCAGCAATTTCTGGGCTTTTCT

Homo sapiens        TTCCTTAGAAGGCCTCTCTTCTCTGTGTTCCTAGAGACTCTGGCTTTAATAGAACGGAAGTCTAT-GCCTCACATGGGCTGAGCATGCAAGAACCCTCTCTCAGTAGCCTCCTTAGAAGC
Loxodonta africana  TTCCCTAAAAGGCCTCTCCTCTCTGTGTTCATAGAGACTCTGGCTTTAATAAAACGGAAGCCGGTGGCCCCGAATGGGCTGAGCATGCAAGAA-CCTCTCTCAGTAGCCTCCTTAGAAGT

Homo sapiens        AGGCTGATGA-AAATAGTCACAATGGGTCAGAACTTGGAACCCTGGGGTGACCAGTGCTAGCATGGTTTTCTAACTGCAGCTCTTTGCACACACTGGGTTTTGCAAGCAATTGAATGATA
Loxodonta africana  AGGCTGATGATGAATGGTAATGCCGGACTGGAACTTGGAACCCAGGAGTGCCCAGTACCAGAATGGTTTTCTTGTTGTAGCTGTTCGCACACACTGGGTTTTGCAAGCAATTGGGTGATA

Homo sapiens        TGAACTCTGAATGCCATTGCAGCTAGAGAAAGTTTGGAAGGAGACACACCCAACTGTGAAC----AGACAGAGGAATGGGACTT----GCGGGGAATGGGGGGTGGG------------
Loxodonta africana  TGAACTCTAAATGCCGTTGCAGCTAGAAAAAGTTTGGAAGGAGACACACCCAACTGCTAACACAGAGAGAGAGAAATGGGACTTGGAGGCAGGGGTGGGGTGGTGGGGGAGGCCGAGGGT

Homo sapiens        ----GCATTTTCAGGAGTTTAGCTTTGTCTGTACGGAGGATTTCAAGTCTTTATCCTAAGAATATATTCCTA-GATTACTTATGGAATTACTAAACATAAATAAAAGTTAGAGAAAG-AA
Loxodonta africana  TGTTGTAGTCTCAGGACATTATTTTTATTTATAT----TATTTGAAGTTTTTATGCTGACGATATATTCCTAGGATTACTTGTGCAATTATTCAACATAAATGGTAGTTAGAAAAAACAA

Homo sapiens        GAAATTATTTTTAAAAACAAACCAAAAATAAAACAC--------------------------------AAAATA-AAGTGGTACAACCATTAAGCTTGTCGGGGTAGAAAGATTGCTTGG
Loxodonta africana  GAAAAAAATTAAAAATACAATATAAAAGCAAGCCATTGGAAACATATTTTAGAGGTTGCTGCCAATTAAAGATACAAGTGGCGCAACCATTAAGCTTGTTGGGGGAGAAAGGTTGCTTGG

Homo sapiens        GTCGGGGGGTGGATGTCCGGGATTCCTAGAGAGAGCTTGCAAGGAGAGGAGTGAAAAGGGCTTGAGAAAGAACCCTAGGGAAACATATCTTTAAAAAAAATAAAATT------AACTTTT
Loxodonta africana  GT----GAGGGGATGTTGGAGATCCCT--AGAAAGCATGCAGGAAAAGGAGTGTAGAGGGCTGAAGAGACAATTCTAGGGAAACACACCTTTAAAAAACAAAGAACTTCAAAGAAGTTTC

Homo sapiens        ATTAGTTTTTTTCTTTTC-----------GTATTATGGGTCCATTATAGACCAATGAGAAAATACAGATAAGGAAAATGAAAATTAAAAATCCCCTCTAATC-CCATCATTCAACTGTGG
Loxodonta africana  ATGGTTTTTTTCTTCTTCTTCTTATGAATGTAATATGGGTCCATTATAGAGAACGGAGAAAATTCAGATAAGAAAAACGAAAATAAAAAATCCTCTTTAATCTCCACCATTTGGCTATGG

Homo sapiens        TCACTGTTGGCGTTTTGGTATATATCCTTCCAGA-------TTTTTTTTCTATGCAGAAATCTACATATTTATCTTTATGATAATCAGATCCTCATGTACACATTAGAATA-TATTTTCA
Loxodonta africana  TCATTGTTAACATTTTGGTATATATCCTTTCATACTTTATTTTTTTTTTCTATGCAAAAATATGCACCTTTATTTT----ATAAAC--ATCATTAGGTCCACCCTATAATGTTATTTTCA

Homo sapiens        ATGGCTACAATAGTGGCTGCTACAATTGCACCATGATTCGTTTAATCATTAAACAAGTTGTGTTCTAACATTAGACACTTAGGTTATTTCCAAAATTGTTGCATTAGAAATAAACAACAG
Loxodonta africana  ATGACTACATTATTGGCTACTATGGATTCACCAATATTCAACTGATCGTTAGATGAATTGTATT-TACTATTGGGCATTTAGGTGGTTTCCAATTTTTTATTATC----ATAAACAGCAG

Homo sapiens        TGCCCTGTAGCTGTGTGACATTGGGAAAGCTAGTTAACCTCTCTGT-TCCTCTGTTCTCATCTGGAAAATGAAGGTGCTAATAGAACCTACCTCGTAGTA-TGTTGTGAGTGTCAAATGA
Loxodonta africana  TGCGTCCTAGCTGTGTGATCTTGAGAAGGTTTGTTAACCTCTCTGTGTACTGGTTCCTCATCTGGGACATAAAAGTGATAATAATACCTCCCTCACAGGGTTGTTGTGAGCCTCACACGA

Homo sapiens        GATCATATAGGTAAAGCACTTAGCACAGGGCCTGGCACAGTAAACGTTAGCTTTAATTGTTAGGTGTGTTAATAATAATAATAGTAGTTGTTGTGAGGCCATTGTTAATAGAATGGCT--
Loxodonta africana  GATCATGCATGTAAAGCATT-----------TGACACAGTAAATGCTAACTTCTATCATTAGCTA---TAATAATAATAATAGTTGCTGTTGCTACGCCATTGGTAATAGAGTGGTTGA

Homo sapiens        ATTCTGTGACTTTTTTTTTTTTTTTTTTTTTTTTGCTTTGAAGGGAGAGGAGAGCG--ACTGGAATTTTTCTTTT-GAGTAGTATGCTATAGTGAGCTCTGGAGTCACACAGAGCTGTGT
Loxodonta africana  ATAGAGTGG----------TTGTGACAAAATGTTGTTTAGAGAAGAGGGGCGGGTGATACTGAAATTTTTCTTTTGGAGCAGTATGCTATAGGGAGCTCTGGAGTCAGCCAGAGCTGTGT

Homo sapiens        GCCTTTGGTCATGTCAATTCATCTCATGGAGGCTCAGTTTCCTTACCTGTATACTGAGAATTTAAAGGACCTAGCTCACAGGGTAATTGTCAAGATCAAATGAGATAATGCTGGTGAAGC
Loxodonta africana  G-CTTTGTTTCTGTCTGTTCATCTCACAGAGACTCAGTTTCCCTATCTGGAAACTGAGCATTCAAAAGCCTTAGCTCACAGGGTAGTTGTGAGGCCCAAAGGAGATAATGCTTGTGAAGC

Homo sapiens        CCTCAGCCCCATTCTTGGCATATGGGAAGAGTTCAAGCAATATCAGTTGTTGTTGTT---ATTATTACTACAAGGATTCAGCCAT------TTGTAGTAAGAAAGTTTCCTTGCTTTGTG
Loxodonta africana  TCACA---CCACTCTTGGTGTATGGGAAGAGTATGATCAATGTCAGTCGCTATTATGATAATGATTCCTACAGTGATTCATTCATCAGTAATGGTAATGAGAAAGTTTCCTTGCCTGGTG

Homo sapiens        GTGTGGGGATGTAGGGGA---GATGGCAGTGAGAAGAGTACTCCGA--GTTTCTAATCACTTGTCTGATTACAAAGAGATAAAA-----------ATATTTGGAAACAGAATGGCCAG
Loxodonta africana  TAGTGGGGGTGGTGGGGAAGCGGTGGCAGTGAGTGGGGTGCTTGGAGTGTTTTTAACCACTTGTCTGATTACAAAGAGAGGAAATGTTAAGGACCTGGGTTTTGGAACCAGGCGGATGTG

Homo sapiens        GGCTGAAATCTGGGATTTAAAAAAAATTATTATTATTATTTTATGTGACCGTGGTCACATAGACTTCTCCAAGCCCATTTCCTCATGTATAAAATGGGAATGATCTCAGTGCCTACCTTA
Loxodonta africana  GACTCAAATCTGGGCTTTGCCA---------TTTGCTAGCTC-CGTGACCTTAGTCGCATA-ACTTCTCTAAGCCTATTTCCTCATTTCTAAGGTGGGAATGCTGACAGTGCCTGTCTTA

Homo sapiens        CAGGTGTTGCTGGGAGGTCGGAATTATATAATGCATATGTAGTGCTTAGCCCAAGTGCTCAGTTGTTGTTGGTCGTTAATTATG---------AGGTTTATAACGACCTGAATGCCTCCC
Loxodonta africana  CGGGGATTGATGTGAGGTCTGAATGATATAATGCATATATAGTGCTTAGCCCAAGGGCTTAATTATTGCTGGTTGCTAGTTAAGAGTGTTACAAGGTTTATAATGACCTGAATGTCTTCT

Homo sapiens        TT-----------GTCAAACTCTACATCCCTATACCTAGGCTACTTGAACTTCACCTGGAGGCTTTGCCCTATCCAAGGCTCAGGGACCAATTCTCCCCTTAGACTGAGGGTTTCTCCCC
Loxodonta africana  CTCCCCCAACTGCACCAAACTCTAAGTCCTCATATCT-GACTACTTAAACTTCACCTGAAGACTTTGCCCTCTCTAAGGTTCAGGAATCGATTCTGCCCCTAGTCTGGGGTTTTA-----

Homo sapiens        TTAGACTGAGTGCACCAGTTCAGGAAAACTGGAGTA------CTCTGGTAAACCTTTCTGCTAGCTCTACTCTGACCTTGTGCCCCAGTTCTAGAAAGCAGGCTGTAGCCATGTTTGTAT
Loxodonta africana  -------GGGGATACTGATTCA-GAAAGTTGGAAGAGTTGGCATCAAGTAAACCGTTCTGGTAGTTCTTCTCTGGCCTTGAGCCCCGGTTCTAGAAATCAGGCTTTAGCCCTGTTTCT-T

Homo sapiens        GAACAAGCCCTCAGCTGGTATGCTCAAGGCTGGTGTGCTTTTCTTGCTAGCTGTCTGAAATCCATGGAGCCTTTCTGTTGAATCCTAGCCCTATGGCAGGGATACCGGTCGCTGGATTTT
Loxodonta africana  GAGCAAGCCTTCACCTGGTCTGCTCAAGACTGGTGTGCTTTTCTTGCCAGCCTTCTGAAATCTCTGGAGTCTTGCTGTTGAATTCTAGCCCCGTGGCAGCAACACTGGTTGCCAGATTT-

Homo sapiens        TCTCCGTGTCTGTGACCCTGGGGTAAATTG---------TCTTCGTTATGATCACCATTACAGGGCTCTATTAAGGTGTTTGCTTGCCAGCCCCCACTGTCCA-TCCTCCCAAACCCT-C
Loxodonta africana  --------CCTG-----CAGGGGCAAATTGGCTGTTTTTTTTTTTTAATCATTATCTTTGCAGGGCTCTTTTAAGGTGTTTGCCCCCAAGTACCCACTGCCCATTCCCCCCAAACCCTGG

Homo sapiens        TTTCCCTGCACCTGAACCTCTTTGAACGTTTCTGATGCTGCTCCACTCAATCTCCAGCCTCAGTTTCTTTCCCTCGAGCCCTCTGCC----TGTGCTTCCATGTCATATCAGTTGCAGTG
Loxodonta africana  TCTCTGTGCACCTGAACCTCTTTGAACTTTGCTGCCACTACTCCACTCAACCACCACTCCCAGATCGCTTAGCTCTAACCCCCTGCCTGCATGTGCTT-CATGTCACACCAGTTGCAGCG

Homo sapiens        CCTGGGTCCCAGGACTCCTGTGTCTGTTCAACTGCAGAGGGCACGTATCTTGTTTCCTGAGCCAGGACCCTTTTCATCTCATCTTTTTAATTGTGTCAATCTCCTCTTTTCTTTG-----
Loxodonta africana  CCTAACTCCCAGGACTCTCATGCCTGGCCAACTGCAGAAGGTATG--TCTCATTTCCTAAGCCAGAACCCTGTCAGTCTCATCTAGTTAATTGTGGGACTCTTCTCTTTTCTGTGGGACA

Homo sapiens        ----------GGCAGAGTGGGGT-GGGGCATAGGCTGGTGGGACTGTTGGGGGCGACAGGGAAGAGGGGTGGAGTTGCATAGGCCAAGTGCTAAGGCGGAGCTTCTGGAGGACTGTCAAG
Loxodonta africana  GCCAGAGGCTGGCATAGTGAGGTGGGGGCGTAGGC-AATGGAACTGTCGGGGGTGAGGGGAAAGAGGGGTGGAATTGCCTAGGCCAAGTGCTCAAGTGGAGCTTCTGGAGGGCTGTCAAA

Homo sapiens        TGAAAGGCGGCCAGAGGCAAATGTCAGAGCCTATATAGAGGCAGGAATCTGGGACCAGAGAGACAGAGCAAGCAGTAGCAAGTCAGGAGCAGTGGGGCGGGTGGGCGGGCAGGCTGGCTG
Loxodonta africana  TGAAAGGCAGCCAGAGGCAAATGTCAGAGCCTATATAGAGGCAGGAATCTGGGACCCGAGAGACAGAGCAAGCACTAGCAAGTTGGGAACAGTGGGGCGGGTGGGCGGGCAGGCTAGCTG

Homo sapiens        GC----GGGC---AGGGGGGCGTGGCAGGGGATAGCAAAGGGAGATTGCAGAGAACAGCTGTTGGTACTGGGTCTTACTTTAATGAACTGGCTGGGCACGAGGTATAAGTTAATTGGCCA
Loxodonta africana  GCTGGAGGGCAGGAGGGGGGCGTGGCAGGGGATAGCTGAGGGAGATTGCAGTGAACAGCTGCTGGTTTTGGGTCTTACTTTAATGAACTGGCTGGGCATGGGGCATAAGTAAATTGGCCA

Homo sapiens        GAGTGAAAGAGCTAGGGGCGCAAAGGATACTCTCTCCCTCTTCTCCGTCACTGGCAAGGTAAGATCTTTCTGGGTTTTGCCTGGTGGGAAACTGTGGCAT-GTTCAGATATTTTTCTGAA
Loxodonta africana  GACTCAAAAAGCTAGGGGCGCAAAGAACACTCCCTTCCTCTTCTCCCTGACTAGCAAGGTAAGATCTTCCTGGGTTTTGCCTGGTGGGAAACTATGGCATAGTTCAGAGA-TTTTCTGAA

Homo sapiens        GATGGGAGGTGGAGGTCTATGGCTGCTTTTTTGACTCATCTTTC--CCATTGCACCAATTATTACAATTATTACAATAACCAGGGTGTCAGCAGGAGGCCAGCATTTCTTTTATGACAAC
```

```
Loxodonta africana  GATGGGAGGTGGGCTTCTAGGACTACTCTTTTGACTCATATTTTTGTCATTGCAC---------CAAGCGTTAGAGTAACCAACGAGTCAGAAGGAGGCCAG-----------------

Homo sapiens        CCATCTTGGGGTTGTCCAGGCTGGAGACAGAGGCAGGGGTCTCGGTAGTGTGACTGAG-TAAGGGGGAGAG------------GTGGATCTTTATAGCACCTTGGTGAAGAGTAGGGGGC
Loxodonta africana  -CTTCTTGGGGTTGTCCAAGCTGGAGATAGAGGCTGGGGTCTTGGTAATGTGACTAAGTTAAGTGGGAGAGGGGGCAGACCAAGTGAATCATTATAACACAGAGGTGAAGAGTAGGAGGC

Homo sapiens        TCTGGAACTGGATGGACCCAGGAATCTATGTGACTGCAGGCAAACTATTAAGCTTCTCTGGGTCTGAATTTTCTCATTTGTAAGATAAAGGAAGAGTACAACCGAAATCTCTTAGTGTTG
Loxodonta africana  TCTGAAGTGAGACAGACCTGGGAATCGATGTGGGTGCTGGTAAATTATTAAGCTTTTCTGGGCCTTAGTTTTCTCATTTGTACAAGGAAGAAATAGTACTACTAAAATCTCTTAGTGTTG

Homo sapiens        CTATGGGAACCACATGAGATGATACATGTAAAGTGCCTAGCAGAGTGTTTGCCAGTCAGTGCATGCTAAAGAAATGTTCACTGTTAGCGTTTCTGTTGCAGGTTTTCTCATTA---TTAT
Loxodonta africana  CTGCAGGGACCCAATGAGATGATGCATGAGAAGTGCCTAGCACGGTGCTTGCCAGTCAGTCCAATCTCAAGAAATGTTAGCTGTTGGTATTTCTGTTGCTAGCGTTACCATTATTCTTAT

Homo sapiens        GGAATC-ACAATTCCATGAGGTTGATGACTTTTGTGACATTGTCCCAAAGTAGCAAGTTTTCTGCTTTTGGCATGGGCAGGCTGTGGGGACATGTCAACATTGTGTAATATTTGCATTGG
Loxodonta africana  GGAGGCAAAAATGACATGAGATTGATGGCTTTTGTGAAATTGTCCCAAAGTAGCAAGGCTCCCATTTTTGGCATGGGGAGGCTG----------------CTATAGGATGTGTGGATTGG

Homo sapiens        CTTCCAAGCCGTGTGTCCAAAAGCCCCTTCT-GGGCTGCTTATTGGAGCAGGCTGGAGGGGTGAGGCTCT-GGATTGCCCACCCCCATGGCATCAGAGAAACTTGAAACTTGGACATGCC
Loxodonta africana  TTTCCAAGCTGTGCGCCAAAGAACCGTGTCTGGGGCTGCTTTGGGGAGTGAATCAGAGGGATAAGACTCCAGGATTGCCCACCCCCATGTCACCAGAGAAATTCGAAATTTGTTTGCTTT

Homo sapiens        AGACACTGGGCTTTAAGTGACCCATGATCTGGGATGTCAGAGAAATAATTGAATGCAAGCTCAGGGATGCCAAACACTAGGCAACCCCCAGGAGGGGGTCAGAGTCAATGCTATACAGTT
Loxodonta africana  ATAGACCGGGCTTCAAGTGAACCATGGTCTAGGATGTCATAGAAATAAATGAAAGCAACCTTAGAGATACCAAAGGTTAGGCAACCCTGCAGAGAGAGTGAAAGTTAAAAGCATACAGTT

Homo sapiens        TGTTACTGTTGCAGTGACCCTATTTTAGCCTTGACCCTGGCTAATCTGAGAAAGCTTGTAAAATGGGGATAATAATAT---CTATTGCCCAGGAGATTGTAAGACCCAAATGGGATAATG
Loxodonta africana  TGTTACTGTCATGGTGACCCTATTTTAAGCCCTACCCT-GCTAATCTGAGAAAGCTGGT-AAATGAGGATAATAGTATTACCTATTACGCAGGATGTTGTGAGATGCAAGTGGGATAAAG

Homo sapiens        CTTACAAAGGCCCTCACGTTATAGGAAGTTGTGACAGTTGTCTAAAGTACAGTGCCTGGCACATACTACGTGAAGCAATAAATAAATAGTAGTAATGGGGTGGTGGTTGCTGTTATTAAT
Loxodonta africana  CTTGTAAAGCCC-----------------------------------TACAGTGCCTAGCACATACTAAGTG-----ACCAATAAATGGGAGTAAT--AGTGGTCATTGTTGATAATAAT

Homo sapiens        AAGAATAATGAAAATAA------TAAGCAGAATTTTCAGTTCATGGAATGTTGCTCTCAGTCATTGATTAGATAAATGTCTCCCTTTTCCTGAGAACATTCAGGGCCATCAGAGTAGTAA
Loxodonta africana  AATATTAAAAAAAAAAAAAAACCCAAACAGGAATTTTGGTTCATGGAATGCTGCTCCCAGTCATTGATCGGACAAATACCTTCCTTTTCCTGAGGACATTCAGGACCATCAGAGTAGTAA

Homo sapiens        AATGAGGATTAAACCACTGGCAGAATTCAATGAGCCCCAGGAACTAATAATTATCCTGGGGCAAAGGACTTT---CCCATTCAGGCTTGCCAGTACCTTGGCTGGCTTGTGAGTACTGCC
Loxodonta africana  AATGAGGGTTAAACCACTGTCAGGACTCAGTGAGCCCTAGGTATTAATGATTATCCAGGAGCAAAGAGCTTTCAACCCACAC-----TGTCAGTACC-TGGCTGCCT--TGAGTGATGTG

Homo sapiens        GTGGGATAGAATACAGGCCAGGAATCCGCATTTCTCACATTTGGCCAGAAGGTGGTGCTGCTGCTTTATTTGAGTCAGGACTGCCCA-GGCTCCTGGCTGCGGAGGCTGCTGGCGTTTAT
Loxodonta africana  GTGGGACAGAACATAGCCCAGAAGCCTGCACTTCTTGAAGTTGGCCAGAAGGTGGTGCTGCTGCTTCATTTGAGCCAGTACTAACCAGGGCTCAGGGCTGCTGAGGTTG-GGACATTTAC

Homo sapiens        AGCATTTACAAACTGAGAGGCTGGGTTG---------------GTTTGAAAAGGATAGAGGCAGAAGGGACATTTGCTCCAGCTCCAAAGTTGTTCATTGCTTCACAGCTTCTTTAATCT
Loxodonta africana  AGCATTTATAAACTGAGAGGTTGTGTTGTGACGGGGTGAATTGGTTTGAAAAGGGTAGAGGAAGAAGGGACATTTGCTCTGGCTTCAAATTAGTTCATTACTGCCCAGCCTCTTTAA-CT

Homo sapiens        GATAGCCTCACCTTCAATGCCATATACCTGACAACGCATAAAAACAGGTCTCCACCATGAACAATTTGTCTGTTTCCAAATCTTCTTGATACTGGCTTTTGACCTCTAAACTTGTTTCTT
Loxodonta africana  GATAGGCCCATCTCCAATGCTGCATAGCTGACAACACAGAAAAACAGGTCTCTAGGATGAATCATTTGTCTATTTCCCAATCTTCTTGATAATGCCTTTTGACTGCTAAACTTGTTTCCT

Homo sapiens        GTTTACAGATTTTCTTGCCTCCTGGGCT-----------------------------------CCCTCCACACTATCCTGTGGCTGTGGCAGATATGTCAATACCCTTACATTCACTGCG
Loxodonta africana  GTTTACAGATTTCCTTGCCGCTTGAGCTTCAGCAGGACCCCTGATATCATCACGTTAGGGCGTCCCTCCACGCTGTCCTTTGGCCATGGCAGATATGTCAATGACCTTACATTCACTGCG

Homo sapiens        ATTCAACAATACAATGAGGGAGGAAAATGTTGAGCCCCAAAACAAGCAGATGGCCTTCTGTAGACCAATGACAGAGACCAGAGCAGACGTACAGATTCTGCATTCTCATGTACAGTTGCC
Loxodonta africana  ATTCAACAATATGCTGAGGGAGGAAAATGCCGACCCCCAAAACAGGGGGACGACCTTCTCTAGACCAGTGACAGAAACCAGAACGGAGGTCCAAATTCTGCCTTCTCCTGCAGAGTTGCC

Homo sapiens        TATAGTCTCAACTTCAGCCTCAGACCCTGGAGGGACATCCACACAGTTGATGACATCTCCAGTCTTTGACACCATGTCTGCACCTCTAATGGGAGTACCAAACTCTGGAGCATTGTCCCC
Loxodonta africana  CATAGCCTCAACTTCAGCTTCAGACCCTGGAGGGATGTCCACACAGCTAATGACATCCCCAGCCTTTGACACCATGTCTGCACCTCTAATGGGAGCACCAGACTCTGAAGCACTGTCCCC

Homo sapiens        ACCCCTAATGCCAGCCTCAGACTCTGGGGCACTTTCCCCATTGCTAATGCCAGCCTCAGACTCTGGGGCACTGTCCCCATTGCTAATGCCAGCCTTAGATTCTGGAACATTGTCCCCATT
Loxodonta africana  ACCGCTATTGCCAACCTCGGACTCTGGGACACTGTCCCCATTACTAATGCCATCCTCAGATTCTGGGACACTGTCCCCATTGCTAATGCCAGCCTCAGATTCTGGAGTGCTCTCCCCATT

Homo sapiens        GCTGTCCACTTCAGAGTATGGGGTAATGTCCCCAGGGATGATGACAATTCCTGATTTTGGAACGATGTCCGCAACGCTAATGGTAGCACCAGATTCTGCAGAGATATCACCATTGGCAAT
Loxodonta africana  GCTATCCACTTCAGACTATGGGTTAATGTCCCCAGGGCTGATGACAATTCCTGACTTTGGAACGATGTCCACAGCACTAATGGCAGCACCAGATTCTGGAGAGATATCACCATTGGCGAT

Homo sapiens        GCCAGCTCCATCCTCTGGAGTGGTGTGTACACCTATAATGAGCACTTCATCCTCTGAGGCAATGTCCACACCATTAATGCTAGCCCCAGATTCTGGAGAGTTATCCCCAATTCTAATGCA
Loxodonta africana  GCCAGCTTCATCTTCTGGAGTGCTGTCTACACCTATAATGAGCACCCCATCCTCTGAAGCAATGTCCACACCATTAATGCTACCCTCAGATCCTGGAGAGATATCCCCACTGCTTATGCC

Homo sapiens        AGATATGAATCCTGGAGTGATGTCTACACAGCCAGTGCCAGCTCCCAGCTCTGAGGCAATGTCCCCATTGCAAATTACAGATGAAGACACCGAAGCAATGTCCAAAGTGCTAATGACTGC
Loxodonta africana  AGATATAAACCCTGGAGGGACATCCGCCCAGCAAATGACATCTCCAGGCTCTGAAGCAATGTCCCCTTTGCAAATTACAGACGAAGACACTGAAGCAATGTCCAAAGTGCTAATGACTGC

Homo sapiens        TCTAGCCTCTGGAGAGATATCTTCGCTGCTAATGTCAGGCACGGACTCTGAAGCAATATCCTCACTGATAATGTCAGCTGTAGCTTCTGGAGGTACATCACCCCAGCCAACAAGCACCCA
Loxodonta africana  TCTAGCCTCTGGAGAGATCTCTTCCCTGCTAATGTCAGGCACAGACTCTGAAGCAATATCCTCACTGATAATGTCAGCCCTAGCTTCGGGAACAATGCCAACCCAGCCAATGAGCACCCA

Homo sapiens        AAACTCTGGGGGAATACCTACCCCGCTCATGTCAGATCTAGACTCTGGAATAATGTCATCGCTTTTAATGTCATCTCCAGGCTCTGAAGTAATGTCCACACCGCTACTGTCAGTCCCAGA
Loxodonta africana  AGACTCTGGGGGAATGCCCACCCCAGTAATGTCCGGCCCGGACTCCAGAGTAATGTCTTCACTGCTAACATCAGCTCCAGGCTCCGGAGCAATGTCCACACCACTACTGTCAGTTCCAGA

Homo sapiens        TGCTGGAGAAATGTCCACATTACCAAAGCCAGCTCCAGATGCTGAAGCAATGTCCCCAGCACTAATGACGGCCCTACCCTCTGGAGTGATGCCCACCCAAACGATGCCAGCCCCAGGCTC
Loxodonta africana  TGCTGGAGAAATGTCCACATTACTAAAGCCAACCCCAGGCTCCGAAGCAGTGTCCCCACTGCTAATGACTGCTGTAGCCTCTGGAGGGATGCCAGCCCAGCTAACGCCAGGCCAAGGTTC

Homo sapiens        TGGGGCGATGTCCCCATGGTCAACACAAAATGTAGACTCTGAAATGATGTCTAATCCGCCAGTGAGAGCAACAGCCTCTGGGGTGATGTCTGCACCACCAGTAAGAGCTTTAGATTCTGG
Loxodonta africana  TGGATTGATGTCCCCACTGTTA---CAAAATCTAGACTCTGGAATCATGTCTACTCCGCCAATGAGAGCAACAGCCTCCGGAGTGACGTCCACATTGCCAATGAGAGCCTCAGATTCTGG

Homo sapiens        AGCAATGTCCACACCGCTAATGGGAGCCCCAGCCTCTGGAAATATGTCTACATTGCAAAAGACAGTTCCAGCCTCTGGAGCCATGACCACCTCACTGATGACAGTCCCAAGCTCTGGAGT
Loxodonta africana  AGCCATGTCCACACCACTAATGAGAGCCACAGCCTCTGGACATACGTCTACATTGCCAAAGACAGTCCCAGCTTCTGGAGCAGTGTCCACCCCACTGATGACAGTCACAACATCTGAAGC

Homo sapiens        GATGTCCACAGAGCAAATGTCAGCCACAGCCTCTAGAGTAATGTCCGCACAGTTAACAATGGCCAAAACTTCTGGAGCAATGCCCACAGGCTCTATGAAAGCCGTGGCAAAACAATACAA
Loxodonta africana  AATGTCCACCGAGAAAATGTCAGCCACAGCCTCTAGAGTGGTATCCACACAGTCAACAATGGCCAAAAATTCTGGAGCAATATCCACAGGGTTTATGAAAGCC----------------

Homo sapiens        GAGAGCCACAGCCTCTGGAAAGATGTCCACGCCACTGAGGAGAGCTCCAACTTCTGGAGCAATGTCCACCCAACCAGTTACGGCAACAGCCTCTGAAACAATGTCCATGCCACAATTGAC
Loxodonta africana  -------ACAGGCAATGGAGCAAAGTCCATGCAGTGGACAAGAGCCTCAGTCTCTGGAGTGATGTCCCCACCTTTAATGAGAGCCACAGCCCCTGAAACAGTGTCCATGTCACAATTTAC

Homo sapiens        AGTCCCAGCCTCTGGATCAATGTCCATGCTGCAAATGAGAGCCCCTGTCTCTGAAGCAATGTCCATGCCACAAATGAGAACCATGGCCTCAGGATTGACATCTGCAGCACAGATGAAAGC
Loxodonta africana  AGCCCCAGCCTCTGGATCGATGTCCACACTGCAAATGAGAGCCCCAGACTCTGGAGCAACGTCCATGTCACAAATGAGAGCCACAGCCTCAGGAGTGATGTCGACATCACAAATGAGAGC

Homo sapiens        CATGACTTCTGGAGCAATGTCCACCCCACTAATGACAGCCCAAACCTCTGGATCAACATCCACGCTGTTAATGAGAGACACAGCCTCAGGAGTGATGTCCTGTCCACAAATGAGATCTCT
Loxodonta africana  CACGGCCTCCGGAGCAATGTCCATTCCACTAACGTCAGCCAAAGCCTCAGGGTCGATGCCCACACTGTTAACGAGAGATACAAACTTGGGAGTAATTTCTATGCCACAAATGAGAGCTAC

Homo sapiens        GGCCTCTGGAGCATTGTCCAAGCCACTAATGACACCCAAAGCCTCAGGAACGATGTTCACGGAGAAAATGACAACCACAGCTTCTGAAGCAATGCCCACACTGCTAATGAGAGACACAGT
Loxodonta africana  GGCCTCTGGAACATCATCCAAGCCACTAATGACATCCAAAGTCTCTGGAGAAATGTTCACACAGCAAATGACAACCACAACTTCTGGAGAGGTATCTACAACACTAATGAGAGACACAGC

Homo sapiens        TTCTGGAGCTCTGTCCATGCCGCAAATGACAGACACAGCCTCTGGAGGGTTGTCTGCATCGCTAATGAGAGACACAGCTTCTGGAGCAATGTCCACATCACAAATGACAGCCACAGTCTC
Loxodonta africana  TTCTAGAGGGATGTCCATGCCACAAATGATATCCACGGCCTCTGGAGGGATGTCCAGGTCGCTAATGAGAGCCCCAGCCTCGGGAGTGATTCCCACCCCACAAATGACAGCCACAGCCTC

Homo sapiens        TGGAGGGATGTCCATGCCACTAATGAGAGCTCAAGACCCAGGAGTAATGCCTGCCTCACTAATGAGAGCCAAAGTGTCTGGAAAGATGCTCAGTCAGCCAATGAGCACCCAAGATCCTGG
Loxodonta africana  TGGAGCTATGTCCATGCCACTAATGAGAGC---------------------------------------CACAGCCTCTGGAAAGGTGTCCAGTCAGCCAATGAGTGCTCAAGATTCTGG

Homo sapiens        AGGGATGTCCATGTCGCCCATGAAGTCCATGACCGCTGGAGGGATGCAGATGAATTCCCCAACCTCTGATGTGATGTCCACACCAACAGTGAGAGCCTGGACCTCTGAAACAATGTCCAC
Loxodonta africana  AGGGATGTCCACATCACTCATGAGATCCATGACCTGTGGAGGAATG------------------------------TCTACACTGCAGCCGAGAGCCCCAGCCTCTGAAGTGATGTCCGC

Homo sapiens        ACCACTAATGAGAACCTCAGACCCTGGAGAGAGGCCCTCACTGCTCACAAGAGCTTCATCCTCTGGAGAGATGTCCCTACCACTAATGAGAGCTCCAGCTTCTGGAGAGATAGCCACGCC
Loxodonta africana  ACCACTAATGAGAGCCTCAGAGTCTGGAGAGATGCCCAAAGTGCTCATGAGAGGGCCATCATCTGGAGAAATGTCTCCACCACAAATGAGAGCCTCAGCTTCTGGAGAGATATCTACACC

Homo sapiens        TCTGAGATCCCCAGCTTATGGAGCCATGTCTGCTCCACAAATGACAGCCACAGCCTCTGGAATGATGTCATCCATGCCACAAGTGAAGGCTCCCATCTCTGGAGCAATGTCCATGCCACT
Loxodonta africana  ------------AGCTTATGGAGTGATGTCCACTCCACAAATGACTTCCACAGCCTCTGGAATGATGTC---CATGACACAAATGAGAGTTCCAGTCTCTGAAGTGATGTCCACACCACT

Homo sapiens        AACAAGATCCACAGCCTCTGGAGGGATGTCCATGCCACTGATGAGAGCCCCAGACTCTAGAGTGACATCCACATCACAAATGATGCCCACAGCTTCTGGAGACATGTGCACACTACCAGT
Loxodonta africana  AATGAGATCCACAGCCTCTGGAGCTATGTCTATACCTCAGATGA---------------------CATCCATG..............................................

Homo sapiens        GCGAGCCCCAGCCTCTGGAGGGGTGTCCTCACCACTAGTAAGAGCTCCAGCCTCTGGAACTATGTCCACACCACTAAGGAGACCCTCAGCCTGTGAGACTGTGTCCACAGAGTTAATGAG
Loxodonta africana  ..........GCCCCTGGAGGAATGTCCATGCCGATGATAAGACCTCCCAGCTCTGGAACAATGTCCACACCACAAATGATGCCCACAGCTTCTGGAGGGATGTGCACTGATTTCATGAG

Homo sapiens        AGCTTCAGCCTCTGGACATATGTCCACTGCACAAACAACAGCCATGGTCTCTGGAGGGATGTCCAAGCCATTAATGAGAGCCCCGGCCTCTGGAACAATGCCCATGCCTTTAATGTCAGC
Loxodonta africana  AACTCCAGCCTCTGGAAATATGTACATGGCACAAACAACAGCCATGGCCTCTGGAGGGATGTCCATGCCACTAATGAGAGCCACAGACTCTGGAACAATGGCCACGCCATTAATGTCAGC

Homo sapiens        CATGGCTTCTGGAGAGATGTCTATGCCGCTAATGGAAACCATGGCCTCTGGAGCAACGTCCACATTGCAAACCAGTGTTGCGAACTCTAGATCTATGTCCTTGTCACAAACAACATATAC
Loxodonta africana  CATGGCTTCTGGAGAGATGTCTAAGCCACTAATGAGAGCCCCAGCCCCGGGAGCAATGTCCACGCTGCAAACAAGAGCCCCAAGCTTTGGATCTATGTCGACACCGCAAATGACATACAC

Homo sapiens        CGTGTCTGGAAGGATGGCCACAGCGCCAATTAGAGCCTCTGCTTCTGGAGCAAGGTCCACATCATTTATGAGAGCCTCAGTTTCTGGATCGATGCCCATGCCACTACCAAGAGCCACAGC
Loxodonta africana  AGCTTCTAGAGGGATGTCCACATTACCAACGAGAGCCTCAGCTTCTGGAGCAATGTCCAC---------------------------------------GCCACTACTGAGAGCCACAGT

Homo sapiens        CTCTGGATGTGGCATGGGTATGTCCATGCCACAAATGACAGCCACAGACTCTAGAGGGATGTCCACACCACTAATGAGGGCCTCAGGCCCTGGAACAATGTCCACACCACAG--------
```

```
Loxodonta africana  CTCTGGA---------GGGATGTCCATGCCACAAATGACAGCCATGGCTTCTGGAGGGCTGTCCAGGCCGCTGATGAGAGCCCCAGCCTCGGGAGCAATGTCCACAACCCAAGTGGAAGC

Homo sapiens        -ACAGCCTTTGGAGTGATGTCCACTCCGGAAATCAAAGCCACAGACTCTGGAGAGGCATCCACCTCTCACATTAACATCACAGCCTCTGGATCAAAGCCCACATCGCACATGACTGCCAC
Loxodonta africana  CACAGCCTCTAGAGTGATGTCCAACCCAGAAACCAAAACCACAGACTCTGGAAAGACATCCACCTGTCACATCAACACTACATCCTCTGGATCAACGTCCACACCATGTGCGACAGCCAC

Homo sapiens        AACTCCTGAAACTGCGAAACCACCACCAAAGGAAGTGCCATCCTTCGGAATGTTGACCCCAGCACTCTGCTACCTCTTAGAAGAGCAGGAAGCAGCCCGGGGCTCATGCTCTGTGGAGGA
Loxodonta africana  AAGCCCTGAAATGAAGAACCCACCACCAAAGGAAGTCCGATCCTTTGGAATGTTGACCCCAGCACTCTGTTACCTCTTAGAAGAACAGGAAGCAGCCCGAGGTTCATGCCCTGTGGAAGA

Homo sapiens        AGAGATGGAGATTGATGAGGAGAAGCAAATGAAGGGGTTTTTGGACGATTCAGAGAGAATGGCATTTCTGGTATCTCTTCATCTGGGAGCAGCAGAGAGGTGGTTCATCTTGCAAATGGA
Loxodonta africana  GGAGATGGAGATTGATGAGGAGAAGCAGATGAAGTGTTTTTTGGACGATTCGGAGAAAATGGCATTTCTGGTGTCTCTTCATTTGGGGGCAGCAGAGAGGTGGTCCATCTTGCAGATGGA

Homo sapiens        GGTAGGAGAACCTCTCTCACATGAAAATAAATCTTTCCTGAGAAGATCCCAGGGCATATATGACTCCCTATCTGAGATAGACATCCTCAGTGCTGTTCTTTGCCATCCCAAACAGGGCCA
Loxodonta africana  GGTAGGAAACCCCCTCTCAGGTGCAAATAAATCTTTCCTGAGAAGATCACAAGGCTTATATGAATCCTTATCTGAGGTAGACATCCTCAGTGCTGTTCTTTGCCATCCCAAGCAAGGCCA

Homo sapiens        AAAGTCAGTCAGGCAGTATGCCACTGACTTCCTGCTGCTGGCCCGACATTTGTCTTGGTCTGATGCCATTCTACGGACCAGGTTTCTGGAAGGACTCTCAGAAGCTGTTACCACCAAAAT
Loxodonta africana  GAAGTCAGTCAGGCAGTATGCCACTGACTTTCTACTGCTGGCCCGACATTTGTCTTGGTCTGATGCCATTTTACGGACCAGGTTTCTGGAAGGGCTCTCAGAAGCTGTTGCCACCAAAAT

Homo sapiens        GGGTCGGATCTTCCTGAAGGTGGCTGGCAGCCTAAAGGAGCTGATAGACAGGTCTCTGTACACCGAGTGCCAGCTGGCTGAAGAGAAGGATTCCCCAGGCAACTCAAGCCAGGTTCTGCC
Loxodonta africana  GGGCCGGATCTTCCTGAAGGTTGCCAGCAGCTTAAAGGAGCTGATAGACAGGTCTCTCTACACTGAGTGCCAGCTGGCTGAAGAGAAGGATTCGCCAGGCAACTCAAGCCAGGATTTGCC

Homo sapiens        AACAGCCTGTAAGCGGAATAATGAGGAGGCCATGGGGAATGAACTGAGCTCTCAGCAGCAGACTGAGGAGGTAATGAGGGGGAAATCCGCTGAAGGTTTTTG-------AGCAGAGAAAT
Loxodonta africana  GTCAGCCTGTAAGCGGAATAATGAGGAGGCAATGGAGAATGAATTGAACTCTCATCAGCAGACTGAAGAGGTAATGATGAGGGAAAACACTCAAGTTTTTTGTTTTTTTAGCAGAAAAAA

Homo sapiens        GAGGAGGTCACGACAGGGAATACATCCTGCTTCCTTGAGTAGAATTGGGAAGAGGCAAGGGGGAGGCATGGAGCTCCTAGACCATTAAACCCCA----GCAGTTTCATCAAGTGAGCATG
Loxodonta africana  GA----------------------------------ACTAGAATAAGGAAGGGGCAATGGGGAGGCGTTGAGGTCCCAGACTA-TAAACCCCACACTGGAGTTTCATC-AGTGATCACG

Homo sapiens        GCTCTGCAGTTATTTTGGGTATATTAACTACTTTGAGGAGCAATGTCCTGCCCAAGTTACTGAGAACGTGGGATAGTTGAAGTACTAATTCTTCTCATTATGAGGGAACTGGATGGGCTC
Loxodonta africana  GCTACACAGTTATTTTGGGTGTATTAACCATTTTGAGGGGCAATGCCCTGCCCAAATTACTTAGGGACTGGGATGGTTAAAGAACTAAATCTG---ATTATGAGGGAATTGGCTGGGCTC

Homo sapiens        AGTTCCAATTCTCCCCTTGTTCCAAGTCCAATTCTAATAAATGGGT----CTTTTGGGAGCTGGGGCCCATCTACAG-CAACAT-GTGTTGTGGAGTCAGGTCACTAGGGAATTAGAATA
Loxodonta africana  AGCCCCAGTTCTCCACTTATTCCAAGTTCAGTTCTAAGGAAGCAGTATTCATTTTGGGAGGTGGGTCCCACTTATAGCCCATATGGTGTTATGGAATCAGGTCATTAGGGAATTTGGTTA

Homo sapiens        GCCTACTCCTTGGTCACACTGTATGCAAG-TGAGATAAATTAGCAAAGTGTCTTGATCTGCTAAGTCACAGAACTTATCCAGGCAGAACACCCGTTGCCATAGTAAGTGTGGCTCTTACC
Loxodonta africana  GCCCAGCCTTTGGTCTCCCTTTGTACAAGTTAAGATAAATTAGCCAAGTTCCTTGATCTGCTAA-TTGCTGAATTTATTCAGGCAGAACACCTCTTGTCACAGTAAGTGTGGTTTTTACT

Homo sapiens        TCTGTTGTCTTTTTCTCACTCCCAGCACCAGCATGTTTCCAAACGCTGTTACTACCTGAAAGAGCATGGAGACCCCCAAGAAGGTCTTCATGATCACCTTGGACAGAGCACAGGCCATCA
Loxodonta africana  GCTGCTGTGTTTTTCTTTCTACTAGCACCAGCATGTTCCCAAACGCTGTTACTACCTGAAAGAGCATGGAGACCCCCAAGAAGGTCTTCACGATCACCTTCGACAGAGCACAGGC---CA

Homo sapiens        TCAGAAGGCCCATACCAACAAGTAAAACTCCATGGAATCTTCTCCTGTGATATCTGACTCGACCGCTAACTGGAGGACTGGTTCCTGGACCCATTCCATTAAACTACATTATAAACCTTG
Loxodonta africana  TCCGAAGGCCCCCACTGACAAGTAATGCTCAATGGATTCTTCTCCTGTGGTATCCGACTCTGCTGCTAACTTGAGGACTGGTTTCTGGACCCATCCCATTCAATTACGTCATAAACCTTG

Homo sapiens        TTGCCTTCACCCTTCAGCCTCCCGCTCCAGGCACATCCCACCTTACCTCTCACTTGGCTGATTTGACCTTCTCTTTCA-CCCACATGCCTAAGACCTGCCCTGACAATCAGAGTAGGGAC
Loxodonta africana  TTGCCTTCTGCCTTCAGCTTCCCCATCCAGGCACATCCCACCCTACCTGCCACTTAGCTGACTTGACCTTATCCTTCACCCCACATGCCTATGACCTGCCCTGACAATCAGAAATCAGAA

Homo sapiens        TACAAGAGTGTATGGTGTATGAGGTTCTGAACTCCCATCACCACCGAGGCCAGTCCTAGTTCTTGGTCCAGGGAGAAGTCTAGGCAGGATAAATCATATCTTACCTCAAAAAACCCTTAG
Loxodonta africana  TATGAGAGTATATGGTATATGAAGTTCTGAACTCCCATCGCCATCAAGACTAGTCCTAGTTCTTGGTCCAGGAAGCAGTCTAGGCAGGAGAAATCATGTCCTACCTCAAAAATACCTCAG

Homo sapiens        CGTATCTTGCCATGTGTCAGCAGGCAAACTTGAGGAAGCCCTGGTTCTATTTCACCTGGCAGGGGAGCCTATAAGGAGAGCGATGCCCACTCCTATACCACCCTTGTAACCCAGTAGACT
Loxodonta africana  AGTGTCTTGCCACATGTCATCAGGCAAACTTGAGGAGACCCTGGCCCCACTTAACCTGGCAGGGGAGCATGTAAGGAGAGTGATACCCATTCCTATACCACCCTTTTAACCCAGTAGACT

Homo sapiens        TGCCCC-ATATGCCCCAACTCATAGCACCT-AAAATGGGTCTCCAGGGTCCTTGCCCTGTTTATAGTATCCATCCTGAACCATTCGTTTTGACCCATAATAATAGTGATA-GTATAATGG
Loxodonta africana  TGCCCCTATATGCCACAACTCATAGCACCTAAAAATAGGCCTCCAGGGCCCTTGCCCTGTTTATAGCCTCCATGCTGAACCATTCGTTTTGACCCATAACAATAACAATACGAGTAATGG

Homo sapiens        CTACCAGTTATTGATAATGTATCATGTGACAGGCATTGGGCTAAACTCTTGACAAGCACCATCTCACTACATTCTTACAGTAACACAACTTGTTCTTGTGTTGTCAGCTTCTGACCTCTT
Loxodonta africana  CTAACATTTGTTGATTATGTGCTGTGTGACAGCTACTGGGCTAAACTCATGACAAGCATCATTTCATTGCATTCTTAAATTAATCCAACTTGTTCTTGTGTTGTCAGTTTCTGACTTCTT

Homo sapiens        GATCTGTTGTTTATGGCCTTTGTCTTGTCCCTCAGTGGGGGTTCTCTAGCTCTGACCTCTGGGTGACCCCCTCAGTTGTTGATTGCTCTCACTCCCCTTGGCTGTTGTGCTTCATGGCCA
Loxodonta africana  GATC---TGTTTATGACCCTTGTCTTGTCCCTAAGTGTCAAATCTCT-GTTCTGACCTCTGAGTGACTCCCTCGGTTATTAATTTCCCTCACTCCCCTTGGCTATTGTGCTTCACGGCCA

Homo sapiens        GCACTCTTGATGAGGGTCTTGGTCTATAAACAGGCATAGATAGCTAGAACATAATGTGAGCAGTGTTTGCCTTCAGGTGGTGGGGTTATGGGTAGTTTTT-ATTTTCTTCCTTCTACTTC
Loxodonta africana  GCACTCTTGATAGGGGTCTTGGTCTCTAAACAGGCATAGATA-TTAGAACATAATATGAGCAGTGTTTGCCTTCAGGTGGTGGGCTTTGGATGTTTTTTAATTCTCTTCTTTCTACTTC

Homo sapiens        TCTATACATTTCAGATTCTCTACAGTAAGCATGTGTTACATTTGTAATTATAAAAGTAAGTAAACTTCATTTATGAAAAAAGAGTTCATTGTGGCTCCTAGAATTTGATTTACTGAGTCC
Loxodonta africana  TCTGTACTTTCCAGATTCTCTATAGTGAGCATGTATTACATTTGTAATCATAAAAGGAAATAAAATTCATTTGCGAAAAAAAAATTAATAGTGGCTAATAGAATTTGATTTATTGAGTCC

Homo sapiens        TTTATTCAACCAATATTAGTTATGTAACTTATGTCAGCTACTCTTTGGGGGACAGACGGATAAGAATCCTGCTGATATGAACATTCCATTCCAATGGAGTGGGGAGAACAAGAACACACT
Loxodonta africana  TTCATTCGATCAATATTTATTATCTAAAATGTGCCAGCTACTCTTTTAGGAACAGACAGAAAAGAATC---------------TCCAATTGCGGCGGTGGGGGGAG---GGGGGCAGAC-

Homo sapiens        GAACTTAGATTGGAATTGTGAAGGCTGTGAAGAGAAGAAATCAGGACAATGGGGAATTTGATGCAAATAAGACCAATTTATGTATTCAATAGTAGTCCATATAATTGAGTTACTCAAAGA
Loxodonta africana  -------------AAT-----AAGCTACAAAGACAATAAATCAGGAGGATGGGGAATTTGACACCCACGGGACCGATTTATTTATTCAATAATACTCCATGTAGGTGGGTTACTCAAAGA

Homo sapiens        TCTTTCAACAATAGTAGGAGAGACATGATTAGAAACTTATTTTATATAGTTAAATTGGCAACAGTTTGGAGACAGGATGGAGGAAGGG-ATCAGAGAGCAGCT--GAGCCCTCTACTGCA
Loxodonta africana  TGTTTTAAAAACAGTAGAAGTGACATGATTAGAAACATACCTTAGGAAGCTAACTTGGCAACAGTTTGGAGGCAGGATGGAGGAAGGATGTCAGAGGCCAGTTAGGAGCCC-CTATGACA

Homo sapiens        ATAGTCCAGGTAACTGACTGGCTAGGTGACTCAATCTAGGGTGGTGGCAGCAGAGATGATAATTCAGGAACAG---AGACCTAACCTTCTCTGAGGACTCAAGTCCACTGCTCCAGGACT
Loxodonta africana  ATAATCCAGGTGACTGG------------------CTAGGGTGGTGGCAGTGGGGATGGAAAGACAGGGACAGATCAAACCTCAAGTCTTCTGGTAGCTC----TCTCTG----AGGACT

Homo sapiens        CAAGTCCACTGCTCCATTACCCTAAACTTTGTGTCTGATTTCAGGAAGAGGAAAATGATACTCAGGAAAGAC--AGATTGGCAGATAAATC
Loxodonta africana  GAAGTCTGCTGCTCTATTACTCTAAATATTGGGTCTGATTCCAGGAAGAGGAAAATGATTTTAGGAAGAACAAAGATTGGCAAACAAATT

Homo sapiens  AAACCTCAAAGGGAGTG--TAAAATGCAGAGTGTTGT-CAACTTATCACACCAAGAATATTTTTTTGCACCTTTTTAGTTTGGAAGGGTCAAATTGAGATGACAGAGGAATAGTGGATAA
Bos taurus    AAACCTCAAATGGAACGATTTCAATTTATAGGCATATATAACTTTTTTC----------TTTTTTTGCACTTTTTAGGTTTGAAAGGGACACATTGGGATGGCAGAGCAGTAGTGACTGG

Homo sapiens  GAGCTATAGTGCTATTAAATTACATTCTAGAGTTGGGAAAATCGAGACCCATAATCCTAGGAATTATTAATGATAAGAGCTAACACTTTCTGAGTGCTTACCACATGCCAGGCCCTATAC
Bos taurus    AGGCCATAATGCTGCTAAATTACATTCTA---------AAATCAAGACCGGTAATCCTAGGATTT-TTAATGATAAGAATCAATACTTTTAGAGCATTTACCATGGGCCAGGCCCTGTAC

Homo sapiens  TGAATGCATCATGTCCATTTTCTCATTTGATCCTGACAA-CCACCTGTGAGGCCTCATTTTGAAGACAAGCAAGCTGAGGCTCAGACCT-TGGATTTGTCCAAGGTCACTCAGTTGGTCA
Bos taurus    TGAACGCTTCATGTCCATTTTCTCATTTGTTCCTGAAAAACCGCCTGTGAGGCCTCATTTGACAGATTAGCAAACTGAGGCTCAGAGAGATTGATTTGTTCAAGGTTATCTAGTTGGTCA

Homo sapiens  TTTACAGAGCCCTGATTCAAACTCTCTTTTGCTTGATTACAAAGCCATTGCTCTAAAAGATTAGATTATACTGTCTGTATTTGCTTCTGGGAGCTTATGGAATTCACTCCTATCTGTTCT
Bos taurus    TTTGCAGAGCCTAGATTCATACTCAATTTTGCTTGATGGCAAAGCCATTATTATTAAAGATCAGACTCTTCTGACTCTATCTGCTTTGGGGATTTCATGGAGTCTACTCCAATCTGTCCT

Homo sapiens  CACAAGGAACTCT-ATTGGGTTTTGAAAAAGGCTAAAGAATTGAAAAGAAACTTTGTAGGCTGGGAAAC------------ATAGTGTCCTTTGAGCAGTGAGATAGGCTAAGCTCAGAG
Bos taurus    CATAATAAACTCTTATTGGGCTTTGAAAGAAGCTGAAGAGTCGGGAAGAAACTTTGTGAGTCAGAAAGCTAGTAAAAGCAGCCAGTGTCCTGTGAACAATGAGGTAGACCAAGCTTAGAG

Homo sapiens  GACTGGGCTCTGGAATGTGGAAAGGAGACGTGCCCACTGGAGAGCAAGTCAGAACTTTCGTGAGCCCTGCTCCAGGTCCCAGGAGAAAGTGCCAGCCATGAGGACTGGAGTATTGCCTGG
Bos taurus    GACAGGGCTTGGGAAACTGGATGGCAGAAGCGCCCACTGGGGAACAACTAAGGGCTTCTCTGAACCCTGCTTTAGGCCCAAGGAAGAAGTCACAACCATGGGCGTTGAAGTTCTGCTTGG

Homo sapiens  TAAGGAGAAGATTATTATTTTATCTAACACGTTCTTCTATAACGCTCTATATGCTAGGCACTGGTCTGCACTGCTCTAAGTACTTTATAAGTATTAACTCCTTTACATCTTATAACACTT
Bos taurus    CAAGG------CCATTGTTTTACCTAACACACTCTTCTGTAATGCTCTTTATGCCAGACACTGGTCTCCATGGCTT--AGTACTTTACAAACATGAACTCACTTAAGTCTTATAGCAACT

Homo sapiens  CCCTGGAAATAGGCATTATTATTGCCCCCATAATACATATGAGTAAACTGAAGTTC-----AGAGAT---GTTAAATAACTTGTTCAAGGTCACATAGCTGGTTAAGTGCCAGAGCCAGG
Bos taurus    CCATGGAAATTAGTACTATTATTATCCCCATATGATAGATGAGTAAACTGAAGTTCAATTTAGAAATTACATTGAGTAACTTGTCCAAAATCACACAGCTGG-TAAGT------GCCAGG

Homo sapiens  CTTTGAACCTAGGCAGTCTGGATCTAGTGGCTGTGTTATACTGCTTCGCAACAGAA--ACTTGATTTTTCTTTCTTCTGTGTTTATCATCCTTTCTCAATCACTCCTGGGCAAGTCTACT
Bos taurus    CTTTGAACCTAGGCAGTCTAGATCTAATGGCTGTGTTATATTGCCTCTCAGTAGAAATATTTGATTTTTCTGCCTCTTGTGCTTGACATCCTTTTTCAACAACTCCTGGACAAGTCTACT

Homo sapiens  TTGCTAGCAAATTTCCCTTACCCCTTTGGCTTTTTTTTCACCCATGAACCCTCTAATGTTTTCTGCAGCTTCTTTGAGCTCATTCTTTATCTTCCACACTGCCTCTTTTGGCATTACTCA
Bos taurus    TTGCTGGTGAATTTCCCTTACCCTACTGGCTTTTCCACCAACCATGA-----------CTTTCTGCAGTGTCC---------TTTTTCATCTTTTACACTACCTCCTTCGGCATTATTCT

Homo sapiens  TGAACGACATGCTCTTTTCATCTGCCAAATATTTTTTCCATAAATTATCCCATCTCAACTTGGGAAGTCACAATGGGAAGTATTGTTCCTATTTTGTGAATGAGGCAACTAAGGCAAGCA
Bos taurus    TGAACAACATGCTCTTGTCACCCGCCAAATAATTGTTTCATAAATGGTCCCATCTCATCTT-GGAAGTGAGGATGTAAAGTGTTGCTCCTATTTTGTGAATGACGGAACCAAGGAAAGCA

Homo sapiens  ACTTTCCTGAAGTCTCACACAACCATCTAGTGGCAAAGTGCACT--AGAATCCAGATTCTCTGATTCTGAATCTAATGTTCTTGTCCATTGCACCATCTGATGGAATTTAACTATCTATT
Bos taurus    AATTGCCTAATATCTCTTACAATTATCTAGTGGCAAAGTGAACTAGAGAATCCAGGTTCTCTGACTCTTAATCCAGTGATCTTTTCTTTTGTACCATCAAATGGGTTTCACATATCCATT

Homo sapiens  CATTAATTAATTTATTC--------ATTAATATTGTAAAAATATACTGAGTGTCTTACTGTATGGTAAGGCCCTGGGTG----CAAAAGTCGGTATAAAAATTGTTGCTCTTAAGCAACA
Bos taurus    CATTTATTCATCTATTCCTCCACTTATTCATATTATAACAATGAATTAAGTA-CCTACTGTATGCAAAGGCACTAGATGGGAAGGAAGGTAGATATAAAAATTCTTGCTCTTAAGGACCT

Homo sapiens  GACAACACAGA-GAGATAAGGTCTCACCATAAAAAGTTAAATAGCAGCACA--------TATACTAAGTGCCAAATGAGTGGTCTGGTCACATCATACAAGAATTTTGAGGTTGGATATG
Bos taurus    GACAACACAACTGAGATAAAATCTCACCATTGAAAGTTGAATGACAACACACAGGTACATATGCTAAGTGCCAAATGGGTGATCTACTTAGGTCATATGGGAATTTTGAGGCTGGGAATG

Homo sapiens  TAGCATCAAAGACTGCTTTATCAGAAAGGTGAGACTTGAACTTGGCATAGAAGGGTACTTAGAACTTAGAGAAGAGAATGGAGCAGCACTTTCCAAACTCTGGGAATGGTGTGCATTAAA
```

Bos taurus TAGCAACATAGACAGCTTTATCAAGAAGGTGGGTCTTGAACTTGGCATAGAAGTGCACTTAAA-------GAAAAGGATGGAACAGAACTTCCCAGGATGAGGGAGTTGTGTGCACTAAG

Homo sapiens GCAGTCCTTCACAACCTTTATTGTGTCATGGCATGCAG--------AGAAAAGCATAGTATTTGTAAAGCACACTAGGCTGAATGGAGGAAGCAGTTTGACATCTAGAGGCAAGCATTCT
Bos taurus GCAGCGCTTCCTAACTTTTATTGTAGCA---CACATAGTATTTGTAAGAAAAGCATAGTATTTGTAAAG----------TGGATGGAGGAAGCAGTTTCA-GCCCAGAGGCAAGTGACC-

Homo sapiens AGGAGTTTCAACAGCACTGTCTCTGGGCTCAGTTGAGGACAGAGAAGAAATCTCATAGCCTCAGACACTTGTAATATGTTTGGGAAGGTCTGCACTAGGTATTGGGGCACACTGTGGTC
Bos taurus AAAAGTTTCAACTATACTGCACCTG------------------------------------AGACACCTGTAACACTCTTGGGAAGGTCTACACTAAGTTATCGGGACAAGCTATGGTC

Homo sapiens ATGGTGGGTCTAAAGACCACTGACAAGCCCACAGGGATGAAATGAGGGGAATTTCCAGAGAAGCCATAGTAGATACCATTGGAAAGAAAAGTTATGGGCAAATGATGAAGGGCTTTTGAA
Bos taurus ATGGTGGGTGTAAAGACTGCTGACAAGGCCACAGAGATAAAATGGAGGGACTTCACAGAATAACCATGGCAGATACCATTGGAAAG-AAGGTTGTGGCCAAGTGATGAAAGGTTTTTGAA

Homo sapiens TTTTATTCTGTGGACCTACAGAATAGGTAGGCAAAATATGCATAGCACTGTTTTAGAAAGATTCCACTGGTTTTCATATGGGAGATTAACTAGAGGGAAGAAGAGTAGTCAAGAGGCTAT
Bos taurus TTTTATTCTGTGGACCTACTAA------AGGTGAAA----------AGTGTTTTTAAAAGATCCCGCTGATATCAGTATGGAAGACAGAGTAGAGGAATGGAAGGTAGTTAGAAGACTAT

Homo sapiens TACCATTGTCCAGGTGGGAGGTGATGTTATCTTGAACTAGATTGATAGTAATAGAGACAGAGAATGGTGGACAGAATTGAGTTATATTTTGGAGGTAAAAATGTCAGGGTTTGGTAATAG
Bos taurus TACTGTTGTTCATATGGAGGATGA-----------------TGGTGGTAACAGAGACTGAGAAAAG-GGGCAGAATTGAGTTATATTTAGGAGCTAAAAGTGACAAGGCTTGATAAT-G

Homo sapiens AATTGCATAATGGAGAGTAGAAGAAAGGGAGGTGACAAGGAGGATTTCAAATTTTCAAGTTTAAGCAAATGCTTATGAATAGTGATGCCATTCTTTAAGATAGAGAGCCCAGGAGAAGTA
Bos taurus GATTGCAT-ATGGAAAGTAGGAGAAATGGAGAAGATAAGGATAATTTTCAGGTTTCAAACTTGAACAAATAAGTATGAATGGTGATGTTATTCTTTATGATGGCAACCCCAGGAGAAATG

Homo sapiens GAAAGATTTTTTTGGAGGGGAAGGACAAATGAGATGATGAGTTTCAAGTTGACATTTGGAGGCAAGGAAACCAGTTAGGAGGCTATTGTAAAA-GCCAAGTAAGACAAGGTGAGATCCTG
Bos taurus GAGGGA-TTTTTTGGAGGGGAAGGACAGATGAGAAGATGAGTTTCAACTTGACATTTAGAGTCAAGGAAGCCAATTAAGTGGCCATTGTAAAAGGTCAAGTGAGACAGAACGAGATCCTG

Homo sapiens GACTACAATGGTAGCAGTGGGAATGGAAGGAAAATGGTGAATTGAGAGACATTATACAGGAAGAATTGATAGGATTTTGTCACGGACTGACTTCTGGAGTTGAAAGGTAGAGGAAGGCTT
Bos taurus GGTTAGGACAGTAGCAGTGGGAATGGAAAGTAAATGTTGGATTGAGAAAC---ATAAAGGAAGAATTGATAGGATTTTGACATGCACTAAA-TCTAGAATTGATAGGTAGAGGAAGACTT

Homo sapiens GAAGCCTAAGATATCTTCTGTCCTTTATCTTCTTAGTGATGCTCCGATCT-TCGTGGCTTAATATACTATCTATTTGCTGATGAGTCACAAATTTG-TATCTCCATAC------------
Bos taurus TAAGCCTTGGATCTCTTC-------TCTCTCCTTGGTGATGCTTCTATTTCTCATGGCTCAAAATACCGTCTATAAGCGGATGACTCCTAAATGTACTATCTCCAGCCTGAACCCTCCAT

Homo sapiens -TTTTATGCCCAATTGCCTGCTGGACATATCAACTTGCATGCCTAATAGGTATCTCCAACTTACCATGTTCAAAACTG------------CCCCTGCCCACA--------------ACCC
Bos taurus ATTTCATAACCAATTGCCTGCTTGACATGTCACCTTGGATGCCTAGTAGGCATCTTAAACTTAACACATCCAAAACTGAGCTCTTAATTCCCCCTGCCTCCACCCCTACCCTGGACACTC

Homo sapiens CCAACAAAACCTGCTGCACTCACAAAACCTGCTCCTCACAGTCTTCCCCGTCTCCC---TAAATACCAATTTCATTCTTCCGGTTGCTCAGGCGGAAGACCTAGGAATCAATCTTGATTT
Bos taurus CCACCTAAACTTGCTCCACTTATCAAATCTGCTCCTCACTGTCTTCCCCATCTCATGGGTAAATGGTAACTTCATTCTTCCAGTCACTTGGGCTGAAGACCTTAGGATCATTCTTGATTC

Homo sapiens TT--CCCTCTTTCATATCCTACATTTATTTGATCAGCAAATCTTATTGGTTCTACCTTCAGAATATATCTAGAATCTGACCACTTTTTCATTGCTACTATCCTGGTTTATGTCACAATGA
Bos taurus TTTACTTTCTTTCACATCGCACATTTATTCTACCATCAAATCTTGTCGG-TCCATCTCTGAAATATGCCTAGAATTCGACTAC--TTTCATCA-------CCTGGTTTAAGTCACAATGA

Homo sapiens TCTGCTTCCTGGATTATATTGCACTAGCCTCTCAACTGTTCTCCTTGCTTCTGTTCTTGCCACCATCACTCAAGCCTATTATTCTCTCACTGTTTTTTGGAACCAGGATAATTTTATT-A
Bos taurus TCCATATTCAGAATTATAT---------CTCCCAACCCTTTTCCTTGCTCCTGGCTTGCTGCCCTCATCCAAGTCTATTATTCCCAT-CTTAATTTGAGGGCTAGACTAATCTTATTAA

Homo sapiens TGTGAATGTCAGATCATGTCACTCCTCTGCTCAAAACTCCTCCAATGGCTTCCCATCTGACACTCAGTAACACAAAACCCCTTATAATGGCCCATAATGCTGTATGCAACCCAGATATT
Bos taurus TATGAAAGTCAGACTAGGTCACTCCTCTACTCAAAA---CCCCAGTGACATCCTGTCTCACACATAATAATAGTAAAACTCCTTACAATGGCCCACTATGCTGTATACAACCCAAATATC

Homo sapiens GACAT---------ACTCCTGTCCTTCAGGTGATGGATCAAATATCCC--ACTTGGTAAAATCTTCTCTGATTACCATATTTAGTATTGCAAGCGCCTTCTCGCCATTCTCTATCTGCCT
Bos taurus AATATAACTCACTCACTCACCTCCTTCAAGTGGTGGATCAGACATCCCATACTTGGTGAGATCTTCTCTGACAGCCTGGTTAAAGTTGTAATTTTCCCCTCCACATTCTCCATCTGCCT

Homo sapiens TCCATGTTTTA--TTTCTTTATACTTTGAATACTAGCAGATGTTATATGTTTATCTATTTATTTGTTTATCATCTATCTCCTTCTGCTAGAACAGGAGCTGGAAAACTTTTTCTGTAAAG
Bos taurus TTCTGGCTTTCTTTTTCTTTATACTGAGACACTACTTGACATTATATGTTT.........................CCTCTCCTGCTAGAATAAGGGCCAGCAAACTTTTTCTCTAAAG

Homo sapiens GTCCAGATAATAAATATTTTAGGCTTTGTGAGCCACGTAATCTTTAACACAACTACCTACCTCTATCATTGTTATACAAAAGCTGCCATAGATGA-------------GCATGGCTGTGT
Bos taurus GAATAGATAGTAAATATTTTAGGCTTTGTGGGCCAGTGAGTTTCCAACACAAATACCCAACTCTGT---TGTTACACAGAAATAGTCATAGATGATACATCAAGAGATGTATGGCTGTGT

Homo sapiens TCCAATAAAACTGTATTTACCAAAACAGGGAGCGGGCTAGATTTAGCTAATGGGCCATAGTGTACTGACCCCTGCTAGAATGTAAATTCCAGGAGGGCAAGGACTTTTGTTTACTGCTGT
Bos taurus CTCAGTAATGCT--ATTTACCAAAACAAGTGATGGGCAGAATTTGTCCATGAGCCATACTGT--TGACTCCTACTAGAATGTAAGTTCCAGGAAGGCAAGGATTTTTGTTTACTTTTAT

Homo sapiens ATCTCCAACATCTAGAATAGTACCTGGCATATAGTAGGCACATGATTTATTTT-ACTGAATGAATGCATCAGTGGGAGATTGGAAAGCTGTGGAATAAAAAATATATATATATAACAGAG
Bos taurus ATTCCCGACACCCAGAACAGTGCCTGGCATGTTATAGGCACCCAATATATTTTTGTTGAAGGAAAGTATCAGTGGGGGATAGGAGA-CTGGGAGGTTGGGA-----TAGATATCATAGAC

Homo sapiens CCATTGATGGTGCAGAATCCTGAACAAAATCTAGCTTTGGATGGGTTTGCAGCATATCAACTCTCCTTCCTTCTTTTTCCCCTCATCCCTACACTAAAACTTAA-GCTTGTTATATAAAG
Bos taurus CCATTGATGGTGCAGAATGTTTAGCAACAACTGATTCTGGCTAAGTTTCCAGCATACCAATTCTCCTTCCTCCCTTCCCCTGTCATCCCTACACTACTACTTAAAGAATGTCATATGATG

Homo sapiens TAAAAGTTGCTTTCATTGCCTGTGCAATTCTCCCAATGTGTGAAATTTCATCTGACTTCCTTTAATGTTGTCATTTGTCTATTTAACCCTCCTGACTCAGACTCCCTCTAATAACAATAA
Bos taurus TGAAAGTTACATCCATTGCCTGTGCAGCGCCCCTGATGTGTGAAATTTCATCTGACTTCCTTTAATGTTATCATTTGTCTATGTGATCCTCCTGACTCAGAC--ACTCTAATAACAATAA

Homo sapiens TAACTCAAATACTCTGTACTAAAACCAGAACGCTGAAATCAGTATTTTTAATTAATTTGAAACAACTGCTAGCAATAGCTTCTATTGACAACTCTACGCTTTTCAACACTTAATCATATT
Bos taurus TAACTCAAGTGCTCTGTACTAAAACCAGAACGCCAGAATCAGTATTTTTAATTAATTTAAGACAACTGCTAGGAATAGCTTCTGTTGACAATGCTACACTTTTCAACACTTTATCATGTT

Homo sapiens CTGAAGTACTCCCAGAAGCTGGGCGACTGTTACTCTTATGATATGAGATGATATTTTCCTTCCATGCATTCAATGTGAATGGTTTGTTCTGAAAAACTTCAGCTTTCATGTACTCCTAAT
Bos taurus TTGGAGTACTCTCTGAAGCTGAGCAACTGTTACTGTTATGATATGAAATGTTATTTTCCATCCATGCATTCAGCTTGACTGGTTTGTTCTGATAAACTTCAGTTTTCAAGTACTCCCAAC

Homo sapiens GGTAAGAATCACATGGTAGGAAATATGATAAATGAAGTATTTACCAGTTCTTACTGACTGTTCTTTCATACTTCCTTGGATCTCTTACAGGCACTAAAGATATCCTGTAGTTATATGACT
Bos taurus AGTAGGAATCACATGATAAGAGACATGGTAAATGAAGTATTCACCCGTCCTTACTGACTCTTCTCCCATCCTTCTCTGAATTTCTTATAGGCACTAAAGATTTCCTGGAGTTATATGTCT

Homo sapiens TCCTATTGAAAATGCACTTTCACTCATTCATTATTTTATATAGTCTTCCACACATTGCTAACATTTACCAAACATTCA-CTACGTATGTGGTATTGTGCTGGGGGATTTATATATATTAA
Bos taurus TCCTGTTGAAAATGTACGTTCACTTATTTATTATTTCATATAGTCCTTCAGTCATTGCAAACATTTACTGAGCATTCAGTTATGTATATGGTACTGTCCTAAGGGCTTTATTCATATTAA

Homo sapiens TTCATTTAATTCTTATAAAACACCTAAGTGATATATAC------TATCATAATTTTATAGATGAAGGAACTGAAGTACTAAAAGCTGAAGTAATTTGCTCAAGGCCCCACAACCAGCAGA
Bos taurus CTCATTTAATCCTTATGAAACATCTAGGAGCTAAGTACCACTATTACCATTGTTTTATAGATGAGGGAATTGAGGTCCCAAGAGCTGCAGTAATTTTCTCAAGGCCCCACAGCTAGCAAA

Homo sapiens AAAGGAAGCTAGCATTTCAACTCAGGTCTGCCCAACTCCAGAGCCAAGATTTATCTATTGTTTTTGTCACCTCAACAAATATTTACCATATGCCATGCAGCACACTAGGCAGTACCTAC
Bos taurus AAGGGGAGCTAGGATTTAAATCCAGGTCTGCCTGACTCCAGGGTCAAGATT----TACTGTCTTTCCTCATCT.......TATTTACCATATGCCATAAAGTATACAAAACAGAATCTAT

Homo sapiens AAAGATGAATAGGGCATGATCTCCTTGGCCTTCCAGAGGTAACACTAGTTGGGGCTATATGGGTGGGAATGGAG--TGTCAGATGCAGAAGCTAAGGAATTTTCTCTTTACAAACAGGTG
Bos taurus AAGGATGAATAGGGCACAATCTCCTTTGTCTCCCAGACCTAATACTGGCTGGGGCTGTGTGGGTGGGAATGTAGGATATCAGGACAGAGATGAAAGAATTTTCTGTTTATAAACAGGTT

Homo sapiens GGTGAGAAAGTAGACACAGCACTGTGAATTTGTGGAGTTTTGAAAAATG-GACAGAGGGCTACAGACTGAGAGGGAATAGGGCCACCAACTCTAGAATTAAGGTCAACTGATAGGGTAGA
Bos taurus GGTGAGAACATAGACATAGCTCTATGAATTCACAGGTCTCTGAAAATGGAGACAGAGGGATACAGATTAAAAGGGAAGAGGGCCCCCAACTCTAGAGCTCAGGACAACTAATA----AGA

Homo sapiens AACATGCCATTCTCAGGGGCTTAAGCTGACAAAAGAAATTGCCCACTGTAGAAATGAGATTGGCTGCCCACAGAAAAG-----------------TATTTACTGAT-AGATAAGGGACCT
Bos taurus AAAATACCACGCTGGAGGACTTAAACTGATAGGA-AAGTTGCCCACTGCAGAAATGGGATTGGTTGCTCACACACAAAAAAAAAAAAAAAACAACCATGTACTGATTAGATAAGTGACCT

Homo sapiens CCCTATGTGTCATTAAGGAGTAATGCTCTAAAATGTAATCAGTGGAAAA-CACAACTGTAGCCTTGGAAATGCTATGGCTGGGAAAGTCCAT-ATAACTAAGACGGGGTTCATGATATTG
Bos taurus CCCTATATGTCATTAAGAGGCAATGCTCTAAAGTGTAAACAGTGGAAAAACCCTACTGTAGCCTTTCGAATGCTATGGCTGG-AAGGCCCATCAAAGCTAAGAAGGGATTTATGATAT--

Homo sapiens TGACATGTACAAACAAGTTATTTGACCATTATAGAGAGAAAAATAAAAATAATTAGAAAGAGAAGTTGCTTTTATTACTCTGTGCCCTGGCCTACTGACACATTTATTGACTTTCTGATA
Bos taurus -GATATGGCTAAGTAAGCTATTTAACCATTATCAAAA--CAAATAAAAATAATTAGAAAGA--AGTTGTTTCTCTCATGCCGTGCT-TGACCTGGTGATGTATTTACTGTCTTTCTGATA

Homo sapiens GGGTGATGCATGGATGCATCAGACTGAATAAACTACATGACTCCATCTCTTCAGATCATCCCACACCATATACT-ATCTCTTCACTCACCAGTAATAACAGTCACCAT-------ATAAC
Bos taurus GGG-AATGGATGGTTGCATCAGGCTGTATAAACTATACGACTCCATCTTTTCAAAACATCCCACAC--TCTACTGACTTCTTCACTCACCAGTAACAACAGCCACCTTGTACTAAATAAT

Homo sapiens AAGTGCCTACA--GAGTCAAACACTTTACATAAATTATCTTATTTAATCCTCAAGGCAACTCTATGATATCATAGAATTCTATTATTAGTTCCATTTTCAAATGACGGAACTGAGGCTCA
Bos taurus AAGCACCTACTCTAAACCGAGCACTTTATATAAATGAACTGACTT--CTCCCACAGTAACCCTATGACCTCATAGGTTTCTCTAGTTAGCCCTGTTTTCAGATGAGGGAACTGAGGCTCA

Homo sapiens GAGAGGTTATGTGACTTA----AGCCCATAAAACGGAATGAGTGGTATATTAGTTTTCTGTGACTACCATAACAAATTGCCACAAACTTGGTGGCTTAAAACAGCAGATATTTATACTTT
Bos taurus CAGAGGTAATGTGGCTTACACAAGTCCATAAAACTAAATGAGCA--ATACTAATTTCCATGGCTACTGTA-CAAATTACCACGAATTTAGTGGCTTGAAACAACAGAAATGCATTCTCT

Homo sapiens CACAGTTCTGGAGGCCAGAAGTTCAAAATCAAGGTGTTGGCAGGGCCATGCTCCCTACAAAGGCTCTAGGGGAGATTCTGTTCCTTGCCTCTTC-------CTAGCGGTTCCAGGTGTTG
Bos taurus CACAGTTCTGGAGGCCAAAAATCTGAGATCAAGGCATTGGCAGGGCCATGCTTCCTCCAAAGTCTCTAGGGGAGATTGCATTCCTTGTTTCTTCTTAGTTTCTGGTGGCTGCAGACTTAG

Homo sapiens CTTGGCTTG-TGGTAGCATCATTCCAATCCCTGCCTCTGTCTTCAGATGGGCTTCTCCCTTGTCTCTGTGCCCTGTCACTTATAAGGACACTTATCATTGGATTTACAGCCCACCCTAAT
Bos taurus CTTGACTTACTAGCTGCATTATTCCCACCTCTGCCTCTATCTTCACGTGACCTTCTCCCCTATGTCT-----CTTTCTATTTTAAGGACTTTCATCATTGGATTTAGAGCCCACCCTAAT

Homo sapiens CCAGGATGATCTCATGTAGACATCCTTATCTTACATACATCAGCAAAGAGTCATATTCTAAATAAGTTCACAT--TTTGCAGTTCCAGTTGGATGTAACTTTTGGGGGCGGTGACACAAT
Bos taurus CCAG-CTGACCTCATATAGAGATCCTTACCTTAATTACATCTGCAAAGACCCTTATCCCAAATCAGATCACATATTCTGAGGTTCCAATTGGACATATCTGTTTGTAG----AACAGCAT

Homo sapiens TCAACCCACTATGTATAGCCTAGCTGGGACCCTAGCCTA--TCTG----ACTCCAAAACTCTGGCTCTTTCCACTAACTAAAGCAGGCTGCTCCCACCAAACAAACAGCCTTTTGTCTT-
Bos taurus TCAACTCACTACAGGTAGCCTAGCTGGGATTCTAACCTAGATCTGGATGACTCTAAAATCTTGGCTCTTTTCACTAA----ACCAGGCTGCCTCCACCAAA-----AGGCGTTTGCTTTG

Homo sapiens -----TATTTGGTTTCCTAAGTAGATTTCCTACTGGGAT--------------------------------------TTATCAAGATGAATTAATAAATTGACATTTTGGCTTCCAAAT
Bos taurus CCTTTTATTTGGTTTCCTAAGTAGATTTCCTACTGGGAAGAGGACGCACTTGTTGACATAGGTTTGCTTTGTAATTAATTATCAAGATGAATTAATAAATTGACATTTTGGTTTCCAAAA

Homo sapiens TATTGCCTTATAAGTTTAGGTTGTGTGAAATTTGTCCCCTAGAGCCTGATCAATCCCTGGCTAAAATGGAAAAAATTGAGTTTTGGAACCTGTGAAGCTGATGAATGGATCCTGAATAGG

```
Bos taurus    CATTTCTTTGTAACTTTAGATTGTGTGAAATGTGTCCCCCAGAGCTTAATCATTCCCTGGTTAAAACTGAAAAAATTGAGTTTTAGAACCTGTGAAACTGAGGATTGTATCTTGG-----

Homo sapiens  ACCAAGGTCCATTTAGAGTATAAGCATCCCACAGCCTGCCAGCCATACTAATTTCTTTACTATATATGTGACCATAATATAATGGGATAGCATAATATTCTAAGTCTACCGAAGAATGTC
Bos taurus    --CAAGATCCCTTCAGGATAGAAGCATCTCACAGCCTACAATCCATGTCCATTTGTTTACCATGTAAGAGACCACACTA--ATGAAATGCCATAAATTTATGAGTATACAGAAGAATTTC

Homo sapiens  TAATCATCTAAGAGCTTACAGGGGGAATTTTTGTG-TACCTTAATATTATATCC-AGAAATATTCCCTGTA-GTGCCAACTATATTTGTTCCTGAGCCTCAGCTCCCTGCATCAGGTCAC
Bos taurus    TAATTACTTAAAAGCTTCTAGGAGGAATTTGGATTCTACCTCAATGTGATGTCCCAGAAAGGTTCCTCATAAGAACCTTCTATGTTTGCTCCCGAGCCTTGGCCCCCATCATCCAAACAT

Homo sapiens  AAGGAAG---ATGTCTCTAGGAGTTCATTATCACATTAAGACAAGGATTCTGTAGGAAAA-TGGGAATCTAGAAACAGTAGGGACT-----------------------TCCT------G
Bos taurus    AGGGAAGGAGATGCCTTTAAGAGTTCTTTATCATATTAAGATGAG-ATTCAGTAGAAAAAATAGGGATCCAGAAACAGTAGAAACTCAGTTTCGAACCCCCAACCTGCTTCCTAAATTAG

Homo sapiens  TACCTCTACAT---CAGTGTCTTTGC-----------------------------AGGGGAATTGCCCTAGCTGACCAGTTGACACTGGCC-TGCTCACACCTTAATTATGAATAGAATA
Bos taurus    TATTTCTACATATTCAGAGTCCTTGCTAAGTTAATAGGCAATGTGAGTCTCAAAGAGAGGGATGGTCTCTGCAGAACAACTGCCCCCAGCCCTGCTAACACCTTGATTAGGAAGAGAATA

Homo sapiens  TTCCCAGGGATGTCTTTCTCAGATTGTGGTCAGTTTGTGGGCCGCCTATTGTGGTGGTCCACTGAGTGACACCTGGGGAGCTAGTTAAAAATGTCAGTTTCCTGGTCCTTTATAGCAGAC
Bos taurus    ACCTTTGCAATGTGTTTATCAGGTTGTGATCAGTCGGTGGACCACCCATAGCGATGGACCACTGAGTGTAACCAAGGGAGCTTGTTAAGAGTGCAAACTTCCTGGCCCT......CGGGC

Homo sapiens  TTACTGAGTTAGTATATCTGAAGATAGGGT-CAGGAGCCTGCATTTTAAAACAAGATTCTGGAATAATTCTGATACATATTCAAGTTTGAGAACCATTGCCTTCTGGTGAAATCAGGCTT
Bos taurus    TTACTGAGTTAGTATTTCTGAAGATAGAGTACAGGAACCTACCTTTTAAAACAGGTAACTGGAATAATTCTGAGATACACTTAGGTTTAAGGACCATGGCATTCTGGTGAAATCGGGCTT

Homo sapiens  CCATGACGTTGAAATACAGAATAAACTCCAAATGTTCATTTACAATTCCAGCCAATAATCACTTACCTTATTGCATTCTTGTAGCGTGATCTATATGCTTTAGTTCATGTGACAATATCA
Bos taurus    CCACAGCAGTGAAACACAGATTCAACTCTAAATGTTCATTTACAATTCCAGCCAATAACCAATTACAATATTGCATTCTTACAGCATGATCTGTAGACTTTAATTTGTGTGACAATATCA

Homo sapiens  TTTCATATGGCTTAATTTAAATGAAAGCAAAGGCAGTTATGGCAAGATTTCTTAATCCTGCTCTCCTGAGACAATTTACATAATAACTTTTGGGGAGAATGCAATAATCTCTCTCTAATA
Bos taurus    TTTTATATGGCTTGATTTAAATCAAA------ACAGTTATGGCAAAATTTATTAATCCTGCTCTCCTGAGACAATTTATGTAATAAATTTTGAGGAGAATACAATAA----CCTCTACTA

Homo sapiens  TCTTAAACTGATCTCTAAG-GTTCCTTTTGGCTCCAACATTCTGATTCTATCAGTGTTCCTAGGGCTTCAAATCTGTCTGTGCAAAACACATTGCTTGTTACAGACTAAACCTATTTTCC
Bos taurus    TCTTAACTTGATCTCTAAATGTCCCTTTCAGCT-CAACATTC-AATTCTATGAA-GTTCCTAGGGCTTCAAATCTGTTTGTCACAAACACATCACTCATTACTGACTAAACTTATTTCTC

Homo sapiens  TTCTTCCAGGCAACAAGTTTCTGAAGAAAACTGGTTGCTCATGATCTTTCTGGG--------TGTTTTCTAATAATATATACCTTCTCACAATATTATTGCAAAGAGTGAGCGAGGTGAT
Bos taurus    TTCTTCCAGGCAACAAGTTTCTGATGAACACTGGTTACTGGTGATTTTCCTGAATTTTTTTTTTTTTTTTAATAACAGCTACCTTCTCACCATGTTATTGTGAAGAGTGAGTGAGAAAAT

Homo sapiens  CTATGCAAAGTGCCAAGCATAGTGCCTGACACAAGGAAGTGGTTAGTAAATATGCATGCTGCTTCCAGTGCATTGTGACAATACTATCTCTTGAATTATTGAAAATATAACATCAGGAAA
Bos taurus    ATATGTGAAACGCCAAGCAGAGTGCCTGACTCAAGAAAGTGGTTAATAAATGTTCATATCACCTTCTGTGCACTGACACAATAATATCTCTTAAATTATTGACGAGATAACATGGGAGAG

Homo sapiens  CTTCAGCTGAATGAAAACTAGGGAATTAGCTGTATTGACAATATTAGG--TTTATTTGGCTTTAGGGGAACTAGATAGAACCGAAATCTCTGCAGACTGGCTCAGCTGCAGAATAGAGAG
Bos taurus    CTGCAGATGAATGAAAATGAGGGAATCAGCTGTACTGACAATATCAGGGTTTTGAGGGGCTGTCAGGGAACTGGCTAGAACCCCAGTCTCCAAGGGCAAGATCAGCTGCAG-ACAGTGAG

Homo sapiens  ATGGCCCGGGGAGGACATGTGTTGGTTCCTGCCATGCATATTGTCTTGGAGAAAAGCTGCTATGACTCTGGCCCAACAAAGGTTTTATCCTAAAAGTCAGCTTCCAATCCCCAACATGAT
Bos taurus    ATGGCTTGGGGAGG----GTGTGAGTTCCTGCAACACACACCACCTTGGAGAAAGGCTGCAGTGAGT-----------GAGGTTTTGGCCTAAAACTCAGGTTCTAATCTGCAACATGAT

Homo sapiens  TCCTAAGCTAGCATTTCTCAAAATGTATTCCATGGAGTACTAGTTTCTGAGGATTGA-----------------TGTTGATA--GTATTACAT---AAAGAGCTCCATGAACAAGCAATG
Bos taurus    TCCTAACTTAGTGTTTCTCAAAGT-TGTTCCACAGAATATTAGTCCTTGGGGATGGACTAATTCATATTCATCTTATGAATAAGATGTTACACAAGAAAGAGCCCTAGGAACAGGCAAAG

Homo sapiens  TTACAAACCACTGGATTAGTCAAATGTGGTTTCCTGGCTGCGAGAATATTCAGAGTCCTTAATAGGTCAACAGGTAATGTGAATCTCCA-AAAACTAATGGGGTATACCGGATTTGAAAA
Bos taurus    TTGTGAACTGCTGGATTAGTCAAATTGGATTTCCCAACTACAGGAATATTCAGAATCCTTGATATGTTAACAGGCAATGTGGATTCCTATGAGAAGGTTGGGGAACACAGGGTTTTCAAG

Homo sapiens  TTTTTGTGACCTCACTCACAGAGATGGTGGTGAGGGACTAGTGGTTCTGAAAAATGCAATTATGAGAACACTACTCCAAAATGACTAGTG-------ACTTGCTAACAAGAATGGACACT
Bos taurus    GTTTTGTGACTTCACTTACAGGGGTGATGAT---------GTGGTTTTGAGAAA--CAATCGTGAGAACACTGCT-CAAGGCCACTGGTGCCCTCCTACTTGGTAACAGGAATGGCTGCT

Homo sapiens  TTTCAGCCCTTACATGATAAAAGTT---AAGAGAACAATTACGTGCTAAACTATAGAGTACTGTCCCTCAGCCCCCTAGTGTCTCCATTTTCTTCTTTGATCAGCTTAGATTCTATAACC
Bos taurus    TTTTAGCCCTTTCATGACAAAAGTTAACAAGAGAACACTTATGTGCTGAACTGT-----------TCTCAGTCCCCTGGTGTCATCATTGTCTTCTTTGTACAGCTTAGATCCTATGTGC

Homo sapiens  ATCCTTAAAGTCACTGTTTAGCATACACCCTAGAT-TCCCTAGGCTCTCTCTGCTTTCATGTGATTTGAATAACAGACATCCAATCGTTGTTAAATCCTACTCTC--TTTATTCCCAACC
Bos taurus    ACCATTATATTCACTGTTTGGCACACACCCTATATCTCCCTTGCCTCTCTCCACCCCCATCTTAGTTGAGCAACAGACACCTAATCCTGGTTAAATCCAACTCTCTATCTATTCCAAACC

Homo sapiens  TGCCCCAGCAGCCGAATGAGATTGGATAAAAGCTTATAACCGTGCTAACCAGTCTCGCTTTAGATTCAGGACCACTAACTTCAAATGGGCCCTTAGTACTATCTGCTAACTTCACTACAT
Bos taurus    TGCCCCAGCAGCTGAACAAGATTAGA-AAATTCTTAAAACCATGCTAACTAGTCTCACTTTCAATTCATGACCATTAACCTCAAGTGGGCCCTTCATGCTACCCTCCAATTTTCCTACAT

Homo sapiens  TCATTTCATCCATTCATTCTTGAATGCTCCTGGGTGGCTTTTTTACATCTTTCCTT--------------------------------TCCTCA-------AACTTCCGACACTTGT---
Bos taurus    TTTCTTCATTCAACCGTTCTTGAACTCTCTCTGATGGCTGTTTTGCGTCTTTCCTTCTGTAGGGGATCTTACTGCCCCAGGGATTGAATCCTCATCTCTTGCATTTCCTGCATTGGCAGA

Homo sapiens  -----------------------------------------TTCTACATTTTCCC-----------------------------------TTTCAGCTGCT-ACCTTGTTTCCTATTTCA
Bos taurus    TAGATTCTTTACCACTGAGCCACTGGGGAAGCCCAGTGGCTGTTTTACACCTTCCCTATCCTCCTCCCAAACCCCTTCCCCTATCCTCATTCTCAGCTGCTGACTCTGCTTCTCATTTCA

Homo sapiens  CTGAGAAAATAGAAGTTGTCAGAAAAGA----------ACTCCCACC------------AATCTATTTGTATTTGTATCCATA--------------------AACTCTGCCTTCCCTCT
Bos taurus    CTGAGAAAATAAAAAATGTCAGAAGAGAACAAGTGCAGACTCTTACCATGGTATCTGCAAACCTACTTGTATCTCTATCCACATACCCTGCTTCCTGCTTGTCAACCTTGCCTTCCTGCT

Homo sapiens  TGTCACTGTGAGCGAACCATCTGGGATTCCACTGAATGCTGACTCCTCTACTTATGCACTAAATTTTATTTCTTTTTGCCTAATCAAGGTCATTACTCAAGTCA-TTCACTCTTCCCTCA
Bos taurus    TGTCACTAGGAATGAACTGTCTGTAATTCCACCAATGGCTGACTCCTTCACTTGTGTGCTAGATCTTTCCCCTTTTGAACTTACCAAGGTCATGACTCTAGTCATTTTTCTCTCCCTCTA

Homo sapiens  TGGCAT---CAATTTTTCTTTCATTGAA-CAACATCTTCAATGTT------TAAAAAATCTGTTCTCTTCACCCAAGTCATAGCCCCATTTCTCT-----CCTCCTCAGAGCAAAAATTC
Bos taurus    TGACATCAGCAATTCTTCTCTCACCAAACCAACATCTCCAATGTTAAGAAACAAAACAAAACTTTTCTTTGCCTCCGCTACAGCCCCATTTTTTTTTCTCTCTCCTTAAAGCAAAA----

Homo sapiens  TGGAATGAGTTGTCTCTGCTCATTCGTTCCAATTTCTCTCCTCTCATTTTCTCTTGAATCCTCTCCAGTCAGGCTTTTGCCATCATCACTCCACCAAAATGATTCATATCAAGGTCAACA
Bos taurus    -----------------------------CATATTTTCTCCTACTAGTCTCTCTTGAACCCTCTCCAATCAGGCTTTTGCCCCCAGCACTCCACCAGAATGATTCATGTCAAGGTCAATA

Homo sapiens  ATATCTCCTCAGTATTAAATTCAATGGTCATTTTACTGTTCTCCTTTTTTTTTCGCCCAACAGCGGCATTTGACACAGTTTATCACTCTCTCCTTGAAACACTTTTTTCTTCCACTTGGC
Bos taurus    GTATCTCCTCAGTGTTAAATACAATGATCCTTTTTCTGTTCTCATTTTATTTG-ACTCAACAGCAGCATTTGACACAGTTGATCACTCCCTTCTTGAAACAATTTGTT----CACTTGGC

Homo sapiens  TTCCAGGACACCA---TGTAATTCTTGGCTCTTTCTACTTCACTAGCTACTATTTCTTAATATCCTTTACAGGTATGTCTTCATTTTCATGATAACTAGGTTGTCTGATTGGCATCTCAA
Bos taurus    TTCTGAGACACCACTCTGTATTGGTT--CTCCTCCTTCTTTGCTAGCTACTCTTTCTCAGTATCCTTTGCTGGGTTCTCCTCATCTTTCTAACCATTAGGCTGTCTAATTGGCCACTCAA

Homo sapiens  CTTGAAATTATCCAAGGTCAAAGTGACTATTTTCCTTATACCTGCTTCTCCCACAGCCTTCTTTATCCATTCTTCTGGTTATTTAGGCCAAAGCCTTGGAGTCTGACTTGA---CTCCCT
Bos taurus    CTCAAAATTCTCCAAAACCAAAATGACTATTTTCCTCAGACCTGTTTATCCCACAGTCTTCTTTA-CCATGTTTCTAGAATTTCAGGCCAAA--CTTAGAGCCTTCCTCGACACCTCCCA

Homo sapiens  TTTTCTCTCACACTCCAGATCCTATCTATCAGCAATACTTTCAGCTGAATAGTTAAAAATTTATTTGTTTAGAAACAGCATCTCACACTGTCACCCAGGCTGGAATGGAGTGGCACAATC
Bos taurus    TTTTCTCACACCTCACAG----TATCAATCAGTGATACTGTGGGTTGAGTCTTTCAAAA...........................................................

Homo sapiens  ATAGCTCACTATAACTTCAGACTCCTGGGCTCAAGGGATCCTCCCACCTCAGATTTCCCAGTAGTTGGAACTACAGGTACTCACCACAGTGCTGGCTAATTATTATTATTATTATTATTA
Bos taurus    ..........................................................................................................................

Homo sapiens  TTATTATTATTATTATTATTATTTTGTAGAAACAGATGTCTTGCTTTGTTGCCCAGGCTGGTCTTGAACTTCTGGCCTAAAGTTATTTTCCCACCATGGCCTCTCAAATTGTTGGGATTA
Bos taurus    ..........................................................................................................................

Homo sapiens  CAGGTGTGAGCCACTGCACCTGGCCTAAAAGTATATTTAGAATCTGAACACTTCTCTACTGCTATAACCTTAATCCAAGCCAGCATCATTTCTTACTTGGATCATTACATAATCCATTAA
Bos taurus    ..........................................................................................................................

Homo sapiens  ACATGTAAATCAGATCATGTCATGCTCCTGCTCAAAACCGTCCGTGGCCTTTTATATTGTTCACAATAAAAGCCAAAGTCTTTAGAGTGACCTACAAGATTCTACATGATCTACCTTCAT
Bos taurus    ..........................................................................................................................

Homo sapiens  TCCCATTACCTCTCTGACCTCATGCCCTGTCACTCTCCTCTTTGATTACTGAGATCAGCCATATGGCTTACTTGCTGTTTCTCAGACATACCAAGAAAACTCCCAGAGGTCCATTGCTTT
Bos taurus    ..........................................................................................................................

Homo sapiens  TCTCTCTAACTAGGATGTTTCTTCCTCATAGAGTGTCATGCTTATTCACTTGCTTCTGTCAAATCTCTGTGCAAATGACACTTTATCAGTGATACTTTCTTTATCATCTTATTTAAAAAT
Bos taurus    ..........................................................................................................................

Homo sapiens  TGCCACTCTCTGCATGCTTCCTACAACTTTACTCTGCTTAATTTTTTTCCATAGTACTTGTCATTTTCTGATATATTAATATTGATTTGTTTGTTTACTGTCTATCTTCACCACTAGAAT
Bos taurus    ..........................................................................................................................

Homo sapiens  ATAAGCTTCAGGAAGGCAGGGATTTTATTTTGTTGCTGCTGTATCCCTAGTGTGTAATGTGGTAGCTGGCACATAATAGGTGCTCAATAAATTTCAAATTATTATAATAAAGAATGATTC
Bos taurus    ..........................................................................................................................

Homo sapiens  CAAGTGCAATAGGAATTCCAAGATTCCTGCCATCTTCAGATGGACCCAGATATCCTTCATCATCTGCAGCAATCCCCCACCCAGTGCCAAACTCTGTGACACTGCATGTCTGGTAGTGAG
Bos taurus    ..........................................................................................................................

Homo sapiens  TTTAGTCTTTACATGAGGACTTCCAGGAAGTTCCATAGATTCTTAAGCAAAGTGCTCCATTATTGAAGCTATTTAACTCCTAGAAAGATCTTTGTGGCTGTGTGCGGTGGCTCACGCCTG
Bos taurus    ..........................................................................................................................

Homo sapiens  TAATCCCAGCACTTTGGGAGGCTGAGGTGGGCAGATCAGGAAGTCAGGGAATTGAGACCATCCTGGCTAACACAGTGAAACCCCGTCTCTACTAAAAATAGAAAAAATTAACCGGGTGTG
Bos taurus    ..........................................................................................................................

Homo sapiens  GTGGCACGCTCCTGTGGTCCCAGCTACTCGGTAGGCTGAGACAGGAGAATTGCTTGAACCCGGGGGGTGGAGGTTGCAGTGAGCAGAGATGGCACCACTGCACTACAGCCTGGGCGACAG
```

```
Bos taurus    ........................................................................................................................

Homo sapiens  AGCAAGATTCTGTCTCAGTAAAAGAAAAAAAGAAAAAAAGAAAAAGAAAGATTTTTGCCAAGGGTCCTTTTCTGATACATAACATTTTTTGAATAGGTATTAGGTGTACACGATACAAAG
Bos taurus    ........................................................................................................................

Homo sapiens  TGCCAAATGTTCAAAGAGCAAGTAGTGAAAAGTATATTCTACCACTCCCACTGCTACCAACTTATTCCAAGCCACCCACATTTCTAAACTGGAATTATAGCACAATTTTTTTAGAAACAG
Bos taurus    ........................................................................................................................

Homo sapiens  AAGTTAGATGATGTCGCTCCTCTGCTAACATCAGCCTGGGGCATGTCATCACACTCAAGGTAAAAGCTAGAGTCTCCCTCTGCCCCCAGCCACCAAGATCCCTTCCTGGAGGCAAACACT
Bos taurus    ........................................................................................................................

Homo sapiens  CTTACTAGTTTATTGTGAATTTTTTAGAGATAGCATGTATATTATACACACATATCTCCATGTGTATAATTCATTTTTAAACTGTACACACTATTTTTTATATTTCTTTTTTTCTCACTT
Bos taurus    ........................................................................................................................

Homo sapiens  TATTCTACCTCATTCTTTTTAATAGCTACAGTTGTATCAGTATGGCTACTGTATTCTCCAGAGATTCTTAGTCTGGAGTCCATATATGGGTTTCAATGGGTCTATGAATCTCCTGAAAGT
Bos taurus    ........................................................................................................................

Homo sapiens  GCATGTTGACACTTGTGTGCGTGTGTACATGTATATCTTTTAGCAGAGCAGGCCCATATCTTTGATCAGATTGAAAGGAATCTGAGACCCAAAATGAGTAAGAAACCACTATATTGAGTC
Bos taurus    ........................................................................................................................

Homo sapiens  TGAATCTGCCTTCCTGAAACACCTACCCTTTAGTCCTAATTCCACTGTTTGGAGCAGTTCTGTCAGGACCTGGTACATAGAGGACATACAACAGGTAACTTGTGAAATGGATGAAGGGAC
Bos taurus    ........................................................................................................................

Homo sapiens  AACATAAAATGAATCTATTCTGTCTCTCTCTTTTTAAAATTTTTAAATTTTTATTTATTATTATTTTTTAAAGACAGAGTATCACTCTTGTCACCCAGGCTGGAGTGCAATGGCACAATA
Bos taurus    ........................................................................................................................

Homo sapiens  TTGGTTCACTGAAAACTTCCACTCCCAGGTTCAAGCAATTGTCCTGCCTCAGCCTCCCCAGTAGCTAGGATTACAGGCACCTGCCACCATGCCCAGCTAATTTTTGTATTTTTAGTAGAG
Bos taurus    ........................................................................................................................

Homo sapiens  ACATGGTTTCACCATGTTGGCCAGGCTGGTCACAAACTCCTGACCTCATGTGATCCACCCGCCTAGGCCTCCCAAAGTGCTGGGATTACAAGCGTGAGCCACTGCGCCCGGCCTATTCTC
Bos taurus    .................................................................................................................TACTCCC

Homo sapiens  TCTTCTTTATGTTAGCTTTTGGTTAGCCTGAGGAAACT-ATCATAT---TCTAAGTCATCCCTTTTCTAAAGTTAATTACTTAATTTTCTTAACTTTTCCTAATTTGACATGGTTTCCAA
Bos taurus    TCTTCTCTGTGCTAGCTTTTGGTTGGCTTGGAGAAAGTCATTATGTCCTTCTAAATCATCTCTTCTGCAAGGCAAA--------------AACT---CTTAATTTGTCATGGTTTCCAA

Homo sapiens  ACCTGTAGGCTTTCCCT--------ACATGCTGTAATGTGGGT-TTCTTCCTCAAATGCCATCGG-GGCAAGCTGGTAATTTA-AAAAAGTGAAGTGGATCAGATGTGAGACAACGGCAA
Bos taurus    ACCCACCTGCTTTGCTCTCCCCGGGACAAGCCCTGATATGGATGTTCTCCTCCACATGTCTTCAGTGGAAAGTAGATACTTTTTAAAAACTGAGGTAGACCAGTTGTGAGACAATGGCAA

Homo sapiens  GTGGTGGGACCTGTGGAGTCCACATGGCT-----AAGATGTTCACATTCAAACTCACATATAACACATCCATAACCAAACAAAAAATATTTTCAGGCCAGTTTTGGACTATGGGCAGGCA
Bos taurus    GTAGTGGGACCTGTGGAGTCCATGTAGTTGTCTAAAGATATTCACATTCAAACTCG--------------------AGACAAAACATATTTACAGGTCCATTTTGTCCCGTGAGCTGACA

Homo sapiens  GCTTGCAAATATAAAAATTTGGACCTCGTACAGTTTGTTGATACTCTTTTCACATCTTCTGCACTCTATCAATTCACTTTTGTCCTCCCC-ATAGGCCTGACAATTTAATCAAACTAAGG
Bos taurus    GTTTGCAAATCTA----TTTGGAACTCATACAGTTTGGCAACACTCCTTTCACATTTCCTCACTCCATGACCTCACCTTTGTCCTCCCCCATAAGCCCAACTATTTAGTCACACAAGAC

Homo sapiens  TAGTGCCTGCATCTCAAATACCCCTTAAGATTCCTTGCTATTATTCCTTTGCCAATATGGCTCTGTCTGTATAAAATGCCTTTGTGGAATATTCTCTACTTGCCCCTCAAGATCCAC-TT
Bos taurus    TATGCTCTGCTTCTCAAATACCCCTTGTGATCTCTTGCTCTTATTCCTTTACCCATATGGCGCTCTCTGCATCAAACGCCTATTCGGAATAGTCCCTATTTGTCTCTCCAGATCCACTTT

Homo sapiens  TTAAAAATAG--ACTATTTTTTAGAGCAGTTTTAGGTTAACAGCAAAATTGAGAGGAACATACAGAGAGTTCCCGT-TAGCCCCTAAAGATCCAGTTTTCACCCTTCTTCATCCTCTTTG
Bos taurus    TTAAAAATAGTTTTTATTTTTTAGAGAAGTTCTAGGCTCACAGAAAAATTGAGAGGAACATATAGAGAGTTTCCGGATACCCTCAGTGGATCCACTTTTCCCCCTTTTCCACTCTTTCCC

Homo sapiens  TGTACCCCCCGAGACTGACCTTTATAGACTGCATTTAAAAACATATTTTTAGTTATTGTGGGTACATAGTAGGTGTATATATTTTTGGGGTACATGAGATGTTCTGATACAGGCATGCAA
Bos taurus    TGTACCTCTAGAGGCTGACCTTTGTGGACTGTATT.....................................................................................

Homo sapiens  TGTGAAATAAACACATCATGAAGAATGGGGTATCCATACCCTCAAGCATTTACTCACTGAGTCGCAAACAATCCAATTACAGTCCTTAAATTATTTTTAAATGTACAGTTATCACTGGCT
Bos taurus    ........................................................................................................................

Homo sapiens  ATAGTCACCCTGTTGTGCTATCAAATAGCAGATCTTATTCATTCTATTTTTTGTACCCATTGACCATCCCCAACTCCCCCCAGCCCCCCACTACCTTCCCAGCCTCTGGTAACCATATGA
Bos taurus    ........................................................................................................................

Homo sapiens  ATTGGCTTCCTTGTCCTCTGAATTCTTCTTGGATATGGCCAATGTAAGGCACTAACAGGGGACTGGAGATCAGAGACAGGTTAAAGTCTGCGTATTTATTCTCCTGGCTCCCTCTTTATG
Bos taurus    ................................................................GGAGACCAGAAGCAGATTAAAATCTGAATATTTATTCTCCTATCCCCCTCTCTGTG

Homo sapiens  GAGTGGGTGCAGCTTGGCTGAATCCTGTTAGAAGGATCTGCTGTGAGCCACAGACTTTTTTTTGGCAGTCATCTTCTAAAGCTATGGCTACTCTGTCTAGGTTCTGGTTAACTGCTTTCT
Bos taurus    GAGTGGTAGCAGTTTGGTTGAGTCCTTCTAGAAGAATCTAACATAGGCCACAGACTTTTTTT-GGCAGCCATCTTTTATAGCTACAGCTACTCTTTCTGGGTTCTGAT-AACTGTTCCTT

Homo sapiens  CCCCTTGGCTTTTCAGGTCTCAGGGTGGTAAGTGCTCCCTGGGGTGCTTCACCATCCTTT-TTGCTTTCCCTTAACAATGCTCACACTGTTATAAACAGTCCGTTCATTAAACTCCACTC
Bos taurus    CCTCTTGGCCTTTCAGGTATAAAGATGGTAAAGGCTCCCTGGGCTGTCTCACCATCCTTTCTTGATTTCCCTTAACGCTGCTCACATGGTTGTAAACAGTCCCATCATTAAACTCCCCTC

Homo sapiens  ACTT-CCCTGTTG-AATATGCTCTCAATATCCTACTAGATCCTTTACTGTTACCAATGCCTTCAACTCCCTTGTGTACTTGGTGTACTCCCCATTGTTAAGGTCCCAGCTCCTAGGACAA
Bos taurus    ATGTATTCTGTTTCAGTATGCTGTTTGTTTCCTACTAGACCCTTGACTGCCACCA-TGCCCTGG-------TGTCTATTCGGTGAACTCCACTTTGTTCAGGTCCCAGCTCCCC------

Homo sapiens  TGTGGTGCATTCCTAGATATCACTCCCTTTGAGAAAGTTAGCTATTCCCCCACCTCTTTGAGAACATTTCTTGCAACACTCTG------------TGT--------TTAGATCTTGGGCT
Bos taurus    TGTGAGGCTTCCCTGAGTGTCCTTCCTTCTGAGAAAGTTAGATATTTCCCCAGCTCCTTGAGAACATTTCTTACAGCACTCTGATTATTTATGCATGTCTCTCCCATTAGATCTTGAGCT

Homo sapiens  TCTTATGATTGGGAATGAAGCCTATTCGTTATTGTT--------TCCTGGCCAAGTAGATGTTTGGTGAGTATTGGTTATCTTTTTTGCTTTGTGAGGAGGATATAAAATCAAGGAAGGA
Bos taurus    TCTTATGGCAGGGAATGAAGCTTATTCATCATTATTGTCTCAGCTCCTGGCCAAGCGCATACTTGGTACATGCTGGTTATCTTTTCTTTTGGAGAGAAAGGGGATGAAGTTGGAGAGGGA

Homo sapiens  GGATATTTGCTTTTTATTTCTTTCAAATTATTTGATTTTAAAAGAAATGCCTACACATTATTTTTTAAA----AATTAAAATGAAAAGAAAAAAGTAGTAAAAGTGGCACCGTTCTTCAC
Bos taurus    G--TGTTTGTTTTTCTCTTTATTCAAATTATGTATTTTTAGAAGAAATGCCTACCTATTATTTTTTAAATTATAACTAAAGGGAAAAGAGTGAAGTA---AAAGT----CTATCCCTCAT

Homo sapiens  ATCCGTGCTGGAAGTAACAGTGGTAATGGTTTTTGTTGTGCATCCTTCCAGTATCTTTT-TATGCACATAAAGGCTTAAGTTTGTGTACATAAAATGTAGACTCACATATTTCTCTGCTC
Bos taurus    ATGCATGCTAGAATTAGTCATAGTAATGGTCTGTGGTGGGCATCCTCCCAGTACCTTTTCTGTGTACAAAAATGTACATATTTATATATACACAATATAGACACCCAT----CTCTGTTT

Homo sapiens  CTTGCTTTTTTTTTAACTTAACAACACACCTGTCT---TTCTATATCAGCCAATGTAAGTATACAGCCCTACTTCATTATGTTTTATGGCTGCATTGTATTTCATTTGATGAATTTACCA
Bos taurus    CTTGCTTTTTTTC--ACTTAACAATGCAACAGTCTGTATTCTATGTCAGCTCATATAGGTATGCAGACCTACTTCATTAT-TTTTATAGCTGAATTGTATTCCATTTGAAAAATTTATCA

Homo sapiens  TAATTTATTTAACAAG-CCTCTATTATTAGCTAGTT---TATCTAATATTT-TCACTGTTAAAAGCAACACAACTTAAAACACAGAGACATACTGGATCAAATTTAAGCCCAAAAATATA
Bos taurus    TAATTTATTTAACAAATCCCCTATTATTGGACAGTTAGTTATCTAATATTGGTCCATATTAGAAGCAATGATTCTCAAAACACAGGGACACACTGCATAAAATTTAA-CCACAAAATATA

Homo sapiens  TGGCTGAGCTCCAGAAGCAAGAAAGGAAAACTGTCAGATTCCAGAGACAAGAG-AAAACTCACAGTCACAGCAATGAGCATAAATTGACATTTCACCACTCACAGGAAAAATGGGACCAG
Bos taurus    TAGCTGAACTCAAGAAGCAAGAAAGGAAAACTTCCAGATACCAGAAACAAAAGTAGAACTCAAAACCCTAGCAATGAACACAAATTGAAGTGTCACAACTCACAGGGATAATGGAACCAG

Homo sapiens  GTATATTCCTTAAAGGATGAGATTATAAGTCCTCTGTAGGGCTTAGAATTGAGTTAGAATTGTGCTTCCCTCATAAAACAAAGAGTAGAAGGATGCTGCCCCATTTATTATTTAAAAGCA
Bos taurus    ATAAGCACCTTGGAGACTGAGGTTTGGGGTCCTCCAAAGGGCTTGGAATTGACTTAGACCTGTGCTTCCTGCACAAAACAAGGAGCAGTGGAGACCTGCCTCATTTATGAATTGAAACCA

Homo sapiens  GATTTCCCAAATAGTCAGGGGTACAATAATAACAAATCTTATTTAGTACTTACTATGTGCCAAGCATTGTTCTACATGATTTACATGTATTTATTTATTTAACCATCACAGAAACCCTAT
Bos taurus    CCTTTGTCAATTAGGCAGATGCACAATAATAACAAATCTTGATCAGTACACACAATGTGCCAGGTACTGTTCTATATATTTTGCATGTGTTAATTTCTTTAATCTTCACA-AAACCCCAT

Homo sapiens  GATATTAGGTACTATTATAAACCCCATTTTATAAGTAGGGAGTAAAACGTTTAAGTAAC----------------TTGCGCAGCTAGGAGGTGGCAGCTCTAAAATTCAGACTCAGGCAT
Bos taurus    TAAGGGAGGTACTGTTATAACCCCCATTTTACAAATAAGGGAGAAAATGTTTAAGTATTAATAAATTGCTTAAGGTTACACTGCTAGGAAGTAGTAGGTATAAGCTTCAGACCCAGGTAT

Homo sapiens  CCTGACTCCAGGACCATGCCTGTAAGTAAATGTGCTATATTGCTTCTCTACAAACAGGATGTAGGTCATCTTCCTTAGGATGAGG-TGGAAATAAACTCACCCATAAGAAGTTTAAAAC-
Bos taurus    CCTGATTCCAGTATCGTGCATGAAAGTGAATATACTATACTACGTCACTACAAATAAGATGTAGGCCATCTTCCGTAGGACAAGGGTGAAAATAAACTCATCTCTAAGAAGTTTAAAATG

Homo sapiens  TTTAAACCTGAGCTGCACACTAGTATGAAACCAAAATTCACAGTACCCACATAATGCAGAAACCCTGGGGTGAGAACTTAACATAAAACCTGGCTCAGAGCAGGA-----ACACAATGAA
Bos taurus    TTTAAACCTGAGCTACACACTGGTATGAGACCCAAATTCATAATGCCCATGTATTTCAGAAATCCTGAGCTGAGACCTTTACATAAAAACTAACTCAGAGTAAAATCCTCACAGAATGTA

Homo sapiens  ACTCTAGCAGAGGC--------------AAATGAAAAACCGCCTGTAGA--CCCACCTCCATAACTC-------------------------AGAGTCCAATTC------TTACGGA---
Bos taurus    ACTCCTGTAGAAGATAAGCTCACATAGAAAATTATAAAGCACATGAAGAAGCCCGTTACAATAAAATTTAGTCAGTATACACAAGAAATGGGAGAATTCAGTCCAAGGAATTATGGACAA

Homo sapiens  --------------------------------------AGGGTTCTAACAGAAAACAGTTCCTGCAGA-------------AGATAAGCTCACATGCAAAAAA---ATGATAAAGCACA
Bos taurus    TACAGCAATCCAAAAATACTTCAAAATATTGTATATTTAAAGGTTTAAAGAGGAAAAAACTCCAGAATACATATATATATATATATATATATATATGTACATCATATATAATATGGTATA

Homo sapiens  TG---------------------------------AGGAAATTGACTGGAATG------------------------AGTTAG--------TTGGTATATA------------------
Bos taurus    TGTTATATATGGACTTCCTGGTGGCTCAGATGGTAAAGAATCTGCTTGCAATGTGGGAGACCTGGGTCAGATCCCTGAGTTAGGAAGACTATACGTATATATGTGTGTGTGTGTGTGT

Homo sapiens  -------------------CAAGAAATGGGAGAATTATGT-------------------CCCAAGAAACTACAGATAATACAGTAATCTGAAAGGACTTTATTTTTTGGGTTTTTCTCAT
Bos taurus    GTGTGTGTGTATATTCCATCAAAGAATGGATGGAATATATACTTCATATATATGGGATTCCTTGGTGGCT-CAGACAGTA-AGGAATCTG..............................

Homo sapiens  CTTTTTGTAGCCCTCATGTATGAAAGAACTTTAAAATGTTGTATGTTTGAAGGTTTCAAGATGTAAAAAAGGAACATAATACATAAAGCAAGAGCACAACTTTCCTCGGTGTGTGTACAC
Bos taurus    ........................................................................................................................

Homo sapiens  CAAATAAATAGGCCAGAAATGAAGAATATAGAAAATATGGGATGAGCATTGTAAGATTTTAGTTCTAGCCCCAATGAAATATTTAAAAAGTCAAATGACAGTTTAAACTACAAATAAGTT
```

```
Bos taurus    .AAAGAAAAGTTCTAGAAATGAAGACTCTAGAAAATATGGGATGATCGTTGGAAGACATTAGTTCTCATCCCATTTAAACATT-ACAAATTCAACTGACAGGTTAAGCTACAAATAAGGC

Homo sapiens  ATGAGTGAAGAGAGAACAAATGGAATAACAAATAGAGCCGAGATAATCATAGAATATAGCATGGCACTATAAAAATGCTAAATATGAAAGGGAAAATAGAAGACACACATGATATA----
Bos taurus    ATGGATGAGGGAAGAATTAGTGAAATAA---ATAAAGCTGAGATATTCATAAAATATAGAACAACACTATAAAAATGCTAAACATGAAAGAGAAATTAGAAGATACAGATGATAGATAGA

Homo sapiens  ACAAGAAACTTCACATTCATCTAATA-AAGCTCCAGAA--------AAAAAATAGGAAGAGATAATATTCAAAGAGAAATAGCTGATAATTTTCTAAAACTGAACAGGACATGAGTCCTG
Bos taurus    ATAAGAAGCTCCACATACATATAGCAGAAATTCCAGAAGAGGAAAAAAAAAAACAGGAAAAAGTAATGTTTGAAGAGAAATGGCTGTGAATTTCTCAGAATT-CATAAGACACGAGTCCTC

Homo sapiens  AGATTAAAACCATGTACCTAGTTCTAAGCAGAATAAA----GACAAATTGATACCCTGTAGATGCAACAGGGTGAAACTGTAGAACATCAAAGATAAAATGAAAATCTTGAAACCTACTA
Bos taurus    AGGTTAAAATCCTGTACCTAATCCTAAACAGAATAAATGAAGACAAATGGATAACTTCTAGATGCATCATGATGGAACTTCAGGACATCAAGGATAAAAGGAAAATCTCAAAACTTACTA

Homo sapiens  TAGACAAACAATGTTGCAAAATATTTCTGTAGAATAGATTCCTAAAA-TGGAATTTCTGGGCCAGGGTATATGCACATTAAAAAAA--TGAATATGTTTTAAATGCATGAGTGAGTGAAT
Bos taurus    GAGACAAATACTACTGCATGGTATTTCTGTGGGATAAATTCTTAAAGGTGGAATTTCTGGAACAGAGGATAAGCACTTTGAAAAGAGATGGATGTGTTTGAAATGAATGAGTGAGTGAAT

Homo sapiens  TAATGAGTCTA-----GTAGTATTTCTCTCAATCTGGACAT-ATATTTATTTTAGTACAGCCTGAGATTGGATCCCCCAGCTATCTATTTATTTATATATTTGTGACTGCATCACCACTA
Bos taurus    TAATGAGCCAGTGGAGGCATTATTTCTCTCAATCTAGGCATGATATTTCCTTTAAAATAGCCTGAGATTGTATCTCCCAGCTATCCATCTATTTATACATTTGCAACTGCATCACTACTA

Homo sapiens  TTGATTTCTATTGAGCATAAGACCAATTAAAATCCCCAGGACTTTTTCAAATGAAAGGCTGTCAAATGAGGTCCTCTTTAACATGTACTTAGGCAATTAATCTTTAAAACTTAGAAGTAG
Bos taurus    TTGATTCATATTGAGCAGGAGGCCAATTAAAACCCCCAGGACTTTTTCAAATGAAGGGCTGTCAAATGCGTCCTTCTCTAGCATGTACTTAGGCAATTAATTTTTAAAACTCGGATGTAG

Homo sapiens  GGCTATTAAGACATTGGTGAGATCACTGAGAGCTAGAGCTGTTAAAGAAGGTTTCATAAAGGAAGTAGGACTTAGGCTGCA---------------TAGAATTTATTTTCGGTGGAGGGT
Bos taurus    AACTATTTGGTTGCTGGTGAGATCATCGAGAGCTGGAGCCATTAAAGCAGGCTTCTTATAGGAAGTAAGACTTCAGCTGCAGAGAAGTTCCATAAGTAGGATTTATTTTAGATAGAAGGG

Homo sapiens  GCAAAGGACAGCTTCTATGTTTGTGGGATGGAGGTAAGAAGAGTGATAAGGTGCATGAATAGACCATAGGATAGGAAATGAGCTTGGGGTGGATATGTGGAGGGTGGGTGAGTAAAGAGA
Bos taurus    ATACAAGAGAGCTTCCAGGTTTGTGGGATGGAGCTAACAGGAGTGATGAGGTGCATGAATAAGCCATAGGCTAGGAAATGAGCTTGGGGTGAATGTGTGAAAGTGAGGGGAGTAAAGAGA

Homo sapiens  TAAGGTTCAAGGGAAGGGGTGGGGCAAGATTATGGAGGGCCTAAGAAGCCAGGGAGAGTGTCTGCATATGATATTATACATGGGCAATTGGAAGTCCCTTCAGCCTTTT-TAAAGGAAAT
Bos taurus    ----GTTCAAAGGCAAGG-TGGGAATAGATTATGGAGGGCCTGGAAAGCCAGGTGGTACGTTTGGACTTGATAT----------CAATTGGGAGCCACTTCAGCGTTTTAGAAAGGAAAT

Homo sapiens  TATATGTTTTCTGACTGGCCAATGAAAGGCTAACTAACCCAGGAAAGAGCAAGAAGGCTGGGTGAAGTAATGCAGGCCCCTAGTTCTCCTGTTTCTGGGTTACTGGATAGGTTATATAG-
Bos taurus    TATAT--TATCTGGCCAGCCAAAGAAACATGAACTCATCGTGGAAGGAGTAAGGAGACTGGGTGAAATCAT-----------------------------TGCTA-ACAGGTTATCCAGA

Homo sapiens  CAAGTAGGCAAGCTTAGCAAACAGCTCAGGGACACTAGGGTGACTTGATGTTAAACTGCCTGACTAGTGAGAATTAAATGTAATAAAACTGGAATTGCAAGACTAAAAATGGTATTTAGG
Bos taurus    CAAGTAGTCAAGCTCATGAAACAGGTCAGAGACACTGGGGTGACTTGATGTTGAACCAACAGACTAGTGGAAACTATGT-TAACCAAACTATCA--GCAAGGCTGAAACTGGTGTTTAGA

Homo sapiens  CAGAATAATCT-ACTAGCAAATTCAGGATTCTTTAGAGTTGAGTGGGAAATATAAGAAAGAAAGAAAGATGCTGGATGCAGGAGAACAAGAGATGGAATGAGAAAGTGGAATATACACTG
Bos taurus    AAGAACAGTCTTAATAGCAAA--CAACATGCTTTAGAG---------------------------------CTGGATGCAGGAGAGCAAGAGACAGAAGTGAAGAGTGGCATATCTATAA

Homo sapiens  GAATGAGCAACA--GGAAACTTTAGTTCTAGTCTCAGCTCTACC-CCTACTAACCGCACAACTTTGTGCAGATTACTTAAACATTGAGGCTAGTTTCCTCCTCTGCAAACAACCTAGAAT
Bos taurus    GAATAAGCATTATTAGAAATTCTTATTCTAGTGCTGGCTCTACCTCCTGCTCATCACA--------TGCAGATTATTTAAACATTGAGGTTCGTTTCCTCATCTGCAAATCTACTGGAAT

Homo sapiens  ACAA--------------ACTCTAAAGGGTGGTAACCAGATCTGCCATGTTCATCATTGTATCTCTAGCCACTGGTACAGGGCCTAGAACGTAGTAGGGGCTCAGTAAATATTGGATGAA
Bos taurus    ATAAGTTCTAGACTCGGGAGTCTAGAGAGTTGTAACCAGTTCTGCTTTATTCACC---------CTAACCCCTAGTATAGGGCCTGGCAGATAGTAGAGCCTCACTAGATAGTGGTTGAA

Homo sapiens  TAGATGAATGAATGAATGAATGAATGAATCAATCAATCAATCAATTAGAGCCTGCCCTGCCTGCCTCACAGAGTTATTATAAACTTCAAATGAAATCAGGGATGGGGAAATATTACATGA
Bos taurus    TAGATGAATGAATGAACAAAGGAATGAATGAATGAACTAAT--------GCCTGCCCTGCCTGCTTCATGGAGTTATTGTAAAATTCAGATGAGATCAGGAATGGGAAGTTACTACATGA

Homo sapiens  ACTTAAAACACTGAGCAATCCGATGGTAAAATACTACAGCTACTACTCACAATTATTATTTTAAAAATTAAATTAGAATGCTATTTGTAGTACTTTAAGGGTGAAATCATAAGTGGGCTA
Bos taurus    AATTAAAACACTAAACAATCCTATGGTAAAATACTATAGCTACTACTGAGAAGTTTATTTTTTAAAATTAAATTAGAAGACTGGTTAGAGTACATTAAAGGTAAAATGAATGGTGGGCTA

Homo sapiens  ATGATTTCAAGTTTTCTTAGCATGGAAAGTAAGAAGAAAAGGAAAAGTATAAGACATTTTGAGTG-------------AGGTAAATAAAACATCAGAATCTGTGTTCTGAACCATGTTCT
Bos taurus    AGGATATCAAGTTTTCTTAGCATGGAAAGTAAGAAGAATAGCAAAAATGCAAGACACTTCAAGTGATTTTCTCAATTTAGGTAAAGAACACATCAGGATCTGTGTTCTGTACCGTGTTCT

Homo sapiens  GTCATTTGCATTCAATGTGCTTATGGTAGGGCTAATATTGATGTCAGTTATAGTTAGCATTCATTGACTACATACAGTAATGCTATGTCCTGTCTGTCCTTAAAAAAATAGAACACCCTT
Bos taurus    GTCCTTTGCACTCAATGTGTTCATGGTAGGGGTAATAATGGTGTTAATTACAGTTAACATTCTTTGACTACGTACAAT-GTGCTATGTCCTATCGGTCCTTAAAAAA--AAAAAACCCTG

Homo sapiens  CAAAGAAGGTAGTCACATTCTCATTTTAGTGATGTGGAAACTGAGATTCAGAAAGGTTAAATGACTTGCTTTTAAAACCATGGTAAGGTGGTTTCAGAACCAGACATTTTCTCCCAGATA
Bos taurus    CAAAGAAGGTAATTACATTCCCATTTTAGAGATGAGGAAACTGAGGTTTAGAGAGGTGAAATGACTTGC--TTAGAGCCATAATGAGGAGGCTGAAGAG--------TTTGTC-------

Homo sapiens  TTTTTGTCTCTGAAGCCAGTGGGAAAAAAACTGCAGATAAATAGATTTCTTTACACCATAAGGATGCCTTTGCTAAGAGACAGC--AATTCAAAGAAGGAATGGGCTGCCTCCTGAGCTA
Bos taurus    TGTCTGTCTCTGAAGCCAGTGGG-AAGAAGCTGAGAGTAAACAGATTTCTTCACACCATAAGGATGTCCTCGCTAGGAGACAGAGGAGTCCAGTGATGGAATGGACTGCCTCCTGAGCTA

Homo sapiens  ATGAGTTTCTCATCACTAGAAGCAGGATCGCTATGTAATTTGCAGGGCCCAGTGCAAAATGAAATGGCCTTATGTCCAGGAAGCTGGTCTTGGCTGGTAGTTTGCAAGCAGAAGTTAGCC
Bos taurus    ATGAGTTTCTCTTCATTGGAAGCAGAATTAGCACCTAATTTGCTGGGCCCAGCACAAAATGAAATAGCCATATGCCCATGAAGCAAGCTCTGACTGGAAATCTTCAAGCACGAGTGAGA-

Homo sapiens  TGCCTCCAGACAGGGATGTCAGAGTGAGGCGTCAACATGAGCTGATGCACAGGACCAGATGGCCTTAATCTGAGACACCATGGCTCTATTCTATTAGCCCAAGTGAGTTAAGGGAGCATC
Bos taurus    -GCCTCTAGACAGGGATGTTAGAGAGAAGAGTCGACAGGAGCTGAGGCACTGGAAAAGATGACCTTAATCTGAG-------------ATTCTATTTCCCAAATGGATTAGGGGAGCTTC

Homo sapiens  CCAAGG----TAGCTGACATGACACCTTAGAGG-CTTCAGATGAGAGAGAATTCTGCAAGAGGATTTGGAGAGAGCCATGGTGCCATGATCATCTGGGGCACT-GAAAACATGCTCTGCC
Bos taurus    CTGAGGTGGTTGGCTGACATGATACCTCAAAGGCCTTCAGATGAGGGAGAATTCTACAGGGCGA--TGCAGAGATCCATGGTGCTGTAATCATCTGGAGTACCAAAAAACATGGTCTGCC

Homo sapiens  CTATGAGGCAGCACCAACACTCAGAAGAAAACAAAAAAGAAACTCTTTTAGTGGAGGTGAAGTGTAATGAAAAGAATTAGACAGATGTGGGTTCAAATCCTAGCTCTGGCAATTACTAGC
Bos taurus    CTGTCAGGAAGCACGAACATTCAGGAGAAACCAAAAAAGAAACTCTTTTAGTGGTGGTGAAGCATCATAGAAAGGAT----CAGATGTGGGTTCAAATTCTCGCTCTGCCTCTTCCTAGC

Homo sapiens  TAGCTATTTGCCTTTGAACAAATCACTTAAACCTCCCTGCGACTGTTCTGTCATCTAGAATATGATCACAGTACCTGCCTTGCAGAGGTGTGGGGA------------AGGAGGCAAGTC
Bos taurus    TAG-TGTATGCTTTTCGAC-AGTCACTTAAAACT--CTGACACTGTTCTCTCACCTACAATATGACCATAGTGCCTTCCTTGCTGGGTTGTGGTGAGATTCCATTGAGATGATTCAAGTC

Homo sapiens  AAATGTATCTGGCACTGGGTAAGCCCTCCATAAATGTTAACTGTTGGATGTGTTTATAAACCACTGGGAGTGGGGACGGTATGACGTTGGGTCATTTGCCTTTCCTTGAGCACTTCATGG
Bos taurus    AAGTGCATCTGGCACTTGATAAGTCCTCAATGAATATTAGTCATT--ATTTCTTT--------CTGTTACTGAAGGAGGTATGACATTTGGTCATTTGCTTCTCCTTGAGCACTTCTTGA

Homo sapiens  ACG-------------AATAGCCAAATCTGCCGGA-CCCACCTAGGGGCATGGAAACCACTTCCCGCTCTAGTGCTACTAACTACAGCACTGCAGTAGTGGTAGGCAGTGGGTGAAAGTG
Bos taurus    GCTTCTCGTTGAGCTCAGTAGATAAGTCTTTCAGATCCCACCTCAGGACACAGAAACTCCTTCCCACTTGGGTCTTCCTAATCACAACCCGATACTGGCAGTGGGCTGTGGGTGAATGTA

Homo sapiens  GAGTGGGGCCACTAGTAGACAGTAATAACAATAAACTCTCCAGGAGAACAGGGCCCTCAAGCTTTTCCCCTAGCCTTGAAGATAC----TTACAGGAGATAACTGCCCAAGTCCAATCTT
Bos taurus    GAACAGAGCTGCTAGTGGACAGTATT---AATGAACCTTCAAGGGAAATAACACTCTCAAGGTTTTCCCTCA-CTCTGAAGATATTCCATTACAGAAGACAGCTATTTAAATCCAATCTT

Homo sapiens  GGAATCCTGCTAGTTGAGTGTTTTACCCACAAGGACATTTCATGAGATCAGGACTAAGAATAAAATTCACTTCTGCCCAGTAGCGCTATATGCATTAGTCACTTGGAATAGCAACAATCT
Bos taurus    GAAATCCTGCCAGGAGAATGTTTCACCTACGCTGACTTTTTACAGGATCAGTAC---GAATAAAATTCACTCCTGCCAGGCAA-ACTATATGTGTTAACCACTTGGGAGAGGAAGAACCT

Homo sapiens  TAATATCTTTCTATTTGTCCGCAAACCCCACCATCCCACCTCTTTAAAATTCCGTGAAATGGCCAGAATAGCGAAACACCGATACCTACAGTCTTCCAGGAACTTCATTGTCCCTTATGC
Bos taurus    TAATACCTCTCTATTTGCCTGTAAGACC--TCTGTCTACTTCTTCATAATTCTGTGAAATAGCCAGCATAGCAGAGCACAGATGCCCAGAGTTTTCTAGGAACTTCATTGAGCCTTACGC

Homo sapiens  AACATAGAAGGAATGGTATGAGAACTAAGAGAACATTGCAGTAAATCACTGAATTTCTTGGCTTTCTTTTTTTCTTTTAATAATAAAAAGCACTC-CAATCAAACTATTAATTAACT-TG
Bos taurus    AGTACAGAAAGAACGGAATGAAAACTAAGGGAACATTGCAGTAAGTCACTGAATAGCTTGGCTTCTTTTTTGTTTTTC--TTTTAAACAACACCCAAAAGCAAACCATTAATCAACTGGG

Homo sapiens  TAAAAGGCTCGAACTATTATGTACTATTCCCCGCCC----ATGTCCATGTGCTGCACAGTATTTTCTTACTCAGGACTGGTGGAACATTTCTTTTGCTTTTGTTTG-AAAAATTACTTTT
Bos taurus    CAAAAGGCTGGAACTATTATATACTAATCCCCACCCCTCTATGTCCATGTGCTGCAGAGTATTTTCTTACTCAGGACAGGTGGAACATTTCTTTTGCTTCTGTTTGAAAAAAATTCTTTT

Homo sapiens  TATTTGGCCTTGGGCTGCTATCATAACTTCTGTTTTCACTAGATTGCATAAGCCATACATCTGCACACATTTTTGATTTGTACAGGGAGTTTGAATTTTAATATCTACCTCCATCTCAAT
Bos taurus    CATTTGGCCTTGGGCTCATTGGATAACTTCCCTTTCCACTAGATTACATAAGTCATATATCATCAT-CAATTTTGATTTGTACAGGGAGTCTGGATTTTAATATCTACCCTCACCTCT..

Homo sapiens  CCCAGCACTTTGGGAGGCCGAGGCGGGCGGATCACGAGGTCAGGAGATCGAGACCATCCTGGCTAACACGGTGAAACCCCGTCTCTACTAAAAATACAAAAAATTAGCCGGGCGTGGTAG
Bos taurus    ........................................................................................................................

Homo sapiens  CGGGCCCCTGTAGTCCCAGCTACTCGGGAGGCTGAGGCAGGAGAATGGCGTGAACCCGGGAGGCGGAGCTTGCAGTGAGCCGAGATCGCGCCACTGCACTCCAGCCTGGGCGACAGAGCG
Bos taurus    ........................................................................................................................

Homo sapiens  AGACTCCGTCTCAAAAAAAAAAAAAAAAAAAAAAAAATATCTACCTCCATCTCAAAAACCTCTCATCTTTTGTCCTTTTCTTCAAGATTCTGATTTCCTTGTGGACATTCATTTTGATA-C
Bos taurus    .....................................................AGTATCTCTCCATTTTTGTCCTTTAGTTTAGGAGCCTGGTTTCCTTGTGGACATTCATTTTGAAATC

Homo sapiens  TTACATAAATCTTGCGTTATTCTGCCCA-TTTTTTTGGCTACAA---GGCTCAGCTTTGGGGCTGTAAATGTGAAATATTTCCATCATAACCTATCTTTGGGTA--TGGGAAGGTCCTTA
Bos taurus    TGACATAC--------TTATCCTGTTCAGTTTCTTCTGCCATGGTGTGGCACAGTTCGGTGGCCATAAATATGAAATATGTCCATTATGACTTACCTTTGGGTAATGGGGGAAGTCCTTA

Homo sapiens  ATGCTGGAAGTTCATTGAATAATTGCTTATTTTAATAGATGTTTATTTTCATTCACATTTCTCCCTCCACTCCATCCCTTGGGGCAGTACCCTAGTTCAGTTCTTCATCTACTCTCTCCT
Bos taurus    ATGTTGGAGA------GAGAAACAG--------AGTAGA---------------------------------------TTGTGGAAG----------------TTCACTGAATGTTTATT

Homo sapiens  TGACTGTTGCGATAGCCTCCTAAGCAGCCACTCCACATCCAATCTCCCCATATCCAAAGTATCCTAAACATAGCTACCAGTCATCTCTCTAAACCACAGCTTTTGGCAAAAGATCCTT-G
Bos taurus    TAACT-----GATGGCC-------CTACTCTTTCACATCC--CTTCCCTGCCTCCCA--TTCCCTAAGCATAGCTGCCAGTCATCTCTATAAGCCTCAGCCTTTGGCTAATGACCTTTAG

Homo sapiens  AAGTTTCCCTTTTGCCTAATGAGTAACGTACTAGCTCCTCATCCTGGCATTCGAAGCTCTCTAT-GTTTATATGTTTGCCTATCTTTCTTTTCTAGCTTCTTTCCCATTACTTGTCTTCA
Bos taurus    TGGCCACCCCTTTACCTGCTGGGTAATGTACAAACTCCTCATCCTGGCACTCAAGGCTCTCCACAGAATATGTGCTCACCGATCTTCCTTTCCAGCCTTCTCTGCCTTTCCTCTTTTGCA

Homo sapiens  TATAGCTTTAGCTTTTGCCAAACT--ACCATTCTTTGTTTTCAGAGCACACCCTATACTTTCCAGCATCAGTGTTTCTTTCACCTGGAATTAACTGCCTGCCACAGAGATCTCCGTTCAT
```

```
Bos taurus     CACAGCTTTGGTTTCAGCCAAACCTGACCACTTCTTGTTTCCAGAGCACACCCTCTGCTCTCCCACTTCAGTGTTTCTCCCACCTGGAATTAACTCCCCTCCACCAAATTCTCTATTCAC

Homo sapiens   CAGCCTATTCATTCTTTAATGCCCAAGACACATGCCACCAATTTCATTTGAATGGTCCTTAGAGACACTGAAATGGCGCCTGCTGTACACCTGTCCACCCCGACCCCCGCTGCCCACTGC
Bos taurus     AATCTTATTCATTCTTTAGTGTCCATGTCAAATGTCTCCTACTTTAT.........................................................................

Homo sapiens   TGGCTCTTCCAGTCTTGTGAAACATTAACCTCTCCTAACAGCAAGCACAGCTTCTCCATGGAGAAGACACTGAAATGGCACCTGCTGGACACCCGTCCACCCCCCACCCCTGCTGCCCAC
Bos taurus     ...................................................TTCTCCA-GGAAGACACATTGGAGTGGT-CCTTAGAGACACTGGCTCGGTGGCTGGG--TGCCTCCCAT

Homo sapiens   TGCTGCGTCTGCCAGTCTCGTGAAACATTAACCTCTCCTAACAGCAAGCACAGCTTCTCCATGGGGCCTGCTCACTCTGGCCCTCTGGTCCCACCATTTTTCTCACTACTCAAATAAAGT
Bos taurus     GGCTGGCTCTGCCAATCTGCCAGAGCATCA-CTTTTCTTAAGAGCAAGCACAGCTTCTTCGCATCCCCTGCTCACTCTGGCTTTCTGTTCCTGTCACTTTGCTCACCACTCAGATGACAT

Homo sapiens   TTGGAGACCTTTCCCTGATTTT---TTAAGAACATGCTAGCTCTTTCATGATACTCGTTATCATACCACCCCCATCTGCTTTTCAGTGTACCTTTCAATTGCTCTCTAGTGCTCCATTCT
Bos taurus     TTTGAG-CCCCTCACTGGTTTTCTCTTAACAATAGGACTGCTCTTTCACAGTACTCTGTATCACACCACCCCCATCCACTTTTCACTGCACCTTTGAATTGTTCTCTAGTGTTG-ATTCT

Homo sapiens   TGATTGTTTTCCATTTCTATTATAGCTTTCAAGGAGAGTCTTTACTTGGCCTACTTTCTTAGTAAATGGTTTGGTGAGATTCTGATTTTCATCTGATATTAGTTCTTTCTCCAAGAGGGA
Bos taurus     TGATTGCTTTCCATTTCTCTGATAGCTTTCAGGGAGACACTTTCCATGGTCTACTTTCTTAGTAAATGGTTTAGTGAGATTCTGATTTTCATCTGATGTTAATTCTCAGTT-------GA

Homo sapiens   CAGAGAAAAATGAAAAAAATATCTTGCCTTCAGTTATTCTTTTCATATTCTTTGAAATTCTAAGCCACAGAAGTCTTCTGTTAGGCTGCTACCTTCATTTTAAGAGACTC------TTCC
Bos taurus     AAGACAAAGCCAAAAAGAGGATC-TGCTTTCAGCTGTTCTTTT-----------AAATTATAAGTCATGGAAGTCTTCTGTCAGGTTACCGCCTTTCTGTCAAGAGATTCTTCACTTTCT

Homo sapiens   CTAAATGACAAGTTGTGACTTCAGAACTAGAGAACATGGCAGCCTCTCATGGCTTTCTGCTCAGTCCCATTTTTCTTTTCTGGATCCACTCTGGGCCTTCTTTAAAGTGACTCAACTTCC
Bos taurus     CTTAGTGATAACTTGTGACTTGAGAACTAGAGAACACTGTAGCCCACCGCGGCTTTCTGCATAGTCCTCTTTTGCTCCTTTGGGTCCACTCTGGGCCTCCCTTTGAGTGACCCAACTTCC

Homo sapiens   CAAGCATGTGATTTTACGTATTTTAAAAACAAGTCTGGTTTTTTCAACTCATTCTGAAATGTGGCTGCCAAAGTAAATA--CCCAACCCTCT-----CTAACACATACATACCCTCTTTC
Bos taurus     CTGACATCCGA-TTGACTTATTTTGAAAATAAGTCTTGTTTCCTCAACTCCCTCTGAAATGTAATTGCTGAAGTAAATAACCCCACCCCGCTGCCCCACAACACATATATACTGTCTCTG

Homo sapiens   ACACACACATGCACATGCACATGT------------------AAACATACTCCTGAAGGATGAGGAAAGCAATGGTAAATGGGGAAGCAAAAAAATACCATTTTTCATTTAGACAGACTT
Bos taurus     TCACACACATGCATGTGCACATGTTTGAACATGGGCACATACAGACACACTCTTGACG-------------------------------------TACTATTCTTCTTTTAAATAGACTG

Homo sapiens   CCCAATACCGATGAACTAAAATTCAGGGATTTGTGATTTGAAATGCATGGATGGCCTTTGAGTGGTCTTTTCAAATCCCCTAAATATATCAATTTAATAACTTATTGAGCATCTACCATT
Bos taurus     TCTGATATTGATGAATTAAAATTCAAGAATTTCTAATGCAAAATGCATGGATGG-CTTTGGGTGGTCTGTG-AAGCCCGTGAAACATAGCCATTTGATAATTTATTGAGCATCTACTATA

Homo sapiens   TGATGAGTGCTGT-GTAGATGTATCTATATATATCATAAAATTTATTTCTTGCTACAACTTTTGGAAGAGATTATTATAATCCTCATTTACAGATGAGGAAACTGAGTCTCTGTGAGAGG
Bos taurus     TGATAGATATTGTGGTAGATGTTGGTAAACAT---ATATAATATATTCCTCACTGTAAGTTTGAGAATAGATTATTATCATC--CACTTACTGATGAAG-ACTTTTTTCTCAGTGATGGA

Homo sapiens   AAATGATTTGCCCAAGATCCC-------------------------------------------TCAGGCAAT----AAGTAGTAAATCTGACTTTGAAACACAGGTCTGATTCCTTA
Bos taurus     AAATGACTGGCCCAAGATCCCACATGCCATCTGGCCAAGGGGAAAAAAGAAAAGAAGGCACCAAGATCATGCAATCAGAAAGTAGTAAATCTGAGTTGGAAAGCCAGGT-TGATTCCTTA

Homo sapiens   TCCCCATCAGATTTCCCAGGCAT----------TCTGGTACAGGTGGTGTTTCAACAGATGGGAATGGGGCAGGGTGAGAAAGATAGTATGAATAA---------AGGT------ACAGA
Bos taurus     CCATCATCAGCTT--CCAGGCACAAAGGAGCAGTCGATAGCAGTTGATGTTTCAAAAGACGAGAGTGGGGCAAAGTAAGAAAGCTAGAATGAATAATATGACTAGAAGTATTGAAACAAA

Homo sapiens   CATGGGAAAATGTAGCTACACATAGGACAGTAGTAGGAATTAAGGTTGCAAAATTGTGTTAGGGACAGAACACAAAAGGATTAGAAACTTGAACTTCCTTCCATTGTCCTGAAGGGACAA
Bos taurus     CATGGGAAAGTGTAGCTGCATGTACAAGAGTAGTAAGAGTTGAGGTCGTCAAATT-TGTTGGGGACAGAACATACAAGCTTTAGAAATTTGAACTTCTTTCCACCAACCTTATGAAGCAA

Homo sapiens   TAAAAGATTTTGTAGTAGGAAAACAATATAATCAGAGAGCCAAATCAAGAATGCAATCCAATTTACAATAGCCATACAAAAAAAATTAAATACCTAAGAATACATCTAACCAAAGAGGTG
Bos taurus     TGACAGATTTTGGAGTCAGACAATAGTATAATCA......................................................................................

Homo sapiens   AAAGATCTCTACAGGGAGAATTAACATACACTGCTGAGAGAAATCAGAGGCAACACAGACAAATGGAAAAACATTCCACACTCATTAATTAGAAGAATCAATATTGTTAAAATGGCCATA
Bos taurus     ........................................................................................................................

Homo sapiens   CTGCTCAAAGCAATCTACAGATTCAACGCTATTCCTATCAAATAACCAATGTCACTTTTCACAGATTTAAAAAAAGATTTTAAAATTCATATGGAATTTAAAACGAGCCCAAATAGTCAA
Bos taurus     ........................................................................................................................

Homo sapiens   AGTAAACCTAAGCCAAAAGAACAAAGCTGGAGGCATCACATTACCCAACTTCAAACTATACTACAAGGCTACAGGAACCAAAACAGCATGTTGCTGGTACAGAAAAAGACATATAGACCA
Bos taurus     ........................................................................................................................

Homo sapiens   ATGCAACAGGATAGAGAATGCAGAAATAAAGCCACACACCTACAACCAACTAGTCTTTGACAAAGTTGATAAAAAATAAGCAACAGGGACAGGACTTTCTATTCAATAAATGGTGCTGGG
Bos taurus     ........................................................................................................................

Homo sapiens   ATAACTGGCATATGCAGAAGATTGAAACTGGACCTCTGGCTATCACCATATATACAAAAATTAACTCAAGATTGATTAAAGACTTAAATGTAAGACTTCAAACTATAAAAACTCCAGAAG
Bos taurus     ........................................................................................................................

Homo sapiens   GAAACCTGGGAAATATCCTTTGGACATCAGCCTAGGCAAAGAATTTATGACTGAGTCATCAAAAGCAATTGAAACAAAACCAAAAATTGACAGGTGGGATCTAATTAAACCAAAGAGCTT
Bos taurus     ........................................................................................................................

Homo sapiens   CTGCACAGCAAAAGAAAATATCGACAGGCACCCTACAGAACTGGATAAAATATTCATAAACTATGCATCCAACAAAGGACTAATATCCAGCATCTATAAGGAACTTAAATCAACAAGCAA
Bos taurus     ........................................................................................................................

Homo sapiens   AAAACAACCCCATTGAAAAGAGGGCAAAGGACATGAACAGACGCTTTTCAAAAGAAGACATACAAGTGGCCAAAAGACATGAAAAAATGCTCATCATTACTAATCATCAGAGAAATGTGA
Bos taurus     ........................................................................................................................

Homo sapiens   ATCAAAACCACAGTGAGATACCATCTCACACCAGTCAGAATGGCTATTATTAAAACGTCAAAAACAGCAGATGTTGAAGAGGTTGCAGTGAAAAGGGAATGTGTATACATTGCTGGTGGC
Bos taurus     ........................................................................................................................

Homo sapiens   AATGTAAGTTAGTTCAGCCCCTGTGGAAAGCAGTTAAAGATTTCTCAAAGATCTAAAAATAGAAATACCATTTGATCCAGCAGTCCTCTTACTGGGTATATATCCAAAGGAAAATAAACC
Bos taurus     ........................................................................................................................

Homo sapiens   ATTCTACCAAAAAGACACCTGCACTTGTATGTTCATCGCAGCACTATTCACAACAGTAAAGACATGGAATCAACATAGGTGCCCATTGATAGTGGATCGGATAAAGAAAATGTACACGTA
Bos taurus     ........................................................................................................................

Homo sapiens   CACCATGGAATACTACCCAGCCATAAAAATGAACAAAATCATGTCCTTTATGGGAACATGGATGCAGCTGAAGGCCATTATCCTAAGCAAATTAATGCAAAAAAAAATCACATATTCTCA
Bos taurus     ........................................................................................................................

Homo sapiens   CTTATAAGTGGGAGCTATACCTTGGGTACACATGGACATAAAGATGAGAACAATAGACATTAAGGACTCCAAAAACTATCTGGGTGATGGAATCAATAGAAGCCCAAACCTCAGCATCAT
Bos taurus     ........................................................................................................................

Homo sapiens   GCAGTACACCCTTGTAATAAACCTGTACATGTACCCCCTGAATCTAAAATAAAAATTAAAAAAAATTTAAACAGCTGCATTTTAGACAGATTAATCTAATTCAAGTCTGAAT--------
Bos taurus     ................................................................................TTTAGGAAGATTAATCTGATTCAGGTCTGAATTCACGCCT

Homo sapiens   ---CTATGCTGTTGACAGTATTTTT-----AAAAATTCTTTCCTAAGAATTCTTTATTCACTCCAGGCCTATGCTTTGGTCAGAATTCTCTCTTTGTTTTTCAATACTTTAGTGAAAATT
Bos taurus     CCTCTATGCTGTTGATAGTATTTTTTTTAGAAAAATTCTTTGCTAAGAATTATGTATTC----CATACCTGTGCTTTGGTTAGAATTCTCTCTTTGTTTTTAAATACTGTAGCAAAAATT

Homo sapiens   CAGAAGGCCATGTGGAAAAAA-----ACAGAGGAAACAAACCACACACAAACACAAGATAATTATATACAAGTCTGGGAAGAAAGAGCACAATGGGTCATGACGAACTTTATTGAACAGA
Bos taurus     CAGAAGGCTATGTGGAAAAAAACAGAGACAGAAGAAACGAACCACATGCAAACACAAGATAATTACACACAAGTCTGGGAAGAAAGAGCACAGTGGGTCATGACGAACTTTATGGAACAGA

Homo sapiens   AGGCGAATAAGCAACGTTCCAATTGTACAGGAAAAAAAAAAACCCAGCAACCCAAACACCACCACCAACAATAAGAAACAAAGGCAGCCAGGATCTGGGAGACATGGATGAAGCTTGTGAA
Bos taurus     GTGTGAGTAAGCAGTGTACCAATCATAAAGGAAATAAA-----CAGCAACCAAAACACCACCATCGACAACAAGAAACAAAGGCCACCGGGGTCTGGGAGACAGGAATGAGGGTGGCAGA

Homo sapiens   AATGACAC--------------------TTTCTTAAAAGAGCAGAAGAGAAACTAAAAAGGATGAAGGGGAGAACCATGGATTTGAGGGTTAAACTAAAAG-GAAAAATAGAAAGGAAAT
Bos taurus     AATGGCACTTTTAGACTTTCTGATTCCTTTTCTTAAAAGAGCAGAAAAGAAACTACAAAGAAGGCAAGGGAGAACCATGGAGTTGCGCGCTAGACTAAATGAGAAAAATAGAAAAGAAAT

Homo sapiens   AGGAAGGAAGGGTGTAACTCATAGCTGGTTTTGATATATCATCCCAGTCTGACAACCAAGGCAGTCTCCCT-GCATTAGAAGGCCATGCTAAAAGTAGATGA--GTTGTAAACAAGCCTC
Bos taurus     AAGAAGGAAGGATATAACCTGTAGCTAGTTTCG-TATGTCAGCCCACTCAGACAACCAACGCAAGCTCACTGGCATTAGACAGCCATATTGAAAGTAGATAAGTGTCATAAACAAATGTC

Homo sapiens   CCACTGGTCATTAGTTTGGCAAATTAGTGGGTTATCTCTTAGAGTCCTCTCTTTTTCTCCTTTCCCTTTCCTCTGCATTCAATTTGTCACTCAT-TCCTACAAGTCTACCCCAGAAGGGT
Bos taurus     CTGCTGGTAATTAATTTGTCAAATTAGTCAGCTATCTCCTAGAGTCCTCGC-----------------TCCTCTATATTCATTTGGTCACCAAGATGTTATATGTCTACCTCAGAAGGCT

Homo sapiens   TGCCTGAATCAAAGCCCTTCTCCCCATTTCCACTCACGGGGCCACTGTGTTAATTAAATTCCATGTCGTTTCTCAGCTGGACCAGGCTTCCTGCCTCTAATTCGTTTCTTCCTCCCTCTA
Bos taurus     GTCCTGACTCAAAGCCCTTCTCCTGACTCCCGCTCACAGGGCCACTGTGTTAATTAAATTCCATATCCTCTCTGATCTGGGCC----------------ATTAGTCTCTTCCTCCCTCTA

Homo sapiens   ATTGAGGCTTAGATCTGCCCCAGTATTGAGCTTCCT-ATACTCTCTTGCTTATAAACCTTCTTTGGTGGCCCATGGCCTATAAGGTACAGTAGAACTCACTCAGTTTTACATGATGCAGT
Bos taurus     GTTCAGGCTTCAATCTGTCTCAAGAGTGAGCTTTCTAATACTTCCCTGCTTATCAACTTTCTGTGGTGGACCATTGCCTATGGGACATAGCAGAATTCCCTCAGTTTCGCACAATGACTT

Homo sapiens   GGGAGTGGAATCAAAGGGTTCCCCTAGACCCATGGATGAAGAGACGACTAAGGAGCTAAGTCTGGGATGAGAAACTCTTGCAAAAAGCCAACTAATAGCTTTTTCCTCCTCATCTCAGAA
Bos taurus     GGTAA-AGAACCAAAGGATTCCCCCAGACTAATAGATGAAGGGACAACTAAGGAACTATGTCTGGGGTGAGAAACTCTTAAAACAAGCCATCTAGTAGCTTTTCCCTCCAGATATCAG-A

Homo sapiens   CCACCCCTCTTCCCAGAGTGTGTGTATA---------CAAGATGAGTTACTTAAGATTGCACCCAACCTGATGGTAGATTGGAGAAGGCTATCTGTC--CCTTTCTCAGTTGAATAT-AT
Bos taurus     TCATCTTTCTTTCTAGTATGTGTGTATAGGGTGAGTGTGCATTGAGTAACTTCTGCTTGCACTCAACTTAATGGTAGGTTGGAGAAGGGTATCTGTCTGTCTCTCTCAAATGAATCTGAG

Homo sapiens   GAGGGTCTTCTTCCCAGGAGGCTTGCCCACACCAGTCTTCTTTCCCACCATGGCCCATCCCCTTTCCAAAGAGTATAAACTTCATACCCTCCTCCCTTCAAGAACTCCTATCTTTGCTTA
Bos taurus     GCAGATATCCTTGTTA----------------CAGTC-TCTTTCCCACCAGGGCCCATGTCCTTTCCAGAGAGTATACTCTTCCAACCTTCCTGCCCCCAAGAATTCTTATCCTTGCTTA

Homo sapiens   GCAAAATTTGTTTCCCAAGCTTGGAGAGAGGAAAGGGGCCTTTTTCCAAATCAGGGGTAAA-TAAAAAGTGGCAGAATCTTACAGTAGACATCATATTAGAGCTCATTTTTATGAATT-C
```

```
Bos taurus    G-AAAACTCATTTCCCAAGCTTAGAGAAAGGAAA-GGACTCTTCAAAAAATCAGGGGTGAATTCAAAAGGGGGAGAATCTCCCAGTAGACATCATATTGGAGCTCATTTTTTGGTATCAA

Homo sapiens  TTCTACTGTATATAAGG-TCCTCACTTTCTTATTACTCAGCCTACCACTCCAGTTTCATCTTCCACTTTTACTGCTTCTCCCCACCCCTTCACAATCCTGACATACCCTAAACTCCAGTC
Bos taurus    TTCTACTGTGCACAAGGCCCCTCCCACTCTGATTACTTAGCTCACTCCTCCAGTCTCATCTTCCACTTTCACTGCTGCT-CCCACCCTTTCATAATCCTGACATATTCTAGATACTAGTC

Homo sapiens  ATACTGAACTTTCCAACATTCCTCAGATAAATCAAGCCTTT-GCAGAAATTCATGCACAAGCTGTTCCTTCTGCTTAGAACATCCTTACTCTTCTTATCTCGCCAGTAAATTCCTACTCT
Bos taurus    ATGCTGAACTTTTCAACATTCCTCAGATAAACTAGGCCTTCAACAGAAGTTCATGCACATGTTGTTCCCTCTGCCCAGAACACCCTTACTCCTCTTATCTCACCAGTAAATTCCTTCCCT

Homo sapiens  CCTTTAAGACCCTGCTCAACTATTGCCTCTTCTGGGAACTCTTTCCAGACTGTTCCAGATAGAATTAATTA-ATCGTCCTTTGAGCTCTCATAGCATTTTTCCATATCCCTTTGATAGCA
Bos taurus    CCTTTAAGACCCTGCACAACTATTTCATCCTGTGGGAAACCTCTCCCGACTGCCCCAGATAGAATTCATGACCTTCTCCTTTGGGTTCCCATAGCATTTTTTCCAACTCTTATTATAGCA

Homo sapiens  CCATCCTTATCATGGCAAAACAATATAACAACTAAAGGGATGGGCTCTGGAGGCAGACTGCCTGGGTTTGAGTTCTTACTCTGCCACTTATAA-GCTATGTGATGTTGAGCCAATTACTT
Bos taurus    CCATACTTATCATGGAAATGCAATGTAATGAGTAGAAGGCTAGGCTCTGGAGGCATACTGCCTGGTTTTGAGTCCTGATGGTGCTACATATGATGCTATGTGATGTTGGGCCAATTATTT

Homo sapiens  CGCCTTTCTTTGCTTCAGCTTCGTCATGTATAAAACAAAATAAATAAGATGATACTTGTAAAACCTTTAGAACAGAACCTGACACATTGCAAATGTTAGTA---AATATTATACATCATC
Bos taurus    CACCTCCTTTTGTCTCAGTTTTCTCGCCTATAAACTGGAATAAAGGAGATAATACTTATAAAGCCCTGAGAACAGAACCTGTCAAATTGTAAATGTTGGTAGTTGACATCATACATCATC

Homo sapiens  ATTTCTTCTATTGTTTTATTGTTATCGTATATATAACCTACTTGTATATGTAATATACATGTGTTATTA-TATAGCACTATTATAGCATTACTTAACTTTTGCTGTGTCTGTGTCTTGTC
Bos taurus    ATCTCTTGTAATGTTGTATTATTCCTATATATATAGCATACTTGTATACACAGTATATTTAATTATTATTCTATTATTCTCATAGCATT-TAAAACCTTTTATGTGTATATATCTTGAC

Homo sapiens  TTCCAAGCTGGTTTAT-AGTGTTTTGAGGGCAGAAACCTTGTATTACTTTCTCCCCCATAGTGCCTGGTGTTAAGTATGGGCTCATTAAGTATTTGTTGGGGTCAGTATATTATACTGAA
Bos taurus    ACTCAA-CCAGTTTATAAGTATTTTGAGGAGAGAAGACTTGTATTACCTTCTCCCACATAGTACCTCGTG-TAAGTATAAGCTCATTAAATATTTGTTGAAGGCAGTATGTTGTAGTGGG

Homo sapiens  AAAAGCACTGAAATCTGAGAGATTTGGGTTTGGATCTTGATTCTGCTCATAATTGTTGTATGATCTCAATCTTCCTGAGCCTCAGTTTCCTCATCTATAAAATGAGGATGATAATAAGAG
Bos taurus    AAAAGCA---------------------TTAGAATCTTGACTCTGTTCTTAGTTATTGTATGATCTCAATCTCCCTGAGCCTCAGTTTCCTCATCTGTAAAATGAGGATGATAATAAGTG

Homo sapiens  TACCTACCTCACAGGGATATTTTAAGAATTAAATAACTTAGTACATGTAAAACCTAGCACATAATAACATAATAAGAACTCACTAAATGGTTTTTATTATCAGCTTTGTATCCCTTTGCA
Bos taurus    TACCTACCCCATAGGGATGCTTTAAGGATTCAATAAGTCAG------------------CATAGT-ACATGGTAAACATTCACTAAATGGTTGGTATTATCAACTTTGTATTTCCTTGCA

Homo sapiens  TTTAACTTAATAAATACTTT--------TACTTGA-------------------------------ATGAAT-------GAACCCAGCCATTTTCTGGAACTTGAGGATCAAGGGAATCC
Bos taurus    TTTATTTTTTAAAAAACTTTTTATTTTATACTGGAGCACAGGCAATTAACACTATGATAGTTTCAGGTGAATAGCAAAGGGACTCAGCCAT..........................TCC

Homo sapiens  AGGCAGA--TACTCTATTTGTTCCTAAGAATCCAGAGGAAAATGTGAGTCATCTCCATTCTGTATCATGGCTCACTCTGAACTGGGTCACCATCCTCCAGAAGGTGACAAATGTGGCTGG
Bos taurus    AGATAGGATTATTCCATATCCTCGTAGGAATCAAGAGGAAAATGTGAGTCA--TCCATTCTGCTTCCTGGGTCA--CTGAACTGGGGCACCATCTCACAGACAGCAACAGACATGGCCAA

Homo sapiens  CTGAGTCACTATAAGAGCTTTTCTGTAAGGATTTGCCCGAACCCCCCACCATAG---CTCATTGGACATAGTATCTTTCATATTAGGTGCGAGGTGCATACCCTTCCTGAAATGGGCAGA
Bos taurus    CTGATTCACAGTAAAGTCCTTTCTATAAGA--TTACCCTAAAACACTACTTCTGTCCCTCATTGGACACAGTCTCTCCCATGCTATGTACAGGTTACGTACTCTTCCTATGATGGGCAGA

Homo sapiens  ATAAAGAGGTGCAAAACTCCTGGGGAATCAGAGTGGCTATGAATTCACTCCT------CTTTCTTA---TTCTGTTGCCTCTTCCCTTTGTTTGGTTTATTTGCTAATCAACTTTGCCCA
Bos taurus    AGAAAGAGTTA-AAAACCACAGCAGAGCCA----GTCTATAAATCCACTCCTCTTGTGCTTTCTTAGGCTTGCGTAGGCTTTTCCCT--------TTTATTTGCTAATTAACTTTCCCTC

Homo sapiens  CACTCACATCTAGTGTAGCCCAAGTCTGTGCAGAGAAGACACTTTGAAAGCCTAAGACTATCATAGTTCCAGCAGAAT-AATAGAAAAATAACAAGTGTTGACTATTCAGTTCTGATGTA
Bos taurus    CACTCTCTTCCAGTGTAGCCCATGCCTGTGC-----AGACTCTCT------------TCATCATGGCTCCAGCAGAATAAATGGAGAGATGACAAGCATTGACTATTTGGGTCCAATATA

Homo sapiens  GCACTGTATTGAAGAACATAACAT----TCTAGATCCAGATGGCCTGAATTTGAATCCCAGCTCCACCACCAACTAGCTGTGAGACCTCAGGCAAATCACTTAACGTTTGCATCCTTTCA
Bos taurus    GCACTGCATTGAAGAATGTGACACTCTATCTAGACTGAGATGGCCTGAATCAGAGACCCACCTCCACCAC---TTAG-TGTGAGAGCTCAGGCTAATCACTTAATGTTTGTGTAACTCCA

Homo sapiens  TTTCTCCATCTATCAAATAGAAACAATAAAAGTACTTACCTACCTCTTAGGGTTTTGTGAGGGTTAAATGAGTTAATACATACAGAACACTTAAAAGAATGTCTGGCACATAGTAGGTGC
Bos taurus    GTTCCCCATCTGTCAAATAGGGACAATGAAAA----TACCTTCCTCCTAGGGTCTTGTGAGGATTAAATGGGTTAGTACATCTAGGACACTTAGAAGAGTGT----CACACAATAGGTGC

Homo sapiens  TATATAAATATTAGCTTTTAAAAACAGCTTTA------TTGAGATATAATTCATCTATGTGAATTATATCTCAATAAATTCACCTATTTAAAGTGTACAGTTCAGTCATTTTTAGTATTT
Bos taurus    TATATAAATATCAGCTATT-ATCATAATTTTAACAACTTTGAGACAAAAATTCACTATAAAA---------CCATTAATTCACCCACTGAAAGTGTACAGTTCTGTGATTTTCATATAGT

Homo sapiens  CACAGAGTTGTGCAACCATCACCACAATCTAATTTTTAGAAAATATTCATTACCCCAAAAAGGAATCTCATACCCACTGGTAGCCGTTTCCATCCCTGAAGCTCCCTCTCCCCCTTCCCA
Bos taurus    TACAAAGCTGTGTAACCATTATCACAATCTAA-CTTTGTAACCTTTTCATCACCCC-AAAAGGAACCCTGTACCCATTAGCAGTCATTCTCATCCCTGA---------TTCCCTTCCTCA

Homo sapiens  GCTCCAGGTAATCACTAACCTACTTTCTGTCTGTATAGATTTGCCTCTTCTGGACATTTTATATAAATGAACTCATACAATAGGTGGTCTTCTGTGACTAGCTTCTCTCCCTTAGCATAA
Bos taurus    GCCTCAAGCAATCACTAGTTTACTTTCTG------TAGTTTTGCCTCTTCTGGACATTTTATATAAATGGGCTCATATAATAGGTGATTTTTTGTGGCTAAATTATTTCACTTAGCATA.

Homo sapiens  CTGTCTTTTAAAAAATTGCAACTTCCATTTTAGATTCAGGGTGTACATGTCCAGGTTTGCTACATGGGTATATTGCATGATGCTGAGGTTTGGGGTACAGCTGATCCCATCACCCAGACA
Bos taurus    ........................................................................................................................

Homo sapiens  GTGAGCATCCAATAGGTAGTTTTTCGGGCCTTGTCCACCTCCCTTCCTCCCCTGTCTAGTAGTCTCCAGTGTCTATCGTTCCCATATTTATGTCCATGTGTACCCAGTGTTTAGCTCCCA
Bos taurus    ........................................................................................................................

Homo sapiens  CTTACAAGTGAGAACATAGCATAATGTTTTTAAGGTTCATCCATGTTGTA-TATATATCAGAGCTTACTTCCTTTTTAAGGCTGAATAATAGTTCATTATATGAATATATCATATTTTGT
Bos taurus    ..........................TTTTTGAGGTTCATCTAGGTTATAGCATGTATCAGTACTTCATTGTTTTTT--TGCCAAATAATATTTCATTGTATGGATATACCATATTTTAT

Homo sapiens  TTTTCCATTTATCAGTTGATTGACATTTTGGATTGTTTCTA-TCTTTTGGCTAGTATGAATAACACTGCTATCAATATTCATTTACAAGTTTTTGTGTAAAGATATGTTTTTAAATCTCT
Bos taurus    TTATCCATTCATCATTCGATTGACATTTGGGTTGTTTTCCATTCTTTTTGTTATTATGAGTAATGCTGCTATCAACTTTCATTTACAAGTTTTTGTGTGATGCTATGTTTTCAGTTCTCT

Homo sapiens  TGGGTATATATCTGGGAGTGGAATTGCTGGATCATATGGTAACACTATGTTTAATGGTTTGAGGAACCACCAAACTATTTTCCAAAGAGCCTGCACTATTTAAAATTCCCTCCAGTAGTG
Bos taurus    TCACTGTATCCCTAGGAGTGGAATTGCTGGGTCATATGGTA------------------TGAGGAATTGCCAAACTGTATCCCATAGCAGCTATACCATTTTACATTCCAGCTAGAGGTG

Homo sapiens  GGAATGATGGTTTCAATTTTTCCACATCTTCATCAACACTTGTTATTTTATTTTATTTTTATTTTATTAAATTATTATTTTTATTATATTTATTTTATTTTTTATTTATTTTTTATTATA
Bos taurus    GGAATGAGACTTCCAATTTCTCCACATCTTCATCACCACTTGTTATT----------------------AGCTGT---------------------------------CTATCTATTAAA

Homo sapiens  GCCATCCTACTGTGTGTAAAGTAGTATCTCATTGTGGTTTTGATTTGCATTTCTCTGATGGTTAATGAGGTTGAGTATCTTTTCATGTGTTTATTGACCATTCTTATGTCTACTTTGGAG
Bos taurus    GCCATCCTAAAAGGTATGAAGTGGTATCTCATTGTGA-TTTGATTTTCAGTTCCCTGATGGCTAATCATAT----TATCTCTTCAGTTGCTTATTGGCCATTCTTACATCTTCTTTGGAG

Homo sapiens  AAATGTCCCTACAAATTCTTTGCCCATTTTTAAATTGGGTTGTC-------------------------ATTTTATTACTGAGTTGTA---------------------------------
Bos taurus    AAATGTCTATTCAACTCCTTTGCACA-TTTAAGATTAAGTTGTCTTCTGATTTTTTATCTATTTTTAAAAATTTATTCTTTAATTGGAGGAAAATTGCTTTACAATGTTGTGTTGGTTTC

Homo sapiens  --------------ACAGTTC--------TTATATA-----------------------------------TTCTGCATGCAAATCTCTTGTCAGATATATGTTTGTGAATATTCTGTCA
Bos taurus    TGCTATACAACCACACAATTCAGCCATAATTATACATATATCACCTCCCCCCTTATTGAGCTTTAATAGTTTTTTATATACAAGTCCTTTGTTATATGTAGGTTTGCATATATTTCCTC-

Homo sapiens  TTC------------TGTGTAATTTTTTTTCATTTTCTTGAAGCTATTATTATTAAATAGAAATTTGTAAGTTCGCATATAATAAAGATGACAGATTGTATTAGAAAGAATGAGATCTAA
Bos taurus    -TCAAGAAAATGAAAAGCATGATATCTTTTCACTTTCTTGAAGCTATTGTTATTAAATAGACATTTACAAGTTCACATATGATAAAGAGCACAGACTGCAGAGGGGAAGATGAGATCTAA

Homo sapiens  AA-TCACCTGATGCA--CCCTATGGTACACTACCCTGCTC--CTGCACTCCTTTGAAATTAAATTCAAAACAGATCAAGCTTCTCCTCTGTGCCAGGCACTGTGGGGGAATAAGACCTAA
Bos taurus    AAGCCAGCTGAGAGATGCTCTTAAGTACCCTACCCTTCCCTACTGTGCTCCTTTGAAATTAAATTCAAAGCATATTAAGCTTCTACTCTGTTCTAGGCACTGT-AAGGAATAAAACCTGG

Homo sapiens  CCTCTATTCTCAAAGAGCTTCATAACACA----GAAGAGAAGATGCAAACATGTGGAAACTTAAATAATTATAACATGTTTAAATAGCAGTTCAGGACCACAACAGAAAAGATGCCACAG
Bos taurus    ACTCTATCCTCAAAGAGCTTACTAACTTAGAGGGAAGAGGAGATGCAAACTTGTGGAAACTTAAATAATTATAACATGTTTAAATAGCAGTTCAAGACCACAATAGAAAAGATGCCACAA

Homo sapiens  AGCACTATATGATTAATTTCCAAATGAATTGCATGGAAAAGAATTGCTATGGGAACTCAA--GGGAGGGA--GAAAGCACTTGGGGCTGAGGTGGTCAGGGGAAGCTTTAAGGAGAAGGG
Bos taurus    GGCACGGTGTGATTAATTGCCAAATGAATTGCATGGAAAATAATTGCTATGGGAACTCAAAGGGGAGGGAGTGAGAGCACTTGGGGCTGCAGCGGTCAGGGAAAGCTTTAAGGAGGAGGG

Homo sapiens  ATCTAAGCAGTCTGTAATTAACTACCGCCATGCCTGTGTAATACTAAACAAAGACTTGGAGAG-AAAAAAATATTGACTGTTTTTGAGGTGAGGCATCTTAGTGATTAAATTGATACCAC
Bos taurus    GTGTAAGC--TCTATAATTAACTGCGGCCATGCCTGTGTAATACTAAACAAAGACTTGGGGAGAAAAAAAAATATTGACTCTGTTTGAAGGGAGGCATCTTAATGATTAAACTGATACCAT

Homo sapiens  CATTTCTTTTCAACCTCTGCAGGAATCCCCACAGTCCCACCTTTCCAATGAAGACTTACGTGGCCTCTGTCTAGTCCAGAGGTTACCAATTAACAAAGTTAACAAGCCACATCTGGCTTA
Bos taurus    CACTTAATTTCAACCTCAGCAAAATTCTCCGCAATTTCATCTTCCCTATGAAGACTTACATGGCCTCTGTATAGTCCTGAGGTTACCAATTAGCAATA-TAACAAGCTGCATCTGGCTCA

Homo sapiens  CAGACACGTTTGGTTTGGTCCAATT------------AAAATATTAGAACTAGCTGCTAAAACATTTAACTGGACAAATTTCACATAAAATATTAATTTCCAGTTTCTCTAGAAAAATCA
Bos taurus    CAGATACATTTTGTTTGGTCCAGTTTAAAAAAAAAAAAAAACTGTACAGTTAGCTGCAAAAAGTTGTAA-TGGGCAAGTTTCACATAAAATATGGATTTCTC-TTTGTTTGGAAAAATTG

Homo sapiens  GAAGACCTGGCTACCCTGCATTGGAACATGCATTCTCTGGGACCATGGCCAATGGCAACCCTGCTTGCACATGCAGAA--TTCAGTTTGAGACTTCCTAGAATCATAGTTCTTAGACTAA
Bos taurus    GAAGACTTGGCAACCCTCGGTCGGAGCATGTATGCTCTAGGAACATGGTTCATTTCACCCCCATGTGCACCTGTGGACTTTTTAGTTTGAGATCTCCTAGATTCAGAGGACTTAGACTGA

Homo sapiens  AAGGTATCTTAAGAGTGCATCTG-TTTCTATTCCCTGACTTTGGGAATGTATGGTATTATAATATTACCCTCAATTTACAACTTGATTTAGAATATCAACTAAAACCCACAGAGAATTTG
Bos taurus    AAGCTACCTTAAGAGTGCATCTGCTTTTTGTTCCCTGCCTTTGGGCATGTATGGTAC-----CATTACCCTTAATTTAC----------AGAGTGTCAATTAAAACCCATAGAGGTTGAG

Homo sapiens  ACTTGCTGCAGGCCACAGGGCTATTAACTGGAAGAGGGAGGGCTAAAACCCATGTTCTCTACTTCTCAACGG--------------------AATGTTCTTTCAACA--ATCTGTCTTTA
Bos taurus    ACTTGCCCAAGATCACAGAAATATTAAGTGAAAGAGAGAGAACTATAACCCACATTTTCTGCTCTTTGGATGTTCAGCTCATAGATGTTTTCAATGTTCTTTCAACAACATATTTTTTTC

Homo sapiens  CAGTCTGGATGCATATTGTTTAGTAGCATTCTTCTGGCACTTCCTAATCACTGCTTTGTGTTGTTGGACGATTTTTGATATGATCGTTATCTCCCTCTCTCTTTTTTTTTTTTTTTTTT
Bos taurus    CAGTCTATATGCCTATTGCTTAGTTGCAGTCTTCTGGGACTTTCTGATCACTACTTTGTTCTGTTGGACAGTTTTTAATATGATAAATGTTTCTCT.........................

Homo sapiens  TTTGAGACGGTGTCTCACTCTGTCGCCCAGGCTGGAGTGCGGTGGTGCGATCTTGGCTCACTGCAAGCTCCGCCTCCCGGGTTCACGCCATTCTCCTGCCTCAGCCTCCCAAGTAGCTGG
Bos taurus    ........................................................................................................................

Homo sapiens  GACAACAGGCGCCCGCCATCACGCCCAGCTAATTTTCTTTTTTTGTATTTTTAGTAGAGACGGGGTTTCATCATGTTAGCCAGGATGGTCTCGATCTCCTGACCTCATGATCTGCCCGCC
```

```
Bos taurus     ........................................................................................................................

Homo sapiens   TCAGCCTCCCAAAGTGTTGGGATTACAGGCGTGAGCCACCGCCCAGCCTGATCATTATCTCTCTTAAATGACATTGTAACATCCCAAACTGCAAAGGGGCCCACATCAAGTTTTTCTATG
Bos taurus     ........................................................................................................................

Homo sapiens   AAAGCCCATGACGTGGAGCACAATGCTTTGTACTAAATAGGCACTCAGGTGGTATTTGT-----TATTGATTGATAGGATTCTGAAGAACTCAGTATGTAACCATCCTTCCAAACCTGGG
Bos taurus     ..................................................GATATTTGTCAGTTTACTGATCAATAGGATTGTGAAGAATTCACTGTGTAACCTCCCTTCAAAACTTGGG

Homo sapiens   TGTGTTAAAATGTGTTCATTGTTCAGAGACAAAG--GTCTGGTACCTTTAAGAAGTCTCAGCTTTGCAATGAGTTTGTGGGGCA-GCTGCTTGGCATAGATACTGGGGAGGGACAGGGAG
Bos taurus     TGTGTTAAAATGTGCTTACTGTTTAGAGACAAATATGTCTGTTACCTTTAAGAAGTTTCAGCTTTGCTATGACTTGGTGGGGCAAGCTGCTTGCCATGGATACTGGGGAGGGGTAGGGAG

Homo sapiens   GAGATCATTATGTAGGCACATGGCGCTAGGATGTACCTTACTTTGTCTTGGCTGTTTTCTGAATGTTAAGAGGAAGGATGAAATAATAGCAGATATAGGAAAAGTTATTTAAAGCCTTGC
Bos taurus     GAGATT-TTATGTAGGCACATGTCCCTGGCATGTACCTTATTTTGTCTTGGCTGTTTTCTGAGTGACAAGAGAAAAAATGAAATGATAGCAGATATAGGGAAA-TGGTTTATAGCCTTGC

Homo sapiens   CTTAGA---TCAGTGGTTCTCAGGCGTGAGGATGCATCAAAAGCCCCTGGAGGGCTTCTTAAAACACAGATTGCTATATCCCACCCCTGAGTTTCTGGTTCAGTAGGTCTGAAGTGAGTG
Bos taurus     CTTAGAAGATCAGTGGGTCTCAG-----------------AATCCCCTGGAGGGCTTGTTAAAACACAGATCATTG-AGCCTGCCCCAGAATTTCTGACTCAATAGTTCTGAAGTAAGTG

Homo sapiens   GGGTCCACAAATATGCATTTCTAACAAGTTCTCAGGTGATGCTGATGCTCCTGGTCCAGGGACCACACTTTAAGAACCATTGCTCTG------CCAAGTAGTTGGCTCAAGAG--TAGCT
Bos taurus     AGGCCCCAAAATATGCATTTCTGACAAGTTCCCAGGTGATGGTGATGCCACTGACCCAGGGCCCACATTTTAAGAACCATTGCCTTAGATTAACAAGGTAGTTGTCTCAAGAGAGCAGCT

Homo sapiens   AATTAGAGAAATCTGAGCACAGTTAGCAACTGGAAGCAAAAAGTCCATGAGGAAACTGTGTGATGTACGTAGTGGGGAGAGTGTGAACTGGCTAGGAAACCAGAATGGTCAGATTCCAGT
Bos taurus     AACTAGAAAAATCCGAGTAAAAGAAACCAGAAGGAAAAAAAAGACCATGGGTAAAGCATGTGATAGATGCAGTGGGGAGAATGTGAACTGGCCAGGAAATCAGAATGGCTGGCTTCCAGT

Homo sapiens   ACCAGGGGTATCATATCTTTTTAAGCAGTGTCCAAA--GAATAAGTATATTTTAATTCACATATTCCTGATTCATTTTACTAGTGTTTCCCAAAATGTGCACTGTGGAACCCTAGAAGAT
Bos taurus     ACCAG-----CAATACTTTCTTAAGCAGTGTCCAAACATAGTAAGTACAATTTCATTTACACGTTCCTGATTTATTTTACAAGTGTTTTTCAAATTACACACTATGGAGCCCTACAAGAT

Homo sapiens   ATTAATAGGTAGGCAATGAGATTAAAAAGGATCCATAGAGAAA-GATGTTGGAACATTTTGAATTAAAGAAAATTGAACAGGATTCTTTAC-----GGCTTCTAAGAGCCTTTAAAATGT
Bos taurus     GTTAATAGGCATTCAATGGGAT-AAAAAAGATCCATAGTTAAATAAGGCTGGAAATTTTTGCGTAAAATAAAATTGAACAGGGTTCTTTACACCAAGTCCTCTCAGAGCCTTTCAAATGC

Homo sapiens   GAATGAATGAATGCACTGCAAATCTCCAAGGAAATATAGAATATGCAGTATTTATTTTATTAATTTATGCAGAATATTCAGAATTAGTTGACCAAAGATCCTTCGTTAACACTTGGCTGT
Bos taurus     TAAGCAA------------AGATCTCCAAGGGAAGATAGAATATGCTGGATCT----------------------CTCAAACTTTACAAACTAAGGATCCTTTACTAATACCTGGTTTT

Homo sapiens   TTTTTT---------GTAAAATGTTGTTTAGGAAACCTTCATTGGTTTACACCTGAAATGCCACAATATTTTATCAATAGATGAGGTTGCTTGCAACAGTGGAAACAGCACTGCACTGGG
Bos taurus     TTTTTTTTTTTCCTGGAGAATATGGCTTGAGAAACATTCATTGGTTTACATCC-AAATGCCAAGACATCTTATCAACGAATGAGGCTGCTTGTAACAATGAACACAGCATTGGAGTGGG

Homo sapiens   AGTCAAAACATCTGGGATTTTGTTCTGTCTCTGCCACTTAGGTCTGCCACAAGTTGGCTTGCTCCCATGAAAAAGATGGGTATGATTGAAATTTATCATCCCTTTGCAAATGGTAGCAGC
Bos taurus     AGTCAAAATATCTGGGATTTTGTCCTGTTTCTGCCACTTCACTCTGCCACAAGTTGACTTGTTCCCACGAT---GATGGGTGTGACTGAAATTTATCATTCCCTTGCAAATGGGGGAAGC

Homo sapiens   CAGTAGCTCCACCCAAAATAAGTAAATAAAATCTCACACAATCAATTTTTTTAAATCTAATTTACCATACCCTTAAGGTTATTCAATCTTTTTGTTTTTTTTTTCTCATGTTACCTTGTG
Bos taurus     CAGTGGCCCCAGCCAGAACAGACATTCACACACACACACATGCA---------------------CACAC---------------ATTTTTTTTCTTTTTATCTGGAATTCAACATGCCC

Homo sapiens   TAACAGGTATTCAACCTTCAAAGCCAGAAATATGAGAAGCCTTATGCTGAGAGTTTGATAGAAAGGAAGGGAGGAAGGGAGAAAGTAGAAAGGGAGAAAAGGAAGAAGAGAGGAAGGGAT
Bos taurus     TGAAGAATATTCAATCTTCAAAGCCAGAAGGGTAAGAAACCTTATGCTGAGAGTTTGATAGGAAGGAAGGAAA...............................................

Homo sapiens   GAAAGGAGGAAAGGCGGGGCAGGGAGGAAAGGAAGAACAGCAGAAAGAAGGAAGGAAGGAAAAATAAGAAGTCAGACAGTCAGGTCATTGCAGACCATGATTCCATCTGAAAAGAAAAGG
Bos taurus     .............................................................................AGACAGGCCATTGCAGGCCATGATTCCAACTGGAATGAACAGG

Homo sapiens   CTGAGAAGCATAGACACATCCTGTGTTTATTTGATTCCTGGCTCTAGGAGAAAGTTTTATTTCACCAAGGACA--GAGTCAGCTCGCTTGTTTACCACAGAGTATTGGAAGTGTGTGAAG
Bos taurus     CTGAGAAATGTAGGCACATCCTGTGTTTATTTGATTCCTGGCTCTTAGGGAAAGTTTTGTTTCACCAAGGACACTGAGTCAGCTTGTTTGTTTACCATAGAGCATTG---------GAAG

Homo sapiens   TGTGTTTGTCCCCCAATTAACCATTAGT-----TGTTTTTTTTTCACTTGAACAAGATACAAACTTGACCCAATGGAGAAACATTGCAAGTTGTATGTGGGAGGATGCCATAAAATATCT
Bos taurus     TGTGTCTGTCCCCCAATTAATCATTCTTTGTTGTGTTTTTTTTCCACTTGAACAAAACACAAACTTAACCCAATGGAGAAACACTGCAAGTTGGATGTGGCAGGATACTGC---ATTTTT

Homo sapiens   ACATTGATATTTAAAT----AAAATACCTGCCTACATTAAACGATC-TTTGAAAAGAAACTGATGGGATTCAAGGCTGAATTATATTTACTGAATTCCTACAAGGTACATAAATATATGC
Bos taurus     ACATTGATATATATATATAAAAATCATGTGCCCACATTAAACAATCATTTGAAAAGAAAATGATGGGATTTAAGATTCAATAATATTTGCTGAATTTCTACAAGGTACATAAATATC--C

Homo sapiens   AACATGGAGTCCCTTGTAGATCCATGATGAAATCATATTTCTTTTAAACACAAGGGTCAATTCAAGTTAAAAAATAAAATAAATGTTTACAAGCTTTCTGCCTTTTTAAATTTCAAAGT
Bos taurus     AAAATGGGGTCCCTTATACATCTCTGTGAAAATTAAA---AAAAAAAAAAAAGAGTCAGTTCAGGTT-AAAAATA----GAATGTTTATAAGTTTTCTGTTGCATTTTTATTTCAAAGT

Homo sapiens   GAAAATAATATTTAATTGCATTTAAATATATACATATATTTTAATAACTCTGATTACCTATTTGATTGCATAAAAGAATGAC-GAGAACATCAACACTGCTTTTAGCCACAATATTTGAG
Bos taurus     GCAAATACTATTTAATAGCATACA-------ATATATATTCTAATA--TAAGATGACCTATTTGATTGCATAGAAGAATGACAAAAAACATCAGCACTCCTTTTAACTAAAATATTTGAG

Homo sapiens   AAGTAGCAGC-CAGAAGTGTTTTTTTAAATATTTTATCTTGAAATCAGATCTCATGT---------ACCTAGGTGAACTGAATATGCCCAGAGGATATCAGGGGACTCTCCACTACTGTA
Bos taurus     AAGTAACAACTCAGAAGTAGCTTAC-ATATATTTTCTCTTGAAATTAAATCTCCTGTTTATCTTGCAATTGGGTGAACTGAATATGGCCAAAATGTCTCAGGAGATTCCCTACTGCTATA

Homo sapiens   GATCCAA-AAAACAGAGGGCTAAATAGAAATTCCACTTGCTGTCATCCAGCAAAATAGAGATGGCTTTGCTGAGTCCTTAGATCAT-GTTTAGTTTCAGGAGGGGTTGAGCTGTAATGAC
Bos taurus     GACCCCCCAAAGCACAGGGCTGAAAAGAAGTGGTAGAGGCTACCATACAGCACAATAGAAATGTCTCTGTTGAGTTCTTGGATCATTGTCTAGATGCTGGAGAGGTTGAGCTGTATGGAC

Homo sapiens   TAAATTATCTGGTCACTCTAACCAAGCAGCTAATTAATCCAGTCAAACAAACTTATCAGTCATGA-----AGGTGAAATTGTTTTTTCATCTAGAATTTGATCATAAGGGCTTTCAAGAA
Bos taurus     TAAATT----GGTCCCTCTAACCTAGCAG-------------TCAAACTACCTTATCAGTCAAGACAAGAAGGTGAACTTGGCTTTTCATTTAGATTTTGATCTTTA-----TTCAAGAT

Homo sapiens   GGAAGCTCCTTGAGTCTCCCTTGTAAACCCTTTCTGCAGTATGCCTTTCTTTCCCACGTCTGTTGAAAATTATTTATTCTTGAACCAGCTTCAGTGGTGAACAGCAGCTGGTTGCTACCC
Bos taurus     AGAAGCTTCGTAAGTCTCTCTTATAAATCCTTTTTATGG---GCCTTTCTTTCCCATGGCTGTTGAAAGTTATTTACTCTTGAGCCAGCCTCAGTCATGAACAGCAGATGGCCGCCACTC

Homo sapiens   TCTCATACCCATTTTTTGTCATCTGGAACATGGTTATTCAGCGTAGCTTCATCTTCAAAAGGTGCCTGATTTACTCTGGGATTGGGAATGGAGAGAATAATTTTGAAGCTCTTTCTCTTT
Bos taurus     TCTTCTCTCCACATTT-GTCAACTGGAACTGAGTTATTCTGAGTAGTCTCAACTTCAAAAGAAGATTGGTTCACTCTCAAGTGGGAAGTGCAGAGAATCCCCATGAAGCTCTTTCCCTTC

Homo sapiens   GCCTGTTTCCTTCCTTCCATGACTTTGGGTTATTTTCTAGTGATCTTCAGTATTAAAAGGCATACTGACAGCTCTTTTCTCCCAGGCTGATTTTCAGCTTGTCTACTGAGCTTTTTTGGG
Bos taurus     GCCT----CTTGCCTTCTATAATCTTAAGTTACTTTCTATTGATCTGCAGTTCTAAAATGTGTGCTGAAAGATTTGCTCTTCTGGGCTACTTTTCACTGTTTCTGCCGGGCTTTTCTGGG

Homo sapiens   TTTGGTTTTATGGAATGAAATCCCCTTGTTTGGTTTTCCCCACATTCACACTGAGGCTGTGAATGTCCACCAACTCAATTCTGAGCTACAATTGAGTAGTGTATTAAGAATACATGTGAC
Bos taurus     TTTGTTTTTGTAGAATGAAATCACGTTGTTTGGTTTTCCACACATTCACACTGAGGCTGTGAATGTCCACTAACTCAATTCAGAGCTACAATTGAATAGTGTATTAAGTGTCCATGTGAC

Homo sapiens   TGCCACATGGGCCCTGGACTGCTGAATTCATGGTCAAATCATTGTTTTCCC-AACCATCATGCTTGGGGGCACCACATCAGCCTACAGTGAACATGGATTAATAAGTGCTGATTTAGAGT
Bos taurus     GGCCTCATGGGCCCTGGTCTGCTGAATTCATGGGCAAGTCATTGTTTCCCCTAACCACCATGTTCAGGGGCACCCTCCCAGGCTATAGCAAACATGGGTTAATCAGTGCTGACTTAGACT

Homo sapiens   AAATAGTTTTCTGGTAAGTGGT---ACATTGAAGATTTCTCTTCCCCTTTCTATGATTTG---TATAGTAGCCACAATTTTCTATATCATTATTGCAGCTTCCCAGTACTACTTTATGTA
Bos taurus     CAGTATTTTTCTGATAAGTGGTGTAATACTGATGATTTCTCCTCCACCTTTGCTGTTTTGTGTTATAGTAGCCACATTTTCCTACATCATTATGGCAGCTT...................

Homo sapiens   GAAATCTTGCACATTCCTACCTCTGGCTTTTGCTCAGGCTACGACTCTATGGCAGTGGCAGAACCGTTTTAGGAATGTCTTCTGCTGTAATTTTCTCTTTGTCCCACCACTTCCACTTTA
Bos taurus     ....................................GGGCTCCAACTCCATGGCAGTGGCAGAACCATTTAGGAATGTCTTCTGCCACAGTTTCTCCTTTGTCCCACCCCTTCCTTTTTC

Homo sapiens   AACTTCT---------GGTGTATTCCCTAATCAAAGAGGCATCTAAGTTGTCTTTAGTGAGAAACCATCTATATATACAAAGCCATAACATGTGGATGAAAAGGACATACTGTCAACTCT
Bos taurus     AACTTCTCATTGTTTCCATATATTCTTTCGTAACAGAGGCATCCAAGTTG--TTTAGTGAGAAGCCATCTATATACACAAAGCCATAACATGTGAATGGAAAGAAAATACTCTGAACCCT

Homo sapiens   CTTGTGCCAGACACCCAGATGTTGGCAAGTAGTACTTGGACCGAACAGCATAGTCGTTGA-----------ACAGCATCATCTTCCCCTTTTTAATTCATCCAGATGTTTTGGCATCAC
Bos taurus     TTCAGGCCAAACACCCAGATGTTGGCAAGTAGTATATGGACCAAACAGACTAGTCAATGACTATATCAATGACAATATCACCTCCTCCATTTTT-ATTCATCCATATCTGTTGACATCAT

Homo sapiens   AGACACATAGCCAGATTTCATCCCTGATTTTGTCAAAGGCTTAAAAAAATGTGAAAAAATAATATGCTTCTCCTCTGATGAACAATTACCAATGAA--CTCTGTTCAAAAGGG-AAAACA
Bos taurus     GCATACCCAGCCAATTTTTACCCGTGATTTTGTCAGAGACTGAAAAAAAATATGGAAAATATTATGCTTCTCCTCTGATGAACAATTATCAATGAACTCTCTGTTCAGAAGAGCGACAGA

Homo sapiens   TAATGTTTCATTTA-AAAGACCAATAATATTGAATTAAGATAAAGTTCCCATGAGAGTATA-TTTTACATTCTCATGAATCACGGATGAGTTTATTATGATCCATGCTTGTATGAAAGAC
Bos taurus     TAATGTTTCACTTAGAAAGACCAATAATGCTGAATTAAGATGAAGTTCCCATGGGAGTATGTTTTTACATTCTTGTGAATCATAGATGAGTTTATTATGATCCATACTAGTAGGAAAGAA

Homo sapiens   TGACAAATAAATTCATATTGATTTGACATTTAAAATAGCATGTCTAAAAATGTACAGCATCACATTAAGTATCAAGTAATTATTCAACTATCAGCTTCAAATGTACATGGGAGAAAAT-G
Bos taurus     TGACAAATGAATTCATATTGTTTATTCATTTAAAATAACATGTCTAAAAATGTACAGCATCACATTAAATATCAGGCAGTTATTCGTCTACTAACTCTGAACGTGAGTGGAAGGAAATGG

Homo sapiens   GGTCCAGAACCCAAAA-TGTAGTGAAAGATTTAAATGCTCAAATTTCTACCACAGGTTTTTTTTTTTTTTTTTTTTTGAGACAAGGTCTCACTCTATCACCCAGTCTGTGTGCAATGGT
Bos taurus     GGTCTAAAACCTGAAATTGTAGTGGAAGATTTAAGTGTTCAGCTTCCTAC......................................................................

Homo sapiens   GTAATCATGGCTCACTGCAGCCTCAAACTCCTGGGCTCGAGTGATCCTCCCACCTCAGCCTCCCAAGTAGTTAGGACTTACAGTCATGTGCCGTCATGCCTGGCTAATTTATTTTATTTT
Bos taurus     ........................................................................................................................

Homo sapiens   TATTTTTGTAGAGACAAGGCCTTGCTTTGTTGTCCAGGTTGGTCTTGAACTCCTGGCCTCAAGCAGTCCTCTCACTTCGGCCTCCCAAAGGACTAGGATTATAGGCGTGAGCCACTGTGC
Bos taurus     ........................................................................................................................

Homo sapiens   CCAGCCTTCTACCACAATTTTTAAAGAAGCCAATACCATACATTAATCCATTTACAAGGGCTCACGCAAGAGTGCCTTTTGAGCCTGTGTTTGACAGGAAGTTTCTATTCAGATCATTGT
Bos taurus     .............................................................................TTTTAATCTGCGTTTGATTGGAAATATATAATCAGATCATTTT

Homo sapiens   GCCTAGAGTATATAGAGGGGTTACAGCAAAGTGCTGTACTTTGGGTTACAGTAAAGTGACTCAATTGATGTACCAAGATATTGATCATTTACACCCTT--------TAGG----------
Bos taurus     GCTTGGAGTAGACAGAAGGTAT----------------CTGTGAGTTACAGCAAAGCGACCTGATTGATGTACCAAGCTCTTGATTTCTGTGACCCTCAGAGAGACTAGGAGGGGCCTGA

Homo sapiens   -----------CCTGAAAAATATACTTTTCTTTGTGTTCCATGAAACTAAAAAGGCAGGGAAGCATTAAAGAGACAGCAATAAAAAGATTTGATGGTATGTATATGTGAGTTTGAGTGTT
```

```
Bos taurus     GAACCCTGGCACCTTAAAAATATAGTCTCCTTTGTTTTCATGAAATTAAAGAGGCTGGGAAGCAATGAGGAGGAACAGTCAAAAAGATGTGATGTGATGTGTGTACCTGAGTGTGTGTG

Homo sapiens   TGAGCTGGCGTTGTGAGTTTGTGGGTGGATTAGAAAGCATATGGCTAAAGACATGTGCAACAGATGCAAGGGCCTGGGCTTTGGCCTTCCCTTTGAAACTTCTTTATGGGGACATGTGCT
Bos taurus     TGAGCTGGGGTGGGGG--TTATGCGTGCCTTGGA----------------------------------GAGCCCAGGCCTTGGTCTACCTTCTGCAGCTTCTTTACGGGGGCCTGTGTT

Homo sapiens   -------GCCTTGCTTCCTGGATTAAGACCACATTTGCACAATTTGCACATTGTCCCTC------TTGGGCCTGCTTCCCACCGGAGACTGCATCTTCCACAAAAACAAGGCAAGAAGTA
Bos taurus     CGCAGGAAACTTGCTTCCTGGATTAAGACCACATTTGTGCTATTTGCACATTGCCCCTCTAATTTTTGGGCCTGCCTCTGACAAGAGACCACATCTCCCATGGAAACAGGG-AGACAGTA

Homo sapiens   AAGAACTTAAGAGAGCTGATTGTGAAGTCCAACTACATGGGTTCAAATCCTGGTACCATCATTAATTAGCTGGGTCATTTTGGACCACTTAACTTCCAGGACCCTCATTTTCCTCATCTA
Bos taurus     GAGAGCCTA--AAAGCTTGGCCTGAAGTCC-ACTGCCTGGGTTCAAACAGTGGTACCATCACTTACTAGCTGGATCATCTTGGGCCGGTTAACTTCCTTGAACCTTA-CTTCCTTGCCAA

Homo sapiens   CACAAAGGGCTAATAACAGTATTTACCTCATAGTTGT----------------------TATGTAAAGTGCTTATCCCAGAGCCTGGCAGAGAGCAGACACTAGTTGATCATTATGATT
Bos taurus     TAAAAAGGGCTAA-AACAGTACATACTTCATAGTTGTGGTGAGGAATAAATGAATAAAGGCATGTAAACTGCTTATCCCAGTGCTTGATACAGTGC.......................

Homo sapiens   ATTGAGCAATTACTATGTACCAGGTGCTTTATTTTCATCTCTGATCCTCACAACAGCCCTACAAGGCAGATACTATTATCTTCAGTTTACAAGTGAAGAGGCTGAAGCTCAGTGAGATAA
Bos taurus     ........................................................................................................................

Homo sapiens   AATACACTGCTCAAGGTCACACAACCAGGAAGAGCAGAAACCAGGCTCCAAGTCCCTCTGTCCCTCACTCCTAAGGCCTGTGCTATTTGCCATATCATCATCCCACTGGTGCCTCTAAAA
Bos taurus     ........................................................................................................................

Homo sapiens   GTTTCTTCTTCTAGGGTTCATCCCTCTTCTCTCTTCATCCTCCTCTTGGCCTACCCTGGCCTGAGAACTGCTTGGCACAATCCTATACAGCAGGTCCTTGAATAACATTGTTTTGTTCAA
Bos taurus     ..TTCCTCTTCTAGGAATCATCCCTGTTCTCTGCTCATCCAGCCAATGGCCTACCTTGGCCTGAGAGGGGTTTACCACAAAGCTA..................................

Homo sapiens   AGCCATTTAGTTATCACGTTAATGGGGAAAATTTGGTTTTCCCCAGGGCCACTGTCCATGGACAGGATGGCGTCCTGACCAGGGTGGTTCCCACCTTTTGTGCTCCTTGTGCTGCCAGGA
Bos taurus     ........................................................................................................................

Homo sapiens   TGGGCTCCGGCCACCTGTGACCCTGAACTGGAATCAGCGGGTTAGAAAATAACTGAATGAGTGAATACAAATTATTGGAAAATGAAAACTCGTAAAGTACACAATGACCATACGAATCCA
Bos taurus     ........................................................................................................................

Homo sapiens   TGATAATAAATGACACGATAGGAAAGCGCTCAATGAGCGCTTTTGTGATTCTTCTGGAACTGCTGGGTGGCAGGAGGTGCTCCTTATGATTTTGCTTTGCAAATGTTTATTCCTTGACCT
Bos taurus     ........................................................................................................................

Homo sapiens   GACCCGCCATTGCTACAACCGCTGTCACTCATTGATTCACCAAAAATTAGGCAAATAATTCTCTTACTTGTTTTTATTAATCTTTCCTAAATGTATGTGTAGCTCACACTTATTGTTAAT
Bos taurus     ........................................................................................................................

Homo sapiens   GTTTAATTAACTTATTAATGTTTAATATTAGAAGTGTTTGGGTGGTTTTTGTTTGTTTGTTTTTTTGTTTGTTTTTGAGACAGGGTCTCACTCCTCTCGCCCAGGCTGGAGTGCAGTGGC
Bos taurus     ........................................................................................................................

Homo sapiens   GCAATCACGGCTCACTGCAGCCTCAACCTCCCAGGATTCAGGTGATCCTCCCACCTCAGCCTCCTGAGTAGCTGTGACTACAGACGTGTGCCACCATGCCTGGCTAAATTTTTGTATTTT
Bos taurus     ........................................................................................................................

Homo sapiens   TAGTAGAGACAGGGTTTCACCATGTTGCCCAGGCTGGTATTTGGATCTTTATTTAGAAGTTTGGTGATATTTTTGTGACCAGAAATATGCTGTGGGTACTTAACTCTTGTTTATATCAAT
Bos taurus     ........................................................................................................................

Homo sapiens   TAGCCTACAGTAAAATTAGTTTTGTTATTCGTTGTTTCACTTAAAGTCCAGTTCCCAAGAACCTGTCAAGGACGTTCAGTGAGGACTTACTGTACTCAGAGACAGCAGAGAGCAAAGGGC
Bos taurus     ..........................................................................................CTTGGAGACAGTGG-GAGTCAAGGGC

Homo sapiens   AAGAAGGCTGGTTGGAGGACGAGGAGGCCTAGAGCCCCTTGCCTGCTTTTGCACATGCATCTGCTCCTGGTGGATGACTCACTTAACCTCTCCGTGCTTCTGCCTCTTCAACTGGAAAAT
Bos taurus     AAGAATGCTGGTGGGAGGATGGGGA-------------------------CATGTGAGTTGGACCACAGTGGGTGATACACTTTAGCTCTCCGAGCTTGTATCCCTTCACATGGAAAGT

Homo sapiens   GGAATCTGTAAGTTGCAGCACTAG----CTTCTGTGACATTCTACTAGGCCTCCAGGAAGAGGAGGCCATCCCTCTATATGTCCAGTCAGCATATGGGGAACAGTGGGG--CTGTGTGTG
Bos taurus     GGAATCTGCAAACTGTAGGTCAAGGTTGCCTCTGGGACATTTTGCTTGGCCTTCAGGGAGAGCGGGCCATCCCTCAGTATGTCCAGTCAGCATATGCTGAGCAGCTGGGGCCTGCCTGTG

Homo sapiens   ACTCACCCTG-TGGTGTGCCAGCCTCAGGCTTATACTAGCCTAGGCTTATACTCCCAGATCCTGAGCTAGCAGCTGGAGCTTTGGGATCTGGAACTGTTACAGGGCCAAAGAACCTGCTT
Bos taurus     ATTCATTCAGCTGGCGGG--GGCC---------CACTATCCCAGGCTCATAGTCCCAGATGCTGAGTTATTGGCTGGGGTTTCTGGATCTGGAACAGTT-CAGGACCAAAGAGCCTGCTC

Homo sapiens   AGCTTCAGGGCTGCTTCTAGATAAACTGCCAGGATGTGGGCAAAGCATTTGTCATGGAAGGTGGGGAGTGTATGTAGGTTGTTTCAGGGGCTGCTTTGTGGGAAG----CCAAACCTTTT
Bos taurus     CAGTCAGGTGCAACTTCTAGAT-AACTACTGGGATCTGGGCAAAGTATGTG----GTCAGGCAGAGAATGTGT-TGGGCTGCTTCAGGGG-TGCTTTATTGGAATTAGCCTGAAACCTCT

Homo sapiens   GGCAATGGGCTCAGAACTGGCTTTCCTTTTTGGGTGTCCTCTAAATTCCGAGGGTGCCCCCACACCAGAGGGTCTCCACTT---CTGCCATCCCTAA------CCTAGTGGGGAGATGAA
Bos taurus     GGT-GTGAGCTCAGAACTGGGTTTCTTTCTTGAGTGTCCTGTAAAATCCTAGTGC-CCCTCAGGCCAAAGGGTTTCCACCTCACCGGGGGCCCCTGGATTCTCCCTGGTGAAAACGTGCA

Homo sapiens   TCTGATCTTTTGTCTCAAAGCCCACATTAAAGAGGACCATTGCATGCTTCATCACCAGGCTGTGACTCCCAG--ACCTCAACTCATGTCTTTTTCCCTC----TTCACCAGGGCAGAGGC
Bos taurus     CGGGGTGTTTTGCCTCAGGGCCCATGCTCAAGGGGAGCCCTGAGCTCTGGATGATGAGGCTATGACTCAGAGGTACTTCAACTCCTGTTCCTTCTCCGCACCTCCCACCCAGGCAGAGAA

Homo sapiens   TAGACTGTTTCCTCTCCTCTCCCTGAACAATAGCAGTGCCCAGATTTTGGAGGGCATACCGCCTACCTGGCACTGTGCTAAGTACTTTGCCTGCATTTCCTTGGTAATCCTCATGTAATC
Bos taurus     TAAACTCTTTATTCTCCTGTCCCTCAGCAAGAGCAGTGCCTGGGTTTGGGAGAGCTTACCTTGAACCTGGAACCATG-TAAGCAC---------TTTCTTCACTAACCCTCACGTGACC

Homo sapiens   CTACAAGGGAAGTACCATTTTGTCACGATTTAACAGAAGAGGATACAAAAGCTCAGAGCCTGGAGGTGACTTGCTCAAA-GTCACATTGATAGTAAG-TGGTAAAGCCGGGACTGAAATA
Bos taurus     CTGTGAGGGAAGTGCCATTTTGTCATGATTTTACAGAAGAGGCAATGGAAGCTCAGAGCATTGAGGTGATGTGCTCAAAGGTCACATTGGTAGTCAGTTGTGGAGTCTGGATTCAAATA

Homo sapiens   CAG----GTCTAACTTTTGAGACAACCTTTTTTCTTTTTCTTTTTTTTCTTTTCAATTACATAATCCTAATCCTGTGCGTGTTTGAAGGGGAGAGACAGGGGAGGGAGTGAGATGGCAA
Bos taurus     CAGGTCTGTCTAACTTTTGAAAT-GCCTCATTGC---------TCATGTGATTTCAATGACA------CAATCCTGGACAAGTGTGAGGGAGAGGGACAGGCAAGGGGGGGAAAGGGCAA

Homo sapiens   TTTGCTTGTGACCTAAGGAGGCCC----------TGAAATCTCCTGGGAATGAGGAT--GGGGGACCTACATGAGTTGCCCAGGGCACTGGTAATGCAGCCCACACCAGCAGTCAGTGAT
Bos taurus     TTTGCTTGAGACTTCAGAGTGTCCCATGGAAGCTCAGAATCTCCTTGGAGTTAGGATCGGGTGGGACTTTGTGAGTTGCTCAGGGCACTGGAAAGGCAGCCCACCCCAGCTTGCAGTGGT

Homo sapiens   CTCCAACATGGAGGGCTCCAGGCCTGCCTAGCTGCTGGCTGCTACTG--GAGGCCAGAAAAAGACAGCTCCAGTGTCCTTCTAGGCTGGGGGTGGTTCTTGCCTCCAGGGCTTATGGAAG
Bos taurus     ---------------------CCTCCCTAGCCATTGGCTACAGCAGCACAGGTCA-CAAAAGAGAGCTTCAGTGCCCTTTT-----------------------AGGGATCATGGAAG

Homo sapiens   AACTTATCAGACAAAACTCTGCCCTTTCAAGCAGAA----------------------------AAGTACTTTACAAAGAACATAGAGAGGTGAAGTGGGTACTTTTCAATTTTTGCCT
Bos taurus     AATTCATCAGAAAGCACTCTGGCCTTTCAAGCAGAAAGTGGCATCAGAGGCATCAGAGACATCGGAGGCAATTTAGAAAGAACCTAGATAGATGAGGTGGACATTTGTCACTTTTT-TCT

Homo sapiens   GATTATCTAATATCTAAATGCTTTTTCTTCACTTTGGAATCCTGACCTGACCTGTTTTGTGCCCTGTCCACCCACCACCACTGAGTCAAAGCTCAGGGCCTGGCATCCAGTATTAGTCA-
Bos taurus     GGCCACCTAATATTGAAA-GCCTTTCCTCTGTTTGGGACCCCTGACCTGGTCTCTTTTGTGCCTCTTGTACCTACCACTGTTGAGTCAAACACAGGGCCTGGTAGCCAGTATTACTCCA

Homo sapiens   -TACCTATTTGTTGCCCGAATGAATGGGGCTGAACCCTGGTCTGACTCTAAAATACATGCTTGAAAAAAATTATTATGGAAATTTTCAAACACATACACAAAAGTAAAAAATATAGTATA
Bos taurus     TTACACATTTATTGCTTAAATAACTGGGGTCAAACTCAGGTCTGACTCTAAAACACATGCTTT-AAAAATTTATTATGAAAATTTTCATAC----AGACAGAAGTAGA----ATAGTATA

Homo sapiens   ATGAACCCCCAAGTATCCATCACCCAGATTCAACATCTATCATGATT--TTCCATGTTAGCTTTACCTATCTCTCTTTCTTTTTCCCTCTT----------------------------
Bos taurus     AT.AATCCCTGTGTACTCATTACC-AGATTCAATAGCTATCAAGATTATTTTCACGTTAGCTTTATTTATCTTT--TTCTTTTTCTCTTTTCTTTTAAGGCCTTGCCTTAATATTTGGAAG

Homo sapiens   ----------------ATGCTTTACCCC-ACATGTTTCATTATGCAACTCAAAAA--TAGGGATATTTTTATATACAATGACGAAATTATTATCCCCCCCATCGCCAAACTAACAATAA
Bos taurus     GAAATCCCAGGTGTTATATCATTTACCTCTACATACTTCATGACACATCTAAAACAATTAGGGATATTTTCATATCCAGTGACAGTATTACCATTGCCCCTA--ACCAAACTAACAACAA

Homo sapiens   TTCCCTTCAAACCATGCTCTTGAGCACTACACTGGAGTTCCTCCATTCTACTTTCTTTCCCAGTCCCCAACAGGAAGCATTTCTCAGGCATCTGCTCTTGCACCTCCTCAGAGCTGGAAT
Bos taurus     ---CTTTAAAACCATGCTCTTGAGCACTACAGGCTAGCTTCTCCATTCTGCTTTC----CCAGCCTCCAGCAGGAAGCATTTCTCAGGCATCTGCTCT-GTTCCTCCTTGAAGCTGGAAT

Homo sapiens   TACAGTTTGATGTGTATTGGGTACTCACAATGTGCCCTTTTAGGTAAGCAGGGGGCCAGATTCCCCTCTCCCTGTTTTACAAAGAAGAATGGCGACACACAGCTATTAAGTGCCTTGTAC
Bos taurus     TACAGTTTGATGTGTATTGGGTACTCACAATGTGTCCCTCTTAGGTAAGCAGGGGGCCTAACTGCCCTCTGTCTGTTTTC--------------------------------------TCC

Homo sapiens   AAGGTCACACAGTCAATTAGTGGCTGATAAATGGGACTCAAGCACTGTGGCTGGGTCTATACAGCCCCAAATACAGTTTGCAAGGAAATTAAATGAAGGGACTCGTGTCCTGGGGACAGT
Bos taurus     AAGGTCACGCAGTCAGTTAGTAGCTGATGAATGAGACTCAAGCACAGCAGCTGGGTCTATACAGCCCCAAACACAGTTTGCAATGAGATGAAGCAAAGGGACCTCAGTCCTAGGGACAGT

Homo sapiens   ACCGTGTGTTTTCTGTTGCTGTTGAAGTCATTGCACTTAGC-------------------------------CCAT-------CAGGACAGCCTGATTGATGGGATCAGTTGGACTCCT
Bos taurus     CCTGCAT------TGTTTCTGTTGAAATCCTGCCACTCAGCATCAAGCAAGTCCTTCGTCTGGAACAGTTTGATCATGACTGTTCAGGACTGCCTGATTGAGGGGCTCCGTGGGGCTCCT

Homo sapiens   ATGTTTGTTTTCCT-CATCTGACTGGATTGCAACAATCACCTACAGGCAGGCTGGCCACACCCGTGAGTTTTTCTCTTCTTTTTTTCCATGTTCCTGGAGCTGACTC---------CGGA
Bos taurus     AGGGCTGTTCTCCTTCATCTGATTGGAATGTGTCCGCCGCCTACAGCCAGGCCTGCCACAACCTGGAACTTCTCTCCTCTTCTTTCCC-TGTTC-TAGAGCTGATTCAGAGGAGGGCGGG

Homo sapiens   GCCCACATTTCTCCTGTCTTTCTAGGCTTGGACTTGCTCTTCTGCCAAAGCAGATGACTCCCAGAGCAGCCACAGTCTGGGACAGGGCAAATGTGAGAAGCAGAAGGACTAGCTTAACTGC
Bos taurus     GTGCTCATCTTTCTTCTCTTTCTAGGCTTTGACTTGCTTTCTGCCAAAGCAGAGGCTC--AGAGCA-CCACAGGTCAAGACAGTGCAAATTCGAGAAGCAGTAAAACTAGCTCCGCTGC

Homo sapiens   TTTTCTGTTCCTGCCTCCTTCTTGTTCCAAAGACTTACTCTTAGATGCTTGGCTCTCCTGAGCTTGATCTCCATGTCTAATCTCTAGACCTCTTCCACTCCCCT-GATATCCCACCCAC
Bos taurus     TTTTCTGTTCTTGCTCCCTTCTTTTTTCA--GACTTTACTTAGGTCACCAGGCTCTCCTGAGCTTGACCTCCATTTCTAATTCCTCAAACCTCTTCCACTCCCCTTAATCTCCCACCCAG

Homo sapiens   ATTTTCCAAGTAAGTCTCTCAGAGCCTTGTATTCCAAAATACAAACTGGCTAGTGGAAAGAAGGAATCATTGCTGTTGGCTAGTGGATGACTGATCAGTCTGAGTCCAACCAGGAAAATA
Bos taurus     ATTTCCCAGATAAGTCTCTCAGAGCATTGCATTCCAAAATGAAAATTGACTAGTGCAGAGCAAGAATCCTGGCTCTGGGCCGGTGGAGGGTTGATCAGTCTG-GTCCAACTGGGACAACA

Homo sapiens   GAAAT----CACTGAGTATTTATTTAAGGGAAAAGGGATTCAGTGCAGGGAATTGATCACACAGGTGATGGAAAAGCTGTGATGCCAAGCAGAGGACAATGAAGCAACCTAGAGATTAGT
Bos taurus     GGAATGAATCACTGAGTATTTA----AGGGAGAAGGAATTCAGTGCAGGGAATTGGTTACCCAGCTGATGAGAGAGCTTCAGTGCCAAGCCAAGGACAATGAGGCTACCCAGAGATTAGC

Homo sapiens   CACAGCAGGGAGCTGCTACCATTCCTTGGCTAGAACCTCAGG-GCTGGGACACTTAGTAGAAACTTGAGCCACAGTAACCTGGTCAGCAGTGCCAGAGCCACAAAGAGACACAGTCACTG
```

```
Bos taurus    CACAGCAAGAAGCTCCTAC---------ACCAGAAGTGAGGGAGCTAGGTTGCTCGGTGGAAACTGGAGCCACA---------------------------AGAGAGACACAGTCACTG

Homo sapiens  CTGGGGACACCACATGAAGCAGAGGGGGAGGGAGAGAAATACCCTGGTTTCTCTCTCCCTTTTGCTTTCTAATATCCTAACAGTGCCTTCCATTGGCCAAACCCATCAGAGCCTGGGGAA
Bos taurus    TGGGGGACATCACATGAGGCAGAGAGGGAAGGAGAGAAGTAGCCCAGCTTTGTTCTCCCTTCTGTTTTCCAA-GTCCTACCAGTGCCTTCCATTGGCCAAACCCACCAGGACCTGGGAAA

Homo sapiens  CAGCCTGCAAAGGCCAAC--------CTGCCCCATCAG--CTTCCCTGCTACAAG---AGCAGAGATGTGAAAAGGAAGTAATGGACTTGGGGGGCAAACAGGCCTGGGGACAGCA---
Bos taurus    CAGCCTTCAGGGGTCCACTCATCCCCCTCCCCTCTCTGTCCTCTCCTGCTACAATGTCAGCAGAGAAGGGAAGAGGAAGGGATGGATT---GTGGGCAAACAGGCCTGGGAGCAGCAAGA

Homo sapiens  -----CAGAT----------CTCCTCCTCCCTGCCACCCCCAACTAACTTATTTGGATATGTCAGGGTCTATTTGGAAACAGTGCATTTACTCAACAAAATGATTCTAAAGCTGACAAGA
Bos taurus    AACTACTGATTGGTTATATACTCCTCCTTCC-GCCCCCCACCTCCAACGTATTTGGATCTAACAGGGTCTGTCTGGAAAAAGTGCTTTTACCCAA-------------AGCCAACAAGA

Homo sapiens  AAG--ACCCTTAAGGAAAAGGGTGTGGGTGGAGCACGAACCCGCTTCCTTGACTA----------CCTGCAAGTCTTAGCATCCAGATTCTAGCCCAGTCCTCTTATTTCACTTCCTCCT
Bos taurus    AAGTGGGTTGTAAGTTGGAGGGTGGGAGTGGAGCTTGAACCTGCTTCCTTGACTAGCTTCCTTGACCTGCAAGTCTTA---TCCAGATTCTAGCT-GGTTCTCTTATTTCGCCTCCCCCT

Homo sapiens  GCATGCTCCAT----CCCTCCTCC-TCCCACGCATAGGCTGACTCCAACAAAGGCAGGCCGGCCATTTTGCTTTCTGGCAATGTGTGATGCATTCATTGGCTCCCAGGACTGTGTAAACC
Bos taurus    GCACGCACCCTTCTGCCCTCCTCCGCCCCACACACACTCAGAGTCCAACAAAGATAGGTC----ACTTTGCTTTCTGGCAACGTGTGACACATTTATTAGATCCTAGGACCATGTAAAAC

Homo sapiens  CAACCTTCTTGCCTGCAGGGTTTCAGCTTGGCAGCCAGGCTCTGAACTGCAGTGCTGTCAGGCAGGTGAACCCGCTGGCACCACAATCCAGAAAACATATCTCCTGATTCCTCAGGTGTT
Bos taurus    CAACCTTCTTGCTGGGAGGGTTTTGGCTTGGCAGCAAGGCTCTGAATTACAGTGCTGTCAGGCTGGTGAACCTGCTGGCATCGCAGTCCAGAAAACATACCTCCTGATTCTCCAGGTGTT

Homo sapiens  CTACACGTTTGAATGTGTAGATGAAGGGAGATGGCTGGGAGGGGGAAAAGAAAGACATCAAACTATTTATGTGCTTTCCCACACC---TTATTTCATTTACTCTCCACAACTCCTCAAGG
Bos taurus    CAGCGAGTTTGAAGGTGTAGGTGAAGGGAGATGCCTGGGAGGGGCAAAAGAAAAGCACCGAAC----TACATGCTTT-CCACACCTCTTTATCTCATTTTATCTTCACAGTCTCCCAAGG

Homo sapiens  CAGATGCCCTTCTTCCCA------TTAAAAAATAAATTTTTAAATCTTAGAATACTTTTAGATTTACAGGACAGTTGAAAAGAGAGTACAGAGAATCCCTATGCATCCTTCACTCAGTTT
Bos taurus    TAATTGCCCTTCTGCCTATTTTTTTTAAATAAGAAAAATTTAAACTTTGGAATAATTCTAGATTTACACAACAGTTGCAAGGA-------------------------TAACTCAGTTT

Homo sapiens  CTCCTGATGTTAACATCTTACAAAATCATCTTTTGTCTGTTTTTACAAATGATGAAACTGGGGCTCAGAGAGGTT-AGTGACTTGCCCAAGGTCATAGAACTAGGACACAGCAGAAGAAA
Bos taurus    CCCCCGATGTTAACACCTTACATAACCATCTCAGGTCTGTTTTTACAAATGAGAAAACGGGGGCTCAGAGAGGTTAAGTGATTTGTCCAAGGTCACAGAGCAAGGACACAGCAGAAGAAA

Homo sapiens  CAGGCATAAATCCAGTTAGATCTTAACTGTATCACACCACCAAGTTGAATTATTTGTTTCCGACTCATTATATTGGAAGTCACTCTCTGAACCTTGAACCCTTCACTTCACCTCATAGTT
Bos taurus    CAGACATAAATCCAGGTCGCTCTT-ATTGCCTTACCTCTTTGGGTTGAATTATTTGTTTCCATCTCATTAGGTGGGAAGTCACTCTCTGATCCCGGAGCCCTTCACTTCACCTTGGAGTT

Homo sapiens  GGGTTGTAAACATTATCAGAGAGGATTTCTTCCATAGATCCATAGTATGTTAACACTAAAAGATACATCTAAGACCATTTAATCTAGGGAAGGCAAATAAGTTTCATTTCATTTGCCGAC
Bos taurus    GACTTGCAAACTTTATCAGAAGGCACTTCTTCCACAGAGCCACAGAATGGTAGCACTAAAACATATGTCTGAGGCTGTTTAATACAGGGAAGGCAAATAAACTTCATTTCAGTTGCCAAC

Homo sapiens  TTTAATTGAGCAGTAGAGGCTGTTTGGAATTCCGTGCTGAGAATGATTCTAAATCTATGTTTTTGCTCATTAGGAAAAAAATGCTATGACTGATTATTGATACCTGCCATATATGTTGAC
Bos taurus    TTTAATTGAGCAGTAGAGGCTATTTGGGATCCGGCATTGAGAATGATTCTG----------TTTGCTCCTCAGG-AACAAGTGCTGTGATTGATTATTGATGCCTGTCTTGAATGTTGAT

Homo sapiens  TAGGAGAGTGGTGGAACTCATGTTACCTATTAGCCGTTTCTCAGTGAA-----------------------------------------TGTCTCTGCCTCATTAGTGGTACAGCTAA
Bos taurus    TAGAAGACTGGAGGAAGTCATGTGATGTACTAGCCACCCTTCAGCAAATCTCCTCATTTTACAGAGTGTCTGAGGTCAAGAGATGAGACAGTGTCTTTGCCTCATTAGTAGTGCAGCTCA

Homo sapiens  GAACAGAACTCAGGCTCCTGACTCATGGTCTCTTGTTCTTTCTGGTTTACAATGGACTCACAATAACAACAATAATATAATACAGTGTATCACATAATGAGTGTCTACTCACTGCCAGGC
Bos taurus    GAACATAACCTAGGCTCCCGACTCAGAGCCTTGGGTTCTTTCTGCCTTACAATGGACCCGCAGTAACAATAATAATA----AC----TACCACTTGATGAGTGTCTGCTCACTGCCGGGC

Homo sapiens  ACTCTCCTAAGCACATTCCACTTTCTGTACCTTACTTTTAATTCTCAGAACAACCCTTCAAGGTAGGCACATTCAACC--CCAAACTTAGAAATGCAGAAGCCTAGGCTCAGTGAATTT-
Bos taurus    A-----CAAAGCACCTTCCAATTTCCATTCCTTACCTTCAATCCTCACAACAACCCTGTGAGGGCGGTATGCTCAACCCACCAGGCTCAGTACTGGAGAAGCCTAGACTTGGGGAAGTTA

Homo sapiens  TAAAAACTTACCTAAGGTCCACAGTGAGCAAGTGGTAAAACTGGAATTTAAACCTACGCTGGTCTGGCTCC-AAAGCCTAAACCCTGTGGTCTCACTTCCTGCAAACACAATGTTGAATT
Bos taurus    AAAAAACTTGCCTAAGGCCCACAGCAAGCAAGTACTAAAGCTGAAATTTAAACAGAGACCAGTCTGGCTCCAAAAGTGTAAAACCTGGGTTCTGACTTCCTACAGACACAAGGTTGCATT

Homo sapiens  TGCGCTC-------ACAGCAGGTGGAGATGACCCTGGTCCCACTGCTTTGATTAAGACTAAACTGGAGTGAAAAA------ATCATTTTTGTTCTTACTAATTTTCTTAAAAAGATTTGT
Bos taurus    TGCCCTCACAACCTACAGCAGGTGGAGATGACCCTGGTCACATTGACTTGGTTAGCATTAAACTACACTAAAAAAAACCCCATCATTTTTGTTCTTACTAATTTTC-TAAGAACATTTGC

Homo sapiens  TCACAGTTCCTTACTTAGATGGAACTGATTTTTTCGGGCTGTCTCATATTAGATGTGATACAAATTGACATGTTAGGAGAGCTGATGTGAAAGGCGACCTGGTTCACTTACAAATAAATG
Bos taurus    TTGCAGGCCCTTAGCTAGATGGAACTGATTTGTTTGGGCTGTTTCATGTTAGATGTGTTAAAAATTGACATGTTAGGAGAGCTGACGTGAAAGGTGACCTGGTTCCCTTGCAAATAAATG

Homo sapiens  ATTAATATAATCTCATTGTGCGGCTAGCCTGCATTACTGATTCTCTCACTTGGGAGGCTCAGAAAAACAACAGGTCAGACAATAAAACCCATGCTAGCATAAGACTATAGTGTGGGATGA
Bos taurus    ATTAATATAACCTCATTGTGTGGCGAGACT--ATTACTGATTCTCTC----GGGAGGCTTAGAAAAACAACAGGTCAGACAATAATATCCATGCTAGCATCAGATGATAGTGTGGGATGA

Homo sapiens  CCATGGATGAGTCAGGGAGAATAAGAAGATAATTAACTAAGAGGGAGTTCTATGGAGTTCGAAGATAA-TTAAAAAATGTTCCTTCTCTATCCTAAGAATATCTGACATTAT-TAGCGAA
Bos taurus    CCATGGATGAGTCAGGG-GGTTCAGAAGATAATTAACAAAGAGGGAGCTCTATGGAGTCCAAAGAGAATTTAAAAAACGTTCCTTCTCTACCCT--GACTATCTGAGATTATATAGCAAA

Homo sapiens  GGCAATGAATAAGATAAGGGGAAGAGGTGAGTTAATTAGAATTATGAGATACAACATTTTAAAGAAATTGACAGGGATCACATTTCAACGGAAAGACATTCAATTCAACCGACTTCATTA
Bos taurus    GGCAATGAATAGGAGAGGGGGAAAAGGTGGATTCATTCAAGTGATTCGCTAAGACATTTTAAAGAAATTGACAGGGATCAAATTTCAAGGGGAATACATTCAATTCAACTGACCATGTTA

Homo sapiens  TAGCAATTCAACATTGATTAGGGACATGTTATGTGTGTGGCTGTGCTCTGTGTGCTTAGAGGACTACTGAGATGCTGTAATCTTCCTCCCTGACATTCTACTGAATAGATACCCAAGTTT
Bos taurus    CAGCAACTCAACAGTGATTAGGGATAGATGA--CGTGTGGCTGTGGCCTACACACTTAGGG----------------------CAGCTATGACAAT------ACTATACACCCAAGTTT

Homo sapiens  TGGCAGCGCAGTAAATAATCTCTTACACACACATTTGGGTGCAATTTCTAAAATTGTTTCTGCAAAATAGTATTATGTGCCACTGAGTAGATATCTTGTATGCCAGATATTACTGTTCCT
Bos taurus    TAGCAGGATAGTGGAGAATTTCTTCTCTATAGATATGGATACACGTTAGAAAAACGCTTCTGTGAAACAGTATTATGTGCCAATGTATATGTATCTTGTATGCCATATATTACTGTCAAT

Homo sapiens  GCCAGGCTAAATGTTCAAACAACATGGAAGCCTCAGTGATAGCCAATCCATTATTTATCTACCTATATTCACTTCAGTTGCCAAAACTATTCTAAATATAAGGAATCCCTTCCTTAGAGG
Bos taurus    GTCAGGCTAAATTTTCAAATAGCATGGAAGTCTCTGTAATAGCAAATACATCGATTATCCACCTATAGTCACTTCAGTGGCCAAAATTATTCTAAATATGAGGAGTCCCTTCC-TAAAGA

Homo sapiens  CCCCAATATGGCTTTCCGTATTG----TTCTGAGTCCTTTCTAACTGGTTAATGCCCATGCCATATTGGTTATCATAAATTTTGAATAGCATCTTTTCTCTGCTCAGAAGTGTCGCTCAG
Bos taurus    CCCCATCGTGACCTTATTTTTTATGGCTCCGGGGTCTTTTCAAAATGTTTCATGCCCATGCCACACTGGTTGTTATAAATCTTGAATGTCATC-TCTCTTGGCTCAGAGGTGTCACTCAA

Homo sapiens  TAATACCCTACCTCTTCTGACTCCTCCTACAGTGTAATCTCTCACCAGTCCTCACTACATTCTGAACTGATGTTCCAGCTCCACTTAAATCTGTCATGTTTTGTTCCTTAACATATGCTA
Bos taurus    GAGTCCTCTACCCCATCTGACTCCTCCTCCAGGGTAATTTCTCACTGATCCTCCC--------------TGTTCCAGCTCCATCTAAACCTGCCCTGTTCTGTTACTCAACATATGCTA

Homo sapiens  TTCCCTTTACTTGGAATACACTCTCTTTCCTTGTCTGCCTAACAATTGCTGCTTGTCTCGCAAA------------TATTTTCTCTGTGAAGCTTTCTCAGTGCCCTTAGGTACCCTCAT
Bos taurus    TTCTCTTCCCTTGGAATATACTTTCTTTTTCTGTCTGCCTGACAACTGCTACTTGTCCTTCAAGTTCTACCTCAGCTTTCTTCTTTGGGAAGGTTTCTCAGTACCCTCAGGAGTCTTCCC

Homo sapiens  GGCCCTCCTGAATTTATCTCTATTATAATGTTTTATATTTTATGGCAAGAGTTTGTTGGATCTTTGCCCACTTGAATGTGAGCCCCTTGAGAACAGGGACTAT--TTTATTT--------
Bos taurus    CGCCCTCTTGGATCTACCACTTTTATAATGTGTTCCATCAAGTGGCTGGAGTATGTTGCAGCTTTGCCCACTAGAATATGAGTCCCTTGAGAACAAGGACTGTTATTTATTTATTTATTT

Homo sapiens  ------ATGTCTGAGTCTCTAGGGAACAAAAGAGGGATTCAGGGCTTATGTGGTAAATGAATCAATGAATGCTCCCAGTTCTTCGTCATCTTTAGAGGCTACTTCCAGGGCTTTTAAATT
Bos taurus    ATTTTCATGTGTGACTCTCCAGGATA-GAAGGAGAGGTTCAGGGCTTGCATGTTGAATGAATTAATGAAAG-TCCCAATT-------ATCTTCAGAAGCTATTTCCAGGGCTTTCAAATT

Homo sapiens  TGGACTCCCTTTTCTTCCAAGAGGTGGTGAGAGCAGGGTAGGTGGACATGATATTTAATGAATTTCTTTTAAGTGCCAGACATTGTGCTGAACCATTTACACAAACAATCTCACGTCTCA
Bos taurus    GGGTCTCCCTTTTCTGCCAAGAGGTGGTAAGAACTGGCCAGATGGATTTGATACTTAATGAGTTTCTATTAAGTACCAGGCATTGTGCTGAACATTTTGCATGCACGATCTCATGTCTGA

Homo sapiens  AGTCGATGTTTTTA-TTTTCATCCTCGTTTTACAGATGAAAGTGGAAAATTCAG----------------------------ACCAGTGACT------TGCCACA-------------
Bos taurus    AAGAGATGTTTTTAATGTCCATCTGTACTTCACAAATGTTGTTG-----TTCAGTCACTAAGTCATGTCCCACTCTTTGCGACCCCATGGACTACAGCATGCCACACTTCGCTGTCCTTC

Homo sapiens  ------------------TTCAGAGGAGTAAC------AAAGATACATAGCAAGTAGGCTGCAAAGCTAGGATTTGAACGTAGGCTCCAATACCTGCATCAGCTCCCTTGCACTGTGCAC
Bos taurus    ATGATGAGGAAATGTAAATTCAGAGTAGTGACTTGCCCAGGGTCATGTTGCTGGGAAGTTGCAAAGCCATGATTTGAACCTAGGCTGT----------TGGTCTCCAGTGC---------

Homo sapiens  TCTTCCCTCGCTTGTGTGTATGGTGAGCAGAGTCAGCTCTGGGCAA------AATGGTGAAATAAGCCAAGTTTGTCTTTAGTGTCTTCAGACTCCCAGTGTGTCTCGGGGACTCAGTAC
Bos taurus    -CTTCTCTCCTTTTTGTGTATGGAGGGTGGGGCCTCTTCTGGGTAATACAGTGATGGTGACATAAGTCAAGCTTGTCTTTGATGTCTTCAGGCTTGTAAGGTGTCTCAGGGACTCTGCGC

Homo sapiens  TACAGTGCCACCTACATGCTGCAACACCTACTATGGCTCCTAC-AGGCAGCCCATCCTGCTGAGAGCTTC-TCCTTCCTAACTCCTCTAAGACCTTGACCTCTGGTTTGTGTTTTCTGAA
Bos taurus    CACAGTGTTACCCACCC-CTGCAGCCACTACTATGGGGCCTACCAGGTAGCCCATCCCATTGGGAGCTTTGTCCTTTGCAACTCCTCTGAGACTTTGACTTCTGGGCTGTG---------

Homo sapiens  GGTAAACTGCCTTAACCTAACCTTTTCCAGAGCACATATTATATTTATTGGTGCCTTCCCAGAAAGCAATCTTTAGCTTAGGAGCAGGTAGCTCCCACGGGAGGCATGTAGGTGTT----
Bos taurus    ----------------CTAAACTTTTCCAGAGCACATGTT-TACATGTT-------------AATGCAATCTTTAACCCAGGAGCAAGCGGCCCTTGTGTAAGCTGTGGGGGGGTCGGGG

Homo sapiens  -----------TCTGTGTTGTGGTAAG---GGGAAGGGGTTG-TCTGGGGGCCTCTATCAAGATACACCATGGAGGCTTCTCCTACAACCAAAGGGTGTTTGGCCCCTTTCTCAGATGGC
Bos taurus    GGCGGGCGGGCTCTG-GTTATGGGGAGTGGGGGTACAGGTTGCTCTGGGCATCTCTTTCAACAGACAT-GTGGGGGCCCCTCCCACATTCTCAAGGTGCTTGGTGCCTACCTCGGAGGGC

Homo sapiens  TGAGGGCCCTGGAGTCAGATCAATAAGCATTTGAATCTTGGCTCTGCCATTC----ACTGATTGCATGATTTCCCACAGTTAATCTCAGTGAGCCTTCGTTTCCTCCCCTGTAAAATGGA
Bos taurus    TGAGAGCTCTGGAGTCAGACCAACAGGTGTTTCAATCT-GGCTTCACCATTCCAGGATGAATTGTGTCATTGGTCACAAATAACCTCATTGAGCCTCCCATTCCCCTTTTGTCACATGGA

Homo sapiens  TCTAATAATAGTTCTCATCTCCTAGACGTGTTGTGAGGATGACATGAGATAACACACTTACGGTGTCCAGTACAAAATAAGCATGTA------------------AAAATGTTAACTGT
Bos taurus    TCTCATAATACTTCTCATCTCCTGGGGAGGTTGTGAGGATGACATGAGACAACACAGCAACTGTGTGTAGTACAAAATAAGCCTGTAGTACAAAATAATCACTTGAAAAATGTGAAATCT

Homo sapiens  TCCCTTAAATCAATTTTTAGTAGTCTTCTGCCTCACCCCCATA---ACCATCCAGGTCTCTCTTCGCTTCCTTGTTTCTCTTTTCCTCTTCTCACCAACAAGTCTCTTTCTTCAAAGTCT
Bos taurus    TTCCTGAAATCTGTTTCTACTGG----CTCCCCCACCCCCACCCCTGCCATCTAGGTTTCCCTTC----CCCCGTTGCTTCTTTCCTTGCCTCACCAACAGGTATCCTTTTGGAAACTCT

Homo sapiens  CCCTTTCCTTTCCCAACTCCCTCCCACCAGTACGCTCAACCTTTGG--CAACCCTCTGTCCCACACTTTCCAGGTC-AATCCCAATTTCAGATAATCTCTCTTGATATCA-AACTGCATG
Bos taurus    TCCCTTCCTTTCCCAG-TGGCTCCCTCTGGCATCCTCAACCTTTGGAACAACTAGCCGTC--AGAGTTCCTGGGGCTGATCCCGTTTCTGGATATTCTCTCCTGCTGTCTTGAGTGCACG

Homo sapiens  ACCTGATTTGAATCGGAAAACAGAGTGAAACAGAGTCCCTTGCTCTGAGGAATGTTGCTATGGTCTATCTTAGATCAAGAAGACAAGAGCTGCCTTGGCAGCCCTACATCATAATCTGAT
```

```
Bos taurus    AGCTGATTTGAATT------CAGA---AAGCAGAGTTCATTCCTGCGAGGAGTGTCACCACAGTCCATCTTACATTCAAATGGTGAAGGCTGACTTGATGGCCCCACGTCGTTATCTGAT

Homo sapiens  GAGCATGAAGAAGGCT---CACCTTGCCTATAAATATTTGTTTTTTGCCTATGAGTGATGCCAGAAACCGTCATGGTCGGCATACAATTCCTTTGAGTCAAATTGCAA-GGTTTGTCTGT
Bos taurus    GGGTGTTGAGAAGGCCTCCCCCCTCGGACCCAGAGACTTCCTTCTGGCCTGTGAGTAATGCCACTATCTCT-ACCATCAGTGGACGATCCCTTTGAGTCCAACTGCCCTGGGTGGTCTGG

Homo sapiens  CAGGGTTGCCTTGGTAAGGCAGGGTTATTATTTATATCTGAGTGCAAATTCCTTGATCAGAGCCTTTCCTGTCAGGGAGAGGAGACGGAAGGGTTCCAGGGAGCCTACTCCCCACGGAAG
Bos taurus    GGGGAATGGCTGGGCCAGGCAGGGTTGCTTTGTGTATGTGAGTGCAGATTTTTTGATCAGAGCCTTTCCTGTCAGGGGGAGGGGAGGAAAAGGTTAGAGGGAGCTGACTCCCCAGGGAAG

Homo sapiens  AGGAAGGCTGTGCTAGCATGGATGGGGTATGTGAGTGTGGGAGCTCAGCTGACTGAGAGCTGTGTCCCCGGCTCCATTTTGCCTAGGGGCGAGCCCTGTGTGTTGCAACAGATGTGTGCT
Bos taurus    AGGAAAGCCAGGCAGGCAGGGGTGGGGCGTGGGGGTGTGGGAAATCAGCTGATAGAAGGCTGTGTCTCCGGCACAGTTTTGCCTAGGG-TGGGCCCTGTGTATTGCAACAGATGTGAACT

Homo sapiens  TTGGAATCACCAAGGTTGGGAAGCCAGGCCTCAGAGGGAGTGGCTGGAGGCTGATCTCATCCTGAAATGATGTGGGCTTGGATTCCCAGAGGGTGGAGAGGAGGACCAGTCCAGAGAGAA
Bos taurus    TTGGAATCACCAAGGTTGGGAAGCCAGGCCTCAGAGGGAGTGGTTGGAGGCTGAGCTCATCCTGAAATGATGTGGGCTTGAATTCCTGGAGGGCAGAGAGGAGGACCAG-----GGAGGA

Homo sapiens  GGACATGCTTTTGGAGGGAGGTCTAAAGACAAAAAGAGGGCTTTAGTTATTTTTTGAGCTTAGGTGTAGGTGATGGTTGGGGGTAAGTGA----CAAAAGAAAATAGGTCCTGAAAGGGG
Bos taurus    GGTCCTACTTT---GGGGGTATCTAAAGACAGAAAGAGAGTCTTGGT-GTCTTTCATCTCTACGTGTAAGTGACACCTGTGGATAGGTGGGTCGTAGTAGAAAATGCCCCCTGAAGGGTG

Homo sapiens  GGTGTCTCCTTTC-ATGATTAGGAACAGAAGAATGAACTCAAGGACTGCCCCCTTTTCTTCAGGAAGCAAACAACTTCTGAGGGTGTGACAGGGTTCACTGGGTGTTCCCCTTTAAGGGA
Bos taurus    GGGGTCTCCTTTTTCTGACTGCTAAGAGGAGACTTAGCTGAAGGACTGCCCCCTTTCCTTTGGAAAGGAAACAACTTCTGAGGGTGTGGCAGGGTTCACTGGGTGTTCCCCCTCAAGGGA

Homo sapiens  GGTTAGCCCCTACGTGAAGCTGCTTGGCTGGTAGAGGATAATTGGGTGTTCCCCAATCCAGATAAGCAGAAGCTCAGGTGCCCCTCCCTGTAGGCTTTTCAGCTTTCTTTATCACTTGCT
Bos taurus    GGTTACCCCCTAAGTGAGGGTGCTTGGCTGGTAGAGGGTAACTAGGTGTTCCTCACCCCACAGAAGAAGGCGCTTCAGTGCTCCTCCCTGTAGGCTTTTCA-----CTTTGTCTCTT---

Homo sapiens  AGGCAGGCCCTGCCATCCTTGGGAGTAGGTATGAGAGAGGGAGTTGAGAGGCCAGGCTTTCCCTGCCCCTGTTCTTCACAGAACACAGCTGAAAAAGGAAGGCCAGAGATGTGACACCCC
Bos taurus    -GACGTGCACCGCCATCTACCACAGCAGGTAT---------------------------------------------GAACACAGCTTAAAAAGGAAGGTCAAAGATGT----CCCC

Homo sapiens  CCAGCCC----CAGGGATTCTACCACACATTGCAAACCTCACAG-AGGATGCTTATTCTACAAAATTTGTGGGCATATTCATAGGTACTATGGAGCTAAATAATTTGAA-------AACA
Bos taurus    TCAACCCCCTTCAGTGGTTCTACCACACCCTGTGGACCCCACTGAAGGATGCCGATTCTG-AAATTTTGTGGCCTTGCCCGTAGGGACCAAGGGGCCAAATATTTGAAATCATCATAACC

Homo sapiens  ATAACAAAACCCATTTACTGATTATTTGG------TTAATAAAGACCTCATTCCTTCAAAGTATGTTGCTAGGAGCCACTGAAAGTGGACTTGACCTGAGGAGCAGCAAGGAGCTTCCTC
Bos taurus    ATAACAAACACCATTGGCTGACCATTTGGGATGCCTTACCAGGGGCTACTTT--------ATATAT-------AGGCCCTGAATGTGGGCTTGACCTGAGGGATAATGAGG-TTTTCCCA

Homo sapiens  CTGCTGTATACACAGACACACACAGACAGACAGACAGACACACACACACGCATGCGCGCACACA------CATGCACATGCCC------------------GGATCCTTCTCC----AA
Bos taurus    CTGCTTCACGCAC--ACACACACACACACACACACACACACACACACACACGCACGCACACATGCATGCATGCACATGCCCACACAAACACACACACACAGAGTCAAGTTCCTCGAAA

Homo sapiens  ACACGTCCATGATACCAGGGCAGCAGAATTGGGGCACATTAGAGACACAGGCTCTGCCCACATTCTGAAGCTCCCTGTCATTCCCAGGTCTTGGTGCTTGGCCAGACTCCCCTTACCCAG
Bos taurus    ACATGTCCACAGTCCTGAGGCCACAGA------GCACGTTAGAGACACATTCTCTGCCTGCAGTCAAAAG--TCCCGTTTTTCCCAAGTCCTGCTGCTAGACCCGACTCGGTGCACACAG

Homo sapiens  ACTCATAGCCTCATAGCTATCATTCCAAAACCCCAAAAGCTCTGGAAACTGAGAGCC-TTTGTACGTTTATGACAAACTCATTTGGCAGCGCAACTTGAACTGACATGAGGTTATTTATA
Bos taurus    ATACACATTCCCATATCCATCCTTCCCAAGCCCC-AAAGCTCTGAAAACTGAGGGCCTTTTGTAAGTTTAAGGTAAATTCAGTTGGCAGCCAAACCTGCGCTGGCATGAGGCTATTTATA

Homo sapiens  GTCTTTATTTTTCACACCCTGTGTGAATATTCATGTGTTTTTGCTCCAGAAATATCAATGTACTTGATTACTGGGTACTGCCTCAGACCCCACTGGAGGTGATATATA----TGTGCTGT
Bos taurus    GTCTTTTTCTATCACACCCTGTGTGAATATTCATGTGTTTTTGCTCCTGAAATATCAATGTACTTGATTGCTGGATGCTGTCTCAGACCCCACTGGAAGTGACATATAATATTCTGCTAT

Homo sapiens  ATACACCCAATCGCCTTTTAAAAGTCCAAAGCATTCTGAATTCCTTCAATTATACTAAGCCTCCCACTGTCTTATAATGATCTGTTTGGCATGTCTC------------CCTCATTAGC
Bos taurus    AGATCCCCTCTGGCTTTCTTAAAATTCAAAGCATTCCAAATTCCTTCTATTATAGAACGCACCCCACTGTCTTGTAATGATCTATTTA--ATGTCCCTAAACAACAACAACAACATTGGC

Homo sapiens  CTGTGAACTTCTTCTCCAGTGCTCAGCACACAGTCCAGCATGGGGTGGAAGCTCGACTAATGTTTTAGGAATGAAGAACGAACAGTCTTTAGTGATTCCGGAGAC---TTGTTGTCATGT
Bos taurus    CTGTGAA---CTTCTCCAGGACCCAGCACATGGTCCAACATGTG----------------CTTTTAGGACTTAAGAATAAATGGCCTTTAGTGTTGAAGGAGATGCATTGTTGGGAAGT

Homo sapiens  TCTTTCTTACATCTAAACTAAAGTACGTCATGCTACCATTCGTTTATTCTGTGCCATCAATAGCGAGGAAG-AACAGTTGGTCAGGCTCCTTCA----TTTTTTTTCCTCTTGAGGCTAA
Bos taurus    TCTTTCTTACATCTAACCTAAAGTATCTCATGCTGCCATTCATGTACTGCATGCCATTGATAGCAAGGAAGAAACAGCTGGTTA-ACGCCTTCATTTTTTTTTTTTTCTCTTGGGACTAA

Homo sapiens  ATCGTCCTTATAATCCTAATACCTTTAACCTTTTCCCCTAGATCCTCTTTTCCAATCCCTTAATCGTCTTCATTGCTGTCTTCTGAAGTCTTTACAGGTTCTTTCCTTCCGAAATTGTGG
Bos taurus    ATCGTCCTTCTAATCCTAATTCCTTTCACCTTTTCCCCTAGATCCTCTTTTCCAATCCCTTAATCATCTTCACGGCTATCTTCCAAAGCCTTTACAGGTTCTTTCATTCTGAAATTGTGG

Homo sapiens  CCTAGTGCTTAGGATAGGGCTGCAAAGAGCCCAGCTATTACACATAATAGGCCTCTGGTTTCTATGGTG--CTTTTCCGATCAGTTTCATTTGGCTGGGCTAAGAAGCTTTGCCTTTAAA
Bos taurus    CCTAGCACTTAGGACTTGGCTGCAAAGAGCTCGGCCATTATATGTAATAGGTCTCCCGTTTCTATGGTGAACATTTCCAATCAGTTTCATTTGGCTGGTCTAAGAAGCTTTGCCTTTTAA

Homo sapiens  AATACACTCGCAGGACAAACTCCAGGGG-CATGAACAACTTTCACCTGAGCATGCTGGTTGGTAATAGGAAGGGAAAATAGCACTGTCTGATCATAAGAAACTGTAATTAC-GAAAGCTG
Bos taurus    AATACACTTACAGGGCAAACTCAAGGGGCCATGAATGATTTTCTCCCAAGTGTGCTGGTTGGTAAAAAGCAGGGAAAGCAGCGCAATCTCACCAGAAGAAACTGTAATTACAGAAAGTTG

Homo sapiens  AAAGCAACAGGTAACACATGCAGTAGGCACCTGTATACAAAAACCACTGTTGATAATTACGGT-ACCTCAGCCTATTA-TTTTTACCTTGGGAGAACCGGCCTTGTTTGTGCGCATCATG
Bos taurus    AAAGTAACAGGTGACATATGTAATAGGAACCTGCATGCAAAAAGCACTGTTGATAATTACAGTGACCTCAACCTGCCATTTTTTACTTTGGGAGAACCAGCCTTGCTGGTAC------CG

Homo sapiens  TGCCTTTTCTCTCATAAGGTTGCTAAAGGCCATTTCTGACCAC----TTCCCATTAAATTCAGGGAGTGAACCTGACTCTGTCAGGGTTGCTG--AAAATCACCGGATTAAGTTAAACTC
Bos taurus    TGCCTTTTCTCTAACAAGGTTGCTGAAGGCCATTTCCTACCACTTATTTCCCACTAAATGCAGGGAGTGAGCCTGACTTGGGCTGGGGTGATTGCAAAACCACTGGCTTAAGTTAAACTC

Homo sapiens  ACTAATTAAGCCTGTTAGACTTCCTGTAACTCACGATTTTTCTTGGCATAAGTAAAAACATTTCACTCAGAGGCAAAATGTGTTATATTAATAACCAGGAAACAAGAAAATACACATAGT
Bos taurus    ........................................................................................................................

Homo sapiens  AGCATTTTCGGAAGCAAATTCTGTCATCCGCATTCTGTTGAAACGGCCATTGTCATTTCTCCTAATAGCCTAAAGTGTTTACTTTTGGGGTGGAAACTATACATACTGTGATGGAGATTC
Bos taurus    ........................................................................................................................

Homo sapiens  AGAGATACGCTTTTGTCAGTTAAATTCGAAAAATATATTCAGGTGTGCTTGTTGTGTCCCCCCCCCCCCCAAGCATGAGTCAGAGCCTTTCATGGGGTTTTGTGTAAATGTATTTTCTAA
Bos taurus    ........................................................................................................................

Homo sapiens  GAGGTGCTGTTGGGATTTTGCATACAGGTCATTCTTTTTAAAAAGGTCACGTTGCAATTTTTCTATCCTTTCTTGTGTTGTTATCCCTCAGGGAAGAAGATGTAGAATGCCAAATTGCAA
Bos taurus    ........................................................................................................................

Homo sapiens  AGGGAGCTTGTAAAGAGGGGAACATTAAAATTGGATAACCAAGAGCTCCCAGAGAGGGCCTCAATGGCTGAAAGGAGCCCCTTCTGAGCAAATGGGTAGCAGCGTAAAAGCCAAATTAAC
Bos taurus    ........................................................................................................................

Homo sapiens  CTGGGAGGAGAAGGTATGGAACTTTTGGAGAAACACTTTGTTTACCTCACCATCTTACCATATCCAGCTGGCTGTTTCTCCAAGAATGTTAGTGCTCCAGAAGGGAGGGAAACCTGTCCC
Bos taurus    ........................................................................................................................

Homo sapiens  CACTGCTAATAGAAGAGGCTGAAATTTTCTTTCAGAGTTTCCAGATGTCCTTGGCAAATGCTAGCAATAGTCATTCTCTCTCTTTTAATAATTATAATAAGCTGCTTTTTAAGGGAACAT
Bos taurus    ........................................................................................................................

Homo sapiens  TAACTGTGTGGTAGGCTCTATGCTAAGTGTTTTATATAAGGTATCTCTATTAATCTTCACTATGATCCTTCCAAAAAGGTATTGCTATTACCCTCAATTTCCATAGGAGGAAACGGGCCC
Bos taurus    ........................................................................................................................

Homo sapiens  AAAGAAGTTAGACAACCTGGGCAAGGTCACCCAGCTAGTAAGTGGAGGAGCCAGAACTTAAACGCAGATCCACCTGACACCAACACCTGGTTCTGAACCATGGTGTGCAAGTCCTTGTAA
Bos taurus    .................................................................................................................CTTGTAA

Homo sapiens  AGTCAAACAGTCTCTTTAATAAGCTTGACTATATTTTCTCTTT--------TACCAAATCACAGAACATCTGGTCTGCTTAATTTTATGAGACTGTAATTACAGCAATGAAAAGGATGTT
Bos taurus    AGCCAAATAGCCATCTCAATAAGCTCAACTGTCTCCACCCCCCACCCCCACCACCAAATAGCAGAATATTTGGGTTGCTTGATTTTGTGAGACAATGATTACAGCAACTGAA-GGATGTT

Homo sapiens  AGCCTACTCTGAAAACCCTAGCTGAAGGGCTTTCTATTGCTTTGTTTAGAGATTTGTTCTTTTCTCTCCACCTCCACCCCCGACTCCCCAAATTGGATAGTAAGTTCTTCAAGGAAGGTT
Bos taurus    CACCTACTCTGAAAGACCTAGCTGACGGGCTTTCTATTGCTTTA-TCAGGAACTGGTTCTCCCCATCCCGCCACAACTCTC--CTC------TTGGATAGCAACCAC-GAAGGGAAGGTT

Homo sapiens  CTGGAAAGTGGTTTTTTTTTTTTCTTCAAGTTCTGTGTTACATGTGCAGAACTTGCAGTTTGGTTACTTAGGTATACAAGTGCCATGGTGGTTTGCTGCACCCATCTACCCGTCACCTAC
Bos taurus    CTAGA...................................................................................................................

Homo sapiens  ATTAGGTATTTCTCCTAATGCTATCCCTCCCCTAGCCCCCCACGCCCCACAGGCCCCGGTGTGTGATGTTCCCCTCTCTGCTTCCATGTGTTCTCATTGTTCAACTCCCACTTATGAGTG
Bos taurus    ........................................................................................................................

Homo sapiens  AGGACATGCGGTGTTTGGTTTTCTGATCTTGCGATAGTTTGCTGAGAATGATGGTTTCCAGCTTCATCCATGTCCCTGCAAAGGACGTGAACTCATTCTTTTTTATGGCTGCATAATATT
Bos taurus    ........................................................................................................................

Homo sapiens  CCATGGTGTATGTGTGCCACATTTTCTTTATCCAGTCTATCATTGATGGACATTTGGGTTGGTTCCAAGTCTTTGCTATTGTGAATAGTGCCGCAATAAACATAGGTGGGCATGTGTCTT
Bos taurus    ........................................................................................................................

Homo sapiens  TATAGCAGCATGATTTGTAATCCTTTGTGTATATGCCCAGTAATGGGATCGCTGGGTCAAATGGTATTTCTAGTTCTAGATCCTTGAAGAATCTCCAGTGTCTTCCACGATATTTGAACT
Bos taurus    ........................................................................................................................

Homo sapiens  AATTTACACACCCACCAACAGTGTAAAAGCATTCCTCTTTTTGGTTCTGGAAAGTCTTATGCT----CCTCTGCTTTCTATAGTTCTCATAGTGCCTGGCATGTAGTGTGTGCTCCATAA
Bos taurus    .............................................................CTGATTCCTCTGCTTTCTATAGCCTGCACAGTGCCACACACATAGTACGTGCTCAATAA

Homo sapiens  ATATTGACTGATTGATGAATGTGTGGATGTTTCATGCAATAGTTTTTGGGTTGTTCAACACCATTTCTTAATGCTAAGCAAACAGGTTAGAACCTTCAAGATGTCTGTTGTCTTAATCTA
Bos taurus    ATATTGATCAATAAACAAATGTCTGGACATTCCCTTCAGTGGTTTCTGGGTTCTTCAAGATCCTTTGTTAATGCTAAGCAAACAG-TTAGAACCTTCAAGATGTCTGTTGCCCTGATCTA

Homo sapiens  GTTGCTTGCACTGAGGTAACTTTTTAATATCATCTCTGCACAGGAGCTGAATGCCTTCTTTGGCACCTGGATGATTGATTGTGTTTTCTCCTTTTCCTCGGAAAGCTCTGGTATTGCGCA
```

```
Bos taurus     ACTGCTTGTACTGAGATGACTTTTTAGTATCATCTATGTACAGGAGCTGAATGTACTATTTGACACATGGATGATTGATTGTGTTTTTCCCTTTTCCTCCAAAACCTTTGGCACCATGCA

Homo sapiens   AACGCTGCTTTAGGTGATTAACAATGCCGCCAACTTCACCAGTCACCTTTGCAGACAATTCTTGAATTTTATAGGAAAGTGGGAACCTG--TTTAAGTATAGTTTCTAGTTAATTCTGCT
Bos taurus     GACACAGCTT-AGGTGATTAACCATGCAGCCAACTTCACCAGACACTGATTCAGACAA----------------GAAAGTGGGTGCCGTATTTTAACGATAGTTCCTAATTAATCCCGCT

Homo sapiens   TCCCCATCCTGGTTTGCTCAATG-CTACATAAAAGATTTTCTTTGTGGAATGAGACAGAATAGAAAAAATAAAA--------ATACATTCTCAATGGCTGGGTGAAGAGAGTCCTTGAGA
Bos taurus     CCCCAGTTCTGGTCTGCTCAATGACTCCTTCTCAGATAGTGTTTGTGAGATGAAACAGGATAGGAAAAAAAAAATACTTAAAAAGCATTCTTTTGGCCTGGGTGAAAAGAGCGCTGAAGA

Homo sapiens   TTTTACCAGCTTGGTATTTCTCA-CTGTTGGAACTTCAACCCAGGATGCAGATTCATCCTAGTGAGCCTCACAGGAAACTCTGCCTTGGTCTAGAAGATACAGTGCAAATATATAGGATA
Bos taurus     TTTTCCCAGCTTATTACTTCTCAGCAAATGGAACTTCAAGCCAG-ATGCAGACCCATCCTGCTCAGCCTCATATAAACTTCTAACTTGGTCTGGAAGATAAAGTGGAAACATGCAGAATG

Homo sapiens   GGACAGGTAAGGTCAGCAGAAGCCACTTTTCAGCACATACCATGTGATTGCTAGAATAATACAAATAACAACCATTTACTAGCAGATTACTATGTGGCAGGTACTGTGCTAAGCATTTTC
Bos taurus     GGAGAAGTAGAGTCAGCAGAAGCCAGTGTGCAGTGCACTCCATGTGATTCCCAGAATAGGAACAAAAACTATCATTTTCGGGTGGATTGCTGTGTGCCAGGCAGGCTACTAAACATCTTA

Homo sapiens   CAGACATTCATT-----------------------------------------ATTGTTGCCATTTTTCCAGGTGAGCAAACTGCAGCTCAGAGAGTCTAAGTGAATTTCTTTGTCTTTA
Bos taurus     TAAGCATTCATTTCATTTCATCTCTCAAGGACACTGTGGAGGTAGGTACTATTATTATCGCCGTTTTTCCAAATGAACAAACTGCCGCTCAGAGAGTCTAAGTGAA..............

Homo sapiens   AGCAGTATGAGATAGACCCTGGACTTGAACTCAGGTCCATCTGATTCCAGAGTACATAGTCCTTTTGCTACAGTGCTTTGCAGGGAGCTGTGCTGGCTTCTCAAATGGTTTGGGGCTATC
Bos taurus     ...............................................................................................GCTTCTCAAGTCGCCCAGAG-----

Homo sapiens   TGTGTGAGGCTGACAAGAAAACTATTTAAAGGCTGGTAGAAGAGGGCCCTAACAGAAGGTCTGACTCTCATTTTCTTTCTATTTGCAGAAGATAATTCTTTCCTGTTTCAATATCAGAAG
Bos taurus     ----TGGGGATGGAGGGGAA------------CTGGGAGCTGAGGG.....................................................................AGGGG

Homo sapiens   GGGATCTAGCTGAAGGCAAAAGGCCACAGGGTTCTGGTGAGGCACAAGTTCAAACCTTGCTGTAGTTGGACCTGTTCTACTGACTATCAACGGCCTGGGTCTGTGGGTGATCATAGTGTC
Bos taurus     GGG-----------------------CAGGGCT.......................................................................................

Homo sapiens   AGGGGGCTGGAGATTGGAAGTTCAGAGTGGAAGAGCTGGCATATGCCGCAAAATGCACCCTGTTGGGATGGAGCAGGAAACTCAACTGCAG---CTGAGTTTAGTGGGG-CACCCAAAGA
Bos taurus     ...GAGCTG-------------CAGAGCTGAAGGGCGGCCATCTGCAAGTAGTGGCACTCT--TGGCATGGCAAAGGAGGCTCTGCTGCGGGTGCCGAGTTCAGCGGGGGCACCTGAAAG

Homo sapiens   GAGTGGCAACAACAGGTATCAATTTAGAGAAGCACTTGGCTTTGGTGAAAGCAGAAGTCCTGCCCAGCCTAGCTGAACTGAGCAGGAGCAGGTGAGGCTTAGGAGTCCTGCCAGGTGCTA
Bos taurus     GAGTGGGAGCAACAGGTTGTAATCCAGACAAGCAGTCGCCTCCGGTGGAAGCAAA-------------CTAGCTGAACTGATCAAGAGGGCTTGAAGCTTATGAGTCCCGTCAGACAGTG

Homo sapiens   GTAGCAAGGATGGGACTCTAGTCCTTGGAGGCAAGGGTGAAGGAGTTGGGAGCCTGGGAAGAACCAGACAAAATCCCAGTTCCACAGAAACTGAATGACT-AGACTGGTGGCTCTCAACT
Bos taurus     GTAGCAAGGATTGGACTCCATTCCTTGGGTGCAAGGGTGAAGGGGATGAGGATTTGGGGAGAATCAGACAAAGCTCTAGTTCTATGGAAACGGAGACACTTAGCCTGATGACGCGAAACT

Homo sapiens   GAGACAATACTGCCCCCTAGGGG-ACTTTTGGAAAATGCAAGAGGGTATTCAGAGGACTGGAAATGGGGATGCT------------------AGATGTTCTCCAACGCCCACGACAGACT
Bos taurus     GAGCCAATACTGCCCCCTAGGGGGACTTTGGGGAAATGTGTGAGGTCATTTAGTGGACAAGAAATGGAGATGCCTCACGTCCTGGACTGCCCAGATGTTCTCCAGCACCCAGGACAGTCT

Homo sapiens   TGCAAAATGAAAAATAGTCTAGCATCCCGAATAATTTTTGAATGTTCTGCTGGACTTCCATGGAGCTGAAAACTGCTTTTCTAATTATCTAAACTTTTACTATAAATGTTTTATACCTAA
Bos taurus     TGCAAAATGAAGACTAGTCCAACATCCTATATGATTTTCAAATGTCCTGTTGGACTTCCGTGGAGGTGGAAATTCTTCTTATAATGATCAGAACGTGTACTGTAAATATTTTACATATAT

Homo sapiens   GCATAAAGGGTTTTGCTTCCCCTGCCCCGACCCCACAGTTTTAATCAACTATTCTGTAAAGATATGGAAGATT--ACATGGTT--------ACAGAGGATCACACAGACTCATTCACCAT
Bos taurus     GTACAAAAAGTTTGGCTTCC--TGCCTCCACAC-ACAGTTTTAATGAA-TCTTGTGTAAATAGAGGGAAGATGGAACTTTGTTTTCTCCTCCCAGAGGTTTATCCACAGTCGTTCACCAT

Homo sapiens   TTCAGGAAATCATGT--CAACAATTGTGATGTTGCTTTTGGAATTAA----------TCAATATAACATATCAGTTTGTGTCCATAACTGTTGCATT-CATGGGGATTCTATATAAAGGC
Bos taurus     TTCAA-AAATCACGTGGTAACAGTAGTGATGTTGCTTTTGGAATTAGAGTCGTCTGTGCAACATCCCTTATCAGTTTGTGCC------TGTTGCATTTCGTGGGGCTTCTCGATGAAGGC

Homo sapiens   ACAAGCTTCTGACTACTTCATTTAATCATTCAATGTAGTCCTGTGTGAGCATTTACAGAGTTAAATACATAGTGTTTTATTATGTATTGCTTT-ATTTTCTCTTTATGTGGCAGGACATA
Bos taurus     GCAAGTCTCTGCCTACTTCATTTAGTCTTTCACTGTCATCCTATGTAAGTATTTACATATTTAAATACATTGTCGTTGATTATGGATTATTTTCATTTCTTCTTTGTGTGGAAGGACACT

Homo sapiens   TAGCTATCTTTTTGAACTTATGCGTGTAGGTAGGTTATAGTACCTAGAAATTTAATTTCAGGATAGTAAAGGGGT---------------------------------------------
Bos taurus     TCTTGATCTTTTGGAACTTATGTGTATATGCAGGTTATTATAGCTATGATTTTAATTTCAGGATAATAAAAGGGTATGACGGGCTTCCCCAGTGTCTCAGCAGGTTCCTGCCTGCAACAC

Homo sapiens   --GAAGCAAAAGCAGATTTATTATAAAAGAGAGGATGTTAAGTCTGATAAGGTTGAACACCACTGTGCTAGCCTGATGGCTTCGTGAGGGCCAGGACCATGT--GTTTTGCTTCCGTTTG
Bos taurus     AGGAGACAAAGATACATTTATGATAAAAAGAGAAGTTTCGATTCTGATAAGATTGATATTTACTGTGCTAGCCTCTTGGCTTCCCGGGGGCCAAGACCATGTCTGTTTTGCTTCGATTTG

Homo sapiens   GCCTAATTAACTGAG----------------GTGCTTA----------------TAAATAATGAATAATGAATGTGGATGCAAGCACACAGCTGGAG--TCAGGCATGTAGGTCAGCATA
Bos taurus     GCCTAATTAACCTAGTGCCAGGTCAGAGTAGGTGCTTAGTAAATAATGGGCAACTGAAGAATGAGTAATAAATGTGGACTCAAACCTAGAGCCAGAGAGTCAGGGATCTAGGGCAGCAGA

Homo sapiens   GTCGGGGTATATAAAACTGGCAGGAGATGGGGACTTTTTTTTTTGAGACGGGGACTTTTGATCACCAAAAGAAGGCAGGATCCCAGTTATCAGAAGAGAAACCATAGTTCAGAGTTTGCC
Bos taurus     GTTGGGGGATA--AGAGTGGCAGTAGATGGGGGCTCTT---------------------GATCACTGAAAGAGGGCAGGTGTCC-GCTATGAAAGGAGGACTC-TAATTCAGAGCTTTCC

Homo sapiens   TCAAAGCTAGAGCGTCCTAATGTCAAGGCCCAGGGCTTCCTACAGTCCTCTTATCTTGAGACATGCTGGAGCCAAGGGACTGACGTCAGATGGTCCCAGTGGTGAAGAGAGTTGTGTTTA
Bos taurus     CCAAAGCTAG--TGTCCTGATGTCAAGGCCCAGTGCTTCTTACAGGTCTCTGGGCCTGAATCATGCTTGGGTCCAGGGACTTGACTCAGATGGTACCAGTGGTGGAGAGAGAAGTGTTTA

Homo sapiens   CATTCTCACTTGCTGGTATCAATCGTAGGGGCCCAAAGACCTCACTACCTAAAAAGAGGGCTGTTTTCCAGCTTAGCTGTACCTCTTTGCATTGTTTTGTCTGG-CCAAGACAGAGAGGA
Bos taurus     CGCTCTTACTT-TTGGTACCAACCTCAAAAGCCATAAGACCTCACCAATTAAA--GAAGGCTATTCCCTAGCATGGCTGTACCTCTCTGCACTGTGTGACCTGGGCTGAGGTGGAGAGGA

Homo sapiens   TACCAGGGAAAATGAGGCTCGAGTATTGTACGTATAATCTGTCAATGCCCTCCATGCCCCCATTAGGATCTCAGGCTGTCTTGGCCTTCAGGCAGATCCAGCTCCCCCATTTTTCTGTTG
Bos taurus     CTCCAGGGTATGGGAGCCTCAAGTGCGGTCCCTAAAATACATCTGTGTCCTCGGTA--------GGGGTGTCAGGCTGCCTTGAACTTCACACAGATCTAGTTCCCC-ATTTTCCTGTTG

Homo sapiens   TTGCTGCTGCTGTTTTTTTTTTTTCACTCCCCTGATCCCATCAATAATACGTGTACTCCTTCCTTCTGGGTCCTGTCCTCAAAGGTAACTGCTAAATATCTGACAAAGGAATGGCTAAAG
Bos taurus     TTGCTACTGTTGCTTTTCTCTTTCCTAGTCCCCTTCTCCAACAATAAGGTGTGTACCTTTTCCTCTTGGCTTCTGTGCTCAAAG--------------TCTAACCAGTGGGTGGTGGAAT

Homo sapiens   CCTTCAGGGCTCCTGATAAGAGAATGGGCTCCCACTTAGCAAGAAGGGCAGGGGGC------AGCACCTCACTTAGAGATATGG---TACATAAGTACAGAGGGAGGTATTGAACCACCT
Bos taurus     GCTTCAGGGCTCTTGATGAGCAAGTAT-CCCTGACTCTGCATGGGGTTGGGGAGGCGAAGGGAGCATCCCTCTTAGAGATAAAAAAATACAGAAATGCTGAGGGAGGTATCAAACCAGCT

Homo sapiens   GTTGTCAGGAGCAGAAGGGACCCTGAGGTCATCTGGTTCACTAGATCCCAAACCTGGCTGTACAATGGAATTACCAGGAATGCTGTTGAAATACAGATGCAGTGTGGCTCCTAATCCTCA
Bos taurus     AGCGTCAGAGGTTGAAGTGGCCTTGGGGTCATCTGATTCACTGGATCAGAAACCTGCCGGTACAGTGGCATCACCGGGAGTTCTGTTGACATACAGATATAGTG--ACCCCTAAAACTGA

Homo sapiens   GA--GTCTGATTCAGTAAGTCCGAGATGAGGACTTGAATTATCTGCATATTATTAACAGTATCACTGGTGATTTTGATGAGGAACCCAGTTAGGGAAACCACTCAGACTGACTTTCTCGT
Bos taurus     GATAATCTGACTCAGGATGTCTGAG-TTAGGGCTTGAA--ATTTGCACATCAGTAA--GTATCACTGGTGATTCTGATGAGGAGCTTGGTGAGGGAGTCCACTCAGACTGA---TCTCAT

Homo sapiens   TTTATGGGTAGCCAGCTGAGTCACCGACAGGGATGATGACTCGCCTCGGGTCACAGAGCAAGTCATGGCAGAGTTGGAAATGGAACTCAGGGTTTTTCCTCCCCTGTATTGGTGGTGGGA
Bos taurus     TTTGCTGGAAAGTACCCAAGTCTCAGAGAGGGGCAGTGATTTACCCTAGGTCACACAGCAAGTCATGGCAGAGTTGGAGCTAAAAATCGGGTCTCTT--TCCACTGTGTTGGCGGTGGGA

Homo sapiens   CGATCTGACATTA----------------------TTAGTGTGGGAAGAAGGACCTTAAATTTCACTGTACAAGCT-GCTGCTCACTCTGCCTAGAGGTAGAGGACAGA-----GGGCAT
Bos taurus     ATATCTGGAGTCAGTGTGACGGGTTGGAGTGATCCTTGGCATGGGAAGAACCGCTTTAAATCTCACTGCAGAGGCCAGCTGCCCACCCTGCCTATAGCTGGGGGACAGATCCGAGGCCAC

Homo sapiens   CTTAAAGGTTGTTTTTCGTTATAATAAGCAGTACATTTACTTCAGAGTTTGGAAGTGAATCAACAAAATACCCATCAAGATTAGTAGGACAAGAGGAACTCGCCAATATCTTTGACGATG
Bos taurus     C----AGGTTGCCTTGCATTATCGCAAGCAGTAAATTCACTGCTGAGTTTTGGAGAGAAGCAACAAAATACCCATCAAGGTTAGTAGGATGATAAGAATGTGCTAATGTGTTTGGCTGTG

Homo sapiens   TCTATCCATAGAAAGCCAAGTGAAATAATGGAGGTAATTATCCACCTATTTTCTTTGAACACTTTGACCGAAAATCTCCTGGTAACTGAGGGAAAATAGGAGCTTTTAAA----AATTGT
Bos taurus     TCTATGCACGGAAAGCCGAGCGAAATAATGGTGACAATTATCCACCTATTTTGTTCAAACACTTTGACCAAAAAGCTCCTGGGAACTGAGGGAAAATATGA---TTTAAACGAAAAATGT

Homo sapiens   TGTCTCAAGTAGCCTGAATGACTTCAACTCCCACTTTACTTTTCTTTTTCTGTGGAGATGCTCTCCCAGATGCTTGTCTCCTGGGGGTAGCAGCTTTCCACAGACAAAATGTCCACAGCT
Bos taurus     TGCCTCATGTAGCCAGAATGACAT-AACTCCCACTTCACATGTCTGTTTCCCTGGAGACCCCTTCCTGCATGCCTGCCTGCTCGTGGT--------------GAGACCACGTGCACAGCC

Homo sapiens   TTGGGAAGCATGGAGGGAGAAGG--------------GAAACATTAAGTGAAATGGGATGTCAGGTCAAGCTGCGTTGGTGGTGATGTAGTCACCTGTCGTTTCAAGATAACCTTTTGGA
Bos taurus     CCAGGAAATGTGGACGGAGAAGGCAAACCCTGTATAGGAGACAACCCGTGAAATGGAATATTAGGCCAAGTGCCAT----GGAGATGTGCTC-CTTGTCCTTGGAAGATGACCTTTTAGA

Homo sapiens   TCCTAGATATGTCGTAGCTGGGGCCTTGGGACAAGTCCCAGCAGCCTCCAAGCCACAAGCTTATCATTTTGGCGCAATTCAAGGCAGCCTCCTTGAAAAGCTCC-ACGATGGCAGCTGCT
Bos taurus     TTCTAGAGCTGTTCTAGCCAGGGCCTCAGAACAAATCCCAGAAGTCTCCAAACCACCAGCTT-------TGGCCCAATTTGAGGCAGCCCCCTCAGAAGGCTCCCAGGACAGCAGCTGCT

Homo sapiens   CCATCAGCAACTGGATGAAAACAATGCCAAATCTCTTTATTTAAAATTAGGCACTTCCTATTTTGGTTCCTAAA-TGGCAAATCTATCTGCTCTCTGCCTCCAGTCTTCTGTGTCCAAAG
Bos taurus     CTGTCAGCCGCCGAATGGAAACAAAGCCAGATTTCTTTATTTAGAAC-AGACACTTTCTATTTTGATTCTTAAAGTGGCAAATCTATCTGTCCTCTGC--------------AGCCCAAA

Homo sapiens   CTGCCTTAATGTGAGGTATTTCAGCAGCGATCAGCAAGTGTCTTCCTATTTATAAGCAGCAGAGCTGCCATGGGTCACTGC---ACTTGTATCTCTGAGCAAGAGAAGTCAGGGGGCCTA
Bos taurus     CTGCCTTA-TGTCAGGTAGCT-AGTACAGATCAGCAGGTGTTTGCCTGTTGATAAGTCGCAGAGCCACTTCGGACCGCTGCTGCATGCCTATCTCTGAGCCAGGG--GGCAGGGGGCTTA

Homo sapiens   TTGGAAAGGAAAAATACT-GTGGCCTCCGACAGTGAACCTTGGCGTGGGTCAGGGAAGACTGTTCAAGTGCACCAGAGAATGGAGCTGTGATGAGTTCAGTTTTGTATCACTGGCATGGA
Bos taurus     CTAGAAAGGGAAAGCGCCCTTGGCAGCAGACAGTGAGTCCCGGCCCCGGTCAGGGAGGGTGGATCCAGTGCAGTGATAGATGGAGCTGTGGTGAGCTCTATCCCACATTGCTGCCATGGG

Homo sapiens   AGTCAGT---------AGCCTCAAGATCTGGGAAGAGTTTGGTCACCACTAATTTGCTACATGACCCTGGGCAAGAGTCGTCCCTTCTCTGGGCTTCAGCCTCACCATTTCTGGGGAAAA
Bos taurus     AGTCAGCCACCCTTCAGACCTCACAGTCCTGCTCGATTCTGG-CACTGCTAATTTGCTATGTGACCCTGAGCAAGGCTATTCCTTTCTGTGGACCTCG--CTGGACCCTCCTGGGGAAGA

Homo sapiens   GGATGCACCATTTAATACATGGTGCCATTTACTGTGACAACCGGCCTGCCACC----AAAATATAAAGCTGAATCTTTATCTCACTCCTTATTCCAAAATATAATCCAATTAGGTCAGA-
Bos taurus     GGACACACAGTTTAACCCGTGGGGCCATTTACTGTGACAAGTGGCCTGCCACTGGAAAAAAAATCAAGCTGAATTTCTGCCTCCCTCTTTATTTCTAAATAGATCGCATCTTAGTCAAAG

Homo sapiens   -TTTAGATGTTAAAAAGAGAAACCATTAAAAGATTAGAAGAAGGCATAAGAGAAT------------ATTGAGGTAGGGAGTCTTTGCAAAGCAACGAGAAA-CTGTAGAATCTATGAAG
Bos taurus     CTGTCGATTTTCAAAAGAGACACCATTGAAAGATTGGAAGAAAGCATAGGAAAATTAAAAAAAAAAAATGGAGGTGGGAAGTCTTTGGAAAGTAACAAAAAATCCCTAGGAGCTATAAAG

Homo sapiens   AAAAAGAATGATAGATTTGACT---ATGCAAAAATTTAACAACCTGAGTATGGTGAAAGATATCTCATGCAAAGTGGAAAGGCAACATATAAACCAGGATAAAGTCACAACATAGAAGGA
```

```
Bos taurus    AAAAAGAATGACAGATTTGCCTGGAAAAAAAAAAATGAACAGCTTTGCTATGGCAAAAGATATCTCATGCAAAGTTGAAAGACAAAGGACAAACCAGGATAAAGTCA--------AAGGC

Homo sapiens  AGACAATGGTTTATTATTCAGAATACTCCACCAGCTCCAGTGTAAGAAGTGCATTATTTTCTTTACAGCACTTACCATGATTTACAATGACATGATTTAGCTACTTGTTTTTCTATTATT
Bos taurus    AGACAGAGGTTTATTATTCATAATGTTTCAAGAGCTCCGTTATAGGATGTGTGCT--CTTCTTTACATCACTCACCATGACCTGCAATTACATTATTTAATGATTTGCTTGTTATCTGTT

Homo sapiens  TCCTCTGCTTACATGTTAGCTTCATGAAAGCTC-AGGTTTTGCTTACTCACTATTATATTTCTAGTGCTAGACTGGTGCCTGGCATACAGTAGGTACTCAATAAATACTTGTTGAAGGAA
Bos taurus    TCCTCTACCTATGTGTTTGCTTCATGAAAGCTGGAAGCTTTGCTTATTCACCGCTGTATTCCTAGTTCTAG-----TATCTGGCACACAGAAGGTGCTCAATAAATACTCATTGACAGAT

Homo sapiens  GGAATAAACGAGCTTCTGTAGACCAATGAGAAGATAATGAACAATTCACTAGAAAAATGCTCAAAGAATAATGAACAGGCAATTCACAGAGGAAAGGCAAAGGGCCTATGCCCTATTAAA
Bos taurus    TGAAGGAGGGAGCTTCTATAGATCAATGAGAACATGATGAACAGTCCACTAAAAAAATAGTCAAAGAATAATGAACAGGGAATCC-CAGAGGAAACACAAATGGTTTATGCCCTATGAAA

Homo sapiens  AGGAACACCAA--TCAGTAATAATTAAGAA--ATGCAAATTAAAACAATATTGAGATGATTCAGATGTCAGCTTTTACCTCTCAAATTGGCAAAAATTTAAAAGGTCACTAAAACCCAGG
Bos taurus    AGGTTC-CCAACCTCAGTAATAATTAATAATAATGCAAATTACAACAGTGTTGAGAGAGCTGAAATGTT---TCTCACCTTTCAGATTGGCAGAAATTTAAAAGGTTGCTAAAATCCAGC

Homo sapiens  GTTTGTGAGAATCTGGTGAAACAG-CACACTCATTCACTCTTGATGCACATCT-----------------GGAGGGCAACTGGAAATGTTATTTGAGTCAGCAATTCCTGTCTTAGGAGT
Bos taurus    GTTTGTGGGGCTATGGTGAAATGGGCACATGTATTCATTCTTGGTGTACATCTAAATTAGCACAATTCCTGGGGAGCAGCTTGAAATATTATTTGACCCAGCAAGAGTTCTTTTAGGAGT

Homo sapiens  TTACCTTTGCAAAATGCTCCATGTATAAGGATGTTCATTTAAACATTATTTGTAAATAGGAGCAAATTGAAAA--AACTAAATGACCTCGGTGAAGGACTGGT-----------------
Bos taurus    TTAGCCGTTTGAAATACTCCATGAATA-GGATCTTCATTTAAACATTATTTGTAAATAGTGAAAATTTGGAAATAACCCAAATGATGTCTGTGGAGGGCTGGTGTCATAATTTATAATTT

Homo sapiens  -TAAATCATTTATAATACATCCCAATAATGACATACTAAGCATTACTATGCTAATGATTAAGAGTTTGAGCCTTG--GTTAGACTGCCTAGGTTTGACTTCCTCCTCTAGCTTTTATGAG
Bos taurus    GTAAATAATTTATAGCATGTCCAAATAGTGGCAGACCAAGCTTCATTATGCTAATGATTAAGAATTTGGGCTCTGTGGTCAGATGACCTAGGTCTGACTTCCTCCTCTAGCCCTTGTGAG

Homo sapiens  CTGTGTGACTTCGACCAAGTTTCTAA-CCTCTCTGTGCCGT----TTCTCATCTGTAAAATGGGAAAAGTAACAAT----------------TCATGAAGTTGTGAAGAGTAAATGAGAT
Bos taurus    CTGTGTGACCTTGCCCAAGTTCCTAAACTTCTCTGTGCCTCAGTGGTCTCATCTATAGCATGGAAATGATAATAATAAAAACATTACCTCCTTCATAACATTGTGAAGAGTAAATGAGAC

Homo sapiens  AATTCATATGA----------AATATTCAGATAGGACCTGGCACAGAGCATTTAATAATTGCTACACATTACTATTCCAACTACTAGTATAATGATGACAATGACGATGTAGCCACAAAT
Bos taurus    AACCCCTATGATGGCTTATGAAATATT----TAGTACCTGGCACAGAGCATTCAATAACTACTACATAGCATTATCCCTACTACTACTGTAATGATGACAGTGACAATGTAGCCATAAAT

Homo sapiens  AAAGACAAGCTAGATCTTTAAATACTGATGTGCAA---------------------------------------TGATATCTCCAATATAGTATTGTGATAAAAGCAAGTTGCAGAAGAGT
Bos taurus    AAGGACAAGTGAGATCTTTATATATTGATATCCATAGCTAGATCTTTGTATACAGATATAATTTATATATGATTGATGTCTCCAATATAATATTGTGATAAAAGCAAGTTGCAGGAAAAT

Homo sapiens  AAGTATAGTAGGATCTGATTTTTGTTAAGGGTATTATATGTTACACATACAGGAAAAGACTGGAAGGATAGACAGACTGTCAACA----------CTGGGGAGTAGAATAGGAGAGGTAG
Bos taurus    CAGTATAATAGGATCTGATTTTCGTTAAGGATATTATTTGTTATTCATA-GGAAAAAATCTGGAAGGATAGACAGATTGTTAACAGTGGTTACCTCTGGGGAAAGGAATGGG-GAGGTGG

Homo sapiens  ATTTTCCCTTGAGCAATAATTAGAGTTGTTTGAAACTAGCATGTGCTACTTTTATAATAAACAAAAGATGTAAAGGGAAAAAAAAGCTCCGTTTGGAATTCCGAGGACAGTTAATCCCAC
Bos taurus    ATTTTCACTTCAGTAATGATTAGAATTGCCTGAAACAAGCATGTGTTACTTTTATAATAAACAAAAGATGTAAAGGGAAAAGA--GCTTCATTTGGAAGTCTGAGGACAGTTAATCCCAC

Homo sapiens  CAGGCTTCACATCCCCCAGTTCTTGGCCACAGCCCAAACGTTAAGCCTCCCTGCTCGTCATCTGTCCTCTCCCATACCATCCTCATACTGCTCAGTGCTCCCAT-CCCTGCCACCCC-GA
Bos taurus    CAAGCCTCACATCCCCCCTTCCTTGGCTACAGCCCAAACGT-GAGTCTCCCTGCCCATCATTTGTCCTCTCCCACACCGTCCCCATACTGCTCTGCACTCCCGTTCCCCGCAACCCCTGA

Homo sapiens  CTCATCCACCTCCCCACTACCGCCCTCTCTCACTCTTCTCAGTACCCTCTGGAAG----CCCCCACCTTGC------AGTAAACAATTCCCCCTACACCCTGGATAAATTTTACAGAGCA
Bos taurus    CTCCTCCATCTCACTACCACTGCCCTTTCCTACTCTTCTCTGTACCCTCTGGAAAGCCCCCCCACCCCGCCCCCACGGGAAACAATTCCCTCTTCACTCCAAATAAGCTCCGCAGAACA

Homo sapiens  CTATTGTCTACCTCCGGGCCTGGAAGTGCTGAAACCTGAGTCTCCACGGCGGATCTCACGGCTTTGCCACCAGCGCCCTCTAGTGGACACTGTCCACACTCCCACACTACAGGCACCCTA
Bos taurus    CTGTTCTCTACCTCTGCGCCTGCAAGTGCTGAAACCTGAGTCTCCACGGAGGGGCTCACGGCTTTGCCTGCAGCGCCCTCTGGTGGACACTTCTCACACTCCTCTACCACAGACACCCTA

Homo sapiens  GGGCTGGGAGGTGGGGTCTGTGCTCTAGCTTCCCCTGTTTGCTTCCAGGCTGTTAGTCCTCCGTCCTCAGCCAAGAACTCCTCCTTCTCATGCCATCTGGCTAC----------------
Bos taurus    GGGCTGGGAGGTGGGGTCTGTGCTCTGGCTTCCCCTGTTTGCGTCCAGATCGTTACTCCTCCG-CTTCTGCCAAGAACTCCTCCTTCTGAAGCCATCTGGCTACAGCACCCTCTTCTTTT

Homo sapiens  ---TTCTCTCCTCGTTGACGCTTCTTTATAACCATCCCCTCCTGGTCAACCCCCCCCCACCCCCACATTCAACGAAGAGTTCGACATCTGGCTCATTGTCTTCTTCTCCACCCCAAGTCC
Bos taurus    TTCTCCTCTCCTCATCGACTCTTCTTTTTTAGCCACCCATCC-----------CCCTCACCC----ATTCAAGGAAGAGTTCGACATCTGGCTCACTGTCTTCTTCTCCACCCCAAGTCC

Homo sapiens  TGACATCATTCTGGGTGAGTTGGACATCCGTGATCCATGTACAGGACACACTCTCATCCTAGCCTTACCATG-------CTTCATCATCAGTCAGAAC--------------CCCCTAC
Bos taurus    TGACATCATCCTGGGTGAGTTG-ACATCCATGATCCATGTGCAGGACACACCTCCATTATAGCCATACCATGTTTCATCCTTCATCATCCGTCAGAATTGCTCCATCTCAGAGCCCCCAC

Homo sapiens  CATCTACACCTTGCTGGCTGGCCTCAACGCTCTTCTTTTTACCTCAGGAGGCTCTCCAGTTTTTTGAGCCCCCTCCATCCATTTCTTCCATCCCATCAGCCCCTTTCACCTTACTACCTT
Bos taurus    CATCCATCTCCTGCCCACCGACCTCAAAGCTCATCCTTT-GCCTCAAGAGGATCTCCAGGTTCTTGAGTCACTTCACTCC------CCCACCCCATTAGCCCTTCTAGCTTTTCTTCCTC

Homo sapiens  CCCTCTCCATCCTGGAACATGATGATGATGATGATAAGAATAACAACT-AATATGTATGTGCCAGGCATATACTACCCATCTTATGACATCATTTCAGTAATCTCATGTTATCCTCACAA
Bos taurus    CTCTCTCCATCCTTGAA---AATGATGATGATGTTAAAAATAATAATTGAATATGTATGTGGCAGGCACATACTAGGCATCTTA---CATCATTTCAATAACCTCATGTAATTCTCACAG

Homo sapiens  TAACTTTATGAGGAAGATACTATTGTGGTT-----TTCCTTTTAATGAGGTGACTGAGGCTCAGAGAGGTTGAGTGACTTACCCAAAGTCCCACAGC-----------AGAGATGAGATT
Bos taurus    TAACTTTATGAGACAGAGACTGTTGTTATTATTATTTTTTTTTAATGAGGTGACTGGGGCTCAGAGAGGTTGAGTGACTTTCCCAAAGTCCCCCAGCTAGTTCAGGACAGAGATGAGATT

Homo sapiens  TGAAGTCAGGTCTGTTTGCTT-------------------TCAGAATCTTGCGTATATCCCCAATTCCCTTGCCCTGTGTCTTTCTGCTGCATCCATGAGGCAAAATCCTAAGTCTGGAT
Bos taurus    TGAAGCCAACTCTGTTTGCCTAACTCCTGGAATGTAGACTTCAGGATCTTGCCTATATCCTGAACTCCCTTGCCCTGTGTCTTTCTGACACATCCACTAGGCAAAAACCCAAGCCTGGAT

Homo sapiens  AAATTTAACTGACAGTTTCCTCCTCTGTGCTTATGCCCAGATGGAGGAACATTGCTGGAGAAAATCCCATCACTTTGTTGGTTAGTCCCACCAGAAAGTCATAGTTTCCAACCTCAGCGG
Bos taurus    AAATTTAACTGACAGCCTCCTCCTCTGTGCTTACACCCAGATGGAGGAGCACTGCTGGAGAAAATCCCATCACTTTGTTGATTAGTCCCACTCAAAACTCATAGTTTCCAACCTCAGCTG

Homo sapiens  GGCCCTCAACTCAGCTAAACAATCCTTCTAAGTAACTGTCCTGACAATAGCACTTGCAAACTTTCTCCACTGCCCTCTGGAGTCAGACTG-TCTGGGTTAGAGTC-TGCTTTGGCCACTT
Bos taurus    GGCCCTCGACTTGGCTAAACAATCCTTCTAAGTAACTGTCCTGACAACGGCACTTTCAAACTTTCTCCACTGCCCTTTGGAGTCAGACTGATCTGGGTTAGAATCCTGTTTCAGCCACTT

Homo sapiens  CCATGCTCCATACCATGCCAGCCTTTAAGAAAGGGTGAAGTGGAGGAAAAAGGATGAGGGGAAAGGGGAAGTGATATGTATCGAGTGTCTTCTAGGTACCAGCCACTGTGCCAGGTGCTT
Bos taurus    TCATGCTCCATACCGTGCCAGCCTTTAAGGAAGGGTGAGGTGGAAGACAAGGGAATGAGGGGAAGGGGAAGAAACATGTCTTGAGTGTCTTCTGGGTGCCAGGCACTGCGCCAGGTGCTT

Homo sapiens  TGCATGCAGTGTTTCATTTAATCCTGCCAGCAGCCCTGCAAGGGCAATACT-CTTGTTATTTTTGCTTTACAGAGGAGGAAGCTGAGGCACAGACAGGTTAAGTCACTCACTAACGTGAC
Bos taurus    TGCATGCAGTATCTCATCTAGTCCTCCCAGCAGCTCCACAAGGGAGATACTATTTTTTATCCTTGCTTTACAATAGAGC---CTAAGGCACAGAGAGGTTAAGTCACCTACTAAGGTGAC

Homo sapiens  ACAATTTGTATGTGGAGAAGCCATCTCATTCGTTTAGACCTTCCTTATCTTGATGAATTATTGTGACAAGCTGATAAATGGGTCCCCTGTTA---CCTATCTCATGCCCTACAATCTACC
Bos taurus    TCAGAGTGTAAGTGGAGAAGCCATCTCAGTCATTTAGACCCTCCTTATCTTGATCGATGACTCTGATAGCCTGCTAACCTGTCCCCCTGTTCTTCCTTAGCTCATATCCTTCCATCTAGC

Homo sapiens  TTCCATTCTGTTGCTGGGACAATCTTTCTAAAATGCACATCTGACAGTGCTACTACTCTGCTTAAA----GTCCTTCCAGAGCTT-CCCCTCAGTCTTCAGAATGAAACAGAAACCTTTG
Bos taurus    CTCCATTCTGTTGCTGGGACATTCTTCCTCAGTTGTGTATCCGACTGTGCTACTACTGTGTTTAAATTCAATCTTTCCCAAGCTTTCCCCATGGTTTTCACAATGAAACAGAAACCTTGA

Homo sapiens  ATCTTAATGATTTGGCCCTGT----CCTTCCTTTCTCACTGCATCCACTACCACTTGCCCATG---CAGTCACACAGTACTATTTTTGGCCACCTGATGACACCTTGCTTTCTTCTGAAG
Bos taurus    CTCTTAATGAGTTGCCCCCCCACCCCTTTCCTTTC---CTGTGTCCACTGCCATCTGCCTAGGAGCCAGTCATTCTGTACTATTTTTGGCTACCTGATGACACTTTGTTTTCTCCTAACT

Homo sapiens  AAAGAAGAAAGCATATATATATATATATTCTCTGCCT------CCCCATCCTC-----CACACCCTTTCCTGCCCACTTTAAATGCTCCTCTTCTGTGC-TTTCACAGCATTCATG--TTTA
Bos taurus    ----------TCATGTCTTTGCATGTGCTCTGTCTGATCTCTCCCATCCCCACTCCCCCTCCCCTTCCTGCCCACCTTTAATGCTTCTCTTCTGTGCAGTTCACAGCATTTATGCATTGA

Homo sapiens  TCCCAGTGTGTTATAATTTCCTGCTGTCTTCTCTGTTTCCCCCATTAAACTGGAAGCATCTTGAGAACTGGGATTGTGCCTTAGTCATCTCTGTAC-----TCCCCAGGGCCGTAGCACC
Bos taurus    TCACGGTGCACTCTAATTTCCTGCTGACTGGTCTGTTTCTCCCGCTAAACTGGGAGCTGCTTGAGGGCGGGGACTGTGATTTAGTCATCTCGGTCTCGCGATCCCCAGGGC-GTAGCACC

Homo sapiens  AGAGCCATGGTTAAATCTATGTTTGTTGAATGACTGAATTAATGAATGAATAGAGTGAATGAGAGAAGAAAAAGCTCTCACTTGGAAAGTTTAGTGACATGAGCTGTATGGTATCTGATT
Bos taurus    AGAGCC-TGGTTCAATCCAGGTTTGTCGAATGGCTGACTGACAGAATGAATGGAGTGCATGAGGGAGGCAAAAGCCCTCACTTGGAAAGTTTAGTGACATGAGCTGTGTGGTATCTGGTT

Homo sapiens  CTTGGCTGAAGCTGCTGCAGTTTTTCCCTATGACTACAGTGCTATAAAAATATGTCTGCACATGGATAAAGACTGGAAGAGAACTTTCCACACAAAACTAAGAGTTGTGTTAGAGTGGTG
Bos taurus    CTTGGCTGAAGCTACTGCAGTTTTTCCCTATGACTACAGTGCTGTATAAATATGTCTGCACGTGGATAAAGACTGGAAGAGAACTTTCCA----AAATTAAGAGTTGTGTTAGAGTGGTG

Homo sapiens  AGT-GATGGATGCTTTTTTC----------CCCATTTTCAAAATGTTCTTTAGTGCTGCTTTTGGATTGTTTTGGCAATGTAGCAGACATTGTAGAAATGGAATTTGGGGCTCCCATGGA
Bos taurus    AGTTGATGGATGCTTTTTTTTTTTTCCCCTCCTGTTTTCAAAATGTTCTTTAGTGGTGCTTTCGGTGAGTTTTG..............................................

Homo sapiens  GGAATCGACAACCAATATGTATTGATGCCCAGTGTACTCTGCTGGGTTCTGGGTGCTGTGGGGAGATGGGTGGGTAAAGGCGACTCATTCTGACTCAGGTTTGCATGGTCAGGAATCTTG
Bos taurus    ..................................................................................ATTCATTCTGATTCTGGTTCACATGGCCAGGGGTCTTG

Homo sapiens  CCAATTACGTTGGATTTGCTGGAATAAAAAGATAACTTTCAAGAATTTTCCAAGCTCCTGGGTTGAGGTATTTCTTTTCTCCCAAAGCTGGGCCGCAAACAAAGATGGCTTTTGTTTGCA
Bos taurus    CCAGATTGGTTGGATTTGTTGGAATAAAATGAGAACTTTCAAGAATTTTCCAAGCTTCTGGGTTAAGGTATTT--TCCCTCCCAAAGCTGGGCAGCAAACAAAGATGGTGTTTGTTTGCA

Homo sapiens  GTAAAATACTCATTCCTTCAATT-------TTGTCCTGCAAAGTGACTTCTATAGCTTTGGGAGTCAGGTTTGCTGAGTGGGGAAACTGATTGTCCATGCTTCATTGATTTAGAAATGAC
Bos taurus    GTAAAATACTCATTCCTTCAATTATTTTTTTTGTCCTGCAA---GACTTCTGCAACTTTGGGTG--AGGTTTACTGAATGGGGAAACAAATCGCCCATGTTT--GTGGTTTGGAAATGAC

Homo sapiens  CAAAAATGTTTGCCTTTTCTGGCTCTTGAA-TAAAATGTTTAGGAATTTCATTGGCAAAATGATGAAATTGTTCCCTTTACTGCTGCAAGACTTCCAAAACTATATATCGTGGGTTAACT
Bos taurus    CAAAAATGTTTCCCTTTTCTGGCTCTTGAAAGAAAATGTTTAGGGGTTTCATTGGCAAAAGGATGAAATTGTTCC-TTTACTGCGGCAGGATTTCCAAAACTACATATTGTGTGTTAAC-

Homo sapiens  TAACCCAGAAGAGGCAAAAAAAAAA--AAAAAATGGGAAGC----AGACTCAGATGATTAACACTCTGCCTTAATTGGGTGCTTACTCACTCTGCCGTGGCAAGGCACTGTGTTCTGGAC
Bos taurus    ----CCAGAAGAGGCAAAAAAATAATTAGGAAGTAGCACTCTCCAAGATTCAGATGATGAGCACTCTACCTTTGTTGGGTGCTAACTCACTCTTCTG-GGTCATGCCGGGTGTTCAGGAC

Homo sapiens  TTTGGAGATATCTCATTTAAAACTCTCAACAGCCTGTGGGTAAGGGACTGTTATTGTTCTCATGGTAGAGATGGTCAAACTGAGTTTCATAGAGGCTCAGCTCTGAAATCATGAGACCAG
Bos taurus    TTTGGTACTATCTCATTTAAAAGTAGAAGCAGGCTTTGGGGTGGGGACTATTAGTATCCCCACGTTAGAGGTGATGAAACTGAGGTTCAAAGAGAGTCAG--------TCATGAGACTGG

Homo sapiens  GATTTGAAGCCGAGTTCAGCTGGTTCCATGGGCACACACTAACCATTTGGCAACAGTGCCTCTAGACTAGTCTGGCTACAAAGGGACAGAGGTATGTATTTATTTGATTTAACGAACCG
```

```
Bos taurus    GCTTGGAAGCCGGGTTCAGCTAGTTCCACAGGTAGGCTCTAAC-ACTTGAACACCCTCCCTCTGGGCTGATCTTGCTACAGAGATACAGAGATACCCATTCATTTTTATTTAACAAATAG

Homo sapiens  TTGTTTAATGTTTACCATGTCCCGGGCACATGGTAACTGCTTTAAAAATGTTAGATTATTTACTTCTCATAGACAACCCTGTGAGGAGGTACTATTATTAATATCATATGACAGGTGGGA
Bos taurus    TTGTATGGTGTTT-CCATATACAGGGCACATGGCAAGTGCTTTACAAAGATTGGGTTATTTGACTTTTGTTGACAGCTCTGTGAGGAGGTACTATTACTGGCACCTTGAGATAGATGAGA

Homo sapiens  AAATGGAGGCACAGGGGTGTTGAATCACTTGCCCGGGGTCACTCAGGCTAGCATGTGACAGAGCTAGAGTTACAATCCAAGTGGCCATGTTCAGGTGTGTGGCCCATTACCTTGACTCTG
Bos taurus    AAACAGAAACACTGGGAGGTTAAATCACTTGCCTAGGGTCA-------------------------GAGATACAGTGTGGGCAGCCATGTTCTGGTTT---------CACTTTGACCCTG

Homo sapiens  TGCTGGTTAGCATGCTACCCCCTGTGCCCTGTCTGCATAGCTCTGGCTCAGAAACACTGAAGACATGGGCCTCTTTTTGTAGTTTTGTAACAGATTTGTACTT---------TTTAAAGT
Bos taurus    TGCTGGGCAGCATGCTATTCCCTGGGCCCTGTCTACAAAGCTTTGGCTCAGAAACACTGAAGACATGGGCCTCTT---GTAGTTTGAAAGAAGATTTGTATTTATTCATTTTTTTGCAGT

Homo sapiens  ATTTTCCACCATTAAAGCCATACGCTCTGCTTCTAAGCGTCCTGTGGGGTTGGTGGGAAGGGTGAAAACATACTCCTGTCCTTCTATAGCAAGACTAGCTTAGAGGCAAGGTGGCCCAGT
Bos taurus    ATTTTTCCTCAGTAAAGCTGTGTTTTCT--TCCCGGGGATCTTGAGG------TGGGAGGGATAAATACATACCTCTGTCCTTTTGGAGCAAGACTCACCTAGTGGCAAGGTTGCAAAAT

Homo sapiens  GGTTAGAGCATGCCCATGGTGCCAGT-GAACTCAGCTTCAAATCCTGGCCTTATGATTTCAGAGCTGAACCTCTCTGAATGTCTGTATCACTATCTTTGCTAAAGGAAGAGGAAGCTTTC
Bos taurus    GGTTAGAGTTTTTCTGTGACACCAGTTAAACTCTGCTTTGAAGCCCACTCTTGTGATTTCAGAAGCTAACCTCTTTGAACCTCAGTTTCACCATCTCTGCGGAAGGGGAAGGAAACTTTT

Homo sapiens  TCCCGATATCTTCATTTACTTCCAATTTTCGGTTTTT--GGGGGAGAGCTGGGTTTCCTCCACAGCTAGGCGCTGGGTGG-CGAAGGGCTTTTGGACTGTC-----------------A
Bos taurus    TCCTGACACCTTCACTTCTGTCAATTTTTCAGTGTCTCAGGGGAAGAAGGGGGTTTCCTCAACTGTTAGACCCTGGATGGGTGAGGGACTTTTGAGGTGTCCCCTCAGGAGTCCAAGCCA

Homo sapiens  CTACTTTTCATTCGACAGAGACTTCAGGATAGCCCCCTTCAAAGGCAGTCTGTTTGGGGTCATCAGGAACAGACAGAGTGCTCTTCCCTACCGCACTCATCCCACCTAGTGCCATCTATC
Bos taurus    TCTCTTCTCATTCAGCAGAGACTTCAGGAGAGCATCTTTCCAAGGCAGTTTGCTCAGAG--------AGCAGACAGTGGGCTCTTCCCTACCTGACTTGTTCTGCCAGG----------C

Homo sapiens  CTTACCCACTCTGAATCCCTCCTCTCGGCAGGCAC--TGCCTCATTCCCCAGAGCCAGGCCTAGACAATGGCGGCTGCTCAGCTTGGAGGGAGGGGGAGT-GAAGAGGCAGCAGGCCCAA
Bos taurus    TCGTCCCACT-----TCCCACC------CAGCCACCCTGATTCAGTCCCCAGAGCCCT-TCGAGACAATGGCGGCAGCTCGGCTTGAAGGGAGGGGGAGTCGAAGAGGCAGCAAGCCCAA

Homo sapiens  CCTGCCAGCTTGAAAGCTTCATTTGACAGGGGAGGAGGCACATCTGGTTTGGGTTTTCAATTCAATTCAACAATGATTTATTGTGGGTCTGCCAGCTGCAGAGGTTCTGGGCTAGGCTAG
Bos taurus    CCCACCAGCTTGAAAGCTTCATTTGACAGGGGAGGAGGCACATCTGGTTTGGGTTTTCAATTCAATTCAACAATGATTTATTGTGGGCCTGCCAGCTGCAGAGGTTCTGGGCTAGGCTAG

Homo sapiens  GCAGCAAGAGATGCAAAGATAAGTAAAATATTGTCTGTGCCCTTAAAGGAGTTTTCTAGGTTGTAGGGGTGTGCAGACGCAAATGAGCATTTTTTTTTTATGCGATAAGCCCTGTGCTTA
Bos taurus    GCAGGGAGAGATGCAAAGATAAGTCAAATATTATCTGTGCCCTTAAAGGAATTTTCTAGGTTGTAGCAGAGCACAGACCTGAATGAGC----CATGTTTATGTGATGGGTCCTGTACTGA

Homo sapiens  GCAGTTTACCTTGCCTACATTATTATGTCTTTGCATCTTCCCAGCAGGTTTTGTGATGGAGGTGGGTAAACTATTATCACTTCATTTTATACACATGAGGAAACAGAGCCTCAGAGTGGA
Bos taurus    GGGGTTTACCT-GCATACAGCAC---ATCTTTGAACCTCCCCAACAGGTTCTGTGATGG--GCAGAGAAACTGTGCTTACTT--TGTTGTACACATAAGGAAACTGGGCCTCAGAAAGGA

Homo sapiens  AGAGACTGGCCCAAGGTCACATTTTTAAGAATGGACCAAACCAGTTCTGGAACT--------------------------------------ATTACATTCTTCTGCTCCCCAGAGGTGA
Bos taurus    AGTGACTGGCCAAAGGTCACACAGTTAGAAAGTGATGGAACCGGGCCTGGAACTTAGATGAATCTGGTTCCGAAGCTCACACACTCTTAACCATGATTTCACTCTGCTTCCCAGAGATGA

Homo sapiens  TGAAGATGATGATGATGACAAAAAACACTTACTGAGCTCTGGAGGCCAAGTCTTGTGCTATGCAGGCTGCAGATAT---TATTTTACTGGCAACTTCCAACATCCCATTGAGGCTGATGT
Bos taurus    TGAAGATGATGACGA------GTAGCTCTCACTGAGCTCCCTTGACCAAATCGTGTACTGACTGGGCTGCAGATATAATTATTTTATTGGCACCTTCCAATATCCCATTGAGGCGG--GT

Homo sapiens  TGTTATCCCTTTTTCTAGAAGAGAAAACGGAGGTAGCCTTGGGAAGTGATGGAGTTGGAATTTGAAACCAGGTCATT-CCAGTCCACCAGCCTGTACCATAAAAGAGGTAGACACAGGGT
Bos taurus    TGTTAACCCCTTTTCCATAAGAGAAAAT--AGGT-TCTTAGGGAAGTGATGGAGTTGGGCTTTGAACCTAGGATATTTCCAGTCTGCCAACCTATAGCCTGAAAGAGGTAGACACAGGCT

Homo sapiens  GCCATG----TTCAGGGGCAAGAGCAATGAATTGTGCCTGCAAAAGCTTCATGCCAGAGATGGCATTTACATTTGGCCTTGAAGGGTGAGATGGGGTTATGATTGGTAGAGTTGGGAGAG
Bos taurus    GTCATGGGGGCTCAGGGACGAGG-------------------------------CAGGGATGGCATTTAGATTTGACCTTGAAGGGTGAGTTGGGGTTTTGAAAGGTAGGATTGGGAGAG

Homo sapiens  AGGGCATCCCAGTCAGAAGGGATATTCTGAGCAAAGAACCCAAAGAGATGACTTGTGGAGCATTTTGATTGAGA-GGCTGTAGGTTCATCCGGCATGCAGGCCGGGGACTCTGGGTGTAG
Bos taurus    AGCGCCTCATGGTCAGA-GGGATATTATGAACAAAGA---------GATGAACTGTGGGGCATTTTGATCATGAAGGCTGCAGGTTCATCCAGTTCGTGGCAGCGTACCCTGGGTATAG

Homo sapiens  AGGAACAATGTGAAGGAAACCAGAGATACGCCTGATGGTCTAACACATTTTTGTCAAGTTGAGGTTCTTATTTTTTTAATTAAGAGCAGGTACTCTGCCCCAAAGGCTTTTCAAATGATA
Bos taurus    GGGAGCAGTGGGAAGGAAACTGAA--TACCCTTGATGGTGT--TCAAATTTTGTCAAGTTGAAGTTCTTGTTTTTC-AATTAAGAGCAGCTCCTCTACCCAAAAGGCTTTTCAAATGATA

Homo sapiens  CTATCTTGCAACCCATGAGCAAACACACTCGAGTCTCTCACTGTGTAAATTCTACTGTGCTGAGAGCTGCGGTGAATACAAAGAAAACAACTCAATTCCTCAAGTGTGTGCCGATGGCC
Bos taurus    CTATCATGTAACCAATGAGCAAAAGCACAGGAGACTCTCACTGTGTATATACTCCTGGGCTGAGAGCAGAGAAGAAGACTGAAGAAAGCAACGTGGTTCCTCCAATGTGTGCTGAGGGAC

Homo sapiens  CCCGTGTGGTTGAGGAGGCAAGATACACACAATCAGGAAAAGCAGCAGCTAGCATAGGCAGTTAGTGATCAGGGCCAAATGAGTTTTTCCAGCCGTCAGATTTCAAGGAACATGAACGAG
Bos taurus    CCTGTATGGTTGGAGAGTCAAGATATGCACA-CCAGAAAAAGCAGCAGCCAGCCCAGGCAATTTGTGATCAGGGCCAAATGAGTCTTCCCAACTGTCAAATTTCAAATAACATGAAGGAG

Homo sapiens  AGGAAGGCCGGCAAGCTTAAGGCCGGGTGGGGCAATCAGTGTAGCTAGCATTAAGGGCTCTGGCCAGGCCTCCCTTCCCCAAACTGCCACGGTCTCCAACCATGTCTTGACCTCCCAGAG
Bos taurus    AGGAAGGCTGGCAAACTTAAGACCAGGTAGAGCTACCAGTGTTGCTGGCATGGAGGGCTCTGTGTAGGCCTCCCTTCCTCAAACCACGGCTGCCCCCATCTCTGTCTTGACCTCCAGGAG

Homo sapiens  TTTGTTAGGGAAGATGGGACCTTATGGGATCAGAGACAGCGCATGGGAGCTGGGAAACGGAGCCGAATACCTGATATTCCTGATCCTCCAATTTTCTACACTTATTCTCATTTCCTGGCA
Bos taurus    TTTATTAGGGAAGATGGGACTGTACTGGGTTAGAGACAACCCGTGGGGGCTGGGAAACAAGGCCCGGCACCCGATATTTCTGATCCTCCAATTTTCTACACTTAGCCTCAATTCCTGGCA

Homo sapiens  GCTCCTCCAGCTGCTGAATTGTATGTGTATGTGTGAGCCGTATTTTAAATTAAGCAGTGAGTTCTTTCAGCTCATCATAAACATGCTAAAAAGCTTCAGCCGCGGCCTGAGCCCACCCCC
Bos taurus    GCTCCCCCAGCTGCTGAATTGTGTGTGTATGTGTGAGCGGTATTTTAAATTAAGCAGTGAGTGCTTTCAGCTCATCATAAACATGCTAAAAAGCTTCAGCCACGGCCTGAGCCCACCCCC

Homo sapiens  GCCCTTTACACACACAGGCAGCAGCTGGGGCCACTCGACCCCACACTCTAATGTGCATTGCCTATGACTCACCAACAAAAACGACTGTATGTTTCAGTCTCCCTGGGTAGCTGATTCCTA
Bos taurus    GCCCCTTACACTCA--GGCGGCAGCTGGGGCCACTCGACCCTACACTCTAATGCGTGTTGCCTATGACTCACCAACGAAAAAGACTATATGTTTCAGTCTTGCTGGGTAGCTGATTCCTA

Homo sapiens  GTTGTGGTGGGAAATCTCAGTCCGATTT-GGAAGGCAAAGGGCCTCAGCCCCGCCCTACTGACTGTGAATTCTACAACATATGGAGCGCCTACCTTGTGCAAGGCATTGTGGGTAAGTCC
Bos taurus    GTTGTGGTGGGAAATCTCAGTCCGATTTTGGAAGGCAGTGGGCCTCAGCCCCGCCCTGCAGACTGACAATTCTACAACATATGGAGCGCCTACTGTGTGCAAGGCATTGTGGGTAAGTCT

Homo sapiens  ATTCATCTCTCTGGGCTTTCCATTTCCTCAGCTTCAAAATGCATGTACTAATCCTGCCGGCTTTACCTCCCTCCTAGGGCTCTGTGTCTCAGCCACACTGGTCTTTCAATTTCTGCAACA
Bos taurus    GTTAATCTCTCTGGGCTTTCCATGCCCTCA--TTCAGAATGCATTGACTAATTCTGTCTGCTTTACCTCCCTACTGGGGCTGCATGTCTCAGCCATGCTGTTCTTTTGATTTCTGGAACA

Homo sapiens  TTCTC--GGTCTTTTCTCGCCCCAGGGCCTTTGTTCGTGCT------CCTGCCTGGACTGCCCTTCCTCTTGCTTTTGGCATGGAAGGCTCCTTCCTAACCTTTCAGTCTCAGCTTAA-C
Bos taurus    TTCCCAAGGTCTTTTGCTGCCCCAGGGCCTTTGTGCATGCTATTCCTTCTGCCTGGACTGCC-TTGCTCTTGCTTTTGACATGGGAGGCTTCATCTTAACCTTTCGGTCTCGGCTCAAGT

Homo sapiens  GTTACCTCCTGAGAAGAGCCTTCTCTGACCACCCACAGTTAGATGCCTCCTGTTCCTCTCTTACATTTCTTTTATTAGCAATGCTTCCTTAACCCCATTTACTGCTCTGGGTAATTATGT
Bos taurus    GTCACCTCCTGAGATGTGCCTTCTGCAACCACCTCCAGTTAATTCCCTCCTA----------ATATTCCTCTTATTGCCTATAGCTCCTTAATCCCACTTACCTCTCTGTGTAATT----

Homo sapiens  CTATTTATATTTGTCTATTTATGGTATGTCTCTCCCACTGGAATGTAAGCTCCAAGAGGACAGGGACCTTGTCTGTGTCACTCAGCAATGTGTCCCTCTCCTCTAGAGTGGTGCTTGGCA
Bos taurus    CTGTTTCTATGTATCTATTTAGAGTCTGTCTTTCCTGTTGGAATGTAAACTCCAGGAGGACAGGGACCTTGTCTTTATCACTTGGTAATATGTGCTTCTCATCTAGATGGGTGCTTGGCA

Homo sapiens  CATAGTAGGCTGACATAAGCAAAACCATCTGGAAAATATTTTCTTCCTGAGGTTCTTGCCAGACATATGGTTGCTGAGGGAAAAA-TTATGGCAGAACTGTGTCATTCCATA-GGAAAAA
Bos taurus    CATAGTAGGCTGACATAAACAAAACCATCTGGGAAATATTTTCTCCCTGAGGTTTTTGCCAGACATATGGTTGCTGAGGAAAAAAATCATGACAGAACTGTGTCATTCCAAATGAAAAAA

Homo sapiens  AAATCAGGCTAAAAGGGAAGAGTTTGTTGAAAACAGTTCTGTTTTAGAAACCAGTCCTTGGAGTCTCATCGCCAGACACAGTCTTATGAAA-TTAAACAAAAGAAATAAAAAGCATAAAA
Bos taurus    AAATCAGGCTGAA-GGGAGGAGTTTGTTGAAAGCAGTTTTGTTTTAGAAACTGGTCCTTGGAGTCTTATCACCAGACACAGTCTTACGAAAGTTAAACAAAAGGAACAGAAAGCACAGGA

Homo sapiens  AGCTAAGTTTTTATTGGGTATTTACTATGTGCCAGGAACTGTTCTAAGTGTTCTACACAGATTATCTCATTTAATCCTCATCATAACCCAATGACGTCAACTCTATTATTAGCT---CCA
Bos taurus    AGCTAACTTTTTACTGGGGGCTTACTGTGCGTCAGGAGCTGTTCTAAGTGTTCTAGGGAGATTATCTCATTTAATCCTCATAATAACCCAATGATGTCAATTCTATTATTAGCCACCCCA

Homo sapiens  TTTAACAGATGAAGGAATTGAGGCTCTGGGATGTTAAGAAAAGTGCCCAAGGTCACACAGCTAGCAAGAGACAGAACCAAAAGTTGAACTCAAGGCAGTCTGAGTCCAAGTCCACAAACT
Bos taurus    TTTTACAGATGAGGGAGTTGAGGCCCTGAGATGTTAAGCAAAGTGCCCAAGGTCATGTATCTAGCGAGAGACAGAACCAAAAGTTGAACCCAGGGCCATCTGAGGCTGGG------AACT

Homo sapiens  AAATCATCTATGCCACC-TGCAGCCCCCACCTCATGTTACCCTACACCTCCCACGCCACCCCCAGGCACGATTCTACTACAGCACTGGCCACATTGCAGAACAGGGGTTTGTTTACTTGT
Bos taurus    GAATCATGCATGCCCCGGTGCAGCCCCCAA------------------------GCTACCC-------------------------GGCCACAGCTCTGATCAA---------------

Homo sapiens  CTGTCCTTCCTACTAGACGAGGAGCTTCTCAAGTGCAGAGACTGGATCTTATTCATTTTTGCTTCCCCTGTGCCTGGAAGAGAGCTTGTTCTGGAATAGCTAACCAAGGAATATTCGTTG
Bos taurus    CAATCATTCCCACTAGACTGGGAGCTTCTCAAGTTCAAGGACTCTGTCTTCTTCATTTTTTCTTCCCCTGTGTCTAGAATAGAGCCTGTTCCAAAACAGGTA-CCAGTCAATATTTGTTG

Homo sapiens  AATGAGCAAATGAGTGAGGGAGTAAGTGAATCCATATTCCTTAATATTTTCAGGCTTCAGGCTTATGGGCTCATGTGTATTTCCCTGG-------TCAAGCATAGCACTGGGCACAAGG-
Bos taurus    A----GTAAATGAATAAGGGAGGTAGTGAATCCCTGTTCTTTTATTTTCTCAGGCG-CAGGCTTATGGGTTCATGTTTATTTCCCTGGCTTGCATTAAAATGTAGCACTGATGACAAGGT

Homo sapiens  GTATTCTGGTGCAAGAGGTGCCTCTCACCACAGGGTACAAGAAGGACTAGCTCTGGCTGGGTAAACCAAAGCAGTGTTCCACAGAGCAAGGGGCTTGATCCAGAAGGTTTCATTTTGTCA
Bos taurus    GTGTTCTGATGTGAGAGGTCCCTCTCCCTGTGGGATACAGGGGGGACTGGCGCTGACTGAGCAA-CCTCGGCAGCGTTTC-TAGAGCAAGGCGCTTGATTGTAAGAGTTTCATTTTGTCG

Homo sapiens  TCGGTCTAAAAAGTAGCTGGACCTGTTTAGGACTTAAACAAACTTTCAGTGTTCCAATCCCATCTCAAATCCTTTGCAGAATGAGGCAGGGTGTGGGGAGGTGGTGGTGGGGCAGTTATA
Bos taurus    TCCATCTGAAAAGTAGCTGGACCTGTTTAGGACTTAAACAAACTTTCAGTTTTCCAATCCGATTTCAAATCCTTTGCAGAAGGAGGCAGAGTGAGGGGGAGGAGCAGGGGAG-AGTTATA

Homo sapiens  AATAAACAAATACCCACAAACCGGCTGGTTTTTTAAACAGAATTTGCTTCAGTCAGTGCTGCACCACATTTTCCCAACTGGCCTCCGTCGTGAGAACAAGTGGGAACTTTCATACTGAAT
Bos taurus    AATAAACAAATACCCACAAAACGGCTGGTTTTTTAAACAGAATTTGCTTCAATCAGCGCTGCGCCACATTTTCCCAACTGGCCTCCATTATGAGAACAAGTGGGAACTTTCATACTGAAT

Homo sapiens  GGCTTTATTTTTATT--CTGACCAGATGAATGGCAGGAGTTTTGGCTCGGGAGGCAGGTTTCCGATCCTCAGCACACAGTTTGCTGCCCTACTGTGCCCCGTCCATCACACACAGCGCCC
Bos taurus    GGCTTTATTTTTTTTTCCTGACCAGATGAATGGTAGAAGTTTTGGCTTGGGAGGCAAATTTCAAACCCTCGGCACACAGTTTGCTGCCCTACTGTGCCCCGTCCGTCACACACAGTGCTC

Homo sapiens  TGCACAACATACCCAGTCATTGCATCTACTGCCTCAGCCCTTGAAAATAATTGGGTCATTGTTTTATTACACCAGCGAGAGAGCTGCAAGCCTGTTAGGATTATGTCATTGATATTTGCG
Bos taurus    TTCACAGCCTACCTAGTCATCACATCTGCTGCTTCAGCCCTGCAAAATAATTGGGTCATTGTTTTATTACACCAGTGAGAGAGCTGCGAGTCTGTTAGGATTATGTCATTGACATTTGCG

Homo sapiens  TGAAAAGGCAATCATGATATGCTGGTCATTTAACACAGCTGGGAATGCAACTGTGTTGTCATTGTTTAAGGTTCTCATCCAGCCCCTCTGACTTTTTTGCACAGCCCAGCCCGTGAAAAT
```

```
Bos taurus     TGAAAAGGCAATCATGATATGCTGGTCATTTCACACAGCTGGGAATGCAACTCTGTTGCTATTGTGTGAGGTTCTCATCCAACCCCTCTGATTTCGTGTCACAGCCCAACAAGTGTCAAT

Homo sapiens   GGATCACTCTGATGTATGTCACCTTTAAAATGCTTTCTAGCCCTTCAAGGGACCAAGCAGGATTTAACTCTTTTCATGCACTTGGACTTTGCTGCTTGTAGGCACAGCAGTCTGAAGGTG
Bos taurus     GGATTGCCCTGATGTCTGTCACCTTCCAGGGGTTTTCTCATCCTTCAAAAGACTAAGCAGGATCTAACTCTTTTCAGGCACTTGGACTTTTTGGCTTGTACGCACAGAGGGCTGAAGGTG

Homo sapiens   AGAGATGCTTGCTTTTCATACTTGTGGGAGTTGTACACCCTAGTGGCAGGATGCTGCCATACAGTGTCATTTGTTCACTAACTGCTGATCAGAGCCAAGGACAATTTTGGCTCAAGAACA
Bos taurus     AGAGATGCCTGCTTTTCCTGCGTGT-GGAGGTGTATACTCTAGTGGCGGGATTCTGTGATACAATGTCATTTGTTCACTGACTGCTAATTGGAGCCAAGGACAGTTTTGGCCGGAGAACA

Homo sapiens   AATGAGACCTAA---TGTCTGTGAGGGAAGACTATGTAGATAAGTCACGGGTCAGAGTTGTGTTGCGACTTGTATTGTGTGCTAGTTTCAGTAGGACTTGTGCTTTCATTGCAATTGTCA
Bos taurus     AATGATGGCTAACGTTGTCGGTGGGGGAGGACTATTTAGACAATTTATGACTCGGAGTTTTGTGGTGATTTCTATTGTGTGTTCCTTTCAGTAGGACCCGTGCTTTGCTTGCAGTTGTCA

Homo sapiens   GTATGCTTAAGCAGGGGTCTCTGCTAATGAAATTCGAGCATATTTGCATTTATCGACAGGTATTACTGATGGTAGAACATCTTATTTCCACACAAGGCAATGAGTAACATTGATTATAGA
Bos taurus     GTGTGCTTAAATGCGGGTCTCTGTGAGTGTCATTCAAGCCTATTTGCACTTATGAACAGGTATTAATGATGGCAGAGCATCTCAGTTGCAAACAAGGAAATGACTAGCACTGATTATAGA

Homo sapiens   AGTGTAAACAAAGCTCAAATTCATGTGCCAATTTGTCTATTTCAACTCATCGATCCTCTTCTTCCACTGGCATGCTAGCCTGAGTTTTATGGCATAAGGCCATTAAAGTATTCTTCAAAG
Bos taurus     AGTATACACAGAGCCCAAATTCATGTGCCAATTCTGCTATTGCAACTCACTGATCTTTTTTCCCCACTAGTGTTCTAGCTGGAGTTTTATGGTGTGTGGCCATTAACGTACTCTTCAAAG

Homo sapiens   GAATCTGTTGCAGACACCTGTTCATCCCTCTTCTGGTCCCGGTCCTTGCCAAACCAGCTCTGCCTTTGCCAACCACCTTGAGGTGTGACAGGAAAAGCTGACCCAATAAGGGAGTCAACT
Bos taurus     GAATCTGTCATTGATACCTGTCCATCCCTCTTCTGGTCCCTGTCCTTGCCAAACTGGCCCTTC-----CCAGCCACCTTGAGGTGTGAGAGGTACAGGCAGCCCAATAAGGTAGTCAGCT

Homo sapiens   GAAGGGATGGAGTGGCTTAGATGCAAATGGCCAAATCCCAATGGCCATGGCTATAAGGAACACAGGTTCCTGAAGTTTTCTTGGCAACTAGCTCTAACTGCTTTGCCTTCTGAGTGCTTT
Bos taurus     GAAGAGATATAGTGGTTTACATGCAAATCTCCTAATCCCCTTTGCCATGACTATAAAGAACTCAGGTTCCTAAATTTTTCTTGGCAGCTGATTCCAAGTG------CCTCTGGGAGCTTT

Homo sapiens   GGTATTTGTTTTATTCTAAGTTCCCAGTTTTGATCTATTTTTGCCTTATGGCTGCAGATTCCCTTTCTGGAAAATGGGGCTCTGGCTGGGATCAAATCAGAGAACACACCCAGACTTGGC
Bos taurus     GGTATTTGTACTGTTCCTGGTTCCTGCTTTTGAT----------CTTAT-ACTGCAGTTTCCCTTTCTGTAAAATGGGGCTCTTGCTGCGATCAAATAAGAGAAGTTACCCAGAGCTGGC

Homo sapiens   ATAGTGCCT----GGCACAGAGTAAGTGCTCAA-CAAATGGTAGCTGTTGTCTCTTTTTCCCTGTCCAGGTTTCTTCTGGTTCTGTTCTCTGTACCTCCAGTATTGGCCCAGAACCAGGA
Bos taurus     ACAGTGCCTGATGGGGAGAGAGGAAGTGCTCAAGCAATAAGTCCTTGCCATCTCCTTTTTCCTGTCCTGGCTTCTTCTGGTTTTGTTTGCTGTACCTCTGATATTGGCCCTGGACCATGG

Homo sapiens   ACTGTTCCCTCCCCTGACTGGCAAGTCAGATTACCTGGCCCTCAGACAAGCTGGGCTCCCTTTTCTGGCAGCCTTTTGTTGTCCTT-ACCTGTTGCCATGTGCCACTCAGTGTAACCACA
Bos taurus     CCTGAACCCTCCCCTGACTGGCAAGCCAGGTCCTCCACTCCTCAGACAATCCAAGCCCCCTTTTCTGGCGGCTTTCTGTAGTCCTTAACCTGTCGCCATGCGGCACTCAGTGTAACCACG

Homo sapiens   GGCAGACCTCTGCCAATTGCTGACATTAACCTATTGAATCCTGGCAGAGAAGAATAAGTTTGCAGACTGGAATTTGCATGTCTGGGCCAGATACGAGACCAGGAGGCAAAGCCATATTTA
Bos taurus     GGCAGACCTCTGCCAGCTGCTGTCATTAACCTGTTGAATTCTGGCAGGGAAGAATCAGTTTCCAGACAGGAATTTGCACGCTGGGGCCAGATACAAGACCAGGAGGCCAAGTCATATTTA

Homo sapiens   CCTGTTCTCCCCAGTTGTG-GCAAATCACCCACATTCTGGTTAAAACCAGATGTTTCACACCCATGACTGCAATGCCACACATCAAACAGTTGCTTACAAAGAACACAAAGTGGTTTTCA
Bos taurus     CTTGCCTCCCCTAGTTGTGAGCCAGTCACCCACATTCTGGTTACAACCAGATGTTTCACACCCATGACTGCAATACCAC---CCAAACGGTTGCT-------AACACAGAGTGGTTTTCA

Homo sapiens   C-ATCGCAAATGTAACTTGCAGGTCACTTCTCAAATTTCCTCGCAGCAGCTACCATGCCTGGGTATTTTTCCCTTAAAAATGACAACCTTGTTGGGTCTCTTGTAGGGACTTTCATTTGG
Bos taurus     CTATCAGAAACGTAACCTGTGTGTCACTTCTCAAATTT-CTTAAACCAGCTACCACATCTGGGTACTTTTCCCTTGAAAATGACCACCTTGTTGGGCGTCTTGTAGGAATTTTAACTTGG

Homo sapiens   AAGCCCTGGGAGAACTTATATGCTCTTTCCAAGAGGGTCTCTGATGGCTTTAATTCACATGGAGTTAATATATTGGAAAAGA-AAAGAGATCTTCCCCAAACTCACCTGCTCCTTGAAAA
Bos taurus     AAGCGCTGAGAGTGCT--TATGCTCTTTACAAAGGGTTCCCTGATTACTTTCATTGTCAGGGAGTTAAAATATCGGAGAAGATAATGAGATCTTCCCCAGGCTCACCTGCTTTTTGGAAA

Homo sapiens   TGCTATTCTGTTGCCTTGGCGAACAGCCAATCCAAACA-TTAAAAA---------------------AGAGATAGAGAATGCAGGAATCCTAAATGCTATTCTCAGTGCATGTCCAAACA
Bos taurus     TGCTCTTCAGTTGCCTTGGCAAACAGCCAATCCAAACAGTTAAAAAAAAAAAAAAAAGAGAGAGAGAGAGAGAGAGAGAATGCAGGAATCCTGAATGCCATTCTTGGTGCAAGCCCAAACA

Homo sapiens   AGAACTGGGGGCTGAGGG---GCAAAGAACAGAAAGAGGAGAAAAG--AGTTTTTGTAGCCTAGGCCGTGAGTTGATC-------------------CCACCCCGTCATGTCCTGTACCC
Bos taurus     AGAA--GAGGGCAGGGGGTGTGGGCACCACAGAAAGTGGAGAATAGGTGGTTTTTGCATCCTAGGCTGGGAGCTGATCTCTGCCTGTAGCCCCCTCGTCCCCCCATCATGTCCGGTCTCT

Homo sapiens   TTCACGCTCATAAAGAAGACAGAGGAGGCCGGGCGCGGTGTCTCATGCCTGTAATCCCAGCACTTTGGGTGGCCGAGGCAGGCGGATCACCTGAGGTCAGGTGTTGGAGACCAGCCTGGC
Bos taurus     CCTGGACTCATAAAGAGGAC....................................................................................................

Homo sapiens   CAACATGGCGAAACCCTGTCCCTACTAAAAATACAAAAATTAGCCGGGGGTGGTGGAACATGCCTGTAATCCCAGCTACTCGGGAGTCTGAGACAGGAGAATTGCTTGAACCTGGGAGGT
Bos taurus     ........................................................................................................................

Homo sapiens   TGAGGTTGCAGTGAGCCAAGATTGTGCCATTGCACTCCAACCTGGGCAACAAGAGTGAAACTCGGTCTCAAAAAAAAAAAAAGAAAAAGAAAAAGAAAGAGGAAGGAGAAGGAGAAGAAG
Bos taurus     ........................................................................................................................

Homo sapiens   GAGAAGGAGAAGGAGAGAAGAAGAAGGAGAAGGAGAACGAGAAGATG-TGGTGGCATCATGAC-------TTCTCTGCTAGGGTTAATTGCTCAGCCTGAGAGGCTCTCCAGGCTGGCAG
Bos taurus     ............................................ATGATGGTGACATCATGACAGCAGACTCCCCTGTCAGAGTCAGCCACTCAGCCCAAGAGGTTTTCCAGTCTGGCAG

Homo sapiens   GTCACACTAGAGCCAAGAGTGAAGGGTCCATTCAGTAAGGTCACTGCCATGATCAGTTCTCAACCACTTTAACTCAGTTTTTCTGTTGCTGCTTTGT------TGGTTAGTGGTTTGTTT
Bos taurus     GTCACCTCAGGGCCAAGAGTAAAAGGTCTATCCAGTGAGTTCTCTGCCATGATTGGGTTTCAACTGCTTAGATT---TTTTTCTTTTTCTGCTTTGGCTTCAATGGTTATTGGTGTATGT

Homo sapiens   GTTTCTACCCATTTGGGAGAAGGGTAGGAAAGGAACTAAGGTGATCAACTATTCCATATGTGTCAGGCCCTGTACTTGTCTTTTCAAACACTTATTCACACTCTGAGGGACTCTCTCAGG
Bos taurus     GCTTCTACACATTTGGGAGGGG------------CTGACATGACCAACTATTCTGTGTGT---AGGTGTCAAATTTGTACTTTTCAATGTTTTATCACATTCTCAGAGACCCTCTCTGT

Homo sapiens   TCCAGCAACTGTGTAGCTATGATGTGAAATAGCAGCTTCTCTCTATGCATTCACTGGGAATGGTTTGTTCTGATCACACAATTTTTTCCAGTTCTTCCGACGTTAGGTGTTGCATGGTAA
Bos taurus     TCCTGCAACTGCAAGATTGTGCTTTGAAACTG--GCTTCTTTCTCTGCATTCACTGGGAATGGTTTATTCTGATAACGTTAAATTC-CCACTGTTTCCAACATGAGGTATTACATCAGAA

Homo sapiens   GAAACAGGTGGTTAATTATTGAGACAGTTCTTATTTGGAATTGTCTCTTCCTCCTTCTCCTCTCAGGCAGATGCTAAAGGATGTCTGTAGTTT-TTTCATGGCATTTTGCTAAAGATGCA
Bos taurus     GAGACAGGTGGTTAAGTGTTTAACCAGCTTTTACTGGGAACTCTCTTTTCCTCCTTCAGATCTCTTGTAGATGCTAAAAGGTGTCTGGAGTTTCTTTCATGCCATTTTATTGAAAATTCA

Homo sapiens   CATTCATTAATTCATCAAATAGCTTTTTTTGAGACATTATTATGAGTGAGGTGTTGTGCTATGATATGCTTTATATACCATATCTTATTTCATCCTCACCACAAAGCTATGAGGTAGGTA
Bos taurus     TATTCACTAATTCA---AACAGATTTTTT-GAG---TCATTATGTGTGAGGTACTGTACTATGATATGCTTTATATCCCATACCTTATTTCATCCTCACCACAAAGCAGTAAGGGAAGTA

Homo sapiens   CTATCATTATCTTAATTTTACAA-TGAAGAGACTGAGGCTCAGGAAACATAATGCAAGGTCACACTCAGTTAATAAGTTGAGAGCTAGGATTCAAACCCAGGTTTATCTGACTCAGTTAC
Bos taurus     CTGTCATTATCTTAATTTGACAAACAAAGAGAC--AGGCTCAGGGAACATAATGCAAGATCACAC--AATCTATATGTGGGGAGGTGGGATTCAAACCCAGGCTTATCTGATGCAACAGC

Homo sapiens   ACTACACTTCCTCCACATAGCACATTGTGGGCCCGTCACAGCTCTTGAGGACAAAATTCATCCTTAGAAAAGTTTTCTTACTCCACTTTTTGCTGAT-------ACTACTGCTGCTGTTG
Bos taurus     ACTATACTGCCTCCACATAGCATATTTCAGGCCTCTCACAGCCCTTGAGAACAAAATGAATCCTTGACAAAAATGCCCTACTCTACATTTCGCTAATCGTTAAAATTACTACTGCTATTA

Homo sapiens   CTGCTGCTACTACTATTACTACTACTGCTACTGTTTCTCCTCCTCTATTTATCCAGAACTGCATTTTTCCAGCACTCCTAGGTGCTTTGAACCCAGCTACCAGTTGTAGAGTTCAAACCC
Bos taurus     CTACTGCCACTGCTGCTATGGTTACTGGTGCTATTAT-----------------------CATTTTCCCAGCACAGGCATGTGCTTTATACCTAGCTGCCAGTTGTAGAGTTCAAGCCT

Homo sapiens   AGGCCTGCATGAGTCCCTTGCAC--------------------------------------------CCATTCTACTTTTCTGCACTGCTTGTAATGTAACACAGGAGGTATGGGACATC
Bos taurus     AGGCTCATATGAGTCCCCTGCATTGGAAGGTGGATTCTTAACCACTGGATCGCCAAGGAAGTCCCCGCCATTCCACCTTTTTGCACTGCTTGGAATAACACCCAGAAGATGGAAGACATA

Homo sapiens   CATAACTCAGGTAGGTAATTGACTTCAAGCTTAAC-------ATTCACTCACCAATCATTTATTGAGCACATATTAGATAAGCAACCTATGGCTCCTGCCCTGGAGTTGTTCATAAGACC
Bos taurus     CACTCCTCAGGTAGGTTACTGACTGCTGGCTCAACTACCAGCATTCACCCAT-----GGTTAGTGAGCA--TGTCAGATGAATGATATATAGCTCTTGCCCTTGAGTTGCTTGTAAGACC

Homo sapiens   TTCCAGGCAGACAGCTATGGGACGAATACACTTGAATTTATTAAATCTACCTCAGGCAAAAAACCAAGCATGTAATTATAAGTAGTATATCAGAGTATGTGTAAATGGGGTTCTGTAAAA
Bos taurus     TTCTGGGTAGACACATACGGGAAGTACTCATTTAAATGTATTACATTTACCTGTGGTGGAAAAC-AAGCATG-ATCTGTAATTCGTATGCTAGACTATGTGTAAATGGGAA-CTGGAAAG

Homo sapiens   TGTTTGAAAGAGGCAGGCCTGGGTTAGTGGTGAGAACAAGGATCAAGAAGGCTCTGCCACCAAATAACAACATGAGTGTTGTCCCGTTAATCTCTCTGGGCTTCTGTTTCCAGATCAAGG
Bos taurus     GGCTTGAAAGAGACTGGACTAGTTTAATAGTAAGAATAAGGAACAGGAAGACTCCTAAACTAACTAGCTAC-------CTGTGCCCTTAACCTCTCTGGGCTTCTGTTTCCCCAGCCAGG

Homo sapiens   ACCTCAAAATAATAGTCATAGTCACCATTTATTGAGTGCTTTACTGTTAACCAGGCAGTATGCTAGGTGGTTTTCATTTAGTCTTGACAACAGT-ATTCTCGCTTTCCCTTTTCTACAGC
Bos taurus     GCCCCAAAATAATAGTCATAGCTACCATTTATTAGGTACTTTATTGTGCACCAGGCACCCTCCTAAGTGGTCTCCATGTAGTCTTGAAGACCGTTATTCTCAAGTTCCCTTCTCTGGAGC

Homo sapiens   TGAGACTCAGAGACATTAGGTAATGT---CTAGGTCACACAGCTCTTCAGTGACCAGTTGTTTCTATGTC-AGAATCCTCTTATAATCACTGAGTTGTATCGCTTCCCTTGCCTCCTGCT
Bos taurus     TCAGGCTCAGAGACTGTAGGCAATATGTCTTAGGTCACATAGCTCTTCAGTGGCTGATCATGTCTATCTCTAGAATCATCTCA-GGCCACTTACTTGCATCGCCTCCCTTACCTCCTGAC

Homo sapiens   C-----CCAAGTCAAGTATTCTCTACTATGTCGCCTGAAAATTAAAATTAAAATCAAGGTTAATCTTGTTCAACTGAGCCAAGAGGATAAACATTAGAAATAGCAATTAGTAGGTAGAAA
Bos taurus     TGAACTCCAAGGCAAGTGTGTTCTACTCTGTTGCCTTTAAATGAAAATTAAAATCAAGGAGAGTCTTGTTCAGCCGAGCCAAGAGGATAAACATTCGAAATAGCAATTAGTAGGTAGAAA

Homo sapiens   TGTTGCCAGGAAGAGCTATACAAAAGCATAGAAAGGCAACCAAGGGAGTTAATGATG---GGGGTGTGGCAGGGGTTGCATGTTGATGGGTAATTGGATCCTGGCCAGCTCTTACCTTCT
Bos taurus     TGTTGCCAGGAAGAGCTATACAAAAGCACAGAAAGACACCCAAGGGAGTTAACAATGTCGGGGGTGCGGCGGCGGTTGCACGTTGATGTTTAATTGGATCCCTGCCAGCCCTTGCCTTCC

Homo sapiens   TCCTTCACCTTCCCACTCCGCAATTTCTCAGGCTTCTCTAGAGAGCACAACCCACCTAGAGACAGCCTGTGTTGCCACAGGAGGGCACCAGGCCAGGAATGGCAGGCCTAGGGCAGTGGG
Bos taurus     TCTTTCACCTTCCTGCTCCGTAATTTCTCAGGTTTCTCTAGAGAGCACACCCCACCTAGGGGTAGCCAGAGCCACCACAGGAGGGCACCAGGCCAGGATTGGCAGGCCTAGGGCAGTTGG

Homo sapiens   GCTCTGGGCTGGGAACCATAAATAACCACTTTAATGAAAACCAGCTTTGGCCAAAAGCCTTATCATTTCTTAAGAGGTTCTGTGCTTCTTCTAATGACCTAGTTACTTGAGAAAGGCCTA
Bos taurus     GCTCTGGGCTGGAAACCATAAATAACAACTTTAATGAAAGGCAGCTTTGGCCAAGAGCCCTGTCATTTCTTAAGAGATTCTGGGCCTCATCTATGGACCTAGTTACTTGAGAAAGGCCCT

Homo sapiens   GCC-TAACATATACAACAACAATAAT--------AGTTGCCCTATAGTGAGTACTTACTATGGGCCAGGGCCTGTGCTAAACACT------CACTAATTCATTTAATCCTCACACAGCCC
Bos taurus     GCCATAGTATATATAACAAGGATAATGATATAACAGTTGCCCTTTAGTGAGCATTTACTATGGGCCAGGCCCT-TGCTGAACACTTCCAGACACTAACTCATTTAACCCTTAAGTAGCCC

Homo sapiens   TACAGGAGCAAGTACTATTATTTCCCCCACGTTACAGAGCAGGCATCAGGCTGAGAGAGGCTAAGTCATTTGCTCAAGATCATATAGCTGGTTGGAGCTGGAATTTGAAGCCTGAAGCAT
Bos taurus     TATGAAAACAACTGTTATGGTTTCTCTCATTTTCTATAGGAGGCGTCAGGCTCATAGAGGCTAAGTAACTTGCTCAAGTTC--ATAGCAAATGGGAGCTAGAATTTGAAGCCTGCAGCAT

Homo sapiens   CTAGTTCCAGGGCCTGAGCTCTTTAATTCCTATATTTAATGAATGTCTGTCGAAGGAATAAACAGATGAATGAATAGACAAATGT----ATTCATTTCATTTCACTTTAACAAACATTTA
```

```
Bos taurus    CTGGTTTGGGGGCTAGAGCTC-TTAATTCCTGTATTTAATAAATGTCTGCTAAAGGAATAAACAGATGAATGAATGGACAAATGTATTAATTCATTTCACTTTACTTTAACAAACATTTA

Homo sapiens  TTGAGTGCTCTGTGTCAGCTCCTGTGCTAGGCATTGAGGATTTATAAGTTTAGTGCGTGCGTGCGCGCACACACACACACACACAAACGCACACACACACACACACACATCCTGCATGGC
Bos taurus    TTGAGTGCTCTGTGTCAGCCCCTAGGCTGGGCATTGAGGATTCTAATGTT--------------------------AAACATTTAAACACAGGCACACCCACTC----------CATGGC

Homo sapiens  CCTTGCTCTCCAGGGGTTCACAAAAGCATTCTCTATTGTGGCTTGATTTCTTCTGGAGAGAAGTAATTTGAATGTCTTGAACAGCAGTTTTTAATGATGCTATTAAATTTAACCTAAAGC
Bos taurus    CCCTG---TGCACGAGCTCACAAAAGAGCTCTCTAATGTGGCATGATTTCTTCTGGGGAAAAGTAATTCGAGTTTCTTGAACAGCAGTTTTTAATGATGCTATTAAATTTAACCCAAAGC

Homo sapiens  GGGGGTTTTGCACCGGTGAGCAAAGTGTTTCAAATGAGAATGTGTCTTTGATTAATCATCGATTCAGCCAGAGAATAGTTACTACCAG-AATGGAGCTGCAACTCCTATTATATTAGCTA
Bos taurus    AGGGGTTGTGCACCGGTTAGCGAAAAGTTTCAAATGAGAAAGTGTCTTTGATTAGTCATCAATTCAGCCAGAGAATGGTTACTATCAGAAATGGAGCTACAACTCTGATCACATTAACTA

Homo sapiens  ACTCTTCTTCCATCTATCATGTCTCTATCTTGTCGAACCCTCCCACGATACAAAAGTTATCTGTACCCTAATGGACAATTATCCCATCCATCATTCCTGCTGGTCTTGTTGATTGCTGGT
Bos taurus    ACTCTTCTTCCATCTATCATGTCTATATGCTGTCGAACCCTCCCACGGTACAGAAATTATCTGTACTCTAATGGACAATTATCCCATCCATCAT--CTCCTGGTCCTGCTGATTGCTGGC

Homo sapiens  AGTCTGCTTGCCCACGGAGGAGACTTACAAATAGTCATGACCAGTGACGCACTGGAAAACTGCTCATTTTTAGACATTTTAAATCTATGTAGAATGAATTATTTTTCAGGCTGTAC-TTA
Bos taurus    TCTCTACTTGCCCACGGAAGGGAGTTATAAGTAGTCACGACCGATGATGCACTGGAAAATTGCTCATTTTTACACATTTCAAATCCATGCAGAATGAATGTTTTTGCAGGCTGCCCTTTA

Homo sapiens  AGCTGCATTTGCTTTGTTGGTTATGTAAGAAGAGA-TATGAATTCCTTACCTATGCGTGAGACATGGCGTC--TGAATATTTTTAAGTTCCTGTGGGCAGCAGCCCTATACATATTTGTT
Bos taurus    AGCTACATTTGTTTCCCTGGTTATATAAGAGCAGGTTATGAATTCTTTACCTGTATGTGAAATGTGGCCACTGTGAACATTCCAAAACCCCTCTAGGCAGCAACCCTATAAATA----TT

Homo sapiens  AAAATGATGAAAGAATAAAGTAAACATACCTAGAGTAATTCCAGAGGAGCAGGCTTCATACCTGGAGCTCGGGTGGGTGGCCA-TCATGTCAGGGTAGTCACCGTCTTCCTTTTCCTTTC
Bos taurus    AAAATAACGAATGAATG----AAACACACCTGAGGTTATAGCAGAGGTGTATGCCCGGTACCTTGGGCTTGGGCAGCTAGTCAGTCATGTCAGTGTGATCACTGTCTTTCTTTTCTTTTC

Homo sapiens  TAAGTACTTTGAGGAGAATCCCAAGTTGATTAGGGTCAAGGTGAGGACTCATGGTGCTGAAAATAGGGATTGAGTGGACAATGAGAGATGCCTGTTGAATTCCCTGGGACTATTGCTATG
Bos taurus    CGGGCATTTTGAAGAGAATCCCAAGTTGATTAGGGTCGAGGTAATGACTCATGATGCTGAAAGCAGGGGTTAAGTGGACAGTGAGAAATGCCTGTTAAATTCTCTGAAATGATTACTATG

Homo sapiens  AGATTTGAGGGCAGAAATGCCCCCCTCTCCCATGTCCTACCCTAAGCCTGGTAACACATTTAACTTTTTGTTACAGTAACTACCATGCCTAGTAAAACTGGAGTCACTGGTTGCCCAGGG
Bos taurus    ATATTTGAGGATAGAAATGCCCCGCTCCCCAACTGCTCAACCCAAGTCCGGTAATACCTCTAACTTTTGATTGCAGTAACCAACATGCCCAGTGCAATCAGACTCACCAGTTGCCCAAGG

Homo sapiens  CAAAAACCCAAGAGTTGTAGTTCACTCATCTTTATTTCTCACCTCTACATTCAGCCAGTCACTAAGCCCTGCCAAGTATATTTCCTAAGCATTCCTTGCATGTCACCTCTTTTCTCCATT
Bos taurus    CAAAAAGCCTAGAGTTGTTTTTCCTGCATCCTTATTTCTCACCTTTATGTTCAGCCAGTCAATAAGCCCTGCCAAGCTTACTTCCTAAGCGTTCCTCAAATCTGACC-CATTTCTCCATT

Homo sapiens  CCTACTGCCAGGGCTTTAGTTCAGGACTCCTCCCACCTCCAGACATCTCTTGCCTTGATTCCTACAATACCTTCTGGTTTTCTCACCTTCTCTCCTTAGTCCTCTGTGACCATTCTCCCC
Bos taurus    TCTCCTGCCAGTGCTTTAGTTCATGACT-------TCCTCAGTCTTCTCTTGCCTTGATTCCCTCAGTAGCTTCTTGTTTCCTCACCTCTCATTTTC--CCCTTGGTGATGATTCTCCTC

Homo sapiens  AAAGTGGCCTGAGTGATCTTTCAAACATGCAAATCAAATCATTTCACACCTCCATCCCCT------------------CCACTTAACACT-----------------------TGTAAAT
Bos taurus    AAAGTGGTCTGAACTATCTTTCCAAGAGGCAGGTCAGACCATTTCCCACCACCATCCTCTGGAGAAGGGAATGGCAACCCACTTAACATTTCTTTTCTTTCTTCCACTGAAGTGTAACT

Homo sapiens  AGCCTCACAACACCTGAGACTTGAGGCTCTCAAAATGTGGGTCCTGGGGCCAGCAACACCAGCATCTCTGGCAACTTCTTAAAGTTAGAGTACGTACTCATTAGAGTACCTACTGAATCA
Bos taurus    CACTTTACAATACCTGAGACCAGAAGCTCTCAAAGTGT-AGTCATGGGGCCAGCAGCCACAGCATCTCTGAAAACCT------GTTAAAGTAGGTACTTCTTAGAG-ACCTACTGAATGA

Homo sapiens  GAAACTCTGAGAGTGCTCACCTGACTCTGTGTTTAAC-AGCCCTACAGGTGATTCTGATGAGCACTCAAATTTGAGAACTGGCACCCTAGAGCCATGGTTCTCAATCCTGGCTGCACATT
Bos taurus    GAAACTCAGGGTACACCACCCCCCATTTGGGTCCGACAAGCCCTCTGGGTGATTCTTATGAGTGCTCAGATTT-AGACCTCCCACCCTAAAGCCCTGGTTCACAATTCTGCTTGCACATT

Homo sapiens  CAAATAAATGGAGGGAGCTTTTAAAAGTTCTAATGCCTCTCCTGCAGAGTAATTAAATC--AGTTTCTAGGGGTGGGATCCAGTCATAAGTATTGTTTTTAAAGTTCCCCAGGTGCTTCT
Bos taurus    AGTATAAACCTGGGGAGCTTTTAAACATTCCAACACCTCCCCAATGGGCTTATTAAATCTGAGTCCCTGGGAGTGGGAGCTATTCATCCGTGTT-TTTTAAGAGTTCCCCAGGTGATTTC

Homo sapiens  AATATACAGTCAAGGCTGAGAACTACTGCTCTATAGGATGAAGTCCAAACTCCTTAGCTTGGCATTTAAGGTCTATCTCTTTGGTCATAACCACCCCCAACACTATGCTCTTGCT-TGTT
Bos taurus    AGTTGGCAGTCAAGGCTGACGACTACTGTTCTTCAAGATGAAGTCCCAA---------------------------CTCTTCGGTCCTGTCTCCCC--------ACGCTCCTTCTATCCT

Homo sapiens  CATTCTAGAATTCAATCACAGGGAGTCATTTGCAATTCTCTTAGAGCCACACATGGTCTCTTACCTCTGTGCATTTGCACACATCATTCCCACTGCTGTGCCCATCCCTTCCTCCCTCCC
Bos taurus    CCTTCTAG-----AATCATAGGGAATCATTTACACTTCTCTGAG-GCCACA----GTCTCTTATCTCTGAGCATCTGCACACATCTCCCCCCTT------CCCGCCCCTCCCTCTCCCCC

Homo sapiens  TTCACCCAGCTAACACCATCTCATCTACAACACATAGCTCAGAAGACACCTCTAAGGAACAGTTTTCCCCG-----CCTGTCCAGTCTGGGTTAGATGCCCCTCCTCTGTACTCCCACAG
Bos taurus    ---------CTGATGCCTTCTTGTCTACAACACAGAGATCAGAAGACACCTCTACAGAAGAGCCTTCCCTGATCCCCCCTCCCTGTCTGGGTGAGATGCCTCTCCCCTGAACACCCACAG

Homo sapiens  CGTCCACAACATAGCACTGGATACATTCTCTTGTTCTTCTCTGGTTAGTTGTCTACTTTCTCTACCAGTCTGAGAGCTAGGATCCCCA---------CCTGGCACACAGTAGATGTTCCA
Bos taurus    CATCCACATTACAGCACTTTCCCCATCATCTCGTTATTCTCTGCTTAGTTGTCTGCTTCCTCTAGCAGCCTGAGAACAGGGACTCTCAAGGACAGTGCCTGGCACATAGTAGACGTGGCA

Homo sapiens  TGATTCTGTACATCCTGTTCCCTCTTTTTGGAGTGACTTTCTGTGCCTTGTCTGTGG-----------------CCTTCAGGATGCAGCTCAGATATCACTTCTTG---GGAATCTCAGA
Bos taurus    TGATCTTACACATTCTATGCCCTCTTCCTGGGTTGCTTGCCTGTACCTTGTCCATGGGGTGAATTCCTATACCACCTTCAACATGCAGCTCAGATATCGCATCTTGGTAGAAATCTCTGA

Homo sapiens  ATTCTCTGTCT-------GGACCCTTTCTCTAGGCTCCCGCAACAATCTAAATGTAATCTGATCCTAGCCCTTAGCACACAGGGCTATCAGGCTGCCATAGTCTGTTTACTTATCTGCCT
Bos taurus    ATCCCCTGTCTGGGTTAGGGACTTTTTCTCTGGGTTCCCACAACACTCTCACCTTAATCTGGTCCTGGCCCTTAGCACATGG--------GGTGGTCATGGTCTGTTTACTTATTTGTCT

Homo sapiens  CCCTCACTGAACTGTAAGTAACTTGAGGTTAGGGACAGAGCCTTGTACATTCCTGTATCCCAGCTCCTAGCACAAAGACTCACACATTATAGGTGCTCAGTAGCCATAACCATCTGCAGA
Bos taurus    CCTT------GCTGGGAGCAGCTTGAGGTCAGGGGTGGGGCCGTGTGCAACCCCAAATCTCAGCCCCTAGCACTGTGCCTAGCACATCTTATGTGCTCAG------TAAACATCTGGAGA

Homo sapiens  ACGAATAACTGAATCA-ATGATGACCTTTTCCTCTTCTCCATATCATCAGCCACTATTTCTGTCCAGGCCCCCGTTGCTTTATTGCAATAGCCTTCTGACTATTTCCCATCTCTCCTTTC
Bos taurus    ATGGGTAACTGAGTAATGTGAAGACCTTTTC--CTTCTCCATGTCTGCTGTCACTATTCCTGTCTGGACCATGGTTGCTTTATTGCAATAGTGCCCTGACTAATCCTCATCTCTGCTTTC

Homo sapiens  TCTGATGCCATCTGAACACTCCCCCCCTCTCCA----------AG------ATGTGGCTTTCTAAAACCACAGTCACCCTATCCCTCCCCTGCTCAAACACTTTCAGTCATTCCTCATTG
Bos taurus    TCCAGT-CCGTCTGTTACCTGCCTCCCCCACCACCACCCACCCAGTTTCCCACGTGGCTTTCTAAAACCACAGTCACCACATCCCTCTTGGACTCAAACACTTACAGTGATTCCTCATTG

Homo sapiens  TCTGCAGGGCAAATATCAAAGTGGAATTCCAGATCCTTCCTTCCCAAGAGTGGTCTGCCTTCCCAACCATACCTCCCCCCATTCCCC--TATGGAATACCAGCCACATCATCTGAGGGT-
Bos taurus    TCTGCAGAGCAGATATCAGAGTGGCATTCAAGG----TCCTTCCCAACAGT-GCCCGTCTTCCT-GCCATA-CTCTCCTCATTGCCCTATATATAGGACTGGTTATACCATTTGTGGGTG

Homo sapiens  ---CCTGGTGCAAAACGAAAATGTGGAACCCCGTGTTAAAATATA-ATCAAGGATTTCAAGATAGCAGCAAAGGAGAGCATTAAACCAAGCAT----AGGGCTCTTCTGACTTCAGGGTC
Bos taurus    GGGTCTGGGGCAAAATGAAAATGTAGAGTCCCCTTGTAAAAAATGTATTCAGATGTTCAAGACAGCAAC--AGCAGCACATTCAACCAAGCATGGTGGGGGCCCTTGTGAGTGCTAGGTC

Homo sapiens  CGATGCGACGGCACAGGTCTGCCCCTACGTGTATCCAGCGCACTGGCCAAATGCACTTGCTCCTCCTTCAACTGCTCTGTAGGTTCACTTTCTCCACGGCTTTTGTCAGGCTGTT-CCAT
Bos taurus    CTGTGCTTGGGCACAGGCCTGCCCCTGCATATAGCCAGTGTGCTGGCCGGATGAATTCACTGTTCCCTAAACTGCTGTGTGGGCTCATTTTCTCCATAATGTTGGTCAGGCTGTTCCCAT

Homo sapiens  CTCTTCTACCCCACTAGCCAATTCAAAGCTTGTCTTCCCTGTAGATCCA------CCCTGTTCTTCAAGAATTGCTCAATTCTCATCTCCTACTGTTCCAGACCACAGTAGTTTCTACCA
Bos taurus    CTCTTCTACTGCTGTCACCACTCCAAAGCTCCTCTTCCCCACAGATCTGCCTCTTCCCTGTCCTTTGGG--------------TACCTCCTACCATTTCAGACCACAGTAGTTTCTACCA

Homo sapiens  CACAGCCTGATATTAATAATTATTTTTATATGTGTGTAGTCTTGCCTGTCTACTGAATTTTAAGCTTCTTGAGGGCAAGGACTCTATTTTGTTCATCTTTTT--TTTTTATAGACTTAGC
Bos taurus    CACAACCTGAGAGCCATGATTATTTT--TCTGTATGTAGTCATGTCTCTGTACTGACTTTTAAGCTACTTGAGGGCAGAGATACTCTTGTGTTCATCTTGGCAGTTTCTACAGATCTAAC

Homo sapiens  ATAGTACTTTGTAATCATCTCACTT-GTTGAATGACTCAGGGAAGAAATAATACAACTCCAGTCACTGAGTTTGCCAAAAAACAGTAGACACTGCAAACCTGATTTAAATCCCTCATTAT
Bos taurus    A-AGTGCTTTGTACTCATCTCAGTCAATTGACTGACTCAGGGAAAATATGGCCCAGCTCCAGTAACCAAGTGTGCCAAAAAACATTAGACACTGAGAACCTGACTTGAGTCCCTCGTGAT

Homo sapiens  TAGCTATGCAACATTGAAGGAGTCACCTC--------ACTTCTCTTTGCCATCTATAAAACGATGATACTTAGAGAAAAAGTGTTTTTTTTTTTTTTTGTTTTGTTTTTTTGGTTTTTTT
Bos taurus    TAGCTGTGTAATATTGAAAGAGTCACTTCCTTCAAAGCCCTAGCTTTGCCCTCTACAAAA-GATGATACTT...............................................

Homo sapiens  GTTTTTACAGAGGCTTGCTCTCTTGCCCAGGCTGGAGTACAGTGGCATGCAATCTCGGCTCACTGCAACCTCCGCCTCCTGGGTTCAAGTGATTCTCCTGCCTCAGCCTCCCAAGTAGCT
Bos taurus    ........................................................................................................................

Homo sapiens  GGGATTACGGGCCCATGCCACCATGCCCTGCTAATTTTGTATTTTTAGTAGAGATGGGGTTTCACCATGTTGGCCAGGCTGGTCTTGAACTCCTGACCTCAAGTGTTCCACCTGTCTCGG
Bos taurus    ........................................................................................................................

Homo sapiens  CCTCCCAAAGTGCTGGGATTACAGGCGTTGAGCCACCATGCCTGGCTAGAAAAAGTAATTTTTTGTTAGTATCTTGTTCTTTGGCAATGTTTTCAAAATC---TTTTGTTTTTTAATACA
Bos taurus    ....................................................AATTATTTTCTGCTAGTATCTTGTCCTCTGCTAATGTTTTCAAAATCCTCTTTTGTTTTTTAATGCA

Homo sapiens  TGTAAAACTTAT-----TTTATATTTTATATCACCTAATTGCAATACCTGAGGTCTTTGAAGACCTGATTCTGTTTTCATTGTTTCTGGCTTCATCGTGTTCTTTGTAATTTTGGATTAT
Bos taurus    CCTAAAATTTTTAAAAATACATATTTTATATGACATAATTGCAATATCTGAA--TCTTGAGGATCTGATTCTGTTTCTGTTTTTTCTGGCTTCATGGTGTGCTTTGTAATTTTGGATTAT

Homo sapiens  GAGTTCATGTTCAACCTGACTTTATCCTTGGTAATCCTATGAAAACTGGGTGGTGGGCATGTTCTTCCAGGGAGGATTTGTGTTTGGTTCTTCCAAGTGCATCAGGGCACTATTAATCCA
Bos taurus    GAGTTCATGTTCAGCCAGACTCTATTGATGGGAATCC----GGAAGCAGATGGAAGGTGTGTTCCTCCAGTAAGAATTTGTGTTTGCTTCTTCCAGGTGCTTCAGGGCATTGTTAACCCA

Homo sapiens  GGAGTTCTTCCAGTGAATTGTTTAGTCCCAAAGGCAAGGGCCATGTCTTCTTTGGCTATATGATCCCAGGGCTTAACTCAGGGACTTGTAGATGCTCAACATATATTTGCTGATTGAGTG
Bos taurus    GCAATACTTTGAGTGAACTGTTTATTCCCAAACACAAGAGCTGTGCCTTATTTGTCTATATGATCCAAG-------CTTAG---CTTGTAGATGCTCAATGTGGATTTGTTGATTGAATG

Homo sapiens  GATGAATGAATGTGGTACTTTACCGCTTCTTTTGGAAGCCCAATAAGAATTGTGACCTCTGGTATTAACAAAGAACAGTAGGCACATCAGGAAAATAATAATTACTAACATTTATTGAGC
Bos taurus    GATGAATGAATAAGAC-CTCTATGAGTTTTTTCTGAAGCCCAATAAGAACTCTAATCTCTGGTGGTAACAAAGGACAGTAGGTGCATTAGGAAAATAAT---TACTAACATTTATTGAGT

Homo sapiens  AATGATTATGTGTCAGGTATCATTCTAAGTGCTTTGTGTGTATTAACTCATTTAATTCTCACTATGATGCTGTGAGGTAGGTTACTATTATTATCTTCACATTACAGAGGAGGCACCAGA
Bos taurus    ACT---TATGTGCCAGGTATCATTCTTAGTGCACCGTGTGTATTAATTCACTTGATTCTCACTATAACACTGTGAGGTAGGT-ACTATTGTTGTCTTCATTTTACAGAGGAGGC------

Homo sapiens  CTCAGACAGGCTGAATAACTTGGCCAAGGTCACATAGCTAGTGAATGGCGGAACACAGATATGAACCCAGG---------CACCCTAGAGACACATCTACTTACCACCTTGTATTGGAGA
Bos taurus    -TCAGACAGGCTGAGTAACTTGGCCAAGGTCACACAGCTAGTGAATGGTGGAACTCAGATATGAACCCAGAGATACATCCCACCCTGAAGATACATTTAACCACCATCTTATATTGAAGG

Homo sapiens  AGAGGTCAACAGTAGTGACAGAAGCAATA-GAGTGGAAATTTTCAGTACTGCCAAA---ACTATGAGAGCCATATAGTCCACACCAGGCATGAAAAGGAAGCTGGCATGGTCTTGTGTTG
```

```
Bos taurus    AGAGAGGAATAGTCATGACAGTAGCAGTAAGAGTAGAAAATTTCCATACTGTTGGAGGGAACTCAGGAGTCACCCAGGCCACACCAGGCATGGAGGGAAACATGGCATAATCTTGTATCA

Homo sapiens  TCAGTTAACCCTTGTATATAGGGTTTTCATTTCCTAACTAGAGAAGGAGGAGTTTGAAAGCACGAACCATGAATTACAGCTCTTTGAAATGCTCAACATAGATTTGCTGAGTGAATAAAT
Bos taurus    TCAGTTAACCCTTGTATGTAGG-CTTCCCCGCCCCAGCTAGAATAGGAGCAGTTTGAAGGCAGGAACAATGGTTTACAACTCATTGAAATGTTTCAAAGATATTTGCTGAATGAACAAAT

Homo sapiens  GACTGTTTTGAAATT---------AAACACAGGAGTGCTTTGAATAAATTACTTTGCATCTTGTATGTCAAACTGAAATTGGTGAGACATCTTTCACCTTGTGTTAAAAACTAAAAATCC
Bos taurus    GAAT---TTAAAATTCATTTAAGTAAACACATGAATCCTTTAAATAAAGTATATCACATGTTACATGTCAGAC--AAACTGGCGATAAAGATTTCACTTTACTTTAAAATTTGAAAATGC

Homo sapiens  CTAGATGTGGTCATAACATGGCTGGTTATAATGACACCTTGCATTTGTACTTGTGTATGATACTCAGGACTCCCTGAGAAAAGGAATGAAGAAACTTGGATTTTGCCTTCATAGGCCCTG
Bos taurus    CTAGATGTGACCATAACACAGTCTGGTACAGTCACACCTTACATTTGTCTCAGCGCATGCCACTTAGGACCCTTCGGGAAA--GAAGGAAGAAACTTGGATTCCACCTTCATAGGCCCTG

Homo sapiens  AGGCTGTCAGGAACCAAGGGCCACTGTGTTTGTAATCCATTCCTTTGGCTGCTCTCCAAATCTAGGAAGACCTGGAGGAAAGGGGGGCTTTATTTTCT---ATCTTTGCCAAGATCAGGA
Bos taurus    AGGCTGTCAGGAACCAAAGGCCACTGTATTTGTAACCCATCCCTT-GGCTGCTATCCAGATCTAGGAGGGCCTGAAGGAAAGAGGGGCTTTATTTTCTCTTGTCCTTGCCAAGGCTAGGA

Homo sapiens  AATAATTTG----TGAACTCTCAGTAAACATTCTGGGGGCCTTCCCTCTAACACTGCTTTGGTGGAATAATATCTCCAGTTTTCACCAATCTTGGTTCAATATTTACCTGGGGGTTTGCA
Bos taurus    AAGAATTTGAGTGTGGACTCTCAGTAAACATTCTGGG--TCTTCCCTCCAGCACTGGTCTGATGGAATGAAATCCCCAGTCTCTACCAATCTTGGCTCAATATTTACCTGGGGGTTTGCA

Homo sapiens  GTGATCATGGGGACAGGGCTCCAACATGAAGTGAAGGTTCATGAGAAGGAAAATGAGGGGAAGGCTGTGTGTGTGTGTGTGTGTGTG-TGTGTGTGTGCTGGCTGAGGGATGAAGGTGGG
Bos taurus    GTGAGCCTGGGGACAGGGTTCCAGAATGAAGAGGAATG----GAGTTGGGGAGTGAGGGGAGGGGTGTGTGTGTGTGTATGTGTGTACTGTGTGAGGAATGGC--AGGCGTGG-GGTGGG

Homo sapiens  AGGAATACCAGGATTCTGAAGCAATGAGAGTGGGATTGGGAGAGAGAGCTCATGTTGAAGAAGAAATAACTCACCAGTTCACATGGGCTCCTTTTCAAGGGTTTTTCTTTATATAAACAA
Bos taurus    TGG--TACCAGG-TTGCAAGGCAATGAGAGTGAGGTCTGGACAGAGAG----TGTTGGAGAAGAAATAACTCCTTAGTACATACTGGTTCCCTTCCCAGGACTTCCCCTTGCTGAAACAA

Homo sapiens  TTTGCCTGGTTCCAAT--TAGAAGCCCACATT-AGGCAGTTAGCTCCAATTTGGCAGTTCATTCTTAATTTGGCTTTCCTTATGGGTGGGAG--GGAGGGCTGACAGTTAAAAGGTTGAT
Bos taurus    CTTGCCTGATCCTCATGTTAGAAGCTCAGATTGAGGCAGTTAGCCACC------CAG---GTTCCCAGCTTGACTTTCCTTATGGGTGTGTGTGGAGGGGGTGATGATTAAGAGG-----

Homo sapiens  GGGAAAAACCTTGGTATCCTGGTGTGAAGACAGACCCTGGGATCAGAAGGTTGCCCTGATGACTTTGCCAATGTGTTCATTGCCCCTGTCCTGCTGGGGCGCATACAGCAGGCCCTTCTT
Bos taurus    -----------------------GTGAAGAGAGGCTCTGGGAATAGGAGTTTGCCCC-ATGACTGTGCTGATATATTCATTGCCTCTATCCTGCTAGTGGACACACAGCAAGCCCTTCCC

Homo sapiens  TCTCATACGCCCTTGACAGACCAGGACTCACTGCTAGAAAACCCCTGGGGAAGTCATTGCTGAAGACTCTAGTGTGGGGCACATTAGGTTTGAGGAGCAGGGGAAACATTCAGGCAGCTG
Bos taurus    TCTCCTACTCCCTGGGCAGACCAGGACTCAGTGCTAGAAAACCCTTGGGGACGTCATTGCCAAAGAATGTAATATGGAGCCCGTTGGATCTGAGGAGCATGAGCAACATTCAGGCAGCTG

Homo sapiens  GCAAGGTCTGGGCTACCAGGAGAGATTTGGGAGTTAGAAGCATATTAGGTGAGAGTTGACACTGTCCAAAGGGAGTATATAGAAAGAAAAGCAGAGGAAAGGGCTCATGGAGTCATTACA
Bos taurus    GCAGGGTCTTGGCTG--GAAAGAGATATGGAAGTTAGGAGCATGT-AGGTGAGAGTTGAAACTGCCCAAAGGGAGTACATAGAAAGAAAAGCAGAGGAAAGGGCTTGCGGAGGTATTATG

Homo sapiens  GCCTGTGAGGTGGGAACAGGGTTTGGGAGTACTAAGGGTTTGGGGAGGGTTTATGTGTTTATTCACCCATTCATTTATTCAACATTTCTTGAGCTTTATGGCAGATGCTGAGTT-GAAGA
Bos taurus    GTCTGTGAAGTGGGAACAGGACATGCGAGTCATA--GGTTTGAAGAGCCTTCGTGTATTCATCCATCCATCCATTCATTCAACGTTTACTGAGCTTTGTGCCAGATACCAAGTTTGAAGA

Homo sapiens  GGAAGCTGGGGATTTAAGAGGGAGAAGAGTTAAGAGGGGATCCCTGGGTGGATCCAGATGAAGGAGAAGGGAGGGGCTAAGTGCCTAGGCTCGCTCTTGCCAGGAGAGCACATCTGTTAA
Bos taurus    AGAAGTTGGGGATTCAAGAGGGAGAAGGGTTAAGAGGGGACCCCTGGGTGGATTCAGATGAAGGAGAAGGGAGGGGCAGAGTTGCTGGGTTCTCTCCTGCTAGGAGAGCAGGTCTGTTAA

Homo sapiens  CACAAAGCTGCATCTGTATCTCAGCCAGCTCTCTT-CCATTTCATCTCCTCCTCTCTACCCACACTTGCAAGACGGGGAGCCACATGGCGGCTTCCTACTCCCTTGTACCACTACACCCC
Bos taurus    CACAAAGCTGCATCTGCCTCCCAGCCTGCTCCCCCACCCCCGCACCTCCTCCTGTCTTCTCATCCACACAAG--------CCACATGGTGGCTTTCTATTGCCCTGTACC---------C

Homo sapiens  CTGCCGGGTTCTTTCTAGGCATTTAGCTGTGGCATCAGCAGCACCCCTTCTGAGAGATCAGGCAATCCCCAAGGGTGAG-TGGTAGTGGTAGTGGTGGGGAGGGTTGATAGCTTCGAGAG
Bos taurus    CTTCCACGTTCTTTCTGGGCACTTAACTGTGGCGCCAGCAGCACCCCTACTGAGAGCTCAGGCAATCTCCAGGGGCGGGCTGGTAGGGGCGGTG-CGGGGAGGGATGACAGTTTGGAAAA

Homo sapiens  AA-GAGC----TGGAGAAGGCGCTGTCTGGCTGTAATTAGCCCCAGCTGCTGTTGTGAGGCAGCGTTGGGGGCCGTCAGGAGTGTACATGCATGTGTATGAACCTGTGAGCGTGGCACCT
Bos taurus    AAAGAGCCGGATGGAAAAGGAGCTGTCTGTCTGTAATTAGCTCCAGCTGCTGTTGCGCGGCAGCGCTGGGGGC-GTCAGGAGTGTGAATGCATGCGGATGAACCTGCGTGCGTGGCTCCT

Homo sapiens  CATTGCTATCACTCTATTTTTCACTGTATTGTGTGCTGGCTGCAGCCTCGCTGCTCTGAAACCCCTCCTGTCCCTGAGGATCCCGAAAGACAGAGTGTTCCACAGGCAGCACCAGCTTGA
Bos taurus    CATTGCTATCACT--ATTTTTCACCGTCTTGTGTGCTGGCTTCAGCCTCGCAGCTCTGCAACCTCTCCTGTCCCCGAAGGTCCCAAAGGACAGAGTGTTCCACAGGCAGCACCAGCTTGA

Homo sapiens  ATCACTAATGCTATTAAGCAGGAGGACAACAAATATAGGCCCCCATATGTCCTGGATATGCAAGTCCCTGCTCTCTCTTTCCCCACCCCTGCTTCCTGAATGTTTCCAAGTGAGTGAAGT
Bos taurus    ATCACTAACGCTGCTAAGCAGAAGGACAACAAATCTAGGCCCCCATATGTCCTGGATACACGAGCCCTTGCTCTCTCC--CTCCACCCACGCTTCCTGAATGTTTCCTACTGACTGAAG-

Homo sapiens  CCCAGCACTTGAAATTAGCAACACCTCACTTCTGCACAGCCTTTTGGCAAAGAGCTTTCACAGCTGCCATGTCATGCATCCTCAGACGCTTCTAGGAGAAGCATTTTATAGGTGAGGACC
Bos taurus    CCCAGCACTTGAAATTAACAACACCTTAA-----------CTTCTGGCAAAGAGCTTTCACAGCTGGCAGGCTGTGCCTCCTCAGACAGCCAGAGGGGCAGCATTTTACAGGTGAGGACC

Homo sapiens  CTGTGGTTCAGGGAGGTGGAGAGATTTGCACAGGCTAACCCAGACAGTCAGTGGCAGACTTGTGTCCAGGAAGCAAGTCCTCGACCTCCTGGCCTGGTGGCCCATCCCCAGCAGAGCACT
Bos taurus    CCGAGACCCAGGCAAGCAGGGAGATTGGCGCAGGGTCACCTGGCCAGTTGGTGGCAGAGCTGTGTCTGGGATGCA-------------------GGTGGCCAGTCCCCAGCAGGGCGCT

Homo sapiens  GCCAAGAGGGCTGCTCCCGGGTGGCTCCAGCTGCTGCTGTCTGTGAGCTCGTGAGAACCTGTCAGCTGTGGCTCCAAGACCTGCCTCAGTGATTGCTGAAAACGCTGAGAGTGCTGAAAA
Bos taurus    GCCAAGAGGGCTGATACCGGGTGGCGCTGGCT-TTGCTCTCTGTG--CTCCCGAGAATCTGGAAATCACGACTCCAAGACCTGGCTCAGCAACTGATGAAATC-----------------

Homo sapiens  CCAGGACC-CTCAAGCCGAGCCTGACGTCACATAGTTGATTGAGTACCTGCTATGTGCCCACCACTGTGCTGGTGCTTATGGGAGAC-AAAACAGGGGTAGTGCATAGCCCGTATCCTCA
Bos taurus    -TAGGACCTCTGAGGCTGAGTCCA--GACTAAGAGCTTATTTAGAGCCTGCCGCGTGCCCACCACTGAGCCAGTGCTTATGGTGGCCTGAAACAGGGGTCCAGCATAGCCCCTGCTCTTA

Homo sapiens  AAGAAGTGATAACTTGAATGGGGAAACAACTCTTAGATACCAAGTCCAAAGGCAATTCCAAGCACTTCTCTCTCTTCCACTAAGCAGTCTGTTGTCAGTGACAATGACACAGGAACCCCT
Bos taurus    AAGAAATGACAACCTGAATGGAGTGACTGAGCTTAGATACTAAGTCCAAAGG-AATCCCAAGGGCTTCCCTCTTTTCTGCTAACCAGTCTGTTACCAGGGACCATAACTCAGAGACCCCT

Homo sapiens  TCTGAATTCATCTGTCTTAT--CGCAGGCCTGGGGTAGGAGAGGATGGGAGGATGACTAGGAAGGG-TAATGAGGAAGGGCAGGGAGATGGGGGAAGGAAGGGAAAAACTGGAGCTCCTA
Bos taurus    GC---ATTTACCTCCCTCCTGACCCAGCCCTG-------------TAGAGTGATGGGTAGCAAGGGCTAAGGGTGAAGGATAGGGAAGATGGGGGAAGAAAGGAAAAACTAGAGTTCCTT

Homo sapiens  ATAAACACCAGGCACATTGTGAAGTTTATATGTTATACATAGGTCATTTGTATTATGCTTATCATACATGTACATGAATATATACACCCAAACACATACACAAACACATACACAATCACT
Bos taurus    CTAGGTGTCAGGCA--GTGTGCTTGATACATGTTATAAATATGTCATCTAAAT-----------------------GACATCTATATCTTTATACATAAACACATGCATACCTAACC---

Homo sapiens  TTCCAGGGATTGACTCATCTATTTGGCTCACTCTCAATCATGAAAAGAGCTTAGGACAGTGAC--------ACTTTTAGAAAGCCCTGAATGAGTGTTAACTAGCCTTTCTTCTTATCAT
Bos taurus    ---------------------------TGACTCTCATCCATATAAAGAGTTTAAGACAGTGTCTGGTAGGTATTAATAGAAAGCACCAAGAGGATGCTAACTATTCTTCCTTCATATCAT

Homo sapiens  GATCATCTTCATTGTCATTATCGGCATATTATATAAATTATCTCATTTATTTCTTCTAACTACAGGACTTAGCAACTATTATTTTTATTTCCAGTTTACAAATGAGGAAACCAGTATCTT
Bos taurus    CGCCATCTTCTTTATCATTATC-----------TACATTATCTCAGTTAATCCTTCTAACTCTGGGAGGTAGCAACTATTCTTTTCATGATGCACCTAAAAATAGGGAAGCCAAGAGTTT

Homo sapiens  ATTTTATCCAAGCTTGTGGGTACTGAGAATATAAAGAGGAATTCAATATGGTTCTA--GGCAAACTTTGGGGTCTTCCCTATCTAGTGGGAGGGACCGAGGCACACATAAGCCCTCATGG
Bos taurus    ATTGTATTCAA-----------CTGAGAATGCAAATGAGAACCCAATACCTTTGTATTGGTAGCCTTTGGGGACCTCTCCATCTCGCAGGACTAAT-GGGGCACACATAAATACTCATGG

Homo sapiens  TCCAATAGGTGCTGTGGTAGAGGAACGTA----------------------------TCTGG-----GGAAGTTGAGGAAGTCTCCACGAATGAAGGAGGCTTGAGCAGCATCTTGAGGG
Bos taurus    TACAGC-TGTGCTGTGGTCGAAGGGAGCAGCATCACCCGGGCACTGATGGTAAGTGATCTGGTATCCAGAAGTT-AGGTGGGCTTTTCAGATGAAGGACATTTGAGCTGAATTTTGAGGG

Homo sapiens  ATGGCAGGTTATGTCAGGAGGAACAGTGGAGGCAGGGGAAGACATTCCAGGCAAAGTGAGCAGTATTTGTGTGACAGGGCCTAGTGCATGTTTCAAAGGGCAGGA---------------
Bos taurus    ATGGAGGATTTGGCCAGGAGGAGCAGTGGAGGGAGGGGAAGGACATTTGGGCAGAGGGAGCAGTATTTGCATGAAAGAGCCT-------GTTCCAGAGGGCGGGAGGATGGTTGGGGTGG

Homo sapiens  --------------------CAATTGTAGCTGGG----TGCACCACTGGCAGCAGCAGGAGGGGAGTGGACGAGTATTTGGGGTCAGCT-ATTCAGAGCCCTATTGGCCAAGCTGAAGGT
Bos taurus    CGTGGGGGTGGTGGTGGGGGTGATTGGGGTTGGGGTGGTGCGCAATTGGCAGCAGCAGAGGGGG--TGGAAAGGTGGTTGGGAGCAGCTTATACTAGGCTTCGTTGGCCA-GCTGAGTTT

Homo sapiens  T-----TTGTTCTGTGAGCAAGAGGGAGCCACTGGAGCTTTTTATTAAGGCAGATTCGGGACAGACTAGGAAGACAGTCCTTACCAGGGTGTTGAAAGATTGGAGGGAAGAGAGGCCAGC
Bos taurus    TGCACCTTGTTCTGTGAGCAAAGGGGC-----------------TCAAAGTGGGTT...............................................................

Homo sapiens  TGCATGCGGACCAATTGGGAGACCATCTCCAGGGTCTGGGCAAGAAGTGGAACAGGCTCTGAACCACGACCGTGGCAGTGAGAGCCGAGAGGAGGGGATGACTCAAGGCACATGTCAACA
Bos taurus    .......AGACCGATTGGGAAGCCATCTCAAGG-------CATGAAGTGGCAGGAGCTCTGGCTCAGGAGTGTG-CAGTGGGAGC-------AGGAAACCATTCCAGGGACATGTCA---

Homo sapiens  GGTAGAAACCTCAGGGCTGGCTGTCATGGGGT--CAAGGAGAGGGAAAAGGTGACAAGGACCCTGAGGTTCCAGCTTGGGTCCGTGGTGATACTGTCAACTGAGGCAGGGAACACAAGAC
Bos taurus    --CAGAAACCACAGGGCTAA-TGTTGTGGAGTATTAAGGAGAGAGAAAAGGTGACAATGACCCTGAGCTTCCAGCTCGGGCCCCTGGTGTTGCTGTTTACAGAGACAGGG----------

Homo sapiens  TGTTAGTAGAGAGGGAGAGTGAGGGGAGACAGATGACAAGGTCTGTTTGGACTTGCTGGGTTTGGAGTGCTGGA------TAGCGGACATGTCCAGCAAGAAACTGGGAATCTTGGCTTT
Bos taurus    --TTGGCAGGG-----GCATGAGTGGGGACAGGCGACAAGTTCAGTG-GGACTCGTAGGGTGTGGGTTGCCAGTGGGACTTAGTGGACATATCCAGCAGGAAGTGGGG-----------

Homo sapiens  GAGCCCAGCTGAGACATGGGGGCTGGAAAGCCACCACAGTC-AAGGGATATTAGTTGACCTCAGGGGACTGGATGGGGTTTCTCCTCTCCCATTTTACTCATCCAGGGCAAGGGCTAGGG
Bos taurus    ---------TGAGAGCTGAGGGCTGAGAACCCACCAGGGTCTAAGTGGCATTAGCTGACCCTGTGGGACCGGATGGAGTATCTCTTGTCCCGTTT-ACTCCTACAGGACAAGGGCTGGGG

Homo sapiens  ACAGAGCCAGGTGAATACCAGTATTTAATGAGGGAGGTGGTGGGGGACACCAGCCCGGAGTGAGAAGACACTCTTCTTTGGACCTTCCCAGAAACATCATCCCTTTCCCCTCATGATCAT
Bos taurus    ACAGAGCCAGGGGAGCCCCAGCATGAAAGGGGTGTGATGGCGGGG-ACACTGGCCCAGGGTGAGAAGACAC--------AGCCCCTTCCAGAAACCCCGTCCCTT-CTCCTCAAGACCAG

Homo sapiens  GGTCCTTGTTCCAGGGCTGCACTCAGGCCAGTGACAGAAGGAAGGGAGGGACAGAGGGAGAAAGGGAGGAAAGAAGGGAGGGAAGGAGGGAGGGGGGAGGAAAGAAGGGAAGGAAGGAGG
Bos taurus    TGTCCCTTTTCCATGGCTATGCTCCCACCAGTGA.....................................................................................

Homo sapiens  GAGGGGGGAGGAGGAAGGAGGGAGGGAGGGGGAAGAAGGAGGGAGTGAGGGAGGAAGGAAAGGGAGGAAGGAGGGAAGGAGGGAGGGAGGAGCTCTGTGCACAGAACTCATGCCTGCACA
Bos taurus    ..............................................................................AGGGAGGTGCTCTGTGTGCAGAACTCATGTC-ACACG

Homo sapiens  GGGGCCATGAAGCAAGGACAGGACAGTCACTTCAGCTCTGGAGTTCTTTAGTGTGGGAGTATCTTCTAACAAAACCTAATAAATTAACAGCTGTGGGTTTGGGGCAGCAAGGACAAAGAC
Bos taurus    GGGG---TGAGCCATGGAAAGGACAATCACTTCAGCTTTTGGGTTCTTTGGTG-GGGCGTGTCTTCTGATGAAATCCAATAAATGAACCACCAGGGATTTGGGACAGAAAGGACAAATGC

Homo sapiens  CACAACTCTACAGTGACCCCTGGAGCACTCAGTGCCACTGACTGCGTCTCCACTGCTGCAGAGCTGGGAGCACATTCATGACAGCATTTTTCAAGCTCCTGTCTGTGCAAACCACAGCTG
```

```
Bos taurus    AAGGGCTATATGATGACAGCTGGAGCATTCAGTGCAAACAACCGAGTATTCAGTGCTGCAGAACTGGAAGCACATTCACGAGGGCATTTCTAAAGATCTACTCTGTGCAGACTACCATGG

Homo sapiens  GTCTCCTAGGAGTCCTAACACTTTAATTAGACCTAGCAGGTCCCAGAACAAAATCCCATAATCCC-CAAATCTATAGCAACAAGTTAGGCATGAGAGTTTTAGTTACATTCTGGATGTAA
Bos taurus    GTCTCCTAGGAGCCCTAAAGCTTCAATTAGACCTAGTGGGTCCCCTAACCAAATCCCATAATACCATACATCTAAAGCCCCAAGTTAGTCATGAAAGTTTCAGTTACACTCTGAATGTAA

Homo sapiens  GAGAACCCTAACTGCCACTTTCTTTCTGAGTCTCCAAATGACCTTTGACCAATCTCTTTACCAGAAATATTTATCTAGAATGGCCTTGGTCTCTGACACCTTGAGGACTTCTACTGATTT
Bos taurus    GAGACCCCAAACTGCCA-------------------------------------TCTTGAC---AGATATTT------------------------------------------GATTT

Homo sapiens  CTCTGTTAAATCCCTGGAACCACACCAATAATAGTTCTGAACATTTTACACTCATCATCTCATTTATTCCTCACAAATATTATAAACCCTGTTTATATATATGTGTAATATTTATAGTAA
Bos taurus    AT-TGTTAAATTCCTGGGACCGCATCAGTAGTGGTTCTGAACACTTTACTCACATGAACTCACTTATTCCTTATGAATATTATAGACCCTGTTTGTGTGTGTGTGTAATATTAATAGTAA

Homo sapiens  CAAGAGTAGCAGAT-AT-TTTGCTGAGG-------GGAGGC-----ATGGTTCTACACAGGTTACATGAAGTAATTCATAATTCCACATGAACAGCACTATGAGG----TATTATTACTG
Bos taurus    CAATCATAGCATATCATGTTTCCTGAGAACTTAATGTAGGCCCAGCATGGTTCTAAACACATTAGACGAAATAATTCCTAATTCCACATGCACAACACTGTGAGGGGGGTGTTATTACTA

Homo sapiens  TCTCCATTTCACAGGCAAAGAG-CAAATCACAGAAAGAATGAGTAAGTTGTTCAAGGTCACACAGCCTATAAGAGGCA-------------AAGCCTGCCGGTGTGGCTCCACTGTGCT
Bos taurus    TCTCCATTTTACAGGTAAAGAAACAAATCACAGAAAGA----TTAAGTTGCTCAAGGTCATACAGCTTATAGGAGGCCTGGGTTGTGAGTCTAAGTCTACCAGTGTGGCTTCAGCATGCT

Homo sapiens  ATTAACCAGTAAGGTCTTCTATAGCCCCAACTAGCTCAAAATGGTTCTAAATGAAGAATCGCTGTGCGTTTTGCACCTCAGACTGCTAGCATCGCTGATTTAGGTCTGGAGTTTAGATAA
Bos taurus    ATTAACCAGTAAGGCCTTCTAGAGCCTCTAGTAGCTCAGAACACCTCTAGATAAGAGTTCTCTGTCCATTTAGCACTTCAGATTGGTAGCATCAATGATTTTGATCTGCAGTTCAGATAT

Homo sapiens  GTGAAACTACTCAGGGTCTTTGTGAAGTAT-----------------CTTTAGAGCTGCTGCTTGGACAAGACTGGGTCAGGACTTCGAAGGCATTTCCATGTGCT--CCTGG-----A
Bos taurus    TTAAAACTGCTCCGGGTCTTTGTGAAAAAAACAAAAACAAAAACAAAACCTAGGAGCTGCTGCCTGAATGAGA---AGTCAGGATTCAGAAGACATTTACACATGCTTTCCTGGGTAAGA

Homo sapiens  GAGTCAGGATGTAGGCTGGGATTCATTATGCGACCC--AGCTGTGTGACTTTCCTCCCCACCCCTTACCCCCATTCACCAAGATAGAATGCACTGTTTCTTAAGGTGTGGCTTAAGGAGA
Bos taurus    GAGTCAGGATGGAGGCTGGGGCTGGTTATAGGAATCGGAGTCCAGCGATCTTCCTCCCCATCCCCTGCCCTCATTCCACAAGATGGAGCACCACGTTTCTTAAGGTGTGGCTCAAGGAAC

Homo sapiens  GCTCTTTAAACATGCAGATTCTAGGGCTGAAGCACAAACCCA----CTCTGTATCTTTATAGAGATAACTGCTGGAATATGCATTTTAGTAAACTCTCAGGCTGAATTACTTTGAGAA--
Bos taurus    GCTTTTTAAACATGCAGATTCCAGGGATGAACCCCAAGCCCAGACACTCTGTATCTCTATGGAGATTATAGCTGGAATATGCATTTGAGCCAACTCCTGGACTGAAGTAGTTCCACAACT

Homo sapiens  ----TCATCAGAGTCTACTTTGACAGGATATAGGCTTGTCTTCTGATTCTAGAAGTACCATAAGCCTAACTAGGGATGCTTCCCACTCCTCTTTCTTGCTGAGCAGAGAAGCAAGGGGGA
Bos taurus    GGTCTCACTTAAGTCT-CTGAGACTGGA----AGCCTGTCTGCTAATTCTACAGGTACCCAAAGTGTGGCTAGGGGGGCTTCCTCCGCAGCCCCCTTGCCAAGCAGA--ATCAGGGAGGG

Homo sapiens  CTGGAATGCAGAGAACATGCTGAGGGCATGCCTGCACAGAAAGCTGAGCTCCTT---GTCACCACATACCTTGAAAGAGTGGACTCTGTATCCCCAGGAGAGGATGGGGAGGGACAGAGA
Bos taurus    CTTGAATGCAGAGAGCACGCTGAGGGCATGCCTGCACAGAAAGCAGAGCTCTTTTTGCCACCACACCCCTTGAAAGAGAG-----TGAATCCTCAGGAGTGGGCGGGGAGGGGGA-AGA

Homo sapiens  GAGAAAGCTATGGAAAGCCTCTGGAAGCCTTAGTACCCTGGGGCCCAGACAAGGGAGTCCCCTGA---GGGAGGTAGAGAGTGGGGCTGTTGGAGGAGGAGAGCAGCTATGGGCCAGCAG
Bos taurus    GAGGAAGTGAAGGAAATCCTCTTGAAGCTGAAGCACCTTGGGGCCCAGACAACGGGATGTCCTGAAGGGGGAGGGAGGATGGGAGACAGAGGTCGGGGGTGTGTGCTGGTGGGTCAGCAG

Homo sapiens  CCTGAGGAGCTGAGAGTCACAGTGGGAGTGACTGTGGCTTCAGAGTGATGCCATAGCTGCAGTGAGCTGTACAGGAACCAGAGACCCAAAGTTTTCTGTAGGGCTTCTTTTGCTTTGTT-
Bos taurus    CCACAGGATCAGAGAGCCACAGTGCGAGTGGCCATGGCTAGGTATTGATGCTGCAGTTGCAGTCAGCTTTACAGGAACCAGAGATTTGAAATTTTAGGTGGCACTCCTTTCCTTTTGTTG

Homo sapiens  AGTGCTTCAGGAGTTGGAT-----------------------------------CCTTCTGGACCCTGATCTCCCAGAAGTGATTCCATTTCGCCTCTAGGAGGTGGGTAGCAAAGCTCA
Bos taurus    AGTGCTTTAAGAGTTGGACATGACTGAGCCTCTGTACTCTCTAGGGAGCTTCACCCTTCTGGACCCCACCCTCCCAGAAATAACTCCAGCTCATCACTAGGAAAGGAGTAGCAAAGCTCA

Homo sapiens  GTCTGTGGCTGGGGAAAGCCAAGGAAGTGATTTCACGAATCTTTAGCAGTGAATCCACCTATTTAGTTAGC-AGGGGATGGAGCAGAT-CAGAACTAGCGAGAGGAAAGGCTTAGGAATG
Bos taurus    GTCTGTGGTTGAGGAAAGCCAAGAAAATGATTGCATGGATCCCTAATGCTGAATCTGTCTAGTTAGCTAGCTGGGGGGTGGGGTGGATATAAAATCAGTGAGAGGAAAGGCTTGGGAAAG

Homo sapiens  CTCTC---TAGTGGGACTGAGAAGAAAAGGTGAGAGGGTGACATTAACTGGAGGAGATGGTGAGCCCCTGACCCCTGCAGTTGTGAGACTTGCACTGAGTGGGATGATCTGCTGCTATAA
Bos taurus    CTCTCATCTGGTGGGACCGGGAAGCAAAGGAGGAAGAGAGCTACT---TTAAGGAAATGGTAA---------CCCTGATGGTGGTAGATCTGCCCTGGGCAGGACTTCCTGCTGCTGTAA

Homo sapiens  CCTATCCTCAGGCAATGAAGCATA-AGCAGAGAGCCAGGAGTGTTAATCATTGCTGATTTTTATTAAGCATTTCCTTTGTACTGGACACTGTACTGGGCTGTACATGCATCATCACATTC
Bos taurus    CCCATTCTCAGATGATGGTTCATACAGCAGAGAGCTGGAAATCTTAATCGTGGCTGATTTTTGTGAAATATTTATTTCGTACCTGGCACTATGCTGGGCCTTACATGCATCACCTCATT-

Homo sapiens  AGTCTTCACAACATTATATGATAGCTCCTATTA---CTGTGCCCATTTTACAGATGAGGTTTAGATAGGTTATACCACTTGCCCAAGGCCACACAACTAGTAAGTAGCAGAGCCAGAATT
Bos taurus    -----TCATGACACTCTTTGATAGATCCTACTAATATTGTGCCCATTTTACAGATGAGGCTGAGAGAGGTTATATGACTTGCCCAAGGCCATGCAGCTATGGAGTGGCAGAGCCAGGATT

Homo sapiens  TGCACCCAAGTGGTTTTAATCC-AAACTGTGTCCACTAGGCCTTAGGAAGGAGCCCACATAGAGCTTTGGGGATGCTGCATTCTTGACCTGTGGCTGTAGGTGGGCAACTCCTCTTCATA
Bos taurus    TCAGCCCAGGTAGTCTTACTCCCAAACTCTGTTTATTAGGCCCTAGGAAGGG-----TGTAGGGATATGGGGATGCCTTCCCCTGGGCCTGTGGTTCTGCATGGGCAACCTCTCTTTGCA

Homo sapiens  CCTTATAGCTCTTGGTAAATGAAGTGAATCACTGTATCTACCATGAATCTCCTTGGGAATGAGTTCAATGCGGGTATCAGAGGAAGCAAAGGGTGGGAAGGGGGTCTCAAAAGTCTAAAT
Bos taurus    TCTGATAGCTTCTGGTGAACAAGGTGAGTGACTGTATCTATCATGAATTCCCTTGGGTATGAGTTCCCA------ATCCAAGGAA---CAGGTCGGGCAGAGGCTCC---AAGGCTAAGT

Homo sapiens  CTTAGTGTGGTCCCCTCCTTTGACCTACTCTCTCTGGGCTGAAGAAATGCCAGGATTGAGGGGGCAGCAGATTGTTAGATCTGTTTG-TTCCATCTACTGAGGCCTCATAACATTTTTGG
Bos taurus    CCCTATGTGATCCGCTCCTTTGATCCACCCTCT-TGGGCTGGAGAAATGCCGGGGCTGAGGGGTCC-CAGAGTATTTGATCTGTTTGATTCTTTGTACCAAGGCCTCTTCACATTTTTGA

Homo sapiens  AG--CCCTTGTTCAGTGACTCAGGTAATTCAGCCTGAGGGTACCTTTGCAGGGAGTTCCCCTATCTCTTATCCTGTGTTAGTACATGAGATTATTCCTCCTTCTTGTACCCCAGTTACAT
Bos taurus    GAGTCCCTAGTTCAGTGGCTCAGGCAACTGAGCCCGGGGGTGCCTTTGCAGGGAACCCTTCTATCTCCTATCCCACTGTAGCAGATGAGCTCCTTCCTCTTCCCTGT-CCCCAGGTTCAT

Homo sapiens  TAAGTG------GGTCTTATTCCCCAAATCCGTAGTCTCTGCCCCAATAAGTTGCCT-GTACCCCATTTTAGCAAGAATAGAATCTTCCCTTTCTGCCAACAGAGCTTCTTGGCACTTT-
Bos taurus    TAAATGCGTCTTTGTCTTGTTACCCAAATTCTGAGTCTCACCTTTAGTAAGTTGTCTTGTACTTCACTTTCATGAGAAC-----TTTCCCTTTTTGCCAATGGACTCTCTTGGTCCTTTT

Homo sapiens  GGCCTTG-CCTGGTTCCTGCTTGCAGACTCCTGTTCTGAGGCTCATGTTTGTACCTAGAATGATTTCATGTTCCTCCTCTCTCTTTGTCCCTCTATCAGAACCTGCTTTCACGATGGGAG
Bos taurus    GGCCTTGACCTAGGTCCTGCTTCCAG-CTCCTGCTCTGAGGTTTATCTCTGTACCTACAGTAATCTCATGTTCCTGCACTCTCTTCTCTCTGCTATCAGAGT-TGCCATCAGGATGGGAT

Homo sapiens  AT------------TTAAGAGGCTGATCCTCCCAAGAGGTTGGCATTTTAACTGATGCCAAAGCCCTGAGTCAAAAGAATTATATACTTAAAAATTTTCAAGCCCTTGGGAGCGGAT--
Bos taurus    ACATCCAGGTGAGTCTTAACAGATTGACCCTCCCAAGAAGTTGGCATTTTAACTGGTGTCAAAGCCCCATATCAAAGAAATTATGGACTTAAGAATTTTCAAGCCCTTGGGTGGGGATAG

Homo sapiens  GGAGAGATTGGAAAGGCCAATGGAACATTCTTAATGCTCCTGGAATGCCACAAGGTGGACATAAAATTCAGGCTTCTGCCAAGACAGGACTTGGATTATTTTTGTTATAATC----AGAA
Bos taurus    GGAGAGATTTGGAAGGGCAAGGGAATGTTCTTAATACTCCTGGAATACCATGAAGTAGACATCAAATTCAGGCTACCACCAGGCCAGAAGCTGGCTTATTTTTTTTAGAATCCATCACAT

Homo sapiens  TCCGTAATCTGCCCCCACCC---CAGCACTCAGGGCTTTGA-TCAAAAGTCTTCCAGACAATGAACCTCTATGATCCAAGTATCATTTTCTCAGAGCTCC-TTAGAAGGAGGAGAGCTAA
Bos taurus    CGAATGAACTCCCTCCTCTCACTCACCACTCAAGGTTTTGACTAAAAAGTCTTCCAGATAATGAACTTCTATGATGCAAGCATCATTTTCTCAGAGCTCTGTTGGAAGGA------CTGC

Homo sapiens  GAAACTTCATGAATTACCAAGGACCAAGCAAACAGGCGGTAGTGAAGTCAGAAACAAATTCCTCCCGAGACTAGTTTTGTCCTATAGCCTGAAAACAAATCCGGTCTCCACCAGACATAA
Bos taurus    TAAGCTCCATGAGTTACCAAAGACCACACAAACAA-TAGTAGTCAAAGCAAAGCCAAATTCCTCCTGAGACTAGTTTCTTTCTATAACCTGAGAACAAATCCAATCTCCACCCTAGACAG

Homo sapiens  GGTGGAATTCCTGTCTTGATTTTTTTTTTAGCACTGATATTAACATTTTTCTTCTCAAATCCTCTTCAGAACCAGCCCTTTTAG---AACACAAATTAGAAGCTGTTTATTTTGTCAATC
Bos taurus    GGTGATTTTCCTCTTTTCATTTCTCT---GGCACTGATATGAGCCCTCTTCTCCTCTAATCTTCCTCAGA-------CTCTTAGCAGAATACAAATGAGAAGCAGCTGATTTTGCCAGGC

Homo sapiens  AGAGAAACTGAAGGCAGCCAGCCTTGGCCAAAGGAACATCTTATCCTTTGCCTTTGCTAAGGACAATGTTCCTTCTATTGTGCTCTGTGGAAATAAAAGGGCAAAATATGCTTGCCTTCT
Bos taurus    TGAGAAATTGAAAGTAGCCAGTTTTG-------------------CTTTTTCTCTGCCAAAGACAGTGTTCCTTCTGTGCTGCTATATACGGAAAAAAGGGCAAGACCTTCTGACCTTGT

Homo sapiens  TTTATTCTTATTCATGGCTGA--GGGTCAT-ATGAAAGTTCAGAGAAGAGCACAGTTTTTAGTATGGGTCGTGGCAGGTTT-TCCAGATGGCTTCCTAGCCAAGATATTTCTCCCGCTCA
Bos taurus    TATGTTCTTATTCATGACCCATCAAGTCATTAGGAAAGCCTAGAG--GGACACTGTTTTTAGTATGGATCCTGGTAAGTTTATCCAGATAATTTCCTATCAAAGGCATCCCTCACACTCG

Homo sapiens  CCTCCAAACAAGTTATGTACCCCTAAGAATGATTTGGTCTCAGTGTTAGGGGAAGAGGGTAGGTAGCAGGGCCTGGCTTTGTCATCCTGGGCTATTTTGAGGGGACTTCAGCAGTCCCCG
Bos taurus    CCTCCAAACATGTCCTGTGCCC-TAAGAATGCTTTGGTGTTGG--------------GGCAGGTAACAGGGGTGGGCTCTGTCATCTAGGACTCTTTGGAG------------------

Homo sapiens  ACAGAAGGACAAGAGGGGCTGATTCTCCCTTTGTGAGCAGCCATTGGAGCTGCTTCAGGGACTGACAAGCCGGCCAGAGACAATGTCC--TCTCTCAGGAAAGCAGTAAAAACATCAGAA
Bos taurus    --------------------------CCCT-----------CGTTTGAGCTGCTTCAGGGACTGACAAACTGGCCAGAGACTATGTCCAGTGTCTCAGGAAAGCAGTAAA----TAAAAG

Homo sapiens  ATGCTCCTTTCCTGGGGAGCCCTTAGTTATTTTGGCCTCTCTTTCCATTAAAATAGGAAAGCATTGAAAGGGACCAT-TCGTGGGATAGCGTGGAGCCTCCC-CATTGTGGCAGCATTTC
Bos taurus    CTCCTCCTTTCCTGGGGAGCCCATGTCTATTTTGGCCTCTCTTTCCATCAGACTGGGAAGATGTTGAAAAGGGCCATCTCGCGGCAGAGCAGGGAGCTTGCAGTGTGGTGGCTGCGTTTC

Homo sapiens  TAACTGCGTTGAGAATTAACCATATTGCTCTT--CCTTTTTACCATGGAGGAGTTTCTAGATTTTTATTTATACCCTACCGCTTAAAACATCTTTCACTGTTTCAGCAAATTGCAGCATA
Bos taurus    TAATTGGGCTGACTATCAGCCCAAGCCCCACTGCCCTTTTCAACACAGAGGAGTTGCTAAATTCT-ATTTCAACTGTCCTGTTTA---CATCTTGCCCCGTTTCAGCAATTTGCTTTGTA

Homo sapiens  AAAGATGGAGAAGCCAGCAGGGGCTGTGCTCACAATAATAGCTGTGGTTGCATTTAAGACATTCAAATGCCAAGGGTGCACTTCTCCCATTAAGTCCAACATAAGAAATCAGGTGTGCAC
Bos taurus    AAAGATGGAGAAGCCAGCAGTGACTGTACCCACAACAGTAGCTGAGGTCACATTTAAGAAATTCAAATGCTCAGGGCGCGCTTCTCCCATTAAGTCCAACTCGAGCCATTGGATGTGCAC

Homo sapiens  AACATTGATGAACAGAAGACCCCAAAGGACAGGT-GGAGTTTCTGGTATGCTGTGGTGGGGGCGGTGGGGTGGTGAGCTTGGGAAAGAACGAACAGGCATTTTCATTTTCCAAACTAAGA
Bos taurus    AGCACTGCTCAACAGAAGACCCCCGAAGGCTGTTTGGCATTTCTAGTATGCTGGGG-------------------GTTTGGGAAAGAACGAAAAGGCATTTTCATTTTCCGAACTCAGA

Homo sapiens  AGCAGTTTCGGGCGAAGGGGAGCCGTGGGGATGTCAGAAATTCTTCTAACGCCATGGACCTGCCCACACCTGAGTCAGATAAAGATGACCTTCCCTAGGCCCTGCTTTCCTCTCCCCCTC
Bos taurus    AGCACTTCCGGGGGTGGGGGAG-------GGTGTCAGGAATTCTTCCAAAGTCATGGGCCTGCCCACACCTGACT--------GATGACCTACCCCAGGCCCT-------TCCCTCCCTG

Homo sapiens  CCC-----AAAAGACAGCATGAGCATGAGTAAGCCG-GGTACTTACAGGTGCAGAAGAATTTGGAACAGGAAAATGGAGCTTACAGGCTCCCGAATGCCTGCCGAGTCAGTTGCATCAGA
Bos taurus    CCCCAAATAAAAGACAGTATGAGTGACAATGAGCCCCGGTACTTACAGGTGCAGAGGATTTTCGGACAGAAAAATGGAGAAGGCAGGTTCCTGAATGCCTGCTGAGTCAGTTGCATCAGC

Homo sapiens  TTAGGAAGTGATGATCCTAAGAATAAGATGTTTGGAAAATCTTGTCCCCCGAAAGCCCCCTGCTTTGTCTTCTCAGCCCACATCCAAACTCAGACCACCCCC-----------AGGATGT
Bos taurus    TTAGGAGGTGATGATTGTAAAAATAAGATGTTTGGAAAGTCTTGTTGCCC------TCTCCTCTTGTTTTTTTCAGTCCATATCCAAACTCAGATCACCCCCTCTACCTCCCCAGGACAT

Homo sapiens  CTCTCATCCTGCCAGACTGCCTGGTGCCTCCGTGCCCCTTACCGTGAGGGTGCTGGCAGCCACACTGCTTCTTTGAGCCTGTGGCCCGGCAAGGTAGTCAGGCTCATGGGATTAGGTTGG
```

```
Bos taurus    CT---GTCTTTCCTGGCTGCCTGGCGCCTCCATACCCCTTCTCGTGAGGGTGCTGGCAGCCACACTGCCTCTTGGAGCCTGTGGCCCAGCAAGGTAGTCAGGCTCCTGGGATTAGGCTGG

Homo sapiens  CAGGAGCGACTGTCCTCTAAACGATTAGCCCAGAGAAAGAGAGAGAGAGAGAGCAAGCAAACCCGAAAACGGGTCCAGGGTAGAGAGCTCCAGGTCCTTAGCATGTTCCTGGCGTATTCC
Bos taurus    CAAGAATGACTGTCCTCCAAACGATCAGCCCAGAGGAAGAGAGAGCG------CAAGCAAACCCGAAAACAGGTCCAGGATGGAGAGCTCCTAGTGCTGAGCGTGTTTCTGGCGTATTCC

Homo sapiens  AAAAGTGGCATCCACAGCAGAGAAAGTGTCCCAAAATAGCTCAGGCTGCCAATGCCTTGCTGAAACCTGAGAAAGAATTGCTGAATTTAAAGATCTGGGTCGCTGTGGGTGTGTGATCAA
Bos taurus    GAAAGTGGCATCCACAGCAGAGAAAGTGTCCCAAAATAGCTCAGGCTGCCAATGCCTCGCTGAAACCTGAGAAAGAATTGCTGAATTCAAAGATCTGGGTCGCTGTGGGTGTGTGATCAA

Homo sapiens  AGTGCAGATTTGTTGGTAGGAGGAACTCAAGCCATGAGCTTAGGAGTGACATGAGGTCACTTGGGTCACGGCCTGCCTTCTTGTTTCCAACTTGCTGAAGTTGGTGGCAGTGTCAGAGAG
Bos taurus    AGTGCGGATTTGTTGGTAGGAGGAACTCGAGCCATGAGCTTAGGAGAGACGTGAGGTCACCTGGGGCATGGCCTGCCTTCTCTCTTCCAACTTGGTTAAGTGGCTGGTGGTGTCAGAAAG

Homo sapiens  CTCCTCTGTGGTGAGGCAGGGGTTTGGAAAGAGGTCCTAAGGATGCTGTTTTCATGCCCAGGTCTCTGATGCGCTAACAATATGAAATAGCAGTGCCCTGTTTTCTGCATTTCAAACAAA
Bos taurus    CTCCCCTGTGCAGAGTTGGGGGCT-GGCAGGAGTTCCTGGGGATGCTGTTCTCATTCCCAGGTCTCTGATGCACCGACAGTATGAAATAGCAGTGCTCTATTTTCTGCATTTCCAACATG

Homo sapiens  GGAACTGATACTATGCAAAGGTTTCTTTAGGTTATTGCTCCATGTCTGATGAAATTCCCTTTTCAGTAGATTTTTCATTTCTTTTGGCCTTTGGCATAAGTAAATGTCAGCTTCTTAAAA
Bos taurus    GGAACTGATACTGTGCAAAGGTTTCTTTAAGATGCTGCTCCATAACTGATGTAATTCCCCTTCCAGTAGATTTTTCATTTCTTTTGACCTTTGACA-AAATAAATGTCAGCGCCTAAAAA

Homo sapiens  ATCAACAGAGAAGAGCAAACAGTAGGGTCTCTGTGATTTTCTTGGGATTTGAGAAATCTTCCCTCCCTAGCTTCTAACTGGATCATGTCTGTACATTGCTGCCTGTGGTCCTGGAAAGGA
Bos taurus    ATCAACAGAGAGGGGCAAACAGTAGGGTCTCTGTGATTTTCTTGGGATTTGAGAAATCTCTCCTCCCTGGCTTCTGACTGGATGGTGTTTATACAATGCTGCCTATGGTACTGGGAAGGC

Homo sapiens  CGAGGTCCACCCAGACATGCTGTCTCATTTTTATATTTGCTGAGATCTTGGGACACTCTCCTTTCATCAGGTATGTCAAAAACCTTAGGATTGTCTGCAGAGAGTGTGGTTCAGTATGTG
Bos taurus    TGAGGTCCACTCAGTCACGTGGTCTCATTTTGATATTTGC-AAGATCTTGGGTCATTCTCCTTTAATCAGGTGTGTTGAAA-TTTTAGGATTGTCTGCAGAGAGTGTGGGTCAGTATGTG

Homo sapiens  GAAAGGTCAGCATATGTCATTCTTGGACTGGCCCTGGGGTTTAAGAAGAAAAA-GCCCAGAATTTGGTTTCCAATTCCAGCTCTGCCTGTCTTTTGCTGTATGAATTTGGGCAAGTCACT
Bos taurus    AAAAGTATAGCACACATCTCCCTTGGATCAGCCTTGGAGTTTAATAAGAAAAACGCAGAGCATTTGGTTTCTGGCTCTAGATCTGCCTGTCTTTTGCTGAGTGATCTGGGACAAGTCACT

Homo sapiens  TCTCTTCTCTGGACCTCAGTTTCCTCTTCTGTAAACTGAAGACAATAATCTTCATTCTAATTCTAACCCAAATGTTGTAAAGAGTAACTCAGAGAGTTGAAAGAATGCTATAAAGGGCAG
Bos taurus    TCTCTTCGCTGGATCTCAGG----------GTAAAATGAAGACAGTAATCCCCATTCTAATCCTAACCCAAATGTCGTGAAGATTAATGCCAAGGGTCACAAGAATGCTACTAGGGGGAG

Homo sapiens  GGTTTGGCACTCTCAGTAAGTGGTGACATTTTTCAGTTCTTCAAAGGTCCCTGATTGGTAGCCACTACCTGCATCCACATGCCTATGGCGAGCCCAAGACATCCCACAGTACTAGGCACT
Bos taurus    GGTTTGTCTCTCTCAGTATTTGACGGCAGGCTTCAGTTCCTTAGGGGACCCTGACTGGTAGCC------TGTATCAACACGCCTACAGTGAGCCCAGTGCATACCGCAGCACTAGGCACT

Homo sapiens  ATGAGCTTCATGTCTAGGAGTCTAAATAGTTTCCACTTTCA-GTTGTGGGAATTTTGGCAAGTAGCTCAATGCTTAGTAGCTCAGTTTTCCCATCTGTCAAGTGGGTATACAAACTAATA
Bos taurus    AGGGGCTTCATGCATAGGGGTCAACGTTATGTCCAGTTCCAAGCTGTAGGACTGTCGATCAGTTGCTTAGTGCCCAGGAGTTCAGTTTTCCCATCTGTCAAGTGGGTATACAAATTAATA

Homo sapiens  ATAATACAGGTCCACAATCCCTTATCTGAAACTGTTGGAGCTAGACATGTTTTACAGTTCAAAA-------------------TATTTGGATTTTAGAAAAGCAATGTGGTAGATATAAC
Bos taurus    ATAATACAGGTCTACAATCCTTTATCTGAAACCATTGAGACTACATATGTTCTGGATTTCATGAGTTTTTTTTTTTTTTTTTTTTTTTAGATTTTAGTAAAGCAATATGGTACCTATAAC

Homo sapiens  ATATATATCACACCTTAATGAGATCCAAGATAA-TACCCAGTGATCAAACTCACTAATATTTCTAAATGGGATAAATGAATAGCCTCATGTCAATTCAGGCCCAGTTTTGCCACCAAATG
Bos taurus    ATATATAATACCCCTTG-TGGAATCTGGGGCAAGTATTCTGTCATCAATCACACTAATATTTCTCAATGGGATAAATGAATAGCCTCAAAACAATTCAGGCCCAGTTTGGCTACCATATG

Homo sapiens  AGCTTGCCACAGAATTTCAGTTTTCAGAAGCTTTGGGATATCAGAGAGGCACATAGGAAATTGTGGACAATGAAGGAAGTTAATACTTA-----------------TTTCGTGCATGTA
Bos taurus    AGCTTGCCACAAAATTTCAGTTTTCAGAATCCTTTGGATGTCAAAAAGCCAGGTAAGAAATTGTGGACAATGAAAGAAGCTAATACTTAGCTACTAAACTACGTTTCTTTAGTGTGTGCC

Homo sapiens  ATGTACCAGGCATTGTTCTAAG-------TATACATTAACTCATTTAAGTGGCCCAATAAGCCTGTGAGGTAAGCAGTATTATTATTATCATC---CCCATTTCATAGATGAGAAAACTG
Bos taurus    ATGTGCGAGGTATGGTTCTAAGCAGTTTGTACACATTGACTGATTTAGTTGGGCCAATAACCCTATAAGGTGGGCATTATTATTATCACCATCATGCCCATTTTACAGATGAAGAAACAG

Homo sapiens  AGATTCAGAAGTTAAATCACTTGCCCAGGGTCCCAAAGCCAGTTAGGAGCAATGCTGGGATTCCAACCCACACAGTTCAGC-------------TCTTATCCACCTTCTCTGCCTAACC
Bos taurus    GCTCAGAGAGGTTAAATCACCTGAGCAGAGTCCCAAAGCCAGTGAGGGGCAAAACTGGGATTCTAACCCAGACAGTTCAGCCTAAGAATCTGTGCTCTTAACCATCTTCTGTACAAGCTC

Homo sapiens  CATGGGGTTAAATTGAGGCTTTAAATGCCTACAGGGACTGAGCAGGTCGCTG--AAAAAGTAAAGGTGGCCTTATGGGTACTGAGGCAAACTGGTGAGCACGTGCCTCACCTACAGGGAG
Bos taurus    GATGGGGTTAAAATGAGGCTTCAAAGGCCCACAGGGGCCAAGCAAGTCACTGTGAAAAAGTCA--GTGGC-TGGTGGGTACTGAGGCAAAATGGTGAGCCAGTGGTCCATCTACAAGGAG

Homo sapiens  CAGGTTGTTGAGCTCTGGCTGATTGTTGCTATATTGGAATTTGGATCGAAGTCTTTCTCTCTCTCTCTCTCTCTCTCTCTGTGTGTGTGTGTGTGTGTGTGTGTGTGTGTGTGTGTGT
Bos taurus    CAGCTTACCCAGCTCCACCTGACTGTTCCCATCTCAGAACCAGGATCTGAGTTTT-----------CTGTTTTTCCCGCATTATGTAGGTG............................

Homo sapiens  GTTTCAGAAAAGCCAGAAATCCAGCTTTCTATCTGAAATTTCCTGACTTTTAAATGTTGGCAATTAATTTTAAAAAATGTTTAAAACGTTGTACGGGTCAAATAAACTCTGTCTGCAGTC
Bos taurus    ..................................AAATCTCCTGACTTTCAAATGTTAGCAAT--ACTTTAAAAAATGGCAAAAACATTGTAGGGGTCAAATAAAATCCTTCTACAGTC

Homo sapiens  TGAATTTAGCCTGTGGCTGCCAGTTTGCAAGCTCTGCAATGAGTAAATAGATGTGAAAGTGCTTTGGAAACTTAAAAACGTGCTCTAGAAACAGAAGAGTCTAATT-TTAGGGTTATA--
Bos taurus    TGAATCCAGCCTGTGGCTGCTAGTTTACAGACTCTGTGATGAGGAAATAGATGTGAAAGAGCTTTGAAAACTGAAGAACGTGTTCTAGACACAGAC-AGTCTCATTCTTAGGGTTAAGGT

Homo sapiens  --TCTTGTTTTTTCCTTGACTGTGGTCTAGTTTTTTAAAAACTTGTATTCCCGACCTGATGGAGATGAAAAGTCAGAATTAAATAGAGACTTCAGTGAACTTGAGTACTTCATTCCTTTG
Bos taurus    ATTCTTTTTTTTTCCTTCACCGTG-TCTAGTTTTTTGAAAACTGGTATTCCTGGCCTGATGGAGATGAAAAGTAGGAATTAAATAGAGACTTCAGTGAACTTGAATGCTTCATTCCTTTG

Homo sapiens  GCAGGCTGGATAAATGAGAAGCCTAGGTGTACTGGATGGACTAAGCCTCAGAATTCTTGCCACACCCACAAGCCCCTTCTAAGGAGTTTCCATCCACAGACCCTAGCTGAGGGAAGTAGC
Bos taurus    GCAGGCTGGATAAATGAGAAGCCTAGGTGTACTGGATGGGCTAAGCCTCAGAATTCCTGCCACACCCATAGGCCCCTTCTAAAGAGTTTCCATCCGCAGACCCTAGCCGAGGGAAGGAGC

Homo sapiens  AGTTTCTTATGAATAGGTGACTATTCTTGGTACC-ACGTGACCCTGCTTCCCCCAGC-AATAGCCACTTGAGCCAGGAGAGGGCATCTGATCCATGAGCAGTCACTCTATAGGCTGGCAA
Bos taurus    AGTTTCTTATGAATAGGTGGCTATGTTTGGTACCCACGTGACCCTGCTTCTCCCTGCCAATAGCCGACTGAGCCAGGAGAGGGCATCTGATCCAAGAGCAGTCACTCTATAGGCTGGCAA

Homo sapiens  ACAGCCTATGAAGGAACCTGGGATGAGAACTTTGCCCAGCAAGTACAGTT---ACTGAATTTGGACCAATGAGAGTTTCTCTCTTGGGGAGTTTGAATACCAGACCTAGAAAGAGTGAGC
Bos taurus    GTTGCCTAGGAGGGAACGTGGGATGAGAACTTTGCCCAACAAGCACAGTGGTGACTAAATTTGGACCAATGAGAGTCTCTCGCTTGGGGAGCTTGAATACCAGACCTAGAAAGAGTGAGC

Homo sapiens  AATTGGCAACAGGGGCAGAAGCTGAACACAGAGAAGGCAGGCAGCAGGGACCCCGGATAAGCAGGACCAGGAGAGATTACTGAACTTCAACTATGTGCCAGATTCTTTACACGTTGTTT-
Bos taurus    AGTTGGCAACAGGGGCAGAAT---------------ACAGGCAGCAGGGCTCACAAATAAGCAGGCCCAGGAGAGGTGACTGAGCTTCAACTAGGTGCCAGACCCTTGACGCATGTTTTG

Homo sapiens  TGTTTAAAACATTCAAAGAACATGCGAGGTAGGTACTGTTATTATGCCTGTTTTACAGATGAGGAAAC------------TGAGGTGAACTAACTTGAAGTATTGAGGTTCAAGCCCAGG
Bos taurus    TGTTTAAAACATTCAAAGATCCTGTGAGGTAGGTACCGTTATTATCCCTGTTTTACAGAAAAGATAACAGGCCAGTGCTGGGAGTTGGAAT--CCTGGTTTATTTGATATTAAATTCAGG

Homo sapiens  --------------------CCTGT------------TTGA--------------------------------------------AGTTAAAATGA-----------------------
Bos taurus    ATGGAAGGGATACATGTATACCTGTGGCTGATTCATATTGATGTATGGCAGAAACCATCACAATATTGTGAAGCAATTATCCTCCAATTAAAATAAATTAGTTAATTAAAAATAATATCC

Homo sapiens  -GAGTGTAGGAACCCCAAAGCCAGTGGTTCTGAAACTCTAG--TGTTTCAGAATCACTTGGGTTAAA-TGCAGGTTCCTTGGCGTCACCCCAGACCTATATTGAATCTGACTCTTTGTGG
Bos taurus    AGGGTGTCCAGATCCCTAAGCCAGCTGTTCTTAAACTCTAGAGGGTTTCAGAATCACTTGGGTCAAAATGCAGATTCCTCGGCCTCACTCTGGACCTAC--TGAGTTTGACTCTCTGAAT

Homo sapiens  GTAGAGTCTCCCTCCCC--CTACAAATCTGTGTTTTAGCAGTTTTCCCAGG-GAGCACTTGATGCA--GGGTTTCCCCACCACACTTTTATTTTGAGAAATGCAGTTTCAGAGGGTATGG
Bos taurus    GTGGAGCCCCACCTCCCAACTCTGCATCTGCATTTTAACAGTCCTCACAGAAGAATGACTAATGCAAAGGGCTTCCCAACCACACTTA------GAGAAATGCAGTTTGAGGGAGTATGG

Homo sapiens  CAAGTACTGTGTACTGTGCTACCTTTACCTACACAACCAAGGTCAATGTCAACAGATGTGGTCAAATTCCAATTTAAAGTCTTTTGGAAATGGGGCCAATTATGTAAAGAAGAGGGCATT
Bos taurus    CAGGTACTGTAAACTGCTCTGACTTCACCTTCAGAAATAAGGTCAAGGTCAATAGATGAGGTCAAAGTCCAATTGACGCTCTTTTGGAAATAGGGCTGATTCTACAAATATGAGGACATT

Homo sapiens  AGTTTGTTCTCTTCTCTCATCAAGGAAGTCTACATTATTACCAGTTATTTGAGAGAACAG---AAGATTGGGATGAATGCTTAGTA-GTTGTAAACACGATCGGATCTGCACTTCAG---
Bos taurus    AGTCTGTCCTCTTTTCTAATTCAAGAAGTATCCACTGACACCAGTTACCTGAGGAAAGATGGCAGGCCTGAGATGAAGGCTCAGCACTTTGTAGAGAAAAACCTACCTGTTCTTCAGCAG

Homo sapiens  GGTGGGTGCAAAGCTAGCATATGTTCACTAAGCATTTTCCATGAGGTGCGTAAACTGTAGAAACTCAGTGCTCTGGCAACAAAGAACATGAGAGAAAATTACCAAATGCAGATAGACATA
Bos taurus    GCAGAGCTCATAGCAGCCATACCGTCACTAAGCATTTTCCACAAAGAGCACACATTGCAGAGACTTCCGGTATTGGCAGCGAAGAACAGGAGAGAAAATGCCCAAAGGCAGAGACATAGA

Homo sapiens  TTGTAAACCGTGGAGCTTAAAA-TGTAAACCTACTTGCTAGGCTGATCATAGAGGTTCTTCTCAGCCCCAGAGAATTTGTGTAAGAATCCTGTCCATGCCTTTATTTACACCATTGATTA
Bos taurus    CTATAAACCATGGGATTTAAAAATGTAAATCTACTTCCTAGGCCAAACATAGAGATTCTCCTCAGCGCTAGAATAGTTGTGTAAAAGATCCATCTATGCTCTTCTTCACACCATTGATGA

Homo sapiens  AAAATGCTAATCAGGAGAGGATCAAGGCTATGGACCTCCTTCTAGGTTGTTGCAGAACTCCCACTCTGGCAATGGTATTCAGCTGGTTTTGAATTTTTCTGC-TTTAAGAACTATCCCTG
Bos taurus    GAGG-ATTGAACAGGATGGGACCAAAGTTAGAAGCTGCCTTCTAGATTGTTGTAGAAC-CCCAGTCTGGCTATAGTATTGAGCCAATTGAGAATTCATTTGCCTTTAGGAACTCTCCCTG

Homo sapiens  GGAAGACTAGTG---GTCTGAAGTTTGCTTGGAGAGTCTCAAGCCGGAAACTGATTTGTGGTCCAAATGCTTAATGGCATCAGACACTTATTCCTACAAGAGAGGCATGTGGTCCAAGGG
Bos taurus    GAGAGACCAGTAATAGTCTGAAGGCTGCTTGGAGTGTCTCAAGTCAGAAACTGGTTTGTGAGCCAAATGCTTAATGGCATCAGGCAAATATTCCTACAGGACAGTTGGGTGGTCCAAGGG

Homo sapiens  GTGGCACAGGTCAAATGTGATGGCCAAGTCCCGCCTCAGCAGGGCTTGAATCCGCACTTGGCAACTTTTATTTCTCTC-TGTCCATTCCTGTGGATACTTTATCTGTGTTAATGTCCTCA
Bos taurus    ATGGCAAAAGTCAAATGTGCTGGCCAAGTCCTGCC-CAGCAGGGCTTGAATTTGCACTTGGCAAGTTCTATTTCTCTCATGCTCCTTTATGTGGACACTTTATGTATGTTAATATCCTCA

Homo sapiens  TTTAGGCTAAGGTCAAACTCTGTTCTTCTCTCTTCTGGCCAGAAAGACTTCCTGTTTTACAGGTATATGATCTTATCTGTGTTGATACCTTAACTGTGAGTGATTCCCTAAAGCAGGTCC
Bos taurus    CTTAGGCTAAGGTCAAACTCCGTTCTTCCCAGTTCTGGCCAGAAAGATTTCCTGTCTTACAGGTATCTGATCTTATCTTTGTTGATATTTTCACTGTGAGGGATTCCCTAAAGAAGGTCT

Homo sapiens  TTGGACAAGTGCCCATCCTCCAGGAAAATTGCAGCAGTCCACCG-TGCAATGAGAAAGCCTGAGGACTAGGTAGTAAATGTTTCGCAGAGAAGAGAAATCTATTTCAT-----GTATTAT
Bos taurus    TTGGATAAATGCCCATCCTCCAGGAAAATTATTGCAGTCCTCGGGTGCAATGAGAAAGCCTGAGGACGATGTAGCCAGTGTTCTGTA---AAGCGAAACCTATTTCACTGAAAGAACTGT

Homo sapiens  TCTTTATTCTGAGGTTATTTCCTTCTCTTTTGGTGTTGAAAGGAAGCCCCTTCTTTTTAGAAAATGATGATTTGTGGTTGTTCTGT-TTCTTAATGATTTTACTTGGGAAAATAAAAATC
Bos taurus    CCTTTATTCTGAGATTGTTTGCTTCTCTTTTTGTGTTTAAAGAAAACCCTTTCT----AGGAAATGATAATTTGAGGTTGTTTTGTCTTGCTTGTTCCTTTATTTGGAGAAATAAAAACC

Homo sapiens  TGGAAGTTCAATATCAGTC--CCTGAAATTTTGGGTTGCATTAGCTGCAAGTCT-GGGAACCTGGTCTAATT---TTGAGTGTTCTGCTAGTACCTCTAGGTACCTCCTATTGCCACTTG
Bos taurus    TGGCAACCTACTGTCAGTCTCCCCAGATTTTTGGTTTGTGATAGCTGCCAGTCTTGGGAACCTGGCTTAATTGGTTTCAGT--TCTGCTAGAACCTCTAGGTACCTTCTATCACCACTTC

Homo sapiens  TGTTCTAAGTGCTTATAAATTACCTGCTTCGCAATCTGCAGCAGAATCTCTCCGAGGATTGACATCAAGCTTACAGCTTTGGAGTTTACGGTATACTGGTCTGTAGTTTCCTTATTTAAG
```

```
Bos taurus     CATTCTAAGTGCTTATAAATTACCTGCTTCATAATCTGCGGCAGAATCTCTCCAAGGATTGACATCAAGCTTACAGCTCTGGAGTTTACAGTATACTGGTCTGTAGTTTCCTTCTTTAAG

Homo sapiens   AAAATTGGGACACTGCCCATTTGCAGTCCTCTGGTATCCCTCCTGTTCTCTCTGATTCCACAGATCCTTCAGACCGGCACCTAATGCAAGGCAGTTCCGTTTGCTCTTCTTATAATGTTC
Bos taurus     AAAATTGGGATGCTGCCCATGTGCAATCCTTTGATATCCCTCCTGTTCTCTCCGATTGCACAGG---------ACAGCAGCTAATGCAAGGCAGTTCCGTTTGCTCTTTTTATAATGTTT

Homo sapiens   TTTTCCCTATAGAGTTGGACATGCATATACATGTTGTTTGGGGAATTTGCAGCACATCCAATGAAAGCATAATTCCCAACTAACAGTCAAATCCTTTTCTTTGGAGCTACCCAGAACACA
Bos taurus     TTTTCCCTATAGAGTTGGACATGCATATACATGTTGTTTGGGGAATTTGCAGCACATCCAATGAAAGTGTAATTCCCAACTAACAGTCAAATCCTTCTCTTTGGAGCTACCCAGAACACA

Homo sapiens   CCTACCCTCTCTTCCATATGACAGCCCTTCAGAGACTTGAAGACAGTGTGTGACTCTCCTGTGAGTTAAGAGCACTGGCTGAGTCAGACAGACCCGAGTGACAAGCCCAGCTTCGCTCCC
Bos taurus     CCTACCCTCTCTTCCATATGACAGCCCTTCAGAGACTCGAAGACAGTGTGTGACTCTCCTTTGAGTTAAGAGCACCAGCTGAGTCAGACAGTCGCGAGTGACAAGCCCAGCTTCACCCCC

Homo sapiens   TCTCAGCGGTGAGAGCCAAGGTAAGTTACTTCATTTTAATTTCTCTCTAGTTCAGTTCTTT-------AGCTGATTTAGAAAATGGGAGT-AAAAAAGCCACTTTGTTTGTTTATCGTTC
Bos taurus     TCTCGGCTGTGGGAGCCCAGGTGAGTTACTTGATTTTATTTTCTCTCCACTTGTTTTTTTTTTTTTTAAGCTGATTTATAAAATTAGGGTTAAAAAAACCACTTCATTGGTTTGTTGTGT

Homo sapiens   AATAAATAAGATGAGGTATGTAAAGTGTTTAACATAGTGCCTGGAATAGTAAGCACTCAGTAAATGCTAGCTATTTCCCCCTCTTTATTTATAAAGAGCC-GGTGCGCATTTTAAGTGTC
Bos taurus     GATAAATGATGTGATATATGTAAAGAGCTTAACCCAGTGCCTGGCAGAGTGAGGATTCCGTGAATGCAAGCTCTTTCCCCCT-TCGATTTATAAAGAGCCAGGTGGGCATTTTAAGCATC

Homo sapiens   AGGGCTGTGGGGGAGGAAGGAAGTGGGGAGGGCTTCTACTTCCCTGAATGATCAAGGAAGGAAGAAGATCCTAAAGTAGACCAGGTATTTCTTTAGCCTTAGGTTGTCTGTTTTCAGTTA
Bos taurus     AAGGCTATTGGAGA-------AGTGGGGTGAGC------------AATGGGGTGAGGGAGGTGGCAGACCCTCAAGTAGATCAAGTCTTTCTCTACCCTGAGGTTGTCTCTTTTCTGTTA

Homo sapiens   TCTGGATGCCTATTTGCTAGGACTTG--GGGAAAGAATATTGTTGAGAATCTAGGGAAAGGCTTATTTTCAACAGCTGGAAAGCTATTTGTTGACATATTGAACCGTCTCTTGTTTATTC
Bos taurus     TCTTAATGCTTGCTTGCTAGTGCCGGCAGGGAGAGGCTATCATGCAGAATCTCAGGAAAAGTTTATTTTCAAGAGCTGGAGAGCTATTTGTTGACATATTGAACTGTCTCTTGTTTATTC

Homo sapiens   TTATTTCCCTGAGCAGCTAGCCATGCAATCCCCTAAACTCAATTACCTTTTGTGAGGCGGCAGGAGCATGACACCTCCTA---GCCGTATTGATCAGTGCAGACTGGGTGGGACGTGAGG
Bos taurus     TTATTTCCCTGAGCAGCTAGCC-----------CAAACTCAATTACCTTTTGTGAGGCAGCAGGCATGTGACACTTCCTACTAGCTGTATTGATCAATGTAGACTGGGCCAGACGTGAGA

Homo sapiens   GACCTGAGAGATGCTAAGCGGTAAATTCTGGGCTTTTCCTTCCTTAGAAGGCCTCTCTTCTCTGTGTTCCTAGAGACTCTGGCTTTAATAGAACGGAAGTCTATGCCTCACATGGGCTGA
Bos taurus     GATCTGAGA--TGCTAAGCAGCAGTTTCTGGGCTTTTCCTTCCTTAGTAGGCCTCCCCTCTCTGTGTCTATAGAGACACTGGCACGAACAAAATGAAAATCTGGGACCCAAATGGCCTGA

Homo sapiens   GCATGCAAGAACCCTCTCTCAGTAGCCTCCTTAGAAGCAGGCTGATGAA-AATAGTCACAATGGGTCAGAACTTGGAACCCTGGGGTGACCAGTGCTAGCATGGTTTTCTAACTGCAGCT
Bos taurus     GCCTGCAAGCACTCTTGCTAGATAGCCACCTTAGAAGACGGCTGATGATTAATGGTCACAATGAACCAGAACTTGAACTCCTGGA-TGTCCAGAGTCAGAGTGGTTTTCTAACAGGAGCT

Homo sapiens   CTTTGCACACACTGGGTTTTGCAAGCAATTGAATGATATGAACTCTGAATGCCATTGCAGCTAGAGAAAGTTTGGAAGGAGACACACCCAACTGTGAACAGACAGAGGAATGGGACTTGC
Bos taurus     CTTTGCACACCCTGGGTTTTGCAAGCAATTGCATGATATGAACTCCAAATGCCATTGCAGCTAGAAAAAGTTTGGAAGGAGACACACCCAACTGTGAACAGACAGAGGAATGGAACTTGA

Homo sapiens   GGGGAATGGG--GGGTGGGGCATTTTCAGGAGTTTAGCTTTGTCTGTACGGAGGATTTCAAGTCTTTATCCTAAGAATATATTCCTAGATTACTTATGGAATTACTAAACATAAATAAAA
Bos taurus     GGGGATGGGGTGGGGTGGGGTAGGTGCAGGA--CTGGCTTTATCTGTAGGTAGGATTTCAAGTTTTTATCCTGAGAATATATTCCTAGATTACTTGTGTAATTCTTAGGCATAAATAAAA

Homo sapiens   GTTAGAGAAAGAAGAAATT--ATTTTTAAAAACAAACCAAAAATAAAACACAAAATAA---------------------------------------------AGTGGTACAACCATTAA
Bos taurus     GT-AGAAAAATAAGAAATTGGATTTTTAAAAAGTAAT----GATAAAAAATAAAATAAGCCATTAGAGACAGTTTTCAGAGGTCACTACTGACCAAGGATCTCAGTGATACAAGAATTAA

Homo sapiens   GCTTGTCGGGGTAGAAAGATTGCTTGGGTCGGGGGGTGGATGTCCGGGATTCCTAGAGAGAGCTTGCAAGGAGAGGAGTGAAAAGGGCTTGAGAAAGAACCCTAGGGAAACATATCTTT-
Bos taurus     GCCTATTGGAGGAGAAAGGTTACTTGGGT----GGGGAGATGTTGGGGGCCCTTAGAGAGAG--------GAGAGGAG-----AGGGCTAGAGATAGAACTGTAGAGGAACATACCTTTT

Homo sapiens   AAAAAAAATAAAATTAACTTTTATTAGTTTTTTTCTTTTCGTATTATGGGTCCATTATAGACCAATGAGAAAATACAGATAAGGAAAATGAAAATTAAAAATCCCCTCTAATCCCATCAT
Bos taurus     AAAAGAAAGAAAAATA-----------TTTTTTTCTTCTAATACAATAATATTTATATGGACAGATGAGAGAATACAGCTAAGAAAAAC-AAAAGTAAAAATTCTCTTTAATCCAGTCAC

Homo sapiens   TCAACTGTGGTCACTGTTGGCGTTTTGGTATATATCCTTCCAGA-----TTTTTTTTCTATGCAGAAATCTACATATTTATCTTTATGATAATCAGATCCTCATGTACACATTAGAATAT
Bos taurus     TCAGGTATGGTCACTGTTAACATTTTGGTATACATCTTTCCAAACTGTATTTTTTTTTCTCTGCAAAAATATACATATTTAT-TTTATAAAAATAAGATACTCATTTTCAC----------

Homo sapiens   ATTTTCAATGGCTACAATAGTGGCTGCTACAATTGCACCATGATTCGTTTAATCATTAAACAAGTTGTGTTCTAACATTAGACACTTAGGTTATTTCCAAAATTGTTGCATTAGAAATAA
Bos taurus     --------------------TGGACACTGGAGATGTACAATGATTTGTTTAATCATTCAGTGGATTGGGTTCTAATGCTGG-CATTTAGGTAATTTCCAAGTTTTTATGATCA----TAA

Homo sapiens   ACAACAGTGCCCTGTAGCTGTGTGACATTGGGAAAGCTAGTTAACCTCTCTGTTCCTCTGTTCTCATCTGGAAAATGAAGGTGCTAATAGAACCTACCTCGTAGTAT-GTTGTGAGTGTC
Bos taurus     ACAACAGTGCTTTCTGGCTATGTGACCTTGGCAAAGCTAACTAATCTCTCTGTGCCTC-------ATCTG-AAAATGAGAGTGATAATA---CCTACCTCATAGGGCAGCTGTGAGCAGC

Homo sapiens   AAATGAGATCATATAGGTAAAGCACTTAGCACAGGGCCTGGCACAGTAAACGTTAGCTTTAATTGTTAGGTGTGTTAATAATAATAATAGTAGTTGTTGTGAGGCCATTGTTAATAGAAT
Bos taurus     AAATGAGATTTTGCATGTATAGTGCTTAGCACAGTGCCTGGCACAGTAAGTGTTAGCTGTTATTATTAAGTATAATAATAATAATAATAATAATAATTGT-----TGTTGTT--------

Homo sapiens   GGCTATTCTGTGACTTTTTTTTTTTTTTTTTTTTTTTTTGCTTTGAAGGGAGAGGAGAGCGACTGGAA-TTTTTCTTTTGAGTAGTATGCTATAGTGAGCTCTGGAGTCACACAGAGCTGT
Bos taurus     GGGTTCCCTGTGA----------------GTACTTTTTGGTTTAGGGTAGGGGGAGAGTGACTGGAAGTTTTCCCTTTGAGTAGTATACTACAGTGAGTTCTGGAGTCAAATGGAGCTGT

Homo sapiens   GTGCCTTTGGTCATGTCAATTCATCTCATGGAGGCTCAGTTTCCTTACCTGTATACTGAGAATTTAAAGGACCTAGCTCACAGGGTAATTGTCAAGATCAAATGAGATAATGCTGGTGAA
Bos taurus     ATGGTTTTGGTCATGTCAGTTCACTTCACCGAGACTCAGTTTTCTTATCTGTAAACTGAGAATTAAAAACACCTAGCTTACAGGATAGATGTTGGGATCAAATGAGAGAATGCATGTACA

Homo sapiens   GCCCTCAGCCCCATTCTTGGCATATGGGAAGAGTTCAAGCAATATCAGTTGTTGTTGTTATTATTACTAC---------------AAGGATTCAGCCATTTGTAGTAAGAAAGTTTCCTT
Bos taurus     ACCCTCAGCGTAGTTCTTGGCATGTAGCAAGAGTTCCAA----GTCAGTTGGTATTATTATTATTACTATTATTATTATCACAATGATGATTCAGCCATTAGT-----------------

Homo sapiens   GCTTTGTGGTGTGGGGATGTAGGGGAGATGGCAGTGAGAAGAGTACTCCGAG----TTTCTAATCACTTGTCTGATTACAAAGAGATAAAAATA------TTTTGGAAACAGAATGGCCA
Bos taurus     ------------GGGGGTA---GGAAGAGCACGGTG------GTACTTGGGGAGTGTTTCTAATCACTTGTCTGTTTACCAAGAGAGGAAATTAGTCTGATTCTGGAATCAGACTAACTT

Homo sapiens   GGGCTGAAATCTGGGATTTAAAAAAAATTATTATTATTATTTTATGTGACCGTGGTCACATAGACTTCTCCAAGCCCATTTCCTCATGTATAAAATGGGAATGATCTCAGTGCCTACCTT
Bos taurus     GGGCTCATATCAGGGCTTTG----------CCATGTGCGGGTTCTATGATATTGGTTACA---ATTTCTCCAAGCACCTCTCACCATTTGTAAAATGGGAATGGTATTAGTGCCTACCTT

Homo sapiens   ACAGGTGTTGCTGGGAGGTCGGAATTATATAATGCATATGTAGTGCTTAGCCCAAGTGCTCAGTTGTTGTTGGTCGTTAATTAT---------------------GAGGTTTATAACGAC
Bos taurus     GCAGGTGTGGCCATGAGGTGTGAAGGATATAAAACAAGTGTGGTGCTTAGCCTGAGTGCCCAGGTATTGTGGGGTGATAATTATCTTTGGTTGTTGGAGCATGGGGAGGTTTATAACAAG

Homo sapiens   CTGAATGCCTCCCTTGTCAAACTCTACATCCCTA-TACCTAGGCTACTTGAACTTCACCTGGAGGCTTTGCCCTATCCAAGGCTCAGGGACCAATTCTCCCCTTAGACTGAGGGTTTCTC
Bos taurus     CTGAATGCCTCCCCC--CAGCCCCTAGATTCTGAGTCCCTGGGGTACTTCAACTTCATCTAAAGGCTTTGCCCTATTCGGGGCTCAGGGATCATTTCTGCCCTTAGTATAAGGGTTG---

Homo sapiens   CCCTTAGACTGAGTGCACCA-GTTCAGGAAAACTGGAGTACTCTGGTAAACCTTTCTGCTAGCTCTACTCTGACCTTGTGCCCCAGTTCTAGAAAGCAGGCTGTAGCCATGTTTGTATGA
Bos taurus     ----------GAGGGCTCCTTATTCAGAAGG---------CTCTGGCAAACCTTTTTGTTAATTTTACTCTGATCTTGTGCCCCAGTTCTAGAAAGCAGGCTAAAGTCCTGCTTCTATGA

Homo sapiens   ACAAGCCCTCAGCTGGTATGCTCAAGGCTGGTGTGCTTTTCTTGCTAGCTGTCTGAAATCCATGGAGCCTTTCTGTTGAATCCTAGCCCTATGGCAGGGATACCGGTCGCTGGATTTTTC
Bos taurus     GCAAGCTCTCACCTGGTATGCTTAAGATTGGCATGCTTTTCTTGCCAGTTGTCTGGATGCCCTGGAGCCTCATTGTTGAATTCTAACCCTATGGCAGGGTAGCTGGTTGCAAGATTTCCC

Homo sapiens   TCCGTGTCTGTGACCCTGGGGTAAATTGTCTTCGTTATGATCACCATTACAGGGCTCTATTAAGGTGTTTGCTTGCCAGCCCCCACTGTCCATCCTCCCAAACCCTC-TTTCCCTGCACC
Bos taurus     ACTGAGTCTCTGACACTAGGGCCAGTTGGCTTTTTAGTTGTCACCATTGCTGGGCTCTCTTAAG----------------CCCCGTGGCCCATCCTTCCAAACCCTGGTTTACCCGCACC

Homo sapiens   TGAACCTCTTTGAACGTTTCTGATGCTGCTCCACTCAATCTCCAGCCTCAGTTTCTTTCCCTCGAGCCCTCTGCCTGTGCTTCCATGTCATATCAGTTGCAGTGCCTGGGTCCCAGGACT
Bos taurus     TGAACCC------ACTCTTCT-CCACTGCTCCACTCAGCCTCCAGCCCTG------TTCCCTCTATCCC-CTACCTGCTCTTCCACGGCCCACTGGTGGTAGCGCCTGGCTCCCACGACC

Homo sapiens   CCTGTGTCTGTTCAACTGCAGAGGGCACGTATCTTGTTTCCTGAGCCAGGACCCTTTTCATCTCATCTTTTTAATTGTGTCAATCTCCTCTTTTCTTTGGG-----CAGAGTGGGGTGGG
Bos taurus     CTGGTGTCCACTCAGCCACAGAGAGAATGT--CTCATTTCCTGAGCCAAGACCCTTTCGGTCTCATCTCTTTACTTGTGTGACTCTCCTCTTTCCTTTGGGATGGCCAGAGGTGGGCAGA

Homo sapiens   GCATAGGCTGGTGGGACTGTTGGGGGCGACAGGGAAGAGGGGTGGAGTTGCATAGGCCAAGTGCTAAGGCGGAGCTTCTGGAGGACTGTCAAGTGAAAGGCGGCCAGAGGCAAATGTCAG
Bos taurus     G--TGGCCTGGTGCAGCTACTGGGGGCGA-GGGGAAGAAGGGTGGAGTTGCATAGGCCAAGTGCTAAAGTGGAGCTTCTGGAGGGCTGTCAAGTGAAACGCGGCCAGAGGCAAATGTCAG

Homo sapiens   AGCCTATATAGAGGCAGGAATCTGGGACCAGAGAGACAGAGCAAGCAGTAGCAAGTCAGGAGCAGTGGGGCGGGTGGGCGGG--------CAGGCTGGCTGGCGGGCAGG---GGGGCGT
Bos taurus     AGCCTATATAGAGGCAGGAATCTGGGACCAGAGAGACAGAGCAAGCACTAGCAAGTCAGGAGCAGTGGGGCGGGTGGGCGGGTGGGCAGGCTGGCTGGCTGGCGGGCAGGAGGGGGGCGT

Homo sapiens   GGCAGGGGATAGCAAAGGGAGATTGCAGAGAACAGCTGTTGGTACTGGGTCTTACTTTAATGAACTGGCTGGGCACGAGGTATAAGTTAATTGGCCAGAGTGAAAGAGCTAGGGGCGCAA
Bos taurus     GGCAGGGGATAGCTAAGGGAGATTGCAGAGAACAGCTGCTGGTACTGGGTCTTACTTTAATGAACTGGCTGGGCATGGGGCATAAGTAAATTGGCCAGACCAAAACAACTAGGGGCGCAA

Homo sapiens   AGGATACTCTCTCCCTCTTCTCCGTCACTGGCAAGGTAAGATCTTTCTGGGTTTTGCCTGGTGGGAAACTGTGGCATGTTCAGATATTTTTCTGAAGATGGG--AGGTGGAGGT------
Bos taurus     AGGACATACTCCCTCTCTTCTCCCTGACTGGCAAGGTAAGATCTTTCCGGGTTTTGCCTGGTGGGAAACTATGGCATGTTCAGGGATTTTTCTGAAGATGTGGTAGGTGGGGGTGGTCAG

Homo sapiens   CTATGGCTGCTTTTTTGACTCATCTTTCCCATTGCACCAATTATTACAATTATTACAATAACCAGGGTGTCAGCAGGAGGCCAGCATTTCTTTTATGACAACCCATCTTGGGGTTGTCCA
Bos taurus     CTACGGCTTCCCTTTTGACCCATCTTTCCCATTGCACCAATTATTAGAGT---------AACCACGGTCCTAACATGAGGCCAGTGTTTC------------------------------

Homo sapiens   GGCTGGAGACAGAGGCAGGGGTCTCGGTAGTGTGACTGAGTAAGGGGGAGAGGTGGATCTTTATAGCACCTTGGTGAAGAGTAGGGGGCTCTGGAACTGGATGGACCCAGGAATCTATGT
Bos taurus     ------AGATA---------------------------------------AGGTGGATCATTATAGCACAGCAGAGAAGAGTAGGGGTCTCTGGAGTTGGGTGGGCCTGGGAATCCAAGT

Homo sapiens   GACTGCAGGCAAACTATTAAGCTTCTCTGGGTCTGAATTTTCTCATTTGTAAGATAA--------AGGAAGA----------GTACAACCGAAATCTCTTAGTGTTGCTATGGGAACCAC
Bos taurus     GACTGCAGGCAAATTATGAAGCCTCTCTGGATCTTAGTTTTCTCCTTTGTAAGATGAGGATTTTGAGGATGACCGTCACTGAGGATGACCAAAATCTCTCAGTGTTGCTGTGGGGACCAA

Homo sapiens   ATGAGATGATACATGTAAAGTGCCTAGCAGAGTGTTTGCCAGTCAGTGCATGCTAAAGAAATGTTCA-CTGTTAGCGTTTCTGTTGCAGGTTTTCTCA---TTATTATGGAATCACAATT
Bos taurus     GTAAGAGAGTGCATGTAAAGTGCCCAGTAGGGTACTTGCCAGTCATCATATGCTCAAGAGATGTTGAGCATTTGGCACTTCTGTTGCTAGCATTCTCAATATTATTATGGAGGCACG-CT

Homo sapiens   CCATGAGGTTGATGACTTTTGTGACATTGTCCCAAAGTAGCAAGTTTTCTGCTTTTGGCATGGGCAGGCTGTGGGGACATGTCAACATTGTGTAATATTTGCATTGGCTTCCAAGCCGTG
Bos taurus     GCACCAGATTGATGAGTTTTATGACATTGTCCCAAATGAGCAAGTGTTCCACTGGGGGCATGAGCAGGCTGAGGGGACATGTCAACATTGTGTCATGTGTGTGTTAGTTTCCAAGTTCTG

Homo sapiens   TGTCCAAAAGCCCCTTCTG-GGCTGCTTATTGGAGCAGGCTGGAGGGGTGAGGCTCTGGA-TTGCCCACCC-------CCATGGCATCAGAGAAACTTGAAACTTGGACATGCCAGACAC
```

```
Bos taurus    TGCCAAGAAGCCCCATTGGAGGCTGCTTATGGGAGGGGGCTGGAGGGGTGAGGCTCTGGGCTTGCCCACTCACTCTTACCCAGTTGTCAGAGAAGCTTGAAGTTTGTACTTCCTATAGAC

Homo sapiens  TGGGCTTTAAGTGACCCATGATCTGGGATGTCAGAGAAATAATTGAATGCAAGCTCAGGGATGCCAAACACTAGGCAACCCCCAGGAGGGGGTCAGAGTCAATGCTATACAGTTTGTTAC
Bos taurus    TGAGCTTCAAGTGAACCATGATCTAGGACATCAGAGAGATAACTGAATGTAGCCTTAGGGATGCCAAAGGCTAGGCAACCCCAAGGAGTAAGTGATAGTCAAAGACAAACAGTTT-CTAG

Homo sapiens  TGTTGCAGTGACCCTATTTTAGCCTTGACCCTGGCTAATCTGAGAAAGCTTGTAAAATGGGGATAATAATATCTATTGCCCAGGAGATTGTAAGACCCAAATGGGATAATGCTTACAAAG
Bos taurus    TGTTTCAGTGGTCTTATTCTAACCCCAATCCCAACTAATCTGAGTGCACTGGTAAAATGGGAATAATACGACT-------------ATTGTAAGATTCCAATGTG-TAATGCTT------

Homo sapiens  GCCCTCACGTTATAGGAAGTTGTGACAGTTGTCTAAAGTACAGTGCCTGGCACATACTACGTGAAGCAATAAATAAATAGTAGTAATGGGGTGGTGGTTGCTGTTATTAATAAGAATAAT
Bos taurus    ----CTACATCCTGGTACACTG---CAGATG-CTAAAGTGTACTGCCTTGAACACACTAGGTGA-GCAGTAGGTGG-TAGTAGTA-----GTGGTGATTTTTGTTATTAAAAATCAAAAT

Homo sapiens  GAAAATAATAAGCAGAATTTTCAGTTCATGGAATGTTGCTCTCAGTCATTGATTAGATAAATGTCTCCCTTTTCCTGAGAA---CATTCAGGGCCATCAGAGTAGTAAAATGAGGATTAA
Bos taurus    GT---TAATAAACAGGAATTTCAGTTTGTGGAATGCTACTCTCA----TTGGTCAGTTAAATGCCTTCCTTTTCCTGAGGAAGACATTTAGGTCCATTAGAGTAGTAAAATGGGGATTAA

Homo sapiens  ACCACTGGCAGAATTCAATGAGCCCCAGGAACTAATAATTATCCTGGGGCAAAGGACTTTCCCATTCAGGCTTGCCAGTACCTTGGCTGGCTTGTGAGTACTGCC--GTGGGA-TAGAAT
Bos taurus    AGCATTAGCAGGACTCAGTGAGCACTAGGAACTAATAGTTATCCTGGGGCGAAAGGTGTTCTCATCCAGGC---------CCTTGGCTGGCTTGCTAGTGCTGTATGGTGGGAAGAGAGC

Homo sapiens  ACAGGCCAGGAATCCGCATTTCTCACATTTGGCCAGAAGGTGGTGCTGCTGCTTTATTTGAGTCAGGACTGCCCAGGCTCCTGGCTGCGGAGGCTGCTGGCGTTTATAGCATTTACAAAC
Bos taurus    GTAGCTCAGGAACCTGCACTTCTCACAATTTTCCAGAAGGTGGTGCTGCTGTTTCATTTCAACCAGGACCTCCCAGGCTCTGGGCTGCTGAAGTTGCCAGTGTTGATAGCTTTTATAAAC

Homo sapiens  TGAGAGGCTG-----------------GGTTGGTTTGAAAAGGATAGAGGCAGAAGGGACATTTGCTCCAGCTCCAAAGTTGTTCATTGCTTCACAGCTTCTTTAATCTGATAGCCTCAC
Bos taurus    TGAGTGTGTGTGCCGTGTGACTGAGCAGGTTGGTTTGAAAAGGATAGAGGTGGAAAGGATAGTTGCTCCAGCTTCGAATTTGTTTAATGCCATACAGCTTCTTTAACCTGAGAGGCCCAT

Homo sapiens  CTTCAATGCCATATACCTGACAA-CGCATAAAAACAGGTCTCCACCATGAACAATTTGTCTGTTTCCAAATCTTCTTGATACTGGCTTTTGACCTCTAAACTTGTTTCTTGTTTACAGAT
Bos taurus    CTCTGATGCCACAGAGCCAGCAAACACATAAGA---GGTCTCCATCACGAGTCATTTGTCTATTTCCCCATTTTTTTGAT-TTGCCTTTTGACTGCTAAACTTGTTTCCTGTTTACAGAT

Homo sapiens  TTTCTTGCCTCCTGGGCTCC------------------------------------CTCCACACTATCCTGTGGCTGTGGCAGATATGTCAATACCCTTACATTCACTGCGATTCAACAAT
Bos taurus    TTTCTTGCCACTTGGGCTTCAGCAGGACCCCTGATATAATTGCTTTAGAGGTTCTCTACACACTGTCCTTTGGCCATGGCAGATACATCAATACCCTTAAATTCACTGCAGTTCAGCAAT

Homo sapiens  ACAATGAGGGAGGAAAATGTTGAGCCCCAAAACAAGCAGATGGCCTTCTGTAGACCAATGACAGAGACCAGAGCAGACGTACAGATTCTGCATTCTCATGTACAGTTGCCTATAGTCTCA
Bos taurus    GTGCTGAGGGAGGAAAATGGCGACACCCAAAATAGGGAGATGACCTTCTCTGGACCAACGCAGGAGAACACAGCAGAAGGCCAAATTCTGCCTTCTAGTGTCCAG---CCTATGGTCTCA

Homo sapiens  ACTTCAGCCTCAGACCCTGGAGGGACATCCACACAGTTGATGACATCTCCAGTCTTTGACACCATGTCTGCACCTCTAATGGGAGTACCAAACTCTGGAGC-------------------
Bos taurus    ACTTCAGCATCAGACCCTGGAGGGATGTCCACACAGCTGATGACATCCCCAGCCTTTGACACCATGGCCACACCTCTAATGGGAACAGCAAACTCTGGAGCTGTGTCTTCACCGCTCCTG

Homo sapiens  --------------ATTGTCCCCACCCCTAATGCCAGCCTCAGACTCTGGGGCACTTTCCCCATTGCTAATGCCAGCCTCAGACTCTGGGGCACTGTCCCCATTGCTAATGCCAGCCTTA
Bos taurus    TCAGACTCTGGGGCACTGTCCCCATTGTTAATGCCAACTTCAGATTCAGAGGCTCTGTCCCCATTGTTAATGCCAACCTCAGACTCTGGGATGCTTTCCCCATTGCTAATGCCAGCCTCA

Homo sapiens  GATTCTGGAACATTGTCCCCATTGCTGTCCACTTCAGAGTATGGGGTAATGTCCCCAGGGATGATGACAATTCCTGATTTTGGAACGATGTCCGCAACGCTAATGGTAGCACCAGATTCT
Bos taurus    GATTCTGGAACATTGTCTCCATTGCTGTCCACTTCAGAGTATGGATTAATGTCCCCAGGGCTGATGA---TTCCTGACTTTGGAACAATGTCCACAGCACTAACGGCAACACCAGATTCT

Homo sapiens  GCAGAGATATCACCATTGGCAATGCCAGCTCCATCCTCTGGAGTGGTGTGTACACCTATAATGAGCACTTCATCCTCTGAGGCAATGTCCACACCATTAATGCTAGCCCCAGATTCTGGA
Bos taurus    GAAGACATATCACCACTGGCAATGTCAGCC----------------------------------------TCATCCTCTGAAGCAATGGCCACACCATTGATGCTAGCCCCAGATTCTGGA

Homo sapiens  GAGTTATCCCCAATTCTAATGCAAGATATGAATCCTGGAGTGATGTCTACACAGCCAGTGCCAGCTCCCAGCTCTGAGGCAATGTCCCCATTGCAAATTACAGATGAAGACACCGAAGCA
Bos taurus    GAGCTCTCCCCACTGCTAATGCCAGATATTAACCCCAGAGAGATGTCCACACAGCCAACGCCAGCTCCAGGCTTTGAAGGAATGTCACCATTGCAAATTACAGATGAAGACACTGAAGCA

Homo sapiens  ATGTCCAAAGTGCTAATGACTGCTCTAGCCTCTGGAGAGATATCTTCGCTGCTAATGTCAGGCACGGACTCTGAAGCAATATCCTCACTGATAATGTCAGCTGTAGCTTCTGGAGGTACA
Bos taurus    ATGTCCAAAGTGCTAATGACTGCCCTGGTCTCTGGAGAGATCTCTTCACTGCTCATGTCAGACACAGACTCTGAAGCGATCTCCTCACTGATAATGTCAGCCCTAACTTCTGAAGCAATG

Homo sapiens  TCACCCCAGCCAACAAGCACCCAAAACTCTGGGGGAATACCTACCCCGCTCATGTCAGATCTAGACTCTGGAATAATGTCATCGCTTTTAATGTCATCTCCAGGCTCTGAAGTAATGTCC
Bos taurus    TCCACCCAGCTAACGAGCACCCAAGATTCTGGGGAAATGTCCACCCAGCTAATGTCAGGCCCAGACTCTGCAGTACAGTCTTTACCACTAACAACAGCTCCAGGCTCCGGAGCCATATCC

Homo sapiens  ACACCGCTACTGTCAGTCCCAGATGCTGGAGAAATGTCCACATTACCAAAGCCAGCTCCAGATGCTGAAGCAATGTCCCCAGCACTAATGACGGCCCTACCCTCTGGAGTGATGCCCACC
Bos taurus    TCACCACTCCTGTCAGTCCCCGAGGCTAGAGAAATGCCCATCTTGTCAAAGCCAGCCCCAGATGCTGAGGCAGTGTCCCCACTGCTAATGATGGCTCTGAGCTCTGGAGGGATGCCCACC

Homo sapiens  CAAACGATGCCAGCCCCAGGCTCTGGGGCGATGTCCCCATGGTCAACACAAAATGTAGACTCTGAAATGATGTCTAATCCGCCAGTGAGAGCAACAGCCTCTGGGGTGATGTCTGCACCA
Bos taurus    CAGCTGATGCCAGCCCAAAGCTCTGGAGCTATGTCCTCACAGTTAACACAAAATCTAGATTCTCAGATTGTGTCTACTCCACCAACGAGAGCAACAGCCTCCGGGGAGATGTCTGCTTCG

Homo sapiens  CCAGTAAGAGCTTTAGATTCTGGAGCAATGTCCACACCGCTAATGGGAGCCCCAGCCTCTGGAAATATGTCTACATTGCAAAAGACAGTTCCAGCCTCTGGAGCCATGACCACCTCACTG
Bos taurus    TCAGCGAGAGCCTCAGATTCTGGAGCAAGGTCGACACTGCGAATGAGAACCCCAGCCTCTGGCAATATGTCCACGCTGCTAAAGACAGTCCCAGACTCTGCAGCACTGTCCACCCCACTG

Homo sapiens  ATGACAGTCCCAAGCTCTGGAGTGATGTCCACAGAGCAAATGTCAGCCACAGCCTCTAGAGTAATGTCCGCACAGTTAACAATGGCCAAAACTTCTGGAGCAATGCCCACAGGCTCTATG
Bos taurus    ATGACAGCCACAACCTCTGGAACCATGTCCGCAGAGCCAGTGCCAACTGCAGCCTCTAGAGGAATGTCCACACACTTAACAACGGCTCAAATTTCTGGAGCCACATCCACGGGCTTTATG

Homo sapiens  AAAGCCGTGGCAAAACAATACAAGAGAGCCACAGCCTCTGGAAAGATGTCCACGCCACTGAGGAGAGCTCCAACTTCTGGAGCAATGTCCACCCAACCAGTTACGGCAACAGCCTCTGAA
Bos taurus    AAAGTC------------------------CCAGCCAGTGGGGCAAAGTCCACACCACGAATGAGAGCTCCAGCTTTGGGGGCAATGTCCATCCCACCCAGCACAGCCCCCATCTCTGAA

Homo sapiens  ACAATGTCCATGCCACAATTGACAGTCCCAGCCTCTGGATCAATGTCCATGCTGCAAATGAGAGCCCCTGTCTCTGAAGCAATGTCCATGCCACAAATGAGAACCATGGCCTCAGGATTG
Bos taurus    ACAGTATCTATGCTACAAATGACCACCCCAGCCTCTGGGTCAGTGCCCACACTGCAAATAAAAGCTCCTGTCTCTGGAGCAACATCTAGGTCACAAAGGAGAGCCACAGCCTTGAGGGTG

Homo sapiens  ACATCTGCAGCACAGATGAAAGCCATGACTTCTGGAGCAATGTCCACCCCACTAATGACAGCCCAAACCTCTGGATCAACATCCACGCTGTTAATGAGAGACACAGCCTCAGGAGTGATG
Bos taurus    ACAGCCGGACCACAAATGAGAGCC------TCTGGAGCCATGTCCACCCCACAGATGACAGCCAAAGCCTCTGGATCCATGTCCTCACTATTAACGAGAGACACAGCCCCGGGAGTGATG

Homo sapiens  TCCTGTCCACAAATGAGATCTCTGGCCTCTGGAGCATTGTCCAAGCCACTAATGACACCCAAAGCCTCAGGAACGATGTTCACGGAGAAAATGACAACCACAGCTTCTGAAGCAATGCCC
Bos taurus    TCCGTGCCGCTGATGAAAGCTACAGCCTCAGGAGCATTGTCCAAGCCACTAACAACAACCAAAGCCTCAGAAGCAATGTTCATGCAGCAAATGACAACTGCAGCTTCTGGAGAGATCTCC

Homo sapiens  ACACTGCTAATGAGAGACACAGTTTCTGGAGCTCTGTCCATGCCGCAAATGACAGACACAGCCTCTGGAGGGTTGTCTGCATCGCTAATGAGAGACACAGCTTCTGGAGCAATGTCCACA
Bos taurus    ACAATGCTAATGAGAGACATAGCTTCTGGAGCCATGTCCATGCCACAGATGACAGACACTGCCTCTGCCGGGATGTCCACACCACTAATGAGAGCCCCAGCCTCTGGAGACGTGTCCACA

Homo sapiens  TCACAAATGACAGCCACAGTCTCTGGAGGGATGTCCATGCCACTAATGAGAGCTCAAGACCCAGGAGTAATGCCTGCCTCACTAATGAGAGCCAAAGTGTCTGGAAAGATGCTCAGTCAG
Bos taurus    CCACAAATGACAGCCGCAGCCTCTGGAAGCATGTCCGTGCCCCTAATGAGAGCCCCGGGCCCTGGAACCATGCCCACAGTGCTAATGAGATCCACAGTCTCTGGAAAGATGCCCAGTCAG

Homo sapiens  CCAATGAGCACCCAAGATCCTGGAGGGATGTCCATGTCGCCCATGAAGTCCATGACCGCTGGAGGGATG---------CAGATGAATTCCCCAACCTCTGATGTGATGTCCACACCAACA
Bos taurus    CCAATAGGCACTCAAGACTTTGGGGGGATGTCCATGTCGTTCAAAAGATTCATGACCTCTGGAGGGATGTCCACACTGCAGATGCGAGCCCCAGCCTCTGAAGTGATGTCCACACCAAAA

Homo sapiens  GTGAGAGCCTGGACCTCTGAAACAATGTCCACACCACTAATGAGAACCTCAGACCCTGGAGAGAGGCCCTCACTGCTCACAAGAGCTTCATCCTCTGGAGAGATGTCCCTACCACTAATG
Bos taurus    ATTAGAGCCCCAAGCTTTGGGGCAGTATCCACACCCCTGATGAGAGCCTCAAACCCTGGAGAGGTGTCCACACTTCTCACAAGAGCTTCATCCTCTGGAGAGATGTCCTCAGCACCAACG

Homo sapiens  AGAGCTCCAGCTTCTGGAGAGATAGCCACG---CCTCTGAGATCCCCAGCTTATGGAGCCATGTCTGCTCCACAAATGACAGCCACAGCCTCTGGAATGATGTCATCCATGCCACAAGTG
Bos taurus    AGATCCCCAACTTCTGGGGAGATAGCCACCACCCCTCTGAGAGCCCCAGCTTATGGAGCAATGTCTACTCCGCAAAGAACAGCCACAGCCTCTGGCATGATGTC---CATGCCACAAATG

Homo sapiens  AAGGCTCCCATCTCTGGAGCAATGTCCATGCCACTAACAAGATCCACAGCCTCTGGAGGGATGTCCA-----------------------------------------TGCCACTGATGAGA
Bos taurus    AGGGCTCCAATCTCTGGGCCGATGTCCACACCACTCATGAGATCCACAGCCTCTGGAGTGATGTCCACGCCTCAAATGACAGCTGTGGCCTCTGGAGGGGTGCCCATGCCATTGTTGAGG

Homo sapiens  GCCCCAGACTCTAGAGTGACATCCACATCACAAATGATGCCCACAGCTTCTGGAGACATGTGCACACTACCAGTGCGAGCCCCAGCCTCTGGAGGGGTGTCCTCACCACTAGTAAGAGCT
Bos taurus    GCCCCAGCCCCTGGAGCAACATCTGCACCACAGATGCCCACAGCAGCATCTGCAGAGGTGTGCACGCTATCCATGCGAGCCCCCAGCTTGGGAGTGATGTCCCCGCCATTATTAAGGGCT

Homo sapiens  CCAGCCTCTGGAACTATGTCCACACCACTAAGGAGACCCTCAGCCTGTGAGACTGTGTCCACAGAGTTAATGAGAGCTTCAGCCTCTGGACATATGTCCACTGCACAAACAACAGCCATG
Bos taurus    CCAGTCTCTGGAATTATGTGCACACCCCCAAGGAGACCCTCAGTCTCTGAAGATGTGTCCACAGAGTTCATGAGAGGTCCAGCCTCTGGAAAGATGGCCACCGCACAAACCACAGCCATG

Homo sapiens  GTCTCTGGAGGGATGTCCAAGCCATTAATGAGAGCCCCGGCCTCTGGAACAATGCCCATGCCTTTAATGTCAGCCATGGCTTCTGGAGAGATGTCTATGCCGCTAATGGAAACCATGGCC
Bos taurus    GCCTCGGGAGGGATGTCCAAGCCATCCGTGAGAGCTGTGGCCTCTGGAGTAACACCCATGCCACTGATGTCTGCCATGGCTTCTGGAGAGACATCCATACCACTAATGAAAAGCCTGTCA

Homo sapiens  TCTGGAGCAACGTCCACATTGCAAACCAGTGTTGCGAACTCTAGATCTATGTCCTTGTCACAAACAACATATACCGTGTCTGGAAGGATGGCCACAGCGCCAATTAGAGCCTCTGCTTCT
Bos taurus    TCTGGAGCAATGTCCACACTGCAAACAAGAGTTGTGAGTTCTGGATCAACGTCCTTGCCACAAACAACATACACAACCTCAGGAAAGATGTCCACACCACTGATGAGAGCCTCGGCTTCC

Homo sapiens  GGAGCAAGGTCCACATCATTTATGAGAGCCTCAGTTTCTGGATCGATGCCCATGCCACTACCAAGAGCCACAGCCTCTGGATGTGGCATGGGTATGTCCATGCCACAAATGACAGCCACA
Bos taurus    AGAGCCACATCCACACCACTTATGAGAGCCTCAGCTTCTGGAATGATGTCCATGCCACTTCTAAGACCCATATCCTCTGGG---------------------------------------

Homo sapiens  GACTCTAGAGGGATGTCCACACCACTAATGAGGGCCTCAGGCCCTGGAACAATGTCCACACCACA---GACAGCCTTTGGAGTGATGTCCACTCCGGAAATCAAAGCCACAGACTCTGGA
Bos taurus    ---------GGGATGTCCATGCCACTAATGAGGGCCACAGTCCCAGGAGCCATGTTCGCATCCCAAATGGCAGCCCCTGGAATGATGTCCACCTTGGGAATCAAAGCCACAGACTCTGGG

Homo sapiens  GAGGCATCCACCTCTCACATTAACATCACAGCCTCTGGATCAAAGCCCACATCGCACATGACTGCCACAACTCCTGAAACTGCGAAACCACCACCAAAGGAAGTGCCATCCTTCGGAATG
Bos taurus    GAAACATCTGCCCCTCGCATCAATGTCACAGCCTCGGGATCAAAGTCCACACCACATGCGACGACCACCCCCCCCAAAACAGTAAAGCCACCATCAGAGGAAGTCCCATCTTTTGGCATG

Homo sapiens  TTGACCCCAGCACTCTGCTACCTCTTAGAAGAGCAGGAAGCAGCCCGGGGCTCATGCTCTGTGGAGGAAGAGATGGAGATTGATGAGGAGAAGCAAATGAAGGGGTTTTTGGACGATTCA
Bos taurus    CTGACCCCAGCACTCTGTTACCTCTTAGAAGAACAAGAAGCAGCCCGGGGTTCATGCCCTGTGGAAGAGGAGGTGGAGATTGATGAGGAGAAGCAAATGAAGGGATTTTTGAATGATTCA

Homo sapiens  GAGAGAATGGCATTTCTGGTATCTCTTCATCTGGGAGCAGCAGAGAGGTGGTTCATCTTGCAAATGGAGGTAGGAGAACCTCTCTCACATGAAAATAAATCTTTCCTGAGAAGATCCCAG
Bos taurus    GAGAAAATGGCTTTTCTGGTGTCTCTTCATCTGGGGGCAGCAGAGAGGTGGTCCATCTTGCAGATGGAGGTAGGAAACCCCCTCACCAGTGAAGATAAATCTTTCCTGAGAAGATCCCAG

Homo sapiens  GGCATATATGACTCCCTATCTGAGATAGACATCCTCAGTGCTGTTCTTTGCCATCCCAAACAGGGCCAAAAGTCAGTCAGGCAGTATGCCACTGACTTCCTGCTGCTGGCCCGACATTTG
```

```
Bos taurus     GGCTTATATGACTCCCTGTCTGAAATTGACATCCTCAGTGCAGTTCTCTGCCATCCCAAGCAGGGCCAGAAGTCGGTCAGGCAGTATGCCACTGACTTCCTTCTGCTGGCCCGACATTTG

Homo sapiens   TCTTGGTCTGATGCCATTCTACGGACCAGGTTTCTGGAAGGACTCTCAGAAGCTGTTACCACCAAAATGGGTCGGATCTTCCTGAAGGTGGCTGGCAGCCTAAAGGAGCTGATAGACAGG
Bos taurus     TCTTGGTCTGATGCCATTCTGCGGACCAGGTTCCTGGAAGGACTCTCTGAAGCTGTTGCCACCAAAATGGGCCGGATCTTCCTGAAGGTGGCTGGCAGCCTAAAGGAGCTGATAGATAGG

Homo sapiens   TCTCTGTACACCGAGTGCCAGCTGGCTGAAGAGAAGGATTCCCCAGGCAACTCAAGCCAGGTTCTGCCAACAGCCTGTAAGCGGAATAATGAGGAGGCCATGGGGAATGAACTGAGCTCT
Bos taurus     TCTCTCTATACCGAGTGCCAGCTGGCCGGAGAGAAGAATTCCCGAGGCAGCTCCAGCCAGGTTCTGACATCACCCTGTAAGCGGAATAGTGAGGAAGCAATGGAGAATGAACTGAACCCC

Homo sapiens   CAGCAGCAGACTGAGGAGGTAATGAGGGGGAAATCCGCTGAAGGTTTTTGAGCAGAGAAATGAGGAGGTCACGACAGGGAATACATCCTGCTTCCTTGAGTAGAATTGGGAAGAGGCAAG
Bos taurus     CAGCAGCAGACCGAGGAGGTAATGATGGGGAAACACACTCAAGGTTTCTGAATAGAAAATTGAAG-----------------GCATCCTGCTCCTTTATGTAGACTAAGGAAGAGGT-TG

Homo sapiens   GGGGAGGCATGGAGCTCCTAGACCATTAAACCCCAGC----AGTTTCATCAAGTGAGCATGGCTCTGCAGTTATTTTGGGTATATTAACT------------ACTTTGAGGAGCAATGTC
Bos taurus     GGGGAGGCATTGAGCTCCTAGACCA-TAAGCCTCAACCTGGAATTTCATC-AGCGGGCCTGGTTAGGCCCTTGTTTTGGGCATATTAACCCAAACTCCATTAACTTTGTGGAGCCTTGCC

Homo sapiens   CTGCCCAAGTTACTGAGAACGTGGGATAGTTGAAGTACTAATTCTTCTCATTATGAGGGAACTGGATGGGCTCAGTTCCAATTCT---CCCCTTGTTCCAAGTCCAATTCTAATAAATGG
Bos taurus     CTACCTAAGATACTCAGGGTTTGGGGGTGGTAAAGGTCTAAATCTTC------TGAGGGGACTGGTTGGGCTCAGTCCCAATTTGCTACTACTGATTCCAGGTCCAGTTCTAAAAGAGGG

Homo sapiens   GTC---TTTTGGGAGCTGGGGCCCATCTACAGCAACATGTGTTGTGGAGTCAGGTCACTAGGGAATTAGAATAGCCTACTCCTTGGTCACAC--TGTATGCAAGTGAGATAAATTAGCAA
Bos taurus     GTCATGTTTTGGAGGCTGGGTCCCACCTACAGCCACATGTGTTGTAGACCCAGGTCACTAGGGAATTTGACCAGTCTAGTCCTTGGTCACACTTTTTATACAAATAAGGTACGTTGGCCA

Homo sapiens   AGTGTCTTGATCTGCTAAGTCACAGAACTTATCCAGGCAGAACACCCGTTGCCATAGTAAGTGTGGCTCTTACCTCTGTTG---------TCTTTTTCTCACTCCCAGCACCAGCATGTT
Bos taurus     AGTTCCTTGACATGTTAAGTGATTGAGATTATCCAGGTGGAACCCCACTTGTCATAGTAAGCATGGCTCTTACATTTGCTGTTTTTGTTTTTTTTTTCTCACTCCCAGCACCAGCATGTT

Homo sapiens   TCCAAACGCTGTTACTACCTGAAAGAGCATGGAGACCCCCAAGAAGGTCTTCATGATCACCTTGGACAGAGCACAGGCCATCATCAGAAGGCCCATACCAACAAGTAAAACTCCATGGAA
Bos taurus     CCCAAACGCTGTTACTACCTGAAAGAGCATGAAGACCCTCAAGAGGGTCTGCACGACCACCTTCGAAAGAGCACAGGC---CATCAGAAGGCCCCCACTAACAAGTAATGCTACCTGGAC

Homo sapiens   TCTTCTCCTGTGATATCTGACTCGACCGCTAACTGGAGGACTGGTTCCTGGACCCATTCCATTAAACTACATTA-TAAACCTTGTTGCC-TTCACCCTTCAGCCTCCCGCTCCAGGCACA
Bos taurus     TCTTCTCCTGTGATATCCAACTTGGCTGCTAACTTGAGGACTGGTTCCTAGACTCATTCCATGCAATTACATCATTAGAACTTGTGGCCTTTCCCCCTTCAGCACCCC-CTCCAGGTGCA

Homo sapiens   TCCCACCTTACCTCTCACTTGGCTGATTTGACCTTCTCTTTCA-CCCACATGCCTAAGACCTGCCCTGACAATCAGAGTAGGGACTACAAGAGTGTATGGTGTATGAGGTTCTGAACTCC
Bos taurus     TCCCACCCTATCCCTCACTTGGTTGACTTGACCTTCTCTTTCACCCCACATGCCTTTAACCTGCCCCAACAATCAGAACGTGGACTTCAAGAATGTTCAATGTATGAGGTTCTGAACTCC

Homo sapiens   CATCACCACCGAGGCCAGTCCTAGTTCTTGGTCCAGGGAGAAGTCTAGGCAGGATAAATCATATCTTACCTCAAAAAACCCTTAGCGTATCTTGCCATGTGTCAGCAGGCAAACTTGAGG
Bos taurus     CATTACCAACAAGGACAGTGCTAATTCTTGGTCCAGGGAGAAGTCTAGGCAGGAGAAACTATGTCCTACCTCAAAAACACCTTAGAGTGTCTTGCCACATGTCATCAGGCAAACTTGAGG

Homo sapiens   AAGCCCTGGTTCTATTTCACCTGGCAGGGGAGCCTATAAGGAGAGCGATGCCCACTCCTATACCACCCTTGTAACCCAGTAGACTTG-CCCCATATGCCCCAACTCATAGCACCTAAAAT
Bos taurus     AAGCCCTGGTCCCATTTCACCTGGCAAGGGAGCCCATAAGGAGAGTGATGCCCACTCCAATACCATCCTTTTCATCCAGTATACTTGTCCCTATATGCCCCAACTCATAGCACTTAAATT

Homo sapiens   GGGTCTCCAGGGTCCTTGCCCTGTTTATAGTATCCATCCTGAACCATTCGTTTTGACCCATAATAATAGTGATAGTATAATGGCTACCAGTTATTGATAATGTATCATGTGACAGGCATT
Bos taurus     TGGTCTCCAAGGCCCTTGTCCTGTTTATAGTCTCCATCCTGAACCATTCGTTTTGACCCATAGTAATAATCTTAG--TAATAGCTAATGTTTATTGTTTATGTACTATGTGACAGATAGT

Homo sapiens   GGGCTAAACTCTTGACAAGCACCATCTCACTACATTCTTACAGTAACACAACTTGTTCTTGTGTTGTCAGCTTCTGACCTCTTGATCTGTTGTTTATGGCCTTTGTCTTGTCCCTCAGTG
Bos taurus     GGGCTAAACTCTTGACAAGCATCATCTCACTACATTCTTACCATAACCCAACTTGTTCTTCTGTTGTCAACTTCTGAAGTCTTGATCTATTGTAAGTGGCCATTGTCTTGTCCCTTAGTG

Homo sapiens   GGGGTTCTCTAGCTCTGACCTCTGGGTGACCCCCTCAGTTGTTGATTGCTCTCACTCCCCTTGGCTGTTGTGCTTCATGGCCAGCACTCTTGATGAGGGTCTTGGTCTATAAACAGGCAT
Bos taurus     TGAAATTTCTAGCTCTGACCTTTGGGTGACCTCCTCAGTTGTTAATTGCCCTCACTCCCCTTGGCTATTGTGCTTCGTGGCCAGCACTCTTGATAAAGATCTTGGTCTTTAAACAGGCAT

Homo sapiens   AGATAGCTAGAACATAATGTGAGCAGTGTTTGCCTTCAGGTGGTGGGGTTATGGGTAGTTTTTATTTTCTTCCTTCTACTTCTCTATACATTTCAGATTCTCTACAGTAAGCATGTGTTA
Bos taurus     AGA----TAGAACATAATGTGAACAGTGTTTGCCTTAATGTGGTGGGATTATGGGT-GTTTTAATGTTCT---TTTTACTTCTCTGTACTTTCCAGTTTCTCTACAGTAAGCATGTATTA

Homo sapiens   CATTTGTAATTATAAAAGTAAGTAAACTTCATTTATG-AAAAAAGAGTTCATTGTGGCTCCTAGAATTTGATTTACTGAGTCCTTTATTCAACCAATATTAGTTATGTAACTTATGTCAG
Bos taurus     CATTTGTAATCGTTAAAGTAAATAAAGTTCATTTATGAAAAAAAGAATTAACTGTGGCTAACAGAATTTGATTTATTGAGTCCTTCATTCAGTCAATATTTATTATCTAACAT-TGTCAG

Homo sapiens   CTACTCTTTGGGGGACAGACGGATAAGAATCCTGCTGATATGAACATTCCATTCCAATGGAGTGGGGAG----------AACAAGAACACACTGAACT----TAGATTGGAATT---GTG
Bos taurus     CCACTGTTTTGGGGACAGACAGATAAGAACATTGATCAAACGGAAACTTCATTCCAACAGAAGAGGGAGACAGACAATAAGTAAGAAAACACATAAGTAAGATAGGTTGAAATCGTGGTG

Homo sapiens   AAGGCTGTGAAGAGAAGAAATCAGGACAATGGGGAATTTGATGCAAATAAGACCAATTTATGTATTCAATAGTAGTCCATATAATTGAGTTACTCAAAGATCTTTCAACAATAGTAGGAG
Bos taurus     AAGGCTATAAAGACAGGAAATAAGGATAATGGGGAATTTGATACTAACAGAGCAAATTTATTTATTCAATAATAGTCCATGTAATTGGGTTACTCAAAGATCTTTTAAAAACAGTAGGAG

Homo sapiens   AGACATGATTAGAAACTTATTTTATATAGTTAAATTGGCAACAGTTTGGAGACAGG-------------ATGGAGG---AAGGGATCAGA-------GAGCAGCTGAGCCCT-------C
Bos taurus     TGACATGATTAGAAACACATTTTAGAAAACTAACTTGG--ACAG--AGGAGCCTGGCAGGCTATAGTCCATGGGGTTGCAAAGAGTCGGACATGACCGAGTGCCTGAGCACGGCAGCAAG

Homo sapiens   TACTGCAATAGTCCAGGTAACTGACTGGCTAGGTGACTCAATCTAGGGTGGTGGCAGCAGAGATGATAATTCAGGAACAGAGACCTAACCTTCTCTGAGGACTCAAGTCCACTGCTCCAG
Bos taurus     CACTGCAATAGTACAGGTGACTGACTCTTCAGATGACTCAATCTAGGGTCAGGGCAGTGGGGATGAAAAG--AGGCACAGAGACCAAATCTTCTCTG--------------------AG

Homo sapiens   GACTCAAGTCCACTGCTCCATTACCCTAAACTTTGTGTCTGATTTCAGGAAGAGGAAAATGATACTCAGGAAAG---ACAGATTGGCAGATAAATC
Bos taurus     GGTTCAAGTCTGCTGCTCCATTACCTTAAACATTGGATCTGATTCCAGGAAGAGGAAAATGATTCTTGGAAAAAAACAAAGATTAGCAGACAAATT


Homo sapiens   AAACCTCAAAGGGAGTGTAAAATGCAGAGTGTTGTCAACTTA-TCACACCAAGAATATTTTTTTGCACCTTTTTAGTTTGGAAGGGTCAAATTGAGATGACAGAGGAATAGTGGATAAGA
Mus musculus   AAACAACAAGTGGAGTACAAAATGGTGAGTGTTTTCAATTTACTGGCCCCAGGAATATTTTT--ACCCCTTTGAAGGTTAGAAAGGGCAAATGGAGAAAACAGGGGAATAGTGGATG---

Homo sapiens   GCTATAGTGCTATTAAATTACATTCTAGAGTTGGGAAAATCGAGACCCATAATCCTAGGAATTATTAATGATAAGAGCTAACACTTTCTGAGTGCTTACCACATGCCAGGCCCTATACTG
Mus musculus   GCCATTGT--------------------AGCAGGCAAAATCTAGTGCCATAATCATAGCTGTCATTAACAGTAAGTGCTAACATTTCTAAAGGGCTTACCATGTGCAACACCCTTAGGAA

Homo sapiens   AATGCATCATGTCCATTTTCTCATTTGATC----CTGACAACCACCTGTGAGGCCTCATTTTGAAGACAAGCAAGCTGAGGCTCAGACCTTGGATTTGTCCAAGGTCACTCAGTTGGTCA
Mus musculus   ATTGCCTTACATCCATTTCCTCCTTTGATCGTGCCTGACAACTACCTATGAGGTCTCATTTTGCAGACCAG--AGT---------------------------GGTCACACAGTTGATCT

Homo sapiens   TTTACAGAGCCCTGATTCAAACTCTCTTTTGCTTGATTACAAAGCCATTGCTCTAAAAGATTAGATTATACTGTCTGTATTTGCTTCTGGGAGCTTATGGAATTCACTCCTATCTGTTCT
Mus musculus   TTGACT---------TTCAAACTCCCTTTGACTTGATTGTAGGGACATTGC-----------------------CTATACTTGCTTTTAGTGATTT-TGGAATTCATGGTTATCTGTCCT

Homo sapiens   CACAAGGAAC----TCTATTGGGTTTTGAAAAAGGCTAAAGAATTGAAAAGAAACTTTGTAGGCTGGGAAACATAGTGTCCTTTGAGCAGTGAGATAGGCTAAGCTCAGAGGACTGGGCT
Mus musculus   TACAATGGACCGACTCTACTGGGCTCTGAAACGGGCTAAAGCATGGAGAAGACATTTTGCCAGCTGGGAGATATA--------------------TAGGCTAAGCT---AAGATTTGCTT

Homo sapiens   CTGGAATGTGGAAAGGAGACGTGCCCACTGGAGAGCAAGTCAGAACTTTCGTGAGCCCTGCTCCAGGTCCCAGGAGAAAGTGCCAGCCATGAGGACTGGAGTATTGCCTGGTAAGGAGAA
Mus musculus   CTGGAACTTGCAGTGG---TGTGACCACTAGAGTGCAACTCAGAGCTTATGTG-------------GCTCAAAGAGAAAGTGCCAGTCAAGGTGA-TGGAATGCT..............

Homo sapiens   GATTATTATTTTATCTAACACGTTCTTCTATAACGCTCTATATGCTAGGCACTGGTCTGCACTGCTCTAAGTACTTTATAAGTATTAACTCCTTTACATCTTATAACACTTCCCTGGAAA
Mus musculus   ..................................................................TTTGAAGGCTTTGAAATGATTAGCACTTTTCAATCTTGTAACAAATCCCTGGAAA

Homo sapiens   TAGGCATTATTATTGCCCCCATAATACATATGAGTAAACTGAAGTTCAGAGATGTTAAATAACTTGTTCAAGGTCACATAGCTGGTTAAGTGCCAGAGCCAGGCTTTGAACCTAGGCAGT
Mus musculus   TTGGTACTACTGTTATCCT-ATAATACATATGATCTAACTGAAGTTCAGAGATGTTAATTTCCATGTCTGCATTTG--TACCTCGGGAA-----AGAAACAGAGTGTG-------GCAGT

Homo sapiens   CTGGATCTAGTGGCTGTGTTATACTGCTTCGCA--ACAGAAACTTGATTTTTCTTTCTTCTGTGTTTATCATCCTTTCTCAATCACTCCTGGGCAAGTC----TACTTTGCTAGCAAATT
Mus musculus   CAGAATATAGTGGCTCTGTTATATTGCCTCTCAGGACAGAGGCTTGAATTTCCTTTTTCTGGTGCTT----TTCCTTCTCGTCTATATCTTAGCAAGTAAGTGTGCTGTGCTAGCAAATT

Homo sapiens   TCCCTTACCCCTTTGGCTTTTTTTTCACCCATGAACCCTCTAATGTTTTCTGCAGCTTCTTTGAGCTCATTCTTTATCTTCCACACTGCCTCTTTTGGCATTACTCATGAACGACATGCT
Mus musculus   TCCCCAAACCCCGTGGCTTTTATATCATTAGTGACTTCCCATTTGGTTTCTTCATTATTCTGGGGCTCCTTTATCATCCTTCATCCTGGTTCATTTGGCATTACTTATGGCAGACACCTT

Homo sapiens   CTTTTCATCTGCCAAATATTTTT----TCCATAAATTATCCCATCTCAACTTGGGA--------AGTCACAATGGAAGTATTGTTCCTATTTTGTGAATGAGGCAACTAAGGCAAGCAA
Mus musculus   CTTTTCACATCCCTAATTTTTTTCTTTTTCATAAATGATTCCATTTTGACTTGGGACGTGTCTTAGTCACAATTAGAAGTTTGGTTTGTATTACATGAATGAAAGAAGAATGGTGAGCAG

Homo sapiens   CTTTCCTGAAGTCTCACACA----ACCATCTAGTGGCAAAGTGCACTAGAATCCAGATTCTCTGATTCTGAATCTAATGTTCTTGTCCATTGCACCATCTGATGGAAT---TTAACT-AT
Mus musculus   TTTGCCTAATGTCTCATACATATAACCCTTTAGTGGCAACATGAATTAGGCTCTAGGTTCTCTCATTATCAATCCAATGTTCTTTTCCATTGGATCATCTAAGAGAATGCATGAACTTAT

Homo sapiens   CTATTCATTAATTAATTTATTCATTAATATTGTAAAAATATACTGAGTGTCTTACTGTATGGTAAGGCCCT-----GGGTGCAAAAGTCGGTATAAAAATTGTTGCTCTTAAGCAACAGA
Mus musculus   TTATTTAACTACTCGTTTATCTGCTACTATTATAACAACACATTGCGT---TTATTATATA-CAAGGCACTTGGGGGGGTAAGAAGGTCATTAATAAAACTGTTTCC---AAGGTTCTGA

Homo sapiens   CAACAC-AGAGAGATAAGGTCTCACCATAAAAAGTTAAATAGCAGCACATATACTAAGTGCCAAATGAGTGGTCTGGTCACATCATACAAGAATTTTGAGGTTGGATATGTAGCATCAAA
Mus musculus   TAACACGAGTGAGATGATGTCTCACAAGAAAGAGCTGAATAACATCACA----------GTCACATA---GACCTGCTCAGGTCATATAGGATTTTTGAGAGTGGATACATAGCATCAAA

Homo sapiens   GACTGCTTTATCAGAAAGGTGAGACTTGAACTTGGCATAGAAGGGTACTTAGAACTTAGAGAAGAGAAT-GGAGCAGCACTTTCCAAACTCTGGGAATGGTGTGCATTAAAGCAGTCCTT
Mus musculus   GAC--CTTCAGTAGGAAGGCG-GTTTTGAATTTGGCATATGAGGGTGTGAAGAAGTGAGAGAAAGGAATTGAAGCAACACTTTCCAAGTT---------GTGGCCACTAATGCAGTCTGT

Homo sapiens   CACAACCTTTATTGTGTCATGGCATGCAGAGAAAAGCATAGTATTTGTAAAGCACACTAGGCTGAATGGAGGAAGCAGTTTGACATCT-AGAGGCAAGCATTCTAGGAGTTTCAACAGCA
Mus musculus   ATCAATATTTGTCATGCCATT-TGGGCAGATGGAAGCGTAATATTTGCAAGGCATGCTGGG-TCAGTGGAGGAA-TAATCTTACACCTCAGAGGCAAGCATTCCAGGAATTTCAGCTGCA

Homo sapiens   CTGTCTCTGGGCTCAGTTGAGGACAGAGAAGAAATCTCA-TAGCCTCAGACACTTGTAATATGTTTGGGAAGGTCTGCACTAGGTATTGGGGGCACACTGTGGTCATGGTGGGTCTAAAG
Mus musculus   GTGTGTTTACGCTCAGTTGAGTACAGAAGAGAAGACTCAAGGGTTTCAGATACTTATGAGAATCTCATGATGGTCTGCACCAAGTTATGGCTG-----TGTGGTCAATGTGGGTATA---

Homo sapiens   ACCACTGACAAGCCCACAGGGATGAAATGAGGGGAATTTCCAGA-----GAAGCCATAGTAGATACCATTGGAAAGAAAAGTTATGGGCAAATGATGAAGGGCTTTTGAATTTTATTCTG
Mus musculus   -CTACTAACATGGTCATGGAGGTGAAATGGTGGTCATTTCTGGAAGAGTGATGTTATGGCAAATATGATTAGAGAGAAAATTTATAGTGAGATAATGA-GGATTTTTGAATTTTATTCTG
```

```
Homo sapiens  TGGACCTACAGAA-TAGGTAGGCAAAATATGCATAGCACTGTTTTAGAAAGATTCCACTGGTTTTCATATGGGAGATTAACTAGAGGGAAGAAGAGTAGTCAAGAGGCTATTACCATTGT
Mus musculus  TGAGAAAAGAGAAATAGGTAGATAAAATACACGTAACATTAGTTTAGGAAGGTCTTTTTGTCACTAGCATAAATGATAGACTGTAGGGAGTAAAAGTAGTTGGTGGGTTACTACCATCTT

Homo sapiens  CCAGGTGGGAGGTGATGTTATCTTGAACTAGATTGATAGTAATAGAGACAGAGAATGGTGGACAGAATTGAGTTATATTTTGGAGGT-AAAAATGTCAGGGTTTGGTAATAGAATTGCAT
Mus musculus  CCATATGATACATTATGGTATCTTGGTCTAGAGAGGCTATAACAGAAGAAAAAAATGGTAGACAGAATAGAGTTATATTGTGGAAGTGAAAAATGATATGGTTGGGGAACAGACTCACAT

Homo sapiens  AATGGAGAGTAGAAGAAA------------GGGAGGTGACAAGGAGGATTTCAAATTTTCAAGTTTAAGCAAATGCTTATGAAT--------AGTGATGCCATTCTTTAA---------G
Mus musculus  ATTGGAAGGAACAAGAAATTTATGTAGCTTGGTAAGAGAAAAGGGCGATTCCCAGGTTTCAAATTTGAACAAATGACTATAAATGAGGAGGAAGTATTGCATTGCTGTACATATGGGGTG

Homo sapiens  ATAGAGAGC-----CCAGGAGAAGT-----------------------------AGAAAGATTTT---TTTGGAGGGGAAGGACAAATGAGATGATGAGTTTCAAGTTGACATTTGGAGG
Mus musculus  ACAGAGGACAATGTTCAGGAGAAGCCTTTACTTGCTGAGCCATCTCACTGGTCCAGAAAGATTTTATTTTTTCATGCGCAAGACAAATGGAATGATGAGTTTCAAGCTGAAACTTAGAGA

Homo sapiens  CAAGGAAA-CCAGTTAGGAGGCTATTGTAAAAG-CCAAGTAAGACAAGGTGAGATCCTGGACTACAATGGTAGCAGTGGGAATGGAAGGAAAATGGTGAATTGAGAGACATTATACAGGA
Mus musculus  CAAAACAATCCAATTAGAACACCATTGCAAAGGGCCCGGCCAGGCAGTATGAAGCACTAGATTGGAAAGATAACTGTGAGAATGGGAAGAAAATGCTGGATTAAAATGTGTCATGAATGA

Homo sapiens  AGAATTGATAGGATTTTGTCACGGACTGACTTCTGGAGTTGAAAGGTAGAGGAAGGCTTGAAGCCTAAGATATCTTCTGTCCTTTATCTTCTTAGTGATGCTCCGATCTTCGTGGCTTAA
Mus musculus  AGACTTGACAAGATTTTGTCTTGGACTAACT-ATAGAGTTGCAAGATAGAGAATATCTGGAAGCCTCAGATCTCTTCTGTTCTTCA-----------AAGCATCCATTCTCATGGCTTGA

Homo sapiens  TATACTATCTATTTGCTGATGAGTCACAAATTT----GTATCT---CCATACTTTTATGCCCAATTGCCTGC------TGGACATATCAACTTGCATGCCTAATAGGTATCTCCAACTTA
Mus musculus  GATACTATTTTTATCCTAATGATTCCCAGATTTAGCGGCACTCAGACCACACTCTCTTGGCCAACTGCCTTCCTTTTATGTACACATGAAGTTTTATGCCTGATAAACATCTACCA--TC

Homo sapiens  CCATGTTCAAAACTG--CCCCTGCCCACAACCCCCAACAAAACCTGCTGCACTCACAAAACCTGCTCCTCACAGTCTTCCCCGTCTCCCTAAATACCAATTTCATTCTTCCGGTTGCTCA
Mus musculus  CCATTTTCAAAACTGAATGCTTGGTTTCTAGCCCCA-----------------------------TCCTCA--GTCTTC------GCACTGAATGTTGACCATATTCTCCCAGTGGCTCA

Homo sapiens  GGCGGAAGACCTAGGAATCAATCTTGATTTTTCCCT--CTTTCATATCCTACATTTATTTGATCAGCAAATCTTATTGGTTCTACCTTCAGAATATATCTAGAATCTGACCACTTTT---
Mus musculus  GGCAGAAGACCTAGGAGTTAGCCTTGATTTTTCTTTTTCTTTCACATCCTTCATTTATTCTCCTGGAAAATCTT---GGTTTTGCCTTAGACAGTTATCTAGAATGT-ACCATTTTTATC

Homo sapiens  ----TCATTGCTACTATCCTGGTTTATGTCACAATGATCTG-CTTCCTGGATTATATTGCACTAGCCTCTCAACTGTTCTCCTTGCTTCTGTTCTTGCCACCATCACTCAAGCCTATTAT
Mus musculus  ACTTCCATTGCTACTCTCCTGATTCATATCATAATGATCTATCTTTTTGGAGTATAGTATACTGGCTTCCCATGTGGTTTTGCTCCTT-TGTCTTTGCTACCTTCATCAAAGTCTATTAT

Homo sapiens  TCTCTCACTGTTTTTTGGAACCAGGATAATTTTATTATGTGAATGTCAGATCATGTCACTCCTCTGCTCAAAACTCCTCCAATGGCTTCCCATCTGACACTCAGTAACAACAAAACCCCT
Mus musculus  TTCTTTATTGCCTTTAGGAAGCAGATGGATGTTGTTATATGAAAGTCAGAC---GTCATTCCTCTGCTCAAA-CCCCTTCAATG-CCTCTCATCTTACACATAGTGATAGCAAATCTCCT

Homo sapiens  TATAATGGCCCATAATGC-TGTATGCA-------------ACCCAGATATTGACATACTCCTGTCCTTCAGGTGATGGATCAA------ATATCCC------ACTTGGTAAAATCTT---
Mus musculus  TTCAAGGACCCATTGCGCGCGCACGCACACACACACACACACACACATACACACACGCACTCGTGCATCATGATATAACTCATTCCTTTACATCCTTTGGGAGTTAGATGAAATATTGCT

Homo sapiens  CTCTGATTACCATATTTAGTATTGCAAGCGCCTTCT-CGCCATTCTCTATCTGCCTTCCATGTTTTATTTCTTTATACTTTGAATACTAGCAGATGTTATATGTTTATCTATTTATTTGT
Mus musculus  TTCTGACTGTCATATTTGAATTGGCAAGTGCTTGATACTTCATTATCTAATTATCTTCCA-GTTTTATTTCTCTATACTTTAAAAACAACTTCTCATTATGTAATTGTCTATTT--TTG-

Homo sapiens  TTATCATCTATCTCCTTCTGCTAGAACAGGAGCTGGAAAACTTTTTCTGTAAAGGTCCA-GATAATAAATATTTTAGGCTTTGTGAGCCACGTAATCTTTAACACAACTACCTACCTCTA
Mus musculus  -----GACTCTCTGATTCTGCTGGAACAGGTC...........TTTTTGTAAATATTTATGATAGGAAACATTTCAGGTATTTCGAAT-ATCCACTCTCTAACTTAACTGCTTAACTTTG

Homo sapiens  TCATTGTTATACAAAAGCTGCCATAGATGA--------------GCATGGCTGTGTTCCAATAAAACTGTATTTA----------------------CCAAAACAGGGAGCGGGCTAGAT
Mus musculus  TTATTGATATAGAAAAGCATCCACAAATAATATACATGAGAGTGATATGTTGATGTTTCAGTAAGACTGTATTTGTATAAGCTTTGTATAAGCAAAGCTATAATAGGTGACAGGCCAGAT

Homo sapiens  TTAGCTAATGGGCCATAGTGTACTGACCCCTGCTAGAATGTAAATTCCAGGAGGGCAAG-GACTTTTGTTTACTGCTGTATCTCCAACA----TCTAGAATAGTACCTGGCATATAGTAG
Mus musculus  TCAAATGTTGGGAGCTAATGTGCTGATGCTTACTATAGTATAAATTCTAGAAAGACAAAAGAGTTTTGTTTACTACCACATCTCCAACAATTGTTGAGAACGGT-TCTGTCACATAGTAG

Homo sapiens  GCACATGATTTATTTTACTGAATGAATGCATCAGTGGGAGATTGGAAAGCTGTGGAATAAAAAATATATATATATAACAGAGCCATTGATGGTGCAGAATCCTGAACAAAATCTAGCTTT
Mus musculus  GCACCTGATATAGTTTA--AAATTATCAAAGTAATGG-AGACTGAAGACCTGCAGAATCAAAACAAT-----TATAACAGCAGCTCTGATGGCACAGAATCTTGAGCAAGAATGGGCTTT

Homo sapiens  GGATGGGTTTGCAGCATATCAACTCTCCTTCCTTCTTTTTCCCCTCATCCCTACACTAAAACTTAAGCTTGT---TATATAAAGTAAAAGTTGCTTTCATTGCCTGTGCAATTCTCCCAA
Mus musculus  GGTTAGGTTTATAGCATATCAGCTCTCCTTCCTCCCATCCCTTCTCATCCCTACACTATCACTTAAGCATGTCTGTGAAGTAAATAAAAGTCGTTTTCATTGCCTATGCAACTCTCCTGG

Homo sapiens  TGTGTGAAATTTCATCTGACTTCCTTTAATGTTGTCATTTGTCTATTTAACCCTCCTGACTCAGACTCCCTCTAATAACAATAATAACTCAAATACTCTGTACTAAAACCAGAACGCTGA
Mus musculus  TCTGTGAAATTTCATTTGACTTCCTTTAATGTTGTCATTTGTCTACTCAACCCTCCTGACTCAGACTC--TCTAATAACAATAATAACTCAAGTGATCTGCACTAAAACCAGAATGCTGG

Homo sapiens  AATCAGTATTTTT-AATTAATTTGAAACAACTGCTAGCAATAGCTTCTATTGACAACTCTACGCTTTTCAACACTTAATCATATTCTGAAGTACTCCCAGAAGCTGGGCGACTGTTACTC
Mus musculus  AATCGGTGTTTTTTAATTAATCTAAAGCAAGTGTTAGCAATAGCTCCTATTGACAACTCTACAGTTTTTAACACTTTGGCACATTTTGAAGGACTCTCAGAAGTTAAGCAACTGTTACTG

Homo sapiens  TTATGATATGAGATGATATTTTCCTTCCATGCATTCAATGTGAATGGTTTGTTCTGAAAAACTTCAGCTTTCATGTACTCCTAATGGTAAGAATCACATGGTAGGAAATATGATAAATGA
Mus musculus  TTATGATATGAGGTGTTATTTTCCTTCCACACATGTGATGTGAATGGTTTGTCCTGATAAACATTCGCTTTCAAATACTCCCAACAGTAGGAAACATGTGCTAAGAAATATGCTAAATTA

Homo sapiens  AGTATTTACCAGTTCTTACTGACTGTTCTTTCATACTTCCTTGGATCTCTTACAGGCACTAAAGATATCCTGTAGTTATATGACTTCC-TATTGAAAATGCACTTTCACTCATTCATTAT
Mus musculus  AGTATCCATCAGTTATT------------------TTTCACTGGGTCTCTTACAGGCTCTAAAGTTTTCTTGAGGTTATATACCTACCTTGTTGAAAAGTCTCTCAAACTCATTTACTAA

Homo sapiens  TTTATATAGTCTTCCACACATTGCTAACATTTACCAAACATTCACTACGTATGTGGTATTGTGCTGGGGGATTTATATATATTAATTCATTTAATTCTTATAAAACACCTAAGTGA---T
Mus musculus  ATCATATGGTCTTTCACATGTTGCTAGAATCTAAGGAGCCTTCACTATGTATGTGGTGATATGCAGAGGG-TTTTCATTGACTAGCCCATTTAATTCTTATTAAGTATCTGGGGGAGAGC

Homo sapiens  ATA-TACTATCATAATTTTATAGATGAAGGAA--CTGAAGTACTAAAAGCTGAAGTAATTTGCTCAAGGCCCCACAACCAGCAGAAAAGGAAGCTAGCATTTCAACTCAGGTCTGCCCAA
Mus musculus  ATATTAATATCATTATTCTACTGATGAGAGGGCTCTTAAGTGCCAAAACCTAAAATAATTTGCTTCAGCCTGCACAGCCCGCA.....................................

Homo sapiens  CTCCAGAGCCAAGATTTATCTATTGTTTTTTGTCACCTCAACAAATATTTACCATATGCCATGCAGCACACTAGGCAGTACCTACAAAGATGAATAGGGCATGATCTCCTTGGCCTTCCA
Mus musculus  ........................................................................................................................

Homo sapiens  GAGGTAACACTAGTTGGGGCTATATGGGTGGGAATGGAGTGTCAGATGCAGAAGCTAAGGAATTTTCTCTTTACAAACAGGTGGGTGAGAAAGTAGACACAGCACTGTGAATTTGTGGAG
Mus musculus  ........................................................AAGGAATTTTCTATTTACAAAGAAGTTGTTGAGGAAGTGGGTGTGGCGCTATGAGTT--CAGGG

Homo sapiens  TTTTGAAAAATGGACAGA--------GGGCTACAGACTGAGAGGGAATAGGGCCACC----AACTCTAGAATTAAGGTCAACTGATAGGGTAGAAACATGCCATTCTCAGGGGCTTAAGC
Mus musculus  TCCTGAAAAACAAAAATGGTGCCTATGGACTAAAGACTTAAAAGG-ATATGACACTCATTTAATTCTAGACTTTGGAGTAACAAGTA-GGCAAGAGCATGCAATTCTAACGTACTTCAGA

Homo sapiens  TGA-CAAAAGAAATTGCCCACTGTAGAAATGAGATTGGCTGCCCACAGAAAAGTATTTACTGATAGATAAGGGACCTCCCTATGTGTCATTAAGGAGTAATGCTCTAAAATGTAATCAGT
Mus musculus  TGATTATAAAAACTTTCCCCCCGTGGAAATGGGATAAGCTGCCCACAGGAACATGTTTACT----AATCAAGAACCTCCCTCTGTGTCAATAAACTGCATTGCTT---AATGTAGTCACT

Homo sapiens  GGAAAACACAACTGTAGCCTTGGAAATGCTATGGCTGGGAAAGTCCATAT--AACTAAGACGGGGTTCATGATATTGTGACATGTACAAACAAGTTATTTGACCATTATAGAGAGAAAAA
Mus musculus  GGGAAACAGCACTGTGGCTTTGGAAGGGCTGTGGCT-GGAAAGCCCATAGAAAACTATGACAGAGGTC---ATATCATTACATGTGCAAACAAGTTACTTGGCCCTTGTAGAAACAAACA

Homo sapiens  TAAAAATAATTAGAAAGAGAAGTTGCTTTTATTACTCTGTGCCCTGGCCTACTGACACATTTATTGACTTTCTGATAGGGTGATGCATGGATGCATCAGACTGAATAAACTACATGACTC
Mus musculus  TAAAAATAATTAGAGGGAGAAGTTGTCTTTCTTCGTCTGTGCCCCGACTTGCTGGTGCATTTACTGTCTTCTTGATAGGGGGATGAATCGTTGTGCCAGACTGCATAAACTGTATGACTC

Homo sapiens  CATCTCTTCAGATCATCCCACACCATATACTATCTCTTCACTCACCAGTAATAACAGTCACCATATAACA---AGTGCCTACAGAGTCAAACACTTTACATAAATTATCTTATTTAATCC
Mus musculus  CATCTTTTCAGATCATTTCACATCCTATACCATCTCTTCAGGCATCAGTAACAGCAGCCAGCACATAATAAGGAGTCTTCTCAGAGACAAGCAATTTATATGAATTATCTCATTTCATCA

Homo sapiens  TCAAGGCAACTCTATGATATCATAGAATTCTATTATTAGTTCCATTTTCAAATGACGGAACTGAGGCTCAGAGAGGTTATGTGACTT----AAGCCCATAAAACGGAATGAGTGGTATAT
Mus musculus  TCACAGTTAGCCTGTGATTTCATAGGCGTTTATTAGGAGCCCCAGTTTCTGATGAGTGGGCTGAAGCTCAGAAAAGTTATCTGAGTTACCCAAGCAACTAAAATGACATGGAAGTTATAT

Homo sapiens  TAGTTTTCTGTGACTACCATAACAAATTGCCACAAACTTGGTGGCTTAAAACAGCAGATATTTATACTTTCACAGTTCTGGAGGCCAGAAGTTCAAAATCAAGGTGTTGGCAGGGCCATG
Mus musculus  GAGTTTTCTGTGACTGCTAGAACAAAGGGCTCCAAACTC--TGGCTGAAAACAGCAGCCATTCACTC-TTCACAGGTCT-------AGGAGTCTAAGATCATGGTGTTGGTGACACCATG

Homo sapiens  CTCCCTACAAAGGCTCTA-----GGGGAGATTCTGTTCCTTGCCTCTTCCTAGC-------GGTTCCAGGTGTTGCTTGGCTTGTGGTAGCATCATTCCAATCCCTGCCTCTGTCTTCAG
Mus musculus  GAGTCTTTAAAAACTCAAGGGGGGGGGGGATTCTGTTCCATGCCTTTTCTTAACCTTTGTTAGGTCCCAATATTGCTTGGCTGGAG------------TAATGTCTGCCACCATTTTCAC

Homo sapiens  ATGGGCTTCTCCCTTGTCTCTGTGCCCTGTCACTTATAAGGACACTTATCATTGGATTTACAGCCCACCCTAATCCAGGATGATCTCATGTAGACATCCTTATCTTACATACATCAGCAA
Mus musculus  AAGATCTCTTTCTTGGTATCGATGTTTTCTGCCTCATGAGGACAC---CCATTGAATTTAAGTTTGCCTCTAATAC-TGATAGTACCA-ATACAGATCCTTACTGTAATTTTATCTGCAA

Homo sapiens  AGAGTCATATTCTAAATAAGTTCACATTTTGCAGTTCCAGTTGGATGTAACTTTTGGGGGCGGTGACACAATTCAACCCACTATGTATAGCCTAGCTGGGACCCTAGCCTATCTGACTCC
Mus musculus  AGATGACTATTCCAA-----------------AGCCCCGGTTGGGCATATCTTT-----GTGGAAACACCCTTTAA-TCAGTACAAGTCACCTAGACAG--CCAATATTTATTTGTTTGT

Homo sapiens  AAAACTCTGGCTCTTTCCACTAACTAAAGCAGGCTGCTCCCACCAAACAAACAGCCTTTTGTCTTTATTTGGTTTCCTAAGTAGATTTCCTACTGGGA--------------------TT
Mus musculus  AAGATGTTGGA-CTTTCCACTGGAGTAGGCAGGCTGTCTCTAC----GGAACAGTCTTTTGCGATTATTTGGTTTCCTCAGTATATTTCCTACAGTGAAGATTGACATGAGTGAACTTTA

Homo sapiens  TATCAAGATGAATTAATAAATTGACATTTTGGCTTCCAAATTATTGCCTTATAAGTTTAGGTTGTGTGAAATTTGTCCCCTAGAGCCTGATCAATCCCTGGCTAAAATGGAAAAAATTGA
Mus musculus  TATCAAGATGAATTAATAAGCTGAT-TTTTGGCTTCTGAAATATTGCTGCAAGTGTTT-TATTGTATTCAGCGTGTCCTCCAGAGTTTGATCATTCCCTGGCTAAAACTGACAAAATTTT

Homo sapiens  GTTTTGGAACCTGTGAAGCTGATGAATGGATCCTGAAT-AGGACCAAGGTCCATTTAGAGTATAAGCATCCCACAGCCTGCCAGCCATACTAATTTCTTTACTATATATGTGACCATAAT
Mus musculus  TATTTGAAATTTGCAAAATTGAGGATTGGCCTCTGTATAAAGACCAATATTCCTTTGAAGTTTGGACATACCAAAGGCCTCTGGCCATGGCCATGTCTTTAGTATGTAAATGGCTATGAA

Homo sapiens  ATAATGGGATAGCATAATATTCTAAGTCTACCGAAGAATGTCTAATCATCTAAGAGCTTACAGGGGGAATTTTTGTG--TACCTTAATATTATATCCAGAAATATTCCCTGTAGTGCCAA
Mus musculus  TGAATGAAATATCCAAATTTTTGATGAC---------ATTTCTAATCATT--AAGGCTTACAGG----ACTTTTATACCCACCTATGTATTATATACCTAAG-ATTCCTCATAGCACCTA

Homo sapiens  CTATATTTGTTCCTGAGCCTCAGCTCCCTGCATCAGGTCACAAGGAAGATGTCTCTAGGAGTTCATTATCACATTAAGACAAGGATTCTGTAG--GAAAATGGGAATCTAGAAACAGTAG
Mus musculus  TACTGTGAATTTCCAGACCT-AGCTAGCTACTTCAAGTTACAGAAAAGATTTATCTAAGCACGCATTATCACCTTAAGATGAAGATGCTTTAGAAAAAAATGGAAATACAGAAACA----
```

```
Homo sapiens  GGACTTCCTGTACCTCTACATCAGTGTCTTTGCAGGGGAATTGCCCTAGCTGACCAGTTGACACTGGCCTGCTCACACCTTAATTATGAATAGAATATTCCAGGGATGTCTTTCTCAGA
Mus musculus  GGACTTCCTATGCCTTTGAGTTGGTGTGAGTGCAGGGCAATTGGCCCAGCTGACCATTCAACACTGACTGTCTAATACCTTGATTATGAAAATAATATTCCTAGAGATGTGTTTCTTCAA

Homo sapiens  TTGTGGTCAGTTTGTGGGCCGCCTATTGTGGTGGTCCACTGAGTGACACCTGGGGAGCTAGTTAAAAATGTCAGTTTCCTGGTCCTTTATAGCAGACTTACTGAGTTAG-TATATCTGAA
Mus musculus  ------TCAAT----------------------TCCATAGGGTTTCATCTAGGGAACTTGTTAAAAATGTAAACTCCCTGCT-TTTAATATCAAATTCACTGAATTAGTTACCTCTGAA

Homo sapiens  GATAGGG--TCAGGAGCCTGCATTTTAAAACAAGATTCTGGAATAATTCTGATACATATTCAAGTTTGAGAACCATTGCCTTCTGGTGAAATCAGGCTTCCATGACGTTGAAATACAGAA
Mus musculus  GATGGGGATCTAGGAATCTGTTCTTTAAAGCCAGTAACATGAGTAATTCTGATATATATTTGAGTTTGAGAATCACTGTATTCTATTGAAATCAGCCTTCCATGACATTGAAACACAGAT

Homo sapiens  TAAACT-CCAAATGTTCATTTACAATTCCAGCCAATAATCACTTACCTTATTGCATTCTTGTAGCGTGATCTATATGCTTTAGTTCATGTGACAATATCATTTCATATGGCTTAATTTAA
Mus musculus  TCAACTCCCAAACGTTCATTTACAATCTTAGTCAATAACTAATGATAGTATTGTATCAGTACATCATGAAGTATGGGCTTTAGTTCTTATGAT--TATCATTTCACGTGGTTTTGTTTAA

Homo sapiens  ATGAAAGCAAAGGCAGTTATGGCAAGATTTCTTAATCCTGCTCTCCTGAGACAATTTACATAATAACTTTT---------GGGGAGAATGCAATAATCTCTCTCTAATATCTTAAACTGA
Mus musculus  ATCAAAGCAAA-ATAGTTGTGGCAAAA-TTCTTATTCTCACAATTCTAAAGCAATGTATGTAATAGGTTTTTTTTTGGGGGGGGGGGCTACAATAACCACTCCCTAATATCTTGAATTAA

Homo sapiens  TCTCTAAGGTTCCTTTTGGCTCCAACATTCTGATTCTATCAGTGTTCCTAGGGCTTCAAATCTGTCTGTGCAAAACACATTGCTTGTTACAGACTAAACCTATTTTCCTTCTTCCAGGCA
Mus musculus  TCTCTAAAGTCTCTTTCTACTCCAATATTCTGATCCTAGAAATATTCCTGGGGCTTTAAATACATTTGTAAGAAACACATTCTTTGCTGCAAAAGAGACATATTTCCCTTCT---GGGTA

Homo sapiens  ACAAGTTTCTGAAGAAAACTGGTTGCTCATGATCTTTCTGGGTGTTTTCTAATAATATATACCTTCTCACAATATTATTGCAAAGAGTGAGCGAGGTGATCTATGCAAAGTGCCAAGCAT
Mus musculus  ATGAGTTTCTTAAAAGGA---------------------GAATGTTTTCTCATAACATATTCCTTCTT----TAGTACTTTGAAGAGT----GAGACAATATATGCACA-TGCCAAGTAT

Homo sapiens  A--GTGCCTGACACAAGGAAGTGGTTAGTAAATATGCATGCTGCTTCCAGTGCATTGTGACAATACTATCTCTTGAATTATTGAAAATATAACATCAGGAAACTTCAGCTGAATGAAAAC
Mus musculus  CTTGTGTCTGATACAAGGAAGTGGTTAATAAATGTTCGTGCTGCCTCTGCTACACCGCTACAAGAATATCTCTTGAATTATTGAAAGTATAAAATTGGAGAGCTGCCTCCGAGTGAAAAC

Homo sapiens  TAGGGAATTAGCTGTATTGACAATATTAGGTTTATTTGGCTTTAGGGGAACTAGATAGAACCGAAATCTCTGCAGACTGGCTCAGCTGCAGAATAGAGAGATGGCCCGGGGAGGACATGT
Mus musculus  TAGGGAATCAGCTGCATTGACAATATTGTGTTTATCTGACTTTAGGCAAACAAGATAGAACCCACATCTCTGTAGTCTGGCTCAGTGACAGAATAGTGAGTTAGTTTGGGAAAGACATGA

Homo sapiens  GTTGGTTCCTGCCATGCATATTGTCTT-------GGAGAAAAGCTGCTATGACTCTGGCCCAACAAAGGTTTTATCCTAAAAGTCAGCTTCCAATCCCCAACATGATTCCTAAGCTAGCA
Mus musculus  GTTG----CTG----ACACATTTTCTTTTAGGGGGGAAAAAAGCTGTGGTGACTCCGACCCAGCAAATGTCTTGTCCTAAAACGCAGTTTCTAATCCCCAACATACTTTCTGAATTAACA

Homo sapiens  TTTCTCAAAATGTATTCCATGGAGTACTAGTTTCTGAGGATTGATGTTGATAG-TATTACATAAA-GAGCTCCATGAACAAGCAATGTTACAAACCACTGGATTAGTCAAATGTGGTTTC
Mus musculus  TTTCTCAGAGTGCATTCCATGGTGTACTAGTCTCTGGGAATGGACCTTGATAGTTTTTACAGAAATGAGCTCTGCGAACAAGTAATGTTGTCAACCATTGCATGAGTC-AATATGTTTTC

Homo sapiens  CTGGCTGCGAGAATATTCAGAGTCCTTAATAGGTCAACAGGTAATGTGAATCTCC-AAAAACTAATGGGGTATACCGGATTTGAAAATTT---TTGTGACCTCACTCACAGAGATGGTGG
Mus musculus  CCAGCTGCCAGGATATTTGGAGTCCTTACTATGTTAACTGGTAATATGAACCTCCTAGAAGAGGCTGGAGCACACTG--TTCTAACATTTTACTTTTTCTCTTAGTCCCAAGGATAGTGG

Homo sapiens  TGAGGGACTAGTGGTTCTGAAAAATGCAATTATGAGAACACTACTCCAAAATGACTAGTG------ACTTGCTAACAAGAATGGACACTTTTCAGCCCTTACATGATAAAA----GTTAA
Mus musculus  TCAAGGACTAG----TTTGAGAAACACAATTATGAGAAAACTGCTCTAAGGTAAGTAGCGAGTCCTATTTGCTAAGAAATAAGGATGCTTTT-AGACCATAATTGATAAAAAAGGAACAA

Homo sapiens  GAGAACAATTACGTGCTAAACTATAGAGTACTGTCCCTCAGCCCCCTAGTGTCTCCATTTTCTTCTTTG--ATCAGCTTAGATTCTATAACCATCCTTAAAGTCAC---TGTTTAGCATA
Mus musculus  GAAACGAATTACCTCCTGAATGCTAGAGCTCTGTCCTTCAGCCTCACAGTAGTCCCACTTTCCTCTTTGCACACAGCTCAGATTCTTTACCCATCACTACAGTCACTTGTGTCT------

Homo sapiens  CACCCTAGATTCCCTAGGCTCTCTCTGCTTTCATGTGATTTGAATAACAGACATCCAATCGTTGTTAAATCCTACTCTCTTTATTCCCAACCTGCCCCAGCAGCCGAATGAGATTGGATA
Mus musculus  CTCCTTAGACCACTCTGGC-CTCTCTGCCTTCATAAGA..................................................................................

Homo sapiens  AAAGCTTATAACCGTGCTAACCAGTCTCGCTTTAGATTCAGGACCACTAACTTCAAATGGGCCCTTAGTACTATCTGCTAACTTCACTACATTCATTTCATCCATTCATTCTTGAATGCT
Mus musculus  ......................................................AAATG.............................................................

Homo sapiens  CCTGGGTGGCTTTTTTACATCTTTCCTTTCCTCAAACTTCCGACACTTGTTTCTACATTTTCCCTTTCAGCTGCTACCTTGTTTCCTATTTCACTGAGAAAATAGAAGTTGTCAGAAAAG
Mus musculus  ...........................................................................ACCCTGCTTTCTGCCTCATGGGGTAAACAGAAGCAGTCAGAAAAG

Homo sapiens  AACTC---CCACCAATCTATTTGTATTTGTATCCATAAACTCTGCCTTCCCTCTTGTCACTGTGAGCGAACCATCTGGGATTCCACTGAATGC-TGACTCCTCTACTTATGCACTAAATT
Mus musculus  AGCCTTCACTACCACTGTCTTCATAGCTGTA-CCACAAGCTCTGCCTCTTCTCTTCTCA----------------------------GCATGCTTGATCCCTCTATTTGGATACTACAAT

Homo sapiens  TTATTTCTTTTTGCCTAATCAAGGTCATTACTCAAGTCATTCACTCTTCCCTCATGGCATCAAT---TTTTCTTTCA--TTGAACAACATCTT----------------CAATGTTTAAA
Mus musculus  CC-CCCCTTTTTATTTAGTGAAGGGCATTGATCTAATCAC---CTTGCATCCTGTGACATCAACACATTTTCTATTATTTTAATCAACATCCTAACCTGGAACAAAAACCAAGATTTAAA

Homo sapiens  AAATCTGTTCTCTTCACCCAAGTCATAGCCCCATTTCTCTCCTCCTCAGAGCAAAAATTCTGGAATGAGTTGTCTCTGCTCATTCGTTCCAATTTCTCTCCTCTCATTTTCTCTTGAATC
Mus musculus  AAACTGTTTCCCCTTA---AAG---CAGCTCCATTTCTCTTCCCCTCAAAGCAAAAATGCTGGAATGAGTTATCTCAGCTCATTGGTCCCAGTTTCTCTTTTGTCTTCTTCATTTGAGCC

Homo sapiens  CTCTCCAGTCAGGCTTTTGCCATCATCACTCCACCAAAATGATTCATATCAAGGTCAACAAT---ATCTCCTCAGTATTAAATTCAATGGTCATTTTACTGTTCTCCTTTTTTTTTCGCC
Mus musculus  CTAGCTAATCAAACATATGCCA--------CCACATGGAAGACTCACACCTTAGCTAACAAGGAAATCTCCTTAGTGTTAAATCCAATGATCATTTCTCTA------CTTTTATTTGATC

Homo sapiens  CAACAGCGGCATTTGA---CACAGTTTATCACTCTCTCCTTGAAACACTTTTTTCTTCCACTTGGCTTCCAGGACACCATGTAATTCTTGG-CTCTTTCTACTTCACTAGCTACTATTTC
Mus musculus  CAACAGTAGCACTTAATATCATTGCTTAACACTGATCCC------------CATCCCCCACTTGACTTCTGGAACTTCACATATTTTAGGGTCTCCTCTCACTTAAAAACCTAGTAT...

Homo sapiens  TTAATATCCTTTACAGGTATGTCTTCATTTTCATGATAACTAGGTTGTCTGATTGGCATCTCAACTTGAAATTATCCAAGGTCAAAGTGACTATTTTCCTTATACCTGCTTCTCCCACAG
Mus musculus  ........................................................................................................................

Homo sapiens  CCTTCTTTATCCATTCTTCTGGTTATTTAGGCCAAAGCCTTGGAGTCTGACTTGACTCCCTTTTTCTCTCACACTCCAGATCCTATCTATCAGCAATACTTTCAGCTGAATAGTTAAAAA
Mus musculus  ........................................................................................................................

Homo sapiens  TTTATTTGTTTAGAAACAGCATCTCACACTGTCACCCAGGCTGGAATGGAGTGGCACAATCATAGCTCACTATAACTTCAGACTCCTGGGCTCAAGGGATCCTCCCACCTCAGATTTCCC
Mus musculus  ........................................................................................................................

Homo sapiens  AGTAGTTGGAACTACAGGTACTCACCACAGTGCTGGCTAATTATTATTATTATTATTATTATTATTATTATTATTATTATTATTTGTAGAAACAGATGTCTTGCTTTGTTGCCCAGGCT
Mus musculus  ........................................................................................................................

Homo sapiens  GGTCTTGAACTTCTGGCCTAAAGTTATTTTCCCACCATGGCCTCTCAAATTGTTGGGATTACAGGTGTGAGCCACTGCACCTGGCCTAAAAGTATATTTAGAATCTGAACACTTCTCTAC
Mus musculus  ........................................................................................................................

Homo sapiens  TGCTATAACCTTAATCCAAGCCAGCATCATTTCTTACTTGGATCATTACATAATCCATTAAACATGTAAATCAGATCATGTCATGCTCCTGCTCAAAACCGTCCGTGGCCTTTTATATTG
Mus musculus  ........................................................................................................................

Homo sapiens  TTCACAATAAAAGCCAAAGTCTTTAGAGTGACCTACAAGATTCTACATGATCTACCTTCATTCCCATTACCTCTCTGACCTCATGCCCTGTCACTCTCCTCTTTGATTACTGAGATCAGC
Mus musculus  ........................................................................................................................

Homo sapiens  CATATGGCTTACTTGCTGTTTCTCAGACATACCAAGAAAACTCCCAGAGGTCCATTGCTTTTCTCTCTAACTAGGATGTTTCTTCCTCATAGAGTGTCATGCTTATTCACTTGCTTCTGT
Mus musculus  ........................................................................................................................

Homo sapiens  CAAATCTCTGTGCAAATGACACTTTATCAGTGATACTTTCTTTATCATCTTATTTAAAAATTGCCACTCTCTGCATGCTTCCTACAACTTTACTCTGCTTAATTTTTTTCCATAGTACTT
Mus musculus  ........................................................................................................................

Homo sapiens  GTCATTTTCTGATATATTAATATTGATTTGTTTGTTTACTGTCTATCTTCACCACTAGAATATAAGCTTCAGGAAGGCAGGGATTTTATTTTGTTGCTGCTGTATCCCTAGTGTGTAATG
Mus musculus  ........................................................................................................................

Homo sapiens  TGGTAGCTGGCACATAATAGGTGCTCAATAAATTTCAAATTATTATAATAAAGAATGATTCCAAGTGCAATAGGAATTCCAAGATTCCTGCCATCTTCAGATGGACCCAGATATCCTTCA
Mus musculus  ........................................................................................................................

Homo sapiens  TCATCTGCAGCAATCCCCCACCCAGTGCCAAACTCTGTGACACTGCATGTCTGGTAGTGAGTTTAGTCTTTACATGAGGACTTCCAGGAAGTTCCATAGATTCTTAAGCAAAGTGCTCCA
Mus musculus  ........................................................................................................................

Homo sapiens  TTATTGAAGCTATTTAACTCCTAGAAAGATCTTTGTGGCTGTGTGCGGTGGCTCACGCCTGTAATCCCAGCACTTTGGGAGGCTGAGGTGGGCAGATCAGGAAGTCAGGGAATTGAGACC
Mus musculus  ........................................................................................................................

Homo sapiens  ATCCTGGCTAACACAGTGAAACCCCGTCTCTACTAAAAATAGAAAAAATTAACCGGGTGTGGTGGCACGCTCCTGTGGTCCCAGCTACTCGGTAGGCTGAGACAGGAGAATTGCTTGAAC
Mus musculus  ........................................................................................................................

Homo sapiens  CCGGGGGGTGGAGGTTGCAGTGAGCAGAGATGGCACCACTGCACTACAGCCTGGGCGACAGAGCAAGATTCTGTCTCAGTAAAAGAAAAAAAGAAAAAAAGAAAAAGAAAGATTTTTGCC
Mus musculus  ........................................................................................................................

Homo sapiens  AAGGGTCCTTTTCTGATACATAACATTTTTTGAATAGGTATTAGGTGTACACGATACAAAGTGCCAAATGTTCAAAGAGCAAGTAGTGAAAAGTATATTCTACCACTCCCACTGCTACCA
Mus musculus  ........................................................................................................................

Homo sapiens  ACTTATTCCAAGCCACCCACATTTCTAAACTGGAATTATAGCACAATTTTTTTAGAAACAGAAGTTAGATGATGTCGCTCCTCTGCTAACATCAGCCTGGGGCATGTCATCACACTCAAG
Mus musculus  ........................................................................................................................

Homo sapiens  GTAAAAGCTAGAGTCTCCCTCTGCCCCCAGCCACCAAGATCCCTTCCTGGAGGCAAACACTCTTACTAGTTTATTGTGAATTTTTTAGAGATAGCATGTATATTATACACACATATCTCC
Mus musculus  ........................................................................................................................

Homo sapiens  ATGTGTATAATTCATTTTTAAACTGTACACACTATTTTTTATATTTCTTTTTTTCTCACTTTATTCTACCTCATTCTTTTTAATAGCTACAGTTGTATCAGTATGGCTACTGTATTCTCC
Mus musculus  ........................................................................................................................
```

Homo sapiens AGAGATTCTTAGTCTGGAGTCCATATATGGGTTTCAATGGGTCTATGAATCTCCTGAAAGTGCATGTTGACACTTGTGTGCGTGTGTACATGTATATCTTTTAGCAGAGCAGGCCCATAT
Mus musculus ....

Homo sapiens CTTTGATCAGATTGAAAGGAATCTGAGACCCAAAATGAGTAAGAAACCACTATATTGAGTCTGAATCTGCCTTCCTGAAACACCTACCCTTTAGTCCTAATTCCACTGTTTGGAGCAGTT
Mus musculus ....

Homo sapiens CTGTCAGGACCTGGTACATAGAGGACATACAACAGGTAACTTGTGAAATGGATGAAGGGACAACATAAAATGAATCTATTCTGTCTCTCTCTTTTTAAAATTTTTAAATTTTTATTTATT
Mus musculus ....

Homo sapiens ATTATTTTTTAAAGACAGAGTATCACTCTTGTCACCCAGGCTGGAGTGCAATGGCACAATATTGGTTCACTGAAAACTTCCACTCCCAGGTTCAAGCAATTGTCCTGCCTCAGCCTCCCC
Mus musculus ....

Homo sapiens AGTAGCTAGGATTACAGGCACCTGCCACCATGCCCAGCTAATTTTTGTATTTTTAGTAGAGACATGGTTTCACCATGTTGGCCAGGCTGGTCACAAACTCCTGACCTCATGTGATCCACC
Mus musculus ....

Homo sapiens CGCCTAGGCCTCCCAAAGTGCTGGGATTACAAGCGTGAGCCACTGCGCCCGGCCTATTCTCTCTTCTTTATGTTAGCTTTTGGTTAGCCTGAGGAAACTATCATATTCTAAGTCATCCCT
Mus musculus ....

Homo sapiens TTTCTAAAGTTAATTACTTAATTTTCTTAACTTTTCCTAATTTGACATGGTTTCCAAACCTGTAGGCTTTCCCTACATGCTGTAATGTGGGTTTCTTCCTCAAATGCCATCGGGGCAAGC
Mus musculus ....

Homo sapiens TGGTAATTTAAAAAAGTGAAGTGGATCAGATGTGAGACAACGGCAAGTGGTGGGACCTGTGGAGTCCACATGGCTAAGATGTTCACATTCAAACTCACATATAACACATCCATAACCAAA
Mus musculus ....

Homo sapiens CAAAAAATATTTTCAGGCCAGTTTTGGACTATGGGCAGGCAGCTTGCAAATATAAAAATTTGGACCTCGTACAGTTTGTTGATACTCTTTTCACATCTTCTGCACTCTATCAATTCACTT
Mus musculus ....

Homo sapiens TTGTCCTCCCCATAGGCCTGACAATTTAATCAAACTAAGGTAGTGCCTGCATCTCAAATACCCCTTAAGATTCCTTGCTATTATTCCTTTGCCAATATGGCTCTGTCTGTATAAAATGCC
Mus musculus ....

Homo sapiens TTTGTGGAATATTCTCTACTTGCCCCTCAAGATCCACTTTTAAAAATAGACTATTTTTTAGAGCAGTTTTAGGTTAACAGCAAAATTGAGAGGAACATACAGAGAGTTCCCGTTAGCCCC
Mus musculus ....

Homo sapiens TAAAGATCCAGTTTTCACCCTTCTTCATCCTCTTTGTGTACCCCCCGAGACTGACCTTTATAGACTGCATTTAAAAACATATTTTTAGTTATTGTGGGTACATAGTAGGTGTATATATTT
Mus musculus ....

Homo sapiens TTGGGGTACATGAGATGTTCTGATACAGGCATGCAATGTGAAATAAACACATCATGAAGAATGGGGTATCCATACCCTCAAGCATTTACTCACTGAGTCGCAAACAATCCAATTACAGTC
Mus musculus ....

Homo sapiens CTTAAATTATTTTTAAATGTACAGTTATCACTGGCTATAGTCACCCTGTTGTGCTATCAAATAGCAGATCTTATTCATTCTATTTTTTGTACCCATTGACCATCCCCAACTCCCCCCAGC
Mus musculus ....

Homo sapiens CCCCCACTACCTTCCCAGCCTCTGGTAACCATATGAATTGGCTTCCTTGTCCTCTGAATTCTTCTTGGATATGGCCAATGTAAGGCACTAACAGGGGACTGGAGATCAGAGACAGGTTAA
Mus musculus ....

Homo sapiens AGTCTGCGTATTTATTCTCCTGGCTCCCTCTTTATGGAGTGGGTGCAGCTTGGCTGAATCCTGTTAGAAGGATCTGCTGTGAGCCACAGACTTTTTTTTGGCAGTCATCTTCTAAAGCTA
Mus musculus ....

Homo sapiens TGGCTACTCTGTCTAGGTTCTGGTTAACTGCTTTCTCCCCTTGGCTTTTCAGGTCTCAGGGTGGTAAGTGCTCCCTGGGGTGCTTCACCATCCTTTTTGCTTTCCCTTAACAATGCTCAC
Mus musculus ....

Homo sapiens ACTGTTATAAACAGTCCGTTCATTAAACTCCACTCACTTCCCTGTTGAATATGCTCTCAATATCCTACTAGATCCTTTACTGTTACCAATGCCTTCAACTCCCTTGTGTACTTGGTGTAC
Mus musculus ....

Homo sapiens TCCCCATTGTTAAGGTCCCAGCTCCTAGGACAATGTGGTGCATTCCTAGATATCACTCCCTTTGAGAAAGTTAGCTATTCCCCCACCTCTTTGAGAACATTTCTTGCAACACTCTGTGTT
Mus musculus ....

Homo sapiens TAGATCTTGGGCTTCTTATGATTGGGAATGAAGCCTATTCGTTATTGTTTCCTGGCCAAGTAGATGTTTGGTGAGTATTGGTTATCTTTTTTGCTTTGTGAGGAGGATATAAAATCAAGG
Mus musculus ....

Homo sapiens AAGGAGGATATTTGCTTTTTATTTCTTTCAAATTATTTGATTTTAAAAGAAATGCCTACACATTATTTTTTAAAAATTAAAATGAAAAGAAAAAAGTAGTAAAAGTGGCACCGTTCTTCA
Mus musculus ....

Homo sapiens CATCCGTGCTGGAAGTAACAGTGGTAATGGTTTTTGTTGTGCATCCTTCCAGTATCTTTTTATGCACATAAAGGCTTAAGTTTGTGTACATAAAATGTAGACTCACATATTTCTCTGCTC
Mus musculus ....

Homo sapiens CTTGCTTTTTTTTTAACTTAACAACACACCTGTCTTTCTATATCAGCCAATGTAAGTATACAGCCCTACTTCATTATGTTTTATGGCTGCATTGTATTTCATTTGATGAATTTACCATAA
Mus musculus ....

Homo sapiens TTTATTTAACAAGCCTCTATTATTAGCTAGTTTATCTAATATTTTCACTGTTAAAAGCAACACAACTTAAAACACAGAGACATACTGGATCAAATTTAAGCCCAAAAATATATGGCTGAG
Mus musculus ....

Homo sapiens CTCCAGAAGCAAGAAAGGAAAACTGTCAGATTCCAGAGACAAGAGAAAACTCACAGTCACAGCAATGAGCATAAATTGACATTTCACCACTCACAGGAAAAATGGGACCAGGTATATTCC
Mus musculus ....

Homo sapiens TTAAAGGATGAGATTATAAGTCCTCTGTAGGGCTTAGAATTGAGTTAGAATTGTGCTTCCCTCATAAAACAAAGAGTAGAAGGATGCTGCCCCATTTATTATTTAAAAGCAGATTTCCCA
Mus musculus ....

Homo sapiens AATAGTCAGGGGTACAATAATAACAAATCTTATTTAGTACTTACTATGTGCCAAGCATTGTTCTACATGATTTACATGTATTTATTTATTTAACCATCACAGAAACCCTATGATATTAGG
Mus musculus ....

Homo sapiens TACTATTATAAACCCCATTTTATAAGTAGGGAGTAAAACGTTTAAGTAACTTGCGCAGCTAGGAGGTGGCAGCTCTAAAATTCAGACTCAGGCATCCTGACTCCAGGACCATGCCTGTAA
Mus musculus ....

Homo sapiens GTAAATGTGCTATATTGCTTCTCTACAAACAGGATGTAGGTCATCTTCCTTAGGATGAGGTGGAAATAAACTCACCCATAAGAAGTTTAAAACTTTAAACCTGAGCTGCACACTAGTATG
Mus musculus ....

Homo sapiens AAACCAAAATTCACAGTACCCACATAATGCAGAAACCCTGGGGTGAGAACTTAACATAAAACCTGGCTCAGAGCAGGAACACAATGAAACTCTAGCAGAGGCAAATGAAAAACCGCCTGT
Mus musculus ....

Homo sapiens AGACCCACCTCCATAACTCAGAGTCCAATTCTTACGGAAGGGTTCTAACAGAAAACAGTTCCTGCAGAAGATAAGCTCACATGCAAAAAAATGATAAAGCACATGAGGAAATTGACTGGA
Mus musculus ....TCTAACAGGCAAAACTCTCTTGGGAGACT--TCTCACATGCAAAAT--TGATAAAGCTTGCGAGGAGACACACTAGA

Homo sapiens AT--GAGTTAGTTGGTATATACAAGAAATGGGAGAATTATGTCCCAAGAAACTACAGATAATACAGTAATCTGAAAGGACTTTATTTTTTGGGTTTTTCTCATCTTTTTGTAGCCCTCAT
Mus musculus ATAAGAGTTAGTTGCTACATACATCAAAGAAAAGACTTTGGTTCCAATGAACTACCGGTGATCGAGTAAT..

Homo sapiens GTATGAAAGAACTTTAAAATGTTGTATGTTTGAAGGTTTCAAGATGTAAAAAAGGAACATAATACATAAAGCAAGAGCACAACTTTCCTCGGTGTGTGTACACCAAATAAATAGGCCAGA
Mus musculus ..AGGAAATGATGTTAAGATATCATATCTCTAAAGATACCAA---------CTGGAGCACAGTATGTAACTCAAGAGCACAACTC------ATGTGTTTGCGT-AAATGCTTACCTCAGA

Homo sapiens AAT-------------GAAGAATATAGAAAATATGGGATGAGCATTGTAAGATTTTAGTTCTAGCCCCAATGAAATATTTAAAAAGTCAAATGACAGTTTAAACTACAAATAAGTTATG
Mus musculus AATATTGTGGTATTTCTGAGGACTATATACTATACACAATAAGTACAGGAAGACCTTACCTCTGTTGCAACTGAAATGTTTTAAAAGTTGATTAAAAGGCTAAACTACAAATA-----TG

Homo sapiens AGTGAAGAGAGAACAAATGGAATAACAAATAGAGCCGAGATAATCATAGAATATAGCATGGCACTATAAAAATGCTAAATATGAAAGGGAAAATAGAAGACACACATGATATAACAAGAA
Mus musculus ACTCAGGAGAGAACAAGCAAAATA-CAAATTGAGTTGAGAGCATCATAGAACATAGCACAGCACTGTGGAA-CACTACACAGTAATCAGAACAGAGAGAACACGCATAAACTCCATAGAA

Homo sapiens ACTTCACATTCATCTAATAAAGCTCCAGAAAAA-----AAATAGGAAGAGATAATATTCAAAGAGAAATAGCTGATAATTTTCTAAAACTGAACAGGACATGAGTCCTGAGATTAAAACC
Mus musculus CCTAAGCCCTACAGGAAGAGAACATGAAAAGAAGAGAAAAACAAGAAGAGAAATGGCTCATAGGAAGATAGCTAACAGTTTTCCAGAAAGCAAGAAAACATAAATCCTTAGGTTAATACT

Homo sapiens ATGTACCTAGTTCTAAGCAGAATAAA----GACAAATTGATACCCTGTAGATGCAACAGGGTGAAACTGTAGAACATCAAAGATAAAATGAAAATCTTGAAACCTACTATAGACAAACAA
Mus musculus ATATATCAGGTCCTAATTAGAATAAACCAAGACAAGTCAATACCCTGGGCATGCATCATG-TGTAACTG----------AAGAGACAGGGAAAATACTGAAGTCTGCTAGAAACAAACAC

Homo sapiens TGTTGCAAAATATTTCTGTAGAATAGATT-CCTAAAATGGAATTTCTGGGCCAGGGTATATGCACATTAAAAAAATGAATATGTTTTAAATGCATGAGTGAGTGAATTAATGAGTCTAGT
Mus musculus GGAAGCAAAGCATTTCTGTAGCATGGATTACTTGAAGTGGAATGTCTGGAACAAGGGATACAAACATCAATACAATGGATAAGATTAAAATG----------TGATTTGTGGATTTTAAT

Homo sapiens AGTATTTCTCTCAATCTGGACATATATTTATTTTAGTACAGCCTGAGATTGGATCCCCC---AGCTATCTATTTATTTATATATTTGTGACTGCATCACCACTATTGATTTCTATTGAGC
Mus musculus AATATTTCTCTCAGTCTAAAAACATATTTGTTTTAGTACAGTCTGTGATGGCACCCCCCCCCAGCTATCCATTTATTTATATGCTTGCAACTGCATTACCACTATTGATTCATATTGAGC

Homo sapiens ATAAGACCAATTAAAATCCCCAGGACTTTTTCAAATGAAAGGCTGTCAAATGAGGTCCTCTTTAACATGTACTTAGGCAATTAATCTTTAAAACTTAGAAGTAGGGCTATTAAGACATTG
Mus musculus ATGAGGCCAATTAAAATCCCCAGGTCTTTTTCAGATGAAAGGCTATCAAATGAGGTCCTCTTCAGTATGGACCTAGGCAATTAAAGTTTAAAATACAAATGCTGGACTATTCATATATTG

Homo sapiens GTGAGATCACTGAGAGCTAGAGCTGTTAAAGAAGGTTTCATAAAGGAAGTAGGACTTAGGCTGCATAGAATTTATTTTCGGTGGAGGGTGCAAAGGACAGCTTCTATGTTTGTGGGATGG
Mus musculus GAGTCATCCTTGACAGTTTGAGTTATTTTCAAAAGTTCCTTAAAGGAACTAG-ACTTGAGCTGCATAGA-TTTATTTTAAGCTGGGGATAGAGAGGAGAGTT-----------GGGGTGG

```
Homo sapiens  AGGTAAGAAGAGTGATAAGGTGCATGAATAGACCATAGGATAGGAAATGAGCTTGGGGTGGATATGTGGAGGGTGGGTGAGTAAAGAGATAAGGTTCAAGGGAAGGGGTGGGGCAAGATT
Mus musculus  AGGCAAGAGGAGCAATAAGGTATTCGAGGCAGTTGTAGGGTAGGAGAGGAATATAGAGTAGATACAAGGAGGGTGG----------AGATAATGT------------GTG---------T

Homo sapiens  ATGGAGGGCCTAAGAAGCCAGGGAGAGTGTCTGCATATGATATTATACATGGGCAATTGGAAGTCCCTTCAGCCTTTTTAAAGGAAATTATATGTTTTCTGACTGGCCAATGAAAGGCTA
Mus musculus  GTGGAGGG........................................................................AAAGATATCA---------------GTTAAGGGGAGGGTG

Homo sapiens  ACTAACCCAGGAAAGAGCAAGAAGGCTGGGTGAAGTAATGCA-GGCCCCTAGTTCTCCTGTTTCTGGGTTACTGGATAGGTTATATAGC-AAGTAGGCAAGCTTAGCAAACAGCTCAGGG
Mus musculus  A--GACCTA--AAGGAGCAAGGAAG------------ACACAAGGCTTCTAGTCCCCCTGCTTCGTGGT-ACTGACCAGATCATACAGTTAAATAGTGTGGCTTAGCTAATAGCACAGGG

Homo sapiens  ACA------CTAGGGTGACTTGATGT-TAAACTGCCTGACTAGTGAGAATTAAATGTAATAAAACTGGAATTGCAAGACTAAAAATGGTATTTAGGCAGAATAATCTACTAGCAAATTCA
Mus musculus  ACAGGGACACTGGAGTG--CTGACGCATCACCCGTCCAACTA---AATATTATGTATAATAAAACTTGAATGG-AAAACTAAAAGGGGTGTTCTAAAAGTATAATCCAATAGCAAATTCA

Homo sapiens  GGATTCTTTAGAGTTGAGTGGGAAATATAAGAAAGAAAGAAAGATGCTGGATGCA-GGAGAACAAGAGATGGAATGA-GAAAGTGGAATATACACTGGAATGAGCAACAGGAAACTTTAG
Mus musculus  AGATTCTTTAAAGCAAAGAAGGAAGTATAAAAAAGGAAGA----TGTTGAATACATGGGAAACTAAACACAGACAGACGAGAGAGGAATATACATCACAGTAAACAACAGCAAACCTTAG

Homo sapiens  TTCTAGTCTCAGCTCTACCCCT-ACTAACCGCACAACTTTGTGCAGATTACTTAAACATTGAGGCTAGTTTCCTCCTCTGCAAACAACCTAGAATACAAACTCTAAAGGGTGGTAACCAG
Mus musculus  TTCTAGTCTGAGTTCTACCTCTTACAAACCACGCAACTT-GTACAG----------------------TTTCCTTATCAGTGACTATCCTTGAAATGGAGCTCCAGAGGGTTGAAACTAG

Homo sapiens  ATCTGC-----------------CATGTTCATCATTGTATCTCTAGCCACTGGTACAGGGCCTAGAACGTAGTAGGGGCTCAGTAAATATTGGATGAATAGATGAATGAATGAATGAATG
Mus musculus  GTCTGTGGTTTGTTTTCTTTTTTCTTTTCCATTGCTCTATCCTCAGTTCCAACGATAAGGTCTAGCATATAGTCAGGGCACAGCAAACATTCATTAAATACATAAATGAATGAATGAT--

Homo sapiens  AATGAATCAATCAATCAATCAATTAGAGCCTGCCCTGCCTGCCTCACAGAGTTATTATAAACTTCAAATGAAATCAGGGATGGGGAAATATTACATGAACTTAAAACACTGAGCAATCCG
Mus musculus  ---------------------------GCCAGTCCTGCCTCATGCATGGAGTCACTGTGACCTTCAAATGAAATTAGAGATGAGAAAATATTACAGGAAATGAAAACACTGAGCAATCTT

Homo sapiens  ATGGTAAAATACTACAGCTACTACTCACAATTATTATTTTAAAAATTAAATTAGAATGCTATTTGTAGTACTTTAAGGGTGAAATCATAAGTGGGCTAATGATTTCAAGT--TTTCTTAG
Mus musculus  TCAGAAAAGCACTACATGTACTACTGAGAATTCTT--TTTCCAAGTTAAATTATGAAGTCATTTGCAGTTCTTTAAGGGTGAAATGGTAAGTGGGACAATATTTTCTAGTCTTTTTTTAG

Homo sapiens  CATGGAAAGTAAGAAGAAAAGGAAAAGTATAAGACATTTTGAGTGAGGTAAATAAAACATCAGAATCTGTGTTCTGAACCATGTTCTGTCATTTGCATTCAATGTGCTTATGGTAGGGCT
Mus musculus  CATGGAAAAGAACAAGAATGGTGAAAGTTTAAAGCACTTCAAGTGAGGCGAGGCACACACCAAGATCTGT--------------TCTCTCATTGTTACCCAATGATCTGATGCTAGGACT

Homo sapiens  AATATTGATGTCAGTTATAGTTAGCATTCATTGACTACATACAGTAATGCTATGTCCTGTCTGTCCTTAAAAAAATAGAACACCCTTCAAAGAAGGTAGTCACATTCTCATTTTAGTGAT
Mus musculus  TACAATGATGTTAATTAGAGTT----------GACCACACAAAATG-TGTTAGG-CCTATCTGCTATTTTAAAAAAGAAAAGTCTTACAAAGACAGTAATATTATTCCCATTTGAG--GC

Homo sapiens  GTGGAAACTGAGATTCAGAAAGGTTAAATGACTTGCTTTTAAAACCATGGTAAGGTGGTTTCAGAACCAGACATTTTCTCCCAGATATTTTTGTCTCTGAAGCCAGTGGGAAAAAAACTG
Mus musculus  AAGAAAATGAAGGTTCAGAAAAGTTAAATGACTTG--TTTAAAACGAAGATGAAAAGGTTGTAAAGACAAAC---TTATCTCAGGTTCTCTTGTCTCTGATGTCAGTGGGAAGAAAA-TA

Homo sapiens  CAGATAAATAGATTTCTTTACACCATAAGGATGCCTTTGCTAAGAGACAGCAATTCAAAGAAGGAATGGGCTGCCTCCTGAGCTAATGAGTTTCTCATCACTAGAAGCAGGATCGCTATG
Mus musculus  CAGATAAACAGGCTTCTACACACCATAAAGATGCCTTCATTAAGAGACA--CATTAAAAGGTGGAA-------CATGTTGGTCTAACAAGTTTCTCATCACT-GAAGCAGGGTTACTACA

Homo sapiens  TAATTTGCAGGGCCCAGTGCAAAATGAAATGGCCTTATGTCCAGGAAGCTGGTCTTGGCTGGTAGTTTGCAAGCAGAAGTTAGCCTGCCTCCAGACAGGGATGTCAGAGTGAGGCGTCAA
Mus musculus  TAATTTACCAAGCTCAGTGAAAAATGAAATGACCCCATGGACATGAAGTCAGGTCTCACTAGATGTTTTTGAG--AAATTCCCCCAGCCTCTGAACAGGGATGTTAAACAGAAATG--AG

Homo sapiens  CATGAGCTGATGCACAGGACCAGATGGCCTTAATCTGAGACACCATGGCTCTATTCTATTAGCCCAAGTGAGTTAAGGGAGCATCCCAAGGTAGCTGACATGACACCTTAGAGGCTTC-A
Mus musculus  TCTGAGCTGACACATAGGTGGAGACACCTTTAAATTGAGACTTTGTGGTCCTATACAAGTGGTCCAAATGGGTTAAGGGCACTTCCTAGGGTTGTTGACATGATACCTTAGAGGTAACTG

Homo sapiens  GATGAGAGAGAATTCTGCAAGAGGATTT-GGAGAGAGCCATGGTGCCATGATCATCTGGGGCACTGAAAACATGCTCTGCCCTATGAGGCAGCACCAACACTCAGAAGAAAACAAAAAAG
Mus musculus  GATGAGAAAAAATTCTTCAAGAGAACTCCGAAGAGATCAAGGGTGCTATAATC-TCTGGGATACTACAAACA---------------GAGAGCGTAAGAGCCCAGTACAAAC---ACAGC

Homo sapiens  AAACTCTTTTAGTGGAGGTGAAGTGTAATGAAAAGAATTAGACAGATGTGGGTTCAAATCCTAGCTCTGGCAATTACTAGCTAGCTATTTGCCTTTGAACAAATCACTTAAACCTCCCTG
Mus musculus  AAACACTTTTAGT---------------------------------TGTG--------------CTCTGAGACTTATTAGCTAAG-------CTTTGAAGAAATCATAGAAACCTGCCCC

Homo sapiens  CGACTGTTCTGTCATCTAGAATATGATCACAGTACCTGCCTTGCAGAGGTGTGGGGAAGG--------AGGCAAGTCAAATG----------------------------------TATC
Mus musculus  AACCTAGTCTCTCACTTTGAATATGACCACAGTACCTGCCTTACAATGTTGGGGTAAAGGCATCAATGAGACAAGTCTAATGGCTGCTTTTGTTGCATTTAGTTTCCCAAAGAGATATA

Homo sapiens  TGGCACTGGGTAAGCCCTCCATAAATGTTAA--------------------------------CTGTTGGATGTGTTTATAAAC---------CACTGGGAGTGGGGAC-GGTATGACGT
Mus musculus  TAAAAAAGTGTCTGCTCACCTTAGATGGGGAAGAGTTCTACAATACCATACAATACAATACATCTGTTTGGGGAGTTTGTGTTCATTTGTTAGCAC-GGAAGAGCAGACAGGCAAGACAT

Homo sapiens  T-----------------GGGTCATTTGC----CTTTCCTTGAGCACTTCATG--GACGA------------ATAGCCAAATCTGCCGGACCCACCTAGGGG-CATGGAAA-CCACTTC
Mus musculus  TTAACCCATTCTCATTAGAGAGGCATTCACAACTCTTCCCTTGAACAAGTCATGTTGACAATATCGCAAAGACAAAGTCAAGTCAGCCAG-CCCATCTAGAGGACACAGAAAGCCTTTTT

Homo sapiens  CCGCTCTAGTGCTACTAACTACAGCACTGCAGTAGTGGTAGGCAGTGGGTGAAAGTGGAGTGGGGCCACTAGTAGACAGTAATAACAATAAACTCTCCAGGAGAACAGGGCCCTCAAGCT
Mus musculus  CCATGCTAGTTCTA---AGTCCTGCACAATGTCAGTGGCTGACTATGATTTAAAGTGAAGGGAGTCTTCTAGTGG---GTACTAATAACAAACCTTCAAGGAGAATGAAGTTGACAAGGA

Homo sapiens  TTTCCCCTAGCCTTGAAGATACTT----ACAGGAGATAACTGCCCAAGTCCAATCTTGGAATCCTGCTAGTTGAGTGTTTTACCCACAAGGACATTTCATGAGATCAGGACTAAGAATAA
Mus musculus  TTTCCCCTCATCCTGGAGACACTCCCTCACTGAAGAGAACAGTT-AAATCCTTTCTTGGAATTCTTCCAGGGAAGTGTTTTTCTTATACTGATTTTCACAGAATGAGAACTAAGAATAA

Homo sapiens  AATTCACTTCT-GCCCAGTAGCGCTATATGCATTAGTCACTTGGAATAGCAACAATCTTAATATCTTTCTATTTGTCCGCAAACCCCACCATCCCACCTCTTTAAAATTC---CGTGAAA
Mus musculus  AATTCACTCCCAGCCCAGCATAGCTATATGTGGTAGGTACTTGAAATAGCAATAATCTTACCACCTTT------GTCTGAAAGACCCC---TCCCACCTCCTGATAACTCTTACGTGCAA

Homo sapiens  TGGCCAGAATAGCGAAACACCGATACCTACAGTCTTCCAGGAACTTCATTGTCCCTTATGCAACATAGAAGGAATGGTATGAGAACTAAGAGAACATTGCAGTAAATCACTGAATTTCTT
Mus musculus  TGACCTGAATCGTGGAGCACAGATACCCAGAGTTTTCCAAGAACTCTATTGTCCTTTACGAAGCCCAGAAAGAATGGTACACGACCTAAAGGAACATTAGAGTAAATCACTGGGTAGCCT

Homo sapiens  GGCTTTCTTTTTTTCTTTTAATAAT-----AAAAAGCACTCCAA--TCAAACTATTAATTAACTTGT--AAAAGGCTCGAACTATTATGTACTATTC---------CCCGCCCATGTCCA
Mus musculus  GGC-TTCTTTTCTACTTTTAATAGTAATAAAAAAAATACATTGACCTCAAATCATTAATTAACTGAGCAAAAAGGCCGTAACTATTATGTACAAGCCTCCCACCCACCCTCTCACGTCCA

Homo sapiens  TGTGCTGCACAGTATTTTCTTACTCAGGACTGGTGGAACATTTCTTTTGCTTTTGTTTGAAAAAT--TACTTTTTATTTGGCCTTGGGCTGCTATCATAACTTCTGTTTTCACTAGATTG
Mus musculus  CATGTTGCGTTGCAGTCTCTGATTTGGGATGGGTGGAAAGTTTCTTTTGCTTTTCTTTGAAAAACCATACTTTTTATTTGGACTCAGGATGATCTCATATCTTGTCTTTTCACTAGGTTA

Homo sapiens  CATAAGCCATAC----ATCTGCACACATTTT---TGATTTGTACAGGGAGTTTGAATTTTAA---TATCTACCTCCATCTCAATCCCAGCACTTTGGGAGGCCGAGGCGGGCGGATCACG
Mus musculus  CATCAGTCATACATCTATCTGCACACATAGTCCGTGTTTTGCGCGGGGATTTGAATTTTTAATGTATATTCCTTC.............................................

Homo sapiens  AGGTCAGGAGATCGAGACCATCCTGGCTAACACGGTGAAACCCCGTCTCTACTAAAAATACAAAAAATTAGCCGGGCGTGGTAGCGGGCCCCTGTAGTCCCAGCTACTCGGGAGGCTGAG
Mus musculus  ........................................................................................................................

Homo sapiens  GCAGGAGAATGGCGTGAACCCGGGAGGCGGAGCTTGCAGTGAGCCGAGATCGCGCCACTGCACTCCAGCCTGGGCGACAGAGCGAGACTCCGTCTCAAAAAAAAAAAAAAAAAAAAAAAA
Mus musculus  ........................................................................................................................

Homo sapiens  TATCTACCTCCATCTCAAAAACCTCTCATCTTTTGTCCTTTTCTTCAAGAT-TCTGATTTCCTTGTGGACATTCATTTTGATA-CTTACATAAATCTTGCGTTATTCTGCCCATTTTTTT
Mus musculus  .........CCAGTTCTGATATCTCCCCACCTTTGTCTTTAATAGCAACATCTTTTGTTTCCTTGTGGA----CATCTTGAGATCTTATATA--------TTTATTCTGTCTAGTGTTTA

Homo sapiens  GGCT-ACAAGGCTCAGCTTTGGGGCTGTAAATGTGAAATATTTCCATCATAACCTATCTTT-----GGGTAT---------GGGAAGGTCC----TTAATGCTGGAAGTTCATTGAATAA
Mus musculus  GATTCACAAGGCTCAGTTTTGGCACCATAAATATGGAATACTTCCCACGTTATCTTCCTTTGATTCGGGTATTTAAGTTGAAGGGAGAAACAGAGTAGATAATGGAAGTTCATGACCTAA

Homo sapiens  TTGCTTATTTTAATAGATGTTTA---TTTTCATTCACATTTCTCCCTCCACTCCATCCCTTGGGGCAGTACCCTAGTTCAGTTCTTCATCTACTCTCTCCTTGACTGTTGCGATAGCCTC
Mus musculus  TTACTGATTTTAACACATCTTCAGTCTTTCCATCCCGGTGTCACTCAC--------------------TATACTAGTTCAGCTCTTCATTTCTTCTTGCCTGGACTGTTGCTATAGCCTG

Homo sapiens  CTAAGCAGCCACTCCACATCCAATCTCCCCATATCCAAAGTATCCTAAACATAGCTACCAGTCATCTCTCTAAACCACAGCTTTTGGCAAAAGATCCTTGAAGTTTCCCTTTTGCCTAAT
Mus musculus  CTAACAAGTTACTGCATGCTCAGCCTCCTTCTCTTCAAATTATCCGAAACACAGCTATTGATCATCTGTCTAAACCACAGCTCTTAAGTAAAGATCCT-----------------TACT

Homo sapiens  GAGTAACGTACTAGCTCCTCATCCTGGCATTCGAAGCTCTCTATGTTTATATGTTTGCCTATCTTTCTTTTCTAGCTTCTTTCCCATTACTTGTCTTCATATAGCTTTAGCTTTTGCCAA
Mus musculus  GAGTAATAGAC--ACACTTCATGTTGGCATTCAAGGCTTTGCTTGGTTATGTAATTAC----------------AAGTCTTTCCTGTTGTCGTTCTGCATA-----TTAGCCTGAGGCAA

Homo sapiens  ACT--ACCATTCTTTGTTTTCAGAGCACACCCTATACTTTCCAGCATCAGTGTTTCTTTCACCTGGAATTAACTGCCTGCCACAGAGATCTCCGTTCATCAGCCTATTCATTCTTTAATG
Mus musculus  ATTTGACCACTCCTCATTTCCAGAGGAAACT------------GCTCTGATGATT-TTCCACTTTGAATAAGTTTCACTCAAGAGATA-CTCTGGTCACCATATTTTTCTTCCTTCCCTT

Homo sapiens  CCCAAGACACATGCCACCAATTTCATTTGAATGGTCCTTAGAGACACTGAAATGGCGCCTGCTGTACACCTGTCCACCCCGACCCCCGCTGCCCACTGCTGGCTCTTCCAGTCTTGTGAA
Mus musculus  CCC.....................................................................................................................

Homo sapiens  ACATTAACCTCTCCTAACAGCAAGCACAGCTTCTCCATGGAGAAGACACTGAAATGGCACCTGCTGGACACCCGTCCACCCCCCACCCCTGCTGCCCACTGCTGCGTCTGCCAGTCTCGT
Mus musculus  ........................................................................................................................

Homo sapiens  GAAACATTAACCTCTCCTAACAGCAAGCACAGCTTCTCCATGGGGC-CTGCTCACTCTGGCCCTCTGGTCCCACCATTTTTCTCACTACTCAAATAAAGTTTGGAGA-CCTTTCCCTGAT
Mus musculus  ................CTAATGGCACACAGAGC-----CAAGGATCTCTGCTCACTTTGCCTCTTGGCTCCTGTCATCTTTTTCAACACTCAGATGAAGTTTGGAGAACCCCTCCCTGGT

Homo sapiens  TTT---TTAAGAACATGCTAGCTC--TTTCATGATA-----CTCGTTATCATACCACCCCCATCTGCTTTTCAGTGT-ACCTTTCAATTGCTCTCTAGTGCTCCATTCTTGATTGTTTTC
Mus musculus  TCTCCTTTAACCACAGCCTTTTTTCTTTTCACAATAGCATGCTCCACCTGGCACCAGCCTCAG-TCCCTCTCACCATCGCCTTTGAATTGCTCTCTAGTATTCCAGTCTTGATTGTCTTC

Homo sapiens  CATTTCTATTATAGCTTTCAAGGAGAGTCTTTACTTGGCCTACTTTCTTAGTAAATGGTTTGGTGAGATTCTGATTTTCATCTGATATTAGTTCTTTCTCCAAGAGGGACAGAGAAAAAT
Mus musculus  CACCTCTACGATAGCTTCTAGGGAGAGCCTTCCCTTGGCCTACTTTCTTAGTGAATGCTTCGGTAGAATTTTGATTTTCATCTAATATTAACTCTTTCTCTATGAAGGACAGAGGAAAAG

Homo sapiens  --GAAAAAAATATCTTGCCTTCAGTTATTCTTTTCATATTCTTTGAAATTCTAAGCCACAGAA-GTCTTCTGTTAGGCTGCTACCTTCATTTTAAGAGACTCTTCCCTAAATGACAAGTT
Mus musculus  AAGGAGAAAGAGTGCTGCTTTCAGCTGTTCT----------TTTAAAATCGTCAGGCACAGAAGGGCTTCTGTCACGTTGTCACCTTTATTTCAAGCTA--TTTTCTTCACTGCTCAGTT
```

```
Homo sapiens  GTGACTTCAGAACTAGAGAACATGGCAGCCTCTCATGGCTTTCTGCTCAGTCCCATTTTTCTTTTCTG--GATCCACTCTGGGCCTTCTTTAAAGTGACTCAACTTCCCAAGCATGTGAT
Mus musculus  GAGACTTCAAACCGAGAGAACACTGCAGTCCCTGGTGGCTCTCTACACAGTTTTCTTTTTCTTTTTTAGTGTTTTTCCATGGGTCCTCTTTAAAATGACC--ACTTCACATGCACTTTAT

Homo sapiens  TTTACGTATTTTAAAAACAAGTCTGGTTTTTTCAACTCATTCTGAAATGTGGCTGCCAAAGTAAATACCC---AACCCTCTCTAACACATACATACCCTCTTTCACACACACATGCACAT
Mus musculus  TCCACTTACTTGGAAAATATAACTTCTTTTCTTGACTCCCTTTGAAATGTAGTCATGGAAATAAATACCCGCTGCCCTTCTTTAGCACAAACTCACACACTCTCAAACAC----GCACAG

Homo sapiens  GCACATGTAAACATACTCCTGAAGGATGAGGAAAGCAATGGTAAATGGGGAAGCAAAAAAATACCATTTTTCATTTAGACAGACTTCCCAATACCGATGAACTAAAATTCAGGGATTTGT
Mus musculus  GCAATTATAAA-ACACCCTTGAAGAATGAGGAAAGCCATGGTAAACAGGGA--CAAAAAAATGTAGTTTTTCATTTAGATAGACTTCCAAATAGTAATGAAAGAAAGGCCAGGGCTTTGC

Homo sapiens  GATTTGAAATGCATGGATGGCCTTTGAGTGGTCTTTTCAAATCCCCT-AAATATATCAATTTAATAACTTATTGAGCATCTACCATTTGATGAGTGCTGTGTAGATGTATCTATATATAT
Mus musculus  CATTTGAAATGTATAGGGAACCTTTGCAT-----------TCTCCTAAAATGTATCCAT----TAACATAGAAAGCATTTACTACATGGTGGGCACCATGCTGCTATTGCTGTATATGC

Homo sapiens  CATAAAATTTATTTCTTGCTACAACTTTTGGAAGAGATTATTATAATCCTCATTTACAGATGAGGAAAC-------TGAGTCTCTGTGAGAGGAAATGATTTGCCCAAGATCCCTCAGGC
Mus musculus  CACATTGTTATTCTTTCAGTATGTTCTCGAGAAGATATCATTATGAGCCTCATTGACATATGAGAACATTGACATATGAATCTGCTCCAGAAGAAATTACTTGCCCAACTTTGCTCACTC

Homo sapiens  AATAAGTAGTAAATCTGACTTTGAAACACAGGTCTGATTCCTTATCCCCATCAGATTTCCCAGGCATTCTGGTACAGG-TGGTGTTTCAACAGATGGGAATGGGGCAGGGTGAGAAAGAT
Mus musculus  AGTAAGAGAAAAAT-----------GCATGTGCTTG-----TTAACAGCACCATGTCTTCCAG--ATCCAAGGATTGGTTGGCATTGTGGCACATGGGGATAAGGCAGGGTGGTAAAGGG

Homo sapiens  AGTAT------GAATAAAGGTACAGACATGGGAAAATGTAGC-TACACATAGGACAGTAGTAGGAATTAAGGTTGCAAAATTGTGTTAGGGACAGAACACAAAAGGATTAGAAACTTGAA
Mus musculus  GGGATGGCAAGGAGTAAGAGTGTAGACATGAGCAAGCACAGCTTAATCATCAGAAAACAGTAGGAGTTGTAATTGCAACAT---------ACAAGAATCCATAAGATTTAGAAATATGAC

Homo sapiens  CTTCCTTCCATTGTCCTGAAGGG-----ACAATAAAAGATTTTGTAGTAGGAAAACAATATAATCAGAGAGCCAAATCAAGAATGCAATCCAATTTACAATAGCCATACAAAAAAAATTA
Mus musculus  CTCCTTTCCATTGACCCAACAGAACAGCACAGTAAAGGGCTCTGGAGTGAG......................................................................

Homo sapiens  AATACCTAAGAATACATCTAACCAAAGAGGTGAAAGATCTCTACAGGGAGAATTAACATACACTGCTGAGAGAAATCAGAGGCAACACAGACAAATGGAAAAACATTCCACACTCATTAA
Mus musculus  ........................................................................................................................

Homo sapiens  TTAGAAGAATCAATATTGTTAAAATGGCCATACTGCTCAAAGCAATCTACAGATTCAACGCTATTCCTATCAAATAACCAATGTCACTTTTCACAGATTTAAAAAAAGATTTTAAAATTC
Mus musculus  ........................................................................................................................

Homo sapiens  ATATGGAATTTAAAACGAGCCCAAATAGTCAAAGTAAACCTAAGCCAAAAGAACAAAGCTGGAGGCATCACATTACCCAACTTCAAACTATACTACAAGGCTACAGGAACCAAAACAGCA
Mus musculus  ........................................................................................................................

Homo sapiens  TGTTGCTGGTACAGAAAAAGACATATAGACCAATGCAACAGGATAGAGAATGCAGAAATAAAGCCACACACCTACAACCAACTAGTCTTTGACAAAGTTGATAAAAAATAAGCAACAGGG
Mus musculus  ........................................................................................................................

Homo sapiens  ACAGGACTTTCTATTCAATAAATGGTGCTGGGATAACTGGCATATGCAGAAGATTGAAACTGGACCTCTGGCTATCACCATATATACAAAAATTAACTCAAGATTGATTAAAGACTTAAA
Mus musculus  ........................................................................................................................

Homo sapiens  TGTAAGACTTCAAACTATAAAAACTCCAGAAGGAAACCTGGGAAATATCCTTTGGACATCAGCCTAGGCAAAGAATTTATGACTGAGTCATCAAAAGCAATTGAAACAAAACCAAAAATT
Mus musculus  ........................................................................................................................

Homo sapiens  GACAGGTGGGATCTAATTAAACCAAAGAGCTTCTGCACAGCAAAAGAAAATATCGACAGGCACCCTACAGAACTGGATAAAATATTCATAAACTATGCATCCAACAAAGGACTAATATCC
Mus musculus  ........................................................................................................................

Homo sapiens  AGCATCTATAAGGAACTTAAATCAACAAGCAAAAAACAACCCCATTGAAAAGAGGGCAAAGGACATGAACAGACGCTTTTCAAAAGAAGACATACAAGTGGCCAAAAGACATGAAAAAAT
Mus musculus  ........................................................................................................................

Homo sapiens  GCTCATCATTACTAATCATCAGAGAAATGTGAATCAAAACCACAGTGAGATACCATCTCACACCAGTCAGAATGGCTATTATTAAAACGTCAAAAACAGCAGATGTTGAAGAGGTTGCAG
Mus musculus  ........................................................................................................................

Homo sapiens  TGAAAAGGGAATGTGTATACATTGCTGGTGGCAATGTAAGTTAGTTCAGCCCCTGTGGAAAGCAGTTAAAGATTTCTCAAAGATCTAAAAATAGAAATACCATTTGATCCAGCAGTCCTC
Mus musculus  ........................................................................................................................

Homo sapiens  TTACTGGGTATATATCCAAAGGAAAATAAACCATTCTACCAAAAAGACACCTGCACTTGTATGTTCATCGCAGCACTATTCACAACAGTAAAGACATGGAATCAACATAGGTGCCCATTG
Mus musculus  ........................................................................................................................

Homo sapiens  ATAGTGGATCGGATAAAGAAAATGTACACGTACACCATGGAATACTACCCAGCCATAAAAATGAACAAAATCATGTCCTTTATGGGAACATGGATGCAGCTGAAGGCCATTATCCTAAGC
Mus musculus  ........................................................................................................................

Homo sapiens  AAATTAATGCAAAAAAAAATCACATATTCTCACTTATAAGTGGGAGCTATACCTTGGGTACACATGGACATAAAGATGAGAACAATAGACATTAAGGACTCCAAAAACTATCTGGGTGAT
Mus musculus  ........................................................................................................................

Homo sapiens  GGAATCAATAGAAGCCCAAACCTCAGCATCATGCAGTACACCCTTGTAATAAACCTGTACATGTACCCCCTGAATCTAAAATAAAAATTAAAAAAAATTTAAACAGCTGCATTTTAGACA
Mus musculus  ........................................................................................................AGATAGACTTTAAGGA

Homo sapiens  GATTAATCTAATTCAAGTCTGAATCTATG-----------CTGTTGACAGTATTTT----------------------------TAAAAATTCTTTCCTAAGAATTCTTTATTCACTCCA
Mus musculus  GATAAATATGACTCAGTACTGAATCCATGGTTTCTTTTTGCTATTGATAGTATTTAAAACAAAACAGAACAAAACAAAACAAAACAAAACTTGTTTATTAATAATTCTGTATTCAACCCA

Homo sapiens  GGCCTATGCTTTGGTCAGAATTCTCTCTTTGTTTTTCAATACTTTAGTGAAAATTCAGAAGGCCATGTGGAAAAAAACAGAGGAAACAAACCACACACAAACACAAGATAATTATATACA
Mus musculus  AGCTTTTGCTTTGGTACAAATTCT----TTGTTTTTACATATTTTCGTCAAAATTTAGAAGGCCACATGAAAAAGAACAAAAGGAACAAACCACACATAAACACAAGATAATTGTGTTCA

Homo sapiens  AGTCTGGGAAGAAAGAGCACAATGGGTCATGACGAACTTTATTGAACAGAAGGCGAATAAGCAACGTTCCAATTGTACAGGAAAAAAAAAAACCCAGCAACCCAAACACCACCACCAACAA
Mus musculus  AGTCCAGGAATAAAGATCACAGTGGGTCATGACAAACATTATTGAATGGAGTGTGAGTAAGCTGTGTCCCTGTTGTAAAGGGAAGAAA----CTAGCAACAAAAACACC-CCCTTAGTCG

Homo sapiens  TAAGAAACAAAGGCAGCCAGGATCTGGGAGACATGGATGAAGCTTGTGAAAATG---------------------ACACTTTCTTAAAAGAGCAGAAGAGAAACTAAAAAGGATGAAGG
Mus musculus  CAAGAAACAAAGGCAGCCCAGATCTGCGAGGAACTCATGCAGGTTGTGCAGACATGGCACTTTTGCATCTACTGGTTCACTTTCTTCTAGAAGCAGGGGA-AAACTGAAACAGAGGCACT

Homo sapiens  GGAGAACCATGGATTTGAGGGTTAAACTAAA--AGGAAAAATAGAAAGGAAATAGGAAGGAAGGGTGTAACTCATAGCTGGTTTTGATATATCATCCCAGTCTGACAACCAAGGCAGTCT
Mus musculus  GGAGAACCATGGAACTGAAGGGTAGACAAAATGGGGTAAAATAG---GGAAATTCAGAGGGAAGCTACAATATAGACCTGGCCTGGGTATAGCTATCCAGTTAGAGAA---------GCT

Homo sapiens  CCCTG--CATTAGAAGG----CCATGCT--AAAAGTAGATGA-----GTTGTAAACAAGCCTCCCACTGGTCATTAGTTTGGCAAATTAGTGGGTTATCTCTTAGAGTCCTCTCTTTTTC
Mus musculus  CACTGACCATTAACAGGGACAGCGTACTACAAAAGTATATGAAGATTGGGGGGAAAAA------CACTGGTAATTAGTTTGCCAAACTTGTTGACTATTTC-GAGAGTCTTCTCTAACTT

Homo sapiens  TCCTTTCCCTTTCCTCTGCATTCAATTTGTC-ACTCATTCCTACAAGTCTACCCCAGAAGGGTTG-CCTGAATCAAAGCCCTTCTCCCCATTTCCACTCACGGGGCCACTGTGTTAATTA
Mus musculus  ACTCTCTTCCATCTTTCAAACTCAATTTGTCTTCATATTTATGCTTGTCTACTTCTGAAGGGGTATCCTGAACCAATGCTCTCTTCCTTCTCCCCACTCAGAGGAGCAGCATGTTAAACA

Homo sapiens  AATTCCATGTCGTTTCTCAGCTGGACCAGGCTTCCTGCCTCTAATTCGTTTCTTCCTCCCTCTAATTGAGGCTTAGATCTGCCCCAGTATTGAGCTTCCTATACTCTCTTGCTTATAAAC
Mus musculus  A----TATGTTCTCCCTTACCTGGATCATATTTTCTGTCCCCA----GTTCCCCAGTTCCTCTAGTGTGGGCTCCAGTTT---TTAAGTTTGAGTCTTCTAATATGTTTGGCTTATAAAG

Homo sapiens  CTTCTTTGGTGGCCCATGGCCTATAAGGTACAGTAGAACTCACTCAGTTTTACATGATGCAGTGGGAGTGGAATCAAAGGGTTCCCCTAGACCCATGGATGAAGAGACGACTAAGGAGCT
Mus musculus  CTTGTCTGACAGCCCAGAGCATACGGAAGACCATGGAACTTCCTTAGATCTGAATGATGTAAAGGCAAAACAAGCAAAGGATACTTCTGATC---TGGAGGATATGCTGACTAAGGAGCT

Homo sapiens  AAGTCTGGGATGAGAAACTCTTGCAAAAAGCCAACTAATAGCTTTTTCCTCCTCATCTCAGAACCACCCCTCTTCCCAGAGTGTGTGTA-------------TACAAGATGAGTT-ACT
Mus musculus  ACAACTAGGATGAGAAACTTTTA-ATACACATGAATACTACCTTTTCCATTCTAATCTCAGCACTACCCTTCTTCCAAGAGCTTGTGTAGAGTGTGTGTGGGCTGGGGGATGGGTTAACT

Homo sapiens  TAAGATTGCACCCAACCTGATGGTAGATTGGAGAAGGCTATCTGTCCCTTTCTCAGTTGAATATATGAGGGTCTTCTTCCCAGGAGGCTTGCCCACACCAGTCTTCTTTCCCACCATGGC
Mus musculus  GTTTCTTGTACTTAACCTATGTGTATCTTGGGGAGGGGTGGCTATTCCTCTATCAACTGAATCTATAA--GCCCCCCTCCCAGGAGGCTGCCCCATTACAGCCTTCTTTCTCACCATGGT

Homo sapiens  CCATCCCCTTTCCAAAGAGTATAAACTTCATACC--------CTCCTCCCTTCAAGAACTCCTATCTTTGCTTAGC----AAAATTTGTTTCCCAAGCTTGGAGAGAGGAAAGGGGCCTT
Mus musculus  CCATCCCCTTTCCAAAAAGTATATCCTTCACTCAAACTTCAGCTCCCCAGATCAAGACTTCTAGTGCTTGCCTAGCTCTTAAAACTCATCCCATACACGTGGAGAAAGAAAGGGGATCTT

Homo sapiens  TTTCCAAATCAGGGGTAAATAAAAAGTGGCAGAATCTTACAGTAGACATCATATTAGAGCTCATTT--TTATGAATTCTTCTACTGTATATAAGGTCCT-CACTTTCTTATTACTCAGCC
Mus musculus  TTTCAAAAGCAGGGGTGAGCAATGGGTGGTGAAATATTGTAGTAGACATCATATTAAACATCATTTAAAAATGGATTCTTCTACCCTACATAAGGCTTTGTCCTCTCTGATTGCCTAGCT

Homo sapiens  TACCACTCCAGTTTCATCTTCCACTTTTACTGCTTCTCCCCACCCCTTCACAATCCTGACATACCCTAAACTCCAGTCATACTGAACTTTCCAACATTCCTCAGATAAATCAAGCCTTTG
Mus musculus  AACAGCACCAGCCTTGGGTTGTGTTCTCAGTG-------------CTTCATAAGTTTCCCCTACCTTAGACTCCAGGCATGCTCAACTTTTCAACTTTCCTCAGACAAACCAGGCCTTTG

Homo sapiens  CAGAAATTCATGCACAAGCTGTTCCTTCTGCTTAGAACATCCTTACTCTTCTTATCTCGCCAGTAAATTCCTACTCTCCTTTAAGACCCTGCTCAACTATTGCCTCTTCTGGGAACTCTT
Mus musculus  AAAACGTTCACAAACACAATGTTCCTTCTTCACAGAACACCCTTACTCCTCTTATGTT-CTAGGAAATTCCTATCTTTCTTTAAGATTCCATACAA--GTTGCTTCCT-----AACGTTT

Homo sapiens  TCCAGACTGTTCCAGATAGAAT-TAATTAATCG-TCCTTTGAGCTCTCATAGCATTTTTCCATATCCCTTTGATAGCACCATCCTTA-TCATGGCAAAACAATATAACAACTAAAGGGAT
Mus musculus  CCCA-ACTATGCCATGGAGAATGTAATGCCTCTCTCCTTTCAGTACTTGGACTGTTTTT-TGTACTCTAATGATAGAACCATACTCATTTGTGGCAGA-----GTACTGACTAAATGGAT

Homo sapiens  GGGCTCTGGAGGCAGACTGCCTGGGTTTGAGTTCTTACTCTGCCACTTATAAGCTATGTGATGTTGAGCC-------AATTACTTCGCCTTTC----TTTGCTTCAGCTT-CGTCATGTA
Mus musculus  GGGGATT-AAGGGAGATTGTTT-------------------------------TATCCGGTGCTGGACCAAATCCTAATAGCTTTACCTTTCTGGTTTTGGTTTGGTTTGCTTTATTTT

Homo sapiens  TAAAACAA-----AATAAATAAGATGATACTTGTAAAACCTTTAGAACAGAACCTGACACATTGCAAATGTTAGTAAATATTATACATCATCATTTCTTCTATTGTTTTATTGTTATCGT
Mus musculus  TTTTTCAATTTTCAGTACATGAGATT---CTTGTGAAGTCTTTAGAATAG--TCAGTAATATTAGAAAATGTGATCAATATTGGACATAAAATGTTC-----TTGTTTTATTGCTACCAT

Homo sapiens  ATATATAACCTACTTGTATATGTAATATACATGTGTTATT-ATATAGCACTATTATAGCATTACTTAACTTTTGCTGTGTCTGTGTCTTGTCTTCCAAGCTGGTTTAT-AGTGTTTTGAG
Mus musculus  AGATG-----TACTTGTATGCATACTACATACGTGTTGCCAATATTACA----TACAGTGTTGTATAACTTTTATGGTGTTTATATCTTATCTCCTCAATTGGCTTGCAAGGGTTTTGAG
```

```
Homo sapiens  GGCAGAAACCTTGTATTACTTTCTCCCCCATAGTGCCTGGTGTTAAGTATGGGCTCATTAAGTATTTGTTGGGGTCAGTATATTATACT--GAAAAAAGCACTGAAATCTGAGAGATTTG
Mus musculus  TATAGGACACCTTTATCATCCTCTCTTTCATAGTGCCTTGTG-TAAGTGTGGAATCATTAAATGTATGCCAGAGGCAGTATATAACAGTAGAAAAAAACCACTCCAACCTGGTAGATTTG

Homo sapiens  GGTTTGGATCTTGATTCTGCTCATAATTGTTGTATGATCTCAATCTTCCTGAGCCTCAGTTTCCTCATCTATAAAATGAGG-ATGATAATAAGAGTACCTACCTCACAGGGATATTTTAA
Mus musculus  GGTTCCAATTC---CTCTATTCCTAATTGTTGTATGTGTTTATTCTTCCT-AGTGAAAGTTCCCTCATCGCGAAAATAAAGTGTGATAATAGTAGTACGTACCTCATATGAATGTTCTAA

Homo sapiens  GAATTAAATAACTTAGTACATGTAAAACCTAGCACATAATAACATAATAAGAACTCACTAAATGGTTTTTATTATCAGCTTTGTATCCCTTTGCATTTAACTTAATAAATACTTTTACTT
Mus musculus  AGAATAAATAAATCGGTATATGTAGAGCTCAAC--------ACATAACAGGTACATCCTAAGTGATTGCTAGTATCAACTCACTTTCCCTTTACATCTAGATTAATAAATGCTTGCTCCT

Homo sapiens  G-----AATGAATGAACCCAGCCATTT------TCTGGAACTTGAGGATCAAGGGAATCCAGGCAGATACTCTATTTGTTCCTAAGAATCCAGAGGAAAA--TGTGAGTCATCTCCATTC
Mus musculus  GGGGCTGGGGAGCGAGCTCAGAGATTTAGGCGCTTCCGAGTCTGAAGATCAAGAAGATCCAAGCTGAC-------CTACTCCAAAGAATCCAGGGGAGAAACCGTGAATCATCTTCATTC

Homo sapiens  TGTATCATGGCTCACTCTGAACTGGGTCACCATCCTCCAGAAGGTGACAAATGTGGCTGGCTGAGTCACTATAAGAGCTTTTCT--GTAAGGATTTGCCCGAACCCCCCACCATAGCTCA
Mus musculus  TGTTTCATAGGTCACTCT-AACCAGGACACCATCATACAGAAGGCAACAGATGTAGCCAGCTGAGTCACTATAAGAACTTTTCTTAAGAAAAAATTGCCCTAGCTCCC-ACTATGGCTCG

Homo sapiens  TTGGACATAGTATCTTTCATATTAGGTGCGAGGT-GCATACCCTTCCTGAAATGGGCAGAATAAAG-AGGTGCAAAACTCCTGGGGAATCAGAGTGGCTATGAATTCACTCCTCTTTCTT
Mus musculus  CTGGACTCAGTGCCTCTCATACTAAGGACTAGATTATGTACTCTTGCTATGACAAGTGGAAAAAGGCAGTTATACAACTACTGAAAAGCTTGAGTGTTTATGAATCTACCCGT--TTCTG

Homo sapiens  ATTCTGTTGCCTCTTCCCTTTGTTTGGTTTATTTGCTAATCAACTTTGCCCACACTCACATCTAGTGTAGCCCAAGTCTGTGCAGAGAAGACACTTTGAAAGC-CTAAGACTATCATAGT
Mus musculus  CTTCAGCTTCTATTTCCATTT--------TAATCACTAATTAACCTTCCAGATATTCACCTCCAGTGTA--CTGTACCGATGAAGAAAAGACACACTGAAAGTGCTAAACTTAT------

Homo sapiens  TCCAGCA--GAATAATAGAAA-AATAACAAGTGTTGACTATTCAGTTCTGATGTAGCACTGTATTGAAGAACATAACATTCTAGATCCAGATGGCCTGAATTTGAATCCCAGCTCCACCA
Mus musculus  TCCATTATGGAATTAGAGGAGCAATAACAAA--------ATCCAG----GATAATATACTGGGTTGAA---CATTCTGCTTTAAACCCAAATGGCTTGGGTTGGAAGGTTTGTTCTACCA

Homo sapiens  CCAACTAGCTGTGAGACCTCAGGCAAATCACTTAACGTTTGCATCCTTTCATTTCTCCATCTATCAAATAGAAACAATAAAAGTA--CTTACCTACCTCTTAGGGTTTTGTGAGGGTTAA
Mus musculus  CTTACTTGTAGCAAGCCCTTGAATGG-----TTAAACTTTCTGTGCATTCATTTCTCCAGATAGTAAAT-GTAACATAAACATTAGCCATTCCTATTTTT-------------------

Homo sapiens  ATGAGTTAATACATACAGAACACTTAAAAGAATGTCTGGCACATAGTAGGTGCTATATAAATATTAGCTTTTAAAAACAGCTTTATTGAGATATAATTCATCTATGTGAATTATATCTCA
Mus musculus  ------------ATATGAACCACTTC--------TTTAACAGCTTCAAGGTG----ACAAAAATGCACATGACAAAATA-------CGAGACTTAATTCAT------------------

Homo sapiens  ATAAATTCACCTATTTAAAGTGTACAGTTCAGTCATTTTTAGTA---TTTCACAGAGTTGTGCAACCATCACCACAATCTAATTTTTAGAAAATATTCATTACCCCAAAAAGGAATCTCA
Mus musculus  -------------TTTACGGGACACTGCTTAGTTGCTTTTAGCAGACTTTTGTGT--TTGTGCAACCGTCAATAC-----------TGGAAGGTCTTCATCACTCCAGAAAGGAATGGTG

Homo sapiens  TACCCACTGGTAGCCGTTTCCATCCCTGAAGCTCCCTCTCCCCCTTCCCAGCTCCAGGTAATCACTAACCTACTTTCTGTCTGTATAGATTTGCCTCTTCTGGACATTTTATATAAATGA
Mus musculus  TATATGCTGAAGACCATTCTTGTCTCTGTGG-TCTTTCCATACCCTGCCAGCCCTGAGGAACCACT--------TTCTGTCTCTATGGGGTTTCCTCTTCTTGCCATTTTACATAAATGG

Homo sapiens  ACTCATACAATAGGTGGTCTTCTGTGACTAGCTTCTCTCCCTTAGCATAACTGTCTTTTAAAAAATTGCAACTTCCATTTTAGATTCAGGGTGTACATGTCCAGGTTTGCTACATGGGTA
Mus musculus  ACCCAGCCGTTAGATGATCTTTTGTGACAAGCATCTTTCATTCAAAAAAA---------AAAAAA.......................................................

Homo sapiens  TATTGCATGATGCTGAGGTTTGGGGTACAGCTGATCCCATCACCCAGACAGTGAGCATCCAATAGGTAGTTTTTCGGGCCTTGTCCACCTCCCTTCCTCCCCTGTCTAGTAGTCTCCAGT
Mus musculus  ........................................................................................................................

Homo sapiens  GTCTATCGTTCCCATATTTATGTCCATGTGTACCCAGTGTTTAGCTCCCACTTACAAGTGAGAACATAGCATAATGTTTTTAAGGTTCATCCATGTTGTA-TATATATCAGAGCTTACTT
Mus musculus  ...........................................................................ATTTTAAACATTTATCCTTAATGTAGCACATCTCAAAGTTT----

Homo sapiens  CCTTTTTAAGGCTGAATAATAGTTCATTATATGAATATATCATATTTTGTTTTTCCATTTATCAGTTGATTGACATTTTGGATTGTTTCTATCTTT-TGGCTAGTATGAATAACACTGCT
Mus musculus  TCCTTTCATGGATGAATGATAGTTCACTATAT----ATATCACATTTTATTTATCCAATCATAAATTGATTAAA--TTAGGGCTATTTCTAGTTTTATGACTATTATGAATAATGTTGCT

Homo sapiens  ATCAATATTCATTTACAAGTTTTTGTGTAAAGATATGTTTTTAAATCTCTTGGGTATATATCTGGGAGTGGAATTGCTGGATCATATGGTAACACTATGTTTAATGGTTTGAGGAACCAC
Mus musculus  ATGAGTGTTTCTTTACAAGTTTGTGTGTGAAGGTATGTTTTCAG-TCCTTTGGGTGTATACCCAGGAGTCAAGTTGCTGAATCACGTGGTAACATGGTGTCTAAT---TTGAAGGACTGC

Homo sapiens  CAAACTATTTTCCAAAGAGCCTGCACTATTTAAAATTCCCTCCAGTAGTGGGAATGAT----GGTTTCAATTTTTCCACAT--CTTCATCAACACTTGTTATTTTATTTTATTTTTATTT
Mus musculus  CAAATTGTTTTCCAAATAGGCCACACCATTTTAATTTCCATCCAGTTATGGGAATGAATGAAGGTTGTAATTTTTTAAAATTTCTTCACCAATACTTGTTGTTTT--------------

Homo sapiens  TATTAAATTATTATTTTTATTATATTTATTTTATTTTTTATTTATTTTTTATTATAGCCATCCTACTGTGTGTAAAGTAGTATCTCATTGTGGTTTTGATTTGCATTTCTCTGATGGTTA
Mus musculus  --------TATTGTTTCT----------------------------TTTATTATACCCACCCTAGAGGGTGTAAAATAGTATCCCACTGTGGTTTTGATCTGTATTTCCCTGATGGCTA

Homo sapiens  ATGAGGTTGAGTATCTTTT-CATGTGTTTATTGACCATTCTTATGTCTACTTTGGAGAAATGTCCCTACAAATTCTTTGCCCATTTTTAAATTGGG----TTGTCATTTTATTACTGAGT
Mus musculus  ATGAGGTTGCACATAACTCATATGTGCTTATTGGCTATTCTCATGTCTTCTTTGGAGAACTGCCTATTCAAATCCTTTGTCTAATATTCAGTTGAGCTATTTGTCCTTTTATGATTTAGC

Homo sapiens  TGTAACAGTTCTTATATATTCTGCATGCAAATCTCTTGTCAGATATATGTTTGTGAATATTCTGT-CATTCTGTGTAATT-TTTTTTCATTTTCTTGAAGCTATTATTATTAAATAGAAA
Mus musculus  TTTAATGATTACTGTAT--TCTGAATACAAGTCCTCTG-CAGATACACAGTTGTGAATATTTTGAACATTTTGTATGATTGTCTTTTAACTTTCTTGAAGCTGTTATTGCTAAGTAAAAA

Homo sapiens  TTTGTAAGTTCGCATATAATAAAGATGACAGATTGTATTAGAAAGAATGAGATCTAAAA-----TCACCTGATGCACCCTATGGTACACTACCCTGCTC--CTGCACTCCTTTGAAATTA
Mus musculus  GTTACAAGTACACATATAATAAGGG-GGTGAACTGTAGTCAAAAGGATGAGATCAAAAAATTGGTTAGAAGATGT--CCTTAGGTACACTCTCTTGCTCTACAGCCTTCCATTGAAACTA

Homo sapiens  AATTCAAAACAGATCAAGCTTCTCCTCTGTGCCAGGCACTGTGGGGGAATAAGACCTAACCTCTATTCTCAAAGAGCTTCATAACACAGA----AGAGAAGATGCAAACATGTGGAAACT
Mus musculus  AATCTAAAACAGATGTAGCTTCCTTTCTGTGCTGGGCACTGTGGGG-AATAAGAGTTTGCTTCTATCCTCAAAGAGCTCACTAACTCAGAGGGAAGAGAAGAGGCAAACATGTGGAAACT

Homo sapiens  TAAATAATTATAACATGTTTAAATAGCAGTTCAGGACCACAACAGAAAAGATGCCACAGAGCACTA---TATGATTAATTTCCAAATGAATTGCATGGAAAAGAATTGCTATGGGAACTC
Mus musculus  TAAATAATTATAACATATTTAAATAGCAGTTCAAGACCACAATAGAAAAGATGACACAAGGCACCACTGTGTGATTAATTGCCAAATGAATTGCACGGAAAGTAATTGCCATGGGAACTC

Homo sapiens  AAGGGAGGGAGAAAGCACTTGGGGCTGAGGTGGTCAGGGGAAGCTTTAAGGAGAAGGGATCTAAGCAGTCTGTAATTAACTACCGCCATGCCTGTGTAATACTAAACAAAGACTTGGAGA
Mus musculus  AACAGAGGAAGAGAGCACT-GGGGCTAGCGCGGTCAGGGAAAACTTTAAGGAGGAGGGATCTAAGCAGTCTATAATTGACTACAGCCGTGCCTGTCTAATACTAAACAAAGACTTGGAGA

Homo sapiens  GA---AAAAAATATTGACTGTTTTTGAGGTGAGGCATCTTAGTGATTAAATTGATACCACCATTTCTTTTCAACCTCTGCAGGAATCCCCACAGTCCCACCTTTCCAATGAAGACTTACG
Mus musculus  GAGAAAAAAAAATATCGACTCTGTTGGAGGTGAAGTACTTTAATGATTAAATTGATAGCAGTATTTCATTTTAACTTCAGCGGGAATTTTCATAGCCCAACCTTCCTCATGGAGAGTTGTG

Homo sapiens  TGGCCTCTGTCTAGTCCAGAGGTTACCAATTAACAAAGTTAACAAGCCACATCTGGCTTACAGACACGTTTGGTTTGGTCCAATTAAAATATTAGAACTAGCTGCTAAAACATTT--AAC
Mus musculus  TGGCCTCTGTTTAGTCCAGAGGTGAACAATTAGC-GAGACAACCAGCCACATCTGGCTCACAGACCTATTTAATGTGGTCCAATTAAAAGAATAGAATGAGCTG---AAATTTTTTCCAT

Homo sapiens  TGGACAAATTTCACATAAAATATT-----AATTTCCAGTTTCTCTAGAAAAATCAGAAGACCTGGCTACCCTGCATTGG--AACATGCATTCTCTGGGACCATGGCCAATGGCAACCCTG
Mus musculus  AGGCCAATTTTCATATGAAATATTTAGAAAAATGACATTTTCCTAAAAATGACAGAAAGATCTGGCAATTCTGAGATAGATAGAATTCACTCTCCAGGTCCATAGTCAATTCCATTCCTA

Homo sapiens  CTTGCACATGCAGAATTCAGTTTGAGACTTCCTAGAATCATAGTTCTTAGACTAAAAGGTATCTTAAGAGTGCATCTGTTTCTATTCCCTGACTTTGGGAATGTATGGTATTATAATATT
Mus musculus  GTT--------------------AGACTT-CTAGAATCATGGGTCATAAATTGAAAGGTACCACAAGTGTACATCTGTTTCCATTTCCTGGTTTTGGAATTGTATGGTAT-----TATT

Homo sapiens  ACCCTCAATTTACAACTTGATTTAGAATATCAACTAAAACCCACAGAGAATTTGACTTGCTGCAGGCCACAGGGCTATTAACTGGAAGAGGGAGGGCTAAAACCCATGTTCTCTACTTCT
Mus musculus  ACTTTTAATTTA----------TAGATTATTAACGAAATCC...............................................................................

Homo sapiens  CAACGGAATGTTCTTTCAACAATCTGTCTTTACAGTCTGGATGCATATTGTTTAGTAGCATTCTTCTGGCACTTCCTAATCACTGCTTTGTGTTGTTGGACGATTTTTGATATGATCGTT
Mus musculus  ........................................................................................................................

Homo sapiens  ATCTCCCTCTCTCTTTTTTTTTTTTTTTTTTTTTTGAGACGGTGTCTCACTCTGTCGCCCAGGCTGGAGTGCGGTGGTGCGATCTTGGCTCACTGCAAGCTCCGCCTCCCGGGTTCACGC
Mus musculus  ........................................................................................................................

Homo sapiens  CATTCTCCTGCCTCAGCCTCCCAAGTAGCTGGGACAACAGGCGCCCGCCATCACGCCCAGCTAATTTTCTTTTTTTGTATTTTTAGTAGAGACGGGGTTTCATCATGTTAGCCAGGATGG
Mus musculus  ........................................................................................................................

Homo sapiens  TCTCGATCTCCTGACCTCATGATCTGCCCGCCTCAGCCTCCCAAAGTGTTGGGATTACAGGCGTGAGCCACCGCCCAGCCTGATCATTATCTCTCTTAAATGACATTGTAACATCCCAAA
Mus musculus  ........................................................................................................................

Homo sapiens  CTGCAAAGGGGCCCACATCAAGTTTTTCTATGAAAGCCCATGACGTGGAGCACAATGCTTTGTACTAAATAGGCACTCAGGTGGTATTTGTTATTGATTGATAGGATTCTGAAGAACTCA
Mus musculus  ........................................................................................................................

Homo sapiens  GTATGTAACCATCCTTCCAAACCTGGGTGTGTTAAAATGTGTTCATTGTTCAGAGACAAAGGTCTGGTACCTTTAAGAAGTCTCAGCTTTGCAATGAGTTTGTGGGGCAGCTGCTTGGCA
Mus musculus  ........................................................................................................................

Homo sapiens  TAGATACTGGGGAGGGACAGGGAGGAGATCATTA----TGTAGGCACATGGCGCTAGGATGTACCTTACTTTGTCTTGGCTGTTTTCTGAATGTTAAGAGGAAGGATGAAATAATAGCAG
Mus musculus  ..........GAAAGGACAGAGAGACGGCTATCAATCACACAGGTGTATATTACTGGATAGGACCTTGCTTTCTCTT--CAGTATTTTGAGTGATAAAAG-----ATGAAATAATAG---

Homo sapiens  ATATAGGAAAAGTTATTTAAAGCCTTGCCTTAGATCAGTGGTTCTCAGGCGTGAGGATGCATCAAAAGCCCCTGGAGGGCTTCTTAAAACACAGATTGCTATATCCCACCCCTGAGTTTC
Mus musculus  ------------------AAATTCTTG----AGGTCAGTGATTC---------AAAAAACATCAGAGCCTACTGGAGTGCTAGTCAGG-CATTGACTGCTATATCCTCCCACAGGGTTTC

Homo sapiens  TGGTTCAGTAGGTCTGAAGTGAGTGGGGTCCACAAATATGCATTTCTAACAAGTTCTCAGGTG--ATGCTGATGCTCCTGGTCCAGGGACCACACTTTAAGAACCATTGCTCTG------
Mus musculus  TAGTTCAGTAGATTGAAAGTGAGTAGGGTACAAGAATGTGCGTCTCTAACATGG----AGGTAGCATACTGTT--TGTTGGTCTAGGAACTACACCTTAAGAACAATGACTCTGAGGTAA

Homo sapiens  CCAAGTAGTTGGCTCAAGAGTAGCTAATTAGAGAAATCTGAGCA-CAGTTAGCAACTGGAAGCAAAAAGTCCATGAGGAAACTGTGTGATGTACGTAGTGGGGAGAGTGTGAACTGGCTA
Mus musculus  CAGGAGAGCTGTATAAAGAGTAGGTAATTTGAGACGTCTGAGCATCTGTTAGCAACAGGCATTTTAAAA--------GAAAGTCTGTGCTATAGGTAGCAGGGAGATCAAGAACTGGCCA

Homo sapiens  GGAAACCAGAATGGTCAGATTCCAGTACCAGGGGTATCATATCTTTTTAAGCAGTGTCCAAAGAATAAGTATATTTTAATTCACATATTCCTGATTCATTTTACTAGTGTTTCC----CA
Mus musculus  GGAACCCAGAGGAAATAATTTCCAATACCAGGGGAATCATATCTTTTTAAACAGCATCCA--GAACACAGTTAGTTTGACTCACATGTTCCTGATG-ACTTTACTAGCGTTTCCACCTCA
```

```
Homo sapiens  A-AATGTGCACTGTGGAACCCTAGAA-GATATTAATAGGTAGGCAATGAGAT--TAAAAAGGATCCATAGAGAAAGATGTTGGAACATTTTGAATTAAAGAAAATTGAACAGGATTCTTT
Mus musculus  GTCAGTTTCAGTGGGGAATCTTCTCTTGATGTCATTGGGCATTCAGGAAGATAAGAGAAAGTATCCACAGTGAAAGAAGTGAGAACATTCTAAATTCAG---ATTTG--TGGAGTTCTTT

Homo sapiens  ACGGCTTCTAAGAGCCTTTAAAATGTGAATGAATG--AATGCACTGCAAATCTCCA-----------------AGGAAAT-------------------ATAGAATATGCAGTATTTATT
Mus musculus  GCTGC----------------AATCTGACTCAATGTGAATGCAGTACCAGTCTCCACCAAAAGAAAAAAAAAAAAAGAAATGAAGGAAGGAAGGAAGAAAAAAGAATGTGTGCCATTTTAT

Homo sapiens  TTATTAATTTATGCAGAATATTCAGAATTAGTTGACCAAAGATCC-----------TTCGTTAACACTTGGCTGTTTTTTTGTAAAATGTTGTTTAGGAAACCTTCATTGGTTTACACCT
Mus musculus  CTATGACT-----CACTTTATTCAGACTCAGTCGGCCAAGCATCCCTTATGAATTCTTTATTAACACCTAACTAGCTTTTTGTAGAACACGGTATAAGAAATCTTGAT------GTATCC

Homo sapiens  GAAATGCCACAATATTTTATCAATAGATGAGGTTGCTTGCAACAGTGGAAACAGCACTGCACTGGGAGTCAAAACATCTGGGATTTTGTTCTGTCTCTGCCACTTAGGTCTGCCACAAGT
Mus musculus  AGAGTGCCCAAATACTTTACGAATGGGCAAGGATGCTTATAACAAT--AAAGAGCA-----------------------------TCATTCTTTCT........................

Homo sapiens  TGGCTTGCTCCCATGAAAAAGATGGGTATGATTGAAATTTATCATCCCTTTGCAAATGGTAGCAGCCAGTAGCTCCACCCAAAATAAGTAAATAAAATCTCACACAATCAATTTT---TT
Mus musculus  ................................TGAACCATGACATCT-------------------------------------TAA--AAAAAAAAATCGTACAAAACACATTTAAAGA

Homo sapiens  TAAATCTAATTTACCATACCCTTAAGGTTATTCAATCTTTTTGTTTTTTTTTTCTCATGTTACCTTGTGTAACAGGTATTCAACCTTCAAAGCCAGAAATATGAGAAGCCTTATGCTGAG
Mus musculus  TAGCTCAAATTCAACATGCCCAAAGGAATATTCTGTCTT------------------------------------------------CCAAGTCAGGTAGGTGAGAAGCCTTATGTTGAG

Homo sapiens  AGTTTGATAGAAAGGAAGGGAGGAAGGGAGAAAGTAGAAAGGGAGAAAAGGAAGAAGAGAGGAAGGGATGAAAGGAGGAAAGGCGGGGCAGGGAGGAAAGGAAGAACAGCAGAAAGAAGG
Mus musculus  AGTT---------------GAGGAACTGAG-----------------------------------GGAAGAAAGCAGGTAAAG------------------------AACAAAAGGAAGG

Homo sapiens  AAGGAAGGAAAAATAAGAAGTCAGACAGTCAGGTCATTGCAGACCATGATTCCATCTGAAAAGAAAAGGCTGAGAAGCATAGACACATCCTGTGTTTATTTGATTCCTGGCTCTAGGAGA
Mus musculus  GAAGAAAGCAAAA----------GGCAGACAGGCCATTG--------------------AAAGACCAGGCTGAGAAGCA--GGCACATTCTGTGTTTATTTTATCCCTGGCACTCAAAGA

Homo sapiens  AAGTTTTATTTCACCAAGGACA--GAGTCAGCTCGCTTGTTTACCACAGAGTATTGGAAGTGTGTGAAGTGTGTTTGTCCCCCAATTAACCATTAGTTGTTTTTTTTTCACTTGAACAAG
Mus musculus  AAGCTGTGTTTTATCAAGGACACCAATTCAGTTTGTTTGCTTATCACCAAGTACTGGACGTGTGT---------TTATACCTCAATCA-------------TTTTTTTCTCTTGAATGAG

Homo sapiens  ATACAAACTTGACCCAATGGAGAAACATTGC-AAGTTGTATGTGGGAGGATGCCATAAAATATCTACATTGATATTTAAATAAAATACCTGCCTACATTAAACGA-TCTTTGAAAAGAAA
Mus musculus  ACAGAACCTGGAATCAACAGACAGACAATATTAAGCTGCATAT-------TGTCACAAACCATCCACGTTGATATACACATAAATTGCGTGACCACATTAGACAACTGTGTGAAAAGAAA

Homo sapiens  CTGATGGGATTCAAGGCTGAATTA----TATTTACTGAATTCCTACAAGGTACATAAATATA------------TGCAACATGGAGTCCC-----------------------TTGTAGA
Mus musculus  CTGCGTAGGTTTAAAATTCAGTGACTACTTCTTTCTGAATCGCTGGGAGGCACATAAACACAGAGGCTCTTCATTGTACAGTGAAGTTCCAATATCATTAAAGCCAGTGTAAGTTGAAAA

Homo sapiens  TCCATGATGAAATCATATT------------TCTTTTAAACAC---------------------AAGGGTCAATT-CAAGTTAAAAAATAAAATAAATGTTTACAAGCTTTC----TGCC
Mus musculus  TATGTCATAAA--CACATTGAAGACAGCCAACCTTCTAAACACCGTAGCCTAGCAACAAGGCACAAGGGTCAATTACTAGCCCAGGAA-AAGATCAACATT--CAAACTTACAGTATGGC

Homo sapiens  TTTTTTAAATTT-----------------------------CAAAGTGAAAATAATATTTAATTGCATTTAAATATATACATATATTTTAATAACTCTGATTACCTATTTGATTGCATAA
Mus musculus  TTTATTGAATTTGTATCATTTCTATATCATTGTGAAGCCTGCATTTTGAAAATCAGACCATCCTGAATTGGAGATCATC--TGTAGTCTCATAACTCTGATTACCTATTTGATTGCAGAA

Homo sapiens  AAGAATGACGAGAACATCAACACTGCTTTTAGCCACAATATTTGAGAAGTAGCAGC-CAGAAGTGTT-TTTTTAAATATTTTATCTTGAAATCAGATCTCATGT---------ACCTAGG
Mus musculus  AA---TGACAAGAACATCAGTCCTGCCTCGAAGTCAAATATTTGAGAAGCAGTAACTCAGAAGTCTCATTTTTTTATCTTTCATTTTCAAATCAGACCTCAGGTGGATCTTGAATCTATG

Homo sapiens  TGAACTGAATATGCCC-AGAGGATATCAGGGGACTCTCCACTACTGTAGATCCAAAAAACAGAGGGCTAAATAGAAATTCCACTTGCTGTCATCCAGCAAAATAGAGATGGCTTTGCTGA
Mus musculus  TGAACTGAATATGTCCGAGGGGGTGTCGGGGAACCCTCTGCTGCTGTAGACCCAGAAAGCAAAGCGCTCAGCAGAAATGACACTTGCTGTGCTCCAGGAAGAAAGCAAAACCCCTGTTGT

Homo sapiens  GTCCTTAGATCATGTTTAGTTTCAGGAGGGGTTGAGCTGTAATGACTAAATTATCTGGTCACTCTAACCAAGCAGCTAATTAATCCAGTCAAACAAACTTAT-------------CAGTC
Mus musculus  GTTTGAAAATCACCTTCCCACTTAGGAGGTGCTGAACTCTATTGGCCAAATTGTCTGATTACTCTAGTTAAGTACCCGATTAAGCCAGTCAAACAGCATGATTAAGAAGGTAATCCAGTT

Homo sapiens  ATGAAG------------------------------------------------GTGAAATTGTTTTTT------------CATCTAGAA-------------------TTTGATCATAA
Mus musculus  CTAAGGAGACTGAGGCAGGAGGGTCACAAGTTTGAGGTCAACCTGGGCTATATAGTGAGGCAGTGTTCTAAAAAAAACAAACAGCTAAAAAGACGAAAGTGAAATTGATTTTGATCGGGA

Homo sapiens  GGGCTTTCAAGAAGGAAGCTCCTTGAGTCTCCCTTGTAAACCCTTTCTGCAGTATGCCTTTCTTTCCCACGTCTGTTGAAAATTATTTATTCTTGAACCAGCTTCAGTGGTGAACAGCAG
Mus musculus  GGGCTTTCAAGACAAAGGCGGTCAGCATCTCTCTTATAAATC---TCTGCAGTATGCCTTTCTTTCTCTGGAGAGCTCACCGTCTTTCACTCTCCAGTCAGCCTTGGTGGCAGAAGACTG

Homo sapiens  CTGGTTGC-------TACCCTCTCATAC--CCATTTTTTGTCATCTGGAACA-TGGTTATTCAGCGTAGCTT-CATCTTCAAAAGGTGCCTGATTTACTCTGGGATTGGGAATGGAGAGA
Mus musculus  CTGTCTGCGCCTCTCCACCCCCGCTTGCTGCCTTTATTCTTCAGCTGGAACAATGGTTATTCAGAGTAGCCCACAACTTCAAAAGGAGTCCGATTTACTTTGGGATGTAGACTGGAGAGA

Homo sapiens  ATAATTTTGAAGCTCTTTCTCTTTGCCTGTTTCCTTCCTTCCATGACTTTGGGTTATTTTCTAGTGATCTTCAGTATTAAAAGGCATACTGACAGCTCTTTTCTCCCAGGCTGATTTTCA
Mus musculus  ATCATTTTGAAGCCCTTTCTCTCTGCCTATTTCCTTCCTTGTACAATTTGCTGTTATTTTCTATGGATCTGCGGCTTGAAAGGGC-------------TGCTCTTCCGGGCTGCTCTTCA

Homo sapiens  GCTTGTCTACTGAGCTTTTTTGGGTTTGGTTTTATGGAATGAAATCCCCTTGTTTGGTTTTCCCCACATTCACACTGAGGCTGTGAATGTCCACCAACTCAATTCTGAGCTACAATTGAG
Mus musculus  G-TTGCCT---GAGCTGTTGTGGGTTTGCTTTGATAAAGGGAAATCCCCTTGTTTTGCCGCCCCCCCCCCC-CACT----CTCTGCCTGGCTACTAACTCAATTCAGAGTTACAATTGAA

Homo sapiens  TAGTGTATTAAGAATACATGTGACTGCCACATGGGCCCTGGACTGCTGAATTCATGGTCAAATCATTGTTTTCCCAACCATCATGCTTGGGGGCA----CCACATCAGCCTACAGTGAAC
Mus musculus  TAATGTGTTAAGAGTGGGTGTGGCTGCCATGTGGCCCTCTGTCC-CTGGATTCCTGGGCCAGGCATTGCTTCCCCAAACATCTCACTCAGGACTGACTGTCACATTAACCTCTGAAGAGT

Homo sapiens  ATGGATTAATAAGTGCTGATTTAGAGTAAATAGTTTTCTGGTAAGTGGTACATTGAAGATTTCTC-TTCCCCTTTCTATGATTTGTATAGTAGCCACAATTTTCTATATCATTATTGCAG
Mus musculus  GTAGATTAGTAAATCCTCACCTAGACTAAACAATTCTCTGCCAAGTGATACACTGTTGATTTCTCACGCCCCTTCCTGTGTTCTTGATAATAGGAAGGATCC-ATCCACCATTGCTGAAG

Homo sapiens  CTTCCCAGTACTACTTTATGTAGAAATCTTGCACATTCCTACCTCTGGCTTTTGCTCAGGCTACGACTCTATGGCAGTGGCAGAACCGTTTTAGGAATGTCTTCTGCTGTAATTTTCTCT
Mus musculus  GCTCCTAATGTTGCTTTC......................................................................................................

Homo sapiens  TTGTCCCACCACTTCCACTTTAAACTTCTGGTGTATTCCCTAATCAAAGAGGCATCTAAGTTGTCTTTAGTGAGAAACCATCTATATATACAAAGCCATAACATGTGGATGAAAAGGACA
Mus musculus  .....................................................................................................CATGTGGACAAAAAGAA-A

Homo sapiens  TACTGTCAACTCTCTTGTGCCAGACACCCAGATGTTGGCAAGTAGTACTTGGACCGAACAGCATAGTCGTTGAACAGCATCATCTTCCCCTTTTTAATTCATCCAGATGTTTTGGCATC
Mus musculus  TAC-ATCACCCCTCTAGCATCACATACTTAGATATTGACAAGAAGTACTTGGACC-AATAGGCTGGTCCCCAAATGACATCATCTT--------TGTAGTCATCTACAAATTTTCACATC

Homo sapiens  ACAGACACATAGCCAGATTTCATCCCTGATTTTGTCAAAGGCTTAAAAAAA------------TGTGAAAAAATAATATGCTTCTCCTCTGATGAACAATTACCAATGAACTC--TGTTC
Mus musculus  AGGCAAACATGGCTAATTTTTACTCTTGATTTTGTCAAGTGCAAACGAAACCAAAACACAACCTGTGAAAAC-TGTTATTCTTGCCCTCTGCTGGGTCATTACCAATGAATTACATGCTC

Homo sapiens  AAAA-GGGAAAACA--TAATGTTTCATTT-AAAAGACCAATAATATTGAATTAAGATAAAGTTCCCATGAGAGTATATTTTACATTCTCATGAATCACGGATGAGTTTATTATGATCCAT
Mus musculus  AGAATGGGAAAACAGATAATGTTTCATTTTAAAAGACCAATAATATTGAATTAAAATAAAATTTCCGTGACAGTATATTTTACATTTTCATGAATCACGAATGAGTTTATTATGATTCAT

Homo sapiens  GCTTGTATGAAAGACTGACAAATAAATTCATATTGATTTGACATTTAAAATAGCATGTCTAAAAATGTACAGCATCACATTAAGTATCAAGTAATTATTCAACTATCAGCTTCAAATGTA
Mus musculus  GCTTGCAGGAAAGAATGACAAATGAATTCATATTGATTACACATTTGAAATAATGTGTCTAAAAAATGACA-----ATGTGAATTGTCAGGCAATTATTCGTCTATCAGC-TCAAATGTA

Homo sapiens  CATGGGAGAAAATGGGTCCAGAACCCAAAATGTAGTGAAAGATTTAAATGCTCAAATTTCTACCACAGGTTTTTTTTTTTTTTTTTTTTTTGAGACAAGGTCTCACTCTATCACCCAGTC
Mus musculus  TGTGGAAGAAAAAGAATCCCAAACTCAAAATACAGTAAAAGATTAAGA........................................................................

Homo sapiens  TGTGTGCAATGGTGTAATCATGGCTCACTGCAGCCTCAAACTCCTGGGCTCGAGTGATCCTCCCACCTCAGCCTCCCAAGTAGTTAGGACTTACAGTCATGTGCCGTCATGCCTGGCTAA
Mus musculus  ........................................................................................................................

Homo sapiens  TTTATTTTATTTTTATTTTTGTAGAGACAAGGCCTTGCTTTGTTGTCCAGGTTGGTCTTGAACTCCTGGCCTCAAGCAGTCCTCTCACTTCGGCCTCCCAAAGGACTAGGATTATAGGCG
Mus musculus  ........................................................................................................................

Homo sapiens  TGAGCCACTGTGCCCAGCCTTCTACCACAATTTTTAAAGAAGCCAATAC-CATACATTAATCCATTTACAAGGGCTCACGCAAGAGTGCCTTTTGAGCCTGTGTTTGACAGGAAGTTTCT
Mus musculus  ........................CCAAGAACTCTTAAGGAGCTAAAACTCTTACATTAATCTCTCCATGAGGATGCATGCTGAAATTCCTCTTGAGTATGCGTTGGATTGGAAATCTAT

Homo sapiens  ATTCAGATCATTGTGCCTAGAGTATATAGAGGGGTTACAGCAAAGTGCTGTACTTTGGGTTACAGTAAAGTGACTCAATTGATGTACCAAGATATTGATCATTTAC--------------
Mus musculus  ATTTAGGGCATTAGATCTGTAGGATATAG------------AAAAGGCT----TGCGGTTTAGAGCAAAGTGACTCGGTTGTCATGTCATAAGACTTATCATTCAACTCTGAGTGCCTGG

Homo sapiens  ---------------------ACCCTTTAGGCCTGAAAAATATACTTTTCTTTGTGTTCCATGAAACTAAAAAGGCAGGGAAGCATTAAAGAGACAGCAATAAAAAGATTTGATGGTATG
Mus musculus  AAGAGGCCTGAGTCAGAAAGGGCCCTTGAAAACTTGAACGTAGATGTTTCCTTGTGACTCAGGAAATTAAGCAGACATGG--------------------GCAAAAGACTGAGCTGTAGG

Homo sapiens  TATA-TGTGAGTTTGAGTGTT-TGAGCTGGCGTTGTGAGTTTGTGGGTGGATTAGAAAGCATATGGCTAAAGACATGTGCAACAGATGCAAGGGCCTGGGCTTTGGCCTTCCCTTTGAAA
Mus musculus  CATGCTGATAGTATGCGAGCTGTGAACAA-----GGGACTGTATGGATGGACTAGGAGTCATCAGGCTAAAGAGA----CAACAGATGAGAAGGACTGGGATTTGGACTG-GGTTTGAAA

Homo sapiens  CTTCTTTATGGGGACATGTGCT-----GCCTTGCTTCCTGGATTAAGACCACATTTGCACAATTTGCACATTGTCCC-----TCTTGGGCCTGCTTCCCACCGGAGACTGC----ATCTT
Mus musculus  CTTCTTGATAGAGGCATATGCTTAAACAAATGGCTTCCTGGATTAAATCTACATCAGCCGCATTTATACATTGTGCTTTGAGTCCCAGGCCTGCCTCCTA--GAAGTACACATAGATCTC

Homo sapiens  CCACAAAAACAAGGCAAGAAGTAAAGAACTTAAGAGAGCTGATTGTGAAGTCCAACTACATGG----GTTCAAATCCTGGTACCATCAT---TAATTAGCTGGGTCATTTTGGACCACTT
Mus musculus  CTATGAAGGCAGG--AACAGTTTGAAAATGGAAGACGTCTGGGCATGAAGTCC-----CATGGCCTTGTTTAAAGACTGGTACTGGCATGGGTAGGTGGCTCAGT...............

Homo sapiens  AACTTCCAGGACCCTCATTTTCCTCATCTACACAAAGGGCTAATAACAGTATTTACCTCATAGTTGTTATGTAAAGTGCTTATCCCAGAGCCTGGCAGAGAGCAGACACTAGTTGATCAT
Mus musculus  ................................................................................................................TTGGCCAT

Homo sapiens  TATGATTATTGAGCAATTACTATGTACCAGGTGCTTTATTTTCATCTCTGATCCTCACAACAGCCCTACAAGGCAGATACTATTATCT-TCAGTTTACAAGTGAAGAGGCTGAAGCTCAG
Mus musculus  GGTGATTAGTAAGCACCGGTTGTTTACCAGGTGTTTTATCTTTGTCTTTGATCTTCACAACAACCCTATAAGACAGAAACAATTCTTTCTCCATTTACAGGAGAATGG------------

Homo sapiens  TGAGATAAAATACACTGCTCAAGGTCACACAACCAGGAAGAGCAGAAACCAGGCTC----CAAGTCCCTCTGTCCCTCACTCCTAAGGCCTGTGCTATTTGCCATATCATCATCCCACTG
Mus musculus  ----------------GCCCAAGGTCACATAATGAAGAAAAGCAGGAACAAGGCTTGAAAAAAATCCCTCTCTTCCTTATTCCCAAGGCTCACACTCCTCAACA---CATCACTCTGTCA
```

```
Homo sapiens  GTGCCTCTAAAAGTTTCTTCTT-----------------CTAGGGTTCATCCCTCT-------------------------------TCTCTCTTCATCCTCCTCTTGGCCTACCCTGGC
Mus musculus  AATCTTCTAAAGTTTACTTTGTGTCATAGAAAAGAATAGCCATGTTACACTGCTCTGGAAAAGGGGCAGTCTGTTTCCAGTGATCACTATTTTCTCACCCTCCCCCTAGCCTTCCCTCGC

Homo sapiens  CTGAGAACTGCTTGGCACAATCCTATACAGCAGGTCCTTGAATAACATTGTTTTGTTCAAAGCCATTTAGTTATCACGTTAATGGGGAAAATTTGGTTTTCCCCAGGGCCACTGTCCATG
Mus musculus  CTGAGCACT--TTGTAACAATCTT................................................................................................

Homo sapiens  GACAGGATGGCGTCCTGACCAGGGTGGTTCCCACCTTTTGTGCTCCTTGTGCTGCCAGGATGGGCTCCGGCCACCTGTGACCCTGAACTGGAATCAGCGGGTTAGAAAATAACTGAATGA
Mus musculus  ........................................................................................................................

Homo sapiens  GTGAATACAAATTATTGGAAAATGAAAACTCGTAAAGTACACAATGACCATACGAATCCATGATAATAAATGACACGATAGGAAAGCGCTCAATGAGCGCTTTTGTGATTCTTCTGGAAC
Mus musculus  ........................................................................................................................

Homo sapiens  TGCTGGGTGGCAGGAGGTGCTCCTTATGATTTTGCTTTGCAAATGTTTATTCCTTGACCTGACCCGCCATTGCTACAACCGCTGTCACTCATTGATTCACCAAAAATTAGGCAAATAATT
Mus musculus  ........................................................................................................................

Homo sapiens  CTCTTACTTGTTTTTATTAATCTTTCCTAAATGTATGTGTAGCTCACACTTATTGTTAATGTTTAATTAACTTATTAATGTTTAATATTAGAAGTGTTTGGGTGGTTTTTGTTTGTTTGT
Mus musculus  ........................................................................................................................

Homo sapiens  TTTTTTGTTTGTTTTTGAGACAGGGTCTCACTCCTCTCGCCCAGGCTGGAGTGCAGTGGCGCAATCACGGCTCACTGCAGCCTCAACCTCCCAGGATTCAGGTGATCCTCCCACCTCAGC
Mus musculus  ........................................................................................................................

Homo sapiens  CTCCTGAGTAGCTGTGACTACAGACGTGTGCCACCATGCCTGGCTAAATTTTTGTATTTTTAGTAGAGACAGGGTTTCACCATGTTGCCCAGGCTGGTATTTGGATCTTTATTTAGAAGT
Mus musculus  ........................................................................................................................

Homo sapiens  TTGGTGATATTTTTGTGACCAGAAATATGCTGTGGGTACTTAACTCTTGTTTATATCAATTAGCCTACAGTAAAATTAGTTTTGTTATTCGTTGTTTCACTTAAAGTCCAGTTCCCAAGA
Mus musculus  ........................................................................................................................

Homo sapiens  ACCTGTCAAGGACGTTCAGTGAGGACTTACTGTACTCAGAGACAGCAGAGAGCAAAGGGCAAGAAGGCTGGTTGGAGGACGAGGAGGCCTAGAGCCCCTTGCCTGCTTTTGCACATGCAT
Mus musculus  ....................................CAGAGACAATGTGG------GGGAGAGAAAGATGG---GAGGATGAGGAGGCCTGCTGCCACTTGCCAGCTCTTGCACATGACT

Homo sapiens  CTGCTCCTGGTGGATGACTCACTTAACCTCTCCGTGCTTCTGCCTCTTCAACTGGAAAATGGAATCTGTAAGTTGCAGCAC-----TAGCTTCTGTGACATTCTACTAGGCCTCCAGGAA
Mus musculus  TAGGCCAGAGTGCACGATCCACTCTACCTCTCTGTGCTTCTGCCTCTTCCCCTGGAAAATGTAATCTGTAAACTACGGCGCCCGAGTTGCTTTTGTGGTGTTTTGCTTAGCCTCCAGGAA

Homo sapiens  GA----GGAGGCCATCCCTCTATATGTCCAGTCAGCATATGGGGAACAGT---GGGGCTGTGTGTGACTCACCCTGTGGTGTGCCAGCCTCAGGCTTATACTAGCCTAGGCTTATACT--
Mus musculus  GGAAGGGAAGGCTGACTCTTTATAAGTGCACATAGCACATGCAGTGCAGTTGGGGGGCTATGTGTCATTCA----GCTGCTTGCCTGCC-CAGCTTTAGGTTGTCCCAGGATTATTCTAA

Homo sapiens  -CCCAGATCCTGAGCTAGCAGCTGGAGCTTTGGGATCTGGAACTGTTACAGGGCCAAAGAACCTGCTTAGCTTCAGGGCTGCTTCTAGATAAACTGCCAGGATGTGGGCAAAGCATTTGT
Mus musculus  GTCCAGATTCTGAGCTAGTAGCTAAAGCTTTGAGATCAAGTTCAGTTACAGGGTTAAAGAACCTGTTCAATTCCAGGGCTCCTTTTGTGTAA-CTCTCAGTATGTAGATGAAGCATCTGA

Homo sapiens  CATGGAAGGTGGGGAGTGTATGTAGGTTGTTTCAGGGGCTGCTTTGTGGGAAGCCAAACCT-------TTTGGCAATGGGCTCAGAACTGGCTTTCCTTTTTGGGTGTCCTCTAAATTCC
Mus musculus  CCTGAGAGGGGAGGAGT-TGTGTGGGGTGGCTTCAGGGCTG---TGTTGGAAAC---ACCTGGAAATACTTGGCA---GGGTCCACATTGGCTTTTCTTCTTGAGTGTCTTCTAAAATCC

Homo sapiens  GAGGGTGCCCCCACACCAGAGGGTCTCCACTTCTGCCATCCCTAACCTAGTGG------GGAGATGAATCTGATCTTTTGTCTCAAAGCCCACATTAAAGAGGACCATTGCATGCTTCAT
Mus musculus  TAGGGTGCTTCCACATCAGAGAATCTCCATTTTTTTTTTTTTGGACTCTCTGGTCTCTGGGAACTGAACCATATCTTTTACCCCAGGACCCATAATAATGAGGACCCCTTCAAGGTGAAC

Homo sapiens  CACCAGGCTGTGACTCCCAGAC-CTCAACTCATGTCTTTTTCCCTCTTCACCAGGGCAGAGGCTAGACTGTTTCCTCTCCTCTCCCTGAACAATAGCAGTGCCCAGATTTTGGAGGGCAT
Mus musculus  CCTCAAGCCCTGCCTCAAAGATACTCATCTTATGTCTTGTTCCCTCTTCACCATGGTTGAAGATATAGGCTTGTTGCCTCTTTCCTTTAATAATTGCAGTGCCTGAGTTCTCCATGACAT

Homo sapiens  ACCGCC---------------TACCTGGCACTGTGCTAAGTACTTTGCCTGC------ATTTCCTTGGTAATCCTC--ATGTAAT-CCTACAA----GGGAAGTACCATTTTGTCACGAT
Mus musculus  AAAGCTATGTTGTGTACCTTATACCAGGTACAATGCTGAGTCATCTGCCGGTTAATCAACCTATTAATTAATCCGCCTATTTAATGCTTACAATTCAGGGAGGGATGA----ATCTCGCC

Homo sapiens  TTAACAGAAG-----AGGATACAAAAGCTCAGAGCCTGGAGGTGACTTGCTCAAAGTCACATTGATAGTAAGTGGTAA-AGCCGGGACTGAAATACAG--GTCTAACTTTTGAGACAACC
Mus musculus  TTTACAGAAGGAAGGAGGAACAGGGAACATAAAGCCTAGCGGTGATTTGCTCAGAGTCACCATGTCACTAAATGGAAGTAGCTGGAGTCAGTTCACAGCTGTGTAACTTTTG--------

Homo sapiens  TTTTTTCTTTTTCTTTTTTTTCTATTTCAATTACATAATCCTAATCCTGTGCGTGTTTGAAGGGGAGAGACAGGGGAGGGAGTGAGATGGCAATTTGCTTGTGACCTAAGGAGGCCCTGA
Mus musculus  ----------------CCTCCTGACTTCTATTACACAAGTCT------GTGCAGCCTTGAAGGAAATAAACAGGGGAGGGAGATAGGCAGCGATTTGCTTGTGATTTAAGGAGATCTT-A

Homo sapiens  AATCTCCTGGGAATGAGGATGGGGGACCTACATGAGTTGCCCAGGGCACTGGTAATGCAGCCC-ACACCAGCAGTCAGTGATCTCCAACATGGAGGGCTCCAGGCCTGCCTAGCTGCTGG
Mus musculus  AATCTCCTGGGCAGGAGAATAGG---CCTAGGTGAGGTGCTCGGGGTGCTAGGAATGCAGCCCCGTATCTGGTTTCCATGGTACAGCCTGTGGAGGTCTTGAGACCTGCTTAGTCGTTGG

Homo sapiens  CTGCTACTGGAGGCCAGAAAAAGACAGCTCCAGTGTCCTTCTAGGCTGGGGGTGGTTCTTGCCTCCAGGGCTTATGGAAGAACTTATCAGACAAAACTCTGCCCTTTCAAGCAGAAA-AG
Mus musculus  CTACAGC--------AGAAGAGG----------------TCTAAGCAGGGGGTACCTTTTATCTGCAGAGAGTAAGGA-GAAGTTATCAGAGAACCTTCTGGTCTT-CAAGCAGAAAGAA

Homo sapiens  TACTTTACAAAGAA-----------------------CATAGAGAGGTGAAGTGGGTACTTTTCA-----ATTTTTGCCTGA----------------------------------TTAT
Mus musculus  TAATAGAAAAGGAAAAATGGACATTTATTTTTCTGGCCATGGAACATTTAAATGATCTCTCTTCCTCCTTCTCTCTGTCTGTCTCTGTCTCTCTCTCTGTTTCCCTCCCTCTACCTTTCT

Homo sapiens  CTAATATCTAAATGCTTTTTCTTC--ACTTTGGAATCCTGACCTGACCTGTTTTGTGCCCTGTCCACCCACCACCACTGAGTCAAAGCTCAGGGCCTGGCATCCAGTATTAGTCATACCT
Mus musculus  CTAATAGCTAAATGCTTTTTTTTCCTACTTGGGAATAAT--CTTGACCCTTTTTTT-TCTTATCCTCCTATTGCTACTGAGTCAAAGTTCAAGGCTT-----CAAATATTTTCATCATCT

Homo sapiens  ATTTGTTGCCCGAATGAATGGGGCTGAACCCTGGTCTGACTCTAAAATACATGCTTGAAAAAAATTATTATGGAAATTTTCAAACACATACACAAAAGTAAAAAATATAGTATAATGAAC
Mus musculus  AAGTGTT---TGAATAAATGGAGCTAAACCGAGATCTGACTCTCAAATATATGCTTTCAAAAG--TATTATA-AAATATTAAGACACA------AGAGTAGTAGA-ATAATATTCTAAAC

Homo sapiens  CCCCAA-GTATCCATCACCCAGATTCAACATCTATCATGAT-----------TTTCCATGTTAGCTTTACCTATCTCTCTT----------TCTTTTTCCCTCTTATGCTTTACCCCACA
Mus musculus  CCCTAATATACCCATC---CATATTCAGCAACTATCAAGATCAACGGCCTCTTTTTCTTTTTCTCTTTGCCTAAATATTTTGAAAAAAAAATCATAGACACTCTCTCATTTATCCCTAAA

Homo sapiens  TGTTTCATTATGCAACTCAAAAA---TAGGGATATTTTTATATACAATGACGA-----AATTATTATCCCCCCCATCGCCAAACTAACAATAATTCCCTTCAAACCATGCTCTTGAGCAC
Mus musculus  TGCT-CAGTATTCATCTTAAATAAGCTGAGCATATTTTCATAACTAATGGCAATTGTTCATAACTAATGCCCTAAGTAC---ACTAACAAGAATCCCTTTCTAACCATGCTGTTGAGCAC

Homo sapiens  TACACTGGAGTTCCT-CCATTCTACTTTCTTTCCCAGTCCCCAACAGGAAGCA---TTTCTCAGGCATCTGCT-CTTGCACCTC-CTCAGAGCTGGAATTACAGTTTGATGTGTATTGGG
Mus musculus  AAAACT----CTCCTACCTTTCTAGCTTCTGCACCCGCTCCCA-TAGGAAGCAGAACTTCATGGGCATCTTTTTCTTGCACCCAACTCAAAGCTGGAATTGAGGCTTGTTGTGCACT---

Homo sapiens  TACTCACAATGTGCCCTTTTAGGTAAGCAGGGGGCCAGATTCCCCTCTCCCTGTTTTACAAAGAAGAATGGCG-ACACACAGCTATTAAGTGCCTTGTACAAGGTCACACAGTCAATTAG
Mus musculus  ------CAATGTGCCCTTTTAGGTAAGCAGAGGATGAGGCTCCCCTCGCCCAGTTTCACAAAGAGAAATGGAGGAGACGGCGCTTTTAAGTGCTTTGCACAAGACTACACAGTCAATTAG

Homo sapiens  TGGCTGATAAATGGGACTCAAGCACTGTGGCTGGGTCTATACAGCCCCAAATACAGTTTGCAAGGAAATTAAATGAAGGGACTCGTGTCCTGGGGACAGTACCGTGTGTTTTCTGTTGCT
Mus musculus  TGGCTGATGAATGGGACTCAAGTGCTATGGCTAGGTCTATGCAGTCCCAAACACAATTTGCAAGGAAATTAAGTGAAGG--CTCCTGTCCTTAGGACGGTAGTGCATGGCTTCCGTTGCT

Homo sapiens  GTTGAAGTCATTGC-ACTTAGCC---------------------------------------CATCAGGACAGCCTGATTGATGGGATCAGTTGGACTCCTATGTTTGTTTTCCTCATCT
Mus musculus  ATTGAAGTCATTGCCATTTAGCCTTAAGCAACTTCTTTCCCTGGGGCAGCCTGACAATGGTCCATTAGGACAGGCTGATTGATAGGATCGGTTGGGCTCCTATGTTTGTTTTC-------

Homo sapiens  GACTGGATTGCAACAATC---ACCTACAGGCAGGC---TGGCCACACCCGTGAGTTTTTCTCTTCTTTTTTTCCATGTTCCTGGAGCTGACTCCGGAGCCCACATTTCTCCTGTCTTTCT
Mus musculus  --------TGCCAAAGCCTCAACTTAAAGACAGGAAAATGATTTCTCCTATG-GCCCCTCTCTTCTTTTCTTC----TTCCTGGACTGGGCTCTGGGGCCCTCATCTCTCCCCTCCTTTT

Homo sapiens  AGGCTTGGACTTGCTCTCTGCCAAAGCAGATGACTCCCAGAGCAGCCACAGTCTGGGACAGGGCAAATGTGAGAAGCAGAAGGACTAGCTTAACTGCTTTTCTGTTCCTGCCTCCTTCTT
Mus musculus  AAGCCTAGACCTGCTCT-------------GGAGTCCTAGGGCATGCA-----GGGACCAGGCCAAGCACAAGGAGCAGCAGGGCTAGTATAGCTGCTTTTCAGATTTACTCCCTCCTC

Homo sapiens  GTTCCAAAGACTTACTCTTAGATGCTTGGCTCTCCTGAGCTTGATCTCCATGTCTAATTCTCTAGACCTCTTCCACTCCCCTGATATCCCACCCACATTTTCCAAGTAAGTCTCTCAGAG
Mus musculus  ATTTCCAAGATGTCTCCTTAGATGCCCCACCCTCCCGAGGTTGCCCTCCATGTCAAATTCCCGAGGCCTTTTCTACTCCCTTGATCACATATCCAAATTTTCTACCTAATCATCGGAAAG

Homo sapiens  CCTTGTATTCCAAAATACAAACTGGCTAGTGG----------------------AAAGAAGGAATCATTGCTGTTGGCTAGTGGATGACTGATCAGTCTGAGTCCAACCAGGAAAATAGA
Mus musculus  TCTTGAGTTCCAAATTGAAACTTGTCTGGGGGGTTGGGGGGAAGGCAGGGGGGCAGGGAAAGGGTTTGGGCTATTGGGTAAGAAGAGATTGATAATTTTGGGTCCAACTCATCGTGCAGA

Homo sapiens  AATCACTGAGTATTTATTTAAGGGAAAAGGGATTCAGTGCAGGGAATTGATCACACAGGTGATGGAAAA---GCTGTGATGC-CAAGCAGAGGACAATGAAGCAACCTAGAG-ATTAGTC
Mus musculus  AT---CTGAGTGTTCC----AGGGGCTCCAGACGCAAAGCAGGGCATTAGCAACACAGATGATAAACAACTAACTGGGAAGCATAATCTCA----AATTTATTATCAGAGAGGAGGGGTC

Homo sapiens  ACAGCAGGGAGCTGCTACCATTCCTTGGCTAGAACCTCAGGGCTGGGACACTTAGTAGAAACTTGAGCCA-CAGTAACCTGGTCAGCAGTGCCAGAGCCACAAAGA--GACACAGTCACT
Mus musculus  AAACCAGGG----GCTACCACCCCTATGCTAAAACCTT------------CTTCGGGTTAGATTATGATATCAGTAGCCTGATCAGGAAT--CTGAGTCACAAAAAAGGGCTCCGTCACT

Homo sapiens  GCTGGGGA-CACCACATGAAGCAGAGGGGGAGGGAGAGAAATACCCTGGTTTCTCTCTCCCTTTTGCTTTCTAATATCCTAACAGTGCCTTCCAT---TGGCCAAACCCATCAGAGCCTG
Mus musculus  GCTTAGGAGCACCAAACAAGGTAGAAAAGGAGAGAGGATAAAACATTGCTTTCCTAATCCCCCA-GCTTTCCAGTGTCTTACCTTTCCCATCAAAGGATGCCCAAACCCACCAGA--CTA

Homo sapiens  GGGAACAGCCTGCAAAGGCCAACCTGCCCCATCAGCTTCCCTGCTACAAGAG----CAGAGATGTGAAAAGGAAGTAATGGACTTGGGGGGGCAAACAGGCCTGGGGACAGCACAGATCT
Mus musculus  AGAAACAGTCTACAAAGGCTATCCTACCCAGACC-CTTCAATGTTTCAATAGGCATCAGAAAAGAGTAAAGGAAGAGATGAA-------GGACTAAGGGTGCTTAGGACAGCACTGGTCT

Homo sapiens  CCTC---CTCCCTGCCACCCC--CAACTAACTTATTTGGATATGTCAGGGTCTATTTGGAAACAGTGCATTTACTCAACAAAATGATTCTAAAGCTGACAAGAAAGACCCTTAAGGAAAA
Mus musculus  CCTCTTGCCTCCTTCCACCTCACCTACTAACATACTAGGCTATCTCTAGGTCCATCTGGAGATTGTGCTTTTGCCCAAAGGAATGATGATAGAGTTGACGTTAAAGCGTTTT--------

Homo sapiens  GGGTGTGGGTGGAGCACGAACCCGCTTCCTTGACTACCTGCAAGTCTTAGCATCCAGATTCTAGCCCAGTCCTCTTATTTCACTTCCTCCTGCATGCTCCATCCCTCCTCCTCCCACGCA
Mus musculus  ---------------------CCATATGATTGTCTATGGTCACATCTCATCATCTGGATTTTACTCCAGTTCGCCTATTTCAC---CACTTACATTCCCCACCCCCAACACTAAC---CA

Homo sapiens  TAGGCTGACTCCAACAAAGGCAGGCCGGCCATTTTGCTTTCTGGCAATGTGTGATGCATTCATTGGCTCCCAGGACTGTGTAAACCCAACCTTCTTGCCTGCAGGGTTTCAGCTTGGCAG
Mus musculus  TAG-CAGAGGTCAATAAAGACAGACAGACCGGTTTGCTTCCTGGCCATGTGTGATACCTTCATTGGCTCTAAGGA------GGAGCAGACCTTCTTGCCTTCTGGGCTTCCGCTTGGCAA
```

```
Homo sapiens  CCAGGCTCTGAA--CTGCAGTGCTGTCAGGCAGGTGAACCCGCTGGCACCACAATCCAGAAAACATATCTCCTGATTCCTCAGGTGTTCTACACGTTTGAATGTGTAGATGAAGGGAGAT
Mus musculus  CAAGGCTCTGGAAGCCGCAATGCTGTCAGACTGGTCAACCTGCTTGCATCACAGTCCAGCAAACAGATGTCCTGATTCATTCGGTGTTCTATAAATTTGAATGTGTAGGTAAAGGGAGGT

Homo sapiens  GGCTGGGAGGGGGAAAAGAAAGACATCAAAC-------TATTTATGTG-------CTTTCCCACACCTTATTTCATTTACTCTCCACAACTCCTCAAGGCAGATGCCCTTCTTCCCATTA
Mus musculus  CTCCAGAGGGGACATATGAAGGA-GTCAAATAAAGGAACACATAAGGGAGCCAGACTTTCCC-------ATTTAAGTTGCTCTCCACACTTCTTCAAGGTAGTTTCCTTTTTGCCCATTT

Homo sapiens  AAAAATAAATTTTTAAATCTTAGAATACTTTTAGATTTACAGGACAGTTGAAAAGAGAGTACAGAGAATCCCTATGCATCCTTCACTCAGTTTCTCCTGATGTTAACATCTTACAAAATC
Mus musculus  TGAAATAAGCTTTTAGACTTCAGTGTAATTTTAGGCCTATGGAGCCATTGTAATGGCAGTACATGCCATTCTTCC-TATTCTTCATCCGATTTCCCACCATGTTAACA...TGCACAACC

Homo sapiens  ATCTTTTGTCTGTTTTTACAAATGATGAAACTGGGGCTCAGAGAGGTTAG-TGACTTGCCCAAGGTCATAGAACTAGGACACAGCAGAAGAAACAGGCATAAATCCAGTTAGATCTTAAC
Mus musculus  ATCTTTTAACTAATTTCCCCAATGAGGAACCTGGGCCTTAGAGAGGTCAGGCGACTTGCCAGTAGTCCCAGAGCTCAGACACAACTGAAGGCACAAACATAAATCCAGGTGGATCTTAG-

Homo sapiens  TGTATCACACCACCAAGTTGAATTATTTGTTTCCGACTCATTATATTGGAAGTCACTCTCTGAACCTTGAACCCTTCACTTCACCTCATAGTTGGGTTGTAAACATTATCAGAGAGGATT
Mus musculus  TGTGTCACAC-AC--AGCTGAATTGTCGGCTTGCATCTCCTTACACAGGAAGCCACTCTCTGAACTCAGAACCCATTGTCTCA-------GTTGAGTTGCCAACTTTATGAGAGGGCACT

Homo sapiens  TCTTCCATAGATCCATAGTATGTTAACACTAAAAGATACATCTAAGACCATTTAATCTAGGGAAGGCAAATAAGTTTCATTTCATTTGCCGACTTTAATTGAGCAGTAGAGGCTGTTTGG
Mus musculus  CTTTCCACAGAGCCATATAACGCCTGTCCTGAAGGGTACATCTGAGTGCATTTAATTGAAAGAAGGCAGAGAAGCTTCATCTCATTTGCCAACTTTAATTGTTCAAGGGAAGCTAATTGG

Homo sapiens  AATTCCGTGCTGAGAATGATTCTAAATCTATGTTTTTGCTCATTAGGAAAAAAATGCTATGACTGATTATTGATACCTGCCATATATGTTGACTAGGAGAGTGGTGGAACTCATGTTACC
Mus musculus  AAGCCAGTATTGAAAACGATTTGAGGCCCATGCTTTTGCTCATTAGGGATGAG-TGTTGTTACTGATTATCGG-----------GTGTTGAAGAGAACACTGGTAGAA-TCCTGTCATC

Homo sapiens  TATTAGCCGTTTCTCAGT----------------------------------------GAATGTCTCTGCCTCATTAGTGGTACAGCTAAGAACAGAACTCAGGCTCCTGACTCATG
Mus musculus  AATTATCTATCCATCAGCAGTTCTTATTTTATAGACAAGGTTTCTGGAGGTGCAGCGACAGGACAATCTCCGGCCTATTCGTGGGACAGCTAAGAACAGAACTAGGGCTCCAGACTCATA

Homo sapiens  GTCTCTTGTTCTTTCTGGTTTACAATGGACTCACAATAACAACAATAATATAATACAGTGTATCACATAATGAGTGTCTACTCACTGCCAGGCACTCTCCTAAGCACATTCCACTTTCTG
Mus musculus  GTTTTACATGTACTCTGCAGTAGAATGGACTGGAGATGGCAA---TAACATAAC------CATAGCTTAACAAGTGTCCAGTTACTGCCTGGCCCCAGACTCATCATTTTCCA-TATGTA

Homo sapiens  TACCTTACTTTTAATTCTCAGAACAACCCTTCAAGGTAGGCACATTCAACCCCAAACTTAGAAATGCAG-----------AAGCCTAGGCTCAGTG--------------AATTTTAAA
Mus musculus  TGCATTATCTTAACTCCCCTGGAC---CCTGCCTAGTTGAGAGTTTGATCCCTTGATTTATTAATTCAGGGAACAAAGCAAAAACAAAACTCAAAGGAACAAACAAACAAAAAACCCACA

Homo sapiens  AACTTACCTAAGGTCCACAGTGAGCAAGTGGTAAAACTGGAATTTAAACCTACGCTGGTCTGGCTCCAAAGCCTAAACCCTGTGGTCTCACTTCCTGCAAAC--ACAATGTTGAATTTGC
Mus musculus  CACTTACCAAAGGGCCACGGGAAACAAATGGGAAAGCTGGAATTTAAATGGGAGCTGGTCACATCCCAAAGCTGAACCACTGGCCTCTCACCTCTTACAAACTGACA-------------

Homo sapiens  GCTCACAGCAGGTGGAGATGACCCTGGTCCCACTGCTTTGATTAAGACTAAACTGGAGTGAAAAAATCATTTTTGTTCTT-ACTAATTTTCTTAAAAAGATTTGTTCACAGTTCCTTACT
Mus musculus  GCCTACAGCAGGCAGTGGTAGCCCTGGCTCCACCTAT---ACTGAGTTGGGACTAAATAAAATAAAACCTTTTTGTTTTTCACTAATTTTCTCAACAAGATTTGCTAGCAGGCCCTTACC

Homo sapiens  TAGATGGAACTGATTTTTTCGGGCTGTCTCATATTAGATGTGATACAAATTGACATGTTAGGAGAGCTGATGTGAAAGGCGACCTGGTTCACTTACAAATAAATGATTAATATAATCTCA
Mus musculus  TAAGTAGAACTGATTTCTTCCGCTTATTTTGTATGAAATGTGCTAAAAATTGACATGGTAGGAGAGCTGATGTGAAAGGTGACCTGGTTCACTTACAAATAAATGATTAATATAACCTCA

Homo sapiens  TTGTGCGGCTAGCCTGCATTACTGATTCTCTCACTTGGGAGGCTCAGAAAAACAACAGGTCAGACAATAAAACCCATGCTAGCATAAGACTATAGTGTGGGATGACCATGGATGAGTCAG
Mus musculus  TTGTGAGGCTACTCTGCCTTACTGATTCTCTCATACGGGAGGAACAGAAAAACAATGGGTCATGCCATAAAATCTATACTAGCCTGGGATCATAGTGTGGGA-GGTCATGGATGAGTCAG

Homo sapiens  GGAGAATAAGAAGATAATTAACTAAGAGGGAGTTCTATGGAGTTCGAAGATAA-TTAAAAAATGTTCCTTCTCTATCCTAAGAATATCTGACATTATTAGCGAAGGCAATGAATAAGATA
Mus musculus  AGAGAAGCAGAAGATAATTAATGAAGAGAGCATTCCACAGAGCTTAAAAAGAATTTAGAAAACGTCCATTCGTTAATCTAAGGATATCCGGCATTTTTAACACAGGCAGTGAAC-AGATA

Homo sapiens  AGGGGAAGAGGTGAGTTAATTAGAATTATGAGATACAACATTTTAAAGAAATTGACAGGGATCACATTTCAACGGAAAGACATTCAATTCAACCGACTTCATTATAGCAATTCAACATTG
Mus musculus  GGAGGAAAACAAAAGCTAATTAGAATTATTAAGCACCATGTTTAAAATAAATTGACAGAGATCAAATTTCAAAGGGAAGACATTTAATTCAACTGACTTTGTTATACCTATTCAACATTG

Homo sapiens  ATTAGGGACATGTTATGTGTGTGGCTGTGCTCTGTGTGCTTAGAGGA-CTACTGAGATGCTGTAATCTTCCTCCCTGACATTCTACTGAATAGATACCCAAGTTTTGGCAGCGCAGTAAA
Mus musculus  GTGAGAGACATGTTATGTATGTGGCTGTAGCCTTTGTGCTTAGGGGAGTTGCTAAGATTCTCCACTCTCCCTCCCAGGGAATACTCTGAATAGA----------TTGG----GTAGTAAA

Homo sapiens  TAATCTCTTACACACACATTTGGGTGCAATTTCTAAAATTGTTTCTGCAAAATAGTAT-TATGTGCCACTG-AGTAGATATCTTGTATGCCAGAT-----------------ATTACTGT
Mus musculus  GATTCTCTGATATACAGATATGTGTGAAATTTGTAAAACTGATTCTGAAAAATGGTGTGTGTGTGCCAAGGTTATGCATATTTTATGGGACCGGTGAAATAGGGCCGATGGAATGTCTGT

Homo sapiens  TCCTGCCAGGCTAAATGTTCAAACAACATGGAAGCCTCAGTGATAGCCAATCCATTATTTATCTACCTATATTCACTTCAGTTGCCAAAACTATTCTAAATATAAGGAATCCCTTCCTTA
Mus musculus  TT---TCTGGCCAAATGTTCAAGCAACATAGAAGCCTCAGTAGTGACAAATATATTAGTTATCTCCTTATA-----TTCAGTTACAAAAATTGTTCTAAGTCTAAGAAAT..........

Homo sapiens  GAGGCCCCAATATGGCTTTCCGTATTGTTCTGAGTCCTTTCTAACTGGTTAATGCCCATGCCATATTGGTTATCATAAATTTTGAATAGCATCTTTTCTCTGCTCAGAAGTGTCGCTCAG
Mus musculus  ........................................................................................................................

Homo sapiens  TAATACCCTACCTCTTCTGACTCCTCCTACAGTGTAATCTCTCACCAGTCCTCACTACATTCTGAACTGATGTTCCAGCTCCACTTAAATCTGTCATGTTTTGTTCCTTAACATATGCTA
Mus musculus  ........................................................................................................................

Homo sapiens  TTCCCTTTACTTGGAATACACTCTCTTTCCTTGTCTGCCTAACAATTGCTGCTTGTCTCGCAAATATTTTCTCTGTGAAGCTTTCTCAGTGCCCTTAGGTACCCTCATGGCCCTCCTGAA
Mus musculus  ........................................................................................................................

Homo sapiens  TTTATCTCTATTATAATGTTTTATATTTTATGGCAAGAGTTTGTTGGATCTTTGCCCACTTGAATGTGAGCCCCTTGAGAACAGGGACTATTTTATTTATGTCTGAGTCTCTAGGGAACA
Mus musculus  ........................................................................................................................

Homo sapiens  AAAGAGGGATTCAGGGCTTATGTGGTAAATGAATCAATGAATGCTCCCAGTTCTTCGTCATCTTTAGAGGCTACTTCCAGGGCTTTTAAATTTGGACTCCCTTTTCTTCCAAGAGGTGGT
Mus musculus  ........................................................................................................................

Homo sapiens  GAGAGCAGGGTAGGTGGACATGATATTTAATGAATTTCTTTTAAGTGCCAGACATTGTGCTGAACCATTTACACAAACAATCTCACGTCTCAAGTCGATGTTTTTATTTTCATCCTCGTT
Mus musculus  ........................................................................................................................

Homo sapiens  TTACAGATGAAAGTGGAAAATTCAGACCAGTGACTTGCCACATTCAGAGGAGTAACAAAGATACATAGCAAGTAGGCTGCAAAGCTAGGATTTGAACGT-----AGGCTCCAATACCTGC
Mus musculus  ..........................................................AGACACATAGCTAGTCGGTTAGAAAGCCAGGGCCTGGACTTCTATAACTCTCCAGGGCCTGT

Homo sapiens  ATCAGCTCCCTTGCACTGTGCACTCTTCCCTCGCTTGTGTGTATGGTGAGCAGAGT-CAGCTCTGGGCAAAATGGTGAAA----TAAGCCAAGTTTGTCTTTAGTGTCTTCAGACTCCCA
Mus musculus  GTTCGTT---GTGCCCCG-GCACTTCTGCCTTCAGTATCAGTGTGGAACACAAAGGGCACTTCTGGGAAAAACAGTGAAAGAAGTAAGTTGAACTTGTCTTGTCTGCCTCCAGACTCCAA

Homo sapiens  GTGTGTCTCGGGGACTCAGTACTACAGTGCC--ACCTACATGCTGCAACACCTACTATGGCTCCTACAGGCAGCCCATCCTGCTGAGAGCTTCTCCTTCCTAACTCCTCTAAGACCTTGA
Mus musculus  TCATATCTCACAGACTTCGTGCCATGTTACCCTGCCCATAAGCCACCTC-CCCACAGTGGGT-------GTGGTTCCACCTTCTCAGAGCTTCTCCTTCTCAATTCCTCTAAGCCCTTG.

Homo sapiens  CCTCTGGTTTGTGTTTTCTGAAGGTAAACTGCCTTAACCTAACCTTTTCCAGAGCACATATTATATTTATTGGTGCCTTCCCAGAAAGCAATCTTTAGCTTAGGAGCAGGTAGCTCCCAC
Mus musculus  ........................................................................................................................

Homo sapiens  GGGAGGCATGTAGGTGTTTCTGTGTTGTGGTAAGGGGAAGGGGTTGTCTGGGGGCCTCTATCAAGATACACCATGGAGGCTTCTCCTACAACCAAAGGGTGTTTGGCCCCTTTCTCAGAT
Mus musculus  .......ATGTGGATGATTTAACACCCTGGTAAGGGTAATGGTCTATCTGAGGACTATTCCCGAGCTATACCAAAGACGATCCTCTTAGAAGCACAAGCTGCTTGGCTCCTT----AGAT

Homo sapiens  GGCTGAGGGCCCTGGAGTCAGATCAATAAGCATTTGAATCTTGGCTCTGCCATTCACTGATTGCATGATT---------TCCCACAGTTAATCTCAGTGAGCCTTCGTTTCCTCCCCTGT
Mus musculus  ACCTCGCATGTTTACCATCTGATC----GGTATGTGAGCCCAGCTTCTACAATTGATCTGTGGTGTGATTTGCTCCGAATTCCACAACTA----CAGTGCTCCTGTATATTCCTCCTTGT

Homo sapiens  AAAATGGATCTAATAATAGTTCTCATCTCCTAGACGTGTTGTGAG-GATGACATGAGATAACACACTTACGGTG-TCCAGTACAAAATAAGCATGTAAAAATGTTAACTGTTCCCTTAAA
Mus musculus  AAAATCGATTTGATAAGAATTCTCAGCTCATTGAGCAGCTGTGGGAGAGGCTATGAAATAACACAGCTGCAAAACTGGAGTATAAAACGAACACGGAAAA--GTTCACGATTCTCTCCAA

Homo sapiens  TCAATTTTTAGTAGTCTTCTGCCTCACCCCCATAACCATCCAGGTCTCTCTT---------CGCTTCCTTGTTTCTCTTTTCCTCTTCTCACCAACAAGTCTCTTTCTTCAAAGTCTCCC
Mus musculus  GCCATTGTTACTCGCCATCTGCTTC-CCCCTACTGACATCCACATTTCTCTCGCTCGATGCTGCTTCCCTCACT-TCGTTTTTTCCCCCCACCAGCACATCCTTGCTTT--AAGTCTCGT

Homo sapiens  TTTCCTTTCCCAACTCCCTCCCACCAGTACGCTCAACCTTTGGCAACCCTCTGTCCCACACTTTCCAGGTCAATCCCAATTTCAGATAATCTCTCTTGATATCAAACTGCATGACCTGAT
Mus musculus  CTTCCTTCCCTT-----------CCAGTACATCAGGCCCTTGGCATCTGTATATCC-AGTCTTTCCAGGTCCATCCCCATTCCAGATAATCTGTCCAGATGTCTGACTTTAGAACCTGGT

Homo sapiens  TTGAATCGGAAAACAGAGTGAAACAGAGTCCCTTGCTCTGAGGAATGTTGCTATGGTCTATCTTAGATCAAGAAGACAAGAGCTGCCTTGGCAGCCCTACATC-ATAATCTGATGAGCAT
Mus musculus  TTGTATTCTGAAA---------ACAGGCTCCATTCCTCTGAGGACTGTTCCCATGGTTCATCTTACATCAAGAAGGCTAGAGATGACTTGGTGGCCCTCCATCTATAACCCAGAGGACAC

Homo sapiens  GAAGAAGGCTCACCTTGCCTATAAATATTTGTTTTTTGC---CTATGAGTGATGCCAGAAACCGTCATGGTCGGCATACAATTCCTTTGAGTCAAATTGCAAGGTTTG-TCTGTCAGGGT
Mus musculus  CAGAAAGAC--ATCCAATCTACAAATATCTTTTTTTTTTAAGTTGTAAAGGATGCCAGGAACT-CCATCATCACTATACAATTCATTTGAATTCGATTGCAAGGGGTGGTCTGGCAGGGG

Homo sapiens  TGCCTTGGTAAGGCAGGGTTATTATTTATATCTGAGTGCAAATTCCTTGATCAGAGCCTTTCCTGTCAGGGAGAGGAGACGGAAGGGTTCCAGGGAGCCTACTCCCCACGGAAGAGGAAG
Mus musculus  TGCCTCTGTAACGCAATGTTATTATTTATATCACACTGAAAATTCCTTTATCACAGCCTTTCCTGGCAGCGAGAAGGGAGGGAAAGGTTCCAGGGAGTTGGCTCCCCAAGGAAGAAGAAG

Homo sapiens  GCTGTGCTAGCATGGATGGGGTATGTGAGTGTGGGAGCTCAGCTGACTGAGAGCTGTGTCCCCGGCTCCATTTTGCCTAGGGGCGAGCCCTGTGTGTTGCAACAGATGTGTGCTTTGGAA
Mus musculus  GCAGTGCGATCAGGGGTGGGGTGCGGGGGTGTGGGAAATCAGCAGACTAAAGACCGTGTCCCCAGCTCAGTTTTGCCTA-GGGTGGGCCCTTTGTGTTGCAACAGATGTGTGCTTTGGAA

Homo sapiens  TCACCAAGGTTGGGAAGCCAGGCCTCAG--------AGGGAGTGGCTGGAGGCTGATCTCATCCTGAAATGATGTGGGCTTGGATTCCCAGAGGGTGGAGAGGAGGACCAGTCCAGAGAG
Mus musculus  TTACTGGGGCTGGGAAGGCAGGCCTCGGAGGGAGGAAGGGAGTGGTTCTAGACTGAGCTCATCCTCAAATGATGTGGGCTCAGATTCCTGGAGGGCGGAGCAGGAGACTGGGCCTAAGGG

Homo sapiens  ----AAGGACATGCTTTTGGAGGGAGGTCTAAAGACAAAAAGAGGGCTTTAGTTATTTTTTGAGCTT--AGGTGTAGGTGAT--GGTTGGGGGTAAGTGACAAAAGAAAATAGGTCCTGA
Mus musculus  GAAAGAAGACGAGCTTTTGCATGAGG--CTAAGGTTGAACAGATTGCTTCGGT--TTGTTTAAGCTCAGAGATGGAGGAGATGGGGTGGGGGGTAGGTGGGAGAAGAAAATGATGCTTCA

Homo sapiens  AAGGGGGGTGTCTCCTTTCATGATTAGGAACAGAAGAATGAACTCAAGGACTGCCCCCTTTTCTTCAGGAAGCAAACAACTTCTGAGGGTGTGACAGGGTTCACTGGGTGTTCCCCTTTA
Mus musculus  TAGGA----GTCTTCTT-----------AGTAGAAGACCAACCCTATAGAAAGCGTCCTTTTTCTCAGAAAACAAACAACTTCCGAGGGTGTGGCTGAGTTGACTAGGTGTTTCCCCACC
```

```
Homo sapiens  AGGGAGGTTAGCCCCTACGTGAAGCTGCTTGGCTGGTAGAGGATAATTGGGTGTTCCCCAATCCAGATAAGCAGAAGCTCAGGTGCCCCTCCCTGTAGGCTTTTCAGC------TTTCTT
Mus musculus  AGGGAAGTT-GTCCCTTTGTGAAGCTGCCCAGCCAGTAGAGGGTAACTGGGTGTCCCCCAGTCCTGATAACCAGAAGGT--GCTGACCCTCCATGTGGGGTTTTCAGCCTTTTTTTTTCT

Homo sapiens  TATCACTTGCTAGGCAGGCCCTGCCATCCTTGGGAGTAGGTATGAGAGAGGGAGTTGAGAGGCCAGGCTTTCCCTGCCC-CTGTTCTTCACAGAACACAGCTGAAAAGGAAGGCCAGAG
Mus musculus  TATCACCTCAT----AGGCCCTGTCATCTTCTGGACGCTGTGTGAGAGAGG-------GGGACCAGGCTCTCTCGACCCTCAGCTTTTGCAGAACAC----------TAAAGGCCAGAG

Homo sapiens  ATGTGACACCCCCC----AGCCCCAGGGATTCTACCACACATTGCAAACCTCACA-GAGGATGCTTATTCTACAAAATTTGTGGGCATATTCATAGGTACTATGGAGCTAAATAATTTGA
Mus musculus  ACATGAAGTCTCTCACAGAAACATAGTGATTCTATCCCAT-TGAGAAGTCCCACATGAAGGTGTTTATTCTGGAAAGTTCACAAATGTGTTCCTAGGAAATCTAGGACAAAAT-GTTTGA

Homo sapiens  AAACA----ATAACAAAACCCATTTACT-GATTATTTGGTTAATAAAGACCTCATTCCTTCAAAGTATGTTGCTAGGAGCCACTGAAAGTGGACTTGACCTGAGGAGCAGCAAGGAGCTT
Mus musculus  AATCATTTGACAACACAACCTACTAATTGGAGAGTTTGATTACAAAAGGGCTTATTCCTTTGAGACATGTGGGCAGGGA.........................................

Homo sapiens  CCTCCTGCTGTATACACAGACACACACAGAC--AGACAGACAGACACACACACACGCATGCGCG---------------------------------------CACACACATGCACATGC
Mus musculus  ........TGTGTATGCGCACATGTACAGACAAAGACAGAGACACACATACACACACATGAGAGACAGAGAAAGAGATAGAGAGACAGACACAGCGAAAGAGACAGACAGATACACATAG

Homo sapiens  CCGGATCCTTCTCCA-----------AACACGTCCATGATACCAG-GGCAGCAGAATTGGGGCACATTAGAGAC----------ACAGGCTCTGCCCACATTCTGAAGCTCCCTGTCATT
Mus musculus  AGAGA--------CAGAGAGAGACAGAGCACACTCTGGGTTGCAGAGGCAGCAGAGCTGGTGTACATTAGAGGCATTTAGAGGGACATACTCTACCCACATACTGCAGCTGTCCAGCATT

Homo sapiens  CCCAGGTCTTGGTGCTTGGCCAGACTCCCCTTACCCAGACTCATAGCCTCATAGCTATCATTCCAAAACCCCAAAAGCTCTGGA-AACTGAGAGCCTTTGTACGTTTATGACAAACTCAT
Mus musculus  CCCAGG----------------------------------------CCCGTATCTGTGATTCCAAACTTAAAAGGGTCCTGAACAAATGAGAGCCTTTATAAGTTGATGACAAACTCAT

Homo sapiens  TTGGCAGCGCAACTTGAACTGACATGAGGTTATTTATAGTCTTTATTTTTCACACCCTGTGTGAATATTCATGTGTTTTTGCTCCAGAAATATCAATGTACTTGATTACTGGGTACTGCC
Mus musculus  TTGGCAGCCGCACTTGAGCTGGTATGAGACTCCGGATGCTCTTTGTTAATCCCACCCTGGGTGAACACTTCTGTATTTTTGCTCGAGAAATACCTAGGTATTTGATGCCTGGGT--TACT

Homo sapiens  TCAGACCCCACTGGAGGTGATATA---TATGTGCTGTATACACCCAATCGCCTTTTAAAAGTCCAAAGCATTCTGAATTCCTTCAATTATACTAAGCCTCCCACTGTCTT---ATAATGA
Mus musculus  TCAGACTCTACTAGAGAAGGTACATACTATATGCTGTAC-CACACAGTCTCCTTGGAACAAGCGCACGCATTTTGAGTTCTTTCAGTTATAGCACACATACCCCTTTCTTCTGATGATGA

Homo sapiens  TCTGTTTGGCATGTCTCCCTCATTAGCCTGTGAACTTCTTCTCCAGTGCTCAGCACACAGTCCAGCATGGGGTGGAAGCTCGACTAATGTTTTAGGAATGAAGAACGAACAGTCTTTAGT
Mus musculus  TCAGGTCTGTGGCTCTCTCCCATTTACCCTGGGAGGTCCACCACAGCCTGGCGTATATAGTC---------------------------------GAATGACGAATGAA...........

Homo sapiens  GATTCCGGAGACTTGTTGTCATGTTCTTTCTTACATCTAAACTAAAGTACGTCATGCTACCATTCGTTTATTCTGTGCCATCAATAGCGAGGAAGAACAGTTGGTCAGGCTCCTTCATTT
Mus musculus  ........................................................................................................................

Homo sapiens  TTTTTCCTCTTGAGGCTAAATCGTCCTTATAATCCTAATACCTTTAACCTTTTCCCCTAGATCCTCTTTTCCAATCCCTTAATCGTCTTCATTGCTGTCTTCTGAAGTCTTTACAGGTTC
Mus musculus  ........................................................................................................................

Homo sapiens  TTTCCTTCCGAAATTGTGGCCTAGTGCTTAGGATAGGGCTGCAAAGAGCCCAGCTATTACACATAATAGGCCTCTGGTTTCTATGGTGCTTTTCCGATCAGTTTCATTTGGCTGGGCTAA
Mus musculus  .............................................................................TTTCAGTTGCACTTT-----------------GTCTGGCCTAA

Homo sapiens  GAAGCTTTGCCTTT-AAAAATACACTCGCAGGACAAACTC-CAGGGGCATGAACAACTTTCACCTGAGCATGCTGGTTGGTAATAGGAAGGGAAAATAGCACTGTCTGATCATAAGAAAC
Mus musculus  GAAGCATTGCCTTTAAAAAATACACTTAGGGGGTAAGCCCATAAGGCCATGAATGACTTTCTCCTGAGCATGCTGACTGGCAGTAGGAAGGGAAAAGAGCACTGTCTGATTGTAAGACAC

Homo sapiens  TG-TAATTAC-GAAAGCTGAAAGCAACAGGTAACACATGCAGTAGGCACCTGTATACAAAAACCACTGTTGATAATTACGGTACCTCAGCCTATTATTTTTACCTTGGGAGAACCGGCCT
Mus musculus  TATTAATTCCAGAAAGCTGAAAGCAATAGGTAACACTTGCAATGGGATCCTGAATGAAACAACCACTATAGGTAATTCGAGCACATCAGCCTGTTATTTTTATCTCAGGAGAACCAGCCC

Homo sapiens  TGTTTGTGCGCATCATGTGCCT---TTTCTCTCATAAGGTTGCTAAAGGCCATTTCTGACCAC----TTCCCATTAAATTCAGGGAGTGAACCTGACTCTGTCAGGGTTGCTG-AAAATC
Mus musculus  TATGTATACACATCA-GTCCCTCACCCACTCTCACGTAGCCCTTAAAGGCCACTTCTGATGACTTAGTTCTTATTAACTGCAGGT-GTGAGCCTTAGTCCATCAGGGTTGCTGCAAAACC

Homo sapiens  ACCGGATTAAGTTAAA---CTCACTAATTAAGCCTGTTAGACTTCCTGTAACTCACGATTTTTCTTGGCATAAGTAAAAACATTTCACTCAGAGGCAAAATGTGTTATATTAATAACC-A
Mus musculus  ACTGGATTAAGTGAAACTCCTCACTAATTAGGCCTCTTAGACTTCCTGTAAGTCATGCTTTTTTTTGGCAGAAGTCAAAACATTTGACTCAGAGGCAAAATGTATTATATCACTATCCAA

Homo sapiens  GGAAACAAGAAAATACACATAGTAGCATTTTCGGAAGCA-AATTCTGTCATCCGCATTCTGTTGAAACGGCCATTGTCATTTCTCCTAATAGCCTAAAGTGTTTACTTTTGGGGTGGAAA
Mus musculus  GAAAACAACAAAACGCATGCGGCAGCACTTTAGGGAGCACAATCCCGTCATCTGCAAGGTGTTGGAATTACCCTT---ACTTCTCATAGCAGCCCCAAGCATTTCCTTCTGGGGTGGGAA

Homo sapiens  CTAT--ACATACTGTGATGGAGATTCAGAGATACGCTTTTGTCAGTTAAATTCGAAAAATATATTCAGGTGTGCTTGTTGTGTCCCCCCCCCCCCCAAGCATGAGTCAGAGCCTTTCATG
Mus musculus  CTATCGGAACACTCTGATGTGGATCTGGAGATAAGTCTTTGCCATTACAAT--GGAAAATCTATTCA-CTCTGCTTAT--------------CTTAACTGTGAGTCAGAGCCTTTTGTG

Homo sapiens  GGGTTTTGTGTAAATGTATTTTCTAAGAGGTGCTGT-----TGGGATTTTGC-----ATACAGGTCATTCTTTTTAAAAAGGTCACGTTGCAATTTTTCTATCCTTTCTTGTGTTGTTAT
Mus musculus  AGGTTTTATGTACGTGCATTTTTCTCAAGGTGCTGTGTGTGTGTGTTTTTCCTCTTAATACAGGTCATTCATTTGAAAAGGTCACATTATAATTTCTCTAGCCTTTCCTGAGCTA--AT

Homo sapiens  CCCTCAGGGAAGAAGATGTAGAATGC-CAAATTGCAAAGGGAGCTTGTAAAGAGGGGAAC---ATTAAAATTGGATAACCAAGAGCTCCCAGAGA-GGGCCTCAATGGCTGAAAGGAGCC
Mus musculus  CCCCCGGGAAAGACAATGTAGGACACGGGAACTGCAGAGGGAACGTGTAAAGGGAGGAATAAGATTGAAATTGAGGCA-CAGGAGCTCACAGCGAGGGGCCACAGTAG-TGAAAGG----

Homo sapiens  CCTTCTGAGCAAATGGGTAGCAGCGTAAAAGCCAAATTAACCTGGGAGGAGAAGGTATGGAACTTTTGGAGAAACACTTTGTTTACCTCACCATCTTACCATATCCAGCTGGCTGTTTCT
Mus musculus  --------GCAAAAGATTGGCAGCA-GAAACCCAGCTTAACCTATG-GGATAGGGAATAG------------ACACTTCCTGTGCCTTGCCATTTTACTGGGCTCCACTGGAAG-GTCC

Homo sapiens  CCAAGAATGTTAGTGCTCCAGAAGGGAGGGAAACCTGTCCCCACTGCTAATAGAAGAGGCTGAAATTTTCTTTCAGAGTTTCCAGATGTCCTTGGCAAATGCTAGCAATAGTCATTCTCT
Mus musculus  CCAGGAACACTGGTACTCCACAGAGGGAGGATCCTTGCCCCTATTGCTAGTATGAAAGGGAAGAATTTGCTTTCAGAGGCTCTGGATATTTCAGTCAATGCTAACGATGGTCATTTTTT

Homo sapiens  C--TCTTTTAATAATTATAATAAGCTGCTTTTTAAGGGAACATTAACTGTGTGGTAGGCTCTATGCTAAGTGTTTTATATAAGGTATCTCTATTAATCTTCACTATGATCCTTCCAAAAA
Mus musculus  TTGTCCTTTAATGATTATGGGAATCTAAGTTTTAC-AAAACATTGGCT-TGCAGTCAGCTCCATGCCGAGGGATTTACATGAGGCATCTCTTTTAACCACCATGACAGTCCTTTAAAGAA

Homo sapiens  GGTATTGCTATTACCCTCAATTTCCATAGGAGGAAACGGGCCCAAAGAAGTTAGACAACCTGGGCAAGGTCACCCAGCTAGTAAGTGGAGGAGCCAGAACTTAAACGCAGATCCACCTGA
Mus musculus  GGGGTCATTACTGTCTTCTGTGTTCACAGGGGGGAACTGGTTCAGAGAGGGTGGGCATCCCGACCCATGCTAAGCAGCTAGGAAATGTAAG-----------------TGATCCTCCTAA

Homo sapiens  CACCAA-----CACCTGGTTCT-GAACCATGGTGTGCAAGTCCTTGTAAAGTCAAACAGTCTCTTTAATAAGCTTGACTATATTTTCTCTTTTACCAAATCACAGAACATCTGGTCTGCT
Mus musculus  TGTCAAAAAATAACCTGGTCCTGGAATCACGCTGTGTAAGTCCTTGAGAAGT----CAGTCACCATAATAAACCTGACTGTGTTTTTCCTCTTACCAAATAGCAGAACATCTGGCCTGCT

Homo sapiens  TAATTTTATGAGACTGTAATTACAGCAATGAAAAGGATGTTAGCCTACTCTGAAAACCCTAGCTGAAGGGCTTTCTATTGCTTTGTTTAGAGATTTGTTC-------------------
Mus musculus  TGACTTTATGAGATTGTAAATACAGCAACTAAAAGCATG--------------------AGCTGAACAACTTTCCATTGCTTTGGATTGAGTTTTTTTTTTCTACTGCTTTGAACTGAG

Homo sapiens  -TTTTCTCTCCACCTCCACCCCCGACTCCC-CAAATTGGATAGTAAGTTCTTCAAGGAAGGTTCTGGAAAGTGGTTTTTTTTTTTTTCTTCAAGTTCTGTGTTACATGTGCAGAACTTGCA
Mus musculus  ATTTTTTTTCCCCCTCCTTCCTCCTGTCCCATCCACTGGACAGTGAGTTCTTCATGGGAAG...........................................................

Homo sapiens  GTTTGGTTACTTAGGTATACAAGTGCCATGGTGGTTTGCTGCACCCATCTACCCGTCACCTACATTAGGTATTTCTCCTAATGCTATCCCTCCCCTAGCCCCCCACGCCCCACAGGCCCC
Mus musculus  ........................................................................................................................

Homo sapiens  GGTGTGTGATGTTCCCCTCTCTGCTTCCATGTGTTCTCATTGTTCAACTCCCACTTATGAGTGAGGACATGCGGTGTTTGGTTTTCTGATCTTGCGATAGTTTGCTGAGAATGATGGTTT
Mus musculus  ........................................................................................................................

Homo sapiens  CCAGCTTCATCCATGTCCCTGCAAAGGACGTGAACTCATTCTTTTTTATGGCTGCATAATATTCCATGGTGTATGTGTGCCACATTTTCTTTATCCAGTCTATCATTGATGGACATTTGG
Mus musculus  ........................................................................................................................

Homo sapiens  GTTGGTTCCAAGTCTTTGCTATTGTGAATAGTGCCGCAATAAACATAGGTGGGCATGTGTCTTTATAGCAGCATGATTTGTAATCCTTTGTGTATATGCCCAGTAATGGGATCGCTGGGT
Mus musculus  ........................................................................................................................

Homo sapiens  CAAATGGTATTTCTAGTTCTAGATCCTTGAAGAATCTCCAGTGTCTTCCACGATATTTGAACTAATTTACACACCCACCAACAGTGTAAAAGCATTCCTCTTTTTGGTTCTGGAAAGTCT
Mus musculus  ...................................................................................................................AATGT

Homo sapiens  TATGCTCCTCTGCTTTCTATAGTTCTCATAGTGCCTGGCATGTAGTGTGTGCTCCATAAATATTGACTGATTGATGAATGTGTGGATGTTTCATGCAATAGTTTTTGGGTTGTT----CA
Mus musculus  TGTACTCTTCTGCTTTCTACATCCCTCAGAGTGTCAGGTACATAGTACATGCTCAATAAATATCGACTGACTGATGAATGTGTGGCCGTCCTGGTCAATTGTTTCTGGGTTTTTTTTTCA

Homo sapiens  ACACCATTTCTTAATGCTAAGCAAACAGGTTAGAACCTTCAAGATGTCTGTTGTCTTAATCTAGTTGCTTGCACTGAGG-TAACTTTTTAATATCATCTCTGCACAGGAGCTGAATGCCT
Mus musculus  AGACCCTTTCTTGATGCCAAGCAGGCAGGTTAGGACCTTAAAGGTGTCTGTTGCCTCAATCTAGTTGCT-GTACTGAGGATGACTTTTTAATATTGTGTCCGCACGGGAGCCGACCACTT

Homo sapiens  TCTTTGGCACCTGGATGATTGATTGTGTTTTCTCCTTTTCCTCGGAAAGCTCTGGTATTGCGCAAACGCTGCTTTAGGTGATTAACAATGCCGCCAACTTCACCAGTCACCTTTGCAGAC
Mus musculus  TCTTCGGCACCTGGATGATTGATGGTGTTTTCCCCTTT-CTTACAAAAGCTCTGGCACTGCACAGACACCGCTTGTGGTGATTAACAACGCAGCCAACCTCA---GTCCTCTCTGCAGAC

Homo sapiens  AATTCTTGAATTTTATAG-GAAAGTGGGAACCTGTTTAAGTATAGTTTCTAGTTAATTCTGCTTCCCCATCCTGGTTTGCTCAATG-CTACATAAAAGATTTTCTTTGTGGAATGAGACA
Mus musculus  ATGTCTTGAATTCTATAGAGAAGGAGGGGTTTCGTTTAAGGATAGTTCCTAGTTAACTGTACGCCTCCAGCCTG-----CTTGATGGCTACATCTCAGAATTGCTTTATGGAACGAAACA

Homo sapiens  GAATAGAAAAAATAAAAATACATT---------CTCAATGGCTGGGTGAAGAGAGTCCTTGAGATTTTACCAGCTTGGTATTTCTCACTGT-TGGAACTTCAACCCAGGATGCAGATTCA
Mus musculus  GGATTGTTTGTTTGTTTGTTTCTTTGTTTAAGTCTTGATGGCTGGGTGAAGAG------------CTTACCAATCCAGCATTTCTGATTGTGTGGTATTTTAAACCAGGGTGTGGATGCA

Homo sapiens  TCCTAGTGAGCCTCACAGGAAACT-CTGCCTTGGTCTAGAAGATACAGTGCAAATATATAGGATAGGACAGGTAAGGTCAGCAGAAGCCACTTTTCAGCACATACCATGTGATTGCTAGA
Mus musculus  TCTTGGTCAGTGTCATTAAAAACTTCAGTCTCGGTCTAGACATCATA-TGAAAATGTACAAGGCGGGGCAGGCAGGTCAAGTCT--GCCAGATTTTAGCACATACCATTTGCTTCCCAGA

Homo sapiens  ATAATACAAATAACAACCATT-----TACTAGCAGATTACTATGTGGCAGGT---------ACTGTGCTAAGCATTTTCCAGACATTCATTATTGTTGCCATTTTTCCAGGTGAGCAAAC
Mus musculus  ATCAGA------ACAACTGTGAGGGGTGGTGGTGGGGCG-TGTGTTGGAGATTGTCTATACACTGTATGAAGCATTTTATA-----TCATTATT----------TTCCAAATTAGCAAAT

Homo sapiens  T-----------GCAGC-TCAGAGAGTCT--------------------------------AAGTGAATTTCTTTGTCTTTAAGCAGTA-------------TGAGATAGACCCTGGACT
Mus musculus  GAAACTCTCTAAGCAGCATCAGATAGACCCAGAACTACAACACCTCCCCCATCCGATTCTCAGGCAAATATTCTTGTCTTCACTCTGTAGCCCTGGCTGTCCTGGAACTCACTCTGGTCT
```

```
Homo sapiens  TGAACTCAGG--TCCATCTGATTCC------------------AGAGTA--------------CATAGTCCTTTTG------------------CTACAGTGCTTTGCAGGGAGCTGTGC
Mus musculus  CAAACTCAGAGATACCTCTGATGCCTCTGCCTCTCTAGTGCTAGGATTAAAGGTGTGAGCCACCATTTTCTTTCTGGCAGGCAAATACTCTTGACTATAATGCCCTGCAAGGAGCTGGGC

Homo sapiens  TGGCTTCTCAAATGGTTTGGGGCTATCTGTGTGAGGCTGACAAGAAAACTATTTAAAGGCTGGTAGAAGAGGGCCCTAACAGAAGGTCTGACTCTCATTTTCTTTCTATTTGCAGAAGAT
Mus musculus  CGCTTTCTCA----GTTAAGGGGT---TTTGTGATGTGGA------------------GCTGGTAGAAGAAACAGCCAAGAGTGGATTACACTCT----GTTTTGGTATTTTCAGAAGGG

Homo sapiens  AATTCTTTCCTGTTTCAATATCAGAAGGGGATCTAGCTGAAGGCAAAAGGCCACAGGGTTCTGGTGAGGCACAAGTTCAAACCTTGCTGTAGTTGGACCTGTTCTACTGACTATCAACGG
Mus musculus  GATTGACTGTTATTTCA-----------GATCCTAGCTACAGA-------------------AGTGAGGCACAAGTTCAAACCTTG---TGGCTGCATTTGTTCTACTGATTCTCAACAG

Homo sapiens  CCTGGG--TCTGTGGGTGATCATAGTGTCAGGGGGCTGGAGATTGGAAGTTCAGAGTGGAAGAGCTGGCATATGCCGCAAAATGCACCCTGTTGGGATGGAGCAGGAAACT--CAACTGC
Mus musculus  GTTGGGAGTCTGGAGGCAGTGGTGGTA--------CAGGTGGTCAAAGGTTCA-AGTGGAAA--------------GTGAAGGGCACC-TCTTGGCACTGGGCCGGATAATAGCTATAGA

Homo sapiens  AGCTGAGTTTAGTGGGGCACCCAAAGAGAGTGGCAACAACAGGTATCAATTTAGAGAAGCACTTGGCTTTGGTGAAAGCAGAAGTCCTGCCCAGCCTAGCTGAACTGAGCAGGAGCAGGT
Mus musculus  GGCTGACTTCAATAGGGTGTTTCA-GAGACTAGCAAC--CAGAGTGCAACCCAGAA--------GGCTGTGATGGAAGGAGCAACCATT---------------------GGAAGTGCT

Homo sapiens  GAGGCTTAGGAGTCCTGCCAGGTGCTAGTA---GCAAGGATGGGACTCTAGTCCTTGGAGGCAAGGGTGAAGGAGTTGGGAGCCTGGGAAGAACCAGACAAAATCCCAGTTCCACAGAAA
Mus musculus  GGAGCTTAGGAATCTCATTAGATACAAGGATAAACAAGGACTGAACTCCAGACAGAGGAGGATAGGGTG---------GGAGCCTGGCCAGAACCAGGTAAAGGCCCTATCCTCCCAGAA

Homo sapiens  CTGAATGACTAGACTGGTGGCTCTCAACTGAGACAATACTGCCCCCTAG-GGGACTTTTGGAAAATGCAAGAGGGTATTCAGAGGACTGGAAATGGGGATGCTAGATGTTCTCCAACGCC
Mus musculus  GTGAACTGCTACACTGG---CTCTCAACTGAGACAATACTGCCCCCTAGAGGGATCTTTGGAAACTACATGAGGGTATTTAATGTCTCAGAAATG-------------------------

Homo sapiens  CACGACAGACTTGCAAAATGAAAAATAGTCTAGCATCCCGAATAATTTTTGAATGTTCTGCTGGACTTCCATGGAGCTGAAAACTGCTTTTCTAATTATCTAAACTTTTACTATAAATGT
Mus musculus  ----------------ACCAAAAAAATAGTC-AACATCTCACATGGGTTTTGAGTA--------GATTTCCACTGATGTGAAAATCCAATTTATAATCATGTTACCT----CAACTGCTTT

Homo sapiens  TTTATACCTAAGCATAAAGGGTTTTGCTTCCCCTGCCCCGACCCCACAGTTTTAAT-CAACTATTCTGTAAAGATATGGAAGATTACATGGTT--ACAGAGGATCACACAGACTCATTCA
Mus musculus  TTTTTACAGAACCACAAAGGGTTTTT---------------------GTGTTAGTGCAAAGATTATG--AAGAGATGGAATAAGGGACTGTTTGCCCCAGGTTGACCTTGAGTCATCCG

Homo sapiens  CCATTTCAGGAAA---TCATGTCAACAATTGTGATGTT--GCTTTTGGAATTAATCAATATAACATATC------AGTTTGTGTCCATAACTGTTGCATTCATGGGGATTCTATATAAAG
Mus musculus  T--TTTCAGACAACGGTCCTGTCAACAGTTGTGCTGTTTGGCAATGAGAGCTA-TCAATACAACATACCTCTACTCATTTGTGTTTGCAGCCA---CATCCACCGGGATTTAATGTAAAA

Homo sapiens  GCACAAGCTTCTGACTACTTCATTTAATCATTCAATGTAGTCCTG----TGTGAGCATTTACAGAGTTAAATACATAGTGTTTTATTATGTATTGCTTT-ATTTTCTCTTTATGTGGCAG
Mus musculus  GCACACACTTCTGTCCTTGTCATTCAGTCTTTCTATGTGGGATTGATTGTGCAAGCATTTATA---TGCCAAACACAGTATTTTCTTATGGGTTGCTTTCATTTCCTCTTTCTGTGG---

Homo sapiens  GACATATAGCTATCTTTTTGAACTTATGCGTGTAGGTAGGTTATAGTACCTAGAAATTTAATTTCAGGATAGTAAAGGGGTGAAGCAAAAGCAGAT-----------TTATTATAAAAGA
Mus musculus  -ATCCTCATTTGTGTCTTTGAACTTGAG------GGTAGAGTATAACAGCTATATATTTAATTTGGGGACAGAGATGGAATGAAACAAATTTACATGAGGGTTTGGGTTTTTATTTGTGT

Homo sapiens  GAGGATGT-------TAAGTCTGATAAGGTTG-AACACCACT--GTGCTAG-----CCTGATGGCTTCGTGAGGGCCAGGACCATG--TGTTTTGCTTCCGTTTGGCCTAATTAACTGAG
Mus musculus  GTGTGTGTGTGTGTGTGAGTGGGGTGGGGTAGCGATGCCAGTAAGGGTCAGAACTACTTGTTGGCTTTCTG-GGGCTGGGATTGTGGCTGCTCTGCTTTGGCTTGGCCTAGTTAACCGAG

Homo sapiens  G----------------TGC-TTATAAATAATG---------------AATAATGAATGTGGATGCAAGCACACAGCTGGAGTCAGGCATGTAGGTCAGCATAGTCGGGGTATATAAAAC
Mus musculus  GACTGGGCTGGAGGAGATGCATGGTAAATAGTGGGCAAAAGGATGAGGACTAACTAGCGTGGCCTCAAGCACGGGTCTAGACC-----------ATCATCATGGTGGGAACATCCAGACA

Homo sapiens  TGGCAGGAGATGGGGACTTTTTTTTTTGAGACGGGGACTTTTGATCACCAAAAGAAGGCAGGATCCCAGTTATCAGAAGAGAAACCATAGTTCAGAGTTTGCCTCAAAGCTAGAGCGTCC
Mus musculus  TATCCTGAGATGTGGATCAT-----------------------------AGAGCCAGGCAGGATTCTGGACATCAGAAGAAAGATCCTAATGCAGATGTTTCCTCGTTGCTAGACTATCC

Homo sapiens  TAATGTCAAGGCCCAGGGCTTCCTACAGTCCTCTTATCTTGAGACATGCTGGAGCCAAGGGACTGACGTCAGATGGTCCCAGTGGTGAAGAGAGTTGTGTTTACATTCTCACTTGCTGGT
Mus musculus  TGATGCCAAAGCTCAGTGGATCGTACAGTCCCGTTATCTCAAGCTGTACTGGGGTCAAGAGACTGAGCTCAAATGGTGCCAGTGGTGGATAGAGGGATAGTT--ATGCTGACAGGCCGCT

Homo sapiens  ATCAATCGTAGGGGCCCAAAGAC-CTCACTACCTAAAAAGAGGGCTGTTTTCCAGCTTAGCTGTACCTCTTTGCATTGTTTTGTCT-GGCCAAGACAGAGAGGATACCAGGGAAAATGAG
Mus musculus  AGCAGGCCTCGGAGGCCTCAGACACTGATGACTTGGCTAAAGG--TACTTCCCTGTTCAGCTAAATCCCTTTCCATTGTTTCATTTAGGCCACGGTTGAGGAGACTCCAGGGACA--GGG

Homo sapiens  GC-TCGAGTATTGTACGTATAATCTGTCAATGCCCTCCATGCCCCCATTAGGATCTCAGGCTGTCTTGGCCTTCAGGCAGATCCAGCTCCCCCATTTTTCTGTTGTTGCTGCTGCTGTTT
Mus musculus  GCATCAAGTTCTGTCTTTTCAATGTGTCAA--CTTTCTATGACCCTAGTAGGTCCTCAGCTCAGTTTGGTCCTCCAAAAGAGCCAGTCCTTCCA-GCTCCCATTGTCATTCCTGCTGTTT

Homo sapiens  TTTTTTTTTCACTCCCCTGATCCCATCAATAATACGTGTACTCCTTCCTTCTGGGTCCTGTCCTCAAAGGTAACTGCTAAATATCTGACAAAGGAATGGCTAAAGCCTTCAGGGCTCCTG
Mus musculus  CACAGCCTTGACCTTCCTCATC..................................................................................................

Homo sapiens  ATAAGAGAATGGGCTCCCACTTAGCAAGAAGGGCAGGGGGCAGCACCTCACTTAGAGATATGGTACATAAGTACAGAGGGAGGTATTGAACCACCTGTTGTCAGGAGCAGAAGGGACCCT
Mus musculus  ........................................................................................................................

Homo sapiens  GAGGTCATCTGGTTCACTAGATCCCAAACCTGGCTGTACAATGGAATTACCAGGAATGCTGTTGAAATACAGATGCAGTGTGGCTCCTAATCCTCAGAGTCTGATTCAGTAAGTCCGAGA
Mus musculus  ........................................................................................................................

Homo sapiens  TGAGGACTTGAATTATCTGCATATTATTAACAGTATCACTGGTGATTTTGATGAGGAACCCAGTTAGGGAAACCACTCAGACTGACTTTCTCGTTTTATGGGTAGCCAGCTGAGTCACCG
Mus musculus  ........................................................................................................................

Homo sapiens  ACAGGGATGATGACTCGCCTCGGGTCACAGAGCAAGTCATGGCAGAGTTGGAAATGGAACTCAGGGTTTTTCCTCCCCTGTATTGGTGGTGGGACGATCTGACATTATTAGTGTGGGAAG
Mus musculus  ..............................................................................................CAATAAGACACCATTAA-GCGGGATG

Homo sapiens  AAGGACCTTAAATTTCACTGTACAAGCTGCTGCTCACTCTGCCTAGAGGTAGAGGACAGAGGGCATCTTAAAGGTTGTTTTTCGTTATAATAAGCAGTACATTTACTTCAGAGTTTGGAA
Mus musculus  AAGGACCTTCAGGTTCTGGGTAGAAGCTGCTGCCCACTCTGCCTGCAGTTGGAGGATTGAAGAGATGATCAAAGTCGCTCTACCTTACTATAAGCAATAAATTGACTTCAGCGACTGGGA

Homo sapiens  GTGAATCAACAAAATACCCATCAAGATTAGTAGGACAAGAGGAACTCGCCAATATCTTTGACGATGTCTATCCATAGAAAGCCAAGTGAAATAATGGAGGTAATTATCCACCTATTTTCT
Mus musculus  GAGAAGCAGCAACATATCAGCTGGGGATGGTGGAGCAA---ATACTTACTGACATGTCTGGCTGTGTTTTTACATGGAAAGCTGAGTGAAATGATGGAGGCGCTCAGACACCTCTTTTGT

Homo sapiens  TTGAACACTTTGACCGAAAATCTCCTGGTAACTGAGGGAAAATAGGAGCTTTTAAAAATTGTTGTCTCAAGTAGCCTGAATGACTTCAACTCCCACTTTACTTTTCTTTTT--CTGTGGA
Mus musculus  TTAAGCA-TTCAGCTGAGAACCTCCCGGGAACCGAGACAAAGTGTGAGCTT---AAGAGCGCTGTTTCAAGGAGCCTGAATGATTTCAAGTCCCAGTTCCCTTTCCTTGTTGCCCTTGGG

Homo sapiens  GATGCTCTCCCAGATGCTTGTCTCCTGGG--GGTAGCAGCTTTCCACAGACAAAATGTCCACAGCTTTGGGAAGCATGGAGGGAGAAGGGAAAC--------ATTAAGTGAAATGGGATG
Mus musculus  ATCCCTGGTGTGGGTGCCTCTCCTCCGGGTAGGTGACAT-----------GAAGATGCCTACATCCTCCAGAAATGTGTAACCAGATGGGTGCCTTGGCGCTATCAAATGTAAGGGACAA

Homo sapiens  TCAGGTCAAGCTGCGTTGGTGGTGATGTAGTCACCTGTCGTTTCAAGATAACCTTTTGGATCCTAGATATGTCGTAGCTGGGGCCTTGGGACAAGTCCCAGCAGCCTCC-AAGCCACAAG
Mus musculus  CCAAGCCAAGCTGCATT...........................AAGAAAATCTCTTGGATCCTAGGCCTCTTCTAACTGAGACCTTGGGACAAGTCCTTGCAGCCTCTGAAGCCCCCAA

Homo sapiens  CTTATCATTTTGGCGCAATTCAAGGCAGCCTCCTTGAAAAGCTCCACG-ATGGCAGCTGCTCCATCAGCAACTGGATGAAAACAATGCCAAATCTCTTTATTTAAAATTAGGCACTTCCT
Mus musculus  CTTATCTCTTTGACATCATTCCAGGCAG-CTCCGTGAAAGGCCCTTAGCATGGCAGATGCCCTATCAGCCACTGAAGGCAAACAGTGCAACATTTCTTTCTTT-AGACTAGACATGTGCT

Homo sapiens  ATTTTGGTTCCTAAATGGCAAATCTATCTGCTCTCTGCCTCCAGTCTTCTGTGTCCAAAGCTGCCTTAATGTGAGGTATTTCAGCAGCGATCAGCAAGTGTCTTCCTATTTATAAGCAGC
Mus musculus  ATTGTGGTACCCACAAAGCTGCTCTAGC--CACTCTAACCCCATACTTCGGTGACCTACGTTGCCTCAATGTGACACATTTCAACAGGGATCCATGGGTGTC-TCCTTTTTGTGTTTTG-

Homo sapiens  AGAGCTGCCATGGGTCACTGCACTTGTATCTCTGAGCAAGAGAAGTCAGGGGGCCTATTGGAAAGGAAAAATACTGTGGCCTCCGACAGTGAACCTTGGCGTGGGTCAGGGAAGACT---
Mus musculus  -----TCTTTTGATTCACTGAACTTCTTGCTCTTCAGGAGAGATGCCA--GGTCTCAGTGCAAAGGAAAGGTGTAATGG---CTGCTAATGAATCATGGTGTGTGGCAGGCAAGGGCGAG

Homo sapiens  --------------GTTCAAGTGCACCAGAGAATGGAGCTGTGATGAGTT-----CAGTTTTGTATCACTGGCATGGAAGTCAGTAGCCTCAAGATCTGGGAAGAGTTTGGTCACCACTA
Mus musculus  CAGGGAATGTTGAAGTTCAAGGGCATCAAGTGATAGAGCCCTAAC-AGTTATATACATTCATATGGCAATGTCACGG-AGTGAAGGATGTTGGCGTCTGTGAACACTTCTGTCACCACTG

Homo sapiens  ATTTGCTACATGACCCTGGGCAAGAGTCGTCCCTTCTCTGGGCTTCAGCCTCACCATTTCTGGGGAAAAGGATGCACCATTTAATACATGGTGCCATTTACTGTGACAACCGGCCTGCCA
Mus musculus  ATTTG----ATAGTCCCATGCAAAGTTC---------CGGGGCCTCACACGTACCATGTCTGTGGGAAAGGATTTGTCATTTTGTCCTTGGCATCATTTACTGTAGCAACCACTCCAGCA

Homo sapiens  CCAAAATATAAAGCTGAATCTTTATCTCACTCCTTATTCCAAAATATAATCCAATTAGGTCAGATTTAGATGTTAAAAAGAGAAACCATTAAAAGATTAGAAGAAGGCATAAGAGAATAT
Mus musculus  TTCAAAAAGAGAGCTGAGTTTGTATTGCATTCTTT.....................................................................................

Homo sapiens  TGAGGTAGGGAGTCTTTGCAAAGCAACGAGAAACTGTAGAATCTATGAAGAAAAAGAATGATAGATTTGACTATGCAAAAATTTAACAACCTGAGTATGGTGAAAGATATCTCATGCAAA
Mus musculus  ........................................................................................................................

Homo sapiens  GTGGAAAGGCAACATATAAACCAGGATAAAGTCACAACATAGAAGGAAGACAATGGTTTATTATTCAGAATACTCCACCAGCTCCAGTGTAAGAAGTGCATTATTTTCTTTACAGCACTT
Mus musculus  ...............................................................................AGCTCTATTACAAGAAGTGCCCTCTTTCCTTTAGCCCATCT

Homo sapiens  ACCATGATTTACAATGACATGATTTAGCTACTTGTTTTTCTATTATTTCCTCTGCTTACAT----GTTAGCTTCATGAAAGCTCAGGTTTTGCTTACTCACTATTATATTTCTAGTGCTA
Mus musculus  ACAGCGATGTGAAATTGCACAGGTGA-TTATTTGCTTGCTTATTATCTACTTCCCATACATTCAAATCGACTTCATGAAGGATGAGATTTCACTTCGCCACTACCATAGTCCTTGTGCTA

Homo sapiens  GACTGGTGCCTGGCATACAGTAGGTACTCAATAAATACTTGTTGAAGGAAGGAATAAACGAGCTTCTGTAGACCAATGAGAAGATAATGAACAATTCACTAGAAAAATGCTCAAAGAATA
Mus musculus  GGATAGTGCCTGGTAT-----------TTGAGAAAGGAGTGTTGAGGGAATGGATGAGAGAGCTCTTGTAGATTATTGTAATACCGATACGCAATCTACTAGCAGTATACTAAAAAAAAA

Homo sapiens  ATGAACA-GGCAATTCACAGAGGAAAGGCAAAGGGCCTATGCCCTATTAAAAGGAACACCAATCAGTAATAATTAAGAAATGCAAATTAAAACAATATTGAGATGATTCAGATGTCAGCT
Mus musculus  AAAAACACAGCAAAAATAAAAACACACAAAAAATTTTTATGCCATTTGAAAAGGTACGTAAAACACTAATAATTAAGAGATGCAAATTAAGACAGCGTCAAGGTGATTGACATGCCA-TT

Homo sapiens  TTTACCTCTCAAATTGGCAAAAATTTAAAAG---GTCACTAAAACCCAGGGTTTGTGAGAATCTGGTGA-AACA-GCACACTCATTCACTCTTGATGCACATCTGGA---GGGCAACT--
Mus musculus  TTCATCTTTCAAATGGGCCAAATTAAAAAAAAAAGTTGCAAAAGCCTAGGCTTTGTGAGGATGTGGTAACAACAAGCACACCCACGTACGCGTGGTGCACATCTGAATAAGAACAACTTC

Homo sapiens  -----GGAAATGTTATTTGAGTCAGCAATTCCTGTCTTAGG------------------AGTTTACCTTTG-----------------------CAAAATGCTCCATGTATAAGGATG--
Mus musculus  CAGATGGAAATGTCATACT-GTCAAAATTCTCCATGTTAAGTATTGCTTGCTCACTAAAAGATTACTTGTAAACAGACATAAAATCGAAATAACCAAAATG-GCCATGCATGGTGGCGCA
```

```
Homo sapiens  -----TTCATTTAAACATT--------------------ATTTGTAAATAGGAG-------------CAAATTGA---------------------------------------AAA
Mus musculus  CGCCTTTAATCCCAGCACTTGGGAGGCAGAGGCAGGTGGGTTTCTGAGTTCGAGGCCAGCCTGGTCTACAGAGTGAGTTCCAGGACAGCCAGGGCTACACAGAGAAACCCTGTCTCGAAA

Homo sapiens  AACTAAATGACCTCGGTGAAGGACTGGTTAAATCATTTATAATACATCCCAATAATGACATACTAAGCATTACTATGCTAATGATTAAGAGTTTGAGCCTTGGTTAGACTG---------
Mus musculus  AACCAAATGATGTGAGTAACGATTTGGTTAACTCATT-ATAGCATATCTAAATAGCACAGTCATACACTTTTTTGTGATTCCAAGGAAGAACTTGGGCTTGGGTAAGACTGAATCTCCAG

Homo sapiens  --------------------CCTAGGTTTGACTTCCTCCTCTAGCTTTTATGAGCTGTGTGACTTCGACCAAGTTTCTAACCTCTCTGTGCCGTTTCTCATCTGTAAAA--TGGGAAAA-
Mus musculus  AACCAAGGTAAAGCAGATACCCTAGGTT.........................................ATGTTTCTAAACACAACTTGAT-TTTCTCACCACCAAAAACGGGGAGAAT

Homo sapiens  -GTAACAATTC------ATGAAGTTGTGAAGAGTAAATGAGATAAT--TCATATGAAATATTCAGATAGGACCTGGCACAGAGCATTTA----ATAATTGCTACACATTACTATTCCAAC
Mus musculus  TGTCATAAATCTCCACCATGAGGTTATGAAGAGCAAATGAGACAGTGCTCACAGTGGATGCTCTGGTGACAACTAGCACGGAGCAGCTAGCTAATAGTTGCTATCCAGGTCTATTCTAGT

Homo sapiens  TA--CTAGTATAATGATGACAATGACGATGTAGCCACAAATAAAGACAAGCTAGATCTTTAAATACTGATGTGCAATGATATCTCCAATATAGTATTGTGATAAAAGCAAGTTGCAGAAG
Mus musculus  TAGACTACGAT--TGGTGACAATGACAGCATCTCCCCAAATAAGGACAAGCTTGATCTTTGTACATGGACAGGCA--GAGATGCTCAATAGAGCATTGTGACAAAAATCA----------

Homo sapiens  AGTAAGTATAGTAGGATCTGATTTTTGTTAAGGGTATTATATGTTACACATACAGGAAAAGACTGGAAGGATAGACAGACTGTCAACACTGGGGAGTAGAATAGGAGAGGTAGATTTTCC
Mus musculus  ------CATTGTAGGCTATGATTTCTGTTAAGGCTATTG-------CACGAACAG------------------------CTCTTGCTTTTGGCAAACAGGCTCCAGGAGATAGATTTTCA

Homo sapiens  CTTGAGCAATAATTAGAGTTGTTTGAAACTAGCATGTGCTACTTTTATAATAAACAAAAGATGTAAAG--GGAAAAAAAAGCTCCGTTTGGAATTCCGAGGACAGTTAATCCCACCAGGC
Mus musculus  CCTTAGTAATGATTAGACTCCTTTGAGACAAGCATGTGTTAGTTTTATAATAAACAAAAGATGTAAAGAGGGGGACAAAATCTCCATTTATAACTTCAAGGACAGTTAATCCCACCAGGC

Homo sapiens  TTCACATCCCCCAGTTCTTGGCCACAGCCCAAACGTTAAGCCTCCCTGCTCGTCATCTGTCCTCTCCCATACCATCCTCATACTGCTCAGTGCTCCCATCCCTGCCACCCCGACTCATCC
Mus musculus  TTCACATCCCCCTGTCCTTGGCTACAGCCCAAGTGCTAAGCCTTCCTGTCCTTGGCCCGTTCTCTCCCCT-TCATCCTCCCACTGCAC-------------------CCCCGACCCATCG

Homo sapiens  ACCTCCCCACTACCGCCCTCTCTCACTCTTCTCAGTACCCTCTGGAAGC----CCCCACCTTGCAGTAAACAATTCCCCCTACACCCTGGATAAATTTT---ACAGAGCACTATT--GTC
Mus musculus  AATTCCCCACTACTGCCCCTTTCCACTCCTCTCAGTGCCCTCTAGAAGTGTCACCCCAC--------AATCGACTCCCTGTTTACTCGGGATAAACTTTCCTACACAACAATCTTAGGTC

Homo sapiens  TACCTCCGGGCCTGGAAGTGCTGAAACCTGAGTCTCCACGGCGGATCTCACGGCTTTGCCACCAGCGCCCTCTAGTGGACACTGTCCACACTCCCACACTACAGGCACCCTAGGGCTGGG
Mus musculus  TGCCTTCCCTTCTGGAAGTGCTGAAACCTGAGTCGCGCCACCGAGT-TCACAGCTTTGCAGGCAGCGCCCTCTAGTGGCCACTTCCCGCACTCCTCCACTACAGGCACCCTGGGGCTGGG

Homo sapiens  AGGTGGGGTCTGTGCTCTAGCTTCCCCTGTTTGCTTCCAGGCTGTT-AGTCCTCCGTCCTCAGCCAAGAACTCCTCCTTCTCATGCCATCTGGCTAC---------TTCTCTCCTCGTTG
Mus musculus  AGGTGGGGTCTGTGCTCCAGCTTCCCCTGTCTGCTTCCAGATCGTTTAGTTCTCCATCCTCCGCCAAGAACTCCTCCTTCTAATGCCATCTGGCTACAGCACCCTCTTCTCTCCTCGTCG

Homo sapiens  ACGCTTCTTTATAACCATCCCCTCCTGGTCAACCCCCCCCCACCCCCACATTCAACGAAGAGTTCGACATCTGGCTCATTGTCTTCTTCTCCACCCCAAGTCCTGACATCATTCTGGGTG
Mus musculus  GCTCTTCTTT--------------TTGGTCAACCCCCC---ACTCCCACATTCAAGGAAGAGTTCAACATCTGGCTCACTGTCTTCTTCTCCACCCCAAGTCCTGACATCAATATTGGTG

Homo sapiens  AGTTGGACATCCGTGATCCATGTACAGGACACACTCTCATCCTAGCCTTACCATGCTTCATCATCAGTCAGAACCCCCTACCATCTACACCTTGCTGGCTGGCCTCAACGCTCTTCTTTT
Mus musculus  AGTTTAACATCCATGGTCCATGTGCGGGACACACCTCCCTTGTAGCCTTACCATGCTTCATCATCAGCTAGAACCTCCT-CCATCCACATCCTGTCTGCTGACCTCAAAGCTCCTCTCAT

Homo sapiens  TACCTCAGGAGGCTCTCCAGTTTTTTGAGCCCCCTCCATCCATTTCTTCCATCCCATCAGCCCCTTTCACCTTACTACCTTCCCTCTCCATCCTGGAACATGATGATGATGATGATAAGA
Mus musculus  TACCTCAGGAGGTTTGCCAGGCGTTTGCAGCTCCTCCATCCATGTTCTC-ATTCCATCAAACCCTCTTACCTTACTTGCTTCCCCCTTCATTCTCGAAAATCACCA-----ACGGTAAGA

Homo sapiens  ATAACAACTAATATGTATGTGCCAGGCATATACTACCCATCTTATGACATCATTTCAGTAATCTCATGTTATCCTCACAATAACTTTATGAGGAAGATACTATTGTGGTTTTCCTTTTAA
Mus musculus  ACATTAGTGAATATCTATGTGCCAGACACATAATAAGCTTATGATGCCATCATTTCTATATTTTAATGCCATCCTCACAATCATTGTATGAGACAG---CTATTGTTGTTCTTTATAAAA

Homo sapiens  TGAGGTGACTGAGGCTCAGAGAGGTTGAGTGACTTACCCAAAGTCCCACAG-----------CAGAGATGAGATTTGAAGTCAGGTCTGTTTGCTT-----------------------
Mus musculus  TGAGATTGCTGGGGATCAGAGAGGTTGAGTGACTTACCTAAAGTCCAACAGTCAGGTTGCGGCAGAAATGAGATTGGAGG------CTGCGTGCTTCAATCTAACTCAAGGAACACGGAC

Homo sapiens  -TCAGAATCTTGCGTATATCCCCAATTCCCTTGCCCTGTGTCTTTCTGCTGCATCCATGAGGCAAAATCCTAAGTCTGGATAAATTTAACTGACAGTTTCCTCCTCTGTGCTTATGCCCA
Mus musculus  TTCAAGATTTTGCCTATTTTGACAACTCCTTCGTC---------------ACATCCATGAGGCAAAATCCAAAGTCTGGATAAATTTAACTGATCGTCTCCTCCTT--------------

Homo sapiens  GATGGAGGAACATTGCTGGAGAAAATCCCATCACTTTGTTGGTTAGTCCCACCAGAAAGTCATAGTTTCCAACCTCAGCGGGGCCCTCAACTCAGCTAAACAATCCTTCTAAGTAACTGT
Mus musculus  GACAGAGGAA----GCTGGA----------TCACTTTGTTAATTAGTCCCAGTAGGAAGTCACAGTTTCCCACCTCTGTGGGGCCCTTGGCTCATCTAAAGAATCCTC---AGTAGTTGT

Homo sapiens  CCTGACAATAGCACTTGCAAACTTTCTCCACTGCCCTCTGGAGTCAGACTGTCTGGGTTAGAGTCTGCTTTGGCCACTTCCATGCTCCATACCATGCCAGCCTTTAAGAAAGGGTGAAGT
Mus musculus  CCTGACAATGGCATTTTCAAACTTTCTCCACTGCCCTCTGGAGTGAGATGGTCT-----------TGTCTCAGCCACTTCCATGCTTAATAGTGTAGCAGGCTTTAAGGAAGGGTCAGGA

Homo sapiens  GGAGGA--AAAAGGATGAGGGGAAAGGGGAAGTGATATGTATCGAGTGTCTTCTAGGTACCAGCCACTGTGCCAGGTGCTTTGCATGCAGTGTTTCATTTAATCCTGCCAGCAGCCCTGC
Mus musculus  GAAGGACAGAGAGGATGAAGGGAA-GTAATAGTGCCATGGATTGAGCGTCTCCTATA-ACAAGCCTCTGGGACAGGTGCTTTGCCTGCAGCATCTTACAGAATCCTCCTAGCAGCTCCAC

Homo sapiens  A-AGGGCAATACTCTTGTTATTTTTGCTTTACAGAGGAGGAAGCTGAGGCACAGACAGGTTAAGTCACTCACTAACGTG-----------------------------ACACAATTTGTA
Mus musculus  AGAGAGCGATGTTGTTGTGATTCTTACTGTACAGAGAAGGAGACAGAGGCACAGAGAGGTGAAG-CGCTTGCTCGGGGGTGGGGTGGGGGTGGCGGAGGCGGGTGGAGATATGGTTTGTA

Homo sapiens  TGTGGAGAAGCCATCTCATTCGTTTAGACCTTCCTTATCTTGATGAATTATTGTGACAAG----CTGATAAATGGGTCCCCTGTTACCTATCTCATGCCCTACAATCTACCTTCCATTCT
Mus musculus  AGTAGAGAGGTCACA----------AAACCCTTC--ATCTTGAGAGATTACTGTGATTTGTCCTCCGTTAA---------CTGTTAACTATCTCACACCCTCCAGTCTGGCTGCAAGCAT

Homo sapiens  GTTGCTGGGACAATCTTTCTAAAATGCACATCTGACAGTGCTACTACTCTGCTTAAAGTCCTTCCAGAGCTTCCCCTCAGTCTTCAGAATGAAACAGAAACCTTTGATCTTAATGATTTG
Mus musculus  ATTGCTCGGACCACCTTCCCAAAATGAGCATCT-------------TCCTGCT-------CTTCCAAACCATTGCC-CAGCCTTCCCGATGCCCAAGAAATCTTTGATCTTGCCA-----

Homo sapiens  GCCCTGTCCTTCCTTTCTCACTGCATCCACTACCACTTGCCCATGCAGTCACACAGTACTATTTTTGGCCACCTGATGACACCTTGCTTTCTTCTGAAGAAAGAAGAAAGCATATATATA
Mus musculus  -------CCTTTCTTTCTTACTACACCCATTGCCACCTCCCTAGA-------ACAGGATTACTTTGGGCTACCCAACAACACCCTGTTTTCCTCTG-----------------------

Homo sapiens  TATATTCTCTGCC--TCCCCATCCTCCACAC-CCTTTCCTGCCCACTTTAAATGCTCCTCTTCTGTGCTTTCA--CAGCATT--CATGTTTATCCCAGTGTGTTATAATTTCCTGCTGTC
Mus musculus  ----CCCTCCACCCTTTCCTGTCTTCAGTACTCCTTTCCAGTCTCCCTTAAATGCACCTCTTCTGTGCTTTTATGCAGTATTTACACATTGATTCCAGCATCCTGTCATTTCCCGCTGAC

Homo sapiens  TTCTCT-GTTTCCCCCATTAAACTGGAAGCATCTTGAGAACTGGGATTGTGCCTTAGTCATCTCTGTACTCCCCAGGGCCGTAGCACCAGAGCCATGGTTAAATCTATGTTTGTTGAATG
Mus musculus  TTTTCTTGTTTCCTTCAATAAACAGGGAGCCGCTTAAAGGTGGGTACTGTGCCTTTGTTAACTCAGTACTCTCCAGGGC-ATAGCACTCGGGGCTGCTTAAAACCCATGTTTGTTGACTG

Homo sapiens  ACTGAATTAATGAATGAATAGAGTGAATGAGAGAAGAAAAAGCTCTCACTTGGAAAGTTTAGTGACATGAGCTGTATGGTATCTGATTCTTGGCTGAAGCTGCTGCAGTTTTTCCCTATG
Mus musculus  ACTGACTGAATGAATGAATGGAGTGAACGAGGGAGAAGAAAGCCCTCACTTGGAAAGTTTAGTGACATGAGCTGTATGGTATCTGGCTCTTGGCTGGAGTTGCTGCAGTTTTTCCTTATG

Homo sapiens  ACTACAGTGCTATAAAAATATGTCTGCACATGGATAAAGACTGGAAGAGAA-CTTTCCACACAAAACTAAGAGTTGTGTTAGAGTGGTGAGT-GATGGATGCTTTTTT--------CCCC
Mus musculus  ACTACAGTGCTGTAAAAATATGTCTGCACGTGGATAAAGACTGCAGGAGAAGTTTTCCACACAAA-TTAAGAGTTGTGTTAGCGTGGTGAGTTGATGGATGTTTTTTTATTTCCCTTCCT

Homo sapiens  ATTTTCAAAATGTTCTTTAGTGCTGCTTTTGGATTGTTTTGGCAATGTAGCAGACATTGTAGAAAT-GGAATTTGGGGCTCCCATGGAGGAATCGACAACCAATATGTATTGATGCCCAG
Mus musculus  GTTTGCAAAATGTTCTTTAGCACGACTTTGCGAGTGCTTTGGCCAAGTGGCCGACATCATAGAAGCAGGAGTCTGGGGCTGTCATGGAGGGATCGACAGTCAATATGTATTGATGCCCAC

Homo sapiens  TGTACTCTGCT---GGGTTCTGGGTGCTGTGGGGAGATGGGTGGGTAAAGGCGACTCATTCTGACTCAGGTTTGCATGGTCAGGAATCTTGCCAATTACGTTGGATTTGCTGGAATAAAA
Mus musculus  TAGACTCTCCTCCTGGGTTCTGGGTGCCGTGGGGAGACAAGTGGGTAGAGGTGGCTCATCCTGACTCAGGTTCACATGTCCAGAAACCTTGCCAATTTTGTTGGATTTGCTGGAATAAAA

Homo sapiens  AGATAACTTTCAAGAATTTTCCAAGCTCCTGGGTTGAGGTATTTCTTTT----CTCCCAAAGCTGGGCCGCAAACAAAGATGGCTTTTGTTTGCAGTAAAATACTCATTCCTTCAATTTT
Mus musculus  TGAGAACTTTCAAGAATTTTCCAAGCTCCTGGGTTGAGGTATTTTTTTTTCCTCTCCTAAAGTTGTGCAGCAAACAAAGATGGCTTTTGTTTGCAGTAAAACACTCATTCCTTCAATTTT

Homo sapiens  GTCCTGCAAAGTGACTTCTATAGCTT---------------------------------------------TGGGAGTCAGGTTTGCTGAGTGGGGAAACTGATTGTCCATGCTTCATTG
Mus musculus  ATATTGCAGAGTGACTTCTGCTGCTGGGGTGGTGCTGGTGGTGGTGGTGCTGGTGGTGGTGGTGGTAGTGGTGGTGGTGAGGTTTGCTAAACAGGAAAACGAACTGCCCATACTTGGTTG

Homo sapiens  ATTTAGAAATGACCAAAAATGTTTGCCT-TTTCTGGCTCTTGAATAAAATGTTTAGGAATTTCATTGGCAAAATGATGAAATTGTTCCCTTTACTGCTGCAAGACTTCCAAAACTATATA
Mus musculus  GTACAGAGGTGACTGAAATGTTTTCCCTCTTTCTGGCTCCTGAATAACATGTTTAGGAATTTCATTGGCAAAATGATGAAATTGTTCA-TTTACTGTGGCAGGATTTCCAAAGCTACATG

Homo sapiens  TCGTGGGTTAACTTAACCCAGAAGAGGCAAAAAAAAAAAAAAAAATGGGAAGCAG---------ACTCAGATGATTAACACTCTGCCTTAATTGGGTGCTTACTCACTCTGCCGT------
Mus musculus  CCGTGAGTTAAC-----CCAGAAGA----AAAGGGGGAAAAATCGGGGTGGCAGTTCCCAAAGATTCAGCGGATGAGTGCTCTGCTTTTATTGGGTACTTCCTCACCCGCACGCACGCCC

Homo sapiens  GGCAA----GGCACTGTGTTCTGGACTTTGGAGATATCTCATTTAAAACTCTCAACAG------CCTGTGGGTAAGGGACTGTTATTGTTCTCATGGTAGAGATGGTCAAACTGAGTTTC
Mus musculus  GGCACTGCTGGCATTGTGCTTGGGAATGTGGGGGTACCTCATTTAAACCCTGGAGTGATGTTTTCCTG-------GAGATTCTCCTTAGCCCCATGTTAGAGACTGGAAAACTGAGCTTC

Homo sapiens  ATAGAGGCTCAGCTCTGAAATCATGAGACCAGGATTTGAAGCCGAGTTCAGCTGGTTCCATGGGCACACACTAACCATTTGGCAACAGTGCCTCTAGACTAGTCTGGCTACAAAGGGACA
Mus musculus  A----GATTTACTTGGAAATTCTGAGACCAGGGTTCAAAGGCCAGCTCAGCTGG--CCAT-------CTTTAACCATTTGGCAACCTTGCCTCTCCACGAGTGTACCCGTAGAGGAGCT

Homo sapiens  GAGGTATGTATTTAT--TTTGATTTAACGAACCGTTGTTTAATGTTTACCATGTCCCGGGCACATGGTAACTGCTTTAAAAATGTTAGATTATTTACTTCTCATAGACAACCCTGTGAGG
Mus musculus  GCGGTATACATTTATCTTTTCAGTTAACAAACAGTT---TAGTGTTTCCCATGTGCCGTGCATGTGGCCAGGGTTTTATAAACACTAGTTCATTTAGTCCTTAAAG-----CCTGTG-TG

Homo sapiens  AGGTACTATTATTAATATCATATGACAGGTGGGAAAATGGAGGCACAGGGGTGTTGAATCACTTGCCCGGGGTCACTCAGGCTAGCATGTGACAGAGCTAGAGTTACAATCCAAGTGGCC
Mus musculus  AAGTACTATTATTAATTCCATGTGAGTGGTGGGATCACAGAGGCAGAGAGGT--TAAGTCACTTGCTAGGGATCAGGAAAGCTAGCATGTGACGAAGCCAGAGTCACCATCTAGTCTGCC

Homo sapiens  ATGTTCAGGTGTGTGGCCCATTACCTTGACTCTGTGCTGGTTAGCATGCTACCCCCTGTGCCCTGTCTGCATAGCTCTGGCTCAGAAACACTGAAGACATGGGCCTCTTTTTGTAGTTTT
Mus musculus  GTGTTCTAGGGTCTGCTTCTTAACCTTGATCTTGTGTTGCATAACA-----------------------CATAGGTTCCTCTCCAACACAC-----------------------------

Homo sapiens  GTAACAGATTTGTACTTTTTAAAGTATTTTCCACCATTAAAGCCATACGCTCTGCTTCTAAGCGTCCTGTGGGGTTGGTGGGAAGGGTGAAAACATACTCCTGTCCTTCTATAGCAAGAC
Mus musculus  ATCCCCAGCCTGTACCTTTCAA.............................................GTGGGGGTGG--GGGATGGTAAATGCCATCTTCTGTACC---AAGGCAAGAG

Homo sapiens  TAGC-TTAGAGGCAAGGTGGCCCAGTGGTTAGAGCATGCCCATGGTGCCAGTGAACTCAGCTTCAAATCCTGGCCTTATGATTTCAGAGCTGAACCTCTCTGAATGTCTGTATCACTATC
Mus musculus  TGGCCTTAGCGGCAGGTAGCTTCTGTGAATAAGG---------------ACTGGGCTCGGATTGGAAGTGGAGTGTTCAGTTTTCAGAGCTGAGCCTCTCCAAAGCTTGGTTCCAC----
```

```
Homo sapiens  TTTGCTAAAGGAAGAGGAAG-CTTTCTCCCGATATCTTCAT--------TTACTTCCAATTTTCGGT-TTTTGGGGGAGAGCTGGGTTTCCTCCACAGCTAGGCGCTGGGTGGC-GAAGG
Mus musculus  ------AGATAAAGAAGAGGTCCTTCACCAGAGCCCTTCATGGATTGAACTGGTGTGATTTTTCAGTGTTTTAAAGGCAAGGCGGGCTTCATTTGCGGCT---TGCTGGGCTCCTGAAGG

Homo sapiens  GCTTTTGGACTGTCACTACTTTTCATT-CGACA-GAGACTTCAGGATAGCCCCCTTCAAAGGCAGTCTGTTTGGGGTCATCAGGAACAGACAGAGTGCTCTTCCCTACCGC---ACTCAT
Mus musculus  GCTTTTGGTTTGTCTCCTCTTTTTGTTTCAACGCGAGACGCCAAGATAGTGCCTTTCCAAGGTAATTTGCATAAGGTACACAGGGAGGGA--GAGTGGCCTTCCCTCCCCCTCGGCTCAC

Homo sapiens  CCCACCTAGTGCCATCTATCCTTACCCACTCTGAATCCCTCCTCTCGGCAGGCACTGCCTCATTCCCCAGAGCCAGGCCTAGACAATGGCGGCTGCTCAGCTTGGAGGGAGGGGGAGTGA
Mus musculus  ACCTCTCATTCCTACCTTTCCCAACAGGCCC------------------AGTCACAGCCTTGTTCCCTAGAGCCAAGCCTAGACAATGGTGGCAGCTCGGCT-GGATGGAGGAGGAG--G

Homo sapiens  AGAGGCAGCAGGCCCAACCTGCCAGCTTGAAAGCTTCATTTGACAGGGGAGGAGGCACATCTGGTTTGGGTTTTCAATTCAATTCAACAATGATTTATTGTGGGTCTGCCAGCTGCAGAG
Mus musculus  AGTAGTAGCAGGCCCAGCCCACCAGCTTGAAAGCTTCATCTGACAGGGGAGGAGGCACATCTGGTTTGGGTTTTCAATTCAATTCAACAATGATTTATTGTAGGCCTGCCAGCTGCAGAG

Homo sapiens  GTTCTGGGCTAGGCTAGGCAGCAAGAGATGCAAAGAT-AAGTAAAATATTGTCTGTGCCCTTAAAGGAGTTTTCTAGGTTGTAGGGGTGTGCAGACGCAAATGAGCATTTTTTTTTTATG
Mus musculus  GTTCTGGGCTCAGCTAGGCGGCA-GAGATTCGAAGATCAAGAAAAATCTTGTCTGTGCCCTTAAAGGAGTTTGCTGGGTCCTACAGCTGTGTAGACCCGG--GAACAACTTTT----ATG

Homo sapiens  CGATAAGCCCTGTGCTTAGCAGTTTACCTTGCCTACATTATTATGTCTTTGCATCTTCCCAGCAGGTTTTGTGATGGAGGTGGGTAAACTATTATCACTTCATTTTATACACATGAGGAA
Mus musculus  GGACAAACTC---------------GCCTTGCGTCCGTGTT---GCCTTTGAACCTTCCCAACATGGTCTGTGATGGGGAAGGGT---TTTTGGTTACTTCCACTT-TACATGTGAGGAA

Homo sapiens  ACAGAGCCTCAGAGTGGAAGAGACTGGCCCAAGGTCACATTTTTAAGAATGGACCAAACCAGTTCTGGAAC-----------------------------TATTA------CATTCTTCT
Mus musculus  AGTGGGCCTCGGAGAGGAAGTGACTGGCCCAAGGTCATGTACTTAGGAAGTGACCACACCAGCCCTGGAACCTAGGCGAGACTGCTTCTCAAGTTTGCAGTTTTAACTGTTCATTTGGCT

Homo sapiens  GCTCCCCAGAGGTGATGAAGATGATGATGATGACAAAAAACACTTACT---GAGCTCTGGAGGCCAAGTCTTGTGCTATGCAGGCTGCAGATATTATTTTACTGGCAACTTCCAACATCC
Mus musculus  CCTTCCCAGAAACAATGAAGACGA---------CACTAAGTACTTTTTTGTGTGCTCTGGAGGTCCTGTCCTGGGCTGAGAAGGCTGTGGATGTCCTTCCTTGGGTCACCT-----GGCC

Homo sapiens  CATTGAGGCTGATGTTGTTATCCCTTTTTCTAGAAGAGAAAACGGAGGTAGCCTTGGGAAGTGATGGAGTTGGAATTTGAAACCAGGTCATTCCAGTCCACCAGCCTGTACCATAAAAGA
Mus musculus  CATTGAGGCTGTTGCTGGCATTCTCTTTTTCAGAG-----CATGGATGTTATTTTGGGAAGAGATGGAGCTGAGATCTGGAACTGGGTTATTCTAGTTCATCGATGTGTATCACAAACGC

Homo sapiens  GGTAGACACAGGGTGCCATGTTCAGGGGCAAGAGCAATGAATTGTGCCTGCAAAAGCTTCAT-GCCAGAGATGGCATTTACATTTGGCCTTGAAGGGTGAGATGGGGTTATGATTGGTAG
Mus musculus  CATAGAAGGGAGGAAGGGTGCTTAGGGCTCAG---------------CTGGGAGAGCTTCATAGCTCTCCATGGC-------TTTGGCTTTTATGGGCAAATAGATATTCGTACAGGTAG

Homo sapiens  AGTTGGGAGAGAGGGCATCCCAGTCAGAAGGGATATTCTGAGCAAAGAACCCAAAGAGATGACTTGTGGAGCATTTTGATTGAGAGGCTGTAGGTTCATCCGGCATGCAGGCCGGGGACT
Mus musculus  TTTTGGCAGAGACCGTTTTCTGGTCAGAAGGAATGTTATGAACAAGAGGCTCAACGTGATGAAC-ACATAGCATCATGACTGACAGGCTAC............................

Homo sapiens  CTGGGTGTAGAGGAACAATGTGAAGGAAACCAGAGATACGCCTGATGGTCTAACACATTTTTGTCAAGTTGAGGTTCTTATTTTTTTAATTAAGAGCAGGTACTCTGCCCC-AAAGGCTT
Mus musculus  ....................................................CATCTCTTTGTCGAGATGATGATCATGCCCTTCTG-TTACGACCAGGTACTCAATCCAGAAAAGCTT

Homo sapiens  TTCAAATGATAC-----TATCTTGCAACCCATGAGCAAACACACTCGAGTCTCTCACTGTGTAAATTCTACTGTGCTGAGAGCTGC---GGTGAATACAAAAGAAAACAACTCAATTCCT
Mus musculus  TTCAGAGGACACACGGTCATGCTATTGTCCGTGAGCC------------TCTCTCTGTGTACAAAGGCTTCTGCCAAGAGAGCAGCTAGAAGAAACACAAAAGAAGGCT-CTCAGT-CCT

Homo sapiens  CAAGTGTGTGCCGATGGCCCCCGTGTGGTTGAGGAGGCAAGATACACACAATCAGGAAAAGCAGCAGCTAGCATAGGCAGTTAGTGATCAGGGCCAAATGAGTTTTTCCAGCCGTCAGAT
Mus musculus  TGAGCATGTGATGGGGGCCCCCGTGTGGTTGGGGAGACAAGATACACACC-CCGGAAAGAGCAGCAGCCAGCCTAGGCAGTCAGCAATCTGGACCAAATGAGTTTTCCCAACTGTCAGAT

Homo sapiens  TTCAAGGAACATGAACG-AGAGGAAGGCCGGCAAGCTTAAGGCCGGGTGGGGCAATCAGTGTAGCTAGCATTAAGGGCTCTGGCCAGGCCTCCCTTCCCCAAACTGCCACGGTCTCCAAC
Mus musculus  TTCAAGGAGCGTGGATGCAGAGGAAGGCAGGCGAGCTGAAGACCGGGTGGGGCTACCAGTGTGGCTAGGCTGGAGGCTTCTGCAGAGGTCTCTGTTTTCCAAACTGCTGCTGTCCCCATC

Homo sapiens  CATGTCTTGACCTCCCAGAGTTTGTTAGGGAAGATGGGACCTTATGGGATCAGAGACAGCGCATGGGAGCTGGGAAACGGAGCCGAATACCTGATATTCCTGATCC-TCCAATTTTCTAC
Mus musculus  TATGTCTTGACTTTCAGGAGTTTGTTCCAGGAGATGAGACTCGATGGGATCACAGAGAGCCCTTGGGAGCTGGGAAATAGGGCTGGGTACCTGATATTCCTGATCCCTCCAATTTTCTAC

Homo sapiens  ACTTATTCTCATTTCCTGGCAGCTCCTCCAGCTGCTGAATTGTATGTGTATGTGTGAGCCGTATTTTAAATTAAGCAGTGAGTTCTTTCAGCTCATCATAAACATGCTAAAAAGCTTCAG
Mus musculus  ACTTAGCCTCAATTCCTGGCAGCTCCCCCAGCTGCTGAATTGTATGTGTATGTGTGAGCGGTATTTTAAATTAAGCAGTGAGTGCTTTCAGCTCATCATAAACATGCTAAAAAGCTTCGG

Homo sapiens  CCGCGGCCTGAGCCCACCCCCGCCCTTTACACACACAGGCAGCAGCTGGGGCCACTCGACCCCACACTCTAATGTGCATTGCCTATGACTCACCAAC----------------AAAAACG
Mus musculus  CCCCAGCCTGAGCCCACCCCCGCCCCTT--GCACACAGGCAGCAGCTGGGGCCACTCGACCCCACACTCTAATGCGCATTGCCTATGACTCACCACCACTACCAACAACAACAAAAAAAG

Homo sapiens  ACTGTATGTTTCAGTCTCCCTGGGTAGCTGATTCCTAGTTGTGGTGGGAAATCTCAGTCCG-ATTTGGAAGGCAAAGGGCCTCAGCCCCGCCCTACTGACTGTGAATTCTACAACATATG
Mus musculus  ACTGTATGTTTCAGTCTCCCTGGGTAGCTGATTCCTAGTTCTGGTGGGAAATCTCACTTCGAATTTGGAAAGCAAAGGGCCTGAGCCCCGCCCTACTGGCTGAGCTGTCTGCAGTATATG

Homo sapiens  GAGCGCCTACCTTGTGCAAGGCATTGTGGGTAAGTCCATTCATCTCTCTGGGCTTTCCATTTCCTCAGCTTCAAAATGCATGTACTAATCCTGCCGGCTTTACCTCCCTCCTAGGGCTCT
Mus musculus  GAGCGCCTACTGTGTGCGAGGCATTGTGGGTAAGTCTATTAATCTTTCCGGGCTTT-------CTCAGCTTCAATATGCATGCAGTCTTCTTGCTTGTTTTACCTCCCTGCCAGGGACCA

Homo sapiens  GTGTCTCA---GCCACACTGGTCTTTC----AATTTCTGCAACATTCTC--GGTCTTTTCTCGCCCCAGGGCCTTTGTTCG---TGCTCCTGCCTGGACTGCCCTTCCTCTTGCTTTTGG
Mus musculus  TTATTGCACGCACGACACTGGTCTTTCAGTTAGTTTCTGGAACACTCCCAAAGTCTTTTCCTGCCCCAGGGCCTTTGTGCATACTGCTTTTGCCTGGATTGTCTTTGCTCTTGCTTCTGG

Homo sapiens  CATGGAAGGCTCCTTCCTAACCTTTCAGTCTCAGCT---TAACGTTACCTCCTGAGAAGAG---------CCTTCTCTGACCACCCACAGTTAGATGCCTCCTGTTCCTCTCTTACATTT
Mus musculus  CATGGGAGGATTCATCTTAACCTTTGGTTCTCAACTTTGTAATGTTA-CTCTAAAGATGAGGCTTTTTTTCCCTTTCTTGCTAGCAATAGTTA------TCTCAGTCCCTTCTTA-ATAT

Homo sapiens  CTTTTATTAGCAATGCTTCCTTAACCCCATTTACTGCTCTGGGTAATTATGTCTATTTATATTTGTCTATTTATGGTATGTCTCTCCCA-CTGGAATGTAAGCTCCAAGAGGACAGGGAC
Mus musculus  TTCTTAATCG--------------------TTATCTCTCTGGGT-ATTTTGCCTGTTTATA-TTGTCTGCTTATGGTATGTTTCACCCATTTGGAATGAAACCTCTAAGAGGCCAGGGGC

Homo sapiens  CTTGTCTGTGTCACTCAGCAATGTG-TCCCTCTCCTCTAGAGTGGTGCTTGGCACATAGTAGGCTGACATAAGCAAAACCATCTGGAAAATATTTTCTTCCTGAGGTTCTTGCCAGACAT
Mus musculus  CTTGTCTCCATGCCTTAGCAATGTGCCCCCTCTCATCTAGAATGGTGTTTGGCACATAGTAGGCTGACATAAACAAAACCATCTGGGAAATATTTTCTCTCTGAGACTCTTGCCAGACAT

Homo sapiens  ATGGTTGCTGAGGGAAAAATTATGGCAGAACTGTGTCATTCCATAGGAAAAAAAA-----------------TCAGGCTAAAAGGGAAGAGTTTGTTGAAAACAGTTCTGTTTTAGAAAC
Mus musculus  ATGGTTGCTGAGGGAAAGATCATGGCAGATCTGTGTCATTCCATATGAAAGAAAAAAAAAAAAAAAGAAGAAGTAGGACTGGATGGGAGGAGTTTGTTGAAAACAATTCTGTTTTAGAAAC

Homo sapiens  CAGTCCTTGGAGTCTCATCGCCAGACACAGTCTTATGAAATTAAACAAAAGAAATAAAAAGCAT-AAAAAGCTAA---GTTTTTATTGGGTATTTACTATGTGCCAGGAACTGTTCTAAG
Mus musculus  CGGTCCTTAGAGTCTCATCCCTAGACACAGTCTTATGAAATTAAACAAAAGGAATAAAAAGCATAAAAAAGATCACTTGTTTTTATTGGGTGCTTAGTGTGTGCCAGGAACTGTTCTCAG

Homo sapiens  TGTTCTACACAGATTATCTCATTTAATCCTCATCATAACCCAATGACGTCAACT-CTATTATTAGCTCCATTTAACAGATGAAGGAATTGAGGCTCTGGGATGTTAAGAAAAGTGCCCAA
Mus musculus  T-TTCTGTACCGATTATCTCATTTAATCCCCATAATAAGCCAGCCACGTCAGGTCCTATTATTAGCCCCATTTAACAGATGAGGGAATTGAGGCTCTAAGGGGAGAAGCAAAGTGCCTAA

Homo sapiens  GGTCACACAGCTAGCAAGAGACAGAACCAAAAGTTGAAC--TCAAGGCAGTCTGAGTCCAAGTCCACAAACTAAATCATCTATGCCACCTGCAGCCCCCACCTCATGTTACCC--TACAC
Mus musculus  GGCCACGTAGTTATGGAGAGGCAAACCCAAAAGATGAACAGTCAGAGCAGTGTGTG------------ACTTAAATTCTCCACACCCCTTGAAGCCCCCATACCACCGTAGCAAGTGTGT

Homo sapiens  CTCCCACGCCACCCCCAGGCACGATTCTA---------------------------CTACAGCACTGGCCACAT------TGCAG-----------------------------AACAGGG
Mus musculus  CTCCCAGAGCG----TGAGCAGAATTCTACATTTCTTTCTGTGGTGGGGGTGGCACCCAGGGCCCTGTGCCCATTGAGCATGCAGTATACCACTAAGCTCCCTTGTCTTTGACAGCAATG

Homo sapiens  GTTTGTTT------------------ACTTGTCTGTCCTTCCTACTAGACGAGGAGCTTCTCAAGTGCAGAGACTGGATCTTATTCATTTTTGCTTCCCCTGTGCCTGGAAGAGAGCTTG
Mus musculus  TTTCTTTTTTCTTTTTCTTTTTTTCAACCTGGCTGTCCTTCTCA-TGGACTTGGAGCTTCCCGAGTCCAGCGACTGAGATTTCTTC-----CAGTTCCCCT------------GAACTTG

Homo sapiens  TTCTGGAATAGCTAACCAAGGAATATTCGTTGAATGAGCAAA--TGAGTGAGGGAGTAAGTGAATCCATATT-CCTTAATATTTTCAGGCTTCAGGCTTATGGGCTCATGTGTATTTCCC
Mus musculus  TTCCAGAATAGGT--CCCGTCAATATTTGTGGATGGAATGAAACCTCATAAGGGAGTGAGTGGGTCGCTGTTACCTTCTTTTCTTCGGGCTTCGGCATTACAGGTTTCTGTTTATTTCCC

Homo sapiens  TGGTCAAGCATAGCACTGGGCACAAGGGTATTCTGGTGCAAGAGGTGCCTCTCACCACAGGGTACAAGAAGGACTAGCTCTGGCTGGGTAAACCAAAGCAGTGTTCCACAGAGCAAGGGG
Mus musculus  TGGTGCAGGGTAGCACTGGGGACCAAAGTGGTCTGGGGGAAGGAGTCTCTCTGACCACAGGGTACAAGGAGCACCAGTTCTGCTTAGGCCAAC-----CTGTGTTTTTAAGAGCACAGTG

Homo sapiens  CTTGATCCAGAAGGTTTCATTTTGTCATCGGTCTAAAAAGTAGCTGGACCTGTTTAGGACTTAAACAAACTTTCAGTGTTCCAATCCCATCTCAAATCCTTTGCAGAATGAGGCAGGGTG
Mus musculus  CTTGATCCAAAGGATTTCATTTTGTCGTCAGTCTAAAAAGTGGCCGGACCTGTTTAGGACTTAAACAAACTTTCAGTGTTCAAATCCTATCTCAAATCCTTTGCAGAACTACGCAGGGTG

Homo sapiens  TGGGGAGGTGGTGGTGGGGCAGTTATAAATAAACAAATACCCACAAACCGGCTGGTTTTTTAAACAGAATTTGCTTCAGTCAGTGCTGCACCACATTTTCCCAACTGGCCTCCGTCGTGA
Mus musculus  TGGGGAGGCAGAGAGAGGGGAGGTATAAATAAACAAATACCCACAAACTGGCTGGCTTTTTAAACAGAATTTGCTTCAATCAGTGCTGT------------------GCCTCCGTCGTGA

Homo sapiens  GAACAAGTGGGAACTTTCATACTGAATGGCTTTA--------------TTTTTATTCTGACCAGATGAATGGCAGGAGTTTTGGCTCGGGAGGCAGGTTTCCGATCCTCAGCACACAGTT
Mus musculus  GAACAAGTGGGAACTTTCATACTGAATGGTTTTAGCCCCCCCCCCACTCCCCCTCCCCGGCCAGATGAATGGCAG--TTTTTGGCTGGGGAGGCAGGTTTCCAATCC-CAGCCCACAGTT

Homo sapiens  TGCTGCCCTACTGTGCCCCGTCCATCACACACAGCGCCCTGCACAACATACCCAGTCATTGCATCTACTGCCTCA--GCCCTTGAAAATAATTGGGTCATTGTTTTATTACACCAGCGAG
Mus musculus  AGCTGCCCTACTGTGCCCCATCCATCACACACAGGGCTCTGAACGACACACCCAGTCGTTGTATCTGCTGCTCCAGGGCTCTCAGAAATAATTGGGTCATTGTTTCATTACACTAGCGAG

Homo sapiens  AGAGCTGCAAGCCTGTTAGGATTATGTCATTGATATTTGCGTGAAAAGGCAATCATGATATGCTGGTCATTTAACACAGCTGGGAATGCAACTGTGTTGTCATTGTTTAAGGTTCTCATC
Mus musculus  AGGGCTGCGAGCCCGTTCGGATTATGTCACTGATATTTTCGTGAAAAGGCAATCATGATATGCTGTTCATTGAACACAGCTGGGAATGCAACTCCGTT---ATTATTTGAGGT-------

Homo sapiens  CAGCCCCTCTGACTTTTTTGCACAGCCCAGCCCGTGAAAATGGATCACTCTGATGTATGT--------CACCTTTAAAATGCTTTCTAGCCCTTCAAGGGACCAAGCAGGATTTAACTCT
Mus musculus  -----CCTCCACCGTTGCTAAATAGCCCGGCCACTGTGAATGCATCTCCGTGTTGCTTGCTTGCCTGGCACCTTTGCAACACCTCCTCATGCGTTGAAAGCCCAAGCAGGATAGAACTTT

Homo sapiens  TTTCATGCACTTGGACTTTGCTGCTTGTAGGCACAGCAGTCTGAAGGTGAGAGATGCTTGCTTTTCATACTTGTGGGAGTTGTACAC--CCTAGTGGCAGGATGCTGCCATACAGTGTCA
Mus musculus  GTTC-----------CCTTA---------------------------------------GCTTTCCATGTGCAAAGGAGCCATGCACTGTCTAGTGGCAGAAGGCTGGAAGCCAGGGTTG

Homo sapiens  TTTGTTCACTAACTGCTGATCAGAGCCAAGGACAATTTTGGCTCAAGAACAAATGAGACC--TAATGTCTGTGAGGGAAGACTATGTAGATAAGTCACGGGTCAGAGTTGTGTTGCGACT
Mus musculus  TTTGGCCTTTAACTGCTGACTGGAGCCAAGGACAGTGCTGGCTCAGGAACAGAGAGTGTTGGTGATATTTGTGGGACAAGATTCGGCAGATAGGTCATACATCAGGGTTCAGCTGTGCCT

Homo sapiens  TGTATTGTGTGCTAGTTTCAGTAGGACTTGTGCTTTCATTGCAATTGTCAGTATGCTTAAGCAGGGGTCTCTGCTAATGAAATTCGAGCATATTTGCATTTATCGACAGGTATTACTGAT
Mus musculus  TCTCGTGAGTGCTAGTGTCAGCAGAGCCTGGGCCTTCCTTGTAGTGGCTGTGAAACTTGAGCCAG------------------TCAAGAATATTTGTGCTTATCAGCAAATAGCCTT---
```

Homo sapiens GGTAGAACATCTTATTTCCACACAAGGCAATGAGTAACATTGATTATAGAAGTGTAAACAAAGCTCAAATTCATGTGCCAATTTGTCTATTTCAACTCATCGATCCTCTTCTTCCACTGG
Mus musculus ----GCAAATTATTCTTCTAAATAGGGACTTTATTTGTTTGGATTATAGAAGCATAAACAGAGGCCAGGTTTGGATGCTATCTTTCTTATTACAGCCCATCAGTTTCCCTCTCCTACTGC

Homo sapiens CATGCTAGCCTGAGTTTTATGGCATAAGGCCATTAAAGTATTCTTCAAAGGAATCTGTTGCAGACACCTGTTCATCCCTCTTCTGGTCCCGGTCCTTGCCAAACCAGCTCTGC--CTTTG
Mus musculus TGCGCTAGCTGGAATTTTAGGGCATGTGGCCATTACCATACTCTTTAAAGGAATCTGTGGTAGGCACCTGTCCATTCCCCTTCTGGCTTCAGTGTTTGCCAAACCAGCACTGTAGCCTTG

Homo sapiens CCAACCACCTTGAGGTGTGACAGGAAAAGCTGACCCAATAAGG-GAGTCAACTGAAGGGATGGAGTGGCTTAGATGCAAATGGCCAAATCCCAATGGCCATGGCTATAAGGAACACAGGT
Mus musculus CCAGCCACCTTGAGGTGTGACAGCCAGAGCTGGCTTGATAAAGCAAGTTTGCCAAAAGGATGGAGTGGCTCAGATGCAAATCTTCAAGTCCCAGTCACCATAGCTCTAAAGAGCTCAGAT

Homo sapiens TCCTGAAGTTTTCTTGGCAACTAGCTCTAACTGCTTTGCCTTCTGAGTGCTTTGGTATTTGTTTTATTCTAAGTTCCCAGTTTTGATCTATTTTTGCCTTATGGCTGCA----GATTCCC
Mus musculus TCCTGAATGTTTGTTGGCAATTGACTCCAACCACTTTGCCTTCAAAGCATTTTGGACTTCGTTTTGTTCCTGGTTTCTGCTTTGGCTCTGTCTTTTCTTTCTGGCTGTAGTTTGTTTCCC

Homo sapiens TTTCTGGAAAATGGGGCTCTGGCTGGGATCAAATCAGAGAACACACCCAGACTTGGCATAGTGCCTGGCACAGAGTAAGTGCTCAACAAATGGTAGCTGTTGTCTCTTTTTCCCTGTCCA
Mus musculus TTTCTGTAAAATGGGGTTCCTGCTAGGAGCAAATAAGAAAACACA----------------GGTCTGGCATGGAGTAAGTGCTCAATAAATGGTAGCTGTCAACTCCTTTGCCCTGTCCA

Homo sapiens GGTTTCTTCTGGTTCTGTTCTCTGTACCTCCAGTATTGGCCCAGAACCAGGAACTGTTCCCTCCCCTGACTGGCAAGTCAGATTACCTGGCCCTCAGACAAGCTGGGCTCCCTTTTCTGG
Mus musculus GACTGCTTCTGGCTCTGTTCTCTGTATTTCTAATAGTGGCCT-GGATCAGGGCCTATTAACATTGCTGATTGGCAGCCTAGGTCATCTGCCCCTCAGACAAGTTGGGCGTTCCTCTCTGG

Homo sapiens CAGCCTTTTGTTGTCCTTACCTGTTGCCATGTGCCACTCAGTG-TAACCACAGGCAGACCTCTGCCAATTGCTGACATTAACCTATTGAATCCTGGCAGAGAAGAATAAGTTTGCAGACT
Mus musculus CAGCATTCTGTTGTCATTACCTGTCGCTGTAACACACTCACTGTTAACCACACGTGGACGTCTGCCAGCTGCTGACATTAACCTATCAAATCCTGGTA---CCTAATAAGTTTGCAGACA

Homo sapiens GGAATTTGCATGTCTGGGCCAGATACGAGACCAGGAGGCAAAGCCATATTTACCTGTTC-TCCCCAGTTGTG-GCAAATCACCCACATTCTGGTTAAA-------------ACCAGATG
Mus musculus GGAATTTGCATGTCGGGGCCAAATATAAGACTAGGAGGCAAAGCTATATTTACTTTCCCTTTCCCAGTTGTGAACAGATCACCCATATTCTGGTTAAAAAAAAAAAAAAACCCAACAGATG

Homo sapiens TTTCACACCCATGACTGCAATGCCACACATCAAACAGTTGCTTACAAAGAACACAAAGTGGTTTTCACATCGCAAATGTAACTTGCAGGTCACTTCTCAAATTTCCTCGCAGCAGCTACC
Mus musculus TTTCACACCCATGACTACAATGCAACTTAGTAAATAATTGATTACAAAGA--GCAGAGTGCTTTAGTAATCAGAAATGTAACCTCCAGGTCACTTCTC-AATTCCCTAAAAGTGGCTAC-

Homo sapiens ATGCCTGGGTATTTTTCCCTTAAAAATGACAACCTTGTTGGGTCTCTTGTAGGGACTTTCATTTGGAAGCCCTGGGAGAACTTATATGCTCTTTCCAAGAGGGTCTCTGATGGCTTTAAT
Mus musculus -TACATGGGTAGTTTTCGTTTGAAAAGGACGGTCTTGTTTGTTATGAAAAATGGGTTTTAATTGGGAAGATCATGAAGCACTTATATATTCTAGGGGCGGGGGT-TCTGATTGCCTTACA

Homo sapiens TCACATGGAGTTAATATATTGGAAAAGAAAAGAGATCTTCCCCAAACTCACCTGCTC-CTTGAAAATGCTATTCTGTTGCCTTGGCGAACAGCCAATCCAAACATTAAAAAAGAGATAGA
Mus musculus TTGCAGGGAGTTCAGATAGTGGAGAAGA---------------TAACTCACCTGCTCTTTTGAAACTGCTGCTTAAGCACCTTGGCAGATGGCCAATCCAAAAATACAGACAGAGACAGG

Homo sapiens GAATGCAGGAATCCTAAATGCTATTCTCAGTGCATGTCCAAACAAGAACTGGGGGCTGAGGGGCAAAGAACAGA----AAGAGGAGAAAAGAGTTTTTGTAGCCTAGGCCGTGAGTTGAT
Mus musculus CAATGCAGGAATCTTGACAGCTTATAAAGGTTCGTGCCCAAACAAGAATAGGG---TAAGGGGGAAAGCACAGAAGAGAAGACAAGACAAGACTTTTGGCGGCCTTGGTTTTAAGCTAAC

Homo sapiens CCCACCCCGT-CATGTCCTGTACCCTTCACGCTCATAAAGAAGACAGAGGAGGCCGGGCGCGGTGTCTCATGCCTGTAATCCCAGCACTTTGGGTGGCCGAGGCAGGCGGATCACCTGAG
Mus musculus GCCATCGCACACAAATCTTGTTCCCTATATGGTC--AAAGAGGAC...

Homo sapiens GTCAGGTGTTGGAGACCAGCCTGGCCAACATGGCGAAACCCTGTCCCTACTAAAAATACAAAAATTAGCCGGGGGTGGTGGAACATGCCTGTAATCCCAGCTACTCGGGAGTCTGAGACA
Mus musculus ....

Homo sapiens GGAGAATTGCTTGAACCTGGGAGGTTGAGGTTGCAGTGAGCCAAGATTGTGCCATTGCACTCCAACCTGGGCAACAAGAGTGAAACTCGGTCTCAAAAAAAAAAAAAGAAAAAGAAAAAG
Mus musculus ....

Homo sapiens AAAGAGGAAGGAGAAGGAGAAGAAGGAGAAGGAGAAGGAGAGAAGAAGAAGGAGAAGGAGAACGAGAAGATG--TGGTGGCATCATGAC-------TTCTCTGCTAGGGTTAATTGCTCA
Mus musculus ....AGAAGATGCGTGGTGGCATGGTAACAACAGAGTTCCCTGCCAGGGTCAGTTGGTCA

Homo sapiens GCCTGAGAGGCTCTCCAGGCTGG-CAGGTCACACTAGAGCCAAGAGTGAAGGGTCCATTCAGTAAGGTCACTGCCATGATCAGTTCTCAACCACTTTAACTCAGTTTTTCTGTTGCTGCT
Mus musculus GTCTGAAAGCTTTGCTGGCCTGGGAAGGCCACA---AGGCAAAGGCCACCGGATCAGTCTAGT----TTGCTACCAGGGTTGGTTATCACGCACATTGGATTTTTTTTTCT----CTTCT

Homo sapiens TTGTTGG--TTAGTGG------TTTGTTTGTTTCTACCCATTTGGGAGAAGGGTAGGAAAGGAACTAAGGTG--ATCAACTATTCCATATGTGTCAGGCCCTGTACTTGTCTTTTCAAAC
Mus musculus CTTGTGGATTTGGTGGCGATCCTTTGCTTGAGTCTCCTTGTTGGCGAGCAGGGTAGGGAAGGAA--AAGGTACAATGAGCCACTCTGTACTTTCTCG--CTTGTGCTTGTCTTTGCAG-C

Homo sapiens ACTTATTCACACTCTGAGGGACTCTCTCAGGTCCAGCAACTGTGTAGCTATGATGTGAAATAGCAGCTTCTCTCTATGCATTCACTGGGAATGGTTTGTTCTGATCACACAATTTTTTCC
Mus musculus ACTCCTTCCTAATCTGGGAGACTCTC--AGGTCAATGAGCTGTGTGGTTTCCATGGGCAAAGCAGGCCAGTTTCTATGCACTTACTGGGAATGGTTGGCTCTTGTCAAGCCAATTTT-CT

Homo sapiens AGTTCTTCCGACGTTAGGTGTTGCATGGTAAGAAACAGGTGGTTAATTATTGAGACAGTTCTTATTTGGAATTGTCTCTTCCTCCTTCTCCTCTCAG-------GCAGATGCTAAAGGAT
Mus musculus GTTGTTTTCAGTGTCAGATGTTCTATGCTAAGATGCAGGAGCTTAATTACAGAACTAGCTCTTTTTTGTAATTGCCTCTACCTCCTTCTTCTTCCAGATTTAATGCAGATGCTAAAGGTA

Homo sapiens GTCTGTAGTTT-TTTCATGGCATTTTGCTAAAGATGCACATTCATTAATTCATCAAATAGCTTTTTTTGAGACATTATTATGAGTGAGGTGTTGTGCTATGATATGCTTTATATACCATA
Mus musculus GTCTGTCGTTTCTTTCATGCCATTTTACAGAAAATACCCATGCATTAACACATCAAATAGATTTTCTGAGTCCCTTATTACAGATGAAGTGCTGTGCTACAGTGTACTTTTTACGTCGTA

Homo sapiens TCTTATTTCATCCTCACCACAAAGCTATGAGGTAGGTACTATCATTATCTTAATTTTACAATGAAGAGACTGAGGCTCAGGAAACATAATGCAAGGTCACACTCAGTTAATAAGTTGAGA
Mus musculus TCTTATGCTATCCTCACCA--GAGTTGTGAGATGGGTGCTATCATTATCTTAATTTTACAACGAAAACACTGAGGCCCAGAAAGCATAATACGCAGTCACA---AGTTCACAAATGGAGA

Homo sapiens GCTAGGATTCAAACCCAGGTTTATCTGACTCAGTTACACTACACTTCCTCCACATAGCACATTGTGGCCCGTCACAGCTCTTGAGGACAAAATTCATCC-TTAGAAAAGTTTTCTTACT
Mus musculus GCTGAGATTCAAACCTGGGTTTTTCTGCCTCAATTACAATACATTTTCTCCATACAGTGCATTTTAGCTACCCAGTATTCCTTGAGGACAACATTCATCTGTTGGCAGAATTTCCCTGTT

Homo sapiens CCACTTTTTGCTGATACTACTGCTGCTGTTGCTGCTGCTACTA-CTATTACTACTACTGCTACTGTTTCTCCTCCTCTATTTATCCAGAACTGCATTTTTCCAGCACTCCTAGGTGCTTT
Mus musculus CTGCCTCTTTCCCTTTCTCCTCCTGCCCCTTCCTCTCCTCCAATCCCTTTCCTCTTCTACTTCTCTCCCCTCTCCTTCTTTCCTCCTACTCTCTCCTCTTCCCCCTCCCCTTCGTCTTTT

Homo sapiens ---GAACC--CAGCTACC------------AGTTGTAGAGTTCAAACCCAGGC----------CTGCATGAGTCCCTTG
Mus musculus CCTTCTCCCCTTCGTCCTTATCTCATCCATCATTTTGTTTATTGGGACTGGCTGCTACCTCATACCCTTCTAATGACAGAGCTCAAAGCCATCTGCTCTCACCACTGTGCGAATTCCATT

Homo sapiens CACCCATTCTACTTTTCTGCACTGCTTGTAATGTAACACAGGAGGTATGGGACATCCATAACTCAGGTAGGTAATTGACT--TCAAGCTTAACATTCACTCACCAATCATTTATTGAGCA
Mus musculus ------TTCTTTTTGTCTATATTGCTTGGCATATGCCATGTAAAATGGGGGACATTGGCAACTTGCATTAGTTACTGGCCAATATAGCTGAACATTC---CACCAAACATGAACTGAGTA

Homo sapiens CATATTAGATAAGCAACCTATGGCTCCTGCCCTGGAGTTGTTCATAAGACCTTCCAGGCAGACAGCTATGGGACGAATACACTTGAATTTATTAAATCTACCTCAGGCAAAAAACCAAGC
Mus musculus CACATCAGCTGGCCACGCTGTAGTCCTTGCTCTTCAGTGGGTCATAAGAGCTTGAAGGAAGACAGACACTGGAGACACAAATTTGAACTTACTGCAATGTGTTTGATCTAAAAGCCAAGC

Homo sapiens ATG----TAATTATAAGTAGTATATCAGAGTATGTGTAAATGGGGTTCTGTAAAATGTTTGAAAGAGGCAGGCCTGGGTTAGTGGTGAGAACAAGGATCAAGAAGGCTCTGCCACCAAAT
Mus musculus ATGACCTTGGTGTTGAGT-GTATATCAGGATATGTGTAGACAAGGA-CTGTAAAAGGTTTGAAAGAGGT--GCTCAGG----------AGGCAGAGGTCAAAAAA...............

Homo sapiens AACAACATGAGTGTTGTCCCGTTAATCTCTCTGGGCTTCTGTTTCCAGATCAAGGACCTCAAAATAATAGTCATAGTCACCATTTATTGAGTGCTTTACTGTTAACCAGGCAGTATGCTA
Mus musculus ....TTCTGT--CAAGTCTGAGGACCCC..

Homo sapiens GGTGGTTTTCATTTAGTCTTGACAACAGTATTCTCGCTTTCCCTTTTCTACAGCTGAGACTCAGAGACATTAGGTAATGTCTAGGTCACACAGCTCTTCAGTGACCAGTTGTTTCTATGT
Mus musculus ....

Homo sapiens CAGAATCCTCTTATAATCACTGAGTTGTATCGCTTCCCTTGCCTCCTGCTCCCAAGTCAAGTATTCTCTACTATGTCGCCTGAAAATTAAAATTAAAATCAAGGTTAATCTTGTTCAACT
Mus musculus ....

Homo sapiens GAGCCAAGAGGATAAACATTAGAAATAGCAATTAGTAGGTAGAAATGTTGCCAGGAAGAGCTATACAAAAGCATAGAAAGGCAACCAAGGGAGTTAATGATG--GGGGTGTGGCAGGGGT
Mus musculus ....AGGCTAGGAAAGTGCTCAAGTCAGTTGCCTATGATGAAGTGTGAT---GGT

Homo sapiens TGCATGTTGATGGGTAATTGGATCCTGGCCAGCTCTTACCTTCTTCCTTCACCTTCCCACTCCGCAATTTCTCAGGCTTCTCTAGAGAGCACAACCCACCTAGAGACAGCCTGTGTTGCC
Mus musculus TGTGCATTAATGGTTGCCTGAAT--TTACTGTCTTTTTCCTTCCTTCTCTGCCTCCTAGCTCCACATTTTCTCAGGCTTCTCTGGAAAGCA-AGTCCATCTTGAGCCAGCTTGGGCTGCC

Homo sapiens ACAGGAGGGCACCAGGCCAGGAATGGCAGGCCTAGGGCAGTGGGGCTCTGGGCTGGGAACCATAAATAACCACTTTAATGAAAACCAGCTTTGGCCAAAAGC---CTTATCATTTCTTAA
Mus musculus ACAGG------------------GGTAGGCTTAGGGCAGTGGTGTGTTG--CTGAGGAC-ATAAATAAGCACATTGATGAAAACCAGCTTTGGCCAAAAAAATACTCATCATTTCTCGA

Homo sapiens GAGGTTCTGTGCTTCTTCTAATGACCTAGTTACTTGAGAAAGGCCTAGCCTAACATATACAACAACAATAATAGTTGCCCTATAGTGAGTACTTACTATGGGCCAGGGCCTGTGCTAAAC
Mus musculus GATATTTGATGCCCCTTAGAGTGACCCAGTTACTTGAGAGAAGCCTAGCATCACATATGCA-CAAGGACAA--GTTGTCATTTAGTGAGCACCTACTA---------------------

Homo sapiens ACTCACTAATTCATTTAATCCTCACACAGCCCTACAGGAGCAAGTACTATTATTTCCCCCACGTTACAGAGCAGGCATCAGGCTGAGAGAGGCTAAGTCATTTGCTCAAGATCATATAGC
Mus musculus ACTTACCAA--------GTCCTTACTCAATAATACA----CAAATA-------TCCCCCCACTTTATGCAGTAGGTATCAGGTTTAGAAAGAGTCA-TTATTTTTCCAGGTGGGTTCAGC

Homo sapiens TGGTTGGAGCTGGAATTTGAAGCCTGAAGCATCTAGTTCCAGGGCCTGAGCTCTTTAATTCCTATATTTAATGAATGTCTGTCGAAGGAATAAA----CAGATGAATGAATAGACAAATG
Mus musculus TAGTTAGAGTTGTAATTTGAAGCCCAAAGCCTGGATTTCCAGGGCCAGAGCT-GTTAATTCCTCCATGTAATGAATGTCTGTTGAAGAAAGAAAGAAGCAGATGGATGAAAGGACATGTG

Homo sapiens TATTCATTTCATTTCACTTTAACAAACATTTATTGAGTGCTCTGTGTCAGCTCCTGTGCTAGGCATTGAGGATTTATAAGTTTAGTGCGTGCGTGCGCGCACACACACACACACACAAAC
Mus musculus TATTCATT-AACTTCACTTTAATAAACATTTATCAAATGCTCTGTGTCAGCCCCTATGCCAGACATGAA--------------AGCACGCACGCACGCACACACACACACACACACACAC

Homo sapiens GCACACACACACACACACACATCCTGCATGGCCCTTGCTCTCCAGGGGTTCACAAAAGCATTCTCTATTGTGGCTTGATTTCTTCTGGAGAGAAGTAATTTGAATGTCTTGAACAGCAGT
Mus musculus ACACACACACACACACACACACACTACATGGCATCTACTATAAAGGAGCTCACAAAACCACTTTTTACTATAGCATGACTG----TGGATAGAAGTAATTTGAGTTTCTTGAATAGCAGT

Homo sapiens TTTTAATGATGCTA---TTAAATTTAA------------------------------CCTAAAGCGGGGG-TTTTGCACCGGTGAGCAAAGTGTTTCAAATGAGAATGTGTCTTTGATTA
Mus musculus TTTTAATGATGCTAAGTCTCAGTTTCAGACCCCACCTCTATTTCTTTCTCTCTCCTTTCTGTAGTGGAGGATTGTGCTCCCACCTGCAAGG-GTCTCAAATGA--------------TTA

Homo sapiens ATCATCGATTCAGCCAGAGAATAGTTA----CTACCAGAATGGAGCTGCAACTCCTATTATATTAGCTAACTCTTCTTCCATCTATCATGTCTCTATCTTGTCGAACCCTCCCACGATAC
Mus musculus ATCATCAATTCAACCACAGCATACATAGCCTTTAACA-AACAGCCCTGCAACTCCTATCACGTTAGTTAACTCTTCTTCCATTTATCATGTGTCTACTTTGTTGAATTCTTCC-TAGTAT

Homo sapiens AAAAGTTATCTG-TACCCTAATGGACAATTATCCCATCCATCATTCCTGCTGGTCTTGTTGATTGCTGGTAGTCTGCTTGCCCACGGAGGAGACTTACAAATAGTCATGACCAGTGACGC
Mus musculus GAAAGTTATCTGTTACCCTAATGGACAATTATCCCATCCATCACTTCTGCTGGTATGGCTGGTCCCTAGCAGTCTTCTT---CATAGAGGAGGCTTAGACATAGTCGCAGACA-TGATAG

Homo sapiens ACTGGAAAACTGCTCA-TTTTTAGACATTTTAAATCTATGTAGAATGAATTATTTTTCAGGCTGTAC-TTAAGCTGCATTTGCTTTGTTGGTTATGTAAGAAGA-GATATGAATTCCTTA
Mus musculus ACTGGAAAATTGCTCAGTTTTTGTACATTTTTCATCCATGCAGAATGAATTCCTTTGCACGCTGTCCTTTAAGCTGTGTTTGTTTCATTAGTTATATAAGGGCATGTTTCAAATTCTTCG

Homo sapiens CCTATGCGTGAGACATGGC----------------------------GTCTGAATATTTTTAAGTTCCTGTGGGCAGCAGCCCTATACATATTTGTTAAAAT-------GATGAAAGAAT
Mus musculus CCTGGATGAGAAACATATCTTTCCATTGGCTTGTTTTTGTAGACACGGTCTCACTATTTTCAAATTCCTCTAGGCAATAGCATTCTACAACTTTGTTAAAATAGGAATGGATGAATGGAT

Homo sapiens AAA-----------GTAAACATACCTAGAGTAATTCCAGAGGAGCAGGCTTCATACCTGGAGCTCGGGTGGGTGGCCATCATGTCAGGGTAGTCACCGTCTTCCTTTTCCTTTCTAAGTA
Mus musculus GGATGGATAATTGCATAAGCATTCTTAGGGGTATAGCAGAGAAGCATGTTTTGTACCCAGGGACCAGGTGGCTGATCTTCCTGTCGGGGAGATGGCCG-CTTCCCTTTACTTTGCATGCA

Homo sapiens CTTTGAGGAGAATCCCAAGTTGATTAGGGTCAAGGTGAGGACTCATGGTGCTGAAAATAGGGATTGAGTGGACAATGAGAGATGCCTGTTGAATTCCCTGGGACTATTGCTATGAGATTT
Mus musculus TTTGGATGGGACTCCCAAGTTGAATATGGTTAAAGTGAAG-CTCATCGTACGAACAGTAGAGAAT---------------GTTGCCACTTGAAATATCTGAGATTCTCACTAC-AGCCAT

Homo sapiens GAGGGCAGAAATGCCCCCCTCTCCCATGTCCTACCCTAAGCCT----GGTAACACATTTAACTTTTTGTTACAGTAACTACCATGCCTAGTAAAACTGGAGTCACTGGTTGCCCAGGGCA
Mus musculus GTGGGTAGAAATGCTCTCCTCCCCTATGTCC--ATTTAAGCCTGCTAGCTAACACATTACACTTGTCTTT..

Homo sapiens AAAACCCAAGAGTTGTAGTTCACTCATCTTTATTTCTCACCTCTACATTCAGCCAGTCACTAAGCCCTGCCAAGTATATTTCCTAAGCATTCCTTGCATGTCACCTCTTTTCTCCATTCC
Mus musculus ....

Homo sapiens TACTGCCAGGGCTTTAGTTCAGGACTCCTCCCACCTCCAGACATCTCTTGCCTTGATTCCTACAATACCTTCTGGTTTTCTCACCTTCTCTCCTTAGTCCTCTGTGACCATTCTCCCCAA
Mus musculus ....TAACCAGTATCCCCAG

Homo sapiens AGTGGCCTGAGTGATCTTTCAAACATGCAA-ATCAAATCATTTCACACCTCCATCCCCTCCACTTAACACTTGTAAATAGCCTCACAACACCTGAGACTTGAGGCTCTCAAAATGTGGGT
Mus musculus AG----CTGTGTCTCTAGTTGCATATGTAACATAAGATCATTTC----------------TACTTAATCCTTGCAAATGGATTTTCAACATCTGAAACTAGAGGCTCTCAGAGTG-AGAT

Homo sapiens CCTGGGGCCAGCAACACCAGCATCTCTGGCAACTTCTTAAAGTTAGAGTACGTACTCATTAGAGTACCTACTGAATCAGAAACTCTGAGAGTGCTCACCTGACTCTGTGTTTAACAGCCC
Mus musculus CCTAGGAGCAATAGCACCAGCCATTCTGACAACTTGTCTGAGTTA-------------------------CTGAATCAGATATAGGGAGTGTCCACCTCTGACT-TATATTTAACAAACC

Homo sapiens TAC-AGGTGATTCTGATGAGCACTCAAATTTGAGAACTGGCACCCTAGAGCCATGGTTCTCAATCCTGGCTGCACATTCAAATAAATGGAGGGAGCTTTT-AAAAGTTCTAATGCCTCTC
Mus musculus TTCTAGGTGATTTTAATAAGTACTAAAATT-GAGAACTTGCCTCCCTGA------------AGTTGTGACTGCACACTAGAACAAACTGGAAAAGCTTTTTAAAATTTCCAGTGCCTTTC

Homo sapiens CTGCAGAGTAATTAAATCAG-TTTCTAGGGGTGGGATCCAGTCATAAGTATTGTTTTTAAAGTTCCCCAGGTGCTTCTAATATACAGTCAAGGCTGAGAACTACTGCTCTATAGGATGAA
Mus musculus CCACAGACGAATTAAATCAGAGTTCCTGGTGTAGGATCCAGTCATCAATATT----TTAAAGCTTTTTAGGTGATTCTAATATGGAATGAAAGCTGAGAGCTACTGTA----AGTATGAA

Homo sapiens GTCCAAACTCCTTAGCTTGGCAT-TTAAGGTCTATCTCTTTGGTCATAACCACCCCCAACACTATGCTCTTGCTTGTTCATTCTAGAATTCAATCACAGGGAGTCATTTGCAATTCTCTT
Mus musculus GTCCAAAATCCTTAACTTGGCTTCTTAAGGCCTATTTCCCTGGTCAAATCTTTCC--------ATGCTC-TACTCAGTCATTCTGGAATTTCATCACACAACACCATTAAGAATTGCCTT

Homo sapiens AGAGCCACACATGGTCTCTTACCTCTGTGCATTTGCACACATCATTCCCACTGCTGTGCCCATCCCTTCCTCCCTCCCTTCACCCAGCTAACACCATCTCATCTACAACACATAGCTCAG
Mus musculus AGAGTTATGCAT------------------ATAGGCACACATCGCATGCAGCGTCTAGCCTTTACTCCCCTGCCTCTCTTTACCTAGCTGATATTTTCTCATCCACAGAAAATGATTC--

Homo sapiens AAGACACCTCTAAGGAACAGTTTTCCCCGCCTGTCCAGTCTGGGTTAGATGCCCCTCCTCTGTACTCCCACAGCGTCCACAACATAGCACTGGATACATTCTCTTGTTCTTCTCTGGTTA
Mus musculus ------CTTTTGTGGACGATACCTACCTGACCTACCACCCCAAACTGTAGGGGTGTCTACAGTGGT--CATATCACTCACTACCTG-----TGGTATGTCATCTTGTTATCCTCTGGTTC

Homo sapiens GTTGTCTACTTTCTCTACCAGTCTGA----------GAGCTAGGATCCCCA-CCTGGCACACAG-----------TAGATGTTCCATGAT---TCTGTACATCCTGTTCCCTCTTTTTGG
Mus musculus ATTGT---CTTTCTTTACCAGCTTGAGCTGGGATGGGGGCTATGATTCTCAGTCTACAATACAGTATCCAGCTAATAGTTGGTTCCTAGTAGGTTTCGATATTTGGTTGGCTCTCCCTGG

Homo sapiens AGT-------GACTTTCTGTGCCTTG---------------TCTGTGGC--CTTCAGGATGCAGCTCAGATATCACTTCTTGGGAATCTCAGAATTCTCTGTCTGG-------ACCCTTT
Mus musculus GGGATGGGGAGGTGTTCTTAGCTTTGCTCATCAGGTAGAGTTCTATTCCAGTTTCAAAGTGCAGCCTAGATGGTGCTCTCTGAG--TTTTAAAAT--TCTGTCTGGGTTAGGTACCCTTC

Homo sapiens CTCTAGGCTCCCGCAACAATCTAAATGTAATCTGATCCTAGCCCTTAGCACACAGGGCTATCAGGCTGCCATAGTCTGTTTACTTATCTGCCTCCCTCACTGAACTGTAAGTAACTTGAG
Mus musculus CTTGAGGC..

Homo sapiens GTTAGGGACAGAGCCTTGTACATTCCTGTATCCCAGCTCCTAGCACAAAGACTCACACATTATAGGTGCTCAGTAGCCATAACCATCTGCAGAACGAATAACTGAATCAATGATGACCTT
Mus musculus ....

Homo sapiens TTCCTCTTCTCCATATCATCAGCCACTATTTCTGTCCAGGCCCCCGTTGCTTTATTGCAATAGCCTTCTGACTATTTCCCATCTCTCCTTTCTCTGATGCCATCTGAACACTCCCCCCT
Mus musculus ....

Homo sapiens CTCCAAGATGTGGCTTTCTAAAACCACAGTCACCCTATCCCTCCCCTGCTCAAACACTTTCAGTCATTCCTCATTGTCTGCAGGGCAAATATCAAAGTGGAATTCCAGATCCTTCCTTCC
Mus musculus ....

Homo sapiens CAAGAGTGGTCTGCCTTCCCAACCATACCTCCCCCATTCCCCTATGGAATACCAGCCACATCATCTGAGGGTCCTGGTGCAAAACGAAAATGTGGAACCCCGTGTTAAAATATAATCAA
Mus musculus ....

Homo sapiens GGATTTCAAGATAGCAGCAAAGGAGAGCATTAAACCAAGCATAGGGCTCTTCTGACTTCAGGGTCCGATGCGACGGCACAGGTCTGCCCCTACGTGTATCCAGCGCACTGGCCAAATGCA
Mus musculus ....

Homo sapiens CTTGCTCCTCCTTCAACTGCTCTGTAGGTTCACTTTCTCCACGGCTTTTGTCAGGCTGTTCCATCTCTTCTACCCCACTAGCCAATTCAAAGCTTGTCTTCCCTGTAGATCCACCCTGTT
Mus musculus ....

Homo sapiens CTTCAAGAATTGCTCAATTCTCATCTCCTACTGTTCCAGACCACAGTAGTTTCTACCACACAGCCTGATATTAATAATTATTTTTATATGTGTGTAGTCTTGCCTGTCTACTGAATTTTA
Mus musculus ....

Homo sapiens AGCTTCTTGAGGGCAAGGACTCTATTTTGTTCATCTTTTTTTTTTATAGACTTAGCATAGTACTTTGTAATCATCTCACTTGTTGAATGACTCAGGGAAGAAATAATACAACTCCAGTCA
Mus musculus ....

Homo sapiens CTGAGTTTGCCAAAAAACAGTAGACACTGCAAACCTGATTTAAATCCCTCATTATTAGCTATGCAACATTGAAGGAGTCACCTCACTTCTCTTTGCCATCTATAAAACGATGATACTTAG
Mus musculus ....

Homo sapiens AGAAAAAGTGTTTTTTTTTTTTTTGTTTTGTTTTTTTGGTTTTTTTTGTTTTTACAGAGGCTTGCTCTCTTGCCCAGGCTGGAGTACAGTGGCATGCAATCTCGGCTCACTGCAACCTCC
Mus musculus ....

Homo sapiens GCCTCCTGGGTTCAAGTGATTCTCCTGCCTCAGCCTCCCAAGTAGCTGGGATTACGGGCCCATGCCACCATGCCCTGCTAATTTTGTATTTTTAGTAGAGATGGGGTTTCACCATGTTGG
Mus musculus ....

Homo sapiens CCAGGCTGGTCTTGAACTCCTGACCTCAAGTGTTCCACCTGTCTCGGCCTCCCAAAGTGCTGGGATTACAGGCGTTGAGCCACCATGCCTGGCTAGAAAAAGTA-ATTTTTTGTTAGTAT
Mus musculus ....AGAAAAGTTATATTTTGTGCTAGTAT

Homo sapiens CTTGTTCTTTGGCAATGTTTTCAAAATCTTTTGTTTTTTAATACATGTAAAACTTATTTTATATTTTATATCACCTAATTGCAATACCTGAGGTCTTTGAAGACCTGATTCTGTTTTCAT
Mus musculus CTTCTTCTATGCTAATGCTTGCAAAGGTTTTTGTTTTATAATGCCTTTAAAACATACTTCATTTTTC------CACAATTGCGATACCTGAGGTCTTTGAGGATCTAATTCTGTTTTCCT

Homo sapiens TGTTTCTGGCTTCATCGTGTTCTTTGTAATTTTGGATTATGAGTTCATGTTCAACCTGACTTTATCCTTGGTAATCCTATGAAAACTGGGTGGTGGGCATGTTCTTCCAGGGAGGATTTG
Mus musculus TGTCCCTGACTTCATTGTATGTTTTGTAATTTTTGATTATGAGTTCATGTTCAGCCAGACTTTA-CCTTAGTAATCCTACGAAAACTGGTTTGAGGGTGTGTTTCTCCAGGGAGGATTTG

Homo sapiens TGTTTGGTTCTTCCAAGTGCATCAGGGCACTATTAATCCAGGAGTTCTTCCAGTGAATTGTTTAGTCCCAAAGGCAAGGGCCATGTCTTCTTTGGCTATATGATCCCAGGGCTTAACTCA
Mus musculus TGTTTGCTTCATCTAGATGCTTCAGGGCACTATTAATCCAGCACTGCTTGGGAGGAATTCTTTAGTCCGAAAGGCAGGGTTCATATGTTCTTTGTTTATATAACTCCAATGCTTAGCTCA

Homo sapiens GGGACTTGTAGATGCTCAACATATATTTGCTGATTGAGTGGATGAATGAATGTGGTACTTTACC-GCTTCTTTTGGAAGCCCAATAAGAATTGTGACCTCTGGTATTAACAAAGAACAGT
Mus musculus GGAACTTTTGGGCATTCAGCATGTATTCGTTGATTGAACAGATGAGTAAATAGGGCACTTTCTCTGTTTTTCTTGGAGACCCAGTGAGAAATATAACCTGCAGTGCTAGCAAAGGTCAGT

Homo sapiens AGGCACATCAGGAAAATAATAATTACTAACATTTATTGAGCAATGATTATGTGTCAGGTATCATTCTAAGTGCTTTGTGTGTATTAACTCATTTAATTCTCACTATGATGCTGTGAGGTA
Mus musculus AGATCTAGCATGAAAATGGGAATTGCTAGCA...

Homo sapiens GGTTACTATTATTATCTTCACATTAC---AGAGGAGGCACCAGACTCAGACAGGCTGAATAACTTGGCCAAGGTCACATAGCTAGTGAATGGCGGAACACAGATATGAACCCAGGCACCC
Mus musculus .......ATAACCATCTTTCTATTTCCTGAGGAGGTGCACCAGGTGCAGGCAGACTGAGAATCTTAGGCAAAGTCATACAGTCAGAGAACGGAAGAACTTTAGTGAGAGCTCAGGACCTC

Homo sapiens TAGAGACACATCTACTTACCACCTTGTAT--TGGAGAAGAGGTCAACAGTAGTGA---CAGAAGCAATAGAGTGGAAATTTTCAGTACTGCCAAA---ACTATGAGAGCCATATAGT---
Mus musculus TAGAAGTACA---------------GAATCGTGGATGAGAGCACACTAGCAGTGAGTGCCAGGGCAATGGGACGATACATTTCAGTACTATGAAAGGGACT-TCAGTGCTACATAATAAG

Homo sapiens CCACACCAGGCATGAAAAGGAAGCTGGCATGGTCTTGTGTTGTCAGTTAACCCT-------TGTATATAGGGTTTTCATTTCCTAACTAGAGAAGGAGGAGTTTGAAAGCACGAACCATG
Mus musculus TCATATGAAGCACAGGATGGAACTTGACATGGTCTTTTATTATGAACAACACCAGTAATAATGTCTGAATGTTGTCTGCCTACCAGCAAGAAAAGGAACAGGTTGAAGGAAGGAACCACT

Homo sapiens AATTACAGCTCTTTGAAATGCTCAACATAGATTTGCTGAGTGAATAAATGACTGTTTTGAAATTAAACACAGGAGTGCTTTGAATAAATTACTTTGCATCTTGTATGTCAAACTGAAATT
Mus musculus GAATATTGGTCTTTATTTGACTCAGTATAGATTTGCTAAGTGAA--------CGTTTTGAAGTTAAATGT--GAGCGCTTTGAACAAGTTACATCCTGTGTT-TATGCCAAACTGAGATT

Homo sapiens GGTGAGACATCTTTCACCTTGTGTTAAAAAC--TAAAAATCCCTAGATGTGGTCATAACATGGCTGGTTATAATGACACCTTGCATTTGTACTTGTGTATGATACTCAGGACTCCCTGAG
Mus musculus ACTGATGAATCTTTCACTTTGCATTGAAAACATGAGAAACGTCTAGATGTGACTGTTACATTGACGG-CGTACTGACACCTTTCA--------TGTGCGTACTCCTAAGG-CTCCCATAA

Homo sapiens AAAAGGAATGAAGAAACTTGGATTTTGCCTTCATAGGCCCTGAGGCTGTCAGGAACCA-AGGGCCACTGTGTTTGTAATCCATTCCTTTGG-----CTGCTCTCCAAATCTAGGAAGACC
Mus musculus AAGAGGAAGGAAGAAACTTGGCTTCTACTATCTTCAGCCCT-AGGTTGTCAGAAAACACAAGGCAACT-------TA--CCATTTTTTAAAAACCCTACTATTCAAATTTAGGAGGGCC

```
Homo sapiens  TGGAGGAAAGGGGGGCTTTATTTTCT---ATCTTTGCCAAGATCAGGAAATAATTTG----TGAACTCTCAGTAAACATTCTGGGGGCCTTCCCTCTAACACTGCTTTGGTGGAATAATA
Mus musculus  TGGAGGAGAGGGGGCCCTTCTTTACTCTTATCCTTGCCAGAACCACAGAAGAATTTGAATGTGGGGTCTCAATAAACAGCCTGGGT-CGTCAGCTCCAGCACTCCTCCAATG-AATGAAA

Homo sapiens  TCTCCAGTTTTCACCAATCTTGGTTCAATATTTACCTG------GGGGTTTGCAGTGATCATGGGGACAGGGCTCCAACATGAAGTGAA----GGTTCA----TGAGAAGGAAAATGAGG
Mus musculus  CCTCCATATCCTACCAGGCTTGATTAAGTACTTACTTAATGCCTGGGGGCTGCAGTGAGCTTAGAGCCAGGGCTCTAAAGTGCAGAGGAATGGGGCTCAGGAGTGAGATAGAATCTGAGG

Homo sapiens  GGAAGGCTGTGTGTGTGTGTGTGTGTGTGTGTGTGTGTGCTGGCTGAGGGATGAAGGTGGGAGGAATACCAGGATTCTGAAGCAATGAGAGTGGGATTGGGAGAGAGAGCTCATGTTGAA
Mus musculus  TG----CTGGGTGAGGG-------------------ATGGTGGCCATAGGAAGGTGGCAGTGGCAATGCCAGAGCTGCAAAATAGTGAG---TGGATTAGGGAAGGGGGATC---TTGAA

Homo sapiens  GAAGAAATAACTCACCAGTTCACATGGGCTCCTTTTCAAG---GGTTTTTC-------TTTATATAAACAATTTGCCTGGTTCCAATTAGAAGCCCACA--TTAGGCAGTTAGCTCCAAT
Mus musculus  GAAGGAACAATACAC---TTTAGGTCAGTTCCTTTTTAAGACAGCCCCCTCCCAGCCTTTGCCAGAATCAATTTGCCTGGCTCTAGTTTTAGATTCACAGTTTGGGCGGTTAGTACCAGT

Homo sapiens  TTGGCAGTTCATTCTTAATTTGGCTTTCCTTATGGGTGGGAGGGAGGGCTGACAGTTAAAAGGTTGATGGGAAAAACCTTGGTATCCTGGTGTGAAGACAGACCCTGGGATCAGAAGGTT
Mus musculus  TTGGTGGTTAGTTTCCAACTTGACCATTCTTGTTG-TGTGTGGGGCAGCAG-----TGAGAGGATGAAGGGGAGAAATACGTCCCTCTCCAGTGAACAGAGGCCCTGGTATCAGGAATTT

Homo sapiens  GCCCTGATGACTTTGCCAATGTGTTCATTGCCCCTGTCCTGCTGGGGCGCATACAGCAGGCCCTTCTTTCTCATACGCCCTTG----ACAGACCAGGACTCACTGCTAGAAA---ACCCC
Mus musculus  GCCTTA------------------------------TTCTTCTAGGAGCTATGTAGTGGGTC--TCTCACGAATGTGCCCCTGTCTGGCAGATCATGCCTCATCATTAGAAATCCATCCC

Homo sapiens  TGGGGAAGTCATTGCTGAAGACTCTAGTGTGGGGCACATTAGGTTTGAGGAGCAGGGGAAACATTCAGGCAGCTGGCAAGGTCTGGGCTACCAGGAGAGATTTGGGAGTTAGAAGCATAT
Mus musculus  GGACAAGGTTATTATTGGAGACATAAATGAGGGCCATTTTGAGTCTGAGGAGCACCAGGAACATTCAGACAGCTGGAGCGGG-TGTACGTCTGAAAGAGATTGGGTACTTGGGAGCAC-T

Homo sapiens  TAGGTGAGAGTTGACACTGTCCAAAGGGAGTATATAGAAAGAAAAGCAGAGGAAAGGGCTCATGGAGTCATTACAGCCTGT-GAGGTGGGAA-CAGGGTTTGGGAGTACTAAGGGTTTGG
Mus musculus  TAGGTGAGAGTTGAAAGTGTCCAAAGAGAGTACATAGACAG----GCAGAAGAAAGGGCTTATGCAGCTATTATAGTCTGCAGGGGTGGGAAATAAAGCTTGGGAGTTGTAAGGATTTGG

Homo sapiens  GGAGGGTTTATGTGTTTATTCACCCATTCATTTATTCAACATTTCTTGAGCTTTATGGCAGATGCTGAGTTGAAGAGGAAGCTGGGGATTTAAGAGGGAGAAGAGTTAAGAGGGGATCCC
Mus musculus  TGAGGGTACATGTGTTCATTCATCCATCCATTCATTCAACATTTATTAAGCTTTATGCCAGATATAGAATTGGAGAGGAAGTTGGGGATTTAAGAGGGAGAAGGGTTGAGAGGGGATTCC

Homo sapiens  TGGGTGGATCCAGATGAAGGAGAAGGGAGGGGCTAAGTGCCTAGGCTCGCTCTTGCCAGGAGAGCACATCTGTTAACACAAAGCTGCATCTGTATCTCAGCCAGCTCTCTTCCAT--TTC
Mus musculus  TGGGTGGATCCAGATGAAGGAGAAGGGAGGAGCTAAGTGCCTGGGCTCCCGCCTGCCAGGAGAGCACATCTGTTAACACAAAGCTGCATCTGTATTTGAGCCAGCTCTCTTCCTTGCTCC

Homo sapiens  ATCTCCTCCTCTCTACCCACACTTGCAAGACGGGGAGCCACATGGCGG-CTTCCTACTCCCTTGTACCACTACACCCCCT--------GCCGGGTTCTTTCTAGGCATTTAGCTGTGGCA
Mus musculus  ATCCCCTCCTTTTTACCCACCCT---AAGACAGGGAGCCACATGGCGGGCTTCCTACTTCCCCCGAACACCCCCCCCCCCAGTGCCTCGCAGTGTTCTTTCTAGGCACTTAGTTGTGTCT

Homo sapiens  TCAGCAGCACCCCTTCTGAGAGATCAGGCAATCCCCAAGGGTGAGT-----------GGTAGTGGTAGTGGTGGGGAGGGTTGATAGCTTCGAGAGAAGAGCTGGAGAAGGCGCTGT---
Mus musculus  CCAGCA--CCCCCTCCCAAGAGCTCAATCAACTCCCAGAGGCGGGTCGGGTCGGGGTGGTAGTGTTGGTGGTAGGGAGGGTTGACAGTTTTGAGAGAAGAGCCAGAGAAGGCGCTGTTTG

Homo sapiens  -CTGGCTGTAATTAGCCCCAGCTGCTGTTGTGAGGCAGCGTTGGGGGCCGTCAGGAGTGTACATGCATGTGTATGAACCTGTGAGCGTGGCACCTCATTGCTATCACTCTATTTTTCACT
Mus musculus  TCTGTCTGTAATTAGCTCCAGCTGCTGTTCCGAAGCAGCGTTGGGGGCTGTGAGGAGTGCGAATGCATGCCTATGAGCCTGTGTGCGTGGCGCCTCATCGCTATCGCTCTATTTCTCACT

Homo sapiens  GTATTGTGTGCTGGCTGCAGCCTCGCTGCTCTGAAACCCCTCCTGTCCCTGAGGATCCCGAAAGACAGAGTGTTCCACAGGCAGCACCAGCTTGAATCACTAATGCTATTAAGCAGGAGG
Mus musculus  GGATTGCACGCTGGCTGCAGCCCTGCTGCTCCGCAACCCCTCCTGTCCCCAAGGATCCCAAAGGACAGAGTGTTCCACAGGCTGCACCAGCTTGATTCGTTAATGCTGCTAAGCAGAAGG

Homo sapiens  ACAACAAATATAGGCCCCCATATGTCCTGGATATGCAAGTCCCTGCTCTCTCTTTCCCCACCCCTGCTTCCTGAATGTTTCCAAGTGAGTGAAGTCCCAGCACTTGAAATTAGCAACACC
Mus musculus  ACAACAAATATAGGCCCCCATATGTCCTGGATACACAAGCCCCGGCTCTCT--TCCCCCACACCCTCTTCCTGAGTGTTTCCTGGTGAGTGAAGCCTCAGCACTTGAAATTAGCAACACT

Homo sapiens  TCACTTCTGCACAGCCTTTTGGCAAAGAGCTTTCACAGCTGCCATGTCATGCAT----CCTCAGAC-GCTTCTAGGAGAAGCATTTTATAGGTGAGGACCCTGTGGTTCAGGGAGGTGGA
Mus musculus  TCGCTTCTGCGCAGCCTTTTGGCAAGGAACTTTTGCAGCTGCCCTGCCATGCATGCATTCCCAGACAGCCCGGAGGGGCAGCATTATACAGGTGAGGACACGGAGGCCCAAGGAAGTGGA

Homo sapiens  GAGATTTGCACAGGCTAACCCAGACAGTCAGTGGCAGACT-TGTGTCCAGGAAGCAAGTCCTCGACCTCCTGGCCTGGTGGCCCATCCCCAGCAGAGCACTGCCAAGAGGGCTGCTCCCG
Mus musculus  GAGATTTGCATCTGGTTACCCTGATGGT-GGTGGTAGGATACATCTCTGGGGTAGGAGTTCATGGGTTCCTGGCCTGGCGGCACACCCTCATTAAAGTGCAGCCAAAAGGGCTTTTCCTG

Homo sapiens  GGTGGCTCCAGCTGCTGCTGTCTGTGAGCTCGTGAGAACCTGTCAGCTGTGGCTCCAAGACCTG----CCTCAGTGATTG-CTGAAAACGCTGAGAGTGCTGAAAACCAGGACCCTCAAG
Mus musculus  GGTGGCTCTAGCTGTTGCTGTCTATCTGTTCATGAGAACCTGTTAGCTATAA-TCTAGCATCTGTCTGCCTCCGTCATCAACTGAAAATGCTGATGGTGCTGATAACTCGGAATCCTAAG

Homo sapiens  CCGAGCCTGACGTCACATAGTTGATTGAGTACCTGCTATGTGCCCACCACTGTGCTGGTGCTTATGGGAGACA-AAACAGGGGTAGTGCATAGCCCGTATCCTCAAAGAAGTGATAACTT
Mus musculus  GCCAAC------------GATTCATTTAGTGCATGTCACATGCTTACCACAGTGCTGGTACTTTTGGGGGACACAAACTGCAGTACAATATCGACTCTGTCCTTGAAGACATGGTAACTT

Homo sapiens  GAATGGGGAAACAACTC-TTAGATACCAAGTCCAAAGGCAATTCCAAGCACTTCTCTCTCTTCCACTAAGCAGTCTGTTGTCAGTGACAATGACACAGGAACCCCTTCTGAATTCATCTG
Mus musculus  AAATCAGGAGAATACTCCTTAGATGCCAACTCCAAAGGTAACACCCATGGATTCTCTTT--TGCATTAATCAGTCCGTTCTCATGGCAAATGAAGCAGA.....................

Homo sapiens  TCTTATCGCAGGCCTGGGGTAGGAGAGGATGGGAGGATGACTAGG-AAGGGTAATGAGGAAGGGCAGGGAGATGGGGGAAGGAAGGGAAAAACTGGAGCTCCTAATAAACACCAGGCACA
Mus musculus  ..............................GAGAGGACGGCTAGGTCAGCGTAATGA---AGGGTAGAAAAATGAATGAAAGACAGGAAAAAACAGAGTTCCCACTAAATATC-GGCATA

Homo sapiens  TTGTGAAGTTTATATGTTATACATAGGTCATTTGTATTATGCTTATCATA---CATGTACATGAATATATACACCCAAACACATACACAAACACATACACAATCACTTTCCAGGGATTGA
Mus musculus  GTGTGGAGTAGGTATTTTGAATATATGTCATCTACACTATATTCATAGTAACACACAGACACACACACACACACACACACACACACACACACACACACATAGCCATAGAGCATGGAGTAA

Homo sapiens  CTCATCTATTTGGCTCACTCTCAA--TCATGAAAAGAGCTTAGGACAGTGAC--ACTTTTAGAAAGCCCTGAATGAGTGTTAACTAGCCTTTCTTCTTATCATGATCATCTTCATTGTCA
Mus musculus  CCCTTTGATTTTAACTATTTTCAAAGCCACATGAAGAGCTTAGAATGTTTTCTGACCCTTACAAAGCACTGAATGGATGCTGAGTATTCTGTCT-CTTATC----TTACCTGCACCA---

Homo sapiens  TTATCGGCATATTATATAAATTATCTCATTTATTTCTTCTAACTACAGGACTTAGCAACTATTATTTTTATTTCCAGTTTACAAATGAGGAAACCAGTATCTTATTTTATCCAAGCTTGT
Mus musculus  --------ATATTATCTACACCACTTTACTCATTCTTTATAATTCCAG----------CTATCATTTGTATTGCCAGATTGCAGACAAGGAAACCAGTATTTTATTGTAAACAATCCAA-

Homo sapiens  GGGTACTGAGAATATAAAGAGGAATTCAATATGGTTCTAGGCAAACTTTGGGGTCTTCCCTATCTAGTGGGAGGGACCGAGGCACACATAAGCCCTCATGGTCCAATAGGTGCTGTGGTA
Mus musculus  AGGTTCAGAGAATACACACAATCATCTAGCATATTCCTAGCTAGACTTTGGGGACCTATTCATCTCCCGGGGTGCAG-GGGTCACACATAGAACCTTATGGACTAGTGGTTGCTGTTGTA

Homo sapiens  GAGGAA----------------------------------------CGTATCTGGGGAAGTTGAGGAAGTCTCCACGAATGAAGGAGGCTTGAGCAGCATCTTGAGGGATGGCAGGTTA
Mus musculus  AAAACAAATGTCCTATTCATGGGGCAGTGGTTGATGGTGACTGACCTCATACCTGAGGAAGTTAGCGAAGACATAAAGA-TGAAGGGGG-TTCAGCTGGAACCTGAGGAATGGAGATTTT

Homo sapiens  TGTCAGGAGGAACAGTGGAGGCAGGGGAAGACATTCCAGGCAAAGTGAGCA-GTATTTGTGTGACAGGGCCTAGTGCATGTTTCAAAGGGCAGGACAATTGTAGCTGGGTGCACCACTGG
Mus musculus  TGCCAAAAACAGTAATGGAGG-AACATAGGACACCTCAGGCAGAGCGATTATGTCTTTGTG----AAAGTCTGGGGCATTT-----------AGGCATTT---------------ACTGG

Homo sapiens  CAGCAGCAGGAGGGGAGTGGACGAG-TATTTGGGGTCAGCTATTCAGAGCCCTATTGGCCAAGCTGAAGGTTTTGTTCTGTGAGCAAGAGGGAGCCACTGGAGCTTTTTATTAAGGCAGA
Mus musculus  AGTGAGGTGGAGGTGAGTGGGGGAGGTGGCTGGAGTCAGTTATCCAGAGCTTCCTTGACCAAGATGA-GCTTTTGTTCTGTGAGCAATAAGGAACTAATGAAGGCTTCT-----------

Homo sapiens  TTCGGGACAGACTAGGAAGACAGTCCT----TACCAGGGTGT-TGAAAGATTGGAGGGAAGAGAGGCCAGCTGCATGCGGACCAATTGGGAGACCATCTCCAGGGTCTGGGCAAGAAGTG
Mus musculus  ---------GAGGAAGAAGACAGTTTTACAGCACCAGGGTGAAGGAAAAGCTGGAGAGGAGGGAGCCTCAAGGCAGGCTGACCA------------TCTGATTGGTCCAGGCAAGAAAGG

Homo sapiens  GAACAGGCTCTGAACCA-CGACCGTGGCAGTGAGAGCCGAGAGGAGGGGATGACTCAA-----------------GGC------ACATGTCAACAGGTAGAAACCTCAGGGCTGGCTGTC
Mus musculus  CAAT-TTCTCTGGATCAGAGACTGTGACAGTAAGCTCTGAGAGGAGGGGATGGCTTCATAATGTCTCATGTCAGTGGCTCCCTTACATGTCAGCATGTGGAAATCACAGGGCTGGGTGTC

Homo sapiens  ATGGGGTCAAGGAGAG---GGAAAAGGTGACAAGGACCCTGAGGTTCCAGCTTGGGTCCGTGGTGATACTGTCAACTGAGGCAGGGAACACAAGACTGTTAGTAGAGAGGGAGAGTGAGG
Mus musculus  AGTGGGTCAAGGGGGTTGTGGGGGATGTGACAATGATCCTGACATTCCAGCTTGGGCCTGTGGTGACACTGTCAGTGGAGACAAGGAACACA----TGTAGGTAG-------GAGTGATG

Homo sapiens  GGAGACAGATGACAAGGTCTGTTTGGACTTGCTGGGTTTGGAGTGCTGGATAG---------CGGACATGTCCAGCAAGAAACTGGGAATCTTGGCTTTGAGCCCAGCTGAGACATGGGG
Mus musculus  GTAAA-AGATGACA-GGCCAGCTTGGACATGTGAGCTTGTGGGTGCCAGTGGGGCTTCTAGGTGGCTGTGTATAGCAGTAAGAGGAGAATCTTGGCCTAGAGCCCAG-----GCAAGGGA

Homo sapiens  GCTGGAAAGCCACCACAGTCAAGGGATATTAGTTGACCTCAGGGGACTGGATGGGGTTTCTCCTCTCCCATTTTACTCATCCAGGGCAAGGGCTAGGGA-CAGAGCCAGGTGAATACCAG
Mus musculus  G----------------GCCAGATGGTACTGGTGGACCTCA------TTGTTTTATTTTCTCTCCTCCTACTTTACACTTCTAGGGCCTGGACCTGGAAGCACAGGCGGGT---------

Homo sapiens  TATTTAATGAGGGAGGTGGTGGGGGACACCAGCCC---GGAGTGAGAAGACACTCTTCTTTGGACCTTCCCAGAAACATCATCCCTTTCCCCTCATGATCATGGTCCTTGTTCCAGGGCT
Mus musculus  ----------------------GGGCCTCCAGCTCTAAGGAGTGGGGTGATGTGCTTTTT--ACTCTTCCCAGAAACCTTATCTGATCTCCCTCAAGGCCAGAATCTTCCTTTAAGGGCC

Homo sapiens  GCA-CTCAGGCCAGTGACAGAAGGAAGGGAGGGACAGAGGGAGAAAGGGAGGAAAGAAGGGAGGGAAGGAGGGAGGGGGGAGGAAAGAAGGGAAGGAAGGAGGGAGGGGGGAGGAGGAAG
Mus musculus  ATGGCTTTGGCCACCATTGGAAGGGAAGATGACAATCAGGCATGTAGGTA......................................................................

Homo sapiens  GAGGGAGGGAGGGGGAAGAAGGAGGGAGTGAGGGAGGAAGGAAAGGGAGGAAGGAGGGAAGGAGGGAGGGAGGAGCTCTGTGCACAGAACTCATGCCTGCACAGGGGCCATGAAGCAAGG
Mus musculus  ........................................................................................................................

Homo sapiens  ACAGGACAGTCACTTCAGCTCTGGAGTTCTTTAGTGTGGGAGTATCTTCTAACAAAACCTAATAAATTAACAGCTG---TGGGTTTGGGGCAGCAAGGACAAAGACCACAACTCTACAGT
Mus musculus  .....................TGAGGTGCTATGTTGTAGTGATAT--------------TACCAAATCAATAGCCCCACTGCATTTGGGGCCTAAATGAGAAAGGCCACAAGGACATGAT

Homo sapiens  GACCCCTGGAGCACTCAGTGCCACTGACTGCGTCTCCACTGCTGCAGAGCTGGGAGCACATT----------------------------CATGACAGCATTTTTCAAGCTCCTGTCTGT
Mus musculus  GGTAGCTGGAGCCTTCTATGCTAGAAA-----GCTCCATT----------TGGGAGTACATCATAGAGGGTTTATTTTTATTTTATTTTATTTAAAAAAATTTTT--AGATCCAGGCTGT

Homo sapiens  GCAAACCACAGCTGGTCTCCTAGGAGTCCTAACACTTTAATTAGACCTAGCAGGTCCCAGAACAAAATCCCATAATCCC-CAAATCTATAGCAACAAGTTAGGCATGAGAGTTTTAGTTA
Mus musculus  GCAA-CTTCAGCTGGTCTCCTCAGAGTCCTAAAACTTTAATTAGACCCAGCAGGTCCTAGAACCAAATCCCATAATCCCATAAATCTAATGCAACAAGTTAAGGATGAGAGTCTAAGTCA

Homo sapiens  CATTCTG-GATGTAAGAGAACCCTAACTGCCACTTTCTTTCTGAGTCTCCAAATGACCTTTGACCAATCTCTTTACCAGAAATATTTATCTAGAATGGCCTTGGTCTCTGACA---CCTT
Mus musculus  AACTCTTTAATGTAAGCAACCCAAAACTACCATCTTCATTCTGGGTCTTTGACTGACTCTTGGCCAATGTCTCCTCCCAAAATAGTTATCCAACATGACATTTTTCTCTTATAGGATTCT

Homo sapiens  GAGGACTTCTACTGATTTCTCTGTTAAAT-CCCTGGAACCACACCAATAATAGT--TCTGAACATTTT--ACACTCATCATCTCATTTATTCCTCACAAATA------------TTATAA
Mus musculus  TAGAACATTTACAGAATTCTATTTTAAAGATCCCAGAGCCATACTTATAATAGTATTCTGAATATTTTTCACATATATCAACTCATTTATTCTGTGTGGATAGTAGCTAGCTAGTTAGAT
```

```
Homo sapiens  ACCCTGTTTATATATATGTGTAATATTTATAGTAACAAGAGTAGCAGATATT--TTGCTGA-----------GGGGAGGCATGGTTCTACACAGGTTACATGAAGTAATTCATAATTCC
Mus musculus  AGCTAGTTTGTGTGTGTGTGTGATGTTTATAAGAAGAAGAACCATAGATTTTGCTTGCTAAATGCTTAATGTTGGCCAGGCACAGTTTTCAACATATTACATGAAGTAATTCATAATTCC

Homo sapiens  ACATGAACAGCACTATGAGGTAT----------TATTACTGTCTCCATTTCACAGGCAAAGAGCAAATCACAGAAAGAATGAGTAAGTTGTTCAAGGTCACACAGCCTATAAGAGGCAAA
Mus musculus  ACATGAACAACATGATGAGGCAGGTATTCCCTGTGTTATCATTTTCATTTCAAAAATGATGAGCAAATCATGGG-------------TCTTCTAGCCTACGAAGCCTA-----GGCTAA

Homo sapiens  GCC----------TGCCGGTGTGGCTCCACTGTGCTATTAACCAGTAAGGTCTTCTATAGCCCCAACTAGCTCAAAATGGTTCTAAATGAAGAATCGCTGTGCGTTTTGCACCTCAGACT
Mus musculus  CCCCATCCAACTCTGCCACTTTGGCTACAGTGCATTATCAACCACCAAGATCTTCTACAGCTCCAACTAGCTCCAAACAACTCTTGATGAGGA--CTCTATCCATTTTGCATCTCGTA-T

Homo sapiens  GCTAGCATCGCTGATTTAGGTCTGGAGTTTAGATAAGTGAAACTACTCAGGGTCTTTGTGAAGTATCTTTAGAGCTGCTGCTTGGACAAGACTGGGTCAGGACTTCGAAGGCATTTCCAT
Mus musculus  GATGGCAACACTGATTTAGACCTTCAAGCTCCAAATCTGAAACTGCTTAAGGGTTAGGGGAAGCAGTCTGGGAGCTGCTGCTTGGACAAGACAAGGCCAGCACTCAGGAGGCACTGACAC

Homo sapiens  GTGCTCCTGGAGAGTCAGGAT-GTAGGCTGGGATTCATTATGCGACCCAGCTGTGTGACTTTCCTCCCCACCCCTTACCCCCATTCACCAAGATAGAATGCACTGTTTCTTAAGGTGTGG
Mus musculus  AGGTTTCTGGAGCCTCAGGGTTGAAGGCTG..........................................................................................

Homo sapiens  CTTAAGGAGAGCTCTTTAAACATGCAGATTCTAGGGCTGAAGCACAAACCCA--CTCTGTATCTTTATAGAGATAACTGCTGGAA-TATGCATTTTAGTAAACTCTCAGGCTGAATTACT
Mus musculus  ...AAGGAGAGCTTTAGAAACATGCAGATTCCAGGGCTGAGGCCCAAACCCAGACACTGTATTTCTATGGAGATAGTTTTTTAAAGTATGTCTCTTAGCCAACTCCCAG----AATTAGT

Homo sapiens  TTGAGAATCATCAGAGTCTACTTTG--ACAGGATATAGGCTTGTCTTCTGATTCTAGAAGTAC--CATAAGCCTAACTAGGGATGCTTCCCACTCCTCTTTCTTGCTGAGCAGAGAAGCA
Mus musculus  TCAAGAAACACTGCCATTTCGTTTGTGAGAAGATGTAGGTCTGTCTTCTGATTTGAGAGATACTGCCTGGGGCTAGCTAGGGAGGGTTCCTTCTCCTCCGTTGTG--GAGCAGAGACACA

Homo sapiens  AGGGGGACTGGAATGCAGAGAACATGCTGAGGGCATGCCTGCACAGAAAGCTGAGCTCC----TTGTCACCACATACCTTGAAAGAGTGGACTCTGTATCCCCAGGAGAGGATGGGGAGG
Mus musculus  GGGAGGGCCAGAAAGCAGAGGGCACAATGAGGGCATGCCTGCACAGAAAGTAGAGCTCCCCCTTTATCACCACACCCCTTACAAGAGTGAACTCT--CTCCCCAGAAAAGCACAGGGAGA

Homo sapiens  GACAGAGAGAGAAAGCTATGGAAAGCCTCTGGAAGCCTTAGTACCCTGGGGCCCAGACAAGGGAGTCCCCTGAGGGAGGTAGAGAGTGGGGCTGTTGGAGGAGGAGAGCAGCTATGGGCC
Mus musculus  AGCAGAGAGAGGAAGCTGAGGGAAGCTGCC---------AGTACCCTGGGGTGCAGAAAAGGGACTCCTCTCAGGGATG------------CTGTTGGGAGAAGGGAGT-GCAGTGGTCT

Homo sapiens  AGCAGCCTGAGGAGCTGAGAGTCACAGTGGGAGTGACTGTGGCTTCAGAGTGATGCCATAGCTGCAGTGAGCTGTACAGGAACCAGAGACCCAAAGTTTTCTGTAGGGCTTCTTTTGCTT
Mus musculus  AGCAGCC----------------ACAGT-----------------CATAGGGAATCCCCAGC---------CTGTGC-----CCAGTTGTCTTGAGGTGACAGTGGGGCTCCTTTTCCCT

Homo sapiens  TGTTAGTGCTTCAGGAGTTGGATCCTTCTGGACCCTGATCTCCCAGAAGTGATTCCATTTCGCCTCTAGGAGGTGGGTAGCAAAGCTCAGTCTGTGGCTGGGGAAAGCCAAGGAAGTGAT
Mus musculus  TCTTGGGCCATACAGAGCTGCACTCTACTGGACCC-ACTGGGCCAGAATTGATTTCATG------ATAGGGAATGGGAGACAAAGCTCATTTGGTG---------AGCCAAGAGAAAGAC

Homo sapiens  TTCACGAATCTTTAGCAGTGAATCCACCTATTTAGTTAGCAGGG-GATGGAGCAGAT-CAGAACTAGCGAGAGGAAAGGCTTAGGAATGCT---CTCTAGTGGGACTGAGAAGAAAAGGT
Mus musculus  TTCATGAACCTCTAGCAGCAATCCCGACTGTTTAGTTAGTTGGGAGATGGAGGGGATGCAGAAGCAGTGGGAGGAAAGGCTT------GCTTTACTCTAGGGATCTTGGCAGGAAAGGGG

Homo sapiens  GAGAGGGTGACATT--AACTGGAGGAGA-TGGTGAGCCCCTGACCCCTGCAGTTGTGAGACTTGCACTGAGTGGGATGATCT-----GCTGCTATAACCTATCCTCAGGCAATGAAGCAT
Mus musculus  GGAAGAGAACCATTTTAAACGAAGGAAACTGATGAGCCAGCAACCCGTAGTGCTATTAGAAGTGCACCGAGGGGGACCACCTTGACTGCGGCTGCAGGACATTCTCGGATGATGACGCAG

Homo sapiens  A-AGCAGAGAGCCAGGAGTGTTAATCATTGCTGATTTTTATTAAGCATTTCCTTTGTACTGGACACTGTACTGGGCTGTACATGCATCATCACATTCAGTCTTCACAACATTATATGATA
Mus musculus  AGAGCAGAAAGCAGGGAATGTTCATCATTGCTGACTTTT-TTAAACACATCCTTCGTACTAGGAACTGTTCAGGGCTCCACAGGCATTGTTGTATTTAGCTTTTATGACACTCT--GACA

Homo sapiens  GCTCCT---ATTACTGTGCCCATTTTACAGATGAGGTTTAGATAGGTTATACCACTTGCCCAAGGCCACACAACTAGTAAGTAGCAGAGCCAGAATTTGCACCCAAGTGGTTTTAATCCA
Mus musculus  TATCCTGTCATTACTGTGTCCACTTGGCAGATGAGACTAAAATCCGGTCCATGACTTGCCCAGGGCCACCCAGCTAGGAAGTAGAAGGGCTAAGGTTTGAACTCAGGTGCTCCTATTC--

Homo sapiens  AACTGTGTCCACTAGGCCTTAGGAAGGAGCCCACATAGAGCTTTGGGGATGCTGCATTCTTGACCTGTGGCTGTAGGTGGGCAACTCCTCTTCATACCTTATAGCTCTTGGTAAATGAAG
Mus musculus  -----TGTTTGCCAAGGTCTACAAAGGAGCC----------------------------TTGACTTGTGGCTGTGCTGAGGTAACCTCTCTATGTACCTTATGGCTGTTGGCAAGCGAA-

Homo sapiens  TGAATCACTGTATCTACCATGAATCTCCTTGGGAATGAGTTCAATGCGGGTATCAGAGGAAGCAAAGGGTGGGAAGGGGGTCTCAAAAGTCTAAATCTTAGTGTGGTCCCCTCCTTTGAC
Mus musculus  TGTGCCATCCAATTTACCATGAAACTCCTTAAAAGTATGCTTAATATTGGGTGCAGAGTAGAGAAACGGTTGGAA----------CAAGT----------GTGTGTGCCTCTCTTTTGAT

Homo sapiens  CTACTCTCTCTGGGCTGAAGAAATGCCAGGATTGAGGGGGCAGCAGATTGTTAGATCTGTTT-GTTCCATCTACTGAGGCCTCATAACATTTTTGGAGCCCT-TGTTCAGTGACTCAGGT
Mus musculus  TT---------------------------GAATGGAGGGGTGGCAGGGTACTAGTTTTATTTCATTTCTTATCCTGAGGCGCCTTCACAATTTTGGCGGCCTATGTTCAGTGGCTTGGGT

Homo sapiens  AATTCAGCCTGAGGGTACCTTTGCAGGGAGTTCCCCTATCTCTTATCCTGTGTTAGTACATGAGATTATTCCTCCTTCTTGTACCCCAGTTACATTAAGTGGGTCT-TATTCCCCAAATC
Mus musculus  AACTCCCTCTGCAGATAAACTTGCAAG-AGTCCTCCCATCTATTAATCCATTCCTCTAGATGAGATT-------CTTTCTGGACCCCAATTTTATTAAGTAGATCCCTATTCACCAAA-C

Homo sapiens  CGTAGTCTCTGCCCCAATAAGTTGCCT-GTACCCCATTTTAGCAAGAATAGAATCTTCCCTTTCTGCCAACAGAGCTTCTTGGCACTTTGGCCTT--GCCTGGTTCCTGCTTGCA-GACT
Mus musculus  TTTAGCCTCTGCTTCAATAGTTTGTCTTGCACGCCATTTTCGCAAGACTAGAGCTCCCTCTTTGTGTCTAGAGGGCTTCTCGGTGCTTCTGATTGTAATCTGGCTTCTGCCTCCCTGGTT

Homo sapiens  CCTGTTCTGAGGCTCATGTT--TGTACCTAGAATGATTTCATGTTCCTCCTCTCTCTTTGTCCCTCTATCAGAACCTGCTTTCACGATGGGA-------------GATTTAAGAGGCTGA
Mus musculus  TCTGATCCAAGGGTCATTTTCATGTACCGATCTTACCTTC--GCTCCTTTTTGCTCTTCTACCCTCTGTCAAAATCGACTGTCAGAATGGGATTTACCCAAATGAGCTTTAACAGGCTGA

Homo sapiens  TCCTCCCAAGAGGTTGGCATTTTAACTGATGCCAAAGCCCTGAGTCAAAAGAATTATATACTTAAAAATTTTCAAGCCCTTGGGAGCGGATGGAGAG-ATTGGAAAGGCCAATGG-AACA
Mus musculus  CCCTCCCAGGAGGTTGGCATTTTAACTGATGCCAAAGCCCTGGGTTAAAGGAATTATGTACTTAAGACTTTCCAAGTCCCTGGGAGTGGATAGGGAGGATTTAGAAGGCCAGGGGGAACA

Homo sapiens  TTCTTAATGCTCCTGGAATGCCACAAGGTGGACATAAAATTCAGGCTTCTGCCAAGACAGGACTTGGATTATTTTTGTTATAATCAGAATCCGTAATCTGCCCCCACCCCAGCACTCAGG
Mus musculus  TTTTTAATGCTCTTGGAATGCCACAAGGTAGACATGAGATTCAGGCTAC.......................................................CACTACCACTCAAG

Homo sapiens  GCTTTGATCAAAAGTCTTCCAGACAATGAACCTCTATGATCCAAGTATCATTTTCTCAGAGCTCCTTAGAAGGAGGAGAGCTAAGAAACTTCATGAATTACCAAGGACCAAGCAAACAGG
Mus musculus  GCTCTGCAACAAAGCCTTCCTGACAGTGAACTTCTGGGACCCAAGCGGCATTTACTCAGAGCTCCTCACAAAGTG----------AGACTTCACAAAATCCCGAAGACCATACCAACAGG

Homo sapiens  CGGTAGTGAAGTCAGAAACAAATTCCTCCCGAGACTAGTTTTGTCCTATAGCCTGAAAACAAATCCGGTCTCCACCAGACATAAGGTGGAATTCCTGTCTTGATTTTTTTTTTAGCACTG
Mus musculus  CAGCAGCCAAGGGGAAAACAAGTGCCTTTGGAGGCCAGCTTCTTTCTGTACACCGAGAAATAAACCTTGTTCTACTATCCACAAAGTACGTGTC---TCTTCAGCATTTCTGCAGTCCAG

Homo sapiens  ATATTAACATTTTTCTTCTCAAATCCTCTTCAGAACCAGCCCT---TTTAGAACACAAATTAGAAGCTGTTTATTTTGTCAATCAGAGAAACTGAAGGCAGCCAGCCTTGGCCAAAGGAA
Mus musculus  ATAGCAACAGCGAT------AACCCCACTTCATACCCAGCACTGACTGTAGAACTCAGC----AAGCTTTTGACTTGGCTAGTCACAGACACTGAAG-TAGCCAGCCT-GATTCAAGCAA

Homo sapiens  CATCTTATCCTTTGCCTTTGCT-AAGGACAATGTTCCTTCTATTGTGCTCTGTGGAAATAAAAGGGCAAAATATGCTTGCCTTCTTTTATTCTTATTCATGGCTGAG---GGTCATATGA
Mus musculus  CATCTTATGCTTTTTCTTCTCTTAAGGGCAGTGCTGCATCTGT--------ATGGAAGTCA------------------------CCCATTCTTATTTGTGGCTGAGCACGTTATTACAA

Homo sapiens  AAGTTCAGAGAAGAGCACAGTTTTTAGTATGGGTCGTGGCAGGTTTTCCAGATGGCTTCCTAGCCAAGATATTTCTCCCGCTCACCTCCAAACAAGTTATGTACCCCTAAGAATGATTTG
Mus musculus  AAGCTCAGA--------TGGACATTAGGATATA----GGTAGACACTTCAACTGGCTTCCTAGCAAAGACAGATTTCACTCTTACCTCCCAAGGAATAAAGTACTT-TATGGGTGATTTG

Homo sapiens  GTCTCAGTGTTAGGGGAAGAGGGTAGGTAGCAGGGCCTGGCTTTGTCATCCTGGGCTATTTTGAGGGGACTTCAGCAGTCCCCGACAGAAGGACA-AGAGGGGCTGATTCTCCCT-----
Mus musculus  GTCTTGGTGTTGAGAGAAGGGGTGTGTGTGTAGGACCTGGCTTTACCATCTTGGGATATTTTCTGGAAACTCCTGCAGGCCCCTAAAGTAGGCAACAGAAGGGCCGACTCTCTCTGTCTC

Homo sapiens  --------------------TTGTGAGCAGCCATTGGAGCTGCTTCAGGGACTGACAAGCCGGCCAGAGACAATGTCC--TCTCTCAGGAAAGCAGTAAAAACATCAGAAATGCTCCTTT
Mus musculus  TGTCTCTTTCTCTCTCTTTCCTTTAGGCAGCCATTGGAACTGCTTCAGGGACTGTTAAGCTGCCTGGAAACAGTGTCCAGTCTCTCAGTAAAGTGGTAAAAGCATCAGAAATGCTCCTTT

Homo sapiens  CCTGGGGAGCCCTTAGTTATTTTGGCCTCTCTTTCCATTAAAATAGGAAAGCATTGAAAGGGACCAT-TCGTGGGATAGCGTGGAGCCTCCCCATTGTGGCAGCATTTCTAACTGCGTTG
Mus musculus  CCTGGGGAGCTAGCAGCTATTTTGGCCTCTCTTTCCATTAAAATATGAAGGCATTGAAAGGGACCATCTCGCTGCCTAGCAGGAAGCCTCCCCATTGTGGCAGCATTTCTAACTGCGTTG

Homo sapiens  AGAATTAACCATATTGCTCTTCCTTTTTACCATGGAGGAGTTTCTAGATTTTTATTTATACCCTACCGCTTAAAACATCTTTCACTGTTTCAGCAAATTGCAGCATAAAAGATGGAGAAG
Mus musculus  AGAATCAACCACATCGCTCTTCCCTTTTACCATGGAGGAGTTTCTAGATTCTCATTTATACCCTCCCGCTTACAACATCTTTCACAGTTTCAGCAAATTACTGCATAAAAGATGGAGAAA

Homo sapiens  CCAGCAGGGGCTGTGCTCACAATAATAGCTGTGGTTGCATTTAAGACATTCAAATGCCAAGGGTGCACTTCTCCCATTAAGTCCAACATAAGAAA------TCAGGTGTGCACAACATTG
Mus musculus  CCAGCAGGGACTGTGCCCACAATAGTAGATGTGGATGCATTTAAGAAATTCAAATGCCAAGGGTACACTCTTGCCATTAAATCCAACATGAGAAAAAAAAAATCAGGTGTGAACAGTACTG

Homo sapiens  ATGAACAGAAGACCCCAAAGGACAGGTGGAGTTTCTGGTATGCTGTGGTGGGGGCGGTGGGGTGGTGAGCTTGGGAAAGAACGAACAGGCATTTTCATTTTCCAAACTAAGAAGCAGTTT
Mus musculus  ATGAACAAAGTACCCCAAAGCATAGATGGGGTTTCTGTTATGCTAGAGGGAGG----------------CTTAGGAAAGGAC--AGAAGCATTTTCAGTTTTCAAGCTAAGAAGCAGTTT

Homo sapiens  ------CGGGCGAAGGGGAGCCGTGGGG-ATGTCAGAAATTCTTCTAACGCCATGGACCTGCCCACACCTGAGTCAGATAAAGATGACCTTCCCTAGGCCCTGCTTTCCTCTCCCCCTCC
Mus musculus  GGTGGGCAGGGGAAGGGGTGGGGGGGGTCTGACAGACATTCTTCCAAATCCTCGGACTTGCCCACACCTGAATCAGATAATGATGACCATCTCTATACTCTTCCCTCCTCTCCCCCTCC

Homo sapiens  CCAAAAGACAGCATGAGCATGAGTAAGCCG-GGTACTTACAGGTGCAGAAGAATTTGGAACAGGAAAATGGAGCTTACAGGCTCCCGAATGCCTGCCGAGTCAGTTGCATCAGATTAGGA
Mus musculus  CCAGAAGTCAGCGTGAACATCAATGAGCCCCGGTACTTACAGGTGCAGAAGATTTTAGAAGAGGAAAATGGAGCTCACAGGCTCCTGAATGCCTGCCGAGTCAGTTGCATCAGATTGGAA

Homo sapiens  AGTGATGATCC----TAAGAATAAGATGTTTGG------AAAATCTTGT----------CCCCCGAAAGCCCCCTGCTTTGTCTTCTCAGCCCACATCCAAACTCAGACCACCCCCAGGA
Mus musculus  AGTGATGATCGATCGTAGGGATAAGATGTTTGGGGGAATAAAACCTCTCATTTTCCCTACCCCCACACCCTCCTCATCTTGTCTTCTCAGCCTGCATCCAAACTTAGACCA---------

Homo sapiens  TGTCTCTCATCCTGCCAGACTGCCTGGTGCCTCCGTGCCCCTTACCGTGAGGGTGCTGGCAGCCACACTGCTTCTTTGAGCCTGTGGCCCGGCAAGGTAGTCAGGCTCATGGGATTAGGT
Mus musculus  ---------TCCTGTCAGGCTGCTTGGTGCCTTCATATCCCTTACCGTGAGGGTGCTGGCAGGCACATTGCTGCTTGTAGCCTGTGGCTGGGCAAGGTAGTCAGGCCTCTGGGATTAGGT

Homo sapiens  TGGCAGGAGCGACTGTCCTCTAAACGATTAGC-------------------------------------CCAGAGAAAGAGAGAGAGAGAGAGCAAGCAAACCCGAAAACGGGTCCAG
Mus musculus  TGGCAGGCGTGATTGTCCTCCAAATACTCAGCGGGGGAGGGAGAGAGAGAGAGAGAGAGAGAGAGAGAGAGAGAGAGAGAGAGAGAGAGAGAGCGCAAACCCCCAAACAGGTCCAG

Homo sapiens  GGTAGAGAGCTCCAGGTCCTTAGCATGTTCCTGGCGTATTCCAAAAGTGGCATCCACAGCAGAGAAAGTGTCCCAAAATAGCTCAGGCTGCCAATGCCTTGCTGAAACCTGAGAAAGAAT
Mus musculus  GATAGAGAGCTCCAGGTCCTTAGCATGTTCCTGGCGTATTCCGAATGTGGCATCCACAGCAGAGAAAGTGTCCCAAAATAGCTCAGGCTTCCAATGCCTTGCTGAAACCTGAGAAAGAAT

Homo sapiens  TGCTGAATTTAAAGATCTGGGTCGCTGTGGGTGTGTGATCAAAGTGCAGATTTGTTGGTAGGAGGAACTCAAGCCATGAGCTTAGGAGTGACATGAGGTCACTTGGGTCACGGCCTGCCT
Mus musculus  TGCTGAATTCAAAGATCTGGGTCGCTGTGGGTGTGTGATCAAAGT-CAGATTTGTTGGTAGCAGGAACCCAAGCCATGAGCTTAGGAGGGACACGAGGTCACCCGGGTTATAGCCTGCCT
```

```
Homo sapiens  TCTTGTTTCCAACTTGCTGAAGTTGGTGGCAGTGTCAGAGAGCTCCTCTGTGGTGAGGCAGGGGT-TTGGAAAGAGGTCCTAAGGATGCTGTTTTCATGCCCAGGTCTCTGATGCGCTAA
Mus musculus  TCTTGTTTCCAACTTG-TCAACTTGGAGTTGCAGTCACAACCCTCCTCTGTGCTATGGCAGGGGGGCTACAAAGAGGTCCTGGGCATGCTGCTCTCATGTGCCAATCTCTGATGCACTAA

Homo sapiens  CAATATGAAATAGCAGTGCCCTGTTTTCTGCATTTCAAACAAAGGAACTGATACTATGCAAAGGTTTCTTTAGGTTATTGCTCCATGTCTGATGAAATTCCCTTTTCAGTAGATTTTTCA
Mus musculus  CAATATGAAATAACCGTGCTTTGCTTTCTGCATTTCAAACAAGGGAACTGATAGTGTGCAAAGGTTTCTTTAACATATTGCTTCATGACTGATGAAATTCCCTCTCCAGTAGATTTTTCA

Homo sapiens  TTTCTTTTGGCCTTTGGCATAAGTAAATGTCAGCTTCTTAAAAATCAACAGAGAAGAGCAAACAGTAGGGTCTCTGTGATTTT-CTTGGGATTTGAGAAATCTTCCCTCCCTAGCTTCTA
Mus musculus  TTTCTTTTGGCCTTTGGCATAAGTAAATGTCAGCTTCTTAAAAATCAACAGAGAAGAGCAAACAGTAGGGTCTCTGTGATTTTTCTTGGGATTTGAGAAATCTCCCCTCCCTGGCTTCTG

Homo sapiens  ACTGGATCATGTCTGTACATTGCTGCCTGTGGTCCTGGAAAGGACGAGGTCCACCCAGACATGCTGTCTCATTTTTATATTTGCTGAGATCTTGGGACACTCTCCTTTCATCAGGTATGT
Mus musculus  ACTGGATGGTGTCTGCACATTGCTGCTTATGGAACAGGGAAGGATGAGGTCCACCCAGACATGCTGTCTCATTTTGATATTTGCTGAGATCTTGGGAAATTCTCCTTTCATCAGGTATGT

Homo sapiens  CAAAAACCTTAGGATTGTCTGCAGAGAGTGTGGTTCAGTATGTGGAAAGGTCAGCATATGTCATTCTTGGACTGGCCCTGGGGTTTAAGAAGAAAAAGCCCAGAATTTGGTTTCCAATTC
Mus musculus  TGCAAACTTTAGGACTGTCTGCGGAGTGTGAGGGTCAGTATGTTAAAAGGTCAGTGGATGTCATGGCTGGACTGGCCCTGGGGTTTA-GAAGGAAAAGCACAGAAATAGATTTTGAGTTT

Homo sapiens  CAGCTCTGCCT--GTCTTTTGCTGTATGAATTTGGGCAAGTCACTTCTCTTCTCTGGACCTCAGTTTCCTCTTCTGT-AAACTGAAGACAATAATCTTCATTCTAATTCTAACCCAAATG
Mus musculus  CAGTTCTGCCTTTATTTTTTGGTGTGTGAATTTGAGTAAATTGCTTCTCTTCCCTGGGCCTCAGTTTCCTCTTCTGTTAAAAAGGATACAATAATGCACACCCTAACTTTATCCCAATAG

Homo sapiens  T-TGTAAAGAGTAA------CTCAGAGAGTTGAAAGAATGCTATAAAGGGCAGGGTTTGGCACTCTCAGTAAGTGGTGACATTTTTCAGTTCTTCAA-AGGTCCCTGATTGGTAGCCACT
Mus musculus  TATATAGAAAATAAAATGAACATAGAGAGGTGAAGGAATGCTACTAAGGGGAAG------------AGTAAATGGGGATAGCGTTTAGTTCTTCAGGAGATCCCTTTGTGGTAGCTGCT

Homo sapiens  ACCTGCATCCACATGCCTATGGCGAGCCCAAGACATCCCACAGTACTAGGCACTATGAGCTTCATGTCTAGGAGTCTAAATAGTTTCCACTTTCA-GTTGTGGGAATTTTGGCAAGTAGC
Mus musculus  ATCTATATCAATACGCCTATTGAAACATCAGGGCGTACTAGGATACTAGACAGGATGGACTTCATGTTTAGTGCTCCAAATTGCTTCTGCTTTTAAGTCACGCGGCTTGGGGAAGGGTTC

Homo sapiens  TCAATGCTTAG----TAGCTCAGTTTTCCCATCTGTCAAGTGGGTATACAAACTAATAATAATACAGGTCCACAATCCCTTATCTGAAACTGTTGGAGCTAGACATGTTTTACAGTTCAA
Mus musculus  TTGCTATTCTGGAGTTAGCTTGATTTTACCATCTGCTGGGGAGGTATACAAACTAGTCATAATAAA---------------------------------------TGTTTTGTAGTTCCA

Homo sapiens  AATATTT----------GGATTTTAGAAAAGCAATGTGGTAGATATAACATATAT-----ATCACACCTTAATGAGATCCAAGATAATACCCAGTGATCAAACTCACTAATATTTCTAAA
Mus musculus  AATTTTTTTGGGGGGGGGCGCTTTTGTAAAGTAATTTGGTGCATAAAATATATACTAACCAACACCCGCTAATGGAATCTGGAAGAGTACCTTGTGA--AAACACATCAATATTTCAAAA

Homo sapiens  TGGGATAAATGAATAGCCTCATGTCAATTCAGGCCCAGTTTTGCCACCAAATGAGCTTGCCACAGAA-TTTCAGTTTTCAGAAGCTTTG-GGATATCAGAGAGGCACATA-GGAAATTGT
Mus musculus  TGGAATAAATAGCTTAGTATCTGTCAGTCCAGGACCAATTTTGCTACTAAATGAGCTTGCCACAAAAATGTTAGCTTTCAGAAACTTTTCAGATGTCAGAGAGGCACATAAGGAAACTGT

Homo sapiens  GGACAATGAAGGAAGTTAATACTTATTTCGTGCATGTAATGTACCAGGCATT--------GTTCTAAGTATACATTAACTCATTTAAGTGGCCCAATAAGCCTGTGAGGTAAGCAGTATT
Mus musculus  AGGCCAGGGTAGAAATTGATAGATATTTAATGCTCCTAATTTTCCAGCTAGTCATCCTGTGTTGTAAGTATGTATTTACTCATTTAGTTGCCCCAATATGTCTTTGAGTTGGG---TGAT

Homo sapiens  ATTATTATCATCCCCATTTCATAGATGAGAAAACTGAGATTCAGAAG--TTAAATCACTTGCCCAGGGTCCCAAAGCCAGTTAG-GAGCAATGCTGGGATTCCAACC-----------CA
Mus musculus  ATTATTATGACTCCCATTTTATAGACGACGAAAAGAAAAGTCAGAGGGGTTGGGAAACTTGTCCAGAGTCCCAAAGCAAGGTAATGAGCAAAGTGGAGATTTCAACCGGGTTGGGTTTCA

Homo sapiens  CACAGTTCAGCTCTTATCCACCTTCTCTGCCTAACCCATGGGGTTAAATTGAGGCTTTAAATGCCTACAGGGACTGAGCAGGTCGC--TGAAAAAGTAAAGGTGGCCTTATGGGTACTGA
Mus musculus  GAATGTGTGGTCTTTATTATTTTTATCCACTTAATCCAAGGGACTCAAATGGGGCTTCAGAAACCCACAGGGGCCAAGTAGGTCACCATGAGAAAGGAAAGGTGGCTGGGAGGATAC---

Homo sapiens  GGCAAACTGGTGAGCACGTGCCTCACCTACAGGGAGCAGGTTGTTGAGCTCTGGCTGATTGTTGCTATATTGGAATTTGGATCGAAGTCTTTCTCTCTCTCTCTCTCTCTCTCTCTCTGT
Mus musculus  --------AGTGAGCTCATGCCTCATCCACAGGCTACAGTCTTTTTGGTCCCAGCTGACGCTTGTCCGGATGGAATGAGGATCTAAATCGCTTTTGTTTTCCC.................

Homo sapiens  GTGTGTGTGTGTGTGTGTGTGTGTGTGTGTGTGTGTGTGTTTCAGAAAAGCCAGAAATCCAGCTTTCTATCTGAAATTTCCTGACTTTTAAATGTTGGCAATTAATTTTAAAAAATGTTTAA
Mus musculus  ........................................CAGAGAAGCCAGAAATCCAGGGTTTTATGGGAAATCTCCTGACTTTTAAGTGTTGGCAACTAATT--AAAAAATGTGAAA

Homo sapiens  AA-CGTTGTACGGGTCAAATAAACTCTGTCTGCAGTCTGAATTTAGCCTGTGGCTGCCAGTTTGCAAGCTCTGCAATGAGTAAATAGATGTGAAAGTGCTTTGGAAACTTAAAAACGTGC
Mus musculus  AAACTTTGTGTGGGTCAAATAAAATCTGTCTGCAGTCTGGATCTAGCCTGTGGCTGCCAGTCTGTAAACTCTGCAAAGAGGAAATGGACGTGAAAGTGCTTTGGAAACTGAAAAATGTGT

Homo sapiens  TCTAGAAACAGAAGAGTCTAATTTTAGGGTTATATCTTGTTTTTTCCTTGACTGTGGTCTAG---------------------TTTTTAAAAACTTGTATTCCCGACCTGATGGAGAT
Mus musculus  TCTAGAAACAG----GTCTCTTTTTAGGGTTAAGTTTT-TCCCCTTCTTAGCTGTGGTCTAGGGTTTTTTTTTTCTAGTTTTTTTTTTTTTTAAACTGCTGTTTCTGGCCTGGTGGAGAT

Homo sapiens  GAAAAGTCAGAATTAAATAGAGACTTCAGTGAACTTGAGTACTTCATTCCTTTGGCAGGCTGGATAAATGAGAAGCCTAGGTGTACTGGATGGACTAAGCCTCAGAATTCTTGCCACACC
Mus musculus  GAAAAGTTGGAATTAAAAAGAGACTTCAGTTAACTTGAATGCCTCATTACCTTGGCAGGCTGGATAAATGAGAAGCCTAGGTGTACTGGACGGGCTAAGCCTCAGAATTCCTGCCACACC

Homo sapiens  CACAAGCCCCTTCTAAGGAGTTTCCATCCACAGACCCTAGCTGAGGGAAGTAGCAGTTTCTTATGAATAGGTGACTATTCTTGGTACCACGTGACCCTGCTT----CCCCCAGCAATAGC
Mus musculus  CACGGGCCCCTTCTAAGGAGTTTCTATCCACAGACCCTAGCTGAGGAAAGCGGCAGTTTCTTATGAATAGGTGGCTATGTTTGGTACCACGGGATTCTGCCTTCCACCCCCAACTCCAAG

Homo sapiens  C--ACTTGAGCCAGGAGAGGGCATCTGATCCATGAGCAGTCACTCTATAGGCTGGCAAACAGCCTATGAAGGAACCTGGGATGAGAACTTTGCCCAGCAAGTACAGTTACTGAATTTGGA
Mus musculus  CTGATTTGAGCAGGGAATGGGCACCTGATCTTTGAGCACACATTCTATAGGCTGGCAAACAACCTATGATGGAACCAGCGATGACAACTTTGCCCAAAGAGCACA--------ACTTGGA

Homo sapiens  CCAATGAGAGTTTCTCTCTTGGGGAGTTTGAATACCAGACCTAGAAAGAGTGAGCAATTG-GCAACAG----GGGCAGAAGCTGAACACAGAGAAGGCAGGCAGCAGGGACCCCGGATAA
Mus musculus  CCAATGAGAATCTCTCTCTTGGGGAGTTTGAATACCAGACCTCAAAAGAGTGAGCAGTTCCGCAACAGAACAGAGCAGAAGCTGAGCCGAGAGGAGGCAGGCTGCAGAG--TTTGGTGAA

Homo sapiens  G-CAGGACCAGGAGAGATTACTGAACTTCAACT-ATGTGCCAGAT--TCTTTACACGTTGTTTTGTTTAAAACATTCAAAGAACATGCGAGGTAGGTACTGTTATTATGCCTGTTTTACA
Mus musculus  GACACAGCGAAGGGAGCTTACTGAGCTGCAACTCATGTGCCAGATACTCTATACATGTTGTTTTCTTTAAAGCTCTTAAAGAG-GTATGAGATAGGT-CTGGGATTATTC----------

Homo sapiens  GATGAGGA-AACTGAGGTGAACTAACTTGAAGTATTGAGGTTCAAGCCCAGGCCTGTTTGAAGTTAAAATGAGAG----------------------------------------TGT
Mus musculus  ----AGAATAATTGAG---AAGTGACTAAAAGTACTGGGGTTCCAGCTCAGGCTTGTATGATGGTACAATCAGAGAACATGGCCTATCTGGGCTGCGTCACACTTGTAATCCCAGAATGT

Homo sapiens  AGGAACCCCAAAGC----CAGTGGTTCTGAAACTCTAGTG-TTTCAGAATCACTTGGGTTAAA-TGCAGGTTCCTTGGCGTCACCCCAGACCTATATTGAATCTGACTCTTTGTGGGTAG
Mus musculus  AGAAACCCCCGATCAGAGCAATGGCCCTTAAGCTCAAGGGATTTGGGAATCA--TGGGTTAAACTGCACATTTCTTGGTCTCACTTCTTA--TTTCCTAAGTCTGACTCTTTGAAAATGG

Homo sapiens  AGTCTCCCTCCCCCT---ACAAATCTGTGTTTTAGCAGTTTTCCCAGGGAGCACTTGATGCAGGGTTTCCCCACCACACTTTTATTTTGAGAAATGCAGTTTCAGAGGGTATGGCAAGTA
Mus musculus  AGCCCCTCTCCCCATTGAATGAATGTGCATTTTTGTAGTCCTCCTCTGGGGAGATGGATGCGCAGGCTCTCCATTAGTCATTTCTCTTGAGAAATGCAGTTCTAGAGGATGTAGCAAGCA

Homo sapiens  C---TGTGTACTGTGCTACCTTTACCTACACAACCAAGGTCAATGTCAACAGATGTGGTCAAATTCCAATTTAAAGTCTTTTGGAAATGGGGCC-AATTATGTAAAGAAGAGGGCATTAG
Mus musculus  AGTGTGTGTACTGTGTGGTGGTCATCTTCAGAAACAAGGTCAATGTCAATAGACATAGTCAAATTGCAATTTAAGAGCACTTAGAAAGGAGGCTTGACTCTGCAAATAGGAGA-CACCAG

Homo sapiens  TTTGTTCTCTTCTCTCATCAAGGAAGTCTACATTATTACCAGTTATTTGAGAGAACAG---AAGATTGGGATGAATGCTTAGTAGTTGTAAACACGATCGGATCTGCACTTCAGGGTGGG
Mus musculus  TCTCTCCTCTCTGCTCATCACGGGACTGTCCAATGGCATGGATCATTTAAAAGCTGAGGGCAGGTATGGGCTGGTTGCTCAGCCATTGTGTAAAGTAAGAGATCTGTTCT----------

Homo sapiens  TGCAAAGCTAGCATATGTTCACTAAG-CATTTTCCATGAGGTGCGTAAACTGTAGAAACTCAGTGCTCTGGCAACAAAGAACATGAGAGAAAATTACCAAATGCAGATAGACATATTGTA
Mus musculus  --CAGAGTGAGCATATGGTCTGTAAGGCTTTTTACGTGAGAAGCTCAAACTGCTGGAACTTAGTAAACTGGCAACAAAGAACATGAGAGAAAATGACCAAATGCAGACAGACATATTGTA

Homo sapiens  AACCGTGGAGCTTAAAATGTAAACCTACTTGCTAGGCTGATCATAGAGGTTCTTCTCAGCCCCAGAGAATTTGTGTAAGAAT-CCTGTCCATGCCTTTATTTACACCATTGATTAAAAAT
Mus musculus  AACCACGGGACTTCAAATGTAAACCTTTCTGCTAGGCT---------------TCTCAACTCTAGAAAAGCTGTGTAAGAATTCCCATCAGTGCCTTTATTTACAGCATATGTAAAACGT

Homo sapiens  GCTAATCAGGAGAGGATCAAGGCTATGGACCTCCTTCTAGG--TTGTTGCAGAACTCCCAC-------TCTGGCAA-TGGTATTCAGCTGGTTTTGAATTTTTCTGCTTT--AAGAACTA
Mus musculus  GCAA--CGGGAGAGGAGTCCAGCTAGAGAAATCCTTCTAGACCTAGCCCCCCACCCCCCACCCCCGGCTCTGGAAACTGGTATTTTGCCTGTTTTGTATTCTTCTGCTTTTAAAAAGTCG

Homo sapiens  TCCCTGGGAAGAC--TAGTGGTCTGAAGTTTGCTTGGAGAGTCTCAAGCCGGAAACTGATTTGTGGTCCAAATGCTTAATGGCATCAGACACTTATTCCTACAAGAGAGGCATGTGGTCC
Mus musculus  ACCCTGGGAAGACAGTAGTAGTTTGAAGGTTGCTTGGAGATCATCAGGTCAGAAACTAATTTGTGGTGTAAATGCTTAGTGGCATCAGGCACTTACTCGTACAAGGGAGCTGTTTGGTCC

Homo sapiens  AAGGGGTGGCACAGGTCAAATGTGATGGCCAAGTCCCGCCTCAGCAGGGCTTGAATCCGCACTTGGCAACTTTTATTTCTCTCTGTCCATTCCTGTGGATAC----TTTATCTGTGTTAA
Mus musculus  AAGGGGTAGCACAGGTCAAATGTCTTGGCCAAGTTCTGCCTCAGCAGGGCTTTCCTTCCTACTTGGCAACTTTTCTTTCTGT-GACTTGTTCCTGTGGCTACGTTCTTTATCTATGCTTA

Homo sapiens  TGTCCTCATTTAGGCTAAGGTCAAACTCTGTTCTTCTCTCTTCTGGCCAGAAAGACTTCCTGTTTTACAGGTATATGATCTTATCTGTGTTGATACCTTAACTGTGAGTGATTCCCTAAA
Mus musculus  TGTCCTTATGTAGGCTAAGGTCAAATTCT--TCTCTTCTCTAATGGCCAGAAAGACTTCCTGTCTTACAGGTATTGGATCTTATATATGTCGATGTTTTAACTG--AGTGGCTCCCTAAA

Homo sapiens  GCAGGTCCTTGGACAAGTGCCCATCCTCC-AGGAAAATTGCAGCAGT--------------------CCA--CCGTGCAATGAGAAAGCCTGAGGACTAGGTAGTAAATGTTTCGCAGA
Mus musculus  GCAGGTCCTTGGACAAGTTCCCATCCCCCCAGGAATATTACAGTAGTGTGCCCCGCCCCGCCCCCCCCCCATCCCCTGCTAGGAGAAAGCCTGAGGACTGTGTAGTAAATGATTTGTAAA

Homo sapiens  GAAGAGAAATCTATTTCATGT-------ATTATTCTTTATTCTGAGGTTATTTCCTTCTCTTTTGGTGT-TGAAAGGAAGCCCCTTCTTTTTAGAAAATGATGATTTGTGGTTGTTCTGT
Mus musculus  GAGGAGACAAACGTATTGTGTTGAAAGGACTGTCTTTTATTCTGAGATTATTTCCTTTTCTTGTGGTGTCTTAAAGAAAGCCCTTTCTCTTTAGGAAATGATAATTTGGGGTTGTTTTGT

Homo sapiens  TTCTTAATGATTTTACTTGGGAAAATAAAAATCTGGAAGTTCAATATCAGTCCCTGAAATTTTGGG--TTGCATTAGCTGCAA---GTCTGGGAACCTGGTCTAATTTTGAGTGTTCTGC
Mus musculus  TTCTCAATGCCTTTCCTTGGAGAAAAGAAAATAGAGTAACCCAAAGTCAGTCCCCCAGTTTTGGGGCTTTGCAATCGCTATAAATAGTCTGGGAGCTTGGTCTCATTTGTAGAGTCCTGT

Homo sapiens  TAGTACCTCTAGGTACCTCCTATTGCCACTTGTGTTCTAAGTGCTTATAAATTACCTGCTTCGCAATCTGCAGCAGAATCTCTCCGAGGATTGACATCAAGCTTACAGCTTTGGAGTTTA
Mus musculus  GAGTACCTCTAGGTACCTCCTGATGCCACTTACATCGTAAGTGCTTATAAATTACCTGCTTCATAATCTGCAGCAGAATCTCTCTAAGGATTGACATCAAGCTTACAGCTCTGGAGTTTA

Homo sapiens  CGGTATACTGGTCTGTAGTTTCCTTATTTAAGAAAATTGGGACACTGCCCATTTGCAGTCCTCTGGTATCCCTCCTGTTCTCTCTGATTCCACAGATCCTTCAGACCGGCACCTAATGCA
Mus musculus  CAGAACACCGGTATGTAGTTTCCTTATTTAAGAAAATTGGGACACTGCCCATTTGCAGTCCTCTCGTATCCCCCCTGTTCTTTCTGATTCCACAGATCCTTCAGGACGGCACCTAATGTA

Homo sapiens  AGGCAGTTCCGTTT--GCTCTTCTTATAATGTTCTTTTCCCTATAGAGTTGGACATGCATATACATGTTGTTTGGGG-AATTTGCAGCACATCCAATGAAAGCATAATTCCCAACTAACA
Mus musculus  AGGCAGTTCCGTTTTTGCTCTTCTTAGAGTGTTCTTTTCCCTATAGAGTTGGACATGCGTGTACATGTTGTTTGGGGGAATTTGCAGCACATCCGATGAAAGCATAATTCCCAACTAACA

Homo sapiens  GTCAAATCCTTTTCTTTGGAGCTACCCAGAACACACCTACCCTCTCTTCCATATGACAGCCCTTCAGAGACTTGAAGACAGTGTGTGACTCTCCTGTGAGTTAAGAGCACTGGCTGAGTC
Mus musculus  GTGAAATCCTTTTCTTTGGAGCTACCCAGAACACACCTACCATCTCTTCCATATGACAGCCCTTCAGAGACTCGAAGACAGTGTGTGACTCTCCTGTGAGCTAAGAGCACTGGCTGAGTC
```

```
Homo sapiens  AGACAGACCCGAGTGACAAGCCCAGCTTCGCTCCCTCTCAGCGGTGAGAGCCAAGGTAAGTTACTTCATTTTAATTTCTCTCTAGTTCAGTTCTTTAGCTGATTTAGAAAATGGGAGTAA
Mus musculus  AGACAGACTCGAGTGACAAGCCCAGCTTCACTCCCTGTCAGCTGTAACATCCAAGGTAAGTTACTGTATTTTAATTTTTCTCCACTTCTGTTCTGTGGCTGATTTCTAAAATGGGGGCAA

Homo sapiens  AAAAGCCACTTTGTTTGTTTATCGTTCAATAAATAAGATGAGGTATGTAAAGTGTTTAACATAGTGCCTGGAATAGTAAGCACTCAGTAAATGCTAGCTATT-TCCCCCTCTTTATTTAT
Mus musculus  CTCCTGCACTTTCTTCGTATTTTGTGCGATGAAAACAGTGT---------AATGTTTAGTGCAGTGTCTGGCATAGCAAGAACTTAGTAAATGCTAGCCATTCTCTACCACTTCATTTAC

Homo sapiens  AAAGAGCCGG-TGCGCATTTTAAGTGTCAGGGCTGTGGGGGAGGAAGGAAGTGGG---GAGGGCTTCTACTTCCCTGAATGATCAAG---GAAGGAAGAAGATCCTAAAGTAGACCAGGT
Mus musculus  AAAGAACCGGGTGGGCATTTCAAGTGTCAAGGCTGATGGAGAACAAAAGTGTGTGTGTGTGTGTGTGTGTGCGCGCGCGCGCGCTCGCGCGTGAAGGTAGAATACCCCAAACTAGA---GGT

Homo sapiens  ATTTCTTTAGCCTTAGGTTGTCTGTTTTCAGTTATCTGGATGCCTATTTGCTAGGACTTGGGGAAAGAATATTGTTGAGAATCTAGGGAAAGGCTTATTTTCAACAGCTGGAAAGCTATT
Mus musculus  TGATCTTTAAGCTTTG-----CTGCTTTCAGGTAACTGGGTATGTGTTCAGTACAGCCGGGGGAGA---TATTATTGGGAATCTAGAGGCAGGCTTATTTTCAACAGCTGGGAAGCTATT

Homo sapiens  TGTTGACATATTGAACCGTCTCTTGTTTATTCTTATTTCCCTGAGCAGCTAGCCATGCAATCCCCTAAACTCAATTACCTTTTGTGAGGCGGCAGGAGCATGACACCTCCTAGCCGTATT
Mus musculus  TGTTGACATATTGAACAGTCTCTTGTTTATTCTTATTTCCCCAAGC---TAGCCATGCAGTCCCCCAAACTCAATTACCTCTGGCAAGGCGGCAGGAGCTCGACATCT---AGCTGTATT

Homo sapiens  GATCAGTGCAGACTGGGTGGGACGTGAGGGACCTGAGAGATGCTAAGCGGTAAATTCTGGGCTTTTCCTTCCTTAGAAGGCCTCTCTTCTCTGTGTTCCTAGAGACTCTGGCTTTAATAG
Mus musculus  GATCAGTGCAGACTGGGTGGGACGTGAGGGATCTGAGAGCTGTTAAGTAGCAATTTCTGGACTTTTCCTGCCTTAGGAGGTCCTCCTTTTCTGTAATCCTAGAGACTTT----TTAATAA

Homo sapiens  AACGGAAGTCTATGCCTCACATGGGCTGAGCATGCAAGAACCCTCTCTCAGTAGCCTCCTTAGAAGCAGGCTGATGAAAATAGTCACAATGGGTCAGAACTTGGAACCCTGGGGTGACCA
Mus musculus  AGCAGAAGTCTGTGCCCCA-ATGGGCCAAGCATTCAAGAGCCCTCTCTCATTAGGCTTCTTGGAGGCAAACTGTTGAAAATTGTCACAGTGGACCAAAACATGAAAGCCTGGAGTGGCCA

Homo sapiens  GTGCTAGCATGGTTTTCTAACTGCAGCTCTTTGCACACACTGGGTTTTG---------CAAGCAATTGAATGATATGAACTCTGAATGCCATTGCAGCTAGAGAAAGTTTGGAAGGAGAC
Mus musculus  G------AATGGTTTTCTAGCTGTAGCTTTTTGCATACACTGGGCTTTTTTTCTTCTTCTAGCAATTGCATGATATAAACTCTGAATACCATTGCAAGTAGAGAAAGTTTGGAAGGAGAC

Homo sapiens  ACACCCAACTGTGAACAGACAGAGGAATGGGACTTGCGGGGAATGGGGGGTGGGGCATTTTCAGGAGTTTAGCTTTGTCTGTACGGAGGATTTCAAGTCTTTA-TCCTAAGAATATATTC
Mus musculus  ACACCCAACTCTGAGCAGACGGAGCAGTGGGACTTGCGGGGAA-GCGGGGTGGAGTAGTCTCAGGACTTT-----TATCTGGACTGAG-ATTTCAAGTTTTTAATCCTAGGAAAAGTTTC

Homo sapiens  CTAGATTACTTATGGAATTACTAAACATAAATAAAAGTTAGAGAAAGAAGAAATTATTTTTAAAAACAAACCAAAAATAA-------AACACAAAATA----------------------
Mus musculus  CTAGATTACTTGCA----TATTAAACCCCGATAAAAGTTAGAAGTA--------TTTTTTAAAATATAAACCAAGAAAAACTGAATAAAAGCAAGCCACCGGAGATACGATTTTAGAGGT

Homo sapiens  ---------------------AAGTGGTACAACCATTAAGCTTGTCGGGGTAGAAAGATTGCTTGGGTCGGGGGGTGGATGTCCGGGATTCCTAGAGAGAGCTTGCAAGGAGAGGAGTGA
Mus musculus  CACTTTGGAAGGGGAAGGCCCAAGTAGTGCAACTGTTAAGCTTGTTGGAGGAGAAAGGTTGCTTGTG--AGGGAAT---TTTGGGGGGTCCCTTGGGAGTACATGCA----GAAGACTGT

Homo sapiens  AAAGGGCTTGAGAAAGAACCCTAGGGAAACA--TATCTTTAAAAAAAATAAAATTAACTTTTATTAGTTTTTTTCTTTTCG------TATTATGGGTCCATTATAGACCAATGAGAAAAT
Mus musculus  GAAGGGCTAGACATAGATCCATAGGGAATCAGTCATGTTTAAAACTGG--AAACTAATTTTTGTT---TCTTTTCTTTGCAAATAAATATAATGGAAGGACTTTAGACAAATTAGAAACT

Homo sapiens  ACAGATA-AGGAAAATGAAAATTAAAAATCCCCTCTAATCCCATCATTCAACTGTGGTCACTGTTGGCGTTTTGGTATA------------------------------------TATC
Mus musculus  GTAGGTACAGAAAAATGAAAAT-AAAAATGCCATGTAATTCTACAGCTCGAGTCTGGTCACTGTTAATATTTTGATATGCTTTTCTATACTAACTTTTGGGAGGGGTAAATATAATTATA

Homo sapiens  CTTCCA------------------------------------GATTTTTTTTCTATGCAGAAATCTACATATTTATCTTTATGATAATCAGATCCT-----CATGTACACATTAGAAT
Mus musculus  ATTTCATAAAGTATTATTAAAATATGTACATTTAAAACAATTGTGGTTTTCTTCTTGTGCAGAAACAGGCACATTTAT-TTTATTGTAAAAGGATCTTGTAATTAAGCTCACATTAGATT

Homo sapiens  ATA-TTTTCAATGGCTACAATAGTGGCTGCTACAATTGCACCATGATTCGTTTAATCATTAAACAAGTTGTGTTCTAACATTAGACACTTAGGTTATTTCCAAAATTGTTGCATTAGAAA
Mus musculus  TTAACTCTTAATAGATGTGTTAG-GGCTGCTA-AATTGTACCCTGGCTCATTTAGTCATTAAGCAA-TTGTGTTCTAACATCAGGTATTGGGTTATTTCCAAC-TTTTTTTGTTCGCAA

Homo sapiens  TAAACAACAGTGCCCTGTAGCTGTGTGACATTGGGAAAGCTAGTTAACCTCTCTGTTCCTCT--GTTCTCATCTGGAAAATG-AAGGTGCTAATAGAACCTACCTCGTAG-TATGTTGTG
Mus musculus  -AGACAACTGAACAGTGTAGCTGGGTGACATTTGAAATGTTGGTTAGCATCTCTGTGCTTTTAGGTTCTCATCTGGATAATGGAAGCTGATAATAGCACCTGCCTCATAAAGATGCTTTA

Homo sapiens  AGTGTCAAATGAGATCATATAGGTAAAGCACTTAGCACAGGGCCTGGCACAGTAAACGTTAGCTTTAATTGTTAGGT-----------------GTGTTAATAAT-------------
Mus musculus  ----TTAGATGAAATCAAATAAGTAAAATGTTTAGTATAGTACCTGACTCAGTAATTGTTAACTTTAATCATTAGGTACAATAGCAATAGTAACTGTGGTTATAATGAATCCATTATTAA

Homo sapiens  AATAATAG-----------------------------TAGTTGTTGTGAGGCCATTGTTAA---TAGAATGGCTATTCTGTGACTTTTTTTTTTTTTTTTTTTTTTTTTTGCTTTGAAGGGA
Mus musculus  AATAATGGGTTTTCCTTCCAAATATTTATTATTATTATCATTGTTATGAGGACATTGTGAACCTTAGAATGGGTTTTCTATGACCGTTTTTCTGTTTTTTTTTTCT---CATTCAACGGA

Homo sapiens  GAGGAGAGCGACTGGAA-TTTTTCTTTTGAGTAGTATGCTATAGTGAGCTCTGGAGTCACACAGAGCTGTGTGCCTTTGGTCATGTCAATTCATCTCATGGAGGCTCAGTTTCCTTACCT
Mus musculus  GACG-----GATTAGAAACTTTGTTTTTGAGCAGTA----------AACTCT--AGTCCTGCAGAATTGTGCACATTTAGTTTTTTGGGG....CCTGTGGAAGCTCAACTTTATTATCA

Homo sapiens  GTATACTGAGAATTTAAAGGACCTAGCTCACAGGGTAATTGTCAAGATCAAATGAGATAATGCTGGTGAAGCCCTCAGCCCCATTCTTGGCATATGGGAAGAGT--TCAAG------CAA
Mus musculus  GCAAACTGAGAGCTACAAACCTCTAGCTCAAAGGGTAATTCTGTAGAGCAAATAACAGAAGACTTCTGAAGCCCTTAGCACAATTTTTGACTTA-GAGAAGAGTATTCAAGGCAAGGTAT

Homo sapiens  TATCA--------GTTGTTGTTGTTATTATTACTACAAGGATTCAGCCATTGTAGTAAGAAAGTTTCCTTGCTTTGTGGTGTGGGGATGTAGGGGAGATGGCAGTGAGAAGAGTACTCC
Mus musculus  TATTATGACTGCCACTACTGTCACCATGGCTGCTACTAAAATTTAACCACTCGTGGCAAGAACATTTCTTTGCTTGGTGGGGTGGAGATCCAG..........................

Homo sapiens  GAGTTTCTAATCACTTGTCTGATTACAAAGAGATAAAAATATTTTGGAAACAGAATGGCCAGGGCTGAAATCTGGGATTTAAAAAAAATTATTATTATTATTTTATGTGACCGTGGTCAC
Mus musculus  ........................................................................................................................

Homo sapiens  ATAGACTTCTCCAAGCCCATTTCCTCATGTATAAAATGGGAATGATCTCAGTGCCTACCTTACAGGTGTTGCTGGGAGGTCGGAATTATATAATGCATATGTAGTGCTTAGCCCAAGTGC
Mus musculus  ........................................................................................................................

Homo sapiens  TCAGTTGTTGTTGGTCGTTAATTATGAGGTTTATAACGACCTGAATGCCTCCCTTGTCAAACTCTACATCCCTATACCTAGGCTACTTGAACTTCACCTGGAGGCTTTGCCCTATCCAAG
Mus musculus  ........................................................................................................................

Homo sapiens  GCTCAGGGACCAATTCTCCCCTTAGACTGAGGGTTTCTCCCCTTAGACTGAGTGCACCAGTTCAGGAAAACTGGAGTACTCTGGTAAACCTTTCTGCTAGCTCTACTCTGACCTTGTGCC
Mus musculus  ..............................................................CAGGAAAACT--------CTGGTAA-TTATTCTG-TACTTCTACTATGACCTCAAGCC

Homo sapiens  CCAGTTCTAGAAAGCAGGCTGTAGCCATGTTGTATGAACAAG-CCCTCAGCTGGTATGCTCAAGGCTGGTGTGCTTTTCTTGCTAGCTGTCTGAAATCCATGGAGCCTTTCTGTTGAAT
Mus musculus  CTCCTTCTAGACTATAGGCTTCTCCTTTGTTTATATGAAAGGGATCTCATGGGTAGGCTCAAGTCTAACATTCTTGCCTT-----CTGTTTGAAACCTATTGAGCCTC-ATGTTGAAT

Homo sapiens  CCTAGCCCTATGGCAGGGATACCGGTCGCTGGATTTTTCTCCGTGTCTGTGACCCTGGGGTAAATTGTCTTCGTTA----TGATCACCATTACAGGGCTCTATTAAGGTGTTTGCTTGCC
Mus musculus  CCTAGCTTTTCAGAATGGACACTGGCTGCCAGATTT--------GTCTTTGTCATTGGGGCATAATGGCTT--TTAACATTGCTCATGACTATGGGTGGCTATTAAGGTATTTACTTTCC

Homo sapiens  AGCCCCCACTGTCCATCCTC--CCAAACCCTCTTTCCCTGCACCTGAACCTCTTTGAACGTTTCTGATGCTGCTCCACTCAATCTCCAGCCTCAGTTTCTTTCCCTCGAGCCCTCTGCCT
Mus musculus  AGCCACTACCACCCAGTCTCTTCCCACCCTGCTTTACC----CATGAACCTCCTTGAGTTTCACGGATGTTACTTCTCAGGATCCTCAGTCCCAGT--CCTTACCTCTAACACTGAGCCA

Homo sapiens  GTGCTTCCATGTCATATCAGTTGCAGTGCCTGGGTCCCAGGACTCCTGTGTCTGTTCAACTGCAGAGGGCACGTA--TCTTGTTTCCTGAGCCAGGACCCTTTTCATCTCATCTTTTTAA
Mus musculus  GTACTTCTTGGTTTGGCCAGCTGCCTTGTCTAGGATCCAGAGTAGCTGTGCCTGCT-GGCTGCAGAGAGCATGCACGTTTCATTTCCAGAGTGAGGACACTGTCTGGTTCATCTCTTTAG

Homo sapiens  TTGTGTCAATCTCCTCTTTTCTTTGGGCAGAGTGGGGTGGGGCAT---------AGGCTGGTGGGACTGTTGGGGGCGACAGGGAAGAGGGGTGGAGTTGCATAGGCCAAGTGCTAAGGC
Mus musculus  TTGTGATGGTTTCTTCTCTT--TGGGGTAGCCAGAGGTGGG-CATCGTGGTGGGAAGCTTGTGGTATTATTGGAGGCAAGGGGCAAAGGGGTGGGACCCGCAGAGGCCAAGAGCTAAAGT

Homo sapiens  GGAGCTTCTGGAGGACTGTCAAGTGAAAGGCGGCCAGAGGCAAATGTCAGAGCCTATATAGAGGCAGGAATCTGGGACCAGAGAGACAGAGCAAGCAGTAGCAAGTCAGGAGCAGTGGGG
Mus musculus  AGAGCTTCTGGAGGGCTGACAAGAGAAAGGCGGCCAGAGGCAAATGTCAGAGCCTATATAGAGGCAGGAATCTGGGACCTGAGAGCCAGAACATGCACTAGCAAGTCAAGAGCATTGGGG

Homo sapiens  CGGGTGGGCGGGC----AGGCTGGCTGGCGGGCA---GGGGGGCGTGGCAGGGGATAGCAAAGGGAGATTGCAGAGAACAGCTGTTGGTACTGGGTCTTACTTTAATGAACTGGCTGGGC
Mus musculus  CCGGTGGGCGGGCCAACAGGCTGGCTGGCGGGCAAGAGGGGGGTGTGGCATGGGATAGCTAAGGGAGATTGCAGAGAACAGCTGCTAGCATTGGACCTTGCTGTAATGAACTGGC-----

Homo sapiens  ACGAGGTATAAGTTAATTGGCCAGAGTGAAAGAGCTAGGGGCGCAAAGGATACTCTCTCCCTCTTCTCCGTCACTGGCAAGGTAAGATCTTTCTGGGTTTTGCCTGGTGGGAAACTGTGG
Mus musculus  ACAGGGTATAAGTCAATTGGTCAGACCAAAAGAGCTACAG-TGGAAAGGACACTATG--ACTCTTTTCTGTCACTGGTAAGGTAAGAACTTTCTGAGTTGTGACTCGTGGAAAATTGTGC

Homo sapiens  CATGTTCAGATATTTTTCTGAAGATGGGAGGTGGAGGTCTATGGCTGCTTTTTTGA---CTCATCTTTCCCATTGCACCAATTATTACAATTATTACAATAACCAGGTGTCAGCAGGAG
Mus musculus  TGCACACAGGGATTAAT-TGATGGTGAAAGGTGGAATTGCAGGATTGATTTTTTGGGGGCCAATCTTTCTCATGGCATGAATTATGA------TTAGGCTAACTGAAGTGTTTGAAGGAC

Homo sapiens  GCCAGCATTTCTTTTATGACAACCCATCTTGGGGTTGTCCAGGCTGGAGACAGAGGCAGGGGTCTCGGTAGTGTGACTGAGTAAGGGGGAGAGGTGGATCTTTATAGCACCTTGGTGAAG
Mus musculus  -CTAGTATTTCTTTTATGATGCCTTACCTTAGAGTTTCCTAGTC------------------------AATGGGATTGAG-GACTGAAAGAAGCAAATCATTACAGCCTATTAGTGAGG

Homo sapiens  AGTAGGGGGCTCTGGAACTGGATGGACCCAGGAATCTATGTGACTGCAGGCAAACTATTAAGCTTCTCTGGGTCTGAATTTTCTCATTTGTAAGATAAAGGAAGAGTACAACCGAAATCT
Mus musculus  AGTAGGGGGCTGAAGAATTGGATGGGCCTGAGAATCTTTCAAACCACACACAAATGATGAAATTTCTGTGAGTCTTCACTCTCCCA-------------GAGGATTGC--CCAGCATCT

Homo sapiens  CTTAGTG-TTGCTATGGGAACCACATGAGATGATACATGTAAAGTGCCTAGCAGAGTGTTTGCCAGTCAGTGCATGCTAAAGAAATGTTCACTGTTAGCGTTTCTGTTGCAGGTTTTCTC
Mus musculus  CTGAGGGGTTGCTGTG-----TAGATGTTACAAGGCAGGTAAAGTGCCTAGCAGAA------CTAATGAGTGCA------AGAAATGCTTGCTGTTGGTAATTCTGCTCTTAGTGTTCCA

Homo sapiens  ATTATTATGGAATCACAATTCCATGAGGTTGATGACTTTTGTGACATTGTCCCAAAGTAGCAAGTTTTCTGCTTTTGG--CATGGGCAGGCTGTGGGGACATGTCAACATTGTGTAATAT
Mus musculus  ATTGTTACAGAGGCCCAAAGCCACGAAGTTGATGCTTTCTTTGACATTGCCCCAAAGTAGCCAGTCTTCTTTTTTAGGGCCATGATCAGGTTACAGGAACATGTTAATCTTAAGTAATGT

Homo sapiens  TTG--CATTGGCTTCCAAGCCGTGTGTCCAAAAGCCCCTTCTG-GGCTGCTTATTGGAGCAGGCTGGAGGGGTGAGGCTCTG-GATTGCCCACCCCCAT------GGCATCAGAGAAACT
Mus musculus  GTATACATGGGTTTCCAGCTCGTGTGCCAGTGCACCCCTTTTGTGGCTGCTTTGGGAAACAGGTTAGAGAGGTGAGGCTATGAGAGTGGCCAGCCCCCTTCTTAGGTCAACAGGAGAATT

Homo sapiens  TGAAACTTGGACATGCCAGACACTGGGCTTTAAGTGACCCATGATCTGGGATGTCAGAGAAATAATTGAATGCAAGCTCAGGGATGCCAAAC-ACTAGGCAACCCCCAGGAGGGGGT-CA
Mus musculus  TGAAATTTCTGCATACTGGAGAATAAGCTGCCAGTGATCTCTAGGCTAGGATGTCAGACAAACAACTACACGCAATGTCAGGGATACCAAAAGATCAAGTAACTCCAAGGAAAGGGTGAA

Homo sapiens  GAGTCAATGCTATACAGTTTGTTACTGTTGCAGTGACCCTATTTTAGCCTTGACCCTGGCTAATCTGAGAAAGCTTGTAAAATGGGGATAATAATATCTATTGCCCAGGAGATTGTAAGA
Mus musculus  GAGGCAAAA-----------GATACCATCACAGTGTTCCTATTTTAGATGCAACTCTTGTTAATCTTAGAAAACTGGTACAACAGGCATAGTAATA-CTACT------------TAAGG
```

```
Homo sapiens  CCCAAATGGGATAATGCTTACAAAGGCCCTCACGTTATAGGAAGTTGTGACAGTTGTCTAAAGT-ACAGTGCCTGG---------CACATACTACGTGAAGCAATAAATAAATAGTAGTA
Mus musculus  TCCAAATAGAACCAGGCTTGTAATGTCATGCACATCATAGGCTGTTT--AAAGTTCTGTGAAGTTAAAGTTCTCTGTCATCCACACACCTACTACGT-AAGCAATAA-------GTAGGA

Homo sapiens  ATGGGGTGGTGGTTGCTGTTATTAATAAGAATAATGAAAATAATAAGCAGAATTTTCAGTTCATGGAATGTTGCTCTCAGTCATTGATTAGATAAATGTCTCCCTTTTCCTGAGAA---C
Mus musculus  TTGTAGCTGTGG---CTGTTAATAATAATGATGATGTTAATATTAAGCAAGGTTTT-GCTTTGCAGAATGCTATTCTTAGTCATTGATCAGATAAATGTGCTATGTTTCCTGAGAAAGAC

Homo sapiens  ATTCAGGGCCATCAGAGTAGTAAAATGAGGATTAAACCACTGGCAGAATTCAATGAGCCCCAGGAACTAATAATTATCCTGGGGCAAAGGACTTTCCCATTCAGGCTTGCCAGTACCTTG
Mus musculus  ATTAA------TAATACTAGTAAAATCTGGGTTAAACCATTGGTGGGATTCAATGACCATTAGGAACCAATAATTATCATGGAGTACAGGGCTCTTTCATTCCTGCCTACCAGTACCTAG

Homo sapiens  GCTGGCTTGTGAGTACTGCCGTGGGATAGAATACAGGCCAGGAATCCGCATTTCTCACATTTGGCCAGAAGGTGGTGCTGCTGCTTTATTTGAGTCAGGACTGCCCAGGCTCCTGGCTGC
Mus musculus  GTTGACTTG---------CCTTTGAATA--ACTTAGGCAAAGAATCTGAACTTCTTGTAATTGGCCAGAAGGTGGTGCTGCTGACTCATTTGAGACAGGGCTTCTCTGGCTCT-------

Homo sapiens  GGAGGCTGCT---GGCGTTTATAGCATTTACAAACTGAGAGGC-----------TGGGTTGGTTTGAAA-AGGATAGAGGCAGAAGGGACATTTGCTCCAGCTCCAAAGTTGTTCATTGC
Mus musculus  GGAGCCCACTATAGGCATTTATAA-ATATGGAAGCTGTGCTGTATAACTGGGTATGGGTTGGTTTAAAAGAGGACAGAGGTTGGAAGGATAGTTGCTTCAGCTTTGGGGATATTTGTTGC

Homo sapiens  TTCACAGCTTCTTTAATCTGATAGCCTCACCTTCAATGCCATATACCTGACAACGCATAAAAACAGGTCTCCACCATGAACAATTTG--TCTGTTTCCAAATCTTCTTGATACTGGCTTT
Mus musculus  TATATATTTTCTTCAAACTGATAGACCCACTTTCAAAGCCATATATGTGACAACTCATAAAAGCAG----CCTCTATGAATCATTGGAATATATTTCTAAA----------ATTGGTTTT

Homo sapiens  TGACCTCTAAACTTGTTTCTTGTTTACAGATTTTCTTGCCTCCTGGGCTCCCTC-----------------CACA---------------CTATCCTGTGGCTGTGGCAGATATGTCAAT
Mus musculus  TGACCTTTAATCTTTCCTCTTGTTTACAGATTTCATTGATTTGTGGGTTTCATCAGTGCCTCTGATGTCAACACATTAGAGTCTGCAACCCTGCCTTGTAGCCATGGCAGATATGTCAAT

Homo sapiens  ACCCTTACATTCACTGCGATTCAACAATACAATGAGGGAGGAAAATGTTGAGCCCCAAAACAAGCAGATGGCCTTCTGTAGACCAATGACAGAGACCAGAGCAGACGTACAGATTCTGCA
Mus musculus  ACCATTACATTCACTACGCTTCAACAATATGATGAAGGAGGAAAATGGTGACCCTCAAAACAGAGGGGCAACTTTCTCTAGACCAATGACAGAGACCAGAGCAGAAGTTCAAATCCTGCA

Homo sapiens  TTCTCATGTACAGTTGCCTATAGTCTCAACTTCAGCCTCAGACCCTGGAGGGACATCCACACAGTTGATGACATCTCCAGTCTTTGACACCATGTCTGCACCTCTAATGGGAGTACCAAA
Mus musculus  TTCACAGTTGCAGTTGCCTGTTGTTTCGACTTCAGCCTCAGATCTCGAAGGGACATCTACACAGCTGATGACATCGCCAGGCTTTGACAGTCTGTCCACACCTCTCATGGGAGCACCACA

Homo sapiens  CTCTGGAGCATTGTCCCCACCCCTAATGCCAGCCTCAGACTCTGGGGCACTTTCCCCATTGCTAATGCCAGCCTCAGA---------------------------------------CTC
Mus musculus  CTCTGGGACGTTGTCCCCACCACTGATGTCAGCCTCAGACTCTGGGACACTGTCCCCACTGCTAATGCCGGCCTCAGATTCGGGAACACTATCCCCACTCCTAATGCCAGTTTCAGACTC

Homo sapiens  TGGGGCACTGTCCCCATTGCTAATGCCAGCCTTAGATTCTGGAACATTGTCCCCATTGCTGTCCACTTCAGAGTATGGGGTAATGTCCCCAGGGATGATGACAATTCCTGATTTTGGAAC
Mus musculus  TGGAACACTGTCTCCACTGCTAATGCCAGCCTCAGATTCAGGAACACTGTCCCCACTGTTGTCTACTTCAGACTATGGGTTAATGTCCCCAGGGATGATGTCAATACCTGATTTTGGGAC

Homo sapiens  GATGTCCGCAACGCTAATGGTAGCACCAGATTCTGCAGAGATATCACCATTGGCAATGCCAGCTCCATCCTCTGGAGTGGTGTGTACACCTATAATGAGCACTTCATCCTCTGAGGCAAT
Mus musculus  AATGTC---ATCACTAATGGCAGCCCCAGATTCTGCAGAGATATCACCATTGGCAATGCCAATTCAGTCCTCTGGAGTGATATCTGCACCTATAATGAGCACTTCATCCTCCGAGGCA--

Homo sapiens  GTCCACACCATTAATGCTAGCCCCAGATTCTGGAGAGTTATCCCCAATTCTAATGCAAGATATGAATCCTGGAGTGATGTCTACACAGCCAGTGCCAGCTCCCAGCTCTGAGGCAATGTC
Mus musculus  -------TCGTTAATGCTAGGATCAGATCCTGGTGAGATATCCCCACTCCTGATTCCAGATATGAACCCTGGAGTGACATCCACACCACCAATGACAGCTCCAGGCTCAGAAGCAATGTC

Homo sapiens  CCCATTGCAAATTACAGATGAAGACACCGAAGCAATGTCCAAAGTGCTAATGACTGCTCTAGCCTCTGGAGAGATATCTTCGCTGCTAATGTCAGGCACGGACTCTGAAGCAATATCCTC
Mus musculus  CCCACTGCAAATTACAGATGAGGACACAGAAGCAATGTCCAAAGTGCTAATGACGGCTCTAGCTTCTGGAGAGATATCATCACTGCTAATGTCAGGCACAGACTCTGAAGCGATATCTTC

Homo sapiens  ACTGATAATGTCAGCTGTAGCTTCTGGAGGTACATCACCCCAGCCAACAAGCACCCAAAACTCTGGGGGAATACCTACCCCGCTCATGTCAGATCTAGACTCTGGAATAATGTCATCGCT
Mus musculus  TCTGATCATGTCAGCTCTAGCTTCTGGGGAAACACCAGCCCAGCCAACAAATCCTCCAGAGTCTGAAGGGATACCCACTGTGCTCATGTCAGGCTCAGACTCTGCAGTCATGTCTTCACT

Homo sapiens  TTTAATGTCATCTCCAGGCTCTGAAGTAATGTCCACACCGCTACTGTCAGTCCCAGATGCTGGAGAAATGTCCACATTACCAAAGCCAGCTCCAGATGCTGAAGCAATGTCCCCAGCACT
Mus musculus  GCCTATGCCAGTTTCTGGCTCTGGAGCAATGCCTACACCACTCCTGTCAATCCCAGATGCTGGAGAAATAGCCACATTACCAAAGCCAGTCCCAGATGTTGAGGCAATGTCCCCACTGTT

Homo sapiens  AATGACGGCCCTACCCTCTGGAGTGATGCCCACCCAAACGATGCCAGCCCCAGGCTCTGGGGCGATGTCCCCATGGTCAACACAAAATGTAGACTCTGAAATGATGTCTAATCCGCCAGT
Mus musculus  AATGACAGCCCTCACCTCTACAGTGATGCCCAGTCAACTGATTTCAGCATCTAGTTCTGGAGTGATGTCCCCAGATACAACACAAAATATCAACTCTGAGGTCATGTCTGCTCCACCAAT

Homo sapiens  GAGAGCAACAGCCTCTGGGGTGATGTCTGCACCACCAGTAAGAGCTTTAGATTCTGGAGCAATGTCCACACCGCTAATGGGAGCCCCAGCCTCTGGAAATATGTCTACATTGCAAAAGAC
Mus musculus  AAGAGTTTCATCCACTGGATTGATGTCCACACTACCTGTGAGAGCTTCTGATACAGCGGCAACACCCATACAGCTAATGAGAGTCCCTGCCTCTGGAAATATGTCTACATCACAAAAGAC

Homo sapiens  AGTTCCAGCCTCTGGAGCCATGACCACCTCACTGATGACAGTCCCAAGCTCTGGAGTGATGTCCACAGAGCAAATGTCAGCCACAGCCTCTAGAGTAATGTCCGCACAGTTAACAATGGC
Mus musculus  AGTTCCAGTCTCTGGATCAATGTCCACTCCACTGATGGCTGTCACAAGCCCTGGAGCAATATTCACAGAACAAATGCCAAGCACAGCCTCTGGTACAATGTCTACACATTTAACAATGCC

Homo sapiens  CAAAACTTCTGGAGCAATGCCCACAGGCTCTATGAAAGCCGTGGCAAAACAATACAAGAGAGCCACAGCCTCTGGAAAGATGTCCACGCCACTGAGGAGAGCTCCAACTTCTGGAGCAAT
Mus musculus  CCAAACACCTGGAACAATGCCCATAGGTTTTATGAAGTCCACATCAAA-----------------------TGGAGCCGTTTCTGCACAGCAAATTAGATGTTCAGTTTCTGGGATGAT

Homo sapiens  GTCCACCCAACCAGTTACGGCAACAGCCTCTGAAACAATGTCCATGCCACAATTGACAGTCCCAGCCTCTGGATCAATGTCCATGCTGCAAATGAGAGCCCCTGTCTCTGAAGCAATGTC
Mus musculus  GTCCACACAGCCAGTTATAGCTACAGCCTCTGAAATAATGTCTGTGTCACAATCAACAGTCCCAACCTCTGGGTCAGTGTCCACACAGAAAACCCGAGCTCCTGTCTCTGGACCAATGTC

Homo sapiens  CATGCCACAAATGAGAACCATGGCCTCAGGATTGACATCTGCAGCACAGATGAAAGCCATGACTTCTGGAGCAATGTCCACCCCACTAATGACAGCCCAAACCTCTGGATCAACATCCAC
Mus musculus  TACAACCCAAATAAGAACCACAGCCTCAGCGTTGACATCTACACCACAAATGAGAGCCACAGCCTCCGGGACAATGTCTATCCCACTGATGACAGCCAAAACCTCTGGATCAGCATCCAC

Homo sapiens  GCTGTTAATGAGAGACACAGCCTCAGGAGTGATGTCCTGTCCACAAATGAGATCTCTGGCCTCTGGAGCATTGTCCAAGCCACTAATGACACCCAAAGCCTCAGGAACGATGTTCACGGA
Mus musculus  ACTGTTAATGAGAGACACAGCCTCAGGAGTGATATCCGTGCCACAGATGAGAGCTCCAGGCTCTGGAACAGTGTCCAAGCCACTAATGACATCCAAAGCTTCTGGA---ATGTTCATGCA

Homo sapiens  GAAAATGACAACCACAGCTTCTGAAGCAATGCCCACACTGCTAATGAGAGACACAGTTTCTGGAGCTCTGTCCATGCCGCAAATGACAGACACAGCCTCTGGAGGGTTGTCTGCATCGCT
Mus musculus  GCAAATGACAACTGCAGCTTTTGGAGCAACGCCTACATCGCTAATGAGAGACACAGCTTCTGGAGGTCTGTCCATGCCACAGATGACAGATCCAGCCTCAGGAGGCATGTCCACACTGCT

Homo sapiens  AATGAGAGACACAGCTTCTGGAGCAATGTCCACATCACAAATGACAGCCACAGTCTCTGGAGGGATGTCCATGCCACTAATGAGAGCTCAAGACCCAGGAGTAATGCCTGCCTCACTAAT
Mus musculus  AACAAGAGCCACAGCTTCTGGAACAAAGTCTACATCACAGATGACAGCCACCACCTCTGGAGGCATTTTCATGCCTCAAACAAGACTCTCAGGTCCTGGAGCAACATCCACACCACTAAT

Homo sapiens  GAGAGCCAAAGTGTCTGGAAAGATGCTCAGTCAGCCAATGAGCACCCAAGATCCTGGAGGGATGTCCATGTCGCCCATGAAGTCCATGACCGCTGGAGGGATGCAGATGAATTCCCCAAC
Mus musculus  GAGAGCCACAGCCTCTGAGAAGATGCCTAGTCAGGCAATGAACATTCAAGACTCTGGAGGAGTGTCCACACCGCTCATGAGACCTGTTGCCTTGGGAGGGGTGCA---------------

Homo sapiens  CTCTGATGTGATGTCCACACCAACAGTGAGAGCCTGGACCTCTGAAACAATGTCCACACCACTAATGAGAACCTCAGACCCTGGAGAGAGGCCCTCACTGCTCACAAGAGCTTCATCCTC
Mus musculus  -----------------------AATGAGATCCCAGGGCTCTGGAACAATGTCCACACCCCTATTGAGAGCCTCGGACTCATCAGAGATGAGTATGTTGCTCACAAAAGCCCCATCCTC

Homo sapiens  TGGAGAGATGTCCCTACCACTAATGAGAGCTCCAGCTTCTGGAGAGATAGCCACGCCTCTGAGATCCCCAGCTTATGGAGCCATGTCTGCTCCACAAATGACAGCCACAGCCTCTGGAAT
Mus musculus  TGGAGAGCGGCCTCTGCTATTAGTGAGACCTCCAGCTTCAGGAGAGATAGCTCCACATTCGAGAACCCCAGTTTATGGGACAATATCTGCTCCACACATGACGACCACAGCCTCAGGAGT

Homo sapiens  GATGTCATCCATGCCACAAGTGAAGGCTCCCATCTCTGGAGCAATGTCCATGCCACTAACAAGATCCACAGCCTCTGGAGGGATGTCCATGCCACTGATGAGAGCCCCAGACTCTAGAGT
Mus musculus  GATG---ACCATGTCACCTATGAAGACTTCTGTTCCTGTATCAGAGTCGGCAACTCTCCTGAGACCCACAGATTCAGGAGTGATGTCCATACCACTGACGAGAACACCAGCTTCTAGAGC

Homo sapiens  GACATCCACATCACAAATGATGCCCACAGCTTCTGGAGACATGTGCACACTACCAGTGCGAGCCCCAGCCTCTGGAGGGGTGTCCTCACCACTAGTAAGAGCTCCAGCCTCTGGAACTAT
Mus musculus  AAAGTCCAGACCACAAATGG---CCACAGCTTGTGGAGACATGTGTCCACTCCCAGTGCGAGCTCCAGCCACTGCAGGGATCTCTCCATCACCAGTCAGATCACCAGCCTCTTCCACCTT

Homo sapiens  GTCCACACCACTAAGGAGACCCTCAGCCTGTGAGACTGTGTCCACAGAGTTAATGAGAGCTTCAGCCTCTGGACATATGTCCACTGCACAAACAACAGCCATGGTCTCTGGAGGGATGTC
Mus musculus  GTCTACACTACTTAGGAGACCCTCTG---ATGGAGCTGTGACTGCAGAGTTAGAGAGAGTTCTAGGCC---------------CTGCACAATTTGCAGCTATGACCCCGGGAGAGATGTC

Homo sapiens  CAAGCCATTAATGAGAGCCCCGGCCTCTGGAACAATGCCCATGCCTTTAATGTCAGCCATGGCTTCTGGAGAGATGTCTATGCCGCTAATGGAAACCATGGCCTCTGGAGCAACGTCCAC
Mus musculus  CAAGCCACTGATGAGGGCTTCAGCCCCTGGAACCACAACCATGCCTTTGATGTCCCCCATGACTTCTGGAGAGATGTCTATGCCACTAATGAAAACCACGCCCTCTGGGACAATGTCAAC

Homo sapiens  ATTGCAAACCAGTGTTGCGAACTCTAGATCTATGTCCTTGTCACAAACAACATATACCGTGTCTGGAAGGATGGCCACAGCGCCAATTAGAGCCTCTGCTTCTGGAGCAAGGTCCACATC
Mus musculus  TCTACAAACCAAAGTCATGAGCTCTAGAGCTACATCCTTACCACAGCCAAGAAATGCAGCTTCTGGGGTAATAGCTAACCCCCCACAGAGAGCCCCAGCCTCTGGAGCAGGGTCTACACC

Homo sapiens  ATTTATGAGAGCCTCAGTTTCTGGATCGATGCCCATGCCACTACCAAGAGCCACAGCCTCTGGATGTGGCATGGGTATGTCCATGCCACAAATGACAGCCACAGACTCTAGAGGGATGTC
Mus musculus  ACTTATGAGAGTCTCAGGTTCAGGAATGATGTCCACGCCACTATTGGGGGCTACAGCTTCAGGA---------GGAATGTCCATGCCACAAATGGCACCTCCAACCTCTGGAGATATGTT

Homo sapiens  CACACCACTAATGAGGGCCTCAGGCCCTGGAACAATGTCCACACCACAGACAGCCTTTGGAGTGATGTCCACTCCGGAAATCAAAGCCACAGACTCTGGAGAGGCATCCACCTCTCACAT
Mus musculus  CTCACCACTAATGAGGTCCCCGGCACCTGGAATAATGTCCACACCACAAACGGCCTTTGGAATGACACCCACTCTGAACGTCAAAGCCACAGATTCGGGAGAGGCATCTACCTCTCACAC

Homo sapiens  TAACATCACAGCCTCTGGATCAAAGCCCACATCGCACATGACTGCCACAACTCCTGAAACTGCGAAACCACCACCAAAGGAAGTGCCATCCTTCGGAATGTTGACCCCAGCACTCTGCTA
Mus musculus  CAGATTCACAGCCCCTGGCTCAAAGAGCACACCACACATGACCAGCACAGCCCCTGAAATGAAGACCCCCCCACCAAAGGAAGTGCCGTCCTTTGGTATGTTGACCCCAGCACTCTGTTA

Homo sapiens  CCTCTTAGAAGAGCAGGAAGCAGCCCGGGGCTCATGCTCTGTGGAGGAAGAGAT---GGAGATTGATGAGGAGAAGCAAATGAAGGGGTTTTTGGACGATTCAGAGAGAATGGCATTTCT
Mus musculus  CCTCTTAGAAGAGCAAGAAGCAGCCCGGGGCTCATCCTCTGTGGAGGAAGATGCAGAGGAGATTGATGAGGAGAAACAAATGAAGGGGTTTTTAGATGATTCTGAGAAAATGGCATTTCT

Homo sapiens  GGTATCTCTTCATCTGGGAGCAGCAGAGAGGTGGTTCATCTTGCAAATGGAGGTAGGAGAACCTCTCTCACATGAAAATAAATCTTTCCTGAGAAGATCCCAGGGCATATATGACTCCCT
Mus musculus  GGTGTCTCTTCATCTGGGGGCAGCAGAGAGGTGGTCCATCTTGCAAATGGAGGTAGGAAATCCCATCTCCAGTGATAATAAAGCTTTCTTGAGAAGATCACAGGGCTTATATGACTCCCT

Homo sapiens  ATCTGAGATAGACATCCTCAGTGCTGTTCTTTGCCATCCCAAACAGGGCCAAAAGTCAGTCAGGCAGTATGCCACTGACTTCCTGCTGCTGGCCCGACATTTGTCTTGGTCTGATGCCAT
Mus musculus  ATCTGAGATAGACATCCTAAGTGCTGTTCTTTGCCATCCCAAGCAGGGCAAAAAGTCAGTAAGGCAGTATGCCACTGACTTCCTGCTGTTGGCCCGACATTTGTCTTGGTCTGATGCCAT

Homo sapiens  TCTACGGACCAGGTTTCTGGAAGGACTCTCAGAAGCTGTTACCACCAAAATGGGTCGGATCTTCCTGAAGGTGGCTGGCAGCCTAAAGGAGCTGATAGACAGGTCTCTGTACACCGAGTG
Mus musculus  TCTACGGACCAGGTTTCTGGAAGGACTCTCAGAAGCTGTTACCACCAAAATGGGCCGTATCTTCCTGAAGGTGGCCGGCAGCCTAAAGGAGCTGATAGATAGGTCTCTGTATACTGAGTG

Homo sapiens  CCAGCTGGCTGAAGAGAAGGATTCCCCAGGCAACTCAAGCCAGGTTCTGCCAACAGCCTGTAAGCGGAATAATGAGGAGGCCATGGGGAATGAACTGAGCTCTCAGCAGCAGACTGAGGA
Mus musculus  CCAGCTGGCTGAAGAGAAGGATTCTTCAGGCAACTCAAACCAAGTTGTGCCAACATCCTGTAAGCGGAACAATGAGGAGGCCATGGAGAATGAACTGGGCTCTCAGCAGCAGACTGAGGA
```

```
Homo sapiens  GGTAATGAGGGGGAAATCCGCTGAAGGTTTTTGAGCAGAGAAATGAGGAGGTCACGACAGGGAAT--ACATCC-TGCTTCCTTGAGTAGAATT-GGGAAGAGGCAAGGGGGAGGCATGGA
Mus musculus  GGTAATCAAGGGGACATATGCTCATGGTTTCTGAGTAGAAAAATGAGGAAGTAATGATTAGGGATTAACACCCATAGATTTTCAGGTAGAGTTAAGGAAGAGGCAAGAAGGAGGTACTGA

Homo sapiens  GCTCCTAGACCATTAAACCCCAGC----AGTTTCATCAAGTGAGCATGGCTCTGCAGTTATTTTGGGTATATTAACTACTTTGAGGAGCAATGTCCTGCCCAAGTTACTGAGAACGTGGG
Mus musculus  GTTCCTAGACCATAAAAGTCCAATGTAGAATTCTACC-AGTAAGTATGGATATGAAGTTATTTTGCTTCTATTAGCCATTTTCAGAAACAAAGTCTAGCTCAGGTTACTGAAGGGG----

Homo sapiens  ATAGTTGAAGTACTAATTCTTCTCATTATGAGGGAACTGGATGGGCTCAGTTCCAATTCTCCCCTTGTTCCAAGTCCAATTCTAATAAATGGGTCTTTTGGGAGCTGGGGCCCATCTACA
Mus musculus  ------------------CTCCAGATCATAAGGAAACAGTAC--ACTCAATCCCAACTCTTTGTGTATTGCAAATCCAGTTGAGAGAGAGAGATCTTTTATGGATTGATTTCTACTTATA

Homo sapiens  GCAACATGTGTTGTGGAGTCAGGTCACTAGGGAATTAGAATAGCCTACTCCTTGGTCACACTGTATGCAAGTGAGATAAATTAGCAAAGTGTCTTGATCTGCTAAGTCACAGAACTTATC
Mus musculus  GCCACATGTGTTATAGATGCAAGTCACTAGGAGACCTCATTAATTTAACACCTGGCCATACTTTTTCCATGTCTGATCAATTA-CTCAGTTCTTTGACTTGGCCGATTACTGAACTTAAT

Homo sapiens  CAGGCAGAACACCCGTTGCCATAGTAAGTGTGGCTCTTACCTCTGTTGTCTTTTTCTCACTCCCAGCACCAGCATGTTTCCAAACGCTGTTACTACCTGAAAGAGCATGGAGACCCCCAA
Mus musculus  CAGATACAAAGTCTTTGGTCATAGGAAGTGTGATTCTTACTTATGCCTTTCCCCCCTTTCTTATAGCACCAGCATGTTCCCAAACGCTGTTACTACTTGAAAGAACATGGAGACCCCCAG

Homo sapiens  GAAGGTCTTCATGATCACCTTGGACAGAGCACAGGCCATCATCAGAAGGCCCATACCAACAAGTAAAACTCCATGGAATCTTCTCCTGTGATATCTGACTCGACCGCTAACTGGAGGACT
Mus musculus  GAAAGTCTTCATGACCACCTTCGACAGAGTGCAGGC---CTTCCGAAGGCCCCTACAAACAAGTAGTACTCCTTGGAATCTT---ATGTGATTTCTGACTTGGCTGCTAATTTGAGGACT

Homo sapiens  GGTTCCTGGACCCATTCCATTAAACTACATTATAAACCTTGTTGCCTTCACCCTTCAGCCTCCCGCTCCAGGCACATCCCACCTTACCTCTCACTTGGCTGATTTGACCTTCTCTTTCAC
Mus musculus  GGTCCCTGGACCCATTCCATTCAACTACATTGTAAAACTTGTTGCCTTCACCCTCCAGCCTTCCTCTCCAGGCACATCCCACTTTATCTCCCTTTTGGTTGCCTTGACATTTTCTTTCAC

Homo sapiens  CCACATGCCTAAGACCTGCCCTGACAATCAGAGTAGGGACTACAAGAGTGTATGGTGTATGAGGTTCTGAACTCCCATCA---CCACCGAGGCCAGTCCTAGTTCTTGGTCCAGGGAGAA
Mus musculus  CTACATGCCTGTGACCTGCCCTGACAATCAGTGT--GGACTATATGAGTGTCTGGTGTATGAGGTTCTGAACTCCCATTATTGTCATTAAGGCCAGTTCCAATTCTTGGTCCAGGGAGAA

Homo sapiens  GTCTAGGCAGGATAAATCATATCTTACCTCAAAAAACCCTTAGCGTATCTTGCCATGTGTCAGCAGGCAAACTTGAGGAAGCCCTGGTTCTATTTCACCTGGCAGGGGAGCCTATAAGGA
Mus musculus  GTCTAGGCAGGAGAAATCATGTCCTACCTCAAAACA-CCTTAGAGTGTCTTGCCATGTGTCATCAGGCAAACTTGAGGAAACCCTGGTACTATTCCACCTGGCAGGGGAGCCTATAAAGA

Homo sapiens  GAGCGATGCCCACTCCTATACCACCCTTGTAACCCAGTAGACTTGCCC-CATATGCCCCAACTCATAGCACCTAAAATGGGTCTCCAGGGTCCTTGCCCTGTTTATAGTATCCATCCTGA
Mus musculus  GAGAGATACCCACTCCTATACCATCCTTTCAAACCAGTAGACTTGCCCTTATATGCCCCAATTCAAAGTATCT-AAATGGGTCTCCAGGGCCTTTGCCCTGTTAATAGTCTCCATCCTAA

Homo sapiens  ACCATTCGTTTTGACCCA------TAATAATAGTGATAGTATAATGGCTACCA---------------------------GTTATTGATAATGTATCATGTGACAGGCATTGGGCTAAA
Mus musculus  ACCATTTGTTTTGACCCATGATATTAATAGTAGTGATAG--TAATGGCTAACAATGTGGTGTTGTTGGTACTTGCCATGTGATTGTTGGTAATGTGCCATGTGATAAGTACTGGGCTAAA

Homo sapiens  CTCTTGACAAGCACCATCTCACTACATTCTTACAGTAACACAACTTGTTCTTGTGTTGTCAGCTTCTGACCTCTTGATCTGTTGTTTATGGCCTTTGTCTTGTCCCTCAGTGGGGGTTCT
Mus musculus  CCTTTGACAAACACCATCTCACTACAGTGTTATAGTAACCTACCTTGTTCTTGTGTTGTTAGCTTCTGATCTCTTGATCTGTTGCTTATGGCC-----CTTGTTCTTCAGTGTGGAATCT

Homo sapiens  CTAGCTCTGACCTCTGGGTGACCCCCTCAGTTGTTGATTGCTCTCACTCCCCTTGGCTGTTGTGCTTCATGGCCAGCACTCTTGATGAGGGTCTTGGTCTATAAACAGGCATAGATAGCT
Mus musculus  CTAGCTATGACCTCTGGGTGATCCCCTCAGTTGTTAAGTACCCTTGCTCCCCTTGGCTGTTGTGCTTCATGGCCAACACTCTTCATAAGGGCCTTGGTGTCTAAACAGGTGTAGGTAGTT

Homo sapiens  AGAACATAATGTGAGCAGTGTTTGCCTTCAGGTGGTGGGGTTATGGGTAGTTTTTATTTTCTTCCTTCTACTTCTCTATACATTTCAGATTCTCTACAGTAAGCATGTGTTACATTTGTA
Mus musculus  AGAACATAATATGAACAGTGTTTGCCTTCAGGTGGT-GGGTTATGGGTATTTTTTATGTTCTTTTTACTG-TTCTCTGTACTTTCCAGATTGACCACAGTAAGCATGTATTACATTTGTA

Homo sapiens  ATTATAAAAGTAAGTAAACTTCATTTATGAAAAAAGAGTTCATTGTGGCTCCTAGAATTTGATTTACTGAGTCCTTTA----TTCAACCAATATT------------------------
Mus musculus  ATCATAAA---AAATAAAGGTTATTTGTGAAAAATGAATTAACTGTAGCCTTTAGAATTTGATTTATTGAGTTCTTGAATATTTTAACCTTTATTTTATTTTGTGTGTTTGTGTGTGACA

Homo sapiens  -------AGTTATG--------------------------TAACTTATGTCAGCTACTCTTTGGGGGACAGACGGATAAGAATCCTGCTGATATGAACATTCCATTCCAATGGAGTGGG
Mus musculus  GGGAAAGGGTGATGCACAATGCATATGTAGAGGCAAAAAGATAACTTGTGTTGGCTACTGTTTGGATGACA----GTTAAGACCTTTTCTTATATTAAAATTCCACCCAAATTAAGTTAG

Homo sapiens  GAGAACAAGAACACACTGAACTTAGATTGGAATTGTGAAGGCTG--------TGAAGAGAAGAAATCAGGACAATGGGGAATTTGATGCAAATAAGACCAATTTATGTATTCAATAGTAG
Mus musculus  GAGAGAAACAACAAGCAAGAC-CACATGTAAAAAGAGACAGATACATTAGCTTGAAGACGAGAAA-CAGAAAAATAAGAAAATTGATATAAGTAGGACCAATTTATGTTTTCAATAATAG

Homo sapiens  TCCATATAATTGAGTTACTCAAAGATCTTTCAACAATAGTAGGAGAGACATGATTAGAAACTTATTTTATATAGTTAAATTGGCAACAGTTTGGAGACAGGATGGAGGAAGGGATCAGAG
Mus musculus  TCAGTGTAG------TATTCAAAGATATTTTAGAAATAGAAGGAATGATTAAATTTGAAAACATTTCTAATT--CTAGATTGGTGACAGTTTGGAGGCAGGGTGGAAGAAGAGGTCAGAG

Homo sapiens  AGCAGCTGAGCCCTCTACTGCAATAGTCCAGGTAACTGACTGGCTAGGTGACTCAATCTAGGGTGGTGGCAGCAGAGATGATAATTCAGGAACAGAGACCTAACCTTCTCTGAGGACTCA
Mus musculus  -GCAGCTGGGGCCTCTGGTCCAATAGTCTAGGAGATTGGTTGGCTGAATGACCTA--CTGTGGTGGAGGAAACA-----------CCAAGGACAGAACCCAAAGCCTCTCTG--------

Homo sapiens  AGTCCACTGCTCCAGGACTCAAGTCCACTGCTCCATTACCCTAAACTTTGTGTCTGATTTCAGGAAGAGGAAAATGATACTCAGGAAAGAC--AGATTGGCAGATAAATC
Mus musculus  ---------------------AGTCTGCTGCTCCATTACCCTAAACATTGGACCTGATTCAAGGAAGAGGGAAATTATTCTCAGGCAAAGCAAAGGCTGGCAGACACTTT
```

Rgag4

```
>chromosome:GRCh37:X:71346358:71352358:-1
GCATAACTTATGGCCCTGCCTCAGTCTCTGTCCTTGTTTCCCCATGGGCTACTGAGCTGA
AAAAAGGGGCTGGGGATGGGGGAAACAGCCCTGGAAATAGCCTCTTTCAGAATCATTAG
GCTGAAAGGTGAAAGCTGGAAACTGCTTTGTCGATTGTTTTTAAACTCTCTGTCTTTATC
TTGCTGGTCCCTGTCCTCTTTCTTCTTTGCCTCCCTCCTGCCGCTGCGCATCTCGCTTCT
GGGCTCCGGCCGCAGCCCCCCTCCCTCTCGGTACCTTGCAACAGGCTCTGGAGATTGAGC
TGCGCCTCCCGAAGCAGCTCCTCTACACTCGGGGGCCTGCCCGAGGAGAGGAACACGTTC
ACTGGCTGTCGCCATGACGACCTCGAGTGGGCAGTCGCTGTCGCATTTTCCCGACCCGAG
TTAGCTGCAGCTAGGGAAGGAGAGAGTCAGTGAGAGGCGCAGGCGAGCTGGAGGGGAGCG
GCGCGCGGCGGCCAGTGGGCGCCCGCGGTTCCCCAAGGCCCGCCCCTGCGTGGGGTGGGG
GGAGTGCGAGGCTGCGCTTAAAAGGCGGGGAATCGCCGGCCGCAAGATTTTTCCTCGACA
GATCCTGCCGCAGTCCGCTCCCTCCCGCTCCGCTCGCCCCTGCCCGCCCGCCCGCCCGGT
CGCGCGGTCGCCGCCGCCACCGTGTCGCCCGCATCCTCCGCCTGGCGCCTCCGGGCCCCG
TGCCTCCGCCCGCCCTCTCCGGCCCCGGCGACGAGGTAACGCGCCCTGGGCCGAGCCGCC
GGGCCCTCCGCCCAGAGCGCCGCCCTCCGAGCTCGCCCCGGTGGGTGTGGCCCGGCCTGG
CCCGTGCCCCGCTGCCGCACTTCGGTCCCTCCCGAGCCCCGCCGCCGCATTTCGGCCTCC
TCCGGCCGCCCGTAGTTCCAGTTCCCCTTTCCCCACCACGGAGCCCCAGGGCCCCTGGGC
TTGTCGAGATGTCTGAGGCGTCAGGAAATCTCAACAGCCTCCGCATGGCGAATGTGGCCC
TGCGCGAAGAATTAAATGCCCTTCGCGGGGAGAATGCCAATTTGGGCCTCCAGCTCGGGA
GAGCCCTGGCCGAGGTTAATTCCTTGCGGGGCAATGTCTCCAGCTACATCCGCTGGCCAG
TACCCATAGTGCCCGTCCTTGCGGAGGAGAACTTGGAGTTCGCGCTCAGTGAGATCGAAG
TCATTCCCGGGGGAGAACTGCCCTTCTTGTGCCGGCCTCCCCCACGCGCGGAGCCCGACT
GTATCTCGGATGATCTTTTGATTAACGTGATCCAGGATCGCAGCACCCCCGACGGGCCCG
CTGACCCCCCTCTGCTGCCCATCCCGCCCCCGCCGGCGCTGCCTCCGCCCGCGTCAAAGG
AACCGCCCCCGCAACCCCCTCTGGCACCGCTGGAGCGGCCTGAGATAGAGCCCTTTTCAG
GAGACCCAGTCTACCTGGCTGAATTCCTGATGCAGCTAGAGACCTTCATAGCCGACCATG
AGGTTCATTTCCCCGGGGGCGCCGAGCGGGTGGCCTTTCTGATCTCCTTTTTCACTGGCG
AAGCCAAGGACTGGGCCATCTCGGTCACCCAGGAAGGAAGCCCCTTGCATGCCAACTTCC
CGCGCTTCCTGGATGAAATCCGTAAGGAATTCTGTGGCCCCATCCCCCCACGTGTGGCTA
AAAAGGCCATTCGCAAGCTCAAGCAGGGCCACTGTACCCTCGGCAGCTATGCAGATGCTT
TTCAGTTCCTGGCCCAATTCTTGTCTTGGGATGACTGCCGCCTCCAAAACCAATTCCTCA
AAGGCCTGTCGGAATTCTTCCGCAAAGAGCTCTTATGGTCAACAGAAATGGCCGACCTGG
ATGAGCTGATTCTCGAATGTGTGGAGATAGAAAGAAAAGTGCGCGTTCCCAAGCCTATCC
CGCTCCCTGGAGTTCGAAATATCATCTTCCCTTTTGCTCCGAGTCCCAATGAGGAAGAAA
GTGAAGATGAAGAGTACTACAGTGAAGATGAAGACCAAGAGGCACGCAGGCACAGGCTTC
ACTCCAAGGACCAGAGGAAGCGCATGAGGGCTTTTCAGCAAGAGATGAAGGAGAAGGAGG
AGGAGGAGATGAAGAAGGAGGAAGAAATGAAGAAGAAGGAAGAGAAGGAGGAGGAGGAGG
AAGAGGAAATGAAACAGAAAGAGGAGGAGGAGGAGATAAGGAACAAGAATGAGGAGGAAG
GAGAGAGTAAGGATGAAGAAGATGAAGATGAGGATGGGGGCCAGAAACCAGAAGGAGAGC
CACAGCAGGATCCAGGGACTGAGGAGACCTATGGTGAGGTGGAGGAGGAGCCACTGGATG
AGGCCCAAGATGATGACCTAGATGAGCTGATGGAGATGGAGCCGACCTTTGTTCACGCGT
CATCCCAGACTTCTGGCCCCACAAGTGGTTACCACGCCGAAAACTTCCTGGGTGCATCAC
CTCCCATAATACAGCCTAGCAGACGGAGGAACCAGAATCGAGTCCCACTTCTGGAAGGCC
TTCCAGGCACCAATTCACCATTCTACAGCTCCCCCCAACTGATTCGCCGCACAGGTCGTT
TAGGACAACGCCAAGTTCGAAGACGCCCCCCTGTGCTTTTCCGCCTCACTCCGAGACAGG
GGGGCCACCGAGCTGCTCGTGGCCGAATTCGAGTTTGAGTGCTGGGGCCAGATGGCCACC
CAGAACACACCCTTCTTTTGCATCCCCCGCCCCTGCTATTGCTGCCATACCTGCCCCATT
TGCTGACCAGGACTTCAGGGAGTTTCAGACCTGCAGAACCTGAAGAAGACCTGCAGAATT
CCAGACCATCTTGCAACCGTGACCAGAGAGCGACGTGTGCCCAACCAGGGCCACCTGGGA
AAGGAGGGATCAGCAATAGCTGCCCTCTTGGCATGTACTCTGCTCAGTCCGGCCACGAGC
TGGAGAGCAAGTTTCTGGGCCTGTTCCCATAAATGAACTGGTCACCCTCTTGTATTTCCC
CTCTGGTTGTTCCTAACCCTCACCTCTCCTGCGTTTCTTCACCCTATGTTCTACAGTTTT
ATTCTCTGACTGACCCACCAGGACTTCACCCAATGCCTCACTGATCTGCCCCAATGAGCA
AGAGGACAGGCTACCCTGAACTCCTAAAGCCACTGAGGCCAGTTGCTAGATATAATCGAT
GGTGAGCAGGATGGTATCCAGAAATGTCCCTAGAATCCTACACTCATTTCATGCCACACC
TCCAGCAGAGGGCCTGGAAGAGGACCCGACATCTAGCCCAGTGAGAGTGTGCAAAGCCAC
ATGTCAAATGGACAACTTGACGGAGATTTGCCCTTGACTGACTGTGCAGGCTGGTTCCAC
```

```
TTACAGCACTTGAGTTTACCTTTGCACAGATTCCTGTATCAGGGCTCTTGGACTGACTTG
CCACACATCTGGAGCCCTTTCCTCTCACCACATTGCCCTCCTGGGCACCCCCGGTGGACA
GCGCCCAACATTGCAGGCTCCACAACTTTCCTTGATGTTGCCTAAAGGGTAGCCAGTTAG
GGTTCCGGAGAGGAGAAGGAGGAAGAAGAGGAGGAGGAGAAAGAAGCAGCAACGATCATT
CTGCCATTGTAAAGAGTGACTCTTTCCTTAGGGATGGTGGTCTTCTCCCCTTGCCAGGCC
TTGGACTGCAGCCCCCAGTCAAAGGTCTTTGCAGGACACTTTCTATCCTTGGAGTTGCAG
AGGAGGCCTGATTGGCCATTCCTTCTGGGACAGACAGAGAGAGGGCCCATGGCCTGCTCT
GAGCTCCCTTCAATTTCAAGGAACCCTTAGTTCTGAGAGGAGAGAGGAAGGCTGCACCTA
CCGTGGCCTCACAGCTGGGACCCCTCATGGGGGCCAACCCCTGGCCTGCTGCTGTGCTTC
AGTGACACAGTAAAGCTTAAGAGGGTGGCTGCCTCCTAGGAACTCCTGGGACTTCTAGGT
ACAAACTCAGGTGCCCTTATAAAGAGACCCACTGCTTGCCTTGAGTCCAGAAAGCACTGC
TTCTGCAGTGAAAAACACACACTTACTATGTCAGTTTTGGCCATAAAATGCCAGCCAAAT
CCACTGCTGGCCTGAACTGCCCCTGGACACTGGACTTTCTGCACAGTGACTGGCAGCAGA
GCAAGTGCAGCCAGGGTTTGTGGCCAAAGGGAAGAAAGCATAGTAAATTCAACCTCTGTT
CCTCATGGGAGTCTGACCGATAATCTCAGGCGGACCTTAGGATCTTGGGCCTGGAGGCAA
GAGAAAAAAGCTGGAGGAAGAGGAGTCGCAGAGAAACCCAGTCCTCTGACTGGTTTCTGG
TGAGGTCTCTATTCATCAGTGGGAGGTAAGATGCCCTTTTCATTAGCGCTTTTGTTTCTC
AGCCTGGGGATCAGTGGATTTGGATTCCTATAACCTGTTGCTACCTTTTAAAAAGATCCC
CTCTCTCCAAAGAGAAAAGTAATACATAATCATTAAAGAAAGTTTGGAAAATAGAGAAAA
CTATGAGGACAAAAACCCCCCAGCCATTCAGAGTCCCACCACACAGAGGCAAGCACTATT
AACACTTTGCTGCTTTCCTTTCTAGTGTGTTCTTGCTATGTCTTGTCAAAGCTGAGATCA
CCCTGCCAGTTCTTGATCAGCAGGATTTAAAATGGCTGCCACCTGTGCAGTCATGCATAC
TGAATAATTCATTTAAGCATTCTCCTAATTTGTTGGACCTTTCAATTGTCTGGTTTTCCC
CATCCCCACCAGGGCAGGAGTAAACAACTTTGTAAAGAAAATTTTGTCTGTTTTTCAGCT
GATTTTATCTGATTTCCTCAAGAGTGCATTCCAGAAGGGGACCTGCTATTCTTCGAGGGG
ACCTGCTATTCTTCGAGGCAGTGGACCCTGTTTGCTGATGGTCAACACTCTGGCCCTGCT
GCCCCATGTCCCTGTGGAGAAGTGTGAAGGACGGGTGCCTTAAACCCACACTTCCAGGGA
CCCTTCAGCACAGGTCCTGCCTGTTCCCATGGTGCTTCTCACCCTGAACCCTCAGAGCTA
CAGCCAGGGATAGAGCAGATGAGCAGTGAGCTGGTTTCCACCCTCTGGGGCAGGAGGGGC
TTGGGCTTCCCTTTGACCCTCTTCCTTCCTTTCCCAGATCTTCAATATTTGTTGTGATGT
AACAGCCATAAGAGCTGTAAGAGGAACTGTGGGCTTTGGGAGCTTTCTTGGGTTGAAAGA
GGGGTGGGGGGAGGGGATGCATGCAGGGGTATGAAGGCAGCCCAAGGTCTCCCAGAGGCT
CGGGAGGAAACCCTTTTGGAATACATGTGTTATTGTTGCATTACTGTGTTGTGAAGATAA
GAAGCTGCTCTGTATGCTAAAGCTTTCTCTCCTCAATAAAAATATAAAGGGTGTGTAAAG
ATTGTGCCTCTTTGTTATTGAGCAAGCATAGATGGGTACAGAGTAGGCATAGGGTACTTC
CAAGGTCCCCTGCCGGAGTCAACCCTACCCTGAAGTCTTGAAATCCTTAGACAAGGAAAC
CAAGGGCCGGTGAGGCTCAAGGGACCTGTCTACAGTTCTGTGCATTTGTGGATGGAAGAA
ATTGTTGGGAGGGAAGTGGAAGCACTGGGCATCTTGAAAAATAGCATTCGTTCTCCCGGG
GACTATGGGTGGCCCAGCCCTACAGGCACGCACGGGCATGCATGGGACTTTGAGTGAAGG
AAGAGAAAGAGGCCAGGTTGGCTGGGGAGGGCCCTTCCGCTTTTTGGCACTAGGTGGCGC
TATGGGATACCAGTGAGTTAGCTGGTGATGAATCACAAGTCCCCAGAGCAGTCCATGTAA
GAGGCCCTCCAAGCAAGATCTCATGGCTCCTCACGGACTGTGGGGTCAGAAGAAACTGAA
TCCCATTCTGGGATTTGCCCAAGTTTCCCAGCATGAATTGGACTTTTTTTTTTTTCCAGG
GGAAGTGGGTGGGAACATTCATTAAGCCTCTGCTGCTTTTGAGTGCTGTGCCAGGCACTT
C
```

```
Homo sapiens        GCATAACTTATGGCCCTGCCTCAGTCTCTGTCCTTGTTTCCCCAT----------GGGCT----ACTGAGCTGAAAAAAGGGGCTGGGGATGGGGGGAAACAGCCCTGGAAATAGCCTCT
Loxodonta africana  GCCTGAGCCATTGACCTGCTTCAGCTTCTGTCCTTGTTTCCCCATTACGCCACCAGGGCTCCTAACTTAACTGAAAAAAGGGG--GGGCA----GGGAAACACCGGTGGAAACGG--GCT

Homo sapiens        TTCAGAATCATTAGGCTGAAAGGTGAAAGCTGGAAACTG----CTTTGTCGAT-----TGTTTTTAA--ACTCTCTGTCTTTATCTTGCTGGTCCCTGTCCTCTTTCTTCTTTGCCTCCC
Loxodonta africana  TTTAGAATCATTAGCCTAGCAGGTGCAAGGCGGAAAATGTGTGCTTTGTTTTTCACTATGTTTGTAATTGCTCTCTCTTTTGCTCTTGC-AGTCCCTGTCCTCTTTCTTCGCTGCCTCCC

Homo sapiens        TCCTGCCGCTGC----------GCATCTCGCTTCTG-----GGCTCCGGCCGCAGCCCCCCTCCCTCTCGGTACCTTGCAACAGGCTCTGGAGATTGAGCTGCGCCTCCCGAAGCAGCTC
Loxodonta africana  TCCAGCCGCTGCTGCGAAGGACGCTTCTCACCGCCGCCGCCGGCTC-AGCCGCAGCCCCCCTCCCTCTCAGTACCTTGCAACAGGCTCTGGAGATTGAGCTGCGCCTCGTGAAGCAGCTC

Homo sapiens        CTCTACACTCGGGGGCCTGCCCGAGGAGAGGAACACGTTCACTGGCTGTCGCCATGACGACCTCGAGTGGGCAGTCGCTGTCGCATTTTCCCGACCCGAGTTAGCTGCAGCTAGGGAAGG
Loxodonta africana  CTCTACACTCGGGGGCCTGCCTGAAGAGAGGAACACATTCGCTGGCTGCCGCCATGACGACCTCGAGTGGGCAGTCGCTGTCGCATTTTCCTGACCCGAGTTAGCTGCAGCTAAG-AAGG

Homo sapiens        AGAGAGTCAGTGAGAGGCGCAGGCGAGCTGGAGG-GGAGCGGCGCGCGGCGGCCAGTGGGCGCCCGCGGTTCCCCAAGG-----CCCGCCCCTGCGTGGGGTGGGGGGAGTGCGAGGCTG
Loxodonta africana  AGAGAGTCAGTGAGAGGCGCTGCCGA--CGGAGGCGGGGCGGCGC-CGG--GCCAGTGGGCGCGCGCG---CCGCAAGGTGAGGCCCGCCC-TGCGCGGCGCGGGCCGGGGGCGAGGGGG

Homo sapiens        CGCTTAAAAGGCGGGGAATCGCCGGCCGCAAGATT-TTTCCTCGACAGATCCTGCCGCAGTCCGCTCCCTCCCGCTCCGCTCGCCCCTGCCCGCCCGCCCGCCCGGTCGCGCGGTCGCCG
Loxodonta africana  C-CTTAAAAGGCGGAGGCTCGGCGGCTCCAGGAGGAGTTCCTCGACAGATCTTGCCGCGGTT-GTTCCCTGCCGCTTCACCCGCCGCCGCCCCGCCGCTCC-----TCGC-CGGCCGCCG

Homo sapiens        CCGCCACCGTGTCGCCCGCATCCTCCGCCT-GGCGCCTCCGGGCCCCGTGCCTCCGCCCGCCCT-CTCCGGCCCCGGCGACGAGGTAACGCGCCCTGGGCCGAGCCGCCGGGCCCTCCGC
Loxodonta africana  CCGAGCCCGCG------GGCTCCTCCGCCTCGGCCCTACCCGGCTTCGCGCCGCCGCCCGCCCCGCTCCGGCTTCGGCGACGAGGTAACGCGCCCCGGGTCGCGCCGCGGGGCCCTCGGC

Homo sapiens        CCAGAGCGCCGCCCTCCGAGCTCGCCCCGGTGGGTGTGGCCCGGCCTGGCCCGTGCCCCGCTGCCGCACTTCGGTCCCTCCCGAGCCCCGCCGCCGCATTTCGGCCTCCTCCGGCCGCCC
Loxodonta africana  ACA---CTCCGCGCCGCAGTGGCCGCCTGGTGGGTGTGGCCCGGCCCGG-----------------------GTCTCTCCTGCGCCCCGACGCCGC------------CGCCGCCGTCC

Homo sapiens        GTAGTTCCAGTTCCCCTTTCCCCACC--ACGGAGCCCCAGGGCCCCTGGGCTTGTCGAGATGTCTGAGGCGTCAGGAAATCTCAACAGCCTCCGCATGGCGAATGTGGCCCTGCGCGAAG
Loxodonta africana  GTG----CGGCCCCTCGCTCCCCGCCCCGCGCAGTCCCAGGGCGA-GGAGCCAGTGAAGATGTCCGAGGCGGCCGGAAATCTCAATAGCCTCCGCATGGCAAATGTAGCCCTGCGAGAAG

Homo sapiens        AATTAAATGCCCTTCGCGGGGAGAATGCCAATTTGGGCCTCCAGCTCGGGAGAGCCCTGGCCGAGGTTAATTCCTTGCGGGGCAATGTCTCCAGCTACATCCGCTGGCCAGTACCCATAG
Loxodonta africana  AATTAAATGCCCTTCGCGGGGAGAATGCCAATTTGGGCCTTCAGCTTGGAAAAGCCCTGGCAGAGGTTAACTCCTTGCGGGGCAATGTCTCGAGCTACATGAGCTGGCCCGTGCCCATAG

Homo sapiens        TGCCCGTCCTTGCGGAGGAGAACTTGGAGTTCGCGCTCAGTGAGATCGAAGTCATTCCCGGGGGAGAACTGCCCTTCTTGTGCCGGCCTCCCCCACGCGCGGAGCCCGACTGTATCTCGG
Loxodonta africana  TGCCCGTCCTGGCTGAGGAGAATTTTGAGTTCCCGCTCAATGAGATCGATGCCATTCCCGAGAGAGAATTGCCCTTCTTATGCTGGTCTCCCCCACGTGGGGAGCCCGAGCATATCCCAG

Homo sapiens        ATGATCTTTTGATTAACGTGATCCAGGATCGCAGCACCCCCGACGGGCCCGCTGACCCCCCTCTGCTGCCCATCCCGCCCCCGCCGGCGCTGCCTCCGCCCGCGTCAAAGGAACCGCCCC
Loxodonta africana  ATGAACTGCTGGTTAATGTGATCCAGGATTGCGGCACCCCTGACGGCACCAGCGACCCCCCTCTGCTGCCCAGCCTGCCACCTCCGGCGCTGCCTGCGCCAGAGCCAAAGGAGACGCCCC

Homo sapiens        CGCAACCCCCTCTGG------------CACCGCTGGAGCGGCCTGAGATAGAGCCCTTTTCAGGAGACCCAGTCTACCTGGCTGAATTCCTGATGCAGCTAGAGACCTTCATAGCCGACC
Loxodonta africana  CGCAGCCCCCTCTGCAGCCCCCTCTGCCATCGCTGGAGCGGCCTGAGATGGAGTCCTTTTCAGGCGACCCAGTCTACCTTCCTGAATTCCTGATGCAACTAGAGACCTTCATAGTTAACC

Homo sapiens        ATGAGGTTCATTTCCCCGGGGGCGCCGAGCGGGTGGCCTTTCTGATCTCCTTTTTCACTGGCGAAGCCAAGGACTGGGCCATCTCGGTCACCCAGGAAGGAAGCCCCTTGCATGCCAACT
Loxodonta africana  ATGAGGATCGTTTCCCGGGGGGCGCCGAGCAGGTGGCCTTTCTGATCTCCTTTTTCACAGGTGCAGCCAAGGATTGGGCCATCTCAGTTACCCAGGAAGGAAGCCCCCTCCATGCCAACT

Homo sapiens        TCCCGCGCTTCCTGGATGAAATCCGTAAGGAATTCTGTGGCCCCATCCCCCCACGTGTGGCTAAAAAGGCCATTCGCAAGCTCAAGCAGGGCCACTGTACCCTCGGCAGCTATGCAGATG
Loxodonta africana  TCCCGCGCTTCCTGGATGAAATCCGTAAGGAATTCTGTGGCCCAATCCCCCCACGTGTGGCTAAAAAGGCCATCCAAAAGCTCAAGCAGGGAGACTGTACTATCGGCAGCTATGCAGATG

Homo sapiens        CTTTTCAGTTCCTGGCCCAATTCTTGTCTTGGGATGACTGCCGCCTCCAAAACCAATTCCTCAAAGGCCTGTCGGAATTCTTCCGCAAAGAGCTCTTATGGTCAACAGAAATGGCCGACC
Loxodonta africana  CTTTTCAGTTCCTGGCCCAGTTCTTGTCTTGGGATGACTGCCGTCTCCAACGCCAGTTTATCAAAGGGCTGTCAGATTTTTTCTGCAAGGAGCTCTTATGGTCACCCGAAATGGTGGACC

Homo sapiens        TGGATGAGCTGATTCTCGAATGTGTGGAGATAGAAAGAAAAGTGCGCGTTCCCAAGCCTATCCCGCTCCCTGGAGTTCGAAATATCATCTTCCCTTTTGCTCCGAGTCCCAATGAGGAAG
Loxodonta africana  TGGATGAGCTGATTCTCGAATGTATGGAGATAGAAAGAAGAGTGCGTGGCCCCAAGCCAGTCCTGCTCCCTGGGGTTCGAAATATCTTCCTCCCTTTTGCTGCTAACCCTAATGATGATG

Homo sapiens        AAAGTGAAGATGAAGAGTACTACAGTGAAGATGAAGACCAAGAGGCACGCAGGCACAGGCTTCACTCCAAGGACCAGAGGAAGCGCATGAGGGCTTTTCAGCAAGAGATGAAGGAGAAGG
Loxodonta africana  AAAGTGAAGATGAGGAGTTCTACAGTGAGGATGAAGATGGAGAGGCATGCAAAAGCAGGCATTACCTGAAGGACCAGTGCAGGTGCATGAGGGCTTTTCCGCAAGAGATGA.........

Homo sapiens        AGGAGGAGGAGATGAAGAAGGAGGAAGAAATGAAGAAGAAGGAAGAGAAGGAGGAGGAGGAGGAAGAGGAAATGAAACAGAAAGAGGAGGAGGAGGAGATAAGGAACAAGAATGAGGAGG
Loxodonta africana  .........................................................................AGGAGGAAGAGGAGATGAGAAAGAAAAGTGAGGATG

Homo sapiens        AAGGAGAGAGTAAGGATGAAGAAGATGAAGATGAGGATGGGGGCCAGAAACCAGAAGGAGAGCCACAGCAGGATCCAGGGACTGAGGAGACCTATGGTGAGGTGGAGGAGGAGCCACTGG
Loxodonta africana  AAGATGAGAGTAAGGATGAGAAA---AAAGATGAGGATGGGGGCCAGGAGACAGAA------------CAGGAGCAAGAGACAGAGGAGGTCTGTGGTGAGGT-----------------

Homo sapiens        ATGAGGCCCAAGATGATGACCTAGATGAGCTGATGGAGATGGAGCCGACCTTTGTTCACGCGTCATCCCAGACTTCTGGCCCCACAAGTGGTTACCACGCCGAAAACTTCCTGGGTGCAT
Loxodonta africana  -------CCAGGATGATGACTTAGATGAGCTAATGGAGATGGAAGAGCCCATTGCCCATACTTCATCCCAGGCTTCTGGCTCCACAAGTGGTAACCACGCTGAAAATTTCCTAAATGCCT

Homo sapiens        CACCTCCCATAATACAGCCTAGCAGACGGAGGAACCAGAATCGAGTCCCACTTCTGGAAGGCCTTCCAGGCACCAATTCACCATTCTACAGCTCCCCCCAACTGATTCGCCGCACAGGTC
Loxodonta africana  CACTGCCCATAATACAGCCTGGTAGACGGAGGAACCAGAATCGAGTCCCACTTCTGGAGGGCCTCCCCGGTACCAATTCACCGTACTACAGTTCTCCACCAGTGACTCACCGTGCAGGTC

Homo sapiens        GTTTAGGACAACGCCAAGTTCGAAGACGCCCCCCTGTGCTTTTCCGCCTCACTCCGAGACAGGGGGGCCACCGAGCTGCTCGTGGCCGAATTCGAGTTTGAGTGCTGGGGCCAGATGGCC
Loxodonta africana  GCTTGGGGCAACGCCAAATTAGAAGACGCCCCCCAGTGCTATTCCGCCTCACCCCAAGACAGGGGGGCCACCGAGCTGCTCGTGGCCGCATTCGTGTGTGAGTACTGAAGCAAGATGGCC

Homo sapiens        ACCCAGAACACACCCTTCTTTTGCATCCCCCGCCCCTGCTATTGCTGCCATACCTGCCCCATTTGCTGACCAGGACTTCAGGGAGTTTCAGACCTGCAGAACCTGAAGAAG---------
Loxodonta africana  ACTCAGAACACACCCTCC---TGGATTCCCTGCCCCTGCTATTGCCGCAACACCTTCCCTATCTGTTGACCAGTACTTAAGGGAGTTCCAGACCTGCAGAACCTGGAGAAGACACACAGA

Homo sapiens        -------ACCTGCAGAATTCCAGACCATCTTGCAACCGTGACCAGAGAGCGACGTGTGCCCAACCAGGGCCACCTGGGAAAGGAGGGATCAGCAATAGCTGCCCTCTTGGCATGTACTCT
Loxodonta africana  ACTGAAAACTTGCAGAACTCCAGACCATCTTGCAAGCATGACTGAGGAGTGACATGGGCCCAACCAAGGCCATCTGGGAAAGGAGAGATCAGCAATCGCTGTCCCCTTGGAATGTACTCT
```

```
Homo sapiens        GCTCAGTCCGGCCACGAGCTGGAGAGCAAGTTTCTGGGCCTGTTCCCATAAATGAACTGGTCACCCTCTTGTATTTCCCCTCTGGTTGTTCCTAACCCTCACCTCTCCTGCGTTTCTTCA
Loxodonta africana  ------------------------------TTCCTGGGCCTACTCCCATAAGTGAACTGGTCACTCTCTTGTATTTCACCTCTGGTCGTTCCTAACCCTCACTTCTCCTGCATTTATTCA

Homo sapiens        CCCTATGTTCTACAGTTTTATTCTCTGACTGACCCACCAGGACTTCACCCAATGCCTCACTGATCTGCCCCAATGAGCAAGAGGACAGGCTACCCTGAACTCCTAAAGCCACTGAGGCCA
Loxodonta africana  CCCTGTGTTCTCTGGTTTTATTCTCTGACTGGCTCACGGGGACTTCACCCACTGCCTCACTCATCTGCCCCAGTGATCTAAAGGACAGGCTA-----------------CACTGAGACCG

Homo sapiens        GTTGCTAGATATAATCGATGGTGAGCAGGATGGTATCCAGAAATGTCCCTAGAATCCTACACTCATTTCATGCCACACCTCCAGCAGAGGGCCTGGAAGAGGACCCGACATCTAGCCCAG
Loxodonta africana  GTTACTAGATACGGTTAACTGAGAACAGGATATTATCAAGACATATACCTGGAAGCTTACACTTGTCTCAAGCCACGCCTCCAGCAGAAGG............................

Homo sapiens        TGAGAGTGTGCAAAGCCACATGTCAAATGGACAACTTGACGGAGATTTGCCCTTGACTGACTGTGCAGGCTGGTTCCACTTACAGCACTTGAGTTTACCTTTGCACAGATTCCTGTATCA
Loxodonta africana  .....................GTCAAAAGGACAATGTAGTTGAGATTTGCCCTTAACTGATCGTGCAAGCTGGTCTC-TTTCCAGCACTTGACTTTATCTTCCTACAGACTCCTGTATCA

Homo sapiens        GGGCTCTTGGACTGACTTGCCACACATCTGGAGCCCTTTCCTCTCACCACATTGCCCTCCTGGGCACCCCCGGTGGACAGCGCCCAACATTGCAGGCTCCACAACTTTCCTTGATGTTGC
Loxodonta africana  GTGCCCTTGGACCAGCTTGCCAAAC--CTGGGGCCCTCTCCTCTCACCACCTTGCCCTCATGGGCATCCCTGGTGGGCATAGCTCAATGTTGCAGGCGCT---ACTTTTCTTGATATTGC

Homo sapiens        CTAAAGGGTAGCCAGTTAGGGTTCCGGAGAGGAGAAGGAGGAAGAAGAGGAGGAGGAGAAAGAAGCAGCAACGATCATTCTGCCATTGTAAAGAGTGACTCTTTCCTTAGGGATGGTGGT
Loxodonta africana  CTGAAGCGTAGGTGGCT-----------GGGTGGAAGGTGG-----GAGGAAGAGGGGAAAGAAGCAACTAT--TCAATTTTACATTGTAAGGAGTGACTCCTTCCTTAGGGACTGTAGT

Homo sapiens        CTTCTCCCCTTGCCAGGCCTTGGACTGCAGCCCCCAGTCAAAGGTCTTTGCAGGACACTTTCTATCCTTGGAGTTGCAGAGGAGGCCTGATTGGCCATTCCTTCTGGGACAGAC----AG
Loxodonta africana  CTCCTCCCTGGGTCAGGCCTTGGACTGCAGCCCCCAGCCAAAGGTCTTTGCAGGAGACTTTTCATCGTTGGGCTTCCAAAGGAGGCCTAACTGGCCTTTCCTTCCCAGATAGCCAAAGAG

Homo sapiens        AGAGAGGGCCCATGGCCTGCTC-TGAGCTC--CCTTCAATTTCAAGGAACCCTTAGTTCTGAGAGGAGAGAGGA-------------AGGCTGCACCTACCGTGGCCTCACAGCTGGGAC
Loxodonta africana  AGAGAGGGCCCGAGACCTGCTCTTTAACTCATCCTTCAACTTAAAGGATTTCTTAGTTGGAAAAGGAGAGAGGGAGTTTCATTCCACAGACTGTACCTACTGCAGCCTCGCAGCTGGGGC

Homo sapiens        -CCCTCATG----------GGGGCCAACCCCTGGCCTGCTGCTGTGCTTCAGTGACACAGTAAAGCTTAAGAGGGTGGCTGCCTCCTAGGAACTCCTGGGACTTCTAGGTACAAACTCAG
Loxodonta africana  ATTTTCATGCCTTTCACACGGAATCAACCCGTGACCTTTTGCTGTGCTTCAATTAAAC----AAGCTTAAGAGGGTGACTGCCCACTATTAACTCTTGGGACTTCTAGGTAGAAACATAG

Homo sapiens        GTGCCCTTATAAAGAGACCCACTGCTTGCCTTGAGTCCAGAAAGCACTGCTTCTGCAGTGA-AAAACACACACTTACTATGTCAGTTTTGGCCATAAAATGCCAGCCAAATCCACTGCTG
Loxodonta africana  GGG--CTTATAAAGAGACACACTGCTTGCCTTGAGTCCAGAAAACACCATTTCTGCAGTGAGAAAACACACATTTACTGTGTCAGCTATAACCACAAAATGCCAGCCAAATCCACTGCTG

Homo sapiens        GCCTGAACTGCCCCTGGACACTGGACTTTCTGCACAGTGACTGGCAGCAGAGCAAGTGCAGCC--AGGGTTTGTGGCCAAAGGGAAGAAAGCATAGTAAATTCAACCTCTGTTCCTCATG
Loxodonta africana  GCCTGAACTGCCCTTTGACACTGACCTTGCAGTACAATGCCTGGCAGCGGAGCAAGTGCAGCCAAAGTGTTTGTGACCAGACGGAAGAAAAC--AGTAAATTAAGCCTCCGTTCCTCACT

Homo sapiens        GGAGTCTGACCGATAATCTCAGGCGGACCTTAGGATCTTGGGCCTGGAGGCAAGAGAAAAAAGCTGGAGGAAGAGGAGTCGCAGAGAAACCCAGTCCTCTGACTGGTTTCTGGTGAGGTC
Loxodonta africana  GGAGTCTGACAGATAACCTCAGCCCAGCCTTAGGATTTTGAACCTGGAGGCAAGAGGAAGGAGCTGGAGGAAGAGGAGTCACAGGGAAACCCAGTCCTCTGGCTGGTTTCTGGTAAGGTC

Homo sapiens        TCTATTCATCAGTGGGAGGTAAGATGCCCTTTTCATTAGCGCTTTT-----GTTTCTCAGCCTGGGGATCAGTGGATTTGGATTCCTATAACCTGTTGCTACCTTTTAAAAAGATCCCCT
Loxodonta africana  TCTATTCACCCCTGGGAGGTAAGAAGCCCTTT--------GTTTCTCACACATTTCTCAGCCTGGGGGTCGGTGAAGCTGGGTTCCTAGAGCCTATTGCTACCTTTTAGAAAT-CATCTT

Homo sapiens        CTCTCCAAAGAGAAAAGTAATACATAATCATTAAAGAAAGTTTGGAAAATAGAGAAAACTATGAGGACA----AAAACCCCCCAGCCATTCAGAGTCCCACCACACAGAGGCAAGCACTA
Loxodonta africana  CCCTTCAAAGGTACAAATAATACATAATCATTGTTGAAAATTTGGAAAATAGAGAAAATGATGAGGACGAAACAAAATCCTCAAAACATTCAGAATCCCACTGTACAGATGCGAGCAATA

Homo sapiens        TTAACACTTTGCTGCTTTCCTTTCTAGTGTGTTCTTGCTATGTCTTGTCAAAGCTGAGATCACCCTGCCAGTTCTTGATCAGCAGGATTTAAAATGGCTGCCACCTGTGCAGTCATGCAT
Loxodonta africana  TTAACACTTTGCTGCCTTTCTTTACAGTGTATCCTTCCTGTATCTTTTCAGAGCTGAGATCATTCTGCAAACTCTTGATCCACAGGATTTAAAATGGCTGCCACCTATGCAGTTATGCAT

Homo sapiens        ACTGAATAATTCATTTAAGCATTCTCCTAATTTGTTGGACCTTTCAAT--TGTCTGGTTTTCCCCATCCCCACCAGGGCAGGAGTAAACAACTTTGTAAAGAAAATTTTGTCTGTTTTTC
Loxodonta africana  GCAGGATA----ATTTAAGCGTTCTCCTGATTTGTCAGACATTGCAGTTGTGTCTGGTTTCCCCCACCCCGAACAGTGCAGGAGTAAACAACCTTGTAAAGAAAATTTTGTCTGTTTTTC

Homo sapiens        AGCTGATTTTATCTGATTTCCTCAAGAGTGCATTCCAGAAGGGGACCTGCTATTCTTCGAGGGGACCTGCTATTCTTCGAGGCAGTGGACCCTGTTTGCTGATGGTCAACACTCTGGCCC
Loxodonta africana  AG----------CTTATTTCCTCAGGAGAGAGTTCCGGA--------------------AGGCGAACTGCTATTCCTCAAGGCTGTGGACCTTGTTTGCTGATGGTCTACCCACTGGCCC

Homo sapiens        TGCTGCCCCATGTCCCTG-TGGAGAAGTGTGAAGGACGGGTGCCTTAAACCCACA---------CTTCCAGGGACCCTTCAGCACAGGTCCTGCCTGTTCCCATGGTG------------
Loxodonta africana  TGCCG-CCTGTGTCCTGGCTGGAGAAGTGCAAAGAACAGGTGCCTTGCACCCCCACTTCCCCTGCTTCCAGGGACCCCTCAGCACAGGCTCTGCCTGTTGCCGTAGTGCTTCTCACCATG

Homo sapiens        ----CTTCTCACCCTGAACCCTCAGAGCTACAGCCAGGGATAGAGCAGATGAGCAGTGAGCTGGTTTCC-ACCCTCTGGGGCAGGAGGGGCTTGGGCTTCCCTTTGACCCTCTTCCTTCC
Loxodonta africana  AACTCTTCTCACCATGAACTCTCGTAGCCTCAGCCAGGAGAGGCACGTATGAGTACTGAGCTGATTTCTGGCCCTTTGGGGGCAGATGGGCTTGGACTTTGC-TTGACCCCTCTTCGCCC

Homo sapiens        TTTCCCAGATCTTCAATATTTGTTGTGATGTAACAGCCATAAGAGCTGTAAGAGGAACTGTGGGCTTTGGGAGCTTTCTTGGGTTGAAAGAG------GGGTGGGGGGAGG--GGATGCA
Loxodonta africana  TTTCCCAGATATTCAATATTTGTTGTGACGTAACAGCCACAGGAGCTGTAAGAGGAACTATGGGCTTTGGGAGCTTTCTTGGGTTGAAAGAAGTAGGTGGGTTGGAGGGGGTCGGATGTA

Homo sapiens        TGCAGGGGTATGAAGGCAGCCCAAGGTCTCCCAGAGGCTCGGGAGGAAACCCTTTTGGAATACATG-------TGTTATTGTTGCATTACTGTGTTGTGAAGATAAGAAGCTGCTCTGTA
Loxodonta africana  TGCAGGGGTAGGAAGGCAGCCCAAGGGTTCTCAGAGGCTTGGGAGGAAACCAGTTTGAAATAAACGTGTTAACTGTTATTGTTGTATTACTGTGTTGTGAGGGTAAGAAATTG---TGTA

Homo sapiens        TGCTAAAGCTTTCTCTCCTCAATAAAAATATAAAGGGTGTGTAAAGATTGTGCCTCTTT--GTTATTGAGCAAGCATAGATGGGTACAGAGTAGGCATAGGGTACTTCCAAGGTCCCCTG
Loxodonta africana  TGCTAAAGCTTTCT---CTCAATAAAAATATAAAGGGTGTGTAAAGACTGTGTCTTTCTGAGTTATTGAGCAAGCATAAAGGGGCACCCAGTAGGCATCAGGCACTTCCTAGGCTCCCTG

Homo sapiens        CCGGAGTCAACCCTACCCTGAAGTCTTGAAATCCTTAGACAAGGAAACCAAGGGCCGGTGAGGCTCAAGGGACCTGTCTACAGTTCTGTGCA---TTTGTGGATGGAAGAAATTGTTGGG
Loxodonta africana  CCTGAATCAACTGTAGCCTGAATCCTTGCAATCCTTAGATGAGGAAACTCA-GGCCATTGAGGCTCAAGGGACCTGCCTACAGTCCT-TGCACATTTTGTGGACAGCAGAAGTTGTTGAG

Homo sapiens        AGGGAAGTGGAAGCACTGGGCATCTTGAAAAATAGCATTCGTTCTCC------------CGGGGACTATGGGTGGCCCAGCCCTAC-----------------------------AGGCA
Loxodonta africana  AGGG----------ACTGGGCATCTTGAAAAATAGCATTAATTCTCCATTTATTGAAATATGAAGGTATGGGTGGCCCAGCCCTACCATCCTACGAGAGTCCCCCCACCCCCGCAACGCA

Homo sapiens        CGCACGGGCATGCATG-------------GGACTTTGAGTGAAGGAAGAGAAAGAGGCCAGGTTGGCTGGGGAGGGCCCTTCCGCTTTTTGGCACTAGGTGGCGCTATGGGATACCAGTG
Loxodonta africana  CACACACAGACACATGCGCGCGCACACAAGGACTTTGAGTAAAGGGAGGGA-------------GGTCAGGCAGGGCCCTTCCACTTTTTGGCACCAGGTGGTGCTGTGGGCCACCAGAG

Homo sapiens        AGTTAGCTGGTGATGAATCACAA------GTCCCCAGAGCAGTCCATGTAAGAGGCCCTCCA--------AGCAAGATCTCATGGCTCCTCACGGACTGTGGGGTCAGAAGAAACTGAAT
Loxodonta africana  AGCGAGCTGGTGAAGATTTTTAAACTCCGGACCCCAGAGTAGCCCCCTT-AGAGCCCTGCCACCCGCCCTAGCTAGACTTCACTG-----------CTGTGGAGTCAGAAGAAACTGAAT

Homo sapiens        CCCATTCTGGGATTTGCCCAAGTTT-CCCAGCATGAATTGG---ACTTTTTTTTTTTTCCAGGGGAAGTGGGTGGGAACATTCATTAAGCCTCTGCT---GCTTTTGAGTGCTGTGCCAG
Loxodonta africana  CCCGTTAT-GGATTTGATCAAGTTTCCCCAACAATAATTTGTAAAGTTTTTTTTCCCCCTTGGGAAAATGGGTGAGAATGCTTATTGAGCCCCTACTTAAGGTTCCAGGTGCCTTGCCAA

Homo sapiens        GCACTTC
Loxodonta africana  GCACTTC
```

```
Homo sapiens          GCATAACTTATGGCCCTGCCTCAGTCTCTGTCCTTGTTTCCCCATGGGCTACTGAGCTGAAAAAAGGGGCTGGGGATGGGGGAAACAGCCCTGGAAATAGCCTCTTTCAGAATCATTAG
Dasypus novemcinctus  GCTTGACCCACTGCCCTGCCTCAGCCTCTGACCT---------------TACTGAACTGAAAA----GGGCGGGACCGGGGAAATACAGC--------------CTTTTAGAATCATTCG

Homo sapiens          GCTGAAAGGTGAAAGCTGGAAACTG----------------CTTTGTCGAT-TGTTTTTAA---------ACTCTCTGTCTTTATCTTGCTGGTCCCTGTCCTCTTTCTTCTTTGCCTCC
Dasypus novemcinctus  TCTGGAAGGCGAAAGATGGAAAATGTGAGGGTGTTTCCTCTCCTCGCCAGTATGTTTTTAATTATTTCCCCCCCTCCCCTTTTCTCTTGTTTGTCCCTATC..................

Homo sapiens          CTCCTGCCGCTGCGCATCTCGCTTCTGGGCTCCGGCCGCAGCCCCCCTCCCTCTCGGTACCTTGCAACAGGCTCTGGAGATTGAGCTGCGCCTCCCGAAGCAGCTCCTCTACACTCGGGG
Dasypus novemcinctus  ........................................................................................................................

Homo sapiens          GCCTGCCCGAGGAGAGGAACACGTTCACTGGCTGTCGCCATGACGACCTCGAGTGGGCAGTCGCTGTCGCATTTTCCCGACCCGAGTTAGCTGCAGCTAGGGAAGGAGAGAGTCAGTGAG
Dasypus novemcinctus  .........................................TGACGACCTCGAGTGGGCAGTCGCTGTCACATATTCCCTACCCGAGTTAGCTGCAGCTAGGGAAGGAGAGAGTGAGTGAG

Homo sapiens          AGGCGCAGGCGAGCTGGAGGGGAGCGGCGCGCGGCGGCCAGTGGGCGCCCGCGGTTCCCCAAGGCCCGCCC---CTGCGTGGGGTGGGG--GGAGTGCGAGGCTGCGCTTAAAAGGCGGG
Dasypus novemcinctus  AGGCGC-GGCGAGC---GGGGGAGCGGCGCGCGGCGGCCAGTAGGCTCCTGCCATTCTGCAAGGCCCGCCCGGTCCGGTGGAAGTGGGGTGGGAGTGGGGACCTACGCTTAAAAGGCGGG

Homo sapiens          G-AATCGCCGGCCGCAAGATTTTT-CCTCGACAGATCCTGCCGCAGTCCGCTCCCTCCCGCTCCGCTCGCCCCTGCCCGCCCGCCCGCCCGGTCGCGCGGTCGCCGCCGCCACCGTGTCG
Dasypus novemcinctus  GGAATAACCTCCCGCAAGATTTTTTCCTCGACAGATCCTGCCGC----------------TTCCTCTCACTCCTGCC-------------------------ACCGCCGCC---------

Homo sapiens          CCCGCATCCTCCGCCTGGCGCCTCCGGGCCCCGTGCCTCCGCCCGCCCTCTCCGGCCCCGGCGACGAGGTAACGCGCCCTGGGCCGAGCCGCCGGGCCCTC-----------CGCCCAGA
Dasypus novemcinctus  -----------------GCGCCTCTGTGCACCG--CCGCCGCCTGCCCTCTCGGGCCTCTGGGACG--GTAACCGGCCTTGGGTGGAGCCGCTGG-CCCTCGGCGGCACCGGCACGCGGC

Homo sapiens          GCGCCGCCCTCCGAGCTCGCCCCGGTGGGTGTGGCCCGGCCTGG----------CCCGTGCCCCGCTGCCGCACTTCGGTCCCT-CCCGAGCCCCGCCGCCGCATTTCGGCCTCCTCCGG
Dasypus novemcinctus  GCCCAGCCATCTGCGGCCGCCCTGGTGGGTGTGGTCCGGCCTGGTGGCCCCTCTCGCGTTTACCGTCGCCGCACGTCGGTCCCCGCCAGAGTCCCGCCGCCGCATTTCGGCTTT-----G

Homo sapiens          CCGCCCGTAGTTCCAGTTCCCCTTTCCCCACCACGGAGCCCCAGGGCCCCTGG----GCTTGTCGAGATGTCTGAGGCGTCAGGAAATCTCAACAGCCTCCGCATGGCGAATGTGGCCCT
Dasypus novemcinctus  CCTCTCGCGGCCCC--TTGCCTTCTCTCC----TAGATCCCGGGGGCCCTTGGACGAGCTAGTCACAATGTCCGAGGCTGCAGGAAATGTCAATAGCCTCCGCCTGGCGAATGTAGCCTT

Homo sapiens          GCGCGAAGAATTAAATGCCCTTCGCGGGGAGAATGCCAATTTGGGCCTCCAGCTCGGGAGAGCCCTGGCCGAGGTTAATTCCTTGCGGGGCAATGTCTCCAGCTACATCCGCTGGCCAGT
Dasypus novemcinctus  GGGGGAAGAATTAAATGCCCTGCGCAGGGAGAATGCCAATTTGGGCCTCTAGCTCGGGAGAGCCCTGGCCGAGGTTAATTCCCTACAGGGCAACATCTCGAGCTACAATCGCTGGCCGGT

Homo sapiens          ACCCATAGTGCCCGTCCTTGCGGAGGAGAACTTGGAGTTCGCGCTCAGTGAGATCGAAGTCATTCCCGGGGGAGAACTGCCCTTCTTGTGCCGGCCTCCCCCACGCGCGGAGCCCGACTG
Dasypus novemcinctus  GCCTCTACTGCCCGTGCTCTCCGAGGA----------------------------GATGCCGTTCCCGAGGGAGAGCTGCCCTTCTTGTGCTGGTCTGCCCCCCGCACGGAGCCTGAGTA

Homo sapiens          TATCTCGGATGATCTTTTGATTAACGTGATCCAGGATCGCAGCACCCCCGACGGGCCCGCTGACCCCCCTCTGCTGCCCATCCCGCCCCCGCCGGCGCTGCCTCCGCCCGCGTCAAAGGA
Dasypus novemcinctus  TATCTCCGATGAACTGTTGATTAGCTTGATCCAGGATTGTGGCACCCCGGATGGGCCCATCGACCCTCCTCTGCTGCCTATCCCGTCCCCGCCTGGGCTGCCTCCGCGCCTGTTGAAGG-

Homo sapiens          ACCGCCCCCGCAACCCCCTCTGGCACCGCTGGAGCGGCCTGAGATAGAGCCCTTTTCAGGAGACCCAGTCTACCTGGCTGAATTCCTGATGCAGCTAGAGACCTTCATAGCCGACCATGA
```

```
Dasypus novemcinctus  -----------AGCCCCCTCTTCCCCCTACAGACCGGCCCGAGATTGAGCCGTTTTCAGGCGACCCCGTCTACCTGGCTGAGTT-CTGATGCAGCAAGAGAACTTCAGAGTCCACCATGA

Homo sapiens          GGTTCATTTCCCCGGGGGCGCCGAGCG-------------GGTGGCCTTTCTGATCTCCTTTTTCACTGGCGAAGCCAAGGACTGGGCCATCTCGGTCACCCAGGAAGGAAGCCCCTTGC
Dasypus novemcinctus  GGACCATTTCCGCGGGGCCGCTGAGCGGGGCGGGGGGGCCGGTGG-CTTTCTGATCTCCTTTTCCGCTGGGGAAGCCAAGGACCGAGCCATCTCAGTCACCCAGGAAGGAAGCCCACTGC

Homo sapiens          ATGCCAACTTCCCGCGCTTCCTGGATGAAATCCGTAAGGAATTCTGTGGCCCCATCCCCCCACGTGTGGCTAAAAAGGCCATTCGCAAGCTCAAGCAGGGCCACTGTACCCTCGGCAGCT
Dasypus novemcinctus  ATACCAACTTCCCGCACTTCCTTGATGAAATCCCTAAGGAATTCTGTGACTCCATCCCCCCACGTGTAACCCAAAAGGCCATCCGTAAGCTCAAGCAGGA-GACTCAACCCTTGGCAGCT

Homo sapiens          ATGCAGATGCTTTTCAGTTCCTGGCCCAATTCTTGTCTTGGGATGACTGCCGCCTCCAAAACCAATTCCTCAAAGGCCTGTCGGAATTCTTCCGCAAAGAGCTCTTATGGTCAACAGAAA
Dasypus novemcinctus  ATGC....................................................................................................................

Homo sapiens          TGGCCGACCTGGATGAGCTGATTCTCGAATGTGTGGAGATAGAAAGAAAAGTGCGCGTTCCCAAGCCTATCCCGCTCCCTGGAGTTCGAAATATCATCTTCCCTTTTGCTCCGAGTCCCA
Dasypus novemcinctus  ............................................AAGAAAGTGTGTGTGCCCAAGCCAATCCCACTCCCTGGGGTCTGCCATATCTTCTTCCCTTTTGCTGCAAACTTTA

Homo sapiens          ATGAGGAAGAAAGTGAAGATGAAGAGTACTACAGTGAAGATGAAGACCAAGAGGCACGCAGGCACAGGCTTCACTCCAAGGACCAGAGGAAGCGCATGAGGGCTTTTCAGCAAGAGATGA
Dasypus novemcinctus  ACGACATTGAAAGTGAATATGAAGAGTAAGACAGTGACGACCAAGATGAAGAGGCACGCAGGCTCAGGCTTTACCTGAAGAACTGGTACAGATGCGTGACGGCTTTTCGTCAAGAGATGA

Homo sapiens          AGGAGAAGGAGGAGGAGGAGATGAAGAAGGAGGAAGAAATGAAGAAGAAGGAAGAGAAGGAGGAGGAGGAGGAAGAGGAAATGAAACAGAAAGAGGAGGAGGAGGAGATAAGGAACAAGA
Dasypus novemcinctus  GGGAGAAGGAGGAGGAGGAG---------------GAGATGAGGAAGAAAGAGGAAGAGATGAGGCAGAAGGAGGAGGAGATAAGGTGGAA---------------GAATATGAAGAAGA

Homo sapiens          ATGAGGAGGAAGGAGAGAGTAAGGATG--AAGAAGATGAAGATGAGGATGGGGGCCAGAAACCAGAAGGAGAGCCACAGCAGGATCCAGGGACTGAGGAGACCTATGGTGAGGTGGAGGA
Dasypus novemcinctus  ATGAGGATAAAGATGGGAATAAGGATGGAAAAAAAATGAAGATGAGGATGGGAGCCAAGAACCAGAGCAGGAGCCAGAGCAGGAGCAAAAGATGGAGGAGGCTCATGGTGAGG-------

Homo sapiens          GGAGCCACTGGATGAGGCCCAAGATGATGACCTAGATGAGCTGATGGAGATGGAGCCGACCTTTGTTCACGCGTCATCCCAGACTTCTGGCCCCACAAGTGGTTACCACGCCGAAAACTT
Dasypus novemcinctus  -----------------CCCAGGATGATGGTCT-GATGAGCTGATGCAGATGGAGGTGTCCATTGCTCATACTTCTTCCCAGATTTCTGGCTCCACAAGTGGCAACCATGCTGAAAATTT

Homo sapiens          CCTGGGTGCATCACCTCCCATAATACAGCCTAGCAGACGGAGGAACCAGAATCGAGTCCCACTTCTGGAAGGCCTTCCAGGCACCAATTCACCATTCTACAGCTCCCCCCAACTGATTCG
Dasypus novemcinctus  CCTAGGTGCATCATCTCTCATAATACAGCCTGATAGAAGGAGGAGCCAGAATCAAGTACCACTTCTGGAGGGCCTTCCGGGTACCAATTCGCCCTTCTACAGCTCCCCACCCCTGATTCA

Homo sapiens          CCGCACAGGTCGTTTAGGACAACGCCAAGTTCGAAGACGCCCCCCTGTGCTTTTCCGCCTCACTCCGAGACAGGGGGGCCACCGAGCTGCTCGTGGCCGAATTCGAGTTTGAGTGCTGGG
Dasypus novemcinctus  CCATGCAAGTCGCTTGGGGCAACACCAAATT---AGAAGCCCCCCGGTGCTTTTCTGACTCACTCCGAGGCAGGGGGGACACAGAGCTGCTCGCGGTAGAATTCATGTCTGAGTGCTGGG

Homo sapiens          GCCAGATGGCCACCCAGAACACACCCTTCTTTTGCATCCCCCGCCCCTGCTATTGCTGCCATACCTGCCCCATTTGCTGACCAGGACTTCAGGGAGTTTCAGACCTGCAGAACCTGAA--
Dasypus novemcinctus  GCGAGATGGCCATCCAGAATACACCCTTCTACTGCATTTCCCACCCCTGTTAT---TGCCACACCTGCCCTATCTGCTGACCAGTACTTAAGGGAGTTCCAGACCTGCAGAACCAGAAGA

Homo sapiens          -----------GAAGACCTGCAGAATTCCAGACCATCTTGCAACCGTGACCAGAGAGCGACGTGTG-CCCAACCAGGGCCACCTG-GGAAAGGAGGGATCAGCAATAGCTGCCCTCTTGG
Dasypus novemcinctus  CCTGCACAACTGAAGACCTGCAGAGTTCCAGATCATCTCACAACTGTGACCCAGGAGTGACATGTGCCCCAACTGAGGTGACCTGAGAAAAGGAAGGATCAGTGTTAGCTGTCCTCTTGT

Homo sapiens          CATGTACTCTGCTCAGTCCGGCCACGAGCTGGAGAGCAAGTTTCTGGGCCTGTTCCCATAAATGAACTGGTCACCCTCTTGTATTTCCCCTCTGGTTGTTCCTAACCCTCACCTCTCCTG
Dasypus novemcinctus  CATGTGCCTTGCTCAGTACTGCCGTGAGCCAGAGAGAAGGTTTCTGGGCCTGTTCCCCTAACTGAACTGGTTACCCTCTTGAATGTCACCTCTCAGAGTTCCTAACCCTCACCTCTCCTG

Homo sapiens          CGTTTCTTCACCCTATGTTCTACAGTTTTATTC-TCTGACTGACCCACCAGGACTTCACCCAATGCCTCACTGATCTGCCCCAATGAGCAAGAGGACAGGCTACCCTGAACTCCTAAAGC
Dasypus novemcinctus  CATTTATTCACCCTGTGTTCTATGGTGTTATCCTTCTAATTGGTTCACTAGGACTTCATCCAATGCCTCACTGATCTGCCCCAATGATCTAAAGGACAGGTGACACTGAACTTCTAAAAC

Homo sapiens          CACTGAGGCCAGTTGCTAGATATAATCGATGGTGAGCAGGATGGTATCCAGAAATGTCCCTAGAATCC-TACACTCATTTCATGCCACACCTCCAGCAGA-GGGCCTGGAAGAGGACCCG
Dasypus novemcinctus  CACCAAAG-CAGTTACTAGATAGAATCCAC--TGAGCAGGATGGTATTAAGAAATATACGTAGGAGCCTTGCACTTATCTCAGGTCATACTTCCAGCAGACAGGCCTCAGTGTAAATCTG

Homo sapiens          ACATCTAGCCCAGTGAGAGTGTGCAAAGCCACATGTCAAATGGACAACTTGACGGAGATTTGCCCTTGACTGACTGTGCAGGCTGGTTCCACTTACAGCACTTGAGTTTACCTTTGCACA
Dasypus novemcinctus  A------------TGAGAGTGTGTAAAGTCACAGGTCAAATGGATGACTTGGTAGAGATTTGTCCTTAATAGACTGTGGAAGGTGATTCCACTTTCAGAACTTTAATTTACCTTCCCACA

Homo sapiens          GATTCCTGTATCAGGGCTCTTGGACTGACTTGCCACACATCTGGAGCCCTTTCCTCTCACCACATTGCCCTCC-TGGGCACCCCCGGTGGACAGCGCCCAACATTGCAGGCTCCACAACT
Dasypus novemcinctus  GACTCGT-------GGCCCTCAGAACTACTTA------ATCCGGGATCCTCTTCTCTTCCCATGCTGCCCCCCATGGACATCTCTGGTTGACAG-GCCC-ACAATGCAGACTCTACCCCT

Homo sapiens          T-TCCTTGATGTTGCCTAAAGGGTAGCCAGTTAGGGTTCCGGAGAGGAGAAGGAGGAAGAAGAGGAGGAGGAGAAAGAAGCAGCAACG-ATCATTCTGCCATTGTAAAGAGTGACTCTTT
Dasypus novemcinctus  TCTCCTTGTGATTGCCT-AAGGGTAGGTGGTTGGGT------------------GGGGGAGGAGGAGAAGGAGAAAGGAGAAGCAACGTCTCAGTTCTAGATAGTAAGGTGTGACTCCTC

Homo sapiens          CCTTAGGGATGGTGGTCTTCTCCCCTT-GCCAGGCCTTGGACTGCAGCCCCCAGTCAAAGGTCTTTGCAGGACACTTTCTATCCTTGGAGTTGCAGAGGAGGCCTGATTGGCCATTCCTT
Dasypus novemcinctus  CCTTAGGGCCTCTGGTCTCCTCCCCTTGGCCAGGCCTTGGACCTTAGTTCCTAGCCAAAGGTCTTCCTAGAAGACTTTCCAACATTGGGGTTCCAGAGGAGGCCTAGTTGGCC-TTTCTT

Homo sapiens          CTGGGACAGACAGAGA----GAGGGCCCATGGCCTGCTCTG-AGCT--CCCTTCAATTTCAAGGAACCCTTAGTTCTGAGAGGAGAGAGGAAGG---------------CTGCACCTACC
Dasypus novemcinctus  CCCAGACAGTCAAAGAGACTGAGGGTCCAAGACCTGCTCTTCAACTTGCCCTTCAACTTAAAGGATTCCTGAGTTCTGAGAGGAGAGACAAAGGTTCACTCTACAGAGACTGCACCCACT

Homo sapiens          GTGGCCTCACA-GCTGGGAC--CCCT----------CATGGGGGCCAACCCCTGGCCTGCTGCTGTGCTTCAGTGACACAGTAAAGCTTAAGAGGGTGGCTGCCTCCTAGGAACTCCT--
Dasypus novemcinctus  GCAGCCTCACCTGCCAGGACAGCCTTGTGTTTATTCCATGGGGGTCAGTCCTTGACTTTTTGCTGTGTTTCGATTAGACAGTGAGGCTTCAGAGGGTGACTGCCTCCTATTAACTCTTAT

Homo sapiens          --GGGACTTCTAGGTACAAACTCAGGTGCCCTTATAAAGAGACCCACTGCTTGCCTTGAGTCCAGAAAGCACTGCTTCTGCAGTGAAAAACACACACTTACTATGTCAGTTTTGGCCATA
Dasypus novemcinctus  AGGTACCTTATAGGTACCAACAGAGGTGCCCTTATAAAGAGGCTTGCTGCTTACCTTGGGTCCAGAAAA---------GGCAATGAAAAATACACACTTA------------TAGCCACA

Homo sapiens          AAATGCCAGCCAAATCCACTGCTGGCCTGAACTGCCCCTGGACACTGGACTTTC-TGCACAGTGACTGGCAGCAGAGCAAGTGCAGCC--AGGGTTTGTGGCCAAAGGGAAGAAAGCATA
Dasypus novemcinctus  AAATGCCAACCAAATCCACTGCTGGCCTGAGCTTCCCTTTGACATTGAGCTTTCAAGCATAGTGCTTGGCACCTGGGCAAGTGCAGCCCAAGTATCTGTGACCAGAGGGAAGAAAACAGA

Homo sapiens          GTAAATTCAACCTCTGTTCCTCATGGGAGTCTGACCGATAATCTCAGGCGGACCTTAGGATCTTGGGCCTGGAGGCAAGAGAAAAAAGCTGGAGGAAGAGGAGTCGCAGAGAAACCCAGT
Dasypus novemcinctus  GTAAGTTAAGCCTCAGTTCCTCACTGGAGTCTGACAGATAAATTCAGACCATTCTTAGGATCCTGAGCCTGGAGGCAAGAGAAAGGAGCTGGAGGAGGAAGGGTCATAGAAGAACCCAGT

Homo sapiens          CCTCTGACTGGTTTCTGGTGAGGTCTCTATTCATCAGTGGGAGGTAAGATGCCCTTTTCATTAGCGCTTTTGTTTCTCAGCCTGGGGATCA-GTGGATTTGGATTCCTATAACCTGTTGC
Dasypus novemcinctus  CCAATGGCTAGTTTCTGGTGAGGTCTCTATTCATCATTGGGAGGTAAGATGCCCTTTTGTTTATCAC--ACATTTCTCAGTTTGGGGGGCATGTGTGTGTGTAAAATGGCTTCCGGCTGC

Homo sapiens          TACCTTTTAAAAAGATCCCCTCTCTCCAAAGAGAAAAGTAATACATAATCATTAAAGAAAGTTTGGAAAATAGAGAAAACTATGAG-----------------GACAAAAACCCCCCAGC
Dasypus novemcinctus  TACCTTTT-----------CCCCTGCTGAAGATAAAAGCAATATATGATCATTAGAGAAAATTTGGAAAATAGAGAAAACTATAGGAACAAACCAAGTAAACAAACAAAAAGACCCCAAA

Homo sapiens          CATTCAGAGTCCCACCACACAGAGGCAAGCACTATTAACACTTTGCTGCTTTCCTTTCTAGTGTGTTCTTGCTATGTCTTGTCAAAGCTGAGATCACCCTGCCAGTTCTTGATCAGCAGG
Dasypus novemcinctus  CATTCAGAATCCCACCACACAGGCATGAGCATTAGTAACACTTTGTGGCCTTTCTTTCTAGCCTATCCTCCCTGAGTCTTTTCAATGCTGAGCTCATCCTGCAAGGTTTCAATCAGCAGG

Homo sapiens          ATTTAAAATGGCTGCCACCTGTGCAGTCATGCATACTGAATAATTCATTTAAGCATTCTCCTAATTTGTTGGACCTTTCAAT--TGTCTGGTTTTCCCCATCCC--CACCAGGGCAGGAG
Dasypus novemcinctus  TTTTAAAATGGCTGCCACCTCTGCAGTCACACATACAGAAAAATTTCTTTAAGCATTCTTGGAATTTGTGGACCCTTTCACTTCTGTCTGGTT---CCCAACCCATCAGCAGTACAGGAG

Homo sapiens          TAAACAACTTTGTAAAGAAAATTTTGTCTGTTTTTCAGCTGATTTTATCTGATTTCCTCAAGAGTGCATTCCAGAAGGGGACCTGCTATTCTTCGAGGGGACCTGCTATTCTTCGAGGCA
Dasypus novemcinctus  TAAACAACCTGGTAAAGAAAA---TGTCT...........................................................................................

Homo sapiens          GTGGACCCTGTTTGCTGATGGTCAACACTCTGGCCCTGCTGCCCCATGTCCCTGTGGAGAAGTGTGAAGGACGGGTGCCTTAAACCCACACTTCCAGGGACCCTTCAGCACAGGTCCTGC
Dasypus novemcinctus  ........................................................................................TACTTCCAGGGATGCTTCAGCAAAGGCCCTGC

Homo sapiens          CTGTTCCCATGGTGCTTCTCACCCTGAACCCTCAGAGCTACAGCCAGGGATAGAGCAGATGAGCAGTGAGCTGGTTTCC-ACCCTCTGGGGCAGGAGGGGCTTGGGCTTCCCTTTGACCC
Dasypus novemcinctus  CTTTTTCCATGGTGCTTCTCACCCTGAACCCT--------CGGCCAGGAAAGGATCATAGGAGCAGTGAGCTGGTTTCTGGCCCTCTGGGGGTAGGGGAGCATGGGCTTCCCTTTA--CC

Homo sapiens          TCTTCCTTCCTTTCCCAGATCTTCAATATTTGTTGTGATGTAACAGCCATAAGAGCTGTAAGAGGAACTGTGGGCTTTGGGAGCTTTCTTGGGTTGAAAGA----GGGGTGGGGGGAGGG
Dasypus novemcinctus  TCCTCCTCCCTTTCTCAGATCTTCCATATTTATTGTGATGTAACAGCCACAAGAGCTGTAAGAGGAACTACGGGTTTTGGGTACTTTCCTGGGCTGAAAGAGTTTGGGGTGGGAGTGGGG

Homo sapiens          GATGCATGCAGGGGTATGAAGGCAGCCCAAGGTCTCCCAGAGGCTCGGGAGGAAACCCTTTTGGAATACA--------------TGTGTTATTGTTGCATTACTGTGTTGTGAAGATAAG
Dasypus novemcinctus  GATGCAGGCAGGGGTATGAAGGCAGCCCAAGAATTCCCAGTCACTCAGGAGGACACTGGTTTGAAATAAATGTGTTCTTGTTATTGTGTTATTGTTGTATGACTGTGTTGTGAAGATA--

Homo sapiens          AAGCTGCTCTGTATGCTAAAGCTTTCTCTCCTCAATAA-AAATATAAAGGGTGTGTAAAGATTGTGCCTCTTTG--TTATTGAGCAAG-CATAGATGGGTACAGAGTAGGCATAGGGTAC
Dasypus novemcinctus  -----GCACAGTATGCTAAAGCTTCCATTTCTCAGTAAGAAATATAAAGGGTGTGTAAAGACTGTGTGTCTCTGACTTATTGAGCAAGCCATAGATAGGTGTCCAGTAGGCATCGAACAC

Homo sapiens          TTCCAAGGTCCCCTGCCGGAGTCAACCCTACCCTGAAGTCTTGAAATCCTTAGACAAGGAAACCAAGGGCCGGTGAGGCTCAAGGGACCTGTCTACAGTTCTG---TGCATTTGTGGATG
Dasypus novemcinctus  CAACTAGGCACCCTACCTGAATCAACCGTAGCCCCCATCTTTGAAATCCTTAGATGAGGAAACTAAAGCCCAGTGAGGCTCAAAGGACCTGCCTACAGTTCCACACCACATTTGTGCATG

Homo sapiens          GAAGAAATTGTTGGGAGGGAAGTGGAAGCACTGGGCATCTTGAAAAATAGCATTCGTTCTCC-----CGGGGAC-------TATGGGTGGCCCAGCCCTACAGGC--------------
Dasypus novemcinctus  ATAGAAGTGCTTGGGAGGGAAATGGAAGGACTGGGCACCTTGAAAAATAGCCTTAGTTCTCCAGTTTCTGGTATGTAAATGTATGAGTGGCCCAGCCCTATCGTCCTGTGACAGCCCTCT

Homo sapiens          --------------ACGCACGGGCATGCATGGGACTT-----------TGAGTGAAGGAAGAGAAAGAGGCCAGGT---TGGCTGGGGAGGGCCCTTCCGCTTTTTGGCACTAGGTGGCG
Dasypus novemcinctus  CCCCCAAAAAACATATACACACACACACCTGGGACCTATGAAGGGGAATGAGTGAAGGA--AGGATTAAGTCAGGCAGGTGGGTAGGGAGGGCTCTTCCACTTTGTGCCACTAGGTGGTG

Homo sapiens          CTATGGGATACCAGTGAGTTAGCTGG------------TGATGAATCACAAGTCCCCAGAGCAGTCCATGTAAGAGGCCCTCCAAGCAAGATCTCATGGCTCCTCACGGACTGTGGGGTC
Dasypus novemcinctus  GTATGGGACACCAGTGAGCTAGCTGGATTTTTTTTTTTTTTTTGAACGCCATTCCCCATATCTATCCATAT-AGAGCCCCTTCCAGAACGCCCTCTCTGTTATTCA--GTCTGTGGGGTC

Homo sapiens          AGAAGAAACTGAATCCCATTCTGGGATTTGCCCAAG-TTTCCCAGC-ATGAATTGGACTTTTTTTTTTTTCCAGGGGAAGTGGGTGGGAACATTCATTAAGCCTCTGCTGCTTTTGAGTG
Dasypus novemcinctus  GGAAGAAACTGAAACCCATTCTAGGATTTGCTCAAGTTTTCTCAGCAATGAATTGCAC--ATTTTTTTCCCCTGGGGAAATGGGTGGGATCCCTTATTGAACCCCTATTAGGTACCAGGG

Homo sapiens          CTGTGCCAGGCACTTC
Dasypus novemcinctus  CTGTGCCAGGCACTTC
```

```
Homo sapiens  GCATAACTTATGGCCCTGCCTCAGTCTCTGTCCTTGTTTCCCC-ATGGGCTACTGAGCTGAAAAAAGGGGCTGGGGATGGGGGAAACAGCCCTGGAAATAGCCTCTTTCAGAATCATTA
Mus musculus  GCTTGACC-ACTACCCTGTCTCAGCCTGTATCCTTTCTCCCTTTATGGACTACTGAACCAAAAAGGGAGG---GAGGAAGGGAGCAACAGCACTGGAAACAGAATGTT--AGCATCATCA

Homo sapiens  GGCTGAAAGGTGAAAGCTGGAAACTGCTTTGTCGATTGTTTTTAAACTCTCTGTCTTTATCTTGCTGGTCCCTGTCCTCTTTCTTCTTTGCCTCCCTCCTGCCGCTGCGCATCTCGCTTC
Mus musculus  GTCTGGAAGGTGAAAAGAGAGA-------------TGGTTTGGAACTGTTTTCTTTTCTTTTGCTGCTCCCCAT-----TTCTTCTTTGCCTCCCTCCTGC---TGCGCAGCT-GCCTC

Homo sapiens  TGGGCTCCGGCCGCAGCCCCCCTCCCTCTCGGTACCTTGCAACAGGCTCTGGAGATTGAGCTGCGCCTCCCGAAGCAGCTCCTCTACACTCGGGGGCCTGCCCGAGGAGAGGAACACGTT
Mus musculus  TAAG--------GCAGCCCCCCACCCTCGCAGTACCTTGCAACAGGCTCTGGAGATTGAGCTGCGCCTCGCGAAGCAGTTCCTCTACACTCGGGGGCCTGCCCGAGGAGAGGAACACGTT

Homo sapiens  CACTGGCTGTCGCCATGACGACCTCGAGTGGGCAGTCGCTGTCGCATTTTCCCGACCCGAGTTAGCTGCAGCTAGGGAAGGAGAGAGTCAGTGAGAGGCGCAGGCGAGCTGGAGGGGAGC
Mus musculus  CACTGGCTGTCGCCATGACGACCTCGAGTGGGCAGTCGCTGTCGCATTTTCCCGACCCGAGTTAGCTGCAGCTAGGGAAGGAGAGAGTGAGTGAGAG-CGCAGCCG-------------

Homo sapiens  GGCGCGCGGCGGCCAGTGGGCGCCCGCGGTTCCCCAAGGCCCGCCCCTGCGTGGGGTGGGGGGAGTGCGAGGCTGCGCTTAAAAGGCGGG--GAATCGCCGGCCGCAAGATTTTTCCTCG
Mus musculus  ------CGGGAGCCAGTGGGCTCCCTCGGTTCCCGAAGGCCCGCCCCTGCG-------GGGGGCGCGCGTAGCTGAGCTTAAAAGGCGAGAGGGATCGCTGTCTGCAAGATTTTTCCTCG

Homo sapiens  ACAGATCCTGCCGCAGTCCGCTCCCTCCCGCTCCGCTCGCCCCTGCCCGCCCGCCCGCCCGGTCGCGCGGTCGCCGCCGCCACCGTGTCGCCCGCATCCTCCGCCTGGCGCCTCCGGGCC
Mus musculus  ACAGATCCTGCCGCAGTCCCTTCCCTCCTACTCTGCCTGCCTCTGTTGGC------------------GTCACCTCTGCCAGCGTCTCGTCTGTCTCCTGCGCCTGCCGCCTCCAGGTC

Homo sapiens  CCGTGCCTCCGCCCGCCCTCTC--CGGCCCCGGCGACGAGGTAACGCGCCCTGGGCCGAGCCGCCGGGCCCTCCGCCCAGAGCGCCGCCCTCCGAGCTCGCCCCGGTGGGTGTGGCCCGG
Mus musculus  CCGAGCTGCTGCCTGCTCTCTCTCCGACCCCTGCCACGAGGTAACGCGTCCCGGACCGAGCC----------------------------------------CCTGTGGGTGTGGCCCGG

Homo sapiens  CCTGGCCCGTGCC--------CCGCTGCCGCACTTCGGTCCCTCCCGAGCCCCGCCGCCGCATTTCGGCCTCCTCCGGCCGCCCGTAGTTCCAGTTCCCCTTTCCCCACCACGGAGC-CC
Mus musculus  GCTCGCTTGCGCCGGGTCGCCCTGTCGCCGCACGTTGGTCTCCTCCCACGCCCACCTCCTCATTTCTGCCTCCTCCTGCACTCCCCA----TAACTCCCTACCCCCAAGTGCCCAGCACT

Homo sapiens  CAGGGCCCCTGGG-CTTGTCGAGATGTCTGAGGCGTCAGGAAATCTCAACAGCCTCCGCATGGCGAATGTGGCCCTGCGCGAAGAATTAAATGCCCTTCGCGGGGAGAATGCCAATTTGG
Mus musculus  CAAAGCCCCAGGAACTTGTCACAATGTCTGAAGCGGCGGGAAATCTCAATAGCCTCCGCTTGGCGAATGTGGCCCTGCGAGAGGAGTTAAATGCCCTTCGAGGAGAGAATGTCCAGCTGG

Homo sapiens  GCCTCCAGCTCGGGAGAGCCCTGGCCGAGGTTAATTCCTTGCGGGGCAATGTCTCCAGCTACATCCGCTGGCCAGTACCCATAGTGCCCGTCCTTGCGGAGGAGAACTTGGAGTTCGCGC
Mus musculus  GCCTGCAGCTCGGAAGAGCCTTGGCCGAGGTTAATTCCTTGCGGGGCAATGTCTCGAGCTACATCCGCTGGCCCATGCCCATAGTACCGGTCCTTGCCGAGGAGAACTTTGAGTTCCTGC

Homo sapiens  TCAGTGAGATCGAAGTCATTCCCGGGGGAGAAC-----------------TGCCCTTCTTGTGCCGGCCTCCCCCACGCGCGGAGCCCGACTGTATCTCGGATGATCTTTTGATTAACG
Mus musculus  TCAATGAGACTGATCCCACTCCTGAGGAGGAGGAAGAGGAGGAGGAGGAGGTGCCTTTCCTGTGCTGGCCTCCTCCACGCACAGATCCCGAGTACGTCTCAGATGATCTTTTGATTAATG

Homo sapiens  TGATCCAGGATCGCAGCACCCCCGACGGGCCCGCTGACCCCCCTCTGCTGCCCATCCCGCCCCCGCCGGCGCTGCCTCCGCCCGCGTCAAAGGAACCGCCCCCGCAACCCCCTCTGGCAC
Mus musculus  TGGTCCAGGACTATACCAACCCCGACGGGTCCAGTGACCCTCCTCTGTCACCCAGCCCGTCCCAGCCGGAACTGCACTCCCCCATGTTAAAGGAGCCAACCTTTGAGTTCCTTTTGCCAC

Homo sapiens  CGCTGGAGCGGCCTGAGATAGAGCCCTTTTCAGGAGACCCAGTCTACCTGGCTGAATTCCTGATGCAGCTAGAGACCTTCATAGCCGACCATGAGGTTCATTTCCCCGGGGGCGCCGAGC
Mus musculus  CGCTGGAGCGGCCAGACATAGAACCCTTTTCAGGCGACCCAGTGTACCTGGCTGAATTTCTGATGCAGCTAGAGACTTTCATAGCAGATCACGAGGATCACTTCCCTGGGGGCGCGGAAC

Homo sapiens  GGGTGGCCTTTCTGATCTCCTTTTTCACTGGCGAAGCCAAGGACTGGGCCATCTCGGTCACCCAGGAAGGAAGCCCCTTGCATGCCAACTTCCCGCGCTTCCTGGATGAAATCCGTAAGG
Mus musculus  GGGTGGCATTTCTCATCTCCTTTTTCACAGGGGAAGCGAGAGACTGGGCTATCTCAGTCACTCAGGAAGGAAGCTCCTTGCATGCCAACTTCCCGCGCTTCCTGGATGAAATCCGTAAGG

Homo sapiens  AATTCTGTGGCCCCATCCCCCCACGTGTGGCTAAAAAGGCCATTCGCAAGCTCAAGCAGGGCCACTGTACCCTCGGCAGCTATGCAGATGCTTTTCAGTTCCTGGCCCAATTCTTGTCTT
Mus musculus  AATTCTGTGGCCCCATCCCCTCACGCGTGGCTAAAAAGGCCATACGCAAGCTCAAGCAGGGAAACTGTACCCTGGGCAGCTATGCAGATGCTTTTCAGTTCTTGGCCCAGTTCTTGTCTT

Homo sapiens  GGGATGACTGCCGCCTCCAAAACCAATTCCTCAAAGGCCTGTCGGAATTCTTCCGCAAAGAGCTCTTATGGTCAACAGAAATGGCCGACCTGGATGAGCTGATTCTCGAATGTGTGGAGA
Mus musculus  GGGATGACTGTCGCCTCCAAAACCAATTTCTCAAAGGCTTGTCAGAAATTTTTCGAAAGGAACTCTTATGGTCAACTGAAGTGGCCGACCTGGATGAGCTGATTCTTGAGTGTGTCAAGA

Homo sapiens  TAGAAAGAAAAGTGCGCGTTCCCAAGCCTATCCCGCTCCCTGGAGTTCGAAATATCATCTTCCCTTTTGCTCCGAGTCCCAATGAGGAAGAAAGTGAAGATGAAGAGTACTACAGTGAA-
Mus musculus  TAGAAAGGAAAGTGCGTGTCCCCAAGACAGCCTCGCTCACTGGAGTTCAAAATAGCTGCTGCCCCTTTGCACTAATTCCTAATGAGGATGAAAATGAAGGTGTGGAGTTCTACAGTGAAA

Homo sapiens  -----GATGAAGACCAAGAGGCACGCAGGCACAGGCTTCACTCCAAGGACCAGAGGAAGCGCATGAGGGCTTTTCAGCAAGAGATGAAGGAGAAGGAGGAGGAGGAGATGAAGAAGGAGG
Mus musculus  ATGAGGGTGAGGGTGAAGAGGCAGGCGGGTACAGGCTTTATCTGAAGGATCAGAGGCAGCACATGACAGCTTTCCCACAAGAGATGAGGGAGGAAGAGGAGGAGATGAGGAAAGAGGAAG

Homo sapiens  AAGAAATGAAGAAGAAGGAAGAGAAGGAGGAG--------------------------------------------GAGGAGGAAGAGGAAATGAAACAGAAAGAGGAGGAGGAGGAGA
Mus musculus  ACGAGATGGAGGATGAGGAGGATGAGGACGAGGATGAGGATTATGAATTTGAGGAGGAGGATGAAGACGACGACGACGAGGAGGAAGAGGAGGAGGAAGAGGAGGAGGAAGACAAGGAGG

Homo sapiens  TAAGGAACAAGAATGAGGAGGAAGGAGAGAGTAAG---GATGAAGAAGATGAAGATGAGGATGGGGGCCAGAAACCAGAAGGAGAGCCACAGCAGGATCCAGGGACTGAGGAGACCTATG
Mus musculus  AGGAGATGAAGAATGAGGATAGCGATGAGAATAAGTATGAAGAAGAAGATGAAGTTATCGTTAGGGTCCTGGAGCCAGAGCAGGAGCAAGAGAGGGA---GGAGATAGAGCACGAGCATG

Homo sapiens  GTGAGGTGGAGGAGGA-------GCCACTGGA--------TGAGGCCCAAGATGATGACCTAGAT---GAGCTGATGGAGATG---GAGCCGACCTTTGTTCACGCGTCATCCCAGACTT
Mus musculus  TGTATGTGCACGAGCACATACACGCCCATGTGCACACACTTGCTGCCCACCACCACGGCCTACATGGAGAGCTCATGGTGATGGACGAACCTGTCCTTGTTGACACGTCAACCCAGACCA

Homo sapiens  CTGGCCCCACAAGTGGTTACCACGCCGAAAACTTCCTGGGTGCATCACCTCCCATAATACAGCCTAGCAGACGGAGGAACCAGAATCGAGTCCCACTTCTGGAAGGCCTTCCAGGCACCA
Mus musculus  TTAGCTCCGCCATAGGCTACCATGCTGAAAATTACCTGGGGGTATCCCCTTCTGTAATGCATTCTAGCAGACAGAGGAGCCAGAATCGAGTCCCACTTCTGGAGGGCCTTCCCGGGACCA

Homo sapiens  ATTCACCATTCTACAGCTCCCCCCAACTGATTCGCCGCACAGGTCGTTTAGGACAACGCCAAGTTCGAAGACGCCCCCCTGTGCTTTTCCGCCTCACTCCGAGACAGGGGGGCCACCGAG
Mus musculus  ATTCATCCTTCTACAGCCCGCCACCACTGATGCGCCATGCAGGTCGCTTGGGGCAACGCCAAATGCGAAGATGTCCCTCAGTGCTCTTCTGCCTCACTCCTAGGCAGGGGGGCCACCGAG

Homo sapiens  CTGCTCGTGGCCGAATTCGAGTTTGAGTGCT-GGGGCCAGATGGCCACCCAGAACACACCCTTCTTTTGCATCCCCCGCCCCTGCTATTGCTGCCATACCTGCCCCATTTGCTGACCAGG
Mus musculus  CTACTCAAGGTCGAATTCGAGTGTGAACACTGGGGGTGAGTTGGCCACGGGGAACACACCCTTCCCCTGCTTCCCTTGCCTCTGCTATTGCTGCTGTACGTGCCCCATCTACTGACCAGT

Homo sapiens  ACTTCAGGGAGTTTCAGACCTGCAGAA---CCTGAAGAAGACCTGCAGAATTCCAGACCATCTTGCAACCGTGACCAG-AGAGCGACGTGTGCCCAACCAGGGCCACCTGGGAAAGGAGG
Mus musculus  CCGTACGAAAGCCCCAGACTGGAGGAAGACCCCAAAGAAGACCTGCAGAATTCCAGATTGTTTCGCAACCACAACCAGAAGAGTGAAAAGTGCCCAACCAGGGCCATCCAGGAAAGGAGA

Homo sapiens  GATCAGCAAT-AGCTGCCCTCTTGGCATGTACTCTGCTCAGTCCGGCCAC-GAGCTGGAGAGCAAGTTTCTGGGCCTGTTCCCATAAATGA-ACTGGTCACCCTCTTGTATTTCCCCTCT
Mus musculus  GGTCAGGACTCAACAGTCCTCTTGCTGTGTACTCGGCTCAGTCCTCGCACAAAGTTGCAAAGCAAGTCTCTTGGCCTGTTCTTGTAAATGGCACTGGTCACCTTTGT---CTCCAACTCT

Homo sapiens  GGTTGTTCCTAACCCTCACCTCTCCTGCGTTTCTTCACCCTATGTTCTACAGTTTTATTCTCTGACTGACCCACCAGGACTTCA-CCCAATGCCTCACTGATCTGCC----CCAATGAGC
Mus musculus  AGTTGTCCCCAATCTTCGCCTCTCCTGTGCTTCTTTGCCCTGTGTTCTACAGTGTTATCCT----------------GACTTTACCCCAATGCCTGAGTGATCTGCCCCAACCAATGATC

Homo sapiens  AAGAGG-ACAGGCTACCCTGAACTCCTAAAGCCACTGAGGCCAGTTGCTAGATATAATCGATGGTGAGCAGGATGGTATCCAGAAATGTCCCTAGAATCCTACACTCATTTCATGCCACA
Mus musculus  TAAAGGCATTAGAT---CTAAACTCTTAATGCT-CTGAGGCCACTTACTAGACA---------ACGAGCAGGATGACACCAAGAAATA------------TGTACTC-------GCCATA

Homo sapiens  CCTCCAGCAG-AGGGCCTGGAAGAGGACCCGACATCTAGCCCAGTGAGAGTGTGCAAAGCCACATGTCAAATGGACAACTTGACGGAGATTTGCCCTT-----------------GACTG
Mus musculus  TTTCCAGCAGAAGGGCCTGAAAGGGGAACCTACACCTAGAGCAGCGAGAACCTGCAAAGCCATGAGTGCAATGGACAACTTGACTGAGGTTTACTCTCTTTGGGGGCGGGAGGGGGGATG

Homo sapiens  ACTGTGCAGGCTGGTTCCACTTACAGCACTTGAGTTTACCTTTGCA-CAGATTCCTGTATCAGGGCTCTTGGACTGACTTGCCACACATCTGGAGCCCTTTCCTCTCACCACATTGCCCT
Mus musculus  GC-GGGCAAACAGACCTCACTTTGAGTGCTGGGGTTTACCTTCCCCTCAGACTCCTGCCTCAGGACCTCTGCACAGACTT----------------------------------------

Homo sapiens  CCTGGGCACCCCCGGTGGACAGCGCCCAACATTGCAGGCTCCACAACTTTCCTTGATGTTGCCTAAAGGGTAGCCAGTTAGGGTTCCGGAGAGGAGAAGGAGGAAGAAGAGGAGGAGGAG
Mus musculus  --TGGCTAATTCGG-----------------------------------CCTTCATGTTGCCTACAGGACTGGCATT------------------AGGGGGAGGAGGAGGCAGAGGTG

Homo sapiens  AAAGAAGCAGCAACGATCATTCTGCCATTGTAAAGAGTGACTCTTTCCTTAGGGATGGTGGTCTTCTCCCCTTGCCAGGCCTT-GGACTGCAGCCCCCAGTCAAAGGTCTTTGCAGGACA
Mus musculus  GCTGGAGCACCATAGTTCAGTTTGACATCCTAAGGAGTGAATCTTGCTTTAGGAACAGGG---CCCTCTTCTGGCTAGGCTTTAAGACTGCAG-TCCCAGCCAGAGGCCTCTG-AGGAGA

Homo sapiens  CTTTCTATCCTTGGAGTTGCAGAGGAGGCCTGATTGGCCATTCCTTCTGGGACAGACAGAGAGAGGGCCCATGGCCTGCTCTGAGCTCCCTTCAATTTCAAGGAACCCTTAGTTCTGAGA
Mus musculus  CTG--TGTCCTCTAGGTTGTGGGGGTAGCCCTGCTGGCC-----------------------------------CACTCTTTAAGC-CCCTCTGACTCTAAGGAGTCCTTAGAACTGAAA

Homo sapiens  GGAGAGAGGAAGGCTGCACCTACCG----TGGCCTCACAGCTG----------------------GGACCCCTCATG-GGGGCCAACCCCT----GGCCTGCTGCTGTGCTTCAGTGACA
Mus musculus  GGAGAAAACAGGGCTGGCTCTGCAGAGACTGGACTCATTGCAGCATTGCTGGCCAACACCCCTATGGTTCTCACGTGAGGGGCAAACCTCTCCTAGGCCTGTTGTTGAACTTCAGTTAAC

Homo sapiens  CAGTAAAGCTTAAGAGGGTGGCTGCCTCCTAGGAACTCCTGGGACTTC-TAGGTACAAACTCAGGTGCCCTTATAAAGAGACCCACTGCTTGC-CTTGAGTCCA-GAAAGCACTGCTTCT
Mus musculus  CAGTGTGGCTTAAGA-GGCGACTGCCTCCTGCACACTCCTGGGGCTTCTTGGGCACCAACTCAGGTGCCCCTAGTCAAAGACCTGCTGTTCACTTTTGGGTCCAGGAAAATACTGCTGCT

Homo sapiens  GCAGTGAAAAACACACACTTACTATGTCAGTTTTGGCCATAAAATGCCAGCCAAATCCACTGCTGGCCTGAACTGCCCCTGGACACTGGACTTTCTGCACAGTGACTGGCAGCAGAGC-A
Mus musculus  GCAGTGGGAAGTACACATGTATTATGTCCATTCTGGCCACAAAATGGCAGCCTGGTTCACTGCTGGCCCAAGTTGTTCCTGGACATTGAACTTGCTGCACGGTGCCTAGCAGCTGGGCAA

Homo sapiens  AGTGCAGCCAGGGTT---TGTGGCCAAAGGGAAGAAAGCATAGTAAATTCAACCTCTGTTCCTCATGGGAGTCTGA--CCGATAATCTCAGGCGGACCTTAGGATCTTGGGCCTGGAGGC
Mus musculus  AGTGGAGCCCAAGTTGTCCGTGGTCAGCAGGAAGAAAGC--AGCAAAT--GGTGTCTCTTCCTCATTGGAGTCTGAAGATGAGAACCC------AGCCCTAGAGTTT--GAACTTAAGAC

Homo sapiens  AAGAGAAAAAAGCTGGAGGAAGAGGAGTCGCAGAGAAACCCAGTCCTCTGACTGGTTTCTGGTGAGGTCTCTATTCATCAGTGGGAGGTAAGATGCCCTTTTCATTAGCGCTTTTGTTTC
Mus musculus  CAGAGAAGGAA-CTTGAGGAAGAGGAGTCCTAGACAGACCTAGTCCTCAGACTGGTTAACGGTGAGCTCTCTATTCATCAGCAAGAGGTAAGGGGCCTTTTTGTTTAGCACTTCCATTTC

Homo sapiens  TCAGCCTGGGGATCAGTGGATTTGGATTCCTATAACCTGTTGCTACCTTTTAAAAAGATCCCCTCTCTCCAAAGAGAAAAG-TAATACATAATCATTAAAGAAAGTTTGGAAAATAGAGA
Mus musculus  TT-GCCTGAAGATCAGTCGAATTGGGTTCCTATAGCCTGCGGCTACCTTTTACCATGGTTTC----CTCCAAAGAGAGAAGTTAACGTATAGTCATTATGGGAAATATGGGAAGTAGTGA

Homo sapiens  AAACTATGAGGACAAAA-----ACCCCCCAGCCATTCAGAGTCCCACCACACAGAGGCAAGCACTATTAACACTTTGCTGCTTTCCTTTCTAGTGTGTTCTTGCTATGTCTTGTCAAAGC
Mus musculus  ATGCCATAAGGACAAAATAGCTTCCTCCCGACCATTCAGAACCTCGCCACACAGAGGCA-------------------GCATTTCTTTCT------------------TTCTTCAAGG

Homo sapiens  TGAGATCACCCTGCCAGTTCTTGATCAGCAGGATTTAAAATGGCTGCCACCTGTGCAGTCATGCATACTGAATAATTCATTTAAGCATTCTCCTA----ATTTGTTGGACCTTTCAATTG
Mus musculus  TGAGGTCATCCTGCTGGAGGGCCAGCAGCAGGCCTGAAAATGGCTGTCATCTGTGTG-------ACGCAGAATCATTT-------CACCTTCCTGAGTCAGTTGTTAGACATTTCAGTTG
```

```
Homo sapiens  TCTGGTTTTCCCCATCCCCACCAGGGCAGGAG--TAAACAACTTTGTAAAGA----AAATTTTGTCTGTTTTTCAGCTGATTTTATCTGATTTCCTCAAGAGTGCATTCCAGAAGGGGAC
Mus musculus  TTT-GCCTTGCCCATTCCCACCAAGGCAGGAGGATAGGGAACCATGTGAAGACTTTGTTTTGTGTCTCTTTTTCAG----------CTTATTTCCTCAAGCGAGAATTCCAGAAGGGAA-

Homo sapiens  CTGCTATTCTTCGAGGGGACCTGCTATTCTTCGAGGCAGTGGACCCTGTTTGCTGATGGTCAACACTCTGGCCCTGCTGCCCCATGTCCCTGTGGAGAAGTGTGAAGGACGGGTGCCTTA
Mus musculus  ------------------AACT-CTATTCCTTGAAGCTATGGACCCTGTCTGCTGAT-GCCAACATACTGGTCACGCTG-CCTGTGTCCTTGTGGAGAAGCGCAAGAAGCAGGTGTCTTG

Homo sapiens  AACCCACACTTCCAGGGACCCTTC------AGCACAGGTCCTGCCTGTTCCCATGGTGCTTCTCACCCTGAACCCTCAGAGCTACAGCCAGGGATA--GAGCAGATGAGCAGTGAGCTGG
Mus musculus  TACCTCAGATTCCAGAGATCCCCCTTGGCACACACAGGCTTTGCCTGTCTTCACGGTGCTTCTTAGTCTGAATGCTTGTGGCCCTGACCAGGGAGACCAAGCACATGAGCACTAAACTGG

Homo sapiens  TTTCC--ACCCTCTGGGGCAGGAGGGGCTTGGGCTTCCCTTTGACCCTCTTCCTTCCTTTCCCAGATCTTCAATATTTGTTGTGATGTAACAGCCATAAGAGCTGTAAGAGGAACTGTGG
Mus musculus  CCTGTGGGCCTCTGGGGGCAGAGGGGCCTTGAACTTCCTTT--AACCTTTTCCCTTCTTTCCCAGTTCTTCAATATTTGTTGTGATGTGACAGCTACAGGAGCTGTCCGA---------A

Homo sapiens  GCTTTGGGAGCTTTCTTGGGTTGAAAGA---GGGGTGGGGGGAGGGGATGCATGCAGGGGTATGAAGGCAGCCCAAGGTCTCCCAGAGGCTCGGGAGGAA----------ACCCTTTTGG
Mus musculus  GCTCTGAGTGCCTCCTTGGGTTGAGGGGGGTGGGGGTGGGGGTGGGGATGCATGCA-GGGTACAAGGGTGATCCAAAGACACCCGGAGGCTCCAGAGAAACAAATGTGTTACTGTTTT--

Homo sapiens  AATACATGTGTTATTGTTGCATTACTGTGTTGTGAAGATAAGAAGCTGCTCTGTATGCTAAAGCTTTCTCTCCTCAATAAAA-ATATAAAGGGTGTGTAAAGATTGTGCC--TCTTTGTT
Mus musculus  --TAC---TGTTACTGTTGTATTATTGTGCTGTGAAGAT---AAATTGTTCTGTATGCTAAAGCTTTCTCTCCTCAATAAAACAAATAAAGGGTGTGTAAAGATTGTGTCTGTCTGTGTT

Homo sapiens  ATTGAGCAAGCATAGATGGGTACAGAGTAGGCATAGGGTACTTCCAAGGTCCCCTGCCGGAGTCAACCCTACCCTGAAGTCTTGAAATCCTTAGACAAGGAAACCAAGGGCCGGTGAGGC
Mus musculus  ACTG----AACATAGACGGGGACCCAAGATGC-----TTACTTCGCAGGCACCTTAGCTGA-TCGGTGCTAGCCTGAAACCTTGACGTCCAGCAACAA------CAAG------------

Homo sapiens  TCAAGGGACCTGTCTACAGTTCTGTGCATTTGTGGATGGAAGAAATTGTTGGGAGGGAAGTGGAAGCACTGGGCATCTTGAAAAATAGCATTCGTTCTCCCGGGGACTATGGGTGGCCCA
Mus musculus  ----------------CAGTTCT---------TGGAAGGAGG.......................................................................

Homo sapiens  GCCCTACAGGCACGCACGGGCATGCATGGGACTTTGAGTGAAGGAAGAGAAAGAGGCCAGGTTGGCTGGGGAGGGCCCTTCCGCTTTTTGGCACTAGGTGGCGCTATGGGATACCAGTGA
Mus musculus  .................................GAGTGAAGGCAGG---------CAGGGTGGCGAGAAAGCGCCCTTCCACTTTGGGACACTAGATGGTGCTATGGGATTCCAGTGA

Homo sapiens  GTTAGCTGGTGATGAATCACAAGTCCCCAGAGCAGTCCATGTAAGAGGCCCTCCAAGCAAGATCTCATGGCTCCTCACGGACTGTGGGGTCAGAAGAAACTGAATCC-CATTCTGGGATT
Mus musculus  AGTAGCTG-----GAAC--CAAATTCCCAGAGCAGGCCAAGTAGGG-------TCAGCAAGACCTCCCTGCTTCTTAGAGAGTGTGGAGTCAGAAAGAACTGCACCCTCATTCTGGGACT

Homo sapiens  TGCCCAAGTTTCCCAG-CATGAATTGGACTTTTTTTTTTTTCCAGGGGAAGTGGGTGGGAACATTCATTAAGCCTCTGCTGCTTTTGAGTGCTGTGCCAGGCACTTC
Mus musculus  GGCTTAGCTTTCTCGGACATGAG-TGAACGTTTTTGTTTCCCC-------------------TCTGCTGAGCCT-TCCTGCTTGCGAGTGCTGGACTAAGCCCTGA
```

```
Homo sapiens            GCATAACTTATGGCCCTGCCTCAGTCTCTGTCCTTGTTTCCCCATGGGCTACTGAGCTGAAAAAAGG--GGCTGGGGATGGGGGG------AAACAGCCCTGGAAATAGCCTCTTTCAGA
Canis lupus familiaris  GCTTGACCAATA-CCCTGCTTCTGGCTCAGTCCCT-TCCCCTCCTGGCCTCCTGAACTGAAAAAGAGAGGGGTGGGGAGGGGGGAGTGAGTAAACAGCTGGAGAAATGGAGCCCTCTCGA

Homo sapiens            ATCATTAGGCTGAAAGGTGAAA-GCTGGAAACTGC------TTTGTCGATTGTTTTTAAACTCTCTGTCTTT----ATCTTGCTGGTCCCTGTCCTCTTTCTTCTTTGCCTCCCTCCTGC
Canis lupus familiaris  AGCTT----CTGGAAGGTAAAAAGATGGCAAACGTGTTTTGTTTTTCAGTGGGGTTTAAACTGTTCTTTTTTTCCTCTCTCGCTGGCCCCTGTCCTCTTTCTTCTTCGCCTCCCTCCAGC

Homo sapiens            CGCTGCGCATCTCGCTTCTGGGCTCCGGCCGCAGCCCCCCTCCCTCTCGGTACCTTGCAACAGGCTCTGGAGATTGAGCTGCGCCTCCCGAAGCAGCTCCTCTACACTCGGGGGCCTGCC
Canis lupus familiaris  CGCTGCGCATCTCGCCTGTGGGCTCCGGCCGCAGCCCCCCTCCCTCTCAGTACCTTGCAACAGGCTCTGGAGATTGAGCTGCGCCTCTCGAAGCAGCTCCTCTACACTCGGGGGCCTGCC

Homo sapiens            CGAGGAGAGGAACACGTTCACTGGCTGTCGCCATGACGACCTCGAGTGGGCAGTCGCTGTCGCATTTTCCCGACCCGAGTTAGCTGCAGCTAGGGAAGGAGAGAGTCAGTGAGAGGCGCA
Canis lupus familiaris  CGAGGAGAGGAACACGTTCACTGGCTGTCGCCATGACGACCTCGAGTGGGCAGTCGCTGTCGCATTTTCCCGACCCGAGTTAGCTGCAGCTAGGGAAGGAGAGAGTCAGTGAGAAGCGCA

Homo sapiens            GGCGAGCTGGAGGGGAGCGGCGCGCGGC---GGCCAGTGGGCGCCCGCGGTTCCCCAAGGCCCGCCCCTGCGTGGGGTGGGGGG-AGTGCGAGGCTGCGCTTAAAAGGCGGGGAATCGCC
Canis lupus familiaris  GGCGGG--GGGAGGGTAGGGGGGGCAGCCCGGGCCACTGGGCGCCTGCGGATCCCTAAGACCCGCCCCAGCCGGGGGTGGGGGGCGGCGCGAGTCTGCGCTTAAAAGGCGGGGACTCGTC

Homo sapiens            GGCCGCAAGATTTTTCCTCGACAGATCCTGCCGCAGTCCGCT-CCCTCCCGCTCCGCTCGCCCCTGCCCGCCCGCCCGCCCGGTCGCGCGGTCGCCGCCGCCACCGTGTCGCCCGCATCC
Canis lupus familiaris  AGCCGCAAGATTTTTCCTCGACAGATCCTGCCGCAGTCTGCTTCCCTCCTGCCCCGCCAGCCTCCGCCCGCCC---------------CGCCTGCCGCC-------TGCCGCCCGCGTCC

Homo sapiens            TCCGCCTGGCGCCTCCGGGCCCCGTGCCTCCGCCCGCCCTCTCCGGCCCCGGC-------GACGAGGTAACGCGCCCTG-GGCCGAGCCGCCGGGCCCTCCGCCCAGAGCGCC-GCCCTC
Canis lupus familiaris  TCTGCCCGGCACCTCCCGGCTAC-CGCCGCCGCCGGCCCTCTCCGATCCCCGATCCCCCGGACGAGGTAAAGCGCCCCGCGGTCCAGCCGCTGGGCCCTCCAGCCGGAGCGCCCGCCCTC

Homo sapiens            CGAGCTCGCCCCGGTGGGTGTGGCCCGGCCTGGCCCGTGCCCCGCTGCCGCA---CTTCGGTCCCTCCC-------GAGCCCCGCCGCCGCATTTCGGCCTCCTCCGGCCGCCCGTAGTT
Canis lupus familiaris  CGGTCTCGCCCCAGTGGGTGTGGCTCGGCCTCTCCCGCGCCCCGCCACCGCGGCCCTCCGATCCCCTCCCCCCTCCGCGCCCAGCTGCCGCATTTCAGCCTCCTCTCGCGGCCTCCAGCT

Homo sapiens            CCAGTTCCCC----TTTCCCCACCACGGAGCCCCAGGGCCCCTGGGCTTGTCGAGATGTCTGAGGCGTCAGGAAATCTCAACAGCCTCCGCATGGCGAATGTGGCCCTGCGCGAAGAATT
Canis lupus familiaris  CCCCACCCCCACCCTCGCCCCCGCCCTCAGACCCAGGGCCCCCGAGTTAATCCTAATGTCCGAGGCCGCAGGAAATCTAAACAGCCTGCGCATGGCGAACGTGGCCCTGCGAGAAGAATT

Homo sapiens            AAATGCCCTTCGCGGGGAGAATGCCAATTTGGGCCTCCAGCTCGGGAGAGCCCTGGCCGAGGTTAATTCCTTGCGGGGCAATGTCTCCAGCTACATCCGCTGGCCAGTACCCATAGTGCC
Canis lupus familiaris  AAATGCTCTTCGCGGGGAGAATGCCAATTTGGGCCTTCAGCTCGGAAGAGCTCTAGCCGAGGTTAATTCCTTGCGGGGCAACGTCTCGAGCTACATCCGCTGGCCAGTGCCGGTGGTGCC

Homo sapiens            CGTCCTTGCGGAGGAGAACTTGGAGTTCGCGCTCAGTGAGATCGAAGTCATTCCCGGGGGAGAACTGCCCTTCTTGTGCCGGCCTCCCCCACGCGCGGAGCCCGACTGTATCTCGGATGA
Canis lupus familiaris  CGTCCTTGCCGAGGAGAACTTTGAGTTCCCGCTCAGCGAGATCGACGCAGTTCCCGAGGGGGAGCTGCCGTTCTTGTGCTGGCCTCCCCCTCGCGCGGAGCCCGAGTACGCCTCGGACGA

Homo sapiens            TCTTTTGATTAACGTGATCCAGGATCGCAGCACCCCCGACGGGCCCGCTGACCCCCCTCTGCTGCCCATCCCGCCCCCGCCGGCGCTGCCTCCGCCCGCGTCAAAGGAACCGCCCCCGCA
Canis lupus familiaris  ACTGTTGATTAGCGTGATCCAGGATTGCAGTACCTCCGATGCGCCGGTCGACCCTCCCCCGCTGCCCATCCCGCCCCCGCTGGCGCTGCCCCCGCCCCCAGCCAAGGAGCTGCCCCCGCA

Homo sapiens            ACCCCCTCTGGCACCGCTGGAGCGGCCTGAGATAGAGCCCTTTTCAGGAGACCCAGTCTACCTGGCTGAATTCCTGATGCAGCTAGAGACCTTCATAGCCGACCATGAGGTTCATTTCCC
Canis lupus familiaris  GCCTCCTCTGCCGCCGCTGGAGCGGCCTGAGCTAGAGCCCTTCTCGGGCGACCCAGTCTACCTGGCTGAATTCCTGATGCAGCTAGAGACCTTCATAGCTGACCATGAGGATCATTTCCC

Homo sapiens            CGGGGGCGCCGAGCGGGTGGCCTTTCTGATCTCCTTTTTCACTGGCGAAGCCAAGGACTGGGCCATCTCGGTCACCCAGGAAGGAAGCCCCTTGCATGCCAACTTCCCGCGCTTCCTGGA
Canis lupus familiaris  CGGGGGCGCCGAACGGGTGGCCTTTCTGATCTCTTTTTTCGCGGGCGAAGCCAAGGACTGGGCCATCTCAGTCACCCGGGAAGGAAGCCCCCTGCGAGCCAACTTCCCACGCTTCCTGGA

Homo sapiens            TGAAATCCGTAAGGAATTCTGTGGCCCCATCCCCCCACGTGTGGCTAAAAAGGCCATTCGCAAGCTCAAGCAGGGCCACTGTACCCTCGGCAGCTATGCAGATGCTTTTCAGTTCCTGGC
Canis lupus familiaris  TGAGATCCGCAAGGAATTCTGCGGCCCCATCCCCCCAAGCGTGGCTAAAAAGGCCATCCGCAAGCTCAAGCAGGGGGACTGTACCCTGGGCAGCTATGCGGATGCTTTTCAGTTCCTGGC

Homo sapiens            CCAATTCTTGTCTTGGGATGACTGCCGCCTCCAAAACCAATTCCTCAAAGGCCTGTCGGAATTCTTCCGCAAAGAGCTCTTATGGTCAACAGAAATGGCCGACCTGGATGAGCTGATTCT
Canis lupus familiaris  CCAGTTCTTGTCTTGGGATGACTGCCGCCTTCAAAACCAGTTCCTTAAAGGCCTGTCTGAATTCTTCCGCAAGGAGCTCTTATGGTCCACTGAAATGGCCGACCTGGACGAGCTGATTCT

Homo sapiens            CGAATGTGTGGAGATAGAAAGAAAAGTGCGCGTTCCCAAGCCTATCCCGCTCCCTGGAGTTCGAAATATCATCTTCCCTTTTGCTCCGAGTCCCAATGAGGAAGAAAGTGAAGATGAAGA
Canis lupus familiaris  CGAATGTGTGGAGATAGAAAGAAAAGTGCGTGTCCCCAAGCCAATCCCGCTCCCTGGGGTTCGCAACATCTTCTTCCCTTTTGCTGCGGACCCTAATAACGATGACAGTGATGATGAAGA

Homo sapiens            GTACTACAGTGAAGATGAAGACCAAGAGGCACGCAGGCACAGGCTTCACTCCAAGGACCAGAGGAAGCGCATGAGGGCTTTTCAGCAAGAGATGAAGGAGAAGGAGGAGGAGGAGATGAA
Canis lupus familiaris  GTACTACAGT---GACGAAGATGAAGAGACACGCAGGCGCAGGCTTTATGACCGGGAACAGCGGAGGCGCATGAGAGCTATTCAGCAGGAGGCGAGGGAGGAGGAAGAAAAGAGGAAGAA

Homo sapiens            GAAGGAGGAAGAAATGAAGAAGAAGGAAGAGAAGGAGGAGGAGGAGGAAGAGGAAATGAAACAGAAAGAGGAGGAGGAGGAGATAAGGAACAAGAATGAGGAGGAAGGAGAGAGTAAGGA
Canis lupus familiaris  GGAGGAAGAGAAGAGGAAGAAGCAGGAAGAGATGAGGAAGAAGCAGCAGGAGGAGATGAGGAAGAAGCAGCAGGAGGAGATGAGGAAGAAACAGGAAGAGAAGGAAGAGGAAGATGATGA

Homo sapiens            TGAAGAAGATGAAGATGAGGATGGGGGC-----------------------CAGAAACCAGAAGGAGAG-------------CCACAGCAGGATCCAG----GGACTGAGGAGACCTATG
Canis lupus familiaris  GGACGAGGAGGTGGACGTGGATGGGGACGTGGATGTAGAGCTGATCAAGGATGAAGACGAGAATAAGGGCGAACGTTTGGGCCCTCTGCCAGAGCCAGACCCGGACC-AGGAGCCAGAGC

Homo sapiens            GTGAGGTGGAGGAGGAGCCACTGGATGAGGCCCAAGATGATGACCTAGATGAGCTGATGGAGATGGAGCCGACCTTTGTTCACGCGTCATCCCAGACTTCTGGCCCCACAAGTGGTTACC
Canis lupus familiaris  CCGAGCCCGAGCCGGAGTCAGAGGATGAAACCCAAGATGATGACCTCGATGAGATGATGGAGATGGAACCCACTGTTGCCCACGCTTCATCCCAGACCACTGGGTTC---------TACC

Homo sapiens            ACGCCGAAAACTTCCTGGGTGCATCACCTCCCATAATACAGCCTAGCAGACGGAGGAACCAGAATCGAGTCCCACTTCTGGAAGGCCTTCCAGGCACCAATTCACCATTCTACAGCTCCC
Canis lupus familiaris  A---TGAGAATTTCCTGGATGCCTCGCCCCCCATAATCCAGCCCAGCAGGAGGAGGAACCAGAATCGAGTCCCACTTCTGGAGGGCCTTCCAGGTACCAATTCACCCTTCTATAGTTCGC

Homo sapiens            CCCAACTGATTCGCCGCACAGGTCGTTTAGGACAACGCCAAGTTCGAAGACGCCCCCCTGTGCTTTTCCGCCTCACTCCGAGACAGGGGGGCCACCGAGCTGCTCGTGGCCGAATTCGAG
Canis lupus familiaris  CGCCACTGATTCGCCGTGCAGGCCGCCTGGGGCAACGCCAAATTCGAAGACGTCCCCCAGTGCTATTCCGCCTCACCCCGAGACAGGGGGGCCACCGGGCCGCACGTGGCCGAATTCGCG

Homo sapiens            TTTGAGT-GCTGGGGCCAGATGGCCACCCAGAACACACCCTTCTTTTGCATCCCCCGCCCCTGCTATTGCTGCCATACCTGCCCCATTTGCTGACCAGGACTTCAGGGAGTTTCAGACCT
Canis lupus familiaris  TGTGAGTGGCTGGGGCAAGCTCACCACACAGAACACACCCTTCTACTGCGTCCCCTGCCCCTGCTATTGCTGCCACACCTGCCCCATTTGCTGACCAGTACTTGAGGGAGTTCCAGACCC

Homo sapiens            GCAGAACCTGAAG-AAGACCTGCAGAATTCCAGACCATCTTGCAACCGTGACCA-----------GAGAGCGACGTGTGCCCAACCAGGGCCACCTGGGAAAGGAGGGATCAGCAATAGC
Canis lupus familiaris  ATAGAGCCCGAAGAAAGACCTACAGAGCTCCAGATCGTATTGCAGCCATGACCAGGGAGTGACACGGGAGTGACACGTGTCCAGCCAAGGCCACCTGCGAAGGAAGGGATCAGCAATAGT

Homo sapiens            TGCCCTCTTGGCATGTACTCTGCTCAGTC-CGGCCACGAGCTGGAGAGCAAGTTTCTGGGCCTGTTCCCATAAATGAACTGGTCACCCTCTTGTA-TTTCCCCTCTGGTTGTTCCTAACC
Canis lupus familiaris  TGTCCCCTTGGAGTGCACTGTGCTCAATCTTTGCCATGAACTAGAGAGCAGGTTTCTGGGCCTGTTCCCATAAAAGAACTGGTCACCCTCTTGTATTTTCCCCTCTAGTTGTTCCTAACC

Homo sapiens            CTCACCTCTCCTGCGTTTCTTCACCCTATGTTCTACAGTTTTATTCTCTGACTGACCCACCAGGACTTCACCCAATGCCTCACTGATCTGCCCCAATGAGCAAGAGGACAGGCTACCCTG
Canis lupus familiaris  CTCACCTCTCCTGCGTTTATTCATCCTGTGTCCTACGGTTTTATCCTCTGACTGGCTCGCCAGGACTTCACCCAATGCCTCACTGACCTGCCCCAGTGAGAGAAAGGACAGGCTACACCG

Homo sapiens            AACTCCTAAAGCCACTGAGGCCAGTTGCTAGATATAATCGATGGTGAGCAGGATGGTATCCAGAAATGTCCCTAGAATCCTACACTCATTTCATGCCACACC----TCCAGCAG-AGGGC
Canis lupus familiaris  AACTCGTAAAGCCACTGAGGCCACTTACTAGATAGAATCAACTGAAAGCGGGATGGTATCAAGAGATAT-ATGAAAAGCCTACACTTGTTTCAAGCTCCCCCCCTTTCTGGCAGAAGGGC

Homo sapiens            CTGGAAGAGGACCCGACATCTAGCCCAGTGAGAGTGTGCAAAGCCACATGTCAAATGGACAACTTGACGGAGATTTGCCCTTGACTGACTGTGCAGGCTGGTTCCACTTACAGCACTTGA
Canis lupus familiaris  CCTGAGGAGAACATTGCATCCAGCCCAGGAAGTGTGCACAAAGCCACACGTCAGATAGACACCATGGCTGAGATATGCTTTTGATTGACTGACCG---------------AGCAACTGG
```

```
Homo sapiens            GTTTACCTTTGCACAGATTCCTGTATCAGGGCTCTTGGACTGACTTGCCACACATCTGGAGCCCTTTCCTCTCACCACATTGCCCTCCTGGGCACCCCCGGTGGACAGCGCCCAACATTG
Canis lupus familiaris  TTCCTCCTCCCCACAGACTCCTGTAGCGGGGCCCTTGGGTCAGCT--CTCCAAGTCCGGGACCCTCGCCTCTTACTAAATTGCCTCCCTGGGCATCCCCAGCAGACGAAGCCCAGTGTTA

Homo sapiens            CAGGCTCCACAACTTTCCTTGATGTTGCCTAAAGGGTAGCCAGTTAGGGTTCCGGAGAGGAGAAGGAGGAAGAAGAGGAGGAGGAGAAAGAAGCAGCAACGA-TCATTCTGCCATTGTAA
Canis lupus familiaris  CAGGCTCTCACCCTTTTCTTGATGTTGCCTAAAAGGTGGAGGAGGAGA-----GGAGGGGGGCGGGTGGCGGCAGTGGAGGAGGAAACAGCAGCAGCAACGACTCAGCTTGACATTGTAA

Homo sapiens            AGAGTGACTCTTTCCTTAGGGATGGTGGTCTTCTCCCCTTGCCAGGCCTTGGACTGCAGCCCCCAGTCAAAGGTCTTTGCAGGACACTTTCTATCCTTGGAGTTGCAGAGGAGGCCTGAT
Canis lupus familiaris  GGAGTGAATCTTTCCTTAGGGATGGTGGTCTCCTTCCCTGGCTAGACCTTGGACTGCAGCCCCCAGCCAAAGGTCTTTGCAGGAGCCTTGCCATCCTAAAGATTCCAGAAGAAGCATCAT

Homo sapiens            TGGCCATTCCTTCTGGGAC------------AGACAGAGAGAG----GGCCCATGGCCTGCTCTGAGCTCCCTTCAATTTCAAGGAACCCTTAGTTCTGAGAGGAGAGAGGAAGGCTGCA
Canis lupus familiaris  TGGCCTTTCTTCCCTGGACTTCCAGAGATTGAGAGAGAGAGAGAGCCAGCCCAAGCTCTGCTCT---TTGACATTGACTTAAAGGATTCCTTGGTTCTGAGAGGAGAGAGGAAGATTTTG

Homo sapiens            CCTACCGTGGCCTCACAGCTGGGACCCCTC-----------------ATGGGGGCCAACCCCTGGCCTGCTGCTGTGCTTCAGTGACACAGTAAAGCTTAAGAGGGTGGCTGCCTCCTAG
Canis lupus familiaris  TCTAC---AGCCTTGCAGCTGGGACATCTTCATGTTTCTCATATGGGGTGGGAGGCGAGCCATGGCCTGGTGCTGGGCTTCGGTTAGACAACGAGACTTAAGAGGGTGACTGCCTCCTAG

Homo sapiens            GAACTCCTGGGACTTCTAGGTACAAACTCAGGTGCCCTTATAAAGAGACCCACTGCTTGCCTTGAGTCCAGAAAGCACTGCTTCTGCAGTGAAAAACACACACTTACTATGTCAGTTTTG
Canis lupus familiaris  TAACTCCTGGGACTTCTGGATACAAACCCAGGGACCCTCAT--AGAGTCCTGCTGCTTGTCTTGGGTCCAGAACACACTGCTTTTGCAGTGAAAAATACAC----ACTATGTCA-CTCTG

Homo sapiens            GCCATAAAATGCCAGCCAAATCCACTGCTGGCCTGAACTGCCCCTGGACACTGGACTTTCTGCACAGTGACTGGCAGCAGAGCAAGTGCAGCCAGGGT---TTGTGGCCAAAGGGAAGAA
Canis lupus familiaris  GCCACAGAATGCCAGCCAAATACATTGCTGGCCTGAACTG-CCCTCAACACTGAGCTTCCTGCACAGTGCCTGGCAGCTGGGCAAGTGCAGCCAAAATGTCTGGTGGCCAGAGGGAAGAA

Homo sapiens            AGCATAGTAAATTCAACCTCTGTTCCTCATGGGAGTCTGACCGATAATCTCAGGCGGACCTTAGGATCTTGGGCCTGGAGGCAAGAGAAAAAAGCTGGAGGAAGAGGAGTCGCAGAGAAA
Canis lupus familiaris  AAC--AGTAAATTATGCCTCTGTTCCTCACCGGAGTCTGACAGATAACCTCAGCCCAGCCTTAGGATTTTGAGCCTGGAGGCAAGAGGAAGGCGCTGGAAGAAGAGGAGTAACAGAGAAA

Homo sapiens            CCCAGTCCTCTGACTGGTTTCTGGTGAGGTCTCTATTCATCAGTGGGAGGTAAGATGCCCTTTTCATTAGCGCTTTTGTTTCTCAGCCTGGGGATCAGTGGATTTGGATTCCTATAACCT
Canis lupus familiaris  TCCAGTCCTCTGGCTGGTTTCTGGTGAGGTCTCTATTCATCAGTGGGAGGTAAGAAGCCCTTCTGTTTAAAAC--ACACTTCTCAGCCCAGGGATCAGTGGAGCTGGGTTCCTATAATCT

Homo sapiens            GTTGCTACCTTTTAAAAAGATCCCCTC---TCTCCAAAGAGAAAAGTAATACATAATCATTAAAGAAAGTTTGGAAAATAGAGAAAACTAT-GAGGACAAAAACCCCCCAGCCATTCAGA
Canis lupus familiaris  GTTGTTACCTTTTAAGAAAGGCTCCCCACTTCTCTAAGGAGAAAAGTGACAGATAATCATAATAGAAAATTTGGAAAATAGAGAAAATTATAAAGGACAAACACCCCCCAGGCATTCAGA

Homo sapiens            GTCCCACCACACAGAGGCAAGCACTATTAACACTTTGCTGCTTTCCTTTCTAGTGTGTTCTTGCTATGTCTTGTCAAAGCTGAGATCACCCTGCCAGTTCTTGATCAGCAGGATTTAAAA
Canis lupus familiaris  ATCTGGGCACACAGATGCAAGTACTATTAACACTTTGTTGCTTTTCTTTCTAGCATGTTCTC----TGTCTTTTCGAAGCTGAGATCATCCTGTAAGCTCTTGATCAGCAGGATTTAAAA

Homo sapiens            TGGCTGCCACCTGTGCAGTCATGCATACTGAATAATTCATTTAAGCATTCTCCTAATTTGTTGGACCTTTCAATTGTCTGGTTTTCCCCATCCCCACCAGGGCAGGAGTAAACAACTTTG
Canis lupus familiaris  TGGCTGCCACCTATGCAGTCATGCATACTGAGTAATTTATTTAAGCAGTCTCCTGATTCGTTGCACATTTCAATTGCCTGGTTTTCTCCATTCCCACCAGTACAGGAGTCAACAACCCTG

Homo sapiens            TAAAGAAAATTTTGTCTGTTTTTCAGCTGATTTTATCTGATTTCCTCAAGAGTGCATTCCAGAAGGGGACCTGCTATTCTTCGAGGGGACCTGCTATTCTTCGAGGCAGTGGACCCTGTT
Canis lupus familiaris  TAAAGAAAATTTTGTCTGTTTTTCAGCTG-----------TTTCCTCAGGAGAGAGTTCCAGAAGGGGAACTGCTGTCCT----------------------AGGCTGTGGACCCTGTT

Homo sapiens            TGCTGATGGTCAACACTCTGGCCCTGCTGCCCCATGTCCCTG-TGGAGAAGTGTGAAGGACGGGTGCCTTAAACCCACACTTCCAGGGACCCTTCAGCACAGGTCCTGCCTGTTCCCATG
Canis lupus familiaris  TGCTGATGGTCGACACACTGGCCCTGCTG-CCCATGTCCCTGCCAGAGAAGTGCAAAGGACAGGTGCCTTGCACCCCGACCTCCAGGGACCCTTCAGCACAGGCCCTG-CTGTTCCCATG

Homo sapiens            GTGCTTCTCACCCTGAACCCTCAGAGCTACAGCCAGGGATAGAGCAGATGAGCAGTGAGCTGGTTTC-CACCCTCTG---------------GGGCAGGAGGGGCTTGGGCTTCCCTTTG
Canis lupus familiaris  GTGCTTCTCACCCTGAGCTCCTGTAGCCTCAGCCAGGGAAAGAGTGGATGAGCAGTGAGCTGGTTTCTTGCCCTCCAGTGGCAGCGGGGAGCGGGGGGGGGGGGTTGAGCTTCCCTTTG

Homo sapiens            ACCCTCTT-CCTTCCTTTCCCAGATCTTCAATATTTGTTGTGATGTAACAGCCATAAGAGCTGTAAGAGGAACTGTGGGCTTTGGGAGCTTTCTTGGGTTGAAAGAGGGGTGGGGGGAGG
Canis lupus familiaris  ACCCTCTTCCCCCCCTTTCCCAGATCTTCAATATTTGTTGTGATGTAACAGCTACAAGAGCTGTAAGAGGAACTATGGGCTTTGGGTGTTTTCTTGGGTTGAAA-AGGGATGGGGGGAGG

Homo sapiens            GG-------ATGCATGCAGGGGTATGAAGGCAGCCCAAGGTCTCCCAGAGGCTCGGGAGGAAACCCTTTTGGAATACA----------------TGTGTTATTGTTGCATTACTGTGTTG
Canis lupus familiaris  GGGGGGGAAATGCATGCAGGGGTATGAAGGCAGCCCAAGGATTCCCAGAGACTCGGGAGGAAACCTGTTTGAAACAAAAATGTGTTATTGTTATTGTGTTATTGTTGCATTACTGTGTTG

Homo sapiens            TGAAGATAAGAAGCTGCTCTGTATGCTAAAGCTTTCTCTCCTCAATAAAAATATAAAGGGTGTGTAAAGATTGTGCCTCTTTG--TTATTGAGCAAGCATAGATGGGTACAGAGTAGGCA
Canis lupus familiaris  TGAAGAT---AAATTGTTCTGTATGCTAAAGCTT--TCTCCTCAATAAAAATATAAAGGGTGTGTAAAGACCATGTCTCTCTGGCTTATTGGGCAAACACAGGTGGGCACTCAATAGCCA

Homo sapiens            TAGGGTACTTCCAAGGTCCCCTGCCGGAGTCAACCCTACCCTGAAGTCTTGAAATCCTTAGACAAGGAAACCAAGGGCCGGTGAGGCTCAAGGGACCTGTCTACAGTTCTGTGCATTTGT
Canis lupus familiaris  TGGGGCATTTCCCAGGCACCTGG----------------CCTGAAGCCTTGCAATCCTTAGATGAGGCAATTAAGGCACAGCAAGGCTCAAGGGACCTGCCTAGAGTTCTACGCACTTGT

Homo sapiens            GGATGGAAGAAATTGTTGGGAGGGAAGTGGAAGCACTGGG-CATCTTGAAAAATAGCATTCGTTCTCC-----CGGGGAC-------TATGGGTGGCCCAGCCCTACAGGCACGCACGGG
Canis lupus familiaris  GAATGGCAG---TTGTTGGGAGGGAGGTAAAAAGACTGAGTCATCTTGAAAAATAGCATTAGTTCTCCACTTACTGGGATATCAAGGTATGGATGGCCCCATCCTACACACACACACGCG

Homo sapiens            CATGCATGGG----------ACTTTGAGTGAAGGAAGAGAAAGAGGCCAGGTTGGCTGGGGAGGGCCCTTCCGCTTTTTGGCACTAGGTGGCGCTATGGGATACCAGTGAGTTAGCTGGT
Canis lupus familiaris  CACGCACATGCGCGTCCACAGCTC----TGAAAGAAGTGGGGAAGGC-----------GGTAGGGCCCTTCCACTTTGGGGCACTAGGTGGTGCTACTGTACACCACTGAGCTAGATGCT

Homo sapiens            GA------TGAATCACAAGTCCCCAGAGCAGTCCATGTAAGAGGCCCTCCAAGCAAGATCTCATGGCTCCTCACGGACTGTGGGGTCAGAAGAAACTGAATCCCATTCTGGGATTTGCCC
Canis lupus familiaris  GCAGATTTTTAAATACACCTGCCCAGAGCGGTTCACA-GAGAGACCCTC--AGCAAAATTTCACAGCTCCTCACAGACTGTAGGGTTAGAAGAC----CATCCCATTCTTGGATTTGCTA

Homo sapiens            AAGTTTCCCAGC-ATGAATTGGACTTTTTTTTTTTCCAGGGGAAGTGGGTGGGAACATTCATTAAGCCTCT-----GCTGCTTTTGAGTGCTGTGCCAGGCACTTC
Canis lupus familiaris  ACGTTTCCCAGCTATGGATTGGAA---TTTCTTACCCCTGGGACAGCGGGTGGG-ATGTTCATTGAGCCCCCTACAAGTTAGGTGCCAGTGTTACGCCAGGCCCTTC
```

ZCCHC12

>chromosome:GRCh37:X:117957153:117961531:1
GTGAGCCGCCCGCCTCGGCCTCCCAAAGTGCTGGGATTTCAGGCGTGAGCCACCATTCCC
GGCCAGAAGACTGCATTTCTAACAAGCTCCCCCAAATGACTCTGATGTCTTGCCCCCATA
CTTGAATGGGTATTGGTTGAGAGCCTTGTTCTCCAGCTCTCCTCTCTGTTAAATGGACAT
ACTGCTACTTCTTTCACAGTAAAAGAGGAGATGTGTGTGAAAGCCTGTTTCCGCAATGCA
CGTGCTAATGCTCTTCCCGCCCCTTCCCCTCTTCCCCGTCTTTTCCCTATACCAAGGTGA
AGAACTTTTATTATTCACTTGATTTTGGACCCACTTCGTGTTTAATGCACCTGGCCTAAG
ATGCAGAGTTGAGCCCAGGGAGAAGGGACGGGTTGGGGGAGGAAGAAAGAGTCGCAAATT
CCCAGGCAGATTGGCTCCATCGGTCTAATCAAGCTGCACTTGACGCTCCGCGGGCCTGCT
GCGCCCCCGCCCCCTCAGGCGAAAACTCGCCTCTGCCCAAGGCTGTGCGGGGCGTCCGCC
CCCGCACTTCGCTGATTGGCCGCACTAGCCCGCTCGTCACGCTCTTTTGTCTCAGCTGGC
AGAGGATAAAAGCCGCCGCGGCTGCCTTAGGAACGGCGCTGCCTCGTCTCTGCTACCCCT
GGTTGGGCGGCCCTGCGAAGCAGCTCCTTCGGGCAGCCCCGGGTCGCTTAGCGGCCAAGG
AGGCTTCAGTTCTTTGCCGCCTGCAAGGCGGAGACCAGAAGGCGGAATCCACAGCTGGCG
ACGCGGGAGCATCTGCTGTCCACCAGCGGAGCACAGGTAAAGAATGGGGGTGTGATTGCC
CGGCGGGGAGAGGGGTGGGGGAAGACCCACCAGTACTGGGCGGCTCCCAGCTTGGAGAAT
CGGTGACTGTGAGGCAGGGGTAGGAGTACTCTCGTGGCCCGGCTGCGCTGAGGGAGGAGG
TGGGGTTCACCTGGATACTGCGGGCTAGGTAGGATTTGCCTGGATTTGGGGGTGGGAGGA
GAGGCGAGGAGTAGCGGGGGGAGGAATCTCGAGCTGTTTGCTTCGAACATCCTTTGCATC
TCCTCATCTCTTTCTGGGAGGAATATGTGGGTGGGTGTGTCCAACTCCTATTCCCTTTCT
CCGTGGCAG**GCCATCAAAGCCGCATCTGAACTTGAATTCTGTGCAGCTGATTGCAGAGCT**
GGTAGGCCGACGGCTTCCCGGGGGAAATGTAATGCTGGAGGGTGGGGGGCTGGTCTCTGG
CTTATAGTCAGGAGGGGGCAGAGAGAATTTTTCGCCTGGCAACAGCGGAGGTGGTGGGTA
GCAGGATGACGGGTGAAGCTGCTTCTAACCCTTCCCTTCTCTGGCCTTCTCCGCTGTGGC
GCAATCTTGAAACCTCAAG**GACCCGGATCTGCGACCCCCTGTGGACAGAGGTTGACCGTA**
CCCCGGAGAGGAGCTTTCTCACGGAGGGCACTGGTTGCAGAGGCTGGAAGTGAAATAAAG
ACGCGCTCTTGTTTCAGAGTTCGTGTAAGAATCTGAGAAATAACAAGGGGGACGGGCGTG
GGGGGCACTTTGAGAAGGGGGTGTGGTTGAGACCAGAGACCATGAGGTTAATGAGATCTT
ATTTTATTTCCACAAATCTGCGATCATTGTACTCTATTTGGAGAGCCCAATATCAGCTCC
AGACACTGCTACACCTAGAATTTGAAGCAACAGTTACCGTCCCTCTTGGAGGACTGGTGG
GAGGGATGGATGTGGCTGCAAAAAGCACCTTGCTAGCACGCAGGCATCGGGTAGCTCTGG
GAGGTTGTAAGTGTCAATCTCCTACAGGATTATTTTTAGGGTAGTGGGCAAATATAAAAT
GTACTGCAGGATATTCAGGTAGGAAGAGAATGCAGCTGCAGCCCGCTCCCTTATTAACCA
GCCCCCCTTTCTTTTTTACAG**CCCCTGCTGAGATAGGAAGGCAGAGCCACCTCCTCTCCT**
CTCCCACCTGCAGATTAAGCTTTTCTAAAAAGCCTAGGCATCTTCTTATATTCAGATACC
CTATCGTCGTCAGTCATGGCTAGCATCATTGCACGTGTCGGTAACAGCCGGCGGCTGAAT
GCACCCTTGCCGCCTTGGGCCCATTCCATGCTGAGGTCCCTGGGGAGAAGTCTCGGTCCT
ATAATGGCCAGCATGGCAGACAGAAACATGAAGTTGTTCTCGGGGAGGGTGGTGCCAGCC
CAAGGGGAAGAAACCTTTGAAAACTGGCTGACCCAAGTCAATGGCGTCCTGCCAGATTGG
AATATGTCTGAGGAGGAAAAGCTCAAGCGCTTGATGAAAACCCTTAGGGGCCCTGCCCGC
GAGGTCATGCGTGTGCTTCAGGCGACCAACCCTAACCTAAGTGTGGCAGATTTCTTGCGA
GCCATGAAATTGGTGTTTGGGGAGTCTGAAAGCAGTGTGACTGCCCATGGTAAATTTTTT
AACACCCTACAAGCTCAAGGGGAGAAAGCCTCCCTTTATGTGATCCGTTTAGAGGTGCAG
CTCCAGAACGCTATTCAGGCAGGCATTATAGCTGAGAAAGATGCAAACCGGACTCGCTTG
CAGCAGCTCCTTTTAGGCGGTGAGCTGAGTAGGGACCTCCGACTCAGACTTAAGGATTTT
CTCAGGATGTATGCAAATGAGCAGGAGCGGCTTCCCAACTTTCTGGAGTTAATCAGAATG
GTAAGGGAGGAAGAGGATTGGGATGATGCTTTTATTAAACGGAAGCGTCCAAAAAGGTCT
GAGTCAATGGTGGAGAGGGCAGTCAGCCCTGTGGCATTTCAGGGCTCCCCACCGATAGTG

```
ATCGGCAGTGCTGACTGCAATGTGATAGAGATAGATGATACCCTCGACGACTCCGATGAG
GATGTGATCCTGGTGGAGTCTCAGGACCCTCCACTTCCATCCTGGGGTGCCCCTCCCCTC
AGAGACAGGGCCAGACCTCAGGATGAAGTGCTGGTCATTGATTCCCCCCACAATTCCAGG
GCTCAGTTTCCTTCCACCAGTGGTGGTTCTGGCTATAAGAATAACGGTCCTGGGGAGATG
CGTAGAGCCAGGAAGCGAAAACACACAATCCGCTGTTCGTATTGTGGTGAGGAAGGCCAC
TCAAAAGAAACCTGTGACAACGAGAGTGACAAGGCCCAGGTTTTTGAGAATTTGATCATC
ACTCTCCAGGAGCTGACCCATACTGAGATGGAGAGGTCAAGAGTGGCCCCTGGCGAATAC
AATGACTTCTCTGAGCCACTGTAAGGACCACCCCCAGGTTTCAGTGAACCCTTACCTAT
ATTCAGCATCCAGTAGTGGGAAAACTGGGGTGGGGGTGGGGGTGGGACTTCTAACTGCAT
GAATTAATCCACAAAGCGGCTATCTTTTGGGGTGGAGTAGAAAGGGTCTTGGATACCAGC
ACATTGGAGGGAGATAGCCTGACCTCTGTCCTTGCTCCTTCTCCCTGCAGCCTACGGGTC
TGTTTTCTGTGTGTGCCCATTTCCTTGACAGCTTTATTCTTTGTGAAAGTGGTATAATTT
ATTGTTAAATATTTGAACAATAAAAAAGGTACAAAAAGTGAAGTACAAATTACCCAAATC
TCTCCACCCTTATATAATCATTGTCAACCCTTTGATGAGTGATATTTCCCTATACCTATG
TACCCAGATAGATATATGCATAGATAAAAGTGATGAAATATAAGTGCTGTTCTATCTGTA
TTTTTTCACCAAACAATATATGTTGTGAGCTTCTATGTCAATAAATATATATATCAGCAT
CTGCCTTAATGTGTCTGTGCTGTTAGGAAGAATTTTTAAAAAAGAAAATATAGTAAAAAA
CTGTAAAGGGGGGTTGTACTCTGAGGTGGGATTTTTAACCGCATTTTGCAAATGAAAAAA
AATAAATGGGCTGAGATACTTCCCAAAGGTAAAAGGACAGAGCCCCCTTACCACAACTAA
CCTGTCCCTTCGCTCAGAGAGCCAAATTGTGACACCCACTTGTTGACAAAACACCAGTGC
TTTCTTCTCTGATATACCCAGGAGACTGATGGGGGATGGGGGAGTCCGGTGCTGAGGTAA
TAGCCCAATATGGGGATTCCAGGGGCTCCATTTCTGTTCACTTTGGGGATGGGCAGTTCT
TCATCTGGGCAGATTCCTTAGATTGTTGTTGCTGTTGCTGCCTCTGGACAACGTCCTGGT
CACAACAAGGAATCTCTCCTGTGGGCTGACACCACCTTCTGCCCCCTCCCCAGTTAAGGA
AAAGAGAGAGCAGCCACTCATGGGAGCTCAGCATTAGCAGGCTCAACTCCTGACGGCGCC
AGAGCCTCCCCTATAAACTCTGAAGCTATGCGCTGGAGGCCACCGGCAGCCCACGGACT
```

```
Homo sapiens          GTGAGCCGCCCGCCTCGGCCTCCCAAAGTGCTGGGATTTCAGGCGTGAGCCACCATTCCCGGCCAGAAGACTGCATTTCTAACAAGCTCCCCCAAATGACTCTGATGT-------CTTGC
Dasypus novemcinctus  .....................................................................GCATTTCTAACAAGGTTCCCCAAATGATTGTGATGCACCCAGCCTTGC

Homo sapiens          CCCCATACTTGAATGGGTATTGGTTGAGAGCCTTGTTCTCCAGCTCTCCTCTCTGTTAAATGGACATACTGCTACTTCTTTCACAGTAAAAGAGGAGATGTGTGTGAAAGCCTGTTTCCG
Dasypus novemcinctus  ACCCACACTTTAGTGAGTACTGGTTAAGAGCCTTGGTGCTTAATTCTCCTCTTGGTGG---GGACATACTGCCACTTCA------------------------GCGCATGCTCAACAGCA

Homo sapiens          CAATGCACGTGCTAATGCTCTTCCCGCCCCTTCCCCTCTTCCCCGTCTTTTCCCTATACCAAGGTGAAGAACTTTTATTATTCACTTGATTTTGGACCCACTTCGTGTTTAATGCA-CCT
Dasypus novemcinctus  CACAGAAGGAGGTAATTCATTTT-------TTCCCCTTTTCCCC----------TATATAAAGGTGAGGAACTTTTATTGATCACTTGATTTCGGCCCCAC------CATAAGGCATCCA

Homo sapiens          GGCCTAAGATGCAGAGTTGAGCCCAGGGAGAAGGGACGGGTTGGGGGAGGAAGAAAGAGTCGCAAATTCCCAGGCAGATTGGCTCCATCGGTCTAATCAAGCTGCACTTGACGCTCCGCG
Dasypus novemcinctus  GACTCAAAACGCGGAGT......................................................................................................

Homo sapiens          GGCCTGCTGCGCCCCCGCCCCCTCAGGCGAAAACTCGCCTCTGCCCAAGGCTGTGCGGGGCGTCCGCCCCCGCACTTCGCTGATTGGCCGCACTAGCCCGCTCGTCACGCTCTTTTGTCT
Dasypus novemcinctus  .......................................................................................................................

Homo sapiens          CAGCTGGCAGAGGATAAAAGCCGCCGCGGCTGCCTTAGGAACGGCGCTGCCTCGTCTCTGCTACCCCTGGTTGGGCGGCCCTGCGAAGCAGCTCCTTCGGGCAGCCCCGGGTCGCTTAGC
Dasypus novemcinctus  .......................................................................................................................

Homo sapiens          GGCCAAGGAGGCTTCAGTTCTTTGCCGCCTGCAAGGCGGAGACCAGAAGGCGGAATCCACAGCTGGCGACGCGGGAGCATCTGCTGTCCACCAGCGGAGCACAGGTAAAGAATGGGGGTG
Dasypus novemcinctus  ...........................................................................................................AGAAAGGGGGCG

Homo sapiens          TGATTGCCCGGCGGGGAGAGGGGTGGGGGAAGACCCACCAGTACTGGGCGGCTCCCAGCTTGGAGAATCGGTGACTGTGAGGCAGGGGTAGGAGTACTCTCGTGGCCCGGCTGCGCTGAG
Dasypus novemcinctus  TGATGGCCCGGCGCTGAGAGCGGAGAGAGAAGTCCCGCAGGCCCTGGGCTACTCGT-GCTTGGCCACTCGATAACCGCGAGGCTGGGCCAGGGGCACTCTCGTAGCCTGGCTCCGCTGCG

Homo sapiens          GGAGGAGGTGGGGTTCACCTGGATACTGCGGGCTAGGTAGGATTTGCCTGGATTTGGG----------------------------GGTGGGAGGAGAGGCGAGGAGTAGCGGGGGGAGG
Dasypus novemcinctus  GGAGGAGGTGGGGCTCGACTGTATGCTGCCGAAGAGGTAG-ATTTGCCTGGATTGAGGCAGGGGGGTGTTGGTAGTGCATTTTCGTGGGGGGGGGGGGGCCCTGAAAGGCAGGGGGA--

Homo sapiens          AATCTCGAGCTGTTTGCTTCGAACATCCTTTGCATCTCCTCATCTCTTTCTGGG-AGGAATATGTGGGTGGGTGTGTCCAACTCCTATTCCCTTTCTCCGTGGCAGGCCATCAAAGCCGC
Dasypus novemcinctus  -ATCCCAAACTGTCTGACGTGAACGCCCCTCTCCTCTCCTCATCTCTTCCTGGGGAGGAATATGTGGGTGTGCATGTCCAATCCCTTTTCCCTTTTTCCGCGGCAGGCCGTCAAAGCCG-

Homo sapiens          ATCTGAACTTGAATTCTGTGCAGCTGATTGCAGAGCTGGTAGGCCGACGGCTTCCCGGGGGAAA-TGTAATGCTGGAGGGTGGGGGGCTGGTCTCTGGCTTA-TAGTCAGGAGGGGGCAG
Dasypus novemcinctus  -----------ACTCCTGCGTGGCCGATTGCAGAGCTGGTAGGCCGACGGTTTCCGGGGGGGAAGTGTAGTT---GGGGGAAGCGGGCAGCACTTCGGCTTGGCATTCAGGTGGG---AG

Homo sapiens          AGAGAATTTTTCGCCTGGCAACAGCGGAGGTGGTGGGTAGCAGGATGACGGGTGAAGCTGCTTCTAACCCTTC-----CCTTCTCTGGCCTTCTCCGCTGTGGCGCAATCTTGAAACCTC
Dasypus novemcinctus  AGAGAATTTTCTGCCTGGCGACTTCGGAGGGAGGGGGGGCGGGAGGAGCGAGGAAGCTCCTTCAGACGCTTCGGTTCCTTTCTCTGACCG---CCGCTGCAGCGCAGGCTTGACGCCCT

Homo sapiens          AAGGACCCGGATCTGCGACCCCCTGTGGACAGAGGTTGACCGTACCCCGGAGAGGAGCTTTCTCACGGAGGGCACTGGTTGCAGAGGCTGGAAGTGAAATAAAGACGCGCTCTTGTTTCA
Dasypus novemcinctus  ATGGACCCGGATCC---ACCCGGGGTGGACCGAGGCCCACCACACCCCCTGGCG-AGCTTTCACCC--AGGGCGCTG-TGCCAGGGGCTGCAAGTGAAATAAAGGGTCGTGCTGGTTCCT

Homo sapiens          GAGTTCGTGTAAGAATCTGAGAAATAACAAGGGGGACGGGCGTGGGGGGCACTTTGAGAAGGGGGTGTGGTTGAGACCAGAGACCATGAGGTTAATGAGATCTTATTTTATTTCCACAAA
Dasypus novemcinctus  GAACTTACGTAAGAATCGGAGAG-------------------------------------GGGGGTGTGGTTGAGGCCC-------TGCGGTTAATGAGATCTTATTT-ATTCTCACAAA

Homo sapiens          TCTGCGA--------TCATTGTACTCTATTTGGAGAGCCCAATATCAGCTCCAGACACTGCTACACCTAGAATTTGAAGCAACAGTTACCGTCCCTCTTGGAGGACTGGTGGGAGGGA-T
Dasypus novemcinctus  TCCGTGAGACTTAGGCCACTGCGCTCTATCCTGAGAGCCCAATATTGGCTTCGCACACAATGACACCTAAAAGGCAAAGTAACAGTTGCCGTCCCTCTTGGATGATTGGCGGGAGGGAAT

Homo sapiens          GGATGTGGCTGCAAAAAGCACCTTGCTAGCACGCAGGCATCGGGTAGCTCTGGGAGGTTGTAAGTGTCAATCTCCTACAGGATTATTTTTAGGGTAGTGGGCAAATATAAAATGTACTGC
Dasypus novemcinctus  GGATGTGGCTCTGAAAGGCGCTCCACTGGCAC-----------------CTGG-------TAATTGCTAGTCTTCTGC-TGCTTATTTTAAGGGCATTGGGCAGATATAAAATGTACTGC

Homo sapiens          AGGATATTCAGGTAGGAAGAGAATGCAGCTGCAG---------------CCCGCTCCCTTATTAACCAGCCCCCCTTTCTTTTT----TACAGCCCCTGCTGAGATA-----GGAAGGCA
Dasypus novemcinctus  AGGGTATTTAGGACGGGAGAGAATGTGGCTGCAGATGGTTTTCCTGCAGCTTGCTACCTTATTGATCTTTTCTTTTTCTTTTTTGCTTTACAATCCCTGTTGAGAGATACAAGGAAGGCA

Homo sapiens          GAGCCACCTCCTCTCCT--CTCCCACCTGCAGATTAAGCTTTTCTAAAAAGCCTAGGCATCTTCTTATATTCAGATACCCTATCGTCGTCAGTCATGGCTAGCATCATT-GCACGTGTCG
Dasypus novemcinctus  GAGCCAAAGGCCTTTGGGGATTCCTCCTGCAGATTAGGCTTTTCTAAAAATCCTGAGCATCTTGTTGTATTCAGATAGGTGATCATTGTCGATCATGGCAAGCATTATTTGCACATGTGG

Homo sapiens          GTAACAGCCGGCGGCTGAATGCACCCTTGCCGCCTTGGGCCCATTCCATGCTGAGGTCCCTGGGGAGAAGTCTCGGTCCTATAATGGCCAGCATGGCAGACAGAAACATGAAGTTGTTCT
Dasypus novemcinctus  GTAACAGCAGGCAGGGAAATGCACCCTTGCTGCCTTGGGCCCATTCCATGCTGAGGTCCCTGGGGAGGAGTCTTGGTCCTTTAATAGGCAACATGACTGGGAAAAACATGAAGTTGTTTT

Homo sapiens          CGGGGAGGGTGGTGCCAGCCCAAGGGGAAGAAACCTTTGAAAACTGGCTGACCCAAGTCAATGGCGTCCTGCCAGATTGGAATATGTCTGAGGAGGAAAAGCTCAAGCGCTTGATGAAAA
Dasypus novemcinctus  CCGGGAAGGTAGTGCCAGTCCAGGGGGAAGAAACCTTTGAAAACTGGCTGATCCAAGTCAGTGGGATCCTGCCAGATTGAAATATGTCTGAGGAGGAAAAGCTCAAGTGCTTGATGAAAA

Homo sapiens          CCCTTAGGGGCCCTGCCCGCGAGGTCATGCGTGTGCTTCAGGCGACCAACCCTAACCTAAGTGTGGCAGATTTCTTGCGAGCCATGAAATTGGTGTTTGGGGAGTCTGAAAGCAGTGTGA
Dasypus novemcinctus  CTCTTAGGGGCCCTGCCTGGGACATCATGTGATTGCTTCCGGCAGCCAACCCCACCTTAAGTGTGGCTGATTTCTTGCAGGCAATGAAATTGGTGTTTGCGGAGTCTGAAAGCAGTGTGA

Homo sapiens          CTGCCCATGGTAAATTTTTT--AACACCCTACAAGCTCAAGGGGAGAAAGCCTCCCTTTATGTGATCCGTTTAGAGGTGCAGCTCCAGAACGCTATTCAGGCAGGCATTATAGCTGAGAA
Dasypus novemcinctus  CTGCCCATGGTAAATTTTTTTTAACACCTTGCAGGTTCAGGGGGAGAAAACTTCCCTTAATGTGATCTGTTTAGAGGTGCAGCTCCAGAATGCTATTCAGGCTGGGATTATACCTGAGAA

Homo sapiens          AGATGCAAACCGGACTCGCTTGCAGCAGCTCCTTTTAGGCGGTGAGCTGAGTAGGGACCTCCGACTCAGACTTAAGGATTTTCTCAGGATGTATGCAAATGAGCAGGAGCGGCTTCCCAA
Dasypus novemcinctus  AGATGCAAACCAGACTCGCCTGCACCAGCTCCTTTTAGGGGCTGAACTGACTAGGAACCTGCACTTCAGGCTTAAGGATCTTCTCGGGATGTATGCAAATGAGCAGGGGCAGCTTCCCAG

Homo sapiens          CTTTCTGGAGTTAATCAGAATGGTAAGGGAGGAAGAGGATTGGGATGATGCTTTTATTAAACGGAAGCGTCCAAAAAGGTCTGAGTCAATGGTGGAGAGGGCAGTCAGCCCTGTGGCATT
Dasypus novemcinctus  TTTCCTGGAGGTAATCAGGATGATAACAGAGGAAGAGGATTGGGATGGCAATTTTATTAAACGGAAGCGACCCACAAGATC------------AGAGAGAGGGGCCAGCCCTGTGGCATT

Homo sapiens          TCAGGGCTCCCCACCGATAGTGATCGGCAGTGCTGACTGCAATGTGATAGAGATAGATGATACCCTCGACGACTCCGATGAGGATGTGATCCTGGTGGAGTCTCAGGACCCTCCACTTCC
Dasypus novemcinctus  TCAGGACCCCCTGATGACAGCCATGGGCAGTGCTGACTGCAATGTGATAGAGATAGATGACTC----------------GAGGATGTGATCCTGGTGAGATCTTGGGACCCTCTACTTTC

Homo sapiens          ATCCTGGGGTGCC----CCTCCCCTCA---------GAGACAGGGCCAGACCTCAGGATGAAGTGCTGGTCATTGATTCCCCCCACAATTCCAGGGCTCAGTTTCCTTCCACCAGTGGTG
Dasypus novemcinctus  ATCCTCGAACCCCACCCCCACCCCCCACCCCCACCCGATGCAGGGCCAGACCCCAGGATCATATGCTGCTCATTGATTCCCTCAGAAGTTCCTGGGCCCAATCTCCTTCCACCAGTGCTG

Homo sapiens          GTTCTGGCTATAAGAATAACGGTCCTGGGGAGATGCGTAGAGCCAGGAAGCGAAAACACACAATCCGCTGTTCGTATTGTGGTGAGGAAGGCCACTCAAAAGAAACCTGTGACAACGAGA
Dasypus novemcinctus  GTTCTGGATATAAGCAAGATGGTCTTGGGAATAAGTATAGAGCCAGGAAGCAAAAATACACAATCCACTGTTCATACTGTAGTGAGGAGGGCCACTCCAAAGACACCTGTGACAATGAGA

Homo sapiens          GTGACAAGGCCCAGGTTTTTGAGAATTTGATCATCACTCTCCAGGAGCTGACCCATACTGAGATGGAGAGGTCAAGAGTGGCCCCTGGCGAATACAATGACTTCTCTGAGCCACTGTAAG
Dasypus novemcinctus  GCAACCAGGCCCAGGTGTTTGAGAATCTGCTTATCACTCTGCAGGAACTGATTAAAGCAAAGGAGGAAGGGTCAAAAGAGGCCCCTGG------------------------CTGTA--

Homo sapiens          GGACCACCCCCAGGTTTCAGTGAACCCTTACCTATATTCAGCATCCAGTAGTGGGAAAACTGGGGTGGGGGTGGGGGTGGGACTTCTAACTGCATGAATTAATCCACAAAGCGGCTATCT
Dasypus novemcinctus  ------------------AATGAACATTTACCCATATTCAGAGCCTGGTGATGAGAAAACTGGAG----------------------AATTGCATGAATTAATCCACAAAGGGGCTTTAT

Homo sapiens          TTTGGGGTGGAGTAGAAAGGGTCTTGGATACCAGCACATTGGAGGGAGATAGCCTGACCTCTGTCCTTGCTCCTTCTCCCTGCAGCCTACGGGTCTGTTTTCTGTGTGTGCCCATTTCCT
Dasypus novemcinctus  TTTGGAGAAGAGGAGAACAGGCCTTGGATACCAGCAAACC--AAGGGGATGGCCTAATCTCTATTCCTGCTCTATCCCAC-------------CCTGTTC------TGTGTTTATTTCCT

Homo sapiens          TGACAGCTTTA--TTCTTTGTGAAAGTGGTATAATTTATTGTTAAATATTTGAACAATAAAAAAGGTACAAAAAGTGAAGTACAAATTACCCAAATCTCTCCACCCTTATATAATCATTG
Dasypus novemcinctus  TGATGGCTTTATTTTCTTTGTGAA----GTCTAACT--CTGTTAAATGTTC--ACAAAGATAAAAATATAAAAACTAAAGTAAAAATGACCTTGA-------ACACCTATATAGCTATGG

Homo sapiens          TCAACCCTTTGATGAGT---------GATATTTCCCTATACCTATGTACCCAGATAGATATATGCATAGATAAAAGTGATGAAATATAAGTGCTGTTCTAT---CTGTATTTTTTCACCA
Dasypus novemcinctus  TCAACCCTTTGATGAATGTCCTTCTAGATATTTCCCTATATCTTC---------TAGATATATGTATAGATTAAAGTGATCAAATATACATGTTGCTCTATTTGCTGTATTATTTCACCA
```

```
Homo sapiens          AACAATATATGTTGTGAGC----TTCTATGTCAATAAATATATATATCAGCATCTGCCTTAATGTGTCTGTGCTGTTA-----GGAAGAATTTTTAAAAAAGAAAAT--------ATAGT
Dasypus novemcinctus  AATAATTTATGTTGTAGACCTCTTTCTATGTAAATGA--ATATATATCAGCATCTATTTTAATGTCTCTGTGATATTATATTCGGAAAAAGCAAAGGAAAAAGAAATGAAAATAAATAGT

Homo sapiens          -----AAAAAACTGTAAAGGGGGG-TTGTACTCTGAGGTGGGATT------------TTTA--ACCGCATTTTGCAAATGAAAAAAAAT-------AAATGGGCTGAGATACTTCCCAA
Dasypus novemcinctus  TTAAAAAAAAGCAATAAAGAGGGAATTATACCCTGAGGTGGGGTTGGTGCAGTTTTTTTTTATCACCTCATTTTACAGATGGGGAAAGGGAAGACGAAATGGGCTGAAATATCCCCCAA

Homo sapiens          AGGTAAAAGGACAGAGCCCCCTTACCACAACTAACCTGTCCCTTCGCTCAGAGAGCCAAATTGTGACACCCACTTGTTGACAAAACACCAGTGCTTTCTTCTCTGATATA-CCCAGGAGA
Dasypus novemcinctus  AAGTACAGGAGCAGGGCCGC-TTACCACAACCGACCTGTCCCTTAGGTCAGAGGGTCAAACTAGAGCGCCCACTAGTTGACAATACCCCAATGCTTTCTTCTCTAATGTATCCCAGAGGA

Homo sapiens          CTGATGGGGGATGGGGGAGTCCGGTGCTGAGGTAATAGCCCAATATGGGGATTCCAGGGGCTCCATTTCTGTTCACTTTGGGGATGGGCAGTTCTTC---ATCTGGGCAGATTCCTTAGA
Dasypus novemcinctus  GTGC--GACGCTGGGAGAGTCTGATGCTGAGGT-------------------------------ATTCCTATTCACTCTGGGGAGCAATAGCTCTCCCTTCTCTGGGTAAATTCTTTAGA

Homo sapiens          TTGTTGTTGCTGTTGCTGCCTCTGGACAACGTCCTGGTCACAACAAGGAATCTCTCCTGTGGGCTGACACCACCTTCTGCCCCCTCCCCAGTTAAGGAAAAGAGAG-AGCAGCCACTCAT
Dasypus novemcinctus  ------TTGCTGCTGCTGCTTCAGGACAACATCCTGGTCACAAAAA-------------------GATTCTACCCTCTGCCTCTTCCCCATTTAAGGAGAGGGGAGAAGCAACTACTTAT

Homo sapiens          GGGAGCTCAGCATTAGCAGGCTCAACTCCTGACGGCGCCAGAGCCTC-CCCTATAAACTCTGAAGCTATGCGCTGGAGGCCACCGGCAGCCCACGGACT
Dasypus novemcinctus  GGGAATTCAGCACCTAAAGGCT--GTTTCTGATTGCATCCAAGCCTCACCCAGTAAACTCTGAAGCTACATGCTAGAGG-CATGGGCAGCCCAAGGACT


Homo sapiens        GTGAGCCGCCCGCCTCGGCCTCCCAAAGTGCTGGGATTTCAGGCGTGAGCCACCATTCCCGGCCAGAAGACTGCATTTCTAACAAGCTCCCCCAAATGACTCTGATGT-------CTTGC
Loxodonta africana  ......................................................................CTGCACTTCCAACCAGCTCCCCAAAATGATTCTGATGCAGCCTGCCTTGC

Homo sapiens        CCCCATACTTGAATGGGTATTGGTTGAGAGCCTTGTTCTCCAGCTCTCCTCTCTGTTAAATGGACATACTGCTACTTCTTTCACAG------TAAAAGAGGAGATGTGTGTGAAAGCCTG
Loxodonta africana  ACCTATACTTTAGTGGATACTGAATAAGAGCCTTGGTGCTTCAATCCACTCTCTGTTAAACGAAAATACTACCACTTCTTGCACAGGGCTGCTGAGAATAAAGAGGTTCGTGCGAGCCTA

Homo sapiens        TTTCCGCAATGCACGTGCTAATGCTCTTCCCGCCCCTTCCCCTCTTCCCCGTCTTTTCCCTATACCAAGGTGAAGAACTTTT-ATTATTCACTTGATTTTGGACCCACTTCGTGTTTAAT
Loxodonta africana  TTTGTACCCAGAAACCGCTAGTGCTC--CCCATCCCGCCC---------------TGCCCTATACAAAGGTAAGGAACTTTTTATTGTTCGGCTGATTTCGGTCCCACCTCTTGTTTAAG

Homo sapiens        GCACCTGGCCTAAGA-TGCAGAGTTGAGCCCAGGGAGAAGGGACGGGTTGGGGGAGGAA------GAAAGAGTCGCAAATTCCCAGGCAGATTG-GCTCCATCGGTCTAAT-----CAAG
Loxodonta africana  GCCCCCGAATTCAGAGCGCGGATTTAAGCCAAGGGAGAAAGGGAGAGGAAGGGAAGCGGCGGTTGGGGAGAGTCTCCAACTCCTGAGTAAATTGCGAATCGGCGGGTGGGTGATTCCAAG

Homo sapiens        CTGCACTTGACGCTCCGCGGGCCTGCTGCGCCCCCGCCCCCTCAGGCGAAAACTCGCCTCTGCCCAAGGCTGTGCGGGGCGTCCGCCCCCGCACTTCGCTGATTGGCCGCACTAGCCCGC
Loxodonta africana  CTGCGCTTGATGCCTCTCGG------------CCCGCCCCGT------------------------GGCTGCGCGGAGAGCCCGCCCCTGCGCCCCGCTGATTGGCTGTACCGCCCCGC

Homo sapiens        TCGTCACGCTCTTTTGTCTCAGCTGGCAGAGGATAAAAGCCGCCGCGGCTGCCTTAGGAACGGCGCTGCCTCGTCTCTGCTACCCCTGGTTGGGCGGCCCTGCGAAGCAGCTCCTTCGGG
Loxodonta africana  GCGTCACGCTCTTTTGTCTCAGTGGGCAGAGGCTAAAAGGCG--GCGTCTGGCGTGGGAACCTCGCTGCCTCTACTTGGCTACCCCTT-CTGTGCGGCCGTGGGAAGCGGCTCGGGCGCG

Homo sapiens        CAGCCCCGGGTCGCTT--AGCGGCCAAGGAGGCTTCAGTTCTTTGCCGCCTGCAAGGCGGAGACCAGAAGGCGGAATCCACAGCTGGCGACGCGGGAGCATCTGCTGTCCACCAGCGGAG
Loxodonta africana  CAGCTCGGGTTTGCTCGGAGCGGCAGAGGAGGCGCCCGTCTTTTTCCCCTTGCAGGGCGGCGGAC-----GCGGACTC-ACAGCTACCGACGCCTGGGCATCCGTTGTCCATCAGCAG-G

Homo sapiens        CACAGGTAAAGAATGGGGGTGTGATTGCCCGGCGGGGAGAGGGGTGGGGGAAGACCCACCAGTACTGGGCGGCTCCCAGCTTGGAGAATCGGTGACTGTGAGGCAGGGGTAGGAGTACTC
Loxodonta africana  TACAGGTAGGGGA-AGGGGCGCGATTGCCCGGCGCGGAGAG--------------------TCCAGGGCGGCTCCCCGCT-----------------------GGGGAAGGAGAATTC

Homo sapiens        TCGTGGCCCGGCTGCGCTGAGGGAGGAGGTGGGGTTCACCTGGATACTGCGGGCTAGGTAGGATTTGCCTGGATTTGGGGGTGGGAG----------GAGAGGCGAGGAGTAGCGGGGGG
Loxodonta africana  GCGTGGCCGGGCTGCACCGAGGGAGGAGGTAGGGTGCCACTGGATGCTGCCGAATGGGTAGGATTTGCCTGGATT-GAGGGTGGGGGAGCATTTCGGGGGGGGTTGGAAGGGTGGAGGGT

Homo sapiens        AGGAATCTCGAGCTGTTTGCTTCGAACATCCTTTGCATCTCCTCATCTCTTTCTGGGAGGAATATGTGGGTGGGTGTGTCCAACTCCTATTCCCTTTCTCCGTGGCAGGCCATCAAAGCC
Loxodonta africana  TGAAATCGCAAACTG------------------------CCTCATCTCTTCTTGGGGAGAATATGGGGGTGTGAGTGTCCAACTCGTTTTCCCTTTCTCCGTGGCAGGTCATCGAAGCC

Homo sapiens        GCATCTGAACTTGAATTCTGTGCAGCTGATTGCAGAGCTGGTAGGCCGACGGCTTCCCGGGGGAAATGTAATGCTGGAGGGTGGGGGGCTGGTCTCTGGCTTATAGTCAGGAGGGGGCAG
Loxodonta africana  AAAGCCGAACTTGAAATCCGTGTAGCTGGTTGCAGAGCTGGTAGGCCCACGGTTTCCCTGGGGGCGTGTAA--------AGGGGGGGGTCG--CTTTGGCTGGTATTCAGCAGGTGGGAG

Homo sapiens        AGAGAATTTTTCGCCTGGCAACAGCGGAGGTGGT-GGGTAGCAGGATG---ACGGGTGAAGCTGCTTCTAACCCTTCCCTTCTCTGGCCT--TCTCCGCTGTGGCGCAATCTTGAAACCT
Loxodonta africana  AGGAAAATTTCCACCTGGCGACAGCGGGGGCGACCGGGGAGGGGGGTGGTGAAGGATGAGGCTGCTATTGACCCTTCCCTTCCCTGCTCTGTCCGCCGCCGTGGCGCAGTCTTGACGCCC

Homo sapiens        CAAGGACCCGGATCTGCGACCCCCTGTGGACAGAGGTTGACCGTACCCCGGAGAGGAGCTTTCTCACGGAGGGCACTGGTTGCAGAGGCTGGAAGTGAAATAAAGACGCGCTCTTGTTTC
Loxodonta africana  TGAGGACCCCGATCGGCGACCCTTAGTGGACAGAGGCCGACTGGACCCCGGAGGCGAGATTTCGCA------GCGCTGACTGCAGAGGCTGGAACTGAAACAAAGGCGTGCACTTGTTCC

Homo sapiens        AGAGTTCGTGTAAGAATCTGAGAAATAACAAGGGGGACGGGCGTGGGGGGCACTTTGAGAAGGGGGTGTGGTTGAGACCAGAGACCATGAGGTTAATGAGATCTTATTTTATTTCCACAA
Loxodonta africana  TGAGATCCTGTAAGCATC--AGAAATAACGAGGAAAAC--------------TTTGAAAAGGGGGTGTGGTGGAGACGTTAGACCATGAGATTAATAAGATCTTATTT-ATTTCCACAA

Homo sapiens        ATCTGC--------GATCATTGTACTCTATTTGGAGAGCCCAATATCAGCTCCAGACACTGCTACACCTAGAATTTGAAGCAACAGTTACCGTCCCTCTTGGAGGACTGGTGGGAGG-GA
Loxodonta africana  ATCTGTCAGTCTTAGGCCATGGCACCCTCCTTAGAGAGCCCAGTATCAGCCCCACACAGGATTACACCTAGAATTCGAAGCAACAGTTGTCCTCCCTCTTGGAGGACTGGAGGGAGGAGA

Homo sapiens        TGGATGTGGCTGCAAAAAGCACCTTGCTAGCACGCAGGCATCGGGTAGCTCTGGGAGGTTGTAAGTGTCAATCTCCTACAGGATTATTTTTAGGGTAGTGGGCAAATATAAAATGTACTG
Loxodonta africana  TGAATGTGGCTCTGAAAAGAACCT-ACTGCCAAAGGGACACTGGCTGTTGCTATGAGGTTGTCATTGTC---CTTCTGCGGGGCT-TTCTTAGTGCAGTGGGCAGATATAAAATGCAGTG

Homo sapiens        CAGGATATTCAGGTAGGAAGAGAAT------GCAG---------CTGCAGCCCGCTCCCTTATTAACCA---GCCCCCCTTTCTTTTTT----ACAGCCCCTGCTGAGATA---GGAAGG
Loxodonta africana  CAGGGTATTCAGGAAGGGAGAAAATTGGGCTGCAGATTGCTACCCTGTAGTCTGCTGCTTTATTGACCCCCCGCCTCCTTTTTTTTTTTCTTTACAGTCCCTACTGAGATACAAGGAAGG

Homo sapiens        CAGAGCCACCTCCTCTCCTCT------CCCACCTGCAGATTAAGCTTTTCTAAAAAGCCTAGGCATCTTCTTATATTCAGATACCCTATCGTCGTCAGTCATGGCTAGCATCATTGCACG
Loxodonta africana  CAGAGTCCTGCACATTGCTTTGGAGATTCCTCCTGCAGATTAGGCTTCTCTGAAAAGCCTGAGCATTTTGTTACCTTCAGATAGCCAGTCATTGTCAGTCATGGGTAGCATCTTTGCACA

Homo sapiens        TGTCGGTAACAGCCGGCGGCTGAATGCACCCTTGCCGCCTTGGGCCCATTCCATGCTGAGGTCCCTGGGGAGAAGTCTCGGTCCTATAATGGCCAGCATGGCAGACAGAAACATGAAGTT
Loxodonta africana  TGTGTTTAACAGCCGGGGCCGGAACGCACGCTTGTCACCTTGGGCCCATTCCATGCTGAGGTCCCTGGGGAGGAGCCTCGGTCCTTTAACGGCCAACTTGGCAGAGAGGAACCTGAAGTT

Homo sapiens        GTTCTCGGGGAGGGTGGTGCCAGCCCAAGGGGAAGAAACCTTTGAAAACTGGCTGACCCAAGTCAATGGCGTCCTGCCAGATTGGAATATGTCTGAGGAGGAAAAGCTCAAGCGCTTGAT
Loxodonta africana  GTTTTCAGGGAGGGTGGTGCCAGACCAGGTGGAAGAAACCTTTGAAAACTGGCTGATCCAAGTCAATGGGGTTTTGCCAGATTGGAATACCTCTGAGGAGGAAAAGCTCAAGCGCTTGAT

Homo sapiens        GAAAACCCTTA---GGGGCCCTGCCCGCGAGGTCATGCGTGTGCTTCAGGCGACCAACCCTAACCTAAGTGTGGCAGATTTCTTGCGAGCCATGAAATTGGTGTTTGGGGAGTCTGAAAG
Loxodonta africana  GAAAACCCTTACTTAGGGCCCTGCCAGGGAGGCCATGGGTTTGCTCCAGGCAGCCAACTCCAGCCTAAGTGTGGCAGGTTCCTTGCAGGCCATGAAACTGGTGTTTGGGGAGTCTAAAAG

Homo sapiens        CAGTGTGACTGCCCATGGTAAATTTTTTAACACCCTACAAGCTCAAGGGGAGAAAGCCTCCCTTTATGTGATCCGTTTAGAGGTGCAGCTCCAGAACGCTATTCAGGCAGGCATTATAGC
Loxodonta africana  CAGTGTGACCACCCATGGTAAGTTTTTTAACACCTGGCAGTTGCAAGGGGAGAAAGCATCCCTTTATGTGATCCCTTTAGAGGTGCAACTCCAGAATGCTATTCAAGCAGGGAGCATATC

Homo sapiens        TGAGAAAGATGCAAACCGGACTCGCTTGCAGCAGCTCCTTTTAGGCGGTGAGCTGAGTAGGGACCTCCGACTCAGACTTAAGGATTTTCTCAGGATGTATGCAAATGAGCAGGAGCGGCT
Loxodonta africana  TGAGAAAGATGCAAATCAGACTCTCCTGCCCCAGCTCCTTTGAGGGGCTGAGCTAAACAGGGATCTGC-CTTTAGGCTTAAGGATCTTCTCAGGATGTATGCGAATGAGCAAGGGCAGCT

Homo sapiens        TCCCAACTTTCTGGAGTTAATCAGAATGGTAAGGGAGGAAGAGGATTGGGATGATGCTTTTATTAAACGGAAGCGTCCAAAAAGGTCTGAGTCAATGGTGGAGAGGGCAGTCAGCCCTGT
Loxodonta africana  GCC----------GAATTAATCAGGGTGATAAAGACAGAAGAGGATTGGGATGACACTTTT--TAAACAGAAGCGGCCCAAAAGATCTGAGCCAATCCTGGAGAGGGCATCCAACCCTGT

Homo sapiens        GGCATTTCAGGGCTCCCCACCGATAGTGATCGGCAGTGCTGACTGCAATGTGATAGAGATAGATGATACCCTCGACGACTCCGATGAGGATGTGATCCTGGTGGAGTCTCAGGACCCTCC
Loxodonta africana  GGCATTTCAGGCCCCCCAGTTGATAGCAATTGGCAATGTTAACTGCAACATGATACAGATAGAAGATACCCTCCATGACTCAGGTAAGGATGTGATCCTGGTGGAGCCTCAGGACCCTCC

Homo sapiens        ACTTCCATCCTGGGGTGCCCCTCCCCTCAGAGACAGGGCCAGACCTCAGGATGAAGTGCTGGTCATTGATTCCCCCCACAATTCCAGGGCTCAGTTTCCTTCCACCAGTGGTGGTTCTGG
Loxodonta africana  ACTTTCATCCACAGCTGCTCCTCCCCCTAGATGCAGGGCCAGACCTCAGGATGAGGTGCTGGTCAGTGATTCCCCCAACAGTTCCTGGGCCTAGTCTTCTTCCACCAGTGGTAGTTCTGG

Homo sapiens        CTATAAGAATAACGGTCCTGGGGAGATGCGTAGAGCCAGGAAGCGAAAACACACAATCCGCTGTTCGTATTGTGGTGAGGAAGGCCACTCAAAAGAAACCTGTGACAACGAGAGTGACAA
Loxodonta africana  GCATAAGAATGATGGTCCTGGGGATAAGTGTAGAGCCAGGAAGTGAAAATACACAACCCACTGTTCATATTGTAGCAAGGAGGACCATACAAAAGAAACATGTGTCAGTGAGAGGAATAC

Homo sapiens        GGCCCAGGTTTTTGAGAATTTGATCATCACTCTCCAGGAGCTGACCCATACTGAGATGGAGAGGTCAAGAGTGGCCCCTGGCGAATACAATGACTTCTCTGAGCCACTGTAAGGGACCA-
Loxodonta africana  GGCCTAGGTGTTTGAGAATCTGATCATCACTGTGCAGGAGCTGA........GAGGAGGAGGAGCTAAAACAGGTCTGTGGTGAACACAGTGACCCCTCTGAGCCACAGAGAGGTGCTAA

Homo sapiens        CCCCCAGGTTTCAGTGAACCCTTACCTATATTCAGCATCCAGTAGTGGGAAAACTGGGGTGGGGGTGGGGGTGGGACTTCTAACTGCATGAATTAATCCACAAAGCGGCTATCTTTTGGG
Loxodonta africana  CTCCCAGCCCTAAATGAACCCTTACCTATATTCAGAGTCCAGTGGTGGGAAGACTAGGGAGGGG-------------TTCTCATTGCATGCATTAATCCACAGAGGAACTTTTTTTTGGG

Homo sapiens        GTGGAGTAGAAAGGGTCTTGGATACCAGCACATTGGAGGGAGATAGCCTGACCTCTGT--CCTTGCTCCTTCTCCCTGCA-GCCTACGGGTCTGTTTTCTGTGTGTGCCCATTTCCTTGA
Loxodonta africana  GTGGAGGAGAAAAAGTCTTGGAT-CCAGCACAGTGGAGGGGGGTGGGCTGACCTCTGTGTCCCTGCTCC--CCCACTGCCTGTCTAAGGGTCTAGTTTCTGTGTTT----ATTTCCTTGA

Homo sapiens        CAGCTTTATTCTTTGTGAAAGTGGTATAATTTATTGTTAAATATTTGAACAATAAAAAAGGTACAAAAAGTGAAGTACAAATTACCCAAATCTCTCCACCCTTATATAATCATTGTCAAC
Loxodonta africana  TGGCTTTACTCTGTTTGAAAGCAGTACAAGT------------------------GCAAG--ACAAAAACGAAAGTAGAAACTGCTGAAACC-CTCCACCCTTACATAACCATTGTCAAC

Homo sapiens        CCTTTGATGAGTG---------ATATTTCCCTATACCTATGTACCCAGATAGATATATGCATAGATAAAAGTGATGAAATATAAGTGCTGTTCTATC---TGTATTTTTTCACCAAACAA
Loxodonta africana  CCTTTGATAAATGCCCTTCTAGATACTTCCCTATACCTGTGTATCCACATAGATTTATGTGTAGATAAAAGTTATCAAATATAAGTGCTGTTCTATACTGTGTATTTTTTCACCAAACAA

Homo sapiens        TATATGTTGTGAGCTTCTATGTCAATAAATA--TATATATCAGCATCTGCCTTAATGTGTCTGTGCTGTTA------GGAAGAATTTTTAAAAAAGAAA--------ATATAGTAAAAAA
Loxodonta africana  TATATGTTGTATGCTTCTTTCTTTACCAATAAATATATATCAGCATTGACTTTAATGTCTCCGTGGTATTATTTTAGGGAGAAGTAAAGGAAAAAGAAATAAGAAAAATATAATAAAAAG

Homo sapiens        CTGTAAAGGGGGGTTGTACTCTGAGGTGGGATTTTTAACCG----CATTTTGCAAAT--GAAAAAAAATAAATGGG-----CTGAGATACTTCCCAAAGGTAAAAGGACAGAGCCCCCTT
Loxodonta africana  CTGCAAAGAGGGGTCATACCCTGAGGTGAGGTTTTTTATCAATTTCATTCTACAAACAAGGAAAAGAGAAGATGAAACACCTTGTAATATTCCCCAAAGGTAAAAGGAAAAGAGCCCCTT
```

```
Homo sapiens        ACCACAACTAACCTGTCCCTTCGCTCAGAGAGCCAAATTGTGACACCCACTTGTTGACAAAACACCAGTGCTTTCTTCTCTGATATACCCAGGAGACTGATGGGGGATGGGGGAGTCCGG
Loxodonta africana  ACCACAACTGACCTGACCCT-AGGTCATAGAGCCAAACTACGGCATTCATTAGTTGACAGTGCTCCAATATTTTCTTCTCTAATATATCCAGAGGA-----GTCTGACGGTGGAGTCTGC

Homo sapiens        TGCTGAGGTAATAGCCCAATATGGGGATTCCAGGGGCTCCATTTCTGTTCACTTTGGGGATGGGCAGTTCTTC---ATCTGGGCAGATTCCTTAGATTGTTGTTGCTGTTGCTGCCTCTG
Loxodonta africana  TGCCAAGGTAGTAGTTCTACATGGGGATTTCAGGGGCTTGATTTCTGTTCACTTTGCAAAAAGGCTGCTCTCCCTTCTTTGGGTGGATTTCTTAGA------TTGCTGCTGCTGCTTCTG

Homo sapiens        GACAACGTCCTGGTCACAACAAGGAATCTC-TCCTGTGGGCTGACACC-ACCTTCTGCCCCC-------TCCCCAGTTAAGGAAAAGAGAGAGCAGCCACTCATGGGAGCTCAGCATTAG
Loxodonta africana  GACAGTATCCTGGTCACAACAAGGAATCTCTTCCTATCCTCTGGCCCCTACCCCCCACCCCCACCCCCGCCCCCAATTAAGGAAAGGAAAGAGCAGCTACTCATGGCAGCTCAGTGTCAG

Homo sapiens        CAGGCTCAACTCCTGACGGCGCCAGAGCCTCCCCTATAAACTCTGAAGCTATGCGCTGGAGGCCACCGGCAGCCCACGGACT
Loxodonta africana  GAGGCTCAGGTCCCTAAGCCGC--------ACCCTGAAAACCCTGTAGCTGCATACTAGAGAGCACAGGTAGCCGGAGGACT


Homo sapiens       GTGAGCCGCCCGCCTCGGCCTCCCAAAGTGCTGGGATTTCAGGCGTGAGCCACCATTCCCGGCCAGAAGACTGCATTTCTAACAAGCTCCCCCAAATGACTCTGATGTCTTGCCCCCATA
Procavia capensis  .......................................................................................................................

Homo sapiens       CTTGAATGGGTATTGGTTGAGAGCCTTGTTCTCCAGCTCTCCTCTCTGTTAAATGGACA-TACTGCTACTTCTTTCACAGT---------AAAAGAGGAGATGTGTGTGAAAGCCTGTTT
Procavia capensis  ....................................TTTCCTCTCT-CTAACTGGGCAAGACTGCTACTGCCTTCACAAGGCTGCTGAGAAGAAATGAGATGTCCCCGCAAGCGTATTT

Homo sapiens       CCGCAATGCACGTGCT-----AATGCTCTTCCCGCCCCTTCCCCTCTTCCCCGTCTTTTCCCTATACCAAGGTGAAGAACTTTTATTATTCACTTGATTTTGGACCCACTTCGTGTTTAA
Procavia capensis  GCATACAGAAAGTGTTCAGTAAATGCTCTTCCTTCC-------------------TTCTCCCTATACAAAGGTGAGGAACTGTCATTGTTCATTTGATTTCTGTCGCACCTCCTGTTGGA

Homo sapiens       TGCACCTGGCCT-AAGATGCAGAGTTGAGCCCAGGGAGAAGGGACGGGTTGGGGGAGGAAGAAAGAGTCGCAAATTCCCAGGCAGATTGGCTCCATCGGTCTAATCA----------AGC
Procavia capensis  GGCACCTGGTCTCAAAGCGCAGAGGAGAGTCGAGGGGGAAAAG---------GAGAGGGAGAA---GCAGCATACTCACGGATAAAATG--CCAATCGGCCCGATCCCTTGCTTCTCAGC

Homo sapiens       TGCACTTGACGCTC------------CGCGGGCCTGCTGCGCCCCCGCCCCCTCAGGCGAAAACTCGCCTCTGC-----------------CCAAGGCTGTGCGGGGCGTCCGCCCCCGC
Procavia capensis  TGCACTTGACACTGTACCGGCTCCCGCGCGGGTCTGCAGCAGCTCCCGCCGCTCGCTC-----CTCCTTTTTGCTTGCTTTCTGGGGCCCCCCGAGGCGATCCAGGA-GCCCGCCCTCGC

Homo sapiens       ACTTCGCTGATTGGCCGCACTAGCCCGCTC-GTCACGCTCTTTTGTCTCAGCTGGCAGAGGATAAAAGCCGCCGCGGCTGCCTTAGGAACGGCGCTGCCTCGTCTCTGCTACCCCTGGTT
Procavia capensis  GCCCCTCTGATTGGCTGTACCACCCCGCTCTGTCACGCTCTTTTGTCTCAGTGGGTAGAGGCTAAAAGCAGCGGCGGCTGCCGTGGGAACCGCGTTGCCTCCACTCGGCTACTT-TGGCT

Homo sapiens       GGGCGGCCCTGCGAAGCAGCTCCTTCGGGCAGCCCCGGGTCGCTT---AGCGGCCAAGGAGGCTTCAGTTCTTTGCCGCCTGCAAG---GCGGAGACCAGAAGGCGGAATCCACAGCTGG
Procavia capensis  GTGCGGCGGTGCGAAGCAGCTCAGGCGTGCGGCCCCGGGTAACTTCCTAGTTACCGAGGAGGCGCCCGTCTTTCTCTCTTTGCAGGACGGCGGCGGCCAGGACGCGGACTCCACAGCTAT

Homo sapiens       CGACGCGGGAGCATCTGCTGTCCACCAGCGGAGCACAGGTAAAGAATGGGGGTGTGATTGCCCGGCGGGGAGAGGGGTGGGGGAAGACCCACCAGTACTGGGCGGCTCCCAGCTTGGAGA
Procavia capensis  AGATGC---------CGTTCTCCATCACCAGGTC-CAGGTAGAGAA-AGGGGTGCGATTGCCTAGCA--------------------CCGAAGGTGCTGGGTGGCTCCCCGCTTGGGGC

Homo sapiens       ATCGGTGACTGTGAGGCAGGGGTAGGAGTACTCTCGTGGCCCGGCTGCGCTGAGGGAGGAGGTGGGGTTCACCTGGATACTGCGGGCTAGGTAGGATTTGCCTGGATT-----TGGGGG-
Procavia capensis  A----------------------AAGAGCACTCCAGTAGCTGGGCTGCA--GAGAGAGGAGGTAGGGTTCAACTGGATGCTGCCGAATGGGTAGGGTTTGCCTGAATTGAGAATGGGGGA

Homo sapiens       -----TGGGAGGAGAGGCGAG-----GAGTAGCGGGGGGAGGAATCTCGAGCTGTTTGCTTCGAACATCCTTTGCATCTCCTCATCTCTTTCTGGGAGGAATATGTGGGTGGGTGTGTCC
Procavia capensis  GAATTTGGGGGCAGGGGCAGGCTTGGAAAGATGGAGGGTTGAAATCGCGAACTGCT-GCCTTAAACCCCTTTTGCCTCTGCTCATCTCGTCCTGGAGAAAATATGTGAATGG------CC

Homo sapiens       AACTCCTATTCCCTTTCTCCGTGGCAGGCCATCAAAGCCGCATCTGAACTTGAATTCTGTGCAGCTGATTGCAGAGCTGGTAGGCCGACGGCTTCCCGGGG-----------GAAATGTA
Procavia capensis  GGCTCATTTTCCCTTTCTCCGTGACAGGTCATCGAAGCCAAAGCCGAAGTTAAGCTCCGTGTGGCTGATTTCAGAGCTGGTAGGCCCACGGTTCCTCGGGGAGGGGGGCGGTGGGATTTA

Homo sapiens       ATGCTGGAGGGTGGGGGGCTGGTCTCTGGCTTATAGTCAGGAGGGGGCAGAGAGAATTTTTCGCCTGGCAACAGCGGAGGTGGTGGGTAGCAGGATGACGGG-------TGAAGCTGCTT
Procavia capensis  ATGGAGGAGGG------ACTCG-CTTTGGCTTGCATTCAGCAGGTGGGAGTGGAAATTTTTCACCTGGCGACAGCAGAGGTGACTGGGATGGGGGTGGGGAGGTGGGAATGAGGCTGCCC

Homo sapiens       CTAACCCTTCCCTTCTCTGGCCTTCTCCGCTGTGGCGCAATCTTGAAACCT-CAAGGACCCGGATCTGCGACCCCCTGTGGACAGAGGTTGACCGTACCCCGGAGAGGAGCTTTCTCACG
Procavia capensis  TT-------------------TTCTCTACCACAGTGCAGTCTTGGACGCTGTGAGGACCCTAATCCGCGAACCTCAGTGGACAGAGGCCTACAGGACCTTGAGGCTG-GATTTCTCA--

Homo sapiens       GAGGGCACTGGTTG----CAGAGGCTGGAAGTGAAATAAAGACGCGCTCTTGTTTCAGAGTTCGTGTAAGAATCTGAGAAATAACAAGGGGGACGGGCGTGGGGGGCACTTTGAGAAGGG
Procavia capensis  ----GCACTGACTGAAGGCAGAGGCTGGAACTGAAACAAAGGCGTACACTTGTTCCTGAGATCACGTAAGAATC--AGAAAT--------------------GAGAATTTTGAGAAGGG

Homo sapiens       GGTGTGGTTGAGACCAGAGACCATGAGGTTAATGAGATCTTATTTTATTTCCACAAATCTGC--------GATCATTGTACTCTATTTGGAGAGCCCAATATCAGCTCCAGACACTGCTA
Procavia capensis  GGTGTGGTGGAGACGTTAGGCCTTGAGATTAATGAGATTTTATTT-ATTTTCACAAATCTATCAGATTTAGACCGTGACACTCTACTTAGAGAGCCCAGTCTCAGCTCCACACACCATTA

Homo sapiens       CACCTAGAATTTGAAGCAACAGTTACCGTCCCTCTTGGAGGACTGG-TGGGAGGGATGGATGTGGCTGCAAAAAGCACCTTGCTAGCACGCAGGCATCGGGTAGCTCTGGGAGGTTGTAA
Procavia capensis  CACCTAGAATGTGAAGCAACAGTTGCCCTCTGTATTGGAGGATTAGCTCGCGGAGATAAATGCGGCTCTGAAAAGCACCGTACTGGCCAAGGGACACTGGCTATTGCTCTGAGGCCGTAG

Homo sapiens       GTGTCAATCTCCTACAGGATTATTTTTAGGGTAGTGGGCAAATATAAAATGTACTGCAGGATATTCAGGTAGGAAGAGAATGCAGCTGCAG---------------CCCGCTCCCTTATT
Procavia capensis  TTGTCTTTTTCTTGCAGGGC-GTTTTTAGTGCAGTGGGCAGATACAAAATGCAGTGTAGGGTATTCAGAAAGGGAGAGCACTGGGCTGCAGATTGCCACTGTGTAGCCTGCTACTTTACT

Homo sapiens       AACCAGCCCCCCTTTCTTT--------TTTACAGCCCCTGCTGAGATA---GGAAGGCAGAGCC-------ACCTCCTCTCCTCTCCCACCTGCAGATTAAGCTTTTCTAAAAAGCCTAG
Procavia capensis  GACCCCCTTCCCCTTTTTTCCCTTTTCTTTACAGTTCCTACTGAGATACAAGGGAGGCAGAGCCCTGGAACATTGCTTTGGAGATTCCTGGTGCAAACTAGGCTTTTCTGCAAAACCTGA

Homo sapiens       GCATCTTCTTATATTCAGATACCCTATCGTCGTCAGTCATGGCTAGCATCATTGCACGTGTCGGTAACAGCCGGCGGCTGAATGCACCCTTGCCGCCTTGGGCCCATTCCATGCTGAGGT
Procavia capensis  GCATTTTGTTATATTCAGATAACCAGTCATTGTCAGTCATGGCTAGCATCATTGCACATATGTTGAACAGTCGGGGCCAGAATGCACCCTTGCCACCTTGGGCCCATTCCATGCTGAGGT

Homo sapiens       CCCTGGGGAGAAGTCTCGGTCCTATAATGGCCAGCATGGCAGACAGAAACATGAAGTTGTTCTCGGGGAGGGTGGTGCCAGCCCAAGGGGAAGAAACCTTTGAAAACTGGCTGACCCAAG
Procavia capensis  CCCTGGGGAGGAGCCTTGGTCCCTTAATGGCCCACTTGGCAGAGAGAAACATAAAGTTGTTCTCGGGAAGGGTGGTGCCAGTCCAGGGGGAAGAAACCTTTGAAAACTGGCTGAATCAAG

Homo sapiens       TCAATGGCGTCCTGCCAGATTGGAATATGTCTGAGGAGGAAAAGCTCAAGCGCTTGATGAAAACCCTTAGGGGCCCTGCCCGCGAGGTCATGCGTGTGCTTCAGGCGACCAACCCTAACC
Procavia capensis  TCAATGGGATCTTGCCAGATTGGAATACCTCTGAGGAGGAAAAACTCAAGCGCTTGATGCAGACCCTTAGGGGCCCTGCCAGGGAGGTCATGCGCTTGCTTCAGGCAGCCAACCCCAGCC

Homo sapiens       TAAGTGTGGCAGATTTCTTGCGAGCCATGAAATTGGTGTTTGGGGAGTCTGAAAGCAGTGTGACTGCCCATGGTAAATTTTTTAACACCCTACAAGCTCAAGGGGAGAAAGCCTCCCTTT
Procavia capensis  TAAGTGTGGCAGATTTCTTGCGGGCCATGAAACTGGTGTTTGGGGAGTCTGAAAGTAGCGTGACTGCCCATGGTAAATTTTTTAACACCCTGCAGGCGCAAGGAGAGAAAACATCCCTGT

Homo sapiens       ATGTGATCCGTTTAGAGGTGCAGCTCCAGAACGCTATTCAGGCAGGCATTATAGCTGAGAAAGATGCAAACCGGACTCGCTTGCAGCAGCTCCTTTTAGGCGGTGAGCTGAGTAGGGACC
Procavia capensis  ATGTGATCCGTTTAGAGGTGCAGCTCCAGAATGCTATTCAGGCAGGAGTCGTAGCTGAGAAAGATGCAAACCAGACTCGCTTGCACCAACTCCTTCTAGGGGCTGAGCTGAACAGGGACC

Homo sapiens       TCCGACTCAGACTTAAGGATTTTCTCAGGATGTATGCAAATGAGCAGGAGCGGCTTCCCAACTTTCTGGAGTTAATCAGAATGGTAAGGGAGGAAGAGGATTGGGATGATGCTTTTATTA
Procavia capensis  TGCGCTTCAGGCTTAAGGAGCTTCTCAGGATGTATGCAAATGAGCAGGAGTGTCTTCCCGGTTTCCTGGAGCTAATCAGAATGATAAGGGCTGAAGAGGATTGGGATGGCACTTTTATTA

Homo sapiens       AACGGAAGCGTCCAAAAAGGTCTGAGTCAATGGTGGAGAGGGCAGTCAGCCCTGTGGCATTTCAGGGCTCCCCACCGATAGTGATCGGCAGTGCTGACTGCAATGTGATAGAGATAGATG
Procavia capensis  AACGAAAGCGACCCAAAAGATCTGAGTCAGTTGTGGAGAGGGCAGCTAACCCTGTGGCATCGCAGGGCCTGCAGCCGATAGTGACTGGCAATGCTAATTGCAACTTGATAGAGATCGATG

Homo sapiens       ATACCCTCGACGACTCCGATGAGGATGTGATCCTGGTGGAGTCTCAGGACCCTCCACTTCCATCCTGGGGTGCCCCTCCCCTCAGAGACAGGGCCAGACCTCAGGATGAAGTGCTGGTCA
Procavia capensis  ATTCCCTTGATGATTCAGATGAGGATGTGATCTTGGTGGAGCCTCAGGACCCTCCAGTCATATCGGTGGCTTCTCCTCCCCCTGTGTGCAGGGCCAGACCTCAGGATCAGGTGCTGGTCA

Homo sapiens       TTGATTCCCCCCACAATTCCAGGGCTCAGTTTCCTTCCACCAGTGGTGGTTCTGGCTATAAGAATAACGGTCCTGGGGAGATGCGTAGAGCCAGGAAGCGAAAACACACAATCCGCTGTT
Procavia capensis  ATGATTCCCCCAACAATTCCTGGGCCCGGTTTCCTTCCACCAGTGGTGGTTCTGGGCATCAGAGTGATGGTCCTGGGGATAAGCGTAAGGCCAGGAAGCGAAAATACACAATCCGCTGTT

Homo sapiens       CGTATTGTGGTGAGGAAGGCCACTCAAAAGAAACCTGTGACAACGAGAGTGACAAGGCCCAGGTTTTTGAGAATTTGATCATCACTCTCCAGGAGCTGACCCATACTGAGATGGAGAGGT
Procavia capensis  TATTTTGTGGTGAAGAGGGCCACACAAAAGAAATTTGTG---GTGAAAACAACAAAGCCCAGGTTTTTGAGAATCTCATTATCACCCTTCAGGAACTGACACATACAGAGGAGGAGGAGA

Homo sapiens       CAAGAGTGGCCCCTGGCGAATACAATGACTTCTCTGAGCCACTGTAAGGGACCA-CCCCCAGGTTTCAGTGAACCCTTACCTATATTCAGCATCCAGTAGTGGGAAAACTGGGGTGGGGG
Procavia capensis  CAAAAGAGGTCTCTGGTGGACGCAATGACCTCTCTGAGCTGCGGAAAGGAGCTAGCCCCCAGCCCTAAATGAACCGTTGTCTATATTTAGCGTACAGTGGGGGGAAGACTGGGGAGGGG-

Homo sapiens       TGGGGGTGGGACTTCTAACTGCATGAATTAATCCACAAAGCGGCTATCTTTTGGGGTGGAGTAGAAAGGGTCTTGGATACCAGCACATTGGAGGGAGATAGCCTGACC--TCTGTCCTTG
Procavia capensis  ------------TTTTCATTGCATGCATTAATCCTCAGAGCAACTTTCTTTTGGGGTGGAGGACAAAAAGCTTTTGATGCCAGCACAGGGGAGGGGAATGGCCTGACCTTTCTGTCTCTG

Homo sapiens       CTCCTTCTCCCTGCAGCCTACGGGTCTGTTTTCTGTGTGTGCCCATTTCCTTGACAGCTTTA--TTCTTTGTGAAAGTGGTATAATTTATTGTTAAATATTTGAACAATAAAAAAGGTAC
Procavia capensis  CTC--TCTCCCT---ACCTAAGGGTCCATTTTCTGTGTGTGTCTAATTCTTTGATGGGTTTACTCTCTTTGTGAAACTGGTGCAA-------------------------------GTAC

Homo sapiens       AAAAAGTGAAGTACAAATTACCCAAATCTCTCCACCCTTATATAATCATTGTCAACCCTTTGATGAGT---------GATATTTCCCTATACCTATGTACCCAGATAGATATATGCATAG
Procavia capensis  AAAAACTAATGTAC-AGCTACCTGAAACCCTCCACCCATATATAATCATTGTCAAGCCTTTGATGAATGCCTTTCTAGATACTTCCCTATACCTGTGTACCCAGATAGATTTATGTATAG

Homo sapiens       AT-AAAAGTGATGAAAT-ATAAGTGCTGTTCTAT-CTGTAT-TTTTTCACCAAACAATATATGTTGTGAGCTTCTATGTCAATAAATATATATATCAGCATCTGCCTTAATGTGTCTGTG
Procavia capensis  ATAAAAAGTGATCAAATAAAAAGTGCTGTTGTATATTGTGTATTTTTCACCAAA.......................................CAGCATCTATATTAATGTCTCTGCG

Homo sapiens       CTG-----TTAGGAAGAATTTTTAAAAAA----GAAAATATAGTAAAAAACTGTAAAGGGGGG----TTGTACTCTGAGGTGGGATTT---------------------TTAACCGCATT
Procavia capensis  GTATTACTTTAGTAAAAAGTAAAAAGAAATGAGGAAAATATCCTAAAAAGCTGCAAAGAAGGATTACTTACACTCTGAGGTGAGTTTTTGTTTGTTTGTTTGTTTTTAATCAATCTCATT

Homo sapiens       TTGCAAATGAAAAAA--AATAAATGGGCTGA----GATACTTCCCAAAGGTAAAAGGACAGAGCCCCCTTACCACAACTAACCTGTCCCTTCGCTCAGAGAGCCAAATTGTGACACCCAC
Procavia capensis  CTATAGGCAAAGAAAGGAGAAGACGGAATGCTCGTAATATCCCCCGAAGTTAAATGTGGAAGAGACCCTTACTGCAGCTGACCTGAACC-TAGGTCACAGAGCCAAA-TGAGACAACCAT

Homo sapiens       TTGTTGACAAAACACCAGTGCTTTCTTCTCTGATATACCCAGGAGACTGATGGGGGATGGGGGAGTCCGGTGCTGAGGTAATAGCCCAATATGGGGATTCCAGGGGCTCCATTTCTGTTC
Procavia capensis  TAGTTGACATAGGTCCAATATTTTCCTCTCTAACGTATCCAGAGGA-----GTCTGGTGGTGGCATTTGGTTCTGAGGTGATAGCTCTACATGAGGATTCTAGGGGCTCAATTTCTGTTC

Homo sapiens       ACTTTG----GGGATGGGCAGTTCTTCATCTGGGCAGATTCCTTAGATTGTTGTTGCTGTTGCTGCCTCTGGACAACGTCCTGGTCACAACAAGGAATCTCTCCTGTGGGCTGACACCAC
Procavia capensis  CCTTTGCTCAGAGATGGGCAATTCTTTCTCTGGGTGGATTCTTCAGA------TTGCTGCTGTTGCTTCTGGAAGGTATCCTGATCACAACAAGGAATCACTCCTGTCAGTTGACACTAT
```

```
Homo sapiens       CTTCTGC------------CCCCT-----CCCCAGTTAAGGAAAAGAGAGAGCAGCCACTCATGGGAGCTCAGCATTAGCAGGCTCAACTCCTGACGGCGCCAGAGCCTC-CCCTATAAA
Procavia capensis  CTTCTGCTGACTGCCTACTCCCCTCCAGCCCCCAATTCAGGAAAGGAATGAGGAGCTACTCAGGGCAGCTCAGCACCAGGAGGTTCAACTCCTGCTAGCAACTAAACCTCACCATAAAAC

Homo sapiens       CTCTGAAGCTATGCGCT-GGAGGCCACCGGCAGCCCACGGACT
Procavia capensis  CCTTGTGGCTACATAATCAGATAGCACAGGCAGCTGGAGGACT
```

```
Homo sapiens  GTGAGCCGCCCGCCTCGGCCTCCCAAAGTGCTGGGATTTCAGGCGTGAGCCACCATTCCCGGCCAGAAGACTGCATTTCTAACAAGCTCCCCCAAATGACTCTGAT-----GTCTTGCCC
Bos taurus    ..............................................................AGGAATCTGCATTTGTAACAAGCACCCCAAAATGATTGAGGCAGCCAGCCTTGCCC

Homo sapiens  CCATACTTGAATGGGTATTGGTTGA-GAGCCTTGTTCTCCAGCTCTCCTCTCTGTTAAATGGACA---TACTGCTACTTCTTTCACAGTAAAAGAGGAGAT-------GTGTGTGAAAGC
Bos taurus    ACATCTTTTCGTGAGTATTGGTTAAAGAGTCTGAGGGCCCAAGTGCCCTCACTGTTAAATGGACGCAGTACTGCCACCTTCTTCATAGTAAAAAAAAAAAAAAAAAAAAGTCTGTGCAAGT

Homo sapiens  CTGTTTCCGCAATGCACGTGCTAATGCTCTTCCCGCCCCTTCCCCTCTTCCCCGTCTTTTCCCTATACCAAGGTGAAGAACTTTTATTATTCACTTGATTTTGGACCCACTTCGTGTTTA
Bos taurus    -TATTTGCACAAAGAAAACGCTAACGCTTTTATTTTCCACTCCCCTA-----------------ATACAAAGGTGAAGAACTTTTATTGCTTATTTGATTTGGATTCCGCCTCATGTTTA

Homo sapiens  ATGCACCTGGC-CTAAGATGCAGAGTTGAGCCCAGGGAGAAGGGACGGGTTGGGGGAGGAAGAAAGAGTCGCAAATTCCCAGGCAGATTGGCTCCATCGGTCT----AATCAAGCTGCAC
Bos taurus    ATGCACCTGGCTCCAAGACCCAAAGTTGAGCCGACAGAGA-----------GAGGGAGAAGGGAAGACTCGAGAA--------------AACTCCTCCAGTTCCCACAACCAAGTCGCAC

Homo sapiens  TTGACGCTCCGCGGGCCTGCTGC-----GCCCCCGCCC-CCTCAGGCGAAAACTCGCCTCT-GCCCAAGGCTGTGCGGGGCGTCCGCCCCCGCACTTCGCTGATTGGCCGCACTAGCCCG
Bos taurus    TTGACCTCGCGGGGACCTGCTGCTTATCGCCACCCCCCACCGCCGGCGCACATTTGTATCCAGCCCTAGGCTGCGCGGCAAGCCCGCCCCCGCGCCCGGTTGATTGGCCGCGCCCGCCCG

Homo sapiens  CTCGTCACGCTCTTTTGTCTCAGCTGGCAGAGGATAAAAGCCGCCGCGGCTGCCTTAGGAACGGCGCTGCCTCGTCTCTGCTACCCCTGGTTGGGCGGCCCTGCGAAGCAGCTCCTTCGG
Bos taurus    CCCGTCACGCTCTTTTGTCTCGGTTAGCTGAGGATAAAAGCTGCCGCGGCTGCCGTGGGAACCGTGCTGTCTCGACTCGGCTACCAGTCTCAGGGCGGCAGTGCAAAGCAGCTTCATCGG

Homo sapiens  GCAGCCCCGGGTCGCTTAGCGGCCAAGGAGGCTTCAGTTCTTTGCCGCCTGCAAG---GCGGAGACCAGAAGGCGGAATCCACAGCTGGCGACGCGGGAGCATCTGCTGTCCACCAGCGG
Bos taurus    GTGGTCCCGGCTAGCTCAGCGGCTGAGGAGGCGCCCATCCTTTTCC-CCTGTAGGACCGCGGAGACC-GAAGGCGGAG-ACGCAGCTGTCAACGCCAGAGCGTCCGCTGCCCACCAGCCC

Homo sapiens  AGCACAGGTAAAGAA-TGGGGGTGTGATTGCCCGGCGGGGAGAGGGGTGGGGGAAGACCCACCAGTACTGGGCGGCTCCCAGCTTGGAGAATCGGTGACTGTGAGGCAGGGGTAGGAGTA
Bos taurus    AACACAGGTAAAGAAAGGGGGGCAAGATTGCCCCGCGCGGAGAGAGGAGGGGGAAGCCCTACCAGGACTGG-CGGCTCCCAGTTTAGGGAATCGAGGGTTGTGCGGCCGGGGAAGGAGCC

Homo sapiens  CTCTCGTGGCCCGGCTGCGCTGAGGGAGGAGGTGGGGTTCACCTGGATACTGCGGGCTAGGTAGGATTTGCCTGGATTT-----GGGGGTG-------GGAGGAGAGGCGAGGAG-----
Bos taurus    CTCCCGCGGCCTGGCTGCGCTGAGGGAGGAGGTGGGGTTCCACTGGATGCTGCCGAATGGGTAGGATTTGCCCGGATTGAGAAAGGGGGCGTTTTCAGGGGGAGGTGGCAAGGGGCGCGT

Homo sapiens  -TAGCGGGGGGAGGAATCTCGAGCTGTTTGCTTCGAACATCCTTTGCATCTCCTCATCTCTTTC--TGGGAGGAATATGTGGGTGGGTGTGTCCAACTCCTATTCCCTTTCTCCGTGGCA
Bos taurus    TTGGGAGGTGGGAGAATCGCAAACTG----CCTTGAAGCCCCTTTGCATCTCCTCATCCCTTCCGGCGGGAGGAATATGTGGGTGTGCGTCTCCAACTCCGATTCTCTTTCTCGGTGGCA

Homo sapiens  GGCCATCAAAGCCGCATCTGAACTTGAATTCTGTGCAGCTGATTGCAGAGCTGGTAGGCCGACGGCTTCCC-GGGGGAAATGTAATGCTGGAGGGTGGGGGGCTGGTCTCTGGCTTATAG
Bos taurus    GGCCACCAGAACCGAAGCCGAACTTGAATTCCGTGCGGCTGATTACAGAGCTGGTAGGACGACGATTTCCCCGGGGGAAATGGAATG------GGGGGGCGCCT--TCTTTGGCTTGTAT

Homo sapiens  TCAGGAGGGGGCAGAGAGAATTTTTCGCCTGGCAACAGCGGAGGTGGTGGGT------------AGCAGGATGACGGGTGAAGCTGCTTCTAACCCTTCCCTTCTCT----GGCCTTCTC
Bos taurus    TCAGAAGGTGGGAGAAGGAGTTTTCCACCTGGCTACTGCGGAGGCCGCGGGGTGGGGAGGAAGAAGCAGAATGAGGAATGAGGCTGTTTCTGACCCTTCCCCTCCCTTCCAAGCC--CTC

Homo sapiens  CGCTGTGGCGCAATCTTGAAACCTCAAGGACCCGGATCTGCGACCCCCTGTGGACAGAGGTTGACCGTACCCCGGAGAGGAGCTTTCTCACGGAGGGCACTGGTTGCAGAGGCTGGAAGT
Bos taurus    TGCGGCGGCGCAGTCTTGACGCCCTAAGGTCCTGGATCGGCGACCCCCGGTGGACAGCGATCAGTTGAAGCCCGGAGGCGAGCTTTCCCACGGAAGCCACCGGTTGCAGAAGCTGGAAGT

Homo sapiens  GAAATAAAGACGCGCTCTTGTTTCAGAGTTCGTGTAAGAATCTGAGAAATAACAAGGGGGACGGGCGTGGGGGGCACTTTGAGAAGGGGGTGTGGTTGAGACCAGAGACCATGAGGTTAA
Bos taurus    GAAATAAAGGCGCGCACTTGTTCCTGAGCTCCTGTAAGAATCTGAGAAATAGC---GGGGCGGGGTGGGGGGTGGACTTTTAGAAGGGGGTGTGGT-GAGACCGGAAACCAAGAGGTTAA

Homo sapiens  TGAGATCTTATTTTATTTCCACAAATCTGCGATCATTGTACTCTATTTGGAGAGCCCAATATCAGCTCCAGACACTGCTACACCTAGAATTTGAAGCAACAGTTACCGTCCCTCTTGGAG
Bos taurus    TGAGAGCTTATTT-ATTCCCACAAATCTGTGACCGCTGTGCTCTATTTGAGAGCCCACTATCCTCTCCATACACTGTTACACTTAGAA------GCAACAGTTGCCGTCCCTCTTGAAG

Homo sapiens  GA-CTGGTGGGAGGGATGGATGTGGCTGCAAAAAGCACCTTGCTAGCACGCAGGCATCGGGTAGCTCTGGGAGGTTGTAAGT----GTCAATCTCCTACAGGATTATTTTTAGGGTAGTG
Bos taurus    GAACCGAGGGAAGGGATAGAAGTGGCTGTAAAAAGCATCCTGCTAACACG-AGACACTGGCTGGCTCCGTGAGGTTGTAATTAATTGCCAGTCTCCTGCAGGAT-ATTTTTAGGGTAGTG

Homo sapiens  GGCAAATATAAAATGTACTGCAGGATATTCAGGTAGGAAGAGAATGCAGCTGCAGCCCGCTCCC--TTATTAACCAGCCCCCCTT---TCTTTTTTACAGCCCCTGCTGAGATAGGAAGG
Bos taurus    GGCAGATATAAAATGTACTGTAGGATATTCAGGAAGGGAGAAAAT--AACC----CCTGCTCCCGTTTTTTGTTTTGTTTCGTTTGTTTCTTTTTTACAGTTCCTACTGAGACAGGAGGA

Homo sapiens  C---AGAGCCACCTCCTCTCCTCTCCCACCTGCAGATTAAGCTTTTCTAAAAAGCCTAGGCATCTTCTTA-TATTCAGATACCCTATCGTCGTCAGTCATGGCTAGCATCATTGCACGTG
Bos taurus    CGACAGAACC--------CTCCAGCACGCCTGCAGATTAGGCTTTTTGTAAAAGCCTGAGAATCTTGTTAACATTTAGAGACCCAGTCATCATCCGTCATGACTAGCATCATTGCACGCA

Homo sapiens  TCGGTAACAGCCGGCGGCTGAATGCACCCTTGCCGCCTTGGGCCCATTCCATGCTGAGGTCCCTGGGGAGAAGTCTCGGTCCTATAATGGCCAGCATGGCAGACAGAAACATGAAGTTGT
Bos taurus    TGAGTAACAGCCGGCGGCAGAACACATCCTTGCCACCTTGGGCCCATTCTATGCTAAGGTCTCTGGAAAGGAGTCTCGGTCCTTTAATGGCCATCCTGGCAGAGAGAAACCTGAAATTGT

Homo sapiens  TCTCGGGGAGGGTGGTGCCAGCCCAAGGGGAAGAAACCTTTGAAAACTGGCTGACCCAAGTCAATGGCGTCCTGCCAGATTGGAATATGTCTGAGGAGGAAAAGCTCAAGCGCTTGATGA
Bos taurus    TCTCAGGGAGGGTGGTGCCAGCCCAGGGGGAAGAAACTTTTGAGAACTGGCTGATCCAAGTCAATGAGGTCTTGCCAGATTGGAATATGTCTGAAGAAGAAAAGCTCAGGCGCTTAATTA

Homo sapiens  AAACCCTTAGGGGCCCTGCCCGCGAGGTCATGCGTGTGCTTCAGGCGACCAACCCTAACCTAAGTGTGGCAGATTTCTTGCGAGCCATGAAATTGGTGTTTGGGGAGTCTGAAAGCAGTG
Bos taurus    AAACCCTGAGGGGCCCTGCGCGGGAGGTGATGCTTTTGCTGCAGGCGGCCAACCCCAACCTCAGCGTGGCAGATTTCTTGCACGCCATGAAACTGGTGTTTGGGGAGTCTGAAAGCAGTG

Homo sapiens  TGACTGCCCATGGTAAATTTTTTAACACCCTACAAGCTCAAGGGGAGAAAGCCTCCCTTTATGTGATCCGTTTAGAGGTGCAGCTCCAGAACGCTATTCAGGCAGGCATTATAGCTGAGA
Bos taurus    TGACTGCTCATAGTAAATTTTTTAACACCCTGCAGGCGCAAGGGGAGAAAGCCTCCCTTTATGTGATCCGTTTAGAGGTGCAGCTCCAGAATGCTATTCAGGCAGGGATCATAGCTCAGA

Homo sapiens  AAGATGCAAACCGGACTCGCTTGCAGCAGCTCCTTTTAGGCGGTGAGCTGAGTAGGGACCTCCGACTCAGACTTAAGGATTTTCTCAGGATGTATGCAAATGAGCAGGAGCGGCTTCCCA
Bos taurus    AAGATGCCAACCAGTCTCGCCTGCACCAGTTCCTTTTAGGGGCTGAGCTGAATGGGGACCTGCGCTTCAGGCTGAAGAATCTTCTCAGGATGTATGCAAATGAGCAGGAGCGTCTCCCCA

Homo sapiens  ACTTTCTGGAGTTAATCAGAATGGTAAGGGAGGAAGAGGATTGGGATGATGCTTTTATTAAACGGAAGCGTCCAAAAAGGTCTGAGTCAATGGTGGAGAGGGCAGTCAGCCCTGTGGCAT
Bos taurus    GTTTCCTGGAGTTAATCAGGATGATAAGGGAGGAAGAGGATTGGGATGACATTTTCATTAAACAGAAGCGGGCCAAGAGATCTGAGTCAGTGGTGGCAAGGGCAACTAGGCCAGTGGCAT

Homo sapiens  TTCAGGGCTCCCCACCGATAGTGATCGGCAGTGCTGACTGCAATGTGATAGAGATAGATGATACCCTCGACGACTCCGATGAGGATGTGATCCTGGTGG---AGTCTCAGGACCCTCCAC
Bos taurus    TTGGGGGCTCCCCACCCATAGTGATTGATAATAAAGACTGTAACGTGATTGAGATAGATGATACCCCTGATGACTCAGATGAGGATGTGATCCTGGTGGGGGAGTCTCAGGACCTTCCAC

Homo sapiens  TTCCATCCTGGGGTGCCCCTCCCCTCAGAGACAGGGCCAGACCTCAGGATGAAGTGCTGGTCATTGATTCCCCCCACAATTCCAGGGCTCAGTTTCCTTCCACCAGTGGTGGTTCTGGCT
Bos taurus    GTTCATTCTCGGATTCTCCTCCCTCCAGACGCAGGGCCAGACCTCAGGATCAAGTGCTAATCATTGATTCCCCCAACAATTCCCAGTTTCCATCTCCTTGCACCAGTGGGGGTTCTGGGT

Homo sapiens  ATAAGAATAACGGTCCTGGGGAGATGCGTAGAGCCAGGAAGCGAAAACACACAATCCGCTGTTCGTATTGTGGTGAGGAAGGCCACTCAAAAGAAACCTGTGACAACGAGAGTGACAAGG
Bos taurus    ATAAGAATGATGGTCCTGGGAATATGCGTAGAACCAGGAAGCGAAAACACACAATTCGCTGTTCATATTGTGGTGAAGAGGGCCACTCAAAGGAAACCTGTGACAATGAGAGCAACAAGG

Homo sapiens  CCCAGGTTTTTGAGAATTTGATCATCACTCTCCAGGAGCTGACCCATACTGAGATGGAGAGGTCAAGAGTGGCCCCTGGCGAATACAATGACTTCTCTGAGCCACTGTAAGGACCA-CC
Bos taurus    CCCAGATGTTTGAAAATCTGATCATCACCCTGCAGGAGCTGGCACATACCGA---GGAGAGGTCAAGAGAGGCCCCTGTCGAACCCAGCGACCCTTGTGAGCTACAGTGAGGTGCTAGCC

Homo sapiens  CCCAGGTTTCAGTGAACCCTTACCTATATTCAGCATCCAGTAGTGGGAAAACTGGGGTGGGGGTGGGGGTGGGACTTCTAACTGCATGAATTAATCCACAAAGCGGCTATCTTTTGGGGT
Bos taurus    CCCAGCTTTAAGTGAACCCTAACCTATATGCAGAGTCCAGCAATGGGAAAAATGG---GGGGGAGAGGATG---CTTCTAATCACATAAATTAATCCACAAAGCAACTTTCTTTTGGGGT

Homo sapiens  GGAGTAGAAAGGGTCTTGGATACCAGCACATTGGAGGGAGATAGCCTGACCTCTGTCCTTGCTCCTTCT-CCCTGCAGCCTACGGGTCTGTTTTCTGTGTGTGCCCATTTCCTTGACAGC
Bos taurus    GGAGGATGGAGGGTCTTGAATATTGGTATAGTGGGGGGAGATGGCCTGACATCCATCCTTGCTACTAATCCCCTGCTGCCTAAGGGTCTACCCTCTGCGTGCCCCCCTTTCCTTGATGCT

Homo sapiens  TTTAT--TCTTTGTGAAAGTGGTATAATTTATTGTTAAATATTTGAACAATAAAAA-AGGTACAAAAAGTGAAGTACAAATTACCCAAATCTCTCC-ACCCTTATATAATCATTGTCAAC
Bos taurus    TTTATTCTCTTTGTGAAAGTTATATAATTCATTGTTAAATATTAAAACAATAAAGACAAATGCAAA-----ATATACAAATTACCCCAAACCCTCCAACCCTTAAATAACCATTGTCAAC

Homo sapiens  CCTTTGATGAGT---------GATATTTCCCTATACCTATGTACCCAGATAGATATATGCATAGATAAAAGTGATGAAATATAAGTGCTGTTCTA---TCTGTATTTTTTCACCAAACAA
Bos taurus    CCTTTGATGAATGTCCTTCTAGGTAGTTCCCTTTACCTATGTACCCAGATAGATATATGTATAGATAAGAGTGAGCAAATATAAGTGTTGTTCTAAAGTCTGTATTTTTTCAGCAAATAA

Homo sapiens  TATATGTTGTGAGCTTCTATGTCAATAAAT--ATATATATCAGCATCTGCCTTAATGTGTCTGTGCTGTT--------------AGGAAGAATTTTTAAAAAAGAAAATATAGTAAAAAA
Bos taurus    TATATGTTCTGAGCTTCTTTCTAGATCAACAAATAAACAGCAGCAGCTAGCTTACTGTCTGTGTATTATTTTAAGGGGAAGTAAAGGAAAAA---GGAATAAGAAAAATACAGTAAAAAG

Homo sapiens  CTGTAAAGG--GGGGTTGTACTCTGAGGTGGGATTT---TTAACCGCATTTTGCAAATGAAAAAA-------AATAAATGGGCTGAGATACTTCCCAAAGGTAAAAGGACAGAGCCCCCT
Bos taurus    CTATAAAGGGTGGGGTTATACACTGAGGTGGGGTTTTGATTAACTTCACTTTGCAGATGAGGAAAAGGGAAGAAGAAATGGGCTGAAATCTTCCCCAGAGGTAAATGGACAGAG-CCCCT

Homo sapiens  TACCACAACTAACCTGTCCCTTCGCTCAGAGAGCCAAATTGTGACACCCACTTGTTGACAAAACACCAGTGCTTTCTTCTCTGATATACCCAGGAGA--CTGATGGGGGATGGGGGAGTC
Bos taurus    TACCACCACTGACCTGTCCCTTAGGTCACAGAGCCAAACTGTGGCACCCACTAGTTGACAA---CTTAAGGCTTTCTTCTCTAAGGTATCCAGAAGATTCTGACAG----CGGGGGAGGC

Homo sapiens  CGGTGCTGAGGTAATAGCCCAATATGGGGATTCCAGGGGCTCCATTTCTGTTCACTTTGGGGATGGGCAGTTCTTC---ATCTGGGCAGATTCCTTAGATTGTTGTTGCTGTTGCTGCCT
Bos taurus    TGGTGCTGAGGTAATAGCCCAAACGGAGGATACCAGGGG-TCCATTCCTGATTACTTTGGGGAGGGGCAGCTCTCCCTGCTCTGGGTGGGTTTTTGACATCGCTGTTGCCATGGCTTC--

Homo sapiens  CTGGACAACGTCCTGGTCACAACAAGGAATCTCTCCTGTGGGCTGA------CACCACCTTCTGCCCCCTCCCCAGTTAAGGAAAAGAGAGAGCAGCCACTCATGGGAGCTCAGCATTAG
Bos taurus    ----ACCCCTTCCAGGTTACAATGGAAATCTCTCCTGTGGGCTGATGCGGCCCCTACCCCACCCCACTCCCCCAGTTAAGG-AAGGAGAGAGAAGCTACTGGTGGGAGC-CAGCGTCAG

Homo sapiens  CAGGCTCAACTCCTGACGGCGCCAGAGCCTCCCCTATAAACTCTGAAGCTATGCGCTGGAGGCCACCGGCAGCCCACGGACT
Bos taurus    GGGGCTCAG---------------------CCCCCAGAAGCCCTGAAGTTACATGTTAGAGGTCACTGGCAGCCCAATGACT
```

```
Homo sapiens  GTGAGCCGCCCGCCTCGGCCTCCCAAAGTGCTGGGATTTCAGGCGTGAGCCACCATTCCCGGCCAGAAGACTGCATTTCTAACAAGCTCCCCCAAATGACTCTGATGTCTTGCCCCCATA
Mus musculus  ..........................................................................................................................

Homo sapiens  CTTGAATGGGTATTGGTTGAGAGCCTTGTTCTCCAGCTCTCCTCTCTGTTAAATGGACATACTGCTACTTCTTTCACAGTAAAAGAGGAGATGTGTGTGAAAGCCTGTTTCCGCAATGCA
Mus musculus  ......................................CTCCTCTCTGTTAAATGGGTGCACTGCTACGTTTT.................................................

Homo sapiens  CGTGCTAATGCTCTTCCCGCCCCTTCCCCTCTTCCCCGTCTTTTCCCTATACCAAGGTGAAGAACTTTTATTATTC-ACTTGATTTTGGACCCACTTCGTGTTTAATGCAC--CTGGCCT
Mus musculus  ................................................................CTTTTACTGTTTTACCTGATTTGGGTCCCACTTTGCCATACATACACATCTGGCCT

Homo sapiens  -AAGATGCAGAGTTGAGCCCAGGGAGAA-------------GGGACGGGTTGGGGGAGGAAGAAAGAGTCGCAAAT-TCCCAGGCAGATTGGCTCCATCGGTCTAATCAAGCTGCACTTG
Mus musculus  CGAGACACAATTTTGGGCCAAGAGAGAACACAAAAGCAGAAGGGAAGAGTGGAGGGAAAGAGTCACAATCGCAGGGGTGCAATGTAGTTTAGCTCCCTAAA-CTCGCCCAGCACCACTGG

Homo sapiens  ACGCTCCGCGGGCCTGCTGC-------------GCCCCCGCCCCCTCAGGCGAAAACTCGCCTCTGCCCAAGGCTGTG-----CGGGGCGT------CCGCCCCCGCACTTCGCTGATTG
Mus musculus  ---CTACCTGAGCCTGTTGCATACCCTCCTCCCGCTTCCGCCCCCC..........CTTTCCCCCACCCGAACCCTTGGGCTTTGGGCCGCTAGAAGCCGCCCCCGCACTCCACTGATTG

Homo sapiens  GCCGCACTAGCCCGCTCGTCACGCTCTTTTGTCTCAGCTGGCAGAGGATAAAAGCCGCCGCGGCTGCCTTAGGAACGGCGCTGCCTCGTCTCTGCTACCCCTGGTTGGGCGGCCCTGCGA
Mus musculus  GCTCCGCTGGCCAGCTCGTCACACTCTTTTGTGTCAGTAGGCTGCTGATAAAAGCT-TTGCAGCTGCCTTGGAAACTGCGCTATTCGAATCCGGTTACCTGTGGGTAAGTAGCTGGGTGA

Homo sapiens  AGCAGCTCCTTCGGGCAGCCCCGGGTCGCTTAGCGGCCAAGGAGGCTTCAGTTCTTTGCCGCCTGCAAGGCGGAGACCAGAAGGCGGAATCCACAGCTGGCGACGCGGGAGCATCTGCTG
Mus musculus  AGCAGCTCTTTCTGGTAGCTTCGAGTCGCCTAGCGGCTCCGGAGGTGCCAGCACTTTTCTCCCCG--------GGACTCACAGGCAGAATCCATAGCTGAAGACACCACAGCAGCCTCTG

Homo sapiens  TCCACCAGCGGAGCACAGGT----------------AAAGAATGGGG-GTGTGATTGCCCGGCGGGGAGA---------------GGGGTGGGGG----AAGACCCACCAGTACTGGGCG
Mus musculus  GCTACCAGTGGATCACAGGTTTTAAAACGGGGGGGGGGGGGATGGGATGGGTGATTCCTTGGTGTGGAAAAGGAATGGGGCGCGCGGGGTGGGGGGCAGAGAAGCCCTCGGGCATAGGCC

Homo sapiens  GCTCCCAGCTTGGAGAATCGGTGACTGTGAGGCAGGGGTAGGAGTACTCTCGTGGCCCGGCTGCGCTGAGGGAGGAGGTGGGGTTCACCTGGATACTGCGGGCTAGGTAGGATTTGCCTG
Mus musculus  GCTCCTGCCTTGGAGAATCTGCAATTGCGACGCTGGGGGAGGTAAGGTGCGTTAGCATGACCTCACTGGGAG------------TCACCTGGAAGATGCCA-CTTGGTAGGATTTGCCTG

Homo sapiens  GATTTGG---GGGTGGGAGGAGAGGCGA---GGAGTA-GCGGGGGGAGGAATCTCGAGCTGTTTGCTTCGAACATCCTTTGCATCTCCTCATCTCTTTCTGGGAGGAATATGTGGGTGGG
Mus musculus  GCCAGGGAGGGGGTAGGGGAAGAGGCCAACTGGAGGATGACGGAGAAGCAATTT-----TGTTTGCCTTAAACCCCCTATGTATCTCCTCTAATTTCTCTGGG--TACTGTGGGGGTGGG

Homo sapiens  TGTGTCCAACTCCTATTCCCTTTCTCCGTGGCAGGCCATCAAAGCCGCATCTGAACTTGAATTCTGTGCAGCTGATTGCAGAGCTGG-TAGGCCGACGGCTTCCCGGG--GGAAATGTA-
Mus musculus  TGTGTCCAACTCTTATTCTGTTTTACAGTAGCAGCCCATCAAAGCGGAATCTGAACTTGAATTTGGTGCAGCTGGTTGTAGAGCTGGGTAGGATAATGGTATCTAGGGATGCAAATATGT

Homo sapiens  ---ATGCTGGAGGGTGGGGGGCTGGTCTCTGGCTTATAGTCAGGAGGGGGCAGAGAGAATTTTTCGCCTGGCAACAGC---------------------GGAGGTGGTGGG--------T
Mus musculus  GGGGTTTTGGGGGGTAGGGGATCG--TTTTGTATTGTATTCAGAAGGTGGGAGAGGGAATTTTCTACCTAATGACAGCAGAAATGGGCGGGAGAAAGAGGGAGGGGGAGGGAGGAACAGG

Homo sapiens  AGCAGGATGACGGGTGAAGCTGCTTCTAACCCTTCCCTTCTCTGGCCTTCTCCGCTGTGGCGCAATCTTGAAACCTCAAGGACCCGGATCTGCGACCCCCTGTGGACAGAGGTT----GA
Mus musculus  AGAAGGATGACGACGGAGTATTTTTCTGACCTTTCTTTT-----GCCTCCTCCAATGTGGCGCAGTCTTGA--------GCATCAGGATCTGAGACCCCCAGTCGATCGAGGTCCATCAA

Homo sapiens  CCGTACCCCGGAGAGGAGCTTTCTCACGG-----AGGGCACTGGTTGCAGAGGCTGGAAGTGAAATAAAGACGCGCTCTTGTTTCAGAGTTCGTGTAAGAATCTGAGAAATAACAAGGGG
Mus musculus  CTGGACCCTGGAGGCAGGCTTTCTCACGGGAGGGAGGACACAGGTTGCAGAAGCTGGAAATA-------------CTCCAGTTTCCCAGCTGATGTAAGAATCTGATAAATCACAGAGGG

Homo sapiens  G----------ACGGGCGTGGGG----------------------GGCACTTTGAGAAGGGGGTGTGGTTGAGACCAGAGACCATGAGGTTAATGAGATCTTATTTTATTTCC-ACAAAT
Mus musculus  GGGGGGGTGGTATGGGGGTGGGGTGGGGGGAAGTGGGTGGTCCAGGGAGTTTTGAGAGGGGCTTGCAGTTAAGACCAGAGATCATGAAGTTAATGACGTCTCTTTTTAACCCCTGCGAAT

Homo sapiens  CTGCGATCATTGTACTCTATTTGGAGAGCCCAATATCAGCTCCAGACACTGCTACACCTAGAATTTGAAGCAACAGTTACCGTCCCTCTTGGAGGACTGGTGGGAGGG-ATGGATG---T
Mus musculus  TTGTGACCATTGTGC---ATTT------CCACAAGTAAAATCCAGAA-----------------TGGAAGCAACAGTTGCCCTCCCTCTCACAGGACTGGCGGGAGGGTATGGATGACGT

Homo sapiens  GGCTGCAAAAAGCACCTTGCTAGCACGCAGGCATCGGGTAGCTCTGGGAGGTTGTAAGTGTCAATCTCCTA--CAGGATTATTTTTAGGGTAGTGGGCAAATATAAAATGTACTGCAGGA
Mus musculus  GGCTATAAAAAACAC--TGTTAGTATTGAGACATCAGCTAGCTCCAGGAGGCTGCAACTGC---TCTCCTAGCCCGTATT----TTAGGACAGTGGACAAATATA----------TGGGG

Homo sapiens  TATTCAGGTAGGAAGAGAATGCAGCTGCAGCCCGCTCCCTTATTAA-----CCAGCCCCCCTTTCTTTTTTACAGCCCC-TGCTGAGATAGGAAGGCAGAGCCACCTCCTCTCCTCTCCC
Mus musculus  TATTGAGGCA--CAGTAATTACAGCTGGAGCCTGCTCCCTTATTAAATTCTCCTCTCCCTTTTGCTTTTTTGCAGCCCCCTACTGAGATTGGAGA------------CTGCTGCACTGC-

Homo sapiens  ACCTGCAGATTAAGCTTTT-----------CTAAAAAGCCTAGGCATCTTCTTATATTCAGATACCCTATC---GTCGTCAGTCATGGCTAGCATCATTGCACGTGTCGGTAACAGCCGG
Mus musculus  ---TGAAGATAAAGCGTTTTTGTTTTGTTTCTAAAACGCCTTG--AACTTCGTGTATTAAGATATCCTATCACTGCTGTCAGACATGGCTAGCATCCTTTCACGTTTGGGTAGCAGCCGG

Homo sapiens  CGGCTGAATGCACCCTTGCCGCCTTGGGCCCATTCCATGCTGAGGTCCCTGGGGAGAAGTCTCGGTCCTATAATGGCCAGCATGGCAGACAGAAACATGAAGTTGTTCTCGGGGAGGGTG
Mus musculus  GGGCAGAACTCACCCCTGCCACCTTGGGCTCATTCCATGCTGAGATCCCTGGGGAGGAGTCTTGGCCCTTTAATGGCCAGCATGGCAGAGAGAAACATGAGGTTGTTCTCTGGGAGGGCA

Homo sapiens  GTGCCAGCCCAAGGGGAAGAAACCTTTGAAAACTGGCTGACCCAAGTCAATGGCGTCCTGCCAGATTGGAATATGTCTGAGGAGGAAAAGCTCAAGCGCTTGATGAAAACCCTTAGGGGC
Mus musculus  GAGCCAGCCCAGGGGGAAGAAACCTTTGAAAACTGGCTGAGCCAAGTAACTGGGGTGCTGCCTGATTGGCATATGCCTGAGGAGGAAAAGGTCAGACGTCTAATGAGAACCCTTAGAGGC

Homo sapiens  CCTGCCCGCGAGGTCATGCGTGTGCTTCAGGCGACCAACCCTAACCTAAGTGTGGCAGATTTCTTGCGAGCCATGAAATTGGTGTTTGGGGAGTCTGAAAGCAGTGTGACTGCCCATGGT
Mus musculus  CCTGCCCGCGAGGTCATGCGTTTGCTCCAAGCTGCCAATCCTGGCCTAGATGTAGAGGATTTTTTGCGGGCGATGAAACTGGTCTTTGGGGAGTCTGAGAGCAGTGTGACAGCCCATAGT

Homo sapiens  AAATTTTTTAACACCCTACAAGCTCAAGGGGAGAAAGCCTCCCTTTATGTGATCCGTTTAGAGGTGCAGCTCCAGAACGCTATTCAGGCAGGCATTATAGCTGAGAAAGATGCAAACCGG
Mus musculus  AAATTTGTTAACACTGTTCAGGAACATGGAGAGAAACCATCCCTGTATGTGATCCGTTTAGAGGTGCAGCTGCAGAATGCTATCCAGGCAGGGGTGTTTGCTGAGAGAGAGGCAAACCAG

Homo sapiens  ACTCGCTTGCAGCAGCTCCTTTTAGGCGGTGAGCTGAGTAGGGACCTCCGACTCAGACTTAAGGATTTCTCAGGATGTATGCAAATGAGCAGGAGCGGCTTCCCAACTTTCTGGAGTTA
Mus musculus  GCTCGCCTGCATCAGCTCCTGGTAGGAGCTGAGATGAGTACAGACCTTCGATTCAGGCTTAAGAATCTTCTCCGGGTATATGCAAATGAGCCAGAGCGCCTTCCCAATTTCCTAGAGTTA

Homo sapiens  ATCAGAATGGTAAGGGAGGAAGAGGATTGGGATGATGCTTTTATTAAACGGAAGCGTCCAAAAAGGTCTGAGTCAATGGTGGAGAGGGCAGTCAGCCCTGTGGCATTTCAGGGCTCCCCA
Mus musculus  ATCAGGATGATAAGGGAGGAAGAGGAATGGGAGGAGACTTTTATTAATCCAAAGCGGCCCAGAAGAGCCGAGTCAGTGATGGAGAGGGCACTCAGCCCTATGGCATTTCAGAGCTCCCCA

Homo sapiens  CCGATAGTGATCGGCAGTGCTGACTGCAATGTGATAGAGATAGATGATACCCTCGACGACTCCGATGAGGATGTGATCCTGGTGGAGTCTCAGGACCCTCCACTTCCATCCTGGGGTGCC
Mus musculus  CCAATAATGATCAGCAGTATCGACTGCAATGTGATCGAGATAGATGACTCTCCTGATGATTCGGATGAGGATGTGATCTTGGTGGAGCCTGAGGACCCACCACTGCCATCCTCAAGTGCC

Homo sapiens  CCTCCCCTCAGAGACAGGGCCAGACCTCAGGATGAAGTGCTGGTCATTGATTCCCCCCACAATTCCAGGGCTCAGTTTCCTTCCACCAGTGGTGGTTCTGGCTATAAGAATAAC---GGT
Mus musculus  TCTTCCTTCCTAGGCAGGGCTGTATCTGAGGACCAAGTGCTGGTCATTGAGTCCCCAAACATTTTTGAGATCCAGGCTCCTTCCACTAGCAGTGGTGCTGGGAGAAAGAACAATCGGGGT

Homo sapiens  CCTGGGGAGATGCGTAGAGCCAGGAAGCGAAAACACACAATCCGCTGTTCGTATTGTGGTGAGGAAGGCCACTCAAAAGAAACCTGTGACAACGAGAGTGACAAGGCCCAGGTTTTTGAG
Mus musculus  TTTGGGGAGCTGCGTAGAGCCAGGAAGCGCAAACATACGATCCATTGTTCCCACTGTGGTGAGGAGGGCCACTCAAAAGAAACCTGTGACAATGAGAGTGACAAGGGCCAGGTTTTTGAG

Homo sapiens  AATTTGATCATCACTCTCCAGGAGCTGACCCATACTGAGATGGAGAGGTCAAGAGTGGCCCCTGGCGAATACAATGACTTCTCTGAGCCACTGTAAGGGACCAC-CCCCAGGTTTCAGTG
Mus musculus  AATCTGATCATCACCCTGCAGGAGCTGACACATGCAGA---GGAGAGGGCAAGGGGGGCCCCTGGTGAACCCATTGGCCTATCTGAACCACAGTAAGATGCGACTCCCCAGCCTTAAATT

Homo sapiens  AACCCTTACCTATATTCAGCATCCAGTAGTGGGAAAACTGGGGTGGGGGTGGGGGTGGGACTTCTAACTGCATGAATTAATCCACAAAGCGGCTATCTTTTGGGGTGGAGTAGAAAGGGT
Mus musculus  AACCCTTACCCGTATTAAATGTTTAACGGTGGAAAAGTTG----------TGGGGACAGATTTCTAATTGCATGAACAAATCCACAAAGTGGCTTTCTTTGGGGGTGG---AGAAAGGGT

Homo sapiens  CTTGGATACCAGCACATTGGAGGGAGATAGCCTGACCTCTGTCCTTGCTCCTTCTCCCTGCAGCCTACGGGTCTGTTTTCTGTGTGTGCCCATTTCCTTGACAGCTTTAT--TCTTTGTG
Mus musculus  CCCGAATACCAGTACAGTAAAGAGAGGTGGCCTGACCTATGATCGTGTTCCCATTCCCCACTGCTTAACAGTCTATTTTCTGTGTGAACCTCTCTCCTTGGTGGCTTTGTTCTCTTTGTG

Homo sapiens  AAAGTGGTATAATTTATTGTTAAATATTTGAACAATAAAAAAGGTACAAAAAGTGAAGTACAAATTACCCAAATCTCTCCACCCTTATATAATCATTGTCAACCCTTTGATGAGT-----
Mus musculus  AAAGTGGTATTGTTCATTGTTCAATCTTCA---AGTAAGAAAATTATTAAA----AAGTACAAATTACCCAGACCTCTCTA-CCTTGTGAAACAATTGTCAGCCCTTTGGTGCCTATCCT

Homo sapiens  ----GATATTTCCCTATACCTATGTACCCAGA-TAGATATATGCATAGATAAAAGTGATGAAATATAAGTGCTGTTCTAT---CTGTATTTTTTCACCAAACAATATATGTTGTGAGCTT
Mus musculus  TCTAAATATTTCTCTATATCTGTGTTCCTAGATTAGAAATATGTATAGACGAAAGTGATCAAATAGAAGTGTTGTTCTATATGCTGTATTTTTTCACCAA--AACGTATGTTGTGGCCTT

Homo sapiens  CTATGTCAATAAAT------ATATATATCAGCATCTGCCTTAATGTGTCTGTGCTGTTAGGAAGAATTTTT------------AAAAAAGAAAATATAGTAAAAAACTGTAAAGGGG-GG
Mus musculus  CTTTGTCAATAAATATATACATATATGTCAGCATCTACTTTAATGTCTCTGTGGTATTA--------TTTTAGTAAGCAAAGGAAGAAGAAAAATACAATGAGAAGGTATAAAGGGGTGA

Homo sapiens  TTGTACTCTGAGGTGGGAT---TTTTAACCGCAT------TTTGCAAATGAAAAAAAATAAATGGGCTGAG-ATACTTCCCAAAGGTAAAAGGACAGAGCCCCCTTACCACAACTAACCT
Mus musculus  AAGTACCCTTGGAAGTGTTTTGTTTTAAACAAACTTTTGATTTGCAGATAAGAAAAAAGATATAGGCTGAATAAATCTCCCAAAG--CAAAGGATA-----CCTTTGCCACAGTTGAATG

Homo sapiens  GTCCCTTCGCTCAGAGAGCCAAATTGTGACACCCACTTGTTGACAAAACACCAGTGCTTTCTTCTCTGATATACC----CAGGAGACTGA-------------TGGGGGATGGGGG---A
Mus musculus  G---CTGCCCCCACAGAGTCAAATTATGGCCTATACTTATTGAAAAATCTTCATTGCTTTCTT---TAATATTCCAGAGCAGTGTGCTGGGCGGCAGCAGCAGCGGGGGATGGGAGGGCA

Homo sapiens  GTCCGGTGCTGAG-----GTAATAGCCCAATATGGGGATTCCAGGGGCTCCATTTCTGTTCACTTTGGGGATGGGCAGTTCTTCATCTGGGCAGATTCCTTAGATTGTTGTTGCTGTTGC
Mus musculus  GTCCAGTGCTGAGCTGAGGTAGTAGCCTAACATGGGAATTCCAGTGATTCAATTTGTGCTCATGTTGGCAA-GGGCATTCCATCCTTTGGGTAGCATTTTAAAATCTCTGCTATTGTTGC

Homo sapiens  TGCCTCTGGACAACGTCCTGGTCACAACAAGGAATCTCTCCTGTGGGCTGACACCACCTTCTGCCCCCTCCCCAGTTA-AGGAAAAGAGAGAGCAGCCACTCATGGGAGCTCAGCATTAG
Mus musculus  CG---------GGCATCCTAG-------------TCTCTTCTGTGGTCTGATACTACCCTCTGCTGCGTTCTGGATTACAGAAAAAAAGATAGCAGTTGCT--------CTTAGCATCAG

Homo sapiens  CAGGCTCAACTCCTGACGGCGCCAGAGCCTCCCCTATAAACTCTGAAGCTATGCGCTGGAGGCCACCGGCAGCCCACGGACT
Mus musculus  CAGGCTCTGCTCCTGACGGTG-----GCCT--------AATACTTAGTCTGTAAAATATAGGATATAGG-AGCCCAAGGACC


Homo sapiens          GTGAGCCGCCCGCCTCGGCCTCCCAAAGTGCTGGGATTTCAGGCGTGAGCCACCATTCCCGGCCAGAAGACTGCATTTCTAACAAGCTCCCCCAAATGACTCTGATGT-------CTTGC
Oryctolagus cuniculus  .............................................................AGAAATCTGCATTTCTAACAAGCTCCCCAAAATAATTCTGATGTAGCCAGCTTTGC
```

```
Homo sapiens           CCCCATACTTGAATGGGTATTGGTTGAGAGCCTTGTT-CTCCAGCTCTCCTCTCTGTTAAATGGACATACTGCTACTTCTTTCACAGTAAAAGAGGAGATGTGTGTGAAAGCCTGTTTCC
Oryctolagus cuniculus  ACCTAT---TGAGTGAAGACTGGTTAAGAGCCTTGGTGTTCCAAGTGCCTTCTCTATTAAATGAGCATACCGCCAC--CTTCTACACTCCTAAAAGAGATGTCTATGCAAACCTGTTTGC

Homo sapiens           GCAATGCACGTGCTAATGCTCTTCCCGCCCCTTCCCCTCTTCCCCGTCTTTTCCCTAT--ACCAAGGTGAAGAACTTTTATTATTCACTTGATTTTGGACCCACTTCGTGTTTAATGCAC
Oryctolagus cuniculus  ACAAGGAAAGAGCTGATGCC------------TCCCTCCTCATCCGGCTTCCCTCTATGTACCAAGGTGAAGAACTTTTACTGTTCAGTTTATTTCGGTCCCACCTCGTGTTTAGTGTAC

Homo sapiens           CTGGCCT-AAGATGCAGAGTTGAGCCCAGGGAGAA----------------GGGACGGGTTGGGGGAGGAAGAAAGAGTCGCAAATTCCC-----------AGGCAGATTGGCTCCATCG
Oryctolagus cuniculus  CTGGCCTCGAGACACAGAGTTGTGCCAAGGGAGAAAATGGAGAGGGAGAAGGGAAAGGG--GGGAGAAGGAGAAAGAGTCGCAAATGCTAGCTTGTGTAAAACGCAAATCGGCTC-----

Homo sapiens           GTCTAATCAAGCTGCACTTGACGCTCCGCGGGCCTGCTGCGCC------------------------CCCGCCCCCTCAGGCGAAAACTCGCCTCTGCCCAAGGCTGTGCGGGGCGTCCG
Oryctolagus cuniculus  ---------AGCTGCGCTTGACGCCTGGAGGGCTTGTTGCGCCGGTGTTCCCCCCTCACCTCCCCCCCCCCCCCCCCCGCCAGAGGATTTGCCTGGGGCCCAGGCTGTGCCGCGAGCCCG

Homo sapiens           CCCCCGCACTTCGCTGATTGGCCGCACTAGCCCGCTCGTCACGCTCTTTTGTCTCAGCTGGCAGAGGATAAAAGCCGCCGCGGCTGCCTTAGGAACGGCGCTGCCTCGTCTCTGCTACCC
Oryctolagus cuniculus  CCTCCACGATCCGCTGATTGGCCGCGTTGGCCCACTTGTCAAGCTCTTTTGTCTCAGCGGGCAGAGGATAAAAGCAGCAGCCGCTGCCTTGGGAACCGCGCTGCCTCAATTCGGCTACCC

Homo sapiens           CTGGTTGGGCGGCCCTGCGAAGCAGCTCCTTCGGGCAGCCCCGGGTCGCTTAGCGGCCAAGGAGGCTTCAGTTCTTTGCCGCCTGCAAGGC---GGAGACCAGAAGGCGGAATCCA--CA
Oryctolagus cuniculus  CTTGCCGGGCTGCCCTGCGAAGCAGCTCGGGCGGGCAACTCCGGGTCGCTTAGTGGCCGAGGAGCCGCTAGTCCTTTTCC-CATGCGCGGCCGCGGAGATCTGCAAGAGGGATCCATCCA

Homo sapiens           GCTGGCGACGCGGGAGCATCTGCTGTCCACCAGCGGAGCACAGGTAAAGAATGGGGG----TGTGATTGCCCGGCGGGGAGAGGGGTGGGGGAAGACCCACCAGTACTGGGCGGCTCCCA
Oryctolagus cuniculus  G-TAAAAAAGCGA-AGCACCCGCTGCCCACCAGCAGAGCGCAGGTAAAGAAAAGCGGGCTCCGGGATTGCCTAGCGCG-AGAGGGGAGGGAGAAGTGCCGCCTGGACTGGGCGGCTCCCA

Homo sapiens           GCTTGGAGAATCGGTGACTGTGAGGCAGGGGTAGGAGTACTCTCGTGGCCCGGCTGCGCTGAGGGAGGAGGTGGGGTTCACCTGGATACTGCGGGCTAGGTAGGATTTGCCTGGATTTGG
Oryctolagus cuniculus  TCGTGGAGAATCGGTAACTGCGAAGCTGGGG--AGAGAACTCCCATGGGCCGGCTGCACCGAGGGAGGAGGTGGGGTTCACCTGGGTACTGCCCACTGGGTAGGATTTACCTCGATTGCG

Homo sapiens           GGGTGGGAGGAGAGGCGAGGAGTAGCGGGGGGAGGAATCTCGAGCTGTTTGCTTCGAACATCCTTTGCATCTCCTCATCTCTTTCTGGGAGG-AATATGTGGGTGGGTGTGTCCAACTCC
Oryctolagus cuniculus  GGTGGGGAAGGGCGGTGGGGCATAA-------ACAAATTGTGTGTC--TTGAATTGAATCCCCTTTGCATCTTTTAATCTCTTCCCGAGAGGGAAGAGATGAGCGGGTGTGTTTAACTCG

Homo sapiens           TATTCCCTTTCTCCGTGGCAGGCCATCAAAGCCGCATCTGAACTTGAATTCTGTGCAGCTGATTGCAGAGCTGGTAGGCCGACGGCTTCCCGGGG----------------GAAATGTAA
Oryctolagus cuniculus  TGTTCTCTTTCTCCGTAGCAACCCAGCCAAGCTGAATCCGAGGTTGAATTCTATGCGGCAGGTTGCAGAGCTGGTAGGCCATCGGTTTCCGGGGGTGGGGTGGGGTGGGGGGAAATGTAA

Homo sapiens           TGCTGGAGGGTGGGGGGCTGGTCTCTGGCTTATAGTCAGGAGGGGGCAGAGAGAATTTTTCGCCTGGCAACAGCGGAGGTGG----------------------TGGGTAGCAGGATGAC
Oryctolagus cuniculus  TG----AGGGTGGGGGTGGGGCGTTTGGCTTGTATTCCCCAGTTGGAAGAGGGAATTTTCCCCTAGCCAACAGAGGCGGGGGGGGGGGGGGAGTGGGTTGTGGGTGGGTGGTAGGATGAC

Homo sapiens           GGGTGAAGCTGCTTCTAACCCTTC---CCTTCTCTGGCCTTCTCCGCTGTGGCGCAATCTTGAAACCTCAAGGACCCGGATCTGCGACCCCCTGTGGACAGAGGTTGACCGTACCCCGGA
Oryctolagus cuniculus  GAGTGAGGCCGCTTCTGACCCTTCTCTCCCTCCCTCTCCTCCTCCGCTGTGGCGCAGTTTTGACGCCCCGTGGCTCTGAATCTGTGACCCCCAGTGGACTGAGGTCCACCATATCCCC-A

Homo sapiens           GAGGAGCTTTCTCA-CGGAGGGCACTGGTTGCAGAGGCTGGAAGTGAAATAAAGACGCGCTCTTGTTTCAGAGTTCGTGTAAGAA-TCTGAGAAATAACAAGGGGGACGGGCGTGGG---
Oryctolagus cuniculus  GGCAAGCTTTCTCAACGGAGGCCACCGGTTGGAGAGGCTGGAACTGAAATCAAGGCACACTCTTATTTGCGATCTCCTGTAAGAAATCCTGCAAACAACTGAGGGGGCGGGGAGGGGTGA

Homo sapiens           ----GGGCACTTTGAGAAGGGGGTGTGGTTGAGACCAGAGACCATGAGGTTAATGAGATCTTATTTTATTTCCACAAATCTGCGATCATTGTACTCTATTTGGAGAGCCCAATATCAGCT
Oryctolagus cuniculus  AGGGAGAGGCTCTGGGAAGGGGGTATGGTTGAGACCAGAGATCACGAAGTTAATATGGTACTATTTTATTCTCATACACCTGGGACCATCGTACCCCATTTTGAGAGCCCACTCTTGGCT

Homo sapiens           CCAGACACTGCTACACCTAGAATTTGAAGCAACAGTTACCGTCCCTCTTGGAGGACTGGTGGGA-GGGATGGATGTGGCTGCAAAAAGCACCTTGCTAGCACGCAGGCATCGGGTAGCTC
Oryctolagus cuniculus  C----------------TGGAATGGGAAGCAACAGTTGCCATCCCTCTTGAAGGACTGGTGGGAAGGGATGGATGTCGTCGCGAAGAGCACCATGCCTGCACGCGGCCG-------GCTC

Homo sapiens           TGGGAGGTTGTAAGTGTCAATCTCCTACAGGATTATTTTTAGGGTAGTGGGCAAATATAAAATGTACTGCAGGATATTCAGGTAGGAAGAGAATGCAGCTGCAGCCCGCTCCCTTATTAA
Oryctolagus cuniculus  CAGGAGACT---AATTTCAGTTTCCTGCAGGGTAACTTC-AGGGCGGTGGGAAAATGAGAAATATACTGCAGGATTTTCAGGTAGGGAGAGAATGCAACCGCAGCCTGCTCCCCTACTAA

Homo sapiens           CCAGCC----CCCCTTTCTTTTTT-ACAGCCCCTGCTGAG---ATAGGAAGGCAGAGC--------CACCTCCTCTCCTCTCCCACCTGCAGATTAAGCTTTTCTAAAAAGCCTAGGCAT
Oryctolagus cuniculus  CCGCCCGCCACCTTTTTCTTTTTTCACAGCTAACGCGGAGGAAAGAGGAAGGCAGAGCTCCCCGCCCCCCTCCTGCCCCCGCACACCTGTAGATTCGGTTTTTCCTGAAAGCCTGAGCAT

Homo sapiens           CTTCTTATATTCAGATACCCTATCGTCGTC-------AGTCATGGCTAGCATCATTGCACGTGTCGGTAACAGCCGGCGGCTGAATGCACCCTTGCCGCCTTGGGCCCATTCCATGCTGA
Oryctolagus cuniculus  CTTGTTATATTCAGATACCCTGTCGTCGTCGTCAGTCAGTCATGGCTAGCATCCTTGCACATGTGGGTAACAGCCGGCGGCAGAATGCACCCTTGCCGCCTTGGGCCCATTCCATGCTGA

Homo sapiens           GGTCCCTGGGGAGAAGTCTCGGTCCTATAATGGCCAGCATGGCAGACAGAAACATGAAGTTGTTCTCGGGGAGGGTGGTGCCAGCCCAAGGGGAAGAAACCTTTGAAAACTGGCTGACCC
Oryctolagus cuniculus  GGTCCCTGGGGCGGAGTCTCGGTCCTTTAATGGCCCACATGGCAGAGAAAAACATGAAGCTGTTCTCGGGAAGGGCGGTGCCAGCCCAGGGGGAGGAAACCTTTGAAAACTGGCTGATCC

Homo sapiens           AAGTCAATGGCGTCCTGCCAGATTGGAATATGTCTGAGGAGGAAAAGCTCAAGCGCTTGATGAAAACCCTTAGGGGCCCTGCCCGCGAGGTCATGCGTGTGCTTCAGGCGACCAACCCTA
Oryctolagus cuniculus  AAGTCAATGGGGTCCTGCCAGACTGGAATATGTCCGAGGAGGAAAAACTCAAGCGCTTGATGAAAACCCTTAGGGGCCCTGCCCGGGAGGTCATGCATCTGCTCCAGGCGGCCAATCCTA

Homo sapiens           ACCTAAGTGTGGCAGATTTCTTGCGAGCCATGAAATTGGTGTTTGGGGAGTCTGAAAGCAGTGTGACTGCCCATGGTAAATTTTTTAACACCCTACAAGCTCAAGGGGAGAAAGCCTCCC
Oryctolagus cuniculus  ATCTAAGCGTGGCGGATTTCTTGCGGGCCATGAAATTGGTGTTTGGCGAATCTGAAAGCAGTGTGACTGCCCATGGTAAATTTTTTAACACCCTGCAGGCGCAGGGGGAGAAAGCCTCCC

Homo sapiens           TTTATGTGATCCGTTTAGAGGTGCAGCTCCAGAACGCTATTCAGGCAGGCATTATAGCTGAGAAAGATGCAAACCGGACTCGCTTGCAGCAGCTCCTTTTAGGCGGTGAGCTGAGTAGGG
Oryctolagus cuniculus  TTTATGTGATCCGTTTAGAGGTGCAGCTGCAGAATGCCATCCAGGCAGGCATCCTAGCTCAGAAAGATGCAAACCAGATTCGCCTGCACCAGCTCCTTGCTGGAGCTGAGCTGAACAGGG

Homo sapiens           ACCTCCGACTCAGACTTAAGGATTTTCTCAGGATGTATGCAAATGAGCAGGAGCGGCTTCCCAACTTTCTGGAGTTAATCAGAATGGTAAGGGAGGAAGAGGATTGGGATGATGCTTTTA
Oryctolagus cuniculus  ACCTGCGATTCAGGCTTAAAGGTCTTCTCAGGATGTATGCCAATGAGCAGGAGCATCTTCCCAATTTCCTGGAGTTAATCAGGATGATAAGGGAGGAGGAGGACTGGGATGACACGTTCA

Homo sapiens           TTAAACGGAAGCGTCCAAAAAGGTCTGAGTCAATGGTGGAGAGGGCAGTCAGCCCTGTGGCATTTCAGGGCTCCCCACCGATAGTGATCGGCAGTGCTGACTGCAATGTGATAGAGATAG
Oryctolagus cuniculus  TTAGACGGAAGCGTCCCAAAAGATCTGAGTCCATGGTGGAGAGGGCAGCCAGCCCTGTGGCATTTCAGGACTCCTCCCCCATCGTGATCAGCAGCACCGGCTGCAACGTGATAGAGATAG

Homo sapiens           ATGATACCCTCGACGACTCCGATGAGGATGTGATCCTGGTGGAGTCTCAGGACCCTCCACTTCCATCCTGGGGTGCCCCTCCCCTCAGAGACAGGGCCAGACCTCAGGATGAAGTGCTGG
Oryctolagus cuniculus  ATGATACGCTGGATGACTCAGATGAGGATGTGATCCTGGTGGAGTCTGAGGA------------------------CCCTCCCCTCAGAGGCAGGGCCAGATCTCAGGATCAGGTGCTGG

Homo sapiens           TCATTGATTCCCCCCACAATTCCAGGGCTCAGTTTCCTTCCACCAGTGGTGGTTCTGGCTATAAGAATAACGGTCCTGGGGAGATGCGTAGAGCCAGGAAGCGAAAACACACAATCCGCT
Oryctolagus cuniculus  TCATTGATTCCCCCCACACTTCGGGGCTTCAGTTGCCTTCCACTAGTGTTGGTTCTGGCCATAAGAATACTGGTCCTGGAGATGTGCGTAGAACCAGGAAGCGGAAGTGCACGATCCGCT

Homo sapiens           GTTCGTATTGTGGTGAGGAAGGCCACTCAAAAGAAACCTGTGACAACGAGAGTGACAAGGCCCAGGTTTTTGAGAATTTGATCATCACTCTCCAGGAGCTGACCCATACTGAGATGGAGA
Oryctolagus cuniculus  GTTCCTACTGTGGCGAGGAGGGCCACTCTAAAGAAACCTGTGACAATGAGAGCAACAAGGCCCAGGTTTTCGAGAACCTGATCATCACCCTGCAGGAGCTGACACATGCAGAAGAGGAGG

Homo sapiens           GGTCAAGAGTGGCCCCTGGCGAATACAATGACTTCTCTGAGCCACTGTAAGGGACCA-CCCCCAGGTTTCAGTGAACCCTTACCTATATTCAGCATCCAGTAGTGGGAAAACTGGGGTGG
Oryctolagus cuniculus  GGTCAGGAGTTGCACCTGATGAACACAGTGACCCCTCTGAGTTACAGTAAGCTGCTAGCCTCCTGCCTTAAATGCACCCTTACCTATATCCACAGTCCAATGATGGGAAAACTGG-----

Homo sapiens           GGGTGGGGGTGGGACTTCTAACTGCATGAATTAATCCACAAAGCGGCTATCTTTTGGGGTGGAG--TAGAAAGGGTCTTGGATACCAGCACATTGGAGGGAGATAGCCTGACCTCTGTCC
Oryctolagus cuniculus  ----AGGGAGGGAACTTCTGTTTGCATGAATTAATCCACAGAATGACTTTCTTTCGGGGTGCAGGAGAGAAAGGGTC-TGAATACCAGCACAATGGAAAGGGATGG-CTGACCTCTGTCC

Homo sapiens           TTGCTCCTTCTCCCTGCAGCCTACGGGTCTGTTTT-CTGTGTGTGCCCATTTCCTTGACAGCTTTAT-----TCTTTGTGAAAGTGGTATAATTTATTGTTAAATATTTGAACAATA-AA
Oryctolagus cuniculus  CTGCTCCTTCTCCTCACTGCTTATGGGTCTATTTTCCTGTGTGTGCACATTTCCTTGATGGCTTTATTATTATCTTTGTGGAAGTGGTATAACTCATTGTTAAATCTTCAAATACTAGAG

Homo sapiens           AAAGGTACAAAAAGTGAAGTACAAAT--TACCCAAATCTCTCCACCCTTATATAATCATTGTCAACCCTTTGATGAGT---------GATATTTCCCTATACCTATGTACCCAGATAGAT
Oryctolagus cuniculus  AAAAGTACAAA-----AGGTACAGATTATACCCAAACT--------------------------CCCTTTGCTGACTCTCCTTCCAGATATTTCCCTATACCTATATACCCAGTTAGAT

Homo sapiens           ATATGCATAGATAAAAGTGATGAAATATAAGTGCTGTTCTA---TCTGTATTTTTTCACCAAACAATATATGTTGTGA----GCTTCTATGTCAATAAATATATATATCAGCATCTGCCT
Oryctolagus cuniculus  A----CATAGATAGAAGTGATCAAATATAAGTGCTGTTCTATACTCTGTATTTTTTCACCAAATGATATATGTTGTGACCTTACTTCTATGTCAATAA--ATATATATCAGCATCTATTT

Homo sapiens           TAATGTGTCTG----TGCTGTTAGGAAGAATTTTTAAAAAAG------AAAATATAGTA-AAAAACTGTAAAGGGG-GGTTGTACTCTGAGGTGGGATTTTTAAC------CGCATTTTG
Oryctolagus cuniculus  TAATGTCTCTGGTATTATTGTAGGAAGAAGTAAATGAAAAAGCAATAAAAAATATAATACAAAAACTATAAAGGGGAGATCATTCTCTGAGGTCAGTTTTTTTTTTTATTACACTCTTTG

Homo sapiens           CAAATGAAAAAA-------AATAAATGGGCTGAGATACTTCCCAAAGGTAAAAGGACAGAGCCCCCTTACCACAACTAACCTGTCCCTTCGCTCAGAGAGCCAAATTGTGACACCCACTT
Oryctolagus cuniculus  AAGATGAGGAAAATGGAAGAAAAAGTGGGCCAAAAGATTTCCCAAAGGTAAAGGGACACAGCCCCTTTAACACAACTGATCTGTCCCCCAGCTCATAGAGCTACATTATGGCACTTACTC

Homo sapiens           GTTGACAAAACACCAGTGCTTTCTTCTCTG-ATATACCCAGGAGACTGATGGGGGATGGGGGAGTCCGGTGCTGAGGTAATAGCCCAATATGGGGATTCCAGGGGCTCCATTTCTGTTCA
Oryctolagus cuniculus  ACGGACAATGCCCCAGGGCTTTCTTCTCTGTACACCCACAGGACTCTGATGGTGGGTGGAGGAATCGGGTGCCCAGGTAATAGTCCAACCTCAGGACTTTGGGGGCTCCATTCCTGTTTA

Homo sapiens           CTTTGGGGATGGGCAGTTCTTC----ATCTGGGCAGATTCCTTAGATTGTTGTTGCTGTTGCTGCCTCTGG--ACAACGTCCTGGTCAC-----AACAAGGAATCTCTCCTGTGGGCTGA
Oryctolagus cuniculus  CGTTGGGGAGGGGCAGTTCTTCCTGGGTGTGGGTAGGTTCCGTA---CGTTGCTGCTGCTGCTGCTCCTGGTCACAACAGCCTGGTCACGAATGAACAGGAG---------GTGGGAAGA

Homo sapiens           CACCACCTTCTGCCCCCTCCCCAGTTAAGGAAAAGAGAGAGCAGCCACTCATGGGAGCTCAGCATTAGCAGGCTCAACTCCTGACGGCGCCAGAGCCTCCCCTATAAACTCTGAAGCTAT
Oryctolagus cuniculus  TCTCAGTCTCTGTCTT--------TCAAATAAAAAAGTGCGAACACA-TCATG-----------TTAGCAGA...............................................

Homo sapiens           GCGCTGGAGGCCACCGGCAGCCCACGGACT
Oryctolagus cuniculus  ..............................


Homo sapiens      GTGAGCCGCCCGCCTCGGCCTCCCAAAGTGCTGGGATTTCAGGCGTGAGCCACCATTCCCGGCCAGAAGACTGCATTTCTAACAAGCTCCCCCAAATGACTCT-GATG-------TCTTG
Tupaia belangeri  .....................................................................................CTCCCCGAAATAATTTTTGATGCATCCAGTCTCT

Homo sapiens      CCCCCATACTTGAATGGGTATTGGTTGAGAGCCTTG-TTCTCCAGCTCTCCTCTCTGTTAAATGG--ACATACTGCTACTTCTTTCACAGTAAAAGAGGAGATGTGTGTGAAAGCCTGTT
Tupaia belangeri  CCCCTATA-GTATGTGAGTATTGGTTAAGAGGCTCGAGGCTCCAAGT----TCCCTGTTAATAGGTGACATGCTGCCACCTACTTCACAATAAAAAC-AGTTGTCTATGCAAGC-TACT

Homo sapiens      TCCGCAATGCACGTGCTAATGCTCTTCCCGCCCCTTCCCCTCTTCCCCGTCTTTTCCCTATACCAAGGTGAAGAACTTTTATTATTCACTTGATTTTGGACCCACTTCGTGTTTAATGCA
```

```
Tupaia belangeri  TGCGTAACGACAGCGCTAACGTTCT--------ATTCCCCCCCCCCCCGTCTCCCCCC----------------------------------ATTTCAGTCCCGTCTCGTGTTTAATGCA

Homo sapiens      CCTGGCCTAAGATGCAGAGTTGAGCCCAGGGAGAAGGG--------------ACGGGTTGGGGGAGGAAGAAAGAGTCGCAAATTCCCAGG-------CAGATTGGCTCCATCGGTCTAA
Tupaia belangeri  CCTGACCTCAAA-ACAGAGTCAAGTCAAGGGAGAAGGGGAGATGGAGAAGGGAAGGGTGGAGAGAGGGAGAACGAGTCGCCAGCTCTCCGGTAAAACGCAAATTGGCTCAGTCA--CTAG

Homo sapiens      TCAAGCT-GCACTTGACGCTCCGCGGGCCTGCTGCGCCCCCGCCCCCTCAGGCGAAAACTCGCCTC-TGCCCAAGGCTGTGCGGGGCGTCCGCCCCCGCACTTCGCTGATTGGCCGCACT
Tupaia belangeri  CCCTGCCAGCACTTGTGACCGCGCGGGTCTTCTGCGCACCCACCCCCTTGGGCATGCACCGACCTCAGGGCCCAGACCGCTCGGGGAGCCCGCCCCCACACTCCGCTGATTGGCCCCACT

Homo sapiens      AGCCCGCTCGTCACGCTCTTTTGTCTCAGCTGGCAGAGGATAAAAGCCGCCGCGGCTGCCTTAGGAACGGCGCTGCCTCGTCTCTGCTACCCCTGGTTGGGCGGCCCTGCGAAGCAGCTC
Tupaia belangeri  GGTCCGCTCGTCATGCTCTTTTGTCTCGGCTGGCAGAGGATAAAATCTGC-GTGGCTGTCGTGGGAACCGCGCTGCCTCTACTTGGCCACCCTTAACTGGACCGCCGGGCGAAGCGGCTC

Homo sapiens      CTTCGGGCAGCCCCGGG-TCGCTTAGCGGCCAAGGAGGCTTCAGTTCTTTGCCGCCTGCAAGGC---GGAGACCAGAAGGCGGAATCCACAGCTGGCGACGCGGGAGCATCTGCTGTCCA
Tupaia belangeri  GGTCGGGCAGCCCCGGGGTCGCTGAGCCGCCAGAGAAGCGCCCATCCTCTCCC--CTGCAGGGCCGCGGAGATCGGAAGGCGGGCTCCGCAACAGTGGACGCCGGAGGCTCCGCTGCTCG

Homo sapiens      CCAGCGGAGCACAG-----GTAAAGAATGGGGGTGTGATTGCCCGGCGGGGAGAGGGGTGGGGGAAGACCCACCAGTACTGGGCGGCTCCCAGCTTGGAGAATCGGTGACTGTGAGGCAG
Tupaia belangeri  CCAGCGCAGCGCAGCGCAGGTAAAGCGAGGGGGCGCGATTGGCCGGCGCGGAGAGGCGAGAGGAAAGCCTCGCGGGTACTGGGCAGCTCCGAGCTTGGAGAACCGGTTACTGCGAGGCAG

Homo sapiens      GGGTAGGAGTACTCTCGT-------GGCCCGGCTGCGCTGAGGGAGGAGGTGGGGTTCACCTGGATACTGCGGGCTAGGTAGGATTTGCCTGGATTTGGGGGTGGAGGAGAGGCGAGGA
Tupaia belangeri  GGAAAGGAGCACTCCCGTGGGCCGCGGGCCGGCTGTGCTGAGGGAGGAGGTGTGGTTCACCTAGATGCTGCCATCTGGGGAGGATTTGCCTGGATTCGCGGATG---AGAGAGGCGAGGG

Homo sapiens      GTAGCGGGGGGAGGAATCTCGAGCTGTTTGCTTCGAACATCCTTTGCATCTCCTCATCTCTTTCT-GGGAGGAATAT----GTGGGTGGGTGTGTCCAACTCCTATTCCCTTTCTCCGTG
Tupaia belangeri  GCAGGGGGTGCAGGAATCGGAAGCTTTTTGCCATGAACG-CCTTTGCATCTTCTCCCTTCTTCCTGGGGAGGAATATGTGGGTGGGTGGGTGTGTCCAGCTCCTGGTCCGTTTCTCCGTG

Homo sapiens      GCAGGCCATCAAAGCCGCATCTGAACTTGAATTCTGTGC--------AGCTGATTGCAGAGCTGGTAGGCCGACGGCTTCCCGGGGGAAATGTAATGCTGGAGGGTGGGGGGCTGGTCTC
Tupaia belangeri  GCCGACCGTCAAAGCCGAATCCAAACTTGAGTTTTTTGCGGGGGGGGGGGGGGTGCGGAGCTCGTAGGCCAATGATTTCCCAAGGGAACTTTAA---TGGAGGGGGAGAGAGTCG-CTT

Homo sapiens      TGGCTTATAGT--CAGGAGGGGGCAGAGAGAATTTTTCGCCTGGCAACAGCGGAGGTGGTGGGTAGCAGGA--TGACGGGTGAAGCTGCTTCTAACCCTTCCCTTCTCTGGCCTTCTCCG
Tupaia belangeri  TAACTTGTATTTATAGGAGGGAG-AGAGGGAATTTTGTGCTTGGCCACAATTGGGGCGGTGGACGAGAGGAGTCTAAGAATGA-----CTTCTGACTCTCCCCTATCCTGCC---CTCCG

Homo sapiens      CTGTGGCGCAATCTTGAAACCTCAAGGACCCGGATCTGCGACCCCCTGTGGACAGAGGTTGACCGTACCCCGGAGAGGAGCTTTCTCACGGAGGGCACTGGTTGCAGAGGCTGGAAGTGA
Tupaia belangeri  CTGTGACGCAGTCTTGTTGCCCTGAGGCCTGGAATCTGCGACCCTCTGTGGACACTGGTCGACCAGA-TCTGGAGTCGAGCTTTCTCA-GGAGAATACCGGTGGCAGAGGCTGGAAGTGA

Homo sapiens      AATAAAGACGCGCTCTTGTTTCAGAGTTCGTGTAAGAATCTGAGAAATAACAAGGGGGACGGGCGTGGGGGGCACTTTGAGAAGGGGGTGTGGTTGAGACCAGAGACCATGAGGTTAATG
Tupaia belangeri  AATAAAAGCACGCTCTTGCTTCCGAGCTCATGTAAGAATCTGAATAAT---------------CGGTGGGGAGACTGAGATGAGAGGGTGTGATTGAGACCGGAGACTACACGGTTAATA

Homo sapiens      AGATCTTATTTTATTTCCACAAATCTGCGA----------------------------TCATTGTACTCTATTTGGAGAGCCCAATATCAGCTCCAGACACTGCTACACCTAGAATTTG
Tupaia belangeri  GGATCTTATTTTATTACCAAAGATCCATGAGACATAACCCATTGAGCTCTATTTTTTTTTTTTTTGAGCTCTATTTTGATAGCCCAATCGTGGCTGCAAACACCAGTTCTACTAAAATTTA

Homo sapiens      AAGCAACAGTTACCGTCCCT-CTTGGAGGACTGGTGGG-AGGGATGGATGTGGCTGCAAAAAGCACCTTGCTAGCACGCAGGCATCGGGTAGCTCTGGGAGGTTGTAAGTGTCAATCTCC
Tupaia belangeri  AAGCAATAGTCATAGCCCTTCCTTGGAAGACGGGAAAGTGGGGATGGGTGTA-----AAAGATAAGCT---TAGAACAGGGACACCTGCTAGCCCCTGCGGGTTGCAGTTGCCCATCTTC

Homo sapiens      TACAGGATTATTTTTAGGGTAGTGGGCAA-ATATAAAATGTACTGCAGGATATTCAGGTAGGAAGAGAATGCAGCTGCAGCCCGCTCCCTTATTAA-----CCAGCCCCCCTTTCTTT--
Tupaia belangeri  CACAGGG-TATTTTTAGGGTAGTGGGCAATATAAAATATACACAGCAGGTTATGCAGGAAGGTGGAGACTG--ACTGCAGACTGCTTCCCTCCTAGGTCTTACAGACCTTTTTATTTTTT

Homo sapiens      ------TTTACAGCCCCTGCTGAGAT---AGGAAGGCAGAGCCACCTCCTCTCCTCTCCCACCTGCAGATTAAGCTTTTCTAAAAAGCCTAGGCATCTTCTTATATTCAGATACCC----
Tupaia belangeri  GTGTTCTTTACAGTCCCTACTGAAATACAAGTAAGGCAAAGAC.........................ATTATGCTTTTCTAAAAGCCTAGAACTTCTTGTTATACTCAGATACCCTGAA

Homo sapiens      TATCGTCGTCAGTCATGGCTAGCATCATTGCACGTGTCGGTAACAGCCGGCGGCTGAATGCACCCTTGCCGCCTTGGGCCCATTCCATGCTGAGGTCCCTGGGGAGAAGTCTCGGTCCTA
Tupaia belangeri  TATAGATGTCTTTCATGGCTAGCATCATTGCACGTGTGGGTAACAGCCAGGG---GAATGCACCCTTGCCCCCTTGGGCCCATTCCATGTTGAGGTCTCTGCAGAG-AGTCTT-GTCATT

Homo sapiens      TAATGGCCAGCATGGCAGACAGAAACATGAAGTTGTTCTCGGGGAGGGTGGTGCCAGCCCAAGGGGAAGAAACCTTTGAAAACTGGCTGACCCAAGTCAATGGCGTCCTGCCAGATTGGA
Tupaia belangeri  TAAT-GTCAACAT-GCAGAAAGAAACATG-AGTTGTTCTCAGG--AGGTAGTTTCAGCCCA...........................................................

Homo sapiens      ATATGTCTGAGGAGGAAAAGCTCAAGCGCTTGATGAAAACCCTTAGGGGCCCTGCCCGCGAGGTCATGCGTGTGCTTCAGGCGACCAACCCTAACCTAAGTGTGGCAGATTTCTTGCGAG
Tupaia belangeri  ......................................................................................................TGGCAGATTTTTTGCGGG

Homo sapiens      CCATGAAATTGGTGTTTGGGGAGTCTGAAAGCAGTGTGACTGCCCATGGTAAATTTTTTAACACCCTACAAGCTCAAGGGGAGAAAGCCTCCCTTTATGTGATCCGTTTAGA-GGTGCAG
Tupaia belangeri  ACATGAAACTGACGTTTGGCGAGTCTGAAAGCAGTGTGACAGCTCATGGTAAATTTTTTAACACTCTGCAGGCACAAGGGGAGAAAGCCTCCCTTTATATGATCCATTTAGAAGGTGCAG

Homo sapiens      CTCCAGAACGCTATTCAGGCAGGCATTATAGCTGAGAAAGATGCAAACCGGACTCGCTTGCAGCAGCTCCTTTTAGGCGGTGAGCTGAGTAGGGACCTCCGACTCAGACTTAAGGATTTT
Tupaia belangeri  CTCCAGAATGCTATTCAGACAGGCATCCTGGCTGAGAAAGATGCAAACCAGACTCGCCTGCACCAGCTCCTTTTAGGCGCTGAGATGACTAGGGACCTGTATTTCAGGCTTAAG---CTT

Homo sapiens      CTCAGGATGTATGCAAATGAGCAGGAGCGGCTTCCCAACTTTCTGGAGTTAATCAGAATGGTAAGGGAGGAAGAGGATTGGGATGATGCTTTTATTAAACGGAAGCGTCCAAAAAGGTCT
Tupaia belangeri  CTCAGGATGTACACAAAAGATAAGGGGCATCTTCCTAGCTTCCTGGAGTTAGTCAGGATGATCAGGGAGGAGGAGGATTGGGATGACACTTTTATTAAACGGAAGCGGCCCAGAAGATCT

Homo sapiens      GAGTCAATGGTGGAGAGGGCAGTCAGCCCTGTGGCATTTCAGGGCTCCCCACCGATAGTGATCGGCAGTGCTGACTGCAATGTGATAGAGATAGATGATACCCTCGACGACTCCGATGAG
Tupaia belangeri  GAGTCAATGGTGGAGAGGGCAGCCAGCCCCGTGGCGTTTCAGGGCTCCCTGCCAATAGCAATTGACGGTGCCGACTGCAACGTGATAGAGATAGATGATACACTGGATGACTCCGAAGAG

Homo sapiens      GATGTGATCCTGGTGGAGTCTCAGGACCCTCCACTTCCATCCTGGGGTGCCCCTCCCCTCAGAGACAGGGCCAGACCTCAGGATGAAGTGCTGGTCATTGATTCCCCCCACAATTCCAGG
Tupaia belangeri  GACGTGATCCTGGTGGAGTCTCAGGACCCTCCGCTTTCATCCTCTGGTGACCCTCTCTTCAGAGGCAGGGCCAGGTCCAGGGATCGAGTACTGCTCATTGATTCCCCTAATAATTCTGGG

Homo sapiens      GCTCAGTTTCCTTCCACCAGTGGTGGTTCTGGCTATAAGAATAACGGTCCTGGGGAGATGCGTAGAGCCAGGAAGCGAAAACACACAATCCGCTGTTCGTATTGTGGTGAGGAAGGCCAC
Tupaia belangeri  GCTCAGTCTCCTTCCACCAGTGGTGGTTCAGGGTATAAGAATGATGCTCCTATAGATGGGCGTAGAGTCAGGAAGCGGAAGTACACAATCTGCTGTTCATATTGTGGCGAGGAGGGCCAC

Homo sapiens      TCAAAAGAAACCTGTGACAACGAG---AGTGACAAGGCCCAGGTTTTTGAGAATTTGATCATCACTCTCCAGGAGCTGACCCATACTGAGATGGAGAGGTCAAGAGTGGCCCCTGGCGAA
Tupaia belangeri  TCAAAAGAAACCTGTGACAATGAGAGCAGCAGCAGGGCCCAGGTTTTTGAGAATCTGATCATCACCCTGCAGGAGCTGACACACACTGA---AGAGGGGTCAAGAGAGGCCCCTGGTGAA

Homo sapiens      TACAATGACTTCTCTGAGCCACTGTAAGGGACCACCCCCAGGTTTCAGTGAACCCTTACCTATATTCAGCATCCAGTAGTGGGAAAACTGGGGTGGGGGTGGGGGTGGGACTTCTAACTG
Tupaia belangeri  TTAAGTGACCCCTCTGAGCCATAGTAAGGTGCTAGCCCCAGCCGTAAGTGA------------------ATCCAGCGATAGGAAAACTGGGGTGAG---------AGGGTTTCTACTTG

Homo sapiens      CATGAATTAATCCACAAAGCGGCTATCTTTTGGGGTGGAGTAGAAAGGGTCTTGGATACCAGCACATTGGAGGGAGATAGCCTGACCTCTGTCCTTGCTCCTTCTCCCTGCAGCCTACGG
Tupaia belangeri  CATGAATTAATACACAAAGCAACTTTCTTTTGGGATGG----GGAAGAGTTTTGAATGCCAGCACAGTGGAGGGAGATGGCCTGACTCATGTCCTTGTTCCCTGGCCCAGCTGCCTAAGG

Homo sapiens      GTCTGTTTTCTGTGTGTGCCCATTTCCTTGACAGCTTTAT--TCTTTGTGAAAGTGGTATAATTTATTGTTAAATATTTGAACAATAAAAAAGGTACAAAAAGTGAAGTACAAATTACCC
Tupaia belangeri  GTCTATTTCCTGTGTGTGCCCATTT-CTTGATGGCTTTATTCTCTTTGTGAAAGTGGCATAATTCAATGTGCAGTCTTCAAATAGAGAAAAA--TATAAAAAGTAAAGTACGAATTCCCC

Homo sapiens      AAATCTCTCCACCCTTATATAATCATTGTCAACCCTTTGATGAGT---------GATATTTCCCTATACCTATGTACCCAGATAGATATATGCATAGATAAAAGTGATGAAATATAAGTG
Tupaia belangeri  CCAACTCTTCATCCTTATATTTCCATTGTCAATGCTTTGATGAATGTCCTTCTAGATATTTCTCTATACCTATCTATCCAGATAGTTATATGTATAGATAAAAGTGATCAAATATAAGTG

Homo sapiens      CTGTTCTA---TCTGTATTTTTTCACCAAACAATATATGTTGTGAGC----TTCTATGTCAATAAATATATATATCAGCATCTGCCTTAATGTGTCTGTGCTG-----TTAGGAAGAATT
Tupaia belangeri  CTGTTCTATACTCTGTATTTTTTCACCAAACAATATATGTCGTGGGCCTCTTTCTATGTCAATAAATATATATATAAGCATCTATTTTAATGCCTCTGTGGTATTATTTTAGGAGTAAGT

Homo sapiens      TTTAAAAAAGAAAATATAGTAAAAAACTGTAAAGGGGG--GTTGTACTCTGAGGTGGGATTT---TTAACCGCATTTTGCAAATGAAAAAAAATAAATG------GGCTGAGATACTTCC
Tupaia belangeri  AAAGGAAAAAGAAATAACAACGAAAGCTATAAAGGGGAGTGTGGTACTCAGAGGTGGGATTTTCATTAACCTCATTTTGCAGATGAAGAAAAAGGAAGAAGAAATGGTTGAAATATTCCC

Homo sapiens      CAAAGGTAAAAGGACAGAGCCCCCTTACCACAACTAACCTGTCCCTTCGCTCAGAGAGCCAAATTGTGACACCCACTTGTTGACAAA-ACACCAGTGCTTTCTTCTCTGATATACCCAGG
Tupaia belangeri  CAAATGTAAAGGGACAGAGCCCCTTTAACACAACTTACCAGTCCCTTAGCTCATAAAGCCAAATTATGGCACCGACTAGTTGACAAATACGCCAACGCTTTCTTCTCTAATGTGTCCAGA

Homo sapiens      AGACTGATGGGGGATGGGGGAGTCCGGTGCTGAGGTAATAGCCCAATATGGGGATTCCAGGGGCTCCATTTCTGTTCACTTTGGGGATGGCAGTTCT---TCATCTGGGCAGATTCCTT
Tupaia belangeri  GGAAT--CCAGAGGTGGAGAGATCTGGGG-TGAGGTAACAGCCCAACATGAAGATTCCAGGGTCTCTATTCCTGTTCACTTTGGGGAGGGTCACTTTTTACTCCTCAGGATGGGTTCCTT

Homo sapiens      AGATTGTTGTTGCTGTTGCTGCCTCTGGACAACGTCCTGGTCACAACAAGGAATCTCTCCTGTGGGCTGACACCACCTTCTGCCCCCTCCC-CAGTTAAGGAAAAGAGAGAGCAGCCACT
Tupaia belangeri  AAA------TTTCAGCTGCTGCTTCCTGCTAACAACCACGTCACAGCCTAAAATCTGTCCCGTGGCTTGGCATTTCCCTTTGCCTTCTTCCATGAATAAGGAAAGGGGAAAGCAGCTACT

Homo sapiens      CATGGGAGCTCAGCATTAGCAGGCTCAACTCCTGACGGCGCCAGAGCCTCCCCTATAAACTCTGAAGCTATGCGCTGGAGGCCACCGGCAGCCCACGGACT
Tupaia belangeri  CACAGGAACTCAGCAC------------------AAGGCTTCAG-------CCTGTAAACTCTGACACTATGT-CTAGCGCACATAGGCAGCCTACGGACT


Homo sapiens       GTGAGCCGCCCGCCTCGGCCTCCCAAAGTGCTGGGATTTCAGGCGTGAGCCACCATTCCCGGCCAGAAGACTGCATTTCTAACAAGCTCCCCCAAATGACTCTGATG-------TCTTGC
Otolemur garnettii ................................................................................AACAAGCTCCCCTAAATGATTATGATGCAACCAGTCTTGA

Homo sapiens       CCCCATACTTGAATGGGTAT---TGGTTGAGAGCCTTGTTC-TCCAGCTCTCCTCTCTGTTAAATGGACATACTGCTACTTCTTTCACAGTAAAAGAGGAGATGTGTGTGAAAGCCTGTT
Otolemur garnettii ACCTGTACTTGAGAGTGAATGTTTATTTAAGAGCCTTGGTGGTCCAAGCACCATCTCTTTTAAAAGGACGTAATGCCTCCTCCTTCGC---------GGAGCTGGCTGTGGAAGCTTATT

Homo sapiens       TCCGCAATGCACGTGCTAATGCTCTTCCCGCCCCTTCCCCTCTTCCCCGTCTTTTCCCTATACCAAGGTGAAGAACTTTTATTATTCACTTGATTTTGGACCCACTTCGTGTTTAATGCA
Otolemur garnettii A-------AAAAGCTCTAACGCTCTT--------------TCTCCCCCAT-TTTCCCCTATACAAAGATGGGGAACTTTTATTGCGTACTTGATTTTCGTCGCACCATATTTTTT-TTTA

Homo sapiens       CCTGGCCT-AAGATGCAGAGTTGAGCCCAGGGAGAA----GGGACGGGTTGGG--------------GGAGGAAGAAAGAGTCGCAAATTCCC-------AGGCAGATTGGCTCCATCGG
Otolemur garnettii CCTGGTCTCAAGATGCGGAGCTGAGTCAAGGGAGAAACGGGGGAAGGGTAGGGTGGGCGCTGGAGGAAGGGGAGGAAAGAGTCGCAAACTCCCTGGTAACAGGCAAATTGGCTCAGCAGG

Homo sapiens       TCTAATCAAGCTGCACTTGACGCTCCGCGGGCCTGCTGCGCCCCCGCCCCCTCAGGCGAAAACTCGCCTCTGCCCAAGGCTGTGCGGGGCGTCCGCCCCCGCACTTCGCTGATTGGCCGC
Otolemur garnettii CCAACCCAAGATGCACTTGACGCTGCGCGGTCCAGCAGCGCCCCG--CCCCTCGGACGAAT-CTCGCCTCTGGC-GCTGCTGCGCGGGGAGCCCGCCCC-GCACTCCGCTGATTGGCTGC
```

```
Homo sapiens        ACTAGCCCGCTCGTCACGCTCTTTTGTCTCAGCTGGCAGAGGATAAAAGCCGCCGCGGCTGCCTTAGGAACGGCGCTGCCTCGTCTCTGCTA-CCCCTGGTTGGGCGGCCCTGCGAAGCA
Otolemur garnettii  AACGGCC-GCTCCTCACGCTCTTTTGTCTCAGCGGGCGGAGGATAAAAGCCTCCGTAGCTGCCTTGGGAACTGCGCTGCCTCGACTCGGCTACCCCCTCGCTGGGCCGCCGTGCGAAGCA

Homo sapiens        GCTCCTTCGGGCAGCCCCGGGTCGCTTAGCGGCCAAGGAGGCTTCAGTTCTTTGCCGCCTGCAAG---GCGGAGACCAGAAGGCGGAATCCACAGCTGGCGACGCGGGAGCATCTGCTGT
Otolemur garnettii  GCTCCCTCGAGCAGCCCCGGGTCGCTTAGCGGCTGAGGCTGCGCGGGTCCTTT--CTCCTTCAGGGCCGCGGAGACTGGAAGGTGGACTCCACAGCT-GTGGTGCCGGAGCTTTAGCTGC

Homo sapiens        CCACCAGCGGAGCACAGGT----AAAGAATGGGGGTGTGATTGCCCGGCGGGGAGAGGGGTGGGGGAAGACCCACCAGTACTGGGCGGCTCCCAGCTTGGAGAATCGGTGACTGTGAGGC
Otolemur garnettii  CCGCCAGCGCGGCGCAGGTAAAAAAAAAAAAAGGTGTGCAGTTGCCCAGCGTGGACGGGGGAGGAGGAAG-CCCGCCGGTGCTGGGCGGCTCCGGGTTTGGAGAATCCGT--TCTCGAGGC

Homo sapiens        AGGGGTAGGAGTACTCTCGTGGCCCGGCTGCGCTGAGGGAGGAGGTGGGGTTCACCTGGATACTGCGGGCTAGGTAGGATTTGCCTGGATTTGGGGGTGGGAGGAGAGGCGAGGA--GTA
Otolemur garnettii  AGGGA-AAGAGCGCTCCCGCGGCCCTGCTGTGCCAAAGGAGGAGGTGAGGCTCACCTGAATGCTGCCCACTGAGTGAGATTTGCCTGAATTGGGGGCGGGGGCGCGTTTCCAGGGAGGTA

Homo sapiens        GCGGGGGGAGGAA--TCTCGAGCTGTTTGCTTCGAACATCCTTTGCATCTCCTCATCTCTTTCTGGGAGGAATATGTGGGTGGG--TGTGTCCAACTCCTATTCCCTTTCTCCGTGGCAG
Otolemur garnettii  GCAAGGGGCGGGAGGTCTTGAACCCCTT-------------TTTGCATCTCCTCATCTCTCCT----GGGAATATGGAAGCGGGAGGGTATCCGACTTCTATTCCCTTTCTCCCTGGCAG

Homo sapiens        GCCATCAAAGCCGCATCTGAACTTGAATTCTGTGCAGCTGATTGCAGAGC-TGGTAGGCCGACGGCTTCCCGGGGGAAATGTAATGCTGGAGGGTGGGGGGCTGGTCTCTGGCTTATAGT
Otolemur garnettii  GTCATCAAAGCTGAATCCGAACTTGAATTCTGTACAGCTGATTGCAGAGCTTGGTAGGCCGACGGTTTCCCATAGGAAATGTAA----------AGAGGGGC---GCTTTGGTTCATAGT

Homo sapiens        CAGGAGGGGGCAGAGAGAATTTTTCGCCTGGCAACA------GCGGAGGTGGTGGG------TAGCAGGATGACGGGTGAAGCTGCTTCTAACCCTTCCCTTCTCTGGCCTTCTCCGCTG
Otolemur garnettii  CAGAAGGGGGCAGAGGAAATTTTCTGCCTGGGGACAATGAAGACGGGGGTGGGGGGGAAGATTAGCAGGATGACGAATGAGGCTGCTTCTGACCCTTCCTTTCCCTTCCCTGTGCTGCAG

Homo sapiens        TGGCGCAATCTTGAAACCTCAAGGACCCGGATCTGCGACCCCCTGTGGACAGAGGTTGACCGTACCCCGGAGAGGAGCTTTCTCACGGAGGGCACTGGTTGCAGAGGCTGGAAGTGAAAT
Otolemur garnettii  AGGCACAGTCTTCAACCCCTGATGACAGGGATCTGCGACCCCCACGGGACAGAGGTTGACC-TATCCCGGAGGCGAGTTTTCTCACAAAGCGCACCTGTTGCAGAGGCTGGAAGTGAAAT

Homo sapiens        AAAGAC-GCGCTCTTGTTTCAGAGTTCGTGTAAGAATCTGAGAAATAACAAGGGGGACGGGCGTGGGGGGCACTTTGAGAAGGGGGTGTGGTTGAGACCAGAGACCATGAGGTTAATGAG
Otolemur garnettii  AAAGGCTGAAGGCTAGTTTCCGAGCT......................................................AGGGGGTGTGGTTGAGACCTGAGACTACGAGGTTAATGAA

Homo sapiens        ATCTTATTTTATTTCCACAAATCTGCGATCATTGTACTCTATTTGGAGAGCCCAATATCAGCTCCAGACACTGCTACACCTAGAATTTGAAGCAACAGTTACCGTCCCTCTTGGAGGACT
Otolemur garnettii  ATATTATTTTATCCCCACAAGTCTGTGACCATTGTGCTCTCTTTTGAGAGCCCAATATAGGTTCCATGTATT--CATACCTAGAATTTGAAGCAACAGTT---GTCCCTCTTGAAGGACT

Homo sapiens        GGTGGGAGGG--ATGGATGTGGCTGCAAAAAGCACCTTGCTAGCACGCAGGCATCGGGTAGCTCTGGGAGGTTGTAAGTGTCAATCTCCTACAGGATTATTTTTAGGGTAGTGGGCAAAT
Otolemur garnettii  GGCAGGAGGGGGATGGAGGGGGCTGCAAAAGCCCCTTTGCTGGCACGCACTCAC----TAGCTTCAGGAGATTGTATCTACTTATCTTTTGCAGGGTAGTTTT---AGTAGTGGGCAAAA

Homo sapiens        ATAAAATGTACTGCAGGATATTCAGGTAGGAAGAGAATGCAGCTGCAGCCCGCTCCCTTATT--AACCAGCCCCCCTTTC--TTTTTTACAGCCCCTGCTGAGATAGGA---AGGCAGAG
Otolemur garnettii  AACAAAC-TACTGCAGGATATT-AAGTAGGA-GAGAATGCGGCTGCAGCCTGCTCCCTTTTTTGACCCCCCCCCCCTTTTCTTTTTTTACAGCCTCTACTGAAATAGGAGGAAGGAAGAG

Homo sapiens        CCACCTCCTCTCCTCTCCCACCTGCAGATTAAGCTTTTCTAAAAAGCCTAGGCATCTTCTTATATTCAGATACCCTATCGTCGTCAGTCATGGCTAGCATCATTGCACGTGTCGGTAACA
Otolemur garnettii  CCACC-CTTCTCCCTGAACACCTGCAGATTAGGCTTTTCTAAAAAGCCTGAGCATCTTGTTATATTCAGACAGCTTGTCATCCTTAGTCATGGCTAGCCTCATTGCACGTGTGGGTAACA

Homo sapiens        GCCGGCGGCTGAATGCACCCTTGCCGCCTTGGGCCCATTCCATGCTGAGGTCCCTGGGGAGAAGTCTCGGTCCTATAATGGCCAGCATGGCAGACAGAAACATGAAGTTGTTCTCGGGGA
Otolemur garnettii  GCCGTGGGCAGAACGCGCCCTTGCCGCCTTGGGCGCATTCTATGCTGAGGTCCCTGGGGAGGAGTCTTGGTCCTTTAATGGCCAACATGGCAGAAAGAAACATGAAGTTGTTCTCAGGGA

Homo sapiens        GGGTGGTGCCAGCCCAAGGGGAAGAAACCTTTGAAAACTGGCTGACCCAAGTCAATGGCGTCCTGCCAGATTGGAATATGTCTGAGGAGGAAAAGCTCAAGCGCTTGATGAAAACCCTTA
Otolemur garnettii  ATGTAGTGCCAGCCCAGGGGGAGGAAACCTTTGAAAACTGGCTGACCCAAGTCAATGGGGTCCTGCCAGATTGGAATATGTCTGAGGAGGAGAAGCTCAAGCGCTTGATGAAAACCCTTA

Homo sapiens        GGGGCCCTGCCCGCGAGGTCATGCGTGTGCTTCAGGCGACCAACCCTAACCTAAGTGTGGCAGATTTCTTGCGAGCCATGAAATTGGTGTTTGGGGAGTCTGAAAGCAGTGTGACTGCCC
Otolemur garnettii  GGGGCCCTGCCCGGGAGGTCATGCGTTTGCTTCAGGCAGCCAACCCCAACTTAAGTGTGGCAGATTTCTTGAGGGCCATGAAATTGGTGTTTGGGGAATCTGAAAGCAGTGTTACTGCCC

Homo sapiens        ATGGTAAATTTTTTAACACCCTACAAGCTCAAGGGGAGAAAGCCTCCCTTTATGTGATCCGTTTAGAGGTGCAGCTCCAGAACGCTATTCAGGCAGGCATTATAGCTGAGAAAGATGCAA
Otolemur garnettii  ATGGTAAATTTTTTAACACCCTGCAGGCGCAAGGGGAGAAAGCCTCCCTTTATGTGATCCGTTTAGAGGTGCAGCTCCAGAATGCTATTCAGGCAGGCATCCTAGCTGAAAAAGATGCCA

Homo sapiens        ACCGGACTCGCTTGCAGCAGCTCCTTTTAGGCGGTGAGCTGAGTAGGGACCTCCGACTCAGACTTAAGGATTTTCTCAGGATGTATGCAAATGAGCAGGAGCGGCTTCCCAACTTTCTGG
Otolemur garnettii  ACCAGACCCGCCTGCAGCAGCTCCTTTTAGGGGCTGAGCTGAATAGGGACCTGCGCTTCAGGATTAAGGAACTTCTCAGGGTGTATGCAAATGAGCAGGAGCACCTTCCTAATTTCCTAG

Homo sapiens        AGTTAATCAGAATGGTAAGGGAGGAAGAGGATTGGGATGATGCTTTTATTAAACGGAAGCGTCCAAAAAGGTCTGAGTCAATGGTGGAGAGGGCAGTCAGCCCTGTGGCATTTCAGGGCT
Otolemur garnettii  AGTTAATCAAAATGATAAGGCAGGAAGAGGATTGGGATGACACCTTTATTAAACGGAAGAGGCCCAAAAGGTCTGAGTCACTGGTGGATAGGGCATCTAGCCCTGTGTTGTTTCAGGGCC

Homo sapiens        CCCCACC-GATAGTGATCGGCAGTGCTGACTGCAATGTGATAGAGATAGATGATACCCTCGACGACTCCGATGAGGATGTGATCCTGGTGGAGTCTCAGGACCCTCCACTTCCATCCTGG
Otolemur garnettii  CCCCATTTGATGGCCAATGGCAGTGCTGACTGCAACGTGATAGAGATAGATGATAGCCTGGATGACTCAGATGAGGATGTGATCTTGGTGGAGGCTCAGGATCCTCCACTT---------

Homo sapiens        GGTGCCCCTCCCCTCAGAGACAGGGCCAGACCTCAGGATGAAGTGCTGGTCATTGATTCCCCCCACAATTCCAGGGCTCAGTTTCCTTCCACCAGTGGTGGTTCTGGCTATAAGAATAAC
Otolemur garnettii  GGTGCCCCTTCCTTCAGAGATGAGGCCAGATCTCAGGATCAAGTGCTGTTTGTTGATTCCACCAACAATTATGTGCCTCAGGTTGCTTCCTCCAGTGATGGTTCTGGGCATAAGAATGAT

Homo sapiens        GGTCCTGGGGAGATGCGTAGAGCCAGGAAGCGAAAACACACAATCCGCTGTTCGTATTGTGGTGAGGAAGGCCACTCAAAAGAAACCTGTGACAACGAGAGTGACAAGGCCCAGGTTTTT
Otolemur garnettii  GGTCCTGGGGATGTGCGTAGAGGCAGGAAGCGAAAATACACAATCCGCTGCTCATATTGTGGGGAGGAGGGACACTCAAAGGAAACATGTGGCAATGGGAGCAACAAGGGCCAGGTTTTT

Homo sapiens        GAGAATTTGATCATCACTCTCCAGGAGCTGACCCATACTGAGATGGAGAGGTCAAGAGTGGCCCCTGGCGAATACAATGACTTCTCTGAGCCACTGTAAGG-GACCACCCCCAGGTTTCA
Otolemur garnettii  GAGAACCTGATCATCACCCTGCAGGAGCTGACCAATCCTGAGGAGGAGGGGTCA------------GGTGCACACCATGACCTCTTTGAGGCAGAGGAAGGTGATAGTCGCCAGTTTTAA

Homo sapiens        GTGAACCCTTACCTATATTCAGCATCCAGTAGTGGGAAAACTGGGGTGGGGGTGGGGGTGGGACTTCTAACTGCATGAATTAATCCACAAAGCGGCTATCTTTTGGGGTGGAGTAGAAAG
Otolemur garnettii  ATGAGCCCTTATCTATATTCAGAGTCCAATGATGGGAAAACTGGGGGGGGG-----------CTTCTAATTGCATGAGTCATTCCACAAAGCGGCTATCTTTTGGGGTGGAGAAG--AG

Homo sapiens        GGTCTTGGATACCAGCACATTGGAGGGAGATAGCCTGACCTCTGTCCTTGCTCCTTCTCCCTGCAGCCTACGGGTCTGTTTTCTGTGTGTGCCCATTTCCTTGACAGCTTTAT--TCTTT
Otolemur garnettii  GGTCTTGGATACCAGCATAGTTGAGGGAGATGGCCTGACCTCTGCCCTTGCTTTGTCACCCCGCTGCCTATGGCTCTATCTTCTGTGTATGCCCATTTTCCTGATGGCTTTATTCTCTTT

Homo sapiens        GTGAAAGTGGTATAATTTATTGTTAAATATTTGAACAATAAAAAAGGTACAAAAAGTGAAGTACAAATTACCCAAATCTCTCCACCCTTATATAATCATTGTCAACCCTTTGATGAGT--
Otolemur garnettii  GTGAAAGTGGTACCATTCATTGTTAAATCTTC-----------AAAGTACAAAAAGTAA-----AAATTACCCCAAACTCTCCACCCTTACATAACTATTGTCAACCCTTTGATGAGCAT

Homo sapiens        -------GATATTTCCCTATACCTATGTACCC----AGATAGATATATGCATAGATAAAAGTGATGAAATATAAGTGCTGTTCTA---TCTGTATTTTTTCACCAAACAATATATGTTGT
Otolemur garnettii  CCTTCTAGATATTTCCCTATACCAGTGTATCCAGATAGATAGATATATGTATAGATGAAAGT----AAATATAAGTGCTGTTCTATACTCTGTATTTTTTCACCAAACAATATATGTTGT

Homo sapiens        GAGC----TTCTATGTCAATAAATATATATATCAGCATCTGCCTTAATGTGTCTGTGCTG-----TTAGGAAGAATTTTTAAAAAAGAAAATATAGTAAAAAACTGTAAAGGG-GGGTTG
Otolemur garnettii  GAGCTGCTTTCTATGTCAATAA----ATATATCAGCATTGATTTTAATGTCTCTGTGGTATTATTTTAGGAAG--------AAATAAAAAATATAGTGAAAAGCTATAAAGGGTGGGTGA

Homo sapiens        TACTCTGAGGTGGGATTT---TTAACCGCATTTTGCAAATGAAAAAAAATAAA--------TGGGCTGAGATACTTCCCAAAGGTAAAAGGACAGAGCCCCCTTACCACAACTAACCTGT
Otolemur garnettii  CACTCTGAGGTGGGGTTTTTGTTAAGCTCATTTTGCAGGTGAAGAAAAGGGAAGAAGAAGTTGGGCTGAAATA-TTTCCAAAGGCAAAAGGACAGAGTCCCC--ACCACGACTGATGTGT

Homo sapiens        -CCCTTCGCTCAGAGAGCCAAATTGTGACACCCACTTGTTGACAAAACACCAGTGCTTTCTTCTCTGATATACCCAGGAGACTG-----ATGGGGGATGGGGGAGTCCGGTGCTGAGGTA
Otolemur garnettii  CCCCCTAGGTTTTAGAGCCCACCTCTG-CACTCACTAGTTAATAATGTCCCAATG-TTTTTTCTCGAATGTATCCTGAGAAGTGTGATCACGGGGGGCGGGG-------------GGCA

Homo sapiens        ATAGCCCAATATGGGGATTCCAGGGGCTCCATTTCTGTTCACTTTGGGGATGGGCAGTTCTTCATCTGGGCAGATTCCTTAGATTGTTGTTGCTGTTGCTGCCTCTGGACAACGTCCTGG
Otolemur garnettii  AATACAGAAAATGGGTATCCCGGAGACTCTATTCCTGTTCACTTTAGGAAGAGGCA................................................................

Homo sapiens        TCACAACAAGGAATCTCTCCTGTGGGCTGACACCACCTTCTGCCCCCTCCCCAGTTAAGGAAAAGAGAGAGCAGCCACTCATGGGAGCTCAGCATTAGCAGGCTCAACTCCTGACGGCGC
Otolemur garnettii  ..........................................CCCCTCCCCCAAGCGAGGAAAGGAAAGAGCAGCTGCTCCTGGAGGTTCAGAGCCAAGAGGCTCAGCACCTGGCAATTT

Homo sapiens        CAGAGCCTCCCCTATAAACTCTGAAGCTATGCGCTGGAGGCCACCGGCAGCCCACGGACT
Otolemur garnettii  CTATACCTAACTTTGTAGACCTGTAGCTATATAATAGA......................
```

ZCCHC18

to je za ZCCHC12

Homo sapiens >
chromosome:GRCh37:X:117957153:117961531:1
Loxodonta africana >
supercontig:loxAfr3:scaffold_32:12108492:12110481:-1
supercontig:loxAfr3:scaffold_32:28025187:28025482:-1
supercontig:loxAfr3:scaffold_32:28023671:28024410:-1
supercontig:loxAfr3:scaffold_32:12107284:12108446:-1

to je za ZCCHC18

Homo sapiens >
chromosome:GRCh37:X:103356222:103361133:1
Loxodonta africana >
supercontig:loxAfr3:scaffold_32:28026176:28028226:-1
supercontig:loxAfr3:scaffold_32:28025739:28025990:-1

```
supercontig:loxAfr3:scaffold_32:28025187:28025631:-1
supercontig:loxAfr3:scaffold_32:28023045:28024410:-1
supercontig:loxAfr3:scaffold_32:12107531:12107799:-1
supercontig:loxAfr3:scaffold_32:12106820:12107090:-1
```

```
>chromosome:GRCh37:X:103356222:103361133:1
GAAAGAAAGAAAAGTACCAAGAAGAGTTTCCTCTTCTGTACAATGGGCACCATAAAGGTT
GCCCTCATCTTCCCTCAGCCCTCTCCCCACTACCTCCTCACCCCCTCCCTCAGCACTATT
ATCACATTGAGACTGCTGTTGAGATGGTTTGAGACTACCTGTGCAAAAATACTCTCCAGG
CAGCGCTGCAGGGCAACGAATTAGCATCTAGAGAGCAACAGACGGTGCTAGGTTGGATGC
TGTAACTTATTTGCTGCAGGATACAGGCCAAAACTAGTAACCTGTCTCTCTCTGGCTCAG
TTTCTTCATCTGTGAAATGGAGATAATGGTTCTTGCTCACCAATTCTATGCAATCCCAAG
TTGGTCCTCCCATCTGGCTTTGGTTTTTCTCCACTATCCCTTAATCTGACAGAATAGAGG
AGTGCTGGCAAGGGGCAGCCTTTGAAGTCAGACATGGTGACTTGGGTGTGAGCTCTTAAC
TCTGCACTTCCTAACTGTACTGCCTGGGTGCTTCAGTCCTTTCCTGTTAAATGGGCACAC
TACTACCTCCTTCACACGGCTGCTGAGAAGAGGAAAGGAGATGTCCATGCAGGCGCCTCT
GCACACAGAAAGCACTCAGTAAACGCTGTTTCCCCGCTTCCCCCTATACAAAGGTGAGGA
ACTCTCATTGTTCACTTGGTTTCTGTCCCACCTCGTGTTTAAGGTATCCGGTCTAAAAGC
CCGGAGGAGAGCCGAGGTGTAAGGGGATAGGGAGAAGGGAAGAGATGGGTTAGCAGCAAA
CTCACGGATGAAAGGGAAATGGGCCCAGTCCGTGCGTCCGAGCTGCTGACGCCGTGCCGC
CTCCCGCGCCTGTCTGCCGCAGCCCCCGCCCGCCGGCCCGCCGGCCAGCCGGCCCGCTCC
CCCTTCTTGCTAGCAGTAGCTTCGGGGCCGGAGGCTGTGCGGCGAGGCCGCCCCCTCGCC
CCGCTGATTGGCCGCGTCGCCCGGCCCGTCACGCTCTTTTGTCTCAGTCGCCAGAGACTG
AAAGCAACCGCGGCTGCCCGGAGGGCCGAACTGGAGGGTGGGCGCAGCCTTGGCTGCCTT
GGGAACCGTGCTGCCTCGACTCGGCTACCCCTCGCTGGGCGGTCGTGCGCACTCGCTAGG
TCGGGCAGCCCCGGGTCGCTTAGCGGCCGAGGAGGCGGCAGAGATCCCGTTCCCCTGCAG
AGCTACGGGGACCGGAAGGCGAAACAGAGCTGTGGACGCGGGAGCACATGCTGCCCACCA
GTGGAGCACAAGTAAAGAAAGAACGCGCGATTGCCCGGCGCGGAGAGGGGTGGGGGAAGC
CCCGCCAGGACTGGGCGGCGCCCGGCTTGGAGAATCAGTAACTGCGAGGCAGGGGATGGA
GCACTCCCTTGGCCTGGTTGCGCTGAGGAAGGAGTTGTGGTTCATCTGGATGCTGCCGAC
TGGATAGGATTTAGGATTTGCCTGGATTAAGGTGGGTGGGGGTGTTGTCGTAGGTCGGGG
GTGTTGTTGGGTGTCGGGGGCGGGGTGTGTTCGGGGAGGTGGCAAGGGGCGGGAGGAGAC
GATCGCAAGCTGTCTTGAACCCCTTTTGCATCTCCTCATCTCTTCCTGGGAAGGAATATG
AGGGTGGGAGGTTATCCAACTTCTAGTCTCTTTCTCCCTGGCAGGTCATCAAAGCCGAAT
CCGAACTTGAATTCTGTGCGGCGGATTGCAGAGCTGGTAGGGCGAGGGTCTCCCGGAGGA
AATGTAAGGGGGATGCGCGGGAGGGCAGGAGGCACTTTGGCTTGCGTTCAGAAGATGGGA
GAGGGAATTTTCCGCCTGGCGACAGTGGAGGCGGTGGAGGGGAGGTATCAGGTTTTCAGA
TGAAGCTGTTTCCAACCCTTCCTTTCCCTCCTTCGCGGCACAGTCTTGAGGCCCTGAGGA
CTGGGATCTGCGACACCCAGTGGACAAAAGTCGGCCGTACCCCAGAGGCTAATTTTCTCA
CGGCAGGCACCTGTTGCAGAGGCTGCACGTAAAATAAAGGCGAACGCTAGTTTCCGAGCT
CATGTAAGAATCTGAGAAATAACGGGGGAAACGTTGACAAGGGGGTGTGATTGAGACCTG
AGGCCATGAGGTTAATGGTTTCTTATTATCATAAATCTGTGACACACAACCATTGAGTTC
CGTTTTGAGGGCCCAGTATCAGCTCCCAACACTATGTCATCTAGAATTAGAAGCAACAGT
TGTCGCCCCTCCTGGAGTACAGGCAGGAGGGGATGGATGTGGCTCTGAAAAACTACCTAC
TAGCCCAGGGACACCTGCTAGCTCTGGAATGTTGTAATTGCCAGTCTCCTGCAGGGTATT
GTTAAGGCACTGGGCAGATATAAAATGCACTGCAAGGCTATTCAGGAAGATAGAGAATGC
TACTGCAGACTGCTTCCCCACAGCCCACTATCTTATTAACCTTTTTTACTTTCCTTAGAA
TCCCTACTGAAATATAAGGCAGGCACAGCCCAGGAACGTTGCTTTGGAGAATCCTGCAGA
TAAGGCTTTTCCAAAAAGCGCGAGCATCTTGTTGTATTCAGATACCCTATCGTCGTCAGT
CATGGCTAGCATCACTGCGTGTGTGGGTAACAGCAGGCAGCAGAATGCACCTTTGCCGCC
TTGGGCCCATTCCATGTTGAGGTCTCTGGGGAGGAGTCTCTGTCCTTTAGTGGTCAAAAT
GGCAGAGAGAAACATGAAGTTGTTCTCAGGAAGAGTGGTGCCAGCCCAGGGGAAAGAAAC
CTTTGAAAACTGGCTGATCCAAGTCAATGAGGTCCTGCCAGATTGGAGTATGTCTGAGGA
GGAAAAACTCAAGCGCTTGATGAAAACACTTAGGGGCCCTGCCCGGGAGGTCATGCGTTT
GCTTCAGGCGGCCAACCCCAACCTAAGTGTAGCAGATTTCTTGCGGGCAATGAAATTGGT
GTTTGGGGAGTCTGAAAGCAGTGTGACTGCCCATGGTAAATTTTTTAACACCCTGCAGGC
ACAAGGGGAGAAAGCCTCCCTTTATGTGATCCGTTTAGAGGTGCAGCTCCAGAATGCTAT
TCAGGCAGGCATCCTAGCTGAGAAAGATGCAAACCAGACTCGCTTGCAACAGCTTCTTTT
AGGCGCTGAGCTGAATAGGGACCTGCGCTTCAGGCTTAAGCATCTTCTCAGGATGTATGC
AAATAAGCAGGAGCGGCTTCCCAATTTCCTGGAGTTAATCAAGATGATAAGGGAGGAAGA
GGATTGGGATGATGCTTTTATTAAACGGAAGCGGCCGAAAAGGTCTGAGCCAATAATGGA
GAGGGCAGCCAGCCCTGTGGCATTTCAGGGCGCCCAGCCAATAGCAATCAGCAGTGCTGA
CTGTAACTGCAACGTGATAGAAATAGATGATACCCTTGATGACTCTGATGAGGATGTGAT
CCTGGTGGTGTCTCTGTACCCTTCACTGACACCTACAGGTGCCCCTCCCTTCAGAGGAAG
AGCCAGACCTCTGGATCAAGTGCTGGTTATTGATTCCCCCAACAATTCTGGGGCTCAGTC
TCTTTCTACCAGTGGTGGTTCTGGGTATAAGAATGATGGTCCTGGGAATATTCGTAGAGC
CAGGAAGCGAAAATACACAACCCGCTGTTCATATTGTGGGGAGGAGGGCCACTCAAAAGA
AACCTGTGACAATGAGAGCAACAAGGCCCAGGTTTTTGAGAATCTGATCATCACCCTGCA
GGAGCTGACACATACAGAGGAGAGGTCAAAAGAGGTCCCTGGAGAACACAGTGATGCTTC
TGAGCCACAGTAAGGATCTAGTCCAGCCCTAAATGAGTCCTTGACTGTATTCAGAGTCTG
GTAATGGGAATAACAGGAGAGGGGGGTGGGTTTCTAACTGCATGAATTAATCCACAAAGC
AGTTTTCCTTTGGGAAGGAGAAGAGGTCTTGCATACCAGCACAGTGGATGGGGAATGGTG
TGACCTCTGTCCCTGCTCTCTTCTCTGCTGGCTTAAGGGTCTATTCTCCATGTGTCTATT
TCTTTGATGACTTTATACTTTTTATGAAAGGAGCATCTTTTCATTAAACCTTCAAAAATA
AAGGAAGATACAAAAACTAAAGTACAAATTACCTGAAACTCAGCACCCTTTTGTAACCAT
TGTCAGCCCTTTGGTGAATGTCCTTCCAGATATCTCCCTGTACCTGTGTACCCAGATAGA
TATATGTATAGATAAGAGTGACCAAATATAAGTGCTGTTCTATACTGTGTATTTTTCACC
AAATAATGTATCTCGTGGACTTCTATGTCGATGAATATATATAAATATCTAACTTCATGT
CTCTGTGTGATGTTACTTTTAGAAAGATAAAGAAAAATGAAACTAAATATAGAAGCTATA
AGGGGGGGTCATACCATGAGGAGGGGTTTTTATCGACCTCATTTATAGGAAAGGAGAAAG
GAAGCTGAAATGGACTGAATATCTCCCATACAACCCCTTAACACAACTGACCTGTCTCCT
AGGTCATAGAGCCAACCCCTGCACCCATGAGTTAATAATGTTCCAATGTTTTCTTCTTGA
ACATATCATGAGACATCAGATGGGGAACGGGGGGGGACAAAAATTGAAAATGGGTATTC
CAGAGGCTCCATTCCTGTTTGTATTGGGAAGGGACAACTACTCCTGCCCGATCTCTTGAC
CTCAGATTAGAGGGTGATAAAAGATGACATGTGACCTCTATCCCTGAGGGTCCCAAGTTT
CTGGTAGGTACAGTAGTCCCTATCTTCACGAAGAGGTCAAAACTGGCAAGGAGCAGGGGG
GAATGATTTCATTTAAGGCAAGGAGTGGAGTGAAATGAATGCAGACCTGAGGACCTGAGG
GAGTTTGCCTGGGAGGAAAGAGGGGGTCCCTGGGCTGACAAGACAGGGTGTG
```

```
Homo sapiens        GAAAGAAAGAAAAGTACCAAGAAGAGTTTCCTCTTCTGTACAATGGGCACCATAAAGGTTGCCCTCATCTTCCCTCAGCCCTCTCCCCACTACCTCCTCACCCCCTCCCTCAGCACTATT
Loxodonta africana  ..................................................................................................CACTCTACTTCACAGCACT---

Homo sapiens        ATCACATTGAGACTGCTGTTGAGATGGTTTGAGACTACCTGTGCAAAAATA---CTCTCCAGGCAGCGCTGCAGGGCAACGAATTAGCATCTAGAGAGCAACAGACG---GTGCTAGGTT
Loxodonta africana  ---------AGGCAGCCACTGAAATCGTTTGATGTCACATACATAAAAAAAAAGTTTTCTAGGCA---------------GAGTTTGCATTTGGAG---AACAGACTTGCGTGTAAAATC

Homo sapiens        GGATGCTGTAACTTATTTGCTGCAGGATACAGGCCAAAACTAGTAACCTGTCTCTCTCTGGCTCAGTTTCTTCATCTGTGAAATGGAGATAATGGTTCTTGCTCACCAATTCTATGCAAT
Loxodonta africana  AAATGGTGGTATTTATATACTGTATGATGTAGGACAAGACGGGTAAC--------CTCTGTGTCTGTTTCCTCATCTGTATAATGGACATAATTTTCCTAGTTCACTACTTCTACATAGC

Homo sapiens        CCCAAGTTGGTCCTCCCAT-CTGGCTTTGGTTTTTCTCCACTATCCCTTAATCTGACAGAATAGAGGAGTGCTGGCAAGGGGCAGCCTTTGAAGTCAGACATGGTGACTTGGGTGTGAGC
Loxodonta africana  CCCAAGTGGGCCCTCCCATCCCCGCTTTGGCTTTTCTCCACTCACCCTCAACCTGGCAGTAGTGAGGAGTGTTTACAAGGGGCAGCCTTTGAAGTCAGACATGTCGACTTGGGTTTGAGC

Homo sapiens        TCTTAACTCTGCACTTCCTAACTGTACTGCCTGGGTGCTTCAG-TCCTTTCCTGTTAAATGGGCACACTACTACCTCCTTCACACGGCTGCTGAGAAGAGGAAAGGAGATGTCCATGCAG
Loxodonta africana  TCTCAGCTCTGGACTTCCTAACCGTACTGCCTGGATGCTTAAGTTTCCTCTCTGTTAAATGGTCAGGCTGGTACCGCCTTCACAAGACTGCTGAAAA---GAAATGAGACGTCCCTGGAA

Homo sapiens        GCGCCTCTGCACACAGAAAGCACTCAGTAAACGCTGTTTCCCCGCTTCCCCCTATACAAAGGTGAGGAACTCTCATTGTTCACTTGGTTTCTGTCCCACCTCGTGTTTAAGGTATCCGGT
Loxodonta africana  GCGCATCTGCGCACA-AAAGTGCTCAGTAAATGCTTCTTCCTCCCTTCTCCCTATACAAAGGTGAGGAACTCTTATTGTTCACTTGATTTCTGACCCACCTCGTGTTGAAGGCACATGGT

Homo sapiens        CTAAAAGCCCGGAGGAGAGCCGAGGTGTAAGGGGATAGGGAGAAGGGAAGAGATGGGTTAGCAGCAAACTCACGGATGAAAGGGAAATGGGCCCAGTCCGTGCGTCCGAGCTG---CTGA
Loxodonta africana  CTCAAAGCGCGGAGGAGAGCCGAGGAAGAAGGAGAGAGGGAGAAAGGAAACGAAGGGGAAGCAGCACACTCACGGATAAAATGCAGATCGGTCCGGTCCGTGCTTCTCAGCTGAACTAGA

Homo sapiens        CGCCGTGCCGCCTCCCGCGCCTGTCTGC--CGCAGCCCCCGCCCGCCGGCCCGCCGGCCAGCCGGCCCGCTCCC-CCTTCTTGCTAGCAGTAGCTTCGGGGCCGGAGGCTGTGCGGCGAG
Loxodonta africana  CGCCGCACCGACTCCTGCACGGGTCTGCAGCACAGCCCCGGCC---------------------GCTCGCTCCCTCCTTTTTGCTAGCTTT---CTGGGGGCCGGAGGCC-CGCGAGGAG

Homo sapiens        GCCGCCCCCTCGCCCCGCTGATTGGCCGCGTCGCCCGGCCCGTCACGCTCTTTTGTCTCAGTCGCCAGAGACTGAAAGCAACCGCGGCTGCCCGGAGGGCCGAACTGGAGGGTGGGCGCA
Loxodonta africana  CCCGCCCCCGCGTCCCGCTGATTGGCTGCG-CGCCCGGCTCGTCGCGCTCTCCTGTCTCAATGACCAAAGAATCAAAGCAGCCGGGGCC-CTGGGAGGGCGGAACAGGCGGGCAGAGCCC

Homo sapiens        GCCTTGGCTGCCTTGGGAACCGTGCTGCCTCGACTCGGCTACCCCTCGCTGGGCGGTCGTGCGCACTCGCTAGGTCGGGCAGCCCCGGGTCGCTTAG----CGGCCGAGGAGGCGGCAGA
Loxodonta africana  GCCTC-GCTATCCAGGGCACCTCGCCGCCTCCACTCGGCT-CCTCTCGCTGGGCGGCCGCGCGGAGCCGCTCGGTCAGGCAGCCTCGGGTAGCTTAGCGGTCGGCCGATGAGGTGCCAGG

Homo sapiens        GATCCCGTTCCCCTGCAGAGCTACGGGGACCGGAAGGCGAA--ACAGAGCTGTGGACGCGGGAGCACATGCTGCCCACCAGTGGAGCACAAGTAAAGAAAGAACGCGCGATTGCCCGGCG
```

```
Loxodonta africana  ---CCTTATCCCGTGCAGGGCGGCGGAGGCCGGAAGGTGGACTGCATAGCTGTCGACGCCTGCGCATCCGCTGCCCACCCGCGGATCACAGGTAAAGGG-----GCGCGACTGCCCGGCG

Homo sapiens        CGGAGAGGGGTGGGGGAAGCCCCGCCAGGACTGGGCGGCGCCCGGCTTGGAGAATCAGTAACTGCGAGGCAGGGGATGGAGCACTCCCTTGGCCTGGTTGCGCTGAGGAAGGAGTTGTGG
Loxodonta africana  CCCAGAGAGGAGCGGGGCGCCCCGCCAGTACTGCG----------------GGATTGATAACCCTGAGGCAG-----------------------GGCTGCATCCAGGGAGGAGGTGAAA

Homo sapiens        TTCATCTGGATGCTGCCGACTGGATAGGATTTAGGATTTGCCTGGATTAAGGTGGGTGGGGGTGTTGTCGTAGGTCGGGGGTGTTGTTGGGTGTCGGGGGCGGGGTGTGTTCGGGGAGGT
Loxodonta africana  TACAACTAGATGCTGCCGAATGGGTAGGATTTA-------CTTGGATT--GATGAGTGG-----------------------------------------GGAGCATTTTCAGGGAGGA

Homo sapiens        GGCAAGGGGCGGGAGGAGACGATCGCAAGCTGTCTTGAACCCCTTTTGCATCTCCTCATCTCTTCCTGGGAAGGAATATGAGGGTGGGAGGTTATCCAACTTCTAGTCTCTTTCTCCCTG
Loxodonta africana  TT---GGGGCAAGAGGA----ACTGCAAGCTGT------CCCCCTTTGC------------CTTCCTGGGGAGGAATATGCGGGTGTGCG--TGTCCAACTTCAGTTCCCTTTCTCCCTG

Homo sapiens        GCAGGTCATCAAAGCCGAATCCGAACTTGAATTCTGTGCGGCGGATTGCAGAGCTGGTAGGGCGAGGGTCTCCCGGAGGAAATGTAAGGGGGATGCGCGGGAGGGCAGGAGGCACTTTGG
Loxodonta africana  GCAGGCCATCAGAGCCGAATCCGAACTTGAATTCTGTGTGGCTGATTGCAGAGCTGGTAGGCCCACGGTTTCCCGG-GGGAATGTAA---------TTGAGGGGGC----GTCACTTTGG

Homo sapiens        CTTGCGTTCAGAAGATGGGAGAGGGAATTTTCCGCCTGGCGACAGTGG---AGGCGGTGGAGGGGAGGTATCAGGTTTTCAGATGAAGCTGTTTCCAACCCTTCCTTTCCCTCCTTCGCG
Loxodonta africana  CTGGTATTCAGAAGGTGGAAGAGGAAGTTTTCCTCTTGACGACAGCGGAGTAGGGGGTTGGTTGTGGGGATAAGG------GATGAGTCTGCTTGTGACCCTTCCCTGC------TGGCG

Homo sapiens        GCACAGTCTTGAGGCCCTGAGGACTGGGATCTGCGACACCCAGTGGACAAAAGTCGGCCGTACCCCAGAGGCTAATTTTCTCACGGCAGGCACCTGTTGCAGAGGCTGCACGTAAAATAA
Loxodonta africana  GCGCAGCCTTGACGCCCTGAGGACCCGGATTGGCAACCGTCGATGGACAGAGGCTTACCATACCCCAGAGGCGACCGTTCTTAGGAAGGGTGCCAGTTACAGAGGCCGGAAGTGAAATGA

Homo sapiens        AGGCGAACGCTAGTTTCCGAGCTCATGT-AAGAATCTGAGAAATAACGGGGGAAACGTTGACAAGGGGGGTGTGATTGAGACCTGAGGCCATGAGGTTAATGG----TTTCTTATTATCAT
Loxodonta africana  AGGCTTGTGCTTGTTCCCAAGATCATGTAAAGAATCTGAGAGATAAT-GTGGAAACTTTGCGAAGGGGGGTGTGGTTAAGACGTGAGACCATGACATTAATGAGATCTTATTTATTCCCAT

Homo sapiens        AAATCTGTG-ACACACAACCATTGAGTTCCGTTTTGAGGGCCCAGTATCAGCTCCCAACACTATGTCATCTAGAATTAGAAGCAACAGTTGTCG-CCCCTCCTGGAGTACAGGCAGGAGG
Loxodonta africana  GAATCTGTGAACTTTAGACCATTGAGCTCTATTTTGAAAGCCCAATATCAGCTCCGCACAATATTTCACCCAGAAGTC-AAGCAACAGTAGTCGTCCTTTTCTGGAAGACTGGCAGGAAG

Homo sapiens        GGATGGATGTGGCT--CTGAAAAACTA-CCTACTAGCCCAGGGACACCTGCTAGCTCTGGAATGTTGTAATTGCCAGTCTCCTGCAGGGTATTGTTAAGGCACTGGGCAGATATAAAATG
Loxodonta africana  GGCTGTATGTGGCTGGCTCCAAAAGTACCCTGCTGGTGCAGGGACACCCACCAGCTCCTTGAAGTTGTAATTGCTGTTCTCCTGCAGGGTATTTTAGGACTTTAGGCAGTTATAAAATA

Homo sapiens        CACTGCAAGGCTATTCAGGAAGATAGAGAATGCTACTGCAGACTGCTTCCCCACAGCCCACTATCTTATTAACCTTTTTTACTTTCCTTAGAATCCCTA-CTGAAATATAAGGCAGGCAC
Loxodonta africana  TACTGTAGCG-TATTCAGAA----AGAGGATGTGGCTGCAGACTGCTTTCCTGCAGCCTGCTTACTGGTTTTTTTTTTTTTTTTCCATTAG--TCCCTATTTGAGATACAAGGAAGGCAG

Homo sapiens        AGCCCAGGAACGTTGCTTTGGAGAATC---CTGCAGATAAGGCTTTTCCAAAAAGCGCGAGCATCTTGTTGTAT----------------------------TCAGATACCCTATCGTCG
Loxodonta africana  AGTCCAGGAACAATGCTTTGGGGATTCCTTCTGCAGATTA-GCTTTTTTGAAGAGCCAGAGCATCTTGCTAGATAGACGATTATTGTCAGTTATGGCTAGCGTCGGAAATCCTGTCGCTA

Homo sapiens        TCAGTC-----------------------------------------ATGGCTAGCATCACTGCGTGTGTGGGTAACAGCAGGCAGCAGAATGCACCTTTGCCGCCTTGGGCCCATTCCA
Loxodonta africana  TGAGTCGGAATCAACTTGAAGGCAACAGGTTTGGTTTTTTGGTTTTTATGGCTAGCATCATTGCACATATGGGTAATAGCCAGCAGCAGAACACACCTTTAATGCCTTGGGTCCATTCCA

Homo sapiens        TGTTGAGGTCTCTGGGGAGGAGTCTCTGTCCTTTAGTGGTCAAAATGGCAGAGAGAAACATGAAGTTGTTCTCAGGAAGAGTGGTGCCAGCCCAGGGGAAAGAAACCTTTGAAAACTGGC
Loxodonta africana  TGCTGAAGTCCCTGGGGAAAAGCCTCGGTCCTTTAACGGCCAACTTGGCAGAGAGGAACCTGAAGTTGTTTTCAGGGAGGGTGGTGCCAGACCAGGTGGAAGAAACCTTTGAAAACTGGC

Homo sapiens        TGATCCAAGTCAATGAGGTCCTGCCAGATTGGAGTATGTCTGAGGAGGAAAAACTCAAGCGCTTGATGAAAACACTTA---GGGGCCCTGCCCGGGAGGTCATGCGTTTGCTTCAGGCGG
Loxodonta africana  TGATCCAAGTCAATGGGGTTTTGCCAGATTGGAATACCTCTGAGGAGGAAAAGCTCAAGCGCTTGATGAAAACCCTTACTTAGGGCCCTGCCAGGGAGGCCATGGGTTTGCTCCAGGCAG

Homo sapiens        CCAACCCCAACCTAAGTGTAGCAGATTTCTTGCGGGCAATGAAATTGGTGTTTGGGGAGTCTGAAAGCAGTGTGACTGCCCATGGTAAATTTTTTAACACCCTGCAGGCACAAGGGGAGA
Loxodonta africana  CCAACTCCAGCCTAAGTGTGGCAGGTTCCTTGCAGGCCATGAAACTGGTGTTTGGGGAGTCTAAAAGCAGTGTGACCACCCATGGTAAGTTTTTTAACACCTGGCAGTTGCAAGGGGAGA

Homo sapiens        AAGCCTCCCTTTATGTGATCCGTTTAGAGGTGCAGCTCCAGAATGCTATTCAGGCAGGCATCCTAGCTGAGAAAGATGCAAACCAGACTCGCTTGCAACAGCTTCTTTTAGGCGCTGAGC
Loxodonta africana  AAGCATCCCTTTATGTGATCCCTTTAGAGGTGCAACTCCAGAATGCTATTCAAGCAGGGAGCATATCTGAGAAAGATGCAAATCAGACTCTCCTGCCCCAGCTCCTTTGAGGGGCTGAGC

Homo sapiens        TGAATAGGGACCTGCGCTTCAGGCTTAAGCATCTTCTCAGGATGTATGCAAATAAGCAGGAGCGGCTTCCCAATTTCCTGGAGTTAATCAAGATGATAAGGGAGGAAGAGGATTGGGATG
Loxodonta africana  TAAACAGGGATCTGC-CTTTAGGCTTAAGGATCTTCTCAGGATGTATGCGAATGAGCAAGGGCAGCTGCC----------GAATTAATCAGGGTGATAAAGACAGAAGAGGATTGGGATG

Homo sapiens        ATGCTTTTATTAAACGGAAGCGGCCGAAAAGGTCTGAGCCAATAATGGAGAGGGCAGCCAGCCCTGTGGCATTTCAGGGCGCCCAGCCAATAGCAATCAGCAGTGCTGACTGTAACTGCA
Loxodonta africana  ACACTTTT--TAAACAGAAGCGGCCCAAAAGATCTGAGCCAATCCTGGAGAGGGCATCCAACCCTGTGGCATTTCAGGCCCCCCAGTTGATAGCAATTGGCAATGTT------AACTGCA

Homo sapiens        ACGTGATAGAAATAGATGATACCCTTGATGACTCTGATGAGGATGTGATCCTGGTGGTGTCTCTGTACCCTTCACTGACACCTACAGGTGCCCCTCCCTTCAGAGGAAGAGCCAGACCTC
Loxodonta africana  ACATGATACAGATAGAAGATACCCTCCATGACTCAGGTAAGGATGTGATCCTGGTGGAGCCTCAGGACCCTCCACTTTCATCCACAGCTGCTCCTCCCCCTAGATGCAGGGCCAGACCTC

Homo sapiens        TGGATCAAGTGCTGGTTATTGATTCCCCCAACAATTCTGGGGCTCAGTCTCTTTCTACCAGTGGTGGTTCTGGGTATAAGAATGATGGTCCTGGGAATATTCGTAGAGCCAGGAAGCGAA
Loxodonta africana  AGGATGAGGTGCTGGTCAGTGATTCCCCCAACAGTTCCTGGGCCTAGTCTTCTTCCACCAGTGGTAGTTCTGGGCATAAGAATGATGGTCCTGGGGATAAGTGTAGAGCCAGGAAGTGAA

Homo sapiens        AATACACAACCCGCTGTTCATATTGTGGGGAGGAGGGCCACTCAAAAGAAACCTGTGACAATGAGAGCAACAAGGCCCAGGTTTTGAGAATCTGATCATCACCCTGCAGGAGCTGACAC
Loxodonta africana  AATACACAACCCACTGTTCATATTGTAGCAAGGAGGACCATACAAAAGAAACATGTGTCAGTGAGAGGAATACGGCCTAGGTGTTTGAGAATCTGATCATCACTGTGCAGGAGCTGA---

Homo sapiens        ATACAGAGGAGAGGTCAAAAGAGGTCCCTGGAGAACACAGTGATGCTTCTGAGCCACAGTAAGGATCTAG--TCCAGCCCTAAATGAGTCCTTGACTGTATTCAGAGTCTGGTAATGGGA
Loxodonta africana  ---------------GAAAAGAAGTCCCCGGAGAAGACAATAA-GCCCTTGAGCCACAGTGAGGTGCTAGACCCCAACCCTAAATGAACCCTTAATTGTTCATAGAGTCCAGT-------

Homo sapiens        ATAACAGGAGAGGGGGGTGGGTTTCTAACTGCATGAATTAATCCACAAAGCAGTTTTCCTTTGGGAAG---GAGAAGAGGTCTTGCATACCAGCACAGTGGATGGGGAATGGTGTGACCT
Loxodonta africana  ------GGAACTGGGGGTGGGTT----------TGAATTAATCCACAAAGCATTTTTCTTTGGGGGAGGATGAGAATAGGTCTTGGATACCAGCACAGTGGA-GGAGGATGGCCTGACTT

Homo sapiens        CTGTCCCTGCTCTCTTCTCTGCTGGCTTAAGGGTC----TATTCTCCATGTGTCTATTTCTTTGATGACTTTATACTTTTTATGAAAGGAGCAT--CTTTTCATTAAACCTTCAAAAATA
Loxodonta africana  CTGTCCCTGCTCTCTCCCCCGCCTGCCTAAGAGTCTATTTATTCTCTGTGTGTTTGTTTCCCTGATGACTTTACTCTCTTGAAGAAAGGAGTATAGCTGTTTGTTAAACCTTGAAAACC-

Homo sapiens        AAGGAAGATACAAAAACTAAAGTACAAATTACCTGAAACTCAGCACCCTTTTGTAACCATTGTCAGCCCTTTGGTGAATGTCCTTCCAGATATCTCCCTGTACCTGTGTACCCAGATAGA
Loxodonta africana  ---TAAAACACAAAAACTTAAGTACCAACTACCTGAAACCCTCCACACATATATAACCATTGTCAACCCTTTGATGAATGTCCT---AGATATTTACCTA-ACCTTTGTACTCAGATAGG

Homo sapiens        -TATATGTATAGATAAGAGTGACCAAATATAAGTGCTGTTCTATACTGTGTAT--------------TTTTCACCAAATAATGTATCTCGTGGACTTC---TATGTCGATGAATATATAT
Loxodonta africana  CCACGTGATCAAATTAAAGTG-----------GTGGTGTTCTACACTCTGTATTTTCTTTTTGTTCTTTTTACCAAATAATGT-----GTGGACTTCTTTTATGTCAATGAATATATAT

Homo sapiens        AAATATCTAACTTCATGTCTCTGTGTGATGTTACTTTTAGAAAG--ATAAAGAAAAATGAAACTAAATATAGAAGCTATAAGGGGGGGTCATACCATGAGGAGGGGTTTTT-ATCGACCT
Loxodonta africana  TAGCATCTA---TAATGTCTCTGTGTTATAGTACTTTTAGGAAGAAATAAAGGAAAATAA--------ATAAAAGCTATAATTGGGGGTCTGATCATGGGGTGAGGTTTTTTATCAATTT

Homo sapiens        CATT-TATAGGAAAGGAGAAAGGAAGCTGAAATGGACTG-AATATCTCCCATAC-------------AACCCCTTAACACAACTGACCTGTCTCCTAGGTCATAGAGCCAACCC-CTGCA
Loxodonta africana  CATTCTACAAACAAGGAAAAGAGAAGATGAAACACCTTGTAATATTCCCCAAAGGTAAAAGGAAAAGAGCCCCTTACCACAACTGACCTGAC-CCTAGGTCATAGAGCCAAACTACGGCA

Homo sapiens        CCCATGAGTTAATAATGTTCCAATGTTTTCTTCTTGAACATATCATGAGACATCAGATGGGGAA---CGGGGGGGGGACAAAAATTGAAAATGGGTATTCCAGAGGCTCCATTCCTGTTT
Loxodonta africana  TTCATTAGTTGACAGTGCTCCAATATTTTCTTCTCTAATATATCCAGAGGAGTCTGACGGTGGAGTCTGCTGCCAAGGTAGTAGTTCTACATGGGGATTTCAGGGGCTTGATTTCTGTTC

Homo sapiens        GTATTGGGAAGGGACAACTACTCCTGCCCGATCTCTTGACCTCAGATTAGAGGGTGATAAAAGATGACATGTG--ACCTCTATCCCTGAGGGTCCCAA-GTTTCTGGTAGGTACAGTAGT
Loxodonta africana  ACTTTGCAAAAGGGCACCTTCTCCTGCTCTATCTCCTGACCTCATATAGGAGGGTTATAAAAGAGGACATGTGTCATCTCAGTTCCCGAGGGACCCTATATTTCTTGCAGGTGCTTAAGC

Homo sapiens        CCCTATCTTCACGAAGAGGTCAAAACTGGCAAGGA--GCAGGGGGGAATG----ATTTCATTTAAGGCA-------AGGAGTGGAGTGAAATGAATGC-------AGACCTGAGGACCTG
Loxodonta africana  CCCTATTCTTGTGA--------------GCAGGGAAGGCTGGGGGGTATGTGTGACTTCATTTAAAGCGGTGGGGGAGGGGGAGAATGAAATCAATGCCCTATGCGGACCTGAGGACCTG

Homo sapiens        AGGGAGTTTGCCTGGGAGGAAAGAGGGGGTCCCTGGGCTGACAA--GACAGGGTGTG
Loxodonta africana  AGAGAGTTTGTCATGCAGGCAA-CGGGTTCCACTGGGCTGAGAGAGGACAGGGCGTG


Mus musculus  ATTTTGGCTTCTCTCCACTAAGCCTTAATCTGACACTAGAAAGGGGGTGTTTAAAGGGTGTAACCCTTGAGGCTAGATGCCAGGGCTGGGGTGAGAACTGTTAATTCTGTGCTTCCTACC
Homo sapiens  GCTTTGGTTTTTCTCCACTATCCCTTAATCTGACAGAATAGAGGAG-TGCTGGCAAGGGGCAGCCTTTGAAGTCAGACATGGTGACTTGGGTGTGAGCTCTTAACTCTGCACTTCCTAAC

Mus musculus  CATACTGCCTAGGTGCTTACATCCTCCTCTCTGTTAAATGGGTGCACTGCTACGTTTTAGCGCAAGACTGATGAGAAGAATACGTGAGATGTCCGTGCACGCGCACAGGACACACGGAAA
Homo sapiens  TGTACTGCCTGGGTGCTTCAGTCCT--TTCCTGTTAAATGGGCACACTACTACCTCCT-TCACACGGCTGCTGAGAAGAGGAAAGGAGATGTCCATGCAGGCGCCTCTG-CACACAGAAA

Mus musculus  GAGCTCAGCAAATGCTAT---CCCCTTCCCCCCTATACAAAGGTGAAGAACTCTCATTGTTCACTTGGTTTCGGTCCCACCTCGTGTTTAAGGCATCTGGTCGCAAAGCATCGGAGGGGA
Homo sapiens  GCACTCAGTAAACGCTGTTTCCCCGCTTCCCCCTATACAAAGGTGAGGAACTCTCATTGTTCACTTGGTTTCTGTCCCACCTCGTGTTTAAGGTATCCGGTCTAAAAGC-CCGGAGGAGA

Mus musculus  GCCAAAGCAGAAGGCGATAGGAAGAAGGGAAGAGAGGGGA-AGCTGCAAACTCACGAATGAAATGTAAATGGGCCAGGTCCGTGAGTCCAAGCTGCACTTGACGCCGCGCCCGCTCCCGC
Homo sapiens  GCCGAGGTGTAAGGGGATAGGGAGAAGGGAAGAGATGGGTTAGCAGCAAACTCACGGATGAAAGGGAAATGGGCCCAGTCCGTGCGTCCGAGCTGC---TGACGCCGTGCCGCCTCCCGC

Mus musculus  GCCAGTCTGCCGCAGCCCCCTCTAGCAGGT--------------------TCCCCCCTCTGGCAGGCATTAGCATCCTGGCCC-AGGCTTTGCAGGGAGGCCGCCCCTGCGAACAGCTGA
Homo sapiens  GCCTGTCTGCCGCAGCCCCCGCCCGCCGGCCCGCCGGCCAGCCGGCCCGCTCCCCCTTCTTGCTAGCAGTAGCTTCGGGGCCGGAGGCTGTGCGGCGAGGCCGCCCCCTCGCCCCGCTGA

Mus musculus  TTGGCCACGCCACTACCTTCGTCACTCTCTTTTGTCTCAATGGGCAGAGACTAAAAGCCGCCGCTGCTGCTCTGCAGGCTGAAATTGAGGGCGGGGGCAGCTATAGCTGCCTTGGAAACC
Homo sapiens  TTGGCCGCGTCGCCCGGCCCGTCACGCTCTTTTGTCTCAGTCGCCAGAGACTGAAAGCAACCGCGGCTGCCCGGAGGGCCGAACTGGAGGGTGGGCGCAGCCTTGGCTGCCTTGGGAACC

Mus musculus  GCG--GCTGCGACTCTGCTACCCCTGGCTGGGCGACCCTGCAGAGAGCCTAACCCAGACAGCTCTGAG--GCT-----------GAGGCGCCA-----TCCTTTCCTCTGCAAGGCCTAC
Homo sapiens  GTGCTGCCTCGACTCGGCTACCCCTCGCTGGGCGGTCGTGCGCACTCGCTAGGTCGGGCAGCCCCGGGTCGCTTAGCGGCCGAGGAGGCGGCAGAGATCCCGTTCCCCTGCAGAGC-TAC

Mus musculus  G---------AGGTGAGACAGCTCTTCGCA-GCAGACCCTGAGCATCCTCTGCTCATCTACGAAG-----GTAAAGGGAG---GTGCAACTGCATAATGCGGAGATGGGAGGATGAAACC
Homo sapiens  GGGGACCGGAAGGCGAAACAGAGCTGTGGACGCGG-----GAGCACATGCTGCCCACCAGTGGAGCACAAGTAAAGAAAGAACGCGCGATTGCCCGGCGCGGAGAGGGGTGGGGGAAGCC
```

```
Mus musculus  TTAATA-----------------TTTTGAGCTTCCGAATTGGCAGCTTCGAGGCAAGGAAGTGAACACCCTAGTGGTTGGGCTATATGGTGGTATGAGGTGAGTTTCCT----------
Homo sapiens  CCACCAGGACTGGGCGGCGCCCGGCTTGGA------GAATCAGTAACTGCGAGGCAGGGGATGGAGCACTCCCATGGCCTGGTTGCGCTGAGAAAGGAGTTGTGGCTCATCTGGATGCTG

Mus musculus  -------------ACCTAGGATTTGCCTAGATTGGAGAGG--GGACTTTTTTTC-----------------TTCCAGAAAGGTAGCAAAAGACAATGAGAATGATTCACAATCCGT----
Homo sapiens  CCGACTGGGTAGGATTTATGATTTGCCTGGATTGAAGTGGGCGGGGGTGTTGTCGGGCGTCGGGGGCGGGTGTTCGGGGAGGTGGCAAGGGGCGGGAGAAGACGATCGCAAGCTGTCTTG

Mus musculus  ------TTGTCCTTTCTCTCATCTCTTCGGGAATAGAAATACGGAGGTGGGAGGGTATTGCAATTTCTCTTCTTTTTCTCCATAGCAGCTCATCAAAGCCGATTTCAAACGTGAATTCTG
Homo sapiens  AACCCCTTTTGCATCTCCTCATCTCTTCCTGGGAAGGAATATGGGGGTGGGAGGTCATC-CAACTTCTAGTCCCTTTCTCTCTGGCAGGTCATCAAAGCCGAATCCGAATTTGAATTCTG

Mus musculus  TGTGACTGATTGCAGAGCTGGTAGGCCGCTGTTTTCCCCGGGTGAAATGGATTGGGGGAGGGGTGGTC-----CCCATTGGCTTTTATTCTGAAGGTAGGGT------TTTTCCTCCTGG
Homo sapiens  TGCGGCTGATTGCAGAGCTGGTAGGCCGACGGTTT-CCCGCAGGAAATGTACGGGGGGCGGGAGTGCAGGAGGCACTTTGGCTTGTGTTCAGAAGATGGGAGAGGGAATTTTCCGCCTGG

Mus musculus  CGACAATGGCGCCTGTGAAGAAATGAT---------------GAGACTGCTGACGTCTCAGACCCTTCTCCTCCCTTCCTTTTGCTGTGGCGCAGTTTAGACTAGAGGCCCAGGGCACCT
Homo sapiens  CGACAGAGGAGGCGGTGGAGGGGAGGTATCAGGTTTTCAGAAGAGGCTGCT-----------CCCAACCCTTCCTTTCCTTCCTTCGCGGCACAGT-----CTTGAGGCCCTGAGGACTG

Mus musculus  GGATCGGAGAGCCT--GCGGTCAGAGATCAATCCTACCCGGACAAGAGCTA------TTGCGGAAAGCACCTGTTGCAGAGAGTAGGCTTGAAATAAAGGTGGAAGCCAGTTTCTGAGCT
Homo sapiens  GGATCTGCGACCCCCAGTGGACAAAAGTCGACTCTACCC---CAGAGGCTAATTTTCTCAAGGCAGGCACCTGTTG-AGAGGCTGCAAGTAAAATACAGGGGAACGCTAGTTTCCGAGCT

Mus musculus  CTGGTAAGAATCCTAGAAAAGGGGGAGGGGGCAGAGGGCGGGAGGGGGCAGAGGGCGGGGGGGGGGAGGAACTTGCTACCTAATAGATTTAAGGCCTTACTTATTAACATAATTCTGTAA
Homo sapiens  CATGTAAGAATCTGAGAAATAACGGGGGACGTTGACAAGGGGGTGTGATTGAGACCTGAGGCCATGAGG----------TTAATGGTTT----------CTTATTATCATAAATCTGTGA

Mus musculus  GGTTTACTCTACTGCCCTTTA-------TTTGAGAGCCTAGTAGGGGCTCCAAAGGTTGTTGTAGCTAGAATTCCAAGCAACAGTTGTTGCCTCTCCTAGAGAACAGGCAGTGGGGTGGG
Homo sapiens  -------CACACAACCATTGAGTTCCGTTTTGAGGGCCCAGTATCAGCTCCCAACACTATGTCATCTAGAATTAGAAGCAACAGTTGTCGCCCCTCCTGGAGTACAGGCAG---------

Mus musculus  TGGGGCGCAGTAGAGAGGGAGGAGATGTGACT--GACAAAGGCCCCACTAGCACACAAAAA--TTATTGCTCCTCAAGGTTGGGAAATGGCAGTTTCCTGCTGAGTAGTTTTAGGGCAGT
Homo sapiens  --------------GAGGGGATGGATGTGGCTCTGAAAAACTACCTACTAGCCCAGGGACACCTGCTAGCTCTGGAATGTTGT-AATTGCCAGTCTCCTGCAGGGTATTGTTAAGGCACT

Mus musculus  GGGCAAATAT--------------------TCAAGATGGTAGAAAATGTGATTAGAGGCTTGTTCCCAA--GTCTATTGCGTTATTGAATTTTTGTTTTGTCTTGTTTTGTTTTACAGTT
Homo sapiens  GGGCAGATATAAAATGCACTGCAAGGCTATTCAGGAAGATAGAGAATGCTACTGCAGACTGCTTCCCCACAGCCCACTATCTTATTAACCTTTTTTACTTTCCT---------TAGAATC

Mus musculus  TCTACCCAAACACAAGGAAGAGACTGCTGGGGAACGTTCCTTTGGAGGTTTCTGCAGATTGGACTGCTTTTGTTTGCAGAAAGTCCAGGCGTCTTGT---ATTCAGATATCCAATTGTTG
Homo sapiens  CCTACTGAAATATAAGGCAGGCACAGCCCAGGAACGTTGCTTTGGAGAATCCTGCAGATAAGGCT-------TTTCCAAAAAGCGCGAGCATCTTGTTGTATTCAGATACCCTATCGTCG

Mus musculus  TCAGGCATGGCTAGCCTCCTTGCTAGGATGGGTAACAGTCGCCGGCAGAATGCAGCTTTTATGCCTTTTGCCCATTCCATGCTGAGGGCCCTGGGGAGGAGCCTTGGTCCTTTGATTGCC
Homo sapiens  TCAGTCATGGCTAGCATCACTGCGTGTGTGGGTAACAGCAGGCAGCAGAATGCACCTTTGCCGCCTTGGGCCCATTCCATGTTGAGGTCTCTGGGGAGGAGTCTCTGTCCTTTAGTGGTC

Mus musculus  AACATTGCAGAGAGAAACATACAGTCGTTCTCTGGGAGGGCAGAGCTAGGACCCGGGGAAGAAACCTTTGAAAACTGGCTAAGCCAAGTCCATGAGGTCCTGCCAGATTGGCCTATGTCT
Homo sapiens  AAAATGGCAGAGAGAAACATGAAGTTGTTCTCAGGAAGAGTGGTGCCAGCCCAGGGGAAAGAAACCTTTGAAAACTGGCTGATCCAAGTCAATGAGGTCCTGCCAGATTGGAGTATGTCT

Mus musculus  GAAGAAGAAAAGATCAAGCGCCTGATGAGAACCCTTAGGGGCCCTGCCCGGGAGGCCATGCGTTTGTTCCAGGCTGACAATCCCAATCTAAATGTCGCAGAATTTTTGCGGGCAATGAAG
Homo sapiens  GAGGAGGAAAAACTCAAGCGCTTGATGAAAACACTTAGGGGCCCTGCCCGGGAGGTCATGCGTTTGCTTCAGGCGGCCAACCCCAACCTAAGTGTAGCAGATTTCTTGCGGGCAATGAAA

Mus musculus  CTGCTCTTTGGAGCGTCTGAGAGCAGTATAACAGCGCATGGCAAATTTTTAAGCACCCTGCAGGCACAGGGAGAGAAACCATCCCTGTATGTGATCCGTTTAGAGGTGCAGCTACAGAAT
Homo sapiens  TTGGTGTTTGGGGAGTCTGAAAGCAGTGTGACTGCCCATGGTAAATTTTTTAACACCCTGCAGGCACAAGGGGAGAAAGCCTCCCTTTATGTGATCCGTTTAGAGGTGCAGCTCCAGAAT

Mus musculus  GCTATCCAGGCAGGGGTCCTTCCTCAGAGCGAGGCAAACAGGACTCGCCTGCACCAGCTCCTTGTAGGGGCTGAGCTGAGTAGGGAGCTGCGCACCAAGCTTAAGGGGCTTCTCCAAATG
Homo sapiens  GCTATTCAGGCAGGCATCCTAGCTGAGAAAGATGCAAACCAGACTCGCTTGCAACAGCTTCTTTTAGGCGCTGAGCTGAATAGGGACCTGCGCTTCAGGCTTAAGCATCTTCTCAGGATG

Mus musculus  CATGCGCATAATGAGCAGGAAAACCTTCCTGACTTCCTGGAGTTGATCAGGATGATAAGGGAGGAAGAGGATTGGGATGAGACATTCCTTAGAAATAAGCGGCCCAGAAGATCTGAGACA
Homo sapiens  TATGCA---AATAAGCAGGAGCGGCTTCCCAATTTCCTGGAGTTAATCAAGATGATAAGGGAGGAAGAGGATTGGGATGATGCTTTTATTAAACGGAAGCGGCCGAAAAGGTCTGAGCCA

Mus musculus  GTGATGGAGCGGGCAGCCAGCCCTGTGGTCTTTCAAGGCTCCCTGCCTATAGTGATAGGCAGTGCCGACTGT------AATGTGATAGAGATAGATGATTCCCAGGATGATTCAGATGAA
Homo sapiens  ATAATGGAGAGGGCAGCCAGCCCTGTGGCATTTCAGGGCGCCCAGCCAATAGCAATCAGCAGTGCTGACTGTAACTGCAACGTGATAGAAATAGATGATACCCTTGATGACTCTGATGAG

Mus musculus  GATGTGATCCTGGTGGAGCCCGAGGACCCTCCACTCTCATCCCCAGGTGCCTCATCCCTCCGAGGCACAGCCAGTACTCAGGAAGAGATGCTGATTATTGAGTCCCCTGATGATTTTGAT
Homo sapiens  GATGTGATCCTGGTGGTGTCTCTGTACCCTTCACTGACACCTACAGGTGCCCCTCCCTTCAGAGGAAGAGCCAGACCTCTGGATCAAGTGCTGGTTATTGATTCCCCCAACAATTCTGGG

Mus musculus  GAGGAGTCTCCTTCCACTAGCAGTGGTTCTGGGCAAAGGAATAACGGGCCTGGGGATCTAGGTAGAACCAGAAAGCGCAAGTACCCAATCCGCTGTCCTCATTGTGGTGAAGAGGGCCAC
Homo sapiens  GCTCAGTCTCTTTCTACCAGTGGTGGTTCTGGGTATAAGAATGATGGTCCTGGGAATATTCGTAGAGCCAGGAAGCGAAAATACACAACCCGCTGTTCATATTGTGGGGAGGAGGGCCAC

Mus musculus  GCCAAAGAAACCTGTGACAATACCAGCAACAAGGGCCAGGTTTTTGAGAATTTGATAGTCACTCTGCAGGAGCTGACTCACAT---GGAGAGACCCAAA--------------------
Homo sapiens  TCAAAAGAAACCTGTGACAATGAGAGCAACAAGGCCCAGGTTTTTGAGAATCTGATCATCACCCTGCAGGAGCTGACACATACAGAGGAGAGGTCAAAAGAGGTCCCTGGAGAACACAGT

Mus musculus  ---CCCTCTGTACCATACTAAGGTGTGACCCTCGGCCCCAGATCAGTGTTTGAATCTCCTCAGAAGCTAATTCTGGAGGAAAAAG--GGATGGGGTGGGCTGCTGACATTAAGGAATTCA
Homo sapiens  GATGCTTCTGAGCCACAGTAAGGATCTAGTC-CAGCCCTAAATGAGTCCTTGACTGTATTCAGAGTCTGGTAATGGGAATAACAGGAGAGGGGGGTGGGTTTCTAAC-TGCATGAATTAA

Mus musculus  TTTAAAACAAGCTTTTTCTTTGGAGAAGAAAAAGCAAACTGGGGTACCA-TGCAACAGA-GAGGCATGGGCCCACATCATTCCTTGCTCATTCCATTAACTGCTTAATGGTTGTTTCTCT
Homo sapiens  TCCACAAAGCAGTTTTCCTTTGG-GAAGGAGAAGAGGTCTTGCATACCAGCACAGTGGATGGGGAATGGTGTGACCTCTGTCCCTGCTCTCTTCTCTGCTGGCTTAAGGGTCTATTCTCC

Mus musculus  GTGTGTC--TTTCTCTGGTGATTTTAATCTATCCGTG-AAGTGGTACCTTT---TTGAACCTTCCAAATGAAATTAAAAAGAAAAAAACTAAAGTGTGCATTACCTGAAAAATCTCACCC
Homo sapiens  ATGTGTCTATTTCTTTGATGACTTTATACTTTTTATGAAAGGAGCATCTTTTCATTAAACCTTCAAAAATAAA-GGAAGATACAAAAACTAAAGTACAAATTACCTGAAACTCAGCACCC

Mus musculus  TG-------AATTGTCAGCCCCTTGGTGAATGTCCTGC---GTATTTCCCTGTACCTGTGTGCCTCGGT--ATATATGTATTGATAAAAGTGATCACATATAAGTGCTGTTCTATAGTAT
Homo sapiens  TTTTGTAACCATTGTCAGCCCTTTGGTGAATGTCCTTCCAGATATCTCCCTGTACCTGTGTACCCAGATAGATATATGTATAGATAAGAGTGACCAAATATAAGTGCTGTTCTATACTGT

Mus musculus  GTATTTTTTCATCAGGCAGTATATATTGTGGACTTCTAAATAGATGA--ATATATAACCATCTAATGTCATGCATACTATAT-------CTTTGGTGAAGACAAATAAAAACTGAAACTA
Homo sapiens  GTA-TTTTTCACCAAATAATGTATCTCGTGGACTTCTATGTCGATGAATATATATAAATATCTAACTTCATGTCT-CTGTGTGATGTTACTTTTAGAAAGATAAAGAAAAA-TGAAACTA

Mus musculus  ATTGTAACAACTAC-------------------GGCAGGGGTGTT--------CTATTTA-AAGATAGGAG--GGGAAGTTGAAATGAGCTATATCTCTTTTCAAC-ACCTGTTGTAATT
Homo sapiens  AATATAGAAGCTATAAGGGGGGGTCATACCATGAGGAGGGGTTTTTATCGACCTCATTTATAGGAAAGGAGAAAGGAAGCTGAAATGGACTGAATATCTCCCATACAACCCCTTAACACA

Mus musculus  ACTGGCCTGCCTCCTAGTTCATACAGCCA---TCTGCACCTACTAGT---TAGGGTCTCAATG-----TCCTCAACCATAT--TGAGAAATC----------------------------
Homo sapiens  ACTGACCTGTCTCCTAGGTCATAGAGCCAACCCCTGCACCCATGAGTTAATAATGTTCCAATGTTTTCTTCTTGAACATATCATGAGACATCAGATGGGGAACGGGGGGGGGACAAAAAT

Mus musculus  TGACCATGG----CCCATAAGAGGAGCCACCCCTGTCTGG-------------------TTCTCCCTCACGTCTCAGCCTGCAATCAGAAGCTG----AAGCTGACCTCTGGTATCTGTC
Homo sapiens  TGAAAATGGGTATTCC---AGAGGCTCCATTCCTGTTTGTATTGGGAAGGGACAACTACTCCTGCCCGATCTCTTGACCTCAGATTAGAGGGTGATAAAAGATGACATGTGACCTCTATC

Mus musculus  TTTGAGCATTC-----TTCTTGTGGGTACATTAGTC-----TTTTACTGAGAAAAAAAAA---------------AAAGAATGACTGCATATAAGGCTGACAGCAGAGTGTAAATGAATG
Homo sapiens  CCTGAGGGTCCCAAGTTTCTGGTAGGTACAGTAGTCCCTATCTTCACGAAGAGGTCAAAACTGGCAAGGAGCAGGGGGGAATGATTTCATTTAAGGCAAGGAGTGGAGTG-AAATGAATG

Mus musculus  CCAATCTGAGGATCTGAAACATTTGTAAAAGATTAATTTTTATGTGAAGTATAGGACTTTTTGCCTGCATGTATTATGAGCCTACAGAGGCCAGAAGATGATGTCAGATCCCGTGTACCT
Homo sapiens  CAGACCTGAGGACCTGA.......................................................................................................

Mus musculus  GGAGTTTGTAAGTCACCATATGGGTAGGTACTGTGAACAAAATCTAGGTCTTTGAGGGCATAAAGTGCTCTTGTCTGATGAAATACCTCTGCGGCTGCAGTCTGAATCATTTTACAATAT
Homo sapiens  ........................................................................................................................

Mus musculus  AACAGGGTTTGCAGAGGTCTGTCTGTGAAGAGGGTGTACAGCACTGGTAGCTGGCATGTCAGGGACAGAAGTAGAGATCAGGTGCTACATGCAGGTTATACAGCCTTCTTGGGGATTCTG
Homo sapiens  ..............................AGGGTGTGCAGCAGCCAGAGCTGGCACCTCAGGGAAAGGAGCACAGA...........................................

Mus musculus  CACTCACATCTGGCCTTCGAGTGCCTAGAATCACCCTTCTTGGGTGGTCCTGCCTGGCCTCTTATCC----TGTTCCATCCCTACCCCCACAATATCATTTTGTACTC---CCCCTTTAC
Homo sapiens  ...........................................................TCCTATCCCCCTTGCTCCATCCATTTTATCACAGTCTCACTTTACCACCACTCCCCTTCCC

Mus musculus  TGAAGCCACACTCATTTCCTGGT-GATAGAGTT---GGCTCACACCCTTTTGTGTTTTCTGCCTATGACCATTTCCCCTGCTCCCAGCACTTCATGTTGTCCTAAAGCCT----------
Homo sapiens  TGGAGCCACACTCACCTCTTGGTGGAGGGAGATGGAGGCTCA----GCTTCATGTGCTCTGCCCATCACCACCTCCCCCTCCTCC-GCATTCCATGATGGCCTTAAGCCTCAGCCTCCTT

Mus musculus  -----TCAGGGCCAGAAGGAGAGTAGAAGG--CCCTGTGTTCCTGAGAT--------AGAGCTTGCCAGGGTCAAAGGTTCAAGGGGGAGGGACATTTCTTACATCTTTCTCAAATAATT
Homo sapiens  AATGCTCATGGCC---AGTAGACTGGGAGGGCCCCTCTGCCTCTGACCTTTTAGCTGAGAACTGAGCAGGAGTAAAGGTCCAA----GAGACCCACTTTTTGCATCCCTCTGGGGCACCT

Mus musculus  CTGGGCACTTGGCAGTCATATCCATATTTGAATAGTCAAAATGGGCCCCTCCCCTACCTCTTCCTGCCTAGGTCACTCAGGCCTACAGAGTTACAGGGTCCTCAACC---------TTCT
Homo sapiens  CT-GGCTCCTGGCAGCCACCTCCATCTCACCACAGGCAAAGAGGACCCCACCACCCACGCAGGAGGC--AGCTTCATCAAGCCCCCAAAGCCACAGGGTCCCCCAACACTACCTGGCTCT

Mus musculus  TTCAGATTTATAGCTCTTACCTTTCTCTATAATCAAATCTACCCCAATAG----------------------CCAGAGTATAAGATCATTTCTGCACGGGCTGCTCATCTTTTCAT-CTT
Homo sapiens  TACAGCCCCCTA-TTCCTCCCTACCTCTCCCA--AAGTCTATCCTTGGAGGGGATTTGCCCACTCATCTCTCCAAGATTAGAATATAGTTCCC---CTTGCTGCTCACCTTTTCTCAAAT

Mus musculus  TTACTCTAT--------TTGGGGGGTATTTCCCCAGAC------TTACAGGTTCCATTAGAAAGTAGCTAGCTTTAAGA-AGAAATATATGCTATACCCACACTGCCATCCTTCTT---G
Homo sapiens  TCACTCTATAAATGCTATTGAATG--ATTTACCTAGATATGCTGTCATAATTTCTTTCTGAAACCAGGTAGAGTGAAAAGAGGAATAAATGCTACACCTACCC--CCATCCTCCTTTTCA

Mus musculus  CTGCTATTACCTTGGAGGTCCAGTGTGGACTGCTGATTGGAAAAAGCTATAGCTTTTATATTCTGTAGCACATCCTCTTGTCTTCCTTTCCCATCCT---TTCAACTATACAAACCTGAG
Homo sapiens  CTGGAGTTAGCTTGGAGGTTCA----AGATCATTACTTTGAAA------------CCCTGCTGTGTAATTCATCCCCTTTCCTTCC-CCCCCATCCTGTCCTCTGTTGTGTAAACCTGAA

Mus musculus  GGTGTGTGAATTTTCATCTCACCAAAAATGGTGCAAAGCTCATGGTTTCTATTTTACTTTATGGGACACTGAGTTTTCCTTTATTGAAGAAACATGTACAGACTTCATGTGTGACAACTG
Homo sapiens  ACTGTCTAAATTTTTACTTCACC-AAAATGATGCAAAAGTGAGACTTTTATTTCATTTTGTGTCACACTG-GCTTTCCTTTATTCAAGAAGTATGTA.......................
```

```
Mus musculus  GCCTAGGACCTAGTACCTGTCACCCTTTTAGTGGCTGCTGAGCCCTGCCAACTACCTAGTGCTTGCTGCTGCCCTTAAGGCCATGCCCTGGAGCTCTTTGATAAAGCAGCCCTAAACGTG
Homo sapiens  ........................................................................................................................

Mus musculus  CTGCAGCCTCCAGAGTTTAGAAATGAAAAAATTTTATCTTTAGCTTCAACCTTGAAGACACTTCTGTTCTTTAAAGCTTTAAGGATCACAGTTCCTATCTGGCTATTTTACAATGAAATC
Homo sapiens  ........................................................................................................................

Mus musculus  TTATATTTGAAGATGCTGTTGGAATGTCCAATAGAGACAGCTATTTTTATGCTGCAATTATTGCTGTTCACGTGGTTCTGGCCCTGTTTGTCTATGTGGCCTGGAATGAAGGCTCATGGT
Homo sapiens  ........................................................................................................................

Mus musculus  GGCATGGAGGCAAACAGGACTAAAGTGAACATCACCTTTTGATCACATGAAATTTATTTTTGGAATATGTTAAACACTTCTGGGATATTGATAATAAATATATTGTTTGAATAAAGTTGA
Homo sapiens  ........................................................................................................................

Mus musculus  AAGGACATGTTTAAAAATAAAGAACTATGTACAAACTGGGTACAGTGGTATACACCTTTAATTCCAGCACTCTGGAAGCAAGAGGCAAGTGGATCTCTATGAGTTCAAGGCCAACATGCT
Homo sapiens  ........................................................................................................................

Mus musculus  CTACAGAGTGAGTTCCAGGACATCCTGGGGTGACATGGAGAAACCCTGTCTCAAAATAATAGAAATAAAAACTAAAAAGAGAACTATGTCTGAGGAGAAAGCCTGGCATTTCTGCCTTCA
Homo sapiens  ........................................................................................................................

Mus musculus  TTCTGTGTTCGTCTCCTTTTCCTTCCTGTTACCTGTTTACCTCTGCACATAGGCATGCATGTAAATCACTCAAGGAATGCCGCTGTCCATTCTTTCATCCCTTTTTATGGCTAAAGGCAT
Homo sapiens  ........................................................................................................................

Mus musculus  AGAGTAAATCAGTGTCCTAATCCTACAGTCACCATGGATTGTAGGAACAGCAGAAGCTCACTAGAAAGCCGGTCCTTCAGATGAGGGCAAGTCTCTATGACAGTACAGCTGTATGCTAGT
Homo sapiens  ........................................................................................................................

Mus musculus  AGGGAAATAATCAACATCAGCGAACCAGATTGACTCTCTCAAGCAATTACATACAGCAATAAGGACTTCATCAGACCACCCTGCAGGGCTGGGACTGAGGTGATATGATAAACTAGGCCA
Homo sapiens  ........................................................................................................................

Mus musculus  CACTCAGAGGAGGCCTGCTTTAATTACACATCTCCCGC
Homo sapiens  ......................................


Homo sapiens           GAAAGAAAGAAAAGTACCAAGAAGAGTTTCCTCTTCTGTACAATGGGCACCATAAAGGTTGCCCTCATCTTCCCTCAGCCCTCTCCCCACTACCTCCTCACCCCCTCCCTCAGCACTATT
Dasypus novemcinctus   --------GAAAGGTGCC----TTAGTTTCCTCTTCTGAACAACGAGTGCCATAAAAGTTGCCTTCCTCCTCTCTCCACTCTTTCTCCAC----------------------------C

Homo sapiens           ATCACATTGAGACTGCTGTTGAGATGGTTTGAGACTACCTGTGCAAAAATACTCTCCAGGCAGCGCTGCAGGGCAACGAATTAGCATCTAGAGAGCAACAGAC---GGTGCTAGGTTGGA
Dasypus novemcinctus   ATCACACAAAGGCTGCTATTGAGATAGTTTGAGACCATGTATGTAAAATTGAATTCTAGGCCATGAAGCAGGGCAGAGCGT--GCATCTGGAG---AACAGACCTGGGTGCAAAACCGAG

Homo sapiens           TGCTGTAACTTATTTGCTGCAGGATACAGGCCAAAACTAGTAACCTGTCTCTCTCTGGCTCAGTTTCTTCATCTGTGAAATGGAGATAATGGTTC-TTGCTCACCAATTCTATGCAATCC
Dasypus novemcinctus   TGCTGATAATTATATGCTGCATGATGTACAGCAAGATGCATAACCT------------CTCAGTTTCCTTATCTGTGAAATGGAGGTAATAGTCCATTGTTCACTAATTCTAT--AGTCC

Homo sapiens           CAAGTTGGTCCTCCCAT-CTGGCTTTGGTTTTTCTCCACTATCCCTTAATCTGACAGAATAGAGGAGTGCTGGC-AAGGGGCAGCCTTTGAAGTCAGACATGGTGACTTGGGTGTGAGCT
Dasypus novemcinctus   CAAGTAGGCCCTCCCATCCCAGCTTTGGCTGCTCTCTACTACCCCTTAATCTGGCAGTAGAGAGGAGCGTTTACAAAGGGGCAGTC----AAGT--------GGGACTTGGATGTGCGCT

Homo sapiens           CTTAACTCTGCACTTCCTAACTGTACTGCCTGGGTGCTTCAG-TCCTTTCCTGTTAAAT-GGGCACACTACTACC-TCCTTCACACGGCTGCTGAGAAGAGGAAAGGAGATGTCCATGCA
Dasypus novemcinctus   CTTAGTTCTGCACTTCCTAACCACTGTGCCTGGATGCTAAAGTTCCCTCTCTATTAAATGGGGCATACTGCTACCTTCCTTCACAAGGCTGCTGAGAGGATTAAATGCAAGTTCCCTGCA

Homo sapiens           GGCGCCTCTGCACACAGAAAGCACTCAGTAAACGCTGTTTCCCCGCTTCCCCCTATACAAAGGTGAGGAACTCTCATTGTTCACTTGGTTTCTGTCCCACCTCGTGTTTAAGGTATCCGG
Dasypus novemcinctus   TGCTCAACAGCACACAGAAGGAG----GTAATTCATTTTTTCCCCTTTTCCCCTATATAAAGGTGAGGAACTTTTATTGATCACTTGATTTCGGCCCCACC------ATAAGGCATCCAG

Homo sapiens           TCTAAAAGCCCGGAGGAGAGCCGAGGTGTAAGGGGATAGGGAGAAGGGAAGAGATGGGTTAGCAGCAAACTCACGGATGAAAGGGAAATGGGCCCAGTCCGTGCGTCCGAGCTGCTGACG
Dasypus novemcinctus   ACTCAAAACGCGGAG.........................................................................................................

Homo sapiens           CCGTGCCGCCTCCCGCGCCTGTCTGCCGCAGCCCCCGCCCGCCGGCCCGCCGGCCAGCCGGCCCGCTCCCCCTTCTTGCTAGCAGTAGCTTCGGGGCCGGAGGCTGTGCGGCGAGGCCGC
Dasypus novemcinctus   ........................................................................................................................

Homo sapiens           CCCCTCGCCCCGCTGATTGGCCGCGTCGCCCGGCCCGTCACGCTCTTTTGTCTCAGTCGCCAGAGACTGAAAGCAACCGCGGCTGCCCGGAGGGCCGAACTGGAGGGTGGGCGCAGCCTT
Dasypus novemcinctus   ........................................................................................................................

Homo sapiens           GGCTGCCTTGGGAACCGTGCTGCCTCGACTCGGCTACCCCTCGCTGGGCGGTCGTGCGCACTCGCTAGGTCGGGCAGCCCCGGGTCGCTTAGCGGCCGAGGAGGCGGCAGAGATCCCGTT
Dasypus novemcinctus   ........................................................................................................................

Homo sapiens           CCCCTGCAGAGCTACGGGGACCGGAAGGCGAAACAGAGCTGTGGACGCGGGAGCACATGCTGCCCACCAGTGGAGCACAAGTAAAGAAAGAACGCGCGATTGCCCGGCGCGGAGAGGGGT
Dasypus novemcinctus   ....................................................................................AGAAAGGGGGCGTGATGGCCCGGCGCTGAGAGCGGA

Homo sapiens           GGGGGAAGCCCCGCCAGGACTGGGCGGCGCCCGGCTTGGAGAATCAGTAACTGCGAGGCAGGGGATGGAGCACTCCCTTGGCCTGGTTGCGCTGAGGAAGGAGTTGTGGTTCATCTGGAT
Dasypus novemcinctus   GAGAGAAGTCCCGCAGGCCCTGGGCTACTCGTG-CTTGGCCACTCGATAACCGCGAGGCTGGGCCAGGGGCACTCTCGTAGCCTGGCTCCGCTGCGGGAGGAGGTGGGGCTCGACTGTAT

Homo sapiens           GCTGCCGACTGGATAGGATTTAGGATTTGCCTGGATTAAGGTGGGTGGGGGTGTTGTCGTAGGTCGGGGGTGTTGTTGGGTGTCGGGGGCGGGGTGTGTTCGGGGAGGTGGCAAGGGGCG
Dasypus novemcinctus   GCTGCCGAAGAGGTAG--------ATTTGCCTGGATTGAGGCAGG--GGGGTGTTG---------------GTAGTGCATTTTCGTGGGGGGGG--------GGGGGGCCCTGAAAGGCA

Homo sapiens           GGAGGAGACGATCGCAAGCTGTCT----TGAACCCCTTTTGCATCTCCTCATCTCTTCCTGGGAAGGAATATGAGGGTGGGAGGTTATCCAACTTCTAGTCTCTTTCTCCCTGGCAGGTC
Dasypus novemcinctus   GGGGGA----ATCCCAAACTGTCTGACGTGAACGCCCCTCTCCTCTCCTCATCTCTTCCTGGGGAGGAATATGTGGGTGTGCA--TGTCCAATCCCTTTTCCCTTTTTCCGCGGCAGGCC

Homo sapiens           ATCAAAGCCGAATCCGAACTTGAATTCTGTGCGGCGGATTGCAGAGCTGGTAGGGCGAGGGTCTCCCGGAGGAAA-TGTAAGGGGGATGCGCGGGAGGGCAGGAGGCACTTTGGCTTG-C
Dasypus novemcinctus   GTCAAAGCCGACTCC------------TGCGTGGCCGATTGCAGAGCTGGTAGGCCGACGGTTTCCGGGGGGGAAGTGTAGTTGGG--------GGAAGCGGGCAGCACTTCGGCTTGGC

Homo sapiens           GTTCAGAAGATGGGAGAGGGAATTTTCCGCCTGGCGACAGTGGAGGCGGTGGAGGGGAGGTATCAGGTTTTCAGATGAAGCTGTTTCCAACCCTTCCTTTCCCTCCTTCG----------
Dasypus novemcinctus   ATTCAGG---TGGGAGAGAGAATTTTCTGCCTGGCGACTTCGGAGGGAGGGGAGGGGGGGC.............AGGAAGCTCCTTCAGACGCTTCGGTTCCTTTCTCTGACCGCCGCTG

Homo sapiens           CGGCACAGTCTTGAGGCCCTGAGGACTGGGATCTGCGACACCCAG--TGGACAAAAGTCGGCCGTACCCCAGAGGCTAATTTTCTCACGGCAGGCACCTGTTGCAGAGGCTGCACGTAAA
Dasypus novemcinctus   CAGCGCAGGCTTGACGCCCTATGGACCCGGATC-----CACCCGGGGTGGACCGAGGCCCACCACACCCCCT-GGCGAGCTTTCACCCAG--GGCGC-TGTGCCAGGGGCTGCAAGTGAA

Homo sapiens           ATAAAGGCGAACGCTAGTTTCCGAGCTCATGTAAGAATCTGAGAAATAACGGGGGAAACGTTGACAAGGGGGTGTGATTGAGACCTGAGGCCATGAGGTTAATGG----TTTCTTATTAT
Dasypus novemcinctus   ATAAAGGGTCGTGCTGGTTCCTGAACTTACGTAAGAATCGGAGAG---------------------GGGGGTGTGGTTGAG-------GCCCTGCGGTTAATGAGATCTTATTTATTCT

Homo sapiens           CATAAATCTGTGACACACAA-CCATTGAGTTCCGTTTTGAGGGCCCAGTATCAGCTCCCAACACTATGTCATCTAGAATTAGAAGCAACAGTTGTCGCCCCTCCTGGAGTACAGGCAGGA
Dasypus novemcinctus   CACAAATCCGTGAGACTTAGGCCACTGCGCTCTATCCTGAGAGCCCAATATTGGCTTCGCACACAATGACACCTAAAAGGCAAAGTAACAGTTGCCGTCCCTCTTGGATGATTGGCGGGA

Homo sapiens           GGGGATGGATGTGGCTCTGAAAAACTACCTACTAGCCCAGGGACACCTGCTAGCTCTGGAATGTTGTAATTGCCAGTCTCCTGCAGGGTATTGTTAAGGCACTGGGCAGATATAAAATGC
Dasypus novemcinctus   GGGAATGGATGTGGCTCTGAAAGGCGCTCCACTGGC--------ACCTG----------------GTAATTGCTAGTCTTCTGCTGCTTATTTTAAGGGCATTGGGCAGATATAAAATGT

Homo sapiens           ACTGCAAGGCTATTCAGGAAGATAGAGAATGCTACTGCAGACTGCTTCCCCACAGCCCACTATCTTATTAACCTTTT-------TTACTTTCCTTAGAATCCCTACTGAAA--TATAAGG
Dasypus novemcinctus   ACTGCAGGG-TATTTAGGACGGGAGAGAATGTGGCTGCAGATGGTTTTCCTGCAGCTTGCTACCTTATTGATCTTTTCTTTTTCTTTTTTGCTTTACAATCCCTGTTGAGAGATACAAGG

Homo sapiens           CAGGCACAGCCCAGGAACGTTGCTTTGGAGA---ATCCTGCAGATAAGGCTTTTCCAAAAAGCGCGAGCATCTTGTTGTATTCAGATACCCTATCGTCGTCAGTCATGGCTAGCATCACT
Dasypus novemcinctus   AAGGCAGAGCCAAAGGCC-----TTTGGGGATTCCTCCTGCAGATTAGGCTTTTCTAAAAATCCTGAGCATCTTGTTGTATTCAGATAGGTGATCATTGTCGATCATGGCAAGCATTATT

Homo sapiens           -GCGTGTGTGGGTAACAGCAGGCAGCAGAATGCACCTTTGCCGCCTTGGGCCCATTCCATGTTGAGGTCTCTGGGGAGGAGTCTCTGTCCTTTAGTGGTCAAAATGGCAGAGAGAAACAT
Dasypus novemcinctus   TGCACATGTGGGTAACAGCAGGCAGGGAAATGCACCCTTGCTGCCTTGGGCCCATTCCATGCTGAGGTCCCTGGGGAGGAGTCTTGGTCCTTTAATAGGCAACATGACTGGGAAAAACAT

Homo sapiens           GAAGTTGTTCTCAGGAAGAGTGGTGCCAGCCCAGGGGAAAGAAACCTTTGAAAACTGGCTGATCCAAGTCAATGAGGTCCTGCCAGATTGGAGTATGTCTGAGGAGGAAAAACTCAAGCG
Dasypus novemcinctus   GAAGTTGTTTTCCGGGAAGGTAGTGCCAGTCCAGGGGGAAGAAACCTTTGAAAACTGGCTGATCCAAGTCAGTGGGATCCTGCCAGATTGAAATATGTCTGAGGAGGAAAAGCTCAAGTG

Homo sapiens           CTTGATGAAAACACTTAGGGGCCCTGCCCGGGAGGTCATGCGTTTGCTTCAGGCGGCCAACCCCAACCTAAGTGTAGCAGATTTCTTGCGGGCAATGAAATTGGTGTTTGGGGAGTCTGA
Dasypus novemcinctus   CTTGATGAAAACTCTTAGGGGCCCTGCCTGGGACATCATGTGATTGCTTCCGGCAGCCAACCCCACCTTAAGTGTGGCTGATTTCTTGCAGGCAATGAAATTGGTGTTTGCGGAGTCTGA

Homo sapiens           AAGCAGTGTGACTGCCCATGGTAAA--TTTTTTAACACCCTGCAGGCACAAGGGGAGAAAGCCTCCCTTTATGTGATCCGTTTAGAGGTGCAGCTCCAGAATGCTATTCAGGCAGGCATC
Dasypus novemcinctus   AAGCAGTGTGACTGCCCATGGTAAATTTTTTTTAACACCTTGCAGGTTCAGGGGGAGAAAACTTCCCTTAATGTGATCTGTTTAGAGGTGCAGCTCCAGAATGCTATTCAGGCTGGGATT

Homo sapiens           CTAGCTGAGAAAGATGCAAACCAGACTCGCTTGCAACAGCTTCTTTTAGGCGCTGAGCTGAATAGGGACCTGCGCTTCAGGCTTAAGCATCTTCTCAGGATGTATGCAAATAAGCAGGAG
Dasypus novemcinctus   ATACCTGAGAAAGATGCAAACCAGACTCGCCTGCACCAGCTCCTTTTAGGGGCTGAACTGACTAGGAACCTGCACTTCAGGCTTAAGGATCTTCTCGGGATGTATGCAAATGAGCAGGGG

Homo sapiens           CGGCTTCCCAATTTCCTGGAGTTAATCAAGATGATAAGGGAGGAAGAGGATTGGGATGATGCTTTTATTAAACGGAAGCGGCCGAAAAGGTCTGAGCCAATAATGGAGAGGGCAGCCAGC
Dasypus novemcinctus   CAGCTTCCCAGTTTCCTGGAGGTAATCAGGATGATAACAGAGGAAGAGGATTGGGATGGCAATTTTATTAAACGGAAGCGACCCACAAGATC------------AGAGAGAGGGGCCAGC

Homo sapiens           CCTGTGGCATTTCAGGGCGCCCAGCCAATAGCAATCAGCAGTGCTGACTGTAACTGCAACGTGATAGAAATAGATGATACCCTTGATGACTCTGATGAGGATGTGATCCTGGTGGTGTCT
Dasypus novemcinctus   CCTGTGGCATTTCAGGACCCCCTGATGACAGCCATGGGCAGTGCTG------ACTGCAATGTGATAGAGATAGATGACTC----------------GAGGATGTGATCCTGGTGAGATCT

Homo sapiens           CTGTACCCTTCACT---------GA----CACCTACAGGTGCCCCTCCCTTCAGAGGAAGAGCCAGACCTCTGGATCAAGTGCTGGTTATTGATTCCCCCAACAATTCTGGGGCTCAGTC
Dasypus novemcinctus   TGGGACCCTCTACTTTCATCCTCGAACCCCACCCCCACCCCCCACCCCCACCCGATGCAGGGCCAGACCCCAGGATCATATGCTGCTCATTGATTCCCTCAGAAGTTCCTGGGCCCAATC

Homo sapiens           TCTTTCTACCAGTGGTGGTTCTGGGTATAAGAATGATGGTCCTGGGAATATTCGTAGAGCCAGGAAGCGAAAATACACAACCCGCTGTTCATATTGTGGGGAGGAGGGCCACTCAAAAGA
```

```
Dasypus novemcinctus  TCCTTCCACCAGTGCTGGTTCTGGATATAAGCAAGATGGTCTTGGGAATAAGTATAGAGCCAGGAAGCAAAAATACACAATCCACTGTTCATACTGTAGTGAGGAGGGCCACTCCAAAGA

Homo sapiens          AACCTGTGACAATGAGAGCAACAAGGCCCAGGTTTTTGAGAATCTGATCATCACCCTGCAGGAGCTGACACATACA---GAGGAGAGGTCAAAAGAGGTCCCTGGAGAACACAGTGATGC
Dasypus novemcinctus  CACCTGTGACAATGAGAGCAACCAGGCCCAGGTGTTTGAGAATCTGCTTATCACTCTGCAGGAACTGATTAAAGCAAAGGAGGAAGGGTCAAAAGAGGCCCCTGGTGAACCCAATGACTC

Homo sapiens          TTCTGAGCCACAGTAAGGATCTA-GTCCAGCCCTAAATGAGTCCTTGACTGTATTCAGAGTCTGGTAATGGGAATAACAGGAGAGGGGGGTGGGTTTCTAACTGCATGAATTAATCCACA
Dasypus novemcinctus  CTCTGAGCAGCAGTGAGATGCTAGGCCCTGCTGTAAATGAACATTTACCCATATTCAGAGCCTGGTGATGAGAA-AACTGGAG----------------AATTGCATGAATTAATCCACA

Homo sapiens          AAGCAGTTTTCCTTTGG---GAAGGAGAAGAGGTCTTGCATACCAGCACAGTGGATGGGGAATGGTGTGACCTCTGTCCCTGCTCTCTTCTCTGCTGGCTTAAGGGTCTATTCTCCATGT
Dasypus novemcinctus  AAGGGGCTTTATTTTGGAGAAGAGGAGAACAGGCCTTGGATACCAGCAAACCA---AGGGGATGGCCTAATCTCTATTCCTGCTCTATCCCACCCTG-------------TTC----TGT

Homo sapiens          GTCTATTTCTTTGATGACTTTATACTTTTTATGAAAGGAGCATCTTTTCATTAAACCTTCAAAAATAAAGGAAGATACAAAAACTAAAGTACAAATTACCTGAAACTCAGCACCCTTTTG
Dasypus novemcinctus  GTTTATTTCCTTGATGGCTTTATTTTCTTTGTGA----AGTCTAACTCTGTTAAATGTTCACAAAGATA--AAAATATAAAAACTAAAGTAAAAATGACCTTGA-------ACACCTATA

Homo sapiens          TAACCATTGTCAGCCCTTTGGTGAATGTCCTTCCAGATATCTCCCTGTACCTGTGTACCCAGATAGATATATGTATAGATAAGAGTGACCAAATATAAGTGCTGTTCTATACTGTGTATT
Dasypus novemcinctus  TAGCTATGGTCAACCCTTTGATGAATGTCCTTCTAGATATTTCCCTATATCTTC---------TAGATATATGTATAGATTAAAGTGATCAAATATACATGTTGCTCTATTTGCTGTATT

Homo sapiens          -TTTCACCAAATAATGTATCTCGTGGAC----TTCTATGTCGATGAATATATATAAATATCTAACTTCATGTCTCTGTGTGATGTTACTTTTAG--AAAGATAAAGAAA-AATGAAACTA
Dasypus novemcinctus  ATTTCACCAAATAATTTATGTTGTAGACCTCTTTCTATGTAAATGAATATATATCAGCATCTATTTTAATGTCTCTGTGTAATACTACTTTTAGGAAAAAATAAAGGAACAATAAAGCTA

Homo sapiens          AATATAGAAGCTATA-----AGGGGGGGTCATACCATGAGGAGGGGTTTTTATCGACCTCATTTATAGGAAAGGAGAAAGGAAGCTGAAATGGACTGAATATCTCCCATACAACCCCTTA
Dasypus novemcinctus  TCATAAGAAGGTACACTGGTGGGGGAGGTCATGCCGTGAGGTGGAGTTTTCACC---CCATTTTATAAATGAGGGAAAGGGAA----------ACCAATTATCTCCCAATGAGCCCATTA

Homo sapiens          ACACAACTGACCTGTCTCCTAGGTCATAGAGCCAACCCCTGCACCCATGAGTTAATAATGTTCCAATGTTTTCTTCTTGAACATATCATGAGACATCAGATGGGGAACGGGGGGGGGACA
Dasypus novemcinctus  CCATAACCGACCTCTCTCCGAGGTCATATAGCCAAACTCTGTGCCCAC---CTAATATTGTCCCAATGCTTGCTTCTCAGAGGAGTCATGA.............................

Homo sapiens          AAAATTGAAAATGGGTATTCCAGAGGCTCCATTCCTGTTTGTATTGGGAAGGGACAACTACTCCTGCCCGATCTCTTGACCTCAGATTAGAGGGTGATAAAA-GATGACA--TGTGACCT
Dasypus novemcinctus  ...........................................................ACTCCTGTCCCATTTTCTGGCCTCAGATTGGAGATTGATAAAAAGAGGGCAAGTGTGACCT

Homo sapiens          CTATCCCTGAGGGTCCCAAGTTTCTGGTAGGTACAGTAGTCCCTATCTTCACGAAGAGGTCAAAACTGGCAAGGAGCAGGGGGGAATGATTTCATTTAAGGCAAGGAGTGG--AGTGAAA
Dasypus novemcinctus  CAGACACTGGGGGTCCCATGTTTCTTGTAGGTACATTAGTCCCTATCCTTGTGAGCAGGACTAGACTAGT-GTGTGTGTGGGGGGGTGACTTCCTTTAAGGCAGGTAGCAGATAGTGAAA

Homo sapiens          TGAA--TGCAGACCTGAGGACCTGAGGGAGTTTGCCTGGGAGGAAAGAGGGGGTCCCTGGGCTGACAAGACAGGGTGTG
Dasypus novemcinctus  TGAATGTGCAGATCTGAGAACCCAAGGTTGTTTATCAAGAAGGAAAT-GTACTCTACTGGGCTGAGAGGACA--GTATG
```

```
Homo sapiens  GAAAGAAAGAAAAGTACCAAGAAGAGTTTCCTCTTCTGTACAATGGGCACCATAAAGGTTGCCCTCATCTTCCCTCAGCCCTCTCCCCACTACCTCCTCACCCCCTCCCTCAGCACTATT
Bos taurus    --------------------------TTTCTTATTCTGTACAATGGGTACCATACAAGTTTCCTACCTCTTCCCCTACCCCTCTTTGCGTTGCCTCTCCAAGCCCTCCTTCAGCAC----

Homo sapiens  ATCACATTGAGACTGCTGTTGAGATGGTTTGAGACTACCTGTGCAAAAA-----TACTCTCCAGGCAGCGCTGCAGGGCAACGAATTAGCATCTAGAGAGCAACAGACGGTGCTAGGTTG
Bos taurus    -----ACTAAGACTGCAGCTGAGCTGGTTTAAGACCACTCATGTAAAAAAAAAATGCCCTCTAGGCAGCTCAGATCGGCAAAAAGTTGGTATTTGGAAAACAACAGA----GTAAAACCG

Homo sapiens  GATGCTGTAACTTATTTGCTGCAGGATACAGGCCAAAACTAGTAACCTGTCTCTCTCTGGCTCAGTTTCTTCATCTGTGAAATGGAGATAATGGTTCTTGCTCACCAATTCTATGCAATC
Bos taurus    GATGCTGATATTTATTTGCTGTGTGATGTGGGGCAAGCCGCCTAACCT---------TGTGCCTGTTTCCTCATCTGAAAAATGGAGATAATAGTCGTTGCTCACTAATTCTATATAGTT

Homo sapiens  CCAAGTTGGTCCTCCCATCT-------GGCTTTGGTTTTTCTCCACTATCCCTTAATCTGACAGAATAGAGGAGTGCTGGCAAGGGGCAGCCTTTGAAGTCAGACATGGTGACTTGGGTG
Bos taurus    CCAAATAGGCCTTCCCATCTCCACTTTGGCTTTGGCTTTTCTCAGCTAACCCTCAGTCTGAGAGTAGAGAGGAGT------AGGGGGCAGCCTTTGAAATCAGACTTGTAGACTTGAGTG

Homo sapiens  TGAGCTCTTAACTCTGCACTTCCTAACTGTACTGCCTGGGTGCTTCAGTCCTTTC-CTGTTAAATGGGCACACTACTACCTCCTTCACACGGCTGCTGAGAAGAGGAAAGGAGATGTCCA
Bos taurus    TGAGCTCTTACCTCTGCACTTCCTAAGAATAGTGACTGGGTGCTTAAGTCCCCTCTCTGTTAAGTGGGCATACTGCTACCTCCTTCGCAAGGCTGTTGAAAATA---AACGAGTTGTCCG

Homo sapiens  TGCAGGCGCCTCTGCACACAGAAAGCACTCAGTAAACGCTGTTTCCCCGCTTCCCCCTATACAAAGGTGAGGAACTCTCATTGTTCACTTGGTTTCTGTCCCACCTCGTGTTTAAGGTAT
Bos taurus    TGCAAGCCCATTTGCACGTAGAAAGCGCTCAGTAAATGCTTTTGCCCC--TTTCCTCTGTACAAAGGTGAGGAACTTTCACTGTTCGCTTGATGTCTGTCCCACCTCGTGTTGAAGGCAT

Homo sapiens  CCGGTCTAAAAGCCCGGAGGAGAGCCGAGGTGTAAGGGGATAGGGAGAAGGGAAGAGATGGGTTAGCAGCAAACTCACGGATGAAAGGGAAATGGCCCAGTCCGTGCGTCCGAGCTGC-
Bos taurus    CTGGTCTCAAAGCGTGGAGGA---CACAGGTGGAAGAGGTGAAAGAGAAAGAAAGCGAGGGGCAAGCAGCAAACTCACGG-TAAAATGCCAATCGGCAGGGTCTGAGAGTCCGAGCTGCA

Homo sapiens  --TGACGCCGTGCCGCCTCCCGCGCCTGTCTGCCGCAGCCCCCGCCCGCCGGCCCGCCGGCCAGCCGGCCCGCTCCCCCTTCTTGCTAGCAGTAGCTTCGGGGCCGGAGGCTGTGCGGCG
Bos taurus    CTTGACACCGCACCGGCTTCCGCGCCTGTGTGCCGCAGCCCCCGCC-------------------GGCCTGCTACTCCTTTTTGCTAGCAATGGCC-CTGAGCAGGAGGCTGCGCGGGG

Homo sapiens  AGGCCGCCCCCTCGCCCCGCTGATTGGCCGCGTCGCCCGGCCCGTCACGCTCTTTTGTCTCAGTCGCCAGAGACTGAAAGCAACCGCGGCTGCCCGGAGGGCCGAACTGGAGGGTGGGCG
Bos taurus    AGGCCGCCCCCTCGCCCCGCTGATTGGCCTCGCCAACCGACTCATCATTCTCTTT-GTCTCAAACCGCAGAGGTTAAAAGAAGCGGCGGCTGCTGGGAGCTACCAGCGAGTGGGCGGGGG

Homo sapiens  CAGCCTTGGCTGCCTTGGGAACCGTGCTGCCTCGACTCGGCTACCCCTCGCTGGGCGGTCGTGCGCACTCGCTAGGTCGGGCAGCCCCGGGTCGCTTAGCGGCCGAGGAGGCGGCAGAG-
Bos taurus    CGGCCGCTGCTGCCTTGGGAACCGAGCTGCTTCGACCCAGCTACCCAAAGCTGGGTGA--------AGAGGCTCCGTCTGGAGGCTCTGAGTTGGTTAGCGGTAGAGGAGGCGGCAGAGG

Homo sapiens  -------ATCCCGTTCCCCTGCAGAGCTACGGGGACCGGAAGGCGAAA--CAGAGCTGTGGACGCGGGAGCACATGCTGCCCACCAGTGGAGCACAAGTAAAGAAAGAACGCGCGATTGC
Bos taurus    AGGCGTCATCCTTTTTCCCTGCAGGGCCACGGCGACCGGAAGGCGGCATCCACACCTGTCCTCGCCGGAGCATTCGCTGTCCACCGGCCGAGCACA-GTAACGAAAAAGGGCGCGATTGC

Homo sapiens  CCGGCGCGGAGAGGGGTGGGGGAAGCCCCGCCAGGACTGGGCGGCGC-CCGGCTTGGAGAATCAGTAA--CTGCGAGGCAGGGGATGGAGCACTCCCTTGGCCTGGTTGCGCTGAGGAAG
Bos taurus    CTGTCCCGGAGAAAGGAGGGAAGAGCTCCATCAGGATTGGGCGGCTCTCCTGTTTACCAAATCGCGAGGTCTGAAAGGCAGCCGAAGGAGCACTCCCGCGGCCCGGCTGCGCAGAGACAG

Homo sapiens  GAGTTGTGGTTCATCTGGATGCTGCCGACTGGATAGGATTTAGGATTTGCCTGGATTAAGGTGGGTGGGGGTGTTGTCGTAGGTCGGGGGTGTTGTTGGGTGTCGGGGGCGGGGTGTGTT
Bos taurus    GAGGTG-GGTTCTACTGAATGATGCCAAATGGGTAGGATTT-------GCCTAGATT--GACAGGAGAACGTTTT---------CAGGGGGATAGTTAGG------GGGCGGG-------

Homo sapiens  CGGGGAGGTGGCAAGGGGCGGGAGGAGACGATCGCAAGCTGTCT----TGAACCCCTTTTGCATCTCCTCATCTCTTCCTGGGAAGGAATATGAGGGTGGGAGGTTATCCAACTTCTAGT
Bos taurus    ------------AAGCAGCGGG-------GATCGCAGGCTGTCTGCCTTGAACCCCCTTTGCATCTCCTGATCCCTTCCTGGTGAGGCATATGTGGGCGTGTG--TGTCCAACTCCCATT

Homo sapiens  CTCTTTCTCCCTGGCAGGTCATCAAAGCCGAATCCGAACTTGAATTCTGTGCGGCGGATTGCAGAGCTGGTAGGGCGAGGGTCTCCCGGAGGAAATGTAAGGGGGATGCGCGGGAGGGCA
Bos taurus    CCCTCTCTCCGTGGCAGACCACCAAAGTCGAATTCGAACTCGATTTCTGTGCGGCTGATTGCGGAGCTGGTAGGACAACGATTTCCCGGAGTGGAT-TCAGGGGTATGTAATAGAGGGT-

Homo sapiens  GGAGGCACTTTGGCTTGCGTTCAGAAGATGGGAGAGGGAATTTTCCGCCTGGCGACAGTGGAGGCGGTGGAGGGGAGGTATCAGGTTTTCAGATGAAGCTGTTTCCAACCCTTCCTTTCC
Bos taurus    -----CACTTTGGCTTGTATTCACAAGGTGGGAGAGGGAATTCTCCGTCTGGCTACTGTGGAGG--GAGGGGTCGAGGGAGCGAGATGAGGGATGAAACTCCTTCTAACCCTTCCCTTCC

Homo sapiens  CTCCTTCGCGGCACAGTCT-TGAGGCCCTGAGGACTGGGATCTGCGACACCCAGTGGACAAAAGTCGGCCGTACCCCAGAGGCTAATTTTCTCACGGCAGGCACCTGTTGCAGAGGCTGC
Bos taurus    CTGCCCTGCTTTGCCCTCCGCCATGGCCTGAAGACTGGGATCTTCAATCTCGCGTGGACAGAGGTCAACC-TACCTCGGAATCCAGCTTTCTAACT-CAAGCTCTTGTTG----------

Homo sapiens  ACGTAAAATAAAGGCGAACGCTAGTTTCCGAGCTCATGTAAGAATCTGAGAAATAACGGGGGAAACGTTGACAAGGGGGTGTGATTGAGACCTGAGGCCATGAGGTTAATGG-----TTT
Bos taurus    ---TGAAATAAAGGCGCGCACTTGTTTCCAAGCTCATATAGGAATCTGAGAAATAACGGGGGAAACTTTGAGAAGGGGGTGTGGTTGAGACCTGAAACCGTCAGTTTCATGGAGATCTTA

Homo sapiens  CTTATTATCATAAATCTGTGACACACA-ACCATTGAGTTCCGTTTTGAGGGCCCAGTATCAGCTCCCAACACTATGTCATCTAGAATTAGAAGCAACAGTTGTCGCCCCTCCTGGAGTAC
Bos taurus    TTTGCTACCACAAATCTGTGAGACTTAGACCACTGCTCTCTATTTTGAGAGCCCAATATGGGCTCTAAGCACTATTTCACCTAGAATTCTAAGCCGCTGTTGCCTTCCTTCCTGGAGGAT

Homo sapiens  AGGCAGGAGGGGATGGATGTGGCTCTGAAAAACTACCTACTAGCCCAGGGACACCTGCTAGCTCTGGAATGTTGTAATTGCCAGTCTCCTGCAGGGTATTGTTAAGGCACTGGGCAGATA
Bos taurus    TCTCGGGAGAGGATAGTTGCAGATCTGAAAAGCACCCTGC-AGCACGGGAACACCCATTAACTCCTGAAGGTTGTAATTGCCAGTTTCCTGCAGAATATTTTCAGGGCAGTAGCCGAATG

Homo sapiens  TAAAATGCACTGCAAGGCTATTCAGGAAGATAGAGAATGCTACTGCAGACTGCTTCCCCACAGCCCACTATCTTATTAACCTTTTTAC------TTTCCTTAGAATCCCTACTGAAATA
Bos taurus    GAAAATGTACCCGCAGGGTGTTCAGGAACGTAAAGGAAGCGACCGCAAACTGCTTCAT-GCAGTCTACTACCTTACTAACCTTTTCCCCCCTCCTTTTCTTTACAGACCCTACTGAGATA

Homo sapiens  TAAGGCAGGCACAGCCCAGGAACGTTGCTTTGGAGA---ATCCTGCAGATAAGGCTTTTCCAAAAAGCGCGAGCATCTTGTTGTATTCAGATACCCTATCGTCGTCAGTCATGGCTAGCA
Bos taurus    CAAGGAAGATGGAGCCCAGGAACGTTGCTTTGGGGATTTCTCCTGCAGATCAGGCTTTTCTAGAAAGGCTGAGCATTTTGTTACATTCATATAGACGATCATTGTCAGTCATGGCCAGCA

Homo sapiens  TCACTGCGTGTGTGGGTAACAGCAGGCAGCAGAATGCACCTTTGCCGCCTTGGGCCCATTCCATGTTGAGGTCTCTGGGGAGGA------GTCTCTGTCCTTTAGTGGTCAAAATGGCAG
Bos taurus    TCATTGCACGTGTGGGTAACAACTGGCAGCAGAATACAGCCTTGCCACCCTGGGCCCGTTCCATGTTGAGGTCCCTGAGGGGGAAGGGGAGTCTTGGTCCTTCAAGGGTCAACATGGCAG

Homo sapiens  AGAGAAACATGAAGTTGTTCTCAGGAAGAGTGGTGCCAGCCCAGGGGAAAGAAACCTTTGAAAACTGGCTGATCCAAGTCAATGAGGTCCTGCCAGATTGGAGTATGTCTGAGGAGGAAA
Bos taurus    AAGGAAAGATGAAATTGTTCTCCGGGAGGGTGGTGCCAGCCCAGAAGGAGGAAACCTTTGAAAACTGGCTGATACAAGTCAATGGGGCCCTGCCAGATTGGAATATGTCTGAATAGGAAA

Homo sapiens  AACTCAAGCGCTTGATGAAAACACTTAGGGGCCCTGCCCGGGAGGTCATGCGTTTGCTTCAGGCGGCCAACCCCAACCTAAGTGTAGCAGATTTCTTGCGGGCAATGAAATTGGTGTTTG
Bos taurus    AGCTCAAGCACTTGATGAAAACCCTGAGGGGCCCCACACGGGAGGTGATGCGTTTACTGCAGACAGCCAACCCCAACCTCAGTGTGGCAAATTTCTTGTGTGCCATGAAGTTGGTGTT-G

Homo sapiens  GGGAGTCTGAAAGCAGTGTGACTGCCCATGGTAAATTTTTTAACACCCTGCAGGCACAAGGGGAGAAAGCCTCCCTTTATGTGATCCGTTTAGAGGTGCAGCTCCAGAATGCTATTCAGG
Bos taurus    GGGATTCTGAAAGCAGTGTCACTGCCCATGGCAAATTTTTTAACACCTTGCAGGCACAAGGGGAGAAAGCCTCCCTTTGTGTGATCCGTTTAGAGGTGCAGCTCCAGAACGCTATTCAGG

Homo sapiens  CAGGCATCCTAGCTGAGAAAGATGCAAACCAGACTCGCTTGCAACAGCTTCTTTTAGGCGCTGAGCTGAATAGGGACCTGCGCTTCAGGCTTAAGCATCTTCTCAGGATGTATGCAAATA
Bos taurus    CTGGGATCATAGCTGAGAAAGATGCAAATCAGACACAGCTGCACCAGCTCCTTTTAGGGGCTGAGCTGAATGGGGACCTGTTCTTCAGGCTGAAGAATCTTCTCAGGATGTATGCAAATG

Homo sapiens  AGCAGGAGCGGCTTCCCAATTTCCTGGAGTTAATCAAGATGATAAGGGAGGAAGAGGATTGGGATGATGCTTTTATTAAACGGAAGCGGCCGAAAAGGTCTGAGCCAATAATGGAGAGGG
Bos taurus    AGCAGGAGTGTCTCCCCAATTTCCTGGAGTTAATCAGGACGATAAGGGAGGAAGAGGATTGTAATGACACTT----------GAAGCGGCCCAAAAGATCTGAGCTAATCATGGAGACGG

Homo sapiens  CAGCCAGCCCTGTGGCATTTCAGGGCGCCCAGCCAATAGCAATCAGCAGTGCTGACTGTAACTGCAACGTGATAGAAATAGATGATACCCTTGATGACTCTGATGAGGATGTGATCCTGG
Bos taurus    CAGTAAGCCCTGTGGCATTTCAGGGCCCCCAGGCAATAGCCATCAGCACTGCTGATTG------CAATGTGATAGAAGTAGATGATACCCTTAATGACTCACACGAGGATGTGATCCTGG
```

```
Homo sapiens  TGGTGTCTCTGTACCCTTCACTGACACCTACAGGTGCCCCTCCCTTCAGAGGAAGAGCCAGACCTCTGGATCAAGTGCTGGTTATTGATTCCCCCAACAATTCTGGGGCTCAGTCTCTTT
Bos taurus    TGGAGTCTCAGAACCCTCCACTTACATCCATAGGTGCCCCTCCCCTCAGGGTCAGGGCCAGACTTCAGGATCAAATACTGGTCATTGATTCCCTAAACAGTTCTCAGGCTCAATCTCCTT

Homo sapiens  CTACCAGTGGTGGTTCTGGGTATAAGAATGATGGTCCTGGGAATATTCGTAGAGCCAGGAAGCGAAAATACACAACCCGCTGTTCATATTGTGGGGAGGAGGGCCACTCAAAAGAAACCT
Bos taurus    CTACCAGTGGTGGTTCTGCATATAACAATGATGGTCCTGGGGATATATGTAGAACCAGGAAGAGAAAATATACTATCCACTGTTCATACTGTGGTGAGGAGGGCCACTCAAAGGAAACCA

Homo sapiens  GTGACAATGAGAGCAACAAGGCCCAGGT-TTTTGAGAATCTGATCATCACCCTGCAGGAGCTGACACATACAGAGGAGAGG---TCAAAAGAGGTCCCTGGAGAACACAGTGATGCTTCT
Bos taurus    GTGACAGTAAGAGCAACAAGGCCCAGGTGTTTTGAGAATCTGATCATCACCCTGCAAAAGCTGACACATACAGAGGAGGAGACATCAAAAGAGATCCCAGGCAAGCACAGTGACCTCTCC

Homo sapiens  GAGCCACAGTAAGGATCTAG-TCCAGCCCTAAATGAGTCCTTGACTGTATTCAGAGTCTGGTAATGGGAATAAC-AGGAGAGG--GGGGTGGGTTTCTAACTGCATGAATTAATCCACAA
Bos taurus    GAGCCACAGTAATGTGCTAGACCCAGCCCTAAGTGAACCCTTAACTGTATTCAGAGACTGCTGATGGGGAAAACTAGGGTAGGCTGGGGGAGGCTTCTATGTGCATGAATTAATCCACAA

Homo sapiens  AGCAGTTTTCCTTTGGGAAGGAGAAGAGGTCTTGCATACCAGCACAGTGGATGGGGAATGGTGTGACCTCTGTCCCTGCTCTCTTCTCTGCTGGCTTAAGGGTCTATTCTCCATGTGTCT
Bos taurus    AGTGGCTTTCTTTGGGGA---AGAAGAGATTGTAGATACCAGCAAAAAGAA-----GACAGCCTGACCTCTGTCCCTGCTTCTCCCTCCATCTGCCTAAGGGTCTATG---TGTGTGTCT

Homo sapiens  ATTTCTTTGATGACTTTATACTTTTTATGAAAGGAG--CATCTTTTCATTAAACCTTCAAAAA-TAAAGGAAGATACAAAAACTAAAGTACAAATTACCTGAAACTCAGCACCCTTTTGT
Bos taurus    ATTTCCTTGATGATTGTGTTCCTTCTGTGAAAGGAGTACAAGGTATTGTTAAACTTTCAAAGACCGAAGGAAACTACAAAAACTGAAGTGCAAATTACCTGAAACTCCCCACCCTTGCAT

Homo sapiens  AACCATTGTCAGCCCTTTGGTGAATGTCCTTCCAGATATCTCCCTGTACCTGTGTACCCAGATAGATA-TATGTATAGATAAGAGTGACCAAATATAAGTGCTGTTCTATACTGTGTA-T
Bos taurus    AACCGTTGTCAGTCCTTGGATGAATGGCCTTCTAGATATCTCCCTATTCCTGTGTACCAAGATAGATATTATATACAGATAAAAGTGATCAAATATAAGTGTTATTCTATGCTCTGTATT

Homo sapiens  TTTTCACCAAATAATGTATCTCGTGGACTTCTATGTCGATGAATATATAT-AAATATCTAACTTCATGTCTCTGTGTGATGTTACTTTTAGAAAGATA-AAGAAAAAT------GAAACT
Bos taurus    TTTTCACCAAACAATATATGTTGTGGACTTTTATGTCAATTCATAAGCATCAAGTAT---GCTTGCTGTCTCTGTGTGAGATTACTTTTAGGAATACAGAAGGAAAATAATAAGAAAACT

Homo sapiens  AAATATAGAAGCTATAAGGGGGGG----TCATACCATGAGGAGGGGTTTTTATCGACCTCATTTATAGGAAAGGAGAAAGGAAGCTGAAATGGACTGAATATCTCCCATACAACCCCTTA
Bos taurus    AAATACAGAAGCTCTAAATGGGGGGAACTCATACTGTGGAG------TTTTATCAACCTCATTTACAGATGAGGAAAATGGAAGCTGAAAGGAGC----TATCTCCCAAAGAGTCCCCTA

Homo sapiens  ACACAACTGACCTGTCTCCTA--------------------------GGTCATAGAGCCA-ACCC------CTGCACCCATGAGTTAATAATGTTCCAATGTTTTCTTCTTGAACATAT
Bos taurus    TCACAACTGACCTATCTCCCAATGCAGGGGCCCTGGGTTTCAATTCCTGGTCAGGGAACTATATCCCATATGCCACAACGAAGAGTTAACAATGCCCCAGTGCTTTCTTAGCTAATACAT

Homo sapiens  CATGAGACATCAGATGGGGAAC-GGGGGGGGGACAAAAATTGAAAATGGGTATTCCAGAGGCTCCATTCCTGTTTGTATTGGGAAGGGACAACTACTCCTGCCCGATCTCTTGACCTCAG
Bos taurus    CTTGAGAAATCTGATGACAGACAGGGGAGGGGGCAATAATAGGAAATGGGTATTTCAGGGGCTCCATTCCTGTTCACTTTGGGAAGGGGCGGTGTTCCCTTCCC...............

Homo sapiens  ATTAGAGGGTGATAAAAGATGACATGTGACCTCTATCCCTGAGGGTCCCAAGTTTCTGGTAGGTACAGTAGTCCCTATCTTCACGAAGAGGTCAAAACTGGCAAGGAGCAGGGGGGAATG
Bos taurus    ........................................................................................................................

Homo sapiens  ATTTCATTTAAGGCAAGGAGTGGAGTGAAATGAATGCAGACCTGAGGACCTGAGGGAGTTTGCCTGGGAGGAAAGAGGGGGTCCCTGGGCTGACAAGACAGGGTGTG
Bos taurus    .............CAAGAAGAAATGAATGGTCAGTGCAGATCTGAGAACCTGAGGGAGTT-GTCAGGAAGGAACTAGGGG..........TAAGAGGACAGGGTGTG
```

```
Mus musculus              ATTTTGGCTTCTCTCCACTAAGCCTTAATCTGACACTAGAAAGGGGGTGTTTAAAGGGTGTAACCCTTGAGGCTAGATGCCAGGGCTGGGGTGAGAACTGTTAATTCTGTGCTTCCTACC
Canis lupus familiaris    ACTTTGGCTTTTCTCCACTAACTTTCAGTCTGAGAGTATAGAGAAG-TGTTCAGAAGGGGGAGCCTTCGAAATCAGATATGTCGACTTGGGTGTGAGCTCTTAGTTTTGCACTTCCTAAC

Mus musculus              CATACTGCCTAGGTGCTTACATCCTCCTCTCTGTTAAATGGGTGCACTGCTACGTTTTAGCGCAAGACTGATGAGAAGAATACGTGAGATGTCCGTGCACGCGCACAGGACACACGGAAA
Canis lupus familiaris    CACACTGCTTGAATGCTGAAGTGC-CCTCTCTGTTAAACCGGCACACCGCTATCTCCTC-CACAGGGCTGCTCAGAAGAATAAATGAGACGTCCGTGCAAACGCATTCG-CAAGCAGAAA

Mus musculus              GAGCTCAGCAAATGCTATC-CCCTTCCCCCCTATACAAAGGTGAAGAACTCTCATTGTTCACTTGGTTTCGGTCCCACCTCGTGTTTAAGGCATCTGGTCGCAAAGC---ATCGGAGGGG
Canis lupus familiaris    GTACTCAGTAAATACTTTCTCCCCTTCCCGCTACACAGAAGTGAGGGGCTCTTATTGTTCACTTGATTTCTGTCCCACCCCGTGTTTAAGGCATCTGGTCTCAAAGAGCGAAGGGAGGAG

Mus musculus              AGCCAAAGCAGAAGGCGATAGGAAGAAGGGAAGAGA-GGGGAAGCTGCAAACTCACGAATGAAATGTAAATGGGCCAGGTCCGTGAGTCCAAGCTGCACTTGACGCCGCGCCCGCTCCCG
Canis lupus familiaris    AGTCGAGATGGAAGGGGTGAGGGAGAAAGAATGAGAAGGGGAAGCAGCAAACTCACGGGTAAAATGTAAATCGGCCCGGTCGGTGCGTCTGAGCTGCACTTGACACCGCGCTGGCTCCCG

Mus musculus              CGCCAGTCTGCCGCAGCCCCCTCTAGCAGGTTCCCCCCTCTGGCAGGCATTAGCATCCTGGCCC--AGGCTTTGCAGGGAGGCCGCCCCTGCGAACAGCTGATTGGCCACGCCACTACCT
Canis lupus familiaris    TGCCCGTGTGCCGCAGCCCCCGCCAGCTGGCTCCCCCCTTTTGCTAGCAATGGCCTCGGGGGCCGGAGGCTGAGAGGGGAGGCCTCCCCCGCGCCCCGCTGATTGGCCGCGCCGCCCCGC

Mus musculus              TCGTCACTCTCTTTTGTCTCAATGGGCAGAGACTAAAAGCCGCCGCTGCTGCTCTGCAGGCTGAAATTGAGGGCGGGGGCAGCTATAGCTGCCTTGGAAACCGCGGCTGC--GACTCTGC
Canis lupus familiaris    TCATCAAGCTCTATTGTGTCAATGGGCAGAGGCTAACCGAAGCGGCGGCTGCTGGGAGGGCCCAGCCGGTGGGCGGGGGCGGCCGCGGCTGCCTTGGGAACCGCG-CTGCTAGACTTGGC

Mus musculus              TACCCCTGGCTGGGCGACCCTGCAGAG-AGCCTAACCCAGACAGCTCT-------------GAGGCTGAGGCGCCATCCTTT-CCTCTGCAAGGCCTACGAGGTGAGACAGC--TCTTCG
Canis lupus familiaris    TACCCCTTGCTGGGCGGCTGTGGGGAGCAGTTCCATCCAG-CAGCTCCCGGTCGCTTGGCGGCGGAGGAGGCGTCATCCTTTTCCCCTGCAGGCCCGCGGAGACTGGAAGGCGGCATTCA

Mus musculus              CAGCAGACC----CTGAGCATCCTCTGCTCATCTACGAAG-----GTAA---------------------AGGGAGGTGCAACTGCATAATGCGGAGATGGGAGGATGAAACCTTA---
Canis lupus familiaris    CAGCTGTCCAAGCCTGAGCATCCGCTGCTCACCCGCGGAGCGCAGGTAACAAAAAAAAGGCGGGCGGGGTGGGGGGGACGCGATAGCCCAGCACGGAGAGAGGAAGTGGAAGCTTTACCT

Mus musculus              ATATTTTGAGCTTCC---------GAATTGGCAGCTTCGAGGCAAG----GAAGTGAACACCCTAGTGGTTGGGCTATATGGTGGTATGAGGTGAGTTTCCTACC--------------
Canis lupus familiaris    GTACTTGGCGGCTCCCCACTTGGGGAATCGGGAACTGCGAGGTTGGTTGAGGATGGAGCAATCCCGTGGCCCCGCGGTGCTGAGGGAGGGGGTGGGGTTTTTGCTGGATACAGCTAATGG

Mus musculus              -TAGGATTTGCCTAGATTGGAGAGGGGA----CTTTTTTTCTTCCAGAAAGGTAGCAAAAGACAATGAGAATG-ATTCACAATCCGTTTGT--------CCTTTC-----TCTCATCTCT
Canis lupus familiaris    GTAGGATTTGCCTGAATTGAAAGGGGGAGGGGCGTTTCT-------GAGGGGTCATAGG-GACAAGGAGGAGATAATCACAAGCTGTCTGCTTTGAACCCCTTTTGCATTTTCCAACTCT

Mus musculus              TCGGGAATAGAAATACGGAGGTGGGAGGGTATTGCAATTTCTCTTCTTTTTCTCCATAGCAGCTCATCAAAGCCGATTTCAAACGTGAATTCTGTGTGACTGATTGCAGAGCTGGTAGGC
Canis lupus familiaris    TTCCGGGGAGGAATATGTGGGTGTGCGTGT---TCAACTCTTATTCCCTTTCTCCGTGGTAGGCCATCAAAGCCAAATCCGAACTTGAATTCTGTGCGGCTTATTGCGGAGCGGATAAGC

Mus musculus              CGCTGTTTTCCCCGGGTGAAATGGATTGGGGGAGGGGTGGTCCCCATTGGCTTTTATTCTGAAGGT------AGGGTTTTTCCTCCTGGCGACAATGGCGCC-----------TGTGAAG
Canis lupus familiaris    CAATGATTTCCCAGGG-GACATGTAATGGAAGGGG--------TCACTGACTTGTATTCAAGAGGTGGGAGAAGGAATTTTCCGTCTGGCTACAGCGGAGACGGGAGTGAGGGTGGGGAG

Mus musculus              A----------AATGATGAGACTGCTGACGTCTCAGACCCTTCTCCTCCCTTCCTTTTGCTGTGGCGCAGTTTAGACTAGAGGCCCAGGGCACCTGGATCGGAGAGCCT--GCGGTCAGA
Canis lupus familiaris    ACAGCAGAATGAGAGATAAGGCTGCTTCTGACTCTT-CCCTTCTCTGTCCTGCCCAACAAGACGAAGCAGTCTTGACT-----CCCTGAGGACTGGGATCTGCAAACCCCAGTGGACAGA

Mus musculus              GATCAATCCTACCCGGA---CAAGAGCTATTGCGGAAAGCACCTGTTGCAGAGAGTAGGCTTGAAATAAAGGTGGAAGCCAGTTTCTGAGCTCTGGTA--AGAATCCTAGAAAAGGGGGA
Canis lupus familiaris    AGTCGA-CCTACCCTGAAGGCGAGCTTTCTCATGGAGGGCACCTGTTGCAGAGGCTGGAAGTGAAATTAAGGCATACACTTGTTTCCGAGTTTACGTTTTATAATCCAAGAATTGAGGGA

Mus musculus              GGGGGCAGAGGGCGGGAGGGGGCAGAGGGCGGGG--------------------GGGGGGAGGAACTTGCTACCTAATAGATTT-----AAGGCCTTACTTATTAACATAATTCTGTAA
Canis lupus familiaris    GGAATC-----TCGGAGAAGGGTGCATGGTTGAGATCTTTAATTTTCAAATATTTGAGTATAG----TTGACATCCAGTGATTTTTACCTGATTTCTTACTTATTATCACAAAACTGTGA

Mus musculus              GGTTTACTCTACTGCCCTTTATTT-GAGAGCCTAGTAGG-GGCTCCAAAGGTTGTTGTAGCTAGAATTCCAAGCAACAGTTGTTGCCTCTCCTAGAGAACAGGCAGTGGGGTGGGTGGGG
Canis lupus familiaris    GATTTAGACCATTGTACTCTATTTTGAGAGCCCAACAAACAACTCCACACACTAGTTCCCCTAGAATTCAAAGCAACAGTTGCCCTCCCTCCTGGAGGACTGGCTG-------------

Mus musculus              CGCAGTAGAGAGGGAGGAGATGTGACTGACAAAGGCCCCACTAGCACACAAAAATT--ATTGCTCCTCAAGGTTGGGAAATGGCAGTTTCCTGCTGAGTAGTTTTAGGGCAGTGGGCAAA
Canis lupus familiaris    ---------GAAGGGATGGATGTAACT---------CCTGCTAGCACAAGGACACCCAGTAGCTCCAGAAAGTTCT-AACTGCCAATTTCCTGCAGGATATTTTTACGGCAGTGAGCAAA

Mus musculus              TAT-------------------TCAAGATGGTAGAAAATGTGATTAGAGGCTTGTTCCCA--AGTCTATTGCGTTATTGAATTTTTGTTTTGTCTTGTTTTGTTTTACAGTTTCTACCCA
Canis lupus familiaris    TATAAAATGTACTGCAAGGTATTCAGGGAAGTAGA----GTGACTGCAGACCACTTCCCGGCAATCTACTACCTTATTGACCTTTTTTA----CTCCTTTTTCTTTACAGTCACTATTGA

Mus musculus              AACACAAGGAAGAGACTGCTGGGGAACGTTCCTTTGGAGG---TTTCTGCAGATTGGACTGCTTTTGTTTGCAGAAAGTCCAGGCGTCTTGT---ATTCAGATA--------------T
Canis lupus familiaris    GATACAAG-AAGCCAGAGCCCAGGAGCATTGCTTTGGGGAATCCTCCTACAGATTAGGCT-------TTTCTAAAAAGCCTGAGCATCTTGTTATATTCAGATAGAAGACCGTCATCCAT

Mus musculus              CCA--------ATTGTTGTCAGGCATGGCTAGCCTCCTTGCTAGGATGGGTAACAGTCGCCGGCAGAATGCAGCTTTTATGCCTTTTGCCCATTCCATGCTGAGGGCCCTGGGGAGGAGC
Canis lupus familiaris    CCATCAAGGCTAGCGTCATCCATCAAGGCTAGCATCGTTGCACATGTGGGTAATAGCCGGCGGCAAAACACACCCTTGCCGCCTTGGGCCCATTCAATTTTGAGGTCCATGGGAAGGAGT

Mus musculus              CTTGGTCCTTTGATTGCCAACATTGCAGAGAGAAACATACAGTCGTTCTCTGGGAGGGCAGAGCTAGGACCCGGGGAAGAAACCTTTGAAAACTGGCTAAGCCAAGTCCATGAGGTCCTG
Canis lupus familiaris    CTTCGTCCTTTAAGGGTCAACATGGCAGAAAGAAATGTGAAGTTGTTCTCTGGGAGAGTGGTGCCAGCCCAAGGGGAAGAAACCTTTGAAACCTGGCTGATCCAGGTCATTGGGGTCCTG

Mus musculus              CCAGATTGGCCTATGTCTGAAGAAGAAAAGATCAAGCGCCTGATGAGAACCCTTAGGGGCCCTGCCCGGGAGGCCATGCGTTTGTTCCAGGCTGACAATCCCAATCTAAATGTCGCAGAA
Canis lupus familiaris    CCTGTTTGGAATATGTCTGAGGAGGAAAAGCTCGAGTGCTTGATGAACACCCTTAGGGGCCCTGCCAGGGAGGTCATGCGTTTGCTCCAGGCCACCAACCCCAATCTAAGTG--------

Mus musculus              TTTTTGCGGGCAATGAAGCTGCTCTTTGGAGCGTCTGAGAGCAGTATAACAGCGCATGGCAAATTTTTAAGCACCCTGCAGGCACAGGGAGAGAAACCATCCCTGTATGTGATCCGTTTA
Canis lupus familiaris    -------GGGCAATGAAATTGATGTTTGGGGAGTCTGAAAGCAAGGTCACTGCCCATGGGAAATTTTTTAACACTTTGCAAGCACAAGGGGAGAAAACCTCCCTTTATGTGATCCGTTTA

Mus musculus              GAGGTGCAGCTACAGAATGCTATCCAGGCAGGGGTCCTTCCTCAGAGCGAGGCAAACAGGACTCGCCTGCACCAGCTCCTTGTAGGGGCTGAGCTGAGTAGGGAGCTGCGCACCAAGCTT
Canis lupus familiaris    GAAGTACAGCTCCAGAATGCTATTCAGGCAGGGATCATAGCTCAGAAAGATGCAAACCAGATCCGTCTGCACCAGCTCCTTTTAGGAGCTAAGCTAAATAGGGACCTGCGCTTTAGGCTG

Mus musculus              AAGGGGCTTCTCCAAATGCATGCGCATAATGAGCAGGAAAACCTTCCTGACTTCCTGGAGTTGATCAGGATGATAAGGGAGGAAGAGGATTGGGATGAGACATTCCTTAGAAATAAGCGG
Canis lupus familiaris    AAA---CATCTCAGGATGTATGCA---AATGATCAGGAGCATCCTCCTAATTTCTTGGAGTTAATCAAGATGATAAAGCAGGAAGAGGATTGGAGTGACACTTTTATGAAACCAAAGTGG

Mus musculus              CCCAGAAGATCTGAGACAGTGATGGAGCGGGCAGCCAGCCCTGTGGTCTTTCAAGGCTCCCTGCCTATAGTGATAGGCAGTGCCGACTGTAATGTGATAGAGATAGATGATTCCCAGGAT
Canis lupus familiaris    CCCAAAAGGTCTGAGCTAATCTTGGAGAGGGCAGCAAGCCCTGTAGCATTTCAGAGCCCCCAGCCAATAGCAATCAGTAGTGCTGACTGCAACGTGAAAGAGGTAGATGATACCCTTGAC

Mus musculus              GATTCAGATGAAGATGTGATCCTGGTGGAGCCCGAGGACCCTCCACTCTCATCCCCAGGTGCCTCATCCCTCCGAGGCACAGCCAGTACTCAGGAAGAGATGCTGATTATTGAGTCCCCT
Canis lupus familiaris    GACTCAGATGAGGATGTGATTCTAGTAGAATCTCAGGACCCTCTACTTACACTTATGGATGCCCCTCCCCTTAGAGGCAGTGCCAGACCTCTAGATCAAATACTGGTCATTGATTCCTCC
```

```
Mus musculus              GATGATTTTGATGAGGAGTCTCCTTCCACTAGCAGTGGTTCTGGGCAAAGGAATAACGGGCCTGGGGATCTAGGTAGAACCAGAAAGCGCAAGTACCCAATCCGCTGTCCTCATTGTGGT
Canis lupus familiaris    AACAGCTCCTCAACTCAATCTCCTTCCACCAGTGGGGGTTCTGGGTATAAGAATAATGGTCCTGGGGATATACATAGAGCCAGAAAGCGAAAATACACAATCTTTTGTTCATATTGTGGT

Mus musculus              GAAGAGGGCCACGCCAAAGAAACCTGTGACAATACCAGCAACAAGGGCCAGGTTTTGAGAATTTGATAGTCACTCTGCAGGAGCTGACTCACAT------GGAGAGACCCAAACCCTCT
Canis lupus familiaris    GAGGAAAGTCACCCAAAGGAAACCTGCAACAGTGAGAGCCACAAGGCCCAGGTGTTTGAGAATCTGATCATCACTCTGCAGGAGCTGACACATATAGAGAAGGAGAGGTCAAAA------

Mus musculus              GTACCATACTAAGGTGTGACCCTCGGCCCCAGAT-CAGTGTTTGAATCTCCTCAGAAGCTAATTCTGGAGGAAAAAGGGATGGGGTGGGCTGCTGACATTAAGGAATTCATTTAAAACAA
Canis lupus familiaris    ---------------GTGGGCCTTGG---CAGACACAATG-----ACCTCTCTGGACCTCAAGTAAGGTGCTAGAATGAGCCAAGTGAACCCTTAAC-----------------------

Mus musculus              GCTTTTTCTTTGGAGAAGAAAAAGCAAACTGGGGTACCATGCAACAGAGAGGCATGGGCCCACATCATTCCTTGCTCATTCCATTAACTGCTTAATGGTTGTTTCTCTGTGTGTCTTTCT
Canis lupus familiaris    ----TGTTTTCAGATGGAAAAA------------------CAGTGGGGGGGGGGGAGTAGACTTCTTTC---------TGCATTAA.................................

Mus musculus              CTGGTGATTTTAATCTATCCGTGAAGTGGTACCTTTTTGAACCTTCCAAATGAAATTAAAAAGAAAAAAACTAAAGTGTGCATTACCTGAAAAATCTCACCCTGA-------ATTGTCAG
Canis lupus familiaris    ......................AAAGTGG-------------CTTTCA--------CAAAATGAAGGAAAATACAA-----ATTACCTGAAACTCTCCAACCTCATATAAACATTCCTAG

Mus musculus              CCCCTTGGTGAATGTCCTGC---GTATTTCCCTGTACCTGTGTGCCTCGGT--ATATATGTATTGATAAAAGTGATCACATATAAGTGCTGTTCTATAGTATGTATTTTTTCATCAGGCA
Canis lupus familiaris    GCCTTTGATGAATGCCCTTCTAGATATTTCCCTGTAACTGTGTACCTAGATAGACATATGTATAGATAAAAGTGATCAAATATAAGTGCTGTTCTATG--CTGTTATTTTTCACCAAATA

Mus musculus              GTATATATTGTGGACTTCTAAATAGATGAATATATAACCATC-----TAATGTC---ATGCATACTATATCTTTGGTGAAGACAAAT--AAAAACTGAAACTAATTGTAACAACTACGGC
Canis lupus familiaris    ATATTTGTTGTGGAGTTCGATGTCAAT--ATATATAAGCATCAAGTTCAATGTCTCTGTGTGTGATGTTACTTTTGGGAATATAGAGGAAAAAATAAGAACCAAATATAGAAGCTCTAAT

Mus musculus              AGGGGTG------------------------------TTCTATTTAAAGATAGG---AGGGGAAGTTGAAATGAGCTATA--TCTCTTTTCAACACCTGTTGTAATTACTGGCCTGCCTC
Canis lupus familiaris    GGGGGAGGGTCATACTGTGAGGTGGAGTTTTTACCAACCTCATTTAAAGATGAGGAAAAAGGAAGCTGAAATGGGCTGAAATTCTCCCAAGAAGGCCCTTTATCACAACTGACCTGTCTC

Mus musculus              CTAGTTCATACAGCCATCTGCACCTACTAGT---TAGGGTCTCAATGTC-------CTCAACCATATTGAGAAATCTGACCATGGCCCATAAGAGGAGCCACCCCTGTCTGGTTCTCCCT
Canis lupus familiaris    CTAGGTCAGGATCAAATCTGTACCCACTAGTTAATAATATTCCAATGCTTGCCTCGCTAATGCATCCTGAGAAAAGTGATGATGG...................................

Mus musculus              CACGTCTCAGCCTGCAATCAGAAGCTGAAGCTGACCTCTGGTATCTGTCTTTGAGCATTCTTCTTGTGGGTACATTAGTCTTTTACTGAGAAAAAAAAAAAAAGAATGACTGCATATAAGG
Canis lupus familiaris    ........................................................................................................................

Mus musculus              CTGACAGCAGAGTGTAAATGAATGCCAATCTGAGGATCTGAAACATTTGTAAAAGATTAATTTTTATGTGAAGTATAGGACTTTTTGCCTGCATGTATTATGAGCCTACAGAGGCCAGAA
Canis lupus familiaris    ........................................................................................................................

Mus musculus              GATGATGTCAGATCCCGTGTACCTGGAGTTTGTAAGTCACCATATGGGTAGGTACTGTGAACAAAATCTAGGTCTTTGAGGGCATAAAGTGCTCTTGTCTGATGAAATACCTCTGCGGCT
Canis lupus familiaris    ........................................................................................................................

Mus musculus              GCAGTCTGAATCATTTTACAATATAACAGGGTTTGCAGAGGTCTGTCTGTGAAGAGGGTGTACAGCACTGGTAGCTGGCATGTCAGGGACAGAAGTAGAGATCAGGTGCTACATGCAGGT
Canis lupus familiaris    .....................................................................................GAGGGAGGAATAGAAGCCAAGTAATGCAAGAAGCC

Mus musculus              TAT-ACAGCCTTCTTGGGGATTCTGCACTCACATCTGGCCTTCGAGTGC--------CTAGAATCACCCTTCTTGGGTGGTCCTGCCTGGCCTCTTA-TCCTGTTCCATCCCTACCCCCA
Canis lupus familiaris    CATTGCAGCCTTCTCTGGGCTGCCACATCCAAAACCTGCCATGAAGTGCTTCTCCTTCCAGAATTGTCCCTATCAGTT---CTTCCCTGGCTTCTTGCTCCTGCTCCCTCCCTAACCCCA

Mus musculus              CAATATCATTTTGTA--CTCCCCCTTTACTGAAGCCACACTCATTTCCT--------GGTGATAGAGTTGGCTCACACCCTTTTGTGTTTTCTGCCTATGACCATTTCCCCTGCTCCCAG
Canis lupus familiaris    TAACCTAACCTTACAGGCTCCCCCAC-----AAGCCACACACTCTCTCTCAGTGGAGGGGACTAGAG---GCTCAGTCTCT-----GTGCTCTGCCAACATCC--CTCCCCTATGCCTAG

Mus musculus              CACTTCATGTTGTCCTAAAGC-------------CTTCAGGGCCAGAAGGAGAGTAGAAGGCCCTGTGTTCCTGAGAT----------AGAGCTTGCCAGGGTCAAAGGTTCAAGGGGGA
Canis lupus familiaris    CACCCCATGTTTGCCTCAAGCTTCACCTCCTTAACCTCATGGCCAGAAGGAGACTGGCAGGGCCCTTGTGCCTCAGATCCTTTAGCTGAGAACTGACCAGAAGCAAAGGCTCATGAGTGA

Mus musculus              GGGACATTTCTTACATCTTTCTCAAATAATTCTGGGCACTTGGCAGTCATATCCATATTTGAATAGTCAAAATGGGCCCC-TCCCCTACCTCTTCCTGCCTAGGTC-ACTCAGGCCTACA
Canis lupus familiaris    TGAACACTCTTTGCATCTCTTTGGGGCACCCCTGGGACCCTGGAAGCCACCCTCATCTCTCCCAAGCCAAAGGGAACCCCCTCCCCCACC----CCAGGCAAGCTCCACTCAGGCCCTCA

Mus musculus              GAGTTACAGG-GTCCTCAAC-----------CTTCTTTCAGATTTATAGCTCTTACCTTTCTCTATAATCAAATCTACCCCAATAGCCAGAG---------------TATAAGATCATTT
Canis lupus familiaris    AAGCCACAGGAGCCCCCAACACTTGACAGCACTTAATTCCTTTCTATGTCTCCTATTCCTCCCTTTCTTTAAATCTTCCC---TTGCAAGGGATCTCTTCTATCGTATATTAAAGTATCA

Mus musculus              CTGCACGGGCTGCTCATCTTTTCATC-TTTTACTCTAT------TTGGGGGGTA-TTTCCCCAGACTTACAGGTT------------CCATTAGAAAGTAGCTAGCTTTAAGA-AGAAAT
Canis lupus familiaris    CTCCTCTTTCTGCTCACCTTTTCTCAATTTACTCTATAAATGCTTGTTGAATAATTTACCCAAATAAATAGTTTATGCTGTCATTTCTTTCTGGGACCAGGTAGAATAAAAAGAAAAAT

Mus musculus              ATATGCTATACCCAC-ACTGCCATCCTTCTT-GCTGCTATTACCTTGGAGGTCCAGTGTGGACTGCTGATTGGAAAAAGCTATAGCTTTTATATTCTGTAGCACATCCTCTTGTCTT-CC
Canis lupus familiaris    AAATGTGACACCCACCACTGCCCTTTTTTTCCACTGGCATTAGCTTGGAGGTTCAGTATAGACCATTACTTTGTAAAA------------CTGCTCTATGATTTATCCCCATATCTTCCC

Mus musculus              TTTCCCATCCTTTCAACTATAC---AAACCTGAGGGTGTGTGAATTTTCATCTCACCAAAAATGGTGCAAAGCTCATGGTTTCTATTTTACTTTATGGGACACTGAGTTTTCCTTTATTG
Canis lupus familiaris    TCCCCCATCCTCTTCTTTGTCCTGTACACTTAAAATTGTCTG-ATTTTTTTTTCACC-AAAATGATGCACACTTGATACTT--TATTTCACTTTGTGTAACATTGGCTTTTCCAGTATTC

Mus musculus              AAGAAACATGTACAGACTTCATGTGTGACAACTGGCCTAGGACCTAGTACCTGTCACCCTTTTAGTGGCTGCTGAGCCCTGCCAACTACCTAGTGCTTGCTGCTGCCCTTAAGGCCATGC
Canis lupus familiaris    AGGACATATGTA............................................................................................................

Mus musculus              CCTGGAGCTCTTTGATAAAGCAGCCCTAAACGTGCTGCAGCCTCCAGAGTTTAGAAATGAAAAAATTTTATCTTTAGCTTCAACCTTGAAGACACTTCTGTTCTTTAAAGCTTTAAGGAT
Canis lupus familiaris    ........................................................................................................................

Mus musculus              CACAGTTCCTATCTGGCTATTTTACAATGAAATCTTATATTTGAAGATGCTGTTGGAATGTCCAATAGAGACAGCTATTTTTATGCTGCAATTATTGCTGTTCACGTGGTTCTGGCCCTG
Canis lupus familiaris    ........................................................................................................................

Mus musculus              TTTGTCTATGTGGCCTGGAATGAAGGCTCATGGTGGCATGGAGGCAAACAGGACTAAAGTGAACATCACCTTTTGATCACATGAAATTTATTTTTGGAATATGTTAAACACTTCTGGGAT
Canis lupus familiaris    ........................................................................................................................

Mus musculus              ATTGATAATAAATATATTGTTTGAATAAAGTTGAAAGGACATGTTTAAAAATAAAGAACTATGTACAAACTGGGTACAGTGGTATACACCTTTAATTCCAGCACTCTGGAAGCAAGAGGC
Canis lupus familiaris    ........................................................................................................................

Mus musculus              AAGTGGATCTCTATGAGTTCAAGGCCAACATGCTCTACAGAGTGAGTTCCAGGACATCCTGGGGTGACATGGAGAAACCCTGTCTCAAAATAATAGAAATAAAAACTAAAAAGAGAACTA
Canis lupus familiaris    ........................................................................................................................

Mus musculus              TGTCTGAGGAGAAAGCCTGGCATTTCTGCCTTCATTCTGTGTTCGTCTCCTTTTCCTTCCTGTTACCTGTTTACCTCTGCACATAGGCATGCATGTAAATCACTCAAGGAATGCCGCTGT
Canis lupus familiaris    ........................................................................................................................

Mus musculus              CCATTCTTTCATCCCTTTTTATGGCTAAAGGCATAGAGTAAATCAGTGTCCTAATCCTACAGTCACCATGGATTGTAGGAACAGCAGAAGCTCACTAGAAAGCCGGTCCTTCAGATGAGG
Canis lupus familiaris    ........................................................................................................................

Mus musculus              GCAAGTCTCTATGACAGTACAGCTGTATGCTAGTAGGGAAATAATCAACATCAGCGAACCAGATTGACTCTCTCAAGCAATTACATACAGCAATAAGGACTTCATCAGACCACCCTGCAG
Canis lupus familiaris    ........................................................................................................................

Mus musculus              GGCTGGGACTGAGGTGATATGATAAACTAGGCCACACTCAGAGGAGGCCTGCTTTAATTACACATCTCCCGC
Canis lupus familiaris    ........................................................................
```

ZCCHC5

```
>chromosome:GRCh37:X:77910966:77915425:-1
GTTCAGGGAAGCATGAGATCTTTGGTCCAGGACATTCAGAAAGAGCCAGGGACTCTGTAA
GACTGCTATAAGTACCCACCAACACAATTGGTCTCCCTGACATTTGTACCCTTTTATACC
TTTTCTAGATCTGTCTGTGGCAAATTTTCCAAGAAAACATTGCAAGGAAAAACCCTAGGG
ACTGAATAGGTAGTAAGCAAGGGAAGGGGCTGAGGGTTTGCAGGCTGTTCCCTGAGTGAG
AAGTAAACAAATGGCCCAATTTGGACAGAGTAAGAATAGGTTAGATGAGACAGACATAAA
AAATCACATCCCAAACCACTGTGGTCATTCAAAAATGTTTGCAACTTTTCACAAGGGCAG
TGGTATTACTCACAATTTCCTGGCCAAATATTCATTAAAGTAACCAAGGACTGAGTACAT
GAAGCTGCTTCACATCAGCACAGCATCCTACTTTCCTTGCAGCTCTGAACTGTGAGGTAC
TGTCAAAAAACTCTTCCACACTTTAAAAAGCTATGTTACAGAGGAAAGTTGGAATGTGTT
CTGGGAGTGGGTGTGTTTGCTTGAGTTCAGTGTGTGCAGAAAAGGCAAGCCTCCACAGCC
ATTTACACATTTCCCTCCTCAGCAATGGGTACACAGACAGCCAAGCTCCTGGACTAAGTC
TCTGAATGGTAACGTACATTTATAAAGATTATTTTCTTAACTATTTTGAGGTATTTTATA
AATAATATGCATGTGTCTTCTGAGGGCATATATATATATATATATATATATATTTAGTCGAC
TTTTGCTTGTTTTCTCCTGAGGCTGGTCTTGTGTGGTTACTTATGTGGTATGATTTCTGA
GGTGTCAGAAAGAGCTCCCTCCTACTTCCTACCACCCTAGTCACCCAAAAATATCTCTTG
GGCTGGCCCACCGCAGAGCCTAAAATTCCCACATTGCTCAAACATTTGATTTAGTCACTG
AAAGCAGCTGTTAAAATCCAAATGTTACATTGGGATATTAGGCAGATATTCAAGATTAAA
GACTATATAAAACAGAAAATAATATTAAAAAGTTGCTTTTGATATAATGTTAAGTTTCAA
AGGATAAATGCAAAATGACATGTGCAGTTTGGGACAGATGCTGTTCTTAAAATGTCGTGG
AGTCAGATCCAGCATATATACCCCAGCTCATGAACATAAGCCTATGGTAAAAGAAACCCA
GCATTGTTGGGCAGATTTTGTTTTATGTTGAATGTGTGCTTTCCACAGTTTCTGATCCTC
AGCTCCCACTCTCTTCGCAGTGTATCTGACACTTCCCCTTACTGAGCTCCGGAGGCTCCT
TCTTGCCGAGCTCCGGAGGCTCCCAGTGACCTCTTGATAATCAGCCTTGGCGGGGCCCAA
ATACCACCCCTTGGAAAACTCACTGAGCCTGACATCTGCCTGTAAGCAGCTTTCAGGAAG
```

```
GATCTCACAAACACCCACCCAATCTCTCTTGGTAGCAAATTGTCAAATCATACCCATTTC
CTCAAAAGATGGTGGAGGACTTAGCAGCCTCCTATATTGTTCTGAAATTGGAGAATGAAA
TTCGGCAGGCTCAAGTGCAGTGGCTGATGGAAGAAAATGCTGCTCTCCAGGCCCAGATCC
CAGAGCTTCAGAAGTCCCAAGCAGCCAAGGAGTATGATCTACTCCGAAAGTCCTCAGAGG
CCAAGGAGCCCCAGAAGCTCCCAGAGCACATGAATCCCCCAGCAGCCTGGGAGGCCCAAA
AGACCCCAGAGTTCAAGGAACCCCAGAAGCCACCAGAGCCACAGGATCTTCTACCCTGGG
AACCCCCAGCAGCCTGGGAACTCCAGGAGGCCCCAGCAGCCCCAGAGTCCCTGGCGCCTC
CAGCAACCCGGGAGTCCCAGAAACCTCCAATGGCCCATGAGATCCCAACAGTCTTGGAGG
GCCAGGGGCCTGCAAACACCCAGGATGCCACAATAGCCCAAGAGCCCAAGAATTCAGAAC
CCCAGGATCCCCCAAATATTGAGAAGCCCCAGGAGGCACCAGAATACCAGGAAACTGCAG
CACAGCTAGAGTTCCTTGAGCTTCCACCTCCCCAGGAGCCTCTGGAGCCTTCAAATGCCC
AGGAATTCCTAGAACTCTCAGCAGCCCAGGAGTCCCTGGAGGGTCTAATAGTTGTGGAGA
CATCAGCAGCTTCAGAGTTTCCACAGGCCCCTATTGGGTTAGAGGCTACAGATTTCCCCC
TGCAATACACTTTAACCTTCAGTGGAGATTCCCAGAAGCTTCCTGAGTTCCTGGTTCAGC
TGTATAGTTACATGAGAGTCAGAGGGCACCTGTATCCCACTGAAGCAGCCCTGGTGAGCT
TTGTTGGCAATTGCTTCTCAGGTAGGGCAGGATGGTGGTTCCAGCTCTTACTGGATATCC
AAAGCCCCTTACTGGAGCAATGTGAAAGTTTCATACCTGTGCTCCAGGATACTTTTGATA
ATCCAGAAAACATGAAAGATGCCAACCAGTGCATCCATCAACTCTGCCAAGGGGAGGGTC
ATGTAGCAACCCACTTCCACCTCATTGCTCAAGAGCTGAACTGGGATGAAAGCACTCTCT
GGATCCAATTTCAAGAAGGTCTTGCCAGTTCTATACAAGATGAACTGTCTCACACAAGCC
CAGCCACCAACCTATCTGATCTCATCACTCAGTGCATCAGCCTGGAAGAGAAGCCTGATC
CCAATCCATTAGGGAAAAGTTCCTCTGCAGAGGGAGATGGACCCGAGAGTCCACCAGCTG
AAAACCAACCTATGCAGGCTGCAATCAACTGCCCACACATCAGTGAAGCTGAATGGGTCC
GTTGGCACAAAGGCCGCTTATGCCTCTACTGTGGTTATCCTGGTCATTTTGCCAGAGATT
GCCCTGTCAAGCCTCATCAGGCCCTGCAGGCGGGAAACATCCAGGCCTGCCAGTAAGGGG
GACCCCACGTGGGCCAATCTACAAATATGCGTCCCCCTCTCTTCCAATATCCATGTCTCA
TACACTATGGCTTACTGTTGAAATCCAACTGGGAAGAAGATAATTCTTTGAGCAAGCAAT
GGTAGATTCAGGCTCCTACAGAAACAGCATTGATCATACTGTGGTTCTTCGAGAGAAGCT
GCCCATCCGCAGTAATATCTTCCCTCTGATGCTGGAAACTGTCGACGGCCATCCACTTAT
TAATGGACCCATAACTAAGGAAACATCACCTGTCCAAGTTCAAATTGGAAACCATGTTGA
AGAGCTCCAGTTTGACATTATTCATGCACCACGATACCCTCTGATTATTGGAATCCATTG
GCTTGAGACACATGACCCAAACATAGAATGGAGTACCCGCACTGTGTCCTTTCTATCACG
TTATTGTCACTACAATTGCTTCAGGCACAGGTGGAATAGAAGAAATCGTGATGAAATAAT
TTCTGGGTAGATCCTGTGTCCTAGTGACTGTTCCTGGCAACCTGTCTTCTGTTACCTCAG
CTGTCATCAGCATTTGCTGCTCCCTGAATCTTCTACTGAACATCTTGCTATGTACTAAGG
CTACCTGTACAACGACTCTGCCTCTTAGATGATAGACTGAACCTGATGACTTTGAGCTGC
TTTTTTTTTTTTCACTAACTCTGCATGCCTCTATCTCAGAACTCTGCATCCTGCGGAACT
GTTTTAATTACAATTATACTAACAATACACATGAGAATAGATATTAGAAACTAATAATGA
AAAAGCATAGCTGTTAAACTGACTTGATTTTTTTCTTATCAATATTTTCTATATCACAAA
AATGGCAACATCTTTTACATAAAGAAAATAAGTTTGTAAGTGATATTTCTTAAAAGAATC
AATAAATATTAGCAATTTCTACTTGTGTTTGAGTTATTTCTTGGCTTAGCTAGAGGAAGA
GATAATGAACCAGTAAATTGGGAGGGAGAGAGGGAATTAGAAAATGTTCAATGTTTAGGG
AGGCTCCTAAACATTCCGGGTGTAGTGATAGGTCTGCAGATGATGGAGAAATGATGTAGA
GGGTAAGATGGAGAGAGTAGGGAATGGTTAGGGCTGAAAGTGAGAGTAGGGAATGGTTAG
GGCTGAAAGTAAGTGAGGTATGGAGGGATTAGGGGAAAGGTAGAGGTAAGATTGAGAAGA
GGGTTTGGGGGAGATTGGGAGGAGGTTGAAGAGGGTTTAGAGGAGGGTTGGGGTGCTGAG
AGGTAGAATCTCCAGGTGAGGTGTGAGGAGTGGTCTAAGCAGAACCTAATGCTTTTCCTC
TGTGTTTTCAGCTTTCCCTTAGGAGCCTCTCTCATTGAGGCTGCTAAAAGCGGACTCTTT
CCCTCATTTAGACAAATGTAGACAAAATATCAAATAAATGATTATAACCCTAGAAGGTAA
TAGGGAAGGGGCTTACCATTCATTGGACCACTAATATGTTCAGCTACAATGTCAGACATT
TAACACACATTAATCATCTC
```

```
Homo sapiens         GTTCAGGGAAGCATGAGATCTTTGGTCCAGGACATTCAGAAAGAGCCAGGGACTCTGTAAGACTGCTATAAGTACCCACCAACACAATTGGTCTCCCTGACATTTGTACCCTTTTATACC
Choloepus hoffmanni  GCTCAGGGAAGCATGATAATCCTGATTCAGGGCATTCAGAATGATCCAGGGGCCCTGCAAGACTGCTGAATGTACCCATCAACAAAATTGGCCTCCCCCACGATTTTAGTCTATTAGACC

Homo sapiens         TTTTCTAGATCTGTCTGTGGCAAATTTTCCAAGAAAACATTGCAAGGAAAAACCCTAGGGACTGAATAGGTAGTAAGCAAGGGAAGGGGCTGAGGGTTTGCAGGCTGTTCCCTGAGTGAG
Choloepus hoffmanni  CTGCCTAGATCTGTCTATGG-AACTGTTCCAA........................................................................................

Homo sapiens         AAGTAAACAAATGGCCCAATTTGGACAGAGTAAGAATAGGTTAGATGAGACAGACATAAAAAATCACATCCCAAACCACTGTGGTCATTCAAAAATGTTTGCAACTTTTCACAAGGGCAG
Choloepus hoffmanni  ........................................................................................................................

Homo sapiens         TGGTATTACTCACAATTTCCTGGCCAAATATTCATTAAAGTAACCAAGGACTGAGTACATGAAGCTGCTTCACATCAGCACAGCATCCTACTTTCCTTGCAGCTCTGAACTGTGAGGTAC
Choloepus hoffmanni  ........................................................................................................................

Homo sapiens         TGTCAAAAAACTCTTCCACACTTTAAAAAGCTATGTTACAGAGGAAAGTTGGAATGTGTTCTGGGAGTGGGTGTGTTTGCTTGAGTTCAGTGTGTGCAGAAAAGGCAAGCCTCCACAGCC
Choloepus hoffmanni  ........................................................................................................................

Homo sapiens         ATTTACACATTTCCCTCCTCAGCAATGGGTACACAGACAGCCAAGCTCCTGGACTAAGTCTCTGAATGGTAACGTACATTTATAAAGATTATTTTCTTAACTATTTTGAGGTATTTTATA
Choloepus hoffmanni  ...............................................CCTGGACTGGGTCTCTGAATGGTAACGTATATTTATAAAGATTATCTTCCTTACTTATTTCAGACATTTTACA

Homo sapiens         AATAATATGCATGTGTCTTCTGAGGGCATATATATATATATAT----ATATATATTTAGTCGACTTTTGCTTGTTTTCTCCTGAGGCTGGTCTTGTGTGGTTACTTATGTGGTATGATTT
Choloepus hoffmanni  AATAATATGCATGTGTCTAGTGAGCATACATATATATATATATCACAATATATACATTGTTCTTTTTTGCT.................................................

Homo sapiens         CTGAGGTGTCAGAAAGAGCTCCCTCCTACTTCCTACCACCCTAGTCACCCAAAAATATCTCTTGGGCTGGCCCACCGCAGAGCCTAAAATTCCCACATTGCTCAAACATTTGATTTAGTC
Choloepus hoffmanni  ........................................................................................................................

Homo sapiens         ACTGAAAGCAGCTGTTAAAATCCAAATGTTACATTGGGATATTAGGCAGATATTCAAGATTAAAGACTATATAAAACAGAAAATAATATTAAAAAGTTGCTTTTGATATAATGTTAAGTT
Choloepus hoffmanni  ........................................................................................................................

Homo sapiens         TCAAAGGATAAATGCAAAATGACATGTGCAGTTTGGGACAGATGCTGTTCTTAAAATGTCGTGGAGTCAGATCCAGCATATATACCCCAGCTCATGAACATAAGCCTATGGTAAAAGAAA
Choloepus hoffmanni  ........................................................................................................................

Homo sapiens         CCCAGCATTGTTGGGCAGATTTTGTTTTATGTTGAATGTGTGCTTTCCACAGTTTCTGATCCTCAGCTCCCACTCTCTTCGCAGTGTATCTGACACTTCCCCTTACTGAGCTCCGGAGGC
Choloepus hoffmanni  ........................................................................................................................

Homo sapiens         TCCTTCTTGCCGAGCTCCGGAGGCTCCCAGTGACCTCTTGATAATCAGCCTTGGCGGGGCCCAAATACCACCCCTTGGAAAACTCACTGAGCCTGACATCTGCCTGTAAGCAGCTTTCAG
Choloepus hoffmanni  ........................................................................................................................

Homo sapiens         GAAGGATCTCACAAACACCCACCCAATCTCTCTTGGTAGCAAATTGTCAAATCATACCCATTTCCTCAAAAGATGGTGGAGGACTTAGCAGCCTCCTATATTGTTCTGAAATTGGAGAAT
Choloepus hoffmanni  ..................................................................................................................GAGAAT

Homo sapiens         GAAATTCGGCAGGCTCAAGTGCAGTGGCTGATGGAAGAAAATGCTGCTCTCCAGGCCCAGATCCCAGAGCTTCAGAAGTCCCAAGCAGCCAAGGAGTATGATCTACTCCGAAAGTCCTCA
Choloepus hoffmanni  CTGATTCTACAGCCACAAGTGCAGCACCTGAATGAGGAAAATACT--TCCCCAAG.................................................................

Homo sapiens         GAGGCCAAGGAGCCCCAGAAGCTCCCAGAGCACATGAATCCCCCAGCAGCCTGGGAGGCCCAAAAGACCCCAGAGTTCAAGGAACCCCAGAAGCCACCAGAGCCACAGGATCTTCTACCC
Choloepus hoffmanni  ........................................................................................................................

Homo sapiens         TGGGAACCCCCAGCAGCCTGGGAACTCCAGGAGGCCCCAGCAGCCCCAGAGTCCCTGGCGCCTCCAGCAACCCGGGAGTCCCAGAAACCTCCAATGGCCCATGAGATCCCAACAGTCTTG
Choloepus hoffmanni  ........................................................................................................................

Homo sapiens         GAGGGCCAGGGGCCTGCAAACACCCAGGATGCCACAATAGCCCAAGAGCCCAAGAATTCAGAACCCCAGGATCCCCCAAATATTGAGAAGCCCCAGGAGGCACCAGAATACCAGGAAACT
Choloepus hoffmanni  ........................................................................................................................

Homo sapiens         GCAGCACAGCTAGAGTTCCTTGAGCTTCCACCTCCCCAGGAGCCTCTGGAGCCTTCAAATGCCCAGGAATTCCTAGAACTCTCAGCAGCCCAGGAGTCCCTGGAGGGTCTAATAGTTGTG
Choloepus hoffmanni  ........................................................................................................................

Homo sapiens         GAGACATCAGCAGCTTCAGAGTTTCCACAGGCCCCTATTGGGTTAGAGGCTACAGATTTCCCCCTGCAATACACTTTAACCTTCAGTGGAGATTCCCAGAAGCTTCCTGAGTTCCTGGTT
Choloepus hoffmanni  ........................................................................................................................

Homo sapiens         CAGCTGTATAGTTACATGAGAGTCAGAGGGCACCTGTATCCCACTGAAGCAGCCCTGGTGAGCTTTGTTGGCAATTGCTTCTCAGGTAGGGCAGGATGGTGGTTCCAGCTCTTACTGGAT
Choloepus hoffmanni  ........................................................................................................................

Homo sapiens         ATCCAAAGCCCCTTACTGGAGCAATGTGAAAGTTTCATACCTGTGCTCCAGGATACTTTTGATAA-TCCAGAAAACATGAAAGATGCCAACCAGTGCATCCATCAACTCTGCCAAGGGGA
Choloepus hoffmanni  ..CTAGAGTACTTTGCTGAAGCAATATGAAAACTTCATTCCTGAGTACCAGCAGTCATTTGGCAAACCCACAAAACAGGAAATGAACCCTGTGGTGAATGC--TAAGGTTGACAAAGGTA

Homo sapiens         GGG-------------TCATGTAGCAACCCACTTCCACCTCATTGCTCAAGAGCTGAACTGGGATGAAAGCACTCTCTGGATCCAATTTCAAGAAGGTCTTGCCAGTTCTATACAAGAT
Choloepus hoffmanni  ATGACTCCTCTTTGCCATTATGTTAC----CATTTCCAGTTCCTTGCTCAAAATTTGAGCTATAATGAAACCACTATGTAAGATCCACTCCAAGAGGGACTGGCTGGTCCTGTCTGTGAT

Homo sapiens         GAACTGTCTCACACAAGCCCAGCCACCAACCTATCTGATCTCATCACTCAGTGCATCAGCCTGGAAGAGAA------------GCCTGATCCCAATCCATTAGGGAAAAGTTCCTCTGC
Choloepus hoffmanni  GAAATGAGTGGCAAGAATATGGACA---ATCCCCCAGACCTGATCACTCGGTGTATTAAGTTGGACAAAAAAATGTAGTGATGGGCCAGAGC----TCCTTCAGTCAGAGGTCCAGCTCC
```

```
Homo sapiens        AGAGGGAGATGGACCCGA---------------------------GAGTCCACCAGCTGAAAACCAACCTATGCAGGCTGCAA-----TCAACTGCCCACACATCAGTGAAGCTGAATG
Choloepus hoffmanni CAAGATCAACTTCCCTGATCTACCACCAATACCTCTCCAGCACCACAGGTACACTACCCAAGGAAAAACCTGTATAG-TTGCAGGAAGGCCAGCTGCTTC----TCACTACAGCT-AAGG

Homo sapiens        GGTCCGTTGGCACAAAGGCCGCTTATGCCTCTACTGTGGTTATCCTGGTCATTTTGCCAGAGATTGCCCTGTCAAGCCTCATCAGGCCCTGCAGGCGGGAAACATCCAGGCCTGCCAGTA
Choloepus hoffmanni AGCCCACCAACAAGAAACCTAATTTTGCTCGTACTGCAGCCAGGCTGATCATTTCACAAGAGATTGCCTCACCAAGCATTCTCGAGCCCCAGCAAAAACAAATAACCCAGCTTGCCAGTA

Homo sapiens        AGGGGGACCCCACGTGGGCCAATCTACAAATATGCGTCCCCCTCTCTTCCAATATCCATGTCTCATACACTATGGCTTACTGTTGAAATCCAACTGGGAAGAAGATAATTCTTTGAGCAA
Choloepus hoffmanni A-GAGGAGCTCAGGCAAGTCACTTTCCAACT-TATATCCCTCTTACTTCCAGCCTTACTGATTTATATCCTGC-ACCAACTTTTAAAATCCAGAAGGGGAGACACACCCTGTTCGTCCGA

Homo sapiens        GCAATGGTAGATTCAGGCTCCTACAGAAACAGCATTGATCATACTGTGGTTCTTCGAGAGAAGCTGCCCATCCGCAGTAATATCTTCCCTCTGATGCTGGAAACTGTCGACGGCCATCCA
Choloepus hoffmanni GCCATGATCGATTCTGGTGCTTCTGGCAACTTCATTGATCACGAATACGTTGCTCAAAATGGAATTCCTCTAAGAGTTAAGGATTGGCCAATACTTGTGGAAGCAATTGATGGACGCCC-

Homo sapiens        CTTATTAATGGACCCA--TAACTAAGGAA-ACATCACCTGTCCAAGTTCAAATTGGAAACCATGTTGAAGAGCTCCAGTTTGACATTATTCATGCACCACGATACCCTCTGATTATTGGA
Choloepus hoffmanni CATAGCATCGGGCCCAGTTGTCCACGAAACACATGACCTGAT--AGTTGACATGGGAGATCACCGTGAGGTGCTGTCATTTGACGTGACTCAGTCTCCATTCTTTCCCGTGGTCCTCGGG

Homo sapiens        ATCCATTGGCTTGAGACACATGACCCAAACATAGAATGGAGTACCCGCACTGTGTCCTTTCTATCACGTTATTGTCACTACAATTGCTTCAGGCACAGGTGGAATAGAAGAAATCGTGAT
Choloepus hoffmanni GTTCGCTGGCTGAGCACACATGATCCCAACATTACTTGGAGCACCCGATCTATCGTCTTTGATTCTGAATATTGTCGTTACCACTGCCGGATGTACA.....................

Homo sapiens        GAAATAATTTCTGGGTAGATCCTGTGTCCTAGTGACTGTTCCTGGCAACCTGTCTTCTGTTACCTCAGCTGTCATCAGCATTTGCTGCTCCCTGAATCTTCTACTGAACATCTTGCTATG
Choloepus hoffmanni ........................................................................................................................

Homo sapiens        TACTAAGGCTACCTGTACAACGACTCTGCCTCTTAGATGATAGACTGAACCTGATGACTTTGAGCTGCTTTTTTTTTTTTCACTAACTCTGCATGCCTCTATCTCAGAACTCTGCATCCT
Choloepus hoffmanni ........................................................................................................................

Homo sapiens        GCGGAACTGTTTTAATTACAATTATACTAACAATACACATGAGAATAGATATTAGAAACTAATAATGAAAAAGCATAGCTGTTAAACTGACTTGATTTTTTTCTTATCAATATTTTCTAT
Choloepus hoffmanni ........................................................................................................................

Homo sapiens        ATCACAAAAATGGCAACATCTTTTACATAAAGAAAATAAGTTTGTAAGTGATATTTCTTAAAAGAATCAATAAATATTAGCAATTTCTACTTGTGTTTGAGTTATTTCTTGGCTTAGCTA
Choloepus hoffmanni ........................................................................................................................

Homo sapiens        GAGGAAGAGATAATGAACCAGTAAATTGGGAGGGAGAGAGGGAATTAGAAAATGTTCAATGTTTAGGGAGGCTCCTAAACATTCCGGGTGTAGTGATAGGTCTGCAGATGATGGAGAAAT
Choloepus hoffmanni ........................................................................................................................

Homo sapiens        GATGTAGAGGGTAAGATGGAGAGAGTAGGGAATGGTTAGGGCTGAAAGTGAGAGTAGGGAATGGTTAGGGCTGAAAGTAAGTGAGGTATGGAGGGATTAGGGGAAAGGTAGAGGTAAGAT
Choloepus hoffmanni ........................................................................................................................

Homo sapiens        TGAGAAGAGGGTTTGGGGGAGATTGGGAGGAGGTTGAAGAGGGTTTAGAGGAGGGTTGGGGTGCTGAGAGGTAGAATCTCCAGGTGAGGTGTGAGGAGTGGTCTAAGCAGAACCTAATGC
Choloepus hoffmanni ........................................................................................................................

Homo sapiens        TTTTCCTCTGTGTTTTCAGCTTTCCCTTAGGAGCCTCTCTCATTGAGGCTGCTAAAAGCGGACTCTTTCCCTCATTTAGACAAATGTAGACAAAATATCAAATAAATGATTATAACCCTA
Choloepus hoffmanni ........................................................................................................................

Homo sapiens        GAAGGTAATAGGGAAGGGGCTTACCATTCATTGGACCACTAATATGTTCAGCTACAATGTCAGACATTTAACACACATTAATCATCTC
Choloepus hoffmanni ........................................................................................
```

```
Homo sapiens        GTTCAGGGAAGCATGAGA--TCTTTGGTC--CAGGACATTCAGAAAGAGCCAGGGACTCTGTAAGACTGCTATAAGTACCCACCAACACAATTGGTCTCCCTGACATTTGTACCCTTTTA
Loxodonta africana  GATTAGGAAACCATGAGAACTCTTGGGTTGTTAGGGCATACAGAAGGAACCCAAGGCCCTGCAAAAGTGTTGCAAGTAACCATCAACAAAGTTGGCCTTCCCCACAGTCTTACCCTGTTA

Homo sapiens        TACCTTTTCTAGATCTGTCTGTGGCAAATTTTCCAAGAAAACATTGCAAGGAAAAACCCTAGGGACTGAATAGGTAGTAAGCAAGGGAAGGGGCTGAGGGTTTGCAGGCTGTTCCCTGAG
Loxodonta africana  GACCCTGCCTAGATCTATCCATGGTAAAGTTTTCAAGAAAACATTGCAGGGAGAGACCCTAGGGACTAAGTAGGTGG---GTGGGAGAAGGGGCTGAGG-----CAGGGTACTCTCTAAG

Homo sapiens        TGAGAAGTAAACAAATGGCCCAATTTGGACAGAGTAAGAATAGGTTAGATGAGACAGACATAAAAAATCACATCCCAAACCACTGTGGTCATTCAAAAATGTTTGCAACTTTTCACAAGG
Loxodonta africana  TGAGAATTAAAGAAATGCTTCATTGTGGATGGAGTGAGAA-AGGTTAAATGAGACATACA----AAATAAAGTCCCAAACCACTGTGGTCATTCATAAATGTTTGTAACTTTTCACAAGG

Homo sapiens        GCAGTGGTATTACTCACAATTTCCTGGCCAAATATTCATTAAAGTAACCAAGGACTGAGTACATGAAGCTGCTTCACATCAGCACAGCATCCTACTTTCCTTGCAGCTCTGAACTGTGAG
Loxodonta africana  GCAGTGGTATTACTCCCAATTTCCTGGCCAAATATTTATTAAAGTAACCAAGGACTGAGTACATGAGGCTACCTCACATCAGCACAGCATCCTACTTTCCTTGAGACTCTAAATTGTGAG

Homo sapiens        GTACTGTCAAAAAAC----TCTTCCACACTTTAAAAAGCTATGTTACAGAGGAAAGTTGGAATGTGTTCTGGGAGTGGGTGTGTTTGCTTGAGTTCAGTGTGTGCAGAAAAGGCAAGCCT
Loxodonta africana  GTACCATTAAAAAAAAAAAGTGTCCACCCTTGAAATAGCTACGTTACAGAGTAGAGTTGGAACGTGTTCTGGGAGTGGGTGTGTTTG----AGTTCAGTGTGTGCAGACAAGGCAAGCGT

Homo sapiens        CCACAGCCATTTACACATTTCCCTCCTCAGCAATGGGTACACAGACAGCCAAGCTCCTGGACTAAGTCTCTGAATGGTAACGTACATTTATAAAGATTATTTTCTTAACTATTTTGAGGT
Loxodonta africana  CCACAACCATTCACACACTTCCCTCCTCAGTGATGGTTGTACAGACAGCTAAGCTCCTGGACTACGGCTCTGAATGGTAACATATACTTATAAAGATTATTTTCTTAAATATTTTGAGGA

Homo sapiens        ATTTTATAAATAATATGCATGTGTCTTCTGAGGGCATATATATATATATATATATATATTTAGT---------------------------------CGACTTTTGCTTGTTTTCTCCT
Loxodonta africana  ATTTTATAAATAATATGCATGTGTCTTCTGAGCATATATATGCATGTATGTGTGTATGTATAACTTTTGCTTGTTTTCTGTACACACACACACGCCTGCAACATTTGTGTGTTTTCTCCC

Homo sapiens        GAGGCTGGTCTTGTGTGGTTACTTATGTGGTATGATTTCTGAGGTGTCAGAAAGAGCTCCCTCCTACTTCCTACCACCCTAGTCACCCAAAAATATCTCTTGGGCTGGCCCACCGCAGAG
Loxodonta africana  GAGGCTGATCTTATGCGTTTACTTACATGGTATAATTTCTGAGGTGTCAGAAAGGGTTCCCCTCCACCTCCTACC---CCAGTTACCAAAAAATAT-TCTTGGGTTGGCCCATTACAGGG

Homo sapiens        CCTAAAATTCCCACATTGCTCAAACATTTGATTTAGTCACTGAAAGCAGCTGTTAAAATCCAAATGTTACATTGGGATATTAGGCAGATATTCAAGATTAAAGACTATATAAAACAGAAA
Loxodonta africana  CCTAAAATCCCCACGTTGCCCAAACATATGATTTAGTCATTGAAAACTGCTGTTAAA-TCCA---TTAACTTTTGGATGTTAAGCAGCCATTAGAAATTAAAGACTATGTAAAACAGAGG

Homo sapiens        ATAATATTAAAAAGTTGCTTTTGATATAATGTTAAGTTTCAAAGGATAAATGCAAAATGACATGTGCAGTTTGGGACAGATGCTGTTCTTAAAATGTCGTGGAGTCAGATCCAGCATATA
Loxodonta africana  AGAATATGGAGAAATTGCTTCTGATATAATGTTAACTTTTGAAAGA---ATGCAAAACGACACACATAGTGTGGGTCAGGTGCTGTTCTTAAAATTCCACGGGACCAGAGCCAGCAAATA

Homo sapiens        TACCCCAGCTCATGAACATAAGCCTATGGTAAAAGAAACCCAGCATTGTTGGGCAGATTT-----TGTTTTATGTTGAATGTGTGCTTTCCACAGTTTCTGATCCTCAGCTCCC-ACTCT
Loxodonta africana  TGTCCCAGCTCATGAACTTGAGCTCTTGATGAAAGAAACTCAGCACTGTCAGGCAAATTTAATTCTGATTTATTTTAAATGAGTACTTTCCACAGTTTCTGATCCTCAGCTCCCCACTCT

Homo sapiens        CTTCGCAGTGTATCTGACACTTCCCCTTACTGAGCTCCGGAGGCTCCTTCTTGCCGAGCTCCGGAGGCTCCCAGTGACCTCTTGATAATCAGCCTTGGCGGGGCCCAAATACC-ACCCCT
Loxodonta africana  CTTTACAGTGTTTCTGACACCTCCCTTT-----------------------GCTGAGCTCTGGAGGCTCCCAGTGACCTCCTGATAATCAGCCTTGACAAAACCCAAGTCCCCACCCCT

Homo sapiens        TGGAAAACTCACTGAGCCTGACATCTGCCTGTAAGCAGCTTTCAGGAAGGATCTCACAAACACCCACCCAATCTCTCTTGGTAGCAAATTGTCAAATCATACCCATTTCCTCAAAAGATG
Loxodonta africana  TGGAAAACTCACAGAGCCTGACATCTGCCTGTGAGCAACTTTTAGGAGAGACCTCACAAAAGCCCATGCAATCACTTTTGGCAGCAAACTGTCATACCAAGTCCATTTCCTCAAAAGATG

Homo sapiens        GTGGAGGACTTAGCAGCCTCCTATATTGTTCTGAAATTGGAGAATGAAATTCGGCAGGCTCAAGTGCAGTGGCTGATGGAAGAAAATGCTGCTCTCCAGGCCCAGATCCCAGAGCTTCAG
Loxodonta africana  GTGGAGGACTTAACAGCCTCCTATAATGCTCTGAAATTGGAGAATGAAATTCTGCAGGTCCATGTGCAATGGCTTATGGAAGAAAATGCTGCCCTCCAGTCACAAATCCCAGAGCTCCAG

Homo sapiens        AAGTCCCAAGCAGCCAAGGAGTATGATCTACTCCGAAAGTCCTCAGAGGCCAAGGAGCCCCAGAAGCTCCCAGAGCACATGAATCCCCCAGCAGCCTGGGAGGCCCAAAAGACCCCAGAG
Loxodonta africana  AAGCCCTGAGCAGCCAAAAAGGATGAACTACTCCAGAAGCCCTCAGAGGTCCAGAATCCCCAGCAACCTTCAGAACCCCTGGGTCCCCCAGCAGCCTAGAAACCCCAAGAGCCCCTAAAG

Homo sapiens        TTCAAGGAACCCCAGAAGCCACCAGAGCCACAGGATCTTC---TACCCTGGGAACCCCCAGCAGCCTGGGAACTCCAGGAGGCCCCAGCAGCCCCAGAG---------TCCCTGGCGCCT
Loxodonta africana  GTCAAAAAGTCCCTGAAGCCCACAGAGTCCCAGGACTTTCCAGCAGCCTGGCAGCACCCAGCAGCCCAGAAGCCCCAGAAGACTCCAATGGCCCAGGAGACTCAGAAACCCCTGGATGCC

Homo sapiens        CCAGCAACCCGGGAGTCCCAGAAACCTCCAATGGCCCATGAGATCCCAACAGTCTTGGAGGGCCAGGGGCCTGCAAACACCCAGGATGCCACAATAGCCCAAGAGCCCAAGAATTCAGAA
Loxodonta africana  CCAGCAGTCCAGGATCCCCAGGAGCTTCCAATGGCCCATGAGCTCCCAGCAGCTTGGGAGGCTCAAGAACCCCCAGAAACCTAAGATGCACCAGCAGTCCGTGAGCCCCAGAATTCAGAA

Homo sapiens        CCCCAGGATCCCCCAAATATTGAGAAGCCCCAGGAGGCACCAGAATACCAGGAAACTGCAGCACAGCTAGAGTTCCTTGAGCTTCCACCTCCCCAGGAGCCTCTGGAGCCTTCAAATGCC
Loxodonta africana  TCTCAGGATCCCCCAAATGCCCAGGAGTCCCAGGAGGCACTGGCATACCAGGAGACCTTGGCACACCTGGAGCCCCTTGAGCTACCAGGTCCCCAGAAGCCCCTGGATCCCTCAGTTGCT

Homo sapiens        CAGGAATTCCTAGAACTCTCAGCAGCCCAGGAGTCCCTGGAGGGTCTAATAGTTGTGGAGACATCAGCAGCTTCAGAGTTTCCACAGGCCCCTATTGGGTTAGAGGCTACAGATTTCCCC
Loxodonta africana  CAAAAGTCCCTAGAGATCTCTGCACTCCAGGATCCCCTGGACAGCCTAATAGCTGTGAAGATATCAGTAGCTCCAGAGTTCCCACAGGGTCCCAGTGGGCTAAAGACTGCAGCTTTTTCC

Homo sapiens        CTGCAATACACTTTAACCTTCAGTGGAGATTCCCAGAAGCTTCCTGAGTTCCTGGTTCAGCTGTATAGTTACATGAGAGTCAGAGGGCACCTGTATCCCACT-GAAGCAGCCCTGGTGAG
Loxodonta africana  CTAGAATACCCTTTTGTCTTCAGTGTACATGTCCAGAAGCTTTCTGAGTTCCTGGTTCAACTGACCAATTACATGAGAGTCAGAGGACACCTGTATCCCACCAAAAGCAGCCTTGGTGAG

Homo sapiens        CTTTGTTGGCAATTGCTTCTCAGGTAGGGCAGGATGGTGGTTCCAGCTCTTACTGGATATCCAAAGCCCCTTACTGGAGCAATGTGAAAGTTTCATACCTGTGCTCCAGGATACTTTTGA
Loxodonta africana  TTTCGTCGGCAAGCACTTCCCGGGTGAGGCAGGAAGTTGGTTCCAGCCCTTAATATATATCCAAAGCCCCTTGCTGGAGCAATTTGAAAGTTTCATACCAGTGCTCCAGGATACTTTCAA

Homo sapiens        TAATCCAGAAAACATGAAAGATGCCAACCAGTGCATCCATCAACTCTGCCAAGGGGAGGGTCATGT---------AGCAACCCACTTCCACCTCATTGCTCAAGAGCTGAACTGGGATGA
Loxodonta africana  CAATCCAGAAAACATACAAGATGCCAGCCACTGCATCCCTCAGCTCTGCCAAGGGGAGCACCCTGTTCACCAGTACACAACCCACTTCCATCTCATTGCTCAAGAACTAAACAGGGATGA

Homo sapiens        AAGCACTCTCTGGATCCAATTTCAAGAAGGTCTTGCCAGTTCTATACAAGATGAACTGTCTCACACAAGCCCAGCCACCAACCTATCTGATCTCATCACTCAGTGCATCAGCCTGGAAGA
Loxodonta africana  AAGTACTCTCTGTATTCAATTTCAGGAAGGGCTTTCCAGTTCTATCCTATATGAACTGTCTCACACAAGCCCAGCCACCAACTTATTTGATCTGATCACCCAATGCATCAGCCTGGAAGA

Homo sapiens        ------------GAAGCCTGATCCCAATCCATTAGGGAAAAGTTCCTCTGCAGAGGGAGATGGACCCGAGAGTCCACCAGCTGAAAACCAACCTATGCAGGCTGCAATCAACTGCCCACA
Loxodonta africana  CAACCTAAGTGGTAAGCCTGATCCCAGTCCATCTGGAGCAAGCCCTTCTGAA----GAGATAGTCCTGAGAGCCCACCAGTTGAAAACCAGCCTGTGCAGAGTACAAGCCACCACCCACC

Homo sapiens        CATCAGTGAAGCTGAATGGGTCCGTTGGCACAAAGGCCGCTTATGCCTCTACTGTGGTTATCCTGGTCATTTTGCCAGAGATTGCCCTGTCAAGCCTCATCAGGCCCTGCAGGCGGGAAA
Loxodonta africana  GCTCAGTGAAGCTGAACAGGCCTGCCGCTTTGAAGGCCACTAGTGCCTCTACTGTGGTTATCCTGGTCATTTTCCCAGAGATTGCCCTATTAAACCTCATCGCATCCAACAAGTGGGAAA

Homo sapiens        CATCCAGGCCTGCCAGTAAGGGGGACCCCACGTGGGCCAATCTACAAATATGCGTCCCCCTCTCTTCCAATATCCATGTCTCATACACTATGGCTTACTGTTGAAATCCAACTGGGAAGA
```

```
Loxodonta africana  CATTGAGGCCCGGTGGTAAGGGGGACCCTGGCCAGGCCAATCTACAAATATGCGTACCCCTCTCTTCCAGTATCCATCTCCCATACCCTATGGCTAACTGTTGAAATTTGATTGGGAAGG

Homo sapiens        AGATAATTCTTTGAGCAAGCAATGGTAGATTCAGGCTCCTACAGAAACAGCATTGATCATACTGTGGTTCTTCGAGAGAAGCTGCCCATCCGCAGTAATATCTTCCCTCTGATGCTGGAA
Loxodonta africana  CGATAATTCTTTGAGCAAGCGATGGTGTATTCAGGCT-CTACAGAAACAGCATCGACCATTCTGTGGCTCTTCGAGAGAAGCTGCACATCCCTAATAACGTAGTCCCTCTGATGCTCGAA

Homo sapiens        ACTGTCGACGGCCATCCACTTATTAATGGACCCATAACTAAGGAAACATCACCTGTCCAAGTTCAAATTGGAAACCATGTTGAAGAGCTCCAGTTTGACATTATTCATGCACCACGATAC
Loxodonta africana  ACCTTTGATGGCCGTCCACTGATCGATGGACCCATAACAAAAGAAACACCACCTGTTAAAGTTAGAATTGGAAACCATATTGAAGAGCTCCAGTTTCACATAATTCATGCTCCAAGGTAC

Homo sapiens        CCTCTGATTATTGGAATCCATTGGCTTGAGACACATGACCCAAACATAGAATGGAGTACCCGCACTGTGTCCTTTCTATCACGTTATTGTCACTACAATTGCTTCAGGCACAGGTGGAAT
Loxodonta africana  CCTCTGATTCTTGGAATCCACTGGCTAAAGACACATGACCCAAACATAGAGTGGATTACTAGCACTGTGTCCTTTCCATCCCGTTATTACTGCTACAATTGCTTCAGACACAGATGGAAT

Homo sapiens        AGAAGAAATCGTGATGAAATAATTTCTGGGTAGATCCTGTGTCCTAGTGAC--------------------TGTTCCTGGCAACCTGTCTTCTGTTACCTCAGCTGTCATCAGCATTTGC
Loxodonta africana  AGAAGACGTCGTGATGAAATAATTGCTGGGTAGATCCTGCATCCTAGTGACATCCTACTCAGAATCTTGTTTGGCCCTGGCATCCCTTCTTCTATTACCTCAGCTGTCAACTACATTTGC

Homo sapiens        TGCTCCCTGAATCTTCTACTGAACATCTTGCTATGTACTAAGGCTACCTGTACAACGACTCTGCCTCTTAGATGATAGACTGAACCTGATGACTTTGAGCTGCTTTTTTTTTTTTCACTA
Loxodonta africana  TCTTCCCCTAATCTTCCACTGAACCTCT-------------GGCTATCTGTACAATGGCCCTACCTCTTGAATCATGGACTGAACCTGATGACTTTGAGCTGC-----TTTTTCTCACCA

Homo sapiens        ACTCTGCATGCCTCTATCTCAGAACTCTGCATCCTGCGGAACTGTTTTAATTACAA-TTATACTAACAATACACATGAGAATAGATATTAGAAACTAATAATGAAAAAGCATAGCTGTTA
Loxodonta africana  ATCCTGCATGCCCCCCTCTCTGAACCCTTCATTATGTGGGGATATTTTAATTATAATTTATACTAACAATATGCATAAGAATAGATATTAGAAACTAACACTGAAAAAGTGTAGTTGTTA

Homo sapiens        AACTGACTTGATTTTTTTCTTATCAATATTTTCTATATCACAAAAATGGCAACATCTTTTACATAAAGAAAATAAGTTTGTAAGTGATATTTCTTAAAAGAATCAATAAATATTAGCAAT
Loxodonta africana  GACTAATCTGA-TTTTTTCTTATCAACAATTCCTTTATCAC---AATTGCAACATCTTTTCAATAACAAAAATAAATTTATAAGTG--ATTTTTTAA-------AATAAATATTGACAAT

Homo sapiens        TTCTACTTGTGTTTGAGTTATTTCTTGGCTTAGCTAGAGGAAGAGA-TAATGAACCAGTAAATTGGGAGGGAGAG-------AGGGAATTAGAAAATGTTCAATGTTTAGGGAGGCTCCT
Loxodonta africana  TTTTACTTGTGTTTGAATTATTTCTTGGCTTAGCTGAGAGAAGAGACTAGGGGACATGCAAATTGAGAGGGAGGTAAGAAGCAAGGGAATGGAAAAGGCTCAGAGTTTGAGTGGAGCTCT

Homo sapiens        AAACATTCCGGGTGTAGTGATAGGTCTGCAGATGATGGAGAAATGATGTAGAGGGTAAGATGGAGAGAGTAGGGAATGGTTAGGGCTGAAAGTGAGAGTAGGGAATGGTTAGGGCTGAAA
Loxodonta africana  AAGCATTCCAAAGGTAGTGA--------CAGACTAAGGA--------------------------AGCGTAG----TGACAAGGACT---AGGGAAGGTGGGGGAGG---------GAGA

Homo sapiens        GTAAGTGAGGTATGGAGGGATTAGGGGAAAGGTAGAGGTAAGATTGAGAAGAGGGTTTGGGGGAGATTGGGAGGAGGTTGAAGAGGGTTTAGAG-GAGGGTTGGGG--TGCTGAGAGGTA
Loxodonta africana  TTGGGAGAGCTATGAAGGGATTAGTGGGAAAGTGGAGAAAAGGTTGAGAAGAAAGTTTGGGGGAGGTTGGGAGGAGGTTGGAGAAGGTTCAGGGAGAAGGCTGAGGATTGCTGAG..GTA

Homo sapiens        GAATCTCCAGGTGAGGTGTGAGGAG---TGGTCTAAGCAGAACCTAATGCTTTTCCTCTGTGTTTTCAGCTTTCCCTTAGGAGCCTCTCTCATTGAGGCTGCTAAAAGCGGACTCTTTCC
Loxodonta africana  GCATCTCTAGCT-AGATGCAAGAAAGGCTGGTCCAAGGCGACCCTAATGCTTTTCTTCTGCATTTTCAATGTTCCCTTGGGATTCTCTCTCTTTGAGGCCAATTAAAGGGGATTCATCCT

Homo sapiens        CTCATTTAGACAAATGTAGACAAAATATCAAATAAATGATTATAACCCTAGAAGGTAATAGGGAAGGGGCTTACCATTCATTGGACCACTAATATGTT-CAGCTACAATGTCAGACATTT
Loxodonta africana  CTCACTTACACAAATG----------ATCAAATAGATGATCATAAGCCTAGAATGATATAAGG--GAAACTTACCATGAGTTGAATCCTTACTATGTTCCAGCTATAATACTAGGCATGT

Homo sapiens        AACACACATTAATCATCTC
Loxodonta africana  TACA--------------C


Homo sapiens       GTTCAGGGAAGCATGAGATCTTTGG----------------------------------------------TCCAGGACATTCAGAAAGAGCCAGGGACTCTGTAAGACTGCTATAAGT
Procavia capensis  AATCAGGAAACCATGAGAACTCTTGGGTGACTAGCATATATGACATCTGATAATTAAGTGTGCAATTGTCATTCCAGAATATACAGAAGTAACTCAAGGCCCTGCAAG--TGTTGCGAGT

Homo sapiens       ACCCACCAACACAATTGGTCTCCCTGACATTTGTACCCTTTTATACCTTTTCTAGATCTGTCTGTGGCAAATTTTCCAAGAAAACATTGCAAGGAAAAACCCTAGGGACTGAATAGGTAG
Procavia capensis  ACCCATCAACAGAGTTGGTCTACCTCACAGTCTCACCCTGTTAAGTCAGGCCTAGAACTATCCAACATCAAATTATCAAGAAAAGATTGCAATGAGAAAGCACAGGGACTAATTAGATGG

Homo sapiens       TAAGCAAGGGAAGGGGCTGAGGGTTTGCAGGCTGTTCCCTGAGTGAGAAGTAAACAAATGGCCCAATTTGGACAGAGTAAGAATAGGTTAGATGAGACAGACATAAAAAATCACATCCCA
Procavia capensis  ---ACAGGGGAAGGGACTAAGG-----TGAACTACTCTATAAGTGAGAATTGAACCAATAATTCATTGTGGGTAGAGTAAGAA-AGGTTAAATGAAAGAGGCATACAAAATCAAGTCCCA

Homo sapiens       AACCACTGTGGTCATTCAAAAATGTTTGCAACTTTTCACAAGGGCAGTGGTATTACTCACAATTTCCTGGCCAAATATTCATTAAAGTAACCAAGGACTGAGTACATGAAGCTGCTTCAC
Procavia capensis  AACCAGTGTTTGCA-------ATGTTTGTAACTTTTCACAAGGGCAGTGGTATTCCTC.CAATTTCCTAGCTAAATATTAATTAAGGTAGCTGAAGACTGCGTACATGAATCTACCTTGC

Homo sapiens       ATCAGCACAGCATCCTACTTTCCTTGCAGCTCTGAACTGTGAGGTACTGTCAAAAAACTCTTCCACACTTTAAAAAGCTATGTTACAGAGGAAAGTTGGAATGTGTTCTGGGAGTGGGTG
Procavia capensis  ATCAGCACAATATCCTACTTTATTTGAGACTGTACACTATGATGCACTGTCAAAAAGAAATTCCACTCTTTAAATAGCTACATTACAGAGTAGAGTTGAAACATGTTCTAGGAGTG----

Homo sapiens       TGTTTGCTTGAGTTCAGTGTGTGCAGAAAAGGCAAGCCTCCACAGCCATTTACACATTTCCCTCCTCAGCAATGGGTACACAGACAGCCAAGCTCCTGGACTAAGTCTCTGAATGGTAAC
Procavia capensis  TGTTTGTTTGAGTTCAGTGTATACAGAAAAG-CAAGCCTCCACAGCCATTCA----------TCCTCAGGGATGGCTGCACAG----CCGAGCTCCGGGACTAAGACTCTGAATGGTAAC

Homo sapiens       GTACATTTATAAAGATTATTTTCTTAACTATTTTGAGGTATTTTATAAATAATATGCATGTGTCTTCTGAGGGCATATATATATATATAT-------ATATATATTTAGTCGACTTTTGC
Procavia capensis  GTTTATCTATAAAGATTATTT-CGTAACTATTTTGTGGAATTTTAGAAATAATATGCATGTGTCTTCTGAGTATATATATATGTATGTATGTTTTACATATGTATAACATTTGCCTTTGC

Homo sapiens       TTGTTTTCTCCTGAGGCTGGTCTTGTGTGGTTACTTATGTGGTATGATTTCTGAGGTGTCAGAAAGAGCTCCCTCCTACTTCCTACCACCCTAGTCACCCAAAAATATCTCTTGGGCTGG
Procavia capensis  TTGTTTTCTCCTGAGGCTAATCTTACATGGT-ACTTACGTGGTATAATTTCTAAGGTGTCAAGAAGGGCTCCCCTCCACCTCCTACT---CTAGTTACCCAAAAATATTTCTTACTTTGG

Homo sapiens       CCCACCGCAGAGCCTAAAATTCCCACATTGCTCAAACATTTGATTTAGTCACTGAAAGCAGCTGTTAAAATCCAAATGTTA-CATTGGGATATTAGGCAGATATTCAAGATTAAAGACTA
Procavia capensis  CCCA--GTAGAGCCTAAAACTCCCACATTGCCCAAACATATGATTTAGTCATTGGAAACTGCTGTTAAAATCAAAGTGTTAACATTTGGATGTTAAGCAGCTGTTAAAAATTAAAGGCTG

Homo sapiens       TATAAAACAGAAAA-TAATATTAAA-AAGTTGCTTTT--GATATAATGTTAAGTTTCAAAGGATAAATGCAAAATGACATGTGCAGTTTGGGACAGATGCTGTTCTTAAAATGTCGTGGA
Procavia capensis  GGTAAAACAAAAAAAGAATATGGGAGAAATTGTTTTTCTGATGTAATGTTAACTTTTTAAAGG--AATGCAAAATGACATACATAGGGTGGGTCAGGCAGTGTTCTTAAAATTTCATGGG

Homo sapiens       GTCAGATCCAGCATATATACCCCAGCTCATGAACATAAGCCTATGGTAAAAGAAACCCAGCATTGTTGGGCAGATTT-----TGTTTTATGTTGAATGTGTGCTTTCCACAGTTTCTGAT
Procavia capensis  ACCAGAGCCAGCAAATATGCTGCTGCTCATGAACTTCAGCTCTTGATGAAAGAAACTCAGCACTGTTAAGCAGATTTAAATTTAATTTATTTAAATGAATGCTT-CTGCAGTTTCTGAT

Homo sapiens       CCTCAGCTCCCACTCTCTTCGCAGTGTATCTGACACTTCCCCTTACTGAGCTCCGGAGGCTCCTTCTTGCCGAGCTCCGGAGGCTCCCAGTGACCTCTTGATAATCAGCCTTGGCGGGGC
Procavia capensis  CCTCAGCTTCTGATCTCTTTACAGCATATCTGACACCTCCCTTT-----------------------GCTGAGATTCAGAGGCTCCCAGTGACTTCCTTGCAATCAGCTTTGGCAAGAC

Homo sapiens       CCAAATACCACCCCTTGGAAAACTCACTGAGCCTGACATCTGCCTGTAAGCAGCTTTCAGGAAGGATCTCACAAACACCCACCCAATCTCTCTTGGTAGCAAATTGTCAAATCATACCCA
Procavia capensis  CTATATCCCCACCCTTTGAAAACTCATGAGGTCTGATATTGGCCTGTGAGAAACTTTGGGGAAAGACGTCACGAGAGCCCATTTAATCACT-TTGGCAGCAAACTCTCACACTAAGTCCA

Homo sapiens       TTTCCTCAAAAGATGGTGGAGGACTTAGCAGCCTCCTATATTGTTCTGAAATTGGAGAATGAAATTCGGCAGGCTCAAGTGCAGTGGCTGATGGAAGAAAATGCTGCTCTCCAGGCCCAG
Procavia capensis  TTTCCTCAAAAGACAGTAGAAGACTTAATAGCCTCCTATATTTCTCTGAAACTGGAGAATGAAATTCTTCAGGCCCATATGCAAACGCTTTCAGAAGAAAATGCTGCCCTCTAGTCATTA

Homo sapiens       ATCCCAGAGCTTCAGAAGTCCCAAGCAGCCAAGGAGTATGATCTACTCCGAAAGTCCTCAGAGGCCAAGGAGCCCCAGAAGCTCCCAGAGCACATGAATCCCCCAGCAGCCTGGGAGGCC
Procavia capensis  ATCCCAGAGCTCTAGAAACTGCAAGAAGCCAAAAAGTATAAACTTCTTTAAAAGCCTTCAGAGATCCAGGATCCCCAGCAGCCCTAAGAACCCCTGGATCTCTCAGAAGCCTGTAAACCC

Homo sapiens       CAAAAGACCCCAGAGTTCAAGGAACCCCAGAAGCCACCAGAGCCACAGGATCTTCTAC---CCTGGGAACCCCCAGCAGCCTGGGAACTCCAGGAGGCCCCAGCAGCCCCAGA-GTCCCT
Procavia capensis  CAAGAGCCCCTATAGGCCAAGAAGTCCCAGAAGCCCACAGAGTTCCAGGACCTCCCAGAAGCCTGGGA-------GCACTCTGAAGACTACA--ATGTCCCAGGAGAGCCAGAAGCCCCT

Homo sapiens       GGCGCCTCCAGCAACCCGGGAGTCCCAGAAACCTCCAATGGCCCATGAGATCCCAACAGTCTTGGAGGGCC--------------AGGGGCCTGCAAAC--------------------
Procavia capensis  GAACCCTCCAGTAGTCCAGGACCTCCAGGAGCTTCTAGTGGCCCATGA-ATCCCTCTAGGCCAAGGAAGTCTGTAAGTCTCCAAAGGAGGCCCACAATCCCTCGGCAACAAAAGAGGTTC

Homo sapiens       --------------ACCCAGGATGCCACAATAGCCCAAGAGCCCAAGAATTCAGAACCCCAGGATCCCCCAAATATTGAGAAGCCCCAGGAGGCACCAGAATACCAGGAAACTGCAGCAC
Procavia capensis  AAGAATGCCTAGAAACCTACAATTCTCCAGTAGTCTGTGAGACCCGCAATTCAGAACCCCAGGATTCCACAAACACCCAAGAGTCCCAGGAGGCACTAGCATACCAAGAGAACTTGGCAT

Homo sapiens       AGCTAGAGTTCCTTGAGCTTCCACCTCCCCAGGAGCCTCTGGAGCCTTCAAATGCCCAGGAATTCCTAGAACTCTCAGCAGCCCAGGAGTCCCTGGAGGGTCTAATAGTTGTGGAGACAT
Procavia capensis  ACCGGGAGCCCTCTGAGCTACTAGCTTCCCAGGAGCCGCTGGATCCCTCAGATGCTCAGGAGTTCCTGGAGCTCTCAGTACCCCAGGAGACCCTGGACAACCTAATAGATACCAAAGTAT

Homo sapiens       CAGCAGCTTCAGAGTTTCCACAGGCCCCTATTGGGTTAGAGGCTACAGATTTCCCCCTGCAATACACTTTAACCTTCAGTGGAGATTCCCAGAAGCTTCCTGAGTTCCTGGTTCAGCTGT
Procavia capensis  CAATTGCTCTGGAGTTCCCACAGTGTTC-AGTGGTTTACAGGCGGCAGCTTTTCCCCTAGAATACCACTTAGTCTTCAGTGAAGATCTCCAGAAGCTTCCTGAGTTCCTGGTTCAACTGA

Homo sapiens       ATAGTTACATGAGAGTCAGAGGGCACCTGTATCCCACTGAAGCAGCCCTGGTGAGCTTTGTTGGCAATTGCTTCTCAGGTAGGGCAGGATGGTGGTTCCAGCTCTTACTGGATATCCAAA
Procavia capensis  ATAATTACATGAGAGTCAGAAGGCATTTGTATCTCACCAAAGCAGCCTTGGTGAGTTTCACTGGCAAGCACTTTTCAGGTGAGTCAGGAAGTTGGTTCCAGACTTTAGTATATATTCAAA

Homo sapiens       GCCCCTTACTGGAGCAATGTGAAAGTTTCATACCTGTGCTCCAGGATACTTTTGATAATCCAGAAAACATGAAAGATGCCAACCAGTGCATCCATCAACTCTGCCAAGGGGAGGGTCATG
Procavia capensis  GCCC-GTATGGAATCAATGTGAAAGTTTCATACAAGTGCTCTAGGATACTTTTGACAATCCAGAAATTGTACAAGATACAAACCACCACACCCATAGGCTCTGCCAAGGGGAGGACCCTG

Homo sapiens       T---------AGCAACCCACTTCCACCTCATTGCTCAAGAGCTGAACTGGGATGAAAGCACTCTCTGGATCCAATTTCAAGAAGGTCTTGCCAGTTCTATACAAGATGAACTGTCTCACA
Procavia capensis  TTTACCAGTACACAACCCACTTTCACCTCATTGTTCAAGAGCTAAACAGAGATGAAAGTACTCTTTGT-TTCAGTTTCAGGAAGGGTGTTCCAGTTCTAT...................

Homo sapiens       CAAGCCCAGCCACCAACCTATCTGATCTCATCACTCAGTGCATCAGCCTGGAAGAGAAGCCTGATCCCAATCCATTAGGGAAAAGTTCCTCTGCAGAGGGAGATGGACCCGAGAGTCCAC
Procavia capensis  ...........................................................CCAAATCCATCAGGAACAAGCCCCTCTAAAGAGAACAACAGTCCTGAGAGCCCAC

Homo sapiens       CAGCTGAAAACCAACCTATGCAGGCTGCAATCAACTGCCCACACATCAGTGAAGCTGAATGGGTCCGTTGGCACAAAGGCCGCTTATGCCTCTACTGTGGTTATCCTGGTCATTTTGCCA
Procavia capensis  CAATTGAAAACCA---------GAGTGCAAGTCACCACCTACCCCTCAGTGAAGCTGACTGGGCCCACTACCTTGAAGTTCACTTGTGCTTCTACTGTGTCCATCCTGGTCATTTTGCCA

Homo sapiens       GAGATTGCCCTGTCAAGCCTCATCAGGCCCTGCAGGCGGGAAACATCCAGGCCTGCCAGTAAGGGGGACCCCACGTGGGCCAATCTACAAATATGCGTCCCCCTCTCTTCCAATATCCAT
Procavia capensis  GAGATTGCCTAATTAAACTTTATTGTGTCTAATAAGCATGAAACATCAAGGCCTGGCAGTAAGGGACACCCTTAGCAGGCCAGTCTACAAATACGCATCCCCCTCTTTTCTAATATCCAT

Homo sapiens       GTCTCATACACTATGGCTTACTGTTGAAATCCAACTGGGAAGAAGATAATTCTTTGAGCAAGCAATGGTAGATTCAGGCTCCTACAGAAACAGCATTGATCATACTGTGGTTCTTCGAGA
Procavia capensis  GTCCCATAT-CCATGGCTAACAGCTGAAATTCAACTG---AGAAGGTAATTCTTTGAGCAAGCACCAGTAAATTCAGGCTCCTACAAACACAGCATTGACCATTCTGTGATTCTTTGAGA
```

```
Homo sapiens       GAAGCTGCCCATCCGCAGTAATATCTTCCCTCTGATGCTGGAAACTGTCGACGGCCATCCACTTATTAATGGACCCATAACTAAGGAAACATCACCTGTCCAAGTTCAAATTGGAAACCA
Procavia capensis  GAAGCTACCTGTCCACAGCAACATATGCCCTCTAATGGTTGAAACCTTTGATGGCCATCCAGTGATTAATGGACCCATAACTATGGAAACACCACTTGTTGAAGCTAAAATTGGAGACCA

Homo sapiens       TGTTGAAGAGCTCCAGTTTGACATTATTCATGCACCACGATACCCTCTGATTATTGGAATCCATTGGCTTGAGACACATGACCCAAACATAGAATGGAGTACCCGCACTGTGTCCTTTCT
Procavia capensis  TATTGAAAAGCTCCAGTTTGACACACTTCATACTCCAATGTACCCTCTGATTCTTGGAATCCAGTGGCTTGAGACACATGACCAAAACATAGAGTGGAATACTTGCACTGTATCTTTTCT

Homo sapiens       ATCACGTTATTGTCACTACAATTGCTTCAGGCACAGGTGGAATAGAAGAAATCGTGATGAAATAATTTCTGGGTAGATCCTGTGTCCTAGTGACTGTTCCTGGCAACCTGTCTTCTGTTA
Procavia capensis  ATC-CATTATTGTCACTCCAATTGTTTCAGACACAAGTAGGATAGAAAATGTTGTGGTGAAATAATGGCTGGCTAGACCCTGTATCCTAGTGACTGCTCCTGGCATCCCTTCTTCTGTTA

Homo sapiens       CCTCAGCTGTCATCAGCATTTGCTGCTCCCTGAATCTTCTACTGAACATCTTGCTATGTACTAAGGCTACCTGTACAACGA---CTCTGCCTCTTAGATGATAGACTGAACCTGATGACT
Procavia capensis  CCTCATCTGTCAACAGCATTTGCTGATCCTGTCACCTTCTACCAGACCTTT-------------AGTTACCTGTACAAGAGGCCTCCTGCCTCTTGAGTCATGGACTCAACCTGATGACT

Homo sapiens       TTGAGCTGCTTTTTTTTTTTTCACTAACTCTGCATGCCTCTATCTCAGAACTCTGCATCCTGCGGAACTGTTTTAATTACAATT-ATACTAACAATACACATGAGAATAGATATTAGAAA
Procavia capensis  TAGAGCTGC-----TTTTTTTCATGAATCTTGAACATCCCCCTCTCTGAACCCTTCACTATGTGGAGATATTTTAATTATAATTCATACTAACAATATTAATAAGAATAGATATTAAAAA

Homo sapiens       CTAATAATGAAAAAGCATAGCTGTTAAACTGACTTGATTTTTTTCTTATCAATATTTTCTATATCACAAAAATGGCAACATCTTTTACATAAAGAAAATAAGTTTGTAAGTGATATTTCT
Procavia capensis  CTGACCCTGAAAAAGTCTCATTGTTAGAA-----TAATTTTTTTCTTATCAACATTTTCTTCATCAC---AATTGTACTGTCTTTTCAATAAGGAAAATAAATTAGCAAGTGATACTTC-

Homo sapiens       TAAAAGAATCAATAAATATTAGCAATTTCTACTTGTGTTTGAGTTATTTCTTGGCTTAGCTAGAGGAAG-AGATAATGAACCAGTAAATTGGGAGGGAGAGAGGGAATTAGAAAATG---
Procavia capensis  -AAAA-----AGTAAGTACTGGAAATATTTACTTACATTTGAATCATTTATAGGTTTAGCTAAGTGAAGTGGGTAGCGTACAAGCAAACTGAGAAGGAGAGAAGAAGCAAGGGAATGGAC

Homo sapiens       ----TTCAATGTTTAGGGAGGCTCCTAAACATTCCGGGTGTAGTGATAGGTCTGCAGATGATGGAGAAATGATGTAGAGGGTAAGATGGAGAGAGTAGGGAATGGTTAGGGCTGAAAGTG
Procavia capensis  AAGATCCAGTGTTTGTGTGGAGCCCTAAGCATTCCAAAGATAAGGACAGG----------------------------------------AACTAGGGAA-GGGTAGTGGTAA-----

Homo sapiens       AGAGTAGGGAATGGTTAGGGCTGAAAGTAAGTGAGGTATGGAGGGATTAGGGGAAAGGTAGAGGTAAGATTGAGAAGAGGGTTTGGGGGAGATTGGGAGGAGGTTGAAGAGGGTTTAGAG
Procavia capensis  AGACTGGGGAAGGGATGGAG---------AGGGAGATATG------TTAGTGAGGAAGCAGAGGAAAGGTTGAAAAGAGAGTTTGCAGGAGGTTGGGAAGACTTTGGAAAAGGTTCAGGG

Homo sapiens       GAG-GGTTGGGGTGCTGAGAGGTAGAATCTCCAGGTGAGGTGTGAG---GAGTGGTCTAAGCAGAACCTAATGCTTTTCCTCTGTGTTTTCAGCTTTC-CCTTAGGAGCCTCTCTCATTG
Procavia capensis  GGGCTGTTGGGG------GAGGTGGAACCTCCAGCTAAGATGAGAGAAGGGGTGGTCCAAGCACACCTTAACACTT-----CTGTGTTTTCAGCATCCGCTTTGGGAGACTCTCT--TTG

Homo sapiens       AGGCTGCTAAAAGCGGACTCTTTCCCTCATTTAGACAAATGTAGACAAAATATCAAATAAATGATTATAACCCTAGAAGGTAATAGGGAAGGGGCTTACCATTCATTGGACCACTAATAT
Procavia capensis  AGACCACTTAAAGAGAATTCATCCCCTTGCTTATACAAATA----------ATCAAATAAATGATCAGAAGCCTAGAA................TTGCCATTAGTAAAACCGTTACTAT

Homo sapiens       GTTC-AGCTACAATGTCAGACATTTAACACACATTAATCATCTC
Procavia capensis  GTTCCAGCTACAATACAAAGTGTGTTACACA-ATATATTTTCT-
```

```
Homo sapiens  GTTCAGGGAAGCATGAGATCTTTGGTCCAGGACATTCAGAAAGAGCCAGGGACTCTGTAAGACTGCTATAAGTACCCACCAACACAATTGGTCTCCCTGACATTTGTACCCTTTTATACC
Bos taurus    GTTCAGGGAAAGATAAGAACCCTGGGCCAAGACATTCAGAGCAAGCCAAGGACCTTGCAAGACTGTTACAAGTACCCTTCAATAAAGTTGGCCTCCCACAAAATCTTACCCTATTATACC

Homo sapiens  TTTTCTAGATCTGTCTGTGGCAAATTTTCCAAGAAAACATTGCAAGGAAAAACCCTAGGGACTGAATAGGTAGTAAGCAAGG--GAAGGGGCTGAGGGTTTGCAGGCTGTTCCCTGAGTG
Bos taurus    TTGTCCAGGTCTGTCCATGGTAAAATTCCCAAGAAAACATTACAGGGAGGGACACTAGGAACAGAGTAAGTGGGGGGCGGGGCGGCGGGGACTAAGGTGTTGCAGGCTGCTCTCTGAGTA

Homo sapiens  AGAAGTAAACAAATGGCCCAATTTGGACAGAGTAAGAATAGGTTAGATGAGACAGACATAAAAAATCACATCCCAAACCACTGTGGTCATTCAAAAATGTTTGCAACTTTTCACAAGGGC
Bos taurus    AGAATTCAACAAACAGCCCAGTGTGGACAGAGTGATGGGAGGTTATATGAGACAGATATAAAAAAATAAACTCCAAAACTACTGTGGTCATTCAGAAATGTTTGCAACTTTTCACAAGGGC

Homo sapiens  AGTGGTATTACTCACAATTTCCTGGCCAAATATTCATTAAAGTAACCAAGGACTGAGTACATGAAGCTGCTTCACATCAGCACAGCATCCTACTTTCCTTGCAGCTCTGAACTGTGAGGT
Bos taurus    AGTGGCATTACTCCCAGTTTCCTGGCCAAATATTTATTAAAGTAACCAAGGACTGAGTACATGAGGCTGCCTCACATCAGTGCCGCATCCTACTTTCTTTGTGGCTCTAAACTGAGAGGT

Homo sapiens  ACTGTCAAAA----------AACTCTTCCACACTTTAAAAAGCTATGTTACAGAGGAAAGTTGGAATGTGTTCTGGGAGTGGGTGTGTTTGCTTGAGTTCAGTGTGTGCAGAAAAGGCAA
Bos taurus    ACTGTCAAAAGAAATAAATAAATTCTTCCACACTCAAAATAGCTATGTTACAGAGGAGAGCTGGAATGTGTTCTTGGAGTGGGTATGTTTGTTTGAGTTCAGTGTATGCAGAAAAGACAA

Homo sapiens  GCCTCCACAGCCATTTA----CACATTTCCCTCCTCAGCAATGGGTACACAGACAGCCAAGCTCCTGGACTAAGTCTCTGAATGGTAACGTACATTTATAAAGATTATTTTCTTAACTAT
Bos taurus    GCCTCCACAGCCATTCATTCACATGCTTCCCTTCTCTGTGACAGGTATGCAGACAGTGGAGCTCTTGGACTAAGTCTCTGAATGGTAATGTACATTTATAAATATTATTTTCCTAACTAT

Homo sapiens  TTTGAGGTATTTTATAAATAATATGCATGTGTCTTCTGAGGGCATATATATATATATATATATATATTTAGTCGACTTTTGCTTGTTTTCTCCTGAGGCTGGTCTTGTGTGGTTACTTAT
Bos taurus    TTTGAGGTATTTTATAAATAATGCACATGTGTCTGCTGA----------------ACATATATATATTTAGTAAAATTTTGCTTGTTTGTTCCTGAGACTAATCTTGTGTGTTTACTTAG

Homo sapiens  GTGGTATGATTTCTGAGGTGTCAGAAAGAGCTCCCTCCTACTTCCTACCACCCTAGTCACCCAAAAATATCTCTTGGGCTGGCCCACCGCAGAGCCTAAAATTCCCACATTGCTCAAACA
Bos taurus    GTGGTATGATTTCTGAGATGTCAGAAAG--CTCCCCCCTACCTCTTACC---CCAGCCACCCAAAAATATCTCTTGGGTTTGCCCAC-ACATAACTTAAAATCTCCACATTGTTCAAACA

Homo sapiens  TTTGATTTAGTCACTGAAAGCAGCTGTTAAAATCCAAATGTTACATTGGGATATTAG-GCAGATATTCAAGATTAAAGACTATATAAAACAGAAAATAATATTAAAAAGTTGCTTTTGAT
Bos taurus    TTTGATTTAGTCATTCAAAGCAGCTGTTAAAATCCAAGTGTTACATTGGGAAATTAAAGTAGACATTAGAAATTAAATACTATATAAAACAGGAAAGAATAGGGGGAAATTGCTTCTATT

Homo sapiens  ATAATGTTAAGTTTCAAAGGATAAATGCAAAATGACATGTGCAGTTTGGGACAGATGCTGTTCTTAAAATGTCGTGGAGTCAGATCCAGCATATATACCCCAGCTCATGAACATAAGCCT
Bos taurus    ATAATGCTCAGTTTCAAAAGAAGAATGCAAAATAA-ATACACAGTCTTGATCAGATGTTGT-CTTAAGATGTCATGAGGTCAGAGCTAGCATATATGACCCAGCTCATGAATATAAGCC-

Homo sapiens  ATGGTAAAAGAAACCCAGCATTGTTGGGCAGATTTTGTT-----TTATGTTGAATGTGTGCTTTCCACAGTTTCTGATCCTCAGCTCCCACTCTCTTCGCAGTGTATCTGACACTTCCCC
Bos taurus    ----------------AGCACCGTCAGGCAACTTTTCTTATTATTTATTTCTAATGAGTGCTTTCCACAGTTTCTGATCCTCAGATCACACTCTCTTCACAGTGTATCTAATATCTCCCT

Homo sapiens  TTACTGAGCTCCGGAGGCTCCTTCTTGCCGAGCTCCGGAGGCTCCCAGTGACCTCTTGATAATCAGCCTTGGCGGGGCCCAAATACC-ACCCCTTGGAAAACTCACTGAGCCTGACATCT
Bos taurus    TTTCTGAGCTCCAGAGGCT-------------------------------------TGTTAATCAATTTTGGTGGGACACAGATCCCCATTTTCTGGAAAGCTCACGGAGCCTGATATCT

Homo sapiens  GCCTGTAAGCAGCTTTCAGGAAGGATCTCACAAACACCCACCCAATCTCTCTTGGTAGCAAATTGTCAAATCATACCCATTTCCTCAAAAGATGGTGGAGGACTTAGCAGCCTCCTATAT
Bos taurus    GCCTGTGAGGAGCTTTTAGAAAG-ACCTCACAAGCACTCACCTA--CTCTCTCAGCAGCAAACTGCCAAACCAACCTCATTTCCTTAAAAGATGATGGAGGACTTAGCAGCATCCTATAT

Homo sapiens  TGTTCTGAAATTGGAGAATGAAATTCGGCAGGCTCAAGTGCAGTGGCTGATGGAAGAAAATGCTGCTCTCCAGGCCCAGATCCCAGAGCTTCAGAAGTCCCAAGCAGCCAAGGAGTATGA
Bos taurus    TGCTCTGAAATCAGAGAATGAAATTCTGCAGGCTCAAGT-CAGCAGCTGATGGAGGAAAATGCTGCCTTGCAGTCCCTCATTCAAGAACTCCAGAAGCCCCAAGCAGCCAAGGAGGATGA

Homo sapiens  TCTACTCCGAAAGTCCTCAGAGGCCAAGGAGCCCCAGAAGCTCCCAGAGCACA-TGAATCCCCCAGCAGCCTGGGAGGCCCAAAAGACCCCAGAGTTCAAGGAACCCCAGAAGCCACCAG
Bos taurus    ACCACTCC-----------------AGGAGACCCAGAGACTCCCAGAGCCCCCTGGATTTTCTAGCAGTCTTGGAACCCCAAGAGCCCCCAGATGTCGAGGAGTCCCA-AAGCCCAGAG

Homo sapiens  AGCCACAGGATCTTCTACCCTGGGAACCCCCAGCAGCCTGGGAACTCCA---------GGAGGCCCCAGCAGCCCCAGAGTCCCTGGCGCCTCCAGCAACCCGGGAGTCCCAGAAACCTC
Bos taurus    AGTCTCAGGACCTCCAAACCTGGGAGACCTCAGCATTTCAGGATCCCCAAAAGTCCTTGGAGCCCCTAGCATCTCCAGGGTGCCTGGAGCCCCCAGCAACCCAGGAGCCTCAGAAGCTCC

Homo sapiens  CAATGGCCCATGAGATCCCAACAGTCTTGGAGGGCCAGGGGCCTGCAAACACCCAGGATGCCACAATAGCCCAAGAGCCCAAGAATTCAGAACCCCAGGATCCCCCAAATATTGAGAAGC
Bos taurus    CAATGGCCCAGGAGAGCCTACCAGGCTCGGAGGATCAGAAGTCCCCAGATACCCAGAATGCCCCAGCAGGCCAGGAGCCCCAGAAATTAGAGCCCTGGCACCCCTCAAATGCCCAGGAGC

Homo sapiens  CCCAGGAGGCACCAGAATACCAGGAAACTGCAGCACAGCTAGAGTTCCTTGAGCTTCCACCTCCCCAGGAGCCTCTGGAGCCTTCAAATGCCCAGGAATTCCTAGAACTCTCAGCAGCCC
Bos taurus    TACAGGAGGCACCAAAATGTCAAGATACCTCTAGACACATGGAATTGTTTGAGTTTCCTGCTCCCCAGGAGCTCCAGGATCCTAAACATGCCCAGGAATTCCTAGGACTCATAACATCCA

Homo sapiens  AGGAGTCCCTGGAGGGTCTAATAGTTGTGGAGACATCAGCAGCTTCAGAGTTTCCACAGGCCCCTATTGGGTTAGAGGCTACAGATTTCCCCCTGCAATACACTTTAACCTTCAGTGGAG
Bos taurus    AGGAGTCCCTGGACAGTCTAACAGCTGCTGAAACAACAACAGCTTCAGAGTTTCCACAGTCTTCCAATGAGTTAAAGGATGAAGCTTTCCTCCTAGAATATTCTTTAGCCTTCAGTAGGG

Homo sapiens  ATTCCCAGAAGCTTCCTGAGTTCCTGGTTCAGCTGTATAGTTACATGAGAGTCAGAGGGCACCTGTATCCCACTGAAGCAGCCCTGGTGAGCTTTGTTGGCAATTGCTTCTCAGGTAGGG
Bos taurus    ATACCCAGAAGATTCCTGAGTTTCTGGTTCAATTGAAGAGTTCCATGAGAGTCAGAGGGTGCTTGTATCCCACCAAAGCAGCTCTGGTAAGCTTTGTTGGCAACTACTTCTTAGGTAAAG

Homo sapiens  CAGGA---------------TGGTGGTTCCAGCTCTTACTGGATATCCAAAGCCCCTTACTGGAGCAATGTGAAAGTTTCATACCTGTGCTCCAGGATACTTTTGATAATCCAGAAAAC
Bos taurus    AAGAAAAGTTGTTCCAGCTGTTAGTGGTTCCAGCTGTTAGCAGATATCCAAAGCCCCCTGCTGGAGCAATTTGAAAGTTTCATGCAAGTGCTTCAGGATACTTTTGACAATCCAGAAAAC

Homo sapiens  ATGAAAGATGCCAACCAGTGCATCCATCAACTCTGCCAAGGGGAGGGTCATGTA---------GCAACCCACTTCCACCTCATTGCTCAAGAGCTGAACTGGGATGAAAGCACTCTCTGG
Bos taurus    ATGGAAGATGCCAACCACCACATCCCTAAGCTCTGCCAACAAGAAGACCATGTCCACCAGTATGCAACCCACTTCCACCTCATTATTCAAGAACAAAATTTGGGTGAAAGCATTCTCTGA

Homo sapiens  ATCCAATTTCAAGAAGGTCTTGCCAGTTCTATACAAGATGAACTGTCTCACACAAGCCCAGCCACCAACCTATCTGATCTCATCACTCAGTGCATCAGCCTGGAAGAGAAGC--------
Bos taurus    ATCCAATTTCAGGAAGGGTTTGCCAGTTCTATCTGAAGTGAACTGTCTCACACAAGCCCAGCTACCAATCTGTCTGATCTGATCACCCAATGCATCTCCCTAGAAGAGAAGCTAAGTGGT

Homo sapiens  ----CTGATCCCAATCCATTAGGGAAAAGTTCCTCTGCAGAGGGAGATGGACCCGAGAGTCCACCAGCTGAAAACCAACCTATGCAGGCTGCAATCAACTGCCCACACATCAGTGAAGCT
Bos taurus    AAACCTGATCCAAGTCCACAAGATGCAAGTCCCTCTGAGGAGAGAGCTGGACTCAAGAGTTTACCAGCTGAAAACCAGCCAGTGCAGGCTTCAAGTTGTCACCAACACCTCAATGAAGCT

Homo sapiens  GAATGGGTCCGTTGGCACAAAGGCCGCTTATGCCTCTACTGTGGTTATCCTGGTCATTTTGCCAGAGATTGCCCTGTCAAGCCTCATCAGGCCCTGCAGGCGGGAAACATCCAGGCCTGC
Bos taurus    GATCGAGCCCGCTGCCGTGAAGGCCACTTGTGCCTCTACTGTGGACATCCTGGTCATTTCACCAGAGACTGCCCTGTCAAGCTTCATCATGCCCAGCAGGTGGGAAACATCGAGGCCCTG

Homo sapiens  CAGTAAGGGGGACCCCACGTGGGCCAATCTACAAATATGCGTCCCCCTCTCTTCCAATATCCATGTCTCATACACTATGGCTTACTGTTGAAATCCAACTGGGAAGAAGATAATTCTTTG
Bos taurus    TGGTAAGGGGGAACCCGGGCAGGCCAATCTACAAATATGCATCCCCCTCACTTCCAGTATCCATATTTCATACACTATGGTTTACCGTTGAAATCCGACCGGGAAGACGACAGCTCTTTG

Homo sapiens  AGCAAGCAATGGTAGATTCAGGCTCCTACAGAAACAGCATTGATCATACTGTGGTTCTTCGAGAGAAGCTGCCCATCCGCAGTAATATCTTCCCTCTGATGCTGGAAACTGTCGACGGCC
Bos taurus    AGCAAGCTACAGTGGATTCAGGCTCCTACAGAAACAGCATTGACCATTCTGTGGTTCCTCAAGAGAAGCTGCCCATTCGCAGTAACATCTTTTCTCTGATGCTGGAAACTGTAGATGGCC

Homo sapiens  ATCCACTTATTAATGGACCCATAACTAAGGAAACATCACCTGTCCAAGTTCAAATTGGAAACCATGTTGAAGAGCTCCAGTTTGACATTATTCATGCACCACGATACCCTCTGATTATTG
Bos taurus    ATACATTGATTAATGGACCTATAACCAAGGAAACACCACCTGTTGAAGTTAAAATTGGAAACCATGTTGAAGAGCTCCAGTTTGACATTATTCATGCACCAAGGTACCTTCTGATTCTTG
```

```
Homo sapiens   GAATCCATTGGCTTGAGACACATGACCCAAACATAGAATGGAGTACCCGCACTGTGTCCTTTCTATCACGTTATTGTCACTACAATTGCTTCAGGCACAGGTGGAATAGAAGAAATCGTG
Bos taurus     GAATCCA----CTTGAAACACATGATCCTAACATCGAGTAGAGTACCCACACTATATCTTTTACATCCTGTTATTGTCACTACAATTGCTTCAGGCACAGGTGGAATAGAAGACATCATG

Homo sapiens   ATGAAATAATTTCTGGGTAGATCCTGTGTCCTAGTGACTGTTCCTGGCAACCTGTCTTCTGTTACCTCAGCTGTCATCAGCATTTGCTGCTCCCTGAATCTTCTACTGAACATCTTGCTA
Bos taurus     ATGAAATAATTGCTGGGTAGATTCTTTGTCCTAGTGACTACTCCAAGAAACCTATCTTTTGTAACCTCAGCTGTCATCAGCATTTGCTGCTCCCAGAATCTTCCACTGAATCTCTGGCAA

Homo sapiens   TGTACTAAGGCTACCTGTACAACGACTCTGCCTCTTAGATGATAGACTGAACCTGATGACTTTGAGCTGCTTTTTTTTTTTTCACTAACTCTGCATGCCTCTATCTCAGAACTCTGCATC
Bos taurus     TGAACTAAGGCTACCTGTACAACAACCCTGCCTC--GGATTGTGAACTGAACCTGATAACTTTGAATTAC----ATTTTTTTCACCAATCCTTCATGCCCCCTTCTTTGAACCCTACATT

Homo sapiens   -CTGCGGAACTGTTTTAATTACAA-TTATACTAACAATACACATGAGAATAGATATTAGAAACTAATAATGAAAAAGCATAGCTGTTAAACTGACTTGATTTTTTTCTTATCAATATTTT
Bos taurus     AATGTGGGCCTGTTTTAATTACAATTTATTCTAACAATATGCATGA----AGATAATAGAAACTAACATCTAAAAA-TGTAGTTGTTATATTGACTTGATTTTTTCCTTATCAAGATTTT

Homo sapiens   CTATATCACAAAAATGGCAACATCTTTTACATAAAGAAAATAAGTTTGTAAGTGATATTTCTTAAAAGAATCAATAAATATTAGCAATTTCTACTTGTGTTTGAGTTATTTCTTGGCTTA
Bos taurus     CTTTATCACAACAATTGCAGCATATTTTCAATAAAGAAAATAAATTTGCAAGTGA---TTTTTTAAAAAACCAATAAACTAGTGCAAATTCTACTAGTGTTTTAATTATTTCTTGGTTTA

Homo sapiens   GCTAGAGGAAGAGATAATGAACCAGTAAATTGGGAGGGAGAGAGGGAATTAGAAAATGTTCAATGT--TTAGGGAGGCT-----CCTAAACATTCCGGGTGTAGTGATAGGTCTGCAGAT
Bos taurus     GCTGAGTGAATGAATAAGACACAAGTAAATTAGGAGGGAGAGAGGAAAGTAGGAAGGGAGGGAGGGAATTGAAAAGTCTCAAAGCTTGAACATTCCAGGAGTAGTGACATGTCAGTGAGT

Homo sapiens   GATGGAGAAATGATGTAGAGGGTAAGATGGAGAGAGTAG-GGAATGGTTAGGGCTGAAAGTGAGAGTAGGGAATGGTTAGGGCTGAAAGTAAGTGAGGTATGGAGGGATTAGGGGAAAGG
Bos taurus     GATGGG--------GTACAGG--AGGATGGACAGAGCAGAGGACTGGTTGG-------AGTGGAGGTAG-----------------ACTGAATGAGAAATGGAGGGATTAGAGAGAGGA

Homo sapiens   TAGAGGTAAGATTGAGAAGAGGGTTTGGGGGAGATTGGGAGGAGGTTGAAGAGGGTTTAGAGGAGGGTTGGGG--TGCTGAGAGGTAGAATCTCCAGGTGAGGTGTGAGGAGTGGTCTAA
Bos taurus     TAGAGGGAAAGTTTAGAAGAGGGTTT-GGGAAAATTAGAAGGAGGTTGAAAAGGATTCAGATGAGAGTTAGGAGCTGCTGAGAG----------CAGG---GGTGAGAGGAAGGGT....

Homo sapiens   GCAGAACCTAATGCTTTTCCTCTGTGTTTTCAGCTTTCCCTTAGGAGCCTCTCTCATTGAGGCTGCTAAAAGCGGACTCTTTCCCTCATTTAGACAAATGTAGACAAAATATCAAATAAA
Bos taurus     ........................................................................................................................

Homo sapiens   TGATTATAACCCTAGAAGGTAATAGGGAAGGGGCTTACCATTCATTGGACCACTAATATGTTC-AGCTACAATGTCAGACATTTAACACACATTAATCATCTC
Bos taurus     .......AACCCTAGAAGGAGACAGGTGAGGAATTTACCATTCATTGAGCCTGTAATATCTTCCAGCAACAATGTCTGGCATGTTATGCACATTAATTATTTA
```

```
Homo sapiens   GTTCAGGGAAGCATGAGATCTTTGGTCCAGGACATTCAGAAAGAGCCAGGGACTCTGTA--AGACTGCTATAAGTACCCACCAACACAATTGGTCTCCCTGACATTTGTACCCTTTTATA
Mus musculus   GCTCAGGTGGGCGGAAGAACCCTGGGCAGGTATATTCACAATCAGCCAAGTGCTTTTTACAAGATAGCCA---------ATCAAAA--GTTTGCCTCTCTGACATCCTTACTCTATTATA

Homo sapiens   CCTTTTCTAGATCTGTCTGTGGCAAATTTTCCAAGAAAACATTGCAAGGAAAAACCCTAGGGACTGAATAGGTAGTAAGCAAGGGAAGGGGCTGAGGGT-TTGCAGGCTGTTCCCTGAGT
Mus musculus   AGTTTCCTAGATCTGTCCACGGTGAAGATTCTAAAAAAGTATTTCAAGGTGGGACACTCGGGACTGAGTAGGTAG---ACAGGAGGAAGGACTGAGGGGGCTGTAGTTTGCTCTCTAAGT

Homo sapiens   GAGAAGTAAACAAATGGCCCAATTTGGACAGAGTAAGAATAGGTTAGATGAGACAGACATAAAAAA-TCACATCCCAAACCACTGTGGTCATTCAAAAATGTTTGCAACTTTTCACAAGG
Mus musculus   AAGAATTAAACAAACGTCTCAGTGTGGATAGAGTACGGGTAAGTTACGTGAAACAGACATAAAAAAATCAAATCCCAAACCACTGCGGTCATTCAGAAATGTTTGCAACTTTTCACAAGG

Homo sapiens   GCAGTGGTATTACTCACAATTTCCTGGCCAAATATTCATTAAAGTAACCAAGGACTGAGTACATGAAGCTGCTTCACATCAGCACAGCATCCTACTTTCCTTGCAGCTCTGAACTGTGAG
Mus musculus   GCAGTGGTATTACTCCCAATTTCCTGACCAAATATTTATTAAAGTAACCAAGGACTGAGTGCACGAAGCTGCCTCACATTGGCGCAGCATCCTACTTTCCTTCTAGCTCGGAACTGTGAG

Homo sapiens   GTACTGTCAAAAAA----------------------------CTCTTCCACACTTTAAAAAGCTATGTTACAGAGGAAAGTTGGAATGTGTTCTGGGAGTGGGTGTGTTTGCTTGAGTTC
Mus musculus   GTACTGTCAAAAGAGAGAGAGAGAGAGAGAGAAAAAAAAAAACCACTCTTCCATACTCAAAATAGCTACGTTACAGAGGAGAGTTGGAACGTGTTCTGGGAGTGGGTGTGTTTGTTTGAGTTC

Homo sapiens   AGTGTGTGCAGAAAAGGCAAGCCTCCACAGCCATTTACACATTTCCCTCCTCAGCAATGGGTACACAGACAGCCAAGCTCCTGGACTAAGTCTCTGAATGGTAACGTACATTTATAAAGA
Mus musculus   AGTGTGTGCAGAAAAGGCAAGCCTCCGCAGCCATTCACACACTTCTCTCCTCAGCGATGGCTATGCAGACAGCAGAGCTCCAGCCCTACATCTCTAAATGGTAATGTACATCTATAAAGA

Homo sapiens   TTATTTTCTTAACTATTTTGAGGTATTTTATAAATAATATGCATGTGTCTTCTGAGGGCATATATATATATA----------TATATATATATTTAGTCGACTTTTGCTTGTTTTCTCCT
Mus musculus   GGATTTTGCTAACTATTTTGAAGCATTTTATAAATAATATGCATGTGCCTGCTTAATATGTATATATGTATGCTCATTTATTTATTTATTTATTTATTCAACTTTTGCTTGCTTTCTCCC

Homo sapiens   GAGGCTGGTCTTGTGTGGTTACTTATGTGGTATGATTTCTGAGGTGTCAGAAAGAGCTCCCTC----CTACTTCCTACCACCCTAGTCACCCAAAAATATCTCTTGGGCTGGCCCACCGC
Mus musculus   GTGTCTGATTGTGTGTGGTTACTTGTGTGGTATGATTTCTGAGGTGGGAGAAAGAGCACCCCCACCCCCAATTCCTATG---CTAGTCACTCAAAA-TATCTCTTGTGTTAGTCCTCC--

Homo sapiens   AGAGCCTAAAATTCCCACATTGCTC-AAACATTTGATTTAGTCACTGAAAGCAGCTGTTAAAATCCAAATGTTACATTGGGATATTAGGCAGATATTCAAGATTAAAGACTATATAAAAC
Mus musculus   CAAACCTAAAATCCCCATAGCGCTCTAAACATCTGATTTGGTCGCTGAAAGCAGCTGCTAACATCCAAATGTTACATTGAAATATTAGGTAGACACTAGAAATTAAAAACGACTTAAAAC

Homo sapiens   AGAAAATAATATTAAAAAGTTGCTTTTGATATAATGTTAAGTTTCAAAGGATAAATGCAAAATGACATGTGCAGTTTGGGACAGATGCTGTTCTTAAAATGTCGTGGAGTCAGATCCAGC
Mus musculus   A--AAGGCATATGGGGCAATTGTTTCTGATATAGTGTGAAGTTTCAAA----AAATGCAAACTGACATGTACAGTCTGGGTCATGTGCT---TGTAAAATGTCATGAGGTCAGGGCAAAT

Homo sapiens   ATATATACCCCAGCTCATGAACATAAGCCTATGGTAAAAGAAACCCAGCATTGTTGGGCAGATTTTGTT---TTATGTTGAATGTGTGCTTTCCACAGTTTCTGATCCTCAGCTCCCACT
Mus musculus   AAATATACTCCAGTTCATGAACATGAGCTTGTGGTAAAAGAAAGCCAGCAGAGTTGGACAGATTTTGTTGATTTATATAGAATGAGTGCTTTCCACAGTTTCTGATCCTCAGCTCCCTCT

Homo sapiens   CTCTTCGCAGTGTATCTGACACTTCCCCTTACTGAGCTCCGGAGGCTCCTTCTTGCCGAGCTCCGGAGGCTCCCAGTGACCTCTTGATAATCAGCCTTGGCGGGGCCCAAATACC-ACCC
Mus musculus   CTCTTCACAGTGTGTCTCACACTTCCCGGTGCTGAGCTCTA------------------------AGGCTCCCAGTGACCTCTTGATAATCAGCCTTGGCTGGGTCCAAATCCCCACCC

Homo sapiens   CTTGGAAAACTCACTGAGCCTGACATCTG----CCTGTAAGCAGCTTTCAGGAAGGATCTCACAAACACCCACCCAATCTCTCTTGGTAGCAAATTGTCAAATCATACCCATTTCCTCAA
Mus musculus   CTTGGAAAAGTCACGGAGCCTGACGTCTGTCTGCCTGTGAGCCGCTTTCAGCAAAGACCTCACAAGCAGCTACTCAATCTCTCTTGGTAGCAAGTTGCCAAACCAGGCCCGTTTCCTCAA

Homo sapiens   AAGATGGTGGAGGACTTAGCAGCCTCCTATATTGTTCTGAAATTGGAGAATGAAATTCGGCAGGCTCAAGTGCAGTGGCTGATGGAAGAAAATGCTGCTCTCCAGGCCCAGATCCCAGAG
Mus musculus   AAGATGGTAGAGGACTTAGCAGCTTCCTATGTTACTCTGAAATTGGAGAATGAAATTCTGCAGGCTCAAGTGAAAAGGCTCATGGAAGAAAATGCTGCCCTCCAGGCCCAGATACCAGAG

Homo sapiens   CTTCAGAAGTCCCAAGCAGCCAAGGAGTATGATCTACTCCGAAAGTCCTCAGAGGCCAAGGAGCCCCAGAAGCTCCCAGAG-------------------CACAT--------GAATCCC
Mus musculus   CTCCAGAAATCCGGAGCAGTCAAAGAGCATGAACCACTCCGAAAGCCCTCAGAGGCCCAAGAGCCCCCAGAGTCCCCAGAGTTCCCAGCAGCCAGGGAGTCACAGAACCCCTGGGAACCC

Homo sapiens   CCAGCA--------GCCTGGGAGGCCCA-AAAGA---------------------------------------------CCCCAGAGTTCAAGGAACCCCAGAAGCCACCAGAGCCACAG
Mus musculus   CCAGCAACCACAGAGCCCGGGGAGCCCACAAAGATCAGAGAGCCCAGGGAACCCTCAGCCATCAGTGAGCTCAGGGAACCCCCAGAGATCAAGGAGCCCCAGGAGCCCCCAGAGCCTCAG

Homo sapiens   GATCTTCTA------------CCCTGGGAACCCCCAG---------------CAGCCTGGGAACT--------CCAGGAGGCCCCAGCAGCCCCAGA-GTCCCTGGCGCCTCCAGCAACC
Mus musculus   GAGCTTCCAATGGTCAAGGAATCCTGGGAGCCCCCAGAGGCCCTGGATTCCACAGCCTGGAAGCCACCAGCAGTCAAGGAATCCCAGGAGATCCAGAAGGCTCTGGAGTCCCCAGCAACC

Homo sapiens   CGGGAGTCCCAGAAACCTCCAATGGCCCATGAGATCCCAACAGTCTTGGAGGGCCAGGGGCCTGCAAACACCCAGGATGCCACAATAGCCCAAGAGCCCAAGAATTCAGAACCCCAGGAT
Mus musculus   CAGAGGCCCCAGGCGTCCCCAAGGGGTTATGATCTCCCAGCAGTCTGTGAGG---CGGAGCCCACAGACAACCAGGGTACCCCAGCCCTCAAGGAGCTTCAGAGTCCACAGCTCCACAAC

Homo sapiens   CCCCCAAATATTGAGAAGCCCCAGGAGGCACCAGAATACCAGGAAACTGCAGCACAGCTAGAGTTCCTTGAGCTTCCACCTCCCCAGGAGCCTCTGGAGCCT-----------------
Mus musculus   CCTACAAATGATGAGGAGTCTCAGACGGTTCCAGAATACCAGGAGACCTCATCACAGCTGGAGCCCCTTGAGCATCCACCTCCCCAGGAGACTCTGGAGCCTCGGGTGCCCCAGGAGCCC

Homo sapiens   ---------TCAAATGCCCAGGAATTCCTAGAACTCTCAGCAGCCCAGGAGTCCCTGGAGGGTCTAATAGTTGTGGAGACATCAGCAGCTTCAGAGTTTCCACAGGCCCCTATTGGGTTA
Mus musculus   CTGGACCCCTCAGATGCCGAGGAGTTTCTAGAACTCTCAGTACCTGAAGAGTCCCTAGAGGGCCTGATAGTTGCGAGAACAGGAACAGA------------ACAGGCCCAGTGTGAATTA

Homo sapiens   GAGGCTACAGATTTCCCCCTGCAATACACTTTAACCTTCAGTGGAGATTCCCAGAAGCTTCCTGAGTTCCTGGTTCAGCTGTATAGTTACATGAGAGTCAGAGGGCACCTGTATCCCACT
Mus musculus   GAGGCTACAACTTTGCCCCTAGAGTACCCTTTGGCCTTCAGTGAGGATTTTCAGAAACTCTCTGAGTTTTTAGTTCAGTTGACCAGTTACCTGAGAAGCAGAGGG------TATCCCACT

Homo sapiens   GAAGCAGCCCTGGTGAGCTTTGTTGGCAATTGCTTCTCAGGTAGGGCAGGATGGTGGTTCCAGCTCTTACTGGATATCCAAAGCCCCTTACTGGAGCAATGTGAAAGTTTCATACCTGTG
Mus musculus   GAAGCGGCTCTAGTGAGCTTTGTTGGCAGCTTCTTCTCAGGTGAGGCGGGAAGGATGTTCCAGCCCTTATTAGATAGCCAACCCCCCCTGGTGGAGCAATTTGAAAGGCTCCTGCGAGCG

Homo sapiens   CTCCAGGATACTTTTGATAATCCAGAAAACATGAAAGATGCCAACCAGTGCATCCATCAACTCTGCCAAGGGGAGGGTCATGT----AG-----CAACCCACTTCCACCTCATTGCTCAA
Mus musculus   CTCCAAGATACTTTTGACAATCCAGAAAGCTTGGAAGTTGCTAACCAGGGCCTCCCTCAACTTCGCCAAGGGGAAGGCCTTGCTCCCAGGTACTCAACCCGCTTCCATCTCATTGCGCAA

Homo sapiens   GAGCTGAACTGGGATGAAAGCACTCTCTGGATCCAATTTCAAGAAGGTCTTGCCAGTTCTATACAAGATGAACTGTCTCACACAAGCCCAGCCACCAACCTATCTGATCTCATCACTCAG
Mus musculus   GAGTTAGATTTGGGTGAAAGCACGCTCTGCATCCAATTTCAAGAAGAGCTTGCCAGTTCTATTCAAAATGAACTGTCTTGCACAAGTCCAGCCACCAACCTATCTGATGTGATCATTGAG

Homo sapiens   TGCATCAGCCTGGAAGAGAAGCC------------TGATCCCAATCCATTAGGGAAAAGTTCCTCTGCAGAGGGAGATGGACCCGAGAGTCCACCAGCTGAAAACCAACCTATGCAGGCT
Mus musculus   TGTGTCACCCTAGAAGAGAAGGCAAGTGGCGGGGTTGATTCCAGTTCATCA---------TCGTCTGAAGAGGAAAATGGGTCCGAGGGTCCTCCCACTGAAAACCAACCTGTTCAAGCT

Homo sapiens   GCAATCAACTGCCCACACATCAGTGAAGCTGAATGGGTCCGTTGGCACAAAGGCCGCTTATGCCTCTACTGTGGTTATCCTGGTCATTTTGCCAGAGATTGCCCTGTCAAGCCTCATCAG
Mus musculus   ACAAGTAATCGCCCCCACCTCAGCGAGGCTGAACGGGCAAGACGCCGCGAAGGCCACTTGTGCCTCTACTGTGGCCATCCAGGTCATTTTGCCAGAGACTGCCCTGTCAAGCCTCATCGT

Homo sapiens   GCCCTGCAGGCGGGAAACATCCAGGCCTGCCAGTAAGGGGGACCCCACGTGGGCCAATCTACAAATATGCGTCCCCCTCTCTTCCAATATCCATGTCTCATACACTATGGCTTACTGTTG
Mus musculus   GTCCAGCAGGCGGGAAACATGGAGGCCCGGCGGTAAGGGGGATCCCGGGCGGGCCAATCTACAAATACGCGTCCCCCTCTCTTCCCATATCTATGTCCCGTACCCTGTGGCTAACTGTCG

Homo sapiens   AAATCCAACTGGGAAGAAGATAATTCTTTGAGCAAGCAATGGTAGATTCAGGCTCCTACAGAAACAGCATTGATCATACTGTGGTTCTTCGAGAGAAGCTGCCCATCCGCAGTAATATCT
Mus musculus   AGATTAGGCTTGGAAGACGGCAATTCTTTGAGCAAGCAATGGTGGATTCAGGCTCCTACAGAAACAGCATCGACCGCTCTGTGGTTCTTCGAGAGAAGATACCAATCCGCAATAATATCT

Homo sapiens   TCCCTCTGATGCTGGAAACTGTCGACGGCCATCCACTTATTAATGGACCCATAACTAAGGAAACATCACCTGTCCAAGTTCAAATTGGAAACCATGTTGAAGAGCTCCAGTTTGACATTA
Mus musculus   TCCCTCTTATGCTTGAAACGGTCGATGGGCGTCCGCTGATTAATGGACCCATTACCAAAGAAACCCCACCTGTTGAAGTAAAAATTGGAAACCACGTTGAAGAGCTCCAGTTTGACATTA

Homo sapiens   TTCATGCACCACGATACCCTCTGATTATTGGAATCCATTGGCTTGAGACACATGACCCAAACATAGAATGGAGTACCCGCACTGTGTCCTTTCTATCACGTTATTGTCACTACAATTGCT
Mus musculus   TTCATGCACCACGCTACCCTCTGATTCTTGGAATCCATTGGCTTGAGACCCATGACCCAAACATCGAATGGAGTACCCGAACTGTGTCATTTCCATCGCATTACTGTCATTACAATTGCT

Homo sapiens   TCAGGCACAGGTGGAATAGAAGAAATCGTGATGAAATAATTTCTGGGTAGATCCTGTGTCCTAGTGACTGTTCCTGGCAACCTGTCTTCTGTTACCTCAGCTGTCATCAGCATTTGCTGC
```

```
Mus musculus  TCAGGCACAGGTGGAATCGAAGACGGCGTGATGAAATAATTGCTGGTTAGATGGTGGGTCCTAGTGAC---TTCTGGCCATCTGTCTTCTGTTACCTCAGCTGTCATCAGCATTTGCTGC

Homo sapiens  TCCCTGAATCTTCTACTGAACATCTTGCTATGTACTAAGGCTACCTGTACAACGACTCTGCCTCTTAGATGATAGACTGAACCTGATGACTTTGAGCTGCTTTTTTTTTTTTCACTAACT
Mus musculus  TCTCTGAATCTTTCACTGAACCTCTGGCTATGAACGAAGGCTACCTGTACAACGACGCTGCCTCTTGGATGATGGACTGAACCTGACGACTTTGAGCTGC-----TTTTCCTCACCAACC

Homo sapiens  CTGCATGCCTCTATCTCAGAACTCTGCATCCTGCGGAACTG-----TTTTAATTACAATTAT-ACTAACAATACACATGAGAATAGATATTAGAAACTAATAATGAAAAAGCATAGCTGT
Mus musculus  TTGCATGCCTCCCTCTTTGAGCCCTGCATTATGTAGGGCTGTTTTTTTTTAATCGCAATTCTCACTAACGATATGCATGCGAATAGATATTAGAAACTAATACTGAAAAC-TGTAGTTGT

Homo sapiens  TAAACTGACTTGATTTTTTTCTTATCA----------ATATTTTCTATATCACAAAAATGGCAACATCTTTTACATAAAGAAAATAAGTTTGTAAGTGATATTTCTTAAAAGAATCAATA
Mus musculus  TAGACCGATTTTGTTACTTTCTAGTCAAAATTACCACATATTTTCTG------------------GTTTTTTTTTCAATAAAAGCAATTTTCAAGTG-TATTTTTC--AGTAATCAATA

Homo sapiens  AATATTAGCAATTTCTACTTGTGTTTG--AGTTATTTCTTGGCTTAGCTAGAGGAAGAGATAATGAACCAGTAAATTGGGAGGGAGAGAGGGAATTAGAAAATGTTCAATGTTTAGGGAG
Mus musculus  AACAATGGGAAGTGTTACTTGTGTTTGTTAATTATTTATTGGGTTAGCTGAGGAAGGGGGGAGGGGGCCACTAAGTTTAGAGGAGGGGAGAGGG-------------------AGGGAG

Homo sapiens  GCTCCTAAACATTCCGGGTGTAGTGATAGGTCTGCAGATGATGGAGAAATGATGTAGAGGGTAAGATGGAGAGAGTAGGGAATGGTTAGGGCTGAAAGTGAGAGTAGGGAATGGTTAGGG
Mus musculus  G----TAAAGTGTGAGAGTGAAAGGGTGGG------AATAATGGGGGAA----GGGGTGGGAAGGATTAAGGAGGGAGGGAG-GAAAAGAGGAGGAAGTGAAAATGGAGA------AGGG

Homo sapiens  CTGAAAGTAAGTGAGGTATGGAGGGATTAGGGGAAAGGTAGAGGTAAGATTGAGAAGAGGGTTTGGGGGAGATTGGGAGGAGGTTGAAGAGGGTTTAGAGGAGGGTTGGGGTGCTGAGAG
Mus musculus  GTGAAGGGAAGGGAGAAGAGAAAGAGAGAAGGGAGAGGTGAGGGAGAGATTTGGGGAAAGCTTAAGG.....................................................

Homo sapiens  GTAGAATCTCCAGGTGAGGTGTGAGGAGTGGTCTAAGCAGAACCTAATGCTTTTCCTCTGTGTTTTCAGCTTTCCCTTAGGAGCCTCTCTCATTGAGGCTGCTAAAAGCGGACTCTTTCC
Mus musculus  ................AAGGGGAAGAAGTGATCCATGCCCATTTTAAC---TTTCTTCTGTGTTCTCAACTTCTCCCTTGGAATTTCT----TTGAAGATGCATGAATGGCAGTGTT---

Homo sapiens  CTCATTTAGACAAATGTAGACAAAATATCAAATAAATGATTATAACCCTAGAAGGTAATAGGGAAGGGGCTTACCATTCATTGGACCACTAATATGTTCAGCTACAATGTCAGACATTTA
Mus musculus  ----------CTAACATAGGTGA----TCAAATAAA---------------------------AAGAGCTCCCATTTCCTT---------------------------TTGGCATCTT

Homo sapiens  ACACACATTAATCATCTC
Mus musculus  ACACACATTAATCATCTC
```

LDOC1L

```
>chromosome:GRCh37:22:44887852:44894778:-1
GCAGTGGCTCACGCCTGTAATCCCAGAACTTTGGGAGGCCAAGGCAGGCGGATGCCCTGA
GGTCAGGAGTTGGAGACTGGCCAGGCCAACAAGGTGAAACCCCGTCTCTACTAAAAATAC
AAAAATTAGCCGGGCATGGTAGCACGCGCCTGTAATCCCAGTTACTTGGGTGGCTGAGGC
AGAAGAATCGCTTGAACCCGGGAGGCGGAGGTTGCAGTGACTCAGGATCGCGCCACTGCA
CTCCAGCCTAGGCGACAGAGCGAGACTCCGTCTCAAAAAAAAAAAAAAAATAAGAAGAACA
GTAAATCATCAAAAAAATAAACCCAATGACAACCACAGAAAGAGCCTTGCAAGCCCTCTT
GGGAGGAATGACTGGGAGAACACCAGGTACCCGCCCTGATGAAGACGCCGCAGCAACTAC
AGTCTTCCCCGATTGGCAGCAGCCCGAGTTGGGGGGTGGGGGGGGGTGGGACTTTGTGCA
TCACTTCCGGGGGCGGGGCAGCGGTCGTCTCCGCACGCGCGGGAGCGGTCGGTATCCATG
GCAACCAGCGGGGCCAGCCTAAGGCCCGCCCGCCACCGCCACCGCGCCGGCCCCGCCCGC
ACAAAGGCCGCGCGGTGACCCGCCCTCTGCCGCGACCCCGGCCCCGCCCCTGCCCCGGCC
CCGGCCTGGCCCGGTCCCTGCACCTGCCGGAGGCCGCCCGCCGCCATCGCCAGCGCCGTC
GCTTGGCTGCGCTGACCGGGAAGCAGTTCTCGGCGGTCCGCGCGGTGATTCATCGGCCTG
GCCGCGTCCCCCGCGCGGGCCATCTTGGTCACGGCCGGCGGCGGACAGAGCCGCGGAGGA
ACCGCGAGACCCCAGGTACGCGTGCCCCCGGCCCCCTGCCCGGCCGGGCCCCGCCACTGT
GGTGGCCCCCAGGCCCGCGGCCGCCAAGGAGCAGCCCCCTTTGTTCTCCCCCGGGCGGGC
GCGGGGCGGGCGCCGCGGGGCTTTGTGCAGCCTCGCCACCCACGCGCGGGCCCTCCACGC
CGCCCCTCACCCTCCTGCCCGCTAGTCGCAACTGAAACCTTGAAAACATCTTTTCTTAAT
CCTCCAGGGCCCAGAGCAGGTCCAGCCACCCGTCTGGCTCCGTAGTCCGCGAAGTCTGCA
GTAAAGGAGCCGCGTGGGACCGGGGCCGAAGTCCCTAGGGCCTGGCACCGCGTGCCCCTC
TCTCGGGCCCTCGGCTTGGGGGTCTCGCAGCGCCCTGCCGTCGTGCACCCCACACTGCCC
CCATTTCTACAGGGGCCCACCTGGTCACCACACCCTCTTGGTCCACCCGCGGATCTCAGC
GTGTGGCTGACCTCTGGCCAGCATGGTGCAGCCGCAGACGTCCAAAGCTGAAAGCCCAGC
CTTGGCAGCGTCTCCGAATGCCCAGATGGATGACGTTATTGACACCCTGACCTCCCTGCG
CCTCACCAACTCGGCGCTGAGGCGGGAGGCTTCCACCCTGCGGGCGGAGAAGGCCAATCT
CACCAACATGCTGGAGAGCGTGATGGCAGAGCTGACCTTGTTACGCACCAGGGCGCGGAT
CCCGGGGGCTCTGCAGATCACCCCGCCCATCTCCTCAATTACTTCAAACGGGACTCGACC
CATGACCACACCTCCAACCTCTCTGCCCGAGCCCTTTTCCGGGGACCCAGGCCGGTTGGC
GGGGTTCCTGATGCAGATGGACAGATTCATGATCTTCCAGGCCTCCCGCTTCCCGGGTGA
GGCCGAGCGTGTGGCCTTCCTTGTGTCTCGACTGACTGGGGAGGCGGAGAAGTGGGCTAT
CCCCCACATGCAACCTGACAGCCCCTTGCGCAACAACTATCAGGGGTTCCTGGCAGAGTT
GCGGAGAACCTACAAGTCTCCGCTCCGGCATGCGCGGCGCGCCCAAATCAGGAAGACTTC
TGCCTCTAATAGGGCTGTGCGAGAGAGGCAGATGCTCTGCCGCCAGCTGGCCTCTGCGGG
CACGGGGCCTTGCCCAGTGCATCCAGCTTCCAACGGGACTAGTCCAGCGCCAGCCCTGCC
TGCCCGAGCACGGAATCTTTAAGAATCCGCCAGCACTTGGTAGCGTCTGCAGCCACCCAG
GTAGCATACGCTCTTTGCTGTGTAGAAGAAATGCCCATACGACAGCTTTGCCCCTGTTTG
AAGACCTCCCTTCTTGCCTCTCCAGACGTGTTCCCCGAGGAGATCTTCCTTCCGTCCTTC
CTGGCGCCCTGGTTGCCCACCTTGCCGTGCTTCCTCTTACGTGCTAGCTTTGTACCTATC
GCTCACTGCATGCTCGCCTCCCTCTTGCTGGCATCCCGGCCTGTTTCAATGACTACCGCT
CTGCTACTTAGGCACAGGGACTCCGCCGCACGCTGACGGACCACGAGGGCTGACCCCTTC
CAGCCTGACTTGGTTCATGGAGGCTCCTACTCTGCCCTCTCCAAGCTCCCCTGGCGGCTC
CCCACCTGGTTGCCCAGTTCCTATTGATGAGCTCTGGACAGAAAGATGCCCGTTTGGCCA
GGCTGGTGGCTTGATGGGTGTACCTGGAGAGGGGGTCTGGCTTCCTGCCCAAGATGCCTC
CCAGCCCTGCCAGGGCCCGGTGCAGCGGGCAGGGCCTCATCTGTGCTGTAGTGGTCGAGT
GGTTGCTGCAAGGAGCGTAGTTCTGCCATGTCTGGGGGCCAGGTTCCACTCTGCACATGA
ATATGCAGTCTGGGAGGCCCCACTGCTCTCACTGGGAAGGACCAATGTTGCACCTCTGTT
AATGCCTGACTTCAGCTGCTGGTGTTCTGATGGAGCCAGAGGCTTGGGGAATCTGGAACT
TGCCTGCTAAATAAGGTCGTGGTGGACTCTCAGCCATTGGGCAGGTCTATCAGGCTGCAG
GTTCCTACACACCCACGCCTGAGGGTCATAGCAGGCTAAGGGTGGATACCAGCGACTCCC
TTTGCTGCCCAGGATCTCCATGGGCAGTGCCACAGCGGCTGATGCTCAGTCACTCCTGCT
TCTACCCCCTGTCACTGATGGCGAGCCTTGCCCAGCTTGAGACCTGTTCCCATCTCTATT
CAGGTGCCATGTGGCCTTCACTGCAGCCCTGCAGCCACCCACGCACCATCTGTGGGTCTC
CAAAGGCACCTTGTAGCATGTACTCCCCGTGCCTGGGCAATCAGATGGGCTGCCTTTGTC
CAAGGGAAAACAGACTCCCTTCGGGAAACATCCTTAAGCACTTAAGGCCGGGGGGGGTGT
CTGCCTCTGGCAACCCAGCCAGGGTCTTGGTGGCATTTGTAAAAGCAAAGAGCTGTGGAC
TGCCGTGGTCCTAGTGTGGTGACAATGCAGCACTGGCATGCATGTCCTCCTTCTGAAGGA
CCTCATCCTTCCTCACAGGGGGATGACCAAGAAATCATTTTGTGGCTGAGTTTGGCCACG
CCCTTTGGACTGTGCTGTTCCGCCATATTTCAATGCCAAATGAACCACATTGACATGACC
TGGACCATAGGGCTTCCTATCCTGGGCTCAGCTGCCCCTGTCTGAAGGGTCTTGGCTTGA
TTGCAGAAGGACAACCTCCGCACCCACCTAAAGACATGTATATGTCTTGGGATCCCAGAG
ATTGGGTCCTTGGGCCTGGCTTCTTAAGAGTTTTGATGATGCTGGGAAAAGTGACTGCGA
TTCTGAAGAACCGCTGCCTTGCAAGGTCAAGGACATTCAGTGGTTGCTGGGGTCCGCAGA
CTACTGCCACCCACTCACCATCAACTCTGTTAGCCCAATTGCCCTGCTGAACAACTGCCT
GAATACAGGCTTTAGGTTCCCCTGGACTCCAGCCAAGGCTGTTCAGGTGGGACCATGGTG
CTCTTTAAGCGTGATCGGAGGGAAGACACACAGCAGGGCCACCATTCCATGAATGGGAGG
TGTACAGATCACTTTCTCTTTGTGCTCAGTTCTGTTCTGTCTCCAGCAGCTATATTGGTA
AGACTAGTACCTGCCAGGGAGAGGTGCCCCCAAGTGAAGGGGTACAGTGGCACCTGGGAA
AAGGCACCTGGAAGGTTTCCATGTGGCCCAGCCCAGCATGGAAGCAGGGTGGGAACTCTG
CTGTGTCGCCAGCGCTCACTCTACTCGAGTGGCTTTTTGAAAGCCCTACCATGTCTGTGT
CAGGCCTGTGCTGCTTCACATCCTACAGCTGCCTAGGAAAGGCCGGCCACGCTCCCTGTC
CACACACTCCCTGTCCACACACTCCCTGTCCACACACTCCCTGTCCACAACTGCAGCCGG
GCCCTCTGCCTATGGGCACCCAATCCAAGCAGCTGCTCCACCTTTGTTTGGCATGGTGAT
TTGTGTTTTTTCTCTTGGTGCTTATGTGTGTGGGCTTGGACGAGTGCTGGTATGCACTT
AGGACCTTCTTGATAGCTCCCTGCACTTTGGAACACGGAGCAGATGAGAGAGGGTCGGGG
CTTGCCCTCCACCTTGGACTTGGAAGAAGCCCACATTGGAGAGGTGAGGACCCCATGGTG
GCTCTAGTGGAAGATACGTTAGTCTCCAGCTAAGGAGGATGAGGCGCAGCCCCAGAGGGA
GACCTCAGTGATAGGGGATCAGGCTAAGAAAGTGGGGGAAGGGAGATGCTTTGTACATAT
TTTGGGGTTATAATTTCTCTAAATTTTAGGAGAACGGGTATTGATTGATAAAAGGGACAG
GCAGTAGTGTTCAACAGTGCATGTGAAGGAAAGTTCTGTTTTCCATGGTTTTGACATTCT
TTGGACTGTATTGTGACTGCTGTCTGGTCCACATGGTACCCCTTTGGTAAGTAGGCTTCA
GTGCATACCAGGGTATCACTGGAGATGGGAGTTAGTGAAGGGGTGACTCCCTGGCCTAGT
ATAGTGTGACCCTGGGACTAACTTAATGTCCTAAAGCATTTTGGTGACTTCTAGGGAATA
GCAAAGACCTATTTCATTGTCCCCAGGTAAGTATGTGATGAGCAATGAGGAGGAGTGGAA
AACAAAACCCAGAAAGTGCGGCAGGACCAGCCTGACGCACACGCTCCTGTTGTCATGGCA
GACAGCCGCCTTGGGTGGGCACCACCCTGGCAGTTCCAGCCTGTAGGGGAGTGAAGGGAC
ATGGCTGAGCTGGGCATGTGCTGAGGTTGACTTAGGGAACAAGCCCTGGGATTGGACAAA
AGGGCCCATGCTGCAGCCACTGACTGGGGGCAGAGCTCTGGGTGGAAGAGGGAAGAGATC
CTAATGGAGGCGCCTCCATCTGCAACCACAGTTGTAAGGCTCATGGCACCTCTGCTTGGA
AAGCACTGGTTTAGGGACTTAGAGAGGTAGGCACAAGGTGGGTCTCCTGGGTAAGGGAAG
```

**CAAGAGCAGACTGTTGGGCCAACAGGAGAAGCTCCCCAGAGTAGGGGAGAAGGTTGGGGT
GTAGGGCCTTCCACGTGGAACAGACAGCCCCTGTGTCTCTGTCTCTTGGGGACCTGAGTT
TGGGTGGGGTGGCAGTTGGCACAGCGCAGATGCGGTAGAGATGGGAGGAAACCCAGCTCC
TCACTTCCGTGTGCCTCATGCCTTTGCATACACAAGCACCAAACCTACTAGGTCTTCTCA
TTACCCATGTAAACCACATGTTAGATAAATTTTTGCAAGTAGAGGAAAGAAGGAAATAAA
ACATCACATTTTGGTGTCTCTCAGGCTTTCCCCCCCAACTATGGTTTCTTTGCTTTTTGT
TTTAACATAGTTTTGTTGCTGTCTTCTGTAATGATACAGTTTTGTGCAGCTGTTTTCACT
TAGCATATCGTGGGCATCTCCCCTTATGATTACTAAATATTTTATTTTGGAGTGGCTGTG
TACTCTCCCATTGACTAGATGGACCATTGTGCCAGTTGCCAATCACTAATGCTGTTACTA
ACTTTTCAGTTATAAATTGATGAATATCTTTGTGCACAGGCTGTTTCCCAATGTCAAGTT
ATTAGGGTAGACTCCAGGAGGTGGGATTCTTCAACTAAAGAATATGAAAACCTTTGAGGC
TTTTACTACATATTGACAAAATGGTTTCCGGAAATATTTGTATCCCCTTACACTGCCACC
AGCAAGGATAAACATGTCCATCTTGCCCGTATTGGGAATTATCATCTGGCTAAATATTTG
CTAATTTGATAATGAAAAAATAGCATCGTGTTTCAGTTGGCATTTCACTGACTTCTAGCA
CGGTTGAACATCTTTCATGTGGAGCGATTGTATTTCCTCCTTTGTGGATTGTCAGTGTCC
TTTGCTCTATCTTCTGGGGTCAGATAAATTTGTATGAGCTCGGTATATATTAAAGATATT
AACCTGGTGTGTGTCATATTTGCTACA**GCTGTTTCTGGGTTTTTGTGTGTTTTATCTTAT
TGGAATTTTGACATACATAGTCGATCTGTCTCCACAGCACTGTGTCTGCTAGACAAGAAC
ACAAGGCAGGCAGGTGGATTTTGTCTGTTTTGTTCATGGCCGTAGCACTAGCACCTATGA
CAATGCCTGGCACGTGATAGGTACTCAGTACACTTCTGTTGAATTAGTTACTCCAGTGGA
TCTGCAACGCCTTGGGATCCACGGCACGTGATAAGCACTCAGTACACGTTTGTTGAGTTA
GTTACTCTGATGGATCTGCAACACATTGGGATCCACACATGGATCCAGCAGTCCGCGCTC
CAGCTCACAGGCCGAGGCTCTCACACTCCAGAGCTCGCAGGCCGAGGCTCTCACACTCCA
GAGCTCGCAGGCCGAGGCTCTCACACATTTGTCTCAGGGTCCCCTTGCACTATAAGAAC
TCATTGAGGGCCTCAAACAGCTTTTGCTTATATAGGTTTTCTCTATCAATATTTACCATA
TTATACATTTAAACAGAATTTTAAAATAATTATTTGGAAAATAAAAACACTGTTAAACAA
CATTTTAAATAAAAAAAACTATATTTT

```
Homo sapiens          GCAGTGGCTCACGCCTGTAATCCCAGAACTTTGGGAGGCCAAGGCAGGCGGATGCCCTGAGGTCAGGAGTTGGAGACTGGCCAGGCCAACAAGGTGAAACCCCGTCTCTACTAAAAATAC
Dasypus novemcinctus  ........................................................................................................................

Homo sapiens          AAAAATTAGCCGGGCATGGTAGCACGCGCCTGTAATCCCAGTTACTTGGGTGGCTGAGGCAGAAGAATCGCTTGAACCCGGGAGGCGGAGGTTGCAGTGACTCAGGATCGCGCCACTGCA
Dasypus novemcinctus  ........................................................................................................................

Homo sapiens          CTCCAGCCTAGGCGACAGAGCGAGACTCCGTCTCAAAAAAAAAAAAAAAATAAGAAGAACAGTAAATCATCAAAAAAATAAACCCAATGACAACCACAGAAAGAGCCTTGCAAGCCCTCTT
Dasypus novemcinctus  ........................................................................................................................

Homo sapiens          GGGAGGAATGACTGGGAGAACACCAGGTACCCGCCCTGATGAAGACGCCGCAGCAACTACAGTCTTCCCCGATTGGCAGCAGCCCGAGTTGGGGGGTGGGGGGGGGTGGGACTTTGTGCA
Dasypus novemcinctus  ........................................................................................................................

Homo sapiens          TCACTTCCGGGGGCGGGGCAGCGGTCGTCTCCGCACGCGCGGGAGCGGTCGGTATCCATGGCAACCAGCGGGGCCAGCCTAAGGCCCGCCCGCCACCGCCACCGCGCCGGCCCCGCCCGC
Dasypus novemcinctus  ........................................................................................................................

Homo sapiens          ACAAAGGCCGCGCGGTGACCCGCCCTCTGCCGCGACCCCGGCCCCGCCCCTGCCCCGGCCCCGGCCTGGCCCGGTCCCTGCACCTGCCGGAGGCCGCCCGCCGCCATCGCCAGCGCCGTC
Dasypus novemcinctus  ........................................................................................................................

Homo sapiens          GCTTGGCTGCGCTGACCGGGAAGCAGTTCTCGGCGGTCCGCGCGGTGATTCATCGGCCTGGCCGCGTCCCCCGCGCGGGCCATCTTGGTCACGGCCGGCGGCGGACAGAGCCGCGGAGGA
Dasypus novemcinctus  ...............................................................................CCATCTTGGTCGCGGCCGGC---------AGCCGCGGAGGA

Homo sapiens          A-CCGCG-AGACCCCAGGTACGCGTGCC--------CCCGGCCCCCTGC-CCGGCCGGGCCCCGCCACTGTGGTGGCCCCCAGGCCCGCGGCCGCCAAGGAGCAG--CCCCCTTTGTTCT
Dasypus novemcinctus  AGCCGCGGAGGCTGCAGGTACGCGCGTCTGCCGCCGCTCTGCTCCCGGCGCTCGCCGTGGCCCCCCGCCGTGGC--CCCCCGCTCCCGCCGC-----AGGTGCAGGGTGCCCTTTGTTCT

Homo sapiens          CCC--CCGGGCGGGCGCGGGGCGGGCGCCGCGGGGCTTT-GTGC-AGCCTCGCCACCCACGCGCGGGCCCTCCACGCCGCCCCTCACCCTCCT-----GCCCGCTAGTCGCAACTGAAAC
Dasypus novemcinctus  GCAGGCGGGATGGGCCCGGGGCGG-CGCTGGGGCGCCTTCATGCCGGCCTTGCCTCCCGCCTTCGG----CCCACCCCGCACCCTGCTCTTCTTTCTAGTTCCCTTTCCTCAATCGAAAC

Homo sapiens          CTTGA---AAACATC------TTTTCTTAATCCTCCAGGGCCCAGAGCAGGTCCAGCCACCCGTCTGGCT--CCGTAGTCCG-CGAAGTCTGCAGTAAAGGAGCCGCGTG--GGACCGGG
Dasypus novemcinctus  CTTCATTTAAGTTTTGTTTTGTTTTTTAAATTCCTCAGGATCTAGGACACA-CCAGCCTCCTGTCCAGCTGCCCTTGGCCTGGAGAAGTCTGTGGTCAAGTTGCTGCACCCCGGAGTCAA

Homo sapiens          GCCGAAGTCCCTAGGGCCTGGCACCGCGTGCC-----CCTCTCTCGGGCCCT------------CGGCTTGGG--GGTCTCGCAGC------------GCCCTGCCGTCGTGCACCCC
Dasypus novemcinctus  GCTTAGATCCTGAAGGGCCCCCACCGCCTGCCGCGACCCCATCCTGGGACCTTCCCCACACCTCGCCGCCTGTGTTGGTCTCTAGGCCTCTTCAGCACTTGCCCTCTTGTTGAGTCTTCT

Homo sapiens          ACACTGCCCCCATTTCTACA----------------------GGGGCCCACCTGGTCACCACACCCTCTTGGTCCACCCGCGGATCTCAGCGTGTGG---------------------
Dasypus novemcinctus  CAGCTACTCTCTTGTCTTTACTCCTTCCATCCTCTCCACCTTCTGGATATCTCTGGGTCTCACCCCTTTTTAG--CACCCCAGTTTCTCAGATTGGAGGTTACCCATATCCCCCTTGACC

Homo sapiens          -----------CTGA-----CCTCTGGCCAGCATGGTGCAGCCGCAGACGTCCAAAGCTGAAAGCCCAGCCTTGGCAGCGTCTCCGAATGCCCAGATGGATGACGTTATTGACACCCTGA
Dasypus novemcinctus  CCTCCTTTGGCCTGAGCCCTCCTCTGGTCAACATGGTCCAACCCCAGACAGCAAAAGCTGAAACCGCAGCCACGGCAGCTTCTACCAGTGCCCCAATGGACGATGTCATCGACACTCTGA

Homo sapiens          CCTCCCTGCGCCTCACCAACTCGGCGCTGAGGCGGGAGGCTTCCACCCTGCGGGCGGAGAAGGCCAATCTCACCAACATGCTGGAGAGCGTGATGGCAGAGCTGACCTTGTTACGCACCA
Dasypus novemcinctus  CCTCCCTGCGGCTCACCAACTCGGCGCTGAGGCGGGAGGCGTCCACCCTGCGGGCGGAAAAGGCCAGCCTCACCAGCATGCTGGAGAGCGTGGTGGCCGAGCTGACCTTGCTGCGCGCCA

Homo sapiens          GGGCGCGGATCCCGGGGGCTCTGCAGATCACCCCGCCCATCTCCTCAATTACTTCAAACGGGACTCGACCCATGACCACACCTCCAACCTCTCTGCCCGAGCCCTTTTCCGGGGACCCAG
Dasypus novemcinctus  GAGCCCGGGTCCCGGGGGCTCTGCAGATCACCCCTCCCGTCTCTGCCATGACTTCCGGTGGGACTCGGCCGATGACCACCCCGCCGACCTCTCTGCCTGAGCCCTTCTCGGGAGACCCCG

Homo sapiens          GCCGGTTGGCGGGGTTCCTGATGCAGATGGACAGATTCATGATCTTCCAGGCCTCCCGCTTCCCGGGTGAGGCCGAGCGTGTGGCCTTCCTTGTGTCTCGACTGACTGGGGAGGCGGAGA
Dasypus novemcinctus  GGCAGTTGGCGGGGTTCTTGATGCAGATGGACAGGTTCATGATCTTCCAGGCCTCCCGGTTCCC-AGCGAGGCCGAGCGAGTGGCGTTCCTCGTGTCGCGGCTGACGGGGGAGGCAGAGA

Homo sapiens          AGTGGGCTATCCCCCACATGCAACCTGACAGCCCCTTGCGCAACAACTATCAGGGGTTCCTGGCAGAGTTGCGGAGAACCTACAAGTCTCCGCT-CCGGCATGCGCGGCGCGCCCAAATC
Dasypus novemcinctus  GGTGGGCCGTCCCCCACATGCAGC.............................................................AGTCTCCTTTTCCCATATGCTTGGGG-GCCCAAATC

Homo sapiens          AGGAAGACTTC-TGCCTCTAATAGGGC-TGTGCGA-GAGAGGCAGATGCTCTGCCGC-CAGCTGGCCTCTGCGGGCACGGGGCCTTG-CCCAGTGCATCCAGCTTCCAACGGGACTAGTC
Dasypus novemcinctus  AGAAAGGCTTTGTGCCTCCAATAGGGGGTGTGCAAAGAACAGCAGGAGCTGTGCCACGAAGCTGGCTGCCATGGGCACAGGGCCGTGTCCCCGTGCATCCAGCCTCAGATGGGACCAGCC

Homo sapiens          CAGCGCCAGCCCTGCCTGCCCGAGCACGGAATCTTTAAGAATCCGCCAGCACT-TGGTAGCGTCTGCAGCCACCCAGGTAGCATACGCTCTTTGCTGTGTAGAAGAAATGCCCATACGAC
Dasypus novemcinctus  CAGCTCCAACCCTGCTCATCCAAGCACAGAACCTTTAAGAACCCGCCCCCACCCTGGCAGTGCCTGGATCC---TAGGTAGCAGCCA-TCTTTGCTGCACAGAAGAAATGTCCATACAAC

Homo sapiens          AGCTTTGCCCCTGTTTGAAGACCTCCCTTCTTGCCTCTCCAGACGTGTTCCCCGAGGAGATCTTCCTTCCGTCCTTCCTGGCGCCCTGGTTG--CCCACCTTGCCGTGCTTC---CTCTT
Dasypus novemcinctus  AGCATAGCTC-----------------TTTCTACCTCTACAGACCTCTTCCATAAGGAGATCTTCCTTCCATCCCTCTTGG------GGATGACCCCCCCTTACTGTGCTTCTTGCTCTT

Homo sapiens          ACGTGCTAGCTTTGTACCTATCGCTCACTGCATGCTCGCCTCCCTCTTGCTGGCATCCCGGCCTGTTTCAATGACTACCGCTCTGCTACTTAGGCACAGGGACTCCGCCGCACGCTGACG
Dasypus novemcinctus  ACATACTGGCTTTGTACCTGCAGCCCAATGCATGCCTCCCTC--TCTTGCCAGCAGCCCCAGCTCTTCTAAGGACTATGCCTCTGCTGTTCAGTCCCATG-ACTCTACAACAGGCTGACG

Homo sapiens          GACCACGAGGGCTGACCCCTTCCAGCCTGACTTGGTTCATGGAGGCTCCTACTCTGCCCTCTCCAAGCTCCCCTGGCGGCTCCCCACCTGGTTGCCCAGTTCCTATTGATGAGCTCTGGA
Dasypus novemcinctus  GACCATCTGGGCTGGCCCCTCCCAGCCTCACTAGATCCATGGATGCTTCCATGCCATCCTCTGCA-GCCTCCAGGGCAGCTCCC---------------------TGATGAGCTCTGGA

Homo sapiens          CAGAAAGATGCCCGTTTGGCCAGGCTGGTGGCTTGATGGGTGTACCTGGAGAGGGGGTCTGGCTTCCTGCCCA-AGATGCCTCCCAGCCCTGCCAGGGCCCGGTGCAGCGGGCAGGGCCT
Dasypus novemcinctus  CAGAGAG--GCCAGTGTGGCCAAGGTGGTGGCTCGATGTGTGAATCTGGACATGGGGTCTGGTTTCCTGCTCACAGATGGTCACCAGCTCAGCTAGGACCCAG---------AAGGCCCT

Homo sapiens          CATCTGTGCTGT----AGTGGTCGAGTGGTTGCTGCAAGGAGCGTAGTTCTGCCATGTCTGGGGGCCAGGTTCCACTCTGCACATGAATATGCAGTCTGGGAGGCCCCACTGCTCTCACT
Dasypus novemcinctus  CAACCCTGCTGTCCATAGTGATCAAA--------GCTAGGAGTGTGGGTTTAGTCTTTCTGGGCAGCAGGCTTCACTATGTACATGAAT------TCCAGTGTGTCCTGCTCCTGCC-CT

Homo sapiens          GGGAAGGACC-AATGTTGCA---------------------------CCTCTGTTAATGCCTGACTTCAGCTGCTGGTGTTCTGATGGAGCCAGAGGCTTGGGGAATCTGGAACTTGCCT
Dasypus novemcinctus  GGGAAAGACCTAATGCTGCAGCTAAAGGAAGAGTCCCCTGGACACTTCTTCTGTTAATGCTTGGCTTTGCCTGGCAGTGTTTAGATAGCTGAAGGAATTTCAGGAAATTGGAACCTCTCT

Homo sapiens          GCTAAATAAGGTCGTGGTGGACTCTCAGCCATTGGGCAGGTCTATCA----------GGCTGCAGGTTCCTACACACCCACGCCTGAGGGTCATAGCAGGCTAAGG-GTGGATACCAGCG
Dasypus novemcinctus  GCCAAATAAGGTCCTTGCAGACTTGGGGCCACTGGGCAGCAACATCAAGTGAACAGAGGCAGCAGACCCCTATGT--CCAAGCCTGGAACTGACCACAGGCCAAGTCATGGACCCTGGTG

Homo sapiens          ACTCCCTTTGCTGCCCAGGATCTCCATGGGCAGTGCCACAGCGGCTGATGCTCAGTCACTCCTGCTTCTACCCCCTGTCACTGATGGCGAGCCTTGCCCAGCTTGAGACCTGTTCCCATC
Dasypus novemcinctus  GCTCACTTCTTTACCC-GGATCTCAC------------CAGCTGCTAGTGTTCAGTCACCCCTTCCTCTGCGCCCCATGCTCCCAGCTGGACCTTGCCCAGCTTGACACCTATTCCCTTC

Homo sapiens          TCTATTCAGGTGCCATGTGGCCTTCACTGCAGCCCTGCAGCCACCCACGCACCATCTGTGGGTCTCCAAAGGCACCTTGTAGCATGTACTCCCCGTGCCTGGGC----AATCAGATGGGC
Dasypus novemcinctus  CACATTCAGGTGGTATGTGGTCCTCACACAGCCCCTTCAGCCACCCAC--ATCATCTGGGG--CTCCAGGACCTCCATGTAGCATGCCCTCCCAGTGCCTGGGCTGGCCATCAGCTAGCC

Homo sapiens          TGCCTTTGTCCAAGGGAAAACAGAC-----------------------TCCCTTC-GGGAAACATCCTTAAGCACTTAAGGCCGGGGGGGGTGTCTGCCTCTGGCAACCC-AGCCAGGGT
Dasypus novemcinctus  AGCCTGCACACAAAGAAACATGTACCTTCTCTTATAGCTGGGGCCCTATCTCTTCTGGGATATATCATGAAGCA-GTAAGGC--AGGTGGATGTCTGCCTCTGGCACCCCTAGCCAGTGC

Homo sapiens          CTTGGTGGCATTTGTAAAAGCAAAGAGCTGTGGACTGCCGTGGTCCTAGTGTGGTGACAATGCAGCACTGGCATGCATGTCCTCCTTCTGAAGGACCTCATC------------------
Dasypus novemcinctus  TTTGGTGACATTTGTAAAGGGGAAGAGCTATGGACTGCCTCAGTCTCAA---GGTGACAGGGCAGGGCTGGCTCTCCTATCATAATTTGGAGGGACATCTTCATGGTGGGTGGTCTTGGG

Homo sapiens          CTTCCTCACAGGGGGATGACCAAGAAATCATTTTGTGGCTGAGTTTGGCCACGCCCTTTGGACTGTGCTG-TTCCGCCATATTTCAATGCCAAATGAACCACATTGACATGACCTGGACC
Dasypus novemcinctus  CTGCCTCGCAGAGAGTGGACTAAGAGACCGCTTCTAAGTGAGACTTGGCCAAGCCTTTTGAACCATGATGATCCCGTCATCTTTCAATGCCAAATGAACCACATTTCCATGACC--ACCC

Homo sapiens          ATAGGGCTTCCTATCCTGGGCTCAGCTGCCCCTGTCTGAAGGGTCTTGGCTTGATTGCAGAAGGACAACCTCCGCACCCACCTAAAGACATGTATATGTCTTGGGAT-CCCAGAGATTGG
```

```
Dasypus novemcinctus  AGA-------CCACCCTGGGGTCAACTACT........................TGCAGAGGGACAATCTCTGCACCAACCCAGAGACTTAGCGATTAC--GGGATACCCGGAGGGTGG

Homo sapiens          GTCCTTGGGCCTGGCTTCT----TAAGAGTTTTGATGATGCTGGGAAAAGTGACTGCGATTCTGAAGA-ACCGCTGCCTTGCAAGGTCAAGGACATTCAGTGGTTGCTGGGGTCCGCAGA
Dasypus novemcinctus  GTGCCAGGATCCATCCTCTCTTTTAAGAGTTTTGATGATGCTGGGAA--GTGACTATGTTCCTGAAGCTCCCACTGCCTTGTGAGGTCGAGGCTGTTCAGTGGACGCTGGGGTACGTGGC

Homo sapiens          CTACTGCCACCCACTCACCATCAACTCTGTTAGCCCAATTGCCCTGCTGAACAACTGCCTGAATACAGGCTTTAGGTTCCCCTGGACTCCAGCCAAGGCTGTTCAGGTGGGACCATGGTG
Dasypus novemcinctus  CCACTGCCACCTGCTCGTTGTAAACCCTGTTAGCCCAGGTGATCCGCTGAACAACTGCCTGATTGCAGGCCTCAGGTTCCCCT-GCTTCCAGTCAAGGCTGTGCAGGTGGGACCACAGTG

Homo sapiens          CTCTTTAAGCGTGATCGGAGGGAAGACACACAGCAGGGCCACCATTCCATGAATGGG-----AGGTGTACAGATCACTTTCTCTTTGTGCTCAGTTCTGTTCTGTCTCCAGCAGCTATAT
Dasypus novemcinctus  CTCATTACATGTGCAGGGAGGAGGGACC----------CCCCTGTCCGGGGCCTGGGGCTGCAGGTACACAGCTCGCTTTCTCCTTATGCTCAATTCTCTTCTGGCTGTAGTAGCTGCAT

Homo sapiens          TGGTAAGACTAGTACCTGCCAGGGAGAGGTGCCCCCAAGTGAAGGGGTACAGTGGCACCTGGGAAAAGGCACCTGGAAGGTTTCCATGTGGCCCAGCCCAGCATGGAAGCAGGGTGGGAA
Dasypus novemcinctus  TGGTACGATGTGTACCTGTCAGGTAGAGGTGCTCACAGATGAAAGGGCACAGTGGCCCTGGAGAGGAGGC................................................

Homo sapiens          CTCTGCTGTGTCGCCAGCGCTCACTCTACTCGAGTGGCTTTTTGAAAGCCCTACCATGTCTGTGTCAGGC-CTGTGCTGCTTCACATCCTACAGCTGCCTAGGAAAGGCCGGCCACGCTC
Dasypus novemcinctus  ..CTGCTGTG---------------------------GTTTTGAAAATGCCCCCATGCTTGGACTGGGAGCTGTGTT--TCCGAGTTGTGCTGCTGC--AGGCAGGGCCAGTCGCA...

Homo sapiens          CCTGTCCACACACTCCCTGTCCACACACTCCCTGTCCACACACTCCCTGT-CCACAACTGCAGCCGGGCCCTCTGCCTATGGGCACCCAATCCAAGCAGCTGCTCCACCTTTGTTTGGC-
Dasypus novemcinctus  ...........................................TGTGCCCCGGCTG--GCTGGCCTCCCTGCCGGGAGGGGGCC------TGCTGCTGCTCTGTCTC--TTTGGCT

Homo sapiens          ATGGTGAT----TTGTGTTTTTTCTCTTGGTGCTTATGTGTGTGGGCTTGGG-----ACGAGTGCTGGTATGCACTTAGGACCTTCTTGATAGCTCCCTGCA-CTTTGGAACACGGAGCA
Dasypus novemcinctus  TTGGTTATTCCATTGTGCTGCATCTCTTGGTGTTTACCTTTGTGGGCTCTGGGCCTAACCAGTGCGG---------TGGTACCTTCGTGATACCTTCTTACAGAGGTGAGAAATGGAGCC

Homo sapiens          GATGAGAGAGG-----GTCGGGGCTTGCCCTCCACCTTGGACTTGGAAGAAGCCCACATTGGAGAGGTGAGGACCCCATGGTGGCTCTAGTGGAAGATACGTTAGTCTCCAGCTAAGGAG
Dasypus novemcinctus  CATGAGAGAGGTCCTAGCCAGAG--------CCACCTC------------AGTCCCCAGCGGGACGGTCTGAGACCCAGGCTGGCC----------------------------------

Homo sapiens          GATGAGGCGCAGCCCCAGAGGGAGACCTCAGTGATAGGGGATCAGGCTAAGAAAGTGGGGGAAGGGAGATGCTTTGTACATATTTTGGGGTTATAATTTCTCTAAATTTTAGGAGAACGG
Dasypus novemcinctus  ---------CAGCCCCAGGGTGAGACCAGAGTGGCCACGGCTCTAGCT------------GAAGACGAATG-TGCGCACAGATTTGGGGGTTCATCTTTTTCTAAG-CCTAAGAGAGCAG

Homo sapiens          GTATTGATTGATAAAAGGGACAGGCAGTAGTGTTCAACAGTGCATGTGAAGGAAAGTTCTGTTTTCCATGGTTTTGAC--ATTCTTTGGACTGTATTGTGACTGCTGTCTGGTCCACATG
Dasypus novemcinctus  AGGT--ATTAATAAAAGGAACAGGATGT------------TTTAGGTGG--------------TTGCACGGCATCGGCCTATTACCTGCCCTGTTTTGTGACTGCT-TCCTGTCCACGAG

Homo sapiens          GTACCCCTTTGGTAAGTAGGCTTCAGTGCATACCAGGGTATCACTGGAGATGGGAGTTAGTGAAGGGGTGACTCCCTGGCCTAGTATAGTGTGACCCTGGGACTAACTTAATGTCCTAAA
Dasypus novemcinctus  GTACCCACCTGGTAAGTAAGCTTCAG-GCACAGCAGGGTGTAATTTGAGGTGGGATTTAG-ATAGGGGTGTCCCCCTGGCCTGA---GGTGTGCCCCTGGGATTAGAGTCATGGCCTAAA

Homo sapiens          GCATTTTGGTGACTTCTAGGGAATAGCAAAGACCTATTTCATTGTCCCCAGGTAAGTATGTGATGAGCAATGAGGAGGAGTGGAAAACAAAACCCAGAAAGTGCGGCAGGACCAGCCTGA
Dasypus novemcinctus  GCATTTCAGGGACTCCTGGGAAGCAGCAAAGA-CGGCCTCATGGGCCCCGGATAAGCGTGTG--GCACAGTGGGAGGGC------------------------TGGCAGG--TGGCCTGG

Homo sapiens          CGC----ACACGCTCCTGTTGTC-ATGGCAGACAGCCGCCTTGGGTGGGCACCACCCTGGCAGTTCCAGCCTGTAGGGGAGTGAAGGGACATGGCTGAGCTGGGCATGTGCTGAGGTTGA
Dasypus novemcinctus  TGATGGGCCAAGCTCCTGGCGTCGGTGACGGAC---------GCGTGGCCGTCACCT------------------GGGGA----------------GAGTTGGACACACACTT-TGCTGA

Homo sapiens          CTTAGGGAACAAGCCCTGGGATTGGACAAAAGGGCCCATGCTGCAGCCACTGACTGGGGGCAGAGCTCTGGGTGGAAGAGGGAAGAGATCCTAATGGAGGCGCCTCCATCT-GCAACCAC
Dasypus novemcinctus  C---GGGAGCGAGCCCCCTGATTGTGTCGGGGGACCCAGGCTGCAGCGCCTG------------------------CGAGGG---------TAGCAAGGGCCCTCGCGTCTGGCCCCCGG

Homo sapiens          AGTTGTAAGGCTCATGGCACCTCTGCTTGGAAAGCACTGGTTTAGGGACTTAGAGAGGTAGGCACAAGGTGGGTCTCCTGGGTAAGGGAAGCA-AGAGCAGACTGTTGGGCCAA-CAGGA
Dasypus novemcinctus  ACCTGTATGGGTCCT-----------CTGGGAGGCACTGGTTGGGGGACTTACAGATGAAAAGGCGAGACGGG-CTCCTGGGGAGCGGAAGTGCAGAGCGGATAGTCAGGCCAAGCAGGA

Homo sapiens          GAAGCTCCCCAGAGTAG---GGGAGAAGGTTGGGGTGTAGGGCCTTCCACGTGGAACAGACAGCCCCTGTGTCTCTGTCTCTTGGGGACCTGAGTTTGGGTGGGGTGGCAGTTGGCACA-
Dasypus novemcinctus  AAGCCTACTGGGAGGAGCTCGGGCTCGAGCTAGGATTCCTGAGCCTCCAGGGAGTGTAAG---------------------TGGGGCCCTG-GCCTGGACATCCGGGCTGGTGGTGCGT

Homo sapiens          GCGCAGATGCGGTAGAGATGGGAGGAAACCCAGCTCCTCACTTCCGTGTGCCTCATGCCTTTGCATACACAAGCACCA-AACCTACTAGGTCTTCTCATTACCCATGTAAACC-ACATGT
Dasypus novemcinctus  GCGCAGCAGCTTTGGAGATGT--GGAGGCTCAGCTCCAGTCTCAACCATCCCTAACACCTGTCTGTG---AAGTAACAGAACGTGCTGTGTCTTTTCATTATAAAAGTAAACCTTCACAG

Homo sapiens          TAGATAAATTTTTGCAAGTAGAGGAAAGAAGGAAATAAAACATCACATTTTGGTGTCTCT--CAGGCTTT------CCCCCCCAACTATGGTTTCTTTGCTTTTTGTTTTAACATAGTTT
Dasypus novemcinctus  TATAGAAATTTTGGAAAATAGAGGAAAGAAAAAAGAAAAAT-TAACATTTTGGTGTCTTTTCTAGCCTTTTTGTTGTTCTTCCAATTATGGTTTCTCTGCCTTTTGTCCAAACACACTTG

Homo sapiens          TGTTGCTGTCTTCTGTAATGATACAGTTTTGTGCAGCTGTTTTCACTTAGCATATCGTGGGCATCTCCCCTTATGATTACTAAATATTTTATTTTGGAGTGGCTGTGTACTCTCCCATTG
Dasypus novemcinctus  TGATGGCACCTTCTGT----ATTTAGTTTGGTGTAGCTGTTTTCACTATGCCAGTCACGGGCCTTTCCCCAGGTCTTTACTA---GTTTTAGTTGGAAATGACTCTATGCTTATCCATGC

Homo sapiens          ACTAGATGGACCATTGTGCCAGTTGCCAATCACTAATGCTGTTACTAACTTTTCAGTTATAAATTGATGAATATCTTTGTGCAC-AGGCTGTTTCCCAATGTCAAGTTA-----TTAGGG
Dasypus novemcinctus  CATAGACACACACTAGTGCTGACTGTTGGTCACCGCTGCTGGCACTGGGCTTTCTCTTA-AAATAGATGA------CTGTGCATAAAGCTTTTCCCCAATGTGAAGTTGTTTGCTTAGGC

Homo sapiens          TAGACTCCAGGAGGTGGGATTCTTCAACTAAAGAATATGAAAACCTTTGAGGCTTTTACTACATATTGACAAAATGGTTTCCGGAAATATTTGTATCCCCTTACACTGCCACCAGCAAGG
Dasypus novemcinctus  TAGATTCCCAGAGGTGGGAAGATTGAGTTAAAGAGTATGAAAAGTTTTGAGGTTCTCAATACATATTGACAAAATGGTTTCCAGCTATATTTATATCAGTTTGGACTGCCACCAGCAAGG

Homo sapiens          ATAAACATGTCCATCTTGCCCGTATTGGGAATTATCATCTGGCTAAATATTTGCTAATTTGATAATGAAAAAATAGCATCGTGTTTCAGTTGGCATTTCACTGACTTCTAGCACGGTTGA
Dasypus novemcinctus  CTGAGTGTGCCCATC--GACC--ACTGCACATTGTCATCTGGATAAATTCTTAC-------------------------------------CATTTCTTTGGTTTCTAGCA--GTTGA

Homo sapiens          ACATC-TTTCATGTGGAGCGATTGTATTTCCTCCTTTGTGGATTGTCAGTGTCCTTTGCTC-TATCTTCTGGGGTCAGATAAATTTGTATGAGCTCGGTATATATTAAAGATATTAACCT
Dasypus novemcinctus  ACATCTTTTTATATTAAGCAGCTGTATTTCCTATTTTGTGGATTGTTGGTGTCCTGTGGTCTTATATACTGGGGTCTGATCAATTTGTATGAACTCCTCATATATTAAAGACATTAACCT

Homo sapiens          GGTGTGTGTCATATTTGCTACAGCTGTTTCTGGGTTTTTGTGTGTTTTATCTTATTGGAATTTTGACATACATAGTCGATCTGTCTCCACAGCACTGTGTCTGCTAGACAAGAACACAAG
Dasypus novemcinctus  GTTGAGTGTTGTATTTACTGAAG.................................................................................................

Homo sapiens          GCAGGCAGGTGGATTTTGTCTGTTTTGTTCATGGCCGTAGCACTAGCACCTATGACAATGCCTGGCACGTGATAGGTACTCAGTACACTTCTGTTGAATTAGTTACTCCAGTGGATCTGC
Dasypus novemcinctus  ........................................................................................................................

Homo sapiens          AACGCCTTGGGATCCACGGCACGTGATAAGCACTCAGTACACGTTTGTTGAGTTAGTTACTCTGATGGATCTGCAACACATTGGGATCCACACATGGATCCAGCAGTCCGCGCTCCAGCT
Dasypus novemcinctus  ........................................................................................................................

Homo sapiens          CACAGGCCGAGGCTCTCACACTCCAGAGCTCGCAGGCCGAGGCTCTCACACTCCAGAGCTCGCAGGCCGAGGCTCTCACACATTTTGTCTCAGGGTCCCCTTGCACTATAAGAACTCATT
Dasypus novemcinctus  ........................................................................................................................

Homo sapiens          GAGGGCCTCAAACAGCTTTTGCTTATATAGGTTTTCTCTATCAATATTTACCATATTATACATTTAAACAGAATTTTAAAATAATTATTTGGAAAATAAAAACACTGTTAAACAACATTT
Dasypus novemcinctus  ........................................................................................................................

Homo sapiens          TAAATAAAAAAAACTATATTTT
Dasypus novemcinctus  ......................


Homo sapiens        GCAGTGGCTCACGCCTGTAATCCCAGAACTTTGGGAGGCCAAGGCAGGCGGATGCCCTGAGGTCAGGAGTTGGAGACTGGCCAGGCCAACAAGGTGAAACCCCGTCTCTACTAAAAATAC
Loxodonta africana  ........................................................................................................................

Homo sapiens        AAAAATTAGCCGGGCATGGTAGCACGCGCCTGTAATCCCAGTTACTTGGGTGGCTGAGGCAGAAGAATCGCTTGAACCCGGGAGGCGGAGGTTGCAGTGACTCAGGATCGCGCCACTGCA
Loxodonta africana  ........................................................................................................................

Homo sapiens        CTCCAGCCTAGGCGACAGAGCGAGACTCCGTCTCAAAAAAAAAAAAAAATAAGAAGAACAGTAAATCATCAAAAAAATAAACCCAATGACAACCACAGAAAGAGCCTTGCAAGCCCTCTT
Loxodonta africana  ......................................................................AAGAAAATATTTACAATA-----CACA-AAACAGCTTTGCAAGCTGTCTT

Homo sapiens        GGGAGGAATGACTGGGAGAACACCAGGTAC-CCGCCCTGA----TGAAGACGCCGCAGCAACTACAGTCTTCCCCGATTGGC---------AGCAGCCCGAGTTGGGGGGT-------GG
Loxodonta africana  GTAAAGAATCAAAGGAAAAAAAGGAGGGAAGACGCCCAGAGTAGTAAAGAAGGAAC-GTAGGCACAGCGATCACTGATTGGCTGCGGGAGGAGCGGCTCGAGGAGGCGGGGCTGCGCGGA

Homo sapiens        GGGGGGGTGGGACTTTGTGCATCACTTCCGGGGGCGGGGCAGCGGTCGTCTCCGCACGCGCGGGAGCGGTCGGTATCCATGGCAACCAGCGGGGCCAGCCTAAGGCCCGCCCGCCACCGC
Loxodonta africana  GTGGGGGCGGGGCCAAGTGCACCACTTCCGGGG-CGGGGCCTCGGCCTCCGCCGCGCGCTCGGTGGAGGACCGTCTCCATGGCAACCAGCAGGGCCCGCGCCAGGCCCTCCCGTCCCCGC

Homo sapiens        CACCGCGCCGGCCCCGCCCGCACAAAGGCCGCGCGGTGACCCGCCCTCTGCCGCGACCCCGGCCCCGCCCCTGCCCCGGCCCCGGCCTGGCCCGGTCCCTGCACCTGCCGG--AGGCCGC
Loxodonta africana  CCCCGCGCCGGGCCCGCCCGCCCTGAGGCCGCTCGG-GGCTGGGCCT---CCCCGACCCCCGACCCGGTCCCGGTCCCGCTTCTGCTGGAGGCCGCGCCGCCGCCTGCCCGCCCGTCCAC

Homo sapiens        CCGCCGCCATCGCCAGCGCCGTCGCTTGGCTGCGCTGACCGGGAAGCAGTTCT---CGGCGGTCCGCGCGGTGATTCATCGGCCTGGCCGCGTCCCCCGCGCGGGCCATCTTGGTCACGG
Loxodonta africana  CCGCCGCCATCA-------CGCCGCCCGGCTGC-CTGATCCGGAAGCAGTTCTGGCCGGCCGCCGCCGCGGTGA-CCATCTTGGTGGCCGCGT--------CGGGCCATCTTGG------

Homo sapiens        CCGGCGGCGGACAGAGCCGCGGA-GGAACCGCGAGACCCCAGGTACGCG--TGCCCCCGGCCCCCTGCCCGGCCGGGCCCCGCCACTGTGGTGGCCCCCAGGCCCGCGGCCGCCA-AGGA
Loxodonta africana  -CGGCGGCGGACGGAGGCGCGGAGGGAGCCGCGGAGACTCAGGTACGCGACCGCCCGCGGCCCCC----------GGCCCCGCCGCCGTTGTCGTCC---------CGGCCGTTGCAGGT

Homo sapiens        GCAGCCCCCTTTGTTCTCCCCCGGGCGGGCGCGGGGCGGGCGCCGCGGGGCTTTGTGCAGCCTCGCCACCCACGCGCGGGCCCTCCACGCCGCCCCTCACCCTCCTGCCCGCTAGTCGCA
Loxodonta africana  GCCGTCTCTTTGGTTCTGTCCCTGCCGGGC-CGAATCGG--GCTGCGGGATTTTG.................................................................

Homo sapiens        ACTGAAACCTTGAAAACATCTTTTCTTAATCCTCCAGGGCCCAGAGCAGGTCCAGCCACCCGTCTGGCTCCGTAGTCCGCGAAGTCTGCAGTAAAGGAGCCGCGTGGGACCGGGGCC---
Loxodonta africana  ...........................................................................................GGGACCCTGTGGGACCGGGCCCTTC

Homo sapiens        GAAGTCCCTAGGGCCTGGCACCGCGTGCCCCTCTCTCGGGCCCTCGGCTTGGGGGTCTCGCAGCGCCCTGCCGTCGTGCACCCCACACTGCCCCCA-TTTCTACAGGGGCCCACCT----
Loxodonta africana  GGAGCCCTCAGG------CTCCATCTGACCAGCCTTC------TCGTTT----GATCTCATCA-GCCACTCTACTCTGAACCCCACCCTTCCCCCGGTTTCTGTTTGGGTCAACCCCTAT
```

```
Homo sapiens        ----------------------GGTCACCACACCCTCTTGGTCCACCCGCGG--ATCTCAGCGTGTGGCTGACCTCTGGCCAGCATGGTGCAGCCGCAGACGTCCAAAGCTGAAAGCCCA
Loxodonta africana  CTGAGGGTTACTTAGATTTGGGGCTCCCTGTATCTCCCCCATCCGACCACAGCAGCCTCCGTGTTTGGCTAACCTCCAGCCAACATGGTCCAACCCCAGACCTCAAAGCTGAAACCCCA

Homo sapiens        GCCTTGGCAGCGTCTCCGAATGCCCAGATGGATGACGTTATTGACACCCTGACCTCCCTGCGCCTCACCAACTCGGCGCTGAGGCGGGAGGCTTCCACCCTGCGGGCGGAGAAGGCCAAT
Loxodonta africana  GCCCCCGCAGCACCTACAGACGGCCACGTGGAGGATGTCATCGACACCCTGACCTCCCTGCGCCTCACAAACTCCGCGCTGCGGCGGGAGGCCTCCACCCTGCGGGCCGAGAAGGCCAAT

Homo sapiens        CTCACCAACATGCTGGAGAGCGTGATGGCAGAGCTGACCTTGTTACGCACCAGGGCGCGGATCCCGGGGGCTCTGCAGATCACCCCGCCCATCTCCTCAATTACTTCAAACGGGACTCGA
Loxodonta africana  CTCACCAACATGCTGGAGAGCGTGATGGCAGAGCTGACCCTGCTGCGCACCAGGGCGCGAATTCCGGGCGCTCTGCAGATCACTCCACCCATCTCTGCCATCACCTCCAACGGGGCTCGG

Homo sapiens        CCCATGACCACACCTCCAACCTCTCTGCCCGAGCCCTTTTCCGGGGACCCAGGCCGGTTGGCGGGGTTCCTGATGCAGATGGACAGATTCATGATCTTCCAGGCCTCCCGCTTCCCGGGT
Loxodonta africana  CCCATGACCACGCCCCCCACCTCTCTGCCCGAGCCCTTTTCTGGAGACCCCGGTCAGTTGGCGGGCTTCTTGATGCAGATGGACCGGTTCATGATCTTCCAGGCCTCCCGCTTCCCCGGC

Homo sapiens        GAGGCCGAGCGTGTGGCCTTCCTTGTGTCTCGACTGACTGGGGAGGCGGAGAAGTGGGCTATCCCCCACATGCAACCTGACAGCCCCTTGCGCAACAACTATCAGGGGTTCCTGGCAGAG
Loxodonta africana  GAGGCGGAGCGAGTGGCCTTCCTGGTGTCGCGTCTGACTGGGGAGGCAGAGAAGTGGGCCATTCCCCACATGCAGCCAGACAGCCCCTTGCGAAACAACTACCAGGGGTTCCTGGCAGAG

Homo sapiens        TTGCGGAGAACCTACAAGTCTCCGCTCCGGCATGCGCGGCGCGCCCAAATCAGGAAGACTTCTGCCTCTAATAGGGCTGTGCGAGAGAGGCAGATGCTCTGCCGCCAGCTGGCCTCTGCG
Loxodonta africana  CTGCGGAGAACCTACAAGTCTCCACTCCGGCACGCACGGCGCGCCCAAATCCGGAAGACTTCCGCCTCTAACAGGGCTGTGAAAGAGCGGCAGATGCTGAGCCGCCAGCTGGCGGCCGCG

Homo sapiens        GGCACGGGGCCTTGCCCAGTGCATCCAGCTTCCAACGGGACTAGTCCAGCGCCAGCCCTGCCTGCCCGAGCACGGAATCTTTAAGAATCCGCCAGCACTTGGTAGCGTCTGCAGCCACCC
Loxodonta africana  GGTACCGGGGCCTGCCCTGTGCACCCGGCCTCCAACGGGACTAGTCCCGCTCCAGCCCTGCCCACACGAGCACGGAACGTTTAAGAACCCGCCA--------------------CCACTC

Homo sapiens        AGGTAGCATACGCTCTTTGCTGTGTAGAAGAAATGCCCATACGACAGCTTTGCCCCTGTTTGAAGACCTCCCTTCTTGCCTCTCCAGACGTGTTCCCCGAGGAGATCTTCCTTCCGTCCT
Loxodonta africana  AGGTAGCCCGCACCCTTTGCTGCGTAGAGGAAATATCTGCACAACAGCATAGCCCTTGTTTGAAGAAC-ACCTTCCTGCCTGTCCGGTCCTGTTCCAGAAGAAGATCT---GTCCACACC

Homo sapiens        TCCTGGCGCCCTGGTTGCCCACCTTGCCGTGCTTCCTCTT---ACGTGCTAGCTTTGTACCTATCGCTCACTGCATGCTCGCCTCCCTCTTGCTGGCATCCCGGCCTGTTTCAATGACTA
Loxodonta africana  TGTGGGTGCCCTCATC-CCCACTGTGTTGCGCTTCCCATTGTGATGTGCTGGCTTTGTACCTACAGCTCAATGCACGTTTCCCT--CTGTTGCTTGGTCCCCCAGCCGTTCTGATGACGG

Homo sapiens        CCGCTCTGCTACTTAGGCACAGGGACTCCGCCGCACGCTGACGGACCACGAGGGCTGACCCCTTCCAGCCTGACTTGGTTCATGGAGGCTCCTACTCTGCCCTCTCCAAGCTCCCCTGGC
Loxodonta africana  CGACTCTGCTGCCCAGTGACACTGATGCCACAACATGCTTGCGAACCACTTGGGCTGACTCAGTCCA----------------------------------CCCCTGGAGCCCCCAGGGC

Homo sapiens        GGCTCCCCACCTGGTTGCCCAGTTCCTATTGATGAGCTCTGGACAGAAAGATGCCCGTTTGGCCAGGCTGGTGGCTTGATGGGTGTACCTGGAGAGGGGGTCTGGCTTCCTGCCCAAGAT
Loxodonta africana  AGCTCTTCACCTGAGTGCCCTGTTCTCACAGAGGACCTCTGGACAGAGCTAAGCCTGTTGGGCCAAGCTGGTGGCTCGTTGTATGTACCTGGAGAGGAGGTCTGGCTTCCTGCCCA----

Homo sapiens        GCCTCCCAGCCCTGCCAGGGCCCGGTGCAGCGGGCAGGGCCTCATCTGTGCTGTAGTGGTCGAGTGG--TTGCTGCAAGGAGCGTAGTTCTGCCATGTCTGGGGGCCAGGTTCCACTCTG
Loxodonta africana  -------GGCCCTGCT----CCC-------------------------------AGTGGCCACGTGACCCCTCTGCTAGGAGTGGGGTTGTAACATATCTGGGGG-CAAGCTTCGCTTTG

Homo sapiens        CACATGAATATGCAGTCTGGGAGGCCCCACTGCTCTCACTGG------GAAGGACC-AATGTTGCA-----------------------------CCTCTGTTAATGCCTGACTTCAGCT
Loxodonta africana  CACATGAATACACAGTCCAGTACACCCA---GTCCTTCCTGGCATGTAGAAAGACCTGATAGTGCAGCGAAAGGGACAGCCCCCTGGACGAGGTGCCTCTGGCAAGGCGAGTGGT-ATCT

Homo sapiens        GCTGGTGTTCTGATGGAGCCAGAGGCTTGGGGAATCTGGAACTTG-CCTGCTAAATAAGGTCGTGGTGGACTCTCAGCCATTGGGCAGGTCTATCAGGCTGCAGGTTCCTACACACCCAC
Loxodonta africana  GGCCATGTTCCGATAGCTTCAGGGCTTTTGGGGGTCTGGAACCTGACCTGCTACTTAAGGGCGTTGTGGACTTTGGGCCA---------------------------CATGCGTGTCCCC

Homo sapiens        GCCTGAGGGTCATAGCAGGCTAAGGGTGGATACCAGCGACTC---CCTTTGCTGCCCAGGATCTCCATGGGCAGTGCCACAGCGGCTGATGCTCAGTCACTCCTGCTTCTACCC-----C
Loxodonta africana  ACCTGAGACTCAT-GCACGCTGAGGG-GGACATTGGTGACTCACACTTTCCCACCCTGGGGTTCCCGTGGGAACTACCACAA-GGTGCTTGTTCAGCTCCCCCTCCTCCTTGCCCCACAC

Homo sapiens        CTGTCACTGATGGCGAGCCTTGCCCAGCTTGAGACCTGTTCCCATCTCTATTCAGGTGCCATGTGGCCTTCACTGCAGCC-CTGCAGCCACCCACGCACCATCTGTGGGTCTCCAAAGGC
Loxodonta africana  CAGTTGCCATTGCTGAGCCTTGCCTGGCTTGAGACCCATTCTCGTCTTCATTCAGGCCATCTGCAGCCCTCCCCTCAGCCTCTGCAGCTGC---CACACTGTCTAGG----TCCAGA-GC

Homo sapiens        ACCTTGTAGCATGTACTCCCCGTGCCTGGGCAA---TCAGATGGGCTGCCTTTGTCCAAGGGAAAACAGACTCCCTTCGGGAAACATCCTTAAGCACTTAAGGCCGGGGGGGGTGTCTGC
Loxodonta africana  CCCTCCAAGCTTGCCCTCCCAGGGCCTGGGCTGCCCTCAGGTGTCCAGCCTCCGTGTAAGG--AAACGTGCTCCCTCTTTT--ACATGGCTCAGCATGCAAGGT--GGGGAGGCGTCTGC

Homo sapiens        CTCTGGCAA-CCCAGCCAGGGTCTTGGTGGCATTTGTAAAAGCAAAGAGCTGTGGACTGCCGTGGTCCTAGTGTGGTGACAATGCAGCACTGGCATGCATGTCCTCCTTCTGAAGGA---
Loxodonta africana  CTTTGGCAACCCCAGCCAGGGCCTCGGTAACATTTTTCA----AAAGAGCAGTGGACTACC-TCGTCCTAATGTG--------------TTGGTGCCCTTGTCCTAATTCTGAGGGACAA

Homo sapiens        -------------------------CCTCATCCTTCCTCACAGGGGGATGACCAAGAAATCATTTTGTGGCTGAG-TTTGGCCACGCCCTTTGGACTGTGCTG-TTCCGCCATATTTCAA
Loxodonta africana  TGCTATCCCTCTTAGTGGCGGACAGCCTTGTGCTTCCTCACAGGGG--TGACCAGGAAACCATATCCTAAAGGGGACTTGGCCA-ACCCTTTGGACTGTGATGCTCCCGCCGTATTTCAA

Homo sapiens        TGCCAAATGAACCACATTGAC---ATGACCTGGACCATA---------GGGCTTCCTATCCTGGGCTCAGCTGCCCCT-GTCTGAAGGGTCTTGGCTTGATTGCAGAAGGACAACCTCCG
Loxodonta africana  TGCCAAATGAACCACATTCACGCAACGGCCTGGACCATGGGGGGAGGTGGGTGTCTTACCCTAGGTTCATCTGCCCCTGGTCCAGACGGTCCTGGCTCAACTGCAGAGGAGCAGCCTCTG

Homo sapiens        CACCCACCTAAAGACATGTATATGTCTTGGGAT-CCCAGAGATTGGGTCCTTGGGCCTG-----GCTTCTTAAGAGTTTTGATGATGCTGGGAAAAGTGACTGCGATTCTGAAG-AACCG
Loxodonta africana  C-CCCATCCAAAGAC----ACATGTCTTGAGGTACCCAGAGGTTGGGTGCTAGGACCCGTCCTCTCTTCCTGAGAGTTTTGATGATGCTGGGAGAAGTGACTGTGTTCCTGAAGCAACCA

Homo sapiens        CTGCCTTGCAAGGTCAAGGACATTCAGTGGTTGCTGGGGTCCGCAGACTACTGCCACCCACTCACCATCAACTCTGTTAGCCCAATTGCCCTGCTGAACAACTGCCTGAATACAGGCTTT
Loxodonta africana  CTGCCTCACGCAGTCAAGGGCGTTT----GTTGCTGGGGT--GTGGACCACAGCCACCGGCTTATTGTGAACTCCGTTAGCCCAATTGCCCTGCCGAACAACTGCCTGAAC-CAGGCTTC

Homo sapiens        AGGTTCCCCTGGACTCCAGCCAAGGCTGTTCAGGTGGGACCATGGTGCTCTTTAAGCGTGATCGGAGGGAAGACACACA--GCAGGGCCACCATTCCATGAATGGGAGGTGTACAGATCA
Loxodonta africana  AGGTTCCCCT-GACTGCAGCCGAGGCTG--CAG--GGGACCGTGGTGCTCATT--GCGGGAATGGAGAAAGGATGCATAGGGCGGGGGGACT--------GACTGGAGGCACACAGCTCA

Homo sapiens        CTTTCTCTTTGTGCTCAGTTCTGTTCTGTCTCCAGCAGCTATATTGGTAAGACTAGTACCTGCCAGGGAGAGGTGCCCCCAAGTGAAGGGGTACAGTGGCACCTGGG-------------
Loxodonta africana  CTTTCTCTTTGTGCTCGGT---------TCTCCAGCAGCTCTGTTGGTAGAACGTGTACCTGCCAGGTGGAGGTGCTCTGGGATGAA--GGTCCAGTGGCACCCGGGGAAAGTTTGGCCT

Homo sapiens        ---------AAAAGGCACCTGGAAGGTTTCCATGTGGCCCAGCCCAGCATGGAAGCAGGGTGGGAACTCTGCTGTGTCGCCAGCGCTCACTCTACTCGAGTGGCTTTTTGAAAGCCCTAC
Loxodonta africana  GCAGGGGGTAAAAGACAGCCTGAATGCTTCCGGGTGGCCTAGCCAGTCAGGAGACCCAGCGTGGGCCTCTGCTGTGTTGCCCACCCTGGGTCTGC-CAAGTGGCGTTTAGAAAGTGCTGG

Homo sapiens        CATGTC--------------TGTGTCAGGCCTGTGCTGCTTCACATCCTACAGCTGCCTAGGAAAGGCCGGCCACGCTCCCTGTCCACACACTCCCTGTCCACACACTCCCTGTCCA--C
Loxodonta africana  CATGTTTGCCTTGGGAGCTGTGTTTTAGGCTCGTGCTACCACGTATATCAAAA----CCAGAA------------------------------------CCTCACATGCCAGGGCTGGCC

Homo sapiens        ACACTCCCTGTCCACAACTGCAGCCGGGCCCTCTGCCTATGGGC------ACCCAATCCAAGCAGCTGCTCCACCTTTGTTTGGCATGGTGATT----TGTGTTTTTTCTCTTGGTGCTT
Loxodonta africana  ATCCTCCCTGCCCATGACCATGACCAAGCCCATTG--TAATGGCTGGTAGTGCCAGGCCAAGC-------------TCAGTGGGTTTGGTACCTCCACTGTGTTTTGTCTCTTGGTGTTT

Homo sapiens        ATGTGTGTGGGCTTGGGACGAGTGCTGGTATGCACTTAGGACCTTCTTGATAGCTCCCTG-CACTTTGGAACACGGAGCAGATGAGAGAGGGTCGGGGCTTGCCCTCCACCTTGGACTTG
Loxodonta africana  ACGTGTGTGAGCTGGGGACTGGTACTGTTGTACATTTGTGACCTTCTTGATGGCTTCCTACCACTTTGGGAAGTGAAGCAGATGAGAGAGGGTCAGGGGTTGCCCTCCACACTGGCCTGG

Homo sapiens        GAAGAAGCCCACATTGGA-GAGGTGAGGACCCCATGGTGGCTCTAGTGGAAGATACGTTAGTCTCCAGCTAAGGAGGATGAG-----------GCGCAGCCCCAGAGGGAGACCTCAGTG
Loxodonta africana  AAAGAAGTCCCCATTGGAGGAGCTGAGGAACCTATTGTGGTTCTGGTGGACAACATCGCAGGCCCCAGCAGAAGAGGATGAGACCTGAGCTCCACGCAGCCCCAGAGTGGGAC--CAGTG

Homo sapiens        ATAGGGGA--TCAGGCTAAGAAAGT---GGGGGAAGGGAGATGCTTTGTACATATTTTGGGGTTATAATTTCTCTAAATTTTAGGAGAACGGGTATTGATTGATAAAAG--GGACAGGCA
Loxodonta africana  ACTGGGAGACTCACGCTGAAGAAGCCAGTGGGGAGGGGAGATGCTTTGTACATA-TTCGGGGTTATAATTTCTGTAAA-TTTAAGAGAACAGGTATTGATTTATAAAAGACAGGTGGTTA

Homo sapiens        GTAGTGTTCAACAGTGCATGTGAAGGAAAGTTCTGT---TTTCCATGGTTTTGACATTCTTTGGACTGTATTGTGACTGCTGTCTGGTCCACATGGTACCCC-TTTGGTAAGTAGGCTTC
Loxodonta africana  GTAGTGTTAAACAATGCATGTGAGGGAAAGTTCTGGGTATTTTCATGATATTGACATTACTTGGGCTGTGT--TGATGTGTTTCTGGTCCACGTGACTCCCTATGTGGTAAGTGACCTTT

Homo sapiens        AGTGCATACCAGGGTATCACTGGAGATGGGAGTTAGTG-AAGGGGTGACTCCCTGGCCTAGTATAGTG--TGACCCTGGGACTAACTTA-----ATGTCCTAAAGCATTTTGGTGACTTC
Loxodonta africana  GTGGCATCACA-GACCTGATTTGAGACGGGATCTAGAATACAGGGCGA----CTGGCCTAAGACAGAGCATGACCGTGGGATTAATATACAGTCATGTCCTAAAGCGTGTC-GTGACTTC

Homo sapiens        TAGGGAATAGCAAAGACCTATTTCATTGTCCCCAGGTAAGTATGTGATGAGCAATGAGGAGGAGTGGAAAACAAAACCCAGAAAGTGCGGCAGGACCAGCCTGACGCACACGCTCC--TG
Loxodonta africana  T-GGAAACAACACAGCCCCACTTCCTTGGTTCCA-GTAAGTATGTGATG-TTAATGACGAGGGGGGAAAAGTA-----------------CCTGACTTCCCTGATGCACGTGCCCCTGTG

Homo sapiens        TTGTCATGGCAGACAGCCGCCTTGGGTGGGCACCACCCTGGCAGTTCCAGCCTGTAGGGGAGTGAAGGGACATGGCTGAGCTGGGCATGTGCTGAGGTTGAC-TTAGGGAACAAGCCCTG
Loxodonta africana  TTGGGGGAGCAGACGCACCCCC---ATGACCTTGGTCCTGGCAGTT-GGGCCCCTAGGCGAGGGAACAAATGGGGCAGTGTTGGGCATACACTTGGGTTAACGGAGGGCGCAAGCCTTC

Homo sapiens        GGATTGGACAAAAGGGCCCATGCTGCAGCCACTGACTGGGGGCAGAGCTCTGGGTGGAAGAGGGAAGAGATCCTAA--------------TGGAGGCGCC-TCCATCTGCAACCACAGTT
Loxodonta africana  TGGTTGTATCAAAGGACACGCTTGGGGGCCACTT-CTGGGGGTGAGGTTTTGGTGGGAAGAGGGGACAGATCCAAAGGCACCTTCCCTTGTGGAAGCCCCTTCTGTTAGCACCCTGAGGT

Homo sapiens        GTAAGGCTCATGGCACCTCTGCTTGGAAAGCACTGGTTTAGGGACTTAGAGAGGTAGGCACAAGGTGGGTCTCCTGGGTAAGGGAAGCA-AGAGCAGACTGTTGGGCC-AACAGGAGAAG
Loxodonta africana  GTAAGG------------------GGA--------GTGTAGGGGTTCAGGGATGAAGACACGGGATGCGGCTCTTTGGGAAAGGAAGTGTCGAACAGGCTGTTGGGTCAAACGGAAAAAG

Homo sapiens        CTCCCCAGAGTAGGGGAGAAGGTTGGGGTGTAGGGC-----CTTCCACGTGGAA-------CAGACAGCCCCTGTGTCTCT-------GTCTCTTGGGGACCTGAGTTTGGGTGGGGTGG
Loxodonta africana  CTTCTCCGTGGAGGGGAGAAGAC--GGGCTTAGGGCAGATACTGCCGCCCGGTAGCGGGTGTAAATGGACCTGCGGTCTCCTATCACGTCCTTTTGGGGTCCTGACACC--GTGTGGTGG

Homo sapiens        CAGTTGGCACAGCGCA-GATGCGGTAGAGATGGGAGGAAACCCAGCTCCTCACTTCCGTGTGCCTCA----TGCCTTTGCATACACAAGCACCAAACCTACTAGGTCTTCTCATTACCCA
Loxodonta africana  CGGTGGGCCTGGCGGGTGCAGCGGAGGAGACTGGAGGCAAGACGGCTCCTC----CTGTGAGCGTCACCACCACCTGCCTGGGAACAAGTGACAGA-GTGCTAGCTCTGTTCACTATAGA

Homo sapiens        TG-TAAACCACATGTTAGATAAATTTTTGCAAGTAGAGGAAAGAAGGAAATAAAACATCACATTTTGGTGTCTCT-----CAGG-----------CTTTCCCCCCCAACTATGGTTTCTT
Loxodonta africana  CGTTAACCCACACGTTACAGAAATTTTGCAGACCAGAGGAAAGAGAAAAGGAAAAATAAACACGGTGGTGTCTCTCCTTCCAGGTCCGCCGCCTCCCGCCCGCTCCAGTCATAATTGCTT

Homo sapiens        TGCTTTTTGTTTTAACATAGTTTTGTTGCTGTCTTCTGTAATGATACAGTTTTGTGCAGCTGTTTTCACTTAGCATATCGTGGGCATCTCCCCTTATGATTACTAAATATTTTATTTTGG
Loxodonta africana  TGCTTTTTGTTTTCCTGTAGTTGTGATGCTACCTTCTGTCATT-CGCAGT-------AGCTGTCTCCTGCGAACACTCCCCAGGCGTCTCGCCGTGTTGTCACCA---GCTTTCTTTTGG

Homo sapiens        AGTGGCTGTGTACTCTCCCATTGACTAGATGGACCATTGTGCCAGTTGCCAATCACTAATGCTGTTACTAACTTTTCAGTTATAAATTGATGAATATCTTTGTGCAC-AGGCTGTTTCCC
Loxodonta africana  AATGGCCACGCACTGTTCCTGTCAGGAGACGGGCA-----GCAGGCCGGCCGACCCCAGTGCTGCTGTCGTCTC-----TCGTAAACAGGTGACCCTCTCTGTGCATAAAGCTGTTCCCC
```

```
Homo sapiens        AATGTCAAGTTA---TTAGGGTAGACTCCAGGAGGTGGGATTCTTCAACTAAAGAATATGAAAACCTTTGAGGCTTTTACTACATATTG----ACAAAATGGTTTCCGGAAATATTTGTA
Loxodonta africana  AGTGTCAGGTTACCCTTAGGCTGGGTTCCCAGAGGTGGGGTTACAGAAGTCAGGAGTAT-AAAACTTTTGAAGCACTTATTACGTATCGGAAAAAAAAAAGGTTTCCAGAAAAGTTTGTA

Homo sapiens        TCCCCTTACACTGCCACCAGCAAGGATAAACATG--------TCCATCTTGCCCGTATTGGGAATTATCATCTGGCTAAATATTTGCTAATTTGATAATGAAAAAATAGCATCGTGTTTC
Loxodonta africana  TCCATTTCCACTGCCGCCAGCGAAGATGAACCCGGCGACCTCCCCACCTTGCCGGTACGAGGTCTTATCATCTGGCTAAAGATTTGCCGACTTGGTAATGACGAATGGTACTTGAGCTTC

Homo sapiens        AGTTGGCATTTCACTGACTTCTAGCACGGTTGAACATCTTT-CATGTGGAGCGATTGTATTTCCTCCTTTGTGGATTGTCAGTGTCCTTTGCT-CTATCTTCTGGGGTCAGATAAATTTG
Loxodonta africana  AGTTTGCATCTCTTTGGCGTCTAGCAAAGTTGACCATCTTTCCGTGGTAAGCAGCTGTGTTTCCCATTCTGTGGGTCGGCAGTGTCCATCACTCCTGTCTGCTCGGGTCTGGTACATCTG

Homo sapiens        TATGAGCTCGGTATATATT-AAAGATATTAACCTGGTGTGTGTCATATTTGCTACAGCTGTTTCTGGGTTTTTG--------TGTGTTTTATCTTATTGGAATTTTGA--CATACATAGT
Loxodonta africana  CAGGAGCGCGTTATATATTAAAAGATGTTAACCTGTTGTGTGTCATATTCGCTGCCAGTGTTTCCAGCTTTTTAATGTTTTCTGTGTGTTAACTTACCGAAGTTTTGACCCATGTGTGGT

Homo sapiens        CGATCTGTCTC---CACAGCACTGTGTCTGCTA--------GACAAGAACACAAGGCAGGCAGGTGGATTTTGTCTGTTTTGTTCATGGCCGTAGC----------ACTAGCACCTATGA
Loxodonta africana  GGATGATTCCCTGACTTAGCGCTGTGGCCACCGACGTTAGCAGCAATAACA------AGGACGGCCTGCTTTGTTGGCCTTGTTCCCGGCTCTCTCCGGCTCTCTTATTAGCACCCGTAA

Homo sapiens        CAATGCCTGGCACGTGATAGGTACTCAGTACACTTCTGTTGAATTAGTTACTCCAGTGGATCTGCAACGCCTTGGGATCCACGGCACGTGATAAGCACTCAGTACACGTTTGTTGAGTTA
Loxodonta africana  CAGTGCCTGGCACGTAGTAGGTG-----------GCTGGCCAACCAGTTGCAGGAATGGATGTGTTCCTGCTGGG.............................................

Homo sapiens        GTTACTCTGATGGATCTGCAACACATTGGGATCCACACATGGATCCAGCAGTCCGCGCTCCAGCTCACAGGCCGAGGCTCTCACACTCCAGAGCTCGCAGGCCGAGGCTCTCACACTCCA
Loxodonta africana  ........................................................................................................................

Homo sapiens        GAGCTCGCAGGCCGAGGCTCTCACACATTTTGTCTCAGGGTCCCCTTGCACTAT-AAGAACTCATTGAGGGCCTCAAACAGCTTTTGCTTATATAGGTTTTCTCTATCAATATTTACCAT
Loxodonta africana  ..........................ATTCACTCTCGGGATCCCTTTGCCCTCCGAAAAAATCATTGAGGACC-CAGAGAGCTTTCGCTTAGGTAGCTTCTATCTATCGATATTGACCAC

Homo sapiens        ATTATACATTTAAACAG----AATTTTAAAATAATTATTTGGAAA------------ATAAAAACACTGTTAAACAACATTTTAAATAAAAAAAACTATATTTT
Loxodonta africana  ATTAGAAATGAAAATGGAGAAAGCTTTAAAACGTTAATTTGAAAAGAAACTCATTACGTATCAACATAGATAA--CACATTTTTATGAAAAAAAACTATTTTCT


Homo sapiens        GCAGTGGCTCACGCCTGTAATCCCAGAACTTTGGGAGGCCAAGGCAGGCGGATGCCCTGAGGTCAGGAGTTGGAGACTGGCCAGGCCAACAAGGTGAAACCCCGTCTCTACTAAAAATAC
Procavia capensis   ........................................................................................................................

Homo sapiens        AAAAATTAGCCGGGCATGGTAGCACGCGCCTGTAATCCCAGTTACTTGGGTGGCTGAGGCAGAAGAATCGCTTGAACCCGGGAGGCGGAGGTTGCAGTGACTCAGGATCGCGCCACTGCA
Procavia capensis   ........................................................................................................................

Homo sapiens        CTCCAGCCTAGGCGACAGAGCGAGACTCCGTCTCAAAAAAAAAAAAAAATAAGAAGAACAGTAAATCATCAAAAAAATAAACCCAATGACAACCACAGAAAGAGCCTTGCAAGCCCTCTT
Procavia capensis   ................................................................................................AAGAAGAACTTCACAAGCAGTCTT

Homo sapiens        GGGAGGAATGACTGGGAGAACACCAGGTACCCGCCCTGATGAAGACGCCGCAGCAACTACAGTCTTCCCCGATTGGCAGCAGCCCGAGTTGGGG-GGTG--GGGGGGGGTGGGACTTTGT
Procavia capensis   AAGAAGAAGCAAAGAGAAGGGACT--GCGACCACCTTAGTGAAAAAG----------TAAAAGACGCACGGGCGAGCACCGATTCCTGTTGGGATGGTGCTGGGGGAGGCGGGGCTGCGC

Homo sapiens        GC-------------------ATCACTTCCGGGGGCGGGGCAGCGGTCGTCTCCGCACGCGCGGGAGCGGTCGGTATCCATGGCAACCAGCGGGGCCAGCCTAAGGCCCGCCCGCCACC
Procavia capensis   ACCTTGGGGGCGGGGCCAAAACACTACTTCCGGGG-CGGGGCCTCAGCCTCCGCTGCGCGCTCCAGGGCAGGTGGTCACCATGGCAACCAGCAAGGCCCGCTCTACGCCTCCTTGTCCCC

Homo sapiens        GCCACCGCGCCGGCCCCGCCCGCACAAAGGCCGCGCGGTGACCCGCCCT------CTGCCGCGACCCCGGCCCCGCCCCTGCCCCGGCCCCGGCCTGGCCCGGTCCCTGCACCTGCCGGA
Procavia capensis   ACGGCCG--------------------GGCTACACCGTGACACGACCTCCTGCGCTGCCGACACAGTGGTCCCGCGTCTGCTGGAGGCTGTGC----CACCGCCCGCCCGCCTGT----

Homo sapiens        GGCCGCCCGCCGCCATCGCCAGCGCCGTCGCTTGGCTGCGCTGACCGGGAAGCAGTTCTCGGCGG-----TCCGCGCGGTGATTCATCGGCCTGGCCGCGTCCCCCGCG-CGGGCCATCT
Procavia capensis   --CCTCCCGCCGCCACCG-------CGCCGTTCAGCTGC-CTGATCCGGAAGCGCTTCTGGGCGGGCGACGCCGCGCGG------ACCATCTTGGTCG------CCGCATCGGGCCATCT

Homo sapiens        TGGTCACGGCCGGCGGCGGACAGAGCCGCGGAGGAA-CCGCGAGACCCCAGGTACGCG---TGCCCCCGGCCCCCTGCCCGGCCGGGCCCCGCCACTGTGGTGGCCCCCAGGC-CCGCGG
Procavia capensis   TGGTCTC-GCCGGAG-------GAACCGCGGAGGAAGGCGCGGAGACCCAGGTACGCGACCGTCCCCCAGACCCTGGCCC-------CGCCGCCACTGTCGCCCACCCTTGTCGCCCCGG

Homo sapiens        CCGCCA-AGGAGCAGCCCCCTTTGTTCTCCCCCGGGCGGGCGCGGGGCG-GGCGCCGCGGGGCTTTGTGCAGCCTCGCCACCCACGCGCGGGCCCTCCAC--GCCGCCCCTCACCCTCCT
Procavia capensis   CCGCCGCAGGTGCCACCACCTTTGTTCGTCCGCGGCCGGGC-TGGGCCGCGGGGACCCCGGGCTTTGTGTG--CCAGCCCCCAGCCCCCGGACCATTCACTGTCTGCCCC---CCGTTCC

Homo sapiens        GCCCGCTAGTCGCAACTGAAACCTTGAAAACATCTTTTCTTAATCCTCCAGGGCCCAGAGCAGGTCCAGCCACCCGTCTGGCTCCGTAGTCCGCGAAGTCTGCAGTAAAGGAGCCGCGTG
Procavia capensis   GTCGGCTAGCT----------CTTTTTCAAATTATTCTTTCAATCCTTCA-GGCCCAGAGACAGTCTA-CTGCCTGTCTAAC-CTGCCCGCCGTGCAAGGCGCAG----AGAGGCGTCCC

Homo sapiens        GGACCGGGGCCGAAGTCCCTAG-GGCCTGGCACCGCGTGC-----------CCCTCTCTCGGGCCCTCGGCTTGGGGGTCTCGCAGCGCCCTGCCGTCGTGCACCCCACACTGCCCCCA-
Procavia capensis   GGAGCGAGGCATCGATCCAGAGAGGCCTGGCCCTTCGCGCTCTTCGCGGCACCATCTGCCCTGCCTTCTGGTTGGGTGTCAT-CAGC----TTCCACCCTGACTCCCACCCTTCCCCCAA

Homo sapiens        -----------------------------TTTCTACAGGGGCCCACCTGGTCAC-----CACACCCTCTTGGTCCACCCGCG--GATCTCAGCGTGTGGCTGACCTCTGGCCAGCATGGT
Procavia capensis   TTTCTCTTTAGGTCAACCCCTGTTTTAGGCTTCTTTAGATTTTCACCTCTTCACACCCCCACATCCTCCTCACCCCCCCACATCAGTCTCAGACTTGGGCTCACCTTCAGCCAACATGGT

Homo sapiens        GCAGCCGCAGACGTCCAAAGCTGAAAGCCCAGCCTTGGCAGCGTCTCCGAATGCCCAGATGGATGACGTTATTGACACCCTGACCTCCCTGCGCCTCACCAACTCGGCGCTGAGGCGGGA
Procavia capensis   CCAGCCCCAGACGTCAAAAGCTGAAAACCCGGCCTCCTCGGCGTCTGCAAACGGCCAAATGGAGGCTGTGATCGACACCCTGACCTCCCTGCGCCTCACCAACTCGGCGCTGCGGCGGGA

Homo sapiens        GGCTTCCACCCTGCGGGCGGAGAAGGCCAATCTCACCAACATGCTGGAGAGCGTGATGGCAGAGCTGACCTTGTTACGCACCAGGGCGCGGATCCCGGGGGCTCTGCAGATCACCCCGCC
Procavia capensis   GGCCTCCACCCTGCGCGCGGAGAAGGCCAACCTCACCAGCATGCTGGAGAGTGTGATGGCCGAGCTGACCTTGCTCCGCACCAGGGCGCGAATCCCGGGCGCGCTGCAGATCACTCCACC

Homo sapiens        CATCTCCTCAATTACTTCAAACGGGACTCGACCCATGACCACACCTCCAACCTCTCTGCCCGAGCCCTTTTCCGGGGACCCAGGCCGGTTGGCGGGGTTCCTGATGCAGATGGACAGATT
Procavia capensis   CATCTCTGCCATCACGTCCAACGGGACCCGGCCGATGACCACCCCTCCCACCTCTCTCCCCGAGCCCTTCTCCGGAGACCCCGGCCAGTTGGCCGGGTTCTTGATGCAGATGGACAGGTT

Homo sapiens        CATGATCTTCCAGGCCTCCCGCTTCCCGGGTGAGGCCGAGCGTGTGGCCTTCCTTGTGTCTCGACTGACTGGGGAGGCGGAGAAGTGGGCTATCCCCCACATGCAACCTGACAGCCCCTT
Procavia capensis   CATGATCTTTCAGGCTTCCCGCTTCCCCGGCGAGACAGAGCGAGTGGCCTTCCTGGTGTCTCGTCTGACTGGGGAGGCCGAGAAGTGGGCCATCCCCCACATGCAGCCGGACAGCCCCTT

Homo sapiens        GCGCAACAACTATCAGGGGTTCCTGGCAGAGTTGCGGAGAACCTACAAGTCTCCGCTCCGGCATGCGCGGCGCGCCCAAATCAGGAAGACTTCTGCCTCTAATAGGGCTGTGCGAGAGAG
Procavia capensis   GCGAAACGACTACCAAGGCTTTCTGGCCGAGTTGCGCAGAACGTACAAGTCTCCGCTGCGACAAGCACGGCGCGCGCAAATCCGGAAGGCTTCGGCCTCCAATCGGGCTGTGCGCGAGCG

Homo sapiens        GCAGATGCTCTGCCGCCAGCTGGCCTCTGCGGGCACGGGGCCTTGCCCAGTGCATCCAGCTTCCAACGGGACTAGTCCAGCGCCAGCCCTGCCTGCCCGAGCACGGAATCTTTAAGAATC
Procavia capensis   GCAGGTGCTGTGCCGCCAGCTGGCTGCAGCGGGTACAGGGCCCTGCCCGGTGCATCCTGCCTCTAGCGGGACTCATCCAGCGCCAGCCCTGCCCACGCGAGCACGGAACATTTAAGAA--

Homo sapiens        CGCCAGCACTTGGTAGCGTCTGCAGCCACCCAGGTAGCATACGCTCTTTGCTGTGTAGAAGAAATGCCCATACGACAGCTTTGCCCCTGTTTGAAGACCTCCCTTCTTGCCTCTCCAGAC
Procavia capensis   -----------------CCTACTGCCCGCCTGGTGGCCCGCGACCTTTGCTGCGCAGACAAAATATCTGCACAGCATTATAGCCCTTCCTTGAAGAACACCTCTCCCGCCTCTCCGGTC

Homo sapiens        GTGTTCCCCGAGGAGATCTTCCTTCCGTC----CTTCCTGGCGCCCTGGT-----------------TGCCCACCTTGCCGTGCTTCCTCTTACGTGCTAG----CTTTGTACCTATCGC
Procavia capensis   CTCTTCCAGAAGGAGATGTCTTTCCAGCCAGCACTTGCGGGGACCCTCACGCCCATGCCCACGCCCACGCCCACCTTGCCATGCTTCCCATTGTTTTCCGGTTGCCTTTGCACCCACAGC

Homo sapiens        TCACTGCATGCTCGCCTCCCTCTTGCTGGCATCCCGGCCTGTTTCAATGACTACCGCTCTGCTACTTAGGCACAGGGACTCCGCCGCACGCTG----------ACGGACCACGAGGGCTG
Procavia capensis   -CGCTGCATGTCTCCCTCTCTGTTGCTTGCAACGCCACGTCTCCTGATGACTACA---CTGCCGCCTGTTCACAG---CTGTGCCGCAGCCTGGGCTGGAGCTGTGAAGTGCCTGGGCTG

Homo sapiens        ACCCCTTCCAGCCTGACTTGGTTCATGGAGGCTCCTACTCTGCCCTCTCCAAGCTCCCCTGGCGGCTCCCCACCTGGTTGCCCAGTTCCTATTGATGAGCTCTGGACAGAAAGATGCCCG
Procavia capensis   GCCCCGTCTGG--------AATTCACGGAGGGGCCAGTACCTCCCACTGT-AGCCCGCAGGGCAGGCGCTCCGCTGTGTGCCCTGTTCCCGTGGGGGACCTCTGGACAGAGTTAAGCCTG

Homo sapiens        TTTGGCCAGGCTGGTGGCTTGATGGGTGTACCTGGAGAGGGGGTCTGGCTTCCTGCCCAAGATGCCTCCCAGCCCTGCCAGGGCCCGGTGCAGCGGGCAG-GGCCTCATCTGTGCTGTAG
Procavia capensis   TTTGGCCAAGCTTGT-GCTCGTCGTGTGTACCAGAGGAGAGGAACCGGCCT---------------------CCTGCCAGGGCCC--TGCTCCCCGCAGCCACCTAGTCCCTTCGCTAG

Homo sapiens        TGGTCGAGTGGTTGCTGCAAGGAGCGTAGTTCTGCCATGTCTGGGGGCCAGGTTCCACTCTGCACATGAATATGCAGTCTGGGAGGCCCCACTGCTCTCA----CTGGGAAGGACCAATG
Procavia capensis   GAGTGGAGCTGTAGC---------------------CGTTTGAGGGGCAAGCTGCACTCTGCACGTGAACACACGGTCCTGTGTGCCCAGTCCCTCCTGGCTCCTGGAAAGACCCTGTG

Homo sapiens        TTGCA---------------CCTCTGTTAATGCCTGACTTCAGCTGCTGGTGTTCTGATGGAGCCAGAGGCTTGGGGAATCTGGAACTTGCCTGCTAAATAAGGTCGTGGTGGACTCTCA
Procavia capensis   GTGCAGAGCAAGGGAGAGACCCCTCAAGGACCTCTGGCT----------GTGTGCTTGTAGCTTCAGA-CCTTTGGGAATCTGGAACTTGGCTGCC-AGTGAGGGCATTGTGGACTTTGG

Homo sapiens        GCCATTGGGCAGGTCTATCAGGCT----------GCAGGTTCCTACACACCCACGCCTGAGGGTCATAGCAGGCTAAGGGTGGATACCAGCGACT--CCCTTTGCTGCCCAGGATCTCCA
Procavia capensis   ACCACTGTCTGGCTGCATCAAGCTGACCCAGACAGCAGACTCACACGCGTCT-CACTTGAGACTCCT-GCAGCTGATGGAGGGACATTGGGGGCTTACACTTTCCCACCC..........

Homo sapiens        TGGGCAGTGCCACAGCGGCTGATGCTCAGTCACTCCTGCTTCTACCCCCTGTCACTGATGGCGAGCCTTGCCCAGCTTGAGACCTGTTCCCATCTCTATTCAGGTGCCATGTGGCCTTCA
Procavia capensis   .............................................................CAGGCTTCGTCGAGCTTGAGCCCTGTCCCCGCCTTCATTTGTGCTGTCTGCTGCCGTGC

Homo sapiens        CTGCAGCCC-TGCAGCCACCCACGCACCATCTGTGGGTCTCCAAAGGCACCTTGTAGCATGTACTCCCCGTGCCTGG--GCAATCAGATGGGCTGCCTTTGTCC---AAGGGAAAACAGA
Procavia capensis   TCTCAGCCTGTGTGGTTACC---ATGCCATCTCTAGGTCCACA---GCCTCTCCAGGCTGGACCATCCAGTGCCTGGCTGCCATCAGGTGTCCTGTGTCCGGCCTGTAAGGGAACATATT

Homo sapiens        CTCCCTTCGGGAAACATCCTTAAGCACTTAAGGCCGGGGGGGGTGTCTGCCTCTGGCAACCCAGCCAGGGTCTTGGTGGCATTTGTAAAAGCAAAGAGCTGTGGACTGCCGTGGTCCTAG
Procavia capensis   CCCTCTT------ACATAACTCAGCATGCAGGGT--GGCGAGGCAGCTGCCCCTGGCCACCCAGGCCGGGCCACGATGGCATCTGTCAAAGAAAAGAGCAGGGGACTGCCGCA-TCTGAG

Homo sapiens        TGTG-----------GTGACAATGCAGCACTGGCATGCATGTCCTCCTTCTGAAGGA---CCTCATCCTTCCTCACAGGGGGATGACCAAGAAATCATTTTGTGGCTGAG-TTTGGCCAC
Procavia capensis   TGTGTCAGCCCTCTTGTCCTAATTCGGAGGGGCAAAGCCATCCCTCTCACTGGAAGATAGCCTCGTGCCTCCTCACAGGG---TGACGAAGAAGCCGCATTCTAAAGAAGACTTGGCTGA

Homo sapiens        GCCCTTTGGACTGTGCTGTTCC-GCCATATTTCAATGCCAAATGAACCACATTGACATGAC---CTGGACCATAGGG--------CTTCCTATCCTGGGCTCAGCTGCCCCTG-TCTGAA
Procavia capensis   GCCCCTTAGACCATGATGCTCCTGCCACGTTTCAGTACCGAGTGAACTGCGTTCATGTGACTGCCCGGACTGTGAGGGGTGGTGGTGTCTTAGCCTGGGTTCCTCTGCCTCTGGTTCAGA

Homo sapiens        GGGTCTTGGCTTGATTGCAGAAGG-ACAACCTCCGCACCCACCTAAAGACATGTATATGTCTTGGGAT-CCCAGAGATTGGGTCCTTGGGCCTGGCTTCTT-----AAGAGTTTTGATGA
Procavia capensis   CAGTCCTGGTGTGACTACAGAGGGGACAGCATCAGC-CCTAACCAGAGACACGT----GTCTTGAGATACTCTGAGCTTGGGTCCTGGGACCTACCCTCTTTAGCTAAGAGTTTCAGTGC
```

```
Homo sapiens        TGCTGGGAAAAGTGACTGCGATTCTGAAG-AACCGCTGCCTTGCAAGGTCAAGGACATTCAGTGGTTGCTGGGGTCCGCAGACTACTGCCACCCACTCACCATCAACTCTGTTAGCCCAA
Procavia capensis   TGCTGGGAGAGGTGACTG---TTCTGAATTGGCCACTGCCTCGGCGGGTAGGGG-----TGCTGGTTGCTGGGGCGTGGGGACCA--GGTACCCA-TCAGTGTGAGCTCTGTTAGTCCGG

Homo sapiens        TTGCCCTGCTGAACAACTGCCTGAATACAGGCTTTAGGTTCCCCTGGACTCCAGCCAAGGCTGTTCAGGTGGGACCATGGTGCTCTTTAAGCGTGATCGGAGGGAAGACACACAGCAGGG
Procavia capensis   TCGCCCTGCCGGGCAGCTGCCTGAACACAG-CGTCAGGT--CCCGTGATGCCAGCCGAGGCTGTGCCCGTGGG-CCACAATGCCCAGTAAGCAGGAATGG-GAGAGGGTTCAGGGT-GGG

Homo sapiens        CCACCATTCCATGAATGGGAGGTGTACAGATCACTTTCTCTTTGTGCTCAGTTCTGTTCTGTCTCCAGCAGCTATATTGGTAAGACTAGTACCTGCCAGGGAGAGGTGCCCCCAAGTGAA
Procavia capensis   CTGTGGGGCTCTGA--TGGGGGCGCAGTGTGCCCTTCCTGTCTGGGCTGCGTTCT---------CCAGCTGCTGGGTTGGTGGGATGTGTACGTACCAGGTGTAGGTGCTCT-GGGTGAA

Homo sapiens        GGGGTACAGTGGCACCTGGGAAAAGG---------------------CACCTGGAAGGTTTCCATGTGGCCCAGCCCAGCATGGAAGCAGGGTG-GGAACTCTGCTGTGTCGCCAGCGCT
Procavia capensis   GG--CCCAGTGACTCTTGGGGAAGGGTGGCCTGCAGGGGCTCAAAGACAACCTGAAAGCCTCCATGTGGCCCAGTCAGGGAAGGAAACCCAGCATGGTCCTCTGCCGTGTCACCACC-CT

Homo sapiens        CACTCTACTCGAGTGGCTTTTTGAAAGCCCTACCATGTCTGTGTCAGGCCTGTGCTGCTTCACATCCTACAGCTGCCTAGGAAAGGCCGGCCACGCTCCCTGTCCACACACTCCCTGTCC
Procavia capensis   CGGTCGGCT-GAGTGGTGTGTAGGAAGTGCCAGTGTGTTTG-----------------------CCTGGAGTTGTGTTTTAGAGTCGTGCCAC------------CACAC-----GTAA

Homo sapiens        ACACACTCC----CTGTCCACACACTCCCTGTCCACAACTGCAGCCGGGCCCTCTGCCTATGGGCACCCAATCCAAGCAGCTGCT---------CCACCTTTGTTTGGCATGGTGATTTG
Procavia capensis   AGGAGCGCCAAGGCTGTCCAT------CCTGCCCAGGACTGT-GCCAGGCCCACCGC------------------AGTGGCTGGTAGTGCCGGGCCACGCTCATCGGCTGTGGTGGCTTG
```
[truncated: 1,647,851 more chars]
